# Mendelian randomization analysis reveals causal associations of serum metabolites with sepsis and 28-day mortality

**DOI:** 10.1038/s41598-024-58160-1

**Published:** 2024-05-21

**Authors:** Guoqing Jing, Jing Zuo, Zhi Liu, Huifan Liu, Miao Cheng, Min Yuan, Hailong Gong, Xiaojing Wu, Xuemin Song

**Affiliations:** 1 Research Centre of Anesthesiology and Critical Care Medicine, Zhongnan Hospital of Wuhan University, Wuhan, Hubei China; 2 Department of Pediatrics, Children’s Digital Health and Data Center, Zhongnan Hospital of Wuhan University, Wuhan, Hubei China; 3 Jingmen Central Hospital, Jingmen, Hubei China; 4 Department of Anesthesiology, Renmin Hospital of Wuhan University, Wuhan, Hubei China

**Keywords:** Genetics, Immunology, Diseases, Medical research

## Abstract

Metabolic disorder has been found to be an important factor in the pathogenesis and progression of sepsis. However, the causation of such an association between serum metabolites and sepsis has not been established. We conducted a two-sample Mendelian randomization (MR) study. A genome-wide association study of 486 human serum metabolites was used as the exposure, whereas sepsis and sepsis mortality within 28 days were set as the outcomes. In MR analysis, 6 serum metabolites were identified to be associated with an increased risk of sepsis, and 6 serum metabolites were found to be related to a reduced risk of sepsis. Furthermore, there were 9 metabolites positively associated with sepsis-related mortality, and 8 metabolites were negatively correlated with sepsis mortality. In addition, “glycolysis/gluconeogenesis” (*p* = 0.001), and “pyruvate metabolism” (*p* = 0.042) two metabolic pathways were associated with the incidence of sepsis. This MR study suggested that serum metabolites played significant roles in the pathogenesis of sepsis, which may provide helpful biomarkers for early disease diagnosis, therapeutic interventions, and prognostic assessments for sepsis.

## Introduction

Sepsis is a life-threatening organ dysfunction caused by a dysregulated host immune response triggered by infection^1^. In a report about sepsis worldwide, the incidence of hospital-treated adult sepsis was 189 cases per 100,000 persons, which is likely higher in low-income and middle-income countries owing to the high infection rate and backward medical environment^2^. Another research based on analyzing death records to calculate mortality related to sepsis and estimated the global incidence of sepsis was 677.5 cases per 100,000 persons worldwide^3^. In fact, sepsis incidence and mortality varied substantially across regions, and the global incidence of sepsis is likely to be higher than estimated. The number of sepsis diagnoses continues to rise year by year despite advancements in critical care and antimicrobial therapy, making it a leading cause of mortality among ICU patients^4^. Therefore, finding accurate and effective sepsis biomarkers is of great significance to patient treatment and prognosis. According to previous research, C-reactive protein (CRP)^5^, procalcitonin (PCT)^6^, pancreatic stone protein (PSP)^7^, interleukin 6 (IL-6)^8^, high-mobility group box 1 (HMGB1)^9^, soluble triggering receptor expressed on myeloid cell-1 (sTREM-1)^10^ are commonly used for early identification and severity assessment of sepsis, and serum amyloid A protein (SAA)^11^, HMGB1, adrenomedullin (ADM)^12^, programmed death-1 (PD-1)^13^, endothelial cell specific molecule-1 (ESM-1)^14^, plasminogen activator inhibitor 1 (PAI-1)^15^, lncRNA CASC2^16^ are important predictors of sepsis-related death.

In recent years, there has been an increasing body of evidence supporting the notion that metabolic reprogramming is a crucial condition contributing to immune dysregulation in sepsis^17^. Cellular metabolism within the body is highly heterogeneous, dynamic, and plastic at different stages of sepsis. Raymond et al. have revealed robust and reproducible metabolic differences in host responses to sepsis despite significant inter-individual metabolic heterogeneity between septic and non-infected SIRS patients and between sepsis survivors and non-survivors^18^. The study identified 63 metabolites that differed between sepsis patients and non-infected SIRS patients, with lower concentrations of succinate, citrate, glycerol, glycerol-3-phosphate, phosphate, 21 amino acids and their catabolites, 12 glycerophospholipids, and glycerophosphoethanolamines, as well as 6 acylcarnitines in the plasma of sepsis patients. The differences were more pronounced between sepsis survivors (within 28 days) and non-survivors, with significant increases in 17 amino acid breakdown products, 16 acylcarnitines, 11 nucleotide breakdown products, 5 glycolytic and citric acid cycle components (citrate, succinate, pyruvate, dihydroxyacetone, and phosphate), and 4 free fatty acids in the non-survivor group. This study, in line with several other investigations, collectively underscores the significant role of metabolites in the pathogenesis and prognosis of sepsis^19,20^. Nevertheless, our understanding of the causal relationships between specific serum metabolites and the occurrence and mortality of sepsis remains highly limited.

Research on the association between blood metabolites and the occurrence and prognosis of sepsis primarily relies on observational studies and basic experiments. However, observational studies are susceptible to confounding factors^21^, and the results of basic experiments are challenging to validate in natural populations due to expensive prices, time-consuming, complex ethical issues, and so on^22^. To overcome these limitations, we employed a Mendelian Randomization (MR) research approach, an application of instrumental variables (IVs) analysis, that aims to test a causal hypothesis in non-experimental data. In an MR analysis, genetic variation, commonly single nucleotide polymorphism (SNP), is used as an IV for assuming risk factors. The principle of MR is based on Mendel’s second law of independent segregation of genetic alleles when DNA is transmitted from parents to offspring at gamete formation, and utilizes the random allocation characteristics of genotypes to phenotypes in nature for causal inference, therefore overcoming the limitations of residual confounding and reverse causation in conventional observational studies^23,24^. MR, as a genetic epidemiological method, has become a widely used approach to explore the potential causal relationships between a modifiable exposure and a clinically relevant outcome^25^. Accumulating evidence has proven the reliability of MR. For instance, Thorkildsen et al.^26^ have demonstrated insomnia is potentially causally associated with the risk of sepsis through MR analysis. In addition, You et al.^27^ have confirmed the specific intestinal flora that had a causal relationship with the risk and prognosis of sepsis at the level of gene prediction.

Some MR studies have been performed to explore the relationship between exposure and sepsis. However, the main focus was single exposures or common exposure factors, such as polyunsaturated fatty acids^28^, vitamins^29^, body mass index^30^, and iron status^31^. Few studies have focused on blood metabolites and sepsis and mortality. In this study, we performed a two-sample MR approach to assess the causal relationship between 486 human blood metabolites and sepsis occurrence and mortality risk to provide a deeper understanding of the pathogenesis of sepsis.

## Materials and methods

### Study design

**Figure 1:**
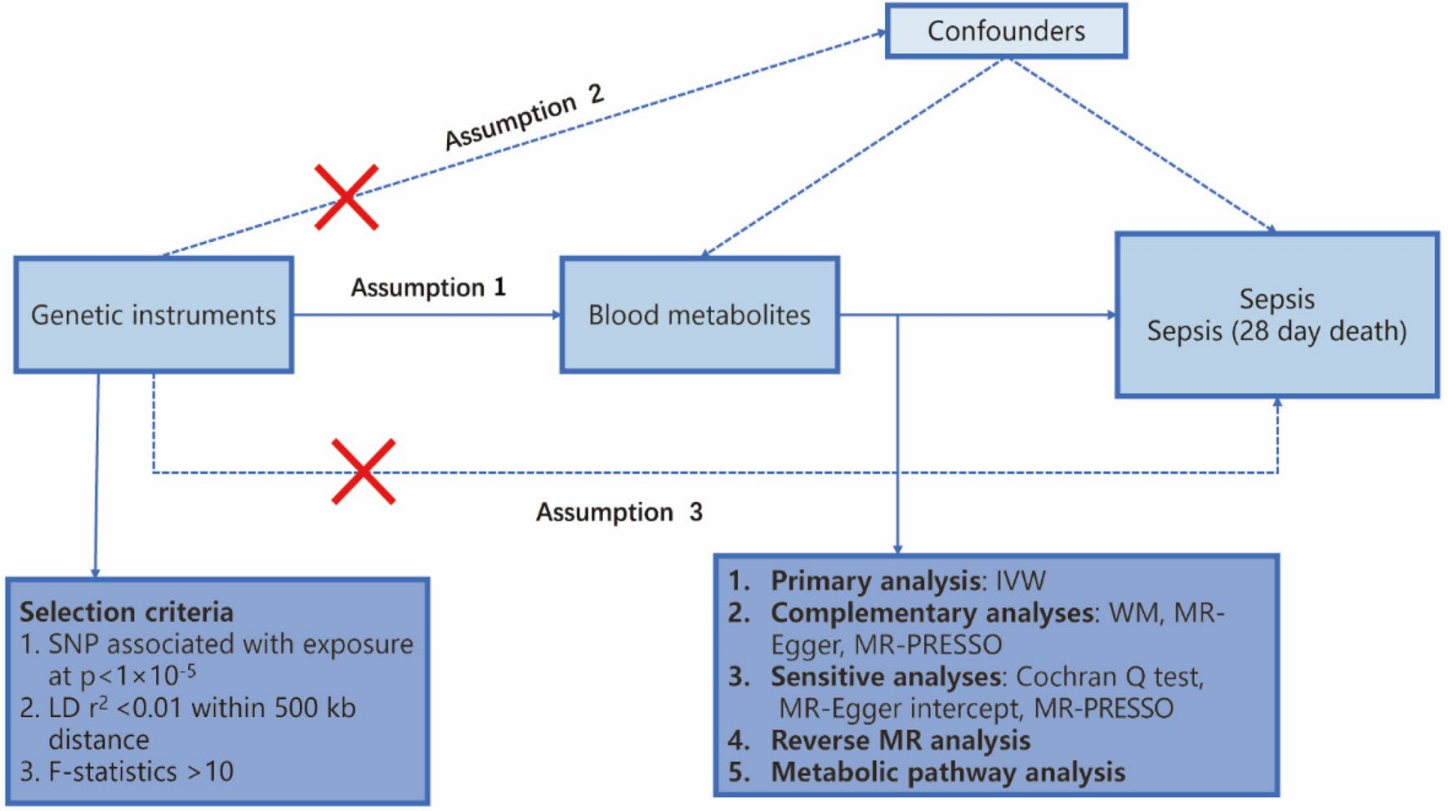
Overview of this Mendelian randomization (MR) analysis. Assumption 1: The genetic instruments are directly associated with the exposure. Assumption 2: Genetic instruments have no impact on the outcome and are independent of known and unknown confounders. Assumption 3: Genetic instruments are unrelated to the outcome and affect the outcome entirely through exposure.

We assessed the causal relationship between human serum metabolites and sepsis using an MR design with two distinct samples. MR studies rely on three fundamental assumptions: (1) the genetic instruments used must be directly associated with the exposure (serum metabolites), (2) these instruments should have no impact on the outcome (sepsis and sepsis mortality within 28 days) and should be independent of known and unknown confounders, and (3) the effect of the instruments on the outcome (sepsis and sepsis mortality within 28 days) is entirely mediated through the exposure (serum metabolites).To prevent sample overlap, we obtained genetic information for metabolites and sepsis separately from independent GWAS datasets. An overview of this study is presented in Fig. 1.

### GWAS data and cohort characteristics

The serum metabolite GWAS data were obtained from the study by Shin et al.^32^ which is the most comprehensive analysis of human metabolites to date, and its complete summary statistics are publicly available through the Metabolomics GWAS server (http://metabolomics.helmholtz-muenchen.de/gwas/). Plasma or serum samples from 7824 adult individuals from two European population studies (the Twins UK cohort and KORA F4 cohort) were collected and analyzed for metabolites using liquid chromatography and gas chromatography coupled to tandem mass spectrometry. A total of 486 metabolites were measured by the MS (Metabolon) platform and GWAS analysis was performed on the HapMap2-based imputed genotype dataset. After data processing, 2.1 million single nucleotide polymorphisms (SNPs) for 486 metabolites were identified, including 309 known and 177 unknown metabolites, respectively. According to the Kyoto Encyclopedia of Genes and Genomes (KEGG) database, 309 known metabolites can be divided into eight broad metabolite groups: amino acids, carbohydrates, cofactors and vitamins, energy, lipids, nucleosides, peptides, and xenobiotic metabolism. Meanwhile, the chemical properties of another 177 unknown metabolites have yet to be conclusively determined.

The GWAS data for sepsis and 28-day mortality after sepsis were sourced from the UK Biobank (http://www.nealelab.is/uk-biobank/). These datasets encompassed a combined total of 486,484 European adult volunteers, with 11,643 sepsis patients and 474,841 controls (IEU GWAS ID: ieu-b-4980), as well as 1896 sepsis patients who succumbed within 28 days and 484,588 controls (IEU GWAS ID: ieu-b-5086). Sepsis cases were identified by the International Classification of Diseases (ICD) 10th revision codes A02, A39, A40, and A41, in line with definitions used in recent literature^33^.

### Selection of SNPs

SNPs selected as IVs should satisfy three MR assumptions: relevance, independence, and exclusion restriction. The selection of SNPs in this study was based on the following criteria: (1) SNPs significantly associated with risk factors at the genome-wide level (*p* < 1 × 10^−5^); (2) SNPs were mutually independent (linkage disequilibrium with r^2^ < 0.01 within a 500 kb range). The statistical power of SNPs depends on their explanatory power for the phenotype (*R*^*2*^), which can be assessed through the strength of the correlation between SNPs and the phenotype.* R*^2^ refers to the proportion of variance in the exposure explained by the genetic variant. It gives an indication of the strength of the genetic instrument, with higher values suggesting a stronger instrument. Its formula is as follows^34^. $R2=\frac{2\times EAF\times \left(1-EAF\right)\times beta2}{2\times EAF\times \left(1-EAF\right)\times beta2+2\times EAF\times \left(1-EAF\right)\times N\times se(beta)2}$ In the formula, EAF stands for Effect Allele Frequency, that is, the frequency with which the effect allele appears in the population. beta is the effect size of the SNP on the metabolite and is a measure of the strength and direction of the association between the SNP and the metabolite. se(beta) is the standard error of the effect size and it represents the precision of the estimated effect size. N is the sample size. The above indicators collectively determine the IV’s strength and relevance. The *F*-statistic can reflect the strength of the correlation between SNPs and phenotypes, and its formula is as follows^35^. $F=\frac{N-K-1}{K}\times \frac{R2}{1-R2}$ Where N represents the sample size, K represents the number of SNPs. The magnitude of the *F*-statistic decreases with an increase in the number of SNPs, while it increases with an increase in the sample size and the explanatory power of SNPs on the exposure. In order to remove the weak instruments, we calculated the *F* statistics for each IV and excluded those with an *F*-statistic lower than 10^36^.

### MR and sensitivity analysis

Considering that the random-effects inverse variance weighting (IVW) can provide the most accurate assessment, assuming that all SNPs are valid instruments, we employed the IVW method as the primary analysis to evaluate the causal relationship between serum metabolites and sepsis and sepsis mortality within 28 days with *p* < 0.05.

To obtain more reliable results, we applied weighted median (WM), MR-Egger, and MR-PRESSO, the other three additional methods, as complementary analyses to further assess metabolites with significant estimates (IVW derived *p* < 0.05). The WM method provides a reliable estimate that the compelling IV accounts for more than 50% of the weight^37^, and the MR-Egger method can supply unbiased estimations under the strength of IVs independent of direct effect^38^. Moreover, MR-PRESSO can check and rectify horizontal pleiotropic outliers, thus providing correct assessments^39^.

Sensitivity analysis is crucial because it examines horizontal pleiotropy and heterogeneity, which may severely violate MR estimates. Horizontal pleiotropy can be observed when the IVs affect the outcome via pathways other than the exposure of interest. Therefore, we conducted several methods, including the Cochran Q test, MR-Egger intercept test, and MR-PRESSO, to detect and correct for heterogeneity and pleiotropy. The Cochran’s Q test identified heterogeneity among used SNPs estimates, with *p* < 0.05 indicating potential heterogeneity. We employed the MR-Egger intercept test to evaluate the possibility of horizontal pleiotropy, with *p* < 0.05 indicating possible pleiotropic effects. Furthermore, this study used the MR-PRESSO global test to evaluate the presence of horizontal pleiotropy, and *p* < 0.05 suggested the presence of horizontal pleiotropy.

In summary, we rigorously screened serum metabolites with potential causal effects on sepsis and sepsis mortality within 28 days via multiple criteria: (1) The *p*-value of the IVW method was significant (*p* < 0.05). (2) There is no heterogeneity and horizontal pleiotropy in MR results. (3) The directions of the four MR methods are consistent.

### Reverse MR analysis

To evaluate the causal relationship between serum metabolites and sepsis and sepsis mortality within 28 days, we also performed reverse MR analysis on serum metabolites causally related to sepsis and sepsis mortality within 28 days in the forward MR analysis. The methods and settings used are consistent with forward MR.

### Metabolic pathway analysis

Based on the analysis of known metabolite pathways and referencing the KEGG database, we conducted a metabolic pathway analysis using MetaboAnalyst 5.0 (https://www.metaboanalyst.ca/) to investigate the causal associations of metabolites identified through MR analysis with sepsis incidence and sepsis-related 28-day mortality^40,41^. This study only analyzed metabolites that passed the suggested association threshold by the IVW method (*p* < 0.05). Pathways were considered significantly enriched if *p* < 0.05 and the impact score > 0.1.

### Ethics approval and consent to participate

This study contains human participants collected from several studies conducted by previous researches. All participants gave informed consent in all corresponding original studies. Our study is based on the large-scale GWAS dataset rather than individual-level data. Hence, ethical approval is not applicable.

## Results

### The selection of instrumental variables

For each metabolite, we extracted genetic variants as IVs to test their causal relationship with the outcomes. SNP and their *F*-values, *R*^*2*^, for all IVs used in the forward MR, are provided in Supplementary Tables 1 and 2. A total of 9077 SNPs for Sepsis and 8651 SNPs for sepsis mortality within 28 days were finally included in the analysis. The number of SNPs for each metabolite ranged from 7 to 37. The minimum *F* statistic of these IVs was 17.41, indicating that all IVs were sufficiently compelling for the MR analysis of the 486 metabolites.

### Causal effects of serum metabolites on sepsis and sepsis mortality within 28 days

#### Sepsis

**Figure 2:**
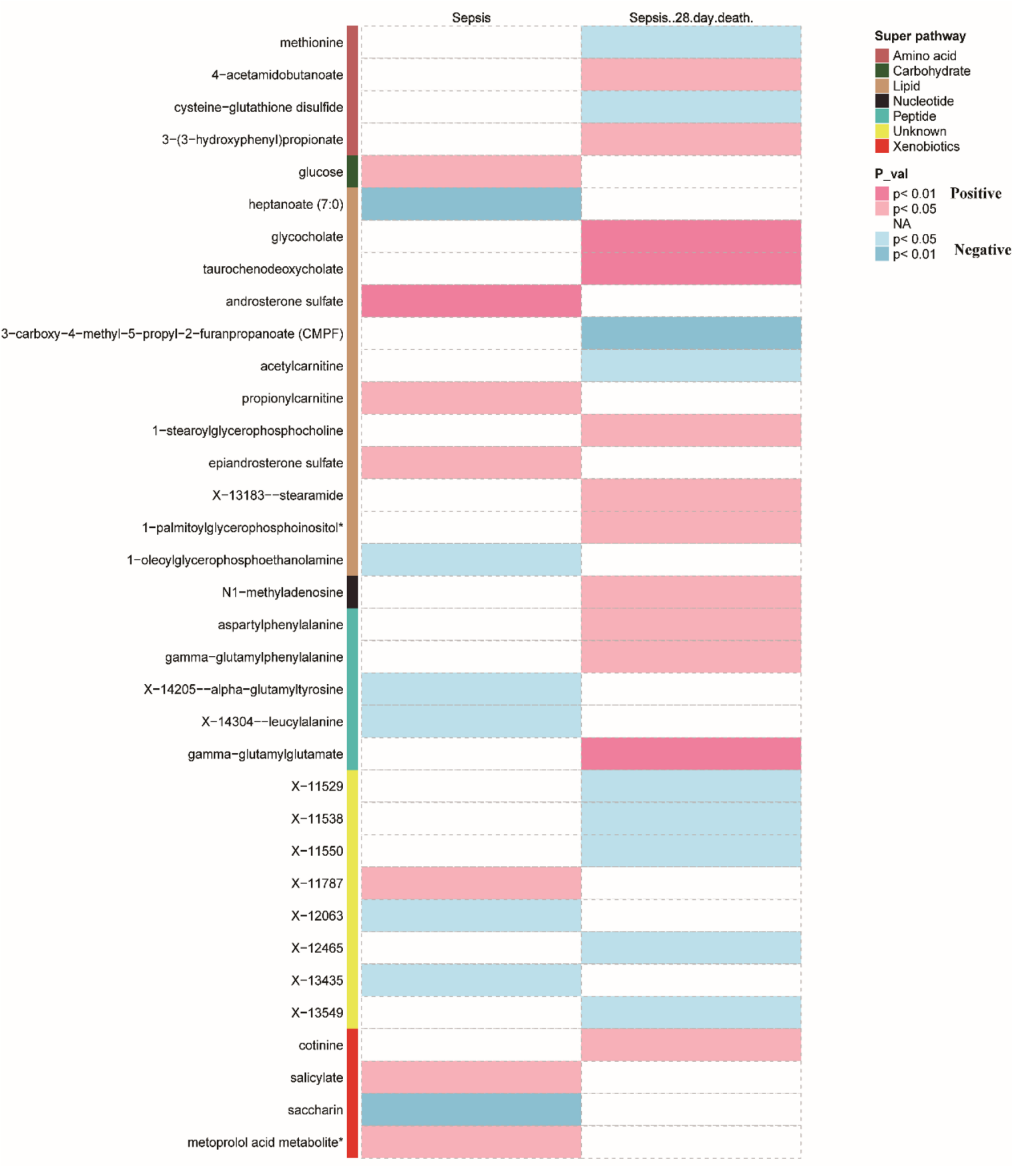
Causal association heatmap of serum metabolites with sepsis and 28-day mortality in sepsis by IVW analysis. Rows in the figure represent different serum metabolites, while columns depict two outcomes: sepsis and 28-day mortality in sepsis. Different metabolites are presented in distinct colors, with pink and blue indicating positive and negative factors, respectively. Deeper colors on the heatmap signify higher significance.

**Figure 3:**
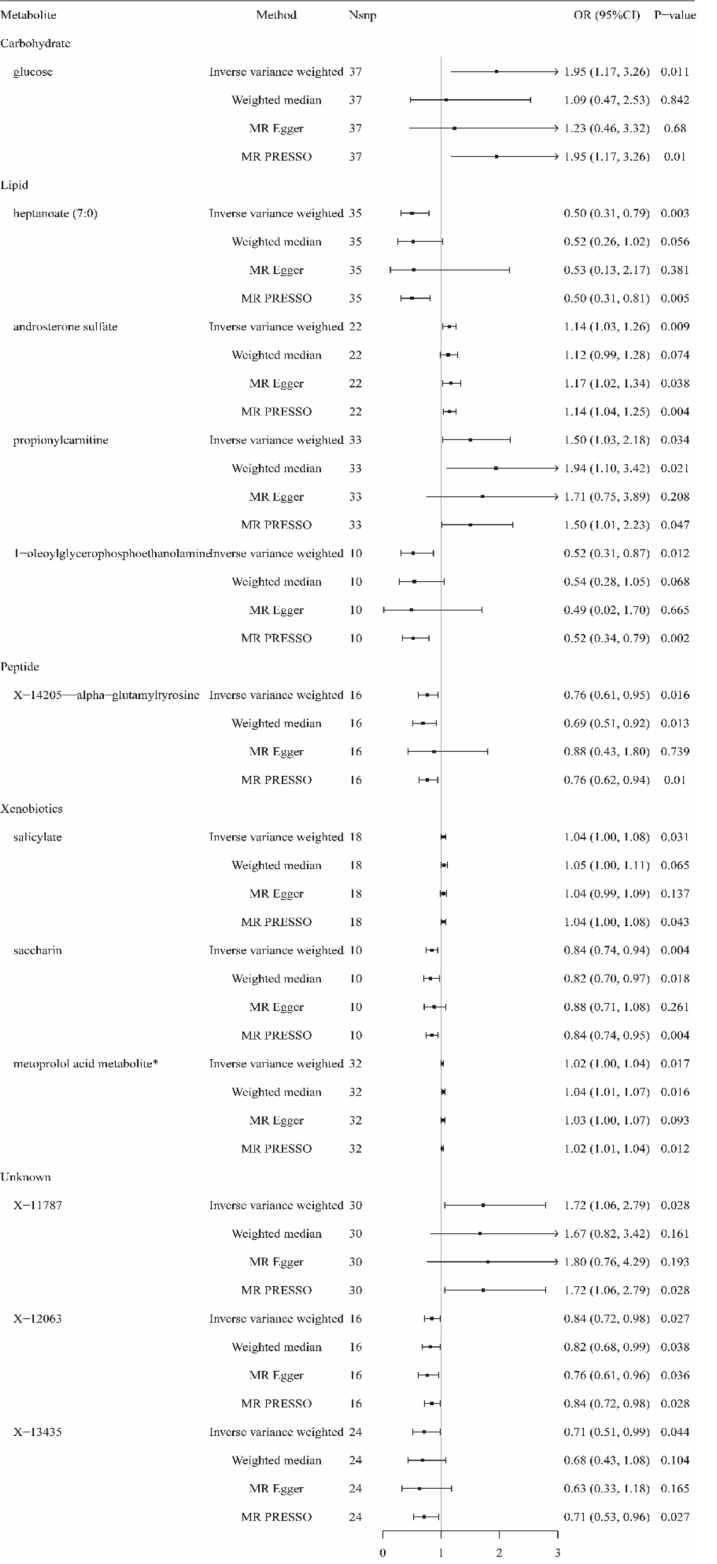
Forest plot for the causal effect of metabolites on the risk of sepsis by IVW, Weighted median, MR-PRESSO, and MR-Egger methods. Nsnp, number of SNP; OR, odds ratio; CI, confidence interval.

Using the above serum metabolites as IVs, we estimated causal associations between the 486 serum metabolites and sepsis, and a total of 14 metabolites were identified to have causal associations with sepsis (IVW method, *p* < 0.05) (Fig. 2). Combined with complementary analysis methods (WM, MR-Egger, and MR-PRESSO), we removed two metabolites (epiandrosterone sulfate and X-14304-leucylalanine) because of inconsistent with the direction of IVW, and 12 metabolites were finally obtained that were causally related to sepsis. These metabolites include 9 known metabolites with established structures and functions and 3 unknown metabolites. The known metabolites are derived from metabolic pathways such as carbohydrate, lipid, peptide, and xenobiotics. Among these, glucose (OR 1.95, 95% CI 1.17–3.26, *p* = 0.011), androsterone sulfate (OR 1.14, 95% CI 1.03–1.26, *p* = 0.009), propionylcarnitine (OR 1.50, 95% CI 1.03–2.18, *p* = 0.034), salicylate (OR 1.04, 95% CI 1.00–1.08, *p* = 0.031), metoprolol acid metabolite (OR 1.02, 95% CI 1.00–1.04, *p* = 0.017) and X-11787 (OR 1.72, 95% CI 1.06–2.79, *p* = 0.028) were positively associated with sepsis. While, heptanoate (7:0) (OR 0.50, 95% CI 0.31–0.79, *p* = 0.003), 1-oleoylglycerophosphoethanolamine (OR 0.52, 95% CI 0.31–0.87, *p* = 0.012), X-14205-alpha-glutamyl tyrosine (OR 0.76, 95% CI 0.61–0.95, *p* = 0.016), saccharin (OR 0.84, 95% CI 0.74–0.94, *p* = 0.004), X-12063 (OR 0.84, 95% CI 0.72–0.98, *p* = 0.027) and X-13435 (OR 0.71, 95% CI 0.51–0.99, *p* = 0.044) were negatively associated with sepsis (Fig. 3).

#### Sepsis mortality within 28 days

**Figure 4:**
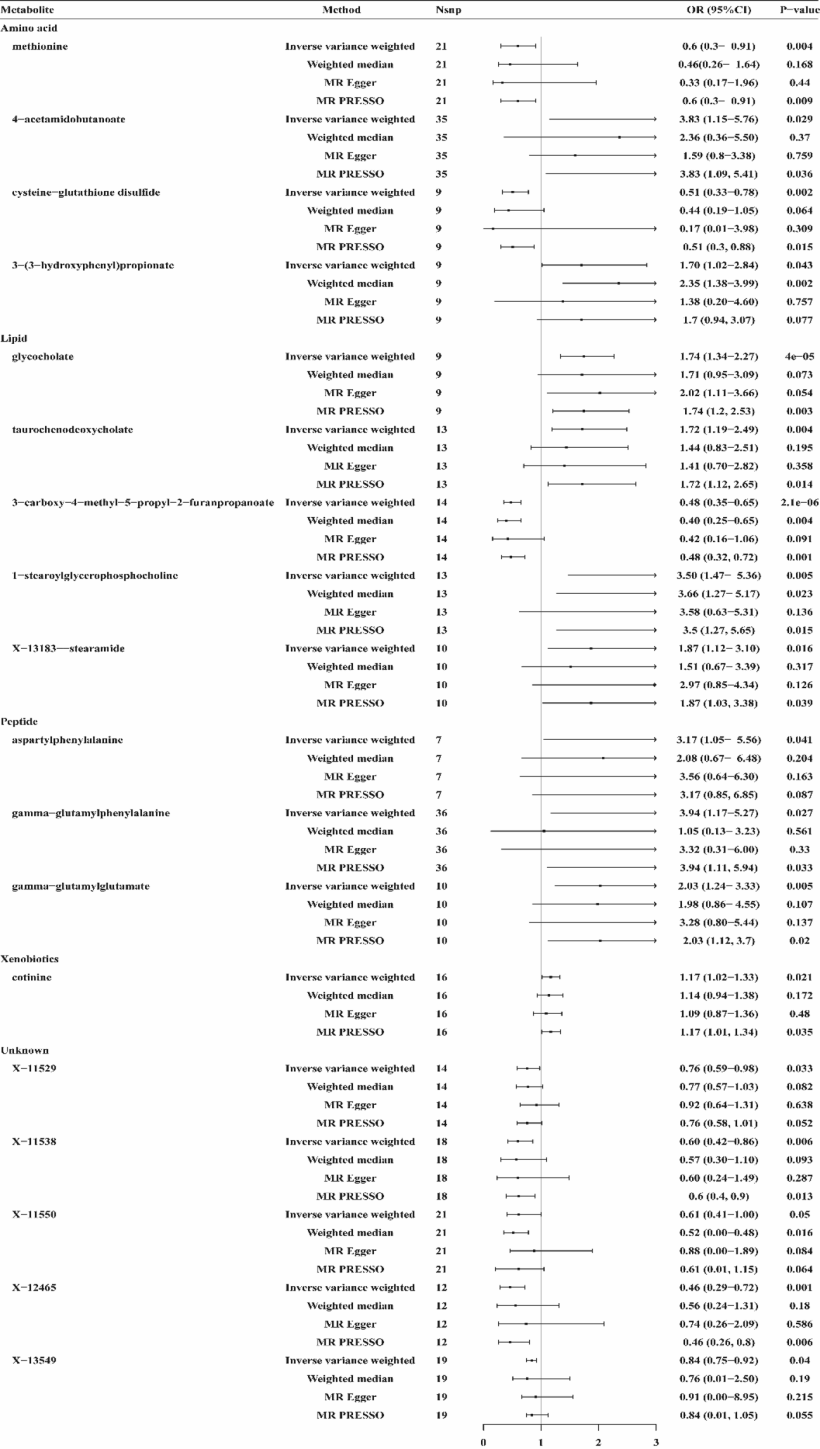
Forest plot for the causal effect of metabolites on the risk of sepsis mortality within 28 days by IVW, Weighted median, MR-PRESSO, and MR-Egger methods. Nsnp, number of SNP; OR, odds ratio; CI, confidence interval.

This study identified 21 causal relationships between the serum metabolites and sepsis mortality within 28 days (IVW method, *p* < 0.05) (Fig. 2). By utilizing WM, MR-Egger, and MR-PRESSO analysis, three candidate metabolites, including acetylcarnitine, 1-palmitoylglycerophosphoinositol, and N1-methyladenosine inconsistent with the direction of IVW, were excluded, and 18 metabolites were finally identified as having a causal relationship with 28-day death in sepsis. These metabolites include 13 known metabolites with established structures and functions and 5 unknown metabolites. The known metabolites are derived from various metabolic pathways such as amino acid, lipid, peptide, and xenobiotics. Genetically predicted 10 serum metabolites were associated with a increased risk of sepsis mortality in 28 days, including 4-acetamidobutanoate (OR 3.83, 95% CI 1.15–5.76, *p* = 0.029), 3-(3-hydroxyphenyl)propionate (OR 1.70, 95% CI 1.02–2.84, *p* = 0.043), glycocholate (OR 1.74, 95% CI 1.34–2.27, *p* = 4E-05), taurochenodeoxycholate (OR 1.72, 95% CI 1.19–2.49, *p* = 0.004), 1-stearoylglycerophosphocholine (OR 3.50, 95% CI 1.47–5.36, *p* = 0.005), X-13183-stearamide (OR 1.87, 95% CI 1.12–3.10, *p* = 0.016), aspartylphenylalanine (OR 3.17, 95% CI 1.05–5.56, *p* = 0.041), gamma-glutamylphenylalanine (OR 3.94, 95% CI 1.17–5.27, *p* = 0.027), gamma-glutamylglutamate (OR 2.03, 95% CI 1.24–3.33, *p* = 0.005), cotinine (OR 1.17, 95% CI 1.02–1.33, *p* = 0.021). Conversely, methionine (OR 0.6, 95% CI 0.3–0.91, *p* = 0.004), cysteine–glutathione disulfide (OR 0.51, 95% CI 0.33–0.78, *p* = 0.002), 3-carboxy-4-methyl-5-propyl-2-furanpropanoate (CMPF) (OR 0.48, 95% CI 0.35–0.65, *p* = 2.1E-06), X-11529 (OR 0.76, 95% CI 0.59–0.98, *p* = 0.033), X-11538 (OR 0.60, 95% CI 0.42–0.86, *p* = 0.006), X-11550 (OR 0.61, 95% CI 0.41–1.00, *p* = 0.05), X-12465 (OR 0.46, 95% CI 0.29–0.72, *p* = 0.001), X-13549 (OR 0.84, 95% CI 0.75–0.92, *p* = 0.04) were related with a decreased risk of 28-day death in sepsis (Fig. 4).

Since the *p*-value threshold is artificially prescribed, it only represents a low false positive result. It cannot guarantee an accurate result, no matter how small the *p*-value is. In addition, *p* < 0.05 is a relatively loose threshold, and we need to implement multiple tests to eliminate false positives by modifying the *p*-value threshold. The formula for Bonferroni correction is p*(1/486), where *p* is the original threshold, and 486 is the total number of tests. After Bonferroni correction, only glycocholate and CMPF passed this criterion; all other metabolites were nominally significant.

### Sensitivity analysis

Although the IVW method is highly effective in inferring causal relationships between exposures and disease outcomes, it is well known to be susceptible to weak instrument bias. Therefore, we further carried out sensitivity and pleiotropy analyses to evaluate the robustness of the causal relationship. As shown in Table 1, results from Cochran’s Q test suggested that no apparent heterogeneity was found in the selected SNPs (*p* > 0.05) except for 3-(3-hydroxyphenyl)propionate (*p* = 0.041), which serves as a positive factor for mortality within 28 days in sepsis. Moreover, both the MR-PRESSO test and MR-Egger interception test did not detect any signs of horizontal pleiotropy (*p* > 0.05). These findings indicate that the levels of heterogeneity and pleiotropy are not pronounced, supporting the robustness of the estimated causal relationships between these serum metabolites and the occurrence and mortality of sepsis.

**Table 1:** Sensitivity analysis results of serum metabolites on sepsis and sepsis mortality within 28 days by MR-PRESSO, Cochrane’s Q test, and MR-Egger methods.

Outcome	Metabolite	Super-pathway	*P*_Global_test_	*P*_Heterogeneity_	*P*_Intercept_
Sepsis	glucose	Carbohydrate	0.628	0.647	0.295
	heptanoate (7:0)	Lipid	0.627	0.579	0.934
	androsterone sulfate	Lipid	0.894	0.880	0.634
	propionylcarnitine	Lipid	0.400	0.383	0.718
	1-oleoylglycerophosphoethanolamine	Lipid	0.961	0.954	0.977
	X-14205-alpha-glutamyltyrosine	Peptide	0.837	0.807	0.678
	X-11787	Unknown	0.658	0.623	0.898
	X-12063	Unknown	0.725	0.644	0.275
	X-13435	Unknown	0.832	0.823	0.656
	salicylate	Xenobiotics	0.569	0.483	0.965
	saccharin	Xenobiotics	0.859	0.813	0.594
	metoprolol acid metabolite*	Xenobiotics	0.791	0.782	0.659
Sepsis (28 day death)	cotinine	Xenobiotics	0.579	0.541	0.466
	methionine	Amino acid	0.926	0.915	0.874
	4-acetamidobutanoate	Amino acid	0.423	0.393	0.527
	cysteine-glutathione disulfide	Amino acid	0.907	0.908	0.512
	3-(3-hydroxyphenyl)propionate	Amino acid	0.063	0.041	0.830
	glycocholate	Lipid	0.939	0.927	0.517
	taurochenodeoxycholate	Lipid	0.719	0.662	0.489
	3-carboxy-4-methyl-5-propyl-2-furanpropanoate (CMPF)	Lipid	0.817	0.746	0.759
	1-stearoylglycerophosphocholine	Lipid	0.855	0.826	0.475
	X-13183-stearamide	Lipid	0.657	0.639	0.436
	aspartylphenylalanine	Peptide	0.133	0.085	0.419
	gamma-glutamylphenylalanine	Peptide	0.400	0.405	0.871
	gamma-glutamylglutamate	Peptide	0.687	0.590	0.491
	X-11529	Unknown	0.576	0.420	0.170
	X-11538	Unknown	0.949	0.932	0.988
	X-11550	Unknown	0.306	0.278	0.194
	X-12465	Unknown	0.927	0.912	0.274
	X-13549	Unknown	0.189	0.179	0.534

### Reverse causation between metabolites and sepsis-related phenotypes

To obtain robust MR evidence, we performed a reverse causal effect analysis on the sepsis-related phenotypes on the identified candidate metabolites. In the IVW model, none of the sepsis and sepsis mortality within 28 days had opposite effects on these candidate metabolites, further supported by the other three MR models. Reverse MR is detailed in Supplementary Tables 3 and 4.

### Metabolic pathway analysis

The results of metabolic pathway analysis (Table 2) reveal three metabolic pathways associated with the risk of sepsis, namely Glycolysis/Gluconeogenesis (*p* < 0.001), and Pyruvate metabolism (*p* = 0.042). Additionally, we did not observed any pathway is involved in sepsis mortality within 28 days.

**Table 2:** Metabolic pathway analysis.

Trait	Pathway name	Total	Expected	Hits	Impact	*P*-value
Sepsis	Glycolysis/gluconeogenesis	26	0.050	2	0.101	0.001
Sepsis	Pyruvate metabolism	22	0.043	1	0.207	0.042

## Discussion

In this study, we firstly explored causal relationships between 486 human blood metabolites and risk of sepsis and mortality within 28 days by utilizing the MR causal effects model. We identified 12 blood metabolites that were causally related to sepsis and these metabolites may influence sepsis development through pathways such as glycolysis/gluconeogenesis, and pyruvate metabolism. Among the 12 blood metabolites, in addition to unknown metabolites and reported glucose^42^ and propionylcarnitine^43^ as risk factors for sepsis, we also discovered that elevated levels of salicylate, metoprolol acid metabolite, and androsterone sulfate were associated with an increased risk of sepsis. While, elevated levels of heptanoate (7:0), 1-oleoylglycerophosphoeth-anolamine, alpha-glutamyltyrosine, and saccharin could reduce the risk of sepsis. Meanwhile, we found 17 blood metabolites that were causally associated with 28-day mortality in sepsis. In addition to the previously reported associations between taurochenodeoxycholate^44^, stearamide^45^, cotinine^46^, methionine^47^, CMPF^48^ and sepsis mortality, we also discovered that 4-acetamidobutanoate, glycocholate, 1-stearoylglycerophosphocholine, aspartylphenylalanine, gamma-glutamylphenylalanine, gamma-glutamylglutamate and cysteine-glutathione disulfide is causally associated with 28-day mortality in sepsis. These results may provide biomarkers for early diagnosis of sepsis and targets for timely treatment of sepsis.

Through MR research, we establish causal relationships between 9 metabolites and sepsis, of which glucose has been intensively studied in sepsis. Recently, Jiang et al. established a predictive model for 47,185 septic patients in a retrospective observational cohort study, and they identified glucose levels as a significant risk factor for sepsis^42^. We further added genetic evidence for the role of glucose in sepsis. Neugebauer et al.^43^ conducted a retrospective analysis of 406 patients and found that the serum concentrations of most acylcarnitines including propionylcarnitine were altered in sepsis compared with systemic inflammatory response syndrome, which is also consistent with our research results. Surprisingly, salicylate and metoprolol acid metabolite were identified as the risk factors for sepsis in our study. Salicylate in the human body mainly comes from the intake of external drugs, especially aspirin. Although multiple studies have shown that aspirin can reduce sepsis progression by inhibiting the release of inflammatory factors and platelet aggregation, a growing number of studies have shown that the use of aspirin as a primary prevention strategy does not reduce the burden of sepsis^49^. What is more, studies have reported that the use of aspirin in critically ill patients can increase the incidence of sepsis^50,51^. The mechanism may be that aspirin irreversibly inhibit platelet function, hindering its activation and surface expression of adhesion molecules, thereby forming microvascular thrombus and causing ischemia, leading to tissue damage and multiple organ dysfunction syndrome. This seemingly contradictory result highlights the complexity of sepsis pathogenesis and requires further studies to explain and validate. Marika et al. discovered significant changes in androgen metabolism when analyzing novel potential molecular biomarkers of sepsis through untargeted metabolites. Among them, androsterone sulfate is significantly increased in sepsis patients with complicated febrile neutropenia^52^, and our study provided further evidence to support the role of androgen metabolism in sepsis. However, further research is needed to explore the mechanism of androsterone sulfate in sepsis.

We also found that elevated levels of 1-oleoylglycerophosphoeth-anolamine, alpha-glutamyltyrosine, heptanoate (7:0), and saccharin exhibited protective roles against sepsis. 1-oleoylglycerophosphoeth-anolamine is an important component of phosphatidylethanolamine (PE). PE is the main phospholipid in mammalian cell membranes and can attenuate inflammatory responses and increase cell survival^53^. Studies have shown that pretreatment with phosphatidylethanolamine diethylene triamine pentaacetate (PE-DTPA) inhibits LPS-induced TNF-α production in human myeloid cells, leading to NF-κB activation^54^. Therefore, we hypothesized that 1-oleoylglycerolphosphoethanolamine may affect the development of sepsis by regulating inflammatory responses. Inflammatory mechanisms play a key role in the pathogenesis of sepsis caused by changes in metabolic phenotype. Immune cells activated by inflammation, require large amounts of energy to perform their functions, which increases the body’s demand for glucose and fatty acids. This increased energy demand can alter an individual’s metabolic status, leading to changes in glucose metabolism and lipid metabolism. Cytokines released during inflammation, such as TNF-α, IL-1, and IL-6, not only play a role in the immune response but also directly or indirectly affect metabolic pathways. For example, TNF-α is thought to be involved in the development of insulin resistance, where dysregulation of glucose metabolism may promote the development of sepsis^55^. Increased production of ROS caused by inflammation can cause cell damage and impairment of energy metabolism. The role of oxidative stress in inflammation links reductions in red blood cell oxygenation and energy production, factors that can influence the development of sepsis. Research also shows that the gut microbiome is closely connected to the host’s metabolic and immune systems. Intestinal barrier damage and bacterial translocation during inflammation can further affect metabolic status, promote the production of inflammatory factors, and aggravate the process of sepsis^56^. In sepsis, there is a bidirectional relationship between altered metabolic phenotypes and inflammation. Inflammation leads to metabolic abnormalities, and these metabolic abnormalities can in turn exacerbate inflammation, forming a vicious cycle. This cycle may be one of the key drivers in the development of sepsis, triggering systemic inflammatory response and multiorgan failure.

As a tyrosine-containing dipeptide, alpha-glutamyltyrosine has high antioxidant properties and can improve oxidative stress in the pathogenesis of sepsis^57^. Heptanoate (7:0) is a derivative of heptanoic acid. The exogenous addition of medium-chain fatty acids (including heptanoic acid) could increase mitochondrial respiratory capacity under starvation and inflammation and improve mitochondrial dysfunction in sepsis^58^. Mitochondrial dysfunction plays a crucial role in the pathogenesis of sepsis. Inflammatory mechanisms and alterations in metabolic pathways contribute significantly to this dysfunction, leading to the development and progression of sepsis. Inflammatory cytokines elevate ROS production, overwhelming mitochondrial antioxidant defenses. Excessive ROS can damage mitochondrial DNA, proteins, and lipids, impairing the electron transport chain and ATP production. Furthermore, the energetic dysfunction extends to alterations in substrate utilization and metabolic reprogramming. Sepsis induces a shift from oxidative phosphorylation to aerobic glycolysis in immune cells upon activation, contributing to lactate release into the blood. This metabolic condition, resembling starvation, is characterized by the breakdown of protein, carbohydrate, and fat reserves, leading to further mitochondrial injury and organ dysfunction. The lipolysis-related rise in free fatty acid concentrations and the impaired fatty acid oxidation in the mitochondria are particularly noteworthy, contributing to a metabolic deficit that is associated with sepsis-related cardiomyopathy and increased hospital mortality^59^. Therefore, heptanoate (7:0) may play a protective role in sepsis by restoring mitochondrial function and providing energy to the body in inflammatory conditions. Studies have shown that saccharin intake in mice can change the composition of the intestinal microbiome, reduce bacterial load and increase the relative abundance of bacteroidetes, thereby alleviating intestinal inflammatory response and playing a protective role in dextran sodium sulfate-induced experimental colitis, which is also consistent with the results that saccharin can reduce the occurrence of sepsis in our study^60^.

Our study on metabolic pathway analysis suggests the involvement of glucose metabolism pathways such as glycolysis/gluconeogenesis, and pyruvate metabolism in the occurrence of sepsis in the population, with glycolysis being the most prominent. During the hyperinflammatory phase of sepsis, the expression of genes related to glycolysis in monocytes/macrophages increases; while during the immune tolerance phase, glycolysis levels decrease^61^. Tannahill et al. found that LPS stimulation of mouse BMDMs could increase the expression of Glut1 and HK3, increase glycolysis, and promote IL-1β expression, while using 2-deoxy-D-glucose (2-DG) to inhibit glycolysis in BMDMs significantly reduced IL-1β production^62^. In addition, metabolomic and transcriptomic analysis of polymorphonuclear neutrophils isolated from sepsis patients showed that glycolysis and the Warburg effect were significantly altered and downregulation of LDHA mediated by the PI3K/Akt-HIF-1α pathway caused inhibition of glycolysis, leading to neutrophil immunosuppression during sepsis^63^. During pyruvate metabolism, when pyruvate is transferred to mitochondria, pyruvate dehydrogenase complex (PDHC) oxidizes pyruvate to acetyl-CoA, thereby accelerating aerobic oxidation. Clinical studies have shown that PDHC activity in peripheral blood mononuclear cells of sepsis patients is significantly lower than that of healthy controls, and this reduced activity may affect the prognosis of patients with sepsis^64^. Targeted activation of PDHC can not only regulate glucose metabolism and inhibit lactate accumulation in septic cells but can also significantly restore TCA metabolite levels to control levels and improve liver function in sepsis^65^. Our findings further confirm the roles of glycolysis/gluconeogenesis and pyruvate metabolism in the occurrence and development of sepsis.

Given the persistently high mortality rate among sepsis patients, researchers have been investigating the risk factors for sepsis-related mortality. Our study demonstrates that serum metabolites, such as glycocholate and taurochenodeoxycholate, are identified as risk factors for 28-day mortality in sepsis. Taurochenodeoxycholate, formed by combining free primary bile acids in the liver with taurine is also considered closely related to inflammatory responses and the regulation of immune cells^66,67^. Recently, Long et al.^44^ conducted a metabolomic analysis of fecal samples from 23 sepsis patients. The data revealed a significant increase in taurine levels during the third week of illness in sepsis patients who ultimately succumbed, compared to the first week. A linear model depicted that higher concentrations of taurine-conjugated bile acids constitute a risk factor for sepsis mortality. Additionally, the blood metabolites of membrane synthesis (1-stearoylglycerophosphocholine) and amino acid metabolism (aspartylphenylalanine, gamma-glutamyl phenylalanine, gamma-glutamyl glutamate) closely correlate with the 28-day mortality risk factors for sepsis. 1-stearoylglycerophosphocholine is synthesized from stearic acid and glycerophosphocholine, both contributing to membrane formation. As the cellular signaling platform, the composition and physicochemical properties of the membrane, including membrane fluidity and potential, can cooperatively regulate immune cell signaling and receptor functions (including T cell receptors and B cell receptors). Both palmitic acid and stearic acid are saturated fatty acids that can reduce membrane fluidity, thereby increasing susceptibility to diseases^68,69^. In addition, circulatory failure secondary to shock is the most common direct cause of sepsis-related death. Angiotensin-converting enzyme (ACE) is a critical enzyme that converts angiotensin I to angiotensin II, participating in the regulation of cardiovascular functions, including blood pressure^70^. Therefore, as a product of ACE-catalyzed peptide cleavage, aspartate phenylalanine may be involved in septic shock. Dynamic plasma lipidomic analysis revealed that stearamide was increased significantly in patients with sepsis after cardiopulmonary bypass cardiovascular surgery, accompanied by high mortality^45^. Plasma cotinine is a nicotine metabolite. Romina et al. have shown that increased neutrophil extracellular traps (NETs) formation may be a trigger for sepsis in smokers and cotinine could effectively induce the formation of NETs, which may be the main reason why cotinine aggravates the death of patients with sepsis^46^.

Our study also suggests a connection between metabolic-induced liver dysfunction and susceptibility to circulatory failure, resulting in a higher risk of mortality within 28 days in sepsis. Traditionally, abnormal gamma-glutamyl transferase (GGT) has been seen as an indicator of liver dysfunction in critically ill patients, linked to increased mortality and longer hospital stays^71^. Thomson et al.^72^ found that among 263 ICU patients without pre-existing liver or biliary diseases, 61% had abnormal liver function upon admission and abnormal GGT significantly increased the risk of death within 30 days of admission. Our results indicate that gamma-glutamyl phenylalanine and gamma-glutamyl glutamate, associated with GGT, constitute high-risk factors for 28-day mortality in sepsis. In addition, studies have shown that plasma 4-acetamidobutanoate levels are elevated in patients with severe cirrhosis, suggesting that 4-acetamidobutanoate is associated with liver dysfunction in humans^73^. In fact, liver dysfunction plays an important role in sepsis caused by alteration of bile acids metabolism. Liver malfunction significantly alters bile acids metabolism during sepsis, affecting both the liver’s function and its communication with the gut, known as the gut-liver axis. In sepsis, liver dysfunction can lead to increased levels of bile acids, which in turn activate inflammatory and oxidative stress pathways, potentially resulting in cellular damage such as apoptosis, necrosis, fibrosis, and cirrhosis. The farnesoid X receptor (FXR) and G protein-coupled receptors like TGR5 are key mediators in the regulatory actions of bile acids, influencing various metabolic processes including lipid and glucose homeostasis^74,75^. The interaction between bile acids and the liver, underscores a complex regulatory network that is significantly impacted during sepsis. Understanding this network is crucial for identifying potential therapeutic targets aimed at mitigating liver injury and improving outcomes in septic patients. Therefore, we speculate that the reason for the increased mortality in sepsis caused by glycocholate and taurochenodeoxycholate may be related to liver dysfunction.

In contrast, methionine serves as a protective factor for 28-day mortality in sepsis. Methionine is enzymatically converted to S-adenosylmethionine (SAM), and S-adenosylhomocysteine (SAH) is the by-product of SAM transmethylation reactions. An observational study showed that SAM and SAH were significantly higher in patients who died from sepsis than in those who survived^47^. Our results also demonstrate a strong causal relationship between CMPF and reduced sepsis mortality within 28 days. CMPF is a major furan fatty acid metabolite whose role in disease has been controversial. Some studies have shown that CMPF can increase the production of ROS in human kidney cells and induce kidney injury^76^. However, there was also literature showing that higher CMPF levels were associated with a reduced risk of all-cause death^48^ and periodontitis^77^. Consistent with our study, Wei et al. found that (CMPF) is causally related to a reduced risk of sepsis in an MR study.

Beyond the known constituents and functions mentioned above, this study also suggests that 8 unknown metabolites are associated with sepsis occurrence and mortality. Most of these metabolites appear to serve as protective factors in the development and outcome of sepsis. Further identification, analysis, and exploration of the relationship between these unknown metabolites and sepsis may offer new insights into diagnosing and treating sepsis.

Our study employed four causal inference MR models to enhance the reliability of research findings. Among these, the IVW model serves as the primary method in MR research by averaging the effects of multiple genetic variants to improve the robustness of estimates. Each MR method is designed with unique assumptions and advantages. The observed heterogeneity in p values ​​may stem from the different assumptions and mechanisms used by these methods to account for pleiotropic and invalid instruments. Despite these differences, the consistency in the direction of effects across all methods enhances the credibility of our findings. This consistency suggests that our results are robust to the various forms of bias that each method is designed to address. Furthermore, MR-PRESSO, Cochran’s Q statistic, and MR-Egger models were utilized to detect heterogeneity and horizontal pleiotropy among genetic variants. Additionally, bidirectional MR analysis confirmed causal inferences between metabolites and the occurrence of sepsis and 28-day mortality.

While our study offers valuable insights into the potential causal relationships between metabolites and sepsis outcomes, we acknowledge several limitations that are inherent to our research design and methodology. Firstly, since all samples in this study are of European descent, caution should be exercised when extrapolating the results to other populations. Secondly, the SNP selection employed a *p*-value threshold of 1 × 10^−5^, resulting in a limited number of selected SNPs. This may only account for partial exposure variations and could impact the statistical power of causal estimation. Thirdly, in view of the complexity and multi-factor etiology of sepsis as a disease, we prioritized establishing a broad understanding of potential causal relationships and did not perform an in-depth analysis of the impact of confounding on the exposure-outcome relationship by subgroups defined by age, gender, or comorbidities. Future studies are needed to build on our findings by incorporating stratified analyses and multivariable adjustments to elaborate how different subgroups (based on age, gender, or underlying health conditions) exhibit different causal relationships between metabolites and sepsis outcomes.

In summary, this MR study provides preliminary evidence of a causal relationship between blood metabolites and the occurrence of sepsis, as well as the risk of death within 28 days. The study results underscore the involvement of cellular glycolysis and energy-related serum metabolite heterogeneity in the susceptibility to sepsis formation. Additionally, metabolites related to liver function impairment demonstrate a causal relationship with sepsis-related mortality within 28 days, highlighting the susceptibility to sepsis occurrence and death based on different metabolite phenotypes.

### Supplementary Information

Supplementary Table 1.

Supplementary Table 2.

Supplementary Table 3.

Supplementary Table 4.

## Data Availability

The datasets used during the current study are publicly available. The data for serum metabolite can be found here: http://metabolomics.helmholtz-muenchen.de/gwas/. The GWAS data for sepsis and 28-day mortality after sepsis were sourced from the UK Biobank: http://www.nealelab.is/uk-biobank/.
