# Supplementary material for: Mendelian randomization analysis reveals causal associations of serum metabolites with sepsis and 28-day mortality
Source: Sci Rep. 2024 May 21;14:11551. doi: 10.1038/s41598-024-58160-1 (PMC11109149; doi:10.1038/s41598-024-58160-1)
Supplement: Supplementary file 1 — Supplementary Table 1. [file 41598_2024_58160_MOESM1_ESM.pdf]

Supplementary table 1. The characteristics of SNPs and their genetic associations with known metabolites and sepsis.

	SNP	effect_allel	other_allel	effect_allel	other_allel	beta.expos	beta.outco	eaf.exposu	eaf.outcon	remove	palindromi	ambiguous	id.outcome	se.outcom	pval.outcor	samplesize	outcome	mr_keep.o	pval_origin	data_sourc	se.exposur	pval.expos	samplesize	exposure	mr_keep.e	pval_origin	id.exposure	action	mr_keep	R2	F
6203	rs7096654	T	C	T	C	0.2537	-0.01644	0.6211	0.61027	FALSE	FALSE	FALSE	DEG58p	0.01407	0.242633	486484	sepsis	TRUE	reported	textfile	0.0047	1.00E-200	7500	M33507.m	TRUE	reported	ycykD0	2	TRUE	0.279795	2912.926
2095	rs1188521	A	G	A	G	0.1518	1.75E-06	0.7254	0.699474	FALSE	FALSE	FALSE	MN85S3	0.014958	0.999906	486484	sepsis	TRUE	reported	textfile	0.0042	1.00E-200	7574	M15630.m	TRUE	reported	FUih5R	2	TRUE	0.147101	1305.961
7568	rs316020	A	G	A	G	-0.1799	-0.01573	0.0937	0.103666	FALSE	FALSE	FALSE	Ot2ZAn	0.022473	0.484009	486484	sepsis	TRUE	reported	textfile	0.0053	1.00E-200	7119	M34481.m	TRUE	reported	tpyFcK	2	TRUE	0.139298	1151.83
1419	rs887829	T	C	T	C	0.1131	-0.00314	0.3411	0.316055	FALSE	FALSE	FALSE	33ptwk	0.014686	0.830494	486484	sepsis	TRUE	reported	textfile	0.0041	2.50E-168	6252	M02137.m	TRUE	reported	SQY1na	2	TRUE	0.108507	760.709
7919	rs3738934	T	C	T	C	-0.1056	0.00326	0.6231	0.629287	FALSE	FALSE	FALSE	8qXKkX	0.014153	0.817842	486484	sepsis	TRUE	reported	textfile	0.0043	1.21E-134	6234	M35189.m	TRUE	reported	Nt6oaC	2	TRUE	0.08821	602.9087
2111	rs7594485	T	C	T	C	-0.1043	0.004852	0.285	0.281148	FALSE	FALSE	FALSE	MN85S3	0.015177	0.749192	486484	sepsis	TRUE	reported	textfile	0.0043	1.03E-132	7574	M15630.m	TRUE	reported	FUih5R	2	TRUE	0.07208	588.1892
5016	rs1020689	T	C	T	C	0.0399	0.018737	0.78	0.779049	FALSE	FALSE	FALSE	BNnOvt	0.016429	0.2541	486484	sepsis	TRUE	reported	textfile	0.0017	7.74E-123	7362	M33132.m	TRUE	reported	rHcCPG	2	TRUE	0.069617	550.7189
4692	rs4148324	T	G	T	G	-0.1091	0.002557	0.6706	0.684664	FALSE	FALSE	FALSE	MV108h	0.014691	0.861827	486484	sepsis	TRUE	reported	textfile	0.0048	1.52E-116	6642	M32758.m	TRUE	reported	btjNXQ	2	TRUE	0.072167	516.4595
3614	rs1027804	A	G	A	G	-0.3609	-0.04087	0.0419	0.059726	FALSE	FALSE	FALSE	gq4ISJ	0.028822	0.156229	486484	sepsis	TRUE	reported	textfile	0.016	8.82E-113	7338	M31591.m	TRUE	reported	wjwl4J	2	TRUE	0.06484	508.6457
5947	rs1024245	A	G	A	G	0.2212	-0.04929	0.9335	0.93212	FALSE	FALSE	FALSE	mDZrGw	0.027174	0.069701	486484	sepsis	TRUE	reported	textfile	0.0099	1.67E-109	6797	M33415.m	TRUE	reported	MzEP7R	2	TRUE	0.068423	499.0822
928	rs1255072	T	C	T	C	0.0637	0.009577	0.9233	0.91425	FALSE	FALSE	FALSE	tv99UN	0.024439	0.695136	486484	sepsis	TRUE	reported	textfile	0.0029	5.97E-110	7354	M01494.m	TRUE	reported	osDclp	2	TRUE	0.061569	482.3527
3730	rs1116152	T	C	T	C	0.0703	0.007759	0.6983	0.69812	FALSE	FALSE	FALSE	k16oIH	0.014871	0.601837	486484	sepsis	TRUE	reported	textfile	0.0033	6.42E-100	7340	M32328.m	TRUE	reported	TQA3sZ	2	TRUE	0.058228	453.6954
1141	rs938554	C	G	C	G	-0.0348	0.003821	0.2256	0.218593	FALSE	TRUE	FALSE	nlqUuV	0.016519	0.817079	486484	sepsis	TRUE	reported	textfile	0.0017	5.85E-93	7371	M01604.m	TRUE	reported	Uro9nC	2	TRUE	0.053792	418.9313
8898	rs1472631	A	G	A	G	0.0366	-0.00227	0.4919	0.487561	FALSE	FALSE	FALSE	eLhsld	0.013676	0.868205	486484	sepsis	TRUE	reported	textfile	0.0018	1.85E-88	6552	M37058.m	TRUE	reported	RSeWE5	2	TRUE	0.059357	413.3182
3038	rs7157785	T	G	T	G	0.0681	-0.00527	0.1649	0.160732	FALSE	FALSE	FALSE	FJZEYz	0.0186	0.776966	486484	sepsis	TRUE	reported	textfile	0.0034	7.23E-87	7284	M21630.m	TRUE	reported	s6Pshg	2	TRUE	0.052201	401.0672
4868	rs1871395	A	G	A	G	-0.1101	0.020673	0.8385	0.847702	FALSE	FALSE	FALSE	KBJVQO	0.019069	0.278318	486484	sepsis	TRUE	reported	textfile	0.0055	1.06E-89	7355	M32855.m	TRUE	reported	Xea7Fn	2	TRUE	0.051669	400.6186
614	rs174548	C	G	C	G	0.0488	-0.02225	0.7005	0.686724	FALSE	TRUE	FALSE	BYGzq4	0.014732	0.130925	486484	sepsis	TRUE	reported	textfile	0.0025	1.43E-84	7367	M01110.m	TRUE	reported	oZiXqP	2	TRUE	0.049178	380.927
6711	rs7552404	A	G	A	G	0.0746	0.009026	0.6984	0.694948	FALSE	FALSE	FALSE	bzT5yX	0.014825	0.542622	486484	sepsis	TRUE	reported	textfile	0.004	1.27E-77	7345	M33936.m	TRUE	reported	W7BO8n	2	TRUE	0.045214	347.7278
6841	rs1198147	T	C	T	C	-0.1844	-0.03447	0.0861	0.086296	FALSE	FALSE	FALSE	aWy2Uj	0.024403	0.157759	486484	sepsis	TRUE	reported	textfile	0.01	2.80E-75	7326	M33973.m	TRUE	reported	SUSE3b	2	TRUE	0.044356	339.9408
7904	rs1231726	A	G	A	G	-0.1377	0.020631	0.8337	0.847892	FALSE	FALSE	FALSE	AhsT9A	0.019077	0.279487	486484	sepsis	TRUE	reported	textfile	0.0079	2.09E-67	5953	M35187.m	TRUE	reported	uLU0Fy	2	TRUE	0.048558	303.7161
5030	rs1934963	T	C	T	C	0.0293	0.017991	0.8021	0.800605	FALSE	FALSE	FALSE	BNnOvt	0.017107	0.292946	486484	sepsis	TRUE	reported	textfile	0.0017	8.69E-65	7362	M33132.m	TRUE	reported	rHcCPG	2	TRUE	0.038785	296.9747
1894	rs1235619	A	G	A	G	0.0274	0.011037	0.8362	0.832956	FALSE	FALSE	FALSE	v2KE89	0.018314	0.546733	486484	sepsis	TRUE	reported	textfile	0.0016	3.69E-63	7349	M15500.m	TRUE	reported	to2t7H	2	TRUE	0.038374	293.1858
4085	rs9333029	A	G	A	G	-0.0695	-0.00345	0.8669	0.868114	FALSE	FALSE	FALSE	TyHtdh	0.020184	0.864448	486484	sepsis	TRUE	reported	textfile	0.0042	1.52E-61	7358	M32497.m	TRUE	reported	H3KmLR	2	TRUE	0.035879	273.7493
7393	rs4921914	T	C	T	C	0.0879	-0.00641	0.7848	0.777478	FALSE	FALSE	FALSE	AW6M5u	0.016476	0.697374	486484	sepsis	TRUE	reported	textfile	0.0054	1.09E-60	5195	M34389.m	TRUE	reported	6ZYYQK	2	TRUE	0.048529	264.864
1340	rs2518802	A	C	A	C	-0.0534	0.019347	0.903	0.902567	FALSE	FALSE	FALSE	LDUJbh	0.023044	0.401157	486484	sepsis	TRUE	reported	textfile	0.0033	4.15E-59	7367	M01898.m	TRUE	reported	JRSLeH	2	TRUE	0.034324	261.7802
1014	rs721399	T	C	T	C	-0.0289	0.011196	0.7176	0.71737	FALSE	FALSE	FALSE	jfkZbM	0.015195	0.461245	486484	sepsis	TRUE	reported	textfile	0.0018	3.95E-58	6523	M01558.m	TRUE	reported	LFZvMG	2	TRUE	0.038016	257.7018
4874	rs4149034	A	G	A	G	-0.0649	-0.00187	0.3353	0.338028	FALSE	FALSE	FALSE	KBJVQO	0.014434	0.896717	486484	sepsis	TRUE	reported	textfile	0.0042	4.03E-55	7355	M32855.m	TRUE	reported	Xea7Fn	2	TRUE	0.031444	238.7111
6208	rs1046996	A	G	A	G	0.1024	-0.01244	0.2526	0.252821	FALSE	FALSE	FALSE	FQfT2o	0.015804	0.431174	486484	sepsis	TRUE	reported	textfile	0.0068	1.12E-51	2759	M33508.m	TRUE	reported	JtbOy2	2	TRUE	0.07595	226.6038
6217	rs4488133	A	T	A	T	0.0985	-0.01457	0.7014	0.65566	FALSE	TRUE	FALSE	FQfT2o	0.014385	0.311013	486484	sepsis	TRUE	reported	textfile	0.0067	1.26E-48	2759	M33508.m	TRUE	reported	JtbOy2	2	TRUE	0.072647	215.9772
7505	rs4253311	A	G	A	G	-0.1412	0.001176	0.4869	0.487409	FALSE	FALSE	FALSE	1aXQWs	0.013672	0.931456	486484	sepsis	TRUE	reported	textfile	0.0097	3.56E-48	4452	M34420.m	TRUE	reported	FOqhHW	2	TRUE	0.045434	211.8024
1346	rs5747934	T	C	T	C	0.0715	-0.01222	0.0468	0.043358	FALSE	FALSE	FALSE	LDUJbh	0.033711	0.716977	486484	sepsis	TRUE	reported	textfile	0.0051	2.00E-44	7367	M01898.m	TRUE	reported	JRSLeH	2	TRUE	0.025986	196.496
8241	rs8012	A	G	A	G	0.0374	0.009662	0.4414	0.451946	FALSE	FALSE	FALSE	4YAWhA	0.013522	0.474917	486484	sepsis	TRUE	reported	textfile	0.0027	6.36E-45	7256	M35439.m	TRUE	reported	bSSC4Z	2	TRUE	0.025762	191.8209
2894	rs7570971	A	C	A	C	-0.0373	-0.0227	0.3255	0.283862	FALSE	FALSE	FALSE	mUzn1R	0.015566	0.144785	486484	sepsis	TRUE	reported	textfile	0.0027	7.86E-45	7301	M20675.m	TRUE	reported	XwY04K	2	TRUE	0.025474	190.7968
7449	rs1195056	A	C	A	C	0.0362	0.017034	0.5231	0.524588	FALSE	FALSE	FALSE	7ubZow	0.013682	0.213131	486484	sepsis	TRUE	reported	textfile	0.0027	2.31E-41	7344	M34407.m	TRUE	reported	LRsXN2	2	TRUE	0.023892	179.7096
5082	rs3099557	A	G	A	G	-0.1484	0.02745	0.8504	0.852602	FALSE	FALSE	FALSE	VzfWsT	0.019271	0.154322	486484	sepsis	TRUE	reported	textfile	0.0113	1.83E-39	4817	M33144.m	TRUE	reported	ZtQP3u	2	TRUE	0.034567	172.3973
3383	rs4902243	A	G	A	G	-0.0442	0.004622	0.8624	0.839571	FALSE	FALSE	FALSE	O2JtzH	0.018617	0.803934	486484	sepsis	TRUE	reported	textfile	0.0035	3.41E-37	7347	M27278.m	TRUE	reported	fxCIPW	2	TRUE	0.021246	159.4374
4320	rs174535	T	C	T	C	-0.044	-0.02158	0.6677	0.649782	FALSE	FALSE	FALSE	EOAssJ	0.014337	0.132353	486484	sepsis	TRUE	reported	textfile	0.0035	2.82E-36	7369	M32635.m	TRUE	reported	X1mTjy	2	TRUE	0.020996	157.9979
3924	rs4766962	A	T	A	T	0.0516	0.006731	0.6544	0.659994	FALSE	TRUE	FALSE	BrQzHt	0.014405	0.640304	486484	sepsis	TRUE	reported	textfile	0.0043	1.70E-33	7796	M32412.m	TRUE	reported	eVvYVX	2	TRUE	0.018136	143.9631
6712	rs8396	T	C	T	C	0.0478	0.024961	0.6972	0.697287	FALSE	FALSE	FALSE	bzT5yX	0.014849	0.09276	486484	sepsis	TRUE	reported	textfile	0.0041	1.38E-31	7345	M33936.m	TRUE	reported	W7BO8n	2	TRUE	0.018169	135.8845
8242	rs8056893	A	C	A	C	-0.0302	-0.01949	0.7336	0.732887	FALSE	FALSE	FALSE	4YAWhA	0.015481	0.208072	486484	sepsis	TRUE	reported	textfile	0.0026	2.23E-30	7256	M35439.m	TRUE	reported	bSSC4Z	2	TRUE	0.018254	134.88
3930	rs7303401	A	T	A	T	-0.0692	-0.00633	0.1111	0.109882	FALSE	TRUE	FALSE	BrQzHt	0.021926	0.772691	486484	sepsis	TRUE	reported	textfile	0.006	1.38E-30	7796	M32412.m	TRUE	reported	eVvYVX	2	TRUE	0.016776	132.9837
2875	rs1246580	A	G	A	G	-0.0364	-0.02975	0.31	0.276149	FALSE	FALSE	FALSE	mUzn1R	0.015889	0.061183	486484	sepsis	TRUE	reported	textfile	0.0032	3.47E-29	7301	M20675.m	TRUE	reported	XwY04K	2	TRUE	0.017414	129.3552
6721	rs2403254	T	C	T	C	-0.0409	0.01382	0.5505	0.579221	FALSE	FALSE	FALSE	B6lZly	0.013886	0.319616	486484	sepsis	TRUE	reported	textfile	0.0036	1.26E-30	7222	M33937.m	TRUE	reported	qFPzjh	2	TRUE	0.017559	129.0391
8928	rs2405522	A	G	A	G	-0.1264	-0.01396	0.1672	0.169924	FALSE	FALSE	FALSE	ns6PV1	0.018179	0.442698	486484	sepsis	TRUE	reported	textfile	0.0113	4.49E-29	7014	M37097.m	TRUE	reported	ZYmYt4	2	TRUE	0.017526	125.0874
3692	rs1163251	T	C	T	C	0.0188	-0.00769	0.6	0.594142	FALSE	FALSE	FALSE	agPgtF	0.014058	0.584604	486484	sepsis	TRUE	reported	textfile	0.0017	7.05E-27	7349	M32315.m	TRUE	reported	hMRjcV	2	TRUE	0.016369	122.2643
3920	rs2708104	T	G	T	G	0.0464	-0.01098	0.3881	0.38549	FALSE	FALSE	FALSE	BrQzHt	0.014108	0.436286	486484	sepsis	TRUE	reported	textfile	0.0042	1.26E-28	7796	M32412.m	TRUE	reported	eVvYVX	2	TRUE	0.015414	122.0186
8230	rs1337574	T	C	T	C	0.0297	0.001144	0.7801	0.774476	FALSE	FALSE	FALSE	4YAWhA	0.01638	0.944321	486484	sepsis	TRUE	reported	textfile	0.0027	2.80E-28	7256	M35439.m	TRUE	reported	bSSC4Z	2	TRUE	0.016402	120.9666
7412	rs1495741	A	G	A	G	0.057	-0.00836	0.7841	0.779473	FALSE	FALSE	FALSE	7i5VUS	0.016454	0.611429	486484	sepsis	TRUE	reported	textfile	0.0052	1.04E-27	5208	M34395.m	TRUE	reported	64hEaL	2	TRUE	0.022551	120.1092
4486	rs1260290	C	G	C	G	-0.0368	0.002645	0.1717	0.180734	FALSE	TRUE	FALSE	1wNJ1E	0.017766	0.881664	486484	sepsis	TRUE	reported	textfile	0.0034	1.15E-26	7363	M32709.m	TRUE	reported	zihBHg	2	TRUE	0.015661	117.117
6619	rs6685187	A	G	A	G	-0.0432	0.007337	0.4088	0.389975	FALSE	FALSE	FALSE	2plv9p	0.014011	0.60055	486484	sepsis	TRUE	reported	textfile	0.0041	2.88E-26	7334	M33884.m	TRUE	reported	AoFcqC	2	TRUE	0.014912	110.9894
4396	rs273913	T	C	T	C	0.0261	0.016484	0.3822	0.374367	FALSE	FALSE	FALSE	UJygVd	0.014101	0.242417	486484	sepsis	TRUE	reported	textfile	0.0025	1.08E-25	7361	M32654.m	TRUE	reported	kv9R0W	2	TRUE	0.014591	108.964
4411	rs1161333	A	G	A	G	0.0374	0.005836	0.5532	0.549787	FALSE	FALSE	FALSE	NzBiVA	0.013727	0.670754	486484	sepsis	TRUE	reported	textfile	0.0036	2.23E-25	7354	M32672.m	TRUE	reported	YLIhHJ	2	TRUE	0.014464	107.8997
1813	rs8051149	A	G	A	G	0.0258	0.014181	0.2127	0.245418	FALSE	FALSE	FALSE	JEcCRZ	0.015966	0.374433	486484	sepsis	TRUE	reported	textfile	0.0025	9.07E-26	7368	M15140.m	TRUE	reported	xYCzKP	2	TRUE	0.014249	106.4735
4985	rs174550	T	C	T	C	0.0347	-0.02215	0.6676	0.656005	FALSE	FALSE	FALSE	AjJi2i	0.014389	0.123679	486484	sepsis	TRUE	reported	textfile	0.0034	6.79E-24	7330	M32980.m	TRUE	reported	gVSm9e	2	TRUE	0.014011	104.1316
4082	rs7556052	A	T	A	T	-0.0346	0.010255	0.6721	0.910181	FALSE	TRUE	FALSE	TyHtdh	0.024794	0.679165	486484	sepsis	TRUE	reported	textfile	0.0034	5.50E-24	7358	M32497.m	TRUE	reported	H3KmLR	2	TRUE	0.013879	103.5324
6610	rs2271316	C	G	C	G	0.1133	-0.01569	0.3822	0.40342	FALSE	TRUE	FALSE	VfOuo8	0.013992	0.262031	486484	sepsis	TRUE	reported	textfile	0.0113	1.79E-23	2725	M33883.m	TRUE	reported	BDe0Nk	2	TRUE	0.03558	100.4579
6446	rs8002180	T	C	T	C	-0.0239	-0.00869	0.7203	0.724916	FALSE	FALSE	FALSE	QCw5dg	0.015293	0.569945	486484	sepsis	TRUE	reported	textfile	0.0024	8.23E-23	6279	M33666.m	TRUE	reported	zYHmzg	2	TRUE	0.015548	99.13682
6061	rs2065396	C	G	C	G	0.035	0.03444	0.3289	0.328774	FALSE	TRUE	FALSE	P4BuMD	0.014553	0.017952	486484	sepsis	TRUE	reported	textfile	0.0036	2.77E-22	7365	M33441.m	TRUE	reported	kqrc1d	2	TRUE	0.012671	94.49594
7907	rs4149032	T	C	T	C	-0.0621	-0.0013	0.3324	0.338222	FALSE	FALSE	FALSE	AhsT9A	0.014431	0.928475	486484	sepsis	TRUE	reported	textfile	0.0064	4.61E-22	5953	M35187.m	TRUE	reported	uLU0Fy	2	TRUE	0.015569	94.119
8235	rs1764197	T	C	T	C	-0.0252	0.010048	0.6608	0.652367	FALSE	FALSE	FALSE	4YAWhA	0.014425	0.486081	486484	sepsis	TRUE	reported	textfile	0.0026	3.35E-22	7256	M35439.m	TRUE	reported	bSSC4Z	2	TRUE	0.012781	93.91494
5051	rs4253252	T	G	T	G	-0.0862	0.001969	0.4803	0.487908	FALSE	FALSE	FALSE	JLCSne	0.013657	0.885348	486484	sepsis	TRUE	reported	textfile	0.0089	2.83E-22	2391	M33137.m	TRUE	reported	LGKYXK	2	TRUE	0.037752	93.72838
2008	rs6479648	A	G	A	G	-0.0087	-0.00763	0.4199	0.413597	FALSE	FALSE	FALSE	v2KE89	0.013911	0.583129	486484	sepsis	TRUE	reported	textfile	9.00E-04	8.40E-22	7349	M15500.m	TRUE	reported	to2t7H	2	TRUE	0.012556	93.41901
2631	rs174556	T	C	T	C	-0.0356	0.023914	0.299	0.30722	FALSE	FALSE	FALSE	UxuT5B	0.01482	0.106596	486484	sepsis	TRUE	reported	textfile	0.0037	1.97E-22	7367	M18467.m	TRUE	reported	eGQj6o	2	TRUE	0.01241	92.55047
3098	rs4784054	A	G	A	G	0.0349	-0.01566	0.1063	0.099208	FALSE	FALSE	FALSE	mr0awv	0.022874	0.493702	486484	sepsis	TRUE	reported	textfile	0.0037	2.31E-21	5702	M22130.m	TRUE	reported	Y2TfWQ	2	TRUE	0.015364	88.93957
3862	rs4787294	A	T	A	T	0.075	-0.04032	0.9278	0.934916	FALSE	TRUE	FALSE	8gg1zS	0.027763	0.146394	486484	sepsis	TRUE	reported	textfile	0.008	9.64E-21	6119	M32379.m	TRUE	reported	uZWtuS	2	TRUE	0.01416	87.8619
4418	rs1727943	A	G	A	G	0.059	-0.00336	0.0953	0.106244	FALSE	FALSE	FALSE	NzBiVA	0.022143	0.879267	486484	sepsis	TRUE	reported	textfile	0.0063	1.25E-20	7354	M32672.m	TRUE	reported	YLIhHJ	2	TRUE	0.011786	87.68086
6962	rs4675874	A	G	A	G	-0.0243	0.018623	0.7094	0.71145	FALSE	FALSE	FALSE	7Cw2XZ	0.015109	0.217743	486484	sepsis	TRUE	reported	textfile	0.0026	3.13E-20	7049	M34123.m	TRUE	reported	vrgMI3	2	TRUE	0.01224	87.32581
8792	rs1282970	A	G	A	G	-0.042	-0.02317	0.1927	0.184971	FALSE	FALSE	FALSE	0YKkSS	0.017573	0.187376	486484	sepsis	TRUE	reported	textfile	0.0045	9.49E-21	6876	M36754.m	TRUE	reported	cf9IBu	2	TRUE	0.01251	87.08577
3884	rs1394678	T	C	T	C	-0.0354	0.006222	0.2785	0.282022	FALSE	FALSE	FALSE	bmXxuz	0.015332	0.684889	486484	sepsis	TRUE	reported	textfile	0.0038	1.70E-20	7358	M32405.m	TRUE	reported	A9BFZF	2	TRUE	0.011657	86.76034
6441	rs6804368	A	G	A	G	0.0221	0.003926	0.5714	0.559719	FALSE	FALSE	FALSE	QCw5dg	0.013796	0.775992	486484	sepsis	TRUE	reported	textfile	0.0024	8.17E-21	6279	M33666.m	TRUE	reported	zYHmzg	2	TRUE	0.013324	84.76639
3117	rs4351	A	G	A	G	-0.0455	0.008926	0.4754	0.479486	FALSE	FALSE	FALSE	a9KeDe	0.013739	0.51591	486484	sepsis	TRUE	reported	textfile	0.005	1.24E-19	3838	M22175.m	TRUE	reported	shg5gu	2	TRUE	0.021121	82.76685
251	rs1440581	T	C	T	C	-0.0081	0.007	0.4592	0.467697	FALSE	FALSE	FALSE	tbRL8J	0.013681	0.608899	486484	sepsis	TRUE	reported	textfile	9.00E-04	1.44E-19	7351	M00060.m	TRUE	reported	oOvRKT	2	TRUE	0.010899	80.97796
3621	rs2572023	A	G	A	G	-0.0576	0.010309	0.4687	0.476254	FALSE	FALSE	FALSE	gq4ISJ	0.013694	0.451586	486484	sepsis	TRUE	reported	textfile	0.0064	4.18E-19	7338	M31591.m	TRUE	reported	wjwl4J	2	TRUE	0.010918	80.97792
1527	rs1687639	T	C	T	C	0.0304	-0.01467	0.8993	0.894471	FALSE	FALSE	FALSE	YvgTeW	0.022271	0.509977	486484	sepsis	TRUE	reported	textfile	0.0034	1.49E-19	7357	M03141.m	TRUE	reported	srKjSc	2	TRUE	0.01075	79.9229
6530	rs968567	T	C	T	C	0.04	0.011032	0.1798	0.187234	FALSE	FALSE	FALSE	OKIXJ8	0.017523	0.528981	486484	sepsis	TRUE	reported	textfile	0.0045	2.84E-19	7362	M33821.m	TRUE	reported	XGkaXH	2	TRUE	0.010618	78.99088
4719	rs7604035	T	C	T	C	0.031	-0.02731	0.3261	0.33177	FALSE	FALSE	FALSE	L2WFM1	0.014557	0.060602	486484	sepsis	TRUE	reported	textfile	0.0035	9.39E-19	7313	M32761.m	TRUE	reported	5GHzph	2	TRUE	0.010613	78.42752
2112	rs869927	A	G	A	G	-0.0522	0.061648	0.1385	0.136001	FALSE	FALSE	FALSE	MN85S3	0.020254	0.002336	486484	sepsis	TRUE	reported	textfile	0.0059	1.04E-18	7574	M15630.m	TRUE	reported	FUih5R	2	TRUE	0.010229	78.25684
4	rs2657879	A	G	A	G	0.015	0.010195	0.8179	0.817272	FALSE	FALSE	FALSE	0cebDu	0.017639	0.563273	486484	sepsis	TRUE	reported	textfile	0.0017	6.15E-18	7372	M00053.m	TRUE	reported	i8iuxX	2	TRUE	0.01045	77.83355
1976	rs419291	T	C	T	C	0.0079	0.01909	0.3667	0.368831	FALSE	FALSE	FALSE	v2KE89	0.014219	0.179413	486484	sepsis	TRUE	reported	textfile	9.00E-04	3.10E-18	7349	M15500.m	TRUE	reported	to2t7H	2	TRUE	0.010376	77.02841
1796	rs3184504	T	C	T	C	0.0149	-0.00695	0.4851	0.482729	FALSE	FALSE	FALSE	JEcCRZ	0.013678	0.611378	486484	sepsis	TRUE	reported	textfile	0.0017	6.05E-18	7368	M15140.m	TRUE	reported	xYCzKP	2	TRUE	0.010319	76.79922
3056	rs493519	T	C	T	C	-0.0358	0.015618	0.1252	0.124372	FALSE	FALSE	FALSE	H6vcCG	0.020713	0.450822	486484	sepsis	TRUE	reported	textfile	0.0041	1.32E-18	6180	M22030.m	TRUE	reported	XetVX7	2	TRUE	0.012187	76.21804
6982	rs8736	T	C	T	C	-0.0227	-0.03175	0.4379	0.43758	FALSE	FALSE	FALSE	ZlvYs5	0.013842	0.02182	486484	sepsis	TRUE	reported	textfile	0.0026	8.45E-18	7351	M34214.m	TRUE	reported	qU57S1	2	TRUE	0.010263	76.20559
8889	rs1098821	A	G	A	G	-0.0164	0.010384	0.3883	0.396208	FALSE	FALSE	FALSE	eLhsld	0.014081	0.46086	486484	sepsis	TRUE	reported	textfile	0.0019	1.36E-18	6552	M37058.m	TRUE	reported	RSeWE5	2	TRUE	0.011243	74.48141
4945	rs174675	T	C	T	C	0.0155	0.01285	0.2812	0.281659	FALSE	FALSE	FALSE	bKmDMa	0.01522	0.398513	486484	sepsis	TRUE	reported	textfile	0.0018	2.28E-18	7344	M32910.m	TRUE	reported	nAuPzO	2	TRUE	0.009996	74.13104
7617	rs2035647	A	G	A	G	-0.0292	0.006476	0.2052	0.207803	FALSE	FALSE	FALSE	kVfKqT	0.01686	0.700895	486484	sepsis	TRUE	reported	textfile	0.0034	2.18E-17	7322	M34527.m	TRUE	reported	s4ti92	2	TRUE	0.009973	73.73764
1741	rs2187375	A	G	A	G	-0.0221	0.015179	0.1651	0.17578	FALSE	FALSE	FALSE	nFLpAP	0.018023	0.399673	486484	sepsis	TRUE	reported	textfile	0.0026	2.65E-17	7356	M12774.m	TRUE	reported	yOSQTj	2	TRUE	0.009726	72.23036
4603	rs6151429	T	C	T	C	-0.0289	0.048321	0.9291	0.921487	FALSE	FALSE	FALSE	Pzplqu	0.025415	0.057269	486484	sepsis	TRUE	reported	textfile	0.0034	6.51E-18	7318	M32740.m	TRUE	reported	FjEBS5	2	TRUE	0.009776	72.23025
6843	rs474229	T	G	T	G	0.0492	0.001632	0.6017	0.590034	FALSE	FALSE	FALSE	aWy2Uj	0.013926	0.906719	486484	sepsis	TRUE	reported	textfile	0.0058	3.51E-17	7326	M33973.m	TRUE	reported	SUSE3b	2	TRUE	0.009727	71.93755
4718	rs6735065	C	G	C	G	0.0678	-0.01243	0.953	0.93674	FALSE	TRUE	FALSE	L2WFM1	0.0283	0.660593	486484	sepsis	TRUE	reported	textfile	0.008	1.99E-17	7313	M32761.m	TRUE	reported	5GHzph	2	TRUE	0.009726	71.80598
3112	rs8101881	T	C	T	C	0.0325	-0.01138	0.613	0.602566	FALSE	FALSE	FALSE	ApMZhy	0.013973	0.415591	486484	sepsis	TRUE	reported	textfile	0.0039	1.43E-16	3950	M22138.m	TRUE	reported	LiJliF	2	TRUE	0.017277	69.40928
3036	rs4814176	T	C	T	C	0.0216	-0.01731	0.3938	0.392493	FALSE	FALSE	FALSE	FJZEYz	0.014014	0.216862	486484	sepsis	TRUE	reported	textfile	0.0026	2.97E-17	7284	M21630.m	TRUE	reported	s6Pshg	2	TRUE	0.009386	68.9988
8212	rs2270968	T	G	T	G	0.0307	0.017889	0.2634	0.261096	FALSE	FALSE	FALSE	aY1HfB	0.015555	0.250146	486484	sepsis	TRUE	reported	textfile	0.0037	1.31E-16	5306	M35433.m	TRUE	reported	H5O4i2	2	TRUE	0.012809	68.81919
3991	rs7727544	T	C	T	C	-0.0148	-0.0214	0.5553	0.558604	FALSE	FALSE	FALSE	XdJbEU	0.013759	0.119797	486484	sepsis	TRUE	reported	textfile	0.0018	6.61E-16	7364	M32452.m	TRUE	reported	V5xWKi	2	TRUE	0.009097	67.58658
1919	rs1466788	A	G	A	G	-0.0074	-0.01315	0.4068	0.414557	FALSE	FALSE	FALSE	v2KE89	0.013877	0.34318	486484	sepsis	TRUE	reported	textfile	9.00E-04	3.05E-16	7349	M15500.m	TRUE	reported	to2t7H	2	TRUE	0.009115	67.58654
5953	rs1323055	T	C	T	C	-0.1701	0.015496	0.016	0.019966	FALSE	FALSE	FALSE	mDZrGw	0.04894	0.751524	486484	sepsis	TRUE	reported	textfile	0.0209	4.31E-16	6797	M33415.m	TRUE	reported	MzEP7R	2	TRUE	0.009651	66.21986
4227	rs1934955	A	G	A	G	-0.0301	0.004049	0.713	0.7187	FALSE	FALSE	FALSE	FDy32d	0.015183	0.789703	486484	sepsis	TRUE	reported	textfile	0.0037	3.33E-16	7369	M32587.m	TRUE	reported	S10k1h	2	TRUE	0.008901	66.16246
561	rs131794	A	C	A	C	0.0146	-0.01838	0.2074	0.209311	FALSE	FALSE	FALSE	fNqkCW	0.016763	0.272802	486484	sepsis	TRUE	reported	textfile	0.0018	4.37E-16	7354	M00606.m	TRUE	reported	g9uwOn	2	TRUE	0.008867	65.77223
1515	rs6459467	A	G	A	G	0.0202	-0.01611	0.3888	0.383205	FALSE	FALSE	FALSE	rzkxmh	0.014081	0.252612	486484	sepsis	TRUE	reported	textfile	0.0025	1.93E-16	6941	M03127.m	TRUE	reported	3IrzhA	2	TRUE	0.009318	65.26759
6828	rs174601	T	C	T	C	-0.0336	0.029263	0.3398	0.374872	FALSE	FALSE	FALSE	ZPJCMx	0.014115	0.038152	486484	sepsis	TRUE	reported	textfile	0.0042	7.93E-16	7330	M33969.m	TRUE	reported	mLJPAI	2	TRUE	0.008656	63.98254
3901	rs1161131	A	T	A	T	0.0463	-0.03143	0.8579	0.839423	FALSE	TRUE	FALSE	BrQzHt	0.018679	0.09247	486484	sepsis	TRUE	reported	textfile	0.0058	1.65E-15	7796	M32412.m	TRUE	reported	eVvYVX	2	TRUE	0.008108	63.70809
2884	rs3800993	T	C	T	C	0.0287	-0.02742	0.1697	0.175045	FALSE	FALSE	FALSE	mUzn1R	0.017999	0.12762	486484	sepsis	TRUE	reported	textfile	0.0036	1.53E-15	7301	M20675.m	TRUE	reported	XwY04K	2	TRUE	0.00863	63.53892
6444	rs7775554	A	C	A	C	0.019	0.013946	0.5279	0.524786	FALSE	FALSE	FALSE	QCw5dg	0.013683	0.30809	486484	sepsis	TRUE	reported	textfile	0.0024	1.35E-15	6279	M33666.m	TRUE	reported	zYHmzg	2	TRUE	0.009883	62.65365
1342	rs416659	C	G	C	G	0.0268	-0.0257	0.7277	0.710495	FALSE	TRUE	FALSE	LDUJbh	0.01514	0.089596	486484	sepsis	TRUE	reported	textfile	0.0034	4.66E-15	7367	M01898.m	TRUE	reported	JRSLeH	2	TRUE	0.008363	62.11462
1757	rs964184	C	G	C	G	-0.0252	0.000763	0.8617	0.86637	FALSE	TRUE	FALSE	nFLpAP	0.02008	0.969689	486484	sepsis	TRUE	reported	textfile	0.0032	7.95E-15	7356	M12774.m	TRUE	reported	yOSQTj	2	TRUE	0.00836	61.99876
8707	rs7822232	A	C	A	C	0.0299	0.009553	0.9203	0.914804	FALSE	FALSE	FALSE	Bg4VnB	0.024506	0.696665	486484	sepsis	TRUE	reported	textfile	0.0038	5.12E-15	7092	M36552.m	TRUE	reported	6zdDpC	2	TRUE	0.008654	61.89459
1751	rs6679002	T	G	T	G	0.0141	-0.02853	0.5914	0.593821	FALSE	FALSE	FALSE	nFLpAP	0.013946	0.04076	486484	sepsis	TRUE	reported	textfile	0.0018	2.16E-15	7356	M12774.m	TRUE	reported	yOSQTj	2	TRUE	0.008273	61.34443
5159	rs6664483	T	G	T	G	1.1464	-0.00533	0.9803	0.951187	FALSE	FALSE	FALSE	L2DJzK	0.03164	0.866232	486484	sepsis	TRUE	reported	textfile	0.1447	2.32E-15	76	M33173.m	TRUE	reported	hRG70D	2	TRUE	0.452322	61.11579
1791	rs1763023	A	G	A	G	0.0131	-0.00848	0.4183	0.408736	FALSE	FALSE	FALSE	JEcCRZ	0.013913	0.542031	486484	sepsis	TRUE	reported	textfile	0.0017	3.41E-14	7368	M15140.m	TRUE	reported	xYCzKP	2	TRUE	0.007995	59.3645
8360	rs6663731	A	T	A	T	0.0624	-0.00297	0.8857	0.867839	FALSE	TRUE	FALSE	6VVCe4	0.020197	0.883257	486484	sepsis	TRUE	reported	textfile	0.0081	1.39E-14	5629	M35669.m	TRUE	reported	I0fk8X	2	TRUE	0.010433	59.32596
4403	rs6897597	T	C	T	C	-0.02	-0.01538	0.2368	0.242198	FALSE	FALSE	FALSE	UJygVd	0.015982	0.336053	486484	sepsis	TRUE	reported	textfile	0.0026	1.10E-14	7361	M32654.m	TRUE	reported	kv9R0W	2	TRUE	0.007974	59.15552
648	rs603424	A	G	A	G	0.0292	0.008091	0.1865	0.173202	FALSE	FALSE	FALSE	FfmsRf	0.018088	0.654627	486484	sepsis	TRUE	reported	textfile	0.0038	1.56E-14	7349	M01121.m	TRUE	reported	9R12A7	2	TRUE	0.007971	59.03102
4479	rs1005390	T	G	T	G	0.0261	-0.00435	0.7998	0.792417	FALSE	FALSE	FALSE	1wNJ1E	0.016989	0.797851	486484	sepsis	TRUE	reported	textfile	0.0034	2.50E-14	7363	M32709.m	TRUE	reported	zihBHg	2	TRUE	0.00794	58.91219
8236	rs246234	C	G	C	G	-0.0199	-0.00922	0.2958	0.303591	FALSE	TRUE	FALSE	4YAWhA	0.014894	0.535751	486484	sepsis	TRUE	reported	textfile	0.0026	2.56E-14	7256	M35439.m	TRUE	reported	bSSC4Z	2	TRUE	0.008009	58.56521
3707	rs4947534	T	C	T	C	-0.0183	-0.01156	0.2479	0.248025	FALSE	FALSE	FALSE	agPgtF	0.015881	0.466804	486484	sepsis	TRUE	reported	textfile	0.0024	1.96E-14	7349	M32315.m	TRUE	reported	hMRjcV	2	TRUE	0.007849	58.1248
3902	rs1171617	T	G	T	G	0.0358	-0.00907	0.7771	0.767566	FALSE	FALSE	FALSE	BrQzHt	0.016207	0.575755	486484	sepsis	TRUE	reported	textfile	0.0047	1.61E-14	7796	M32412.m	TRUE	reported	eVvYVX	2	TRUE	0.007387	58.00413
721	rs9400467	T	C	T	C	-0.0121	-0.00492	0.7059	0.709744	FALSE	FALSE	FALSE	H9I3s3	0.01529	0.747603	486484	sepsis	TRUE	reported	textfile	0.0016	6.54E-14	7358	M01299.m	TRUE	reported	kXWyUq	2	TRUE	0.007713	57.17586
7912	rs1509821	T	C	T	C	0.0574	-0.00642	0.1091	0.108902	FALSE	FALSE	FALSE	8qXKkX	0.022086	0.771278	486484	sepsis	TRUE	reported	textfile	0.0076	5.29E-14	6234	M35189.m	TRUE	reported	Nt6oaC	2	TRUE	0.009067	57.02394
4091	rs174538	A	G	A	G	-0.0264	0.019151	0.3025	0.315646	FALSE	FALSE	FALSE	j2JYxt	0.014705	0.192813	486484	sepsis	TRUE	reported	textfile	0.0035	8.90E-14	7373	M32504.m	TRUE	reported	poO7a5	2	TRUE	0.007658	56.87926
3945	rs1176152	T	C	T	C	-0.0675	-0.0354	0.0736	0.086688	FALSE	FALSE	FALSE	N6UQnQ	0.024246	0.144261	486484	sepsis	TRUE	reported	textfile	0.009	6.70E-14	7346	M32425.m	TRUE	reported	FEcGHY	2	TRUE	0.007599	56.23469
4136	rs2071426	T	C	T	C	0.0547	0.001852	0.7107	0.718055	FALSE	FALSE	FALSE	pLh7sk	0.015164	0.902787	486484	sepsis	TRUE	reported	textfile	0.0073	6.87E-14	7256	M32549.m	TRUE	reported	RSQOnp	2	TRUE	0.007679	56.13183
513	rs6968554	A	G	A	G	0.0644	0.003577	0.3756	0.366672	FALSE	FALSE	FALSE	ZOMIXT	0.014196	0.801078	486484	sepsis	TRUE	reported	textfile	0.0086	8.68E-14	7274	M00569.m	TRUE	reported	kZGouU	2	TRUE	0.00765	56.0603
198	rs1686824	C	G	C	G	0.0097	-9.37E-05	0.2113	0.206694	FALSE	TRUE	FALSE	FbLTng	0.016858	0.995564	486484	sepsis	TRUE	reported	textfile	0.0013	1.78E-14	7355	M00059.m	TRUE	reported	KK7HbM	2	TRUE	0.007513	55.65942
6189	rs1365	A	C	A	C	0.0752	-0.02288	0.9458	0.942435	FALSE	FALSE	FALSE	DEG58p	0.029518	0.438287	486484	sepsis	TRUE	reported	textfile	0.0101	1.16E-13	7500	M33507.m	TRUE	reported	ycykD0	2	TRUE	0.007337	55.42135
5015	rs7061710	C	G	C	G	0.0453	0.013641	0.2282	0.220637	FALSE	TRUE	FALSE	URCVGm	0.016467	0.407482	486484	sepsis	TRUE	reported	textfile	0.0061	7.52E-14	2773	M33131.m	TRUE	reported	4rQ2gs	2	TRUE	0.0195	55.10911
4560	rs4680	A	G	A	G	-0.0436	-0.00865	0.5065	0.514871	FALSE	FALSE	FALSE	KfoRxi	0.013679	0.527252	486484	sepsis	TRUE	reported	textfile	0.0059	1.31E-13	6000	M32735.m	TRUE	reported	UDRXAp	2	TRUE	0.00902	54.59139
6143	rs4687717	T	C	T	C	0.0133	-0.00948	0.4248	0.422068	FALSE	FALSE	FALSE	MiSJly	0.013891	0.495165	486484	sepsis	TRUE	reported	textfile	0.0018	1.07E-13	7307	M33477.m	TRUE	reported	WwL1hq	2	TRUE	0.007416	54.58074
1418	rs838705	A	G	A	G	0.0266	0.01025	0.6134	0.606656	FALSE	FALSE	FALSE	33ptwk	0.013987	0.463669	486484	sepsis	TRUE	reported	textfile	0.0036	1.23E-13	6252	M02137.m	TRUE	reported	SQY1na	2	TRUE	0.008657	54.57821
4842	rs1761746	T	C	T	C	0.0827	-0.01912	0.9017	0.889038	FALSE	FALSE	FALSE	jvlpBt	0.02168	0.377744	486484	sepsis	TRUE	reported	textfile	0.0112	2.01E-13	6250	M32846.m	TRUE	reported	RAXcz5	2	TRUE	0.008648	54.50495
4995	rs2731672	T	C	T	C	-0.081	-0.01888	0.257	0.255166	FALSE	FALSE	FALSE	OsqE1P	0.015673	0.22828	486484	sepsis	TRUE	reported	textfile	0.011	1.79E-13	3659	M33084.m	TRUE	reported	VZYh6f	2	TRUE	0.014603	54.1935
2024	rs7098081	A	G	A	G	0.0184	0.045183	0.0469	0.050101	FALSE	FALSE	FALSE	v2KE89	0.031329	0.149246	486484	sepsis	TRUE	reported	textfile	0.0025	3.10E-13	7349	M15500.m	TRUE	reported	to2t7H	2	TRUE	0.007317	54.15486
2822	rs4808136	A	G	A	G	0.0132	0.007065	0.3392	0.351506	FALSE	FALSE	FALSE	Sx5Uhq	0.014308	0.621465	486484	sepsis	TRUE	reported	textfile	0.0018	5.42E-14	7354	M19934.m	TRUE	reported	DijYTE	2	TRUE	0.00726	53.76315
1046	rs170149	A	G	A	G	0.0256	-0.03709	0.074	0.073876	FALSE	FALSE	FALSE	tRtQ2e	0.026175	0.156445	486484	sepsis	TRUE	reported	textfile	0.0035	1.42E-13	7364	M01564.m	TRUE	reported	Tbg0zM	2	TRUE	0.007213	53.48425
4483	rs1110173	A	T	A	T	-0.0553	-0.0409	0.9414	0.921699	FALSE	TRUE	FALSE	1wNJ1E	0.025388	0.107215	486484	sepsis	TRUE	reported	textfile	0.0076	3.28E-13	7363	M32709.m	TRUE	reported	zihBHg	2	TRUE	0.007139	52.93039
6062	rs2404602	A	G	A	G	0.0259	0.000234	0.4398	0.436121	FALSE	FALSE	FALSE	P4BuMD	0.013764	0.986457	486484	sepsis	TRUE	reported	textfile	0.0036	3.56E-13	7365	M33441.m	TRUE	reported	kqrc1d	2	TRUE	0.006979	51.74598
3657	rs4625783	T	C	T	C	-0.0187	0.010861	0.4268	0.408413	FALSE	FALSE	FALSE	fnjup6	0.013909	0.434895	486484	sepsis	TRUE	reported	textfile	0.0026	3.90E-13	7346	M32197.m	TRUE	reported	oQzaMk	2	TRUE	0.006993	51.71521
4760	rs2734331	A	G	A	G	0.0726	0.044285	0.9651	0.968741	FALSE	FALSE	FALSE	RDfDIS	0.039211	0.258727	486484	sepsis	TRUE	reported	textfile	0.0101	6.56E-13	6857	M32787.m	TRUE	reported	Hq6nGt	2	TRUE	0.007479	51.65398
7508	rs5030062	A	C	A	C	-0.0718	0.013088	0.6147	0.628077	FALSE	FALSE	FALSE	1aXQWs	0.014119	0.353922	486484	sepsis	TRUE	reported	textfile	0.01	5.94E-13	4452	M34420.m	TRUE	reported	FOqhHW	2	TRUE	0.011447	51.52924
4188	rs1853207	T	C	T	C	0.1085	-0.03336	0.0598	0.063202	FALSE	FALSE	FALSE	TxOCzG	0.028055	0.234396	486484	sepsis	TRUE	reported	textfile	0.0152	9.58E-13	6970	M32564.m	TRUE	reported	9JP6ND	2	TRUE	0.007257	50.93868
2821	rs4788439	T	C	T	C	-0.0271	0.028634	0.0772	0.067708	FALSE	FALSE	FALSE	Sx5Uhq	0.027297	0.294178	486484	sepsis	TRUE	reported	textfile	0.0038	6.59E-13	7354	M19934.m	TRUE	reported	DijYTE	2	TRUE	0.006868	50.84559
4274	rs3761097	T	C	T	C	0.057	0.02869	0.053	0.052456	FALSE	FALSE	FALSE	ylV4bp	0.031179	0.357493	486484	sepsis	TRUE	reported	textfile	0.008	1.04E-12	5575	M32632.m	TRUE	reported	hqHJoU	2	TRUE	0.009024	50.74741
8264	rs2041073	T	C	T	C	0.0178	0.007835	0.5366	0.535603	FALSE	FALSE	FALSE	TdsGCZ	0.013682	0.566875	486484	sepsis	TRUE	reported	textfile	0.0025	3.41E-13	6883	M35464.m	TRUE	reported	sOspot	2	TRUE	0.007311	50.67967
2061	rs9842133	T	C	T	C	0.0064	0.012383	0.6649	0.664733	FALSE	FALSE	FALSE	v2KE89	0.014533	0.394179	486484	sepsis	TRUE	reported	textfile	9.00E-04	4.20E-12	7349	M15500.m	TRUE	reported	to2t7H	2	TRUE	0.006834	50.55414
3951	rs296396	T	C	T	C	-0.0462	0.024783	0.1646	0.1616	FALSE	FALSE	FALSE	N6UQnQ	0.018631	0.183458	486484	sepsis	TRUE	reported	textfile	0.0065	1.17E-12	7346	M32425.m	TRUE	reported	FEcGHY	2	TRUE	0.00683	50.50554
657	rs494562	A	G	A	G	-0.1182	0.031625	0.8988	0.904536	FALSE	FALSE	FALSE	7BPGuQ	0.023292	0.174538	486484	sepsis	TRUE	reported	textfile	0.0167	1.34E-12	2639	M01123.m	TRUE	reported	WV2hVn	2	TRUE	0.018629	50.05791
2572	rs15676	A	G	A	G	0.0191	0.009593	0.7123	0.715932	FALSE	FALSE	FALSE	KHS91f	0.01521	0.528229	486484	sepsis	TRUE	reported	textfile	0.0027	1.33E-12	6939	M18349.m	TRUE	reported	PeVGil	2	TRUE	0.00716	50.0281
6010	rs1122815	A	T	A	T	0.61	0.000204	0.9654	0.950015	FALSE	TRUE	FALSE	33ojbt	0.031644	0.994849	486484	sepsis	TRUE	reported	textfile	0.0865	1.72E-12	1391	M33423.m	TRUE	reported	ivEtvl	2	TRUE	0.034518	49.65953
4275	rs4327428	A	C	A	C	-0.031	-0.0012	0.1117	0.120156	FALSE	FALSE	FALSE	ylV4bp	0.02099	0.954308	486484	sepsis	TRUE	reported	textfile	0.0044	2.81E-12	7337	M32632.m	TRUE	reported	hqHJoU	2	TRUE	0.00672	49.6249
4822	rs2007084	A	G	A	G	-0.0683	-0.03246	0.091	0.086702	FALSE	FALSE	FALSE	B6WchP	0.024741	0.189561	486484	sepsis	TRUE	reported	textfile	0.0097	1.77E-12	7255	M32836.m	TRUE	reported	7UzUw0	2	TRUE	0.006787	49.56535
3678	rs274567	T	C	T	C	0.0176	0.018886	0.3709	0.366626	FALSE	FALSE	FALSE	rGHJcj	0.014165	0.182427	486484	sepsis	TRUE	reported	textfile	0.0025	2.85E-12	7356	M32198.m	TRUE	reported	6P1Nno	2	TRUE	0.006692	49.54812
7102	rs7568884	T	C	T	C	-0.0183	-0.01495	0.2496	0.241596	FALSE	FALSE	FALSE	pxNICA	0.016215	0.356459	486484	sepsis	TRUE	reported	textfile	0.0026	5.39E-12	6994	M34306.m	TRUE	reported	xk5b9M	2	TRUE	0.007033	49.52577
6718	rs1214104	T	C	T	C	-0.0253	0.007357	0.4692	0.469097	FALSE	FALSE	FALSE	B6lZly	0.01373	0.592055	486484	sepsis	TRUE	reported	textfile	0.0036	1.77E-12	7222	M33937.m	TRUE	reported	qFPzjh	2	TRUE	0.006792	49.37598
7874	rs5760492	A	G	A	G	-0.0492	-0.00972	0.333	0.344431	FALSE	FALSE	FALSE	r428eQ	0.014391	0.499598	486484	sepsis	TRUE	reported	textfile	0.007	2.34E-12	1982	M35159.m	TRUE	reported	3r5zQo	2	TRUE	0.024319	49.35097
539	rs937813	T	C	T	C	0.0271	0.011209	0.9025	0.889884	FALSE	FALSE	FALSE	wXMWHy	0.022018	0.6107	486484	sepsis	TRUE	reported	textfile	0.0039	2.65E-12	7345	M00584.m	TRUE	reported	7CKn2b	2	TRUE	0.006531	48.27153
2917	rs7542172	A	G	A	G	-0.0236	0.000845	0.1535	0.196427	FALSE	FALSE	FALSE	FBDEQi	0.017317	0.961091	486484	sepsis	TRUE	reported	textfile	0.0034	1.98E-12	7327	M20699.m	TRUE	reported	nvNIlp	2	TRUE	0.006533	48.16678
7144	rs707922	T	G	T	G	0.2854	-0.02123	0.04	0.051157	FALSE	FALSE	FALSE	de8tA6	0.03118	0.496029	486484	sepsis	TRUE	reported	textfile	0.0406	2.12E-12	75	M34322.m	TRUE	reported	sXPPBO	2	TRUE	0.397177	48.09694
5956	rs2417971	A	G	A	G	-0.0401	-0.0094	0.2843	0.291706	FALSE	FALSE	FALSE	mDZrGw	0.015107	0.533588	486484	sepsis	TRUE	reported	textfile	0.0058	3.83E-12	6797	M33415.m	TRUE	reported	MzEP7R	2	TRUE	0.006983	47.78647
2317	rs1030420	T	C	T	C	-0.0221	0.00804	0.1884	0.184521	FALSE	FALSE	FALSE	IZXwpR	0.017654	0.648803	486484	sepsis	TRUE	reported	textfile	0.0032	1.02E-11	6549	M16822.m	TRUE	reported	Zx9k2V	2	TRUE	0.00723	47.68172
8666	rs4986894	T	C	T	C	-0.0421	-0.03321	0.838	0.851041	FALSE	FALSE	FALSE	BFEGyF	0.01918	0.083318	486484	sepsis	TRUE	reported	textfile	0.0061	5.55E-12	6486	M36399.m	TRUE	reported	nhT4FM	2	TRUE	0.00729	47.61794
3620	rs182420	T	C	T	C	0.051	-0.02008	0.7553	0.757661	FALSE	FALSE	FALSE	gq4ISJ	0.016072	0.21164	486484	sepsis	TRUE	reported	textfile	0.0074	6.24E-12	7338	M31591.m	TRUE	reported	wjwl4J	2	TRUE	0.006431	47.48523
44	rs1312225	T	C	T	C	0.0062	0.01069	0.5542	0.572215	FALSE	FALSE	FALSE	xmGuol	0.013898	0.441801	486484	sepsis	TRUE	reported	textfile	9.00E-04	8.95E-12	7355	M00054.m	TRUE	reported	GW50Ri	2	TRUE	0.006411	47.44389
5948	rs1121592	T	C	T	C	-0.0343	0.016851	0.54	0.541704	FALSE	FALSE	FALSE	mDZrGw	0.013719	0.219334	486484	sepsis	TRUE	reported	textfile	0.005	6.38E-12	6797	M33415.m	TRUE	reported	MzEP7R	2	TRUE	0.006876	47.04575
6206	rs942800	A	C	A	C	0.0362	-0.00745	0.4265	0.440165	FALSE	FALSE	FALSE	DEG58p	0.01444	0.60568	486484	sepsis	TRUE	reported	textfile	0.0053	1.06E-11	7500	M33507.m	TRUE	reported	ycykD0	2	TRUE	0.006182	46.63904
6205	rs754586	T	C	T	C	-0.0341	-0.00863	0.4128	0.397088	FALSE	FALSE	FALSE	DEG58p	0.014097	0.54048	486484	sepsis	TRUE	reported	textfile	0.005	1.35E-11	7500	M33507.m	TRUE	reported	ycykD0	2	TRUE	0.006163	46.5
444	rs1498694	A	G	A	G	0.0075	-0.00768	0.4035	0.390573	FALSE	FALSE	FALSE	gcJMTM	0.014	0.583299	486484	sepsis	TRUE	reported	textfile	0.0011	7.34E-11	7354	M00064.m	TRUE	reported	g2hUXe	2	TRUE	0.006282	46.47496
6956	rs2235649	T	C	T	C	-0.0184	0.008714	0.2159	0.223707	FALSE	FALSE	FALSE	7Cw2XZ	0.016384	0.5948	486484	sepsis	TRUE	reported	textfile	0.0027	1.95E-11	7049	M34123.m	TRUE	reported	vrgMI3	2	TRUE	0.006545	46.42852
3738	rs272869	A	G	A	G	0.0224	0.019382	0.3706	0.367942	FALSE	FALSE	FALSE	k16oIH	0.014155	0.170902	486484	sepsis	TRUE	reported	textfile	0.0033	1.40E-11	7340	M32328.m	TRUE	reported	TQA3sZ	2	TRUE	0.006238	46.06274
4862	rs1050587	A	G	A	G	0.0298	0.001569	0.2696	0.280358	FALSE	FALSE	FALSE	KBJVQO	0.015242	0.917991	486484	sepsis	TRUE	reported	textfile	0.0044	9.16E-12	7355	M32855.m	TRUE	reported	Xea7Fn	2	TRUE	0.006198	45.85736
4388	rs1717158	T	G	T	G	0.0331	0.017641	0.943	0.970317	FALSE	FALSE	FALSE	UJygVd	0.040371	0.662133	486484	sepsis	TRUE	reported	textfile	0.0049	2.06E-11	7361	M32654.m	TRUE	reported	kv9R0W	2	TRUE	0.006161	45.61901
2164	rs1164758	A	G	A	G	0.0303	-0.00137	0.7151	0.718851	FALSE	FALSE	FALSE	XCBNPg	0.015312	0.928664	486484	sepsis	TRUE	reported	textfile	0.0045	2.85E-11	5829	M15749.m	TRUE	reported	dKBewD	2	TRUE	0.007718	45.32222
2491	rs9632722	A	G	A	G	0.0571	0.02757	0.9087	0.910834	FALSE	FALSE	FALSE	SrfyMU	0.02403	0.251243	486484	sepsis	TRUE	reported	textfile	0.0085	1.52E-11	7226	M17807.m	TRUE	reported	0zrJsu	2	TRUE	0.006206	45.11429
6673	rs2547231	A	C	A	C	0.0409	-0.02537	0.833	0.833993	FALSE	FALSE	FALSE	6iZSOI	0.018394	0.167895	486484	sepsis	TRUE	reported	textfile	0.0061	2.82E-11	4533	M33901.m	TRUE	reported	ApKaGe	2	TRUE	0.00982	44.93609
3391	rs1182518	A	G	A	G	0.0761	-0.01553	0.0766	0.06734	FALSE	FALSE	FALSE	frOZiy	0.02718	0.567782	486484	sepsis	TRUE	reported	textfile	0.0114	2.93E-11	2759	M27447.m	TRUE	reported	2z9fSv	2	TRUE	0.015895	44.52918
6065	rs4886484	C	G	C	G	-0.024	-5.57E-05	0.6217	0.62062	FALSE	TRUE	FALSE	P4BuMD	0.014089	0.996843	486484	sepsis	TRUE	reported	textfile	0.0036	2.69E-11	7365	M33441.m	TRUE	reported	kqrc1d	2	TRUE	0.005998	44.43238
6265	rs2160860	A	T	A	T	0.012	-0.01412	0.6141	0.617882	FALSE	TRUE	FALSE	MOonP9	0.014148	0.318215	486484	sepsis	TRUE	reported	textfile	0.0018	7.35E-11	7066	M33515.m	TRUE	reported	226GuP	2	TRUE	0.006251	44.43186
4307	rs7499892	T	C	T	C	-0.02	0.007646	0.175	0.178341	FALSE	FALSE	FALSE	0tjjPC	0.017861	0.668592	486484	sepsis	TRUE	reported	textfile	0.003	2.62E-11	5598	M32634.m	TRUE	reported	nJJFkX	2	TRUE	0.007877	44.42857
6509	rs601338	A	G	A	G	0.0413	0.001797	0.4316	0.507835	FALSE	FALSE	FALSE	dIuFOH	0.013711	0.895752	486484	sepsis	TRUE	reported	textfile	0.0062	2.52E-11	3845	M33801.m	TRUE	reported	JCXy06	2	TRUE	0.011409	44.34971
3732	rs1171615	T	C	T	C	0.0246	-0.00925	0.7778	0.768972	FALSE	FALSE	FALSE	k16oIH	0.016246	0.568992	486484	sepsis	TRUE	reported	textfile	0.0037	4.05E-11	7340	M32328.m	TRUE	reported	TQA3sZ	2	TRUE	0.005986	44.19248
2889	rs4954573	T	G	T	G	0.0219	0.015496	0.7291	0.757222	FALSE	FALSE	FALSE	mUzn1R	0.016192	0.338575	486484	sepsis	TRUE	reported	textfile	0.0033	2.53E-11	7301	M20675.m	TRUE	reported	XwY04K	2	TRUE	0.005996	44.02926
2392	rs1698667	A	G	A	G	-0.1548	-0.06874	0.0614	0.021255	FALSE	FALSE	FALSE	NBJ2Do	0.047643	0.149096	486484	sepsis	TRUE	reported	textfile	0.0224	5.22E-12	25	M17799.m	TRUE	reported	Uz0WgI	2	TRUE	0.656395	43.93733
8438	rs6498540	A	G	A	G	0.0172	-0.01613	0.7042	0.698603	FALSE	FALSE	FALSE	Nvt7pI	0.014897	0.278881	486484	sepsis	TRUE	reported	textfile	0.0026	2.55E-11	7358	M35718.m	TRUE	reported	WkA5Gq	2	TRUE	0.005913	43.75142
2465	rs9999552	A	G	A	G	0.1464	-0.02193	0.9321	0.891376	FALSE	FALSE	FALSE	NBJ2Do	0.021973	0.318268	486484	sepsis	TRUE	reported	textfile	0.0213	5.70E-12	25	M17799.m	TRUE	reported	Uz0WgI	2	TRUE	0.653938	43.46211
8901	rs1780688	T	C	T	C	-0.0224	0.011653	0.8806	0.882445	FALSE	FALSE	FALSE	eLhsld	0.021242	0.583297	486484	sepsis	TRUE	reported	textfile	0.0034	7.15E-11	6552	M37058.m	TRUE	reported	RSeWE5	2	TRUE	0.006581	43.39159
2898	rs1053941	T	G	T	G	-0.0171	-0.00207	0.2263	0.233145	FALSE	FALSE	FALSE	FBDEQi	0.016165	0.898068	486484	sepsis	TRUE	reported	textfile	0.0026	4.99E-11	7327	M20699.m	TRUE	reported	nvNIlp	2	TRUE	0.005869	43.24411
3457	rs1167525	A	G	A	G	0.0697	0.013537	0.0885	0.093019	FALSE	FALSE	FALSE	jRejFm	0.023591	0.566071	486484	sepsis	TRUE	reported	textfile	0.0106	5.81E-11	6393	M27716.m	TRUE	reported	tg7Rvj	2	TRUE	0.006718	43.2233
1382	rs1935815	A	T	A	T	0.0111	-0.0119	0.3513	0.349273	FALSE	TRUE	FALSE	KrWhws	0.014344	0.406873	486484	sepsis	TRUE	reported	textfile	0.0017	1.55E-10	7325	M02132.m	TRUE	reported	7pzpfK	2	TRUE	0.005787	42.62158
2405	rs1896703	T	G	T	G	-0.1538	-0.00524	0.0817	0.071178	FALSE	FALSE	FALSE	NBJ2Do	0.026817	0.845069	486484	sepsis	TRUE	reported	textfile	0.0226	9.67E-12	25	M17799.m	TRUE	reported	Uz0WgI	2	TRUE	0.649429	42.60726
1126	rs1173760	A	G	A	G	0.0104	-0.00014	0.6847	0.697225	FALSE	FALSE	FALSE	nlqUuV	0.014923	0.992363	486484	sepsis	TRUE	reported	textfile	0.0016	1.34E-10	7371	M01604.m	TRUE	reported	Uro9nC	2	TRUE	0.005699	42.23854
3815	rs1002246	T	C	T	C	-0.0117	-0.01503	0.452	0.444355	FALSE	FALSE	FALSE	tRX2u0	0.013785	0.275642	486484	sepsis	TRUE	reported	textfile	0.0018	4.55E-11	7365	M32348.m	TRUE	reported	ruBgwQ	2	TRUE	0.005704	42.23853
3129	rs1327884	A	G	A	G	-0.013	0.012626	0.73	0.736365	FALSE	FALSE	FALSE	JiXR2t	0.015559	0.417083	486484	sepsis	TRUE	reported	textfile	0.002	2.79E-11	6535	M22177.m	TRUE	reported	joi6p6	2	TRUE	0.006424	42.23707
7468	rs272881	A	G	A	G	-0.0214	-0.01691	0.617	0.625153	FALSE	FALSE	FALSE	kasr9a	0.014097	0.230346	486484	sepsis	TRUE	reported	textfile	0.0033	1.11E-10	6768	M34409.m	TRUE	reported	3VaBw3	2	TRUE	0.006175	42.04083
1534	rs567754	T	C	T	C	0.0162	-0.00193	0.3247	0.340305	FALSE	FALSE	FALSE	YvgTeW	0.014453	0.893539	486484	sepsis	TRUE	reported	textfile	0.0025	4.96E-11	7357	M03141.m	TRUE	reported	srKjSc	2	TRUE	0.005675	41.97898
5767	rs7962263	T	C	T	C	-0.0317	-0.01193	0.2686	0.282798	FALSE	FALSE	FALSE	n16uO2	0.015151	0.430987	486484	sepsis	TRUE	reported	textfile	0.0049	7.86E-11	4409	M33250.m	TRUE	reported	9dhI67	2	TRUE	0.009403	41.83399
4394	rs2291428	C	G	C	G	-0.0168	-0.00898	0.2351	0.241829	FALSE	TRUE	FALSE	UJygVd	0.015977	0.573908	486484	sepsis	TRUE	reported	textfile	0.0026	8.71E-11	7361	M32654.m	TRUE	reported	kv9R0W	2	TRUE	0.00564	41.74014
13	rs1016522	A	G	A	G	0.0058	0.019506	0.5803	0.575669	FALSE	FALSE	FALSE	xmGuol	0.013877	0.159818	486484	sepsis	TRUE	reported	textfile	9.00E-04	1.59E-10	7355	M00054.m	TRUE	reported	GW50Ri	2	TRUE	0.005615	41.51957
510	rs2472297	T	C	T	C	-0.0668	0.004997	0.2362	0.261592	FALSE	FALSE	FALSE	ZOMIXT	0.015539	0.747784	486484	sepsis	TRUE	reported	textfile	0.0104	1.50E-10	7274	M00569.m	TRUE	reported	kZGouU	2	TRUE	0.00564	41.24457
8100	rs1224602	A	G	A	G	0.0115	0.002517	0.2666	0.268367	FALSE	FALSE	FALSE	nNbXBS	0.015511	0.871068	486484	sepsis	TRUE	reported	textfile	0.0018	1.05E-10	5768	M35326.m	TRUE	reported	UNDorn	2	TRUE	0.007027	40.80375
2479	rs2199680	A	T	A	T	-0.0415	0.013218	0.8328	0.839848	FALSE	TRUE	FALSE	SrfyMU	0.018841	0.482948	486484	sepsis	TRUE	reported	textfile	0.0065	2.17E-10	7226	M17807.m	TRUE	reported	0zrJsu	2	TRUE	0.00561	40.75203
950	rs1395	A	G	A	G	0.0172	0.005188	0.7034	0.695829	FALSE	FALSE	FALSE	tWoDq4	0.014821	0.726308	486484	sepsis	TRUE	reported	textfile	0.0027	8.14E-11	7160	M01508.m	TRUE	reported	6ltDw0	2	TRUE	0.005636	40.57028
5439	rs3816015	A	T	A	T	-0.1041	-0.02011	0.3923	0.408994	FALSE	TRUE	FALSE	q32d4E	0.013915	0.148497	486484	sepsis	TRUE	reported	textfile	0.0164	2.20E-10	1017	M33178.m	TRUE	reported	cS3BwT	2	TRUE	0.038108	40.21229
3637	rs1175428	A	G	A	G	0.0488	-0.0107	0.4322	0.419466	FALSE	FALSE	FALSE	Vq2efp	0.013826	0.438976	486484	sepsis	TRUE	reported	textfile	0.0077	2.25E-10	7363	M31787.m	TRUE	reported	krl4Tw	2	TRUE	0.005426	40.15505
1067	rs835154	A	G	A	G	-0.0114	0.006469	0.5753	0.5557	FALSE	FALSE	FALSE	tRtQ2e	0.013852	0.640481	486484	sepsis	TRUE	reported	textfile	0.0018	1.15E-10	7364	M01564.m	TRUE	reported	Tbg0zM	2	TRUE	0.005417	40.10022
111	rs4306882	T	G	T	G	-0.0057	0.020459	0.6153	0.609648	FALSE	FALSE	FALSE	xmGuol	0.014044	0.145165	486484	sepsis	TRUE	reported	textfile	9.00E-04	2.52E-10	7355	M00054.m	TRUE	reported	GW50Ri	2	TRUE	0.005424	40.1002
1971	rs3736438	T	G	T	G	-0.0057	0.015876	0.3889	0.388232	FALSE	FALSE	FALSE	v2KE89	0.014019	0.25742	486484	sepsis	TRUE	reported	textfile	9.00E-04	3.19E-10	7349	M15500.m	TRUE	reported	to2t7H	2	TRUE	0.005428	40.1002

4606	rs651007	T	C	T	C	-0.0152	-0.03077	0.2007	0.207276	FALSE	FALSE	FALSE	Pzplqu	0.016862	0.068053	486484	sepsis	TRUE	reported	textfile	0.0024	2.78E-10	7318	M32740.m	TRUE	reported	FjEBS5	2	TRUE	0.005451	40.10015
3183	rs273914	A	T	A	T	0.0209	0.016687	0.3691	0.374818	FALSE	TRUE	FALSE	gvZCBj	0.014097	0.236523	486484	sepsis	TRUE	reported	textfile	0.0033	4.02E-10	7259	M22189.m	TRUE	reported	GKqg2h	2	TRUE	0.005495	40.10006
3417	rs7809234	A	T	A	T	0.0285	-0.01576	0.8755	0.882505	FALSE	TRUE	FALSE	VU62LG	0.021269	0.458675	486484	sepsis	TRUE	reported	textfile	0.0045	1.60E-10	7180	M27513.m	TRUE	reported	0didOx	2	TRUE	0.005555	40.09994
1533	rs495360	A	C	A	C	-0.019	0.002456	0.497	0.501258	FALSE	FALSE	FALSE	YvgTeW	0.013876	0.859508	486484	sepsis	TRUE	reported	textfile	0.003	2.40E-10	5598	M03141.m	TRUE	reported	srKjSc	2	TRUE	0.007114	40.09678
6009	rs1042327(	T	G	T	G	1.0018	0.008164	0.9333	0.951285	FALSE	FALSE	FALSE	33ojbt	0.032291	0.800394	486484	sepsis	TRUE	reported	textfile	0.1561	1.38E-10	60	M33423.m	TRUE	reported	ivEtvl	2	TRUE	0.407036	39.81374
7459	rs4706020	A	G	A	G	-0.0214	-0.01004	0.3834	0.378721	FALSE	FALSE	FALSE	7ubZow	0.01416	0.478387	486484	sepsis	TRUE	reported	textfile	0.0034	3.80E-10	7344	M34407.m	TRUE	reported	LRsXN2	2	TRUE	0.005365	39.60513
4383	rs1007003	A	G	A	G	-0.0409	0.002373	0.0297	0.028865	FALSE	FALSE	FALSE	UJygVd	0.040823	0.953649	486484	sepsis	TRUE	reported	textfile	0.0065	4.30E-10	7361	M32654.m	TRUE	reported	kv9R0W	2	TRUE	0.00535	39.58238
6849	rs1165209	A	G	A	G	-0.0226	0.011083	0.5531	0.566604	FALSE	FALSE	FALSE	2pJy8f	0.013778	0.421185	486484	sepsis	TRUE	reported	textfile	0.0036	5.13E-10	7132	M34040.m	TRUE	reported	zuJC1r	2	TRUE	0.005496	39.39944
5154	rs4951117	A	T	A	T	-0.4779	-0.03118	0.0855	0.104557	FALSE	TRUE	FALSE	L2DJzK	0.022392	0.163831	486484	sepsis	TRUE	reported	textfile	0.0752	2.05E-10	76	M33173.m	TRUE	reported	hRG70D	2	TRUE	0.347004	39.32389
4628	rs556339	T	C	T	C	0.0382	-0.0219	0.2533	0.260572	FALSE	FALSE	FALSE	0oA38w	0.015531	0.158472	486484	sepsis	TRUE	reported	textfile	0.0061	4.83E-10	7356	M32753.m	TRUE	reported	dQUaUX	2	TRUE	0.005303	39.20568
429	rs445925	A	G	A	G	-0.025	0.013494	0.079	0.108393	FALSE	FALSE	FALSE	cTsNj6	0.021901	0.5378	486484	sepsis	TRUE	reported	textfile	0.004	4.11E-10	5602	M00063.m	TRUE	reported	7KYTrQ	2	TRUE	0.006925	39.04855
4729	rs4149081	A	G	A	G	0.068	-0.01154	0.1794	0.162129	FALSE	FALSE	FALSE	fz63r4	0.018543	0.533722	486484	sepsis	TRUE	reported	textfile	0.0109	5.22E-10	2537	M32762.m	TRUE	reported	kgxlBo	2	TRUE	0.015109	38.8886
7352	rs2309461	T	C	T	C	-0.2016	0.000972	0.7222	0.667121	FALSE	FALSE	FALSE	mm9ksf	0.014572	0.94683	486484	sepsis	TRUE	reported	textfile	0.0305	3.95E-11	18	M34365.m	TRUE	reported	AVwEqD	2	TRUE	0.708218	38.8355
152	rs6935961	A	G	A	G	-0.0056	-0.00733	0.5578	0.556347	FALSE	FALSE	FALSE	xmGuol	0.013768	0.594462	486484	sepsis	TRUE	reported	textfile	9.00E-04	3.75E-10	7355	M00054.m	TRUE	reported	GW50Ri	2	TRUE	0.005236	38.70552
2347	rs1001508(	A	C	A	C	0.1094	-0.00945	0.9289	0.910201	FALSE	FALSE	FALSE	NBJ2Do	0.023932	0.692824	486484	sepsis	TRUE	reported	textfile	0.0176	5.05E-10	1739	M17799.m	TRUE	reported	Uz0Wgl	2	TRUE	0.021735	38.59309
4134	rs1165196	A	G	A	G	-0.0409	0.011255	0.549	0.566584	FALSE	FALSE	FALSE	pLh7sk	0.013775	0.413905	486484	sepsis	TRUE	reported	textfile	0.0066	4.80E-10	7256	M32549.m	TRUE	reported	RSQOnp	2	TRUE	0.005265	38.39185
3737	rs2070630	C	G	C	G	-0.021	-0.026	0.2948	0.302967	FALSE	TRUE	FALSE	k16olH	0.014935	0.081653	486484	sepsis	TRUE	reported	textfile	0.0034	6.76E-10	7340	M32328.m	TRUE	reported	TQA3sZ	2	TRUE	0.005171	38.13839
5959	rs6544713	T	C	T	C	-0.0314	-0.00636	0.3211	0.32316	FALSE	FALSE	FALSE	mDZrGw	0.014597	0.663071	486484	sepsis	TRUE	reported	textfile	0.0051	8.97E-10	6797	M33415.m	TRUE	reported	MzEP7R	2	TRUE	0.005546	37.8958
778	rs7503429	A	C	A	C	-0.016	0.011967	0.4255	0.418614	FALSE	FALSE	FALSE	tYvbNc	0.01401	0.392993	486484	sepsis	TRUE	reported	textfile	0.0026	1.64E-09	6946	M01303.m	TRUE	reported	hT3gsE	2	TRUE	0.005422	37.85892
6715	rs924135	A	T	A	T	-0.0209	-0.00524	0.384	0.388339	FALSE	TRUE	FALSE	bzT5yX	0.014021	0.70842	486484	sepsis	TRUE	reported	textfile	0.0034	1.09E-09	7345	M33936.m	TRUE	reported	W7BO8n	2	TRUE	0.005118	37.77604
4321	rs2070895	A	G	A	G	0.0257	-0.0162	0.21	0.219377	FALSE	FALSE	FALSE	EOAssJ	0.016506	0.326248	486484	sepsis	TRUE	reported	textfile	0.0042	1.22E-09	7369	M32635.m	TRUE	reported	X1mTjy	2	TRUE	0.005055	37.43258
1066	rs807670	A	C	A	C	0.011	-0.0032	0.421	0.432011	FALSE	FALSE	FALSE	tRtQ2e	0.013877	0.81747	486484	sepsis	TRUE	reported	textfile	0.0018	6.84E-10	7364	M01564.m	TRUE	reported	Tbg0zM	2	TRUE	0.005046	37.33554
2018	rs6862024	A	G	A	G	-0.0055	-0.02069	0.3691	0.371176	FALSE	FALSE	FALSE	v2KE89	0.014167	0.144172	486484	sepsis	TRUE	reported	textfile	9.00E-04	8.99E-10	7349	M15500.m	TRUE	reported	to2t7H	2	TRUE	0.005056	37.33552
6043	rs4838294	A	G	A	G	-1.8595	-0.07996	0.0167	0.011761	FALSE	FALSE	FALSE	33ojbt	0.063013	0.204465	486484	sepsis	TRUE	reported	textfile	0.2997	5.49E-10	60	M33423.m	TRUE	reported	ivEtvl	2	TRUE	0.39084	37.21308
4267	rs1809148	A	G	A	G	-0.0262	0.014595	0.136	0.130981	FALSE	FALSE	FALSE	ylV4bp	0.020214	0.470266	486484	sepsis	TRUE	reported	textfile	0.0043	9.51E-10	7337	M32632.m	TRUE	reported	hqHJoU	2	TRUE	0.005034	37.11481
6026	rs1713299!	T	G	T	G	-1.8613	0.016628	0.0177	0.038808	FALSE	FALSE	FALSE	33ojbt	0.055243	0.763416	486484	sepsis	TRUE	reported	textfile	0.3006	5.95E-10	60	M33423.m	TRUE	reported	ivEtvl	2	TRUE	0.389873	37.06223
7012	rs7969341	A	G	A	G	-0.0213	0.01185	0.833	0.837659	FALSE	FALSE	FALSE	ePdfmg	0.018542	0.522761	486484	sepsis	TRUE	reported	textfile	0.0035	1.01E-09	7347	M34244.m	TRUE	reported	l9nzfX	2	TRUE	0.005016	37.02584
6280	rs4843718	A	G	A	G	0.0158	0.014604	0.2413	0.245192	FALSE	FALSE	FALSE	MOonP9	0.015972	0.36054	486484	sepsis	TRUE	reported	textfile	0.0026	1.71E-09	7066	M33515.m	TRUE	reported	226GuP	2	TRUE	0.005199	36.91854
1779	rs1008593!	T	C	T	C	-0.0103	0.012595	0.3782	0.373392	FALSE	FALSE	FALSE	JEcCRZ	0.014143	0.373158	486484	sepsis	TRUE	reported	textfile	0.0017	3.33E-09	7368	M15140.m	TRUE	reported	xYCzKP	2	TRUE	0.004958	36.69938
8213	rs274570	T	C	T	C	0.0224	0.026268	0.2962	0.293331	FALSE	FALSE	FALSE	aY1HfB	0.015014	0.080197	486484	sepsis	TRUE	reported	textfile	0.0037	1.78E-09	5306	M35433.m	TRUE	reported	H5O4i2	2	TRUE	0.00686	36.63776
4740	rs1050967	C	G	C	G	-0.0471	0.012447	0.1624	0.151929	FALSE	TRUE	FALSE	oQPvO3	0.019048	0.513479	486484	sepsis	TRUE	reported	textfile	0.0078	1.78E-09	7330	M32786.m	TRUE	reported	Th0GcB	2	TRUE	0.00495	36.45307
5958	rs4917639	A	C	A	C	-0.0362	0.019208	0.8015	0.801852	FALSE	FALSE	FALSE	mDZrGw	0.017136	0.262317	486484	sepsis	TRUE	reported	textfile	0.006	1.39E-09	6797	M33415.m	TRUE	reported	MzEP7R	2	TRUE	0.005327	36.3904
2368	rs1181904(	T	C	T	C	-0.1237	0.033919	0.0689	0.037004	FALSE	FALSE	FALSE	NBJ2Do	0.036408	0.35153	486484	sepsis	TRUE	reported	textfile	0.0205	1.68E-09	1739	M17799.m	TRUE	reported	Uz0Wgl	2	TRUE	0.020508	36.36905
6749	rs4691536	A	G	A	G	0.0247	-0.01777	0.3688	0.382599	FALSE	FALSE	FALSE	NZVg9N	0.014083	0.206892	486484	sepsis	TRUE	reported	textfile	0.0041	1.62E-09	7320	M33941.m	TRUE	reported	kRWNNK	2	TRUE	0.004934	36.28336
5127	rs1242635	T	G	T	G	-1.192	0.013824	0.0132	0.008723	FALSE	FALSE	FALSE	L2DJzK	0.073428	0.850672	486484	sepsis	TRUE	reported	textfile	0.1953	1.04E-09	76	M33173.m	TRUE	reported	hRG70D	2	TRUE	0.328929	36.27155
3921	rs278136	T	C	T	C	-0.0259	0.020463	0.234	0.231581	FALSE	FALSE	FALSE	BrQzHt	0.016231	0.207385	486484	sepsis	TRUE	reported	textfile	0.0043	1.96E-09	7796	M32412.m	TRUE	reported	eVvYVX	2	TRUE	0.004632	36.2703
6192	rs1713453	A	G	A	G	-0.0408	0.00777	0.1563	0.153546	FALSE	FALSE	FALSE	DEG58p	0.018975	0.682186	486484	sepsis	TRUE	reported	textfile	0.0068	1.52E-09	7500	M33507.m	TRUE	reported	ycykD0	2	TRUE	0.004777	35.9904
432	rs602633	T	G	T	G	-0.0102	-0.02621	0.2163	0.217297	FALSE	FALSE	FALSE	cTsNj6	0.016558	0.113423	486484	sepsis	TRUE	reported	textfile	0.0017	5.02E-09	7365	M00063.m	TRUE	reported	7KYTrQ	2	TRUE	0.004864	35.99022
4095	rs1046801	T	C	T	C	0.0108	-0.01509	0.289	0.295989	FALSE	FALSE	FALSE	4rtLd0	0.014965	0.313123	486484	sepsis	TRUE	reported	textfile	0.0018	3.45E-09	7350	M32518.m	TRUE	reported	j7f1ER	2	TRUE	0.004874	35.9902
2027	rs735315	T	C	T	C	0.0054	0.021988	0.4943	0.496814	FALSE	FALSE	FALSE	v2KE89	0.013741	0.10956	486484	sepsis	TRUE	reported	textfile	9.00E-04	1.87E-09	7349	M15500.m	TRUE	reported	to2t7H	2	TRUE	0.004875	35.9902
8226	rs1259788(	T	G	T	G	0.0156	-0.00524	0.3363	0.330182	FALSE	FALSE	FALSE	4YAWhA	0.014294	0.714169	486484	sepsis	TRUE	reported	textfile	0.0026	3.78E-09	7256	M35439.m	TRUE	reported	bSSC4Z	2	TRUE	0.004937	35.99008
92	rs284191	A	G	A	G	-0.006	0.010226	0.615	0.617284	FALSE	FALSE	FALSE	xmGuol	0.014119	0.468884	486484	sepsis	TRUE	reported	textfile	0.001	1.97E-09	5592	M00054.m	TRUE	reported	GW50Ri	2	TRUE	0.006397	35.98712
291	rs2713737	A	G	A	G	-0.006	-0.00892	0.44	0.439028	FALSE	FALSE	FALSE	tbRL8J	0.013894	0.521046	486484	sepsis	TRUE	reported	textfile	0.001	1.97E-09	5589	M00060.m	TRUE	reported	oOvRKT	2	TRUE	0.0064	35.98712
7856	rs7203065	T	G	T	G	0.0114	-0.02279	0.3934	0.389998	FALSE	FALSE	FALSE	h5whIS	0.014328	0.111773	486484	sepsis	TRUE	reported	textfile	0.0019	2.00E-09	4900	M35137.m	TRUE	reported	xVITKF	2	TRUE	0.007293	35.98531
7216	rs1912320	A	G	A	G	0.3974	-0.0411	0.0355	0.034393	FALSE	FALSE	FALSE	i1uJ7T	0.037667	0.275183	486484	sepsis	TRUE	reported	textfile	0.0662	1.98E-09	535	M34338.m	TRUE	reported	pi3mKW	2	TRUE	0.063107	35.90155
2091	rs1017562(	A	G	A	G	-0.0305	-0.01654	0.8013	0.795857	FALSE	FALSE	FALSE	MN85S3	0.01693	0.328571	486484	sepsis	TRUE	reported	textfile	0.0051	1.88E-09	7574	M15630.m	TRUE	reported	FUih5R	2	TRUE	0.0047	35.75565
4678	rs3732218	A	G	A	G	-0.0532	0.006257	0.0805	0.082397	FALSE	FALSE	FALSE	xaapK0	0.024915	0.801704	486484	sepsis	TRUE	reported	textfile	0.0089	2.77E-09	7250	M32757.m	TRUE	reported	64n1Z4	2	TRUE	0.004904	35.72099
7339	rs1269734(	T	C	T	C	-0.2064	-0.00321	0.8333	0.788994	FALSE	FALSE	FALSE	mm9ksf	0.016719	0.847848	486484	sepsis	TRUE	reported	textfile	0.0326	2.54E-10	18	M34365.m	TRUE	reported	AVwEqD	2	TRUE	0.69011	35.6313
3980	rs2715311	A	G	A	G	-0.0107	-0.01797	0.3884	0.385011	FALSE	FALSE	FALSE	XdJbEU	0.014063	0.201354	486484	sepsis	TRUE	reported	textfile	0.0018	6.57E-09	7364	M32452.m	TRUE	reported	V5xWKi	2	TRUE	0.004776	35.32682
7059	rs3017098	A	G	A	G	0.0107	0.003911	0.5517	0.553483	FALSE	FALSE	FALSE	uthruC	0.01376	0.776226	486484	sepsis	TRUE	reported	textfile	0.0018	1.48E-09	7316	M34283.m	TRUE	reported	ftTuRU	2	TRUE	0.004807	35.32676
6997	rs1077835	A	G	A	G	-0.0208	0.017555	0.7976	0.779568	FALSE	FALSE	FALSE	ePdfmg	0.0165	0.287361	486484	sepsis	TRUE	reported	textfile	0.0035	1.82E-09	7347	M34244.m	TRUE	reported	l9nzfX	2	TRUE	0.004784	35.30794
6850	rs1466245	T	C	T	C	0.0481	0.025172	0.0511	0.052168	FALSE	FALSE	FALSE	2pJy8f	0.030811	0.413944	486484	sepsis	TRUE	reported	textfile	0.0081	2.78E-09	7132	M34040.m	TRUE	reported	zuJC1r	2	TRUE	0.00492	35.25318
7221	rs2830801	A	G	A	G	-1.072	-0.00563	0.023	0.115068	FALSE	FALSE	FALSE	i1uJ7T	0.02147	0.793274	486484	sepsis	TRUE	reported	textfile	0.179	2.11E-09	115	M34338.m	TRUE	reported	pi3mKW	2	TRUE	0.237734	35.24229
4765	rs6929324	T	G	T	G	-0.0896	-0.08327	0.0166	0.012603	FALSE	FALSE	FALSE	RDfDIS	0.061368	0.174834	486484	sepsis	TRUE	reported	textfile	0.0151	3.25E-09	6857	M32787.m	TRUE	reported	Hq6nGt	2	TRUE	0.005109	35.19941
1403	rs1017016(	T	C	T	C	0.0338	-0.01669	0.5675	0.625457	FALSE	FALSE	FALSE	33ptwk	0.014119	0.237144	486484	sepsis	TRUE	reported	textfile	0.0057	2.71E-09	6252	M02137.m	TRUE	reported	SQY1na	2	TRUE	0.005593	35.15157
7108	rs1470777	T	C	T	C	-0.0515	-0.00748	0.5972	0.603158	FALSE	FALSE	FALSE	DfLd6z	0.014039	0.593997	486484	sepsis	TRUE	reported	textfile	0.0087	3.73E-09	1766	M34314.m	TRUE	reported	OgloAQ	2	TRUE	0.019456	35.00127
3808	rs4870490	A	G	A	G	0.0857	-0.00377	0.066	0.072314	FALSE	FALSE	FALSE	HOq2gb	0.026388	0.886517	486484	sepsis	TRUE	reported	textfile	0.0145	3.14E-09	6687	M32346.m	TRUE	reported	Ss5q17	2	TRUE	0.005197	34.92173
7646	rs279941	A	C	A	C	-0.0478	-0.02249	0.1171	0.122154	FALSE	FALSE	FALSE	wrTMYM	0.020833	0.280361	486484	sepsis	TRUE	reported	textfile	0.0081	4.31E-09	5856	M34533.m	TRUE	reported	qIRVku	2	TRUE	0.005912	34.81268
161	rs7463805	T	C	T	C	-0.0053	0.001899	0.5213	0.511221	FALSE	FALSE	FALSE	xmGuol	0.013717	0.889889	486484	sepsis	TRUE	reported	textfile	9.00E-04	4.60E-09	7355	M00054.m	TRUE	reported	GW50Ri	2	TRUE	0.004693	34.66958
219	rs1092301(	A	G	A	G	0.0053	0.021616	0.6191	0.62302	FALSE	FALSE	FALSE	tbRL8J	0.014114	0.125625	486484	sepsis	TRUE	reported	textfile	9.00E-04	2.88E-09	7351	M00060.m	TRUE	reported	oOvRKT	2	TRUE	0.004695	34.66958
360	rs7647029	A	G	A	G	0.0053	-0.03021	0.6479	0.639897	FALSE	FALSE	FALSE	tbRL8J	0.014286	0.034479	486484	sepsis	TRUE	reported	textfile	9.00E-04	1.02E-08	7351	M00060.m	TRUE	reported	oOvRKT	2	TRUE	0.004695	34.66958
1903	rs1270939	T	G	T	G	0.0053	0.001868	0.6286	0.632092	FALSE	FALSE	FALSE	v2KE89	0.014221	0.895477	486484	sepsis	TRUE	reported	textfile	9.00E-04	6.41E-09	7349	M15500.m	TRUE	reported	to2t7H	2	TRUE	0.004697	34.66957
4976	rs9318225	T	C	T	C	-0.0106	-0.00501	0.6829	0.684825	FALSE	FALSE	FALSE	bKmDMa	0.01472	0.733351	486484	sepsis	TRUE	reported	textfile	0.0018	1.95E-09	7344	M32910.m	TRUE	reported	nAuPzO	2	TRUE	0.0047	34.66957
6119	rs1026376(	T	C	T	C	-0.0106	0.001797	0.2442	0.246096	FALSE	FALSE	FALSE	MiSJly	0.015858	0.909767	486484	sepsis	TRUE	reported	textfile	0.0018	7.47E-09	7307	M33477.m	TRUE	reported	WwL1hq	2	TRUE	0.004724	34.66952
1648	rs1094564	A	T	A	T	0.0212	-0.00358	0.1287	0.135836	FALSE	TRUE	FALSE	YzX5ZD	0.020017	0.858168	486484	sepsis	TRUE	reported	textfile	0.0036	4.29E-09	6864	M12129.m	TRUE	reported	6tMfaW	2	TRUE	0.005027	34.66891
1359	rs1016452	A	G	A	G	-0.01	0.012308	0.6775	0.669782	FALSE	FALSE	FALSE	KrWhws	0.014556	0.397774	486484	sepsis	TRUE	reported	textfile	0.0017	1.06E-08	7325	M02132.m	TRUE	reported	7pzpfK	2	TRUE	0.004702	34.59263
3623	rs4236541	T	G	T	G	0.0423	0.006805	0.7287	0.735283	FALSE	FALSE	FALSE	gq4ISJ	0.015526	0.661147	486484	sepsis	TRUE	reported	textfile	0.0072	5.09E-09	7338	M31591.m	TRUE	reported	wjwl4J	2	TRUE	0.004682	34.50622
7145	rs726389	T	C	T	C	-0.31	-0.05438	0.9733	0.977712	FALSE	FALSE	FALSE	de8tA6	0.046864	0.245927	486484	sepsis	TRUE	reported	textfile	0.0521	2.71E-09	75	M34322.m	TRUE	reported	sXPPBO	2	TRUE	0.320675	34.45954
4796	rs1264510	A	G	A	G	0.0463	0.013902	0.1473	0.152392	FALSE	FALSE	FALSE	9qWhFj	0.019059	0.465748	486484	sepsis	TRUE	reported	textfile	0.0079	4.61E-09	6200	M32808.m	TRUE	reported	MQcOSi	2	TRUE	0.00551	34.33742
2897	rs9756306	A	G	A	G	-0.0158	0.000136	0.3332	0.324404	FALSE	FALSE	FALSE	mUzn1R	0.014599	0.992553	486484	sepsis	TRUE	reported	textfile	0.0027	3.64E-09	7301	M20675.m	TRUE	reported	XwY04K	2	TRUE	0.004668	34.23479
4781	rs1705769(	T	C	T	C	0.0831	-0.04521	0.0555	0.05084	FALSE	FALSE	FALSE	MmZZoM	0.031131	0.146387	486484	sepsis	TRUE	reported	textfile	0.0142	5.00E-09	4274	M32800.m	TRUE	reported	70YSCO	2	TRUE	0.007949	34.2312
4691	rs3820744	T	C	T	C	-0.0433	-0.01344	0.9084	0.907302	FALSE	FALSE	FALSE	MV108h	0.023646	0.569837	486484	sepsis	TRUE	reported	textfile	0.0074	4.28E-09	6642	M32758.m	TRUE	reported	btjNXQ	2	TRUE	0.005128	34.228
7614	rs1166660	T	C	T	C	0.0234	0.003124	0.2406	0.236421	FALSE	FALSE	FALSE	kVfKqT	0.016297	0.847993	486484	sepsis	TRUE	reported	textfile	0.004	6.96E-09	7322	M34527.m	TRUE	reported	s4ti92	2	TRUE	0.004652	34.21315
4417	rs1600760	A	T	A	T	-0.0216	-0.01215	0.663	0.673632	FALSE	TRUE	FALSE	NzBiVA	0.014564	0.404002	486484	sepsis	TRUE	reported	textfile	0.0037	5.12E-09	7354	M32672.m	TRUE	reported	YLlhHJ	2	TRUE	0.004613	34.07108
5160	rs690764	A	G	A	G	-0.6782	0.001785	0.0574	0.071812	FALSE	FALSE	FALSE	L2DJzK	0.026557	0.946404	486484	sepsis	TRUE	reported	textfile	0.1147	3.36E-09	76	M33173.m	TRUE	reported	hRG70D	2	TRUE	0.315077	34.04136
8402	rs9404466	T	C	T	C	0.0105	-0.00921	0.2671	0.273739	FALSE	FALSE	FALSE	SCzh0A	0.015323	0.547751	486484	sepsis	TRUE	reported	textfile	0.0018	1.26E-08	7348	M35675.m	TRUE	reported	n1SREk	2	TRUE	0.00461	34.01852
5988	rs2263065	T	G	T	G	-0.0105	-0.01666	0.4417	0.519497	FALSE	FALSE	FALSE	3KsoXq	0.013747	0.225494	486484	sepsis	TRUE	reported	textfile	0.0018	3.56E-09	7306	M33422.m	TRUE	reported	aNr6Ug	2	TRUE	0.004636	34.01846
7616	rs1761493	A	G	A	G	0.0204	0.003766	0.782	0.777585	FALSE	FALSE	FALSE	kVfKqT	0.016624	0.820758	486484	sepsis	TRUE	reported	textfile	0.0035	5.67E-09	7322	M34527.m	TRUE	reported	s4ti92	2	TRUE	0.004618	33.96297
6227	rs6430553	T	C	T	C	-0.0204	-0.01544	0.4007	0.371084	FALSE	FALSE	FALSE	8zUBbU	0.014261	0.279042	486484	sepsis	TRUE	reported	textfile	0.0035	4.81E-09	7044	M33509.m	TRUE	reported	1mO9m8	2	TRUE	0.0048	33.9626
2842	rs1343165	T	C	T	C	0.0099	-0.01487	0.7174	0.692255	FALSE	FALSE	FALSE	Y5qWBm	0.01489	0.318109	486484	sepsis	TRUE	reported	textfile	0.0017	4.34E-09	7325	M20489.m	TRUE	reported	f2g9Su	2	TRUE	0.004608	33.90424
1290	rs1093881!	T	G	T	G	0.0099	0.007692	0.5932	0.587326	FALSE	FALSE	FALSE	WQdpmo	0.01388	0.579447	486484	sepsis	TRUE	reported	textfile	0.0017	8.38E-09	7136	M01769.m	TRUE	reported	BkptOK	2	TRUE	0.00473	33.90399
2367	rs1180319	T	C	T	C	0.1002	-0.01553	0.9061	0.917584	FALSE	FALSE	FALSE	NBJ2Do	0.024895	0.53279	486484	sepsis	TRUE	reported	textfile	0.0172	6.10E-09	1739	M17799.m	TRUE	reported	Uz0Wgl	2	TRUE	0.019142	33.89837
2481	rs296381	T	C	T	C	0.039	-0.02129	0.8282	0.835977	FALSE	FALSE	FALSE	SrfyMU	0.018492	0.249654	486484	sepsis	TRUE	reported	textfile	0.0067	4.83E-09	7226	M17807.m	TRUE	reported	0zrJsu	2	TRUE	0.004667	33.87345
4212	rs920812	T	C	T	C	0.0256	0.034229	0.326	0.328569	FALSE	FALSE	FALSE	rdKSjG	0.014554	0.018684	486484	sepsis	TRUE	reported	textfile	0.0044	8.19E-09	7322	M32578.m	TRUE	reported	RAzSLK	2	TRUE	0.004602	33.84199
3451	rs7948073	T	G	T	G	-0.0256	0.029309	0.5702	0.568527	FALSE	FALSE	FALSE	J1I92u	0.013795	0.033616	486484	sepsis	TRUE	reported	textfile	0.0044	4.60E-09	6715	M27710.m	TRUE	reported	9qjplZ	2	TRUE	0.005016	33.84116
4800	rs4149026	A	C	A	C	0.036	0.015121	0.6863	0.694115	FALSE	FALSE	FALSE	9qWhFj	0.014959	0.312096	486484	sepsis	TRUE	reported	textfile	0.0062	5.67E-09	6200	M32808.m	TRUE	reported	MQcOSi	2	TRUE	0.005408	33.704
5731	rs3738544	T	C	T	C	0.0203	0.008987	0.8071	0.803494	FALSE	FALSE	FALSE	YDQaWa	0.017267	0.602759	486484	sepsis	TRUE	reported	textfile	0.0035	8.05E-09	7063	M33228.m	TRUE	reported	BmYg8r	2	TRUE	0.00474	33.63047
6564	rs1535	A	G	A	G	-0.0203	-0.0263	0.662	0.65351	FALSE	FALSE	FALSE	aTDqRQ	0.014366	0.067114	486484	sepsis	TRUE	reported	textfile	0.0035	6.15E-09	6506	M33871.m	TRUE	reported	Lqimdw	2	TRUE	0.005144	33.62966
6022	rs1684815!	A	G	A	G	-1.7999	-0.0514	0.0199	0.009966	FALSE	FALSE	FALSE	33ojbt	0.068444	0.452652	486484	sepsis	TRUE	reported	textfile	0.3054	3.79E-09	60	M33423.m	TRUE	reported	ivEtvl	2	TRUE	0.36665	33.5765
7569	rs3799344	T	C	T	C	0.0197	-0.01183	0.4519	0.43657	FALSE	FALSE	FALSE	Ot2ZAn	0.013758	0.390033	486484	sepsis	TRUE	reported	textfile	0.0034	8.94E-09	7119	M34481.m	TRUE	reported	tpyFcK	2	TRUE	0.004694	33.56237
1352	rs7288481	A	G	A	G	-0.0139	0.001113	0.2805	0.268543	FALSE	FALSE	FALSE	LDUJbh	0.015575	0.943006	486484	sepsis	TRUE	reported	textfile	0.0024	7.27E-09	7367	M01898.m	TRUE	reported	JRSLeH	2	TRUE	0.004533	33.5343
3871	rs1083801	A	G	A	G	-0.0139	0.001847	0.7066	0.725819	FALSE	FALSE	FALSE	bwkKtK	0.015821	0.90708	486484	sepsis	TRUE	reported	textfile	0.0024	8.78E-09	7307	M32393.m	TRUE	reported	RvZzAy	2	TRUE	0.00457	33.53422
7732	rs1339995	A	C	A	C	0.011	-0.00728	0.4714	0.458232	FALSE	FALSE	FALSE	HyMNYM	0.013712	0.595573	486484	sepsis	TRUE	reported	textfile	0.0019	1.63E-08	5885	M34912.m	TRUE	reported	Tl8Hcw	2	TRUE	0.005663	33.50661
6664	rs1084178!	A	C	A	C	0.0301	0.010324	0.7347	0.718232	FALSE	FALSE	FALSE	6iZSOl	0.01517	0.496136	486484	sepsis	TRUE	reported	textfile	0.0052	5.78E-09	4533	M33901.m	TRUE	reported	ApKaGe	2	TRUE	0.007337	33.4915
55	rs1559063	C	G	C	G	0.0052	0.012157	0.6209	0.618648	FALSE	TRUE	FALSE	xmGuol	0.01407	0.387562	486484	sepsis	TRUE	reported	textfile	9.00E-04	7.82E-09	7355	M00054.m	TRUE	reported	GW50Ri	2	TRUE	0.004518	33.37364
337	rs6735596	A	C	A	C	-0.0052	-0.02272	0.6267	0.62053	FALSE	FALSE	FALSE	tbRL8J	0.014078	0.106528	486484	sepsis	TRUE	reported	textfile	9.00E-04	1.15E-08	7351	M00060.m	TRUE	reported	oOvRKT	2	TRUE	0.004521	33.37363
4337	rs1219181	T	G	T	G	0.0104	-0.00492	0.684	0.681355	FALSE	FALSE	FALSE	6VSoQQ	0.014689	0.737746	486484	sepsis	TRUE	reported	textfile	0.0018	1.66E-08	7229	M32644.m	TRUE	reported	oC2AV0	2	TRUE	0.004597	33.37348
8019	rs1006989(	A	C	A	C	0.0104	-0.03037	0.1781	0.182629	FALSE	FALSE	FALSE	SwiqSi	0.017683	0.085874	486484	sepsis	TRUE	reported	textfile	0.0018	1.42E-08	7217	M35270.m	TRUE	reported	21ZIsQ	2	TRUE	0.004604	33.37346
1495	rs9940990	A	T	A	T	-0.0104	-0.00975	0.3545	0.358067	FALSE	TRUE	FALSE	pkb876	0.014252	0.493914	486484	sepsis	TRUE	reported	textfile	0.0018	4.75E-09	7037	M02734.m	TRUE	reported	0LCMVI	2	TRUE	0.004721	33.37323
1044	rs1509123	T	C	T	C	-0.0248	-0.01778	0.0476	0.062718	FALSE	FALSE	FALSE	tRtQ2e	0.028266	0.529325	486484	sepsis	TRUE	reported	textfile	0.0043	5.20E-09	7364	M01564.m	TRUE	reported	Tbg0zM	2	TRUE	0.004497	33.25435
1853	rs4970834	T	C	T	C	-0.0098	-0.03237	0.1821	0.186662	FALSE	FALSE	FALSE	S1eWts	0.017641	0.066559	486484	sepsis	TRUE	reported	textfile	0.0017	1.06E-08	7345	M15488.m	TRUE	reported	511Kl9	2	TRUE	0.004504	33.22279
7621	rs430200	T	G	T	G	0.0201	-0.01522	0.7744	0.77917	FALSE	FALSE	FALSE	kVfKqT	0.016503	0.356327	486484	sepsis	TRUE	reported	textfile	0.0035	6.45E-09	7322	M34527.m	TRUE	reported	s4ti92	2	TRUE	0.004484	32.9714
7861	rs7749940	A	C	A	C	0.0109	-0.0152	0.3736	0.36025	FALSE	FALSE	FALSE	h5whIS	0.014242	0.285921	486484	sepsis	TRUE	reported	textfile	0.0019	8.30E-09	4900	M35137.m	TRUE	reported	xVITKF	2	TRUE	0.006672	32.89792
5055	rs698078	A	G	A	G	-0.0636	0.009972	0.5789	0.596094	FALSE	FALSE	FALSE	JLCSne	0.013909	0.473416	486484	sepsis	TRUE	reported	textfile	0.0111	1.02E-08	2391	M33137.m	TRUE	reported	LGKYXK	2	TRUE	0.013545	32.80234
4970	rs7707010	A	G	A	G	0.0252	0.022079	0.9582	0.95836	FALSE	FALSE	FALSE	bKmDMa	0.034324	0.520058	486484	sepsis	TRUE	reported	textfile	0.0044	9.61E-09	7344	M32910.m	TRUE	reported	nAuPzO	2	TRUE	0.004447	32.79272
1188	rs1015340!	A	G	A	G	-0.0103	0.019452	0.5342	0.538748	FALSE	FALSE	FALSE	Ckdgh1	0.013755	0.157292	486484	sepsis	TRUE	reported	textfile	0.0018	9.27E-09	7355	M01644.m	TRUE	reported	nKxz7B	2	TRUE	0.004432	32.73492
7199	rs1232699(	A	C	A	C	-1.038	0.000727	0.011	0.036351	FALSE	FALSE	FALSE	i1uJ7T	0.038106	0.984771	486484	sepsis	TRUE	reported	textfile	0.18	8.09E-09	115	M34338.m	TRUE	reported	pi3mKW	2	TRUE	0.224307	32.67611
6584	rs1711533(	T	C	T	C	-0.6712	0.018539	0.9866	0.973685	FALSE	FALSE	FALSE	XO3rXq	0.043961	0.673229	486484	sepsis	TRUE	reported	textfile	0.1168	9.04E-09	176	M33877.m	TRUE	reported	p2sETg	2	TRUE	0.157988	32.64787
1125	rs1173032(	T	C	T	C	0.0097	0.007531	0.6665	0.644844	FALSE	FALSE	FALSE	nlqUuV	0.014841	0.611813	486484	sepsis	TRUE	reported	textfile	0.0017	7.08E-09	7371	M01604.m	TRUE	reported	Uro9nC	2	TRUE	0.004397	32.54826
4955	rs2686184	A	G	A	G	-0.0097	0.009145	0.408	0.407878	FALSE	FALSE	FALSE	bKmDMa	0.013928	0.511454	486484	sepsis	TRUE	reported	textfile	0.0017	1.89E-08	7344	M32910.m	TRUE	reported	nAuPzO	2	TRUE	0.004414	32.54823
5135	rs1296083!	T	C	T	C	-1.1492	-0.01911	0.0132	0.03284	FALSE	FALSE	FALSE	L2DJzK	0.038456	0.619274	486484	sepsis	TRUE	reported	textfile	0.1988	7.40E-09	76	M33173.m	TRUE	reported	hRG70D	2	TRUE	0.305405	32.53693
8221	rs1108583!	T	C	T	C	0.0154	-0.0035	0.2018	0.203646	FALSE	FALSE	FALSE	4YAWhA	0.017089	0.837631	486484	sepsis	TRUE	reported	textfile	0.0027	1.04E-08	7256	M35439.m	TRUE	reported	bSSC4Z	2	TRUE	0.004463	32.52327
6353	rs8082167	T	C	T	C	0.4457	-0.01497	0.0288	0.022316	FALSE	FALSE	FALSE	qLUY00	0.047531	0.75283	486484	sepsis	TRUE	reported	textfile	0.0774	8.49E-09	102	M33610.m	TRUE	reported	8cAJbB	2	TRUE	0.245334	32.50897
3918	rs2469211	A	T	A	T	-0.0342	0.009432	0.8921	0.89144	FALSE	TRUE	FALSE	BrQzHt	0.022004	0.668178	486484	sepsis	TRUE	reported	textfile	0.006	1.06E-08	7796	M32412.m	TRUE	reported	eVvYVX	2	TRUE	0.00415	32.48166
7269	rs4001461	T	C	T	C	0.0513	0.011291	0.4402	0.444794	FALSE	FALSE	FALSE	P5Xm4S	0.013744	0.411336	486484	sepsis	TRUE	reported	textfile	0.009	1.39E-08	3491	M34350.m	TRUE	reported	YU4x0J	2	TRUE	0.009221	32.47139
2650	rs2912546	T	C	T	C	0.2679	-0.01642	0.0501	0.040697	FALSE	FALSE	FALSE	9GoQdh	0.035205	0.64097	486484	sepsis	TRUE	reported	textfile	0.0469	1.15E-08	257	M18474.m	TRUE	reported	p6Dq8p	2	TRUE	0.112657	32.37478
3282	rs1800775	A	C	A	C	0.0108	-0.02977	0.4986	0.487001	FALSE	FALSE	FALSE	vYWPe9	0.013669	0.029436	486484	sepsis	TRUE	reported	textfile	0.0019	7.96E-09	7340	M25459.m	TRUE	reported	VvJ6Vx	2	TRUE	0.004383	32.30145
8461	rs2708257	A	C	A	C	-0.0108	-0.02475	0.6508	0.647175	FALSE	FALSE	FALSE	uMajV6	0.014337	0.084326	486484	sepsis	TRUE	reported	textfile	0.0019	2.17E-08	6604	M35754.m	TRUE	reported	b8QB7S	2	TRUE	0.004869	32.30046
8917	rs6703518	T	G	T	G	-0.0108	-0.00492	0.4273	0.425424	FALSE	FALSE	FALSE	eLhsld	0.01383	0.721827	486484	sepsis	TRUE	reported	textfile	0.0019	5.85E-09	6552	M37058.m	TRUE	reported	RSeWE5	2	TRUE	0.004907	32.30039
3166	rs711995	T	C	T	C	0.0108	-0.00396	0.4481	0.447732	FALSE	FALSE	FALSE	JiXR2t	0.013832	0.774617	486484	sepsis	TRUE	reported	textfile	0.0019	2.70E-08	6535	M22177.m	TRUE	reported	joi6p6	2	TRUE	0.00492	32.30036
7636	rs1101997(	C	G	C	G	0.1331	-0.05028	0.0717	0.058637	FALSE	TRUE	FALSE	wrTMYM	0.029131	0.08433	486484	sepsis	TRUE	reported	textfile	0.0234	1.36E-08	1212	M34533.m	TRUE	reported	qIRVku	2	TRUE	0.026	32.30034
1462	rs1219271	A	T	A	T	0.0142	0.022761	0.1412	0.145803	FALSE	TRUE	FALSE	pkb876	0.019385	0.240321	486484	sepsis	TRUE	reported	textfile	0.0025	2.10E-08	7037	M02734.m	TRUE	reported	0LCMVI	2	TRUE	0.004564	32.25323
6057	rs9921315	A	G	A	G	0.7395	0.010629	0.8729	0.86161	FALSE	FALSE	FALSE	33ojbt	0.019899	0.59325	486484	sepsis	TRUE	reported	textfile	0.1281	7.82E-09	60	M33423.m	TRUE	reported	ivEtvl	2	TRUE	0.35709	32.21476
6524	rs3198697	T	C	T	C	0.0193	-0.01093	0.4174	0.407569	FALSE	FALSE	FALSE	OKIXJ8	0.013897	0.431741	486484	sepsis	TRUE	reported	textfile	0.0034	1.65E-08	7362	M33821.m	TRUE	reported	XGkaXH	2	TRUE	0.004358	32.21356
7706	rs8022857	T	C	T	C	0.0772	-0.02301	0.9764	0.972759	FALSE	FALSE	FALSE	2eJlPM	0.042554	0.588677	486484	sepsis	TRUE	reported	textfile	0.0136	1.45E-08	6381	M34761.m	TRUE	reported	druKtT	2	TRUE	0.005024	32.21222
4679	rs4715354	A	G	A	G	0.0278	0.01096	0.478	0.483689	FALSE	FALSE	FALSE	xaapK0	0.01368	0.423021	486484	sepsis	TRUE	reported	textfile	0.0049	1.66E-08	7250	M32757.m	TRUE	reported	64n1Z4	2	TRUE	0.00442	32.17938
7211	rs1689678	A	G	A	G	1.024	-0.00175	0.989	0.99122	FALSE	FALSE	FALSE	i1uJ7T	0.074104	0.98114	486484	sepsis	TRUE	reported	textfile	0.179	1.06E-08	115	M34338.m	TRUE	reported	pi3mKW	2	TRUE	0.221532	32.15692
2770	rs3783200	A	C	A	C	-0.0414	0.006773	0.6859	0.679317	FALSE	FALSE	FALSE	JWylTj	0.014654	0.643972	486484	sepsis	TRUE	reported	textfile	0.0073	1.51E-08	1643	M19396.m	TRUE	reported	6S7kzt	2	TRUE	0.0192	32.12373
2097	rs1341501	T	C	T	C	0.034	-0.0083	0.69	0.715439	FALSE	FALSE	FALSE	MN85S3	0.017371	0.63262	486484	sepsis	TRUE	reported	textfile	0.006	1.46E-08	7574	M15630.m	TRUE	reported	FUih5R	2	TRUE	0.004222	32.10263
3754	rs2370998	A	G	A	G	-0.0136	0.006527	0.5725	0.559124	FALSE	FALSE	FALSE	OZLdls	0.013812	0.636538	486484	sepsis	TRUE	reported	textfile	0.0024	1.56E-08	7356	M32338.m	TRUE	reported	s2itmB	2	TRUE	0.004346	32.10238
105	rs38271	A	G	A	G	-0.0051	-0.00778	0.5925	0.594601	FALSE	FALSE	FALSE	xmGuol	0.013936	0.57689	486484	sepsis	TRUE	reported	textfile	9.00E-04	1.19E-08	7355	M00054.m	TRUE	reported	GW50Ri	2	TRUE	0.004347	32.10238
132	rs603446	T	C	T	C	0.0051	-0.00339	0.4448	0.453206	FALSE	FALSE	FALSE	xmGuol	0.01372	0.804934	486484	sepsis	TRUE	reported	textfile	9.00E-04	1.38E-08	7355	M00054.m	TRUE	reported	GW50Ri	2	TRUE	0.004347	32.10238
70	rs2111118	T	C	T	C	0.0051	0.008689	0.6207	0.624241	FALSE	FALSE	FALSE	xmGuol	0.014109	0.53801	486484	sepsis	TRUE	reported	textfile	9.00E-04	1.21E-08	7355	M00054.m	TRUE	reported	GW50Ri	2	TRUE	0.004347	32.10238
338	rs6737109	T	C	T	C	0.0051	-0.02311	0.5761	0.580037	FALSE	FALSE	FALSE	tbRL8J	0.013857	0.095313	486484	sepsis	TRUE	reported	textfile	9.00E-04	1.45E-08	7351	M00060.m	TRUE	reported	oOvRKT	2	TRUE	0.004349	32.10237
340	rs6820011	T	G	T	G	-0.0051	-0.00231	0.5152	0.506483	FALSE	FALSE	FALSE	tbRL8J	0.013718	0.866256	486484	sepsis	TRUE	reported	textfile	9.00E-04	1.18E-08	7351	M00060.m	TRUE	reported	oOvRKT	2	TRUE	0.004349	32.10237
1881	rs1162095	A	G	A	G	-0.0051	-0.02409	0.4767	0.483434	FALSE	FALSE	FALSE	v2KE89	0.013722	0.07913	486484	sepsis	TRUE	reported	textfile	9.00E-04	1.63E-08	7349	M15500.m	TRUE	reported	to2t7H	2	TRUE	0.00435	32.10237
1882	rs1162097	A	G	A	G	-0.0051	0.005239	0.6326	0.626106	FALSE	FALSE	FALSE	v2KE89	0.014266	0.713429	486484	sepsis	TRUE	reported	textfile	9.00E-04	2.35E-08	7349	M15500.m	TRUE	reported	to2t7H	2	TRUE	0.00435	32.10237
1492	rs875740	A	C	A	C	0.0102	-0.0114	0.6656	0.665835	FALSE	FALSE	FALSE	pkb876	0.014491	0.431632	486484	sepsis	TRUE	reported	textfile	0.0018	6.35E-09	7037	M02734.m	TRUE	reported	0LCMVI	2	TRUE	0.004542	32.10198
8922	rs8060756	A	G	A	G	0.0102	0.006949	0.3703	0.371881	FALSE	FALSE	FALSE	eLhsld	0.014153	0.623423	486484	sepsis	TRUE	reported	textfile	0.0018	4.07E-08	6552	M37058.m	TRUE	reported	RSeWE5	2	TRUE	0.004877	32.10131
2426	rs4768766	A	C	A	C	-0.1376	-0.00401	0.0417	0.044174	FALSE	FALSE	FALSE	NBJ2Do	0.033573	0.90502	486484	sepsis	TRUE	reported	textfile	0.0243	1.58E-08	1739	M17799.m	TRUE	reported	Uz0Wgl	2	TRUE	0.018105	32.02761
8244	rs1028074	T	C	T	C	-0.0838	-0.00532	0.8585	0.852839	FALSE	FALSE	FALSE	G4plxe	0.019349	0.78328	486484	sepsis	TRUE	reported	textfile	0.0148	1.52E-08	1383	M35451.m	TRUE	reported	hNOVMI	2	TRUE	0.022656	32.01372
6310	rs1233521	T	C	T	C	0.323	-0.06771	0.013	0.034581	FALSE	FALSE	FALSE	5B5LTX	0.036493	0.063527	486484	sepsis	TRUE	reported	textfile	0.057	1.46E-08	296	M33609.m	TRUE	reported	m0eM6N	2	TRUE	0.097867	31.89414
6199	rs4919234	T	G	T	G	0.0288	-0.00563	0.3296	0.31577	FALSE	FALSE	FALSE	DEG58p	0.014781	0.70321	486484	sepsis	TRUE	reported	textfile	0.0051	1.52E-08	7500	M33507.m	TRUE	reported	ycykD0	2	TRUE	0.004234	31.88077
698	rs1272867(	T	C	T	C	-0.0096	0.007985	0.2003	0.218978	FALSE	FALSE	FALSE	H9I3s3	0.016514	0.628744	486484	sepsis	TRUE	reported	textfile	0.0017	2.19E-08	7358	M01299.m	TRUE	reported	kXWyUq	2	TRUE	0.004315	31.88061
1374	rs1509820	A	G	A	G	0.0096	-0.00639	0.5299	0.542114	FALSE	FALSE	FALSE	KrWhws	0.013723	0.641371	486484	sepsis	TRUE	reported	textfile	0.0017	1.75E-08	7325	M02132.m	TRUE	reported	7pzpfK	2	TRUE	0.004335	31.88057
7995	rs2121073	T	C	T	C	-0.0293	0.000327	0.1209	0.122112	FALSE	FALSE	FALSE	Xrodum	0.020881	0.98751	486484	sepsis	TRUE	reported	textfile	0.0052	2.15E-08	7291	M35255.m	TRUE	reported	T8t1tl	2	TRUE	0.004336	31.74018
8299	rs1218973(	T	C	T	C	-0.0642	-0.02102	0.8963	0.89088	FALSE	FALSE	FALSE	HELgy7	0.021978	0.338984	486484	sepsis	TRUE	reported	textfile	0.0114	1.56E-08	5035	M35551.m	TRUE	reported	7x7uUn	2	TRUE	0.006259	31.70208
5738	rs748196	T	G	T	G	0.0152	0.027609	0.4596	0.465569	FALSE	FALSE	FALSE	YDQaWa	0.013725	0.044264	486484	sepsis	TRUE	reported	textfile	0.0027	1.70E-08	7063	M33228.m	TRUE	reported	BmYg8r	2	TRUE	0.004467	31.68376
1571	rs8043217	A	G	A	G	0.009	0.014101	0.524	0.517314	FALSE	FALSE	FALSE	Ke0sNh	0.01379	0.306522	486484	sepsis	TRUE	reported	textfile	0.0016	2.79E-08	7799	M10642.m	TRUE	reported	ZlNwVb	2	TRUE	0.004041	31.63251
898	rs1272528	T	C	T	C	0.0315	0.003209	0.1606	0.251219	FALSE	FALSE	FALSE	I6zQnf	0.015741	0.838464	486484	sepsis	TRUE	reported	textfile	0.0056	1.72E-08	7345	M01444.m	TRUE	reported	llzalm	2	TRUE	0.004289	31.63201
6746	rs2062541	A	G	A	G	0.0225	0.004044	0.6144	0.612586	FALSE	FALSE	FALSE	NZVg9N	0.014035	0.773239	486484	sepsis	TRUE	reported	textfile	0.004	2.89E-08	7320	M33941.m	TRUE	reported	kRWNNK	2	TRUE	0.004304	31.63198
3869	rs1572603	A	G	A	G	-0.036	-0.00104	0.13	0.122924	FALSE	FALSE	FALSE	7LphBB	0.020802	0.960243	486484	sepsis	TRUE	reported	textfile	0.0064	1.46E-08	5130	M32388.m	TRUE	reported	5oj12c	2	TRUE	0.00613	31.62829
2395	rs1709809	T	C	T	C	-0.0961	0.015265	0.0738	0.097764	FALSE	FALSE	FALSE	NBJ2Do	0.023058	0.507958	486484	sepsis	TRUE	reported	textfile	0.0171	1.93E-08	1739	M17799.m	TRUE	reported	Uz0Wgl	2	TRUE	0.017838	31.54676
3048	rs1116011	T	C	T	C	-0.0146	0.015576	0.7128	0.694214	FALSE	FALSE	FALSE	H6vcCG	0.014832	0.293651	486484	sepsis	TRUE	reported	textfile	0.0026	2.34E-08	6180	M22030.m	TRUE	reported	XetVX7	2	TRUE	0.005076	31.52234
7204	rs1296746(	T	C	T	C	1.008	-0.01174	0.987	0.962323	FALSE	FALSE	FALSE	i1uJ7T	0.038015	0.757424	486484	sepsis	TRUE	reported	textfile	0.178	1.49E-08	115	M34338.m	TRUE	reported	pi3mKW	2	TRUE	0.218052	31.51096
1058	rs6507399	A	G	A	G	-0.0101	-0.0243	0.6713	0.664269	FALSE	FALSE	FALSE	tRtQ2e	0.014522	0.094263	486484	sepsis	TRUE	reported	textfile	0.0018	1.70E-08	7364	M01564.m	TRUE	reported	Tbg0zM	2	TRUE	0.004257	31.47602
1068	rs868395	T	G	T	G	0.0101	0.006679	0.6653	0.660404	FALSE	FALSE	FALSE	tRtQ2e	0.01447	0.644371	486484	sepsis	TRUE	reported	textfile	0.0018	2.03E-08	7364	M01564.m	TRUE	reported	Tbg0zM	2	TRUE	0.004257	31.47602
1217	rs864428	T	C	T	C	0.0101	-0.00588	0.4058	0.412814	FALSE	FALSE	FALSE	Ckdgh1	0.013913	0.672526	486484	sepsis	TRUE	reported	textfile	0.0018	2.99E-08	7355	M01644.m	TRUE	reported	nKxz7B	2	TRUE	0.004262	31.47601
1494	rs956966	A	G	A	G	-0.0101	-0.00509	0.4858	0.478082	FALSE	FALSE	FALSE	pkb876	0.013685	0.710151	486484	sepsis	TRUE	reported	textfile	0.0018	1.48E-08	7037	M02734.m	TRUE	reported	0LCMVI	2	TRUE	0.004454	31.47562
705	rs174408	A	G	A	G	0.0129	0.031069	0.149	0.150129	FALSE	FALSE	FALSE	H9I3s3	0.019173	0.105136	486484	sepsis	TRUE	reported	textfile	0.0023	1.41E-08	7358	M01299.m	TRUE	reported	kXWyUq	2	TRUE	0.004257	31.44892
2431	rs600711	A	G	A	G	0.1004	0.015362	0.9334	0.916259	FALSE	FALSE	FALSE	NBJ2Do	0.024759	0.534968	486484	sepsis	TRUE	reported	textfile	0.0179	2.08E-08	1739	M17799.m	TRUE	reported	Uz0Wgl	2	TRUE	0.017769	31.42401
4825	rs7535263	A	G	A	G	-0.0443	0.003761	0.435	0.404243	FALSE	FALSE	FALSE	B6WchP	0.01391	0.78689	486484	sepsis	TRUE	reported	textfile	0.0079	2.20E-08	1711	M32836.m	TRUE	reported	7UzUw0	2	TRUE	0.018047	31.40836
5065	rs3923630	A	G	A	G	-0.028	-0.00883	0.333	0.331723	FALSE	FALSE	FALSE	nHZXjp	0.014499	0.54253	486484	sepsis	TRUE	reported	textfile	0.005	2.14E-08	5568	M33138.m	TRUE	reported	ccoK1F	2	TRUE	0.005601	31.34874
6327	rs1042548(	A	G	A	G	0.5394	-0.05183	0.0157	0.010996	FALSE	FALSE	FALSE	qLUY00	0.065793	0.430796	486484	sepsis	TRUE	reported	textfile	0.0954	1.59E-08	102	M33610.m	TRUE	reported	8cAJbB	2	TRUE	0.238628	31.34187
568	rs532545	T	C	T	C	-0.0095	-0.01038	0.3065	0.340111	FALSE	FALSE	FALSE	fNqkCW	0.014417	0.471555	486484	sepsis	TRUE	reported	textfile	0.0017	3.41E-08	7354	M00606.m	TRUE	reported	g9uwOn	2	TRUE	0.004228	31.21988
2441	rs7454011	T	G	T	G	-0.1313	0.000929	0.0448	0.052731	FALSE	FALSE	FALSE	NBJ2Do	0.030661	0.97583	486484	sepsis	TRUE	reported	textfile	0.0235	2.34E-08	1739	M17799.m	TRUE	reported	Uz0Wgl	2	TRUE	0.017635	31.18128
4381	rs9424148	A	C	A	C	-0.0201	-0.01856	0.7912	0.78401	FALSE	FALSE	FALSE	SPhl52	0.016643	0.264729	486484	sepsis	TRUE	reported	textfile	0.0036	1.61E-08	5211	M32651.m	TRUE	reported	V0Bnpj	2	TRUE	0.005947	31.16165
3190	rs1093830	T	C	T	C	-0.0106	-0.00415	0.4663	0.469713	FALSE	FALSE	FALSE	UfLkBq	0.013687	0.761551	486484	sepsis	TRUE	reported	textfile	0.0019	1.48E-08	7329	M22481.m	TRUE	reported	kWMdiT	2	TRUE	0.004229	31.11616
5922	rs1173635	C	G	C	G	0.516	-0.00266	0.0263	0.012893	FALSE	TRUE	FALSE	veBhfS	0.061254	0.965405	486484	sepsis	TRUE	reported	textfile	0.092	2.02E-08	171	M33391.m	TRUE	reported	Gfjy5a	2	TRUE	0.155378	31.08954
1789	rs1692489	A	T	A	T	0.0814	-0.03131	0.024	0.023955	FALSE	TRUE	FALSE	JEcCRZ	0.044697	0.483583	486484	sepsis	TRUE	reported	textfile	0.0146	2.33E-08	1764	M15140.m	TRUE	reported	xYCzKP	2	TRUE	0.017316	31.0492
1850	rs2799635	T	C	T	C	0.0089	-0.01621	0.3644	0.359149	FALSE	FALSE	FALSE	S1eWts	0.014249	0.255305	486484	sepsis	TRUE	reported	textfile	0.0016	1.96E-08	7345	M15488.m	TRUE	reported	511Kl9	2	TRUE	0.004195	30.93298
1640	rs6140831	A	C	A	C	0.0089	-0.01696	0.5484	0.555286	FALSE	FALSE	FALSE	n5rDuT	0.013762	0.217768	486484	sepsis	TRUE	reported	textfile	0.0016	5.40E-08	7081	M12067.m	TRUE	reported	ZQLTeR	2	TRUE	0.004351	30.93267
2953	rs1012025	A	G	A	G	-0.0484	-0.01428	0.8189	0.825398	FALSE	FALSE	FALSE	zndu0r	0.018094	0.429865	486484	sepsis	TRUE	reported	textfile	0.0087	3.03E-08	3422	M21049.m	TRUE	reported	2XpPDU	2	TRUE	0.008963	30.93131
5790	rs1681529	A	G	A	G	0.4453	0.018841	0.0182	0.020203	FALSE	FALSE	FALSE	lUkr5t	0.049019	0.700706	486484	sepsis	TRUE	reported	textfile	0.0799	2.49E-08	474	M33359.m	TRUE	reported	DjliwS	2	TRUE	0.061499	30.92968
4077	rs6077678	A	G	A	G	-0.0228	-0.02026	0.848	0.832232	FALSE	FALSE	FALSE	TyHtdh	0.018345	0.269318	486484	sepsis	TRUE	reported	textfile	0.0041	3.38E-08	7358	M32497.m	TRUE	reported	H3KmLR	2	TRUE	0.004185	30.91604
4440	rs6867478	A	G	A	G	0.0695	0.10494	0.0139	0.011277	FALSE	FALSE	FALSE	WGPdpq	0.065592	0.109624	486484	sepsis	TRUE	reported	textfile	0.0125	2.97E-08	1756	M32675.m	TRUE	reported	u0eUAG	2	TRUE	0.0173	30.87839
8563	rs1165165	T	C	T	C	0.0439	-0.02547	0.2392	0.240954	FALSE	FALSE	FALSE	Yjjldt	0.015961	0.110554	486484	sepsis	TRUE	reported	textfile	0.0079	2.37E-08	7054	M36098.m	TRUE	reported	U2ORsF	2	TRUE	0.004359	30.87107
2429	rs4905451	T	C	T	C	-0.1551	0.01651	0.0399	0.017529	FALSE	FALSE	FALSE	NBJ2Do	0.052725	0.75418	486484	sepsis	TRUE	reported	textfile	0.0279	2.57E-08	1739	M17799.m	TRUE	reported	Uz0Wgl	2	TRUE	0.017461	30.86849
9048	rs633695	A	G	A	G	-0.01	0.010685	0.7183	0.707193	FALSE	FALSE	FALSE	df0MCM	0.015077	0.478519	486484	sepsis	TRUE	reported	textfile	0.0018	2.47E-08	7366	M37506.m	TRUE	reported	iUfr4u	2	TRUE	0.004173	30.85582
1604	rs1938479	T	C	T	C	-0.01	-0.01154	0.6445	0.645299	FALSE	FALSE	FALSE	zP3C2v	0.0143	0.419584	486484	sepsis	TRUE	reported	textfile	0.0018	1.78E-08	7356	M12035.m	TRUE	reported	1Pooe3	2	TRUE	0.004178	30.85581
47	rs1373962	T	C	T	C	0.005	-0.01179	0.5973	0.594481	FALSE	FALSE	FALSE	xmGuol	0.013929	0.397265	486484	sepsis	TRUE	reported	textfile	9.00E-04	2.71E-08	7355	M00054.m	TRUE	reported	GW50Ri	2	TRUE	0.004179	30.8558
125	rs4958379	A	G	A	G	-0.005	-0.00737	0.433	0.431617	FALSE	FALSE	FALSE	xmGuol	0.013873	0.595485	486484	sepsis	TRUE	reported	textfile	9.00E-04	2.18E-08	7355	M00054.m	TRUE	reported	GW50Ri	2	TRUE	0.004179	30.8558
156	rs710580	A	C	A	C	-0.005	0.000452	0.3566	0.372877	FALSE	FALSE	FALSE	xmGuol	0.014103	0.974407	486484	sepsis	TRUE	reported	textfile	9.00E-04	3.57E-08	7355	M00054.m	TRUE	reported	GW50Ri	2	TRUE	0.004179	30.8558
163	rs7584842	T	C	T	C	-0.005	-0.01624	0.5377	0.544598	FALSE	FALSE	FALSE	xmGuol	0.013883	0.242215	486484	sepsis	TRUE	reported	textfile	9.00E-04	4.15E-08	7355	M00054.m	TRUE	reported	GW50Ri	2	TRUE	0.004179	30.8558
185	rs9511152	A	G	A	G	-0.005	-0.01748	0.559	0.58113	FALSE	FALSE	FALSE	xmGuol	0.013874	0.207815	486484	sepsis	TRUE	reported	textfile	9.00E-04	2.99E-08	7355	M00054.m	TRUE	reported	GW50Ri	2	TRUE	0.004179	30.8558
192	rs972459	T	C	T	C	0.005	0.017635	0.5787	0.584668	FALSE	FALSE	FALSE	xmGuol	0.013877	0.203785	486484	sepsis	TRUE	reported	textfile	9.00E-04	1.97E-08	7355	M00054.m	TRUE	reported	GW50Ri	2	TRUE	0.004179	30.8558
1909	rs1318251	A	T	A	T	-0.005	-0.00734	0.3806	0.389217	FALSE	TRUE	FALSE	v2KE89	0.013977	0.599438	486484	sepsis	TRUE	reported	textfile	9.00E-04	3.36E-08	7349	M15500.m	TRUE	reported	to2t7H	2	TRUE	0.004182	30.8558
1938	rs2114713	T	G	T	G	-0.005	0.017416	0.58	0.582119	FALSE	FALSE	FALSE	v2KE89	0.013896	0.210085	486484	sepsis	TRUE	reported	textfile	9.00E-04	2.67E-08	7349	M15500.m	TRUE	reported	to2t7H	2	TRUE	0.004182	30.8558
1945	rs2279014	T	C	T	C	-0.005	-0.00574	0.367	0.372748	FALSE	FALSE	FALSE	v2KE89	0.014121	0.684285	486484	sepsis	TRUE	reported	textfile	9.00E-04	4.86E-08	7349	M15500.m	TRUE	reported	to2t7H	2	TRUE	0.004182	30.8558
1255	rs9563502	A	G	A	G	-0.01	-0.01012	0.7115	0.713546	FALSE	FALSE	FALSE	V50rWC	0.015094	0.502654	486484	sepsis	TRUE	reported	textfile	0.0018	2.56E-08	7346	M01645.m	TRUE	reported	yN3gpQ	2	TRUE	0.004184	30.85579
5993	rs2817817	A	G	A	G	-0.01	0.007716	0.2809	0.30403	FALSE	FALSE	FALSE	3KsoXq	0.014985	0.60664	486484	sepsis	TRUE	reported	textfile	0.0018	2.14E-08	7306	M33422.m	TRUE	reported	aNr6Ug	2	TRUE	0.004207	30.85575
8051	rs795213	A	G	A	G	0.01	0.016572	0.3339	0.367606	FALSE	FALSE	FALSE	SwiqSi	0.014256	0.245069	486484	sepsis	TRUE	reported	textfile	0.0018	2.95E-08	7217	M35270.m	TRUE	reported	21ZIsQ	2	TRUE	0.004258	30.85564
7403	rs2163783	T	C	T	C	-0.03	-0.01647	0.2984	0.295205	FALSE	FALSE	FALSE	XfpKv0	0.015642	0.292234	486484	sepsis	TRUE	reported	textfile	0.0054	2.98E-08	5561	M34390.m	TRUE	reported	mmOsbK	2	TRUE	0.005519	30.8531
4831	rs1683366(	A	C	A	C	0.0211	0.008296	0.3149	0.309231	FALSE	FALSE	FALSE	yLDcUj	0.014822	0.575681	486484	sepsis	TRUE	reported	textfile	0.0038	2.91E-08	6753	M32838.m	TRUE	reported	5dQgAN	2	TRUE	0.004545	30.82259
7203	rs1276971	T	C	T	C	-1.008	-0.06038	0.011	0.052588	FALSE	FALSE	FALSE	i1uJ7T	0.035498	0.088969	486484	sepsis	TRUE	reported	textfile	0.18	2.14E-08	115	M34338.m	TRUE	reported	pi3mKW	2	TRUE	0.214266	30.81461
3416	rs6497490	T	G	T	G	-0.0305	-0.00319	0.8823	0.865078	FALSE	FALSE	FALSE	VU62LG	0.021112	0.879933	486484	sepsis	TRUE	reported	textfile	0.0055	3.49E-08	7180	M27513.m	TRUE	reported	0didOx	2	TRUE	0.004265	30.7435
3085	rs4941615	T	C	T	C	0.0133	0.027558	0.2119	0.208617	FALSE	FALSE	FALSE	esOP8m	0.016841	0.101772	486484	sepsis	TRUE	reported	textfile	0.0024	4.54E-08	7329	M22116.m	TRUE	reported	fDMh7Z	2	TRUE	0.004173	30.70169
2118	rs315988	T	C	T	C	0.0072	-0.01312	0.2367	0.239914	FALSE	FALSE	FALSE	t7SQ8V	0.016002	0.412119	486484	sepsis	TRUE	reported	textfile	0.0013	1.08E-08	7365	M15650.m	TRUE	reported	QQT7qA	2	TRUE	0.004148	30.66623
7453	rs2291449	A	G	A	G	-0.0288	0.004761	0.9135	0.910424	FALSE	FALSE	FALSE	7ubZow	0.023958	0.842464	486484	sepsis	TRUE	reported	textfile	0.0052	2.35E-08	7344	M34407.m	TRUE	reported	LRsXN2	2	TRUE	0.004159	30.6662
2371	rs1232797(	A	G	A	G	0.1079	0.012592	0.9239	0.934357	FALSE	FALSE	FALSE	NBJ2Do	0.027809	0.650703	486484	sepsis	TRUE	reported	textfile	0.0195	3.28E-08	1739	M17799.m	TRUE	reported	Uz0Wgl	2	TRUE	0.017302	30.58256
564	rs2686796	T	C	T	C	0.0094	0.023354	0.4524	0.439978	FALSE	FALSE	FALSE	fNqkCW	0.013784	0.090192	486484	sepsis	TRUE	reported	textfile	0.0017	1.89E-08	7354	M00606.m	TRUE	reported	g9uwOn	2	TRUE	0.00414	30.56608
6983	rs9326061	C	G	C	G	-0.0188	-0.01945	0.8497	0.851916	FALSE	TRUE	FALSE	ZlvYs5	0.019246	0.312278	486484	sepsis	TRUE	reported	textfile	0.0034	4.39E-08	7351	M34214.m	TRUE	reported	qU57S1	2	TRUE	0.004142	30.56608
753	rs320485	T	C	T	C	0.0094	0.015076	0.1324	0.136228	FALSE	FALSE	FALSE	RHWptG	0.019958	0.450012	486484	sepsis	TRUE	reported	textfile	0.0017	4.97E-08	7347	M01302.m	TRUE	reported	z1OLfr	2	TRUE	0.004144	30.56607
1681	rs1013258(	T	G	T	G	0.0094	-0.00144	0.1734	0.173495	FALSE	FALSE	FALSE	f01en0	0.018083	0.936689	486484	sepsis	TRUE	reported	textfile	0.0017	5.67E-08	7314	M12593.m	TRUE	reported	EAsM6U	2	TRUE	0.004163	30.56603
8925	rs1318497	A	T	A	T	-0.0763	0.026113	0.103	0.115561	FALSE	TRUE	FALSE	ns6PV1	0.021398	0.222337	486484	sepsis	TRUE	reported	textfile	0.0138	3.29E-08	7014	M37097.m	TRUE	reported	ZYmYt4	2	TRUE	0.004339	30.56096
3379	rs3762660	T	G	T	G	0.0199	0.01118	0.8935	0.888773	FALSE	FALSE	FALSE	O2JtzH	0.021749	0.607212	486484	sepsis	TRUE	reported	textfile	0.0036	2.68E-08	7347	M27278.m	TRUE	reported	fxCIPW	2	TRUE	0.004142	30.54801
8613	rs2881913	A	C	A	C	0.0315	-0.01649	0.449	0.443444	FALSE	FALSE	FALSE	oHM5TD	0.013769	0.231138	486484	sepsis	TRUE	reported	textfile	0.0057	3.99E-08	1780	M36131.m	TRUE	reported	Xw6wmg	2	TRUE	0.016868	30.50585

6135	rs2391217	A	C	A	C	0.0149	-0.03586	0.1069	0.108711	FALSE	FALSE	FALSE	MiSJly	0.021964	0.102499	486484	sepsis	TRUE	reported	textfile	0.0027	4.88E-08	7307	M33477.m	TRUE	reported	WwL1hq	2	TRUE	0.00415	30.44571
8218	rs1059264	T	C	T	C	-0.0149	0.020191	0.3254	0.336355	FALSE	FALSE	FALSE	4YAWhA	0.014444	0.162164	486484	sepsis	TRUE	reported	textfile	0.0027	5.57E-08	7256	M35439.m	TRUE	reported	bSSC4Z	2	TRUE	0.00418	30.44565
1440	rs1751956	T	G	T	G	0.016	0.016818	0.8647	0.857251	FALSE	FALSE	FALSE	1M4gqj	0.019547	0.38959	486484	sepsis	TRUE	reported	textfile	0.0029	2.35E-08	7226	M02730.m	TRUE	reported	RWyYEM	2	TRUE	0.004195	30.43153
6044	rs6818342	T	C	T	C	-0.849	-0.00036	0.0593	0.042414	FALSE	FALSE	FALSE	33ojbt	0.03431	0.991599	486484	sepsis	TRUE	reported	textfile	0.1515	2.12E-08	60	M33423.m	TRUE	reported	ivEtvl	2	TRUE	0.343576	30.35756
3575	rs2277503	A	G	A	G	0.1667	0.037274	0.0502	0.045102	FALSE	FALSE	FALSE	F1s6qg	0.032908	0.257352	486484	sepsis	TRUE	reported	textfile	0.0302	3.43E-08	522	M31536.m	TRUE	reported	Zm40GX	2	TRUE	0.05515	30.3522
6318	rs1899124	T	C	T	C	0.304	-0.00344	0.051	0.902431	FALSE	FALSE	FALSE	5B5LTX	0.023001	0.880955	486484	sepsis	TRUE	reported	textfile	0.055	3.25E-08	296	M33609.m	TRUE	reported	m0eM6N	2	TRUE	0.093556	30.34432
1246	rs6441206	A	G	A	G	0.0099	-0.00701	0.4638	0.477931	FALSE	FALSE	FALSE	V50rWC	0.013667	0.607903	486484	sepsis	TRUE	reported	textfile	0.0018	2.09E-08	7346	M01645.m	TRUE	reported	yN3gpQ	2	TRUE	0.004101	30.24176
4305	rs6593265	A	C	A	C	0.011	-0.00185	0.311	0.310218	FALSE	FALSE	FALSE	0tjjPC	0.014827	0.900595	486484	sepsis	TRUE	reported	textfile	0.002	3.80E-08	5598	M32634.m	TRUE	reported	nJJFkX	2	TRUE	0.005375	30.23919
6864	rs1107617	A	G	A	G	0.011	-0.00858	0.845	0.821907	FALSE	FALSE	FALSE	kFWZjs	0.017892	0.63155	486484	sepsis	TRUE	reported	textfile	0.002	3.80E-08	5594	M34062.m	TRUE	reported	eLONsg	2	TRUE	0.005378	30.23918
4112	rs2935461	A	G	A	G	-0.011	-0.01766	0.81	0.809251	FALSE	FALSE	FALSE	4rtLd0	0.017407	0.310412	486484	sepsis	TRUE	reported	textfile	0.002	3.80E-08	5584	M32518.m	TRUE	reported	j7f1ER	2	TRUE	0.005388	30.23917
2126	rs1108439	T	C	T	C	-0.011	0.012616	0.544	0.549258	FALSE	FALSE	FALSE	TEupfd	0.013673	0.356182	486484	sepsis	TRUE	reported	textfile	0.002	3.80E-08	5566	M15676.m	TRUE	reported	NXiaPC	2	TRUE	0.005405	30.23913
2751	rs6053126	A	G	A	G	-0.022	-0.01423	0.253	0.344616	FALSE	FALSE	FALSE	qFAbee	0.01449	0.325985	486484	sepsis	TRUE	reported	textfile	0.004	3.80E-08	5275	M19364.m	TRUE	reported	tZgXNL	2	TRUE	0.005702	30.23853
8908	rs2686513	T	C	T	C	-0.011	0.013042	0.552	0.553263	FALSE	FALSE	FALSE	eLhsld	0.013745	0.342676	486484	sepsis	TRUE	reported	textfile	0.002	3.80E-08	5018	M37058.m	TRUE	reported	RSeWE5	2	TRUE	0.005992	30.23794
6113	rs7036192	A	T	A	T	0.0835	-0.00353	0.9301	0.9404	FALSE	TRUE	FALSE	a0osmD	0.029019	0.903221	486484	sepsis	TRUE	reported	textfile	0.0152	3.50E-08	1118	M33453.m	TRUE	reported	CePFnK	2	TRUE	0.026283	30.12369
7782	rs4797105	C	G	C	G	0.0406	0.004031	0.7968	0.766799	FALSE	TRUE	FALSE	15BqPa	0.016382	0.805618	486484	sepsis	TRUE	reported	textfile	0.0074	3.59E-08	7364	M35126.m	TRUE	reported	zvDzP2	2	TRUE	0.004071	30.09336
3017	rs6662425	C	G	C	G	0.0148	0.001945	0.6385	0.613568	FALSE	TRUE	FALSE	gxupFu	0.01409	0.890227	486484	sepsis	TRUE	reported	textfile	0.0027	3.51E-08	6556	M21188.m	TRUE	reported	gwwXh2	2	TRUE	0.004562	30.03747
7924	rs992029	T	C	T	C	-0.0422	-0.00138	0.0981	0.097047	FALSE	FALSE	FALSE	8qXKkX	0.02311	0.952218	486484	sepsis	TRUE	reported	textfile	0.0077	3.93E-08	6234	M35189.m	TRUE	reported	Nt6oaC	2	TRUE	0.004795	30.02646
7808	rs9594738	T	C	T	C	0.0137	0.010819	0.4782	0.486275	FALSE	FALSE	FALSE	cQ5TvO	0.013678	0.428954	486484	sepsis	TRUE	reported	textfile	0.0025	4.53E-08	7344	M35127.m	TRUE	reported	BVxkfZ	2	TRUE	0.004072	30.02222
2815	rs4131290	A	C	A	C	-0.0126	-0.0353	0.702	0.711383	FALSE	FALSE	FALSE	Sx5Uhq	0.015231	0.020479	486484	sepsis	TRUE	reported	textfile	0.0023	7.47E-08	7354	M19934.m	TRUE	reported	DijYTE	2	TRUE	0.004064	30.00318
3962	rs2182325	A	G	A	G	-0.0378	-0.01967	0.2818	0.349675	FALSE	FALSE	FALSE	sKCkQs	0.014352	0.170474	486484	sepsis	TRUE	reported	textfile	0.0069	4.34E-08	5080	M32445.m	TRUE	reported	egPvJc	2	TRUE	0.005873	29.99953
6728	rs893971	A	G	A	G	-0.0197	0.010625	0.5882	0.600922	FALSE	FALSE	FALSE	B6lZly	0.013922	0.44534	486484	sepsis	TRUE	reported	textfile	0.0036	4.05E-08	7222	M33937.m	TRUE	reported	qFPzjh	2	TRUE	0.004129	29.93692
3830	rs1864774	T	C	T	C	0.0093	0.024264	0.586	0.581461	FALSE	FALSE	FALSE	tRX2u0	0.01385	0.07979	486484	sepsis	TRUE	reported	textfile	0.0017	1.15E-07	7365	M32348.m	TRUE	reported	ruBgwQ	2	TRUE	0.004047	29.91921
5923	rs1250831	A	T	A	T	-0.4363	0.044686	0.9577	0.926865	FALSE	TRUE	FALSE	veBhfS	0.026273	0.088975	486484	sepsis	TRUE	reported	textfile	0.0793	3.71E-08	171	M33391.m	TRUE	reported	Gfjy5a	2	TRUE	0.150398	29.91677
4739	rs9348689	T	G	T	G	0.0339	0.017259	0.5165	0.524885	FALSE	FALSE	FALSE	Do3Q7g	0.013977	0.216921	486484	sepsis	TRUE	reported	textfile	0.0062	4.41E-08	6150	M32769.m	TRUE	reported	ys2LKp	2	TRUE	0.004838	29.88648
873	rs6887589	A	G	A	G	-0.0142	0.001444	0.7787	0.782892	FALSE	FALSE	FALSE	Vg5OOR	0.016702	0.931107	486484	sepsis	TRUE	reported	textfile	0.0026	4.33E-08	7063	M01361.m	TRUE	reported	XnrS00	2	TRUE	0.004205	29.81996
1586	rs339707	T	G	T	G	-0.0142	-0.03241	0.6768	0.66738	FALSE	FALSE	FALSE	7QN2kA	0.01457	0.026129	486484	sepsis	TRUE	reported	textfile	0.0026	6.35E-08	5656	M12017.m	TRUE	reported	52YQ1d	2	TRUE	0.005246	29.81785
7206	rs1309455	A	G	A	G	0.975	0.039273	0.985	0.948687	FALSE	FALSE	FALSE	i1uJ7T	0.030925	0.204107	486484	sepsis	TRUE	reported	textfile	0.177	3.62E-08	115	M34338.m	TRUE	reported	pi3mKW	2	TRUE	0.20877	29.81558
587	rs7115681	T	C	T	C	-0.0131	0.024663	0.4668	0.409764	FALSE	FALSE	FALSE	ZoqU2j	0.014044	0.079051	486484	sepsis	TRUE	reported	textfile	0.0024	8.29E-08	7333	M01105.m	TRUE	reported	2WxSOH	2	TRUE	0.004046	29.78528
6920	rs2928923	A	G	A	G	0.4932	-0.07757	0.979	0.973313	FALSE	FALSE	FALSE	SvfSvH	0.042994	0.071187	486484	sepsis	TRUE	reported	textfile	0.0901	4.38E-08	282	M34109.m	TRUE	reported	7OSbiu	2	TRUE	0.096049	29.75127
4450	rs1277892	A	C	A	C	0.0371	0.01121	0.2745	0.27816	FALSE	FALSE	FALSE	g2RNym	0.015289	0.46341	486484	sepsis	TRUE	reported	textfile	0.0068	6.05E-08	2575	M32691.m	TRUE	reported	35eo6p	2	TRUE	0.011428	29.74353
3733	rs1175399	A	G	A	G	-0.0229	-0.00656	0.1568	0.17093	FALSE	FALSE	FALSE	k16oIH	0.018186	0.718229	486484	sepsis	TRUE	reported	textfile	0.0042	7.24E-08	7340	M32328.m	TRUE	reported	TQA3sZ	2	TRUE	0.004034	29.72036
5245	rs1212279	A	G	A	G	-0.1811	-0.02559	0.038	0.027773	FALSE	FALSE	FALSE	q32d4E	0.041607	0.53849	486484	sepsis	TRUE	reported	textfile	0.0332	4.87E-08	1017	M33178.m	TRUE	reported	cS3BwT	2	TRUE	0.028426	29.69654
642	rs8192870	T	G	T	G	-0.0365	0.026375	0.3498	0.342035	FALSE	FALSE	FALSE	F6Dn8H	0.014403	0.067073	486484	sepsis	TRUE	reported	textfile	0.0067	3.99E-08	4934	M01114.m	TRUE	reported	dVHatZ	2	TRUE	0.005979	29.66607
1330	rs1180288	A	T	A	T	0.0098	0.014907	0.3634	0.354428	FALSE	TRUE	FALSE	LDUJbh	0.014291	0.296907	486484	sepsis	TRUE	reported	textfile	0.0018	2.33E-08	7367	M01898.m	TRUE	reported	JRSLeH	2	TRUE	0.004007	29.63393
6059	rs1146579	T	C	T	C	-0.0196	0.016319	0.6674	0.662041	FALSE	FALSE	FALSE	P4BuMD	0.014446	0.258647	486484	sepsis	TRUE	reported	textfile	0.0036	5.78E-08	7365	M33441.m	TRUE	reported	kqrc1d	2	TRUE	0.004009	29.63393
3973	rs1389939	C	G	C	G	0.0098	0.008152	0.4085	0.417072	FALSE	TRUE	FALSE	XdJbEU	0.013885	0.557145	486484	sepsis	TRUE	reported	textfile	0.0018	1.13E-07	7364	M32452.m	TRUE	reported	V5xWKi	2	TRUE	0.004009	29.63392
1758	rs9855331	A	T	A	T	0.0147	-0.00407	0.7473	0.720137	FALSE	TRUE	FALSE	nFLpAP	0.015261	0.789776	486484	sepsis	TRUE	reported	textfile	0.0027	3.81E-08	7356	M12774.m	TRUE	reported	yOSQTj	2	TRUE	0.004013	29.63392
53	rs154246	T	C	T	C	-0.0049	-0.01609	0.6103	0.620378	FALSE	FALSE	FALSE	xmGuol	0.014068	0.252784	486484	sepsis	TRUE	reported	textfile	9.00E-04	6.70E-08	7355	M00054.m	TRUE	reported	GW50Ri	2	TRUE	0.004014	29.63391
59	rs1697901	A	G	A	G	0.0049	0.021421	0.3501	0.357057	FALSE	FALSE	FALSE	xmGuol	0.014439	0.13792	486484	sepsis	TRUE	reported	textfile	9.00E-04	6.31E-08	7355	M00054.m	TRUE	reported	GW50Ri	2	TRUE	0.004014	29.63391
89	rs2798184	T	C	T	C	-0.0049	0.020585	0.4314	0.428102	FALSE	FALSE	FALSE	xmGuol	0.01393	0.139476	486484	sepsis	TRUE	reported	textfile	9.00E-04	6.37E-08	7355	M00054.m	TRUE	reported	GW50Ri	2	TRUE	0.004014	29.63391
114	rs4615256	A	G	A	G	0.0049	0.005673	0.5327	0.52837	FALSE	FALSE	FALSE	xmGuol	0.013716	0.679154	486484	sepsis	TRUE	reported	textfile	9.00E-04	4.99E-08	7355	M00054.m	TRUE	reported	GW50Ri	2	TRUE	0.004014	29.63391
138	rs6480970	A	G	A	G	-0.0049	0.016699	0.6159	0.606311	FALSE	FALSE	FALSE	xmGuol	0.014047	0.234509	486484	sepsis	TRUE	reported	textfile	9.00E-04	4.29E-08	7355	M00054.m	TRUE	reported	GW50Ri	2	TRUE	0.004014	29.63391
169	rs8021242	T	C	T	C	0.0049	0.011968	0.5899	0.583544	FALSE	FALSE	FALSE	xmGuol	0.013892	0.388942	486484	sepsis	TRUE	reported	textfile	9.00E-04	5.18E-08	7355	M00054.m	TRUE	reported	GW50Ri	2	TRUE	0.004014	29.63391
1192	rs1084819	A	G	A	G	-0.0098	0.020621	0.5584	0.555714	FALSE	FALSE	FALSE	Ckdgh1	0.013739	0.133389	486484	sepsis	TRUE	reported	textfile	0.0018	5.25E-08	7355	M01644.m	TRUE	reported	nKxz7B	2	TRUE	0.004014	29.63391
252	rs1463301	T	C	T	C	-0.0049	-0.01646	0.5742	0.579952	FALSE	FALSE	FALSE	tbRL8J	0.013853	0.2347	486484	sepsis	TRUE	reported	textfile	9.00E-04	7.49E-08	7351	M00060.m	TRUE	reported	oOvRKT	2	TRUE	0.004016	29.63391
262	rs1733198	A	G	A	G	0.0049	-0.00708	0.6308	0.622084	FALSE	FALSE	FALSE	tbRL8J	0.014091	0.615195	486484	sepsis	TRUE	reported	textfile	9.00E-04	7.62E-08	7351	M00060.m	TRUE	reported	oOvRKT	2	TRUE	0.004016	29.63391
341	rs6820313	T	G	T	G	0.0049	0.007606	0.5169	0.517975	FALSE	FALSE	FALSE	tbRL8J	0.013761	0.580457	486484	sepsis	TRUE	reported	textfile	9.00E-04	4.65E-08	7351	M00060.m	TRUE	reported	oOvRKT	2	TRUE	0.004016	29.63391
366	rs7740614	A	G	A	G	-0.0049	-0.01839	0.4838	0.486488	FALSE	FALSE	FALSE	tbRL8J	0.013734	0.180648	486484	sepsis	TRUE	reported	textfile	9.00E-04	4.75E-08	7351	M00060.m	TRUE	reported	oOvRKT	2	TRUE	0.004016	29.63391
371	rs7915	A	C	A	C	-0.0049	0.012484	0.6374	0.627082	FALSE	FALSE	FALSE	tbRL8J	0.014138	0.377238	486484	sepsis	TRUE	reported	textfile	9.00E-04	5.82E-08	7351	M00060.m	TRUE	reported	oOvRKT	2	TRUE	0.004016	29.63391
1927	rs1610649	T	C	T	C	-0.0049	-5.77E-05	0.6073	0.608115	FALSE	FALSE	FALSE	v2KE89	0.014	0.996713	486484	sepsis	TRUE	reported	textfile	9.00E-04	7.92E-08	7349	M15500.m	TRUE	reported	to2t7H	2	TRUE	0.004017	29.63391
1951	rs2396004	A	G	A	G	0.0049	-0.0162	0.4389	0.434229	FALSE	FALSE	FALSE	v2KE89	0.013765	0.239138	486484	sepsis	TRUE	reported	textfile	9.00E-04	4.32E-08	7349	M15500.m	TRUE	reported	to2t7H	2	TRUE	0.004017	29.63391
1970	rs3128061	T	G	T	G	-0.0049	-0.00944	0.3975	0.420342	FALSE	FALSE	FALSE	v2KE89	0.013868	0.496121	486484	sepsis	TRUE	reported	textfile	9.00E-04	5.74E-08	7349	M15500.m	TRUE	reported	to2t7H	2	TRUE	0.004017	29.63391
1983	rs459625	T	C	T	C	-0.0049	0.00207	0.4343	0.431164	FALSE	FALSE	FALSE	v2KE89	0.013857	0.881273	486484	sepsis	TRUE	reported	textfile	9.00E-04	5.18E-08	7349	M15500.m	TRUE	reported	to2t7H	2	TRUE	0.004017	29.63391
2064	rs9942113	A	C	A	C	-0.0049	0.007801	0.3764	0.372792	FALSE	FALSE	FALSE	v2KE89	0.014126	0.580797	486484	sepsis	TRUE	reported	textfile	9.00E-04	6.00E-08	7349	M15500.m	TRUE	reported	to2t7H	2	TRUE	0.004017	29.63391
8389	rs4132537	A	G	A	G	-0.0098	0.004269	0.4952	0.514623	FALSE	FALSE	FALSE	SCzh0A	0.013706	0.755423	486484	sepsis	TRUE	reported	textfile	0.0018	8.29E-08	7348	M35675.m	TRUE	reported	n1SREk	2	TRUE	0.004018	29.63391
2472	rs1390947	A	C	A	C	-0.0272	0.006157	0.4471	0.450785	FALSE	FALSE	FALSE	SrfyMU	0.013786	0.655125	486484	sepsis	TRUE	reported	textfile	0.005	5.72E-08	7226	M17807.m	TRUE	reported	0zrJsu	2	TRUE	0.004079	29.58541
3828	rs1766896	A	C	A	C	0.0326	-0.03765	0.0383	0.049554	FALSE	FALSE	FALSE	tRX2u0	0.031512	0.23222	486484	sepsis	TRUE	reported	textfile	0.006	5.33E-08	7365	M32348.m	TRUE	reported	ruBgwQ	2	TRUE	0.003992	29.51309
3050	rs1163502	A	G	A	G	-0.0152	-0.01707	0.508	0.514561	FALSE	FALSE	FALSE	H6vcCG	0.013729	0.213744	486484	sepsis	TRUE	reported	textfile	0.0028	4.25E-08	6180	M22030.m	TRUE	reported	XetVX7	2	TRUE	0.004746	29.45985
4455	rs457273	A	G	A	G	-0.0597	-0.01981	0.8172	0.846916	FALSE	FALSE	FALSE	g2RNym	0.019167	0.301353	486484	sepsis	TRUE	reported	textfile	0.011	5.34E-08	1675	M32691.m	TRUE	reported	35eo6p	2	TRUE	0.017281	29.42012
2394	rs1706941	A	G	A	G	-0.1552	-0.00686	0.0383	0.021688	FALSE	FALSE	FALSE	NBJ2Do	0.047211	0.884418	486484	sepsis	TRUE	reported	textfile	0.0286	5.66E-08	1739	M17799.m	TRUE	reported	Uz0Wgl	2	TRUE	0.016652	29.41383
7441	rs1944577	T	C	T	C	-0.0733	-0.02559	0.2625	0.253079	FALSE	FALSE	FALSE	otAubi	0.015736	0.103901	486484	sepsis	TRUE	reported	textfile	0.0135	5.42E-08	777	M34404.m	TRUE	reported	SAZaNF	2	TRUE	0.036555	29.40499
1522	rs1215182	T	C	T	C	-0.0141	-0.00272	0.8159	0.809735	FALSE	FALSE	FALSE	YvgTeW	0.017454	0.876313	486484	sepsis	TRUE	reported	textfile	0.0026	6.35E-08	7357	M03141.m	TRUE	reported	srKjSc	2	TRUE	0.003982	29.40177
8124	rs622034	T	C	T	C	0.0141	0.002599	0.128	0.121834	FALSE	FALSE	FALSE	nNbXBS	0.02098	0.901404	486484	sepsis	TRUE	reported	textfile	0.0026	4.20E-08	5768	M35326.m	TRUE	reported	UNDorn	2	TRUE	0.005073	29.39957
3127	rs1299626	C	G	C	G	-0.0103	-0.00632	0.6148	0.594706	FALSE	TRUE	FALSE	JiXR2t	0.014019	0.652143	486484	sepsis	TRUE	reported	textfile	0.0019	1.23E-07	6535	M22177.m	TRUE	reported	joi6p6	2	TRUE	0.004477	29.37882
3169	rs7541235	T	G	T	G	-0.0103	0.016597	0.6521	0.644471	FALSE	FALSE	FALSE	JiXR2t	0.014391	0.24881	486484	sepsis	TRUE	reported	textfile	0.0019	1.15E-07	6535	M22177.m	TRUE	reported	joi6p6	2	TRUE	0.004477	29.37882
5038	rs739626	A	C	A	C	0.013	0.047457	0.0935	0.117727	FALSE	FALSE	FALSE	BNnOvt	0.021269	0.025665	486484	sepsis	TRUE	reported	textfile	0.0024	7.96E-08	7362	M33132.m	TRUE	reported	rHcCPG	2	TRUE	0.00397	29.33231
6437	rs2980782	C	G	C	G	-0.013	-0.02484	0.3307	0.34671	FALSE	TRUE	FALSE	QCw5dg	0.014341	0.083247	486484	sepsis	TRUE	reported	textfile	0.0024	6.71E-08	6279	M33666.m	TRUE	reported	zYHmzg	2	TRUE	0.004651	29.33093
2440	rs7423047	T	C	T	C	-0.1451	0.038243	0.0375	0.031168	FALSE	FALSE	FALSE	NBJ2Do	0.03936	0.331236	486484	sepsis	TRUE	reported	textfile	0.0268	6.31E-08	1739	M17799.m	TRUE	reported	Uz0Wgl	2	TRUE	0.016577	29.27962
1928	rs1706523	T	C	T	C	-0.0092	0.021909	0.094	0.086151	FALSE	FALSE	FALSE	v2KE89	0.024392	0.369066	486484	sepsis	TRUE	reported	textfile	0.0017	7.16E-08	7349	M15500.m	TRUE	reported	to2t7H	2	TRUE	0.003969	29.27923
2848	rs2497587	A	G	A	G	-0.0092	-0.01825	0.1887	0.190286	FALSE	FALSE	FALSE	Y5qWBm	0.017455	0.295904	486484	sepsis	TRUE	reported	textfile	0.0017	1.30E-07	7325	M20489.m	TRUE	reported	f2g9Su	2	TRUE	0.003982	29.2792
8854	rs6107895	A	G	A	G	-0.0249	-0.00221	0.1405	0.148499	FALSE	FALSE	FALSE	XIHeTV	0.019276	0.90886	486484	sepsis	TRUE	reported	textfile	0.0046	6.18E-08	1764	M36808.m	TRUE	reported	37Vi2D	2	TRUE	0.016339	29.26782
3225	rs1186547	A	G	A	G	-0.0146	0.011766	0.506	0.493652	FALSE	FALSE	FALSE	I25GHB	0.013661	0.389085	486484	sepsis	TRUE	reported	textfile	0.0027	6.71E-08	7184	M22548.m	TRUE	reported	eBmTQD	2	TRUE	0.004054	29.23191
7607	rs6541298	A	G	A	G	0.8157	0.001588	0.0117	0.015629	FALSE	FALSE	FALSE	PIYt5g	0.054667	0.976823	486484	sepsis	TRUE	reported	textfile	0.1497	5.09E-08	128	M34516.m	TRUE	reported	jX41tf	2	TRUE	0.188283	29.22657
7093	rs1452253	A	C	A	C	0.0173	-0.00065	0.6045	0.549642	FALSE	FALSE	FALSE	pxNICA	0.013902	0.962639	486484	sepsis	TRUE	reported	textfile	0.0032	6.60E-08	6994	M34306.m	TRUE	reported	xk5b9M	2	TRUE	0.004162	29.21918
8588	rs2819047	A	G	A	G	-0.0373	-0.00056	0.2125	0.199108	FALSE	FALSE	FALSE	iuJmRL	0.017185	0.974097	486484	sepsis	TRUE	reported	textfile	0.0069	5.82E-08	7311	M36103.m	TRUE	reported	jfJbVw	2	TRUE	0.003981	29.21465
6041	rs4516063	T	G	T	G	0.8886	-0.03665	0.9154	0.903789	FALSE	FALSE	FALSE	33ojbt	0.023306	0.115865	486484	sepsis	TRUE	reported	textfile	0.1617	3.92E-08	60	M33423.m	TRUE	reported	ivEtvl	2	TRUE	0.334804	29.19237
6581	rs1699435	T	G	T	G	-0.4144	-0.03097	0.9773	0.978857	FALSE	FALSE	FALSE	XO3rXq	0.047512	0.514517	486484	sepsis	TRUE	reported	textfile	0.0763	5.60E-08	176	M33877.m	TRUE	reported	p2sETg	2	TRUE	0.143543	29.16265
2925	rs1710134	A	G	A	G	-0.0189	-0.00966	0.7777	0.764104	FALSE	FALSE	FALSE	fV4qul	0.016179	0.550309	486484	sepsis	TRUE	reported	textfile	0.0035	5.64E-08	7366	M21044.m	TRUE	reported	hbdBQJ	2	TRUE	0.003943	29.15208
2083	rs4487482	A	G	A	G	0.0081	0.013829	0.8039	0.803596	FALSE	FALSE	FALSE	NPVH5j	0.017283	0.423596	486484	sepsis	TRUE	reported	textfile	0.0015	1.82E-07	7310	M15506.m	TRUE	reported	r7r3vM	2	TRUE	0.003973	29.15202
6211	rs1229587	A	G	A	G	0.2913	-0.00769	0.018	0.015039	FALSE	FALSE	FALSE	FQfT2o	0.05625	0.891258	486484	sepsis	TRUE	reported	textfile	0.0539	6.67E-08	972	M33508.m	TRUE	reported	JtbOy2	2	TRUE	0.029173	29.14801
4746	rs7495382	T	G	T	G	-0.0448	0.032595	0.2056	0.22372	FALSE	FALSE	FALSE	oQPvO3	0.016452	0.047564	486484	sepsis	TRUE	reported	textfile	0.0083	7.19E-08	7330	M32786.m	TRUE	reported	Th0GcB	2	TRUE	0.003959	29.12603
6178	rs7942396	T	C	T	C	-0.048	0.017859	0.0664	0.073275	FALSE	FALSE	FALSE	O4Ty2l	0.026335	0.497672	486484	sepsis	TRUE	reported	textfile	0.0089	7.68E-08	5107	M33488.m	TRUE	reported	RGtF84	2	TRUE	0.005663	29.07585
8980	rs4398451	T	C	T	C	-0.0345	-0.02118	0.5546	0.54838	FALSE	FALSE	FALSE	snOepl	0.013746	0.123333	486484	sepsis	TRUE	reported	textfile	0.0064	7.26E-08	7355	M37202.m	TRUE	reported	PQx272	2	TRUE	0.003935	29.05094
6823	rs2279501	C	G	C	G	0.0221	-0.00347	0.5933	0.603268	FALSE	TRUE	FALSE	wedECv	0.01399	0.804001	486484	sepsis	TRUE	reported	textfile	0.0041	5.60E-08	7322	M33968.m	TRUE	reported	GxGxRG	2	TRUE	0.003952	29.04679
3576	rs2838057	A	C	A	C	-0.1987	0.015025	0.9626	0.967143	FALSE	FALSE	FALSE	F1s6qg	0.038459	0.696033	486484	sepsis	TRUE	reported	textfile	0.0368	6.91E-08	522	M31536.m	TRUE	reported	Zm40GX	2	TRUE	0.052897	29.04243
4297	rs2098462	A	G	A	G	0.0097	0.016211	0.7432	0.746835	FALSE	FALSE	FALSE	0tjjPC	0.015781	0.304299	486484	sepsis	TRUE	reported	textfile	0.0018	1.04E-07	7362	M32634.m	TRUE	reported	nJJFkX	2	TRUE	0.003929	29.03223
5034	rs4900819	T	C	T	C	-0.0097	-0.02709	0.8744	0.871601	FALSE	FALSE	FALSE	BNnOvt	0.020448	0.185151	486484	sepsis	TRUE	reported	textfile	0.0018	6.88E-08	7362	M33132.m	TRUE	reported	rHcCPG	2	TRUE	0.003929	29.03223
4056	rs8059670	T	C	T	C	-0.0097	4.48E-05	0.4437	0.456107	FALSE	FALSE	FALSE	SiYGYU	0.013717	0.997392	486484	sepsis	TRUE	reported	textfile	0.0018	3.72E-08	7355	M32492.m	TRUE	reported	GfQvpU	2	TRUE	0.003933	29.03223
4936	rs1041280	T	C	T	C	0.0097	-0.00219	0.3119	0.316942	FALSE	FALSE	FALSE	bKmDMa	0.014672	0.881212	486484	sepsis	TRUE	reported	textfile	0.0018	4.42E-08	7344	M32910.m	TRUE	reported	nAuPzO	2	TRUE	0.003939	29.03221
1392	rs682103	T	C	T	C	-0.0097	0.029592	0.7055	0.703762	FALSE	FALSE	FALSE	KrWhws	0.015062	0.049458	486484	sepsis	TRUE	reported	textfile	0.0018	4.26E-08	7325	M02132.m	TRUE	reported	7pzpfK	2	TRUE	0.003949	29.03219
6158	rs9555127	A	G	A	G	-0.0097	0.001806	0.3316	0.342323	FALSE	FALSE	FALSE	MiSJly	0.014452	0.900538	486484	sepsis	TRUE	reported	textfile	0.0018	7.00E-08	7307	M33477.m	TRUE	reported	WwL1hq	2	TRUE	0.003959	29.03217
1107	rs1114413	T	G	T	G	0.0097	0.020777	0.1375	0.141422	FALSE	FALSE	FALSE	yahiGZ	0.019608	0.289334	486484	sepsis	TRUE	reported	textfile	0.0018	4.86E-08	7271	M01585.m	TRUE	reported	dwZ7vX	2	TRUE	0.003978	29.03214
1015	rs7228099	T	C	T	C	-0.0097	-0.0164	0.4364	0.432448	FALSE	FALSE	FALSE	jfkZbM	0.013838	0.235845	486484	sepsis	TRUE	reported	textfile	0.0018	3.61E-08	6523	M01558.m	TRUE	reported	LFZvMG	2	TRUE	0.004432	29.03122
7210	rs1683176	A	G	A	G	0.44	-0.04505	0.988	0.991793	FALSE	FALSE	FALSE	i1uJ7T	0.077299	0.560037	486484	sepsis	TRUE	reported	textfile	0.081	5.57E-08	115	M34338.m	TRUE	reported	pi3mKW	2	TRUE	0.204195	28.99452
8319	rs1533302	A	G	A	G	-0.014	-0.01098	0.4472	0.445389	FALSE	FALSE	FALSE	o0GzT1	0.013859	0.428189	486484	sepsis	TRUE	reported	textfile	0.0026	1.09E-07	7317	M35631.m	TRUE	reported	G1XFx0	2	TRUE	0.003947	28.98616
7977	rs1314948	A	G	A	G	0.014	-0.01548	0.5433	0.538521	FALSE	FALSE	FALSE	t02AUT	0.013744	0.259972	486484	sepsis	TRUE	reported	textfile	0.0026	1.24E-07	7100	M35254.m	TRUE	reported	uCIJaT	2	TRUE	0.004067	28.98592
772	rs26724	A	G	A	G	0.014	0.025912	0.3507	0.354682	FALSE	FALSE	FALSE	tYvbNc	0.014277	0.06953	486484	sepsis	TRUE	reported	textfile	0.0026	1.26E-07	6946	M01303.m	TRUE	reported	hT3gsE	2	TRUE	0.004157	28.98573
2739	rs1021152	A	G	A	G	-0.014	-0.03087	0.4195	0.423896	FALSE	FALSE	FALSE	qFAbee	0.013897	0.026302	486484	sepsis	TRUE	reported	textfile	0.0026	5.39E-08	6929	M19364.m	TRUE	reported	tZgXNL	2	TRUE	0.004167	28.98571
2740	rs1021452	T	C	T	C	0.014	0.018054	0.642	0.627483	FALSE	FALSE	FALSE	qFAbee	0.014158	0.202265	486484	sepsis	TRUE	reported	textfile	0.0026	7.83E-08	6929	M19364.m	TRUE	reported	tZgXNL	2	TRUE	0.004167	28.98571
5688	rs155146	T	C	T	C	-0.4768	-0.02623	0.9834	0.987092	FALSE	FALSE	FALSE	KxtFnC	0.060918	0.666744	486484	sepsis	TRUE	reported	textfile	0.0885	7.24E-08	930	M33203.m	TRUE	reported	Uz0wKU	2	TRUE	0.030266	28.9635
8462	rs3110907	C	G	C	G	-0.0199	0.041911	0.8988	0.86727	FALSE	TRUE	FALSE	uMajV6	0.020201	0.038015	486484	sepsis	TRUE	reported	textfile	0.0037	1.04E-07	6604	M35754.m	TRUE	reported	b8QB7S	2	TRUE	0.004361	28.91819
3726	rs2037458	A	G	A	G	0.0188	-0.01064	0.6676	0.666818	FALSE	FALSE	FALSE	Z55Bsv	0.014494	0.463047	486484	sepsis	TRUE	reported	textfile	0.0035	8.78E-08	7357	M32322.m	TRUE	reported	6FDRcB	2	TRUE	0.003906	28.8444
5099	rs1246710	A	G	A	G	0.0188	0.002704	0.1697	0.169787	FALSE	FALSE	FALSE	Uiz8VU	0.018189	0.881803	486484	sepsis	TRUE	reported	textfile	0.0035	8.38E-08	6404	M33163.m	TRUE	reported	ky0Bda	2	TRUE	0.004485	28.84323
2397	rs1730340	C	G	C	G	0.1424	0.01332	0.9617	0.94781	FALSE	TRUE	FALSE	NBJ2Do	0.03132	0.670632	486484	sepsis	TRUE	reported	textfile	0.0265	7.46E-08	1739	M17799.m	TRUE	reported	Uz0Wgl	2	TRUE	0.016333	28.84221
4634	rs1405215	A	G	A	G	0.0623	0.005031	0.165	0.17387	FALSE	FALSE	FALSE	DfakIt	0.018082	0.780838	486484	sepsis	TRUE	reported	textfile	0.0116	8.00E-08	6379	M32754.m	TRUE	reported	y21cDm	2	TRUE	0.004501	28.83526
2705	rs7607746	T	C	T	C	-0.0333	0.017375	0.1582	0.183926	FALSE	FALSE	FALSE	XsQHZK	0.017639	0.3246	486484	sepsis	TRUE	reported	textfile	0.0062	8.35E-08	1748	M18929.m	TRUE	reported	k8R7hN	2	TRUE	0.016235	28.81429
3963	rs3525171	A	C	A	C	-0.0865	-0.056	0.1112	0.10398	FALSE	FALSE	FALSE	sKCkQs	0.022353	0.012239	486484	sepsis	TRUE	reported	textfile	0.0161	8.30E-08	1120	M32445.m	TRUE	reported	egPvJc	2	TRUE	0.025125	28.81405
3203	rs3104893	T	C	T	C	-0.0102	-0.01396	0.5566	0.560877	FALSE	FALSE	FALSE	UfLkBq	0.013764	0.310601	486484	sepsis	TRUE	reported	textfile	0.0019	6.03E-08	7329	M22481.m	TRUE	reported	kWMdiT	2	TRUE	0.003917	28.81208
7785	rs629187	A	G	A	G	-0.0327	-0.01647	0.8438	0.847052	FALSE	FALSE	FALSE	15BqPa	0.018981	0.385611	486484	sepsis	TRUE	reported	textfile	0.0061	1.03E-07	7364	M35126.m	TRUE	reported	zvDzP2	2	TRUE	0.003887	28.72883
7552	rs1075621	T	C	T	C	0.0268	0.00312	0.2358	0.231857	FALSE	FALSE	FALSE	APSWAU	0.016177	0.84708	486484	sepsis	TRUE	reported	textfile	0.005	6.63E-08	6144	M34469.m	TRUE	reported	frF3RX	2	TRUE	0.004654	28.72025
5725	rs1246367	A	G	A	G	0.015	0.001632	0.6888	0.686852	FALSE	FALSE	FALSE	YDQaWa	0.01473	0.911782	486484	sepsis	TRUE	reported	textfile	0.0028	5.14E-08	7063	M33228.m	TRUE	reported	BmYg8r	2	TRUE	0.004047	28.69085
4365	rs1703546	T	C	T	C	-0.015	0.007533	0.3092	0.33235	FALSE	FALSE	FALSE	SPhI52	0.014465	0.602514	486484	sepsis	TRUE	reported	textfile	0.0028	1.20E-07	5211	M32651.m	TRUE	reported	V0Bnpj	2	TRUE	0.005477	28.68796
4630	rs939885	A	G	A	G	0.0316	-0.01815	0.4911	0.484186	FALSE	FALSE	FALSE	0oA38w	0.013665	0.184192	486484	sepsis	TRUE	reported	textfile	0.0059	6.88E-08	7356	M32753.m	TRUE	reported	dQUaUX	2	TRUE	0.003885	28.67821
3424	rs1095432	T	C	T	C	0.2208	-0.01036	0.026	0.019491	FALSE	FALSE	FALSE	HHQf8w	0.04989	0.835466	486484	sepsis	TRUE	reported	textfile	0.0412	8.46E-08	1309	M27531.m	TRUE	reported	3vTluK	2	TRUE	0.02147	28.67739
2801	rs1265238	A	G	A	G	0.0091	-0.01926	0.6121	0.619256	FALSE	FALSE	FALSE	Sx5Uhq	0.014265	0.176901	486484	sepsis	TRUE	reported	textfile	0.0017	1.57E-07	7354	M19934.m	TRUE	reported	DijYTE	2	TRUE	0.003881	28.64619
8164	rs1251276	T	C	T	C	0.0091	-0.00766	0.7813	0.80825	FALSE	FALSE	FALSE	8UhtPK	0.017546	0.662589	486484	sepsis	TRUE	reported	textfile	0.0017	9.07E-08	7345	M35397.m	TRUE	reported	rvVJba	2	TRUE	0.003886	28.64618
4968	rs6824211	A	G	A	G	-0.0091	0.01533	0.3182	0.312687	FALSE	FALSE	FALSE	bKmDMa	0.014881	0.302905	486484	sepsis	TRUE	reported	textfile	0.0017	2.16E-07	7344	M32910.m	TRUE	reported	nAuPzO	2	TRUE	0.003887	28.64618
1363	rs1085807	T	C	T	C	-0.0091	0.002538	0.4061	0.403767	FALSE	FALSE	FALSE	KrWhws	0.014005	0.856185	486484	sepsis	TRUE	reported	textfile	0.0017	1.12E-07	7325	M02132.m	TRUE	reported	7pzpfK	2	TRUE	0.003897	28.64616
4609	rs6928245	A	G	A	G	0.0091	0.005222	0.3637	0.363118	FALSE	FALSE	FALSE	Pzplqu	0.014259	0.714187	486484	sepsis	TRUE	reported	textfile	0.0017	1.79E-07	7318	M32740.m	TRUE	reported	FjEBS5	2	TRUE	0.0039	28.64615
5845	rs944243	A	G	A	G	-0.0091	-0.01968	0.3604	0.363317	FALSE	FALSE	FALSE	R5joEf	0.01432	0.16937	486484	sepsis	TRUE	reported	textfile	0.0017	1.48E-07	7118	M33380.m	TRUE	reported	kRvbfZ	2	TRUE	0.004009	28.64593
1639	rs4519482	A	T	A	T	-0.0091	0.013722	0.291	0.335168	FALSE	TRUE	FALSE	n5rDuT	0.014558	0.345886	486484	sepsis	TRUE	reported	textfile	0.0017	8.79E-08	7081	M12067.m	TRUE	reported	ZQLTeR	2	TRUE	0.00403	28.64589
4228	rs2241480	T	C	T	C	0.0198	-0.01087	0.2872	0.287173	FALSE	FALSE	FALSE	FDy32d	0.015129	0.472456	486484	sepsis	TRUE	reported	textfile	0.0037	7.72E-08	7369	M32587.m	TRUE	reported	S10k1h	2	TRUE	0.003871	28.62919
8486	rs1961334	A	G	A	G	-0.0198	-0.00543	0.4773	0.468908	FALSE	FALSE	FALSE	ABkZ7x	0.013708	0.691949	486484	sepsis	TRUE	reported	textfile	0.0037	6.03E-08	6960	M35854.m	TRUE	reported	wc0CeK	2	TRUE	0.004098	28.62873
4344	rs334698	C	G	C	G	-0.0246	0.059968	0.0445	0.045693	FALSE	TRUE	FALSE	6VSoQQ	0.032989	0.069092	486484	sepsis	TRUE	reported	textfile	0.0046	7.72E-08	7229	M32644.m	TRUE	reported	oC2AV0	2	TRUE	0.003941	28.59133
1351	rs7208186	T	C	T	C	0.0139	0.005249	0.8619	0.840971	FALSE	FALSE	FALSE	LDUJbh	0.018681	0.778725	486484	sepsis	TRUE	reported	textfile	0.0026	6.26E-08	7367	M01898.m	TRUE	reported	JRSLeH	2	TRUE	0.003865	28.5736
2903	rs1731817	A	G	A	G	0.0139	-0.00698	0.1785	0.185684	FALSE	FALSE	FALSE	FBDEQi	0.017763	0.694178	486484	sepsis	TRUE	reported	textfile	0.0026	1.28E-07	7327	M20699.m	TRUE	reported	nvNIlp	2	TRUE	0.003886	28.57356
3233	rs710719	C	G	C	G	-0.0139	0.005328	0.3209	0.317661	FALSE	TRUE	FALSE	I25GHB	0.014695	0.716912	486484	sepsis	TRUE	reported	textfile	0.0026	5.58E-08	7184	M22548.m	TRUE	reported	eBmTQD	2	TRUE	0.003963	28.5734
766	rs1089938	A	G	A	G	0.0139	0.014489	0.3819	0.377903	FALSE	FALSE	FALSE	tYvbNc	0.014252	0.309323	486484	sepsis	TRUE	reported	textfile	0.0026	1.27E-07	6946	M01303.m	TRUE	reported	hT3gsE	2	TRUE	0.004098	28.57313
1435	rs1043257	A	G	A	G	0.0738	0.01301	0.0145	0.021814	FALSE	FALSE	FALSE	1M4gqj	0.047497	0.784153	486484	sepsis	TRUE	reported	textfile	0.0138	9.50E-08	1766	M02730.m	TRUE	reported	RWyYEM	2	TRUE	0.015936	28.56686
6655	rs2116833	A	G	A	G	-0.0155	0.033017	0.35	0.354154	FALSE	FALSE	FALSE	REeQiq	0.014513	0.022903	486484	sepsis	TRUE	reported	textfile	0.0029	1.05E-07	6004	M33892.m	TRUE	reported	cVOa3u	2	TRUE	0.004735	28.55767
8545	rs1479400	T	C	T	C	0.5062	0.018069	0.8279	0.855413	FALSE	FALSE	FALSE	aQhCav	0.019435	0.352523	486484	sepsis	TRUE	reported	textfile	0.094	7.19E-08	122	M36097.m	TRUE	reported	9DJS9j	2	TRUE	0.19205	28.52397
974	rs1097762	T	C	T	C	0.8146	0.040706	0.0168	0.02104	FALSE	FALSE	FALSE	xSRIl4	0.048067	0.397068	486484	sepsis	TRUE	reported	textfile	0.1524	9.06E-08	482	M01515.m	TRUE	reported	juZ5GE	2	TRUE	0.055958	28.45202
2931	rs4670465	T	G	T	G	-0.0144	0.026611	0.5498	0.541709	FALSE	FALSE	FALSE	fV4qul	0.01372	0.052434	486484	sepsis	TRUE	reported	textfile	0.0027	6.37E-08	7366	M21044.m	TRUE	reported	hbdBQJ	2	TRUE	0.003847	28.43672
417	rs1776525	A	G	A	G	0.0096	0.009721	0.1845	0.18921	FALSE	FALSE	FALSE	cTsNj6	0.017594	0.580581	486484	sepsis	TRUE	reported	textfile	0.0018	8.27E-08	7365	M00063.m	TRUE	reported	7KYTrQ	2	TRUE	0.003847	28.43672
4283	rs1020066	T	G	T	G	0.0096	-0.00058	0.7374	0.735476	FALSE	FALSE	FALSE	0tjjPC	0.015547	0.970013	486484	sepsis	TRUE	reported	textfile	0.0018	1.44E-07	7362	M32634.m	TRUE	reported	nJJFkX	2	TRUE	0.003849	28.43672
451	rs1217510	A	C	A	C	0.0048	-0.01165	0.4153	0.429966	FALSE	FALSE	FALSE	cl8ucN	0.013833	0.399652	486484	sepsis	TRUE	reported	textfile	9.00E-04	4.86E-07	7361	M00513.m	TRUE	reported	Ian0Q8	2	TRUE	0.003849	28.43672
83	rs2658761	T	C	T	C	-0.0048	0.010255	0.4343	0.436236	FALSE	FALSE	FALSE	xmGuol	0.013814	0.457867	486484	sepsis	TRUE	reported	textfile	9.00E-04	6.75E-08	7355	M00054.m	TRUE	reported	GW50Ri	2	TRUE	0.003852	28.43671
94	rs295142	A	G	A	G	-0.0048	-0.00411	0.5909	0.600658	FALSE	FALSE	FALSE	xmGuol	0.013959	0.768638	486484	sepsis	TRUE	reported	textfile	9.00E-04	1.54E-07	7355	M00054.m	TRUE	reported	GW50Ri	2	TRUE	0.003852	28.43671
124	rs4926571	T	C	T	C	-0.0048	0.007631	0.5231	0.533092	FALSE	FALSE	FALSE	xmGuol	0.013821	0.58085	486484	sepsis	TRUE	reported	textfile	9.00E-04	9.03E-08	7355	M00054.m	TRUE	reported	GW50Ri	2	TRUE	0.003852	28.43671
149	rs6826247	T	C	T	C	0.0048	-0.02884	0.5071	0.509922	FALSE	FALSE	FALSE	xmGuol	0.013721	0.035537	486484	sepsis	TRUE	reported	textfile	9.00E-04	8.80E-08	7355	M00054.m	TRUE	reported	GW50Ri	2	TRUE	0.003852	28.43671
180	rs9315440	A	G	A	G	0.0048	-0.00343	0.5843	0.57781	FALSE	FALSE	FALSE	xmGuol	0.013824	0.804051	486484	sepsis	TRUE	reported	textfile	9.00E-04	6.71E-08	7355	M00054.m	TRUE	reported	GW50Ri	2	TRUE	0.003852	28.43671
6765	rs2490289	T	C	T	C	0.0096	-0.03391	0.2222	0.229001	FALSE	FALSE	FALSE	EIf6MQ	0.016476	0.039585	486484	sepsis	TRUE	reported	textfile	0.0018	1.53E-07	7354	M33955.m	TRUE	reported	Lxn4RF	2	TRUE	0.003853	28.43671
6781	rs8052052	T	G	T	G	-0.0096	0.001243	0.2037	0.199206	FALSE	FALSE	FALSE	EIf6MQ	0.017116	0.942119	486484	sepsis	TRUE	reported	textfile	0.0018	1.86E-07	7354	M33955.m	TRUE	reported	Lxn4RF	2	TRUE	0.003853	28.43671
209	rs1043991	T	C	T	C	0.0048	-0.00296	0.6375	0.625513	FALSE	FALSE	FALSE	tbRL8J	0.014092	0.833584	486484	sepsis	TRUE	reported	textfile	9.00E-04	8.33E-08	7351	M00060.m	TRUE	reported	oOvRKT	2	TRUE	0.003855	28.43671
214	rs1076394	A	G	A	G	0.0048	0.010152	0.3654	0.373453	FALSE	FALSE	FALSE	tbRL8J	0.014192	0.474384	486484	sepsis	TRUE	reported	textfile	9.00E-04	1.17E-07	7351	M00060.m	TRUE	reported	oOvRKT	2	TRUE	0.003855	28.43671
253	rs1541245	T	C	T	C	-0.0048	-0.0158	0.5318	0.52296	FALSE	FALSE	FALSE	tbRL8J	0.01372	0.249422	486484	sepsis	TRUE	reported	textfile	9.00E-04	7.99E-08	7351	M00060.m	TRUE	reported	oOvRKT	2	TRUE	0.003855	28.43671
256	rs1654495	A	C	A	C	0.0048	0.016561	0.5709	0.55807	FALSE	FALSE	FALSE	tbRL8J	0.013816	0.230669	486484	sepsis	TRUE	reported	textfile	9.00E-04	1.04E-07	7351	M00060.m	TRUE	reported	oOvRKT	2	TRUE	0.003855	28.43671
270	rs1952935	A	C	A	C	-0.0048	-0.00341	0.5806	0.585009	FALSE	FALSE	FALSE	tbRL8J	0.013923	0.806269	486484	sepsis	TRUE	reported	textfile	9.00E-04	1.07E-07	7351	M00060.m	TRUE	reported	oOvRKT	2	TRUE	0.003855	28.43671
282	rs242082	T	C	T	C	0.0048	0.015526	0.3867	0.389483	FALSE	FALSE	FALSE	tbRL8J	0.014086	0.270353	486484	sepsis	TRUE	reported	textfile	9.00E-04	1.38E-07	7351	M00060.m	TRUE	reported	oOvRKT	2	TRUE	0.003855	28.43671
284	rs2465250	A	C	A	C	0.0048	0.013572	0.5503	0.540415	FALSE	FALSE	FALSE	tbRL8J	0.013712	0.322304	486484	sepsis	TRUE	reported	textfile	9.00E-04	7.76E-08	7351	M00060.m	TRUE	reported	oOvRKT	2	TRUE	0.003855	28.43671
285	rs2471650	T	C	T	C	-0.0048	0.009285	0.5252	0.524594	FALSE	FALSE	FALSE	tbRL8J	0.013742	0.499229	486484	sepsis	TRUE	reported	textfile	9.00E-04	1.16E-07	7351	M00060.m	TRUE	reported	oOvRKT	2	TRUE	0.003855	28.43671
287	rs248065	A	G	A	G	-0.0048	-0.0156	0.5081	0.504241	FALSE	FALSE	FALSE	tbRL8J	0.013689	0.254613	486484	sepsis	TRUE	reported	textfile	9.00E-04	1.13E-07	7351	M00060.m	TRUE	reported	oOvRKT	2	TRUE	0.003855	28.43671
292	rs2722276	A	G	A	G	0.0048	0.014346	0.523	0.526219	FALSE	FALSE	FALSE	tbRL8J	0.013739	0.29642	486484	sepsis	TRUE	reported	textfile	9.00E-04	8.70E-08	7351	M00060.m	TRUE	reported	oOvRKT	2	TRUE	0.003855	28.43671
298	rs3102519	A	G	A	G	0.0048	-0.00691	0.375	0.379059	FALSE	FALSE	FALSE	tbRL8J	0.014098	0.6242	486484	sepsis	TRUE	reported	textfile	9.00E-04	7.83E-08	7351	M00060.m	TRUE	reported	oOvRKT	2	TRUE	0.003855	28.43671
315	rs4758270	A	G	A	G	-0.0048	-0.01945	0.6483	0.648976	FALSE	FALSE	FALSE	tbRL8J	0.014353	0.175487	486484	sepsis	TRUE	reported	textfile	9.00E-04	2.01E-07	7351	M00060.m	TRUE	reported	oOvRKT	2	TRUE	0.003855	28.43671
356	rs7584322	A	C	A	C	-0.0048	-0.00215	0.4742	0.47369	FALSE	FALSE	FALSE	tbRL8J	0.01376	0.875626	486484	sepsis	TRUE	reported	textfile	9.00E-04	7.03E-08	7351	M00060.m	TRUE	reported	oOvRKT	2	TRUE	0.003855	28.43671
1865	rs1078455	T	C	T	C	-0.0048	0.009652	0.4845	0.489351	FALSE	FALSE	FALSE	v2KE89	0.013687	0.480714	486484	sepsis	TRUE	reported	textfile	9.00E-04	1.06E-07	7349	M15500.m	TRUE	reported	to2t7H	2	TRUE	0.003856	28.4367
1869	rs1082058	T	G	T	G	0.0048	0.017459	0.5616	0.557899	FALSE	FALSE	FALSE	v2KE89	0.013782	0.205227	486484	sepsis	TRUE	reported	textfile	9.00E-04	1.64E-07	7349	M15500.m	TRUE	reported	to2t7H	2	TRUE	0.003856	28.4367
1918	rs1449844	C	G	C	G	-0.0048	0.02202	0.4023	0.414163	FALSE	TRUE	FALSE	v2KE89	0.013908	0.113371	486484	sepsis	TRUE	reported	textfile	9.00E-04	9.23E-08	7349	M15500.m	TRUE	reported	to2t7H	2	TRUE	0.003856	28.4367
1920	rs1479614	A	G	A	G	-0.0048	0.00153	0.6092	0.601759	FALSE	FALSE	FALSE	v2KE89	0.014008	0.91303	486484	sepsis	TRUE	reported	textfile	9.00E-04	9.93E-08	7349	M15500.m	TRUE	reported	to2t7H	2	TRUE	0.003856	28.4367
1932	rs1925611	A	G	A	G	-0.0048	-0.01396	0.4682	0.458392	FALSE	FALSE	FALSE	v2KE89	0.014108	0.322396	486484	sepsis	TRUE	reported	textfile	9.00E-04	2.00E-07	7349	M15500.m	TRUE	reported	to2t7H	2	TRUE	0.003856	28.4367
2025	rs7162803	A	G	A	G	0.0048	-0.02432	0.5029	0.49624	FALSE	FALSE	FALSE	v2KE89	0.013683	0.075445	486484	sepsis	TRUE	reported	textfile	9.00E-04	8.50E-08	7349	M15500.m	TRUE	reported	to2t7H	2	TRUE	0.003856	28.4367
1229	rs1294060	A	C	A	C	-0.0096	-0.00071	0.6059	0.610077	FALSE	FALSE	FALSE	V50rWC	0.014086	0.960067	486484	sepsis	TRUE	reported	textfile	0.0018	1.82E-07	7346	M01645.m	TRUE	reported	yN3gpQ	2	TRUE	0.003857	28.4367
2849	rs2501894	A	G	A	G	-0.0096	-0.0021	0.1704	0.168111	FALSE	FALSE	FALSE	Y5qWBm	0.018294	0.90857	486484	sepsis	TRUE	reported	textfile	0.0018	6.03E-08	7325	M20489.m	TRUE	reported	f2g9Su	2	TRUE	0.003868	28.43668
4348	rs6466580	T	C	T	C	-0.0096	0.039971	0.6573	0.651137	FALSE	FALSE	FALSE	6VSoQQ	0.014319	0.005246	486484	sepsis	TRUE	reported	textfile	0.0018	1.41E-07	7229	M32644.m	TRUE	reported	oC2AV0	2	TRUE	0.003919	28.43657
7284	rs1329833	T	C	T	C	0.0096	-0.01757	0.6276	0.636652	FALSE	FALSE	FALSE	Vcz4EA	0.014199	0.215866	486484	sepsis	TRUE	reported	textfile	0.0018	1.05E-07	6755	M34359.m	TRUE	reported	aUSo2J	2	TRUE	0.004193	28.43602
7306	rs4905134	A	G	A	G	-0.0096	-0.0044	0.5097	0.505696	FALSE	FALSE	FALSE	Vcz4EA	0.013745	0.748738	486484	sepsis	TRUE	reported	textfile	0.0018	6.15E-08	6755	M34359.m	TRUE	reported	aUSo2J	2	TRUE	0.004193	28.43602
7635	rs1050586	T	G	T	G	-0.0288	0.015252	0.665	0.660561	FALSE	FALSE	FALSE	wrTMYM	0.014423	0.290306	486484	sepsis	TRUE	reported	textfile	0.0054	9.92E-08	5856	M34533.m	TRUE	reported	qIRVku	2	TRUE	0.004834	28.43473
2304	rs1781619	T	C	T	C	0.016	-0.03673	0.751	0.739444	FALSE	FALSE	FALSE	ZzUW8U	0.015613	0.018654	486484	sepsis	TRUE	reported	textfile	0.003	9.64E-08	5288	M16821.m	TRUE	reported	g2xs3i	2	TRUE	0.00535	28.43369
1508	rs275721	A	C	A	C	-0.016	0.026349	0.509	0.503189	FALSE	FALSE	FALSE	rzkxmh	0.013686	0.054193	486484	sepsis	TRUE	reported	textfile	0.003	9.64E-08	5196	M03127.m	TRUE	reported	3lrzhA	2	TRUE	0.005444	28.4335
7190	rs894127	A	G	A	G	0.0208	-0.00694	0.1321	0.145218	FALSE	FALSE	FALSE	2hlepp	0.019511	0.722169	486484	sepsis	TRUE	reported	textfile	0.0039	1.23E-07	5081	M34336.m	TRUE	reported	XyK8mO	2	TRUE	0.005567	28.43325
6105	rs1748431	C	G	C	G	0.0618	0.06411	0.0326	0.045311	FALSE	TRUE	FALSE	a0osmD	0.032815	0.050735	486484	sepsis	TRUE	reported	textfile	0.0116	1.07E-07	5717	M33453.m	TRUE	reported	CePFnK	2	TRUE	0.00494	28.37325
7507	rs4899048	A	G	A	G	-0.0959	-0.04156	0.0941	0.084575	FALSE	FALSE	FALSE	1aXQWs	0.024597	0.091126	486484	sepsis	TRUE	reported	textfile	0.018	9.37E-08	4452	M34420.m	TRUE	reported	FOqhHW	2	TRUE	0.006335	28.37246
7268	rs3917538	A	G	A	G	-0.0586	0.014522	0.2133	0.229851	FALSE	FALSE	FALSE	P5Xm4S	0.01624	0.371226	486484	sepsis	TRUE	reported	textfile	0.011	9.75E-08	3491	M34350.m	TRUE	reported	YU4x0J	2	TRUE	0.008064	28.36358
2434	rs6559174	A	C	A	C	0.1289	0.030574	0.9569	0.946997	FALSE	FALSE	FALSE	NBJ2Do	0.030686	0.319088	486484	sepsis	TRUE	reported	textfile	0.0242	9.59E-08	1739	M17799.m	TRUE	reported	Uz0Wgl	2	TRUE	0.016053	28.3384
7888	rs1703172	C	G	C	G	-0.0181	0.012132	0.8162	0.822412	FALSE	TRUE	FALSE	pU8zps	0.017853	0.496782	486484	sepsis	TRUE	reported	textfile	0.0034	7.68E-08	7350	M35186.m	TRUE	reported	Oc2IOF	2	TRUE	0.003841	28.33225
6477	rs2292787	A	G	A	G	-0.6793	-0.00115	0.0326	0.017509	FALSE	FALSE	FALSE	ecwVg8	0.052187	0.982362	486484	sepsis	TRUE	reported	textfile	0.126	6.93E-08	79	M33751.m	TRUE	reported	NnvbIQ	2	TRUE	0.268964	28.32995
3535	rs281499	A	G	A	G	0.0149	0.01836	0.5873	0.571516	FALSE	FALSE	FALSE	rXzSlH	0.013869	0.185574	486484	sepsis	TRUE	reported	textfile	0.0028	7.82E-08	6795	M30805.m	TRUE	reported	7wg9QH	2	TRUE	0.00415	28.30927
8356	rs2554887	A	T	A	T	0.0282	-0.00512	0.683	0.680261	FALSE	TRUE	FALSE	6VVCe4	0.014696	0.72749	486484	sepsis	TRUE	reported	textfile	0.0053	1.29E-07	5629	M35669.m	TRUE	reported	I0fk8X	2	TRUE	0.005004	28.30037
856	rs6453591	A	T	A	T	0.0133	0.00875	0.771	0.757363	FALSE	TRUE	FALSE	1tFtaf	0.01596	0.583522	486484	sepsis	TRUE	reported	textfile	0.0025	8.55E-08	7323	M01359.m	TRUE	reported	qq3q6E	2	TRUE	0.00385	28.29467
1315	rs7814638	A	G	A	G	-0.0133	0.012041	0.141	0.142969	FALSE	FALSE	FALSE	WQdpmo	0.019508	0.537055	486484	sepsis	TRUE	reported	textfile	0.0025	1.23E-07	7136	M01769.m	TRUE	reported	BkptOK	2	TRUE	0.00395	28.29447
2900	rs1262913	T	G	T	G	0.0234	-0.00564	0.0631	0.066448	FALSE	FALSE	FALSE	FBDEQi	0.027456	0.837141	486484	sepsis	TRUE	reported	textfile	0.0044	1.29E-07	7327	M20699.m	TRUE	reported	nvNIlp	2	TRUE	0.003845	28.27534
3302	rs1294679	A	G	A	G	0.0234	-0.02009	0.8939	0.885946	FALSE	FALSE	FALSE	nqmBts	0.021583	0.35197	486484	sepsis	TRUE	reported	textfile	0.0044	8.83E-08	6388	M25599.m	TRUE	reported	VkwSh1	2	TRUE	0.004408	28.2742
6845	rs7078211	A	G	A	G	-0.0404	-0.00756	0.8445	0.846308	FALSE	FALSE	FALSE	aWy2Uj	0.018953	0.689898	486484	sepsis	TRUE	reported	textfile	0.0076	1.26E-07	7326	M33973.m	TRUE	reported	SUSE3b	2	TRUE	0.003842	28.2499
3133	rs1630999	C	G	C	G	-0.0101	0.005358	0.4049	0.390765	FALSE	TRUE	FALSE	JiXR2t	0.014045	0.702831	486484	sepsis	TRUE	reported	textfile	0.0019	1.93E-07	6535	M22177.m	TRUE	reported	joi6p6	2	TRUE	0.004305	28.24897
7726	rs1188026	T	C	T	C	-0.0335	-0.01027	0.2898	0.296224	FALSE	FALSE	FALSE	HyMNYM	0.014958	0.492486	486484	sepsis	TRUE	reported	textfile	0.0063	1.30E-07	1060	M34912.m	TRUE	reported	TI8Hcw	2	TRUE	0.025982	28.22203
661	rs1002471	A	G	A	G	0.0085	-0.01268	0.3772	0.367194	FALSE	FALSE	FALSE	p4oCVP	0.014183	0.371483	486484	sepsis	TRUE	reported	textfile	0.0016	7.50E-08	7352	M01125.m	TRUE	reported	OwD5re	2	TRUE	0.003824	28.21498
7251	rs1050970	C	G	C	G	-0.1004	-0.01751	0.9525	0.95136	FALSE	TRUE	FALSE	4t4W6t	0.031954	0.583686	486484	sepsis	TRUE	reported	textfile	0.0189	1.09E-07	5270	M34344.m	TRUE	reported	Rz9mCt	2	TRUE	0.005326	28.20843
3820	rs1187926	A	G	A	G	-0.0138	-0.03351	0.17	0.16875	FALSE	FALSE	FALSE	tRX2u0	0.018307	0.067208	486484	sepsis	TRUE	reported	textfile	0.0026	8.70E-08	7365	M32348.m	TRUE	reported	ruBgwQ	2	TRUE	0.00381	28.16395
5093	rs1076223	A	G	A	G	-0.0138	0.015987	0.3787	0.377605	FALSE	FALSE	FALSE	Uiz8VU	0.014197	0.260159	486484	sepsis	TRUE	reported	textfile	0.0026	1.68E-07	6404	M33163.m	TRUE	reported	ky0Bda	2	TRUE	0.00438	28.1628
7215	rs1779696	T	C	T	C	0.202	0.024448	0.9439	0.930536	FALSE	FALSE	FALSE	i1uJ7T	0.026912	0.363643	486484	sepsis	TRUE	reported	textfile	0.038	1.06E-07	535	M34338.m	TRUE	reported	pi3mKW	2	TRUE	0.050168	28.15198
3972	rs1220235	T	C	T	C	0.0191	0.010669	0.9123	0.902127	FALSE	FALSE	FALSE	XdJbEU	0.022984	0.642498	486484	sepsis	TRUE	reported	textfile	0.0036	1.14E-07	7364	M32452.m	TRUE	reported	V5xWKi	2	TRUE	0.003808	28.14127
1650	rs1185260	A	G	A	G	-0.0191	0.016801	0.1225	0.123442	FALSE	FALSE	FALSE	YzX5ZD	0.020779	0.418758	486484	sepsis	TRUE	reported	textfile	0.0036	1.14E-07	6864	M12129.m	TRUE	reported	6tMfaW	2	TRUE	0.004084	28.14072
4755	rs176400	A	G	A	G	0.0191	-0.02466	0.331	0.320254	FALSE	FALSE	FALSE	RDfDIS	0.014684	0.093029	486484	sepsis	TRUE	reported	textfile	0.0036	1.20E-07	6857	M32787.m	TRUE	reported	Hq6nGt	2	TRUE	0.004088	28.14071
2728	rs1709612	A	G	A	G	0.0435	0.006801	0.9614	0.958921	FALSE	FALSE	FALSE	H4yqSK	0.034369	0.84313	486484	sepsis	TRUE	reported	textfile	0.0082	1.06E-07	1746	M19362.m	TRUE	reported	w2PDEO	2	TRUE	0.015862	28.1095
6321	rs542663	T	C	T	C	-0.0674	-0.00131	0.8073	0.815146	FALSE	FALSE	FALSE	5B5LTX	0.0176	0.940606	486484	sepsis	TRUE	reported	textfile	0.0127	1.12E-07	824	M33609.m	TRUE	reported	m0eM6N	2	TRUE	0.033051	28.09681
2149	rs7761964	T	C	T	C	0.0175	-0.00571	0.6305	0.623564	FALSE	FALSE	FALSE	TEupfd	0.014101	0.685436	486484	sepsis	TRUE	reported	textfile	0.0033	1.71E-07	1765	M15676.m	TRUE	reported	NXiaPC	2	TRUE	0.015683	28.09026
3154	rs4878628	T	C	T	C	0.0106	-0.03276	0.2753	0.271546	FALSE	FALSE	FALSE	JiXR2t	0.015403	0.033411	486484	sepsis	TRUE	reported	textfile	0.002	6.18E-08	6535	M22177.m	TRUE	reported	joi6p6	2	TRUE	0.00428	28.0814
7664	rs705795	T	C	T	C	0.0551	-0.01589	0.2579	0.266021	FALSE	FALSE	FALSE	z8S1DZ	0.015669	0.31064	486484	sepsis	TRUE	reported	textfile	0.0104	1.13E-07	4515	M34535.m	TRUE	reported	FNLxxh	2	TRUE	0.006179	28.05719
4011	rs1748974	A	T	A	T	0.0143	0.008471	0.1166	0.117618	FALSE	TRUE	FALSE	V9AtId	0.0212	0.689473	486484	sepsis	TRUE	reported	textfile	0.0027	1.53E-07	7363	M32489.m	TRUE	reported	8B8S8a	2	TRUE	0.003795	28.04314
460	rs2289804	A	G	A	G	-0.0143	0.02096	0.9343	0.93512	FALSE	FALSE	FALSE	cl8ucN	0.027774	0.450448	486484	sepsis	TRUE	reported	textfile	0.0027	1.25E-07	7361	M00513.m	TRUE	reported	Ian0Q8	2	TRUE	0.003796	28.04313
555	rs1103350	A	G	A	G	0.0143	-0.0028	0.9307	0.92809	FALSE	FALSE	FALSE	fNqkCW	0.026731	0.916641	486484	sepsis	TRUE	reported	textfile	0.0027	9.46E-08	7354	M00606.m	TRUE	reported	g9uwOn	2	TRUE	0.0038	28.04313
8927	rs2255908	A	G	A	G	0.0466	0.00167	0.6525	0.646732	FALSE	FALSE	FALSE	ns6PV1	0.014365	0.90748	486484	sepsis	TRUE	reported	textfile	0.0088	1.04E-07	7014	M37097.m	TRUE	reported	ZYmYt4	2	TRUE	0.003982	28.03384
399	rs1049354	A	G	A	G	-0.009	-0.00587	0.7989	0.801287	FALSE	FALSE	FALSE	cTsNj6	0.017147	0.731938	486484	sepsis	TRUE	reported	textfile	0.0017	2.27E-07	7365	M00063.m	TRUE	reported	7KYTrQ	2	TRUE	0.003791	28.02007
2128	rs1161586	A	G	A	G	-0.009	0.000804	0.3917	0.376308	FALSE	FALSE	FALSE	TEupfd	0.014102	0.954529	486484	sepsis	TRUE	reported	textfile	0.0017	1.34E-07	7331	M15676.m	TRUE	reported	NXiaPC	2	TRUE	0.003809	28.02004
4590	rs2038410	T	C	T	C	-0.009	-0.0181	0.4395	0.444539	FALSE	FALSE	FALSE	Pzplqu	0.013782	0.189021	486484	sepsis	TRUE	reported	textfile	0.0017	1.65E-07	7318	M32740.m	TRUE	reported	FjEBS5	2	TRUE	0.003815	28.02002
2726	rs1655155	T	C	T	C	-0.009	-0.0016	0.5977	0.60913	FALSE	FALSE	FALSE	H4yqSK	0.014275	0.910659	486484	sepsis	TRUE	reported	textfile	0.0017	1.51E-07	7182	M19362.m	TRUE	reported	w2PDEO	2	TRUE	0.003887	28.01988

7258	rs7524781	A	G	A	G	0.1192	0.011063	0.1738	0.170513	FALSE	FALSE	FALSE	4t4W6t	0.018158	0.542348	486484	sepsis	TRUE	reported	textfile	0.0225	1.20E-07	955	M34344.m	TRUE	reported	Rz9mCt	2	TRUE	0.02855	28.00767
6395	rs1014242	A	C	A	C	-0.0328	0.019219	0.6825	0.67167	FALSE	FALSE	FALSE	Z1h3mT	0.014685	0.190609	486484	sepsis	TRUE	reported	textfile	0.0062	1.33E-07	5328	M33652.m	TRUE	reported	nszuTQ	2	TRUE	0.005225	27.97701
3118	rs495828	T	G	T	G	-0.0312	-0.03136	0.217	0.207952	FALSE	FALSE	FALSE	a9KeDe	0.016779	0.061635	486484	sepsis	TRUE	reported	textfile	0.0059	1.43E-07	3838	M22175.m	TRUE	reported	shg5gu	2	TRUE	0.007233	27.94981
4887	rs1289375	T	C	T	C	0.1566	-0.04278	0.9005	0.842101	FALSE	FALSE	FALSE	ocaywK	0.023933	0.073879	486484	sepsis	TRUE	reported	textfile	0.0296	1.26E-07	1162	M32863.m	TRUE	reported	WH6KGr	2	TRUE	0.023521	27.94164
1766	rs1321675	A	G	A	G	0.0555	0.12513	0.0117	0.015076	FALSE	FALSE	FALSE	wM5dkT	0.055991	0.025428	486484	sepsis	TRUE	reported	textfile	0.0105	1.31E-07	7352	M15122.m	TRUE	reported	CBpa4t	2	TRUE	0.003786	27.93118
3500	rs7095787	A	T	A	T	0.0185	0.026936	0.788	0.775657	FALSE	TRUE	FALSE	Awafb0	0.016507	0.102724	486484	sepsis	TRUE	reported	textfile	0.0035	1.58E-07	5562	M27728.m	TRUE	reported	Lfj8L0	2	TRUE	0.004998	27.92873
8930	rs3828671	A	G	A	G	-0.1357	0.075455	0.0276	0.028874	FALSE	FALSE	FALSE	ns6PV1	0.041162	0.066785	486484	sepsis	TRUE	reported	textfile	0.0257	1.24E-07	7014	M37097.m	TRUE	reported	ZYmYt4	2	TRUE	0.003959	27.87209
943	rs9964014	T	C	T	C	-0.0132	-0.00684	0.1115	0.139808	FALSE	FALSE	FALSE	tv99UN	0.020238	0.735216	486484	sepsis	TRUE	reported	textfile	0.0025	1.80E-07	7354	M01494.m	TRUE	reported	osDclp	2	TRUE	0.003777	27.87082
1486	rs5010651	T	C	T	C	-0.0132	-0.00201	0.8332	0.680879	FALSE	FALSE	FALSE	pkb876	0.014664	0.891153	486484	sepsis	TRUE	reported	textfile	0.0025	1.77E-07	7037	M02734.m	TRUE	reported	0LCMVI	2	TRUE	0.003946	27.87048
8099	rs1204979	A	G	A	G	0.0132	-0.01322	0.1285	0.121903	FALSE	FALSE	FALSE	nNbXBS	0.020871	0.526377	486484	sepsis	TRUE	reported	textfile	0.0025	1.99E-07	5768	M35326.m	TRUE	reported	UNDorn	2	TRUE	0.00481	27.86873
4788	rs9953591	A	T	A	T	0.0681	0.010249	0.0705	0.079449	FALSE	TRUE	FALSE	MmZZoM	0.025428	0.686906	486484	sepsis	TRUE	reported	textfile	0.0129	1.40E-07	4274	M32800.m	TRUE	reported	70YSCO	2	TRUE	0.006478	27.85554
1792	rs21327	A	G	A	G	0.0095	-0.01806	0.286	0.28648	FALSE	FALSE	FALSE	JEcCRZ	0.015103	0.231778	486484	sepsis	TRUE	reported	textfile	0.0018	1.25E-07	7368	M15140.m	TRUE	reported	xYCzKP	2	TRUE	0.003766	27.84738
5020	rs1118796	A	G	A	G	0.0095	0.013126	0.1222	0.130403	FALSE	FALSE	FALSE	BNnOvt	0.02035	0.518903	486484	sepsis	TRUE	reported	textfile	0.0018	2.05E-07	7362	M33132.m	TRUE	reported	rHcCPG	2	TRUE	0.003769	27.84737
8405	rs9543004	T	C	T	C	-0.0095	0.000793	0.6185	0.627053	FALSE	FALSE	FALSE	SCzh0A	0.014244	0.955616	486484	sepsis	TRUE	reported	textfile	0.0018	2.16E-07	7348	M35675.m	TRUE	reported	n1SREk	2	TRUE	0.003777	27.84736
1846	rs1211923	T	C	T	C	0.0095	-0.00165	0.2374	0.234153	FALSE	FALSE	FALSE	S1eWts	0.016522	0.920465	486484	sepsis	TRUE	reported	textfile	0.0018	7.11E-08	7345	M15488.m	TRUE	reported	511KI9	2	TRUE	0.003778	27.84735
4959	rs350729	T	G	T	G	-0.0095	0.00365	0.313	0.305088	FALSE	FALSE	FALSE	bKmDMa	0.014885	0.806287	486484	sepsis	TRUE	reported	textfile	0.0018	6.90E-08	7344	M32910.m	TRUE	reported	nAuPzO	2	TRUE	0.003779	27.84735
4573	rs1091668	A	T	A	T	0.0095	0.026147	0.6493	0.632213	FALSE	TRUE	FALSE	Pzplqu	0.014285	0.067189	486484	sepsis	TRUE	reported	textfile	0.0018	8.65E-08	7318	M32740.m	TRUE	reported	FjEBS5	2	TRUE	0.003792	27.84733
6126	rs1124111	A	G	A	G	0.0095	-0.01328	0.7231	0.716373	FALSE	FALSE	FALSE	MiSJly	0.015175	0.381447	486484	sepsis	TRUE	reported	textfile	0.0018	2.15E-07	7307	M33477.m	TRUE	reported	WwL1hq	2	TRUE	0.003798	27.84731
6002	rs7111143	A	G	A	G	-0.0095	0.008465	0.2847	0.332102	FALSE	FALSE	FALSE	3KsoXq	0.014707	0.564906	486484	sepsis	TRUE	reported	textfile	0.0018	5.35E-08	7306	M33422.m	TRUE	reported	aNr6Ug	2	TRUE	0.003798	27.84731
6257	rs1166040	T	C	T	C	0.0095	-0.00259	0.604	0.597895	FALSE	FALSE	FALSE	MOonP9	0.013941	0.852472	486484	sepsis	TRUE	reported	textfile	0.0018	2.21E-07	7066	M33515.m	TRUE	reported	226GuP	2	TRUE	0.003927	27.84705
6282	rs4902201	A	G	A	G	0.0095	-0.03219	0.3616	0.370225	FALSE	FALSE	FALSE	MOonP9	0.014247	0.023861	486484	sepsis	TRUE	reported	textfile	0.0018	2.21E-07	7066	M33515.m	TRUE	reported	226GuP	2	TRUE	0.003927	27.84705
1019	rs9940567	T	C	T	C	0.0095	-0.02492	0.7319	0.734741	FALSE	FALSE	FALSE	jfkZbM	0.015496	0.107865	486484	sepsis	TRUE	reported	textfile	0.0018	1.57E-07	6523	M01558.m	TRUE	reported	LFZvMG	2	TRUE	0.004252	27.8464
8304	rs7171302	C	G	C	G	-0.0438	-0.01911	0.2838	0.295231	FALSE	TRUE	FALSE	HELgy7	0.015094	0.205567	486484	sepsis	TRUE	reported	textfile	0.0083	1.54E-07	5035	M35551.m	TRUE	reported	7x7uUn	2	TRUE	0.0055	27.83681
3315	rs6688960	A	G	A	G	-0.1911	-0.04275	0.9856	0.984296	FALSE	FALSE	FALSE	nqmBts	0.056524	0.449453	486484	sepsis	TRUE	reported	textfile	0.0362	1.34E-07	1358	M25599.m	TRUE	reported	VkwSh1	2	TRUE	0.020109	27.82686
8	rs780093	T	C	T	C	-0.0058	-0.00404	0.3995	0.38476	FALSE	FALSE	FALSE	0cebDu	0.014044	0.773508	486484	sepsis	TRUE	reported	textfile	0.0011	1.93E-07	7372	M00053.m	TRUE	reported	i8iuxX	2	TRUE	0.003757	27.79411
7794	rs1192055	A	G	A	G	0.0174	0.003748	0.218	0.218949	FALSE	FALSE	FALSE	cQ5TvO	0.016621	0.821592	486484	sepsis	TRUE	reported	textfile	0.0033	1.63E-07	7344	M35127.m	TRUE	reported	BVxkfZ	2	TRUE	0.003771	27.79408
5140	rs1702200	A	G	A	G	-0.7015	-0.04106	0.0339	0.014367	FALSE	FALSE	FALSE	L2DJzK	0.057825	0.477674	486484	sepsis	TRUE	reported	textfile	0.1313	9.16E-08	76	M33173.m	TRUE	reported	hRG70D	2	TRUE	0.273038	27.79355
3423	rs1086501	A	G	A	G	0.0453	0.020248	0.1816	0.188581	FALSE	FALSE	FALSE	HHQf8w	0.017497	0.247182	486484	sepsis	TRUE	reported	textfile	0.0086	1.36E-07	5673	M27531.m	TRUE	reported	3vTluK	2	TRUE	0.004867	27.73616
2071	rs1165621	T	C	T	C	-0.0079	-0.03124	0.498	0.525293	FALSE	FALSE	FALSE	NPVH5j	0.01369	0.022493	486484	sepsis	TRUE	reported	textfile	0.0015	7.48E-08	7310	M15506.m	TRUE	reported	r7r3vM	2	TRUE	0.00378	27.73019
7176	rs1341995	T	C	T	C	-0.0158	-0.02524	0.7317	0.720031	FALSE	FALSE	FALSE	2hlepp	0.015217	0.097225	486484	sepsis	TRUE	reported	textfile	0.003	9.57E-08	5081	M34336.m	TRUE	reported	XyK8mO	2	TRUE	0.005429	27.72686
4667	rs1203824	A	G	A	G	0.0258	0.002425	0.4301	0.437906	FALSE	FALSE	FALSE	xaapK0	0.013808	0.860611	486484	sepsis	TRUE	reported	textfile	0.0049	1.84E-07	7250	M32757.m	TRUE	reported	64n1Z4	2	TRUE	0.003809	27.7158
4646	rs1000491	A	G	A	G	0.0179	-0.00339	0.5631	0.564363	FALSE	FALSE	FALSE	SyAFR2	0.0138	0.805833	486484	sepsis	TRUE	reported	textfile	0.0034	1.62E-07	6658	M32755.m	TRUE	reported	prUo74	2	TRUE	0.004146	27.7088
4903	rs1245254	A	T	A	T	-0.01	-0.00011	0.8568	0.844489	FALSE	TRUE	FALSE	VqcRGX	0.01896	0.995449	486484	sepsis	TRUE	reported	textfile	0.0019	7.64E-08	7329	M32867.m	TRUE	reported	psuFJy	2	TRUE	0.003765	27.69327
3143	rs2514587	A	G	A	G	0.01	-0.02843	0.6223	0.627679	FALSE	FALSE	FALSE	JiXR2t	0.014303	0.046869	486484	sepsis	TRUE	reported	textfile	0.0019	3.00E-07	6535	M22177.m	TRUE	reported	joi6p6	2	TRUE	0.004221	27.69235
7858	rs7446715	A	G	A	G	-0.01	0.00679	0.6732	0.657081	FALSE	FALSE	FALSE	h5whIS	0.014394	0.637093	486484	sepsis	TRUE	reported	textfile	0.0019	1.81E-07	4900	M35137.m	TRUE	reported	xVITKF	2	TRUE	0.005621	27.68952
3747	rs11578	T	C	T	C	0.0142	-0.01155	0.2919	0.290093	FALSE	FALSE	FALSE	OZLdls	0.015017	0.441838	486484	sepsis	TRUE	reported	textfile	0.0027	1.93E-07	7356	M32338.m	TRUE	reported	s2itmB	2	TRUE	0.003746	27.65229
3010	rs2014576	A	G	A	G	-0.0142	0.003642	0.4568	0.449484	FALSE	FALSE	FALSE	gxupFu	0.013742	0.79099	486484	sepsis	TRUE	reported	textfile	0.0027	1.15E-07	6556	M21188.m	TRUE	reported	gwwXh2	2	TRUE	0.004201	27.65137
7249	rs9667032	T	G	T	G	0.3286	-0.03692	0.0244	0.031179	FALSE	FALSE	FALSE	RIcE3J	0.039329	0.347868	486484	sepsis	TRUE	reported	textfile	0.0624	1.38E-07	633	M34339.m	TRUE	reported	QQrwdm	2	TRUE	0.04197	27.64341
916	rs1192521	A	T	A	T	-0.0163	0.00115	0.7755	0.730302	FALSE	TRUE	FALSE	rVMAPf	0.015476	0.940748	486484	sepsis	TRUE	reported	textfile	0.0031	1.40E-07	7307	M01493.m	TRUE	reported	siVqDi	2	TRUE	0.003769	27.63968
6550	rs542320	A	T	A	T	0.0853	-0.00727	0.3812	0.398427	FALSE	TRUE	FALSE	zmDnD7	0.014106	0.606457	486484	sepsis	TRUE	reported	textfile	0.0162	1.52E-07	471	M33835.m	TRUE	reported	MLBiKM	2	TRUE	0.055591	27.60705
3704	rs3828090	A	G	A	G	0.0126	-0.01317	0.2682	0.277079	FALSE	FALSE	FALSE	agPgtF	0.015443	0.393911	486484	sepsis	TRUE	reported	textfile	0.0024	9.97E-08	7349	M32315.m	TRUE	reported	hMRjcV	2	TRUE	0.003736	27.555
4690	rs3133577	T	C	T	C	-0.0252	-0.00732	0.6707	0.667203	FALSE	FALSE	FALSE	MV108h	0.014625	0.616772	486484	sepsis	TRUE	reported	textfile	0.0048	1.98E-07	6642	M32758.m	TRUE	reported	btjNXQ	2	TRUE	0.004133	27.5542
1414	rs6823453	A	G	A	G	0.0231	-0.00041	0.2233	0.229804	FALSE	FALSE	FALSE	33ptwk	0.016245	0.97994	486484	sepsis	TRUE	reported	textfile	0.0044	1.78E-07	6252	M02137.m	TRUE	reported	SQY1na	2	TRUE	0.004389	27.55368
4135	rs1224843	T	C	T	C	0.0467	-0.01193	0.8404	0.847722	FALSE	FALSE	FALSE	pLh7sk	0.019025	0.530768	486484	sepsis	TRUE	reported	textfile	0.0089	1.53E-07	7256	M32549.m	TRUE	reported	RSQOnp	2	TRUE	0.00378	27.52542
2700	rs1799405	A	T	A	T	0.032	0.063675	0.9643	0.952511	FALSE	TRUE	FALSE	XsQHZK	0.032156	0.047678	486484	sepsis	TRUE	reported	textfile	0.0061	1.81E-07	7286	M18929.m	TRUE	reported	k8R7hN	2	TRUE	0.003763	27.51193
7594	rs1046301	A	G	A	G	-0.796	0.074346	0.9883	0.98664	FALSE	FALSE	FALSE	PlYt5g	0.059088	0.208311	486484	sepsis	TRUE	reported	textfile	0.1506	1.24E-07	128	M34516.m	TRUE	reported	jX41tf	2	TRUE	0.179154	27.50026
5476	rs4805151	A	G	A	G	-0.2498	0.053643	0.0347	0.054451	FALSE	FALSE	FALSE	q32d4E	0.030267	0.076341	486484	sepsis	TRUE	reported	textfile	0.0476	1.50E-07	1017	M33178.m	TRUE	reported	cS3BwT	2	TRUE	0.026366	27.48629
3691	rs1092389	T	C	T	C	-0.0388	0.026442	0.9802	0.979721	FALSE	FALSE	FALSE	agPgtF	0.049012	0.589544	486484	sepsis	TRUE	reported	textfile	0.0074	1.82E-07	7349	M32315.m	TRUE	reported	hMRjcV	2	TRUE	0.003727	27.48412
7445	rs9945857	A	G	A	G	0.1688	0.005447	0.0947	0.112796	FALSE	FALSE	FALSE	otAubi	0.021599	0.800907	486484	sepsis	TRUE	reported	textfile	0.0321	1.51E-07	314	M34404.m	TRUE	reported	SAZaNF	2	TRUE	0.080938	27.4764
8076	rs1051423	A	G	A	G	0.1745	-0.03265	0.04	0.041181	FALSE	FALSE	FALSE	z65cF2	0.034431	0.342979	486484	sepsis	TRUE	reported	textfile	0.0332	1.49E-07	349	M35322.m	TRUE	reported	3GUSWk	2	TRUE	0.073351	27.46748
5780	rs2320173	T	C	T	C	-0.0493	0.006955	0.3067	0.332196	FALSE	FALSE	FALSE	eT2We0	0.014735	0.636927	486484	sepsis	TRUE	reported	textfile	0.0094	1.35E-07	1273	M33353.m	TRUE	reported	uZx90m	2	TRUE	0.021151	27.46346
7353	rs2402511	A	G	A	G	0.2034	0.032766	0.3786	0.398857	FALSE	FALSE	FALSE	mm9ksf	0.014003	0.019283	486484	sepsis	TRUE	reported	textfile	0.0366	2.82E-08	18	M34365.m	TRUE	reported	AVwEqD	2	TRUE	0.631785	27.45284
433	rs6438950	C	G	C	G	0.0131	0.01639	0.9036	0.905934	FALSE	TRUE	FALSE	cTsNj6	0.023582	0.48704	486484	sepsis	TRUE	reported	textfile	0.0025	1.54E-07	7365	M00063.m	TRUE	reported	7KYTrQ	2	TRUE	0.003714	27.45014
6439	rs6047134	A	G	A	G	-0.0131	0.011179	0.2672	0.277795	FALSE	FALSE	FALSE	QCw5dg	0.015334	0.465955	486484	sepsis	TRUE	reported	textfile	0.0025	8.93E-08	6279	M33666.m	TRUE	reported	zYHmzg	2	TRUE	0.004354	27.44885
660	rs9359674	A	T	A	T	0.1142	-0.00192	0.0565	0.058117	FALSE	TRUE	FALSE	7BPGuQ	0.029315	0.947686	486484	sepsis	TRUE	reported	textfile	0.0218	1.57E-07	2639	M01123.m	TRUE	reported	WV2hVn	2	TRUE	0.010292	27.42142
3903	rs1183313	A	G	A	G	0.0508	-0.01001	0.0443	0.053058	FALSE	FALSE	FALSE	BrQzHt	0.030706	0.744312	486484	sepsis	TRUE	reported	textfile	0.0097	1.87E-07	7796	M32412.m	TRUE	reported	eVvYVX	2	TRUE	0.003506	27.42032
9017	rs1018051	T	C	T	C	0.0089	-0.01861	0.4607	0.445759	FALSE	FALSE	FALSE	df0MCM	0.01382	0.178229	486484	sepsis	TRUE	reported	textfile	0.0017	3.75E-07	7366	M37506.m	TRUE	reported	iUfr4u	2	TRUE	0.003707	27.40086
2828	rs7544659	A	G	A	G	0.0089	0.008697	0.4048	0.387307	FALSE	FALSE	FALSE	Sx5Uhq	0.014053	0.536022	486484	sepsis	TRUE	reported	textfile	0.0017	3.13E-07	7354	M19934.m	TRUE	reported	DijYTE	2	TRUE	0.003713	27.40085
784	rs1113389	A	G	A	G	0.0089	0.00387	0.4427	0.437868	FALSE	FALSE	FALSE	dyT79I	0.013946	0.781406	486484	sepsis	TRUE	reported	textfile	0.0017	1.56E-07	7352	M01336.m	TRUE	reported	OemVJ2	2	TRUE	0.003714	27.40085
1296	rs1724833	T	C	T	C	-0.0089	0.010988	0.5848	0.578463	FALSE	FALSE	FALSE	WQdpmo	0.013871	0.428272	486484	sepsis	TRUE	reported	textfile	0.0017	1.70E-07	7136	M01769.m	TRUE	reported	BkptOK	2	TRUE	0.003826	27.40062
1459	rs1116545	T	C	T	C	0.0089	0.016827	0.5583	0.569018	FALSE	FALSE	FALSE	pkb876	0.013839	0.224026	486484	sepsis	TRUE	reported	textfile	0.0017	2.79E-07	7037	M02734.m	TRUE	reported	0LCMVI	2	TRUE	0.00388	27.40051
1473	rs1864151	A	C	A	C	0.0089	0.003991	0.5575	0.558514	FALSE	FALSE	FALSE	pkb876	0.013758	0.771783	486484	sepsis	TRUE	reported	textfile	0.0017	2.40E-07	7037	M02734.m	TRUE	reported	0LCMVI	2	TRUE	0.00388	27.40051
8127	rs6912426	A	G	A	G	-0.0089	0.009891	0.3535	0.35276	FALSE	FALSE	FALSE	nNbXBS	0.014428	0.493006	486484	sepsis	TRUE	reported	textfile	0.0017	3.19E-07	5768	M35326.m	TRUE	reported	UNDorn	2	TRUE	0.004729	27.3988
2711	rs1049107	T	C	T	C	0.122	0.014583	0.0175	0.021814	FALSE	FALSE	FALSE	89XKxw	0.046979	0.756239	486484	sepsis	TRUE	reported	textfile	0.0233	1.66E-07	1738	M19324.m	TRUE	reported	gryJuD	2	TRUE	0.01553	27.38469
1734	rs1082224	T	C	T	C	-0.0136	0.002667	0.8488	0.845546	FALSE	FALSE	FALSE	nFLpAP	0.018984	0.888273	486484	sepsis	TRUE	reported	textfile	0.0026	2.84E-07	7356	M12774.m	TRUE	reported	yOSQTj	2	TRUE	0.003706	27.35351
1770	rs2501242	T	G	T	G	-0.0136	-0.0326	0.223	0.230583	FALSE	FALSE	FALSE	wM5dkT	0.016346	0.046133	486484	sepsis	TRUE	reported	textfile	0.0026	1.84E-07	7352	M15122.m	TRUE	reported	CBpa4t	2	TRUE	0.003708	27.3535
4914	rs862226	T	G	T	G	0.0068	0.000346	0.2555	0.262859	FALSE	FALSE	FALSE	VqcRGX	0.01552	0.982188	486484	sepsis	TRUE	reported	textfile	0.0013	4.34E-07	7329	M32867.m	TRUE	reported	psuFJy	2	TRUE	0.003719	27.35348
7788	rs879092	A	T	A	T	0.0434	0.032487	0.9154	0.90644	FALSE	TRUE	FALSE	15BqPa	0.023516	0.167132	486484	sepsis	TRUE	reported	textfile	0.0083	1.56E-07	7364	M35126.m	TRUE	reported	zvDzP2	2	TRUE	0.003699	27.33413
2375	rs1300781	A	G	A	G	-0.114	0.006242	0.0685	0.130085	FALSE	FALSE	FALSE	NBJ2Do	0.020671	0.762672	486484	sepsis	TRUE	reported	textfile	0.0218	1.62E-07	1739	M17799.m	TRUE	reported	Uz0WgI	2	TRUE	0.015482	27.31473
7566	rs1320667	T	C	T	C	-0.0737	-0.02948	0.0161	0.016682	FALSE	FALSE	FALSE	Ot2ZAn	0.054533	0.588812	486484	sepsis	TRUE	reported	textfile	0.0141	1.67E-07	7119	M34481.m	TRUE	reported	tpyFcK	2	TRUE	0.003823	27.31333
6423	rs6502323	A	T	A	T	0.0434	-0.01054	0.2996	0.335103	FALSE	TRUE	FALSE	F7mjRv	0.014505	0.467337	486484	sepsis	TRUE	reported	textfile	0.0083	1.56E-07	1860	M33658.m	TRUE	reported	frG0Zf	2	TRUE	0.014487	27.31216
2450	rs7785951	T	G	T	G	-0.1396	0.0143	0.0389	0.04409	FALSE	FALSE	FALSE	NBJ2Do	0.033443	0.668946	486484	sepsis	TRUE	reported	textfile	0.0267	1.67E-07	1739	M17799.m	TRUE	reported	Uz0WgI	2	TRUE	0.015477	27.3054
2422	rs4454728	A	G	A	G	-0.1672	0.077265	0.0408	0.03211	FALSE	FALSE	FALSE	NBJ2Do	0.039562	0.050816	486484	sepsis	TRUE	reported	textfile	0.0307	5.21E-08	25	M17799.m	TRUE	reported	Uz0WgI	2	TRUE	0.542641	27.28875
2385	rs1682838	T	C	T	C	-0.165	0.115857	0.0405	0.009534	FALSE	FALSE	FALSE	NBJ2Do	0.070546	0.10053	486484	sepsis	TRUE	reported	textfile	0.0303	5.01E-08	25	M17799.m	TRUE	reported	Uz0WgI	2	TRUE	0.542577	27.28164
3848	rs6972344	T	C	T	C	0.0094	-0.01064	0.3962	0.416189	FALSE	FALSE	FALSE	tRX2u0	0.013884	0.443364	486484	sepsis	TRUE	reported	textfile	0.0018	1.11E-07	7365	M32348.m	TRUE	reported	ruBgwQ	2	TRUE	0.003689	27.2642
3983	rs3829681	C	G	C	G	-0.0141	-0.01247	0.8259	0.806366	FALSE	TRUE	FALSE	XdJbEU	0.017324	0.471696	486484	sepsis	TRUE	reported	textfile	0.0027	1.68E-07	7364	M32452.m	TRUE	reported	V5xWKi	2	TRUE	0.00369	27.2642
3992	rs8181126	T	C	T	C	0.0094	0.021522	0.4188	0.419959	FALSE	FALSE	FALSE	XdJbEU	0.013854	0.120315	486484	sepsis	TRUE	reported	textfile	0.0018	2.73E-07	7364	M32452.m	TRUE	reported	V5xWKi	2	TRUE	0.00369	27.2642
5036	rs666276	T	C	T	C	-0.0094	0.032598	0.1213	0.11658	FALSE	FALSE	FALSE	BNnOvt	0.021286	0.125665	486484	sepsis	TRUE	reported	textfile	0.0018	2.19E-07	7362	M33132.m	TRUE	reported	rHcCPG	2	TRUE	0.003691	27.2642
6885	rs6749150	A	G	A	G	0.0094	-0.00638	0.8699	0.831073	FALSE	FALSE	FALSE	kFWZjs	0.018214	0.726078	486484	sepsis	TRUE	reported	textfile	0.0018	3.52E-07	7362	M34062.m	TRUE	reported	eLONsg	2	TRUE	0.003691	27.2642
1598	rs1189546	T	G	T	G	0.0094	-0.00654	0.3807	0.379397	FALSE	FALSE	FALSE	zP3C2v	0.01414	0.643799	486484	sepsis	TRUE	reported	textfile	0.0018	8.43E-08	7356	M12035.m	TRUE	reported	1Pooe3	2	TRUE	0.003694	27.26419
56	rs1663501	A	G	A	G	-0.0047	0.020585	0.4634	0.454681	FALSE	FALSE	FALSE	xmGuol	0.013721	0.13354	486484	sepsis	TRUE	reported	textfile	9.00E-04	2.44E-07	7355	M00054.m	TRUE	reported	GW50Ri	2	TRUE	0.003694	27.26419
64	rs1995960	A	G	A	G	-0.0047	-0.00645	0.5949	0.601923	FALSE	FALSE	FALSE	xmGuol	0.013978	0.644549	486484	sepsis	TRUE	reported	textfile	9.00E-04	1.63E-07	7355	M00054.m	TRUE	reported	GW50Ri	2	TRUE	0.003694	27.26419
66	rs201262	T	G	T	G	0.0047	0.011392	0.5563	0.570191	FALSE	FALSE	FALSE	xmGuol	0.013818	0.409686	486484	sepsis	TRUE	reported	textfile	9.00E-04	1.36E-07	7355	M00054.m	TRUE	reported	GW50Ri	2	TRUE	0.003694	27.26419
157	rs7109477	A	G	A	G	-0.0047	-0.00764	0.4588	0.449037	FALSE	FALSE	FALSE	xmGuol	0.013703	0.577076	486484	sepsis	TRUE	reported	textfile	9.00E-04	1.89E-07	7355	M00054.m	TRUE	reported	GW50Ri	2	TRUE	0.003694	27.26419
165	rs7638383	A	G	A	G	0.0047	-0.01008	0.3984	0.401311	FALSE	FALSE	FALSE	xmGuol	0.013928	0.469337	486484	sepsis	TRUE	reported	textfile	9.00E-04	1.64E-07	7355	M00054.m	TRUE	reported	GW50Ri	2	TRUE	0.003694	27.26419
173	rs860916	A	G	A	G	-0.0047	0.02373	0.4232	0.417302	FALSE	FALSE	FALSE	xmGuol	0.013856	0.086778	486484	sepsis	TRUE	reported	textfile	9.00E-04	1.51E-07	7355	M00054.m	TRUE	reported	GW50Ri	2	TRUE	0.003694	27.26419
569	rs6988242	T	G	T	G	0.0094	0.012569	0.769	0.718969	FALSE	FALSE	FALSE	fNqkCW	0.015582	0.41986	486484	sepsis	TRUE	reported	textfile	0.0018	1.31E-07	7354	M00606.m	TRUE	reported	g9uwOn	2	TRUE	0.003695	27.26419
1777	rs9374302	A	G	A	G	-0.0141	-0.00425	0.1744	0.175583	FALSE	FALSE	FALSE	wM5dkT	0.018014	0.813698	486484	sepsis	TRUE	reported	textfile	0.0027	1.72E-07	7352	M15122.m	TRUE	reported	CBpa4t	2	TRUE	0.003696	27.26419
245	rs1314757	A	G	A	G	-0.0047	-0.01184	0.5809	0.58404	FALSE	FALSE	FALSE	tbRL8J	0.013918	0.395078	486484	sepsis	TRUE	reported	textfile	9.00E-04	1.99E-07	7351	M00060.m	TRUE	reported	oOvRKT	2	TRUE	0.003696	27.26419
267	rs1862710	A	G	A	G	0.0047	-0.01664	0.5489	0.554919	FALSE	FALSE	FALSE	tbRL8J	0.013722	0.225305	486484	sepsis	TRUE	reported	textfile	9.00E-04	1.17E-07	7351	M00060.m	TRUE	reported	oOvRKT	2	TRUE	0.003696	27.26419
283	rs2454043	A	C	A	C	-0.0047	-0.0102	0.588	0.586279	FALSE	FALSE	FALSE	tbRL8J	0.013978	0.465359	486484	sepsis	TRUE	reported	textfile	9.00E-04	1.85E-07	7351	M00060.m	TRUE	reported	oOvRKT	2	TRUE	0.003696	27.26419
314	rs4741887	A	G	A	G	-0.0047	-0.01039	0.4584	0.46471	FALSE	FALSE	FALSE	tbRL8J	0.013705	0.448253	486484	sepsis	TRUE	reported	textfile	9.00E-04	1.68E-07	7351	M00060.m	TRUE	reported	oOvRKT	2	TRUE	0.003696	27.26419
316	rs4767788	A	G	A	G	0.0047	0.016594	0.4189	0.419645	FALSE	FALSE	FALSE	tbRL8J	0.013823	0.229967	486484	sepsis	TRUE	reported	textfile	9.00E-04	5.74E-07	7351	M00060.m	TRUE	reported	oOvRKT	2	TRUE	0.003696	27.26419
317	rs477992	A	G	A	G	-0.0047	-0.00468	0.3228	0.320749	FALSE	FALSE	FALSE	tbRL8J	0.014606	0.74865	486484	sepsis	TRUE	reported	textfile	9.00E-04	2.00E-07	7351	M00060.m	TRUE	reported	oOvRKT	2	TRUE	0.003696	27.26419
318	rs4795552	A	G	A	G	-0.0047	0.016705	0.3894	0.389303	FALSE	FALSE	FALSE	tbRL8J	0.01407	0.235093	486484	sepsis	TRUE	reported	textfile	9.00E-04	1.55E-07	7351	M00060.m	TRUE	reported	oOvRKT	2	TRUE	0.003696	27.26419
368	rs7825809	T	C	T	C	-0.0047	-9.68E-06	0.4319	0.422111	FALSE	FALSE	FALSE	tbRL8J	0.013843	0.999442	486484	sepsis	TRUE	reported	textfile	9.00E-04	1.55E-07	7351	M00060.m	TRUE	reported	oOvRKT	2	TRUE	0.003696	27.26419
384	rs911981	A	G	A	G	-0.0047	-0.00388	0.5283	0.533397	FALSE	FALSE	FALSE	tbRL8J	0.013763	0.778241	486484	sepsis	TRUE	reported	textfile	9.00E-04	2.40E-07	7351	M00060.m	TRUE	reported	oOvRKT	2	TRUE	0.003696	27.26419
1867	rs1080252	T	G	T	G	-0.0047	0.012874	0.4544	0.446706	FALSE	FALSE	FALSE	v2KE89	0.013763	0.349575	486484	sepsis	TRUE	reported	textfile	9.00E-04	1.95E-07	7349	M15500.m	TRUE	reported	to2t7H	2	TRUE	0.003697	27.26418
1877	rs1122321	A	G	A	G	0.0047	-0.01572	0.4713	0.480001	FALSE	FALSE	FALSE	v2KE89	0.013698	0.251253	486484	sepsis	TRUE	reported	textfile	9.00E-04	1.71E-07	7349	M15500.m	TRUE	reported	to2t7H	2	TRUE	0.003697	27.26418
1914	rs140572	A	C	A	C	0.0047	-0.00435	0.5115	0.508008	FALSE	FALSE	FALSE	v2KE89	0.01368	0.750749	486484	sepsis	TRUE	reported	textfile	9.00E-04	2.19E-07	7349	M15500.m	TRUE	reported	to2t7H	2	TRUE	0.003697	27.26418
1929	rs1737034	A	C	A	C	-0.0047	-0.01497	0.6374	0.641775	FALSE	FALSE	FALSE	v2KE89	0.014283	0.294622	486484	sepsis	TRUE	reported	textfile	9.00E-04	1.66E-07	7349	M15500.m	TRUE	reported	to2t7H	2	TRUE	0.003697	27.26418
1942	rs2170082	A	G	A	G	-0.0047	-0.01677	0.5174	0.523864	FALSE	FALSE	FALSE	v2KE89	0.013658	0.219428	486484	sepsis	TRUE	reported	textfile	9.00E-04	2.16E-07	7349	M15500.m	TRUE	reported	to2t7H	2	TRUE	0.003697	27.26418
1950	rs2394298	A	G	A	G	0.0047	0.006508	0.5879	0.583393	FALSE	FALSE	FALSE	v2KE89	0.013861	0.638672	486484	sepsis	TRUE	reported	textfile	9.00E-04	1.51E-07	7349	M15500.m	TRUE	reported	to2t7H	2	TRUE	0.003697	27.26418
2044	rs844016	T	C	T	C	0.0047	0.007229	0.5766	0.576941	FALSE	FALSE	FALSE	v2KE89	0.013845	0.601541	486484	sepsis	TRUE	reported	textfile	9.00E-04	2.22E-07	7349	M15500.m	TRUE	reported	to2t7H	2	TRUE	0.003697	27.26418
2047	rs899584	T	C	T	C	-0.0047	-0.00713	0.356	0.362158	FALSE	FALSE	FALSE	v2KE89	0.014263	0.617177	486484	sepsis	TRUE	reported	textfile	9.00E-04	2.89E-07	7349	M15500.m	TRUE	reported	to2t7H	2	TRUE	0.003697	27.26418
2049	rs9313491	C	G	C	G	0.0047	0.009874	0.643	0.631459	FALSE	TRUE	FALSE	v2KE89	0.014198	0.486799	486484	sepsis	TRUE	reported	textfile	9.00E-04	6.09E-07	7349	M15500.m	TRUE	reported	to2t7H	2	TRUE	0.003697	27.26418
2060	rs9838540	A	C	A	C	0.0047	-0.00628	0.6102	0.617406	FALSE	FALSE	FALSE	v2KE89	0.014045	0.655004	486484	sepsis	TRUE	reported	textfile	9.00E-04	1.80E-07	7349	M15500.m	TRUE	reported	to2t7H	2	TRUE	0.003697	27.26418
4437	rs3800014	A	C	A	C	-0.0094	-0.04743	0.1334	0.138381	FALSE	FALSE	FALSE	WGPdpq	0.019772	0.016451	486484	sepsis	TRUE	reported	textfile	0.0018	2.68E-07	7338	M32675.m	TRUE	reported	u0eUAG	2	TRUE	0.003703	27.26417
6076	rs1730463	A	T	A	T	-0.0094	0.014553	0.8349	0.826106	FALSE	TRUE	FALSE	P8n0HP	0.01811	0.421649	486484	sepsis	TRUE	reported	textfile	0.0018	8.64E-08	7336	M33442.m	TRUE	reported	cljMTI	2	TRUE	0.003704	27.26417
3195	rs1769965	T	C	T	C	0.0141	0.020647	0.2136	0.219274	FALSE	FALSE	FALSE	UfLkBq	0.016692	0.216092	486484	sepsis	TRUE	reported	textfile	0.0027	2.28E-07	7329	M22481.m	TRUE	reported	kWMdiT	2	TRUE	0.003707	27.26416
2507	rs2555231	T	C	T	C	-0.0094	-0.02414	0.6247	0.617039	FALSE	FALSE	FALSE	LwUYua	0.014125	0.087484	486484	sepsis	TRUE	reported	textfile	0.0018	3.22E-07	7315	M17945.m	TRUE	reported	SUN8dc	2	TRUE	0.003714	27.26415
5886	rs4700085	T	C	T	C	0.0094	-0.01751	0.3754	0.354544	FALSE	FALSE	FALSE	tqW0ld	0.014287	0.220399	486484	sepsis	TRUE	reported	textfile	0.0018	2.98E-07	7315	M33389.m	TRUE	reported	Sv7EyV	2	TRUE	0.003714	27.26415
2219	rs4852620	A	G	A	G	0.0141	0.019375	0.1096	0.118917	FALSE	FALSE	FALSE	Bf6uMK	0.021094	0.358338	486484	sepsis	TRUE	reported	textfile	0.0027	1.73E-07	7308	M15778.m	TRUE	reported	jowM2N	2	TRUE	0.003718	27.26414
6130	rs1302272	A	G	A	G	0.0094	-0.00879	0.3471	0.360071	FALSE	FALSE	FALSE	MiSJly	0.014266	0.537623	486484	sepsis	TRUE	reported	textfile	0.0018	2.46E-07	7307	M33477.m	TRUE	reported	WwL1hq	2	TRUE	0.003718	27.26414
1623	rs1122530	A	G	A	G	-0.0094	0.008872	0.8519	0.842435	FALSE	FALSE	FALSE	n5rDuT	0.018823	0.63741	486484	sepsis	TRUE	reported	textfile	0.0018	1.49E-07	7081	M12067.m	TRUE	reported	ZQLTeR	2	TRUE	0.003837	27.2639
5740	rs823554	A	T	A	T	-0.0188	-0.00525	0.8205	0.801881	FALSE	TRUE	FALSE	YDQaWa	0.017321	0.761726	486484	sepsis	TRUE	reported	textfile	0.0036	2.45E-07	7063	M33228.m	TRUE	reported	BmYg8r	2	TRUE	0.003846	27.26388
7299	rs2861609	A	G	A	G	-0.0094	-0.03198	0.3031	0.298569	FALSE	FALSE	FALSE	Vcz4EA	0.014965	0.032592	486484	sepsis	TRUE	reported	textfile	0.0018	1.63E-07	6755	M34359.m	TRUE	reported	aUSo2J	2	TRUE	0.004021	27.26353
7300	rs2881130	A	G	A	G	-0.0094	0.036784	0.3385	0.339239	FALSE	FALSE	FALSE	Vcz4EA	0.014421	0.01075	486484	sepsis	TRUE	reported	textfile	0.0018	1.25E-07	6755	M34359.m	TRUE	reported	aUSo2J	2	TRUE	0.004021	27.26353
991	rs1113643	T	C	T	C	0.0094	0.012195	0.2769	0.278633	FALSE	FALSE	FALSE	jfkZbM	0.015314	0.425833	486484	sepsis	TRUE	reported	textfile	0.0018	2.24E-07	6523	M01558.m	TRUE	reported	LFZvMG	2	TRUE	0.004163	27.26324
994	rs1280717	A	G	A	G	0.0094	-0.02506	0.2512	0.241103	FALSE	FALSE	FALSE	jfkZbM	0.015929	0.11566	486484	sepsis	TRUE	reported	textfile	0.0018	2.01E-07	6523	M01558.m	TRUE	reported	LFZvMG	2	TRUE	0.004163	27.26324
1021	rs1024570	T	C	T	C	-0.0663	-0.047	0.0181	0.019778	FALSE	FALSE	FALSE	haWxZg	0.050561	0.352584	486484	sepsis	TRUE	reported	textfile	0.0127	1.95E-07	7276	M01561.m	TRUE	reported	5wm9R3	2	TRUE	0.003732	27.24584
2417	rs2895167	T	C	T	C	-0.1627	-0.08301	0.0414	0.068393	FALSE	FALSE	FALSE	NBJ2Do	0.027195	0.002271	486484	sepsis	TRUE	reported	textfile	0.0299	5.15E-08	25	M17799.m	TRUE	reported	Uz0WgI	2	TRUE	0.542205	27.24084
4762	rs3740393	C	G	C	G	-0.0261	0.027656	0.1428	0.13742	FALSE	TRUE	FALSE	RDfDIS	0.019904	0.164696	486484	sepsis	TRUE	reported	textfile	0.005	1.59E-07	6857	M32787.m	TRUE	reported	Hq6nGt	2	TRUE	0.003958	27.24045
3434	rs7276515	T	C	T	C	-0.0454	0.003242	0.8161	0.807473	FALSE	FALSE	FALSE	HHQf8w	0.017416	0.852319	486484	sepsis	TRUE	reported	textfile	0.0087	2.00E-07	5673	M27531.m	TRUE	reported	3vTluK	2	TRUE	0.004777	27.222
1438	rs1273784	T	C	T	C	0.012	0.011741	0.7976	0.796649	FALSE	FALSE	FALSE	1M4gqj	0.016988	0.489494	486484	sepsis	TRUE	reported	textfile	0.0023	1.96E-07	7226	M02730.m	TRUE	reported	RWyYEM	2	TRUE	0.003753	27.21364
6466	rs8085059	A	G	A	G	-0.1427	0.009302	0.4278	0.433575	FALSE	FALSE	FALSE	yLohrN	0.01394	0.504568	486484	sepsis	TRUE	reported	textfile	0.0273	1.69E-07	477	M33683.m	TRUE	reported	mvhAZ4	2	TRUE	0.054177	27.20808
6182	rs1118955	T	C	T	C	0.0944	0.003404	0.9816	0.980655	FALSE	FALSE	FALSE	DEG58p	0.050914	0.946694	486484	sepsis	TRUE	reported	textfile	0.0181	1.76E-07	7500	M33507.m	TRUE	reported	ycykD0	2	TRUE	0.003614	27.19387
2458	rs9341191	T	C	T	C	-0.1636	0.03657	0.0476	0.059594	FALSE	FALSE	FALSE	NBJ2Do	0.028885	0.205493	486484	sepsis	TRUE	reported	textfile	0.0301	5.68E-08	25	M17799.m	TRUE	reported	Uz0WgI	2	TRUE	0.541634	27.17825
2442	rs7616688	A	G	A	G	-0.163	-0.0197	0.0408	0.04767	FALSE	FALSE	FALSE	NBJ2Do	0.032083	0.539154	486484	sepsis	TRUE	reported	textfile	0.03	5.50E-08	25	M17799.m	TRUE	reported	Uz0WgI	2	TRUE	0.541462	27.15942
4406	rs9382940	T	C	T	C	0.0172	0.023471	0.1616	0.173184	FALSE	FALSE	FALSE	UJygVd	0.018212	0.197492	486484	sepsis	TRUE	reported	textfile	0.0033	2.31E-07	7361	M32654.m	TRUE	reported	kv9R0W	2	TRUE	0.003677	27.15883
3254	rs1123692	C	G	C	G	-0.0172	-0.02209	0.1592	0.158164	FALSE	TRUE	FALSE	ZNPBqT	0.018722	0.23798	486484	sepsis	TRUE	reported	textfile	0.0033	1.50E-07	7256	M24074.m	TRUE	reported	8pPtOI	2	TRUE	0.00373	27.15872
5906	rs9822180	T	C	T	C	0.0099	-0.00586	0.7155	0.718795	FALSE	FALSE	FALSE	tqW0ld	0.015207	0.700101	486484	sepsis	TRUE	reported	textfile	0.0019	2.58E-07	7315	M33389.m	TRUE	reported	Sv7EyV	2	TRUE	0.003698	27.14216
3813	rs9297619	A	G	A	G	0.1272	0.002787	0.0979	0.089761	FALSE	FALSE	FALSE	HOq2gb	0.023909	0.90721	486484	sepsis	TRUE	reported	textfile	0.0244	1.93E-07	1565	M32346.m	TRUE	reported	Ss5q17	2	TRUE	0.017069	27.14184
8447	rs1214542	A	G	A	G	0.0099	0.000229	0.3757	0.368595	FALSE	FALSE	FALSE	uMajV6	0.01416	0.987077	486484	sepsis	TRUE	reported	textfile	0.0019	2.22E-07	6604	M35754.m	TRUE	reported	b8QB7S	2	TRUE	0.004094	27.14136
4528	rs2170489	A	G	A	G	-0.0099	-0.02184	0.4041	0.405736	FALSE	FALSE	FALSE	r0qJaM	0.013934	0.117087	486484	sepsis	TRUE	reported	textfile	0.0019	1.57E-07	6497	M32729.m	TRUE	reported	vYv5FI	2	TRUE	0.004161	27.14123
4543	rs7819333	C	G	C	G	-0.0099	0.008464	0.3311	0.327824	FALSE	TRUE	FALSE	r0qJaM	0.014616	0.562517	486484	sepsis	TRUE	reported	textfile	0.0019	1.45E-07	6497	M32729.m	TRUE	reported	vYv5FI	2	TRUE	0.004161	27.14123
7843	rs550328	A	G	A	G	0.0099	-0.0075	0.2687	0.27544	FALSE	FALSE	FALSE	h5whIS	0.015336	0.624817	486484	sepsis	TRUE	reported	textfile	0.0019	2.54E-07	4900	M35137.m	TRUE	reported	xVITKF	2	TRUE	0.00551	27.1385
7988	rs1046315	T	C	T	C	-0.0224	-0.02535	0.2297	0.268815	FALSE	FALSE	FALSE	Xrodum	0.015393	0.099623	486484	sepsis	TRUE	reported	textfile	0.0043	2.39E-07	7291	M35255.m	TRUE	reported	T8t1tl	2	TRUE	0.003708	27.12939
3995	rs9450092	A	G	A	G	0.037	0.033004	0.886	0.899433	FALSE	FALSE	FALSE	XdJbEU	0.022654	0.145162	486484	sepsis	TRUE	reported	textfile	0.0071	2.11E-07	1766	M32452.m	TRUE	reported	V5xWKi	2	TRUE	0.015145	27.12655
1075	rs1106141	A	G	A	G	-0.0125	0.01665	0.6428	0.648699	FALSE	FALSE	FALSE	JLtEiZ	0.014232	0.242056	486484	sepsis	TRUE	reported	textfile	0.0024	1.76E-07	7333	M01572.m	TRUE	reported	TSSe28	2	TRUE	0.003686	27.11934
6350	rs7870108	T	G	T	G	0.6206	-0.00477	0.0102	0.015185	FALSE	FALSE	FALSE	qLUY00	0.061124	0.93785	486484	sepsis	TRUE	reported	textfile	0.118	1.46E-07	102	M33610.m	TRUE	reported	8cAJbB	2	TRUE	0.21333	27.11811
2356	rs1098878	T	C	T	C	-0.1623	0.018704	0.04	0.084994	FALSE	FALSE	FALSE	NBJ2Do	0.02459	0.446869	486484	sepsis	TRUE	reported	textfile	0.0299	5.62E-08	25	M17799.m	TRUE	reported	Uz0WgI	2	TRUE	0.540983	27.10706
2357	rs1099886	A	G	A	G	0.1623	-0.00784	0.96	0.874188	FALSE	FALSE	FALSE	NBJ2Do	0.020642	0.704172	486484	sepsis	TRUE	reported	textfile	0.0299	5.62E-08	25	M17799.m	TRUE	reported	Uz0WgI	2	TRUE	0.540983	27.10706
2358	rs1112968	T	C	T	C	0.1623	-0.04111	0.96	0.977007	FALSE	FALSE	FALSE	NBJ2Do	0.048788	0.399489	486484	sepsis	TRUE	reported	textfile	0.0299	5.62E-08	25	M17799.m	TRUE	reported	Uz0WgI	2	TRUE	0.540983	27.10706
2360	rs1116172	A	C	A	C	-0.1623	-0.04785	0.04	0.013582	FALSE	FALSE	FALSE	NBJ2Do	0.05996	0.424843	486484	sepsis	TRUE	reported	textfile	0.0299	5.62E-08	25	M17799.m	TRUE	reported	Uz0WgI	2	TRUE	0.540983	27.10706
2372	rs1262255	T	C	T	C	-0.1623	-0.03351	0.04	0.059918	FALSE	FALSE	FALSE	NBJ2Do	0.028773	0.244165	486484	sepsis	TRUE	reported	textfile	0.0299	5.62E-08	25	M17799.m	TRUE	reported	Uz0WgI	2	TRUE	0.540983	27.10706
2373	rs1268474	A	G	A	G	0.1623	0.013521	0.96	0.964912	FALSE	FALSE	FALSE	NBJ2Do	0.037196	0.716229	486484	sepsis	TRUE	reported	textfile	0.0299	5.62E-08	25	M17799.m	TRUE	reported	Uz0WgI	2	TRUE	0.540983	27.10706
2384	rs1608368	T	C	T	C	-0.1623	0.043485	0.04	0.057543	FALSE	FALSE	FALSE	NBJ2Do	0.029651	0.1425	486484	sepsis	TRUE	reported	textfile	0.0299	5.62E-08	25	M17799.m	TRUE	reported	Uz0WgI	2	TRUE	0.540983	27.10706
2404	rs187090	T	C	T	C	-0.1623	0.062278	0.04	0.013762	FALSE	FALSE	FALSE	NBJ2Do	0.058922	0.290531	486484	sepsis	TRUE	reported	textfile	0.0299	5.62E-08	25	M17799.m	TRUE	reported	Uz0WgI	2	TRUE	0.540983	27.10706
2423	rs4488147	T	C	T	C	-0.1623	-0.01381	0.04	0.035229	FALSE	FALSE	FALSE	NBJ2Do	0.036958	0.708693	486484	sepsis	TRUE	reported	textfile	0.0299	5.62E-08	25	M17799.m	TRUE	reported	Uz0WgI	2	TRUE	0.540983	27.10706
2456	rs836533	A	T	A	T	0.1623	-0.0068	0.96	0.989964	FALSE	TRUE	FALSE	NBJ2Do	0.069195	0.921768	486484	sepsis	TRUE	reported	textfile	0.0299	5.62E-08	25	M17799.m	TRUE	reported	Uz0WgI	2	TRUE	0.540983	27.10706
4656	rs4866873	T	C	T	C	0.0521	-0.02708	0.8725	0.863898	FALSE	FALSE	FALSE	SyAFR2	0.020031	0.176369	486484	sepsis	TRUE	reported	textfile	0.01	1.71E-07	1435	M32755.m	TRUE	reported	prUo74	2	TRUE	0.018565	27.10627
1339	rs2185865	A	C	A	C	0.0177	-0.03549	0.9245	0.930425	FALSE	FALSE	FALSE	LDUJbh	0.026827	0.185866	486484	sepsis	TRUE	reported	textfile	0.0034	1.70E-07	7367	M01898.m	TRUE	reported	JRSLeH	2	TRUE	0.003665	27.09385
8667	rs967942	T	C	T	C	0.0255	-0.01634	0.2883	0.302116	FALSE	FALSE	FALSE	BFEGyF	0.014923	0.273483	486484	sepsis	TRUE	reported	textfile	0.0049	1.64E-07	6486	M36399.m	TRUE	reported	nhT4FM	2	TRUE	0.004158	27.07411
4737	rs4633	T	C	T	C	0.0307	-0.01043	0.5168	0.515271	FALSE	FALSE	FALSE	Do3Q7g	0.013728	0.447582	486484	sepsis	TRUE	reported	textfile	0.0059	1.84E-07	6150	M32769.m	TRUE	reported	ys2LKp	2	TRUE	0.004383	27.06646
3119	rs4968748	A	G	A	G	-0.0307	-0.01287	0.232	0.223073	FALSE	FALSE	FALSE	a9KeDe	0.016433	0.43336	486484	sepsis	TRUE	reported	textfile	0.0059	1.83E-07	3838	M22175.m	TRUE	reported	shg5gu	2	TRUE	0.007005	27.06116
2553	rs1686540	A	T	A	T	-0.0385	0.013876	0.7722	0.787862	FALSE	TRUE	FALSE	NxZhaQ	0.016778	0.408209	486484	sepsis	TRUE	reported	textfile	0.0074	1.78E-07	5984	M18283.m	TRUE	reported	5Pi7r3	2	TRUE	0.004503	27.05907
2560	rs2726698	C	G	C	G	-0.0791	0.036293	0.6936	0.847632	FALSE	TRUE	FALSE	WfyP8n	0.019116	0.057618	486484	sepsis	TRUE	reported	textfile	0.0152	2.12E-07	1452	M18335.m	TRUE	reported	mjIjpe	2	TRUE	0.018309	27.04377
607	rs1024196	A	G	A	G	0.0182	-0.0393	0.0952	0.099684	FALSE	FALSE	FALSE	BYGzq4	0.022875	0.085826	486484	sepsis	TRUE	reported	textfile	0.0035	2.25E-07	7367	M01110.m	TRUE	reported	oZiXqP	2	TRUE	0.003657	27.03266
6782	rs9318232	T	C	T	C	-0.0104	-0.00458	0.2312	0.237039	FALSE	FALSE	FALSE	Elf6MQ	0.016069	0.775593	486484	sepsis	TRUE	reported	textfile	0.002	1.05E-07	7354	M33955.m	TRUE	reported	Lxn4RF	2	TRUE	0.003663	27.03265
3076	rs1074150	A	T	A	T	-0.026	0.035168	0.041	0.043222	FALSE	TRUE	FALSE	esOP8m	0.033614	0.295455	486484	sepsis	TRUE	reported	textfile	0.005	2.57E-07	7329	M22116.m	TRUE	reported	fDMh7Z	2	TRUE	0.003676	27.03262
3229	rs4073054	A	C	A	C	0.013	-0.05542	0.6227	0.627017	FALSE	FALSE	FALSE	I25GHB	0.014114	8.61E-05	486484	sepsis	TRUE	reported	textfile	0.0025	2.74E-07	7184	M22548.m	TRUE	reported	eBmTQD	2	TRUE	0.00375	27.03247
860	rs1103689	T	C	T	C	-0.013	0.021807	0.3592	0.366326	FALSE	FALSE	FALSE	Vg5OOR	0.014199	0.124589	486484	sepsis	TRUE	reported	textfile	0.0025	3.01E-07	7063	M01361.m	TRUE	reported	XnrS00	2	TRUE	0.003814	27.03234
2352	rs1081618	A	G	A	G	0.1493	-0.00817	0.9623	0.949775	FALSE	FALSE	FALSE	NBJ2Do	0.031522	0.795537	486484	sepsis	TRUE	reported	textfile	0.0287	1.97E-07	1739	M17799.m	TRUE	reported	Uz0WgI	2	TRUE	0.015323	27.03062
7248	rs862029	A	G	A	G	0.2654	0.070738	0.0214	0.011835	FALSE	FALSE	FALSE	RIcE3J	0.065865	0.282823	486484	sepsis	TRUE	reported	textfile	0.051	1.92E-07	633	M34339.m	TRUE	reported	QQrwdm	2	TRUE	0.041026	26.99524
4810	rs2433679	A	G	A	G	0.0213	0.030023	0.8868	0.901555	FALSE	FALSE	FALSE	bJwLV5	0.022939	0.190599	486484	sepsis	TRUE	reported	textfile	0.0041	1.65E-07	7063	M32814.m	TRUE	reported	qte0qp	2	TRUE	0.003807	26.98165
7556	rs1258042	A	G	A	G	0.0431	0.009686	0.9345	0.932383	FALSE	FALSE	FALSE	APSWAU	0.027288	0.72261	486484	sepsis	TRUE	reported	textfile	0.0083	2.20E-07	6144	M34469.m	TRUE	reported	frF3RX	2	TRUE	0.00437	26.95609
2746	rs2846556	T	G	T	G	-0.0135	0.012149	0.3337	0.330295	FALSE	FALSE	FALSE	qFAbee	0.014606	0.405521	486484	sepsis	TRUE	reported	textfile	0.0026	2.03E-07	6929	M19364.m	TRUE	reported	tZgXNL	2	TRUE	0.003876	26.95228
5100	rs1336659	A	G	A	G	0.0135	-0.00141	0.5079	0.521129	FALSE	FALSE	FALSE	Uiz8VU	0.013822	0.918877	486484	sepsis	TRUE	reported	textfile	0.0026	3.01E-07	6404	M33163.m	TRUE	reported	ky0Bda	2	TRUE	0.004192	26.95164
8531	rs7697282	T	C	T	C	0.0997	0.052453	0.9785	0.979081	FALSE	FALSE	FALSE	gM7v2m	0.048043	0.274917	486484	sepsis	TRUE	reported	textfile	0.0192	1.95E-07	2651	M36009.m	TRUE	reported	6Xcypd	2	TRUE	0.010069	26.94388
7117	rs1048576	T	C	T	C	-0.2299	0.044052	0.9733	0.956035	FALSE	FALSE	FALSE	de8tA6	0.03331	0.186004	486484	sepsis	TRUE	reported	textfile	0.0437	1.48E-07	75	M34322.m	TRUE	reported	sXPPBO	2	TRUE	0.269552	26.9387
493	rs1683077	C	G	C	G	-0.5244	-0.01872	0.0197	0.024296	FALSE	TRUE	FALSE	RtjZPn	0.044576	0.674548	486484	sepsis	TRUE	reported	textfile	0.1007	1.92E-07	283	M00553.m	TRUE	reported	C3OvsI	2	TRUE	0.087446	26.9269
3870	rs7499321	T	C	T	C	0.0192	-0.00227	0.3347	0.355779	FALSE	FALSE	FALSE	7LphBB	0.014295	0.87402	486484	sepsis	TRUE	reported	textfile	0.0037	2.14E-07	6066	M32388.m	TRUE	reported	5oj12c	2	TRUE	0.004419	26.91881
1854	rs7288834	T	G	T	G	-0.0083	-0.00644	0.3986	0.461114	FALSE	FALSE	FALSE	S1eWts	0.013734	0.639224	486484	sepsis	TRUE	reported	textfile	0.0016	1.51E-07	7345	M15488.m	TRUE	reported	511KI9	2	TRUE	0.00365	26.90283
2089	rs8033100	T	C	T	C	-0.0083	0.014573	0.8565	0.855227	FALSE	FALSE	FALSE	NPVH5j	0.019422	0.453063	486484	sepsis	TRUE	reported	textfile	0.0016	4.94E-07	7310	M15506.m	TRUE	reported	r7r3vM	2	TRUE	0.003668	26.90279
4216	rs1173658	T	C	T	C	-0.0332	-0.00023	0.7281	0.725707	FALSE	FALSE	FALSE	aS6krA	0.015316	0.98777	486484	sepsis	TRUE	reported	textfile	0.0064	2.19E-07	7303	M32586.m	TRUE	reported	g9cBDI	2	TRUE	0.003671	26.90279
1161	rs2246012	T	C	T	C	-0.0166	-0.01924	0.8388	0.833936	FALSE	FALSE	FALSE	cSBFJ3	0.018343	0.294237	486484	sepsis	TRUE	reported	textfile	0.0032	2.23E-07	7104	M01638.m	TRUE	reported	sUkjpE	2	TRUE	0.003774	26.90258
4145	rs1319847	A	G	A	G	0.042	-0.02266	0.0945	0.094248	FALSE	FALSE	FALSE	CrVaiQ	0.023331	0.331333	486484	sepsis	TRUE	reported	textfile	0.0081	2.24E-07	7361	M32553.m	TRUE	reported	HsKdAI	2	TRUE	0.003639	26.87884
6255	rs1102290	A	G	A	G	-0.014	0.015728	0.154	0.14822	FALSE	FALSE	FALSE	MOonP9	0.019239	0.413642	486484	sepsis	TRUE	reported	textfile	0.0027	1.86E-07	7066	M33515.m	TRUE	reported	226GuP	2	TRUE	0.003791	26.87854
5736	rs6784447	A	G	A	G	0.014	-0.00871	0.3405	0.341233	FALSE	FALSE	FALSE	YDQaWa	0.014476	0.547184	486484	sepsis	TRUE	reported	textfile	0.0027	2.91E-07	7063	M33228.m	TRUE	reported	BmYg8r	2	TRUE	0.003792	26.87853
2123	rs1008258	A	T	A	T	-0.0259	0.036184	0.0409	0.043192	FALSE	TRUE	FALSE	TEupfd	0.03364	0.282089	486484	sepsis	TRUE	reported	textfile	0.005	1.88E-07	7331	M15676.m	TRUE	reported	NXiaPC	2	TRUE	0.003647	26.82508
5124	rs1113719	A	G	A	G	-0.7694	0.03615	0.0263	0.021429	FALSE	FALSE	FALSE	L2DJzK	0.047087	0.442646	486484	sepsis	TRUE	reported	textfile	0.1466	1.53E-07	76	M33173.m	TRUE	reported	hRG70D	2	TRUE	0.266017	26.81974
2459	rs945493	A	G	A	G	-0.1625	-0.01145	0.0433	0.091733	FALSE	FALSE	FALSE	NBJ2Do	0.023768	0.629957	486484	sepsis	TRUE	reported	textfile	0.0301	6.45E-08	25	M17799.m	TRUE	reported	Uz0WgI	2	TRUE	0.538282	26.814
3530	rs1311508	A	G	A	G	0.0145	0.021023	0.792	0.779277	FALSE	FALSE	FALSE	rXzSIH	0.016544	0.203806	486484	sepsis	TRUE	reported	textfile	0.0028	3.10E-07	6795	M30805.m	TRUE	reported	7wg9QH	2	TRUE	0.003931	26.80971
2174	rs9355342	A	G	A	G	0.1456	-0.02422	0.9692	0.963615	FALSE	FALSE	FALSE	XCBNPg	0.03828	0.526908	486484	sepsis	TRUE	reported	textfile	0.0281	2.19E-07	1259	M15749.m	TRUE	reported	dKBewD	2	TRUE	0.02088	26.80524
5966	rs1060467	A	G	A	G	0.0233	0.004488	0.5903	0.578805	FALSE	FALSE	FALSE	YCswLG	0.013862	0.746134	486484	sepsis	TRUE	reported	textfile	0.0045	2.61E-07	5822	M33420.m	TRUE	reported	WH7xfc	2	TRUE	0.004584	26.80017
6171	rs328833	A	G	A	G	0.0233	-0.00028	0.6759	0.678505	FALSE	FALSE	FALSE	O4Ty2I	0.014633	0.984518	486484	sepsis	TRUE	reported	textfile	0.0045	2.72E-07	5107	M33488.m	TRUE	reported	RGtF84	2	TRUE	0.005222	26.79888
1618	rs7809262	A	G	A	G	-0.0088	-0.00848	0.425	0.406468	FALSE	FALSE	FALSE	zP3C2v	0.013918	0.542298	486484	sepsis	TRUE	reported	textfile	0.0017	4.41E-07	7356	M12035.m	TRUE	reported	1Pooe3	2	TRUE	0.003629	26.78856
6079	rs3853497	T	C	T	C	-0.0088	-0.02194	0.2686	0.251972	FALSE	FALSE	FALSE	P8n0HP	0.015823	0.165563	486484	sepsis	TRUE	reported	textfile	0.0017	2.91E-07	7336	M33442.m	TRUE	reported	cljMTI	2	TRUE	0.003639	26.78854
2073	rs1203911	T	C	T	C	-0.0088	0.023655	0.1289	0.132598	FALSE	FALSE	FALSE	NPVH5j	0.02017	0.240865	486484	sepsis	TRUE	reported	textfile	0.0017	1.93E-07	7310	M15506.m	TRUE	reported	r7r3vM	2	TRUE	0.003652	26.78852
1642	rs6967282	A	G	A	G	0.0088	0.015793	0.797	0.779882	FALSE	FALSE	FALSE	n5rDuT	0.016565	0.340417	486484	sepsis	TRUE	reported	textfile	0.0017	3.96E-07	7081	M12067.m	TRUE	reported	ZQLTeR	2	TRUE	0.00377	26.78828
1643	rs7069652	A	C	A	C	-0.0088	0.001888	0.1869	0.190612	FALSE	FALSE	FALSE	n5rDuT	0.017438	0.913803	486484	sepsis	TRUE	reported	textfile	0.0017	4.03E-07	7081	M12067.m	TRUE	reported	ZQLTeR	2	TRUE	0.00377	26.78828
1456	rs1049827	A	G	A	G	0.0088	0.003453	0.6222	0.619128	FALSE	FALSE	FALSE	pkb876	0.014086	0.806359	486484	sepsis	TRUE	reported	textfile	0.0017	4.73E-07	7037	M02734.m	TRUE	reported	0LCMVI	2	TRUE	0.003793	26.78823
1484	rs4572660	T	G	T	G	-0.0088	0.005794	0.6118	0.613978	FALSE	FALSE	FALSE	pkb876	0.014059	0.680238	486484	sepsis	TRUE	reported	textfile	0.0017	4.69E-07	7037	M02734.m	TRUE	reported	0LCMVI	2	TRUE	0.003793	26.78823
7466	rs212051	T	C	T	C	-0.0176	0.012219	0.2796	0.277424	FALSE	FALSE	FALSE	kasr9a	0.015405	0.427652	486484	sepsis	TRUE	reported	textfile	0.0034	3.08E-07	6768	M34409.m	TRUE	reported	3VaBw3	2	TRUE	0.003944	26.78793
6576	rs1322565	A	T	A	T	-0.1616	-0.04625	0.8762	0.855042	FALSE	TRUE	FALSE	XO3rXq	0.019592	0.01823	486484	sepsis	TRUE	reported	textfile	0.0311	2.08E-07	228	M33877.m	TRUE	reported	p2sETg	2	TRUE	0.105882	26.76305
6014	rs1177309	C	G	C	G	0.9721	0.032795	0.9667	0.984336	FALSE	TRUE	FALSE	33ojbt	0.055044	0.551309	486484	sepsis	TRUE	reported	textfile	0.1848	1.45E-07	60	M33423.m	TRUE	reported	ivEtvI	2	TRUE	0.31562	26.74821
8781	rs1209853	T	C	T	C	0.0642	-0.01253	0.0626	0.056889	FALSE	FALSE	FALSE	4bkfvN	0.029445	0.670364	486484	sepsis	TRUE	reported	textfile	0.0124	2.21E-07	922	M36738.m	TRUE	reported	xcqyX2	2	TRUE	0.028252	26.74752
3449	rs4774192	T	C	T	C	0.0709	0.009184	0.1306	0.134538	FALSE	FALSE	FALSE	J1I92u	0.019991	0.645953	486484	sepsis	TRUE	reported	textfile	0.0137	2.06E-07	1510	M27710.m	TRUE	reported	9qjpIZ	2	TRUE	0.017428	26.74704
8502	rs9831949	T	G	T	G	-0.0693	0.031415	0.0277	0.024425	FALSE	FALSE	FALSE	4MCEPy	0.044167	0.476913	486484	sepsis	TRUE	reported	textfile	0.0134	2.38E-07	6901	M35977.m	TRUE	reported	B3kU6y	2	TRUE	0.003861	26.73813
3725	rs1087977	T	C	T	C	-0.0181	0.010882	0.3441	0.347641	FALSE	FALSE	FALSE	Z55Bsv	0.014398	0.449782	486484	sepsis	TRUE	reported	textfile	0.0035	2.59E-07	7357	M32322.m	TRUE	reported	6FDRcB	2	TRUE	0.003622	26.7364
4357	rs9936831	A	T	A	T	0.0212	0.047189	0.9266	0.889986	FALSE	TRUE	FALSE	6VSoQQ	0.021999	0.031948	486484	sepsis	TRUE	reported	textfile	0.0041	2.88E-07	7229	M32644.m	TRUE	reported	oC2AV0	2	TRUE	0.003685	26.72907
2331	rs8112502	A	G	A	G	-0.0212	-0.00482	0.0918	0.087086	FALSE	FALSE	FALSE	IZXwpR	0.024327	0.84298	486484	sepsis	TRUE	reported	textfile	0.0041	2.64E-07	6549	M16822.m	TRUE	reported	Zx9k2V	2	TRUE	0.004066	26.7283
1070	rs910616	T	C	T	C	-0.0093	0.017505	0.4991	0.500328	FALSE	FALSE	FALSE	tRtQ2e	0.013673	0.200478	486484	sepsis	TRUE	reported	textfile	0.0018	1.97E-07	7364	M01564.m	TRUE	reported	Tbg0zM	2	TRUE	0.003612	26.68719
741	rs6587731	T	G	T	G	-0.0093	-0.02476	0.1626	0.166798	FALSE	FALSE	FALSE	9u0SC8	0.018774	0.187255	486484	sepsis	TRUE	reported	textfile	0.0018	1.96E-07	7363	M01301.m	TRUE	reported	H95uec	2	TRUE	0.003612	26.68719
4300	rs3740779	A	G	A	G	0.0093	0.009938	0.3688	0.383381	FALSE	FALSE	FALSE	0tjjPC	0.014276	0.486355	486484	sepsis	TRUE	reported	textfile	0.0018	2.97E-07	7362	M32634.m	TRUE	reported	nJJFkX	2	TRUE	0.003613	26.68719
6888	rs9973286	C	G	C	G	-0.0093	0.009292	0.8771	0.864646	FALSE	TRUE	FALSE	kFWZjs	0.020116	0.64415	486484	sepsis	TRUE	reported	textfile	0.0018	3.23E-07	7362	M34062.m	TRUE	reported	eLONsg	2	TRUE	0.003613	26.68719

1540	rs9462856	T	C	T	C	-0.0093	0.002389	0.4711	0.460895	FALSE	FALSE	FALSE	YvgTeW	0.013705	0.861643	486484	sepsis	TRUE	reported	textfile	0.0018	2.00E-07	7357	M03141.m	TRUE	reported	srKjSc	2	TRUE	0.003615	26.68719
1595	rs1084820	T	C	T	C	0.0093	-0.02372	0.5451	0.548172	FALSE	FALSE	FALSE	zP3C2v	0.013712	0.083601	486484	sepsis	TRUE	reported	textfile	0.0018	1.26E-07	7356	M12035.m	TRUE	reported	1Pooe3	2	TRUE	0.003616	26.68719
1615	rs7136574	T	C	T	C	0.0093	0.005803	0.4345	0.422651	FALSE	FALSE	FALSE	zP3C2v	0.013834	0.67486	486484	sepsis	TRUE	reported	textfile	0.0018	1.33E-07	7356	M12035.m	TRUE	reported	1Pooe3	2	TRUE	0.003616	26.68719
1199	rs1752058	A	G	A	G	0.0093	0.008258	0.332	0.331505	FALSE	FALSE	FALSE	Ckdgh1	0.01453	0.569803	486484	sepsis	TRUE	reported	textfile	0.0018	3.60E-07	7355	M01644.m	TRUE	reported	nKxz7B	2	TRUE	0.003616	26.68719
1212	rs630782	T	C	T	C	0.0093	0.009191	0.4713	0.459751	FALSE	FALSE	FALSE	Ckdgh1	0.013715	0.502737	486484	sepsis	TRUE	reported	textfile	0.0018	2.32E-07	7355	M01644.m	TRUE	reported	nKxz7B	2	TRUE	0.003616	26.68719
4029	rs1004733	A	G	A	G	-0.0093	-0.01592	0.6773	0.673037	FALSE	FALSE	FALSE	SiYGYU	0.014584	0.275117	486484	sepsis	TRUE	reported	textfile	0.0018	2.43E-07	7355	M32492.m	TRUE	reported	GfQvpU	2	TRUE	0.003616	26.68719
1240	rs4327548	A	G	A	G	-0.0093	-0.00294	0.2904	0.282351	FALSE	FALSE	FALSE	V50rWC	0.015195	0.846448	486484	sepsis	TRUE	reported	textfile	0.0018	2.16E-07	7346	M01645.m	TRUE	reported	yN3gpQ	2	TRUE	0.003621	26.68718
3791	rs703462	C	G	C	G	0.0093	-0.00723	0.3569	0.352398	FALSE	TRUE	FALSE	NW1aaR	0.014308	0.613286	486484	sepsis	TRUE	reported	textfile	0.0018	1.06E-07	7344	M32339.m	TRUE	reported	pBPkAB	2	TRUE	0.003622	26.68717
3295	rs7523745	T	C	T	C	-0.0093	-0.01129	0.4559	0.461125	FALSE	FALSE	FALSE	vYWPe9	0.013713	0.410197	486484	sepsis	TRUE	reported	textfile	0.0018	3.42E-07	7340	M25459.m	TRUE	reported	VvJ6Vx	2	TRUE	0.003624	26.68717
4592	rs2371276	T	C	T	C	-0.0093	-0.00197	0.4783	0.475598	FALSE	FALSE	FALSE	Pzplqu	0.013854	0.886699	486484	sepsis	TRUE	reported	textfile	0.0018	1.91E-07	7318	M32740.m	TRUE	reported	FjEBS5	2	TRUE	0.003635	26.68715
4601	rs529833	A	G	A	G	-0.0093	-0.004	0.6913	0.695234	FALSE	FALSE	FALSE	Pzplqu	0.01486	0.787539	486484	sepsis	TRUE	reported	textfile	0.0018	1.32E-07	7318	M32740.m	TRUE	reported	FjEBS5	2	TRUE	0.003635	26.68715
7052	rs1536873	A	G	A	G	-0.0093	0.000256	0.4478	0.46899	FALSE	FALSE	FALSE	uthruC	0.013785	0.985168	486484	sepsis	TRUE	reported	textfile	0.0018	1.67E-07	7316	M34283.m	TRUE	reported	ftTuRU	2	TRUE	0.003636	26.68715
6275	rs4634107	T	G	T	G	-0.0093	0.01916	0.5163	0.527821	FALSE	FALSE	FALSE	MOonP9	0.013707	0.16217	486484	sepsis	TRUE	reported	textfile	0.0018	3.25E-07	7066	M33515.m	TRUE	reported	226GuP	2	TRUE	0.003764	26.68689
6284	rs6536380	T	C	T	C	0.0093	-0.00726	0.6346	0.630786	FALSE	FALSE	FALSE	MOonP9	0.014235	0.610167	486484	sepsis	TRUE	reported	textfile	0.0018	4.49E-07	7066	M33515.m	TRUE	reported	226GuP	2	TRUE	0.003764	26.68689
6954	rs2187448	T	C	T	C	-0.0186	-0.00151	0.1296	0.126111	FALSE	FALSE	FALSE	7Cw2XZ	0.020584	0.941361	486484	sepsis	TRUE	reported	textfile	0.0036	1.79E-07	7049	M34123.m	TRUE	reported	vrgMI3	2	TRUE	0.003773	26.68687
779	rs912170	A	G	A	G	-0.0186	-0.02353	0.8773	0.879174	FALSE	FALSE	FALSE	tYvbNc	0.021006	0.262623	486484	sepsis	TRUE	reported	textfile	0.0036	2.09E-07	6946	M01303.m	TRUE	reported	hT3gsE	2	TRUE	0.003828	26.68676
7292	rs1905351	T	C	T	C	0.0093	0.009081	0.3924	0.40245	FALSE	FALSE	FALSE	Vcz4EA	0.01399	0.516267	486484	sepsis	TRUE	reported	textfile	0.0018	1.38E-07	6755	M34359.m	TRUE	reported	aUSo2J	2	TRUE	0.003936	26.68654
7705	rs633290	A	G	A	G	-0.0217	0.017822	0.6791	0.67558	FALSE	FALSE	FALSE	2eJlPM	0.014613	0.22263	486484	sepsis	TRUE	reported	textfile	0.0042	2.13E-07	6381	M34761.m	TRUE	reported	druKtT	2	TRUE	0.004166	26.68608
7182	rs4533654	T	G	T	G	-0.0155	-0.01278	0.5883	0.601491	FALSE	FALSE	FALSE	2hlepp	0.014077	0.363928	486484	sepsis	TRUE	reported	textfile	0.003	1.61E-07	5081	M34336.m	TRUE	reported	XyK8mO	2	TRUE	0.005226	26.68394
4869	rs1899256	C	G	C	G	0.0377	0.050079	0.9149	0.915157	FALSE	TRUE	FALSE	KBJVQO	0.024507	0.041011	486484	sepsis	TRUE	reported	textfile	0.0073	2.61E-07	7355	M32855.m	TRUE	reported	Xea7Fn	2	TRUE	0.003613	26.66361
8653	rs2874702	T	C	T	C	-0.0496	0.00679	0.4189	0.406372	FALSE	FALSE	FALSE	J6i46i	0.013917	0.625616	486484	sepsis	TRUE	reported	textfile	0.0096	2.47E-07	1529	M36376.m	TRUE	reported	IdddPp	2	TRUE	0.017159	26.65953
2485	rs6603803	A	G	A	G	-0.0253	-0.0166	0.4879	0.492489	FALSE	FALSE	FALSE	SrfyMU	0.013666	0.224402	486484	sepsis	TRUE	reported	textfile	0.0049	2.93E-07	7226	M17807.m	TRUE	reported	0zrJsu	2	TRUE	0.003676	26.65193
8923	rs1007983	T	G	T	G	-0.1332	-0.0124	0.0301	0.02847	FALSE	FALSE	FALSE	ns6PV1	0.04123	0.763574	486484	sepsis	TRUE	reported	textfile	0.0258	2.33E-07	7014	M37097.m	TRUE	reported	ZYmYt4	2	TRUE	0.003786	26.64681
7935	rs2326537	A	T	A	T	-0.1064	0.06695	0.0294	0.041108	FALSE	TRUE	FALSE	kFTWXY	0.03446	0.052037	486484	sepsis	TRUE	reported	textfile	0.0206	2.43E-07	1525	M35193.m	TRUE	reported	cjJvJl	2	TRUE	0.017193	26.64274
938	rs5764925	A	G	A	G	-0.016	0.008789	0.0772	0.086068	FALSE	FALSE	FALSE	tv99UN	0.024697	0.72193	486484	sepsis	TRUE	reported	textfile	0.0031	1.91E-07	7354	M01494.m	TRUE	reported	osDclp	2	TRUE	0.003609	26.63167
1550	rs1258682	T	C	T	C	0.0129	-0.02744	0.1222	0.120333	FALSE	FALSE	FALSE	Ke0sNh	0.021167	0.194903	486484	sepsis	TRUE	reported	textfile	0.0025	1.62E-07	7799	M10642.m	TRUE	reported	ZINwVb	2	TRUE	0.003402	26.61877
2134	rs1687022	A	G	A	G	0.0129	0.044525	0.1456	0.14317	FALSE	FALSE	FALSE	TEupfd	0.019605	0.023142	486484	sepsis	TRUE	reported	textfile	0.0025	3.23E-07	7331	M15676.m	TRUE	reported	NXiaPC	2	TRUE	0.003619	26.61834
1517	rs7644390	A	T	A	T	0.0129	0.002727	0.3478	0.354162	FALSE	TRUE	FALSE	rzkxmh	0.014334	0.849124	486484	sepsis	TRUE	reported	textfile	0.0025	1.76E-07	6941	M03127.m	TRUE	reported	3lrzhA	2	TRUE	0.003821	26.61793
5131	rs1263280	T	C	T	C	-0.783	-0.00452	0.0407	0.031519	FALSE	FALSE	FALSE	L2DJzK	0.039081	0.907863	486484	sepsis	TRUE	reported	textfile	0.1498	1.73E-07	76	M33173.m	TRUE	reported	hRG70D	2	TRUE	0.26443	26.60223
2207	rs247616	T	C	T	C	0.0098	-0.02821	0.322	0.32435	FALSE	FALSE	FALSE	Bf6uMK	0.014605	0.053401	486484	sepsis	TRUE	reported	textfile	0.0019	1.32E-07	7308	M15778.m	TRUE	reported	jowM2N	2	TRUE	0.003627	26.5966
4335	rs1189652	A	G	A	G	-0.0098	0.013312	0.3492	0.340575	FALSE	FALSE	FALSE	6VSoQQ	0.014456	0.357116	486484	sepsis	TRUE	reported	textfile	0.0019	1.28E-07	7229	M32644.m	TRUE	reported	oC2AV0	2	TRUE	0.003667	26.59652
6256	rs1110277	A	G	A	G	-0.0098	0.020843	0.675	0.661621	FALSE	FALSE	FALSE	MOonP9	0.014454	0.149294	486484	sepsis	TRUE	reported	textfile	0.0019	1.47E-07	7066	M33515.m	TRUE	reported	226GuP	2	TRUE	0.003751	26.59635
8911	rs327737	T	C	T	C	0.0098	-0.01896	0.577	0.577215	FALSE	FALSE	FALSE	eLhsld	0.0142	0.181832	486484	sepsis	TRUE	reported	textfile	0.0019	1.18E-07	6552	M37058.m	TRUE	reported	RSeWE5	2	TRUE	0.004044	26.59576
3147	rs328370	T	G	T	G	0.0098	-0.01665	0.722	0.706978	FALSE	FALSE	FALSE	JiXR2t	0.0151	0.270168	486484	sepsis	TRUE	reported	textfile	0.0019	5.30E-07	6535	M22177.m	TRUE	reported	joi6p6	2	TRUE	0.004054	26.59574
5138	rs1689278	C	G	C	G	-0.6355	-0.06938	0.04	0.019054	FALSE	TRUE	FALSE	L2DJzK	0.050415	0.16878	486484	sepsis	TRUE	reported	textfile	0.1216	1.73E-07	76	M33173.m	TRUE	reported	hRG70D	2	TRUE	0.264369	26.5939
7855	rs7179088	T	C	T	C	0.0098	-0.00072	0.3056	0.302467	FALSE	FALSE	FALSE	h5whlS	0.014912	0.961693	486484	sepsis	TRUE	reported	textfile	0.0019	2.86E-07	4900	M35137.m	TRUE	reported	xVITKF	2	TRUE	0.0054	26.59302
6187	rs1246087	T	C	T	C	0.0263	-0.01229	0.6114	0.605659	FALSE	FALSE	FALSE	DEG58p	0.013986	0.37957	486484	sepsis	TRUE	reported	textfile	0.0051	1.85E-07	7500	M33507.m	TRUE	reported	ycykD0	2	TRUE	0.003533	26.58614
420	rs2130712	A	C	A	C	0.0165	0.012684	0.92	0.918891	FALSE	FALSE	FALSE	cTsNj6	0.025001	0.611929	486484	sepsis	TRUE	reported	textfile	0.0032	2.43E-07	7365	M00063.m	TRUE	reported	7KYTrQ	2	TRUE	0.003597	26.57969
7332	rs1196522	A	C	A	C	0.0965	-0.02504	0.3865	0.381115	FALSE	FALSE	FALSE	mm9ksf	0.014122	0.076213	486484	sepsis	TRUE	reported	textfile	0.0187	2.53E-07	954	M34365.m	TRUE	reported	AVwEqD	2	TRUE	0.027156	26.57419
8072	rs7249061	T	C	T	C	0.0201	-0.01402	0.5464	0.538413	FALSE	FALSE	FALSE	7NkUCa	0.013724	0.306867	486484	sepsis	TRUE	reported	textfile	0.0039	2.62E-07	7359	M35320.m	TRUE	reported	GixRsK	2	TRUE	0.003596	26.55491
1576	rs1466522	T	C	T	C	-0.0067	0.037375	0.7645	0.763784	FALSE	FALSE	FALSE	lxzgXW	0.016097	0.020238	486484	sepsis	TRUE	reported	textfile	0.0013	3.15E-07	7341	M11438.m	TRUE	reported	ZjFyyS	2	TRUE	0.003605	26.55489
7692	rs9806159	T	G	T	G	-0.05	-0.00436	0.1395	0.141805	FALSE	FALSE	FALSE	5FAxbZ	0.019608	0.824155	486484	sepsis	TRUE	reported	textfile	0.0097	2.56E-07	1560	M34674.m	TRUE	reported	TDQqa8	2	TRUE	0.016747	26.53624
8281	rs1704783	T	G	T	G	0.0768	0.006002	0.9243	0.928201	FALSE	FALSE	FALSE	J6o4xC	0.026535	0.821043	486484	sepsis	TRUE	reported	textfile	0.0149	2.51E-07	1640	M35472.m	TRUE	reported	3XBcu9	2	TRUE	0.015941	26.53505
590	rs1077122	A	G	A	G	0.0237	-0.00878	0.78	0.773514	FALSE	FALSE	FALSE	KVK33e	0.016249	0.589052	486484	sepsis	TRUE	reported	textfile	0.0046	2.87E-07	5344	M01107.m	TRUE	reported	eAyR8w	2	TRUE	0.004943	26.53496
2995	rs2529488	T	C	T	C	-0.0376	0.007573	0.8627	0.861338	FALSE	FALSE	FALSE	xmzJ5F	0.019761	0.70153	486484	sepsis	TRUE	reported	textfile	0.0073	2.32E-07	5436	M21184.m	TRUE	reported	xgwhDp	2	TRUE	0.004857	26.51979
2433	rs6506569	T	C	T	C	0.0541	-0.01192	0.5349	0.522147	FALSE	FALSE	FALSE	NBJ2Do	0.014392	0.407378	486484	sepsis	TRUE	reported	textfile	0.0105	2.40E-07	1739	M17799.m	TRUE	reported	Uz0Wgl	2	TRUE	0.015036	26.5165
3683	rs662138	C	G	C	G	0.0139	-0.00026	0.8297	0.814014	FALSE	TRUE	FALSE	rGHJcj	0.017557	0.987965	486484	sepsis	TRUE	reported	textfile	0.0027	2.95E-07	7356	M32198.m	TRUE	reported	6P1Nno	2	TRUE	0.00359	26.49622
3508	rs9471691	T	C	T	C	0.0139	-0.01016	0.6461	0.648412	FALSE	FALSE	FALSE	Awafb0	0.014349	0.479018	486484	sepsis	TRUE	reported	textfile	0.0027	2.77E-07	5562	M27728.m	TRUE	reported	Lfj8L0	2	TRUE	0.004742	26.4939
1635	rs1723332	A	G	A	G	-0.0175	0.019622	0.9519	0.962019	FALSE	FALSE	FALSE	n5rDuT	0.035743	0.58303	486484	sepsis	TRUE	reported	textfile	0.0034	3.74E-07	7081	M12067.m	TRUE	reported	ZQLTeR	2	TRUE	0.003727	26.48473
686	rs1709295	T	G	T	G	-0.0175	-0.05311	0.1024	0.109991	FALSE	FALSE	FALSE	aT1Fw1	0.022001	0.015771	486484	sepsis	TRUE	reported	textfile	0.0034	3.39E-07	5687	M01284.m	TRUE	reported	mbTe8H	2	TRUE	0.004637	26.4829
5171	rs1004469	A	G	A	G	0.3085	-0.01054	0.9784	0.98343	FALSE	FALSE	FALSE	q32d4E	0.053618	0.844093	486484	sepsis	TRUE	reported	textfile	0.0599	2.58E-07	1017	M33178.m	TRUE	reported	cS3BwT	2	TRUE	0.025419	26.47292
5050	rs2937754	T	C	T	C	-0.0597	0.003079	0.8251	0.838226	FALSE	FALSE	FALSE	JLCSne	0.018641	0.868827	486484	sepsis	TRUE	reported	textfile	0.0116	2.91E-07	2391	M33137.m	TRUE	reported	LGKYXK	2	TRUE	0.010956	26.46484
202	rs7014133	A	G	A	G	-0.0561	0.007236	0.0119	0.009312	FALSE	FALSE	FALSE	FbLTng	0.072284	0.920264	486484	sepsis	TRUE	reported	textfile	0.0109	2.74E-07	1762	M00059.m	TRUE	reported	KK7HbM	2	TRUE	0.014811	26.45937
7519	rs1703308	T	C	T	C	-0.0484	0.006733	0.3485	0.317396	FALSE	FALSE	FALSE	rp1LGA	0.014691	0.646703	486484	sepsis	TRUE	reported	textfile	0.0094	3.02E-07	997	M34441.m	TRUE	reported	SU47tz	2	TRUE	0.025903	26.45836
7464	rs9775485	T	C	T	C	-0.0144	-0.01219	0.5852	0.570899	FALSE	FALSE	FALSE	7ubZow	0.01381	0.377308	486484	sepsis	TRUE	reported	textfile	0.0028	2.01E-07	7344	M34407.m	TRUE	reported	LRsXN2	2	TRUE	0.003589	26.44178
8027	rs1240394	A	C	A	C	-0.0144	0.031124	0.0874	0.095911	FALSE	FALSE	FALSE	SwiqSi	0.023224	0.180206	486484	sepsis	TRUE	reported	textfile	0.0028	1.59E-07	7217	M35270.m	TRUE	reported	21ZIsQ	2	TRUE	0.003651	26.44165
3411	rs289999	T	C	T	C	-0.018	0.008861	0.6577	0.667709	FALSE	FALSE	FALSE	VU62LG	0.01471	0.546915	486484	sepsis	TRUE	reported	textfile	0.0035	1.91E-07	7180	M27513.m	TRUE	reported	0didOx	2	TRUE	0.00367	26.44161
2496	rs1115060	T	C	T	C	-0.072	-0.07859	0.989	0.983502	FALSE	FALSE	FALSE	LwUYua	0.054094	0.146274	486484	sepsis	TRUE	reported	textfile	0.014	2.71E-07	5554	M17945.m	TRUE	reported	SUN8dc	2	TRUE	0.00474	26.43946
4148	rs1690178	A	C	A	C	0.0365	-0.00053	0.1157	0.136691	FALSE	FALSE	FALSE	CrVaiQ	0.019829	0.978529	486484	sepsis	TRUE	reported	textfile	0.0071	2.64E-07	7361	M32553.m	TRUE	reported	HsKdAI	2	TRUE	0.003577	26.42111
4657	rs4963728	T	C	T	C	-0.0257	-0.01707	0.1436	0.140814	FALSE	FALSE	FALSE	SyAFR2	0.019743	0.387201	486484	sepsis	TRUE	reported	textfile	0.005	2.59E-07	6658	M32755.m	TRUE	reported	prUo74	2	TRUE	0.003952	26.41166
8259	rs6542859	T	C	T	C	0.1378	0.046153	0.0339	0.047932	FALSE	FALSE	FALSE	G4plxe	0.03281	0.159521	486484	sepsis	TRUE	reported	textfile	0.0268	2.68E-07	1383	M35451.m	TRUE	reported	hNOVMI	2	TRUE	0.018758	26.39978
4824	rs4253236	T	C	T	C	-0.037	-0.01039	0.362	0.367998	FALSE	FALSE	FALSE	B6WchP	0.014154	0.46282	486484	sepsis	TRUE	reported	textfile	0.0072	2.61E-07	5544	M32836.m	TRUE	reported	7UzUw0	2	TRUE	0.004741	26.39865
4235	rs1448663	A	G	A	G	0.0334	-0.02804	0.1756	0.195988	FALSE	FALSE	FALSE	wWEMr1	0.017482	0.108682	486484	sepsis	TRUE	reported	textfile	0.0065	3.47E-07	5955	M32593.m	TRUE	reported	Mh2g4i	2	TRUE	0.004414	26.39492
7238	rs1270117	T	G	T	G	-0.11	0.017059	0.9455	0.951157	FALSE	FALSE	FALSE	RlcE3J	0.031731	0.59084	486484	sepsis	TRUE	reported	textfile	0.0214	2.59E-07	1649	M34339.m	TRUE	reported	QQrwdm	2	TRUE	0.01577	26.38948
8940	rs1322179	T	C	T	C	0.0411	0.031503	0.1489	0.148882	FALSE	FALSE	FALSE	mXJP3h	0.019207	0.100963	486484	sepsis	TRUE	reported	textfile	0.008	3.09E-07	4520	M37104.m	TRUE	reported	HRZBak	2	TRUE	0.005805	26.38223
3616	rs1474102	T	C	T	C	-0.0339	-0.00405	0.337	0.344112	FALSE	FALSE	FALSE	gq4lSJ	0.014466	0.77934	486484	sepsis	TRUE	reported	textfile	0.0066	3.32E-07	7338	M31591.m	TRUE	reported	wjwI4J	2	TRUE	0.003582	26.37504
6857	rs6689147	T	C	T	C	0.0226	-0.03709	0.2377	0.238077	FALSE	FALSE	FALSE	2pJy8f	0.01612	0.02141	486484	sepsis	TRUE	reported	textfile	0.0044	3.86E-07	7132	M34040.m	TRUE	reported	zuJC1r	2	TRUE	0.003686	26.37483
7668	rs1104291	T	C	T	C	0.0226	0.017998	0.2479	0.245292	FALSE	FALSE	FALSE	iqkM9B	0.015997	0.260534	486484	sepsis	TRUE	reported	textfile	0.0044	2.80E-07	4800	M34539.m	TRUE	reported	5nFq6f	2	TRUE	0.005466	26.37124
6764	rs2273534	T	C	T	C	0.019	0.000289	0.947	0.945415	FALSE	FALSE	FALSE	Elf6MQ	0.03007	0.992341	486484	sepsis	TRUE	reported	textfile	0.0037	4.20E-07	7354	M33955.m	TRUE	reported	Lxn4RF	2	TRUE	0.003573	26.36244
9068	rs193538	T	G	T	G	0.019	0.01041	0.7047	0.6974	FALSE	FALSE	FALSE	0l8lKx	0.014901	0.484794	486484	sepsis	TRUE	reported	textfile	0.0037	2.27E-07	7219	M38178.m	TRUE	reported	bWs4mL	2	TRUE	0.00364	26.36231
3964	rs4668464	A	G	A	G	0.0272	0.005799	0.3316	0.340981	FALSE	FALSE	FALSE	sKCkQs	0.014411	0.687371	486484	sepsis	TRUE	reported	textfile	0.0053	2.93E-07	5080	M32445.m	TRUE	reported	egPvJc	2	TRUE	0.005158	26.32783
926	rs1214358	A	G	A	G	0.0118	0.032597	0.1428	0.177098	FALSE	FALSE	FALSE	tv99UN	0.018208	0.073406	486484	sepsis	TRUE	reported	textfile	0.0023	3.38E-07	7354	M01494.m	TRUE	reported	osDclp	2	TRUE	0.003566	26.3142
5938	rs1684440	A	G	A	G	-0.1401	0.001471	0.9409	0.93862	FALSE	FALSE	FALSE	yepzrL	0.028616	0.958992	486484	sepsis	TRUE	reported	textfile	0.0273	2.97E-07	1094	M33408.m	TRUE	reported	SY4CoS	2	TRUE	0.023507	26.28792
1546	rs1166419	T	C	T	C	-0.0082	-0.02453	0.592	0.606212	FALSE	FALSE	FALSE	Ke0sNh	0.013969	0.079108	486484	sepsis	TRUE	reported	textfile	0.0016	6.47E-07	7799	M10642.m	TRUE	reported	ZINwVb	2	TRUE	0.003357	26.25889
1113	rs266742	T	G	T	G	0.0082	-0.00688	0.7307	0.675196	FALSE	FALSE	FALSE	yahiGZ	0.014601	0.637666	486484	sepsis	TRUE	reported	textfile	0.0016	5.68E-07	7271	M01585.m	TRUE	reported	dwZ7vX	2	TRUE	0.003599	26.2584
8698	rs2028012	T	C	T	C	-0.0123	-0.01837	0.1636	0.164743	FALSE	FALSE	FALSE	Bg4VnB	0.018442	0.319197	486484	sepsis	TRUE	reported	textfile	0.0024	4.78E-07	7092	M36552.m	TRUE	reported	6zdDpC	2	TRUE	0.00369	26.25822
8296	rs1008297	T	C	T	C	-0.0451	-0.01564	0.1881	0.181407	FALSE	FALSE	FALSE	HELgy7	0.017785	0.379201	486484	sepsis	TRUE	reported	textfile	0.0088	2.99E-07	5035	M35551.m	TRUE	reported	7x7uUn	2	TRUE	0.00519	26.25519
4642	rs6415698	A	C	A	C	0.0548	-0.00919	0.7774	0.778634	FALSE	FALSE	FALSE	Dfaklt	0.016545	0.578453	486484	sepsis	TRUE	reported	textfile	0.0107	3.10E-07	6379	M32754.m	TRUE	reported	y21cDm	2	TRUE	0.004095	26.22149
2996	rs4937147	A	G	A	G	0.0379	-0.0108	0.1341	0.146782	FALSE	FALSE	FALSE	xmzJ5F	0.019406	0.577684	486484	sepsis	TRUE	reported	textfile	0.0074	3.11E-07	5436	M21184.m	TRUE	reported	xgwhDp	2	TRUE	0.004802	26.22136
5800	rs7083972	A	T	A	T	-0.0292	0.008298	0.2206	0.20133	FALSE	TRUE	FALSE	ka9zG7	0.017051	0.626514	486484	sepsis	TRUE	reported	textfile	0.0057	3.63E-07	1378	M33363.m	TRUE	reported	UFTVNc	2	TRUE	0.018688	26.20506
2363	rs1160238	A	G	A	G	-0.1147	0.021049	0.0779	0.047851	FALSE	FALSE	FALSE	NBJ2Do	0.032087	0.511815	486484	sepsis	TRUE	reported	textfile	0.0224	3.20E-07	1739	M17799.m	TRUE	reported	Uz0Wgl	2	TRUE	0.014854	26.18973
1127	rs1174118	T	C	T	C	-0.0087	-0.00117	0.177	0.169875	FALSE	FALSE	FALSE	nlqUuV	0.018338	0.949166	486484	sepsis	TRUE	reported	textfile	0.0017	3.78E-07	7371	M01604.m	TRUE	reported	Uro9nC	2	TRUE	0.003541	26.18321
431	rs4941743	T	C	T	C	0.0087	0.017057	0.2179	0.227874	FALSE	FALSE	FALSE	cTsNj6	0.016378	0.297659	486484	sepsis	TRUE	reported	textfile	0.0017	3.28E-07	7365	M00063.m	TRUE	reported	7KYTrQ	2	TRUE	0.003543	26.1832
2806	rs2160717	T	C	T	C	-0.0087	-0.02597	0.4042	0.41851	FALSE	FALSE	FALSE	Sx5Uhq	0.013854	0.060849	486484	sepsis	TRUE	reported	textfile	0.0017	4.64E-07	7354	M19934.m	TRUE	reported	DijYTE	2	TRUE	0.003549	26.18319
790	rs1293126	T	C	T	C	-0.0087	-0.01224	0.6329	0.631948	FALSE	FALSE	FALSE	dyT79I	0.014179	0.388107	486484	sepsis	TRUE	reported	textfile	0.0017	2.91E-07	7352	M01336.m	TRUE	reported	OemVJ2	2	TRUE	0.00355	26.18319
234	rs1199926	A	T	A	T	-0.0087	0.022859	0.0943	0.092775	FALSE	TRUE	FALSE	tbRL8J	0.023576	0.332268	486484	sepsis	TRUE	reported	textfile	0.0017	6.56E-07	7351	M00060.m	TRUE	reported	oOvRKT	2	TRUE	0.00355	26.18319
8186	rs7551359	A	C	A	C	0.0087	-0.00202	0.7125	0.6941	FALSE	FALSE	FALSE	8UhtPK	0.014915	0.892349	486484	sepsis	TRUE	reported	textfile	0.0017	2.43E-07	7345	M35397.m	TRUE	reported	rvVJba	2	TRUE	0.003553	26.18318
3782	rs465152	A	G	A	G	-0.0087	-0.00686	0.5429	0.541074	FALSE	FALSE	FALSE	NW1aaR	0.013755	0.618043	486484	sepsis	TRUE	reported	textfile	0.0017	6.45E-07	7344	M32339.m	TRUE	reported	pBPkAB	2	TRUE	0.003554	26.18318
1365	rs1090132	A	G	A	G	0.0087	0.012395	0.4313	0.430189	FALSE	FALSE	FALSE	KrWhws	0.01382	0.369756	486484	sepsis	TRUE	reported	textfile	0.0017	4.34E-07	7325	M02132.m	TRUE	reported	7pzpfK	2	TRUE	0.003563	26.18316
2398	rs1748895	T	G	T	G	-0.1116	-0.02566	0.0776	0.051376	FALSE	FALSE	FALSE	NBJ2Do	0.031366	0.413266	486484	sepsis	TRUE	reported	textfile	0.0218	3.19E-07	1739	M17799.m	TRUE	reported	Uz0Wgl	2	TRUE	0.014846	26.17674
3645	rs6947392	T	C	T	C	0.0486	0.000694	0.2858	0.252835	FALSE	FALSE	FALSE	Vq2efp	0.015724	0.964788	486484	sepsis	TRUE	reported	textfile	0.0095	2.81E-07	7363	M31787.m	TRUE	reported	krl4Tw	2	TRUE	0.003542	26.16419
2750	rs505125	C	G	C	G	0.0133	0.013448	0.7328	0.732954	FALSE	TRUE	FALSE	qFAbee	0.015567	0.387628	486484	sepsis	TRUE	reported	textfile	0.0026	5.22E-07	6929	M19364.m	TRUE	reported	tZgXNL	2	TRUE	0.003762	26.15961
2767	rs764428	T	C	T	C	-0.0133	0.023648	0.6245	0.619529	FALSE	FALSE	FALSE	H6txH3	0.014091	0.093304	486484	sepsis	TRUE	reported	textfile	0.0026	2.46E-07	6678	M19368.m	TRUE	reported	b094NF	2	TRUE	0.003903	26.15932
3959	rs1707120	A	G	A	G	-0.0312	-0.01662	0.7844	0.795559	FALSE	FALSE	FALSE	sKCkQs	0.017058	0.329755	486484	sepsis	TRUE	reported	textfile	0.0061	3.92E-07	5080	M32445.m	TRUE	reported	egPvJc	2	TRUE	0.005123	26.15041
3489	rs210331	A	G	A	G	-0.0179	-0.02645	0.8206	0.828912	FALSE	FALSE	FALSE	Awafb0	0.018244	0.147199	486484	sepsis	TRUE	reported	textfile	0.0035	3.53E-07	5562	M27728.m	TRUE	reported	Lfj8L0	2	TRUE	0.004681	26.14651
7997	rs7277840	A	G	A	G	0.0266	0.000813	0.5377	0.531157	FALSE	FALSE	FALSE	Xrodum	0.013878	0.953265	486484	sepsis	TRUE	reported	textfile	0.0052	3.76E-07	1767	M35255.m	TRUE	reported	T8t1tl	2	TRUE	0.014593	26.13754
1273	rs1010874	A	G	A	G	-0.0491	-0.01289	0.0629	0.046417	FALSE	FALSE	FALSE	nvWJJ3	0.032611	0.692537	486484	sepsis	TRUE	reported	textfile	0.0096	3.17E-07	1753	M01712.m	TRUE	reported	1x44lf	2	TRUE	0.014703	26.12912
626	rs9679675	A	C	A	C	0.0092	0.018782	0.4732	0.470515	FALSE	FALSE	FALSE	BYGzq4	0.013695	0.170234	486484	sepsis	TRUE	reported	textfile	0.0018	4.17E-07	7367	M01110.m	TRUE	reported	oZiXqP	2	TRUE	0.003533	26.11636
9050	rs6708367	A	G	A	G	0.0092	-0.0092	0.7078	0.704035	FALSE	FALSE	FALSE	df0MCM	0.014988	0.539144	486484	sepsis	TRUE	reported	textfile	0.0018	3.09E-07	7366	M37506.m	TRUE	reported	iUfr4u	2	TRUE	0.003534	26.11636
9035	rs1789679	A	G	A	G	0.0092	0.008469	0.689	0.689894	FALSE	FALSE	FALSE	df0MCM	0.014792	0.566937	486484	sepsis	TRUE	reported	textfile	0.0018	2.92E-07	7366	M37506.m	TRUE	reported	iUfr4u	2	TRUE	0.003534	26.11636
3819	rs1152592	A	G	A	G	-0.0092	0.01198	0.6183	0.627005	FALSE	FALSE	FALSE	tRX2u0	0.014144	0.396981	486484	sepsis	TRUE	reported	textfile	0.0018	1.57E-07	7365	M32348.m	TRUE	reported	ruBgwQ	2	TRUE	0.003534	26.11636
3838	rs4315324	A	G	A	G	0.0092	-0.01741	0.5036	0.508199	FALSE	FALSE	FALSE	tRX2u0	0.013674	0.202968	486484	sepsis	TRUE	reported	textfile	0.0018	1.39E-07	7365	M32348.m	TRUE	reported	ruBgwQ	2	TRUE	0.003534	26.11636
4299	rs288500	A	G	A	G	-0.0092	0.012895	0.5823	0.594735	FALSE	FALSE	FALSE	0tjjPC	0.013925	0.354403	486484	sepsis	TRUE	reported	textfile	0.0018	2.36E-07	7362	M32634.m	TRUE	reported	nJJFkX	2	TRUE	0.003536	26.11636
4304	rs626787	C	G	C	G	0.0092	-0.03751	0.6639	0.647179	FALSE	TRUE	FALSE	0tjjPC	0.014337	0.008883	486484	sepsis	TRUE	reported	textfile	0.0018	3.80E-07	7362	M32634.m	TRUE	reported	nJJFkX	2	TRUE	0.003536	26.11636
5019	rs1107347	A	G	A	G	-0.0092	-0.00179	0.1492	0.179622	FALSE	FALSE	FALSE	BNnOvt	0.017933	0.920404	486484	sepsis	TRUE	reported	textfile	0.0018	5.55E-07	7362	M33132.m	TRUE	reported	rHcCPG	2	TRUE	0.003536	26.11636
5031	rs2009767	T	C	T	C	0.0092	0.024675	0.8814	0.878218	FALSE	FALSE	FALSE	BNnOvt	0.020997	0.239937	486484	sepsis	TRUE	reported	textfile	0.0018	4.14E-07	7362	M33132.m	TRUE	reported	rHcCPG	2	TRUE	0.003536	26.11636
6515	rs1018724	A	T	A	T	-0.0184	-0.00094	0.7692	0.757521	FALSE	TRUE	FALSE	OKlXJ8	0.016038	0.953203	486484	sepsis	TRUE	reported	textfile	0.0036	2.96E-07	7362	M33821.m	TRUE	reported	XGkaXH	2	TRUE	0.003536	26.11636
26	rs1092710	A	C	A	C	-0.0046	-0.00074	0.3738	0.372671	FALSE	FALSE	FALSE	xmGuol	0.01411	0.958118	486484	sepsis	TRUE	reported	textfile	9.00E-04	2.72E-07	7355	M00054.m	TRUE	reported	GW50Ri	2	TRUE	0.003539	26.11635
31	rs1172086	T	G	T	G	-0.0046	-0.01009	0.6034	0.60546	FALSE	FALSE	FALSE	xmGuol	0.014045	0.472554	486484	sepsis	TRUE	reported	textfile	9.00E-04	3.58E-07	7355	M00054.m	TRUE	reported	GW50Ri	2	TRUE	0.003539	26.11635
36	rs1220605	A	G	A	G	-0.0046	0.010393	0.6312	0.626949	FALSE	FALSE	FALSE	xmGuol	0.01412	0.461733	486484	sepsis	TRUE	reported	textfile	9.00E-04	9.29E-07	7355	M00054.m	TRUE	reported	GW50Ri	2	TRUE	0.003539	26.11635
123	rs4880888	A	G	A	G	-0.0046	0.006613	0.4785	0.487946	FALSE	FALSE	FALSE	xmGuol	0.013688	0.629005	486484	sepsis	TRUE	reported	textfile	9.00E-04	3.24E-07	7355	M00054.m	TRUE	reported	GW50Ri	2	TRUE	0.003539	26.11635
126	rs4972588	A	G	A	G	-0.0046	-0.01356	0.5138	0.512925	FALSE	FALSE	FALSE	xmGuol	0.013672	0.321226	486484	sepsis	TRUE	reported	textfile	9.00E-04	3.74E-07	7355	M00054.m	TRUE	reported	GW50Ri	2	TRUE	0.003539	26.11635
128	rs502654	T	C	T	C	-0.0046	0.003489	0.559	0.552772	FALSE	FALSE	FALSE	xmGuol	0.013835	0.800873	486484	sepsis	TRUE	reported	textfile	9.00E-04	2.60E-07	7355	M00054.m	TRUE	reported	GW50Ri	2	TRUE	0.003539	26.11635
168	rs8006663	A	G	A	G	-0.0046	0.004056	0.4556	0.468163	FALSE	FALSE	FALSE	xmGuol	0.013774	0.768399	486484	sepsis	TRUE	reported	textfile	9.00E-04	3.02E-07	7355	M00054.m	TRUE	reported	GW50Ri	2	TRUE	0.003539	26.11635
182	rs937382	T	C	T	C	-0.0046	-0.00135	0.4466	0.447111	FALSE	FALSE	FALSE	xmGuol	0.013749	0.922071	486484	sepsis	TRUE	reported	textfile	9.00E-04	2.31E-07	7355	M00054.m	TRUE	reported	GW50Ri	2	TRUE	0.003539	26.11635
186	rs9517869	T	C	T	C	-0.0046	-0.0107	0.3661	0.366053	FALSE	FALSE	FALSE	xmGuol	0.014217	0.451671	486484	sepsis	TRUE	reported	textfile	9.00E-04	3.46E-07	7355	M00054.m	TRUE	reported	GW50Ri	2	TRUE	0.003539	26.11635
554	rs1007797	T	C	T	C	-0.0092	0.040077	0.879	0.87685	FALSE	FALSE	FALSE	fNqkCW	0.020816	0.05419	486484	sepsis	TRUE	reported	textfile	0.0018	6.49E-07	7354	M00606.m	TRUE	reported	g9uwOn	2	TRUE	0.00354	26.11635
1767	rs1614303	T	G	T	G	0.0138	0.017337	0.8159	0.816176	FALSE	FALSE	FALSE	wM5dkT	0.017639	0.325666	486484	sepsis	TRUE	reported	textfile	0.0027	2.40E-07	7352	M15122.m	TRUE	reported	CBpa4t	2	TRUE	0.003541	26.11635
210	rs1048737	A	G	A	G	0.0046	0.0114	0.5764	0.599071	FALSE	FALSE	FALSE	tbRL8J	0.013952	0.413896	486484	sepsis	TRUE	reported	textfile	9.00E-04	4.09E-07	7351	M00060.m	TRUE	reported	oOvRKT	2	TRUE	0.003541	26.11635
216	rs1081058	T	C	T	C	0.0046	-0.00766	0.5583	0.550117	FALSE	FALSE	FALSE	tbRL8J	0.013715	0.57635	486484	sepsis	TRUE	reported	textfile	9.00E-04	3.28E-07	7351	M00060.m	TRUE	reported	oOvRKT	2	TRUE	0.003541	26.11635
233	rs1198809	T	C	T	C	-0.0046	0.015615	0.6185	0.623239	FALSE	FALSE	FALSE	tbRL8J	0.014172	0.270524	486484	sepsis	TRUE	reported	textfile	9.00E-04	3.29E-07	7351	M00060.m	TRUE	reported	oOvRKT	2	TRUE	0.003541	26.11635
235	rs1204761	T	G	T	G	0.0046	0.008505	0.3271	0.320841	FALSE	FALSE	FALSE	tbRL8J	0.014734	0.563797	486484	sepsis	TRUE	reported	textfile	9.00E-04	4.25E-07	7351	M00060.m	TRUE	reported	oOvRKT	2	TRUE	0.003541	26.11635
308	rs417144	T	C	T	C	0.0046	0.001467	0.3507	0.340128	FALSE	FALSE	FALSE	tbRL8J	0.014423	0.918969	486484	sepsis	TRUE	reported	textfile	9.00E-04	2.57E-07	7351	M00060.m	TRUE	reported	oOvRKT	2	TRUE	0.003541	26.11635
333	rs6475359	A	G	A	G	-0.0046	0.010799	0.4127	0.424088	FALSE	FALSE	FALSE	tbRL8J	0.014191	0.446664	486484	sepsis	TRUE	reported	textfile	9.00E-04	3.57E-07	7351	M00060.m	TRUE	reported	oOvRKT	2	TRUE	0.003541	26.11635
343	rs6833720	A	G	A	G	-0.0046	-0.01976	0.4766	0.476424	FALSE	FALSE	FALSE	tbRL8J	0.013761	0.150931	486484	sepsis	TRUE	reported	textfile	9.00E-04	2.22E-07	7351	M00060.m	TRUE	reported	oOvRKT	2	TRUE	0.003541	26.11635
363	rs7689825	T	C	T	C	0.0046	-0.00462	0.643	0.634374	FALSE	FALSE	FALSE	tbRL8J	0.014209	0.745181	486484	sepsis	TRUE	reported	textfile	9.00E-04	3.97E-07	7351	M00060.m	TRUE	reported	oOvRKT	2	TRUE	0.003541	26.11635
4105	rs1771823	A	G	A	G	0.0092	-0.0069	0.706	0.708818	FALSE	FALSE	FALSE	4rtLd0	0.015047	0.64656	486484	sepsis	TRUE	reported	textfile	0.0018	4.94E-07	7350	M32518.m	TRUE	reported	j7f1ER	2	TRUE	0.003542	26.11635
1862	rs1027502	T	C	T	C	0.0046	0.010243	0.6522	0.646398	FALSE	FALSE	FALSE	v2KE89	0.014279	0.473125	486484	sepsis	TRUE	reported	textfile	9.00E-04	4.90E-07	7349	M15500.m	TRUE	reported	to2t7H	2	TRUE	0.003542	26.11635
1878	rs1144963	T	C	T	C	-0.0046	0.007634	0.5594	0.554125	FALSE	FALSE	FALSE	v2KE89	0.013873	0.582121	486484	sepsis	TRUE	reported	textfile	9.00E-04	2.48E-07	7349	M15500.m	TRUE	reported	to2t7H	2	TRUE	0.003542	26.11635
1885	rs1170795	A	G	A	G	-0.0046	-0.01597	0.5684	0.568738	FALSE	FALSE	FALSE	v2KE89	0.013804	0.24733	486484	sepsis	TRUE	reported	textfile	9.00E-04	3.93E-07	7349	M15500.m	TRUE	reported	to2t7H	2	TRUE	0.003542	26.11635
1905	rs1293802	T	C	T	C	0.0046	0.009276	0.6104	0.611552	FALSE	FALSE	FALSE	v2KE89	0.014039	0.508774	486484	sepsis	TRUE	reported	textfile	9.00E-04	2.77E-07	7349	M15500.m	TRUE	reported	to2t7H	2	TRUE	0.003542	26.11635
1910	rs131919	T	C	T	C	-0.0046	-0.00189	0.3349	0.33205	FALSE	FALSE	FALSE	v2KE89	0.014695	0.897476	486484	sepsis	TRUE	reported	textfile	9.00E-04	9.01E-07	7349	M15500.m	TRUE	reported	to2t7H	2	TRUE	0.003542	26.11635
1935	rs1981138	T	G	T	G	0.0046	0.019009	0.433	0.440359	FALSE	FALSE	FALSE	v2KE89	0.013833	0.169398	486484	sepsis	TRUE	reported	textfile	9.00E-04	3.31E-07	7349	M15500.m	TRUE	reported	to2t7H	2	TRUE	0.003542	26.11635
1977	rs4284335	A	G	A	G	-0.0046	0.008061	0.4121	0.417381	FALSE	FALSE	FALSE	v2KE89	0.013888	0.561621	486484	sepsis	TRUE	reported	textfile	9.00E-04	3.53E-07	7349	M15500.m	TRUE	reported	to2t7H	2	TRUE	0.003542	26.11635
1989	rs4674438	T	C	T	C	0.0046	0.021052	0.3643	0.36609	FALSE	FALSE	FALSE	v2KE89	0.014241	0.139332	486484	sepsis	TRUE	reported	textfile	9.00E-04	2.93E-07	7349	M15500.m	TRUE	reported	to2t7H	2	TRUE	0.003542	26.11635
2000	rs4888504	T	C	T	C	0.0046	-0.00876	0.4223	0.436172	FALSE	FALSE	FALSE	v2KE89	0.013826	0.526178	486484	sepsis	TRUE	reported	textfile	9.00E-04	2.68E-07	7349	M15500.m	TRUE	reported	to2t7H	2	TRUE	0.003542	26.11635
2032	rs769809	A	G	A	G	0.0046	-0.03118	0.5723	0.573241	FALSE	FALSE	FALSE	v2KE89	0.013824	0.024116	486484	sepsis	TRUE	reported	textfile	9.00E-04	3.50E-07	7349	M15500.m	TRUE	reported	to2t7H	2	TRUE	0.003542	26.11635
2067	rs996408	T	C	T	C	-0.0046	0.008268	0.5159	0.517084	FALSE	FALSE	FALSE	v2KE89	0.013764	0.548035	486484	sepsis	TRUE	reported	textfile	9.00E-04	3.67E-07	7349	M15500.m	TRUE	reported	to2t7H	2	TRUE	0.003542	26.11635
8391	rs4643071	T	C	T	C	-0.0092	0.030331	0.6782	0.666169	FALSE	FALSE	FALSE	SCzh0A	0.014546	0.037054	486484	sepsis	TRUE	reported	textfile	0.0018	5.07E-07	7348	M35675.m	TRUE	reported	n1SREk	2	TRUE	0.003543	26.11635
1245	rs6112257	A	G	A	G	-0.0092	-0.00739	0.6843	0.68039	FALSE	FALSE	FALSE	V50rWC	0.014655	0.614192	486484	sepsis	TRUE	reported	textfile	0.0018	3.39E-07	7346	M01645.m	TRUE	reported	yN3gpQ	2	TRUE	0.003544	26.11634
4940	rs1424092	T	G	T	G	-0.0092	0.026143	0.3648	0.364041	FALSE	FALSE	FALSE	bKmDMa	0.014291	0.067357	486484	sepsis	TRUE	reported	textfile	0.0018	1.78E-07	7344	M32910.m	TRUE	reported	nAuPzO	2	TRUE	0.003545	26.11634
5858	rs1011090	A	G	A	G	-0.0092	0.025675	0.468	0.460597	FALSE	FALSE	FALSE	tqW0ld	0.013721	0.06131	486484	sepsis	TRUE	reported	textfile	0.0018	4.83E-07	7315	M33389.m	TRUE	reported	Sv7EyV	2	TRUE	0.003559	26.11631
2199	rs1263785	A	T	A	T	-0.0138	-0.00957	0.8926	0.870426	FALSE	TRUE	FALSE	Bf6uMK	0.020396	0.638925	486484	sepsis	TRUE	reported	textfile	0.0027	4.33E-07	7308	M15778.m	TRUE	reported	jowM2N	2	TRUE	0.003562	26.11631
2212	rs3765035	A	G	A	G	0.0092	0.002014	0.3438	0.354991	FALSE	FALSE	FALSE	Bf6uMK	0.014364	0.888478	486484	sepsis	TRUE	reported	textfile	0.0018	3.76E-07	7308	M15778.m	TRUE	reported	jowM2N	2	TRUE	0.003562	26.11631
6145	rs4853388	A	G	A	G	-0.0092	-0.02169	0.2394	0.237822	FALSE	FALSE	FALSE	MiSJly	0.016066	0.176936	486484	sepsis	TRUE	reported	textfile	0.0018	5.38E-07	7307	M33477.m	TRUE	reported	WwL1hq	2	TRUE	0.003562	26.11631
6251	rs1008717	T	C	T	C	-0.0092	0.025056	0.6135	0.596977	FALSE	FALSE	FALSE	MOonP9	0.013947	0.072425	486484	sepsis	TRUE	reported	textfile	0.0018	5.13E-07	7066	M33515.m	TRUE	reported	226GuP	2	TRUE	0.003683	26.11606
5741	rs9462177	A	G	A	G	0.0138	0.006873	0.6032	0.599518	FALSE	FALSE	FALSE	YDQaWa	0.013965	0.622618	486484	sepsis	TRUE	reported	textfile	0.0027	4.82E-07	7063	M33228.m	TRUE	reported	BmYg8r	2	TRUE	0.003685	26.11606
2973	rs4774615	A	G	A	G	-0.0184	0.000354	0.8406	0.831705	FALSE	FALSE	FALSE	dOOXpo	0.018304	0.984586	486484	sepsis	TRUE	reported	textfile	0.0036	4.07E-07	6999	M21127.m	TRUE	reported	IdiP4j	2	TRUE	0.003719	26.11599
7928	rs1235299	T	C	T	C	0.0184	0.002228	0.8104	0.812198	FALSE	FALSE	FALSE	kFTWXY	0.01755	0.898962	486484	sepsis	TRUE	reported	textfile	0.0036	2.54E-07	6543	M35193.m	TRUE	reported	cjJvJl	2	TRUE	0.003977	26.11547
7936	rs2378668	A	C	A	C	0.0138	0.01344	0.3623	0.360193	FALSE	FALSE	FALSE	kFTWXY	0.014263	0.346039	486484	sepsis	TRUE	reported	textfile	0.0027	5.04E-07	6543	M35193.m	TRUE	reported	cjJvJl	2	TRUE	0.003977	26.11547
3504	rs757704	A	G	A	G	0.0138	0.012984	0.5037	0.494834	FALSE	FALSE	FALSE	Awafb0	0.013832	0.347888	486484	sepsis	TRUE	reported	textfile	0.0027	2.22E-07	5562	M27728.m	TRUE	reported	Lfj8L0	2	TRUE	0.004675	26.11406
6166	rs2197399	A	G	A	G	-0.023	0.003171	0.5961	0.578521	FALSE	FALSE	FALSE	O4Ty2l	0.013841	0.818769	486484	sepsis	TRUE	reported	textfile	0.0045	2.68E-07	5107	M33488.m	TRUE	reported	RGtF84	2	TRUE	0.005089	26.11323
5083	rs3916092	A	G	A	G	0.0511	0.002639	0.2029	0.208186	FALSE	FALSE	FALSE	VzfWsT	0.017198	0.878038	486484	sepsis	TRUE	reported	textfile	0.01	3.22E-07	4817	M33144.m	TRUE	reported	ZtQP3u	2	TRUE	0.005392	26.10126
1178	rs6713914	T	C	T	C	0.0593	-0.03265	0.5741	0.53945	FALSE	FALSE	FALSE	FgZx4a	0.013701	0.017173	486484	sepsis	TRUE	reported	textfile	0.0116	3.22E-07	1567	M01640.m	TRUE	reported	hNPAJ8	2	TRUE	0.016404	26.09989
2408	rs217763	T	C	T	C	0.0552	-0.01939	0.4911	0.475575	FALSE	FALSE	FALSE	NBJ2Do	0.013724	0.157585	486484	sepsis	TRUE	reported	textfile	0.0108	3.23E-07	1739	M17799.m	TRUE	reported	Uz0Wgl	2	TRUE	0.0148	26.09341
1427	rs2742351	C	G	C	G	-0.0235	0.005862	0.2209	0.279648	FALSE	TRUE	FALSE	ac6RGB	0.0153	0.701604	486484	sepsis	TRUE	reported	textfile	0.0046	3.23E-07	5791	M02342.m	TRUE	reported	dC6Rkp	2	TRUE	0.004487	26.08976
7031	rs923673	A	C	A	C	0.0235	-0.01612	0.7212	0.712673	FALSE	FALSE	FALSE	zMIkYA	0.015165	0.287672	486484	sepsis	TRUE	reported	textfile	0.0046	2.98E-07	4908	M34245.m	TRUE	reported	itCtz8	2	TRUE	0.005289	26.08814
2464	rs997892	A	C	A	C	0.0925	0.04412	0.9349	0.922536	FALSE	FALSE	FALSE	NBJ2Do	0.025845	0.087803	486484	sepsis	TRUE	reported	textfile	0.0181	3.14E-07	1739	M17799.m	TRUE	reported	Uz0Wgl	2	TRUE	0.014796	26.08715
524	rs1167691	A	G	A	G	-0.0429	0.058342	0.0216	0.03425	FALSE	FALSE	FALSE	wXMWHy	0.03908	0.135466	486484	sepsis	TRUE	reported	textfile	0.0084	3.66E-07	7345	M00584.m	TRUE	reported	7CKn2b	2	TRUE	0.003539	26.07581
4757	rs2174474	T	C	T	C	0.0327	-0.01817	0.7012	0.739073	FALSE	FALSE	FALSE	RDfDIS	0.015607	0.244332	486484	sepsis	TRUE	reported	textfile	0.0064	3.67E-07	1593	M32787.m	TRUE	reported	Hq6nGt	2	TRUE	0.016124	26.07294
9024	rs1247804	A	G	A	G	0.0097	0.006878	0.2527	0.253903	FALSE	FALSE	FALSE	df0MCM	0.015794	0.663227	486484	sepsis	TRUE	reported	textfile	0.0019	1.49E-07	7366	M37506.m	TRUE	reported	iUfr4u	2	TRUE	0.003526	26.05664
8384	rs2285688	A	G	A	G	0.0097	0.002992	0.705	0.699786	FALSE	FALSE	FALSE	SCzh0A	0.014977	0.841659	486484	sepsis	TRUE	reported	textfile	0.0019	1.82E-07	7348	M35675.m	TRUE	reported	n1SREk	2	TRUE	0.003535	26.05662
3186	rs1012455	A	G	A	G	-0.0097	-0.00116	0.5085	0.504163	FALSE	FALSE	FALSE	UfLkBq	0.013706	0.932838	486484	sepsis	TRUE	reported	textfile	0.0019	2.16E-07	7329	M22481.m	TRUE	reported	kWMdiT	2	TRUE	0.003544	26.0566
1276	rs1185513	A	G	A	G	-0.0618	0.00995	0.0406	0.037466	FALSE	FALSE	FALSE	nvWJJ3	0.036753	0.786589	486484	sepsis	TRUE	reported	textfile	0.0121	2.99E-07	1753	M01712.m	TRUE	reported	1x44lf	2	TRUE	0.014663	26.05616
3140	rs227375	T	C	T	C	0.0097	0.008421	0.4802	0.489404	FALSE	FALSE	FALSE	JiXR2t	0.013674	0.537969	486484	sepsis	TRUE	reported	textfile	0.0019	5.76E-07	6535	M22177.m	TRUE	reported	joi6p6	2	TRUE	0.003972	26.05574
8093	rs1048875	T	C	T	C	0.0097	-0.01771	0.675	0.679875	FALSE	FALSE	FALSE	nNbXBS	0.01465	0.226626	486484	sepsis	TRUE	reported	textfile	0.0019	2.13E-07	5768	M35326.m	TRUE	reported	UNDorn	2	TRUE	0.004498	26.05467
7814	rs1113199	T	G	T	G	-0.0097	0.004496	0.6998	0.698125	FALSE	FALSE	FALSE	h5whlS	0.014877	0.762502	486484	sepsis	TRUE	reported	textfile	0.0019	4.20E-07	4900	M35137.m	TRUE	reported	xVITKF	2	TRUE	0.005291	26.05307
7829	rs2468526	T	G	T	G	0.0097	0.001357	0.6827	0.684035	FALSE	FALSE	FALSE	h5whlS	0.014715	0.92653	486484	sepsis	TRUE	reported	textfile	0.0019	3.48E-07	4900	M35137.m	TRUE	reported	xVITKF	2	TRUE	0.005291	26.05307
7867	rs9649625	A	C	A	C	-0.0097	0.016598	0.2883	0.285441	FALSE	FALSE	FALSE	h5whlS	0.015236	0.275975	486484	sepsis	TRUE	reported	textfile	0.0019	5.81E-07	4900	M35137.m	TRUE	reported	xVITKF	2	TRUE	0.005291	26.05307
8237	rs3809197	T	C	T	C	0.1072	0.03339	0.0135	0.01151	FALSE	FALSE	FALSE	4YAWhA	0.064806	0.606387	486484	sepsis	TRUE	reported	textfile	0.021	3.25E-07	1718	M35439.m	TRUE	reported	bSSC4Z	2	TRUE	0.014941	26.02826
7923	rs7741438	A	G	A	G	0.0255	-0.01163	0.4744	0.478613	FALSE	FALSE	FALSE	8qXKkX	0.01387	0.401594	486484	sepsis	TRUE	reported	textfile	0.005	3.38E-07	6234	M35189.m	TRUE	reported	Nt6oaC	2	TRUE	0.004155	26.00166
4176	rs4687396	A	C	A	C	-0.1489	0.086517	0.9784	0.988674	FALSE	FALSE	FALSE	Aq7H4p	0.068299	0.205246	486484	sepsis	TRUE	reported	textfile	0.0292	3.34E-07	2091	M32560.m	TRUE	reported	NIzbA7	2	TRUE	0.012283	25.97814
8759	rs1710294	A	G	A	G	0.0836	-0.03774	0.9519	0.954724	FALSE	FALSE	FALSE	KMisdy	0.032903	0.251384	486484	sepsis	TRUE	reported	textfile	0.0164	3.51E-07	3977	M36589.m	TRUE	reported	J1ISTP	2	TRUE	0.006491	25.97206
8161	rs9885127	T	C	T	C	-0.1433	-0.00726	0.9868	0.978075	FALSE	FALSE	FALSE	sRlsb6	0.049126	0.882468	486484	sepsis	TRUE	reported	textfile	0.0281	3.32E-07	1375	M35331.m	TRUE	reported	GPijIC	2	TRUE	0.018563	25.96854
3316	rs6767775	A	G	A	G	-0.0311	-0.02689	0.4648	0.476571	FALSE	FALSE	FALSE	nqmBts	0.013746	0.050423	486484	sepsis	TRUE	reported	textfile	0.0061	4.06E-07	1358	M25599.m	TRUE	reported	VkwSh1	2	TRUE	0.018781	25.955
3386	rs1023792	A	G	A	G	0.0382	0.008692	0.749	0.731091	FALSE	FALSE	FALSE	frOZiy	0.015393	0.572312	486484	sepsis	TRUE	reported	textfile	0.0075	3.11E-07	2759	M27447.m	TRUE	reported	2z9fSv	2	TRUE	0.009315	25.92324
2	rs1697704	T	C	T	C	-0.0056	-0.01309	0.7034	0.708292	FALSE	FALSE	FALSE	0cebDu	0.014989	0.382594	486484	sepsis	TRUE	reported	textfile	0.0011	1.08E-06	7372	M00053.m	TRUE	reported	i8iuxX	2	TRUE	0.003503	25.91032
4064	rs1168060	C	G	C	G	0.0168	-0.0263	0.7095	0.703265	FALSE	TRUE	FALSE	TyHtdh	0.015008	0.079697	486484	sepsis	TRUE	reported	textfile	0.0033	3.48E-07	7358	M32497.m	TRUE	reported	H3KmLR	2	TRUE	0.00351	25.91031
1542	rs1125012	A	G	A	G	-0.0494	0.005159	0.043	0.058544	FALSE	FALSE	FALSE	tApf9z	0.029145	0.85951	486484	sepsis	TRUE	reported	textfile	0.0097	3.83E-07	1748	M03147.m	TRUE	reported	tXdmtV	2	TRUE	0.014621	25.90677
6497	rs1267078	A	G	A	G	-0.1499	0.019461	0.8761	0.885846	FALSE	FALSE	FALSE	HNc7C9	0.021586	0.367302	486484	sepsis	TRUE	reported	textfile	0.0294	3.30E-07	581	M33782.m	TRUE	reported	SAGYGx	2	TRUE	0.042827	25.90664
5776	rs1343863	A	G	A	G	-0.0601	-0.00938	0.7954	0.761733	FALSE	FALSE	FALSE	eT2We0	0.016289	0.564716	486484	sepsis	TRUE	reported	textfile	0.0118	3.80E-07	1273	M33353.m	TRUE	reported	uZx90m	2	TRUE	0.019971	25.90014
2256	rs7711255	A	G	A	G	-0.0173	0.00662	0.7968	0.768625	FALSE	FALSE	FALSE	zxcPox	0.016259	0.683888	486484	sepsis	TRUE	reported	textfile	0.0034	4.92E-07	6782	M15990.m	TRUE	reported	34Vg8F	2	TRUE	0.003803	25.8825
7291	rs1763887	T	C	T	C	0.0173	0.038472	0.9074	0.860692	FALSE	FALSE	FALSE	Vcz4EA	0.019658	0.050336	486484	sepsis	TRUE	reported	textfile	0.0034	2.44E-07	6755	M34359.m	TRUE	reported	aUSo2J	2	TRUE	0.003818	25.88247
7911	rs1300312	T	C	T	C	0.0346	-0.02261	0.1214	0.120968	FALSE	FALSE	FALSE	8qXKkX	0.02105	0.282804	486484	sepsis	TRUE	reported	textfile	0.0068	3.63E-07	6234	M35189.m	TRUE	reported	Nt6oaC	2	TRUE	0.004136	25.88183
2694	rs7809441	A	G	A	G	0.0748	-0.01303	0.0807	0.085485	FALSE	FALSE	FALSE	ATsH6N	0.024689	0.597605	486484	sepsis	TRUE	reported	textfile	0.0147	3.80E-07	3783	M18497.m	TRUE	reported	IGuIRI	2	TRUE	0.006798	25.87849
480	rs1171988	C	G	C	G	-0.0524	-0.01399	0.8445	0.829608	FALSE	TRUE	FALSE	ScJVI8	0.018168	0.441382	486484	sepsis	TRUE	reported	textfile	0.0103	3.86E-07	7371	M00542.m	TRUE	reported	Sa1L04	2	TRUE	0.003499	25.8744
6799	rs1708956	T	C	T	C	0.0468	-0.05861	0.0209	0.03532	FALSE	FALSE	FALSE	OZ0I1H	0.037302	0.116145	486484	sepsis	TRUE	reported	textfile	0.0092	3.39E-07	7364	M33960.m	TRUE	reported	GlPp4w	2	TRUE	0.003502	25.8701
2132	rs1245558	T	C	T	C	0.0117	-0.02589	0.3104	0.379655	FALSE	FALSE	FALSE	TEupfd	0.014185	0.067949	486484	sepsis	TRUE	reported	textfile	0.0023	4.39E-07	7331	M15676.m	TRUE	reported	NXiaPC	2	TRUE	0.003517	25.87007
7535	rs165582	A	G	A	G	0.0234	0.014753	0.1156	0.133224	FALSE	FALSE	FALSE	JJsHvX	0.020157	0.464223	486484	sepsis	TRUE	reported	textfile	0.0046	3.31E-07	5207	M34456.m	TRUE	reported	aklxve	2	TRUE	0.004945	25.86719
8637	rs4949966	T	C	T	C	0.0468	-0.01348	0.874	0.86653	FALSE	FALSE	FALSE	4cZjpQ	0.020168	0.503731	486484	sepsis	TRUE	reported	textfile	0.0092	3.56E-07	2400	M36230.m	TRUE	reported	TPUeNs	2	TRUE	0.010667	25.85556
2961	rs1785007	C	G	C	G	0.1233	0.015176	0.1024	0.09151	FALSE	TRUE	FALSE	zndu0r	0.02374	0.522638	486484	sepsis	TRUE	reported	textfile	0.0242	3.35E-07	413	M21049.m	TRUE	reported	2XpPDU	2	TRUE	0.059139	25.83373
6661	rs6891011	A	G	A	G	-0.0244	-0.03436	0.8862	0.860106	FALSE	FALSE	FALSE	REeQiq	0.019732	0.081589	486484	sepsis	TRUE	reported	textfile	0.0048	3.64E-07	6004	M33892.m	TRUE	reported	cVOa3u	2	TRUE	0.004285	25.83167
1662	rs7446718	A	G	A	G	-0.0417	-0.01096	0.8591	0.834337	FALSE	FALSE	FALSE	YzX5ZD	0.018387	0.551072	486484	sepsis	TRUE	reported	textfile	0.0082	3.83E-07	1586	M12129.m	TRUE	reported	6tMfaW	2	TRUE	0.016044	25.82833
5137	rs162882	C	G	C	G	0.4501	0.007896	0.9145	0.900227	FALSE	TRUE	FALSE	L2DJzK	0.022848	0.729654	486484	sepsis	TRUE	reported	textfile	0.0874	2.57E-07	76	M33173.m	TRUE	reported	hRG70D	2	TRUE	0.258691	25.82339
8246	rs1076659	A	G	A	G	-0.1244	0.020324	0.2938	0.327509	FALSE	FALSE	FALSE	G4plxe	0.014606	0.164091	486484	sepsis	TRUE	reported	textfile	0.0244	3.26E-07	284	M35451.m	TRUE	reported	hNOVMI	2	TRUE	0.083851	25.81023
7366	rs6464160	T	G	T	G	0.1498	0.011426	0.2552	0.305163	FALSE	FALSE	FALSE	mm9ksf	0.014844	0.441453	486484	sepsis	TRUE	reported	textfile	0.0278	6.94E-08	18	M34365.m	TRUE	reported	AVwEqD	2	TRUE	0.617313	25.80961
3974	rs1417985	A	G	A	G	0.0315	-0.05626	0.9726	0.971005	FALSE	FALSE	FALSE	XdJbEU	0.041063	0.170632	486484	sepsis	TRUE	reported	textfile	0.0062	4.65E-07	7364	M32452.m	TRUE	reported	V5xWKi	2	TRUE	0.003493	25.80594
3757	rs329319	A	G	A	G	0.0127	-0.01621	0.4276	0.42825	FALSE	FALSE	FALSE	OZLdls	0.014338	0.258259	486484	sepsis	TRUE	reported	textfile	0.0025	6.50E-07	7356	M32338.m	TRUE	reported	s2itmB	2	TRUE	0.003496	25.79938
581	rs1956129	T	C	T	C	-0.0127	-0.02723	0.7589	0.772197	FALSE	FALSE	FALSE	ZoqU2j	0.016301	0.094774	486484	sepsis	TRUE	reported	textfile	0.0025	3.66E-07	7333	M01105.m	TRUE	reported	2WxSOH	2	TRUE	0.003507	25.79936
8801	rs1167464	C	G	C	G	-0.0381	-0.00027	0.1049	0.122975	FALSE	TRUE	FALSE	QWTooc	0.020798	0.989708	486484	sepsis	TRUE	reported	textfile	0.0075	3.46E-07	3248	M36756.m	TRUE	reported	OLsw6C	2	TRUE	0.007883	25.79051
6696	rs1713747	T	C	T	C	-0.0386	0.010323	0.5085	0.500405	FALSE	FALSE	FALSE	AcRp2F	0.013687	0.450688	486484	sepsis	TRUE	reported	textfile	0.0076	3.86E-07	6756	M33935.m	TRUE	reported	FQCKJy	2	TRUE	0.003804	25.78807
4366	rs1714248	T	G	T	G	0.0391	0.030542	0.0349	0.048895	FALSE	FALSE	FALSE	SPhl52	0.031604	0.333853	486484	sepsis	TRUE	reported	textfile	0.0077	4.07E-07	5211	M32651.m	TRUE	reported	V0Bnpj	2	TRUE	0.004924	25.7754
3969	rs1123954	A	T	A	T	-0.0132	-0.00812	0.2416	0.246501	FALSE	TRUE	FALSE	XdJbEU	0.015847	0.608421	486484	sepsis	TRUE	reported	textfile	0.0026	5.22E-07	7364	M32452.m	TRUE	reported	V5xWKi	2	TRUE	0.003488	25.76815
4482	rs1077177	A	G	A	G	0.0132	0.003876	0.5978	0.596348	FALSE	FALSE	FALSE	1wNJ1E	0.01396	0.781302	486484	sepsis	TRUE	reported	textfile	0.0026	2.91E-07	7363	M32709.m	TRUE	reported	zihBHg	2	TRUE	0.003488	25.76815
4444	rs9566165	T	C	T	C	0.0132	-0.04098	0.0679	0.065771	FALSE	FALSE	FALSE	WGPdpq	0.027667	0.138532	486484	sepsis	TRUE	reported	textfile	0.0026	4.47E-07	7338	M32675.m	TRUE	reported	u0eUAG	2	TRUE	0.0035	25.76812
2319	rs1333292	A	C	A	C	0.0132	-0.03473	0.2301	0.224186	FALSE	FALSE	FALSE	IZXwpR	0.016496	0.03524	486484	sepsis	TRUE	reported	textfile	0.0026	4.76E-07	6549	M16822.m	TRUE	reported	Zx9k2V	2	TRUE	0.00392	25.76728
7326	rs1089202	A	G	A	G	0.1609	-0.03582	0.4724	0.458288	FALSE	FALSE	FALSE	mm9ksf	0.013797	0.009431	486484	sepsis	TRUE	reported	textfile	0.0299	7.01E-08	18	M34365.m	TRUE	reported	AVwEqD	2	TRUE	0.616679	25.74051

9022	rs1178238	T	C	T	C	-0.0137	0.002292	0.1092	0.112483	FALSE	FALSE	FALSE	df0MCM	0.021577	0.915395	486484	sepsis	TRUE	reported	textfile	0.0027	3.09E-07	7366	M37506.m	TRUE	reported	iUfr4u	2	TRUE	0.003483	25.73924
7009	rs2444830	T	C	T	C	0.0137	0.010378	0.6646	0.663869	FALSE	FALSE	FALSE	ePdfmg	0.014425	0.471882	486484	sepsis	TRUE	reported	textfile	0.0027	4.52E-07	7347	M34244.m	TRUE	reported	l9nzfX	2	TRUE	0.003492	25.73922
4262	rs1511782	A	C	A	C	-0.0137	0.020993	0.543	0.533714	FALSE	FALSE	FALSE	ylV4bp	0.013708	0.125648	486484	sepsis	TRUE	reported	textfile	0.0027	3.74E-07	7337	M32632.m	TRUE	reported	hqHJoU	2	TRUE	0.003497	25.73921
5888	rs588136	T	C	T	C	-0.0137	0.013194	0.7976	0.786715	FALSE	FALSE	FALSE	tqW0ld	0.01672	0.430072	486484	sepsis	TRUE	reported	textfile	0.0027	2.79E-07	7315	M33389.m	TRUE	reported	Sv7EyV	2	TRUE	0.003507	25.73919
8224	rs1220202	T	C	T	C	-0.0137	-0.00247	0.7878	0.78863	FALSE	FALSE	FALSE	4YAWhA	0.016896	0.883657	486484	sepsis	TRUE	reported	textfile	0.0027	3.91E-07	7256	M35439.m	TRUE	reported	bSSC4Z	2	TRUE	0.003536	25.73913
3244	rs774211	T	C	T	C	0.0137	0.012228	0.8303	0.833525	FALSE	FALSE	FALSE	COSeSD	0.018325	0.504582	486484	sepsis	TRUE	reported	textfile	0.0027	5.19E-07	6517	M22649.m	TRUE	reported	mnH8z2	2	TRUE	0.003935	25.73833
6375	rs7231703	A	T	A	T	0.035	0.000641	0.3913	0.394624	FALSE	TRUE	FALSE	PAgZZz	0.014019	0.963539	486484	sepsis	TRUE	reported	textfile	0.0069	3.67E-07	4323	M33633.m	TRUE	reported	I8R1KJ	2	TRUE	0.005917	25.71798
4859	rs6723705	A	G	A	G	0.0411	0.012257	0.5871	0.616205	FALSE	FALSE	FALSE	6UwyhO	0.014094	0.38447	486484	sepsis	TRUE	reported	textfile	0.0081	3.46E-07	1756	M32854.m	TRUE	reported	YFrJSY	2	TRUE	0.01445	25.7169
4689	rs1786376	A	G	A	G	-0.0705	0.088455	0.0292	0.029083	FALSE	FALSE	FALSE	MV108h	0.041694	0.033877	486484	sepsis	TRUE	reported	textfile	0.0139	3.80E-07	6642	M32758.m	TRUE	reported	btjNXQ	2	TRUE	0.003858	25.71685
1416	rs741382	T	C	T	C	-0.0472	0.019488	0.2234	0.197862	FALSE	FALSE	FALSE	33ptwk	0.017241	0.258315	486484	sepsis	TRUE	reported	textfile	0.0093	3.74E-07	1175	M02137.m	TRUE	reported	SQY1na	2	TRUE	0.021452	25.71451
3534	rs2016976	T	C	T	C	-0.0142	0.020318	0.2607	0.264548	FALSE	FALSE	FALSE	rXzSlH	0.015526	0.190661	486484	sepsis	TRUE	reported	textfile	0.0028	4.10E-07	6795	M30805.m	TRUE	reported	7wg9QH	2	TRUE	0.003771	25.71182
7118	rs1105943	T	C	T	C	0.3901	0.030092	0.0133	0.012389	FALSE	FALSE	FALSE	de8tA6	0.06535	0.645176	486484	sepsis	TRUE	reported	textfile	0.0759	2.74E-07	75	M34322.m	TRUE	reported	sXPPBO	2	TRUE	0.260472	25.71165
3299	rs1015126	T	C	T	C	0.0142	0.023837	0.3406	0.343971	FALSE	FALSE	FALSE	nqmBts	0.014429	0.098528	486484	sepsis	TRUE	reported	textfile	0.0028	5.77E-07	6388	M25599.m	TRUE	reported	VkwSh1	2	TRUE	0.00401	25.71134
5494	rs6066168	A	G	A	G	-0.3223	0.00375	0.0218	0.028326	FALSE	FALSE	FALSE	q32d4E	0.041432	0.927887	486484	sepsis	TRUE	reported	textfile	0.0635	3.82E-07	1017	M33178.m	TRUE	reported	cS3BwT	2	TRUE	0.024705	25.71096
7549	rs918174	T	C	T	C	-0.0142	-0.02226	0.687	0.690958	FALSE	FALSE	FALSE	JJsHvX	0.015097	0.140402	486484	sepsis	TRUE	reported	textfile	0.0028	5.61E-07	5207	M34456.m	TRUE	reported	aklxve	2	TRUE	0.004915	25.70951
5096	rs1110732	C	G	C	G	0.036	0.041766	0.9536	0.9576	FALSE	TRUE	FALSE	Uiz8VU	0.034018	0.219544	486484	sepsis	TRUE	reported	textfile	0.0071	3.70E-07	6404	M33163.m	TRUE	reported	ky0Bda	2	TRUE	0.003998	25.70116
6642	rs1093836	A	C	A	C	0.0147	-0.0181	0.4014	0.388517	FALSE	FALSE	FALSE	REeQiq	0.014074	0.198474	486484	sepsis	TRUE	reported	textfile	0.0029	4.12E-07	6004	M33892.m	TRUE	reported	cVOa3u	2	TRUE	0.004261	25.68585
2427	rs4849717	A	T	A	T	0.1359	0.022333	0.9643	0.954126	FALSE	TRUE	FALSE	NBJ2Do	0.032851	0.49663	486484	sepsis	TRUE	reported	textfile	0.0268	4.04E-07	1739	M17799.m	TRUE	reported	Uz0Wgl	2	TRUE	0.014571	25.68441
5595	rs9401158	A	G	A	G	-0.2648	0.006443	0.0266	0.08823	FALSE	FALSE	FALSE	q32d4E	0.024279	0.790737	486484	sepsis	TRUE	reported	textfile	0.0522	3.91E-07	1017	M33178.m	TRUE	reported	cS3BwT	2	TRUE	0.024679	25.68266
2382	rs1498721	T	C	T	C	0.1222	0.020832	0.953	0.933942	FALSE	FALSE	FALSE	NBJ2Do	0.027595	0.450303	486484	sepsis	TRUE	reported	textfile	0.0241	3.90E-07	1739	M17799.m	TRUE	reported	Uz0Wgl	2	TRUE	0.014569	25.6808
7189	rs893712	T	C	T	C	-0.0152	0.010292	0.7669	0.758968	FALSE	FALSE	FALSE	2hlepp	0.015992	0.519858	486484	sepsis	TRUE	reported	textfile	0.003	2.99E-07	5081	M34336.m	TRUE	reported	XyK8mO	2	TRUE	0.005027	25.66101
2380	rs1429342	A	G	A	G	0.1439	-0.04555	0.9623	0.987384	FALSE	FALSE	FALSE	NBJ2Do	0.062299	0.464676	486484	sepsis	TRUE	reported	textfile	0.0284	4.15E-07	1739	M17799.m	TRUE	reported	Uz0Wgl	2	TRUE	0.014549	25.64396
3758	rs4761007	T	C	T	C	0.0157	-0.00926	0.7354	0.727683	FALSE	FALSE	FALSE	OZLdls	0.015361	0.546499	486484	sepsis	TRUE	reported	textfile	0.0031	4.13E-07	7356	M32338.m	TRUE	reported	s2itmB	2	TRUE	0.003475	25.64235
9067	rs1339080	A	G	A	G	0.0395	0.022086	0.0557	0.06942	FALSE	FALSE	FALSE	0l8lKx	0.02697	0.412839	486484	sepsis	TRUE	reported	textfile	0.0078	4.12E-07	7219	M38178.m	TRUE	reported	bWs4mL	2	TRUE	0.00354	25.63803
952	rs1708260	T	C	T	C	0.0476	0.0199	0.0193	0.017644	FALSE	FALSE	FALSE	tWoDq4	0.05212	0.7026	486484	sepsis	TRUE	reported	textfile	0.0094	4.08E-07	7160	M01508.m	TRUE	reported	6ltDw0	2	TRUE	0.003569	25.63521
6324	rs7239639	T	C	T	C	-0.254	0.009628	0.938	0.723124	FALSE	FALSE	FALSE	5B5LTX	0.015261	0.528123	486484	sepsis	TRUE	reported	textfile	0.05	3.77E-07	296	M33609.m	TRUE	reported	m0eM6N	2	TRUE	0.080192	25.63203
8252	rs1293588	A	G	A	G	0.381	-0.01499	0.0392	0.039667	FALSE	FALSE	FALSE	G4plxe	0.034948	0.66793	486484	sepsis	TRUE	reported	textfile	0.075	3.72E-07	284	M35451.m	TRUE	reported	hNOVMl	2	TRUE	0.083298	25.62466
7218	rs207703	T	C	T	C	0.2845	-0.06772	0.9769	0.958898	FALSE	FALSE	FALSE	i1uJ7T	0.034666	0.050764	486484	sepsis	TRUE	reported	textfile	0.0561	3.94E-07	535	M34338.m	TRUE	reported	pi3mKW	2	TRUE	0.045866	25.62195
8256	rs2663644	A	G	A	G	0.5247	0.013413	0.0123	0.012985	FALSE	FALSE	FALSE	G4plxe	0.060721	0.825179	486484	sepsis	TRUE	reported	textfile	0.1033	3.81E-07	284	M35451.m	TRUE	reported	hNOVMl	2	TRUE	0.08328	25.61841
5846	rs1052118	T	C	T	C	-0.3993	-0.03274	0.9682	0.964675	FALSE	FALSE	FALSE	Vkec6Y	0.037062	0.377047	486484	sepsis	TRUE	reported	textfile	0.0786	3.71E-07	272	M33384.m	TRUE	reported	eaxeyR	2	TRUE	0.08666	25.61819
4857	rs1042830	T	C	T	C	0.0324	-0.02169	0.6101	0.620065	FALSE	FALSE	FALSE	6UwyhO	0.014149	0.125265	486484	sepsis	TRUE	reported	textfile	0.0064	4.64E-07	2684	M32854.m	TRUE	reported	YFrJSY	2	TRUE	0.009458	25.60981
6333	rs1572704	A	G	A	G	0.3904	0.003978	0.0272	0.028939	FALSE	FALSE	FALSE	qLUY00	0.040828	0.922383	486484	sepsis	TRUE	reported	textfile	0.0764	3.29E-07	102	M33610.m	TRUE	reported	8cAJbB	2	TRUE	0.203819	25.59957
1676	rs4632709	T	C	T	C	-0.1504	-0.00388	0.95	0.943017	FALSE	FALSE	FALSE	IIFBGY	0.029511	0.895288	486484	sepsis	TRUE	reported	textfile	0.0297	4.00E-07	963	M12261.m	TRUE	reported	F5Vlan	2	TRUE	0.025938	25.59056
1780	rs1085731	T	C	T	C	-0.0086	-0.00468	0.3481	0.351072	FALSE	FALSE	FALSE	JEcCRZ	0.01433	0.743745	486484	sepsis	TRUE	reported	textfile	0.0017	9.45E-07	7368	M15140.m	TRUE	reported	xYCzKP	2	TRUE	0.003461	25.58475
1784	rs1164684	A	G	A	G	-0.0086	0.002255	0.5005	0.501563	FALSE	FALSE	FALSE	JEcCRZ	0.013676	0.869027	486484	sepsis	TRUE	reported	textfile	0.0017	6.40E-07	7368	M15140.m	TRUE	reported	xYCzKP	2	TRUE	0.003461	25.58475
9047	rs6043498	T	G	T	G	-0.0172	-0.02187	0.0688	0.078511	FALSE	FALSE	FALSE	df0MCM	0.025346	0.388197	486484	sepsis	TRUE	reported	textfile	0.0034	4.81E-07	7366	M37506.m	TRUE	reported	iUfr4u	2	TRUE	0.003462	25.58475
7883	rs1004436	T	C	T	C	0.0172	0.001502	0.763	0.761149	FALSE	FALSE	FALSE	pU8zps	0.016045	0.925433	486484	sepsis	TRUE	reported	textfile	0.0034	3.26E-07	7350	M35186.m	TRUE	reported	Oc2lOF	2	TRUE	0.00347	25.58473
3712	rs7022371	T	C	T	C	0.0086	-0.00624	0.5008	0.497788	FALSE	FALSE	FALSE	agPgtF	0.013671	0.648276	486484	sepsis	TRUE	reported	textfile	0.0017	6.54E-07	7349	M32315.m	TRUE	reported	hMRjcV	2	TRUE	0.00347	25.58473
8172	rs2090103	A	G	A	G	-0.0086	-0.02416	0.2395	0.224078	FALSE	FALSE	FALSE	8UhtPK	0.016361	0.139689	486484	sepsis	TRUE	reported	textfile	0.0017	3.37E-07	7345	M35397.m	TRUE	reported	rvVJba	2	TRUE	0.003472	25.58473
4967	rs549114	A	G	A	G	0.0086	-0.01202	0.3334	0.333023	FALSE	FALSE	FALSE	bKmDMa	0.014529	0.408106	486484	sepsis	TRUE	reported	textfile	0.0017	7.57E-07	7344	M32910.m	TRUE	reported	nAuPzO	2	TRUE	0.003473	25.58473
1369	rs1270126	A	G	A	G	0.0086	0.01538	0.3919	0.394675	FALSE	FALSE	FALSE	KrWhws	0.013974	0.271032	486484	sepsis	TRUE	reported	textfile	0.0017	7.37E-07	7325	M02132.m	TRUE	reported	7pzpfK	2	TRUE	0.003482	25.58471
1400	rs879126	T	G	T	G	-0.0086	0.000375	0.3286	0.334996	FALSE	FALSE	FALSE	KrWhws	0.014508	0.979381	486484	sepsis	TRUE	reported	textfile	0.0017	7.47E-07	7325	M02132.m	TRUE	reported	7pzpfK	2	TRUE	0.003482	25.58471
4716	rs2954021	A	G	A	G	0.0172	0.018732	0.5064	0.495425	FALSE	FALSE	FALSE	L2WFM1	0.013666	0.170467	486484	sepsis	TRUE	reported	textfile	0.0034	3.45E-07	7313	M32761.m	TRUE	reported	5GHzph	2	TRUE	0.003487	25.5847
1109	rs1554074	A	G	A	G	0.0086	0.038741	0.1925	0.191246	FALSE	FALSE	FALSE	yahiGZ	0.017358	0.025621	486484	sepsis	TRUE	reported	textfile	0.0017	6.39E-07	7271	M01585.m	TRUE	reported	dwZ7vX	2	TRUE	0.003507	25.58466
1308	rs4717222	A	C	A	C	-0.0086	-0.01237	0.4686	0.463741	FALSE	FALSE	FALSE	WQdpmo	0.013684	0.365996	486484	sepsis	TRUE	reported	textfile	0.0017	4.34E-07	7136	M01769.m	TRUE	reported	BkptOK	2	TRUE	0.003573	25.58452
8258	rs2850065	T	C	T	C	0.5411	0.019753	0.0146	0.462603	FALSE	FALSE	FALSE	G4plxe	0.013693	0.149153	486484	sepsis	TRUE	reported	textfile	0.1066	3.87E-07	284	M35451.m	TRUE	reported	hNOVMl	2	TRUE	0.083178	25.58418
679	rs1043011	T	G	T	G	0.0172	-0.01111	0.1722	0.182598	FALSE	FALSE	FALSE	aT1Fw1	0.017646	0.528965	486484	sepsis	TRUE	reported	textfile	0.0034	5.21E-07	5687	M01284.m	TRUE	reported	mbTe8H	2	TRUE	0.00448	25.5827
5691	rs1866724	T	C	T	C	-0.0688	-0.01329	0.7129	0.70999	FALSE	FALSE	FALSE	KxtFnC	0.0151	0.37869	486484	sepsis	TRUE	reported	textfile	0.0136	4.25E-07	3221	M33203.m	TRUE	reported	Uz0wKU	2	TRUE	0.007883	25.5758
3361	rs6012418	A	G	A	G	-0.0177	0.044743	0.0853	0.08379	FALSE	FALSE	FALSE	LY4Gus	0.024756	0.070707	486484	sepsis	TRUE	reported	textfile	0.0035	3.82E-07	7268	M27273.m	TRUE	reported	QKlTBx	2	TRUE	0.003506	25.56766
2351	rs1042523	T	C	T	C	-0.1209	0.02666	0.0447	0.048369	FALSE	FALSE	FALSE	NBJ2Do	0.031813	0.402025	486484	sepsis	TRUE	reported	textfile	0.0239	4.21E-07	1739	M17799.m	TRUE	reported	Uz0Wgl	2	TRUE	0.014502	25.55978
7415	rs202534	A	G	A	G	-0.042	-0.00917	0.5576	0.495962	FALSE	FALSE	FALSE	7i5VUS	0.013675	0.502281	486484	sepsis	TRUE	reported	textfile	0.0083	3.65E-07	1104	M34395.m	TRUE	reported	64hEaL	2	TRUE	0.022668	25.55965
1788	rs1496635	A	C	A	C	-0.0091	-0.00921	0.3407	0.328944	FALSE	FALSE	FALSE	JEcCRZ	0.014523	0.525811	486484	sepsis	TRUE	reported	textfile	0.0018	2.12E-07	7368	M15140.m	TRUE	reported	xYCzKP	2	TRUE	0.003457	25.5517
1060	rs7105815	A	G	A	G	0.0091	0.009275	0.2968	0.29715	FALSE	FALSE	FALSE	tRtQ2e	0.014945	0.53487	486484	sepsis	TRUE	reported	textfile	0.0018	4.93E-07	7364	M01564.m	TRUE	reported	Tbg0zM	2	TRUE	0.003459	25.5517
4009	rs1683282	T	C	T	C	-0.0091	0.009757	0.6632	0.655572	FALSE	FALSE	FALSE	V9Atld	0.01452	0.501579	486484	sepsis	TRUE	reported	textfile	0.0018	5.52E-07	7363	M32489.m	TRUE	reported	8B8S8a	2	TRUE	0.003459	25.5517
4315	rs981735	T	C	T	C	0.0091	0.003242	0.759	0.758285	FALSE	FALSE	FALSE	0tjjPC	0.015959	0.839027	486484	sepsis	TRUE	reported	textfile	0.0018	6.08E-07	7362	M32634.m	TRUE	reported	nJJFkX	2	TRUE	0.00346	25.5517
4047	rs3795579	T	G	T	G	0.0091	-0.02368	0.3479	0.344558	FALSE	FALSE	FALSE	SiYGYU	0.01438	0.0996	486484	sepsis	TRUE	reported	textfile	0.0018	3.69E-07	7355	M32492.m	TRUE	reported	GfQvpU	2	TRUE	0.003463	25.55169
4414	rs1223387	A	G	A	G	-0.0273	-0.01804	0.1383	0.149093	FALSE	FALSE	FALSE	NzBiVA	0.019243	0.348589	486484	sepsis	TRUE	reported	textfile	0.0054	4.46E-07	7354	M32672.m	TRUE	reported	YLlhHJ	2	TRUE	0.003463	25.55169
666	rs1618996	A	G	A	G	0.0091	-0.01527	0.1298	0.133278	FALSE	FALSE	FALSE	p4oCVP	0.020268	0.451341	486484	sepsis	TRUE	reported	textfile	0.0018	3.78E-07	7352	M01125.m	TRUE	reported	OwD5re	2	TRUE	0.003464	25.55169
3277	rs1312792	A	G	A	G	-0.0091	0.007773	0.5059	0.501377	FALSE	FALSE	FALSE	vYWPe9	0.0138	0.573267	486484	sepsis	TRUE	reported	textfile	0.0018	4.90E-07	7340	M25459.m	TRUE	reported	VvJ6Vx	2	TRUE	0.00347	25.55168
3285	rs4697525	T	C	T	C	-0.0091	0.004673	0.546	0.543121	FALSE	FALSE	FALSE	vYWPe9	0.013741	0.733831	486484	sepsis	TRUE	reported	textfile	0.0018	5.60E-07	7340	M25459.m	TRUE	reported	VvJ6Vx	2	TRUE	0.00347	25.55168
7050	rs1338571	A	G	A	G	0.0091	0.003087	0.5069	0.509821	FALSE	FALSE	FALSE	uthruC	0.01368	0.821485	486484	sepsis	TRUE	reported	textfile	0.0018	3.07E-07	7316	M34283.m	TRUE	reported	ftTuRU	2	TRUE	0.003481	25.55165
5979	rs119194	A	C	A	C	-0.0091	0.023225	0.2105	0.216209	FALSE	FALSE	FALSE	3KsoXq	0.016623	0.162366	486484	sepsis	TRUE	reported	textfile	0.0018	4.33E-07	7306	M33422.m	TRUE	reported	aNr6Ug	2	TRUE	0.003486	25.55165
5990	rs2298381	C	G	C	G	-0.0091	0.010658	0.2267	0.209519	FALSE	TRUE	FALSE	3KsoXq	0.016771	0.525094	486484	sepsis	TRUE	reported	textfile	0.0018	5.23E-07	7306	M33422.m	TRUE	reported	aNr6Ug	2	TRUE	0.003486	25.55165
3334	rs2531899	A	G	A	G	-0.0091	0.011493	0.1941	0.196227	FALSE	FALSE	FALSE	m66Eav	0.017152	0.502817	486484	sepsis	TRUE	reported	textfile	0.0018	4.86E-07	7303	M27256.m	TRUE	reported	pl2ECU	2	TRUE	0.003488	25.55164
8025	rs1197907	A	G	A	G	-0.0091	-0.01568	0.7762	0.76344	FALSE	FALSE	FALSE	SwiqSi	0.016086	0.329578	486484	sepsis	TRUE	reported	textfile	0.0018	5.12E-07	7217	M35270.m	TRUE	reported	21ZlsQ	2	TRUE	0.003529	25.55156
8044	rs484080	T	C	T	C	-0.0182	-0.02048	0.0506	0.083159	FALSE	FALSE	FALSE	SwiqSi	0.024688	0.406803	486484	sepsis	TRUE	reported	textfile	0.0036	5.87E-07	7217	M35270.m	TRUE	reported	21ZlsQ	2	TRUE	0.003529	25.55156
8053	rs852030	A	G	A	G	0.0091	0.008358	0.1953	0.199167	FALSE	FALSE	FALSE	SwiqSi	0.017212	0.627233	486484	sepsis	TRUE	reported	textfile	0.0018	6.32E-07	7217	M35270.m	TRUE	reported	21ZlsQ	2	TRUE	0.003529	25.55156
1627	rs1282625	T	C	T	C	0.0091	-0.04652	0.1364	0.144722	FALSE	FALSE	FALSE	n5rDuT	0.019528	0.017202	486484	sepsis	TRUE	reported	textfile	0.0018	7.05E-07	7081	M12067.m	TRUE	reported	ZQLTeR	2	TRUE	0.003596	25.55142
1634	rs1714533	T	C	T	C	-0.0091	-0.00244	0.8525	0.851319	FALSE	FALSE	FALSE	n5rDuT	0.019232	0.899082	486484	sepsis	TRUE	reported	textfile	0.0018	3.58E-07	7081	M12067.m	TRUE	reported	ZQLTeR	2	TRUE	0.003596	25.55142
1636	rs1864609	A	C	A	C	-0.0091	0.009445	0.8606	0.860305	FALSE	FALSE	FALSE	n5rDuT	0.019761	0.632694	486484	sepsis	TRUE	reported	textfile	0.0018	3.27E-07	7081	M12067.m	TRUE	reported	ZQLTeR	2	TRUE	0.003596	25.55142
6267	rs2280821	T	G	T	G	0.0091	0.01029	0.508	0.527502	FALSE	FALSE	FALSE	MOonP9	0.013689	0.452205	486484	sepsis	TRUE	reported	textfile	0.0018	6.54E-07	7066	M33515.m	TRUE	reported	226GuP	2	TRUE	0.003604	25.55141
1489	rs8013373	A	G	A	G	-0.0091	0.00583	0.3428	0.358602	FALSE	FALSE	FALSE	pkb876	0.014275	0.682999	486484	sepsis	TRUE	reported	textfile	0.0018	4.99E-07	7037	M02734.m	TRUE	reported	0LCMVl	2	TRUE	0.003619	25.55138
8856	rs6538186	T	C	T	C	0.0091	0.011321	0.2552	0.310201	FALSE	FALSE	FALSE	XlHeTV	0.014799	0.444291	486484	sepsis	TRUE	reported	textfile	0.0018	1.95E-07	6940	M36808.m	TRUE	reported	37Vi2D	2	TRUE	0.003669	25.55128
7289	rs1757545	A	C	A	C	-0.0091	0.002205	0.3094	0.305519	FALSE	FALSE	FALSE	Vcz4EA	0.01493	0.882595	486484	sepsis	TRUE	reported	textfile	0.0018	3.57E-07	6755	M34359.m	TRUE	reported	aUSo2J	2	TRUE	0.003769	25.55107
5771	rs1205044	A	T	A	T	-0.044	0.001944	0.656	0.637036	FALSE	TRUE	FALSE	eT2We0	0.014231	0.89137	486484	sepsis	TRUE	reported	textfile	0.0087	4.10E-07	1273	M33353.m	TRUE	reported	uZx90m	2	TRUE	0.019697	25.53783
3466	rs1145077	T	G	T	G	0.0187	0.002798	0.3769	0.373291	FALSE	FALSE	FALSE	BQlbxe	0.01413	0.843026	486484	sepsis	TRUE	reported	textfile	0.0037	3.99E-07	7373	M27718.m	TRUE	reported	MnHHJ0	2	TRUE	0.003452	25.53653
2228	rs9394526	A	G	A	G	-0.0187	-0.02649	0.0529	0.057822	FALSE	FALSE	FALSE	Bf6uMK	0.029362	0.366997	486484	sepsis	TRUE	reported	textfile	0.0037	4.28E-07	7308	M15778.m	TRUE	reported	jowM2N	2	TRUE	0.003483	25.53647
4774	rs7520193	A	C	A	C	0.0283	-0.00641	0.1862	0.187765	FALSE	FALSE	FALSE	mOrnK4	0.017558	0.71509	486484	sepsis	TRUE	reported	textfile	0.0056	4.55E-07	6169	M32795.m	TRUE	reported	7EGxuf	2	TRUE	0.004123	25.5303
2500	rs1253667	C	G	C	G	-0.0096	-0.00732	0.3198	0.315654	FALSE	TRUE	FALSE	LwUYua	0.014713	0.618888	486484	sepsis	TRUE	reported	textfile	0.0019	2.04E-07	7315	M17945.m	TRUE	reported	SUN8dc	2	TRUE	0.003478	25.52211
2522	rs9302365	A	C	A	C	-0.0096	0.030451	0.4684	0.474627	FALSE	FALSE	FALSE	LwUYua	0.013717	0.026425	486484	sepsis	TRUE	reported	textfile	0.0019	2.18E-07	7315	M17945.m	TRUE	reported	SUN8dc	2	TRUE	0.003478	25.52211
8454	rs1696215	T	C	T	C	-0.0192	-0.01654	0.9219	0.916287	FALSE	FALSE	FALSE	uMajV6	0.024695	0.50291	486484	sepsis	TRUE	reported	textfile	0.0038	4.57E-07	6604	M35754.m	TRUE	reported	b8QB7S	2	TRUE	0.003851	25.52135
3152	rs4434479	A	G	A	G	0.0096	0.00496	0.2901	0.287868	FALSE	FALSE	FALSE	JiXR2t	0.015191	0.744053	486484	sepsis	TRUE	reported	textfile	0.0019	8.84E-07	6535	M22177.m	TRUE	reported	joi6p6	2	TRUE	0.003891	25.52127
4529	rs2243498	A	G	A	G	0.0096	0.017266	0.4479	0.444079	FALSE	FALSE	FALSE	r0qJaM	0.013846	0.21239	486484	sepsis	TRUE	reported	textfile	0.0019	2.95E-07	6497	M32729.m	TRUE	reported	vYv5Fl	2	TRUE	0.003914	25.52123
6012	rs1158943	T	C	T	C	-1.6514	0.001913	0.025	0.037741	FALSE	FALSE	FALSE	33ojbt	0.035775	0.957366	486484	sepsis	TRUE	reported	textfile	0.3214	2.77E-07	60	M33423.m	TRUE	reported	ivEtvl	2	TRUE	0.30556	25.52052
7845	rs6026567	A	G	A	G	-0.0096	-0.0036	0.5792	0.575419	FALSE	FALSE	FALSE	h5whlS	0.01395	0.796451	486484	sepsis	TRUE	reported	textfile	0.0019	5.87E-07	4900	M35137.m	TRUE	reported	xVITKF	2	TRUE	0.005183	25.51867
2788	rs1293699	A	G	A	G	-0.0192	-0.00821	0.7358	0.748892	FALSE	FALSE	FALSE	ex7hzq	0.015742	0.60189	486484	sepsis	TRUE	reported	textfile	0.0038	5.86E-07	4460	M19415.m	TRUE	reported	jvXWvV	2	TRUE	0.005691	25.51764
4770	rs1766086	A	G	A	G	0.175	0.032546	0.93	0.967172	FALSE	FALSE	FALSE	mOrnK4	0.038491	0.397802	486484	sepsis	TRUE	reported	textfile	0.0346	4.37E-07	798	M32795.m	TRUE	reported	7EGxuf	2	TRUE	0.031061	25.51726
6464	rs6823047	A	C	A	C	-0.0982	-0.01621	0.7299	0.710925	FALSE	FALSE	FALSE	yLohrN	0.015144	0.284385	486484	sepsis	TRUE	reported	textfile	0.0194	3.91E-07	477	M33683.m	TRUE	reported	mvhAZ4	2	TRUE	0.050977	25.51495
6322	rs548027	A	G	A	G	0.2591	-0.03439	0.0208	0.015455	FALSE	FALSE	FALSE	5B5LTX	0.055534	0.535776	486484	sepsis	TRUE	reported	textfile	0.0512	4.17E-07	528	M33609.m	TRUE	reported	m0eM6N	2	TRUE	0.046259	25.51213
8465	rs4852787	A	C	A	C	-0.0101	0.001514	0.4642	0.470365	FALSE	FALSE	FALSE	uMajV6	0.013696	0.911963	486484	sepsis	TRUE	reported	textfile	0.002	2.86E-07	6604	M35754.m	TRUE	reported	b8QB7S	2	TRUE	0.003847	25.49478
6452	rs2957661	T	C	T	C	0.0308	-0.00452	0.3698	0.370917	FALSE	FALSE	FALSE	OFlEwq	0.014175	0.749943	486484	sepsis	TRUE	reported	textfile	0.0061	4.34E-07	5694	M33675.m	TRUE	reported	T2BzXG	2	TRUE	0.004457	25.48527
4393	rs2269340	T	C	T	C	-0.0222	0.020913	0.9246	0.920577	FALSE	FALSE	FALSE	UJygVd	0.025275	0.407984	486484	sepsis	TRUE	reported	textfile	0.0044	3.58E-07	7361	M32654.m	TRUE	reported	kv9R0W	2	TRUE	0.003446	25.44969
1451	rs7068278	T	G	T	G	0.0116	-0.01605	0.1968	0.199327	FALSE	FALSE	FALSE	1M4gqj	0.017123	0.348709	486484	sepsis	TRUE	reported	textfile	0.0023	6.55E-07	7226	M02730.m	TRUE	reported	RWyYEM	2	TRUE	0.003508	25.42963
5111	rs1012601	C	G	C	G	0.056	0.003722	0.8372	0.848971	FALSE	TRUE	FALSE	O6ine1	0.019165	0.845999	486484	sepsis	TRUE	reported	textfile	0.0111	4.87E-07	1751	M33165.m	TRUE	reported	gGsqjW	2	TRUE	0.014328	25.42341
6631	rs335952	T	C	T	C	-0.0494	0.029293	0.1902	0.191294	FALSE	FALSE	FALSE	IDG01N	0.017375	0.091802	486484	sepsis	TRUE	reported	textfile	0.0098	4.05E-07	4999	M33885.m	TRUE	reported	q3gy7x	2	TRUE	0.005057	25.39966
2409	rs2374150	C	G	C	G	-0.1422	-0.01614	0.0418	0.059922	FALSE	TRUE	FALSE	NBJ2Do	0.029282	0.581534	486484	sepsis	TRUE	reported	textfile	0.0282	4.55E-07	1739	M17799.m	TRUE	reported	Uz0Wgl	2	TRUE	0.014411	25.3981
8271	rs398172	A	G	A	G	-0.0126	0.018042	0.3361	0.339449	FALSE	FALSE	FALSE	TdsGCZ	0.014422	0.210933	486484	sepsis	TRUE	reported	textfile	0.0025	4.04E-07	6883	M35464.m	TRUE	reported	sOspot	2	TRUE	0.003677	25.39422
6587	rs2826321	A	G	A	G	-0.5286	-0.03007	0.9847	0.983491	FALSE	FALSE	FALSE	XO3rXq	0.053755	0.575856	486484	sepsis	TRUE	reported	textfile	0.1043	4.00E-07	176	M33877.m	TRUE	reported	p2sETg	2	TRUE	0.127354	25.39348
972	rs1047116	T	C	T	C	0.9362	0.046308	0.0105	0.0815	FALSE	FALSE	FALSE	xSRll4	0.025069	0.064713	486484	sepsis	TRUE	reported	textfile	0.1854	4.43E-07	482	M01515.m	TRUE	reported	juZ5GE	2	TRUE	0.050244	25.39288
2349	rs1015859	T	C	T	C	0.0847	0.012336	0.8882	0.857783	FALSE	FALSE	FALSE	NBJ2Do	0.019564	0.528316	486484	sepsis	TRUE	reported	textfile	0.0168	4.41E-07	1739	M17799.m	TRUE	reported	Uz0Wgl	2	TRUE	0.014406	25.38917
3368	rs1189070	T	C	T	C	-0.0257	-0.01391	0.9322	0.929784	FALSE	FALSE	FALSE	O2JtzH	0.026929	0.605398	486484	sepsis	TRUE	reported	textfile	0.0051	4.83E-07	7347	M27278.m	TRUE	reported	fxClPW	2	TRUE	0.003444	25.38678
8589	rs478259	A	G	A	G	-0.0393	-0.00762	0.858	0.859624	FALSE	FALSE	FALSE	iuJmRL	0.019687	0.698565	486484	sepsis	TRUE	reported	textfile	0.0078	5.27E-07	7311	M36103.m	TRUE	reported	jfJbVw	2	TRUE	0.00346	25.37915
2389	rs1688312	A	G	A	G	-0.1003	-0.00244	0.0886	0.118022	FALSE	FALSE	FALSE	NBJ2Do	0.021159	0.90804	486484	sepsis	TRUE	reported	textfile	0.0199	4.45E-07	1739	M17799.m	TRUE	reported	Uz0Wgl	2	TRUE	0.014398	25.37441
5165	rs7863822	T	C	T	C	-1.0516	0.010539	0.0132	0.025949	FALSE	FALSE	FALSE	L2DJzK	0.043234	0.807421	486484	sepsis	TRUE	reported	textfile	0.206	3.32E-07	76	M33173.m	TRUE	reported	hRG70D	2	TRUE	0.255337	25.37376
7791	rs1095462	T	C	T	C	-0.0252	0.003365	0.3553	0.380898	FALSE	FALSE	FALSE	cQ5TvO	0.014086	0.811205	486484	sepsis	TRUE	reported	textfile	0.005	4.13E-07	1762	M35127.m	TRUE	reported	BVxkfZ	2	TRUE	0.014211	25.37277
3994	rs9399110	T	C	T	C	0.0267	0.017819	0.0497	0.049602	FALSE	FALSE	FALSE	XdJbEU	0.031477	0.571331	486484	sepsis	TRUE	reported	textfile	0.0053	4.29E-07	7364	M32452.m	TRUE	reported	V5xWKi	2	TRUE	0.003434	25.37189
434	rs6511720	T	G	T	G	-0.0136	-0.04528	0.1197	0.118817	FALSE	FALSE	FALSE	cTsNj6	0.021124	0.032067	486484	sepsis	TRUE	reported	textfile	0.0027	3.69E-07	7365	M00063.m	TRUE	reported	7KYTrQ	2	TRUE	0.003433	25.36485
915	rs1170186	A	C	A	C	-0.0136	-0.00552	0.1761	0.192476	FALSE	FALSE	FALSE	rVMAPf	0.017354	0.750411	486484	sepsis	TRUE	reported	textfile	0.0027	2.75E-07	7307	M01493.m	TRUE	reported	siVqDi	2	TRUE	0.00346	25.3648
2968	rs1544542	A	C	A	C	0.0136	-0.01405	0.4642	0.466273	FALSE	FALSE	FALSE	dOOXpo	0.013728	0.306227	486484	sepsis	TRUE	reported	textfile	0.0027	5.40E-07	6999	M21127.m	TRUE	reported	IdiP4j	2	TRUE	0.003612	25.36449
2306	rs1939990	T	C	T	C	-0.0136	-0.00792	0.2409	0.242652	FALSE	FALSE	FALSE	ZzUW8U	0.01595	0.619696	486484	sepsis	TRUE	reported	textfile	0.0027	6.91E-07	6733	M16821.m	TRUE	reported	g2xs3i	2	TRUE	0.003754	25.36421
5925	rs1336286	T	C	T	C	-0.1665	0.000344	0.9493	0.944971	FALSE	FALSE	FALSE	veBhfS	0.030143	0.990905	486484	sepsis	TRUE	reported	textfile	0.033	4.54E-07	528	M33391.m	TRUE	reported	Gfjy5a	2	TRUE	0.045996	25.36019
8526	rs2243168	A	T	A	T	0.0524	0.009073	0.9071	0.915943	FALSE	TRUE	FALSE	gM7v2m	0.024668	0.713019	486484	sepsis	TRUE	reported	textfile	0.0104	4.88E-07	1743	M36009.m	TRUE	reported	6Xcypd	2	TRUE	0.014356	25.35697
3528	rs1252264	T	C	T	C	-0.0141	-0.01502	0.7914	0.723036	FALSE	FALSE	FALSE	rXzSlH	0.015334	0.327456	486484	sepsis	TRUE	reported	textfile	0.0028	7.20E-07	6795	M30805.m	TRUE	reported	7wg9QH	2	TRUE	0.003718	25.35095
3537	rs559135	T	C	T	C	-0.0141	0.001261	0.2066	0.200674	FALSE	FALSE	FALSE	rXzSlH	0.017135	0.941348	486484	sepsis	TRUE	reported	textfile	0.0028	6.72E-07	6795	M30805.m	TRUE	reported	7wg9QH	2	TRUE	0.003718	25.35095
3314	rs6681770	A	G	A	G	-0.0141	0.002629	0.762	0.755445	FALSE	FALSE	FALSE	nqmBts	0.015943	0.869013	486484	sepsis	TRUE	reported	textfile	0.0028	3.91E-07	6388	M25599.m	TRUE	reported	VkwSh1	2	TRUE	0.003954	25.35048
7542	rs6049700	T	G	T	G	-0.0141	0.008052	0.2893	0.291826	FALSE	FALSE	FALSE	JJsHvX	0.015117	0.594291	486484	sepsis	TRUE	reported	textfile	0.0028	4.95E-07	5207	M34456.m	TRUE	reported	aklxve	2	TRUE	0.004846	25.34868
4971	rs7748892	A	G	A	G	0.0287	-0.01517	0.9721	0.965703	FALSE	FALSE	FALSE	bKmDMa	0.038169	0.691116	486484	sepsis	TRUE	reported	textfile	0.0057	4.30E-07	7344	M32910.m	TRUE	reported	nAuPzO	2	TRUE	0.00344	25.3452
7452	rs1687416	A	G	A	G	0.137	-0.07159	0.9858	0.976643	FALSE	FALSE	FALSE	7ubZow	0.045393	0.114755	486484	sepsis	TRUE	reported	textfile	0.0272	4.49E-07	1754	M34407.m	TRUE	reported	LRsXN2	2	TRUE	0.014257	25.34007
4182	rs1018352	C	G	C	G	0.0896	0.007459	0.9442	0.927894	FALSE	TRUE	FALSE	TxOCzG	0.026437	0.77784	486484	sepsis	TRUE	reported	textfile	0.0178	5.09E-07	6970	M32564.m	TRUE	reported	9JP6ND	2	TRUE	0.003622	25.33094
3670	rs1003746	T	G	T	G	0.0302	-0.00826	0.968	0.968863	FALSE	FALSE	FALSE	rGHJcj	0.039449	0.834235	486484	sepsis	TRUE	reported	textfile	0.006	5.97E-07	7356	M32198.m	TRUE	reported	6P1Nno	2	TRUE	0.003432	25.32756
7338	rs1249809	A	G	A	G	-0.1601	-0.01543	0.5278	0.576092	FALSE	FALSE	FALSE	mm9ksf	0.013829	0.264587	486484	sepsis	TRUE	reported	textfile	0.03	9.17E-08	18	M34365.m	TRUE	reported	AVwEqD	2	TRUE	0.612737	25.31557
8870	rs4148987	A	G	A	G	-0.0317	-0.0039	0.3217	0.322065	FALSE	FALSE	FALSE	nWuK1Q	0.01461	0.789536	486484	sepsis	TRUE	reported	textfile	0.0063	4.09E-07	6511	M36850.m	TRUE	reported	2BA0SE	2	TRUE	0.003874	25.31069
1279	rs1288349	T	C	T	C	0.0166	0.00029	0.6656	0.658126	FALSE	FALSE	FALSE	nvWJJ3	0.014458	0.984015	486484	sepsis	TRUE	reported	textfile	0.0033	3.76E-07	7346	M01712.m	TRUE	reported	1x44lf	2	TRUE	0.003433	25.29706
6213	rs1450233	A	T	A	T	-0.0584	0.012811	0.3174	0.318865	FALSE	TRUE	FALSE	FQfT2o	0.014655	0.382033	486484	sepsis	TRUE	reported	textfile	0.0116	4.32E-07	972	M33508.m	TRUE	reported	JtbOy2	2	TRUE	0.025413	25.29386
6316	rs1743324	A	C	A	C	0.328	-0.04852	0.023	0.063803	FALSE	FALSE	FALSE	5B5LTX	0.028305	0.086505	486484	sepsis	TRUE	reported	textfile	0.065	4.51E-07	296	M33609.m	TRUE	reported	m0eM6N	2	TRUE	0.079212	25.29162
2390	rs1694792	A	G	A	G	-0.1419	0.065115	0.0406	0.030897	FALSE	FALSE	FALSE	NBJ2Do	0.039551	0.099692	486484	sepsis	TRUE	reported	textfile	0.0282	4.87E-07	1739	M17799.m	TRUE	reported	Uz0Wgl	2	TRUE	0.014351	25.29105
976	rs1125602	A	G	A	G	-0.2061	0.018089	0.6357	0.632229	FALSE	FALSE	FALSE	xSRll4	0.014193	0.202491	486484	sepsis	TRUE	reported	textfile	0.0409	4.60E-07	482	M01515.m	TRUE	reported	juZ5GE	2	TRUE	0.050046	25.28736
8111	rs2061282	A	G	A	G	-0.0171	0.002589	0.0735	0.079327	FALSE	FALSE	FALSE	nNbXBS	0.025419	0.918882	486484	sepsis	TRUE	reported	textfile	0.0034	5.26E-07	5768	M35326.m	TRUE	reported	UNDorn	2	TRUE	0.004366	25.28621
945	rs1091904	A	C	A	C	0.0176	-0.00849	0.1408	0.142042	FALSE	FALSE	FALSE	tWoDq4	0.019656	0.665728	486484	sepsis	TRUE	reported	textfile	0.0035	6.75E-07	7160	M01508.m	TRUE	reported	6ltDw0	2	TRUE	0.003519	25.27947
8515	rs6751559	T	G	T	G	-0.0357	0.015903	0.8962	0.898578	FALSE	FALSE	FALSE	vHXktL	0.022692	0.483434	486484	sepsis	TRUE	reported	textfile	0.0071	5.48E-07	4529	M35978.m	TRUE	reported	ceap9w	2	TRUE	0.005551	25.27132
1293	rs1173830	T	C	T	C	-0.0171	-0.00917	0.6542	0.625807	FALSE	FALSE	FALSE	WQdpmo	0.014115	0.51575	486484	sepsis	TRUE	reported	textfile	0.0034	6.60E-07	1714	M01769.m	TRUE	reported	BkptOK	2	TRUE	0.014543	25.26547
2870	rs1074643	A	G	A	G	0.0558	-0.11963	0.9808	0.979019	FALSE	FALSE	FALSE	mUzn1R	0.048505	0.013648	486484	sepsis	TRUE	reported	textfile	0.0111	5.10E-07	7301	M20675.m	TRUE	reported	XwY04K	2	TRUE	0.003449	25.26408
2587	rs4905425	A	C	A	C	0.0377	-0.00407	0.5066	0.498649	FALSE	FALSE	FALSE	JGJ9vH	0.013682	0.765958	486484	sepsis	TRUE	reported	textfile	0.0075	5.72E-07	2079	M18357.m	TRUE	reported	cOV2qw	2	TRUE	0.012008	25.24307
7106	rs1003095	T	C	T	C	-0.0704	-0.01621	0.3662	0.389216	FALSE	FALSE	FALSE	DfLd6z	0.014053	0.248769	486484	sepsis	TRUE	reported	textfile	0.014	4.70E-07	979	M34314.m	TRUE	reported	OgloAQ	2	TRUE	0.025179	25.23487
7310	rs6507247	A	G	A	G	-0.0191	-0.00033	0.4643	0.495373	FALSE	FALSE	FALSE	Vcz4EA	0.013676	0.980811	486484	sepsis	TRUE	reported	textfile	0.0038	4.43E-07	1576	M34359.m	TRUE	reported	aUSo2J	2	TRUE	0.015777	25.23179
4761	rs3135377	A	G	A	G	0.0216	-0.01598	0.2078	0.233314	FALSE	FALSE	FALSE	RDfDIS	0.01617	0.323031	486484	sepsis	TRUE	reported	textfile	0.0043	4.57E-07	6857	M32787.m	TRUE	reported	Hq6nGt	2	TRUE	0.003666	25.22574
2775	rs1118853	A	G	A	G	-0.0221	-0.00934	0.8687	0.857298	FALSE	FALSE	FALSE	yYuamw	0.019567	0.633105	486484	sepsis	TRUE	reported	textfile	0.0044	4.85E-07	4844	M19414.m	TRUE	reported	GxSb5G	2	TRUE	0.005181	25.21737
2743	rs1333626	A	C	A	C	-0.0226	0.005064	0.828	0.797032	FALSE	FALSE	FALSE	qFAbee	0.017088	0.766955	486484	sepsis	TRUE	reported	textfile	0.0045	5.91E-07	6929	M19364.m	TRUE	reported	tZgXNL	2	TRUE	0.003627	25.21544
2452	rs8051340	C	G	C	G	0.104	-0.00161	0.9164	0.877513	FALSE	TRUE	FALSE	NBJ2Do	0.020956	0.938674	486484	sepsis	TRUE	reported	textfile	0.0207	5.01E-07	1739	M17799.m	TRUE	reported	Uz0Wgl	2	TRUE	0.014308	25.2131
7346	rs1735789	C	G	C	G	-0.1895	0.005581	0.901	0.831342	FALSE	TRUE	FALSE	mm9ksf	0.018249	0.759733	486484	sepsis	TRUE	reported	textfile	0.0377	4.94E-07	954	M34365.m	TRUE	reported	AVwEqD	2	TRUE	0.025801	25.21299
7021	rs1461941	C	G	C	G	0.0226	-0.01016	0.6294	0.60971	FALSE	TRUE	FALSE	zMlkYA	0.014116	0.471838	486484	sepsis	TRUE	reported	textfile	0.0045	5.01E-07	4908	M34245.m	TRUE	reported	itCtz8	2	TRUE	0.005113	25.21244
8415	rs1764453	C	G	C	G	-0.2588	-0.07398	0.9834	0.99386	FALSE	TRUE	FALSE	02RdNm	0.090195	0.412088	486484	sepsis	TRUE	reported	textfile	0.0515	4.99E-07	1020	M35678.m	TRUE	reported	BWN5JR	2	TRUE	0.02416	25.20355
6608	rs1494950	A	G	A	G	0.1843	-0.0605	0.9762	0.984098	FALSE	FALSE	FALSE	VfOuo8	0.055151	0.272648	486484	sepsis	TRUE	reported	textfile	0.0367	5.31E-07	2725	M33883.m	TRUE	reported	BDe0Nk	2	TRUE	0.00917	25.19995
2477	rs1864468	A	G	A	G	-0.0251	0.004893	0.6161	0.618397	FALSE	FALSE	FALSE	SrfyMU	0.014057	0.727787	486484	sepsis	TRUE	reported	textfile	0.005	5.94E-07	7226	M17807.m	TRUE	reported	0zrJsu	2	TRUE	0.003475	25.19343
2483	rs450940	T	C	T	C	-0.0251	0.007002	0.5116	0.524582	FALSE	FALSE	FALSE	SrfyMU	0.013666	0.60838	486484	sepsis	TRUE	reported	textfile	0.005	5.04E-07	7226	M17807.m	TRUE	reported	0zrJsu	2	TRUE	0.003475	25.19343
5639	rs6904241	T	G	T	G	0.0527	0.021597	0.5433	0.543106	FALSE	FALSE	FALSE	U0d0ue	0.013726	0.115603	486484	sepsis	TRUE	reported	textfile	0.0105	5.36E-07	2616	M33190.m	TRUE	reported	FoODGF	2	TRUE	0.009538	25.17158
4932	rs6912503	A	G	A	G	-0.0311	0.011335	0.8251	0.848382	FALSE	FALSE	FALSE	clhN6v	0.019076	0.552361	486484	sepsis	TRUE	reported	textfile	0.0062	6.16E-07	2148	M32869.m	TRUE	reported	BzloWk	2	TRUE	0.011578	25.13812
2777	rs1202824	T	C	T	C	-0.0567	0.044488	0.0717	0.06601	FALSE	FALSE	FALSE	yYuamw	0.027597	0.10695	486484	sepsis	TRUE	reported	textfile	0.0113	5.73E-07	1188	M19414.m	TRUE	reported	GxSb5G	2	TRUE	0.020753	25.13492
2677	rs4896923	A	G	A	G	0.0361	-0.01215	0.5134	0.515182	FALSE	FALSE	FALSE	s4UTyf	0.013703	0.375115	486484	sepsis	TRUE	reported	textfile	0.0072	5.31E-07	5376	M18494.m	TRUE	reported	ajPzhg	2	TRUE	0.004654	25.12973
7572	rs7761031	A	G	A	G	-0.0406	-0.00146	0.96	0.956179	FALSE	FALSE	FALSE	Ot2ZAn	0.033685	0.965424	486484	sepsis	TRUE	reported	textfile	0.0081	5.87E-07	7119	M34481.m	TRUE	reported	tpyFcK	2	TRUE	0.003517	25.11655
7528	rs1048312	A	G	A	G	0.0346	-0.01854	0.0205	0.020788	FALSE	FALSE	FALSE	zV4uYs	0.048028	0.699442	486484	sepsis	TRUE	reported	textfile	0.0069	5.61E-07	1755	M34453.m	TRUE	reported	nLRRs2	2	TRUE	0.014125	25.11648
8551	rs2956731	T	G	T	G	0.4689	0.013836	0.931	0.923304	FALSE	FALSE	FALSE	aQhCav	0.025795	0.591696	486484	sepsis	TRUE	reported	textfile	0.0934	5.18E-07	488	M36097.m	TRUE	reported	9DJS9j	2	TRUE	0.049111	25.10055
4731	rs625532	A	G	A	G	-0.0561	0.016925	0.1697	0.160403	FALSE	FALSE	FALSE	fz63r4	0.018663	0.364492	486484	sepsis	TRUE	reported	textfile	0.0112	5.31E-07	2537	M32762.m	TRUE	reported	kgxlBo	2	TRUE	0.009793	25.06959
5001	rs7309973	T	C	T	C	-0.0701	-0.04883	0.0502	0.028617	FALSE	FALSE	FALSE	OsqE1P	0.04127	0.236778	486484	sepsis	TRUE	reported	textfile	0.014	5.85E-07	5425	M33084.m	TRUE	reported	VZYh6f	2	TRUE	0.0046	25.06224
6915	rs2002691	A	C	A	C	0.5054	-0.00355	0.9844	0.975291	FALSE	FALSE	FALSE	SvfSvH	0.044072	0.935826	486484	sepsis	TRUE	reported	textfile	0.1006	5.00E-07	282	M34109.m	TRUE	reported	7OSbiu	2	TRUE	0.082148	25.06014
6924	rs6944023	A	C	A	C	-0.9465	-0.00727	0.0219	0.031974	FALSE	FALSE	FALSE	SvfSvH	0.03928	0.853211	486484	sepsis	TRUE	reported	textfile	0.1878	4.67E-07	148	M34109.m	TRUE	reported	7OSbiu	2	TRUE	0.146487	25.0577
8562	rs9961807	T	C	T	C	-0.32	0.000579	0.2187	0.193119	FALSE	FALSE	FALSE	aQhCav	0.017283	0.973276	486484	sepsis	TRUE	reported	textfile	0.0638	5.35E-07	488	M36097.m	TRUE	reported	9DJS9j	2	TRUE	0.049024	25.05388
2155	rs2126638	A	G	A	G	0.0816	0.009411	0.9266	0.919716	FALSE	FALSE	FALSE	uuY1Mi	0.025321	0.710138	486484	sepsis	TRUE	reported	textfile	0.0163	5.78E-07	5502	M15677.m	TRUE	reported	nWMiPu	2	TRUE	0.004534	25.05228
1132	rs1825043	A	G	A	G	0.019	0.012794	0.9571	0.946326	FALSE	FALSE	FALSE	nlqUuV	0.030653	0.676388	486484	sepsis	TRUE	reported	textfile	0.0038	5.26E-07	7371	M01604.m	TRUE	reported	Uro9nC	2	TRUE	0.00338	24.99322
1139	rs7906282	T	G	T	G	0.008	0.00575	0.3075	0.309156	FALSE	FALSE	FALSE	nlqUuV	0.014782	0.697299	486484	sepsis	TRUE	reported	textfile	0.0016	1.11E-06	7371	M01604.m	TRUE	reported	Uro9nC	2	TRUE	0.00338	24.99322
1800	rs4820242	A	G	A	G	-0.0085	3.12E-05	0.6201	0.615143	FALSE	FALSE	FALSE	JEcCRZ	0.014082	0.998233	486484	sepsis	TRUE	reported	textfile	0.0017	9.03E-07	7368	M15140.m	TRUE	reported	xYCzKP	2	TRUE	0.003382	24.99321
1805	rs6815057	A	G	A	G	0.0085	0.001092	0.5786	0.576571	FALSE	FALSE	FALSE	JEcCRZ	0.013999	0.937813	486484	sepsis	TRUE	reported	textfile	0.0017	1.12E-06	7368	M15140.m	TRUE	reported	xYCzKP	2	TRUE	0.003382	24.99321
1327	rs1095409	T	C	T	C	0.013	0.027854	0.1522	0.156494	FALSE	FALSE	FALSE	LDUJbh	0.018831	0.139109	486484	sepsis	TRUE	reported	textfile	0.0026	3.97E-07	7367	M01898.m	TRUE	reported	JRSLeH	2	TRUE	0.003382	24.99321
2934	rs872745	A	C	A	C	-0.0135	-0.01985	0.4114	0.405906	FALSE	FALSE	FALSE	fV4qul	0.013965	0.155173	486484	sepsis	TRUE	reported	textfile	0.0027	4.93E-07	7366	M21044.m	TRUE	reported	hbdBQJ	2	TRUE	0.003382	24.99321
9039	rs2979471	A	T	A	T	-0.009	-0.01248	0.2882	0.300699	FALSE	TRUE	FALSE	df0MCM	0.014969	0.404485	486484	sepsis	TRUE	reported	textfile	0.0018	5.34E-07	7366	M37506.m	TRUE	reported	iUfr4u	2	TRUE	0.003382	24.99321
9042	rs420422	T	C	T	C	-0.009	-0.00013	0.542	0.560988	FALSE	FALSE	FALSE	df0MCM	0.013842	0.99239	486484	sepsis	TRUE	reported	textfile	0.0018	3.01E-07	7366	M37506.m	TRUE	reported	iUfr4u	2	TRUE	0.003382	24.99321
3817	rs1079007	T	C	T	C	-0.009	0.029873	0.611	0.611867	FALSE	FALSE	FALSE	tRX2u0	0.014214	0.035576	486484	sepsis	TRUE	reported	textfile	0.0018	3.15E-07	7365	M32348.m	TRUE	reported	ruBgwQ	2	TRUE	0.003383	24.99321
1033	rs1078730	A	G	A	G	-0.009	0.008762	0.2701	0.272417	FALSE	FALSE	FALSE	tRtQ2e	0.015363	0.568446	486484	sepsis	TRUE	reported	textfile	0.0018	6.04E-07	7364	M01564.m	TRUE	reported	Tbg0zM	2	TRUE	0.003383	24.99321
1063	rs7851531	A	G	A	G	-0.009	0.029749	0.6594	0.633781	FALSE	FALSE	FALSE	tRtQ2e	0.01427	0.037096	486484	sepsis	TRUE	reported	textfile	0.0018	6.47E-07	7364	M01564.m	TRUE	reported	Tbg0zM	2	TRUE	0.003383	24.99321
3993	rs842636	A	G	A	G	-0.009	0.001935	0.4429	0.435417	FALSE	FALSE	FALSE	XdJbEU	0.013753	0.888081	486484	sepsis	TRUE	reported	textfile	0.0018	9.72E-07	7364	M32452.m	TRUE	reported	V5xWKi	2	TRUE	0.003383	24.99321
6798	rs1696871	A	G	A	G	0.0205	0.025078	0.8679	0.87042	FALSE	FALSE	FALSE	OZ0l1H	0.020507	0.22136	486484	sepsis	TRUE	reported	textfile	0.0041	4.65E-07	7364	M33960.m	TRUE	reported	GlPp4w	2	TRUE	0.003383	24.99321
4025	rs6925339	A	G	A	G	0.009	-0.0173	0.3097	0.304697	FALSE	FALSE	FALSE	V9Atld	0.014873	0.244847	486484	sepsis	TRUE	reported	textfile	0.0018	6.67E-07	7363	M32489.m	TRUE	reported	8B8S8a	2	TRUE	0.003384	24.99321
4313	rs7952698	A	G	A	G	0.009	-0.00928	0.2345	0.238165	FALSE	FALSE	FALSE	0tjjPC	0.016038	0.562778	486484	sepsis	TRUE	reported	textfile	0.0018	8.69E-07	7362	M32634.m	TRUE	reported	nJJFkX	2	TRUE	0.003384	24.99321
6516	rs1089911	A	G	A	G	0.026	0.026631	0.8976	0.905195	FALSE	FALSE	FALSE	OKlXJ8	0.023389	0.254854	486484	sepsis	TRUE	reported	textfile	0.0052	4.58E-07	7362	M33821.m	TRUE	reported	XGkaXH	2	TRUE	0.003384	24.99321
6877	rs3729908	T	C	T	C	0.009	0.007924	0.1234	0.116483	FALSE	FALSE	FALSE	kFWZjs	0.021595	0.713669	486484	sepsis	TRUE	reported	textfile	0.0018	9.91E-07	7362	M34062.m	TRUE	reported	eLONsg	2	TRUE	0.003384	24.99321
447	rs1051944	A	G	A	G	-0.0045	-0.00717	0.5338	0.536125	FALSE	FALSE	FALSE	cl8ucN	0.013759	0.602252	486484	sepsis	TRUE	reported	textfile	9.00E-04	1.85E-06	7361	M00513.m	TRUE	reported	Ian0Q8	2	TRUE	0.003385	24.99321
1525	rs1535039	T	C	T	C	0.0175	-0.00158	0.912	0.878248	FALSE	FALSE	FALSE	YvgTeW	0.020897	0.939869	486484	sepsis	TRUE	reported	textfile	0.0035	4.49E-07	7357	M03141.m	TRUE	reported	srKjSc	2	TRUE	0.003387	24.9932
18	rs1078449	A	G	A	G	0.0045	0.00052	0.5375	0.531303	FALSE	FALSE	FALSE	xmGuol	0.013705	0.96974	486484	sepsis	TRUE	reported	textfile	9.00E-04	5.09E-07	7355	M00054.m	TRUE	reported	GW50Ri	2	TRUE	0.003388	24.9932
23	rs1084666	T	C	T	C	0.0045	0.017308	0.4861	0.483873	FALSE	FALSE	FALSE	xmGuol	0.01379	0.209435	486484	sepsis	TRUE	reported	textfile	9.00E-04	1.65E-06	7355	M00054.m	TRUE	reported	GW50Ri	2	TRUE	0.003388	24.9932
39	rs1253393	A	T	A	T	0.0045	-0.01202	0.3887	0.382543	FALSE	TRUE	FALSE	xmGuol	0.014076	0.393336	486484	sepsis	TRUE	reported	textfile	9.00E-04	6.52E-07	7355	M00054.m	TRUE	reported	GW50Ri	2	TRUE	0.003388	24.9932
54	rs1549225	T	C	T	C	0.0045	-0.01046	0.607	0.601646	FALSE	FALSE	FALSE	xmGuol	0.013955	0.453626	486484	sepsis	TRUE	reported	textfile	9.00E-04	6.06E-07	7355	M00054.m	TRUE	reported	GW50Ri	2	TRUE	0.003388	24.9932
65	rs1997238	A	G	A	G	0.0045	-0.02602	0.5117	0.50945	FALSE	FALSE	FALSE	xmGuol	0.013685	0.057294	486484	sepsis	TRUE	reported	textfile	9.00E-04	6.07E-07	7355	M00054.m	TRUE	reported	GW50Ri	2	TRUE	0.003388	24.9932
79	rs2510595	A	G	A	G	-0.0045	-0.00982	0.6113	0.599706	FALSE	FALSE	FALSE	xmGuol	0.01402	0.483445	486484	sepsis	TRUE	reported	textfile	9.00E-04	4.99E-07	7355	M00054.m	TRUE	reported	GW50Ri	2	TRUE	0.003388	24.9932
80	rs2520117	A	G	A	G	0.0045	0.001704	0.4545	0.455817	FALSE	FALSE	FALSE	xmGuol	0.013766	0.901494	486484	sepsis	TRUE	reported	textfile	9.00E-04	5.06E-07	7355	M00054.m	TRUE	reported	GW50Ri	2	TRUE	0.003388	24.9932
84	rs2662269	A	C	A	C	0.0045	-0.00209	0.417	0.40956	FALSE	FALSE	FALSE	xmGuol	0.013907	0.880398	486484	sepsis	TRUE	reported	textfile	9.00E-04	7.12E-07	7355	M00054.m	TRUE	reported	GW50Ri	2	TRUE	0.003388	24.9932
91	rs2834164	A	C	A	C	-0.0045	-0.03195	0.5041	0.496224	FALSE	FALSE	FALSE	xmGuol	0.013657	0.019297	486484	sepsis	TRUE	reported	textfile	9.00E-04	4.76E-07	7355	M00054.m	TRUE	reported	GW50Ri	2	TRUE	0.003388	24.9932
110	rs428185	T	C	T	C	-0.0045	0.012763	0.5851	0.577921	FALSE	FALSE	FALSE	xmGuol	0.013842	0.35651	486484	sepsis	TRUE	reported	textfile	9.00E-04	6.25E-07	7355	M00054.m	TRUE	reported	GW50Ri	2	TRUE	0.003388	24.9932
117	rs4695289	A	G	A	G	-0.0045	-0.00698	0.3489	0.343522	FALSE	FALSE	FALSE	xmGuol	0.014729	0.635391	486484	sepsis	TRUE	reported	textfile	9.00E-04	7.95E-07	7355	M00054.m	TRUE	reported	GW50Ri	2	TRUE	0.003388	24.9932
119	rs4751282	T	C	T	C	0.0045	0.002065	0.5421	0.552864	FALSE	FALSE	FALSE	xmGuol	0.013898	0.881889	486484	sepsis	TRUE	reported	textfile	9.00E-04	7.41E-07	7355	M00054.m	TRUE	reported	GW50Ri	2	TRUE	0.003388	24.9932
127	rs4989308	A	G	A	G	-0.0045	0.008189	0.3746	0.37913	FALSE	FALSE	FALSE	xmGuol	0.014099	0.561358	486484	sepsis	TRUE	reported	textfile	9.00E-04	8.43E-07	7355	M00054.m	TRUE	reported	GW50Ri	2	TRUE	0.003388	24.9932
154	rs7006068	T	C	T	C	0.0045	-0.01436	0.6264	0.621111	FALSE	FALSE	FALSE	xmGuol	0.014129	0.309366	486484	sepsis	TRUE	reported	textfile	9.00E-04	4.80E-07	7355	M00054.m	TRUE	reported	GW50Ri	2	TRUE	0.003388	24.9932
166	rs7810390	A	G	A	G	-0.0045	0.011596	0.5928	0.591016	FALSE	FALSE	FALSE	xmGuol	0.013919	0.4048	486484	sepsis	TRUE	reported	textfile	9.00E-04	5.61E-07	7355	M00054.m	TRUE	reported	GW50Ri	2	TRUE	0.003388	24.9932
172	rs8110890	T	G	T	G	0.0045	-0.00259	0.4741	0.474728	FALSE	FALSE	FALSE	xmGuol	0.013795	0.851169	486484	sepsis	TRUE	reported	textfile	9.00E-04	7.51E-07	7355	M00054.m	TRUE	reported	GW50Ri	2	TRUE	0.003388	24.9932
836	rs4710948	A	G	A	G	-0.0085	-0.0111	0.3382	0.347428	FALSE	FALSE	FALSE	2qgDgB	0.014384	0.440306	486484	sepsis	TRUE	reported	textfile	0.0017	6.94E-07	7355	M01358.m	TRUE	reported	asmXq0	2	TRUE	0.003388	24.9932
3934	rs1172879	T	C	T	C	-0.021	0.018139	0.7845	0.783605	FALSE	FALSE	FALSE	LlOaWH	0.01669	0.277108	486484	sepsis	TRUE	reported	textfile	0.0042	7.15E-07	7355	M32418.m	TRUE	reported	AtHOVw	2	TRUE	0.003388	24.9932
572	rs7948201	T	C	T	C	-0.023	-0.0133	0.9623	0.923357	FALSE	FALSE	FALSE	fNqkCW	0.025768	0.605867	486484	sepsis	TRUE	reported	textfile	0.0046	6.77E-07	7354	M00606.m	TRUE	reported	g9uwOn	2	TRUE	0.003388	24.9932
6779	rs8028440	T	C	T	C	0.0095	0.016962	0.2136	0.215091	FALSE	FALSE	FALSE	Elf6MQ	0.016596	0.306743	486484	sepsis	TRUE	reported	textfile	0.0019	3.15E-07	7354	M33955.m	TRUE	reported	Lxn4RF	2	TRUE	0.003388	24.9932
243	rs1292666	T	C	T	C	0.0045	0.009245	0.4667	0.463271	FALSE	FALSE	FALSE	tbRL8J	0.013778	0.502241	486484	sepsis	TRUE	reported	textfile	9.00E-04	6.44E-07	7351	M00060.m	TRUE	reported	oOvRKT	2	TRUE	0.003389	24.9932
250	rs1428203	T	G	T	G	0.0045	-0.00355	0.4117	0.411322	FALSE	FALSE	FALSE	tbRL8J	0.01391	0.798323	486484	sepsis	TRUE	reported	textfile	9.00E-04	5.51E-07	7351	M00060.m	TRUE	reported	oOvRKT	2	TRUE	0.003389	24.9932
254	rs1558789	A	C	A	C	0.0045	0.001378	0.51	0.507691	FALSE	FALSE	FALSE	tbRL8J	0.013738	0.920103	486484	sepsis	TRUE	reported	textfile	9.00E-04	3.52E-07	7351	M00060.m	TRUE	reported	oOvRKT	2	TRUE	0.003389	24.9932
255	rs1564480	A	G	A	G	0.0045	-0.01518	0.4765	0.471641	FALSE	FALSE	FALSE	tbRL8J	0.013683	0.267273	486484	sepsis	TRUE	reported	textfile	9.00E-04	6.68E-07	7351	M00060.m	TRUE	reported	oOvRKT	2	TRUE	0.003389	24.9932
294	rs2834578	T	C	T	C	0.0045	0.017997	0.3986	0.398924	FALSE	FALSE	FALSE	tbRL8J	0.014016	0.199124	486484	sepsis	TRUE	reported	textfile	9.00E-04	7.24E-07	7351	M00060.m	TRUE	reported	oOvRKT	2	TRUE	0.003389	24.9932
302	rs3802681	T	C	T	C	0.0045	0.021545	0.6144	0.627027	FALSE	FALSE	FALSE	tbRL8J	0.014136	0.127478	486484	sepsis	TRUE	reported	textfile	9.00E-04	5.44E-07	7351	M00060.m	TRUE	reported	oOvRKT	2	TRUE	0.003389	24.9932
303	rs390836	A	T	A	T	0.0045	-0.01561	0.415	0.417154	FALSE	TRUE	FALSE	tbRL8J	0.013884	0.260859	486484	sepsis	TRUE	reported	textfile	9.00E-04	8.06E-07	7351	M00060.m	TRUE	reported	oOvRKT	2	TRUE	0.003389	24.9932
306	rs4077470	T	C	T	C	-0.0045	0.011165	0.5403	0.542297	FALSE	FALSE	FALSE	tbRL8J	0.013714	0.415566	486484	sepsis	TRUE	reported	textfile	9.00E-04	4.65E-07	7351	M00060.m	TRUE	reported	oOvRKT	2	TRUE	0.003389	24.9932
324	rs4935115	A	G	A	G	0.0045	0.016647	0.529	0.539587	FALSE	FALSE	FALSE	tbRL8J	0.013747	0.225908	486484	sepsis	TRUE	reported	textfile	9.00E-04	5.75E-07	7351	M00060.m	TRUE	reported	oOvRKT	2	TRUE	0.003389	24.9932
339	rs679563	A	G	A	G	-0.0045	0.016788	0.4837	0.491278	FALSE	FALSE	FALSE	tbRL8J	0.013637	0.218282	486484	sepsis	TRUE	reported	textfile	9.00E-04	7.13E-07	7351	M00060.m	TRUE	reported	oOvRKT	2	TRUE	0.003389	24.9932

362	rs7685009	A	G	A	G	-0.0045	0.008482	0.6582	0.652168	FALSE	FALSE	FALSE	tbRL8J	0.014329	0.55388	486484	sepsis	TRUE	reported	textfile	9.00E-04	6.44E-07	7351	M00060.m	TRUE	reported	oOvRKT	2	TRUE	0.003389	24.9932
389	rs9403527	T	C	T	C	0.0045	0.009228	0.3456	0.345078	FALSE	FALSE	FALSE	tbRL8J	0.014496	0.524385	486484	sepsis	TRUE	reported	textfile	9.00E-04	9.91E-07	7351	M00060.m	TRUE	reported	oOvRKT	2	TRUE	0.003389	24.9932
4110	rs2434532	A	G	A	G	-0.0095	-0.01146	0.1919	0.184627	FALSE	FALSE	FALSE	4rtLd0	0.017575	0.514468	486484	sepsis	TRUE	reported	textfile	0.0019	2.84E-07	7350	M32518.m	TRUE	reported	j7f1ER	2	TRUE	0.00339	24.9932
4111	rs2831549	T	C	T	C	-0.009	0.013482	0.7077	0.67212	FALSE	FALSE	FALSE	4rtLd0	0.014551	0.354168	486484	sepsis	TRUE	reported	textfile	0.0018	4.90E-07	7350	M32518.m	TRUE	reported	j7f1ER	2	TRUE	0.00339	24.9932
1864	rs1048118	T	G	T	G	0.0045	-0.00929	0.3707	0.377421	FALSE	FALSE	FALSE	v2KE89	0.014099	0.509767	486484	sepsis	TRUE	reported	textfile	9.00E-04	9.69E-07	7349	M15500.m	TRUE	reported	to2t7H	2	TRUE	0.00339	24.9932
1868	rs1081892	T	C	T	C	-0.0045	-0.01834	0.6254	0.616463	FALSE	FALSE	FALSE	v2KE89	0.014056	0.191874	486484	sepsis	TRUE	reported	textfile	9.00E-04	6.08E-07	7349	M15500.m	TRUE	reported	to2t7H	2	TRUE	0.00339	24.9932
1884	rs1163070	C	G	C	G	0.0045	-0.01994	0.407	0.396825	FALSE	TRUE	FALSE	v2KE89	0.014003	0.154396	486484	sepsis	TRUE	reported	textfile	9.00E-04	5.14E-07	7349	M15500.m	TRUE	reported	to2t7H	2	TRUE	0.00339	24.9932
1890	rs1220110	A	G	A	G	-0.0045	0.0187	0.3703	0.367377	FALSE	FALSE	FALSE	v2KE89	0.014365	0.192991	486484	sepsis	TRUE	reported	textfile	9.00E-04	1.14E-06	7349	M15500.m	TRUE	reported	to2t7H	2	TRUE	0.00339	24.9932
1896	rs1246267	A	G	A	G	0.0045	-0.01151	0.591	0.593007	FALSE	FALSE	FALSE	v2KE89	0.013964	0.409888	486484	sepsis	TRUE	reported	textfile	9.00E-04	1.09E-06	7349	M15500.m	TRUE	reported	to2t7H	2	TRUE	0.00339	24.9932
1930	rs1760903	A	G	A	G	-0.0045	0.019622	0.4432	0.443787	FALSE	FALSE	FALSE	v2KE89	0.013797	0.154972	486484	sepsis	TRUE	reported	textfile	9.00E-04	1.01E-06	7349	M15500.m	TRUE	reported	to2t7H	2	TRUE	0.00339	24.9932
1946	rs2292871	A	G	A	G	0.0045	-0.02105	0.5826	0.576329	FALSE	FALSE	FALSE	v2KE89	0.013855	0.128659	486484	sepsis	TRUE	reported	textfile	9.00E-04	7.68E-07	7349	M15500.m	TRUE	reported	to2t7H	2	TRUE	0.00339	24.9932
1961	rs2702023	C	G	C	G	0.0045	-0.01408	0.4025	0.40424	FALSE	TRUE	FALSE	v2KE89	0.013931	0.312098	486484	sepsis	TRUE	reported	textfile	9.00E-04	8.56E-07	7349	M15500.m	TRUE	reported	to2t7H	2	TRUE	0.00339	24.9932
1979	rs4436868	T	C	T	C	0.0045	-0.005	0.4973	0.4845	FALSE	FALSE	FALSE	v2KE89	0.013701	0.715109	486484	sepsis	TRUE	reported	textfile	9.00E-04	4.29E-07	7349	M15500.m	TRUE	reported	to2t7H	2	TRUE	0.00339	24.9932
1982	rs4531372	A	G	A	G	-0.0045	-0.00358	0.4345	0.436795	FALSE	FALSE	FALSE	v2KE89	0.013867	0.796169	486484	sepsis	TRUE	reported	textfile	9.00E-04	7.13E-07	7349	M15500.m	TRUE	reported	to2t7H	2	TRUE	0.00339	24.9932
1999	rs4881523	T	C	T	C	0.0045	0.01054	0.5372	0.53548	FALSE	FALSE	FALSE	v2KE89	0.013713	0.442134	486484	sepsis	TRUE	reported	textfile	9.00E-04	5.95E-07	7349	M15500.m	TRUE	reported	to2t7H	2	TRUE	0.00339	24.9932
2020	rs6908972	T	G	T	G	-0.009	0.013113	0.082	0.085208	FALSE	FALSE	FALSE	v2KE89	0.024482	0.592235	486484	sepsis	TRUE	reported	textfile	0.0018	7.99E-07	7349	M15500.m	TRUE	reported	to2t7H	2	TRUE	0.00339	24.9932
2022	rs7026148	T	C	T	C	-0.0045	0.005026	0.386	0.386958	FALSE	FALSE	FALSE	v2KE89	0.014034	0.720253	486484	sepsis	TRUE	reported	textfile	9.00E-04	4.47E-07	7349	M15500.m	TRUE	reported	to2t7H	2	TRUE	0.00339	24.9932
2052	rs9467703	A	G	A	G	-0.0045	0.016002	0.3745	0.372693	FALSE	FALSE	FALSE	v2KE89	0.014116	0.256954	486484	sepsis	TRUE	reported	textfile	9.00E-04	8.25E-07	7349	M15500.m	TRUE	reported	to2t7H	2	TRUE	0.00339	24.9932
3689	rs1042725	T	C	T	C	-0.0085	0.010626	0.4977	0.494168	FALSE	FALSE	FALSE	agPgtF	0.013674	0.437091	486484	sepsis	TRUE	reported	textfile	0.0017	7.98E-07	7349	M32315.m	TRUE	reported	hMRjcV	2	TRUE	0.00339	24.9932
7485	rs1730076	A	G	A	G	0.022	-0.02764	0.94	0.939413	FALSE	FALSE	FALSE	jAhmnV	0.028663	0.334973	486484	sepsis	TRUE	reported	textfile	0.0044	7.23E-07	7348	M34419.m	TRUE	reported	F2Tg9L	2	TRUE	0.003391	24.9932
7487	rs1979024	T	C	T	C	0.017	-0.0135	0.8685	0.82872	FALSE	FALSE	FALSE	jAhmnV	0.018147	0.456782	486484	sepsis	TRUE	reported	textfile	0.0034	6.92E-07	7348	M34419.m	TRUE	reported	F2Tg9L	2	TRUE	0.003391	24.9932
7491	rs8085108	T	C	T	C	-0.0135	-0.03575	0.1945	0.191288	FALSE	FALSE	FALSE	jAhmnV	0.017383	0.039704	486484	sepsis	TRUE	reported	textfile	0.0027	7.86E-07	7348	M34419.m	TRUE	reported	F2Tg9L	2	TRUE	0.003391	24.9932
746	rs1123210	A	C	A	C	0.0085	0.005832	0.1151	0.112197	FALSE	FALSE	FALSE	RHWptG	0.022022	0.791156	486484	sepsis	TRUE	reported	textfile	0.0017	3.70E-07	7347	M01302.m	TRUE	reported	z1OLfr	2	TRUE	0.003391	24.99319
1250	rs6973824	A	G	A	G	0.009	0.010549	0.3374	0.344941	FALSE	FALSE	FALSE	V50rWC	0.014408	0.464082	486484	sepsis	TRUE	reported	textfile	0.0018	4.02E-07	7346	M01645.m	TRUE	reported	yN3gpQ	2	TRUE	0.003392	24.99319
1230	rs1530439	T	G	T	G	0.009	0.00437	0.2946	0.307643	FALSE	FALSE	FALSE	V50rWC	0.014899	0.769293	486484	sepsis	TRUE	reported	textfile	0.0018	6.08E-07	7346	M01645.m	TRUE	reported	yN3gpQ	2	TRUE	0.003392	24.99319
1233	rs2069151	A	G	A	G	0.009	0.012151	0.7083	0.72055	FALSE	FALSE	FALSE	V50rWC	0.015264	0.426003	486484	sepsis	TRUE	reported	textfile	0.0018	4.68E-07	7346	M01645.m	TRUE	reported	yN3gpQ	2	TRUE	0.003392	24.99319
8175	rs3781638	T	G	T	G	0.008	0.002176	0.5337	0.54374	FALSE	FALSE	FALSE	8UhtPK	0.01374	0.87416	486484	sepsis	TRUE	reported	textfile	0.0016	4.32E-07	7345	M35397.m	TRUE	reported	rvVJba	2	TRUE	0.003392	24.99319
3774	rs1376492	A	T	A	T	0.009	0.011801	0.6524	0.657391	FALSE	TRUE	FALSE	NW1aaR	0.014397	0.412393	486484	sepsis	TRUE	reported	textfile	0.0018	3.59E-07	7344	M32339.m	TRUE	reported	pBPkAB	2	TRUE	0.003393	24.99319
4947	rs1921984	T	C	T	C	-0.0085	-0.01107	0.5754	0.556995	FALSE	FALSE	FALSE	bKmDMa	0.013886	0.425209	486484	sepsis	TRUE	reported	textfile	0.0017	9.00E-07	7344	M32910.m	TRUE	reported	nAuPzO	2	TRUE	0.003393	24.99319
7447	rs1088902	A	G	A	G	-0.0135	-0.0081	0.4193	0.42616	FALSE	FALSE	FALSE	7ubZow	0.013799	0.557164	486484	sepsis	TRUE	reported	textfile	0.0027	7.12E-07	7344	M34407.m	TRUE	reported	LRsXN2	2	TRUE	0.003393	24.99319
3521	rs7216075	A	G	A	G	-0.0215	0.022358	0.2494	0.27307	FALSE	FALSE	FALSE	hxuqls	0.015358	0.14545	486484	sepsis	TRUE	reported	textfile	0.0043	4.39E-07	7336	M27738.m	TRUE	reported	aueeZs	2	TRUE	0.003396	24.99318
6078	rs2465657	A	G	A	G	-0.0085	0.029962	0.1764	0.170886	FALSE	FALSE	FALSE	P8n0HP	0.018173	0.099211	486484	sepsis	TRUE	reported	textfile	0.0017	9.90E-07	7336	M33442.m	TRUE	reported	cljMTI	2	TRUE	0.003396	24.99318
8811	rs1162721	A	T	A	T	0.013	-0.0079	0.7766	0.7639	FALSE	TRUE	FALSE	X2Rol2	0.016115	0.62407	486484	sepsis	TRUE	reported	textfile	0.0026	6.46E-07	7335	M36776.m	TRUE	reported	nikHw1	2	TRUE	0.003397	24.99318
1360	rs1023418	T	C	T	C	0.0085	0.025972	0.3185	0.325101	FALSE	FALSE	FALSE	KrWhws	0.014595	0.075157	486484	sepsis	TRUE	reported	textfile	0.0017	1.07E-06	7325	M02132.m	TRUE	reported	7pzpfK	2	TRUE	0.003401	24.99317
1379	rs1768168	A	G	A	G	0.0085	-0.00374	0.3158	0.329235	FALSE	FALSE	FALSE	KrWhws	0.014592	0.797788	486484	sepsis	TRUE	reported	textfile	0.0017	1.04E-06	7325	M02132.m	TRUE	reported	7pzpfK	2	TRUE	0.003401	24.99317
1364	rs1090104	T	C	T	C	-0.009	0.003927	0.5877	0.596618	FALSE	FALSE	FALSE	KrWhws	0.013999	0.779067	486484	sepsis	TRUE	reported	textfile	0.0018	2.96E-07	7325	M02132.m	TRUE	reported	7pzpfK	2	TRUE	0.003401	24.99317
4575	rs1169187	T	C	T	C	-0.009	-0.03226	0.3084	0.301319	FALSE	FALSE	FALSE	Pzplqu	0.014927	0.030685	486484	sepsis	TRUE	reported	textfile	0.0018	2.83E-07	7318	M32740.m	TRUE	reported	FjEBS5	2	TRUE	0.003405	24.99317
7058	rs2511225	A	C	A	C	0.009	-0.01442	0.6408	0.645581	FALSE	FALSE	FALSE	uthruC	0.014306	0.313321	486484	sepsis	TRUE	reported	textfile	0.0018	5.53E-07	7316	M34283.m	TRUE	reported	ftTuRU	2	TRUE	0.003406	24.99317
2515	rs6905811	T	C	T	C	0.009	-0.01052	0.343	0.339855	FALSE	FALSE	FALSE	LwUYua	0.014428	0.466111	486484	sepsis	TRUE	reported	textfile	0.0018	1.01E-06	7315	M17945.m	TRUE	reported	SUN8dc	2	TRUE	0.003406	24.99316
2519	rs757202	T	C	T	C	-0.009	9.45E-06	0.5096	0.508722	FALSE	FALSE	FALSE	LwUYua	0.013775	0.999453	486484	sepsis	TRUE	reported	textfile	0.0018	8.35E-07	7315	M17945.m	TRUE	reported	SUN8dc	2	TRUE	0.003406	24.99316
5859	rs1045744	T	C	T	C	-0.018	-0.01984	0.0849	0.13225	FALSE	FALSE	FALSE	tqW0ld	0.020178	0.32551	486484	sepsis	TRUE	reported	textfile	0.0036	4.77E-07	7315	M33389.m	TRUE	reported	Sv7EyV	2	TRUE	0.003406	24.99316
5899	rs928177	T	C	T	C	-0.0135	0.001071	0.146	0.169899	FALSE	FALSE	FALSE	tqW0ld	0.018292	0.953314	486484	sepsis	TRUE	reported	textfile	0.0027	7.14E-07	7315	M33389.m	TRUE	reported	Sv7EyV	2	TRUE	0.003406	24.99316
1703	rs9396681	T	C	T	C	-0.012	0.005299	0.9072	0.905996	FALSE	FALSE	FALSE	f01en0	0.023604	0.822369	486484	sepsis	TRUE	reported	textfile	0.0024	6.25E-07	7314	M12593.m	TRUE	reported	EAsM6U	2	TRUE	0.003406	24.99316
2224	rs7561849	A	G	A	G	0.009	-0.00069	0.2168	0.230545	FALSE	FALSE	FALSE	Bf6uMK	0.016376	0.966317	486484	sepsis	TRUE	reported	textfile	0.0018	1.01E-06	7308	M15778.m	TRUE	reported	jowM2N	2	TRUE	0.003409	24.99316
6132	rs191149	A	C	A	C	0.009	-0.01189	0.7214	0.735668	FALSE	FALSE	FALSE	MiSJly	0.015514	0.44337	486484	sepsis	TRUE	reported	textfile	0.0018	9.14E-07	7307	M33477.m	TRUE	reported	WwL1hq	2	TRUE	0.00341	24.99316
6154	rs741301	T	C	T	C	0.009	-0.01479	0.6849	0.682178	FALSE	FALSE	FALSE	MiSJly	0.014707	0.314554	486484	sepsis	TRUE	reported	textfile	0.0018	8.12E-07	7307	M33477.m	TRUE	reported	WwL1hq	2	TRUE	0.00341	24.99316
3333	rs2287011	A	G	A	G	-0.009	0.021723	0.2273	0.235032	FALSE	FALSE	FALSE	m66Eav	0.016405	0.18546	486484	sepsis	TRUE	reported	textfile	0.0018	4.84E-07	7303	M27256.m	TRUE	reported	pl2ECU	2	TRUE	0.003412	24.99315
2896	rs9556974	C	G	C	G	0.0135	0.003834	0.3426	0.346155	FALSE	TRUE	FALSE	mUzn1R	0.014421	0.790318	486484	sepsis	TRUE	reported	textfile	0.0027	5.36E-07	7301	M20675.m	TRUE	reported	XwY04K	2	TRUE	0.003413	24.99315
2699	rs1682870	A	G	A	G	0.0135	0.00671	0.7407	0.737338	FALSE	FALSE	FALSE	XsQHZK	0.015524	0.66554	486484	sepsis	TRUE	reported	textfile	0.0027	9.08E-07	7286	M18929.m	TRUE	reported	k8R7hN	2	TRUE	0.00342	24.99314
2703	rs2806302	T	G	T	G	0.0125	-0.0222	0.688	0.673705	FALSE	FALSE	FALSE	XsQHZK	0.014764	0.132682	486484	sepsis	TRUE	reported	textfile	0.0025	6.71E-07	7286	M18929.m	TRUE	reported	k8R7hN	2	TRUE	0.00342	24.99314
3033	rs307128	A	G	A	G	-0.013	-0.00337	0.681	0.68835	FALSE	FALSE	FALSE	FJZEYz	0.01479	0.819784	486484	sepsis	TRUE	reported	textfile	0.0026	4.33E-07	7284	M21630.m	TRUE	reported	s6Pshg	2	TRUE	0.00342	24.99314
1103	rs1012520	C	G	C	G	0.009	-0.00124	0.8698	0.86373	FALSE	TRUE	FALSE	yahiGZ	0.019993	0.950573	486484	sepsis	TRUE	reported	textfile	0.0018	4.11E-07	7271	M01585.m	TRUE	reported	dwZ7vX	2	TRUE	0.003427	24.99312
1118	rs6690829	T	C	T	C	-0.008	0.017284	0.24	0.25443	FALSE	FALSE	FALSE	yahiGZ	0.01574	0.272152	486484	sepsis	TRUE	reported	textfile	0.0016	1.24E-06	7271	M01585.m	TRUE	reported	dwZ7vX	2	TRUE	0.003427	24.99312
6247	rs935270	A	G	A	G	-0.0135	-0.00862	0.4556	0.448462	FALSE	FALSE	FALSE	vL52Yb	0.013947	0.536637	486484	sepsis	TRUE	reported	textfile	0.0027	4.66E-07	7269	M33510.m	TRUE	reported	JlO9VX	2	TRUE	0.003427	24.99312
7967	rs485868	A	G	A	G	-0.0135	-0.00483	0.7155	0.713335	FALSE	FALSE	FALSE	zb8m4a	0.015162	0.7501	486484	sepsis	TRUE	reported	textfile	0.0027	4.36E-07	7261	M35253.m	TRUE	reported	tHnnnS	2	TRUE	0.003431	24.99311
3544	rs1171316	A	G	A	G	-0.017	0.010827	0.8575	0.841307	FALSE	FALSE	FALSE	8luUON	0.018814	0.564955	486484	sepsis	TRUE	reported	textfile	0.0034	5.63E-07	7257	M31453.m	TRUE	reported	FBs6G4	2	TRUE	0.003433	24.99311
547	rs1319586	A	C	A	C	0.022	0.005104	0.2043	0.204844	FALSE	FALSE	FALSE	QUUN2g	0.016948	0.76331	486484	sepsis	TRUE	reported	textfile	0.0044	4.13E-07	7237	M00599.m	TRUE	reported	vxn2i7	2	TRUE	0.003443	24.99309
4333	rs1165406	T	C	T	C	0.009	-0.02032	0.5837	0.590778	FALSE	FALSE	FALSE	6VSoQQ	0.013971	0.145888	486484	sepsis	TRUE	reported	textfile	0.0018	8.68E-07	7229	M32644.m	TRUE	reported	oC2AV0	2	TRUE	0.003446	24.99308
4350	rs723967	T	C	T	C	0.0095	-0.03184	0.4929	0.487863	FALSE	FALSE	FALSE	6VSoQQ	0.01375	0.020592	486484	sepsis	TRUE	reported	textfile	0.0019	3.27E-07	7229	M32644.m	TRUE	reported	oC2AV0	2	TRUE	0.003446	24.99308
1448	rs4880550	T	C	T	C	0.015	0.002146	0.1292	0.158607	FALSE	FALSE	FALSE	1M4gqj	0.018744	0.908864	486484	sepsis	TRUE	reported	textfile	0.003	6.32E-07	7226	M02730.m	TRUE	reported	RWyYEM	2	TRUE	0.003448	24.99308
8031	rs1776453	A	G	A	G	0.009	-0.01891	0.1973	0.198056	FALSE	FALSE	FALSE	SwiqSi	0.017359	0.276022	486484	sepsis	TRUE	reported	textfile	0.0018	9.31E-07	7217	M35270.m	TRUE	reported	21ZlsQ	2	TRUE	0.003452	24.99307
3227	rs1472923	T	C	T	C	-0.0165	0.034698	0.1656	0.15969	FALSE	FALSE	FALSE	I25GHB	0.018694	0.063442	486484	sepsis	TRUE	reported	textfile	0.0033	8.09E-07	7184	M22548.m	TRUE	reported	eBmTQD	2	TRUE	0.003468	24.99304
2531	rs2059238	A	C	A	C	-0.0435	-0.02947	0.1958	0.214002	FALSE	FALSE	FALSE	4HwTlG	0.016694	0.077521	486484	sepsis	TRUE	reported	textfile	0.0087	6.60E-07	7071	M18254.m	TRUE	reported	F9KrSC	2	TRUE	0.003523	24.99293
6279	rs4823474	T	C	T	C	0.009	0.014036	0.5551	0.550714	FALSE	FALSE	FALSE	MOonP9	0.01379	0.308762	486484	sepsis	TRUE	reported	textfile	0.0018	8.28E-07	7066	M33515.m	TRUE	reported	226GuP	2	TRUE	0.003526	24.99292
2291	rs1159706	T	C	T	C	-0.018	-0.00778	0.7513	0.74811	FALSE	FALSE	FALSE	43Yh3h	0.015757	0.621281	486484	sepsis	TRUE	reported	textfile	0.0036	5.27E-07	7055	M16818.m	TRUE	reported	beR4IT	2	TRUE	0.003531	24.99291
6221	rs1295340	T	C	T	C	0.022	-0.0097	0.1905	0.187168	FALSE	FALSE	FALSE	8zUBbU	0.01752	0.580009	486484	sepsis	TRUE	reported	textfile	0.0044	7.33E-07	7044	M33509.m	TRUE	reported	1mO9m8	2	TRUE	0.003537	24.9929
1468	rs1560792	A	G	A	G	0.009	0.004142	0.3354	0.345131	FALSE	FALSE	FALSE	pkb876	0.014485	0.774888	486484	sepsis	TRUE	reported	textfile	0.0018	3.33E-07	7037	M02734.m	TRUE	reported	0LCMVI	2	TRUE	0.00354	24.99289
8853	rs510573	T	G	T	G	-0.0085	-0.00037	0.6641	0.674735	FALSE	FALSE	FALSE	XlHeTV	0.014772	0.980089	486484	sepsis	TRUE	reported	textfile	0.0017	9.11E-07	6940	M36808.m	TRUE	reported	37Vi2D	2	TRUE	0.003589	24.9928
2566	rs1055689	A	G	A	G	-0.0135	0.000211	0.2969	0.306843	FALSE	FALSE	FALSE	KHS91f	0.01482	0.988652	486484	sepsis	TRUE	reported	textfile	0.0027	4.26E-07	6939	M18349.m	TRUE	reported	PeVGil	2	TRUE	0.00359	24.99279
8139	rs1110027	A	G	A	G	-0.009	0.010248	0.8163	0.789707	FALSE	FALSE	FALSE	lv3pAQ	0.016752	0.540701	486484	sepsis	TRUE	reported	textfile	0.0018	7.74E-07	6920	M35327.m	TRUE	reported	3Vsglp	2	TRUE	0.0036	24.99277
8144	rs1798613	A	G	A	G	0.009	0.000397	0.2257	0.270146	FALSE	FALSE	FALSE	lv3pAQ	0.015394	0.9794	486484	sepsis	TRUE	reported	textfile	0.0018	3.74E-07	6920	M35327.m	TRUE	reported	3Vsglp	2	TRUE	0.0036	24.99277
6693	rs1189639	T	C	T	C	0.122	0.040571	0.0255	0.02999	FALSE	FALSE	FALSE	AcRp2F	0.040002	0.310484	486484	sepsis	TRUE	reported	textfile	0.0244	5.47E-07	6756	M33935.m	TRUE	reported	FQCKJy	2	TRUE	0.003687	24.9926
7275	rs1075206	A	G	A	G	-0.009	0.005493	0.669	0.665661	FALSE	FALSE	FALSE	Vcz4EA	0.014539	0.705554	486484	sepsis	TRUE	reported	textfile	0.0018	4.61E-07	6755	M34359.m	TRUE	reported	aUSo2J	2	TRUE	0.003687	24.9926
7313	rs749514	A	G	A	G	-0.009	0.021347	0.3055	0.307016	FALSE	FALSE	FALSE	Vcz4EA	0.014843	0.150374	486484	sepsis	TRUE	reported	textfile	0.0018	4.71E-07	6755	M34359.m	TRUE	reported	aUSo2J	2	TRUE	0.003687	24.9926
8448	rs1225903	A	G	A	G	0.014	0.010944	0.1453	0.149786	FALSE	FALSE	FALSE	uMajV6	0.019199	0.568647	486484	sepsis	TRUE	reported	textfile	0.0028	9.07E-07	6604	M35754.m	TRUE	reported	b8QB7S	2	TRUE	0.003771	24.99243
3002	rs1073964	A	G	A	G	-0.0135	0.01327	0.4429	0.439096	FALSE	FALSE	FALSE	gxupFu	0.013815	0.336777	486484	sepsis	TRUE	reported	textfile	0.0027	4.06E-07	6556	M21188.m	TRUE	reported	gwwXh2	2	TRUE	0.003799	24.99237
3003	rs1078376	T	G	T	G	0.0135	0.016314	0.7251	0.725855	FALSE	FALSE	FALSE	gxupFu	0.015336	0.287446	486484	sepsis	TRUE	reported	textfile	0.0027	6.63E-07	6556	M21188.m	TRUE	reported	gwwXh2	2	TRUE	0.003799	24.99237
8893	rs1211447	A	G	A	G	-0.009	0.006771	0.4738	0.474264	FALSE	FALSE	FALSE	eLhsld	0.013688	0.620829	486484	sepsis	TRUE	reported	textfile	0.0018	1.22E-06	6552	M37058.m	TRUE	reported	RSeWE5	2	TRUE	0.003801	24.99237
4508	rs1050482	A	G	A	G	-0.0095	0.023499	0.7046	0.701518	FALSE	FALSE	FALSE	r0qJaM	0.01495	0.115997	486484	sepsis	TRUE	reported	textfile	0.0019	5.81E-07	6497	M32729.m	TRUE	reported	vYv5FI	2	TRUE	0.003833	24.9923
4523	rs1735765	T	C	T	C	-0.0095	-0.00546	0.6988	0.698555	FALSE	FALSE	FALSE	r0qJaM	0.0149	0.714177	486484	sepsis	TRUE	reported	textfile	0.0019	5.48E-07	6497	M32729.m	TRUE	reported	vYv5FI	2	TRUE	0.003833	24.9923
4540	rs6791299	A	G	A	G	-0.0095	0.007038	0.534	0.535495	FALSE	FALSE	FALSE	r0qJaM	0.013738	0.608419	486484	sepsis	TRUE	reported	textfile	0.0019	3.71E-07	6497	M32729.m	TRUE	reported	vYv5FI	2	TRUE	0.003833	24.9923
2774	rs789736	T	G	T	G	0.019	-0.01012	0.2464	0.238968	FALSE	FALSE	FALSE	JWylTj	0.016005	0.527309	486484	sepsis	TRUE	reported	textfile	0.0038	4.03E-07	6385	M19396.m	TRUE	reported	6S7kzt	2	TRUE	0.0039	24.99217
3060	rs7934036	T	G	T	G	0.0125	0.011824	0.3475	0.340771	FALSE	FALSE	FALSE	H6vcCG	0.014381	0.410954	486484	sepsis	TRUE	reported	textfile	0.0025	9.76E-07	6180	M22030.m	TRUE	reported	XetVX7	2	TRUE	0.004029	24.99191
4776	rs930609	A	G	A	G	-0.028	-0.05009	0.7886	0.783111	FALSE	FALSE	FALSE	mOrnK4	0.016573	0.002508	486484	sepsis	TRUE	reported	textfile	0.0056	4.98E-07	6169	M32795.m	TRUE	reported	7EGxuf	2	TRUE	0.004036	24.99189
7735	rs1422587	T	C	T	C	0.0095	0.012271	0.3521	0.353302	FALSE	FALSE	FALSE	HyMNYM	0.01431	0.391146	486484	sepsis	TRUE	reported	textfile	0.0019	4.87E-07	5885	M34912.m	TRUE	reported	Tl8Hcw	2	TRUE	0.00423	24.9915
8117	rs4480029	A	C	A	C	0.009	0.004887	0.5598	0.547359	FALSE	FALSE	FALSE	nNbXBS	0.013802	0.723311	486484	sepsis	TRUE	reported	textfile	0.0018	9.94E-07	5768	M35326.m	TRUE	reported	UNDorn	2	TRUE	0.004316	24.99133
8132	rs924321	T	C	T	C	0.0115	-0.00488	0.305	0.318463	FALSE	FALSE	FALSE	nNbXBS	0.014667	0.739175	486484	sepsis	TRUE	reported	textfile	0.0023	5.74E-07	5768	M35326.m	TRUE	reported	UNDorn	2	TRUE	0.004316	24.99133
8133	rs9290704	T	G	T	G	-0.0085	-0.02211	0.5814	0.585315	FALSE	FALSE	FALSE	nNbXBS	0.013954	0.11305	486484	sepsis	TRUE	reported	textfile	0.0017	8.98E-07	5768	M35326.m	TRUE	reported	UNDorn	2	TRUE	0.004316	24.99133
1794	rs2491294	A	G	A	G	-0.01	-0.00786	0.31	0.311805	FALSE	FALSE	FALSE	JEcCRZ	0.014783	0.594856	486484	sepsis	TRUE	reported	textfile	0.002	5.73E-07	5604	M15140.m	TRUE	reported	xYCzKP	2	TRUE	0.004441	24.99108
9041	rs39578	A	G	A	G	-0.01	0.011727	0.729	0.736659	FALSE	FALSE	FALSE	df0MCM	0.015508	0.449557	486484	sepsis	TRUE	reported	textfile	0.002	5.73E-07	5604	M37506.m	TRUE	reported	iUfr4u	2	TRUE	0.004441	24.99108
4312	rs7912716	T	C	T	C	0.015	-0.01136	0.104	0.108705	FALSE	FALSE	FALSE	0tjjPC	0.021925	0.604416	486484	sepsis	TRUE	reported	textfile	0.003	5.73E-07	5598	M32634.m	TRUE	reported	nJJFkX	2	TRUE	0.004446	24.99107
4497	rs4838726	C	G	C	G	0.045	0.003069	0.032	0.031054	FALSE	TRUE	FALSE	1wNJ1E	0.039532	0.938113	486484	sepsis	TRUE	reported	textfile	0.009	5.73E-07	5597	M32709.m	TRUE	reported	zihBHg	2	TRUE	0.004447	24.99107
9	rs1000137	T	C	T	C	-0.005	0.000622	0.475	0.477687	FALSE	FALSE	FALSE	xmGuol	0.013687	0.963764	486484	sepsis	TRUE	reported	textfile	0.001	5.73E-07	5592	M00054.m	TRUE	reported	GW50Ri	2	TRUE	0.004451	24.99106
22	rs1083636	T	C	T	C	0.005	-0.01056	0.607	0.610918	FALSE	FALSE	FALSE	xmGuol	0.014038	0.451878	486484	sepsis	TRUE	reported	textfile	0.001	5.73E-07	5592	M00054.m	TRUE	reported	GW50Ri	2	TRUE	0.004451	24.99106
74	rs2230532	T	C	T	C	0.005	-0.01434	0.349	0.345677	FALSE	FALSE	FALSE	xmGuol	0.014349	0.317612	486484	sepsis	TRUE	reported	textfile	0.001	5.73E-07	5592	M00054.m	TRUE	reported	GW50Ri	2	TRUE	0.004451	24.99106
78	rs2505644	T	C	T	C	0.005	-0.00079	0.558	0.556744	FALSE	FALSE	FALSE	xmGuol	0.013754	0.95447	486484	sepsis	TRUE	reported	textfile	0.001	5.73E-07	5592	M00054.m	TRUE	reported	GW50Ri	2	TRUE	0.004451	24.99106
97	rs2977272	T	C	T	C	0.005	-0.01943	0.341	0.346977	FALSE	FALSE	FALSE	xmGuol	0.014342	0.175491	486484	sepsis	TRUE	reported	textfile	0.001	5.73E-07	5592	M00054.m	TRUE	reported	GW50Ri	2	TRUE	0.004451	24.99106
99	rs341973	T	C	T	C	0.005	0.014161	0.493	0.494256	FALSE	FALSE	FALSE	xmGuol	0.013741	0.30274	486484	sepsis	TRUE	reported	textfile	0.001	5.73E-07	5592	M00054.m	TRUE	reported	GW50Ri	2	TRUE	0.004451	24.99106
116	rs4686980	T	C	T	C	0.005	-0.00355	0.361	0.359752	FALSE	FALSE	FALSE	xmGuol	0.014479	0.806392	486484	sepsis	TRUE	reported	textfile	0.001	5.73E-07	5592	M00054.m	TRUE	reported	GW50Ri	2	TRUE	0.004451	24.99106
130	rs5997110	T	C	T	C	-0.005	-0.00501	0.523	0.51616	FALSE	FALSE	FALSE	xmGuol	0.013729	0.715107	486484	sepsis	TRUE	reported	textfile	0.001	5.73E-07	5592	M00054.m	TRUE	reported	GW50Ri	2	TRUE	0.004451	24.99106
1863	rs1028981	A	G	A	G	-0.005	0.024324	0.385	0.387683	FALSE	FALSE	FALSE	v2KE89	0.014049	0.083393	486484	sepsis	TRUE	reported	textfile	0.001	5.73E-07	5590	M15500.m	TRUE	reported	to2t7H	2	TRUE	0.004452	24.99106
1897	rs12484	T	C	T	C	-0.005	-0.01507	0.427	0.420798	FALSE	FALSE	FALSE	v2KE89	0.013868	0.277048	486484	sepsis	TRUE	reported	textfile	0.001	5.73E-07	5590	M15500.m	TRUE	reported	to2t7H	2	TRUE	0.004452	24.99106
1963	rs2777788	A	G	A	G	-0.005	-0.01827	0.636	0.628553	FALSE	FALSE	FALSE	v2KE89	0.014322	0.202172	486484	sepsis	TRUE	reported	textfile	0.001	5.73E-07	5590	M15500.m	TRUE	reported	to2t7H	2	TRUE	0.004452	24.99106
1967	rs2961253	T	C	T	C	-0.005	-0.00292	0.55	0.552126	FALSE	FALSE	FALSE	v2KE89	0.013733	0.831645	486484	sepsis	TRUE	reported	textfile	0.001	5.73E-07	5590	M15500.m	TRUE	reported	to2t7H	2	TRUE	0.004452	24.99106
1978	rs4305127	T	C	T	C	-0.005	0.002974	0.404	0.403813	FALSE	FALSE	FALSE	v2KE89	0.015045	0.84329	486484	sepsis	TRUE	reported	textfile	0.001	5.73E-07	5590	M15500.m	TRUE	reported	to2t7H	2	TRUE	0.004452	24.99106
2002	rs4940563	A	G	A	G	-0.005	-0.01509	0.526	0.533338	FALSE	FALSE	FALSE	v2KE89	0.013704	0.270804	486484	sepsis	TRUE	reported	textfile	0.001	5.73E-07	5590	M15500.m	TRUE	reported	to2t7H	2	TRUE	0.004452	24.99106
2041	rs8099879	A	C	A	C	0.005	0.011225	0.383	0.374753	FALSE	FALSE	FALSE	v2KE89	0.015134	0.458274	486484	sepsis	TRUE	reported	textfile	0.001	5.73E-07	5590	M15500.m	TRUE	reported	to2t7H	2	TRUE	0.004452	24.99106
203	rs1000428	A	G	A	G	0.005	-0.0093	0.374	0.377414	FALSE	FALSE	FALSE	tbRL8J	0.014132	0.510547	486484	sepsis	TRUE	reported	textfile	0.001	5.73E-07	5589	M00060.m	TRUE	reported	oOvRKT	2	TRUE	0.004453	24.99105
204	rs1003887	T	C	T	C	-0.005	-0.00963	0.651	0.645013	FALSE	FALSE	FALSE	tbRL8J	0.01427	0.499763	486484	sepsis	TRUE	reported	textfile	0.001	5.73E-07	5589	M00060.m	TRUE	reported	oOvRKT	2	TRUE	0.004453	24.99105
205	rs1019018	T	C	T	C	0.005	0.013898	0.662	0.657368	FALSE	FALSE	FALSE	tbRL8J	0.014427	0.33536	486484	sepsis	TRUE	reported	textfile	0.001	5.73E-07	5589	M00060.m	TRUE	reported	oOvRKT	2	TRUE	0.004453	24.99105
296	rs2898645	T	C	T	C	-0.005	0.012339	0.545	0.550173	FALSE	FALSE	FALSE	tbRL8J	0.013751	0.369569	486484	sepsis	TRUE	reported	textfile	0.001	5.73E-07	5589	M00060.m	TRUE	reported	oOvRKT	2	TRUE	0.004453	24.99105
307	rs4148593	A	G	A	G	0.005	0.016374	0.624	0.617911	FALSE	FALSE	FALSE	tbRL8J	0.01408	0.244851	486484	sepsis	TRUE	reported	textfile	0.001	5.73E-07	5589	M00060.m	TRUE	reported	oOvRKT	2	TRUE	0.004453	24.99105
265	rs1818106	A	C	A	C	0.005	-0.01915	0.501	0.501212	FALSE	FALSE	FALSE	tbRL8J	0.013768	0.16424	486484	sepsis	TRUE	reported	textfile	0.001	5.73E-07	5589	M00060.m	TRUE	reported	oOvRKT	2	TRUE	0.004453	24.99105
332	rs6470194	A	G	A	G	0.005	-0.01206	0.406	0.410037	FALSE	FALSE	FALSE	tbRL8J	0.014676	0.411134	486484	sepsis	TRUE	reported	textfile	0.001	5.73E-07	5589	M00060.m	TRUE	reported	oOvRKT	2	TRUE	0.004453	24.99105
672	rs4347830	A	G	A	G	0.01	-0.00905	0.337	0.343257	FALSE	FALSE	FALSE	p4oCVP	0.014424	0.53054	486484	sepsis	TRUE	reported	textfile	0.002	5.73E-07	5588	M01125.m	TRUE	reported	OwD5re	2	TRUE	0.004454	24.99105
796	rs4420815	A	G	A	G	-0.015	-0.02538	0.143	0.15028	FALSE	FALSE	FALSE	dyT79I	0.019105	0.184011	486484	sepsis	TRUE	reported	textfile	0.003	5.73E-07	5586	M01336.m	TRUE	reported	OemVJ2	2	TRUE	0.004456	24.99105
3273	rs1022713	T	C	T	C	0.01	0.024049	0.435	0.437786	FALSE	FALSE	FALSE	vYWPe9	0.013782	0.081001	486484	sepsis	TRUE	reported	textfile	0.002	5.73E-07	5584	M25459.m	TRUE	reported	VvJ6Vx	2	TRUE	0.004457	24.99105
3274	rs1050457	A	G	A	G	-0.01	0.004679	0.531	0.548035	FALSE	FALSE	FALSE	vYWPe9	0.013748	0.733601	486484	sepsis	TRUE	reported	textfile	0.002	5.73E-07	5584	M25459.m	TRUE	reported	VvJ6Vx	2	TRUE	0.004457	24.99105
1855	rs9811522	A	G	A	G	0.01	0.002458	0.174	0.185035	FALSE	FALSE	FALSE	S1eWts	0.017899	0.890791	486484	sepsis	TRUE	reported	textfile	0.002	5.73E-07	5583	M15488.m	TRUE	reported	511Kl9	2	TRUE	0.004458	24.99104
3482	rs1105769	A	C	A	C	-0.0135	-0.01397	0.3077	0.305056	FALSE	FALSE	FALSE	Awafb0	0.014825	0.345946	486484	sepsis	TRUE	reported	textfile	0.0027	7.19E-07	5562	M27728.m	TRUE	reported	Lfj8L0	2	TRUE	0.004475	24.99101
3319	rs1012431	T	C	T	C	-0.01	0.02352	0.235	0.232678	FALSE	FALSE	FALSE	m66Eav	0.016195	0.146428	486484	sepsis	TRUE	reported	textfile	0.002	5.73E-07	5542	M27256.m	TRUE	reported	pl2ECU	2	TRUE	0.004491	24.99098
8216	rs1000347	T	C	T	C	0.02	0.018364	0.112	0.116445	FALSE	FALSE	FALSE	4YAWhA	0.021331	0.389295	486484	sepsis	TRUE	reported	textfile	0.004	5.73E-07	5538	M35439.m	TRUE	reported	bSSC4Z	2	TRUE	0.004494	24.99097
5840	rs8130833	A	G	A	G	-0.01	-0.00603	0.707	0.716733	FALSE	FALSE	FALSE	R5joEf	0.01515	0.690618	486484	sepsis	TRUE	reported	textfile	0.002	5.73E-07	5536	M33380.m	TRUE	reported	kRvbfZ	2	TRUE	0.004496	24.99097
6274	rs4626675	T	C	T	C	0.01	0.00792	0.466	0.471528	FALSE	FALSE	FALSE	MOonP9	0.014217	0.577496	486484	sepsis	TRUE	reported	textfile	0.002	5.73E-07	5382	M33515.m	TRUE	reported	226GuP	2	TRUE	0.004624	24.99071
7101	rs7120732	T	C	T	C	-0.055	-0.04788	0.018	0.019763	FALSE	FALSE	FALSE	pxNICA	0.049353	0.331958	486484	sepsis	TRUE	reported	textfile	0.011	5.73E-07	5321	M34306.m	TRUE	reported	xk5b9M	2	TRUE	0.004676	24.9906
8768	rs1169860	A	G	A	G	-0.0235	-0.01208	0.828	0.81107	FALSE	FALSE	FALSE	7desNF	0.017521	0.490646	486484	sepsis	TRUE	reported	textfile	0.0047	6.70E-07	5292	M36673.m	TRUE	reported	RKbIT4	2	TRUE	0.004702	24.99055
3536	rs4148088	C	G	C	G	0.025	0.022139	0.926	0.918884	FALSE	TRUE	FALSE	rXzSlH	0.025229	0.380216	486484	sepsis	TRUE	reported	textfile	0.005	5.73E-07	5286	M30805.m	TRUE	reported	7wg9QH	2	TRUE	0.004707	24.99054
7844	rs595286	A	G	A	G	-0.0095	-0.01693	0.5466	0.554473	FALSE	FALSE	FALSE	h5whlS	0.013872	0.222423	486484	sepsis	TRUE	reported	textfile	0.0019	8.62E-07	4900	M35137.m	TRUE	reported	xVITKF	2	TRUE	0.005076	24.9898
517	rs526744	T	C	T	C	-0.021	0.020502	0.243	0.244488	FALSE	FALSE	FALSE	cbwEt9	0.016154	0.204373	486484	sepsis	TRUE	reported	textfile	0.0042	7.14E-07	4500	M00575.m	TRUE	reported	hWSEOI	2	TRUE	0.005525	24.98889
5013	rs4363506	T	C	T	C	0.0245	-0.004	0.5678	0.575479	FALSE	FALSE	FALSE	URCVGm	0.013912	0.773829	486484	sepsis	TRUE	reported	textfile	0.0049	6.01E-07	2773	M33131.m	TRUE	reported	4rQ2gs	2	TRUE	0.008935	24.98197
7078	rs1690878	A	G	A	T	-0.0255	0.009939	0.2086	0.211799	FALSE	FALSE	FALSE	sg7nOB	0.016772	0.553438	486484	sepsis	TRUE	reported	textfile	0.0051	5.52E-07	2278	M34289.m	TRUE	reported	KemvlC	2	TRUE	0.010855	24.97805
4915	rs894282	A	T	A	T	0.013	-0.06052	0.0899	0.09346	FALSE	TRUE	FALSE	VqcRGX	0.023579	0.010263	486484	sepsis	TRUE	reported	textfile	0.0026	9.04E-07	1763	M32867.m	TRUE	reported	psuFJy	2	TRUE	0.013982	24.97164
712	rs703014	A	G	A	G	0.0135	-0.00888	0.326	0.329627	FALSE	FALSE	FALSE	H9I3s3	0.014716	0.546268	486484	sepsis	TRUE	reported	textfile	0.0027	7.19E-07	1761	M01299.m	TRUE	reported	kXWyUq	2	TRUE	0.013998	24.97161
7007	rs1822378	T	C	T	C	0.0315	0.002841	0.6721	0.699001	FALSE	FALSE	FALSE	ePdfmg	0.01502	0.849974	486484	sepsis	TRUE	reported	textfile	0.0063	6.37E-07	1761	M34244.m	TRUE	reported	l9nzfX	2	TRUE	0.013998	24.97161
3441	rs9489956	T	C	T	C	0.0395	-0.02882	0.782	0.783185	FALSE	FALSE	FALSE	5dHWFk	0.016622	0.082922	486484	sepsis	TRUE	reported	textfile	0.0079	5.28E-07	1758	M27672.m	TRUE	reported	pEd8kY	2	TRUE	0.014021	24.97156
7156	rs2826581	T	G	T	G	-0.0695	0.012071	0.789	0.775199	FALSE	FALSE	FALSE	PnBT0M	0.016429	0.462498	486484	sepsis	TRUE	reported	textfile	0.0139	5.58E-07	1612	M34327.m	TRUE	reported	VUt4Q7	2	TRUE	0.015272	24.96898
8735	rs1715247	T	C	T	C	0.2502	-0.04756	0.0133	0.039841	FALSE	FALSE	FALSE	ERC6hD	0.035043	0.174674	486484	sepsis	TRUE	reported	textfile	0.05	5.58E-07	589	M36559.m	TRUE	reported	n8waJF	2	TRUE	0.040779	24.95499
8783	rs1491633	A	G	A	G	0.059	0.027242	0.0765	0.073929	FALSE	FALSE	FALSE	4bkfvN	0.026068	0.296015	486484	sepsis	TRUE	reported	textfile	0.0118	5.48E-07	922	M36738.m	TRUE	reported	xcqyX2	2	TRUE	0.026399	24.94577
4803	rs6686996	T	C	T	C	0.0919	0.065512	0.0256	0.024698	FALSE	FALSE	FALSE	9qWhFj	0.044526	0.141208	486484	sepsis	TRUE	reported	textfile	0.0184	5.81E-07	6200	M32808.m	TRUE	reported	MQcOSi	2	TRUE	0.004007	24.93763
4821	rs1715081	A	C	A	C	-0.0714	0.01268	0.0377	0.053668	FALSE	FALSE	FALSE	B6WchP	0.031181	0.684247	486484	sepsis	TRUE	reported	textfile	0.0143	6.11E-07	7255	M32836.m	TRUE	reported	7UzUw0	2	TRUE	0.003424	24.92325
8955	rs328637	A	G	A	G	-0.0864	0.00709	0.1638	0.16458	FALSE	FALSE	FALSE	Ywk2ve	0.018438	0.7006	486484	sepsis	TRUE	reported	textfile	0.0173	6.13E-07	2560	M37112.m	TRUE	reported	EiAdDj	2	TRUE	0.009649	24.92274
6891	rs1258813	C	G	C	G	0.0694	-0.01153	0.0374	0.040532	FALSE	TRUE	FALSE	MN2lbE	0.034869	0.740822	486484	sepsis	TRUE	reported	textfile	0.0139	6.33E-07	4961	M34106.m	TRUE	reported	q4zXEz	2	TRUE	0.005	24.91806
2542	rs219633	A	G	A	G	0.1483	-0.01058	0.121	0.114932	FALSE	FALSE	FALSE	o0CFya	0.021444	0.621593	486484	sepsis	TRUE	reported	textfile	0.0297	5.96E-07	2433	M18281.m	TRUE	reported	pxbsnu	2	TRUE	0.010144	24.91221
6606	rs138832	A	G	A	G	0.0779	0.022919	0.7832	0.783963	FALSE	FALSE	FALSE	VfOuo8	0.016645	0.168521	486484	sepsis	TRUE	reported	textfile	0.0156	6.18E-07	1742	M33883.m	TRUE	reported	BDe0Nk	2	TRUE	0.014113	24.90731
2841	rs129986	T	C	T	C	-0.0724	-0.00615	0.983	0.971146	FALSE	FALSE	FALSE	Y5qWBm	0.040776	0.880108	486484	sepsis	TRUE	reported	textfile	0.0145	5.55E-07	1754	M20489.m	TRUE	reported	f2g9Su	2	TRUE	0.014015	24.90265
965	rs7458704	T	G	T	G	-0.0544	-0.03944	0.984	0.97943	FALSE	FALSE	FALSE	tWoDq4	0.048389	0.415029	486484	sepsis	TRUE	reported	textfile	0.0109	5.60E-07	7160	M01508.m	TRUE	reported	6ltDw0	2	TRUE	0.003467	24.90138
6892	rs1552059	A	G	A	G	0.0519	-0.02025	0.0942	0.113926	FALSE	FALSE	FALSE	MN2lbE	0.021555	0.347547	486484	sepsis	TRUE	reported	textfile	0.0104	6.38E-07	4961	M34106.m	TRUE	reported	q4zXEz	2	TRUE	0.004995	24.8939
5158	rs6130478	A	G	A	G	0.8585	-0.06304	0.9803	0.980055	FALSE	FALSE	FALSE	L2DJzK	0.049027	0.198476	486484	sepsis	TRUE	reported	textfile	0.1698	4.28E-07	76	M33173.m	TRUE	reported	hRG70D	2	TRUE	0.251693	24.88991
5643	rs1168794	T	G	T	G	-0.0484	0.007604	0.5576	0.55444	FALSE	FALSE	FALSE	BFsMtm	0.013815	0.582036	486484	sepsis	TRUE	reported	textfile	0.0097	6.03E-07	5716	M33192.m	TRUE	reported	NAJN3D	2	TRUE	0.004337	24.8883
8971	rs9602130	A	C	A	C	0.0579	-0.00666	0.3642	0.368688	FALSE	FALSE	FALSE	2iAvlt	0.014215	0.63923	486484	sepsis	TRUE	reported	textfile	0.0116	5.72E-07	1745	M37190.m	TRUE	reported	hdOuHe	2	TRUE	0.014076	24.88531
4888	rs1748316	A	G	A	G	-0.0544	0.01681	0.6475	0.643341	FALSE	FALSE	FALSE	ocaywK	0.014333	0.240883	486484	sepsis	TRUE	reported	textfile	0.0109	6.58E-07	1988	M32863.m	TRUE	reported	WH6KGr	2	TRUE	0.012374	24.88328
8524	rs1695174	A	G	A	G	0.0449	0.013461	0.1023	0.110509	FALSE	FALSE	FALSE	gM7v2m	0.021868	0.538192	486484	sepsis	TRUE	reported	textfile	0.009	6.09E-07	2651	M36009.m	TRUE	reported	6Xcypd	2	TRUE	0.009301	24.87024
5677	rs7808781	A	G	A	G	0.1777	-0.01912	0.1358	0.129259	FALSE	FALSE	FALSE	AAOSvG	0.020761	0.357117	486484	sepsis	TRUE	reported	textfile	0.0356	5.98E-07	996	M33197.m	TRUE	reported	aAmcal	2	TRUE	0.024405	24.86577
8532	rs1076503	T	C	T	C	-0.0798	-0.0295	0.1892	0.193596	FALSE	FALSE	FALSE	3PHxzL	0.017374	0.08952	486484	sepsis	TRUE	reported	textfile	0.016	6.34E-07	3939	M36095.m	TRUE	reported	CRZVSB	2	TRUE	0.006275	24.86253
6417	rs1007401	A	G	A	G	0.0424	0.014621	0.2515	0.26135	FALSE	FALSE	FALSE	F7mjRv	0.015568	0.347643	486484	sepsis	TRUE	reported	textfile	0.0085	5.48E-07	1860	M33658.m	TRUE	reported	frG0Zf	2	TRUE	0.013201	24.85574
6162	rs1114993	A	G	A	G	-0.0349	-0.0168	0.1083	0.108141	FALSE	FALSE	FALSE	O4Ty2I	0.022227	0.449626	486484	sepsis	TRUE	reported	textfile	0.007	6.24E-07	5107	M33488.m	TRUE	reported	RGtF84	2	TRUE	0.004844	24.84761
8750	rs6761802	A	G	A	G	-0.0334	0.014076	0.3637	0.365481	FALSE	FALSE	FALSE	FnYrPc	0.01426	0.323575	486484	sepsis	TRUE	reported	textfile	0.0067	6.53E-07	3346	M36585.m	TRUE	reported	amHtMH	2	TRUE	0.007372	24.83611
2713	rs1184450	C	G	C	G	-0.1381	0.019787	0.9853	0.981734	FALSE	TRUE	FALSE	89XKxw	0.051573	0.70122	486484	sepsis	TRUE	reported	textfile	0.0277	6.28E-07	1738	M19324.m	TRUE	reported	gryJuD	2	TRUE	0.0141	24.8272
3946	rs1212158	A	G	A	G	-0.0289	-0.00446	0.2068	0.229944	FALSE	FALSE	FALSE	N6UQnQ	0.016279	0.783904	486484	sepsis	TRUE	reported	textfile	0.0058	5.43E-07	7346	M32425.m	TRUE	reported	FEcGHY	2	TRUE	0.003368	24.82112
7876	rs1028374	A	G	A	G	0.0264	-0.02416	0.1409	0.152626	FALSE	FALSE	FALSE	mpkL0o	0.019577	0.217198	486484	sepsis	TRUE	reported	textfile	0.0053	5.88E-07	7263	M35160.m	TRUE	reported	MKkvYr	2	TRUE	0.003405	24.80484
2399	rs1750666	T	C	T	C	-0.1171	0.020512	0.0465	0.060203	FALSE	FALSE	FALSE	NBJ2Do	0.02864	0.473871	486484	sepsis	TRUE	reported	textfile	0.0235	6.22E-07	1739	M17799.m	TRUE	reported	Uz0Wgl	2	TRUE	0.014077	24.80152
6792	rs8081778	T	C	T	C	0.0259	-0.01774	0.1587	0.162731	FALSE	FALSE	FALSE	kcZoFT	0.018594	0.339928	486484	sepsis	TRUE	reported	textfile	0.0052	7.06E-07	6998	M33957.m	TRUE	reported	alW1w0	2	TRUE	0.003532	24.80097
6020	rs1515977	A	G	A	G	-0.9112	0.041773	0.0594	0.039977	FALSE	FALSE	FALSE	33ojbt	0.035055	0.233405	486484	sepsis	TRUE	reported	textfile	0.1799	4.06E-07	60	M33423.m	TRUE	reported	ivEtvl	2	TRUE	0.299512	24.79944
6416	rs961787	T	C	T	C	0.0254	0.020671	0.3948	0.396519	FALSE	FALSE	FALSE	5ClPW7	0.013983	0.139336	486484	sepsis	TRUE	reported	textfile	0.0051	5.14E-07	4807	M33653.m	TRUE	reported	H4b2zx	2	TRUE	0.005134	24.79399
8513	rs4665383	C	G	C	G	-0.0254	-0.00863	0.7261	0.743643	FALSE	TRUE	FALSE	vHXktL	0.015661	0.581684	486484	sepsis	TRUE	reported	textfile	0.0051	5.49E-07	4529	M35978.m	TRUE	reported	ceap9w	2	TRUE	0.005447	24.79335
4240	rs2977258	A	G	A	G	0.0244	-0.0269	0.3036	0.309074	FALSE	FALSE	FALSE	wWEMr1	0.014862	0.070272	486484	sepsis	TRUE	reported	textfile	0.0049	5.39E-07	5955	M32593.m	TRUE	reported	Mh2g4i	2	TRUE	0.004147	24.78801
5613	rs974135	T	C	T	C	-0.0912	0.003564	0.2826	0.327035	FALSE	FALSE	FALSE	q32d4E	0.014631	0.807535	486484	sepsis	TRUE	reported	textfile	0.0183	5.84E-07	1017	M33178.m	TRUE	reported	cS3BwT	2	TRUE	0.023839	24.78749
2462	rs96569	A	G	A	G	-0.0846	0.033645	0.125	0.137205	FALSE	FALSE	FALSE	NBJ2Do	0.02323	0.147517	486484	sepsis	TRUE	reported	textfile	0.0163	2.06E-07	25	M17799.m	TRUE	reported	Uz0Wgl	2	TRUE	0.518657	24.78297
6821	rs1771181	T	C	T	C	0.0214	0.016089	0.2564	0.258373	FALSE	FALSE	FALSE	wedECv	0.01572	0.306086	486484	sepsis	TRUE	reported	textfile	0.0043	5.09E-07	7322	M33968.m	TRUE	reported	GxGxRG	2	TRUE	0.003371	24.76122
7667	rs1001046	T	G	T	G	0.0214	-0.02654	0.6845	0.69096	FALSE	FALSE	FALSE	iqkM9B	0.014848	0.073891	486484	sepsis	TRUE	reported	textfile	0.0043	6.53E-07	4800	M34539.m	TRUE	reported	5nFq6f	2	TRUE	0.005134	24.75766
4175	rs4650516	T	C	T	C	-0.0448	-0.00688	0.5865	0.588182	FALSE	FALSE	FALSE	Aq7H4p	0.013899	0.62074	486484	sepsis	TRUE	reported	textfile	0.009	5.75E-07	2091	M32560.m	TRUE	reported	NIzbA7	2	TRUE	0.011711	24.75457
7460	rs7106005	T	C	T	C	0.0204	-0.00025	0.8158	0.797753	FALSE	FALSE	FALSE	7ubZow	0.017086	0.988201	486484	sepsis	TRUE	reported	textfile	0.0041	7.66E-07	7344	M34407.m	TRUE	reported	LRsXN2	2	TRUE	0.00336	24.74995
3574	rs2073779	T	C	T	C	-0.2562	0.042788	0.9759	0.970613	FALSE	FALSE	FALSE	F1s6qg	0.040652	0.292556	486484	sepsis	TRUE	reported	textfile	0.0514	6.09E-07	522	M31536.m	TRUE	reported	Zm40GX	2	TRUE	0.045433	24.74941
6694	rs1248539	A	G	A	G	0.0403	-0.01728	0.3273	0.34007	FALSE	FALSE	FALSE	AcRp2F	0.014463	0.232253	486484	sepsis	TRUE	reported	textfile	0.0081	6.08E-07	6756	M33935.m	TRUE	reported	FQCKJy	2	TRUE	0.003651	24.74637
5919	rs4148622	A	G	A	G	0.0398	0.00701	0.2618	0.266694	FALSE	FALSE	FALSE	iGV9aX	0.015601	0.653186	486484	sepsis	TRUE	reported	textfile	0.008	7.66E-07	5185	M33390.m	TRUE	reported	sLbpnf	2	TRUE	0.004751	24.74108
7774	rs1268073	A	G	A	G	0.206	-0.00348	0.9872	0.987822	FALSE	FALSE	FALSE	15BqPa	0.062882	0.955816	486484	sepsis	TRUE	reported	textfile	0.0414	6.44E-07	1764	M35126.m	TRUE	reported	zvDzP2	2	TRUE	0.013841	24.73097
4829	rs1171660	A	G	A	G	-0.0189	-0.00205	0.6827	0.679364	FALSE	FALSE	FALSE	yLDcUj	0.014665	0.888907	486484	sepsis	TRUE	reported	textfile	0.0038	6.89E-07	6753	M32838.m	TRUE	reported	5dQgAN	2	TRUE	0.00365	24.73021
7213	rs1741548	A	G	A	G	-0.898	-0.00778	0.989	0.892071	FALSE	FALSE	FALSE	i1uJ7T	0.022535	0.729846	486484	sepsis	TRUE	reported	textfile	0.179	5.26E-07	115	M34338.m	TRUE	reported	pi3mKW	2	TRUE	0.179555	24.73018
5784	rs6760934	T	C	T	C	-0.1921	-0.06667	0.9725	0.978203	FALSE	FALSE	FALSE	eT2We0	0.047029	0.156267	486484	sepsis	TRUE	reported	textfile	0.0385	5.91E-07	292	M33353.m	TRUE	reported	uZx90m	2	TRUE	0.078563	24.72569
2876	rs1260353	T	C	T	C	-0.0368	0.036759	0.0394	0.059281	FALSE	FALSE	FALSE	mUzn1R	0.028933	0.203915	486484	sepsis	TRUE	reported	textfile	0.0074	6.62E-07	7301	M20675.m	TRUE	reported	XwY04K	2	TRUE	0.003376	24.72369
498	rs2234233	A	G	A	G	-0.1234	-0.01961	0.135	0.16795	FALSE	FALSE	FALSE	RtjZPn	0.018273	0.283139	486484	sepsis	TRUE	reported	textfile	0.0248	6.38E-07	1233	M00553.m	TRUE	reported	C3Ovsl	2	TRUE	0.019685	24.71849
8307	rs9849918	A	C	A	C	0.0363	-0.00678	0.6538	0.665364	FALSE	FALSE	FALSE	HELgy7	0.014547	0.64136	486484	sepsis	TRUE	reported	textfile	0.0073	7.74E-07	5035	M35551.m	TRUE	reported	7x7uUn	2	TRUE	0.004887	24.71696
2637	rs6468384	A	C	A	C	0.0179	-0.00675	0.5691	0.552655	FALSE	FALSE	FALSE	UxuT5B	0.013736	0.623116	486484	sepsis	TRUE	reported	textfile	0.0036	5.63E-07	7367	M18467.m	TRUE	reported	eGQj6o	2	TRUE	0.003345	24.71628
6168	rs2466085	A	G	A	G	0.1762	0.062012	0.0186	0.012352	FALSE	FALSE	FALSE	O4Ty2I	0.06241	0.320406	486484	sepsis	TRUE	reported	textfile	0.0354	6.70E-07	834	M33488.m	TRUE	reported	RGtF84	2	TRUE	0.028849	24.71511
1177	rs577596	A	G	A	G	-0.0567	0.003413	0.3267	0.347183	FALSE	FALSE	FALSE	FgZx4a	0.014344	0.811944	486484	sepsis	TRUE	reported	textfile	0.0114	6.68E-07	2063	M01640.m	TRUE	reported	hNPAJ8	2	TRUE	0.011849	24.71355
2879	rs1499686	A	G	A	G	-0.0174	0.009231	0.8079	0.81472	FALSE	FALSE	FALSE	mUzn1R	0.017742	0.602859	486484	sepsis	TRUE	reported	textfile	0.0035	7.49E-07	7301	M20675.m	TRUE	reported	XwY04K	2	TRUE	0.003374	24.70833
5006	rs1249168	C	G	C	G	-0.0368	-2.98E-05	0.7659	0.752445	FALSE	TRUE	FALSE	URCVGm	0.015866	0.998503	486484	sepsis	TRUE	reported	textfile	0.0074	7.13E-07	1767	M33131.m	TRUE	reported	4rQ2gs	2	TRUE	0.013803	24.70247
5132	rs1278449	A	T	A	T	0.9912	0.022576	0.9821	0.666449	FALSE	TRUE	FALSE	L2DJzK	0.014516	0.11989	486484	sepsis	TRUE	reported	textfile	0.1968	4.76E-07	76	M33173.m	TRUE	reported	hRG70D	2	TRUE	0.250251	24.69963
503	rs7701941	C	G	C	G	-0.5561	0.062783	0.0194	0.019614	FALSE	TRUE	FALSE	RtjZPn	0.049448	0.204197	486484	sepsis	TRUE	reported	textfile	0.1115	6.10E-07	283	M00553.m	TRUE	reported	C3Ovsl	2	TRUE	0.080795	24.6988
5795	rs1716546	T	C	T	C	0.0358	-0.00633	0.1252	0.224122	FALSE	FALSE	FALSE	ka9zG7	0.016425	0.700079	486484	sepsis	TRUE	reported	textfile	0.0072	7.83E-07	1378	M33363.m	TRUE	reported	UFTVNc	2	TRUE	0.017625	24.68711
5932	rs739484	T	C	T	C	0.1354	0.015592	0.0864	0.118721	FALSE	FALSE	FALSE	veBhfS	0.021321	0.464587	486484	sepsis	TRUE	reported	textfile	0.0272	6.56E-07	528	M33391.m	TRUE	reported	Gfjy5a	2	TRUE	0.044828	24.68604
8978	rs2762353	A	G	A	G	0.0318	-0.01095	0.4552	0.433382	FALSE	FALSE	FALSE	snOepl	0.013778	0.426686	486484	sepsis	TRUE	reported	textfile	0.0064	6.70E-07	7355	M37202.m	TRUE	reported	PQx272	2	TRUE	0.003345	24.68176
4476	rs9457843	T	C	T	C	0.0159	0.012245	0.1218	0.118684	FALSE	FALSE	FALSE	g3iM2w	0.021238	0.564239	486484	sepsis	TRUE	reported	textfile	0.0032	9.53E-07	7308	M32698.m	TRUE	reported	ZQBn0h	2	TRUE	0.003367	24.68172
3640	rs1752156	A	G	A	G	-0.0472	0.016189	0.803	0.797268	FALSE	FALSE	FALSE	Vq2efp	0.017007	0.341163	486484	sepsis	TRUE	reported	textfile	0.0095	6.44E-07	7363	M31787.m	TRUE	reported	krl4Tw	2	TRUE	0.003341	24.6785
4569	rs1261781	T	G	T	G	0.0477	-0.05927	0.0449	0.042326	FALSE	FALSE	FALSE	ILOOFw	0.033958	0.080898	486484	sepsis	TRUE	reported	textfile	0.0096	7.54E-07	1748	M32739.m	TRUE	reported	TpvRBe	2	TRUE	0.013927	24.66023
5346	rs1710604	T	C	T	C	-0.255	-0.04927	0.0221	0.022656	FALSE	FALSE	FALSE	q32d4E	0.046678	0.291202	486484	sepsis	TRUE	reported	textfile	0.0513	6.61E-07	1017	M33178.m	TRUE	reported	cS3BwT	2	TRUE	0.023719	24.65987
3165	rs6919834	T	C	T	C	0.0144	0.034489	0.1237	0.128479	FALSE	FALSE	FALSE	JiXR2t	0.020476	0.092113	486484	sepsis	TRUE	reported	textfile	0.0029	8.27E-07	6535	M22177.m	TRUE	reported	joi6p6	2	TRUE	0.003759	24.64882
1152	rs6763802	T	G	T	G	-0.0576	0.022407	0.0798	0.090804	FALSE	FALSE	FALSE	QtY5Hc	0.023707	0.344574	486484	sepsis	TRUE	reported	textfile	0.0116	6.50E-07	5162	M01605.m	TRUE	reported	Q3iDQX	2	TRUE	0.004754	24.64681
7915	rs1786534	A	G	A	G	-0.0412	0.012659	0.089	0.092975	FALSE	FALSE	FALSE	8qXKkX	0.023631	0.59219	486484	sepsis	TRUE	reported	textfile	0.0083	6.16E-07	6234	M35189.m	TRUE	reported	Nt6oaC	2	TRUE	0.003937	24.63196
4555	rs1689751	A	C	A	C	-0.0412	0.005703	0.1641	0.190447	FALSE	FALSE	FALSE	KfoRxi	0.017364	0.74259	486484	sepsis	TRUE	reported	textfile	0.0083	7.25E-07	6000	M32735.m	TRUE	reported	UDRXAp	2	TRUE	0.00409	24.63165
610	rs1235282	A	G	A	G	-0.0134	-0.0228	0.1506	0.151043	FALSE	FALSE	FALSE	BYGzq4	0.019083	0.232092	486484	sepsis	TRUE	reported	textfile	0.0027	5.84E-07	7367	M01110.m	TRUE	reported	oZiXqP	2	TRUE	0.003332	24.62431
6865	rs1158384	A	C	A	C	0.0134	0.032191	0.9439	0.939713	FALSE	FALSE	FALSE	kFWZjs	0.028799	0.263659	486484	sepsis	TRUE	reported	textfile	0.0027	6.34E-07	7362	M34062.m	TRUE	reported	eLONsg	2	TRUE	0.003335	24.62431
1269	rs7739842	T	G	T	G	0.0134	-0.02663	0.8202	0.810187	FALSE	FALSE	FALSE	gNG3wy	0.017474	0.127543	486484	sepsis	TRUE	reported	textfile	0.0027	8.00E-07	7348	M01670.m	TRUE	reported	t3lsMq	2	TRUE	0.003341	24.6243
7489	rs4789188	A	C	A	C	0.0134	-0.01859	0.1628	0.164975	FALSE	FALSE	FALSE	jAhmnV	0.01847	0.314276	486484	sepsis	TRUE	reported	textfile	0.0027	5.66E-07	7348	M34419.m	TRUE	reported	F2Tg9L	2	TRUE	0.003341	24.6243
8234	rs1760863	A	G	A	G	-0.0134	0.008567	0.2319	0.247986	FALSE	FALSE	FALSE	4YAWhA	0.015857	0.58901	486484	sepsis	TRUE	reported	textfile	0.0027	5.01E-07	7256	M35439.m	TRUE	reported	bSSC4Z	2	TRUE	0.003383	24.62421
1656	rs1738658	A	C	A	C	-0.0134	0.040338	0.2495	0.257242	FALSE	FALSE	FALSE	YzX5ZD	0.01571	0.01024	486484	sepsis	TRUE	reported	textfile	0.0027	9.74E-07	6864	M12129.m	TRUE	reported	6tMfaW	2	TRUE	0.003576	24.62382
3020	rs7504246	A	G	A	G	-0.0134	0.006485	0.2945	0.293604	FALSE	FALSE	FALSE	gxupFu	0.015075	0.667062	486484	sepsis	TRUE	reported	textfile	0.0027	7.62E-07	6556	M21188.m	TRUE	reported	gwwXh2	2	TRUE	0.003743	24.62349

6440	rs6060270	T	G	T	G	0.0134	-0.00784	0.2057	0.211375	FALSE	FALSE	FALSE	QCw5dg	0.016725	0.639308	486484	sepsis	TRUE	reported	textfile	0.0027	8.58E-07	6279	M33666.m	TRUE	reported	zYHmzg	2	TRUE	0.003907	24.62316
3502	rs734567	T	C	T	C	0.0134	0.017692	0.5906	0.596781	FALSE	FALSE	FALSE	Awafb0	0.01398	0.205666	486484	sepsis	TRUE	reported	textfile	0.0027	7.83E-07	5562	M27728.m	TRUE	reported	Lfj8L0	2	TRUE	0.004409	24.62214
5049	rs2926419	T	C	T	C	-0.0551	0.003319	0.2092	0.203479	FALSE	FALSE	FALSE	JLCSne	0.017108	0.846192	486484	sepsis	TRUE	reported	textfile	0.0111	7.12E-07	2391	M33137.m	TRUE	reported	LGKYXK	2	TRUE	0.010201	24.62033
4987	rs6987121	A	G	A	G	-0.0263	0.001608	0.9067	0.902923	FALSE	FALSE	FALSE	AjJi2i	0.023128	0.944581	486484	sepsis	TRUE	reported	textfile	0.0053	7.59E-07	7330	M32980.m	TRUE	reported	gVSm9e	2	TRUE	0.003348	24.61735
2641	rs1026775	T	C	T	C	-0.0591	-0.01307	0.4495	0.448265	FALSE	FALSE	FALSE	9GoQdh	0.013763	0.342318	486484	sepsis	TRUE	reported	textfile	0.0119	6.40E-07	917	M18474.m	TRUE	reported	p6Dq8p	2	TRUE	0.026193	24.6112
6089	rs9633478	A	G	A	G	-0.0129	-0.01166	0.0763	0.071816	FALSE	FALSE	FALSE	P8n0HP	0.026495	0.660005	486484	sepsis	TRUE	reported	textfile	0.0026	5.31E-07	7336	M33442.m	TRUE	reported	cljMTl	2	TRUE	0.003344	24.61015
8960	rs6050798	T	C	T	C	-0.0655	0.000608	0.4924	0.487549	FALSE	FALSE	FALSE	Ywk2ve	0.013733	0.964701	486484	sepsis	TRUE	reported	textfile	0.0132	7.03E-07	2560	M37112.m	TRUE	reported	EiAdDj	2	TRUE	0.009527	24.60341
3179	rs1250154	A	C	A	C	-0.0253	-0.00127	0.8955	0.890469	FALSE	FALSE	FALSE	gvZCBj	0.021935	0.953831	486484	sepsis	TRUE	reported	textfile	0.0051	7.87E-07	7259	M22189.m	TRUE	reported	GKqg2h	2	TRUE	0.003379	24.6026
7798	rs1896198	A	G	A	G	-0.0124	-0.01374	0.416	0.422529	FALSE	FALSE	FALSE	cQ5TvO	0.013902	0.322863	486484	sepsis	TRUE	reported	textfile	0.0025	8.67E-07	7344	M35127.m	TRUE	reported	BVxkfZ	2	TRUE	0.003339	24.5949
7053	rs1734528	T	C	T	C	0.0124	0.011466	0.7364	0.725761	FALSE	FALSE	FALSE	uthruC	0.015289	0.453291	486484	sepsis	TRUE	reported	textfile	0.0025	5.07E-07	7316	M34283.m	TRUE	reported	ftTuRU	2	TRUE	0.003351	24.59487
1507	rs2247330	T	C	T	C	0.0124	-0.01419	0.6524	0.648995	FALSE	FALSE	FALSE	rzkxmh	0.014356	0.32278	486484	sepsis	TRUE	reported	textfile	0.0025	4.56E-07	6941	M03127.m	TRUE	reported	3lrzhA	2	TRUE	0.003532	24.59451
4684	rs1082359	T	C	T	C	-0.0248	0.001218	0.7417	0.739711	FALSE	FALSE	FALSE	MV108h	0.015628	0.93788	486484	sepsis	TRUE	reported	textfile	0.005	8.42E-07	6642	M32758.m	TRUE	reported	btjNXQ	2	TRUE	0.00369	24.59419
6428	rs1311144	T	C	T	C	0.0124	-0.02159	0.7906	0.793845	FALSE	FALSE	FALSE	QCw5dg	0.016909	0.201713	486484	sepsis	TRUE	reported	textfile	0.0025	1.04E-06	6279	M33666.m	TRUE	reported	zYHmzg	2	TRUE	0.003903	24.59376
3948	rs1307289	A	G	A	G	0.0243	0.026551	0.628	0.633655	FALSE	FALSE	FALSE	N6UQnQ	0.0142	0.061513	486484	sepsis	TRUE	reported	textfile	0.0049	7.95E-07	7346	M32425.m	TRUE	reported	FEcGHY	2	TRUE	0.003337	24.58681
5250	rs1224636	A	C	A	C	0.2665	-0.1215	0.9792	0.978619	FALSE	FALSE	FALSE	q32d4E	0.047126	0.009932	486484	sepsis	TRUE	reported	textfile	0.0537	6.93E-07	1017	M33178.m	TRUE	reported	cS3BwT	2	TRUE	0.023645	24.58051
3572	rs1418200	T	C	T	C	0.2117	-0.04739	0.0408	0.03039	FALSE	FALSE	FALSE	F1s6qg	0.040963	0.247325	486484	sepsis	TRUE	reported	textfile	0.0426	6.63E-07	423	M31536.m	TRUE	reported	Zm40GX	2	TRUE	0.055162	24.579
1133	rs2231142	T	G	T	G	0.0119	0.022697	0.1106	0.113225	FALSE	FALSE	FALSE	nlqUuV	0.021548	0.292211	486484	sepsis	TRUE	reported	textfile	0.0024	8.43E-07	7371	M01604.m	TRUE	reported	Uro9nC	2	TRUE	0.003324	24.5784
8275	rs887733	T	C	T	C	0.0119	-0.01649	0.5524	0.565009	FALSE	FALSE	FALSE	TdsGCZ	0.013797	0.23211	486484	sepsis	TRUE	reported	textfile	0.0024	1.02E-06	6883	M35464.m	TRUE	reported	sOspot	2	TRUE	0.003559	24.57793
8997	rs7183977	T	C	T	C	-0.0233	0.010732	0.6755	0.652885	FALSE	FALSE	FALSE	TfdxtP	0.014445	0.457506	486484	sepsis	TRUE	reported	textfile	0.0047	7.19E-07	7333	M37203.m	TRUE	reported	xicmWr	2	TRUE	0.00334	24.56958
7226	rs4976704	T	C	T	C	-0.905	0.012639	0.989	0.817213	FALSE	FALSE	FALSE	i1uJ7T	0.017685	0.474821	486484	sepsis	TRUE	reported	textfile	0.181	5.73E-07	115	M34338.m	TRUE	reported	pi3mKW	2	TRUE	0.178571	24.56522
7225	rs3750318	T	C	T	C	0.895	-0.00489	0.012	0.030845	FALSE	FALSE	FALSE	i1uJ7T	0.039402	0.901143	486484	sepsis	TRUE	reported	textfile	0.179	5.73E-07	115	M34338.m	TRUE	reported	pi3mKW	2	TRUE	0.178571	24.56522
7592	rs1017988	T	C	T	C	0.2058	0.008133	0.168	0.183841	FALSE	FALSE	FALSE	PlYt5g	0.017727	0.646389	486484	sepsis	TRUE	reported	textfile	0.0412	5.90E-07	128	M34516.m	TRUE	reported	jX41tf	2	TRUE	0.163133	24.56161
3431	rs6957252	T	C	T	C	0.0739	-0.01118	0.6329	0.622183	FALSE	FALSE	FALSE	HHQf8w	0.014118	0.428458	486484	sepsis	TRUE	reported	textfile	0.0149	6.84E-07	1309	M27531.m	TRUE	reported	3vTluK	2	TRUE	0.018446	24.56135
1239	rs369895	A	G	A	G	-0.0114	-0.01205	0.574	0.551415	FALSE	FALSE	FALSE	V50rWC	0.013729	0.380128	486484	sepsis	TRUE	reported	textfile	0.0023	1.08E-06	7346	M01645.m	TRUE	reported	yN3gpQ	2	TRUE	0.003333	24.56042
2147	rs7405977	T	C	T	C	-0.0114	0.008569	0.3918	0.418063	FALSE	FALSE	FALSE	TEupfd	0.013887	0.537191	486484	sepsis	TRUE	reported	textfile	0.0023	1.17E-06	7331	M15676.m	TRUE	reported	NXiaPC	2	TRUE	0.00334	24.56041
6051	rs7628540	T	G	T	G	-0.6303	0.015107	0.1273	0.179158	FALSE	FALSE	FALSE	33ojbt	0.017843	0.397171	486484	sepsis	TRUE	reported	textfile	0.1251	4.67E-07	60	M33423.m	TRUE	reported	ivEtvl	2	TRUE	0.297302	24.53899
2927	rs2067997	T	C	T	C	-0.0213	-0.02001	0.8694	0.862937	FALSE	FALSE	FALSE	fV4quI	0.019945	0.315663	486484	sepsis	TRUE	reported	textfile	0.0043	9.40E-07	7366	M21044.m	TRUE	reported	hbdBQJ	2	TRUE	0.00332	24.53038
3055	rs4631845	A	G	A	G	0.0213	0.001809	0.0851	0.092342	FALSE	FALSE	FALSE	H6vcCG	0.02366	0.939055	486484	sepsis	TRUE	reported	textfile	0.0043	9.87E-07	6180	M22030.m	TRUE	reported	XetVX7	2	TRUE	0.003955	24.52911
6674	rs4402594	T	C	T	C	-0.0451	-0.01482	0.4067	0.404384	FALSE	FALSE	FALSE	6iZSOI	0.013927	0.28714	486484	sepsis	TRUE	reported	textfile	0.0091	7.99E-07	1294	M33901.m	TRUE	reported	ApKaGe	2	TRUE	0.018628	24.52441
7428	rs1250572	T	C	T	C	-0.0917	-0.00715	0.1409	0.219091	FALSE	FALSE	FALSE	NZPe5A	0.016528	0.665474	486484	sepsis	TRUE	reported	textfile	0.0185	7.38E-07	1018	M34400.m	TRUE	reported	OyPwYO	2	TRUE	0.023566	24.52117
3042	rs7522439	A	G	A	G	0.0208	0.063034	0.8977	0.900891	FALSE	FALSE	FALSE	FJZEYz	0.022885	0.005879	486484	sepsis	TRUE	reported	textfile	0.0042	7.34E-07	7284	M21630.m	TRUE	reported	s6Pshg	2	TRUE	0.003356	24.51934
4703	rs1764950	A	G	A	G	-0.0416	-0.02472	0.9227	0.896823	FALSE	FALSE	FALSE	WlwKaF	0.022458	0.271019	486484	sepsis	TRUE	reported	textfile	0.0084	8.11E-07	6712	M32759.m	TRUE	reported	Ub4yxH	2	TRUE	0.003641	24.51877
8550	rs1865661	A	T	A	T	1.4423	0.052943	0.9836	0.979012	FALSE	TRUE	FALSE	aQhCav	0.048692	0.276908	486484	sepsis	TRUE	reported	textfile	0.2889	5.95E-07	122	M36097.m	TRUE	reported	9DJS9j	2	TRUE	0.169638	24.51532
6852	rs2469215	T	C	T	C	-0.0307	-0.00292	0.1051	0.110336	FALSE	FALSE	FALSE	2pJy8f	0.021892	0.893865	486484	sepsis	TRUE	reported	textfile	0.0062	6.70E-07	7132	M34040.m	TRUE	reported	zuJC1r	2	TRUE	0.003426	24.51159
5970	rs5994305	A	G	A	G	0.0307	0.027636	0.8318	0.831409	FALSE	FALSE	FALSE	YCswLG	0.018278	0.130549	486484	sepsis	TRUE	reported	textfile	0.0062	7.15E-07	5822	M33420.m	TRUE	reported	WH7xfc	2	TRUE	0.004194	24.51005
6434	rs1742392	T	C	T	C	0.0203	-0.00851	0.91	0.909823	FALSE	FALSE	FALSE	QCw5dg	0.024031	0.723194	486484	sepsis	TRUE	reported	textfile	0.0041	5.47E-07	6279	M33666.m	TRUE	reported	zYHmzg	2	TRUE	0.003889	24.50677
7929	rs1319751	T	C	T	C	-0.0396	-0.01211	0.0369	0.041499	FALSE	FALSE	FALSE	kFTWXY	0.034385	0.724673	486484	sepsis	TRUE	reported	textfile	0.008	6.63E-07	6543	M35193.m	TRUE	reported	cjJvJl	2	TRUE	0.003731	24.49501
8411	rs1246410	A	G	A	G	0.0297	-0.01462	0.1572	0.16152	FALSE	FALSE	FALSE	02RdNm	0.018665	0.433535	486484	sepsis	TRUE	reported	textfile	0.006	8.24E-07	6447	M35678.m	TRUE	reported	BWN5JR	2	TRUE	0.003786	24.4949
6787	rs1220119	A	T	A	T	-0.0391	0.009837	0.9375	0.929466	FALSE	TRUE	FALSE	kcZoFT	0.026795	0.713523	486484	sepsis	TRUE	reported	textfile	0.0079	7.65E-07	6998	M33957.m	TRUE	reported	alW1w0	2	TRUE	0.003488	24.48923
3651	rs1701017	C	G	C	G	-0.0292	-0.03447	0.9497	0.941798	FALSE	TRUE	FALSE	fnjup6	0.029344	0.240081	486484	sepsis	TRUE	reported	textfile	0.0059	6.61E-07	7346	M32197.m	TRUE	reported	oQzaMk	2	TRUE	0.003323	24.48744
7240	rs1721615	A	T	A	T	-0.1713	-0.00818	0.9738	0.938603	FALSE	TRUE	FALSE	RIcE3J	0.029338	0.780326	486484	sepsis	TRUE	reported	textfile	0.0346	7.13E-07	1649	M34339.m	TRUE	reported	QQrwdm	2	TRUE	0.014647	24.48136
8257	rs2728817	A	T	A	T	0.1986	-0.01985	0.0954	0.090694	FALSE	TRUE	FALSE	G4plxe	0.023968	0.407519	486484	sepsis	TRUE	reported	textfile	0.04	6.95E-07	284	M35451.m	TRUE	reported	hNOVMI	2	TRUE	0.079868	24.47762
1221	rs1003973	A	G	A	G	0.0198	0.010048	0.5787	0.612863	FALSE	FALSE	FALSE	V50rWC	0.014118	0.476639	486484	sepsis	TRUE	reported	textfile	0.004	8.47E-07	1767	M01645.m	TRUE	reported	yN3gpQ	2	TRUE	0.013677	24.47477
6910	rs1304928	A	T	A	T	-0.957	-0.00798	0.011	0.032751	FALSE	TRUE	FALSE	SvfSvH	0.04049	0.843692	486484	sepsis	TRUE	reported	textfile	0.192	6.22E-07	134	M34109.m	TRUE	reported	7OSbiu	2	TRUE	0.156405	24.47319
7374	rs7721259	T	C	T	C	0.2104	0.012418	0.1111	0.118719	FALSE	FALSE	FALSE	mm9ksf	0.021148	0.557066	486484	sepsis	TRUE	reported	textfile	0.0401	1.53E-07	18	M34365.m	TRUE	reported	AVwEqD	2	TRUE	0.604654	24.47091
4424	rs9662967	T	C	T	C	-0.0188	-0.02004	0.6984	0.695126	FALSE	FALSE	FALSE	NzBiVA	0.015558	0.197651	486484	sepsis	TRUE	reported	textfile	0.0038	7.39E-07	7354	M32672.m	TRUE	reported	YLlhHJ	2	TRUE	0.003317	24.4698
3777	rs1710664	T	C	T	C	0.0282	-0.03531	0.0296	0.03032	FALSE	FALSE	FALSE	NW1aaR	0.040151	0.379215	486484	sepsis	TRUE	reported	textfile	0.0057	8.85E-07	7344	M32339.m	TRUE	reported	pBPkAB	2	TRUE	0.003322	24.46979
7040	rs1180125	A	G	A	G	-0.0094	0.022126	0.5459	0.548382	FALSE	FALSE	FALSE	uthruC	0.013796	0.108767	486484	sepsis	TRUE	reported	textfile	0.0019	5.68E-07	7316	M34283.m	TRUE	reported	ftTuRU	2	TRUE	0.003334	24.46976
5864	rs1178671	C	G	C	G	-0.0094	0.024652	0.6543	0.633383	FALSE	TRUE	FALSE	tqW0ld	0.01434	0.085589	486484	sepsis	TRUE	reported	textfile	0.0019	4.26E-07	7315	M33389.m	TRUE	reported	Sv7EyV	2	TRUE	0.003335	24.46976
5995	rs3810252	A	G	A	G	-0.0094	-0.02481	0.8089	0.805003	FALSE	FALSE	FALSE	3KsoXq	0.017191	0.148996	486484	sepsis	TRUE	reported	textfile	0.0019	5.21E-07	7306	M33422.m	TRUE	reported	aNr6Ug	2	TRUE	0.003339	24.46975
4352	rs759287	A	G	A	G	0.0094	-0.00148	0.3082	0.322382	FALSE	FALSE	FALSE	6VSoQQ	0.014663	0.919578	486484	sepsis	TRUE	reported	textfile	0.0019	4.48E-07	7229	M32644.m	TRUE	reported	oC2AV0	2	TRUE	0.003374	24.46968
6259	rs1218924	A	G	A	G	0.0094	-0.00404	0.6201	0.620118	FALSE	FALSE	FALSE	MOonP9	0.0141	0.774719	486484	sepsis	TRUE	reported	textfile	0.0019	3.39E-07	7066	M33515.m	TRUE	reported	226GuP	2	TRUE	0.003452	24.46953
8450	rs1257009	T	C	T	C	-0.0094	0.006777	0.5847	0.58231	FALSE	FALSE	FALSE	uMajV6	0.013855	0.624738	486484	sepsis	TRUE	reported	textfile	0.0019	9.18E-07	6604	M35754.m	TRUE	reported	b8QB7S	2	TRUE	0.003693	24.46904
8463	rs4641528	T	C	T	C	0.0094	0.001654	0.5683	0.569383	FALSE	FALSE	FALSE	uMajV6	0.013792	0.904533	486484	sepsis	TRUE	reported	textfile	0.0019	9.44E-07	6604	M35754.m	TRUE	reported	b8QB7S	2	TRUE	0.003693	24.46904
8464	rs4792953	T	C	T	C	0.0094	0.009151	0.3951	0.395911	FALSE	FALSE	FALSE	uMajV6	0.014009	0.513611	486484	sepsis	TRUE	reported	textfile	0.0019	8.92E-07	6604	M35754.m	TRUE	reported	b8QB7S	2	TRUE	0.003693	24.46904
8910	rs300177	C	G	C	G	-0.0094	-0.02866	0.367	0.366881	FALSE	TRUE	FALSE	eLhsld	0.014178	0.043227	486484	sepsis	TRUE	reported	textfile	0.0019	4.30E-07	6552	M37058.m	TRUE	reported	RSeWE5	2	TRUE	0.003722	24.46898
7725	rs1180210	A	G	A	G	-0.0094	0.002403	0.5682	0.556047	FALSE	FALSE	FALSE	HyMNYM	0.013772	0.861504	486484	sepsis	TRUE	reported	textfile	0.0019	6.80E-07	5885	M34912.m	TRUE	reported	Tl8Hcw	2	TRUE	0.004142	24.46814
7812	rs1096239	T	C	T	C	-0.0094	-0.01148	0.5964	0.583356	FALSE	FALSE	FALSE	h5whlS	0.014122	0.41623	486484	sepsis	TRUE	reported	textfile	0.0019	9.52E-07	4900	M35137.m	TRUE	reported	xVITKF	2	TRUE	0.00497	24.46646
7835	rs3843430	T	C	T	C	-0.0094	-0.00084	0.371	0.375114	FALSE	FALSE	FALSE	h5whlS	0.014195	0.952946	486484	sepsis	TRUE	reported	textfile	0.0019	5.81E-07	4900	M35137.m	TRUE	reported	xVITKF	2	TRUE	0.00497	24.46646
7857	rs744432	A	C	A	C	-0.0094	0.000345	0.4475	0.449258	FALSE	FALSE	FALSE	h5whlS	0.013851	0.980106	486484	sepsis	TRUE	reported	textfile	0.0019	6.34E-07	4900	M35137.m	TRUE	reported	xVITKF	2	TRUE	0.00497	24.46646
7866	rs9297758	A	G	A	G	-0.0094	-0.013	0.3801	0.380943	FALSE	FALSE	FALSE	h5whlS	0.014069	0.355314	486484	sepsis	TRUE	reported	textfile	0.0019	8.02E-07	4900	M35137.m	TRUE	reported	xVITKF	2	TRUE	0.00497	24.46646
2527	rs1234181	T	C	T	C	-0.093	-0.05095	0.9564	0.935492	FALSE	FALSE	FALSE	4HwTlG	0.02789	0.067752	486484	sepsis	TRUE	reported	textfile	0.0188	7.44E-07	7071	M18254.m	TRUE	reported	F9KrSC	2	TRUE	0.003449	24.46399
7933	rs1991108	T	C	T	C	-0.0183	-0.01052	0.1597	0.163236	FALSE	FALSE	FALSE	kFTWXY	0.018502	0.56959	486484	sepsis	TRUE	reported	textfile	0.0037	5.77E-07	6543	M35193.m	TRUE	reported	cjJvJl	2	TRUE	0.003725	24.4549
3105	rs9467632	A	G	A	G	-0.0183	0.017709	0.8773	0.880799	FALSE	FALSE	FALSE	mr0awv	0.021037	0.399906	486484	sepsis	TRUE	reported	textfile	0.0037	8.50E-07	5702	M22130.m	TRUE	reported	Y2TfWQ	2	TRUE	0.004272	24.4538
9052	rs6911080	T	C	T	C	-0.0089	0.013702	0.7145	0.698068	FALSE	FALSE	FALSE	df0MCM	0.014866	0.356693	486484	sepsis	TRUE	reported	textfile	0.0018	5.23E-07	7366	M37506.m	TRUE	reported	iUfr4u	2	TRUE	0.003308	24.44089
1040	rs1313002	T	C	T	C	-0.0089	0.030192	0.4312	0.437398	FALSE	FALSE	FALSE	tRtQ2e	0.013789	0.028557	486484	sepsis	TRUE	reported	textfile	0.0018	1.41E-06	7364	M01564.m	TRUE	reported	Tbg0zM	2	TRUE	0.003309	24.44089
3981	rs3766553	A	G	A	G	0.0089	-0.01268	0.5153	0.519722	FALSE	FALSE	FALSE	XdJbEU	0.013722	0.355552	486484	sepsis	TRUE	reported	textfile	0.0018	1.05E-06	7364	M32452.m	TRUE	reported	V5xWKi	2	TRUE	0.003309	24.44089
733	rs1292795	A	G	A	G	0.0089	-0.0039	0.7248	0.699561	FALSE	FALSE	FALSE	9u0SC8	0.014891	0.793401	486484	sepsis	TRUE	reported	textfile	0.0018	3.76E-07	7363	M01301.m	TRUE	reported	H95uec	2	TRUE	0.003309	24.44089
5039	rs7794651	T	C	T	C	0.0089	0.010674	0.1191	0.120977	FALSE	FALSE	FALSE	BNnOvt	0.021006	0.611352	486484	sepsis	TRUE	reported	textfile	0.0018	7.66E-07	7362	M33132.m	TRUE	reported	rHcCPG	2	TRUE	0.00331	24.44089
4296	rs1997111	A	C	A	C	-0.0089	0.005742	0.3558	0.357673	FALSE	FALSE	FALSE	0tjjPC	0.014232	0.686622	486484	sepsis	TRUE	reported	textfile	0.0018	8.72E-07	7362	M32634.m	TRUE	reported	nJJFkX	2	TRUE	0.00331	24.44089
4311	rs7837038	A	G	A	G	0.0089	-0.02498	0.4808	0.493655	FALSE	FALSE	FALSE	0tjjPC	0.013693	0.068086	486484	sepsis	TRUE	reported	textfile	0.0018	5.54E-07	7362	M32634.m	TRUE	reported	nJJFkX	2	TRUE	0.00331	24.44089
1754	rs782378	A	G	A	G	-0.0089	0.007283	0.5252	0.523462	FALSE	FALSE	FALSE	nFLpAP	0.013678	0.594395	486484	sepsis	TRUE	reported	textfile	0.0018	6.01E-07	7356	M12774.m	TRUE	reported	yOSQTj	2	TRUE	0.003312	24.44088
1214	rs6860349	A	G	A	G	-0.0089	0.013138	0.4108	0.405994	FALSE	FALSE	FALSE	Ckdgh1	0.013915	0.345087	486484	sepsis	TRUE	reported	textfile	0.0018	8.67E-07	7355	M01644.m	TRUE	reported	nKxz7B	2	TRUE	0.003313	24.44088
565	rs2904665	A	G	A	G	-0.0089	-0.01645	0.861	0.861051	FALSE	FALSE	FALSE	fNqkCW	0.019867	0.407667	486484	sepsis	TRUE	reported	textfile	0.0018	9.19E-07	7354	M00606.m	TRUE	reported	g9uwOn	2	TRUE	0.003313	24.44088
2793	rs1008667	T	C	T	C	0.0089	-0.00349	0.2938	0.289078	FALSE	FALSE	FALSE	Sx5Uhq	0.015088	0.81725	486484	sepsis	TRUE	reported	textfile	0.0018	5.26E-07	7354	M19934.m	TRUE	reported	DijYTE	2	TRUE	0.003313	24.44088
2829	rs7837995	T	C	T	C	0.0089	0.01709	0.4485	0.442392	FALSE	FALSE	FALSE	Sx5Uhq	0.013933	0.219986	486484	sepsis	TRUE	reported	textfile	0.0018	4.28E-07	7354	M19934.m	TRUE	reported	DijYTE	2	TRUE	0.003313	24.44088
1226	rs1197872	A	T	A	T	-0.0089	-0.01015	0.6876	0.683833	FALSE	TRUE	FALSE	V50rWC	0.014673	0.489131	486484	sepsis	TRUE	reported	textfile	0.0018	5.18E-07	7346	M01645.m	TRUE	reported	yN3gpQ	2	TRUE	0.003317	24.44087
3772	rs1189399	A	G	A	G	0.0089	0.002227	0.4228	0.426329	FALSE	FALSE	FALSE	NW1aaR	0.014089	0.874391	486484	sepsis	TRUE	reported	textfile	0.0018	8.89E-07	7344	M32339.m	TRUE	reported	pBPkAB	2	TRUE	0.003318	24.44087
4943	rs1558688	T	C	T	C	-0.0089	0.014472	0.2928	0.30135	FALSE	FALSE	FALSE	bKmDMa	0.014958	0.333293	486484	sepsis	TRUE	reported	textfile	0.0018	4.34E-07	7344	M32910.m	TRUE	reported	nAuPzO	2	TRUE	0.003318	24.44087
3286	rs4937602	A	G	A	G	0.0089	-0.01036	0.5448	0.539456	FALSE	FALSE	FALSE	vYWPe9	0.013785	0.452175	486484	sepsis	TRUE	reported	textfile	0.0018	9.63E-07	7340	M25459.m	TRUE	reported	VvJ6Vx	2	TRUE	0.00332	24.44087
2861	rs9363753	T	C	T	C	0.0089	0.013399	0.1754	0.181285	FALSE	FALSE	FALSE	Y5qWBm	0.017823	0.452186	486484	sepsis	TRUE	reported	textfile	0.0018	5.21E-07	7325	M20489.m	TRUE	reported	f2g9Su	2	TRUE	0.003326	24.44086
2193	rs1098145	A	G	A	G	-0.0089	0.011153	0.3039	0.324587	FALSE	FALSE	FALSE	Bf6uMK	0.01459	0.444647	486484	sepsis	TRUE	reported	textfile	0.0018	1.08E-06	7308	M15778.m	TRUE	reported	jowM2N	2	TRUE	0.003334	24.44084
6150	rs6948214	A	T	A	T	0.0089	-0.01312	0.2357	0.236652	FALSE	TRUE	FALSE	MiSJly	0.016079	0.414645	486484	sepsis	TRUE	reported	textfile	0.0018	1.36E-06	7307	M33477.m	TRUE	reported	WwL1hq	2	TRUE	0.003335	24.44084
5981	rs1231258	A	T	A	T	0.0089	0.01068	0.8015	0.794115	FALSE	TRUE	FALSE	3KsoXq	0.016914	0.527763	486484	sepsis	TRUE	reported	textfile	0.0018	9.62E-07	7306	M33422.m	TRUE	reported	aNr6Ug	2	TRUE	0.003335	24.44084
5982	rs1250088	C	G	C	G	-0.0089	0.008076	0.7842	0.782691	FALSE	TRUE	FALSE	3KsoXq	0.016579	0.626185	486484	sepsis	TRUE	reported	textfile	0.0018	7.04E-07	7306	M33422.m	TRUE	reported	aNr6Ug	2	TRUE	0.003335	24.44084
6003	rs738873	T	C	T	C	0.0089	0.017731	0.6892	0.670425	FALSE	FALSE	FALSE	3KsoXq	0.0146	0.224568	486484	sepsis	TRUE	reported	textfile	0.0018	5.02E-07	7306	M33422.m	TRUE	reported	aNr6Ug	2	TRUE	0.003335	24.44084
4330	rs1086997	A	G	A	G	-0.0089	0.011056	0.4381	0.436643	FALSE	FALSE	FALSE	6VSoQQ	0.013819	0.423667	486484	sepsis	TRUE	reported	textfile	0.0018	9.69E-07	7229	M32644.m	TRUE	reported	oC2AV0	2	TRUE	0.00337	24.44077
1646	rs7666900	T	C	T	C	0.0089	0.007333	0.1418	0.148169	FALSE	FALSE	FALSE	n5rDuT	0.01928	0.70368	486484	sepsis	TRUE	reported	textfile	0.0018	6.93E-07	7081	M12067.m	TRUE	reported	ZQLTeR	2	TRUE	0.003441	24.44063
6283	rs4980965	T	C	T	C	-0.0089	0.00455	0.5402	0.539117	FALSE	FALSE	FALSE	MOonP9	0.01379	0.741442	486484	sepsis	TRUE	reported	textfile	0.0018	1.28E-06	7066	M33515.m	TRUE	reported	226GuP	2	TRUE	0.003448	24.44061
8137	rs1029481	A	T	A	T	-0.0089	-0.02396	0.7306	0.689503	FALSE	TRUE	FALSE	lv3pAQ	0.014871	0.107103	486484	sepsis	TRUE	reported	textfile	0.0018	8.81E-07	6920	M35327.m	TRUE	reported	3Vsglp	2	TRUE	0.00352	24.44047
7142	rs6961104	T	C	T	C	-0.247	0.03604	0.9667	0.92463	FALSE	FALSE	FALSE	de8tA6	0.026175	0.168546	486484	sepsis	TRUE	reported	textfile	0.0493	5.56E-07	75	M34322.m	TRUE	reported	sXPPBO	2	TRUE	0.250761	24.43215
7435	rs739163	T	C	T	C	0.0262	-0.00455	0.3656	0.379384	FALSE	FALSE	FALSE	NZPe5A	0.014139	0.747467	486484	sepsis	TRUE	reported	textfile	0.0053	6.09E-07	5689	M34400.m	TRUE	reported	OyPwYO	2	TRUE	0.004277	24.42858
970	rs1040210	T	C	T	C	0.9529	0.087726	0.0165	0.012992	FALSE	FALSE	FALSE	xSRII4	0.059933	0.143266	486484	sepsis	TRUE	reported	textfile	0.1924	7.35E-07	482	M01515.m	TRUE	reported	juZ5GE	2	TRUE	0.048426	24.42748
8809	rs1050167	A	C	A	C	-0.0346	0.075006	0.0254	0.028803	FALSE	FALSE	FALSE	X2Rol2	0.040887	0.066581	486484	sepsis	TRUE	reported	textfile	0.007	7.05E-07	7335	M36776.m	TRUE	reported	nikHw1	2	TRUE	0.00332	24.42518
2330	rs6959584	T	C	T	C	0.0173	-0.02817	0.1278	0.129255	FALSE	FALSE	FALSE	IZXwpR	0.020376	0.166783	486484	sepsis	TRUE	reported	textfile	0.0035	6.10E-07	6549	M16822.m	TRUE	reported	Zx9k2V	2	TRUE	0.003717	24.42438
6579	rs1687578	A	G	A	G	0.5363	-0.01259	0.0114	0.014928	FALSE	FALSE	FALSE	XO3rXq	0.056621	0.824086	486484	sepsis	TRUE	reported	textfile	0.1079	6.74E-07	176	M33877.m	TRUE	reported	p2sETg	2	TRUE	0.123088	24.42358
4137	rs2187662	A	G	A	G	0.0939	-0.01995	0.0332	0.028951	FALSE	FALSE	FALSE	pLh7sk	0.040937	0.626018	486484	sepsis	TRUE	reported	textfile	0.019	7.61E-07	7256	M32549.m	TRUE	reported	RSQOnp	2	TRUE	0.003355	24.41767
5142	rs1709537	A	G	A	G	0.5603	0.065838	0.9671	0.994299	FALSE	FALSE	FALSE	L2DJzK	0.092054	0.474478	486484	sepsis	TRUE	reported	textfile	0.1119	5.45E-07	76	M33173.m	TRUE	reported	hRG70D	2	TRUE	0.248057	24.41177
2407	rs2155977	T	C	T	C	0.0964	0.015102	0.9371	0.978953	FALSE	FALSE	FALSE	NBJ2Do	0.04806	0.753342	486484	sepsis	TRUE	reported	textfile	0.0195	8.18E-07	1739	M17799.m	TRUE	reported	Uz0Wgl	2	TRUE	0.013859	24.41097
4793	rs853725	T	C	T	C	0.044	0.005588	0.5592	0.553934	FALSE	FALSE	FALSE	dwreVz	0.013744	0.684334	486484	sepsis	TRUE	reported	textfile	0.0089	8.62E-07	1600	M32802.m	TRUE	reported	7YCayZ	2	TRUE	0.015046	24.41081
9051	rs6745464	T	C	T	C	-0.0084	0.001349	0.5494	0.542552	FALSE	FALSE	FALSE	df0MCM	0.013736	0.921745	486484	sepsis	TRUE	reported	textfile	0.0017	1.39E-06	7366	M37506.m	TRUE	reported	iUfr4u	2	TRUE	0.003304	24.4086
427	rs3897960	A	T	A	T	-0.0084	0.002844	0.2083	0.205881	FALSE	TRUE	FALSE	cTsNj6	0.016936	0.866648	486484	sepsis	TRUE	reported	textfile	0.0017	1.29E-06	7365	M00063.m	TRUE	reported	7KYTrQ	2	TRUE	0.003304	24.40859
3835	rs37379	A	G	A	G	0.0168	-0.03133	0.0868	0.100332	FALSE	FALSE	FALSE	tRX2u0	0.022868	0.170623	486484	sepsis	TRUE	reported	textfile	0.0034	7.94E-07	7365	M32348.m	TRUE	reported	ruBgwQ	2	TRUE	0.003304	24.40859
718	rs8111703	A	T	A	T	-0.0084	-0.00502	0.2142	0.246211	FALSE	TRUE	FALSE	H9I3s3	0.015911	0.752487	486484	sepsis	TRUE	reported	textfile	0.0017	1.47E-06	7358	M01299.m	TRUE	reported	kXWyUq	2	TRUE	0.003307	24.40859
4069	rs17685	A	G	A	G	-0.0168	-0.00878	0.2781	0.277402	FALSE	FALSE	FALSE	TyHtdh	0.015218	0.563951	486484	sepsis	TRUE	reported	textfile	0.0034	6.44E-07	7358	M32497.m	TRUE	reported	H3KmLR	2	TRUE	0.003307	24.40859
1760	rs998933	T	C	T	C	0.0252	-0.04393	0.9528	0.947879	FALSE	FALSE	FALSE	nFLpAP	0.030849	0.154454	486484	sepsis	TRUE	reported	textfile	0.0051	7.95E-07	7356	M12774.m	TRUE	reported	yOSQTj	2	TRUE	0.003308	24.40859
4620	rs1076131	T	C	T	C	-0.0336	0.00734	0.2803	0.30376	FALSE	FALSE	FALSE	0oA38w	0.014958	0.623656	486484	sepsis	TRUE	reported	textfile	0.0068	7.22E-07	7356	M32753.m	TRUE	reported	dQUaUX	2	TRUE	0.003308	24.40859
826	rs2100884	T	C	T	C	-0.0084	0.000765	0.4375	0.446949	FALSE	FALSE	FALSE	2qgDgB	0.013779	0.955708	486484	sepsis	TRUE	reported	textfile	0.0017	8.60E-07	7355	M01358.m	TRUE	reported	asmXq0	2	TRUE	0.003309	24.40859
3696	rs1299292	A	G	A	G	0.0084	0.002008	0.5789	0.570298	FALSE	FALSE	FALSE	agPgtF	0.013842	0.884663	486484	sepsis	TRUE	reported	textfile	0.0017	1.31E-06	7349	M32315.m	TRUE	reported	hMRjcV	2	TRUE	0.003311	24.40858
3718	rs9874508	A	G	A	G	-0.0084	-0.00378	0.4625	0.454359	FALSE	FALSE	FALSE	agPgtF	0.013778	0.783754	486484	sepsis	TRUE	reported	textfile	0.0017	1.42E-06	7349	M32315.m	TRUE	reported	hMRjcV	2	TRUE	0.003311	24.40858
8174	rs2841549	A	G	A	G	-0.0084	-0.00462	0.762	0.761076	FALSE	FALSE	FALSE	8UhtPK	0.016009	0.77277	486484	sepsis	TRUE	reported	textfile	0.0017	4.47E-07	7345	M35397.m	TRUE	reported	rvVJba	2	TRUE	0.003313	24.40858
8185	rs7546483	C	G	C	G	0.0084	-0.02801	0.7027	0.699938	FALSE	TRUE	FALSE	8UhtPK	0.014946	0.060945	486484	sepsis	TRUE	reported	textfile	0.0017	6.20E-07	7345	M35397.m	TRUE	reported	rvVJba	2	TRUE	0.003313	24.40858
2832	rs1049432	T	C	T	C	-0.0084	-0.0001	0.8102	0.812859	FALSE	FALSE	FALSE	Y5qWBm	0.017555	0.995297	486484	sepsis	TRUE	reported	textfile	0.0017	1.43E-06	7325	M20489.m	TRUE	reported	f2g9Su	2	TRUE	0.003322	24.40856
2833	rs1082869	A	G	A	G	-0.0084	-0.0092	0.7048	0.702922	FALSE	FALSE	FALSE	Y5qWBm	0.015041	0.540863	486484	sepsis	TRUE	reported	textfile	0.0017	6.05E-07	7325	M20489.m	TRUE	reported	f2g9Su	2	TRUE	0.003322	24.40856
4582	rs1289948	A	G	A	G	0.0084	0.003105	0.7034	0.709386	FALSE	FALSE	FALSE	Pzplqu	0.015062	0.836687	486484	sepsis	TRUE	reported	textfile	0.0017	1.59E-06	7318	M32740.m	TRUE	reported	FjEBS5	2	TRUE	0.003325	24.40855
1686	rs1204294	A	G	A	G	-0.0084	-0.00755	0.8045	0.795447	FALSE	FALSE	FALSE	f01en0	0.016952	0.656088	486484	sepsis	TRUE	reported	textfile	0.0017	1.40E-06	7314	M12593.m	TRUE	reported	EAsM6U	2	TRUE	0.003327	24.40855
4712	rs1340306	C	G	C	G	0.0168	-0.02326	0.3866	0.405571	FALSE	TRUE	FALSE	L2WFM1	0.013973	0.095991	486484	sepsis	TRUE	reported	textfile	0.0034	9.92E-07	7313	M32761.m	TRUE	reported	5GHzph	2	TRUE	0.003327	24.40855
507	rs1397829	A	G	A	G	0.042	0.020111	0.5328	0.541572	FALSE	FALSE	FALSE	ZOMIXT	0.013792	0.144787	486484	sepsis	TRUE	reported	textfile	0.0085	7.08E-07	7274	M00569.m	TRUE	reported	kZGouU	2	TRUE	0.003345	24.40851
2732	rs4337809	T	C	T	C	-0.0084	-0.01387	0.45	0.445857	FALSE	FALSE	FALSE	H4yqSK	0.013794	0.314795	486484	sepsis	TRUE	reported	textfile	0.0017	1.22E-06	7182	M19362.m	TRUE	reported	w2PDEO	2	TRUE	0.003388	24.40843
959	rs4269521	A	G	A	G	0.0168	0.018751	0.8431	0.848928	FALSE	FALSE	FALSE	tWoDq4	0.019093	0.326048	486484	sepsis	TRUE	reported	textfile	0.0034	1.03E-06	7160	M01508.m	TRUE	reported	6ltDw0	2	TRUE	0.003398	24.40841
1298	rs2118096	A	C	A	C	0.0084	0.002975	0.6068	0.60519	FALSE	FALSE	FALSE	WQdpmo	0.01405	0.832285	486484	sepsis	TRUE	reported	textfile	0.0017	1.13E-06	7136	M01769.m	TRUE	reported	BkptOK	2	TRUE	0.00341	24.40838
1307	rs4653629	T	C	T	C	-0.0084	-0.00475	0.5584	0.5539	FALSE	FALSE	FALSE	WQdpmo	0.013753	0.729547	486484	sepsis	TRUE	reported	textfile	0.0017	1.25E-06	7136	M01769.m	TRUE	reported	BkptOK	2	TRUE	0.00341	24.40838
1310	rs6017596	T	C	T	C	-0.0084	0.02296	0.5825	0.578685	FALSE	FALSE	FALSE	WQdpmo	0.013896	0.098465	486484	sepsis	TRUE	reported	textfile	0.0017	8.31E-07	7136	M01769.m	TRUE	reported	BkptOK	2	TRUE	0.00341	24.40838
1629	rs1688339	A	C	A	C	-0.0084	-0.00028	0.1921	0.222493	FALSE	FALSE	FALSE	n5rDuT	0.01647	0.986289	486484	sepsis	TRUE	reported	textfile	0.0017	1.20E-06	7081	M12067.m	TRUE	reported	ZQLTeR	2	TRUE	0.003436	24.40833
1463	rs1227610	T	C	T	C	-0.0084	0.034304	0.4352	0.442024	FALSE	FALSE	FALSE	pkb876	0.013773	0.012754	486484	sepsis	TRUE	reported	textfile	0.0017	1.56E-06	7037	M02734.m	TRUE	reported	0LCMVl	2	TRUE	0.003458	24.40829
1479	rs322614	A	G	A	G	0.0084	0.001263	0.3892	0.382842	FALSE	FALSE	FALSE	pkb876	0.014056	0.928409	486484	sepsis	TRUE	reported	textfile	0.0017	1.70E-06	7037	M02734.m	TRUE	reported	0LCMVl	2	TRUE	0.003458	24.40829
8849	rs300585	A	G	A	G	0.0084	0.000669	0.3107	0.337785	FALSE	FALSE	FALSE	XlHeTV	0.014612	0.963467	486484	sepsis	TRUE	reported	textfile	0.0017	1.19E-06	6940	M36808.m	TRUE	reported	37Vi2D	2	TRUE	0.003506	24.40819
8148	rs4307594	T	C	T	C	0.0084	-0.00898	0.6911	0.702166	FALSE	FALSE	FALSE	lv3pAQ	0.014922	0.54748	486484	sepsis	TRUE	reported	textfile	0.0017	9.86E-07	6920	M35327.m	TRUE	reported	3Vsglp	2	TRUE	0.003516	24.40817
6732	rs1285732	T	C	T	C	0.0168	0.013377	0.8752	0.878885	FALSE	FALSE	FALSE	SpG9A1	0.020915	0.522432	486484	sepsis	TRUE	reported	textfile	0.0034	9.75E-07	6502	M33939.m	TRUE	reported	Tudxel	2	TRUE	0.003741	24.40771
7202	rs1270261	A	G	A	G	0.892	-0.01025	0.011	0.030852	FALSE	FALSE	FALSE	i1uJ7T	0.039727	0.796416	486484	sepsis	TRUE	reported	textfile	0.179	6.25E-07	115	M34338.m	TRUE	reported	pi3mKW	2	TRUE	0.177589	24.40081
7371	rs7463317	A	G	A	G	0.2216	0.020497	0.108	0.166398	FALSE	FALSE	FALSE	mm9ksf	0.018375	0.264642	486484	sepsis	TRUE	reported	textfile	0.0423	1.60E-07	18	M34365.m	TRUE	reported	AVwEqD	2	TRUE	0.603914	24.39531
6704	rs1763853	A	G	A	G	-0.0652	0.075822	0.0208	0.019216	FALSE	FALSE	FALSE	bzT5yX	0.049706	0.127158	486484	sepsis	TRUE	reported	textfile	0.0132	8.75E-07	7345	M33936.m	TRUE	reported	W7BO8n	2	TRUE	0.003311	24.39097
4717	rs458582	T	G	T	G	0.0163	-0.0164	0.4996	0.492335	FALSE	FALSE	FALSE	L2WFM1	0.013707	0.231596	486484	sepsis	TRUE	reported	textfile	0.0033	1.04E-06	7313	M32761.m	TRUE	reported	5GHzph	2	TRUE	0.003325	24.39094
2413	rs268125	C	G	C	G	0.1492	-0.00435	0.9647	0.939879	FALSE	TRUE	FALSE	NBJ2Do	0.029158	0.881487	486484	sepsis	TRUE	reported	textfile	0.0302	7.71E-07	1739	M17799.m	TRUE	reported	Uz0Wgl	2	TRUE	0.013841	24.37946
422	rs2407893	A	G	A	G	0.0079	-0.00144	0.427	0.418435	FALSE	FALSE	FALSE	cTsNj6	0.013843	0.917176	486484	sepsis	TRUE	reported	textfile	0.0016	1.29E-06	7365	M00063.m	TRUE	reported	7KYTrQ	2	TRUE	0.003299	24.37229
713	rs7151615	T	G	T	G	-0.0079	0.028544	0.273	0.2747	FALSE	FALSE	FALSE	H9I3s3	0.015285	0.061841	486484	sepsis	TRUE	reported	textfile	0.0016	1.47E-06	7358	M01299.m	TRUE	reported	kXWyUq	2	TRUE	0.003302	24.37228
2086	rs7215931	A	G	A	G	0.0079	0.01175	0.2013	0.215733	FALSE	FALSE	FALSE	NPVH5j	0.016634	0.479964	486484	sepsis	TRUE	reported	textfile	0.0016	1.29E-06	7310	M15506.m	TRUE	reported	r7r3vM	2	TRUE	0.003324	24.37224
5508	rs6534458	T	C	T	C	-0.3044	-0.03003	0.0185	0.01584	FALSE	FALSE	FALSE	q32d4E	0.055228	0.586588	486484	sepsis	TRUE	reported	textfile	0.0616	7.65E-07	1017	M33178.m	TRUE	reported	cS3BwT	2	TRUE	0.023448	24.37098
462	rs394282	A	T	A	T	-0.0163	-0.0421	0.1112	0.06855	FALSE	TRUE	FALSE	cl8ucN	0.027	0.118927	486484	sepsis	TRUE	reported	textfile	0.0033	1.02E-06	1763	M00513.m	TRUE	reported	Ian0Q8	2	TRUE	0.01365	24.36994
8988	rs1250835	T	G	T	G	-0.0232	-0.02457	0.328	0.324276	FALSE	FALSE	FALSE	TfdxtP	0.014633	0.093137	486484	sepsis	TRUE	reported	textfile	0.0047	8.24E-07	7333	M37203.m	TRUE	reported	xicmWr	2	TRUE	0.003312	24.35913
602	rs8108144	T	G	T	G	-0.0232	-0.00389	0.788	0.797373	FALSE	FALSE	FALSE	KVK33e	0.017064	0.819586	486484	sepsis	TRUE	reported	textfile	0.0047	6.44E-07	5344	M01107.m	TRUE	reported	eAyR8w	2	TRUE	0.004539	24.35666
3638	rs1245864	T	C	T	C	0.1377	-0.04826	0.0419	0.067228	FALSE	FALSE	FALSE	Vq2efp	0.028926	0.095266	486484	sepsis	TRUE	reported	textfile	0.0279	7.92E-07	7363	M31787.m	TRUE	reported	krl4Tw	2	TRUE	0.003297	24.35238
3765	rs8082170	T	C	T	C	0.0153	-0.00324	0.1804	0.180396	FALSE	FALSE	FALSE	OZLdls	0.017789	0.855531	486484	sepsis	TRUE	reported	textfile	0.0031	1.07E-06	7356	M32338.m	TRUE	reported	s2itmB	2	TRUE	0.003301	24.35238
3427	rs2484454	A	G	A	G	-0.1096	0.023367	0.8988	0.912658	FALSE	FALSE	FALSE	HHQf8w	0.024296	0.336173	486484	sepsis	TRUE	reported	textfile	0.0222	7.71E-07	1309	M27531.m	TRUE	reported	3vTluK	2	TRUE	0.018279	24.33611
2435	rs665540	T	G	T	G	-0.1368	0.028849	0.118	0.254237	FALSE	FALSE	FALSE	NBJ2Do	0.015686	0.065901	486484	sepsis	TRUE	reported	textfile	0.0266	2.77E-07	25	M17799.m	TRUE	reported	Uz0Wgl	2	TRUE	0.514082	24.33306
807	rs1167446	T	C	T	C	0.0148	-0.00618	0.4181	0.425306	FALSE	FALSE	FALSE	J58DI0	0.013815	0.654371	486484	sepsis	TRUE	reported	textfile	0.003	7.01E-07	7335	M01356.m	TRUE	reported	zk9AMB	2	TRUE	0.003307	24.33114
1420	rs1051605	T	C	T	C	-0.0222	-0.01325	0.1978	0.207372	FALSE	FALSE	FALSE	ac6RGB	0.017729	0.454726	486484	sepsis	TRUE	reported	textfile	0.0045	6.99E-07	5791	M02342.m	TRUE	reported	dC6Rkp	2	TRUE	0.004185	24.32937
1408	rs1891053	A	G	A	G	0.1831	0.11355	0.0145	0.012604	FALSE	FALSE	FALSE	33ptwk	0.061488	0.064789	486484	sepsis	TRUE	reported	textfile	0.0371	8.03E-07	1175	M02137.m	TRUE	reported	SQY1na	2	TRUE	0.020309	24.31583
3452	rs797072	A	G	A	G	0.0217	0.004618	0.5347	0.538018	FALSE	FALSE	FALSE	J1I92u	0.013822	0.73828	486484	sepsis	TRUE	reported	textfile	0.0044	6.28E-07	6715	M27710.m	TRUE	reported	9qjplZ	2	TRUE	0.003609	24.31559
7823	rs1734031	C	G	C	G	0.0217	-0.00871	0.9347	0.882968	FALSE	TRUE	FALSE	h5whlS	0.02133	0.682999	486484	sepsis	TRUE	reported	textfile	0.0044	8.78E-07	4900	M35137.m	TRUE	reported	xVITKF	2	TRUE	0.004939	24.3129
8802	rs1306018	T	C	T	C	-0.0217	0.001581	0.5699	0.565259	FALSE	FALSE	FALSE	QWTooc	0.013797	0.908748	486484	sepsis	TRUE	reported	textfile	0.0044	8.17E-07	3248	M36756.m	TRUE	reported	OLsw6C	2	TRUE	0.007433	24.30785
8441	rs7748665	A	G	A	G	-0.0212	0.001254	0.9206	0.91506	FALSE	FALSE	FALSE	Nvt7pl	0.024786	0.959642	486484	sepsis	TRUE	reported	textfile	0.0043	6.71E-07	7358	M35718.m	TRUE	reported	WkA5Gq	2	TRUE	0.003293	24.30059
1029	rs7238006	T	C	T	C	0.0281	0.027056	0.9262	0.930523	FALSE	FALSE	FALSE	haWxZg	0.027075	0.317644	486484	sepsis	TRUE	reported	textfile	0.0057	6.77E-07	7276	M01561.m	TRUE	reported	5wm9R3	2	TRUE	0.003329	24.29649
6934	rs1412535	T	C	T	C	-0.0281	0.012977	0.5882	0.590211	FALSE	FALSE	FALSE	oNv0vf	0.014028	0.354907	486484	sepsis	TRUE	reported	textfile	0.0057	8.90E-07	5581	M34112.m	TRUE	reported	l0pe0N	2	TRUE	0.004336	24.29446
7570	rs3912161	T	C	T	C	-0.035	0.008126	0.9396	0.941188	FALSE	FALSE	FALSE	Ot2ZAn	0.029896	0.785766	486484	sepsis	TRUE	reported	textfile	0.0071	9.26E-07	7119	M34481.m	TRUE	reported	tpyFcK	2	TRUE	0.003402	24.29391
8788	rs690111	T	G	T	G	-0.037	-0.00151	0.2596	0.253988	FALSE	FALSE	FALSE	4bkfvN	0.015794	0.923791	486484	sepsis	TRUE	reported	textfile	0.0075	8.30E-07	922	M36738.m	TRUE	reported	xcqyX2	2	TRUE	0.025718	24.28498
8977	rs2130589	A	G	A	G	0.0345	-0.00648	0.3137	0.327087	FALSE	FALSE	FALSE	snOepl	0.01459	0.657137	486484	sepsis	TRUE	reported	textfile	0.007	9.18E-07	7355	M37202.m	TRUE	reported	PQx272	2	TRUE	0.003292	24.28421
6736	rs4666270	T	G	T	G	-0.0138	0.01786	0.8392	0.846313	FALSE	FALSE	FALSE	SpG9A1	0.019789	0.366784	486484	sepsis	TRUE	reported	textfile	0.0028	1.02E-06	6502	M33939.m	TRUE	reported	Tudxel	2	TRUE	0.003722	24.28334
4564	rs9308696	A	G	A	G	-0.1558	-0.02736	0.9751	0.973929	FALSE	FALSE	FALSE	KfoRxi	0.043089	0.525419	486484	sepsis	TRUE	reported	textfile	0.0316	8.19E-07	1677	M32735.m	TRUE	reported	UDRXAp	2	TRUE	0.014288	24.27965
1094	rs1688748	A	G	A	G	-0.0991	-0.01771	0.9585	0.95611	FALSE	FALSE	FALSE	K3wtZo	0.034019	0.602637	486484	sepsis	TRUE	reported	textfile	0.0201	8.82E-07	1646	M01573.m	TRUE	reported	0D9CvA	2	TRUE	0.014553	24.2788
2666	rs6585252	C	G	C	G	-0.1538	0.00235	0.9571	0.954365	FALSE	TRUE	FALSE	cDEa3G	0.032929	0.943107	486484	sepsis	TRUE	reported	textfile	0.0312	8.55E-07	1457	M18477.m	TRUE	reported	kxZEfC	2	TRUE	0.016404	24.26649
5007	rs1256546	A	G	A	G	0.034	0.000195	0.8419	0.828233	FALSE	FALSE	FALSE	URCVGm	0.018196	0.991443	486484	sepsis	TRUE	reported	textfile	0.0069	8.34E-07	2773	M33131.m	TRUE	reported	4rQ2gs	2	TRUE	0.00868	24.2631
6723	rs2906973	A	T	A	T	-0.0468	-0.02497	0.9593	0.95534	FALSE	TRUE	FALSE	B6lZly	0.033162	0.451457	486484	sepsis	TRUE	reported	textfile	0.0095	8.26E-07	7222	M33937.m	TRUE	reported	qFPzjh	2	TRUE	0.003349	24.26187
2192	rs1074573	A	G	A	G	0.0133	-0.01766	0.907	0.905153	FALSE	FALSE	FALSE	Bf6uMK	0.0234	0.450476	486484	sepsis	TRUE	reported	textfile	0.0027	1.15E-06	7308	M15778.m	TRUE	reported	jowM2N	2	TRUE	0.003309	24.25811
4327	rs1002820	T	C	T	C	-0.0133	-0.00886	0.1218	0.125677	FALSE	FALSE	FALSE	6VSoQQ	0.020618	0.667529	486484	sepsis	TRUE	reported	textfile	0.0027	8.97E-07	7229	M32644.m	TRUE	reported	oC2AV0	2	TRUE	0.003345	24.25803
7978	rs1440201	C	G	C	G	0.0133	0.017928	0.3764	0.383324	FALSE	TRUE	FALSE	t02AUT	0.014137	0.204736	486484	sepsis	TRUE	reported	textfile	0.0027	6.13E-07	7100	M35254.m	TRUE	reported	uClJaT	2	TRUE	0.003406	24.25791
1630	rs1703163	A	G	A	G	0.0133	-0.06274	0.9284	0.922687	FALSE	FALSE	FALSE	n5rDuT	0.025706	0.014656	486484	sepsis	TRUE	reported	textfile	0.0027	5.69E-07	7081	M12067.m	TRUE	reported	ZQLTeR	2	TRUE	0.003415	24.25789
6963	rs4738400	T	C	T	C	0.0133	-0.01139	0.2947	0.29871	FALSE	FALSE	FALSE	7Cw2XZ	0.014934	0.445545	486484	sepsis	TRUE	reported	textfile	0.0027	6.33E-07	7049	M34123.m	TRUE	reported	vrgMI3	2	TRUE	0.00343	24.25786
1653	rs1295097	T	C	T	C	-0.0133	-0.00907	0.6497	0.644111	FALSE	FALSE	FALSE	YzX5ZD	0.014346	0.527284	486484	sepsis	TRUE	reported	textfile	0.0027	9.84E-07	6864	M12129.m	TRUE	reported	6tMfaW	2	TRUE	0.003523	24.25768
2311	rs4976897	T	C	T	C	0.0133	-0.01353	0.7073	0.707585	FALSE	FALSE	FALSE	ZzUW8U	0.015086	0.36988	486484	sepsis	TRUE	reported	textfile	0.0027	1.16E-06	6733	M16821.m	TRUE	reported	g2xs3i	2	TRUE	0.003591	24.25754
3007	rs1726015	T	C	T	C	-0.0133	0.031044	0.7525	0.751819	FALSE	FALSE	FALSE	gxupFu	0.015867	0.050405	486484	sepsis	TRUE	reported	textfile	0.0027	1.08E-06	6556	M21188.m	TRUE	reported	gwwXh2	2	TRUE	0.003688	24.25734
4359	rs1074468	A	G	A	G	0.0133	-0.01136	0.3319	0.336793	FALSE	FALSE	FALSE	SPhl52	0.01452	0.434066	486484	sepsis	TRUE	reported	textfile	0.0027	1.25E-06	5211	M32651.m	TRUE	reported	V0Bnpj	2	TRUE	0.004635	24.25543
8711	rs1714555	T	C	T	C	-0.033	0.022745	0.951	0.944097	FALSE	FALSE	FALSE	I0G0I1	0.029748	0.44452	486484	sepsis	TRUE	reported	textfile	0.0067	7.79E-07	6464	M36553.m	TRUE	reported	lBltb0	2	TRUE	0.003739	24.25179
2432	rs6070814	T	C	T	C	-0.0813	0.05383	0.1104	0.108718	FALSE	FALSE	FALSE	NBJ2Do	0.022227	0.015444	486484	sepsis	TRUE	reported	textfile	0.0165	7.88E-07	1739	M17799.m	TRUE	reported	Uz0Wgl	2	TRUE	0.013769	24.25009
2892	rs5998272	A	C	A	C	0.0261	-0.00039	0.9257	0.919833	FALSE	FALSE	FALSE	mUzn1R	0.025152	0.987649	486484	sepsis	TRUE	reported	textfile	0.0053	1.08E-06	7301	M20675.m	TRUE	reported	XwY04K	2	TRUE	0.003311	24.24434
6320	rs2801848	T	C	T	C	0.0764	0.018158	0.1289	0.123522	FALSE	FALSE	FALSE	5B5LTX	0.020686	0.380061	486484	sepsis	TRUE	reported	textfile	0.0155	8.58E-07	824	M33609.m	TRUE	reported	m0eM6N	2	TRUE	0.02864	24.23639
6936	rs2705534	T	G	T	G	0.0517	-0.02091	0.9192	0.916772	FALSE	FALSE	FALSE	oNv0vf	0.024797	0.399144	486484	sepsis	TRUE	reported	textfile	0.0105	7.90E-07	5581	M34112.m	TRUE	reported	l0pe0N	2	TRUE	0.004325	24.23521
731	rs1179979	A	G	A	G	-0.0128	-0.01098	0.9214	0.920391	FALSE	FALSE	FALSE	9u0SC8	0.025295	0.664191	486484	sepsis	TRUE	reported	textfile	0.0026	9.49E-07	7363	M01301.m	TRUE	reported	H95uec	2	TRUE	0.003281	24.2301
8444	rs9373682	T	C	T	C	-0.0128	-0.01905	0.2404	0.258705	FALSE	FALSE	FALSE	Nvt7pl	0.015692	0.224788	486484	sepsis	TRUE	reported	textfile	0.0026	1.15E-06	7358	M35718.m	TRUE	reported	WkA5Gq	2	TRUE	0.003283	24.2301
8227	rs1262114	A	G	A	G	-0.0128	-0.00273	0.4155	0.419211	FALSE	FALSE	FALSE	4YAWhA	0.013852	0.843709	486484	sepsis	TRUE	reported	textfile	0.0026	9.23E-07	7256	M35439.m	TRUE	reported	bSSC4Z	2	TRUE	0.003329	24.23001
7342	rs1327041	T	C	T	C	-0.1222	0.058098	0.8578	0.828859	FALSE	FALSE	FALSE	mm9ksf	0.018294	0.001494	486484	sepsis	TRUE	reported	textfile	0.0248	8.00E-07	954	M34365.m	TRUE	reported	AVwEqD	2	TRUE	0.024819	24.22856
8098	rs1187601	T	C	T	C	-0.0128	0.02274	0.1123	0.115567	FALSE	FALSE	FALSE	nNbXBS	0.022029	0.301937	486484	sepsis	TRUE	reported	textfile	0.0026	1.15E-06	5768	M35326.m	TRUE	reported	UNDorn	2	TRUE	0.004184	24.22828
2278	rs1262950	A	G	A	G	0.0128	-0.00166	0.3983	0.399278	FALSE	FALSE	FALSE	mRULgB	0.01402	0.905511	486484	sepsis	TRUE	reported	textfile	0.0026	1.10E-06	4689	M16816.m	TRUE	reported	05lFsX	2	TRUE	0.005142	24.22635
5924	rs1278214	A	G	A	G	0.2983	0.014917	0.0137	0.020669	FALSE	FALSE	FALSE	veBhfS	0.049372	0.762549	486484	sepsis	TRUE	reported	textfile	0.0605	8.10E-07	528	M33391.m	TRUE	reported	Gfjy5a	2	TRUE	0.044016	24.21852
7354	rs2408190	A	G	A	G	-0.0867	-0.00629	0.5271	0.53485	FALSE	FALSE	FALSE	mm9ksf	0.013782	0.648121	486484	sepsis	TRUE	reported	textfile	0.0176	7.87E-07	954	M34365.m	TRUE	reported	AVwEqD	2	TRUE	0.024806	24.21595
6937	rs2821373	T	C	T	C	0.0561	-0.03522	0.9257	0.899272	FALSE	FALSE	FALSE	oNv0vf	0.023085	0.127062	486484	sepsis	TRUE	reported	textfile	0.0114	8.61E-07	5581	M34112.m	TRUE	reported	l0pe0N	2	TRUE	0.00432	24.20808
7075	rs16215	T	C	T	C	0.0694	0.062957	0.9711	0.969358	FALSE	FALSE	FALSE	sg7nOB	0.039621	0.112068	486484	sepsis	TRUE	reported	textfile	0.0141	8.05E-07	2278	M34289.m	TRUE	reported	KemvlC	2	TRUE	0.010523	24.20467
9001	rs1019600	T	C	T	C	-0.032	0.030986	0.8157	0.803946	FALSE	FALSE	FALSE	EZQ1cW	0.017242	0.072315	486484	sepsis	TRUE	reported	textfile	0.0065	7.63E-07	1411	M37253.m	TRUE	reported	ddPpd8	2	TRUE	0.016887	24.20233
1798	rs3789978	A	G	A	G	0.0123	-0.01368	0.8469	0.842616	FALSE	FALSE	FALSE	JEcCRZ	0.018782	0.466441	486484	sepsis	TRUE	reported	textfile	0.0025	1.32E-06	7368	M15140.m	TRUE	reported	xYCzKP	2	TRUE	0.003275	24.19983
8810	rs1159305	T	G	T	G	0.0123	0.008569	0.2479	0.27495	FALSE	FALSE	FALSE	X2Rol2	0.015361	0.576971	486484	sepsis	TRUE	reported	textfile	0.0025	1.12E-06	7335	M36776.m	TRUE	reported	nikHw1	2	TRUE	0.003289	24.1998
5073	rs1712741	A	G	A	G	0.0246	-0.00172	0.8728	0.872002	FALSE	FALSE	FALSE	gqzGN4	0.020444	0.932845	486484	sepsis	TRUE	reported	textfile	0.005	7.54E-07	7319	M33140.m	TRUE	reported	DKGBe9	2	TRUE	0.003296	24.19979
4349	rs7025315	C	G	C	G	0.0123	-0.02929	0.3317	0.354876	FALSE	TRUE	FALSE	6VSoQQ	0.014382	0.041712	486484	sepsis	TRUE	reported	textfile	0.0025	6.54E-07	7229	M32644.m	TRUE	reported	oC2AV0	2	TRUE	0.003337	24.1997
1319	rs9391679	A	C	A	C	0.0123	-0.00239	0.157	0.146203	FALSE	FALSE	FALSE	WQdpmo	0.019333	0.901562	486484	sepsis	TRUE	reported	textfile	0.0025	5.82E-07	7136	M01769.m	TRUE	reported	BkptOK	2	TRUE	0.003381	24.19962
8266	rs2088148	T	G	T	G	0.0123	0.001282	0.341	0.33289	FALSE	FALSE	FALSE	TdsGCZ	0.014498	0.929549	486484	sepsis	TRUE	reported	textfile	0.0025	1.08E-06	6883	M35464.m	TRUE	reported	sOspot	2	TRUE	0.003505	24.19937
4931	rs6769112	T	C	T	C	0.0251	-0.02103	0.2973	0.303755	FALSE	FALSE	FALSE	clhN6v	0.014995	0.160699	486484	sepsis	TRUE	reported	textfile	0.0051	7.28E-07	2148	M32869.m	TRUE	reported	BzloWk	2	TRUE	0.011151	24.19928
2316	rs1009184	T	G	T	G	-0.0123	-0.02333	0.5453	0.550294	FALSE	FALSE	FALSE	IZXwpR	0.013776	0.090303	486484	sepsis	TRUE	reported	textfile	0.0025	6.00E-07	6549	M16822.m	TRUE	reported	Zx9k2V	2	TRUE	0.003683	24.19901
4507	rs1048586	T	C	T	C	0.0123	-0.00258	0.8306	0.853616	FALSE	FALSE	FALSE	r0qJaM	0.01939	0.893999	486484	sepsis	TRUE	reported	textfile	0.0025	1.07E-06	6497	M32729.m	TRUE	reported	vYv5FI	2	TRUE	0.003712	24.19895
8725	rs1104551	T	C	T	C	-0.0379	0.012922	0.1391	0.15243	FALSE	FALSE	FALSE	ERC6hD	0.019069	0.497995	486484	sepsis	TRUE	reported	textfile	0.0077	8.66E-07	1636	M36559.m	TRUE	reported	n8waJF	2	TRUE	0.014592	24.19723
7262	rs1083956	C	G	C	G	-0.0797	-0.0048	0.8384	0.698091	FALSE	TRUE	FALSE	P5Xm4S	0.015659	0.759394	486484	sepsis	TRUE	reported	textfile	0.0162	9.03E-07	3491	M34350.m	TRUE	reported	YU4x0J	2	TRUE	0.006886	24.1901
6308	rs9906465	A	G	A	G	-0.0364	-0.01016	0.9319	0.908078	FALSE	FALSE	FALSE	ORVTMc	0.023647	0.667381	486484	sepsis	TRUE	reported	textfile	0.0074	8.97E-07	7352	M33587.m	TRUE	reported	57AsXW	2	TRUE	0.00328	24.18918
3344	rs723078	C	G	C	G	-0.0182	0.026412	0.9499	0.931307	FALSE	TRUE	FALSE	m66Eav	0.026986	0.327708	486484	sepsis	TRUE	reported	textfile	0.0037	6.84E-07	7303	M27256.m	TRUE	reported	pl2ECU	2	TRUE	0.003302	24.18914
2261	rs4690523	T	C	T	C	0.0182	0.009371	0.5243	0.512978	FALSE	FALSE	FALSE	Pb8tMF	0.013666	0.492867	486484	sepsis	TRUE	reported	textfile	0.0037	1.04E-06	7281	M15996.m	TRUE	reported	O7lDgO	2	TRUE	0.003312	24.18912
6851	rs1933041	A	G	A	G	-0.0182	0.015831	0.6651	0.661217	FALSE	FALSE	FALSE	2pJy8f	0.014457	0.273494	486484	sepsis	TRUE	reported	textfile	0.0037	7.23E-07	7132	M34040.m	TRUE	reported	zuJC1r	2	TRUE	0.003381	24.18898
6370	rs1434384	A	G	A	G	-0.0305	0.006412	0.5567	0.557951	FALSE	FALSE	FALSE	PAgZZz	0.013801	0.642185	486484	sepsis	TRUE	reported	textfile	0.0062	7.15E-07	4323	M33633.m	TRUE	reported	I8R1KJ	2	TRUE	0.005567	24.18886
6990	rs2267575	A	T	A	T	-0.0423	-0.00266	0.8736	0.835942	FALSE	TRUE	FALSE	APOtl6	0.018723	0.887213	486484	sepsis	TRUE	reported	textfile	0.0086	8.73E-07	6987	M34221.m	TRUE	reported	looCDC	2	TRUE	0.003451	24.18575
5914	rs1695882	T	C	T	C	-0.0605	0.039027	0.9132	0.912292	FALSE	FALSE	FALSE	iGV9aX	0.024222	0.107136	486484	sepsis	TRUE	reported	textfile	0.0123	9.48E-07	5185	M33390.m	TRUE	reported	sLbpnf	2	TRUE	0.004644	24.18427
1625	rs1211644	T	C	T	C	-0.0679	-0.00016	0.0144	0.015792	FALSE	FALSE	FALSE	n5rDuT	0.054952	0.997671	486484	sepsis	TRUE	reported	textfile	0.0138	9.28E-07	1747	M12067.m	TRUE	reported	ZQLTeR	2	TRUE	0.013668	24.18154

7776	rs2025277	A	G	A	G	-0.03	-0.00629	0.1872	0.184533	FALSE	FALSE	FALSE	15BqPa	0.017737	0.722793	486484	sepsis	TRUE	reported	textfile	0.0061	7.93E-07	7364	M35126.m	TRUE	reported	zvDzP2	2	TRUE	0.003274	24.18048
4075	rs590582	C	G	C	G	0.03	0.039887	0.9437	0.939579	FALSE	TRUE	FALSE	TyHtdh	0.028823	0.166399	486484	sepsis	TRUE	reported	textfile	0.0061	7.95E-07	7358	M32497.m	TRUE	reported	H3KmLR	2	TRUE	0.003276	24.18047
3403	rs1165019	A	G	A	G	0.03	0.002902	0.0782	0.108759	FALSE	FALSE	FALSE	VU62LG	0.022133	0.895679	486484	sepsis	TRUE	reported	textfile	0.0061	7.59E-07	7180	M27513.m	TRUE	reported	0didOx	2	TRUE	0.003357	24.18031
2958	rs1582961	T	C	T	C	0.0423	0.027677	0.2291	0.24245	FALSE	FALSE	FALSE	zndu0r	0.016634	0.09613	486484	sepsis	TRUE	reported	textfile	0.0086	8.08E-07	3422	M21049.m	TRUE	reported	2XpPDU	2	TRUE	0.00702	24.17853
1258	rs1255291	A	T	A	T	-0.0059	-0.01906	0.2932	0.293046	FALSE	TRUE	FALSE	6RGrj0	0.01504	0.205012	486484	sepsis	TRUE	reported	textfile	0.0012	1.16E-06	7359	M01649.m	TRUE	reported	J0vZCF	2	TRUE	0.003274	24.16704
199	rs2176664	T	C	T	C	-0.0059	0.001055	0.2846	0.284541	FALSE	FALSE	FALSE	FbLTng	0.015326	0.945107	486484	sepsis	TRUE	reported	textfile	0.0012	2.03E-06	7355	M00059.m	TRUE	reported	KK7HbM	2	TRUE	0.003276	24.16704
4980	rs1235957	T	G	T	G	0.0177	-0.01303	0.2419	0.258576	FALSE	FALSE	FALSE	AjJi2i	0.015805	0.40953	486484	sepsis	TRUE	reported	textfile	0.0036	6.26E-07	7330	M32980.m	TRUE	reported	gVSm9e	2	TRUE	0.003287	24.16702
6834	rs6986249	T	C	T	C	-0.0177	0.007512	0.4413	0.447188	FALSE	FALSE	FALSE	ZPJCMx	0.013729	0.584253	486484	sepsis	TRUE	reported	textfile	0.0036	6.30E-07	7330	M33969.m	TRUE	reported	mLJPAI	2	TRUE	0.003287	24.16702
3255	rs1251677	A	G	A	G	0.0118	-0.01201	0.5206	0.506137	FALSE	FALSE	FALSE	ZNPBqT	0.013769	0.383196	486484	sepsis	TRUE	reported	textfile	0.0024	1.26E-06	7256	M24074.m	TRUE	reported	8pPtOI	2	TRUE	0.00332	24.16695
2295	rs4332594	T	C	T	C	-0.0177	-0.02163	0.246	0.244061	FALSE	FALSE	FALSE	43Yh3h	0.015888	0.173391	486484	sepsis	TRUE	reported	textfile	0.0036	1.11E-06	7055	M16818.m	TRUE	reported	beR4IT	2	TRUE	0.003415	24.16676
8529	rs6124466	T	C	T	C	-0.0359	0.00632	0.8283	0.8347	FALSE	FALSE	FALSE	gM7v2m	0.01865	0.734713	486484	sepsis	TRUE	reported	textfile	0.0073	7.67E-07	2651	M36009.m	TRUE	reported	6Xcypd	2	TRUE	0.00904	24.16659
6108	rs2471644	A	G	A	G	0.0177	-0.02198	0.6142	0.61605	FALSE	FALSE	FALSE	a0osmD	0.014062	0.118083	486484	sepsis	TRUE	reported	textfile	0.0036	6.50E-07	5717	M33453.m	TRUE	reported	CePFnK	2	TRUE	0.004211	24.16515
6547	rs1310356	T	C	T	C	-0.0418	0.013912	0.7102	0.700002	FALSE	FALSE	FALSE	zmDnD7	0.015164	0.358937	486484	sepsis	TRUE	reported	textfile	0.0085	9.87E-07	2660	M33835.m	TRUE	reported	MLBiKM	2	TRUE	0.00901	24.16507
6025	rs1703360	A	G	A	G	-1.5889	-0.03563	0.0249	0.0377	FALSE	FALSE	FALSE	33ojbt	0.040566	0.379769	486484	sepsis	TRUE	reported	textfile	0.3178	5.75E-07	60	M33423.m	TRUE	reported	ivEtvl	2	TRUE	0.294092	24.16363
6347	rs7147680	A	G	A	G	0.5941	0.003693	0.0114	0.014506	FALSE	FALSE	FALSE	qLUY00	0.057841	0.949096	486484	sepsis	TRUE	reported	textfile	0.1197	6.94E-07	102	M33610.m	TRUE	reported	8cAJbB	2	TRUE	0.194528	24.15075
2814	rs3872668	C	G	C	G	-0.0177	0.003449	0.3688	0.352272	FALSE	TRUE	FALSE	Sx5Uhq	0.014355	0.810116	486484	sepsis	TRUE	reported	textfile	0.0036	7.34E-07	1759	M19934.m	TRUE	reported	DijYTE	2	TRUE	0.013557	24.14613
6703	rs1146594	A	G	A	G	0.0516	-0.05244	0.0294	0.032227	FALSE	FALSE	FALSE	bzT5yX	0.038796	0.176505	486484	sepsis	TRUE	reported	textfile	0.0105	8.65E-07	7345	M33936.m	TRUE	reported	W7BO8n	2	TRUE	0.003277	24.14363
3401	rs1104328	T	C	T	C	0.057	0.002434	0.0188	0.020272	FALSE	FALSE	FALSE	VU62LG	0.048866	0.960272	486484	sepsis	TRUE	reported	textfile	0.0116	8.02E-07	7180	M27513.m	TRUE	reported	0didOx	2	TRUE	0.003352	24.13864
652	rs1707758	A	G	A	G	0.1032	0.015082	0.9187	0.922638	FALSE	FALSE	FALSE	7BPGuQ	0.025795	0.558762	486484	sepsis	TRUE	reported	textfile	0.021	9.20E-07	2639	M01123.m	TRUE	reported	WV2hVn	2	TRUE	0.009068	24.1319
400	rs1077834	T	C	T	C	-0.0113	0.01761	0.7912	0.780636	FALSE	FALSE	FALSE	cTsNj6	0.016522	0.286494	486484	sepsis	TRUE	reported	textfile	0.0023	6.87E-07	7365	M00063.m	TRUE	reported	7KYTrQ	2	TRUE	0.003267	24.13144
930	rs1301322	C	G	C	G	0.0113	0.013715	0.1794	0.194448	FALSE	TRUE	FALSE	tv99UN	0.017334	0.42884	486484	sepsis	TRUE	reported	textfile	0.0023	7.04E-07	7354	M01494.m	TRUE	reported	osDclp	2	TRUE	0.003272	24.13143
2729	rs2041343	T	C	T	C	0.0113	-0.01725	0.4795	0.503259	FALSE	FALSE	FALSE	H4yqSK	0.013903	0.214688	486484	sepsis	TRUE	reported	textfile	0.0023	6.67E-07	7182	M19362.m	TRUE	reported	w2PDEO	2	TRUE	0.00335	24.13127
8364	rs9262	C	G	C	G	-0.0226	-0.00066	0.5925	0.582669	FALSE	TRUE	FALSE	6VVCe4	0.013857	0.961972	486484	sepsis	TRUE	reported	textfile	0.0046	1.01E-06	5629	M35669.m	TRUE	reported	I0fk8X	2	TRUE	0.00427	24.12942
8744	rs1314234	T	G	T	G	0.0339	0.004543	0.2825	0.279416	FALSE	FALSE	FALSE	FnYrPc	0.015254	0.765842	486484	sepsis	TRUE	reported	textfile	0.0069	8.46E-07	3346	M36585.m	TRUE	reported	amHtMH	2	TRUE	0.007162	24.12357
6329	rs1174740	A	T	A	T	0.2269	-0.00205	0.0366	0.04627	FALSE	TRUE	FALSE	qLUY00	0.032543	0.94974	486484	sepsis	TRUE	reported	textfile	0.0461	8.78E-07	470	M33610.m	TRUE	reported	8cAJbB	2	TRUE	0.049016	24.1221
3829	rs1776347	A	C	A	C	0.0167	0.002588	0.0905	0.097925	FALSE	FALSE	FALSE	tRX2u0	0.023111	0.910823	486484	sepsis	TRUE	reported	textfile	0.0034	9.81E-07	7365	M32348.m	TRUE	reported	ruBgwQ	2	TRUE	0.003265	24.11888
3794	rs7791060	A	G	A	G	-0.0167	0.014661	0.9237	0.924867	FALSE	FALSE	FALSE	NW1aaR	0.025975	0.572473	486484	sepsis	TRUE	reported	textfile	0.0034	6.96E-07	7344	M32339.m	TRUE	reported	pBPkAB	2	TRUE	0.003274	24.11886
7446	rs1052007	A	G	A	G	-0.0167	-0.00912	0.3469	0.329982	FALSE	FALSE	FALSE	7ubZow	0.014544	0.530432	486484	sepsis	TRUE	reported	textfile	0.0034	1.36E-06	7344	M34407.m	TRUE	reported	LRsXN2	2	TRUE	0.003274	24.11886
6904	rs1246268	A	T	A	T	0.95	-0.03618	0.988	0.963544	FALSE	TRUE	FALSE	SvfSvH	0.039238	0.356464	486484	sepsis	TRUE	reported	textfile	0.192	7.50E-07	134	M34109.m	TRUE	reported	7OSbiu	2	TRUE	0.154477	24.11648
2831	rs9947181	C	G	C	G	-0.0275	0.024306	0.0304	0.028902	FALSE	TRUE	FALSE	Sx5Uhq	0.041495	0.558033	486484	sepsis	TRUE	reported	textfile	0.0056	9.24E-07	7354	M19934.m	TRUE	reported	DijYTE	2	TRUE	0.003268	24.10856
2989	rs1707103	A	G	A	G	-0.0329	0.029458	0.8334	0.834023	FALSE	FALSE	FALSE	xmzJ5F	0.018408	0.109541	486484	sepsis	TRUE	reported	textfile	0.0067	8.57E-07	5436	M21184.m	TRUE	reported	xgwhDp	2	TRUE	0.004416	24.10363
7331	rs1192474	A	G	A	G	-0.1786	-0.01002	0.6176	0.57223	FALSE	FALSE	FALSE	mm9ksf	0.013826	0.468491	486484	sepsis	TRUE	reported	textfile	0.0343	1.89E-07	18	M34365.m	TRUE	reported	AVwEqD	2	TRUE	0.601	24.10028
889	rs4109037	A	T	A	T	0.0162	-0.02783	0.146	0.143559	FALSE	TRUE	FALSE	oi7KQI	0.01965	0.156632	486484	sepsis	TRUE	reported	textfile	0.0033	9.49E-07	7362	M01365.m	TRUE	reported	7NWGKA	2	TRUE	0.003263	24.09263
4875	rs7687485	A	T	A	T	-0.0324	0.018895	0.0947	0.092258	FALSE	TRUE	FALSE	KBJVQO	0.023644	0.424204	486484	sepsis	TRUE	reported	textfile	0.0066	8.28E-07	7355	M32855.m	TRUE	reported	Xea7Fn	2	TRUE	0.003266	24.09262
6964	rs523932	T	C	T	C	0.054	0.085026	0.9868	0.983533	FALSE	FALSE	FALSE	7Cw2XZ	0.053704	0.113367	486484	sepsis	TRUE	reported	textfile	0.011	8.30E-07	7049	M34123.m	TRUE	reported	vrgMl3	2	TRUE	0.003407	24.09234
7470	rs1042933	T	C	T	C	0.0329	0.013671	0.2704	0.26442	FALSE	FALSE	FALSE	SqfdcK	0.015697	0.383782	486484	sepsis	TRUE	reported	textfile	0.0067	9.14E-07	1567	M34416.m	TRUE	reported	sRGRIK	2	TRUE	0.015154	24.08172
8959	rs4761693	T	C	T	C	0.0643	-0.00193	0.6193	0.614294	FALSE	FALSE	FALSE	Ywk2ve	0.014025	0.890293	486484	sepsis	TRUE	reported	textfile	0.0131	9.65E-07	2560	M37112.m	TRUE	reported	EiAdDj	2	TRUE	0.009323	24.07354
7066	rs6755681	T	C	T	C	-0.0211	0.026579	0.0501	0.056567	FALSE	FALSE	FALSE	uthruC	0.029574	0.368792	486484	sepsis	TRUE	reported	textfile	0.0043	8.62E-07	7316	M34283.m	TRUE	reported	ftTuRU	2	TRUE	0.00328	24.07184
2117	rs1490672	T	C	T	C	-0.0108	0.009293	0.2361	0.235553	FALSE	FALSE	FALSE	t7SQ8V	0.016101	0.563815	486484	sepsis	TRUE	reported	textfile	0.0022	1.13E-06	1763	M15650.m	TRUE	reported	QQT7qA	2	TRUE	0.013485	24.07183
7960	rs1717637	A	G	A	G	0.0211	-0.0031	0.1078	0.106161	FALSE	FALSE	FALSE	zb8m4a	0.022163	0.888634	486484	sepsis	TRUE	reported	textfile	0.0043	8.44E-07	7261	M35253.m	TRUE	reported	tHnnnS	2	TRUE	0.003305	24.07179
7504	rs2938675	A	C	A	C	0.0476	0.03055	0.3923	0.382829	FALSE	FALSE	FALSE	1aXQWs	0.014087	0.030109	486484	sepsis	TRUE	reported	textfile	0.0097	9.79E-07	4452	M34420.m	TRUE	reported	FOqhHW	2	TRUE	0.00538	24.06996
6599	rs9317012	T	C	T	C	-0.1562	0.040294	0.8991	0.909299	FALSE	FALSE	FALSE	XO3rXq	0.023846	0.091067	486484	sepsis	TRUE	reported	textfile	0.0317	8.49E-07	228	M33877.m	TRUE	reported	p2sETg	2	TRUE	0.096241	24.06673
848	rs1301953	C	G	C	G	0.0157	-0.02483	0.841	0.841827	FALSE	TRUE	FALSE	1tFtaf	0.018852	0.187838	486484	sepsis	TRUE	reported	textfile	0.0032	7.52E-07	7323	M01359.m	TRUE	reported	qq3q6E	2	TRUE	0.003276	24.06471
1322	rs9674799	A	G	A	G	0.0157	-0.00071	0.8784	0.879419	FALSE	FALSE	FALSE	WQdpmo	0.020988	0.973037	486484	sepsis	TRUE	reported	textfile	0.0032	7.47E-07	7136	M01769.m	TRUE	reported	BkptOK	2	TRUE	0.003362	24.06454
4930	rs671307	A	G	A	G	0.0319	-0.03996	0.1553	0.155625	FALSE	FALSE	FALSE	clhN6v	0.018928	0.034767	486484	sepsis	TRUE	reported	textfile	0.0065	7.95E-07	2148	M32869.m	TRUE	reported	BzloWk	2	TRUE	0.011089	24.06302
8290	rs1439737	T	C	T	C	0.0368	0.005026	0.5832	0.596457	FALSE	FALSE	FALSE	vXjGkU	0.013914	0.717938	486484	sepsis	TRUE	reported	textfile	0.0075	1.08E-06	1291	M35527.m	TRUE	reported	oS5qaO	2	TRUE	0.018307	24.03808
2754	rs7415896	A	G	A	G	0.0304	-0.0146	0.0488	0.055302	FALSE	FALSE	FALSE	qFAbee	0.030106	0.62775	486484	sepsis	TRUE	reported	textfile	0.0062	8.77E-07	6929	M19364.m	TRUE	reported	tZgXNL	2	TRUE	0.003458	24.03468
7684	rs1687949	A	C	A	C	0.052	0.041281	0.9479	0.979543	FALSE	FALSE	FALSE	5FAxbZ	0.04859	0.39556	486484	sepsis	TRUE	reported	textfile	0.0106	9.81E-07	1560	M34674.m	TRUE	reported	TDQqa8	2	TRUE	0.015192	24.03465
899	rs1476414	C	G	C	G	0.0397	0.049819	0.0535	0.06866	FALSE	TRUE	FALSE	I6zQnf	0.027588	0.070938	486484	sepsis	TRUE	reported	textfile	0.0081	9.30E-07	7345	M01444.m	TRUE	reported	Ilzalm	2	TRUE	0.00326	24.01556
514	rs946712	A	C	A	C	0.1039	0.018639	0.9559	0.954463	FALSE	FALSE	FALSE	ZOMIXT	0.032724	0.568959	486484	sepsis	TRUE	reported	textfile	0.0212	9.59E-07	7274	M00569.m	TRUE	reported	kZGouU	2	TRUE	0.003291	24.01264
1173	rs1116790	T	C	T	C	0.0804	0.019349	0.8545	0.842405	FALSE	FALSE	FALSE	FgZx4a	0.018931	0.306732	486484	sepsis	TRUE	reported	textfile	0.0164	9.83E-07	2063	M01640.m	TRUE	reported	hNPAJ8	2	TRUE	0.011516	24.01061
2439	rs7144659	A	G	A	G	0.0554	0.021058	0.8457	0.961483	FALSE	FALSE	FALSE	NBJ2Do	0.035789	0.556272	486484	sepsis	TRUE	reported	textfile	0.0113	9.86E-07	1739	M17799.m	TRUE	reported	Uz0Wgl	2	TRUE	0.013633	24.00838
3426	rs1765149	A	G	A	G	0.1034	0.003371	0.9423	0.935551	FALSE	FALSE	FALSE	HHQf8w	0.02766	0.902989	486484	sepsis	TRUE	reported	textfile	0.0211	9.13E-07	5673	M27531.m	TRUE	reported	3vTluK	2	TRUE	0.004215	24.00618
4708	rs7215713	T	C	T	C	0.0245	-0.00734	0.2296	0.225648	FALSE	FALSE	FALSE	WlwKaF	0.016525	0.657048	486484	sepsis	TRUE	reported	textfile	0.005	1.18E-06	6712	M32759.m	TRUE	reported	Ub4yxH	2	TRUE	0.003564	24.00285
5558	rs7755459	A	C	A	C	0.3099	0.056061	0.9804	0.977346	FALSE	FALSE	FALSE	q32d4E	0.045822	0.221155	486484	sepsis	TRUE	reported	textfile	0.0632	9.49E-07	1017	M33178.m	TRUE	reported	cS3BwT	2	TRUE	0.023096	23.99684
5774	rs1305496	A	G	A	G	0.05	-0.01946	0.2239	0.224469	FALSE	FALSE	FALSE	eT2We0	0.016446	0.236613	486484	sepsis	TRUE	reported	textfile	0.0102	9.07E-07	1273	M33353.m	TRUE	reported	uZx90m	2	TRUE	0.018526	23.99147
2103	rs2268365	T	C	T	C	0.0289	0.026909	0.8731	0.877716	FALSE	FALSE	FALSE	MN85S3	0.020938	0.198734	486484	sepsis	TRUE	reported	textfile	0.0059	1.11E-06	7574	M15630.m	TRUE	reported	FUih5R	2	TRUE	0.003158	23.98706
7174	rs1103935	T	C	T	C	0.0338	-0.0153	0.0406	0.048353	FALSE	FALSE	FALSE	2hlepp	0.032145	0.634007	486484	sepsis	TRUE	reported	textfile	0.0069	8.47E-07	5081	M34336.m	TRUE	reported	XyK8mO	2	TRUE	0.0047	23.98635
3450	rs780245	T	C	T	C	0.0289	-0.012	0.1757	0.168224	FALSE	FALSE	FALSE	J1I92u	0.018247	0.510882	486484	sepsis	TRUE	reported	textfile	0.0059	1.15E-06	6715	M27710.m	TRUE	reported	9qjplZ	2	TRUE	0.00356	23.98625
6585	rs1716908	A	G	A	G	0.5339	0.035921	0.0134	0.054508	FALSE	FALSE	FALSE	XO3rXq	0.030709	0.242112	486484	sepsis	TRUE	reported	textfile	0.1084	8.32E-07	176	M33877.m	TRUE	reported	p2sETg	2	TRUE	0.121135	23.98269
4879	rs498275	A	G	A	G	0.0338	0.019856	0.3246	0.338148	FALSE	FALSE	FALSE	MDX5FX	0.014505	0.171039	486484	sepsis	TRUE	reported	textfile	0.0069	9.93E-07	2656	M32857.m	TRUE	reported	moAN2E	2	TRUE	0.008954	23.97773
7201	rs1262616	C	G	C	G	0.2276	0.023154	0.0363	0.046055	FALSE	TRUE	FALSE	i1uJ7T	0.032934	0.482038	486484	sepsis	TRUE	reported	textfile	0.0464	9.37E-07	535	M34338.m	TRUE	reported	pi3mKW	2	TRUE	0.043038	23.97077
3764	rs804895	T	C	T	C	-0.0235	0.035297	0.0781	0.085133	FALSE	FALSE	FALSE	OZLdls	0.024457	0.148957	486484	sepsis	TRUE	reported	textfile	0.0048	8.44E-07	7356	M32338.m	TRUE	reported	s2itmB	2	TRUE	0.003248	23.96267
1165	rs511304	A	C	A	C	0.0191	0.01452	0.4368	0.43912	FALSE	FALSE	FALSE	cSBFJ3	0.014001	0.299709	486484	sepsis	TRUE	reported	textfile	0.0039	1.01E-06	1735	M01638.m	TRUE	reported	sUkjpE	2	TRUE	0.013636	23.95723
6473	rs1684379	T	C	T	C	-0.7079	-0.02417	0.974	0.978435	FALSE	FALSE	FALSE	ecwVg8	0.047541	0.611167	486484	sepsis	TRUE	reported	textfile	0.1428	7.10E-07	79	M33751.m	TRUE	reported	NnvblQ	2	TRUE	0.237265	23.95251
4391	rs1948709	T	C	T	C	0.0186	0.017913	0.8952	0.894734	FALSE	FALSE	FALSE	UJygVd	0.022273	0.421251	486484	sepsis	TRUE	reported	textfile	0.0038	7.72E-07	7361	M32654.m	TRUE	reported	kv9R0W	2	TRUE	0.003244	23.95194
556	rs1160770	T	C	T	C	0.0093	-0.01165	0.8783	0.871592	FALSE	FALSE	FALSE	fNqkCW	0.020437	0.568505	486484	sepsis	TRUE	reported	textfile	0.0019	6.94E-07	7354	M00606.m	TRUE	reported	g9uwOn	2	TRUE	0.003247	23.95193
3191	rs1269420	T	C	T	C	0.0093	-0.00448	0.4344	0.441797	FALSE	FALSE	FALSE	UfLkBq	0.013794	0.745461	486484	sepsis	TRUE	reported	textfile	0.0019	6.54E-07	7329	M22481.m	TRUE	reported	kWMdiT	2	TRUE	0.003258	23.95191
2857	rs741160	A	G	A	G	0.0093	0.005484	0.8235	0.818836	FALSE	FALSE	FALSE	Y5qWBm	0.017757	0.757426	486484	sepsis	TRUE	reported	textfile	0.0019	6.82E-07	7325	M20489.m	TRUE	reported	f2g9Su	2	TRUE	0.00326	23.95191
5873	rs236403	T	C	T	C	-0.0093	-0.00998	0.624	0.614409	FALSE	FALSE	FALSE	tqW0ld	0.014063	0.477702	486484	sepsis	TRUE	reported	textfile	0.0019	5.87E-07	7315	M33389.m	TRUE	reported	Sv7EyV	2	TRUE	0.003265	23.9519
2263	rs7150776	T	C	T	C	0.0279	0.030807	0.1105	0.106147	FALSE	FALSE	FALSE	Pb8tMF	0.022217	0.165552	486484	sepsis	TRUE	reported	textfile	0.0057	9.22E-07	7281	M15996.m	TRUE	reported	O7IDgO	2	TRUE	0.00328	23.95187
8475	rs7862610	A	C	A	C	0.0093	0.005236	0.4276	0.436185	FALSE	FALSE	FALSE	uMajV6	0.013813	0.704669	486484	sepsis	TRUE	reported	textfile	0.0019	1.36E-06	6604	M35754.m	TRUE	reported	b8QB7S	2	TRUE	0.003615	23.95119
3126	rs1287183	A	C	A	C	-0.0093	0.005272	0.4654	0.481307	FALSE	FALSE	FALSE	JiXR2t	0.013945	0.705422	486484	sepsis	TRUE	reported	textfile	0.0019	1.53E-06	6535	M22177.m	TRUE	reported	joi6p6	2	TRUE	0.003653	23.95112
3156	rs571139	A	G	A	G	0.0093	0.011984	0.3354	0.348692	FALSE	FALSE	FALSE	JiXR2t	0.014336	0.403184	486484	sepsis	TRUE	reported	textfile	0.0019	1.62E-06	6535	M22177.m	TRUE	reported	joi6p6	2	TRUE	0.003653	23.95112
7827	rs2267290	C	G	C	G	-0.0093	0.011406	0.6914	0.691852	FALSE	TRUE	FALSE	h5whIS	0.014796	0.44079	486484	sepsis	TRUE	reported	textfile	0.0019	8.92E-07	4900	M35137.m	TRUE	reported	xVITKF	2	TRUE	0.004866	23.94867
4777	rs1025573	T	C	T	C	0.0558	0.007946	0.0859	0.092574	FALSE	FALSE	FALSE	MmZZoM	0.023814	0.73864	486484	sepsis	TRUE	reported	textfile	0.0114	9.52E-07	4274	M32800.m	TRUE	reported	70YSCO	2	TRUE	0.005574	23.94724
5670	rs2493950	T	C	T	C	-0.2434	0.022564	0.9465	0.94568	FALSE	FALSE	FALSE	wHu1Rk	0.030168	0.454497	486484	sepsis	TRUE	reported	textfile	0.0497	9.90E-07	1247	M33195.m	TRUE	reported	yvhnuQ	2	TRUE	0.018871	23.94591
472	rs1340344	A	T	A	T	-0.0235	0.0298	0.8588	0.874969	FALSE	TRUE	FALSE	OW48NY	0.020693	0.149841	486484	sepsis	TRUE	reported	textfile	0.0048	1.02E-06	1761	M00527.m	TRUE	reported	57AP2f	2	TRUE	0.013428	23.94196
2987	rs822406	A	G	A	G	0.1625	-0.02443	0.063	0.091749	FALSE	FALSE	FALSE	Tli4xl	0.02424	0.313623	486484	sepsis	TRUE	reported	textfile	0.0332	1.03E-06	2339	M21151.m	TRUE	reported	xaSytY	2	TRUE	0.010139	23.93641
6235	rs1674726	A	G	A	G	-0.0137	0.033338	0.3501	0.366891	FALSE	FALSE	FALSE	vL52Yb	0.014257	0.019366	486484	sepsis	TRUE	reported	textfile	0.0028	8.82E-07	7269	M33510.m	TRUE	reported	JIO9VX	2	TRUE	0.003283	23.93346
7095	rs21806	T	C	T	C	-0.0137	-0.01155	0.3173	0.339219	FALSE	FALSE	FALSE	pxNICA	0.014466	0.424448	486484	sepsis	TRUE	reported	textfile	0.0028	1.17E-06	6994	M34306.m	TRUE	reported	xk5b9M	2	TRUE	0.003411	23.93321
4380	rs9423399	A	G	A	G	0.0137	0.01485	0.3176	0.32543	FALSE	FALSE	FALSE	SPhI52	0.014564	0.307926	486484	sepsis	TRUE	reported	textfile	0.0028	5.89E-07	5211	M32651.m	TRUE	reported	V0Bnpj	2	TRUE	0.004573	23.93086
2782	rs4415526	T	C	T	C	0.0137	-0.01459	0.4107	0.405276	FALSE	FALSE	FALSE	yYuamw	0.013939	0.295356	486484	sepsis	TRUE	reported	textfile	0.0028	7.70E-07	4844	M19414.m	TRUE	reported	GxSb5G	2	TRUE	0.004918	23.93017
2276	rs1098032	T	C	T	C	0.0411	0.031923	0.9677	0.962168	FALSE	FALSE	FALSE	mRULgB	0.035682	0.370974	486484	sepsis	TRUE	reported	textfile	0.0084	9.21E-07	4689	M16816.m	TRUE	reported	05lFsX	2	TRUE	0.00508	23.92984
6714	rs888860	A	C	A	C	-0.0181	0.000328	0.3513	0.354471	FALSE	FALSE	FALSE	bzT5yX	0.014309	0.9817	486484	sepsis	TRUE	reported	textfile	0.0037	9.39E-07	7345	M33936.m	TRUE	reported	W7BO8n	2	TRUE	0.003248	23.92409
4378	rs728661	T	C	T	C	-0.0181	0.02285	0.8438	0.84937	FALSE	FALSE	FALSE	SPhI52	0.019137	0.232476	486484	sepsis	TRUE	reported	textfile	0.0037	1.28E-06	5211	M32651.m	TRUE	reported	V0Bnpj	2	TRUE	0.004571	23.92142
2617	rs4410790	T	C	T	C	0.0313	0.00174	0.3695	0.36919	FALSE	FALSE	FALSE	OiiDPr	0.01418	0.902355	486484	sepsis	TRUE	reported	textfile	0.0064	8.96E-07	7322	M18392.m	TRUE	reported	ScUw6o	2	TRUE	0.003256	23.91168
7525	rs7923671	A	G	A	G	-0.0538	0.00789	0.0466	0.066829	FALSE	FALSE	FALSE	rp1LGA	0.027348	0.772962	486484	sepsis	TRUE	reported	textfile	0.011	9.82E-07	4442	M34441.m	TRUE	reported	SU47tz	2	TRUE	0.005356	23.91022
5416	rs275362	A	G	A	G	-0.1249	0.009229	0.4565	0.490616	FALSE	FALSE	FALSE	q32d4E	0.01402	0.510337	486484	sepsis	TRUE	reported	textfile	0.0245	3.39E-07	25	M33178.m	TRUE	reported	cS3BwT	2	TRUE	0.5097	23.91005
5003	rs1159324	A	G	A	G	-0.0494	-0.01057	0.937	0.928581	FALSE	FALSE	FALSE	URCVGm	0.026585	0.690925	486484	sepsis	TRUE	reported	textfile	0.0101	1.07E-06	2773	M33131.m	TRUE	reported	4rQ2gs	2	TRUE	0.008553	23.9055
7223	rs3735210	T	C	T	C	-0.873	-0.03654	0.985	0.983562	FALSE	FALSE	FALSE	i1uJ7T	0.053781	0.496865	486484	sepsis	TRUE	reported	textfile	0.177	8.13E-07	115	M34338.m	TRUE	reported	pi3mKW	2	TRUE	0.174601	23.90356
6369	rs1337683	A	G	A	G	-0.0401	0.014443	0.8266	0.83126	FALSE	FALSE	FALSE	PAgZZz	0.01828	0.429463	486484	sepsis	TRUE	reported	textfile	0.0082	1.13E-06	4323	M33633.m	TRUE	reported	I8R1KJ	2	TRUE	0.005501	23.90342
1180	rs6834631	T	G	T	G	0.1306	-0.0537	0.9596	0.956031	FALSE	FALSE	FALSE	FgZx4a	0.033442	0.108351	486484	sepsis	TRUE	reported	textfile	0.0267	1.03E-06	2063	M01640.m	TRUE	reported	hNPAJ8	2	TRUE	0.011465	23.90246
9056	rs9266813	C	G	C	G	-0.0088	0.006477	0.7349	0.742286	FALSE	TRUE	FALSE	df0MCM	0.015668	0.679299	486484	sepsis	TRUE	reported	textfile	0.0018	1.02E-06	7366	M37506.m	TRUE	reported	iUfr4u	2	TRUE	0.003234	23.89474
3832	rs2635351	A	C	A	C	-0.0088	-0.02976	0.5749	0.580481	FALSE	FALSE	FALSE	tRX2u0	0.013955	0.032944	486484	sepsis	TRUE	reported	textfile	0.0018	5.95E-07	7365	M32348.m	TRUE	reported	ruBgwQ	2	TRUE	0.003235	23.89474
3976	rs1500394	T	C	T	C	-0.0088	-0.00258	0.5727	0.575934	FALSE	FALSE	FALSE	XdJbEU	0.014383	0.857865	486484	sepsis	TRUE	reported	textfile	0.0018	1.45E-06	7364	M32452.m	TRUE	reported	V5xWKi	2	TRUE	0.003235	23.89474
742	rs7572149	T	C	T	C	0.0088	-0.01483	0.133	0.132792	FALSE	FALSE	FALSE	9u0SC8	0.020164	0.461948	486484	sepsis	TRUE	reported	textfile	0.0018	1.00E-06	7363	M01301.m	TRUE	reported	H95uec	2	TRUE	0.003236	23.89474
4001	rs1235857	T	C	T	C	-0.0088	-0.00052	0.7176	0.723339	FALSE	FALSE	FALSE	V9AtId	0.015333	0.972737	486484	sepsis	TRUE	reported	textfile	0.0018	1.53E-06	7363	M32489.m	TRUE	reported	8B8S8a	2	TRUE	0.003236	23.89474
448	rs1105432	T	C	T	C	0.0088	-0.00969	0.1072	0.101597	FALSE	FALSE	FALSE	cl8ucN	0.022768	0.670459	486484	sepsis	TRUE	reported	textfile	0.0018	1.46E-06	7361	M00513.m	TRUE	reported	Ian0Q8	2	TRUE	0.003237	23.89474
453	rs1280155	T	C	T	C	0.0088	-0.01769	0.8705	0.867245	FALSE	FALSE	FALSE	cl8ucN	0.020139	0.379658	486484	sepsis	TRUE	reported	textfile	0.0018	9.64E-07	7361	M00513.m	TRUE	reported	Ian0Q8	2	TRUE	0.003237	23.89474
1613	rs6864461	C	G	C	G	0.0088	0.024192	0.3644	0.367272	FALSE	TRUE	FALSE	zP3C2v	0.014164	0.087648	486484	sepsis	TRUE	reported	textfile	0.0018	6.76E-07	7356	M12035.m	TRUE	reported	1Pooe3	2	TRUE	0.003239	23.89474
1742	rs2285943	T	G	T	G	0.0088	-0.01735	0.4941	0.502001	FALSE	FALSE	FALSE	nFLpAP	0.013683	0.204896	486484	sepsis	TRUE	reported	textfile	0.0018	7.60E-07	7356	M12774.m	TRUE	reported	yOSQTj	2	TRUE	0.003239	23.89474
10	rs1000770	T	C	T	C	-0.0044	0.003137	0.6418	0.640743	FALSE	FALSE	FALSE	xmGuol	0.014259	0.825844	486484	sepsis	TRUE	reported	textfile	9.00E-04	1.24E-06	7355	M00054.m	TRUE	reported	GW50Ri	2	TRUE	0.003239	23.89474
25	rs1090078	A	C	A	C	-0.0044	0.001345	0.4158	0.421535	FALSE	FALSE	FALSE	xmGuol	0.013832	0.92254	486484	sepsis	TRUE	reported	textfile	9.00E-04	9.74E-07	7355	M00054.m	TRUE	reported	GW50Ri	2	TRUE	0.003239	23.89474
38	rs1246808	T	C	T	C	-0.0044	-0.02125	0.5436	0.535194	FALSE	FALSE	FALSE	xmGuol	0.01383	0.124418	486484	sepsis	TRUE	reported	textfile	9.00E-04	1.25E-06	7355	M00054.m	TRUE	reported	GW50Ri	2	TRUE	0.003239	23.89474
46	rs1349704	A	G	A	G	0.0044	-0.00543	0.4611	0.45823	FALSE	FALSE	FALSE	xmGuol	0.013733	0.692341	486484	sepsis	TRUE	reported	textfile	9.00E-04	8.31E-07	7355	M00054.m	TRUE	reported	GW50Ri	2	TRUE	0.003239	23.89474
52	rs1519309	T	C	T	C	-0.0044	0.002513	0.6544	0.647523	FALSE	FALSE	FALSE	xmGuol	0.014319	0.860693	486484	sepsis	TRUE	reported	textfile	9.00E-04	1.21E-06	7355	M00054.m	TRUE	reported	GW50Ri	2	TRUE	0.003239	23.89474
63	rs1871053	A	G	A	G	-0.0044	0.010751	0.4514	0.449437	FALSE	FALSE	FALSE	xmGuol	0.0138	0.435966	486484	sepsis	TRUE	reported	textfile	9.00E-04	7.68E-07	7355	M00054.m	TRUE	reported	GW50Ri	2	TRUE	0.003239	23.89474
81	rs2580344	T	C	T	C	0.0044	-0.00832	0.6238	0.632002	FALSE	FALSE	FALSE	xmGuol	0.014241	0.558924	486484	sepsis	TRUE	reported	textfile	9.00E-04	7.98E-07	7355	M00054.m	TRUE	reported	GW50Ri	2	TRUE	0.003239	23.89474
82	rs2604365	T	G	T	G	0.0044	0.024777	0.6371	0.633293	FALSE	FALSE	FALSE	xmGuol	0.014256	0.082226	486484	sepsis	TRUE	reported	textfile	9.00E-04	1.45E-06	7355	M00054.m	TRUE	reported	GW50Ri	2	TRUE	0.003239	23.89474
90	rs2813760	A	G	A	G	0.0044	-0.02308	0.5775	0.587314	FALSE	FALSE	FALSE	xmGuol	0.013989	0.098967	486484	sepsis	TRUE	reported	textfile	9.00E-04	1.19E-06	7355	M00054.m	TRUE	reported	GW50Ri	2	TRUE	0.003239	23.89474
96	rs2970335	A	C	A	C	0.0044	0.024556	0.4039	0.415305	FALSE	FALSE	FALSE	xmGuol	0.013893	0.077147	486484	sepsis	TRUE	reported	textfile	9.00E-04	1.02E-06	7355	M00054.m	TRUE	reported	GW50Ri	2	TRUE	0.003239	23.89474
100	rs3742929	T	C	T	C	-0.0044	0.005068	0.512	0.515793	FALSE	FALSE	FALSE	xmGuol	0.013697	0.711392	486484	sepsis	TRUE	reported	textfile	9.00E-04	1.01E-06	7355	M00054.m	TRUE	reported	GW50Ri	2	TRUE	0.003239	23.89474
113	rs4611118	A	G	A	G	-0.0044	-0.00037	0.609	0.612037	FALSE	FALSE	FALSE	xmGuol	0.014039	0.97912	486484	sepsis	TRUE	reported	textfile	9.00E-04	9.41E-07	7355	M00054.m	TRUE	reported	GW50Ri	2	TRUE	0.003239	23.89474
148	rs682359	T	G	T	G	0.0044	0.026021	0.5812	0.578534	FALSE	FALSE	FALSE	xmGuol	0.013903	0.061256	486484	sepsis	TRUE	reported	textfile	9.00E-04	6.54E-07	7355	M00054.m	TRUE	reported	GW50Ri	2	TRUE	0.003239	23.89474
158	rs719564	A	G	A	G	0.0044	-0.01198	0.6101	0.625714	FALSE	FALSE	FALSE	xmGuol	0.014117	0.396038	486484	sepsis	TRUE	reported	textfile	9.00E-04	1.28E-06	7355	M00054.m	TRUE	reported	GW50Ri	2	TRUE	0.003239	23.89474
159	rs7230740	A	C	A	C	0.0044	-0.00339	0.6655	0.672118	FALSE	FALSE	FALSE	xmGuol	0.014513	0.815238	486484	sepsis	TRUE	reported	textfile	9.00E-04	1.56E-06	7355	M00054.m	TRUE	reported	GW50Ri	2	TRUE	0.003239	23.89474
171	rs8088963	A	G	A	G	0.0044	0.019204	0.6387	0.636583	FALSE	FALSE	FALSE	xmGuol	0.014205	0.176385	486484	sepsis	TRUE	reported	textfile	9.00E-04	8.29E-07	7355	M00054.m	TRUE	reported	GW50Ri	2	TRUE	0.003239	23.89474
176	rs881562	T	G	T	G	0.0044	-0.00143	0.4978	0.506632	FALSE	FALSE	FALSE	xmGuol	0.01389	0.918031	486484	sepsis	TRUE	reported	textfile	9.00E-04	1.45E-06	7355	M00054.m	TRUE	reported	GW50Ri	2	TRUE	0.003239	23.89474
4053	rs7200919	A	G	A	G	-0.0088	-0.01021	0.4101	0.401328	FALSE	FALSE	FALSE	SiYGYU	0.013949	0.464161	486484	sepsis	TRUE	reported	textfile	0.0018	7.75E-07	7355	M32492.m	TRUE	reported	GfQvpU	2	TRUE	0.003239	23.89474
4058	rs9403095	A	C	A	C	-0.0088	0.012818	0.3305	0.331258	FALSE	FALSE	FALSE	SiYGYU	0.014519	0.377314	486484	sepsis	TRUE	reported	textfile	0.0018	9.32E-07	7355	M32492.m	TRUE	reported	GfQvpU	2	TRUE	0.003239	23.89474
566	rs2982907	T	G	T	G	-0.0088	-0.00955	0.1465	0.160726	FALSE	FALSE	FALSE	fNqkCW	0.018582	0.607258	486484	sepsis	TRUE	reported	textfile	0.0018	1.28E-06	7354	M00606.m	TRUE	reported	g9uwOn	2	TRUE	0.00324	23.89473
207	rs1025860	A	G	A	G	0.0044	-0.00071	0.4382	0.453791	FALSE	FALSE	FALSE	tbRL8J	0.013765	0.95877	486484	sepsis	TRUE	reported	textfile	9.00E-04	6.73E-07	7351	M00060.m	TRUE	reported	oOvRKT	2	TRUE	0.003241	23.89473
220	rs1093776	A	G	A	G	0.0044	-0.00562	0.5674	0.559169	FALSE	FALSE	FALSE	tbRL8J	0.013854	0.685223	486484	sepsis	TRUE	reported	textfile	9.00E-04	1.53E-06	7351	M00060.m	TRUE	reported	oOvRKT	2	TRUE	0.003241	23.89473
241	rs1261200	A	G	A	G	0.0044	-0.01536	0.3461	0.338549	FALSE	FALSE	FALSE	tbRL8J	0.014431	0.287167	486484	sepsis	TRUE	reported	textfile	9.00E-04	1.20E-06	7351	M00060.m	TRUE	reported	oOvRKT	2	TRUE	0.003241	23.89473
247	rs1334318	A	G	A	G	0.0044	0.012643	0.3572	0.363527	FALSE	FALSE	FALSE	tbRL8J	0.014217	0.373864	486484	sepsis	TRUE	reported	textfile	9.00E-04	1.01E-06	7351	M00060.m	TRUE	reported	oOvRKT	2	TRUE	0.003241	23.89473
258	rs1677193	A	C	A	C	-0.0044	-0.03549	0.6122	0.607768	FALSE	FALSE	FALSE	tbRL8J	0.014093	0.011802	486484	sepsis	TRUE	reported	textfile	9.00E-04	1.25E-06	7351	M00060.m	TRUE	reported	oOvRKT	2	TRUE	0.003241	23.89473
261	rs1717319	T	C	T	C	-0.0044	0.00695	0.3872	0.384533	FALSE	FALSE	FALSE	tbRL8J	0.014107	0.622269	486484	sepsis	TRUE	reported	textfile	9.00E-04	2.46E-06	7351	M00060.m	TRUE	reported	oOvRKT	2	TRUE	0.003241	23.89473
264	rs1795964	A	G	A	G	-0.0044	0.022298	0.4091	0.418577	FALSE	FALSE	FALSE	tbRL8J	0.014678	0.12872	486484	sepsis	TRUE	reported	textfile	9.00E-04	1.98E-06	7351	M00060.m	TRUE	reported	oOvRKT	2	TRUE	0.003241	23.89473
275	rs2060490	T	C	T	C	-0.0044	-0.00593	0.474	0.473842	FALSE	FALSE	FALSE	tbRL8J	0.013681	0.664684	486484	sepsis	TRUE	reported	textfile	9.00E-04	9.10E-07	7351	M00060.m	TRUE	reported	oOvRKT	2	TRUE	0.003241	23.89473
299	rs3775574	A	G	A	G	-0.0044	-0.00155	0.6047	0.60579	FALSE	FALSE	FALSE	tbRL8J	0.014065	0.911969	486484	sepsis	TRUE	reported	textfile	9.00E-04	3.13E-06	7351	M00060.m	TRUE	reported	oOvRKT	2	TRUE	0.003241	23.89473
300	rs37959	T	C	T	C	0.0044	0.018399	0.3912	0.38572	FALSE	FALSE	FALSE	tbRL8J	0.014059	0.190644	486484	sepsis	TRUE	reported	textfile	9.00E-04	1.71E-06	7351	M00060.m	TRUE	reported	oOvRKT	2	TRUE	0.003241	23.89473
319	rs481781	T	C	T	C	0.0044	-0.00077	0.5409	0.537912	FALSE	FALSE	FALSE	tbRL8J	0.013751	0.955146	486484	sepsis	TRUE	reported	textfile	9.00E-04	9.14E-07	7351	M00060.m	TRUE	reported	oOvRKT	2	TRUE	0.003241	23.89473
365	rs7738860	T	C	T	C	-0.0044	0.004845	0.4518	0.440564	FALSE	FALSE	FALSE	tbRL8J	0.013771	0.72495	486484	sepsis	TRUE	reported	textfile	9.00E-04	1.31E-06	7351	M00060.m	TRUE	reported	oOvRKT	2	TRUE	0.003241	23.89473
367	rs7795093	A	G	A	G	-0.0044	-0.00353	0.3813	0.374036	FALSE	FALSE	FALSE	tbRL8J	0.014265	0.804587	486484	sepsis	TRUE	reported	textfile	9.00E-04	1.47E-06	7351	M00060.m	TRUE	reported	oOvRKT	2	TRUE	0.003241	23.89473
372	rs7923885	A	G	A	G	-0.0044	-0.01424	0.5257	0.521805	FALSE	FALSE	FALSE	tbRL8J	0.013692	0.298489	486484	sepsis	TRUE	reported	textfile	9.00E-04	8.08E-07	7351	M00060.m	TRUE	reported	oOvRKT	2	TRUE	0.003241	23.89473
383	rs900532	T	C	T	C	-0.0044	0.009228	0.4524	0.443278	FALSE	FALSE	FALSE	tbRL8J	0.013756	0.502343	486484	sepsis	TRUE	reported	textfile	9.00E-04	8.99E-07	7351	M00060.m	TRUE	reported	oOvRKT	2	TRUE	0.003241	23.89473
391	rs982790	T	C	T	C	-0.0044	0.01964	0.4106	0.411129	FALSE	FALSE	FALSE	tbRL8J	0.013986	0.160244	486484	sepsis	TRUE	reported	textfile	9.00E-04	3.74E-06	7351	M00060.m	TRUE	reported	oOvRKT	2	TRUE	0.003241	23.89473
4121	rs4841120	T	G	T	G	0.0088	-0.01519	0.714	0.702261	FALSE	FALSE	FALSE	4rtLd0	0.014914	0.308314	486484	sepsis	TRUE	reported	textfile	0.0018	1.47E-06	7350	M32518.m	TRUE	reported	j7f1ER	2	TRUE	0.003241	23.89473
4125	rs6946189	A	G	A	G	-0.0088	0.021978	0.2499	0.254868	FALSE	FALSE	FALSE	4rtLd0	0.015834	0.16513	486484	sepsis	TRUE	reported	textfile	0.0018	1.52E-06	7350	M32518.m	TRUE	reported	j7f1ER	2	TRUE	0.003241	23.89473
1861	rs1023913	T	C	T	C	0.0044	0.006691	0.5959	0.587889	FALSE	FALSE	FALSE	v2KE89	0.013925	0.63084	486484	sepsis	TRUE	reported	textfile	9.00E-04	1.49E-06	7349	M15500.m	TRUE	reported	to2t7H	2	TRUE	0.003242	23.89473
1872	rs1092011	T	G	T	G	0.0044	0.005752	0.5507	0.553418	FALSE	FALSE	FALSE	v2KE89	0.014474	0.691056	486484	sepsis	TRUE	reported	textfile	9.00E-04	1.64E-06	7349	M15500.m	TRUE	reported	to2t7H	2	TRUE	0.003242	23.89473
1886	rs1191502	T	G	T	G	-0.0044	-0.0178	0.402	0.41459	FALSE	FALSE	FALSE	v2KE89	0.013893	0.200086	486484	sepsis	TRUE	reported	textfile	9.00E-04	9.59E-07	7349	M15500.m	TRUE	reported	to2t7H	2	TRUE	0.003242	23.89473
1889	rs1219787	T	C	T	C	-0.0044	-0.0038	0.5976	0.595682	FALSE	FALSE	FALSE	v2KE89	0.013964	0.785686	486484	sepsis	TRUE	reported	textfile	9.00E-04	1.23E-06	7349	M15500.m	TRUE	reported	to2t7H	2	TRUE	0.003242	23.89473
1902	rs1262716	A	G	A	G	0.0044	0.008627	0.3466	0.344455	FALSE	FALSE	FALSE	v2KE89	0.014353	0.547801	486484	sepsis	TRUE	reported	textfile	9.00E-04	3.46E-06	7349	M15500.m	TRUE	reported	to2t7H	2	TRUE	0.003242	23.89473
1916	rs1430435	T	C	T	C	0.0044	-0.00973	0.4932	0.490868	FALSE	FALSE	FALSE	v2KE89	0.013679	0.47674	486484	sepsis	TRUE	reported	textfile	9.00E-04	1.26E-06	7349	M15500.m	TRUE	reported	to2t7H	2	TRUE	0.003242	23.89473
1939	rs2130882	T	C	T	C	0.0044	-0.00843	0.4864	0.490382	FALSE	FALSE	FALSE	v2KE89	0.013668	0.537237	486484	sepsis	TRUE	reported	textfile	9.00E-04	1.11E-06	7349	M15500.m	TRUE	reported	to2t7H	2	TRUE	0.003242	23.89473
1972	rs378999	T	C	T	C	-0.0044	0.006002	0.6369	0.631995	FALSE	FALSE	FALSE	v2KE89	0.014199	0.672475	486484	sepsis	TRUE	reported	textfile	9.00E-04	1.20E-06	7349	M15500.m	TRUE	reported	to2t7H	2	TRUE	0.003242	23.89473
1973	rs3848713	T	C	T	C	-0.0044	-0.00764	0.4065	0.379841	FALSE	FALSE	FALSE	v2KE89	0.014075	0.587337	486484	sepsis	TRUE	reported	textfile	9.00E-04	1.11E-06	7349	M15500.m	TRUE	reported	to2t7H	2	TRUE	0.003242	23.89473
1988	rs4672450	T	C	T	C	0.0044	-0.00771	0.4757	0.478858	FALSE	FALSE	FALSE	v2KE89	0.01376	0.575033	486484	sepsis	TRUE	reported	textfile	9.00E-04	1.36E-06	7349	M15500.m	TRUE	reported	to2t7H	2	TRUE	0.003242	23.89473
1991	rs4713396	A	C	A	C	-0.0044	0.009579	0.4478	0.467044	FALSE	FALSE	FALSE	v2KE89	0.013686	0.48398	486484	sepsis	TRUE	reported	textfile	9.00E-04	1.15E-06	7349	M15500.m	TRUE	reported	to2t7H	2	TRUE	0.003242	23.89473
1992	rs4722438	A	G	A	G	0.0044	0.003385	0.4414	0.453971	FALSE	FALSE	FALSE	v2KE89	0.01375	0.805552	486484	sepsis	TRUE	reported	textfile	9.00E-04	1.16E-06	7349	M15500.m	TRUE	reported	to2t7H	2	TRUE	0.003242	23.89473
1993	rs4764292	T	C	T	C	0.0044	0.016845	0.5797	0.58869	FALSE	FALSE	FALSE	v2KE89	0.01391	0.225887	486484	sepsis	TRUE	reported	textfile	9.00E-04	9.76E-07	7349	M15500.m	TRUE	reported	to2t7H	2	TRUE	0.003242	23.89473
2011	rs6554156	T	C	T	C	0.0044	0.015679	0.4037	0.411186	FALSE	FALSE	FALSE	v2KE89	0.013881	0.258694	486484	sepsis	TRUE	reported	textfile	9.00E-04	1.12E-06	7349	M15500.m	TRUE	reported	to2t7H	2	TRUE	0.003242	23.89473
2012	rs6596361	T	C	T	C	0.0044	-0.00228	0.5945	0.594669	FALSE	FALSE	FALSE	v2KE89	0.01394	0.870128	486484	sepsis	TRUE	reported	textfile	9.00E-04	9.19E-07	7349	M15500.m	TRUE	reported	to2t7H	2	TRUE	0.003242	23.89473
2053	rs9530362	T	C	T	C	-0.0044	-0.00509	0.5726	0.543096	FALSE	FALSE	FALSE	v2KE89	0.013803	0.712464	486484	sepsis	TRUE	reported	textfile	9.00E-04	1.17E-06	7349	M15500.m	TRUE	reported	to2t7H	2	TRUE	0.003242	23.89473
2057	rs977041	A	G	A	G	0.0044	-0.01498	0.4355	0.453746	FALSE	FALSE	FALSE	v2KE89	0.013739	0.275481	486484	sepsis	TRUE	reported	textfile	9.00E-04	1.27E-06	7349	M15500.m	TRUE	reported	to2t7H	2	TRUE	0.003242	23.89473
2062	rs9880736	A	C	A	C	-0.0044	-0.00868	0.5431	0.54697	FALSE	FALSE	FALSE	v2KE89	0.013788	0.528916	486484	sepsis	TRUE	reported	textfile	9.00E-04	1.51E-06	7349	M15500.m	TRUE	reported	to2t7H	2	TRUE	0.003242	23.89473
8392	rs4670599	A	G	A	G	-0.0088	-0.00875	0.5558	0.53382	FALSE	FALSE	FALSE	SCzh0A	0.013795	0.526068	486484	sepsis	TRUE	reported	textfile	0.0018	1.29E-06	7348	M35675.m	TRUE	reported	n1SREk	2	TRUE	0.003242	23.89473
8406	rs9602121	T	C	T	C	-0.0088	0.006508	0.6481	0.623559	FALSE	FALSE	FALSE	SCzh0A	0.014149	0.645546	486484	sepsis	TRUE	reported	textfile	0.0018	1.86E-06	7348	M35675.m	TRUE	reported	n1SREk	2	TRUE	0.003242	23.89473
3778	rs1752013	T	C	T	C	-0.0088	0.016496	0.5973	0.596069	FALSE	FALSE	FALSE	NW1aaR	0.013968	0.237602	486484	sepsis	TRUE	reported	textfile	0.0018	6.85E-07	7344	M32339.m	TRUE	reported	pBPkAB	2	TRUE	0.003244	23.89473
4433	rs1950305	T	C	T	C	0.0088	-0.02265	0.1208	0.117921	FALSE	FALSE	FALSE	WGPdpq	0.021345	0.28857	486484	sepsis	TRUE	reported	textfile	0.0018	1.72E-06	7338	M32675.m	TRUE	reported	u0eUAG	2	TRUE	0.003247	23.89472
6068	rs1051331	A	G	A	G	-0.0088	0.00013	0.8435	0.840307	FALSE	FALSE	FALSE	P8n0HP	0.018746	0.994465	486484	sepsis	TRUE	reported	textfile	0.0018	6.47E-07	7336	M33442.m	TRUE	reported	cljMTI	2	TRUE	0.003247	23.89472
7036	rs1117019	T	C	T	C	0.0088	-0.0285	0.619	0.611084	FALSE	FALSE	FALSE	uthruC	0.014017	0.042056	486484	sepsis	TRUE	reported	textfile	0.0018	5.99E-07	7316	M34283.m	TRUE	reported	ftTuRU	2	TRUE	0.003256	23.8947
2495	rs1089295	A	G	A	G	-0.0088	0.016222	0.4059	0.399992	FALSE	FALSE	FALSE	LwUYua	0.013989	0.246209	486484	sepsis	TRUE	reported	textfile	0.0018	1.66E-06	7315	M17945.m	TRUE	reported	SUN8dc	2	TRUE	0.003257	23.8947
2209	rs310597	A	G	A	G	-0.0088	0.006826	0.6532	0.632415	FALSE	FALSE	FALSE	Bf6uMK	0.014208	0.630927	486484	sepsis	TRUE	reported	textfile	0.0018	1.30E-06	7308	M15778.m	TRUE	reported	jowM2N	2	TRUE	0.00326	23.89469
2215	rs4515826	T	G	T	G	-0.0132	-0.02042	0.1021	0.129927	FALSE	FALSE	FALSE	Bf6uMK	0.020425	0.317338	486484	sepsis	TRUE	reported	textfile	0.0027	1.31E-06	7308	M15778.m	TRUE	reported	jowM2N	2	TRUE	0.00326	23.89469
2220	rs6841005	T	C	T	C	0.0088	-0.01479	0.3264	0.341543	FALSE	FALSE	FALSE	Bf6uMK	0.014383	0.303723	486484	sepsis	TRUE	reported	textfile	0.0018	1.15E-06	7308	M15778.m	TRUE	reported	jowM2N	2	TRUE	0.00326	23.89469
6124	rs1106598	A	G	A	G	-0.0088	0.011704	0.5821	0.583631	FALSE	FALSE	FALSE	MiSJly	0.01387	0.398786	486484	sepsis	TRUE	reported	textfile	0.0018	8.30E-07	7307	M33477.m	TRUE	reported	WwL1hq	2	TRUE	0.00326	23.89469
6125	rs1116054	A	G	A	G	-0.0088	-0.01428	0.2939	0.288941	FALSE	FALSE	FALSE	MiSJly	0.015051	0.342601	486484	sepsis	TRUE	reported	textfile	0.0018	1.54E-06	7307	M33477.m	TRUE	reported	WwL1hq	2	TRUE	0.00326	23.89469
3326	rs1185528	T	C	T	C	-0.0088	0.021377	0.8068	0.801746	FALSE	FALSE	FALSE	m66Eav	0.017134	0.212179	486484	sepsis	TRUE	reported	textfile	0.0018	1.93E-06	7303	M27256.m	TRUE	reported	pl2ECU	2	TRUE	0.003262	23.89469
3553	rs2710833	T	C	T	C	-0.0132	0.00527	0.1979	0.193305	FALSE	FALSE	FALSE	8luUON	0.017272	0.760279	486484	sepsis	TRUE	reported	textfile	0.0027	1.20E-06	7257	M31453.m	TRUE	reported	FBs6G4	2	TRUE	0.003283	23.89465
4340	rs2581354	T	C	T	C	0.0088	0.032129	0.5938	0.5788	FALSE	FALSE	FALSE	6VSoQQ	0.013886	0.020683	486484	sepsis	TRUE	reported	textfile	0.0018	1.37E-06	7229	M32644.m	TRUE	reported	oC2AV0	2	TRUE	0.003295	23.89462
8036	rs2267292	T	C	T	C	-0.0088	-0.00453	0.2242	0.217006	FALSE	FALSE	FALSE	SwiqSi	0.016577	0.784412	486484	sepsis	TRUE	reported	textfile	0.0018	1.03E-06	7217	M35270.m	TRUE	reported	21ZlsQ	2	TRUE	0.003301	23.89461
8039	rs2727985	C	G	C	G	-0.0088	-0.00258	0.7599	0.738407	FALSE	TRUE	FALSE	SwiqSi	0.015557	0.868124	486484	sepsis	TRUE	reported	textfile	0.0018	2.03E-06	7217	M35270.m	TRUE	reported	21ZlsQ	2	TRUE	0.003301	23.89461
1306	rs4470135	A	C	A	C	0.0088	0.000448	0.5203	0.526134	FALSE	FALSE	FALSE	WQdpmo	0.013709	0.973919	486484	sepsis	TRUE	reported	textfile	0.0018	5.17E-07	7136	M01769.m	TRUE	reported	BkptOK	2	TRUE	0.003338	23.89454
5813	rs1121287	C	G	C	G	-0.0088	0.023204	0.7979	0.797042	FALSE	TRUE	FALSE	R5joEf	0.017037	0.1732	486484	sepsis	TRUE	reported	textfile	0.0018	7.82E-07	7118	M33380.m	TRUE	reported	kRvbfZ	2	TRUE	0.003347	23.89452
6252	rs1039386	A	G	A	G	-0.0088	-0.00751	0.5733	0.591112	FALSE	FALSE	FALSE	MOonP9	0.014019	0.591977	486484	sepsis	TRUE	reported	textfile	0.0018	1.36E-06	7066	M33515.m	TRUE	reported	226GuP	2	TRUE	0.003371	23.89447
6253	rs1086802	A	G	A	G	0.0088	-0.01043	0.6116	0.623163	FALSE	FALSE	FALSE	MOonP9	0.014104	0.45976	486484	sepsis	TRUE	reported	textfile	0.0018	1.52E-06	7066	M33515.m	TRUE	reported	226GuP	2	TRUE	0.003371	23.89447
771	rs2288867	T	C	T	C	-0.0132	-0.00938	0.2334	0.247087	FALSE	FALSE	FALSE	tYvbNc	0.015872	0.554544	486484	sepsis	TRUE	reported	textfile	0.0027	1.02E-06	6946	M01303.m	TRUE	reported	hT3gsE	2	TRUE	0.003429	23.89435
8834	rs1079174	A	C	A	C	0.0132	0.026218	0.0747	0.078476	FALSE	FALSE	FALSE	XIHeTV	0.025337	0.300774	486484	sepsis	TRUE	reported	textfile	0.0027	9.72E-07	6940	M36808.m	TRUE	reported	37Vi2D	2	TRUE	0.003432	23.89435
8835	rs1119957	A	G	A	G	0.0088	0.019572	0.2001	0.209427	FALSE	FALSE	FALSE	XIHeTV	0.016848	0.245367	486484	sepsis	TRUE	reported	textfile	0.0018	8.70E-07	6940	M36808.m	TRUE	reported	37Vi2D	2	TRUE	0.003432	23.89435
1663	rs7544738	A	G	A	G	0.0132	-0.00289	0.4114	0.420511	FALSE	FALSE	FALSE	YzX5ZD	0.013871	0.834996	486484	sepsis	TRUE	reported	textfile	0.0027	7.15E-07	6864	M12129.m	TRUE	reported	6tMfaW	2	TRUE	0.00347	23.89427
7290	rs1761768	A	G	A	G	0.0088	-0.00424	0.3771	0.39299	FALSE	FALSE	FALSE	Vcz4EA	0.014108	0.763713	486484	sepsis	TRUE	reported	textfile	0.0018	6.07E-07	6755	M34359.m	TRUE	reported	aUSo2J	2	TRUE	0.003526	23.89416
2309	rs2863060	A	G	A	G	-0.0132	-0.02039	0.2677	0.267564	FALSE	FALSE	FALSE	ZzUW8U	0.015494	0.18808	486484	sepsis	TRUE	reported	textfile	0.0027	1.42E-06	6733	M16821.m	TRUE	reported	g2xs3i	2	TRUE	0.003537	23.89413
990	rs1102338	A	G	A	G	0.0088	0.039772	0.4591	0.543107	FALSE	FALSE	FALSE	jfkZbM	0.013708	0.003716	486484	sepsis	TRUE	reported	textfile	0.0018	5.37E-07	6523	M01558.m	TRUE	reported	LFZvMG	2	TRUE	0.003651	23.89391
1005	rs2290156	C	G	C	G	0.0088	0.011579	0.2806	0.279115	FALSE	TRUE	FALSE	jfkZbM	0.015219	0.446762	486484	sepsis	TRUE	reported	textfile	0.0018	1.05E-06	6523	M01558.m	TRUE	reported	LFZvMG	2	TRUE	0.003651	23.89391
3192	rs1284408	T	G	T	G	-0.0269	0.014752	0.5194	0.525184	FALSE	FALSE	FALSE	UfLkBq	0.013771	0.284035	486484	sepsis	TRUE	reported	textfile	0.0055	7.95E-07	1756	M22481.m	TRUE	reported	kWMdiT	2	TRUE	0.013439	23.89375
4846	rs1240164	A	G	A	G	0.1345	0.01348	0.9861	0.986015	FALSE	FALSE	FALSE	StMkBO	0.058232	0.816943	486484	sepsis	TRUE	reported	textfile	0.0275	1.03E-06	1727	M32847.m	TRUE	reported	uzQb8Q	2	TRUE	0.013662	23.89329
8122	rs565666	T	C	T	C	-0.0088	-0.00367	0.3033	0.311026	FALSE	FALSE	FALSE	nNbXBS	0.014758	0.803504	486484	sepsis	TRUE	reported	textfile	0.0018	5.12E-07	5768	M35326.m	TRUE	reported	UNDorn	2	TRUE	0.004127	23.89295
3096	rs2032995	T	G	T	G	0.0132	-0.01329	0.7853	0.763338	FALSE	FALSE	FALSE	mr0awv	0.016129	0.409918	486484	sepsis	TRUE	reported	textfile	0.0027	1.35E-06	5702	M22130.m	TRUE	reported	Y2TfWQ	2	TRUE	0.004174	23.89285
680	rs1043367	T	C	T	C	0.0264	0.028202	0.9584	0.95825	FALSE	FALSE	FALSE	aT1Fw1	0.034161	0.409051	486484	sepsis	TRUE	reported	textfile	0.0054	1.24E-06	5687	M01284.m	TRUE	reported	mbTe8H	2	TRUE	0.004185	23.89283
2185	rs4338651	A	G	A	G	-0.088	0.005124	0.026	0.026015	FALSE	FALSE	FALSE	UokbxN	0.043137	0.905445	486484	sepsis	TRUE	reported	textfile	0.018	1.01E-06	5599	M15753.m	TRUE	reported	IUGKBi	2	TRUE	0.004251	23.8927
4375	rs6782877	A	G	A	G	0.0132	0.00475	0.349	0.344118	FALSE	FALSE	FALSE	SPhI52	0.014389	0.74132	486484	sepsis	TRUE	reported	textfile	0.0027	1.08E-06	5211	M32651.m	TRUE	reported	V0Bnpj	2	TRUE	0.004566	23.89206
8948	rs8180176	T	C	T	C	0.0264	-0.00323	0.4591	0.453323	FALSE	FALSE	FALSE	mXJP3h	0.013752	0.814051	486484	sepsis	TRUE	reported	textfile	0.0054	8.50E-07	4520	M37104.m	TRUE	reported	HRZBak	2	TRUE	0.00526	23.89066
7518	rs1702447	A	G	A	G	0.022	-0.01291	0.2904	0.293666	FALSE	FALSE	FALSE	rp1LGA	0.015054	0.391044	486484	sepsis	TRUE	reported	textfile	0.0045	8.34E-07	4442	M34441.m	TRUE	reported	SU47tz	2	TRUE	0.005352	23.89047
6420	rs1314675	T	C	T	C	0.1467	0.002351	0.0199	0.02423	FALSE	FALSE	FALSE	F7mjRv	0.044231	0.957602	486484	sepsis	TRUE	reported	textfile	0.03	1.02E-06	1860	M33658.m	TRUE	reported	frG0Zf	2	TRUE	0.012693	23.88639
7242	rs1764606	T	C	T	C	-0.2932	-0.02526	0.9899	0.970988	FALSE	FALSE	FALSE	RIcE3J	0.040849	0.536342	486484	sepsis	TRUE	reported	textfile	0.0599	1.01E-06	633	M34339.m	TRUE	reported	QQrwdm	2	TRUE	0.03647	23.88361
2455	rs831541	T	C	T	C	-0.0567	-0.0147	0.2066	0.207296	FALSE	FALSE	FALSE	NBJ2Do	0.016931	0.385358	486484	sepsis	TRUE	reported	textfile	0.0116	9.87E-07	1739	M17799.m	TRUE	reported	Uz0Wgl	2	TRUE	0.013553	23.86439
6975	rs4521036	A	G	A	G	0.0171	0.001243	0.1388	0.13991	FALSE	FALSE	FALSE	ZlvYs5	0.019719	0.949734	486484	sepsis	TRUE	reported	textfile	0.0035	1.33E-06	7351	M34214.m	TRUE	reported	qU57S1	2	TRUE	0.003237	23.86371
6999	rs1095672	T	C	T	C	-0.0342	0.047619	0.9414	0.958174	FALSE	FALSE	FALSE	ePdfmg	0.034101	0.162584	486484	sepsis	TRUE	reported	textfile	0.007	1.19E-06	7347	M34244.m	TRUE	reported	l9nzfX	2	TRUE	0.003238	23.86371
3741	rs6553778	T	C	T	C	-0.0171	0.014811	0.2295	0.237627	FALSE	FALSE	FALSE	k16olH	0.016048	0.356039	486484	sepsis	TRUE	reported	textfile	0.0035	1.18E-06	7340	M32328.m	TRUE	reported	TQA3sZ	2	TRUE	0.003242	23.8637
1833	rs2517897	A	C	A	C	0.0171	0.018843	0.1141	0.152648	FALSE	FALSE	FALSE	2TaDHm	0.019007	0.32151	486484	sepsis	TRUE	reported	textfile	0.0035	1.23E-06	7337	M15365.m	TRUE	reported	LoLveg	2	TRUE	0.003243	23.8637
6075	rs1315759	T	C	T	C	0.0171	0.028769	0.0447	0.048817	FALSE	FALSE	FALSE	P8n0HP	0.031929	0.367578	486484	sepsis	TRUE	reported	textfile	0.0035	1.17E-06	7336	M33442.m	TRUE	reported	cljMTI	2	TRUE	0.003243	23.8637
6928	rs7970370	T	C	T	C	-0.5726	0.039321	0.0206	0.013654	FALSE	FALSE	FALSE	SvfSvH	0.060429	0.515241	486484	sepsis	TRUE	reported	textfile	0.1168	9.37E-07	282	M34109.m	TRUE	reported	7OSbiu	2	TRUE	0.078532	23.86305
7243	rs3810529	T	C	T	C	0.1173	-0.03527	0.0437	0.043544	FALSE	FALSE	FALSE	RIcE3J	0.033494	0.292291	486484	sepsis	TRUE	reported	textfile	0.024	1.04E-06	1649	M34339.m	TRUE	reported	QQrwdm	2	TRUE	0.014279	23.85868
7480	rs1073744	T	G	T	G	0.0127	0.008684	0.2369	0.245321	FALSE	FALSE	FALSE	jAhmnV	0.015921	0.585438	486484	sepsis	TRUE	reported	textfile	0.0026	1.63E-06	7348	M34419.m	TRUE	reported	F2Tg9L	2	TRUE	0.003237	23.85297

3376	rs1753103	C	G	C	G	0.0127	-0.00592	0.2505	0.257931	FALSE	TRUE	FALSE	O2JtzH	0.015635	0.704943	486484	sepsis	TRUE	reported	textfile	0.0026	1.32E-06	7347	M27278.m	TRUE	reported	fxClPW	2	TRUE	0.003237	23.85297
6085	rs6543592	A	G	A	G	-0.0127	-0.01201	0.9265	0.921589	FALSE	FALSE	FALSE	P8n0HP	0.025429	0.636617	486484	sepsis	TRUE	reported	textfile	0.0026	8.84E-07	7336	M33442.m	TRUE	reported	cljMTl	2	TRUE	0.003242	23.85296
808	rs1178070	T	C	T	C	-0.0127	-0.00505	0.7557	0.756377	FALSE	FALSE	FALSE	J58DI0	0.015888	0.75073	486484	sepsis	TRUE	reported	textfile	0.0026	9.89E-07	7335	M01356.m	TRUE	reported	zk9AMB	2	TRUE	0.003242	23.85296
7051	rs1531507	A	G	A	G	0.0127	0.012925	0.2007	0.208186	FALSE	FALSE	FALSE	uthruC	0.016876	0.443763	486484	sepsis	TRUE	reported	textfile	0.0026	6.67E-07	7316	M34283.m	TRUE	reported	ftTuRU	2	TRUE	0.003251	23.85294
2210	rs3112496	T	C	T	C	0.0127	-0.02937	0.1331	0.115906	FALSE	FALSE	FALSE	Bf6uMK	0.021626	0.174462	486484	sepsis	TRUE	reported	textfile	0.0026	1.13E-06	7308	M15778.m	TRUE	reported	jowM2N	2	TRUE	0.003254	23.85294
3028	rs1520218	A	C	A	C	0.0127	0.004607	0.2558	0.276477	FALSE	FALSE	FALSE	FJZEYz	0.015266	0.762844	486484	sepsis	TRUE	reported	textfile	0.0026	1.39E-06	7284	M21630.m	TRUE	reported	s6Pshg	2	TRUE	0.003265	23.85292
7932	rs1853936	T	C	T	C	0.021	-0.00286	0.1762	0.189932	FALSE	FALSE	FALSE	kFTWXY	0.017422	0.869747	486484	sepsis	TRUE	reported	textfile	0.0043	8.96E-07	6543	M35193.m	TRUE	reported	cjJvJl	2	TRUE	0.003632	23.84344
7702	rs2878733	T	C	T	C	0.021	-0.01217	0.703	0.703463	FALSE	FALSE	FALSE	2eJlPM	0.015171	0.422602	486484	sepsis	TRUE	reported	textfile	0.0043	1.18E-06	6381	M34761.m	TRUE	reported	druKtT	2	TRUE	0.003724	23.84325
3858	rs2574985	A	G	A	G	-0.021	0.010321	0.2764	0.29434	FALSE	FALSE	FALSE	8gg1zS	0.015023	0.492066	486484	sepsis	TRUE	reported	textfile	0.0043	1.27E-06	6119	M32379.m	TRUE	reported	uZWtuS	2	TRUE	0.003883	23.84293
8627	rs7980973	T	G	T	G	-0.0381	-0.02037	0.7914	0.779461	FALSE	FALSE	FALSE	MZ60v7	0.016706	0.222608	486484	sepsis	TRUE	reported	textfile	0.0078	1.10E-06	2432	M36134.m	TRUE	reported	dMQewN	2	TRUE	0.009715	23.83985
8646	rs1374273	A	G	A	G	0.0254	-0.01112	0.6234	0.628609	FALSE	FALSE	FALSE	GgPG6k	0.014289	0.436498	486484	sepsis	TRUE	reported	textfile	0.0052	1.05E-06	1732	M36300.m	TRUE	reported	kT1yso	2	TRUE	0.013588	23.83192
736	rs1894947	T	C	T	C	0.0083	-0.02852	0.1801	0.181927	FALSE	FALSE	FALSE	9u0SC8	0.017789	0.108923	486484	sepsis	TRUE	reported	textfile	0.0017	1.85E-06	7363	M01301.m	TRUE	reported	H95uec	2	TRUE	0.003227	23.8309
884	rs1339301	A	G	A	G	0.0415	0.076795	0.0218	0.028508	FALSE	FALSE	FALSE	oi7KQl	0.041708	0.065586	486484	sepsis	TRUE	reported	textfile	0.0085	1.08E-06	7362	M01365.m	TRUE	reported	7NWGKA	2	TRUE	0.003227	23.83089
5040	rs7992587	T	C	T	C	-0.0083	0.005425	0.2605	0.24964	FALSE	FALSE	FALSE	BNnOvt	0.015967	0.734018	486484	sepsis	TRUE	reported	textfile	0.0017	7.72E-07	7362	M33132.m	TRUE	reported	rHcCPG	2	TRUE	0.003227	23.83089
841	rs727892	A	G	A	G	0.0083	-0.00838	0.5021	0.510813	FALSE	FALSE	FALSE	2qgDgB	0.013789	0.543298	486484	sepsis	TRUE	reported	textfile	0.0017	8.15E-07	7355	M01358.m	TRUE	reported	asmXq0	2	TRUE	0.003231	23.83089
567	rs4286846	C	G	C	G	0.0083	0.000307	0.7232	0.709235	FALSE	TRUE	FALSE	fNqkCW	0.015079	0.983758	486484	sepsis	TRUE	reported	textfile	0.0017	1.26E-06	7354	M00606.m	TRUE	reported	g9uwOn	2	TRUE	0.003231	23.83089
2811	rs2667755	T	C	T	C	-0.0083	-0.00065	0.6304	0.630655	FALSE	FALSE	FALSE	Sx5Uhq	0.014196	0.963212	486484	sepsis	TRUE	reported	textfile	0.0017	1.84E-06	7354	M19934.m	TRUE	reported	DijYTE	2	TRUE	0.003231	23.83089
2820	rs4759824	T	C	T	C	0.0083	0.009237	0.6793	0.678223	FALSE	FALSE	FALSE	Sx5Uhq	0.014659	0.528608	486484	sepsis	TRUE	reported	textfile	0.0017	1.85E-06	7354	M19934.m	TRUE	reported	DijYTE	2	TRUE	0.003231	23.83089
3702	rs2762343	A	G	A	G	0.0083	-0.00972	0.5133	0.527108	FALSE	FALSE	FALSE	agPgtF	0.013699	0.477936	486484	sepsis	TRUE	reported	textfile	0.0017	1.22E-06	7349	M32315.m	TRUE	reported	hMRjcV	2	TRUE	0.003233	23.83088
4439	rs6434188	T	C	T	C	0.0083	-0.00017	0.2169	0.226204	FALSE	FALSE	FALSE	WGPdpq	0.016347	0.991489	486484	sepsis	TRUE	reported	textfile	0.0017	9.92E-07	7338	M32675.m	TRUE	reported	u0eUAG	2	TRUE	0.003238	23.83087
6077	rs2209196	T	G	T	G	-0.0083	-0.02689	0.1924	0.191638	FALSE	FALSE	FALSE	P8n0HP	0.017613	0.126839	486484	sepsis	TRUE	reported	textfile	0.0017	2.21E-06	7336	M33442.m	TRUE	reported	cljMTl	2	TRUE	0.003239	23.83087
2148	rs7648992	T	C	T	C	0.0083	0.024572	0.3947	0.403075	FALSE	FALSE	FALSE	TEupfd	0.014001	0.079261	486484	sepsis	TRUE	reported	textfile	0.0017	1.53E-06	7331	M15676.m	TRUE	reported	NXiaPC	2	TRUE	0.003241	23.83087
3351	rs984326	A	G	A	G	0.0083	0.019945	0.2537	0.2981	FALSE	FALSE	FALSE	m66Eav	0.014963	0.182559	486484	sepsis	TRUE	reported	textfile	0.0017	1.50E-06	7303	M27256.m	TRUE	reported	pl2ECU	2	TRUE	0.003253	23.83084
1287	rs1045465	T	C	T	C	0.0083	-0.02283	0.5849	0.583292	FALSE	FALSE	FALSE	WQdpmo	0.013861	0.099527	486484	sepsis	TRUE	reported	textfile	0.0017	1.64E-06	7136	M01769.m	TRUE	reported	BkptOK	2	TRUE	0.003329	23.83069
1475	rs2250515	T	G	T	G	-0.0083	-0.00684	0.5346	0.536097	FALSE	FALSE	FALSE	pkb876	0.013726	0.618327	486484	sepsis	TRUE	reported	textfile	0.0017	1.60E-06	7037	M02734.m	TRUE	reported	0LCMVl	2	TRUE	0.003376	23.8306
1483	rs4487134	T	G	T	G	-0.0083	0.013081	0.355	0.357351	FALSE	FALSE	FALSE	pkb876	0.014289	0.359961	486484	sepsis	TRUE	reported	textfile	0.0017	1.99E-06	7037	M02734.m	TRUE	reported	0LCMVl	2	TRUE	0.003376	23.8306
2569	rs1164780	A	C	A	C	0.0249	-0.01754	0.0735	0.084232	FALSE	FALSE	FALSE	KHS91f	0.024578	0.475433	486484	sepsis	TRUE	reported	textfile	0.0051	8.57E-07	6939	M18349.m	TRUE	reported	PeVGil	2	TRUE	0.003424	23.8305
8733	rs1439977	T	C	T	C	-0.0254	-0.0216	0.3281	0.327651	FALSE	FALSE	FALSE	ERC6hD	0.014526	0.136997	486484	sepsis	TRUE	reported	textfile	0.0052	1.10E-06	1636	M36559.m	TRUE	reported	n8waJF	2	TRUE	0.014374	23.8303
2320	rs1525549	A	G	A	G	0.0166	0.019583	0.1421	0.14437	FALSE	FALSE	FALSE	IZXwpR	0.019495	0.315137	486484	sepsis	TRUE	reported	textfile	0.0034	1.11E-06	6549	M16822.m	TRUE	reported	Zx9k2V	2	TRUE	0.003627	23.83009
8106	rs1571011	A	C	A	C	-0.0083	0.001427	0.5825	0.585107	FALSE	FALSE	FALSE	nNbXBS	0.013897	0.918233	486484	sepsis	TRUE	reported	textfile	0.0017	2.28E-06	5768	M35326.m	TRUE	reported	UNDorn	2	TRUE	0.004116	23.8291
8678	rs327759	T	C	T	C	0.042	-0.01389	0.2319	0.228196	FALSE	FALSE	FALSE	i4bSdJ	0.016682	0.405107	486484	sepsis	TRUE	reported	textfile	0.0086	1.02E-06	1908	M36468.m	TRUE	reported	qJLuZj	2	TRUE	0.012346	23.82573
8673	rs1297404	T	C	T	C	-0.0503	0.006783	0.2168	0.221676	FALSE	FALSE	FALSE	i4bSdJ	0.016764	0.685758	486484	sepsis	TRUE	reported	textfile	0.0103	1.14E-06	1908	M36468.m	TRUE	reported	qJLuZj	2	TRUE	0.012345	23.82353
2656	rs1710702	T	C	T	C	-0.0781	0.009297	0.9608	0.964696	FALSE	FALSE	FALSE	UtYu7J	0.037202	0.802662	486484	sepsis	TRUE	reported	textfile	0.016	1.08E-06	5624	M18476.m	TRUE	reported	wJx0A4	2	TRUE	0.004219	23.81813
900	rs3010320	A	T	A	T	-0.0205	-0.01093	0.232	0.230852	FALSE	TRUE	FALSE	I6zQnf	0.016227	0.500442	486484	sepsis	TRUE	reported	textfile	0.0042	1.00E-06	7345	M01444.m	TRUE	reported	Ilzalm	2	TRUE	0.003233	23.81721
2580	rs7727858	A	G	A	G	-0.0205	-0.01032	0.8891	0.890052	FALSE	FALSE	FALSE	KHS91f	0.021859	0.636867	486484	sepsis	TRUE	reported	textfile	0.0042	1.08E-06	6939	M18349.m	TRUE	reported	PeVGil	2	TRUE	0.003422	23.81683
4551	rs1252109	T	C	T	C	0.0327	0.001587	0.2679	0.264373	FALSE	FALSE	FALSE	KfoRxi	0.015498	0.918451	486484	sepsis	TRUE	reported	textfile	0.0067	8.88E-07	6000	M32735.m	TRUE	reported	UDRXAp	2	TRUE	0.003954	23.81229
2595	rs1718587	A	G	A	G	-0.0122	-0.01184	0.8515	0.844406	FALSE	FALSE	FALSE	ruvJh6	0.018957	0.532352	486484	sepsis	TRUE	reported	textfile	0.0025	8.68E-07	7354	M18369.m	TRUE	reported	GFmwGk	2	TRUE	0.003228	23.80792
8990	rs1252510	A	G	A	G	0.0244	0.0067	0.771	0.756507	FALSE	FALSE	FALSE	TfdxtP	0.016012	0.675616	486484	sepsis	TRUE	reported	textfile	0.005	8.91E-07	7333	M37203.m	TRUE	reported	xicmWr	2	TRUE	0.003237	23.8079
3260	rs1775647	C	G	C	G	0.0122	0.012376	0.7131	0.705775	FALSE	TRUE	FALSE	ZNPBqT	0.015035	0.410418	486484	sepsis	TRUE	reported	textfile	0.0025	1.20E-06	7256	M24074.m	TRUE	reported	8pPtOl	2	TRUE	0.003271	23.80784
5842	rs870031	A	G	A	G	0.0122	0.004266	0.1164	0.113641	FALSE	FALSE	FALSE	R5joEf	0.021585	0.843335	486484	sepsis	TRUE	reported	textfile	0.0025	1.27E-06	7118	M33380.m	TRUE	reported	kRvbfZ	2	TRUE	0.003335	23.80771
865	rs1738584	A	G	A	G	0.0122	-0.00128	0.6443	0.6612	FALSE	FALSE	FALSE	Vg5OOR	0.014532	0.92977	486484	sepsis	TRUE	reported	textfile	0.0025	1.16E-06	7063	M01361.m	TRUE	reported	XnrS00	2	TRUE	0.00336	23.80766
3444	rs1266123	A	G	A	G	0.0244	-0.0191	0.61	0.599749	FALSE	FALSE	FALSE	J1I92u	0.013957	0.171238	486484	sepsis	TRUE	reported	textfile	0.005	1.26E-06	6715	M27710.m	TRUE	reported	9qjpIZ	2	TRUE	0.003534	23.80731
6827	rs1123651	T	C	T	C	0.0283	0.029134	0.897	0.906281	FALSE	FALSE	FALSE	ZPJCMx	0.023455	0.214183	486484	sepsis	TRUE	reported	textfile	0.0058	1.24E-06	7330	M33969.m	TRUE	reported	mLJPAI	2	TRUE	0.003237	23.80117
7574	rs1719882	A	G	A	G	-0.3408	0.096639	0.9879	0.982673	FALSE	FALSE	FALSE	ElkOlW	0.052796	0.067189	486484	sepsis	TRUE	reported	textfile	0.0698	1.06E-06	1235	M34499.m	TRUE	reported	W5iEAE	2	TRUE	0.018937	23.80041
8537	rs1167079	T	C	T	C	0.5691	-0.00561	0.8964	0.858254	FALSE	FALSE	FALSE	aQhCav	0.019718	0.775986	486484	sepsis	TRUE	reported	textfile	0.1157	8.77E-07	122	M36097.m	TRUE	reported	9DJS9j	2	TRUE	0.165493	23.79753
4074	rs3828128	A	G	A	G	-0.0161	0.001049	0.2536	0.259589	FALSE	FALSE	FALSE	TyHtdh	0.015655	0.946578	486484	sepsis	TRUE	reported	textfile	0.0033	1.35E-06	7358	M32497.m	TRUE	reported	H3KmLR	2	TRUE	0.003224	23.7961
3547	rs1329362	T	C	T	C	0.1054	0.034186	0.0166	0.025995	FALSE	FALSE	FALSE	8luUON	0.043286	0.429657	486484	sepsis	TRUE	reported	textfile	0.0216	1.07E-06	1750	M31453.m	TRUE	reported	FBs6G4	2	TRUE	0.013424	23.78357
1669	rs1124193	A	T	A	T	-0.061	0.019152	0.2705	0.283581	FALSE	TRUE	FALSE	IIFBGY	0.015211	0.207999	486484	sepsis	TRUE	reported	textfile	0.0125	1.05E-06	1543	M12261.m	TRUE	reported	F5Vlan	2	TRUE	0.015199	23.78353
521	rs661454	A	G	A	G	0.0317	-0.03284	0.0848	0.087879	FALSE	FALSE	FALSE	AZraib	0.024147	0.173811	486484	sepsis	TRUE	reported	textfile	0.0065	1.31E-06	7333	M00577.m	TRUE	reported	2tucEZ	2	TRUE	0.003233	23.77789
5191	rs1049511	A	C	A	C	0.3075	-0.00336	0.9818	0.984095	FALSE	FALSE	FALSE	q32d4E	0.054728	0.951058	486484	sepsis	TRUE	reported	textfile	0.063	1.06E-06	1017	M33178.m	TRUE	reported	cS3BwT	2	TRUE	0.022889	23.77685
1413	rs6503066	T	C	T	C	0.0317	-0.0026	0.0984	0.104747	FALSE	FALSE	FALSE	33ptwk	0.022368	0.907498	486484	sepsis	TRUE	reported	textfile	0.0065	9.49E-07	6252	M02137.m	TRUE	reported	SQY1na	2	TRUE	0.00379	23.77677
5612	rs9689446	T	C	T	C	0.1635	-0.0089	0.962	0.959808	FALSE	FALSE	FALSE	q32d4E	0.034905	0.79867	486484	sepsis	TRUE	reported	textfile	0.0335	1.04E-06	1017	M33178.m	TRUE	reported	cS3BwT	2	TRUE	0.022886	23.77338
3798	rs9582849	A	G	A	G	0.02	-0.00147	0.7575	0.885689	FALSE	FALSE	FALSE	NW1aaR	0.025545	0.954223	486484	sepsis	TRUE	reported	textfile	0.0041	1.34E-06	1761	M32339.m	TRUE	reported	pBPkAB	2	TRUE	0.013332	23.76834
8059	rs1386540	T	C	T	C	-0.0361	0.011032	0.3484	0.35494	FALSE	FALSE	FALSE	whAa18	0.014281	0.439818	486484	sepsis	TRUE	reported	textfile	0.0074	1.14E-06	1505	M35305.m	TRUE	reported	e6hvDm	2	TRUE	0.015567	23.76695
2381	rs1462176	T	C	T	C	-0.139	0.010362	0.0469	0.051831	FALSE	FALSE	FALSE	NBJ2Do	0.031454	0.741817	486484	sepsis	TRUE	reported	textfile	0.0285	1.07E-06	1739	M17799.m	TRUE	reported	Uz0Wgl	2	TRUE	0.013494	23.75965
1790	rs1697485	T	C	T	C	-0.0234	0.047648	0.038	0.035906	FALSE	FALSE	FALSE	JEcCRZ	0.036929	0.196969	486484	sepsis	TRUE	reported	textfile	0.0048	1.15E-06	7368	M15140.m	TRUE	reported	xYCzKP	2	TRUE	0.003215	23.75917
471	rs1330523	A	G	A	G	-0.0117	-0.00051	0.2326	0.2237	FALSE	FALSE	FALSE	OW48NY	0.016415	0.975212	486484	sepsis	TRUE	reported	textfile	0.0024	1.56E-06	7365	M00527.m	TRUE	reported	57AP2f	2	TRUE	0.003216	23.75917
792	rs1348656	T	C	T	C	-0.0117	-0.01409	0.798	0.798722	FALSE	FALSE	FALSE	dyT79l	0.017184	0.412237	486484	sepsis	TRUE	reported	textfile	0.0024	9.07E-07	7352	M01336.m	TRUE	reported	OemVJ2	2	TRUE	0.003222	23.75916
3711	rs6740983	T	C	T	C	-0.0117	0.005594	0.7752	0.775912	FALSE	FALSE	FALSE	agPgtF	0.016393	0.732931	486484	sepsis	TRUE	reported	textfile	0.0024	1.21E-06	7349	M32315.m	TRUE	reported	hMRjcV	2	TRUE	0.003223	23.75916
2138	rs2404519	A	G	A	G	0.0117	-0.00715	0.1587	0.174997	FALSE	FALSE	FALSE	TEupfd	0.018156	0.693769	486484	sepsis	TRUE	reported	textfile	0.0024	1.60E-06	7331	M15676.m	TRUE	reported	NXiaPC	2	TRUE	0.003231	23.75914
1687	rs1244477	T	C	T	C	-0.0156	-0.03379	0.0607	0.071423	FALSE	FALSE	FALSE	f01en0	0.027101	0.21249	486484	sepsis	TRUE	reported	textfile	0.0032	1.61E-06	7314	M12593.m	TRUE	reported	EAsM6U	2	TRUE	0.003239	23.75913
3610	rs400120	A	G	A	G	0.0195	0.018357	0.6474	0.627096	FALSE	FALSE	FALSE	2z5KhH	0.014127	0.19378	486484	sepsis	TRUE	reported	textfile	0.004	1.30E-06	7263	M31555.m	TRUE	reported	5kcWr2	2	TRUE	0.003261	23.75908
6611	rs6580981	A	G	A	G	-0.0551	-0.02981	0.4501	0.459406	FALSE	FALSE	FALSE	VfOuo8	0.013782	0.030545	486484	sepsis	TRUE	reported	textfile	0.0113	1.12E-06	2725	M33883.m	TRUE	reported	BDe0Nk	2	TRUE	0.00865	23.75896
5820	rs1363574	A	G	A	G	-0.0195	0.01944	0.9545	0.953255	FALSE	FALSE	FALSE	R5joEf	0.032785	0.553216	486484	sepsis	TRUE	reported	textfile	0.004	1.29E-06	7118	M33380.m	TRUE	reported	kRvbfZ	2	TRUE	0.003328	23.75895
8189	rs1013947	T	G	T	G	-0.0156	0.020652	0.2538	0.282926	FALSE	FALSE	FALSE	iLlpho	0.015455	0.181457	486484	sepsis	TRUE	reported	textfile	0.0032	1.47E-06	7007	M35431.m	TRUE	reported	AAZ5ge	2	TRUE	0.00338	23.75884
3454	rs805936	A	G	A	G	-0.0897	-0.00848	0.9841	0.981144	FALSE	FALSE	FALSE	J1I92u	0.050751	0.867317	486484	sepsis	TRUE	reported	textfile	0.0184	1.16E-06	6715	M27710.m	TRUE	reported	9qjpIZ	2	TRUE	0.003527	23.75855
1924	rs1537385	T	G	T	G	0.0317	-0.03061	0.9609	0.950828	FALSE	FALSE	FALSE	v2KE89	0.031901	0.33729	486484	sepsis	TRUE	reported	textfile	0.0065	1.19E-06	1759	M15500.m	TRUE	reported	to2t7H	2	TRUE	0.013341	23.75734
2825	rs6948277	A	G	A	G	0.0951	-0.00733	0.0135	0.020759	FALSE	FALSE	FALSE	Sx5Uhq	0.048154	0.878984	486484	sepsis	TRUE	reported	textfile	0.0195	1.01E-06	1759	M19934.m	TRUE	reported	DijYTE	2	TRUE	0.013341	23.75734
2187	rs475322	A	C	A	C	0.117	-0.01595	0.016	0.035952	FALSE	FALSE	FALSE	UokbxN	0.038069	0.675271	486484	sepsis	TRUE	reported	textfile	0.024	1.09E-06	5599	M15753.m	TRUE	reported	lUGKBi	2	TRUE	0.004227	23.75714
3107	rs2215015	T	C	T	C	-0.0195	0.015018	0.3426	0.35554	FALSE	FALSE	FALSE	ApMZhy	0.014341	0.294997	486484	sepsis	TRUE	reported	textfile	0.004	1.25E-06	3950	M22138.m	TRUE	reported	LiJliF	2	TRUE	0.005981	23.75359
4894	rs465818	T	C	T	C	-0.2195	0.032075	0.963	0.968701	FALSE	FALSE	FALSE	ocaywK	0.039337	0.414849	486484	sepsis	TRUE	reported	textfile	0.045	1.07E-06	1162	M32863.m	TRUE	reported	WH6KGr	2	TRUE	0.020065	23.75176
8784	rs1724952	T	G	T	G	0.0322	-0.00622	0.3177	0.303825	FALSE	FALSE	FALSE	4bkfvN	0.014892	0.676369	486484	sepsis	TRUE	reported	textfile	0.0066	1.22E-06	922	M36738.m	TRUE	reported	xcqyX2	2	TRUE	0.025167	23.75094
7219	rs2212841	T	C	T	C	0.87	0.1288	0.015	0.018214	FALSE	FALSE	FALSE	i1uJ7T	0.051053	0.011639	486484	sepsis	TRUE	reported	textfile	0.177	8.87E-07	115	M34338.m	TRUE	reported	pi3mKW	2	TRUE	0.173611	23.73956
4786	rs7305915	A	G	A	G	-0.0307	-0.02231	0.3824	0.383082	FALSE	FALSE	FALSE	MmZZoM	0.014074	0.112979	486484	sepsis	TRUE	reported	textfile	0.0063	9.85E-07	4274	M32800.m	TRUE	reported	70YSCO	2	TRUE	0.005525	23.73517
3509	rs9608300	C	G	C	G	-0.137	0.084027	0.0264	0.01433	FALSE	TRUE	FALSE	Awafb0	0.060446	0.164498	486484	sepsis	TRUE	reported	textfile	0.0281	1.06E-06	1227	M27728.m	TRUE	reported	Lfj8L0	2	TRUE	0.019004	23.73122
5802	rs1188168	A	G	A	G	-0.0273	0.030806	0.779	0.809845	FALSE	FALSE	FALSE	JE7L71	0.017377	0.076273	486484	sepsis	TRUE	reported	textfile	0.0056	1.06E-06	1338	M33364.m	TRUE	reported	FdLiEs	2	TRUE	0.017452	23.7301
526	rs1221854	C	G	C	G	-0.019	-0.01208	0.0836	0.11212	FALSE	TRUE	FALSE	wXMWHy	0.02168	0.577467	486484	sepsis	TRUE	reported	textfile	0.0039	1.28E-06	7345	M00584.m	TRUE	reported	7CKn2b	2	TRUE	0.003221	23.72792
1845	rs1184372	T	C	T	C	0.019	0.014633	0.9644	0.959595	FALSE	FALSE	FALSE	S1eWts	0.034902	0.675033	486484	sepsis	TRUE	reported	textfile	0.0039	9.47E-07	7345	M15488.m	TRUE	reported	511Kl9	2	TRUE	0.003221	23.72792
2345	rs7679137	T	C	T	C	-0.2217	-0.0033	0.9857	0.984946	FALSE	FALSE	FALSE	13lycQ	0.056567	0.953416	486484	sepsis	TRUE	reported	textfile	0.0455	1.11E-06	3393	M16823.m	TRUE	reported	u2seZ2	2	TRUE	0.006949	23.72753
3810	rs7793797	A	T	A	T	0.038	-0.0064	0.7018	0.696611	FALSE	TRUE	FALSE	HOq2gb	0.014901	0.66757	486484	sepsis	TRUE	reported	textfile	0.0078	1.26E-06	6687	M32346.m	TRUE	reported	Ss5q17	2	TRUE	0.003537	23.72729
1585	rs1281570	A	G	A	G	0.019	-0.00709	0.2806	0.320689	FALSE	FALSE	FALSE	7QN2kA	0.015162	0.640053	486484	sepsis	TRUE	reported	textfile	0.0039	9.10E-07	5656	M12017.m	TRUE	reported	52YQ1d	2	TRUE	0.004179	23.72599
8660	rs1469456	T	C	T	C	-0.0263	0.023243	0.7562	0.752219	FALSE	FALSE	FALSE	BFEGyF	0.015917	0.144232	486484	sepsis	TRUE	reported	textfile	0.0054	1.36E-06	6486	M36399.m	TRUE	reported	nhT4FM	2	TRUE	0.003644	23.71319
4664	rs944846	T	C	T	C	0.0575	-0.00865	0.0802	0.083371	FALSE	FALSE	FALSE	SyAFR2	0.024724	0.726422	486484	sepsis	TRUE	reported	textfile	0.0118	1.19E-06	1435	M32755.m	TRUE	reported	prUo74	2	TRUE	0.016278	23.71188
8938	rs1246726	T	C	T	C	0.0263	0.010692	0.4786	0.482727	FALSE	FALSE	FALSE	mXJP3h	0.01366	0.433776	486484	sepsis	TRUE	reported	textfile	0.0054	1.09E-06	4520	M37104.m	TRUE	reported	HRZBak	2	TRUE	0.005221	23.71001
2643	rs1173669	A	C	A	C	-0.24	-0.00915	0.9602	0.94654	FALSE	FALSE	FALSE	9GoQdh	0.030743	0.765967	486484	sepsis	TRUE	reported	textfile	0.0491	1.04E-06	257	M18474.m	TRUE	reported	p6Dq8p	2	TRUE	0.085059	23.70645
925	rs1158214	C	G	C	G	-0.0112	0.020294	0.1443	0.157731	FALSE	TRUE	FALSE	tv99UN	0.018778	0.27981	486484	sepsis	TRUE	reported	textfile	0.0023	9.32E-07	7354	M01494.m	TRUE	reported	osDclp	2	TRUE	0.003214	23.70622
6536	rs7151939	T	G	T	G	-0.0224	-0.00287	0.1357	0.137462	FALSE	FALSE	FALSE	DedZqr	0.019861	0.884989	486484	sepsis	TRUE	reported	textfile	0.0046	1.22E-06	7350	M33822.m	TRUE	reported	ciYOdn	2	TRUE	0.003216	23.70621
854	rs4353846	T	C	T	C	-0.0112	-0.01865	0.5643	0.545597	FALSE	FALSE	FALSE	1tFtaf	0.013806	0.176659	486484	sepsis	TRUE	reported	textfile	0.0023	1.70E-06	7323	M01359.m	TRUE	reported	qq3q6E	2	TRUE	0.003228	23.70619
8665	rs4542627	A	T	A	T	-0.0224	-0.0022	0.3909	0.418173	FALSE	TRUE	FALSE	BFEGyF	0.013914	0.8741	486484	sepsis	TRUE	reported	textfile	0.0046	1.50E-06	6486	M36399.m	TRUE	reported	nhT4FM	2	TRUE	0.003643	23.70535
7870	rs1289566	T	C	T	C	-0.0302	0.023454	0.6305	0.637242	FALSE	FALSE	FALSE	r428eQ	0.014279	0.100466	486484	sepsis	TRUE	reported	textfile	0.0062	1.24E-06	1982	M35159.m	TRUE	reported	3r5zQo	2	TRUE	0.011829	23.70238
8514	rs4854073	T	C	T	C	-0.0224	0.027963	0.4114	0.427419	FALSE	FALSE	FALSE	vHXktL	0.01395	0.045015	486484	sepsis	TRUE	reported	textfile	0.0046	1.25E-06	4529	M35978.m	TRUE	reported	ceap9w	2	TRUE	0.005208	23.70219
4795	rs1171135	A	T	A	T	0.0297	0.007367	0.582	0.597319	FALSE	TRUE	FALSE	9qWhFj	0.014113	0.601656	486484	sepsis	TRUE	reported	textfile	0.0061	9.67E-07	6200	M32808.m	TRUE	reported	MQcOSi	2	TRUE	0.003809	23.69808
8413	rs1329347	C	G	C	G	-0.037	-0.02269	0.9111	0.908379	FALSE	TRUE	FALSE	02RdNm	0.023809	0.340536	486484	sepsis	TRUE	reported	textfile	0.0076	1.30E-06	6447	M35678.m	TRUE	reported	BWN5JR	2	TRUE	0.003663	23.69417
6931	rs9929954	T	G	T	G	0.1231	-0.00451	0.7364	0.729135	FALSE	FALSE	FALSE	SvfSvH	0.015602	0.772542	486484	sepsis	TRUE	reported	textfile	0.0252	9.87E-07	282	M34109.m	TRUE	reported	7OSbiu	2	TRUE	0.078017	23.69321
605	rs9834988	T	C	T	C	0.0185	-0.02204	0.5297	0.530723	FALSE	FALSE	FALSE	KVK33e	0.013732	0.108442	486484	sepsis	TRUE	reported	textfile	0.0038	7.98E-07	5344	M01107.m	TRUE	reported	eAyR8w	2	TRUE	0.004416	23.69265
4162	rs1695726	C	G	C	G	-0.0443	-0.01497	0.9068	0.910201	FALSE	TRUE	FALSE	stpu5O	0.023985	0.532665	486484	sepsis	TRUE	reported	textfile	0.0091	1.09E-06	7337	M32557.m	TRUE	reported	0oV8Ss	2	TRUE	0.00322	23.69225
3278	rs1322212	A	G	A	G	-0.1154	0.051507	0.9863	0.931973	FALSE	FALSE	FALSE	vYWPe9	0.027243	0.058669	486484	sepsis	TRUE	reported	textfile	0.0237	1.09E-06	1756	M25459.m	TRUE	reported	VvJ6Vx	2	TRUE	0.013322	23.68209
2302	rs1344014	T	G	T	G	-0.0292	0.060155	0.0579	0.07137	FALSE	FALSE	FALSE	ZzUW8U	0.026482	0.023113	486484	sepsis	TRUE	reported	textfile	0.006	1.29E-06	6733	M16821.m	TRUE	reported	g2xs3i	2	TRUE	0.003505	23.67741
2303	rs1745029	T	G	T	G	-0.0219	-0.00272	0.9051	0.876018	FALSE	FALSE	FALSE	ZzUW8U	0.020779	0.895864	486484	sepsis	TRUE	reported	textfile	0.0045	1.11E-06	6733	M16821.m	TRUE	reported	g2xs3i	2	TRUE	0.003505	23.67741
4844	rs4762836	T	C	T	C	0.0584	-0.00032	0.0748	0.072437	FALSE	FALSE	FALSE	jvlpBt	0.02641	0.99044	486484	sepsis	TRUE	reported	textfile	0.012	1.01E-06	6250	M32846.m	TRUE	reported	RAXcz5	2	TRUE	0.003775	23.67687
7147	rs8014529	A	G	A	G	-0.3141	-0.01311	0.9782	0.405576	FALSE	FALSE	FALSE	de8tA6	0.013922	0.346253	486484	sepsis	TRUE	reported	textfile	0.0637	8.21E-07	75	M34322.m	TRUE	reported	sXPPBO	2	TRUE	0.24482	23.66566
4224	rs1711003	T	C	T	C	-0.0433	0.050027	0.961	0.961597	FALSE	FALSE	FALSE	FDy32d	0.035564	0.159527	486484	sepsis	TRUE	reported	textfile	0.0089	1.18E-06	7369	M32587.m	TRUE	reported	S10k1h	2	TRUE	0.003202	23.66344
5117	rs7648714	A	G	A	G	0.018	0.01988	0.4717	0.474138	FALSE	FALSE	FALSE	O6ine1	0.013776	0.149007	486484	sepsis	TRUE	reported	textfile	0.0037	1.06E-06	7263	M33165.m	TRUE	reported	gGsqjW	2	TRUE	0.003248	23.66039
1490	rs8132248	T	C	T	C	0.018	0.018615	0.0986	0.136558	FALSE	FALSE	FALSE	pkb876	0.020116	0.354766	486484	sepsis	TRUE	reported	textfile	0.0037	1.43E-06	7037	M02734.m	TRUE	reported	0LCMVl	2	TRUE	0.003352	23.66018
7288	rs1478747	A	G	A	G	0.018	-0.01747	0.214	0.236462	FALSE	FALSE	FALSE	Vcz4EA	0.017951	0.330379	486484	sepsis	TRUE	reported	textfile	0.0037	1.27E-06	6755	M34359.m	TRUE	reported	aUSo2J	2	TRUE	0.003491	23.6599
593	rs1271859	T	C	T	C	0.018	-0.01341	0.5178	0.512092	FALSE	FALSE	FALSE	KVK33e	0.013659	0.32606	486484	sepsis	TRUE	reported	textfile	0.0037	1.49E-06	5344	M01107.m	TRUE	reported	eAyR8w	2	TRUE	0.004409	23.65805
659	rs7795118	T	C	T	C	0.0905	-0.01587	0.2305	0.269166	FALSE	FALSE	FALSE	7BPGuQ	0.016197	0.327204	486484	sepsis	TRUE	reported	textfile	0.0186	1.21E-06	2639	M01123.m	TRUE	reported	WV2hVn	2	TRUE	0.008891	23.65604
6424	rs7956425	A	G	A	G	-0.093	-0.01041	0.8522	0.854194	FALSE	FALSE	FALSE	F7mjRv	0.019337	0.59038	486484	sepsis	TRUE	reported	textfile	0.0191	1.21E-06	860	M33658.m	TRUE	reported	frG0Zf	2	TRUE	0.026828	23.6531
3653	rs1738925	T	C	T	C	-0.0214	-0.00271	0.9163	0.917168	FALSE	FALSE	FALSE	fnjup6	0.024854	0.913042	486484	sepsis	TRUE	reported	textfile	0.0044	1.03E-06	7346	M32197.m	TRUE	reported	oQzaMk	2	TRUE	0.00321	23.64852
1446	rs35592	T	C	T	C	0.0107	0.017874	0.7618	0.765565	FALSE	FALSE	FALSE	1M4gqj	0.016107	0.267151	486484	sepsis	TRUE	reported	textfile	0.0022	1.24E-06	7226	M02730.m	TRUE	reported	RWyYEM	2	TRUE	0.003263	23.64841
7067	rs7161226	A	C	A	C	-0.0652	0.044767	0.9809	0.979695	FALSE	FALSE	FALSE	uthruC	0.048528	0.356275	486484	sepsis	TRUE	reported	textfile	0.0134	1.10E-06	1755	M34283.m	TRUE	reported	ftTuRU	2	TRUE	0.01331	23.64778
8603	rs1076417	A	T	A	T	-0.0579	-0.00822	0.9265	0.930832	FALSE	TRUE	FALSE	oHM5TD	0.027157	0.762012	486484	sepsis	TRUE	reported	textfile	0.0119	1.22E-06	1780	M36131.m	TRUE	reported	Xw6wmg	2	TRUE	0.013125	23.64694
7609	rs7655071	C	G	C	G	0.1543	0.027194	0.1519	0.183575	FALSE	TRUE	FALSE	PlYt5g	0.0177	0.124451	486484	sepsis	TRUE	reported	textfile	0.0316	1.04E-06	241	M34516.m	TRUE	reported	jX41tf	2	TRUE	0.090026	23.64496
2551	rs1321208	T	C	T	C	-0.0355	0.027472	0.1913	0.227301	FALSE	FALSE	FALSE	NxZhaQ	0.016314	0.092185	486484	sepsis	TRUE	reported	textfile	0.0073	1.05E-06	5984	M18283.m	TRUE	reported	5Pi7r3	2	TRUE	0.003936	23.641
8362	rs704622	T	C	T	C	-0.3467	-0.09244	0.9863	0.987964	FALSE	FALSE	FALSE	6VVCe4	0.062738	0.140655	486484	sepsis	TRUE	reported	textfile	0.0712	1.13E-06	659	M35669.m	TRUE	reported	l0fk8X	2	TRUE	0.03473	23.63892
7072	rs1153203	A	G	A	G	-0.0535	0.024157	0.9564	0.947882	FALSE	FALSE	FALSE	sg7nOB	0.030803	0.432886	486484	sepsis	TRUE	reported	textfile	0.011	1.17E-06	2278	M34289.m	TRUE	reported	KemvIC	2	TRUE	0.010277	23.63419
4816	rs7095335	A	T	A	T	-0.0141	-0.01022	0.6604	0.653823	FALSE	TRUE	FALSE	bJwLV5	0.014366	0.477019	486484	sepsis	TRUE	reported	textfile	0.0029	1.11E-06	7063	M32814.m	TRUE	reported	qte0qp	2	TRUE	0.003336	23.63302
3571	rs1275921	A	G	A	G	-0.1242	0.014738	0.9097	0.899592	FALSE	FALSE	FALSE	F1s6qg	0.022726	0.516649	486484	sepsis	TRUE	reported	textfile	0.0255	1.05E-06	522	M31536.m	TRUE	reported	Zm40GX	2	TRUE	0.04347	23.63174
7455	rs2963485	T	C	T	C	-0.0175	-0.00682	0.1869	0.189321	FALSE	FALSE	FALSE	7ubZow	0.017452	0.696033	486484	sepsis	TRUE	reported	textfile	0.0036	1.06E-06	7344	M34407.m	TRUE	reported	LRsXN2	2	TRUE	0.003207	23.62397
2293	rs3113185	T	C	T	C	-0.0175	-0.00128	0.6254	0.602714	FALSE	FALSE	FALSE	43Yh3h	0.013969	0.927009	486484	sepsis	TRUE	reported	textfile	0.0036	9.50E-07	7055	M16818.m	TRUE	reported	beR4IT	2	TRUE	0.003338	23.6237
3894	rs8093526	T	C	T	C	0.0355	-0.00425	0.4585	0.457352	FALSE	FALSE	FALSE	bmXxuz	0.013871	0.759117	486484	sepsis	TRUE	reported	textfile	0.0073	1.02E-06	1766	M32405.m	TRUE	reported	A9BFZF	2	TRUE	0.013214	23.62212
5806	rs2062528	C	G	C	G	0.0175	0.027232	0.6616	0.668041	FALSE	TRUE	FALSE	JE7L71	0.014536	0.061017	486484	sepsis	TRUE	reported	textfile	0.0036	9.19E-07	3890	M33364.m	TRUE	reported	FdLiEs	2	TRUE	0.006038	23.61825
3565	rs1007243	T	C	T	C	0.3673	2.29E-05	0.0175	0.042562	FALSE	FALSE	FALSE	F1s6qg	0.033928	0.999462	486484	sepsis	TRUE	reported	textfile	0.0754	1.10E-06	423	M31536.m	TRUE	reported	Zm40GX	2	TRUE	0.053119	23.61788
888	rs3759526	T	C	T	C	-0.0209	-0.0032	0.0806	0.076923	FALSE	FALSE	FALSE	oi7KQl	0.025966	0.901861	486484	sepsis	TRUE	reported	textfile	0.0043	8.93E-07	7362	M01365.m	TRUE	reported	7NWGKA	2	TRUE	0.003199	23.6177
1761	rs1003652	A	G	A	G	-0.0209	0.006806	0.0757	0.075813	FALSE	FALSE	FALSE	wM5dkT	0.025899	0.792717	486484	sepsis	TRUE	reported	textfile	0.0043	9.58E-07	7352	M15122.m	TRUE	reported	CBpa4t	2	TRUE	0.003203	23.61769
3865	rs780241	A	G	A	G	0.0209	-0.01547	0.2584	0.249315	FALSE	FALSE	FALSE	8gg1zS	0.015805	0.327798	486484	sepsis	TRUE	reported	textfile	0.0043	1.24E-06	6119	M32379.m	TRUE	reported	uZWtuS	2	TRUE	0.003846	23.6164
4161	rs1447149	A	G	A	G	-0.0311	0.011255	0.2486	0.234974	FALSE	FALSE	FALSE	stpu5O	0.016169	0.486379	486484	sepsis	TRUE	reported	textfile	0.0064	1.08E-06	7337	M32557.m	TRUE	reported	0oV8Ss	2	TRUE	0.003208	23.60709
1181	rs7112460	T	C	T	C	0.1084	-0.02554	0.0658	0.063011	FALSE	FALSE	FALSE	FgZx4a	0.028085	0.363115	486484	sepsis	TRUE	reported	textfile	0.0223	1.14E-06	2063	M01640.m	TRUE	reported	hNPAJ8	2	TRUE	0.011324	23.60628
4845	rs7150683	A	G	A	G	-0.0379	0.02033	0.2063	0.212069	FALSE	FALSE	FALSE	jvlpBt	0.016764	0.225237	486484	sepsis	TRUE	reported	textfile	0.0078	1.13E-06	6250	M32846.m	TRUE	reported	RAXcz5	2	TRUE	0.003763	23.60208
7320	rs1014637	C	G	C	G	0.1099	-0.05828	0.247	0.18747	FALSE	TRUE	FALSE	mm9ksf	0.017511	0.000875	486484	sepsis	TRUE	reported	textfile	0.0226	1.22E-06	954	M34365.m	TRUE	reported	AVwEqD	2	TRUE	0.024188	23.59756
8739	rs6938391	A	G	A	G	0.0802	0.062712	0.0262	0.03458	FALSE	FALSE	FALSE	ERC6hD	0.037846	0.097512	486484	sepsis	TRUE	reported	textfile	0.0165	1.19E-06	1636	M36559.m	TRUE	reported	n8waJF	2	TRUE	0.014235	23.59661
6348	rs7185611	T	C	T	C	-0.4842	0.102505	0.98	0.989967	FALSE	FALSE	FALSE	qLUY00	0.069805	0.141978	486484	sepsis	TRUE	reported	textfile	0.0987	9.41E-07	102	M33610.m	TRUE	reported	8cAJbB	2	TRUE	0.190904	23.59473
6344	rs6679176	T	C	T	C	-0.3414	0.015688	0.9608	0.829539	FALSE	FALSE	FALSE	qLUY00	0.018176	0.388091	486484	sepsis	TRUE	reported	textfile	0.0696	9.20E-07	102	M33610.m	TRUE	reported	8cAJbB	2	TRUE	0.190866	23.58894
7630	rs1196213	A	G	A	G	0.0753	0.016627	0.037	0.037447	FALSE	FALSE	FALSE	xGQKuv	0.036179	0.645821	486484	sepsis	TRUE	reported	textfile	0.0155	1.12E-06	3813	M34530.m	TRUE	reported	wajzOs	2	TRUE	0.006151	23.58841
4407	rs9551761	A	G	A	G	0.017	-0.04493	0.8809	0.928703	FALSE	FALSE	FALSE	UJygVd	0.026557	0.090659	486484	sepsis	TRUE	reported	textfile	0.0035	1.51E-06	7361	M32654.m	TRUE	reported	kv9R0W	2	TRUE	0.003195	23.58543
4900	rs1109583	A	G	A	G	-0.0068	-0.00594	0.235	0.240825	FALSE	FALSE	FALSE	VqcRGX	0.016053	0.71149	486484	sepsis	TRUE	reported	textfile	0.0014	8.70E-07	7329	M32867.m	TRUE	reported	psuFJy	2	TRUE	0.003209	23.5854
5836	rs7526119	A	G	A	G	-0.0136	-0.00765	0.1692	0.21776	FALSE	FALSE	FALSE	R5joEf	0.016698	0.646784	486484	sepsis	TRUE	reported	textfile	0.0028	1.77E-06	7118	M33380.m	TRUE	reported	kRvbfZ	2	TRUE	0.003303	23.58521
2259	rs7795076	T	C	T	C	-0.017	-0.00582	0.8166	0.830944	FALSE	FALSE	FALSE	zxcPox	0.018296	0.75044	486484	sepsis	TRUE	reported	textfile	0.0035	1.62E-06	6782	M15990.m	TRUE	reported	34Vg8F	2	TRUE	0.003467	23.58488
4661	rs760955	T	C	T	C	0.0238	-0.02335	0.281	0.344649	FALSE	FALSE	FALSE	SyAFR2	0.01477	0.113841	486484	sepsis	TRUE	reported	textfile	0.0049	1.09E-06	6658	M32755.m	TRUE	reported	prUo74	2	TRUE	0.003531	23.58475
5106	rs4130215	A	C	A	C	0.0238	-0.02953	0.1106	0.130813	FALSE	FALSE	FALSE	Uiz8VU	0.020222	0.144198	486484	sepsis	TRUE	reported	textfile	0.0049	1.32E-06	6404	M33163.m	TRUE	reported	ky0Bda	2	TRUE	0.00367	23.58447
7557	rs1264047	C	G	C	G	-0.0204	0.013309	0.413	0.414637	FALSE	TRUE	FALSE	APSWAU	0.013938	0.339628	486484	sepsis	TRUE	reported	textfile	0.0042	1.46E-06	6144	M34469.m	TRUE	reported	frF3RX	2	TRUE	0.003825	23.58416
3093	rs1471834	A	G	A	G	0.0136	0.011279	0.7493	0.75275	FALSE	FALSE	FALSE	mr0awv	0.016144	0.484781	486484	sepsis	TRUE	reported	textfile	0.0028	1.45E-06	5702	M22130.m	TRUE	reported	Y2TfWQ	2	TRUE	0.00412	23.58356
7532	rs1333111	A	G	A	G	-0.0136	-0.00654	0.3146	0.319802	FALSE	FALSE	FALSE	JJsHvX	0.014684	0.655873	486484	sepsis	TRUE	reported	textfile	0.0028	9.84E-07	5207	M34456.m	TRUE	reported	aklxve	2	TRUE	0.00451	23.58278
8496	rs1105315	T	C	T	C	0.102	0.026868	0.012	0.023626	FALSE	FALSE	FALSE	4MCEPy	0.049906	0.590326	486484	sepsis	TRUE	reported	textfile	0.021	1.19E-06	5141	M35977.m	TRUE	reported	B3kU6y	2	TRUE	0.004568	23.58266
7658	rs943087	T	C	T	C	0.0306	0.003901	0.1704	0.182645	FALSE	FALSE	FALSE	8W5cqf	0.017894	0.827411	486484	sepsis	TRUE	reported	textfile	0.0063	1.40E-06	4925	M34534.m	TRUE	reported	JV42Pw	2	TRUE	0.004767	23.58226
8552	rs4387456	A	G	A	G	1.6505	-0.03021	0.9863	0.984879	FALSE	FALSE	FALSE	aQhCav	0.05646	0.592631	486484	sepsis	TRUE	reported	textfile	0.3371	9.78E-07	122	M36097.m	TRUE	reported	9DJS9j	2	TRUE	0.164226	23.57952
5805	rs1713335	A	G	A	G	-0.0403	0.036299	0.9531	0.948531	FALSE	FALSE	FALSE	JE7L71	0.03095	0.240863	486484	sepsis	TRUE	reported	textfile	0.0083	1.07E-06	3890	M33364.m	TRUE	reported	FdLiEs	2	TRUE	0.006024	23.563
6220	rs6781351	A	C	A	C	-0.0792	0.001077	0.8686	0.870734	FALSE	FALSE	FALSE	FQfT2o	0.020388	0.957881	486484	sepsis	TRUE	reported	textfile	0.0163	1.14E-06	972	M33508.m	TRUE	reported	JtbOy2	2	TRUE	0.023713	23.56029
6947	rs9319761	T	G	T	G	-0.0869	0.002713	0.9697	0.949131	FALSE	FALSE	FALSE	oNv0vf	0.031172	0.930654	486484	sepsis	TRUE	reported	textfile	0.0179	1.21E-06	5581	M34112.m	TRUE	reported	l0pe0N	2	TRUE	0.004205	23.56014
2344	rs6008912	A	G	A	G	0.1439	0.004543	0.159	0.192496	FALSE	FALSE	FALSE	13lycQ	0.017465	0.794781	486484	sepsis	TRUE	reported	textfile	0.0296	1.19E-06	604	M16823.m	TRUE	reported	u2seZ2	2	TRUE	0.037656	23.55579
8713	rs1783280	C	G	C	G	0.0233	0.010523	0.1092	0.142088	FALSE	TRUE	FALSE	I0G0I1	0.019794	0.594979	486484	sepsis	TRUE	reported	textfile	0.0048	1.55E-06	6464	M36553.m	TRUE	reported	lBltb0	2	TRUE	0.003632	23.55564
907	rs699474	C	G	C	G	0.1282	0.038493	0.0134	0.011849	FALSE	TRUE	FALSE	I6zQnf	0.063644	0.545302	486484	sepsis	TRUE	reported	textfile	0.0264	1.16E-06	1753	M01444.m	TRUE	reported	Ilzalm	2	TRUE	0.013273	23.55442
4449	rs1262149	A	C	A	C	-0.1005	0.027871	0.0275	0.040778	FALSE	FALSE	FALSE	g2RNym	0.034628	0.420899	486484	sepsis	TRUE	reported	textfile	0.0207	1.27E-06	2575	M32691.m	TRUE	reported	35eo6p	2	TRUE	0.009071	23.55342
7653	rs274559	A	G	A	G	-0.0233	-0.01599	0.6171	0.626867	FALSE	FALSE	FALSE	8W5cqf	0.014108	0.257172	486484	sepsis	TRUE	reported	textfile	0.0048	1.50E-06	4925	M34534.m	TRUE	reported	JV42Pw	2	TRUE	0.004762	23.55337
5755	rs1262932	T	C	T	C	-0.0233	-0.00423	0.7183	0.715208	FALSE	FALSE	FALSE	n16uO2	0.015205	0.78068	486484	sepsis	TRUE	reported	textfile	0.0048	1.44E-06	4409	M33250.m	TRUE	reported	9dhI67	2	TRUE	0.005316	23.55225
6742	rs1003620	T	C	T	C	0.0199	0.027591	0.3421	0.329329	FALSE	FALSE	FALSE	NZVg9N	0.014621	0.059153	486484	sepsis	TRUE	reported	textfile	0.0041	1.49E-06	7320	M33941.m	TRUE	reported	kRWNNK	2	TRUE	0.003208	23.55156
8962	rs1118074	A	G	A	G	0.0398	-0.00986	0.1661	0.165012	FALSE	FALSE	FALSE	2iAvlt	0.019042	0.604685	486484	sepsis	TRUE	reported	textfile	0.0082	1.33E-06	6934	M37190.m	TRUE	reported	hdOuHe	2	TRUE	0.003386	23.55121
2923	rs1247056	T	C	T	C	0.0165	-8.21E-05	0.5681	0.592685	FALSE	FALSE	FALSE	fV4qul	0.014058	0.995343	486484	sepsis	TRUE	reported	textfile	0.0034	1.11E-06	7366	M21044.m	TRUE	reported	hbdBQJ	2	TRUE	0.003187	23.54464
2823	rs5994710	A	G	A	G	-0.0165	0.03286	0.0773	0.078451	FALSE	FALSE	FALSE	Sx5Uhq	0.026437	0.213885	486484	sepsis	TRUE	reported	textfile	0.0034	1.16E-06	7354	M19934.m	TRUE	reported	DijYTE	2	TRUE	0.003192	23.54463
4246	rs1823803	T	C	T	C	-0.0427	-0.00404	0.4614	0.449794	FALSE	FALSE	FALSE	KZ60wl	0.013735	0.768796	486484	sepsis	TRUE	reported	textfile	0.0088	1.22E-06	6904	M32616.m	TRUE	reported	SPDpZi	2	TRUE	0.003399	23.53773
3370	rs1276852	T	C	T	C	-0.0131	-0.06547	0.2137	0.232981	FALSE	FALSE	FALSE	O2JtzH	0.016211	5.38E-05	486484	sepsis	TRUE	reported	textfile	0.0027	1.06E-06	7347	M27278.m	TRUE	reported	fxClPW	2	TRUE	0.003194	23.53406
1829	rs1106213	A	G	A	G	0.0131	-0.01461	0.1724	0.175921	FALSE	FALSE	FALSE	2TaDHm	0.017991	0.416793	486484	sepsis	TRUE	reported	textfile	0.0027	1.46E-06	7337	M15365.m	TRUE	reported	LoLveg	2	TRUE	0.003198	23.53405
1659	rs6477873	T	C	T	C	0.0131	-0.02039	0.6394	0.64593	FALSE	FALSE	FALSE	YzX5ZD	0.014287	0.153497	486484	sepsis	TRUE	reported	textfile	0.0027	9.51E-07	6864	M12129.m	TRUE	reported	6tMfaW	2	TRUE	0.003418	23.53361
2247	rs1923140	A	G	A	G	0.0131	-0.00851	0.5322	0.542375	FALSE	FALSE	FALSE	zxcPox	0.013703	0.53442	486484	sepsis	TRUE	reported	textfile	0.0027	8.57E-07	6782	M15990.m	TRUE	reported	34Vg8F	2	TRUE	0.003459	23.53352
2300	rs1255285	T	C	T	C	0.0131	0.018777	0.7391	0.720496	FALSE	FALSE	FALSE	ZzUW8U	0.014779	0.203883	486484	sepsis	TRUE	reported	textfile	0.0027	1.74E-06	6733	M16821.m	TRUE	reported	g2xs3i	2	TRUE	0.003484	23.53347
3241	rs2280289	A	G	A	G	-0.0131	-0.01885	0.8397	0.839601	FALSE	FALSE	FALSE	COSeSD	0.018574	0.310077	486484	sepsis	TRUE	reported	textfile	0.0027	1.38E-06	6517	M22649.m	TRUE	reported	mnH8z2	2	TRUE	0.003599	23.53324
6738	rs508482	T	G	T	G	0.0131	0.033477	0.8237	0.824343	FALSE	FALSE	FALSE	SpG9A1	0.017981	0.06263	486484	sepsis	TRUE	reported	textfile	0.0027	1.12E-06	6502	M33939.m	TRUE	reported	Tudxel	2	TRUE	0.003607	23.53323
6586	rs244904	A	G	A	G	-0.0916	-0.00024	0.5145	0.4779	FALSE	FALSE	FALSE	XO3rXq	0.013719	0.985908	486484	sepsis	TRUE	reported	textfile	0.0188	1.15E-06	228	M33877.m	TRUE	reported	p2sETg	2	TRUE	0.094303	23.53146
4368	rs1991401	A	G	A	G	0.0131	-0.01262	0.6758	0.687167	FALSE	FALSE	FALSE	SPhl52	0.014826	0.394574	486484	sepsis	TRUE	reported	textfile	0.0027	1.91E-06	5211	M32651.m	TRUE	reported	V0Bnpj	2	TRUE	0.004497	23.53143
4219	rs7855272	A	G	A	G	-0.0359	0.021861	0.2071	0.219424	FALSE	FALSE	FALSE	aS6krA	0.016617	0.188308	486484	sepsis	TRUE	reported	textfile	0.0074	1.14E-06	7303	M32586.m	TRUE	reported	g9cBDl	2	TRUE	0.003212	23.52916
8383	rs2177207	T	C	T	C	-0.0927	-0.05613	0.9801	0.97257	FALSE	FALSE	FALSE	SCzh0A	0.042343	0.184967	486484	sepsis	TRUE	reported	textfile	0.0191	1.21E-06	1764	M35675.m	TRUE	reported	n1SREk	2	TRUE	0.013178	23.52882
7709	rs1175299	T	C	T	C	0.0927	0.002768	0.0666	0.030856	FALSE	FALSE	FALSE	qJW9lg	0.040034	0.944868	486484	sepsis	TRUE	reported	textfile	0.0191	1.26E-06	1763	M34878.m	TRUE	reported	42EOzB	2	TRUE	0.013185	23.5288
7714	rs32281	T	C	T	C	-0.1422	0.012225	0.9844	0.987741	FALSE	FALSE	FALSE	qJW9lg	0.063019	0.846182	486484	sepsis	TRUE	reported	textfile	0.0293	1.22E-06	1763	M34878.m	TRUE	reported	42EOzB	2	TRUE	0.013184	23.52724
8663	rs2311717	A	T	A	T	0.0364	-0.00309	0.6829	0.702579	FALSE	TRUE	FALSE	BFEGyF	0.014969	0.836445	486484	sepsis	TRUE	reported	textfile	0.0075	1.30E-06	1582	M36399.m	TRUE	reported	nhT4FM	2	TRUE	0.014671	23.52507
5676	rs7732078	A	G	A	G	0.0524	0.010199	0.6742	0.666281	FALSE	FALSE	FALSE	AAOSvG	0.014505	0.481959	486484	sepsis	TRUE	reported	textfile	0.0108	1.23E-06	2860	M33197.m	TRUE	reported	aAmcal	2	TRUE	0.008164	23.524
6331	rs1249801	T	C	T	C	0.3027	0.025694	0.0395	0.025204	FALSE	FALSE	FALSE	qLUY00	0.043571	0.555387	486484	sepsis	TRUE	reported	textfile	0.0618	9.79E-07	102	M33610.m	TRUE	reported	8cAJbB	2	TRUE	0.190418	23.52056
379	rs825885	A	G	A	G	-0.0097	0.012369	0.1469	0.200866	FALSE	FALSE	FALSE	tbRL8J	0.017136	0.470404	486484	sepsis	TRUE	reported	textfile	0.002	1.56E-06	7351	M00060.m	TRUE	reported	oOvRKT	2	TRUE	0.00319	23.5161
1428	rs5764174	T	C	T	C	-0.0194	0.01172	0.2445	0.240135	FALSE	FALSE	FALSE	ac6RGB	0.015979	0.463279	486484	sepsis	TRUE	reported	textfile	0.004	9.61E-07	5791	M02342.m	TRUE	reported	dC6Rkp	2	TRUE	0.004045	23.51438
4152	rs4324798	A	G	A	G	0.0354	-0.00259	0.1136	0.117102	FALSE	FALSE	FALSE	CrVaiQ	0.021249	0.902888	486484	sepsis	TRUE	reported	textfile	0.0073	1.08E-06	7361	M32553.m	TRUE	reported	HsKdAl	2	TRUE	0.003184	23.50947
2654	rs1073826	T	C	T	C	0.0354	0.003984	0.1746	0.181542	FALSE	FALSE	FALSE	UtYu7J	0.017712	0.822051	486484	sepsis	TRUE	reported	textfile	0.0073	1.13E-06	5624	M18476.m	TRUE	reported	wJx0A4	2	TRUE	0.004164	23.50749
4206	rs4365292	A	C	A	C	0.0257	-0.0058	0.1924	0.194235	FALSE	FALSE	FALSE	rdKSjG	0.017279	0.737033	486484	sepsis	TRUE	reported	textfile	0.0053	1.51E-06	7322	M32578.m	TRUE	reported	RAzSLK	2	TRUE	0.003201	23.50693
2438	rs7069613	T	C	T	C	0.1271	0.032602	0.9576	0.964891	FALSE	FALSE	FALSE	NBJ2Do	0.03703	0.378641	486484	sepsis	TRUE	reported	textfile	0.0262	1.18E-06	1739	M17799.m	TRUE	reported	Uz0Wgl	2	TRUE	0.013352	23.50654
7404	rs4331333	T	C	T	C	-0.0257	0.015904	0.6194	0.615552	FALSE	FALSE	FALSE	XfpKv0	0.014029	0.256961	486484	sepsis	TRUE	reported	textfile	0.0053	1.16E-06	5561	M34390.m	TRUE	reported	mmOsbK	2	TRUE	0.00421	23.50489
8606	rs1411176	T	C	T	C	-0.0359	0.006276	0.3811	0.381654	FALSE	FALSE	FALSE	oHM5TD	0.014091	0.656056	486484	sepsis	TRUE	reported	textfile	0.0074	1.28E-06	1489	M36131.m	TRUE	reported	Xw6wmg	2	TRUE	0.01556	23.504
2246	rs1731292	A	G	A	G	0.016	0.002418	0.3493	0.348719	FALSE	FALSE	FALSE	zxcPox	0.014339	0.866091	486484	sepsis	TRUE	reported	textfile	0.0033	1.42E-06	6782	M15990.m	TRUE	reported	34Vg8F	2	TRUE	0.003454	23.50087
8285	rs397175	A	G	A	G	0.032	-0.02267	0.1373	0.138466	FALSE	FALSE	FALSE	J6o4xC	0.020209	0.261871	486484	sepsis	TRUE	reported	textfile	0.0066	1.36E-06	6595	M35472.m	TRUE	reported	3XBcu9	2	TRUE	0.003552	23.50068
1431	rs8113773	A	G	A	G	0.016	0.025353	0.5616	0.552873	FALSE	FALSE	FALSE	ac6RGB	0.013848	0.067121	486484	sepsis	TRUE	reported	textfile	0.0033	1.12E-06	5791	M02342.m	TRUE	reported	dC6Rkp	2	TRUE	0.004043	23.49969
3014	rs3741298	T	C	T	C	-0.0325	0.000287	0.782	0.808348	FALSE	FALSE	FALSE	gxupFu	0.017376	0.986819	486484	sepsis	TRUE	reported	textfile	0.0067	1.34E-06	1408	M21188.m	TRUE	reported	gwwXh2	2	TRUE	0.016437	23.49632
48	rs1412972	T	G	T	G	0.0097	-0.0112	0.5006	0.51539	FALSE	FALSE	FALSE	xmGuol	0.013676	0.41271	486484	sepsis	TRUE	reported	textfile	0.002	1.63E-06	1763	M00054.m	TRUE	reported	GW50Ri	2	TRUE	0.013167	23.49582
6637	rs1002384	T	G	T	G	0.0606	-0.06101	0.9859	0.975927	FALSE	FALSE	FALSE	REeQiq	0.044428	0.169705	486484	sepsis	TRUE	reported	textfile	0.0125	1.27E-06	6004	M33892.m	TRUE	reported	cVOa3u	2	TRUE	0.003899	23.49527
7224	rs3749229	T	C	T	C	0.88	-0.00828	0.014	0.096528	FALSE	FALSE	FALSE	i1uJ7T	0.02322	0.72144	486484	sepsis	TRUE	reported	textfile	0.18	1.01E-06	115	M34338.m	TRUE	reported	pi3mKW	2	TRUE	0.172074	23.48556
7440	rs1199720	C	G	C	G	-0.0807	0.011732	0.3709	0.376795	FALSE	TRUE	FALSE	otAubi	0.014099	0.405352	486484	sepsis	TRUE	reported	textfile	0.0166	1.13E-06	314	M34404.m	TRUE	reported	SAZaNF	2	TRUE	0.069998	23.48312
3823	rs1325625	A	G	A	G	-0.0126	0.016287	0.1668	0.159727	FALSE	FALSE	FALSE	tRX2u0	0.018722	0.384347	486484	sepsis	TRUE	reported	textfile	0.0026	8.30E-07	7365	M32348.m	TRUE	reported	ruBgwQ	2	TRUE	0.003179	23.47883
1059	rs6678076	A	G	A	G	-0.0252	-0.0404	0.0339	0.03688	FALSE	FALSE	FALSE	tRtQ2e	0.03665	0.27029	486484	sepsis	TRUE	reported	textfile	0.0052	1.08E-06	7364	M01564.m	TRUE	reported	Tbg0zM	2	TRUE	0.003179	23.47883
1193	rs1295337	A	T	A	T	0.0126	0.007597	0.8162	0.771846	FALSE	TRUE	FALSE	Ckdgh1	0.016391	0.643013	486484	sepsis	TRUE	reported	textfile	0.0026	1.34E-06	7355	M01644.m	TRUE	reported	nKxz7B	2	TRUE	0.003183	23.47882
3665	rs718377	A	G	A	G	-0.0126	0.009017	0.3343	0.333537	FALSE	FALSE	FALSE	fnjup6	0.014515	0.53446	486484	sepsis	TRUE	reported	textfile	0.0026	1.29E-06	7346	M32197.m	TRUE	reported	oQzaMk	2	TRUE	0.003187	23.47881
3668	rs8024	A	C	A	C	0.0126	0.030372	0.2975	0.336218	FALSE	FALSE	FALSE	fnjup6	0.014474	0.035878	486484	sepsis	TRUE	reported	textfile	0.0026	1.58E-06	7346	M32197.m	TRUE	reported	oQzaMk	2	TRUE	0.003187	23.47881
4912	rs7829432	T	C	T	C	0.0063	-0.00741	0.346	0.349934	FALSE	FALSE	FALSE	VqcRGX	0.014423	0.607265	486484	sepsis	TRUE	reported	textfile	0.0013	1.14E-06	7329	M32867.m	TRUE	reported	psuFJy	2	TRUE	0.003194	23.4788
4470	rs295893	T	C	T	C	-0.0126	0.026519	0.8293	0.818439	FALSE	FALSE	FALSE	g3iM2w	0.017812	0.136532	486484	sepsis	TRUE	reported	textfile	0.0026	1.50E-06	7308	M32698.m	TRUE	reported	ZQBn0h	2	TRUE	0.003203	23.47878
3320	rs1046720	A	G	A	G	-0.0126	0.051862	0.9094	0.903476	FALSE	FALSE	FALSE	m66Eav	0.023382	0.026553	486484	sepsis	TRUE	reported	textfile	0.0026	1.77E-06	7303	M27256.m	TRUE	reported	pl2ECU	2	TRUE	0.003206	23.47878
2888	rs4791715	T	G	T	G	-0.0126	-0.01092	0.594	0.57943	FALSE	FALSE	FALSE	mUzn1R	0.013872	0.431338	486484	sepsis	TRUE	reported	textfile	0.0026	1.66E-06	7301	M20675.m	TRUE	reported	XwY04K	2	TRUE	0.003206	23.47877
875	rs7791503	T	C	T	C	0.0126	-0.02534	0.269	0.26897	FALSE	FALSE	FALSE	Vg5OOR	0.0155	0.102124	486484	sepsis	TRUE	reported	textfile	0.0026	9.65E-07	7063	M01361.m	TRUE	reported	XnrS00	2	TRUE	0.003314	23.47856
9075	rs3851552	T	C	T	C	0.0126	-0.0055	0.6232	0.620089	FALSE	FALSE	FALSE	C7PD8T	0.014362	0.701565	486484	sepsis	TRUE	reported	textfile	0.0026	1.47E-06	6942	M38768.m	TRUE	reported	ZA3QM7	2	TRUE	0.003372	23.47844
7482	rs1198147	A	C	A	C	-0.0286	0.024454	0.824	0.831381	FALSE	FALSE	FALSE	jAhmnV	0.018341	0.182435	486484	sepsis	TRUE	reported	textfile	0.0059	1.48E-06	1761	M34419.m	TRUE	reported	F2Tg9L	2	TRUE	0.013168	23.47116
5116	rs2298423	T	G	T	G	0.0761	0.026914	0.9286	0.924511	FALSE	FALSE	FALSE	O6ine1	0.026114	0.302718	486484	sepsis	TRUE	reported	textfile	0.0157	1.24E-06	1751	M33165.m	TRUE	reported	gGsqjW	2	TRUE	0.01324	23.46787
7334	rs1205151	A	G	A	G	0.1668	-0.02897	0.0791	0.091212	FALSE	FALSE	FALSE	mm9ksf	0.024044	0.228265	486484	sepsis	TRUE	reported	textfile	0.0344	1.24E-06	954	M34365.m	TRUE	reported	AVwEqD	2	TRUE	0.024052	23.46193
7025	rs2912035	T	G	T	G	0.0218	0.019303	0.6198	0.606475	FALSE	FALSE	FALSE	zMIkYA	0.014119	0.171569	486484	sepsis	TRUE	reported	textfile	0.0045	1.30E-06	4908	M34245.m	TRUE	reported	itCtz8	2	TRUE	0.004759	23.45908

694	rs1003362	T	C	T	C	0.0126	-0.02076	0.6115	0.648733	FALSE	FALSE	FALSE	H9I3s3	0.014473	0.151496	486484	sepsis	TRUE	reported	textfile	0.0026	1.35E-06	1761	M01299.m	TRUE	reported	kXWyUq	2	TRUE	0.013161	23.45853
4425	rs1051173	C	G	C	G	-0.0189	0.00978	0.8576	0.885417	FALSE	TRUE	FALSE	WGPdpq	0.021469	0.648709	486484	sepsis	TRUE	reported	textfile	0.0039	1.66E-06	1756	M32675.m	TRUE	reported	u0eUAG	2	TRUE	0.013198	23.45846
2403	rs1792131	A	G	A	G	0.1008	-0.0007	0.9141	0.876929	FALSE	FALSE	FALSE	NBJ2Do	0.020902	0.97327	486484	sepsis	TRUE	reported	textfile	0.0208	1.21E-06	1739	M17799.m	TRUE	reported	Uz0Wgl	2	TRUE	0.013325	23.4582
8935	rs9651696	T	C	T	C	-0.0756	-0.00059	0.556	0.538	FALSE	FALSE	FALSE	ns6PV1	0.013768	0.965742	486484	sepsis	TRUE	reported	textfile	0.0156	1.20E-06	1672	M37097.m	TRUE	reported	ZYmYt4	2	TRUE	0.013852	23.45711
5917	rs1746325	T	C	T	C	0.1105	-0.04798	0.0671	0.06123	FALSE	FALSE	FALSE	iGV9aX	0.028582	0.09322	486484	sepsis	TRUE	reported	textfile	0.0228	1.31E-06	1464	M33390.m	TRUE	reported	sLbpnf	2	TRUE	0.015791	23.45639
6087	rs8026775	A	G	A	G	-0.0155	0.013094	0.068	0.063096	FALSE	FALSE	FALSE	P8n0HP	0.028038	0.640504	486484	sepsis	TRUE	reported	textfile	0.0032	1.16E-06	7336	M33442.m	TRUE	reported	cljMTI	2	TRUE	0.003188	23.45552
6922	rs6745020	A	G	A	G	-0.932	0.01223	0.011	0.015581	FALSE	FALSE	FALSE	SvfSvH	0.056033	0.827219	486484	sepsis	TRUE	reported	textfile	0.191	1.06E-06	134	M34109.m	TRUE	reported	7OSbiu	2	TRUE	0.150879	23.45493
6465	rs6930522	A	G	A	G	-0.0509	-0.01912	0.6305	0.635032	FALSE	FALSE	FALSE	yLohrN	0.014222	0.178888	486484	sepsis	TRUE	reported	textfile	0.0105	1.18E-06	1027	M33683.m	TRUE	reported	mvhAZ4	2	TRUE	0.02237	23.45365
7938	rs4536931	C	G	C	G	-0.0494	0.013077	0.0288	0.057238	FALSE	TRUE	FALSE	kFTWXY	0.029411	0.656576	486484	sepsis	TRUE	reported	textfile	0.0102	1.37E-06	6543	M35193.m	TRUE	reported	cjJvJl	2	TRUE	0.003572	23.44881
6394	rs9375094	T	C	T	C	-0.0678	0.010263	0.0286	0.031033	FALSE	FALSE	FALSE	eBEF1t	0.039502	0.795005	486484	sepsis	TRUE	reported	textfile	0.014	1.28E-06	6109	M33638.m	TRUE	reported	CRf2lt	2	TRUE	0.003824	23.44559
8399	rs7152842	A	G	A	G	0.0092	0.000879	0.7508	0.747111	FALSE	FALSE	FALSE	SCzh0A	0.015726	0.955434	486484	sepsis	TRUE	reported	textfile	0.0019	6.13E-07	7348	M35675.m	TRUE	reported	n1SREk	2	TRUE	0.003181	23.4396
5866	rs1253906	T	C	T	C	0.0092	-0.00973	0.3111	0.314766	FALSE	FALSE	FALSE	tqW0ld	0.014697	0.507979	486484	sepsis	TRUE	reported	textfile	0.0019	9.03E-07	7315	M33389.m	TRUE	reported	Sv7EyV	2	TRUE	0.003195	23.43957
8445	rs1100886	A	G	A	G	-0.0092	0.011828	0.4749	0.471499	FALSE	FALSE	FALSE	uMajV6	0.0137	0.387914	486484	sepsis	TRUE	reported	textfile	0.0019	1.53E-06	6604	M35754.m	TRUE	reported	b8QB7S	2	TRUE	0.003538	23.43888
8474	rs7814471	A	G	A	G	-0.0092	0.01263	0.4642	0.452787	FALSE	FALSE	FALSE	uMajV6	0.013994	0.366781	486484	sepsis	TRUE	reported	textfile	0.0019	1.78E-06	6604	M35754.m	TRUE	reported	b8QB7S	2	TRUE	0.003538	23.43888
8477	rs8103504	T	C	T	C	0.0092	0.018348	0.5809	0.577907	FALSE	FALSE	FALSE	uMajV6	0.013849	0.185216	486484	sepsis	TRUE	reported	textfile	0.0019	1.62E-06	6604	M35754.m	TRUE	reported	b8QB7S	2	TRUE	0.003538	23.43888
8884	rs1016145	T	C	T	C	0.0092	-0.0222	0.5665	0.571995	FALSE	FALSE	FALSE	eLhsld	0.013811	0.107935	486484	sepsis	TRUE	reported	textfile	0.0019	2.03E-06	6552	M37058.m	TRUE	reported	RSeWE5	2	TRUE	0.003566	23.43883
3157	rs598236	T	C	T	C	-0.0092	-0.00495	0.7138	0.711145	FALSE	FALSE	FALSE	JiXR2t	0.01516	0.743846	486484	sepsis	TRUE	reported	textfile	0.0019	2.28E-06	6535	M22177.m	TRUE	reported	joi6p6	2	TRUE	0.003575	23.43881
3173	rs8180794	A	G	A	G	0.0092	0.009222	0.4133	0.395009	FALSE	FALSE	FALSE	JiXR2t	0.013974	0.509291	486484	sepsis	TRUE	reported	textfile	0.0019	2.02E-06	6535	M22177.m	TRUE	reported	joi6p6	2	TRUE	0.003575	23.43881
4506	rs1012798	T	C	T	C	0.0092	0.002662	0.4763	0.47959	FALSE	FALSE	FALSE	r0qJaM	0.01383	0.847386	486484	sepsis	TRUE	reported	textfile	0.0019	1.13E-06	6497	M32729.m	TRUE	reported	vYv5FI	2	TRUE	0.003596	23.43877
4536	rs4542981	A	C	A	C	-0.0092	-0.01352	0.6707	0.673525	FALSE	FALSE	FALSE	r0qJaM	0.014558	0.353217	486484	sepsis	TRUE	reported	textfile	0.0019	1.24E-06	6497	M32729.m	TRUE	reported	vYv5FI	2	TRUE	0.003596	23.43877
7721	rs1113071	C	G	C	G	-0.0092	-0.01693	0.4027	0.41224	FALSE	TRUE	FALSE	HyMNYM	0.013892	0.222916	486484	sepsis	TRUE	reported	textfile	0.0019	1.55E-06	5885	M34912.m	TRUE	reported	Tl8Hcw	2	TRUE	0.003968	23.43802
7723	rs1160659	A	C	A	C	-0.0092	0.001726	0.3632	0.374925	FALSE	FALSE	FALSE	HyMNYM	0.014116	0.902687	486484	sepsis	TRUE	reported	textfile	0.0019	1.11E-06	5885	M34912.m	TRUE	reported	Tl8Hcw	2	TRUE	0.003968	23.43802
7751	rs6796650	A	G	A	G	-0.0092	0.00293	0.5569	0.54814	FALSE	FALSE	FALSE	HyMNYM	0.013757	0.831337	486484	sepsis	TRUE	reported	textfile	0.0019	1.17E-06	5885	M34912.m	TRUE	reported	Tl8Hcw	2	TRUE	0.003968	23.43802
7752	rs6834482	T	C	T	C	-0.0092	0.002213	0.6503	0.648276	FALSE	FALSE	FALSE	HyMNYM	0.014412	0.877961	486484	sepsis	TRUE	reported	textfile	0.0019	1.45E-06	5885	M34912.m	TRUE	reported	Tl8Hcw	2	TRUE	0.003968	23.43802
7830	rs2928949	T	C	T	C	0.0092	-0.0335	0.6858	0.667928	FALSE	FALSE	FALSE	h5whIS	0.014547	0.021263	486484	sepsis	TRUE	reported	textfile	0.0019	1.52E-06	4900	M35137.m	TRUE	reported	xVITKF	2	TRUE	0.004762	23.43641
6987	rs1196656	C	G	C	G	0.0305	0.044007	0.8182	0.776378	FALSE	TRUE	FALSE	APOtl6	0.016567	0.007899	486484	sepsis	TRUE	reported	textfile	0.0063	1.31E-06	6987	M34221.m	TRUE	reported	looCDC	2	TRUE	0.003343	23.43118
8422	rs7523522	T	C	T	C	-0.0213	0.02018	0.6548	0.647295	FALSE	FALSE	FALSE	02RdNm	0.014361	0.159985	486484	sepsis	TRUE	reported	textfile	0.0044	1.32E-06	6447	M35678.m	TRUE	reported	BWN5JR	2	TRUE	0.003622	23.42713
8300	rs1716788	T	C	T	C	-0.0852	0.076294	0.9594	0.955801	FALSE	FALSE	FALSE	HELgy7	0.03351	0.0228	486484	sepsis	TRUE	reported	textfile	0.0176	1.30E-06	5035	M35551.m	TRUE	reported	7x7uUn	2	TRUE	0.004633	23.42509
6671	rs1854521	A	G	A	G	0.0213	-0.00941	0.4968	0.493425	FALSE	FALSE	FALSE	6iZSOI	0.013684	0.49155	486484	sepsis	TRUE	reported	textfile	0.0044	1.17E-06	4533	M33901.m	TRUE	reported	ApKaGe	2	TRUE	0.005143	23.42406
405	rs1161093	T	C	T	C	-0.0121	-0.04601	0.0989	0.097501	FALSE	FALSE	FALSE	cTsNj6	0.023009	0.045548	486484	sepsis	TRUE	reported	textfile	0.0025	1.34E-06	7365	M00063.m	TRUE	reported	7KYTrQ	2	TRUE	0.003171	23.41924
6804	rs6977605	A	C	A	C	-0.0242	0.045097	0.9311	0.918515	FALSE	FALSE	FALSE	OZ0I1H	0.024992	0.071154	486484	sepsis	TRUE	reported	textfile	0.005	1.67E-06	7364	M33960.m	TRUE	reported	GlPp4w	2	TRUE	0.003171	23.41924
1089	rs6896132	C	G	C	G	0.0121	-1.51E-05	0.219	0.223159	FALSE	TRUE	FALSE	JLtEiZ	0.016428	0.999267	486484	sepsis	TRUE	reported	textfile	0.0025	1.80E-06	7333	M01572.m	TRUE	reported	TSSe28	2	TRUE	0.003184	23.41921
6001	rs6417039	A	G	A	G	-0.0121	-0.01123	0.1602	0.198813	FALSE	FALSE	FALSE	3KsoXq	0.017643	0.524597	486484	sepsis	TRUE	reported	textfile	0.0025	1.11E-06	7306	M33422.m	TRUE	reported	aNr6Ug	2	TRUE	0.003196	23.41919
1294	rs1328020	A	C	A	C	0.0121	-0.01062	0.805	0.725999	FALSE	FALSE	FALSE	WQdpmo	0.015574	0.495111	486484	sepsis	TRUE	reported	textfile	0.0025	2.02E-06	7136	M01769.m	TRUE	reported	BkptOK	2	TRUE	0.003272	23.41903
8282	rs1728046	T	C	T	C	-0.0242	0.016074	0.2358	0.231247	FALSE	FALSE	FALSE	J6o4xC	0.016233	0.322101	486484	sepsis	TRUE	reported	textfile	0.005	1.31E-06	6595	M35472.m	TRUE	reported	3XBcu9	2	TRUE	0.003539	23.4185
2393	rs1702522	A	T	A	T	-0.0944	-0.03461	0.0832	0.101029	FALSE	TRUE	FALSE	NBJ2Do	0.022724	0.127789	486484	sepsis	TRUE	reported	textfile	0.0195	1.32E-06	1739	M17799.m	TRUE	reported	Uz0Wgl	2	TRUE	0.013297	23.40858
519	rs2456584	A	G	A	G	-0.0179	0.002726	0.5393	0.536706	FALSE	FALSE	FALSE	AZraib	0.013676	0.842017	486484	sepsis	TRUE	reported	textfile	0.0037	1.64E-06	7333	M00577.m	TRUE	reported	2tucEZ	2	TRUE	0.003182	23.39829
5655	rs1241361	T	C	T	C	-0.2541	-0.0207	0.9528	0.955385	FALSE	FALSE	FALSE	mod1fD	0.03551	0.559858	486484	sepsis	TRUE	reported	textfile	0.0525	1.29E-06	1586	M33194.m	TRUE	reported	fSFVUj	2	TRUE	0.014555	23.39606
2114	rs9610113	A	G	A	G	-0.0208	-0.00631	0.3153	0.314188	FALSE	FALSE	FALSE	MN85S3	0.014739	0.668601	486484	sepsis	TRUE	reported	textfile	0.0043	1.25E-06	7574	M15630.m	TRUE	reported	FUih5R	2	TRUE	0.00308	23.39242
5931	rs427619	T	C	T	C	0.3498	-0.0086	0.0263	0.022933	FALSE	FALSE	FALSE	veBhfS	0.045969	0.851576	486484	sepsis	TRUE	reported	textfile	0.0719	1.15E-06	171	M33391.m	TRUE	reported	Gfjy5a	2	TRUE	0.121586	23.39227
6615	rs1188325	C	G	C	G	0.0208	0.001792	0.3339	0.341896	FALSE	TRUE	FALSE	2plv9p	0.014468	0.901403	486484	sepsis	TRUE	reported	textfile	0.0043	1.05E-06	7334	M33884.m	TRUE	reported	AoFcqC	2	TRUE	0.00318	23.39221
3608	rs1927938	T	C	T	C	-0.0237	0.008188	0.154	0.163131	FALSE	FALSE	FALSE	2z5KhH	0.018514	0.658296	486484	sepsis	TRUE	reported	textfile	0.0049	1.39E-06	7263	M31555.m	TRUE	reported	5kcWr2	2	TRUE	0.003211	23.38756
8681	rs7211140	A	G	A	G	0.0358	0.008593	0.4598	0.459005	FALSE	FALSE	FALSE	i4bSdJ	0.01383	0.534391	486484	sepsis	TRUE	reported	textfile	0.0074	1.41E-06	1908	M36468.m	TRUE	reported	qJLuZj	2	TRUE	0.012118	23.38014
2681	rs7568568	T	C	T	C	0.1481	0.014111	0.9251	0.913703	FALSE	FALSE	FALSE	s4UTyf	0.024378	0.562707	486484	sepsis	TRUE	reported	textfile	0.0306	1.31E-06	1040	M18494.m	TRUE	reported	ajPzhg	2	TRUE	0.022027	23.37929
1156	rs1111498	T	C	T	C	-0.0324	-0.00151	0.0439	0.044885	FALSE	FALSE	FALSE	cSBFJ3	0.03299	0.963393	486484	sepsis	TRUE	reported	textfile	0.0067	1.41E-06	7104	M01638.m	TRUE	reported	sUkjpE	2	TRUE	0.003281	23.37858
1631	rs170631	C	G	C	G	-0.0208	0.022556	0.8913	0.902549	FALSE	TRUE	FALSE	n5rDuT	0.022845	0.323471	486484	sepsis	TRUE	reported	textfile	0.0043	1.78E-06	1747	M12067.m	TRUE	reported	ZQLTeR	2	TRUE	0.013217	23.37181
502	rs7482087	T	C	T	C	-0.5162	0.017721	0.0254	0.032948	FALSE	FALSE	FALSE	RtjZPn	0.038287	0.643475	486484	sepsis	TRUE	reported	textfile	0.1064	1.24E-06	283	M00553.m	TRUE	reported	C3Ovsl	2	TRUE	0.076784	23.37075
5671	rs1011894	T	C	T	C	0.1001	0.002468	0.0815	0.109156	FALSE	FALSE	FALSE	AAOSvG	0.02269	0.913371	486484	sepsis	TRUE	reported	textfile	0.0207	1.31E-06	2860	M33197.m	TRUE	reported	aAmcal	2	TRUE	0.00811	23.36811
7500	rs1734645	T	C	T	C	-0.1344	0.081252	0.9542	0.948623	FALSE	FALSE	FALSE	1aXQWs	0.030952	0.008661	486484	sepsis	TRUE	reported	textfile	0.0278	1.30E-06	4452	M34420.m	TRUE	reported	FOqhHW	2	TRUE	0.005223	23.3622
3675	rs1655902	A	G	A	G	-0.0295	-0.00623	0.8197	0.82953	FALSE	FALSE	FALSE	rGHJcj	0.017979	0.728945	486484	sepsis	TRUE	reported	textfile	0.0061	1.29E-06	1760	M32198.m	TRUE	reported	6P1Nno	2	TRUE	0.013114	23.36095
5756	rs1343232	T	C	T	C	-0.0933	0.082006	0.016	0.016692	FALSE	FALSE	FALSE	n16uO2	0.053501	0.125322	486484	sepsis	TRUE	reported	textfile	0.0193	1.27E-06	4409	M33250.m	TRUE	reported	9dhI67	2	TRUE	0.005272	23.35886
3584	rs7165202	T	C	T	C	-0.1564	-0.01827	0.9469	0.952018	FALSE	FALSE	FALSE	F1s6qg	0.031836	0.566061	486484	sepsis	TRUE	reported	textfile	0.0323	1.29E-06	522	M31536.m	TRUE	reported	Zm40GX	2	TRUE	0.042985	23.35615
6	rs7078003	T	C	T	C	0.0087	-0.01979	0.1654	0.178694	FALSE	FALSE	FALSE	0cebDu	0.017848	0.267452	486484	sepsis	TRUE	reported	textfile	0.0018	1.84E-06	7372	M00053.m	TRUE	reported	i8iuxX	2	TRUE	0.003159	23.35477
1328	rs1107596	T	C	T	C	-0.0087	-0.01292	0.6699	0.672887	FALSE	FALSE	FALSE	LDUJbh	0.014609	0.376603	486484	sepsis	TRUE	reported	textfile	0.0018	8.67E-07	7367	M01898.m	TRUE	reported	JRSLeH	2	TRUE	0.003161	23.35477
1331	rs1184806	A	G	A	G	0.0087	-0.00723	0.5861	0.582722	FALSE	FALSE	FALSE	LDUJbh	0.013904	0.603205	486484	sepsis	TRUE	reported	textfile	0.0018	7.14E-07	7367	M01898.m	TRUE	reported	JRSLeH	2	TRUE	0.003161	23.35477
3843	rs597467	T	C	T	C	0.0087	0.005987	0.3784	0.385738	FALSE	FALSE	FALSE	tRX2u0	0.014038	0.669755	486484	sepsis	TRUE	reported	textfile	0.0018	8.06E-07	7365	M32348.m	TRUE	reported	ruBgwQ	2	TRUE	0.003162	23.35477
3850	rs755403	T	C	T	C	0.0087	-0.0035	0.5969	0.596543	FALSE	FALSE	FALSE	tRX2u0	0.014127	0.804278	486484	sepsis	TRUE	reported	textfile	0.0018	9.52E-07	7365	M32348.m	TRUE	reported	ruBgwQ	2	TRUE	0.003162	23.35477
1049	rs2236299	A	G	A	G	0.0087	0.018838	0.3065	0.295546	FALSE	FALSE	FALSE	tRtQ2e	0.015013	0.209545	486484	sepsis	TRUE	reported	textfile	0.0018	1.69E-06	7364	M01564.m	TRUE	reported	Tbg0zM	2	TRUE	0.003162	23.35477
735	rs1573544	T	C	T	C	0.0087	0.02838	0.1484	0.158108	FALSE	FALSE	FALSE	9u0SC8	0.01872	0.129516	486484	sepsis	TRUE	reported	textfile	0.0018	9.64E-07	7363	M01301.m	TRUE	reported	H95uec	2	TRUE	0.003163	23.35477
4017	rs331721	T	C	T	C	0.0087	0.000941	0.7235	0.725919	FALSE	FALSE	FALSE	V9Atld	0.015328	0.95105	486484	sepsis	TRUE	reported	textfile	0.0018	1.85E-06	7363	M32489.m	TRUE	reported	8B8S8a	2	TRUE	0.003163	23.35477
4301	rs382543	T	C	T	C	0.0087	-0.0329	0.2625	0.289315	FALSE	FALSE	FALSE	0tjjPC	0.015153	0.029938	486484	sepsis	TRUE	reported	textfile	0.0018	1.94E-06	7362	M32634.m	TRUE	reported	nJJFkX	2	TRUE	0.003163	23.35476
6878	rs3764387	A	G	A	G	-0.0087	0.002147	0.1754	0.210662	FALSE	FALSE	FALSE	kFWZjs	0.016771	0.898126	486484	sepsis	TRUE	reported	textfile	0.0018	1.61E-06	7362	M34062.m	TRUE	reported	eLONsg	2	TRUE	0.003163	23.35476
1259	rs3762466	T	C	T	C	0.0058	-0.01062	0.6819	0.66897	FALSE	FALSE	FALSE	6RGrj0	0.014523	0.464655	486484	sepsis	TRUE	reported	textfile	0.0012	1.06E-06	7359	M01649.m	TRUE	reported	J0vZCF	2	TRUE	0.003164	23.35476
1739	rs1929672	T	C	T	C	0.0087	-0.02879	0.5201	0.525756	FALSE	FALSE	FALSE	nFLpAP	0.013697	0.035531	486484	sepsis	TRUE	reported	textfile	0.0018	1.15E-06	7356	M12774.m	TRUE	reported	yOSQTj	2	TRUE	0.003166	23.35476
3756	rs3012739	A	G	A	G	0.0116	0.002512	0.4834	0.480064	FALSE	FALSE	FALSE	OZLdls	0.013727	0.854818	486484	sepsis	TRUE	reported	textfile	0.0024	2.13E-06	7356	M32338.m	TRUE	reported	s2itmB	2	TRUE	0.003166	23.35476
1187	rs1009287	T	C	T	C	0.0087	0.008177	0.4173	0.411429	FALSE	FALSE	FALSE	Ckdgh1	0.013907	0.556528	486484	sepsis	TRUE	reported	textfile	0.0018	1.25E-06	7355	M01644.m	TRUE	reported	nKxz7B	2	TRUE	0.003166	23.35476
1220	rs990054	T	C	T	C	-0.0087	-0.00789	0.5955	0.597836	FALSE	FALSE	FALSE	Ckdgh1	0.014059	0.574476	486484	sepsis	TRUE	reported	textfile	0.0018	1.33E-06	7355	M01644.m	TRUE	reported	nKxz7B	2	TRUE	0.003166	23.35476
4044	rs2704668	T	C	T	C	-0.0087	0.013699	0.6202	0.614874	FALSE	FALSE	FALSE	SiYGYU	0.014133	0.332395	486484	sepsis	TRUE	reported	textfile	0.0018	8.77E-07	7355	M32492.m	TRUE	reported	GfQvpU	2	TRUE	0.003166	23.35476
6769	rs3851441	C	G	C	G	-0.0087	-0.02805	0.2195	0.218305	FALSE	TRUE	FALSE	Elf6MQ	0.016569	0.090498	486484	sepsis	TRUE	reported	textfile	0.0018	2.30E-06	7354	M33955.m	TRUE	reported	Lxn4RF	2	TRUE	0.003167	23.35476
785	rs1119363	T	C	T	C	0.0087	0.005601	0.6724	0.673596	FALSE	FALSE	FALSE	dyT79I	0.014706	0.70331	486484	sepsis	TRUE	reported	textfile	0.0018	7.34E-07	7352	M01336.m	TRUE	reported	OemVJ2	2	TRUE	0.003167	23.35476
326	rs6003916	T	C	T	C	0.0087	0.00021	0.8966	0.888786	FALSE	FALSE	FALSE	tbRL8J	0.021752	0.992305	486484	sepsis	TRUE	reported	textfile	0.0018	1.06E-06	7351	M00060.m	TRUE	reported	oOvRKT	2	TRUE	0.003168	23.35476
8382	rs2167730	T	C	T	C	-0.0087	0.035346	0.6541	0.666266	FALSE	FALSE	FALSE	SCzh0A	0.014509	0.014847	486484	sepsis	TRUE	reported	textfile	0.0018	2.32E-06	7348	M35675.m	TRUE	reported	n1SREk	2	TRUE	0.003169	23.35475
8396	rs6564157	T	C	T	C	-0.0087	-0.00362	0.4074	0.440392	FALSE	FALSE	FALSE	SCzh0A	0.013787	0.792635	486484	sepsis	TRUE	reported	textfile	0.0018	2.12E-06	7348	M35675.m	TRUE	reported	n1SREk	2	TRUE	0.003169	23.35475
1227	rs1246605	A	G	A	G	0.0087	-0.00225	0.402	0.422036	FALSE	FALSE	FALSE	V50rWC	0.013856	0.8708	486484	sepsis	TRUE	reported	textfile	0.0018	8.44E-07	7346	M01645.m	TRUE	reported	yN3gpQ	2	TRUE	0.00317	23.35475
3770	rs1165255	T	C	T	C	0.0087	0.024231	0.3265	0.327568	FALSE	FALSE	FALSE	NW1aaR	0.014561	0.096079	486484	sepsis	TRUE	reported	textfile	0.0018	1.52E-06	7344	M32339.m	TRUE	reported	pBPkAB	2	TRUE	0.003171	23.35475
3787	rs651158	A	G	A	G	0.0087	0.011861	0.6393	0.652698	FALSE	FALSE	FALSE	NW1aaR	0.014335	0.408017	486484	sepsis	TRUE	reported	textfile	0.0018	8.12E-07	7344	M32339.m	TRUE	reported	pBPkAB	2	TRUE	0.003171	23.35475
4956	rs282145	T	C	T	C	0.0087	0.002583	0.6353	0.649234	FALSE	FALSE	FALSE	bKmDMa	0.014433	0.857966	486484	sepsis	TRUE	reported	textfile	0.0018	7.18E-07	7344	M32910.m	TRUE	reported	nAuPzO	2	TRUE	0.003171	23.35475
4965	rs4983559	A	G	A	G	-0.0087	0.002233	0.6109	0.613581	FALSE	FALSE	FALSE	bKmDMa	0.014049	0.873717	486484	sepsis	TRUE	reported	textfile	0.0018	1.35E-06	7344	M32910.m	TRUE	reported	nAuPzO	2	TRUE	0.003171	23.35475
3289	rs6475805	T	C	T	C	-0.0087	0.004591	0.514	0.524556	FALSE	FALSE	FALSE	vYWPe9	0.013685	0.737272	486484	sepsis	TRUE	reported	textfile	0.0018	1.81E-06	7340	M25459.m	TRUE	reported	VvJ6Vx	2	TRUE	0.003173	23.35475
4261	rs1474920	T	G	T	G	-0.0174	-0.01186	0.1861	0.199175	FALSE	FALSE	FALSE	ylV4bp	0.017229	0.491179	486484	sepsis	TRUE	reported	textfile	0.0036	1.46E-06	7337	M32632.m	TRUE	reported	hqHJoU	2	TRUE	0.003174	23.35474
6080	rs4129608	A	G	A	G	0.0087	-0.01117	0.8509	0.852255	FALSE	FALSE	FALSE	P8n0HP	0.019298	0.562552	486484	sepsis	TRUE	reported	textfile	0.0018	7.16E-07	7336	M33442.m	TRUE	reported	cljMTI	2	TRUE	0.003174	23.35474
8683	rs1262611	T	C	T	C	-0.0058	0.007228	0.2385	0.241767	FALSE	FALSE	FALSE	X2e8qc	0.016079	0.653043	486484	sepsis	TRUE	reported	textfile	0.0012	9.78E-07	7330	M36515.m	TRUE	reported	OaWQSH	2	TRUE	0.003177	23.35474
3087	rs791623	T	G	T	G	-0.0116	0.013159	0.2041	0.198185	FALSE	FALSE	FALSE	esOP8m	0.017141	0.442681	486484	sepsis	TRUE	reported	textfile	0.0024	1.68E-06	7329	M22116.m	TRUE	reported	fDMh7Z	2	TRUE	0.003177	23.35474
2494	rs1084140	A	G	A	G	-0.0087	-0.00719	0.3963	0.384954	FALSE	FALSE	FALSE	LwUYua	0.01407	0.609203	486484	sepsis	TRUE	reported	textfile	0.0018	2.27E-06	7315	M17945.m	TRUE	reported	SUN8dc	2	TRUE	0.003183	23.35472
2512	rs6033014	T	C	T	C	-0.0087	-0.01189	0.3688	0.377754	FALSE	FALSE	FALSE	LwUYua	0.015435	0.441272	486484	sepsis	TRUE	reported	textfile	0.0018	2.60E-06	7315	M17945.m	TRUE	reported	SUN8dc	2	TRUE	0.003183	23.35472
2514	rs629487	T	G	T	G	-0.0087	-0.00809	0.5697	0.568423	FALSE	FALSE	FALSE	LwUYua	0.013785	0.557481	486484	sepsis	TRUE	reported	textfile	0.0018	2.21E-06	7315	M17945.m	TRUE	reported	SUN8dc	2	TRUE	0.003183	23.35472
2222	rs7283154	T	C	T	C	-0.0087	-0.00203	0.2516	0.248387	FALSE	FALSE	FALSE	Bf6uMK	0.015828	0.898013	486484	sepsis	TRUE	reported	textfile	0.0018	2.32E-06	7308	M15778.m	TRUE	reported	jowM2N	2	TRUE	0.003186	23.35472
2225	rs8008040	T	C	T	C	-0.0087	-0.01379	0.7276	0.710754	FALSE	FALSE	FALSE	Bf6uMK	0.015067	0.359956	486484	sepsis	TRUE	reported	textfile	0.0018	2.06E-06	7308	M15778.m	TRUE	reported	jowM2N	2	TRUE	0.003186	23.35472
6139	rs398462	T	C	T	C	0.0087	0.004245	0.5177	0.547671	FALSE	FALSE	FALSE	MiSJly	0.013804	0.758426	486484	sepsis	TRUE	reported	textfile	0.0018	1.39E-06	7307	M33477.m	TRUE	reported	WwL1hq	2	TRUE	0.003187	23.35472
5992	rs2758886	A	G	A	G	0.0087	-0.00511	0.2863	0.29755	FALSE	FALSE	FALSE	3KsoXq	0.014917	0.731885	486484	sepsis	TRUE	reported	textfile	0.0018	7.46E-07	7306	M33422.m	TRUE	reported	aNr6Ug	2	TRUE	0.003187	23.35472
5994	rs2874279	A	G	A	G	-0.0087	0.019356	0.7386	0.73899	FALSE	FALSE	FALSE	3KsoXq	0.015613	0.215075	486484	sepsis	TRUE	reported	textfile	0.0018	1.02E-06	7306	M33422.m	TRUE	reported	aNr6Ug	2	TRUE	0.003187	23.35472
3328	rs1323688	T	C	T	C	0.0087	0.00248	0.2268	0.223705	FALSE	FALSE	FALSE	m66Eav	0.016435	0.880077	486484	sepsis	TRUE	reported	textfile	0.0018	6.90E-07	7303	M27256.m	TRUE	reported	pl2ECU	2	TRUE	0.003189	23.35471
3336	rs2876814	T	G	T	G	-0.0087	0.007501	0.185	0.18583	FALSE	FALSE	FALSE	m66Eav	0.017861	0.674488	486484	sepsis	TRUE	reported	textfile	0.0018	1.08E-06	7303	M27256.m	TRUE	reported	pl2ECU	2	TRUE	0.003189	23.35471
3252	rs1081787	T	C	T	C	0.0116	0.007741	0.5666	0.55299	FALSE	FALSE	FALSE	ZNPBqT	0.013799	0.574827	486484	sepsis	TRUE	reported	textfile	0.0024	1.46E-06	7256	M24074.m	TRUE	reported	8pPtOI	2	TRUE	0.003209	23.35467
6266	rs2278908	T	C	T	C	0.0087	0.001037	0.359	0.374158	FALSE	FALSE	FALSE	MOonP9	0.014254	0.942013	486484	sepsis	TRUE	reported	textfile	0.0018	2.55E-06	7066	M33515.m	TRUE	reported	226GuP	2	TRUE	0.003295	23.3545
6276	rs4669966	A	G	A	G	0.0087	-0.0064	0.5646	0.565276	FALSE	FALSE	FALSE	MOonP9	0.013881	0.644932	486484	sepsis	TRUE	reported	textfile	0.0018	1.86E-06	7066	M33515.m	TRUE	reported	226GuP	2	TRUE	0.003295	23.3545
6292	rs9972361	A	G	A	G	-0.0087	-0.00094	0.3351	0.340635	FALSE	FALSE	FALSE	MOonP9	0.014446	0.947896	486484	sepsis	TRUE	reported	textfile	0.0018	2.32E-06	7066	M33515.m	TRUE	reported	226GuP	2	TRUE	0.003295	23.3545
1455	rs1001373	A	G	A	G	0.0087	0.006744	0.6839	0.670447	FALSE	FALSE	FALSE	pkb876	0.014596	0.644067	486484	sepsis	TRUE	reported	textfile	0.0018	7.90E-07	7037	M02734.m	TRUE	reported	0LCMVI	2	TRUE	0.003309	23.35447
5592	rs9389954	T	C	T	C	0.119	-0.02248	0.8466	0.824599	FALSE	FALSE	FALSE	q32d4E	0.018017	0.212214	486484	sepsis	TRUE	reported	textfile	0.0246	1.30E-06	1017	M33178.m	TRUE	reported	cS3BwT	2	TRUE	0.022492	23.3544
8833	rs1049465	T	G	T	G	-0.0087	-0.00446	0.8351	0.834707	FALSE	FALSE	FALSE	XlHeTV	0.018405	0.808697	486484	sepsis	TRUE	reported	textfile	0.0018	2.04E-06	6940	M36808.m	TRUE	reported	37Vi2D	2	TRUE	0.003355	23.35438
8150	rs6477329	A	T	A	T	0.0087	-0.02831	0.6832	0.653466	FALSE	TRUE	FALSE	lv3pAQ	0.014399	0.049298	486484	sepsis	TRUE	reported	textfile	0.0018	9.49E-07	6920	M35327.m	TRUE	reported	3Vsglp	2	TRUE	0.003365	23.35436
7317	rs973634	T	C	T	C	0.0087	-0.00819	0.505	0.523651	FALSE	FALSE	FALSE	Vcz4EA	0.013709	0.550356	486484	sepsis	TRUE	reported	textfile	0.0018	1.00E-06	6755	M34359.m	TRUE	reported	aUSo2J	2	TRUE	0.003446	23.35419
8896	rs1254802	C	G	C	G	-0.0087	0.005398	0.4	0.400794	FALSE	TRUE	FALSE	eLhsld	0.013956	0.698927	486484	sepsis	TRUE	reported	textfile	0.0018	2.68E-06	6552	M37058.m	TRUE	reported	RSeWE5	2	TRUE	0.003553	23.35398
2327	rs504899	T	C	T	C	-0.0232	0.015955	0.0734	0.082263	FALSE	FALSE	FALSE	IZXwpR	0.024868	0.521136	486484	sepsis	TRUE	reported	textfile	0.0048	1.42E-06	6549	M16822.m	TRUE	reported	Zx9k2V	2	TRUE	0.003554	23.35398
3301	rs1266549	A	G	A	G	0.029	0.006749	0.9441	0.935015	FALSE	FALSE	FALSE	nqmBts	0.027932	0.809071	486484	sepsis	TRUE	reported	textfile	0.006	1.28E-06	6388	M25599.m	TRUE	reported	VkwSh1	2	TRUE	0.003644	23.3538
8097	rs1165327	T	G	T	G	-0.0087	-0.00099	0.721	0.703574	FALSE	FALSE	FALSE	nNbXBS	0.014961	0.947309	486484	sepsis	TRUE	reported	textfile	0.0018	1.05E-06	5768	M35326.m	TRUE	reported	UNDorn	2	TRUE	0.004034	23.35301
8135	rs9951062	A	G	A	G	0.0087	-0.04004	0.7122	0.712471	FALSE	FALSE	FALSE	nNbXBS	0.015112	0.008059	486484	sepsis	TRUE	reported	textfile	0.0018	1.05E-06	5768	M35326.m	TRUE	reported	UNDorn	2	TRUE	0.004034	23.35301
3070	rs333063	T	C	T	C	0.029	-0.01927	0.0879	0.118535	FALSE	FALSE	FALSE	e3izea	0.021128	0.361735	486484	sepsis	TRUE	reported	textfile	0.006	1.49E-06	5676	M22032.m	TRUE	reported	zODKFI	2	TRUE	0.004099	23.35288
7016	rs1051273	C	G	C	G	0.0261	0.013019	0.8047	0.788791	FALSE	TRUE	FALSE	zMIkYA	0.016852	0.439796	486484	sepsis	TRUE	reported	textfile	0.0054	1.15E-06	4908	M34245.m	TRUE	reported	itCtz8	2	TRUE	0.004737	23.35159
8832	rs8031716	T	C	T	C	-0.0145	0.029369	0.555	0.549138	FALSE	FALSE	FALSE	zuPm7L	0.014005	0.035993	486484	sepsis	TRUE	reported	textfile	0.003	1.45E-06	4317	M36802.m	TRUE	reported	cn9d0n	2	TRUE	0.005382	23.35029
6907	rs1270253	A	G	A	G	0.925	0.037429	0.986	0.910412	FALSE	FALSE	FALSE	SvfSvH	0.023947	0.118053	486484	sepsis	TRUE	reported	textfile	0.19	1.13E-06	134	M34109.m	TRUE	reported	7OSbiu	2	TRUE	0.150294	23.34777
7579	rs1002490	A	C	A	C	0.0377	0.000168	0.1598	0.149506	FALSE	FALSE	FALSE	SbjFMF	0.019229	0.993011	486484	sepsis	TRUE	reported	textfile	0.0078	1.30E-06	3239	M34513.m	TRUE	reported	HVYiKM	2	TRUE	0.007161	23.34669
3292	rs7160179	C	G	C	G	-0.0701	-0.02947	0.9807	0.849335	FALSE	TRUE	FALSE	vYWPe9	0.019116	0.123117	486484	sepsis	TRUE	reported	textfile	0.0145	1.28E-06	1756	M25459.m	TRUE	reported	VvJ6Vx	2	TRUE	0.013135	23.3456
1679	rs7961079	A	G	A	G	0.0527	-0.02283	0.6241	0.620876	FALSE	FALSE	FALSE	IIFBGY	0.01409	0.105208	486484	sepsis	TRUE	reported	textfile	0.0109	1.46E-06	1543	M12261.m	TRUE	reported	F5VIan	2	TRUE	0.014924	23.3456
2449	rs7755387	T	G	T	G	-0.1252	0.013825	0.0397	0.060277	FALSE	FALSE	FALSE	NBJ2Do	0.028774	0.630896	486484	sepsis	TRUE	reported	textfile	0.0259	1.30E-06	1739	M17799.m	TRUE	reported	Uz0Wgl	2	TRUE	0.013259	23.34046
488	rs8000457	T	C	T	C	-0.2093	-0.00193	0.9661	0.928496	FALSE	FALSE	FALSE	ScJVI8	0.02658	0.942199	486484	sepsis	TRUE	reported	textfile	0.0433	1.35E-06	1766	M00542.m	TRUE	reported	Sa1L04	2	TRUE	0.013058	23.33837
7195	rs1157359	A	G	A	G	-0.3896	-0.11479	0.0204	0.020513	FALSE	FALSE	FALSE	i1uJ7T	0.048845	0.018767	486484	sepsis	TRUE	reported	textfile	0.0805	1.32E-06	535	M34338.m	TRUE	reported	pi3mKW	2	TRUE	0.041945	23.33563
4552	rs1278781	T	C	T	C	-0.0372	0.000476	0.814	0.804802	FALSE	FALSE	FALSE	KfoRxi	0.01732	0.978063	486484	sepsis	TRUE	reported	textfile	0.0077	1.23E-06	6000	M32735.m	TRUE	reported	UDRXAp	2	TRUE	0.003875	23.33241
3357	rs1714188	C	G	C	G	-0.0314	-0.02403	0.9716	0.970256	FALSE	TRUE	FALSE	LY4Gus	0.040261	0.550623	486484	sepsis	TRUE	reported	textfile	0.0065	1.13E-06	7268	M27273.m	TRUE	reported	QKlTBx	2	TRUE	0.003201	23.32991
1147	rs1711837	T	C	T	C	0.0343	-0.00542	0.2298	0.227243	FALSE	FALSE	FALSE	QtY5Hc	0.016335	0.740258	486484	sepsis	TRUE	reported	textfile	0.0071	1.44E-06	5162	M01605.m	TRUE	reported	Q3iDQX	2	TRUE	0.004501	23.32938
6917	rs2582067	A	G	A	G	0.91	0.036271	0.977	0.845912	FALSE	FALSE	FALSE	SvfSvH	0.020167	0.072101	486484	sepsis	TRUE	reported	textfile	0.187	1.14E-06	134	M34109.m	TRUE	reported	7OSbiu	2	TRUE	0.150183	23.32753
4204	rs1884917	C	G	C	G	-0.0256	0.008635	0.2335	0.22644	FALSE	TRUE	FALSE	rdKSjG	0.016412	0.598786	486484	sepsis	TRUE	reported	textfile	0.0053	1.51E-06	7322	M32578.m	TRUE	reported	RAzSLK	2	TRUE	0.003176	23.32435
1826	rs7184992	T	C	T	C	0.0807	-0.00908	0.3003	0.305354	FALSE	FALSE	FALSE	Yub5KV	0.014833	0.540469	486484	sepsis	TRUE	reported	textfile	0.0167	1.31E-06	1522	M15335.m	TRUE	reported	mcnlNa	2	TRUE	0.015111	23.32078
8087	rs385748	T	C	T	C	-0.3443	-0.01231	0.9894	0.98673	FALSE	FALSE	FALSE	z65cF2	0.060141	0.837783	486484	sepsis	TRUE	reported	textfile	0.0711	1.28E-06	349	M35322.m	TRUE	reported	3GUSWk	2	TRUE	0.06296	23.31519
921	rs6975921	A	G	A	G	0.0343	0.042632	0.9095	0.919455	FALSE	FALSE	FALSE	rVMAPf	0.025196	0.090646	486484	sepsis	TRUE	reported	textfile	0.0071	1.25E-06	1766	M01493.m	TRUE	reported	siVqDi	2	TRUE	0.013043	23.31199
8367	rs1173142	C	G	C	G	0.0169	0.01694	0.9058	0.901716	FALSE	TRUE	FALSE	SCzh0A	0.022959	0.460616	486484	sepsis	TRUE	reported	textfile	0.0035	1.69E-06	7348	M35675.m	TRUE	reported	n1SREk	2	TRUE	0.003163	23.30876
2410	rs2473138	A	G	A	G	0.1053	0.042869	0.9395	0.909382	FALSE	FALSE	FALSE	NBJ2Do	0.023794	0.071591	486484	sepsis	TRUE	reported	textfile	0.0218	1.42E-06	1739	M17799.m	TRUE	reported	Uz0Wgl	2	TRUE	0.013239	23.30473
1548	rs1202639	T	C	T	C	-0.014	-0.00793	0.8893	0.876115	FALSE	FALSE	FALSE	Ke0sNh	0.020731	0.702061	486484	sepsis	TRUE	reported	textfile	0.0029	9.43E-07	7799	M10642.m	TRUE	reported	ZINwVb	2	TRUE	0.002979	23.29961
927	rs1229418	T	C	T	C	0.014	-0.01447	0.0987	0.131913	FALSE	FALSE	FALSE	tv99UN	0.02066	0.483562	486484	sepsis	TRUE	reported	textfile	0.0029	1.07E-06	7354	M01494.m	TRUE	reported	osDclp	2	TRUE	0.003159	23.29925
8724	rs7499206	T	C	T	C	0.014	0.015751	0.29	0.290819	FALSE	FALSE	FALSE	I0G0I1	0.015032	0.294726	486484	sepsis	TRUE	reported	textfile	0.0029	1.40E-06	6464	M36553.m	TRUE	reported	lBltb0	2	TRUE	0.003592	23.29838
7703	rs512251	A	T	A	T	0.028	0.032478	0.8615	0.856318	FALSE	TRUE	FALSE	2eJIPM	0.019502	0.09583	486484	sepsis	TRUE	reported	textfile	0.0058	1.44E-06	6381	M34761.m	TRUE	reported	druKtT	2	TRUE	0.003639	23.29828
6651	rs1746802	T	C	T	C	0.014	-0.01321	0.4198	0.429349	FALSE	FALSE	FALSE	REeQiq	0.013921	0.342763	486484	sepsis	TRUE	reported	textfile	0.0029	1.49E-06	6004	M33892.m	TRUE	reported	cVOa3u	2	TRUE	0.003867	23.29783
6653	rs1995777	T	C	T	C	-0.014	0.011246	0.5594	0.564228	FALSE	FALSE	FALSE	REeQiq	0.014	0.421834	486484	sepsis	TRUE	reported	textfile	0.0029	1.37E-06	6004	M33892.m	TRUE	reported	cVOa3u	2	TRUE	0.003867	23.29783
504	rs9614985	A	G	A	G	-0.0855	-0.00908	0.4177	0.41821	FALSE	FALSE	FALSE	RtjZPn	0.013929	0.51435	486484	sepsis	TRUE	reported	textfile	0.0177	1.28E-06	1233	M00553.m	TRUE	reported	C3Ovsl	2	TRUE	0.018573	23.29596
2176	rs1040090	A	G	A	G	-0.0251	0.011887	0.3089	0.31521	FALSE	FALSE	FALSE	UokbxN	0.014712	0.419085	486484	sepsis	TRUE	reported	textfile	0.0052	1.62E-06	7357	M15753.m	TRUE	reported	lUGKBi	2	TRUE	0.003157	23.29285
8631	rs1712143	A	G	A	G	0.1236	0.005122	0.9807	0.980218	FALSE	FALSE	FALSE	4cZjpQ	0.051405	0.920631	486484	sepsis	TRUE	reported	textfile	0.0256	1.36E-06	2400	M36230.m	TRUE	reported	TPUeNs	2	TRUE	0.009619	23.29137
6901	rs6911308	T	G	T	G	-0.0251	-0.01818	0.5796	0.611689	FALSE	FALSE	FALSE	MN2lbE	0.014067	0.196211	486484	sepsis	TRUE	reported	textfile	0.0052	1.34E-06	4961	M34106.m	TRUE	reported	q4zXEz	2	TRUE	0.004675	23.28979
6929	rs9465476	T	C	T	C	-0.43	0.02276	0.14	0.141731	FALSE	FALSE	FALSE	SvfSvH	0.020604	0.269307	486484	sepsis	TRUE	reported	textfile	0.0885	1.19E-06	148	M34109.m	TRUE	reported	7OSbiu	2	TRUE	0.137567	23.2885
1786	rs1293763	T	C	T	C	-0.0082	0.011888	0.5397	0.538974	FALSE	FALSE	FALSE	JEcCRZ	0.013722	0.386295	486484	sepsis	TRUE	reported	textfile	0.0017	2.28E-06	7368	M15140.m	TRUE	reported	xYCzKP	2	TRUE	0.003148	23.26012
1807	rs739744	T	C	T	C	-0.0082	-0.00399	0.5529	0.557867	FALSE	FALSE	FALSE	JEcCRZ	0.013818	0.772898	486484	sepsis	TRUE	reported	textfile	0.0017	1.88E-06	7368	M15140.m	TRUE	reported	xYCzKP	2	TRUE	0.003148	23.26012
1814	rs886125	A	G	A	G	0.0082	-0.00273	0.463	0.458778	FALSE	FALSE	FALSE	JEcCRZ	0.01372	0.841995	486484	sepsis	TRUE	reported	textfile	0.0017	1.86E-06	7368	M15140.m	TRUE	reported	xYCzKP	2	TRUE	0.003148	23.26012
413	rs1718278	T	C	T	C	-0.0082	-0.00252	0.253	0.265386	FALSE	FALSE	FALSE	cTsNj6	0.015442	0.870141	486484	sepsis	TRUE	reported	textfile	0.0017	2.10E-06	7365	M00063.m	TRUE	reported	7KYTrQ	2	TRUE	0.003149	23.26012
730	rs1165980	C	G	C	G	-0.0082	0.002361	0.1964	0.230471	FALSE	TRUE	FALSE	9u0SC8	0.016393	0.885474	486484	sepsis	TRUE	reported	textfile	0.0017	2.16E-06	7363	M01301.m	TRUE	reported	H95uec	2	TRUE	0.00315	23.26012
743	rs7583413	T	C	T	C	0.0082	0.00997	0.8245	0.806094	FALSE	FALSE	FALSE	9u0SC8	0.017389	0.566394	486484	sepsis	TRUE	reported	textfile	0.0017	2.62E-06	7363	M01301.m	TRUE	reported	H95uec	2	TRUE	0.00315	23.26012
8439	rs732647	A	G	A	G	0.0246	0.006803	0.0598	0.054856	FALSE	FALSE	FALSE	Nvt7pl	0.029974	0.820458	486484	sepsis	TRUE	reported	textfile	0.0051	1.49E-06	7358	M35718.m	TRUE	reported	WkA5Gq	2	TRUE	0.003152	23.26011
1601	rs1447842	A	G	A	G	0.0082	-0.03386	0.5745	0.548543	FALSE	FALSE	FALSE	zP3C2v	0.013749	0.013786	486484	sepsis	TRUE	reported	textfile	0.0017	2.85E-06	7356	M12035.m	TRUE	reported	1Pooe3	2	TRUE	0.003153	23.26011
839	rs6733405	A	G	A	G	-0.0082	0.023526	0.3867	0.377833	FALSE	FALSE	FALSE	2qgDgB	0.01416	0.096614	486484	sepsis	TRUE	reported	textfile	0.0017	2.42E-06	7355	M01358.m	TRUE	reported	asmXq0	2	TRUE	0.003153	23.26011
2600	rs2201453	A	G	A	G	0.0082	0.001634	0.438	0.451009	FALSE	FALSE	FALSE	ruvJh6	0.013744	0.905383	486484	sepsis	TRUE	reported	textfile	0.0017	1.14E-06	7354	M18369.m	TRUE	reported	GFmwGk	2	TRUE	0.003154	23.26011
2603	rs2573124	A	C	A	C	-0.0082	0.002799	0.4579	0.455777	FALSE	FALSE	FALSE	ruvJh6	0.0138	0.83924	486484	sepsis	TRUE	reported	textfile	0.0017	1.13E-06	7354	M18369.m	TRUE	reported	GFmwGk	2	TRUE	0.003154	23.26011
786	rs1122247	T	G	T	G	-0.0082	-0.03811	0.6036	0.607528	FALSE	FALSE	FALSE	dyT79I	0.014052	0.006689	486484	sepsis	TRUE	reported	textfile	0.0017	1.42E-06	7352	M01336.m	TRUE	reported	OemVJ2	2	TRUE	0.003155	23.26011
260	rs1710797	T	C	T	C	-0.0082	-0.02037	0.1068	0.124341	FALSE	FALSE	FALSE	tbRL8J	0.020859	0.328783	486484	sepsis	TRUE	reported	textfile	0.0017	1.50E-06	7351	M00060.m	TRUE	reported	oOvRKT	2	TRUE	0.003155	23.26011
3697	rs1303342	T	C	T	C	0.0082	-0.01187	0.4434	0.430508	FALSE	FALSE	FALSE	agPgtF	0.013802	0.389836	486484	sepsis	TRUE	reported	textfile	0.0017	1.92E-06	7349	M32315.m	TRUE	reported	hMRjcV	2	TRUE	0.003156	23.2601
3706	rs4241779	A	G	A	G	0.0082	0.00912	0.5776	0.564816	FALSE	FALSE	FALSE	agPgtF	0.013763	0.507547	486484	sepsis	TRUE	reported	textfile	0.0017	2.83E-06	7349	M32315.m	TRUE	reported	hMRjcV	2	TRUE	0.003156	23.2601
3708	rs580333	A	G	A	G	0.0082	0.009084	0.4934	0.498021	FALSE	FALSE	FALSE	agPgtF	0.013705	0.507443	486484	sepsis	TRUE	reported	textfile	0.0017	1.81E-06	7349	M32315.m	TRUE	reported	hMRjcV	2	TRUE	0.003156	23.2601
3784	rs4980554	A	G	A	G	0.0082	-0.01428	0.4561	0.455962	FALSE	FALSE	FALSE	NW1aaR	0.013744	0.298791	486484	sepsis	TRUE	reported	textfile	0.0017	2.87E-06	7344	M32339.m	TRUE	reported	pBPkAB	2	TRUE	0.003158	23.2601
1380	rs1782177	T	G	T	G	-0.0082	-0.00741	0.3485	0.35014	FALSE	FALSE	FALSE	KrWhws	0.014348	0.605687	486484	sepsis	TRUE	reported	textfile	0.0017	1.92E-06	7325	M02132.m	TRUE	reported	7pzpfK	2	TRUE	0.003166	23.26008
2855	rs6814228	T	C	T	C	0.0082	0.016301	0.7738	0.770285	FALSE	FALSE	FALSE	Y5qWBm	0.016249	0.315778	486484	sepsis	TRUE	reported	textfile	0.0017	1.99E-06	7325	M20489.m	TRUE	reported	f2g9Su	2	TRUE	0.003166	23.26008
4580	rs1274287	C	G	C	G	-0.0082	-0.02424	0.3923	0.398566	FALSE	TRUE	FALSE	Pzplqu	0.014116	0.085949	486484	sepsis	TRUE	reported	textfile	0.0017	2.70E-06	7318	M32740.m	TRUE	reported	FjEBS5	2	TRUE	0.003169	23.26008
4610	rs7151149	T	C	T	C	-0.0082	0.007597	0.2898	0.298182	FALSE	FALSE	FALSE	Pzplqu	0.014922	0.610699	486484	sepsis	TRUE	reported	textfile	0.0017	3.10E-06	7318	M32740.m	TRUE	reported	FjEBS5	2	TRUE	0.003169	23.26008
1700	rs7914384	T	C	T	C	-0.0082	0.003589	0.1806	0.182776	FALSE	FALSE	FALSE	f01en0	0.017707	0.839391	486484	sepsis	TRUE	reported	textfile	0.0017	1.85E-06	7314	M12593.m	TRUE	reported	EAsM6U	2	TRUE	0.003171	23.26007
6000	rs5751928	A	G	A	G	-0.0082	0.00404	0.4847	0.503127	FALSE	FALSE	FALSE	3KsoXq	0.013682	0.767786	486484	sepsis	TRUE	reported	textfile	0.0017	2.31E-06	7306	M33422.m	TRUE	reported	aNr6Ug	2	TRUE	0.003174	23.26007
2719	rs1211978	A	G	A	G	-0.0082	-0.00311	0.4048	0.405187	FALSE	FALSE	FALSE	H4yqSK	0.013954	0.823566	486484	sepsis	TRUE	reported	textfile	0.0017	2.38E-06	7182	M19362.m	TRUE	reported	w2PDEO	2	TRUE	0.003229	23.25996
1313	rs7661472	A	C	A	C	-0.0082	-0.00497	0.4922	0.496622	FALSE	FALSE	FALSE	WQdpmo	0.013722	0.71732	486484	sepsis	TRUE	reported	textfile	0.0017	1.38E-06	7136	M01769.m	TRUE	reported	BkptOK	2	TRUE	0.00325	23.25992
1641	rs686321	T	C	T	C	0.0082	-0.01357	0.8232	0.811404	FALSE	FALSE	FALSE	n5rDuT	0.017491	0.43799	486484	sepsis	TRUE	reported	textfile	0.0017	2.14E-06	7081	M12067.m	TRUE	reported	ZQLTeR	2	TRUE	0.003275	23.25986
8140	rs1212622	A	G	A	G	0.0082	-0.01599	0.2807	0.305799	FALSE	FALSE	FALSE	lv3pAQ	0.014906	0.283372	486484	sepsis	TRUE	reported	textfile	0.0017	3.01E-06	6920	M35327.m	TRUE	reported	3Vsglp	2	TRUE	0.003351	23.25971
8149	rs450852	A	G	A	G	0.0082	-0.00687	0.3243	0.330242	FALSE	FALSE	FALSE	lv3pAQ	0.014626	0.63875	486484	sepsis	TRUE	reported	textfile	0.0017	1.91E-06	6920	M35327.m	TRUE	reported	3Vsglp	2	TRUE	0.003351	23.25971
2766	rs606114	T	C	T	C	-0.0164	-0.00443	0.1601	0.158333	FALSE	FALSE	FALSE	H6txH3	0.018695	0.812685	486484	sepsis	TRUE	reported	textfile	0.0034	1.48E-06	6678	M19368.m	TRUE	reported	b094NF	2	TRUE	0.003472	23.25947
8003	rs1171256	T	C	T	C	0.0164	-0.02309	0.311	0.318836	FALSE	FALSE	FALSE	Zj5bZH	0.014674	0.115518	486484	sepsis	TRUE	reported	textfile	0.0034	1.50E-06	6538	M35257.m	TRUE	reported	exssAk	2	TRUE	0.003546	23.25932
8095	rs1118890	A	G	A	G	0.0082	-0.03167	0.6306	0.625393	FALSE	FALSE	FALSE	nNbXBS	0.014118	0.024886	486484	sepsis	TRUE	reported	textfile	0.0017	2.77E-06	5768	M35326.m	TRUE	reported	UNDorn	2	TRUE	0.004018	23.25837
8129	rs7327332	T	G	T	G	-0.0082	0.002407	0.6628	0.665672	FALSE	FALSE	FALSE	nNbXBS	0.014491	0.868094	486484	sepsis	TRUE	reported	textfile	0.0017	2.73E-06	5768	M35326.m	TRUE	reported	UNDorn	2	TRUE	0.004018	23.25837
515	rs1242613	A	G	A	G	0.0246	0.012249	0.1446	0.165478	FALSE	FALSE	FALSE	cbwEt9	0.018724	0.513009	486484	sepsis	TRUE	reported	textfile	0.0051	1.68E-06	5502	M00575.m	TRUE	reported	hWSEOI	2	TRUE	0.004211	23.25798
6617	rs2123517	T	C	T	C	0.1158	0.008307	0.023	0.016419	FALSE	FALSE	FALSE	2plv9p	0.054852	0.879619	486484	sepsis	TRUE	reported	textfile	0.024	1.46E-06	1766	M33884.m	TRUE	reported	AoFcqC	2	TRUE	0.013011	23.25426
2616	rs3846406	A	C	A	C	0.0299	0.024282	0.4896	0.497589	FALSE	FALSE	FALSE	OiiDPr	0.013674	0.075758	486484	sepsis	TRUE	reported	textfile	0.0062	1.46E-06	7322	M18392.m	TRUE	reported	ScUw6o	2	TRUE	0.003166	23.25093
2504	rs1690918	T	C	T	C	0.0217	-0.01595	0.0502	0.051568	FALSE	FALSE	FALSE	LwUYua	0.031253	0.609811	486484	sepsis	TRUE	reported	textfile	0.0045	1.39E-06	7315	M17945.m	TRUE	reported	SUN8dc	2	TRUE	0.003169	23.24747
2165	rs1184794	T	C	T	C	-0.0217	0.012118	0.3031	0.31906	FALSE	FALSE	FALSE	XCBNPg	0.014742	0.411063	486484	sepsis	TRUE	reported	textfile	0.0045	1.54E-06	5829	M15749.m	TRUE	reported	dKBewD	2	TRUE	0.003973	23.24585
7587	rs2816252	T	C	T	C	0.082	-0.00902	0.066	0.223581	FALSE	FALSE	FALSE	SbjFMF	0.016721	0.589652	486484	sepsis	TRUE	reported	textfile	0.017	1.41E-06	2168	M34513.m	TRUE	reported	HVYiKM	2	TRUE	0.010618	23.24497
6163	rs1168780	T	C	T	C	-0.0217	-0.01786	0.3033	0.308677	FALSE	FALSE	FALSE	O4Ty2l	0.014813	0.228064	486484	sepsis	TRUE	reported	textfile	0.0045	1.38E-06	5107	M33488.m	TRUE	reported	RGtF84	2	TRUE	0.004533	23.24472
1502	rs1694829	A	T	A	T	-0.041	0.023388	0.0694	0.067807	FALSE	TRUE	FALSE	rzkxmh	0.027436	0.393952	486484	sepsis	TRUE	reported	textfile	0.0085	1.35E-06	1745	M03127.m	TRUE	reported	3lrzhA	2	TRUE	0.013158	23.23977
1453	rs849039	T	G	T	G	-0.0135	-0.00682	0.779	0.74431	FALSE	FALSE	FALSE	1M4gqj	0.015774	0.665511	486484	sepsis	TRUE	reported	textfile	0.0028	1.41E-06	7226	M02730.m	TRUE	reported	RWyYEM	2	TRUE	0.003207	23.23974
8196	rs1709857	A	C	A	C	-0.027	-0.00845	0.9522	0.939871	FALSE	FALSE	FALSE	iLlpho	0.028726	0.768716	486484	sepsis	TRUE	reported	textfile	0.0056	1.54E-06	7007	M35431.m	TRUE	reported	AAZ5ge	2	TRUE	0.003307	23.23954
7511	rs961928	T	C	T	C	0.0487	0.02226	0.3628	0.365674	FALSE	FALSE	FALSE	1aXQWs	0.014466	0.123872	486484	sepsis	TRUE	reported	textfile	0.0101	1.58E-06	4452	M34420.m	TRUE	reported	FOqhHW	2	TRUE	0.005195	23.23914
3308	rs1752600	A	G	A	G	0.0135	-0.01746	0.2579	0.257554	FALSE	FALSE	FALSE	nqmBts	0.015616	0.263565	486484	sepsis	TRUE	reported	textfile	0.0028	1.04E-06	6388	M25599.m	TRUE	reported	VkwSh1	2	TRUE	0.003626	23.2389
7266	rs3093135	A	T	A	T	0.0569	0.007201	0.8288	0.836231	FALSE	TRUE	FALSE	P5Xm4S	0.018466	0.696551	486484	sepsis	TRUE	reported	textfile	0.0118	1.29E-06	3491	M34350.m	TRUE	reported	YU4x0J	2	TRUE	0.006616	23.23869
3074	rs7397059	T	C	T	C	-0.0135	-0.00443	0.5732	0.576755	FALSE	FALSE	FALSE	e3izea	0.013844	0.748868	486484	sepsis	TRUE	reported	textfile	0.0028	1.22E-06	5676	M22032.m	TRUE	reported	zODKFI	2	TRUE	0.004079	23.23798
3680	rs3843502	A	G	A	G	-0.0323	-0.02028	0.0286	0.029436	FALSE	FALSE	FALSE	rGHJcj	0.040573	0.617184	486484	sepsis	TRUE	reported	textfile	0.0067	1.63E-06	7356	M32198.m	TRUE	reported	6P1Nno	2	TRUE	0.00315	23.23471
6945	rs7971484	T	G	T	G	-0.0323	0.001484	0.1909	0.182891	FALSE	FALSE	FALSE	oNv0vf	0.017709	0.933217	486484	sepsis	TRUE	reported	textfile	0.0067	1.54E-06	5581	M34112.m	TRUE	reported	I0pe0N	2	TRUE	0.004147	23.23271
1430	rs7868774	A	G	A	G	-0.0188	-0.01543	0.2709	0.266021	FALSE	FALSE	FALSE	ac6RGB	0.015484	0.318878	486484	sepsis	TRUE	reported	textfile	0.0039	1.78E-06	5791	M02342.m	TRUE	reported	dC6Rkp	2	TRUE	0.003997	23.22932
7599	rs1244181	A	T	A	T	0.2952	0.008809	0.0435	0.040301	FALSE	TRUE	FALSE	PlYt5g	0.035175	0.802261	486484	sepsis	TRUE	reported	textfile	0.061	1.31E-06	241	M34516.m	TRUE	reported	jX41tf	2	TRUE	0.088569	23.2249
5647	rs1705421	A	C	A	C	0.0829	0.009855	0.3934	0.465315	FALSE	FALSE	FALSE	BFsMtm	0.014197	0.487563	486484	sepsis	TRUE	reported	textfile	0.0172	1.54E-06	5716	M33192.m	TRUE	reported	NAJN3D	2	TRUE	0.004048	23.22203
3514	rs1277997	A	G	A	G	0.0347	0.013656	0.9388	0.942136	FALSE	FALSE	FALSE	hxuqls	0.029781	0.646544	486484	sepsis	TRUE	reported	textfile	0.0072	1.26E-06	7336	M27738.m	TRUE	reported	aueeZs	2	TRUE	0.003156	23.22071
4434	rs2101330	A	G	A	G	0.0135	0.017578	0.5266	0.546421	FALSE	FALSE	FALSE	WGPdpq	0.013787	0.202317	486484	sepsis	TRUE	reported	textfile	0.0028	1.08E-06	1756	M32675.m	TRUE	reported	u0eUAG	2	TRUE	0.013065	23.2197
2765	rs4778640	A	G	A	G	0.04	-0.0375	0.9704	0.966054	FALSE	FALSE	FALSE	H6txH3	0.037912	0.322603	486484	sepsis	TRUE	reported	textfile	0.0083	1.39E-06	6678	M19368.m	TRUE	reported	b094NF	2	TRUE	0.003466	23.21848
8675	rs151316	T	C	T	C	0.082	0.007881	0.183	0.198388	FALSE	FALSE	FALSE	i4bSdJ	0.01876	0.674412	486484	sepsis	TRUE	reported	textfile	0.017	1.41E-06	932	M36468.m	TRUE	reported	qJLuZj	2	TRUE	0.024356	23.21651
8583	rs1698451	A	G	A	G	0.202	0.038652	0.9848	0.982134	FALSE	FALSE	FALSE	iuJmRL	0.051359	0.451692	486484	sepsis	TRUE	reported	textfile	0.0419	1.41E-06	1750	M36103.m	TRUE	reported	jfJbVw	2	TRUE	0.013107	23.2155
8204	rs6764615	C	G	C	G	0.0159	0.002522	0.8298	0.838613	FALSE	TRUE	FALSE	iLlpho	0.018648	0.892421	486484	sepsis	TRUE	reported	textfile	0.0033	2.17E-06	7007	M35431.m	TRUE	reported	AAZ5ge	2	TRUE	0.003302	23.20825
4699	rs1156411	A	G	A	G	0.0395	-0.00633	0.9236	0.916566	FALSE	FALSE	FALSE	WlwKaF	0.024666	0.797461	486484	sepsis	TRUE	reported	textfile	0.0082	1.58E-06	6712	M32759.m	TRUE	reported	Ub4yxH	2	TRUE	0.003445	23.19728
4549	rs1019890	A	G	A	G	-0.0342	-0.00616	0.295	0.29926	FALSE	FALSE	FALSE	KfoRxi	0.014991	0.681367	486484	sepsis	TRUE	reported	textfile	0.0071	1.61E-06	6000	M32735.m	TRUE	reported	UDRXAp	2	TRUE	0.003852	23.1948
6234	rs1421106	T	G	T	G	-0.0236	0.028598	0.9228	0.923042	FALSE	FALSE	FALSE	vL52Yb	0.026074	0.272732	486484	sepsis	TRUE	reported	textfile	0.0049	1.79E-06	7269	M33510.m	TRUE	reported	JIO9VX	2	TRUE	0.003181	23.19062
6698	rs6074527	T	C	T	C	0.0419	0.003955	0.7046	0.694152	FALSE	FALSE	FALSE	AcRp2F	0.014836	0.789769	486484	sepsis	TRUE	reported	textfile	0.0087	1.43E-06	6756	M33935.m	TRUE	reported	FQCKJy	2	TRUE	0.003421	23.18788
3472	rs1189197	A	G	A	G	-0.0183	-0.00434	0.3109	0.312081	FALSE	FALSE	FALSE	w7juXi	0.014845	0.769917	486484	sepsis	TRUE	reported	textfile	0.0038	1.35E-06	6830	M27722.m	TRUE	reported	xzWmif	2	TRUE	0.003384	23.18504
3939	rs8088457	A	G	A	G	0.0496	-0.01091	0.0323	0.029937	FALSE	FALSE	FALSE	LlOaWH	0.040247	0.786243	486484	sepsis	TRUE	reported	textfile	0.0103	1.59E-06	7355	M32418.m	TRUE	reported	AtHOVw	2	TRUE	0.003143	23.18306
5781	rs3751035	A	G	A	G	0.0802	0.013832	0.1812	0.199306	FALSE	FALSE	FALSE	eT2We0	0.017102	0.418634	486484	sepsis	TRUE	reported	textfile	0.0166	1.40E-06	292	M33353.m	TRUE	reported	uZx90m	2	TRUE	0.07402	23.18183
7030	rs807741	T	G	T	G	-0.0805	-0.01532	0.0918	0.085035	FALSE	FALSE	FALSE	zMIkYA	0.024468	0.531248	486484	sepsis	TRUE	reported	textfile	0.0167	1.38E-06	788	M34245.m	TRUE	reported	itCtz8	2	TRUE	0.028643	23.17689
6759	rs1235119	A	T	A	T	-0.013	0.017475	0.8949	0.875258	FALSE	TRUE	FALSE	Elf6MQ	0.021243	0.410727	486484	sepsis	TRUE	reported	textfile	0.0027	1.08E-06	7354	M33955.m	TRUE	reported	Lxn4RF	2	TRUE	0.003142	23.17614
6998	rs1095669	T	C	T	C	0.013	0.009307	0.4838	0.490025	FALSE	FALSE	FALSE	ePdfmg	0.014176	0.511466	486484	sepsis	TRUE	reported	textfile	0.0027	1.43E-06	7347	M34244.m	TRUE	reported	l9nzfX	2	TRUE	0.003145	23.17613
3661	rs6691915	A	G	A	G	0.013	0.00848	0.2814	0.289688	FALSE	FALSE	FALSE	fnjup6	0.015129	0.575142	486484	sepsis	TRUE	reported	textfile	0.0027	8.95E-07	7346	M32197.m	TRUE	reported	oQzaMk	2	TRUE	0.003146	23.17613
7457	rs4463295	A	G	A	G	0.013	0.002458	0.5733	0.568537	FALSE	FALSE	FALSE	7ubZow	0.013824	0.858885	486484	sepsis	TRUE	reported	textfile	0.0027	1.60E-06	7344	M34407.m	TRUE	reported	LRsXN2	2	TRUE	0.003147	23.17613

4268	rs2416366	A	C	A	C	0.013	0.002302	0.4649	0.461764	FALSE	FALSE	FALSE	ylV4bp	0.013764	0.86719	486484	sepsis	TRUE	reported	textfile	0.0027	1.17E-06	7337	M32632.m	TRUE	reported	hqHJoU	2	TRUE	0.00315	23.17612
8327	rs2606621	A	G	A	G	-0.013	0.018934	0.2991	0.289492	FALSE	FALSE	FALSE	o0GzT1	0.015082	0.209336	486484	sepsis	TRUE	reported	textfile	0.0027	9.59E-07	7317	M35631.m	TRUE	reported	G1XFx0	2	TRUE	0.003158	23.17611
2874	rs1172236	T	C	T	C	0.013	-0.02476	0.3602	0.347574	FALSE	FALSE	FALSE	mUzn1R	0.01436	0.084629	486484	sepsis	TRUE	reported	textfile	0.0027	1.03E-06	7301	M20675.m	TRUE	reported	XwY04K	2	TRUE	0.003165	23.17609
7954	rs1095161	C	G	C	G	-0.013	0.017636	0.3777	0.384899	FALSE	TRUE	FALSE	zb8m4a	0.014083	0.210471	486484	sepsis	TRUE	reported	textfile	0.0027	1.02E-06	7261	M35253.m	TRUE	reported	tHnnnS	2	TRUE	0.003183	23.17606
3402	rs1124295	A	G	A	G	-0.013	0.008983	0.5528	0.560065	FALSE	FALSE	FALSE	VU62LG	0.013772	0.514212	486484	sepsis	TRUE	reported	textfile	0.0027	1.67E-06	7180	M27513.m	TRUE	reported	0didOx	2	TRUE	0.003218	23.17598
3415	rs4324578	C	G	C	G	0.013	0.000229	0.6112	0.605783	FALSE	TRUE	FALSE	VU62LG	0.014036	0.986995	486484	sepsis	TRUE	reported	textfile	0.0027	1.99E-06	7180	M27513.m	TRUE	reported	0didOx	2	TRUE	0.003218	23.17598
5729	rs203846	T	C	T	C	-0.013	-0.00867	0.6027	0.603101	FALSE	FALSE	FALSE	YDQaWa	0.014015	0.536043	486484	sepsis	TRUE	reported	textfile	0.0027	1.63E-06	7063	M33228.m	TRUE	reported	BmYg8r	2	TRUE	0.003271	23.17588
7086	rs1168101	A	G	A	G	-0.013	-0.00648	0.3403	0.358014	FALSE	FALSE	FALSE	pxNICA	0.014385	0.652561	486484	sepsis	TRUE	reported	textfile	0.0027	1.27E-06	6994	M34306.m	TRUE	reported	xk5b9M	2	TRUE	0.003304	23.17581
7091	rs1299655	C	G	C	G	-0.013	-0.02744	0.7649	0.744053	FALSE	TRUE	FALSE	pxNICA	0.01569	0.080325	486484	sepsis	TRUE	reported	textfile	0.0027	2.15E-06	6994	M34306.m	TRUE	reported	xk5b9M	2	TRUE	0.003304	23.17581
3006	rs1456959	T	C	T	C	0.013	0.009793	0.3972	0.389449	FALSE	FALSE	FALSE	gxupFu	0.014085	0.486891	486484	sepsis	TRUE	reported	textfile	0.0027	1.30E-06	6556	M21188.m	TRUE	reported	gwwXh2	2	TRUE	0.003524	23.17537
3239	rs1929842	C	G	C	G	0.013	-0.02018	0.2049	0.221426	FALSE	TRUE	FALSE	COSeSD	0.016469	0.220446	486484	sepsis	TRUE	reported	textfile	0.0027	1.94E-06	6517	M22649.m	TRUE	reported	mnH8z2	2	TRUE	0.003545	23.17533
3306	rs1309556	T	C	T	C	0.013	0.007761	0.7452	0.747687	FALSE	FALSE	FALSE	nqmBts	0.01578	0.622835	486484	sepsis	TRUE	reported	textfile	0.0027	2.07E-06	6388	M25599.m	TRUE	reported	VkwSh1	2	TRUE	0.003616	23.17518
3095	rs1821685	T	C	T	C	0.013	-0.01837	0.3252	0.354864	FALSE	FALSE	FALSE	mr0awv	0.014278	0.198362	486484	sepsis	TRUE	reported	textfile	0.0027	1.71E-06	5702	M22130.m	TRUE	reported	Y2TfWQ	2	TRUE	0.004049	23.17431
2881	rs1687134	T	C	T	C	0.0289	-0.01018	0.7206	0.722755	FALSE	FALSE	FALSE	mUzn1R	0.015368	0.507862	486484	sepsis	TRUE	reported	textfile	0.006	1.58E-06	1747	M20675.m	TRUE	reported	XwY04K	2	TRUE	0.013106	23.17372
4358	rs1003737	T	C	T	C	0.013	0.013676	0.5465	0.551035	FALSE	FALSE	FALSE	SPhl52	0.013842	0.323141	486484	sepsis	TRUE	reported	textfile	0.0027	1.77E-06	5211	M32651.m	TRUE	reported	V0Bnpj	2	TRUE	0.004429	23.17354
3209	rs5768188	A	G	A	G	0.0467	-0.1102	0.9868	0.985395	FALSE	FALSE	FALSE	UfLkBq	0.058202	0.058311	486484	sepsis	TRUE	reported	textfile	0.0097	1.44E-06	7329	M22481.m	TRUE	reported	kWMdiT	2	TRUE	0.003153	23.17244
2486	rs6900835	T	C	T	C	-0.0284	-0.01842	0.2163	0.220472	FALSE	FALSE	FALSE	SrfyMU	0.016469	0.263252	486484	sepsis	TRUE	reported	textfile	0.0059	1.44E-06	7226	M17807.m	TRUE	reported	0zrJsu	2	TRUE	0.003196	23.16394
6916	rs2255960	C	G	C	G	-0.931	-0.04098	0.01	0.020363	FALSE	TRUE	FALSE	SvfSvH	0.049284	0.40568	486484	sepsis	TRUE	reported	textfile	0.192	1.24E-06	134	M34109.m	TRUE	reported	7OSbiu	2	TRUE	0.149273	23.16147
5765	rs7238314	T	G	T	G	-0.0775	0.055049	0.9792	0.980493	FALSE	FALSE	FALSE	n16uO2	0.049342	0.264568	486484	sepsis	TRUE	reported	textfile	0.0161	1.49E-06	4409	M33250.m	TRUE	reported	9dhl67	2	TRUE	0.005228	23.16086
3633	rs9824080	T	C	T	C	0.0438	0.007884	0.1602	0.168163	FALSE	FALSE	FALSE	gq4lSJ	0.018325	0.667029	486484	sepsis	TRUE	reported	textfile	0.0091	1.62E-06	7338	M31591.m	TRUE	reported	wjwl4J	2	TRUE	0.003147	23.16045
2424	rs463183	A	G	A	G	-0.1164	0.058963	0.0833	0.064157	FALSE	FALSE	FALSE	NBJ2Do	0.027871	0.034383	486484	sepsis	TRUE	reported	textfile	0.0232	4.98E-07	25	M17799.m	TRUE	reported	Uz0Wgl	2	TRUE	0.501721	23.15889
3108	rs2243617	A	G	A	G	-0.0284	0.009623	0.8747	0.864067	FALSE	FALSE	FALSE	ApMZhy	0.020178	0.633426	486484	sepsis	TRUE	reported	textfile	0.0059	1.62E-06	3950	M22138.m	TRUE	reported	LiJliF	2	TRUE	0.005832	23.15862
1551	rs1338027	T	C	T	C	-0.0077	0.02601	0.4149	0.41192	FALSE	FALSE	FALSE	Ke0sNh	0.013865	0.060664	486484	sepsis	TRUE	reported	textfile	0.0016	2.90E-06	7799	M10642.m	TRUE	reported	ZINwVb	2	TRUE	0.002961	23.15422
715	rs7641634	A	G	A	G	-0.0077	0.006871	0.266	0.277966	FALSE	FALSE	FALSE	H9l3s3	0.01525	0.652307	486484	sepsis	TRUE	reported	textfile	0.0016	2.56E-06	7358	M01299.m	TRUE	reported	kXWyUq	2	TRUE	0.003138	23.15386
2014	rs6709816	A	G	A	G	-0.0077	0.019355	0.1494	0.143992	FALSE	FALSE	FALSE	v2KE89	0.019422	0.318988	486484	sepsis	TRUE	reported	textfile	0.0016	1.86E-06	7349	M15500.m	TRUE	reported	to2t7H	2	TRUE	0.003142	23.15385
2081	rs3786284	A	G	A	G	0.0077	-0.05144	0.1481	0.135998	FALSE	FALSE	FALSE	NPVH5j	0.020075	0.010391	486484	sepsis	TRUE	reported	textfile	0.0016	2.04E-06	7310	M15506.m	TRUE	reported	r7r3vM	2	TRUE	0.003158	23.15382
4813	rs550214	A	G	A	G	-0.0154	0.002153	0.2053	0.213764	FALSE	FALSE	FALSE	bJwLV5	0.016754	0.897756	486484	sepsis	TRUE	reported	textfile	0.0032	1.78E-06	7063	M32814.m	TRUE	reported	qte0qp	2	TRUE	0.003268	23.1536
5637	rs6426944	A	G	A	G	-0.0568	0.010494	0.2621	0.26855	FALSE	FALSE	FALSE	U0d0ue	0.015477	0.497747	486484	sepsis	TRUE	reported	textfile	0.0118	1.55E-06	2616	M33190.m	TRUE	reported	FoODGF	2	TRUE	0.008779	23.15264
4892	rs2714775	T	C	T	C	-0.1526	0.024341	0.9695	0.962144	FALSE	FALSE	FALSE	ocaywK	0.035914	0.497927	486484	sepsis	TRUE	reported	textfile	0.0317	1.52E-06	1988	M32863.m	TRUE	reported	WH6KGr	2	TRUE	0.011522	23.15013
5945	rs7512146	T	G	T	G	0.0332	0.015242	0.5108	0.507569	FALSE	FALSE	FALSE	yepzrL	0.01367	0.264854	486484	sepsis	TRUE	reported	textfile	0.0069	1.38E-06	3890	M33408.m	TRUE	reported	SY4CoS	2	TRUE	0.005916	23.13954
6067	rs959046	C	G	C	G	0.0178	-0.01177	0.4	0.396603	FALSE	TRUE	FALSE	P4BuMD	0.013968	0.399247	486484	sepsis	TRUE	reported	textfile	0.0037	1.12E-06	7365	M33441.m	TRUE	reported	kqrc1d	2	TRUE	0.003133	23.13762
4789	rs1017275	T	C	T	C	0.0433	0.017406	0.0898	0.089705	FALSE	FALSE	FALSE	dwreVz	0.024376	0.475193	486484	sepsis	TRUE	reported	textfile	0.009	1.50E-06	4311	M32802.m	TRUE	reported	7YCayZ	2	TRUE	0.005341	23.13605
609	rs1183742	A	G	A	G	0.0385	-0.00413	0.088	0.085967	FALSE	FALSE	FALSE	BYGzq4	0.024394	0.865636	486484	sepsis	TRUE	reported	textfile	0.008	1.52E-06	1766	M01110.m	TRUE	reported	oZiXqP	2	TRUE	0.012945	23.13393
5754	rs1121140	A	C	A	C	0.0279	0.000292	0.7964	0.791664	FALSE	FALSE	FALSE	n16uO2	0.016905	0.986238	486484	sepsis	TRUE	reported	textfile	0.0058	1.45E-06	4409	M33250.m	TRUE	reported	9dhl67	2	TRUE	0.005221	23.12892
4723	rs1117449	A	G	A	G	0.0433	0.003638	0.3526	0.365142	FALSE	FALSE	FALSE	fz63r4	0.014215	0.798018	486484	sepsis	TRUE	reported	textfile	0.009	1.42E-06	2537	M32762.m	TRUE	reported	kgxlBo	2	TRUE	0.009041	23.12854
5918	rs1773598	T	C	T	C	-0.1949	-0.00816	0.9801	0.984327	FALSE	FALSE	FALSE	iGV9aX	0.056937	0.886088	486484	sepsis	TRUE	reported	textfile	0.0405	1.51E-06	1464	M33390.m	TRUE	reported	sLbpnf	2	TRUE	0.015572	23.12703
3421	rs1051897	T	C	T	C	0.0505	0.010415	0.11	0.120119	FALSE	FALSE	FALSE	HHQf8w	0.021055	0.620855	486484	sepsis	TRUE	reported	textfile	0.0105	1.35E-06	5673	M27531.m	TRUE	reported	3vTluK	2	TRUE	0.004061	23.12336
7159	rs898219	T	G	T	G	-0.0664	-0.00115	0.6202	0.627375	FALSE	FALSE	FALSE	PnBT0M	0.014128	0.935081	486484	sepsis	TRUE	reported	textfile	0.0138	1.44E-06	1612	M34327.m	TRUE	reported	VUt4Q7	2	TRUE	0.014159	23.12271
5793	rs7799018	T	C	T	C	-0.0433	0.008406	0.427	0.435208	FALSE	FALSE	FALSE	lUkr5t	0.013801	0.542468	486484	sepsis	TRUE	reported	textfile	0.009	1.62E-06	1863	M33359.m	TRUE	reported	DjliwS	2	TRUE	0.012272	23.12194
8780	rs1104436	C	G	C	G	0.0982	-0.01003	0.9662	0.961071	FALSE	TRUE	FALSE	4bkfvN	0.036002	0.780509	486484	sepsis	TRUE	reported	textfile	0.0204	1.41E-06	922	M36738.m	TRUE	reported	xcqyX2	2	TRUE	0.024516	23.12169
6169	rs3020376	A	G	A	G	0.0544	0.004442	0.7234	0.740932	FALSE	FALSE	FALSE	O4Ty2l	0.015855	0.77936	486484	sepsis	TRUE	reported	textfile	0.0113	1.33E-06	834	M33488.m	TRUE	reported	RGtF84	2	TRUE	0.027038	23.12055
5657	rs1683963	A	G	A	G	0.0957	-0.02808	0.9359	0.928494	FALSE	FALSE	FALSE	mod1fD	0.026635	0.291849	486484	sepsis	TRUE	reported	textfile	0.0199	1.51E-06	5064	M33194.m	TRUE	reported	fSFVUj	2	TRUE	0.004546	23.11778
1134	rs233340	A	G	A	G	-0.0125	-0.0378	0.0707	0.072744	FALSE	FALSE	FALSE	nlqUuV	0.0263	0.150599	486484	sepsis	TRUE	reported	textfile	0.0026	2.54E-06	7371	M01604.m	TRUE	reported	Uro9nC	2	TRUE	0.003126	23.10763
9028	rs1432288	T	G	T	G	0.0125	-0.01482	0.1421	0.13885	FALSE	FALSE	FALSE	df0MCM	0.019888	0.456051	486484	sepsis	TRUE	reported	textfile	0.0026	1.18E-06	7366	M37506.m	TRUE	reported	iUfr4u	2	TRUE	0.003128	23.10763
4063	rs1126502	A	G	A	G	0.0125	-0.00837	0.4733	0.462971	FALSE	FALSE	FALSE	TyHtdh	0.01369	0.541083	486484	sepsis	TRUE	reported	textfile	0.0026	1.85E-06	7358	M32497.m	TRUE	reported	H3KmLR	2	TRUE	0.003131	23.10762
8434	rs1752812	A	G	A	G	-0.0125	0.007621	0.755	0.742849	FALSE	FALSE	FALSE	Nvt7pl	0.015715	0.627704	486484	sepsis	TRUE	reported	textfile	0.0026	2.11E-06	7358	M35718.m	TRUE	reported	WkA5Gq	2	TRUE	0.003131	23.10762
6971	rs1260333	A	G	A	G	0.0125	-0.00459	0.4472	0.43667	FALSE	FALSE	FALSE	ZlvYs5	0.013775	0.739069	486484	sepsis	TRUE	reported	textfile	0.0026	1.26E-06	7351	M34214.m	TRUE	reported	qU57S1	2	TRUE	0.003134	23.10762
6973	rs1725094	C	G	C	G	-0.0125	0.003748	0.4039	0.411501	FALSE	TRUE	FALSE	ZlvYs5	0.013955	0.788274	486484	sepsis	TRUE	reported	textfile	0.0026	1.81E-06	7351	M34214.m	TRUE	reported	qU57S1	2	TRUE	0.003134	23.10762
2913	rs6106307	T	C	T	C	0.0125	-0.01289	0.7398	0.734684	FALSE	FALSE	FALSE	FBDEQi	0.015559	0.407512	486484	sepsis	TRUE	reported	textfile	0.0026	8.85E-07	7327	M20699.m	TRUE	reported	nvNIlp	2	TRUE	0.003145	23.1076
4574	rs1105470	A	G	A	G	-0.0125	0.001876	0.1397	0.151055	FALSE	FALSE	FALSE	Pzplqu	0.019156	0.921986	486484	sepsis	TRUE	reported	textfile	0.0026	1.32E-06	7318	M32740.m	TRUE	reported	FjEBS5	2	TRUE	0.003149	23.10759
8338	rs9854668	A	C	A	C	0.0125	0.021942	0.702	0.690944	FALSE	FALSE	FALSE	o0GzT1	0.014793	0.138011	486484	sepsis	TRUE	reported	textfile	0.0026	2.52E-06	7317	M35631.m	TRUE	reported	G1XFx0	2	TRUE	0.003149	23.10759
4478	rs9809318	T	C	T	C	0.0125	-0.02492	0.7743	0.77694	FALSE	FALSE	FALSE	g3iM2w	0.016515	0.131381	486484	sepsis	TRUE	reported	textfile	0.0026	9.84E-07	7308	M32698.m	TRUE	reported	ZQBn0h	2	TRUE	0.003153	23.10758
918	rs1342408	A	G	A	G	-0.0125	0.014997	0.1735	0.184515	FALSE	FALSE	FALSE	rVMAPf	0.017676	0.3962	486484	sepsis	TRUE	reported	textfile	0.0026	1.80E-06	7307	M01493.m	TRUE	reported	siVqDi	2	TRUE	0.003153	23.10758
3321	rs1048952	A	G	A	G	0.0125	0.031991	0.0955	0.108434	FALSE	FALSE	FALSE	m66Eav	0.022018	0.146243	486484	sepsis	TRUE	reported	textfile	0.0026	1.47E-06	7303	M27256.m	TRUE	reported	pl2ECU	2	TRUE	0.003155	23.10758
3027	rs1325526	T	C	T	C	-0.0125	0.012007	0.2518	0.243204	FALSE	FALSE	FALSE	FJZEYz	0.015936	0.451167	486484	sepsis	TRUE	reported	textfile	0.0026	1.96E-06	7284	M21630.m	TRUE	reported	s6Pshg	2	TRUE	0.003163	23.10756
6240	rs303031	A	G	A	G	-0.0125	-0.02994	0.5388	0.543967	FALSE	FALSE	FALSE	vL52Yb	0.013844	0.030559	486484	sepsis	TRUE	reported	textfile	0.0026	1.72E-06	7269	M33510.m	TRUE	reported	JlO9VX	2	TRUE	0.00317	23.10755
6244	rs6055874	T	C	T	C	0.0125	-0.00542	0.4882	0.487597	FALSE	FALSE	FALSE	vL52Yb	0.013733	0.693047	486484	sepsis	TRUE	reported	textfile	0.0026	2.15E-06	7269	M33510.m	TRUE	reported	JlO9VX	2	TRUE	0.00317	23.10755
6245	rs7327971	A	G	A	G	-0.0125	0.02129	0.3546	0.372212	FALSE	FALSE	FALSE	vL52Yb	0.014531	0.142863	486484	sepsis	TRUE	reported	textfile	0.0026	2.25E-06	7269	M33510.m	TRUE	reported	JlO9VX	2	TRUE	0.00317	23.10755
8240	rs7633111	T	C	T	C	-0.0125	-0.01888	0.5766	0.55449	FALSE	FALSE	FALSE	4YAWhA	0.013829	0.172068	486484	sepsis	TRUE	reported	textfile	0.0026	1.93E-06	7256	M35439.m	TRUE	reported	bSSC4Z	2	TRUE	0.003175	23.10753
955	rs2173615	T	G	T	G	0.0125	0.005787	0.3153	0.30454	FALSE	FALSE	FALSE	tWoDq4	0.014826	0.696319	486484	sepsis	TRUE	reported	textfile	0.0026	1.69E-06	7160	M01508.m	TRUE	reported	6ltDw0	2	TRUE	0.003218	23.10745
1632	rs1709456	A	T	A	T	-0.0125	0.029261	0.9357	0.935824	FALSE	TRUE	FALSE	n5rDuT	0.028123	0.298114	486484	sepsis	TRUE	reported	textfile	0.0026	1.88E-06	7081	M12067.m	TRUE	reported	ZQLTeR	2	TRUE	0.003254	23.10738
5808	rs310104	T	C	T	C	-0.0558	0.030041	0.0428	0.053585	FALSE	FALSE	FALSE	JE7L71	0.030635	0.326787	486484	sepsis	TRUE	reported	textfile	0.0116	1.52E-06	1338	M33364.m	TRUE	reported	FdLiEs	2	TRUE	0.017	23.10483
2286	rs6810964	A	G	A	G	0.0125	-0.00579	0.5495	0.527871	FALSE	FALSE	FALSE	mRULgB	0.013692	0.672181	486484	sepsis	TRUE	reported	textfile	0.0026	2.08E-06	4689	M16816.m	TRUE	reported	05lFsX	2	TRUE	0.004905	23.10405
2298	rs7084948	T	C	T	C	-0.0322	0.071571	0.067	0.061922	FALSE	FALSE	FALSE	43Yh3h	0.028465	0.011925	486484	sepsis	TRUE	reported	textfile	0.0067	1.54E-06	7055	M16818.m	TRUE	reported	beR4IT	2	TRUE	0.003263	23.0908
8682	rs802524	T	C	T	C	0.0875	0.001673	0.9577	0.954122	FALSE	FALSE	FALSE	i4bSdJ	0.0329	0.959454	486484	sepsis	TRUE	reported	textfile	0.0182	1.47E-06	1908	M36468.m	TRUE	reported	qJLuZj	2	TRUE	0.011969	23.08968
1819	rs1696775	T	C	T	C	0.0495	-0.01002	0.3037	0.297349	FALSE	FALSE	FALSE	Yub5KV	0.014956	0.502763	486484	sepsis	TRUE	reported	textfile	0.0103	1.43E-06	5606	M15335.m	TRUE	reported	mcnINa	2	TRUE	0.004103	23.08772
5026	rs1749046	A	G	A	G	-0.0173	-0.03284	0.0364	0.039015	FALSE	FALSE	FALSE	BNnOvt	0.035597	0.356276	486484	sepsis	TRUE	reported	textfile	0.0036	1.22E-06	7362	M33132.m	TRUE	reported	rHcCPG	2	TRUE	0.003127	23.08709
8305	rs7326215	T	C	T	C	0.0495	-0.00925	0.8718	0.876758	FALSE	FALSE	FALSE	HELgy7	0.02079	0.656376	486484	sepsis	TRUE	reported	textfile	0.0103	1.49E-06	5035	M35551.m	TRUE	reported	7x7uUn	2	TRUE	0.004566	23.08678
777	rs726900	T	G	T	G	0.0173	0.001252	0.8497	0.844698	FALSE	FALSE	FALSE	tYvbNc	0.018838	0.947003	486484	sepsis	TRUE	reported	textfile	0.0036	1.59E-06	6946	M01303.m	TRUE	reported	hT3gsE	2	TRUE	0.003314	23.08671
7478	rs6466344	C	G	C	G	-0.0173	-0.02412	0.2679	0.269322	FALSE	TRUE	FALSE	SqfdcK	0.015517	0.120066	486484	sepsis	TRUE	reported	textfile	0.0036	1.22E-06	6929	M34416.m	TRUE	reported	sRGRIK	2	TRUE	0.003322	23.0867
8764	rs4490450	A	G	A	G	0.0346	0.006269	0.6949	0.687523	FALSE	FALSE	FALSE	KMisdy	0.014759	0.671007	486484	sepsis	TRUE	reported	textfile	0.0072	1.47E-06	3977	M36589.m	TRUE	reported	J1ISTP	2	TRUE	0.005773	23.08175
4376	rs6898211	A	C	A	C	0.037	0.015217	0.0348	0.037738	FALSE	FALSE	FALSE	SPhl52	0.035869	0.671396	486484	sepsis	TRUE	reported	textfile	0.0077	1.62E-06	5211	M32651.m	TRUE	reported	V0Bnpj	2	TRUE	0.004411	23.08104
6517	rs1206213	T	C	T	C	-0.0639	0.017699	0.9849	0.980161	FALSE	FALSE	FALSE	OKIXJ8	0.049364	0.719939	486484	sepsis	TRUE	reported	textfile	0.0133	1.49E-06	7362	M33821.m	TRUE	reported	XGkaXH	2	TRUE	0.003126	23.07706
2797	rs1178708	A	G	A	G	0.0221	-0.04122	0.0475	0.055705	FALSE	FALSE	FALSE	Sx5Uhq	0.029882	0.167804	486484	sepsis	TRUE	reported	textfile	0.0046	1.63E-06	7354	M19934.m	TRUE	reported	DijYTE	2	TRUE	0.003129	23.07548
9074	rs3751196	A	G	A	G	0.0221	0.03298	0.0984	0.104603	FALSE	FALSE	FALSE	C7PD8T	0.022416	0.141223	486484	sepsis	TRUE	reported	textfile	0.0046	1.99E-06	6942	M38768.m	TRUE	reported	ZA3QM7	2	TRUE	0.003314	23.07511
2984	rs7133595	A	G	A	G	0.0519	-0.01817	0.3868	0.383755	FALSE	FALSE	FALSE	Tli4xl	0.014067	0.196581	486484	sepsis	TRUE	reported	textfile	0.0108	1.66E-06	2339	M21151.m	TRUE	reported	xaSytY	2	TRUE	0.009777	23.07362
3652	rs1710140	A	G	A	G	0.0245	0.026121	0.0774	0.043533	FALSE	FALSE	FALSE	fnjup6	0.033715	0.438491	486484	sepsis	TRUE	reported	textfile	0.0051	1.85E-06	7346	M32197.m	TRUE	reported	oQzaMk	2	TRUE	0.003132	23.07138
6357	rs1252105	A	C	A	C	-0.0197	0.004516	0.402	0.3816	FALSE	FALSE	FALSE	WWmDGv	0.014041	0.747734	486484	sepsis	TRUE	reported	textfile	0.0041	1.50E-06	2757	M33627.m	TRUE	reported	2kkEru	2	TRUE	0.008304	23.07011
6183	rs1181637	T	C	T	C	0.1273	-0.07778	0.0299	0.015652	FALSE	FALSE	FALSE	DEG58p	0.060962	0.202023	486484	sepsis	TRUE	reported	textfile	0.0265	1.63E-06	7500	M33507.m	TRUE	reported	ycykD0	2	TRUE	0.003067	23.07009
6035	rs2494014	A	G	A	G	-0.1788	-0.01599	0.8251	0.827297	FALSE	FALSE	FALSE	33ojbt	0.018078	0.376349	486484	sepsis	TRUE	reported	textfile	0.0372	1.56E-06	1391	M33423.m	TRUE	reported	ivEtvl	2	TRUE	0.016337	23.06876
2668	rs806784	T	C	T	C	-0.0952	0.001861	0.7156	0.714978	FALSE	FALSE	FALSE	cDEa3G	0.015152	0.902251	486484	sepsis	TRUE	reported	textfile	0.0198	1.48E-06	868	M18477.m	TRUE	reported	kxZEfC	2	TRUE	0.025942	23.06437
6667	rs1305484	A	T	A	T	0.0293	0.011709	0.8357	0.833654	FALSE	TRUE	FALSE	6iZSOI	0.01842	0.525	486484	sepsis	TRUE	reported	textfile	0.0061	1.27E-06	4533	M33901.m	TRUE	reported	ApKaGe	2	TRUE	0.005064	23.06131
4991	rs1263030	T	C	T	C	0.0711	0.006466	0.0603	0.063362	FALSE	FALSE	FALSE	MdUq0m	0.027982	0.817258	486484	sepsis	TRUE	reported	textfile	0.0148	1.65E-06	1462	M33009.m	TRUE	reported	icuhyC	2	TRUE	0.015541	23.04736
8078	rs1101192	T	C	T	C	-0.1477	-0.02259	0.0435	0.037732	FALSE	FALSE	FALSE	z65cF2	0.036194	0.53258	486484	sepsis	TRUE	reported	textfile	0.0307	1.52E-06	448	M35322.m	TRUE	reported	3GUSWk	2	TRUE	0.049128	23.04311
2463	rs9861255	A	G	A	G	0.1156	0.022118	0.92	0.910804	FALSE	FALSE	FALSE	NBJ2Do	0.024069	0.358117	486484	sepsis	TRUE	reported	textfile	0.0231	5.74E-07	25	M17799.m	TRUE	reported	Uz0Wgl	2	TRUE	0.500433	23.03984
1179	rs6826474	T	C	T	C	-0.1383	-0.0086	0.0393	0.028048	FALSE	FALSE	FALSE	FgZx4a	0.04171	0.836589	486484	sepsis	TRUE	reported	textfile	0.0288	1.56E-06	2063	M01640.m	TRUE	reported	hNPAJ8	2	TRUE	0.011054	23.03765
470	rs1193345	A	G	A	G	-0.0216	-0.06063	0.9357	0.934468	FALSE	FALSE	FALSE	OW48NY	0.027632	0.028225	486484	sepsis	TRUE	reported	textfile	0.0045	1.43E-06	7365	M00527.m	TRUE	reported	57AP2f	2	TRUE	0.003119	23.03374
4295	rs1992232	A	G	A	G	0.0216	0.009741	0.958	0.953078	FALSE	FALSE	FALSE	0tjjPC	0.032277	0.762818	486484	sepsis	TRUE	reported	textfile	0.0045	1.41E-06	7362	M32634.m	TRUE	reported	nJJFkX	2	TRUE	0.00312	23.03374
4155	rs7714057	T	C	T	C	0.0456	0.006525	0.0685	0.080125	FALSE	FALSE	FALSE	CrVaiQ	0.025271	0.796244	486484	sepsis	TRUE	reported	textfile	0.0095	1.75E-06	7361	M32553.m	TRUE	reported	HsKdAI	2	TRUE	0.00312	23.03374
1526	rs164372	T	C	T	C	0.012	0.004576	0.8036	0.80088	FALSE	FALSE	FALSE	YvgTeW	0.017144	0.789538	486484	sepsis	TRUE	reported	textfile	0.0025	2.64E-06	7357	M03141.m	TRUE	reported	srKjSc	2	TRUE	0.003122	23.03374
3941	rs9888349	T	C	T	C	-0.0192	0.003013	0.7742	0.7727	FALSE	FALSE	FALSE	LlOaWH	0.016312	0.853455	486484	sepsis	TRUE	reported	textfile	0.004	2.02E-06	7355	M32418.m	TRUE	reported	AtHOVw	2	TRUE	0.003123	23.03373
3384	rs4949008	A	G	A	G	0.012	-0.02317	0.6154	0.613763	FALSE	FALSE	FALSE	O2JtzH	0.014043	0.098974	486484	sepsis	TRUE	reported	textfile	0.0025	2.48E-06	7347	M27278.m	TRUE	reported	fxCIPW	2	TRUE	0.003126	23.03373
4975	rs9315310	T	C	T	C	0.012	-0.00526	0.1535	0.216885	FALSE	FALSE	FALSE	bKmDMa	0.016678	0.752563	486484	sepsis	TRUE	reported	textfile	0.0025	1.84E-06	7344	M32910.m	TRUE	reported	nAuPzO	2	TRUE	0.003127	23.03373
3729	rs1087387	T	G	T	G	-0.0216	0.010768	0.8609	0.860112	FALSE	FALSE	FALSE	k16olH	0.019688	0.584423	486484	sepsis	TRUE	reported	textfile	0.0045	1.40E-06	7340	M32328.m	TRUE	reported	TQA3sZ	2	TRUE	0.003129	23.03372
1078	rs1250778	A	G	A	G	-0.012	-0.00059	0.2395	0.247653	FALSE	FALSE	FALSE	JLtEiZ	0.015856	0.97055	486484	sepsis	TRUE	reported	textfile	0.0025	1.78E-06	7333	M01572.m	TRUE	reported	TSSe28	2	TRUE	0.003132	23.03372
3077	rs1094780	T	C	T	C	-0.012	0.004284	0.8118	0.809992	FALSE	FALSE	FALSE	esOP8m	0.017452	0.806075	486484	sepsis	TRUE	reported	textfile	0.0025	1.39E-06	7329	M22116.m	TRUE	reported	fDMh7Z	2	TRUE	0.003134	23.03371
2838	rs1213422	A	G	A	G	0.012	0.028248	0.0991	0.104332	FALSE	FALSE	FALSE	Y5qWBm	0.022507	0.209453	486484	sepsis	TRUE	reported	textfile	0.0025	1.63E-06	7325	M20489.m	TRUE	reported	f2g9Su	2	TRUE	0.003136	23.03371
2856	rs6883962	T	C	T	C	-0.012	0.011718	0.089	0.093144	FALSE	FALSE	FALSE	Y5qWBm	0.023463	0.617477	486484	sepsis	TRUE	reported	textfile	0.0025	1.63E-06	7325	M20489.m	TRUE	reported	f2g9Su	2	TRUE	0.003136	23.03371
4475	rs6996022	C	G	C	G	-0.012	-0.00407	0.1711	0.169658	FALSE	TRUE	FALSE	g3iM2w	0.018294	0.823979	486484	sepsis	TRUE	reported	textfile	0.0025	2.35E-06	7308	M32698.m	TRUE	reported	ZQBn0h	2	TRUE	0.003143	23.03369
543	rs1121626	T	C	T	C	0.0168	-0.0121	0.6435	0.655398	FALSE	FALSE	FALSE	QUUN2g	0.014392	0.40045	486484	sepsis	TRUE	reported	textfile	0.0035	1.56E-06	7237	M00599.m	TRUE	reported	vxn2i7	2	TRUE	0.003174	23.03363
8703	rs4684437	T	C	T	C	-0.012	0.030905	0.158	0.155491	FALSE	FALSE	FALSE	Bg4VnB	0.0189	0.102	486484	sepsis	TRUE	reported	textfile	0.0025	1.34E-06	7092	M36552.m	TRUE	reported	6zdDpC	2	TRUE	0.003238	23.0335
6288	rs7922903	T	G	T	G	-0.0168	-0.01103	0.0945	0.093034	FALSE	FALSE	FALSE	MOonP9	0.023595	0.64013	486484	sepsis	TRUE	reported	textfile	0.0035	2.10E-06	7066	M33515.m	TRUE	reported	226GuP	2	TRUE	0.00325	23.03348
1487	rs5751902	T	C	T	C	0.012	-0.00334	0.3762	0.380665	FALSE	FALSE	FALSE	pkb876	0.014669	0.81963	486484	sepsis	TRUE	reported	textfile	0.0025	2.38E-06	7037	M02734.m	TRUE	reported	0LCMVI	2	TRUE	0.003263	23.03345
1723	rs1748947	T	G	T	G	-0.0264	-0.01766	0.9114	0.883377	FALSE	FALSE	FALSE	RmWDhQ	0.021286	0.406661	486484	sepsis	TRUE	reported	textfile	0.0055	1.37E-06	7034	M12768.m	TRUE	reported	f0vnEz	2	TRUE	0.003265	23.03345
5952	rs1317415	T	G	T	G	-0.024	-0.0153	0.4549	0.458015	FALSE	FALSE	FALSE	mDZrGw	0.013725	0.264826	486484	sepsis	TRUE	reported	textfile	0.005	1.82E-06	6797	M33415.m	TRUE	reported	MzEP7R	2	TRUE	0.003378	23.03322
3021	rs7746531	T	C	T	C	0.0216	-0.02858	0.9206	0.919571	FALSE	FALSE	FALSE	gxupFu	0.025069	0.254247	486484	sepsis	TRUE	reported	textfile	0.0045	2.00E-06	6556	M21188.m	TRUE	reported	gwwXh2	2	TRUE	0.003502	23.03297
4533	rs2828004	T	G	T	G	0.0216	-0.04998	0.9477	0.945571	FALSE	FALSE	FALSE	r0qJaM	0.029967	0.095374	486484	sepsis	TRUE	reported	textfile	0.0045	1.87E-06	6497	M32729.m	TRUE	reported	vYv5FI	2	TRUE	0.003534	23.03291
4775	rs9277015	A	G	A	G	0.024	0.003432	0.358	0.346857	FALSE	FALSE	FALSE	mOrnK4	0.014342	0.810882	486484	sepsis	TRUE	reported	textfile	0.005	1.59E-06	5371	M32795.m	TRUE	reported	7EGxuf	2	TRUE	0.004271	23.03142
7395	rs6803606	T	C	T	C	-0.0216	0.008464	0.7239	0.718809	FALSE	FALSE	FALSE	AW6M5u	0.015195	0.577504	486484	sepsis	TRUE	reported	textfile	0.0045	2.06E-06	5195	M34389.m	TRUE	reported	6ZYYQK	2	TRUE	0.004415	23.03113
2783	rs477580	A	G	A	G	0.0168	0.01973	0.2397	0.233156	FALSE	FALSE	FALSE	yYuamw	0.016218	0.223792	486484	sepsis	TRUE	reported	textfile	0.0035	1.27E-06	4844	M19414.m	TRUE	reported	GxSb5G	2	TRUE	0.004734	23.03049
5762	rs3093198	A	G	A	G	0.024	0.010045	0.279	0.288233	FALSE	FALSE	FALSE	n16uO2	0.015118	0.50643	486484	sepsis	TRUE	reported	textfile	0.005	1.59E-06	4227	M33250.m	TRUE	reported	9dhl67	2	TRUE	0.005421	23.0291
4487	rs1275547	A	G	A	G	-0.0773	0.024218	0.031	0.042161	FALSE	FALSE	FALSE	1wNJ1E	0.034099	0.477563	486484	sepsis	TRUE	reported	textfile	0.0161	1.59E-06	1766	M32709.m	TRUE	reported	zihBHg	2	TRUE	0.012885	23.02582
2374	rs1298395	A	G	A	G	-0.1301	-0.00871	0.1002	0.12618	FALSE	FALSE	FALSE	NBJ2Do	0.020578	0.672221	486484	sepsis	TRUE	reported	textfile	0.0271	1.60E-06	1739	M17799.m	TRUE	reported	Uz0Wgl	2	TRUE	0.01308	23.02058
8142	rs1712268	A	G	A	G	-0.0216	0.015767	0.1379	0.140935	FALSE	FALSE	FALSE	lv3pAQ	0.019641	0.422113	486484	sepsis	TRUE	reported	textfile	0.0045	1.47E-06	1760	M35327.m	TRUE	reported	3Vsglp	2	TRUE	0.012922	23.01382
594	rs1320934	A	T	A	T	0.1215	0.021518	0.9599	0.938297	FALSE	TRUE	FALSE	KVK33e	0.028445	0.449368	486484	sepsis	TRUE	reported	textfile	0.0253	1.51E-06	736	M01107.m	TRUE	reported	eAyR8w	2	TRUE	0.030383	23.0001
4561	rs591058	T	C	T	C	0.0283	0.011369	0.5062	0.514694	FALSE	FALSE	FALSE	KfoRxi	0.013682	0.405988	486484	sepsis	TRUE	reported	textfile	0.0059	1.74E-06	6000	M32735.m	TRUE	reported	UDRXAp	2	TRUE	0.00382	22.9998
2556	rs6725791	T	C	T	C	0.0259	-0.00088	0.4058	0.412207	FALSE	FALSE	FALSE	NxZhaQ	0.013911	0.949492	486484	sepsis	TRUE	reported	textfile	0.0054	1.81E-06	5984	M18283.m	TRUE	reported	5Pi7r3	2	TRUE	0.00383	22.99677
7526	rs802424	C	G	C	G	-0.048	-0.02258	0.3093	0.305604	FALSE	TRUE	FALSE	rp1LGA	0.014845	0.128284	486484	sepsis	TRUE	reported	textfile	0.01	1.52E-06	997	M34441.m	TRUE	reported	SU47tz	2	TRUE	0.022587	22.99378
7903	rs1216076	A	G	A	G	0.0681	-0.03788	0.9499	0.949174	FALSE	FALSE	FALSE	AhsT9A	0.03125	0.225464	486484	sepsis	TRUE	reported	textfile	0.0142	1.78E-06	5953	M35187.m	TRUE	reported	uLU0Fy	2	TRUE	0.003849	22.99173
4087	rs1201686	C	G	C	G	-0.0422	0.066387	0.0467	0.053295	FALSE	TRUE	FALSE	j2JYxt	0.031374	0.034348	486484	sepsis	TRUE	reported	textfile	0.0088	1.60E-06	7373	M32504.m	TRUE	reported	poO7a5	2	TRUE	0.003109	22.99015
8408	rs1086964	A	G	A	G	-0.0211	0.006954	0.4086	0.409526	FALSE	FALSE	FALSE	02RdNm	0.013903	0.616954	486484	sepsis	TRUE	reported	textfile	0.0044	1.37E-06	6447	M35678.m	TRUE	reported	BWN5JR	2	TRUE	0.003554	22.98925
6385	rs1016651	A	G	A	G	-0.0211	-0.01647	0.6837	0.683177	FALSE	FALSE	FALSE	eBEF1t	0.014759	0.264379	486484	sepsis	TRUE	reported	textfile	0.0044	2.06E-06	6109	M33638.m	TRUE	reported	CRf2lt	2	TRUE	0.00375	22.98886
4745	rs4918797	T	C	T	C	0.0374	-0.01834	0.1997	0.199663	FALSE	FALSE	FALSE	oQPvO3	0.017092	0.283305	486484	sepsis	TRUE	reported	textfile	0.0078	1.56E-06	7330	M32786.m	TRUE	reported	Th0GcB	2	TRUE	0.003127	22.98452
2415	rs2861997	A	G	A	G	0.0897	0.016946	0.9163	0.906519	FALSE	FALSE	FALSE	NBJ2Do	0.023456	0.470015	486484	sepsis	TRUE	reported	textfile	0.0187	1.54E-06	1739	M17799.m	TRUE	reported	Uz0Wgl	2	TRUE	0.013059	22.98275
1671	rs1194903	A	G	A	G	0.0662	-0.00477	0.1629	0.164485	FALSE	FALSE	FALSE	IlFBGY	0.018402	0.795377	486484	sepsis	TRUE	reported	textfile	0.0138	1.52E-06	1543	M12261.m	TRUE	reported	F5Vlan	2	TRUE	0.014695	22.98235
7233	rs1017123	T	C	T	C	0.1402	0.037297	0.0634	0.055727	FALSE	FALSE	FALSE	RlcE3J	0.029857	0.211603	486484	sepsis	TRUE	reported	textfile	0.0292	1.51E-06	633	M34339.m	TRUE	reported	QQrwdm	2	TRUE	0.035139	22.98031
2922	rs1178804	A	G	A	G	0.0163	-0.00681	0.2425	0.252392	FALSE	FALSE	FALSE	fV4qul	0.015816	0.666873	486484	sepsis	TRUE	reported	textfile	0.0034	1.94E-06	7366	M21044.m	TRUE	reported	hbdBQJ	2	TRUE	0.003111	22.97732
6968	rs878013	C	G	C	G	0.0163	-0.01327	0.1689	0.177975	FALSE	TRUE	FALSE	7Cw2XZ	0.017921	0.459068	486484	sepsis	TRUE	reported	textfile	0.0034	1.97E-06	7049	M34123.m	TRUE	reported	vrgMl3	2	TRUE	0.00325	22.97704
4651	rs1860538	T	G	T	G	0.0163	0.000452	0.5561	0.549815	FALSE	FALSE	FALSE	SyAFR2	0.013756	0.973794	486484	sepsis	TRUE	reported	textfile	0.0034	1.67E-06	6658	M32755.m	TRUE	reported	prUo74	2	TRUE	0.00344	22.97666
7584	rs1252111	T	G	T	G	0.0374	0.01328	0.1521	0.152272	FALSE	FALSE	FALSE	SbjFMF	0.019066	0.486104	486484	sepsis	TRUE	reported	textfile	0.0078	1.46E-06	3239	M34513.m	TRUE	reported	HVYiKM	2	TRUE	0.007048	22.9766
8712	rs1765184	A	G	A	G	0.0441	0.043242	0.031	0.032874	FALSE	FALSE	FALSE	l0G0l1	0.038495	0.261312	486484	sepsis	TRUE	reported	textfile	0.0092	1.52E-06	6464	M36553.m	TRUE	reported	lBltb0	2	TRUE	0.003542	22.97032
3142	rs2434210	A	T	A	T	-0.0139	6.35E-05	0.8346	0.82668	FALSE	TRUE	FALSE	JiXR2t	0.018073	0.997195	486484	sepsis	TRUE	reported	textfile	0.0029	1.30E-06	6535	M22177.m	TRUE	reported	joi6p6	2	TRUE	0.003503	22.96681
3160	rs6441104	A	G	A	G	0.0139	0.002275	0.1267	0.122513	FALSE	FALSE	FALSE	JiXR2t	0.020937	0.913473	486484	sepsis	TRUE	reported	textfile	0.0029	1.73E-06	6535	M22177.m	TRUE	reported	joi6p6	2	TRUE	0.003503	22.96681
8546	rs1687071	A	C	A	C	-0.5095	-0.0063	0.0674	0.061052	FALSE	FALSE	FALSE	aQhCav	0.028604	0.825581	486484	sepsis	TRUE	reported	textfile	0.1061	1.55E-06	488	M36097.m	TRUE	reported	9DJS9j	2	TRUE	0.045122	22.9654
3617	rs1695865	T	C	T	C	0.0647	0.010376	0.9423	0.942194	FALSE	FALSE	FALSE	gq4lSJ	0.029278	0.723058	486484	sepsis	TRUE	reported	textfile	0.0135	1.68E-06	7338	M31591.m	TRUE	reported	wjwl4J	2	TRUE	0.00312	22.96268
5773	rs1254491	C	G	C	G	-0.1079	-0.06509	0.0372	0.052975	FALSE	TRUE	FALSE	eT2We0	0.030675	0.033834	486484	sepsis	TRUE	reported	textfile	0.0225	1.72E-06	1273	M33353.m	TRUE	reported	uZx90m	2	TRUE	0.017745	22.96122
6389	rs1165189	A	C	A	C	-0.0254	0.021508	0.7496	0.743675	FALSE	FALSE	FALSE	eBEF1t	0.015631	0.168831	486484	sepsis	TRUE	reported	textfile	0.0053	1.46E-06	6109	M33638.m	TRUE	reported	CRf2lt	2	TRUE	0.003746	22.96008
7088	rs1247510	A	C	A	C	-0.0369	0.013154	0.0269	0.028183	FALSE	FALSE	FALSE	pxNICA	0.041247	0.749801	486484	sepsis	TRUE	reported	textfile	0.0077	1.56E-06	6994	M34306.m	TRUE	reported	xk5b9M	2	TRUE	0.003273	22.95869
5668	rs2298126	A	C	A	C	-0.0484	0.002608	0.3406	0.346166	FALSE	FALSE	FALSE	wHu1Rk	0.014436	0.856649	486484	sepsis	TRUE	reported	textfile	0.0101	1.57E-06	4625	M33195.m	TRUE	reported	yvhnuQ	2	TRUE	0.004941	22.95409
3753	rs229914	A	G	A	G	-0.0115	0.024301	0.5846	0.566774	FALSE	FALSE	FALSE	OZLdls	0.013945	0.081392	486484	sepsis	TRUE	reported	textfile	0.0024	2.40E-06	7356	M32338.m	TRUE	reported	s2itmB	2	TRUE	0.003112	22.95383
8700	rs2997247	T	C	T	C	0.0115	0.001713	0.8295	0.839561	FALSE	FALSE	FALSE	Bg4VnB	0.018626	0.926726	486484	sepsis	TRUE	reported	textfile	0.0024	1.77E-06	7092	M36552.m	TRUE	reported	6zdDpC	2	TRUE	0.003227	22.95359
6431	rs1568846	A	G	A	G	-0.0115	0.025706	0.5288	0.528552	FALSE	FALSE	FALSE	QCw5dg	0.013747	0.061495	486484	sepsis	TRUE	reported	textfile	0.0024	1.74E-06	6279	M33666.m	TRUE	reported	zYHmzg	2	TRUE	0.003643	22.95276
5662	rs6574474	A	G	A	G	0.046	-0.00707	0.5481	0.544129	FALSE	FALSE	FALSE	mod1fD	0.013796	0.608136	486484	sepsis	TRUE	reported	textfile	0.0096	1.52E-06	5064	M33194.m	TRUE	reported	fSFVUj	2	TRUE	0.004514	22.951
4863	rs1099585	A	G	A	G	0.0321	-0.02079	0.8946	0.891404	FALSE	FALSE	FALSE	KBJVQO	0.021913	0.342729	486484	sepsis	TRUE	reported	textfile	0.0067	1.95E-06	7355	M32855.m	TRUE	reported	Xea7Fn	2	TRUE	0.003111	22.94787
8091	rs905176	A	G	A	G	-0.0576	-0.01999	0.3698	0.359771	FALSE	FALSE	FALSE	z65cF2	0.014308	0.162273	486484	sepsis	TRUE	reported	textfile	0.012	1.50E-06	448	M35322.m	TRUE	reported	3GUSWk	2	TRUE	0.048913	22.93714
5123	rs1108271	A	G	A	G	0.3479	-0.02018	0.9531	0.934225	FALSE	FALSE	FALSE	L2DJzK	0.027585	0.464483	486484	sepsis	TRUE	reported	textfile	0.0726	1.68E-06	1574	M33173.m	TRUE	reported	hRG70D	2	TRUE	0.014379	22.93419
4005	rs1333190	T	C	T	C	0.0091	0.025165	0.5895	0.590739	FALSE	FALSE	FALSE	V9AtId	0.014002	0.072307	486484	sepsis	TRUE	reported	textfile	0.0019	8.90E-07	7363	M32489.m	TRUE	reported	8B8S8a	2	TRUE	0.003106	22.93283
4390	rs1776847	T	C	T	C	-0.0273	0.005251	0.0454	0.060797	FALSE	FALSE	FALSE	UJygVd	0.028526	0.853968	486484	sepsis	TRUE	reported	textfile	0.0057	1.50E-06	7361	M32654.m	TRUE	reported	kv9R0W	2	TRUE	0.003107	22.93283
2813	rs2834059	A	G	A	G	-0.0091	-0.01616	0.4831	0.491617	FALSE	FALSE	FALSE	Sx5Uhq	0.013824	0.242522	486484	sepsis	TRUE	reported	textfile	0.0019	9.96E-07	7354	M19934.m	TRUE	reported	DijYTE	2	TRUE	0.00311	22.93282
4120	rs4814243	A	G	A	G	-0.0091	-0.00598	0.8087	0.808367	FALSE	FALSE	FALSE	4rtLd0	0.017442	0.731508	486484	sepsis	TRUE	reported	textfile	0.0019	1.00E-06	7350	M32518.m	TRUE	reported	j7f1ER	2	TRUE	0.003111	22.93282
1236	rs2304767	A	G	A	G	0.0091	-0.01138	0.3402	0.347635	FALSE	FALSE	FALSE	V50rWC	0.014416	0.42969	486484	sepsis	TRUE	reported	textfile	0.0019	1.22E-06	7346	M01645.m	TRUE	reported	yN3gpQ	2	TRUE	0.003113	22.93281
3202	rs2863821	A	G	A	G	-0.0091	-0.01838	0.4839	0.483188	FALSE	FALSE	FALSE	UfLkBq	0.013809	0.183099	486484	sepsis	TRUE	reported	textfile	0.0019	1.41E-06	7329	M22481.m	TRUE	reported	kWMdiT	2	TRUE	0.00312	22.9328
3204	rs3213827	A	G	A	G	-0.0091	0.005154	0.5701	0.574664	FALSE	FALSE	FALSE	UfLkBq	0.013821	0.709236	486484	sepsis	TRUE	reported	textfile	0.0019	1.10E-06	7329	M22481.m	TRUE	reported	kWMdiT	2	TRUE	0.00312	22.9328
2839	rs1253454	A	G	A	G	0.0091	0.000708	0.1791	0.175777	FALSE	FALSE	FALSE	Y5qWBm	0.018105	0.968812	486484	sepsis	TRUE	reported	textfile	0.0019	1.22E-06	7325	M20489.m	TRUE	reported	f2g9Su	2	TRUE	0.003122	22.93279
5865	rs1189802	T	C	T	C	0.0091	-0.00143	0.7118	0.713419	FALSE	FALSE	FALSE	tqW0ld	0.015128	0.924812	486484	sepsis	TRUE	reported	textfile	0.0019	1.06E-06	7315	M33389.m	TRUE	reported	Sv7EyV	2	TRUE	0.003126	22.93279
5894	rs7311763	A	C	A	C	-0.0091	0.003073	0.3591	0.360949	FALSE	FALSE	FALSE	tqW0ld	0.014211	0.82877	486484	sepsis	TRUE	reported	textfile	0.0019	9.26E-07	7315	M33389.m	TRUE	reported	Sv7EyV	2	TRUE	0.003126	22.93279
5898	rs7760528	T	C	T	C	-0.0091	0.024601	0.68	0.679385	FALSE	FALSE	FALSE	tqW0ld	0.014805	0.096574	486484	sepsis	TRUE	reported	textfile	0.0019	9.17E-07	7315	M33389.m	TRUE	reported	Sv7EyV	2	TRUE	0.003126	22.93279
8453	rs165558	T	C	T	C	-0.0091	0.01992	0.5657	0.565689	FALSE	FALSE	FALSE	uMajV6	0.013838	0.149997	486484	sepsis	TRUE	reported	textfile	0.0019	2.00E-06	6604	M35754.m	TRUE	reported	b8QB7S	2	TRUE	0.003461	22.93211
8455	rs1722784	A	G	A	G	-0.0091	-0.00115	0.5093	0.495566	FALSE	FALSE	FALSE	uMajV6	0.013702	0.933103	486484	sepsis	TRUE	reported	textfile	0.0019	2.40E-06	6604	M35754.m	TRUE	reported	b8QB7S	2	TRUE	0.003461	22.93211
8473	rs7520333	A	G	A	G	-0.0091	-0.02225	0.554	0.556576	FALSE	FALSE	FALSE	uMajV6	0.013788	0.106617	486484	sepsis	TRUE	reported	textfile	0.0019	2.30E-06	6604	M35754.m	TRUE	reported	b8QB7S	2	TRUE	0.003461	22.93211
3122	rs1177690	A	G	A	G	-0.0091	-0.01033	0.6567	0.647935	FALSE	FALSE	FALSE	JiXR2t	0.014286	0.469624	486484	sepsis	TRUE	reported	textfile	0.0019	3.21E-06	6535	M22177.m	TRUE	reported	joi6p6	2	TRUE	0.003498	22.93204
3164	rs6874723	T	C	T	C	0.0091	-0.00247	0.3661	0.364997	FALSE	FALSE	FALSE	JiXR2t	0.014276	0.862368	486484	sepsis	TRUE	reported	textfile	0.0019	3.17E-06	6535	M22177.m	TRUE	reported	joi6p6	2	TRUE	0.003498	22.93204
3174	rs892857	T	C	T	C	-0.0091	0.008294	0.6572	0.643722	FALSE	FALSE	FALSE	JiXR2t	0.014419	0.565115	486484	sepsis	TRUE	reported	textfile	0.0019	2.83E-06	6535	M22177.m	TRUE	reported	joi6p6	2	TRUE	0.003498	22.93204
4538	rs6706970	T	C	T	C	-0.0091	-0.00993	0.5982	0.6082	FALSE	FALSE	FALSE	r0qJaM	0.014044	0.479698	486484	sepsis	TRUE	reported	textfile	0.0019	1.25E-06	6497	M32729.m	TRUE	reported	vYv5FI	2	TRUE	0.003518	22.932
7748	rs4674025	T	C	T	C	-0.0091	0.018827	0.4561	0.452513	FALSE	FALSE	FALSE	HyMNYM	0.01386	0.174358	486484	sepsis	TRUE	reported	textfile	0.0019	2.03E-06	5885	M34912.m	TRUE	reported	Tl8Hcw	2	TRUE	0.003883	22.93126
7754	rs7022352	A	G	A	G	0.0091	-0.02414	0.5336	0.547817	FALSE	FALSE	FALSE	HyMNYM	0.013914	0.082787	486484	sepsis	TRUE	reported	textfile	0.0019	1.48E-06	5885	M34912.m	TRUE	reported	Tl8Hcw	2	TRUE	0.003883	22.93126
7872	rs2688	T	G	T	G	0.0321	-0.0007	0.6039	0.606529	FALSE	FALSE	FALSE	r428eQ	0.014021	0.960341	486484	sepsis	TRUE	reported	textfile	0.0067	1.46E-06	1982	M35159.m	TRUE	reported	3r5zQo	2	TRUE	0.011449	22.93095
7815	rs1165700	T	G	T	G	-0.0091	0.022271	0.7079	0.699585	FALSE	FALSE	FALSE	h5whlS	0.014933	0.135839	486484	sepsis	TRUE	reported	textfile	0.0019	2.23E-06	4900	M35137.m	TRUE	reported	xVITKF	2	TRUE	0.00466	22.9297
7833	rs3121910	T	G	T	G	-0.0091	-0.0221	0.7221	0.714461	FALSE	FALSE	FALSE	h5whlS	0.015102	0.14332	486484	sepsis	TRUE	reported	textfile	0.0019	1.99E-06	4900	M35137.m	TRUE	reported	xVITKF	2	TRUE	0.00466	22.9297
7839	rs4398300	A	G	A	G	0.0091	-0.0169	0.6235	0.622029	FALSE	FALSE	FALSE	h5whlS	0.014072	0.229756	486484	sepsis	TRUE	reported	textfile	0.0019	1.84E-06	4900	M35137.m	TRUE	reported	xVITKF	2	TRUE	0.00466	22.9297
7852	rs6871718	T	C	T	C	-0.0091	-0.0086	0.665	0.666504	FALSE	FALSE	FALSE	h5whlS	0.01449	0.553067	486484	sepsis	TRUE	reported	textfile	0.0019	1.75E-06	4900	M35137.m	TRUE	reported	xVITKF	2	TRUE	0.00466	22.9297
7220	rs2390633	A	C	A	C	-0.2461	0.052783	0.0402	0.045378	FALSE	FALSE	FALSE	i1uJ7T	0.032794	0.107499	486484	sepsis	TRUE	reported	textfile	0.0513	1.58E-06	535	M34338.m	TRUE	reported	pi3mKW	2	TRUE	0.041242	22.92778
8579	rs1051174	A	T	A	T	0.068	0.066582	0.9606	0.956638	FALSE	TRUE	FALSE	iuJmRL	0.033531	0.047066	486484	sepsis	TRUE	reported	textfile	0.0142	1.73E-06	7311	M36103.m	TRUE	reported	jfJbVw	2	TRUE	0.003127	22.92568
2790	rs3130663	A	G	A	G	-0.1184	-0.00935	0.9754	0.976419	FALSE	FALSE	FALSE	ex7hzq	0.045259	0.836406	486484	sepsis	TRUE	reported	textfile	0.0247	1.55E-06	855	M19415.m	TRUE	reported	jvXWvV	2	TRUE	0.026171	22.92411
7023	rs168190	T	C	T	C	0.0465	0.012895	0.4522	0.462654	FALSE	FALSE	FALSE	zMlkYA	0.013878	0.352818	486484	sepsis	TRUE	reported	textfile	0.0097	1.81E-06	788	M34245.m	TRUE	reported	itCtz8	2	TRUE	0.028337	22.92233
4747	rs7772658	A	G	A	G	0.0565	-0.01529	0.0782	0.0961	FALSE	FALSE	FALSE	oQPvO3	0.02315	0.509066	486484	sepsis	TRUE	reported	textfile	0.0118	1.68E-06	7330	M32786.m	TRUE	reported	Th0GcB	2	TRUE	0.003118	22.91999
7524	rs7074531	T	C	T	C	0.0249	-0.00908	0.6964	0.704396	FALSE	FALSE	FALSE	rp1LGA	0.015008	0.545297	486484	sepsis	TRUE	reported	textfile	0.0052	1.43E-06	4442	M34441.m	TRUE	reported	SU47tz	2	TRUE	0.005135	22.91904
494	rs1780051	T	G	T	G	-0.1308	-0.03235	0.1085	0.100505	FALSE	FALSE	FALSE	RtjZPn	0.022687	0.153837	486484	sepsis	TRUE	reported	textfile	0.0273	1.58E-06	1233	M00553.m	TRUE	reported	C3Ovsl	2	TRUE	0.018277	22.91845
7038	rs1161475	T	C	T	C	-0.0958	0.0745	0.9887	0.989565	FALSE	FALSE	FALSE	uthruC	0.068104	0.27399	486484	sepsis	TRUE	reported	textfile	0.02	1.60E-06	1755	M34283.m	TRUE	reported	ftTuRU	2	TRUE	0.012905	22.91795
781	rs1011200	A	G	A	G	0.0158	0.006371	0.0782	0.077044	FALSE	FALSE	FALSE	dyT79l	0.02569	0.804154	486484	sepsis	TRUE	reported	textfile	0.0033	1.73E-06	7352	M01336.m	TRUE	reported	OemVJ2	2	TRUE	0.003108	22.91755
8650	rs1192458	A	G	A	G	0.034	0.002227	0.6223	0.606006	FALSE	FALSE	FALSE	J6i46i	0.013952	0.873211	486484	sepsis	TRUE	reported	textfile	0.0071	1.89E-06	2520	M36376.m	TRUE	reported	IdddPp	2	TRUE	0.009018	22.91376
7763	rs1204948	T	G	T	G	0.0273	-0.0303	0.744	0.759402	FALSE	FALSE	FALSE	G4gUxB	0.015984	0.058022	486484	sepsis	TRUE	reported	textfile	0.0057	1.81E-06	1657	M35114.m	TRUE	reported	o79OO5	2	TRUE	0.013655	22.91137
1100	rs7438272	T	C	T	C	0.0589	-0.02771	0.1691	0.155717	FALSE	FALSE	FALSE	K3wtZo	0.019232	0.149701	486484	sepsis	TRUE	reported	textfile	0.0123	1.80E-06	2315	M01573.m	TRUE	reported	0D9CvA	2	TRUE	0.009808	22.91105
6824	rs6544157	A	G	A	G	-0.0292	-0.01752	0.9013	0.895243	FALSE	FALSE	FALSE	wedECv	0.022717	0.44053	486484	sepsis	TRUE	reported	textfile	0.0061	2.01E-06	7322	M33968.m	TRUE	reported	GxGxRG	2	TRUE	0.00312	22.90801
5162	rs7071224	C	G	C	G	0.3778	-0.01814	0.9005	0.8323	FALSE	TRUE	FALSE	L2DJzK	0.01828	0.321154	486484	sepsis	TRUE	reported	textfile	0.0779	1.22E-06	76	M33173.m	TRUE	reported	hRG70D	2	TRUE	0.236339	22.90167
7217	rs1954396	A	G	A	G	-0.2752	-0.03031	0.9704	0.96422	FALSE	FALSE	FALSE	i1uJ7T	0.036717	0.409121	486484	sepsis	TRUE	reported	textfile	0.0574	1.65E-06	535	M34338.m	TRUE	reported	pi3mKW	2	TRUE	0.041195	22.90058
5751	rs7603154	A	G	A	G	0.0134	-0.00264	0.5252	0.5312	FALSE	FALSE	FALSE	bm6x2Z	0.013693	0.847293	486484	sepsis	TRUE	reported	textfile	0.0028	1.18E-06	7364	M33230.m	TRUE	reported	8Ccke3	2	TRUE	0.0031	22.89684
1841	rs874758	A	G	A	G	-0.0134	-0.02355	0.1855	0.180374	FALSE	FALSE	FALSE	2TaDHm	0.017958	0.189713	486484	sepsis	TRUE	reported	textfile	0.0028	1.43E-06	7337	M15365.m	TRUE	reported	LoLveg	2	TRUE	0.003112	22.89682
4218	rs4149000	T	C	T	C	0.0402	-0.00069	0.139	0.129189	FALSE	FALSE	FALSE	aS6krA	0.020738	0.973623	486484	sepsis	TRUE	reported	textfile	0.0084	1.72E-06	7303	M32586.m	TRUE	reported	g9cBDI	2	TRUE	0.003126	22.89679
5782	rs4325604	T	C	T	C	0.0771	-0.02116	0.0727	0.078004	FALSE	FALSE	FALSE	eT2We0	0.025478	0.406309	486484	sepsis	TRUE	reported	textfile	0.0161	1.63E-06	1273	M33353.m	TRUE	reported	uZx90m	2	TRUE	0.017696	22.89677
7700	rs1419916	T	C	T	C	0.0201	-0.02408	0.3141	0.297675	FALSE	FALSE	FALSE	2eJlPM	0.014969	0.107688	486484	sepsis	TRUE	reported	textfile	0.0042	2.27E-06	6381	M34761.m	TRUE	reported	druKtT	2	TRUE	0.003576	22.89588
3496	rs4901357	A	G	A	G	-0.0134	-0.00865	0.3396	0.332157	FALSE	FALSE	FALSE	Awafb0	0.014516	0.551185	486484	sepsis	TRUE	reported	textfile	0.0028	1.25E-06	5562	M27728.m	TRUE	reported	Lfj8L0	2	TRUE	0.004101	22.89483
4447	rs1178091	C	G	C	G	0.1939	-0.0374	0.9873	0.959252	FALSE	TRUE	FALSE	g2RNym	0.03462	0.280014	486484	sepsis	TRUE	reported	textfile	0.0405	1.68E-06	1675	M32691.m	TRUE	reported	35eo6p	2	TRUE	0.0135	22.89426
3465	rs1090012	A	G	A	G	-0.0383	0.005673	0.7361	0.734744	FALSE	FALSE	FALSE	BQlbxe	0.015488	0.714157	486484	sepsis	TRUE	reported	textfile	0.008	1.74E-06	1768	M27718.m	TRUE	reported	MnHHJ0	2	TRUE	0.012798	22.89423
2537	rs1088687	T	C	T	C	-0.1704	-0.00744	0.9072	0.853497	FALSE	FALSE	FALSE	o0CFya	0.019476	0.702537	486484	sepsis	TRUE	reported	textfile	0.0356	1.75E-06	2433	M18281.m	TRUE	reported	pxbsnu	2	TRUE	0.009329	22.89191
5145	rs1923325	T	C	T	C	0.5416	-0.02555	0.9478	0.938875	FALSE	FALSE	FALSE	L2DJzK	0.028557	0.370968	486484	sepsis	TRUE	reported	textfile	0.1117	1.26E-06	76	M33173.m	TRUE	reported	hRG70D	2	TRUE	0.236257	22.89123
1722	rs1698522	T	C	T	C	0.1077	-0.02243	0.9655	0.956246	FALSE	FALSE	FALSE	RmWDhQ	0.034008	0.509617	486484	sepsis	TRUE	reported	textfile	0.0225	1.69E-06	1682	M12768.m	TRUE	reported	f0vnEz	2	TRUE	0.013439	22.88493
6008	rs1011820	C	G	C	G	-0.7381	-0.01182	0.0667	0.100583	FALSE	TRUE	FALSE	33ojbt	0.022856	0.604908	486484	sepsis	TRUE	reported	textfile	0.1517	1.14E-06	60	M33423.m	TRUE	reported	ivEtvl	2	TRUE	0.282926	22.88421
6102	rs1043909	T	C	T	C	-0.0244	-0.00219	0.1912	0.207687	FALSE	FALSE	FALSE	a0osmD	0.016938	0.897289	486484	sepsis	TRUE	reported	textfile	0.0051	2.19E-06	5717	M33453.m	TRUE	reported	CePFnK	2	TRUE	0.003988	22.88165
5631	rs2822277	T	C	T	C	0.0512	0.001725	0.3412	0.323379	FALSE	FALSE	FALSE	U0d0ue	0.014615	0.906041	486484	sepsis	TRUE	reported	textfile	0.0107	1.56E-06	2616	M33190.m	TRUE	reported	FoODGF	2	TRUE	0.008677	22.87917
7996	rs2490288	T	C	T	C	0.0177	-0.0159	0.3319	0.329849	FALSE	FALSE	FALSE	Xrodum	0.014543	0.274132	486484	sepsis	TRUE	reported	textfile	0.0037	1.54E-06	7291	M35255.m	TRUE	reported	T8t1tl	2	TRUE	0.003129	22.87831
3268	rs5995601	A	G	A	G	0.0177	0.000185	0.8544	0.831256	FALSE	FALSE	FALSE	ZNPBqT	0.018306	0.991931	486484	sepsis	TRUE	reported	textfile	0.0037	1.70E-06	7256	M24074.m	TRUE	reported	8pPtOl	2	TRUE	0.003144	22.87828
8491	rs6730977	A	G	A	G	0.0177	-0.0129	0.6251	0.612379	FALSE	FALSE	FALSE	ABkZ7x	0.014069	0.359273	486484	sepsis	TRUE	reported	textfile	0.0037	1.38E-06	6960	M35854.m	TRUE	reported	wc0CeK	2	TRUE	0.003277	22.87801
4133	rs1084414	A	C	A	C	-0.0464	-0.00359	0.8691	0.878229	FALSE	FALSE	FALSE	pLh7sk	0.020955	0.863939	486484	sepsis	TRUE	reported	textfile	0.0097	1.84E-06	7256	M32549.m	TRUE	reported	RSQOnp	2	TRUE	0.003144	22.87561
6748	rs2156436	T	C	T	C	0.1445	0.009784	0.0307	0.024616	FALSE	FALSE	FALSE	NZVg9N	0.046031	0.831685	486484	sepsis	TRUE	reported	textfile	0.0302	1.73E-06	1765	M33941.m	TRUE	reported	kRWNNK	2	TRUE	0.012805	22.86806
8952	rs1560661	T	C	T	C	0.1129	-0.00808	0.6472	0.574315	FALSE	FALSE	FALSE	Ywk2ve	0.014228	0.570188	486484	sepsis	TRUE	reported	textfile	0.0236	1.77E-06	2560	M37112.m	TRUE	reported	EiAdDj	2	TRUE	0.008861	22.8678
8981	rs6588480	A	G	A	G	-0.044	-0.01771	0.1493	0.151946	FALSE	FALSE	FALSE	snOepl	0.019075	0.353134	486484	sepsis	TRUE	reported	textfile	0.0092	1.82E-06	7355	M37202.m	TRUE	reported	PQx272	2	TRUE	0.0031	22.86713

6541	rs7096907	T	C	T	C	-0.022	-0.01595	0.181	0.181586	FALSE	FALSE	FALSE	fVPpJo	0.017801	0.370298	486484	sepsis	TRUE	reported	textfile	0.0046	2.15E-06	6327	M33833.m	TRUE	reported	37N6ck	2	TRUE	0.003602	22.86612
6554	rs1013631	A	G	A	G	-0.0378	0.018518	0.5986	0.600781	FALSE	FALSE	FALSE	58qeH5	0.013964	0.184776	486484	sepsis	TRUE	reported	textfile	0.0079	1.86E-06	1428	M33864.m	TRUE	reported	TBsy8r	2	TRUE	0.01578	22.86234
8584	rs1712534	T	C	T	C	-0.0526	0.051587	0.0709	0.040426	FALSE	FALSE	FALSE	iuJmRL	0.034789	0.138107	486484	sepsis	TRUE	reported	textfile	0.011	1.61E-06	7311	M36103.m	TRUE	reported	jfJbVw	2	TRUE	0.003118	22.85953
2646	rs1252615	A	C	A	C	0.4507	0.046718	0.0139	0.028093	FALSE	FALSE	FALSE	9GoQdh	0.041535	0.260685	486484	sepsis	TRUE	reported	textfile	0.0939	1.61E-06	257	M18474.m	TRUE	reported	p6Dq8p	2	TRUE	0.082267	22.85867
5143	rs1806077	A	G	A	G	-0.4253	0.004693	0.0205	0.034363	FALSE	FALSE	FALSE	L2DJzK	0.038992	0.904204	486484	sepsis	TRUE	reported	textfile	0.0889	1.73E-06	1574	M33173.m	TRUE	reported	hRG70D	2	TRUE	0.014332	22.85783
1237	rs2423542	A	G	A	G	0.0153	-0.01134	0.8036	0.739855	FALSE	FALSE	FALSE	V50rWC	0.01732	0.51275	486484	sepsis	TRUE	reported	textfile	0.0032	1.91E-06	7346	M01645.m	TRUE	reported	yN3gpQ	2	TRUE	0.003102	22.85413
4889	rs1770028	C	G	C	G	0.0617	0.0037	0.5903	0.598794	FALSE	TRUE	FALSE	ocaywK	0.014177	0.794118	486484	sepsis	TRUE	reported	textfile	0.0129	1.77E-06	1988	M32863.m	TRUE	reported	WH6KGr	2	TRUE	0.011376	22.85356
997	rs1538330	T	G	T	G	-0.0306	-0.05895	0.0213	0.023436	FALSE	FALSE	FALSE	jfkZbM	0.045288	0.193004	486484	sepsis	TRUE	reported	textfile	0.0064	1.73E-06	6523	M01558.m	TRUE	reported	LFZvMG	2	TRUE	0.003492	22.85334
5909	rs1101935	T	C	T	C	-0.0459	-0.01241	0.8389	0.837139	FALSE	FALSE	FALSE	iGV9aX	0.018542	0.503426	486484	sepsis	TRUE	reported	textfile	0.0096	1.77E-06	5185	M33390.m	TRUE	reported	sLbpnf	2	TRUE	0.00439	22.85153
6438	rs3124018	A	G	A	G	-0.0196	-0.01788	0.9208	0.917452	FALSE	FALSE	FALSE	QCw5dg	0.025085	0.476034	486484	sepsis	TRUE	reported	textfile	0.0041	1.78E-06	6279	M33666.m	TRUE	reported	zYHmzg	2	TRUE	0.003626	22.84578
7662	rs1174382	T	C	T	C	0.0545	-0.00533	0.2041	0.220603	FALSE	FALSE	FALSE	z8S1DZ	0.016588	0.748133	486484	sepsis	TRUE	reported	textfile	0.0114	1.64E-06	4515	M34535.m	TRUE	reported	FNLxxh	2	TRUE	0.005037	22.84499
3826	rs1682600	T	C	T	C	-0.0239	0.030844	0.9608	0.960345	FALSE	FALSE	FALSE	tRX2u0	0.035	0.378183	486484	sepsis	TRUE	reported	textfile	0.005	1.72E-06	7365	M32348.m	TRUE	reported	ruBgwQ	2	TRUE	0.003093	22.8422
4671	rs1543433	A	G	A	G	0.0239	0.021584	0.366	0.36445	FALSE	FALSE	FALSE	xaapK0	0.014238	0.129526	486484	sepsis	TRUE	reported	textfile	0.005	1.99E-06	7250	M32757.m	TRUE	reported	64n1Z4	2	TRUE	0.003142	22.8421
5066	rs4363657	T	C	T	C	-0.0239	0.010366	0.8302	0.836244	FALSE	FALSE	FALSE	nHZXjp	0.018509	0.575425	486484	sepsis	TRUE	reported	textfile	0.005	1.48E-06	7166	M33138.m	TRUE	reported	ccoK1F	2	TRUE	0.003178	22.84202
5046	rs1263245	T	C	T	C	0.0459	-0.00099	0.6341	0.623732	FALSE	FALSE	FALSE	JLCSne	0.014259	0.944612	486484	sepsis	TRUE	reported	textfile	0.0096	1.93E-06	2391	M33137.m	TRUE	reported	LGKYXK	2	TRUE	0.00947	22.84123
7660	rs1107021	T	C	T	C	0.0435	-0.00561	0.4394	0.440795	FALSE	FALSE	FALSE	z8S1DZ	0.013768	0.683595	486484	sepsis	TRUE	reported	textfile	0.0091	1.55E-06	4515	M34535.m	TRUE	reported	FNLxxh	2	TRUE	0.005036	22.84038
6198	rs4919141	A	G	A	G	0.0282	0.014864	0.7786	0.771427	FALSE	FALSE	FALSE	DEG58p	0.016313	0.36221	486484	sepsis	TRUE	reported	textfile	0.0059	1.73E-06	7500	M33507.m	TRUE	reported	ycykD0	2	TRUE	0.003037	22.83907
2552	rs1392786	T	C	T	C	-0.0282	0.013882	0.4017	0.434235	FALSE	FALSE	FALSE	NxZhaQ	0.013809	0.314752	486484	sepsis	TRUE	reported	textfile	0.0059	1.93E-06	5984	M18283.m	TRUE	reported	5Pi7r3	2	TRUE	0.003803	22.83752
8512	rs3789107	A	C	A	C	0.0636	-0.00141	0.8931	0.884175	FALSE	FALSE	FALSE	vHXktL	0.02224	0.949506	486484	sepsis	TRUE	reported	textfile	0.0133	1.90E-06	1517	M35978.m	TRUE	reported	ceap9w	2	TRUE	0.01485	22.83694
7178	rs1714408	A	T	A	T	-0.0282	-0.02491	0.057	0.063095	FALSE	TRUE	FALSE	2hlepp	0.028059	0.374674	486484	sepsis	TRUE	reported	textfile	0.0059	1.89E-06	5081	M34336.m	TRUE	reported	XyK8mO	2	TRUE	0.004476	22.83617
5912	rs1209223	T	G	T	G	0.1535	-0.00817	0.0315	0.029979	FALSE	FALSE	FALSE	iGV9aX	0.040294	0.839302	486484	sepsis	TRUE	reported	textfile	0.0321	1.69E-06	1464	M33390.m	TRUE	reported	sLbpnf	2	TRUE	0.015379	22.83563
5452	rs4294438	A	G	A	G	0.2511	0.000354	0.9765	0.974859	FALSE	FALSE	FALSE	q32d4E	0.043791	0.993547	486484	sepsis	TRUE	reported	textfile	0.0525	1.72E-06	1017	M33178.m	TRUE	reported	cS3BwT	2	TRUE	0.021999	22.83074
1424	rs1264612	T	C	T	C	-0.0497	0.011404	0.0308	0.019073	FALSE	FALSE	FALSE	ac6RGB	0.050107	0.819957	486484	sepsis	TRUE	reported	textfile	0.0104	1.80E-06	5791	M02342.m	TRUE	reported	dC6Rkp	2	TRUE	0.003928	22.82948
1549	rs1213605	T	C	T	C	-0.0731	-0.08505	0.0129	0.0158	FALSE	FALSE	FALSE	Ke0sNh	0.05673	0.133835	486484	sepsis	TRUE	reported	textfile	0.0153	1.75E-06	7799	M10642.m	TRUE	reported	ZINwVb	2	TRUE	0.002918	22.82131
1123	rs1084431	T	G	T	G	-0.0086	-0.00511	0.1637	0.141524	FALSE	FALSE	FALSE	nlqUuV	0.019585	0.794003	486484	sepsis	TRUE	reported	textfile	0.0018	1.12E-06	7371	M01604.m	TRUE	reported	Uro9nC	2	TRUE	0.003087	22.82097
612	rs1430209	T	C	T	C	-0.0086	-0.02656	0.5313	0.523317	FALSE	FALSE	FALSE	BYGzq4	0.013665	0.051982	486484	sepsis	TRUE	reported	textfile	0.0018	1.87E-06	7367	M01110.m	TRUE	reported	oZiXqP	2	TRUE	0.003089	22.82096
1336	rs175866	T	G	T	G	-0.0086	0.013477	0.5784	0.565951	FALSE	FALSE	FALSE	LDUJbh	0.013774	0.327864	486484	sepsis	TRUE	reported	textfile	0.0018	1.14E-06	7367	M01898.m	TRUE	reported	JRSLeH	2	TRUE	0.003089	22.82096
1345	rs535633	A	G	A	G	-0.0086	-0.01764	0.5847	0.58116	FALSE	FALSE	FALSE	LDUJbh	0.014016	0.208203	486484	sepsis	TRUE	reported	textfile	0.0018	1.05E-06	7367	M01898.m	TRUE	reported	JRSLeH	2	TRUE	0.003089	22.82096
1355	rs893175	A	C	A	C	-0.0086	0.001306	0.5884	0.597017	FALSE	FALSE	FALSE	LDUJbh	0.014103	0.926193	486484	sepsis	TRUE	reported	textfile	0.0018	1.53E-06	7367	M01898.m	TRUE	reported	JRSLeH	2	TRUE	0.003089	22.82096
9045	rs534863	T	C	T	C	-0.0086	0.010986	0.3246	0.306619	FALSE	FALSE	FALSE	df0MCM	0.014803	0.458021	486484	sepsis	TRUE	reported	textfile	0.0018	1.48E-06	7366	M37506.m	TRUE	reported	iUfr4u	2	TRUE	0.003089	22.82096
396	rs1001109	T	C	T	C	0.0086	-0.02269	0.8159	0.811894	FALSE	FALSE	FALSE	cTsNj6	0.017475	0.194065	486484	sepsis	TRUE	reported	textfile	0.0018	2.15E-06	7365	M00063.m	TRUE	reported	7KYTrQ	2	TRUE	0.00309	22.82096
426	rs3802195	T	G	T	G	0.0086	0.000353	0.1886	0.18295	FALSE	FALSE	FALSE	cTsNj6	0.017798	0.984174	486484	sepsis	TRUE	reported	textfile	0.0018	1.13E-06	7365	M00063.m	TRUE	reported	7KYTrQ	2	TRUE	0.00309	22.82096
1038	rs1262582	T	G	T	G	0.0086	0.002212	0.3366	0.345432	FALSE	FALSE	FALSE	tRtQ2e	0.014403	0.877943	486484	sepsis	TRUE	reported	textfile	0.0018	2.06E-06	7364	M01564.m	TRUE	reported	Tbg0zM	2	TRUE	0.00309	22.82096
1051	rs2370136	T	C	T	C	-0.0086	0.007386	0.4875	0.466404	FALSE	FALSE	FALSE	tRtQ2e	0.013691	0.58955	486484	sepsis	TRUE	reported	textfile	0.0018	1.25E-06	7364	M01564.m	TRUE	reported	Tbg0zM	2	TRUE	0.00309	22.82096
727	rs1091791	A	C	A	C	-0.0086	-0.01172	0.8597	0.860563	FALSE	FALSE	FALSE	9u0SC8	0.019723	0.552516	486484	sepsis	TRUE	reported	textfile	0.0018	1.65E-06	7363	M01301.m	TRUE	reported	H95uec	2	TRUE	0.003091	22.82096
744	rs9642758	A	G	A	G	0.0086	-0.0271	0.1578	0.151763	FALSE	FALSE	FALSE	9u0SC8	0.019349	0.161332	486484	sepsis	TRUE	reported	textfile	0.0018	1.28E-06	7363	M01301.m	TRUE	reported	H95uec	2	TRUE	0.003091	22.82096
4027	rs8087127	A	G	A	G	0.0086	0.014538	0.5695	0.573277	FALSE	FALSE	FALSE	V9AtId	0.013814	0.292626	486484	sepsis	TRUE	reported	textfile	0.0018	1.62E-06	7363	M32489.m	TRUE	reported	8B8S8a	2	TRUE	0.003091	22.82096
4306	rs709189	A	G	A	G	-0.0086	-0.00221	0.7218	0.713828	FALSE	FALSE	FALSE	0tjjPC	0.01518	0.884231	486484	sepsis	TRUE	reported	textfile	0.0018	1.60E-06	7362	M32634.m	TRUE	reported	nJJFkX	2	TRUE	0.003091	22.82096
5025	rs1742383	A	G	A	G	0.0086	-0.00684	0.8836	0.882321	FALSE	FALSE	FALSE	BNnOvt	0.021182	0.746732	486484	sepsis	TRUE	reported	textfile	0.0018	3.06E-06	7362	M33132.m	TRUE	reported	rHcCPG	2	TRUE	0.003091	22.82096
5032	rs2924762	T	C	T	C	0.0086	0.007219	0.8777	0.870635	FALSE	FALSE	FALSE	BNnOvt	0.020383	0.723206	486484	sepsis	TRUE	reported	textfile	0.0018	2.25E-06	7362	M33132.m	TRUE	reported	rHcCPG	2	TRUE	0.003091	22.82096
466	rs9312777	T	C	T	C	0.0043	0.029671	0.4788	0.480363	FALSE	FALSE	FALSE	cl8ucN	0.013783	0.031342	486484	sepsis	TRUE	reported	textfile	9.00E-04	4.68E-06	7361	M00513.m	TRUE	reported	lan0Q8	2	TRUE	0.003092	22.82096
12	rs1015795	A	G	A	G	0.0043	0.006039	0.5122	0.512713	FALSE	FALSE	FALSE	xmGuol	0.013677	0.658845	486484	sepsis	TRUE	reported	textfile	9.00E-04	2.26E-06	7355	M00054.m	TRUE	reported	GW50Ri	2	TRUE	0.003094	22.82095
16	rs1049826	T	G	T	G	0.0043	0.005663	0.4674	0.485478	FALSE	FALSE	FALSE	xmGuol	0.013671	0.678719	486484	sepsis	TRUE	reported	textfile	9.00E-04	2.35E-06	7355	M00054.m	TRUE	reported	GW50Ri	2	TRUE	0.003094	22.82095
19	rs1080550	T	C	T	C	0.0043	0.008526	0.6272	0.622354	FALSE	FALSE	FALSE	xmGuol	0.014116	0.545832	486484	sepsis	TRUE	reported	textfile	9.00E-04	2.00E-06	7355	M00054.m	TRUE	reported	GW50Ri	2	TRUE	0.003094	22.82095
20	rs1080732	A	T	A	T	0.0043	0.007242	0.5866	0.591895	FALSE	TRUE	FALSE	xmGuol	0.013929	0.6031	486484	sepsis	TRUE	reported	textfile	9.00E-04	1.97E-06	7355	M00054.m	TRUE	reported	GW50Ri	2	TRUE	0.003094	22.82095
28	rs1120103	A	T	A	T	0.0043	-0.00222	0.6064	0.605256	FALSE	TRUE	FALSE	xmGuol	0.014023	0.87433	486484	sepsis	TRUE	reported	textfile	9.00E-04	1.89E-06	7355	M00054.m	TRUE	reported	GW50Ri	2	TRUE	0.003094	22.82095
37	rs1227876	T	C	T	C	0.0043	0.034327	0.5229	0.512784	FALSE	FALSE	FALSE	xmGuol	0.013702	0.012234	486484	sepsis	TRUE	reported	textfile	9.00E-04	1.56E-06	7355	M00054.m	TRUE	reported	GW50Ri	2	TRUE	0.003094	22.82095
43	rs1305696	T	C	T	C	0.0043	-0.00701	0.4376	0.418294	FALSE	FALSE	FALSE	xmGuol	0.013846	0.612898	486484	sepsis	TRUE	reported	textfile	9.00E-04	5.58E-06	7355	M00054.m	TRUE	reported	GW50Ri	2	TRUE	0.003094	22.82095
68	rs207938	A	G	A	G	-0.0043	0.004305	0.3963	0.40186	FALSE	FALSE	FALSE	xmGuol	0.013956	0.75774	486484	sepsis	TRUE	reported	textfile	9.00E-04	1.32E-06	7355	M00054.m	TRUE	reported	GW50Ri	2	TRUE	0.003094	22.82095
69	rs2091289	A	G	A	G	0.0043	0.019531	0.5943	0.595894	FALSE	FALSE	FALSE	xmGuol	0.013897	0.159881	486484	sepsis	TRUE	reported	textfile	9.00E-04	1.50E-06	7355	M00054.m	TRUE	reported	GW50Ri	2	TRUE	0.003094	22.82095
73	rs2216061	A	C	A	C	0.0043	0.004334	0.512	0.509191	FALSE	FALSE	FALSE	xmGuol	0.013693	0.751595	486484	sepsis	TRUE	reported	textfile	9.00E-04	2.21E-06	7355	M00054.m	TRUE	reported	GW50Ri	2	TRUE	0.003094	22.82095
76	rs2383131	T	C	T	C	-0.0043	0.011802	0.4403	0.439499	FALSE	FALSE	FALSE	xmGuol	0.013785	0.391899	486484	sepsis	TRUE	reported	textfile	9.00E-04	3.05E-06	7355	M00054.m	TRUE	reported	GW50Ri	2	TRUE	0.003094	22.82095
77	rs2484178	T	C	T	C	0.0043	-0.0155	0.6063	0.605478	FALSE	FALSE	FALSE	xmGuol	0.013934	0.265911	486484	sepsis	TRUE	reported	textfile	9.00E-04	1.75E-06	7355	M00054.m	TRUE	reported	GW50Ri	2	TRUE	0.003094	22.82095
85	rs2688858	T	C	T	C	0.0043	0.003428	0.5742	0.582663	FALSE	FALSE	FALSE	xmGuol	0.013923	0.805517	486484	sepsis	TRUE	reported	textfile	9.00E-04	2.84E-06	7355	M00054.m	TRUE	reported	GW50Ri	2	TRUE	0.003094	22.82095
86	rs2717586	A	G	A	G	0.0043	0.00664	0.3962	0.399925	FALSE	FALSE	FALSE	xmGuol	0.014048	0.636455	486484	sepsis	TRUE	reported	textfile	9.00E-04	2.31E-06	7355	M00054.m	TRUE	reported	GW50Ri	2	TRUE	0.003094	22.82095
107	rs4063769	T	C	T	C	-0.0043	0.004025	0.5301	0.533444	FALSE	FALSE	FALSE	xmGuol	0.013734	0.769471	486484	sepsis	TRUE	reported	textfile	9.00E-04	1.30E-06	7355	M00054.m	TRUE	reported	GW50Ri	2	TRUE	0.003094	22.82095
140	rs6534667	C	G	C	G	-0.0043	-0.01347	0.4186	0.408912	FALSE	TRUE	FALSE	xmGuol	0.013893	0.332346	486484	sepsis	TRUE	reported	textfile	9.00E-04	1.49E-06	7355	M00054.m	TRUE	reported	GW50Ri	2	TRUE	0.003094	22.82095
142	rs6586235	A	C	A	C	0.0043	-0.00647	0.4598	0.463526	FALSE	FALSE	FALSE	xmGuol	0.01378	0.638597	486484	sepsis	TRUE	reported	textfile	9.00E-04	1.62E-06	7355	M00054.m	TRUE	reported	GW50Ri	2	TRUE	0.003094	22.82095
147	rs6819555	A	G	A	G	-0.0043	0.007394	0.5862	0.586771	FALSE	FALSE	FALSE	xmGuol	0.013882	0.594278	486484	sepsis	TRUE	reported	textfile	9.00E-04	3.69E-06	7355	M00054.m	TRUE	reported	GW50Ri	2	TRUE	0.003094	22.82095
160	rs7246393	A	G	A	G	0.0043	0.002201	0.5222	0.517799	FALSE	FALSE	FALSE	xmGuol	0.013705	0.872409	486484	sepsis	TRUE	reported	textfile	9.00E-04	1.55E-06	7355	M00054.m	TRUE	reported	GW50Ri	2	TRUE	0.003094	22.82095
162	rs7505177	T	G	T	G	-0.0043	-0.00346	0.4132	0.418399	FALSE	FALSE	FALSE	xmGuol	0.013872	0.803128	486484	sepsis	TRUE	reported	textfile	9.00E-04	5.86E-06	7355	M00054.m	TRUE	reported	GW50Ri	2	TRUE	0.003094	22.82095
175	rs875283	A	G	A	G	-0.0043	0.033875	0.4896	0.490423	FALSE	FALSE	FALSE	xmGuol	0.013766	0.013867	486484	sepsis	TRUE	reported	textfile	9.00E-04	2.34E-06	7355	M00054.m	TRUE	reported	GW50Ri	2	TRUE	0.003094	22.82095
187	rs9523030	T	G	T	G	0.0043	-0.01049	0.6269	0.626803	FALSE	FALSE	FALSE	xmGuol	0.014483	0.468982	486484	sepsis	TRUE	reported	textfile	9.00E-04	5.65E-06	7355	M00054.m	TRUE	reported	GW50Ri	2	TRUE	0.003094	22.82095
190	rs9610322	A	G	A	G	-0.0043	-0.01637	0.5195	0.528354	FALSE	FALSE	FALSE	xmGuol	0.01377	0.234636	486484	sepsis	TRUE	reported	textfile	9.00E-04	1.12E-06	7355	M00054.m	TRUE	reported	GW50Ri	2	TRUE	0.003094	22.82095
1204	rs203591	T	C	T	C	-0.0086	0.025045	0.3697	0.364985	FALSE	FALSE	FALSE	Ckdgh1	0.014212	0.078024	486484	sepsis	TRUE	reported	textfile	0.0018	2.01E-06	7355	M01644.m	TRUE	reported	nKxz7B	2	TRUE	0.003094	22.82095
4035	rs1247803	T	C	T	C	-0.0086	0.01711	0.6526	0.657424	FALSE	FALSE	FALSE	SiYGYU	0.014415	0.235238	486484	sepsis	TRUE	reported	textfile	0.0018	2.79E-06	7355	M32492.m	TRUE	reported	GfQvpU	2	TRUE	0.003094	22.82095
4045	rs2706095	A	G	A	G	-0.0086	-0.00964	0.3359	0.355974	FALSE	FALSE	FALSE	SiYGYU	0.014345	0.501515	486484	sepsis	TRUE	reported	textfile	0.0018	2.38E-06	7355	M32492.m	TRUE	reported	GfQvpU	2	TRUE	0.003094	22.82095
563	rs163425	A	G	A	G	0.0086	0.012188	0.8734	0.870653	FALSE	FALSE	FALSE	fNqkCW	0.020376	0.549746	486484	sepsis	TRUE	reported	textfile	0.0018	2.62E-06	7354	M00606.m	TRUE	reported	g9uwOn	2	TRUE	0.003094	22.82095
2805	rs1944518	T	C	T	C	-0.0086	0.009279	0.3068	0.324905	FALSE	FALSE	FALSE	Sx5Uhq	0.014619	0.525607	486484	sepsis	TRUE	reported	textfile	0.0018	1.28E-06	7354	M19934.m	TRUE	reported	DijYTE	2	TRUE	0.003094	22.82095
6754	rs1081458	A	G	A	G	0.0086	-0.00279	0.3122	0.317622	FALSE	FALSE	FALSE	Elf6MQ	0.014848	0.851011	486484	sepsis	TRUE	reported	textfile	0.0018	1.53E-06	7354	M33955.m	TRUE	reported	Lxn4RF	2	TRUE	0.003094	22.82095
6777	rs7547153	A	G	A	G	-0.0086	-0.01821	0.5893	0.58799	FALSE	FALSE	FALSE	Elf6MQ	0.014011	0.193587	486484	sepsis	TRUE	reported	textfile	0.0018	1.13E-06	7354	M33955.m	TRUE	reported	Lxn4RF	2	TRUE	0.003094	22.82095
221	rs1095441	A	T	A	T	0.0043	0.015536	0.3772	0.359756	FALSE	TRUE	FALSE	tbRL8J	0.014232	0.274989	486484	sepsis	TRUE	reported	textfile	9.00E-04	1.81E-06	7351	M00060.m	TRUE	reported	oOvRKT	2	TRUE	0.003096	22.82095
223	rs1100708	T	G	T	G	0.0043	0.023986	0.3779	0.379086	FALSE	FALSE	FALSE	tbRL8J	0.014071	0.088267	486484	sepsis	TRUE	reported	textfile	9.00E-04	1.84E-06	7351	M00060.m	TRUE	reported	oOvRKT	2	TRUE	0.003096	22.82095
226	rs1112474	A	G	A	G	0.0043	0.018307	0.6354	0.62961	FALSE	FALSE	FALSE	tbRL8J	0.014204	0.197462	486484	sepsis	TRUE	reported	textfile	9.00E-04	1.91E-06	7351	M00060.m	TRUE	reported	oOvRKT	2	TRUE	0.003096	22.82095
229	rs1120304	A	G	A	G	-0.0043	-0.02987	0.4458	0.447084	FALSE	FALSE	FALSE	tbRL8J	0.013743	0.029744	486484	sepsis	TRUE	reported	textfile	9.00E-04	1.95E-06	7351	M00060.m	TRUE	reported	oOvRKT	2	TRUE	0.003096	22.82095
259	rs1705832	T	C	T	C	0.0043	0.000269	0.395	0.39505	FALSE	FALSE	FALSE	tbRL8J	0.013981	0.984656	486484	sepsis	TRUE	reported	textfile	9.00E-04	1.90E-06	7351	M00060.m	TRUE	reported	oOvRKT	2	TRUE	0.003096	22.82095
269	rs1931001	T	G	T	G	-0.0043	0.025871	0.3503	0.345403	FALSE	FALSE	FALSE	tbRL8J	0.014396	0.072322	486484	sepsis	TRUE	reported	textfile	9.00E-04	1.62E-06	7351	M00060.m	TRUE	reported	oOvRKT	2	TRUE	0.003096	22.82095
277	rs2273875	C	G	C	G	0.0043	0.032902	0.37	0.363352	FALSE	TRUE	FALSE	tbRL8J	0.014222	0.020699	486484	sepsis	TRUE	reported	textfile	9.00E-04	1.82E-06	7351	M00060.m	TRUE	reported	oOvRKT	2	TRUE	0.003096	22.82095
293	rs2819322	T	C	T	C	-0.0043	0.002219	0.4589	0.458099	FALSE	FALSE	FALSE	tbRL8J	0.013738	0.871705	486484	sepsis	TRUE	reported	textfile	9.00E-04	1.79E-06	7351	M00060.m	TRUE	reported	oOvRKT	2	TRUE	0.003096	22.82095
325	rs4968791	A	G	A	G	0.0043	0.018002	0.5344	0.53217	FALSE	FALSE	FALSE	tbRL8J	0.013771	0.191148	486484	sepsis	TRUE	reported	textfile	9.00E-04	1.48E-06	7351	M00060.m	TRUE	reported	oOvRKT	2	TRUE	0.003096	22.82095
328	rs6103894	T	G	T	G	-0.0043	0.008882	0.5939	0.605357	FALSE	FALSE	FALSE	tbRL8J	0.014001	0.525833	486484	sepsis	TRUE	reported	textfile	9.00E-04	2.71E-06	7351	M00060.m	TRUE	reported	oOvRKT	2	TRUE	0.003096	22.82095
334	rs6518309	A	C	A	C	0.0043	-0.01384	0.6195	0.607629	FALSE	FALSE	FALSE	tbRL8J	0.014003	0.322822	486484	sepsis	TRUE	reported	textfile	9.00E-04	1.78E-06	7351	M00060.m	TRUE	reported	oOvRKT	2	TRUE	0.003096	22.82095
335	rs6562776	A	G	A	G	-0.0043	0.011732	0.6116	0.60265	FALSE	FALSE	FALSE	tbRL8J	0.013997	0.401919	486484	sepsis	TRUE	reported	textfile	9.00E-04	1.65E-06	7351	M00060.m	TRUE	reported	oOvRKT	2	TRUE	0.003096	22.82095
348	rs720293	A	G	A	G	0.0043	-0.00096	0.4312	0.423747	FALSE	FALSE	FALSE	tbRL8J	0.013863	0.944949	486484	sepsis	TRUE	reported	textfile	9.00E-04	2.53E-06	7351	M00060.m	TRUE	reported	oOvRKT	2	TRUE	0.003096	22.82095
350	rs7301176	T	C	T	C	0.0043	0.001906	0.4603	0.453092	FALSE	FALSE	FALSE	tbRL8J	0.013821	0.890296	486484	sepsis	TRUE	reported	textfile	9.00E-04	1.86E-06	7351	M00060.m	TRUE	reported	oOvRKT	2	TRUE	0.003096	22.82095
369	rs7911876	T	C	T	C	0.0043	0.015389	0.4276	0.428668	FALSE	FALSE	FALSE	tbRL8J	0.014131	0.276124	486484	sepsis	TRUE	reported	textfile	9.00E-04	2.76E-06	7351	M00060.m	TRUE	reported	oOvRKT	2	TRUE	0.003096	22.82095
388	rs9369741	T	G	T	G	0.0043	-0.01607	0.5822	0.581551	FALSE	FALSE	FALSE	tbRL8J	0.013894	0.24748	486484	sepsis	TRUE	reported	textfile	9.00E-04	2.42E-06	7351	M00060.m	TRUE	reported	oOvRKT	2	TRUE	0.003096	22.82095
390	rs979242	T	C	T	C	-0.0043	-0.01957	0.3692	0.373345	FALSE	FALSE	FALSE	tbRL8J	0.014153	0.166856	486484	sepsis	TRUE	reported	textfile	9.00E-04	2.45E-06	7351	M00060.m	TRUE	reported	oOvRKT	2	TRUE	0.003096	22.82095
392	rs9837954	T	C	T	C	-0.0043	0.017248	0.6418	0.636952	FALSE	FALSE	FALSE	tbRL8J	0.014328	0.22866	486484	sepsis	TRUE	reported	textfile	9.00E-04	2.64E-06	7351	M00060.m	TRUE	reported	oOvRKT	2	TRUE	0.003096	22.82095
4106	rs2053595	A	G	A	G	0.0086	-0.00325	0.312	0.342847	FALSE	FALSE	FALSE	4rtLd0	0.014407	0.821397	486484	sepsis	TRUE	reported	textfile	0.0018	2.98E-06	7350	M32518.m	TRUE	reported	j7f1ER	2	TRUE	0.003096	22.82095
1887	rs1193038	T	C	T	C	-0.0043	0.011398	0.4025	0.399667	FALSE	FALSE	FALSE	v2KE89	0.013989	0.415191	486484	sepsis	TRUE	reported	textfile	9.00E-04	2.05E-06	7349	M15500.m	TRUE	reported	to2t7H	2	TRUE	0.003097	22.82095
1892	rs1224525	T	C	T	C	0.0043	0.019528	0.4414	0.443316	FALSE	FALSE	FALSE	v2KE89	0.01378	0.156454	486484	sepsis	TRUE	reported	textfile	9.00E-04	1.92E-06	7349	M15500.m	TRUE	reported	to2t7H	2	TRUE	0.003097	22.82095
1893	rs1225019	A	G	A	G	-0.0043	-0.00902	0.5791	0.585618	FALSE	FALSE	FALSE	v2KE89	0.013892	0.51592	486484	sepsis	TRUE	reported	textfile	9.00E-04	1.59E-06	7349	M15500.m	TRUE	reported	to2t7H	2	TRUE	0.003097	22.82095
1900	rs1251090	T	C	T	C	-0.0043	0.01704	0.5338	0.529376	FALSE	FALSE	FALSE	v2KE89	0.013688	0.213185	486484	sepsis	TRUE	reported	textfile	9.00E-04	1.84E-06	7349	M15500.m	TRUE	reported	to2t7H	2	TRUE	0.003097	22.82095
1908	rs1317661	A	G	A	G	0.0043	0.023384	0.4794	0.475347	FALSE	FALSE	FALSE	v2KE89	0.013671	0.087177	486484	sepsis	TRUE	reported	textfile	9.00E-04	1.86E-06	7349	M15500.m	TRUE	reported	to2t7H	2	TRUE	0.003097	22.82095
1925	rs1544412	T	C	T	C	-0.0043	0.020248	0.3552	0.355826	FALSE	FALSE	FALSE	v2KE89	0.014295	0.156643	486484	sepsis	TRUE	reported	textfile	9.00E-04	2.68E-06	7349	M15500.m	TRUE	reported	to2t7H	2	TRUE	0.003097	22.82095
1933	rs1929425	T	C	T	C	0.0043	-0.0032	0.3937	0.389444	FALSE	FALSE	FALSE	v2KE89	0.014157	0.821325	486484	sepsis	TRUE	reported	textfile	9.00E-04	2.08E-06	7349	M15500.m	TRUE	reported	to2t7H	2	TRUE	0.003097	22.82095
1934	rs1936602	T	C	T	C	0.0043	-0.01541	0.4194	0.422025	FALSE	FALSE	FALSE	v2KE89	0.013944	0.269036	486484	sepsis	TRUE	reported	textfile	9.00E-04	2.53E-06	7349	M15500.m	TRUE	reported	to2t7H	2	TRUE	0.003097	22.82095
1953	rs2451996	A	G	A	G	0.0043	0.007966	0.5799	0.572096	FALSE	FALSE	FALSE	v2KE89	0.013854	0.565291	486484	sepsis	TRUE	reported	textfile	9.00E-04	1.67E-06	7349	M15500.m	TRUE	reported	to2t7H	2	TRUE	0.003097	22.82095
1958	rs2577264	A	G	A	G	0.0043	0.014707	0.3826	0.382091	FALSE	FALSE	FALSE	v2KE89	0.014103	0.297046	486484	sepsis	TRUE	reported	textfile	9.00E-04	2.12E-06	7349	M15500.m	TRUE	reported	to2t7H	2	TRUE	0.003097	22.82095
1964	rs2789599	A	G	A	G	0.0043	-0.01396	0.5029	0.495736	FALSE	FALSE	FALSE	v2KE89	0.013709	0.308665	486484	sepsis	TRUE	reported	textfile	9.00E-04	2.16E-06	7349	M15500.m	TRUE	reported	to2t7H	2	TRUE	0.003097	22.82095
1965	rs288332	A	G	A	G	-0.0043	-0.00071	0.6294	0.630267	FALSE	FALSE	FALSE	v2KE89	0.014152	0.959876	486484	sepsis	TRUE	reported	textfile	9.00E-04	1.83E-06	7349	M15500.m	TRUE	reported	to2t7H	2	TRUE	0.003097	22.82095
1969	rs3127274	T	C	T	C	0.0043	0.002391	0.4172	0.418129	FALSE	FALSE	FALSE	v2KE89	0.013865	0.863108	486484	sepsis	TRUE	reported	textfile	9.00E-04	2.09E-06	7349	M15500.m	TRUE	reported	to2t7H	2	TRUE	0.003097	22.82095
1981	rs4525910	A	T	A	T	-0.0043	-0.01742	0.4052	0.405695	FALSE	TRUE	FALSE	v2KE89	0.013932	0.211295	486484	sepsis	TRUE	reported	textfile	9.00E-04	2.20E-06	7349	M15500.m	TRUE	reported	to2t7H	2	TRUE	0.003097	22.82095
1986	rs4665406	T	C	T	C	-0.0043	0.001609	0.3652	0.367426	FALSE	FALSE	FALSE	v2KE89	0.014204	0.909788	486484	sepsis	TRUE	reported	textfile	9.00E-04	1.94E-06	7349	M15500.m	TRUE	reported	to2t7H	2	TRUE	0.003097	22.82095
1998	rs4861126	C	G	C	G	0.0043	-0.01156	0.411	0.415197	FALSE	TRUE	FALSE	v2KE89	0.01386	0.404429	486484	sepsis	TRUE	reported	textfile	9.00E-04	1.56E-06	7349	M15500.m	TRUE	reported	to2t7H	2	TRUE	0.003097	22.82095
2004	rs549386	T	C	T	C	-0.0043	0.012994	0.6142	0.607058	FALSE	FALSE	FALSE	v2KE89	0.014031	0.354406	486484	sepsis	TRUE	reported	textfile	9.00E-04	1.63E-06	7349	M15500.m	TRUE	reported	to2t7H	2	TRUE	0.003097	22.82095
2010	rs6539588	T	C	T	C	0.0043	-0.00461	0.3875	0.38282	FALSE	FALSE	FALSE	v2KE89	0.014096	0.743765	486484	sepsis	TRUE	reported	textfile	9.00E-04	1.92E-06	7349	M15500.m	TRUE	reported	to2t7H	2	TRUE	0.003097	22.82095
2016	rs6793266	T	C	T	C	-0.0043	-0.00028	0.3866	0.401688	FALSE	FALSE	FALSE	v2KE89	0.014007	0.983935	486484	sepsis	TRUE	reported	textfile	9.00E-04	2.92E-06	7349	M15500.m	TRUE	reported	to2t7H	2	TRUE	0.003097	22.82095
2021	rs6937910	T	C	T	C	-0.0043	0.032461	0.5268	0.517032	FALSE	FALSE	FALSE	v2KE89	0.01373	0.018062	486484	sepsis	TRUE	reported	textfile	9.00E-04	1.41E-06	7349	M15500.m	TRUE	reported	to2t7H	2	TRUE	0.003097	22.82095
2026	rs724752	T	C	T	C	0.0043	0.025181	0.3696	0.374736	FALSE	FALSE	FALSE	v2KE89	0.014115	0.074427	486484	sepsis	TRUE	reported	textfile	9.00E-04	2.65E-06	7349	M15500.m	TRUE	reported	to2t7H	2	TRUE	0.003097	22.82095
2037	rs7929841	T	C	T	C	0.0043	0.0125	0.6502	0.657228	FALSE	FALSE	FALSE	v2KE89	0.014402	0.385423	486484	sepsis	TRUE	reported	textfile	9.00E-04	2.10E-06	7349	M15500.m	TRUE	reported	to2t7H	2	TRUE	0.003097	22.82095
2056	rs968103	T	C	T	C	0.0043	-0.01804	0.4463	0.4586	FALSE	FALSE	FALSE	v2KE89	0.013807	0.191475	486484	sepsis	TRUE	reported	textfile	9.00E-04	1.50E-06	7349	M15500.m	TRUE	reported	to2t7H	2	TRUE	0.003097	22.82095
2065	rs9947578	A	G	A	G	0.0043	-1.24E-05	0.5631	0.568339	FALSE	FALSE	FALSE	v2KE89	0.013895	0.999287	486484	sepsis	TRUE	reported	textfile	9.00E-04	3.94E-06	7349	M15500.m	TRUE	reported	to2t7H	2	TRUE	0.003097	22.82095
2068	rs9997562	T	C	T	C	0.0043	-0.00773	0.5196	0.512616	FALSE	FALSE	FALSE	v2KE89	0.013676	0.572108	486484	sepsis	TRUE	reported	textfile	9.00E-04	1.86E-06	7349	M15500.m	TRUE	reported	to2t7H	2	TRUE	0.003097	22.82095
8374	rs1307330	A	G	A	G	0.0086	-0.01376	0.5896	0.602437	FALSE	FALSE	FALSE	SCzh0A	0.013993	0.325374	486484	sepsis	TRUE	reported	textfile	0.0018	2.70E-06	7348	M35675.m	TRUE	reported	n1SREk	2	TRUE	0.003097	22.82095
8393	rs4935623	A	G	A	G	-0.0086	0.009607	0.4782	0.481908	FALSE	FALSE	FALSE	SCzh0A	0.013685	0.482662	486484	sepsis	TRUE	reported	textfile	0.0018	2.28E-06	7348	M35675.m	TRUE	reported	n1SREk	2	TRUE	0.003097	22.82095
3796	rs890230	T	C	T	C	0.0086	-0.00683	0.4592	0.452164	FALSE	FALSE	FALSE	NW1aaR	0.013709	0.618563	486484	sepsis	TRUE	reported	textfile	0.0018	1.06E-06	7344	M32339.m	TRUE	reported	pBPkAB	2	TRUE	0.003099	22.82094
4948	rs1981409	A	T	A	T	-0.0086	-0.00261	0.6036	0.601302	FALSE	TRUE	FALSE	bKmDMa	0.014544	0.857773	486484	sepsis	TRUE	reported	textfile	0.0018	9.93E-07	7344	M32910.m	TRUE	reported	nAuPzO	2	TRUE	0.003099	22.82094
3631	rs9676475	T	C	T	C	0.0387	-0.00377	0.2123	0.19004	FALSE	FALSE	FALSE	gq4lSJ	0.017506	0.829378	486484	sepsis	TRUE	reported	textfile	0.0081	1.72E-06	7338	M31591.m	TRUE	reported	wjwl4J	2	TRUE	0.003101	22.82094
8686	rs2839579	A	G	A	G	0.0043	0.002197	0.312	0.301576	FALSE	FALSE	FALSE	X2e8qc	0.015019	0.883692	486484	sepsis	TRUE	reported	textfile	9.00E-04	4.45E-06	7330	M36515.m	TRUE	reported	OaWQSH	2	TRUE	0.003105	22.82093
8690	rs8184882	T	C	T	C	0.0043	0.004866	0.6224	0.615242	FALSE	FALSE	FALSE	X2e8qc	0.014025	0.72863	486484	sepsis	TRUE	reported	textfile	9.00E-04	7.27E-06	7330	M36515.m	TRUE	reported	OaWQSH	2	TRUE	0.003105	22.82093
3208	rs4702556	T	C	T	C	0.0129	-0.03533	0.3237	0.446039	FALSE	FALSE	FALSE	UfLkBq	0.01377	0.010287	486484	sepsis	TRUE	reported	textfile	0.0027	1.99E-06	7329	M22481.m	TRUE	reported	kWMdiT	2	TRUE	0.003105	22.82093
1388	rs539904	A	G	A	G	-0.0086	0.016278	0.7056	0.700386	FALSE	FALSE	FALSE	KrWhws	0.015042	0.279166	486484	sepsis	TRUE	reported	textfile	0.0018	1.51E-06	7325	M02132.m	TRUE	reported	7pzpfK	2	TRUE	0.003107	22.82093
1399	rs8021336	A	G	A	G	-0.0086	-0.0147	0.313	0.319689	FALSE	FALSE	FALSE	KrWhws	0.014711	0.317824	486484	sepsis	TRUE	reported	textfile	0.0018	9.89E-07	7325	M02132.m	TRUE	reported	7pzpfK	2	TRUE	0.003107	22.82093
7032	rs1028777	A	C	A	C	-0.0086	-0.01067	0.6523	0.648615	FALSE	FALSE	FALSE	uthruC	0.014322	0.456074	486484	sepsis	TRUE	reported	textfile	0.0018	1.52E-06	7316	M34283.m	TRUE	reported	ftTuRU	2	TRUE	0.00311	22.82092
7033	rs1044446	T	C	T	C	0.0086	-0.00337	0.5177	0.520044	FALSE	FALSE	FALSE	uthruC	0.013677	0.805277	486484	sepsis	TRUE	reported	textfile	0.0018	1.71E-06	7316	M34283.m	TRUE	reported	ftTuRU	2	TRUE	0.00311	22.82092
2493	rs1044395	A	G	A	G	0.0086	-0.00525	0.382	0.378273	FALSE	FALSE	FALSE	LwUYua	0.014132	0.710423	486484	sepsis	TRUE	reported	textfile	0.0018	2.60E-06	7315	M17945.m	TRUE	reported	SUN8dc	2	TRUE	0.003111	22.82092
5857	rs1008970	T	G	T	G	-0.0086	0.003624	0.4652	0.461048	FALSE	FALSE	FALSE	tqW0ld	0.013722	0.791697	486484	sepsis	TRUE	reported	textfile	0.0018	3.15E-06	7315	M33389.m	TRUE	reported	Sv7EyV	2	TRUE	0.003111	22.82092
2206	rs2317834	T	G	T	G	0.0129	-0.00467	0.9041	0.885435	FALSE	FALSE	FALSE	Bf6uMK	0.021522	0.828065	486484	sepsis	TRUE	reported	textfile	0.0027	2.31E-06	7308	M15778.m	TRUE	reported	jowM2N	2	TRUE	0.003114	22.82091
2208	rs310528	A	G	A	G	0.0086	-0.03915	0.2233	0.228205	FALSE	FALSE	FALSE	Bf6uMK	0.016376	0.016802	486484	sepsis	TRUE	reported	textfile	0.0018	2.59E-06	7308	M15778.m	TRUE	reported	jowM2N	2	TRUE	0.003114	22.82091
2223	rs7513165	T	C	T	C	0.0086	-0.00471	0.7526	0.751663	FALSE	FALSE	FALSE	Bf6uMK	0.015908	0.767204	486484	sepsis	TRUE	reported	textfile	0.0018	2.83E-06	7308	M15778.m	TRUE	reported	jowM2N	2	TRUE	0.003114	22.82091
2230	rs969075	T	C	T	C	-0.0086	-0.00137	0.3346	0.335177	FALSE	FALSE	FALSE	Bf6uMK	0.014534	0.924669	486484	sepsis	TRUE	reported	textfile	0.0018	2.63E-06	7308	M15778.m	TRUE	reported	jowM2N	2	TRUE	0.003114	22.82091
6123	rs1105484	A	G	A	G	-0.0086	-0.01916	0.3623	0.366155	FALSE	FALSE	FALSE	MiSJly	0.014228	0.178177	486484	sepsis	TRUE	reported	textfile	0.0018	2.85E-06	7307	M33477.m	TRUE	reported	WwL1hq	2	TRUE	0.003114	22.82091
6133	rs201252	T	C	T	C	0.0086	0.023185	0.5963	0.597853	FALSE	FALSE	FALSE	MiSJly	0.013966	0.096897	486484	sepsis	TRUE	reported	textfile	0.0018	1.61E-06	7307	M33477.m	TRUE	reported	WwL1hq	2	TRUE	0.003114	22.82091
6146	rs612149	A	G	A	G	0.0129	0.01035	0.1538	0.164236	FALSE	FALSE	FALSE	MiSJly	0.018536	0.576571	486484	sepsis	TRUE	reported	textfile	0.0027	2.04E-06	7307	M33477.m	TRUE	reported	WwL1hq	2	TRUE	0.003114	22.82091
5987	rs2241217	T	C	T	C	0.0086	-0.01351	0.7557	0.741043	FALSE	FALSE	FALSE	3KsoXq	0.015838	0.393727	486484	sepsis	TRUE	reported	textfile	0.0018	2.73E-06	7306	M33422.m	TRUE	reported	aNr6Ug	2	TRUE	0.003115	22.82091
3339	rs4832148	T	C	T	C	0.0086	-0.02989	0.8088	0.805246	FALSE	FALSE	FALSE	m66Eav	0.017546	0.088465	486484	sepsis	TRUE	reported	textfile	0.0018	2.65E-06	7303	M27256.m	TRUE	reported	pI2ECU	2	TRUE	0.003116	22.82091
1106	rs1104542	A	G	A	G	0.0086	0.016773	0.1266	0.131372	FALSE	FALSE	FALSE	yahiGZ	0.020354	0.409898	486484	sepsis	TRUE	reported	textfile	0.0018	1.83E-06	7271	M01585.m	TRUE	reported	dwZ7vX	2	TRUE	0.00313	22.82088
4342	rs267583	A	G	A	G	0.0086	-0.00088	0.2882	0.293685	FALSE	FALSE	FALSE	6VSoQQ	0.015061	0.953501	486484	sepsis	TRUE	reported	textfile	0.0018	2.88E-06	7229	M32644.m	TRUE	reported	oC2AV0	2	TRUE	0.003148	22.82085
1441	rs1760243	T	C	T	C	0.0172	0.015932	0.079	0.068106	FALSE	FALSE	FALSE	1M4gqj	0.027211	0.558209	486484	sepsis	TRUE	reported	textfile	0.0036	1.57E-06	7226	M02730.m	TRUE	reported	RWyYEM	2	TRUE	0.003149	22.82084
8045	rs4886189	A	G	A	G	0.0086	0.008614	0.4854	0.503288	FALSE	FALSE	FALSE	SwiqSi	0.013799	0.532447	486484	sepsis	TRUE	reported	textfile	0.0018	1.77E-06	7217	M35270.m	TRUE	reported	21ZlsQ	2	TRUE	0.003153	22.82083
8054	rs9351602	T	C	T	C	-0.0086	-0.01176	0.2627	0.272883	FALSE	FALSE	FALSE	SwiqSi	0.015432	0.445971	486484	sepsis	TRUE	reported	textfile	0.0018	1.36E-06	7217	M35270.m	TRUE	reported	21ZlsQ	2	TRUE	0.003153	22.82083
5831	rs4748632	A	G	A	G	0.0086	0.00681	0.8015	0.788892	FALSE	FALSE	FALSE	R5joEf	0.016806	0.685318	486484	sepsis	TRUE	reported	textfile	0.0018	1.45E-06	7118	M33380.m	TRUE	reported	kRvbfZ	2	TRUE	0.003197	22.82075
1637	rs196885	T	C	T	C	-0.0086	0.004141	0.8501	0.845262	FALSE	FALSE	FALSE	n5rDuT	0.018923	0.826774	486484	sepsis	TRUE	reported	textfile	0.0018	1.22E-06	7081	M12067.m	TRUE	reported	ZQLTeR	2	TRUE	0.003213	22.82071
6254	rs1097688	A	G	A	G	0.0086	-0.01018	0.6202	0.620204	FALSE	FALSE	FALSE	MOonP9	0.014206	0.473656	486484	sepsis	TRUE	reported	textfile	0.0018	2.79E-06	7066	M33515.m	TRUE	reported	226GuP	2	TRUE	0.00322	22.8207
6277	rs4766046	A	C	A	C	-0.0086	-0.01382	0.5752	0.564554	FALSE	FALSE	FALSE	MOonP9	0.013844	0.318292	486484	sepsis	TRUE	reported	textfile	0.0018	2.69E-06	7066	M33515.m	TRUE	reported	226GuP	2	TRUE	0.00322	22.8207
6281	rs4890333	A	G	A	G	-0.0086	0.000158	0.5104	0.503935	FALSE	FALSE	FALSE	MOonP9	0.013663	0.990783	486484	sepsis	TRUE	reported	textfile	0.0018	2.06E-06	7066	M33515.m	TRUE	reported	226GuP	2	TRUE	0.00322	22.8207
6286	rs6764891	A	G	A	G	0.0086	0.016814	0.3475	0.354699	FALSE	FALSE	FALSE	MOonP9	0.014366	0.241832	486484	sepsis	TRUE	reported	textfile	0.0018	2.89E-06	7066	M33515.m	TRUE	reported	226GuP	2	TRUE	0.00322	22.8207
1464	rs1242920	A	G	A	G	0.0086	0.018518	0.3376	0.343571	FALSE	FALSE	FALSE	pkb876	0.014395	0.198307	486484	sepsis	TRUE	reported	textfile	0.0018	1.85E-06	7037	M02734.m	TRUE	reported	0LCMVI	2	TRUE	0.003233	22.82067
8192	rs1100625	T	C	T	C	0.0129	-0.01908	0.201	0.206746	FALSE	FALSE	FALSE	iLlpho	0.016944	0.260203	486484	sepsis	TRUE	reported	textfile	0.0027	1.55E-06	7007	M35431.m	TRUE	reported	AAZ5ge	2	TRUE	0.003247	22.82064
8136	rs1002567	T	G	T	G	-0.0086	-0.00225	0.1997	0.197513	FALSE	FALSE	FALSE	lv3pAQ	0.017289	0.896381	486484	sepsis	TRUE	reported	textfile	0.0018	1.27E-06	6920	M35327.m	TRUE	reported	3Vsglp	2	TRUE	0.003288	22.82056
8153	rs9787087	A	G	A	G	0.0086	-0.01179	0.1893	0.189787	FALSE	FALSE	FALSE	lv3pAQ	0.017464	0.499581	486484	sepsis	TRUE	reported	textfile	0.0018	1.47E-06	6920	M35327.m	TRUE	reported	3Vsglp	2	TRUE	0.003288	22.82056
653	rs1722427	A	G	A	G	0.0951	0.032139	0.0778	0.097738	FALSE	FALSE	FALSE	7BPGuQ	0.023047	0.163167	486484	sepsis	TRUE	reported	textfile	0.0199	1.83E-06	2639	M01123.m	TRUE	reported	WV2hVn	2	TRUE	0.00858	22.82052
7285	rs1338182	C	G	C	G	0.0086	-0.01449	0.6082	0.606574	FALSE	TRUE	FALSE	Vcz4EA	0.013997	0.300648	486484	sepsis	TRUE	reported	textfile	0.0018	1.51E-06	6755	M34359.m	TRUE	reported	aUSo2J	2	TRUE	0.003368	22.8204
2301	rs1335768	T	C	T	C	0.0129	-0.0093	0.2441	0.243488	FALSE	FALSE	FALSE	ZzUW8U	0.015932	0.559268	486484	sepsis	TRUE	reported	textfile	0.0027	1.96E-06	6733	M16821.m	TRUE	reported	g2xs3i	2	TRUE	0.003379	22.82038
3013	rs2865986	A	G	A	G	-0.0129	-0.00124	0.6566	0.65923	FALSE	FALSE	FALSE	gxupFu	0.014416	0.931547	486484	sepsis	TRUE	reported	textfile	0.0027	1.63E-06	6556	M21188.m	TRUE	reported	gwwXh2	2	TRUE	0.00347	22.8202
1006	rs2604548	T	C	T	C	-0.0086	-0.02044	0.6463	0.643628	FALSE	FALSE	FALSE	jfkZbM	0.014335	0.153985	486484	sepsis	TRUE	reported	textfile	0.0018	1.29E-06	6523	M01558.m	TRUE	reported	LFZvMG	2	TRUE	0.003487	22.82016
1012	rs604388	T	C	T	C	0.0086	-0.01986	0.6845	0.67443	FALSE	FALSE	FALSE	jfkZbM	0.014574	0.172915	486484	sepsis	TRUE	reported	textfile	0.0018	1.56E-06	6523	M01558.m	TRUE	reported	LFZvMG	2	TRUE	0.003487	22.82016
5679	rs9656510	A	G	A	G	0.252	0.020589	0.0464	0.025865	FALSE	FALSE	FALSE	AAOSvG	0.04312	0.633015	486484	sepsis	TRUE	reported	textfile	0.0527	1.71E-06	996	M33197.m	TRUE	reported	aAmcal	2	TRUE	0.022442	22.81954
8096	rs1162399	A	G	A	G	-0.0086	0.005912	0.3453	0.338577	FALSE	FALSE	FALSE	nNbXBS	0.014448	0.682391	486484	sepsis	TRUE	reported	textfile	0.0018	9.23E-07	5768	M35326.m	TRUE	reported	UNDorn	2	TRUE	0.003942	22.81925
3092	rs1341513	T	C	T	C	-0.0129	-0.0019	0.2763	0.283863	FALSE	FALSE	FALSE	mr0awv	0.0152	0.90035	486484	sepsis	TRUE	reported	textfile	0.0027	2.90E-06	5702	M22130.m	TRUE	reported	Y2TfWQ	2	TRUE	0.003987	22.81915
3102	rs747902	A	G	A	G	-0.0129	0.001799	0.6122	0.648765	FALSE	FALSE	FALSE	mr0awv	0.014445	0.900872	486484	sepsis	TRUE	reported	textfile	0.0027	2.13E-06	5702	M22130.m	TRUE	reported	Y2TfWQ	2	TRUE	0.003987	22.81915
7399	rs9910557	A	G	A	G	-0.0215	-0.0023	0.4874	0.482973	FALSE	FALSE	FALSE	AW6M5u	0.013712	0.866678	486484	sepsis	TRUE	reported	textfile	0.0045	1.62E-06	5195	M34389.m	TRUE	reported	6ZYYQK	2	TRUE	0.004375	22.81837
4187	rs1341146	T	G	T	G	-0.0406	0.016781	0.734	0.735647	FALSE	FALSE	FALSE	TxOCzG	0.015666	0.284087	486484	sepsis	TRUE	reported	textfile	0.0085	1.66E-06	6970	M32564.m	TRUE	reported	9JP6ND	2	TRUE	0.003263	22.80812
5940	rs1769838	T	C	T	C	0.0406	-0.01264	0.7946	0.783193	FALSE	FALSE	FALSE	yepzrL	0.016571	0.44572	486484	sepsis	TRUE	reported	textfile	0.0085	1.88E-06	3890	M33408.m	TRUE	reported	SY4CoS	2	TRUE	0.005831	22.80294
4910	rs7037165	A	G	A	G	0.0277	-0.06796	0.0124	0.01137	FALSE	FALSE	FALSE	VqcRGX	0.064925	0.295211	486484	sepsis	TRUE	reported	textfile	0.0058	2.03E-06	7329	M32867.m	TRUE	reported	psuFJy	2	TRUE	0.003102	22.80263
627	rs1115989	T	C	T	C	0.032	0.011401	0.3295	0.320157	FALSE	FALSE	FALSE	F6Dn8H	0.014667	0.436958	486484	sepsis	TRUE	reported	textfile	0.0067	1.77E-06	4934	M01114.m	TRUE	reported	dVHatZ	2	TRUE	0.004602	22.80207
3232	rs6890028	T	C	T	C	0.0258	0.012651	0.2604	0.287631	FALSE	FALSE	FALSE	I25GHB	0.015109	0.402405	486484	sepsis	TRUE	reported	textfile	0.0054	1.73E-06	1749	M22548.m	TRUE	reported	eBmTQD	2	TRUE	0.012883	22.80106
8533	rs2163880	A	G	A	G	0.1237	-0.01885	0.9321	0.890901	FALSE	FALSE	FALSE	3PHxzL	0.021919	0.389783	486484	sepsis	TRUE	reported	textfile	0.0259	1.78E-06	3939	M36095.m	TRUE	reported	CRZVSB	2	TRUE	0.005758	22.79918
2695	rs9815713	A	G	A	G	-0.0917	-0.02405	0.9315	0.879743	FALSE	FALSE	FALSE	ATsH6N	0.021344	0.259782	486484	sepsis	TRUE	reported	textfile	0.0192	1.79E-06	3783	M18497.m	TRUE	reported	IGuIRI	2	TRUE	0.005994	22.79851
5134	rs1279781	T	C	T	C	-0.7626	-0.0435	0.0291	0.011793	FALSE	FALSE	FALSE	L2DJzK	0.064752	0.501681	486484	sepsis	TRUE	reported	textfile	0.1576	1.31E-06	76	M33173.m	TRUE	reported	hRG70D	2	TRUE	0.235522	22.79813
6626	rs1167951	A	G	A	G	-0.0702	0.007304	0.0743	0.070895	FALSE	FALSE	FALSE	IDG01N	0.027506	0.790604	486484	sepsis	TRUE	reported	textfile	0.0147	1.89E-06	4999	M33885.m	TRUE	reported	q3gy7x	2	TRUE	0.004541	22.79637
4231	rs6490461	A	C	A	C	0.0401	-0.00902	0.0491	0.067744	FALSE	FALSE	FALSE	FDy32d	0.028299	0.750022	486484	sepsis	TRUE	reported	textfile	0.0084	1.60E-06	7369	M32587.m	TRUE	reported	S10k1h	2	TRUE	0.003083	22.78307
6295	rs1162343	T	C	T	C	-0.0296	0.00577	0.4589	0.460039	FALSE	FALSE	FALSE	VlzZVG	0.013739	0.674531	486484	sepsis	TRUE	reported	textfile	0.0062	1.48E-06	2859	M33531.m	TRUE	reported	XiYjn4	2	TRUE	0.007909	22.77698
7336	rs1219155	T	C	T	C	-0.1094	-0.01233	0.2583	0.247235	FALSE	FALSE	FALSE	mm9ksf	0.015905	0.438231	486484	sepsis	TRUE	reported	textfile	0.0229	1.79E-06	954	M34365.m	TRUE	reported	AVwEqD	2	TRUE	0.023364	22.77468
1547	rs1200170	A	G	A	G	-0.0315	0.029243	0.052	0.036891	FALSE	FALSE	FALSE	Ke0sNh	0.036546	0.423615	486484	sepsis	TRUE	reported	textfile	0.0066	1.89E-06	7799	M10642.m	TRUE	reported	ZINwVb	2	TRUE	0.002912	22.77308
8766	rs7666614	A	G	A	G	-0.0463	0.01212	0.1545	0.142787	FALSE	FALSE	FALSE	KMisdy	0.019561	0.535538	486484	sepsis	TRUE	reported	textfile	0.0097	1.81E-06	3977	M36589.m	TRUE	reported	J1ISTP	2	TRUE	0.005696	22.77194
1543	rs4673167	A	G	A	G	-0.021	-0.00406	0.889	0.901788	FALSE	FALSE	FALSE	tApf9z	0.023111	0.860411	486484	sepsis	TRUE	reported	textfile	0.0044	1.70E-06	6515	M03147.m	TRUE	reported	tXdmtV	2	TRUE	0.003484	22.77193
5690	rs1712218	T	C	T	C	-0.1494	0.008084	0.9553	0.937707	FALSE	FALSE	FALSE	KxtFnC	0.028288	0.775054	486484	sepsis	TRUE	reported	textfile	0.0313	1.82E-06	3221	M33203.m	TRUE	reported	Uz0wKU	2	TRUE	0.007024	22.76894
6332	rs1408509	A	G	A	G	0.0526	0.003805	0.3568	0.340281	FALSE	FALSE	FALSE	qLUY00	0.014427	0.791983	486484	sepsis	TRUE	reported	textfile	0.011	1.78E-06	470	M33610.m	TRUE	reported	8cAJbB	2	TRUE	0.046394	22.76848
8591	rs727276	T	C	T	C	0.1628	0.046691	0.9671	0.963656	FALSE	FALSE	FALSE	iuJmRL	0.03665	0.202673	486484	sepsis	TRUE	reported	textfile	0.0341	1.73E-06	1750	M36103.m	TRUE	reported	jfJbVw	2	TRUE	0.012857	22.76687
4217	rs1996490	A	G	A	G	-0.0272	-0.00479	0.4179	0.423771	FALSE	FALSE	FALSE	aS6krA	0.01383	0.729234	486484	sepsis	TRUE	reported	textfile	0.0057	2.08E-06	7303	M32586.m	TRUE	reported	g9cBDI	2	TRUE	0.003108	22.76508
2461	rs9557045	A	T	A	T	-0.1164	-0.01144	0.087	0.062346	FALSE	TRUE	FALSE	NBJ2Do	0.028418	0.687225	486484	sepsis	TRUE	reported	textfile	0.0234	6.28E-07	25	M17799.m	TRUE	reported	Uz0Wgl	2	TRUE	0.497429	22.76471
2991	rs182119	C	G	C	G	-0.0272	-0.03092	0.7302	0.726627	FALSE	TRUE	FALSE	xmzJ5F	0.015409	0.044812	486484	sepsis	TRUE	reported	textfile	0.0057	1.73E-06	5436	M21184.m	TRUE	reported	xgwhDp	2	TRUE	0.004172	22.76294
8440	rs7615671	T	G	T	G	0.0253	0.009761	0.6922	0.7141	FALSE	FALSE	FALSE	Nvt7pl	0.015163	0.519746	486484	sepsis	TRUE	reported	textfile	0.0053	1.52E-06	1766	M35718.m	TRUE	reported	WkA5Gq	2	TRUE	0.012739	22.76131
3366	rs1103006	T	C	T	C	-0.0167	-0.00473	0.1273	0.141964	FALSE	FALSE	FALSE	O2JtzH	0.019543	0.808739	486484	sepsis	TRUE	reported	textfile	0.0035	2.13E-06	7347	M27278.m	TRUE	reported	fxClPW	2	TRUE	0.003089	22.76033
3736	rs1998881	T	C	T	C	-0.0167	0.006281	0.2091	0.218659	FALSE	FALSE	FALSE	k16olH	0.01654	0.704146	486484	sepsis	TRUE	reported	textfile	0.0035	1.33E-06	7340	M32328.m	TRUE	reported	TQA3sZ	2	TRUE	0.003092	22.76033
8313	rs1470121	A	G	A	G	0.0167	-0.01869	0.3619	0.360887	FALSE	FALSE	FALSE	YZIMbv	0.014224	0.188941	486484	sepsis	TRUE	reported	textfile	0.0035	2.36E-06	7302	M35628.m	TRUE	reported	2aP0wZ	2	TRUE	0.003108	22.76029
3226	rs1316091	A	G	A	G	0.0167	0.025021	0.8729	0.869492	FALSE	FALSE	FALSE	I25GHB	0.020309	0.217937	486484	sepsis	TRUE	reported	textfile	0.0035	2.35E-06	7184	M22548.m	TRUE	reported	eBmTQD	2	TRUE	0.003159	22.76019
5937	rs1609276	C	G	C	G	0.1088	0.00429	0.9727	0.980555	FALSE	TRUE	FALSE	yepzrL	0.049703	0.931222	486484	sepsis	TRUE	reported	textfile	0.0228	1.83E-06	3890	M33408.m	TRUE	reported	SY4CoS	2	TRUE	0.00582	22.75961
1588	rs6592988	T	C	T	C	0.0167	-0.01177	0.1439	0.156867	FALSE	FALSE	FALSE	7QN2kA	0.018889	0.533065	486484	sepsis	TRUE	reported	textfile	0.0035	2.40E-06	5656	M12017.m	TRUE	reported	52YQ1d	2	TRUE	0.004009	22.75848
2540	rs1702178	A	G	A	G	-0.3999	-0.01229	0.9857	0.977182	FALSE	FALSE	FALSE	o0CFya	0.045889	0.788827	486484	sepsis	TRUE	reported	textfile	0.0838	1.84E-06	2433	M18281.m	TRUE	reported	pxbsnu	2	TRUE	0.009273	22.75399
7443	rs7042159	A	C	A	C	0.0683	-0.00195	0.2478	0.261316	FALSE	FALSE	FALSE	otAubi	0.015616	0.900455	486484	sepsis	TRUE	reported	textfile	0.0143	1.71E-06	777	M34404.m	TRUE	reported	SAZaNF	2	TRUE	0.028522	22.75359
6735	rs3891367	T	C	T	C	0.0358	-0.02456	0.8915	0.911794	FALSE	FALSE	FALSE	SpG9A1	0.024162	0.30935	486484	sepsis	TRUE	reported	textfile	0.0075	1.73E-06	1404	M33939.m	TRUE	reported	Tudxel	2	TRUE	0.015969	22.75225
4635	rs1692695	T	G	T	G	-0.052	0.006675	0.1948	0.202665	FALSE	FALSE	FALSE	Dfaklt	0.017006	0.69469	486484	sepsis	TRUE	reported	textfile	0.0109	1.77E-06	6379	M32754.m	TRUE	reported	y21cDm	2	TRUE	0.003555	22.75189
7401	rs1277798	T	C	T	C	-0.0291	0.010683	0.2212	0.21544	FALSE	FALSE	FALSE	XfpKv0	0.016785	0.524499	486484	sepsis	TRUE	reported	textfile	0.0061	2.14E-06	5561	M34390.m	TRUE	reported	mmOsbK	2	TRUE	0.004076	22.74941
7821	rs1698618	C	G	C	G	-0.1275	-0.02252	0.9857	0.98002	FALSE	TRUE	FALSE	h5whlS	0.049144	0.646764	486484	sepsis	TRUE	reported	textfile	0.0267	1.81E-06	819	M35137.m	TRUE	reported	xVITKF	2	TRUE	0.027089	22.74762
5449	rs42441	T	G	T	G	0.2726	0.036485	0.9799	0.978935	FALSE	FALSE	FALSE	q32d4E	0.047552	0.442921	486484	sepsis	TRUE	reported	textfile	0.0571	1.80E-06	1017	M33178.m	TRUE	reported	cS3BwT	2	TRUE	0.02192	22.74702
6488	rs7925072	T	C	T	C	-0.0826	0.006576	0.822	0.823318	FALSE	FALSE	FALSE	ecwVg8	0.017929	0.713794	486484	sepsis	TRUE	reported	textfile	0.0173	1.78E-06	831	M33751.m	TRUE	reported	NnvbIQ	2	TRUE	0.0267	22.74162
4385	rs1158981	A	G	A	G	0.0124	0.015906	0.7885	0.793514	FALSE	FALSE	FALSE	UJygVd	0.016883	0.346128	486484	sepsis	TRUE	reported	textfile	0.0026	2.46E-06	7361	M32654.m	TRUE	reported	kv9R0W	2	TRUE	0.00308	22.73938
1732	rs1018695	T	C	T	C	-0.0124	-0.02201	0.837	0.842953	FALSE	FALSE	FALSE	nFLpAP	0.018822	0.242361	486484	sepsis	TRUE	reported	textfile	0.0026	2.42E-06	7356	M12774.m	TRUE	reported	yOSQTj	2	TRUE	0.003083	22.73938
4034	rs1181567	A	G	A	G	-0.0124	0.009526	0.1429	0.162331	FALSE	FALSE	FALSE	SiYGYU	0.019039	0.616824	486484	sepsis	TRUE	reported	textfile	0.0026	1.93E-06	7355	M32492.m	TRUE	reported	GfQvpU	2	TRUE	0.003083	22.73938
3198	rs2322487	A	T	A	T	-0.0124	0.011296	0.6629	0.628232	FALSE	TRUE	FALSE	UfLkBq	0.014141	0.424426	486484	sepsis	TRUE	reported	textfile	0.0026	2.00E-06	7329	M22481.m	TRUE	reported	kWMdiT	2	TRUE	0.003094	22.73936
1717	rs1094532	A	G	A	G	0.0124	0.008027	0.6184	0.62376	FALSE	FALSE	FALSE	RmWDhQ	0.014112	0.569464	486484	sepsis	TRUE	reported	textfile	0.0026	2.32E-06	7034	M12768.m	TRUE	reported	f0vnEz	2	TRUE	0.003223	22.73909
3161	rs6476317	T	C	T	C	0.0186	-0.00813	0.0729	0.080002	FALSE	FALSE	FALSE	JiXR2t	0.025193	0.747039	486484	sepsis	TRUE	reported	textfile	0.0039	1.68E-06	6535	M22177.m	TRUE	reported	joi6p6	2	TRUE	0.003469	22.7386
4558	rs2814818	A	G	A	G	0.031	0.013189	0.6985	0.689067	FALSE	FALSE	FALSE	KfoRxi	0.014773	0.371955	486484	sepsis	TRUE	reported	textfile	0.0065	2.15E-06	6000	M32735.m	TRUE	reported	UDRXAp	2	TRUE	0.003777	22.73798

2340	rs1757966	T	G	T	G	-0.0601	-0.03864	0.2583	0.274607	FALSE	FALSE	FALSE	13lycQ	0.015359	0.011886	486484	sepsis	TRUE	reported	textfile	0.0126	1.84E-06	3393	M16823.m	TRUE	reported	u2seZ2	2	TRUE	0.006661	22.73797
1584	rs1253851	C	G	C	G	0.0124	0.002719	0.3714	0.390647	FALSE	TRUE	FALSE	7QN2kA	0.013986	0.845869	486484	sepsis	TRUE	reported	textfile	0.0026	2.43E-06	5656	M12017.m	TRUE	reported	52YQ1d	2	TRUE	0.004005	22.73752
1590	rs7751499	A	G	A	G	0.0124	-0.00018	0.684	0.67414	FALSE	FALSE	FALSE	7QN2kA	0.01464	0.990328	486484	sepsis	TRUE	reported	textfile	0.0026	2.57E-06	5656	M12017.m	TRUE	reported	52YQ1d	2	TRUE	0.004005	22.73752
7606	rs6111973	T	C	T	C	0.1379	-0.01824	0.2105	0.208831	FALSE	FALSE	FALSE	PIYt5g	0.016798	0.27757	486484	sepsis	TRUE	reported	textfile	0.0288	1.65E-06	241	M34516.m	TRUE	reported	jX41tf	2	TRUE	0.086868	22.73654
2983	rs6016809	T	C	T	C	-0.1345	-0.01314	0.9615	0.961387	FALSE	FALSE	FALSE	Tli4xl	0.035705	0.712919	486484	sepsis	TRUE	reported	textfile	0.0282	1.82E-06	2339	M21151.m	TRUE	reported	xaSytY	2	TRUE	0.009632	22.72871
7079	rs1821897	C	G	C	G	0.0248	0.002229	0.7792	0.755734	FALSE	TRUE	FALSE	sg7nOB	0.016071	0.889697	486484	sepsis	TRUE	reported	textfile	0.0052	1.49E-06	2278	M34289.m	TRUE	reported	KemvlC	2	TRUE	0.009886	22.72559
6930	rs9558761	C	G	C	G	-0.5296	-0.01037	0.0177	0.026179	FALSE	TRUE	FALSE	SvfSvH	0.043275	0.810588	486484	sepsis	TRUE	reported	textfile	0.1107	1.71E-06	282	M34109.m	TRUE	reported	7OSbiu	2	TRUE	0.075069	22.7253
2683	rs10514470	A	G	A	G	0.0391	0.005982	0.4619	0.453228	FALSE	FALSE	FALSE	ATsH6N	0.013892	0.666742	486484	sepsis	TRUE	reported	textfile	0.0082	1.80E-06	3783	M18497.m	TRUE	reported	lGuIRI	2	TRUE	0.005974	22.72459
6809	rs1489866	T	G	T	G	0.0205	0.006056	0.8208	0.824916	FALSE	FALSE	FALSE	PkclDj	0.017967	0.736088	486484	sepsis	TRUE	reported	textfile	0.0043	1.72E-06	7368	M33961.m	TRUE	reported	ww89FU	2	TRUE	0.003075	22.72233
813	rs281216	T	G	T	G	0.0205	-0.02129	0.9184	0.913609	FALSE	FALSE	FALSE	J58DI0	0.024349	0.381874	486484	sepsis	TRUE	reported	textfile	0.0043	1.70E-06	7335	M01356.m	TRUE	reported	zk9AMB	2	TRUE	0.003089	22.7223
6668	rs16853740	T	C	T	C	0.0739	0.001419	0.9739	0.955396	FALSE	FALSE	FALSE	6iZSOI	0.032992	0.965698	486484	sepsis	TRUE	reported	textfile	0.0155	1.98E-06	4533	M33901.m	TRUE	reported	ApKaGe	2	TRUE	0.00499	22.72133
1405	rs1237160	T	C	T	C	-0.0205	-0.00634	0.7709	0.779881	FALSE	FALSE	FALSE	33ptwk	0.0165	0.70063	486484	sepsis	TRUE	reported	textfile	0.0043	2.18E-06	6252	M02137.m	TRUE	reported	SQY1na	2	TRUE	0.003622	22.72123
2296	rs4862124	A	T	A	T	0.0348	0.030372	0.945	0.882539	FALSE	TRUE	FALSE	43Yh3h	0.021343	0.15472	486484	sepsis	TRUE	reported	textfile	0.0073	1.76E-06	7055	M16818.m	TRUE	reported	beR4IT	2	TRUE	0.003211	22.71902
8671	rs12962960	T	C	T	C	0.0453	0.027877	0.17	0.150733	FALSE	FALSE	FALSE	i4bSdJ	0.01928	0.148204	486484	sepsis	TRUE	reported	textfile	0.0095	1.83E-06	1908	M36468.m	TRUE	reported	qJLuZj	2	TRUE	0.011777	22.71401
5757	rs1720779	T	C	T	C	0.0429	0.02582	0.1399	0.270346	FALSE	FALSE	FALSE	n16uO2	0.015408	0.093776	486484	sepsis	TRUE	reported	textfile	0.009	1.91E-06	4409	M33250.m	TRUE	reported	9dhl67	2	TRUE	0.005127	22.7108
2108	rs7309949	A	G	A	G	-0.132	0.030139	0.0184	0.015071	FALSE	FALSE	FALSE	MN85S3	0.05769	0.601376	486484	sepsis	TRUE	reported	textfile	0.0277	1.93E-06	7574	M15630.m	TRUE	reported	FUih5R	2	TRUE	0.002989	22.7025
1787	rs1426134	C	G	C	G	-0.0081	-0.00442	0.3715	0.368216	FALSE	TRUE	FALSE	JEcCRZ	0.014202	0.755541	486484	sepsis	TRUE	reported	textfile	0.0017	3.84E-06	7368	M15140.m	TRUE	reported	xYCzKP	2	TRUE	0.003072	22.69626
1793	rs2320536	T	C	T	C	0.0081	0.003162	0.4131	0.417406	FALSE	FALSE	FALSE	JEcCRZ	0.01393	0.820446	486484	sepsis	TRUE	reported	textfile	0.0017	3.32E-06	7368	M15140.m	TRUE	reported	xYCzKP	2	TRUE	0.003072	22.69626
9044	rs484061	A	G	A	G	-0.0081	0.009239	0.5129	0.524635	FALSE	FALSE	FALSE	df0MCM	0.013802	0.50321	486484	sepsis	TRUE	reported	textfile	0.0017	3.62E-06	7366	M37506.m	TRUE	reported	iUfr4u	2	TRUE	0.003073	22.69626
436	rs6603141	A	G	A	G	-0.0081	0.041828	0.258	0.263523	FALSE	FALSE	FALSE	cTsNj6	0.015512	0.007007	486484	sepsis	TRUE	reported	textfile	0.0017	2.61E-06	7365	M00063.m	TRUE	reported	7KYTrQ	2	TRUE	0.003073	22.69626
702	rs1532775	A	G	A	G	0.0081	-0.02569	0.7796	0.771092	FALSE	FALSE	FALSE	H9I3s3	0.016274	0.114455	486484	sepsis	TRUE	reported	textfile	0.0017	1.04E-06	7358	M01299.m	TRUE	reported	kXWyUq	2	TRUE	0.003076	22.69625
704	rs17293820	T	G	T	G	0.0081	-0.01189	0.8016	0.801443	FALSE	FALSE	FALSE	H9I3s3	0.017139	0.487756	486484	sepsis	TRUE	reported	textfile	0.0017	2.01E-06	7358	M01299.m	TRUE	reported	kXWyUq	2	TRUE	0.003076	22.69625
707	rs2879560	T	C	T	C	-0.0081	-0.02384	0.2226	0.203576	FALSE	FALSE	FALSE	H9I3s3	0.018405	0.195249	486484	sepsis	TRUE	reported	textfile	0.0017	3.48E-06	7358	M01299.m	TRUE	reported	kXWyUq	2	TRUE	0.003076	22.69625
830	rs2489020	T	C	T	C	-0.0081	-0.01011	0.5262	0.52191	FALSE	FALSE	FALSE	2qgDgB	0.013784	0.46339	486484	sepsis	TRUE	reported	textfile	0.0017	2.30E-06	7355	M01358.m	TRUE	reported	asmXq0	2	TRUE	0.003077	22.69625
2592	rs1318821	A	C	A	C	0.0081	-0.0035	0.4184	0.422765	FALSE	FALSE	FALSE	ruvJh6	0.013895	0.800944	486484	sepsis	TRUE	reported	textfile	0.0017	1.77E-06	7354	M18369.m	TRUE	reported	GFmwGk	2	TRUE	0.003078	22.69625
797	rs4957697	A	G	A	G	-0.0081	-0.02292	0.4208	0.432088	FALSE	FALSE	FALSE	dyT79I	0.013818	0.097205	486484	sepsis	TRUE	reported	textfile	0.0017	1.73E-06	7352	M01336.m	TRUE	reported	OemVJ2	2	TRUE	0.003078	22.69625
8167	rs1877564	T	C	T	C	0.0081	-0.00888	0.7887	0.794634	FALSE	FALSE	FALSE	8UhtPK	0.017013	0.601724	486484	sepsis	TRUE	reported	textfile	0.0017	2.20E-06	7345	M35397.m	TRUE	reported	rvVJba	2	TRUE	0.003081	22.69624
2135	rs1732932	T	C	T	C	0.0081	-0.00471	0.4292	0.43082	FALSE	FALSE	FALSE	TEupfd	0.013797	0.732558	486484	sepsis	TRUE	reported	textfile	0.0017	3.49E-06	7331	M15676.m	TRUE	reported	NXiaPC	2	TRUE	0.003087	22.69623
2141	rs4570993	T	C	T	C	0.0081	-0.01182	0.5322	0.53424	FALSE	FALSE	FALSE	TEupfd	0.013762	0.390278	486484	sepsis	TRUE	reported	textfile	0.0017	2.43E-06	7331	M15676.m	TRUE	reported	NXiaPC	2	TRUE	0.003087	22.69623
2145	rs703147	T	C	T	C	0.0081	-0.03967	0.3801	0.382694	FALSE	FALSE	FALSE	TEupfd	0.014052	0.004752	486484	sepsis	TRUE	reported	textfile	0.0017	2.41E-06	7331	M15676.m	TRUE	reported	NXiaPC	2	TRUE	0.003087	22.69623
1372	rs13267180	A	G	A	G	0.0081	-0.01359	0.4794	0.490879	FALSE	FALSE	FALSE	KrWhws	0.013665	0.320027	486484	sepsis	TRUE	reported	textfile	0.0017	3.78E-06	7325	M02132.m	TRUE	reported	7pzpfK	2	TRUE	0.00309	22.69622
1381	rs1920325	T	C	T	C	-0.0081	-0.00305	0.6425	0.640313	FALSE	FALSE	FALSE	KrWhws	0.014255	0.830813	486484	sepsis	TRUE	reported	textfile	0.0017	3.66E-06	7325	M02132.m	TRUE	reported	7pzpfK	2	TRUE	0.00309	22.69622
2840	rs1291128	A	C	A	C	0.0081	-0.00276	0.7545	0.740524	FALSE	FALSE	FALSE	Y5qWBm	0.015805	0.861615	486484	sepsis	TRUE	reported	textfile	0.0017	2.26E-06	7325	M20489.m	TRUE	reported	f2g9Su	2	TRUE	0.00309	22.69622
2859	rs831831	A	G	A	G	-0.0081	-0.03096	0.2007	0.204745	FALSE	FALSE	FALSE	Y5qWBm	0.016983	0.068297	486484	sepsis	TRUE	reported	textfile	0.0017	3.20E-06	7325	M20489.m	TRUE	reported	f2g9Su	2	TRUE	0.00309	22.69622
4589	rs2029209	A	G	A	G	0.0081	0.00402	0.5491	0.533184	FALSE	FALSE	FALSE	Pzplqu	0.013699	0.769168	486484	sepsis	TRUE	reported	textfile	0.0017	2.08E-06	7318	M32740.m	TRUE	reported	FjEBS5	2	TRUE	0.003093	22.69622
2701	rs2028973	T	C	T	C	0.0162	-0.01511	0.1086	0.108187	FALSE	FALSE	FALSE	XsQHZK	0.021995	0.492085	486484	sepsis	TRUE	reported	textfile	0.0034	2.66E-06	7286	M18929.m	TRUE	reported	k8R7hN	2	TRUE	0.003106	22.69619
1119	rs7178551	T	C	T	C	0.0081	0.027567	0.1545	0.196213	FALSE	FALSE	FALSE	yahiGZ	0.017271	0.110456	486484	sepsis	TRUE	reported	textfile	0.0017	2.66E-06	7271	M01585.m	TRUE	reported	dwZ7vX	2	TRUE	0.003113	22.69618
1303	rs2351174	T	G	T	G	-0.0081	-0.01352	0.6063	0.621704	FALSE	FALSE	FALSE	WQdpmo	0.01411	0.33784	486484	sepsis	TRUE	reported	textfile	0.0017	2.63E-06	7136	M01769.m	TRUE	reported	BkptOK	2	TRUE	0.003171	22.69606
1316	rs7837792	T	C	T	C	-0.0081	0.025463	0.5936	0.591734	FALSE	FALSE	FALSE	WQdpmo	0.014017	0.069279	486484	sepsis	TRUE	reported	textfile	0.0017	2.78E-06	7136	M01769.m	TRUE	reported	BkptOK	2	TRUE	0.003171	22.69606
5839	rs8058227	T	C	T	C	0.0081	-0.0196	0.4024	0.401779	FALSE	FALSE	FALSE	R5joEf	0.014015	0.162038	486484	sepsis	TRUE	reported	textfile	0.0017	1.35E-06	7118	M33380.m	TRUE	reported	kRvbfZ	2	TRUE	0.003179	22.69604
4658	rs4977264	T	C	T	C	-0.0162	-0.016	0.5535	0.549788	FALSE	FALSE	FALSE	SyAFR2	0.013813	0.246723	486484	sepsis	TRUE	reported	textfile	0.0034	2.47E-06	6658	M32755.m	TRUE	reported	prUo74	2	TRUE	0.003398	22.6956
8094	rs1077862	A	C	A	C	0.0081	0.016057	0.3766	0.379695	FALSE	FALSE	FALSE	nNbXBS	0.014153	0.256568	486484	sepsis	TRUE	reported	textfile	0.0017	3.82E-06	5768	M35326.m	TRUE	reported	UNDorn	2	TRUE	0.00392	22.69455
2664	rs2420201	A	G	A	G	0.1473	0.013403	0.0494	0.056618	FALSE	FALSE	FALSE	cDEa3G	0.029585	0.650522	486484	sepsis	TRUE	reported	textfile	0.0309	1.90E-06	1457	M18477.m	TRUE	reported	kxZEfC	2	TRUE	0.015357	22.693
2942	rs1694853	A	G	A	G	-0.0224	-0.02206	0.8805	0.882211	FALSE	FALSE	FALSE	pn11BC	0.021228	0.298666	486484	sepsis	TRUE	reported	textfile	0.0047	2.23E-06	1764	M21047.m	TRUE	reported	KJF8U4	2	TRUE	0.012713	22.6886
2657	rs1780783	A	G	A	G	0.0343	-0.01126	0.8027	0.793742	FALSE	FALSE	FALSE	UtYu7J	0.016945	0.506229	486484	sepsis	TRUE	reported	textfile	0.0072	1.71E-06	5624	M18476.m	TRUE	reported	wJx0A4	2	TRUE	0.004019	22.68657
4681	rs9362410	T	C	T	C	0.0262	0.001357	0.3749	0.390701	FALSE	FALSE	FALSE	xaapK0	0.014053	0.923049	486484	sepsis	TRUE	reported	textfile	0.0055	1.83E-06	7250	M32757.m	TRUE	reported	64n1Z4	2	TRUE	0.00312	22.68597
2528	rs1289589	A	G	A	G	-0.0443	-0.02108	0.8278	0.804617	FALSE	FALSE	FALSE	4HwTlG	0.017226	0.221006	486484	sepsis	TRUE	reported	textfile	0.0093	1.96E-06	7071	M18254.m	TRUE	reported	F9KrSC	2	TRUE	0.003199	22.68395
599	rs1799412	C	G	C	G	-0.0262	0.000991	0.8394	0.815135	FALSE	TRUE	FALSE	KVK33e	0.017574	0.955051	486484	sepsis	TRUE	reported	textfile	0.0055	2.28E-06	5344	M01107.m	TRUE	reported	eAyR8w	2	TRUE	0.004228	22.68374
3931	rs7312642	T	G	T	G	-0.0362	-0.00717	0.0717	0.074922	FALSE	FALSE	FALSE	BrQzHt	0.026037	0.783051	486484	sepsis	TRUE	reported	textfile	0.0076	1.76E-06	7500	M32412.m	TRUE	reported	eVvYVX	2	TRUE	0.003016	22.68162
3206	rs3798343	C	G	C	G	-0.0362	0.004025	0.9771	0.977054	FALSE	TRUE	FALSE	UfLkBq	0.045589	0.929652	486484	sepsis	TRUE	reported	textfile	0.0076	2.07E-06	7329	M22481.m	TRUE	reported	kWMdiT	2	TRUE	0.003086	22.68148
4477	rs9544223	A	C	A	C	-0.0181	0.032374	0.8771	0.807479	FALSE	FALSE	FALSE	g3iM2w	0.017534	0.06484	486484	sepsis	TRUE	reported	textfile	0.0038	2.07E-06	7308	M32698.m	TRUE	reported	ZQBn0h	2	TRUE	0.003095	22.68146
5720	rs9615728	A	G	A	G	0.0543	-0.03374	0.0399	0.06672	FALSE	FALSE	FALSE	XDem1i	0.027309	0.216681	486484	sepsis	TRUE	reported	textfile	0.0114	2.01E-06	4665	M33221.m	TRUE	reported	KBV6FH	2	TRUE	0.00484	22.67795
1023	rs11145330	A	C	A	C	0.0324	-0.02716	0.8908	0.887075	FALSE	FALSE	FALSE	haWxZg	0.021584	0.208247	486484	sepsis	TRUE	reported	textfile	0.0068	1.95E-06	1759	M01561.m	TRUE	reported	5wm9R3	2	TRUE	0.012742	22.67661
497	rs2211372	T	C	T	C	0.3317	-0.00634	0.985	0.984942	FALSE	FALSE	FALSE	RtjZPn	0.056667	0.910977	486484	sepsis	TRUE	reported	textfile	0.0696	1.87E-06	1233	M00553.m	TRUE	reported	C3Ovsl	2	TRUE	0.018088	22.67605
4079	rs7013605	T	C	T	C	0.02	0.022271	0.1199	0.144539	FALSE	FALSE	FALSE	TyHtdh	0.019514	0.253749	486484	sepsis	TRUE	reported	textfile	0.0042	2.42E-06	7358	M32497.m	TRUE	reported	H3KmLR	2	TRUE	0.003072	22.66957
4086	rs998531	A	G	A	G	0.02	0.0228	0.8796	0.870793	FALSE	FALSE	FALSE	TyHtdh	0.02037	0.263021	486484	sepsis	TRUE	reported	textfile	0.0042	1.89E-06	7358	M32497.m	TRUE	reported	H3KmLR	2	TRUE	0.003072	22.66957
4409	rs1132054	T	C	T	C	0.02	0.012093	0.575	0.564896	FALSE	FALSE	FALSE	NzBiVA	0.013794	0.38066	486484	sepsis	TRUE	reported	textfile	0.0042	2.34E-06	5590	M32672.m	TRUE	reported	YLIhHJ	2	TRUE	0.00404	22.66762
3066	rs1237556	A	G	A	G	-0.0386	0.029259	0.2606	0.235121	FALSE	FALSE	FALSE	e3izea	0.016181	0.07057	486484	sepsis	TRUE	reported	textfile	0.0081	2.16E-06	1087	M22032.m	TRUE	reported	zODKFl	2	TRUE	0.020464	22.66756
1353	rs7296105	A	C	A	C	0.0262	-0.0066	0.8773	0.887583	FALSE	FALSE	FALSE	LDUJbh	0.021667	0.760752	486484	sepsis	TRUE	reported	textfile	0.0055	2.35E-06	1766	M01898.m	TRUE	reported	JRSLeH	2	TRUE	0.012686	22.66653
4183	rs1050824	A	G	A	G	0.0519	-0.03034	0.1246	0.106916	FALSE	FALSE	FALSE	TxOCzG	0.022424	0.17602	486484	sepsis	TRUE	reported	textfile	0.0109	1.92E-06	6970	M32564.m	TRUE	reported	9JP6ND	2	TRUE	0.003242	22.66507
7772	rs9513567	C	G	C	G	0.0262	-0.01834	0.2905	0.285561	FALSE	TRUE	FALSE	G4gUxB	0.015155	0.226159	486484	sepsis	TRUE	reported	textfile	0.0055	2.08E-06	1657	M35114.m	TRUE	reported	o79OO5	2	TRUE	0.01351	22.66484
2648	rs1599081	T	G	T	G	-0.0691	0.03774	0.788	0.793984	FALSE	FALSE	FALSE	9GoQdh	0.017039	0.026761	486484	sepsis	TRUE	reported	textfile	0.0145	1.90E-06	917	M18474.m	TRUE	reported	p6Dq8p	2	TRUE	0.024167	22.66062
7994	rs2076060	C	G	C	G	0.0219	0.008647	0.2579	0.2832	FALSE	TRUE	FALSE	Xrodum	0.015157	0.568327	486484	sepsis	TRUE	reported	textfile	0.0046	1.55E-06	7291	M35255.m	TRUE	reported	T8t1tl	2	TRUE	0.003099	22.65966
6921	rs4448374	T	C	T	C	-0.2957	-0.02658	0.0283	0.044799	FALSE	FALSE	FALSE	SvfSvH	0.032901	0.419182	486484	sepsis	TRUE	reported	textfile	0.0619	1.80E-06	282	M34109.m	TRUE	reported	7OSbiu	2	TRUE	0.074865	22.65845
6248	rs9451877	A	G	A	G	-0.0943	0.018506	0.9703	0.975787	FALSE	FALSE	FALSE	vL52Yb	0.044434	0.677057	486484	sepsis	TRUE	reported	textfile	0.0198	1.92E-06	1718	M33510.m	TRUE	reported	JIO9VX	2	TRUE	0.013031	22.6562
7793	rs1136613	A	G	A	G	-0.0781	0.000678	0.0194	0.015697	FALSE	FALSE	FALSE	cQ5TvO	0.054955	0.990153	486484	sepsis	TRUE	reported	textfile	0.0164	2.01E-06	1762	M35127.m	TRUE	reported	BVxkfZ	2	TRUE	0.012707	22.65276
8817	rs6755317	A	G	A	G	0.0357	0.004534	0.9729	0.97571	FALSE	FALSE	FALSE	X2Rol2	0.046155	0.921746	486484	sepsis	TRUE	reported	textfile	0.0075	2.03E-06	7335	M36776.m	TRUE	reported	nikHw1	2	TRUE	0.003079	22.65142
578	rs1502520	T	G	T	G	0.0119	-0.00689	0.7334	0.72829	FALSE	FALSE	FALSE	ZoqU2j	0.015404	0.654487	486484	sepsis	TRUE	reported	textfile	0.0025	1.63E-06	7333	M01105.m	TRUE	reported	2WxSOH	2	TRUE	0.00308	22.65142
3881	rs9894902	A	G	A	G	0.0119	0.023532	0.2086	0.216886	FALSE	FALSE	FALSE	bwkKtK	0.016614	0.156661	486484	sepsis	TRUE	reported	textfile	0.0025	2.35E-06	7307	M32393.m	TRUE	reported	RvZzAy	2	TRUE	0.003091	22.6514
8273	rs4789522	A	C	A	C	0.0119	-0.02047	0.666	0.661128	FALSE	FALSE	FALSE	TdsGCZ	0.014468	0.157027	486484	sepsis	TRUE	reported	textfile	0.0025	2.08E-06	6883	M35464.m	TRUE	reported	sOspot	2	TRUE	0.003281	22.65102
2759	rs2263487	T	C	T	C	-0.0119	-0.02668	0.5184	0.515352	FALSE	FALSE	FALSE	H6txH3	0.01367	0.050984	486484	sepsis	TRUE	reported	textfile	0.0025	3.05E-06	6678	M19368.m	TRUE	reported	b094NF	2	TRUE	0.003381	22.65081
7207	rs1312171	A	C	A	C	-0.3919	0.042261	0.9794	0.971629	FALSE	FALSE	FALSE	i1uJ7T	0.041057	0.303325	486484	sepsis	TRUE	reported	textfile	0.0822	1.86E-06	535	M34338.m	TRUE	reported	pi3mKW	2	TRUE	0.040755	22.64542
8614	rs4403944	T	G	T	G	-0.0638	-0.01852	0.0458	0.055212	FALSE	FALSE	FALSE	oHM5TD	0.030092	0.538285	486484	sepsis	TRUE	reported	textfile	0.0134	2.04E-06	1780	M36131.m	TRUE	reported	Xw6wmg	2	TRUE	0.012575	22.6435
7018	rs1106869	A	G	A	G	-0.0395	-0.01771	0.0667	0.068637	FALSE	FALSE	FALSE	zMIkYA	0.027063	0.512769	486484	sepsis	TRUE	reported	textfile	0.0083	2.06E-06	4908	M34245.m	TRUE	reported	itCtz8	2	TRUE	0.004593	22.6392
5960	rs6719679	A	G	A	G	0.0276	0.001253	0.7751	0.772601	FALSE	FALSE	FALSE	mDZrGw	0.016348	0.938883	486484	sepsis	TRUE	reported	textfile	0.0058	2.20E-06	6797	M33415.m	TRUE	reported	MzEP7R	2	TRUE	0.00332	22.63781
5224	rs1160873	T	G	T	G	0.1062	0.009994	0.8494	0.876813	FALSE	FALSE	FALSE	q32d4E	0.02083	0.631385	486484	sepsis	TRUE	reported	textfile	0.0223	1.82E-06	1017	M33178.m	TRUE	reported	cS3BwT	2	TRUE	0.021814	22.6352
5694	rs7693835	A	G	A	G	-0.1204	-0.02578	0.9106	0.888548	FALSE	FALSE	FALSE	KxtFnC	0.021738	0.23563	486484	sepsis	TRUE	reported	textfile	0.0253	2.01E-06	3221	M33203.m	TRUE	reported	Uz0wKU	2	TRUE	0.006982	22.633
6486	rs7334694	T	C	T	C	-0.0643	0.00132	0.4609	0.458427	FALSE	FALSE	FALSE	ecwVg8	0.013733	0.923426	486484	sepsis	TRUE	reported	textfile	0.0135	1.86E-06	831	M33751.m	TRUE	reported	NnvbIQ	2	TRUE	0.026574	22.63122
683	rs10993160	A	G	A	G	0.0295	-0.03752	0.9587	0.969822	FALSE	FALSE	FALSE	aT1Fw1	0.039957	0.347763	486484	sepsis	TRUE	reported	textfile	0.0062	1.68E-06	5687	M01284.m	TRUE	reported	mbTe8H	2	TRUE	0.003965	22.63122
8772	rs1554057	A	G	A	G	0.0295	-0.00406	0.9059	0.914618	FALSE	FALSE	FALSE	7desNF	0.024476	0.868132	486484	sepsis	TRUE	reported	textfile	0.0062	1.78E-06	5292	M36673.m	TRUE	reported	RKbIT4	2	TRUE	0.00426	22.63062
3639	rs1265532	A	G	A	G	0.0733	-0.01059	0.5227	0.520363	FALSE	FALSE	FALSE	Vq2efp	0.013689	0.439356	486484	sepsis	TRUE	reported	textfile	0.0154	2.02E-06	1760	M31787.m	TRUE	reported	krl4Tw	2	TRUE	0.012709	22.62938
622	rs6568389	T	C	T	C	0.0157	-0.00884	0.8061	0.699348	FALSE	FALSE	FALSE	BYGzq4	0.014925	0.553478	486484	sepsis	TRUE	reported	textfile	0.0033	2.16E-06	7367	M01110.m	TRUE	reported	oZiXqP	2	TRUE	0.003063	22.62838
1599	rs1193770	A	G	A	G	0.0157	0.027988	0.1122	0.116119	FALSE	FALSE	FALSE	zP3C2v	0.021319	0.189253	486484	sepsis	TRUE	reported	textfile	0.0033	2.39E-06	7356	M12035.m	TRUE	reported	1Pooe3	2	TRUE	0.003068	22.62837
4924	rs2324594	T	C	T	C	-0.0357	0.002892	0.1825	0.170587	FALSE	FALSE	FALSE	clhN6v	0.018622	0.876576	486484	sepsis	TRUE	reported	textfile	0.0075	2.16E-06	1550	M32869.m	TRUE	reported	BzloWk	2	TRUE	0.014407	22.62836
6070	rs1104776	T	C	T	C	-0.0157	-0.01176	0.0528	0.060776	FALSE	FALSE	FALSE	P8n0HP	0.028774	0.682672	486484	sepsis	TRUE	reported	textfile	0.0033	1.49E-06	7336	M33442.m	TRUE	reported	cljMTl	2	TRUE	0.003076	22.62836
3459	rs249619	T	C	T	C	-0.0533	-0.00762	0.5601	0.569229	FALSE	FALSE	FALSE	jRejFm	0.013864	0.582786	486484	sepsis	TRUE	reported	textfile	0.0112	1.79E-06	1720	M27716.m	TRUE	reported	tg7Rvj	2	TRUE	0.012996	22.62107
3763	rs7709785	T	C	T	C	-0.0176	0.027538	0.148	0.198644	FALSE	FALSE	FALSE	OZLdls	0.017299	0.111404	486484	sepsis	TRUE	reported	textfile	0.0037	1.99E-06	7356	M32338.m	TRUE	reported	s2itmB	2	TRUE	0.003067	22.62058
4756	rs1939769	A	G	A	G	0.0352	-0.0201	0.0647	0.066517	FALSE	FALSE	FALSE	RDfDIS	0.027507	0.46497	486484	sepsis	TRUE	reported	textfile	0.0074	1.77E-06	6857	M32787.m	TRUE	reported	Hq6nGt	2	TRUE	0.003289	22.62014
8016	rs847391	A	G	A	G	0.039	-0.01529	0.9514	0.933734	FALSE	FALSE	FALSE	Zj5bZH	0.028276	0.588745	486484	sepsis	TRUE	reported	textfile	0.0082	2.16E-06	6538	M35257.m	TRUE	reported	exssAk	2	TRUE	0.003448	22.61354
8929	rs3821142	T	C	T	C	0.0747	-0.01265	0.5584	0.527518	FALSE	FALSE	FALSE	ns6PV1	0.013697	0.355888	486484	sepsis	TRUE	reported	textfile	0.0157	2.09E-06	1672	M37097.m	TRUE	reported	ZYmYt4	2	TRUE	0.013359	22.61112
7128	rs1447600	T	C	T	C	0.11	-0.00134	0.81	0.788793	FALSE	FALSE	FALSE	de8tA6	0.016747	0.936009	486484	sepsis	TRUE	reported	textfile	0.023	1.73E-06	173	M34322.m	TRUE	reported	sXPPBO	2	TRUE	0.116776	22.60891
5734	rs540613	T	C	T	C	0.0214	-0.02038	0.1462	0.193676	FALSE	FALSE	FALSE	YDQaWa	0.017567	0.245906	486484	sepsis	TRUE	reported	textfile	0.0045	1.92E-06	7063	M33228.m	TRUE	reported	BmYg8r	2	TRUE	0.003192	22.6089
1499	rs1159507	T	G	T	G	-0.0214	-0.02327	0.0951	0.090256	FALSE	FALSE	FALSE	rzkxmh	0.024038	0.333025	486484	sepsis	TRUE	reported	textfile	0.0045	1.89E-06	6941	M03127.m	TRUE	reported	3lrzhA	2	TRUE	0.003248	22.60879
6386	rs1086138	A	G	A	G	-0.0214	-0.01536	0.7004	0.697383	FALSE	FALSE	FALSE	eBEF1t	0.014925	0.303296	486484	sepsis	TRUE	reported	textfile	0.0045	1.59E-06	6109	M33638.m	TRUE	reported	CRf2lt	2	TRUE	0.003688	22.6079
7498	rs1708887	A	G	A	G	0.0856	-0.02131	0.9182	0.916764	FALSE	FALSE	FALSE	1aXQWs	0.024757	0.389364	486484	sepsis	TRUE	reported	textfile	0.018	2.06E-06	4452	M34420.m	TRUE	reported	FOqhHW	2	TRUE	0.005054	22.60515
6223	rs1693991	A	T	A	T	-0.0333	0.001637	0.2762	0.288427	FALSE	TRUE	FALSE	8zUBbU	0.015104	0.913694	486484	sepsis	TRUE	reported	textfile	0.007	1.92E-06	1733	M33509.m	TRUE	reported	1mO9m8	2	TRUE	0.01289	22.60429
6034	rs2365913	T	C	T	C	1.0329	-0.00288	0.9519	0.698885	FALSE	FALSE	FALSE	33ojbt	0.014906	0.846854	486484	sepsis	TRUE	reported	textfile	0.2136	1.34E-06	60	M33423.m	TRUE	reported	ivEtvl	2	TRUE	0.280435	22.60429
2716	rs2241216	C	G	C	G	0.0252	0.021492	0.199	0.239909	FALSE	TRUE	FALSE	89XKxw	0.016408	0.190255	486484	sepsis	TRUE	reported	textfile	0.0053	2.32E-06	7251	M19324.m	TRUE	reported	gryJuD	2	TRUE	0.003108	22.6011
8836	rs1123296	T	G	T	G	0.0176	0.004691	0.2475	0.270607	FALSE	FALSE	FALSE	XIHeTV	0.015357	0.760009	486484	sepsis	TRUE	reported	textfile	0.0037	1.60E-06	1764	M36808.m	TRUE	reported	37Vi2D	2	TRUE	0.012665	22.60108
4851	rs2831156	A	G	A	G	-0.0252	0.022095	0.1791	0.180074	FALSE	FALSE	FALSE	StMkBO	0.017768	0.213664	486484	sepsis	TRUE	reported	textfile	0.0053	2.38E-06	6967	M32847.m	TRUE	reported	uzQb8Q	2	TRUE	0.003234	22.60084
3967	rs6834891	T	C	T	C	0.0252	-0.0202	0.6263	0.620761	FALSE	FALSE	FALSE	sKCkQs	0.014155	0.15363	486484	sepsis	TRUE	reported	textfile	0.0053	2.20E-06	5080	M32445.m	TRUE	reported	egPvJc	2	TRUE	0.004431	22.59843
722	rs9497139	A	G	A	G	-0.029	-0.00326	0.0155	0.016868	FALSE	FALSE	FALSE	H9I3s3	0.053253	0.951179	486484	sepsis	TRUE	reported	textfile	0.0061	1.97E-06	7358	M01299.m	TRUE	reported	kXWyUq	2	TRUE	0.003062	22.59531
7107	rs1082686	T	C	T	C	-0.039	0.00259	0.6363	0.635329	FALSE	FALSE	FALSE	DfLd6z	0.014234	0.855627	486484	sepsis	TRUE	reported	textfile	0.0082	1.74E-06	1766	M34314.m	TRUE	reported	OgloAQ	2	TRUE	0.012647	22.59485
7274	rs9538657	A	C	A	C	0.4491	0.064348	0.0228	0.016664	FALSE	FALSE	FALSE	P5Xm4S	0.05731	0.261522	486484	sepsis	TRUE	reported	textfile	0.0943	1.93E-06	525	M34350.m	TRUE	reported	YU4x0J	2	TRUE	0.041413	22.59462
7181	rs2380875	T	G	T	G	0.2035	-0.04584	0.0551	0.066806	FALSE	FALSE	FALSE	2hlepp	0.027894	0.100286	486484	sepsis	TRUE	reported	textfile	0.0427	1.86E-06	369	M34336.m	TRUE	reported	XyK8mO	2	TRUE	0.057984	22.58982
6007	rs1007469	C	G	C	G	0.5873	0.01198	0.8839	0.850323	FALSE	TRUE	FALSE	33ojbt	0.019177	0.532173	486484	sepsis	TRUE	reported	textfile	0.1215	1.33E-06	60	M33423.m	TRUE	reported	ivEtvl	2	TRUE	0.280274	22.58625
1432	rs825278	T	C	T	C	-0.0913	0.039693	0.0201	0.014292	FALSE	FALSE	FALSE	ac6RGB	0.058529	0.497665	486484	sepsis	TRUE	reported	textfile	0.0192	2.06E-06	1741	M02342.m	TRUE	reported	dC6Rkp	2	TRUE	0.012821	22.58603
4865	rs11982	A	G	A	G	0.0385	-0.02126	0.0691	0.075095	FALSE	FALSE	FALSE	KBJVQO	0.025955	0.412821	486484	sepsis	TRUE	reported	textfile	0.0081	2.26E-06	7355	M32855.m	TRUE	reported	Xea7Fn	2	TRUE	0.003062	22.58569
2573	rs1707366	A	G	A	G	0.0856	0.018317	0.023	0.018755	FALSE	FALSE	FALSE	KHS91f	0.05099	0.719432	486484	sepsis	TRUE	reported	textfile	0.018	1.84E-06	1492	M18349.m	TRUE	reported	PeVGil	2	TRUE	0.014931	22.58499
6338	rs321188	A	G	A	G	-0.536	-0.12736	0.9806	0.983844	FALSE	FALSE	FALSE	qLUY00	0.053969	0.018278	486484	sepsis	TRUE	reported	textfile	0.1117	1.59E-06	102	M33610.m	TRUE	reported	8cAJbB	2	TRUE	0.184171	22.57476
8945	rs631791	A	G	A	G	0.0628	0.010777	0.3017	0.315805	FALSE	FALSE	FALSE	mXJP3h	0.014726	0.464247	486484	sepsis	TRUE	reported	textfile	0.0132	2.06E-06	751	M37104.m	TRUE	reported	HRZBak	2	TRUE	0.029257	22.57425
4878	rs1578963	C	G	C	G	0.0846	-0.0439	0.0342	0.037851	FALSE	TRUE	FALSE	MDX5FX	0.035936	0.221837	486484	sepsis	TRUE	reported	textfile	0.0178	1.91E-06	2656	M32857.m	TRUE	reported	moAN2E	2	TRUE	0.008433	22.57218
6309	rs1183014	T	G	T	G	0.088	-0.03301	0.0928	0.102431	FALSE	FALSE	FALSE	5B5LTX	0.022702	0.14596	486484	sepsis	TRUE	reported	textfile	0.0185	2.00E-06	824	M33609.m	TRUE	reported	m0eM6N	2	TRUE	0.026726	22.57182
7198	rs1207101	T	C	T	C	0.1843	0.012919	0.2889	0.323926	FALSE	FALSE	FALSE	i1uJ7T	0.014638	0.377483	486484	sepsis	TRUE	reported	textfile	0.0387	1.96E-06	420	M34338.m	TRUE	reported	pi3mKW	2	TRUE	0.051232	22.57126
4244	rs1083610	C	G	C	G	0.086	0.005069	0.0682	0.079915	FALSE	TRUE	FALSE	KZ60wl	0.025423	0.841966	486484	sepsis	TRUE	reported	textfile	0.0181	1.94E-06	6904	M32616.m	TRUE	reported	SPDpZi	2	TRUE	0.003259	22.56908
8786	rs1959363	T	C	T	C	-0.0447	0.035164	0.1197	0.124569	FALSE	FALSE	FALSE	4bkfvN	0.020702	0.089396	486484	sepsis	TRUE	reported	textfile	0.0094	2.06E-06	922	M36738.m	TRUE	reported	xcqyX2	2	TRUE	0.023939	22.56401
1566	rs6540619	T	C	T	C	0.0076	-0.01284	0.434	0.42855	FALSE	FALSE	FALSE	Ke0sNh	0.013842	0.353628	486484	sepsis	TRUE	reported	textfile	0.0016	3.71E-06	7799	M10642.m	TRUE	reported	ZINwVb	2	TRUE	0.002885	22.55671
4322	rs2828209	T	C	T	C	-0.0361	0.011937	0.051	0.051372	FALSE	FALSE	FALSE	EOAssJ	0.031264	0.702608	486484	sepsis	TRUE	reported	textfile	0.0076	1.78E-06	7369	M32635.m	TRUE	reported	X1mTjy	2	TRUE	0.003052	22.55638
5744	rs1314028	A	G	A	G	0.0133	0.01276	0.5842	0.589147	FALSE	FALSE	FALSE	bm6x2Z	0.013921	0.359347	486484	sepsis	TRUE	reported	textfile	0.0028	1.59E-06	7364	M33230.m	TRUE	reported	8Ccke3	2	TRUE	0.003055	22.55637
6876	rs325614	A	G	A	G	0.0171	0.03626	0.9673	0.962703	FALSE	FALSE	FALSE	kFWZjs	0.036128	0.315554	486484	sepsis	TRUE	reported	textfile	0.0036	2.63E-06	7362	M34062.m	TRUE	reported	eLONsg	2	TRUE	0.003055	22.55637
711	rs6493	A	G	A	G	0.0114	0.004122	0.1195	0.162663	FALSE	FALSE	FALSE	H9I3s3	0.018501	0.823675	486484	sepsis	TRUE	reported	textfile	0.0024	2.35E-06	7358	M01299.m	TRUE	reported	kXWyUq	2	TRUE	0.003057	22.55637
720	rs9327433	T	G	T	G	-0.0076	-0.01449	0.5134	0.495704	FALSE	FALSE	FALSE	H9I3s3	0.013789	0.293366	486484	sepsis	TRUE	reported	textfile	0.0016	2.53E-06	7358	M01299.m	TRUE	reported	kXWyUq	2	TRUE	0.003057	22.55637
146	rs6759991	T	G	T	G	0.0076	-0.02919	0.8623	0.842839	FALSE	FALSE	FALSE	xmGuol	0.018781	0.12008	486484	sepsis	TRUE	reported	textfile	0.0016	2.52E-06	7355	M00054.m	TRUE	reported	GW50Ri	2	TRUE	0.003058	22.55636
6980	rs7303969	T	C	T	C	-0.0171	-0.00263	0.8242	0.819454	FALSE	FALSE	FALSE	ZlvYs5	0.017947	0.883539	486484	sepsis	TRUE	reported	textfile	0.0036	2.64E-06	7351	M34214.m	TRUE	reported	qU57S1	2	TRUE	0.00306	22.55636
644	rs1111812	A	C	A	C	-0.019	0.015545	0.8501	0.854411	FALSE	FALSE	FALSE	FfmsRf	0.019681	0.429618	486484	sepsis	TRUE	reported	textfile	0.004	1.97E-06	7349	M01121.m	TRUE	reported	9R12A7	2	TRUE	0.003061	22.55636
1857	rs9998852	A	G	A	G	0.0076	-0.02857	0.6611	0.641767	FALSE	FALSE	FALSE	S1eWts	0.014346	0.046435	486484	sepsis	TRUE	reported	textfile	0.0016	2.77E-06	7345	M15488.m	TRUE	reported	511Kl9	2	TRUE	0.003062	22.55636
8162	rs1168162	T	G	T	G	-0.0076	-0.00528	0.4034	0.413321	FALSE	FALSE	FALSE	8UhtPK	0.013924	0.704523	486484	sepsis	TRUE	reported	textfile	0.0016	2.99E-06	7345	M35397.m	TRUE	reported	rvVJba	2	TRUE	0.003062	22.55636
8171	rs2075158	T	C	T	C	-0.0076	0.017538	0.3188	0.331652	FALSE	FALSE	FALSE	8UhtPK	0.014525	0.227272	486484	sepsis	TRUE	reported	textfile	0.0016	3.23E-06	7345	M35397.m	TRUE	reported	rvVJba	2	TRUE	0.003062	22.55636
4160	rs1344215	A	G	A	G	0.0418	0.010054	0.4834	0.521405	FALSE	FALSE	FALSE	stpu5O	0.01418	0.478305	486484	sepsis	TRUE	reported	textfile	0.0088	1.76E-06	7337	M32557.m	TRUE	reported	0oV8Ss	2	TRUE	0.003066	22.55635
4904	rs1316154	T	C	T	C	-0.0057	-0.00948	0.5812	0.59337	FALSE	FALSE	FALSE	VqcRGX	0.013967	0.497486	486484	sepsis	TRUE	reported	textfile	0.0012	4.84E-06	7329	M32867.m	TRUE	reported	psuFJy	2	TRUE	0.003069	22.55634
2836	rs1185357	T	C	T	C	0.0076	0.001505	0.6055	0.604885	FALSE	FALSE	FALSE	Y5qWBm	0.014046	0.914648	486484	sepsis	TRUE	reported	textfile	0.0016	3.75E-06	7325	M20489.m	TRUE	reported	f2g9Su	2	TRUE	0.003071	22.55634
1684	rs1171591	T	C	T	C	-0.0076	-0.00619	0.2789	0.306207	FALSE	FALSE	FALSE	f01en0	0.014823	0.676419	486484	sepsis	TRUE	reported	textfile	0.0016	3.77E-06	7314	M12593.m	TRUE	reported	EAsM6U	2	TRUE	0.003075	22.55633
1691	rs1615157	T	C	T	C	0.0076	-0.00058	0.5764	0.592249	FALSE	FALSE	FALSE	f01en0	0.014023	0.967176	486484	sepsis	TRUE	reported	textfile	0.0016	2.22E-06	7314	M12593.m	TRUE	reported	EAsM6U	2	TRUE	0.003075	22.55633
2072	rs1193049	A	G	A	G	-0.0076	0.025332	0.803	0.802222	FALSE	FALSE	FALSE	NPVH5j	0.017166	0.140013	486484	sepsis	TRUE	reported	textfile	0.0016	1.52E-06	7310	M15506.m	TRUE	reported	r7r3vM	2	TRUE	0.003077	22.55633
1121	rs9663814	A	G	A	G	0.0076	0.003653	0.6175	0.59571	FALSE	FALSE	FALSE	yahiGZ	0.014022	0.794489	486484	sepsis	TRUE	reported	textfile	0.0016	1.95E-06	7271	M01585.m	TRUE	reported	dwZ7vX	2	TRUE	0.003093	22.55629
7236	rs1187167	T	C	T	C	0.0537	-0.01542	0.2673	0.282176	FALSE	FALSE	FALSE	RlcE3J	0.01518	0.309776	486484	sepsis	TRUE	reported	textfile	0.0113	2.18E-06	1649	M34339.m	TRUE	reported	QQrwdm	2	TRUE	0.01351	22.55613
3529	rs1272885	A	G	A	G	0.0133	-0.0036	0.5552	0.551328	FALSE	FALSE	FALSE	rXzSlH	0.013866	0.795382	486484	sepsis	TRUE	reported	textfile	0.0028	2.04E-06	6795	M30805.m	TRUE	reported	7wg9QH	2	TRUE	0.003309	22.55586
6563	rs1221004	A	G	A	G	-0.0171	0.000362	0.4239	0.420784	FALSE	FALSE	FALSE	aTDqRQ	0.013842	0.979112	486484	sepsis	TRUE	reported	textfile	0.0036	2.13E-06	6506	M33871.m	TRUE	reported	Lqimdw	2	TRUE	0.003456	22.55556
4804	rs6977159	A	G	A	G	-0.0285	0.00949	0.5935	0.600297	FALSE	FALSE	FALSE	9qWhFj	0.014034	0.498881	486484	sepsis	TRUE	reported	textfile	0.006	2.00E-06	6200	M32808.m	TRUE	reported	MQcOSi	2	TRUE	0.003626	22.55522
2558	rs9854875	A	G	A	G	0.0266	-0.00222	0.3185	0.32466	FALSE	FALSE	FALSE	NxZhaQ	0.014693	0.8798	486484	sepsis	TRUE	reported	textfile	0.0056	2.05E-06	5984	M18283.m	TRUE	reported	5Pi7r3	2	TRUE	0.003756	22.55496
5968	rs261301	T	C	T	C	0.0323	-0.02	0.1314	0.139747	FALSE	FALSE	FALSE	YCswLG	0.019697	0.309945	486484	sepsis	TRUE	reported	textfile	0.0068	2.06E-06	5822	M33420.m	TRUE	reported	WH7xfc	2	TRUE	0.00386	22.55475
6114	rs7075080	A	G	A	G	-0.0171	-0.02519	0.6326	0.629904	FALSE	FALSE	FALSE	a0osmD	0.014159	0.07529	486484	sepsis	TRUE	reported	textfile	0.0036	2.61E-06	5717	M33453.m	TRUE	reported	CePFnK	2	TRUE	0.003931	22.55461
3101	rs6966840	A	G	A	G	0.0133	-0.00335	0.7671	0.748275	FALSE	FALSE	FALSE	mr0awv	0.015825	0.832554	486484	sepsis	TRUE	reported	textfile	0.0028	2.01E-06	5702	M22130.m	TRUE	reported	Y2TfWQ	2	TRUE	0.003941	22.55459
3840	rs4717210	A	G	A	G	0.019	0.008795	0.082	0.085793	FALSE	FALSE	FALSE	tRX2u0	0.024382	0.718307	486484	sepsis	TRUE	reported	textfile	0.004	2.03E-06	5600	M32348.m	TRUE	reported	ruBgwQ	2	TRUE	0.004013	22.55444
2607	rs6470195	A	T	A	T	0.019	-0.00949	0.89	0.832862	FALSE	TRUE	FALSE	ruvJh6	0.019834	0.632233	486484	sepsis	TRUE	reported	textfile	0.004	2.03E-06	5592	M18369.m	TRUE	reported	GFmwGk	2	TRUE	0.004019	22.55443
6974	rs4148998	A	G	A	G	-0.019	-0.00909	0.147	0.142511	FALSE	FALSE	FALSE	ZlvYs5	0.019586	0.642473	486484	sepsis	TRUE	reported	textfile	0.004	2.03E-06	5591	M34214.m	TRUE	reported	qU57S1	2	TRUE	0.004019	22.55443
3742	rs658591	T	G	T	G	-0.019	0.005524	0.798	0.799785	FALSE	FALSE	FALSE	k16oIH	0.017084	0.746456	486484	sepsis	TRUE	reported	textfile	0.004	2.03E-06	5575	M32328.m	TRUE	reported	TQA3sZ	2	TRUE	0.004031	22.55441
2730	rs2389336	A	T	A	T	-0.038	0.059335	0.023	0.027863	FALSE	TRUE	FALSE	H4yqSK	0.041305	0.150855	486484	sepsis	TRUE	reported	textfile	0.008	2.03E-06	5436	M19362.m	TRUE	reported	w2PDEO	2	TRUE	0.004133	22.5542
8664	rs2842707	C	G	C	G	0.057	-0.00019	0.399	0.414038	FALSE	TRUE	FALSE	BFEGyF	0.014832	0.989813	486484	sepsis	TRUE	reported	textfile	0.012	2.03E-06	4904	M36399.m	TRUE	reported	nhT4FM	2	TRUE	0.00458	22.5533
8470	rs6895134	C	G	C	G	0.057	-0.02131	0.011	0.017463	FALSE	TRUE	FALSE	uMajV6	0.052611	0.685394	486484	sepsis	TRUE	reported	textfile	0.012	2.03E-06	4895	M35754.m	TRUE	reported	b8QB7S	2	TRUE	0.004588	22.55328
6565	rs247197	T	C	T	C	-0.019	-0.00903	0.382	0.387947	FALSE	FALSE	FALSE	aTDqRQ	0.01402	0.519643	486484	sepsis	TRUE	reported	textfile	0.004	2.03E-06	4867	M33871.m	TRUE	reported	Lqimdw	2	TRUE	0.004614	22.55323
2792	rs7295482	A	G	A	G	0.0304	0.016703	0.9079	0.89364	FALSE	FALSE	FALSE	ex7hzq	0.022117	0.450107	486484	sepsis	TRUE	reported	textfile	0.0064	1.77E-06	4460	M19415.m	TRUE	reported	jvXWvV	2	TRUE	0.005033	22.55238
6494	rs1106641	A	C	A	C	0.0893	0.044706	0.0516	0.050563	FALSE	FALSE	FALSE	HNc7C9	0.03136	0.153989	486484	sepsis	TRUE	reported	textfile	0.0188	2.11E-06	3962	M33782.m	TRUE	reported	SAGYGx	2	TRUE	0.005662	22.55111
3563	rs241323	A	G	A	G	-0.0794	-0.03158	0.1166	0.105247	FALSE	FALSE	FALSE	IPkHXk	0.022245	0.155713	486484	sepsis	TRUE	reported	textfile	0.0167	1.88E-06	789	M31522.m	TRUE	reported	jdWe3V	2	TRUE	0.027852	22.54788
8972	rs1002131	T	C	T	C	-0.2423	0.045864	0.9892	0.992716	FALSE	FALSE	FALSE	snOepl	0.080698	0.569802	486484	sepsis	TRUE	reported	textfile	0.051	2.01E-06	1763	M37202.m	TRUE	reported	PQx272	2	TRUE	0.012641	22.54621
3581	rs626720	A	G	A	G	0.1018	0.019756	0.1195	0.13094	FALSE	FALSE	FALSE	F1s6qg	0.020401	0.332854	486484	sepsis	TRUE	reported	textfile	0.0214	1.86E-06	522	M31536.m	TRUE	reported	Zm40GX	2	TRUE	0.04155	22.54244
8085	rs2819083	A	G	A	G	0.059	0.029529	0.3744	0.379886	FALSE	FALSE	FALSE	z65cF2	0.014177	0.037266	486484	sepsis	TRUE	reported	textfile	0.0124	1.96E-06	448	M35322.m	TRUE	reported	3GUSWk	2	TRUE	0.048103	22.53811
8612	rs2854152	A	G	A	G	-0.0304	0.027757	0.3025	0.321746	FALSE	FALSE	FALSE	oHM5TD	0.014679	0.058637	486484	sepsis	TRUE	reported	textfile	0.0064	2.09E-06	1780	M36131.m	TRUE	reported	Xw6wmg	2	TRUE	0.012517	22.53715
3040	rs7499814	A	C	A	C	-0.0285	0.022739	0.8177	0.824058	FALSE	FALSE	FALSE	FJZEYz	0.018034	0.20735	486484	sepsis	TRUE	reported	textfile	0.006	2.05E-06	1755	M21630.m	TRUE	reported	s6Pshg	2	TRUE	0.012693	22.53679
2444	rs7692251	A	G	A	G	0.133	0.000591	0.9573	0.955896	FALSE	FALSE	FALSE	NBJ2Do	0.033329	0.985851	486484	sepsis	TRUE	reported	textfile	0.028	1.97E-06	1739	M17799.m	TRUE	reported	Uz0Wgl	2	TRUE	0.012808	22.53655
2712	rs1050108	T	C	T	C	0.0836	0.003686	0.0309	0.038901	FALSE	FALSE	FALSE	89XKxw	0.035273	0.916783	486484	sepsis	TRUE	reported	textfile	0.0176	2.10E-06	1738	M19324.m	TRUE	reported	gryJuD	2	TRUE	0.012816	22.53654
8288	rs1193247	A	G	A	G	0.0361	-0.0265	0.4287	0.437714	FALSE	FALSE	FALSE	vXjGkU	0.013876	0.056114	486484	sepsis	TRUE	reported	textfile	0.0076	2.42E-06	1291	M35527.m	TRUE	reported	oS5qaO	2	TRUE	0.017177	22.52755
6336	rs1759425	A	T	A	T	-0.0742	-0.00926	0.1399	0.172715	FALSE	TRUE	FALSE	qLUY00	0.018099	0.609108	486484	sepsis	TRUE	reported	textfile	0.0156	2.10E-06	470	M33610.m	TRUE	reported	8cAJbB	2	TRUE	0.045924	22.52717
6600	rs1053871	T	C	T	C	0.0527	0.021828	0.615	0.597139	FALSE	FALSE	FALSE	VfOuo8	0.013924	0.116973	486484	sepsis	TRUE	reported	textfile	0.0111	2.14E-06	2725	M33883.m	TRUE	reported	BDe0Nk	2	TRUE	0.008204	22.52456
2445	rs7701924	A	G	A	G	-0.0907	-0.02507	0.0923	0.080625	FALSE	FALSE	FALSE	NBJ2Do	0.025156	0.318881	486484	sepsis	TRUE	reported	textfile	0.0191	2.08E-06	1739	M17799.m	TRUE	reported	Uz0Wgl	2	TRUE	0.012801	22.52413
8621	rs4954715	A	G	A	G	-0.0527	0.027265	0.0853	0.096429	FALSE	FALSE	FALSE	MZ60v7	0.02315	0.238895	486484	sepsis	TRUE	reported	textfile	0.0111	2.28E-06	2432	M36134.m	TRUE	reported	dMQewN	2	TRUE	0.009183	22.52257
2629	rs1258300	T	C	T	C	-0.0318	-0.03167	0.0723	0.083657	FALSE	FALSE	FALSE	UxuT5B	0.024822	0.201968	486484	sepsis	TRUE	reported	textfile	0.0067	1.75E-06	7367	M18467.m	TRUE	reported	eGQj6o	2	TRUE	0.003049	22.52095
4247	rs2104340	C	G	C	G	0.0617	-0.02329	0.2526	0.260376	FALSE	TRUE	FALSE	KZ60wl	0.015562	0.134441	486484	sepsis	TRUE	reported	textfile	0.013	2.16E-06	6904	M32616.m	TRUE	reported	SPDpZi	2	TRUE	0.003252	22.51945
1299	rs219918	C	G	C	G	-0.0299	-0.06305	0.0224	0.021951	FALSE	TRUE	FALSE	WQdpmo	0.04687	0.178587	486484	sepsis	TRUE	reported	textfile	0.0063	2.48E-06	7136	M01769.m	TRUE	reported	BkptOK	2	TRUE	0.003147	22.5185
5475	rs4787041	T	C	T	C	0.1672	-0.0622	0.9626	0.984596	FALSE	FALSE	FALSE	q32d4E	0.056026	0.266887	486484	sepsis	TRUE	reported	textfile	0.0352	2.05E-06	1017	M33178.m	TRUE	reported	cS3BwT	2	TRUE	0.021704	22.51813
944	rs9987070	C	G	C	G	-0.028	-0.0253	0.9808	0.974646	FALSE	TRUE	FALSE	tv99UN	0.043727	0.562805	486484	sepsis	TRUE	reported	textfile	0.0059	2.43E-06	7354	M01494.m	TRUE	reported	osDclp	2	TRUE	0.003053	22.51614
8547	rs1687893	A	T	A	T	-0.444	-0.04476	0.0909	0.036027	FALSE	TRUE	FALSE	aQhCav	0.03697	0.226012	486484	sepsis	TRUE	reported	textfile	0.0928	1.74E-06	122	M36097.m	TRUE	reported	9DJS9j	2	TRUE	0.157989	22.51601
3440	rs9379897	T	C	T	C	-0.0261	0.000189	0.8707	0.868785	FALSE	FALSE	FALSE	5dHWFk	0.02018	0.99251	486484	sepsis	TRUE	reported	textfile	0.0055	2.04E-06	7339	M27672.m	TRUE	reported	pEd8kY	2	TRUE	0.003059	22.5132
5623	rs3780277	A	G	A	G	0.1767	-0.01959	0.0847	0.08781	FALSE	FALSE	FALSE	8QFOKi	0.024847	0.430562	486484	sepsis	TRUE	reported	textfile	0.0372	2.07E-06	868	M33188.m	TRUE	reported	DzRwMK	2	TRUE	0.025335	22.51051
7149	rs9375892	T	G	T	G	-0.0867	0.006638	0.8819	0.942365	FALSE	FALSE	FALSE	de8tA6	0.02924	0.820397	486484	sepsis	TRUE	reported	textfile	0.0182	2.02E-06	248	M34322.m	TRUE	reported	sXPPBO	2	TRUE	0.083834	22.51017
2492	rs9972424	A	G	A	G	-0.0242	0.007819	0.6602	0.653553	FALSE	FALSE	FALSE	SrfyMU	0.014361	0.586095	486484	sepsis	TRUE	reported	textfile	0.0051	2.04E-06	7226	M17807.m	TRUE	reported	0zrJsu	2	TRUE	0.003106	22.50972
7267	rs35620	C	G	C	G	0.056	-0.00981	0.8372	0.836859	FALSE	TRUE	FALSE	P5Xm4S	0.018625	0.598374	486484	sepsis	TRUE	reported	textfile	0.0118	2.23E-06	3491	M34350.m	TRUE	reported	YU4x0J	2	TRUE	0.00641	22.50936
7868	rs1115007	A	G	A	G	0.0394	0.000894	0.7148	0.648529	FALSE	FALSE	FALSE	r428eQ	0.014366	0.950355	486484	sepsis	TRUE	reported	textfile	0.0083	2.38E-06	1630	M35159.m	TRUE	reported	3r5zQo	2	TRUE	0.013636	22.50625
6595	rs7908275	A	C	A	C	-0.3564	0.027579	0.979	0.98635	FALSE	FALSE	FALSE	XO3rXq	0.059569	0.643378	486484	sepsis	TRUE	reported	textfile	0.0747	1.81E-06	176	M33877.m	TRUE	reported	p2sETg	2	TRUE	0.114524	22.50457
4997	rs6029632	A	G	A	G	0.0223	-0.0013	0.3782	0.397661	FALSE	FALSE	FALSE	OsqE1P	0.013966	0.925589	486484	sepsis	TRUE	reported	textfile	0.0047	2.60E-06	5425	M33084.m	TRUE	reported	VZYh6f	2	TRUE	0.004133	22.5037
501	rs7359970	C	G	C	G	0.2147	-0.03672	0.8975	0.911599	FALSE	TRUE	FALSE	RtjZPn	0.024127	0.128003	486484	sepsis	TRUE	reported	textfile	0.0451	1.93E-06	283	M00553.m	TRUE	reported	C3Ovsl	2	TRUE	0.074143	22.50251
8804	rs1942651	T	C	T	C	-0.0242	0.0067	0.6705	0.635164	FALSE	FALSE	FALSE	QWTooc	0.014202	0.637102	486484	sepsis	TRUE	reported	textfile	0.0051	2.14E-06	3248	M36756.m	TRUE	reported	OLsw6C	2	TRUE	0.006885	22.50209
2234	rs1768319	A	G	A	G	-0.0446	0.01669	0.0385	0.043757	FALSE	FALSE	FALSE	cwj8fY	0.033331	0.616552	486484	sepsis	TRUE	reported	textfile	0.0094	1.88E-06	4538	M15958.m	TRUE	reported	dPwsBc	2	TRUE	0.004936	22.50207
6238	rs2328991	C	G	C	G	0.0204	-0.00973	0.1132	0.122048	FALSE	TRUE	FALSE	vL52Yb	0.020882	0.641404	486484	sepsis	TRUE	reported	textfile	0.0043	1.88E-06	7269	M33510.m	TRUE	reported	JIO9VX	2	TRUE	0.003087	22.50111
6678	rs6584331	T	C	T	C	0.0204	0.005395	0.4873	0.475342	FALSE	FALSE	FALSE	6iZSOI	0.01367	0.693116	486484	sepsis	TRUE	reported	textfile	0.0043	2.55E-06	4533	M33901.m	TRUE	reported	ApKaGe	2	TRUE	0.004941	22.49737
630	rs1717523	A	G	A	G	0.0593	-0.03467	0.9348	0.953695	FALSE	FALSE	FALSE	F6Dn8H	0.032771	0.290133	486484	sepsis	TRUE	reported	textfile	0.0125	1.99E-06	4934	M01114.m	TRUE	reported	dVHatZ	2	TRUE	0.004541	22.49641
2684	rs1076656	T	C	T	C	0.0389	0.000342	0.4367	0.449415	FALSE	FALSE	FALSE	ATsH6N	0.01372	0.980128	486484	sepsis	TRUE	reported	textfile	0.0082	1.97E-06	3783	M18497.m	TRUE	reported	lGuIRI	2	TRUE	0.005914	22.49271
2691	rs7108724	A	G	A	G	-0.0389	0.022489	0.6246	0.627702	FALSE	FALSE	FALSE	ATsH6N	0.014159	0.112224	486484	sepsis	TRUE	reported	textfile	0.0082	2.13E-06	3783	M18497.m	TRUE	reported	lGuIRI	2	TRUE	0.005914	22.49271
8522	rs1185800	T	C	T	C	-0.0408	0.004348	0.8867	0.886708	FALSE	FALSE	FALSE	gM7v2m	0.021526	0.839935	486484	sepsis	TRUE	reported	textfile	0.0086	2.28E-06	2651	M36009.m	TRUE	reported	6Xcypd	2	TRUE	0.008419	22.49032
2910	rs254840	A	G	A	G	-0.0166	0.018224	0.0939	0.105694	FALSE	FALSE	FALSE	FBDEQi	0.022185	0.411387	486484	sepsis	TRUE	reported	textfile	0.0035	3.03E-06	7327	M20699.m	TRUE	reported	nvNIlp	2	TRUE	0.003061	22.48855
4647	rs1190591	A	G	A	G	-0.0166	-0.00069	0.5878	0.588043	FALSE	FALSE	FALSE	SyAFR2	0.014021	0.960881	486484	sepsis	TRUE	reported	textfile	0.0035	1.92E-06	6658	M32755.m	TRUE	reported	prUo74	2	TRUE	0.003367	22.48794
2396	rs1722451	A	G	A	G	-0.0911	0.009951	0.0812	0.084306	FALSE	FALSE	FALSE	NBJ2Do	0.025022	0.690858	486484	sepsis	TRUE	reported	textfile	0.0192	2.08E-06	1739	M17799.m	TRUE	reported	Uz0Wgl	2	TRUE	0.012781	22.48716
9012	rs933341	A	G	A	G	0.0166	-0.00698	0.2404	0.245937	FALSE	FALSE	FALSE	EZQ1cW	0.015867	0.659904	486484	sepsis	TRUE	reported	textfile	0.0035	1.56E-06	5932	M37253.m	TRUE	reported	ddPpd8	2	TRUE	0.003778	22.48711
751	rs2418886	A	G	A	G	0.0223	0.005249	0.0489	0.050101	FALSE	FALSE	FALSE	RHWptG	0.031354	0.867035	486484	sepsis	TRUE	reported	textfile	0.0047	1.63E-06	1762	M01302.m	TRUE	reported	z1OLfr	2	TRUE	0.012615	22.48644
7716	rs9344588	T	C	T	C	0.0389	-0.02822	0.1831	0.159185	FALSE	FALSE	FALSE	qJW9lg	0.018831	0.133951	486484	sepsis	TRUE	reported	textfile	0.0082	2.31E-06	2474	M34878.m	TRUE	reported	42EOzB	2	TRUE	0.009014	22.48642
4896	rs7856961	A	G	A	G	-0.0778	0.033249	0.1517	0.174901	FALSE	FALSE	FALSE	ocaywK	0.018104	0.066281	486484	sepsis	TRUE	reported	textfile	0.0164	2.15E-06	1988	M32863.m	TRUE	reported	WH6KGr	2	TRUE	0.011194	22.48197
549	rs2964063	T	C	T	C	-0.0735	-0.06759	0.9853	0.983483	FALSE	FALSE	FALSE	QUUN2g	0.053713	0.208265	486484	sepsis	TRUE	reported	textfile	0.0155	2.25E-06	7237	M00599.m	TRUE	reported	vxn2i7	2	TRUE	0.003097	22.47974
1536	rs7126003	T	C	T	C	0.0185	-0.00848	0.6259	0.628538	FALSE	FALSE	FALSE	YvgTeW	0.014173	0.549693	486484	sepsis	TRUE	reported	textfile	0.0039	2.58E-06	1759	M03141.m	TRUE	reported	srKjSc	2	TRUE	0.012631	22.47606
6496	rs1240513	A	G	A	G	0.0697	-0.00717	0.0929	0.090521	FALSE	FALSE	FALSE	HNc7C9	0.023959	0.764651	486484	sepsis	TRUE	reported	textfile	0.0147	2.06E-06	3962	M33782.m	TRUE	reported	SAGYGx	2	TRUE	0.005642	22.47044
1190	rs1049222	T	C	T	C	-0.0128	0.007522	0.8498	0.852195	FALSE	FALSE	FALSE	Ckdgh1	0.019239	0.695804	486484	sepsis	TRUE	reported	textfile	0.0027	1.60E-06	7355	M01644.m	TRUE	reported	nKxz7B	2	TRUE	0.003046	22.46851
7885	rs1104858	A	G	A	G	-0.0128	-0.01707	0.7449	0.754122	FALSE	FALSE	FALSE	pU8zps	0.015981	0.285526	486484	sepsis	TRUE	reported	textfile	0.0027	1.85E-06	7350	M35186.m	TRUE	reported	Oc2lOF	2	TRUE	0.003048	22.46851
8380	rs1739166	A	G	A	G	0.0128	0.022383	0.1367	0.132969	FALSE	FALSE	FALSE	SCzh0A	0.020137	0.266342	486484	sepsis	TRUE	reported	textfile	0.0027	2.26E-06	7348	M35675.m	TRUE	reported	n1SREk	2	TRUE	0.003049	22.46851
3283	rs2223810	A	C	A	C	-0.0128	0.013374	0.8498	0.844972	FALSE	FALSE	FALSE	vYWPe9	0.018873	0.478551	486484	sepsis	TRUE	reported	textfile	0.0027	2.18E-06	7340	M25459.m	TRUE	reported	VvJ6Vx	2	TRUE	0.003053	22.4685
4280	rs9322744	T	C	T	C	0.0128	-0.01394	0.3958	0.40069	FALSE	FALSE	FALSE	ylV4bp	0.013928	0.317032	486484	sepsis	TRUE	reported	textfile	0.0027	1.91E-06	7337	M32632.m	TRUE	reported	hqHJoU	2	TRUE	0.003054	22.4685
3213	rs7990504	A	C	A	C	0.0128	-0.0029	0.8066	0.804934	FALSE	FALSE	FALSE	UfLkBq	0.017275	0.866484	486484	sepsis	TRUE	reported	textfile	0.0027	3.13E-06	7329	M22481.m	TRUE	reported	kWMdiT	2	TRUE	0.003057	22.46849
3587	rs749429	T	G	T	G	-0.142	0.027413	0.9406	0.933053	FALSE	FALSE	FALSE	F1s6qg	0.027277	0.314907	486484	sepsis	TRUE	reported	textfile	0.0299	2.07E-06	522	M31536.m	TRUE	reported	Zm40GX	2	TRUE	0.041418	22.46814
1665	rs789195	A	G	A	G	-0.0128	0.003881	0.7657	0.767274	FALSE	FALSE	FALSE	YzX5ZD	0.016189	0.810527	486484	sepsis	TRUE	reported	textfile	0.0027	2.53E-06	6864	M12129.m	TRUE	reported	6tMfaW	2	TRUE	0.003264	22.46807

2299	rs1164327	C	G	C	G	-0.0128	-0.017	0.7236	0.718557	FALSE	TRUE	FALSE	ZzUW8U	0.01521	0.263679	486484	sepsis	TRUE	reported	textfile	0.0027	2.04E-06	6733	M16821.m	TRUE	reported	g2xs3i	2	TRUE	0.003327	22.46795
2305	rs1879207	C	G	C	G	0.0128	-0.00783	0.2501	0.263427	FALSE	TRUE	FALSE	ZzUW8U	0.015508	0.613405	486484	sepsis	TRUE	reported	textfile	0.0027	2.73E-06	6733	M16821.m	TRUE	reported	g2xs3i	2	TRUE	0.003327	22.46795
7925	rs1021115	T	G	T	G	-0.0128	0.006331	0.4131	0.403778	FALSE	FALSE	FALSE	kFTWXY	0.013915	0.649157	486484	sepsis	TRUE	reported	textfile	0.0027	2.90E-06	6543	M35193.m	TRUE	reported	cjJvJl	2	TRUE	0.003423	22.46775
3298	rs1007865	T	C	T	C	0.0128	0.00663	0.7163	0.719132	FALSE	FALSE	FALSE	nqmBts	0.015297	0.664705	486484	sepsis	TRUE	reported	textfile	0.0027	2.85E-06	6388	M25599.m	TRUE	reported	VkwSh1	2	TRUE	0.003506	22.46759
3305	rs1307318	C	G	C	G	0.0128	0.00597	0.3048	0.310882	FALSE	TRUE	FALSE	nqmBts	0.014812	0.68689	486484	sepsis	TRUE	reported	textfile	0.0027	3.02E-06	6388	M25599.m	TRUE	reported	VkwSh1	2	TRUE	0.003506	22.46759
5167	rs899311	A	T	A	T	0.4035	-0.00326	0.8971	0.865472	FALSE	TRUE	FALSE	L2DJzK	0.020035	0.870664	486484	sepsis	TRUE	reported	textfile	0.084	1.55E-06	76	M33173.m	TRUE	reported	hRG70D	2	TRUE	0.232899	22.46708
6455	rs6764543	A	G	A	G	-0.0256	-0.00514	0.4829	0.48943	FALSE	FALSE	FALSE	OFlEwq	0.013646	0.706592	486484	sepsis	TRUE	reported	textfile	0.0054	1.94E-06	5694	M33675.m	TRUE	reported	T2BzXG	2	TRUE	0.003932	22.46673
4225	rs1745093	A	G	A	G	-0.0365	0.021187	0.0875	0.126446	FALSE	FALSE	FALSE	FDy32d	0.021342	0.320834	486484	sepsis	TRUE	reported	textfile	0.0077	2.21E-06	7369	M32587.m	TRUE	reported	S10k1h	2	TRUE	0.00304	22.46396
5075	rs429020	A	G	A	G	-0.0365	0.026908	0.0511	0.050987	FALSE	FALSE	FALSE	gqzGN4	0.031231	0.388913	486484	sepsis	TRUE	reported	textfile	0.0077	2.01E-06	7319	M33140.m	TRUE	reported	DKGBe9	2	TRUE	0.003061	22.46392
2526	rs1191565	A	G	A	G	-0.0474	-0.00864	0.1557	0.145862	FALSE	FALSE	FALSE	4HwTlG	0.020089	0.667131	486484	sepsis	TRUE	reported	textfile	0.01	2.22E-06	7071	M18254.m	TRUE	reported	F9KrSC	2	TRUE	0.003167	22.46125
424	rs2870664	T	C	T	C	0.0147	-0.00897	0.4767	0.463005	FALSE	FALSE	FALSE	cTsNj6	0.01371	0.512754	486484	sepsis	TRUE	reported	textfile	0.0031	1.60E-06	1763	M00063.m	TRUE	reported	7KYTrQ	2	TRUE	0.012594	22.46044
8773	rs1757342	A	G	A	G	0.0237	-0.0194	0.1497	0.159681	FALSE	FALSE	FALSE	7desNF	0.018928	0.305297	486484	sepsis	TRUE	reported	textfile	0.005	1.70E-06	5292	M36673.m	TRUE	reported	RKbIT4	2	TRUE	0.004228	22.45911
5714	rs2711484	A	G	A	G	-0.0218	0.001462	0.3034	0.30078	FALSE	FALSE	FALSE	XDem1i	0.014903	0.921837	486484	sepsis	TRUE	reported	textfile	0.0046	1.89E-06	4665	M33221.m	TRUE	reported	KBV6FH	2	TRUE	0.004791	22.44973
3495	rs4860118	T	C	T	C	0.0716	-0.0613	0.9631	0.960554	FALSE	FALSE	FALSE	Awafb0	0.035158	0.081231	486484	sepsis	TRUE	reported	textfile	0.0151	2.04E-06	1227	M27728.m	TRUE	reported	Lfj8L0	2	TRUE	0.017995	22.44728
8066	rs1188998	T	C	T	C	0.0493	0.011966	0.7004	0.649997	FALSE	FALSE	FALSE	7NkUCa	0.014376	0.405204	486484	sepsis	TRUE	reported	textfile	0.0104	2.12E-06	1765	M35320.m	TRUE	reported	GixRsK	2	TRUE	0.012572	22.44578
4180	rs9901192	A	G	A	G	0.1267	0.007194	0.0959	0.110511	FALSE	FALSE	FALSE	Aq7H4p	0.021726	0.740536	486484	sepsis	TRUE	reported	textfile	0.0267	2.00E-06	604	M32560.m	TRUE	reported	NIzbA7	2	TRUE	0.035942	22.44348
3739	rs2915420	T	C	T	C	-0.0199	0.00488	0.1595	0.149259	FALSE	FALSE	FALSE	k16olH	0.019302	0.800409	486484	sepsis	TRUE	reported	textfile	0.0042	2.10E-06	7340	M32328.m	TRUE	reported	TQA3sZ	2	TRUE	0.003049	22.44343
8932	rs4898658	A	C	A	C	-0.0777	-0.00448	0.0682	0.06816	FALSE	FALSE	FALSE	ns6PV1	0.027124	0.868682	486484	sepsis	TRUE	reported	textfile	0.0164	2.05E-06	7014	M37097.m	TRUE	reported	ZYmYt4	2	TRUE	0.00319	22.44039
4319	rs1157445	A	C	A	C	-0.0379	-0.01832	0.0424	0.045401	FALSE	FALSE	FALSE	EOAssJ	0.03337	0.58308	486484	sepsis	TRUE	reported	textfile	0.008	1.96E-06	7369	M32635.m	TRUE	reported	X1mTjy	2	TRUE	0.003036	22.43781
5108	rs7146383	T	C	T	C	0.1237	0.058823	0.0124	0.009175	FALSE	FALSE	FALSE	Uiz8VU	0.072871	0.419543	486484	sepsis	TRUE	reported	textfile	0.0261	2.15E-06	1531	M33163.m	TRUE	reported	ky0Bda	2	TRUE	0.01446	22.43317
2236	rs2133896	T	G	T	G	0.0469	0.004939	0.0416	0.04235	FALSE	FALSE	FALSE	cwj8fY	0.034308	0.885532	486484	sepsis	TRUE	reported	textfile	0.0099	2.36E-06	4538	M15958.m	TRUE	reported	dPwsBc	2	TRUE	0.004921	22.43282
8068	rs4741433	T	G	T	G	0.018	-0.00431	0.438	0.412846	FALSE	FALSE	FALSE	7NkUCa	0.013975	0.757785	486484	sepsis	TRUE	reported	textfile	0.0038	2.64E-06	7359	M35320.m	TRUE	reported	GixRsK	2	TRUE	0.00304	22.43158
3889	rs377483	T	C	T	C	-0.018	-0.02047	0.6051	0.60226	FALSE	FALSE	FALSE	bmXxuz	0.014046	0.144985	486484	sepsis	TRUE	reported	textfile	0.0038	1.60E-06	7358	M32405.m	TRUE	reported	A9BFZF	2	TRUE	0.00304	22.43157
3891	rs6011040	A	G	A	G	-0.018	-0.00838	0.3141	0.332641	FALSE	FALSE	FALSE	bmXxuz	0.014466	0.562466	486484	sepsis	TRUE	reported	textfile	0.0038	2.72E-06	7358	M32405.m	TRUE	reported	A9BFZF	2	TRUE	0.00304	22.43157
3896	rs9636231	A	G	A	G	0.018	-0.01628	0.2952	0.289951	FALSE	FALSE	FALSE	bmXxuz	0.015038	0.27893	486484	sepsis	TRUE	reported	textfile	0.0038	2.13E-06	7358	M32405.m	TRUE	reported	A9BFZF	2	TRUE	0.00304	22.43157
4109	rs2431704	A	C	A	C	-0.009	0.009467	0.8029	0.801818	FALSE	FALSE	FALSE	4rtLd0	0.01714	0.580728	486484	sepsis	TRUE	reported	textfile	0.0019	1.04E-06	7350	M32518.m	TRUE	reported	j7f1ER	2	TRUE	0.003043	22.43157
8386	rs25846	T	C	T	C	-0.009	-0.00462	0.3109	0.310492	FALSE	FALSE	FALSE	SCzh0A	0.014971	0.757619	486484	sepsis	TRUE	reported	textfile	0.0019	1.62E-06	7348	M35675.m	TRUE	reported	n1SREk	2	TRUE	0.003044	22.43157
3193	rs1299549	A	G	A	G	0.009	-0.00452	0.5359	0.535896	FALSE	FALSE	FALSE	UfLkBq	0.013728	0.741879	486484	sepsis	TRUE	reported	textfile	0.0019	1.84E-06	7329	M22481.m	TRUE	reported	kWMdiT	2	TRUE	0.003052	22.43155
3207	rs4279521	A	C	A	C	0.009	-0.01499	0.475	0.47755	FALSE	FALSE	FALSE	UfLkBq	0.01365	0.272058	486484	sepsis	TRUE	reported	textfile	0.0019	1.42E-06	7329	M22481.m	TRUE	reported	kWMdiT	2	TRUE	0.003052	22.43155
3218	rs9668088	T	C	T	C	-0.009	0.027192	0.4178	0.425179	FALSE	FALSE	FALSE	UfLkBq	0.013842	0.049486	486484	sepsis	TRUE	reported	textfile	0.0019	1.28E-06	7329	M22481.m	TRUE	reported	kWMdiT	2	TRUE	0.003052	22.43155
4353	rs8082649	A	G	A	G	0.009	0.006848	0.3275	0.330064	FALSE	FALSE	FALSE	6VSoQQ	0.014569	0.638312	486484	sepsis	TRUE	reported	textfile	0.0019	1.37E-06	7229	M32644.m	TRUE	reported	oC2AV0	2	TRUE	0.003094	22.43147
8030	rs1762563	T	C	T	C	-0.009	-0.04114	0.1889	0.196118	FALSE	FALSE	FALSE	SwiqSi	0.01718	0.016625	486484	sepsis	TRUE	reported	textfile	0.0019	1.32E-06	7217	M35270.m	TRUE	reported	21ZlsQ	2	TRUE	0.003099	22.43146
4184	rs1125853	C	G	C	G	0.045	-0.01931	0.1705	0.178737	FALSE	TRUE	FALSE	TxOCzG	0.017831	0.278818	486484	sepsis	TRUE	reported	textfile	0.0095	2.08E-06	6970	M32564.m	TRUE	reported	9JP6ND	2	TRUE	0.003209	22.43123
7303	rs3875143	A	C	A	C	0.009	0.024134	0.6316	0.632641	FALSE	FALSE	FALSE	Vcz4EA	0.014247	0.090255	486484	sepsis	TRUE	reported	textfile	0.0019	1.76E-06	6755	M34359.m	TRUE	reported	aUSo2J	2	TRUE	0.003311	22.43103
8460	rs2683822	T	C	T	C	-0.009	-0.00768	0.6125	0.62328	FALSE	FALSE	FALSE	uMajV6	0.014095	0.585742	486484	sepsis	TRUE	reported	textfile	0.0019	3.46E-06	6604	M35754.m	TRUE	reported	b8QB7S	2	TRUE	0.003386	22.43088
8466	rs4968679	T	C	T	C	-0.009	-0.01029	0.4992	0.492202	FALSE	FALSE	FALSE	uMajV6	0.013726	0.453446	486484	sepsis	TRUE	reported	textfile	0.0019	3.09E-06	6604	M35754.m	TRUE	reported	b8QB7S	2	TRUE	0.003386	22.43088
8471	rs7036147	T	C	T	C	-0.009	-0.00893	0.4125	0.41125	FALSE	FALSE	FALSE	uMajV6	0.013919	0.521369	486484	sepsis	TRUE	reported	textfile	0.0019	2.86E-06	6604	M35754.m	TRUE	reported	b8QB7S	2	TRUE	0.003386	22.43088
8476	rs791320	T	C	T	C	-0.009	-0.01078	0.5496	0.558812	FALSE	FALSE	FALSE	uMajV6	0.013772	0.433785	486484	sepsis	TRUE	reported	textfile	0.0019	2.57E-06	6604	M35754.m	TRUE	reported	b8QB7S	2	TRUE	0.003386	22.43088
8892	rs1171378	A	G	A	G	-0.009	0.002688	0.6291	0.634827	FALSE	FALSE	FALSE	eLhsld	0.014286	0.850725	486484	sepsis	TRUE	reported	textfile	0.0019	1.70E-06	6552	M37058.m	TRUE	reported	RSeWE5	2	TRUE	0.003413	22.43082
8894	rs1228241	T	C	T	C	0.009	-0.01105	0.475	0.469394	FALSE	FALSE	FALSE	eLhsld	0.013688	0.419669	486484	sepsis	TRUE	reported	textfile	0.0019	1.24E-06	6552	M37058.m	TRUE	reported	RSeWE5	2	TRUE	0.003413	22.43082
2334	rs9604751	A	G	A	G	0.018	0.009206	0.1344	0.207142	FALSE	FALSE	FALSE	IZXwpR	0.017585	0.600606	486484	sepsis	TRUE	reported	textfile	0.0038	2.04E-06	6549	M16822.m	TRUE	reported	Zx9k2V	2	TRUE	0.003414	22.43082
3159	rs625676	T	C	T	C	-0.009	-0.01742	0.6649	0.660966	FALSE	FALSE	FALSE	JiXR2t	0.014448	0.228054	486484	sepsis	TRUE	reported	textfile	0.0019	3.67E-06	6535	M22177.m	TRUE	reported	joi6p6	2	TRUE	0.003422	22.43081
3175	rs922048	A	T	A	T	-0.009	0.016199	0.4049	0.400581	FALSE	TRUE	FALSE	JiXR2t	0.013944	0.245351	486484	sepsis	TRUE	reported	textfile	0.0019	3.33E-06	6535	M22177.m	TRUE	reported	joi6p6	2	TRUE	0.003422	22.43081
4515	rs1225182	A	G	A	G	-0.009	0.008203	0.6878	0.695103	FALSE	FALSE	FALSE	r0qJaM	0.014844	0.580518	486484	sepsis	TRUE	reported	textfile	0.0019	2.08E-06	6497	M32729.m	TRUE	reported	vYv5Fl	2	TRUE	0.003442	22.43077
4521	rs1424532	A	G	A	G	-0.009	-0.01096	0.6678	0.670436	FALSE	FALSE	FALSE	r0qJaM	0.014614	0.453267	486484	sepsis	TRUE	reported	textfile	0.0019	1.65E-06	6497	M32729.m	TRUE	reported	vYv5Fl	2	TRUE	0.003442	22.43077
4527	rs2000813	T	C	T	C	0.009	-0.00141	0.2942	0.297929	FALSE	FALSE	FALSE	r0qJaM	0.014919	0.924617	486484	sepsis	TRUE	reported	textfile	0.0019	2.31E-06	6497	M32729.m	TRUE	reported	vYv5Fl	2	TRUE	0.003442	22.43077
2667	rs7856830	T	C	T	C	-0.0891	0.006018	0.1533	0.151616	FALSE	FALSE	FALSE	cDEa3G	0.019093	0.752624	486484	sepsis	TRUE	reported	textfile	0.0188	2.27E-06	1457	M18477.m	TRUE	reported	kxZEfC	2	TRUE	0.015182	22.43072
8754	rs1045987	C	G	C	G	-0.0559	-0.03464	0.9167	0.911903	FALSE	TRUE	FALSE	KMisdy	0.024099	0.150659	486484	sepsis	TRUE	reported	textfile	0.0118	2.28E-06	3977	M36589.m	TRUE	reported	J1ISTP	2	TRUE	0.005611	22.43061
7724	rs1160752	T	C	T	C	0.009	-0.00167	0.5424	0.537961	FALSE	FALSE	FALSE	HyMNYM	0.013732	0.903288	486484	sepsis	TRUE	reported	textfile	0.0019	1.82E-06	5885	M34912.m	TRUE	reported	TI8Hcw	2	TRUE	0.003798	22.43005
7731	rs1326186	A	G	A	G	-0.009	0.023487	0.4078	0.40073	FALSE	FALSE	FALSE	HyMNYM	0.013927	0.091715	486484	sepsis	TRUE	reported	textfile	0.0019	1.92E-06	5885	M34912.m	TRUE	reported	TI8Hcw	2	TRUE	0.003798	22.43005
7733	rs1347222	T	C	T	C	0.009	-0.00177	0.3688	0.371406	FALSE	FALSE	FALSE	HyMNYM	0.014181	0.900798	486484	sepsis	TRUE	reported	textfile	0.0019	2.01E-06	5885	M34912.m	TRUE	reported	TI8Hcw	2	TRUE	0.003798	22.43005
7740	rs1933830	T	C	T	C	-0.009	0.000738	0.4953	0.491568	FALSE	FALSE	FALSE	HyMNYM	0.013665	0.956952	486484	sepsis	TRUE	reported	textfile	0.0019	1.87E-06	5885	M34912.m	TRUE	reported	TI8Hcw	2	TRUE	0.003798	22.43005
7162	rs1097120	T	C	T	C	-0.0436	0.000503	0.4075	0.419228	FALSE	FALSE	FALSE	VfvTtz	0.013903	0.971164	486484	sepsis	TRUE	reported	textfile	0.0092	2.09E-06	1492	M34329.m	TRUE	reported	egac6r	2	TRUE	0.01483	22.42925
7811	rs1049244	T	G	T	G	0.009	-0.00523	0.6924	0.690003	FALSE	FALSE	FALSE	h5whlS	0.014918	0.725838	486484	sepsis	TRUE	reported	textfile	0.0019	2.31E-06	4900	M35137.m	TRUE	reported	xVITKF	2	TRUE	0.004558	22.42851
7850	rs6788007	T	C	T	C	0.009	0.017475	0.4017	0.394246	FALSE	FALSE	FALSE	h5whlS	0.014041	0.213281	486484	sepsis	TRUE	reported	textfile	0.0019	2.17E-06	4900	M35137.m	TRUE	reported	xVITKF	2	TRUE	0.004558	22.42851
6591	rs4897530	T	C	T	C	0.4842	0.030352	0.0228	0.031009	FALSE	FALSE	FALSE	XO3rXq	0.039493	0.442166	486484	sepsis	TRUE	reported	textfile	0.1018	1.97E-06	228	M33877.m	TRUE	reported	p2sETg	2	TRUE	0.090268	22.42475
3380	rs3849097	T	G	T	G	-0.0161	-0.02282	0.8494	0.800015	FALSE	FALSE	FALSE	O2JtzH	0.017128	0.182801	486484	sepsis	TRUE	reported	textfile	0.0034	1.73E-06	7347	M27278.m	TRUE	reported	fxClPW	2	TRUE	0.003043	22.41691
8179	rs4898845	A	G	A	G	-0.0161	0.024121	0.0654	0.0629	FALSE	FALSE	FALSE	8UhtPK	0.028203	0.392399	486484	sepsis	TRUE	reported	textfile	0.0034	2.03E-06	7345	M35397.m	TRUE	reported	rvVJba	2	TRUE	0.003044	22.4169
2909	rs2404571	A	G	A	G	0.0161	0.024055	0.8739	0.862223	FALSE	FALSE	FALSE	FBDEQi	0.020259	0.235085	486484	sepsis	TRUE	reported	textfile	0.0034	2.92E-06	7327	M20699.m	TRUE	reported	nvNIlp	2	TRUE	0.003051	22.41689
6237	rs1775463	A	G	A	G	-0.0161	0.002567	0.7461	0.737217	FALSE	FALSE	FALSE	vL52Yb	0.015659	0.869786	486484	sepsis	TRUE	reported	textfile	0.0034	2.25E-06	7269	M33510.m	TRUE	reported	JIO9VX	2	TRUE	0.003075	22.41684
9034	rs1773401	A	C	A	C	0.0232	-0.01538	0.9604	0.927064	FALSE	FALSE	FALSE	df0MCM	0.026405	0.560299	486484	sepsis	TRUE	reported	textfile	0.0049	2.03E-06	7366	M37506.m	TRUE	reported	iUfr4u	2	TRUE	0.003034	22.41124
3936	rs1508782	A	C	A	C	0.0232	0.007274	0.1332	0.151905	FALSE	FALSE	FALSE	LlOaWH	0.019138	0.703871	486484	sepsis	TRUE	reported	textfile	0.0049	2.09E-06	7355	M32418.m	TRUE	reported	AtHOVw	2	TRUE	0.003039	22.41123
5342	rs1709322	T	C	T	C	-0.1649	0.063491	0.0368	0.036424	FALSE	FALSE	FALSE	q32d4E	0.036451	0.08154	486484	sepsis	TRUE	reported	textfile	0.0348	2.16E-06	1017	M33178.m	TRUE	reported	cS3BwT	2	TRUE	0.021601	22.40928
3814	rs9551641	T	C	T	C	-0.1241	0.013761	0.0894	0.075677	FALSE	FALSE	FALSE	HOq2gb	0.025947	0.595856	486484	sepsis	TRUE	reported	textfile	0.0262	2.24E-06	1565	M32346.m	TRUE	reported	Ss5q17	2	TRUE	0.014133	22.4071
5317	rs1693698	A	G	A	G	0.2563	-0.06661	0.9823	0.9853	FALSE	FALSE	FALSE	q32d4E	0.056546	0.23883	486484	sepsis	TRUE	reported	textfile	0.0541	2.13E-06	1017	M33178.m	TRUE	reported	cS3BwT	2	TRUE	0.021592	22.39999
2541	rs1951656	T	C	T	C	-0.42	-0.03417	0.986	0.965931	FALSE	FALSE	FALSE	o0CFya	0.037714	0.364939	486484	sepsis	TRUE	reported	textfile	0.0887	2.20E-06	2078	M18281.m	TRUE	reported	pxbsnu	2	TRUE	0.010674	22.39923
4401	rs6817256	T	C	T	C	0.0213	-0.02319	0.0773	0.081099	FALSE	FALSE	FALSE	UJygVd	0.02503	0.35421	486484	sepsis	TRUE	reported	textfile	0.0045	1.99E-06	7361	M32654.m	TRUE	reported	kv9R0W	2	TRUE	0.003034	22.39836
940	rs764206	A	G	A	G	0.0071	0.011933	0.6546	0.657489	FALSE	FALSE	FALSE	tv99UN	0.014407	0.407514	486484	sepsis	TRUE	reported	textfile	0.0015	4.58E-06	7354	M01494.m	TRUE	reported	osDclp	2	TRUE	0.003037	22.39835
5120	rs7943834	C	G	C	G	0.0639	0.116332	0.9792	0.9791	FALSE	TRUE	FALSE	O6ine1	0.048005	0.01538	486484	sepsis	TRUE	reported	textfile	0.0135	2.14E-06	7263	M33165.m	TRUE	reported	gGsqjW	2	TRUE	0.003075	22.39827
3181	rs1715787	A	T	A	T	-0.0284	-0.00244	0.9213	0.904718	FALSE	TRUE	FALSE	gvZCBj	0.023402	0.916837	486484	sepsis	TRUE	reported	textfile	0.006	2.32E-06	7259	M22189.m	TRUE	reported	GKqg2h	2	TRUE	0.003077	22.39827
4797	rs1383432	T	G	T	G	-0.0284	-0.0043	0.6573	0.661995	FALSE	FALSE	FALSE	9qWhFj	0.014428	0.765935	486484	sepsis	TRUE	reported	textfile	0.006	2.36E-06	6200	M32808.m	TRUE	reported	MQcOSi	2	TRUE	0.003601	22.39722
682	rs1079857	T	G	T	G	-0.0142	0.007223	0.2212	0.324571	FALSE	FALSE	FALSE	aT1Fw1	0.014605	0.620932	486484	sepsis	TRUE	reported	textfile	0.003	2.03E-06	5687	M01284.m	TRUE	reported	mbTe8H	2	TRUE	0.003924	22.39657
7184	rs7029730	A	C	A	C	-0.0142	-0.00467	0.2493	0.257582	FALSE	FALSE	FALSE	2hlepp	0.015676	0.765782	486484	sepsis	TRUE	reported	textfile	0.003	1.57E-06	5081	M34336.m	TRUE	reported	XyK8mO	2	TRUE	0.00439	22.39563
5530	rs7089115	A	G	A	G	-0.2733	-0.01514	0.0178	0.023172	FALSE	FALSE	FALSE	q32d4E	0.045934	0.7417	486484	sepsis	TRUE	reported	textfile	0.0577	2.19E-06	1017	M33178.m	TRUE	reported	cS3BwT	2	TRUE	0.021584	22.39096
1291	rs1115509	A	C	A	C	-0.0999	0.011064	0.0138	0.019105	FALSE	FALSE	FALSE	WQdpmo	0.049945	0.824691	486484	sepsis	TRUE	reported	textfile	0.0211	2.13E-06	1714	M01769.m	TRUE	reported	BkptOK	2	TRUE	0.01291	22.39025
7386	rs7975440	T	C	T	C	-0.0885	-0.01698	0.1425	0.161025	FALSE	FALSE	FALSE	iLnkG0	0.018757	0.36523	486484	sepsis	TRUE	reported	textfile	0.0187	2.31E-06	2779	M34384.m	TRUE	reported	JVsZvV	2	TRUE	0.007995	22.38158
2437	rs6801674	T	C	T	C	0.0568	-0.00461	0.595	0.58301	FALSE	FALSE	FALSE	NBJ2Do	0.013862	0.739551	486484	sepsis	TRUE	reported	textfile	0.012	2.16E-06	1739	M17799.m	TRUE	reported	Uz0Wgl	2	TRUE	0.01272	22.37868
872	rs6783294	A	G	A	G	-0.0213	0.005611	0.4281	0.384437	FALSE	FALSE	FALSE	Vg5OOR	0.014054	0.689731	486484	sepsis	TRUE	reported	textfile	0.0045	2.41E-06	1700	M01361.m	TRUE	reported	XnrS00	2	TRUE	0.013008	22.37809
3607	rs1431844	T	C	T	C	0.0563	0.008453	0.0256	0.023344	FALSE	FALSE	FALSE	2z5KhH	0.045325	0.852045	486484	sepsis	TRUE	reported	textfile	0.0119	2.30E-06	7263	M31555.m	TRUE	reported	5kcWr2	2	TRUE	0.003072	22.37707
1096	rs2846365	A	G	A	G	0.0336	-0.00887	0.4476	0.455692	FALSE	FALSE	FALSE	K3wtZo	0.013719	0.517813	486484	sepsis	TRUE	reported	textfile	0.0071	1.98E-06	2315	M01573.m	TRUE	reported	0D9CvA	2	TRUE	0.009581	22.37621
4194	rs2807872	T	C	T	C	-0.0833	-0.00693	0.1863	0.18354	FALSE	FALSE	FALSE	TxOCzG	0.017753	0.696136	486484	sepsis	TRUE	reported	textfile	0.0176	2.11E-06	1718	M32564.m	TRUE	reported	9JP6ND	2	TRUE	0.012871	22.37478
4326	rs9570044	A	C	A	C	0.0246	-0.00519	0.1057	0.116807	FALSE	FALSE	FALSE	EOAssJ	0.02127	0.807272	486484	sepsis	TRUE	reported	textfile	0.0052	2.38E-06	7369	M32635.m	TRUE	reported	X1mTjy	2	TRUE	0.003028	22.3741
9049	rs6589371	T	C	T	C	0.0123	-0.00695	0.1212	0.135406	FALSE	FALSE	FALSE	df0MCM	0.019932	0.727207	486484	sepsis	TRUE	reported	textfile	0.0026	3.24E-06	7366	M37506.m	TRUE	reported	iUfr4u	2	TRUE	0.003029	22.3741
4490	rs1781045	A	G	A	G	-0.0123	0.005621	0.6796	0.671221	FALSE	FALSE	FALSE	1wNJ1E	0.014605	0.70034	486484	sepsis	TRUE	reported	textfile	0.0026	3.16E-06	7363	M32709.m	TRUE	reported	zihBHg	2	TRUE	0.00303	22.3741
4498	rs4961335	A	G	A	G	0.0123	0.007469	0.5531	0.552324	FALSE	FALSE	FALSE	1wNJ1E	0.013803	0.588428	486484	sepsis	TRUE	reported	textfile	0.0026	1.89E-06	7363	M32709.m	TRUE	reported	zihBHg	2	TRUE	0.00303	22.3741
4500	rs7165678	A	G	A	G	-0.0123	-0.00268	0.6088	0.61947	FALSE	FALSE	FALSE	1wNJ1E	0.014063	0.84899	486484	sepsis	TRUE	reported	textfile	0.0026	2.63E-06	7363	M32709.m	TRUE	reported	zihBHg	2	TRUE	0.00303	22.3741
4404	rs7819084	A	G	A	G	0.0123	0.012054	0.2474	0.243832	FALSE	FALSE	FALSE	UJygVd	0.01605	0.452649	486484	sepsis	TRUE	reported	textfile	0.0026	2.45E-06	7361	M32654.m	TRUE	reported	kv9R0W	2	TRUE	0.003031	22.3741
1776	rs806214	A	G	A	G	0.0123	-0.02503	0.2175	0.209465	FALSE	FALSE	FALSE	wM5dkT	0.016792	0.136116	486484	sepsis	TRUE	reported	textfile	0.0026	2.94E-06	7352	M15122.m	TRUE	reported	CBpa4t	2	TRUE	0.003035	22.37409
1264	rs2049664	C	G	C	G	0.0123	-0.00172	0.1916	0.197035	FALSE	TRUE	FALSE	gNG3wy	0.017251	0.920599	486484	sepsis	TRUE	reported	textfile	0.0026	3.01E-06	7348	M01670.m	TRUE	reported	t3lsMq	2	TRUE	0.003037	22.37409
3375	rs1730499	A	G	A	G	0.0123	-0.02375	0.7653	0.765073	FALSE	FALSE	FALSE	O2JtzH	0.016182	0.142169	486484	sepsis	TRUE	reported	textfile	0.0026	3.10E-06	7347	M27278.m	TRUE	reported	fxClPW	2	TRUE	0.003037	22.37409
3649	rs1552425	A	T	A	T	-0.0123	0.021752	0.5806	0.584197	FALSE	TRUE	FALSE	fnjup6	0.01382	0.115494	486484	sepsis	TRUE	reported	textfile	0.0026	1.54E-06	7346	M32197.m	TRUE	reported	oQzaMk	2	TRUE	0.003037	22.37408
576	rs1322502	A	G	A	G	-0.0123	0.004	0.6784	0.651077	FALSE	FALSE	FALSE	ZoqU2j	0.014342	0.780313	486484	sepsis	TRUE	reported	textfile	0.0026	1.40E-06	7333	M01105.m	TRUE	reported	2WxSOH	2	TRUE	0.003043	22.37407
1088	rs6743210	T	C	T	C	0.0123	-0.00416	0.6199	0.60749	FALSE	FALSE	FALSE	JLtEiZ	0.015304	0.785876	486484	sepsis	TRUE	reported	textfile	0.0026	2.21E-06	7333	M01572.m	TRUE	reported	TSSe28	2	TRUE	0.003043	22.37407
8332	rs6799366	T	C	T	C	0.0123	-0.0174	0.3135	0.326621	FALSE	FALSE	FALSE	o0GzT1	0.014649	0.234904	486484	sepsis	TRUE	reported	textfile	0.0026	3.50E-06	7317	M35631.m	TRUE	reported	G1XFx0	2	TRUE	0.003049	22.37406
3034	rs3755750	T	C	T	C	-0.0123	-0.00357	0.3156	0.322533	FALSE	FALSE	FALSE	FJZEYz	0.014688	0.807924	486484	sepsis	TRUE	reported	textfile	0.0026	1.81E-06	7284	M21630.m	TRUE	reported	s6Pshg	2	TRUE	0.003063	22.37403
3360	rs4287862	A	G	A	G	0.0123	-0.00844	0.1739	0.1793	FALSE	FALSE	FALSE	LY4Gus	0.017869	0.636614	486484	sepsis	TRUE	reported	textfile	0.0026	2.16E-06	7268	M27273.m	TRUE	reported	QKlTBx	2	TRUE	0.00307	22.37402
3363	rs7184060	A	G	A	G	0.0123	0.024531	0.1847	0.181215	FALSE	FALSE	FALSE	LY4Gus	0.017855	0.169479	486484	sepsis	TRUE	reported	textfile	0.0026	1.80E-06	7268	M27273.m	TRUE	reported	QKlTBx	2	TRUE	0.00307	22.37402
1713	rs8105499	A	C	A	C	0.0123	0.01664	0.2909	0.312526	FALSE	FALSE	FALSE	Zml9HX	0.014909	0.264366	486484	sepsis	TRUE	reported	textfile	0.0026	1.57E-06	7258	M12626.m	TRUE	reported	9SUabZ	2	TRUE	0.003074	22.37401
866	rs1777339	A	G	A	G	0.0123	-0.00734	0.2915	0.313367	FALSE	FALSE	FALSE	Vg5OOR	0.01468	0.616863	486484	sepsis	TRUE	reported	textfile	0.0026	3.25E-06	7063	M01361.m	TRUE	reported	XnrS00	2	TRUE	0.003159	22.37384
1716	rs1078742	T	C	T	C	-0.0123	-0.002	0.4783	0.482889	FALSE	FALSE	FALSE	RmWDhQ	0.013669	0.883949	486484	sepsis	TRUE	reported	textfile	0.0026	2.98E-06	7034	M12768.m	TRUE	reported	f0vnEz	2	TRUE	0.003172	22.37381
1727	rs6464030	T	C	T	C	-0.0123	0.00781	0.591	0.586726	FALSE	FALSE	FALSE	RmWDhQ	0.013879	0.573613	486484	sepsis	TRUE	reported	textfile	0.0026	2.99E-06	7034	M12768.m	TRUE	reported	f0vnEz	2	TRUE	0.003172	22.37381
8205	rs7946997	T	C	T	C	-0.0123	0.013193	0.2974	0.302657	FALSE	FALSE	FALSE	iLlpho	0.014872	0.375031	486484	sepsis	TRUE	reported	textfile	0.0026	2.97E-06	7007	M35431.m	TRUE	reported	AAZ5ge	2	TRUE	0.003184	22.37379
2570	rs1252652	T	C	T	C	-0.0369	-0.01144	0.0349	0.054212	FALSE	FALSE	FALSE	KHS91f	0.030249	0.705302	486484	sepsis	TRUE	reported	textfile	0.0078	2.55E-06	6939	M18349.m	TRUE	reported	PeVGil	2	TRUE	0.003215	22.37373
2768	rs9494155	A	C	A	C	0.0123	-0.01082	0.5988	0.586152	FALSE	FALSE	FALSE	H6txH3	0.013924	0.437275	486484	sepsis	TRUE	reported	textfile	0.0026	1.99E-06	6678	M19368.m	TRUE	reported	b094NF	2	TRUE	0.00334	22.37347
3235	rs1020807	A	G	A	G	0.0123	0.028183	0.689	0.66301	FALSE	FALSE	FALSE	COSeSD	0.01445	0.051123	486484	sepsis	TRUE	reported	textfile	0.0026	1.51E-06	6517	M22649.m	TRUE	reported	mnH8z2	2	TRUE	0.003422	22.37331
3103	rs7689395	T	C	T	C	-0.0246	0.021345	0.0682	0.094223	FALSE	FALSE	FALSE	mr0awv	0.023409	0.361861	486484	sepsis	TRUE	reported	textfile	0.0052	2.34E-06	5702	M22130.m	TRUE	reported	Y2TfWQ	2	TRUE	0.00391	22.37233
4265	rs1738284	A	G	A	G	0.1202	-0.0515	0.9864	0.985357	FALSE	FALSE	FALSE	ylV4bp	0.056882	0.365252	486484	sepsis	TRUE	reported	textfile	0.0254	2.28E-06	1762	M32632.m	TRUE	reported	hqHJoU	2	TRUE	0.01255	22.36909
2772	rs4998531	T	C	T	C	0.0175	-0.00725	0.7039	0.70652	FALSE	FALSE	FALSE	JWylTj	0.015033	0.629793	486484	sepsis	TRUE	reported	textfile	0.0037	2.32E-06	6385	M19396.m	TRUE	reported	6S7kzt	2	TRUE	0.003491	22.36334
490	rs1244001	C	G	C	G	0.1008	0.043508	0.7673	0.761996	FALSE	TRUE	FALSE	RtjZPn	0.016151	0.007064	486484	sepsis	TRUE	reported	textfile	0.0213	2.18E-06	1233	M00553.m	TRUE	reported	C3Ovsl	2	TRUE	0.017839	22.35923
6095	rs4325575	T	C	T	C	-0.0175	0.00181	0.7167	0.712854	FALSE	FALSE	FALSE	xvzgym	0.015085	0.904475	486484	sepsis	TRUE	reported	textfile	0.0037	2.75E-06	4004	M33443.m	TRUE	reported	KtBPyR	2	TRUE	0.005556	22.35917
6572	rs1171478	T	C	T	C	-0.3581	-0.00888	0.9731	0.988465	FALSE	FALSE	FALSE	XO3rXq	0.06416	0.889931	486484	sepsis	TRUE	reported	textfile	0.0753	1.96E-06	176	M33877.m	TRUE	reported	p2sETg	2	TRUE	0.113869	22.35915
4898	rs982503	T	C	T	C	0.0667	0.009229	0.8001	0.794298	FALSE	FALSE	FALSE	ocaywK	0.016963	0.58638	486484	sepsis	TRUE	reported	textfile	0.0141	2.21E-06	1988	M32863.m	TRUE	reported	WH6KGr	2	TRUE	0.011131	22.35508
4685	rs1093989	T	C	T	C	0.0279	0.015421	0.1641	0.161027	FALSE	FALSE	FALSE	MV108h	0.018757	0.410999	486484	sepsis	TRUE	reported	textfile	0.0059	2.18E-06	6642	M32758.m	TRUE	reported	btjNXQ	2	TRUE	0.003355	22.35494
6820	rs1248441	A	G	A	G	0.0492	0.015193	0.8721	0.864268	FALSE	FALSE	FALSE	wedECv	0.020034	0.448258	486484	sepsis	TRUE	reported	textfile	0.0104	2.04E-06	1751	M33968.m	TRUE	reported	GxGxRG	2	TRUE	0.01262	22.35461
6365	rs9521789	T	C	T	C	0.0175	0.010955	0.6021	0.603892	FALSE	FALSE	FALSE	WWmDGN	0.013997	0.433828	486484	sepsis	TRUE	reported	textfile	0.0037	2.29E-06	2757	M33627.m	TRUE	reported	2kkEru	2	TRUE	0.008049	22.35412
3807	rs2078391	T	G	T	G	-0.0331	0.011267	0.4558	0.452756	FALSE	FALSE	FALSE	HOq2gb	0.01375	0.41255	486484	sepsis	TRUE	reported	textfile	0.007	2.56E-06	6687	M32346.m	TRUE	reported	Ss5q17	2	TRUE	0.003333	22.3527
7674	rs1711005	A	G	A	G	-0.0279	-0.01504	0.1356	0.144373	FALSE	FALSE	FALSE	iqkM9B	0.019653	0.443966	486484	sepsis	TRUE	reported	textfile	0.0059	2.08E-06	4800	M34539.m	TRUE	reported	5nFq6f	2	TRUE	0.004637	22.35236
6677	rs6565488	A	C	A	C	0.0331	0.016021	0.1172	0.138482	FALSE	FALSE	FALSE	6iZSOI	0.019797	0.41834	486484	sepsis	TRUE	reported	textfile	0.007	1.98E-06	4533	M33901.m	TRUE	reported	ApKaGe	2	TRUE	0.004908	22.34952
481	rs1328893	A	C	A	C	0.2488	-0.02176	0.0205	0.014133	FALSE	FALSE	FALSE	ScJVI8	0.058965	0.712161	486484	sepsis	TRUE	reported	textfile	0.0526	2.29E-06	1766	M00542.m	TRUE	reported	Sa1L04	2	TRUE	0.01251	22.34792
3517	rs2303010	T	C	T	C	-0.1471	-0.02111	0.0102	0.009392	FALSE	FALSE	FALSE	hxuqls	0.072236	0.770098	486484	sepsis	TRUE	reported	textfile	0.0311	2.26E-06	1758	M27738.m	TRUE	reported	aueeZs	2	TRUE	0.012566	22.34654
8033	rs197926	A	G	A	G	-0.0175	-0.00624	0.5181	0.499169	FALSE	FALSE	FALSE	SwiqSi	0.013672	0.648067	486484	sepsis	TRUE	reported	textfile	0.0037	2.19E-06	1696	M35270.m	TRUE	reported	21ZlsQ	2	TRUE	0.013018	22.34396
1140	rs872114	A	C	A	C	-0.0208	-0.04428	0.9736	0.970876	FALSE	FALSE	FALSE	nlqUuV	0.040543	0.274779	486484	sepsis	TRUE	reported	textfile	0.0044	2.29E-06	7371	M01604.m	TRUE	reported	Uro9nC	2	TRUE	0.003023	22.34104
465	rs6663614	A	C	A	C	-0.0104	0.008874	0.2085	0.284023	FALSE	FALSE	FALSE	cl8ucN	0.01515	0.558049	486484	sepsis	TRUE	reported	textfile	0.0022	3.06E-06	7361	M00513.m	TRUE	reported	lan0Q8	2	TRUE	0.003027	22.34104
3713	rs7908297	A	G	A	G	0.0156	0.007404	0.1133	0.153704	FALSE	FALSE	FALSE	agPgtF	0.018955	0.696069	486484	sepsis	TRUE	reported	textfile	0.0033	1.56E-06	7349	M32315.m	TRUE	reported	hMRjcV	2	TRUE	0.003032	22.34103
8645	rs1167037	A	G	A	G	-0.0208	-0.00272	0.864	0.871494	FALSE	FALSE	FALSE	GgPG6k	0.020506	0.894627	486484	sepsis	TRUE	reported	textfile	0.0044	2.71E-06	7184	M36300.m	TRUE	reported	kT1yso	2	TRUE	0.003101	22.34089
8190	rs1048483	T	C	T	C	0.0208	0.029544	0.0654	0.065775	FALSE	FALSE	FALSE	iLlpho	0.028088	0.292877	486484	sepsis	TRUE	reported	textfile	0.0044	2.98E-06	7007	M35431.m	TRUE	reported	AAZ5ge	2	TRUE	0.003179	22.34073
996	rs1493031	T	C	T	C	0.0208	-0.01664	0.0434	0.04327	FALSE	FALSE	FALSE	jfkZbM	0.033727	0.621786	486484	sepsis	TRUE	reported	textfile	0.0044	1.96E-06	6523	M01558.m	TRUE	reported	LFZvMG	2	TRUE	0.003414	22.34026
7124	rs1207086	T	C	T	C	-0.1619	-0.0025	0.9323	0.926344	FALSE	FALSE	FALSE	de8tA6	0.026186	0.924027	486484	sepsis	TRUE	reported	textfile	0.0338	1.70E-06	75	M34322.m	TRUE	reported	sXPPBO	2	TRUE	0.234253	22.33171
4687	rs1246135	C	G	C	G	0.0449	-0.01048	0.9375	0.914394	FALSE	TRUE	FALSE	MV108h	0.024366	0.667094	486484	sepsis	TRUE	reported	textfile	0.0095	2.09E-06	6642	M32758.m	TRUE	reported	btjNXQ	2	TRUE	0.003352	22.33133
5628	rs2392757	A	G	A	G	-0.0572	0.041443	0.2235	0.23077	FALSE	FALSE	FALSE	U0d0ue	0.016296	0.010987	486484	sepsis	TRUE	reported	textfile	0.0121	2.12E-06	2616	M33190.m	TRUE	reported	FoODGF	2	TRUE	0.00847	22.33002
6501	rs4107301	A	G	A	G	0.0605	-0.01433	0.1196	0.132322	FALSE	FALSE	FALSE	HNc7C9	0.020196	0.477954	486484	sepsis	TRUE	reported	textfile	0.0128	2.39E-06	3962	M33782.m	TRUE	reported	SAGYGx	2	TRUE	0.005607	22.32912
6592	rs6109922	A	T	A	T	-0.4676	-0.01218	0.9822	0.979643	FALSE	TRUE	FALSE	XO3rXq	0.050403	0.809121	486484	sepsis	TRUE	reported	textfile	0.0984	2.01E-06	176	M33877.m	TRUE	reported	p2sETg	2	TRUE	0.113715	22.3252
2697	rs1106154	A	G	A	G	-0.0241	0.017013	0.95	0.950579	FALSE	FALSE	FALSE	XsQHZK	0.031648	0.590871	486484	sepsis	TRUE	reported	textfile	0.0051	2.11E-06	7286	M18929.m	TRUE	reported	k8R7hN	2	TRUE	0.003055	22.32413
5626	rs1267482	T	G	T	G	-0.0501	0.012997	0.3945	0.414801	FALSE	FALSE	FALSE	U0d0ue	0.013903	0.349893	486484	sepsis	TRUE	reported	textfile	0.0106	2.22E-06	2616	M33190.m	TRUE	reported	FoODGF	2	TRUE	0.008467	22.32192
2361	rs1126541	A	G	A	G	0.0995	-0.0616	0.9118	0.978726	FALSE	FALSE	FALSE	NBJ2Do	0.047208	0.191918	486484	sepsis	TRUE	reported	textfile	0.0202	8.30E-07	25	M17799.m	TRUE	reported	Uz0Wgl	2	TRUE	0.492519	22.3219
8638	rs6070840	A	C	A	C	0.0397	-0.01606	0.2034	0.204427	FALSE	FALSE	FALSE	4cZjpQ	0.016997	0.344851	486484	sepsis	TRUE	reported	textfile	0.0084	2.48E-06	2400	M36230.m	TRUE	reported	TPUeNs	2	TRUE	0.009221	22.31826
1587	rs6442530	T	C	T	C	-0.026	0.00908	0.3254	0.334889	FALSE	FALSE	FALSE	7QN2kA	0.014479	0.530585	486484	sepsis	TRUE	reported	textfile	0.0055	2.75E-06	1452	M12017.m	TRUE	reported	52YQ1d	2	TRUE	0.015157	22.31633
1034	rs1099272	A	C	A	C	0.0326	0.071621	0.0163	0.018717	FALSE	FALSE	FALSE	tRtQ2e	0.050341	0.154813	486484	sepsis	TRUE	reported	textfile	0.0069	2.44E-06	7364	M01564.m	TRUE	reported	Tbg0zM	2	TRUE	0.003022	22.31614
2127	rs1156366	A	G	A	G	0.0657	0.006605	0.0136	0.00953	FALSE	FALSE	FALSE	TEupfd	0.070572	0.925431	486484	sepsis	TRUE	reported	textfile	0.0139	2.28E-06	1765	M15676.m	TRUE	reported	NXiaPC	2	TRUE	0.0125	22.31561
7270	rs4631721	A	G	A	G	0.043	0.029232	0.6423	0.630288	FALSE	FALSE	FALSE	P5Xm4S	0.014178	0.039227	486484	sepsis	TRUE	reported	textfile	0.0091	2.49E-06	3491	M34350.m	TRUE	reported	YU4x0J	2	TRUE	0.006355	22.31543
8214	rs6025080	T	C	T	C	-0.1125	-0.04125	0.9782	0.983443	FALSE	FALSE	FALSE	aY1HfB	0.053905	0.444091	486484	sepsis	TRUE	reported	textfile	0.0238	2.22E-06	1522	M35433.m	TRUE	reported	H5O4i2	2	TRUE	0.014468	22.31414
3121	rs1122208	C	G	C	G	-0.0137	0.010802	0.8851	0.881689	FALSE	TRUE	FALSE	JiXR2t	0.021252	0.61127	486484	sepsis	TRUE	reported	textfile	0.0029	2.73E-06	6535	M22177.m	TRUE	reported	joi6p6	2	TRUE	0.003403	22.31065
9010	rs8176023	A	G	A	G	-0.0411	0.00205	0.9284	0.917609	FALSE	FALSE	FALSE	EZQ1cW	0.025667	0.936348	486484	sepsis	TRUE	reported	textfile	0.0087	2.46E-06	5932	M37253.m	TRUE	reported	ddPpd8	2	TRUE	0.003748	22.30995
3619	rs1763806	T	G	T	G	-0.0359	-0.01087	0.2983	0.286182	FALSE	FALSE	FALSE	gq4lSJ	0.015114	0.472127	486484	sepsis	TRUE	reported	textfile	0.0076	2.12E-06	7338	M31591.m	TRUE	reported	wjwl4J	2	TRUE	0.003032	22.30711
4448	rs1245020	T	C	T	C	-0.0841	0.010408	0.0365	0.021189	FALSE	FALSE	FALSE	g2RNym	0.047636	0.827039	486484	sepsis	TRUE	reported	textfile	0.0178	2.30E-06	2575	M32691.m	TRUE	reported	35eo6p	2	TRUE	0.008595	22.30563
6925	rs7088085	A	G	A	G	0.885	0.10961	0.984	0.985485	FALSE	FALSE	FALSE	SvfSvH	0.057372	0.056066	486484	sepsis	TRUE	reported	textfile	0.186	1.96E-06	134	M34109.m	TRUE	reported	7OSbiu	2	TRUE	0.144531	22.30128
8585	rs2072901	A	G	A	G	0.0392	0.027117	0.8764	0.879089	FALSE	FALSE	FALSE	iuJmRL	0.021036	0.19737	486484	sepsis	TRUE	reported	textfile	0.0083	2.47E-06	7311	M36103.m	TRUE	reported	jfJbVw	2	TRUE	0.003042	22.2996
8668	rs1075399	A	C	A	C	-0.0326	0.013427	0.3849	0.383609	FALSE	FALSE	FALSE	i4bSdJ	0.014115	0.341467	486484	sepsis	TRUE	reported	textfile	0.0069	2.50E-06	1908	M36468.m	TRUE	reported	qJLuZj	2	TRUE	0.011564	22.2988
1265	rs2170700	A	G	A	G	0.0326	0.01198	0.8663	0.896183	FALSE	FALSE	FALSE	gNG3wy	0.022735	0.598232	486484	sepsis	TRUE	reported	textfile	0.0069	2.43E-06	1761	M01670.m	TRUE	reported	t3lsMq	2	TRUE	0.012517	22.29685
8790	rs1173371	T	C	T	C	-0.0704	0.036833	0.0461	0.058433	FALSE	FALSE	FALSE	0YKkSS	0.029228	0.207591	486484	sepsis	TRUE	reported	textfile	0.0149	2.47E-06	1506	M36754.m	TRUE	reported	cf9IBu	2	TRUE	0.014607	22.29439
9027	rs1323664	A	G	A	G	0.0085	0.002095	0.7326	0.718667	FALSE	FALSE	FALSE	df0MCM	0.015272	0.890916	486484	sepsis	TRUE	reported	textfile	0.0018	2.48E-06	7366	M37506.m	TRUE	reported	iUfr4u	2	TRUE	0.003018	22.29333
9053	rs7185774	T	C	T	C	0.0085	-0.01437	0.3408	0.326129	FALSE	FALSE	FALSE	df0MCM	0.014506	0.32191	486484	sepsis	TRUE	reported	textfile	0.0018	1.26E-06	7366	M37506.m	TRUE	reported	iUfr4u	2	TRUE	0.003018	22.29333
9057	rs9572325	T	G	T	G	0.0085	0.03278	0.4286	0.419367	FALSE	FALSE	FALSE	df0MCM	0.014056	0.019696	486484	sepsis	TRUE	reported	textfile	0.0018	1.88E-06	7366	M37506.m	TRUE	reported	iUfr4u	2	TRUE	0.003018	22.29333
435	rs6573766	T	G	T	G	-0.0085	-0.02519	0.8061	0.79868	FALSE	FALSE	FALSE	cTsNj6	0.017075	0.140122	486484	sepsis	TRUE	reported	textfile	0.0018	1.55E-06	7365	M00063.m	TRUE	reported	7KYTrQ	2	TRUE	0.003019	22.29333
3836	rs3961919	A	G	A	G	0.0085	-0.00264	0.4308	0.421181	FALSE	FALSE	FALSE	tRX2u0	0.013979	0.850157	486484	sepsis	TRUE	reported	textfile	0.0018	1.48E-06	7365	M32348.m	TRUE	reported	ruBgwQ	2	TRUE	0.003019	22.29333
3842	rs4815285	A	G	A	G	-0.0085	-0.02638	0.4616	0.460779	FALSE	FALSE	FALSE	tRX2u0	0.013704	0.054235	486484	sepsis	TRUE	reported	textfile	0.0018	1.19E-06	7365	M32348.m	TRUE	reported	ruBgwQ	2	TRUE	0.003019	22.29333
3855	rs9821422	T	G	T	G	-0.0085	0.002372	0.5535	0.558024	FALSE	FALSE	FALSE	tRX2u0	0.01378	0.863315	486484	sepsis	TRUE	reported	textfile	0.0018	1.37E-06	7365	M32348.m	TRUE	reported	ruBgwQ	2	TRUE	0.003019	22.29333
6066	rs7820633	A	G	A	G	-0.017	-0.00778	0.6205	0.628712	FALSE	FALSE	FALSE	P4BuMD	0.014581	0.593827	486484	sepsis	TRUE	reported	textfile	0.0036	2.94E-06	7365	M33441.m	TRUE	reported	kqrc1d	2	TRUE	0.003019	22.29333
1036	rs1125471	A	C	A	C	-0.0085	0.005897	0.2928	0.294132	FALSE	FALSE	FALSE	tRtQ2e	0.01503	0.6948	486484	sepsis	TRUE	reported	textfile	0.0018	4.47E-06	7364	M01564.m	TRUE	reported	Tbg0zM	2	TRUE	0.003019	22.29333
1057	rs6077792	T	G	T	G	-0.0085	-0.03371	0.3	0.304062	FALSE	FALSE	FALSE	tRtQ2e	0.014843	0.023155	486484	sepsis	TRUE	reported	textfile	0.0018	2.82E-06	7364	M01564.m	TRUE	reported	Tbg0zM	2	TRUE	0.003019	22.29333
3970	rs1187389	A	G	A	G	0.0085	-0.01394	0.6166	0.614276	FALSE	FALSE	FALSE	XdJbEU	0.014018	0.320057	486484	sepsis	TRUE	reported	textfile	0.0018	3.34E-06	7364	M32452.m	TRUE	reported	V5xWKi	2	TRUE	0.003019	22.29333
5749	rs569688	T	G	T	G	0.017	-0.0049	0.2186	0.219909	FALSE	FALSE	FALSE	bm6x2Z	0.016512	0.766851	486484	sepsis	TRUE	reported	textfile	0.0036	2.49E-06	7364	M33230.m	TRUE	reported	8Ccke3	2	TRUE	0.003019	22.29333
726	rs1085090	T	C	T	C	0.0085	-0.01124	0.127	0.125837	FALSE	FALSE	FALSE	9u0SC8	0.020721	0.587562	486484	sepsis	TRUE	reported	textfile	0.0018	2.74E-06	7363	M01301.m	TRUE	reported	H95uec	2	TRUE	0.003019	22.29333
728	rs1115113	A	G	A	G	0.0085	-0.02454	0.1379	0.137202	FALSE	FALSE	FALSE	9u0SC8	0.020005	0.219914	486484	sepsis	TRUE	reported	textfile	0.0018	1.79E-06	7363	M01301.m	TRUE	reported	H95uec	2	TRUE	0.003019	22.29333
738	rs3024536	T	C	T	C	-0.0085	0.004715	0.1443	0.144204	FALSE	FALSE	FALSE	9u0SC8	0.019476	0.808687	486484	sepsis	TRUE	reported	textfile	0.0018	1.69E-06	7363	M01301.m	TRUE	reported	H95uec	2	TRUE	0.003019	22.29333
3999	rs1050506	T	C	T	C	-0.0085	-0.01857	0.4005	0.388187	FALSE	FALSE	FALSE	V9AtId	0.014013	0.185058	486484	sepsis	TRUE	reported	textfile	0.0018	1.85E-06	7363	M32489.m	TRUE	reported	8B8S8a	2	TRUE	0.003019	22.29333
4026	rs7617041	A	G	A	G	0.0085	0.018893	0.6691	0.672545	FALSE	FALSE	FALSE	V9AtId	0.014538	0.193757	486484	sepsis	TRUE	reported	textfile	0.0018	2.90E-06	7363	M32489.m	TRUE	reported	8B8S8a	2	TRUE	0.003019	22.29333
4308	rs750658	A	G	A	G	0.0085	0.037935	0.2567	0.258784	FALSE	FALSE	FALSE	0tjjPC	0.015676	0.015525	486484	sepsis	TRUE	reported	textfile	0.0018	2.87E-06	7362	M32634.m	TRUE	reported	nJJFkX	2	TRUE	0.00302	22.29332
5033	rs4521827	A	T	A	T	0.0085	-0.02202	0.1344	0.140934	FALSE	TRUE	FALSE	BNnOvt	0.019813	0.266322	486484	sepsis	TRUE	reported	textfile	0.0018	2.17E-06	7362	M33132.m	TRUE	reported	rHcCPG	2	TRUE	0.00302	22.29332
4398	rs4781555	A	G	A	G	0.017	0.010397	0.8048	0.762425	FALSE	FALSE	FALSE	UJygVd	0.016072	0.517724	486484	sepsis	TRUE	reported	textfile	0.0036	2.03E-06	7361	M32654.m	TRUE	reported	kv9R0W	2	TRUE	0.00302	22.29332
1520	rs1015306	T	G	T	G	0.0085	-0.00188	0.4356	0.444455	FALSE	FALSE	FALSE	YvgTeW	0.013784	0.891341	486484	sepsis	TRUE	reported	textfile	0.0018	2.12E-06	7357	M03141.m	TRUE	reported	srKjSc	2	TRUE	0.003022	22.29332
1609	rs4646880	A	G	A	G	-0.0085	-0.01012	0.5434	0.536683	FALSE	FALSE	FALSE	zP3C2v	0.013753	0.461884	486484	sepsis	TRUE	reported	textfile	0.0018	1.50E-06	7356	M12035.m	TRUE	reported	1Pooe3	2	TRUE	0.003022	22.29332
1196	rs1310909	A	C	A	C	0.0085	-0.00334	0.614	0.608694	FALSE	FALSE	FALSE	Ckdgh1	0.014013	0.81133	486484	sepsis	TRUE	reported	textfile	0.0018	4.25E-06	7355	M01644.m	TRUE	reported	nKxz7B	2	TRUE	0.003023	22.29332
1209	rs4618485	A	G	A	G	-0.0085	0.037134	0.595	0.602764	FALSE	FALSE	FALSE	Ckdgh1	0.014274	0.009282	486484	sepsis	TRUE	reported	textfile	0.0018	3.04E-06	7355	M01644.m	TRUE	reported	nKxz7B	2	TRUE	0.003023	22.29332
1219	rs989248	A	G	A	G	-0.0085	0.0068	0.6475	0.646773	FALSE	FALSE	FALSE	Ckdgh1	0.014334	0.635239	486484	sepsis	TRUE	reported	textfile	0.0018	2.96E-06	7355	M01644.m	TRUE	reported	nKxz7B	2	TRUE	0.003023	22.29332
560	rs1258070	T	C	T	C	0.0085	-0.02731	0.1328	0.134355	FALSE	FALSE	FALSE	fNqkCW	0.02003	0.172743	486484	sepsis	TRUE	reported	textfile	0.0018	4.55E-06	7354	M00606.m	TRUE	reported	g9uwOn	2	TRUE	0.003023	22.29332
6757	rs1096601	A	G	A	G	-0.0085	0.005579	0.2684	0.296246	FALSE	FALSE	FALSE	Elf6MQ	0.01512	0.712138	486484	sepsis	TRUE	reported	textfile	0.0018	3.23E-06	7354	M33955.m	TRUE	reported	Lxn4RF	2	TRUE	0.003023	22.29332
6760	rs1288091	T	C	T	C	-0.0085	0.009148	0.7389	0.735328	FALSE	FALSE	FALSE	Elf6MQ	0.015526	0.555707	486484	sepsis	TRUE	reported	textfile	0.0018	2.45E-06	7354	M33955.m	TRUE	reported	Lxn4RF	2	TRUE	0.003023	22.29332
6761	rs1442583	A	G	A	G	-0.017	-0.0332	0.0594	0.062196	FALSE	FALSE	FALSE	Elf6MQ	0.028305	0.240751	486484	sepsis	TRUE	reported	textfile	0.0036	1.94E-06	7354	M33955.m	TRUE	reported	Lxn4RF	2	TRUE	0.003023	22.29332
6766	rs2748805	T	C	T	C	0.0085	-0.01142	0.3004	0.314287	FALSE	FALSE	FALSE	Elf6MQ	0.014769	0.439537	486484	sepsis	TRUE	reported	textfile	0.0018	2.31E-06	7354	M33955.m	TRUE	reported	Lxn4RF	2	TRUE	0.003023	22.29332
6767	rs353694	A	G	A	G	-0.0085	4.39E-06	0.4518	0.455738	FALSE	FALSE	FALSE	Elf6MQ	0.01378	0.999746	486484	sepsis	TRUE	reported	textfile	0.0018	4.09E-06	7354	M33955.m	TRUE	reported	Lxn4RF	2	TRUE	0.003023	22.29332
678	rs7530270	A	C	A	C	-0.0085	0.002323	0.2	0.273442	FALSE	FALSE	FALSE	p4oCVP	0.015334	0.879571	486484	sepsis	TRUE	reported	textfile	0.0018	1.51E-06	7352	M01125.m	TRUE	reported	OwD5re	2	TRUE	0.003024	22.29332
4113	rs2991721	A	G	A	G	-0.0085	0.026868	0.3833	0.419368	FALSE	FALSE	FALSE	4rtLd0	0.013842	0.052244	486484	sepsis	TRUE	reported	textfile	0.0018	3.43E-06	7350	M32518.m	TRUE	reported	j7f1ER	2	TRUE	0.003025	22.29331
4115	rs35441	T	C	T	C	0.0085	0.004096	0.3871	0.381768	FALSE	FALSE	FALSE	4rtLd0	0.014055	0.770746	486484	sepsis	TRUE	reported	textfile	0.0018	2.12E-06	7350	M32518.m	TRUE	reported	j7f1ER	2	TRUE	0.003025	22.29331
8372	rs1269811	A	G	A	G	0.0085	0.000912	0.3013	0.312614	FALSE	FALSE	FALSE	SCzh0A	0.014708	0.950558	486484	sepsis	TRUE	reported	textfile	0.0018	4.15E-06	7348	M35675.m	TRUE	reported	n1SREk	2	TRUE	0.003026	22.29331
8388	rs2901027	A	T	A	T	-0.0085	0.00777	0.341	0.361409	FALSE	TRUE	FALSE	SCzh0A	0.014304	0.586988	486484	sepsis	TRUE	reported	textfile	0.0018	4.10E-06	7348	M35675.m	TRUE	reported	n1SREk	2	TRUE	0.003026	22.29331
1256	rs9852606	C	G	C	G	-0.0085	-0.00625	0.3771	0.359949	FALSE	TRUE	FALSE	V50rWC	0.014296	0.66204	486484	sepsis	TRUE	reported	textfile	0.0018	1.48E-06	7346	M01645.m	TRUE	reported	yN3gpQ	2	TRUE	0.003026	22.29331
4966	rs5019028	T	G	T	G	0.0085	-0.01298	0.7021	0.699026	FALSE	FALSE	FALSE	bKmDMa	0.014903	0.383625	486484	sepsis	TRUE	reported	textfile	0.0018	2.27E-06	7344	M32910.m	TRUE	reported	nAuPzO	2	TRUE	0.003027	22.29331
4430	rs1345255	A	G	A	G	-0.0085	-0.00236	0.8677	0.863478	FALSE	FALSE	FALSE	WGPdpq	0.019973	0.905782	486484	sepsis	TRUE	reported	textfile	0.0018	2.12E-06	7338	M32675.m	TRUE	reported	u0eUAG	2	TRUE	0.00303	22.2933
6081	rs4697477	T	C	T	C	-0.0085	-0.00487	0.8334	0.827407	FALSE	FALSE	FALSE	P8n0HP	0.01814	0.788304	486484	sepsis	TRUE	reported	textfile	0.0018	2.19E-06	7336	M33442.m	TRUE	reported	cljMTI	2	TRUE	0.003031	22.2933
4596	rs3915879	T	C	T	C	-0.0085	-0.00761	0.3166	0.305554	FALSE	FALSE	FALSE	Pzplqu	0.015425	0.621686	486484	sepsis	TRUE	reported	textfile	0.0018	3.91E-06	7318	M32740.m	TRUE	reported	FjEBS5	2	TRUE	0.003038	22.29329
7037	rs1146975	A	G	A	G	0.0085	0.015465	0.3702	0.37314	FALSE	FALSE	FALSE	uthruC	0.014136	0.273968	486484	sepsis	TRUE	reported	textfile	0.0018	1.55E-06	7316	M34283.m	TRUE	reported	ftTuRU	2	TRUE	0.003039	22.29329
7055	rs188611	A	G	A	G	0.0085	-0.00521	0.4253	0.424053	FALSE	FALSE	FALSE	uthruC	0.013907	0.707952	486484	sepsis	TRUE	reported	textfile	0.0018	1.67E-06	7316	M34283.m	TRUE	reported	ftTuRU	2	TRUE	0.003039	22.29329
2506	rs2272588	A	G	A	G	0.0085	0.007136	0.5093	0.519886	FALSE	FALSE	FALSE	LwUYua	0.01371	0.602708	486484	sepsis	TRUE	reported	textfile	0.0018	4.18E-06	7315	M17945.m	TRUE	reported	SUN8dc	2	TRUE	0.003039	22.29329
5875	rs288514	T	C	T	C	-0.0085	0.012574	0.5866	0.596295	FALSE	FALSE	FALSE	tqW0ld	0.013934	0.366854	486484	sepsis	TRUE	reported	textfile	0.0018	4.14E-06	7315	M33389.m	TRUE	reported	Sv7EyV	2	TRUE	0.003039	22.29329
5889	rs686189	A	G	A	G	-0.0085	0.026287	0.5854	0.56919	FALSE	FALSE	FALSE	tqW0ld	0.013841	0.057531	486484	sepsis	TRUE	reported	textfile	0.0018	3.86E-06	7315	M33389.m	TRUE	reported	Sv7EyV	2	TRUE	0.003039	22.29329
2202	rs1315168	T	C	T	C	0.0085	-0.01622	0.3407	0.370789	FALSE	FALSE	FALSE	Bf6uMK	0.014206	0.25362	486484	sepsis	TRUE	reported	textfile	0.0018	3.81E-06	7308	M15778.m	TRUE	reported	jowM2N	2	TRUE	0.003042	22.29328
2217	rs4741625	A	G	A	G	0.0085	-0.02407	0.2777	0.294579	FALSE	FALSE	FALSE	Bf6uMK	0.015116	0.111278	486484	sepsis	TRUE	reported	textfile	0.0018	3.50E-06	7308	M15778.m	TRUE	reported	jowM2N	2	TRUE	0.003042	22.29328
6155	rs7767693	A	G	A	G	0.0085	0.003311	0.2941	0.296707	FALSE	FALSE	FALSE	MiSJly	0.014999	0.82527	486484	sepsis	TRUE	reported	textfile	0.0018	2.73E-06	7307	M33477.m	TRUE	reported	WwL1hq	2	TRUE	0.003042	22.29328
3330	rs1379560	T	C	T	C	-0.0085	0.018736	0.1931	0.197169	FALSE	FALSE	FALSE	m66Eav	0.017186	0.275608	486484	sepsis	TRUE	reported	textfile	0.0018	2.27E-06	7303	M27256.m	TRUE	reported	pI2ECU	2	TRUE	0.003044	22.29328
3331	rs2159022	T	C	T	C	0.0085	0.005049	0.8241	0.827648	FALSE	FALSE	FALSE	m66Eav	0.018129	0.780604	486484	sepsis	TRUE	reported	textfile	0.0018	1.96E-06	7303	M27256.m	TRUE	reported	pI2ECU	2	TRUE	0.003044	22.29328
3348	rs9518163	A	G	A	G	-0.0085	0.006727	0.7373	0.723407	FALSE	FALSE	FALSE	m66Eav	0.015303	0.660237	486484	sepsis	TRUE	reported	textfile	0.0018	2.37E-06	7303	M27256.m	TRUE	reported	pI2ECU	2	TRUE	0.003044	22.29328
1116	rs6462723	A	G	A	G	-0.0085	0.003829	0.121	0.123393	FALSE	FALSE	FALSE	yahiGZ	0.020862	0.854385	486484	sepsis	TRUE	reported	textfile	0.0018	1.92E-06	7271	M01585.m	TRUE	reported	dwZ7vX	2	TRUE	0.003058	22.29325
4331	rs1092735	C	G	C	G	0.0085	-0.0173	0.3072	0.311132	FALSE	TRUE	FALSE	6VSoQQ	0.014754	0.241001	486484	sepsis	TRUE	reported	textfile	0.0018	4.41E-06	7229	M32644.m	TRUE	reported	oC2AV0	2	TRUE	0.003075	22.29321
8023	rs1118482	A	G	A	G	-0.0255	0.053182	0.0247	0.03166	FALSE	FALSE	FALSE	SwiqSi	0.039068	0.173438	486484	sepsis	TRUE	reported	textfile	0.0054	2.61E-06	7217	M35270.m	TRUE	reported	21ZlsQ	2	TRUE	0.00308	22.2932
8029	rs1331927	C	G	C	G	-0.0085	-0.00965	0.7984	0.792825	FALSE	TRUE	FALSE	SwiqSi	0.016948	0.5692	486484	sepsis	TRUE	reported	textfile	0.0018	3.82E-06	7217	M35270.m	TRUE	reported	21ZlsQ	2	TRUE	0.00308	22.2932
8041	rs3770124	T	C	T	C	-0.0085	-0.01558	0.802	0.800278	FALSE	FALSE	FALSE	SwiqSi	0.017107	0.362525	486484	sepsis	TRUE	reported	textfile	0.0018	3.23E-06	7217	M35270.m	TRUE	reported	21ZlsQ	2	TRUE	0.00308	22.2932

6250	rs1001659	A	C	A	C	-0.0085	0.00802	0.5815	0.578911	FALSE	FALSE	FALSE	MOonP9	0.013841	0.56229	486484	sepsis	TRUE	reported	textfile	0.0018	3.12E-06	7066	M33515.m	TRUE	reported	226GuP	2	TRUE	0.003146	22.29307
8850	rs3797510	C	G	C	G	-0.0085	-0.02679	0.8426	0.843686	FALSE	TRUE	FALSE	XIHeTV	0.018986	0.158179	486484	sepsis	TRUE	reported	textfile	0.0018	2.71E-06	6940	M36808.m	TRUE	reported	37Vi2D	2	TRUE	0.003203	22.29296
8151	rs731908	A	C	A	C	-0.0085	0.010303	0.193	0.198544	FALSE	FALSE	FALSE	Iv3pAQ	0.01722	0.549621	486484	sepsis	TRUE	reported	textfile	0.0018	2.73E-06	6920	M35327.m	TRUE	reported	3Vsglp	2	TRUE	0.003212	22.29294
8797	rs631871	A	G	A	G	-0.017	0.016655	0.5197	0.522219	FALSE	FALSE	FALSE	0YKkSS	0.013746	0.225669	486484	sepsis	TRUE	reported	textfile	0.0036	1.76E-06	6876	M36754.m	TRUE	reported	cf9IBu	2	TRUE	0.003233	22.2929
7577	rs34170	T	C	T	C	0.1342	0.01429	0.9477	0.943781	FALSE	FALSE	FALSE	ElkOlW	0.030969	0.644482	486484	sepsis	TRUE	reported	textfile	0.0284	2.32E-06	1235	M34499.m	TRUE	reported	W5iEAE	2	TRUE	0.017759	22.29279
8897	rs1339703	T	C	T	C	-0.0085	0.003593	0.4316	0.431239	FALSE	FALSE	FALSE	eLhsld	0.013834	0.79508	486484	sepsis	TRUE	reported	textfile	0.0018	4.27E-06	6552	M37058.m	TRUE	reported	RSeWE5	2	TRUE	0.003392	22.29258
988	rs1041603	A	G	A	G	0.0085	0.014917	0.3202	0.31937	FALSE	FALSE	FALSE	jfkZbM	0.014812	0.313885	486484	sepsis	TRUE	reported	textfile	0.0018	3.84E-06	6523	M01558.m	TRUE	reported	LFZvMG	2	TRUE	0.003407	22.29255
1001	rs1812125	T	G	T	G	-0.0085	0.007571	0.7578	0.75771	FALSE	FALSE	FALSE	jfkZbM	0.016038	0.636851	486484	sepsis	TRUE	reported	textfile	0.0018	3.48E-06	6523	M01558.m	TRUE	reported	LFZvMG	2	TRUE	0.003407	22.29255
2388	rs1687468	T	C	T	C	0.1181	-0.07416	0.9573	0.95257	FALSE	FALSE	FALSE	NBJ2Do	0.032427	0.022199	486484	sepsis	TRUE	reported	textfile	0.025	2.38E-06	1739	M17799.m	TRUE	reported	Uz0Wgl	2	TRUE	0.01267	22.29051
4891	rs213190	T	G	T	G	0.0529	0.004136	0.5931	0.576362	FALSE	FALSE	FALSE	ocaywK	0.013871	0.765563	486484	sepsis	TRUE	reported	textfile	0.0112	2.36E-06	1988	M32863.m	TRUE	reported	WH6KGr	2	TRUE	0.011097	22.28631
4400	rs6691848	A	G	A	G	0.0288	0.029205	0.9639	0.96973	FALSE	FALSE	FALSE	UJygVd	0.040009	0.465409	486484	sepsis	TRUE	reported	textfile	0.0061	2.12E-06	7361	M32654.m	TRUE	reported	kv9R0W	2	TRUE	0.003019	22.28473
3377	rs1994851	A	G	A	G	-0.0203	0.004135	0.0758	0.097728	FALSE	FALSE	FALSE	O2JtzH	0.023081	0.857805	486484	sepsis	TRUE	reported	textfile	0.0043	2.69E-06	7347	M27278.m	TRUE	reported	fxClPW	2	TRUE	0.003024	22.28112
542	rs1103873	A	T	A	T	-0.0203	-0.01731	0.7937	0.789415	FALSE	TRUE	FALSE	QUUN2g	0.016715	0.30045	486484	sepsis	TRUE	reported	textfile	0.0043	3.01E-06	7237	M00599.m	TRUE	reported	vxn2i7	2	TRUE	0.00307	22.28102
3864	rs6829935	T	C	T	C	-0.0203	0.014414	0.2937	0.313367	FALSE	FALSE	FALSE	8gg1zS	0.014806	0.330284	486484	sepsis	TRUE	reported	textfile	0.0043	2.57E-06	6119	M32379.m	TRUE	reported	uZWtuS	2	TRUE	0.003629	22.2799
4464	rs1110654	T	C	T	C	-0.0321	0.012209	0.9498	0.952664	FALSE	FALSE	FALSE	g3iM2w	0.03218	0.704393	486484	sepsis	TRUE	reported	textfile	0.0068	2.31E-06	7308	M32698.m	TRUE	reported	ZQBn0h	2	TRUE	0.00304	22.27785
7119	rs1107458	T	C	T	C	-0.3698	-0.0279	0.9867	0.984775	FALSE	FALSE	FALSE	de8tA6	0.056155	0.619273	486484	sepsis	TRUE	reported	textfile	0.0773	1.70E-06	75	M34322.m	TRUE	reported	sXPPBO	2	TRUE	0.233805	22.27596
1261	rs1265481	A	G	A	G	0.0118	0.030182	0.2913	0.344149	FALSE	FALSE	FALSE	gNG3wy	0.014368	0.035671	486484	sepsis	TRUE	reported	textfile	0.0025	3.36E-06	7348	M01670.m	TRUE	reported	t3lsMq	2	TRUE	0.003023	22.27234
3666	rs7204324	A	T	A	T	-0.0236	0.022256	0.9189	0.915782	FALSE	TRUE	FALSE	fnjup6	0.024574	0.365119	486484	sepsis	TRUE	reported	textfile	0.005	2.12E-06	7346	M32197.m	TRUE	reported	oQzaMk	2	TRUE	0.003024	22.27233
2124	rs1050886	A	G	A	G	0.0118	-0.00264	0.8446	0.851789	FALSE	FALSE	FALSE	TEupfd	0.019814	0.893824	486484	sepsis	TRUE	reported	textfile	0.0025	1.64E-06	7331	M15676.m	TRUE	reported	NXiaPC	2	TRUE	0.00303	22.27232
2864	rs9548447	T	G	T	G	0.0118	0.037348	0.9001	0.897413	FALSE	FALSE	FALSE	Y5qWBm	0.02254	0.097524	486484	sepsis	TRUE	reported	textfile	0.0025	2.47E-06	7325	M20489.m	TRUE	reported	f2g9Su	2	TRUE	0.003032	22.27232
8265	rs2067776	T	C	T	C	0.0118	0.025573	0.5198	0.515677	FALSE	FALSE	FALSE	TdsGCZ	0.01368	0.061579	486484	sepsis	TRUE	reported	textfile	0.0025	3.41E-06	6883	M35464.m	TRUE	reported	sOspot	2	TRUE	0.003226	22.27193
2674	rs1851722	A	G	A	G	0.0354	-0.008	0.2338	0.240617	FALSE	FALSE	FALSE	s4UTyf	0.015968	0.616351	486484	sepsis	TRUE	reported	textfile	0.0075	2.01E-06	5376	M18494.m	TRUE	reported	ajPzhg	2	TRUE	0.004127	22.27011
5054	rs6835379	A	G	A	G	0.0439	-0.00773	0.5036	0.507054	FALSE	FALSE	FALSE	JLCSne	0.013828	0.576377	486484	sepsis	TRUE	reported	textfile	0.0093	2.16E-06	2391	M33137.m	TRUE	reported	LGKYXK	2	TRUE	0.009233	22.26382
6940	rs4750514	A	G	A	G	0.0269	0.006895	0.5822	0.589186	FALSE	FALSE	FALSE	oNv0vf	0.013924	0.620495	486484	sepsis	TRUE	reported	textfile	0.0057	2.60E-06	5581	M34112.m	TRUE	reported	l0pe0N	2	TRUE	0.003975	22.26379
4164	rs2834622	T	C	T	C	0.0302	-0.0228	0.2733	0.292487	FALSE	FALSE	FALSE	stpu5O	0.015018	0.129044	486484	sepsis	TRUE	reported	textfile	0.0064	2.34E-06	7337	M32557.m	TRUE	reported	0oV8Ss	2	TRUE	0.003026	22.26053
5144	rs1911951	A	C	A	C	0.203	-0.00842	0.934	0.939075	FALSE	FALSE	FALSE	L2DJzK	0.028644	0.76869	486484	sepsis	TRUE	reported	textfile	0.043	2.35E-06	1498	M33173.m	TRUE	reported	hRG70D	2	TRUE	0.01466	22.25743
2686	rs1207017	T	C	T	C	-0.2041	-0.07115	0.951	0.941058	FALSE	FALSE	FALSE	ATsH6N	0.02901	0.014188	486484	sepsis	TRUE	reported	textfile	0.0432	2.31E-06	698	M18497.m	TRUE	reported	lGuIRI	2	TRUE	0.030988	22.25729
8008	rs1716085	T	G	T	G	0.0335	-0.0654	0.9515	0.966683	FALSE	FALSE	FALSE	Zj5bZH	0.038161	0.086565	486484	sepsis	TRUE	reported	textfile	0.0071	2.05E-06	6538	M35257.m	TRUE	reported	exssAk	2	TRUE	0.003394	22.25564
1027	rs2074731	A	C	A	C	-0.0184	-0.03763	0.1654	0.162066	FALSE	FALSE	FALSE	haWxZg	0.018547	0.042446	486484	sepsis	TRUE	reported	textfile	0.0039	2.31E-06	7276	M01561.m	TRUE	reported	5wm9R3	2	TRUE	0.00305	22.25292
5150	rs4299028	A	G	A	G	-0.9695	0.068561	0.0171	0.007656	FALSE	FALSE	FALSE	L2DJzK	0.079249	0.386969	486484	sepsis	TRUE	reported	textfile	0.2028	1.75E-06	76	M33173.m	TRUE	reported	hRG70D	2	TRUE	0.231188	22.25245
7626	rs1082940	A	G	A	G	0.0434	-0.02422	0.1231	0.129946	FALSE	FALSE	FALSE	xGQKuv	0.020358	0.234252	486484	sepsis	TRUE	reported	textfile	0.0092	2.32E-06	3813	M34530.m	TRUE	reported	wajzOs	2	TRUE	0.005802	22.24211
667	rs1895999	A	G	A	G	-0.0151	0.006061	0.1699	0.165552	FALSE	FALSE	FALSE	p4oCVP	0.018454	0.742598	486484	sepsis	TRUE	reported	textfile	0.0032	3.43E-06	1764	M01125.m	TRUE	reported	OwD5re	2	TRUE	0.012465	22.24136
3552	rs2314026	A	G	A	G	0.0283	0.023598	0.9644	0.961446	FALSE	FALSE	FALSE	8IuUON	0.035822	0.510042	486484	sepsis	TRUE	reported	textfile	0.006	2.53E-06	7257	M31453.m	TRUE	reported	FBs6G4	2	TRUE	0.003056	22.24081
2292	rs1231447	C	G	C	G	0.0283	-0.0076	0.0853	0.087245	FALSE	TRUE	FALSE	43Yh3h	0.024157	0.753094	486484	sepsis	TRUE	reported	textfile	0.006	2.76E-06	7055	M16818.m	TRUE	reported	beR4IT	2	TRUE	0.003143	22.24064
3247	rs1772351	A	G	A	G	-0.0481	0.006355	0.6672	0.653061	FALSE	FALSE	FALSE	jxdGCf	0.014392	0.658792	486484	sepsis	TRUE	reported	textfile	0.0102	2.30E-06	5599	M22842.m	TRUE	reported	f0L3pZ	2	TRUE	0.003956	22.22975
2453	rs8101619	T	C	T	C	-0.0618	0.01505	0.5013	0.423727	FALSE	FALSE	FALSE	NBJ2Do	0.013929	0.279951	486484	sepsis	TRUE	reported	textfile	0.0131	2.28E-06	1739	M17799.m	TRUE	reported	Uz0Wgl	2	TRUE	0.012636	22.22975
6447	rs842456	A	G	A	G	-0.0217	0.008431	0.7803	0.780763	FALSE	FALSE	FALSE	QCw5dg	0.016484	0.609043	486484	sepsis	TRUE	reported	textfile	0.0046	1.76E-06	1764	M33666.m	TRUE	reported	zYHmzg	2	TRUE	0.012458	22.22855
8232	rs1524307	T	C	T	C	0.025	0.009285	0.4846	0.498185	FALSE	FALSE	FALSE	4YAWhA	0.01377	0.500134	486484	sepsis	TRUE	reported	textfile	0.0053	1.96E-06	1718	M35439.m	TRUE	reported	bSSC4Z	2	TRUE	0.012785	22.22401
8779	rs1014551	A	G	A	G	-0.0373	-0.0008	0.7599	0.731208	FALSE	FALSE	FALSE	4bkfvN	0.015423	0.958386	486484	sepsis	TRUE	reported	textfile	0.0079	2.46E-06	648	M36738.m	TRUE	reported	xcqyX2	2	TRUE	0.033258	22.22394
6421	rs1735821	T	C	T	C	0.1099	0.003222	0.0313	0.054657	FALSE	FALSE	FALSE	F7mjRv	0.031256	0.917903	486484	sepsis	TRUE	reported	textfile	0.0233	2.36E-06	1860	M33658.m	TRUE	reported	frG0Zf	2	TRUE	0.01182	22.2237
1184	rs8105491	T	G	T	G	-0.0698	0.01314	0.154	0.151561	FALSE	FALSE	FALSE	FgZx4a	0.019016	0.489588	486484	sepsis	TRUE	reported	textfile	0.0148	2.30E-06	2063	M01640.m	TRUE	reported	hNPAJ8	2	TRUE	0.010667	22.22113
3971	rs1202925	A	C	A	C	0.0132	0.033923	0.1421	0.142278	FALSE	FALSE	FALSE	XdJbEU	0.019544	0.082606	486484	sepsis	TRUE	reported	textfile	0.0028	1.57E-06	7364	M32452.m	TRUE	reported	V5xWKi	2	TRUE	0.003009	22.21845
745	rs1006645	C	G	C	G	0.0165	0.038605	0.0387	0.027272	FALSE	TRUE	FALSE	RHWptG	0.042848	0.367605	486484	sepsis	TRUE	reported	textfile	0.0035	1.93E-06	7347	M01302.m	TRUE	reported	z1OLfr	2	TRUE	0.003016	22.21844
3658	rs4641779	A	C	A	C	0.0165	0.027403	0.1417	0.152256	FALSE	FALSE	FALSE	fnjup6	0.019032	0.14992	486484	sepsis	TRUE	reported	textfile	0.0035	2.37E-06	7346	M32197.m	TRUE	reported	oQzaMk	2	TRUE	0.003016	22.21844
6702	rs1086845	A	T	A	T	-0.0165	0.015008	0.3461	0.354504	FALSE	TRUE	FALSE	bzT5yX	0.014277	0.293176	486484	sepsis	TRUE	reported	textfile	0.0035	1.92E-06	7345	M33936.m	TRUE	reported	W7BO8n	2	TRUE	0.003017	22.21844
3771	rs1170495	A	C	A	C	-0.0231	0.004627	0.0362	0.036267	FALSE	FALSE	FALSE	NW1aaR	0.036868	0.900133	486484	sepsis	TRUE	reported	textfile	0.0049	2.85E-06	7344	M32339.m	TRUE	reported	pBPkAB	2	TRUE	0.003017	22.21844
4982	rs1319220	A	T	A	T	0.0165	-0.0059	0.7555	0.740649	FALSE	TRUE	FALSE	AjJi2i	0.015612	0.705318	486484	sepsis	TRUE	reported	textfile	0.0035	2.34E-06	7330	M32980.m	TRUE	reported	gVSm9e	2	TRUE	0.003023	22.21843
3216	rs942358	T	C	T	C	-0.0132	0.008958	0.1543	0.152758	FALSE	FALSE	FALSE	UfLkBq	0.018997	0.637227	486484	sepsis	TRUE	reported	textfile	0.0028	1.94E-06	7329	M22481.m	TRUE	reported	kWMdiT	2	TRUE	0.003023	22.21842
2077	rs1764274	T	G	T	G	-0.0165	0.011771	0.9518	0.925002	FALSE	FALSE	FALSE	NPVH5j	0.025951	0.650136	486484	sepsis	TRUE	reported	textfile	0.0035	2.68E-06	7310	M15506.m	TRUE	reported	r7r3vM	2	TRUE	0.003031	22.21841
5832	rs4919062	A	C	A	C	0.0198	0.045101	0.9652	0.966948	FALSE	FALSE	FALSE	R5joEf	0.038671	0.243499	486484	sepsis	TRUE	reported	textfile	0.0042	3.17E-06	7118	M33380.m	TRUE	reported	kRvbfZ	2	TRUE	0.003113	22.21825
5735	rs6517062	A	G	A	G	0.0132	-0.03702	0.3577	0.359497	FALSE	FALSE	FALSE	YDQaWa	0.014319	0.00972	486484	sepsis	TRUE	reported	textfile	0.0028	1.57E-06	7063	M33228.m	TRUE	reported	BmYg8r	2	TRUE	0.003137	22.2182
2982	rs3112989	A	G	A	G	-0.0514	0.017663	0.5555	0.542551	FALSE	FALSE	FALSE	Tli4xl	0.013766	0.199439	486484	sepsis	TRUE	reported	textfile	0.0109	2.23E-06	2339	M21151.m	TRUE	reported	xaSytY	2	TRUE	0.009417	22.21783
8472	rs7039918	T	C	T	C	0.0132	0.006236	0.8007	0.788391	FALSE	FALSE	FALSE	uMajV6	0.017049	0.714549	486484	sepsis	TRUE	reported	textfile	0.0028	2.76E-06	6604	M35754.m	TRUE	reported	b8QB7S	2	TRUE	0.003354	22.21776
3004	rs1264104	A	G	A	G	-0.0165	0.008429	0.8189	0.822505	FALSE	FALSE	FALSE	gxupFu	0.017904	0.637785	486484	sepsis	TRUE	reported	textfile	0.0035	2.38E-06	6556	M21188.m	TRUE	reported	gwwXh2	2	TRUE	0.003378	22.21771
1164	rs3011362	A	G	A	G	-0.0349	-0.00093	0.0334	0.043032	FALSE	FALSE	FALSE	cSBFJ3	0.033763	0.977955	486484	sepsis	TRUE	reported	textfile	0.0074	2.37E-06	1735	M01638.m	TRUE	reported	sUkjpE	2	TRUE	0.012658	22.21706
7722	rs1126376	A	G	A	G	0.0132	-0.00351	0.7777	0.762317	FALSE	FALSE	FALSE	HyMNYM	0.016089	0.827125	486484	sepsis	TRUE	reported	textfile	0.0028	2.01E-06	5885	M34912.m	TRUE	reported	Tl8Hcw	2	TRUE	0.003762	22.21694
7641	rs1473500	T	C	T	C	-0.0891	0.008214	0.0202	0.02296	FALSE	FALSE	FALSE	wrTMYM	0.045713	0.857399	486484	sepsis	TRUE	reported	textfile	0.0189	2.36E-06	5856	M34533.m	TRUE	reported	qIRVku	2	TRUE	0.003781	22.2169
4893	rs4447447	A	G	A	G	0.2288	-0.00699	0.0449	0.061338	FALSE	FALSE	FALSE	ocaywK	0.028605	0.806892	486484	sepsis	TRUE	reported	textfile	0.0485	2.36E-06	1162	M32863.m	TRUE	reported	WH6KGr	2	TRUE	0.018792	22.21674
6103	rs1272575	A	T	A	T	-0.0198	0.000212	0.6521	0.647361	FALSE	TRUE	FALSE	a0osmD	0.014322	0.988213	486484	sepsis	TRUE	reported	textfile	0.0042	2.96E-06	5717	M33453.m	TRUE	reported	CePFnK	2	TRUE	0.003872	22.21671
3072	rs4147977	T	C	T	C	-0.0132	0.007923	0.4483	0.457757	FALSE	FALSE	FALSE	e3izea	0.014024	0.572082	486484	sepsis	TRUE	reported	textfile	0.0028	1.82E-06	5676	M22032.m	TRUE	reported	zODKFl	2	TRUE	0.0039	22.21666
3493	rs2982376	T	C	T	C	-0.0132	0.000373	0.4212	0.420025	FALSE	FALSE	FALSE	Awafb0	0.013972	0.978705	486484	sepsis	TRUE	reported	textfile	0.0028	2.93E-06	5562	M27728.m	TRUE	reported	Lfj8L0	2	TRUE	0.00398	22.2165
6175	rs7227090	A	G	A	G	0.0297	-0.00981	0.8661	0.867184	FALSE	FALSE	FALSE	O4Ty2I	0.020165	0.626777	486484	sepsis	TRUE	reported	textfile	0.0063	2.28E-06	5107	M33488.m	TRUE	reported	RGtF84	2	TRUE	0.004333	22.21579
7854	rs6979908	A	G	A	G	-0.0132	0.005778	0.1274	0.145843	FALSE	FALSE	FALSE	h5whIS	0.01946	0.76651	486484	sepsis	TRUE	reported	textfile	0.0028	3.05E-06	4900	M35137.m	TRUE	reported	xVITKF	2	TRUE	0.004515	22.21542
2283	rs1861371	A	G	A	G	0.0165	-0.01371	0.1774	0.182111	FALSE	FALSE	FALSE	mRULgB	0.017767	0.44017	486484	sepsis	TRUE	reported	textfile	0.0035	2.35E-06	4689	M16816.m	TRUE	reported	05lFsX	2	TRUE	0.004717	22.21501
6380	rs1993342	A	C	A	C	-0.0533	-0.00743	0.2758	0.284077	FALSE	FALSE	FALSE	pPr1v0	0.015198	0.625053	486484	sepsis	TRUE	reported	textfile	0.0113	2.45E-06	1155	M33637.m	TRUE	reported	Ide1A7	2	TRUE	0.018899	22.20981
8628	rs1106666	A	C	A	C	-0.0297	0.034256	0.4127	0.426412	FALSE	FALSE	FALSE	4cZjpQ	0.013826	0.013224	486484	sepsis	TRUE	reported	textfile	0.0063	2.18E-06	2400	M36230.m	TRUE	reported	TPUeNs	2	TRUE	0.009175	22.20597
5198	rs1084192	C	G	C	G	0.1066	-0.02056	0.6919	0.624522	FALSE	TRUE	FALSE	q32d4E	0.01412	0.145443	486484	sepsis	TRUE	reported	textfile	0.0226	2.38E-06	1017	M33178.m	TRUE	reported	cS3BwT	2	TRUE	0.021408	22.20458
3985	rs6102466	T	C	T	C	-0.0264	-0.02118	0.7812	0.782022	FALSE	FALSE	FALSE	XdJbEU	0.016542	0.200479	486484	sepsis	TRUE	reported	textfile	0.0056	2.68E-06	1766	M32452.m	TRUE	reported	V5xWKi	2	TRUE	0.012428	22.19932
7873	rs4340260	T	C	T	C	-0.0363	-0.0034	0.4537	0.442275	FALSE	FALSE	FALSE	r428eQ	0.013814	0.805338	486484	sepsis	TRUE	reported	textfile	0.0077	2.67E-06	1630	M35159.m	TRUE	reported	3r5zQo	2	TRUE	0.013451	22.19722
6643	rs1190205	A	C	A	C	0.0622	0.01341	0.986	0.981215	FALSE	FALSE	FALSE	REeQiq	0.050505	0.790615	486484	sepsis	TRUE	reported	textfile	0.0132	2.27E-06	6004	M33892.m	TRUE	reported	cVOa3u	2	TRUE	0.003685	22.19669
7424	rs9907459	T	C	T	C	-0.2136	-0.0279	0.9803	0.949358	FALSE	FALSE	FALSE	7i5VUS	0.031299	0.37263	486484	sepsis	TRUE	reported	textfile	0.0453	2.38E-06	1104	M34395.m	TRUE	reported	64hEaL	2	TRUE	0.019741	22.19313
6545	rs1026162	T	G	T	G	-0.041	0.004555	0.6829	0.66554	FALSE	FALSE	FALSE	zmDnD7	0.01453	0.753926	486484	sepsis	TRUE	reported	textfile	0.0087	2.27E-06	2660	M33835.m	TRUE	reported	MLBiKM	2	TRUE	0.00828	22.19231
6988	rs1717000	C	G	C	G	0.0245	0.036684	0.8052	0.78027	FALSE	TRUE	FALSE	APOtl6	0.016589	0.027012	486484	sepsis	TRUE	reported	textfile	0.0052	2.00E-06	6987	M34221.m	TRUE	reported	looCDC	2	TRUE	0.003167	22.19224
6832	rs4811800	A	G	A	G	-0.0212	0.028879	0.484	0.48757	FALSE	FALSE	FALSE	ZPJCMx	0.013674	0.034692	486484	sepsis	TRUE	reported	textfile	0.0045	2.52E-06	7330	M33969.m	TRUE	reported	mLJPAI	2	TRUE	0.003019	22.18851
6218	rs4899659	C	G	C	G	-0.0311	-0.00119	0.3679	0.369859	FALSE	TRUE	FALSE	FQfT2o	0.014218	0.933516	486484	sepsis	TRUE	reported	textfile	0.0066	2.42E-06	2759	M33508.m	TRUE	reported	JtbOy2	2	TRUE	0.007984	22.18799
6724	rs3125596	A	G	A	G	-0.0542	0.013358	0.9148	0.901076	FALSE	FALSE	FALSE	B6lZly	0.022902	0.559733	486484	sepsis	TRUE	reported	textfile	0.0115	2.39E-06	1759	M33937.m	TRUE	reported	qFPzjh	2	TRUE	0.012471	22.18752
4751	rs9299150	T	C	T	C	0.0542	-0.00171	0.3005	0.363476	FALSE	FALSE	FALSE	oQPvO3	0.014209	0.903948	486484	sepsis	TRUE	reported	textfile	0.0115	2.34E-06	1751	M32786.m	TRUE	reported	Th0GcB	2	TRUE	0.012527	22.18741
5713	rs1865099	A	G	A	G	0.0212	-0.01082	0.6476	0.626699	FALSE	FALSE	FALSE	XDem1i	0.014165	0.444966	486484	sepsis	TRUE	reported	textfile	0.0045	2.60E-06	4665	M33221.m	TRUE	reported	KBV6FH	2	TRUE	0.004735	22.18505
7767	rs4737772	A	C	A	C	0.0952	0.049161	0.0139	0.012163	FALSE	FALSE	FALSE	G4gUxB	0.064144	0.443426	486484	sepsis	TRUE	reported	textfile	0.0202	2.55E-06	1657	M35114.m	TRUE	reported	o79OO5	2	TRUE	0.013227	22.18435
3890	rs4954419	C	G	C	G	-0.0179	2.59E-05	0.7089	0.723279	FALSE	TRUE	FALSE	bmXxuz	0.015417	0.998657	486484	sepsis	TRUE	reported	textfile	0.0038	1.89E-06	7358	M32405.m	TRUE	reported	A9BFZF	2	TRUE	0.003007	22.18303
4850	rs1746184	T	C	T	C	-0.0358	0.055685	0.9096	0.909786	FALSE	FALSE	FALSE	StMkBO	0.024194	0.021358	486484	sepsis	TRUE	reported	textfile	0.0076	2.86E-06	6967	M32847.m	TRUE	reported	uzQb8Q	2	TRUE	0.003175	22.18269
8457	rs2041264	T	C	T	C	-0.0179	0.022132	0.9069	0.900802	FALSE	FALSE	FALSE	uMajV6	0.022825	0.332225	486484	sepsis	TRUE	reported	textfile	0.0038	2.53E-06	6604	M35754.m	TRUE	reported	b8QB7S	2	TRUE	0.003349	22.18234
7359	rs4413146	T	G	T	G	-0.1504	-0.02143	0.8985	0.891938	FALSE	FALSE	FALSE	mm9ksf	0.021978	0.329424	486484	sepsis	TRUE	reported	textfile	0.0319	2.39E-06	954	M34365.m	TRUE	reported	AVwEqD	2	TRUE	0.02277	22.18211
9014	rs1272771	T	C	T	C	-0.1065	0.036353	0.0307	0.039775	FALSE	FALSE	FALSE	uxRXvK	0.035304	0.303142	486484	sepsis	TRUE	reported	textfile	0.0226	2.37E-06	1805	M37459.m	TRUE	reported	m4dgs3	2	TRUE	0.012153	22.18201
8586	rs2280042	A	G	A	G	-0.2177	0.013652	0.0129	0.008421	FALSE	FALSE	FALSE	iuJmRL	0.075865	0.857194	486484	sepsis	TRUE	reported	textfile	0.0462	2.48E-06	1750	M36103.m	TRUE	reported	jfJbVw	2	TRUE	0.012529	22.17871
8795	rs359428	A	G	A	G	-0.0471	0.057318	0.0344	0.04431	FALSE	FALSE	FALSE	0YKkSS	0.033369	0.085854	486484	sepsis	TRUE	reported	textfile	0.01	2.44E-06	6876	M36754.m	TRUE	reported	cf9IBu	2	TRUE	0.003216	22.17765
6493	rs9466463	C	G	C	G	0.1372	0.004876	0.0541	0.061027	FALSE	TRUE	FALSE	ecwVg8	0.028624	0.864747	486484	sepsis	TRUE	reported	textfile	0.0291	2.37E-06	831	M33751.m	TRUE	reported	NnvbIQ	2	TRUE	0.026053	22.17562
7807	rs9528023	T	C	T	C	-0.0146	0.010528	0.2466	0.274778	FALSE	FALSE	FALSE	cQ5TvO	0.015315	0.491805	486484	sepsis	TRUE	reported	textfile	0.0031	3.36E-06	7344	M35127.m	TRUE	reported	BVxkfZ	2	TRUE	0.003011	22.17502
3579	rs3794836	A	G	A	G	0.1656	-0.0117	0.0439	0.03675	FALSE	FALSE	FALSE	F1s6gq	0.036412	0.747894	486484	sepsis	TRUE	reported	textfile	0.0351	2.42E-06	522	M31536.m	TRUE	reported	Zm40GX	2	TRUE	0.040898	22.17376
2447	rs771788	A	G	A	G	-0.0881	0.02232	0.0921	0.07299	FALSE	FALSE	FALSE	NBJ2Do	0.026445	0.398653	486484	sepsis	TRUE	reported	textfile	0.0187	2.52E-06	1739	M17799.m	TRUE	reported	Uz0Wgl	2	TRUE	0.012603	22.17016
489	rs1176413	T	C	T	C	-0.1032	0.029699	0.1873	0.184636	FALSE	FALSE	FALSE	RtjZPn	0.017652	0.092466	486484	sepsis	TRUE	reported	textfile	0.0219	2.55E-06	1233	M00553.m	TRUE	reported	C3Ovsl	2	TRUE	0.017691	22.17002
6397	rs1114802	A	G	A	G	-0.0405	-0.02174	0.8748	0.87254	FALSE	FALSE	FALSE	Z1h3mT	0.020579	0.290884	486484	sepsis	TRUE	reported	textfile	0.0086	2.74E-06	5328	M33652.m	TRUE	reported	nszuTQ	2	TRUE	0.004145	22.1692
8652	rs2174099	C	G	C	G	0.0471	-0.01353	0.1469	0.204329	FALSE	TRUE	FALSE	J6i46i	0.017007	0.426446	486484	sepsis	TRUE	reported	textfile	0.01	2.60E-06	2520	M36376.m	TRUE	reported	IdddPp	2	TRUE	0.008726	22.16649
4643	rs7321884	T	C	T	C	0.1488	0.004642	0.0207	0.016759	FALSE	FALSE	FALSE	Dfaklt	0.053618	0.931008	486484	sepsis	TRUE	reported	textfile	0.0316	2.43E-06	6379	M32754.m	TRUE	reported	y21cDm	2	TRUE	0.003464	22.16642
6028	rs1761630	A	T	A	T	-1.4131	0.018409	0.0236	0.01986	FALSE	TRUE	FALSE	33ojbt	0.049813	0.71171	486484	sepsis	TRUE	reported	textfile	0.2951	1.68E-06	60	M33423.m	TRUE	reported	ivEtvl	2	TRUE	0.2765	22.16584
6629	rs1538388	A	T	A	T	0.0372	-0.02134	0.3972	0.391043	FALSE	TRUE	FALSE	IDG01N	0.013994	0.127296	486484	sepsis	TRUE	reported	textfile	0.0079	2.60E-06	4999	M33885.m	TRUE	reported	q3gy7x	2	TRUE	0.004416	22.1645
8520	rs1160377	A	G	A	G	-0.0292	-0.01604	0.269	0.269775	FALSE	FALSE	FALSE	gM7v2m	0.015485	0.300171	486484	sepsis	TRUE	reported	textfile	0.0062	2.32E-06	2651	M36009.m	TRUE	reported	6Xcypd	2	TRUE	0.008298	22.16433
3762	rs7582822	A	G	A	G	0.0113	0.014969	0.388	0.385761	FALSE	FALSE	FALSE	OZLdls	0.014129	0.289403	486484	sepsis	TRUE	reported	textfile	0.0024	3.84E-06	7356	M32338.m	TRUE	reported	s2itmB	2	TRUE	0.003005	22.16238
1254	rs891531	T	G	T	G	0.0113	-0.01923	0.1846	0.226642	FALSE	FALSE	FALSE	V50rWC	0.016382	0.240559	486484	sepsis	TRUE	reported	textfile	0.0024	3.81E-06	7346	M01645.m	TRUE	reported	yN3gpQ	2	TRUE	0.003009	22.16237
8169	rs1943505	T	C	T	C	0.0113	-0.01348	0.1067	0.104425	FALSE	FALSE	FALSE	8UhtPK	0.022555	0.549943	486484	sepsis	TRUE	reported	textfile	0.0024	3.20E-06	7345	M35397.m	TRUE	reported	rvVJba	2	TRUE	0.003009	22.16237
9000	rs9938618	A	G	A	G	-0.0452	0.031917	0.943	0.945264	FALSE	FALSE	FALSE	TfdxtP	0.030028	0.28782	486484	sepsis	TRUE	reported	textfile	0.0096	2.74E-06	7333	M37203.m	TRUE	reported	xicmWr	2	TRUE	0.003014	22.16236
2837	rs1192164	A	T	A	T	0.0113	0.047501	0.8546	0.789728	FALSE	TRUE	FALSE	Y5qWBm	0.016991	0.005179	486484	sepsis	TRUE	reported	textfile	0.0024	3.44E-06	7325	M20489.m	TRUE	reported	f2g9Su	2	TRUE	0.003017	22.16235
5819	rs1282122	T	C	T	C	0.0226	0.049322	0.0348	0.039387	FALSE	FALSE	FALSE	R5joEf	0.035003	0.158814	486484	sepsis	TRUE	reported	textfile	0.0048	2.11E-06	7118	M33380.m	TRUE	reported	kRvbfZ	2	TRUE	0.003105	22.16217
7663	rs2941589	A	G	A	G	0.0598	0.002702	0.8527	0.846687	FALSE	FALSE	FALSE	z8S1DZ	0.019235	0.888303	486484	sepsis	TRUE	reported	textfile	0.0127	2.58E-06	4515	M34535.m	TRUE	reported	FNLxxh	2	TRUE	0.004887	22.16167
443	rs1005005	T	C	T	C	-0.0325	-0.07012	0.9828	0.982329	FALSE	FALSE	FALSE	gcJMTM	0.052056	0.178009	486484	sepsis	TRUE	reported	textfile	0.0069	2.58E-06	1760	M00064.m	TRUE	reported	g2hUXe	2	TRUE	0.012448	22.16025
2490	rs9552597	A	G	A	G	0.0306	-0.01066	0.1845	0.190696	FALSE	FALSE	FALSE	SrfyMU	0.017409	0.540504	486484	sepsis	TRUE	reported	textfile	0.0065	2.15E-06	7226	M17807.m	TRUE	reported	0zrJsu	2	TRUE	0.003058	22.15623
7832	rs2982694	T	G	T	G	-0.041	-0.00508	0.8716	0.842559	FALSE	FALSE	FALSE	h5whIS	0.018778	0.786816	486484	sepsis	TRUE	reported	textfile	0.0087	2.49E-06	819	M35137.m	TRUE	reported	xVITKF	2	TRUE	0.026401	22.15478
5525	rs6987404	T	C	T	C	-0.163	-0.02135	0.0588	0.059019	FALSE	FALSE	FALSE	q32d4E	0.02904	0.462325	486484	sepsis	TRUE	reported	textfile	0.0346	2.53E-06	1017	M33178.m	TRUE	reported	cS3BwT	2	TRUE	0.021356	22.14971
7139	rs418889	A	G	A	G	0.3821	0.009136	0.0143	0.01816	FALSE	FALSE	FALSE	de8tA6	0.051622	0.859525	486484	sepsis	TRUE	reported	textfile	0.0801	1.86E-06	75	M34322.m	TRUE	reported	sXPPBO	2	TRUE	0.232781	22.14882
1575	rs1040498	A	G	A	G	-0.0744	-0.02627	0.9877	0.981963	FALSE	FALSE	FALSE	lxzgXW	0.051136	0.607434	486484	sepsis	TRUE	reported	textfile	0.0158	2.34E-06	1764	M11438.m	TRUE	reported	ZjFyyS	2	TRUE	0.012414	22.14823
6745	rs1715749	A	G	A	G	0.0353	-0.01549	0.074	0.076041	FALSE	FALSE	FALSE	NZVg9N	0.025712	0.546864	486484	sepsis	TRUE	reported	textfile	0.0075	2.21E-06	7320	M33941.m	TRUE	reported	kRWNNK	2	TRUE	0.003017	22.14666
6312	rs1391470	T	C	T	C	0.2838	0.007927	0.0161	0.019416	FALSE	FALSE	FALSE	5B5LTX	0.049655	0.87316	486484	sepsis	TRUE	reported	textfile	0.0602	2.45E-06	528	M33609.m	TRUE	reported	m0eM6N	2	TRUE	0.040392	22.14031
1573	rs947099	A	G	A	G	-0.008	-0.00352	0.3625	0.353475	FALSE	FALSE	FALSE	Ke0sNh	0.014251	0.804979	486484	sepsis	TRUE	reported	textfile	0.0017	1.22E-06	7799	M10642.m	TRUE	reported	ZlNwVb	2	TRUE	0.002831	22.13965
1795	rs2651516	T	C	T	C	0.008	0.02263	0.6585	0.655452	FALSE	FALSE	FALSE	JEcCRZ	0.014425	0.1167	486484	sepsis	TRUE	reported	textfile	0.0017	4.30E-06	7368	M15140.m	TRUE	reported	xYCzKP	2	TRUE	0.002997	22.13932
1804	rs6770323	T	C	T	C	-0.008	-0.00685	0.4299	0.428736	FALSE	FALSE	FALSE	JEcCRZ	0.013856	0.621145	486484	sepsis	TRUE	reported	textfile	0.0017	3.33E-06	7368	M15140.m	TRUE	reported	xYCzKP	2	TRUE	0.002997	22.13932
1810	rs7619096	A	C	A	C	0.008	-0.00569	0.6886	0.687962	FALSE	FALSE	FALSE	JEcCRZ	0.014779	0.700066	486484	sepsis	TRUE	reported	textfile	0.0017	4.92E-06	7368	M15140.m	TRUE	reported	xYCzKP	2	TRUE	0.002997	22.13932
1816	rs9857268	A	G	A	G	0.008	0.020526	0.411	0.418605	FALSE	FALSE	FALSE	JEcCRZ	0.013853	0.138407	486484	sepsis	TRUE	reported	textfile	0.0017	3.07E-06	7368	M15140.m	TRUE	reported	xYCzKP	2	TRUE	0.002997	22.13932
9040	rs3733593	A	G	A	G	0.008	0.004617	0.4373	0.439084	FALSE	FALSE	FALSE	df0MCM	0.013793	0.737829	486484	sepsis	TRUE	reported	textfile	0.0017	4.66E-06	7366	M37506.m	TRUE	reported	iUfr4u	2	TRUE	0.002997	22.13932
418	rs2026590	T	G	T	G	0.008	-0.00712	0.2176	0.207226	FALSE	FALSE	FALSE	cTsNj6	0.01689	0.673414	486484	sepsis	TRUE	reported	textfile	0.0017	3.76E-06	7365	M00063.m	TRUE	reported	7KYTrQ	2	TRUE	0.002998	22.13932
439	rs810551	C	G	C	G	0.008	0.034631	0.7904	0.780879	FALSE	TRUE	FALSE	cTsNj6	0.016521	0.036065	486484	sepsis	TRUE	reported	textfile	0.0017	4.66E-06	7365	M00063.m	TRUE	reported	7KYTrQ	2	TRUE	0.002998	22.13932
5027	rs1759502	T	C	T	C	-0.008	0.014702	0.1176	0.114773	FALSE	FALSE	FALSE	BNnOvt	0.021517	0.494432	486484	sepsis	TRUE	reported	textfile	0.0017	4.76E-06	7362	M33132.m	TRUE	reported	rHcCPG	2	TRUE	0.002999	22.13931
706	rs1851740	A	C	A	C	-0.008	0.015855	0.185	0.184666	FALSE	FALSE	FALSE	H9I3s3	0.017706	0.370528	486484	sepsis	TRUE	reported	textfile	0.0017	2.74E-06	7358	M01299.m	TRUE	reported	kXWyUq	2	TRUE	0.003001	22.13931
828	rs216283	A	C	A	C	0.008	0.000952	0.4171	0.427589	FALSE	FALSE	FALSE	2qgDgB	0.013959	0.945641	486484	sepsis	TRUE	reported	textfile	0.0017	2.63E-06	7355	M01358.m	TRUE	reported	asmXq0	2	TRUE	0.003002	22.13931
834	rs3863373	A	C	A	C	-0.008	-0.00745	0.3335	0.332215	FALSE	FALSE	FALSE	2qgDgB	0.014499	0.607234	486484	sepsis	TRUE	reported	textfile	0.0017	4.21E-06	7355	M01358.m	TRUE	reported	asmXq0	2	TRUE	0.003002	22.13931
2610	rs6951006	A	G	A	G	-0.008	0.014755	0.4884	0.488455	FALSE	FALSE	FALSE	ruvJh6	0.013903	0.288566	486484	sepsis	TRUE	reported	textfile	0.0017	3.53E-06	7354	M18369.m	TRUE	reported	GFmwGk	2	TRUE	0.003002	22.13931
2614	rs9827101	A	C	A	C	0.008	-0.02803	0.5393	0.517279	FALSE	FALSE	FALSE	ruvJh6	0.013721	0.041038	486484	sepsis	TRUE	reported	textfile	0.0017	2.99E-06	7354	M18369.m	TRUE	reported	GFmwGk	2	TRUE	0.003002	22.13931
2804	rs1764963	T	C	T	C	-0.008	-0.01044	0.654	0.663023	FALSE	FALSE	FALSE	Sx5Uhq	0.014502	0.471385	486484	sepsis	TRUE	reported	textfile	0.0017	4.40E-06	7354	M19934.m	TRUE	reported	DijYTE	2	TRUE	0.003002	22.13931
2826	rs722419	A	C	A	C	0.008	0.018617	0.4468	0.472521	FALSE	FALSE	FALSE	Sx5Uhq	0.013754	0.175877	486484	sepsis	TRUE	reported	textfile	0.0017	4.03E-06	7354	M19934.m	TRUE	reported	DijYTE	2	TRUE	0.003002	22.13931
2830	rs9317855	T	C	T	C	-0.008	-0.02551	0.3513	0.358256	FALSE	FALSE	FALSE	Sx5Uhq	0.014279	0.074012	486484	sepsis	TRUE	reported	textfile	0.0017	4.35E-06	7354	M19934.m	TRUE	reported	DijYTE	2	TRUE	0.003002	22.13931
662	rs1006320	T	C	T	C	0.008	-0.01027	0.1559	0.189025	FALSE	FALSE	FALSE	p4oCVP	0.01854	0.579495	486484	sepsis	TRUE	reported	textfile	0.0017	4.06E-06	7352	M01125.m	TRUE	reported	OwD5re	2	TRUE	0.003003	22.1393
799	rs6540294	A	C	A	C	-0.008	0.027612	0.451	0.450836	FALSE	FALSE	FALSE	dyT79I	0.013786	0.045194	486484	sepsis	TRUE	reported	textfile	0.0017	2.87E-06	7352	M01336.m	TRUE	reported	OemVJ2	2	TRUE	0.003003	22.1393
803	rs9809122	A	G	A	G	-0.008	-0.00204	0.5296	0.524047	FALSE	FALSE	FALSE	dyT79I	0.013687	0.881351	486484	sepsis	TRUE	reported	textfile	0.0017	2.45E-06	7352	M01336.m	TRUE	reported	OemVJ2	2	TRUE	0.003003	22.1393
1917	rs1446555	A	C	A	C	0.008	0.019069	0.9028	0.894254	FALSE	FALSE	FALSE	v2KE89	0.022229	0.39098	486484	sepsis	TRUE	reported	textfile	0.0017	3.21E-06	7349	M15500.m	TRUE	reported	to2t7H	2	TRUE	0.003004	22.1393
2013	rs6602640	T	G	T	G	0.008	-0.0166	0.8847	0.886454	FALSE	FALSE	FALSE	v2KE89	0.021572	0.441665	486484	sepsis	TRUE	reported	textfile	0.0017	2.50E-06	7349	M15500.m	TRUE	reported	to2t7H	2	TRUE	0.003004	22.1393
8166	rs1261383	T	C	T	C	-0.008	-0.0072	0.7597	0.772893	FALSE	FALSE	FALSE	8UhtPK	0.016336	0.659194	486484	sepsis	TRUE	reported	textfile	0.0017	1.93E-06	7345	M35397.m	TRUE	reported	rvVJba	2	TRUE	0.003006	22.1393
4974	rs866142	A	G	A	G	0.008	-0.02395	0.605	0.57677	FALSE	FALSE	FALSE	bKmDMa	0.013838	0.083503	486484	sepsis	TRUE	reported	textfile	0.0017	3.79E-06	7344	M32910.m	TRUE	reported	nAuPzO	2	TRUE	0.003006	22.1393
2144	rs699530	T	G	T	G	-0.008	-0.00775	0.5636	0.55929	FALSE	FALSE	FALSE	TEupfd	0.013851	0.575856	486484	sepsis	TRUE	reported	textfile	0.0017	2.78E-06	7331	M15676.m	TRUE	reported	NXiaPC	2	TRUE	0.003012	22.13929
2150	rs7986369	A	C	A	C	-0.008	0.007576	0.3795	0.381947	FALSE	FALSE	FALSE	TEupfd	0.014046	0.589617	486484	sepsis	TRUE	reported	textfile	0.0017	2.97E-06	7331	M15676.m	TRUE	reported	NXiaPC	2	TRUE	0.003012	22.13929
2834	rs1162333	A	G	A	G	-0.008	-0.02241	0.8018	0.802921	FALSE	FALSE	FALSE	Y5qWBm	0.017891	0.210346	486484	sepsis	TRUE	reported	textfile	0.0017	3.48E-06	7325	M20489.m	TRUE	reported	f2g9Su	2	TRUE	0.003014	22.13928
2862	rs950156	A	G	A	G	-0.008	0.009647	0.2591	0.256784	FALSE	FALSE	FALSE	Y5qWBm	0.015839	0.542479	486484	sepsis	TRUE	reported	textfile	0.0017	2.73E-06	7325	M20489.m	TRUE	reported	f2g9Su	2	TRUE	0.003014	22.13928
4576	rs1177828	A	G	A	G	0.008	0.000638	0.3156	0.322107	FALSE	FALSE	FALSE	Pzplqu	0.014642	0.965268	486484	sepsis	TRUE	reported	textfile	0.0017	5.03E-06	7318	M32740.m	TRUE	reported	FjEBS5	2	TRUE	0.003017	22.13928
4598	rs4562058	T	C	T	C	-0.008	0.007281	0.3093	0.309825	FALSE	FALSE	FALSE	Pzplqu	0.014779	0.622233	486484	sepsis	TRUE	reported	textfile	0.0017	3.88E-06	7318	M32740.m	TRUE	reported	FjEBS5	2	TRUE	0.003017	22.13928
1699	rs7068714	A	C	A	C	0.008	0.01516	0.1774	0.178368	FALSE	FALSE	FALSE	f01en0	0.017828	0.395128	486484	sepsis	TRUE	reported	textfile	0.0017	2.97E-06	7314	M12593.m	TRUE	reported	EAsM6U	2	TRUE	0.003019	22.13927
4715	rs2279399	T	C	T	C	0.016	-0.00238	0.4592	0.44372	FALSE	FALSE	FALSE	L2WFM1	0.013862	0.863702	486484	sepsis	TRUE	reported	textfile	0.0034	3.29E-06	7313	M32761.m	TRUE	reported	5GHzph	2	TRUE	0.003019	22.13927
3337	rs453766	C	G	C	G	0.008	-0.0219	0.7548	0.748061	FALSE	TRUE	FALSE	m66Eav	0.01576	0.16465	486484	sepsis	TRUE	reported	textfile	0.0017	4.23E-06	7303	M27256.m	TRUE	reported	pI2ECU	2	TRUE	0.003023	22.13926
3029	rs2035247	T	G	T	G	-0.016	-0.02096	0.8304	0.823354	FALSE	FALSE	FALSE	FJZEYz	0.017977	0.243706	486484	sepsis	TRUE	reported	textfile	0.0034	1.94E-06	7284	M21630.m	TRUE	reported	s6Pshg	2	TRUE	0.003031	22.13925
3269	rs6832163	A	G	A	G	-0.016	-0.0009	0.2711	0.29616	FALSE	FALSE	FALSE	ZNPBqT	0.015287	0.952996	486484	sepsis	TRUE	reported	textfile	0.0034	3.48E-06	7256	M24074.m	TRUE	reported	8pPtOl	2	TRUE	0.003043	22.13922
3220	rs1054634	A	G	A	G	-0.016	-0.0019	0.8558	0.85715	FALSE	FALSE	FALSE	I25GHB	0.019513	0.922277	486484	sepsis	TRUE	reported	textfile	0.0034	2.60E-06	7184	M22548.m	TRUE	reported	eBmTQD	2	TRUE	0.003073	22.13916
2723	rs1382606	A	G	A	G	0.008	0.005078	0.5044	0.507521	FALSE	FALSE	FALSE	H4yqSK	0.013742	0.711741	486484	sepsis	TRUE	reported	textfile	0.0017	3.11E-06	7182	M19362.m	TRUE	reported	w2PDEO	2	TRUE	0.003074	22.13916
1321	rs960381	A	C	A	C	0.008	0.023873	0.5958	0.588819	FALSE	FALSE	FALSE	WQdpmo	0.013872	0.085253	486484	sepsis	TRUE	reported	textfile	0.0017	3.56E-06	7136	M01769.m	TRUE	reported	BkptOK	2	TRUE	0.003094	22.13912
5833	rs6080978	T	C	T	C	-0.008	-0.00206	0.299	0.298197	FALSE	FALSE	FALSE	R5joEf	0.01528	0.892912	486484	sepsis	TRUE	reported	textfile	0.0017	3.18E-06	7118	M33380.m	TRUE	reported	kRvbfZ	2	TRUE	0.003102	22.13911
5834	rs6605235	T	C	T	C	-0.008	0.012418	0.7072	0.711759	FALSE	FALSE	FALSE	R5joEf	0.01511	0.411171	486484	sepsis	TRUE	reported	textfile	0.0017	3.54E-06	7118	M33380.m	TRUE	reported	kRvbfZ	2	TRUE	0.003102	22.13911
1466	rs1340971	T	C	T	C	-0.008	-0.00151	0.6654	0.670345	FALSE	FALSE	FALSE	pkb876	0.014559	0.917408	486484	sepsis	TRUE	reported	textfile	0.0017	4.66E-06	7037	M02734.m	TRUE	reported	0LCMVl	2	TRUE	0.003137	22.13903
5351	rs1714055	A	G	A	G	0.3375	-0.0546	0.9707	0.943083	FALSE	FALSE	FALSE	q32d4E	0.02946	0.063818	486484	sepsis	TRUE	reported	textfile	0.0688	9.24E-07	25	M33178.m	TRUE	reported	cS3BwT	2	TRUE	0.490463	22.13903
3860	rs2761735	T	C	T	C	-0.024	0.021616	0.8145	0.808048	FALSE	FALSE	FALSE	8gg1zS	0.017522	0.217337	486484	sepsis	TRUE	reported	textfile	0.0051	2.67E-06	6119	M32379.m	TRUE	reported	uZWtuS	2	TRUE	0.003606	22.13809
6675	rs4953276	A	C	A	C	-0.032	0.016839	0.1272	0.13585	FALSE	FALSE	FALSE	6iZSOI	0.020021	0.400321	486484	sepsis	TRUE	reported	textfile	0.0068	2.42E-06	4533	M33901.m	TRUE	reported	ApKaGe	2	TRUE	0.004862	22.13556
4604	rs6429032	T	C	T	C	-0.0273	-0.00072	0.0905	0.112042	FALSE	FALSE	FALSE	Pzplqu	0.02171	0.973418	486484	sepsis	TRUE	reported	textfile	0.0058	2.12E-06	1759	M32740.m	TRUE	reported	FjEBS5	2	TRUE	0.012438	22.12968
6993	rs4955937	A	G	A	G	-0.0207	-0.00574	0.5451	0.541054	FALSE	FALSE	FALSE	APOtl6	0.013804	0.677317	486484	sepsis	TRUE	reported	textfile	0.0044	2.17E-06	6987	M34221.m	TRUE	reported	IooCDC	2	TRUE	0.003158	22.12641
7638	rs1175738	T	C	T	C	0.0998	0.017009	0.1781	0.142635	FALSE	FALSE	FALSE	wrTMYM	0.026765	0.525121	486484	sepsis	TRUE	reported	textfile	0.0212	2.45E-06	1212	M34533.m	TRUE	reported	qIRVku	2	TRUE	0.017956	22.12443
9055	rs7632727	T	C	T	C	0.0127	0.011094	0.8935	0.888767	FALSE	FALSE	FALSE	df0MCM	0.021729	0.609662	486484	sepsis	TRUE	reported	textfile	0.0027	2.01E-06	7366	M37506.m	TRUE	reported	iUfr4u	2	TRUE	0.002995	22.11882
3987	rs6881997	A	G	A	G	-0.0127	0.024424	0.8282	0.824724	FALSE	FALSE	FALSE	XdJbEU	0.018025	0.175428	486484	sepsis	TRUE	reported	textfile	0.0027	2.06E-06	7364	M32452.m	TRUE	reported	V5xWKi	2	TRUE	0.002995	22.11882
8433	rs1491024	A	G	A	G	-0.0127	0.003766	0.7762	0.7763	FALSE	FALSE	FALSE	Nvt7pl	0.016433	0.818741	486484	sepsis	TRUE	reported	textfile	0.0027	2.89E-06	7358	M35718.m	TRUE	reported	WkA5Gq	2	TRUE	0.002998	22.11881
1765	rs1310635	A	T	A	T	-0.0127	0.013548	0.2123	0.219655	FALSE	TRUE	FALSE	wM5dkT	0.016578	0.413815	486484	sepsis	TRUE	reported	textfile	0.0027	2.31E-06	7352	M15122.m	TRUE	reported	CBpa4t	2	TRUE	0.003	22.11881
1775	rs641939	A	G	A	G	0.0127	-0.01275	0.7987	0.790855	FALSE	FALSE	FALSE	wM5dkT	0.016848	0.449323	486484	sepsis	TRUE	reported	textfile	0.0027	2.13E-06	7352	M15122.m	TRUE	reported	CBpa4t	2	TRUE	0.003	22.11881
4099	rs1257796	A	G	A	G	-0.0127	0.04598	0.1103	0.107686	FALSE	FALSE	FALSE	4rtLd0	0.022008	0.036686	486484	sepsis	TRUE	reported	textfile	0.0027	2.85E-06	7350	M32518.m	TRUE	reported	j7f1ER	2	TRUE	0.003001	22.11881
4127	rs7349641	T	C	T	C	0.0127	0.024606	0.8878	0.889003	FALSE	FALSE	FALSE	4rtLd0	0.021838	0.259847	486484	sepsis	TRUE	reported	textfile	0.0027	2.72E-06	7350	M32518.m	TRUE	reported	j7f1ER	2	TRUE	0.003001	22.11881
7486	rs1779448	T	C	T	C	-0.0127	0.032556	0.1987	0.204267	FALSE	FALSE	FALSE	jAhmnV	0.017038	0.05603	486484	sepsis	TRUE	reported	textfile	0.0027	2.29E-06	7348	M34419.m	TRUE	reported	F2Tg9L	2	TRUE	0.003002	22.11881
7004	rs1341296	T	C	T	C	0.0127	-0.0036	0.4596	0.462422	FALSE	FALSE	FALSE	ePdfmg	0.013734	0.793202	486484	sepsis	TRUE	reported	textfile	0.0027	2.79E-06	7347	M34244.m	TRUE	reported	l9nzfX	2	TRUE	0.003002	22.11881
1834	rs333383	A	G	A	G	-0.0127	0.016445	0.8384	0.838968	FALSE	FALSE	FALSE	2TaDHm	0.018641	0.377689	486484	sepsis	TRUE	reported	textfile	0.0027	3.38E-06	7337	M15365.m	TRUE	reported	LoLveg	2	TRUE	0.003006	22.1188
4269	rs2449529	A	G	A	G	-0.0127	-0.01436	0.5572	0.555595	FALSE	FALSE	FALSE	ylV4bp	0.013759	0.296477	486484	sepsis	TRUE	reported	textfile	0.0027	2.76E-06	7337	M32632.m	TRUE	reported	hqHJoU	2	TRUE	0.003006	22.1188
2555	rs4708792	A	G	A	G	-0.0673	0.009165	0.1015	0.105946	FALSE	FALSE	FALSE	NxZhaQ	0.022354	0.681802	486484	sepsis	TRUE	reported	textfile	0.0143	2.61E-06	1448	M18283.m	TRUE	reported	5Pi7r3	2	TRUE	0.015066	22.11861
7986	rs9470238	T	G	T	G	-0.0127	-0.00703	0.3575	0.358651	FALSE	FALSE	FALSE	t02AUT	0.014479	0.627131	486484	sepsis	TRUE	reported	textfile	0.0027	3.89E-06	7100	M35254.m	TRUE	reported	uClJaT	2	TRUE	0.003106	22.1186
2969	rs2378442	A	T	A	T	0.0127	0.018925	0.3445	0.333919	FALSE	TRUE	FALSE	dOOXpo	0.014549	0.193324	486484	sepsis	TRUE	reported	textfile	0.0027	3.98E-06	6999	M21127.m	TRUE	reported	IdiP4j	2	TRUE	0.003151	22.11851
7089	rs1259892	T	G	T	G	-0.0127	-0.00103	0.2613	0.265048	FALSE	FALSE	FALSE	pxNICA	0.015461	0.946682	486484	sepsis	TRUE	reported	textfile	0.0027	2.82E-06	6994	M34306.m	TRUE	reported	xk5b9M	2	TRUE	0.003153	22.1185
7640	rs1351284	A	C	A	C	0.0254	-0.00518	0.6287	0.618017	FALSE	FALSE	FALSE	wrTMYM	0.014062	0.712695	486484	sepsis	TRUE	reported	textfile	0.0054	2.63E-06	5856	M34533.m	TRUE	reported	qIRVku	2	TRUE	0.003764	22.11727
3062	rs1003138	A	G	A	G	0.0254	0.006011	0.9233	0.922318	FALSE	FALSE	FALSE	e3izea	0.025568	0.81413	486484	sepsis	TRUE	reported	textfile	0.0054	3.02E-06	5676	M22032.m	TRUE	reported	zODKFl	2	TRUE	0.003883	22.11703
4371	rs4305648	A	G	A	G	0.0127	-0.0044	0.3639	0.366169	FALSE	FALSE	FALSE	SPhI52	0.014226	0.757209	486484	sepsis	TRUE	reported	textfile	0.0027	2.96E-06	5211	M32651.m	TRUE	reported	V0Bnpj	2	TRUE	0.004228	22.11634
7419	rs7705239	A	G	A	G	0.0254	0.020973	0.8521	0.848872	FALSE	FALSE	FALSE	7i5VUS	0.019108	0.27238	486484	sepsis	TRUE	reported	textfile	0.0054	2.30E-06	5208	M34395.m	TRUE	reported	64hEaL	2	TRUE	0.00423	22.11633
3937	rs4612338	A	G	A	G	-0.0367	0.012532	0.1596	0.157475	FALSE	FALSE	FALSE	LIOaWH	0.018742	0.50373	486484	sepsis	TRUE	reported	textfile	0.0078	2.44E-06	1767	M32418.m	TRUE	reported	AtHOVw	2	TRUE	0.012374	22.11317
6482	rs5998862	A	C	A	C	-0.6716	-0.02122	0.9487	0.947988	FALSE	FALSE	FALSE	ecwVg8	0.031977	0.50686	486484	sepsis	TRUE	reported	textfile	0.141	1.91E-06	79	M33751.m	TRUE	reported	NnvbIQ	2	TRUE	0.223109	22.11296
3262	rs2034360	T	C	T	C	0.0174	0.020879	0.8625	0.84349	FALSE	FALSE	FALSE	ZNPBqT	0.018843	0.267823	486484	sepsis	TRUE	reported	textfile	0.0037	2.73E-06	7256	M24074.m	TRUE	reported	8pPtOl	2	TRUE	0.003039	22.10932
4782	rs4437633	T	G	T	G	-0.065	-0.01297	0.468	0.498471	FALSE	FALSE	FALSE	MmZZoM	0.013726	0.344813	486484	sepsis	TRUE	reported	textfile	0.0138	2.44E-06	562	M32800.m	TRUE	reported	70YSCO	2	TRUE	0.037977	22.10651
8248	rs1181484	T	C	T	C	-0.1595	-0.00505	0.9778	0.934884	FALSE	FALSE	FALSE	G4plxe	0.027791	0.855769	486484	sepsis	TRUE	reported	textfile	0.0339	2.48E-06	1383	M35451.m	TRUE	reported	hNOVMl	2	TRUE	0.015754	22.10515
2671	rs1210449	A	T	A	T	-0.0395	-0.04136	0.1572	0.15689	FALSE	TRUE	FALSE	s4UTyf	0.019034	0.029791	486484	sepsis	TRUE	reported	textfile	0.0084	2.70E-06	5376	M18494.m	TRUE	reported	ajPzhg	2	TRUE	0.004096	22.10416
8995	rs3733475	T	G	T	G	-0.0221	-0.00283	0.3611	0.401898	FALSE	FALSE	FALSE	TfdxtP	0.014016	0.839978	486484	sepsis	TRUE	reported	textfile	0.0047	2.78E-06	7333	M37203.m	TRUE	reported	xicmWr	2	TRUE	0.003006	22.10397
4456	rs6563540	T	G	T	G	0.0428	-0.00787	0.1256	0.12783	FALSE	FALSE	FALSE	g2RNym	0.020499	0.700945	486484	sepsis	TRUE	reported	textfile	0.0091	2.72E-06	2575	M32691.m	TRUE	reported	35eo6p	2	TRUE	0.008518	22.10382
8694	rs1282331	T	C	T	C	-0.0221	0.005198	0.0408	0.040118	FALSE	FALSE	FALSE	Bg4VnB	0.035142	0.882404	486484	sepsis	TRUE	reported	textfile	0.0047	2.28E-06	7092	M36552.m	TRUE	reported	6zdDpC	2	TRUE	0.003108	22.10377
3538	rs6890263	T	C	T	C	0.0221	0.020637	0.9379	0.938288	FALSE	FALSE	FALSE	rXzSlH	0.028511	0.469184	486484	sepsis	TRUE	reported	textfile	0.0047	3.18E-06	6795	M30805.m	TRUE	reported	7wg9QH	2	TRUE	0.003243	22.1035
7921	rs4687148	T	G	T	G	0.0489	-0.00019	0.9265	0.896295	FALSE	FALSE	FALSE	8qXKkX	0.022398	0.993246	486484	sepsis	TRUE	reported	textfile	0.0104	2.65E-06	6234	M35189.m	TRUE	reported	Nt6oaC	2	TRUE	0.003534	22.10099
3390	rs1123667	T	C	T	C	0.0348	-0.00317	0.3167	0.348128	FALSE	FALSE	FALSE	frOZiy	0.014337	0.824989	486484	sepsis	TRUE	reported	textfile	0.0074	2.58E-06	2759	M27447.m	TRUE	reported	2z9fSv	2	TRUE	0.007952	22.09938
6636	rs9839976	A	G	A	G	-0.1468	0.017114	0.0718	0.077312	FALSE	FALSE	FALSE	IDG01N	0.025748	0.506276	486484	sepsis	TRUE	reported	textfile	0.0312	2.53E-06	1100	M33885.m	TRUE	reported	q3gy7x	2	TRUE	0.019729	22.09798
1146	rs1258756	A	G	A	G	0.0456	0.002976	0.1148	0.106645	FALSE	FALSE	FALSE	QtY5Hc	0.022182	0.893258	486484	sepsis	TRUE	reported	textfile	0.0097	2.63E-06	5162	M01605.m	TRUE	reported	Q3iDQX	2	TRUE	0.004263	22.09113
2647	rs1290508	A	G	A	G	-0.0574	0.006	0.4166	0.443855	FALSE	FALSE	FALSE	9GoQdh	0.013816	0.664069	486484	sepsis	TRUE	reported	textfile	0.0122	2.58E-06	917	M18474.m	TRUE	reported	p6Dq8p	2	TRUE	0.023571	22.08797
7553	rs1190579	A	G	A	G	-0.0997	0.012224	0.0405	0.059503	FALSE	FALSE	FALSE	APSWAU	0.028986	0.673242	486484	sepsis	TRUE	reported	textfile	0.0212	2.50E-06	1462	M34469.m	TRUE	reported	frF3RX	2	TRUE	0.014902	22.08636
1773	rs5022234	A	C	A	C	0.0221	0.000404	0.6303	0.674151	FALSE	FALSE	FALSE	wM5dkT	0.014595	0.977897	486484	sepsis	TRUE	reported	textfile	0.0047	2.15E-06	1757	M15122.m	TRUE	reported	CBpa4t	2	TRUE	0.012428	22.08484
980	rs1683260	T	C	T	C	0.2205	0.050532	0.0297	0.040014	FALSE	FALSE	FALSE	xSRIl4	0.034838	0.146924	486484	sepsis	TRUE	reported	textfile	0.0469	2.58E-06	2285	M01515.m	TRUE	reported	juZ5GE	2	TRUE	0.009581	22.08469
6529	rs9321466	C	G	C	G	0.0376	0.036519	0.9628	0.961639	FALSE	TRUE	FALSE	OKIXJ8	0.035839	0.308218	486484	sepsis	TRUE	reported	textfile	0.008	2.74E-06	7362	M33821.m	TRUE	reported	XGkaXH	2	TRUE	0.002992	22.084
1085	rs2907828	C	G	C	G	-0.0188	-0.00565	0.1375	0.175395	FALSE	TRUE	FALSE	JLtEiZ	0.018099	0.755052	486484	sepsis	TRUE	reported	textfile	0.004	2.74E-06	7333	M01572.m	TRUE	reported	TSSe28	2	TRUE	0.003003	22.08398
8687	rs2850161	T	G	T	G	0.0047	-0.01442	0.3664	0.369139	FALSE	FALSE	FALSE	X2e8qc	0.014142	0.307733	486484	sepsis	TRUE	reported	textfile	0.001	4.84E-06	7330	M36515.m	TRUE	reported	OaWQSH	2	TRUE	0.003005	22.08397
3158	rs624947	A	G	A	G	-0.0094	0.026559	0.6784	0.686726	FALSE	FALSE	FALSE	JiXR2t	0.015075	0.078112	486484	sepsis	TRUE	reported	textfile	0.002	1.90E-06	6535	M22177.m	TRUE	reported	joi6p6	2	TRUE	0.003369	22.08324
4799	rs1686888	T	C	T	C	-0.1646	-0.02629	0.9702	0.976855	FALSE	FALSE	FALSE	9qWhFj	0.046709	0.573563	486484	sepsis	TRUE	reported	textfile	0.035	2.55E-06	1298	M32808.m	TRUE	reported	MQcOSi	2	TRUE	0.016754	22.08279
4042	rs2239855	T	C	T	C	-0.047	0.050437	0.986	0.976535	FALSE	FALSE	FALSE	SiYGYU	0.045295	0.26549	486484	sepsis	TRUE	reported	textfile	0.01	2.60E-06	5588	M32492.m	TRUE	reported	GfQvpU	2	TRUE	0.003938	22.08209
8771	rs1390370	A	G	A	G	0.0235	-0.00902	0.8174	0.79732	FALSE	FALSE	FALSE	7desNF	0.017318	0.602524	486484	sepsis	TRUE	reported	textfile	0.005	2.62E-06	5292	M36673.m	TRUE	reported	RKbIT4	2	TRUE	0.004157	22.08165
7172	rs1005464	A	G	A	G	0.0141	0.005368	0.229	0.226225	FALSE	FALSE	FALSE	2hlepp	0.016289	0.741746	486484	sepsis	TRUE	reported	textfile	0.003	2.20E-06	5081	M34336.m	TRUE	reported	XyK8mO	2	TRUE	0.004329	22.0813
5273	rs1338583	A	G	A	G	-0.1364	-0.02061	0.1003	0.123664	FALSE	FALSE	FALSE	q32d4E	0.020905	0.324105	486484	sepsis	TRUE	reported	textfile	0.029	2.61E-06	1017	M33178.m	TRUE	reported	cS3BwT	2	TRUE	0.02129	22.07892
6588	rs3752559	T	G	T	G	0.5132	-0.05961	0.0114	0.019688	FALSE	FALSE	FALSE	XO3rXq	0.048979	0.223567	486484	sepsis	TRUE	reported	textfile	0.1086	2.29E-06	176	M33877.m	TRUE	reported	p2sETg	2	TRUE	0.112596	22.07752
5652	rs6878813	T	C	T	C	0.0672	0.015536	0.1376	0.151943	FALSE	FALSE	FALSE	BFsMtm	0.019081	0.415533	486484	sepsis	TRUE	reported	textfile	0.0143	2.62E-06	5716	M33192.m	TRUE	reported	NAJN3D	2	TRUE	0.003849	22.0757

7951	rs1702260	A	G	A	G	-0.0771	-0.08728	0.9828	0.98771	FALSE	FALSE	FALSE	lvUHnQ	0.062392	0.161834	486484	sepsis	TRUE	reported	textfile	0.0164	2.46E-06	1673	M35240.m	TRUE	reported	VH44Ha	2	TRUE	0.013038	22.07504
8339	rs1246187	A	C	A	C	0.0569	-0.00892	0.2704	0.278979	FALSE	FALSE	FALSE	USZiKY	0.015274	0.559437	486484	sepsis	TRUE	reported	textfile	0.0121	2.70E-06	1098	M35635.m	TRUE	reported	HuVMRL	2	TRUE	0.019742	22.07303
2416	rs2866604	T	C	T	C	0.0644	0.009574	0.8329	0.827811	FALSE	FALSE	FALSE	NBJ2Do	0.018276	0.600381	486484	sepsis	TRUE	reported	textfile	0.0137	2.55E-06	1739	M17799.m	TRUE	reported	Uz0WgI	2	TRUE	0.012547	22.07145
6410	rs1715136	C	G	C	G	-0.0343	0.025531	0.1884	0.195246	FALSE	TRUE	FALSE	5ClPW7	0.017445	0.143331	486484	sepsis	TRUE	reported	textfile	0.0073	2.83E-06	4807	M33653.m	TRUE	reported	H4b2zx	2	TRUE	0.004572	22.06794
6457	rs1121845	A	G	A	G	-0.0442	0.004956	0.5504	0.546519	FALSE	FALSE	FALSE	yLohrN	0.013748	0.718464	486484	sepsis	TRUE	reported	textfile	0.0094	2.49E-06	1027	M33683.m	TRUE	reported	mvhAZ4	2	TRUE	0.021075	22.06695
5683	rs1173719	T	C	T	C	-0.3889	0.040777	0.9824	0.977612	FALSE	FALSE	FALSE	KxtFnC	0.04612	0.376612	486484	sepsis	TRUE	reported	textfile	0.0827	2.59E-06	930	M33203.m	TRUE	reported	Uz0wKU	2	TRUE	0.023226	22.06632
7418	rs4356633	A	C	A	C	-0.0249	0.006445	0.1592	0.154138	FALSE	FALSE	FALSE	7i5VUS	0.018916	0.733312	486484	sepsis	TRUE	reported	textfile	0.0053	2.73E-06	5208	M34395.m	TRUE	reported	64hEaL	2	TRUE	0.00422	22.06379
2624	rs27667	T	G	T	G	-0.0564	0.015371	0.6338	0.628009	FALSE	FALSE	FALSE	eygBBR	0.014119	0.276287	486484	sepsis	TRUE	reported	textfile	0.012	2.65E-06	1640	M18394.m	TRUE	reported	hD79iE	2	TRUE	0.01329	22.06306
3634	rs1015592	T	C	T	C	-0.0404	0.00657	0.7352	0.733574	FALSE	FALSE	FALSE	Vq2efp	0.015484	0.671365	486484	sepsis	TRUE	reported	textfile	0.0086	3.01E-06	7363	M31787.m	TRUE	reported	krl4Tw	2	TRUE	0.002988	22.06215
528	rs1247409	A	G	A	G	-0.0202	-0.00931	0.0649	0.075822	FALSE	FALSE	FALSE	wXMWHy	0.025748	0.717646	486484	sepsis	TRUE	reported	textfile	0.0043	1.97E-06	7345	M00584.m	TRUE	reported	7CKn2b	2	TRUE	0.002996	22.06214
6819	rs1194194	A	G	A	G	-0.0202	0.014927	0.2281	0.237716	FALSE	FALSE	FALSE	wedECv	0.016035	0.351922	486484	sepsis	TRUE	reported	textfile	0.0043	2.82E-06	7322	M33968.m	TRUE	reported	GxGxRG	2	TRUE	0.003005	22.06212
1649	rs1160505	T	C	T	C	-0.0202	-0.05453	0.8787	0.876499	FALSE	FALSE	FALSE	YzX5ZD	0.020812	0.008786	486484	sepsis	TRUE	reported	textfile	0.0043	2.24E-06	6864	M12129.m	TRUE	reported	6tMfaW	2	TRUE	0.003205	22.06171
8341	rs1253725	A	G	A	G	0.0912	0.006262	0.1088	0.184174	FALSE	FALSE	FALSE	USZiKY	0.017812	0.725162	486484	sepsis	TRUE	reported	textfile	0.0194	2.48E-06	1098	M35635.m	TRUE	reported	HuVMRL	2	TRUE	0.01973	22.05944
5004	rs1163364	A	G	A	G	-0.0296	-0.00319	0.3194	0.345806	FALSE	FALSE	FALSE	URCVGm	0.014366	0.824281	486484	sepsis	TRUE	reported	textfile	0.0063	2.35E-06	2773	M33131.m	TRUE	reported	4rQ2gs	2	TRUE	0.007898	22.05916
6836	rs220992	A	G	A	G	0.0155	-0.00744	0.4501	0.466376	FALSE	FALSE	FALSE	qL0cJY	0.01367	0.586022	486484	sepsis	TRUE	reported	textfile	0.0033	2.69E-06	7349	M33971.m	TRUE	reported	wb1N61	2	TRUE	0.002993	22.05552
6086	rs7939695	T	C	T	C	-0.0155	0.011882	0.0489	0.072433	FALSE	FALSE	FALSE	P8n0HP	0.026442	0.653156	486484	sepsis	TRUE	reported	textfile	0.0033	2.76E-06	7336	M33442.m	TRUE	reported	cljMTI	2	TRUE	0.002998	22.05551
4873	rs4141475	A	C	A	C	0.039	-0.01949	0.2691	0.339662	FALSE	FALSE	FALSE	KBJVQO	0.014412	0.176169	486484	sepsis	TRUE	reported	textfile	0.0083	2.65E-06	1763	M32855.m	TRUE	reported	Xea7Fn	2	TRUE	0.012368	22.05363
6414	rs7173557	A	G	A	G	-0.031	0.025991	0.8224	0.820328	FALSE	FALSE	FALSE	5ClPW7	0.017834	0.144998	486484	sepsis	TRUE	reported	textfile	0.0066	3.17E-06	4807	M33653.m	TRUE	reported	H4b2zx	2	TRUE	0.004568	22.05235
2985	rs7628676	A	G	A	G	-0.0653	-0.00911	0.8283	0.896926	FALSE	FALSE	FALSE	Tli4xl	0.022721	0.688462	486484	sepsis	TRUE	reported	textfile	0.0139	2.50E-06	2339	M21151.m	TRUE	reported	xaSytY	2	TRUE	0.009347	22.05085
8534	rs5765217	A	G	A	G	-0.0573	0.004671	0.2914	0.288538	FALSE	FALSE	FALSE	3PHxzL	0.015174	0.758202	486484	sepsis	TRUE	reported	textfile	0.0122	2.73E-06	3939	M36095.m	TRUE	reported	CRZVSB	2	TRUE	0.005569	22.04799
3656	rs3749572	T	C	T	C	0.0808	-0.00812	0.0283	0.027579	FALSE	FALSE	FALSE	fnjup6	0.041944	0.846514	486484	sepsis	TRUE	reported	textfile	0.0172	2.62E-06	1761	M32197.m	TRUE	reported	oQzaMk	2	TRUE	0.012377	22.04308
7971	rs7767262	T	C	T	C	0.0324	0.038997	0.9628	0.96134	FALSE	FALSE	FALSE	zb8m4a	0.035723	0.274988	486484	sepsis	TRUE	reported	textfile	0.0069	2.64E-06	7261	M35253.m	TRUE	reported	tHnnnS	2	TRUE	0.003027	22.04308
2948	rs243180	A	G	A	G	0.0108	-0.00136	0.7695	0.767286	FALSE	FALSE	FALSE	pn11BC	0.016198	0.932921	486484	sepsis	TRUE	reported	textfile	0.0023	3.16E-06	7203	M21047.m	TRUE	reported	KJF8U4	2	TRUE	0.003052	22.04303
6854	rs4434099	T	C	T	C	-0.0216	-0.02319	0.8282	0.826625	FALSE	FALSE	FALSE	2pJy8f	0.018171	0.20184	486484	sepsis	TRUE	reported	textfile	0.0046	2.82E-06	7132	M34040.m	TRUE	reported	zuJC1r	2	TRUE	0.003082	22.04297
595	rs1431793	A	C	A	C	-0.0216	0.010977	0.7526	0.7497	FALSE	FALSE	FALSE	KVK33e	0.015877	0.489317	486484	sepsis	TRUE	reported	textfile	0.0046	2.90E-06	5344	M01107.m	TRUE	reported	eAyR8w	2	TRUE	0.004109	22.0409
4738	rs824764	T	C	T	C	0.0709	-0.01425	0.9563	0.953647	FALSE	FALSE	FALSE	Do3Q7g	0.032431	0.66036	486484	sepsis	TRUE	reported	textfile	0.0151	2.73E-06	6150	M32769.m	TRUE	reported	ys2LKp	2	TRUE	0.003572	22.03928
6504	rs7544128	C	G	C	G	0.0432	0.006243	0.7224	0.712901	FALSE	TRUE	FALSE	HNc7C9	0.015203	0.681328	486484	sepsis	TRUE	reported	textfile	0.0092	2.80E-06	3962	M33782.m	TRUE	reported	SAGYGx	2	TRUE	0.005534	22.03802
6491	rs922025	A	G	A	G	-0.0846	0.00713	0.1942	0.227783	FALSE	FALSE	FALSE	ecwVg8	0.016467	0.665011	486484	sepsis	TRUE	reported	textfile	0.018	2.51E-06	831	M33751.m	TRUE	reported	NnvbIQ	2	TRUE	0.025894	22.03684
4452	rs2244987	A	T	A	T	-0.0479	-0.00394	0.8885	0.873654	FALSE	TRUE	FALSE	g2RNym	0.020714	0.849318	486484	sepsis	TRUE	reported	textfile	0.0102	2.73E-06	2575	M32691.m	TRUE	reported	35eo6p	2	TRUE	0.008492	22.03602
4157	rs1050346	C	G	C	G	-0.0338	0.053728	0.8052	0.806948	FALSE	TRUE	FALSE	stpu5O	0.017394	0.00201	486484	sepsis	TRUE	reported	textfile	0.0072	3.14E-06	7337	M32557.m	TRUE	reported	0oV8Ss	2	TRUE	0.002995	22.0318
7694	rs4680092	T	G	T	G	-0.0169	-0.01267	0.2203	0.217919	FALSE	FALSE	FALSE	4qwHDC	0.016655	0.446753	486484	sepsis	TRUE	reported	textfile	0.0036	2.19E-06	6735	M34732.m	TRUE	reported	dm6aLf	2	TRUE	0.003261	22.03126
8063	rs7800039	A	G	A	G	-0.0169	0.010474	0.3424	0.343284	FALSE	FALSE	FALSE	whAa18	0.014507	0.470298	486484	sepsis	TRUE	reported	textfile	0.0036	2.13E-06	5979	M35305.m	TRUE	reported	e6hvDm	2	TRUE	0.003672	22.03044
8123	rs6054468	T	G	T	G	-0.0169	0.002559	0.0819	0.1239	FALSE	FALSE	FALSE	nNbXBS	0.020715	0.901673	486484	sepsis	TRUE	reported	textfile	0.0036	2.50E-06	5768	M35326.m	TRUE	reported	UNDorn	2	TRUE	0.003806	22.03017
7027	rs311496	T	C	T	C	-0.0338	-0.01586	0.0866	0.152241	FALSE	FALSE	FALSE	zMIkYA	0.019033	0.404704	486484	sepsis	TRUE	reported	textfile	0.0072	3.05E-06	4908	M34245.m	TRUE	reported	itCtz8	2	TRUE	0.00447	22.02883
8549	rs1712697	A	G	A	G	0.4543	-0.01943	0.8598	0.779661	FALSE	FALSE	FALSE	aQhCav	0.016523	0.239581	486484	sepsis	TRUE	reported	textfile	0.0966	2.59E-06	488	M36097.m	TRUE	reported	9DJS9j	2	TRUE	0.043357	22.02661
2941	rs1391874	A	G	A	G	0.023	-0.03517	0.9599	0.956778	FALSE	FALSE	FALSE	pn11BC	0.033613	0.295453	486484	sepsis	TRUE	reported	textfile	0.0049	2.57E-06	7203	M21047.m	TRUE	reported	KJF8U4	2	TRUE	0.003049	22.02637
7713	rs1859570	T	C	T	C	0.1451	0.085146	0.0272	0.015583	FALSE	FALSE	FALSE	qJW9Ig	0.055478	0.124839	486484	sepsis	TRUE	reported	textfile	0.0309	2.65E-06	1763	M34878.m	TRUE	reported	42EOzB	2	TRUE	0.012353	22.02546
7229	rs7954487	C	G	C	G	0.4415	0.036221	0.9869	0.987825	FALSE	TRUE	FALSE	i1uJ7T	0.062959	0.565083	486484	sepsis	TRUE	reported	textfile	0.0939	2.57E-06	535	M34338.m	TRUE	reported	pi3mKW	2	TRUE	0.039682	22.02438
6031	rs2077726	T	G	T	G	-0.1686	0.010976	0.7831	0.765793	FALSE	FALSE	FALSE	33ojbt	0.016796	0.51346	486484	sepsis	TRUE	reported	textfile	0.0359	2.71E-06	1391	M33423.m	TRUE	reported	ivEtvl	2	TRUE	0.015609	22.02426
4993	rs9635387	A	G	A	G	0.0324	0.003995	0.5273	0.51123	FALSE	FALSE	FALSE	MdUq0m	0.013657	0.769907	486484	sepsis	TRUE	reported	textfile	0.0069	2.72E-06	1462	M33009.m	TRUE	reported	icuhyC	2	TRUE	0.014857	22.01899
5799	rs6903432	C	G	C	G	-0.069	0.025858	0.0183	0.033955	FALSE	TRUE	FALSE	ka9zG7	0.037961	0.495756	486484	sepsis	TRUE	reported	textfile	0.0147	2.77E-06	2212	M33363.m	TRUE	reported	UFTVNc	2	TRUE	0.009862	22.01257
1055	rs4589188	A	G	A	G	0.0122	-0.01641	0.2827	0.307226	FALSE	FALSE	FALSE	tRtQ2e	0.015008	0.274309	486484	sepsis	TRUE	reported	textfile	0.0026	3.34E-06	7364	M01564.m	TRUE	reported	Tbg0zM	2	TRUE	0.002981	22.01177
4502	rs7806249	T	C	T	C	0.0122	0.017169	0.6546	0.656704	FALSE	FALSE	FALSE	1wNJ1E	0.0144	0.233143	486484	sepsis	TRUE	reported	textfile	0.0026	2.62E-06	7363	M32709.m	TRUE	reported	zihBHg	2	TRUE	0.002981	22.01177
881	rs1225060	T	C	T	C	0.0122	0.012312	0.751	0.747461	FALSE	FALSE	FALSE	oi7KQI	0.015768	0.434907	486484	sepsis	TRUE	reported	textfile	0.0026	2.02E-06	7362	M01365.m	TRUE	reported	7NWGKA	2	TRUE	0.002982	22.01177
4061	rs1083788	T	G	T	G	-0.0122	0.035844	0.6036	0.5958	FALSE	FALSE	FALSE	TyHtdh	0.013899	0.009913	486484	sepsis	TRUE	reported	textfile	0.0026	4.08E-06	7358	M32497.m	TRUE	reported	H3KmLR	2	TRUE	0.002983	22.01177
4076	rs6062362	A	G	A	G	0.0122	0.001039	0.5705	0.562217	FALSE	FALSE	FALSE	TyHtdh	0.01386	0.940217	486484	sepsis	TRUE	reported	textfile	0.0026	3.38E-06	7358	M32497.m	TRUE	reported	H3KmLR	2	TRUE	0.002983	22.01177
4078	rs6801666	A	G	A	G	-0.0122	-0.01867	0.4209	0.424049	FALSE	FALSE	FALSE	TyHtdh	0.013847	0.177511	486484	sepsis	TRUE	reported	textfile	0.0026	2.89E-06	7358	M32497.m	TRUE	reported	H3KmLR	2	TRUE	0.002983	22.01177
197	rs1527683	T	C	T	C	0.0061	0.010983	0.8	0.781567	FALSE	FALSE	FALSE	FbLTng	0.016596	0.508095	486484	sepsis	TRUE	reported	textfile	0.0013	2.17E-06	7355	M00059.m	TRUE	reported	KK7HbM	2	TRUE	0.002985	22.01176
8982	rs7172197	A	G	A	G	-0.0305	-0.01206	0.5376	0.520489	FALSE	FALSE	FALSE	snOepI	0.013706	0.378824	486484	sepsis	TRUE	reported	textfile	0.0065	3.16E-06	7355	M37202.m	TRUE	reported	PQx272	2	TRUE	0.002985	22.01176
7900	rs7307046	A	G	A	G	0.0122	-0.00641	0.4072	0.416195	FALSE	FALSE	FALSE	pU8zps	0.013859	0.64369	486484	sepsis	TRUE	reported	textfile	0.0026	2.33E-06	7350	M35186.m	TRUE	reported	Oc2lOF	2	TRUE	0.002987	22.01176
1270	rs7990832	A	G	A	G	0.0122	-0.00718	0.1792	0.177391	FALSE	FALSE	FALSE	gNG3wy	0.017915	0.68872	486484	sepsis	TRUE	reported	textfile	0.0026	3.87E-06	7348	M01670.m	TRUE	reported	t3lsMq	2	TRUE	0.002987	22.01176
538	rs7747345	A	T	A	T	0.0122	0.025682	0.8153	0.801616	FALSE	TRUE	FALSE	wXMWHy	0.017112	0.133391	486484	sepsis	TRUE	reported	textfile	0.0026	2.52E-06	7345	M00584.m	TRUE	reported	7CKn2b	2	TRUE	0.002989	22.01176
8330	rs4687841	A	G	A	G	0.0122	0.003976	0.6302	0.625282	FALSE	FALSE	FALSE	o0GzT1	0.014122	0.778295	486484	sepsis	TRUE	reported	textfile	0.0026	3.34E-06	7317	M35631.m	TRUE	reported	G1XFx0	2	TRUE	0.003	22.01173
2498	rs1126380	C	G	C	G	0.0122	0.006021	0.2495	0.267616	FALSE	TRUE	FALSE	LwUYua	0.015494	0.69758	486484	sepsis	TRUE	reported	textfile	0.0026	2.76E-06	7315	M17945.m	TRUE	reported	SUN8dc	2	TRUE	0.003001	22.01173
3031	rs230943	A	C	A	C	-0.0122	0.00443	0.5083	0.490608	FALSE	FALSE	FALSE	FJZEYz	0.013699	0.74642	486484	sepsis	TRUE	reported	textfile	0.0026	1.64E-06	7284	M21630.m	TRUE	reported	s6Pshg	2	TRUE	0.003014	22.01171
5823	rs1764225	T	G	T	G	0.0122	-0.01249	0.0886	0.087597	FALSE	FALSE	FALSE	R5joEf	0.024145	0.60494	486484	sepsis	TRUE	reported	textfile	0.0026	4.06E-06	7118	M33380.m	TRUE	reported	kRvbfZ	2	TRUE	0.003084	22.01156
8559	rs873413	T	C	T	C	0.3898	-0.0051	0.5579	0.605855	FALSE	FALSE	FALSE	aQhCav	0.013973	0.715102	486484	sepsis	TRUE	reported	textfile	0.0824	2.27E-06	122	M36097.m	TRUE	reported	9DJS9j	2	TRUE	0.154998	22.01155
7286	rs1352907	T	C	T	C	0.0122	0.006209	0.1679	0.182768	FALSE	FALSE	FALSE	Vcz4EA	0.017749	0.726464	486484	sepsis	TRUE	reported	textfile	0.0026	4.02E-06	6755	M34359.m	TRUE	reported	aUSo2J	2	TRUE	0.003249	22.01123
2763	rs3112726	T	C	T	C	0.0122	0.026945	0.455	0.465622	FALSE	FALSE	FALSE	H6txH3	0.0138	0.050872	486484	sepsis	TRUE	reported	textfile	0.0026	2.93E-06	6678	M19368.m	TRUE	reported	b094NF	2	TRUE	0.003286	22.01116
3242	rs249462	A	T	A	T	-0.0122	0.020482	0.3223	0.318246	FALSE	TRUE	FALSE	COSeSD	0.014938	0.170344	486484	sepsis	TRUE	reported	textfile	0.0026	2.45E-06	6517	M22649.m	TRUE	reported	mnH8z2	2	TRUE	0.003367	22.01099
5094	rs1091042	T	C	T	C	-0.0122	0.018409	0.4474	0.444302	FALSE	FALSE	FALSE	Uiz8VU	0.013796	0.182066	486484	sepsis	TRUE	reported	textfile	0.0026	3.92E-06	6404	M33163.m	TRUE	reported	ky0Bda	2	TRUE	0.003426	22.01088
692	rs4822129	A	G	A	G	0.0122	-0.00923	0.194	0.196979	FALSE	FALSE	FALSE	aT1Fw1	0.017298	0.593778	486484	sepsis	TRUE	reported	textfile	0.0026	3.76E-06	5687	M01284.m	TRUE	reported	mbTe8H	2	TRUE	0.003857	22.01001
6691	rs1952103	T	G	T	G	-0.0244	0.000358	0.7585	0.774623	FALSE	FALSE	FALSE	23zCgC	0.016331	0.982516	486484	sepsis	TRUE	reported	textfile	0.0052	3.07E-06	5534	M33910.m	TRUE	reported	ACl3CA	2	TRUE	0.003963	22.00979
2613	rs7830772	A	C	A	C	-0.0399	0.030592	0.9671	0.973708	FALSE	FALSE	FALSE	ruvJh6	0.042793	0.474684	486484	sepsis	TRUE	reported	textfile	0.0085	2.95E-06	1762	M18369.m	TRUE	reported	GFmwGk	2	TRUE	0.012351	22.00973
868	rs3900774	T	G	T	G	0.061	-0.0067	0.988	0.977508	FALSE	FALSE	FALSE	Vg5OOR	0.046357	0.885104	486484	sepsis	TRUE	reported	textfile	0.013	2.70E-06	5363	M01361.m	TRUE	reported	XnrS00	2	TRUE	0.004089	22.00954
6628	rs1511938	A	G	A	G	-0.0366	0.001883	0.5175	0.506419	FALSE	FALSE	FALSE	IDG01N	0.013673	0.890483	486484	sepsis	TRUE	reported	textfile	0.0078	2.82E-06	4999	M33885.m	TRUE	reported	q3gy7x	2	TRUE	0.004385	22.00894
4453	rs2932198	A	G	A	G	0.0305	-0.01478	0.3908	0.405693	FALSE	FALSE	FALSE	g2RNym	0.013909	0.287911	486484	sepsis	TRUE	reported	textfile	0.0065	2.50E-06	2575	M32691.m	TRUE	reported	35eo6p	2	TRUE	0.008478	22.00065
5779	rs1836845	T	G	T	G	-0.0399	0.02472	0.6005	0.596993	FALSE	FALSE	FALSE	eT2We0	0.013908	0.075504	486484	sepsis	TRUE	reported	textfile	0.0085	2.73E-06	1273	M33353.m	TRUE	reported	uZx90m	2	TRUE	0.017015	22.00012
7501	rs2390196	A	G	A	G	-0.0699	-0.00055	0.1233	0.12287	FALSE	FALSE	FALSE	1aXQWs	0.020888	0.979064	486484	sepsis	TRUE	reported	textfile	0.0149	2.94E-06	4452	M34420.m	TRUE	reported	FOqhHW	2	TRUE	0.004919	21.99818
7697	rs1083330	A	G	A	G	-0.0258	-0.00099	0.1893	0.239223	FALSE	FALSE	FALSE	2eJlPM	0.016169	0.951197	486484	sepsis	TRUE	reported	textfile	0.0055	2.43E-06	6381	M34761.m	TRUE	reported	druKtT	2	TRUE	0.003437	21.99773
5659	rs4126458	A	G	A	G	0.0887	0.019546	0.5107	0.478773	FALSE	FALSE	FALSE	mod1fD	0.013671	0.152773	486484	sepsis	TRUE	reported	textfile	0.0189	2.83E-06	1586	M33194.m	TRUE	reported	fSFVUj	2	TRUE	0.013697	21.99762
2583	rs1709236	A	G	A	G	-0.0671	-0.02898	0.9096	0.891078	FALSE	FALSE	FALSE	JGJ9vH	0.021886	0.185444	486484	sepsis	TRUE	reported	textfile	0.0143	2.61E-06	2079	M18357.m	TRUE	reported	cOV2qw	2	TRUE	0.01048	21.99657
7689	rs6436677	T	C	T	C	0.1131	-0.00736	0.0189	0.010743	FALSE	FALSE	FALSE	5FAxbZ	0.068685	0.91462	486484	sepsis	TRUE	reported	textfile	0.0241	2.66E-06	1560	M34674.m	TRUE	reported	TDQqa8	2	TRUE	0.013921	21.99551
6017	rs1241718	A	T	A	T	-0.2877	-0.01119	0.0796	0.137556	FALSE	TRUE	FALSE	33ojbt	0.019871	0.573275	486484	sepsis	TRUE	reported	textfile	0.0613	2.71E-06	1391	M33423.m	TRUE	reported	ivEtvl	2	TRUE	0.015589	21.9955
5262	rs1267960	A	G	A	G	-0.1582	-0.03417	0.0561	0.035318	FALSE	FALSE	FALSE	q32d4E	0.037238	0.358796	486484	sepsis	TRUE	reported	textfile	0.0337	2.72E-06	1017	M33178.m	TRUE	reported	cS3BwT	2	TRUE	0.021209	21.9937
2816	rs4399682	A	T	A	T	0.0183	-0.02157	0.7244	0.712577	FALSE	TRUE	FALSE	Sx5Uhq	0.015126	0.153798	486484	sepsis	TRUE	reported	textfile	0.0039	2.06E-06	1759	M19934.m	TRUE	reported	DijYTE	2	TRUE	0.012362	21.99272
3597	rs2274629	A	G	A	G	0.0333	0.020013	0.2873	0.268319	FALSE	FALSE	FALSE	e4G8UU	0.015402	0.193829	486484	sepsis	TRUE	reported	textfile	0.0071	3.01E-06	5229	M31548.m	TRUE	reported	kWkkmH	2	TRUE	0.004189	21.98901
7510	rs754739	A	C	A	C	-0.053	0.012898	0.2707	0.267527	FALSE	FALSE	FALSE	1aXQWs	0.015497	0.405241	486484	sepsis	TRUE	reported	textfile	0.0113	2.79E-06	4452	M34420.m	TRUE	reported	FOqhHW	2	TRUE	0.004917	21.98871
8718	rs4657463	C	G	C	G	0.0136	-0.0154	0.6945	0.701313	FALSE	TRUE	FALSE	I0G0I1	0.014972	0.303769	486484	sepsis	TRUE	reported	textfile	0.0029	2.80E-06	6464	M36553.m	TRUE	reported	lBltb0	2	TRUE	0.003391	21.98606
8721	rs6563831	T	C	T	C	0.0136	-0.02027	0.652	0.647593	FALSE	FALSE	FALSE	I0G0I1	0.014396	0.159099	486484	sepsis	TRUE	reported	textfile	0.0029	2.33E-06	6464	M36553.m	TRUE	reported	lBltb0	2	TRUE	0.003391	21.98606
6656	rs2889961	A	G	A	G	0.0136	0.014668	0.3641	0.361023	FALSE	FALSE	FALSE	REeQiq	0.014267	0.30391	486484	sepsis	TRUE	reported	textfile	0.0029	2.70E-06	6004	M33892.m	TRUE	reported	cVOa3u	2	TRUE	0.00365	21.98554
8277	rs1125923	A	G	A	G	-0.0347	-0.0093	0.8943	0.894546	FALSE	FALSE	FALSE	J6o4xC	0.022475	0.678949	486484	sepsis	TRUE	reported	textfile	0.0074	2.39E-06	6595	M35472.m	TRUE	reported	3XBcu9	2	TRUE	0.003323	21.98183
8618	rs1240400	A	G	A	G	0.2036	-0.013	0.9894	0.938651	FALSE	FALSE	FALSE	MZ60v7	0.029677	0.66141	486484	sepsis	TRUE	reported	textfile	0.0434	2.74E-06	1657	M36134.m	TRUE	reported	dMQewN	2	TRUE	0.013108	21.98121
4208	rs520906	A	G	A	G	0.0211	-0.00021	0.6353	0.633931	FALSE	FALSE	FALSE	rdKSjG	0.014165	0.988205	486484	sepsis	TRUE	reported	textfile	0.0045	2.23E-06	7322	M32578.m	TRUE	reported	RAzSLK	2	TRUE	0.002994	21.97967
2771	rs4884317	A	G	A	G	-0.0211	0.017556	0.211	0.215826	FALSE	FALSE	FALSE	JWylTj	0.017033	0.302684	486484	sepsis	TRUE	reported	textfile	0.0045	2.44E-06	6385	M19396.m	TRUE	reported	6S7kzt	2	TRUE	0.003432	21.97879
3484	rs1234223	C	G	C	G	0.0211	0.016067	0.0908	0.093902	FALSE	TRUE	FALSE	Awafb0	0.023428	0.492838	486484	sepsis	TRUE	reported	textfile	0.0045	3.02E-06	5562	M27728.m	TRUE	reported	Lfj8L0	2	TRUE	0.003937	21.97777
5009	rs2197598	T	C	T	C	-0.0408	-0.00644	0.1051	0.10538	FALSE	FALSE	FALSE	URCVGm	0.022348	0.773079	486484	sepsis	TRUE	reported	textfile	0.0087	2.76E-06	2773	M33131.m	TRUE	reported	4rQ2gs	2	TRUE	0.007869	21.977
6830	rs2427612	T	C	T	C	-0.0286	-0.03113	0.364	0.380043	FALSE	FALSE	FALSE	ZPJCMx	0.01427	0.029138	486484	sepsis	TRUE	reported	textfile	0.0061	3.01E-06	7330	M33969.m	TRUE	reported	mLJPAI	2	TRUE	0.00299	21.97626
2159	rs7151608	A	G	A	G	0.0497	-0.01836	0.8127	0.811498	FALSE	FALSE	FALSE	uuY1Mi	0.017517	0.294625	486484	sepsis	TRUE	reported	textfile	0.0106	2.86E-06	5502	M15677.m	TRUE	reported	nWMiPu	2	TRUE	0.00398	21.97572
4720	rs7841945	A	T	A	T	0.0394	-0.03901	0.8698	0.880862	FALSE	TRUE	FALSE	L2WFM1	0.021114	0.06468	486484	sepsis	TRUE	reported	textfile	0.0084	2.49E-06	1734	M32761.m	TRUE	reported	5GHzph	2	TRUE	0.012529	21.97519
6038	rs4240294	T	C	T	C	-0.3218	-0.00277	0.0413	0.056025	FALSE	FALSE	FALSE	33ojbt	0.029794	0.925894	486484	sepsis	TRUE	reported	textfile	0.0686	2.73E-06	1391	M33423.m	TRUE	reported	ivEtvl	2	TRUE	0.015573	21.97349
4236	rs1714898	C	G	C	G	0.0361	-0.01707	0.0869	0.099533	FALSE	TRUE	FALSE	wWEMr1	0.022782	0.453745	486484	sepsis	TRUE	reported	textfile	0.0077	2.52E-06	5955	M32593.m	TRUE	reported	Mh2g4i	2	TRUE	0.003677	21.97288
8060	rs1507362	T	C	T	C	0.0333	0.007098	0.3901	0.394409	FALSE	FALSE	FALSE	whAa18	0.013974	0.611488	486484	sepsis	TRUE	reported	textfile	0.0071	3.02E-06	1505	M35305.m	TRUE	reported	e6hvDm	2	TRUE	0.014406	21.96819
1552	rs1391933	T	G	T	G	-0.0075	0.012597	0.4399	0.446753	FALSE	FALSE	FALSE	Ke0sNh	0.013778	0.360602	486484	sepsis	TRUE	reported	textfile	0.0016	5.79E-06	7799	M10642.m	TRUE	reported	ZINwVb	2	TRUE	0.002809	21.96702
723	rs9594421	A	T	A	T	0.0075	-0.03036	0.2757	0.294598	FALSE	TRUE	FALSE	H9I3s3	0.015059	0.043785	486484	sepsis	TRUE	reported	textfile	0.0016	4.59E-06	7358	M01299.m	TRUE	reported	kXWyUq	2	TRUE	0.002977	21.96668
932	rs1578743	A	C	A	C	0.0075	-0.01318	0.6068	0.60373	FALSE	FALSE	FALSE	tv99UN	0.014078	0.349232	486484	sepsis	TRUE	reported	textfile	0.0016	1.70E-06	7354	M01494.m	TRUE	reported	osDclp	2	TRUE	0.002979	21.96668
757	rs6071991	T	C	T	C	-0.0075	0.002605	0.1495	0.157929	FALSE	FALSE	FALSE	RHWptG	0.018746	0.889497	486484	sepsis	TRUE	reported	textfile	0.0016	3.81E-06	7347	M01302.m	TRUE	reported	z1OLfr	2	TRUE	0.002982	21.96667
1847	rs1248263	T	C	T	C	0.0075	-0.00114	0.2693	0.293695	FALSE	FALSE	FALSE	S1eWts	0.015089	0.939863	486484	sepsis	TRUE	reported	textfile	0.0016	5.40E-06	7345	M15488.m	TRUE	reported	511KI9	2	TRUE	0.002983	21.96667
2720	rs1229866	C	G	C	G	-0.015	0.005356	0.9	0.895497	FALSE	TRUE	FALSE	H4yqSK	0.022352	0.810612	486484	sepsis	TRUE	reported	textfile	0.0032	2.94E-06	7182	M19362.m	TRUE	reported	w2PDEO	2	TRUE	0.00305	21.96654
6989	rs1767986	T	C	T	C	-0.0525	-0.02578	0.0844	0.11858	FALSE	FALSE	FALSE	APOtl6	0.021111	0.221943	486484	sepsis	TRUE	reported	textfile	0.0112	2.85E-06	6987	M34221.m	TRUE	reported	looCDC	2	TRUE	0.003135	21.96637
1421	rs1088142	T	G	T	G	-0.015	-0.01003	0.5558	0.556435	FALSE	FALSE	FALSE	ac6RGB	0.013755	0.465897	486484	sepsis	TRUE	reported	textfile	0.0032	3.69E-06	5791	M02342.m	TRUE	reported	dC6Rkp	2	TRUE	0.00378	21.96507
7655	rs4836309	T	C	T	C	-0.0225	0.015188	0.5412	0.541793	FALSE	FALSE	FALSE	8W5cqf	0.013723	0.268404	486484	sepsis	TRUE	reported	textfile	0.0048	2.38E-06	4925	M34534.m	TRUE	reported	JV42Pw	2	TRUE	0.004442	21.96373
4644	rs768328	T	C	T	C	-0.0464	-0.01095	0.228	0.225767	FALSE	FALSE	FALSE	Dfaklt	0.016335	0.502533	486484	sepsis	TRUE	reported	textfile	0.0099	3.09E-06	6379	M32754.m	TRUE	reported	y21cDm	2	TRUE	0.003432	21.95985
2722	rs1270339	T	C	T	C	0.0314	0.049898	0.9778	0.974206	FALSE	FALSE	FALSE	H4yqSK	0.043038	0.246296	486484	sepsis	TRUE	reported	textfile	0.0067	3.00E-06	7182	M19362.m	TRUE	reported	w2PDEO	2	TRUE	0.003049	21.9578
3958	rs1260374	T	C	T	C	-0.0389	0.00505	0.88	0.860267	FALSE	FALSE	FALSE	sKCkQs	0.019822	0.798915	486484	sepsis	TRUE	reported	textfile	0.0083	2.95E-06	5080	M32445.m	TRUE	reported	egPvJc	2	TRUE	0.004305	21.95695
548	rs2833657	A	G	A	G	0.0239	0.01754	0.8254	0.825775	FALSE	FALSE	FALSE	QUUN2g	0.017986	0.329452	486484	sepsis	TRUE	reported	textfile	0.0051	3.02E-06	7237	M00599.m	TRUE	reported	vxn2i7	2	TRUE	0.003025	21.9551
5943	rs6717460	A	C	A	C	-0.1646	0.043384	0.962	0.969405	FALSE	FALSE	FALSE	yepzrL	0.039747	0.275048	486484	sepsis	TRUE	reported	textfile	0.0351	2.77E-06	1094	M33408.m	TRUE	reported	SY4CoS	2	TRUE	0.019705	21.95082
2709	rs1808751	A	G	A	G	0.0164	0.013528	0.7375	0.725391	FALSE	FALSE	FALSE	uNhql4	0.015308	0.376869	486484	sepsis	TRUE	reported	textfile	0.0035	3.58E-06	7369	M19323.m	TRUE	reported	h9HAwp	2	TRUE	0.002971	21.94996
4484	rs1180318	T	C	T	C	0.0164	-0.01585	0.8545	0.829442	FALSE	FALSE	FALSE	1wNJ1E	0.018228	0.38454	486484	sepsis	TRUE	reported	textfile	0.0035	3.96E-06	7363	M32709.m	TRUE	reported	zihBHg	2	TRUE	0.002973	21.94995
6526	rs6817243	A	G	A	G	0.0164	-0.00384	0.2809	0.275764	FALSE	FALSE	FALSE	OKIXJ8	0.015349	0.802552	486484	sepsis	TRUE	reported	textfile	0.0035	3.29E-06	7362	M33821.m	TRUE	reported	XGkaXH	2	TRUE	0.002973	21.94995
4384	rs1079209	T	G	T	G	0.0164	0.016493	0.1084	0.114399	FALSE	FALSE	FALSE	UJygVd	0.021476	0.442504	486484	sepsis	TRUE	reported	textfile	0.0035	3.15E-06	7361	M32654.m	TRUE	reported	kv9R0W	2	TRUE	0.002974	21.94995
5072	rs1289336	A	G	A	G	-0.0164	-0.02262	0.7444	0.74546	FALSE	FALSE	FALSE	gqzGN4	0.015709	0.149926	486484	sepsis	TRUE	reported	textfile	0.0035	2.28E-06	7319	M33140.m	TRUE	reported	DKGBe9	2	TRUE	0.002991	21.94992
550	rs3925033	T	G	T	G	-0.0164	0.002357	0.4591	0.44852	FALSE	FALSE	FALSE	QUUN2g	0.013767	0.864048	486484	sepsis	TRUE	reported	textfile	0.0035	2.59E-06	7237	M00599.m	TRUE	reported	vxn2i7	2	TRUE	0.003025	21.94985
947	rs1254456	A	G	A	G	0.0164	0.022827	0.148	0.140807	FALSE	FALSE	FALSE	tWoDq4	0.019633	0.244964	486484	sepsis	TRUE	reported	textfile	0.0035	3.32E-06	7160	M01508.m	TRUE	reported	6ltDw0	2	TRUE	0.003057	21.94979
1644	rs7134822	A	G	A	G	-0.0164	-0.05029	0.9597	0.96034	FALSE	FALSE	FALSE	n5rDuT	0.035485	0.156451	486484	sepsis	TRUE	reported	textfile	0.0035	3.87E-06	7081	M12067.m	TRUE	reported	ZQLTeR	2	TRUE	0.003091	21.94972
8005	rs1316848	T	C	T	C	0.0164	0.015548	0.2029	0.199134	FALSE	FALSE	FALSE	Zj5bZH	0.017115	0.36363	486484	sepsis	TRUE	reported	textfile	0.0035	2.68E-06	6538	M35257.m	TRUE	reported	exssAk	2	TRUE	0.003347	21.9492
8009	rs1770008	T	C	T	C	0.0164	0.004937	0.2073	0.218161	FALSE	FALSE	FALSE	Zj5bZH	0.016672	0.767107	486484	sepsis	TRUE	reported	textfile	0.0035	2.83E-06	6538	M35257.m	TRUE	reported	exssAk	2	TRUE	0.003347	21.9492
201	rs4768741	C	G	C	G	-0.015	0.00399	0.9153	0.913671	FALSE	TRUE	FALSE	FbLTng	0.024339	0.869783	486484	sepsis	TRUE	reported	textfile	0.0032	3.52E-06	1762	M00059.m	TRUE	reported	KK7HbM	2	TRUE	0.012317	21.94772
8523	rs1262265	A	G	A	G	-0.0314	-0.0075	0.7765	0.769357	FALSE	FALSE	FALSE	gM7v2m	0.016387	0.647275	486484	sepsis	TRUE	reported	textfile	0.0067	2.89E-06	2651	M36009.m	TRUE	reported	6Xcypd	2	TRUE	0.008217	21.94734
3806	rs2044048	A	G	A	G	-0.0417	-0.00817	0.2224	0.217251	FALSE	FALSE	FALSE	HOq2gb	0.016577	0.622024	486484	sepsis	TRUE	reported	textfile	0.0089	2.63E-06	6687	M32346.m	TRUE	reported	Ss5q17	2	TRUE	0.003272	21.94634
6368	rs1246172	A	G	A	G	0.0328	0.022769	0.2836	0.300551	FALSE	FALSE	FALSE	PAgZZz	0.014966	0.128181	486484	sepsis	TRUE	reported	textfile	0.007	2.52E-06	4323	M33633.m	TRUE	reported	I8R1KJ	2	TRUE	0.005053	21.94576
3932	rs1009343	A	C	A	C	0.1003	0.028174	0.9622	0.962186	FALSE	FALSE	FALSE	LlOaWH	0.03663	0.441807	486484	sepsis	TRUE	reported	textfile	0.0214	2.69E-06	1767	M32418.m	TRUE	reported	AtHOVw	2	TRUE	0.012279	21.94232
6540	rs685747	C	G	C	G	-0.0431	0.048427	0.9581	0.951113	FALSE	TRUE	FALSE	fVPpJo	0.03183	0.128157	486484	sepsis	TRUE	reported	textfile	0.0092	2.96E-06	6327	M33833.m	TRUE	reported	37N6ck	2	TRUE	0.003457	21.94025
8302	rs4841110	A	G	A	G	0.0342	0.030698	0.5869	0.585984	FALSE	FALSE	FALSE	HELgy7	0.013931	0.027554	486484	sepsis	TRUE	reported	textfile	0.0073	2.60E-06	5035	M35551.m	TRUE	reported	7x7uUn	2	TRUE	0.00434	21.93986
5915	rs1709762	T	C	T	C	-0.104	0.025048	0.9727	0.968957	FALSE	FALSE	FALSE	iGV9aX	0.039821	0.529338	486484	sepsis	TRUE	reported	textfile	0.0222	2.72E-06	5185	M33390.m	TRUE	reported	sLbpnf	2	TRUE	0.004215	21.93781
402	rs1110613	T	G	T	G	-0.0089	0.010103	0.2379	0.244839	FALSE	FALSE	FALSE	cTsNj6	0.016	0.527757	486484	sepsis	TRUE	reported	textfile	0.0019	1.81E-06	7365	M00063.m	TRUE	reported	7KYTrQ	2	TRUE	0.00297	21.93587
3685	rs7332276	T	G	T	G	-0.0178	-0.0043	0.4699	0.526337	FALSE	FALSE	FALSE	rGHJcj	0.014177	0.761835	486484	sepsis	TRUE	reported	textfile	0.0038	3.14E-06	7356	M32198.m	TRUE	reported	6P1Nno	2	TRUE	0.002974	21.93586
1203	rs1889463	T	C	T	C	0.0089	-0.00149	0.628	0.639032	FALSE	FALSE	FALSE	Ckdgh1	0.014792	0.919722	486484	sepsis	TRUE	reported	textfile	0.0019	3.36E-06	7355	M01644.m	TRUE	reported	nKxz7B	2	TRUE	0.002974	21.93586
4103	rs1691607	T	G	T	G	0.0089	-0.02181	0.806	0.797762	FALSE	FALSE	FALSE	4rtLd0	0.017069	0.201343	486484	sepsis	TRUE	reported	textfile	0.0019	2.69E-06	7350	M32518.m	TRUE	reported	j7f1ER	2	TRUE	0.002976	21.93586
8377	rs158896	T	C	T	C	0.0089	0.000824	0.2658	0.261117	FALSE	FALSE	FALSE	SCzh0A	0.015645	0.958003	486484	sepsis	TRUE	reported	textfile	0.0019	1.38E-06	7348	M35675.m	TRUE	reported	n1SREk	2	TRUE	0.002977	21.93586
8403	rs9410293	A	T	A	T	0.0089	-0.01371	0.7111	0.709543	FALSE	TRUE	FALSE	SCzh0A	0.015407	0.373617	486484	sepsis	TRUE	reported	textfile	0.0019	4.67E-06	7348	M35675.m	TRUE	reported	n1SREk	2	TRUE	0.002977	21.93586
3210	rs625842	A	C	A	C	0.0089	-0.00012	0.5072	0.514271	FALSE	FALSE	FALSE	UfLkBq	0.01367	0.993048	486484	sepsis	TRUE	reported	textfile	0.0019	1.85E-06	7329	M22481.m	TRUE	reported	kWMdiT	2	TRUE	0.002985	21.93584
2511	rs3796622	T	C	T	C	-0.0089	0.005735	0.3747	0.362229	FALSE	FALSE	FALSE	LwUYua	0.014193	0.68615	486484	sepsis	TRUE	reported	textfile	0.0019	1.35E-06	7315	M17945.m	TRUE	reported	SUN8dc	2	TRUE	0.002991	21.93583
5867	rs1288410	A	C	A	C	-0.0089	0.010462	0.4607	0.567194	FALSE	FALSE	FALSE	tqW0ld	0.01381	0.448727	486484	sepsis	TRUE	reported	textfile	0.0019	1.84E-06	7315	M33389.m	TRUE	reported	Sv7EyV	2	TRUE	0.002991	21.93583
5879	rs348661	A	T	A	T	0.0089	0.034213	0.3458	0.350188	FALSE	TRUE	FALSE	tqW0ld	0.014341	0.017047	486484	sepsis	TRUE	reported	textfile	0.0019	1.78E-06	7315	M33389.m	TRUE	reported	Sv7EyV	2	TRUE	0.002991	21.93583
5901	rs939291	T	C	T	C	0.0089	0.02245	0.711	0.70015	FALSE	FALSE	FALSE	tqW0ld	0.014989	0.134195	486484	sepsis	TRUE	reported	textfile	0.0019	2.63E-06	7315	M33389.m	TRUE	reported	Sv7EyV	2	TRUE	0.002991	21.93583
2204	rs1687615	T	G	T	G	-0.0089	-0.00913	0.2054	0.207148	FALSE	FALSE	FALSE	Bf6uMK	0.016935	0.589616	486484	sepsis	TRUE	reported	textfile	0.0019	1.65E-06	7308	M15778.m	TRUE	reported	jowM2N	2	TRUE	0.002993	21.93582
6141	rs4505759	T	C	T	C	-0.0089	0.00201	0.3074	0.306477	FALSE	FALSE	FALSE	MiSJly	0.01486	0.892412	486484	sepsis	TRUE	reported	textfile	0.0019	2.36E-06	7307	M33477.m	TRUE	reported	WwL1hq	2	TRUE	0.002994	21.93582
6290	rs8045299	A	G	A	G	0.0089	0.006536	0.3403	0.333528	FALSE	FALSE	FALSE	MOonP9	0.014512	0.652435	486484	sepsis	TRUE	reported	textfile	0.0019	1.81E-06	7066	M33515.m	TRUE	reported	226GuP	2	TRUE	0.003096	21.93562
8495	rs9960323	T	C	T	C	0.0178	0.015334	0.2721	0.283489	FALSE	FALSE	FALSE	ABkZ7x	0.015176	0.312306	486484	sepsis	TRUE	reported	textfile	0.0038	2.37E-06	6960	M35854.m	TRUE	reported	wc0CeK	2	TRUE	0.003143	21.93552
4834	rs981831	T	C	T	C	0.0267	0.005401	0.123	0.121851	FALSE	FALSE	FALSE	yLDcUj	0.020876	0.795873	486484	sepsis	TRUE	reported	textfile	0.0057	2.48E-06	6753	M32838.m	TRUE	reported	5dQgAN	2	TRUE	0.003239	21.93533
8452	rs1322878	A	C	A	C	-0.0089	0.00433	0.4235	0.43118	FALSE	FALSE	FALSE	uMajV6	0.013818	0.754006	486484	sepsis	TRUE	reported	textfile	0.0019	3.32E-06	6604	M35754.m	TRUE	reported	b8QB7S	2	TRUE	0.003312	21.93518
8468	rs6827956	A	G	A	G	0.0089	-0.01794	0.4111	0.423174	FALSE	FALSE	FALSE	uMajV6	0.013863	0.195678	486484	sepsis	TRUE	reported	textfile	0.0019	3.66E-06	6604	M35754.m	TRUE	reported	b8QB7S	2	TRUE	0.003312	21.93518
8887	rs1080903	A	C	A	C	-0.0089	-0.01077	0.6121	0.610671	FALSE	FALSE	FALSE	eLhsld	0.014039	0.442808	486484	sepsis	TRUE	reported	textfile	0.0019	1.75E-06	6552	M37058.m	TRUE	reported	RSeWE5	2	TRUE	0.003338	21.93513
8891	rs1168556	A	G	A	G	0.0089	0.000828	0.6358	0.630969	FALSE	FALSE	FALSE	eLhsld	0.014249	0.953679	486484	sepsis	TRUE	reported	textfile	0.0019	1.47E-06	6552	M37058.m	TRUE	reported	RSeWE5	2	TRUE	0.003338	21.93513
8914	rs4837828	A	G	A	G	-0.0089	0.013239	0.6656	0.66502	FALSE	FALSE	FALSE	eLhsld	0.014476	0.360446	486484	sepsis	TRUE	reported	textfile	0.0019	1.54E-06	6552	M37058.m	TRUE	reported	RSeWE5	2	TRUE	0.003338	21.93513
3163	rs6738212	A	G	A	G	-0.0089	-0.02076	0.3037	0.328941	FALSE	FALSE	FALSE	JiXR2t	0.014541	0.153308	486484	sepsis	TRUE	reported	textfile	0.0019	4.29E-06	6535	M22177.m	TRUE	reported	joi6p6	2	TRUE	0.003346	21.93511
3176	rs9539356	A	C	A	C	0.0089	0.009332	0.7252	0.745016	FALSE	FALSE	FALSE	JiXR2t	0.015688	0.551943	486484	sepsis	TRUE	reported	textfile	0.0019	4.75E-06	6535	M22177.m	TRUE	reported	joi6p6	2	TRUE	0.003346	21.93511
7727	rs1219563	T	C	T	C	-0.0089	0.010021	0.6339	0.62588	FALSE	FALSE	FALSE	HyMNYM	0.014106	0.477444	486484	sepsis	TRUE	reported	textfile	0.0019	2.49E-06	5885	M34912.m	TRUE	reported	Tl8Hcw	2	TRUE	0.003715	21.93437
7756	rs7755681	T	C	T	C	0.0089	0.015308	0.5943	0.574046	FALSE	FALSE	FALSE	HyMNYM	0.013845	0.268887	486484	sepsis	TRUE	reported	textfile	0.0019	2.58E-06	5885	M34912.m	TRUE	reported	Tl8Hcw	2	TRUE	0.003715	21.93437
7761	rs982702	T	C	T	C	-0.0089	-0.0112	0.3591	0.353544	FALSE	FALSE	FALSE	HyMNYM	0.014342	0.435017	486484	sepsis	TRUE	reported	textfile	0.0019	2.49E-06	5885	M34912.m	TRUE	reported	Tl8Hcw	2	TRUE	0.003715	21.93437
7170	rs7655049	T	C	T	C	-0.0553	-0.02263	0.1616	0.159886	FALSE	FALSE	FALSE	VfvTtz	0.018707	0.226371	486484	sepsis	TRUE	reported	textfile	0.0118	2.91E-06	1492	M34329.m	TRUE	reported	egac6r	2	TRUE	0.014507	21.93329
7831	rs2969990	A	T	A	T	0.0089	-0.00941	0.3814	0.389454	FALSE	TRUE	FALSE	h5whlS	0.014041	0.502742	486484	sepsis	TRUE	reported	textfile	0.0019	2.82E-06	4900	M35137.m	TRUE	reported	xVITKF	2	TRUE	0.004458	21.93287
7853	rs696958	A	G	A	G	-0.0089	0.015785	0.3585	0.346586	FALSE	FALSE	FALSE	h5whlS	0.016034	0.32488	486484	sepsis	TRUE	reported	textfile	0.0019	3.05E-06	4900	M35137.m	TRUE	reported	xVITKF	2	TRUE	0.004458	21.93287
6404	rs1016841	C	G	C	G	-0.0267	-0.00022	0.6733	0.682586	FALSE	TRUE	FALSE	5ClPW7	0.014673	0.98821	486484	sepsis	TRUE	reported	textfile	0.0057	2.42E-06	4807	M33653.m	TRUE	reported	H4b2zx	2	TRUE	0.004544	21.9327
2817	rs4431081	T	C	T	C	0.0164	0.025924	0.4481	0.452746	FALSE	FALSE	FALSE	Sx5Uhq	0.01372	0.058821	486484	sepsis	TRUE	reported	textfile	0.0035	2.18E-06	1759	M19934.m	TRUE	reported	DijYTE	2	TRUE	0.012328	21.93095
5946	rs7767760	T	C	T	C	0.0356	0.016063	0.2932	0.297773	FALSE	FALSE	FALSE	yepzrL	0.014979	0.283542	486484	sepsis	TRUE	reported	textfile	0.0076	2.97E-06	3890	M33408.m	TRUE	reported	SY4CoS	2	TRUE	0.005609	21.93055
4461	rs913080	T	C	T	C	-0.0417	-0.01101	0.3661	0.342971	FALSE	FALSE	FALSE	g2RNym	0.014419	0.445156	486484	sepsis	TRUE	reported	textfile	0.0089	2.64E-06	1675	M32691.m	TRUE	reported	35eo6p	2	TRUE	0.012937	21.9267
6174	rs6862546	T	C	T	C	-0.0281	-0.01499	0.1752	0.16818	FALSE	FALSE	FALSE	O4Ty2I	0.018304	0.412838	486484	sepsis	TRUE	reported	textfile	0.006	3.31E-06	5107	M33488.m	TRUE	reported	RGtF84	2	TRUE	0.004276	21.92502
7349	rs1806807	A	G	A	G	-0.1364	0.03406	0.772	0.754608	FALSE	FALSE	FALSE	mm9ksf	0.015988	0.033141	486484	sepsis	TRUE	reported	textfile	0.0291	2.69E-06	954	M34365.m	TRUE	reported	AVwEqD	2	TRUE	0.022512	21.92458
6419	rs1104626	T	C	T	C	-0.0431	-0.00099	0.2995	0.313228	FALSE	FALSE	FALSE	F7mjRv	0.015025	0.947441	486484	sepsis	TRUE	reported	textfile	0.0092	2.53E-06	1860	M33658.m	TRUE	reported	frG0Zf	2	TRUE	0.011662	21.92359
2270	rs2216140	A	G	A	G	-0.1026	-0.04927	0.0341	0.027843	FALSE	FALSE	FALSE	Mjgi2R	0.041815	0.238688	486484	sepsis	TRUE	reported	textfile	0.0219	2.73E-06	1749	M16634.m	TRUE	reported	XO0GV7	2	TRUE	0.012394	21.92348
4919	rs1163690	A	C	A	C	0.0623	-0.04964	0.0306	0.037784	FALSE	FALSE	FALSE	clhN6v	0.036273	0.171177	486484	sepsis	TRUE	reported	textfile	0.0133	2.84E-06	2148	M32869.m	TRUE	reported	BzloWk	2	TRUE	0.010112	21.9214
4895	rs7539884	T	C	T	C	0.0623	0.002057	0.749	0.773435	FALSE	FALSE	FALSE	ocaywK	0.016344	0.899865	486484	sepsis	TRUE	reported	textfile	0.0133	2.67E-06	1988	M32863.m	TRUE	reported	WH6KGr	2	TRUE	0.010917	21.91975
6314	rs1706515	A	G	A	G	0.1289	-0.06513	0.0388	0.03887	FALSE	FALSE	FALSE	5B5LTX	0.035677	0.067913	486484	sepsis	TRUE	reported	textfile	0.0275	2.81E-06	824	M33609.m	TRUE	reported	m0eM6N	2	TRUE	0.025971	21.9172
5018	rs1092071	A	G	A	G	-0.0178	0.023124	0.8234	0.821921	FALSE	FALSE	FALSE	BNnOvt	0.017979	0.19838	486484	sepsis	TRUE	reported	textfile	0.0038	2.27E-06	1762	M33132.m	TRUE	reported	rHcCPG	2	TRUE	0.0123	21.91692
3748	rs1194262	T	G	T	G	0.0712	0.076319	0.9829	0.987334	FALSE	FALSE	FALSE	OZLdls	0.060911	0.210219	486484	sepsis	TRUE	reported	textfile	0.0152	2.68E-06	1760	M32338.m	TRUE	reported	s2itmB	2	TRUE	0.012313	21.91689
2454	rs8118890	A	G	A	G	0.1147	0.028172	0.9185	0.923806	FALSE	FALSE	FALSE	NBJ2Do	0.025813	0.275091	486484	sepsis	TRUE	reported	textfile	0.0235	1.06E-06	25	M17799.m	TRUE	reported	Uz0WgI	2	TRUE	0.487943	21.91689
8577	rs3935503	T	G	T	G	-0.0398	0.025153	0.7581	0.761371	FALSE	FALSE	FALSE	3VcRoA	0.016103	0.118274	486484	sepsis	TRUE	reported	textfile	0.0085	2.66E-06	5696	M36099.m	TRUE	reported	zHE146	2	TRUE	0.003834	21.91673
8624	rs7187550	T	C	T	C	0.0473	0.007126	0.1217	0.116841	FALSE	FALSE	FALSE	MZ60v7	0.021297	0.737926	486484	sepsis	TRUE	reported	textfile	0.0101	2.67E-06	2432	M36134.m	TRUE	reported	dMQewN	2	TRUE	0.008938	21.91403
934	rs2115151	A	T	A	T	0.0103	0.001367	0.8627	0.864621	FALSE	TRUE	FALSE	tv99UN	0.019919	0.945265	486484	sepsis	TRUE	reported	textfile	0.0022	3.75E-06	7354	M01494.m	TRUE	reported	osDclp	2	TRUE	0.002972	21.91346
6069	rs1080985	A	G	A	G	-0.0103	0.021202	0.8089	0.791088	FALSE	FALSE	FALSE	P8n0HP	0.016843	0.208095	486484	sepsis	TRUE	reported	textfile	0.0022	4.16E-06	7336	M33442.m	TRUE	reported	cljMTI	2	TRUE	0.002979	21.91345
3182	rs2485904	A	C	A	C	0.0206	0.004402	0.1564	0.148556	FALSE	FALSE	FALSE	gvZCBj	0.019731	0.823468	486484	sepsis	TRUE	reported	textfile	0.0044	2.88E-06	7259	M22189.m	TRUE	reported	GKqg2h	2	TRUE	0.003011	21.91338
1436	rs1049717	T	C	T	C	0.0103	-0.01437	0.2732	0.266111	FALSE	FALSE	FALSE	1M4gqj	0.015666	0.359093	486484	sepsis	TRUE	reported	textfile	0.0022	4.16E-06	7226	M02730.m	TRUE	reported	RWyYEM	2	TRUE	0.003024	21.91335
1443	rs192598	A	C	A	C	0.0103	-0.03187	0.3185	0.315379	FALSE	FALSE	FALSE	1M4gqj	0.014685	0.029972	486484	sepsis	TRUE	reported	textfile	0.0022	1.99E-06	7226	M02730.m	TRUE	reported	RWyYEM	2	TRUE	0.003024	21.91335
5783	rs6565887	T	C	T	C	-0.0773	-0.00464	0.927	0.92813	FALSE	FALSE	FALSE	eT2We0	0.026574	0.861493	486484	sepsis	TRUE	reported	textfile	0.0165	2.90E-06	1273	M33353.m	TRUE	reported	uZx90m	2	TRUE	0.016949	21.91332
946	rs1171963	A	G	A	G	-0.0206	-0.01143	0.0945	0.098411	FALSE	FALSE	FALSE	tWoDq4	0.023163	0.621646	486484	sepsis	TRUE	reported	textfile	0.0044	3.01E-06	7160	M01508.m	TRUE	reported	6ltDw0	2	TRUE	0.003052	21.9133
8017	rs9405761	A	T	A	T	0.0206	-0.0641	0.8788	0.865015	FALSE	TRUE	FALSE	Zj5bZH	0.020057	0.001393	486484	sepsis	TRUE	reported	textfile	0.0044	2.95E-06	6538	M35257.m	TRUE	reported	exssAk	2	TRUE	0.003341	21.91272
8423	rs9304337	A	G	A	G	0.0206	-0.01451	0.5538	0.555102	FALSE	FALSE	FALSE	02RdNm	0.013773	0.292225	486484	sepsis	TRUE	reported	textfile	0.0044	2.26E-06	6447	M35678.m	TRUE	reported	BWN5JR	2	TRUE	0.003388	21.91262
638	rs696155	A	G	A	G	-0.0412	0.018726	0.1888	0.192488	FALSE	FALSE	FALSE	F6Dn8H	0.017422	0.28243	486484	sepsis	TRUE	reported	textfile	0.0088	2.92E-06	4934	M01114.m	TRUE	reported	dVHatZ	2	TRUE	0.004423	21.91054
8598	rs2357258	A	C	A	C	-0.037	-0.00136	0.6387	0.647154	FALSE	FALSE	FALSE	t5potg	0.014307	0.924282	486484	sepsis	TRUE	reported	textfile	0.0079	2.54E-06	1731	M36115.m	TRUE	reported	SSOHIA	2	TRUE	0.012514	21.91024
6822	rs2194101	A	G	A	G	0.0323	-0.01758	0.8777	0.8569	FALSE	FALSE	FALSE	wedECv	0.019584	0.369497	486484	sepsis	TRUE	reported	textfile	0.0069	3.41E-06	7322	M33968.m	TRUE	reported	GxGxRG	2	TRUE	0.002984	21.90727
6635	rs7718486	T	C	T	C	0.0426	0.022314	0.7269	0.734051	FALSE	FALSE	FALSE	IDG01N	0.015463	0.14901	486484	sepsis	TRUE	reported	textfile	0.0091	2.68E-06	4999	M33885.m	TRUE	reported	q3gy7x	2	TRUE	0.004365	21.90598
1753	rs7520876	A	G	A	G	0.044	-0.04861	0.9879	0.981028	FALSE	FALSE	FALSE	nFLpAP	0.049968	0.330673	486484	sepsis	TRUE	reported	textfile	0.0094	3.15E-06	7356	M12774.m	TRUE	reported	yOSQTj	2	TRUE	0.00297	21.90441
5702	rs4807944	T	C	T	C	0.0206	-0.02859	0.1282	0.147262	FALSE	FALSE	FALSE	1SbQTe	0.019335	0.139295	486484	sepsis	TRUE	reported	textfile	0.0044	3.32E-06	2683	M33204.m	TRUE	reported	x7MBDH	2	TRUE	0.008104	21.90308
4150	rs1764259	C	G	C	G	-0.0337	-0.01674	0.8943	0.909613	FALSE	TRUE	FALSE	CrVaiQ	0.023945	0.484567	486484	sepsis	TRUE	reported	textfile	0.0072	2.67E-06	7361	M32553.m	TRUE	reported	HsKdAI	2	TRUE	0.002967	21.90165
4682	rs9445291	T	C	T	C	-0.0337	-0.00914	0.1411	0.139155	FALSE	FALSE	FALSE	xaapK0	0.019921	0.646542	486484	sepsis	TRUE	reported	textfile	0.0072	3.28E-06	7250	M32757.m	TRUE	reported	64n1Z4	2	TRUE	0.003013	21.90156
7073	rs1163471	A	G	A	G	-0.0206	-0.00252	0.3458	0.351945	FALSE	FALSE	FALSE	sg7nOB	0.014375	0.860991	486484	sepsis	TRUE	reported	textfile	0.0044	2.84E-06	2278	M34289.m	TRUE	reported	KemvIC	2	TRUE	0.009531	21.90018
4570	rs7946041	A	G	A	G	0.0454	0.066837	0.0211	0.024901	FALSE	FALSE	FALSE	ILOOFw	0.043976	0.128551	486484	sepsis	TRUE	reported	textfile	0.0097	2.75E-06	6042	M32739.m	TRUE	reported	TpvRBe	2	TRUE	0.003613	21.89901
6502	rs4695275	A	T	A	T	0.1561	0.008036	0.1015	0.102301	FALSE	TRUE	FALSE	HNc7C9	0.022678	0.723066	486484	sepsis	TRUE	reported	textfile	0.0333	2.73E-06	581	M33782.m	TRUE	reported	SAGYGx	2	TRUE	0.036443	21.89877
1559	rs2938330	T	C	T	C	-0.0117	0.020113	0.8105	0.856821	FALSE	FALSE	FALSE	Ke0sNh	0.01949	0.302074	486484	sepsis	TRUE	reported	textfile	0.0025	2.15E-06	7799	M10642.m	TRUE	reported	ZINwVb	2	TRUE	0.0028	21.89678
3839	rs4671635	A	G	A	G	-0.0117	0.019933	0.2317	0.217848	FALSE	FALSE	FALSE	tRX2u0	0.016552	0.228476	486484	sepsis	TRUE	reported	textfile	0.0025	2.35E-06	7365	M32348.m	TRUE	reported	ruBgwQ	2	TRUE	0.002965	21.89645
3682	rs6583751	C	G	C	G	-0.0117	0.015716	0.5964	0.591266	FALSE	TRUE	FALSE	rGHJcj	0.013945	0.259753	486484	sepsis	TRUE	reported	textfile	0.0025	3.60E-06	7356	M32198.m	TRUE	reported	6P1Nno	2	TRUE	0.002969	21.89645
3694	rs1237812	T	C	T	C	0.0117	0.000269	0.8196	0.822232	FALSE	FALSE	FALSE	agPgtF	0.017889	0.988019	486484	sepsis	TRUE	reported	textfile	0.0025	3.91E-06	7349	M32315.m	TRUE	reported	hMRjcV	2	TRUE	0.002971	21.89644
3943	rs1023682	T	C	T	C	-0.0585	-0.0561	0.9564	0.938653	FALSE	FALSE	FALSE	N6UQnQ	0.028638	0.050097	486484	sepsis	TRUE	reported	textfile	0.0125	2.68E-06	7346	M32425.m	TRUE	reported	FEcGHY	2	TRUE	0.002973	21.89644
4972	rs8038921	A	C	A	C	0.0117	0.029231	0.1345	0.136059	FALSE	FALSE	FALSE	bKmDMa	0.020053	0.144919	486484	sepsis	TRUE	reported	textfile	0.0025	4.67E-06	7344	M32910.m	TRUE	reported	nAuPzO	2	TRUE	0.002973	21.89644
7062	rs4888479	A	G	A	G	0.0117	-0.01145	0.8234	0.82803	FALSE	FALSE	FALSE	uthruC	0.018157	0.528406	486484	sepsis	TRUE	reported	textfile	0.0025	4.24E-06	7316	M34283.m	TRUE	reported	ftTuRU	2	TRUE	0.002985	21.89641
4676	rs2828489	T	C	T	C	0.0234	0.004362	0.6364	0.641205	FALSE	FALSE	FALSE	xaapK0	0.014249	0.759532	486484	sepsis	TRUE	reported	textfile	0.005	2.31E-06	7250	M32757.m	TRUE	reported	64n1Z4	2	TRUE	0.003012	21.89636
2949	rs703654	A	G	A	G	-0.0117	0.006455	0.8216	0.815419	FALSE	FALSE	FALSE	pn11BC	0.017671	0.7149	486484	sepsis	TRUE	reported	textfile	0.0025	2.89E-06	7203	M21047.m	TRUE	reported	KJF8U4	2	TRUE	0.003032	21.89632
3223	rs1115739	T	C	T	C	0.0117	-0.01056	0.5826	0.575961	FALSE	FALSE	FALSE	I25GHB	0.013837	0.445285	486484	sepsis	TRUE	reported	textfile	0.0025	3.69E-06	7184	M22548.m	TRUE	reported	eBmTQD	2	TRUE	0.00304	21.8963
2724	rs1460542	A	C	A	C	0.0117	-0.0242	0.1671	0.169732	FALSE	FALSE	FALSE	H4yqSK	0.018195	0.183511	486484	sepsis	TRUE	reported	textfile	0.0025	2.93E-06	7182	M19362.m	TRUE	reported	w2PDEO	2	TRUE	0.00304	21.8963
5843	rs9370455	T	C	T	C	-0.0117	0.019472	0.8882	0.86007	FALSE	FALSE	FALSE	R5joEf	0.019675	0.32235	486484	sepsis	TRUE	reported	textfile	0.0025	2.35E-06	7118	M33380.m	TRUE	reported	kRvbfZ	2	TRUE	0.003068	21.89625
867	rs3135095	A	G	A	G	-0.0117	-0.0069	0.2993	0.31118	FALSE	FALSE	FALSE	Vg5OOR	0.01477	0.640231	486484	sepsis	TRUE	reported	textfile	0.0025	3.92E-06	7063	M01361.m	TRUE	reported	XnrS00	2	TRUE	0.003091	21.8962
8118	rs4693661	A	G	A	G	0.0117	-0.00053	0.1547	0.156974	FALSE	FALSE	FALSE	nNbXBS	0.0189	0.977767	486484	sepsis	TRUE	reported	textfile	0.0025	3.24E-06	5768	M35326.m	TRUE	reported	UNDorn	2	TRUE	0.003783	21.89481
2994	rs2143069	A	G	A	G	-0.0234	-0.02742	0.4261	0.424167	FALSE	FALSE	FALSE	xmzJ5F	0.014008	0.050263	486484	sepsis	TRUE	reported	textfile	0.005	2.64E-06	5436	M21184.m	TRUE	reported	xgwhDp	2	TRUE	0.004013	21.89434
8872	rs7500975	A	G	A	G	-0.0833	-0.05719	0.0376	0.046253	FALSE	FALSE	FALSE	nWuK1Q	0.033102	0.084039	486484	sepsis	TRUE	reported	textfile	0.0178	2.87E-06	6511	M36850.m	TRUE	reported	2BA0SE	2	TRUE	0.003352	21.89357
60	rs1711435	A	G	A	G	0.0248	-0.03934	0.9874	0.985399	FALSE	FALSE	FALSE	xmGuol	0.056835	0.488832	486484	sepsis	TRUE	reported	textfile	0.0053	3.52E-06	7355	M00054.m	TRUE	reported	GW50Ri	2	TRUE	0.002968	21.88938

1	rs1159660	A	G	A	G	0.0323	-0.02926	0.0236	0.047958	FALSE	FALSE	FALSE	0cebDu	0.032054	0.361325	486484	sepsis	TRUE	reported	textfile	0.0069	2.52E-06	1768	M00053.m	TRUE	reported	i8iuxX	2	TRUE	0.012243	21.88846
1818	rs1148709	A	G	A	G	0.248	0.021548	0.012	0.010444	FALSE	FALSE	FALSE	Yub5KV	0.06825	0.75222	486484	sepsis	TRUE	reported	textfile	0.053	2.88E-06	4084	M15335.m	TRUE	reported	mcnlNa	2	TRUE	0.005333	21.88461
2290	rs1104748	A	T	A	T	-0.044	0.011887	0.131	0.14564	FALSE	TRUE	FALSE	43Yh3h	0.019351	0.539018	486484	sepsis	TRUE	reported	textfile	0.0094	3.14E-06	1670	M16818.m	TRUE	reported	beR4IT	2	TRUE	0.01295	21.88413
2653	rs7546527	A	G	A	G	0.4654	0.054457	0.012	0.017741	FALSE	FALSE	FALSE	9GoQdh	0.052538	0.299951	486484	sepsis	TRUE	reported	textfile	0.0991	2.64E-06	257	M18474.m	TRUE	reported	p6Dq8p	2	TRUE	0.079034	21.88328
3199	rs2671471	A	G	A	G	-0.0131	0.042317	0.1641	0.158395	FALSE	FALSE	FALSE	UfLkBq	0.0188	0.024395	486484	sepsis	TRUE	reported	textfile	0.0028	2.67E-06	7329	M22481.m	TRUE	reported	kWMdiT	2	TRUE	0.002978	21.88306
2581	rs7805747	A	G	A	G	0.0131	-0.00092	0.2679	0.276613	FALSE	FALSE	FALSE	KHS91f	0.015243	0.951698	486484	sepsis	TRUE	reported	textfile	0.0028	3.77E-06	6939	M18349.m	TRUE	reported	PeVGil	2	TRUE	0.003145	21.88272
1664	rs7660017	A	G	A	G	-0.0131	0.031011	0.3331	0.334387	FALSE	FALSE	FALSE	YzX5ZD	0.014509	0.032569	486484	sepsis	TRUE	reported	textfile	0.0028	2.34E-06	6864	M12129.m	TRUE	reported	6tMfaW	2	TRUE	0.003179	21.88265
8340	rs1248083	A	G	A	G	-0.081	0.028659	0.0804	0.08789	FALSE	FALSE	FALSE	USZiKY	0.02424	0.237073	486484	sepsis	TRUE	reported	textfile	0.0173	2.89E-06	1098	M35635.m	TRUE	reported	HuVMRL	2	TRUE	0.019574	21.88195
4778	rs1169078	C	G	C	G	0.051	-0.01414	0.1054	0.126538	FALSE	TRUE	FALSE	MmZZoM	0.020869	0.497931	486484	sepsis	TRUE	reported	textfile	0.0109	3.10E-06	4274	M32800.m	TRUE	reported	70YSCO	2	TRUE	0.005096	21.88185
8357	rs2566347	A	C	A	C	-0.0262	-0.00935	0.2063	0.206069	FALSE	FALSE	FALSE	6VVCe4	0.01693	0.580724	486484	sepsis	TRUE	reported	textfile	0.0056	2.44E-06	5629	M35669.m	TRUE	reported	l0fk8X	2	TRUE	0.003874	21.88125
9009	rs7725624	A	C	A	C	-0.044	-0.02812	0.0865	0.103989	FALSE	FALSE	FALSE	EZQ1cW	0.022319	0.207629	486484	sepsis	TRUE	reported	textfile	0.0094	3.02E-06	1411	M37253.m	TRUE	reported	ddPpd8	2	TRUE	0.015291	21.87931
7474	rs1221918	T	C	T	C	0.0407	-0.00696	0.9516	0.781985	FALSE	FALSE	FALSE	SqfdcK	0.01661	0.675238	486484	sepsis	TRUE	reported	textfile	0.0087	2.67E-06	6929	M34416.m	TRUE	reported	sRGRIK	2	TRUE	0.003149	21.87887
7230	rs8187736	T	C	T	C	0.2254	-0.00122	0.0534	0.049328	FALSE	FALSE	FALSE	i1uJ7T	0.031878	0.969418	486484	sepsis	TRUE	reported	textfile	0.0481	2.73E-06	535	M34338.m	TRUE	reported	pi3mKW	2	TRUE	0.039427	21.87717
7708	rs1110278	A	G	A	G	0.0379	-0.00641	0.1984	0.202729	FALSE	FALSE	FALSE	qJW9lg	0.017132	0.708371	486484	sepsis	TRUE	reported	textfile	0.0081	3.16E-06	2474	M34878.m	TRUE	reported	42EOzB	2	TRUE	0.008772	21.87546
5148	rs3850788	A	G	A	G	-0.3028	0.060143	0.0225	0.027847	FALSE	FALSE	FALSE	L2DJzK	0.042362	0.155682	486484	sepsis	TRUE	reported	textfile	0.0647	2.84E-06	1574	M33173.m	TRUE	reported	hRG70D	2	TRUE	0.013725	21.87515
1504	rs1824583	A	G	A	G	-0.0145	0.020634	0.7906	0.781202	FALSE	FALSE	FALSE	rzkxmh	0.016594	0.213708	486484	sepsis	TRUE	reported	textfile	0.0031	3.38E-06	6941	M03127.m	TRUE	reported	3lrzhA	2	TRUE	0.003142	21.87195
3442	rs1089683	T	C	T	C	0.087	0.047154	0.9822	0.981026	FALSE	FALSE	FALSE	J1l92u	0.050353	0.349033	486484	sepsis	TRUE	reported	textfile	0.0186	3.04E-06	6715	M27710.m	TRUE	reported	9qjpIZ	2	TRUE	0.003248	21.87174
8007	rs1715617	T	C	T	C	0.029	0.029064	0.9129	0.877099	FALSE	FALSE	FALSE	Zj5bZH	0.020809	0.162493	486484	sepsis	TRUE	reported	textfile	0.0062	3.30E-06	6538	M35257.m	TRUE	reported	exssAk	2	TRUE	0.003335	21.87156
6436	rs2567470	T	C	T	C	-0.0248	-0.04788	0.1528	0.139505	FALSE	FALSE	FALSE	QCw5dg	0.019759	0.015387	486484	sepsis	TRUE	reported	textfile	0.0053	2.72E-06	1764	M33666.m	TRUE	reported	zYHmzg	2	TRUE	0.01226	21.87051
2343	rs343836	T	C	T	C	-0.1801	0.00191	0.9796	0.972192	FALSE	FALSE	FALSE	13lycQ	0.041783	0.96354	486484	sepsis	TRUE	reported	textfile	0.0385	2.83E-06	3393	M16823.m	TRUE	reported	u2seZ2	2	TRUE	0.006408	21.87006
6432	rs1696535	A	G	A	G	-0.0304	-0.00719	0.035	0.061796	FALSE	FALSE	FALSE	QCw5dg	0.028539	0.800964	486484	sepsis	TRUE	reported	textfile	0.0065	2.78E-06	6279	M33666.m	TRUE	reported	zYHmzg	2	TRUE	0.003472	21.86664
8866	rs1261199	T	C	T	C	-0.0627	-0.00373	0.245	0.224297	FALSE	FALSE	FALSE	nWuK1Q	0.016381	0.819665	486484	sepsis	TRUE	reported	textfile	0.0134	2.87E-06	1583	M36850.m	TRUE	reported	2BA0SE	2	TRUE	0.013642	21.86636
8684	rs1263025	T	C	T	C	-0.0262	-0.04411	0.0162	0.01692	FALSE	FALSE	FALSE	X2e8qc	0.053142	0.406485	486484	sepsis	TRUE	reported	textfile	0.0056	3.09E-06	1763	M36515.m	TRUE	reported	OaWQSH	2	TRUE	0.012264	21.8642
3662	rs6699507	T	C	T	C	-0.0159	-0.01068	0.818	0.803372	FALSE	FALSE	FALSE	fnjup6	0.017196	0.534451	486484	sepsis	TRUE	reported	textfile	0.0034	3.25E-06	7346	M32197.m	TRUE	reported	oQzaMk	2	TRUE	0.002968	21.86342
7799	rs1930131	T	C	T	C	-0.0159	0.004107	0.821	0.816858	FALSE	FALSE	FALSE	cQ5TvO	0.017729	0.816785	486484	sepsis	TRUE	reported	textfile	0.0034	2.35E-06	7344	M35127.m	TRUE	reported	BVxkfZ	2	TRUE	0.002969	21.86342
1077	rs1172419	A	G	A	G	0.0159	0.00265	0.8734	0.845608	FALSE	FALSE	FALSE	JLtEiZ	0.018945	0.888749	486484	sepsis	TRUE	reported	textfile	0.0034	2.29E-06	7333	M01572.m	TRUE	reported	TSSe28	2	TRUE	0.002973	21.86341
7613	rs1104079	A	T	A	T	0.0159	0.003094	0.7347	0.735226	FALSE	TRUE	FALSE	kVfKqT	0.015457	0.841359	486484	sepsis	TRUE	reported	textfile	0.0034	2.73E-06	7322	M34527.m	TRUE	reported	s4ti92	2	TRUE	0.002978	21.8634
6959	rs3773977	T	C	T	C	-0.0159	0.002536	0.7484	0.727965	FALSE	FALSE	FALSE	7Cw2XZ	0.015759	0.872147	486484	sepsis	TRUE	reported	textfile	0.0034	2.63E-06	7049	M34123.m	TRUE	reported	vrgMI3	2	TRUE	0.003093	21.86317
5954	rs1883349	A	G	A	G	-0.0318	-0.00892	0.1609	0.16332	FALSE	FALSE	FALSE	mDZrGw	0.018477	0.629374	486484	sepsis	TRUE	reported	textfile	0.0068	2.48E-06	6797	M33415.m	TRUE	reported	MzEP7R	2	TRUE	0.003207	21.86294
8933	rs719672	A	C	A	C	0.0917	0.014483	0.8137	0.809574	FALSE	FALSE	FALSE	ns6PV1	0.01745	0.406579	486484	sepsis	TRUE	reported	textfile	0.0196	2.91E-06	1672	M37097.m	TRUE	reported	ZYmYt4	2	TRUE	0.012922	21.86285
8014	rs7588373	A	C	A	C	0.0159	-0.01883	0.7215	0.722744	FALSE	FALSE	FALSE	Zj5bZH	0.015344	0.219774	486484	sepsis	TRUE	reported	textfile	0.0034	2.97E-06	6538	M35257.m	TRUE	reported	exssAk	2	TRUE	0.003334	21.86269
2779	rs1296193	A	C	A	C	0.0159	0.015281	0.7434	0.734211	FALSE	FALSE	FALSE	yYuamw	0.015456	0.32281	486484	sepsis	TRUE	reported	textfile	0.0034	3.77E-06	4844	M19414.m	TRUE	reported	GxSb5G	2	TRUE	0.004494	21.86035
4413	rs1172904	A	G	A	G	0.0173	0.009989	0.6296	0.624479	FALSE	FALSE	FALSE	NzBiVA	0.014186	0.481361	486484	sepsis	TRUE	reported	textfile	0.0037	3.67E-06	7354	M32672.m	TRUE	reported	YLIhHJ	2	TRUE	0.002964	21.856
8740	rs7574665	A	G	A	G	-0.2023	0.019617	0.9858	0.990157	FALSE	FALSE	FALSE	ERC6hD	0.070004	0.779304	486484	sepsis	TRUE	reported	textfile	0.0432	2.79E-06	589	M36559.m	TRUE	reported	n8waJF	2	TRUE	0.035895	21.85481
5164	rs7120657	T	G	T	G	-0.6419	0.009312	0.0329	0.024918	FALSE	FALSE	FALSE	L2DJzK	0.044071	0.832661	486484	sepsis	TRUE	reported	textfile	0.1355	2.17E-06	76	M33173.m	TRUE	reported	hRG70D	2	TRUE	0.22797	21.85115
7237	rs1188240	A	C	A	C	0.0721	0.028831	0.5129	0.509374	FALSE	FALSE	FALSE	RIcE3J	0.013673	0.034981	486484	sepsis	TRUE	reported	textfile	0.0154	2.93E-06	633	M34339.m	TRUE	reported	QQrwdm	2	TRUE	0.033469	21.85017
4156	rs9682960	T	C	T	C	-0.0388	-0.03226	0.8843	0.878798	FALSE	FALSE	FALSE	CrVaiQ	0.020953	0.123695	486484	sepsis	TRUE	reported	textfile	0.0083	3.36E-06	7361	M32553.m	TRUE	reported	HsKdAI	2	TRUE	0.00296	21.84687
484	rs4297577	T	C	T	C	0.0977	0.01788	0.0384	0.052989	FALSE	FALSE	FALSE	ScJVI8	0.030493	0.557634	486484	sepsis	TRUE	reported	textfile	0.0209	2.94E-06	7371	M00542.m	TRUE	reported	Sa1L04	2	TRUE	0.002956	21.84634
4259	rs1162913	T	C	T	C	-0.0201	0.003053	0.1656	0.172492	FALSE	FALSE	FALSE	ylV4bp	0.018173	0.866601	486484	sepsis	TRUE	reported	textfile	0.0043	3.09E-06	7337	M32632.m	TRUE	reported	hqHJoU	2	TRUE	0.002969	21.84423
4279	rs7541494	T	C	T	C	-0.0201	0.021631	0.8786	0.880918	FALSE	FALSE	FALSE	ylV4bp	0.021139	0.306177	486484	sepsis	TRUE	reported	textfile	0.0043	3.45E-06	7337	M32632.m	TRUE	reported	hqHJoU	2	TRUE	0.002969	21.84423
2267	rs1294991	T	C	T	C	-0.0201	-0.00332	0.584	0.575774	FALSE	FALSE	FALSE	Mjgi2R	0.013831	0.810424	486484	sepsis	TRUE	reported	textfile	0.0043	2.36E-06	7064	M16634.m	TRUE	reported	XO0GV7	2	TRUE	0.003084	21.844
7431	rs4358841	A	G	A	G	0.079	0.033575	0.9839	0.983919	FALSE	FALSE	FALSE	NZPe5A	0.054921	0.540982	486484	sepsis	TRUE	reported	textfile	0.0169	3.00E-06	5689	M34400.m	TRUE	reported	OyPwYO	2	TRUE	0.003826	21.84379
4688	rs1326601	A	C	A	C	-0.0201	-0.00129	0.5953	0.579064	FALSE	FALSE	FALSE	MV108h	0.013839	0.925715	486484	sepsis	TRUE	reported	textfile	0.0043	2.35E-06	6642	M32758.m	TRUE	reported	btjNXQ	2	TRUE	0.003279	21.84361
2769	rs1773930	A	G	A	G	-0.0215	-0.03138	0.845	0.803066	FALSE	FALSE	FALSE	JWylTj	0.017133	0.066988	486484	sepsis	TRUE	reported	textfile	0.0046	2.29E-06	6385	M19396.m	TRUE	reported	6S7kzt	2	TRUE	0.00341	21.83862
3629	rs7859457	T	C	T	C	-0.0519	-0.03004	0.3974	0.417204	FALSE	FALSE	FALSE	gq4ISJ	0.013859	0.030202	486484	sepsis	TRUE	reported	textfile	0.0111	2.93E-06	1760	M31591.m	TRUE	reported	wjwl4J	2	TRUE	0.012269	21.8371
7391	rs332176	A	G	A	G	-0.0215	-0.02443	0.7408	0.743758	FALSE	FALSE	FALSE	AW6M5u	0.015637	0.118237	486484	sepsis	TRUE	reported	textfile	0.0046	2.52E-06	5195	M34389.m	TRUE	reported	6ZYYQK	2	TRUE	0.004187	21.83705
789	rs1235345	A	G	A	G	-0.0229	0.00333	0.0373	0.046925	FALSE	FALSE	FALSE	dyT79I	0.032405	0.918144	486484	sepsis	TRUE	reported	textfile	0.0049	3.12E-06	7352	M01336.m	TRUE	reported	OemVJ2	2	TRUE	0.002962	21.83537
5589	rs9363575	T	C	T	C	-0.2507	0.018507	0.0374	0.074012	FALSE	FALSE	FALSE	q32d4E	0.028503	0.516142	486484	sepsis	TRUE	reported	textfile	0.0536	2.85E-06	1017	M33178.m	TRUE	reported	cS3BwT	2	TRUE	0.021058	21.83354
8437	rs4978408	A	G	A	G	-0.0243	0.001289	0.066	0.069424	FALSE	FALSE	FALSE	Nvt7pI	0.026892	0.961775	486484	sepsis	TRUE	reported	textfile	0.0052	3.11E-06	7358	M35718.m	TRUE	reported	WkA5Gq	2	TRUE	0.002959	21.83171
6582	rs1700632	A	G	A	G	-0.1619	0.028133	0.9636	0.962626	FALSE	FALSE	FALSE	XO3rXq	0.036326	0.438659	486484	sepsis	TRUE	reported	textfile	0.0345	2.62E-06	228	M33877.m	TRUE	reported	p2sETg	2	TRUE	0.08808	21.82876
3561	rs1160305	A	T	A	T	0.1122	0.026048	0.0414	0.036855	FALSE	TRUE	FALSE	IPkHXk	0.036577	0.476381	486484	sepsis	TRUE	reported	textfile	0.024	2.84E-06	1558	M31522.m	TRUE	reported	jdWe3V	2	TRUE	0.013834	21.82757
8569	rs4322806	C	G	C	G	0.0813	0.012808	0.037	0.039902	FALSE	TRUE	FALSE	Yjjldt	0.034841	0.713162	486484	sepsis	TRUE	reported	textfile	0.0174	2.94E-06	7054	M36098.m	TRUE	reported	U2ORsF	2	TRUE	0.003085	21.82526
6897	rs2992439	A	G	A	G	-0.0271	-0.00096	0.7289	0.724873	FALSE	FALSE	FALSE	MN2lbE	0.015635	0.950873	486484	sepsis	TRUE	reported	textfile	0.0058	3.01E-06	4961	M34106.m	TRUE	reported	q4zXEz	2	TRUE	0.004381	21.82265
7208	rs1314751	T	C	T	C	0.787	-0.00136	0.028	0.098721	FALSE	FALSE	FALSE	i1uJ7T	0.022926	0.95281	486484	sepsis	TRUE	reported	textfile	0.167	2.45E-06	115	M34338.m	TRUE	reported	pi3mKW	2	TRUE	0.161859	21.82213
6451	rs1428261	T	C	T	C	0.0285	-0.01111	0.6371	0.635909	FALSE	FALSE	FALSE	OFIEwq	0.014328	0.438002	486484	sepsis	TRUE	reported	textfile	0.0061	3.25E-06	5694	M33675.m	TRUE	reported	T2BzXG	2	TRUE	0.003819	21.82114
2618	rs884648	C	G	C	G	-0.0327	0.008726	0.4032	0.414189	FALSE	TRUE	FALSE	OiiDPr	0.01394	0.531364	486484	sepsis	TRUE	reported	textfile	0.007	2.91E-06	7322	M18392.m	TRUE	reported	ScUw6o	2	TRUE	0.002972	21.81628
6060	rs1326231	A	G	A	G	0.0668	0.000796	0.9831	0.976795	FALSE	FALSE	FALSE	P4BuMD	0.04589	0.986166	486484	sepsis	TRUE	reported	textfile	0.0143	3.03E-06	7365	M33441.m	TRUE	reported	kqrc1d	2	TRUE	0.002954	21.81539
2634	rs2068592	A	G	A	G	0.071	-0.01066	0.0144	0.017564	FALSE	FALSE	FALSE	UxuT5B	0.052101	0.837872	486484	sepsis	TRUE	reported	textfile	0.0152	2.96E-06	7367	M18467.m	TRUE	reported	eGQj6o	2	TRUE	0.002953	21.81281
3429	rs6024969	T	C	T	C	-0.3856	0.074924	0.9884	0.988969	FALSE	FALSE	FALSE	HHQf8w	0.065037	0.249319	486484	sepsis	TRUE	reported	textfile	0.0825	2.97E-06	1309	M27531.m	TRUE	reported	3vTluK	2	TRUE	0.016415	21.81233
634	rs2391088	A	G	A	G	-0.0341	-0.01991	0.6873	0.679926	FALSE	FALSE	FALSE	F6Dn8H	0.014653	0.174273	486484	sepsis	TRUE	reported	textfile	0.0073	2.92E-06	4934	M01114.m	TRUE	reported	dVHatZ	2	TRUE	0.004403	21.81157
817	rs9543361	T	C	T	C	-0.0514	-0.04527	0.0422	0.043294	FALSE	FALSE	FALSE	J58DI0	0.033708	0.179242	486484	sepsis	TRUE	reported	textfile	0.011	3.18E-06	1755	M01356.m	TRUE	reported	zk9AMB	2	TRUE	0.012288	21.8095
4423	rs7556040	T	G	T	G	-0.0411	-0.00662	0.9548	0.943421	FALSE	FALSE	FALSE	NzBiVA	0.029705	0.823586	486484	sepsis	TRUE	reported	textfile	0.0088	3.38E-06	7354	M32672.m	TRUE	reported	YLIhHJ	2	TRUE	0.002957	21.80721
3630	rs807019	T	C	T	C	0.0425	0.009236	0.1496	0.156286	FALSE	FALSE	FALSE	gq4ISJ	0.018874	0.624571	486484	sepsis	TRUE	reported	textfile	0.0091	3.18E-06	7338	M31591.m	TRUE	reported	wjwl4J	2	TRUE	0.002964	21.80603
4994	rs2324913	T	C	T	C	-0.0411	0.001014	0.12	0.1009	FALSE	FALSE	FALSE	OsqE1P	0.023263	0.965229	486484	sepsis	TRUE	reported	textfile	0.0088	3.31E-06	5425	M33084.m	TRUE	reported	VZYh6f	2	TRUE	0.004005	21.8051
6475	rs1709163	A	G	A	G	0.173	-0.06048	0.027	0.031628	FALSE	FALSE	FALSE	ecwVg8	0.039094	0.121888	486484	sepsis	TRUE	reported	textfile	0.037	2.93E-06	752	M33751.m	TRUE	reported	NnvbIQ	2	TRUE	0.02825	21.8038
8634	rs2278161	T	C	T	C	-0.0341	-0.01062	0.7661	0.764711	FALSE	FALSE	FALSE	4cZjpQ	0.0161	0.509412	486484	sepsis	TRUE	reported	textfile	0.0073	3.25E-06	2400	M36230.m	TRUE	reported	TPUeNs	2	TRUE	0.00901	21.80223
8732	rs1340847	A	G	A	G	0.0594	0.018327	0.1647	0.182001	FALSE	FALSE	FALSE	ERC6hD	0.017724	0.301142	486484	sepsis	TRUE	reported	textfile	0.0127	2.98E-06	589	M36559.m	TRUE	reported	n8waJF	2	TRUE	0.035811	21.80159
1183	rs808686	A	G	A	G	0.0598	0.010772	0.6086	0.510496	FALSE	FALSE	FALSE	FgZx4a	0.013708	0.431961	486484	sepsis	TRUE	reported	textfile	0.0128	3.01E-06	1567	M01640.m	TRUE	reported	hNPAJ8	2	TRUE	0.013737	21.79856
4925	rs2501628	A	C	A	C	0.0299	0.010461	0.3167	0.309589	FALSE	FALSE	FALSE	clhN6v	0.014772	0.478847	486484	sepsis	TRUE	reported	textfile	0.0064	2.85E-06	1550	M32869.m	TRUE	reported	BzloWk	2	TRUE	0.013886	21.79825
4727	rs2592288	T	C	T	C	-0.092	0.002979	0.9423	0.954738	FALSE	FALSE	FALSE	fz63r4	0.033129	0.928347	486484	sepsis	TRUE	reported	textfile	0.0197	3.15E-06	2537	M32762.m	TRUE	reported	kgxlBo	2	TRUE	0.008523	21.79218
6352	rs8036131	T	G	T	G	0.0608	-0.03883	0.7378	0.758441	FALSE	FALSE	FALSE	qLUY00	0.015969	0.015042	486484	sepsis	TRUE	reported	textfile	0.013	3.15E-06	470	M33610.m	TRUE	reported	8cAJbB	2	TRUE	0.04447	21.78053
7158	rs7036822	A	G	A	G	-0.0663	-0.01547	0.807	0.796753	FALSE	FALSE	FALSE	PnBT0M	0.017079	0.36502	486484	sepsis	TRUE	reported	textfile	0.0142	3.12E-06	1612	M34327.m	TRUE	reported	VUt4Q7	2	TRUE	0.013343	21.77265
619	rs4982495	T	C	T	C	-0.0084	-0.00276	0.4275	0.426609	FALSE	FALSE	FALSE	BYGzq4	0.013843	0.842208	486484	sepsis	TRUE	reported	textfile	0.0018	3.27E-06	7367	M01110.m	TRUE	reported	oZiXqP	2	TRUE	0.002947	21.77187
1323	rs1018309	A	G	A	G	-0.0084	-0.00693	0.6391	0.616611	FALSE	FALSE	FALSE	LDUJbh	0.014103	0.622933	486484	sepsis	TRUE	reported	textfile	0.0018	2.44E-06	7367	M01898.m	TRUE	reported	JRSLeH	2	TRUE	0.002947	21.77187
9023	rs1193885	T	C	T	C	-0.0084	0.005181	0.3657	0.368493	FALSE	FALSE	FALSE	df0MCM	0.014159	0.714401	486484	sepsis	TRUE	reported	textfile	0.0018	1.59E-06	7366	M37506.m	TRUE	reported	iUfr4u	2	TRUE	0.002948	21.77186
2116	rs1266253	A	T	A	T	-0.0056	-0.03448	0.2919	0.301235	FALSE	TRUE	FALSE	t7SQ8V	0.014894	0.020612	486484	sepsis	TRUE	reported	textfile	0.0012	6.66E-06	7365	M15650.m	TRUE	reported	QQT7qA	2	TRUE	0.002948	21.77186
2122	rs6108469	A	G	A	G	0.0056	-0.00534	0.7569	0.751776	FALSE	FALSE	FALSE	t7SQ8V	0.015953	0.738033	486484	sepsis	TRUE	reported	textfile	0.0012	6.36E-06	7365	M15650.m	TRUE	reported	QQT7qA	2	TRUE	0.002948	21.77186
3821	rs1188565	T	C	T	C	-0.0084	-0.00888	0.485	0.476291	FALSE	FALSE	FALSE	tRX2u0	0.013682	0.516363	486484	sepsis	TRUE	reported	textfile	0.0018	1.76E-06	7365	M32348.m	TRUE	reported	ruBgwQ	2	TRUE	0.002948	21.77186
1043	rs1404373	T	C	T	C	-0.0084	0.007402	0.6525	0.648553	FALSE	FALSE	FALSE	tRtQ2e	0.014341	0.605756	486484	sepsis	TRUE	reported	textfile	0.0018	2.92E-06	7364	M01564.m	TRUE	reported	Tbg0zM	2	TRUE	0.002949	21.77186
3986	rs6560147	T	C	T	C	0.0126	0.008183	0.1449	0.139525	FALSE	FALSE	FALSE	XdJbEU	0.019666	0.677322	486484	sepsis	TRUE	reported	textfile	0.0027	4.20E-06	7364	M32452.m	TRUE	reported	V5xWKi	2	TRUE	0.002949	21.77186
3990	rs7587616	A	G	A	G	-0.0084	0.020203	0.4743	0.479246	FALSE	FALSE	FALSE	XdJbEU	0.013723	0.140969	486484	sepsis	TRUE	reported	textfile	0.0018	4.71E-06	7364	M32452.m	TRUE	reported	V5xWKi	2	TRUE	0.002949	21.77186
3636	rs1049383	T	G	T	G	-0.0406	-0.01214	0.7644	0.764681	FALSE	FALSE	FALSE	Vq2efp	0.01613	0.451748	486484	sepsis	TRUE	reported	textfile	0.0087	3.20E-06	7363	M31787.m	TRUE	reported	krl4Tw	2	TRUE	0.002949	21.77186
3998	rs1023115	A	G	A	G	-0.0084	0.021679	0.5301	0.53845	FALSE	FALSE	FALSE	V9AtId	0.013709	0.113801	486484	sepsis	TRUE	reported	textfile	0.0018	2.68E-06	7363	M32489.m	TRUE	reported	8B8S8a	2	TRUE	0.002949	21.77186
4006	rs1360501	T	C	T	C	-0.0084	-0.00265	0.6909	0.682399	FALSE	FALSE	FALSE	V9AtId	0.014683	0.856994	486484	sepsis	TRUE	reported	textfile	0.0018	3.49E-06	7363	M32489.m	TRUE	reported	8B8S8a	2	TRUE	0.002949	21.77186
4284	rs1022407	T	C	T	C	-0.0084	-0.01275	0.2481	0.250547	FALSE	FALSE	FALSE	0tjjPC	0.015847	0.421025	486484	sepsis	TRUE	reported	textfile	0.0018	4.69E-06	7362	M32634.m	TRUE	reported	nJJFkX	2	TRUE	0.002949	21.77186
4292	rs1888108	A	G	A	G	0.0084	0.024174	0.6614	0.645644	FALSE	FALSE	FALSE	0tjjPC	0.014328	0.091567	486484	sepsis	TRUE	reported	textfile	0.0018	3.11E-06	7362	M32634.m	TRUE	reported	nJJFkX	2	TRUE	0.002949	21.77186
4314	rs928563	T	C	T	C	0.0084	-0.00996	0.737	0.741224	FALSE	FALSE	FALSE	0tjjPC	0.015615	0.523629	486484	sepsis	TRUE	reported	textfile	0.0018	3.74E-06	7362	M32634.m	TRUE	reported	nJJFkX	2	TRUE	0.002949	21.77186
5024	rs1716881	T	G	T	G	-0.0084	0.008877	0.134	0.152734	FALSE	FALSE	FALSE	BNnOvt	0.019047	0.641165	486484	sepsis	TRUE	reported	textfile	0.0018	3.68E-06	7362	M33132.m	TRUE	reported	rHcCPG	2	TRUE	0.002949	21.77186
6871	rs1926815	A	C	A	C	-0.0084	0.031045	0.1804	0.220991	FALSE	FALSE	FALSE	kFWZjs	0.016426	0.058767	486484	sepsis	TRUE	reported	textfile	0.0018	1.85E-06	7362	M34062.m	TRUE	reported	eLONsg	2	TRUE	0.002949	21.77186
452	rs1232423	T	C	T	C	0.0042	0.005834	0.5209	0.517075	FALSE	FALSE	FALSE	cl8ucN	0.013669	0.669494	486484	sepsis	TRUE	reported	textfile	9.00E-04	8.68E-06	7361	M00513.m	TRUE	reported	Ian0Q8	2	TRUE	0.00295	21.77186
3887	rs2324600	T	C	T	C	0.0266	0.018322	0.1179	0.119639	FALSE	FALSE	FALSE	bmXxuz	0.021052	0.38413	486484	sepsis	TRUE	reported	textfile	0.0057	3.47E-06	7358	M32405.m	TRUE	reported	A9BFZF	2	TRUE	0.002951	21.77186
1600	rs1229590	A	G	A	G	-0.0084	-0.00435	0.505	0.524709	FALSE	FALSE	FALSE	zP3C2v	0.013728	0.751438	486484	sepsis	TRUE	reported	textfile	0.0018	5.23E-06	7356	M12035.m	TRUE	reported	1Pooe3	2	TRUE	0.002952	21.77186
1619	rs899227	A	G	A	G	-0.0084	-0.00404	0.3958	0.397276	FALSE	FALSE	FALSE	zP3C2v	0.014048	0.773877	486484	sepsis	TRUE	reported	textfile	0.0018	2.97E-06	7356	M12035.m	TRUE	reported	1Pooe3	2	TRUE	0.002952	21.77186
1738	rs1289240	T	C	T	C	-0.0084	0.012915	0.5712	0.576068	FALSE	FALSE	FALSE	nFLpAP	0.013822	0.350102	486484	sepsis	TRUE	reported	textfile	0.0018	2.41E-06	7356	M12774.m	TRUE	reported	yOSQTj	2	TRUE	0.002952	21.77186
1743	rs2451284	T	C	T	C	-0.0084	0.005031	0.4026	0.399269	FALSE	FALSE	FALSE	nFLpAP	0.014051	0.720324	486484	sepsis	TRUE	reported	textfile	0.0018	2.75E-06	7356	M12774.m	TRUE	reported	yOSQTj	2	TRUE	0.002952	21.77186
17	rs1075041	T	G	T	G	0.0042	0.003275	0.3959	0.417226	FALSE	FALSE	FALSE	xmGuol	0.013912	0.813874	486484	sepsis	TRUE	reported	textfile	9.00E-04	2.77E-06	7355	M00054.m	TRUE	reported	GW50Ri	2	TRUE	0.002952	21.77186
21	rs1082205	T	C	T	C	0.0042	-0.01323	0.5141	0.530739	FALSE	FALSE	FALSE	xmGuol	0.013694	0.334012	486484	sepsis	TRUE	reported	textfile	9.00E-04	2.65E-06	7355	M00054.m	TRUE	reported	GW50Ri	2	TRUE	0.002952	21.77186
27	rs1093104	T	C	T	C	-0.0042	-0.00341	0.5756	0.575961	FALSE	FALSE	FALSE	xmGuol	0.013921	0.806312	486484	sepsis	TRUE	reported	textfile	9.00E-04	3.36E-06	7355	M00054.m	TRUE	reported	GW50Ri	2	TRUE	0.002952	21.77186
40	rs1260326	T	C	T	C	0.0042	-0.00771	0.404	0.395705	FALSE	FALSE	FALSE	xmGuol	0.01397	0.581085	486484	sepsis	TRUE	reported	textfile	9.00E-04	2.74E-06	7355	M00054.m	TRUE	reported	GW50Ri	2	TRUE	0.002952	21.77186
42	rs1300809	A	C	A	C	-0.0042	0.002454	0.4038	0.411536	FALSE	FALSE	FALSE	xmGuol	0.013945	0.860313	486484	sepsis	TRUE	reported	textfile	9.00E-04	3.32E-06	7355	M00054.m	TRUE	reported	GW50Ri	2	TRUE	0.002952	21.77186
45	rs1330918	A	C	A	C	0.0042	-0.01322	0.6132	0.607339	FALSE	FALSE	FALSE	xmGuol	0.013981	0.344301	486484	sepsis	TRUE	reported	textfile	9.00E-04	2.21E-06	7355	M00054.m	TRUE	reported	GW50Ri	2	TRUE	0.002952	21.77186
50	rs1501803	T	C	T	C	0.0042	-0.00032	0.4152	0.409709	FALSE	FALSE	FALSE	xmGuol	0.014353	0.982248	486484	sepsis	TRUE	reported	textfile	9.00E-04	4.57E-06	7355	M00054.m	TRUE	reported	GW50Ri	2	TRUE	0.002952	21.77186
51	rs1508513	C	G	C	G	0.0042	0.003387	0.6354	0.638855	FALSE	TRUE	FALSE	xmGuol	0.014252	0.812142	486484	sepsis	TRUE	reported	textfile	9.00E-04	2.36E-06	7355	M00054.m	TRUE	reported	GW50Ri	2	TRUE	0.002952	21.77186
71	rs2126498	T	G	T	G	-0.0042	-0.00554	0.6176	0.601139	FALSE	FALSE	FALSE	xmGuol	0.013988	0.691976	486484	sepsis	TRUE	reported	textfile	9.00E-04	2.84E-06	7355	M00054.m	TRUE	reported	GW50Ri	2	TRUE	0.002952	21.77186
72	rs2178260	T	C	T	C	0.0042	-0.00325	0.4251	0.424139	FALSE	FALSE	FALSE	xmGuol	0.013853	0.814262	486484	sepsis	TRUE	reported	textfile	9.00E-04	3.59E-06	7355	M00054.m	TRUE	reported	GW50Ri	2	TRUE	0.002952	21.77186
95	rs2966079	T	C	T	C	-0.0042	4.55E-05	0.5425	0.549892	FALSE	FALSE	FALSE	xmGuol	0.013716	0.997353	486484	sepsis	TRUE	reported	textfile	9.00E-04	2.53E-06	7355	M00054.m	TRUE	reported	GW50Ri	2	TRUE	0.002952	21.77186
106	rs3927971	T	C	T	C	0.0042	-0.00125	0.5178	0.512394	FALSE	FALSE	FALSE	xmGuol	0.013722	0.927579	486484	sepsis	TRUE	reported	textfile	9.00E-04	8.98E-06	7355	M00054.m	TRUE	reported	GW50Ri	2	TRUE	0.002952	21.77186
115	rs4684599	T	C	T	C	-0.0042	-0.00582	0.6319	0.628567	FALSE	FALSE	FALSE	xmGuol	0.01416	0.680882	486484	sepsis	TRUE	reported	textfile	9.00E-04	2.89E-06	7355	M00054.m	TRUE	reported	GW50Ri	2	TRUE	0.002952	21.77186
129	rs575257	A	G	A	G	-0.0042	-0.0225	0.6005	0.605691	FALSE	FALSE	FALSE	xmGuol	0.013985	0.107651	486484	sepsis	TRUE	reported	textfile	9.00E-04	3.51E-06	7355	M00054.m	TRUE	reported	GW50Ri	2	TRUE	0.002952	21.77186
139	rs6517254	T	C	T	C	-0.0042	-0.01059	0.3971	0.397614	FALSE	FALSE	FALSE	xmGuol	0.013997	0.449154	486484	sepsis	TRUE	reported	textfile	9.00E-04	3.12E-06	7355	M00054.m	TRUE	reported	GW50Ri	2	TRUE	0.002952	21.77186
143	rs6685230	T	C	T	C	-0.0042	-0.02758	0.3976	0.411012	FALSE	FALSE	FALSE	xmGuol	0.014582	0.058537	486484	sepsis	TRUE	reported	textfile	9.00E-04	7.78E-06	7355	M00054.m	TRUE	reported	GW50Ri	2	TRUE	0.002952	21.77186
150	rs688627	T	C	T	C	0.0042	0.010845	0.5878	0.59415	FALSE	FALSE	FALSE	xmGuol	0.013941	0.436608	486484	sepsis	TRUE	reported	textfile	9.00E-04	3.62E-06	7355	M00054.m	TRUE	reported	GW50Ri	2	TRUE	0.002952	21.77186
177	rs893922	A	G	A	G	0.0042	-0.00795	0.366	0.368748	FALSE	FALSE	FALSE	xmGuol	0.014191	0.5755	486484	sepsis	TRUE	reported	textfile	9.00E-04	3.95E-06	7355	M00054.m	TRUE	reported	GW50Ri	2	TRUE	0.002952	21.77186
179	rs928963	A	C	A	C	0.0042	0.006732	0.3985	0.399748	FALSE	FALSE	FALSE	xmGuol	0.014029	0.631322	486484	sepsis	TRUE	reported	textfile	9.00E-04	3.62E-06	7355	M00054.m	TRUE	reported	GW50Ri	2	TRUE	0.002952	21.77186
188	rs9541214	T	C	T	C	-0.0042	0.00219	0.653	0.652646	FALSE	FALSE	FALSE	xmGuol	0.014409	0.879204	486484	sepsis	TRUE	reported	textfile	9.00E-04	3.80E-06	7355	M00054.m	TRUE	reported	GW50Ri	2	TRUE	0.002952	21.77186
193	rs993927	T	G	T	G	0.0042	0.007233	0.3773	0.378752	FALSE	FALSE	FALSE	xmGuol	0.014089	0.607693	486484	sepsis	TRUE	reported	textfile	9.00E-04	2.64E-06	7355	M00054.m	TRUE	reported	GW50Ri	2	TRUE	0.002952	21.77186
196	rs9989564	A	G	A	G	-0.0042	-0.01269	0.5142	0.535082	FALSE	FALSE	FALSE	xmGuol	0.013678	0.353425	486484	sepsis	TRUE	reported	textfile	9.00E-04	3.10E-06	7355	M00054.m	TRUE	reported	GW50Ri	2	TRUE	0.002952	21.77186
819	rs1016016	A	G	A	G	0.0084	-0.00096	0.6329	0.605957	FALSE	FALSE	FALSE	2qgDgB	0.013986	0.945032	486484	sepsis	TRUE	reported	textfile	0.0018	3.78E-06	7355	M01358.m	TRUE	reported	asmXq0	2	TRUE	0.002952	21.77186
1191	rs1074032	T	C	T	C	0.0084	-0.00552	0.5935	0.590704	FALSE	FALSE	FALSE	Ckdgh1	0.013871	0.690863	486484	sepsis	TRUE	reported	textfile	0.0018	4.10E-06	7355	M01644.m	TRUE	reported	nKxz7B	2	TRUE	0.002952	21.77186
4031	rs1076249	A	C	A	C	0.0084	-0.00265	0.3563	0.356868	FALSE	FALSE	FALSE	SiYGYU	0.01428	0.852655	486484	sepsis	TRUE	reported	textfile	0.0018	2.25E-06	7355	M32492.m	TRUE	reported	GfQvpU	2	TRUE	0.002952	21.77186
4043	rs2414622	A	G	A	G	-0.0084	0.0022	0.6434	0.643726	FALSE	FALSE	FALSE	SiYGYU	0.014264	0.877445	486484	sepsis	TRUE	reported	textfile	0.0018	2.93E-06	7355	M32492.m	TRUE	reported	GfQvpU	2	TRUE	0.002952	21.77186
4048	rs4858696	T	C	T	C	-0.0084	0.003914	0.5733	0.562125	FALSE	FALSE	FALSE	SiYGYU	0.013827	0.777118	486484	sepsis	TRUE	reported	textfile	0.0018	2.15E-06	7355	M32492.m	TRUE	reported	GfQvpU	2	TRUE	0.002952	21.77186
4059	rs940597	T	C	T	C	-0.0084	-0.01251	0.3479	0.350514	FALSE	FALSE	FALSE	SiYGYU	0.014394	0.384665	486484	sepsis	TRUE	reported	textfile	0.0018	3.00E-06	7355	M32492.m	TRUE	reported	GfQvpU	2	TRUE	0.002952	21.77186
559	rs1254473	C	G	C	G	-0.0084	0.026771	0.8378	0.834536	FALSE	TRUE	FALSE	fNqkCW	0.018407	0.145833	486484	sepsis	TRUE	reported	textfile	0.0018	2.74E-06	7354	M00606.m	TRUE	reported	g9uwOn	2	TRUE	0.002953	21.77186
929	rs1261178	T	C	T	C	-0.007	0.003207	0.6267	0.646614	FALSE	FALSE	FALSE	tv99UN	0.01431	0.822667	486484	sepsis	TRUE	reported	textfile	0.0015	5.51E-06	7354	M01494.m	TRUE	reported	osDclp	2	TRUE	0.002953	21.77186
936	rs306676	A	G	A	G	0.0112	0.00609	0.1304	0.137508	FALSE	FALSE	FALSE	tv99UN	0.020038	0.761195	486484	sepsis	TRUE	reported	textfile	0.0024	2.48E-06	7354	M01494.m	TRUE	reported	osDclp	2	TRUE	0.002953	21.77186
2597	rs1768345	A	T	A	T	0.0112	-0.00866	0.8525	0.855375	FALSE	TRUE	FALSE	ruvJh6	0.019505	0.657211	486484	sepsis	TRUE	reported	textfile	0.0024	4.14E-06	7354	M18369.m	TRUE	reported	GFmwGk	2	TRUE	0.002953	21.77186
6771	rs4459845	A	C	A	C	0.0084	0.003335	0.8009	0.802465	FALSE	FALSE	FALSE	EIf6MQ	0.01717	0.845994	486484	sepsis	TRUE	reported	textfile	0.0018	5.08E-06	7354	M33955.m	TRUE	reported	Lxn4RF	2	TRUE	0.002953	21.77186
2469	rs6738713	T	C	T	C	-0.0308	-0.02382	0.9295	0.923169	FALSE	FALSE	FALSE	C5wkoN	0.025625	0.352687	486484	sepsis	TRUE	reported	textfile	0.0066	3.38E-06	7353	M17805.m	TRUE	reported	F5VXY0	2	TRUE	0.002953	21.77185
211	rs1050090	A	G	A	G	0.0042	-0.00331	0.3597	0.362908	FALSE	FALSE	FALSE	tbRL8J	0.014207	0.816012	486484	sepsis	TRUE	reported	textfile	9.00E-04	3.55E-06	7351	M00060.m	TRUE	reported	oOvRKT	2	TRUE	0.002954	21.77185
213	rs1076384	A	G	A	G	-0.0042	-0.00528	0.4283	0.422174	FALSE	FALSE	FALSE	tbRL8J	0.013825	0.702645	486484	sepsis	TRUE	reported	textfile	9.00E-04	3.45E-06	7351	M00060.m	TRUE	reported	oOvRKT	2	TRUE	0.002954	21.77185
215	rs1079858	A	G	A	G	-0.0042	-0.02103	0.4216	0.422428	FALSE	FALSE	FALSE	tbRL8J	0.013937	0.131279	486484	sepsis	TRUE	reported	textfile	9.00E-04	3.64E-06	7351	M00060.m	TRUE	reported	oOvRKT	2	TRUE	0.002954	21.77185
227	rs1114451	T	C	T	C	-0.0042	0.006657	0.4497	0.464968	FALSE	FALSE	FALSE	tbRL8J	0.014614	0.64876	486484	sepsis	TRUE	reported	textfile	9.00E-04	8.42E-06	7351	M00060.m	TRUE	reported	oOvRKT	2	TRUE	0.002954	21.77185
232	rs1186894	A	G	A	G	-0.0042	-0.01901	0.6435	0.638807	FALSE	FALSE	FALSE	tbRL8J	0.014251	0.182326	486484	sepsis	TRUE	reported	textfile	9.00E-04	4.63E-06	7351	M00060.m	TRUE	reported	oOvRKT	2	TRUE	0.002954	21.77185
239	rs1249870	A	G	A	G	-0.0042	0.00842	0.5998	0.586073	FALSE	FALSE	FALSE	tbRL8J	0.013927	0.54544	486484	sepsis	TRUE	reported	textfile	9.00E-04	2.86E-06	7351	M00060.m	TRUE	reported	oOvRKT	2	TRUE	0.002954	21.77185
242	rs1278312	T	C	T	C	-0.0042	-0.01487	0.4008	0.409264	FALSE	FALSE	FALSE	tbRL8J	0.013898	0.28461	486484	sepsis	TRUE	reported	textfile	9.00E-04	2.87E-06	7351	M00060.m	TRUE	reported	oOvRKT	2	TRUE	0.002954	21.77185
248	rs1360820	A	G	A	G	-0.0042	-0.00652	0.4963	0.511484	FALSE	FALSE	FALSE	tbRL8J	0.013712	0.634546	486484	sepsis	TRUE	reported	textfile	9.00E-04	2.15E-06	7351	M00060.m	TRUE	reported	oOvRKT	2	TRUE	0.002954	21.77185
263	rs174476	T	C	T	C	-0.0042	-0.00174	0.4174	0.419611	FALSE	FALSE	FALSE	tbRL8J	0.013849	0.900283	486484	sepsis	TRUE	reported	textfile	9.00E-04	5.70E-06	7351	M00060.m	TRUE	reported	oOvRKT	2	TRUE	0.002954	21.77185
279	rs2302423	A	G	A	G	0.0042	0.005929	0.55	0.549172	FALSE	FALSE	FALSE	tbRL8J	0.013717	0.665593	486484	sepsis	TRUE	reported	textfile	9.00E-04	2.82E-06	7351	M00060.m	TRUE	reported	oOvRKT	2	TRUE	0.002954	21.77185
280	rs2357713	T	G	T	G	-0.0042	-0.01036	0.447	0.449775	FALSE	FALSE	FALSE	tbRL8J	0.013864	0.454823	486484	sepsis	TRUE	reported	textfile	9.00E-04	3.00E-06	7351	M00060.m	TRUE	reported	oOvRKT	2	TRUE	0.002954	21.77185
297	rs2933284	T	C	T	C	0.0042	0.022281	0.3898	0.390073	FALSE	FALSE	FALSE	tbRL8J	0.014145	0.115219	486484	sepsis	TRUE	reported	textfile	9.00E-04	2.86E-06	7351	M00060.m	TRUE	reported	oOvRKT	2	TRUE	0.002954	21.77185
301	rs3802644	A	T	A	T	-0.0042	0.010393	0.4006	0.407724	FALSE	TRUE	FALSE	tbRL8J	0.013913	0.455093	486484	sepsis	TRUE	reported	textfile	9.00E-04	2.94E-06	7351	M00060.m	TRUE	reported	oOvRKT	2	TRUE	0.002954	21.77185
312	rs4681618	A	G	A	G	-0.0042	-0.00198	0.5793	0.581743	FALSE	FALSE	FALSE	tbRL8J	0.01386	0.886603	486484	sepsis	TRUE	reported	textfile	9.00E-04	3.15E-06	7351	M00060.m	TRUE	reported	oOvRKT	2	TRUE	0.002954	21.77185
313	rs4726308	T	G	T	G	-0.0042	0.003268	0.3944	0.392993	FALSE	FALSE	FALSE	tbRL8J	0.013989	0.815311	486484	sepsis	TRUE	reported	textfile	9.00E-04	3.35E-06	7351	M00060.m	TRUE	reported	oOvRKT	2	TRUE	0.002954	21.77185
320	rs4875397	A	G	A	G	-0.0042	-0.01348	0.5599	0.564212	FALSE	FALSE	FALSE	tbRL8J	0.01383	0.329723	486484	sepsis	TRUE	reported	textfile	9.00E-04	3.04E-06	7351	M00060.m	TRUE	reported	oOvRKT	2	TRUE	0.002954	21.77185
323	rs4921412	C	G	C	G	-0.0042	0.015192	0.3864	0.390391	FALSE	TRUE	FALSE	tbRL8J	0.013987	0.277407	486484	sepsis	TRUE	reported	textfile	9.00E-04	2.78E-06	7351	M00060.m	TRUE	reported	oOvRKT	2	TRUE	0.002954	21.77185
345	rs6982408	T	G	T	G	-0.0042	-0.02499	0.4435	0.440361	FALSE	FALSE	FALSE	tbRL8J	0.013768	0.069536	486484	sepsis	TRUE	reported	textfile	9.00E-04	2.32E-06	7351	M00060.m	TRUE	reported	oOvRKT	2	TRUE	0.002954	21.77185
351	rs7329192	T	C	T	C	-0.0042	0.025622	0.5277	0.520854	FALSE	FALSE	FALSE	tbRL8J	0.013697	0.061393	486484	sepsis	TRUE	reported	textfile	9.00E-04	3.17E-06	7351	M00060.m	TRUE	reported	oOvRKT	2	TRUE	0.002954	21.77185
354	rs757378	A	T	A	T	-0.0042	-0.00527	0.6095	0.608136	FALSE	TRUE	FALSE	tbRL8J	0.01404	0.707419	486484	sepsis	TRUE	reported	textfile	9.00E-04	3.57E-06	7351	M00060.m	TRUE	reported	oOvRKT	2	TRUE	0.002954	21.77185
357	rs759456	T	G	T	G	0.0042	-0.00731	0.6263	0.636335	FALSE	FALSE	FALSE	tbRL8J	0.014277	0.608506	486484	sepsis	TRUE	reported	textfile	9.00E-04	3.73E-06	7351	M00060.m	TRUE	reported	oOvRKT	2	TRUE	0.002954	21.77185
359	rs7625991	A	C	A	C	-0.0042	-0.0105	0.345	0.356789	FALSE	FALSE	FALSE	tbRL8J	0.014328	0.463505	486484	sepsis	TRUE	reported	textfile	9.00E-04	5.90E-06	7351	M00060.m	TRUE	reported	oOvRKT	2	TRUE	0.002954	21.77185
375	rs8003702	T	G	T	G	0.0042	-0.01358	0.536	0.536253	FALSE	FALSE	FALSE	tbRL8J	0.013738	0.322901	486484	sepsis	TRUE	reported	textfile	9.00E-04	2.45E-06	7351	M00060.m	TRUE	reported	oOvRKT	2	TRUE	0.002954	21.77185
376	rs8012205	A	G	A	G	-0.0042	0.008155	0.4966	0.491627	FALSE	FALSE	FALSE	tbRL8J	0.01372	0.552241	486484	sepsis	TRUE	reported	textfile	9.00E-04	2.56E-06	7351	M00060.m	TRUE	reported	oOvRKT	2	TRUE	0.002954	21.77185
380	rs849327	A	G	A	G	0.0042	0.003239	0.3492	0.342693	FALSE	FALSE	FALSE	tbRL8J	0.014401	0.822055	486484	sepsis	TRUE	reported	textfile	9.00E-04	3.45E-06	7351	M00060.m	TRUE	reported	oOvRKT	2	TRUE	0.002954	21.77185
382	rs899273	T	C	T	C	0.0042	-0.00466	0.5202	0.523539	FALSE	FALSE	FALSE	tbRL8J	0.013714	0.733838	486484	sepsis	TRUE	reported	textfile	9.00E-04	3.33E-06	7351	M00060.m	TRUE	reported	oOvRKT	2	TRUE	0.002954	21.77185
4098	rs1188542	A	T	A	T	0.0084	0.000492	0.2326	0.245114	FALSE	TRUE	FALSE	4rtLd0	0.015939	0.975384	486484	sepsis	TRUE	reported	textfile	0.0018	4.34E-06	7350	M32518.m	TRUE	reported	j7f1ER	2	TRUE	0.002954	21.77185
4101	rs1342020	A	C	A	C	-0.0084	-0.02889	0.7652	0.744007	FALSE	FALSE	FALSE	4rtLd0	0.015752	0.066673	486484	sepsis	TRUE	reported	textfile	0.0018	5.55E-06	7350	M32518.m	TRUE	reported	j7f1ER	2	TRUE	0.002954	21.77185
4114	rs345513	A	G	A	G	-0.0084	-0.01441	0.6132	0.613463	FALSE	FALSE	FALSE	4rtLd0	0.014012	0.303729	486484	sepsis	TRUE	reported	textfile	0.0018	2.86E-06	7350	M32518.m	TRUE	reported	j7f1ER	2	TRUE	0.002954	21.77185
4119	rs4724070	T	C	T	C	0.0084	-0.02833	0.2039	0.19916	FALSE	FALSE	FALSE	4rtLd0	0.017093	0.097391	486484	sepsis	TRUE	reported	textfile	0.0018	4.96E-06	7350	M32518.m	TRUE	reported	j7f1ER	2	TRUE	0.002954	21.77185
4128	rs906771	C	G	C	G	-0.0084	0.004772	0.3769	0.404024	FALSE	TRUE	FALSE	4rtLd0	0.013968	0.732647	486484	sepsis	TRUE	reported	textfile	0.0018	2.90E-06	7350	M32518.m	TRUE	reported	j7f1ER	2	TRUE	0.002954	21.77185
1871	rs1087510	A	G	A	G	-0.0042	0.008751	0.5422	0.542764	FALSE	FALSE	FALSE	v2KE89	0.013863	0.527882	486484	sepsis	TRUE	reported	textfile	9.00E-04	4.63E-06	7349	M15500.m	TRUE	reported	to2t7H	2	TRUE	0.002955	21.77185
1874	rs1108591	T	G	T	G	-0.0042	-0.01584	0.5541	0.548395	FALSE	FALSE	FALSE	v2KE89	0.01375	0.249202	486484	sepsis	TRUE	reported	textfile	9.00E-04	2.88E-06	7349	M15500.m	TRUE	reported	to2t7H	2	TRUE	0.002955	21.77185
1876	rs1114130	T	C	T	C	-0.0042	-0.0086	0.3401	0.340554	FALSE	FALSE	FALSE	v2KE89	0.014468	0.55245	486484	sepsis	TRUE	reported	textfile	9.00E-04	4.28E-06	7349	M15500.m	TRUE	reported	to2t7H	2	TRUE	0.002955	21.77185
1880	rs1160143	A	G	A	G	-0.0042	0.006345	0.3912	0.39571	FALSE	FALSE	FALSE	v2KE89	0.014004	0.65047	486484	sepsis	TRUE	reported	textfile	9.00E-04	2.89E-06	7349	M15500.m	TRUE	reported	to2t7H	2	TRUE	0.002955	21.77185
1898	rs1250070	A	G	A	G	-0.0042	-0.00786	0.3913	0.386085	FALSE	FALSE	FALSE	v2KE89	0.014043	0.575787	486484	sepsis	TRUE	reported	textfile	9.00E-04	8.56E-06	7349	M15500.m	TRUE	reported	to2t7H	2	TRUE	0.002955	21.77185
1915	rs1424937	A	G	A	G	0.0042	0.014831	0.4576	0.453934	FALSE	FALSE	FALSE	v2KE89	0.013744	0.280553	486484	sepsis	TRUE	reported	textfile	9.00E-04	2.32E-06	7349	M15500.m	TRUE	reported	to2t7H	2	TRUE	0.002955	21.77185
1931	rs1769463	A	G	A	G	-0.0042	0.012411	0.6289	0.625036	FALSE	FALSE	FALSE	v2KE89	0.014098	0.378669	486484	sepsis	TRUE	reported	textfile	9.00E-04	7.40E-06	7349	M15500.m	TRUE	reported	to2t7H	2	TRUE	0.002955	21.77185
1949	rs238340	A	C	A	C	-0.0042	-0.01107	0.569	0.588057	FALSE	FALSE	FALSE	v2KE89	0.013885	0.425379	486484	sepsis	TRUE	reported	textfile	9.00E-04	4.76E-06	7349	M15500.m	TRUE	reported	to2t7H	2	TRUE	0.002955	21.77185
1952	rs2432560	T	G	T	G	0.0042	0.029058	0.6276	0.627742	FALSE	FALSE	FALSE	v2KE89	0.014196	0.040665	486484	sepsis	TRUE	reported	textfile	9.00E-04	6.09E-06	7349	M15500.m	TRUE	reported	to2t7H	2	TRUE	0.002955	21.77185
1959	rs2595993	A	C	A	C	-0.0042	0.000849	0.4967	0.498163	FALSE	FALSE	FALSE	v2KE89	0.013684	0.95051	486484	sepsis	TRUE	reported	textfile	9.00E-04	2.88E-06	7349	M15500.m	TRUE	reported	to2t7H	2	TRUE	0.002955	21.77185
1960	rs262273	C	G	C	G	-0.0042	-0.00653	0.3946	0.392633	FALSE	TRUE	FALSE	v2KE89	0.013993	0.640665	486484	sepsis	TRUE	reported	textfile	9.00E-04	2.76E-06	7349	M15500.m	TRUE	reported	to2t7H	2	TRUE	0.002955	21.77185
1966	rs294280	T	C	T	C	0.0042	-0.00357	0.3901	0.385059	FALSE	FALSE	FALSE	v2KE89	0.014129	0.800721	486484	sepsis	TRUE	reported	textfile	9.00E-04	3.43E-06	7349	M15500.m	TRUE	reported	to2t7H	2	TRUE	0.002955	21.77185
2001	rs4933391	A	G	A	G	0.0042	0.018748	0.3768	0.376959	FALSE	FALSE	FALSE	v2KE89	0.014146	0.185068	486484	sepsis	TRUE	reported	textfile	9.00E-04	6.00E-06	7349	M15500.m	TRUE	reported	to2t7H	2	TRUE	0.002955	21.77185
2005	rs561895	T	C	T	C	-0.0042	0.005508	0.6577	0.655841	FALSE	FALSE	FALSE	v2KE89	0.014404	0.702182	486484	sepsis	TRUE	reported	textfile	9.00E-04	6.63E-06	7349	M15500.m	TRUE	reported	to2t7H	2	TRUE	0.002955	21.77185
2007	rs6471514	T	C	T	C	-0.0042	-0.01233	0.5379	0.538389	FALSE	FALSE	FALSE	v2KE89	0.013715	0.368691	486484	sepsis	TRUE	reported	textfile	9.00E-04	2.73E-06	7349	M15500.m	TRUE	reported	to2t7H	2	TRUE	0.002955	21.77185
2009	rs6506736	A	G	A	G	0.0042	-0.01625	0.5205	0.518097	FALSE	FALSE	FALSE	v2KE89	0.013671	0.234504	486484	sepsis	TRUE	reported	textfile	9.00E-04	2.56E-06	7349	M15500.m	TRUE	reported	to2t7H	2	TRUE	0.002955	21.77185
2019	rs6866597	T	G	T	G	0.0042	-0.04032	0.5194	0.517394	FALSE	FALSE	FALSE	v2KE89	0.013887	0.003689	486484	sepsis	TRUE	reported	textfile	9.00E-04	3.56E-06	7349	M15500.m	TRUE	reported	to2t7H	2	TRUE	0.002955	21.77185
2033	rs7732790	A	C	A	C	-0.0042	0.021247	0.59	0.558959	FALSE	FALSE	FALSE	v2KE89	0.013812	0.123978	486484	sepsis	TRUE	reported	textfile	9.00E-04	3.72E-06	7349	M15500.m	TRUE	reported	to2t7H	2	TRUE	0.002955	21.77185
2034	rs7760148	A	G	A	G	-0.0042	-0.02887	0.3554	0.360769	FALSE	FALSE	FALSE	v2KE89	0.014252	0.042766	486484	sepsis	TRUE	reported	textfile	9.00E-04	3.39E-06	7349	M15500.m	TRUE	reported	to2t7H	2	TRUE	0.002955	21.77185
2050	rs9383693	T	C	T	C	-0.0042	-0.02281	0.5534	0.544535	FALSE	FALSE	FALSE	v2KE89	0.013782	0.097879	486484	sepsis	TRUE	reported	textfile	9.00E-04	3.57E-06	7349	M15500.m	TRUE	reported	to2t7H	2	TRUE	0.002955	21.77185
2051	rs9419445	T	C	T	C	0.0042	-0.01466	0.5296	0.530557	FALSE	FALSE	FALSE	v2KE89	0.013771	0.286911	486484	sepsis	TRUE	reported	textfile	9.00E-04	3.46E-06	7349	M15500.m	TRUE	reported	to2t7H	2	TRUE	0.002955	21.77185
2054	rs9557806	A	G	A	G	-0.0042	-0.0057	0.5506	0.569227	FALSE	FALSE	FALSE	v2KE89	0.013879	0.681506	486484	sepsis	TRUE	reported	textfile	9.00E-04	2.40E-06	7349	M15500.m	TRUE	reported	to2t7H	2	TRUE	0.002955	21.77185
2058	rs9784110	T	C	T	C	-0.0042	-0.00657	0.6096	0.609439	FALSE	FALSE	FALSE	v2KE89	0.014066	0.640609	486484	sepsis	TRUE	reported	textfile	9.00E-04	3.97E-06	7349	M15500.m	TRUE	reported	to2t7H	2	TRUE	0.002955	21.77185
2059	rs9819702	T	C	T	C	-0.0042	0.013709	0.3613	0.373791	FALSE	FALSE	FALSE	v2KE89	0.014116	0.331496	486484	sepsis	TRUE	reported	textfile	9.00E-04	5.18E-06	7349	M15500.m	TRUE	reported	to2t7H	2	TRUE	0.002955	21.77185
2066	rs9960949	A	G	A	G	-0.0042	-0.02329	0.4788	0.489937	FALSE	FALSE	FALSE	v2KE89	0.013822	0.092049	486484	sepsis	TRUE	reported	textfile	9.00E-04	8.83E-06	7349	M15500.m	TRUE	reported	to2t7H	2	TRUE	0.002955	21.77185
8390	rs4482740	A	G	A	G	0.0084	-0.00319	0.3507	0.349014	FALSE	FALSE	FALSE	SCzh0A	0.014437	0.825122	486484	sepsis	TRUE	reported	textfile	0.0018	4.53E-06	7348	M35675.m	TRUE	reported	n1SREk	2	TRUE	0.002955	21.77185
1223	rs1158985	T	C	T	C	-0.0084	0.011513	0.3809	0.377178	FALSE	FALSE	FALSE	V50rWC	0.014177	0.416738	486484	sepsis	TRUE	reported	textfile	0.0018	2.22E-06	7346	M01645.m	TRUE	reported	yN3gpQ	2	TRUE	0.002956	21.77185
1249	rs696979	T	C	T	C	0.0084	0.000632	0.6026	0.581469	FALSE	FALSE	FALSE	V50rWC	0.013869	0.963661	486484	sepsis	TRUE	reported	textfile	0.0018	2.05E-06	7346	M01645.m	TRUE	reported	yN3gpQ	2	TRUE	0.002956	21.77185
529	rs1311647	A	G	A	G	-0.0448	0.074867	0.9853	0.983076	FALSE	FALSE	FALSE	wXMWHy	0.05371	0.163345	486484	sepsis	TRUE	reported	textfile	0.0096	2.93E-06	7345	M00584.m	TRUE	reported	7CKn2b	2	TRUE	0.002956	21.77185
3773	rs1289671	A	G	A	G	-0.0084	0.016084	0.6005	0.594908	FALSE	FALSE	FALSE	NW1aaR	0.01393	0.248272	486484	sepsis	TRUE	reported	textfile	0.0018	1.56E-06	7344	M32339.m	TRUE	reported	pBPkAB	2	TRUE	0.002957	21.77185
3792	rs7191435	T	C	T	C	0.0084	0.016883	0.6525	0.650007	FALSE	FALSE	FALSE	NW1aaR	0.014388	0.240621	486484	sepsis	TRUE	reported	textfile	0.0018	2.53E-06	7344	M32339.m	TRUE	reported	pBPkAB	2	TRUE	0.002957	21.77185
3793	rs7712111	A	G	A	G	0.0084	0.016965	0.6383	0.639604	FALSE	FALSE	FALSE	NW1aaR	0.014278	0.234759	486484	sepsis	TRUE	reported	textfile	0.0018	1.63E-06	7344	M32339.m	TRUE	reported	pBPkAB	2	TRUE	0.002957	21.77185
4941	rs1480792	A	G	A	G	0.0084	-0.01272	0.3268	0.327543	FALSE	FALSE	FALSE	bKmDMa	0.0146	0.383651	486484	sepsis	TRUE	reported	textfile	0.0018	1.84E-06	7344	M32910.m	TRUE	reported	nAuPzO	2	TRUE	0.002957	21.77185
7454	rs2852371	A	G	A	G	0.0126	-0.00167	0.5831	0.583634	FALSE	FALSE	FALSE	7ubZow	0.013852	0.904106	486484	sepsis	TRUE	reported	textfile	0.0027	3.97E-06	7344	M34407.m	TRUE	reported	LRsXN2	2	TRUE	0.002957	21.77185
7456	rs383911	A	G	A	G	-0.0126	0.005097	0.5551	0.555345	FALSE	FALSE	FALSE	7ubZow	0.013734	0.710565	486484	sepsis	TRUE	reported	textfile	0.0027	2.98E-06	7344	M34407.m	TRUE	reported	LRsXN2	2	TRUE	0.002957	21.77185
3280	rs1746357	A	G	A	G	0.0084	0.023369	0.4831	0.482094	FALSE	FALSE	FALSE	vYWPe9	0.013707	0.088215	486484	sepsis	TRUE	reported	textfile	0.0018	3.37E-06	7340	M25459.m	TRUE	reported	VvJ6Vx	2	TRUE	0.002958	21.77184
4257	rs1088231	A	G	A	G	-0.0126	0.003101	0.5268	0.515508	FALSE	FALSE	FALSE	ylV4bp	0.013656	0.820359	486484	sepsis	TRUE	reported	textfile	0.0027	2.77E-06	7337	M32632.m	TRUE	reported	hqHJoU	2	TRUE	0.002959	21.77184
3217	rs957671	A	G	A	G	0.0168	0.003155	0.9045	0.907312	FALSE	FALSE	FALSE	UfLkBq	0.023837	0.89471	486484	sepsis	TRUE	reported	textfile	0.0036	3.02E-06	7329	M22481.m	TRUE	reported	kWMdiT	2	TRUE	0.002963	21.77183
2918	rs7632086	C	G	C	G	-0.0126	-0.01767	0.8243	0.812491	FALSE	TRUE	FALSE	FBDEQi	0.017564	0.314439	486484	sepsis	TRUE	reported	textfile	0.0027	2.47E-06	7327	M20699.m	TRUE	reported	nvNIlp	2	TRUE	0.002963	21.77183
1389	rs554710	T	C	T	C	-0.0084	0.027707	0.7074	0.709528	FALSE	FALSE	FALSE	KrWhws	0.015028	0.065236	486484	sepsis	TRUE	reported	textfile	0.0018	1.59E-06	7325	M02132.m	TRUE	reported	7pzpfK	2	TRUE	0.002964	21.77183
2835	rs1178768	T	C	T	C	-0.0084	-0.02469	0.7863	0.779978	FALSE	FALSE	FALSE	Y5qWBm	0.016546	0.135581	486484	sepsis	TRUE	reported	textfile	0.0018	3.52E-06	7325	M20489.m	TRUE	reported	f2g9Su	2	TRUE	0.002964	21.77183
2852	rs4572706	A	G	A	G	-0.0084	0.003273	0.197	0.205212	FALSE	FALSE	FALSE	Y5qWBm	0.017022	0.847535	486484	sepsis	TRUE	reported	textfile	0.0018	1.53E-06	7325	M20489.m	TRUE	reported	f2g9Su	2	TRUE	0.002964	21.77183
5076	rs6948511	T	C	T	C	-0.028	0.00332	0.9206	0.914988	FALSE	FALSE	FALSE	gqzGN4	0.024681	0.893006	486484	sepsis	TRUE	reported	textfile	0.006	3.30E-06	7319	M33140.m	TRUE	reported	DKGBe9	2	TRUE	0.002967	21.77183
4583	rs1309883	A	G	A	G	-0.0084	0.000851	0.7208	0.721739	FALSE	FALSE	FALSE	Pzplqu	0.015267	0.95556	486484	sepsis	TRUE	reported	textfile	0.0018	1.93E-06	7318	M32740.m	TRUE	reported	FjEBS5	2	TRUE	0.002967	21.77183
4600	rs4981021	T	C	T	C	-0.0084	0.002553	0.282	0.273712	FALSE	FALSE	FALSE	Pzplqu	0.015403	0.868344	486484	sepsis	TRUE	reported	textfile	0.0018	1.92E-06	7318	M32740.m	TRUE	reported	FjEBS5	2	TRUE	0.002967	21.77183
4612	rs7560697	T	C	T	C	0.0084	3.69E-05	0.2811	0.270952	FALSE	FALSE	FALSE	Pzplqu	0.015445	0.998091	486484	sepsis	TRUE	reported	textfile	0.0018	1.79E-06	7318	M32740.m	TRUE	reported	FjEBS5	2	TRUE	0.002967	21.77183
8325	rs2305779	A	G	A	G	0.0196	-0.00633	0.1237	0.123644	FALSE	FALSE	FALSE	o0GzT1	0.02092	0.76213	486484	sepsis	TRUE	reported	textfile	0.0042	2.79E-06	7317	M35631.m	TRUE	reported	G1XFx0	2	TRUE	0.002967	21.77183

7061	rs4305952	C	G	C	G	0.0084	0.006343	0.6467	0.644858	FALSE	TRUE	FALSE	uthruC	0.014347	0.658403	486484	sepsis	TRUE	reported	textfile	0.0018	2.65E-06	7316	M34283.m	TRUE	reported	ftTuRU	2	TRUE	0.002968	21.77182
7065	rs6683094	A	G	A	G	0.0084	0.00617	0.6238	0.617196	FALSE	FALSE	FALSE	uthruC	0.014076	0.661117	486484	sepsis	TRUE	reported	textfile	0.0018	2.72E-06	7316	M34283.m	TRUE	reported	ftTuRU	2	TRUE	0.002968	21.77182
5869	rs1337483	T	C	T	C	0.0168	0.024233	0.9021	0.898947	FALSE	FALSE	FALSE	tqW0ld	0.022904	0.290049	486484	sepsis	TRUE	reported	textfile	0.0036	3.30E-06	7315	M33389.m	TRUE	reported	Sv7EyV	2	TRUE	0.002968	21.77182
1693	rs2422370	A	G	A	G	-0.0084	0.011699	0.8094	0.821362	FALSE	FALSE	FALSE	f01en0	0.019527	0.549084	486484	sepsis	TRUE	reported	textfile	0.0018	5.96E-06	7314	M12593.m	TRUE	reported	EAsM6U	2	TRUE	0.002969	21.77182
2069	rs1019407	A	C	A	C	-0.007	-0.00225	0.7918	0.790362	FALSE	FALSE	FALSE	NPVH5j	0.016777	0.893382	486484	sepsis	TRUE	reported	textfile	0.0015	6.33E-06	7310	M15506.m	TRUE	reported	r7r3vM	2	TRUE	0.00297	21.77182
2078	rs2080224	A	G	A	G	-0.0112	0.005246	0.9304	0.925914	FALSE	FALSE	FALSE	NPVH5j	0.026205	0.841321	486484	sepsis	TRUE	reported	textfile	0.0024	1.97E-06	7310	M15506.m	TRUE	reported	r7r3vM	2	TRUE	0.00297	21.77182
2226	rs9314172	C	G	C	G	-0.0084	0.004295	0.6969	0.685175	FALSE	TRUE	FALSE	Bf6uMK	0.014895	0.773065	486484	sepsis	TRUE	reported	textfile	0.0018	4.50E-06	7308	M15778.m	TRUE	reported	jowM2N	2	TRUE	0.002971	21.77182
6137	rs2487521	T	C	T	C	0.0084	0.003236	0.6858	0.680754	FALSE	FALSE	FALSE	MiSJly	0.014644	0.825128	486484	sepsis	TRUE	reported	textfile	0.0018	3.54E-06	7307	M33477.m	TRUE	reported	WwL1hq	2	TRUE	0.002972	21.77182
6144	rs476093	A	C	A	C	-0.0084	0.005157	0.7566	0.751256	FALSE	FALSE	FALSE	MiSJly	0.015789	0.743962	486484	sepsis	TRUE	reported	textfile	0.0018	4.98E-06	7307	M33477.m	TRUE	reported	WwL1hq	2	TRUE	0.002972	21.77182
6159	rs9888676	A	G	A	G	0.0084	0.005747	0.2454	0.249815	FALSE	FALSE	FALSE	MiSJly	0.015859	0.717041	486484	sepsis	TRUE	reported	textfile	0.0018	5.44E-06	7307	M33477.m	TRUE	reported	WwL1hq	2	TRUE	0.002972	21.77182
5984	rs1669715	T	C	T	C	-0.0126	0.027985	0.0915	0.090276	FALSE	FALSE	FALSE	3KsoXq	0.023872	0.241077	486484	sepsis	TRUE	reported	textfile	0.0027	2.30E-06	7306	M33422.m	TRUE	reported	aNr6Ug	2	TRUE	0.002972	21.77182
2878	rs1472578	A	T	A	T	-0.0126	-0.01886	0.6894	0.687349	FALSE	TRUE	FALSE	mUzn1R	0.014746	0.201016	486484	sepsis	TRUE	reported	textfile	0.0027	2.44E-06	7301	M20675.m	TRUE	reported	XwY04K	2	TRUE	0.002974	21.77181
2886	rs4671111	T	C	T	C	-0.0126	-0.01036	0.5592	0.546257	FALSE	FALSE	FALSE	mUzn1R	0.013798	0.452684	486484	sepsis	TRUE	reported	textfile	0.0027	2.00E-06	7301	M20675.m	TRUE	reported	XwY04K	2	TRUE	0.002974	21.77181
1105	rs1098835	T	G	T	G	0.0084	0.071215	0.1244	0.134212	FALSE	FALSE	FALSE	yahiGZ	0.020374	0.000474	486484	sepsis	TRUE	reported	textfile	0.0018	3.09E-06	7271	M01585.m	TRUE	reported	dwZ7vX	2	TRUE	0.002986	21.77179
7879	rs360020	A	G	A	G	0.0196	0.0201	0.6799	0.699029	FALSE	FALSE	FALSE	mpkL0o	0.014936	0.178384	486484	sepsis	TRUE	reported	textfile	0.0042	2.48E-06	7263	M35160.m	TRUE	reported	MKkvYr	2	TRUE	0.002989	21.77178
1712	rs7844733	T	C	T	C	-0.0126	-0.02401	0.1881	0.18937	FALSE	FALSE	FALSE	Zml9HX	0.017458	0.168949	486484	sepsis	TRUE	reported	textfile	0.0027	2.68E-06	7258	M12626.m	TRUE	reported	9SUabZ	2	TRUE	0.002992	21.77178
4329	rs1075833	T	C	T	C	-0.0084	0.011885	0.6855	0.669378	FALSE	FALSE	FALSE	6VSoQQ	0.014565	0.414487	486484	sepsis	TRUE	reported	textfile	0.0018	4.46E-06	7229	M32644.m	TRUE	reported	oC2AV0	2	TRUE	0.003004	21.77175
4339	rs1313204	T	C	T	C	-0.0084	0.01379	0.2928	0.300308	FALSE	FALSE	FALSE	6VSoQQ	0.014952	0.356374	486484	sepsis	TRUE	reported	textfile	0.0018	4.58E-06	7229	M32644.m	TRUE	reported	oC2AV0	2	TRUE	0.003004	21.77175
4341	rs2657761	T	C	T	C	-0.0084	0.003549	0.7123	0.704297	FALSE	FALSE	FALSE	6VSoQQ	0.015046	0.813506	486484	sepsis	TRUE	reported	textfile	0.0018	4.99E-06	7229	M32644.m	TRUE	reported	oC2AV0	2	TRUE	0.003004	21.77175
1439	rs1704186	T	C	T	C	0.0196	0.03205	0.939	0.935871	FALSE	FALSE	FALSE	1M4gqj	0.027876	0.250245	486484	sepsis	TRUE	reported	textfile	0.0042	4.02E-06	7226	M02730.m	TRUE	reported	RWyYEM	2	TRUE	0.003005	21.77175
8024	rs1182282	A	G	A	G	0.0084	0.022641	0.7848	0.786894	FALSE	FALSE	FALSE	SwiqSi	0.016685	0.174792	486484	sepsis	TRUE	reported	textfile	0.0018	4.30E-06	7217	M35270.m	TRUE	reported	21ZlsQ	2	TRUE	0.003008	21.77174
8032	rs1883261	A	G	A	G	0.0084	0.006816	0.4801	0.471898	FALSE	FALSE	FALSE	SwiqSi	0.01369	0.618579	486484	sepsis	TRUE	reported	textfile	0.0018	1.92E-06	7217	M35270.m	TRUE	reported	21ZlsQ	2	TRUE	0.003008	21.77174
8046	rs6559771	C	G	C	G	0.0084	-0.00592	0.775	0.773097	FALSE	TRUE	FALSE	SwiqSi	0.01637	0.717692	486484	sepsis	TRUE	reported	textfile	0.0018	4.92E-06	7217	M35270.m	TRUE	reported	21ZlsQ	2	TRUE	0.003008	21.77174
8049	rs7670227	A	C	A	C	-0.0084	0.00513	0.2569	0.254113	FALSE	FALSE	FALSE	SwiqSi	0.015793	0.745312	486484	sepsis	TRUE	reported	textfile	0.0018	3.66E-06	7217	M35270.m	TRUE	reported	21ZlsQ	2	TRUE	0.003008	21.77174
2951	rs791630	A	G	A	G	-0.0112	0.007138	0.1945	0.189962	FALSE	FALSE	FALSE	pn11BC	0.017439	0.68231	486484	sepsis	TRUE	reported	textfile	0.0024	2.80E-06	7203	M21047.m	TRUE	reported	KJF8U4	2	TRUE	0.003014	21.77173
5064	rs3779259	T	G	T	G	0.0308	-0.0178	0.0881	0.0895	FALSE	FALSE	FALSE	nHZXjp	0.023959	0.457487	486484	sepsis	TRUE	reported	textfile	0.0066	2.98E-06	7166	M33138.m	TRUE	reported	ccoK1F	2	TRUE	0.00303	21.7717
957	rs2967437	A	G	A	G	-0.0126	-0.01994	0.6902	0.686352	FALSE	FALSE	FALSE	tWoDq4	0.014737	0.176092	486484	sepsis	TRUE	reported	textfile	0.0027	1.88E-06	7160	M01508.m	TRUE	reported	6ltDw0	2	TRUE	0.003032	21.77169
6270	rs2715313	A	G	A	G	-0.0084	-0.01656	0.3788	0.383906	FALSE	FALSE	FALSE	MOonP9	0.014064	0.239084	486484	sepsis	TRUE	reported	textfile	0.0018	4.60E-06	7066	M33515.m	TRUE	reported	226GuP	2	TRUE	0.003073	21.77161
6272	rs403018	A	G	A	G	-0.0084	-0.01094	0.6712	0.665009	FALSE	FALSE	FALSE	MOonP9	0.014526	0.451437	486484	sepsis	TRUE	reported	textfile	0.0018	5.36E-06	7066	M33515.m	TRUE	reported	226GuP	2	TRUE	0.003073	21.77161
5728	rs1845546	T	C	T	C	-0.0126	-0.02132	0.3674	0.372329	FALSE	FALSE	FALSE	YDQaWa	0.014165	0.132325	486484	sepsis	TRUE	reported	textfile	0.0027	3.25E-06	7063	M33228.m	TRUE	reported	BmYg8r	2	TRUE	0.003074	21.77161
5732	rs418874	A	G	A	G	0.0126	0.021864	0.6931	0.692075	FALSE	FALSE	FALSE	YDQaWa	0.01482	0.140126	486484	sepsis	TRUE	reported	textfile	0.0027	3.64E-06	7063	M33228.m	TRUE	reported	BmYg8r	2	TRUE	0.003074	21.77161
6958	rs27419	T	C	T	C	-0.0126	-0.01004	0.6409	0.634157	FALSE	FALSE	FALSE	7Cw2XZ	0.014164	0.478363	486484	sepsis	TRUE	reported	textfile	0.0027	2.60E-06	7049	M34123.m	TRUE	reported	vrgMl3	2	TRUE	0.00308	21.7716
4856	rs792222	C	G	C	G	-0.0224	0.020635	0.7494	0.734169	FALSE	TRUE	FALSE	StMkBO	0.015527	0.183855	486484	sepsis	TRUE	reported	textfile	0.0048	3.59E-06	6967	M32847.m	TRUE	reported	uzQb8Q	2	TRUE	0.003116	21.77153
9076	rs4765789	T	C	T	C	-0.0112	0.027211	0.4153	0.415979	FALSE	FALSE	FALSE	C7PD8T	0.013897	0.05022	486484	sepsis	TRUE	reported	textfile	0.0024	3.88E-06	6942	M38768.m	TRUE	reported	ZA3QM7	2	TRUE	0.003127	21.7715
8847	rs2305929	A	G	A	G	0.0084	-0.00775	0.819	0.823263	FALSE	FALSE	FALSE	XIHeTV	0.018006	0.666721	486484	sepsis	TRUE	reported	textfile	0.0018	2.62E-06	6940	M36808.m	TRUE	reported	37Vi2D	2	TRUE	0.003128	21.7715
7473	rs1125006	A	C	A	C	-0.0168	0.025595	0.3131	0.32124	FALSE	FALSE	FALSE	SqfdcK	0.014754	0.082777	486484	sepsis	TRUE	reported	textfile	0.0036	2.47E-06	6929	M34416.m	TRUE	reported	sRGRIK	2	TRUE	0.003133	21.77149
1651	rs1186971	A	G	A	G	-0.0126	0.001329	0.7762	0.774418	FALSE	FALSE	FALSE	YzX5ZD	0.016371	0.935287	486484	sepsis	TRUE	reported	textfile	0.0027	4.25E-06	6864	M12129.m	TRUE	reported	6tMfaW	2	TRUE	0.003163	21.77143
8567	rs2674357	C	G	C	G	-0.0775	-0.0215	0.8039	0.806486	FALSE	TRUE	FALSE	Yjjldt	0.017306	0.214041	486484	sepsis	TRUE	reported	textfile	0.0166	2.96E-06	1725	M36098.m	TRUE	reported	U2ORsF	2	TRUE	0.012478	21.77125
8904	rs2206426	A	G	A	G	-0.0084	-0.02177	0.4177	0.418486	FALSE	FALSE	FALSE	eLhsld	0.013909	0.117543	486484	sepsis	TRUE	reported	textfile	0.0018	4.92E-06	6552	M37058.m	TRUE	reported	RSeWE5	2	TRUE	0.003313	21.77113
8912	rs367213	A	G	A	G	-0.0084	0.005358	0.5133	0.51645	FALSE	FALSE	FALSE	eLhsld	0.013645	0.694541	486484	sepsis	TRUE	reported	textfile	0.0018	4.74E-06	6552	M37058.m	TRUE	reported	RSeWE5	2	TRUE	0.003313	21.77113
8012	rs416324	A	G	A	G	-0.0126	0.006296	0.5573	0.552943	FALSE	FALSE	FALSE	Zj5bZH	0.013781	0.647784	486484	sepsis	TRUE	reported	textfile	0.0027	2.10E-06	6538	M35257.m	TRUE	reported	exssAk	2	TRUE	0.00332	21.77112
8013	rs4270639	A	G	A	G	-0.0168	-0.02344	0.7232	0.716522	FALSE	FALSE	FALSE	Zj5bZH	0.015137	0.121515	486484	sepsis	TRUE	reported	textfile	0.0036	3.78E-06	6538	M35257.m	TRUE	reported	exssAk	2	TRUE	0.00332	21.77112
1007	rs304171	T	C	T	C	-0.0168	-0.02139	0.0624	0.061865	FALSE	FALSE	FALSE	jfkZbM	0.028348	0.450489	486484	sepsis	TRUE	reported	textfile	0.0036	2.17E-06	6523	M01558.m	TRUE	reported	LFZvMG	2	TRUE	0.003328	21.7711
8869	rs2609982	T	C	T	C	-0.0294	0.008062	0.2865	0.283071	FALSE	FALSE	FALSE	nWuK1Q	0.015169	0.595088	486484	sepsis	TRUE	reported	textfile	0.0063	3.10E-06	6511	M36850.m	TRUE	reported	2BA0SE	2	TRUE	0.003334	21.77109
3310	rs2211843	T	G	T	G	-0.0126	0.030897	0.2444	0.236203	FALSE	FALSE	FALSE	nqmBts	0.016198	0.056459	486484	sepsis	TRUE	reported	textfile	0.0027	4.23E-06	6388	M25599.m	TRUE	reported	VkwSh1	2	TRUE	0.003398	21.77096
3868	rs1539549	T	C	T	C	-0.0369	0.009076	0.305	0.311654	FALSE	FALSE	FALSE	7LphBB	0.015556	0.559602	486484	sepsis	TRUE	reported	textfile	0.0079	2.72E-06	936	M32388.m	TRUE	reported	5oj12c	2	TRUE	0.022778	21.77056
7426	rs1108665	T	G	T	G	0.0252	-0.02182	0.2207	0.250122	FALSE	FALSE	FALSE	NZPe5A	0.016509	0.186189	486484	sepsis	TRUE	reported	textfile	0.0054	3.24E-06	5689	M34400.m	TRUE	reported	OyPwYO	2	TRUE	0.003813	21.77012
1583	rs1213178	A	G	A	G	0.0126	0.025455	0.7091	0.695999	FALSE	FALSE	FALSE	7QN2kA	0.014987	0.089411	486484	sepsis	TRUE	reported	textfile	0.0027	2.59E-06	5656	M12017.m	TRUE	reported	52YQ1d	2	TRUE	0.003836	21.77008
8358	rs4551320	T	G	T	G	0.0434	0.010248	0.0759	0.10482	FALSE	FALSE	FALSE	6VVCe4	0.022638	0.650775	486484	sepsis	TRUE	reported	textfile	0.0093	2.88E-06	5629	M35669.m	TRUE	reported	l0fk8X	2	TRUE	0.003854	21.77004
5	rs2748991	T	C	T	C	-0.007	0.008984	0.446	0.452764	FALSE	FALSE	FALSE	0cebDu	0.013723	0.512696	486484	sepsis	TRUE	reported	textfile	0.0015	2.34E-06	5604	M00053.m	TRUE	reported	i8iuxX	2	TRUE	0.003871	21.77001
611	rs1253211	T	C	T	C	-0.028	0.002012	0.961	0.964547	FALSE	FALSE	FALSE	BYGzq4	0.037045	0.956676	486484	sepsis	TRUE	reported	textfile	0.006	3.06E-06	5601	M01110.m	TRUE	reported	oZiXqP	2	TRUE	0.003873	21.77
5023	rs1704400	T	G	T	G	-0.014	-0.04612	0.074	0.079005	FALSE	FALSE	FALSE	BNnOvt	0.025381	0.069167	486484	sepsis	TRUE	reported	textfile	0.003	3.06E-06	5600	M33132.m	TRUE	reported	rHcCPG	2	TRUE	0.003874	21.77
6803	rs6061764	A	G	A	G	-0.014	0.014183	0.314	0.314952	FALSE	FALSE	FALSE	OZ0I1H	0.014738	0.335867	486484	sepsis	TRUE	reported	textfile	0.003	3.06E-06	5597	M33960.m	TRUE	reported	GlPp4w	2	TRUE	0.003876	21.77
445	rs1565225	A	G	A	G	0.007	-0.0322	0.245	0.243404	FALSE	FALSE	FALSE	gcJMTM	0.015931	0.043249	486484	sepsis	TRUE	reported	textfile	0.0015	2.34E-06	5594	M00064.m	TRUE	reported	g2hUXe	2	TRUE	0.003878	21.76999
102	rs3772343	A	C	A	C	0.028	0.104775	0.987	0.972126	FALSE	FALSE	FALSE	xmGuol	0.04157	0.01172	486484	sepsis	TRUE	reported	textfile	0.006	3.06E-06	5592	M00054.m	TRUE	reported	GW50Ri	2	TRUE	0.003879	21.76999
800	rs7565567	A	C	A	C	-0.042	0.028532	0.982	0.97778	FALSE	FALSE	FALSE	dyT79l	0.046819	0.542261	486484	sepsis	TRUE	reported	textfile	0.009	3.06E-06	5586	M01336.m	TRUE	reported	OemVJ2	2	TRUE	0.003883	21.76998
4102	rs1388331	A	G	A	G	0.014	0.022535	0.913	0.916689	FALSE	FALSE	FALSE	4rtLd0	0.024903	0.365525	486484	sepsis	TRUE	reported	textfile	0.003	3.06E-06	5584	M32518.m	TRUE	reported	j7f1ER	2	TRUE	0.003885	21.76998
4938	rs1154147	A	G	A	G	-0.014	-0.02068	0.152	0.146399	FALSE	FALSE	FALSE	bKmDMa	0.019317	0.284454	486484	sepsis	TRUE	reported	textfile	0.003	3.06E-06	5584	M32910.m	TRUE	reported	nAuPzO	2	TRUE	0.003885	21.76998
3079	rs1156408	T	C	T	C	-0.028	-0.02138	0.053	0.075465	FALSE	FALSE	FALSE	esOP8m	0.027289	0.433395	486484	sepsis	TRUE	reported	textfile	0.006	3.06E-06	5563	M22116.m	TRUE	reported	fDMh7Z	2	TRUE	0.003899	21.76995
5733	rs4392177	A	G	A	G	-0.014	-0.0272	0.547	0.566858	FALSE	FALSE	FALSE	YDQaWa	0.013789	0.04851	486484	sepsis	TRUE	reported	textfile	0.003	3.06E-06	5451	M33228.m	TRUE	reported	BmYg8r	2	TRUE	0.003979	21.76979
3410	rs2494523	T	C	T	C	-0.014	0.002182	0.492	0.490814	FALSE	FALSE	FALSE	VU62LG	0.013655	0.873018	486484	sepsis	TRUE	reported	textfile	0.003	3.06E-06	5442	M27513.m	TRUE	reported	0didOx	2	TRUE	0.003986	21.76977
2945	rs1895981	T	C	T	C	0.014	0.008971	0.841	0.841493	FALSE	FALSE	FALSE	pn11BC	0.018689	0.631233	486484	sepsis	TRUE	reported	textfile	0.003	3.06E-06	5439	M21047.m	TRUE	reported	KJF8U4	2	TRUE	0.003988	21.76977
1304	rs2364598	A	C	A	C	-0.014	-0.02007	0.149	0.158604	FALSE	FALSE	FALSE	WQdpmo	0.018734	0.284096	486484	sepsis	TRUE	reported	textfile	0.003	3.06E-06	5422	M01769.m	TRUE	reported	BkptOK	2	TRUE	0.004	21.76974
8202	rs4719207	T	C	T	C	-0.014	-0.00813	0.211	0.216005	FALSE	FALSE	FALSE	iLlpho	0.016604	0.624455	486484	sepsis	TRUE	reported	textfile	0.003	3.06E-06	5312	M35431.m	TRUE	reported	AAZ5ge	2	TRUE	0.004083	21.76958
3591	rs1079970	A	G	A	G	0.0308	-0.00093	0.435	0.460382	FALSE	FALSE	FALSE	e4G8UU	0.013723	0.946029	486484	sepsis	TRUE	reported	textfile	0.0066	2.69E-06	5229	M31548.m	TRUE	reported	kWkkmH	2	TRUE	0.004148	21.76945
3598	rs2327511	A	T	A	T	0.035	-0.01006	0.2333	0.244886	FALSE	TRUE	FALSE	e4G8UU	0.015978	0.528885	486484	sepsis	TRUE	reported	textfile	0.0075	3.44E-06	5229	M31548.m	TRUE	reported	kWkkmH	2	TRUE	0.004148	21.76945
7544	rs6785727	A	G	A	G	0.0126	0.016403	0.5467	0.59428	FALSE	FALSE	FALSE	JJsHvX	0.013942	0.239397	486484	sepsis	TRUE	reported	textfile	0.0027	4.52E-06	5207	M34456.m	TRUE	reported	aklxve	2	TRUE	0.004165	21.76941
3011	rs2049974	A	G	A	G	-0.014	0.034873	0.296	0.291343	FALSE	FALSE	FALSE	gxupFu	0.01505	0.020498	486484	sepsis	TRUE	reported	textfile	0.003	3.06E-06	5148	M21188.m	TRUE	reported	gwwXh2	2	TRUE	0.004213	21.76932
2325	rs4325745	T	C	T	C	-0.014	0.004355	0.596	0.60095	FALSE	FALSE	FALSE	IZXwpR	0.014081	0.757112	486484	sepsis	TRUE	reported	textfile	0.003	3.06E-06	4962	M16822.m	TRUE	reported	Zx9k2V	2	TRUE	0.00437	21.769
2778	rs1215246	T	C	T	C	0.021	-0.01985	0.1557	0.177926	FALSE	FALSE	FALSE	yYuamw	0.018221	0.275929	486484	sepsis	TRUE	reported	textfile	0.0045	3.76E-06	4844	M19414.m	TRUE	reported	GxSb5G	2	TRUE	0.004476	21.76879
7737	rs1740655	T	C	T	C	0.028	0.002147	0.959	0.949227	FALSE	FALSE	FALSE	HyMNYM	0.031111	0.94499	486484	sepsis	TRUE	reported	textfile	0.006	3.06E-06	4825	M34912.m	TRUE	reported	Tl8Hcw	2	TRUE	0.004493	21.76875
5942	rs6685925	A	G	A	G	0.0364	0.040108	0.2447	0.25598	FALSE	FALSE	FALSE	yepzrL	0.015678	0.010523	486484	sepsis	TRUE	reported	textfile	0.0078	2.82E-06	3890	M33408.m	TRUE	reported	SY4CoS	2	TRUE	0.005567	21.76658
7365	rs606242	T	C	T	C	-0.0934	-0.02361	0.6853	0.721699	FALSE	FALSE	FALSE	mm9ksf	0.015249	0.12147	486484	sepsis	TRUE	reported	textfile	0.02	3.00E-06	954	M34365.m	TRUE	reported	AVwEqD	2	TRUE	0.02235	21.76318
825	rs1922419	A	G	A	G	-0.0182	0.004962	0.7807	0.768332	FALSE	FALSE	FALSE	2qgDgB	0.016245	0.760018	486484	sepsis	TRUE	reported	textfile	0.0039	3.37E-06	1765	M01358.m	TRUE	reported	asmXq0	2	TRUE	0.012188	21.7531
3720	rs1357743	T	G	T	G	0.0322	-0.00271	0.5585	0.562263	FALSE	FALSE	FALSE	ZRfYkG	0.013782	0.843893	486484	sepsis	TRUE	reported	textfile	0.0069	2.79E-06	1765	M32319.m	TRUE	reported	phVUPX	2	TRUE	0.012188	21.7531
4906	rs2039906	C	G	C	G	0.0084	0.014532	0.2696	0.261364	FALSE	TRUE	FALSE	VqcRGX	0.015594	0.351381	486484	sepsis	TRUE	reported	textfile	0.0018	1.94E-06	1763	M32867.m	TRUE	reported	psuFJy	2	TRUE	0.012202	21.75307
7450	rs1249425	A	G	A	G	0.0308	-0.01177	0.7684	0.792931	FALSE	FALSE	FALSE	7ubZow	0.016966	0.487714	486484	sepsis	TRUE	reported	textfile	0.0066	2.72E-06	1754	M34407.m	TRUE	reported	LRsXN2	2	TRUE	0.012264	21.75295
3261	rs2007443	A	T	A	T	0.028	-0.03119	0.1321	0.103662	FALSE	TRUE	FALSE	ZNPBqT	0.022421	0.164236	486484	sepsis	TRUE	reported	textfile	0.006	2.87E-06	1751	M24074.m	TRUE	reported	8pPtOl	2	TRUE	0.012285	21.7529
8219	rs1089551	T	C	T	C	-0.0532	-0.07381	0.9515	0.958926	FALSE	FALSE	FALSE	4YAWhA	0.034592	0.03287	486484	sepsis	TRUE	reported	textfile	0.0114	2.90E-06	1718	M35439.m	TRUE	reported	bSSC4Z	2	TRUE	0.012518	21.75243
2525	rs1079172	A	T	A	T	-0.0658	0.001118	0.3242	0.346562	FALSE	TRUE	FALSE	4HwTlG	0.014359	0.937962	486484	sepsis	TRUE	reported	textfile	0.0141	2.96E-06	1659	M18254.m	TRUE	reported	F9KrSC	2	TRUE	0.012957	21.75152
6556	rs1360605	C	G	C	G	0.1092	-0.01371	0.9704	0.970949	FALSE	TRUE	FALSE	58qeH5	0.041081	0.73854	486484	sepsis	TRUE	reported	textfile	0.0234	2.96E-06	1428	M33864.m	TRUE	reported	TBsy8r	2	TRUE	0.015021	21.74728
5156	rs575723	A	G	A	G	-0.9929	-0.01434	0.0135	0.015572	FALSE	FALSE	FALSE	L2DJzK	0.054923	0.793955	486484	sepsis	TRUE	reported	textfile	0.2101	2.29E-06	76	M33173.m	TRUE	reported	hRG70D	2	TRUE	0.227121	21.74588
6546	rs1041323	T	C	T	C	-0.0729	0.012752	0.3811	0.379869	FALSE	FALSE	FALSE	zmDnD7	0.014103	0.365904	486484	sepsis	TRUE	reported	textfile	0.0156	3.00E-06	471	M33835.m	TRUE	reported	MLBiKM	2	TRUE	0.04431	21.74492
5149	rs4279445	A	T	A	T	-0.9923	-0.00852	0.0132	0.024988	FALSE	TRUE	FALSE	L2DJzK	0.043816	0.84574	486484	sepsis	TRUE	reported	textfile	0.21	2.30E-06	76	M33173.m	TRUE	reported	hRG70D	2	TRUE	0.227076	21.7403
3073	rs739167	A	G	A	G	-0.0476	0.013272	0.8667	0.881826	FALSE	FALSE	FALSE	e3izea	0.021222	0.531702	486484	sepsis	TRUE	reported	textfile	0.0102	3.25E-06	1087	M22032.m	TRUE	reported	zODKFl	2	TRUE	0.019641	21.73771
8965	rs1685083	A	G	A	G	0.0429	-0.01549	0.1266	0.128495	FALSE	FALSE	FALSE	2iAvlt	0.020497	0.449707	486484	sepsis	TRUE	reported	textfile	0.0092	3.12E-06	6934	M37190.m	TRUE	reported	hdOuHe	2	TRUE	0.003126	21.7377
8083	rs1715158	A	C	A	C	0.0514	-0.00988	0.6597	0.67895	FALSE	FALSE	FALSE	z65cF2	0.014698	0.501624	486484	sepsis	TRUE	reported	textfile	0.011	3.11E-06	448	M35322.m	TRUE	reported	3GUSWk	2	TRUE	0.046473	21.73691
4144	rs1319504	A	G	A	G	-0.0373	0.023047	0.9137	0.88928	FALSE	FALSE	FALSE	CrVaiQ	0.021732	0.288917	486484	sepsis	TRUE	reported	textfile	0.008	3.59E-06	7361	M32553.m	TRUE	reported	HsKdAl	2	TRUE	0.002945	21.733
6905	rs1248391	T	C	T	C	0.4285	-0.05165	0.9829	0.978141	FALSE	FALSE	FALSE	SvfSvH	0.046928	0.271041	486484	sepsis	TRUE	reported	textfile	0.0916	2.90E-06	282	M34109.m	TRUE	reported	7OSbiu	2	TRUE	0.072012	21.72799
3253	rs1110651	A	G	A	G	-0.0331	0.026115	0.9495	0.94027	FALSE	FALSE	FALSE	ZNPBqT	0.029231	0.371646	486484	sepsis	TRUE	reported	textfile	0.0071	3.52E-06	7256	M24074.m	TRUE	reported	8pPtOl	2	TRUE	0.002986	21.72799
7125	rs1263903	T	C	T	C	-0.1844	-0.03224	0.975	0.963987	FALSE	FALSE	FALSE	de8tA6	0.036747	0.380255	486484	sepsis	TRUE	reported	textfile	0.0394	2.84E-06	248	M34322.m	TRUE	reported	sXPPBO	2	TRUE	0.081156	21.72765
7256	rs2513248	T	C	T	C	0.0662	0.005857	0.083	0.088481	FALSE	FALSE	FALSE	4t4W6t	0.024188	0.808673	486484	sepsis	TRUE	reported	textfile	0.0142	3.01E-06	5270	M34344.m	TRUE	reported	Rz9mCt	2	TRUE	0.004107	21.72573
4397	rs3822311	T	C	T	C	0.0303	-0.01271	0.9654	0.962683	FALSE	FALSE	FALSE	UJygVd	0.035956	0.723674	486484	sepsis	TRUE	reported	textfile	0.0065	3.43E-06	7361	M32654.m	TRUE	reported	kv9R0W	2	TRUE	0.002943	21.72404
575	rs1233689	T	G	T	G	0.0289	0.043764	0.9588	0.945407	FALSE	FALSE	FALSE	ZoqU2j	0.030083	0.145741	486484	sepsis	TRUE	reported	textfile	0.0062	2.68E-06	7333	M01105.m	TRUE	reported	2WxSOH	2	TRUE	0.002954	21.7217
553	rs924483	A	G	A	G	-0.0289	0.029777	0.0916	0.095901	FALSE	FALSE	FALSE	QUUN2g	0.023293	0.20112	486484	sepsis	TRUE	reported	textfile	0.0062	3.25E-06	7237	M00599.m	TRUE	reported	vxn2i7	2	TRUE	0.002993	21.72162
6390	rs1383950	T	C	T	C	0.1548	-0.00168	0.9813	0.985329	FALSE	FALSE	FALSE	eBEF1t	0.056937	0.976403	486484	sepsis	TRUE	reported	textfile	0.0332	3.13E-06	1660	M33638.m	TRUE	reported	CRf2lt	2	TRUE	0.012927	21.71412
7704	rs5997515	T	C	T	C	-0.0508	0.017396	0.0469	0.044027	FALSE	FALSE	FALSE	2eJlPM	0.033432	0.602827	486484	sepsis	TRUE	reported	textfile	0.0109	3.27E-06	6381	M34761.m	TRUE	reported	druKtT	2	TRUE	0.003392	21.71392
2895	rs7915784	A	G	A	G	0.0247	0.005029	0.0662	0.070291	FALSE	FALSE	FALSE	mUzn1R	0.026736	0.85079	486484	sepsis	TRUE	reported	textfile	0.0053	2.65E-06	7301	M20675.m	TRUE	reported	XwY04K	2	TRUE	0.002966	21.71317
9070	rs6971411	A	G	A	G	0.0247	-0.02779	0.1381	0.148043	FALSE	FALSE	FALSE	0l8IKx	0.019236	0.148567	486484	sepsis	TRUE	reported	textfile	0.0053	2.92E-06	7219	M38178.m	TRUE	reported	bWs4mL	2	TRUE	0.003	21.7131
3047	rs1079516	T	C	T	C	-0.0233	0.009453	0.9261	0.924433	FALSE	FALSE	FALSE	H6vcCG	0.02585	0.714598	486484	sepsis	TRUE	reported	textfile	0.005	3.51E-06	6180	M22030.m	TRUE	reported	XetVX7	2	TRUE	0.003502	21.70857
878	rs1000529	A	G	A	G	-0.0634	0.004685	0.0267	0.044906	FALSE	FALSE	FALSE	oi7KQI	0.033169	0.887681	486484	sepsis	TRUE	reported	textfile	0.0136	3.40E-06	1768	M01365.m	TRUE	reported	7NWGKA	2	TRUE	0.012143	21.70747
6847	rs1313219	A	T	A	T	0.0317	-0.01791	0.2154	0.193555	FALSE	TRUE	FALSE	F716PJ	0.017401	0.303335	486484	sepsis	TRUE	reported	textfile	0.0068	3.54E-06	1768	M34035.m	TRUE	reported	mm7VL6	2	TRUE	0.012143	21.70747
535	rs2304684	T	C	T	C	-0.0438	-0.00306	0.0182	0.019486	FALSE	FALSE	FALSE	wXMWHy	0.050966	0.952074	486484	sepsis	TRUE	reported	textfile	0.0094	3.02E-06	7345	M00584.m	TRUE	reported	7CKn2b	2	TRUE	0.002947	21.70572
8022	rs1089230	A	T	A	T	-0.0219	-0.02836	0.0417	0.062443	FALSE	TRUE	FALSE	SwiqSi	0.028299	0.316179	486484	sepsis	TRUE	reported	textfile	0.0047	3.23E-06	7217	M35270.m	TRUE	reported	21ZlsQ	2	TRUE	0.002999	21.70562
8737	rs2954636	T	C	T	C	0.0317	-0.0103	0.1715	0.180838	FALSE	FALSE	FALSE	ERC6hD	0.017764	0.561882	486484	sepsis	TRUE	reported	textfile	0.0068	3.18E-06	1636	M36559.m	TRUE	reported	n8waJF	2	TRUE	0.01311	21.70548
4252	rs7847689	T	C	T	C	0.0438	0.023016	0.7003	0.709555	FALSE	FALSE	FALSE	KZ60wl	0.015143	0.128547	486484	sepsis	TRUE	reported	textfile	0.0094	3.44E-06	6904	M32616.m	TRUE	reported	SPDpZi	2	TRUE	0.003135	21.70534
8658	rs1310060	T	G	T	G	-0.0219	-0.02777	0.3761	0.397863	FALSE	FALSE	FALSE	BFEGyF	0.013969	0.046857	486484	sepsis	TRUE	reported	textfile	0.0047	3.14E-06	6486	M36399.m	TRUE	reported	nhT4FM	2	TRUE	0.003336	21.70494
5785	rs7425298	A	T	A	T	-0.2286	-0.02376	0.9846	0.975184	FALSE	TRUE	FALSE	eT2We0	0.044115	0.590235	486484	sepsis	TRUE	reported	textfile	0.0489	2.88E-06	292	M33353.m	TRUE	reported	uZx90m	2	TRUE	0.069632	21.7045
600	rs2293319	T	C	T	C	-0.0219	0.000138	0.7852	0.781983	FALSE	FALSE	FALSE	KVK33e	0.016566	0.993341	486484	sepsis	TRUE	reported	textfile	0.0047	3.59E-06	5344	M01107.m	TRUE	reported	eAyR8w	2	TRUE	0.004046	21.70351
2370	rs1223845	A	G	A	G	0.1431	0.050121	0.9559	0.954384	FALSE	FALSE	FALSE	NBJ2Do	0.03299	0.128693	486484	sepsis	TRUE	reported	textfile	0.0307	3.09E-06	1739	M17799.m	TRUE	reported	Uz0Wgl	2	TRUE	0.01234	21.70215
4927	rs2899743	C	G	C	G	-0.0261	-0.01573	0.6897	0.672583	FALSE	TRUE	FALSE	clhN6v	0.014665	0.283358	486484	sepsis	TRUE	reported	textfile	0.0056	3.79E-06	2148	M32869.m	TRUE	reported	BzloWk	2	TRUE	0.010012	21.70203
7989	rs1167190	A	C	A	C	0.0205	-0.00118	0.7672	0.76176	FALSE	FALSE	FALSE	Xrodum	0.016073	0.941571	486484	sepsis	TRUE	reported	textfile	0.0044	3.63E-06	7291	M35255.m	TRUE	reported	T8t1tl	2	TRUE	0.002968	21.70117
4882	rs6747014	T	C	T	C	0.048	-0.0251	0.8967	0.903007	FALSE	FALSE	FALSE	MDX5FX	0.023091	0.277049	486484	sepsis	TRUE	reported	textfile	0.0103	3.12E-06	2656	M32857.m	TRUE	reported	moAN2E	2	TRUE	0.00811	21.70106
8407	rs1077012	A	G	A	G	0.0205	0.023041	0.5547	0.568123	FALSE	FALSE	FALSE	02RdNm	0.013789	0.094735	486484	sepsis	TRUE	reported	textfile	0.0044	2.68E-06	6447	M35678.m	TRUE	reported	BWN5JR	2	TRUE	0.003356	21.70039
3867	rs1112615	A	G	A	G	-0.0205	0.014909	0.7711	0.750002	FALSE	FALSE	FALSE	7LphBB	0.015782	0.344827	486484	sepsis	TRUE	reported	textfile	0.0044	3.22E-06	6066	M32388.m	TRUE	reported	5oj12c	2	TRUE	0.003566	21.69997
4823	rs3133745	T	C	T	C	-0.0396	-0.01094	0.1259	0.130506	FALSE	FALSE	FALSE	B6WchP	0.02142	0.609564	486484	sepsis	TRUE	reported	textfile	0.0085	2.71E-06	7255	M32836.m	TRUE	reported	7UzUw0	2	TRUE	0.002983	21.69865
6589	rs3791160	T	C	T	C	0.4005	-0.00817	0.0205	0.029603	FALSE	FALSE	FALSE	XO3rXq	0.04053	0.840313	486484	sepsis	TRUE	reported	textfile	0.0856	2.87E-06	228	M33877.m	TRUE	reported	p2sETg	2	TRUE	0.087601	21.69857
7529	rs6429539	A	G	A	G	-0.0261	-0.02423	0.0308	0.020237	FALSE	FALSE	FALSE	zV4uYs	0.048697	0.61881	486484	sepsis	TRUE	reported	textfile	0.0056	3.54E-06	1755	M34453.m	TRUE	reported	nLRRs2	2	TRUE	0.012226	21.6975
4402	rs6862720	T	C	T	C	0.0191	-0.00271	0.0785	0.082896	FALSE	FALSE	FALSE	UJygVd	0.024725	0.912704	486484	sepsis	TRUE	reported	textfile	0.0041	4.28E-06	7361	M32654.m	TRUE	reported	kv9R0W	2	TRUE	0.00294	21.69607
4040	rs1684443	A	G	A	G	-0.0191	-0.01673	0.9276	0.924316	FALSE	FALSE	FALSE	SiYGYU	0.026127	0.521914	486484	sepsis	TRUE	reported	textfile	0.0041	2.72E-06	7355	M32492.m	TRUE	reported	GfQvpU	2	TRUE	0.002942	21.69606
8996	rs6755484	T	C	T	C	-0.0191	-0.02005	0.4847	0.488378	FALSE	FALSE	FALSE	TfdxtP	0.013707	0.143548	486484	sepsis	TRUE	reported	textfile	0.0041	3.11E-06	7333	M37203.m	TRUE	reported	xicmWr	2	TRUE	0.002951	21.69604
3580	rs4384785	A	G	A	G	0.1372	0.019441	0.1018	0.111287	FALSE	FALSE	FALSE	F1s6qg	0.021838	0.373333	486484	sepsis	TRUE	reported	textfile	0.0294	3.16E-06	522	M31536.m	TRUE	reported	Zm40GX	2	TRUE	0.040049	21.69434
5719	rs8796	T	G	T	G	0.0634	-0.00251	0.1633	0.203821	FALSE	FALSE	FALSE	XDem1i	0.017094	0.883237	486484	sepsis	TRUE	reported	textfile	0.0136	3.21E-06	1142	M33221.m	TRUE	reported	KBV6FH	2	TRUE	0.018674	21.69399
5775	rs130798	A	T	A	T	0.0578	0.031311	0.1603	0.182531	FALSE	TRUE	FALSE	eT2We0	0.017709	0.077044	486484	sepsis	TRUE	reported	textfile	0.0124	2.99E-06	1273	M33353.m	TRUE	reported	uZx90m	2	TRUE	0.016782	21.69349
4886	rs1247994	T	G	T	G	-0.0918	-0.01788	0.9236	0.924344	FALSE	FALSE	FALSE	ocaywK	0.025836	0.488802	486484	sepsis	TRUE	reported	textfile	0.0197	3.39E-06	1988	M32863.m	TRUE	reported	WH6KGr	2	TRUE	0.010805	21.69281
6870	rs17352	T	G	T	G	0.0233	-0.01643	0.8945	0.888189	FALSE	FALSE	FALSE	kFWZjs	0.021705	0.449088	486484	sepsis	TRUE	reported	textfile	0.005	3.13E-06	1768	M34062.m	TRUE	reported	eLONsg	2	TRUE	0.012134	21.69103
4828	rs1113499	T	C	T	C	0.0177	0.020092	0.3569	0.368408	FALSE	FALSE	FALSE	yLDcUj	0.014338	0.161122	486484	sepsis	TRUE	reported	textfile	0.0038	2.96E-06	6753	M32838.m	TRUE	reported	5dQgAN	2	TRUE	0.003203	21.68956
8716	rs3790310	A	T	A	T	0.0177	-0.00278	0.8295	0.828755	FALSE	TRUE	FALSE	l0G0l1	0.018139	0.878101	486484	sepsis	TRUE	reported	textfile	0.0038	3.46E-06	6464	M36553.m	TRUE	reported	lBltb0	2	TRUE	0.003345	21.68927
6641	rs1076483	T	C	T	C	-0.0177	-0.00112	0.788	0.788982	FALSE	FALSE	FALSE	REeQiq	0.016904	0.946992	486484	sepsis	TRUE	reported	textfile	0.0038	3.78E-06	6004	M33892.m	TRUE	reported	cVOa3u	2	TRUE	0.003601	21.68876
2355	rs1097659	T	C	T	C	0.1141	0.027052	0.92	0.965407	FALSE	FALSE	FALSE	NBJ2Do	0.037257	0.467784	486484	sepsis	TRUE	reported	textfile	0.0235	1.16E-06	25	M17799.m	TRUE	reported	Uz0Wgl	2	TRUE	0.485323	21.68819
8342	rs1705941	A	G	A	G	0.1061	0.035148	0.3922	0.371775	FALSE	FALSE	FALSE	USZiKY	0.014196	0.01329	486484	sepsis	TRUE	reported	textfile	0.0227	2.86E-06	274	M35635.m	TRUE	reported	HuVMRL	2	TRUE	0.073844	21.6869
6303	rs1341788	A	G	A	G	0.034	-0.00632	0.9333	0.936055	FALSE	FALSE	FALSE	ORVTMc	0.027878	0.820541	486484	sepsis	TRUE	reported	textfile	0.0073	3.41E-06	7352	M33587.m	TRUE	reported	57AsXW	2	TRUE	0.002942	21.68672
6343	rs601208	T	C	T	C	0.0518	-0.02437	0.6648	0.655522	FALSE	FALSE	FALSE	qLUY00	0.01438	0.090074	486484	sepsis	TRUE	reported	textfile	0.0111	3.04E-06	470	M33610.m	TRUE	reported	8cAJbB	2	TRUE	0.044284	21.68511
2966	rs9521664	T	C	T	C	-0.0708	-0.05799	0.9351	0.93545	FALSE	FALSE	FALSE	zndu0r	0.028568	0.042369	486484	sepsis	TRUE	reported	textfile	0.0152	3.24E-06	3422	M21049.m	TRUE	reported	2XpPDU	2	TRUE	0.0063	21.6833
2621	rs1166005	T	C	T	C	-0.0326	0.044563	0.8153	0.790465	FALSE	FALSE	FALSE	eygBBR	0.016791	0.007954	486484	sepsis	TRUE	reported	textfile	0.007	3.68E-06	6970	M18394.m	TRUE	reported	hD79iE	2	TRUE	0.003102	21.68276
4764	rs6762129	T	C	T	C	0.0163	-0.01591	0.3537	0.35818	FALSE	FALSE	FALSE	RDfDIS	0.014307	0.266007	486484	sepsis	TRUE	reported	textfile	0.0035	3.06E-06	6857	M32787.m	TRUE	reported	Hq6nGt	2	TRUE	0.003153	21.68265
1148	rs2421668	A	G	A	G	0.034	-0.00772	0.644	0.644158	FALSE	FALSE	FALSE	QtY5Hc	0.014261	0.588209	486484	sepsis	TRUE	reported	textfile	0.0073	2.87E-06	4207	M01605.m	TRUE	reported	Q3iDQX	2	TRUE	0.00513	21.68231
2916	rs6591040	T	G	T	G	-0.0205	-0.01586	0.5899	0.59247	FALSE	FALSE	FALSE	FBDEQi	0.013887	0.253509	486484	sepsis	TRUE	reported	textfile	0.0044	3.34E-06	1748	M20699.m	TRUE	reported	nvNllp	2	TRUE	0.012266	21.68229
3046	rs1022117	T	C	T	C	0.0163	0.040692	0.1464	0.156776	FALSE	FALSE	FALSE	H6vcCG	0.018809	0.030504	486484	sepsis	TRUE	reported	textfile	0.0035	2.32E-06	6180	M22030.m	TRUE	reported	XetVX7	2	TRUE	0.003497	21.68196
8089	rs724467	T	C	T	C	0.0602	-0.02191	0.2576	0.242604	FALSE	FALSE	FALSE	z65cF2	0.015987	0.170462	486484	sepsis	TRUE	reported	textfile	0.0129	2.92E-06	448	M35322.m	TRUE	reported	3GUSWk	2	TRUE	0.046358	21.68056
3510	rs1009794	A	G	A	G	0.0475	-0.00703	0.9488	0.923218	FALSE	FALSE	FALSE	hxuqls	0.025908	0.786156	486484	sepsis	TRUE	reported	textfile	0.0102	3.41E-06	7336	M27738.m	TRUE	reported	aueeZs	2	TRUE	0.002947	21.68046
1093	rs1577941	A	G	A	G	0.0531	-0.00352	0.1187	0.148668	FALSE	FALSE	FALSE	K3wtZo	0.019287	0.855111	486484	sepsis	TRUE	reported	textfile	0.0114	3.49E-06	2315	M01573.m	TRUE	reported	0D9CvA	2	TRUE	0.009285	21.67724
2196	rs1183853	A	G	A	G	0.0284	-0.04361	0.0205	0.021107	FALSE	FALSE	FALSE	Bf6uMK	0.04772	0.360831	486484	sepsis	TRUE	reported	textfile	0.0061	3.38E-06	7308	M15778.m	TRUE	reported	jowM2N	2	TRUE	0.002957	21.66996
5635	rs4542650	T	C	T	C	0.0624	0.011418	0.1769	0.176582	FALSE	FALSE	FALSE	U0d0ue	0.017942	0.524549	486484	sepsis	TRUE	reported	textfile	0.0134	3.19E-06	2616	M33190.m	TRUE	reported	FoODGF	2	TRUE	0.008221	21.66843
4820	rs1192325	A	G	A	G	-0.0419	0.056238	0.9158	0.914495	FALSE	FALSE	FALSE	B6WchP	0.024444	0.02141	486484	sepsis	TRUE	reported	textfile	0.009	3.30E-06	7255	M32836.m	TRUE	reported	7UzUw0	2	TRUE	0.002979	21.66822
8355	rs2127434	A	G	A	G	0.0284	-0.00689	0.7956	0.779386	FALSE	FALSE	FALSE	6VVCe4	0.016702	0.680166	486484	sepsis	TRUE	reported	textfile	0.0061	3.23E-06	5629	M35669.m	TRUE	reported	l0fk8X	2	TRUE	0.003836	21.66819
7416	rs3852445	C	G	C	G	0.0284	0.023772	0.1284	0.136613	FALSE	TRUE	FALSE	7i5VUS	0.020216	0.239614	486484	sepsis	TRUE	reported	textfile	0.0061	2.95E-06	5208	M34395.m	TRUE	reported	64hEaL	2	TRUE	0.004145	21.66757
7436	rs9333273	A	G	A	G	0.0554	0.040961	0.0869	0.018331	FALSE	FALSE	FALSE	NZPe5A	0.050617	0.418382	486484	sepsis	TRUE	reported	textfile	0.0119	3.54E-06	5689	M34400.m	TRUE	reported	OyPwYO	2	TRUE	0.003795	21.66571
3954	rs9316766	A	G	A	G	-0.027	0.006266	0.7962	0.797514	FALSE	FALSE	FALSE	N6UQnQ	0.01711	0.714199	486484	sepsis	TRUE	reported	textfile	0.0058	2.82E-06	7346	M32425.m	TRUE	reported	FEcGHY	2	TRUE	0.002941	21.66473
2383	rs1561803	T	G	T	G	0.1304	0.029009	0.9623	0.949987	FALSE	FALSE	FALSE	NBJ2Do	0.031288	0.353829	486484	sepsis	TRUE	reported	textfile	0.028	3.28E-06	1739	M17799.m	TRUE	reported	Uz0Wgl	2	TRUE	0.012318	21.66404
7084	rs1083586	A	G	A	G	0.1318	0.011152	0.9826	0.587851	FALSE	FALSE	FALSE	pxNICA	0.013847	0.420608	486484	sepsis	TRUE	reported	textfile	0.0283	3.31E-06	1673	M34306.m	TRUE	reported	xk5b9M	2	TRUE	0.012799	21.66399
6649	rs1738009	T	C	T	C	-0.0135	-0.00408	0.5345	0.550007	FALSE	FALSE	FALSE	REeQiq	0.013969	0.770375	486484	sepsis	TRUE	reported	textfile	0.0029	2.75E-06	6004	M33892.m	TRUE	reported	cVOa3u	2	TRUE	0.003596	21.66341
6654	rs20301	A	G	A	G	0.0135	0.011761	0.5617	0.557914	FALSE	FALSE	FALSE	REeQiq	0.013758	0.39264	486484	sepsis	TRUE	reported	textfile	0.0029	2.98E-06	6004	M33892.m	TRUE	reported	cVOa3u	2	TRUE	0.003596	21.66341
6495	rs1160835	T	C	T	C	0.2555	0.036663	0.0342	0.032267	FALSE	FALSE	FALSE	HNc7C9	0.038815	0.344887	486484	sepsis	TRUE	reported	textfile	0.0548	3.07E-06	581	M33782.m	TRUE	reported	SAGYGx	2	TRUE	0.036066	21.66323
4714	rs2132551	A	C	A	C	0.054	0.002212	0.964	0.958601	FALSE	FALSE	FALSE	L2WFM1	0.034321	0.948612	486484	sepsis	TRUE	reported	textfile	0.0116	3.56E-06	5579	M32761.m	TRUE	reported	5GHzph	2	TRUE	0.003869	21.66286
1822	rs4404993	T	G	T	G	0.0964	0.029414	0.1848	0.20702	FALSE	FALSE	FALSE	Yub5KV	0.016946	0.082602	486484	sepsis	TRUE	reported	textfile	0.0207	3.22E-06	1522	M15335.m	TRUE	reported	mcnINa	2	TRUE	0.014049	21.6592
2901	rs1269917	A	G	A	G	-0.0461	0.040599	0.9499	0.957632	FALSE	FALSE	FALSE	FBDEQi	0.033899	0.231062	486484	sepsis	TRUE	reported	textfile	0.0099	3.03E-06	1748	M20699.m	TRUE	reported	nvNllp	2	TRUE	0.012253	21.65879
710	rs4863214	T	C	T	C	-0.0149	-0.03067	0.7398	0.688595	FALSE	FALSE	FALSE	H9I3s3	0.014952	0.040267	486484	sepsis	TRUE	reported	textfile	0.0032	3.98E-06	1761	M01299.m	TRUE	reported	kXWyUq	2	TRUE	0.012162	21.65604
8941	rs1748359	T	C	T	C	0.1024	0.029273	0.9827	0.982206	FALSE	FALSE	FALSE	mXJP3h	0.051844	0.572328	486484	sepsis	TRUE	reported	textfile	0.022	3.16E-06	4520	M37104.m	TRUE	reported	HRZBak	2	TRUE	0.00477	21.65521
6793	rs1046508	A	T	A	T	-0.0121	-0.00456	0.2818	0.280587	FALSE	TRUE	FALSE	OZ0I1H	0.015208	0.764226	486484	sepsis	TRUE	reported	textfile	0.0026	4.69E-06	7364	M33960.m	TRUE	reported	GlPp4w	2	TRUE	0.002932	21.6524
4491	rs1801394	A	G	A	G	0.0121	-0.0073	0.4675	0.436014	FALSE	FALSE	FALSE	1wNJ1E	0.013785	0.596554	486484	sepsis	TRUE	reported	textfile	0.0026	3.33E-06	7363	M32709.m	TRUE	reported	zihBHg	2	TRUE	0.002933	21.6524
3687	rs9348257	A	T	A	T	0.0121	-0.0147	0.2629	0.251625	FALSE	TRUE	FALSE	rGHJcj	0.015858	0.353829	486484	sepsis	TRUE	reported	textfile	0.0026	2.74E-06	7356	M32198.m	TRUE	reported	6P1Nno	2	TRUE	0.002936	21.6524
783	rs1024217	A	G	A	G	-0.0121	-0.00322	0.1355	0.139437	FALSE	FALSE	FALSE	dyT79l	0.019762	0.870374	486484	sepsis	TRUE	reported	textfile	0.0026	3.73E-06	7352	M01336.m	TRUE	reported	OemVJ2	2	TRUE	0.002937	21.65239
7886	rs1108167	T	C	T	C	0.0121	0.008611	0.3179	0.340467	FALSE	FALSE	FALSE	pU8zps	0.014494	0.552428	486484	sepsis	TRUE	reported	textfile	0.0026	3.70E-06	7350	M35186.m	TRUE	reported	Oc2lOF	2	TRUE	0.002938	21.65239
530	rs1693280	T	G	T	G	-0.0121	-0.01361	0.1673	0.168115	FALSE	FALSE	FALSE	wXMWHy	0.018369	0.458906	486484	sepsis	TRUE	reported	textfile	0.0026	5.01E-06	7345	M00584.m	TRUE	reported	7CKn2b	2	TRUE	0.00294	21.65239
1393	rs6994363	A	G	A	G	-0.0121	-0.00308	0.8662	0.84654	FALSE	FALSE	FALSE	KrWhws	0.0192	0.872721	486484	sepsis	TRUE	reported	textfile	0.0026	2.50E-06	7325	M02132.m	TRUE	reported	7pzpfK	2	TRUE	0.002948	21.65237
2698	rs1280328	A	G	A	G	-0.0121	-0.00788	0.2119	0.229516	FALSE	FALSE	FALSE	XsQHZK	0.016248	0.627493	486484	sepsis	TRUE	reported	textfile	0.0026	4.31E-06	7286	M18929.m	TRUE	reported	k8R7hN	2	TRUE	0.002964	21.65234
2704	rs4904262	T	C	T	C	-0.0121	0.000976	0.1727	0.173911	FALSE	FALSE	FALSE	XsQHZK	0.018041	0.956853	486484	sepsis	TRUE	reported	textfile	0.0026	4.91E-06	7286	M18929.m	TRUE	reported	k8R7hN	2	TRUE	0.002964	21.65234
7973	rs9293713	A	G	A	G	-0.0242	0.04394	0.0653	0.068287	FALSE	FALSE	FALSE	zb8m4a	0.027334	0.107943	486484	sepsis	TRUE	reported	textfile	0.0052	3.39E-06	7261	M35253.m	TRUE	reported	tHnnnS	2	TRUE	0.002974	21.65232
8223	rs1219213	A	G	A	G	-0.0121	0.028908	0.262	0.276038	FALSE	FALSE	FALSE	4YAWhA	0.015276	0.058431	486484	sepsis	TRUE	reported	textfile	0.0026	4.75E-06	7256	M35439.m	TRUE	reported	bSSC4Z	2	TRUE	0.002976	21.65231
3234	rs805780	A	C	A	C	0.0121	-0.02012	0.7634	0.774561	FALSE	FALSE	FALSE	I25GHB	0.016383	0.21953	486484	sepsis	TRUE	reported	textfile	0.0026	3.47E-06	7184	M22548.m	TRUE	reported	eBmTQD	2	TRUE	0.003006	21.65225
6285	rs6694467	A	G	A	G	0.0121	0.02627	0.8153	0.825973	FALSE	FALSE	FALSE	MOonP9	0.018038	0.145293	486484	sepsis	TRUE	reported	textfile	0.0026	4.44E-06	7066	M33515.m	TRUE	reported	226GuP	2	TRUE	0.003056	21.65215
4809	rs1581762	A	G	A	G	-0.0121	-0.00786	0.4121	0.422327	FALSE	FALSE	FALSE	bJwLV5	0.01389	0.57154	486484	sepsis	TRUE	reported	textfile	0.0026	3.28E-06	7063	M32814.m	TRUE	reported	qte0qp	2	TRUE	0.003057	21.65215
8203	rs4730501	A	G	A	G	-0.0121	0.008267	0.239	0.243408	FALSE	FALSE	FALSE	iLlpho	0.015919	0.603522	486484	sepsis	TRUE	reported	textfile	0.0026	4.18E-06	7007	M35431.m	TRUE	reported	AAZ5ge	2	TRUE	0.003081	21.6521
7314	rs7763535	A	C	A	C	0.0121	-0.00737	0.7642	0.707286	FALSE	FALSE	FALSE	Vcz4EA	0.015036	0.624141	486484	sepsis	TRUE	reported	textfile	0.0026	2.06E-06	6755	M34359.m	TRUE	reported	aUSo2J	2	TRUE	0.003196	21.65187
6733	rs1869289	A	C	A	C	-0.0121	0.025509	0.2164	0.221508	FALSE	FALSE	FALSE	SpG9A1	0.0165	0.122099	486484	sepsis	TRUE	reported	textfile	0.0026	4.31E-06	6502	M33939.m	TRUE	reported	Tudxel	2	TRUE	0.00332	21.65162
5095	rs1093283	A	G	A	G	0.0121	0.003148	0.5337	0.542255	FALSE	FALSE	FALSE	Uiz8VU	0.013722	0.81856	486484	sepsis	TRUE	reported	textfile	0.0026	3.86E-06	6404	M33163.m	TRUE	reported	ky0Bda	2	TRUE	0.003371	21.65152
7357	rs3759903	T	G	T	G	0.1253	-0.03886	0.1612	0.074933	FALSE	FALSE	FALSE	mm9ksf	0.02607	0.136094	486484	sepsis	TRUE	reported	textfile	0.0269	3.09E-06	954	M34365.m	TRUE	reported	AVwEqD	2	TRUE	0.022237	21.65141
5849	rs1690511	A	T	A	T	0.3196	-0.01282	0.0413	0.048982	FALSE	TRUE	FALSE	Vkec6Y	0.031938	0.688079	486484	sepsis	TRUE	reported	textfile	0.0686	3.17E-06	803	M33384.m	TRUE	reported	eaxeyR	2	TRUE	0.026319	21.65122
6462	rs2074116	A	G	A	G	-0.0503	-0.02135	0.7665	0.779447	FALSE	FALSE	FALSE	yLohrN	0.016562	0.197302	486484	sepsis	TRUE	reported	textfile	0.0108	3.61E-06	1027	M33683.m	TRUE	reported	mvhAZ4	2	TRUE	0.020684	21.6492
7789	rs929005	A	T	A	T	-0.0349	0.023542	0.1602	0.221982	FALSE	TRUE	FALSE	15BqPa	0.016752	0.159931	486484	sepsis	TRUE	reported	textfile	0.0075	3.18E-06	7364	M35126.m	TRUE	reported	zvDzP2	2	TRUE	0.002932	21.64763
7715	rs7942717	A	G	A	G	-0.1094	-0.00775	0.9543	0.919133	FALSE	FALSE	FALSE	qJW9lg	0.02536	0.759813	486484	sepsis	TRUE	reported	textfile	0.0235	3.18E-06	1763	M34878.m	TRUE	reported	42EOzB	2	TRUE	0.012143	21.64741
1142	rs9549021	T	C	T	C	-0.0135	-0.00374	0.6112	0.626747	FALSE	FALSE	FALSE	nlqUuV	0.014167	0.791555	486484	sepsis	TRUE	reported	textfile	0.0029	2.94E-06	1767	M01604.m	TRUE	reported	Uro9nC	2	TRUE	0.012115	21.6461
815	rs6486528	C	G	C	G	0.0228	0.018511	0.9236	0.921633	FALSE	TRUE	FALSE	J58DI0	0.025858	0.474073	486484	sepsis	TRUE	reported	textfile	0.0049	3.30E-06	7335	M01356.m	TRUE	reported	zk9AMB	2	TRUE	0.002943	21.64508
5002	rs786272	A	G	A	G	0.0228	0.016959	0.6761	0.662684	FALSE	FALSE	FALSE	OsqE1P	0.014465	0.241027	486484	sepsis	TRUE	reported	textfile	0.0049	3.02E-06	5425	M33084.m	TRUE	reported	VZYh6f	2	TRUE	0.003975	21.643
4291	rs1783633	C	G	C	G	0.0335	-0.08188	0.9808	0.986661	FALSE	TRUE	FALSE	0tjjPC	0.059777	0.170775	486484	sepsis	TRUE	reported	textfile	0.0072	2.99E-06	7362	M32634.m	TRUE	reported	nJJFkX	2	TRUE	0.002932	21.64246
5760	rs2526978	T	C	T	C	-0.0228	0.016725	0.2984	0.309899	FALSE	FALSE	FALSE	n16uO2	0.01474	0.256503	486484	sepsis	TRUE	reported	textfile	0.0049	2.63E-06	4409	M33250.m	TRUE	reported	9dhl67	2	TRUE	0.004887	21.64116
7244	rs610810	T	G	T	G	-0.298	0.05619	0.988	0.98346	FALSE	FALSE	FALSE	RlcE3J	0.053829	0.296558	486484	sepsis	TRUE	reported	textfile	0.064	3.22E-06	1016	M34339.m	TRUE	reported	QQrwdm	2	TRUE	0.020893	21.63799
6021	rs1683482	T	C	T	C	-1.557	-0.00664	0.0179	0.02456	FALSE	FALSE	FALSE	33ojbt	0.045015	0.882708	486484	sepsis	TRUE	reported	textfile	0.3291	2.23E-06	60	M33423.m	TRUE	reported	ivEtvl	2	TRUE	0.271696	21.63706
1158	rs1271426	A	G	A	G	-0.0107	0.025976	0.5733	0.570769	FALSE	FALSE	FALSE	cSBFJ3	0.013877	0.061225	486484	sepsis	TRUE	reported	textfile	0.0023	3.98E-06	7104	M01638.m	TRUE	reported	sUkjpE	2	TRUE	0.003037	21.63663
7099	rs7071570	T	C	T	C	0.0214	-0.02356	0.0701	0.071252	FALSE	FALSE	FALSE	pxNICA	0.026495	0.373888	486484	sepsis	TRUE	reported	textfile	0.0046	2.66E-06	6994	M34306.m	TRUE	reported	xk5b9M	2	TRUE	0.003085	21.63653
2660	rs7837471	A	G	A	G	0.3403	-0.01144	0.015	0.027966	FALSE	FALSE	FALSE	UtYu7J	0.04126	0.781495	486484	sepsis	TRUE	reported	textfile	0.0731	3.27E-06	1157	M18476.m	TRUE	reported	wJx0A4	2	TRUE	0.018386	21.63405
7558	rs1281014	T	C	T	C	0.0754	-0.00711	0.079	0.114251	FALSE	FALSE	FALSE	APSWAU	0.02149	0.740694	486484	sepsis	TRUE	reported	textfile	0.0162	3.35E-06	1462	M34469.m	TRUE	reported	frF3RX	2	TRUE	0.014601	21.63307
8410	rs1207084	A	G	A	G	0.0414	-0.05153	0.0702	0.085161	FALSE	FALSE	FALSE	02RdNm	0.024403	0.03473	486484	sepsis	TRUE	reported	textfile	0.0089	3.16E-06	6447	M35678.m	TRUE	reported	BWN5JR	2	TRUE	0.003345	21.63146
8983	rs7968170	T	C	T	C	-0.04	-0.0132	0.4284	0.458576	FALSE	FALSE	FALSE	snOepl	0.013805	0.338813	486484	sepsis	TRUE	reported	textfile	0.0086	3.16E-06	7355	M37202.m	TRUE	reported	PQx272	2	TRUE	0.002933	21.62743
7893	rs2504453	A	T	A	T	0.02	-0.00081	0.8969	0.886885	FALSE	TRUE	FALSE	pU8zps	0.021643	0.970142	486484	sepsis	TRUE	reported	textfile	0.0043	3.35E-06	7350	M35186.m	TRUE	reported	Oc2lOF	2	TRUE	0.002935	21.62743
4198	rs603677	A	C	A	C	0.12	0.060392	0.9724	0.982439	FALSE	FALSE	FALSE	TxOCzG	0.052469	0.249734	486484	sepsis	TRUE	reported	textfile	0.0258	3.35E-06	6970	M32564.m	TRUE	reported	9JP6ND	2	TRUE	0.003094	21.62711
3859	rs2575043	A	T	A	T	-0.02	0.011337	0.2748	0.281522	FALSE	TRUE	FALSE	8gg1zS	0.015208	0.455977	486484	sepsis	TRUE	reported	textfile	0.0043	3.63E-06	6119	M32379.m	TRUE	reported	uZWtuS	2	TRUE	0.003523	21.62624
2562	rs6955908	T	C	T	C	0.0386	0.004634	0.4325	0.427344	FALSE	FALSE	FALSE	WfyP8n	0.013908	0.739018	486484	sepsis	TRUE	reported	textfile	0.0083	3.50E-06	5899	M18335.m	TRUE	reported	mjljpe	2	TRUE	0.003653	21.62077
67	rs2063601	A	C	A	C	-0.0107	0.013645	0.2487	0.236742	FALSE	FALSE	FALSE	xmGuol	0.016043	0.395035	486484	sepsis	TRUE	reported	textfile	0.0023	5.24E-06	1763	M00054.m	TRUE	reported	GW50Ri	2	TRUE	0.012127	21.61817
2179	rs1246642	A	G	A	G	0.0279	0.028282	0.6945	0.694189	FALSE	FALSE	FALSE	UokbxN	0.014758	0.055306	486484	sepsis	TRUE	reported	textfile	0.006	3.96E-06	7357	M15753.m	TRUE	reported	lUGKBi	2	TRUE	0.00293	21.61662
7803	rs4839333	A	T	A	T	0.0186	-0.0131	0.8828	0.886905	FALSE	TRUE	FALSE	cQ5TvO	0.021568	0.543488	486484	sepsis	TRUE	reported	textfile	0.004	2.91E-06	7344	M35127.m	TRUE	reported	BVxkfZ	2	TRUE	0.002936	21.61661
3628	rs7152358	A	G	A	G	0.0465	0.024341	0.8887	0.879995	FALSE	FALSE	FALSE	gq4lSJ	0.021057	0.247702	486484	sepsis	TRUE	reported	textfile	0.01	3.40E-06	7338	M31591.m	TRUE	reported	wjwl4J	2	TRUE	0.002938	21.61661
6967	rs7483614	T	C	T	C	0.0186	0.009181	0.8665	0.837766	FALSE	FALSE	FALSE	7Cw2XZ	0.018555	0.620749	486484	sepsis	TRUE	reported	textfile	0.004	3.97E-06	7049	M34123.m	TRUE	reported	vrgMl3	2	TRUE	0.003058	21.61637
3586	rs737853	A	G	A	G	-0.0736	-0.02426	0.617	0.530644	FALSE	FALSE	FALSE	F1s6qg	0.013838	0.079623	486484	sepsis	TRUE	reported	textfile	0.0158	3.05E-06	522	M31536.m	TRUE	reported	Zm40GX	2	TRUE	0.03991	21.61595
3125	rs1278732	T	G	T	G	0.0093	-0.01649	0.6649	0.650066	FALSE	FALSE	FALSE	JiXR2t	0.014416	0.252588	486484	sepsis	TRUE	reported	textfile	0.002	1.65E-06	6535	M22177.m	TRUE	reported	joi6p6	2	TRUE	0.003298	21.61588
1429	rs6466265	A	G	A	G	0.0186	0.007143	0.7977	0.802858	FALSE	FALSE	FALSE	ac6RGB	0.017302	0.679707	486484	sepsis	TRUE	reported	textfile	0.004	4.15E-06	5791	M02342.m	TRUE	reported	dC6Rkp	2	TRUE	0.00372	21.61503

953	rs1775180	A	G	A	G	0.0628	0.038165	0.9645	0.96565	FALSE	FALSE	FALSE	tWoDq4	0.037886	0.313773	486484	sepsis	TRUE	reported	textfile	0.0135	3.40E-06	1650	M01508.m	TRUE	reported	6ltDw0	2	TRUE	0.012945	21.6135
8936	rs1046376	T	C	T	C	-0.0372	-0.00244	0.1434	0.145405	FALSE	FALSE	FALSE	mXJP3h	0.019429	0.900143	486484	sepsis	TRUE	reported	textfile	0.008	3.37E-06	4520	M37104.m	TRUE	reported	HRZBak	2	TRUE	0.004761	21.61293
5237	rs1187664	T	C	T	C	-0.348	0.111469	0.0271	0.005743	FALSE	FALSE	FALSE	q32d4E	0.091591	0.223591	486484	sepsis	TRUE	reported	textfile	0.0718	1.23E-06	25	M33178.m	TRUE	reported	cS3BwT	2	TRUE	0.484445	21.61212
3512	rs1191458	T	C	T	C	-0.0307	0.013771	0.3531	0.348842	FALSE	FALSE	FALSE	hxuqls	0.014337	0.336801	486484	sepsis	TRUE	reported	textfile	0.0066	2.89E-06	1758	M27738.m	TRUE	reported	aueeZs	2	TRUE	0.012158	21.61198
4171	rs1195197	A	G	A	G	-0.0493	0.029593	0.352	0.424367	FALSE	FALSE	FALSE	Aq7H4p	0.013969	0.034134	486484	sepsis	TRUE	reported	textfile	0.0106	3.15E-06	2091	M32560.m	TRUE	reported	NIzbA7	2	TRUE	0.010239	21.61058
8726	rs1172292	T	C	T	C	-0.0307	-0.03269	0.8291	0.819389	FALSE	FALSE	FALSE	ERC6hD	0.017805	0.0664	486484	sepsis	TRUE	reported	textfile	0.0066	3.74E-06	1636	M36559.m	TRUE	reported	n8waJF	2	TRUE	0.013053	21.61014
7588	rs2974445	T	C	T	C	0.0279	-0.00399	0.5529	0.550707	FALSE	FALSE	FALSE	SbjFMF	0.013733	0.771305	486484	sepsis	TRUE	reported	textfile	0.006	3.11E-06	3239	M34513.m	TRUE	reported	HVYiKM	2	TRUE	0.006631	21.60915
6672	rs2432026	A	G	A	G	-0.0451	-0.02023	0.0587	0.058453	FALSE	FALSE	FALSE	6iZSOI	0.029161	0.487808	486484	sepsis	TRUE	reported	textfile	0.0097	3.49E-06	4533	M33901.m	TRUE	reported	ApKaGe	2	TRUE	0.004746	21.60817
5606	rs9574766	A	G	A	G	-0.3499	0.014416	0.025	0.046651	FALSE	FALSE	FALSE	q32d4E	0.036487	0.692767	486484	sepsis	TRUE	reported	textfile	0.0722	1.25E-06	25	M33178.m	TRUE	reported	cS3BwT	2	TRUE	0.48439	21.60734
7615	rs1326499	A	G	A	G	-0.0293	0.014782	0.2032	0.19159	FALSE	FALSE	FALSE	kVfKqT	0.017403	0.395659	486484	sepsis	TRUE	reported	textfile	0.0063	3.46E-06	1741	M34527.m	TRUE	reported	s4ti92	2	TRUE	0.012271	21.60503
7696	rs6907935	T	C	T	C	-0.0172	-0.0133	0.6945	0.688897	FALSE	FALSE	FALSE	4qwHDC	0.01476	0.367421	486484	sepsis	TRUE	reported	textfile	0.0037	3.81E-06	6735	M34732.m	TRUE	reported	dm6aLf	2	TRUE	0.003198	21.60352
8719	rs4762693	A	G	A	G	0.0172	-0.00955	0.2859	0.292848	FALSE	FALSE	FALSE	I0G0I1	0.015096	0.527082	486484	sepsis	TRUE	reported	textfile	0.0037	4.07E-06	6464	M36553.m	TRUE	reported	IBltb0	2	TRUE	0.003332	21.60325
3071	rs354468	T	C	T	C	0.0172	-0.00121	0.1829	0.184453	FALSE	FALSE	FALSE	e3izea	0.01764	0.945343	486484	sepsis	TRUE	reported	textfile	0.0037	4.59E-06	5676	M22032.m	TRUE	reported	zODKFI	2	TRUE	0.003793	21.60232
4177	rs4742370	C	G	C	G	-0.0465	0.033889	0.3392	0.342557	FALSE	TRUE	FALSE	Aq7H4p	0.014551	0.019858	486484	sepsis	TRUE	reported	textfile	0.01	3.24E-06	2091	M32560.m	TRUE	reported	NIzbA7	2	TRUE	0.010235	21.60182
5777	rs1681855	T	C	T	C	0.0507	0.006084	0.2032	0.285022	FALSE	FALSE	FALSE	eT2We0	0.015158	0.688142	486484	sepsis	TRUE	reported	textfile	0.0109	3.43E-06	1273	M33353.m	TRUE	reported	uZx90m	2	TRUE	0.016712	21.60131
8963	rs1118657	A	G	A	G	-0.1609	0.040233	0.9729	0.966351	FALSE	FALSE	FALSE	2iAvlt	0.037727	0.286236	486484	sepsis	TRUE	reported	textfile	0.0346	3.40E-06	1745	M37190.m	TRUE	reported	hdOuHe	2	TRUE	0.012241	21.6004
6503	rs7132897	A	T	A	T	0.1811	0.024521	0.0742	0.085068	FALSE	TRUE	FALSE	HNc7C9	0.024549	0.317866	486484	sepsis	TRUE	reported	textfile	0.0389	3.20E-06	581	M33782.m	TRUE	reported	SAGYGx	2	TRUE	0.035963	21.59932
492	rs1605457	T	C	T	C	0.3731	0.00442	0.9643	0.950834	FALSE	FALSE	FALSE	RtjZPn	0.031626	0.888846	486484	sepsis	TRUE	reported	textfile	0.08	3.10E-06	283	M00553.m	TRUE	reported	C3Ovsl	2	TRUE	0.071372	21.59685
7920	rs3742758	A	G	A	G	0.0739	-0.0032	0.0191	0.020926	FALSE	FALSE	FALSE	8qXKkX	0.047676	0.946451	486484	sepsis	TRUE	reported	textfile	0.0159	3.45E-06	6234	M35189.m	TRUE	reported	Nt6oaC	2	TRUE	0.003453	21.5951
978	rs1200508	A	T	A	T	0.1232	-0.02127	0.1814	0.229038	FALSE	TRUE	FALSE	xSRII4	0.016374	0.193849	486484	sepsis	TRUE	reported	textfile	0.0265	3.35E-06	2285	M01515.m	TRUE	reported	juZ5GE	2	TRUE	0.00937	21.59481
8209	rs1194525	T	C	T	C	-0.033	0.036277	0.0592	0.061666	FALSE	FALSE	FALSE	aY1HfB	0.02854	0.2037	486484	sepsis	TRUE	reported	textfile	0.0071	3.82E-06	5306	M35433.m	TRUE	reported	H5O4i2	2	TRUE	0.004055	21.59471
3805	rs1602290	A	G	A	G	0.0488	0.04156	0.1276	0.126831	FALSE	FALSE	FALSE	HOq2gb	0.020496	0.042587	486484	sepsis	TRUE	reported	textfile	0.0105	3.25E-06	6687	M32346.m	TRUE	reported	Ss5q17	2	TRUE	0.00322	21.5939
1565	rs569114	A	G	A	G	-0.0079	0.021223	0.6199	0.626721	FALSE	FALSE	FALSE	Ke0sNh	0.014223	0.135648	486484	sepsis	TRUE	reported	textfile	0.0017	1.99E-06	7799	M10642.m	TRUE	reported	ZINwVb	2	TRUE	0.002761	21.58962
1135	rs235320	A	G	A	G	-0.0079	0.010268	0.8241	0.80565	FALSE	FALSE	FALSE	nlqUuV	0.017275	0.55225	486484	sepsis	TRUE	reported	textfile	0.0017	3.68E-06	7371	M01604.m	TRUE	reported	Uro9nC	2	TRUE	0.002921	21.5893
1326	rs1074785	A	G	A	G	-0.0079	0.002867	0.5243	0.52	FALSE	FALSE	FALSE	LDUJbh	0.013746	0.834765	486484	sepsis	TRUE	reported	textfile	0.0017	6.03E-06	7367	M01898.m	TRUE	reported	JRSLeH	2	TRUE	0.002923	21.58929
9046	rs559346	T	C	T	C	0.0079	0.023372	0.4193	0.421261	FALSE	FALSE	FALSE	df0MCM	0.013844	0.091361	486484	sepsis	TRUE	reported	textfile	0.0017	6.62E-06	7366	M37506.m	TRUE	reported	iUfr4u	2	TRUE	0.002923	21.58929
437	rs6970076	T	C	T	C	-0.0079	-0.00855	0.7679	0.766622	FALSE	FALSE	FALSE	cTsNj6	0.016178	0.597088	486484	sepsis	TRUE	reported	textfile	0.0017	4.37E-06	7365	M00063.m	TRUE	reported	7KYTrQ	2	TRUE	0.002924	21.58929
438	rs7595385	T	C	T	C	0.0237	-0.01756	0.9759	0.957312	FALSE	FALSE	FALSE	cTsNj6	0.034512	0.610972	486484	sepsis	TRUE	reported	textfile	0.0051	3.83E-06	7365	M00063.m	TRUE	reported	7KYTrQ	2	TRUE	0.002924	21.58929
442	rs927961	T	G	T	G	-0.0079	-0.02154	0.7597	0.759188	FALSE	FALSE	FALSE	cTsNj6	0.016012	0.17862	486484	sepsis	TRUE	reported	textfile	0.0017	5.06E-06	7365	M00063.m	TRUE	reported	7KYTrQ	2	TRUE	0.002924	21.58929
740	rs4426541	A	G	A	G	-0.0079	0.000515	0.2229	0.226659	FALSE	FALSE	FALSE	9u0SC8	0.016313	0.974835	486484	sepsis	TRUE	reported	textfile	0.0017	5.40E-06	7363	M01301.m	TRUE	reported	H95uec	2	TRUE	0.002924	21.58929
700	rs1475943	T	C	T	C	0.0079	0.021282	0.2215	0.239415	FALSE	FALSE	FALSE	H9I3s3	0.016097	0.186141	486484	sepsis	TRUE	reported	textfile	0.0017	3.69E-06	7358	M01299.m	TRUE	reported	kXWyUq	2	TRUE	0.002926	21.58929
1747	rs3786248	T	C	T	C	-0.0237	-0.00785	0.954	0.958474	FALSE	FALSE	FALSE	nFLpAP	0.034335	0.819239	486484	sepsis	TRUE	reported	textfile	0.0051	3.42E-06	7356	M12774.m	TRUE	reported	yOSQTj	2	TRUE	0.002927	21.58928
824	rs1662999	T	C	T	C	0.0079	0.010026	0.6325	0.621997	FALSE	FALSE	FALSE	2qgDgB	0.014113	0.477431	486484	sepsis	TRUE	reported	textfile	0.0017	4.21E-06	7355	M01358.m	TRUE	reported	asmXq0	2	TRUE	0.002928	21.58928
827	rs2115366	T	C	T	C	-0.0079	-0.02141	0.4418	0.441553	FALSE	FALSE	FALSE	2qgDgB	0.013804	0.120919	486484	sepsis	TRUE	reported	textfile	0.0017	3.93E-06	7355	M01358.m	TRUE	reported	asmXq0	2	TRUE	0.002928	21.58928
843	rs7996008	A	G	A	G	0.0079	-0.00445	0.2935	0.294869	FALSE	FALSE	FALSE	2qgDgB	0.014991	0.766676	486484	sepsis	TRUE	reported	textfile	0.0017	5.69E-06	7355	M01358.m	TRUE	reported	asmXq0	2	TRUE	0.002928	21.58928
7894	rs35544	A	G	A	G	0.0158	-0.02628	0.8264	0.83526	FALSE	FALSE	FALSE	pU8zps	0.018415	0.15354	486484	sepsis	TRUE	reported	textfile	0.0034	4.26E-06	7350	M35186.m	TRUE	reported	Oc2IOF	2	TRUE	0.00293	21.58928
1956	rs2508305	T	C	T	C	-0.0079	0.037952	0.8912	0.882525	FALSE	FALSE	FALSE	v2KE89	0.02126	0.074239	486484	sepsis	TRUE	reported	textfile	0.0017	4.05E-06	7349	M15500.m	TRUE	reported	to2t7H	2	TRUE	0.00293	21.58928
1260	rs1007518	T	G	T	G	0.0316	-0.02807	0.9687	0.967152	FALSE	FALSE	FALSE	gNG3wy	0.038585	0.466874	486484	sepsis	TRUE	reported	textfile	0.0068	3.44E-06	7348	M01670.m	TRUE	reported	t3lsMq	2	TRUE	0.00293	21.58928
764	rs9598459	A	G	A	G	0.0079	0.000754	0.896	0.892532	FALSE	FALSE	FALSE	RHWptG	0.022021	0.972698	486484	sepsis	TRUE	reported	textfile	0.0017	5.17E-06	7347	M01302.m	TRUE	reported	z1OLfr	2	TRUE	0.002931	21.58928
8178	rs4690099	T	C	T	C	0.0079	-0.01783	0.2323	0.227227	FALSE	FALSE	FALSE	8UhtPK	0.016299	0.273926	486484	sepsis	TRUE	reported	textfile	0.0017	2.72E-06	7345	M35397.m	TRUE	reported	rvVJba	2	TRUE	0.002931	21.58928
8182	rs6963604	A	G	A	G	0.0079	-0.00072	0.2522	0.251183	FALSE	FALSE	FALSE	8UhtPK	0.015808	0.963682	486484	sepsis	TRUE	reported	textfile	0.0017	2.20E-06	7345	M35397.m	TRUE	reported	rvVJba	2	TRUE	0.002931	21.58928
4939	rs1194940	T	C	T	C	-0.0079	-0.04196	0.686	0.689441	FALSE	FALSE	FALSE	bKmDMa	0.014835	0.004678	486484	sepsis	TRUE	reported	textfile	0.0017	6.79E-06	7344	M32910.m	TRUE	reported	nAuPzO	2	TRUE	0.002932	21.58927
4427	rs1110233	A	G	A	G	-0.0079	0.015261	0.1938	0.201014	FALSE	FALSE	FALSE	WGPdpq	0.017034	0.37029	486484	sepsis	TRUE	reported	textfile	0.0017	5.37E-06	7338	M32675.m	TRUE	reported	u0eUAG	2	TRUE	0.002934	21.58927
4432	rs1867017	T	G	T	G	-0.0079	0.007783	0.221	0.254222	FALSE	FALSE	FALSE	WGPdpq	0.015779	0.621853	486484	sepsis	TRUE	reported	textfile	0.0017	2.75E-06	7338	M32675.m	TRUE	reported	u0eUAG	2	TRUE	0.002934	21.58927
2133	rs1261661	A	G	A	G	0.0079	-0.01119	0.5527	0.542412	FALSE	FALSE	FALSE	TEupfd	0.013846	0.418929	486484	sepsis	TRUE	reported	textfile	0.0017	3.83E-06	7331	M15676.m	TRUE	reported	NXiaPC	2	TRUE	0.002937	21.58926
2860	rs888977	T	C	T	C	0.0079	-0.00391	0.2126	0.236452	FALSE	FALSE	FALSE	Y5qWBm	0.016235	0.809578	486484	sepsis	TRUE	reported	textfile	0.0017	4.40E-06	7325	M20489.m	TRUE	reported	f2g9Su	2	TRUE	0.002939	21.58926
2868	rs9927153	T	C	T	C	-0.0079	-0.01413	0.2309	0.233078	FALSE	FALSE	FALSE	Y5qWBm	0.016247	0.38457	486484	sepsis	TRUE	reported	textfile	0.0017	5.47E-06	7325	M20489.m	TRUE	reported	f2g9Su	2	TRUE	0.002939	21.58926
4584	rs1620675	T	G	T	G	0.0079	0.010817	0.3434	0.346705	FALSE	FALSE	FALSE	Pzplqu	0.014357	0.451199	486484	sepsis	TRUE	reported	textfile	0.0017	6.02E-06	7318	M32740.m	TRUE	reported	FjEBS5	2	TRUE	0.002942	21.58925
4594	rs2875878	C	G	C	G	-0.0079	0.014441	0.7045	0.698163	FALSE	TRUE	FALSE	Pzplqu	0.0149	0.332459	486484	sepsis	TRUE	reported	textfile	0.0017	6.17E-06	7318	M32740.m	TRUE	reported	FjEBS5	2	TRUE	0.002942	21.58925
4602	rs569137	A	G	A	G	-0.0079	0.016834	0.3585	0.35685	FALSE	FALSE	FALSE	Pzplqu	0.014353	0.240852	486484	sepsis	TRUE	reported	textfile	0.0017	5.13E-06	7318	M32740.m	TRUE	reported	FjEBS5	2	TRUE	0.002942	21.58925
4608	rs6832662	A	G	A	G	-0.0079	-0.00394	0.4257	0.427474	FALSE	FALSE	FALSE	Pzplqu	0.013809	0.77547	486484	sepsis	TRUE	reported	textfile	0.0017	6.07E-06	7318	M32740.m	TRUE	reported	FjEBS5	2	TRUE	0.002942	21.58925
2523	rs9408014	A	T	A	T	-0.0158	-0.01769	0.8976	0.890712	FALSE	TRUE	FALSE	LwUYua	0.021962	0.420444	486484	sepsis	TRUE	reported	textfile	0.0034	4.10E-06	7315	M17945.m	TRUE	reported	SUN8dc	2	TRUE	0.002943	21.58925
1689	rs1379459	T	C	T	C	0.0079	-0.00234	0.8083	0.782633	FALSE	FALSE	FALSE	f01en0	0.016542	0.887277	486484	sepsis	TRUE	reported	textfile	0.0017	3.14E-06	7314	M12593.m	TRUE	reported	EAsM6U	2	TRUE	0.002944	21.58925
1702	rs905508	T	C	T	C	0.0079	0.011755	0.1655	0.162788	FALSE	FALSE	FALSE	f01en0	0.018565	0.526628	486484	sepsis	TRUE	reported	textfile	0.0017	5.94E-06	7314	M12593.m	TRUE	reported	EAsM6U	2	TRUE	0.002944	21.58925
3323	rs1107270	T	C	T	C	0.0079	0.014794	0.71	0.715048	FALSE	FALSE	FALSE	m66Eav	0.015115	0.327689	486484	sepsis	TRUE	reported	textfile	0.0017	4.44E-06	7303	M27256.m	TRUE	reported	pI2ECU	2	TRUE	0.002948	21.58924
3349	rs9520114	A	G	A	G	0.0079	-0.00142	0.2732	0.311048	FALSE	FALSE	FALSE	m66Eav	0.014838	0.923895	486484	sepsis	TRUE	reported	textfile	0.0017	3.70E-06	7303	M27256.m	TRUE	reported	pI2ECU	2	TRUE	0.002948	21.58924
3352	rs1157606	A	G	A	G	-0.0158	-0.0009	0.1125	0.127611	FALSE	FALSE	FALSE	LY4Gus	0.021023	0.965999	486484	sepsis	TRUE	reported	textfile	0.0034	3.19E-06	7268	M27273.m	TRUE	reported	QKITBx	2	TRUE	0.002962	21.58921
2717	rs1111986	T	C	T	C	-0.0079	0.006464	0.4341	0.43298	FALSE	FALSE	FALSE	H4yqSK	0.013775	0.638883	486484	sepsis	TRUE	reported	textfile	0.0017	5.45E-06	7182	M19362.m	TRUE	reported	w2PDEO	2	TRUE	0.002998	21.58914
2718	rs1185470	A	G	A	G	-0.0079	-0.0292	0.4635	0.468052	FALSE	FALSE	FALSE	H4yqSK	0.013797	0.034342	486484	sepsis	TRUE	reported	textfile	0.0017	4.57E-06	7182	M19362.m	TRUE	reported	w2PDEO	2	TRUE	0.002998	21.58914
2725	rs1602068	T	C	T	C	-0.0079	0.006057	0.5655	0.559933	FALSE	FALSE	FALSE	H4yqSK	0.013825	0.661299	486484	sepsis	TRUE	reported	textfile	0.0017	5.67E-06	7182	M19362.m	TRUE	reported	w2PDEO	2	TRUE	0.002998	21.58914
1309	rs4930767	T	C	T	C	-0.0079	0.013495	0.4862	0.47833	FALSE	FALSE	FALSE	WQdpmo	0.013733	0.325777	486484	sepsis	TRUE	reported	textfile	0.0017	3.69E-06	7136	M01769.m	TRUE	reported	BkptOK	2	TRUE	0.003017	21.5891
8857	rs6688105	T	C	T	C	-0.0079	0.003702	0.703	0.694376	FALSE	FALSE	FALSE	XIHeTV	0.014887	0.803634	486484	sepsis	TRUE	reported	textfile	0.0017	4.86E-06	6940	M36808.m	TRUE	reported	37Vi2D	2	TRUE	0.003102	21.58893
8138	rs1106275	A	G	A	G	-0.0079	-0.00366	0.7472	0.743279	FALSE	FALSE	FALSE	Iv3pAQ	0.015777	0.816378	486484	sepsis	TRUE	reported	textfile	0.0017	5.07E-06	6920	M35327.m	TRUE	reported	3Vsglp	2	TRUE	0.003111	21.58891
4662	rs7702466	A	G	A	G	0.0316	6.44E-05	0.1021	0.127283	FALSE	FALSE	FALSE	SyAFR2	0.02064	0.997509	486484	sepsis	TRUE	reported	textfile	0.0068	3.01E-06	6658	M32755.m	TRUE	reported	prUo74	2	TRUE	0.003233	21.58867
7926	rs1115099	T	C	T	C	-0.0158	0.012649	0.6181	0.588614	FALSE	FALSE	FALSE	kFTWXY	0.013987	0.36579	486484	sepsis	TRUE	reported	textfile	0.0034	3.54E-06	6543	M35193.m	TRUE	reported	cjJvJl	2	TRUE	0.00329	21.58855
5098	rs1221117	A	G	A	G	0.0158	0.010075	0.2158	0.208332	FALSE	FALSE	FALSE	Uiz8VU	0.016945	0.55214	486484	sepsis	TRUE	reported	textfile	0.0034	4.57E-06	6404	M33163.m	TRUE	reported	ky0Bda	2	TRUE	0.003361	21.58841
4637	rs1782572	C	G	C	G	-0.0711	-0.02839	0.0842	0.091687	FALSE	TRUE	FALSE	Dfaklt	0.023662	0.230144	486484	sepsis	TRUE	reported	textfile	0.0153	3.36E-06	6379	M32754.m	TRUE	reported	y21cDm	2	TRUE	0.003374	21.58839
8103	rs1300462	A	C	A	C	0.0079	-0.011	0.5442	0.543156	FALSE	FALSE	FALSE	nNbXBS	0.01369	0.421761	486484	sepsis	TRUE	reported	textfile	0.0017	4.07E-06	5768	M35326.m	TRUE	reported	UNDorn	2	TRUE	0.00373	21.58767
8104	rs1313763	T	C	T	C	0.0079	0.026252	0.3423	0.33915	FALSE	FALSE	FALSE	nNbXBS	0.014446	0.069175	486484	sepsis	TRUE	reported	textfile	0.0017	6.26E-06	5768	M35326.m	TRUE	reported	UNDorn	2	TRUE	0.00373	21.58767
8109	rs1838137	A	G	A	G	0.0079	0.018489	0.6843	0.683111	FALSE	FALSE	FALSE	nNbXBS	0.014702	0.208533	486484	sepsis	TRUE	reported	textfile	0.0017	6.70E-06	5768	M35326.m	TRUE	reported	UNDorn	2	TRUE	0.00373	21.58767
5850	rs1701102	A	G	A	G	-0.1149	0.018875	0.7728	0.786132	FALSE	FALSE	FALSE	Vkec6Y	0.016679	0.257757	486484	sepsis	TRUE	reported	textfile	0.0247	3.30E-06	803	M33384.m	TRUE	reported	eaxeyR	2	TRUE	0.026241	21.58555
4668	rs1251271	C	G	C	G	-0.0423	-0.01062	0.6294	0.629752	FALSE	TRUE	FALSE	xaapK0	0.014208	0.454667	486484	sepsis	TRUE	reported	textfile	0.0091	3.63E-06	1761	M32757.m	TRUE	reported	64n1Z4	2	TRUE	0.012121	21.58263
5081	rs294095	A	G	A	G	-0.086	-0.00027	0.1424	0.131781	FALSE	FALSE	FALSE	VzfWsT	0.020222	0.989171	486484	sepsis	TRUE	reported	textfile	0.0185	3.18E-06	1570	M33144.m	TRUE	reported	ZtQP3u	2	TRUE	0.013577	21.58241
5011	rs2501556	A	G	A	G	-0.0237	0.024047	0.522	0.510799	FALSE	FALSE	FALSE	URCVGm	0.01372	0.079638	486484	sepsis	TRUE	reported	textfile	0.0051	3.48E-06	2773	M33131.m	TRUE	reported	4rQ2gs	2	TRUE	0.007727	21.57958
4706	rs3108908	T	C	T	C	0.0223	0.007371	0.3316	0.332797	FALSE	FALSE	FALSE	WlwKaF	0.014616	0.614048	486484	sepsis	TRUE	reported	textfile	0.0048	3.03E-06	6712	M32759.m	TRUE	reported	Ub4yxH	2	TRUE	0.003205	21.57734
2582	rs940008	A	G	A	G	0.0251	0.010091	0.4416	0.460649	FALSE	FALSE	FALSE	KHS91f	0.013683	0.460838	486484	sepsis	TRUE	reported	textfile	0.0054	2.64E-06	1492	M18349.m	TRUE	reported	PeVGil	2	TRUE	0.014274	21.57632
8946	rs7754899	A	G	A	G	0.059	0.01673	0.0573	0.037773	FALSE	FALSE	FALSE	mXJP3h	0.0358	0.64028	486484	sepsis	TRUE	reported	textfile	0.0127	3.33E-06	4520	M37104.m	TRUE	reported	HRZBak	2	TRUE	0.004752	21.57269
913	rs1090004	T	C	T	C	-0.0144	-0.01357	0.8351	0.78607	FALSE	FALSE	FALSE	rVMAPf	0.016628	0.41454	486484	sepsis	TRUE	reported	textfile	0.0031	3.40E-06	7307	M01493.m	TRUE	reported	siVqDi	2	TRUE	0.002944	21.57162
8864	rs1231385	T	C	T	C	-0.0288	-0.00725	0.513	0.526491	FALSE	FALSE	FALSE	nWuK1Q	0.013716	0.597243	486484	sepsis	TRUE	reported	textfile	0.0062	3.38E-06	6511	M36850.m	TRUE	reported	2BA0SE	2	TRUE	0.003303	21.5709
8677	rs2404867	A	C	A	C	0.053	-0.00835	0.7977	0.797654	FALSE	FALSE	FALSE	i4bSdJ	0.017005	0.623258	486484	sepsis	TRUE	reported	textfile	0.0114	3.14E-06	976	M36468.m	TRUE	reported	qJLuZj	2	TRUE	0.021666	21.57005
7678	rs3751563	A	G	A	G	0.0288	0.0234	0.1086	0.10938	FALSE	FALSE	FALSE	iqkM9B	0.021865	0.284525	486484	sepsis	TRUE	reported	textfile	0.0062	3.03E-06	4800	M34539.m	TRUE	reported	5nFq6f	2	TRUE	0.004475	21.56853
5853	rs308097	A	G	A	G	0.3482	0.061096	0.0377	0.045844	FALSE	FALSE	FALSE	Vkec6Y	0.032761	0.062193	486484	sepsis	TRUE	reported	textfile	0.0747	3.16E-06	272	M33384.m	TRUE	reported	eaxeyR	2	TRUE	0.073973	21.56807
4652	rs2032205	T	C	T	C	-0.0706	-0.00715	0.0171	0.019117	FALSE	FALSE	FALSE	SyAFR2	0.050642	0.887772	486484	sepsis	TRUE	reported	textfile	0.0152	3.19E-06	6658	M32755.m	TRUE	reported	prUo74	2	TRUE	0.00323	21.5671
4147	rs1452040	A	C	A	C	-0.0539	-0.00832	0.7973	0.77348	FALSE	FALSE	FALSE	CrVaiQ	0.016343	0.610851	486484	sepsis	TRUE	reported	textfile	0.0116	3.68E-06	1766	M32553.m	TRUE	reported	HsKdAI	2	TRUE	0.012078	21.56599
6549	rs4530331	A	G	A	G	-0.0367	0.00187	0.3391	0.332104	FALSE	FALSE	FALSE	zmDnD7	0.014498	0.897348	486484	sepsis	TRUE	reported	textfile	0.0079	3.39E-06	2660	M33835.m	TRUE	reported	MLBiKM	2	TRUE	0.008048	21.56509
4107	rs2295308	A	C	A	C	0.0209	-0.03941	0.9608	0.950334	FALSE	FALSE	FALSE	4rtLd0	0.031489	0.210759	486484	sepsis	TRUE	reported	textfile	0.0045	3.07E-06	7350	M32518.m	TRUE	reported	j7f1ER	2	TRUE	0.002926	21.56499
579	rs1691129	A	G	A	G	-0.0209	0.037088	0.0771	0.081958	FALSE	FALSE	FALSE	ZoqU2j	0.024888	0.136172	486484	sepsis	TRUE	reported	textfile	0.0045	4.18E-06	7333	M01105.m	TRUE	reported	2WxSOH	2	TRUE	0.002933	21.56498
7085	rs1124386	T	C	T	C	-0.0209	0.021918	0.0768	0.077701	FALSE	FALSE	FALSE	pxNICA	0.025545	0.390883	486484	sepsis	TRUE	reported	textfile	0.0045	3.22E-06	6994	M34306.m	TRUE	reported	xk5b9M	2	TRUE	0.003075	21.5647
4169	rs1103498	A	G	A	G	-0.1561	-0.04658	0.9783	0.980978	FALSE	FALSE	FALSE	Aq7H4p	0.050277	0.354245	486484	sepsis	TRUE	reported	textfile	0.0336	3.51E-06	2091	M32560.m	TRUE	reported	NIzbA7	2	TRUE	0.010217	21.56312
6325	rs756247	A	T	A	T	-0.1619	-0.01252	0.9396	0.95692	FALSE	TRUE	FALSE	5B5LTX	0.034222	0.714562	486484	sepsis	TRUE	reported	textfile	0.0348	3.24E-06	528	M33609.m	TRUE	reported	m0eM6N	2	TRUE	0.039378	21.5619
2652	rs7279572	T	C	T	C	-0.2727	-0.03066	0.9644	0.973031	FALSE	FALSE	FALSE	9GoQdh	0.042391	0.469559	486484	sepsis	TRUE	reported	textfile	0.0585	3.09E-06	257	M18474.m	TRUE	reported	p6Dq8p	2	TRUE	0.077961	21.56084
4632	rs1243532	C	G	C	G	-0.4219	-0.00535	0.0126	0.010382	FALSE	TRUE	FALSE	Dfaklt	0.069785	0.938921	486484	sepsis	TRUE	reported	textfile	0.0908	3.37E-06	1368	M32754.m	TRUE	reported	y21cDm	2	TRUE	0.015537	21.55817
1386	rs310101	A	G	A	G	0.0367	-0.03114	0.9579	0.946415	FALSE	FALSE	FALSE	KrWhws	0.030638	0.30943	486484	sepsis	TRUE	reported	textfile	0.0079	3.86E-06	1753	M02132.m	TRUE	reported	7pzpfK	2	TRUE	0.012161	21.55669
6299	rs6129197	A	G	A	G	0.0418	0.015896	0.8768	0.847381	FALSE	FALSE	FALSE	VlzZVG	0.019073	0.404621	486484	sepsis	TRUE	reported	textfile	0.009	3.79E-06	2859	M33531.m	TRUE	reported	XiYjn4	2	TRUE	0.007488	21.55577
4179	rs899236	A	G	A	G	-0.0497	0.004899	0.2189	0.212034	FALSE	FALSE	FALSE	Aq7H4p	0.016838	0.771076	486484	sepsis	TRUE	reported	textfile	0.0107	3.60E-06	2091	M32560.m	TRUE	reported	NIzbA7	2	TRUE	0.010213	21.55409
6972	rs1706438	A	G	A	G	-0.0195	0.017755	0.102	0.107365	FALSE	FALSE	FALSE	ZlvYs5	0.022234	0.424557	486484	sepsis	TRUE	reported	textfile	0.0042	4.41E-06	7351	M34214.m	TRUE	reported	qU57S1	2	TRUE	0.002924	21.55026
4899	rs1049215	T	C	T	C	0.0065	0.023408	0.7656	0.766846	FALSE	FALSE	FALSE	VqcRGX	0.016134	0.146827	486484	sepsis	TRUE	reported	textfile	0.0014	1.92E-06	7329	M32867.m	TRUE	reported	psuFJy	2	TRUE	0.002933	21.55024
8050	rs7815445	A	G	A	G	-0.013	-0.00665	0.9218	0.922859	FALSE	FALSE	FALSE	SwiqSi	0.025671	0.795697	486484	sepsis	TRUE	reported	textfile	0.0028	2.42E-06	7217	M35270.m	TRUE	reported	21ZlsQ	2	TRUE	0.002978	21.55015
7987	rs9483731	T	C	T	C	-0.0325	-0.03939	0.0382	0.038364	FALSE	FALSE	FALSE	t02AUT	0.035826	0.271578	486484	sepsis	TRUE	reported	textfile	0.007	3.17E-06	7100	M35254.m	TRUE	reported	uClJaT	2	TRUE	0.003027	21.55005
4649	rs174605	T	G	T	G	0.0195	0.039893	0.284	0.296105	FALSE	FALSE	FALSE	SyAFR2	0.014996	0.007809	486484	sepsis	TRUE	reported	textfile	0.0042	3.07E-06	6658	M32755.m	TRUE	reported	prUo74	2	TRUE	0.003227	21.54965
7746	rs4520895	A	C	A	C	0.013	-0.00201	0.8582	0.854718	FALSE	FALSE	FALSE	HyMNYM	0.019395	0.917447	486484	sepsis	TRUE	reported	textfile	0.0028	4.28E-06	5885	M34912.m	TRUE	reported	TI8Hcw	2	TRUE	0.00365	21.5488
7546	rs7924532	A	T	A	T	-0.013	-0.00135	0.6705	0.665973	FALSE	TRUE	FALSE	JJsHvX	0.014569	0.926368	486484	sepsis	TRUE	reported	textfile	0.0028	2.49E-06	5207	M34456.m	TRUE	reported	aklxve	2	TRUE	0.004123	21.54784
6045	rs6940545	A	C	A	C	1.6279	-0.02839	0.9822	0.845954	FALSE	FALSE	FALSE	33ojbt	0.018982	0.134767	486484	sepsis	TRUE	reported	textfile	0.3448	2.35E-06	60	M33423.m	TRUE	reported	ivEtvl	2	TRUE	0.270876	21.54754
7810	rs1015380	T	C	T	C	0.013	0.015165	0.878	0.884787	FALSE	FALSE	FALSE	h5whlS	0.021674	0.484126	486484	sepsis	TRUE	reported	textfile	0.0028	2.93E-06	4900	M35137.m	TRUE	reported	xVITKF	2	TRUE	0.00438	21.54732
5048	rs1307730	T	C	T	C	-0.0553	-0.00489	0.6692	0.696172	FALSE	FALSE	FALSE	JLCSne	0.014879	0.742595	486484	sepsis	TRUE	reported	textfile	0.0119	3.55E-06	888	M33137.m	TRUE	reported	LGKYXK	2	TRUE	0.023742	21.54652
3522	rs7332927	A	G	A	G	0.1384	0.090314	0.9802	0.987499	FALSE	FALSE	FALSE	hxuqls	0.061787	0.143823	486484	sepsis	TRUE	reported	textfile	0.0298	3.37E-06	1758	M27738.m	TRUE	reported	aueeZs	2	TRUE	0.012121	21.54494
1211	rs6051643	A	G	A	G	0.0441	-0.02607	0.986	0.975482	FALSE	FALSE	FALSE	Ckdgh1	0.045727	0.568547	486484	sepsis	TRUE	reported	textfile	0.0095	3.76E-06	7355	M01644.m	TRUE	reported	nKxz7B	2	TRUE	0.002921	21.54328
7212	rs1692861	T	C	T	C	-0.3934	-0.12013	0.0213	0.017161	FALSE	FALSE	FALSE	i1uJ7T	0.052515	0.022167	486484	sepsis	TRUE	reported	textfile	0.0846	3.28E-06	535	M34338.m	TRUE	reported	pi3mKW	2	TRUE	0.038848	21.54276
5786	rs911685	T	G	T	G	-0.0771	-0.04654	0.9276	0.929728	FALSE	FALSE	FALSE	eT2We0	0.026789	0.082359	486484	sepsis	TRUE	reported	textfile	0.0166	3.20E-06	1273	M33353.m	TRUE	reported	uZx90m	2	TRUE	0.016664	21.53822
2944	rs1752858	A	G	A	G	-0.0246	-0.09128	0.0514	0.072904	FALSE	FALSE	FALSE	pn11BC	0.026263	0.00051	486484	sepsis	TRUE	reported	textfile	0.0053	3.10E-06	7203	M21047.m	TRUE	reported	KJF8U4	2	TRUE	0.002982	21.53763
6900	rs6848587	T	C	T	C	-0.0311	0.009891	0.1655	0.164303	FALSE	FALSE	FALSE	MN2IbE	0.018666	0.596166	486484	sepsis	TRUE	reported	textfile	0.0067	3.70E-06	4961	M34106.m	TRUE	reported	q4zXEz	2	TRUE	0.004324	21.53754
2970	rs2584543	A	G	A	G	0.0246	-0.02896	0.0698	0.070326	FALSE	FALSE	FALSE	dOOXpo	0.026543	0.275285	486484	sepsis	TRUE	reported	textfile	0.0053	3.24E-06	6999	M21127.m	TRUE	reported	IdiP4j	2	TRUE	0.003069	21.53745
1658	rs3778889	T	C	T	C	0.0246	-0.01981	0.0616	0.063331	FALSE	FALSE	FALSE	YzX5ZD	0.028219	0.482677	486484	sepsis	TRUE	reported	textfile	0.0053	3.42E-06	6864	M12129.m	TRUE	reported	6tMfaW	2	TRUE	0.003129	21.53733
7871	rs1762464	C	G	C	G	-0.0325	-0.01291	0.2139	0.224812	FALSE	TRUE	FALSE	r428eQ	0.016344	0.429658	486484	sepsis	TRUE	reported	textfile	0.007	3.81E-06	1982	M35159.m	TRUE	reported	3r5zQo	2	TRUE	0.010759	21.53437
8090	rs748276	A	G	A	G	0.0879	0.014735	0.0832	0.080521	FALSE	FALSE	FALSE	z65cF2	0.025246	0.559463	486484	sepsis	TRUE	reported	textfile	0.0189	3.35E-06	448	M35322.m	TRUE	reported	3GUSWk	2	TRUE	0.046057	21.53332
6296	rs1236848	T	C	T	C	0.0376	0.002962	0.135	0.141814	FALSE	FALSE	FALSE	VlzZVG	0.019682	0.880354	486484	sepsis	TRUE	reported	textfile	0.0081	3.21E-06	2859	M33531.m	TRUE	reported	XiYjn4	2	TRUE	0.00748	21.53286
7596	rs1168639	T	C	T	C	-0.1328	-0.03698	0.8106	0.795609	FALSE	FALSE	FALSE	PlYt5g	0.016988	0.029496	486484	sepsis	TRUE	reported	textfile	0.0285	3.27E-06	241	M34516.m	TRUE	reported	jX41tf	2	TRUE	0.082647	21.53214
6173	rs555096	T	C	T	C	-0.0543	0.006649	0.039	0.034698	FALSE	FALSE	FALSE	O4Ty2I	0.037783	0.860304	486484	sepsis	TRUE	reported	textfile	0.0117	3.86E-06	5107	M33488.m	TRUE	reported	RGtF84	2	TRUE	0.0042	21.53068
6461	rs1711529	T	C	T	C	-0.0966	-0.03577	0.9457	0.933197	FALSE	FALSE	FALSE	yLohrN	0.027405	0.19184	486484	sepsis	TRUE	reported	textfile	0.0208	3.31E-06	1027	M33683.m	TRUE	reported	mvhAZ4	2	TRUE	0.02057	21.52688
1329	rs1178467	T	G	T	G	-0.0116	-0.02775	0.7763	0.755633	FALSE	FALSE	FALSE	LDUJbh	0.015872	0.080379	486484	sepsis	TRUE	reported	textfile	0.0025	3.62E-06	7367	M01898.m	TRUE	reported	JRSLeH	2	TRUE	0.002914	21.52376
4386	rs1213875	A	G	A	G	0.0116	-0.00946	0.3354	0.358201	FALSE	FALSE	FALSE	UJygVd	0.014414	0.511731	486484	sepsis	TRUE	reported	textfile	0.0025	3.60E-06	7361	M32654.m	TRUE	reported	kv9R0W	2	TRUE	0.002916	21.52375
1749	rs4236893	T	C	T	C	-0.0116	-0.03429	0.1865	0.188767	FALSE	FALSE	FALSE	nFLpAP	0.017584	0.051138	486484	sepsis	TRUE	reported	textfile	0.0025	4.78E-06	7356	M12774.m	TRUE	reported	yOSQTj	2	TRUE	0.002918	21.52375
7006	rs1744827	A	G	A	G	0.0232	-0.00956	0.9163	0.892786	FALSE	FALSE	FALSE	ePdfmg	0.022214	0.667065	486484	sepsis	TRUE	reported	textfile	0.005	3.92E-06	7347	M34244.m	TRUE	reported	l9nzfX	2	TRUE	0.002922	21.52374
847	rs1245144	A	G	A	G	-0.0116	0.00409	0.2677	0.271325	FALSE	FALSE	FALSE	1tFtaf	0.015424	0.790853	486484	sepsis	TRUE	reported	textfile	0.0025	2.30E-06	7323	M01359.m	TRUE	reported	qq3q6E	2	TRUE	0.002931	21.52372
1301	rs227421	T	C	T	C	0.0116	0.006363	0.1489	0.155053	FALSE	FALSE	FALSE	WQdpmo	0.018859	0.735829	486484	sepsis	TRUE	reported	textfile	0.0025	4.04E-06	7136	M01769.m	TRUE	reported	BkptOK	2	TRUE	0.003008	21.52357
8274	rs806044	T	G	T	G	-0.0116	0.00808	0.6844	0.667736	FALSE	FALSE	FALSE	TdsGCZ	0.0145	0.577358	486484	sepsis	TRUE	reported	textfile	0.0025	4.07E-06	6883	M35464.m	TRUE	reported	sOspot	2	TRUE	0.003118	21.52334
8731	rs1325513	T	C	T	C	0.0441	-0.00563	0.0791	0.078464	FALSE	FALSE	FALSE	ERC6hD	0.025416	0.824574	486484	sepsis	TRUE	reported	textfile	0.0095	3.24E-06	1636	M36559.m	TRUE	reported	n8waJF	2	TRUE	0.013001	21.5228
8107	rs1744710	A	T	A	T	0.0116	-0.0387	0.8434	0.835359	FALSE	TRUE	FALSE	nNbXBS	0.018416	0.03562	486484	sepsis	TRUE	reported	textfile	0.0025	4.80E-06	5768	M35326.m	TRUE	reported	UNDorn	2	TRUE	0.003719	21.52213
8749	rs6138327	C	G	C	G	-0.1123	0.018772	0.98	0.972651	FALSE	TRUE	FALSE	FnYrPc	0.042268	0.65695	486484	sepsis	TRUE	reported	textfile	0.0242	3.52E-06	3346	M36585.m	TRUE	reported	amHtMH	2	TRUE	0.006395	21.52133
6911	rs1319041	T	G	T	G	0.169	0.011984	0.1061	0.140173	FALSE	FALSE	FALSE	SvfSvH	0.019653	0.542013	486484	sepsis	TRUE	reported	textfile	0.0363	3.14E-06	282	M34109.m	TRUE	reported	7OSbiu	2	TRUE	0.071376	21.52133
4165	rs6692597	A	C	A	C	-0.0515	0.003885	0.0597	0.056721	FALSE	FALSE	FALSE	stpu5O	0.029551	0.8954	486484	sepsis	TRUE	reported	textfile	0.0111	3.24E-06	7337	M32557.m	TRUE	reported	0oV8Ss	2	TRUE	0.002925	21.52039
1035	rs1112072	A	G	A	G	-0.0181	-0.01771	0.4596	0.4403	FALSE	FALSE	FALSE	tRtQ2e	0.013865	0.20151	486484	sepsis	TRUE	reported	textfile	0.0039	4.61E-06	1761	M01564.m	TRUE	reported	Tbg0zM	2	TRUE	0.012083	21.51466
8352	rs1756490	A	G	A	G	-0.045	0.002548	0.9315	0.907277	FALSE	FALSE	FALSE	6VVCe4	0.023514	0.91371	486484	sepsis	TRUE	reported	textfile	0.0097	3.97E-06	5629	M35669.m	TRUE	reported	I0fk8X	2	TRUE	0.003809	21.5143
6210	rs1167627	A	G	A	G	-0.0348	-0.00693	0.2042	0.204526	FALSE	FALSE	FALSE	FQfT2o	0.016908	0.6817	486484	sepsis	TRUE	reported	textfile	0.0075	3.86E-06	2759	M33508.m	TRUE	reported	JtbOy2	2	TRUE	0.007743	21.51399
8086	rs3212446	T	C	T	C	0.0516	-0.03001	0.6284	0.676597	FALSE	FALSE	FALSE	z65cF2	0.014611	0.039989	486484	sepsis	TRUE	reported	textfile	0.0111	3.41E-06	448	M35322.m	TRUE	reported	3GUSWk	2	TRUE	0.046017	21.51346
6064	rs3798189	T	G	T	G	0.0167	0.020823	0.4912	0.496962	FALSE	FALSE	FALSE	P4BuMD	0.013799	0.131306	486484	sepsis	TRUE	reported	textfile	0.0036	3.42E-06	7365	M33441.m	TRUE	reported	kqrc1d	2	TRUE	0.002913	21.51345
6527	rs7181157	C	G	C	G	0.0167	0.016967	0.2136	0.215381	FALSE	TRUE	FALSE	OKIXJ8	0.016591	0.306466	486484	sepsis	TRUE	reported	textfile	0.0036	4.83E-06	7362	M33821.m	TRUE	reported	XGkaXH	2	TRUE	0.002915	21.51344
1840	rs7867387	A	G	A	G	0.0167	-0.00081	0.9051	0.903577	FALSE	FALSE	FALSE	2TaDHm	0.023098	0.971981	486484	sepsis	TRUE	reported	textfile	0.0036	4.16E-06	7337	M15365.m	TRUE	reported	LoLveg	2	TRUE	0.002924	21.51342
5868	rs1299698	A	G	A	G	-0.0167	-0.01695	0.0786	0.084398	FALSE	FALSE	FALSE	tqW0ld	0.024598	0.490751	486484	sepsis	TRUE	reported	textfile	0.0036	3.78E-06	7315	M33389.m	TRUE	reported	Sv7EyV	2	TRUE	0.002933	21.51341
6011	rs1156903	T	C	T	C	-0.9284	-0.01927	0.05	0.022595	FALSE	FALSE	FALSE	33ojbt	0.045918	0.674769	486484	sepsis	TRUE	reported	textfile	0.1968	2.39E-06	60	M33423.m	TRUE	reported	ivEtvl	2	TRUE	0.270558	21.51279
8657	rs1161322	C	G	C	G	0.0334	-0.01578	0.8706	0.871978	FALSE	TRUE	FALSE	BFEGyF	0.020544	0.442322	486484	sepsis	TRUE	reported	textfile	0.0072	4.05E-06	6486	M36399.m	TRUE	reported	nhT4FM	2	TRUE	0.003307	21.51265
8058	rs1221057	T	C	T	C	-0.0167	-0.00326	0.4836	0.46947	FALSE	FALSE	FALSE	whAa18	0.013697	0.811674	486484	sepsis	TRUE	reported	textfile	0.0036	3.06E-06	5979	M35305.m	TRUE	reported	e6hvDm	2	TRUE	0.003586	21.51209
6116	rs883044	C	G	C	G	0.0167	-0.01103	0.6316	0.65197	FALSE	TRUE	FALSE	a0osmD	0.014325	0.44151	486484	sepsis	TRUE	reported	textfile	0.0036	3.23E-06	5717	M33453.m	TRUE	reported	CePFnK	2	TRUE	0.00375	21.51176
3069	rs2059405	A	G	A	G	0.0167	-0.0228	0.798	0.797149	FALSE	FALSE	FALSE	e3izea	0.017009	0.180103	486484	sepsis	TRUE	reported	textfile	0.0036	4.73E-06	5676	M22032.m	TRUE	reported	zODKFI	2	TRUE	0.003777	21.51171
981	rs1686216	T	C	T	C	-0.413	0.007328	0.99	0.989116	FALSE	FALSE	FALSE	xSRII4	0.065648	0.911126	486484	sepsis	TRUE	reported	textfile	0.089	3.48E-06	1803	M01515.m	TRUE	reported	juZ5GE	2	TRUE	0.011802	21.50988
7271	rs516834	T	C	T	C	0.045	-0.02613	0.6097	0.598143	FALSE	FALSE	FALSE	P5Xm4S	0.01393	0.060657	486484	sepsis	TRUE	reported	textfile	0.0097	3.86E-06	3491	M34350.m	TRUE	reported	YU4x0J	2	TRUE	0.006127	21.50962
8827	rs190756	A	G	A	G	-0.0167	0.018644	0.2755	0.277562	FALSE	FALSE	FALSE	zuPm7L	0.015301	0.22302	486484	sepsis	TRUE	reported	textfile	0.0036	3.37E-06	4317	M36802.m	TRUE	reported	cn9d0n	2	TRUE	0.00496	21.50932
649	rs7583745	T	C	T	C	-0.0348	-0.00816	0.884	0.902762	FALSE	FALSE	FALSE	FfmsRf	0.023052	0.723459	486484	sepsis	TRUE	reported	textfile	0.0075	3.06E-06	1768	M01121.m	TRUE	reported	9R12A7	2	TRUE	0.012031	21.50525
9062	rs2672893	A	G	A	G	-0.0218	0.024436	0.1652	0.164581	FALSE	FALSE	FALSE	Wn3UAw	0.018553	0.187795	486484	sepsis	TRUE	reported	textfile	0.0047	4.35E-06	4754	M38150.m	TRUE	reported	IRKGRs	2	TRUE	0.004505	21.50476
6362	rs4696727	T	C	T	C	0.0167	0.015679	0.649	0.638129	FALSE	FALSE	FALSE	WWmDGN	0.014368	0.275164	486484	sepsis	TRUE	reported	textfile	0.0036	2.88E-06	2757	M33627.m	TRUE	reported	2kkEru	2	TRUE	0.007745	21.50368
7914	rs1774357	T	C	T	C	-0.0269	0.002754	0.8014	0.816545	FALSE	FALSE	FALSE	8qXKkX	0.017729	0.876551	486484	sepsis	TRUE	reported	textfile	0.0058	3.74E-06	6234	M35189.m	TRUE	reported	Nt6oaC	2	TRUE	0.003439	21.5035
5638	rs6765892	T	C	T	C	0.1002	0.016817	0.0793	0.098202	FALSE	FALSE	FALSE	U0d0ue	0.023286	0.470174	486484	sepsis	TRUE	reported	textfile	0.0216	3.63E-06	2616	M33190.m	TRUE	reported	FoODGF	2	TRUE	0.008159	21.50284
7380	rs9815685	A	G	A	G	0.1928	-0.02521	0.1775	0.233764	FALSE	FALSE	FALSE	mm9ksf	0.016135	0.118167	486484	sepsis	TRUE	reported	textfile	0.0392	8.95E-07	18	M34365.m	TRUE	reported	AVwEqD	2	TRUE	0.573362	21.50252
2636	rs2364754	A	G	A	G	-0.032	-0.02931	0.0751	0.109802	FALSE	FALSE	FALSE	UxuT5B	0.021881	0.180335	486484	sepsis	TRUE	reported	textfile	0.0069	3.69E-06	7367	M18467.m	TRUE	reported	eGQj6o	2	TRUE	0.002911	21.50225
545	rs1133034	T	C	T	C	0.032	0.006728	0.0757	0.071518	FALSE	FALSE	FALSE	QUUN2g	0.026593	0.800265	486484	sepsis	TRUE	reported	textfile	0.0069	3.91E-06	7237	M00599.m	TRUE	reported	vxn2i7	2	TRUE	0.002963	21.50214
4836	rs1052032	A	G	A	G	-0.032	0.009641	0.6558	0.661213	FALSE	FALSE	FALSE	jvlpBt	0.01445	0.504657	486484	sepsis	TRUE	reported	textfile	0.0069	3.27E-06	6250	M32846.m	TRUE	reported	RAXcz5	2	TRUE	0.003429	21.5012
2342	rs269263	T	C	T	C	-0.0487	-0.01987	0.3981	0.402661	FALSE	FALSE	FALSE	13lycQ	0.013939	0.15406	486484	sepsis	TRUE	reported	textfile	0.0105	3.32E-06	3393	M16823.m	TRUE	reported	u2seZ2	2	TRUE	0.0063	21.49925
6046	rs7123491	A	C	A	C	-1.7152	0.002734	0.016	0.016021	FALSE	FALSE	FALSE	33ojbt	0.05478	0.960192	486484	sepsis	TRUE	reported	textfile	0.3637	2.40E-06	60	M33423.m	TRUE	reported	ivEtvl	2	TRUE	0.270432	21.49907
5281	rs1444113	A	C	A	C	-0.297	-0.05405	0.0191	0.022188	FALSE	FALSE	FALSE	q32d4E	0.04673	0.247408	486484	sepsis	TRUE	reported	textfile	0.064	3.45E-06	1017	M33178.m	TRUE	reported	cS3BwT	2	TRUE	0.020736	21.49305
6913	rs1565932	A	T	A	T	0.2624	-0.0016	0.9122	0.892736	FALSE	TRUE	FALSE	SvfSvH	0.023077	0.944847	486484	sepsis	TRUE	reported	textfile	0.0564	3.34E-06	282	M34109.m	TRUE	reported	7OSbiu	2	TRUE	0.071286	21.49208
6912	rs1339453	A	G	A	G	-0.3573	-0.02497	0.0248	0.02859	FALSE	FALSE	FALSE	SvfSvH	0.040927	0.541768	486484	sepsis	TRUE	reported	textfile	0.0768	3.27E-06	282	M34109.m	TRUE	reported	7OSbiu	2	TRUE	0.071282	21.4908
7790	rs973579	A	G	A	G	-0.0204	0.009665	0.5112	0.500545	FALSE	FALSE	FALSE	15BqPa	0.0137	0.480519	486484	sepsis	TRUE	reported	textfile	0.0044	4.64E-06	7364	M35126.m	TRUE	reported	zvDzP2	2	TRUE	0.002911	21.49003
4624	rs2488751	A	G	A	G	-0.0306	0.017188	0.4539	0.475588	FALSE	FALSE	FALSE	0oA38w	0.01389	0.215925	486484	sepsis	TRUE	reported	textfile	0.0066	3.22E-06	7356	M32753.m	TRUE	reported	dQUaUX	2	TRUE	0.002914	21.49002
6537	rs7737048	T	C	T	C	0.0306	0.000425	0.08	0.094852	FALSE	FALSE	FALSE	DedZqr	0.023246	0.985416	486484	sepsis	TRUE	reported	textfile	0.0066	3.92E-06	7350	M33822.m	TRUE	reported	ciYOdn	2	TRUE	0.002916	21.49002
4270	rs2595191	T	C	T	C	0.0204	0.005315	0.8884	0.888917	FALSE	FALSE	FALSE	ylV4bp	0.021784	0.807226	486484	sepsis	TRUE	reported	textfile	0.0044	2.76E-06	7337	M32632.m	TRUE	reported	hqHJoU	2	TRUE	0.002921	21.49001
3256	rs1258375	A	C	A	C	-0.0255	-0.02585	0.0954	0.161661	FALSE	FALSE	FALSE	ZNPBqT	0.019454	0.183856	486484	sepsis	TRUE	reported	textfile	0.0055	3.65E-06	7256	M24074.m	TRUE	reported	8pPtOI	2	TRUE	0.002954	21.48994
3266	rs4798481	A	G	A	G	-0.0153	0.011872	0.1447	0.13349	FALSE	FALSE	FALSE	ZNPBqT	0.02023	0.557304	486484	sepsis	TRUE	reported	textfile	0.0033	2.96E-06	7256	M24074.m	TRUE	reported	8pPtOI	2	TRUE	0.002954	21.48994
5852	rs2722578	T	G	T	G	0.1411	0.001593	0.1259	0.123526	FALSE	FALSE	FALSE	Vkec6Y	0.020882	0.9392	486484	sepsis	TRUE	reported	textfile	0.0304	3.38E-06	803	M33384.m	TRUE	reported	eaxeyR	2	TRUE	0.026127	21.48938
8418	rs4972811	T	C	T	C	0.0204	0.01418	0.428	0.427762	FALSE	FALSE	FALSE	02RdNm	0.013934	0.308831	486484	sepsis	TRUE	reported	textfile	0.0044	4.22E-06	6447	M35678.m	TRUE	reported	BWN5JR	2	TRUE	0.003323	21.4892
3503	rs7428286	A	G	A	G	-0.0561	0.028395	0.0142	0.020754	FALSE	FALSE	FALSE	Awafb0	0.048108	0.555031	486484	sepsis	TRUE	reported	textfile	0.0121	3.88E-06	5562	M27728.m	TRUE	reported	Lfj8L0	2	TRUE	0.00385	21.48814
3642	rs2813524	A	G	A	G	0.0705	-0.01601	0.6401	0.630318	FALSE	FALSE	FALSE	Vq2efp	0.014134	0.257445	486484	sepsis	TRUE	reported	textfile	0.0152	3.31E-06	1760	M31787.m	TRUE	reported	krl4Tw	2	TRUE	0.012075	21.48806
7409	rs1024417	A	G	A	G	0.0204	-0.00977	0.6552	0.650346	FALSE	FALSE	FALSE	7i5VUS	0.014336	0.495691	486484	sepsis	TRUE	reported	textfile	0.0044	3.73E-06	5208	M34395.m	TRUE	reported	64hEaL	2	TRUE	0.004111	21.48761
5753	rs1104963	A	G	A	G	0.0816	-0.02357	0.9833	0.983792	FALSE	FALSE	FALSE	n16uO2	0.054188	0.663534	486484	sepsis	TRUE	reported	textfile	0.0176	3.41E-06	4409	M33250.m	TRUE	reported	9dhl67	2	TRUE	0.004852	21.48612
8746	rs2130048	T	G	T	G	-0.051	0.003336	0.9023	0.895863	FALSE	FALSE	FALSE	FnYrPc	0.022511	0.882177	486484	sepsis	TRUE	reported	textfile	0.011	3.45E-06	3346	M36585.m	TRUE	reported	amHtMH	2	TRUE	0.006383	21.48302
4780	rs1200042	A	G	A	G	0.3394	0.01426	0.0143	0.04352	FALSE	FALSE	FALSE	MmZZoM	0.033967	0.67461	486484	sepsis	TRUE	reported	textfile	0.0731	3.49E-06	562	M32800.m	TRUE	reported	70YSCO	2	TRUE	0.036941	21.48031
5698	rs1327266	T	C	T	C	0.0357	0.032097	0.9338	0.943974	FALSE	FALSE	FALSE	1SbQTe	0.029924	0.283447	486484	sepsis	TRUE	reported	textfile	0.0077	3.88E-06	2683	M33204.m	TRUE	reported	x7MBDH	2	TRUE	0.007948	21.47984
4104	rs1757974	A	G	A	G	0.0292	-0.03164	0.9713	0.957279	FALSE	FALSE	FALSE	4rtLd0	0.034351	0.357054	486484	sepsis	TRUE	reported	textfile	0.0063	3.87E-06	7350	M32518.m	TRUE	reported	j7f1ER	2	TRUE	0.002914	21.47664
3569	rs1122713	A	G	A	G	-0.4069	-0.03097	0.9845	0.978467	FALSE	FALSE	FALSE	F1s6qg	0.048152	0.520061	486484	sepsis	TRUE	reported	textfile	0.0876	3.40E-06	423	M31536.m	TRUE	reported	Zm40GX	2	TRUE	0.048531	21.4738
3557	rs7127674	A	G	A	G	-0.0482	0.045492	0.0147	0.017555	FALSE	FALSE	FALSE	8luUON	0.051775	0.379589	486484	sepsis	TRUE	reported	textfile	0.0104	3.33E-06	7257	M31453.m	TRUE	reported	FBs6G4	2	TRUE	0.002951	21.47374
948	rs1321284	A	C	A	C	-0.0241	-0.0197	0.9252	0.906697	FALSE	FALSE	FALSE	tWoDq4	0.023516	0.402299	486484	sepsis	TRUE	reported	textfile	0.0052	2.88E-06	7160	M01508.m	TRUE	reported	6ltDw0	2	TRUE	0.002991	21.47366
2428	rs4875362	T	G	T	G	-0.1646	-0.01795	0.0309	0.042172	FALSE	FALSE	FALSE	NBJ2Do	0.033987	0.597317	486484	sepsis	TRUE	reported	textfile	0.0355	3.43E-06	1739	M17799.m	TRUE	reported	Uz0Wgl	2	TRUE	0.012211	21.47352
5090	rs1709334	A	G	A	G	-0.1043	0.026198	0.0344	0.029378	FALSE	FALSE	FALSE	Cira8M	0.040999	0.522825	486484	sepsis	TRUE	reported	textfile	0.0225	3.47E-06	2777	M33150.m	TRUE	reported	AofVV7	2	TRUE	0.007679	21.4729
6896	rs28384	T	C	T	C	0.0241	0.003913	0.2933	0.285373	FALSE	FALSE	FALSE	MN2IbE	0.015299	0.798144	486484	sepsis	TRUE	reported	textfile	0.0052	4.46E-06	4961	M34106.m	TRUE	reported	q4zXEz	2	TRUE	0.004311	21.471
7323	rs1024457	A	G	A	G	-0.2133	-0.0097	0.8206	0.871014	FALSE	FALSE	FALSE	mm9ksf	0.020568	0.637268	486484	sepsis	TRUE	reported	textfile	0.0434	8.89E-07	18	M34365.m	TRUE	reported	AVwEqD	2	TRUE	0.573002	21.47087
7679	rs6802120	T	C	T	C	0.0241	-0.00982	0.1586	0.154964	FALSE	FALSE	FALSE	iqkM9B	0.018929	0.604029	486484	sepsis	TRUE	reported	textfile	0.0052	3.99E-06	4800	M34539.m	TRUE	reported	5nFq6f	2	TRUE	0.004455	21.47071
4876	rs910923	A	G	A	G	0.019	-0.00386	0.5988	0.603938	FALSE	FALSE	FALSE	KBJVQO	0.01413	0.784556	486484	sepsis	TRUE	reported	textfile	0.0041	3.90E-06	7355	M32855.m	TRUE	reported	Xea7Fn	2	TRUE	0.002911	21.46947
645	rs1194003	T	C	T	C	0.019	0.021615	0.842	0.820378	FALSE	FALSE	FALSE	FfmsRf	0.017781	0.224148	486484	sepsis	TRUE	reported	textfile	0.0041	2.82E-06	7349	M01121.m	TRUE	reported	9R12A7	2	TRUE	0.002914	21.46947
2907	rs2165127	A	G	A	G	-0.0751	0.019018	0.0444	0.192337	FALSE	FALSE	FALSE	FBDEQi	0.017777	0.284713	486484	sepsis	TRUE	reported	textfile	0.0162	3.37E-06	1748	M20699.m	TRUE	reported	nvNIlp	2	TRUE	0.012145	21.46608
7781	rs4628823	T	C	T	C	0.0468	0.002762	0.9434	0.947315	FALSE	FALSE	FALSE	15BqPa	0.030782	0.928514	486484	sepsis	TRUE	reported	textfile	0.0101	3.68E-06	7364	M35126.m	TRUE	reported	zvDzP2	2	TRUE	0.002907	21.465
7565	rs1252712	A	C	A	C	0.0607	0.04078	0.9801	0.982227	FALSE	FALSE	FALSE	Ot2ZAn	0.051902	0.432031	486484	sepsis	TRUE	reported	textfile	0.0131	3.83E-06	7119	M34481.m	TRUE	reported	tpyFcK	2	TRUE	0.003007	21.4641
6226	rs537721	T	C	T	C	-0.0417	0.057685	0.9625	0.953047	FALSE	FALSE	FALSE	8zUBbU	0.032458	0.075532	486484	sepsis	TRUE	reported	textfile	0.009	3.89E-06	7044	M33509.m	TRUE	reported	1mO9m8	2	TRUE	0.003038	21.46168
6986	rs1092700	T	C	T	C	0.0278	-0.01065	0.8659	0.856332	FALSE	FALSE	FALSE	APOtl6	0.019512	0.585159	486484	sepsis	TRUE	reported	textfile	0.006	3.24E-06	6987	M34221.m	TRUE	reported	IooCDC	2	TRUE	0.003063	21.46163
6995	rs9569679	T	G	T	G	0.0278	-0.00609	0.149	0.176006	FALSE	FALSE	FALSE	APOtl6	0.017954	0.734357	486484	sepsis	TRUE	reported	textfile	0.006	3.62E-06	6987	M34221.m	TRUE	reported	IooCDC	2	TRUE	0.003063	21.46163
8823	rs1087019	A	G	A	G	-0.019	-0.00615	0.613	0.616567	FALSE	FALSE	FALSE	zuPm7L	0.014037	0.661168	486484	sepsis	TRUE	reported	textfile	0.0041	3.70E-06	2952	M36802.m	TRUE	reported	cn9d0n	2	TRUE	0.007222	21.46076
6478	rs2859567	A	C	A	C	-0.1461	-0.02365	0.947	0.9265	FALSE	FALSE	FALSE	ecwVg8	0.026225	0.367096	486484	sepsis	TRUE	reported	textfile	0.0315	3.52E-06	831	M33751.m	TRUE	reported	NnvbIQ	2	TRUE	0.025234	21.46015
5913	rs1693699	A	G	A	G	0.1488	-0.00477	0.0319	0.029731	FALSE	FALSE	FALSE	iGV9aX	0.040415	0.90602	486484	sepsis	TRUE	reported	textfile	0.0321	3.46E-06	1464	M33390.m	TRUE	reported	sLbpnf	2	TRUE	0.014465	21.45864
3641	rs2125644	A	G	A	G	-0.1928	-0.00548	0.0317	0.031852	FALSE	FALSE	FALSE	Vq2efp	0.039417	0.889472	486484	sepsis	TRUE	reported	textfile	0.0416	3.66E-06	1760	M31787.m	TRUE	reported	krl4Tw	2	TRUE	0.012057	21.45525
8807	rs7048966	C	G	C	G	0.0278	-0.01006	0.1755	0.174812	FALSE	TRUE	FALSE	QWTooc	0.01799	0.576125	486484	sepsis	TRUE	reported	textfile	0.006	3.71E-06	3248	M36756.m	TRUE	reported	OLsw6C	2	TRUE	0.006566	21.45456
4871	rs2226274	T	C	T	C	-0.0403	0.007046	0.9342	0.934299	FALSE	FALSE	FALSE	KBJVQO	0.027802	0.799935	486484	sepsis	TRUE	reported	textfile	0.0087	3.33E-06	7355	M32855.m	TRUE	reported	Xea7Fn	2	TRUE	0.002909	21.45129
8669	rs1178617	A	C	A	C	-0.0519	0.002435	0.1501	0.141375	FALSE	FALSE	FALSE	i4bSdJ	0.01995	0.902862	486484	sepsis	TRUE	reported	textfile	0.0112	3.61E-06	1908	M36468.m	TRUE	reported	qJLuZj	2	TRUE	0.011129	21.45079
505	rs1039293	A	G	A	G	0.0709	0.012863	0.4948	0.470613	FALSE	FALSE	FALSE	ZOMIXT	0.013721	0.348535	486484	sepsis	TRUE	reported	textfile	0.0153	3.39E-06	1713	M00569.m	TRUE	reported	kZGouU	2	TRUE	0.012381	21.44876
1675	rs2032297	C	G	C	G	0.057	-0.01803	0.7857	0.780249	FALSE	TRUE	FALSE	IIFBGY	0.016513	0.274893	486484	sepsis	TRUE	reported	textfile	0.0123	3.85E-06	1543	M12261.m	TRUE	reported	F5Vlan	2	TRUE	0.013727	21.44748
6855	rs4865204	T	C	T	C	-0.0329	0.003493	0.481	0.484756	FALSE	FALSE	FALSE	2pJy8f	0.013674	0.798387	486484	sepsis	TRUE	reported	textfile	0.0071	3.77E-06	1714	M34040.m	TRUE	reported	zuJC1r	2	TRUE	0.012372	21.44707
3467	rs1230164	C	G	C	G	0.0176	-0.01202	0.721	0.718775	FALSE	TRUE	FALSE	BQlbxe	0.015329	0.433068	486484	sepsis	TRUE	reported	textfile	0.0038	3.08E-06	7373	M27718.m	TRUE	reported	MnHHJ0	2	TRUE	0.002901	21.4457
1244	rs4930046	A	G	A	G	0.0088	0.009781	0.4652	0.476961	FALSE	FALSE	FALSE	V50rWC	0.013696	0.475107	486484	sepsis	TRUE	reported	textfile	0.0019	2.72E-06	7346	M01645.m	TRUE	reported	yN3gpQ	2	TRUE	0.002912	21.44568
3219	rs9789183	T	C	T	C	0.0088	0.004408	0.517	0.522354	FALSE	FALSE	FALSE	UfLkBq	0.01373	0.74819	486484	sepsis	TRUE	reported	textfile	0.0019	3.27E-06	7329	M22481.m	TRUE	reported	kWMdiT	2	TRUE	0.002918	21.44567
5884	rs4602028	A	C	A	C	-0.0088	-0.02593	0.3766	0.37664	FALSE	FALSE	FALSE	tqW0ld	0.014184	0.067521	486484	sepsis	TRUE	reported	textfile	0.0019	2.07E-06	7315	M33389.m	TRUE	reported	Sv7EyV	2	TRUE	0.002924	21.44566

6117	rs1011985(	A	G	A	G	0.0088	-0.00736	0.5446	0.541683	FALSE	FALSE	FALSE	MiSJly	0.013991	0.598855	486484	sepsis	TRUE	reported	textfile	0.0019	2.60E-06	7307	M33477.m	TRUE	reported	WwL1hq	2	TRUE	0.002927	21.44565
2946	rs2075383	T	C	T	C	-0.0176	0.000319	0.9371	0.934286	FALSE	FALSE	FALSE	pn11BC	0.027618	0.990788	486484	sepsis	TRUE	reported	textfile	0.0038	3.86E-06	7203	M21047.m	TRUE	reported	KJF8U4	2	TRUE	0.002969	21.44557
8478	rs819909	A	G	A	G	0.0088	-0.02158	0.5236	0.514866	FALSE	FALSE	FALSE	uMajV6	0.013681	0.114738	486484	sepsis	TRUE	reported	textfile	0.0019	4.24E-06	6604	M35754.m	TRUE	reported	b8QB7S	2	TRUE	0.003238	21.44503
8920	rs7177	A	C	A	C	-0.0088	-0.01939	0.5419	0.526944	FALSE	FALSE	FALSE	eLhsld	0.013742	0.158148	486484	sepsis	TRUE	reported	textfile	0.0019	2.74E-06	6552	M37058.m	TRUE	reported	RSeWE5	2	TRUE	0.003263	21.44498
3137	rs1876088	A	C	A	C	-0.0088	-0.00922	0.6596	0.654134	FALSE	FALSE	FALSE	JiXR2t	0.014394	0.521805	486484	sepsis	TRUE	reported	textfile	0.0019	5.77E-06	6535	M22177.m	TRUE	reported	joi6p6	2	TRUE	0.003272	21.44496
3138	rs1998125	T	G	T	G	-0.0088	-0.0095	0.5805	0.550475	FALSE	FALSE	FALSE	JiXR2t	0.013796	0.491088	486484	sepsis	TRUE	reported	textfile	0.0019	5.75E-06	6535	M22177.m	TRUE	reported	joi6p6	2	TRUE	0.003272	21.44496
3145	rs2870925	C	G	C	G	-0.0088	-0.00671	0.6398	0.636872	FALSE	TRUE	FALSE	JiXR2t	0.014231	0.637296	486484	sepsis	TRUE	reported	textfile	0.0019	5.84E-06	6535	M22177.m	TRUE	reported	joi6p6	2	TRUE	0.003272	21.44496
3162	rs6513545	A	G	A	G	0.0088	0.013709	0.6085	0.620885	FALSE	FALSE	FALSE	JiXR2t	0.014127	0.331846	486484	sepsis	TRUE	reported	textfile	0.0019	5.25E-06	6535	M22177.m	TRUE	reported	joi6p6	2	TRUE	0.003272	21.44496
3172	rs7800932	T	G	T	G	0.0088	0.00591	0.4133	0.396588	FALSE	FALSE	FALSE	JiXR2t	0.01398	0.672473	486484	sepsis	TRUE	reported	textfile	0.0019	5.52E-06	6535	M22177.m	TRUE	reported	joi6p6	2	TRUE	0.003272	21.44496
3128	rs1326822	T	C	T	C	-0.0088	0.007663	0.5305	0.526119	FALSE	FALSE	FALSE	JiXR2t	0.013699	0.575903	486484	sepsis	TRUE	reported	textfile	0.0019	6.22E-06	6535	M22177.m	TRUE	reported	joi6p6	2	TRUE	0.003272	21.44496
3155	rs534408	T	G	T	G	0.0088	-0.01901	0.7263	0.718286	FALSE	FALSE	FALSE	JiXR2t	0.01531	0.214437	486484	sepsis	TRUE	reported	textfile	0.0019	6.07E-06	6535	M22177.m	TRUE	reported	joi6p6	2	TRUE	0.003272	21.44496
4510	rs1075383	A	G	A	G	0.0088	-0.00663	0.4657	0.476899	FALSE	FALSE	FALSE	r0qJaM	0.013717	0.628877	486484	sepsis	TRUE	reported	textfile	0.0019	2.49E-06	6497	M32729.m	TRUE	reported	vYv5FI	2	TRUE	0.003291	21.44492
4512	rs1102407	T	C	T	C	-0.0088	-0.02426	0.7057	0.695327	FALSE	FALSE	FALSE	r0qJaM	0.014847	0.102259	486484	sepsis	TRUE	reported	textfile	0.0019	3.28E-06	6497	M32729.m	TRUE	reported	vYv5FI	2	TRUE	0.003291	21.44492
4519	rs1311150	T	C	T	C	0.0088	-0.01287	0.6934	0.703405	FALSE	FALSE	FALSE	r0qJaM	0.015369	0.402186	486484	sepsis	TRUE	reported	textfile	0.0019	4.16E-06	6497	M32729.m	TRUE	reported	vYv5FI	2	TRUE	0.003291	21.44492
4546	rs9322929	T	C	T	C	-0.0088	-0.00893	0.6994	0.693931	FALSE	FALSE	FALSE	r0qJaM	0.014856	0.547586	486484	sepsis	TRUE	reported	textfile	0.0019	3.57E-06	6497	M32729.m	TRUE	reported	vYv5FI	2	TRUE	0.003291	21.44492
4548	rs990482	T	G	T	G	0.0088	0.008448	0.2786	0.273536	FALSE	FALSE	FALSE	r0qJaM	0.015909	0.595414	486484	sepsis	TRUE	reported	textfile	0.0019	4.44E-06	6497	M32729.m	TRUE	reported	vYv5FI	2	TRUE	0.003291	21.44492
7729	rs1228679	A	C	A	C	-0.0088	-0.00939	0.5493	0.516808	FALSE	FALSE	FALSE	HyMNYM	0.013712	0.493324	486484	sepsis	TRUE	reported	textfile	0.0019	3.71E-06	5885	M34912.m	TRUE	reported	TI8Hcw	2	TRUE	0.003632	21.44423
7738	rs1771292	A	G	A	G	0.0088	0.003317	0.3432	0.348026	FALSE	FALSE	FALSE	HyMNYM	0.014339	0.817081	486484	sepsis	TRUE	reported	textfile	0.0019	3.59E-06	5885	M34912.m	TRUE	reported	TI8Hcw	2	TRUE	0.003632	21.44423
7762	rs9845510	T	C	T	C	-0.0088	-0.00534	0.5512	0.557771	FALSE	FALSE	FALSE	HyMNYM	0.0138	0.698541	486484	sepsis	TRUE	reported	textfile	0.0019	2.92E-06	5885	M34912.m	TRUE	reported	TI8Hcw	2	TRUE	0.003632	21.44423
5642	rs1153452	T	C	T	C	-0.0616	0.007528	0.8392	0.843304	FALSE	FALSE	FALSE	BFsMtm	0.019115	0.693697	486484	sepsis	TRUE	reported	textfile	0.0133	3.62E-06	5716	M33192.m	TRUE	reported	NAJN3D	2	TRUE	0.003739	21.44402
7816	rs1249967	T	C	T	C	-0.0088	0.014099	0.3638	0.394479	FALSE	FALSE	FALSE	h5whIS	0.014137	0.318629	486484	sepsis	TRUE	reported	textfile	0.0019	3.70E-06	4900	M35137.m	TRUE	reported	xVITKF	2	TRUE	0.004359	21.44277
7862	rs7770207	T	C	T	C	-0.0088	-0.00808	0.689	0.69587	FALSE	FALSE	FALSE	h5whIS	0.014894	0.587442	486484	sepsis	TRUE	reported	textfile	0.0019	3.94E-06	4900	M35137.m	TRUE	reported	xVITKF	2	TRUE	0.004359	21.44277
5930	rs2927446	A	C	A	C	-0.4392	0.050137	0.9737	0.977493	FALSE	FALSE	FALSE	veBhfS	0.046338	0.279259	486484	sepsis	TRUE	reported	textfile	0.0943	3.24E-06	171	M33391.m	TRUE	reported	Gfjy5a	2	TRUE	0.112574	21.43838
8649	rs6913176	T	C	T	C	0.082	0.00604	0.9804	0.987472	FALSE	FALSE	FALSE	GgPG6k	0.061705	0.922019	486484	sepsis	TRUE	reported	textfile	0.0177	3.80E-06	1732	M36300.m	TRUE	reported	kT1yso	2	TRUE	0.01224	21.43776
5594	rs9398363	T	C	T	C	-0.2359	-0.01169	0.0232	0.031015	FALSE	FALSE	FALSE	q32d4E	0.039423	0.766922	486484	sepsis	TRUE	reported	textfile	0.0509	3.58E-06	1017	M33178.m	TRUE	reported	cS3BwT	2	TRUE	0.020683	21.43707
6716	rs1115159	A	C	A	C	0.0213	0.008064	0.7667	0.757298	FALSE	FALSE	FALSE	B6lZly	0.016231	0.619303	486484	sepsis	TRUE	reported	textfile	0.0046	4.11E-06	7222	M33937.m	TRUE	reported	qFPzjh	2	TRUE	0.00296	21.43499
8359	rs4799429	T	C	T	C	-0.0213	-0.02853	0.48	0.487102	FALSE	FALSE	FALSE	6VVCe4	0.013703	0.037323	486484	sepsis	TRUE	reported	textfile	0.0046	3.27E-06	5629	M35669.m	TRUE	reported	l0fk8X	2	TRUE	0.003795	21.43331
8446	rs1202287	A	G	A	G	-0.0403	0.016471	0.8244	0.85318	FALSE	FALSE	FALSE	uMajV6	0.019372	0.39518	486484	sepsis	TRUE	reported	textfile	0.0087	4.17E-06	1709	M35754.m	TRUE	reported	b8QB7S	2	TRUE	0.0124	21.43202
5716	rs2816826	T	C	T	C	0.0213	0.027877	0.2779	0.279325	FALSE	FALSE	FALSE	XDem1i	0.015251	0.067569	486484	sepsis	TRUE	reported	textfile	0.0046	2.82E-06	4665	M33221.m	TRUE	reported	KBV6FH	2	TRUE	0.004575	21.43173
2655	rs1259011	T	G	T	G	-0.0463	-0.01911	0.9201	0.920205	FALSE	FALSE	FALSE	UtYu7J	0.025241	0.448922	486484	sepsis	TRUE	reported	textfile	0.01	3.84E-06	5624	M18476.m	TRUE	reported	wJx0A4	2	TRUE	0.003797	21.42928
3975	rs1420139	C	G	C	G	-0.0125	-0.01361	0.1735	0.211411	FALSE	TRUE	FALSE	XdJbEU	0.016921	0.421233	486484	sepsis	TRUE	reported	textfile	0.0027	3.28E-06	7364	M32452.m	TRUE	reported	V5xWKi	2	TRUE	0.002902	21.42765
4010	rs1743536	T	C	T	C	-0.0125	0.023884	0.1195	0.111206	FALSE	FALSE	FALSE	V9AtId	0.021715	0.271384	486484	sepsis	TRUE	reported	textfile	0.0027	3.20E-06	7363	M32489.m	TRUE	reported	8B8S8a	2	TRUE	0.002903	21.42765
3672	rs1098941	T	C	T	C	0.0125	-0.00159	0.1927	0.191152	FALSE	FALSE	FALSE	rGHJcj	0.017369	0.927163	486484	sepsis	TRUE	reported	textfile	0.0027	3.82E-06	7356	M32198.m	TRUE	reported	6P1Nno	2	TRUE	0.002905	21.42764
7902	rs9624896	T	C	T	C	-0.0125	0.007578	0.2909	0.316477	FALSE	FALSE	FALSE	pU8zps	0.014786	0.60832	486484	sepsis	TRUE	reported	textfile	0.0027	2.40E-06	7350	M35186.m	TRUE	reported	Oc2lOF	2	TRUE	0.002908	21.42764
3371	rs1296569	T	C	T	C	-0.0125	-0.00501	0.7726	0.770612	FALSE	FALSE	FALSE	O2JtzH	0.016279	0.758045	486484	sepsis	TRUE	reported	textfile	0.0027	2.76E-06	7347	M27278.m	TRUE	reported	fxCIPW	2	TRUE	0.002909	21.42764
3372	rs1311709	A	G	A	G	0.0125	-0.01106	0.1954	0.194846	FALSE	FALSE	FALSE	O2JtzH	0.017455	0.526287	486484	sepsis	TRUE	reported	textfile	0.0027	4.89E-06	7347	M27278.m	TRUE	reported	fxCIPW	2	TRUE	0.002909	21.42764
8334	rs7290374	A	G	A	G	0.0125	-0.01037	0.4047	0.402103	FALSE	FALSE	FALSE	o0GzT1	0.01444	0.472865	486484	sepsis	TRUE	reported	textfile	0.0027	3.64E-06	7317	M35631.m	TRUE	reported	G1XFx0	2	TRUE	0.002921	21.42761
2887	rs4742351	T	C	T	C	-0.0125	0.014629	0.393	0.393506	FALSE	FALSE	FALSE	mUzn1R	0.014061	0.298182	486484	sepsis	TRUE	reported	textfile	0.0027	2.81E-06	7301	M20675.m	TRUE	reported	XwY04K	2	TRUE	0.002927	21.4276
3542	rs1092697	T	C	T	C	-0.0125	0.0157	0.2524	0.257289	FALSE	FALSE	FALSE	8IuUON	0.015614	0.314636	486484	sepsis	TRUE	reported	textfile	0.0027	2.66E-06	7257	M31453.m	TRUE	reported	FBs6G4	2	TRUE	0.002945	21.42756
3400	rs1013612	A	G	A	G	-0.0125	0.001401	0.4965	0.498024	FALSE	FALSE	FALSE	VU62LG	0.013683	0.918468	486484	sepsis	TRUE	reported	textfile	0.0027	4.48E-06	7180	M27513.m	TRUE	reported	0didOx	2	TRUE	0.002976	21.4275
3413	rs4144140	T	C	T	C	0.0125	3.55E-05	0.4524	0.459354	FALSE	FALSE	FALSE	VU62LG	0.013763	0.997944	486484	sepsis	TRUE	reported	textfile	0.0027	4.75E-06	7180	M27513.m	TRUE	reported	0didOx	2	TRUE	0.002976	21.4275
7982	rs2319973	A	C	A	C	0.0125	0.013928	0.3507	0.351381	FALSE	FALSE	FALSE	t02AUT	0.014308	0.330347	486484	sepsis	TRUE	reported	textfile	0.0027	2.82E-06	7100	M35254.m	TRUE	reported	uClJaT	2	TRUE	0.00301	21.42743
5737	rs706041	T	C	T	C	-0.0125	-0.01569	0.3351	0.334347	FALSE	FALSE	FALSE	YDQaWa	0.015433	0.309228	486484	sepsis	TRUE	reported	textfile	0.0027	4.45E-06	7063	M33228.m	TRUE	reported	BmYg8r	2	TRUE	0.003025	21.4274
6966	rs6431170	A	C	A	C	0.0125	-0.01296	0.2833	0.302301	FALSE	FALSE	FALSE	7Cw2XZ	0.014962	0.386313	486484	sepsis	TRUE	reported	textfile	0.0027	3.75E-06	7049	M34123.m	TRUE	reported	vrgMI3	2	TRUE	0.003031	21.42739
2971	rs400511	T	C	T	C	0.0125	0.021796	0.6391	0.631063	FALSE	FALSE	FALSE	dOOXpo	0.014183	0.124339	486484	sepsis	TRUE	reported	textfile	0.0027	4.17E-06	6999	M21127.m	TRUE	reported	IdiP4j	2	TRUE	0.003053	21.42735
2974	rs4821424	A	G	A	G	-0.0125	-0.01377	0.5199	0.513086	FALSE	FALSE	FALSE	dOOXpo	0.013678	0.314195	486484	sepsis	TRUE	reported	textfile	0.0027	3.97E-06	6999	M21127.m	TRUE	reported	IdiP4j	2	TRUE	0.003053	21.42735
7098	rs4622179	T	C	T	C	-0.0125	0.006121	0.7749	0.770552	FALSE	FALSE	FALSE	pxNICA	0.016228	0.706026	486484	sepsis	TRUE	reported	textfile	0.0027	4.73E-06	6994	M34306.m	TRUE	reported	xk5b9M	2	TRUE	0.003055	21.42734
773	rs2869990	T	C	T	C	-0.0125	-0.00954	0.7192	0.714466	FALSE	FALSE	FALSE	tYvbNc	0.01512	0.528253	486484	sepsis	TRUE	reported	textfile	0.0027	2.83E-06	6946	M01303.m	TRUE	reported	hT3gsE	2	TRUE	0.003076	21.4273
8909	rs2705088	T	G	T	G	-0.0125	-0.00684	0.7887	0.777455	FALSE	FALSE	FALSE	eLhsld	0.016505	0.678369	486484	sepsis	TRUE	reported	textfile	0.0027	4.17E-06	6552	M37058.m	TRUE	reported	RSeWE5	2	TRUE	0.003261	21.42693
7939	rs4960438	T	C	T	C	-0.0125	0.004672	0.6378	0.638595	FALSE	FALSE	FALSE	kFTWXY	0.01422	0.742467	486484	sepsis	TRUE	reported	textfile	0.0027	4.75E-06	6543	M35193.m	TRUE	reported	cjJvJl	2	TRUE	0.003265	21.42692
3237	rs1177601	T	C	T	C	0.0125	0.008377	0.8309	0.826374	FALSE	FALSE	FALSE	COSeSD	0.018103	0.643563	486484	sepsis	TRUE	reported	textfile	0.0027	3.52E-06	6517	M22649.m	TRUE	reported	mnH8z2	2	TRUE	0.003278	21.42689
3304	rs1304928	C	G	C	G	0.0125	0.028181	0.6544	0.633085	FALSE	TRUE	FALSE	nqmBts	0.014167	0.046678	486484	sepsis	TRUE	reported	textfile	0.0027	4.13E-06	6388	M25599.m	TRUE	reported	VkwSh1	2	TRUE	0.003344	21.42676
4226	rs1811763	A	G	A	G	-0.025	0.025589	0.144	0.141642	FALSE	FALSE	FALSE	FDy32d	0.019589	0.191452	486484	sepsis	TRUE	reported	textfile	0.0054	3.53E-06	5605	M32587.m	TRUE	reported	S10k1h	2	TRUE	0.003809	21.42582
3483	rs1107824	T	C	T	C	-0.0125	0.020761	0.6743	0.668694	FALSE	FALSE	FALSE	Awafb0	0.014639	0.156145	486484	sepsis	TRUE	reported	textfile	0.0027	3.67E-06	5562	M27728.m	TRUE	reported	Lfj8L0	2	TRUE	0.003839	21.42576
3485	rs1317833	A	G	A	G	-0.0125	-0.00075	0.6631	0.652678	FALSE	FALSE	FALSE	Awafb0	0.014673	0.959098	486484	sepsis	TRUE	reported	textfile	0.0027	4.38E-06	5562	M27728.m	TRUE	reported	Lfj8L0	2	TRUE	0.003839	21.42576
3486	rs1401635	C	G	C	G	-0.0125	0.01815	0.3132	0.319872	FALSE	TRUE	FALSE	Awafb0	0.014655	0.215528	486484	sepsis	TRUE	reported	textfile	0.0027	4.91E-06	5562	M27728.m	TRUE	reported	Lfj8L0	2	TRUE	0.003839	21.42576
3492	rs2253834	T	C	T	C	-0.0125	-0.0046	0.5521	0.553999	FALSE	FALSE	FALSE	Awafb0	0.01375	0.737779	486484	sepsis	TRUE	reported	textfile	0.0027	4.55E-06	5562	M27728.m	TRUE	reported	Lfj8L0	2	TRUE	0.003839	21.42576
4249	rs4149056	T	C	T	C	-0.0537	0.021553	0.8373	0.84838	FALSE	FALSE	FALSE	KZ60wl	0.019062	0.258186	486484	sepsis	TRUE	reported	textfile	0.0116	4.00E-06	6904	M32616.m	TRUE	reported	SPDpZi	2	TRUE	0.003094	21.42431
5798	rs4416818	A	G	A	G	0.0213	0.003833	0.2365	0.250979	FALSE	FALSE	FALSE	ka9zG7	0.01576	0.807832	486484	sepsis	TRUE	reported	textfile	0.0046	2.79E-06	2212	M33363.m	TRUE	reported	UFTVNc	2	TRUE	0.0096	21.42154
5972	rs7038957	T	C	T	C	-0.0287	-0.01835	0.8312	0.831529	FALSE	FALSE	FALSE	YCswLG	0.018281	0.315492	486484	sepsis	TRUE	reported	textfile	0.0062	3.86E-06	5822	M33420.m	TRUE	reported	WH7xfc	2	TRUE	0.003667	21.42058
6469	rs1265852	T	C	T	C	0.1682	0.008835	0.0382	0.038162	FALSE	FALSE	FALSE	ecwVg8	0.035716	0.804633	486484	sepsis	TRUE	reported	textfile	0.0363	3.59E-06	831	M33751.m	TRUE	reported	NnvbIQ	2	TRUE	0.025186	21.41866
5672	rs1292729	A	T	A	T	-0.05	0.015058	0.7061	0.715314	FALSE	TRUE	FALSE	AAOSvG	0.015158	0.320536	486484	sepsis	TRUE	reported	textfile	0.0108	3.99E-06	2860	M33197.m	TRUE	reported	aAmcal	2	TRUE	0.007438	21.41848
7514	rs1231157	T	C	T	C	-0.0287	0.032996	0.8478	0.837092	FALSE	FALSE	FALSE	rp1LGA	0.018651	0.076874	486484	sepsis	TRUE	reported	textfile	0.0062	3.26E-06	4442	M34441.m	TRUE	reported	SU47tz	2	TRUE	0.004801	21.41829
2926	rs1712256	T	C	T	C	-0.0162	0.018805	0.2523	0.266371	FALSE	FALSE	FALSE	fV4qul	0.015481	0.224458	486484	sepsis	TRUE	reported	textfile	0.0035	2.75E-06	7366	M21044.m	TRUE	reported	hbdBQJ	2	TRUE	0.0029	21.41786
4626	rs3746024	T	C	T	C	-0.0324	-0.01046	0.8273	0.824452	FALSE	FALSE	FALSE	0oA38w	0.017971	0.560617	486484	sepsis	TRUE	reported	textfile	0.007	3.46E-06	7356	M32753.m	TRUE	reported	dQUaUX	2	TRUE	0.002904	21.41785
7010	rs3807839	T	C	T	C	0.0162	0.00682	0.2604	0.268577	FALSE	FALSE	FALSE	ePdfmg	0.015499	0.659883	486484	sepsis	TRUE	reported	textfile	0.0035	4.40E-06	7347	M34244.m	TRUE	reported	l9nzfX	2	TRUE	0.002907	21.41784
8818	rs7225312	A	T	A	T	-0.0162	-0.00381	0.8749	0.843382	FALSE	TRUE	FALSE	X2Rol2	0.018777	0.83922	486484	sepsis	TRUE	reported	textfile	0.0035	3.16E-06	7335	M36776.m	TRUE	reported	nikHw1	2	TRUE	0.002912	21.41783
1171	rs7717247	A	C	A	C	0.0162	2.68E-05	0.8061	0.836482	FALSE	FALSE	FALSE	cSBFJ3	0.018561	0.998848	486484	sepsis	TRUE	reported	textfile	0.0035	3.35E-06	7104	M01638.m	TRUE	reported	sUkjpE	2	TRUE	0.003007	21.41764
4197	rs4750519	T	G	T	G	-0.0324	-0.00687	0.4178	0.410805	FALSE	FALSE	FALSE	TxOCzG	0.013924	0.621579	486484	sepsis	TRUE	reported	textfile	0.007	4.10E-06	6970	M32564.m	TRUE	reported	9JP6ND	2	TRUE	0.003064	21.41753
8793	rs1761839	A	G	A	G	0.0162	0.012444	0.5998	0.586552	FALSE	FALSE	FALSE	0YKkSS	0.013897	0.37055	486484	sepsis	TRUE	reported	textfile	0.0035	5.10E-06	6876	M36754.m	TRUE	reported	cf9IBu	2	TRUE	0.003106	21.41744
4754	rs1475961	A	G	A	G	0.0162	0.003474	0.6271	0.630235	FALSE	FALSE	FALSE	RDfDIS	0.014144	0.805967	486484	sepsis	TRUE	reported	textfile	0.0035	2.69E-06	6857	M32787.m	TRUE	reported	Hq6nGt	2	TRUE	0.003115	21.41742
2318	rs1185635	T	C	T	C	-0.0162	0.031081	0.8796	0.880604	FALSE	FALSE	FALSE	IZXwpR	0.021406	0.146511	486484	sepsis	TRUE	reported	textfile	0.0035	2.87E-06	6549	M16822.m	TRUE	reported	Zx9k2V	2	TRUE	0.003261	21.41713
5109	rs896401	T	C	T	C	-0.0162	0.034595	0.7318	0.74569	FALSE	FALSE	FALSE	Uiz8VU	0.016499	0.036017	486484	sepsis	TRUE	reported	textfile	0.0035	3.43E-06	6404	M33163.m	TRUE	reported	ky0Bda	2	TRUE	0.003334	21.41698
4468	rs1707062	T	C	T	C	-0.0426	0.022912	0.0388	0.026573	FALSE	FALSE	FALSE	g3iM2w	0.042957	0.593783	486484	sepsis	TRUE	reported	textfile	0.0092	3.88E-06	1757	M32698.m	TRUE	reported	ZQBn0h	2	TRUE	0.012056	21.41652
1670	rs1153699	T	G	T	G	0.0602	0.007287	0.2385	0.242129	FALSE	FALSE	FALSE	IIFBGY	0.016161	0.652044	486484	sepsis	TRUE	reported	textfile	0.013	3.89E-06	1543	M12261.m	TRUE	reported	F5VIan	2	TRUE	0.013707	21.41623
646	rs1466228	T	C	T	C	-0.0361	-0.01771	0.0348	0.044601	FALSE	FALSE	FALSE	FfmsRf	0.032987	0.591259	486484	sepsis	TRUE	reported	textfile	0.0078	3.21E-06	7349	M01121.m	TRUE	reported	9R12A7	2	TRUE	0.002906	21.41445
2662	rs9914239	A	G	A	G	-0.1056	-0.0333	0.9123	0.922167	FALSE	FALSE	FALSE	UtYu7J	0.02547	0.191129	486484	sepsis	TRUE	reported	textfile	0.0228	3.64E-06	1157	M18476.m	TRUE	reported	wJx0A4	2	TRUE	0.018203	21.41444
7597	rs1189335	A	G	A	G	0.5065	0.035926	0.0161	0.020515	FALSE	FALSE	FALSE	PIYt5g	0.047961	0.453818	486484	sepsis	TRUE	reported	textfile	0.1086	3.08E-06	128	M34516.m	TRUE	reported	jX41tf	2	TRUE	0.145254	21.41213
7896	rs39741	A	G	A	G	-0.0199	0.009079	0.0945	0.103851	FALSE	FALSE	FALSE	pU8zps	0.022525	0.686899	486484	sepsis	TRUE	reported	textfile	0.0043	4.51E-06	7350	M35186.m	TRUE	reported	Oc2lOF	2	TRUE	0.002905	21.4117
4256	rs1026341	A	T	A	T	0.0199	-0.00111	0.8572	0.851164	FALSE	TRUE	FALSE	ylV4bp	0.019195	0.953776	486484	sepsis	TRUE	reported	textfile	0.0043	3.58E-06	7337	M32632.m	TRUE	reported	hqHJoU	2	TRUE	0.002911	21.41168
2939	rs1249813	A	G	A	G	-0.0199	0.022762	0.0744	0.087204	FALSE	FALSE	FALSE	pn11BC	0.024238	0.347685	486484	sepsis	TRUE	reported	textfile	0.0043	4.35E-06	7203	M21047.m	TRUE	reported	KJF8U4	2	TRUE	0.002965	21.41158
8143	rs1765427	A	G	A	G	-0.0199	-0.00818	0.9626	0.963783	FALSE	FALSE	FALSE	lv3pAQ	0.036597	0.823171	486484	sepsis	TRUE	reported	textfile	0.0043	4.01E-06	6920	M35327.m	TRUE	reported	3VsgIp	2	TRUE	0.003085	21.41133
4653	rs2395859	A	G	A	G	0.0199	0.002061	0.8015	0.773625	FALSE	FALSE	FALSE	SyAFR2	0.016332	0.899556	486484	sepsis	TRUE	reported	textfile	0.0043	3.88E-06	6658	M32755.m	TRUE	reported	prUo74	2	TRUE	0.003206	21.41109
6684	rs1048781	A	C	A	C	-0.0199	-0.00655	0.4438	0.441563	FALSE	FALSE	FALSE	23zCgC	0.013797	0.635131	486484	sepsis	TRUE	reported	textfile	0.0043	3.94E-06	5534	M33910.m	TRUE	reported	ACI3CA	2	TRUE	0.003855	21.40978
2986	rs8078549	A	G	A	G	0.1884	0.029011	0.0266	0.02219	FALSE	FALSE	FALSE	Tli4xl	0.047514	0.541476	486484	sepsis	TRUE	reported	textfile	0.0407	3.60E-06	2339	M21151.m	TRUE	reported	xaSytY	2	TRUE	0.009078	21.40925
7778	rs2217851	A	G	A	G	0.0236	-0.00509	0.5917	0.618379	FALSE	FALSE	FALSE	15BqPa	0.014155	0.719148	486484	sepsis	TRUE	reported	textfile	0.0051	3.01E-06	7364	M35126.m	TRUE	reported	zvDzP2	2	TRUE	0.002899	21.40749
6618	rs3770914	T	C	T	C	0.0537	-0.00892	0.8905	0.882648	FALSE	FALSE	FALSE	2plv9p	0.021217	0.674261	486484	sepsis	TRUE	reported	textfile	0.0116	3.94E-06	1766	M33884.m	TRUE	reported	AoFcqC	2	TRUE	0.01199	21.40624
8245	rs1050412	A	G	A	G	-0.0588	0.000643	0.817	0.806477	FALSE	FALSE	FALSE	G4plxe	0.017292	0.97036	486484	sepsis	TRUE	reported	textfile	0.0127	3.76E-06	1383	M35451.m	TRUE	reported	hNOVMI	2	TRUE	0.015263	21.40517
2588	rs7349940	A	T	A	T	-0.0611	-0.00455	0.1269	0.115254	FALSE	TRUE	FALSE	JGJ9vH	0.021862	0.835142	486484	sepsis	TRUE	reported	textfile	0.0132	3.59E-06	2079	M18357.m	TRUE	reported	cOV2qw	2	TRUE	0.010201	21.40507
3624	rs4439540	A	G	A	G	0.0574	-0.00798	0.7407	0.73219	FALSE	FALSE	FALSE	gq4ISJ	0.015424	0.604816	486484	sepsis	TRUE	reported	textfile	0.0124	3.80E-06	1760	M31591.m	TRUE	reported	wjwl4J	2	TRUE	0.012029	21.40359
804	rs1002106	T	C	T	C	0.031	0.000699	0.9664	0.957797	FALSE	FALSE	FALSE	J58DI0	0.03386	0.983525	486484	sepsis	TRUE	reported	textfile	0.0067	4.07E-06	7335	M01356.m	TRUE	reported	zk9AMB	2	TRUE	0.00291	21.40205
4140	rs4726900	T	G	T	G	0.031	-0.02385	0.6601	0.658094	FALSE	FALSE	FALSE	pLh7sk	0.014443	0.098642	486484	sepsis	TRUE	reported	textfile	0.0067	3.89E-06	7256	M32549.m	TRUE	reported	RSQOnp	2	TRUE	0.002942	21.40199
8787	rs4930211	T	G	T	G	0.0477	0.022314	0.9025	0.894495	FALSE	FALSE	FALSE	4bkfvN	0.022209	0.315013	486484	sepsis	TRUE	reported	textfile	0.0103	3.30E-06	922	M36738.m	TRUE	reported	xcqyX2	2	TRUE	0.022732	21.40027
3627	rs6963509	C	G	C	G	-0.0731	-0.0174	0.0423	0.045051	FALSE	TRUE	FALSE	gq4ISJ	0.03291	0.597094	486484	sepsis	TRUE	reported	textfile	0.0158	3.46E-06	7338	M31591.m	TRUE	reported	wjwl4J	2	TRUE	0.002909	21.39943
6342	rs5022652	A	G	A	G	0.0649	0.009151	0.1582	0.15676	FALSE	FALSE	FALSE	qLUY00	0.018835	0.627077	486484	sepsis	TRUE	reported	textfile	0.014	3.84E-06	470	M33610.m	TRUE	reported	8cAJbB	2	TRUE	0.043724	21.3984
4460	rs7997650	A	G	A	G	-0.0509	-0.0006	0.0812	0.088421	FALSE	FALSE	FALSE	g2RNym	0.024141	0.980316	486484	sepsis	TRUE	reported	textfile	0.011	3.78E-06	2575	M32691.m	TRUE	reported	35eo6p	2	TRUE	0.008247	21.39502
7123	rs1187858	C	G	C	G	-0.3746	0.000664	0.986	0.986615	FALSE	TRUE	FALSE	de8tA6	0.059661	0.991117	486484	sepsis	TRUE	reported	textfile	0.0799	2.79E-06	75	M34322.m	TRUE	reported	sXPPBO	2	TRUE	0.22665	21.39457
8765	rs749275	A	C	A	C	0.0495	0.024501	0.1139	0.113536	FALSE	FALSE	FALSE	KMisdy	0.021599	0.256655	486484	sepsis	TRUE	reported	textfile	0.0107	3.54E-06	3977	M36589.m	TRUE	reported	J1ISTP	2	TRUE	0.005352	21.39067
4189	rs1884646	C	G	C	G	-0.087	-0.01854	0.1687	0.164954	FALSE	TRUE	FALSE	TxOCzG	0.018413	0.313893	486484	sepsis	TRUE	reported	textfile	0.0188	3.81E-06	1718	M32564.m	TRUE	reported	9JP6ND	2	TRUE	0.012312	21.3903
7347	rs1740198	A	G	A	G	0.2037	0.049376	0.0527	0.055417	FALSE	FALSE	FALSE	mm9ksf	0.029769	0.09719	486484	sepsis	TRUE	reported	textfile	0.044	3.66E-06	954	M34365.m	TRUE	reported	AVwEqD	2	TRUE	0.021972	21.38776
7265	rs1781901	T	G	T	G	0.0717	-0.00884	0.0876	0.088065	FALSE	FALSE	FALSE	P5Xm4S	0.02423	0.715372	486484	sepsis	TRUE	reported	textfile	0.0155	3.90E-06	3491	M34350.m	TRUE	reported	YU4x0J	2	TRUE	0.006092	21.38583
469	rs1078823	T	C	T	C	-0.0111	0.015731	0.6825	0.658631	FALSE	FALSE	FALSE	OW48NY	0.014521	0.278653	486484	sepsis	TRUE	reported	textfile	0.0024	2.46E-06	7365	M00527.m	TRUE	reported	57AP2f	2	TRUE	0.002896	21.38482
475	rs2242578	C	G	C	G	-0.0111	0.031294	0.6704	0.656826	FALSE	TRUE	FALSE	OW48NY	0.014572	0.03175	486484	sepsis	TRUE	reported	textfile	0.0024	5.08E-06	7365	M00527.m	TRUE	reported	57AP2f	2	TRUE	0.002896	21.38482
3761	rs6951820	A	G	A	G	0.0111	0.005653	0.4338	0.449111	FALSE	FALSE	FALSE	OZLdls	0.013773	0.681474	486484	sepsis	TRUE	reported	textfile	0.0024	4.77E-06	7356	M32338.m	TRUE	reported	s2itmB	2	TRUE	0.002899	21.38481
931	rs1315940	T	G	T	G	-0.0148	-0.01361	0.0652	0.071319	FALSE	FALSE	FALSE	tv99UN	0.026502	0.607579	486484	sepsis	TRUE	reported	textfile	0.0032	2.74E-06	7354	M01494.m	TRUE	reported	osDclp	2	TRUE	0.0029	21.38481
393	rs9889080	A	G	A	G	0.0074	-0.02762	0.1641	0.163474	FALSE	FALSE	FALSE	tbRL8J	0.018478	0.134957	486484	sepsis	TRUE	reported	textfile	0.0016	4.21E-06	7351	M00060.m	TRUE	reported	oOvRKT	2	TRUE	0.002901	21.38481
755	rs487946	T	G	T	G	-0.0074	0.031003	0.8292	0.810715	FALSE	FALSE	FALSE	RHWptG	0.017441	0.075466	486484	sepsis	TRUE	reported	textfile	0.0016	3.10E-06	7347	M01302.m	TRUE	reported	z1OLfr	2	TRUE	0.002903	21.3848
1231	rs1694244	A	G	A	G	-0.0259	0.075523	0.0314	0.033194	FALSE	FALSE	FALSE	V50rWC	0.038429	0.049384	486484	sepsis	TRUE	reported	textfile	0.0056	3.32E-06	7346	M01645.m	TRUE	reported	yN3gpQ	2	TRUE	0.002903	21.3848
1849	rs2044899	A	C	A	C	0.0074	0.00774	0.3787	0.371841	FALSE	FALSE	FALSE	S1eWts	0.014207	0.585874	486484	sepsis	TRUE	reported	textfile	0.0016	3.54E-06	7345	M15488.m	TRUE	reported	511KI9	2	TRUE	0.002904	21.3848
8168	rs1878022	T	C	T	C	-0.0074	0.012271	0.6634	0.665407	FALSE	FALSE	FALSE	8UhtPK	0.014455	0.395931	486484	sepsis	TRUE	reported	textfile	0.0016	6.26E-06	7345	M35397.m	TRUE	reported	rvVJba	2	TRUE	0.002904	21.3848
8170	rs1995087	A	G	A	G	-0.0074	0.027035	0.3435	0.355432	FALSE	FALSE	FALSE	8UhtPK	0.014381	0.060128	486484	sepsis	TRUE	reported	textfile	0.0016	6.10E-06	7345	M35397.m	TRUE	reported	rvVJba	2	TRUE	0.002904	21.3848
1086	rs338526	T	C	T	C	-0.0111	0.003669	0.7181	0.716926	FALSE	FALSE	FALSE	JLtEiZ	0.015185	0.809046	486484	sepsis	TRUE	reported	textfile	0.0024	5.61E-06	7333	M01572.m	TRUE	reported	TSSe28	2	TRUE	0.002909	21.38479
1704	rs9861011	A	G	A	G	-0.0074	0.040319	0.6558	0.657406	FALSE	FALSE	FALSE	f01en0	0.014398	0.005105	486484	sepsis	TRUE	reported	textfile	0.0016	3.80E-06	7314	M12593.m	TRUE	reported	EAsM6U	2	TRUE	0.002916	21.38478
2080	rs2824226	A	G	A	G	0.0074	-0.05995	0.8186	0.81956	FALSE	FALSE	FALSE	NPVH5j	0.018115	0.000936	486484	sepsis	TRUE	reported	textfile	0.0016	3.19E-06	7310	M15506.m	TRUE	reported	r7r3vM	2	TRUE	0.002918	21.38477
2938	rs1190577	A	C	A	C	0.0111	0.021152	0.1708	0.161829	FALSE	FALSE	FALSE	pn11BC	0.018701	0.258021	486484	sepsis	TRUE	reported	textfile	0.0024	3.96E-06	7203	M21047.m	TRUE	reported	KJF8U4	2	TRUE	0.002961	21.38469
2940	rs1251548	T	C	T	C	-0.0148	-0.00624	0.0969	0.102586	FALSE	FALSE	FALSE	pn11BC	0.022576	0.782362	486484	sepsis	TRUE	reported	textfile	0.0032	4.11E-06	7203	M21047.m	TRUE	reported	KJF8U4	2	TRUE	0.002961	21.38469
2733	rs4569257	A	G	A	G	-0.0111	-0.0131	0.4136	0.411984	FALSE	FALSE	FALSE	H4yqSK	0.013994	0.349213	486484	sepsis	TRUE	reported	textfile	0.0024	2.62E-06	7182	M19362.m	TRUE	reported	w2PDEO	2	TRUE	0.00297	21.38467
3461	rs4738080	A	G	A	G	0.0555	0.000872	0.0687	0.068863	FALSE	FALSE	FALSE	jRejFm	0.027043	0.974263	486484	sepsis	TRUE	reported	textfile	0.012	3.44E-06	6393	M27716.m	TRUE	reported	tg7Rvj	2	TRUE	0.003335	21.38393
8633	rs205208	T	C	T	C	-0.0495	0.024959	0.096	0.094524	FALSE	FALSE	FALSE	4cZjpQ	0.023492	0.288031	486484	sepsis	TRUE	reported	textfile	0.0107	4.11E-06	2400	M36230.m	TRUE	reported	TPUeNs	2	TRUE	0.008838	21.3836
684	rs1324247	A	G	A	G	0.0111	-0.00623	0.7395	0.73544	FALSE	FALSE	FALSE	aT1Fw1	0.01556	0.68876	486484	sepsis	TRUE	reported	textfile	0.0024	5.47E-06	5687	M01284.m	TRUE	reported	mbTe8H	2	TRUE	0.003747	21.3831
688	rs202490	T	C	T	C	-0.0111	0.003179	0.3533	0.353535	FALSE	FALSE	FALSE	aT1Fw1	0.014339	0.824555	486484	sepsis	TRUE	reported	textfile	0.0024	4.45E-06	5687	M01284.m	TRUE	reported	mbTe8H	2	TRUE	0.003747	21.3831
6006	rs9805690	C	G	C	G	-0.037	0.090481	0.986	0.981046	FALSE	TRUE	FALSE	3KsoXq	0.050579	0.073631	486484	sepsis	TRUE	reported	textfile	0.008	3.75E-06	5558	M33422.m	TRUE	reported	aNr6Ug	2	TRUE	0.003834	21.38293
8347	rs7110135	C	G	C	G	0.0523	0.005769	0.6932	0.696613	FALSE	TRUE	FALSE	USZiKY	0.01493	0.699214	486484	sepsis	TRUE	reported	textfile	0.0113	4.00E-06	1098	M35635.m	TRUE	reported	HuVMRL	2	TRUE	0.019136	21.38231
8730	rs1315965	T	C	T	C	0.031	-0.01078	0.2052	0.207962	FALSE	FALSE	FALSE	ERC6hD	0.016882	0.52328	486484	sepsis	TRUE	reported	textfile	0.0067	3.34E-06	1636	M36559.m	TRUE	reported	n8waJF	2	TRUE	0.012916	21.38171
5715	rs277311	T	C	T	C	0.0259	0.005345	0.1536	0.154088	FALSE	FALSE	FALSE	XDem1i	0.018964	0.778049	486484	sepsis	TRUE	reported	textfile	0.0056	4.06E-06	4665	M33221.m	TRUE	reported	KBV6FH	2	TRUE	0.004564	21.38145
3681	rs4976950	A	C	A	C	-0.068	-0.03408	0.0296	0.02113	FALSE	FALSE	FALSE	rGHJcj	0.047566	0.473748	486484	sepsis	TRUE	reported	textfile	0.0147	3.66E-06	1760	M32198.m	TRUE	reported	6P1Nno	2	TRUE	0.012012	21.37417
8950	rs1258740	A	C	A	C	-0.0888	-0.0095	0.1352	0.142795	FALSE	FALSE	FALSE	Ywk2ve	0.019614	0.627956	486484	sepsis	TRUE	reported	textfile	0.0192	3.86E-06	2560	M37112.m	TRUE	reported	EiAdDj	2	TRUE	0.008286	21.37391
7273	rs7551986	A	T	A	T	-0.08	0.016127	0.0797	0.073967	FALSE	TRUE	FALSE	P5Xm4S	0.026029	0.535538	486484	sepsis	TRUE	reported	textfile	0.0173	3.80E-06	3491	M34350.m	TRUE	reported	YU4x0J	2	TRUE	0.006088	21.37169
2448	rs7751252	T	C	T	C	0.105	0.008507	0.9491	0.939901	FALSE	FALSE	FALSE	NBJ2Do	0.028832	0.767964	486484	sepsis	TRUE	reported	textfile	0.0227	3.90E-06	1739	M17799.m	TRUE	reported	Uz0WgI	2	TRUE	0.012154	21.37111
7582	rs1234318	A	G	A	G	0.037	0.009669	0.645	0.641082	FALSE	FALSE	FALSE	SbjFMF	0.014216	0.496391	486484	sepsis	TRUE	reported	textfile	0.008	3.75E-06	2168	M34513.m	TRUE	reported	HVYiKM	2	TRUE	0.00977	21.37089
6339	rs357118	A	G	A	G	0.1438	0.007279	0.8019	0.752422	FALSE	FALSE	FALSE	qLUY00	0.015941	0.647961	486484	sepsis	TRUE	reported	textfile	0.0308	3.03E-06	102	M33610.m	TRUE	reported	8cAJbB	2	TRUE	0.176077	21.37057
5916	rs1716669	A	C	A	C	-0.0897	0.02801	0.9655	0.95456	FALSE	FALSE	FALSE	iGV9aX	0.032767	0.392651	486484	sepsis	TRUE	reported	textfile	0.0194	3.96E-06	5185	M33390.m	TRUE	reported	sLbpnf	2	TRUE	0.004106	21.37046
910	rs9295427	A	G	A	G	0.0356	0.000526	0.9408	0.938316	FALSE	FALSE	FALSE	I6zQnf	0.028736	0.985395	486484	sepsis	TRUE	reported	textfile	0.0077	3.81E-06	7345	M01444.m	TRUE	reported	IIzalm	2	TRUE	0.002902	21.36979
8301	rs215934	T	C	T	C	0.043	0.009254	0.1585	0.154061	FALSE	FALSE	FALSE	HELgy7	0.0189	0.624384	486484	sepsis	TRUE	reported	textfile	0.0093	3.58E-06	5035	M35551.m	TRUE	reported	7x7uUn	2	TRUE	0.004228	21.3697
7192	rs1002099	A	G	A	G	-0.1357	0.01595	0.1116	0.108042	FALSE	FALSE	FALSE	i1uJ7T	0.022113	0.470731	486484	sepsis	TRUE	reported	textfile	0.0293	3.69E-06	535	M34338.m	TRUE	reported	pi3mKW	2	TRUE	0.038548	21.36967
5926	rs1431419	T	C	T	C	-0.3534	-0.0021	0.9649	0.951484	FALSE	FALSE	FALSE	veBhfS	0.031824	0.947269	486484	sepsis	TRUE	reported	textfile	0.076	3.27E-06	171	M33391.m	TRUE	reported	Gfjy5a	2	TRUE	0.112253	21.36961
2532	rs29456	T	C	T	C	-0.0356	0.002383	0.2189	0.21518	FALSE	FALSE	FALSE	4HwTIG	0.016679	0.886383	486484	sepsis	TRUE	reported	textfile	0.0077	4.10E-06	7071	M18254.m	TRUE	reported	F9KrSC	2	TRUE	0.003014	21.36957
5636	rs593634	C	G	C	G	0.0763	-0.00242	0.1084	0.11126	FALSE	TRUE	FALSE	U0d0ue	0.021853	0.911867	486484	sepsis	TRUE	reported	textfile	0.0165	3.50E-06	2616	M33190.m	TRUE	reported	FoODGF	2	TRUE	0.008108	21.36727
3957	rs1244366	C	G	C	G	-0.0356	0.012158	0.1372	0.134438	FALSE	TRUE	FALSE	sKCkQs	0.020135	0.545975	486484	sepsis	TRUE	reported	textfile	0.0077	4.40E-06	5080	M32445.m	TRUE	reported	egPvJc	2	TRUE	0.00419	21.3672
4255	rs1018900	T	C	T	C	-0.0703	-0.00893	0.0531	0.075849	FALSE	FALSE	FALSE	ylV4bp	0.025786	0.72912	486484	sepsis	TRUE	reported	textfile	0.0152	3.66E-06	1762	M32632.m	TRUE	reported	hqHJoU	2	TRUE	0.011994	21.36635
5941	rs296429	A	G	A	G	-0.0356	0.005287	0.728	0.736746	FALSE	FALSE	FALSE	yepzrL	0.015545	0.733768	486484	sepsis	TRUE	reported	textfile	0.0077	3.52E-06	3890	M33408.m	TRUE	reported	SY4CoS	2	TRUE	0.005465	21.36462
4992	rs8008553	A	G	A	G	0.0333	0.009531	0.7132	0.733567	FALSE	FALSE	FALSE	MdUq0m	0.015502	0.538676	486484	sepsis	TRUE	reported	textfile	0.0072	3.98E-06	1618	M33009.m	TRUE	reported	icuhyC	2	TRUE	0.013048	21.36418
2518	rs7322113	T	C	T	C	-0.0453	0.01194	0.9889	0.987587	FALSE	FALSE	FALSE	LwUYua	0.061561	0.846215	486484	sepsis	TRUE	reported	textfile	0.0098	3.65E-06	7315	M17945.m	TRUE	reported	SUN8dc	2	TRUE	0.002912	21.36119
7580	rs1083665	A	G	A	G	0.0319	-0.01564	0.2061	0.206185	FALSE	FALSE	FALSE	SbjFMF	0.016903	0.354896	486484	sepsis	TRUE	reported	textfile	0.0069	4.41E-06	3239	M34513.m	TRUE	reported	HVYiKM	2	TRUE	0.006556	21.36067
3582	rs6994108	A	G	A	G	0.2358	-0.01176	0.0236	0.038018	FALSE	FALSE	FALSE	F1s6qg	0.035679	0.741647	486484	sepsis	TRUE	reported	textfile	0.0509	3.68E-06	423	M31536.m	TRUE	reported	Zm40GX	2	TRUE	0.048286	21.35963
6030	rs2028039	A	C	A	C	-1.5512	0.00574	0.0177	0.037251	FALSE	FALSE	FALSE	33ojbt	0.036113	0.873719	486484	sepsis	TRUE	reported	textfile	0.33	2.58E-06	60	M33423.m	TRUE	reported	ivEtvl	2	TRUE	0.269146	21.35917
6607	rs1397793	A	G	A	G	-0.0564	-0.00919	0.6623	0.661071	FALSE	FALSE	FALSE	VfOuo8	0.015009	0.540362	486484	sepsis	TRUE	reported	textfile	0.0122	3.65E-06	2725	M33883.m	TRUE	reported	BDe0Nk	2	TRUE	0.007782	21.35599
8345	rs4796899	T	G	T	G	0.093	-0.02431	0.098	0.10004	FALSE	FALSE	FALSE	USZiKY	0.023562	0.302154	486484	sepsis	TRUE	reported	textfile	0.0201	3.55E-06	824	M35635.m	TRUE	reported	HuVMRL	2	TRUE	0.025323	21.35593
4221	rs1225805	A	G	A	G	0.0171	-0.00732	0.3022	0.299218	FALSE	FALSE	FALSE	FDy32d	0.01495	0.624229	486484	sepsis	TRUE	reported	textfile	0.0037	3.14E-06	7369	M32587.m	TRUE	reported	S10k1h	2	TRUE	0.00289	21.35359
4222	rs1284443	T	C	T	C	-0.0171	0.008554	0.2702	0.266069	FALSE	FALSE	FALSE	FDy32d	0.015521	0.581539	486484	sepsis	TRUE	reported	textfile	0.0037	4.97E-06	7369	M32587.m	TRUE	reported	S10k1h	2	TRUE	0.00289	21.35359
4138	rs2272861	A	G	A	G	-0.0342	0.000773	0.7054	0.692795	FALSE	FALSE	FALSE	pLh7sk	0.014859	0.958488	486484	sepsis	TRUE	reported	textfile	0.0074	3.45E-06	7256	M32549.m	TRUE	reported	RSQOnp	2	TRUE	0.002935	21.3535
6115	rs8102864	A	C	A	C	0.0171	-0.00715	0.5284	0.528479	FALSE	FALSE	FALSE	a0osmD	0.013736	0.602923	486484	sepsis	TRUE	reported	textfile	0.0037	4.56E-06	5717	M33453.m	TRUE	reported	CePFnK	2	TRUE	0.003722	21.35191
5052	rs4581716	A	G	A	G	0.049	0.000436	0.6695	0.615659	FALSE	FALSE	FALSE	JLCSne	0.014035	0.975216	486484	sepsis	TRUE	reported	textfile	0.0106	3.69E-06	2391	M33137.m	TRUE	reported	LGKYXK	2	TRUE	0.008858	21.35094
5908	rs1087332	T	G	T	G	0.0476	-0.02024	0.1828	0.212343	FALSE	FALSE	FALSE	iGV9aX	0.016757	0.227169	486484	sepsis	TRUE	reported	textfile	0.0103	3.68E-06	5185	M33390.m	TRUE	reported	sLbpnf	2	TRUE	0.004102	21.34872
407	rs1264876	A	G	A	G	0.0564	-0.07767	0.9861	0.979926	FALSE	FALSE	FALSE	cTsNj6	0.04882	0.111607	486484	sepsis	TRUE	reported	textfile	0.0122	3.57E-06	1763	M00063.m	TRUE	reported	7KYTrQ	2	TRUE	0.011977	21.34743
6024	rs1696258	T	C	T	C	-1.2674	-0.02374	0.0271	0.047589	FALSE	FALSE	FALSE	33ojbt	0.032249	0.461566	486484	sepsis	TRUE	reported	textfile	0.2697	2.62E-06	60	M33423.m	TRUE	reported	ivEtvl	2	TRUE	0.269036	21.34727
8351	rs1724409	A	C	A	C	-0.0439	-0.00954	0.1095	0.166744	FALSE	FALSE	FALSE	6VVCe4	0.01827	0.601546	486484	sepsis	TRUE	reported	textfile	0.0095	4.00E-06	5629	M35669.m	TRUE	reported	l0fk8X	2	TRUE	0.003779	21.34654
5696	rs1095221	T	C	T	C	0.0282	0.013882	0.1046	0.10709	FALSE	FALSE	FALSE	1SbQTe	0.022106	0.530009	486484	sepsis	TRUE	reported	textfile	0.0061	4.38E-06	1673	M33204.m	TRUE	reported	x7MBDH	2	TRUE	0.012613	21.34613
2999	rs7998013	A	G	A	G	-0.0573	0.029348	0.9344	0.939771	FALSE	FALSE	FALSE	xmzJ5F	0.029545	0.320537	486484	sepsis	TRUE	reported	textfile	0.0124	4.08E-06	5436	M21184.m	TRUE	reported	xgwhDp	2	TRUE	0.003913	21.34549
1505	rs2113305	T	G	T	G	-0.0134	0.034979	0.2651	0.28931	FALSE	FALSE	FALSE	rzkxmh	0.01517	0.021122	486484	sepsis	TRUE	reported	textfile	0.0029	4.95E-06	6941	M03127.m	TRUE	reported	3lrzhA	2	TRUE	0.003067	21.34462
3531	rs1545442	T	C	T	C	0.0134	0.018211	0.8127	0.814758	FALSE	FALSE	FALSE	rXzSlH	0.017655	0.302319	486484	sepsis	TRUE	reported	textfile	0.0029	2.87E-06	6795	M30805.m	TRUE	reported	7wg9QH	2	TRUE	0.003132	21.34449
8723	rs6859143	A	G	A	G	0.0134	-0.01893	0.7032	0.691555	FALSE	FALSE	FALSE	I0G0I1	0.014868	0.202848	486484	sepsis	TRUE	reported	textfile	0.0029	3.55E-06	6464	M36553.m	TRUE	reported	IBltb0	2	TRUE	0.003292	21.34417
6652	rs1861512	T	C	T	C	-0.0134	-0.00528	0.3349	0.337923	FALSE	FALSE	FALSE	REeQiq	0.014492	0.71583	486484	sepsis	TRUE	reported	textfile	0.0029	3.81E-06	6004	M33892.m	TRUE	reported	cVOa3u	2	TRUE	0.003543	21.34366
6659	rs4467622	C	G	C	G	-0.0134	0.009914	0.5956	0.591067	FALSE	TRUE	FALSE	REeQiq	0.013934	0.476771	486484	sepsis	TRUE	reported	textfile	0.0029	3.53E-06	6004	M33892.m	TRUE	reported	cVOa3u	2	TRUE	0.003543	21.34366
5961	rs1007793	T	C	T	C	-0.0402	0.010196	0.1376	0.185438	FALSE	FALSE	FALSE	YCswLG	0.017646	0.563381	486484	sepsis	TRUE	reported	textfile	0.0087	4.08E-06	5822	M33420.m	TRUE	reported	WH7xfc	2	TRUE	0.003654	21.34344
2998	rs7525018	T	G	T	G	-0.0268	-0.02224	0.7223	0.695618	FALSE	FALSE	FALSE	xmzJ5F	0.014947	0.136724	486484	sepsis	TRUE	reported	textfile	0.0058	3.64E-06	5436	M21184.m	TRUE	reported	xgwhDp	2	TRUE	0.003912	21.34292
8101	rs1258789	T	C	T	C	0.0416	-0.04466	0.9619	0.964239	FALSE	FALSE	FALSE	nNbXBS	0.036873	0.225849	486484	sepsis	TRUE	reported	textfile	0.009	3.91E-06	1764	M35326.m	TRUE	reported	UNDorn	2	TRUE	0.011967	21.34071
4881	rs671321	T	G	T	G	0.0305	0.02114	0.5978	0.578613	FALSE	FALSE	FALSE	MDX5FX	0.01384	0.126658	486484	sepsis	TRUE	reported	textfile	0.0066	4.01E-06	2656	M32857.m	TRUE	reported	moAN2E	2	TRUE	0.007976	21.33952
5070	rs1039444	T	C	T	C	0.0231	0.011646	0.8895	0.880522	FALSE	FALSE	FALSE	gqzGN4	0.021144	0.581788	486484	sepsis	TRUE	reported	textfile	0.005	4.76E-06	7319	M33140.m	TRUE	reported	DKGBe9	2	TRUE	0.002908	21.33857
7143	rs7009700	C	G	C	G	-0.1461	0.011951	0.9503	0.946472	FALSE	TRUE	FALSE	de8tA6	0.030317	0.69344	486484	sepsis	TRUE	reported	textfile	0.0315	3.54E-06	248	M34322.m	TRUE	reported	sXPPBO	2	TRUE	0.079818	21.33844
1617	rs7642054	T	C	T	C	-0.0171	-0.00054	0.5887	0.565107	FALSE	FALSE	FALSE	zP3C2v	0.013819	0.96898	486484	sepsis	TRUE	reported	textfile	0.0037	4.64E-06	1766	M12035.m	TRUE	reported	1Pooe3	2	TRUE	0.01195	21.3352
4870	rs2016052	C	G	C	G	0.0194	0.00748	0.6469	0.643262	FALSE	TRUE	FALSE	KBJVQO	0.014329	0.601665	486484	sepsis	TRUE	reported	textfile	0.0042	2.92E-06	7355	M32855.m	TRUE	reported	Xea7Fn	2	TRUE	0.002892	21.3298
6842	rs1419352	C	G	C	G	0.0291	0.000246	0.3905	0.400966	FALSE	TRUE	FALSE	aWy2Uj	0.014019	0.985984	486484	sepsis	TRUE	reported	textfile	0.0063	3.72E-06	7326	M33973.m	TRUE	reported	SUSE3b	2	TRUE	0.002904	21.32978
4693	rs4430526	A	G	A	G	0.0194	0.020906	0.5227	0.523445	FALSE	FALSE	FALSE	MV108h	0.0137	0.127034	486484	sepsis	TRUE	reported	textfile	0.0042	3.72E-06	6642	M32758.m	TRUE	reported	btjNXQ	2	TRUE	0.003202	21.32918
1578	rs6126354	T	C	T	C	-0.0134	-0.01416	0.7906	0.651168	FALSE	FALSE	FALSE	lxzgXW	0.014376	0.324785	486484	sepsis	TRUE	reported	textfile	0.0029	5.46E-06	1764	M11438.m	TRUE	reported	ZjFyyS	2	TRUE	0.011959	21.32657
7081	rs4714588	T	C	T	C	0.0231	0.029511	0.7866	0.808384	FALSE	FALSE	FALSE	sg7nOB	0.01769	0.095277	486484	sepsis	TRUE	reported	textfile	0.005	4.86E-06	2278	M34289.m	TRUE	reported	KemvIC	2	TRUE	0.009283	21.32566
3583	rs7039553	T	C	T	C	-0.0694	0.005451	0.6695	0.681638	FALSE	FALSE	FALSE	F1s6qg	0.014725	0.71122	486484	sepsis	TRUE	reported	textfile	0.015	3.56E-06	522	M31536.m	TRUE	reported	Zm40GX	2	TRUE	0.039392	21.32403
2663	rs1774654	A	G	A	G	-0.0883	0.014533	0.5073	0.505763	FALSE	FALSE	FALSE	cDEa3G	0.013644	0.286817	486484	sepsis	TRUE	reported	textfile	0.0191	3.67E-06	868	M18477.m	TRUE	reported	kxZEfC	2	TRUE	0.024031	21.32322
7534	rs1506682	A	C	A	C	0.0416	-0.01179	0.1331	0.144291	FALSE	FALSE	FALSE	JJsHvX	0.0195	0.545584	486484	sepsis	TRUE	reported	textfile	0.009	3.87E-06	1019	M34456.m	TRUE	reported	aklxve	2	TRUE	0.020536	21.32301
3456	rs1021551	A	G	A	G	0.0499	-0.0036	0.5372	0.519537	FALSE	FALSE	FALSE	jRejFm	0.013679	0.792541	486484	sepsis	TRUE	reported	textfile	0.0108	3.85E-06	1720	M27716.m	TRUE	reported	tg7Rvj	2	TRUE	0.012259	21.323
3599	rs388600	A	C	A	C	0.0448	0.011161	0.868	0.864776	FALSE	FALSE	FALSE	e4G8UU	0.020072	0.578175	486484	sepsis	TRUE	reported	textfile	0.0097	4.00E-06	5229	M31548.m	TRUE	reported	kWkkmH	2	TRUE	0.004063	21.32291
4168	rs883118	A	G	A	G	0.0254	-0.0143	0.6702	0.666781	FALSE	FALSE	FALSE	stpu5O	0.014506	0.32427	486484	sepsis	TRUE	reported	textfile	0.0055	4.22E-06	7337	M32557.m	TRUE	reported	0oV8Ss	2	TRUE	0.002898	21.32179
7676	rs2026781	C	G	C	G	0.0351	0.002305	0.7702	0.734783	FALSE	TRUE	FALSE	iqkM9B	0.015486	0.88168	486484	sepsis	TRUE	reported	textfile	0.0076	3.83E-06	4800	M34539.m	TRUE	reported	5nFq6f	2	TRUE	0.004424	21.32093
1332	rs1224329	A	G	A	G	-0.0157	-0.04109	0.8997	0.917654	FALSE	FALSE	FALSE	LDUJbh	0.024892	0.098805	486484	sepsis	TRUE	reported	textfile	0.0034	4.00E-06	7367	M01898.m	TRUE	reported	JRSLeH	2	TRUE	0.002886	21.31688
3824	rs1328846	T	C	T	C	-0.0157	-0.01665	0.9192	0.918788	FALSE	FALSE	FALSE	tRX2u0	0.025153	0.50803	486484	sepsis	TRUE	reported	textfile	0.0034	5.26E-06	7365	M32348.m	TRUE	reported	ruBgwQ	2	TRUE	0.002887	21.31687
3618	rs1752362	A	T	A	T	0.0471	0.029948	0.8853	0.876695	FALSE	TRUE	FALSE	gq4ISJ	0.020775	0.149425	486484	sepsis	TRUE	reported	textfile	0.0102	3.57E-06	7338	M31591.m	TRUE	reported	wjwl4J	2	TRUE	0.002897	21.31685
2076	rs1749738	T	G	T	G	0.0157	0.004622	0.0722	0.054754	FALSE	FALSE	FALSE	NPVH5j	0.031241	0.882388	486484	sepsis	TRUE	reported	textfile	0.0034	5.34E-06	7310	M15506.m	TRUE	reported	r7r3vM	2	TRUE	0.002908	21.31683
4471	rs2980973	T	C	T	C	-0.0157	0.031218	0.9084	0.903457	FALSE	FALSE	FALSE	g3iM2w	0.023162	0.177724	486484	sepsis	TRUE	reported	textfile	0.0034	4.96E-06	7308	M32698.m	TRUE	reported	ZQBn0h	2	TRUE	0.002909	21.31683
8222	rs1178021	A	G	A	G	0.0157	-0.02864	0.8469	0.816002	FALSE	FALSE	FALSE	4YAWhA	0.017691	0.105418	486484	sepsis	TRUE	reported	textfile	0.0034	3.67E-06	7256	M35439.m	TRUE	reported	bSSC4Z	2	TRUE	0.00293	21.31679
2565	rs1015947	A	G	A	G	0.0157	-0.00527	0.8276	0.806934	FALSE	FALSE	FALSE	KHS91f	0.017314	0.760985	486484	sepsis	TRUE	reported	textfile	0.0034	4.09E-06	6939	M18349.m	TRUE	reported	PeVGiI	2	TRUE	0.003063	21.31652
8010	rs2073234	A	G	A	G	0.0157	-0.0051	0.6908	0.686469	FALSE	FALSE	FALSE	Zj5bZH	0.015006	0.733816	486484	sepsis	TRUE	reported	textfile	0.0034	4.46E-06	6538	M35257.m	TRUE	reported	exssAk	2	TRUE	0.003251	21.31614
6577	rs1329418	A	G	A	G	-0.6236	-0.04059	0.9887	0.994806	FALSE	FALSE	FALSE	XO3rXq	0.097702	0.677844	486484	sepsis	TRUE	reported	textfile	0.1343	3.43E-06	176	M33877.m	TRUE	reported	p2sETg	2	TRUE	0.109134	21.31556
7345	rs1614887	A	G	A	G	0.1758	0.008539	0.4688	0.548205	FALSE	FALSE	FALSE	mm9ksf	0.013721	0.533714	486484	sepsis	TRUE	reported	textfile	0.0359	1.01E-06	18	M34365.m	TRUE	reported	AVwEqD	2	TRUE	0.571224	21.31554
7773	rs9992384	T	G	T	G	0.0157	0.016537	0.2562	0.251451	FALSE	FALSE	FALSE	G4gUxB	0.015796	0.295143	486484	sepsis	TRUE	reported	textfile	0.0034	2.81E-06	5804	M35114.m	TRUE	reported	o79OO5	2	TRUE	0.00366	21.31532
2274	rs1025417	T	C	T	C	-0.0157	-0.03451	0.2329	0.23465	FALSE	FALSE	FALSE	mRULgB	0.016124	0.032345	486484	sepsis	TRUE	reported	textfile	0.0034	3.93E-06	4689	M16816.m	TRUE	reported	05lFsX	2	TRUE	0.004527	21.31357
5629	rs2486001	T	C	T	C	0.0702	0.030227	0.1433	0.135114	FALSE	FALSE	FALSE	U0d0ue	0.020152	0.133629	486484	sepsis	TRUE	reported	textfile	0.0152	3.91E-06	2616	M33190.m	TRUE	reported	FoODGF	2	TRUE	0.008088	21.31351
5673	rs1457182	A	G	A	G	-0.194	-0.08824	0.982	0.980151	FALSE	FALSE	FALSE	AAOSvG	0.049088	0.072239	486484	sepsis	TRUE	reported	textfile	0.042	3.86E-06	1864	M33197.m	TRUE	reported	aAmcal	2	TRUE	0.011317	21.31271
1195	rs1308371	T	C	T	C	0.0194	-0.01333	0.5762	0.549935	FALSE	FALSE	FALSE	Ckdgh1	0.0138	0.334014	486484	sepsis	TRUE	reported	textfile	0.0042	4.84E-06	1766	M01644.m	TRUE	reported	nKxz7B	2	TRUE	0.011937	21.31144
2070	rs1105833	A	G	A	G	-0.0217	0.084171	0.0228	0.020874	FALSE	FALSE	FALSE	NPVH5j	0.047836	0.078482	486484	sepsis	TRUE	reported	textfile	0.0047	4.26E-06	7310	M15506.m	TRUE	reported	r7r3vM	2	TRUE	0.002908	21.31105
4830	rs1303767	A	G	A	G	-0.0217	0.007354	0.7782	0.769411	FALSE	FALSE	FALSE	yLDcUj	0.016312	0.652116	486484	sepsis	TRUE	reported	textfile	0.0047	5.01E-06	6753	M32838.m	TRUE	reported	5dQgAN	2	TRUE	0.003147	21.31057
6346	rs7105058	A	G	A	G	0.0532	0.000451	0.4597	0.474682	FALSE	FALSE	FALSE	qLUY00	0.013682	0.973724	486484	sepsis	TRUE	reported	textfile	0.0115	3.36E-06	470	M33610.m	TRUE	reported	8cAJbB	2	TRUE	0.04355	21.30961
2177	rs1084778	A	G	A	G	0.0277	0.003791	0.2367	0.24897	FALSE	FALSE	FALSE	UokbxN	0.015794	0.810324	486484	sepsis	TRUE	reported	textfile	0.006	4.28E-06	7357	M15753.m	TRUE	reported	IUGKBi	2	TRUE	0.002889	21.30782
6273	rs4509324	A	G	A	G	-0.0277	0.039598	0.9671	0.965915	FALSE	FALSE	FALSE	MOonP9	0.037564	0.291817	486484	sepsis	TRUE	reported	textfile	0.006	3.27E-06	7066	M33515.m	TRUE	reported	226GuP	2	TRUE	0.003007	21.30758
7209	rs1618850	T	G	T	G	-0.0911	-0.00451	0.6732	0.664776	FALSE	FALSE	FALSE	i1uJ7T	0.014481	0.755207	486484	sepsis	TRUE	reported	textfile	0.0197	3.77E-06	535	M34338.m	TRUE	reported	pi3mKW	2	TRUE	0.038435	21.30481
6470	rs1322668	C	G	C	G	-0.0975	-0.02893	0.136	0.160674	FALSE	TRUE	FALSE	ecwVg8	0.01858	0.119492	486484	sepsis	TRUE	reported	textfile	0.0211	3.92E-06	831	M33751.m	TRUE	reported	NnvbIQ	2	TRUE	0.025051	21.30089
5161	rs6912629	C	G	C	G	-0.1016	0.001993	0.3944	0.409847	FALSE	TRUE	FALSE	L2DJzK	0.013904	0.886035	486484	sepsis	TRUE	reported	textfile	0.022	3.97E-06	1574	M33173.m	TRUE	reported	hRG70D	2	TRUE	0.013369	21.3005
7583	rs1241943	T	C	T	C	-0.0277	0.0098	0.3944	0.385926	FALSE	FALSE	FALSE	SbjFMF	0.014043	0.485259	486484	sepsis	TRUE	reported	textfile	0.006	4.05E-06	3239	M34513.m	TRUE	reported	HVYiKM	2	TRUE	0.006537	21.30045

7503	rs2803054	A	C	A	C	-0.0457	-0.00812	0.5973	0.594113	FALSE	FALSE	FALSE	1aXQWs	0.013943	0.560254	486484	sepsis	TRUE	reported	textfile	0.0099	4.23E-06	4452	M34420.m	TRUE	reported	FOqhHW	2	TRUE	0.004764	21.29938
5632	rs2906297	A	G	A	G	0.0554	-0.02024	0.2427	0.239524	FALSE	FALSE	FALSE	U0d0ue	0.016002	0.205901	486484	sepsis	TRUE	reported	textfile	0.012	4.13E-06	2616	M33190.m	TRUE	reported	FoODGF	2	TRUE	0.008082	21.29732
1801	rs511797	A	C	A	C	0.012	0.057022	0.1261	0.120106	FALSE	FALSE	FALSE	JEcCRZ	0.021065	0.00679	486484	sepsis	TRUE	reported	textfile	0.0026	3.77E-06	7368	M15140.m	TRUE	reported	xYCzKP	2	TRUE	0.002883	21.29599
608	rs1051449	T	C	T	C	0.012	-0.00435	0.6782	0.673642	FALSE	FALSE	FALSE	BYGzq4	0.014661	0.766692	486484	sepsis	TRUE	reported	textfile	0.0026	2.86E-06	7367	M01110.m	TRUE	reported	oZiXqP	2	TRUE	0.002883	21.29599
1358	rs9540462	T	G	T	G	-0.012	0.017799	0.8756	0.875662	FALSE	FALSE	FALSE	LDUJbh	0.020726	0.390464	486484	sepsis	TRUE	reported	textfile	0.0026	4.80E-06	7367	M01898.m	TRUE	reported	JRSLeH	2	TRUE	0.002883	21.29599
3822	rs1195490	T	C	T	C	0.012	-0.01823	0.8579	0.849236	FALSE	FALSE	FALSE	tRX2u0	0.019213	0.34276	486484	sepsis	TRUE	reported	textfile	0.0026	3.60E-06	7365	M32348.m	TRUE	reported	ruBgwQ	2	TRUE	0.002884	21.29599
1073	rs9389071	A	G	A	G	-0.012	0.015629	0.1579	0.157894	FALSE	FALSE	FALSE	tRtQ2e	0.018751	0.404568	486484	sepsis	TRUE	reported	textfile	0.0026	5.64E-06	7364	M01564.m	TRUE	reported	Tbg0zM	2	TRUE	0.002884	21.29599
6795	rs1260565	T	C	T	C	0.012	-0.00119	0.3031	0.32036	FALSE	FALSE	FALSE	OZ0I1H	0.014722	0.9358	486484	sepsis	TRUE	reported	textfile	0.0026	5.13E-06	7364	M33960.m	TRUE	reported	GIPp4w	2	TRUE	0.002884	21.29599
6801	rs4736583	A	C	A	C	-0.012	0.005812	0.6256	0.62355	FALSE	FALSE	FALSE	OZ0I1H	0.014105	0.680298	486484	sepsis	TRUE	reported	textfile	0.0026	4.89E-06	7364	M33960.m	TRUE	reported	GIPp4w	2	TRUE	0.002884	21.29599
4003	rs1258298	A	G	A	G	-0.012	-0.00641	0.1673	0.207193	FALSE	FALSE	FALSE	V9AtId	0.016914	0.704558	486484	sepsis	TRUE	reported	textfile	0.0026	4.13E-06	7363	M32489.m	TRUE	reported	8B8S8a	2	TRUE	0.002885	21.29599
4022	rs6711493	A	G	A	G	-0.012	0.032032	0.8695	0.875405	FALSE	FALSE	FALSE	V9AtId	0.021018	0.127505	486484	sepsis	TRUE	reported	textfile	0.0026	3.76E-06	7363	M32489.m	TRUE	reported	8B8S8a	2	TRUE	0.002885	21.29599
4485	rs1184512	C	G	C	G	-0.012	-0.00101	0.4016	0.411006	FALSE	TRUE	FALSE	1wNJ1E	0.013911	0.941981	486484	sepsis	TRUE	reported	textfile	0.0026	4.24E-06	7363	M32709.m	TRUE	reported	zihBHg	2	TRUE	0.002885	21.29599
4492	rs2070554	T	G	T	G	0.012	-0.01718	0.3446	0.338269	FALSE	FALSE	FALSE	1wNJ1E	0.014452	0.234469	486484	sepsis	TRUE	reported	textfile	0.0026	4.92E-06	7363	M32709.m	TRUE	reported	zihBHg	2	TRUE	0.002885	21.29599
4153	rs7047770	A	G	A	G	-0.024	-0.01944	0.6563	0.641912	FALSE	FALSE	FALSE	CrVaiQ	0.014267	0.172993	486484	sepsis	TRUE	reported	textfile	0.0052	4.49E-06	7361	M32553.m	TRUE	reported	HsKdAI	2	TRUE	0.002886	21.29599
1530	rs3797248	A	G	A	G	0.012	-0.01413	0.1651	0.17072	FALSE	FALSE	FALSE	YvgTeW	0.018288	0.43982	486484	sepsis	TRUE	reported	textfile	0.0026	4.62E-06	7357	M03141.m	TRUE	reported	srKjSc	2	TRUE	0.002887	21.29598
668	rs2397972	A	G	A	G	0.012	-0.00937	0.054	0.059562	FALSE	FALSE	FALSE	p4oCVP	0.028849	0.745387	486484	sepsis	TRUE	reported	textfile	0.0026	4.05E-06	7352	M01125.m	TRUE	reported	OwD5re	2	TRUE	0.002889	21.29598
6304	rs2527641	A	G	A	G	-0.018	0.000958	0.5656	0.554702	FALSE	FALSE	FALSE	ORVTMc	0.013737	0.944395	486484	sepsis	TRUE	reported	textfile	0.0039	4.94E-06	7352	M33587.m	TRUE	reported	57AsXW	2	TRUE	0.002889	21.29598
6979	rs7267516	T	C	T	C	-0.012	-0.00776	0.4894	0.495216	FALSE	FALSE	FALSE	ZlvYs5	0.013722	0.571681	486484	sepsis	TRUE	reported	textfile	0.0026	2.60E-06	7351	M34214.m	TRUE	reported	qU57S1	2	TRUE	0.002889	21.29598
6985	rs9997	T	C	T	C	0.012	-0.0017	0.6544	0.648772	FALSE	FALSE	FALSE	ZlvYs5	0.014365	0.905786	486484	sepsis	TRUE	reported	textfile	0.0026	3.89E-06	7351	M34214.m	TRUE	reported	qU57S1	2	TRUE	0.002889	21.29598
7901	rs7599943	T	C	T	C	-0.012	0.023894	0.3473	0.353786	FALSE	FALSE	FALSE	pU8zps	0.014301	0.09475	486484	sepsis	TRUE	reported	textfile	0.0026	5.17E-06	7350	M35186.m	TRUE	reported	Oc2IOF	2	TRUE	0.00289	21.29598
1272	rs980922	C	G	C	G	-0.012	0.022017	0.8056	0.78819	FALSE	TRUE	FALSE	gNG3wy	0.016782	0.189548	486484	sepsis	TRUE	reported	textfile	0.0026	3.57E-06	7348	M01670.m	TRUE	reported	t3lsMq	2	TRUE	0.002891	21.29598
7805	rs737842	A	G	A	G	-0.012	-0.02131	0.5825	0.579934	FALSE	FALSE	FALSE	cQ5TvO	0.013842	0.123661	486484	sepsis	TRUE	reported	textfile	0.0026	3.05E-06	7344	M35127.m	TRUE	reported	BVxkfZ	2	TRUE	0.002892	21.29597
2143	rs6846111	T	C	T	C	-0.012	0.006116	0.1508	0.153926	FALSE	FALSE	FALSE	TEupfd	0.018959	0.747006	486484	sepsis	TRUE	reported	textfile	0.0026	5.46E-06	7331	M15676.m	TRUE	reported	NXiaPC	2	TRUE	0.002897	21.29596
2920	rs9814648	A	G	A	G	0.012	-0.00576	0.2592	0.275632	FALSE	FALSE	FALSE	FBDEQi	0.015396	0.708192	486484	sepsis	TRUE	reported	textfile	0.0026	4.60E-06	7327	M20699.m	TRUE	reported	nvNIIp	2	TRUE	0.002899	21.29596
912	rs1049742	T	C	T	C	0.012	-0.00764	0.8064	0.809973	FALSE	FALSE	FALSE	rVMAPf	0.01751	0.662503	486484	sepsis	TRUE	reported	textfile	0.0026	4.47E-06	7307	M01493.m	TRUE	reported	siVqDi	2	TRUE	0.002907	21.29594
3551	rs1948930	A	G	A	G	0.012	-0.00518	0.297	0.310101	FALSE	FALSE	FALSE	8IuUON	0.014835	0.72711	486484	sepsis	TRUE	reported	textfile	0.0026	4.21E-06	7257	M31453.m	TRUE	reported	FBs6G4	2	TRUE	0.002927	21.2959
8048	rs708496	T	C	T	C	-0.012	0.009123	0.5445	0.615886	FALSE	FALSE	FALSE	SwiqSi	0.014334	0.524492	486484	sepsis	TRUE	reported	textfile	0.0026	4.39E-06	7217	M35270.m	TRUE	reported	21ZIsQ	2	TRUE	0.002943	21.29587
956	rs2526477	A	G	A	G	-0.012	-0.02094	0.609	0.610565	FALSE	FALSE	FALSE	tWoDq4	0.01402	0.135255	486484	sepsis	TRUE	reported	textfile	0.0026	2.99E-06	7160	M01508.m	TRUE	reported	6ltDw0	2	TRUE	0.002966	21.29582
2248	rs1955742	A	T	A	T	-0.012	0.005453	0.599	0.596561	FALSE	TRUE	FALSE	zxcPox	0.013917	0.695173	486484	sepsis	TRUE	reported	textfile	0.0026	6.24E-06	6782	M15990.m	TRUE	reported	34Vg8F	2	TRUE	0.003131	21.29549
5107	rs4453360	A	G	A	G	-0.012	-0.00283	0.4648	0.449473	FALSE	FALSE	FALSE	Uiz8VU	0.013871	0.838139	486484	sepsis	TRUE	reported	textfile	0.0026	4.28E-06	6404	M33163.m	TRUE	reported	ky0Bda	2	TRUE	0.003315	21.29512
7917	rs2048073	A	G	A	G	-0.03	-0.01892	0.1579	0.189123	FALSE	FALSE	FALSE	8qXKkX	0.017623	0.28298	486484	sepsis	TRUE	reported	textfile	0.0065	4.13E-06	6234	M35189.m	TRUE	reported	Nt6oaC	2	TRUE	0.003405	21.29494
5660	rs432097	A	G	A	G	-0.048	0.033476	0.2928	0.29804	FALSE	FALSE	FALSE	mod1fD	0.014936	0.025006	486484	sepsis	TRUE	reported	textfile	0.0104	4.08E-06	5064	M33194.m	TRUE	reported	fSFVUj	2	TRUE	0.004189	21.29336
7795	rs1270382	A	T	A	T	-0.0217	0.022143	0.6236	0.633849	FALSE	TRUE	FALSE	cQ5TvO	0.014184	0.118498	486484	sepsis	TRUE	reported	textfile	0.0047	4.64E-06	1762	M35127.m	TRUE	reported	BVxkfZ	2	TRUE	0.011954	21.29269
1799	rs3809198	A	C	A	C	0.0554	0.030786	0.0199	0.011387	FALSE	FALSE	FALSE	JEcCRZ	0.065096	0.636265	486484	sepsis	TRUE	reported	textfile	0.012	4.28E-06	1764	M15140.m	TRUE	reported	xYCzKP	2	TRUE	0.011938	21.28945
3293	rs7242260	A	C	A	C	0.0277	0.006451	0.8606	0.870494	FALSE	FALSE	FALSE	vYWPe9	0.020402	0.751863	486484	sepsis	TRUE	reported	textfile	0.006	3.52E-06	1756	M25459.m	TRUE	reported	VvJ6Vx	2	TRUE	0.011992	21.28934
901	rs3542146	A	G	A	G	0.0277	-0.02476	0.4033	0.396293	FALSE	FALSE	FALSE	I6zQnf	0.013989	0.07669	486484	sepsis	TRUE	reported	textfile	0.006	4.15E-06	1753	M01444.m	TRUE	reported	IIzaIm	2	TRUE	0.012012	21.28929
2421	rs4434498	A	G	A	G	-0.0554	0.032301	0.5074	0.526955	FALSE	FALSE	FALSE	NBJ2Do	0.013693	0.018325	486484	sepsis	TRUE	reported	textfile	0.012	4.23E-06	1739	M17799.m	TRUE	reported	Uz0WgI	2	TRUE	0.012108	21.2891
5658	rs2959857	A	T	A	T	0.0988	0.008982	0.2731	0.2729	FALSE	TRUE	FALSE	mod1fD	0.015385	0.559349	486484	sepsis	TRUE	reported	textfile	0.0214	3.81E-06	1586	M33194.m	TRUE	reported	fSFVUj	2	TRUE	0.013261	21.28817
7122	rs1181731	T	C	T	C	-0.17	-0.02637	0.9575	0.966745	FALSE	FALSE	FALSE	de8tA6	0.038128	0.489167	486484	sepsis	TRUE	reported	textfile	0.0367	3.61E-06	248	M34322.m	TRUE	reported	sXPPBO	2	TRUE	0.07963	21.2838
2181	rs1343188	T	C	T	C	-0.0263	-0.02507	0.5646	0.537053	FALSE	FALSE	FALSE	UokbxN	0.013756	0.068342	486484	sepsis	TRUE	reported	textfile	0.0057	4.17E-06	7357	M15753.m	TRUE	reported	lUGKBi	2	TRUE	0.002885	21.28353
9077	rs7187181	T	C	T	C	0.0263	-0.01245	0.0635	0.070927	FALSE	FALSE	FALSE	C7PD8T	0.027032	0.645061	486484	sepsis	TRUE	reported	textfile	0.0057	3.34E-06	6942	M38768.m	TRUE	reported	ZA3QM7	2	TRUE	0.003057	21.28319
2171	rs6851666	A	T	A	T	-0.0526	0.00041	0.9632	0.957476	FALSE	TRUE	FALSE	XCBNPg	0.033961	0.990359	486484	sepsis	TRUE	reported	textfile	0.0114	4.06E-06	5829	M15749.m	TRUE	reported	dKBewD	2	TRUE	0.003639	21.28202
2377	rs1304156	T	C	T	C	0.0697	0.01413	0.8472	0.802116	FALSE	FALSE	FALSE	NBJ2Do	0.017196	0.411255	486484	sepsis	TRUE	reported	textfile	0.0151	3.76E-06	1739	M17799.m	TRUE	reported	Uz0WgI	2	TRUE	0.012104	21.28197
5644	rs1233996	C	G	C	G	0.0789	0.011933	0.0852	0.090283	FALSE	TRUE	FALSE	BFsMtm	0.023999	0.619038	486484	sepsis	TRUE	reported	textfile	0.0171	3.71E-06	5716	M33192.m	TRUE	reported	NAJN3D	2	TRUE	0.003711	21.28187
6941	rs4850779	A	G	A	G	-0.0263	0.012596	0.415	0.40632	FALSE	FALSE	FALSE	oNv0vf	0.013897	0.364757	486484	sepsis	TRUE	reported	textfile	0.0057	4.40E-06	5581	M34112.m	TRUE	reported	l0pe0N	2	TRUE	0.0038	21.28169
5410	rs2361394	A	G	A	G	0.1413	-0.02215	0.9584	0.956838	FALSE	FALSE	FALSE	q32d4E	0.034148	0.516506	486484	sepsis	TRUE	reported	textfile	0.0306	3.75E-06	1017	M33178.m	TRUE	reported	cS3BwT	2	TRUE	0.020536	21.28073
5848	rs163589	T	G	T	G	0.6297	-0.05191	0.011	0.017198	FALSE	FALSE	FALSE	Vkec6Y	0.052198	0.319971	486484	sepsis	TRUE	reported	textfile	0.136	3.63E-06	272	M33384.m	TRUE	reported	eaxeyR	2	TRUE	0.073059	21.28063
7783	rs4938200	T	C	T	C	-0.0203	-0.03595	0.4388	0.439258	FALSE	FALSE	FALSE	15BqPa	0.013773	0.009041	486484	sepsis	TRUE	reported	textfile	0.0044	4.31E-06	7364	M35126.m	TRUE	reported	zvDzP2	2	TRUE	0.002882	21.27986
6232	rs1279565	A	T	A	T	-0.0203	0.011594	0.8826	0.891025	FALSE	TRUE	FALSE	vL52Yb	0.021958	0.597489	486484	sepsis	TRUE	reported	textfile	0.0044	3.34E-06	7269	M33510.m	TRUE	reported	JIO9VX	2	TRUE	0.00292	21.27978
3230	rs6078040	T	C	T	C	-0.0203	-0.01785	0.9256	0.914642	FALSE	FALSE	FALSE	I25GHB	0.02451	0.466443	486484	sepsis	TRUE	reported	textfile	0.0044	4.82E-06	7184	M22548.m	TRUE	reported	eBmTQD	2	TRUE	0.002954	21.27971
7228	rs693833	A	C	A	C	0.591	-0.09999	0.983	0.978415	FALSE	FALSE	FALSE	i1uJ7T	0.047422	0.034978	486484	sepsis	TRUE	reported	textfile	0.127	3.26E-06	115	M34338.m	TRUE	reported	pi3mKW	2	TRUE	0.158468	21.27885
6887	rs7167675	C	G	C	G	-0.06	-0.13924	0.9875	0.990575	FALSE	TRUE	FALSE	kFWZjs	0.071478	0.051413	486484	sepsis	TRUE	reported	textfile	0.013	3.68E-06	1768	M34062.m	TRUE	reported	eLONsg	2	TRUE	0.011905	21.27768
8841	rs1258238	T	C	T	C	0.036	0.027379	0.0454	0.046476	FALSE	FALSE	FALSE	XIHeTV	0.03295	0.406016	486484	sepsis	TRUE	reported	textfile	0.0078	3.64E-06	1764	M36808.m	TRUE	reported	37Vi2D	2	TRUE	0.011932	21.27762
4441	rs7010049	A	G	A	G	-0.024	0.037645	0.9352	0.935228	FALSE	FALSE	FALSE	WGPdpq	0.027799	0.175672	486484	sepsis	TRUE	reported	textfile	0.0052	3.53E-06	1756	M32675.m	TRUE	reported	u0eUAG	2	TRUE	0.011985	21.27751
544	rs1125992	A	G	A	G	0.042	-0.01156	0.8139	0.791596	FALSE	FALSE	FALSE	QUUN2g	0.016815	0.491801	486484	sepsis	TRUE	reported	textfile	0.0091	3.92E-06	1723	M00599.m	TRUE	reported	vxn2i7	2	TRUE	0.012212	21.27705
7682	rs9832461	A	G	A	G	-0.0203	0.012238	0.7731	0.776976	FALSE	FALSE	FALSE	iqkM9B	0.01651	0.458537	486484	sepsis	TRUE	reported	textfile	0.0044	4.10E-06	4800	M34539.m	TRUE	reported	5nFq6f	2	TRUE	0.004415	21.27677
8207	rs9642829	A	G	A	G	-0.0346	0.017518	0.9744	0.978297	FALSE	FALSE	FALSE	iLlpho	0.047045	0.709622	486484	sepsis	TRUE	reported	textfile	0.0075	3.61E-06	7007	M35431.m	TRUE	reported	AAZ5ge	2	TRUE	0.003028	21.27677
485	rs4838537	T	C	T	C	0.3226	-0.05701	0.0133	0.028845	FALSE	FALSE	FALSE	ScJVI8	0.041118	0.165563	486484	sepsis	TRUE	reported	textfile	0.0699	3.96E-06	1766	M00542.m	TRUE	reported	Sa1L04	2	TRUE	0.011917	21.27562
5429	rs33469	A	G	A	G	0.1639	-0.05462	0.9553	0.97976	FALSE	FALSE	FALSE	q32d4E	0.049138	0.266337	486484	sepsis	TRUE	reported	textfile	0.0355	4.01E-06	1017	M33178.m	TRUE	reported	cS3BwT	2	TRUE	0.020529	21.27386
2436	rs6783548	T	C	T	C	-0.0779	0.003602	0.6219	0.609655	FALSE	FALSE	FALSE	NBJ2Do	0.014017	0.797214	486484	sepsis	TRUE	reported	textfile	0.0162	1.62E-06	25	M17799.m	TRUE	reported	Uz0WgI	2	TRUE	0.480498	21.27319
7112	rs6597533	A	G	A	G	0.1306	-0.01312	0.9771	0.972775	FALSE	FALSE	FALSE	DfLd6z	0.042238	0.756035	486484	sepsis	TRUE	reported	textfile	0.0283	4.04E-06	1766	M34314.m	TRUE	reported	OgloAQ	2	TRUE	0.011916	21.27264
8799	rs1054812	A	G	A	G	-0.0203	0.011466	0.6283	0.621511	FALSE	FALSE	FALSE	QWTooc	0.014145	0.417607	486484	sepsis	TRUE	reported	textfile	0.0044	4.28E-06	3248	M36756.m	TRUE	reported	OLsw6C	2	TRUE	0.006511	21.27253
8806	rs518385	A	G	A	G	-0.0203	-0.00332	0.5033	0.51385	FALSE	FALSE	FALSE	QWTooc	0.013686	0.80848	486484	sepsis	TRUE	reported	textfile	0.0044	3.29E-06	3248	M36756.m	TRUE	reported	OLsw6C	2	TRUE	0.006511	21.27253
2307	rs2043660	A	T	A	T	0.036	-0.01195	0.8396	0.836739	FALSE	TRUE	FALSE	ZzUW8U	0.018508	0.518646	486484	sepsis	TRUE	reported	textfile	0.0078	4.03E-06	1445	M16821.m	TRUE	reported	g2xs3i	2	TRUE	0.014528	21.27229
4361	rs1099586	A	C	A	C	0.0286	-0.00544	0.0589	0.060439	FALSE	FALSE	FALSE	SPhI52	0.028953	0.850928	486484	sepsis	TRUE	reported	textfile	0.0062	3.92E-06	5211	M32651.m	TRUE	reported	V0Bnpj	2	TRUE	0.004067	21.27071
1831	rs1312063	T	G	T	G	0.0443	-0.02407	0.9213	0.928398	FALSE	FALSE	FALSE	2TaDHm	0.026702	0.367398	486484	sepsis	TRUE	reported	textfile	0.0096	3.96E-06	1760	M15365.m	TRUE	reported	LoLveg	2	TRUE	0.011954	21.27018
7712	rs1696425	T	C	T	C	-0.0669	0.009277	0.9549	0.957044	FALSE	FALSE	FALSE	qJW9lg	0.033764	0.783504	486484	sepsis	TRUE	reported	textfile	0.0145	4.02E-06	2474	M34878.m	TRUE	reported	42EOzB	2	TRUE	0.008531	21.26988
3605	rs1244517	A	C	A	C	-0.0226	-0.00759	0.8312	0.818862	FALSE	FALSE	FALSE	2z5KhH	0.017781	0.669678	486484	sepsis	TRUE	reported	textfile	0.0049	3.61E-06	7263	M31555.m	TRUE	reported	5kcWr2	2	TRUE	0.00292	21.26695
4669	rs1366688	C	G	C	G	0.0226	-0.01256	0.6094	0.602271	FALSE	TRUE	FALSE	xaapK0	0.014193	0.376024	486484	sepsis	TRUE	reported	textfile	0.0049	5.15E-06	7250	M32757.m	TRUE	reported	64n1Z4	2	TRUE	0.002926	21.26693
7604	rs1708348	T	C	T	C	-0.1292	0.001401	0.2175	0.222184	FALSE	FALSE	FALSE	PlYt5g	0.01641	0.931953	486484	sepsis	TRUE	reported	textfile	0.0279	3.63E-06	241	M34516.m	TRUE	reported	jX41tf	2	TRUE	0.081711	21.26657
1048	rs2143327	T	C	T	C	0.0392	0.075054	0.0129	0.018485	FALSE	FALSE	FALSE	tRtQ2e	0.050901	0.14034	486484	sepsis	TRUE	reported	textfile	0.0085	4.28E-06	7364	M01564.m	TRUE	reported	Tbg0zM	2	TRUE	0.00288	21.2626
617	rs3798211	A	C	A	C	-0.0083	-0.01433	0.4247	0.425821	FALSE	FALSE	FALSE	BYGzq4	0.013823	0.299934	486484	sepsis	TRUE	reported	textfile	0.0018	4.58E-06	7367	M01110.m	TRUE	reported	oZiXqP	2	TRUE	0.002878	21.25657
1333	rs1394836	T	C	T	C	0.0083	0.025089	0.6032	0.608805	FALSE	FALSE	FALSE	LDUJbh	0.014051	0.074163	486484	sepsis	TRUE	reported	textfile	0.0018	2.23E-06	7367	M01898.m	TRUE	reported	JRSLeH	2	TRUE	0.002878	21.25657
1338	rs1928871	C	G	C	G	0.0083	0.000325	0.3987	0.402665	FALSE	TRUE	FALSE	LDUJbh	0.013932	0.98139	486484	sepsis	TRUE	reported	textfile	0.0018	2.78E-06	7367	M01898.m	TRUE	reported	JRSLeH	2	TRUE	0.002878	21.25657
1356	rs9360494	T	C	T	C	-0.0083	0.004794	0.5977	0.574755	FALSE	FALSE	FALSE	LDUJbh	0.01383	0.728888	486484	sepsis	TRUE	reported	textfile	0.0018	2.69E-06	7367	M01898.m	TRUE	reported	JRSLeH	2	TRUE	0.002878	21.25657
9029	rs1445447	A	G	A	G	-0.0083	-0.00797	0.7448	0.743124	FALSE	FALSE	FALSE	df0MCM	0.015608	0.609419	486484	sepsis	TRUE	reported	textfile	0.0018	3.91E-06	7366	M37506.m	TRUE	reported	iUfr4u	2	TRUE	0.002878	21.25657
9038	rs2294070	A	T	A	T	-0.0083	0.004409	0.7404	0.736916	FALSE	TRUE	FALSE	df0MCM	0.015541	0.776635	486484	sepsis	TRUE	reported	textfile	0.0018	3.87E-06	7366	M37506.m	TRUE	reported	iUfr4u	2	TRUE	0.002878	21.25657
408	rs1288179	T	G	T	G	-0.0083	0.012884	0.7603	0.745516	FALSE	FALSE	FALSE	cTsNj6	0.015794	0.414658	486484	sepsis	TRUE	reported	textfile	0.0018	5.91E-06	7365	M00063.m	TRUE	reported	7KYTrQ	2	TRUE	0.002879	21.25657
425	rs3747924	T	C	T	C	0.0083	0.012679	0.1781	0.178878	FALSE	FALSE	FALSE	cTsNj6	0.017988	0.480917	486484	sepsis	TRUE	reported	textfile	0.0018	4.24E-06	7365	M00063.m	TRUE	reported	7KYTrQ	2	TRUE	0.002879	21.25657
3818	rs1089800	T	C	T	C	0.0083	0.014841	0.5314	0.53667	FALSE	FALSE	FALSE	tRX2u0	0.013791	0.281887	486484	sepsis	TRUE	reported	textfile	0.0018	2.30E-06	7365	M32348.m	TRUE	reported	ruBgwQ	2	TRUE	0.002879	21.25657
3831	rs192812	T	C	T	C	0.0083	0.003036	0.4356	0.438499	FALSE	FALSE	FALSE	tRX2u0	0.013776	0.825586	486484	sepsis	TRUE	reported	textfile	0.0018	2.89E-06	7365	M32348.m	TRUE	reported	ruBgwQ	2	TRUE	0.002879	21.25657
3854	rs9323955	A	G	A	G	0.0083	-0.01223	0.4396	0.440146	FALSE	FALSE	FALSE	tRX2u0	0.013779	0.374684	486484	sepsis	TRUE	reported	textfile	0.0018	2.70E-06	7365	M32348.m	TRUE	reported	ruBgwQ	2	TRUE	0.002879	21.25657
1041	rs1380634	A	G	A	G	-0.0083	-0.0186	0.7425	0.740988	FALSE	FALSE	FALSE	tRtQ2e	0.015677	0.235447	486484	sepsis	TRUE	reported	textfile	0.0018	5.17E-06	7364	M01564.m	TRUE	reported	Tbg0zM	2	TRUE	0.002879	21.25657
1052	rs2534835	T	G	T	G	-0.0083	-0.00486	0.2407	0.245481	FALSE	FALSE	FALSE	tRtQ2e	0.015936	0.760489	486484	sepsis	TRUE	reported	textfile	0.0018	6.23E-06	7364	M01564.m	TRUE	reported	Tbg0zM	2	TRUE	0.002879	21.25657
1054	rs4235093	A	G	A	G	-0.0083	-0.00559	0.2874	0.292324	FALSE	FALSE	FALSE	tRtQ2e	0.015098	0.7114	486484	sepsis	TRUE	reported	textfile	0.0018	5.04E-06	7364	M01564.m	TRUE	reported	Tbg0zM	2	TRUE	0.002879	21.25657
1061	rs7584253	A	C	A	C	0.0083	-0.0261	0.7047	0.702211	FALSE	FALSE	FALSE	tRtQ2e	0.014995	0.081764	486484	sepsis	TRUE	reported	textfile	0.0018	5.04E-06	7364	M01564.m	TRUE	reported	Tbg0zM	2	TRUE	0.002879	21.25657
1065	rs8017368	A	C	A	C	-0.0083	0.030652	0.6452	0.630534	FALSE	FALSE	FALSE	tRtQ2e	0.014155	0.030349	486484	sepsis	TRUE	reported	textfile	0.0018	3.85E-06	7364	M01564.m	TRUE	reported	Tbg0zM	2	TRUE	0.002879	21.25657
4287	rs1264950	T	G	T	G	0.0083	-0.00558	0.2978	0.302562	FALSE	FALSE	FALSE	0tjjPC	0.014911	0.708101	486484	sepsis	TRUE	reported	textfile	0.0018	5.14E-06	7362	M32634.m	TRUE	reported	nJJFkX	2	TRUE	0.00288	21.25657
5037	rs6969691	A	G	A	G	-0.0083	-0.01983	0.8775	0.88018	FALSE	FALSE	FALSE	BNnOvt	0.02103	0.345615	486484	sepsis	TRUE	reported	textfile	0.0018	5.29E-06	7362	M33132.m	TRUE	reported	rHcCPG	2	TRUE	0.00288	21.25657
6866	rs1265800	T	G	T	G	0.0083	0.006701	0.2091	0.232222	FALSE	FALSE	FALSE	kFWZjs	0.016205	0.679238	486484	sepsis	TRUE	reported	textfile	0.0018	2.66E-06	7362	M34062.m	TRUE	reported	eLONsg	2	TRUE	0.00288	21.25657
6869	rs1715036	A	T	A	T	0.0083	0.007426	0.8838	0.881384	FALSE	TRUE	FALSE	kFWZjs	0.021251	0.726758	486484	sepsis	TRUE	reported	textfile	0.0018	6.17E-06	7362	M34062.m	TRUE	reported	eLONsg	2	TRUE	0.00288	21.25657
6874	rs2162481	T	C	T	C	-0.0083	0.017541	0.1224	0.120591	FALSE	FALSE	FALSE	kFWZjs	0.02099	0.403337	486484	sepsis	TRUE	reported	textfile	0.0018	6.89E-06	7362	M34062.m	TRUE	reported	eLONsg	2	TRUE	0.00288	21.25657
6880	rs4748538	T	C	T	C	-0.0083	0.026341	0.88	0.859842	FALSE	FALSE	FALSE	kFWZjs	0.019823	0.183917	486484	sepsis	TRUE	reported	textfile	0.0018	7.41E-06	7362	M34062.m	TRUE	reported	eLONsg	2	TRUE	0.00288	21.25657
709	rs4808739	T	C	T	C	0.0083	0.005739	0.7578	0.763828	FALSE	FALSE	FALSE	H9I3s3	0.016168	0.722602	486484	sepsis	TRUE	reported	textfile	0.0018	2.42E-06	7358	M01299.m	TRUE	reported	kXWyUq	2	TRUE	0.002881	21.25657
1532	rs4646359	T	C	T	C	-0.0083	-0.02609	0.5297	0.526301	FALSE	FALSE	FALSE	YvgTeW	0.013703	0.056887	486484	sepsis	TRUE	reported	textfile	0.0018	3.20E-06	7357	M03141.m	TRUE	reported	srKjSc	2	TRUE	0.002882	21.25657
1603	rs1846975	A	G	A	G	0.0083	-0.00657	0.3554	0.354343	FALSE	FALSE	FALSE	zP3C2v	0.014338	0.646749	486484	sepsis	TRUE	reported	textfile	0.0018	2.56E-06	7356	M12035.m	TRUE	reported	1Pooe3	2	TRUE	0.002882	21.25656
57	rs1682395	T	G	T	G	-0.0083	0.002487	0.9069	0.902679	FALSE	FALSE	FALSE	xmGuol	0.023044	0.914045	486484	sepsis	TRUE	reported	textfile	0.0018	5.08E-06	7355	M00054.m	TRUE	reported	GW50Ri	2	TRUE	0.002883	21.25656
844	rs954820	A	G	A	G	0.0083	0.002521	0.5619	0.552677	FALSE	FALSE	FALSE	2qgDgB	0.013797	0.85505	486484	sepsis	TRUE	reported	textfile	0.0018	2.57E-06	7355	M01358.m	TRUE	reported	asmXq0	2	TRUE	0.002883	21.25656
1189	rs1026672	T	C	T	C	-0.0083	-0.00996	0.4261	0.406072	FALSE	FALSE	FALSE	Ckdgh1	0.013914	0.474168	486484	sepsis	TRUE	reported	textfile	0.0018	4.00E-06	7355	M01644.m	TRUE	reported	nKxz7B	2	TRUE	0.002883	21.25656
1198	rs1728793	T	C	T	C	0.0083	-0.01276	0.4687	0.464841	FALSE	FALSE	FALSE	Ckdgh1	0.013699	0.351631	486484	sepsis	TRUE	reported	textfile	0.0018	5.24E-06	7355	M01644.m	TRUE	reported	nKxz7B	2	TRUE	0.002883	21.25656
1202	rs1866411	A	C	A	C	-0.0083	0.029255	0.3473	0.34778	FALSE	FALSE	FALSE	Ckdgh1	0.014358	0.041601	486484	sepsis	TRUE	reported	textfile	0.0018	5.23E-06	7355	M01644.m	TRUE	reported	nKxz7B	2	TRUE	0.002883	21.25656
1207	rs2291199	A	G	A	G	0.0083	0.008176	0.5373	0.539105	FALSE	FALSE	FALSE	Ckdgh1	0.013696	0.550521	486484	sepsis	TRUE	reported	textfile	0.0018	5.18E-06	7355	M01644.m	TRUE	reported	nKxz7B	2	TRUE	0.002883	21.25656
4032	rs1110289	A	G	A	G	-0.0083	-0.00541	0.4032	0.400224	FALSE	FALSE	FALSE	SiYGYU	0.013967	0.698271	486484	sepsis	TRUE	reported	textfile	0.0018	2.68E-06	7355	M32492.m	TRUE	reported	GfQvpU	2	TRUE	0.002883	21.25656
4057	rs9384386	T	C	T	C	0.0083	-0.00659	0.3966	0.397958	FALSE	FALSE	FALSE	SiYGYU	0.014019	0.63836	486484	sepsis	TRUE	reported	textfile	0.0018	3.26E-06	7355	M32492.m	TRUE	reported	GfQvpU	2	TRUE	0.002883	21.25656
2799	rs1219123	C	G	C	G	0.0083	0.021722	0.6904	0.673831	FALSE	TRUE	FALSE	Sx5Uhq	0.014603	0.136893	486484	sepsis	TRUE	reported	textfile	0.0018	2.27E-06	7354	M19934.m	TRUE	reported	DijYTE	2	TRUE	0.002883	21.25656
2800	rs1258922	T	C	T	C	0.0083	-0.01921	0.3252	0.330859	FALSE	FALSE	FALSE	Sx5Uhq	0.014557	0.186977	486484	sepsis	TRUE	reported	textfile	0.0018	2.41E-06	7354	M19934.m	TRUE	reported	DijYTE	2	TRUE	0.002883	21.25656
2803	rs1699639	A	T	A	T	-0.0083	-0.00651	0.6946	0.699096	FALSE	TRUE	FALSE	Sx5Uhq	0.014939	0.663185	486484	sepsis	TRUE	reported	textfile	0.0018	3.18E-06	7354	M19934.m	TRUE	reported	DijYTE	2	TRUE	0.002883	21.25656
6773	rs476092	A	C	A	C	0.0083	0.005548	0.2852	0.285782	FALSE	FALSE	FALSE	EIf6MQ	0.015366	0.71806	486484	sepsis	TRUE	reported	textfile	0.0018	3.25E-06	7354	M33955.m	TRUE	reported	Lxn4RF	2	TRUE	0.002883	21.25656
669	rs2470439	A	G	A	G	0.0083	-0.00319	0.11	0.112618	FALSE	FALSE	FALSE	p4oCVP	0.021627	0.882774	486484	sepsis	TRUE	reported	textfile	0.0018	5.69E-06	7352	M01125.m	TRUE	reported	OwD5re	2	TRUE	0.002884	21.25656
671	rs4086666	T	G	T	G	0.0083	0.024466	0.8804	0.873957	FALSE	FALSE	FALSE	p4oCVP	0.020592	0.234792	486484	sepsis	TRUE	reported	textfile	0.0018	2.42E-06	7352	M01125.m	TRUE	reported	OwD5re	2	TRUE	0.002884	21.25656
1768	rs2076799	C	G	C	G	0.0166	0.067973	0.0853	0.069815	FALSE	TRUE	FALSE	wM5dkT	0.026877	0.011437	486484	sepsis	TRUE	reported	textfile	0.0036	3.89E-06	7352	M15122.m	TRUE	reported	CBpa4t	2	TRUE	0.002884	21.25656
4097	rs1161461	A	C	A	C	-0.0083	0.002176	0.7392	0.713895	FALSE	FALSE	FALSE	4rtLd0	0.015177	0.885969	486484	sepsis	TRUE	reported	textfile	0.0018	5.84E-06	7350	M32518.m	TRUE	reported	j7f1ER	2	TRUE	0.002884	21.25656
4123	rs4936882	T	G	T	G	0.0083	-0.00354	0.2446	0.234689	FALSE	FALSE	FALSE	4rtLd0	0.016112	0.826074	486484	sepsis	TRUE	reported	textfile	0.0018	5.44E-06	7350	M32518.m	TRUE	reported	j7f1ER	2	TRUE	0.002884	21.25656
3690	rs1081205	A	G	A	G	-0.0083	0.015225	0.5108	0.505998	FALSE	FALSE	FALSE	agPgtF	0.013654	0.26482	486484	sepsis	TRUE	reported	textfile	0.0018	2.56E-06	7349	M32315.m	TRUE	reported	hMRjcV	2	TRUE	0.002885	21.25656
3699	rs1874795	A	G	A	G	-0.0083	0.02206	0.4303	0.441108	FALSE	FALSE	FALSE	agPgtF	0.013784	0.109502	486484	sepsis	TRUE	reported	textfile	0.0018	2.26E-06	7349	M32315.m	TRUE	reported	hMRjcV	2	TRUE	0.002885	21.25656
8401	rs742914	A	C	A	C	-0.0083	-0.00273	0.7235	0.711074	FALSE	FALSE	FALSE	SCzh0A	0.015134	0.856626	486484	sepsis	TRUE	reported	textfile	0.0018	7.46E-06	7348	M35675.m	TRUE	reported	n1SREk	2	TRUE	0.002885	21.25656
752	rs2780242	T	C	T	C	-0.0083	0.004668	0.1138	0.129229	FALSE	FALSE	FALSE	RHWptG	0.020653	0.821168	486484	sepsis	TRUE	reported	textfile	0.0018	3.85E-06	7347	M01302.m	TRUE	reported	z1OLfr	2	TRUE	0.002886	21.25656
3287	rs575528	A	G	A	G	0.0083	-0.0073	0.5409	0.544812	FALSE	FALSE	FALSE	vYWPe9	0.013716	0.594628	486484	sepsis	TRUE	reported	textfile	0.0018	4.60E-06	7340	M25459.m	TRUE	reported	VvJ6Vx	2	TRUE	0.002888	21.25655
3288	rs5758064	T	C	T	C	-0.0083	0.004206	0.5194	0.523401	FALSE	FALSE	FALSE	vYWPe9	0.013768	0.760016	486484	sepsis	TRUE	reported	textfile	0.0018	3.91E-06	7340	M25459.m	TRUE	reported	VvJ6Vx	2	TRUE	0.002888	21.25655
3511	rs1051218	T	C	T	C	0.0166	-0.00842	0.6502	0.643846	FALSE	FALSE	FALSE	hxuqls	0.014353	0.55725	486484	sepsis	TRUE	reported	textfile	0.0036	3.32E-06	7336	M27738.m	TRUE	reported	aueeZs	2	TRUE	0.00289	21.25655
6088	rs9540840	T	C	T	C	0.0083	-0.00665	0.8471	0.848718	FALSE	FALSE	FALSE	P8n0HP	0.019353	0.731068	486484	sepsis	TRUE	reported	textfile	0.0018	3.82E-06	7336	M33442.m	TRUE	reported	cljMTI	2	TRUE	0.00289	21.25655
4913	rs804854	A	T	A	T	0.0083	-0.06804	0.1417	0.140765	FALSE	TRUE	FALSE	VqcRGX	0.019781	0.000583	486484	sepsis	TRUE	reported	textfile	0.0018	2.68E-06	7329	M32867.m	TRUE	reported	psuFJy	2	TRUE	0.002893	21.25654
4619	rs9397197	A	G	A	G	0.0083	-0.00683	0.2948	0.290721	FALSE	FALSE	FALSE	Pzplqu	0.015041	0.649769	486484	sepsis	TRUE	reported	textfile	0.0018	2.21E-06	7318	M32740.m	TRUE	reported	FjEBS5	2	TRUE	0.002897	21.25653
7035	rs1112600	A	G	A	G	0.0083	-0.00169	0.3729	0.37742	FALSE	FALSE	FALSE	uthruC	0.014578	0.907909	486484	sepsis	TRUE	reported	textfile	0.0018	2.61E-06	7316	M34283.m	TRUE	reported	ftTuRU	2	TRUE	0.002898	21.25653
7044	rs1266396	A	T	A	T	-0.0083	-0.00268	0.3638	0.380275	FALSE	TRUE	FALSE	uthruC	0.014157	0.849937	486484	sepsis	TRUE	reported	textfile	0.0018	3.64E-06	7316	M34283.m	TRUE	reported	ftTuRU	2	TRUE	0.002898	21.25653
7057	rs2372474	A	G	A	G	0.0083	-0.02078	0.4893	0.494839	FALSE	FALSE	FALSE	uthruC	0.013697	0.129286	486484	sepsis	TRUE	reported	textfile	0.0018	2.24E-06	7316	M34283.m	TRUE	reported	ftTuRU	2	TRUE	0.002898	21.25653
2508	rs2589314	A	G	A	G	-0.0083	-0.00408	0.5476	0.517408	FALSE	FALSE	FALSE	LwUYua	0.013691	0.765674	486484	sepsis	TRUE	reported	textfile	0.0018	6.42E-06	7315	M17945.m	TRUE	reported	SUN8dc	2	TRUE	0.002898	21.25653
2510	rs2946398	T	C	T	C	-0.0083	-0.01381	0.3787	0.384535	FALSE	FALSE	FALSE	LwUYua	0.014029	0.325019	486484	sepsis	TRUE	reported	textfile	0.0018	7.64E-06	7315	M17945.m	TRUE	reported	SUN8dc	2	TRUE	0.002898	21.25653
5892	rs707	T	C	T	C	-0.0083	0.012862	0.4558	0.413091	FALSE	FALSE	FALSE	tqW0ld	0.013991	0.357908	486484	sepsis	TRUE	reported	textfile	0.0018	5.95E-06	7315	M33389.m	TRUE	reported	Sv7EyV	2	TRUE	0.002898	21.25653
5897	rs7661478	T	C	T	C	0.0083	0.006364	0.6299	0.637706	FALSE	FALSE	FALSE	tqW0ld	0.014222	0.654556	486484	sepsis	TRUE	reported	textfile	0.0018	6.17E-06	7315	M33389.m	TRUE	reported	Sv7EyV	2	TRUE	0.002898	21.25653
6142	rs4687367	T	C	T	C	0.0083	-0.0036	0.4659	0.470629	FALSE	FALSE	FALSE	MiSJly	0.01377	0.793548	486484	sepsis	TRUE	reported	textfile	0.0018	4.21E-06	7307	M33477.m	TRUE	reported	WwL1hq	2	TRUE	0.002901	21.25653
6156	rs8182062	A	G	A	G	-0.0083	0.021923	0.7075	0.713017	FALSE	FALSE	FALSE	MiSJly	0.015091	0.146287	486484	sepsis	TRUE	reported	textfile	0.0018	4.75E-06	7307	M33477.m	TRUE	reported	WwL1hq	2	TRUE	0.002901	21.25653
6157	rs951062	T	C	T	C	0.0083	0.006576	0.703	0.697015	FALSE	FALSE	FALSE	MiSJly	0.014916	0.659312	486484	sepsis	TRUE	reported	textfile	0.0018	5.68E-06	7307	M33477.m	TRUE	reported	WwL1hq	2	TRUE	0.002901	21.25653
5975	rs1050074	T	C	T	C	0.0083	0.002971	0.7541	0.748203	FALSE	FALSE	FALSE	3KsoXq	0.015802	0.850893	486484	sepsis	TRUE	reported	textfile	0.0018	4.57E-06	7306	M33422.m	TRUE	reported	aNr6Ug	2	TRUE	0.002902	21.25653
5983	rs1503575	A	G	A	G	0.0083	0.004068	0.1815	0.181318	FALSE	FALSE	FALSE	3KsoXq	0.017776	0.81898	486484	sepsis	TRUE	reported	textfile	0.0018	6.18E-06	7306	M33422.m	TRUE	reported	aNr6Ug	2	TRUE	0.002902	21.25653
5991	rs2731506	A	G	A	G	-0.0083	0.003032	0.2438	0.244749	FALSE	FALSE	FALSE	3KsoXq	0.015907	0.848812	486484	sepsis	TRUE	reported	textfile	0.0018	3.78E-06	7306	M33422.m	TRUE	reported	aNr6Ug	2	TRUE	0.002902	21.25653
3340	rs4899091	A	G	A	G	-0.0083	0.01581	0.7678	0.759115	FALSE	FALSE	FALSE	m66Eav	0.016019	0.323666	486484	sepsis	TRUE	reported	textfile	0.0018	2.47E-06	7303	M27256.m	TRUE	reported	pI2ECU	2	TRUE	0.002903	21.25652
1112	rs2596194	A	T	A	T	-0.0083	-0.00295	0.1255	0.136855	FALSE	TRUE	FALSE	yahiGZ	0.020215	0.883957	486484	sepsis	TRUE	reported	textfile	0.0018	2.59E-06	7271	M01585.m	TRUE	reported	dwZ7vX	2	TRUE	0.002916	21.2565
4346	rs4711082	T	C	T	C	-0.0083	-0.01479	0.6337	0.636961	FALSE	FALSE	FALSE	6VSoQQ	0.014211	0.298139	486484	sepsis	TRUE	reported	textfile	0.0018	5.92E-06	7229	M32644.m	TRUE	reported	oC2AV0	2	TRUE	0.002933	21.25646
8026	rs1229658	A	G	A	G	-0.0083	0.002525	0.2306	0.23981	FALSE	FALSE	FALSE	SwiqSi	0.016023	0.874789	486484	sepsis	TRUE	reported	textfile	0.0018	6.13E-06	7217	M35270.m	TRUE	reported	21ZIsQ	2	TRUE	0.002937	21.25645
8035	rs2078875	T	C	T	C	-0.0083	0.011961	0.2436	0.233451	FALSE	FALSE	FALSE	SwiqSi	0.016191	0.460057	486484	sepsis	TRUE	reported	textfile	0.0018	4.46E-06	7217	M35270.m	TRUE	reported	21ZIsQ	2	TRUE	0.002937	21.25645
6719	rs1689205	C	G	C	G	0.0775	0.075438	0.0265	0.010409	FALSE	TRUE	FALSE	B6lZly	0.068157	0.268369	486484	sepsis	TRUE	reported	textfile	0.0168	3.81E-06	1759	M33937.m	TRUE	reported	qFPzjh	2	TRUE	0.011954	21.25645
2734	rs5746800	A	G	A	G	0.0083	0.022585	0.5003	0.516737	FALSE	FALSE	FALSE	H4yqSK	0.013811	0.101999	486484	sepsis	TRUE	reported	textfile	0.0018	2.44E-06	7182	M19362.m	TRUE	reported	w2PDEO	2	TRUE	0.002952	21.25642
5824	rs1874617	A	G	A	G	-0.0083	-0.00852	0.7831	0.772772	FALSE	FALSE	FALSE	R5joEf	0.016316	0.601343	486484	sepsis	TRUE	reported	textfile	0.0018	4.23E-06	7118	M33380.m	TRUE	reported	kRvbfZ	2	TRUE	0.002978	21.25637
5835	rs688608	A	G	A	G	-0.0083	-0.01758	0.778	0.767221	FALSE	FALSE	FALSE	R5joEf	0.016167	0.276984	486484	sepsis	TRUE	reported	textfile	0.0018	2.61E-06	7118	M33380.m	TRUE	reported	kRvbfZ	2	TRUE	0.002978	21.25637
6258	rs1180647	T	C	T	C	0.0083	0.00698	0.3749	0.372522	FALSE	FALSE	FALSE	MOonP9	0.014158	0.621989	486484	sepsis	TRUE	reported	textfile	0.0018	6.10E-06	7066	M33515.m	TRUE	reported	226GuP	2	TRUE	0.003	21.25633
6264	rs2067434	T	C	T	C	-0.0083	-0.00774	0.4351	0.427055	FALSE	FALSE	FALSE	MOonP9	0.013805	0.574847	486484	sepsis	TRUE	reported	textfile	0.0018	5.24E-06	7066	M33515.m	TRUE	reported	226GuP	2	TRUE	0.003	21.25633
6268	rs2494461	A	C	A	C	0.0083	-0.01444	0.6292	0.623608	FALSE	FALSE	FALSE	MOonP9	0.014122	0.306388	486484	sepsis	TRUE	reported	textfile	0.0018	6.27E-06	7066	M33515.m	TRUE	reported	226GuP	2	TRUE	0.003	21.25633
6289	rs792369	A	C	A	C	-0.0083	-0.0041	0.659	0.663447	FALSE	FALSE	FALSE	MOonP9	0.014501	0.777551	486484	sepsis	TRUE	reported	textfile	0.0018	6.65E-06	7066	M33515.m	TRUE	reported	226GuP	2	TRUE	0.003	21.25633
1482	rs4465961	T	C	T	C	0.0083	-0.0112	0.3479	0.353439	FALSE	FALSE	FALSE	pkb876	0.014347	0.434959	486484	sepsis	TRUE	reported	textfile	0.0018	3.12E-06	7037	M02734.m	TRUE	reported	0LCMVI	2	TRUE	0.003012	21.2563
8843	rs1309903	A	C	A	C	0.0083	-0.00294	0.181	0.187838	FALSE	FALSE	FALSE	XIHeTV	0.017704	0.868249	486484	sepsis	TRUE	reported	textfile	0.0018	4.09E-06	6940	M36808.m	TRUE	reported	37Vi2D	2	TRUE	0.003054	21.25622
8848	rs27852	A	C	A	C	-0.0083	0.005962	0.2959	0.296255	FALSE	FALSE	FALSE	XIHeTV	0.014985	0.690739	486484	sepsis	TRUE	reported	textfile	0.0018	2.18E-06	6940	M36808.m	TRUE	reported	37Vi2D	2	TRUE	0.003054	21.25622
8851	rs4518383	A	G	A	G	-0.0083	-0.00754	0.8068	0.802654	FALSE	FALSE	FALSE	XIHeTV	0.017174	0.660429	486484	sepsis	TRUE	reported	textfile	0.0018	3.89E-06	6940	M36808.m	TRUE	reported	37Vi2D	2	TRUE	0.003054	21.25622
8862	rs7580094	A	G	A	G	0.0083	0.011735	0.7814	0.765073	FALSE	FALSE	FALSE	XIHeTV	0.016129	0.466872	486484	sepsis	TRUE	reported	textfile	0.0018	3.46E-06	6940	M36808.m	TRUE	reported	37Vi2D	2	TRUE	0.003054	21.25622
7276	rs1088075	A	G	A	G	-0.0083	0.002172	0.488	0.487885	FALSE	FALSE	FALSE	Vcz4EA	0.013697	0.874016	486484	sepsis	TRUE	reported	textfile	0.0018	2.51E-06	6755	M34359.m	TRUE	reported	aUSo2J	2	TRUE	0.003138	21.25605
7283	rs1321291	A	G	A	G	-0.0083	-0.00678	0.6812	0.682645	FALSE	FALSE	FALSE	Vcz4EA	0.014778	0.646526	486484	sepsis	TRUE	reported	textfile	0.0018	2.92E-06	6755	M34359.m	TRUE	reported	aUSo2J	2	TRUE	0.003138	21.25605
7297	rs2712549	A	G	A	G	0.0083	0.010119	0.472	0.478293	FALSE	FALSE	FALSE	Vcz4EA	0.013668	0.459098	486484	sepsis	TRUE	reported	textfile	0.0018	3.22E-06	6755	M34359.m	TRUE	reported	aUSo2J	2	TRUE	0.003138	21.25605
7302	rs310325	T	C	T	C	-0.0083	0.030598	0.4417	0.447502	FALSE	FALSE	FALSE	Vcz4EA	0.013768	0.026257	486484	sepsis	TRUE	reported	textfile	0.0018	2.68E-06	6755	M34359.m	TRUE	reported	aUSo2J	2	TRUE	0.003138	21.25605
7308	rs501438	C	G	C	G	0.0083	-0.00574	0.3909	0.394851	FALSE	TRUE	FALSE	Vcz4EA	0.013982	0.681234	486484	sepsis	TRUE	reported	textfile	0.0018	2.35E-06	6755	M34359.m	TRUE	reported	aUSo2J	2	TRUE	0.003138	21.25605
1013	rs6500864	A	G	A	G	-0.0083	-0.00503	0.2762	0.279675	FALSE	FALSE	FALSE	jfkZbM	0.015295	0.74221	486484	sepsis	TRUE	reported	textfile	0.0018	4.34E-06	6523	M01558.m	TRUE	reported	LFZvMG	2	TRUE	0.003249	21.25583
5700	rs1741879	T	C	T	C	-0.0535	-0.03088	0.9675	0.922376	FALSE	FALSE	FALSE	1SbQTe	0.025552	0.226903	486484	sepsis	TRUE	reported	textfile	0.0116	4.24E-06	2683	M33204.m	TRUE	reported	x7MBDH	2	TRUE	0.007866	21.25532
8134	rs952108	A	G	A	G	0.0083	-0.01682	0.4705	0.449626	FALSE	FALSE	FALSE	nNbXBS	0.01386	0.224941	486484	sepsis	TRUE	reported	textfile	0.0018	2.14E-06	5768	M35326.m	TRUE	reported	UNDorn	2	TRUE	0.003673	21.25497
7650	rs1290262	C	G	C	G	0.0332	-0.009	0.8789	0.87867	FALSE	TRUE	FALSE	8W5cqf	0.020925	0.667103	486484	sepsis	TRUE	reported	textfile	0.0072	4.27E-06	4925	M34534.m	TRUE	reported	JV42Pw	2	TRUE	0.004299	21.25371
1347	rs6085132	T	C	T	C	-0.0272	0.036445	0.9725	0.952293	FALSE	FALSE	FALSE	LDUJbh	0.032131	0.256686	486484	sepsis	TRUE	reported	textfile	0.0059	4.01E-06	7367	M01898.m	TRUE	reported	JRSLeH	2	TRUE	0.002877	21.24789
7333	rs1200575	A	G	A	G	0.0909	0.020397	0.4099	0.406428	FALSE	FALSE	FALSE	mm9ksf	0.013942	0.14348	486484	sepsis	TRUE	reported	textfile	0.0197	4.02E-06	954	M34365.m	TRUE	reported	AVwEqD	2	TRUE	0.02183	21.24633
5368	rs1747065	A	T	A	T	0.3263	0.047679	0.944	0.969079	FALSE	TRUE	FALSE	q32d4E	0.040882	0.243513	486484	sepsis	TRUE	reported	textfile	0.0679	1.54E-06	25	M33178.m	TRUE	reported	cS3BwT	2	TRUE	0.480182	21.24626
7685	rs1742313	A	G	A	G	0.0498	-0.00931	0.0656	0.072099	FALSE	FALSE	FALSE	5FAxbZ	0.026594	0.726243	486484	sepsis	TRUE	reported	textfile	0.0108	3.75E-06	2545	M34674.m	TRUE	reported	TDQqa8	2	TRUE	0.008285	21.24564
7622	rs4953640	T	C	T	C	0.0189	-0.02038	0.1512	0.168642	FALSE	FALSE	FALSE	kVfKqT	0.018309	0.265692	486484	sepsis	TRUE	reported	textfile	0.0041	4.16E-06	7322	M34527.m	TRUE	reported	s4ti92	2	TRUE	0.002894	21.24405
2930	rs4388837	T	G	T	G	0.1102	0.035023	0.9828	0.98128	FALSE	FALSE	FALSE	fV4qul	0.05068	0.489529	486484	sepsis	TRUE	reported	textfile	0.0239	4.15E-06	1765	M21044.m	TRUE	reported	hbdBQJ	2	TRUE	0.011902	21.23611
8753	rs1013196	A	G	A	G	-0.0295	0.011298	0.6038	0.591269	FALSE	FALSE	FALSE	KMisdy	0.013924	0.417138	486484	sepsis	TRUE	reported	textfile	0.0064	4.05E-06	3977	M36589.m	TRUE	reported	J1ISTP	2	TRUE	0.005314	21.23565
922	rs1001210	T	C	T	C	-0.0106	0.012986	0.8353	0.84115	FALSE	FALSE	FALSE	tv99UN	0.018721	0.487895	486484	sepsis	TRUE	reported	textfile	0.0023	3.80E-06	7354	M01494.m	TRUE	reported	osDclp	2	TRUE	0.00288	21.2343
2075	rs1417497	C	G	C	G	-0.0106	0.037916	0.072	0.066991	FALSE	TRUE	FALSE	NPVH5j	0.027368	0.165925	486484	sepsis	TRUE	reported	textfile	0.0023	5.35E-06	7310	M15506.m	TRUE	reported	r7r3vM	2	TRUE	0.002897	21.23426
1157	rs1153903	A	G	A	G	-0.0106	-0.00819	0.3856	0.401436	FALSE	FALSE	FALSE	cSBFJ3	0.013945	0.556942	486484	sepsis	TRUE	reported	textfile	0.0023	4.19E-06	7104	M01638.m	TRUE	reported	sUkjpE	2	TRUE	0.002981	21.2341
4193	rs2722639	T	C	T	C	-0.0318	-0.00216	0.5475	0.556687	FALSE	FALSE	FALSE	TxOCzG	0.01376	0.875169	486484	sepsis	TRUE	reported	textfile	0.0069	4.43E-06	6970	M32564.m	TRUE	reported	9JP6ND	2	TRUE	0.003038	21.23398
3043	rs897656	A	T	A	T	-0.0521	0.064327	0.0443	0.042174	FALSE	TRUE	FALSE	FJZEYz	0.034009	0.058561	486484	sepsis	TRUE	reported	textfile	0.0113	4.06E-06	1755	M21630.m	TRUE	reported	s6Pshg	2	TRUE	0.011968	21.23359
8743	rs1091613	T	C	T	C	-0.0401	0.052531	0.8277	0.824023	FALSE	FALSE	FALSE	FnYrPc	0.018058	0.003626	486484	sepsis	TRUE	reported	textfile	0.0087	3.86E-06	3346	M36585.m	TRUE	reported	amHtMH	2	TRUE	0.006309	21.23198
2420	rs4141286	C	G	C	G	0.1065	0.030796	0.9514	0.940738	FALSE	TRUE	FALSE	NBJ2Do	0.029162	0.290956	486484	sepsis	TRUE	reported	textfile	0.0231	3.95E-06	1739	M17799.m	TRUE	reported	Uz0WgI	2	TRUE	0.012075	21.23125
5133	rs1279200	A	G	A	G	-0.2024	0.062651	0.0653	0.041162	FALSE	FALSE	FALSE	L2DJzK	0.034544	0.069732	486484	sepsis	TRUE	reported	textfile	0.0439	4.01E-06	1574	M33173.m	TRUE	reported	hRG70D	2	TRUE	0.013325	21.2295
6391	rs2776982	A	C	A	C	0.0447	0.035578	0.053	0.056683	FALSE	FALSE	FALSE	eBEF1t	0.029787	0.232307	486484	sepsis	TRUE	reported	textfile	0.0097	4.52E-06	6109	M33638.m	TRUE	reported	CRf2lt	2	TRUE	0.003464	21.22899
7087	rs1192598	T	C	T	C	-0.0341	0.002087	0.0325	0.053438	FALSE	FALSE	FALSE	pxNICA	0.030703	0.945812	486484	sepsis	TRUE	reported	textfile	0.0074	4.08E-06	6994	M34306.m	TRUE	reported	xk5b9M	2	TRUE	0.003027	21.22859
2484	rs480891	A	G	A	G	-0.0235	0.030432	0.6432	0.6297	FALSE	FALSE	FALSE	SrfyMU	0.014239	0.032578	486484	sepsis	TRUE	reported	textfile	0.0051	4.15E-06	7226	M17807.m	TRUE	reported	0zrJsu	2	TRUE	0.00293	21.22634
7916	rs1947110	A	G	A	G	0.0235	-0.00421	0.284	0.281593	FALSE	FALSE	FALSE	8qXKkX	0.015212	0.781775	486484	sepsis	TRUE	reported	textfile	0.0051	3.34E-06	6234	M35189.m	TRUE	reported	Nt6oaC	2	TRUE	0.003394	21.22541
8778	rs659974	A	G	A	G	0.0235	0.033054	0.1642	0.185505	FALSE	FALSE	FALSE	7desNF	0.017572	0.059972	486484	sepsis	TRUE	reported	textfile	0.0051	4.61E-06	5292	M36673.m	TRUE	reported	RKbIT4	2	TRUE	0.003996	21.22419
8566	rs2581383	C	G	C	G	0.0364	0.024157	0.6655	0.653899	FALSE	TRUE	FALSE	Yjjldt	0.014491	0.09551	486484	sepsis	TRUE	reported	textfile	0.0079	3.72E-06	7054	M36098.m	TRUE	reported	U2ORsF	2	TRUE	0.003001	21.22391
7076	rs1684155	A	C	A	C	-0.0212	-0.02009	0.6765	0.670678	FALSE	FALSE	FALSE	sg7nOB	0.014612	0.169097	486484	sepsis	TRUE	reported	textfile	0.0046	4.48E-06	2278	M34289.m	TRUE	reported	KemvIC	2	TRUE	0.009238	21.22143
3369	rs1233749	T	C	T	C	-0.0129	-0.01424	0.2157	0.232594	FALSE	FALSE	FALSE	O2JtzH	0.016173	0.37876	486484	sepsis	TRUE	reported	textfile	0.0028	3.81E-06	7347	M27278.m	TRUE	reported	fxCIPW	2	TRUE	0.002881	21.21999
1447	rs4587207	A	G	A	G	-0.0129	0.009071	0.8566	0.855717	FALSE	FALSE	FALSE	1M4gqj	0.019495	0.641729	486484	sepsis	TRUE	reported	textfile	0.0028	3.19E-06	7226	M02730.m	TRUE	reported	RWyYEM	2	TRUE	0.002929	21.21989
767	rs1099030	T	C	T	C	-0.0129	-0.01425	0.2309	0.229237	FALSE	FALSE	FALSE	tYvbNc	0.016599	0.39062	486484	sepsis	TRUE	reported	textfile	0.0028	3.94E-06	6946	M01303.m	TRUE	reported	hT3gsE	2	TRUE	0.003047	21.21965
2579	rs7503709	T	C	T	C	-0.0129	0.006757	0.4574	0.457622	FALSE	FALSE	FALSE	KHS91f	0.014548	0.642316	486484	sepsis	TRUE	reported	textfile	0.0028	3.30E-06	6939	M18349.m	TRUE	reported	PeVGil	2	TRUE	0.00305	21.21965
4517	rs1274280	T	C	T	C	0.0129	-0.04515	0.1181	0.117132	FALSE	FALSE	FALSE	r0qJaM	0.022241	0.042371	486484	sepsis	TRUE	reported	textfile	0.0028	4.95E-06	6497	M32729.m	TRUE	reported	vYv5FI	2	TRUE	0.003256	21.21923
7759	rs9459207	A	G	A	G	-0.0258	-0.03762	0.0331	0.034509	FALSE	FALSE	FALSE	HyMNYM	0.037408	0.314516	486484	sepsis	TRUE	reported	textfile	0.0056	3.36E-06	5885	M34912.m	TRUE	reported	TI8Hcw	2	TRUE	0.003594	21.21855
3498	rs552279	A	G	A	G	-0.0129	-0.02228	0.6732	0.676227	FALSE	FALSE	FALSE	Awafb0	0.014762	0.131176	486484	sepsis	TRUE	reported	textfile	0.0028	4.90E-06	5562	M27728.m	TRUE	reported	Lfj8L0	2	TRUE	0.003802	21.21813
3497	rs498494	C	G	C	G	0.0258	-0.03039	0.9184	0.901991	FALSE	TRUE	FALSE	Awafb0	0.023006	0.186461	486484	sepsis	TRUE	reported	textfile	0.0056	4.27E-06	5562	M27728.m	TRUE	reported	Lfj8L0	2	TRUE	0.003802	21.21813
2482	rs4393440	T	C	T	C	-0.129	0.008128	0.987	0.951565	FALSE	FALSE	FALSE	SrfyMU	0.031708	0.797693	486484	sepsis	TRUE	reported	textfile	0.028	4.08E-06	5482	M17807.m	TRUE	reported	0zrJsu	2	TRUE	0.003857	21.21802
4360	rs1095733	A	G	A	G	0.0129	0.00577	0.2929	0.295035	FALSE	FALSE	FALSE	SPhI52	0.015028	0.700994	486484	sepsis	TRUE	reported	textfile	0.0028	4.29E-06	5211	M32651.m	TRUE	reported	V0Bnpj	2	TRUE	0.004057	21.21762
4370	rs3762920	A	G	A	G	0.0129	-0.00822	0.6758	0.683243	FALSE	FALSE	FALSE	SPhI52	0.014745	0.576986	486484	sepsis	TRUE	reported	textfile	0.0028	3.15E-06	5211	M32651.m	TRUE	reported	V0Bnpj	2	TRUE	0.004057	21.21762
6525	rs4504394	T	C	T	C	0.0318	-0.01134	0.2788	0.298642	FALSE	FALSE	FALSE	CKKl38	0.019498	0.44951	486484	sepsis	TRUE	reported	textfile	0.0069	3.97E-06	1764	M36808.m	TRUE	reported	37Vi2D	2	TRUE	0.011898	21.21599
4239	rs2698512	A	G	A	G	-0.0539	0.082036	0.0391	0.04088	FALSE	FALSE	FALSE	wWEMr1	0.034558	0.017602	486484	sepsis	TRUE	reported	textfile	0.0147	4.09E-06	5955	M32593.m	TRUE	reported	Mh2g4i	2	TRUE	0.003551	21.21587
4665	rs9513800	A	C	A	C	-0.041	0.030936	0.0449	0.042822	FALSE	FALSE	FALSE	SyAFR2	0.033746	0.359282	486484	sepsis	TRUE	reported	textfile	0.0089	3.85E-06	6658	M32755.m	TRUE	reported	prUo74	2	TRUE	0.003177	21.21569
5699	rs1649892	T	C	T	C	0.0364	-0.00592	0.0398	0.046265	FALSE	FALSE	FALSE	1SbQTe	0.032761	0.856614	486484	sepsis	TRUE	reported	textfile	0.0223	4.25E-06	2683	M33204.m	TRUE	reported	x7MBDH	2	TRUE	0.007851	21.21411
1674	rs1898906	A	G	A	G	-0.1378	-0.05904	0.0296	0.030023	FALSE	FALSE	FALSE	IIFBGY	0.039997	0.139912	486484	sepsis	TRUE	reported	textfile	0.0099	4.09E-06	1543	M12261.m	TRUE	reported	F5Vlan	2	TRUE	0.013579	21.21254
5403	rs2249531	A	G	A	G	-0.0816	-0.02461	0.5482	0.57593	FALSE	FALSE	FALSE	q32d4E	0.014001	0.078813	486484	sepsis	TRUE	reported	textfile	0.0177	4.19E-06	1017	M33178.m	TRUE	reported	cS3BwT	2	TRUE	0.020471	21.21187
2451	rs7969423	A	G	A	G	-0.1138	0.009177	0.0833	0.170309	FALSE	FALSE	FALSE	NBJ2Do	0.018211	0.614323	486484	sepsis	TRUE	reported	textfile	0.0237	1.56E-06	25	M17799.m	TRUE	reported	Uz0WgI	2	TRUE	0.479776	21.21171
3362	rs6941976	T	C	T	C	0.0447	0.03563	0.0447	0.035202	FALSE	FALSE	FALSE	LY4Gus	0.037243	0.367487	486484	sepsis	TRUE	reported	textfile	0.0097	3.90E-06	1751	M27273.m	TRUE	reported	QKlTBx	2	TRUE	0.011983	21.21169
8937	rs1081350	T	C	T	C	-0.0281	0.023155	0.3812	0.4168	FALSE	FALSE	FALSE	mXJP3h	0.013931	0.096503	486484	sepsis	TRUE	reported	textfile	0.0061	4.23E-06	4520	M37104.m	TRUE	reported	HRZBak	2	TRUE	0.004673	21.21098
1092	rs1205290	A	G	A	G	-0.1115	-0.01947	0.9783	0.976581	FALSE	FALSE	FALSE	K3wtZo	0.045427	0.668222	486484	sepsis	TRUE	reported	textfile	0.0242	4.27E-06	2315	M01573.m	TRUE	reported	0D9CvA	2	TRUE	0.009087	21.21015
9033	rs1753984	A	G	A	G	-0.0152	0.00434	0.0938	0.100621	FALSE	FALSE	FALSE	df0MCM	0.022643	0.847997	486484	sepsis	TRUE	reported	textfile	0.0033	4.80E-06	7366	M37506.m	TRUE	reported	iUfr4u	2	TRUE	0.002872	21.21003

3938	rs4755163	A	G	A	G	0.0152	-0.03078	0.5331	0.544987	FALSE	FALSE	FALSE	LlOaWH	0.013718	0.024854	486484	sepsis	TRUE	reported	textfile	0.0033	3.51E-06	7355	M32418.m	TRUE	reported	AtHOVw	2	TRUE	0.002876	21.21003
811	rs1751156	T	C	T	C	0.0152	-0.02955	0.8423	0.823397	FALSE	FALSE	FALSE	J58DI0	0.018179	0.104051	486484	sepsis	TRUE	reported	textfile	0.0033	5.26E-06	7335	M01356.m	TRUE	reported	zk9AMB	2	TRUE	0.002884	21.21001
574	rs1223808	T	C	T	C	0.0152	0.016179	0.1389	0.138928	FALSE	FALSE	FALSE	ZoqU2j	0.019778	0.413338	486484	sepsis	TRUE	reported	textfile	0.0033	3.01E-06	7333	M01105.m	TRUE	reported	2WxSOH	2	TRUE	0.002885	21.21001
1080	rs1310284	C	G	C	G	-0.0152	0.013071	0.8686	0.876891	FALSE	TRUE	FALSE	JLtEiZ	0.020854	0.530788	486484	sepsis	TRUE	reported	textfile	0.0033	3.13E-06	7333	M01572.m	TRUE	reported	TSSe28	2	TRUE	0.002885	21.21001
3877	rs4661470	T	G	T	G	-0.0152	0.025951	0.1974	0.20563	FALSE	FALSE	FALSE	bwkKtK	0.016874	0.124061	486484	sepsis	TRUE	reported	textfile	0.0033	5.44E-06	7307	M32393.m	TRUE	reported	RvZzAy	2	TRUE	0.002895	21.20999
3878	rs708891	T	C	T	C	-0.0152	-0.03175	0.8739	0.872577	FALSE	FALSE	FALSE	bwkKtK	0.020531	0.121999	486484	sepsis	TRUE	reported	textfile	0.0033	3.80E-06	7307	M32393.m	TRUE	reported	RvZzAy	2	TRUE	0.002895	21.20999
4139	rs3736083	T	C	T	C	0.0304	-0.00451	0.5544	0.555137	FALSE	FALSE	FALSE	pLh7sk	0.013893	0.745216	486484	sepsis	TRUE	reported	textfile	0.0066	3.98E-06	7256	M32549.m	TRUE	reported	RSQOnp	2	TRUE	0.002915	21.20995
1506	rs2145985	T	C	T	C	-0.0152	0.008392	0.16	0.168021	FALSE	FALSE	FALSE	rzkxmh	0.018268	0.645969	486484	sepsis	TRUE	reported	textfile	0.0033	3.91E-06	6941	M03127.m	TRUE	reported	3lrzhA	2	TRUE	0.003047	21.20968
2253	rs633114	T	C	T	C	-0.0152	-0.0054	0.3884	0.395526	FALSE	FALSE	FALSE	zxcPox	0.013982	0.69908	486484	sepsis	TRUE	reported	textfile	0.0033	3.91E-06	6782	M15990.m	TRUE	reported	34Vg8F	2	TRUE	0.003118	21.20954
5130	rs1254457	C	G	C	G	-0.3743	-0.0676	0.0921	0.07022	FALSE	TRUE	FALSE	L2DJzK	0.03029	0.02564	486484	sepsis	TRUE	reported	textfile	0.0802	3.07E-06	76	M33173.m	TRUE	reported	hRG70D	2	TRUE	0.222758	21.20846
689	rs3857856	A	G	A	G	0.0152	0.026697	0.8885	0.892992	FALSE	FALSE	FALSE	aT1Fw1	0.022155	0.228191	486484	sepsis	TRUE	reported	textfile	0.0033	4.93E-06	5687	M01284.m	TRUE	reported	mbTe8H	2	TRUE	0.003717	21.20833
4052	rs7172565	T	C	T	C	0.0705	-0.02211	0.0289	0.025812	FALSE	FALSE	FALSE	SiYGYU	0.043227	0.608952	486484	sepsis	TRUE	reported	textfile	0.0153	3.93E-06	1767	M32492.m	TRUE	reported	GfQvpU	2	TRUE	0.011873	21.20819
7373	rs7693137	T	C	T	C	-0.148	0.017221	0.5556	0.614121	FALSE	FALSE	FALSE	mm9ksf	0.014032	0.219718	486484	sepsis	TRUE	reported	textfile	0.0303	1.02E-06	18	M34365.m	TRUE	reported	AVwEqD	2	TRUE	0.569977	21.20731
6630	rs1810195	A	C	A	C	-0.0608	-0.05042	0.9065	0.908545	FALSE	FALSE	FALSE	IDG01N	0.023649	0.032992	486484	sepsis	TRUE	reported	textfile	0.0132	3.97E-06	4999	M33885.m	TRUE	reported	q3gy7x	2	TRUE	0.004226	21.20731
7589	rs317894	A	G	A	G	-0.0281	0.008208	0.3357	0.342998	FALSE	FALSE	FALSE	SbjFMF	0.014454	0.570133	486484	sepsis	TRUE	reported	textfile	0.0061	3.45E-06	3239	M34513.m	TRUE	reported	HVYiKM	2	TRUE	0.006509	21.20727
3533	rs1769434	A	C	A	C	0.0479	-0.01601	0.9783	0.969937	FALSE	FALSE	FALSE	rXzSlH	0.041021	0.696357	486484	sepsis	TRUE	reported	textfile	0.0104	3.96E-06	6795	M30805.m	TRUE	reported	7wg9QH	2	TRUE	0.003112	21.20687
4151	rs3010305	T	C	T	C	0.0327	0.022328	0.1318	0.13561	FALSE	FALSE	FALSE	CrVaiQ	0.020123	0.267202	486484	sepsis	TRUE	reported	textfile	0.0071	4.44E-06	7361	M32553.m	TRUE	reported	HsKdAI	2	TRUE	0.002873	21.2061
4345	rs3757514	A	T	A	T	-0.0364	0.01337	0.9211	0.896136	FALSE	TRUE	FALSE	6VSoQQ	0.022414	0.550853	486484	sepsis	TRUE	reported	textfile	0.0079	3.71E-06	1766	M32644.m	TRUE	reported	oC2AV0	2	TRUE	0.011879	21.20589
3394	rs4797120	C	G	C	G	-0.0364	-0.01337	0.3947	0.396746	FALSE	TRUE	FALSE	frOZiy	0.013948	0.337785	486484	sepsis	TRUE	reported	textfile	0.0079	4.05E-06	1758	M27447.m	TRUE	reported	2z9fSv	2	TRUE	0.011932	21.20578
4199	rs644396	T	C	T	C	-0.0327	0.000196	0.5465	0.544002	FALSE	FALSE	FALSE	TxOCzG	0.013746	0.98864	486484	sepsis	TRUE	reported	textfile	0.0071	4.45E-06	6970	M32564.m	TRUE	reported	9JP6ND	2	TRUE	0.003034	21.20578
3885	rs1561277	A	C	A	C	0.0175	-0.01824	0.2892	0.254654	FALSE	FALSE	FALSE	bmXxuz	0.016235	0.261296	486484	sepsis	TRUE	reported	textfile	0.0038	4.39E-06	7358	M32405.m	TRUE	reported	A9BFZF	2	TRUE	0.002874	21.20268
4983	rs1689955	T	C	T	C	0.0258	-0.01436	0.4884	0.517247	FALSE	FALSE	FALSE	AjJi2i	0.013694	0.294219	486484	sepsis	TRUE	reported	textfile	0.0056	4.29E-06	1767	M32980.m	TRUE	reported	gVSm9e	2	TRUE	0.01187	21.20174
6646	rs1271618	A	T	A	T	0.0175	-0.02604	0.2885	0.325948	FALSE	TRUE	FALSE	REeQiq	0.014711	0.076658	486484	sepsis	TRUE	reported	textfile	0.0038	3.90E-06	6004	M33892.m	TRUE	reported	cVOa3u	2	TRUE	0.00352	21.20138
7165	rs1204632	A	C	A	C	-0.0493	-0.0017	0.7637	0.760261	FALSE	FALSE	FALSE	VfvTtz	0.016267	0.916636	486484	sepsis	TRUE	reported	textfile	0.0107	4.14E-06	1492	M34329.m	TRUE	reported	egac6r	2	TRUE	0.014029	21.20038
2559	rs1720931	C	G	C	G	0.0723	-0.00449	0.1068	0.143062	FALSE	TRUE	FALSE	WfyP8n	0.020106	0.823344	486484	sepsis	TRUE	reported	textfile	0.0157	4.17E-06	5899	M18335.m	TRUE	reported	mjljpe	2	TRUE	0.003582	21.19971
5102	rs2448148	T	C	T	C	-0.0258	-0.00949	0.6465	0.681517	FALSE	FALSE	FALSE	Uiz8VU	0.014723	0.519379	486484	sepsis	TRUE	reported	textfile	0.0056	4.39E-06	1531	M33163.m	TRUE	reported	ky0Bda	2	TRUE	0.013674	21.19804
2633	rs179976	T	C	T	C	-0.0198	0.022226	0.7581	0.744712	FALSE	FALSE	FALSE	UxuT5B	0.015748	0.158131	486484	sepsis	TRUE	reported	textfile	0.0043	3.92E-06	7367	M18467.m	TRUE	reported	eGQj6o	2	TRUE	0.00287	21.19706
4237	rs1978915	A	C	A	C	0.0198	0.007159	0.506	0.512099	FALSE	FALSE	FALSE	wWEMr1	0.013675	0.6006	486484	sepsis	TRUE	reported	textfile	0.0043	5.48E-06	5955	M32593.m	TRUE	reported	Mh2g4i	2	TRUE	0.003548	21.19569
6408	rs1240416	A	G	A	G	0.0396	-0.01331	0.1106	0.126882	FALSE	FALSE	FALSE	5CIPW7	0.020563	0.517344	486484	sepsis	TRUE	reported	textfile	0.0086	4.16E-06	4807	M33653.m	TRUE	reported	H4b2zx	2	TRUE	0.004391	21.19399
3475	rs4692675	T	C	T	C	0.0861	-0.10999	0.0135	0.023003	FALSE	FALSE	FALSE	w7juXi	0.047083	0.019489	486484	sepsis	TRUE	reported	textfile	0.0187	4.36E-06	6830	M27722.m	TRUE	reported	xzWmif	2	TRUE	0.003094	21.19317
4410	rs1159375	T	C	T	C	0.0663	-0.01505	0.0198	0.056751	FALSE	FALSE	FALSE	NzBiVA	0.029995	0.615964	486484	sepsis	TRUE	reported	textfile	0.0144	4.18E-06	7354	M32672.m	TRUE	reported	YLlhHJ	2	TRUE	0.002874	21.19259
1771	rs2941840	A	G	A	G	0.0221	-0.01079	0.0691	0.091184	FALSE	FALSE	FALSE	wM5dkT	0.023735	0.649464	486484	sepsis	TRUE	reported	textfile	0.0048	3.71E-06	7352	M15122.m	TRUE	reported	CBpa4t	2	TRUE	0.002875	21.19258
7259	rs9561023	A	G	A	G	0.064	-0.02526	0.0941	0.095587	FALSE	FALSE	FALSE	4t4W6t	0.023187	0.275969	486484	sepsis	TRUE	reported	textfile	0.0139	4.19E-06	5270	M34344.m	TRUE	reported	Rz9mCt	2	TRUE	0.004007	21.19169
1519	rs9379061	C	G	C	G	-0.0456	0.041403	0.9562	0.963929	FALSE	TRUE	FALSE	rzkxmh	0.037347	0.267597	486484	sepsis	TRUE	reported	textfile	0.0099	3.92E-06	1745	M03127.m	TRUE	reported	3lrzhA	2	TRUE	0.012012	21.19148
5764	rs4775058	A	G	A	G	0.0663	0.017661	0.0236	0.033469	FALSE	FALSE	FALSE	n16uO2	0.03821	0.643932	486484	sepsis	TRUE	reported	textfile	0.0144	3.85E-06	4409	M33250.m	TRUE	reported	9dhI67	2	TRUE	0.004785	21.18873
6991	rs2275353	A	G	A	G	0.0244	-0.00947	0.1625	0.154335	FALSE	FALSE	FALSE	APOtI6	0.018899	0.616304	486484	sepsis	TRUE	reported	textfile	0.0053	4.51E-06	6987	M34221.m	TRUE	reported	looCDC	2	TRUE	0.003024	21.18866
6458	rs1247058	A	G	A	G	0.405	-0.05544	0.0118	0.019325	FALSE	FALSE	FALSE	yLohrN	0.0501	0.268501	486484	sepsis	TRUE	reported	textfile	0.0878	3.99E-06	477	M33683.m	TRUE	reported	mvhAZ4	2	TRUE	0.042702	21.18831
2693	rs7171216	T	G	T	G	-0.0442	-0.01033	0.4139	0.421929	FALSE	FALSE	FALSE	ATsH6N	0.013826	0.455011	486484	sepsis	TRUE	reported	textfile	0.0096	4.41E-06	3783	M18497.m	TRUE	reported	IGuIRI	2	TRUE	0.005572	21.18714
4704	rs2268181	T	C	T	C	-0.0267	0.010748	0.8405	0.8301	FALSE	FALSE	FALSE	WlwKaF	0.018207	0.554983	486484	sepsis	TRUE	reported	textfile	0.0058	4.78E-06	6712	M32759.m	TRUE	reported	Ub4yxH	2	TRUE	0.003147	21.18542
6942	rs6502243	T	G	T	G	-0.0267	-0.01961	0.5849	0.574092	FALSE	FALSE	FALSE	oNv0vf	0.013818	0.155804	486484	sepsis	TRUE	reported	textfile	0.0058	3.48E-06	5581	M34112.m	TRUE	reported	I0pe0N	2	TRUE	0.003783	21.18414
3000	rs9296444	T	C	T	C	0.0267	-0.0029	0.7828	0.788838	FALSE	FALSE	FALSE	xmzJ5F	0.0169	0.863809	486484	sepsis	TRUE	reported	textfile	0.0058	4.79E-06	5436	M21184.m	TRUE	reported	xgwhDp	2	TRUE	0.003883	21.18394
6406	rs1088181	T	C	T	C	-0.0267	-0.00057	0.5581	0.559803	FALSE	FALSE	FALSE	5CIPW7	0.01383	0.967224	486484	sepsis	TRUE	reported	textfile	0.0058	3.54E-06	4807	M33653.m	TRUE	reported	H4b2zx	2	TRUE	0.004389	21.18292
6543	rs7575509	T	C	T	C	0.035	0.021141	0.4488	0.454736	FALSE	FALSE	FALSE	fVPpJo	0.013719	0.12333	486484	sepsis	TRUE	reported	textfile	0.0076	4.64E-06	1631	M33833.m	TRUE	reported	37N6ck	2	TRUE	0.012836	21.18244
3476	rs6486548	C	G	C	G	-0.0502	-0.01018	0.8143	0.874198	FALSE	TRUE	FALSE	w7juXi	0.02082	0.624988	486484	sepsis	TRUE	reported	textfile	0.0109	4.35E-06	1460	M27722.m	TRUE	reported	xzWmif	2	TRUE	0.01432	21.18162
6838	rs530942	A	G	A	G	-0.0373	0.000472	0.86	0.867548	FALSE	FALSE	FALSE	iVyBC0	0.020186	0.981343	486484	sepsis	TRUE	reported	textfile	0.0081	4.69E-06	1766	M33972.m	TRUE	reported	JyBTbA	2	TRUE	0.011865	21.18144
2796	rs1050371	A	G	A	G	0.0313	-0.0197	0.9815	0.88636	FALSE	FALSE	FALSE	Sx5Uhq	0.021555	0.36083	486484	sepsis	TRUE	reported	textfile	0.0068	4.85E-06	7354	M19934.m	TRUE	reported	DijYTE	2	TRUE	0.002873	21.18131
4565	rs1022039	A	T	A	T	0.0198	-0.01192	0.2991	0.285948	FALSE	TRUE	FALSE	ILOOFw	0.015419	0.439613	486484	sepsis	TRUE	reported	textfile	0.0043	5.47E-06	1748	M32739.m	TRUE	reported	TpvRBe	2	TRUE	0.011984	21.17855
6476	rs2173524	T	G	T	G	0.0622	-0.01172	0.3588	0.344125	FALSE	FALSE	FALSE	ecwVg8	0.014391	0.415617	486484	sepsis	TRUE	reported	textfile	0.0135	4.02E-06	831	M33751.m	TRUE	reported	NnvbIQ	2	TRUE	0.024909	21.17711
8881	rs9561537	A	G	A	G	0.0221	-0.00542	0.5764	0.55729	FALSE	FALSE	FALSE	Nv6gDO	0.01379	0.694094	486484	sepsis	TRUE	reported	textfile	0.0048	4.04E-06	1782	M37004.m	TRUE	reported	RqmQdg	2	TRUE	0.011756	21.17456
4089	rs1693690	A	G	A	G	-0.0709	0.018307	0.954	0.961647	FALSE	FALSE	FALSE	j2JYxt	0.035861	0.609703	486484	sepsis	TRUE	reported	textfile	0.0154	4.09E-06	1768	M32504.m	TRUE	reported	poO7a5	2	TRUE	0.011847	21.17188
7717	rs9924628	A	C	A	C	-0.0313	-0.00346	0.3336	0.329772	FALSE	FALSE	FALSE	qJW9Ig	0.014635	0.813259	486484	sepsis	TRUE	reported	textfile	0.0068	3.56E-06	2474	M34878.m	TRUE	reported	42EOzB	2	TRUE	0.008491	21.16994
3387	rs1050107	A	G	A	G	0.0359	0.01672	0.7994	0.803517	FALSE	FALSE	FALSE	frOZiy	0.017233	0.331917	486484	sepsis	TRUE	reported	textfile	0.0078	4.42E-06	2759	M27447.m	TRUE	reported	2z9fSv	2	TRUE	0.007619	21.16824
4553	rs1316249	A	G	A	G	-0.0566	-0.00983	0.0653	0.072295	FALSE	FALSE	FALSE	KfoRxi	0.026298	0.708676	486484	sepsis	TRUE	reported	textfile	0.0123	4.35E-06	6000	M32735.m	TRUE	reported	UDRXAp	2	TRUE	0.003517	21.1679
8544	rs1323558	T	C	T	C	0.3204	-0.00926	0.227	0.224977	FALSE	FALSE	FALSE	aQhCav	0.016319	0.570366	486484	sepsis	TRUE	reported	textfile	0.0695	4.04E-06	488	M36097.m	TRUE	reported	9DJS9j	2	TRUE	0.041733	21.16566
8558	rs8101059	A	G	A	G	0.2683	0.005056	0.4901	0.523184	FALSE	FALSE	FALSE	aQhCav	0.014108	0.720052	486484	sepsis	TRUE	reported	textfile	0.0582	4.05E-06	488	M36097.m	TRUE	reported	9DJS9j	2	TRUE	0.041731	21.16469
5768	rs1008904	A	C	A	C	-0.2008	-0.00585	0.9474	0.708886	FALSE	FALSE	FALSE	eT2We0	0.015053	0.697631	486484	sepsis	TRUE	reported	textfile	0.0435	3.84E-06	292	M33353.m	TRUE	reported	uZx90m	2	TRUE	0.068011	21.16236
1899	rs1250374	A	C	A	C	-0.0336	0.036781	0.0196	0.018774	FALSE	FALSE	FALSE	v2KE89	0.050932	0.470199	486484	sepsis	TRUE	reported	textfile	0.0073	4.82E-06	1759	M15500.m	TRUE	reported	to2t7H	2	TRUE	0.011901	21.16113
5044	rs1123372	T	C	T	C	-0.0902	-0.02085	0.0578	0.054807	FALSE	FALSE	FALSE	JLCSne	0.030321	0.491668	486484	sepsis	TRUE	reported	textfile	0.0196	4.06E-06	2391	M33137.m	TRUE	reported	LGKYXK	2	TRUE	0.00878	21.16106
6354	rs9833839	T	C	T	C	0.2858	0.137335	0.0127	0.011394	FALSE	FALSE	FALSE	qLUY00	0.065448	0.03587	486484	sepsis	TRUE	reported	textfile	0.062	3.96E-06	470	M33610.m	TRUE	reported	8cAJbB	2	TRUE	0.043255	21.1587
6935	rs1706449	A	T	A	T	0.2812	0.066416	0.0225	0.018382	FALSE	TRUE	FALSE	oNv0vf	0.051224	0.194779	486484	sepsis	TRUE	reported	textfile	0.0611	4.20E-06	1654	M34112.m	TRUE	reported	I0pe0N	2	TRUE	0.012644	21.15547
7231	rs9812186	A	G	A	G	-0.3327	-0.05281	0.9771	0.972097	FALSE	FALSE	FALSE	i1uJ7T	0.041943	0.207998	486484	sepsis	TRUE	reported	textfile	0.0722	4.04E-06	535	M34338.m	TRUE	reported	pi3mKW	2	TRUE	0.038175	21.15459
8346	rs6500859	A	T	A	T	-0.0511	-0.00593	0.699	0.713912	FALSE	TRUE	FALSE	USZiKY	0.015099	0.694591	486484	sepsis	TRUE	reported	textfile	0.0111	4.56E-06	1098	M35635.m	TRUE	reported	HuVMRL	2	TRUE	0.018936	21.15456
397	rs1005596	A	G	A	G	0.0115	0.02692	0.1014	0.107995	FALSE	FALSE	FALSE	cTsNj6	0.022084	0.222852	486484	sepsis	TRUE	reported	textfile	0.0025	4.20E-06	7365	M00063.m	TRUE	reported	7KYTrQ	2	TRUE	0.002865	21.15425
5022	rs1290297	T	C	T	C	-0.0115	0.001562	0.9093	0.903009	FALSE	FALSE	FALSE	BNnOvt	0.023112	0.946111	486484	sepsis	TRUE	reported	textfile	0.0025	4.19E-06	7362	M33132.m	TRUE	reported	rHcCPG	2	TRUE	0.002866	21.15425
6528	rs7659812	A	G	A	G	-0.0161	0.017817	0.5527	0.544393	FALSE	FALSE	FALSE	OKIXJ8	0.013741	0.194752	486484	sepsis	TRUE	reported	textfile	0.0035	3.55E-06	7362	M33821.m	TRUE	reported	XGkaXH	2	TRUE	0.002866	21.15425
6531	rs980142	A	G	A	G	-0.023	0.009798	0.1644	0.215017	FALSE	FALSE	FALSE	OKIXJ8	0.01673	0.558104	486484	sepsis	TRUE	reported	textfile	0.005	4.44E-06	7362	M33821.m	TRUE	reported	XGkaXH	2	TRUE	0.002866	21.15425
4038	rs1325436	T	C	T	C	0.0115	-0.0087	0.1852	0.20939	FALSE	FALSE	FALSE	SiYGYU	0.017009	0.609172	486484	sepsis	TRUE	reported	textfile	0.0025	5.57E-06	7355	M32492.m	TRUE	reported	GfQvpU	2	TRUE	0.002869	21.15425
4041	rs2049791	A	G	A	G	-0.0115	-0.01151	0.7537	0.755922	FALSE	FALSE	FALSE	SiYGYU	0.01592	0.469778	486484	sepsis	TRUE	reported	textfile	0.0025	3.30E-06	7355	M32492.m	TRUE	reported	GfQvpU	2	TRUE	0.002869	21.15425
1883	rs1163013	A	G	A	G	0.0092	-0.02124	0.1395	0.149951	FALSE	FALSE	FALSE	v2KE89	0.019118	0.266634	486484	sepsis	TRUE	reported	textfile	0.002	5.78E-06	7349	M15500.m	TRUE	reported	to2t7H	2	TRUE	0.002871	21.15424
3374	rs1704674	A	G	A	G	0.0161	0.005177	0.1133	0.113832	FALSE	FALSE	FALSE	O2JtzH	0.021474	0.809498	486484	sepsis	TRUE	reported	textfile	0.0035	4.58E-06	7347	M27278.m	TRUE	reported	fxCIPW	2	TRUE	0.002872	21.15424
7005	rs1391971	A	G	A	G	-0.0161	-0.01693	0.1795	0.177132	FALSE	FALSE	FALSE	ePdfmg	0.017909	0.344478	486484	sepsis	TRUE	reported	textfile	0.0035	3.90E-06	7347	M34244.m	TRUE	reported	l9nzfX	2	TRUE	0.002872	21.15424
1232	rs1699045	T	C	T	C	0.0161	0.022952	0.0722	0.083522	FALSE	FALSE	FALSE	V50rWC	0.024698	0.352724	486484	sepsis	TRUE	reported	textfile	0.0035	4.18E-06	7346	M01645.m	TRUE	reported	yN3gpQ	2	TRUE	0.002872	21.15424
909	rs7871735	T	G	T	G	0.0184	0.000882	0.2953	0.29157	FALSE	FALSE	FALSE	I6zQnf	0.015058	0.953313	486484	sepsis	TRUE	reported	textfile	0.004	3.40E-06	7345	M01444.m	TRUE	reported	llzalm	2	TRUE	0.002873	21.15424
3518	rs3766606	T	G	T	G	0.0207	-0.00393	0.1659	0.170781	FALSE	FALSE	FALSE	hxuqls	0.018146	0.828676	486484	sepsis	TRUE	reported	textfile	0.0045	4.93E-06	7336	M27738.m	TRUE	reported	aueeZs	2	TRUE	0.002876	21.15423
6835	rs7142028	A	C	A	C	-0.0161	0.015659	0.5693	0.562377	FALSE	FALSE	FALSE	ZPJCMx	0.01381	0.25686	486484	sepsis	TRUE	reported	textfile	0.0035	4.06E-06	7330	M33969.m	TRUE	reported	mLJPAI	2	TRUE	0.002878	21.15423
2846	rs1857095	T	C	T	C	-0.023	-0.06193	0.9748	0.972959	FALSE	FALSE	FALSE	Y5qWBm	0.042106	0.14131	486484	sepsis	TRUE	reported	textfile	0.005	3.79E-06	7325	M20489.m	TRUE	reported	f2g9Su	2	TRUE	0.00288	21.15422
1701	rs8046494	A	G	A	G	0.0138	0.030442	0.9288	0.920075	FALSE	FALSE	FALSE	f01en0	0.025162	0.226328	486484	sepsis	TRUE	reported	textfile	0.003	4.08E-06	7314	M12593.m	TRUE	reported	EAsM6U	2	TRUE	0.002885	21.15421
4465	rs1246593	A	G	A	G	0.0115	0.013488	0.3006	0.303228	FALSE	FALSE	FALSE	g3iM2w	0.014882	0.364779	486484	sepsis	TRUE	reported	textfile	0.0025	5.13E-06	7308	M32698.m	TRUE	reported	ZQBn0h	2	TRUE	0.002887	21.15421
3880	rs878947	A	G	A	G	-0.0115	0.023951	0.7845	0.782355	FALSE	FALSE	FALSE	bwkKtK	0.016652	0.150347	486484	sepsis	TRUE	reported	textfile	0.0025	5.34E-06	7307	M32393.m	TRUE	reported	RvZzAy	2	TRUE	0.002887	21.15421
3332	rs2213261	T	C	T	C	0.0115	0.015173	0.9061	0.908547	FALSE	FALSE	FALSE	m66Eav	0.023837	0.524417	486484	sepsis	TRUE	reported	textfile	0.0025	5.93E-06	7303	M27256.m	TRUE	reported	pI2ECU	2	TRUE	0.002889	21.15421
3035	rs4719411	T	G	T	G	-0.0115	-0.01584	0.5112	0.523446	FALSE	FALSE	FALSE	FJZEYz	0.013662	0.246437	486484	sepsis	TRUE	reported	textfile	0.0025	5.69E-06	7284	M21630.m	TRUE	reported	s6Pshg	2	TRUE	0.002897	21.15419
506	rs1093589	C	G	C	G	-0.0506	-0.0231	0.8197	0.816205	FALSE	TRUE	FALSE	ZOMIXT	0.017628	0.189988	486484	sepsis	TRUE	reported	textfile	0.011	4.54E-06	7274	M00569.m	TRUE	reported	kZGouU	2	TRUE	0.002901	21.15418
3359	rs2694777	A	G	A	G	0.0138	0.025409	0.8261	0.791095	FALSE	FALSE	FALSE	LY4Gus	0.017342	0.142868	486484	sepsis	TRUE	reported	textfile	0.003	5.60E-06	7268	M27273.m	TRUE	reported	QKlTBx	2	TRUE	0.002903	21.15418
3609	rs2197296	A	G	A	G	0.0207	0.015915	0.2529	0.285355	FALSE	FALSE	FALSE	2z5KhH	0.015258	0.296917	486484	sepsis	TRUE	reported	textfile	0.0045	4.75E-06	7263	M31555.m	TRUE	reported	5kcWr2	2	TRUE	0.002905	21.15417
7966	rs4507280	A	T	A	T	0.0161	-0.01794	0.1488	0.154767	FALSE	TRUE	FALSE	zb8m4a	0.018897	0.342408	486484	sepsis	TRUE	reported	textfile	0.0035	3.49E-06	7261	M35253.m	TRUE	reported	tHnnnS	2	TRUE	0.002906	21.15417
3259	rs1775517	A	T	A	T	-0.0115	-0.01118	0.6786	0.645048	FALSE	TRUE	FALSE	ZNPBqT	0.014282	0.433582	486484	sepsis	TRUE	reported	textfile	0.0025	3.44E-06	7256	M24074.m	TRUE	reported	8pPtOI	2	TRUE	0.002908	21.15417
3263	rs4075958	A	G	A	G	0.0115	0.02482	0.262	0.26302	FALSE	FALSE	FALSE	ZNPBqT	0.015494	0.109175	486484	sepsis	TRUE	reported	textfile	0.0025	5.28E-06	7256	M24074.m	TRUE	reported	8pPtOI	2	TRUE	0.002908	21.15417
1449	rs4981468	A	G	A	G	0.0207	0.000917	0.0509	0.051928	FALSE	FALSE	FALSE	1M4gqj	0.030923	0.976352	486484	sepsis	TRUE	reported	textfile	0.0045	4.76E-06	7226	M02730.m	TRUE	reported	RWyYEM	2	TRUE	0.00292	21.15414
8644	rs1089426	A	C	A	C	0.0161	-0.00548	0.5535	0.567466	FALSE	FALSE	FALSE	GgPG6k	0.01393	0.693892	486484	sepsis	TRUE	reported	textfile	0.0035	4.84E-06	7184	M36300.m	TRUE	reported	kT1yso	2	TRUE	0.002937	21.15411
6858	rs7607175	T	C	T	C	0.0345	0.032898	0.9247	0.915203	FALSE	FALSE	FALSE	2pJy8f	0.024459	0.178612	486484	sepsis	TRUE	reported	textfile	0.0075	4.55E-06	7132	M34040.m	TRUE	reported	zuJC1r	2	TRUE	0.002958	21.15407
8708	rs7910605	A	G	A	G	-0.0138	-0.00372	0.876	0.876248	FALSE	FALSE	FALSE	Bg4VnB	0.020784	0.857971	486484	sepsis	TRUE	reported	textfile	0.003	5.45E-06	7092	M36552.m	TRUE	reported	6zdDpC	2	TRUE	0.002975	21.15403
870	rs6471261	T	G	T	G	0.0115	-0.00325	0.2806	0.291259	FALSE	FALSE	FALSE	Vg5OOR	0.015055	0.828946	486484	sepsis	TRUE	reported	textfile	0.0025	6.16E-06	7063	M01361.m	TRUE	reported	XnrS00	2	TRUE	0.002987	21.15401
6952	rs1725671	T	C	T	C	0.0207	-0.01912	0.0762	0.09011	FALSE	FALSE	FALSE	7Cw2XZ	0.023881	0.423361	486484	sepsis	TRUE	reported	textfile	0.0045	3.70E-06	7049	M34123.m	TRUE	reported	vrgMI3	2	TRUE	0.002993	21.154
6229	rs9648853	T	C	T	C	-0.0161	-0.01438	0.6009	0.602196	FALSE	FALSE	FALSE	8zUBbU	0.014038	0.305558	486484	sepsis	TRUE	reported	textfile	0.0035	3.67E-06	7044	M33509.m	TRUE	reported	1mO9m8	2	TRUE	0.002995	21.15399
8934	rs7582926	T	C	T	C	-0.0437	-0.0186	0.7138	0.711477	FALSE	FALSE	FALSE	ns6PV1	0.015171	0.220224	486484	sepsis	TRUE	reported	textfile	0.0095	3.89E-06	7014	M37097.m	TRUE	reported	ZYmYt4	2	TRUE	0.003008	21.15397
4186	rs1312965	C	G	C	G	0.0437	0.005607	0.8162	0.801758	FALSE	TRUE	FALSE	TxOCzG	0.017155	0.743793	486484	sepsis	TRUE	reported	textfile	0.0095	4.02E-06	6970	M32564.m	TRUE	reported	9JP6ND	2	TRUE	0.003027	21.15393
8262	rs1011502	A	G	A	G	0.0115	-0.00233	0.6952	0.708747	FALSE	FALSE	FALSE	TdsGCZ	0.015033	0.876848	486484	sepsis	TRUE	reported	textfile	0.0025	4.66E-06	6883	M35464.m	TRUE	reported	sOspot	2	TRUE	0.003065	21.15385
7311	rs6687408	C	G	C	G	0.0115	-0.02584	0.828	0.837636	FALSE	TRUE	FALSE	Vcz4EA	0.018568	0.164013	486484	sepsis	TRUE	reported	textfile	0.0025	5.18E-06	6755	M34359.m	TRUE	reported	aUSo2J	2	TRUE	0.003123	21.15374
2321	rs2333040	A	G	A	G	0.0115	0.000788	0.4575	0.485999	FALSE	FALSE	FALSE	IZXwpR	0.013715	0.954167	486484	sepsis	TRUE	reported	textfile	0.0025	4.16E-06	6549	M16822.m	TRUE	reported	Zx9k2V	2	TRUE	0.003221	21.15354
5097	rs1184878	A	T	A	T	-0.0161	0.009362	0.2102	0.198405	FALSE	TRUE	FALSE	Uiz8VU	0.01715	0.585147	486484	sepsis	TRUE	reported	textfile	0.0035	3.16E-06	6404	M33163.m	TRUE	reported	ky0Bda	2	TRUE	0.003293	21.15339
3053	rs2278441	T	G	T	G	0.0115	-0.00694	0.4291	0.413612	FALSE	FALSE	FALSE	H6vcCG	0.013947	0.61867	486484	sepsis	TRUE	reported	textfile	0.0025	5.68E-06	6180	M22030.m	TRUE	reported	XetVX7	2	TRUE	0.003412	21.15315
2550	rs1276483	A	G	A	G	0.0253	-0.00829	0.6005	0.583586	FALSE	FALSE	FALSE	NxZhaQ	0.013924	0.551746	486484	sepsis	TRUE	reported	textfile	0.0055	4.46E-06	5984	M18283.m	TRUE	reported	5Pi7r3	2	TRUE	0.003524	21.15293
5772	rs1244143	A	T	A	T	-0.1298	-0.05647	0.0273	0.022091	FALSE	TRUE	FALSE	eT2We0	0.04672	0.226805	486484	sepsis	TRUE	reported	textfile	0.0282	4.31E-06	1273	M33353.m	TRUE	reported	uZx90m	2	TRUE	0.01637	21.15282
5962	rs1013104	T	C	T	C	-0.0207	-0.01998	0.4354	0.441583	FALSE	FALSE	FALSE	YCswLG	0.013802	0.147834	486484	sepsis	TRUE	reported	textfile	0.0045	3.83E-06	5822	M33420.m	TRUE	reported	WH7xfc	2	TRUE	0.003621	21.15273
6111	rs2746997	A	G	A	G	0.0161	0.006491	0.4486	0.44934	FALSE	FALSE	FALSE	a0osmD	0.013764	0.637189	486484	sepsis	TRUE	reported	textfile	0.0035	6.00E-06	5717	M33453.m	TRUE	reported	CePFnK	2	TRUE	0.003688	21.1526
7786	rs6429216	A	G	A	G	-0.023	-0.01569	0.464	0.46139	FALSE	FALSE	FALSE	15BqPa	0.013691	0.251723	486484	sepsis	TRUE	reported	textfile	0.005	4.23E-06	5600	M35126.m	TRUE	reported	zvDzP2	2	TRUE	0.003764	21.15244
4480	rs1006553	A	G	A	G	0.046	-0.01579	0.025	0.035923	FALSE	FALSE	FALSE	1wNJ1E	0.037895	0.676971	486484	sepsis	TRUE	reported	textfile	0.01	4.23E-06	5597	M32709.m	TRUE	reported	zihBHg	2	TRUE	0.003766	21.15244
4209	rs697387	T	C	T	C	-0.023	-0.0151	0.299	0.299485	FALSE	FALSE	FALSE	rdKSjG	0.016918	0.37207	486484	sepsis	TRUE	reported	textfile	0.005	4.23E-06	5596	M32578.m	TRUE	reported	RAzSLK	2	TRUE	0.003767	21.15244
1774	rs5759809	T	C	T	C	0.069	0.021412	0.011	0.059664	FALSE	FALSE	FALSE	wM5dkT	0.029026	0.460725	486484	sepsis	TRUE	reported	textfile	0.015	4.23E-06	5595	M15122.m	TRUE	reported	CBpa4t	2	TRUE	0.003768	21.15244
1082	rs1708674	T	C	T	C	-0.046	-0.10622	0.02	0.021141	FALSE	FALSE	FALSE	JLtEiZ	0.047644	0.025782	486484	sepsis	TRUE	reported	textfile	0.01	4.23E-06	5585	M01572.m	TRUE	reported	TSSe28	2	TRUE	0.003774	21.15242
8276	rs1098273	A	G	A	G	0.023	0.004001	0.627	0.628119	FALSE	FALSE	FALSE	J6o4xC	0.014164	0.777583	486484	sepsis	TRUE	reported	textfile	0.005	4.23E-06	4955	M35472.m	TRUE	reported	3XBcu9	2	TRUE	0.004252	21.15146
5217	rs1112171	T	C	T	C	0.2845	-0.0319	0.9816	0.959867	FALSE	FALSE	FALSE	q32d4E	0.035004	0.362179	486484	sepsis	TRUE	reported	textfile	0.0618	4.12E-06	1017	M33178.m	TRUE	reported	cS3BwT	2	TRUE	0.020413	21.15109
6106	rs1786356	A	G	A	G	0.023	-0.01385	0.606	0.596289	FALSE	FALSE	FALSE	a0osmD	0.014447	0.337851	486484	sepsis	TRUE	reported	textfile	0.005	4.23E-06	4599	M33453.m	TRUE	reported	CePFnK	2	TRUE	0.00458	21.1508
8829	rs4729705	T	C	T	C	0.0138	0.004325	0.539	0.540746	FALSE	FALSE	FALSE	zuPm7L	0.013721	0.75261	486484	sepsis	TRUE	reported	textfile	0.003	5.06E-06	4317	M36802.m	TRUE	reported	cn9d0n	2	TRUE	0.004878	21.1502
7396	rs704852	A	C	A	C	-0.023	0.019083	0.758	0.769505	FALSE	FALSE	FALSE	AW6M5u	0.018647	0.306127	486484	sepsis	TRUE	reported	textfile	0.005	4.23E-06	4020	M34389.m	TRUE	reported	6ZYYQK	2	TRUE	0.005236	21.14947
6049	rs7279663	T	G	T	G	1.5318	-0.04957	0.9746	0.971825	FALSE	FALSE	FALSE	33ojbt	0.041928	0.23706	486484	sepsis	TRUE	reported	textfile	0.3275	2.90E-06	60	M33423.m	TRUE	reported	ivEtvl	2	TRUE	0.267191	21.14746
1174	rs1306999	T	C	T	C	-0.0506	0.011751	0.3781	0.390328	FALSE	FALSE	FALSE	FgZx4a	0.014024	0.402074	486484	sepsis	TRUE	reported	textfile	0.011	4.44E-06	2063	M01640.m	TRUE	reported	hNPAJ8	2	TRUE	0.010153	21.13949
7675	rs1718724	T	C	T	C	-0.0607	-0.05426	0.9712	0.974079	FALSE	FALSE	FALSE	iqkM9B	0.043372	0.210936	486484	sepsis	TRUE	reported	textfile	0.0132	3.91E-06	4800	M34539.m	TRUE	reported	5nFq6f	2	TRUE	0.004386	21.13725
2245	rs170555	T	C	T	C	-0.0253	-0.01428	0.5607	0.57791	FALSE	FALSE	FALSE	zxcPox	0.013841	0.302134	486484	sepsis	TRUE	reported	textfile	0.0055	3.84E-06	1756	M15990.m	TRUE	reported	34Vg8F	2	TRUE	0.011907	21.1359
7444	rs9415646	T	G	T	G	-0.088	0.011132	0.691	0.683177	FALSE	FALSE	FALSE	otAubi	0.01469	0.448591	486484	sepsis	TRUE	reported	textfile	0.0191	4.00E-06	463	M34404.m	TRUE	reported	SAZaNF	2	TRUE	0.043838	21.13579
5665	rs1256685	T	C	T	C	0.0515	-0.00971	0.7395	0.739576	FALSE	FALSE	FALSE	wHu1Rk	0.015565	0.532825	486484	sepsis	TRUE	reported	textfile	0.0112	4.70E-06	4625	M33195.m	TRUE	reported	yvhnuQ	2	TRUE	0.004551	21.13443
6902	rs1184283	A	G	A	G	0.2985	0.006499	0.9424	0.875603	FALSE	FALSE	FALSE	SvfSvH	0.020747	0.754076	486484	sepsis	TRUE	reported	textfile	0.0647	3.97E-06	282	M34109.m	TRUE	reported	7OSbiu	2	TRUE	0.070182	21.13436
7155	rs1373562	T	C	T	C	0.0713	0.034971	0.1574	0.163639	FALSE	FALSE	FALSE	PnBT0M	0.01856	0.059535	486484	sepsis	TRUE	reported	textfile	0.0155	3.89E-06	1612	M34327.m	TRUE	reported	VUt4Q7	2	TRUE	0.012956	21.13375
7492	rs1007455	A	G	A	G	-0.0492	-0.00602	0.4717	0.481072	FALSE	FALSE	FALSE	1aXQWs	0.01368	0.659654	486484	sepsis	TRUE	reported	textfile	0.0107	4.54E-06	4452	M34420.m	TRUE	reported	FOqhHW	2	TRUE	0.004727	21.13331
2692	rs7139744	T	G	T	G	0.0423	-0.00986	0.3014	0.306841	FALSE	FALSE	FALSE	ATsH6N	0.014812	0.505779	486484	sepsis	TRUE	reported	textfile	0.0092	3.87E-06	3783	M18497.m	TRUE	reported	IGuIRI	2	TRUE	0.005557	21.12883
4159	rs1292053	A	G	A	G	0.0308	-0.00971	0.7386	0.729798	FALSE	FALSE	FALSE	stpu5O	0.016486	0.556076	486484	sepsis	TRUE	reported	textfile	0.0067	4.37E-06	7337	M32557.m	TRUE	reported	0oV8Ss	2	TRUE	0.002872	21.12679
2670	rs1112166	T	C	T	C	0.0308	-0.01473	0.7415	0.738569	FALSE	FALSE	FALSE	s4UTyf	0.01559	0.344887	486484	sepsis	TRUE	reported	textfile	0.0067	3.95E-06	5376	M18494.m	TRUE	reported	ajPzhg	2	TRUE	0.003916	21.12468
3585	rs7315595	T	G	T	G	-0.0838	-0.01448	0.7851	0.77018	FALSE	FALSE	FALSE	F1s6qg	0.016227	0.372182	486484	sepsis	TRUE	reported	textfile	0.0182	3.95E-06	522	M31536.m	TRUE	reported	Zm40GX	2	TRUE	0.039029	21.11923
8593	rs9348951	A	C	A	C	0.0239	0.018023	0.4014	0.414758	FALSE	FALSE	FALSE	iuJmRL	0.01386	0.193488	486484	sepsis	TRUE	reported	textfile	0.0052	5.05E-06	7311	M36103.m	TRUE	reported	jfJbVw	2	TRUE	0.002881	21.11885
6680	rs8024954	A	G	A	G	0.1812	0.017248	0.0304	0.03813	FALSE	FALSE	FALSE	6iZSOI	0.037757	0.647802	486484	sepsis	TRUE	reported	textfile	0.0394	4.33E-06	1294	M33901.m	TRUE	reported	ApKaGe	2	TRUE	0.016082	21.11797
8651	rs1893285	A	G	A	G	-0.0331	0.002847	0.5927	0.617468	FALSE	FALSE	FALSE	J6i46i	0.014204	0.841153	486484	sepsis	TRUE	reported	textfile	0.0072	4.81E-06	2520	M36376.m	TRUE	reported	IdddPp	2	TRUE	0.008317	21.11768
5973	rs7350776	C	G	C	G	0.0239	0.004094	0.697	0.697156	FALSE	TRUE	FALSE	YCswLG	0.014914	0.783673	486484	sepsis	TRUE	reported	textfile	0.0052	3.86E-06	5822	M33420.m	TRUE	reported	WH7xfc	2	TRUE	0.003615	21.11737
8601	rs7992673	T	C	T	C	0.161	-0.03894	0.922	0.787807	FALSE	FALSE	FALSE	t5potg	0.016713	0.019817	486484	sepsis	TRUE	reported	textfile	0.035	4.23E-06	978	M36115.m	TRUE	reported	SSOHIA	2	TRUE	0.021178	21.11673
7337	rs1235702	A	G	A	G	0.1173	0.014445	0.1144	0.138195	FALSE	FALSE	FALSE	mm9ksf	0.019769	0.46496	486484	sepsis	TRUE	reported	textfile	0.0255	4.16E-06	954	M34365.m	TRUE	reported	AVwEqD	2	TRUE	0.021699	21.11564
849	rs1749522	A	G	A	G	0.0216	-0.04389	0.0566	0.055445	FALSE	FALSE	FALSE	1tFtaf	0.029849	0.141492	486484	sepsis	TRUE	reported	textfile	0.0047	5.67E-06	7323	M01359.m	TRUE	reported	qq3q6E	2	TRUE	0.002876	21.1151
7603	rs1695521	A	G	A	G	-0.5039	-0.06627	0.9818	0.993971	FALSE	FALSE	FALSE	PlYt5g	0.089279	0.457934	486484	sepsis	TRUE	reported	textfile	0.1088	3.66E-06	128	M34516.m	TRUE	reported	jX41tf	2	TRUE	0.143527	21.11502
8656	rs1088971	T	C	T	C	0.0216	0.010044	0.3474	0.363142	FALSE	FALSE	FALSE	BFEGyF	0.014237	0.480496	486484	sepsis	TRUE	reported	textfile	0.0047	4.74E-06	6486	M36399.m	TRUE	reported	nhT4FM	2	TRUE	0.003246	21.11436
6685	rs1049161	T	G	T	G	-0.0841	-0.01595	0.9743	0.956853	FALSE	FALSE	FALSE	23zCgC	0.03452	0.643979	486484	sepsis	TRUE	reported	textfile	0.0183	4.32E-06	5534	M33910.m	TRUE	reported	ACI3CA	2	TRUE	0.003802	21.11217
2626	rs755677	T	G	T	G	0.0754	-0.01851	0.87	0.888265	FALSE	FALSE	FALSE	eygBBR	0.022011	0.400269	486484	sepsis	TRUE	reported	textfile	0.0164	4.51E-06	1640	M18394.m	TRUE	reported	hD79iE	2	TRUE	0.012725	21.11179
5172	rs1004644	T	C	T	C	-0.1182	0.020622	0.0771	0.08049	FALSE	FALSE	FALSE	q32d4E	0.025223	0.413595	486484	sepsis	TRUE	reported	textfile	0.0257	4.20E-06	1017	M33178.m	TRUE	reported	cS3BwT	2	TRUE	0.020375	21.11124
5067	rs7916009	A	G	A	G	0.0193	-0.01487	0.6156	0.60067	FALSE	FALSE	FALSE	nHZXjp	0.013996	0.288146	486484	sepsis	TRUE	reported	textfile	0.0042	3.92E-06	7166	M33138.m	TRUE	reported	ccoK1F	2	TRUE	0.002938	21.11032
8975	rs1724350	T	C	T	C	-0.0363	0.015817	0.1932	0.187778	FALSE	FALSE	FALSE	snOepI	0.01749	0.365822	486484	sepsis	TRUE	reported	textfile	0.0079	4.54E-06	7355	M37202.m	TRUE	reported	PQx272	2	TRUE	0.002862	21.1077
2639	rs703811	T	C	T	C	0.017	0.019381	0.7585	0.759279	FALSE	FALSE	FALSE	UxuT5B	0.015959	0.224601	486484	sepsis	TRUE	reported	textfile	0.0037	4.20E-06	7367	M18467.m	TRUE	reported	eGQj6o	2	TRUE	0.002857	21.10457
4412	rs1172763	T	C	T	C	0.017	0.01153	0.2943	0.301895	FALSE	FALSE	FALSE	NzBiVA	0.014961	0.440898	486484	sepsis	TRUE	reported	textfile	0.0037	5.18E-06	7354	M32672.m	TRUE	reported	YLlhHJ	2	TRUE	0.002862	21.10456
8488	rs422249	T	C	T	C	-0.017	0.039089	0.309	0.346819	FALSE	FALSE	FALSE	ABkZ7x	0.014443	0.006802	486484	sepsis	TRUE	reported	textfile	0.0037	5.44E-06	6960	M35854.m	TRUE	reported	wc0CeK	2	TRUE	0.003024	21.10423
7710	rs1384609	T	C	T	C	-0.046	-0.0135	0.474	0.47328	FALSE	FALSE	FALSE	qJW9Ig	0.013679	0.32386	486484	sepsis	TRUE	reported	textfile	0.01	4.23E-06	711	M34878.m	TRUE	reported	42EOzB	2	TRUE	0.028901	21.10048
6351	rs8003714	T	G	T	G	0.4616	0.121874	0.017	0.012003	FALSE	FALSE	FALSE	qLUY00	0.062555	0.051384	486484	sepsis	TRUE	reported	textfile	0.0995	3.46E-06	102	M33610.m	TRUE	reported	8cAJbB	2	TRUE	0.174237	21.10014
7506	rs4743627	A	T	A	T	0.0804	0.009529	0.896	0.906246	FALSE	TRUE	FALSE	1aXQWs	0.023758	0.688361	486484	sepsis	TRUE	reported	textfile	0.0175	4.60E-06	4452	M34420.m	TRUE	reported	FOqhHW	2	TRUE	0.004719	21.09798
4167	rs8071320	T	G	T	G	-0.0294	0.012785	0.2306	0.230133	FALSE	FALSE	FALSE	stpu5O	0.016205	0.430143	486484	sepsis	TRUE	reported	textfile	0.0064	4.16E-06	7337	M32557.m	TRUE	reported	0oV8Ss	2	TRUE	0.002868	21.09679
8349	rs1322106	A	G	A	G	-0.0294	0.004299	0.8289	0.82814	FALSE	FALSE	FALSE	6VVCe4	0.018244	0.81371	486484	sepsis	TRUE	reported	textfile	0.0064	4.78E-06	5629	M35669.m	TRUE	reported	l0fk8X	2	TRUE	0.003735	21.09504
2680	rs7432211	T	C	T	C	0.0294	0.002231	0.5934	0.602984	FALSE	FALSE	FALSE	s4UTyf	0.013952	0.872973	486484	sepsis	TRUE	reported	textfile	0.0064	4.93E-06	5376	M18494.m	TRUE	reported	ajPzhg	2	TRUE	0.00391	21.09469
5059	rs1250078	A	T	A	T	-0.0395	-0.01202	0.9481	0.944405	FALSE	TRUE	FALSE	nHZXjp	0.029934	0.687928	486484	sepsis	TRUE	reported	textfile	0.0086	4.76E-06	7166	M33138.m	TRUE	reported	ccoK1F	2	TRUE	0.002935	21.08997
3804	rs1245609	A	G	A	G	-0.0395	-0.00354	0.7235	0.664904	FALSE	FALSE	FALSE	HOq2gb	0.014492	0.807126	486484	sepsis	TRUE	reported	textfile	0.0086	4.75E-06	6687	M32346.m	TRUE	reported	Ss5q17	2	TRUE	0.003145	21.08955
2628	rs915529	A	G	A	G	-0.1861	-0.0211	0.0187	0.018334	FALSE	FALSE	FALSE	eygBBR	0.051111	0.679725	486484	sepsis	TRUE	reported	textfile	0.0405	4.23E-06	1640	M18394.m	TRUE	reported	hD79iE	2	TRUE	0.012711	21.08884
6583	rs1707339	T	C	T	C	0.4078	-0.04098	0.0276	0.017441	FALSE	FALSE	FALSE	XO3rXq	0.052173	0.432189	486484	sepsis	TRUE	reported	textfile	0.0883	3.87E-06	176	M33877.m	TRUE	reported	p2sETg	2	TRUE	0.108089	21.08675
1324	rs1050129	T	C	T	C	-0.0124	0.016664	0.8715	0.863351	FALSE	FALSE	FALSE	LDUJbh	0.019906	0.402523	486484	sepsis	TRUE	reported	textfile	0.0027	3.11E-06	7367	M01898.m	TRUE	reported	JRSLeH	2	TRUE	0.002855	21.08618
4298	rs284431	A	C	A	C	0.0124	0.043901	0.1069	0.106696	FALSE	FALSE	FALSE	0tjjPC	0.022278	0.04877	486484	sepsis	TRUE	reported	textfile	0.0027	4.58E-06	7362	M32634.m	TRUE	reported	nJJFkX	2	TRUE	0.002857	21.08618
4392	rs2186677	A	G	A	G	-0.0124	0.00725	0.2785	0.297355	FALSE	FALSE	FALSE	UJygVd	0.015392	0.637622	486484	sepsis	TRUE	reported	textfile	0.0027	5.86E-06	7361	M32654.m	TRUE	reported	kv9R0W	2	TRUE	0.002857	21.08618
3673	rs1318437	T	C	T	C	-0.0124	-0.00757	0.1519	0.14694	FALSE	FALSE	FALSE	rGHJcj	0.019307	0.69513	486484	sepsis	TRUE	reported	textfile	0.0027	6.38E-06	7356	M32198.m	TRUE	reported	6P1Nno	2	TRUE	0.002859	21.08617
7899	rs6722402	T	G	T	G	-0.0124	0.010839	0.7106	0.712146	FALSE	FALSE	FALSE	pU8zps	0.015134	0.473868	486484	sepsis	TRUE	reported	textfile	0.0027	2.99E-06	7350	M35186.m	TRUE	reported	Oc2lOF	2	TRUE	0.002861	21.08617
7806	rs748651	T	C	T	C	-0.0124	-0.00357	0.5135	0.525451	FALSE	FALSE	FALSE	cQ5TvO	0.013786	0.795905	486484	sepsis	TRUE	reported	textfile	0.0027	4.06E-06	7344	M35127.m	TRUE	reported	BVxkfZ	2	TRUE	0.002864	21.08616
1837	rs6130362	C	G	C	G	0.0124	0.005735	0.2071	0.221344	FALSE	TRUE	FALSE	2TaDHm	0.016617	0.730012	486484	sepsis	TRUE	reported	textfile	0.0027	5.62E-06	7337	M15365.m	TRUE	reported	LoLveg	2	TRUE	0.002866	21.08616
4271	rs2776859	T	C	T	C	0.0124	-0.00321	0.5308	0.529042	FALSE	FALSE	FALSE	ylV4bp	0.013874	0.817233	486484	sepsis	TRUE	reported	textfile	0.0027	5.68E-06	7337	M32632.m	TRUE	reported	hqHJoU	2	TRUE	0.002866	21.08616
5891	rs6977786	A	G	A	G	-0.0124	0.017157	0.1753	0.212496	FALSE	FALSE	FALSE	tqW0ld	0.016708	0.304484	486484	sepsis	TRUE	reported	textfile	0.0027	3.23E-06	7315	M33389.m	TRUE	reported	Sv7EyV	2	TRUE	0.002875	21.08614
2869	rs1050843	A	G	A	G	-0.0124	-0.0335	0.7069	0.708486	FALSE	FALSE	FALSE	mUzn1R	0.015043	0.025975	486484	sepsis	TRUE	reported	textfile	0.0027	4.07E-06	7301	M20675.m	TRUE	reported	XwY04K	2	TRUE	0.002881	21.08613
6241	rs3783853	A	G	A	G	0.0124	0.006136	0.4188	0.41167	FALSE	FALSE	FALSE	vL52Yb	0.013972	0.660557	486484	sepsis	TRUE	reported	textfile	0.0027	3.27E-06	7269	M33510.m	TRUE	reported	JIO9VX	2	TRUE	0.002893	21.0861
7955	rs1107825	A	G	A	G	0.0124	-0.00733	0.3384	0.345874	FALSE	FALSE	FALSE	zb8m4a	0.014484	0.612673	486484	sepsis	TRUE	reported	textfile	0.0027	3.09E-06	7261	M35253.m	TRUE	reported	tHnnnS	2	TRUE	0.002896	21.0861
7957	rs1215584	T	C	T	C	0.0124	-0.02567	0.6503	0.654831	FALSE	FALSE	FALSE	zb8m4a	0.014652	0.079822	486484	sepsis	TRUE	reported	textfile	0.0027	3.64E-06	7261	M35253.m	TRUE	reported	tHnnnS	2	TRUE	0.002896	21.0861
7963	rs2236434	C	G	C	G	-0.0124	0.026544	0.6878	0.680194	FALSE	TRUE	FALSE	zb8m4a	0.014672	0.070438	486484	sepsis	TRUE	reported	textfile	0.0027	3.71E-06	7261	M35253.m	TRUE	reported	tHnnnS	2	TRUE	0.002896	21.0861
7968	rs4905	A	G	A	G	0.0124	-0.00851	0.7122	0.716094	FALSE	FALSE	FALSE	zb8m4a	0.015177	0.575015	486484	sepsis	TRUE	reported	textfile	0.0027	2.92E-06	7261	M35253.m	TRUE	reported	tHnnnS	2	TRUE	0.002896	21.0861
4338	rs1227200	A	T	A	T	-0.0124	-0.00832	0.8447	0.850229	FALSE	TRUE	FALSE	6VSoQQ	0.019407	0.668085	486484	sepsis	TRUE	reported	textfile	0.0027	3.68E-06	7229	M32644.m	TRUE	reported	oC2AV0	2	TRUE	0.002909	21.08607
6848	rs1020972	A	G	A	G	0.0248	-0.0082	0.1471	0.151774	FALSE	FALSE	FALSE	2pJy8f	0.019196	0.669262	486484	sepsis	TRUE	reported	textfile	0.0054	5.04E-06	7132	M34040.m	TRUE	reported	zuJC1r	2	TRUE	0.002949	21.08599
7105	rs9972792	A	C	A	C	0.0124	0.015469	0.6339	0.645893	FALSE	FALSE	FALSE	pxNICA	0.014324	0.280175	486484	sepsis	TRUE	reported	textfile	0.0027	3.18E-06	6994	M34306.m	TRUE	reported	xk5b9M	2	TRUE	0.003007	21.08588
1654	rs1337278	A	G	A	G	0.0124	0.006475	0.6107	0.616143	FALSE	FALSE	FALSE	YzX5ZD	0.014077	0.645522	486484	sepsis	TRUE	reported	textfile	0.0027	3.68E-06	6864	M12129.m	TRUE	reported	6tMfaW	2	TRUE	0.003063	21.08576
1655	rs1448817	A	G	A	G	0.0124	-0.02588	0.7393	0.752245	FALSE	FALSE	FALSE	YzX5ZD	0.015871	0.102958	486484	sepsis	TRUE	reported	textfile	0.0027	4.56E-06	6864	M12129.m	TRUE	reported	6tMfaW	2	TRUE	0.003063	21.08576
2250	rs2834399	T	G	T	G	0.0124	-0.0066	0.4704	0.466002	FALSE	FALSE	FALSE	zxcPox	0.013816	0.632961	486484	sepsis	TRUE	reported	textfile	0.0027	3.23E-06	6782	M15990.m	TRUE	reported	34Vg8F	2	TRUE	0.0031	21.08569
3016	rs4711151	A	G	A	G	-0.0124	-0.03058	0.3776	0.380961	FALSE	FALSE	FALSE	gxupFu	0.01406	0.029644	486484	sepsis	TRUE	reported	textfile	0.0027	3.50E-06	6556	M21188.m	TRUE	reported	gwwXh2	2	TRUE	0.003207	21.08547
3019	rs7152341	A	G	A	G	0.0124	-0.00668	0.2566	0.250972	FALSE	FALSE	FALSE	gxupFu	0.015781	0.672247	486484	sepsis	TRUE	reported	textfile	0.0027	5.26E-06	6556	M21188.m	TRUE	reported	gwwXh2	2	TRUE	0.003207	21.08547
7937	rs3752192	A	G	A	G	0.0124	-0.01164	0.5526	0.555625	FALSE	FALSE	FALSE	kFTWXY	0.01379	0.398596	486484	sepsis	TRUE	reported	textfile	0.0027	5.05E-06	6543	M35193.m	TRUE	reported	cjJvJl	2	TRUE	0.003213	21.08546
7944	rs7942744	T	C	T	C	0.0124	-0.03359	0.556	0.559875	FALSE	FALSE	FALSE	kFTWXY	0.013776	0.014768	486484	sepsis	TRUE	reported	textfile	0.0027	6.42E-06	6543	M35193.m	TRUE	reported	cjJvJl	2	TRUE	0.003213	21.08546
7707	rs9788090	C	G	C	G	0.0248	0.024923	0.7338	0.74612	FALSE	TRUE	FALSE	2eJlPM	0.016044	0.120338	486484	sepsis	TRUE	reported	textfile	0.0054	5.22E-06	6381	M34761.m	TRUE	reported	druKtT	2	TRUE	0.003295	21.0853
8622	rs6089829	A	G	A	G	0.0294	0.036024	0.3531	0.354137	FALSE	FALSE	FALSE	MZ60v7	0.014286	0.011683	486484	sepsis	TRUE	reported	textfile	0.0064	4.75E-06	2432	M36134.m	TRUE	reported	dMQewN	2	TRUE	0.008602	21.08519
4767	rs1107326	T	C	T	C	0.0248	-0.02931	0.5956	0.575324	FALSE	FALSE	FALSE	mOrnK4	0.013925	0.035285	486484	sepsis	TRUE	reported	textfile	0.0054	5.26E-06	6169	M32795.m	TRUE	reported	7EGxuf	2	TRUE	0.003407	21.08507
7132	rs1761927	A	G	A	G	0.071	-0.02533	0.1542	0.054523	FALSE	FALSE	FALSE	de8tA6	0.030068	0.39949	486484	sepsis	TRUE	reported	textfile	0.0154	4.02E-06	248	M34322.m	TRUE	reported	sXPPBO	2	TRUE	0.078942	21.08428
2157	rs2969612	T	C	T	C	-0.0496	-0.02177	0.1583	0.159536	FALSE	FALSE	FALSE	uuY1Mi	0.018738	0.245239	486484	sepsis	TRUE	reported	textfile	0.0108	4.75E-06	5502	M15677.m	TRUE	reported	nWMiPu	2	TRUE	0.003819	21.08424
4362	rs1116690	A	G	A	G	0.0124	0.008486	0.3594	0.372264	FALSE	FALSE	FALSE	SPhl52	0.014193	0.549908	486484	sepsis	TRUE	reported	textfile	0.0027	5.60E-06	5211	M32651.m	TRUE	reported	V0Bnpj	2	TRUE	0.004031	21.08381
4363	rs1271934	A	G	A	G	0.0124	-0.00184	0.3179	0.317123	FALSE	FALSE	FALSE	SPhl52	0.014711	0.900623	486484	sepsis	TRUE	reported	textfile	0.0027	6.09E-06	5211	M32651.m	TRUE	reported	V0Bnpj	2	TRUE	0.004031	21.08381
4379	rs7924340	T	C	T	C	0.0124	-0.00505	0.4691	0.469048	FALSE	FALSE	FALSE	SPhl52	0.013755	0.713505	486484	sepsis	TRUE	reported	textfile	0.0027	4.63E-06	5211	M32651.m	TRUE	reported	V0Bnpj	2	TRUE	0.004031	21.08381
8555	rs6956438	A	G	A	G	-0.4219	0.011703	0.8991	0.890132	FALSE	FALSE	FALSE	aQhCav	0.022239	0.598718	486484	sepsis	TRUE	reported	textfile	0.0917	4.20E-06	488	M36097.m	TRUE	reported	9DJS9j	2	TRUE	0.041574	21.08127
5910	rs1160269	T	C	T	C	-0.0326	-0.00796	0.4442	0.449055	FALSE	FALSE	FALSE	iGV9aX	0.013732	0.561991	486484	sepsis	TRUE	reported	textfile	0.0071	3.70E-06	5185	M33390.m	TRUE	reported	sLbpnf	2	TRUE	0.00405	21.07419
5645	rs1252900	A	G	A	G	0.146	0.045923	0.9751	0.971914	FALSE	FALSE	FALSE	BFsMtm	0.041492	0.268382	486484	sepsis	TRUE	reported	textfile	0.0318	4.39E-06	5716	M33192.m	TRUE	reported	NAJN3D	2	TRUE	0.003674	21.0717
5746	rs1692364	T	C	T	C	0.0202	0.004194	0.1285	0.125212	FALSE	FALSE	FALSE	bm6x2Z	0.02101	0.841792	486484	sepsis	TRUE	reported	textfile	0.0044	4.57E-06	7364	M33230.m	TRUE	reported	8Ccke3	2	TRUE	0.002854	21.07072
7993	rs1703298	T	G	T	G	0.0303	0.00502	0.103	0.112001	FALSE	FALSE	FALSE	Xrodum	0.022202	0.821132	486484	sepsis	TRUE	reported	textfile	0.0066	4.70E-06	7291	M35255.m	TRUE	reported	T8t1tl	2	TRUE	0.002882	21.07066
3412	rs404632	T	C	T	C	-0.0202	-0.02185	0.1335	0.139767	FALSE	FALSE	FALSE	VU62LG	0.019712	0.267632	486484	sepsis	TRUE	reported	textfile	0.0044	5.36E-06	7180	M27513.m	TRUE	reported	0didOx	2	TRUE	0.002927	21.07058
7567	rs2453573	T	C	T	C	-0.0202	0.013716	0.8177	0.807584	FALSE	FALSE	FALSE	Ot2ZAn	0.017323	0.428505	486484	sepsis	TRUE	reported	textfile	0.0044	3.68E-06	7119	M34481.m	TRUE	reported	tpyFcK	2	TRUE	0.002952	21.07053
4922	rs1326510	T	C	T	C	0.0271	-0.0075	0.6244	0.62366	FALSE	FALSE	FALSE	clhN6v	0.014088	0.594684	486484	sepsis	TRUE	reported	textfile	0.0059	4.73E-06	1550	M32869.m	TRUE	reported	BzloWk	2	TRUE	0.013429	21.07045
8742	rs9931063	A	G	A	G	0.0395	-0.01939	0.8768	0.831544	FALSE	FALSE	FALSE	ERC6hD	0.018269	0.288656	486484	sepsis	TRUE	reported	textfile	0.0086	4.32E-06	1636	M36559.m	TRUE	reported	n8waJF	2	TRUE	0.012731	21.07007
1406	rs1249696	A	G	A	G	0.0202	0.003455	0.7844	0.778484	FALSE	FALSE	FALSE	33ptwk	0.016492	0.834046	486484	sepsis	TRUE	reported	textfile	0.0044	4.28E-06	6252	M02137.m	TRUE	reported	SQY1na	2	TRUE	0.00336	21.0697
3592	rs1156455	A	G	A	G	0.0303	0.014428	0.4808	0.474306	FALSE	FALSE	FALSE	e4G8UU	0.013703	0.292367	486484	sepsis	TRUE	reported	textfile	0.0066	3.87E-06	5229	M31548.m	TRUE	reported	kWkkmH	2	TRUE	0.004015	21.06838
2240	rs7243187	A	C	A	C	-0.0455	0.00459	0.2661	0.271123	FALSE	FALSE	FALSE	cwj8fY	0.015376	0.765286	486484	sepsis	TRUE	reported	textfile	0.0099	3.87E-06	773	M15958.m	TRUE	reported	dPwsBc	2	TRUE	0.026599	21.06819
7513	rs1168366	A	G	A	G	0.0202	-0.00586	0.6195	0.612699	FALSE	FALSE	FALSE	rp1LGA	0.014157	0.678771	486484	sepsis	TRUE	reported	textfile	0.0044	4.38E-06	4442	M34441.m	TRUE	reported	SU47tz	2	TRUE	0.004722	21.06696
6682	rs1027200	T	C	T	C	0.101	-0.0039	0.022	0.097657	FALSE	FALSE	FALSE	23zCgC	0.027641	0.887864	486484	sepsis	TRUE	reported	textfile	0.022	4.41E-06	4351	M33910.m	TRUE	reported	ACI3CA	2	TRUE	0.004821	21.06676
2161	rs9915627	A	C	A	C	0.1117	-0.00066	0.1829	0.188411	FALSE	FALSE	FALSE	uuY1Mi	0.021393	0.975485	486484	sepsis	TRUE	reported	textfile	0.0243	4.29E-06	658	M15677.m	TRUE	reported	nWMiPu	2	TRUE	0.031113	21.0655
3601	rs4957657	T	C	T	C	0.0482	-0.00071	0.1104	0.103087	FALSE	FALSE	FALSE	e4G8UU	0.022784	0.975148	486484	sepsis	TRUE	reported	textfile	0.0105	4.53E-06	5229	M31548.m	TRUE	reported	kWkkmH	2	TRUE	0.004014	21.06441
4419	rs1777689	A	G	A	G	0.0349	-0.02528	0.3028	0.293694	FALSE	FALSE	FALSE	NzBiVA	0.015006	0.092079	486484	sepsis	TRUE	reported	textfile	0.0076	4.02E-06	1764	M32672.m	TRUE	reported	YLlhHJ	2	TRUE	0.011813	21.06352
8620	rs4897622	A	G	A	G	-0.0574	0.013285	0.1147	0.118783	FALSE	FALSE	FALSE	MZ60v7	0.021137	0.529661	486484	sepsis	TRUE	reported	textfile	0.0125	4.10E-06	1657	M36134.m	TRUE	reported	dMQewN	2	TRUE	0.012566	21.06101
4474	rs4857879	A	C	A	C	0.0225	0.039168	0.8382	0.84814	FALSE	FALSE	FALSE	g3iM2w	0.019076	0.040052	486484	sepsis	TRUE	reported	textfile	0.0049	3.56E-06	1757	M32698.m	TRUE	reported	ZQBn0h	2	TRUE	0.011858	21.06096

7442	rs6911611	A	T	A	T	0.1861	0.002631	0.9734	0.970406	FALSE	TRUE	FALSE	otAubi	0.040371	0.948037	486484	sepsis	TRUE	reported	textfile	0.0405	4.25E-06	777	M34404.m	TRUE	reported	SAZaNF	2	TRUE	0.026456	21.06024
3893	rs6957675	T	C	T	C	-0.0179	0.002196	0.2559	0.256918	FALSE	FALSE	FALSE	bmXxuz	0.015796	0.889448	486484	sepsis	TRUE	reported	textfile	0.0039	3.51E-06	7358	M32405.m	TRUE	reported	A9BFZF	2	TRUE	0.002855	21.06002
896	rs1118499	A	T	A	T	0.0179	0.008999	0.2894	0.281194	FALSE	TRUE	FALSE	I6zQnf	0.015391	0.558754	486484	sepsis	TRUE	reported	textfile	0.0039	6.01E-06	7345	M01444.m	TRUE	reported	IIzalm	2	TRUE	0.00286	21.06001
1491	rs848767	T	C	T	C	-0.0179	-0.0069	0.0696	0.076785	FALSE	FALSE	FALSE	pkb876	0.025645	0.787969	486484	sepsis	TRUE	reported	textfile	0.0039	3.46E-06	7037	M02734.m	TRUE	reported	0LCMVI	2	TRUE	0.002985	21.05976
1513	rs4676313	T	C	T	C	0.0179	0.02079	0.8778	0.892913	FALSE	FALSE	FALSE	rzkxmh	0.022103	0.346912	486484	sepsis	TRUE	reported	textfile	0.0039	3.54E-06	6941	M03127.m	TRUE	reported	3IrzhA	2	TRUE	0.003026	21.05968
6418	rs1077957	A	G	A	G	0.0427	0.01485	0.209	0.214016	FALSE	FALSE	FALSE	F7mjRv	0.016676	0.373221	486484	sepsis	TRUE	reported	textfile	0.0093	4.07E-06	1860	M33658.m	TRUE	reported	frG0Zf	2	TRUE	0.011207	21.05827
1098	rs4948051	T	C	T	C	0.0528	-0.00624	0.1328	0.123322	FALSE	FALSE	FALSE	K3wtZo	0.021088	0.767411	486484	sepsis	TRUE	reported	textfile	0.0115	4.84E-06	1646	M01573.m	TRUE	reported	0D9CvA	2	TRUE	0.012645	21.05446
6789	rs6530980	T	C	T	C	0.0491	-0.04106	0.0364	0.034876	FALSE	FALSE	FALSE	kcZoFT	0.037374	0.27198	486484	sepsis	TRUE	reported	textfile	0.0107	4.02E-06	6998	M33957.m	TRUE	reported	aIW1w0	2	TRUE	0.003	21.05093
8727	rs1192152	A	G	A	G	-0.0303	0.011993	0.1844	0.185124	FALSE	FALSE	FALSE	ERC6hD	0.01765	0.496816	486484	sepsis	TRUE	reported	textfile	0.0066	4.99E-06	1636	M36559.m	TRUE	reported	n8waJF	2	TRUE	0.012719	21.05068
3425	rs1252704	T	C	T	C	0.1239	-0.02627	0.0167	0.036437	FALSE	FALSE	FALSE	HHQf8w	0.036505	0.471674	486484	sepsis	TRUE	reported	textfile	0.027	4.61E-06	5673	M27531.m	TRUE	reported	3vTluK	2	TRUE	0.003698	21.05048
7522	rs640631	T	C	T	C	0.0335	0.014082	0.904	0.945066	FALSE	FALSE	FALSE	rp1LGA	0.030059	0.639437	486484	sepsis	TRUE	reported	textfile	0.0073	4.42E-06	4442	M34441.m	TRUE	reported	SU47tz	2	TRUE	0.004719	21.04982
6555	rs1298686	T	C	T	C	0.0404	-0.01623	0.2858	0.295209	FALSE	FALSE	FALSE	58qeH5	0.014998	0.279058	486484	sepsis	TRUE	reported	textfile	0.0088	5.01E-06	1428	M33864.m	TRUE	reported	TBsy8r	2	TRUE	0.014545	21.04693
2687	rs1298445	T	C	T	C	-0.0413	0.010122	0.3058	0.314267	FALSE	FALSE	FALSE	ATsH6N	0.014732	0.492047	486484	sepsis	TRUE	reported	textfile	0.009	4.71E-06	3783	M18497.m	TRUE	reported	IGuIRI	2	TRUE	0.005536	21.04677
1569	rs7735310	T	G	T	G	-0.0078	-0.01235	0.7188	0.71342	FALSE	FALSE	FALSE	Ke0sNh	0.015132	0.414394	486484	sepsis	TRUE	reported	textfile	0.0017	3.38E-06	7799	M10642.m	TRUE	reported	ZINwVb	2	TRUE	0.002692	21.0465
1130	rs1331801	T	C	T	C	-0.0078	0.027211	0.8378	0.809101	FALSE	FALSE	FALSE	nlqUuV	0.017438	0.118664	486484	sepsis	TRUE	reported	textfile	0.0017	8.36E-06	7371	M01604.m	TRUE	reported	Uro9nC	2	TRUE	0.002848	21.04619
398	rs1020731	T	G	T	G	0.0078	0.003082	0.2432	0.247887	FALSE	FALSE	FALSE	cTsNj6	0.015862	0.845922	486484	sepsis	TRUE	reported	textfile	0.0017	4.79E-06	7365	M00063.m	TRUE	reported	7KYTrQ	2	TRUE	0.00285	21.04619
404	rs1124515	A	G	A	G	-0.0078	-0.00217	0.2539	0.24816	FALSE	FALSE	FALSE	cTsNj6	0.015825	0.891137	486484	sepsis	TRUE	reported	textfile	0.0017	6.01E-06	7365	M00063.m	TRUE	reported	7KYTrQ	2	TRUE	0.00285	21.04619
414	rs1729344	T	C	T	C	0.0078	-0.00753	0.7718	0.771873	FALSE	FALSE	FALSE	cTsNj6	0.01628	0.643813	486484	sepsis	TRUE	reported	textfile	0.0017	8.09E-06	7365	M00063.m	TRUE	reported	7KYTrQ	2	TRUE	0.00285	21.04619
415	rs1768356	T	C	T	C	0.0078	-0.0074	0.2146	0.216779	FALSE	FALSE	FALSE	cTsNj6	0.016566	0.6553	486484	sepsis	TRUE	reported	textfile	0.0017	7.57E-06	7365	M00063.m	TRUE	reported	7KYTrQ	2	TRUE	0.00285	21.04619
419	rs2050223	A	G	A	G	-0.0078	0.019976	0.2016	0.200176	FALSE	FALSE	FALSE	cTsNj6	0.017062	0.241663	486484	sepsis	TRUE	reported	textfile	0.0017	7.22E-06	7365	M00063.m	TRUE	reported	7KYTrQ	2	TRUE	0.00285	21.04619
421	rs2259571	T	G	T	G	-0.0078	-0.00463	0.6893	0.652881	FALSE	FALSE	FALSE	cTsNj6	0.014371	0.747126	486484	sepsis	TRUE	reported	textfile	0.0017	2.86E-06	7365	M00063.m	TRUE	reported	7KYTrQ	2	TRUE	0.00285	21.04619
423	rs2869804	A	G	A	G	0.0078	-0.00888	0.2489	0.25263	FALSE	FALSE	FALSE	cTsNj6	0.01585	0.575146	486484	sepsis	TRUE	reported	textfile	0.0017	6.50E-06	7365	M00063.m	TRUE	reported	7KYTrQ	2	TRUE	0.00285	21.04619
737	rs2383208	A	G	A	G	0.0078	0.025118	0.8223	0.821932	FALSE	FALSE	FALSE	9u0SC8	0.017836	0.159058	486484	sepsis	TRUE	reported	textfile	0.0017	8.79E-06	7363	M01301.m	TRUE	reported	H95uec	2	TRUE	0.002851	21.04618
831	rs3087751	A	C	A	C	0.0078	0.01418	0.423	0.44744	FALSE	FALSE	FALSE	2qgDgB	0.013717	0.301266	486484	sepsis	TRUE	reported	textfile	0.0017	5.23E-06	7355	M01358.m	TRUE	reported	asmXq0	2	TRUE	0.002854	21.04618
833	rs3793900	A	C	A	C	-0.0078	-0.01721	0.6337	0.6407	FALSE	FALSE	FALSE	2qgDgB	0.014302	0.228956	486484	sepsis	TRUE	reported	textfile	0.0017	5.73E-06	7355	M01358.m	TRUE	reported	asmXq0	2	TRUE	0.002854	21.04618
842	rs7681360	A	G	A	G	0.0078	-0.00012	0.5825	0.584412	FALSE	FALSE	FALSE	2qgDgB	0.013852	0.993248	486484	sepsis	TRUE	reported	textfile	0.0017	4.19E-06	7355	M01358.m	TRUE	reported	asmXq0	2	TRUE	0.002854	21.04618
2596	rs1765261	A	G	A	G	-0.0078	-0.03326	0.5436	0.541141	FALSE	FALSE	FALSE	ruvJh6	0.013823	0.016135	486484	sepsis	TRUE	reported	textfile	0.0017	7.29E-06	7354	M18369.m	TRUE	reported	GFmwGk	2	TRUE	0.002854	21.04618
2818	rs4541491	A	C	A	C	-0.0078	-0.00165	0.6185	0.61753	FALSE	FALSE	FALSE	Sx5Uhq	0.014061	0.90653	486484	sepsis	TRUE	reported	textfile	0.0017	6.60E-06	7354	M19934.m	TRUE	reported	DijYTE	2	TRUE	0.002854	21.04618
787	rs1214752	A	G	A	G	0.0078	-0.00176	0.4485	0.456497	FALSE	FALSE	FALSE	dyT79I	0.01373	0.897967	486484	sepsis	TRUE	reported	textfile	0.0017	5.92E-06	7352	M01336.m	TRUE	reported	OemVJ2	2	TRUE	0.002855	21.04618
791	rs1335597	A	G	A	G	-0.0078	0.011087	0.5663	0.562125	FALSE	FALSE	FALSE	dyT79I	0.013762	0.42046	486484	sepsis	TRUE	reported	textfile	0.0017	3.94E-06	7352	M01336.m	TRUE	reported	OemVJ2	2	TRUE	0.002855	21.04618
794	rs2931383	T	G	T	G	0.0078	-0.01397	0.5816	0.574748	FALSE	FALSE	FALSE	dyT79I	0.013833	0.312601	486484	sepsis	TRUE	reported	textfile	0.0017	4.68E-06	7352	M01336.m	TRUE	reported	OemVJ2	2	TRUE	0.002855	21.04618
748	rs1683931	T	C	T	C	-0.0078	-0.01198	0.8726	0.867981	FALSE	FALSE	FALSE	RHWptG	0.020211	0.55345	486484	sepsis	TRUE	reported	textfile	0.0017	2.75E-06	7347	M01302.m	TRUE	reported	z1OLfr	2	TRUE	0.002857	21.04617
754	rs3740996	A	G	A	G	0.0078	0.005153	0.1208	0.127081	FALSE	FALSE	FALSE	RHWptG	0.020496	0.801498	486484	sepsis	TRUE	reported	textfile	0.0017	4.33E-06	7347	M01302.m	TRUE	reported	z1OLfr	2	TRUE	0.002857	21.04617
760	rs6960830	T	C	T	C	0.0078	-0.00496	0.8851	0.875592	FALSE	FALSE	FALSE	RHWptG	0.020814	0.811636	486484	sepsis	TRUE	reported	textfile	0.0017	4.16E-06	7347	M01302.m	TRUE	reported	z1OLfr	2	TRUE	0.002857	21.04617
1842	rs1049024	A	G	A	G	0.0078	0.013923	0.8318	0.826663	FALSE	FALSE	FALSE	S1eWts	0.018125	0.442402	486484	sepsis	TRUE	reported	textfile	0.0017	5.17E-06	7345	M15488.m	TRUE	reported	511KI9	2	TRUE	0.002858	21.04617
1852	rs3904667	A	T	A	T	-0.0078	0.007007	0.2059	0.204351	FALSE	TRUE	FALSE	S1eWts	0.016937	0.679069	486484	sepsis	TRUE	reported	textfile	0.0017	3.20E-06	7345	M15488.m	TRUE	reported	511KI9	2	TRUE	0.002858	21.04617
8183	rs6998531	T	C	T	C	0.0078	0.001575	0.2087	0.203556	FALSE	FALSE	FALSE	8UhtPK	0.016952	0.925988	486484	sepsis	TRUE	reported	textfile	0.0017	4.34E-06	7345	M35397.m	TRUE	reported	rvVJba	2	TRUE	0.002858	21.04617
4934	rs1005824	T	C	T	C	-0.0078	0.000546	0.4208	0.42116	FALSE	FALSE	FALSE	bKmDMa	0.014038	0.968972	486484	sepsis	TRUE	reported	textfile	0.0017	5.47E-06	7344	M32910.m	TRUE	reported	nAuPzO	2	TRUE	0.002858	21.04617
4960	rs3856584	T	C	T	C	0.0078	0.018767	0.3934	0.386015	FALSE	FALSE	FALSE	bKmDMa	0.014362	0.191312	486484	sepsis	TRUE	reported	textfile	0.0017	6.62E-06	7344	M32910.m	TRUE	reported	nAuPzO	2	TRUE	0.002858	21.04617
4969	rs7255053	A	C	A	C	0.0078	-0.02265	0.6266	0.635295	FALSE	FALSE	FALSE	bKmDMa	0.014935	0.129402	486484	sepsis	TRUE	reported	textfile	0.0017	6.36E-06	7344	M32910.m	TRUE	reported	nAuPzO	2	TRUE	0.002858	21.04617
4977	rs9371494	A	G	A	G	0.0078	0.00066	0.6805	0.684679	FALSE	FALSE	FALSE	bKmDMa	0.014764	0.964347	486484	sepsis	TRUE	reported	textfile	0.0017	7.38E-06	7344	M32910.m	TRUE	reported	nAuPzO	2	TRUE	0.002858	21.04617
2130	rs1196687	T	C	T	C	0.0078	-0.00529	0.6211	0.614948	FALSE	FALSE	FALSE	TEupfd	0.014047	0.706308	486484	sepsis	TRUE	reported	textfile	0.0017	5.82E-06	7331	M15676.m	TRUE	reported	NXiaPC	2	TRUE	0.002863	21.04616
4743	rs2870709	T	C	T	C	0.039	-0.0227	0.1734	0.192198	FALSE	FALSE	FALSE	oQPvO3	0.017368	0.191236	486484	sepsis	TRUE	reported	textfile	0.0085	4.20E-06	7330	M32786.m	TRUE	reported	Th0GcB	2	TRUE	0.002864	21.04616
1387	rs4768769	T	C	T	C	-0.0078	-0.02772	0.6012	0.592335	FALSE	FALSE	FALSE	KrWhws	0.013904	0.046159	486484	sepsis	TRUE	reported	textfile	0.0017	6.64E-06	7325	M02132.m	TRUE	reported	7pzpfK	2	TRUE	0.002866	21.04616
2845	rs1761664	A	G	A	G	-0.0078	-0.00612	0.763	0.746622	FALSE	FALSE	FALSE	Y5qWBm	0.015798	0.698469	486484	sepsis	TRUE	reported	textfile	0.0017	7.49E-06	7325	M20489.m	TRUE	reported	f2g9Su	2	TRUE	0.002866	21.04616
2851	rs428823	T	C	T	C	0.0078	0.015039	0.795	0.794397	FALSE	FALSE	FALSE	Y5qWBm	0.016885	0.373111	486484	sepsis	TRUE	reported	textfile	0.0017	6.48E-06	7325	M20489.m	TRUE	reported	f2g9Su	2	TRUE	0.002866	21.04616
4579	rs1273066	A	G	A	G	0.0078	0.009195	0.6775	0.681433	FALSE	FALSE	FALSE	Pzplqu	0.014729	0.532427	486484	sepsis	TRUE	reported	textfile	0.0017	7.23E-06	7318	M32740.m	TRUE	reported	FjEBS5	2	TRUE	0.002868	21.04615
4588	rs1992235	A	G	A	G	-0.0078	0.002381	0.3191	0.33093	FALSE	FALSE	FALSE	Pzplqu	0.014569	0.870152	486484	sepsis	TRUE	reported	textfile	0.0017	7.66E-06	7318	M32740.m	TRUE	reported	FjEBS5	2	TRUE	0.002868	21.04615
4607	rs6545816	A	C	A	C	-0.0078	-0.01471	0.4566	0.456945	FALSE	FALSE	FALSE	Pzplqu	0.013696	0.282706	486484	sepsis	TRUE	reported	textfile	0.0017	6.06E-06	7318	M32740.m	TRUE	reported	FjEBS5	2	TRUE	0.002868	21.04615
4611	rs743760	C	G	C	G	-0.0078	0.020404	0.3375	0.348797	FALSE	TRUE	FALSE	Pzplqu	0.014421	0.157101	486484	sepsis	TRUE	reported	textfile	0.0017	7.18E-06	7318	M32740.m	TRUE	reported	FjEBS5	2	TRUE	0.002868	21.04615
4614	rs890498	C	G	C	G	-0.0078	-0.00025	0.6478	0.662217	FALSE	TRUE	FALSE	Pzplqu	0.014481	0.986447	486484	sepsis	TRUE	reported	textfile	0.0017	7.23E-06	7318	M32740.m	TRUE	reported	FjEBS5	2	TRUE	0.002868	21.04615
1694	rs2705962	A	G	A	G	0.0078	-0.01874	0.8068	0.823108	FALSE	FALSE	FALSE	f01en0	0.017953	0.296558	486484	sepsis	TRUE	reported	textfile	0.0017	4.63E-06	7314	M12593.m	TRUE	reported	EAsM6U	2	TRUE	0.00287	21.04615
1695	rs6129758	T	G	T	G	0.0078	0.002847	0.843	0.839804	FALSE	FALSE	FALSE	f01en0	0.018654	0.87869	486484	sepsis	TRUE	reported	textfile	0.0017	7.88E-06	7314	M12593.m	TRUE	reported	EAsM6U	2	TRUE	0.00287	21.04615
3350	rs9836183	T	C	T	C	-0.0078	0.000408	0.2976	0.318458	FALSE	FALSE	FALSE	m66Eav	0.014754	0.977933	486484	sepsis	TRUE	reported	textfile	0.0017	6.82E-06	7303	M27256.m	TRUE	reported	pl2ECU	2	TRUE	0.002874	21.04614
1110	rs2019107	T	C	T	C	-0.0078	0.027643	0.6257	0.584142	FALSE	FALSE	FALSE	yahiGZ	0.013942	0.047405	486484	sepsis	TRUE	reported	textfile	0.0017	6.66E-06	7271	M01585.m	TRUE	reported	dwZ7vX	2	TRUE	0.002887	21.04611
1289	rs1085173	A	G	A	G	0.0078	-0.00822	0.6345	0.645826	FALSE	FALSE	FALSE	WQdpmo	0.014348	0.5665	486484	sepsis	TRUE	reported	textfile	0.0017	6.05E-06	7136	M01769.m	TRUE	reported	BkptOK	2	TRUE	0.002941	21.046
1305	rs3095571	A	G	A	G	0.0078	-0.01326	0.4465	0.439448	FALSE	FALSE	FALSE	WQdpmo	0.013848	0.338215	486484	sepsis	TRUE	reported	textfile	0.0017	6.74E-06	7136	M01769.m	TRUE	reported	BkptOK	2	TRUE	0.002941	21.046
1311	rs6539409	T	G	T	G	-0.0078	0.015934	0.5663	0.564176	FALSE	FALSE	FALSE	WQdpmo	0.013768	0.24714	486484	sepsis	TRUE	reported	textfile	0.0017	4.95E-06	7136	M01769.m	TRUE	reported	BkptOK	2	TRUE	0.002941	21.046
1317	rs7915877	T	C	T	C	-0.0078	0.005035	0.5509	0.567624	FALSE	FALSE	FALSE	WQdpmo	0.01379	0.715007	486484	sepsis	TRUE	reported	textfile	0.0017	4.42E-06	7136	M01769.m	TRUE	reported	BkptOK	2	TRUE	0.002941	21.046
8692	rs1173021	T	C	T	C	-0.0078	0.000196	0.495	0.505273	FALSE	FALSE	FALSE	Bg4VnB	0.013732	0.988633	486484	sepsis	TRUE	reported	textfile	0.0017	3.42E-06	7092	M36552.m	TRUE	reported	6zdDpC	2	TRUE	0.00296	21.04597
8704	rs6535384	A	G	A	G	0.0078	-0.00207	0.5194	0.52253	FALSE	FALSE	FALSE	Bg4VnB	0.013691	0.879684	486484	sepsis	TRUE	reported	textfile	0.0017	4.14E-06	7092	M36552.m	TRUE	reported	6zdDpC	2	TRUE	0.00296	21.04597
1472	rs1772037	T	C	T	C	-0.0078	-0.00306	0.4737	0.472843	FALSE	FALSE	FALSE	pkb876	0.013668	0.822813	486484	sepsis	TRUE	reported	textfile	0.0017	6.33E-06	7037	M02734.m	TRUE	reported	0LCMVI	2	TRUE	0.002983	21.04592
8092	rs1015501	A	G	A	G	-0.0078	-0.01403	0.3436	0.350826	FALSE	FALSE	FALSE	nNbXBS	0.014341	0.328023	486484	sepsis	TRUE	reported	textfile	0.0017	7.23E-06	5768	M35326.m	TRUE	reported	UNDorn	2	TRUE	0.003637	21.0446
8578	rs951918	A	G	A	G	0.0546	0.003835	0.8704	0.850105	FALSE	FALSE	FALSE	3VcRoA	0.019191	0.841616	486484	sepsis	TRUE	reported	textfile	0.0119	4.67E-06	5696	M36099.m	TRUE	reported	zHE146	2	TRUE	0.003682	21.04451
1529	rs2396726	T	C	T	C	-0.0179	-0.00339	0.5395	0.512755	FALSE	FALSE	FALSE	YvgTeW	0.013704	0.804363	486484	sepsis	TRUE	reported	textfile	0.0039	4.52E-06	1759	M03141.m	TRUE	reported	srKjSc	2	TRUE	0.011834	21.04179
8969	rs666518	T	C	T	C	-0.0523	0.028971	0.0891	0.098842	FALSE	FALSE	FALSE	2iAvlt	0.022902	0.205873	486484	sepsis	TRUE	reported	textfile	0.0114	4.24E-06	6934	M37190.m	TRUE	reported	hdOuHe	2	TRUE	0.003026	21.0411
5152	rs4784988	T	C	T	C	-0.8149	-0.03353	0.0216	0.014575	FALSE	FALSE	FALSE	L2DJzK	0.057466	0.559522	486484	sepsis	TRUE	reported	textfile	0.1753	3.35E-06	76	M33173.m	TRUE	reported	hRG70D	2	TRUE	0.221387	21.04083
7972	rs7987737	T	C	T	C	-0.0578	0.007372	0.986	0.987547	FALSE	FALSE	FALSE	zb8m4a	0.061531	0.904631	486484	sepsis	TRUE	reported	textfile	0.0126	4.48E-06	7261	M35253.m	TRUE	reported	tHnnnS	2	TRUE	0.00289	21.03754
8989	rs1252199	A	G	A	G	0.05	0.020783	0.9559	0.955883	FALSE	FALSE	FALSE	TfdxtP	0.03329	0.532418	486484	sepsis	TRUE	reported	textfile	0.0109	4.46E-06	7333	M37203.m	TRUE	reported	xicmWr	2	TRUE	0.002861	21.03626
2087	rs7687295	A	G	A	G	0.0211	-0.02243	0.0249	0.023914	FALSE	FALSE	FALSE	NPVH5j	0.045513	0.622095	486484	sepsis	TRUE	reported	textfile	0.0046	4.32E-06	7310	M15506.m	TRUE	reported	r7r3vM	2	TRUE	0.00287	21.03441
961	rs6034318	A	G	A	G	0.1083	-0.03023	0.013	0.00889	FALSE	FALSE	FALSE	tWoDq4	0.07323	0.679768	486484	sepsis	TRUE	reported	textfile	0.0236	4.52E-06	1650	M01508.m	TRUE	reported	6ltDw0	2	TRUE	0.012602	21.03324
3063	rs1078505	T	G	T	G	0.0211	0.02943	0.1146	0.124114	FALSE	FALSE	FALSE	e3izea	0.020771	0.156521	486484	sepsis	TRUE	reported	textfile	0.0046	4.81E-06	5676	M22032.m	TRUE	reported	zODKFI	2	TRUE	0.003693	21.03276
596	rs1447307	T	G	T	G	-0.0211	0.004007	0.227	0.21696	FALSE	FALSE	FALSE	KVK33e	0.016749	0.810905	486484	sepsis	TRUE	reported	textfile	0.0046	5.69E-06	5344	M01107.m	TRUE	reported	eAyR8w	2	TRUE	0.003922	21.0323
5707	rs1000643	T	C	T	C	-0.0211	0.003754	0.2626	0.262391	FALSE	FALSE	FALSE	XDem1i	0.015555	0.809274	486484	sepsis	TRUE	reported	textfile	0.0046	4.02E-06	4665	M33221.m	TRUE	reported	KBV6FH	2	TRUE	0.00449	21.03115
7980	rs1775140	T	G	T	G	-0.0477	-0.00545	0.0263	0.024871	FALSE	FALSE	FALSE	t02AUT	0.043907	0.901175	486484	sepsis	TRUE	reported	textfile	0.0104	4.52E-06	7100	M35254.m	TRUE	reported	uCIJaT	2	TRUE	0.002954	21.03041
6861	rs1080101	T	C	T	C	0.0156	-0.00335	0.2695	0.303932	FALSE	FALSE	FALSE	kFWZjs	0.014872	0.821953	486484	sepsis	TRUE	reported	textfile	0.0034	3.72E-06	1768	M34062.m	TRUE	reported	eLONsg	2	TRUE	0.011767	21.02809
3892	rs6957421	A	G	A	G	-0.0468	-0.00581	0.1573	0.172709	FALSE	FALSE	FALSE	bmXxuz	0.018087	0.747989	486484	sepsis	TRUE	reported	textfile	0.0102	4.57E-06	1766	M32405.m	TRUE	reported	A9BFZF	2	TRUE	0.01178	21.02806
8701	rs329033	A	C	A	C	-0.0133	0.016454	0.8429	0.844522	FALSE	FALSE	FALSE	Bg4VnB	0.018868	0.383195	486484	sepsis	TRUE	reported	textfile	0.0029	4.22E-06	7092	M36552.m	TRUE	reported	6zdDpC	2	TRUE	0.002957	21.02736
8747	rs2499690	A	G	A	G	-0.0766	-0.01233	0.9593	0.97253	FALSE	FALSE	FALSE	FnYrPc	0.041989	0.769038	486484	sepsis	TRUE	reported	textfile	0.0167	4.37E-06	3346	M36585.m	TRUE	reported	amHtMH	2	TRUE	0.006249	21.0264
4234	rs1341748	T	C	T	C	-0.0266	-0.01313	0.8097	0.798796	FALSE	FALSE	FALSE	wWEMr1	0.017046	0.441153	486484	sepsis	TRUE	reported	textfile	0.0058	5.43E-06	5955	M32593.m	TRUE	reported	Mh2g4i	2	TRUE	0.00352	21.02623
4766	rs1043189	A	G	A	G	-0.0454	-0.03523	0.0582	0.082788	FALSE	FALSE	FALSE	mOrnK4	0.024854	0.156349	486484	sepsis	TRUE	reported	textfile	0.0099	4.92E-06	6169	M32795.m	TRUE	reported	7EGxuf	2	TRUE	0.003397	21.02328
4673	rs1706104	T	C	T	C	-0.0642	0.01508	0.9677	0.966165	FALSE	FALSE	FALSE	xaapK0	0.037994	0.691439	486484	sepsis	TRUE	reported	textfile	0.014	4.71E-06	7250	M32757.m	TRUE	reported	64n1Z4	2	TRUE	0.002892	21.02297
7383	rs2688892	T	C	T	C	0.0688	0.028029	0.2396	0.243128	FALSE	FALSE	FALSE	iLnkG0	0.015987	0.079552	486484	sepsis	TRUE	reported	textfile	0.015	4.47E-06	2779	M34384.m	TRUE	reported	JVsZvV	2	TRUE	0.007513	21.02237
8867	rs1688884	T	C	T	C	0.2982	-0.05265	0.0145	0.01478	FALSE	FALSE	FALSE	nWuK1Q	0.056881	0.354626	486484	sepsis	TRUE	reported	textfile	0.065	4.43E-06	1583	M36850.m	TRUE	reported	2BA0SE	2	TRUE	0.013121	21.02033
3458	rs1328713	A	G	A	G	0.0656	-0.00128	0.8214	0.809836	FALSE	FALSE	FALSE	jRejFm	0.01744	0.941358	486484	sepsis	TRUE	reported	textfile	0.0143	4.80E-06	1720	M27716.m	TRUE	reported	tg7Rvj	2	TRUE	0.012087	21.01988
4158	rs1280416	C	G	C	G	0.0376	-0.00166	0.1235	0.140923	FALSE	TRUE	FALSE	stpu5O	0.019767	0.933058	486484	sepsis	TRUE	reported	textfile	0.0082	5.13E-06	7337	M32557.m	TRUE	reported	0oV8Ss	2	TRUE	0.002858	21.01985
6750	rs6537038	A	G	A	G	-0.0188	-0.00745	0.4014	0.386346	FALSE	FALSE	FALSE	NZVg9N	0.014367	0.603976	486484	sepsis	TRUE	reported	textfile	0.0041	4.93E-06	7320	M33941.m	TRUE	reported	kRWNNK	2	TRUE	0.002864	21.01984
7437	rs1091611	T	C	T	C	-0.1021	0.023253	0.8253	0.819601	FALSE	FALSE	FALSE	otAubi	0.017727	0.189621	486484	sepsis	TRUE	reported	textfile	0.0222	4.24E-06	314	M34404.m	TRUE	reported	SAZaNF	2	TRUE	0.063111	21.01699
3081	rs276166	T	G	T	G	-0.0243	-0.0443	0.1052	0.147483	FALSE	FALSE	FALSE	esOP8m	0.019932	0.026236	486484	sepsis	TRUE	reported	textfile	0.0053	5.33E-06	7329	M22116.m	TRUE	reported	fDMh7Z	2	TRUE	0.00286	21.01562
650	rs10489	A	C	A	C	0.1743	0.042889	0.0243	0.031444	FALSE	FALSE	FALSE	7BPGuQ	0.039524	0.277869	486484	sepsis	TRUE	reported	textfile	0.038	4.60E-06	1731	M01123.m	TRUE	reported	WV2hVn	2	TRUE	0.012008	21.01481
6392	rs3024298	A	C	A	C	0.0729	0.001308	0.9784	0.972733	FALSE	FALSE	FALSE	eBEF1t	0.042117	0.975222	486484	sepsis	TRUE	reported	textfile	0.0159	4.32E-06	6109	M33638.m	TRUE	reported	CRf2It	2	TRUE	0.003429	21.01448
8336	rs7706198	A	G	A	G	-0.0344	-0.01561	0.1782	0.195899	FALSE	FALSE	FALSE	o0GzT1	0.01727	0.366068	486484	sepsis	TRUE	reported	textfile	0.0075	4.55E-06	1750	M35631.m	TRUE	reported	G1XFx0	2	TRUE	0.011879	21.01347
3408	rs2002614	A	G	A	G	0.0651	0.087459	0.0114	0.013168	FALSE	FALSE	FALSE	VU62LG	0.059958	0.144656	486484	sepsis	TRUE	reported	textfile	0.0142	4.55E-06	7180	M27513.m	TRUE	reported	0didOx	2	TRUE	0.002919	21.01185
1677	rs4896370	A	G	A	G	0.0555	-0.03269	0.3542	0.407802	FALSE	FALSE	FALSE	IIFBGY	0.014043	0.019923	486484	sepsis	TRUE	reported	textfile	0.0121	4.19E-06	1543	M12261.m	TRUE	reported	F5Vlan	2	TRUE	0.013451	21.01125
8056	rs1001966	A	G	A	G	-0.0344	-0.00556	0.441	0.437302	FALSE	FALSE	FALSE	whAa18	0.013835	0.687945	486484	sepsis	TRUE	reported	textfile	0.0075	5.21E-06	1505	M35305.m	TRUE	reported	e6hvDm	2	TRUE	0.013786	21.00955
2546	rs735507	A	G	A	G	-0.1128	0.029841	0.8134	0.788652	FALSE	FALSE	FALSE	o0CFya	0.016732	0.074514	486484	sepsis	TRUE	reported	textfile	0.0246	4.60E-06	2433	M18281.m	TRUE	reported	pxbsnu	2	TRUE	0.008568	21.0083
8572	rs9616173	T	C	T	C	-0.0518	-0.02661	0.0924	0.087192	FALSE	FALSE	FALSE	Yjjldt	0.024216	0.271738	486484	sepsis	TRUE	reported	textfile	0.0113	4.33E-06	7054	M36098.m	TRUE	reported	U2ORsF	2	TRUE	0.00297	21.00775
7523	rs6824135	T	C	T	C	-0.0601	0.021238	0.1356	0.120854	FALSE	FALSE	FALSE	rp1LGA	0.02099	0.31162	486484	sepsis	TRUE	reported	textfile	0.0131	4.80E-06	997	M34441.m	TRUE	reported	SU47tz	2	TRUE	0.020675	21.00556
3396	rs5997236	T	C	T	C	0.0298	0.026985	0.4	0.399769	FALSE	FALSE	FALSE	frOZiy	0.01396	0.053234	486484	sepsis	TRUE	reported	textfile	0.0065	5.28E-06	2759	M27447.m	TRUE	reported	2z9fSv	2	TRUE	0.007561	21.00346
7	rs7394051	T	C	T	C	0.0055	0.005973	0.7014	0.711452	FALSE	FALSE	FALSE	0cebDu	0.015102	0.692445	486484	sepsis	TRUE	reported	textfile	0.0012	2.18E-06	7372	M00053.m	TRUE	reported	i8iuxX	2	TRUE	0.002841	21.00125
2635	rs2290140	A	G	A	G	-0.0165	0.003105	0.3486	0.343712	FALSE	FALSE	FALSE	UxuT5B	0.014429	0.829644	486484	sepsis	TRUE	reported	textfile	0.0036	5.80E-06	7367	M18467.m	TRUE	reported	eGQj6o	2	TRUE	0.002843	21.00124
2115	rs1051386	A	G	A	G	0.0055	0.004828	0.2661	0.261722	FALSE	FALSE	FALSE	t7SQ8V	0.015579	0.75666	486484	sepsis	TRUE	reported	textfile	0.0012	7.33E-06	7365	M15650.m	TRUE	reported	QQT7qA	2	TRUE	0.002844	21.00124
2119	rs369402	A	G	A	G	-0.0055	-0.00961	0.3892	0.391335	FALSE	FALSE	FALSE	t7SQ8V	0.014011	0.492724	486484	sepsis	TRUE	reported	textfile	0.0012	5.86E-06	7365	M15650.m	TRUE	reported	QQT7qA	2	TRUE	0.002844	21.00124
1257	rs1018155	A	G	A	G	-0.0055	-0.0245	0.296	0.290306	FALSE	FALSE	FALSE	6RGrj0	0.015048	0.103525	486484	sepsis	TRUE	reported	textfile	0.0012	3.88E-06	7359	M01649.m	TRUE	reported	J0vZCF	2	TRUE	0.002846	21.00124
446	rs2905873	A	C	A	C	0.0055	0.003691	0.6858	0.68569	FALSE	FALSE	FALSE	gcJMTM	0.014765	0.802588	486484	sepsis	TRUE	reported	textfile	0.0012	2.97E-06	7354	M00064.m	TRUE	reported	g2hUXe	2	TRUE	0.002848	21.00123
6768	rs3735474	A	G	A	G	-0.0165	0.027457	0.9341	0.931895	FALSE	FALSE	FALSE	Elf6MQ	0.027172	0.312258	486484	sepsis	TRUE	reported	textfile	0.0036	3.97E-06	7354	M33955.m	TRUE	reported	Lxn4RF	2	TRUE	0.002848	21.00123
3698	rs1715449	A	C	A	C	-0.011	-0.0339	0.1953	0.210964	FALSE	FALSE	FALSE	agPgtF	0.017044	0.046707	486484	sepsis	TRUE	reported	textfile	0.0024	6.29E-06	7349	M32315.m	TRUE	reported	hMRjcV	2	TRUE	0.00285	21.00123
1577	rs2921879	T	C	T	C	-0.0055	0.008941	0.5999	0.610004	FALSE	FALSE	FALSE	lxzgXW	0.014999	0.551088	486484	sepsis	TRUE	reported	textfile	0.0012	8.67E-06	7341	M11438.m	TRUE	reported	ZjFyyS	2	TRUE	0.002853	21.00122
1079	rs1286613	A	C	A	C	0.011	0.008326	0.2805	0.287668	FALSE	FALSE	FALSE	JLtEiZ	0.015129	0.582075	486484	sepsis	TRUE	reported	textfile	0.0024	5.62E-06	7333	M01572.m	TRUE	reported	TSSe28	2	TRUE	0.002857	21.00122
8987	rs1008223	T	C	T	C	-0.0385	0.055707	0.0735	0.071632	FALSE	FALSE	FALSE	TfdxtP	0.026548	0.035875	486484	sepsis	TRUE	reported	textfile	0.0084	4.09E-06	7333	M37203.m	TRUE	reported	xicmWr	2	TRUE	0.002857	21.00122
4587	rs1776158	A	G	A	G	0.011	-0.01185	0.8209	0.80895	FALSE	FALSE	FALSE	Pzplqu	0.017365	0.495145	486484	sepsis	TRUE	reported	textfile	0.0024	6.91E-06	7318	M32740.m	TRUE	reported	FjEBS5	2	TRUE	0.002862	21.0012
920	rs4764034	A	G	A	G	-0.011	0.014014	0.6869	0.698305	FALSE	FALSE	FALSE	rVMAPf	0.014895	0.346791	486484	sepsis	TRUE	reported	textfile	0.0024	7.67E-06	7307	M01493.m	TRUE	reported	siVqDi	2	TRUE	0.002867	21.00119
3324	rs1120115	T	C	T	C	0.022	-0.02785	0.0306	0.035472	FALSE	FALSE	FALSE	m66Eav	0.037131	0.453213	486484	sepsis	TRUE	reported	textfile	0.0048	5.42E-06	7303	M27256.m	TRUE	reported	pl2ECU	2	TRUE	0.002868	21.00119
7477	rs2043422	A	G	A	G	0.0165	-0.03423	0.2928	0.290757	FALSE	FALSE	FALSE	SqfdcK	0.015061	0.023039	486484	sepsis	TRUE	reported	textfile	0.0036	4.06E-06	6929	M34416.m	TRUE	reported	sRGRIK	2	TRUE	0.003023	21.00088
8796	rs3794989	T	C	T	C	0.0165	0.02433	0.3285	0.333407	FALSE	FALSE	FALSE	0YKkSS	0.014485	0.093019	486484	sepsis	TRUE	reported	textfile	0.0036	4.95E-06	6876	M36754.m	TRUE	reported	cf9IBu	2	TRUE	0.003046	21.00083
3307	rs1429219	A	G	A	G	0.033	-0.03877	0.0353	0.033392	FALSE	FALSE	FALSE	nqmBts	0.03888	0.318716	486484	sepsis	TRUE	reported	textfile	0.0072	4.53E-06	6388	M25599.m	TRUE	reported	VkwSh1	2	TRUE	0.003278	21.00037
7077	rs1685327	T	G	T	G	0.0298	0.018905	0.8831	0.876707	FALSE	FALSE	FALSE	sg7nOB	0.020998	0.367937	486484	sepsis	TRUE	reported	textfile	0.0065	5.04E-06	2278	M34289.m	TRUE	reported	KemvIC	2	TRUE	0.009142	21.00024
3057	rs674635	T	C	T	C	0.0275	0.04293	0.0415	0.042184	FALSE	FALSE	FALSE	H6vcCG	0.034033	0.207162	486484	sepsis	TRUE	reported	textfile	0.006	3.96E-06	6180	M22030.m	TRUE	reported	XetVX7	2	TRUE	0.003388	21.00015
1422	rs1095303	T	C	T	C	0.022	0.034791	0.8497	0.844183	FALSE	FALSE	FALSE	ac6RGB	0.018929	0.066065	486484	sepsis	TRUE	reported	textfile	0.0048	4.12E-06	5791	M02342.m	TRUE	reported	dC6Rkp	2	TRUE	0.003614	20.99969
6107	rs1978273	A	G	A	G	-0.0165	0.00082	0.4362	0.452449	FALSE	FALSE	FALSE	a0osmD	0.013702	0.95226	486484	sepsis	TRUE	reported	textfile	0.0036	3.51E-06	5717	M33453.m	TRUE	reported	CePFnK	2	TRUE	0.003661	20.9996
8303	rs6942053	C	G	C	G	0.033	-0.00849	0.4176	0.410875	FALSE	TRUE	FALSE	HELgy7	0.013937	0.542487	486484	sepsis	TRUE	reported	textfile	0.0072	4.77E-06	5035	M35551.m	TRUE	reported	7x7uUn	2	TRUE	0.004155	20.9986
9063	rs6089828	T	C	T	C	-0.0165	-0.03564	0.6506	0.645915	FALSE	FALSE	FALSE	Wn3UAw	0.014289	0.012628	486484	sepsis	TRUE	reported	textfile	0.0036	5.86E-06	4754	M38150.m	TRUE	reported	IRKGRs	2	TRUE	0.004399	20.99811
5241	rs1195793	A	G	A	G	-0.2362	-0.03982	0.0459	0.063176	FALSE	FALSE	FALSE	q32d4E	0.028282	0.159189	486484	sepsis	TRUE	reported	textfile	0.0515	4.58E-06	1017	M33178.m	TRUE	reported	cS3BwT	2	TRUE	0.020264	20.99377
2638	rs7005406	T	C	T	C	0.0353	0.020629	0.8036	0.814562	FALSE	FALSE	FALSE	UxuT5B	0.0177	0.243836	486484	sepsis	TRUE	reported	textfile	0.0077	5.29E-06	1767	M18467.m	TRUE	reported	eGQj6o	2	TRUE	0.011754	20.99308
8500	rs4719788	T	C	T	C	-0.0926	0.010359	0.0317	0.022708	FALSE	FALSE	FALSE	4MCEPy	0.045869	0.821334	486484	sepsis	TRUE	reported	textfile	0.0202	4.59E-06	1760	M35977.m	TRUE	reported	B3kU6y	2	TRUE	0.011799	20.99063
7406	rs8069788	T	C	T	C	-0.0486	0.002843	0.4918	0.481973	FALSE	FALSE	FALSE	XfpKv0	0.013872	0.837607	486484	sepsis	TRUE	reported	textfile	0.0106	4.25E-06	1312	M34390.m	TRUE	reported	mmOsbK	2	TRUE	0.01577	20.98932
3419	rs9319010	A	G	A	G	0.1201	0.051973	0.0156	0.012734	FALSE	FALSE	FALSE	VU62LG	0.061467	0.397808	486484	sepsis	TRUE	reported	textfile	0.0262	4.44E-06	1738	M27513.m	TRUE	reported	0didOx	2	TRUE	0.011946	20.9886
6650	rs1742061	C	G	C	G	0.0307	0.012544	0.0545	0.063345	FALSE	TRUE	FALSE	REeQiq	0.028101	0.655312	486484	sepsis	TRUE	reported	textfile	0.0067	4.16E-06	6004	M33892.m	TRUE	reported	cVOa3u	2	TRUE	0.003485	20.98855
7673	rs1490956	A	G	A	G	0.0362	-0.0098	0.8464	0.814513	FALSE	FALSE	FALSE	iqkM9B	0.019244	0.610685	486484	sepsis	TRUE	reported	textfile	0.0079	4.02E-06	4800	M34539.m	TRUE	reported	5nFq6f	2	TRUE	0.004355	20.98853
5610	rs9672326	T	G	T	G	0.155	-0.03244	0.9622	0.978902	FALSE	FALSE	FALSE	q32d4E	0.047674	0.496272	486484	sepsis	TRUE	reported	textfile	0.0338	4.44E-06	1017	M33178.m	TRUE	reported	cS3BwT	2	TRUE	0.020259	20.98819
2627	rs7927424	A	G	A	G	-0.115	-0.06001	0.0147	0.021186	FALSE	FALSE	FALSE	eygBBR	0.048109	0.212289	486484	sepsis	TRUE	reported	textfile	0.0251	4.79E-06	6970	M18394.m	TRUE	reported	hD79iE	2	TRUE	0.003003	20.98571
3099	rs4799200	A	G	A	G	0.0252	-0.01973	0.0528	0.056035	FALSE	FALSE	FALSE	mr0awv	0.029755	0.50726	486484	sepsis	TRUE	reported	textfile	0.0055	3.79E-06	5702	M22130.m	TRUE	reported	Y2TfWQ	2	TRUE	0.003668	20.98569
8947	rs7829309	A	G	A	G	0.0252	-0.00888	0.424	0.423589	FALSE	FALSE	FALSE	mXJP3h	0.013988	0.52544	486484	sepsis	TRUE	reported	textfile	0.0055	4.07E-06	4520	M37104.m	TRUE	reported	HRZBak	2	TRUE	0.004623	20.98377
924	rs1089051	T	C	T	C	-0.0197	-0.00445	0.0433	0.045729	FALSE	FALSE	FALSE	tv99UN	0.033164	0.893159	486484	sepsis	TRUE	reported	textfile	0.0043	3.45E-06	7354	M01494.m	TRUE	reported	osDclp	2	TRUE	0.002846	20.98348
8874	rs1004587	T	G	T	G	-0.0935	0.002892	0.0213	0.028907	FALSE	FALSE	FALSE	Nv6gDO	0.040752	0.943427	486484	sepsis	TRUE	reported	textfile	0.0204	4.78E-06	1782	M37004.m	TRUE	reported	RqmQdg	2	TRUE	0.011651	20.98337
3978	rs1701542	C	G	C	G	-0.022	-0.02759	0.5942	0.600493	FALSE	TRUE	FALSE	XdJbEU	0.013983	0.048465	486484	sepsis	TRUE	reported	textfile	0.0048	4.10E-06	1766	M32452.m	TRUE	reported	V5xWKi	2	TRUE	0.011755	20.98315
8647	rs1557339	A	C	A	C	0.0275	-0.00457	0.7675	0.750841	FALSE	FALSE	FALSE	GgPG6k	0.015933	0.774077	486484	sepsis	TRUE	reported	textfile	0.006	4.42E-06	1732	M36300.m	TRUE	reported	kT1yso	2	TRUE	0.011983	20.98269
7074	rs1322876	C	G	C	G	-0.0582	-0.01373	0.0663	0.217435	FALSE	TRUE	FALSE	sg7nOB	0.016602	0.408303	486484	sepsis	TRUE	reported	textfile	0.0127	4.71E-06	2278	M34289.m	TRUE	reported	KemvIC	2	TRUE	0.009135	20.98249
1411	rs280194	T	C	T	C	-0.0197	-0.00626	0.7311	0.734181	FALSE	FALSE	FALSE	33ptwk	0.015512	0.686515	486484	sepsis	TRUE	reported	textfile	0.0043	5.77E-06	6252	M02137.m	TRUE	reported	SQY1na	2	TRUE	0.003346	20.98247
7913	rs1693551	T	C	T	C	-0.0197	-0.0165	0.5342	0.537808	FALSE	FALSE	FALSE	8qXKkX	0.013706	0.228649	486484	sepsis	TRUE	reported	textfile	0.0043	4.79E-06	6234	M35189.m	TRUE	reported	Nt6oaC	2	TRUE	0.003356	20.98245
5653	rs9892714	T	C	T	C	0.1324	0.032776	0.031	0.031981	FALSE	FALSE	FALSE	BFsMtm	0.038811	0.39838	486484	sepsis	TRUE	reported	textfile	0.0289	4.67E-06	5716	M33192.m	TRUE	reported	NAJN3D	2	TRUE	0.003658	20.9811
5567	rs7942841	A	G	A	G	-0.0917	0.008386	0.8086	0.800411	FALSE	FALSE	FALSE	q32d4E	0.017629	0.634287	486484	sepsis	TRUE	reported	textfile	0.02	4.42E-06	1017	M33178.m	TRUE	reported	cS3BwT	2	TRUE	0.020252	20.98088
8825	rs1728624	A	G	A	G	0.0197	0.024712	0.7403	0.735932	FALSE	FALSE	FALSE	zuPm7L	0.015495	0.110759	486484	sepsis	TRUE	reported	textfile	0.0043	4.25E-06	4317	M36802.m	TRUE	reported	cn9d0n	2	TRUE	0.004838	20.97946
6686	rs1159426	A	G	A	G	0.0339	-0.00253	0.0971	0.089891	FALSE	FALSE	FALSE	23zCgC	0.024018	0.916116	486484	sepsis	TRUE	reported	textfile	0.0074	5.22E-06	5534	M33910.m	TRUE	reported	ACI3CA	2	TRUE	0.003778	20.97872
851	rs2017823	A	G	A	G	0.0142	0.005357	0.841	0.832639	FALSE	FALSE	FALSE	1tFtaf	0.018286	0.769547	486484	sepsis	TRUE	reported	textfile	0.0031	4.85E-06	7323	M01359.m	TRUE	reported	qq3q6E	2	TRUE	0.002857	20.97658
8510	rs1692839	T	C	T	C	0.0623	0.027779	0.9707	0.97468	FALSE	FALSE	FALSE	vHXktL	0.043408	0.522199	486484	sepsis	TRUE	reported	textfile	0.0136	4.57E-06	4529	M35978.m	TRUE	reported	ceap9w	2	TRUE	0.004612	20.97522
8597	rs1699291	A	G	A	G	0.0646	0.023326	0.0528	0.057414	FALSE	FALSE	FALSE	t5potg	0.029567	0.430159	486484	sepsis	TRUE	reported	textfile	0.0141	4.79E-06	2709	M36115.m	TRUE	reported	SSOHIA	2	TRUE	0.007689	20.9752
2262	rs6854832	T	C	T	C	0.0472	0.007852	0.1513	0.138124	FALSE	FALSE	FALSE	Pb8tMF	0.019803	0.691735	486484	sepsis	TRUE	reported	textfile	0.0103	4.98E-06	1721	M15996.m	TRUE	reported	O7IDgO	2	TRUE	0.012055	20.97512
5146	rs265795	C	G	C	G	-0.2845	0.002804	0.1061	0.044722	FALSE	TRUE	FALSE	L2DJzK	0.033227	0.932759	486484	sepsis	TRUE	reported	textfile	0.0613	3.51E-06	76	M33173.m	TRUE	reported	hRG70D	2	TRUE	0.220832	20.97306
8828	rs327837	A	G	A	G	0.0142	-0.02191	0.376	0.38559	FALSE	FALSE	FALSE	zuPm7L	0.014051	0.118905	486484	sepsis	TRUE	reported	textfile	0.0031	4.52E-06	4317	M36802.m	TRUE	reported	cn9d0n	2	TRUE	0.004837	20.97259
879	rs1016415	A	G	A	G	0.0229	0.014733	0.0577	0.059369	FALSE	FALSE	FALSE	oi7KQI	0.028957	0.610905	486484	sepsis	TRUE	reported	textfile	0.005	4.18E-06	7362	M01365.m	TRUE	reported	7NWGKA	2	TRUE	0.002841	20.9707
7467	rs254488	A	G	A	G	-0.0229	-0.02344	0.1157	0.105241	FALSE	FALSE	FALSE	kasr9a	0.022245	0.292014	486484	sepsis	TRUE	reported	textfile	0.005	4.41E-06	6768	M34409.m	TRUE	reported	3VaBw3	2	TRUE	0.00309	20.9702
7169	rs472038	C	G	C	G	0.111	-0.03038	0.077	0.082054	FALSE	TRUE	FALSE	VfvTtz	0.025007	0.224371	486484	sepsis	TRUE	reported	textfile	0.0242	4.49E-06	613	M34329.m	TRUE	reported	egac6r	2	TRUE	0.033182	20.96988
8154	rs1010813	T	C	T	C	0.0142	-0.00724	0.6059	0.595083	FALSE	FALSE	FALSE	sRlsb6	0.013968	0.604081	486484	sepsis	TRUE	reported	textfile	0.0031	6.45E-06	3007	M35331.m	TRUE	reported	GPijIC	2	TRUE	0.006929	20.96835
4149	rs1762801	A	G	A	G	0.0316	-0.00913	0.1561	0.168712	FALSE	FALSE	FALSE	CrVaiQ	0.018292	0.617691	486484	sepsis	TRUE	reported	textfile	0.0069	4.12E-06	7361	M32553.m	TRUE	reported	HsKdAI	2	TRUE	0.002841	20.96805
5703	rs7151750	C	G	C	G	-0.0142	-0.00548	0.5979	0.60979	FALSE	TRUE	FALSE	1SbQTe	0.014005	0.695838	486484	sepsis	TRUE	reported	textfile	0.0031	4.12E-06	2683	M33204.m	TRUE	reported	x7MBDH	2	TRUE	0.00776	20.96667
4702	rs1688958	C	G	C	G	0.0719	-0.05562	0.0382	0.07979	FALSE	TRUE	FALSE	WlwKaF	0.026076	0.032917	486484	sepsis	TRUE	reported	textfile	0.0157	5.00E-06	6712	M32759.m	TRUE	reported	Ub4yxH	2	TRUE	0.003115	20.96665
6306	rs6465164	A	G	A	G	-0.0394	-0.00203	0.1492	0.156421	FALSE	FALSE	FALSE	ORVTMc	0.018853	0.914109	486484	sepsis	TRUE	reported	textfile	0.0086	5.06E-06	1768	M33587.m	TRUE	reported	57AsXW	2	TRUE	0.011732	20.96544
281	rs2373642	A	G	A	G	-0.0536	-0.00037	0.0138	0.036064	FALSE	FALSE	FALSE	tbRL8J	0.036895	0.992001	486484	sepsis	TRUE	reported	textfile	0.0117	4.72E-06	1762	M00060.m	TRUE	reported	oOvRKT	2	TRUE	0.011771	20.96354
6868	rs1710548	A	G	A	G	0.0087	-0.01125	0.8711	0.868576	FALSE	FALSE	FALSE	kFWZjs	0.020305	0.579609	486484	sepsis	TRUE	reported	textfile	0.0019	3.10E-06	7362	M34062.m	TRUE	reported	eLONsg	2	TRUE	0.00284	20.96106
3882	rs1172181	T	C	T	C	0.0174	-0.00874	0.6543	0.667438	FALSE	FALSE	FALSE	bmXxuz	0.014538	0.547858	486484	sepsis	TRUE	reported	textfile	0.0038	3.42E-06	7358	M32405.m	TRUE	reported	A9BFZF	2	TRUE	0.002841	20.96106
8369	rs1215192	T	C	T	C	-0.0087	0.010433	0.7465	0.746343	FALSE	FALSE	FALSE	SCzh0A	0.015784	0.508638	486484	sepsis	TRUE	reported	textfile	0.0019	3.53E-06	7348	M35675.m	TRUE	reported	n1SREk	2	TRUE	0.002845	20.96105
8397	rs679959	A	G	A	G	0.0087	0.008597	0.3159	0.31671	FALSE	FALSE	FALSE	SCzh0A	0.014773	0.560574	486484	sepsis	TRUE	reported	textfile	0.0019	2.79E-06	7348	M35675.m	TRUE	reported	n1SREk	2	TRUE	0.002845	20.96105
3297	rs884518	A	G	A	G	-0.0087	0.006181	0.5041	0.523873	FALSE	FALSE	FALSE	vYWPe9	0.013684	0.651514	486484	sepsis	TRUE	reported	textfile	0.0019	4.78E-06	7340	M25459.m	TRUE	reported	VvJ6Vx	2	TRUE	0.002848	20.96105
5860	rs1094924	T	C	T	C	0.0087	-0.00211	0.5212	0.509458	FALSE	FALSE	FALSE	tqW0ld	0.013729	0.877711	486484	sepsis	TRUE	reported	textfile	0.0019	3.69E-06	7315	M33389.m	TRUE	reported	Sv7EyV	2	TRUE	0.002858	20.96103
5863	rs1171706	T	C	T	C	-0.0087	-0.00694	0.6324	0.617024	FALSE	FALSE	FALSE	tqW0ld	0.014071	0.621901	486484	sepsis	TRUE	reported	textfile	0.0019	2.94E-06	7315	M33389.m	TRUE	reported	Sv7EyV	2	TRUE	0.002858	20.96103
5877	rs33131	A	G	A	G	-0.0087	-0.00249	0.669	0.660696	FALSE	FALSE	FALSE	tqW0ld	0.014541	0.864063	486484	sepsis	TRUE	reported	textfile	0.0019	3.12E-06	7315	M33389.m	TRUE	reported	Sv7EyV	2	TRUE	0.002858	20.96103
5887	rs52701	T	C	T	C	0.0087	0.013648	0.3727	0.368437	FALSE	FALSE	FALSE	tqW0ld	0.01422	0.337173	486484	sepsis	TRUE	reported	textfile	0.0019	2.69E-06	7315	M33389.m	TRUE	reported	Sv7EyV	2	TRUE	0.002858	20.96103
5896	rs7435399	T	C	T	C	0.0087	0.01365	0.66	0.658797	FALSE	FALSE	FALSE	tqW0ld	0.014464	0.345322	486484	sepsis	TRUE	reported	textfile	0.0019	2.48E-06	7315	M33389.m	TRUE	reported	Sv7EyV	2	TRUE	0.002858	20.96103
5900	rs9375723	T	C	T	C	0.0087	0.020855	0.2664	0.258632	FALSE	FALSE	FALSE	tqW0ld	0.015699	0.184026	486484	sepsis	TRUE	reported	textfile	0.0019	3.39E-06	7315	M33389.m	TRUE	reported	Sv7EyV	2	TRUE	0.002858	20.96103
5904	rs962423	A	C	A	C	-0.0087	0.012088	0.4645	0.469667	FALSE	FALSE	FALSE	tqW0ld	0.013673	0.376651	486484	sepsis	TRUE	reported	textfile	0.0019	4.27E-06	7315	M33389.m	TRUE	reported	Sv7EyV	2	TRUE	0.002858	20.96103
2205	rs1791600	T	C	T	C	-0.0087	0.018328	0.2072	0.201672	FALSE	FALSE	FALSE	Bf6uMK	0.017024	0.281653	486484	sepsis	TRUE	reported	textfile	0.0019	4.09E-06	7308	M15778.m	TRUE	reported	jowM2N	2	TRUE	0.002861	20.96102
6129	rs1274976	A	G	A	G	-0.0087	0.013736	0.271	0.280009	FALSE	FALSE	FALSE	MiSJly	0.015206	0.366362	486484	sepsis	TRUE	reported	textfile	0.0019	5.49E-06	7307	M33477.m	TRUE	reported	WwL1hq	2	TRUE	0.002861	20.96102
6147	rs6135736	A	G	A	G	-0.0087	-0.00499	0.248	0.247043	FALSE	FALSE	FALSE	MiSJly	0.016011	0.755214	486484	sepsis	TRUE	reported	textfile	0.0019	2.78E-06	7307	M33477.m	TRUE	reported	WwL1hq	2	TRUE	0.002861	20.96102
3185	rs6738800	A	G	A	G	0.0261	0.013555	0.1176	0.163401	FALSE	FALSE	FALSE	gvZCBj	0.018761	0.469969	486484	sepsis	TRUE	reported	textfile	0.0057	5.20E-06	7259	M22189.m	TRUE	reported	GKqg2h	2	TRUE	0.00288	20.96098
5837	rs7624812	T	C	T	C	-0.0087	0.025122	0.1734	0.182286	FALSE	FALSE	FALSE	R5joEf	0.01771	0.156027	486484	sepsis	TRUE	reported	textfile	0.0019	2.77E-06	7118	M33380.m	TRUE	reported	kRvbfZ	2	TRUE	0.002937	20.96087
3540	rs8081052	A	G	A	G	0.0174	-0.01959	0.1073	0.109809	FALSE	FALSE	FALSE	rXzSlH	0.021932	0.371634	486484	sepsis	TRUE	reported	textfile	0.0038	4.64E-06	6795	M30805.m	TRUE	reported	7wg9QH	2	TRUE	0.003076	20.96059
8481	rs9563725	T	C	T	C	-0.0087	-0.02715	0.4437	0.45296	FALSE	FALSE	FALSE	uMajV6	0.013723	0.047848	486484	sepsis	TRUE	reported	textfile	0.0019	6.02E-06	6604	M35754.m	TRUE	reported	b8QB7S	2	TRUE	0.003165	20.96041
8482	rs970719	T	C	T	C	0.0087	0.012257	0.4248	0.425079	FALSE	FALSE	FALSE	uMajV6	0.013866	0.376725	486484	sepsis	TRUE	reported	textfile	0.0019	6.44E-06	6604	M35754.m	TRUE	reported	b8QB7S	2	TRUE	0.003165	20.96041
8907	rs267939	T	C	T	C	-0.0087	-0.01152	0.6348	0.6463	FALSE	FALSE	FALSE	eLhsld	0.014319	0.421297	486484	sepsis	TRUE	reported	textfile	0.0019	2.59E-06	6552	M37058.m	TRUE	reported	RSeWE5	2	TRUE	0.00319	20.96036
8916	rs6470127	A	G	A	G	0.0087	0.020576	0.6245	0.613502	FALSE	FALSE	FALSE	eLhsld	0.014044	0.142887	486484	sepsis	TRUE	reported	textfile	0.0019	2.87E-06	6552	M37058.m	TRUE	reported	RSeWE5	2	TRUE	0.00319	20.96036
3120	rs1016245	T	C	T	C	0.0087	0.00349	0.7178	0.717951	FALSE	FALSE	FALSE	JiXR2t	0.015201	0.818413	486484	sepsis	TRUE	reported	textfile	0.0019	8.50E-06	6535	M22177.m	TRUE	reported	joi6p6	2	TRUE	0.003198	20.96034
3139	rs2256490	A	T	A	T	0.0087	0.013466	0.2844	0.29189	FALSE	TRUE	FALSE	JiXR2t	0.015025	0.370148	486484	sepsis	TRUE	reported	textfile	0.0019	7.73E-06	6535	M22177.m	TRUE	reported	joi6p6	2	TRUE	0.003198	20.96034
3144	rs2781537	T	C	T	C	0.0087	-0.00645	0.5547	0.574356	FALSE	FALSE	FALSE	JiXR2t	0.013964	0.643984	486484	sepsis	TRUE	reported	textfile	0.0019	7.72E-06	6535	M22177.m	TRUE	reported	joi6p6	2	TRUE	0.003198	20.96034
3149	rs3845671	C	G	C	G	-0.0087	0.002879	0.4044	0.408374	FALSE	TRUE	FALSE	JiXR2t	0.013906	0.83598	486484	sepsis	TRUE	reported	textfile	0.0019	7.34E-06	6535	M22177.m	TRUE	reported	joi6p6	2	TRUE	0.003198	20.96034
3150	rs4147331	A	G	A	G	0.0087	0.009759	0.393	0.396645	FALSE	FALSE	FALSE	JiXR2t	0.014007	0.485999	486484	sepsis	TRUE	reported	textfile	0.0019	7.47E-06	6535	M22177.m	TRUE	reported	joi6p6	2	TRUE	0.003198	20.96034
3151	rs4298477	T	C	T	C	0.0087	-0.01118	0.534	0.546501	FALSE	FALSE	FALSE	JiXR2t	0.013768	0.416718	486484	sepsis	TRUE	reported	textfile	0.0019	8.40E-06	6535	M22177.m	TRUE	reported	joi6p6	2	TRUE	0.003198	20.96034
3167	rs7194277	C	G	C	G	-0.0087	-0.01165	0.6775	0.676108	FALSE	TRUE	FALSE	JiXR2t	0.014678	0.427196	486484	sepsis	TRUE	reported	textfile	0.0019	8.33E-06	6535	M22177.m	TRUE	reported	joi6p6	2	TRUE	0.003198	20.96034
3170	rs7677906	T	C	T	C	0.0087	0.007366	0.5094	0.506111	FALSE	FALSE	FALSE	JiXR2t	0.0137	0.590776	486484	sepsis	TRUE	reported	textfile	0.0019	7.07E-06	6535	M22177.m	TRUE	reported	joi6p6	2	TRUE	0.003198	20.96034
987	rs1012282	T	G	T	G	0.0087	-0.02922	0.3503	0.334612	FALSE	FALSE	FALSE	jfkZbM	0.014488	0.043708	486484	sepsis	TRUE	reported	textfile	0.0019	2.79E-06	6523	M01558.m	TRUE	reported	LFZvMG	2	TRUE	0.003204	20.96033
4509	rs1074115	T	C	T	C	-0.0087	0.003201	0.3902	0.386214	FALSE	FALSE	FALSE	r0qJaM	0.014042	0.819688	486484	sepsis	TRUE	reported	textfile	0.0019	4.02E-06	6497	M32729.m	TRUE	reported	vYv5FI	2	TRUE	0.003217	20.9603
4516	rs1243588	A	G	A	G	-0.0087	0.015072	0.4175	0.411269	FALSE	FALSE	FALSE	r0qJaM	0.013942	0.279675	486484	sepsis	TRUE	reported	textfile	0.0019	4.04E-06	6497	M32729.m	TRUE	reported	vYv5FI	2	TRUE	0.003217	20.9603
7757	rs922331	T	C	T	C	0.0087	0.029992	0.3698	0.370985	FALSE	FALSE	FALSE	HyMNYM	0.014517	0.038833	486484	sepsis	TRUE	reported	textfile	0.0019	6.14E-06	5885	M34912.m	TRUE	reported	TI8Hcw	2	TRUE	0.00355	20.95963
7813	rs1106609	T	C	T	C	0.0087	-0.00398	0.6756	0.67066	FALSE	FALSE	FALSE	h5whIS	0.014622	0.785568	486484	sepsis	TRUE	reported	textfile	0.0019	4.81E-06	4900	M35137.m	TRUE	reported	xVITKF	2	TRUE	0.004261	20.9582
7819	rs1464136	A	C	A	C	0.0087	0.021377	0.7294	0.7336	FALSE	FALSE	FALSE	h5whIS	0.015438	0.16614	486484	sepsis	TRUE	reported	textfile	0.0019	5.32E-06	4900	M35137.m	TRUE	reported	xVITKF	2	TRUE	0.004261	20.9582
7825	rs2064443	A	G	A	G	-0.0087	-0.02664	0.5417	0.540037	FALSE	FALSE	FALSE	h5whIS	0.013789	0.053345	486484	sepsis	TRUE	reported	textfile	0.0019	4.22E-06	4900	M35137.m	TRUE	reported	xVITKF	2	TRUE	0.004261	20.9582
7826	rs2214626	C	G	C	G	-0.0087	-0.00431	0.6223	0.607942	FALSE	TRUE	FALSE	h5whIS	0.014089	0.759716	486484	sepsis	TRUE	reported	textfile	0.0019	4.28E-06	4900	M35137.m	TRUE	reported	xVITKF	2	TRUE	0.004261	20.9582
7834	rs3801969	T	G	T	G	-0.0087	0.00341	0.4459	0.436985	FALSE	FALSE	FALSE	h5whIS	0.013828	0.805235	486484	sepsis	TRUE	reported	textfile	0.0019	4.72E-06	4900	M35137.m	TRUE	reported	xVITKF	2	TRUE	0.004261	20.9582
7838	rs4326611	T	C	T	C	-0.0087	0.007693	0.4285	0.456629	FALSE	FALSE	FALSE	h5whIS	0.01378	0.576647	486484	sepsis	TRUE	reported	textfile	0.0019	6.06E-06	4900	M35137.m	TRUE	reported	xVITKF	2	TRUE	0.004261	20.9582
7846	rs618791	T	C	T	C	-0.0087	-0.00858	0.6833	0.680107	FALSE	FALSE	FALSE	h5whIS	0.014712	0.559716	486484	sepsis	TRUE	reported	textfile	0.0019	4.42E-06	4900	M35137.m	TRUE	reported	xVITKF	2	TRUE	0.004261	20.9582
7859	rs7505882	T	C	T	C	-0.0087	-0.00035	0.4807	0.491426	FALSE	FALSE	FALSE	h5whIS	0.013779	0.979898	486484	sepsis	TRUE	reported	textfile	0.0019	5.43E-06	4900	M35137.m	TRUE	reported	xVITKF	2	TRUE	0.004261	20.9582
7865	rs820140	T	C	T	C	-0.0087	-0.00157	0.4898	0.48135	FALSE	FALSE	FALSE	h5whIS	0.013819	0.909685	486484	sepsis	TRUE	reported	textfile	0.0019	4.65E-06	4900	M35137.m	TRUE	reported	xVITKF	2	TRUE	0.004261	20.9582
4677	rs2852424	A	G	A	G	0.0554	-0.02639	0.9371	0.92026	FALSE	FALSE	FALSE	xaapK0	0.025244	0.295929	486484	sepsis	TRUE	reported	textfile	0.0121	4.71E-06	7250	M32757.m	TRUE	reported	64n1Z4	2	TRUE	0.002883	20.95699
8798	rs1042565	A	G	A	G	-0.0522	0.061732	0.9597	0.961852	FALSE	FALSE	FALSE	QWTooc	0.035887	0.0854	486484	sepsis	TRUE	reported	textfile	0.0114	4.96E-06	3248	M36756.m	TRUE	reported	OLsw6C	2	TRUE	0.006414	20.95385
3373	rs1314945	T	C	T	C	-0.0293	0.030601	0.0539	0.066042	FALSE	FALSE	FALSE	O2JtzH	0.027857	0.271976	486484	sepsis	TRUE	reported	textfile	0.0064	4.34E-06	7347	M27278.m	TRUE	reported	fxCIPW	2	TRUE	0.002845	20.95352
8596	rs1261709	T	C	T	C	-0.0348	-0.02274	0.4512	0.396502	FALSE	FALSE	FALSE	t5potg	0.014464	0.115872	486484	sepsis	TRUE	reported	textfile	0.0076	4.81E-06	2709	M36115.m	TRUE	reported	SSOHIA	2	TRUE	0.00768	20.95128
8611	rs2254145	A	G	A	G	-0.0316	0.024567	0.2138	0.21433	FALSE	FALSE	FALSE	oHM5TD	0.016672	0.140598	486484	sepsis	TRUE	reported	textfile	0.0069	4.29E-06	1780	M36131.m	TRUE	reported	Xw6wmg	2	TRUE	0.011646	20.95018

7908	rs8063217	T	C	T	C	0.1278	0.010831	0.0594	0.052702	FALSE	FALSE	FALSE	AhsT9A	0.030551	0.722956	486484	sepsis	TRUE	reported	textfile	0.0279	4.70E-06	1254	M35187.m	TRUE	reported	uLU0Fy	2	TRUE	0.016457	20.94885
6098	rs7692407	T	C	T	C	-0.0293	0.006631	0.9336	0.926015	FALSE	FALSE	FALSE	xvzgym	0.026295	0.800912	486484	sepsis	TRUE	reported	textfile	0.0064	5.36E-06	4004	M33443.m	TRUE	reported	KtBPyR	2	TRUE	0.005207	20.94876
5010	rs2408393	T	C	T	C	-0.0403	-0.01988	0.866	0.864225	FALSE	FALSE	FALSE	URCVGm	0.020002	0.320346	486484	sepsis	TRUE	reported	textfile	0.0088	5.01E-06	1767	M33131.m	TRUE	reported	4rQ2gs	2	TRUE	0.01173	20.9485
7398	rs9837363	T	C	T	C	-0.0206	0.015445	0.4628	0.475676	FALSE	FALSE	FALSE	AW6M5u	0.014968	0.30212	486484	sepsis	TRUE	reported	textfile	0.0045	3.60E-06	5195	M34389.m	TRUE	reported	6ZYYQK	2	TRUE	0.004018	20.94798
4459	rs7843564	T	G	T	G	0.0554	-0.00607	0.9307	0.920301	FALSE	FALSE	FALSE	g2RNym	0.025171	0.809298	486484	sepsis	TRUE	reported	textfile	0.0121	4.64E-06	2575	M32691.m	TRUE	reported	35eo6p	2	TRUE	0.008075	20.94649
3539	rs7397233	A	G	A	G	0.098	-0.01189	0.0335	0.137881	FALSE	FALSE	FALSE	rXzSlH	0.019851	0.549199	486484	sepsis	TRUE	reported	textfile	0.0214	4.47E-06	1509	M30805.m	TRUE	reported	7wg9QH	2	TRUE	0.013707	20.94347
2819	rs4659612	A	G	A	G	0.0174	0.01726	0.7371	0.744127	FALSE	FALSE	FALSE	Sx5Uhq	0.015677	0.270909	486484	sepsis	TRUE	reported	textfile	0.0038	5.92E-06	1759	M19934.m	TRUE	reported	DijYTE	2	TRUE	0.011779	20.94292
4481	rs1008342	T	C	T	C	0.0119	-0.01307	0.3043	0.301652	FALSE	FALSE	FALSE	1wNJ1E	0.014884	0.379937	486484	sepsis	TRUE	reported	textfile	0.0026	6.74E-06	7363	M32709.m	TRUE	reported	zihBHg	2	TRUE	0.002837	20.94253
4293	rs1990385	T	C	T	C	0.0119	-0.01121	0.8651	0.845845	FALSE	FALSE	FALSE	0tjjPC	0.018974	0.554676	486484	sepsis	TRUE	reported	textfile	0.0026	4.95E-06	7362	M32634.m	TRUE	reported	nJJFkX	2	TRUE	0.002837	20.94253
6879	rs4394864	T	C	T	C	-0.0119	0.027416	0.0645	0.075732	FALSE	FALSE	FALSE	kFWZjs	0.025874	0.289327	486484	sepsis	TRUE	reported	textfile	0.0026	5.52E-06	7362	M34062.m	TRUE	reported	eLONsg	2	TRUE	0.002837	20.94253
6882	rs559422	A	C	A	C	0.0119	-0.00944	0.9115	0.916768	FALSE	FALSE	FALSE	kFWZjs	0.025238	0.708357	486484	sepsis	TRUE	reported	textfile	0.0026	5.77E-06	7362	M34062.m	TRUE	reported	eLONsg	2	TRUE	0.002837	20.94253
1602	rs1688612	A	G	A	G	-0.0119	-0.00552	0.8616	0.872158	FALSE	FALSE	FALSE	zP3C2v	0.020489	0.78743	486484	sepsis	TRUE	reported	textfile	0.0026	3.71E-06	7356	M12035.m	TRUE	reported	1Pooe3	2	TRUE	0.00284	20.94253
1736	rs1194797	T	C	T	C	-0.0119	9.95E-05	0.1515	0.155741	FALSE	FALSE	FALSE	nFLpAP	0.018816	0.995783	486484	sepsis	TRUE	reported	textfile	0.0026	5.03E-06	7356	M12774.m	TRUE	reported	yOSQTj	2	TRUE	0.00284	20.94253
6977	rs4973862	T	C	T	C	-0.0119	-0.01234	0.6928	0.704173	FALSE	FALSE	FALSE	ZlvYs5	0.014949	0.408999	486484	sepsis	TRUE	reported	textfile	0.0026	5.92E-06	7351	M34214.m	TRUE	reported	qU57S1	2	TRUE	0.002842	20.94253
7884	rs1010377	T	C	T	C	0.0119	0.010957	0.3033	0.288678	FALSE	FALSE	FALSE	pU8zps	0.015153	0.469623	486484	sepsis	TRUE	reported	textfile	0.0026	6.76E-06	7350	M35186.m	TRUE	reported	Oc2lOF	2	TRUE	0.002842	20.94252
7889	rs1715284	T	C	T	C	-0.0119	0.022456	0.7203	0.710468	FALSE	FALSE	FALSE	pU8zps	0.01506	0.13594	486484	sepsis	TRUE	reported	textfile	0.0026	5.93E-06	7350	M35186.m	TRUE	reported	Oc2lOF	2	TRUE	0.002842	20.94252
761	rs7570141	A	G	A	G	-0.0119	0.012755	0.104	0.128492	FALSE	FALSE	FALSE	RHWptG	0.021063	0.544806	486484	sepsis	TRUE	reported	textfile	0.0026	6.21E-06	7347	M01302.m	TRUE	reported	z1OLfr	2	TRUE	0.002843	20.94252
3650	rs1574228	A	G	A	G	0.0119	8.52E-05	0.7061	0.712747	FALSE	FALSE	FALSE	fnjup6	0.015119	0.995502	486484	sepsis	TRUE	reported	textfile	0.0026	6.71E-06	7346	M32197.m	TRUE	reported	oQzaMk	2	TRUE	0.002844	20.94252
4937	rs1148526	A	G	A	G	-0.0119	-0.00416	0.8872	0.88587	FALSE	FALSE	FALSE	bKmDMa	0.021458	0.846174	486484	sepsis	TRUE	reported	textfile	0.0026	6.74E-06	7344	M32910.m	TRUE	reported	nAuPzO	2	TRUE	0.002844	20.94252
3728	rs1009567	T	C	T	C	-0.0238	0.023996	0.9066	0.906815	FALSE	FALSE	FALSE	k16olH	0.023569	0.308615	486484	sepsis	TRUE	reported	textfile	0.0052	4.02E-06	7340	M32328.m	TRUE	reported	TQA3sZ	2	TRUE	0.002846	20.94252
1838	rs7160223	A	T	A	T	-0.0119	0.007262	0.419	0.413414	FALSE	TRUE	FALSE	2TaDHm	0.013987	0.603615	486484	sepsis	TRUE	reported	textfile	0.0026	5.52E-06	7337	M15365.m	TRUE	reported	LoLveg	2	TRUE	0.002847	20.94251
8808	rs1006415	A	C	A	C	-0.0119	0.011748	0.7322	0.722383	FALSE	FALSE	FALSE	X2Rol2	0.01527	0.441678	486484	sepsis	TRUE	reported	textfile	0.0026	3.78E-06	7335	M36776.m	TRUE	reported	nikHw1	2	TRUE	0.002848	20.94251
3078	rs1113667	A	G	A	G	-0.0119	-0.01374	0.715	0.715256	FALSE	FALSE	FALSE	esOP8m	0.015242	0.367484	486484	sepsis	TRUE	reported	textfile	0.0026	7.49E-06	7329	M22116.m	TRUE	reported	fDMh7Z	2	TRUE	0.00285	20.94251
3196	rs2202185	A	G	A	G	-0.0119	-0.00965	0.3139	0.366584	FALSE	FALSE	FALSE	UfLkBq	0.014222	0.49759	486484	sepsis	TRUE	reported	textfile	0.0026	6.16E-06	7329	M22481.m	TRUE	reported	kWMdiT	2	TRUE	0.00285	20.94251
1366	rs1111995	T	C	T	C	0.0119	0.008527	0.1175	0.110685	FALSE	FALSE	FALSE	KrWhws	0.021745	0.694944	486484	sepsis	TRUE	reported	textfile	0.0026	4.36E-06	7325	M02132.m	TRUE	reported	7pzpfK	2	TRUE	0.002852	20.94251
8322	rs184943	A	T	A	T	-0.0119	-0.01734	0.7036	0.701873	FALSE	TRUE	FALSE	o0GzT1	0.014972	0.246673	486484	sepsis	TRUE	reported	textfile	0.0026	7.23E-06	7317	M35631.m	TRUE	reported	G1XFx0	2	TRUE	0.002855	20.9425
8324	rs2278476	T	C	T	C	0.0119	-0.02358	0.4871	0.485654	FALSE	FALSE	FALSE	o0GzT1	0.013642	0.083832	486484	sepsis	TRUE	reported	textfile	0.0026	5.00E-06	7317	M35631.m	TRUE	reported	G1XFx0	2	TRUE	0.002855	20.9425
919	rs2351725	A	T	A	T	-0.0119	0.010772	0.7947	0.784403	FALSE	TRUE	FALSE	rVMAPf	0.016683	0.518466	486484	sepsis	TRUE	reported	textfile	0.0026	5.84E-06	7307	M01493.m	TRUE	reported	siVqDi	2	TRUE	0.002859	20.94249
6160	rs9990565	T	C	T	C	-0.0119	-0.03024	0.1528	0.144658	FALSE	FALSE	FALSE	MiSJly	0.019524	0.121456	486484	sepsis	TRUE	reported	textfile	0.0026	5.48E-06	7307	M33477.m	TRUE	reported	WwL1hq	2	TRUE	0.002859	20.94249
6242	rs4073619	T	G	T	G	-0.0119	-0.01914	0.5405	0.530167	FALSE	FALSE	FALSE	vL52Yb	0.013734	0.163525	486484	sepsis	TRUE	reported	textfile	0.0026	6.83E-06	7269	M33510.m	TRUE	reported	JlO9VX	2	TRUE	0.002874	20.94246
5121	rs9650602	T	C	T	C	-0.0238	-0.00868	0.197	0.253952	FALSE	FALSE	FALSE	O6ine1	0.015966	0.586526	486484	sepsis	TRUE	reported	textfile	0.0052	4.43E-06	7263	M33165.m	TRUE	reported	gGsqjW	2	TRUE	0.002876	20.94246
3558	rs7932167	T	G	T	G	0.0119	-0.00278	0.7711	0.784934	FALSE	FALSE	FALSE	8luUON	0.016666	0.867403	486484	sepsis	TRUE	reported	textfile	0.0026	6.66E-06	7257	M31453.m	TRUE	reported	FBs6G4	2	TRUE	0.002878	20.94245
8239	rs6745507	C	G	C	G	-0.0119	0.013183	0.6155	0.585488	FALSE	TRUE	FALSE	4YAWhA	0.014014	0.346877	486484	sepsis	TRUE	reported	textfile	0.0026	3.65E-06	7256	M35439.m	TRUE	reported	bSSC4Z	2	TRUE	0.002879	20.94245
3221	rs1111778	T	C	T	C	-0.0119	-0.00616	0.6373	0.625209	FALSE	FALSE	FALSE	I25GHB	0.0142	0.664474	486484	sepsis	TRUE	reported	textfile	0.0026	3.10E-06	7184	M22548.m	TRUE	reported	eBmTQD	2	TRUE	0.002907	20.94239
3224	rs1167586	T	C	T	C	0.0119	-0.01604	0.7367	0.746813	FALSE	FALSE	FALSE	I25GHB	0.015711	0.307356	486484	sepsis	TRUE	reported	textfile	0.0026	5.84E-06	7184	M22548.m	TRUE	reported	eBmTQD	2	TRUE	0.002907	20.94239
5817	rs1228381	A	G	A	G	-0.0119	-0.02646	0.1003	0.099521	FALSE	FALSE	FALSE	R5joEf	0.022909	0.248088	486484	sepsis	TRUE	reported	textfile	0.0026	4.01E-06	7118	M33380.m	TRUE	reported	kRvbfZ	2	TRUE	0.002934	20.94234
7976	rs1263632	T	C	T	C	-0.0119	-0.00495	0.4945	0.495685	FALSE	FALSE	FALSE	t02AUT	0.013705	0.71815	486484	sepsis	TRUE	reported	textfile	0.0026	6.16E-06	7100	M35254.m	TRUE	reported	uClJaT	2	TRUE	0.002942	20.94232
876	rs8098317	T	G	T	G	-0.0119	-0.00018	0.7857	0.781102	FALSE	FALSE	FALSE	Vg5OOR	0.01658	0.991483	486484	sepsis	TRUE	reported	textfile	0.0026	6.64E-06	7063	M01361.m	TRUE	reported	XnrS00	2	TRUE	0.002957	20.94229
859	rs1050984	A	G	A	G	-0.0119	-0.0306	0.6879	0.743825	FALSE	FALSE	FALSE	Vg5OOR	0.015836	0.053321	486484	sepsis	TRUE	reported	textfile	0.0026	4.23E-06	7063	M01361.m	TRUE	reported	XnrS00	2	TRUE	0.002957	20.94229
1714	rs1041617	T	G	T	G	0.0119	0.000973	0.592	0.581502	FALSE	FALSE	FALSE	RmWDhQ	0.013839	0.943923	486484	sepsis	TRUE	reported	textfile	0.0026	6.93E-06	7034	M12768.m	TRUE	reported	f0vnEz	2	TRUE	0.002969	20.94227
2742	rs1261411	T	C	T	C	0.0119	-0.00293	0.4052	0.4202	FALSE	FALSE	FALSE	qFAbee	0.013972	0.833665	486484	sepsis	TRUE	reported	textfile	0.0026	3.29E-06	6929	M19364.m	TRUE	reported	tZgXNL	2	TRUE	0.003014	20.94218
2755	rs7864694	A	G	A	G	0.0119	0.014604	0.3364	0.346526	FALSE	FALSE	FALSE	qFAbee	0.014349	0.3088	486484	sepsis	TRUE	reported	textfile	0.0026	4.04E-06	6929	M19364.m	TRUE	reported	tZgXNL	2	TRUE	0.003014	20.94218
2756	rs8071524	A	G	A	G	-0.0119	-0.03021	0.3003	0.307023	FALSE	FALSE	FALSE	qFAbee	0.014881	0.042364	486484	sepsis	TRUE	reported	textfile	0.0026	4.55E-06	6929	M19364.m	TRUE	reported	tZgXNL	2	TRUE	0.003014	20.94218
2764	rs4344410	A	C	A	C	0.0119	0.003774	0.503	0.490723	FALSE	FALSE	FALSE	H6txH3	0.013671	0.782517	486484	sepsis	TRUE	reported	textfile	0.0026	3.52E-06	6678	M19368.m	TRUE	reported	b094NF	2	TRUE	0.003127	20.94195
8419	rs6870033	A	T	A	T	-0.0238	0.005021	0.7179	0.688355	FALSE	TRUE	FALSE	02RdNm	0.014768	0.733848	486484	sepsis	TRUE	reported	textfile	0.0052	4.14E-06	6447	M35678.m	TRUE	reported	BWN5JR	2	TRUE	0.003239	20.94173
3051	rs1277442	A	C	A	C	0.0119	-0.00038	0.3106	0.316018	FALSE	FALSE	FALSE	H6vcCG	0.014697	0.979392	486484	sepsis	TRUE	reported	textfile	0.0026	3.21E-06	6180	M22030.m	TRUE	reported	XetVX7	2	TRUE	0.003378	20.94145
3559	rs9572772	T	G	T	G	-0.0902	-0.06645	0.0129	0.009357	FALSE	FALSE	FALSE	8luUON	0.070874	0.348476	486484	sepsis	TRUE	reported	textfile	0.0197	4.65E-06	1750	M31453.m	TRUE	reported	FBs6G4	2	TRUE	0.011838	20.94035
5500	rs633810	T	C	T	C	-0.1081	-0.02862	0.094	0.116988	FALSE	FALSE	FALSE	q32d4E	0.021415	0.181464	486484	sepsis	TRUE	reported	textfile	0.0236	4.79E-06	1017	M33178.m	TRUE	reported	cS3BwT	2	TRUE	0.020213	20.9398
2280	rs1552422	A	G	A	G	-0.0119	-0.01134	0.5052	0.496895	FALSE	FALSE	FALSE	mRULgB	0.013697	0.407658	486484	sepsis	TRUE	reported	textfile	0.0026	6.00E-06	4689	M16816.m	TRUE	reported	05lFsX	2	TRUE	0.004448	20.93929
6906	rs1266106	T	C	T	C	-0.1644	-0.01212	0.1122	0.123043	FALSE	FALSE	FALSE	SvfSvH	0.021241	0.568248	486484	sepsis	TRUE	reported	textfile	0.0358	4.40E-06	282	M34109.m	TRUE	reported	7OSbiu	2	TRUE	0.069577	20.93854
3545	rs1205212	T	C	T	C	0.0389	-0.00212	0.1205	0.122873	FALSE	FALSE	FALSE	8luUON	0.020786	0.918765	486484	sepsis	TRUE	reported	textfile	0.0085	4.18E-06	7257	M31453.m	TRUE	reported	FBs6G4	2	TRUE	0.002878	20.93831
7180	rs1752155	T	C	T	C	0.3198	-0.07574	0.0166	0.015487	FALSE	FALSE	FALSE	2hlepp	0.055846	0.175022	486484	sepsis	TRUE	reported	textfile	0.0697	4.46E-06	369	M34336.m	TRUE	reported	XyK8mO	2	TRUE	0.053972	20.9378
4489	rs1704780	T	C	T	C	-0.038	0.010509	0.8891	0.878063	FALSE	FALSE	FALSE	1wNJ1E	0.020916	0.615371	486484	sepsis	TRUE	reported	textfile	0.0083	4.12E-06	1766	M32709.m	TRUE	reported	zihBHg	2	TRUE	0.01173	20.93721
7175	rs1105889	T	C	T	C	-0.027	-0.01159	0.0551	0.058264	FALSE	FALSE	FALSE	2hlepp	0.029252	0.69206	486484	sepsis	TRUE	reported	textfile	0.0059	4.76E-06	5081	M34336.m	TRUE	reported	XyK8mO	2	TRUE	0.004105	20.93401
6298	rs1708109	A	G	A	G	0.0357	0.021942	0.8144	0.788167	FALSE	FALSE	FALSE	VlzZVG	0.01679	0.19128	486484	sepsis	TRUE	reported	textfile	0.0078	4.93E-06	2859	M33531.m	TRUE	reported	XiYjn4	2	TRUE	0.007274	20.93357
1783	rs1159304	A	T	A	T	0.0151	-0.01092	0.0919	0.092269	FALSE	TRUE	FALSE	JEcCRZ	0.023713	0.645139	486484	sepsis	TRUE	reported	textfile	0.0033	4.55E-06	7368	M15140.m	TRUE	reported	xYCzKP	2	TRUE	0.002834	20.93187
1283	rs4439706	T	C	T	C	0.0151	-0.00115	0.7156	0.714137	FALSE	FALSE	FALSE	nvWJJ3	0.015218	0.93964	486484	sepsis	TRUE	reported	textfile	0.0033	3.78E-06	7346	M01712.m	TRUE	reported	1x44lf	2	TRUE	0.002842	20.93186
4577	rs1256901	A	T	A	T	-0.0151	-0.01025	0.0734	0.076832	FALSE	TRUE	FALSE	Pzplqu	0.025616	0.688919	486484	sepsis	TRUE	reported	textfile	0.0033	6.39E-06	7318	M32740.m	TRUE	reported	FjEBS5	2	TRUE	0.002853	20.93184
5821	rs1509091	T	C	T	C	0.0151	0.021366	0.9357	0.918906	FALSE	FALSE	FALSE	R5joEf	0.02542	0.400623	486484	sepsis	TRUE	reported	textfile	0.0033	6.30E-06	7118	M33380.m	TRUE	reported	kRvbfZ	2	TRUE	0.002933	20.93167
6511	rs7579137	A	G	A	G	-0.027	-0.01351	0.5493	0.564845	FALSE	FALSE	FALSE	dluFOH	0.013791	0.32717	486484	sepsis	TRUE	reported	textfile	0.0059	4.77E-06	3845	M33801.m	TRUE	reported	JCXy06	2	TRUE	0.005417	20.93136
7701	rs1759720	A	G	A	G	-0.0302	0.030868	0.8973	0.892699	FALSE	FALSE	FALSE	2eJlPM	0.022108	0.162642	486484	sepsis	TRUE	reported	textfile	0.0066	5.01E-06	6381	M34761.m	TRUE	reported	druKtT	2	TRUE	0.003271	20.93099
8295	rs9580319	T	C	T	C	-0.0641	-0.01873	0.9296	0.91618	FALSE	FALSE	FALSE	vXjGkU	0.02469	0.448074	486484	sepsis	TRUE	reported	textfile	0.014	4.44E-06	1291	M35527.m	TRUE	reported	oS5qaO	2	TRUE	0.015979	20.93084
4458	rs7513605	T	C	T	C	0.0389	0.043672	0.1575	0.161011	FALSE	FALSE	FALSE	g2RNym	0.018571	0.018692	486484	sepsis	TRUE	reported	textfile	0.0085	4.10E-06	2575	M32691.m	TRUE	reported	35eo6p	2	TRUE	0.008068	20.92782
6019	rs1462054	A	G	A	G	1.266	0.063163	0.9746	0.987922	FALSE	FALSE	FALSE	33ojbt	0.062568	0.31273	486484	sepsis	TRUE	reported	textfile	0.2721	3.27E-06	60	M33423.m	TRUE	reported	ivEtvl	2	TRUE	0.265135	20.92604
5814	rs1154486	T	C	T	C	0.0183	-0.013	0.943	0.927103	FALSE	FALSE	FALSE	R5joEf	0.026342	0.621654	486484	sepsis	TRUE	reported	textfile	0.004	3.71E-06	7118	M33380.m	TRUE	reported	kRvbfZ	2	TRUE	0.002932	20.92474
4734	rs9751295	A	G	A	G	-0.1428	0.00366	0.9778	0.984689	FALSE	FALSE	FALSE	fz63r4	0.055724	0.947629	486484	sepsis	TRUE	reported	textfile	0.0312	4.68E-06	1672	M32762.m	TRUE	reported	kgxlBo	2	TRUE	0.012374	20.92317
984	rs7701675	A	G	A	G	-0.7943	-0.02228	0.9839	0.987028	FALSE	FALSE	FALSE	xSRlI4	0.061174	0.71572	486484	sepsis	TRUE	reported	textfile	0.1733	4.56E-06	482	M01515.m	TRUE	reported	juZ5GE	2	TRUE	0.041764	20.92022
8643	rs9574536	T	C	T	C	-0.0517	0.013058	0.3565	0.406997	FALSE	FALSE	FALSE	4cZjpQ	0.013928	0.348471	486484	sepsis	TRUE	reported	textfile	0.0113	5.06E-06	2400	M36230.m	TRUE	reported	TPUeNs	2	TRUE	0.008647	20.91521
7801	rs270771	T	C	T	C	-0.0279	-0.02422	0.0551	0.053251	FALSE	FALSE	FALSE	cQ5TvO	0.030384	0.425426	486484	sepsis	TRUE	reported	textfile	0.0061	4.02E-06	7344	M35127.m	TRUE	reported	BVxkfZ	2	TRUE	0.00284	20.91368
8554	rs6829571	A	G	A	G	-0.4173	0.015653	0.7541	0.749295	FALSE	FALSE	FALSE	aQhCav	0.015793	0.321618	486484	sepsis	TRUE	reported	textfile	0.0905	3.95E-06	122	M36097.m	TRUE	reported	9DJS9j	2	TRUE	0.148412	20.91323
4805	rs9881563	T	C	T	C	0.0279	0.009051	0.6543	0.667567	FALSE	FALSE	FALSE	9qWhFj	0.014508	0.532718	486484	sepsis	TRUE	reported	textfile	0.0061	4.60E-06	6200	M32808.m	TRUE	reported	MQcOSi	2	TRUE	0.003363	20.91263
5593	rs9395772	A	G	A	G	-0.2838	-0.08773	0.0176	0.016087	FALSE	FALSE	FALSE	q32d4E	0.054832	0.109593	486484	sepsis	TRUE	reported	textfile	0.062	4.67E-06	1017	M33178.m	TRUE	reported	cS3BwT	2	TRUE	0.020187	20.91156
1667	rs9460599	T	C	T	C	0.0302	-0.01156	0.2443	0.248708	FALSE	FALSE	FALSE	YzX5ZD	0.015966	0.468875	486484	sepsis	TRUE	reported	textfile	0.0066	4.86E-06	1586	M12129.m	TRUE	reported	6tMfaW	2	TRUE	0.013029	20.91115
7350	rs1941075	T	C	T	C	-0.1094	0.022918	0.7538	0.747536	FALSE	FALSE	FALSE	mm9ksf	0.015763	0.145983	486484	sepsis	TRUE	reported	textfile	0.0239	4.71E-06	954	M34365.m	TRUE	reported	AVwEqD	2	TRUE	0.021491	20.90872
664	rs1270743	A	G	A	G	-0.0366	0.051684	0.9639	0.976885	FALSE	FALSE	FALSE	p4oCVP	0.045251	0.253379	486484	sepsis	TRUE	reported	textfile	0.008	4.65E-06	1764	M01125.m	TRUE	reported	OwD5re	2	TRUE	0.011726	20.90689
4771	rs3729621	A	G	A	G	0.0503	-0.02132	0.9564	0.953146	FALSE	FALSE	FALSE	mOrnK4	0.03243	0.510897	486484	sepsis	TRUE	reported	textfile	0.011	4.92E-06	6169	M32795.m	TRUE	reported	7EGxuf	2	TRUE	0.003378	20.90306
7439	rs1188573	A	T	A	T	0.0824	0.001522	0.6877	0.664462	FALSE	TRUE	FALSE	otAubi	0.014496	0.916357	486484	sepsis	TRUE	reported	textfile	0.018	4.69E-06	777	M34404.m	TRUE	reported	SAZaNF	2	TRUE	0.026262	20.90211
1628	rs1408276	A	C	A	C	-0.043	-0.00499	0.955	0.951881	FALSE	FALSE	FALSE	n5rDuT	0.032024	0.876248	486484	sepsis	TRUE	reported	textfile	0.0094	4.59E-06	1747	M12067.m	TRUE	reported	ZQLTeR	2	TRUE	0.011836	20.9018
5854	rs3784457	A	G	A	G	0.2165	-0.01085	0.0539	0.063398	FALSE	FALSE	FALSE	Vkec6Y	0.027978	0.698139	486484	sepsis	TRUE	reported	textfile	0.0473	4.66E-06	803	M33384.m	TRUE	reported	eaxeyR	2	TRUE	0.025427	20.89828
8428	rs1163654	T	G	T	G	0.0192	0.019696	0.1055	0.151903	FALSE	FALSE	FALSE	Nvt7pl	0.019383	0.309568	486484	sepsis	TRUE	reported	textfile	0.0042	3.99E-06	7358	M35718.m	TRUE	reported	WkA5Gq	2	TRUE	0.002832	20.89228
818	rs1015277	T	C	T	C	-0.0224	0.01632	0.0425	0.047425	FALSE	FALSE	FALSE	2qgDgB	0.032238	0.61269	486484	sepsis	TRUE	reported	textfile	0.0049	4.73E-06	7355	M01358.m	TRUE	reported	asmXq0	2	TRUE	0.002833	20.89228
6969	rs1082973	C	G	C	G	0.016	-0.00322	0.8464	0.844595	FALSE	TRUE	FALSE	ZlvYs5	0.018857	0.864265	486484	sepsis	TRUE	reported	textfile	0.0035	4.90E-06	7351	M34214.m	TRUE	reported	qU57S1	2	TRUE	0.002835	20.89227
7481	rs1172908	A	G	A	G	-0.0128	0.011062	0.1591	0.171244	FALSE	FALSE	FALSE	jAhmnV	0.01807	0.540416	486484	sepsis	TRUE	reported	textfile	0.0028	3.43E-06	7348	M34419.m	TRUE	reported	F2Tg9L	2	TRUE	0.002836	20.89227
7484	rs1274582	T	G	T	G	0.0128	0.002778	0.8446	0.839104	FALSE	FALSE	FALSE	jAhmnV	0.018741	0.882143	486484	sepsis	TRUE	reported	textfile	0.0028	4.76E-06	7348	M34419.m	TRUE	reported	F2Tg9L	2	TRUE	0.002836	20.89227
3734	rs1685253	A	G	A	G	0.016	0.013557	0.3193	0.373051	FALSE	FALSE	FALSE	k16olH	0.014266	0.34195	486484	sepsis	TRUE	reported	textfile	0.0035	3.83E-06	7340	M32328.m	TRUE	reported	TQA3sZ	2	TRUE	0.002839	20.89226
8814	rs1428365	T	C	T	C	-0.0192	-0.0148	0.9228	0.92151	FALSE	FALSE	FALSE	X2Rol2	0.025601	0.563075	486484	sepsis	TRUE	reported	textfile	0.0042	4.52E-06	7335	M36776.m	TRUE	reported	nikHw1	2	TRUE	0.002841	20.89226
4916	rs931761	C	G	C	G	0.0064	-0.01194	0.755	0.747582	FALSE	TRUE	FALSE	VqcRGX	0.015726	0.447525	486484	sepsis	TRUE	reported	textfile	0.0014	2.80E-06	7329	M32867.m	TRUE	reported	psuFJy	2	TRUE	0.002843	20.89226
2517	rs7283743	T	C	T	C	-0.0128	-0.01283	0.1245	0.129264	FALSE	FALSE	FALSE	LwUYua	0.020618	0.533653	486484	sepsis	TRUE	reported	textfile	0.0028	5.01E-06	7315	M17945.m	TRUE	reported	SUN8dc	2	TRUE	0.002849	20.89225
8592	rs7332616	T	C	T	C	-0.032	0.007715	0.7928	0.7942	FALSE	FALSE	FALSE	iuJmRL	0.016971	0.649413	486484	sepsis	TRUE	reported	textfile	0.007	5.18E-06	7311	M36103.m	TRUE	reported	jfJbVw	2	TRUE	0.00285	20.89224
4472	rs3770063	A	T	A	T	-0.0256	-0.0257	0.0352	0.034809	FALSE	TRUE	FALSE	g3iM2w	0.037195	0.489547	486484	sepsis	TRUE	reported	textfile	0.0056	5.88E-06	7308	M32698.m	TRUE	reported	ZQBn0h	2	TRUE	0.002851	20.89224
5978	rs1162336	T	C	T	C	0.016	0.015379	0.0594	0.063509	FALSE	FALSE	FALSE	3KsoXq	0.028377	0.587848	486484	sepsis	TRUE	reported	textfile	0.0035	5.60E-06	7306	M33422.m	TRUE	reported	aNr6Ug	2	TRUE	0.002852	20.89224
2882	rs1721249	A	G	A	G	0.016	-0.00171	0.1645	0.169008	FALSE	FALSE	FALSE	mUzn1R	0.018225	0.925114	486484	sepsis	TRUE	reported	textfile	0.0035	5.93E-06	7301	M20675.m	TRUE	reported	XwY04K	2	TRUE	0.002854	20.89223
2893	rs618900	C	G	C	G	-0.016	-0.0192	0.8136	0.81069	FALSE	TRUE	FALSE	mUzn1R	0.017423	0.270453	486484	sepsis	TRUE	reported	textfile	0.0035	6.00E-06	7301	M20675.m	TRUE	reported	XwY04K	2	TRUE	0.002854	20.89223
3554	rs4602889	A	G	A	G	0.016	0.010967	0.8859	0.861876	FALSE	FALSE	FALSE	8luUON	0.020076	0.584864	486484	sepsis	TRUE	reported	textfile	0.0035	3.57E-06	7257	M31453.m	TRUE	reported	FBs6G4	2	TRUE	0.002871	20.8922
1450	rs6488270	A	G	A	G	0.0096	0.010046	0.4076	0.392371	FALSE	FALSE	FALSE	1M4gqj	0.013964	0.471899	486484	sepsis	TRUE	reported	textfile	0.0021	4.95E-06	7226	M02730.m	TRUE	reported	RWyYEM	2	TRUE	0.002884	20.89218
6725	rs3821477	T	C	T	C	-0.0288	-0.02154	0.927	0.933021	FALSE	FALSE	FALSE	B6lZly	0.027379	0.43138	486484	sepsis	TRUE	reported	textfile	0.0063	5.35E-06	7222	M33937.m	TRUE	reported	qFPzjh	2	TRUE	0.002885	20.89217
5068	rs894137	A	G	A	G	-0.0192	0.01865	0.2902	0.290273	FALSE	FALSE	FALSE	nHZXjp	0.015087	0.216398	486484	sepsis	TRUE	reported	textfile	0.0042	4.99E-06	7166	M33138.m	TRUE	reported	ccoK1F	2	TRUE	0.002908	20.89213
967	rs8065435	A	G	A	G	-0.0128	-0.02054	0.327	0.331339	FALSE	FALSE	FALSE	tWoDq4	0.015128	0.174647	486484	sepsis	TRUE	reported	textfile	0.0028	5.03E-06	7160	M01508.m	TRUE	reported	6ltDw0	2	TRUE	0.00291	20.89212
2625	rs6779362	T	C	T	C	-0.0288	-0.02213	0.7633	0.769276	FALSE	FALSE	FALSE	eygBBR	0.016201	0.171917	486484	sepsis	TRUE	reported	textfile	0.0063	5.74E-06	6970	M18394.m	TRUE	reported	hD79iE	2	TRUE	0.002989	20.89196
7479	rs6990912	A	C	A	C	-0.016	0.029312	0.3602	0.356322	FALSE	FALSE	FALSE	SqfdcK	0.014338	0.040921	486484	sepsis	TRUE	reported	textfile	0.0035	4.81E-06	6929	M34416.m	TRUE	reported	sRGRIK	2	TRUE	0.003007	20.89193
4758	rs2236525	T	C	T	C	0.016	0.016775	0.5285	0.531039	FALSE	FALSE	FALSE	RDfDIS	0.013673	0.219886	486484	sepsis	TRUE	reported	textfile	0.0035	4.02E-06	6857	M32787.m	TRUE	reported	Hq6nGt	2	TRUE	0.003038	20.89186
4826	rs1002322	T	C	T	C	-0.0256	0.015661	0.8808	0.88182	FALSE	FALSE	FALSE	yLDcUj	0.021188	0.459804	486484	sepsis	TRUE	reported	textfile	0.0056	5.58E-06	6753	M32838.m	TRUE	reported	5dQgAN	2	TRUE	0.003085	20.89177
3802	rs1105842	A	C	A	C	-0.048	-0.00946	0.1435	0.155539	FALSE	FALSE	FALSE	HOq2gb	0.01902	0.61903	486484	sepsis	TRUE	reported	textfile	0.0105	4.44E-06	6687	M32346.m	TRUE	reported	Ss5q17	2	TRUE	0.003115	20.89171
8279	rs1308427	A	G	A	G	-0.0192	0.017067	0.4129	0.406978	FALSE	FALSE	FALSE	J6o4xC	0.013993	0.222592	486484	sepsis	TRUE	reported	textfile	0.0042	5.54E-06	6595	M35472.m	TRUE	reported	3XBcu9	2	TRUE	0.003159	20.89162
3311	rs2934216	A	G	A	G	-0.0128	0.044072	0.7472	0.737857	FALSE	FALSE	FALSE	nqmBts	0.015557	0.004612	486484	sepsis	TRUE	reported	textfile	0.0028	3.10E-06	6388	M25599.m	TRUE	reported	VkwSh1	2	TRUE	0.003261	20.89142
3049	rs1159893	A	T	A	T	0.016	0.009974	0.1347	0.135494	FALSE	TRUE	FALSE	H6vcCG	0.019928	0.616704	486484	sepsis	TRUE	reported	textfile	0.0035	3.55E-06	6180	M22030.m	TRUE	reported	XetVX7	2	TRUE	0.00337	20.8912
7561	rs8086010	A	C	A	C	-0.0192	0.016412	0.6663	0.649268	FALSE	FALSE	FALSE	APSWAU	0.014329	0.252058	486484	sepsis	TRUE	reported	textfile	0.0042	5.48E-06	6144	M34469.m	TRUE	reported	frF3RX	2	TRUE	0.00339	20.89116
8467	rs6475283	T	C	T	C	0.0343	-0.00994	0.2824	0.274524	FALSE	FALSE	FALSE	uMajV6	0.015366	0.517542	486484	sepsis	TRUE	reported	textfile	0.0075	5.12E-06	1709	M35754.m	TRUE	reported	b8QB7S	2	TRUE	0.01209	20.8909
3104	rs8061077	T	C	T	C	-0.0128	-0.02491	0.2169	0.214822	FALSE	FALSE	FALSE	mr0awv	0.01669	0.135599	486484	sepsis	TRUE	reported	textfile	0.0028	4.06E-06	5702	M22130.m	TRUE	reported	Y2TfWQ	2	TRUE	0.003652	20.89063
7537	rs1800600	A	G	A	G	0.0128	-0.00696	0.6929	0.685766	FALSE	FALSE	FALSE	JJsHvX	0.014811	0.63818	486484	sepsis	TRUE	reported	textfile	0.0028	4.06E-06	5207	M34456.m	TRUE	reported	aklxve	2	TRUE	0.003997	20.88993
7539	rs3800552	A	T	A	T	-0.0128	-0.01121	0.3513	0.353845	FALSE	TRUE	FALSE	JJsHvX	0.01434	0.434297	486484	sepsis	TRUE	reported	textfile	0.0028	3.30E-06	5207	M34456.m	TRUE	reported	aklxve	2	TRUE	0.003997	20.88993
7543	rs6430457	A	C	A	C	-0.0128	0.010498	0.6797	0.67971	FALSE	FALSE	FALSE	JJsHvX	0.014745	0.4765	486484	sepsis	TRUE	reported	textfile	0.0028	3.82E-06	5207	M34456.m	TRUE	reported	aklxve	2	TRUE	0.003997	20.88993
6161	rs1018657	A	G	A	G	0.0288	-0.01256	0.1328	0.13952	FALSE	FALSE	FALSE	O4Ty2l	0.019703	0.523772	486484	sepsis	TRUE	reported	textfile	0.0063	5.10E-06	5107	M33488.m	TRUE	reported	RGtF84	2	TRUE	0.004075	20.88978
7840	rs4659909	A	G	A	G	-0.0128	-0.00033	0.1755	0.24269	FALSE	FALSE	FALSE	h5whlS	0.015961	0.983587	486484	sepsis	TRUE	reported	textfile	0.0028	5.06E-06	4900	M35137.m	TRUE	reported	xVlTKF	2	TRUE	0.004247	20.88943
2784	rs6894183	A	T	A	T	0.016	-0.00378	0.8137	0.812638	FALSE	TRUE	FALSE	yYuamw	0.017504	0.829157	486484	sepsis	TRUE	reported	textfile	0.0035	6.03E-06	4844	M19414.m	TRUE	reported	GxSb5G	2	TRUE	0.004296	20.88933
2287	rs8105530	T	C	T	C	0.0128	0.011077	0.5286	0.520753	FALSE	FALSE	FALSE	mRULgB	0.013687	0.41833	486484	sepsis	TRUE	reported	textfile	0.0028	4.85E-06	4689	M16816.m	TRUE	reported	05lFsX	2	TRUE	0.004437	20.88905
8755	rs1095163	A	C	A	C	0.0288	0.011104	0.4616	0.459866	FALSE	FALSE	FALSE	KMisdy	0.013716	0.418216	486484	sepsis	TRUE	reported	textfile	0.0063	5.68E-06	3977	M36589.m	TRUE	reported	J1lSTP	2	TRUE	0.005227	20.88745
5803	rs1211769	T	C	T	C	0.0192	9.41E-05	0.7484	0.718906	FALSE	FALSE	FALSE	JE7L71	0.015192	0.995057	486484	sepsis	TRUE	reported	textfile	0.0042	5.24E-06	3890	M33364.m	TRUE	reported	FdLiEs	2	TRUE	0.005344	20.88721
2689	rs226212	A	G	A	G	-0.0384	0.013717	0.5696	0.57125	FALSE	FALSE	FALSE	ATsH6N	0.013864	0.322473	486484	sepsis	TRUE	reported	textfile	0.0084	4.84E-06	3783	M18497.m	TRUE	reported	lGuIRl	2	TRUE	0.005494	20.88691
7385	rs4708385	A	G	A	G	-0.0608	-0.00275	0.3704	0.406441	FALSE	FALSE	FALSE	iLnkG0	0.013945	0.843636	486484	sepsis	TRUE	reported	textfile	0.0133	5.20E-06	2779	M34384.m	TRUE	reported	JVsZvV	2	TRUE	0.007464	20.88292
6360	rs1852749	A	T	A	T	-0.0352	-0.01769	0.942	0.946627	FALSE	TRUE	FALSE	WWmDG	0.03077	0.565351	486484	sepsis	TRUE	reported	textfile	0.0077	4.80E-06	2757	M33627.m	TRUE	reported	2kkEru	2	TRUE	0.007523	20.8828
6453	rs4573623	T	C	T	C	-0.0535	0.00135	0.5476	0.556	FALSE	FALSE	FALSE	OFlEwq	0.013809	0.922125	486484	sepsis	TRUE	reported	textfile	0.0117	4.69E-06	1554	M33675.m	TRUE	reported	T2BzXG	2	TRUE	0.013276	20.88221
5147	rs2840962	A	G	A	G	-0.4719	0.00918	0.0467	0.033521	FALSE	FALSE	FALSE	L2DJzK	0.038137	0.809774	486484	sepsis	TRUE	reported	textfile	0.1019	3.65E-06	76	M33173.m	TRUE	reported	hRG70D	2	TRUE	0.220083	20.88189
3577	rs3123713	A	G	A	G	0.114	0.013941	0.0862	0.10475	FALSE	FALSE	FALSE	F1s6qg	0.022348	0.532767	486484	sepsis	TRUE	reported	textfile	0.0249	4.88E-06	522	M31536.m	TRUE	reported	Zm40GX	2	TRUE	0.038605	20.88064
7151	rs1091677	A	T	A	T	0.0837	-0.01663	0.8056	0.813887	FALSE	TRUE	FALSE	PnBT0M	0.017585	0.344191	486484	sepsis	TRUE	reported	textfile	0.0183	5.05E-06	1009	M34327.m	TRUE	reported	VUt4Q7	2	TRUE	0.020312	20.87791
1176	rs2070006	T	C	T	C	0.0512	0.003381	0.3713	0.375133	FALSE	FALSE	FALSE	FgZx4a	0.014087	0.810306	486484	sepsis	TRUE	reported	textfile	0.0112	4.76E-06	2063	M01640.m	TRUE	reported	hNPAJ8	2	TRUE	0.010028	20.8777
6487	rs7665221	T	C	T	C	-0.0796	-0.02363	0.8107	0.813022	FALSE	FALSE	FALSE	ecwVg8	0.017532	0.177649	486484	sepsis	TRUE	reported	textfile	0.0174	4.71E-06	831	M33751.m	TRUE	reported	NnvbIQ	2	TRUE	0.024565	20.87763
2414	rs268424	A	C	A	C	-0.1129	0.024064	0.0941	0.10359	FALSE	FALSE	FALSE	NBJ2Do	0.022566	0.286254	486484	sepsis	TRUE	reported	textfile	0.0237	1.83E-06	25	M17799.m	TRUE	reported	Uz0Wgl	2	TRUE	0.475814	20.87753
8670	rs1192742	A	G	A	G	0.08	0.028954	0.9586	0.96256	FALSE	FALSE	FALSE	i4bSdJ	0.03638	0.4261	486484	sepsis	TRUE	reported	textfile	0.0175	4.77E-06	1908	M36468.m	TRUE	reported	qJLuZj	2	TRUE	0.010834	20.87605
3462	rs4918207	T	C	T	C	0.0361	0.01029	0.4099	0.435895	FALSE	FALSE	FALSE	jRejFm	0.013772	0.45496	486484	sepsis	TRUE	reported	textfile	0.0079	4.36E-06	6393	M27716.m	TRUE	reported	tg7Rvj	2	TRUE	0.003256	20.8749
1808	rs7548008	A	G	A	G	0.016	-0.00989	0.504	0.522682	FALSE	FALSE	FALSE	JEcCRZ	0.013696	0.470119	486484	sepsis	TRUE	reported	textfile	0.0035	3.56E-06	1764	M15140.m	TRUE	reported	xYCzKP	2	TRUE	0.011708	20.87427
478	rs4718532	T	C	T	C	-0.016	0.027515	0.5052	0.532127	FALSE	FALSE	FALSE	OW48NY	0.013682	0.044326	486484	sepsis	TRUE	reported	textfile	0.0035	6.61E-06	1761	M00527.m	TRUE	reported	57AP2f	2	TRUE	0.011728	20.87422
1426	rs2036136	T	C	T	C	-0.0256	0.007568	0.4587	0.432072	FALSE	FALSE	FALSE	ac6RGB	0.01384	0.584511	486484	sepsis	TRUE	reported	textfile	0.0056	5.11E-06	1741	M02342.m	TRUE	reported	dC6Rkp	2	TRUE	0.011861	20.87395
8081	rs1243683	A	G	A	G	0.0522	-0.0057	0.5918	0.597566	FALSE	FALSE	FALSE	z65cF2	0.013955	0.682708	486484	sepsis	TRUE	reported	textfile	0.0114	5.04E-06	448	M35322.m	TRUE	reported	3GUSWk	2	TRUE	0.044708	20.87316
4725	rs1940528	A	G	A	G	-0.0649	0.003902	0.8479	0.800841	FALSE	FALSE	FALSE	fz63r4	0.017343	0.822004	486484	sepsis	TRUE	reported	textfile	0.0142	4.71E-06	2537	M32762.m	TRUE	reported	kgxlBo	2	TRUE	0.008166	20.87229
1238	rs2491274	A	G	A	G	-0.0297	0.052362	0.9732	0.958325	FALSE	FALSE	FALSE	V50rWC	0.034274	0.126579	486484	sepsis	TRUE	reported	textfile	0.0065	4.63E-06	7346	M01645.m	TRUE	reported	yN3gpQ	2	TRUE	0.002834	20.87219
7979	rs1708452	A	G	A	G	0.0233	-0.0237	0.0965	0.090089	FALSE	FALSE	FALSE	t02AUT	0.024332	0.330123	486484	sepsis	TRUE	reported	textfile	0.0051	4.74E-06	7100	M35254.m	TRUE	reported	uClJaT	2	TRUE	0.002931	20.86648
8409	rs1172061	T	C	T	C	0.0233	0.034273	0.2508	0.24451	FALSE	FALSE	FALSE	02RdNm	0.015904	0.031164	486484	sepsis	TRUE	reported	textfile	0.0051	4.86E-06	6447	M35678.m	TRUE	reported	BWN5JR	2	TRUE	0.003227	20.86588
7191	rs9462736	T	C	T	C	0.0742	0.018262	0.3451	0.344891	FALSE	FALSE	FALSE	2hlepp	0.01437	0.203776	486484	sepsis	TRUE	reported	textfile	0.0162	4.91E-06	369	M34336.m	TRUE	reported	XyK8mO	2	TRUE	0.053794	20.86496
6521	rs2482705	A	G	A	G	-0.0201	0.034261	0.1531	0.148678	FALSE	FALSE	FALSE	OKlXJ8	0.019185	0.074121	486484	sepsis	TRUE	reported	textfile	0.0044	4.06E-06	7362	M33821.m	TRUE	reported	XGkaXH	2	TRUE	0.002827	20.86262
6614	rs1171725	A	G	A	G	-0.0201	0.015507	0.6186	0.602984	FALSE	FALSE	FALSE	2plv9p	0.014035	0.269223	486484	sepsis	TRUE	reported	textfile	0.0044	4.33E-06	7334	M33884.m	TRUE	reported	AoFcqC	2	TRUE	0.002837	20.86259
8326	rs2477833	T	C	T	C	-0.0201	0.010493	0.1056	0.12802	FALSE	FALSE	FALSE	o0GzT1	0.020483	0.608466	486484	sepsis	TRUE	reported	textfile	0.0044	4.81E-06	7317	M35631.m	TRUE	reported	G1XFx0	2	TRUE	0.002844	20.86258
6948	rs1041099	A	C	A	C	0.0201	-0.00094	0.9182	0.908509	FALSE	FALSE	FALSE	7Cw2XZ	0.023723	0.968332	486484	sepsis	TRUE	reported	textfile	0.0044	6.06E-06	7049	M34123.m	TRUE	reported	vrgMl3	2	TRUE	0.002952	20.86236
3749	rs1234961	T	C	T	C	0.0338	0.016887	0.0551	0.019253	FALSE	FALSE	FALSE	OZLdls	0.049834	0.734708	486484	sepsis	TRUE	reported	textfile	0.0074	5.62E-06	7356	M32338.m	TRUE	reported	s2itmB	2	TRUE	0.002828	20.857
6532	rs1177340	T	C	T	C	-0.0169	0.006559	0.7497	0.752326	FALSE	FALSE	FALSE	DedZqr	0.015852	0.679052	486484	sepsis	TRUE	reported	textfile	0.0037	5.30E-06	7350	M33822.m	TRUE	reported	ciYOdn	2	TRUE	0.00283	20.857
1028	rs261342	C	G	C	G	-0.0169	0.015917	0.794	0.779179	FALSE	TRUE	FALSE	haWxZg	0.016539	0.335857	486484	sepsis	TRUE	reported	textfile	0.0037	5.41E-06	7276	M01561.m	TRUE	reported	5wm9R3	2	TRUE	0.002859	20.85694
5114	rs163027	A	G	A	G	0.0169	-0.01213	0.5505	0.54942	FALSE	FALSE	FALSE	O6ine1	0.013743	0.377615	486484	sepsis	TRUE	reported	textfile	0.0037	4.45E-06	7263	M33165.m	TRUE	reported	gGsqjW	2	TRUE	0.002864	20.85693
5122	rs986611	C	G	C	G	-0.0169	-0.0185	0.6362	0.641909	FALSE	TRUE	FALSE	O6ine1	0.014268	0.194848	486484	sepsis	TRUE	reported	textfile	0.0037	5.62E-06	7263	M33165.m	TRUE	reported	gGsqjW	2	TRUE	0.002864	20.85693
6722	rs2679896	T	C	T	C	0.0169	-0.00081	0.2902	0.312183	FALSE	FALSE	FALSE	B6lZly	0.014905	0.956408	486484	sepsis	TRUE	reported	textfile	0.0037	3.96E-06	7222	M33937.m	TRUE	reported	qFPzjh	2	TRUE	0.00288	20.8569
1415	rs7045137	A	G	A	G	-0.0169	-0.00599	0.3947	0.393878	FALSE	FALSE	FALSE	33ptwk	0.014025	0.669105	486484	sepsis	TRUE	reported	textfile	0.0037	4.00E-06	6252	M02137.m	TRUE	reported	SQY1na	2	TRUE	0.003326	20.856
7538	rs358374	A	G	A	G	0.0169	-0.00056	0.1773	0.175585	FALSE	FALSE	FALSE	JJsHvX	0.017973	0.975048	486484	sepsis	TRUE	reported	textfile	0.0037	4.96E-06	5207	M34456.m	TRUE	reported	aklxve	2	TRUE	0.003991	20.85466
2791	rs4347230	A	G	A	G	-0.0169	-0.03326	0.3906	0.397989	FALSE	FALSE	FALSE	ex7hzq	0.013959	0.01718	486484	sepsis	TRUE	reported	textfile	0.0037	6.04E-06	4460	M19415.m	TRUE	reported	jvXWvV	2	TRUE	0.004656	20.85332
2129	rs1195460	T	C	T	C	0.0306	0.062596	0.9767	0.977114	FALSE	FALSE	FALSE	TEupfd	0.045954	0.173153	486484	sepsis	TRUE	reported	textfile	0.0067	4.83E-06	7331	M15676.m	TRUE	reported	NXiaPC	2	TRUE	0.002837	20.8533
7293	rs1910775	A	G	A	G	0.0297	0.006083	0.0927	0.09242	FALSE	FALSE	FALSE	Vcz4EA	0.023723	0.797621	486484	sepsis	TRUE	reported	textfile	0.0065	4.34E-06	1576	M34359.m	TRUE	reported	aUSo2J	2	TRUE	0.013074	20.85138
4996	rs3793499	C	G	C	G	-0.0306	0.044843	0.8598	0.870463	FALSE	TRUE	FALSE	OsqE1P	0.020349	0.027543	486484	sepsis	TRUE	reported	textfile	0.0067	5.06E-06	5425	M33084.m	TRUE	reported	VZYh6f	2	TRUE	0.00383	20.8513
1026	rs1532701	A	G	A	G	0.0137	-0.01168	0.5456	0.538158	FALSE	FALSE	FALSE	haWxZg	0.01372	0.394584	486484	sepsis	TRUE	reported	textfile	0.003	5.07E-06	7276	M01561.m	TRUE	reported	5wm9R3	2	TRUE	0.002858	20.84871
8672	rs1296620	A	T	A	T	0.0434	-0.03553	0.8443	0.841359	FALSE	TRUE	FALSE	i4bSdJ	0.018817	0.058968	486484	sepsis	TRUE	reported	textfile	0.0095	5.30E-06	1908	M36468.m	TRUE	reported	qJLuZj	2	TRUE	0.01082	20.84859
1497	rs1087807	A	G	A	G	0.0137	0.003144	0.6887	0.683537	FALSE	FALSE	FALSE	rzkxmh	0.014683	0.830472	486484	sepsis	TRUE	reported	textfile	0.003	7.14E-06	6941	M03127.m	TRUE	reported	3lrzhA	2	TRUE	0.002996	20.84844
6363	rs562210	A	C	A	C	0.0169	0.003683	0.6864	0.70955	FALSE	FALSE	FALSE	WWmDG	0.015063	0.80685	486484	sepsis	TRUE	reported	textfile	0.0037	4.72E-06	2757	M33627.m	TRUE	reported	2kkEru	2	TRUE	0.00751	20.84754
1823	rs488225	A	G	A	G	-0.0411	-0.00911	0.3902	0.394685	FALSE	FALSE	FALSE	Yub5KV	0.013993	0.514936	486484	sepsis	TRUE	reported	textfile	0.009	4.87E-06	5606	M15335.m	TRUE	reported	mcnlNa	2	TRUE	0.003706	20.847
4454	rs3133571	T	C	T	C	-0.0338	-0.01134	0.2559	0.268401	FALSE	FALSE	FALSE	g2RNym	0.015478	0.46388	486484	sepsis	TRUE	reported	textfile	0.0074	5.05E-06	2575	M32691.m	TRUE	reported	35eo6p	2	TRUE	0.008037	20.84647
5522	rs6873660	A	G	A	G	0.3413	0.008319	0.9683	0.94915	FALSE	FALSE	FALSE	q32d4E	0.031149	0.789416	486484	sepsis	TRUE	reported	textfile	0.0717	1.93E-06	25	M33178.m	TRUE	reported	cS3BwT	2	TRUE	0.475436	20.84597
7670	rs1208393	T	C	T	C	0.0274	0.020813	0.1261	0.134774	FALSE	FALSE	FALSE	iqkM9B	0.020198	0.302795	486484	sepsis	TRUE	reported	textfile	0.006	4.50E-06	4800	M34539.m	TRUE	reported	5nFq6f	2	TRUE	0.004326	20.84576
5928	rs194546	A	G	A	G	-0.4891	0.031774	0.9795	0.98208	FALSE	FALSE	FALSE	veBhfS	0.051235	0.535154	486484	sepsis	TRUE	reported	textfile	0.1065	4.37E-06	171	M33391.m	TRUE	reported	Gfjy5a	2	TRUE	0.109797	20.84427
8717	rs4610008	T	C	T	C	-0.0562	-0.01388	0.6607	0.568481	FALSE	FALSE	FALSE	I0G0I1	0.013845	0.316083	486484	sepsis	TRUE	reported	textfile	0.0123	5.20E-06	1260	M36553.m	TRUE	reported	lBltb0	2	TRUE	0.016299	20.84359
7581	rs1104422	C	G	C	G	-0.0274	-0.00659	0.6326	0.623335	FALSE	TRUE	FALSE	SbjFMF	0.01419	0.642534	486484	sepsis	TRUE	reported	textfile	0.006	5.49E-06	3239	M34513.m	TRUE	reported	HVYiKM	2	TRUE	0.006397	20.84157
7344	rs1606973	A	C	A	C	-0.184	-0.01751	0.6944	0.806974	FALSE	FALSE	FALSE	mm9ksf	0.017339	0.31259	486484	sepsis	TRUE	reported	textfile	0.038	1.32E-06	18	M34365.m	TRUE	reported	AVwEqD	2	TRUE	0.5657	20.84087
6033	rs2287296	T	C	T	C	-1.2634	0.002479	0.025	0.02668	FALSE	FALSE	FALSE	33ojbt	0.043126	0.954167	486484	sepsis	TRUE	reported	textfile	0.2721	3.44E-06	60	M33423.m	TRUE	reported	ivEtvl	2	TRUE	0.264334	20.84018
6814	rs7305796	A	G	A	G	0.0338	0.003225	0.8905	0.89469	FALSE	FALSE	FALSE	PkclDj	0.022321	0.885109	486484	sepsis	TRUE	reported	textfile	0.0074	4.30E-06	1765	M33961.m	TRUE	reported	ww89FU	2	TRUE	0.011682	20.83903
2346	rs1000591	T	G	T	G	0.1151	-0.00952	0.9533	0.93743	FALSE	FALSE	FALSE	NBJ2Do	0.028267	0.736405	486484	sepsis	TRUE	reported	textfile	0.0252	5.09E-06	1739	M17799.m	TRUE	reported	Uz0Wgl	2	TRUE	0.011854	20.8377
518	rs7790791	T	C	T	C	0.0594	0.008603	0.0873	0.092717	FALSE	FALSE	FALSE	cbwEt9	0.023676	0.716312	486484	sepsis	TRUE	reported	textfile	0.013	4.67E-06	1002	M00575.m	TRUE	reported	hWSEOl	2	TRUE	0.020411	20.8362
2921	rs1016131	T	G	T	G	0.0315	0.026869	0.9568	0.955503	FALSE	FALSE	FALSE	fV4qul	0.033296	0.419671	486484	sepsis	TRUE	reported	textfile	0.0069	5.10E-06	7366	M21044.m	TRUE	reported	hbdBQJ	2	TRUE	0.002821	20.83555
1160	rs1770653	A	G	A	G	0.0105	0.005153	0.6331	0.631833	FALSE	FALSE	FALSE	cSBFJ3	0.014137	0.715458	486484	sepsis	TRUE	reported	textfile	0.0023	6.97E-06	7104	M01638.m	TRUE	reported	sUkjpE	2	TRUE	0.002925	20.83534
1169	rs720118	T	C	T	C	-0.0105	0.020114	0.6152	0.621928	FALSE	FALSE	FALSE	cSBFJ3	0.014129	0.154545	486484	sepsis	TRUE	reported	textfile	0.0023	4.55E-06	7104	M01638.m	TRUE	reported	sUkjpE	2	TRUE	0.002925	20.83534
4833	rs9318132	A	C	A	C	-0.021	0.010959	0.2664	0.270616	FALSE	FALSE	FALSE	yLDcUj	0.015379	0.476089	486484	sepsis	TRUE	reported	textfile	0.0046	5.27E-06	6753	M32838.m	TRUE	reported	5dQgAN	2	TRUE	0.003077	20.83504
985	rs8178289	A	C	A	C	-0.1064	0.014358	0.8497	0.841043	FALSE	FALSE	FALSE	xSRlI4	0.018679	0.44209	486484	sepsis	TRUE	reported	textfile	0.0233	5.10E-06	2285	M01515.m	TRUE	reported	juZ5GE	2	TRUE	0.009044	20.83489
2249	rs1978450	A	C	A	C	0.1329	-0.00572	0.017	0.151139	FALSE	FALSE	FALSE	zxcPox	0.019078	0.764473	486484	sepsis	TRUE	reported	textfile	0.0291	4.75E-06	1756	M15990.m	TRUE	reported	34Vg8F	2	TRUE	0.011738	20.83383
592	rs1218955	A	T	A	T	0.021	-0.02175	0.7684	0.781983	FALSE	TRUE	FALSE	KVK33e	0.01657	0.18931	486484	sepsis	TRUE	reported	textfile	0.0046	5.06E-06	5344	M01107.m	TRUE	reported	eAyR8w	2	TRUE	0.003885	20.83341
7235	rs1054363	T	G	T	G	0.2377	-0.00437	0.019	0.028887	FALSE	FALSE	FALSE	RlcE3J	0.040935	0.915069	486484	sepsis	TRUE	reported	textfile	0.052	4.84E-06	633	M34339.m	TRUE	reported	QQrwdm	2	TRUE	0.031955	20.82943
1514	rs4776752	A	G	A	G	0.0178	0.014523	0.1512	0.15223	FALSE	FALSE	FALSE	rzkxmh	0.019115	0.4474	486484	sepsis	TRUE	reported	textfile	0.0039	4.23E-06	6941	M03127.m	TRUE	reported	3lrzhA	2	TRUE	0.002992	20.82503
5667	rs1764758	A	G	A	G	0.0744	0.002727	0.1044	0.154588	FALSE	FALSE	FALSE	wHu1Rk	0.019639	0.889546	486484	sepsis	TRUE	reported	textfile	0.0163	4.76E-06	4625	M33195.m	TRUE	reported	yvhnuQ	2	TRUE	0.004484	20.82489
7948	rs1151428	A	G	A	G	-0.0178	-0.0121	0.1545	0.16048	FALSE	FALSE	FALSE	lvUHnQ	0.018588	0.515015	486484	sepsis	TRUE	reported	textfile	0.0039	4.77E-06	5694	M35240.m	TRUE	reported	VH44Ha	2	TRUE	0.003645	20.82372
6938	rs2839011	T	C	T	C	0.0356	-0.02276	0.2016	0.223059	FALSE	FALSE	FALSE	oNv0vf	0.016412	0.165473	486484	sepsis	TRUE	reported	textfile	0.0078	5.33E-06	5581	M34112.m	TRUE	reported	l0pe0N	2	TRUE	0.003719	20.82357
8608	rs1695973	T	C	T	C	-0.0484	0.024564	0.1032	0.127591	FALSE	FALSE	FALSE	oHM5TD	0.020511	0.231064	486484	sepsis	TRUE	reported	textfile	0.0106	5.56E-06	1489	M36131.m	TRUE	reported	Xw6wmg	2	TRUE	0.013808	20.8207
5778	rs1727996	A	G	A	G	-0.1137	-0.01805	0.9618	0.931111	FALSE	FALSE	FALSE	eT2We0	0.02691	0.502429	486484	sepsis	TRUE	reported	textfile	0.0249	4.96E-06	1273	M33353.m	TRUE	reported	uZx90m	2	TRUE	0.016115	20.81802
5173	rs1006053	C	G	C	G	0.153	-0.00183	0.9646	0.984502	FALSE	TRUE	FALSE	q32d4E	0.056437	0.974202	486484	sepsis	TRUE	reported	textfile	0.0335	4.88E-06	1017	M33178.m	TRUE	reported	cS3BwT	2	TRUE	0.020098	20.81797
5804	rs1221631	A	T	A	T	-0.0429	0.015645	0.0351	0.03213	FALSE	TRUE	FALSE	JE7L71	0.038785	0.686671	486484	sepsis	TRUE	reported	textfile	0.0094	5.49E-06	3890	M33364.m	TRUE	reported	FdLiEs	2	TRUE	0.005326	20.81783
1574	rs9529827	T	C	T	C	-0.0397	0.009978	0.9843	0.979332	FALSE	FALSE	FALSE	Ke0sNh	0.049408	0.839956	486484	sepsis	TRUE	reported	textfile	0.0087	5.14E-06	7799	M10642.m	TRUE	reported	ZlNwVb	2	TRUE	0.002663	20.81762
7164	rs1109726	T	C	T	C	0.0557	-0.01826	0.1593	0.144973	FALSE	FALSE	FALSE	VfvTtz	0.019347	0.345191	486484	sepsis	TRUE	reported	textfile	0.0122	5.11E-06	1492	M34329.m	TRUE	reported	egac6r	2	TRUE	0.013778	20.81652
3422	rs1077449	A	G	A	G	0.0397	-0.03355	0.1908	0.20131	FALSE	FALSE	FALSE	HHQf8w	0.01706	0.049232	486484	sepsis	TRUE	reported	textfile	0.0087	5.39E-06	5673	M27531.m	TRUE	reported	3vTluK	2	TRUE	0.003657	20.81562
3435	rs7539563	T	G	T	G	0.0397	-0.00994	0.2058	0.208187	FALSE	FALSE	FALSE	HHQf8w	0.016828	0.5549	486484	sepsis	TRUE	reported	textfile	0.0087	5.47E-06	5673	M27531.m	TRUE	reported	3vTluK	2	TRUE	0.003657	20.81562
4170	rs1105306	T	C	T	C	0.1636	-0.05138	0.0474	0.042844	FALSE	FALSE	FALSE	Aq7H4p	0.033922	0.129834	486484	sepsis	TRUE	reported	textfile	0.0358	4.84E-06	604	M32560.m	TRUE	reported	NlzbA7	2	TRUE	0.03342	20.81422
8595	rs1249475	A	C	A	C	0.0429	0.021966	0.1264	0.124665	FALSE	FALSE	FALSE	t5potg	0.02092	0.293718	486484	sepsis	TRUE	reported	textfile	0.0094	4.94E-06	2709	M36115.m	TRUE	reported	SSOHIA	2	TRUE	0.00763	20.81317
5797	rs2642911	T	G	T	G	0.0178	-0.00167	0.6151	0.61833	FALSE	FALSE	FALSE	ka9zG7	0.014098	0.905761	486484	sepsis	TRUE	reported	textfile	0.0039	4.53E-06	2212	M33363.m	TRUE	reported	UFTVNc	2	TRUE	0.009329	20.8122
8616	rs890554	C	G	C	G	-0.0283	0.014379	0.6895	0.687925	FALSE	TRUE	FALSE	oHM5TD	0.014751	0.329656	486484	sepsis	TRUE	reported	textfile	0.0062	5.75E-06	1780	M36131.m	TRUE	reported	Xw6wmg	2	TRUE	0.01157	20.8114
3471	rs7319920	A	G	A	G	-0.0219	0.011002	0.1522	0.153957	FALSE	FALSE	FALSE	BQlbxe	0.018965	0.561815	486484	sepsis	TRUE	reported	textfile	0.0048	4.39E-06	7373	M27718.m	TRUE	reported	MnHHJ0	2	TRUE	0.002815	20.81076
8308	rs1493534	A	C	A	C	0.0219	-0.01644	0.8645	0.856696	FALSE	FALSE	FALSE	xXnn6V	0.019827	0.40695	486484	sepsis	TRUE	reported	textfile	0.0048	4.97E-06	7364	M35626.m	TRUE	reported	WgWa24	2	TRUE	0.002819	20.81075
941	rs7973508	A	G	A	G	-0.0073	-0.00858	0.2831	0.27595	FALSE	FALSE	FALSE	tv99UN	0.015297	0.574866	486484	sepsis	TRUE	reported	textfile	0.0016	5.40E-06	7354	M01494.m	TRUE	reported	osDclp	2	TRUE	0.002823	20.81075
1280	rs1381274	T	C	T	C	-0.0146	0.0064	0.4672	0.463429	FALSE	FALSE	FALSE	nvWJJ3	0.013677	0.639841	486484	sepsis	TRUE	reported	textfile	0.0032	3.63E-06	7346	M01712.m	TRUE	reported	1x44lf	2	TRUE	0.002826	20.81074
2863	rs9511834	A	C	A	C	-0.0073	0.011956	0.4232	0.427941	FALSE	FALSE	FALSE	Y5qWBm	0.013781	0.385615	486484	sepsis	TRUE	reported	textfile	0.0016	8.94E-06	7325	M20489.m	TRUE	reported	f2g9Su	2	TRUE	0.002834	20.81072
7307	rs4925536	A	G	A	G	0.0146	0.002376	0.8988	0.890866	FALSE	FALSE	FALSE	Vcz4EA	0.022012	0.914055	486484	sepsis	TRUE	reported	textfile	0.0032	7.17E-06	6755	M34359.m	TRUE	reported	aUSo2J	2	TRUE	0.003072	20.81024
7657	rs7162018	A	G	A	G	-0.0219	0.011368	0.4943	0.501233	FALSE	FALSE	FALSE	8W5cqf	0.013748	0.408308	486484	sepsis	TRUE	reported	textfile	0.0048	4.54E-06	4925	M34534.m	TRUE	reported	JV42Pw	2	TRUE	0.004209	20.80795
2232	rs1052038	T	G	T	G	0.0219	-0.02837	0.1733	0.167632	FALSE	FALSE	FALSE	cwj8fY	0.018291	0.120907	486484	sepsis	TRUE	reported	textfile	0.0048	4.92E-06	4538	M15958.m	TRUE	reported	dPwsBc	2	TRUE	0.004566	20.80723
6225	rs281796	A	C	A	C	-0.0356	-0.01914	0.8247	0.827763	FALSE	FALSE	FALSE	8zUBbU	0.018143	0.29154	486484	sepsis	TRUE	reported	textfile	0.0078	5.62E-06	1733	M33509.m	TRUE	reported	1mO9m8	2	TRUE	0.011877	20.80699
8745	rs1736651	A	G	A	G	-0.0584	-0.00341	0.9343	0.933186	FALSE	FALSE	FALSE	FnYrPc	0.02753	0.901441	486484	sepsis	TRUE	reported	textfile	0.0128	4.75E-06	3346	M36585.m	TRUE	reported	amHtMH	2	TRUE	0.006183	20.80396
5060	rs1321237	T	G	T	G	-0.0333	0.006434	0.0741	0.06783	FALSE	FALSE	FALSE	nHZXjp	0.027445	0.814659	486484	sepsis	TRUE	reported	textfile	0.0073	4.61E-06	7166	M33138.m	TRUE	reported	ccoK1F	2	TRUE	0.002895	20.80279
7135	rs197712	T	C	T	C	0.098	-0.00079	0.8636	0.863625	FALSE	FALSE	FALSE	de8tA6	0.019833	0.9684	486484	sepsis	TRUE	reported	textfile	0.0214	4.67E-06	248	M34322.m	TRUE	reported	sXPPBO	2	TRUE	0.077968	20.80214

2473	rs1398802	T	G	T	G	-0.026	0.009145	0.6877	0.673165	FALSE	FALSE	FALSE	SrfyMU	0.014602	0.531142	486484	sepsis	TRUE	reported	textfile	0.0057	6.12E-06	7226	M17807.m	TRUE	reported	0zrJsu	2	TRUE	0.002871	20.80064
8492	rs7586148	T	C	T	C	-0.0324	-0.02342	0.5142	0.484812	FALSE	FALSE	FALSE	ABkZ7x	0.013684	0.087	486484	sepsis	TRUE	reported	textfile	0.0071	5.53E-06	1632	M35854.m	TRUE	reported	wc0CeK	2	TRUE	0.012599	20.79892
9060	rs9922615	T	C	T	C	0.0187	-0.01675	0.9445	0.941627	FALSE	FALSE	FALSE	df0MCM	0.029584	0.571285	486484	sepsis	TRUE	reported	textfile	0.0041	6.28E-06	7366	M37506.m	TRUE	reported	iUfr4u	2	TRUE	0.002816	20.79685
8992	rs1341951	A	G	A	G	-0.0187	-0.00601	0.5995	0.5974	FALSE	FALSE	FALSE	TfdxtP	0.013933	0.666392	486484	sepsis	TRUE	reported	textfile	0.0041	5.78E-06	7333	M37203.m	TRUE	reported	xicmWr	2	TRUE	0.002829	20.79682
8993	rs1476117	A	G	A	G	0.0187	0.006754	0.5513	0.539168	FALSE	FALSE	FALSE	TfdxtP	0.013768	0.623762	486484	sepsis	TRUE	reported	textfile	0.0041	5.86E-06	7333	M37203.m	TRUE	reported	xicmWr	2	TRUE	0.002829	20.79682
1361	rs1047745	T	C	T	C	-0.0374	0.024787	0.0169	0.019821	FALSE	FALSE	FALSE	KrWhws	0.049295	0.615092	486484	sepsis	TRUE	reported	textfile	0.0082	4.65E-06	7325	M02132.m	TRUE	reported	7pzpfK	2	TRUE	0.002832	20.79682
4214	rs1079699	T	C	T	C	0.0374	0.007808	0.865	0.855785	FALSE	FALSE	FALSE	aS6krA	0.019492	0.688717	486484	sepsis	TRUE	reported	textfile	0.0082	4.81E-06	7303	M32586.m	TRUE	reported	g9cBDI	2	TRUE	0.00284	20.7968
3115	rs1697378	A	G	A	G	-0.073	-0.07198	0.036	0.051506	FALSE	FALSE	FALSE	a9KeDe	0.031191	0.021016	486484	sepsis	TRUE	reported	textfile	0.016	5.06E-06	2088	M22175.m	TRUE	reported	shg5gu	2	TRUE	0.009871	20.79647
4659	rs7228173	T	C	T	C	0.0187	-0.02248	0.2694	0.290607	FALSE	FALSE	FALSE	SyAFR2	0.015105	0.136721	486484	sepsis	TRUE	reported	textfile	0.0041	6.11E-06	6658	M32755.m	TRUE	reported	prUo74	2	TRUE	0.003115	20.79625
6551	rs9317917	A	C	A	C	-0.0479	0.014553	0.1628	0.19051	FALSE	FALSE	FALSE	zmDnD7	0.017471	0.404864	486484	sepsis	TRUE	reported	textfile	0.0105	5.30E-06	2660	M33835.m	TRUE	reported	MLBiKM	2	TRUE	0.007763	20.79533
7316	rs8071165	T	G	T	G	-0.1013	-0.00663	0.0115	0.02667	FALSE	FALSE	FALSE	Vcz4EA	0.042605	0.87631	486484	sepsis	TRUE	reported	textfile	0.0222	5.09E-06	1576	M34359.m	TRUE	reported	aUSo2J	2	TRUE	0.013039	20.79512
6499	rs1986163	T	C	T	C	0.264	-0.05903	0.0335	0.023189	FALSE	FALSE	FALSE	HNc7C9	0.045578	0.195259	486484	sepsis	TRUE	reported	textfile	0.0578	4.88E-06	581	M33782.m	TRUE	reported	SAGYGx	2	TRUE	0.034662	20.79001
885	rs1715594	T	C	T	C	0.0698	0.036514	0.9746	0.986758	FALSE	FALSE	FALSE	oi7KQI	0.060257	0.544529	486484	sepsis	TRUE	reported	textfile	0.0153	4.75E-06	1768	M01365.m	TRUE	reported	7NWGKA	2	TRUE	0.011635	20.78914
8160	rs764419	T	G	T	G	-0.0187	0.022667	0.1973	0.208673	FALSE	FALSE	FALSE	sRlsb6	0.016863	0.178888	486484	sepsis	TRUE	reported	textfile	0.0041	4.99E-06	3007	M35331.m	TRUE	reported	GPijIC	2	TRUE	0.00687	20.78866
887	rs314241	T	C	T	C	-0.0114	0.008843	0.4625	0.445918	FALSE	FALSE	FALSE	oi7KQI	0.013831	0.522563	486484	sepsis	TRUE	reported	textfile	0.0025	5.25E-06	7362	M01365.m	TRUE	reported	7NWGKA	2	TRUE	0.002816	20.78795
890	rs453612	T	C	T	C	0.0114	0.00377	0.3549	0.350134	FALSE	FALSE	FALSE	oi7KQI	0.014362	0.792939	486484	sepsis	TRUE	reported	textfile	0.0025	5.85E-06	7362	M01365.m	TRUE	reported	7NWGKA	2	TRUE	0.002816	20.78795
891	rs6467872	A	C	A	C	0.0114	-0.0063	0.6087	0.583851	FALSE	FALSE	FALSE	oi7KQI	0.013961	0.651648	486484	sepsis	TRUE	reported	textfile	0.0025	5.64E-06	7362	M01365.m	TRUE	reported	7NWGKA	2	TRUE	0.002816	20.78795
695	rs1051646	T	C	T	C	-0.0114	0.004084	0.1	0.10414	FALSE	FALSE	FALSE	H9I3s3	0.022695	0.857174	486484	sepsis	TRUE	reported	textfile	0.0025	5.89E-06	7358	M01299.m	TRUE	reported	kXWyUq	2	TRUE	0.002818	20.78795
1608	rs2863202	T	C	T	C	0.0114	-0.0204	0.178	0.171109	FALSE	FALSE	FALSE	zP3C2v	0.018166	0.261475	486484	sepsis	TRUE	reported	textfile	0.0025	6.93E-06	7356	M12035.m	TRUE	reported	1Pooe3	2	TRUE	0.002819	20.78795
3648	rs1285355	A	G	A	G	0.0228	0.02475	0.088	0.094176	FALSE	FALSE	FALSE	fnjup6	0.023422	0.290657	486484	sepsis	TRUE	reported	textfile	0.005	4.63E-06	7346	M32197.m	TRUE	reported	oQzaMk	2	TRUE	0.002823	20.78794
3947	rs1260148	T	C	T	C	-0.0342	-0.00707	0.8888	0.887366	FALSE	FALSE	FALSE	N6UQnQ	0.021798	0.745779	486484	sepsis	TRUE	reported	textfile	0.0075	4.43E-06	7346	M32425.m	TRUE	reported	FEcGHY	2	TRUE	0.002823	20.78794
7796	rs177387	A	G	A	G	-0.0114	-0.00411	0.5143	0.524282	FALSE	FALSE	FALSE	cQ5TvO	0.01385	0.766673	486484	sepsis	TRUE	reported	textfile	0.0025	6.19E-06	7344	M35127.m	TRUE	reported	BVxkfZ	2	TRUE	0.002823	20.78794
7797	rs1812081	T	G	T	G	0.0114	0.009046	0.3963	0.398214	FALSE	FALSE	FALSE	cQ5TvO	0.013953	0.516776	486484	sepsis	TRUE	reported	textfile	0.0025	5.48E-06	7344	M35127.m	TRUE	reported	BVxkfZ	2	TRUE	0.002823	20.78794
8994	rs2295709	T	C	T	C	0.0228	-0.00057	0.7937	0.77923	FALSE	FALSE	FALSE	TfdxtP	0.016453	0.972582	486484	sepsis	TRUE	reported	textfile	0.005	4.81E-06	7333	M37203.m	TRUE	reported	xicmWr	2	TRUE	0.002828	20.78793
3080	rs1741183	A	C	A	C	-0.0114	-0.00545	0.7981	0.795221	FALSE	FALSE	FALSE	esOP8m	0.016987	0.748268	486484	sepsis	TRUE	reported	textfile	0.0025	3.21E-06	7329	M22116.m	TRUE	reported	fDMh7Z	2	TRUE	0.002829	20.78793
846	rs1074661	A	G	A	G	-0.0114	0.012519	0.2333	0.237609	FALSE	FALSE	FALSE	1tFtaf	0.016117	0.437277	486484	sepsis	TRUE	reported	textfile	0.0025	4.05E-06	7323	M01359.m	TRUE	reported	qq3q6E	2	TRUE	0.002831	20.78792
6032	rs2111849	A	C	A	C	-1.5433	-0.00421	0.0175	0.017228	FALSE	FALSE	FALSE	33ojbt	0.053144	0.936857	486484	sepsis	TRUE	reported	textfile	0.3328	3.54E-06	60	M33423.m	TRUE	reported	ivEtvl	2	TRUE	0.263846	20.78791
4463	rs1028578	T	C	T	C	0.0114	0.004106	0.183	0.185788	FALSE	FALSE	FALSE	g3iM2w	0.017582	0.815359	486484	sepsis	TRUE	reported	textfile	0.0025	7.52E-06	7308	M32698.m	TRUE	reported	ZQBn0h	2	TRUE	0.002837	20.78791
4473	rs4537895	A	G	A	G	0.0114	-0.01614	0.7962	0.79409	FALSE	FALSE	FALSE	g3iM2w	0.016983	0.341864	486484	sepsis	TRUE	reported	textfile	0.0025	6.62E-06	7308	M32698.m	TRUE	reported	ZQBn0h	2	TRUE	0.002837	20.78791
1172	rs837763	T	C	T	C	0.0114	-0.01317	0.5528	0.556074	FALSE	FALSE	FALSE	cSBFJ3	0.013745	0.338028	486484	sepsis	TRUE	reported	textfile	0.0025	6.03E-06	7104	M01638.m	TRUE	reported	sUkjpE	2	TRUE	0.002918	20.78775
8268	rs2592126	A	G	A	G	-0.0114	0.016407	0.4137	0.434845	FALSE	FALSE	FALSE	TdsGCZ	0.013795	0.234307	486484	sepsis	TRUE	reported	textfile	0.0025	4.75E-06	6883	M35464.m	TRUE	reported	sOspot	2	TRUE	0.003012	20.78756
3443	rs1247053	T	C	T	C	0.0228	0.015482	0.6097	0.604076	FALSE	FALSE	FALSE	J1I92u	0.013986	0.268292	486484	sepsis	TRUE	reported	textfile	0.005	5.45E-06	6715	M27710.m	TRUE	reported	9qjpIZ	2	TRUE	0.003087	20.78741
2332	rs9393768	A	G	A	G	0.0114	0.003182	0.3579	0.357625	FALSE	FALSE	FALSE	IZXwpR	0.014256	0.82338	486484	sepsis	TRUE	reported	textfile	0.0025	6.24E-06	6549	M16822.m	TRUE	reported	Zx9k2V	2	TRUE	0.003165	20.78725
6430	rs1443751	T	C	T	C	0.0114	-0.03131	0.7823	0.779769	FALSE	FALSE	FALSE	QCw5dg	0.016488	0.057601	486484	sepsis	TRUE	reported	textfile	0.0025	5.36E-06	6279	M33666.m	TRUE	reported	zYHmzg	2	TRUE	0.003301	20.78698
3052	rs2054299	A	G	A	G	-0.0114	-0.01223	0.4546	0.478667	FALSE	FALSE	FALSE	H6vcCG	0.013727	0.372875	486484	sepsis	TRUE	reported	textfile	0.0025	6.30E-06	6180	M22030.m	TRUE	reported	XetVX7	2	TRUE	0.003353	20.78687
3045	rs1015339	T	G	T	G	-0.0114	-0.01873	0.4862	0.483223	FALSE	FALSE	FALSE	H6vcCG	0.013758	0.173281	486484	sepsis	TRUE	reported	textfile	0.0025	6.59E-06	6180	M22030.m	TRUE	reported	XetVX7	2	TRUE	0.003353	20.78687
681	rs1079536	C	G	C	G	0.0114	0.029627	0.6957	0.731785	FALSE	TRUE	FALSE	aT1Fw1	0.015464	0.055375	486484	sepsis	TRUE	reported	textfile	0.0025	3.76E-06	5687	M01284.m	TRUE	reported	mbTe8H	2	TRUE	0.003643	20.78629
8659	rs1312517	C	G	C	G	-0.0406	-0.00376	0.1863	0.195476	FALSE	TRUE	FALSE	BFEGyF	0.017275	0.827866	486484	sepsis	TRUE	reported	textfile	0.0089	5.47E-06	1582	M36399.m	TRUE	reported	nhT4FM	2	TRUE	0.012983	20.78369
2748	rs4280872	A	G	A	G	-0.0269	0.02054	0.0491	0.055617	FALSE	FALSE	FALSE	qFAbee	0.030051	0.494291	486484	sepsis	TRUE	reported	textfile	0.0059	5.48E-06	6929	M19364.m	TRUE	reported	tZgXNL	2	TRUE	0.002991	20.78142
5624	rs1087844	A	G	A	G	0.0944	-0.02278	0.0829	0.081391	FALSE	FALSE	FALSE	U0d0ue	0.025309	0.368077	486484	sepsis	TRUE	reported	textfile	0.0207	5.21E-06	2616	M33190.m	TRUE	reported	FoODGF	2	TRUE	0.007887	20.78122
8610	rs2068007	T	C	T	C	0.0374	0.009507	0.9124	0.953881	FALSE	FALSE	FALSE	oHM5TD	0.033029	0.773464	486484	sepsis	TRUE	reported	textfile	0.0082	5.49E-06	1780	M36131.m	TRUE	reported	Xw6wmg	2	TRUE	0.011552	20.77912
4837	rs1102970	T	C	T	C	-0.0424	-0.01141	0.1475	0.169757	FALSE	FALSE	FALSE	jvIpBt	0.018353	0.534305	486484	sepsis	TRUE	reported	textfile	0.0093	5.34E-06	6250	M32846.m	TRUE	reported	RAXcz5	2	TRUE	0.003315	20.7791
8625	rs7474722	T	C	T	C	-0.0757	0.008959	0.9636	0.965685	FALSE	FALSE	FALSE	MZ60v7	0.037529	0.811321	486484	sepsis	TRUE	reported	textfile	0.0166	5.29E-06	2432	M36134.m	TRUE	reported	dMQewN	2	TRUE	0.008478	20.7787
2238	rs2579931	A	G	A	G	0.0269	0.002769	0.1044	0.10035	FALSE	FALSE	FALSE	cwj8fY	0.022864	0.903618	486484	sepsis	TRUE	reported	textfile	0.0059	4.23E-06	4538	M15958.m	TRUE	reported	dPwsBc	2	TRUE	0.00456	20.77826
8427	rs1084231	T	G	T	G	0.0155	-0.0134	0.1288	0.123422	FALSE	FALSE	FALSE	Nvt7pl	0.020901	0.521501	486484	sepsis	TRUE	reported	textfile	0.0034	5.84E-06	7358	M35718.m	TRUE	reported	WkA5Gq	2	TRUE	0.002817	20.77722
1263	rs1792544	A	G	A	G	-0.0155	-0.01073	0.8963	0.906793	FALSE	FALSE	FALSE	gNG3wy	0.023588	0.649156	486484	sepsis	TRUE	reported	textfile	0.0034	5.83E-06	7348	M01670.m	TRUE	reported	t3lsMq	2	TRUE	0.00282	20.77722
1396	rs7597477	A	G	A	G	-0.0155	0.015855	0.9327	0.932347	FALSE	FALSE	FALSE	KrWhws	0.027175	0.559615	486484	sepsis	TRUE	reported	textfile	0.0034	6.80E-06	7325	M02132.m	TRUE	reported	7pzpfK	2	TRUE	0.002829	20.7772
2872	rs1156470	C	G	C	G	0.0155	0.027556	0.8162	0.799568	FALSE	TRUE	FALSE	mUzn1R	0.017154	0.108202	486484	sepsis	TRUE	reported	textfile	0.0034	6.40E-06	7301	M20675.m	TRUE	reported	XwY04K	2	TRUE	0.002838	20.77718
7282	rs1251697	C	G	C	G	0.0155	0.000474	0.9076	0.878339	FALSE	TRUE	FALSE	Vcz4EA	0.021165	0.982137	486484	sepsis	TRUE	reported	textfile	0.0034	6.47E-06	6755	M34359.m	TRUE	reported	aUSo2J	2	TRUE	0.003067	20.77672
9006	rs6064820	T	C	T	C	0.0155	0.008841	0.7229	0.723601	FALSE	FALSE	FALSE	EZQ1cW	0.015297	0.563316	486484	sepsis	TRUE	reported	textfile	0.0034	4.01E-06	5932	M37253.m	TRUE	reported	ddPpd8	2	TRUE	0.003491	20.77586
1437	rs1115140	A	T	A	T	0.0301	0.027804	0.9444	0.950712	FALSE	TRUE	FALSE	1M4gqj	0.031643	0.379584	486484	sepsis	TRUE	reported	textfile	0.0066	5.61E-06	1766	M02730.m	TRUE	reported	RWyYEM	2	TRUE	0.01164	20.77557
2277	rs1193567	A	G	A	G	-0.0155	0.001664	0.202	0.200887	FALSE	FALSE	FALSE	mRULgB	0.017083	0.922382	486484	sepsis	TRUE	reported	textfile	0.0034	6.80E-06	4689	M16816.m	TRUE	reported	05IFsX	2	TRUE	0.004413	20.77401
8343	rs2459791	T	C	T	C	0.0552	0.029938	0.4055	0.40006	FALSE	FALSE	FALSE	USZiKY	0.014242	0.035548	486484	sepsis	TRUE	reported	textfile	0.0121	5.24E-06	1098	M35635.m	TRUE	reported	HuVMRL	2	TRUE	0.018602	20.77378
862	rs1179116	T	G	T	G	0.0196	0.006862	0.0803	0.102444	FALSE	FALSE	FALSE	Vg5OOR	0.022743	0.762862	486484	sepsis	TRUE	reported	textfile	0.0043	4.35E-06	7063	M01361.m	TRUE	reported	XnrS00	2	TRUE	0.002933	20.77075
7152	rs1220077	A	G	A	G	0.0871	-0.08492	0.0859	0.084185	FALSE	FALSE	FALSE	PnBT0M	0.024892	0.000646	486484	sepsis	TRUE	reported	textfile	0.0191	5.10E-06	1612	M34327.m	TRUE	reported	VUt4Q7	2	TRUE	0.012736	20.76971
8220	rs1091873	A	T	A	T	0.0237	0.105571	0.9478	0.93515	FALSE	TRUE	FALSE	4YAWhA	0.02779	0.000145	486484	sepsis	TRUE	reported	textfile	0.0052	6.02E-06	7256	M35439.m	TRUE	reported	bSSC4Z	2	TRUE	0.002855	20.76683
6567	rs432642	A	T	A	T	-0.0237	-0.01345	0.1441	0.164084	FALSE	TRUE	FALSE	aTDqRQ	0.018481	0.466847	486484	sepsis	TRUE	reported	textfile	0.0052	4.09E-06	6506	M33871.m	TRUE	reported	Lqimdw	2	TRUE	0.003183	20.76617
6737	rs4831776	T	C	T	C	0.0237	0.005645	0.9508	0.952315	FALSE	FALSE	FALSE	SpG9A1	0.032433	0.861816	486484	sepsis	TRUE	reported	textfile	0.0052	6.28E-06	6502	M33939.m	TRUE	reported	Tudxel	2	TRUE	0.003185	20.76617
7168	rs2876587	T	C	T	C	0.0684	0.036648	0.1046	0.114792	FALSE	FALSE	FALSE	VfvTtz	0.021512	0.088461	486484	sepsis	TRUE	reported	textfile	0.015	5.01E-06	1492	M34329.m	TRUE	reported	egac6r	2	TRUE	0.013745	20.76573
8635	rs297192	T	G	T	G	0.031	-0.02559	0.4024	0.411186	FALSE	FALSE	FALSE	4cZjpQ	0.013929	0.066148	486484	sepsis	TRUE	reported	textfile	0.0068	4.54E-06	2400	M36230.m	TRUE	reported	TPUeNs	2	TRUE	0.008585	20.76555
6679	rs6987078	A	G	A	G	0.0237	-0.02714	0.2224	0.223972	FALSE	FALSE	FALSE	6iZSOI	0.016617	0.102459	486484	sepsis	TRUE	reported	textfile	0.0052	5.33E-06	4533	M33901.m	TRUE	reported	ApKaGe	2	TRUE	0.004562	20.76339
4174	rs4630914	T	C	T	C	0.062	0.035895	0.1001	0.109445	FALSE	FALSE	FALSE	Aq7H4p	0.02202	0.103084	486484	sepsis	TRUE	reported	textfile	0.0136	4.90E-06	2091	M32560.m	TRUE	reported	NIzbA7	2	TRUE	0.009841	20.76299
4735	rs1259878	C	G	C	G	0.0278	-0.00058	0.599	0.600043	FALSE	TRUE	FALSE	Do3Q7g	0.01397	0.966922	486484	sepsis	TRUE	reported	textfile	0.0061	4.89E-06	6150	M32769.m	TRUE	reported	ys2LKp	2	TRUE	0.003366	20.76293
631	rs1764134	C	G	C	G	0.0725	-0.01609	0.1639	0.175347	FALSE	TRUE	FALSE	F6Dn8H	0.01805	0.372642	486484	sepsis	TRUE	reported	textfile	0.0159	5.26E-06	1447	M01114.m	TRUE	reported	dVHatZ	2	TRUE	0.014165	20.76257
6399	rs1374486	A	G	A	G	-0.0319	-0.00731	0.2098	0.211876	FALSE	FALSE	FALSE	Z1h3mT	0.016716	0.661913	486484	sepsis	TRUE	reported	textfile	0.007	5.52E-06	5328	M33652.m	TRUE	reported	nszuTQ	2	TRUE	0.003883	20.75976
6729	rs1049243	T	C	T	C	-0.036	-0.00746	0.0203	0.018923	FALSE	FALSE	FALSE	SpG9A1	0.050316	0.882094	486484	sepsis	TRUE	reported	textfile	0.0079	5.15E-06	6502	M33939.m	TRUE	reported	Tudxel	2	TRUE	0.003184	20.75952
6063	rs3134034	T	C	T	C	0.031	0.018821	0.5354	0.531267	FALSE	FALSE	FALSE	P4BuMD	0.013761	0.171413	486484	sepsis	TRUE	reported	textfile	0.0068	4.51E-06	1767	M33441.m	TRUE	reported	kqrc1d	2	TRUE	0.011625	20.75935
2847	rs2159491	T	C	T	C	-0.0155	-0.03654	0.7764	0.756153	FALSE	FALSE	FALSE	Y5qWBm	0.015889	0.021473	486484	sepsis	TRUE	reported	textfile	0.0034	4.44E-06	1754	M20489.m	TRUE	reported	f2g9Su	2	TRUE	0.01171	20.75917
6480	rs4691942	A	G	A	G	-0.2972	0.010398	0.8916	0.855824	FALSE	FALSE	FALSE	ecwVg8	0.019447	0.592882	486484	sepsis	TRUE	reported	textfile	0.0644	3.91E-06	79	M33751.m	TRUE	reported	NnvbIQ	2	TRUE	0.212342	20.75819
2400	rs1755780	T	C	T	C	0.0971	-0.02648	0.9267	0.90644	FALSE	FALSE	FALSE	NBJ2Do	0.02372	0.264224	486484	sepsis	TRUE	reported	textfile	0.0213	5.39E-06	1739	M17799.m	TRUE	reported	Uz0Wgl	2	TRUE	0.011809	20.75771
5684	rs1178367	C	G	C	G	-0.0916	0.027017	0.8364	0.813746	FALSE	TRUE	FALSE	KxtFnC	0.01784	0.129917	486484	sepsis	TRUE	reported	textfile	0.0201	5.45E-06	3221	M33203.m	TRUE	reported	Uz0wKU	2	TRUE	0.006406	20.7553
6376	rs1018664	A	G	A	G	-0.1988	0.06716	0.0229	0.027518	FALSE	FALSE	FALSE	pPr1v0	0.041732	0.107549	486484	sepsis	TRUE	reported	textfile	0.0436	5.12E-06	1155	M33637.m	TRUE	reported	Ide1A7	2	TRUE	0.017682	20.75425
6553	rs1008810	A	G	A	G	-0.0465	-0.0074	0.809	0.80722	FALSE	FALSE	FALSE	58qeH5	0.017456	0.671528	486484	sepsis	TRUE	reported	textfile	0.0102	5.18E-06	1428	M33864.m	TRUE	reported	TBsy8r	2	TRUE	0.014345	20.75376
1335	rs1615586	T	G	T	G	-0.0196	-0.00596	0.7674	0.734763	FALSE	FALSE	FALSE	LDUJbh	0.015507	0.700892	486484	sepsis	TRUE	reported	textfile	0.0043	4.18E-06	1766	M01898.m	TRUE	reported	JRSLeH	2	TRUE	0.011628	20.75311
8623	rs7099968	T	C	T	C	-0.0556	-0.00145	0.0466	0.036129	FALSE	FALSE	FALSE	MZ60v7	0.036881	0.968652	486484	sepsis	TRUE	reported	textfile	0.0122	4.66E-06	2432	M36134.m	TRUE	reported	dMQewN	2	TRUE	0.008468	20.75261
1175	rs1310369	T	G	T	G	-0.0474	-0.00802	0.5359	0.541852	FALSE	FALSE	FALSE	FgZx4a	0.013696	0.558018	486484	sepsis	TRUE	reported	textfile	0.0104	5.20E-06	2063	M01640.m	TRUE	reported	hNPAJ8	2	TRUE	0.009969	20.75242
8530	rs743745	T	G	T	G	0.0319	-0.00775	0.1734	0.166632	FALSE	FALSE	FALSE	gM7v2m	0.018341	0.672651	486484	sepsis	TRUE	reported	textfile	0.007	6.00E-06	2651	M36009.m	TRUE	reported	6Xcypd	2	TRUE	0.007773	20.75188
4071	rs2042807	T	C	T	C	-0.0237	-0.00782	0.573	0.564926	FALSE	FALSE	FALSE	TyHtdh	0.013909	0.573752	486484	sepsis	TRUE	reported	textfile	0.0052	5.40E-06	1767	M32497.m	TRUE	reported	H3KmLR	2	TRUE	0.011619	20.74905
3548	rs1337857	A	C	A	C	-0.0711	0.024832	0.0303	0.040213	FALSE	FALSE	FALSE	8luUON	0.035142	0.479803	486484	sepsis	TRUE	reported	textfile	0.0156	4.81E-06	1750	M31453.m	TRUE	reported	FBs6G4	2	TRUE	0.011731	20.74882
6209	rs1049259	A	C	A	C	0.1605	-0.03069	0.0227	0.014126	FALSE	FALSE	FALSE	FQfT2o	0.058093	0.597314	486484	sepsis	TRUE	reported	textfile	0.0352	5.08E-06	972	M33508.m	TRUE	reported	JtbOy2	2	TRUE	0.020941	20.74771
7082	rs574773	T	C	T	C	-0.036	0.00107	0.0733	0.080763	FALSE	FALSE	FALSE	sg7nOB	0.025049	0.965936	486484	sepsis	TRUE	reported	textfile	0.0079	5.97E-06	2278	M34289.m	TRUE	reported	KemvIC	2	TRUE	0.009034	20.74767
4223	rs1624064	T	C	T	C	-0.0164	-0.01532	0.5678	0.579779	FALSE	FALSE	FALSE	FDy32d	0.013876	0.269635	486484	sepsis	TRUE	reported	textfile	0.0036	6.36E-06	7369	M32587.m	TRUE	reported	S10k1h	2	TRUE	0.002808	20.74745
4325	rs7529462	C	G	C	G	0.0164	-0.00488	0.7318	0.714618	FALSE	TRUE	FALSE	EOAssJ	0.015204	0.748011	486484	sepsis	TRUE	reported	textfile	0.0036	4.93E-06	7369	M32635.m	TRUE	reported	X1mTjy	2	TRUE	0.002808	20.74745
6810	rs1595416	A	G	A	G	0.0164	-0.00445	0.2639	0.253379	FALSE	FALSE	FALSE	PkclDj	0.015762	0.777891	486484	sepsis	TRUE	reported	textfile	0.0036	6.93E-06	7368	M33961.m	TRUE	reported	ww89FU	2	TRUE	0.002809	20.74745
9020	rs1168264	A	G	A	G	0.0082	-0.00181	0.5871	0.570835	FALSE	FALSE	FALSE	df0MCM	0.01386	0.896375	486484	sepsis	TRUE	reported	textfile	0.0018	2.66E-06	7366	M37506.m	TRUE	reported	iUfr4u	2	TRUE	0.00281	20.74745
9043	rs4749219	A	G	A	G	0.0082	0.008407	0.2784	0.285084	FALSE	FALSE	FALSE	df0MCM	0.015153	0.579019	486484	sepsis	TRUE	reported	textfile	0.0018	7.02E-06	7366	M37506.m	TRUE	reported	iUfr4u	2	TRUE	0.00281	20.74745
409	rs1289681	T	C	T	C	-0.0082	-0.01343	0.1853	0.18393	FALSE	FALSE	FALSE	cTsNj6	0.017683	0.447434	486484	sepsis	TRUE	reported	textfile	0.0018	3.52E-06	7365	M00063.m	TRUE	reported	7KYTrQ	2	TRUE	0.00281	20.74745
428	rs4401492	T	G	T	G	0.0082	-0.01084	0.1795	0.186892	FALSE	FALSE	FALSE	cTsNj6	0.017544	0.536466	486484	sepsis	TRUE	reported	textfile	0.0018	4.52E-06	7365	M00063.m	TRUE	reported	7KYTrQ	2	TRUE	0.00281	20.74745
440	rs871748	T	C	T	C	-0.0082	-0.00259	0.182	0.186196	FALSE	FALSE	FALSE	cTsNj6	0.017731	0.883992	486484	sepsis	TRUE	reported	textfile	0.0018	8.56E-06	7365	M00063.m	TRUE	reported	7KYTrQ	2	TRUE	0.00281	20.74745
3816	rs1049128	A	G	A	G	-0.0082	0.003662	0.658	0.647076	FALSE	FALSE	FALSE	tRX2u0	0.014321	0.798186	486484	sepsis	TRUE	reported	textfile	0.0018	3.73E-06	7365	M32348.m	TRUE	reported	ruBgwQ	2	TRUE	0.00281	20.74745
3837	rs405945	T	C	T	C	-0.0082	0.005356	0.4396	0.432693	FALSE	FALSE	FALSE	tRX2u0	0.013797	0.697896	486484	sepsis	TRUE	reported	textfile	0.0018	3.15E-06	7365	M32348.m	TRUE	reported	ruBgwQ	2	TRUE	0.00281	20.74745
3845	rs6486771	T	C	T	C	-0.0082	0.000434	0.3983	0.389625	FALSE	FALSE	FALSE	tRX2u0	0.014015	0.975298	486484	sepsis	TRUE	reported	textfile	0.0018	3.45E-06	7365	M32348.m	TRUE	reported	ruBgwQ	2	TRUE	0.00281	20.74745
1031	rs1004301	T	C	T	C	0.0082	0.009874	0.7113	0.708727	FALSE	FALSE	FALSE	tRtQ2e	0.015133	0.514103	486484	sepsis	TRUE	reported	textfile	0.0018	5.58E-06	7364	M01564.m	TRUE	reported	Tbg0zM	2	TRUE	0.00281	20.74745
1069	rs889128	T	G	T	G	0.0082	-0.0143	0.6665	0.658174	FALSE	FALSE	FALSE	tRtQ2e	0.01441	0.320924	486484	sepsis	TRUE	reported	textfile	0.0018	6.75E-06	7364	M01564.m	TRUE	reported	Tbg0zM	2	TRUE	0.00281	20.74745
1074	rs940697	A	G	A	G	0.0082	-0.01102	0.2701	0.286492	FALSE	FALSE	FALSE	tRtQ2e	0.015136	0.466501	486484	sepsis	TRUE	reported	textfile	0.0018	5.71E-06	7364	M01564.m	TRUE	reported	Tbg0zM	2	TRUE	0.00281	20.74745
3977	rs1613813	A	G	A	G	-0.0082	0.014385	0.5082	0.503663	FALSE	FALSE	FALSE	XdJbEU	0.013709	0.29403	486484	sepsis	TRUE	reported	textfile	0.0018	8.30E-06	7364	M32452.m	TRUE	reported	V5xWKi	2	TRUE	0.00281	20.74745
3988	rs7028146	A	G	A	G	0.0082	0.007456	0.6217	0.616742	FALSE	FALSE	FALSE	XdJbEU	0.014092	0.596766	486484	sepsis	TRUE	reported	textfile	0.0018	7.77E-06	7364	M32452.m	TRUE	reported	V5xWKi	2	TRUE	0.00281	20.74745
3996	rs9503551	A	G	A	G	-0.0164	-0.03312	0.0954	0.111387	FALSE	FALSE	FALSE	XdJbEU	0.022129	0.13453	486484	sepsis	TRUE	reported	textfile	0.0036	3.75E-06	7364	M32452.m	TRUE	reported	V5xWKi	2	TRUE	0.00281	20.74745
724	rs1049143	A	C	A	C	0.0082	-0.01351	0.1435	0.142278	FALSE	FALSE	FALSE	9u0SC8	0.019633	0.491394	486484	sepsis	TRUE	reported	textfile	0.0018	3.80E-06	7363	M01301.m	TRUE	reported	H95uec	2	TRUE	0.002811	20.74745
732	rs1226825	T	C	T	C	-0.0082	0.008181	0.1339	0.132538	FALSE	FALSE	FALSE	9u0SC8	0.020224	0.68582	486484	sepsis	TRUE	reported	textfile	0.0018	5.59E-06	7363	M01301.m	TRUE	reported	H95uec	2	TRUE	0.002811	20.74745
4000	rs1193347	T	G	T	G	-0.0082	0.003293	0.3025	0.314956	FALSE	FALSE	FALSE	V9AtId	0.014697	0.822697	486484	sepsis	TRUE	reported	textfile	0.0018	6.54E-06	7363	M32489.m	TRUE	reported	8B8S8a	2	TRUE	0.002811	20.74745
4002	rs1250171	T	C	T	C	0.0082	-0.00729	0.3105	0.310801	FALSE	FALSE	FALSE	V9AtId	0.015176	0.631069	486484	sepsis	TRUE	reported	textfile	0.0018	6.05E-06	7363	M32489.m	TRUE	reported	8B8S8a	2	TRUE	0.002811	20.74745
4013	rs2325358	T	C	T	C	0.0082	-0.00912	0.7049	0.702553	FALSE	FALSE	FALSE	V9AtId	0.015029	0.543795	486484	sepsis	TRUE	reported	textfile	0.0018	5.59E-06	7363	M32489.m	TRUE	reported	8B8S8a	2	TRUE	0.002811	20.74745
4014	rs2467041	T	C	T	C	-0.0082	0.006354	0.6501	0.646401	FALSE	FALSE	FALSE	V9AtId	0.014273	0.656213	486484	sepsis	TRUE	reported	textfile	0.0018	5.71E-06	7363	M32489.m	TRUE	reported	8B8S8a	2	TRUE	0.002811	20.74745
4019	rs4704495	T	C	T	C	0.0082	-0.00641	0.6216	0.627254	FALSE	FALSE	FALSE	V9AtId	0.014257	0.653182	486484	sepsis	TRUE	reported	textfile	0.0018	4.89E-06	7363	M32489.m	TRUE	reported	8B8S8a	2	TRUE	0.002811	20.74745
4021	rs6498908	T	C	T	C	-0.0082	-0.00076	0.3408	0.327739	FALSE	FALSE	FALSE	V9AtId	0.014636	0.958545	486484	sepsis	TRUE	reported	textfile	0.0018	5.76E-06	7363	M32489.m	TRUE	reported	8B8S8a	2	TRUE	0.002811	20.74745
4282	rs1012972	A	G	A	G	-0.0082	0.007754	0.3124	0.300255	FALSE	FALSE	FALSE	0tjjPC	0.014921	0.603273	486484	sepsis	TRUE	reported	textfile	0.0018	5.95E-06	7362	M32634.m	TRUE	reported	nJJFkX	2	TRUE	0.002811	20.74745
6860	rs1049768	A	G	A	G	0.0082	-0.0132	0.8809	0.884375	FALSE	FALSE	FALSE	kFWZjs	0.021373	0.536848	486484	sepsis	TRUE	reported	textfile	0.0018	9.20E-06	7362	M34062.m	TRUE	reported	eLONsg	2	TRUE	0.002811	20.74745
6873	rs2029490	A	G	A	G	0.0082	-0.00282	0.2811	0.288918	FALSE	FALSE	FALSE	kFWZjs	0.015223	0.852981	486484	sepsis	TRUE	reported	textfile	0.0018	5.60E-06	7362	M34062.m	TRUE	reported	eLONsg	2	TRUE	0.002811	20.74745
6886	rs6898299	T	G	T	G	0.0082	-0.03208	0.1197	0.120921	FALSE	FALSE	FALSE	kFWZjs	0.021005	0.12671	486484	sepsis	TRUE	reported	textfile	0.0018	7.50E-06	7362	M34062.m	TRUE	reported	eLONsg	2	TRUE	0.002811	20.74745
457	rs1753352	T	C	T	C	0.0082	0.03201	0.1534	0.158573	FALSE	FALSE	FALSE	cl8ucN	0.018803	0.088683	486484	sepsis	TRUE	reported	textfile	0.0018	5.04E-06	7361	M00513.m	TRUE	reported	Ian0Q8	2	TRUE	0.002811	20.74745
467	rs9456538	A	G	A	G	-0.0082	-0.0174	0.1493	0.143328	FALSE	FALSE	FALSE	cl8ucN	0.019596	0.374537	486484	sepsis	TRUE	reported	textfile	0.0018	4.20E-06	7361	M00513.m	TRUE	reported	Ian0Q8	2	TRUE	0.002811	20.74745
8436	rs1899398	T	C	T	C	0.0123	0.014864	0.799	0.794528	FALSE	FALSE	FALSE	Nvt7pl	0.016964	0.380919	486484	sepsis	TRUE	reported	textfile	0.0027	4.94E-06	7358	M35718.m	TRUE	reported	WkA5Gq	2	TRUE	0.002813	20.74745
1605	rs2049534	T	C	T	C	-0.0082	0.007462	0.5044	0.507532	FALSE	FALSE	FALSE	zP3C2v	0.014758	0.613126	486484	sepsis	TRUE	reported	textfile	0.0018	3.18E-06	7356	M12035.m	TRUE	reported	1Pooe3	2	TRUE	0.002813	20.74744
1733	rs1073834	T	C	T	C	0.0082	0.000415	0.5346	0.529391	FALSE	FALSE	FALSE	nFLpAP	0.013694	0.975821	486484	sepsis	TRUE	reported	textfile	0.0018	3.70E-06	7356	M12774.m	TRUE	reported	yOSQTj	2	TRUE	0.002813	20.74744
1737	rs1241613	A	C	A	C	-0.0082	-0.02121	0.4756	0.492724	FALSE	FALSE	FALSE	nFLpAP	0.013675	0.120853	486484	sepsis	TRUE	reported	textfile	0.0018	3.44E-06	7356	M12774.m	TRUE	reported	yOSQTj	2	TRUE	0.002813	20.74744
11	rs1013667	A	G	A	G	-0.0041	-0.00953	0.6087	0.610687	FALSE	FALSE	FALSE	xmGuol	0.014013	0.496304	486484	sepsis	TRUE	reported	textfile	9.00E-04	5.67E-06	7355	M00054.m	TRUE	reported	GW50Ri	2	TRUE	0.002814	20.74744
24	rs1088100	T	C	T	C	0.0041	0.02647	0.4824	0.484641	FALSE	FALSE	FALSE	xmGuol	0.013673	0.052883	486484	sepsis	TRUE	reported	textfile	9.00E-04	3.39E-06	7355	M00054.m	TRUE	reported	GW50Ri	2	TRUE	0.002814	20.74744
29	rs1167885	T	C	T	C	0.0041	0.003417	0.4138	0.418686	FALSE	FALSE	FALSE	xmGuol	0.013926	0.806159	486484	sepsis	TRUE	reported	textfile	9.00E-04	5.94E-06	7355	M00054.m	TRUE	reported	GW50Ri	2	TRUE	0.002814	20.74744
30	rs1171967	A	T	A	T	-0.0041	-0.00504	0.3965	0.402494	FALSE	TRUE	FALSE	xmGuol	0.013997	0.718957	486484	sepsis	TRUE	reported	textfile	9.00E-04	5.12E-06	7355	M00054.m	TRUE	reported	GW50Ri	2	TRUE	0.002814	20.74744
34	rs1180241	T	C	T	C	-0.0041	0.0185	0.5816	0.573372	FALSE	FALSE	FALSE	xmGuol	0.013862	0.182032	486484	sepsis	TRUE	reported	textfile	9.00E-04	4.47E-06	7355	M00054.m	TRUE	reported	GW50Ri	2	TRUE	0.002814	20.74744
35	rs1180414	T	C	T	C	0.0041	0.032189	0.4678	0.468027	FALSE	FALSE	FALSE	xmGuol	0.01367	0.018539	486484	sepsis	TRUE	reported	textfile	9.00E-04	4.61E-06	7355	M00054.m	TRUE	reported	GW50Ri	2	TRUE	0.002814	20.74744
61	rs1769824	A	C	A	C	-0.0041	0.024735	0.5682	0.560304	FALSE	FALSE	FALSE	xmGuol	0.013819	0.073467	486484	sepsis	TRUE	reported	textfile	9.00E-04	5.50E-06	7355	M00054.m	TRUE	reported	GW50Ri	2	TRUE	0.002814	20.74744
62	rs1774450	T	C	T	C	-0.0041	-0.00219	0.4582	0.460403	FALSE	FALSE	FALSE	xmGuol	0.013735	0.873202	486484	sepsis	TRUE	reported	textfile	9.00E-04	4.16E-06	7355	M00054.m	TRUE	reported	GW50Ri	2	TRUE	0.002814	20.74744
75	rs2349729	A	G	A	G	-0.0041	-0.01779	0.5141	0.511894	FALSE	FALSE	FALSE	xmGuol	0.013715	0.19471	486484	sepsis	TRUE	reported	textfile	9.00E-04	6.45E-06	7355	M00054.m	TRUE	reported	GW50Ri	2	TRUE	0.002814	20.74744
87	rs2722407	A	G	A	G	0.0041	-0.00418	0.587	0.591968	FALSE	FALSE	FALSE	xmGuol	0.013905	0.763597	486484	sepsis	TRUE	reported	textfile	9.00E-04	6.47E-06	7355	M00054.m	TRUE	reported	GW50Ri	2	TRUE	0.002814	20.74744
101	rs3751181	A	G	A	G	0.0041	0.00074	0.6085	0.606098	FALSE	FALSE	FALSE	xmGuol	0.014051	0.958019	486484	sepsis	TRUE	reported	textfile	9.00E-04	5.19E-06	7355	M00054.m	TRUE	reported	GW50Ri	2	TRUE	0.002814	20.74744
112	rs430727	T	C	T	C	0.0041	-0.00518	0.4628	0.440979	FALSE	FALSE	FALSE	xmGuol	0.01374	0.706059	486484	sepsis	TRUE	reported	textfile	9.00E-04	5.13E-06	7355	M00054.m	TRUE	reported	GW50Ri	2	TRUE	0.002814	20.74744
118	rs4702007	A	G	A	G	-0.0041	0.000875	0.3929	0.390897	FALSE	FALSE	FALSE	xmGuol	0.014045	0.950348	486484	sepsis	TRUE	reported	textfile	9.00E-04	6.11E-06	7355	M00054.m	TRUE	reported	GW50Ri	2	TRUE	0.002814	20.74744
120	rs4755291	A	G	A	G	-0.0041	0.012555	0.4644	0.459976	FALSE	FALSE	FALSE	xmGuol	0.013737	0.36075	486484	sepsis	TRUE	reported	textfile	9.00E-04	5.25E-06	7355	M00054.m	TRUE	reported	GW50Ri	2	TRUE	0.002814	20.74744
121	rs4849291	C	G	C	G	0.0041	0.032172	0.6092	0.61142	FALSE	TRUE	FALSE	xmGuol	0.014027	0.021815	486484	sepsis	TRUE	reported	textfile	9.00E-04	5.93E-06	7355	M00054.m	TRUE	reported	GW50Ri	2	TRUE	0.002814	20.74744
122	rs4870304	A	G	A	G	0.0041	0.028861	0.5823	0.574412	FALSE	FALSE	FALSE	xmGuol	0.013898	0.037838	486484	sepsis	TRUE	reported	textfile	9.00E-04	8.31E-06	7355	M00054.m	TRUE	reported	GW50Ri	2	TRUE	0.002814	20.74744
133	rs6077611	T	G	T	G	-0.0041	-0.00505	0.4794	0.46483	FALSE	FALSE	FALSE	xmGuol	0.013738	0.712979	486484	sepsis	TRUE	reported	textfile	9.00E-04	5.32E-06	7355	M00054.m	TRUE	reported	GW50Ri	2	TRUE	0.002814	20.74744
145	rs6747650	A	G	A	G	-0.0041	-0.01564	0.5825	0.584689	FALSE	FALSE	FALSE	xmGuol	0.01385	0.258842	486484	sepsis	TRUE	reported	textfile	9.00E-04	4.36E-06	7355	M00054.m	TRUE	reported	GW50Ri	2	TRUE	0.002814	20.74744
151	rs6910731	A	G	A	G	0.0041	-0.00545	0.4497	0.439244	FALSE	FALSE	FALSE	xmGuol	0.01383	0.693386	486484	sepsis	TRUE	reported	textfile	9.00E-04	6.68E-06	7355	M00054.m	TRUE	reported	GW50Ri	2	TRUE	0.002814	20.74744
155	rs7009696	T	C	T	C	0.0041	-0.00703	0.6243	0.620712	FALSE	FALSE	FALSE	xmGuol	0.014088	0.617752	486484	sepsis	TRUE	reported	textfile	9.00E-04	6.17E-06	7355	M00054.m	TRUE	reported	GW50Ri	2	TRUE	0.002814	20.74744
167	rs7843149	T	C	T	C	-0.0041	-0.02094	0.3896	0.389678	FALSE	FALSE	FALSE	xmGuol	0.014024	0.135353	486484	sepsis	TRUE	reported	textfile	9.00E-04	6.55E-06	7355	M00054.m	TRUE	reported	GW50Ri	2	TRUE	0.002814	20.74744
174	rs875184	T	C	T	C	-0.0041	-0.00061	0.403	0.410268	FALSE	FALSE	FALSE	xmGuol	0.014038	0.965403	486484	sepsis	TRUE	reported	textfile	9.00E-04	5.69E-06	7355	M00054.m	TRUE	reported	GW50Ri	2	TRUE	0.002814	20.74744
178	rs915281	T	C	T	C	-0.0041	0.008297	0.5076	0.505111	FALSE	FALSE	FALSE	xmGuol	0.013664	0.54372	486484	sepsis	TRUE	reported	textfile	9.00E-04	4.42E-06	7355	M00054.m	TRUE	reported	GW50Ri	2	TRUE	0.002814	20.74744
184	rs9464137	A	C	A	C	0.0041	0.005582	0.5592	0.566808	FALSE	FALSE	FALSE	xmGuol	0.013783	0.685501	486484	sepsis	TRUE	reported	textfile	9.00E-04	4.45E-06	7355	M00054.m	TRUE	reported	GW50Ri	2	TRUE	0.002814	20.74744
189	rs9543391	T	G	T	G	0.0041	-0.01109	0.3993	0.414817	FALSE	FALSE	FALSE	xmGuol	0.013977	0.42736	486484	sepsis	TRUE	reported	textfile	9.00E-04	6.39E-06	7355	M00054.m	TRUE	reported	GW50Ri	2	TRUE	0.002814	20.74744
191	rs9649963	T	C	T	C	0.0041	-0.03068	0.634	0.634741	FALSE	FALSE	FALSE	xmGuol	0.014267	0.031534	486484	sepsis	TRUE	reported	textfile	9.00E-04	5.84E-06	7355	M00054.m	TRUE	reported	GW50Ri	2	TRUE	0.002814	20.74744
1206	rs2091331	A	G	A	G	0.0082	-0.00878	0.4127	0.40564	FALSE	FALSE	FALSE	Ckdgh1	0.013926	0.5286	486484	sepsis	TRUE	reported	textfile	0.0018	5.71E-06	7355	M01644.m	TRUE	reported	nKxz7B	2	TRUE	0.002814	20.74744
1210	rs498266	A	G	A	G	0.0082	-0.00298	0.5852	0.577111	FALSE	FALSE	FALSE	Ckdgh1	0.01386	0.829687	486484	sepsis	TRUE	reported	textfile	0.0018	6.15E-06	7355	M01644.m	TRUE	reported	nKxz7B	2	TRUE	0.002814	20.74744
4046	rs351755	A	G	A	G	-0.0082	0.008265	0.5614	0.562027	FALSE	FALSE	FALSE	SiYGYU	0.013782	0.548716	486484	sepsis	TRUE	reported	textfile	0.0018	3.53E-06	7355	M32492.m	TRUE	reported	GfQvpU	2	TRUE	0.002814	20.74744
4049	rs4898707	T	C	T	C	-0.0082	-0.00933	0.4396	0.439196	FALSE	FALSE	FALSE	SiYGYU	0.013802	0.499066	486484	sepsis	TRUE	reported	textfile	0.0018	4.35E-06	7355	M32492.m	TRUE	reported	GfQvpU	2	TRUE	0.002814	20.74744
4060	rs9924102	A	G	A	G	-0.0082	0.0051	0.6426	0.653288	FALSE	FALSE	FALSE	SiYGYU	0.014384	0.722936	486484	sepsis	TRUE	reported	textfile	0.0018	4.25E-06	7355	M32492.m	TRUE	reported	GfQvpU	2	TRUE	0.002814	20.74744
935	rs2592916	T	C	T	C	-0.0123	0.016755	0.1013	0.095169	FALSE	FALSE	FALSE	tv99UN	0.023288	0.471854	486484	sepsis	TRUE	reported	textfile	0.0027	6.55E-06	7354	M01494.m	TRUE	reported	osDclp	2	TRUE	0.002814	20.74744
2798	rs1193409	A	G	A	G	-0.0082	0.019095	0.7176	0.71614	FALSE	FALSE	FALSE	Sx5Uhq	0.015174	0.20825	486484	sepsis	TRUE	reported	textfile	0.0018	3.07E-06	7354	M19934.m	TRUE	reported	DijYTE	2	TRUE	0.002814	20.74744
4415	rs1267072	A	G	A	G	0.0205	0.012254	0.8147	0.799154	FALSE	FALSE	FALSE	NzBiVA	0.017051	0.472362	486484	sepsis	TRUE	reported	textfile	0.0045	6.25E-06	7354	M32672.m	TRUE	reported	YLIhHJ	2	TRUE	0.002814	20.74744
6778	rs7759176	T	G	T	G	0.0082	0.01107	0.2976	0.28987	FALSE	FALSE	FALSE	Elf6MQ	0.015174	0.465703	486484	sepsis	TRUE	reported	textfile	0.0018	4.35E-06	7354	M33955.m	TRUE	reported	Lxn4RF	2	TRUE	0.002814	20.74744
675	rs6084839	A	C	A	C	-0.0123	-0.03456	0.9446	0.937331	FALSE	FALSE	FALSE	p4oCVP	0.028423	0.22396	486484	sepsis	TRUE	reported	textfile	0.0027	3.99E-06	7352	M01125.m	TRUE	reported	OwD5re	2	TRUE	0.002815	20.74744
1764	rs1233747	T	C	T	C	-0.0123	0.012198	0.8324	0.825317	FALSE	FALSE	FALSE	wM5dkT	0.018038	0.498912	486484	sepsis	TRUE	reported	textfile	0.0027	5.90E-06	7352	M15122.m	TRUE	reported	CBpa4t	2	TRUE	0.002815	20.74744
206	rs1025466	T	C	T	C	-0.0041	-0.02202	0.4062	0.406834	FALSE	FALSE	FALSE	tbRL8J	0.014132	0.119117	486484	sepsis	TRUE	reported	textfile	9.00E-04	6.25E-06	7351	M00060.m	TRUE	reported	oOvRKT	2	TRUE	0.002815	20.74744
222	rs1097368	A	C	A	C	-0.0041	0.003833	0.4002	0.407894	FALSE	FALSE	FALSE	tbRL8J	0.013896	0.782681	486484	sepsis	TRUE	reported	textfile	9.00E-04	6.54E-06	7351	M00060.m	TRUE	reported	oOvRKT	2	TRUE	0.002815	20.74744
224	rs1104999	T	C	T	C	0.0041	-0.00612	0.4822	0.482453	FALSE	FALSE	FALSE	tbRL8J	0.013632	0.653574	486484	sepsis	TRUE	reported	textfile	9.00E-04	3.51E-06	7351	M00060.m	TRUE	reported	oOvRKT	2	TRUE	0.002815	20.74744
228	rs1118362	A	G	A	G	0.0041	0.028091	0.4676	0.466525	FALSE	FALSE	FALSE	tbRL8J	0.013837	0.042346	486484	sepsis	TRUE	reported	textfile	9.00E-04	4.68E-06	7351	M00060.m	TRUE	reported	oOvRKT	2	TRUE	0.002815	20.74744
230	rs1157892	A	C	A	C	0.0041	0.020741	0.4246	0.427091	FALSE	FALSE	FALSE	tbRL8J	0.014034	0.139441	486484	sepsis	TRUE	reported	textfile	9.00E-04	4.75E-06	7351	M00060.m	TRUE	reported	oOvRKT	2	TRUE	0.002815	20.74744
231	rs1165712	T	C	T	C	0.0041	0.00459	0.5604	0.560837	FALSE	FALSE	FALSE	tbRL8J	0.013745	0.738438	486484	sepsis	TRUE	reported	textfile	9.00E-04	5.71E-06	7351	M00060.m	TRUE	reported	oOvRKT	2	TRUE	0.002815	20.74744
238	rs1246854	A	G	A	G	0.0041	0.021722	0.5563	0.551759	FALSE	FALSE	FALSE	tbRL8J	0.013734	0.113724	486484	sepsis	TRUE	reported	textfile	9.00E-04	5.76E-06	7351	M00060.m	TRUE	reported	oOvRKT	2	TRUE	0.002815	20.74744
240	rs1251709	A	G	A	G	0.0041	0.007303	0.422	0.412452	FALSE	FALSE	FALSE	tbRL8J	0.013924	0.599922	486484	sepsis	TRUE	reported	textfile	9.00E-04	8.41E-06	7351	M00060.m	TRUE	reported	oOvRKT	2	TRUE	0.002815	20.74744
246	rs1316507	A	G	A	G	0.0041	-0.00852	0.3821	0.370763	FALSE	FALSE	FALSE	tbRL8J	0.014223	0.549122	486484	sepsis	TRUE	reported	textfile	9.00E-04	8.98E-06	7351	M00060.m	TRUE	reported	oOvRKT	2	TRUE	0.002815	20.74744
257	rs167648	A	C	A	C	-0.0041	0.007129	0.4439	0.454091	FALSE	FALSE	FALSE	tbRL8J	0.013791	0.605185	486484	sepsis	TRUE	reported	textfile	9.00E-04	4.52E-06	7351	M00060.m	TRUE	reported	oOvRKT	2	TRUE	0.002815	20.74744
271	rs1971105	T	G	T	G	-0.0041	-0.00107	0.4305	0.424021	FALSE	FALSE	FALSE	tbRL8J	0.013884	0.938622	486484	sepsis	TRUE	reported	textfile	9.00E-04	6.27E-06	7351	M00060.m	TRUE	reported	oOvRKT	2	TRUE	0.002815	20.74744
273	rs1999120	A	G	A	G	-0.0041	-0.00525	0.5692	0.568411	FALSE	FALSE	FALSE	tbRL8J	0.013838	0.704296	486484	sepsis	TRUE	reported	textfile	9.00E-04	4.04E-06	7351	M00060.m	TRUE	reported	oOvRKT	2	TRUE	0.002815	20.74744
274	rs2057435	A	G	A	G	0.0041	0.008273	0.535	0.547756	FALSE	FALSE	FALSE	tbRL8J	0.013758	0.547626	486484	sepsis	TRUE	reported	textfile	9.00E-04	7.95E-06	7351	M00060.m	TRUE	reported	oOvRKT	2	TRUE	0.002815	20.74744
286	rs2478832	T	C	T	C	-0.0041	0.006065	0.3996	0.396873	FALSE	FALSE	FALSE	tbRL8J	0.014096	0.667005	486484	sepsis	TRUE	reported	textfile	9.00E-04	8.05E-06	7351	M00060.m	TRUE	reported	oOvRKT	2	TRUE	0.002815	20.74744
290	rs2667698	A	C	A	C	0.0041	-0.00222	0.3681	0.366604	FALSE	FALSE	FALSE	tbRL8J	0.014178	0.875298	486484	sepsis	TRUE	reported	textfile	9.00E-04	7.07E-06	7351	M00060.m	TRUE	reported	oOvRKT	2	TRUE	0.002815	20.74744
304	rs3924223	A	G	A	G	-0.0041	0.006327	0.549	0.544258	FALSE	FALSE	FALSE	tbRL8J	0.01376	0.645675	486484	sepsis	TRUE	reported	textfile	9.00E-04	6.18E-06	7351	M00060.m	TRUE	reported	oOvRKT	2	TRUE	0.002815	20.74744
309	rs4276770	A	G	A	G	-0.0041	0.006951	0.3999	0.399316	FALSE	FALSE	FALSE	tbRL8J	0.014062	0.621108	486484	sepsis	TRUE	reported	textfile	9.00E-04	5.11E-06	7351	M00060.m	TRUE	reported	oOvRKT	2	TRUE	0.002815	20.74744
311	rs4531478	A	G	A	G	0.0041	0.01381	0.4713	0.474882	FALSE	FALSE	FALSE	tbRL8J	0.013695	0.31328	486484	sepsis	TRUE	reported	textfile	9.00E-04	4.45E-06	7351	M00060.m	TRUE	reported	oOvRKT	2	TRUE	0.002815	20.74744
321	rs4889294	T	C	T	C	-0.0041	-0.00243	0.5449	0.551547	FALSE	FALSE	FALSE	tbRL8J	0.013714	0.859145	486484	sepsis	TRUE	reported	textfile	9.00E-04	3.65E-06	7351	M00060.m	TRUE	reported	oOvRKT	2	TRUE	0.002815	20.74744
329	rs618001	T	C	T	C	0.0041	0.001181	0.5857	0.585799	FALSE	FALSE	FALSE	tbRL8J	0.013865	0.932099	486484	sepsis	TRUE	reported	textfile	9.00E-04	4.18E-06	7351	M00060.m	TRUE	reported	oOvRKT	2	TRUE	0.002815	20.74744
330	rs631750	T	C	T	C	0.0041	-0.00026	0.3544	0.365826	FALSE	FALSE	FALSE	tbRL8J	0.014206	0.985257	486484	sepsis	TRUE	reported	textfile	9.00E-04	7.51E-06	7351	M00060.m	TRUE	reported	oOvRKT	2	TRUE	0.002815	20.74744
331	rs637828	A	C	A	C	0.0041	0.02126	0.5424	0.534658	FALSE	FALSE	FALSE	tbRL8J	0.013747	0.121984	486484	sepsis	TRUE	reported	textfile	9.00E-04	5.81E-06	7351	M00060.m	TRUE	reported	oOvRKT	2	TRUE	0.002815	20.74744
342	rs6822279	A	C	A	C	-0.0041	-0.00313	0.3969	0.395468	FALSE	FALSE	FALSE	tbRL8J	0.014095	0.824102	486484	sepsis	TRUE	reported	textfile	9.00E-04	4.79E-06	7351	M00060.m	TRUE	reported	oOvRKT	2	TRUE	0.002815	20.74744
344	rs697311	A	G	A	G	0.0041	0.010265	0.3792	0.373905	FALSE	FALSE	FALSE	tbRL8J	0.014174	0.468928	486484	sepsis	TRUE	reported	textfile	9.00E-04	4.58E-06	7351	M00060.m	TRUE	reported	oOvRKT	2	TRUE	0.002815	20.74744
355	rs7574695	T	C	T	C	-0.0041	0.014986	0.4769	0.48425	FALSE	FALSE	FALSE	tbRL8J	0.013694	0.273797	486484	sepsis	TRUE	reported	textfile	9.00E-04	5.97E-06	7351	M00060.m	TRUE	reported	oOvRKT	2	TRUE	0.002815	20.74744
358	rs7622687	A	G	A	G	-0.0041	-0.01497	0.4032	0.413949	FALSE	FALSE	FALSE	tbRL8J	0.013946	0.283032	486484	sepsis	TRUE	reported	textfile	9.00E-04	5.21E-06	7351	M00060.m	TRUE	reported	oOvRKT	2	TRUE	0.002815	20.74744
364	rs769630	T	C	T	C	0.0041	0.006446	0.6016	0.605598	FALSE	FALSE	FALSE	tbRL8J	0.014018	0.645627	486484	sepsis	TRUE	reported	textfile	9.00E-04	5.06E-06	7351	M00060.m	TRUE	reported	oOvRKT	2	TRUE	0.002815	20.74744
370	rs7914851	C	G	C	G	0.0041	-0.01392	0.3443	0.343221	FALSE	TRUE	FALSE	tbRL8J	0.014447	0.335226	486484	sepsis	TRUE	reported	textfile	9.00E-04	6.79E-06	7351	M00060.m	TRUE	reported	oOvRKT	2	TRUE	0.002815	20.74744
378	rs8131355	A	G	A	G	-0.0041	0.021385	0.5488	0.535777	FALSE	FALSE	FALSE	tbRL8J	0.013697	0.118462	486484	sepsis	TRUE	reported	textfile	9.00E-04	5.68E-06	7351	M00060.m	TRUE	reported	oOvRKT	2	TRUE	0.002815	20.74744
381	rs891878	T	G	T	G	-0.0041	0.015081	0.4844	0.491143	FALSE	FALSE	FALSE	tbRL8J	0.01366	0.269596	486484	sepsis	TRUE	reported	textfile	9.00E-04	6.03E-06	7351	M00060.m	TRUE	reported	oOvRKT	2	TRUE	0.002815	20.74744
385	rs923818	A	G	A	G	0.0041	-0.00349	0.5072	0.510598	FALSE	FALSE	FALSE	tbRL8J	0.013716	0.798946	486484	sepsis	TRUE	reported	textfile	9.00E-04	6.23E-06	7351	M00060.m	TRUE	reported	oOvRKT	2	TRUE	0.002815	20.74744
386	rs9292230	T	C	T	C	0.0041	0.004138	0.6218	0.624157	FALSE	FALSE	FALSE	tbRL8J	0.014129	0.769612	486484	sepsis	TRUE	reported	textfile	9.00E-04	4.63E-06	7351	M00060.m	TRUE	reported	oOvRKT	2	TRUE	0.002815	20.74744
394	rs989960	T	C	T	C	0.0041	-0.0141	0.4175	0.422877	FALSE	FALSE	FALSE	tbRL8J	0.013838	0.308214	486484	sepsis	TRUE	reported	textfile	9.00E-04	4.66E-06	7351	M00060.m	TRUE	reported	oOvRKT	2	TRUE	0.002815	20.74744
395	rs9903421	T	C	T	C	-0.0041	-0.00378	0.5633	0.563554	FALSE	FALSE	FALSE	tbRL8J	0.013779	0.783778	486484	sepsis	TRUE	reported	textfile	9.00E-04	7.39E-06	7351	M00060.m	TRUE	reported	oOvRKT	2	TRUE	0.002815	20.74744
4118	rs4516432	T	C	T	C	0.0082	0.008801	0.7795	0.775061	FALSE	FALSE	FALSE	4rtLd0	0.01644	0.592403	486484	sepsis	TRUE	reported	textfile	0.0018	7.61E-06	7350	M32518.m	TRUE	reported	j7f1ER	2	TRUE	0.002816	20.74744
4122	rs4904578	T	C	T	C	0.0082	-0.01197	0.3808	0.404733	FALSE	FALSE	FALSE	4rtLd0	0.013913	0.389752	486484	sepsis	TRUE	reported	textfile	0.0018	6.29E-06	7350	M32518.m	TRUE	reported	j7f1ER	2	TRUE	0.002816	20.74744
7887	rs1161762	T	C	T	C	0.0123	-0.0097	0.7232	0.71816	FALSE	FALSE	FALSE	pU8zps	0.015321	0.526598	486484	sepsis	TRUE	reported	textfile	0.0027	4.02E-06	7350	M35186.m	TRUE	reported	Oc2IOF	2	TRUE	0.002816	20.74744
7892	rs2392820	A	G	A	G	0.0123	0.005673	0.28	0.279729	FALSE	FALSE	FALSE	pU8zps	0.015343	0.711594	486484	sepsis	TRUE	reported	textfile	0.0027	3.86E-06	7350	M35186.m	TRUE	reported	Oc2IOF	2	TRUE	0.002816	20.74744
1858	rs1011136	A	G	A	G	-0.0041	-0.00662	0.5456	0.539584	FALSE	FALSE	FALSE	v2KE89	0.013848	0.632837	486484	sepsis	TRUE	reported	textfile	9.00E-04	4.98E-06	7349	M15500.m	TRUE	reported	to2t7H	2	TRUE	0.002816	20.74744
1860	rs1013663	A	G	A	G	0.0041	-0.02051	0.5453	0.548443	FALSE	FALSE	FALSE	v2KE89	0.013736	0.135431	486484	sepsis	TRUE	reported	textfile	9.00E-04	6.00E-06	7349	M15500.m	TRUE	reported	to2t7H	2	TRUE	0.002816	20.74744
1866	rs1078997	T	C	T	C	-0.0041	0.004356	0.4061	0.403049	FALSE	FALSE	FALSE	v2KE89	0.013946	0.754774	486484	sepsis	TRUE	reported	textfile	9.00E-04	6.63E-06	7349	M15500.m	TRUE	reported	to2t7H	2	TRUE	0.002816	20.74744
1870	rs1086901	T	C	T	C	-0.0041	0.009978	0.5113	0.546785	FALSE	FALSE	FALSE	v2KE89	0.013762	0.468418	486484	sepsis	TRUE	reported	textfile	9.00E-04	5.20E-06	7349	M15500.m	TRUE	reported	to2t7H	2	TRUE	0.002816	20.74744
1879	rs1158985	T	G	T	G	-0.0041	-0.00339	0.3573	0.356818	FALSE	FALSE	FALSE	v2KE89	0.01425	0.8119	486484	sepsis	TRUE	reported	textfile	9.00E-04	5.26E-06	7349	M15500.m	TRUE	reported	to2t7H	2	TRUE	0.002816	20.74744
1904	rs1272536	A	G	A	G	-0.0041	0.00393	0.4844	0.460873	FALSE	FALSE	FALSE	v2KE89	0.013705	0.774307	486484	sepsis	TRUE	reported	textfile	9.00E-04	5.55E-06	7349	M15500.m	TRUE	reported	to2t7H	2	TRUE	0.002816	20.74744
1907	rs1316216	A	G	A	G	-0.0041	-0.00849	0.3813	0.376183	FALSE	FALSE	FALSE	v2KE89	0.01411	0.547178	486484	sepsis	TRUE	reported	textfile	9.00E-04	6.37E-06	7349	M15500.m	TRUE	reported	to2t7H	2	TRUE	0.002816	20.74744
1911	rs1323165	T	C	T	C	0.0041	-0.00414	0.4296	0.432866	FALSE	FALSE	FALSE	v2KE89	0.013813	0.764256	486484	sepsis	TRUE	reported	textfile	9.00E-04	4.78E-06	7349	M15500.m	TRUE	reported	to2t7H	2	TRUE	0.002816	20.74744
1913	rs1368388	A	G	A	G	-0.0041	0.027431	0.4238	0.43458	FALSE	FALSE	FALSE	v2KE89	0.013773	0.046403	486484	sepsis	TRUE	reported	textfile	9.00E-04	4.62E-06	7349	M15500.m	TRUE	reported	to2t7H	2	TRUE	0.002816	20.74744
1923	rs1509657	T	C	T	C	0.0041	0.000709	0.4602	0.467101	FALSE	FALSE	FALSE	v2KE89	0.013681	0.958659	486484	sepsis	TRUE	reported	textfile	9.00E-04	6.28E-06	7349	M15500.m	TRUE	reported	to2t7H	2	TRUE	0.002816	20.74744
1936	rs2000364	T	G	T	G	-0.0041	-0.01244	0.6346	0.636156	FALSE	FALSE	FALSE	v2KE89	0.014314	0.384897	486484	sepsis	TRUE	reported	textfile	9.00E-04	5.72E-06	7349	M15500.m	TRUE	reported	to2t7H	2	TRUE	0.002816	20.74744
1937	rs2068985	C	G	C	G	-0.0041	0.017942	0.3461	0.339932	FALSE	TRUE	FALSE	v2KE89	0.014432	0.213766	486484	sepsis	TRUE	reported	textfile	9.00E-04	6.76E-06	7349	M15500.m	TRUE	reported	to2t7H	2	TRUE	0.002816	20.74744
1940	rs2133813	T	C	T	C	0.0041	0.028468	0.4438	0.44876	FALSE	FALSE	FALSE	v2KE89	0.013741	0.038287	486484	sepsis	TRUE	reported	textfile	9.00E-04	9.47E-06	7349	M15500.m	TRUE	reported	to2t7H	2	TRUE	0.002816	20.74744
1948	rs2362839	A	G	A	G	0.0041	0.003474	0.5974	0.594992	FALSE	FALSE	FALSE	v2KE89	0.013905	0.802732	486484	sepsis	TRUE	reported	textfile	9.00E-04	5.15E-06	7349	M15500.m	TRUE	reported	to2t7H	2	TRUE	0.002816	20.74744
1954	rs246946	T	C	T	C	0.0041	0.018785	0.6832	0.693188	FALSE	FALSE	FALSE	v2KE89	0.014815	0.204814	486484	sepsis	TRUE	reported	textfile	9.00E-04	9.26E-06	7349	M15500.m	TRUE	reported	to2t7H	2	TRUE	0.002816	20.74744
1955	rs2504927	T	C	T	C	-0.0041	-0.00608	0.4347	0.405229	FALSE	FALSE	FALSE	v2KE89	0.013945	0.662705	486484	sepsis	TRUE	reported	textfile	9.00E-04	4.35E-06	7349	M15500.m	TRUE	reported	to2t7H	2	TRUE	0.002816	20.74744

1974	rs3905228	A	C	A	C	0.0041	0.014068	0.4682	0.480063	FALSE	FALSE	FALSE	v2KE89	0.013677	0.303668	486484	sepsis	TRUE	reported	textfile	9.00E-04	8.96E-06	7349	M15500.m	TRUE	reported	to2t7H	2	TRUE	0.002816	20.74744
1975	rs4078258	T	C	T	C	-0.0041	0.013736	0.6332	0.633634	FALSE	FALSE	FALSE	v2KE89	0.014236	0.334617	486484	sepsis	TRUE	reported	textfile	9.00E-04	6.08E-06	7349	M15500.m	TRUE	reported	to2t7H	2	TRUE	0.002816	20.74744
1980	rs4504898	T	C	T	C	-0.0041	-0.00931	0.3869	0.38318	FALSE	FALSE	FALSE	v2KE89	0.014156	0.510733	486484	sepsis	TRUE	reported	textfile	9.00E-04	7.03E-06	7349	M15500.m	TRUE	reported	to2t7H	2	TRUE	0.002816	20.74744
1984	rs4633738	A	C	A	C	0.0041	0.003542	0.4809	0.474212	FALSE	FALSE	FALSE	v2KE89	0.013713	0.796197	486484	sepsis	TRUE	reported	textfile	9.00E-04	5.38E-06	7349	M15500.m	TRUE	reported	to2t7H	2	TRUE	0.002816	20.74744
1990	rs4711152	T	C	T	C	0.0041	-0.03464	0.543	0.544223	FALSE	FALSE	FALSE	v2KE89	0.013736	0.011673	486484	sepsis	TRUE	reported	textfile	9.00E-04	5.06E-06	7349	M15500.m	TRUE	reported	to2t7H	2	TRUE	0.002816	20.74744
1997	rs482275	T	C	T	C	0.0041	0.01575	0.5688	0.56534	FALSE	FALSE	FALSE	v2KE89	0.013886	0.256698	486484	sepsis	TRUE	reported	textfile	9.00E-04	7.61E-06	7349	M15500.m	TRUE	reported	to2t7H	2	TRUE	0.002816	20.74744
2015	rs6721330	A	C	A	C	-0.0041	0.000943	0.5542	0.563837	FALSE	FALSE	FALSE	v2KE89	0.013802	0.945554	486484	sepsis	TRUE	reported	textfile	9.00E-04	4.92E-06	7349	M15500.m	TRUE	reported	to2t7H	2	TRUE	0.002816	20.74744
2017	rs6852374	A	G	A	G	0.0041	-0.01263	0.5054	0.514454	FALSE	FALSE	FALSE	v2KE89	0.013673	0.355789	486484	sepsis	TRUE	reported	textfile	9.00E-04	4.44E-06	7349	M15500.m	TRUE	reported	to2t7H	2	TRUE	0.002816	20.74744
2023	rs7038757	T	C	T	C	0.0041	0.01542	0.6526	0.653241	FALSE	FALSE	FALSE	v2KE89	0.014379	0.283558	486484	sepsis	TRUE	reported	textfile	9.00E-04	6.13E-06	7349	M15500.m	TRUE	reported	to2t7H	2	TRUE	0.002816	20.74744
2028	rs7355681	A	G	A	G	0.0041	0.012411	0.5872	0.595869	FALSE	FALSE	FALSE	v2KE89	0.014	0.37536	486484	sepsis	TRUE	reported	textfile	9.00E-04	4.91E-06	7349	M15500.m	TRUE	reported	to2t7H	2	TRUE	0.002816	20.74744
2039	rs7991989	T	C	T	C	0.0041	-0.00176	0.6115	0.607586	FALSE	FALSE	FALSE	v2KE89	0.013983	0.899758	486484	sepsis	TRUE	reported	textfile	9.00E-04	4.30E-06	7349	M15500.m	TRUE	reported	to2t7H	2	TRUE	0.002816	20.74744
2042	rs8127674	A	G	A	G	-0.0041	0.008652	0.59	0.599468	FALSE	FALSE	FALSE	v2KE89	0.013967	0.535607	486484	sepsis	TRUE	reported	textfile	9.00E-04	5.85E-06	7349	M15500.m	TRUE	reported	to2t7H	2	TRUE	0.002816	20.74744
2043	rs8134847	T	C	T	C	0.0041	0.005894	0.397	0.397201	FALSE	FALSE	FALSE	v2KE89	0.013981	0.673332	486484	sepsis	TRUE	reported	textfile	9.00E-04	8.38E-06	7349	M15500.m	TRUE	reported	to2t7H	2	TRUE	0.002816	20.74744
2045	rs865488	T	C	T	C	-0.0041	-0.02796	0.6815	0.677023	FALSE	FALSE	FALSE	v2KE89	0.014595	0.055372	486484	sepsis	TRUE	reported	textfile	9.00E-04	6.65E-06	7349	M15500.m	TRUE	reported	to2t7H	2	TRUE	0.002816	20.74744
2048	rs923075	T	C	T	C	-0.0041	-0.01898	0.6135	0.621587	FALSE	FALSE	FALSE	v2KE89	0.014077	0.177481	486484	sepsis	TRUE	reported	textfile	9.00E-04	4.81E-06	7349	M15500.m	TRUE	reported	to2t7H	2	TRUE	0.002816	20.74744
2055	rs9613019	T	C	T	C	-0.0041	-0.00217	0.4063	0.403368	FALSE	FALSE	FALSE	v2KE89	0.013999	0.877082	486484	sepsis	TRUE	reported	textfile	9.00E-04	7.42E-06	7349	M15500.m	TRUE	reported	to2t7H	2	TRUE	0.002816	20.74744
2063	rs989681	T	C	T	C	0.0041	0.025409	0.5177	0.508689	FALSE	FALSE	FALSE	v2KE89	0.013754	0.064701	486484	sepsis	TRUE	reported	textfile	9.00E-04	7.36E-06	7349	M15500.m	TRUE	reported	to2t7H	2	TRUE	0.002816	20.74744
8381	rs1880030	A	G	A	G	-0.0082	0.011365	0.4317	0.447209	FALSE	FALSE	FALSE	SCzh0A	0.013718	0.407417	486484	sepsis	TRUE	reported	textfile	0.0018	5.81E-06	7348	M35675.m	TRUE	reported	n1SREk	2	TRUE	0.002816	20.74744
8404	rs9541642	A	G	A	G	-0.0082	0.006747	0.6955	0.685795	FALSE	FALSE	FALSE	SCzh0A	0.014803	0.648555	486484	sepsis	TRUE	reported	textfile	0.0018	8.36E-06	7348	M35675.m	TRUE	reported	n1SREk	2	TRUE	0.002816	20.74744
7001	rs1122871!	A	G	A	G	-0.0123	0.014474	0.6124	0.608963	FALSE	FALSE	FALSE	ePdfmg	0.014001	0.301267	486484	sepsis	TRUE	reported	textfile	0.0027	4.14E-06	7347	M34244.m	TRUE	reported	l9nzfX	2	TRUE	0.002817	20.74744
1248	rs6765113	T	C	T	C	0.0082	-0.00275	0.5408	0.550112	FALSE	FALSE	FALSE	V50rWC	0.013825	0.842561	486484	sepsis	TRUE	reported	textfile	0.0018	8.16E-06	7346	M01645.m	TRUE	reported	yN3gpQ	2	TRUE	0.002817	20.74744
1252	rs7011528	A	C	A	C	-0.0082	-0.01444	0.4515	0.441362	FALSE	FALSE	FALSE	V50rWC	0.013756	0.293748	486484	sepsis	TRUE	reported	textfile	0.0018	4.01E-06	7346	M01645.m	TRUE	reported	yN3gpQ	2	TRUE	0.002817	20.74744
3775	rs1440327	T	C	T	C	-0.0082	0.014075	0.4308	0.432675	FALSE	FALSE	FALSE	NW1aaR	0.014077	0.31738	486484	sepsis	TRUE	reported	textfile	0.0018	6.00E-06	7344	M32339.m	TRUE	reported	pBPkAB	2	TRUE	0.002818	20.74743
3776	rs1566951	T	C	T	C	-0.0082	-0.00049	0.3943	0.391263	FALSE	FALSE	FALSE	NW1aaR	0.013994	0.97225	486484	sepsis	TRUE	reported	textfile	0.0018	4.52E-06	7344	M32339.m	TRUE	reported	pBPkAB	2	TRUE	0.002818	20.74743
3780	rs2064272	A	G	A	G	-0.0082	0.014916	0.346	0.344505	FALSE	FALSE	FALSE	NW1aaR	0.014379	0.29959	486484	sepsis	TRUE	reported	textfile	0.0018	4.49E-06	7344	M32339.m	TRUE	reported	pBPkAB	2	TRUE	0.002818	20.74743
3789	rs6839120	A	G	A	G	0.0082	-0.01337	0.6771	0.671188	FALSE	FALSE	FALSE	NW1aaR	0.014579	0.358981	486484	sepsis	TRUE	reported	textfile	0.0018	4.03E-06	7344	M32339.m	TRUE	reported	pBPkAB	2	TRUE	0.002818	20.74743
4952	rs222836	A	G	A	G	-0.0082	-0.00786	0.5336	0.515715	FALSE	FALSE	FALSE	bKmDMa	0.013663	0.564864	486484	sepsis	TRUE	reported	textfile	0.0018	3.90E-06	7344	M32910.m	TRUE	reported	nAuPzO	2	TRUE	0.002818	20.74743
3275	rs1080288:	A	G	A	G	-0.0082	-0.00502	0.3775	0.365349	FALSE	FALSE	FALSE	vYWPe9	0.014237	0.724608	486484	sepsis	TRUE	reported	textfile	0.0018	6.65E-06	7340	M25459.m	TRUE	reported	VvJ6Vx	2	TRUE	0.002819	20.74743
3291	rs6986204	A	C	A	C	-0.0082	-0.00134	0.4755	0.478597	FALSE	FALSE	FALSE	vYWPe9	0.013706	0.922098	486484	sepsis	TRUE	reported	textfile	0.0018	5.63E-06	7340	M25459.m	TRUE	reported	VvJ6Vx	2	TRUE	0.002819	20.74743
4431	rs1511592	A	C	A	C	-0.0082	-0.00683	0.8679	0.865924	FALSE	FALSE	FALSE	WGPdpq	0.020038	0.733257	486484	sepsis	TRUE	reported	textfile	0.0018	8.34E-06	7338	M32675.m	TRUE	reported	u0eUAG	2	TRUE	0.00282	20.74743
4438	rs41070	T	C	T	C	0.0082	-0.00795	0.8499	0.85599	FALSE	FALSE	FALSE	WGPdpq	0.019454	0.68284	486484	sepsis	TRUE	reported	textfile	0.0018	4.35E-06	7338	M32675.m	TRUE	reported	u0eUAG	2	TRUE	0.00282	20.74743
4442	rs8007741	A	G	A	G	0.0082	0.000603	0.8554	0.857602	FALSE	FALSE	FALSE	WGPdpq	0.019768	0.975681	486484	sepsis	TRUE	reported	textfile	0.0018	5.06E-06	7338	M32675.m	TRUE	reported	u0eUAG	2	TRUE	0.00282	20.74743
1836	rs4816048	C	G	C	G	0.0123	-0.03129	0.1794	0.188808	FALSE	TRUE	FALSE	2TaDHm	0.017535	0.074394	486484	sepsis	TRUE	reported	textfile	0.0027	7.13E-06	7337	M15365.m	TRUE	reported	LoLveg	2	TRUE	0.002821	20.74743
4276	rs4834604	T	C	T	C	-0.0123	-0.00767	0.5456	0.538807	FALSE	FALSE	FALSE	ylV4bp	0.013734	0.576384	486484	sepsis	TRUE	reported	textfile	0.0027	4.92E-06	7337	M32632.m	TRUE	reported	hqHJoU	2	TRUE	0.002821	20.74743
2858	rs7751392	T	G	T	G	0.0082	-0.00488	0.1712	0.169267	FALSE	FALSE	FALSE	Y5qWBm	0.01838	0.790766	486484	sepsis	TRUE	reported	textfile	0.0018	3.84E-06	7325	M20489.m	TRUE	reported	f2g9Su	2	TRUE	0.002825	20.74742
4203	rs1205497	A	G	A	G	-0.0205	-0.00346	0.6371	0.643029	FALSE	FALSE	FALSE	rdKSjG	0.014315	0.808805	486484	sepsis	TRUE	reported	textfile	0.0045	6.09E-06	7322	M32578.m	TRUE	reported	RAzSLK	2	TRUE	0.002826	20.74742
4210	rs759400	T	C	T	C	0.0246	-0.04496	0.8126	0.821862	FALSE	FALSE	FALSE	rdKSjG	0.017908	0.01206	486484	sepsis	TRUE	reported	textfile	0.0054	5.26E-06	7322	M32578.m	TRUE	reported	RAzSLK	2	TRUE	0.002826	20.74742
7042	rs1224117;	T	C	T	C	0.0082	-0.00085	0.695	0.697692	FALSE	FALSE	FALSE	uthruC	0.01498	0.954717	486484	sepsis	TRUE	reported	textfile	0.0018	7.58E-06	7316	M34283.m	TRUE	reported	ftTuRU	2	TRUE	0.002829	20.74741
7048	rs1325912!	T	C	T	C	-0.0082	-0.00162	0.3366	0.330804	FALSE	FALSE	FALSE	uthruC	0.014549	0.91121	486484	sepsis	TRUE	reported	textfile	0.0018	4.02E-06	7316	M34283.m	TRUE	reported	ftTuRU	2	TRUE	0.002829	20.74741
7056	rs2245730	A	C	A	C	-0.0082	0.020257	0.5574	0.564982	FALSE	FALSE	FALSE	uthruC	0.013816	0.142602	486484	sepsis	TRUE	reported	textfile	0.0018	3.82E-06	7316	M34283.m	TRUE	reported	ftTuRU	2	TRUE	0.002829	20.74741
7063	rs4896596	T	C	T	C	0.0082	-0.012	0.4122	0.417617	FALSE	FALSE	FALSE	uthruC	0.013881	0.387255	486484	sepsis	TRUE	reported	textfile	0.0018	3.64E-06	7316	M34283.m	TRUE	reported	ftTuRU	2	TRUE	0.002829	20.74741
2520	rs7652277	T	G	T	G	0.0082	-0.02361	0.4645	0.464459	FALSE	FALSE	FALSE	LwUYua	0.013711	0.085012	486484	sepsis	TRUE	reported	textfile	0.0018	8.00E-06	7315	M17945.m	TRUE	reported	SUN8dc	2	TRUE	0.002829	20.74741
2521	rs913416	T	C	T	C	-0.0082	0.011963	0.5313	0.532412	FALSE	FALSE	FALSE	LwUYua	0.013732	0.383676	486484	sepsis	TRUE	reported	textfile	0.0018	7.52E-06	7315	M17945.m	TRUE	reported	SUN8dc	2	TRUE	0.002829	20.74741
5881	rs394811	A	G	A	G	-0.0082	0.009103	0.399	0.414783	FALSE	FALSE	FALSE	tqW0ld	0.01389	0.512217	486484	sepsis	TRUE	reported	textfile	0.0018	9.89E-06	7315	M33389.m	TRUE	reported	Sv7EyV	2	TRUE	0.002829	20.74741
5902	rs9471904	T	C	T	C	-0.0082	0.013929	0.4795	0.492836	FALSE	FALSE	FALSE	tqW0ld	0.013635	0.306988	486484	sepsis	TRUE	reported	textfile	0.0018	7.40E-06	7315	M33389.m	TRUE	reported	Sv7EyV	2	TRUE	0.002829	20.74741
2197	rs1213278(	T	C	T	C	0.0082	0.010011	0.3331	0.318436	FALSE	FALSE	FALSE	Bf6uMK	0.014657	0.494607	486484	sepsis	TRUE	reported	textfile	0.0018	6.82E-06	7308	M15778.m	TRUE	reported	jowM2N	2	TRUE	0.002832	20.74741
2211	rs3131535	A	G	A	G	-0.0205	-0.03707	0.9652	0.958339	FALSE	FALSE	FALSE	Bf6uMK	0.034233	0.278851	486484	sepsis	TRUE	reported	textfile	0.0045	6.30E-06	7308	M15778.m	TRUE	reported	jowM2N	2	TRUE	0.002832	20.74741
2213	rs4353947	A	G	A	G	-0.0082	0.001067	0.2304	0.234326	FALSE	FALSE	FALSE	Bf6uMK	0.016154	0.947333	486484	sepsis	TRUE	reported	textfile	0.0018	8.70E-06	7308	M15778.m	TRUE	reported	jowM2N	2	TRUE	0.002832	20.74741
6120	rs1076599;	A	C	A	C	0.0082	-0.01539	0.6859	0.681651	FALSE	FALSE	FALSE	MiSJly	0.014702	0.295302	486484	sepsis	TRUE	reported	textfile	0.0018	5.13E-06	7307	M33477.m	TRUE	reported	WwL1hq	2	TRUE	0.002832	20.74741
6127	rs1209617(	A	G	A	G	0.0082	0.006974	0.4016	0.416968	FALSE	FALSE	FALSE	MiSJly	0.013851	0.614619	486484	sepsis	TRUE	reported	textfile	0.0018	4.97E-06	7307	M33477.m	TRUE	reported	WwL1hq	2	TRUE	0.002832	20.74741
6138	rs270144	T	C	T	C	-0.0082	0.00418	0.6901	0.648266	FALSE	FALSE	FALSE	MiSJly	0.014365	0.771085	486484	sepsis	TRUE	reported	textfile	0.0018	6.90E-06	7307	M33477.m	TRUE	reported	WwL1hq	2	TRUE	0.002832	20.74741
6149	rs6434958	C	G	C	G	-0.0123	-0.02406	0.1118	0.113448	FALSE	TRUE	FALSE	MiSJly	0.021568	0.264595	486484	sepsis	TRUE	reported	textfile	0.0027	7.10E-06	7307	M33477.m	TRUE	reported	WwL1hq	2	TRUE	0.002832	20.74741
6152	rs7316944	C	G	C	G	-0.0082	-0.02433	0.2987	0.293875	FALSE	TRUE	FALSE	MiSJly	0.015061	0.106265	486484	sepsis	TRUE	reported	textfile	0.0018	6.25E-06	7307	M33477.m	TRUE	reported	WwL1hq	2	TRUE	0.002832	20.74741
5974	rs1011654:	A	G	A	G	0.0123	0.016158	0.0816	0.078972	FALSE	FALSE	FALSE	3KsoXq	0.025295	0.522947	486484	sepsis	TRUE	reported	textfile	0.0027	6.81E-06	7306	M33422.m	TRUE	reported	aNr6Ug	2	TRUE	0.002833	20.74741
5985	rs1764109	A	G	A	G	0.0082	-0.00566	0.2241	0.218469	FALSE	FALSE	FALSE	3KsoXq	0.01662	0.733606	486484	sepsis	TRUE	reported	textfile	0.0018	7.20E-06	7306	M33422.m	TRUE	reported	aNr6Ug	2	TRUE	0.002833	20.74741
5999	rs4870483	T	G	T	G	0.0082	0.019294	0.7193	0.718352	FALSE	FALSE	FALSE	3KsoXq	0.015187	0.203937	486484	sepsis	TRUE	reported	textfile	0.0018	3.85E-06	7306	M33422.m	TRUE	reported	aNr6Ug	2	TRUE	0.002833	20.74741
3322	rs1106851(	A	C	A	C	-0.0082	0.01287	0.1958	0.190076	FALSE	FALSE	FALSE	m66Eav	0.017523	0.462669	486484	sepsis	TRUE	reported	textfile	0.0018	3.71E-06	7303	M27256.m	TRUE	reported	pl2ECU	2	TRUE	0.002834	20.7474
3329	rs1330292	T	G	T	G	-0.0082	0.015141	0.8039	0.811309	FALSE	FALSE	FALSE	m66Eav	0.017455	0.385699	486484	sepsis	TRUE	reported	textfile	0.0018	4.26E-06	7303	M27256.m	TRUE	reported	pl2ECU	2	TRUE	0.002834	20.7474
3341	rs660240	T	C	T	C	-0.0082	-0.02507	0.2096	0.215159	FALSE	FALSE	FALSE	m66Eav	0.016651	0.132157	486484	sepsis	TRUE	reported	textfile	0.0018	3.61E-06	7303	M27256.m	TRUE	reported	pl2ECU	2	TRUE	0.002834	20.7474
3030	rs2239068	T	C	T	C	0.0123	0.018354	0.7471	0.758181	FALSE	FALSE	FALSE	FJZEYz	0.015992	0.251104	486484	sepsis	TRUE	reported	textfile	0.0027	3.78E-06	7284	M21630.m	TRUE	reported	s6Pshg	2	TRUE	0.002841	20.74739
1111	rs2486925	T	G	T	G	-0.0082	0.011854	0.1462	0.145446	FALSE	FALSE	FALSE	yahiGZ	0.019479	0.542825	486484	sepsis	TRUE	reported	textfile	0.0018	3.47E-06	7271	M01585.m	TRUE	reported	dwZ7vX	2	TRUE	0.002846	20.74738
6243	rs4878060	T	G	T	G	-0.0123	-0.00701	0.6539	0.654398	FALSE	FALSE	FALSE	vL52Yb	0.014666	0.632706	486484	sepsis	TRUE	reported	textfile	0.0027	6.17E-06	7269	M33510.m	TRUE	reported	JlO9VX	2	TRUE	0.002847	20.74738
3272	rs8137744	A	G	A	G	0.0123	0.018604	0.3814	0.406592	FALSE	FALSE	FALSE	ZNPBqT	0.014153	0.18869	486484	sepsis	TRUE	reported	textfile	0.0027	5.55E-06	7256	M24074.m	TRUE	reported	8pPtOl	2	TRUE	0.002852	20.74737
4336	rs1208624'	A	G	A	G	-0.0123	0.002933	0.1209	0.123139	FALSE	FALSE	FALSE	6VSoQQ	0.02082	0.887971	486484	sepsis	TRUE	reported	textfile	0.0027	5.77E-06	7229	M32644.m	TRUE	reported	oC2AV0	2	TRUE	0.002863	20.74734
4343	rs2897863	T	G	T	G	0.0082	0.007888	0.697	0.691447	FALSE	FALSE	FALSE	6VSoQQ	0.014868	0.595731	486484	sepsis	TRUE	reported	textfile	0.0018	7.50E-06	7229	M32644.m	TRUE	reported	oC2AV0	2	TRUE	0.002863	20.74734
4356	rs992837	C	G	C	G	-0.0082	-0.01051	0.6787	0.6752	FALSE	TRUE	FALSE	6VSoQQ	0.014606	0.471938	486484	sepsis	TRUE	reported	textfile	0.0018	8.92E-06	7229	M32644.m	TRUE	reported	oC2AV0	2	TRUE	0.002863	20.74734
9065	rs1091015'	T	C	T	C	-0.0164	0.013732	0.4179	0.422412	FALSE	FALSE	FALSE	0l8lKx	0.013833	0.320861	486484	sepsis	TRUE	reported	textfile	0.0036	6.24E-06	7219	M38178.m	TRUE	reported	bWs4mL	2	TRUE	0.002867	20.74734
8034	rs1994003	A	C	A	C	0.0082	-0.00215	0.1798	0.188078	FALSE	FALSE	FALSE	SwiqSi	0.017446	0.90213	486484	sepsis	TRUE	reported	textfile	0.0018	9.74E-06	7217	M35270.m	TRUE	reported	21ZlsQ	2	TRUE	0.002867	20.74734
8040	rs2834543	T	C	T	C	-0.0082	-0.01471	0.7707	0.76899	FALSE	FALSE	FALSE	SwiqSi	0.016246	0.365255	486484	sepsis	TRUE	reported	textfile	0.0018	7.42E-06	7217	M35270.m	TRUE	reported	21ZlsQ	2	TRUE	0.002867	20.74734
8047	rs6784156	C	G	C	G	-0.0082	0.003565	0.7475	0.753307	FALSE	TRUE	FALSE	SwiqSi	0.015855	0.822092	486484	sepsis	TRUE	reported	textfile	0.0018	5.61E-06	7217	M35270.m	TRUE	reported	21ZlsQ	2	TRUE	0.002867	20.74734
8052	rs8069296	T	C	T	C	0.0082	0.004428	0.7988	0.799004	FALSE	FALSE	FALSE	SwiqSi	0.017058	0.795183	486484	sepsis	TRUE	reported	textfile	0.0018	9.97E-06	7217	M35270.m	TRUE	reported	21ZlsQ	2	TRUE	0.002867	20.74734
5822	rs1694532:	A	T	A	T	-0.0082	0.00295	0.2334	0.230434	FALSE	TRUE	FALSE	R5joEf	0.016244	0.855879	486484	sepsis	TRUE	reported	textfile	0.0018	2.72E-06	7118	M33380.m	TRUE	reported	kRvbfZ	2	TRUE	0.002907	20.74726
5829	rs4517836	T	C	T	C	0.0082	-0.01917	0.7357	0.743296	FALSE	FALSE	FALSE	R5joEf	0.015731	0.223024	486484	sepsis	TRUE	reported	textfile	0.0018	3.00E-06	7118	M33380.m	TRUE	reported	kRvbfZ	2	TRUE	0.002907	20.74726
6260	rs1223104!	A	G	A	G	0.0164	-0.03826	0.922	0.91291	FALSE	FALSE	FALSE	MOonP9	0.024176	0.113508	486484	sepsis	TRUE	reported	textfile	0.0036	4.60E-06	7066	M33515.m	TRUE	reported	226GuP	2	TRUE	0.002928	20.74721
6262	rs1373219	T	C	T	C	0.0082	-0.00634	0.5411	0.503245	FALSE	FALSE	FALSE	MOonP9	0.013693	0.643538	486484	sepsis	TRUE	reported	textfile	0.0018	8.22E-06	7066	M33515.m	TRUE	reported	226GuP	2	TRUE	0.002928	20.74721
6263	rs1924466	A	C	A	C	0.0082	0.024891	0.4544	0.460008	FALSE	FALSE	FALSE	MOonP9	0.013755	0.070369	486484	sepsis	TRUE	reported	textfile	0.0018	8.17E-06	7066	M33515.m	TRUE	reported	226GuP	2	TRUE	0.002928	20.74721
6953	rs202815	A	G	A	G	-0.0123	-0.01509	0.3799	0.383195	FALSE	FALSE	FALSE	7Cw2XZ	0.014121	0.285194	486484	sepsis	TRUE	reported	textfile	0.0027	3.49E-06	7049	M34123.m	TRUE	reported	vrgMl3	2	TRUE	0.002935	20.7472
6228	rs7600589	C	G	C	G	0.0164	0.003232	0.7048	0.699553	FALSE	TRUE	FALSE	8zUBbU	0.01493	0.828592	486484	sepsis	TRUE	reported	textfile	0.0036	5.59E-06	7044	M33509.m	TRUE	reported	1mO9m8	2	TRUE	0.002938	20.74719
1493	rs9310707	A	G	A	G	-0.0082	-0.05299	0.3473	0.357692	FALSE	FALSE	FALSE	pkb876	0.014252	0.000201	486484	sepsis	TRUE	reported	textfile	0.0018	6.38E-06	7037	M02734.m	TRUE	reported	0LCMVI	2	TRUE	0.00294	20.74719
2353	rs1087523:	T	G	T	G	-0.0515	-0.00334	0.2715	0.235476	FALSE	FALSE	FALSE	NBJ2Do	0.016119	0.835911	486484	sepsis	TRUE	reported	textfile	0.0113	5.43E-06	1739	M17799.m	TRUE	reported	Uz0Wgl	2	TRUE	0.011803	20.74712
769	rs1939471	A	G	A	G	-0.0123	-0.01088	0.7209	0.71159	FALSE	FALSE	FALSE	tYvbNc	0.015115	0.471722	486484	sepsis	TRUE	reported	textfile	0.0027	4.06E-06	6946	M01303.m	TRUE	reported	hT3gsE	2	TRUE	0.002979	20.74711
8858	rs6774259	A	G	A	G	-0.0082	0.006816	0.829	0.829674	FALSE	FALSE	FALSE	XlHeTV	0.018241	0.708673	486484	sepsis	TRUE	reported	textfile	0.0018	5.13E-06	6940	M36808.m	TRUE	reported	37Vi2D	2	TRUE	0.002981	20.74711
8861	rs7519669	A	G	A	G	0.0082	0.017377	0.1935	0.190482	FALSE	FALSE	FALSE	XlHeTV	0.017395	0.317799	486484	sepsis	TRUE	reported	textfile	0.0018	4.37E-06	6940	M36808.m	TRUE	reported	37Vi2D	2	TRUE	0.002981	20.74711
2578	rs4719964	A	G	A	G	-0.0123	-0.02828	0.2554	0.261188	FALSE	FALSE	FALSE	KHS91f	0.015619	0.070221	486484	sepsis	TRUE	reported	textfile	0.0027	4.46E-06	6939	M18349.m	TRUE	reported	PeVGil	2	TRUE	0.002982	20.7471
7279	rs1184360(	A	G	A	G	-0.0082	0.000131	0.5105	0.509378	FALSE	FALSE	FALSE	Vcz4EA	0.01369	0.992365	486484	sepsis	TRUE	reported	textfile	0.0018	2.85E-06	6755	M34359.m	TRUE	reported	aUSo2J	2	TRUE	0.003063	20.74694
7304	rs4281086	A	C	A	C	0.0082	0.001333	0.667	0.665593	FALSE	FALSE	FALSE	Vcz4EA	0.014525	0.926904	486484	sepsis	TRUE	reported	textfile	0.0018	4.77E-06	6755	M34359.m	TRUE	reported	aUSo2J	2	TRUE	0.003063	20.74694
7305	rs4664490	C	G	C	G	0.0082	-0.00328	0.3876	0.390021	FALSE	TRUE	FALSE	Vcz4EA	0.014074	0.815877	486484	sepsis	TRUE	reported	textfile	0.0018	4.47E-06	6755	M34359.m	TRUE	reported	aUSo2J	2	TRUE	0.003063	20.74694
2315	rs9926816	A	G	A	G	-0.0164	-0.03785	0.8582	0.856888	FALSE	FALSE	FALSE	ZzUW8U	0.019603	0.053481	486484	sepsis	TRUE	reported	textfile	0.0036	5.19E-06	6733	M16821.m	TRUE	reported	g2xs3i	2	TRUE	0.003073	20.74692
3005	rs1271338	A	G	A	G	0.0123	-0.03988	0.7335	0.736468	FALSE	FALSE	FALSE	gxupFu	0.015568	0.010409	486484	sepsis	TRUE	reported	textfile	0.0027	7.86E-06	6556	M21188.m	TRUE	reported	gwwXh2	2	TRUE	0.003156	20.74676
3018	rs7032741	T	C	T	C	-0.0205	-0.02086	0.0891	0.09988	FALSE	FALSE	FALSE	gxupFu	0.022909	0.362494	486484	sepsis	TRUE	reported	textfile	0.0045	5.12E-06	6556	M21188.m	TRUE	reported	gwwXh2	2	TRUE	0.003156	20.74676
8885	rs1026192	A	C	A	C	-0.0123	-0.01573	0.1617	0.166572	FALSE	FALSE	FALSE	eLhsld	0.018361	0.391617	486484	sepsis	TRUE	reported	textfile	0.0027	6.54E-06	6552	M37058.m	TRUE	reported	RSeWE5	2	TRUE	0.003157	20.74675
8913	rs4548847	A	G	A	G	0.0082	0.003056	0.5209	0.533629	FALSE	FALSE	FALSE	eLhsld	0.013712	0.823619	486484	sepsis	TRUE	reported	textfile	0.0018	9.56E-06	6552	M37058.m	TRUE	reported	RSeWE5	2	TRUE	0.003157	20.74675
7942	rs6817210	A	C	A	C	-0.0123	-0.00777	0.5169	0.52401	FALSE	FALSE	FALSE	kFTWXY	0.013685	0.570268	486484	sepsis	TRUE	reported	textfile	0.0027	6.58E-06	6543	M35193.m	TRUE	reported	cjJvJl	2	TRUE	0.003162	20.74674
8000	rs1023143!	T	C	T	C	-0.0246	-0.03351	0.0747	0.075241	FALSE	FALSE	FALSE	Zj5bZH	0.02611	0.199309	486484	sepsis	TRUE	reported	textfile	0.0054	6.26E-06	6538	M35257.m	TRUE	reported	exssAk	2	TRUE	0.003164	20.74674
995	rs1316441:	T	C	T	C	0.0082	-0.01022	0.7246	0.716074	FALSE	FALSE	FALSE	jfkZbM	0.015183	0.500758	486484	sepsis	TRUE	reported	textfile	0.0018	5.33E-06	6523	M01558.m	TRUE	reported	LFZvMG	2	TRUE	0.003171	20.74672
1010	rs4692012	A	G	A	G	-0.0082	0.011444	0.5389	0.524496	FALSE	FALSE	FALSE	jfkZbM	0.01378	0.406278	486484	sepsis	TRUE	reported	textfile	0.0018	3.35E-06	6523	M01558.m	TRUE	reported	LFZvMG	2	TRUE	0.003171	20.74672
7647	rs4764937	T	G	T	G	-0.0246	-0.00349	0.6365	0.636159	FALSE	FALSE	FALSE	wrTMYM	0.014188	0.805811	486484	sepsis	TRUE	reported	textfile	0.0054	5.87E-06	5856	M34533.m	TRUE	reported	qIRVku	2	TRUE	0.003531	20.746
8105	rs1398096	T	C	T	C	0.0082	-0.00013	0.7377	0.736204	FALSE	FALSE	FALSE	nNbXBS	0.015736	0.993565	486484	sepsis	TRUE	reported	textfile	0.0018	4.41E-06	5768	M35326.m	TRUE	reported	UNDorn	2	TRUE	0.003585	20.74589
8120	rs4803892	T	C	T	C	-0.0082	0.028934	0.7313	0.732591	FALSE	FALSE	FALSE	nNbXBS	0.015598	0.063595	486484	sepsis	TRUE	reported	textfile	0.0018	7.13E-06	5768	M35326.m	TRUE	reported	UNDorn	2	TRUE	0.003585	20.74589
8128	rs7117598	T	C	T	C	0.0082	0.007558	0.6692	0.663599	FALSE	FALSE	FALSE	nNbXBS	0.01461	0.604958	486484	sepsis	TRUE	reported	textfile	0.0018	3.74E-06	5768	M35326.m	TRUE	reported	UNDorn	2	TRUE	0.003585	20.74589
6109	rs2649049	T	C	T	C	-0.0164	0.001034	0.5773	0.567861	FALSE	FALSE	FALSE	a0osmD	0.013797	0.940244	486484	sepsis	TRUE	reported	textfile	0.0036	4.63E-06	5717	M33453.m	TRUE	reported	CePFnK	2	TRUE	0.003617	20.74583
691	rs4516857	C	G	C	G	0.0205	0.000212	0.0991	0.091584	FALSE	TRUE	FALSE	aT1Fw1	0.02389	0.992912	486484	sepsis	TRUE	reported	textfile	0.0045	4.93E-06	5687	M01284.m	TRUE	reported	mbTe8H	2	TRUE	0.003636	20.74579
3250	rs310271	A	G	A	G	-0.0533	0.026705	0.2583	0.27735	FALSE	FALSE	FALSE	jxdGCf	0.015304	0.080988	486484	sepsis	TRUE	reported	textfile	0.0117	5.53E-06	5599	M22842.m	TRUE	reported	f0L3pZ	2	TRUE	0.003693	20.74567
2844	rs1747359(	T	C	T	C	-0.041	0.045435	0.014	0.026709	FALSE	FALSE	FALSE	Y5qWBm	0.042504	0.285089	486484	sepsis	TRUE	reported	textfile	0.009	5.23E-06	5571	M20489.m	TRUE	reported	f2g9Su	2	TRUE	0.003711	20.74564
3494	rs4236964	T	C	T	C	-0.0123	0.003032	0.3256	0.317501	FALSE	FALSE	FALSE	Awafb0	0.014719	0.836808	486484	sepsis	TRUE	reported	textfile	0.0027	5.74E-06	5562	M27728.m	TRUE	reported	Lfj8L0	2	TRUE	0.003717	20.74562
2990	rs1772999!	A	G	A	G	-0.0492	-0.00336	0.0612	0.065082	FALSE	FALSE	FALSE	xmzJ5F	0.027772	0.903807	486484	sepsis	TRUE	reported	textfile	0.0108	5.23E-06	5436	M21184.m	TRUE	reported	xgwhDp	2	TRUE	0.003803	20.74545
8210	rs1289637!	A	T	A	T	0.0205	0.013973	0.1961	0.209979	FALSE	TRUE	FALSE	aY1HfB	0.016825	0.406285	486484	sepsis	TRUE	reported	textfile	0.0045	5.72E-06	5306	M35433.m	TRUE	reported	H5O4i2	2	TRUE	0.003896	20.74526
4367	rs1948260	T	C	T	C	0.0123	0.002474	0.5206	0.519035	FALSE	FALSE	FALSE	SPhl52	0.013687	0.85653	486484	sepsis	TRUE	reported	textfile	0.0027	7.10E-06	5211	M32651.m	TRUE	reported	V0Bnpj	2	TRUE	0.003967	20.74512
4369	rs2555100	C	G	C	G	0.0123	0.026386	0.6638	0.670032	FALSE	TRUE	FALSE	SPhl52	0.014585	0.07044	486484	sepsis	TRUE	reported	textfile	0.0027	6.86E-06	5211	M32651.m	TRUE	reported	V0Bnpj	2	TRUE	0.003967	20.74512
4377	rs6953983	A	G	A	G	0.0123	-0.0146	0.3217	0.3239	FALSE	FALSE	FALSE	SPhl52	0.014676	0.319718	486484	sepsis	TRUE	reported	textfile	0.0027	6.79E-06	5211	M32651.m	TRUE	reported	V0Bnpj	2	TRUE	0.003967	20.74512
7423	rs9846511	A	C	A	C	-0.0205	0.009621	0.2559	0.250097	FALSE	FALSE	FALSE	7i5VUS	0.015844	0.5437	486484	sepsis	TRUE	reported	textfile	0.0045	4.25E-06	5208	M34395.m	TRUE	reported	64hEaL	2	TRUE	0.003969	20.74512
635	rs3752241	C	G	C	G	0.0574	0.016659	0.8684	0.831084	FALSE	TRUE	FALSE	F6Dn8H	0.018393	0.365075	486484	sepsis	TRUE	reported	textfile	0.0126	5.52E-06	4934	M01114.m	TRUE	reported	dVHatZ	2	TRUE	0.004189	20.74467
2785	rs7151771	A	G	A	G	-0.0123	-0.0115	0.605	0.605605	FALSE	FALSE	FALSE	yYuamw	0.014004	0.411706	486484	sepsis	TRUE	reported	textfile	0.0027	5.02E-06	4844	M19414.m	TRUE	reported	GxSb5G	2	TRUE	0.004266	20.74452
500	rs6816298	A	G	A	G	0.1842	-0.00173	0.8254	0.815118	FALSE	FALSE	FALSE	RtjZPn	0.017651	0.922062	486484	sepsis	TRUE	reported	textfile	0.0403	4.76E-06	283	M00553.m	TRUE	reported	C3Ovsl	2	TRUE	0.068747	20.74383
8760	rs1965169	T	C	T	C	0.0848	-0.00636	0.1956	0.180967	FALSE	FALSE	FALSE	KMisdy	0.017799	0.72085	486484	sepsis	TRUE	reported	textfile	0.0186	4.89E-06	952	M36589.m	TRUE	reported	J1lSTP	2	TRUE	0.021367	20.74209
2959	rs1684921!	C	G	C	G	-0.0369	-0.0244	0.7736	0.762489	FALSE	TRUE	FALSE	zndu0r	0.016112	0.129885	486484	sepsis	TRUE	reported	textfile	0.0081	5.57E-06	3422	M21049.m	TRUE	reported	2XpPDU	2	TRUE	0.006028	20.74096
411	rs1433904	T	C	T	C	0.0442	0.004992	0.0228	0.028788	FALSE	FALSE	FALSE	cTsNj6	0.041269	0.903713	486484	sepsis	TRUE	reported	textfile	0.0097	5.06E-06	1763	M00063.m	TRUE	reported	7KYTrQ	2	TRUE	0.01164	20.73997
3600	rs4699296	A	G	A	G	0.0483	-0.00487	0.1693	0.188353	FALSE	FALSE	FALSE	e4G8UU	0.017504	0.780621	486484	sepsis	TRUE	reported	textfile	0.0106	4.78E-06	1764	M31548.m	TRUE	reported	kWkkmH	2	TRUE	0.011633	20.7391
7134	rs1824189	T	C	T	C	-0.0631	-0.00665	0.6979	0.708338	FALSE	FALSE	FALSE	de8tA6	0.015014	0.6579	486484	sepsis	TRUE	reported	textfile	0.0138	5.25E-06	248	M34322.m	TRUE	reported	sXPPBO	2	TRUE	0.07775	20.73882
2239	rs4296931	A	G	A	G	0.1523	0.009684	0.0244	0.046319	FALSE	FALSE	FALSE	cwj8fY	0.032994	0.769132	486484	sepsis	TRUE	reported	textfile	0.0334	5.10E-06	773	M15958.m	TRUE	reported	dPwsBc	2	TRUE	0.026194	20.73871
5675	rs7595056	T	C	T	C	0.0533	-0.03321	0.7661	0.774762	FALSE	FALSE	FALSE	AAOSvG	0.016355	0.042317	486484	sepsis	TRUE	reported	textfile	0.0117	5.38E-06	2860	M33197.m	TRUE	reported	aAmcal	2	TRUE	0.007204	20.73857
8528	rs6060489	A	C	A	C	0.0524	0.01547	0.0753	0.074011	FALSE	FALSE	FALSE	gM7v2m	0.026104	0.55343	486484	sepsis	TRUE	reported	textfile	0.0115	4.89E-06	1743	M36009.m	TRUE	reported	6Xcypd	2	TRUE	0.011771	20.73807
8600	rs7229244	A	C	A	C	-0.0328	-0.01982	0.7276	0.726196	FALSE	FALSE	FALSE	t5potg	0.015312	0.195429	486484	sepsis	TRUE	reported	textfile	0.0072	5.67E-06	2709	M36115.m	TRUE	reported	SSOHIA	2	TRUE	0.007603	20.73776
5965	rs1052084!	A	C	A	C	0.191	0.042648	0.0165	0.012398	FALSE	FALSE	FALSE	YCswLG	0.061413	0.487402	486484	sepsis	TRUE	reported	textfile	0.0419	5.27E-06	977	M33420.m	TRUE	reported	WH7xfc	2	TRUE	0.020826	20.73713
6481	rs4822679	A	G	A	G	-0.934	-0.16419	0.9873	0.997265	FALSE	FALSE	FALSE	ecwVg8	0.136567	0.229251	486484	sepsis	TRUE	reported	textfile	0.2025	3.96E-06	79	M33751.m	TRUE	reported	NnvblQ	2	TRUE	0.212157	20.73516
6484	rs7150140	T	C	T	C	-0.934	-0.0253	0.9873	0.98458	FALSE	FALSE	FALSE	ecwVg8	0.056176	0.652493	486484	sepsis	TRUE	reported	textfile	0.2025	3.96E-06	79	M33751.m	TRUE	reported	NnvblQ	2	TRUE	0.212157	20.73516
6489	rs7934379	T	C	T	C	0.934	-0.00758	0.0127	0.010742	FALSE	FALSE	FALSE	ecwVg8	0.067072	0.910076	486484	sepsis	TRUE	reported	textfile	0.2025	3.96E-06	79	M33751.m	TRUE	reported	NnvblQ	2	TRUE	0.212157	20.73516
6492	rs9451887	A	G	A	G	-0.934	0.058234	0.9873	0.990937	FALSE	FALSE	FALSE	ecwVg8	0.072467	0.421632	486484	sepsis	TRUE	reported	textfile	0.2025	3.96E-06	79	M33751.m	TRUE	reported	NnvblQ	2	TRUE	0.212157	20.73516
5229	rs1167212:	A	G	A	G	-0.2949	-0.05064	0.0233	0.039156	FALSE	FALSE	FALSE	q32d4E	0.036755	0.16826	486484	sepsis	TRUE	reported	textfile	0.0647	5.18E-06	1017	M33178.m	TRUE	reported	cS3BwT	2	TRUE	0.020019	20.73414
8984	rs933775	A	G	A	G	0.0337	-0.00557	0.7146	0.712307	FALSE	FALSE	FALSE	snOepl	0.015144	0.713078	486484	sepsis	TRUE	reported	textfile	0.0074	4.81E-06	7355	M37202.m	TRUE	reported	PQx272	2	TRUE	0.002812	20.73377
6908	rs1293728(	T	C	T	C	-0.568	0.032597	0.0146	0.031438	FALSE	FALSE	FALSE	SvfSvH	0.041028	0.426897	486484	sepsis	TRUE	reported	textfile	0.1243	4.89E-06	282	M34109.m	TRUE	reported	7OSbiu	2	TRUE	0.068942	20.73306
3464	rs7659567	A	G	A	G	0.0337	-0.00802	0.2037	0.206658	FALSE	FALSE	FALSE	jRejFm	0.016893	0.634976	486484	sepsis	TRUE	reported	textfile	0.0074	5.01E-06	6393	M27716.m	TRUE	reported	tg7Rvj	2	TRUE	0.003234	20.73292
7750	rs4976671	C	G	C	G	0.0989	-0.0155	0.9854	0.981261	FALSE	TRUE	FALSE	HyMNYM	0.054257	0.775149	486484	sepsis	TRUE	reported	textfile	0.0217	5.01E-06	1060	M34912.m	TRUE	reported	Tl8Hcw	2	TRUE	0.019219	20.73256
2957	rs1261667	T	G	T	G	0.1084	0.041559	0.9778	0.979255	FALSE	FALSE	FALSE	zndu0r	0.048223	0.388793	486484	sepsis	TRUE	reported	textfile	0.0238	5.05E-06	3422	M21049.m	TRUE	reported	2XpPDU	2	TRUE	0.006026	20.73246
5791	rs2792707	T	C	T	C	0.0984	-0.00109	0.0427	0.042324	FALSE	FALSE	FALSE	lUkr5t	0.033913	0.974456	486484	sepsis	TRUE	reported	textfile	0.0216	5.10E-06	1863	M33359.m	TRUE	reported	DjliwS	2	TRUE	0.011017	20.73081
5483	rs4964992	T	C	T	C	0.0752	0.024975	0.5326	0.486337	FALSE	FALSE	FALSE	q32d4E	0.013665	0.067594	486484	sepsis	TRUE	reported	textfile	0.0165	5.01E-06	1017	M33178.m	TRUE	reported	cS3BwT	2	TRUE	0.020015	20.73065
8943	rs4923384	T	C	T	C	-0.0337	0.024541	0.7967	0.785609	FALSE	FALSE	FALSE	mXJP3h	0.016646	0.14041	486484	sepsis	TRUE	reported	textfile	0.0074	4.90E-06	4520	M37104.m	TRUE	reported	HRZBak	2	TRUE	0.004567	20.73023
2675	rs2992907	T	C	T	C	0.0296	0.014195	0.6664	0.664001	FALSE	FALSE	FALSE	s4UTyf	0.014447	0.325844	486484	sepsis	TRUE	reported	textfile	0.0065	6.16E-06	5376	M18494.m	TRUE	reported	ajPzhg	2	TRUE	0.003843	20.7298
2708	rs1078882:	T	G	T	G	0.0369	0.002212	0.1884	0.155394	FALSE	FALSE	FALSE	uNhql4	0.018878	0.906707	486484	sepsis	TRUE	reported	textfile	0.0081	5.96E-06	1766	M19323.m	TRUE	reported	h9HAwp	2	TRUE	0.011615	20.72958
6535	rs6727350	A	T	A	T	-0.041	-0.00049	0.1525	0.149832	FALSE	TRUE	FALSE	DedZqr	0.019182	0.979739	486484	sepsis	TRUE	reported	textfile	0.009	5.00E-06	1765	M33822.m	TRUE	reported	ciYOdn	2	TRUE	0.011621	20.72957
458	rs1990178	T	C	T	C	-0.0164	-0.01356	0.1943	0.236461	FALSE	FALSE	FALSE	cl8ucN	0.016141	0.400729	486484	sepsis	TRUE	reported	textfile	0.0036	6.27E-06	1763	M00513.m	TRUE	reported	lan0Q8	2	TRUE	0.011635	20.72954
4572	rs1076285	A	G	A	G	-0.0164	0.008033	0.6966	0.732667	FALSE	FALSE	FALSE	Pzplqu	0.015563	0.605761	486484	sepsis	TRUE	reported	textfile	0.0036	6.86E-06	1759	M32740.m	TRUE	reported	FjEBS5	2	TRUE	0.011661	20.72949
4248	rs2402228	A	T	A	T	-0.051	0.018478	0.1901	0.197248	FALSE	TRUE	FALSE	KZ60wl	0.017309	0.28572	486484	sepsis	TRUE	reported	textfile	0.0112	5.27E-06	6904	M32616.m	TRUE	reported	SPDpZi	2	TRUE	0.002994	20.72901
4999	rs6480159	A	G	A	G	0.0255	-0.03764	0.2212	0.2205	FALSE	FALSE	FALSE	OsqE1P	0.016553	0.022986	486484	sepsis	TRUE	reported	textfile	0.0056	5.33E-06	5425	M33084.m	TRUE	reported	VZYh6f	2	TRUE	0.003808	20.72737
2967	rs1214593(	T	C	T	C	0.0287	0.009457	0.5511	0.58926	FALSE	FALSE	FALSE	dOOXpo	0.013894	0.49607	486484	sepsis	TRUE	reported	textfile	0.0063	6.21E-06	1608	M21127.m	TRUE	reported	ldiP4j	2	TRUE	0.012742	20.72727
1820	rs1998053	A	G	A	G	0.0724	-0.00765	0.0855	0.082717	FALSE	FALSE	FALSE	Yub5KV	0.024875	0.758516	486484	sepsis	TRUE	reported	textfile	0.0159	5.60E-06	5606	M15335.m	TRUE	reported	mcnlNa	2	TRUE	0.003685	20.72659
3745	rs1111150:	T	C	T	C	-0.0214	0.047662	0.0757	0.083019	FALSE	FALSE	FALSE	OZLdls	0.024888	0.055484	486484	sepsis	TRUE	reported	textfile	0.0047	5.92E-06	7356	M32338.m	TRUE	reported	s2itmB	2	TRUE	0.00281	20.72592
7034	rs1050360:	T	C	T	C	-0.0214	0.033991	0.9489	0.957165	FALSE	FALSE	FALSE	uthruC	0.03383	0.31501	486484	sepsis	TRUE	reported	textfile	0.0047	6.17E-06	7316	M34283.m	TRUE	reported	ftTuRU	2	TRUE	0.002826	20.72589
6720	rs2378733	A	G	A	G	0.0214	0.030154	0.1266	0.127537	FALSE	FALSE	FALSE	B6lZly	0.020529	0.141876	486484	sepsis	TRUE	reported	textfile	0.0047	5.07E-06	7222	M33937.m	TRUE	reported	qFPzjh	2	TRUE	0.002862	20.72581
8714	rs2581320	T	G	T	G	0.0214	-0.00532	0.8846	0.87866	FALSE	FALSE	FALSE	l0G0l1	0.020899	0.799112	486484	sepsis	TRUE	reported	textfile	0.0047	6.20E-06	6464	M36553.m	TRUE	reported	lBltb0	2	TRUE	0.003197	20.72514
6539	rs1271578!	A	G	A	G	0.0214	-0.00098	0.8197	0.82706	FALSE	FALSE	FALSE	fVPpJo	0.018041	0.956758	486484	sepsis	TRUE	reported	textfile	0.0047	5.16E-06	6327	M33833.m	TRUE	reported	37N6ck	2	TRUE	0.003266	20.725
4773	rs3791660	T	G	T	G	-0.0214	0.001619	0.463	0.461206	FALSE	FALSE	FALSE	mOrnK4	0.013707	0.905966	486484	sepsis	TRUE	reported	textfile	0.0047	4.49E-06	6169	M32795.m	TRUE	reported	7EGxuf	2	TRUE	0.003349	20.72483
5128	rs1251651(	A	T	A	T	0.1608	0.009427	0.0973	0.107061	FALSE	TRUE	FALSE	L2DJzK	0.02211	0.669828	486484	sepsis	TRUE	reported	textfile	0.0353	5.33E-06	1574	M33173.m	TRUE	reported	hRG70D	2	TRUE	0.013012	20.72385
7438	rs1095510!	A	C	A	C	-0.0717	0.000334	0.4839	0.490079	FALSE	FALSE	FALSE	otAubi	0.013713	0.980562	486484	sepsis	TRUE	reported	textfile	0.0157	5.14E-06	314	M34404.m	TRUE	reported	SAZaNF	2	TRUE	0.062285	20.72354
2338	rs1333695!	T	C	T	C	0.1061	0.019046	0.0541	0.069347	FALSE	FALSE	FALSE	13lycQ	0.027061	0.481558	486484	sepsis	TRUE	reported	textfile	0.0233	5.48E-06	3393	M16823.m	TRUE	reported	u2seZ2	2	TRUE	0.006074	20.72349
2640	rs8107558	T	C	T	C	0.0665	0.019536	0.9541	0.937451	FALSE	FALSE	FALSE	UxuT5B	0.028296	0.489934	486484	sepsis	TRUE	reported	textfile	0.0146	5.52E-06	1767	M18467.m	TRUE	reported	eGQj6o	2	TRUE	0.011605	20.72267
8074	rs841225	T	C	T	C	-0.0173	0.026016	0.5927	0.591365	FALSE	FALSE	FALSE	7NkUCa	0.013903	0.061318	486484	sepsis	TRUE	reported	textfile	0.0038	5.37E-06	7359	M35320.m	TRUE	reported	GixRsK	2	TRUE	0.002809	20.72082
8820	rs9830523	T	C	T	C	-0.0346	-0.03587	0.9753	0.972917	FALSE	FALSE	FALSE	X2Rol2	0.042717	0.401104	486484	sepsis	TRUE	reported	textfile	0.0076	5.70E-06	7335	M36776.m	TRUE	reported	nikHw1	2	TRUE	0.002818	20.7208
8487	rs3785236	A	C	A	C	-0.0173	0.043738	0.2425	0.245841	FALSE	FALSE	FALSE	ABkZ7x	0.015972	0.006172	486484	sepsis	TRUE	reported	textfile	0.0038	5.25E-06	6960	M35854.m	TRUE	reported	wc0CeK	2	TRUE	0.002969	20.7205
4732	rs6464572	C	G	C	G	-0.0551	-0.02878	0.8341	0.841698	FALSE	TRUE	FALSE	fz63r4	0.018789	0.125631	486484	sepsis	TRUE	reported	textfile	0.0121	5.65E-06	2537	M32762.m	TRUE	reported	kgxlBo	2	TRUE	0.008107	20.72001
6361	rs3923017	T	C	T	C	-0.0469	-0.00043	0.0439	0.077625	FALSE	FALSE	FALSE	WWmDGM	0.025555	0.986678	486484	sepsis	TRUE	reported	textfile	0.0103	5.31E-06	2757	M33627.m	TRUE	reported	2kkEru	2	TRUE	0.007464	20.71839
7875	rs7218918	A	G	A	G	-0.0296	0.017432	0.4081	0.40718	FALSE	FALSE	FALSE	r428eQ	0.013887	0.209377	486484	sepsis	TRUE	reported	textfile	0.0065	4.63E-06	1982	M35159.m	TRUE	reported	3r5zQo	2	TRUE	0.010355	20.71659
5603	rs9562411	T	G	T	G	0.1795	-0.05736	0.9478	0.95856	FALSE	FALSE	FALSE	q32d4E	0.034277	0.094232	486484	sepsis	TRUE	reported	textfile	0.0394	5.20E-06	1017	M33178.m	TRUE	reported	cS3BwT	2	TRUE	0.020001	20.71484
8504	rs1026725(	T	C	T	C	-0.0305	-0.00117	0.8786	0.866632	FALSE	FALSE	FALSE	vHXktL	0.020103	0.953603	486484	sepsis	TRUE	reported	textfile	0.0067	4.71E-06	4529	M35978.m	TRUE	reported	ceap9w	2	TRUE	0.004555	20.71373
6341	rs4458182	A	T	A	T	-0.0862	0.023358	0.0854	0.078129	FALSE	TRUE	FALSE	qLUY00	0.025518	0.359995	486484	sepsis	TRUE	reported	textfile	0.0189	5.30E-06	470	M33610.m	TRUE	reported	8cAJbB	2	TRUE	0.042382	20.71281
6666	rs1287739:	A	C	A	C	-0.0437	-0.02073	0.8993	0.85975	FALSE	FALSE	FALSE	6iZSOl	0.020927	0.321951	486484	sepsis	TRUE	reported	textfile	0.0096	5.85E-06	4533	M33901.m	TRUE	reported	ApKaGe	2	TRUE	0.00455	20.71232
1501	rs1587235	A	G	A	G	-0.0132	0.022299	0.3312	0.334382	FALSE	FALSE	FALSE	rzkxmh	0.014567	0.125826	486484	sepsis	TRUE	reported	textfile	0.0029	6.47E-06	6941	M03127.m	TRUE	reported	3lrzhA	2	TRUE	0.002976	20.71222
8141	rs1257125!	T	C	T	C	-0.0264	0.024321	0.0192	0.021215	FALSE	FALSE	FALSE	lv3pAQ	0.047185	0.60625	486484	sepsis	TRUE	reported	textfile	0.0058	5.39E-06	6920	M35327.m	TRUE	reported	3Vsglp	2	TRUE	0.002985	20.7122
5089	rs1302548(	A	G	A	G	0.205	0.06541	0.977	0.958783	FALSE	FALSE	FALSE	Cira8M	0.03462	0.058838	486484	sepsis	TRUE	reported	textfile	0.045	5.23E-06	1011	M33150.m	TRUE	reported	AofVV7	2	TRUE	0.020114	20.71203
8722	rs6771638	T	G	T	G	-0.0132	0.003322	0.2911	0.294336	FALSE	FALSE	FALSE	l0G0l1	0.015002	0.824772	486484	sepsis	TRUE	reported	textfile	0.0029	5.38E-06	6464	M36553.m	TRUE	reported	lBltb0	2	TRUE	0.003195	20.71178
2543	rs37058	A	T	A	T	0.1894	0.003355	0.0686	0.12364	FALSE	TRUE	FALSE	o0CFya	0.020909	0.872509	486484	sepsis	TRUE	reported	textfile	0.0416	5.25E-06	2433	M18281.m	TRUE	reported	pxbsnu	2	TRUE	0.008448	20.71172
8641	rs7840544	A	G	A	G	0.0947	0.015718	0.9725	0.975126	FALSE	FALSE	FALSE	4cZjpQ	0.043947	0.7206	486484	sepsis	TRUE	reported	textfile	0.0208	5.39E-06	2400	M36230.m	TRUE	reported	TPUeNs	2	TRUE	0.008563	20.71148
6638	rs1015298:	A	G	A	G	-0.0132	0.001768	0.6622	0.658625	FALSE	FALSE	FALSE	REeQiq	0.014478	0.902823	486484	sepsis	TRUE	reported	textfile	0.0029	5.99E-06	6004	M33892.m	TRUE	reported	cVOa3u	2	TRUE	0.003439	20.71129
6660	rs6882710	A	G	A	G	-0.0132	-0.0213	0.5077	0.507138	FALSE	FALSE	FALSE	REeQiq	0.013671	0.119174	486484	sepsis	TRUE	reported	textfile	0.0029	5.26E-06	6004	M33892.m	TRUE	reported	cVOa3u	2	TRUE	0.003439	20.71129
3420	rs1028060(	A	C	A	C	-0.0396	-0.00777	0.8277	0.818808	FALSE	FALSE	FALSE	HHQf8w	0.017848	0.663181	486484	sepsis	TRUE	reported	textfile	0.0087	4.96E-06	5673	M27531.m	TRUE	reported	3vTluK	2	TRUE	0.003639	20.71089
5935	rs1026362:	T	C	T	C	-0.0569	-0.003	0.9157	0.895475	FALSE	FALSE	FALSE	yepzrL	0.022365	0.893247	486484	sepsis	TRUE	reported	textfile	0.0125	5.34E-06	3890	M33408.m	TRUE	reported	SY4CoS	2	TRUE	0.005298	20.71005
640	rs7269193	A	G	A	G	-0.0528	0.041424	0.9092	0.893958	FALSE	FALSE	FALSE	F6Dn8H	0.022288	0.063084	486484	sepsis	TRUE	reported	textfile	0.0116	5.03E-06	4934	M01114.m	TRUE	reported	dVHatZ	2	TRUE	0.004182	20.70979
7822	rs1711352!	T	G	T	G	-0.0132	-0.0207	0.8744	0.869556	FALSE	FALSE	FALSE	h5whlS	0.02028	0.307348	486484	sepsis	TRUE	reported	textfile	0.0029	3.68E-06	4900	M35137.m	TRUE	reported	xVlTKF	2	TRUE	0.00421	20.70974
9003	rs1179333:	T	C	T	C	0.0619	-0.02434	0.0242	0.052628	FALSE	FALSE	FALSE	EZQ1cW	0.031392	0.43807	486484	sepsis	TRUE	reported	textfile	0.0136	5.09E-06	5932	M37253.m	TRUE	reported	ddPpd8	2	TRUE	0.00348	20.7089
1762	rs1021654!	A	G	A	G	-0.0214	-0.01844	0.4694	0.482719	FALSE	FALSE	FALSE	wM5dkT	0.013753	0.179925	486484	sepsis	TRUE	reported	textfile	0.0047	5.19E-06	1757	M15122.m	TRUE	reported	CBpa4t	2	TRUE	0.011662	20.70795
527	rs1241436(	C	G	C	G	0.0214	-0.01451	0.7696	0.79707	FALSE	TRUE	FALSE	wXMWHy	0.017067	0.395097	486484	sepsis	TRUE	reported	textfile	0.0047	4.89E-06	1754	M00584.m	TRUE	reported	7CKn2b	2	TRUE	0.011682	20.70791
476	rs3785024	A	G	A	G	-0.0223	0.034711	0.9469	0.947041	FALSE	FALSE	FALSE	OW48NY	0.030705	0.258281	486484	sepsis	TRUE	reported	textfile	0.0049	5.56E-06	7365	M00527.m	TRUE	reported	57AP2f	2	TRUE	0.002804	20.70616
5739	rs8127620	C	G	C	G	-0.0223	0.009242	0.1786	0.224237	FALSE	TRUE	FALSE	YDQaWa	0.016507	0.575557	486484	sepsis	TRUE	reported	textfile	0.0049	5.93E-06	7063	M33228.m	TRUE	reported	BmYg8r	2	TRUE	0.002924	20.70592
8540	rs1219969:	A	T	A	T	0.4221	0.003392	0.7393	0.746147	FALSE	TRUE	FALSE	aQhCav	0.01572	0.829148	486484	sepsis	TRUE	reported	textfile	0.092	4.50E-06	122	M36097.m	TRUE	reported	9DJS9j	2	TRUE	0.147152	20.70506
3389	rs1118384(	A	G	A	G	-0.0965	0.038124	0.9518	0.948756	FALSE	FALSE	FALSE	frOZiy	0.031782	0.230323	486484	sepsis	TRUE	reported	textfile	0.0212	5.49E-06	2759	M27447.m	TRUE	reported	2z9fSv	2	TRUE	0.007454	20.70465
6483	rs6758453	T	C	T	C	0.0656	-0.01057	0.4234	0.412	FALSE	FALSE	FALSE	ecwVg8	0.013876	0.446101	486484	sepsis	TRUE	reported	textfile	0.0144	4.99E-06	831	M33751.m	TRUE	reported	NnvblQ	2	TRUE	0.024365	20.70314
7234	rs1049605:	T	C	T	C	-0.0906	-0.00608	0.9228	0.910171	FALSE	FALSE	FALSE	RlcE3J	0.024117	0.801103	486484	sepsis	TRUE	reported	textfile	0.0199	5.42E-06	1649	M34339.m	TRUE	reported	QQrwdm	2	TRUE	0.012414	20.70252
4192	rs2679891	T	G	T	G	0.0719	-0.02239	0.0672	0.076162	FALSE	FALSE	FALSE	TxOCzG	0.02577	0.384961	486484	sepsis	TRUE	reported	textfile	0.0158	5.05E-06	6970	M32564.m	TRUE	reported	9JP6ND	2	TRUE	0.002962	20.70232
2180	rs1266097'	T	G	T	G	0.0405	-0.02301	0.1536	0.171573	FALSE	FALSE	FALSE	UokbxN	0.018819	0.221397	486484	sepsis	TRUE	reported	textfile	0.0089	5.67E-06	7357	M15753.m	TRUE	reported	lUGKBi	2	TRUE	0.002807	20.70198
2747	rs4128864	T	C	T	C	-0.0405	-0.01631	0.0303	0.041666	FALSE	FALSE	FALSE	qFAbee	0.034221	0.633664	486484	sepsis	TRUE	reported	textfile	0.0089	5.00E-06	6929	M19364.m	TRUE	reported	tZgXNL	2	TRUE	0.00298	20.70164
7547	rs7961249	T	C	T	C	-0.0419	-0.0059	0.1505	0.178628	FALSE	FALSE	FALSE	JJsHvX	0.01795	0.742181	486484	sepsis	TRUE	reported	textfile	0.0092	5.18E-06	1019	M34456.m	TRUE	reported	aklxve	2	TRUE	0.019949	20.70137
4513	rs1175552	T	C	T	C	-0.0405	0.005246	0.0152	0.020678	FALSE	FALSE	FALSE	r0qJaM	0.047928	0.912846	486484	sepsis	TRUE	reported	textfile	0.0089	5.70E-06	6497	M32729.m	TRUE	reported	vYv5Fl	2	TRUE	0.003177	20.70124
2354	rs1090067(	C	G	C	G	-0.305	0.037677	0.987	0.985531	FALSE	TRUE	FALSE	NBJ2Do	0.057518	0.512436	486484	sepsis	TRUE	reported	textfile	0.067	5.31E-06	1714	M17799.m	TRUE	reported	Uz0Wgl	2	TRUE	0.011946	20.6987
477	rs4635489	A	G	A	G	0.0364	-0.05195	0.0177	0.019796	FALSE	FALSE	FALSE	OW48NY	0.048952	0.288584	486484	sepsis	TRUE	reported	textfile	0.008	5.66E-06	7365	M00527.m	TRUE	reported	57AP2f	2	TRUE	0.002803	20.69688
6055	rs986842	T	C	T	C	-1.5302	0.010131	0.0167	0.047737	FALSE	FALSE	FALSE	33ojbt	0.033425	0.761807	486484	sepsis	TRUE	reported	textfile	0.3307	3.71E-06	60	M33423.m	TRUE	reported	ivEtvl	2	TRUE	0.262995	20.69687
2184	rs3892034	A	T	A	T	-0.0273	0.005931	0.7703	0.756861	FALSE	TRUE	FALSE	UokbxN	0.016012	0.711091	486484	sepsis	TRUE	reported	textfile	0.006	4.75E-06	7357	M15753.m	TRUE	reported	lUGKBi	2	TRUE	0.002806	20.69687
1888	rs1194263:	A	C	A	C	-0.0182	-0.06365	0.0227	0.023661	FALSE	FALSE	FALSE	v2KE89	0.045011	0.157335	486484	sepsis	TRUE	reported	textfile	0.004	5.55E-06	7349	M15500.m	TRUE	reported	to2t7H	2	TRUE	0.002809	20.69687
1282	rs1491606	A	T	A	T	0.0182	-0.01366	0.2992	0.351995	FALSE	TRUE	FALSE	nvWJJ3	0.014364	0.341477	486484	sepsis	TRUE	reported	textfile	0.004	5.80E-06	7346	M01712.m	TRUE	reported	1x44lf	2	TRUE	0.00281	20.69686
902	rs3915237	T	G	T	G	-0.0182	-0.02363	0.7005	0.690926	FALSE	FALSE	FALSE	l6zQnf	0.014729	0.108567	486484	sepsis	TRUE	reported	textfile	0.004	5.52E-06	7345	M01444.m	TRUE	reported	llzalm	2	TRUE	0.002811	20.69686
1076	rs1161822:	T	C	T	C	-0.0182	-0.01114	0.1008	0.170021	FALSE	FALSE	FALSE	JLtEiZ	0.018227	0.54122	486484	sepsis	TRUE	reported	textfile	0.004	6.00E-06	7333	M01572.m	TRUE	reported	TSSe28	2	TRUE	0.002815	20.69685
2085	rs4784175	A	T	A	T	-0.0091	0.001761	0.8586	0.846128	FALSE	TRUE	FALSE	NPVH5j	0.019099	0.926539	486484	sepsis	TRUE	reported	textfile	0.002	8.64E-06	7310	M15506.m	TRUE	reported	r7r3vM	2	TRUE	0.002824	20.69684
7471	rs1099578:	T	C	T	C	-0.0915	-0.02012	0.9786	0.977433	FALSE	FALSE	FALSE	SqfdcK	0.046376	0.664396	486484	sepsis	TRUE	reported	textfile	0.0201	5.21E-06	1567	M34416.m	TRUE	reported	sRGRIK	2	TRUE	0.013052	20.69643
8949	rs9833886	C	G	C	G	0.2264	0.085955	0.0137	0.020225	FALSE	TRUE	FALSE	mXJP3h	0.049107	0.080059	486484	sepsis	TRUE	reported	textfile	0.0497	5.21E-06	751	M37104.m	TRUE	reported	HRZBak	2	TRUE	0.026888	20.69579
2549	rs1234003:	T	G	T	G	-0.0273	-0.01036	0.4693	0.468914	FALSE	FALSE	FALSE	NxZhaQ	0.0138	0.452685	486484	sepsis	TRUE	reported	textfile	0.006	5.59E-06	5984	M18283.m	TRUE	reported	5Pi7r3	2	TRUE	0.003448	20.69558
8435	rs1816995	A	G	A	G	0.0264	-0.02133	0.7712	0.779863	FALSE	FALSE	FALSE	Nvt7pl	0.016497	0.196081	486484	sepsis	TRUE	reported	textfile	0.0058	5.86E-06	1766	M35718.m	TRUE	reported	WkA5Gq	2	TRUE	0.011596	20.69473
6513	rs9684934	T	G	T	G	0.0951	0.029514	0.0187	0.017887	FALSE	FALSE	FALSE	dIuFOH	0.05205	0.57069	486484	sepsis	TRUE	reported	textfile	0.0209	5.17E-06	3845	M33801.m	TRUE	reported	JCXy06	2	TRUE	0.005356	20.69391
7071	rs1048908	T	C	T	C	0.0223	0.008277	0.7357	0.744667	FALSE	FALSE	FALSE	sg7nOB	0.015686	0.597741	486484	sepsis	TRUE	reported	textfile	0.0049	4.20E-06	2278	M34289.m	TRUE	reported	KemvlC	2	TRUE	0.00901	20.6936

4636	rs1704035	A	G	A	G	0.3091	-0.13777	0.9825	0.984463	FALSE	FALSE	FALSE	DfakIt	0.055287	0.012708	486484	sepsis	TRUE	reported	textfile	0.0679	5.38E-06	1368	M32754.m	TRUE	reported	y21cDm	2	TRUE	0.014923	20.69298
8803	rs1433673	A	G	A	G	0.0428	0.011674	0.3426	0.378342	FALSE	FALSE	FALSE	QWTooc	0.014101	0.407731	486484	sepsis	TRUE	reported	textfile	0.0094	5.33E-06	1054	M36756.m	TRUE	reported	OLsw6C	2	TRUE	0.01929	20.69221
3109	rs4452760	A	G	A	G	-0.0182	0.011991	0.569	0.575352	FALSE	FALSE	FALSE	ApMZhy	0.013827	0.385816	486484	sepsis	TRUE	reported	textfile	0.004	4.33E-06	3950	M22138.m	TRUE	reported	LiJliF	2	TRUE	0.005214	20.69202
3106	rs1261555	A	G	A	G	-0.0569	-0.01021	0.9347	0.936045	FALSE	FALSE	FALSE	ApMZhy	0.027892	0.714338	486484	sepsis	TRUE	reported	textfile	0.0125	5.47E-06	1397	M22138.m	TRUE	reported	LiJliF	2	TRUE	0.014616	20.69104
637	rs4579412	A	C	A	C	-0.0414	-0.0318	0.1469	0.138636	FALSE	FALSE	FALSE	F6Dn8H	0.019819	0.108636	486484	sepsis	TRUE	reported	textfile	0.0091	4.77E-06	4934	M01114.m	TRUE	reported	dVHatZ	2	TRUE	0.004177	20.68911
3567	rs1099571	T	G	T	G	0.1194	-0.03975	0.0683	0.064373	FALSE	FALSE	FALSE	F1s6qg	0.027927	0.154662	486484	sepsis	TRUE	reported	textfile	0.0262	5.23E-06	522	M31536.m	TRUE	reported	Zm40GX	2	TRUE	0.038264	20.68897
3997	rs1001159	T	C	T	C	0.0223	-0.00597	0.8351	0.833627	FALSE	FALSE	FALSE	V9AtId	0.018384	0.745235	486484	sepsis	TRUE	reported	textfile	0.0049	5.43E-06	1767	M32489.m	TRUE	reported	8B8S8a	2	TRUE	0.011586	20.68834
8594	rs1107967	T	C	T	C	-0.0819	-0.00057	0.9555	0.953139	FALSE	FALSE	FALSE	t5potg	0.032422	0.985853	486484	sepsis	TRUE	reported	textfile	0.018	5.27E-06	2709	M36115.m	TRUE	reported	SSOHIA	2	TRUE	0.007584	20.68722
6412	rs2540742	A	G	A	G	-0.0232	-0.00326	0.642	0.636523	FALSE	FALSE	FALSE	5CIPW7	0.014268	0.819379	486484	sepsis	TRUE	reported	textfile	0.0051	5.55E-06	4807	M33653.m	TRUE	reported	H4b2zx	2	TRUE	0.004286	20.68497
5648	rs1706826	T	C	T	C	-0.1633	-0.04386	0.9803	0.97569	FALSE	FALSE	FALSE	BFsMtm	0.04468	0.32628	486484	sepsis	TRUE	reported	textfile	0.0359	5.33E-06	5716	M33192.m	TRUE	reported	NAJN3D	2	TRUE	0.003607	20.68386
7407	rs9305620	A	G	A	G	-0.0487	-0.00708	0.3537	0.377452	FALSE	FALSE	FALSE	XfpKv0	0.014212	0.618396	486484	sepsis	TRUE	reported	textfile	0.0107	5.52E-06	1312	M34390.m	TRUE	reported	mmOsbK	2	TRUE	0.015544	20.68368
3113	rs1214310	T	C	T	C	0.0232	-0.00402	0.5922	0.591332	FALSE	FALSE	FALSE	a9KeDe	0.013882	0.771916	486484	sepsis	TRUE	reported	textfile	0.0051	4.77E-06	3838	M22175.m	TRUE	reported	shg5gu	2	TRUE	0.005363	20.6828
7804	rs645288	C	G	C	G	-0.0141	-0.00483	0.2747	0.266184	FALSE	TRUE	FALSE	cQ5TvO	0.015475	0.754887	486484	sepsis	TRUE	reported	textfile	0.0031	6.45E-06	7344	M35127.m	TRUE	reported	BVxkfZ	2	TRUE	0.002809	20.68219
8679	rs3846241	A	G	A	G	-0.0478	-0.03152	0.404	0.462385	FALSE	FALSE	FALSE	i4bSdJ	0.013773	0.022084	486484	sepsis	TRUE	reported	textfile	0.0105	5.75E-06	976	M36468.m	TRUE	reported	qJLuZj	2	TRUE	0.020792	20.6817
7625	rs1007405	A	G	A	G	-0.0373	-0.01861	0.8297	0.832997	FALSE	FALSE	FALSE	xGQKuv	0.01838	0.311217	486484	sepsis	TRUE	reported	textfile	0.0082	5.24E-06	3813	M34530.m	TRUE	reported	wajzOs	2	TRUE	0.005397	20.68055
2685	rs1081335	A	G	A	G	0.0373	-0.00567	0.4043	0.414599	FALSE	FALSE	FALSE	ATsH6N	0.01388	0.68265	486484	sepsis	TRUE	reported	textfile	0.0082	5.62E-06	3783	M18497.m	TRUE	reported	lGulRI	2	TRUE	0.00544	20.68046
7869	rs1226089	T	C	T	C	-0.0596	0.025265	0.9341	0.931989	FALSE	FALSE	FALSE	r428eQ	0.0272	0.352968	486484	sepsis	TRUE	reported	textfile	0.0131	5.63E-06	1982	M35159.m	TRUE	reported	3r5zQo	2	TRUE	0.010336	20.67814
8736	rs1716328	A	C	A	C	-0.0273	0.012655	0.7217	0.727807	FALSE	FALSE	FALSE	ERC6hD	0.015405	0.411365	486484	sepsis	TRUE	reported	textfile	0.006	4.61E-06	1636	M36559.m	TRUE	reported	n8waJF	2	TRUE	0.012496	20.67719
2544	rs3800052	T	C	T	C	0.1715	0.032333	0.0846	0.096376	FALSE	FALSE	FALSE	o0CFya	0.023629	0.171199	486484	sepsis	TRUE	reported	textfile	0.0377	5.46E-06	2433	M18281.m	TRUE	reported	pxbsnu	2	TRUE	0.008434	20.67704
5788	rs1159425	A	T	A	T	-0.1101	-0.00837	0.0335	0.031999	FALSE	TRUE	FALSE	lUkr5t	0.038739	0.829036	486484	sepsis	TRUE	reported	textfile	0.0242	5.34E-06	1863	M33359.m	TRUE	reported	DjliwS	2	TRUE	0.010988	20.67652
4864	rs1166860	A	G	A	G	-0.0191	0.011523	0.4922	0.523286	FALSE	FALSE	FALSE	KBJVQO	0.01372	0.400991	486484	sepsis	TRUE	reported	textfile	0.0042	5.15E-06	7355	M32855.m	TRUE	reported	Xea7Fn	2	TRUE	0.002804	20.67522
6224	rs1742123	C	G	C	G	-0.0191	0.001961	0.2887	0.304082	FALSE	TRUE	FALSE	8zUBbU	0.015029	0.896164	486484	sepsis	TRUE	reported	textfile	0.0042	6.18E-06	7044	M33509.m	TRUE	reported	1mO9m8	2	TRUE	0.002927	20.67497
1477	rs2504282	A	G	A	G	0.0191	-0.02023	0.9338	0.920344	FALSE	FALSE	FALSE	pkb876	0.025426	0.426256	486484	sepsis	TRUE	reported	textfile	0.0042	6.70E-06	7037	M02734.m	TRUE	reported	0LCMVI	2	TRUE	0.00293	20.67496
4640	rs4919957	A	G	A	G	0.1629	0.006029	0.0653	0.053169	FALSE	FALSE	FALSE	DfakIt	0.03081	0.844857	486484	sepsis	TRUE	reported	textfile	0.0358	5.40E-06	1368	M32754.m	TRUE	reported	y21cDm	2	TRUE	0.01491	20.67477
7551	rs1027574	T	C	T	C	0.0191	-0.0023	0.4335	0.449905	FALSE	FALSE	FALSE	APSWAU	0.01374	0.866865	486484	sepsis	TRUE	reported	textfile	0.0042	4.95E-06	6144	M34469.m	TRUE	reported	frF3RX	2	TRUE	0.003355	20.67411
4568	rs1196661	A	G	A	G	-0.0191	0.019916	0.8665	0.85956	FALSE	FALSE	FALSE	ILOOFw	0.019696	0.311945	486484	sepsis	TRUE	reported	textfile	0.0042	5.89E-06	6042	M32739.m	TRUE	reported	TpvRBe	2	TRUE	0.003411	20.67399
1145	rs1173649	T	C	T	C	0.1146	-0.07772	0.0214	0.04415	FALSE	FALSE	FALSE	QtY5Hc	0.033988	0.022219	486484	sepsis	TRUE	reported	textfile	0.0252	5.56E-06	5162	M01605.m	TRUE	reported	Q3iDQX	2	TRUE	0.00399	20.67283
8348	rs1248584	T	C	T	C	0.102	0.019476	0.0771	0.056937	FALSE	FALSE	FALSE	6VVCe4	0.029986	0.51601	486484	sepsis	TRUE	reported	textfile	0.0224	5.40E-06	659	M35669.m	TRUE	reported	l0fk8X	2	TRUE	0.030505	20.67208
6094	rs4076939	A	C	A	C	0.0191	-0.00251	0.2178	0.221201	FALSE	FALSE	FALSE	xvzgym	0.016461	0.878773	486484	sepsis	TRUE	reported	textfile	0.0042	5.49E-06	4004	M33443.m	TRUE	reported	KtBPyR	2	TRUE	0.005139	20.67051
1412	rs4944238	A	G	A	G	-0.0241	-0.00168	0.8712	0.872998	FALSE	FALSE	FALSE	33ptwk	0.02051	0.93477	486484	sepsis	TRUE	reported	textfile	0.0053	4.88E-06	6252	M02137.m	TRUE	reported	SQY1na	2	TRUE	0.003296	20.67014
5634	rs4233480	A	G	A	G	0.0473	0.002155	0.5412	0.53596	FALSE	FALSE	FALSE	U0d0ue	0.013733	0.875308	486484	sepsis	TRUE	reported	textfile	0.0104	5.43E-06	2616	M33190.m	TRUE	reported	FoODGF	2	TRUE	0.007845	20.66919
8979	rs4334280	A	G	A	G	-0.0291	-0.0007	0.3817	0.386269	FALSE	FALSE	FALSE	snOepl	0.014013	0.960145	486484	sepsis	TRUE	reported	textfile	0.0064	6.10E-06	7355	M37202.m	TRUE	reported	PQx272	2	TRUE	0.002803	20.66845
3180	rs155394	A	G	A	G	-0.0291	-0.03511	0.9262	0.914103	FALSE	FALSE	FALSE	gvZCBj	0.024533	0.152388	486484	sepsis	TRUE	reported	textfile	0.0064	5.40E-06	7259	M22189.m	TRUE	reported	GKqg2h	2	TRUE	0.00284	20.66838
7422	rs8043508	A	G	A	G	-0.0773	-0.02074	0.9848	0.983029	FALSE	FALSE	FALSE	7i5VUS	0.05329	0.697169	486484	sepsis	TRUE	reported	textfile	0.017	5.38E-06	5208	M34395.m	TRUE	reported	64hEaL	2	TRUE	0.003954	20.6678
6212	rs1280976	T	C	T	C	-0.0523	0.003091	0.9163	0.88955	FALSE	FALSE	FALSE	FQfT2o	0.021918	0.887841	486484	sepsis	TRUE	reported	textfile	0.0115	5.70E-06	2759	M33508.m	TRUE	reported	JtbOy2	2	TRUE	0.007441	20.66773
5427	rs3169950	A	G	A	G	0.0901	-0.01456	0.409	0.45075	FALSE	FALSE	FALSE	q32d4E	0.013716	0.288282	486484	sepsis	TRUE	reported	textfile	0.0198	5.32E-06	1017	M33178.m	TRUE	reported	cS3BwT	2	TRUE	0.019955	20.66637
6508	rs1206664	T	C	T	C	-0.0482	-0.03195	0.0891	0.103782	FALSE	FALSE	FALSE	dIuFOH	0.022392	0.153588	486484	sepsis	TRUE	reported	textfile	0.0106	4.94E-06	3845	M33801.m	TRUE	reported	JCXy06	2	TRUE	0.005349	20.666
3593	rs1193324	A	G	A	G	-0.0341	0.018602	0.3129	0.310051	FALSE	FALSE	FALSE	e4G8UU	0.015055	0.216606	486484	sepsis	TRUE	reported	textfile	0.0075	5.31E-06	5229	M31548.m	TRUE	reported	kWkkmH	2	TRUE	0.003938	20.66427
3952	rs356296	A	G	A	G	-0.0882	-0.02217	0.016	0.022665	FALSE	FALSE	FALSE	N6UQnQ	0.046051	0.630183	486484	sepsis	TRUE	reported	textfile	0.0194	5.57E-06	7346	M32425.m	TRUE	reported	FEcGHY	2	TRUE	0.002806	20.66405
8353	rs1766164	C	G	C	G	-0.0441	-0.06332	0.9412	0.940831	FALSE	TRUE	FALSE	6VVCe4	0.029004	0.02902	486484	sepsis	TRUE	reported	textfile	0.0097	5.57E-06	5629	M35669.m	TRUE	reported	l0fk8X	2	TRUE	0.003659	20.66233
1685	rs1187499	T	C	T	C	-0.0523	0.01303	0.0175	0.025031	FALSE	FALSE	FALSE	f01en0	0.043727	0.765711	486484	sepsis	TRUE	reported	textfile	0.0115	5.62E-06	1750	M12593.m	TRUE	reported	EAsM6U	2	TRUE	0.011681	20.65908
1213	rs676094	A	G	A	G	0.0191	-0.01794	0.4178	0.407379	FALSE	FALSE	FALSE	Ckdgh1	0.013911	0.197259	486484	sepsis	TRUE	reported	textfile	0.0042	6.61E-06	1766	M01644.m	TRUE	reported	nKxz7B	2	TRUE	0.011575	20.65742
5199	rs1084253	T	C	T	C	-0.1888	-0.02416	0.04	0.048744	FALSE	FALSE	FALSE	q32d4E	0.03197	0.449853	486484	sepsis	TRUE	reported	textfile	0.0415	5.27E-06	1017	M33178.m	TRUE	reported	cS3BwT	2	TRUE	0.019945	20.65632
1561	rs4436895	C	G	C	G	-0.01	-0.02146	0.252	0.278811	FALSE	TRUE	FALSE	Ke0sNh	0.015297	0.160643	486484	sepsis	TRUE	reported	textfile	0.0022	3.47E-06	7799	M10642.m	TRUE	reported	ZINwVb	2	TRUE	0.002642	20.65586
4072	rs2373319	A	T	A	T	-0.015	0.01164	0.7383	0.725452	FALSE	TRUE	FALSE	TyHtdh	0.015384	0.449285	486484	sepsis	TRUE	reported	textfile	0.0033	5.87E-06	7358	M32497.m	TRUE	reported	H3KmLR	2	TRUE	0.0028	20.65554
853	rs4287543	A	G	A	G	0.015	0.038891	0.8771	0.873709	FALSE	FALSE	FALSE	1tFtaf	0.020953	0.063442	486484	sepsis	TRUE	reported	textfile	0.0033	4.54E-06	7323	M01359.m	TRUE	reported	qq3q6E	2	TRUE	0.002813	20.65551
2877	rs1267706	A	G	A	G	-0.015	-0.02689	0.29	0.335335	FALSE	FALSE	FALSE	mUzn1R	0.014448	0.062712	486484	sepsis	TRUE	reported	textfile	0.0033	5.51E-06	7301	M20675.m	TRUE	reported	XwY04K	2	TRUE	0.002822	20.6555
8968	rs650849	T	G	T	G	0.03	0.005457	0.6496	0.6558	FALSE	FALSE	FALSE	2iAvIt	0.014356	0.703824	486484	sepsis	TRUE	reported	textfile	0.0066	5.64E-06	6934	M37190.m	TRUE	reported	hdOuHe	2	TRUE	0.002971	20.6552
5949	rs1159134	A	T	A	T	-0.045	0.065627	0.0643	0.0622	FALSE	TRUE	FALSE	mDZrGw	0.028314	0.020459	486484	sepsis	TRUE	reported	textfile	0.0099	5.05E-06	6797	M33415.m	TRUE	reported	MzEP7R	2	TRUE	0.003031	20.65508
5951	rs1176828	A	G	A	G	0.05	-0.0086	0.0589	0.06752	FALSE	FALSE	FALSE	mDZrGw	0.027604	0.755358	486484	sepsis	TRUE	reported	textfile	0.011	5.90E-06	6797	M33415.m	TRUE	reported	MzEP7R	2	TRUE	0.003031	20.65508
8412	rs1271423	T	C	T	C	-0.02	-0.01243	0.5232	0.513595	FALSE	FALSE	FALSE	02RdNm	0.01366	0.362895	486484	sepsis	TRUE	reported	textfile	0.0044	7.07E-06	6447	M35678.m	TRUE	reported	BWN5JR	2	TRUE	0.003195	20.65475
9013	rs9544785	A	C	A	C	-0.015	0.024048	0.3614	0.357783	FALSE	FALSE	FALSE	EZQ1cW	0.014472	0.096567	486484	sepsis	TRUE	reported	textfile	0.0033	6.63E-06	5932	M37253.m	TRUE	reported	ddPpd8	2	TRUE	0.003471	20.65419
7645	rs2429426	A	G	A	G	0.025	0.000136	0.7093	0.716143	FALSE	FALSE	FALSE	wrTMYM	0.01518	0.992853	486484	sepsis	TRUE	reported	textfile	0.0055	4.70E-06	5856	M34533.m	TRUE	reported	qIRVku	2	TRUE	0.003516	20.6541
7952	rs1981524	T	C	T	C	0.015	0.015205	0.2032	0.188143	FALSE	FALSE	FALSE	lvUHnQ	0.01753	0.385738	486484	sepsis	TRUE	reported	textfile	0.0033	4.71E-06	5694	M35240.m	TRUE	reported	VH44Ha	2	TRUE	0.003615	20.6539
2661	rs795543	T	C	T	C	-0.025	0.012513	0.4091	0.41155	FALSE	FALSE	FALSE	UtYu7J	0.01387	0.36696	486484	sepsis	TRUE	reported	textfile	0.0055	5.19E-06	5624	M18476.m	TRUE	reported	wJx0A4	2	TRUE	0.00366	20.65381
7520	rs2850377	T	C	T	C	0.02	0.006977	0.41	0.431942	FALSE	FALSE	FALSE	rp1LGA	0.013809	0.613373	486484	sepsis	TRUE	reported	textfile	0.0044	5.52E-06	4442	M34441.m	TRUE	reported	SU47tz	2	TRUE	0.00463	20.65185
6097	rs7690478	T	C	T	C	0.015	0.002624	0.304	0.287803	FALSE	FALSE	FALSE	xvzgym	0.015189	0.862858	486484	sepsis	TRUE	reported	textfile	0.0033	6.43E-06	4004	M33443.m	TRUE	reported	KtBPyR	2	TRUE	0.005134	20.65084
8511	rs1693619	C	G	C	G	-0.1014	-0.00835	0.0247	0.027253	FALSE	TRUE	FALSE	vHXktL	0.041855	0.84181	486484	sepsis	TRUE	reported	textfile	0.0223	5.41E-06	1517	M35978.m	TRUE	reported	ceap9w	2	TRUE	0.013446	20.64872
8247	rs1115525	A	G	A	G	-0.0914	-0.01706	0.066	0.063585	FALSE	FALSE	FALSE	G4plxe	0.028112	0.543983	486484	sepsis	TRUE	reported	textfile	0.0201	5.74E-06	1383	M35451.m	TRUE	reported	hNOVMI	2	TRUE	0.014731	20.6477
8615	rs4486099	A	C	A	C	-0.0391	0.014564	0.1884	0.201612	FALSE	FALSE	FALSE	oHM5TD	0.017126	0.395089	486484	sepsis	TRUE	reported	textfile	0.0086	5.79E-06	1780	M36131.m	TRUE	reported	Xw6wmg	2	TRUE	0.011479	20.64754
5474	rs4786186	A	C	A	C	0.2679	0.022527	0.9792	0.968557	FALSE	FALSE	FALSE	q32d4E	0.039942	0.57276	486484	sepsis	TRUE	reported	textfile	0.0589	5.34E-06	1017	M33178.m	TRUE	reported	cS3BwT	2	TRUE	0.019936	20.64714
8756	rs1168837	T	C	T	C	-0.0655	0.037459	0.4786	0.493246	FALSE	FALSE	FALSE	KMisdy	0.013694	0.006229	486484	sepsis	TRUE	reported	textfile	0.0144	5.31E-06	952	M36589.m	TRUE	reported	J1ISTP	2	TRUE	0.021271	20.6464
5087	rs1264047	T	C	T	C	-0.04	0.011371	0.3034	0.289257	FALSE	FALSE	FALSE	Cira8M	0.01514	0.452614	486484	sepsis	TRUE	reported	textfile	0.0088	5.88E-06	2777	M33150.m	TRUE	reported	AofVV7	2	TRUE	0.007385	20.64628
2744	rs1758386	A	G	A	G	-0.0782	-0.01033	0.0199	0.026152	FALSE	FALSE	FALSE	qFAbee	0.044224	0.815312	486484	sepsis	TRUE	reported	textfile	0.0172	5.72E-06	1654	M19364.m	TRUE	reported	tZgXNL	2	TRUE	0.012343	20.64577
7690	rs6947774	T	C	T	C	0.025	-0.00286	0.3078	0.300328	FALSE	FALSE	FALSE	5FAxbZ	0.014899	0.847511	486484	sepsis	TRUE	reported	textfile	0.0055	6.65E-06	2545	M34674.m	TRUE	reported	TDQqa8	2	TRUE	0.008053	20.64492
5796	rs2416328	T	C	T	C	0.02	-0.00827	0.7592	0.758498	FALSE	FALSE	FALSE	ka9zG7	0.015979	0.60466	486484	sepsis	TRUE	reported	textfile	0.0044	6.35E-06	2212	M33363.m	TRUE	reported	UFTVNc	2	TRUE	0.009254	20.64248
6695	rs1688032	T	G	T	G	0.1136	-0.07673	0.978	0.974829	FALSE	FALSE	FALSE	AcRp2F	0.043851	0.08016	486484	sepsis	TRUE	reported	textfile	0.025	5.76E-06	6756	M33935.m	TRUE	reported	FQCKJy	2	TRUE	0.003047	20.64182
8674	rs1479073	T	C	T	C	0.035	0.001341	0.7131	0.687222	FALSE	FALSE	FALSE	i4bSdJ	0.014822	0.927899	486484	sepsis	TRUE	reported	textfile	0.0077	5.97E-06	1908	M36468.m	TRUE	reported	qJLuZj	2	TRUE	0.010713	20.6395
6690	rs1750335	A	G	A	G	0.0777	-0.02235	0.0288	0.056802	FALSE	FALSE	FALSE	23zCgC	0.02965	0.450913	486484	sepsis	TRUE	reported	textfile	0.0171	5.41E-06	5534	M33910.m	TRUE	reported	ACI3CA	2	TRUE	0.003717	20.6392
5718	rs6921033	A	G	A	G	0.1937	0.100082	0.0116	0.013663	FALSE	FALSE	FALSE	XDem1i	0.059081	0.090271	486484	sepsis	TRUE	reported	textfile	0.0426	5.34E-06	1142	M33221.m	TRUE	reported	KBV6FH	2	TRUE	0.017782	20.63853
3251	rs9510021	A	C	A	C	-0.0468	0.009436	0.5753	0.573272	FALSE	FALSE	FALSE	jxdGCf	0.013842	0.495419	486484	sepsis	TRUE	reported	textfile	0.0103	5.77E-06	5599	M22842.m	TRUE	reported	f0L3pZ	2	TRUE	0.003674	20.63774
8181	rs572132	A	T	A	T	-0.025	0.052726	0.9365	0.926901	FALSE	TRUE	FALSE	8UhtPK	0.026303	0.045013	486484	sepsis	TRUE	reported	textfile	0.0055	5.17E-06	1758	M35397.m	TRUE	reported	rvVJba	2	TRUE	0.011616	20.63765
6863	rs1102974	T	G	T	G	0.0209	0.033296	0.0206	0.021473	FALSE	FALSE	FALSE	kFWZjs	0.04733	0.481747	486484	sepsis	TRUE	reported	textfile	0.0046	5.55E-06	7362	M34062.m	TRUE	reported	eLONsg	2	TRUE	0.002796	20.63759
6136	rs244523	A	T	A	T	-0.02	0.01226	0.2872	0.296087	FALSE	TRUE	FALSE	MiSJly	0.014969	0.412761	486484	sepsis	TRUE	reported	textfile	0.0044	6.53E-06	1734	M33477.m	TRUE	reported	WwL1hq	2	TRUE	0.011775	20.63733
6687	rs1167149	A	G	A	G	0.1123	-0.04083	0.0411	0.051747	FALSE	FALSE	FALSE	23zCgC	0.030942	0.186986	486484	sepsis	TRUE	reported	textfile	0.0247	5.23E-06	1183	M33910.m	TRUE	reported	ACI3CA	2	TRUE	0.017173	20.63625
2535	rs4661452	T	C	T	C	-0.0577	-0.01399	0.9194	0.886299	FALSE	FALSE	FALSE	4HwTlG	0.02177	0.520467	486484	sepsis	TRUE	reported	textfile	0.0127	5.18E-06	7071	M18254.m	TRUE	reported	F9KrSC	2	TRUE	0.002911	20.6358
486	rs6493912	T	C	T	C	-0.0368	-0.0137	0.7431	0.712035	FALSE	FALSE	FALSE	ScJVI8	0.015099	0.364177	486484	sepsis	TRUE	reported	textfile	0.0081	5.36E-06	7371	M00542.m	TRUE	reported	Sa1L04	2	TRUE	0.002792	20.63516
1537	rs7668550	T	G	T	G	-0.0368	0.000907	0.0157	0.016804	FALSE	FALSE	FALSE	YvgTeW	0.052906	0.986327	486484	sepsis	TRUE	reported	textfile	0.0081	5.54E-06	7357	M03141.m	TRUE	reported	srKjSc	2	TRUE	0.002798	20.63514
2406	rs2041389	C	G	C	G	-0.1506	-0.06758	0.05	0.017139	FALSE	TRUE	FALSE	NBJ2Do	0.053176	0.203778	486484	sepsis	TRUE	reported	textfile	0.0318	2.25E-06	25	M17799.m	TRUE	reported	Uz0Wgl	2	TRUE	0.472888	20.634
4496	rs4422759	T	C	T	C	0.0159	0.016772	0.1519	0.154779	FALSE	FALSE	FALSE	1wNJ1E	0.018893	0.374673	486484	sepsis	TRUE	reported	textfile	0.0035	6.73E-06	7363	M32709.m	TRUE	reported	zihBHg	2	TRUE	0.002795	20.63195
6523	rs272461	A	G	A	G	0.0159	0.028264	0.2709	0.272864	FALSE	FALSE	FALSE	OKIXJ8	0.015428	0.066956	486484	sepsis	TRUE	reported	textfile	0.0035	6.26E-06	7362	M33821.m	TRUE	reported	XGkaXH	2	TRUE	0.002795	20.63194
4957	rs2904562	A	T	A	T	0.0159	0.060099	0.9187	0.90635	FALSE	TRUE	FALSE	bKmDMa	0.023648	0.01104	486484	sepsis	TRUE	reported	textfile	0.0035	4.67E-06	7344	M32910.m	TRUE	reported	nAuPzO	2	TRUE	0.002802	20.63193
3037	rs6508364	A	G	A	G	-0.0159	-0.00479	0.1404	0.135578	FALSE	FALSE	FALSE	FJZEYz	0.020126	0.811878	486484	sepsis	TRUE	reported	textfile	0.0035	6.52E-06	7284	M21630.m	TRUE	reported	s6Pshg	2	TRUE	0.002825	20.63188
7956	rs1194999	C	G	C	G	0.0159	0.002232	0.8327	0.834838	FALSE	TRUE	FALSE	zb8m4a	0.018382	0.903341	486484	sepsis	TRUE	reported	textfile	0.0035	5.61E-06	7261	M35253.m	TRUE	reported	tHnnnS	2	TRUE	0.002834	20.63187
7959	rs1272691	T	G	T	G	-0.0159	0.019752	0.1774	0.179856	FALSE	FALSE	FALSE	zb8m4a	0.01799	0.272227	486484	sepsis	TRUE	reported	textfile	0.0035	4.04E-06	7261	M35253.m	TRUE	reported	tHnnnS	2	TRUE	0.002834	20.63187
6957	rs251456	A	G	A	G	0.0159	-0.00382	0.1523	0.147404	FALSE	FALSE	FALSE	7Cw2XZ	0.01936	0.843557	486484	sepsis	TRUE	reported	textfile	0.0035	6.63E-06	7049	M34123.m	TRUE	reported	vrgMl3	2	TRUE	0.002919	20.6317
2577	rs314707	A	G	A	G	-0.0159	0.003002	0.8305	0.835612	FALSE	FALSE	FALSE	KHS91f	0.018485	0.870969	486484	sepsis	TRUE	reported	textfile	0.0035	4.43E-06	6939	M18349.m	TRUE	reported	PeVGil	2	TRUE	0.002965	20.6316
7934	rs2278350	A	C	A	C	-0.0159	0.023472	0.3247	0.347694	FALSE	FALSE	FALSE	kFTWXY	0.014417	0.103509	486484	sepsis	TRUE	reported	textfile	0.0035	4.62E-06	6543	M35193.m	TRUE	reported	cjJvJl	2	TRUE	0.003144	20.63124
2169	rs584630	A	C	A	C	-0.0318	0.025586	0.1052	0.14689	FALSE	FALSE	FALSE	XCBNPg	0.019306	0.185066	486484	sepsis	TRUE	reported	textfile	0.007	6.27E-06	5829	M15749.m	TRUE	reported	dKBewD	2	TRUE	0.003528	20.63047
687	rs1726825	T	C	T	C	-0.0159	-0.02367	0.1349	0.163023	FALSE	FALSE	FALSE	aT1Fw1	0.018546	0.201779	486484	sepsis	TRUE	reported	textfile	0.0035	7.31E-06	5687	M01284.m	TRUE	reported	mbTe8H	2	TRUE	0.003616	20.63029
4744	rs4885169	A	G	A	G	-0.0427	0.00078	0.1078	0.096835	FALSE	FALSE	FALSE	oQPvO3	0.02307	0.973041	486484	sepsis	TRUE	reported	textfile	0.0094	5.17E-06	7330	M32786.m	TRUE	reported	Th0GcB	2	TRUE	0.002807	20.62916
5933	rs8094976	T	C	T	C	0.6419	0.007821	0.0131	0.016557	FALSE	FALSE	FALSE	veBhfS	0.053761	0.88433	486484	sepsis	TRUE	reported	textfile	0.1405	4.93E-06	171	M33391.m	TRUE	reported	Gfjy5a	2	TRUE	0.108785	20.62874
3731	rs1122413	T	C	T	C	-0.0409	0.018539	0.1324	0.165072	FALSE	FALSE	FALSE	k16oIH	0.018406	0.31382	486484	sepsis	TRUE	reported	textfile	0.009	5.31E-06	1765	M32328.m	TRUE	reported	TQA3sZ	2	TRUE	0.011566	20.62857
7573	rs1182294	T	C	T	C	0.0477	-0.00254	0.8079	0.789276	FALSE	FALSE	FALSE	ElkOlW	0.016795	0.879788	486484	sepsis	TRUE	reported	textfile	0.0105	5.69E-06	4296	M34499.m	TRUE	reported	W5iEAE	2	TRUE	0.004781	20.62794
7167	rs1685588	T	G	T	G	0.1068	-0.04371	0.0407	0.042215	FALSE	FALSE	FALSE	VfvTtz	0.034554	0.205914	486484	sepsis	TRUE	reported	textfile	0.0235	5.32E-06	1492	M34329.m	TRUE	reported	egac6r	2	TRUE	0.013654	20.62644
7193	rs1040865	T	C	T	C	0.2407	-0.05598	0.0449	0.071187	FALSE	FALSE	FALSE	i1uJ7T	0.026485	0.034551	486484	sepsis	TRUE	reported	textfile	0.0529	5.25E-06	535	M34338.m	TRUE	reported	pi3mKW	2	TRUE	0.037256	20.62596
979	rs1207572	T	G	T	G	-0.4196	-0.02116	0.9442	0.946801	FALSE	FALSE	FALSE	xSRIl4	0.030525	0.488167	486484	sepsis	TRUE	reported	textfile	0.0922	5.39E-06	482	M01515.m	TRUE	reported	juZ5GE	2	TRUE	0.041199	20.62544
7421	rs7973154	C	G	C	G	-0.0595	0.037346	0.0265	0.030435	FALSE	TRUE	FALSE	7i5VUS	0.040144	0.352218	486484	sepsis	TRUE	reported	textfile	0.0131	5.31E-06	5208	M34395.m	TRUE	reported	64hEaL	2	TRUE	0.003946	20.6217
1563	rs4812805	C	G	C	G	-0.0109	0.043081	0.8421	0.856269	FALSE	TRUE	FALSE	Ke0sNh	0.019563	0.027654	486484	sepsis	TRUE	reported	textfile	0.0024	5.88E-06	7799	M10642.m	TRUE	reported	ZINwVb	2	TRUE	0.002638	20.62145
696	rs1087315	T	C	T	C	-0.0109	0.035907	0.0933	0.090697	FALSE	FALSE	FALSE	H9I3s3	0.023806	0.131477	486484	sepsis	TRUE	reported	textfile	0.0024	8.62E-06	7358	M01299.m	TRUE	reported	kXWyUq	2	TRUE	0.002795	20.62113
708	rs2891733	T	C	T	C	-0.0109	-0.01548	0.8941	0.893981	FALSE	FALSE	FALSE	H9I3s3	0.02229	0.487271	486484	sepsis	TRUE	reported	textfile	0.0024	8.47E-06	7358	M01299.m	TRUE	reported	kXWyUq	2	TRUE	0.002795	20.62113
577	rs1477025	T	C	T	C	0.0109	-0.00095	0.6545	0.657068	FALSE	FALSE	FALSE	ZoqU2j	0.014505	0.947587	486484	sepsis	TRUE	reported	textfile	0.0024	7.63E-06	7333	M01105.m	TRUE	reported	2WxSOH	2	TRUE	0.002805	20.62111
583	rs2242415	A	C	A	C	0.0109	0.016365	0.6233	0.616528	FALSE	FALSE	FALSE	ZoqU2j	0.014199	0.249097	486484	sepsis	TRUE	reported	textfile	0.0024	8.24E-06	7333	M01105.m	TRUE	reported	2WxSOH	2	TRUE	0.002805	20.62111
1087	rs4836200	A	G	A	G	-0.0109	-0.00642	0.6785	0.660664	FALSE	FALSE	FALSE	JLtEiZ	0.014469	0.657429	486484	sepsis	TRUE	reported	textfile	0.0024	5.91E-06	7333	M01572.m	TRUE	reported	TSSe28	2	TRUE	0.002805	20.62111
3084	rs3798221	T	G	T	G	0.0109	-0.0015	0.1914	0.191035	FALSE	FALSE	FALSE	esOP8m	0.017334	0.931184	486484	sepsis	TRUE	reported	textfile	0.0024	8.31E-06	7329	M22116.m	TRUE	reported	fDMh7Z	2	TRUE	0.002807	20.62111
2615	rs2081811	A	G	A	G	0.0327	0.014247	0.7543	0.749757	FALSE	FALSE	FALSE	OiiDPr	0.015828	0.368058	486484	sepsis	TRUE	reported	textfile	0.0072	5.81E-06	7322	M18392.m	TRUE	reported	ScUw6o	2	TRUE	0.002809	20.6211
1688	rs1289648	T	C	T	C	-0.0109	0.041869	0.8919	0.878071	FALSE	FALSE	FALSE	f01en0	0.021012	0.0463	486484	sepsis	TRUE	reported	textfile	0.0024	4.17E-06	7314	M12593.m	TRUE	reported	EAsM6U	2	TRUE	0.002812	20.6211
1024	rs1199243	A	G	A	G	0.0327	0.016248	0.9488	0.94184	FALSE	FALSE	FALSE	haWxZg	0.029551	0.582441	486484	sepsis	TRUE	reported	textfile	0.0072	6.38E-06	7276	M01561.m	TRUE	reported	5wm9R3	2	TRUE	0.002827	20.62107
6790	rs6573597	A	G	A	G	-0.0327	-0.00576	0.0874	0.094406	FALSE	FALSE	FALSE	kcZoFT	0.023351	0.805204	486484	sepsis	TRUE	reported	textfile	0.0072	6.33E-06	6998	M33957.m	TRUE	reported	alW1w0	2	TRUE	0.002939	20.62084
1498	rs113515	C	G	C	G	-0.0109	-0.00708	0.5982	0.605419	FALSE	TRUE	FALSE	rzkxmh	0.013972	0.612161	486484	sepsis	TRUE	reported	textfile	0.0024	8.09E-06	6941	M03127.m	TRUE	reported	3lrzhA	2	TRUE	0.002963	20.62079
1510	rs2939707	A	C	A	C	0.0109	0.007729	0.5698	0.563397	FALSE	FALSE	FALSE	rzkxmh	0.013747	0.573949	486484	sepsis	TRUE	reported	textfile	0.0024	7.39E-06	6941	M03127.m	TRUE	reported	3lrzhA	2	TRUE	0.002963	20.62079
4753	rs1091624	A	T	A	T	-0.0218	-0.02184	0.8305	0.803325	FALSE	TRUE	FALSE	RDfDIS	0.017253	0.205548	486484	sepsis	TRUE	reported	textfile	0.0048	5.10E-06	6857	M32787.m	TRUE	reported	Hq6nGt	2	TRUE	0.002999	20.62072
6429	rs1316002	C	G	C	G	0.0109	0.008702	0.6331	0.629325	FALSE	TRUE	FALSE	QCw5dg	0.014174	0.539236	486484	sepsis	TRUE	reported	textfile	0.0024	4.67E-06	6279	M33666.m	TRUE	reported	zYHmzg	2	TRUE	0.003274	20.62017
4233	rs1116197	C	G	C	G	0.0218	0.005724	0.3812	0.389032	FALSE	TRUE	FALSE	wWEMr1	0.01401	0.682853	486484	sepsis	TRUE	reported	textfile	0.0048	4.81E-06	5955	M32593.m	TRUE	reported	Mh2g4i	2	TRUE	0.003452	20.61981
2789	rs1742246	T	G	T	G	-0.0218	0.001092	0.8375	0.826757	FALSE	FALSE	FALSE	ex7hzq	0.018096	0.951861	486484	sepsis	TRUE	reported	textfile	0.0048	4.62E-06	4460	M19415.m	TRUE	reported	jvXWvV	2	TRUE	0.004604	20.61749
7160	rs1000702	T	G	T	G	-0.0418	7.85E-05	0.4182	0.425886	FALSE	FALSE	FALSE	VfvTtz	0.013807	0.995462	486484	sepsis	TRUE	reported	textfile	0.0092	5.39E-06	1492	M34329.m	TRUE	reported	egac6r	2	TRUE	0.013647	20.61552
4260	rs1274801	A	G	A	G	-0.0277	-0.03046	0.0816	0.100788	FALSE	FALSE	FALSE	ylV4bp	0.02267	0.179109	486484	sepsis	TRUE	reported	textfile	0.0061	5.62E-06	7337	M32632.m	TRUE	reported	hqHJoU	2	TRUE	0.002803	20.61491
509	rs2446427	T	C	T	C	-0.0554	-0.00298	0.8371	0.833308	FALSE	FALSE	FALSE	ZOMIXT	0.01852	0.872342	486484	sepsis	TRUE	reported	textfile	0.0122	5.21E-06	7274	M00569.m	TRUE	reported	kZGouU	2	TRUE	0.002827	20.61486
5674	rs1782668	A	G	A	G	-0.0813	0.019348	0.8905	0.838633	FALSE	FALSE	FALSE	AAOSvG	0.018595	0.298119	486484	sepsis	TRUE	reported	textfile	0.0179	5.29E-06	2860	M33197.m	TRUE	reported	aAmcal	2	TRUE	0.007161	20.61442
2682	rs7848609	T	C	T	C	0.1108	-0.0017	0.0168	0.018375	FALSE	FALSE	FALSE	s4UTyf	0.050869	0.973338	486484	sepsis	TRUE	reported	textfile	0.0244	5.69E-06	5376	M18494.m	TRUE	reported	ajPzhg	2	TRUE	0.003821	20.61286
495	rs1818902	T	C	T	C	0.5718	0.049404	0.9701	0.981133	FALSE	FALSE	FALSE	RtjZPn	0.050113	0.324207	486484	sepsis	TRUE	reported	textfile	0.1255	5.21E-06	283	M00553.m	TRUE	reported	C3Ovsl	2	TRUE	0.06834	20.61203
3398	rs804441	T	C	T	C	-0.0327	-0.00437	0.29	0.292079	FALSE	FALSE	FALSE	frOZiy	0.015042	0.77139	486484	sepsis	TRUE	reported	textfile	0.0072	5.87E-06	2759	M27447.m	TRUE	reported	2z9fSv	2	TRUE	0.007421	20.61178
6231	rs1242993	T	C	T	C	-0.0586	-0.00337	0.9468	0.920611	FALSE	FALSE	FALSE	vL52Yb	0.025335	0.894315	486484	sepsis	TRUE	reported	textfile	0.0129	5.31E-06	1718	M33510.m	TRUE	reported	JIO9VX	2	TRUE	0.011869	20.61152
3468	rs1282074	A	G	A	G	-0.0168	-0.0092	0.5528	0.564957	FALSE	FALSE	FALSE	BQIbxe	0.013811	0.505309	486484	sepsis	TRUE	reported	textfile	0.0037	5.06E-06	7373	M27718.m	TRUE	reported	MnHHJ0	2	TRUE	0.002788	20.61092
3883	rs1317320	A	G	A	G	0.0168	-0.00182	0.5657	0.559348	FALSE	FALSE	FALSE	bmXxuz	0.013784	0.89509	486484	sepsis	TRUE	reported	textfile	0.0037	7.35E-06	7358	M32405.m	TRUE	reported	A9BFZF	2	TRUE	0.002794	20.6109
7999	rs9857115	A	C	A	C	0.0168	0.006862	0.538	0.565103	FALSE	FALSE	FALSE	Xrodum	0.013828	0.619712	486484	sepsis	TRUE	reported	textfile	0.0037	4.24E-06	7291	M35255.m	TRUE	reported	T8t1tl	2	TRUE	0.00282	20.61085
552	rs6895278	T	C	T	C	0.0168	0.015495	0.3354	0.33938	FALSE	FALSE	FALSE	QUUN2g	0.014516	0.285777	486484	sepsis	TRUE	reported	textfile	0.0037	4.13E-06	7237	M00599.m	TRUE	reported	vxn2i7	2	TRUE	0.002841	20.61081
7563	rs1112383	A	G	A	G	-0.0168	0.006988	0.24	0.246856	FALSE	FALSE	FALSE	Ot2ZAn	0.016009	0.662454	486484	sepsis	TRUE	reported	textfile	0.0037	4.24E-06	7119	M34481.m	TRUE	reported	tpyFcK	2	TRUE	0.002888	20.61072
5721	rs980833	T	C	T	C	0.0445	-0.00591	0.9438	0.91941	FALSE	FALSE	FALSE	XDem1i	0.025179	0.814525	486484	sepsis	TRUE	reported	textfile	0.0098	5.99E-06	4665	M33221.m	TRUE	reported	KBV6FH	2	TRUE	0.0044	20.61017
8607	rs1498100	A	T	A	T	0.0318	0.014862	0.3919	0.371279	FALSE	TRUE	FALSE	oHM5TD	0.014169	0.294209	486484	sepsis	TRUE	reported	textfile	0.007	5.06E-06	1489	M36131.m	TRUE	reported	Xw6wmg	2	TRUE	0.013671	20.60983
6893	rs1692488	A	G	A	G	-0.0636	-0.01861	0.0992	0.098204	FALSE	FALSE	FALSE	MN2lbE	0.023053	0.419594	486484	sepsis	TRUE	reported	textfile	0.014	5.95E-06	1481	M34106.m	TRUE	reported	q4zXEz	2	TRUE	0.013743	20.60968
3065	rs1224851	T	C	T	C	0.0168	-0.0168	0.186	0.187982	FALSE	FALSE	FALSE	e3izea	0.017531	0.338003	486484	sepsis	TRUE	reported	textfile	0.0037	5.15E-06	5676	M22032.m	TRUE	reported	zODKFl	2	TRUE	0.003619	20.60924
6890	rs1159459	C	G	C	G	0.0336	-0.01367	0.1287	0.13429	FALSE	TRUE	FALSE	MN2lbE	0.020223	0.499215	486484	sepsis	TRUE	reported	textfile	0.0074	6.10E-06	4961	M34106.m	TRUE	reported	q4zXEz	2	TRUE	0.004139	20.6082
2271	rs7857624	T	C	T	C	0.0395	-0.03931	0.9289	0.899311	FALSE	FALSE	FALSE	Mjgi2R	0.022733	0.083803	486484	sepsis	TRUE	reported	textfile	0.0087	6.22E-06	7064	M16634.m	TRUE	reported	XO0GV7	2	TRUE	0.00291	20.60785
9064	rs749902	T	C	T	C	0.0168	-0.01145	0.3362	0.336695	FALSE	FALSE	FALSE	Wn3UAw	0.014432	0.42742	486484	sepsis	TRUE	reported	textfile	0.0037	4.61E-06	4754	M38150.m	TRUE	reported	IRKGRs	2	TRUE	0.004318	20.60784
3024	rs1221724	A	T	A	T	-0.0227	-0.03598	0.9351	0.936758	FALSE	TRUE	FALSE	FJZEYz	0.028102	0.200385	486484	sepsis	TRUE	reported	textfile	0.005	6.31E-06	7284	M21630.m	TRUE	reported	s6Pshg	2	TRUE	0.002822	20.60594
2489	rs932412	A	C	A	C	-0.0227	0.020388	0.3987	0.389739	FALSE	FALSE	FALSE	SrfyMU	0.014022	0.145935	486484	sepsis	TRUE	reported	textfile	0.005	5.84E-06	7226	M17807.m	TRUE	reported	0zrJsu	2	TRUE	0.002844	20.6059
5057	rs1084960	A	T	A	T	-0.0227	0.00817	0.171	0.182782	FALSE	TRUE	FALSE	nHZXjp	0.017672	0.643842	486484	sepsis	TRUE	reported	textfile	0.005	6.51E-06	7166	M33138.m	TRUE	reported	ccoK1F	2	TRUE	0.002868	20.60585
7922	rs7740539	T	C	T	C	-0.0227	0.016973	0.7104	0.716195	FALSE	FALSE	FALSE	8qXKkX	0.015205	0.264319	486484	sepsis	TRUE	reported	textfile	0.005	6.73E-06	6234	M35189.m	TRUE	reported	Nt6oaC	2	TRUE	0.003295	20.60499
7562	rs9635567	T	C	T	C	0.0227	0.000718	0.7787	0.789028	FALSE	FALSE	FALSE	APSWAU	0.016782	0.965895	486484	sepsis	TRUE	reported	textfile	0.005	5.73E-06	6144	M34469.m	TRUE	reported	frF3RX	2	TRUE	0.003344	20.60489
3200	rs278815	T	C	T	C	-0.1249	0.021549	0.989	0.959083	FALSE	FALSE	FALSE	UfLkBq	0.034605	0.533476	486484	sepsis	TRUE	reported	textfile	0.0275	5.73E-06	1756	M22481.m	TRUE	reported	kWMdiT	2	TRUE	0.011611	20.60462
5748	rs4240475	A	C	A	C	0.0286	0.034178	0.0592	0.059376	FALSE	FALSE	FALSE	bm6x2Z	0.02886	0.236304	486484	sepsis	TRUE	reported	textfile	0.0063	6.21E-06	7364	M33230.m	TRUE	reported	8Ccke3	2	TRUE	0.002791	20.60312
6560	rs6925144	T	C	T	C	0.0527	-0.00572	0.5429	0.489997	FALSE	FALSE	FALSE	58qeH5	0.01369	0.675974	486484	sepsis	TRUE	reported	textfile	0.0116	5.76E-06	1069	M33864.m	TRUE	reported	TBsy8r	2	TRUE	0.018942	20.60117
1404	rs1019595	T	C	T	C	-0.0345	0.020609	0.9326	0.940053	FALSE	FALSE	FALSE	33ptwk	0.028953	0.476588	486484	sepsis	TRUE	reported	textfile	0.0076	5.46E-06	6252	M02137.m	TRUE	reported	SQY1na	2	TRUE	0.003285	20.60023
4563	rs831607	T	C	T	C	-0.0404	0.027487	0.5112	0.51957	FALSE	FALSE	FALSE	KfoRxi	0.013998	0.04957	486484	sepsis	TRUE	reported	textfile	0.0089	6.13E-06	6000	M32735.m	TRUE	reported	UDRXAp	2	TRUE	0.003422	20.59861
4695	rs504026	A	G	A	G	0.1099	0.027405	0.9734	0.970394	FALSE	FALSE	FALSE	MV108h	0.040116	0.494519	486484	sepsis	TRUE	reported	textfile	0.0242	5.53E-06	1612	M32758.m	TRUE	reported	btjNXQ	2	TRUE	0.012632	20.59802
2154	rs1311223	C	G	C	G	0.0404	-0.00068	0.3279	0.324784	FALSE	TRUE	FALSE	uuY1Mi	0.014581	0.962766	486484	sepsis	TRUE	reported	textfile	0.0089	5.90E-06	5502	M15677.m	TRUE	reported	nWMiPu	2	TRUE	0.003731	20.59799
6330	rs1193867	T	G	T	G	0.2083	0.053845	0.0205	0.035525	FALSE	FALSE	FALSE	qLUY00	0.037129	0.146995	486484	sepsis	TRUE	reported	textfile	0.0458	5.38E-06	470	M33610.m	TRUE	reported	8cAJbB	2	TRUE	0.042155	20.5966
8363	rs8192287	T	G	T	G	0.0522	-0.01215	0.0436	0.05978	FALSE	FALSE	FALSE	6VVCe4	0.028888	0.674151	486484	sepsis	TRUE	reported	textfile	0.0115	5.84E-06	5629	M35669.m	TRUE	reported	l0fk8X	2	TRUE	0.003647	20.59638
8318	rs1280552	T	C	T	C	0.0999	-0.00753	0.985	0.986753	FALSE	FALSE	FALSE	o0GzT1	0.059812	0.899787	486484	sepsis	TRUE	reported	textfile	0.022	5.86E-06	1750	M35631.m	TRUE	reported	G1XFx0	2	TRUE	0.011646	20.59629
4787	rs9897258	T	C	T	C	-0.0463	-0.00041	0.8542	0.781447	FALSE	FALSE	FALSE	MmZZoM	0.01653	0.980107	486484	sepsis	TRUE	reported	textfile	0.0102	6.07E-06	4274	M32800.m	TRUE	reported	70YSCO	2	TRUE	0.004798	20.59484
2121	rs6094988	C	G	C	G	0.0059	0.031571	0.234	0.243301	FALSE	TRUE	FALSE	t7SQ8V	0.015965	0.047989	486484	sepsis	TRUE	reported	textfile	0.0013	3.14E-06	7365	M15650.m	TRUE	reported	QQT7qA	2	TRUE	0.002789	20.59204
6794	rs1220235	T	C	T	C	0.0118	0.031148	0.7073	0.705439	FALSE	FALSE	FALSE	OZ0I1H	0.015045	0.038426	486484	sepsis	TRUE	reported	textfile	0.0026	7.14E-06	7364	M33960.m	TRUE	reported	GlPp4w	2	TRUE	0.002789	20.59204
6796	rs1366823	A	G	A	G	0.0118	-0.00761	0.6447	0.653199	FALSE	FALSE	FALSE	OZ0I1H	0.014396	0.596962	486484	sepsis	TRUE	reported	textfile	0.0026	6.94E-06	7364	M33960.m	TRUE	reported	GlPp4w	2	TRUE	0.002789	20.59204
7784	rs551938	A	G	A	G	-0.0236	0.002703	0.2537	0.253607	FALSE	FALSE	FALSE	15BqPa	0.016256	0.867919	486484	sepsis	TRUE	reported	textfile	0.0052	5.63E-06	7364	M35126.m	TRUE	reported	zvDzP2	2	TRUE	0.002789	20.59204
4488	rs1352815	A	C	A	C	-0.0118	0.022185	0.6865	0.664601	FALSE	FALSE	FALSE	1wNJ1E	0.01458	0.128122	486484	sepsis	TRUE	reported	textfile	0.0026	8.16E-06	7363	M32709.m	TRUE	reported	zihBHg	2	TRUE	0.00279	20.59204
886	rs1936396	A	G	A	G	-0.0118	-0.00766	0.2253	0.215495	FALSE	FALSE	FALSE	oi7KQI	0.016685	0.646045	486484	sepsis	TRUE	reported	textfile	0.0026	5.67E-06	7362	M01365.m	TRUE	reported	7NWGKA	2	TRUE	0.00279	20.59204
4065	rs1236046	A	G	A	G	0.0118	0.006742	0.5969	0.601817	FALSE	FALSE	FALSE	TyHtdh	0.013953	0.628954	486484	sepsis	TRUE	reported	textfile	0.0026	5.69E-06	7358	M32497.m	TRUE	reported	H3KmLR	2	TRUE	0.002792	20.59203
4073	rs2968858	T	C	T	C	-0.0118	0.008427	0.5283	0.514711	FALSE	FALSE	FALSE	TyHtdh	0.013763	0.540354	486484	sepsis	TRUE	reported	textfile	0.0026	7.25E-06	7358	M32497.m	TRUE	reported	H3KmLR	2	TRUE	0.002792	20.59203
4083	rs8054389	T	C	T	C	-0.0118	0.011459	0.493	0.49251	FALSE	FALSE	FALSE	TyHtdh	0.013695	0.402746	486484	sepsis	TRUE	reported	textfile	0.0026	6.42E-06	7358	M32497.m	TRUE	reported	H3KmLR	2	TRUE	0.002792	20.59203
1524	rs1256921	A	C	A	C	-0.0118	0.034211	0.1612	0.158935	FALSE	FALSE	FALSE	YvgTeW	0.018701	0.067347	486484	sepsis	TRUE	reported	textfile	0.0026	5.82E-06	7357	M03141.m	TRUE	reported	srKjSc	2	TRUE	0.002792	20.59203
1216	rs8079173	A	G	A	G	0.0118	-0.00314	0.1402	0.138747	FALSE	FALSE	FALSE	Ckdgh1	0.01982	0.873945	486484	sepsis	TRUE	reported	textfile	0.0026	8.93E-06	7355	M01644.m	TRUE	reported	nKxz7B	2	TRUE	0.002793	20.59203
2611	rs7574847	A	G	A	G	-0.0118	-0.01689	0.1481	0.154332	FALSE	FALSE	FALSE	ruvJh6	0.018916	0.371852	486484	sepsis	TRUE	reported	textfile	0.0026	3.98E-06	7354	M18369.m	TRUE	reported	GFmwGk	2	TRUE	0.002793	20.59203
6970	rs1116334	A	G	A	G	0.0118	-0.01492	0.3481	0.336673	FALSE	FALSE	FALSE	ZlvYs5	0.014462	0.302131	486484	sepsis	TRUE	reported	textfile	0.0026	5.79E-06	7351	M34214.m	TRUE	reported	qU57S1	2	TRUE	0.002794	20.59203
4129	rs9322458	A	T	A	T	-0.0118	-0.01539	0.8754	0.854948	FALSE	TRUE	FALSE	4rtLd0	0.0195	0.43001	486484	sepsis	TRUE	reported	textfile	0.0026	7.60E-06	7350	M32518.m	TRUE	reported	j7f1ER	2	TRUE	0.002795	20.59203
3385	rs6498634	T	G	T	G	0.0118	0.030171	0.7656	0.766541	FALSE	FALSE	FALSE	O2JtzH	0.01613	0.061407	486484	sepsis	TRUE	reported	textfile	0.0026	7.87E-06	7347	M27278.m	TRUE	reported	fxCIPW	2	TRUE	0.002796	20.59203
522	rs10736	T	C	T	C	-0.0118	0.00707	0.2011	0.203294	FALSE	FALSE	FALSE	wXMWHy	0.016959	0.676775	486484	sepsis	TRUE	reported	textfile	0.0026	4.73E-06	7345	M00584.m	TRUE	reported	7CKn2b	2	TRUE	0.002796	20.59202
4953	rs2295465	A	C	A	C	-0.0118	-0.00245	0.8798	0.87845	FALSE	FALSE	FALSE	bKmDMa	0.020945	0.906708	486484	sepsis	TRUE	reported	textfile	0.0026	6.65E-06	7344	M32910.m	TRUE	reported	nAuPzO	2	TRUE	0.002797	20.59202
6084	rs5761606	T	G	T	G	0.0118	-0.02896	0.07	0.075848	FALSE	FALSE	FALSE	P8n0HP	0.025944	0.264344	486484	sepsis	TRUE	reported	textfile	0.0026	7.86E-06	7336	M33442.m	TRUE	reported	cljMTI	2	TRUE	0.0028	20.59202
814	rs4472784	T	C	T	C	-0.0118	0.002335	0.3048	0.319807	FALSE	FALSE	FALSE	J58DI0	0.014681	0.873606	486484	sepsis	TRUE	reported	textfile	0.0026	4.78E-06	7335	M01356.m	TRUE	reported	zk9AMB	2	TRUE	0.0028	20.59202
8812	rs1221784	A	G	A	G	0.0118	-0.00967	0.287	0.286633	FALSE	FALSE	FALSE	X2Rol2	0.016655	0.561684	486484	sepsis	TRUE	reported	textfile	0.0026	5.74E-06	7335	M36776.m	TRUE	reported	nikHw1	2	TRUE	0.0028	20.59202
8819	rs9369302	T	C	T	C	0.0118	0.029984	0.7183	0.709983	FALSE	FALSE	FALSE	X2Rol2	0.015083	0.046816	486484	sepsis	TRUE	reported	textfile	0.0026	4.15E-06	7335	M36776.m	TRUE	reported	nikHw1	2	TRUE	0.0028	20.59202
8821	rs9953686	T	C	T	C	0.0118	0.015307	0.8128	0.812105	FALSE	FALSE	FALSE	X2Rol2	0.017495	0.381624	486484	sepsis	TRUE	reported	textfile	0.0026	6.93E-06	7335	M36776.m	TRUE	reported	nikHw1	2	TRUE	0.0028	20.59202
4907	rs2251468	A	C	A	C	-0.0059	0.008373	0.6513	0.661203	FALSE	FALSE	FALSE	VqcRGX	0.014454	0.5624	486484	sepsis	TRUE	reported	textfile	0.0013	3.43E-06	7329	M32867.m	TRUE	reported	psuFJy	2	TRUE	0.002803	20.59201
1368	rs1214717	C	G	C	G	-0.0118	-0.01267	0.1169	0.112193	FALSE	TRUE	FALSE	KrWhws	0.02161	0.557554	486484	sepsis	TRUE	reported	textfile	0.0026	4.76E-06	7325	M02132.m	TRUE	reported	7pzpfK	2	TRUE	0.002804	20.59201
1692	rs1756283	T	C	T	C	0.0118	-0.00396	0.0769	0.077966	FALSE	FALSE	FALSE	f01en0	0.025425	0.876209	486484	sepsis	TRUE	reported	textfile	0.0026	4.17E-06	7314	M12593.m	TRUE	reported	EAsM6U	2	TRUE	0.002808	20.592
6246	rs733017	T	C	T	C	-0.0118	-0.01285	0.6092	0.613134	FALSE	FALSE	FALSE	vL52Yb	0.014209	0.36579	486484	sepsis	TRUE	reported	textfile	0.0026	6.94E-06	7269	M33510.m	TRUE	reported	JIO9VX	2	TRUE	0.002826	20.59197
7962	rs2122506	A	G	A	G	0.0118	-0.0013	0.3253	0.330125	FALSE	FALSE	FALSE	zb8m4a	0.014601	0.928835	486484	sepsis	TRUE	reported	textfile	0.0026	9.05E-06	7261	M35253.m	TRUE	reported	tHnnnS	2	TRUE	0.002829	20.59196
7969	rs6903630	C	G	C	G	-0.0118	-0.02034	0.6277	0.624738	FALSE	TRUE	FALSE	zb8m4a	0.014117	0.149675	486484	sepsis	TRUE	reported	textfile	0.0026	6.98E-06	7261	M35253.m	TRUE	reported	tHnnnS	2	TRUE	0.002829	20.59196
962	rs6078500	T	C	T	C	-0.0118	-0.00498	0.5117	0.493675	FALSE	FALSE	FALSE	tWoDq4	0.013669	0.715652	486484	sepsis	TRUE	reported	textfile	0.0026	4.47E-06	7160	M01508.m	TRUE	reported	6ltDw0	2	TRUE	0.002869	20.59188
966	rs7574257	T	C	T	C	0.0118	-0.0129	0.325	0.33282	FALSE	FALSE	FALSE	tWoDq4	0.014586	0.37634	486484	sepsis	TRUE	reported	textfile	0.0026	7.76E-06	7160	M01508.m	TRUE	reported	6ltDw0	2	TRUE	0.002869	20.59188
1622	rs1083956	C	G	C	G	-0.0177	-0.02741	0.9453	0.931726	FALSE	TRUE	FALSE	n5rDuT	0.027327	0.31583	486484	sepsis	TRUE	reported	textfile	0.0039	6.46E-06	7081	M12067.m	TRUE	reported	ZQLTeR	2	TRUE	0.0029	20.59182
7090	rs1290317	T	C	T	C	0.0118	-0.00748	0.6383	0.629432	FALSE	FALSE	FALSE	pxNICA	0.014254	0.599617	486484	sepsis	TRUE	reported	textfile	0.0026	7.87E-06	6994	M34306.m	TRUE	reported	xk5b9M	2	TRUE	0.002936	20.59174
2741	rs1210012	A	G	A	G	-0.0295	-0.02773	0.0425	0.056033	FALSE	FALSE	FALSE	qFAbee	0.029704	0.350614	486484	sepsis	TRUE	reported	textfile	0.0065	5.23E-06	6929	M19364.m	TRUE	reported	tZgXNL	2	TRUE	0.002964	20.59169
2242	rs1102543	A	G	A	G	-0.0118	-0.02477	0.4231	0.428445	FALSE	FALSE	FALSE	zxcPox	0.013811	0.072902	486484	sepsis	TRUE	reported	textfile	0.0026	8.33E-06	6782	M15990.m	TRUE	reported	34Vg8F	2	TRUE	0.003028	20.59156
2333	rs9525877	A	G	A	G	-0.0118	0.014586	0.7816	0.779838	FALSE	FALSE	FALSE	IZXwpR	0.016528	0.377489	486484	sepsis	TRUE	reported	textfile	0.0026	6.50E-06	6549	M16822.m	TRUE	reported	Zx9k2V	2	TRUE	0.003135	20.59134
5104	rs2463492	T	G	T	G	0.0118	-0.00416	0.4249	0.428673	FALSE	FALSE	FALSE	Uiz8VU	0.013804	0.76338	486484	sepsis	TRUE	reported	textfile	0.0026	6.95E-06	6404	M33163.m	TRUE	reported	ky0Bda	2	TRUE	0.003206	20.5912
7661	rs1107861	C	G	C	G	0.3903	-0.00339	0.0126	0.013322	FALSE	TRUE	FALSE	z8S1DZ	0.060014	0.954937	486484	sepsis	TRUE	reported	textfile	0.0859	5.58E-06	754	M34535.m	TRUE	reported	FNLxxh	2	TRUE	0.026651	20.59004
7183	rs606567	A	G	A	G	0.0177	0.012241	0.8204	0.820695	FALSE	FALSE	FALSE	2hlepp	0.017874	0.493431	486484	sepsis	TRUE	reported	textfile	0.0039	6.60E-06	5081	M34336.m	TRUE	reported	XyK8mO	2	TRUE	0.004037	20.58953
2545	rs7016707	T	G	T	G	0.1439	-0.00019	0.099	0.10391	FALSE	FALSE	FALSE	o0CFya	0.02249	0.993299	486484	sepsis	TRUE	reported	textfile	0.0317	5.81E-06	2433	M18281.m	TRUE	reported	pxbsnu	2	TRUE	0.008398	20.58951
5710	rs1442060	A	G	A	G	-0.0236	-0.01172	0.5118	0.511285	FALSE	FALSE	FALSE	XDem1i	0.013865	0.398039	486484	sepsis	TRUE	reported	textfile	0.0052	6.81E-06	4665	M33221.m	TRUE	reported	KBV6FH	2	TRUE	0.004396	20.5888
6665	rs1286736	A	C	A	C	-0.0236	-0.02353	0.7789	0.77522	FALSE	FALSE	FALSE	6iZSOI	0.016371	0.150622	486484	sepsis	TRUE	reported	textfile	0.0052	5.44E-06	4533	M33901.m	TRUE	reported	ApKaGe	2	TRUE	0.004523	20.58855
7194	rs1099516	A	C	A	C	0.3473	-0.04598	0.9644	0.960324	FALSE	FALSE	FALSE	i1uJ7T	0.035021	0.189209	486484	sepsis	TRUE	reported	textfile	0.0764	5.44E-06	535	M34338.m	TRUE	reported	pi3mKW	2	TRUE	0.037189	20.58715
1474	rs2111718	A	G	A	G	-0.0227	-0.0248	0.8615	0.849813	FALSE	FALSE	FALSE	pkb876	0.019191	0.19633	486484	sepsis	TRUE	reported	textfile	0.005	5.00E-06	1667	M02734.m	TRUE	reported	0LCMVI	2	TRUE	0.012213	20.58687
2289	rs1102221	T	C	T	C	0.0572	0.015802	0.9308	0.932961	FALSE	FALSE	FALSE	43Yh3h	0.027376	0.563788	486484	sepsis	TRUE	reported	textfile	0.0126	5.33E-06	1670	M16818.m	TRUE	reported	beR4IT	2	TRUE	0.01219	20.58404
4416	rs1449167	T	C	T	C	0.0345	-0.01484	0.6415	0.650702	FALSE	FALSE	FALSE	NzBiVA	0.014318	0.299873	486484	sepsis	TRUE	reported	textfile	0.0076	5.43E-06	1764	M32672.m	TRUE	reported	YLIhHJ	2	TRUE	0.011547	20.58346
8306	rs9784679	T	C	T	C	0.0363	-0.00476	0.2789	0.276236	FALSE	FALSE	FALSE	HELgy7	0.015418	0.757467	486484	sepsis	TRUE	reported	textfile	0.008	6.20E-06	5035	M35551.m	TRUE	reported	7x7uUn	2	TRUE	0.004073	20.58073
2258	rs7788421	A	G	A	G	-0.0245	-0.00423	0.9134	0.908112	FALSE	FALSE	FALSE	zxcPox	0.02373	0.858483	486484	sepsis	TRUE	reported	textfile	0.0054	5.18E-06	6782	M15990.m	TRUE	reported	34Vg8F	2	TRUE	0.003026	20.57863
7434	rs4780314	C	G	C	G	-0.0245	0.000551	0.7909	0.787158	FALSE	TRUE	FALSE	NZPe5A	0.016818	0.973844	486484	sepsis	TRUE	reported	textfile	0.0054	5.05E-06	5689	M34400.m	TRUE	reported	OyPwYO	2	TRUE	0.003605	20.57747
1154	rs9573265	T	C	T	C	0.2216	0.048494	0.0158	0.010947	FALSE	FALSE	FALSE	QtY5Hc	0.066201	0.463855	486484	sepsis	TRUE	reported	textfile	0.0488	5.55E-06	955	M01605.m	TRUE	reported	Q3iDQX	2	TRUE	0.021136	20.57735
6164	rs1299909	T	G	T	G	0.0245	0.017748	0.8061	0.800896	FALSE	FALSE	FALSE	O4Ty2l	0.017114	0.299728	486484	sepsis	TRUE	reported	textfile	0.0054	5.92E-06	5107	M33488.m	TRUE	reported	RGtF84	2	TRUE	0.004015	20.57664
8957	rs4331456	T	C	T	C	-0.1664	0.015238	0.2107	0.245674	FALSE	FALSE	FALSE	Ywk2ve	0.01606	0.342722	486484	sepsis	TRUE	reported	textfile	0.0366	5.52E-06	437	M37112.m	TRUE	reported	EiAdDj	2	TRUE	0.045164	20.57559
625	rs8123073	A	G	A	G	-0.0372	-0.01165	0.983	0.979596	FALSE	FALSE	FALSE	BYGzq4	0.048398	0.80979	486484	sepsis	TRUE	reported	textfile	0.0082	5.94E-06	7367	M01110.m	TRUE	reported	oZiXqP	2	TRUE	0.002786	20.57502
6744	rs1293243	T	G	T	G	0.0186	-0.03221	0.7139	0.71847	FALSE	FALSE	FALSE	NZVg9N	0.015197	0.034037	486484	sepsis	TRUE	reported	textfile	0.0041	6.34E-06	7320	M33941.m	TRUE	reported	kRWNNK	2	TRUE	0.002804	20.57498
4586	rs1719079	A	G	A	G	0.0186	0.007984	0.9476	0.928146	FALSE	FALSE	FALSE	Pzplqu	0.026461	0.762871	486484	sepsis	TRUE	reported	textfile	0.0041	4.92E-06	7318	M32740.m	TRUE	reported	FjEBS5	2	TRUE	0.002804	20.57498
1621	rs1052019	A	G	A	G	0.0186	-0.06401	0.0296	0.03276	FALSE	FALSE	FALSE	n5rDuT	0.038607	0.097304	486484	sepsis	TRUE	reported	textfile	0.0041	5.64E-06	7081	M12067.m	TRUE	reported	ZQLTeR	2	TRUE	0.002898	20.57479
1025	rs1404410	C	G	C	G	-0.0236	0.004512	0.7853	0.80147	FALSE	TRUE	FALSE	haWxZg	0.017154	0.792536	486484	sepsis	TRUE	reported	textfile	0.0052	4.57E-06	1759	M01561.m	TRUE	reported	5wm9R3	2	TRUE	0.011574	20.57421

3061	rs967725	A	T	A	T	-0.0372	0.080278	0.0226	0.019916	FALSE	TRUE	FALSE	H6vcCG	0.049483	0.104729	486484	sepsis	TRUE	reported	textfile	0.0082	6.15E-06	6180	M22030.m	TRUE	reported	XetVX7	2	TRUE	0.003319	20.57395
3863	rs4931472	A	G	A	G	-0.0186	-0.01466	0.3954	0.386742	FALSE	FALSE	FALSE	8gg1zS	0.01433	0.306173	486484	sepsis	TRUE	reported	textfile	0.0041	7.30E-06	6119	M32379.m	TRUE	reported	uZWtuS	2	TRUE	0.003352	20.57388
6569	rs6426630	A	G	A	G	-0.0885	-0.0089	0.0286	0.055647	FALSE	FALSE	FALSE	aTDqRQ	0.029846	0.765577	486484	sepsis	TRUE	reported	textfile	0.0195	5.37E-06	1639	M33871.m	TRUE	reported	Lqimdw	2	TRUE	0.012411	20.5725
2688	rs1695509	T	C	T	C	0.3552	-0.03618	0.015	0.017698	FALSE	FALSE	FALSE	ATsH6N	0.051194	0.479738	486484	sepsis	TRUE	reported	textfile	0.0782	5.61E-06	698	M18497.m	TRUE	reported	IGuIRI	2	TRUE	0.02871	20.57246
8485	rs1245110	T	C	T	C	-0.059	0.004769	0.0806	0.075822	FALSE	FALSE	FALSE	ABkZ7x	0.026071	0.854859	486484	sepsis	TRUE	reported	textfile	0.013	5.77E-06	1632	M35854.m	TRUE	reported	wc0CeK	2	TRUE	0.012464	20.57239
7197	rs1172553	A	G	A	G	-0.2122	-0.01588	0.9605	0.955488	FALSE	FALSE	FALSE	i1uJ7T	0.032998	0.630411	486484	sepsis	TRUE	reported	textfile	0.0467	5.43E-06	535	M34338.m	TRUE	reported	pi3mKW	2	TRUE	0.037158	20.56982
6083	rs5749010	A	G	A	G	-0.0127	0.001537	0.0828	0.08095	FALSE	FALSE	FALSE	P8n0HP	0.025463	0.951853	486484	sepsis	TRUE	reported	textfile	0.0028	4.42E-06	7336	M33442.m	TRUE	reported	cljMTI	2	TRUE	0.002797	20.5671
3205	rs3734747	A	G	A	G	-0.0127	0.007399	0.164	0.15821	FALSE	FALSE	FALSE	UfLkBq	0.018787	0.693702	486484	sepsis	TRUE	reported	textfile	0.0028	4.31E-06	7329	M22481.m	TRUE	reported	kWMdiT	2	TRUE	0.002799	20.56709
1367	rs1199300	A	G	A	G	-0.0127	-0.00411	0.3167	0.362275	FALSE	FALSE	FALSE	KrWhws	0.014204	0.772119	486484	sepsis	TRUE	reported	textfile	0.0028	5.24E-06	7325	M02132.m	TRUE	reported	7pzpfK	2	TRUE	0.002801	20.56709
7104	rs9393409	A	G	A	G	-0.0127	0.007981	0.3342	0.328834	FALSE	FALSE	FALSE	pxNICA	0.01458	0.58411	486484	sepsis	TRUE	reported	textfile	0.0028	4.11E-06	6994	M34306.m	TRUE	reported	xk5b9M	2	TRUE	0.002933	20.56682
4525	rs1757091	A	G	A	G	0.0127	0.010336	0.1402	0.163756	FALSE	FALSE	FALSE	r0qJaM	0.018531	0.576999	486484	sepsis	TRUE	reported	textfile	0.0028	4.93E-06	6497	M32729.m	TRUE	reported	vYv5FI	2	TRUE	0.003156	20.56637
2554	rs2035344	A	G	A	G	0.0254	0.01589	0.6893	0.69033	FALSE	FALSE	FALSE	NxZhaQ	0.014893	0.286009	486484	sepsis	TRUE	reported	textfile	0.0056	6.74E-06	5984	M18283.m	TRUE	reported	5Pi7r3	2	TRUE	0.003426	20.56583
3094	rs1704258	T	G	T	G	0.0127	0.007213	0.7446	0.729981	FALSE	FALSE	FALSE	mr0awv	0.015397	0.63947	486484	sepsis	TRUE	reported	textfile	0.0028	4.75E-06	5702	M22130.m	TRUE	reported	Y2TfWQ	2	TRUE	0.003595	20.56549
3064	rs1083164	T	C	T	C	0.0127	-0.01383	0.4756	0.473288	FALSE	FALSE	FALSE	e3izea	0.013797	0.316242	486484	sepsis	TRUE	reported	textfile	0.0028	4.73E-06	5676	M22032.m	TRUE	reported	zODKFI	2	TRUE	0.003611	20.56546
7550	rs9696823	A	G	A	G	-0.0127	0.001543	0.6502	0.651002	FALSE	FALSE	FALSE	JJsHvX	0.014376	0.914534	486484	sepsis	TRUE	reported	textfile	0.0028	4.13E-06	5207	M34456.m	TRUE	reported	aklxve	2	TRUE	0.003935	20.5648
8576	rs1686411	T	C	T	C	0.1474	-0.06578	0.0154	0.034013	FALSE	FALSE	FALSE	3VcRoA	0.037513	0.079522	486484	sepsis	TRUE	reported	textfile	0.0325	5.88E-06	5696	M36099.m	TRUE	reported	zHE146	2	TRUE	0.003598	20.56249
3809	rs7645550	T	C	T	C	-0.0322	0.010308	0.4017	0.400174	FALSE	FALSE	FALSE	HOq2gb	0.013958	0.460187	486484	sepsis	TRUE	reported	textfile	0.0071	5.84E-06	6687	M32346.m	TRUE	reported	Ss5q17	2	TRUE	0.003066	20.56199
8570	rs6552978	A	G	A	G	-0.0735	-0.01256	0.808	0.810042	FALSE	FALSE	FALSE	Yjjldt	0.017426	0.471124	486484	sepsis	TRUE	reported	textfile	0.0162	5.70E-06	1725	M36098.m	TRUE	reported	U2ORsF	2	TRUE	0.011792	20.56084
4395	rs2610815	T	C	T	C	-0.0195	0.01636	0.9355	0.935763	FALSE	FALSE	FALSE	UJygVd	0.027914	0.557835	486484	sepsis	TRUE	reported	textfile	0.0043	5.84E-06	7361	M32654.m	TRUE	reported	kv9R0W	2	TRUE	0.002786	20.55958
774	rs4768795	T	G	T	G	0.0195	0.005134	0.3587	0.432011	FALSE	FALSE	FALSE	tYvbNc	0.013786	0.709575	486484	sepsis	TRUE	reported	textfile	0.0043	6.19E-06	6946	M01303.m	TRUE	reported	hT3gsE	2	TRUE	0.002952	20.55925
8011	rs2627272	T	C	T	C	-0.0195	-0.01303	0.8848	0.883097	FALSE	FALSE	FALSE	Zj5bZH	0.021384	0.542384	486484	sepsis	TRUE	reported	textfile	0.0043	6.49E-06	6538	M35257.m	TRUE	reported	exssAk	2	TRUE	0.003136	20.55888
7698	rs1191963	A	G	A	G	0.0195	0.006782	0.7317	0.732627	FALSE	FALSE	FALSE	2eJlPM	0.015456	0.660788	486484	sepsis	TRUE	reported	textfile	0.0043	5.96E-06	6381	M34761.m	TRUE	reported	druKtT	2	TRUE	0.003213	20.55872
6688	rs1336198	A	G	A	G	0.0195	0.012568	0.4413	0.433724	FALSE	FALSE	FALSE	23zCgC	0.013774	0.361558	486484	sepsis	TRUE	reported	textfile	0.0043	6.53E-06	5534	M33910.m	TRUE	reported	ACI3CA	2	TRUE	0.003702	20.55774
6603	rs1116363	A	C	A	C	0.0508	-0.00152	0.4597	0.448599	FALSE	FALSE	FALSE	VfOuo8	0.013773	0.912087	486484	sepsis	TRUE	reported	textfile	0.0112	5.60E-06	2725	M33883.m	TRUE	reported	BDe0Nk	2	TRUE	0.007493	20.5576
6780	rs804572	T	G	T	G	-0.0372	-0.04223	0.0607	0.064367	FALSE	FALSE	FALSE	Elf6MQ	0.02783	0.129189	486484	sepsis	TRUE	reported	textfile	0.0082	5.72E-06	1764	M33955.m	TRUE	reported	Lxn4RF	2	TRUE	0.011532	20.55727
8680	rs6999153	A	G	A	G	0.0313	-0.01792	0.5597	0.554521	FALSE	FALSE	FALSE	i4bSdJ	0.013807	0.194264	486484	sepsis	TRUE	reported	textfile	0.0069	5.97E-06	1908	M36468.m	TRUE	reported	qJLuZj	2	TRUE	0.01067	20.55583
7659	rs1080168	T	G	T	G	-0.4744	0.009002	0.0209	0.511458	FALSE	FALSE	FALSE	z8S1DZ	0.014163	0.525038	486484	sepsis	TRUE	reported	textfile	0.1045	5.67E-06	754	M34535.m	TRUE	reported	FNLxxh	2	TRUE	0.026606	20.55433
6490	rs843172	T	G	T	G	-0.7724	0.004234	0.9755	0.979764	FALSE	FALSE	FALSE	ecwVg8	0.048683	0.9307	486484	sepsis	TRUE	reported	textfile	0.1682	4.37E-06	79	M33751.m	TRUE	reported	NnvbIQ	2	TRUE	0.210693	20.55399
8878	rs1710313	A	G	A	G	0.044	0.017198	0.0712	0.077164	FALSE	FALSE	FALSE	Nv6gDO	0.025877	0.506302	486484	sepsis	TRUE	reported	textfile	0.0097	5.98E-06	1782	M37004.m	TRUE	reported	RqmQdg	2	TRUE	0.011415	20.55295
6627	rs1205060	C	G	C	G	-0.0662	-0.03587	0.0791	0.07331	FALSE	TRUE	FALSE	IDG01N	0.026425	0.174686	486484	sepsis	TRUE	reported	textfile	0.0146	5.73E-06	4999	M33885.m	TRUE	reported	q3gy7x	2	TRUE	0.004096	20.55117
5980	rs1214427	A	C	A	C	-0.0635	-0.0402	0.0297	0.054909	FALSE	FALSE	FALSE	3KsoXq	0.030169	0.182758	486484	sepsis	TRUE	reported	textfile	0.014	5.46E-06	1748	M33422.m	TRUE	reported	aNr6Ug	2	TRUE	0.011632	20.54917
6367	rs1207117	A	G	A	G	0.0934	-0.02253	0.0265	0.032755	FALSE	FALSE	FALSE	PAgZZz	0.038637	0.559733	486484	sepsis	TRUE	reported	textfile	0.0206	5.58E-06	4323	M33633.m	TRUE	reported	I8R1KJ	2	TRUE	0.004733	20.54747
1557	rs2720692	A	G	A	G	-0.0272	-0.00366	0.0648	0.080402	FALSE	FALSE	FALSE	Ke0sNh	0.025174	0.884541	486484	sepsis	TRUE	reported	textfile	0.006	5.63E-06	7799	M10642.m	TRUE	reported	ZINwVb	2	TRUE	0.002628	20.54584
1564	rs4818244	T	C	T	C	0.0136	0.004515	0.0939	0.127076	FALSE	FALSE	FALSE	Ke0sNh	0.020655	0.826954	486484	sepsis	TRUE	reported	textfile	0.003	6.61E-06	7799	M10642.m	TRUE	reported	ZINwVb	2	TRUE	0.002628	20.54584
5043	rs994713	T	C	T	C	-0.0204	-0.0045	0.0245	0.025141	FALSE	FALSE	FALSE	BNnOvt	0.043902	0.918428	486484	sepsis	TRUE	reported	textfile	0.0045	6.37E-06	7362	M33132.m	TRUE	reported	rHcCPG	2	TRUE	0.002784	20.54553
763	rs9410970	A	C	A	C	-0.0068	0.00453	0.2201	0.215502	FALSE	FALSE	FALSE	RHWptG	0.016717	0.786397	486484	sepsis	TRUE	reported	textfile	0.0015	5.49E-06	7347	M01302.m	TRUE	reported	z1OLfr	2	TRUE	0.002789	20.54552
3358	rs2269713	A	G	A	G	0.034	0.019667	0.9761	0.971097	FALSE	FALSE	FALSE	LY4Gus	0.041101	0.632291	486484	sepsis	TRUE	reported	textfile	0.0075	5.65E-06	7268	M27273.m	TRUE	reported	QKITBx	2	TRUE	0.00282	20.54546
2727	rs1686471	A	T	A	T	0.034	0.017889	0.0172	0.02096	FALSE	TRUE	FALSE	H4yqSK	0.04771	0.707696	486484	sepsis	TRUE	reported	textfile	0.0075	6.76E-06	7182	M19362.m	TRUE	reported	w2PDEO	2	TRUE	0.002853	20.54539
8193	rs1163950	A	G	A	G	-0.034	-0.04351	0.9736	0.947764	FALSE	FALSE	FALSE	iLlpho	0.031026	0.160764	486484	sepsis	TRUE	reported	textfile	0.0075	5.69E-06	7007	M35431.m	TRUE	reported	AAZ5ge	2	TRUE	0.002924	20.54525
1407	rs1786837	A	C	A	C	-0.034	-0.00473	0.932	0.932479	FALSE	FALSE	FALSE	33ptwk	0.027315	0.862644	486484	sepsis	TRUE	reported	textfile	0.0075	6.49E-06	6252	M02137.m	TRUE	reported	SQY1na	2	TRUE	0.003276	20.54454
4798	rs1473993	T	C	T	C	0.0272	0.009315	0.6241	0.595022	FALSE	FALSE	FALSE	9qWhFj	0.013922	0.503465	486484	sepsis	TRUE	reported	textfile	0.006	5.85E-06	6200	M32808.m	TRUE	reported	MQcOSi	2	TRUE	0.003304	20.54448
4801	rs514659	A	C	A	C	0.0272	0.003967	0.6737	0.682658	FALSE	FALSE	FALSE	9qWhFj	0.014643	0.786477	486484	sepsis	TRUE	reported	textfile	0.006	6.61E-06	6200	M32808.m	TRUE	reported	MQcOSi	2	TRUE	0.003304	20.54448
7947	rs1027416	T	G	T	G	-0.0136	0.005562	0.4586	0.455978	FALSE	FALSE	FALSE	lvUHnQ	0.013933	0.689725	486484	sepsis	TRUE	reported	textfile	0.003	5.25E-06	5694	M35240.m	TRUE	reported	VH44Ha	2	TRUE	0.003596	20.54389
2665	rs4338010	T	C	T	C	-0.1084	-0.03193	0.0788	0.088855	FALSE	FALSE	FALSE	cDEa3G	0.024094	0.185117	486484	sepsis	TRUE	reported	textfile	0.0239	5.73E-06	1457	M18477.m	TRUE	reported	kxZEfC	2	TRUE	0.013922	20.54311
6381	rs2171596	A	C	A	C	-0.0204	-0.00443	0.6593	0.655627	FALSE	FALSE	FALSE	pPr1v0	0.014391	0.758111	486484	sepsis	TRUE	reported	textfile	0.0045	5.38E-06	5080	M33637.m	TRUE	reported	Ide1A7	2	TRUE	0.004029	20.54302
7764	rs1243184	T	C	T	C	0.0975	0.03155	0.014	0.010412	FALSE	FALSE	FALSE	G4gUxB	0.067866	0.642009	486484	sepsis	TRUE	reported	textfile	0.0215	6.12E-06	1657	M35114.m	TRUE	reported	o79OO5	2	TRUE	0.012259	20.54035
6463	rs6450591	A	G	A	G	-0.0871	-0.0061	0.0819	0.085605	FALSE	FALSE	FALSE	yLohrN	0.024442	0.802876	486484	sepsis	TRUE	reported	textfile	0.0192	5.77E-06	1027	M33683.m	TRUE	reported	mvhAZ4	2	TRUE	0.019645	20.53938
4650	rs1752555	T	C	T	C	-0.0417	0.033777	0.956	0.951822	FALSE	FALSE	FALSE	SyAFR2	0.031994	0.291092	486484	sepsis	TRUE	reported	textfile	0.0092	6.07E-06	6658	M32755.m	TRUE	reported	prUo74	2	TRUE	0.003076	20.53837
4195	rs2884692	T	C	T	C	-0.0349	0.010554	0.3823	0.377268	FALSE	FALSE	FALSE	TxOCzG	0.014143	0.455536	486484	sepsis	TRUE	reported	textfile	0.0077	6.34E-06	6970	M32564.m	TRUE	reported	9JP6ND	2	TRUE	0.002939	20.53737
6498	rs1722092	T	C	T	C	0.0757	0.01051	0.0707	0.076223	FALSE	FALSE	FALSE	HNc7C9	0.025873	0.684591	486484	sepsis	TRUE	reported	textfile	0.0167	5.72E-06	3962	M33782.m	TRUE	reported	SAGYGx	2	TRUE	0.005159	20.53712
7515	rs1323365	A	G	A	G	0.0485	0.003447	0.9528	0.933439	FALSE	FALSE	FALSE	rp1LGA	0.027602	0.900618	486484	sepsis	TRUE	reported	textfile	0.0107	5.67E-06	4442	M34441.m	TRUE	reported	SU47tz	2	TRUE	0.004604	20.53621
5747	rs3811273	A	G	A	G	-0.0281	0.019235	0.9383	0.926186	FALSE	FALSE	FALSE	bm6x2Z	0.026155	0.462095	486484	sepsis	TRUE	reported	textfile	0.0062	5.13E-06	7364	M33230.m	TRUE	reported	8Ccke3	2	TRUE	0.002782	20.53578
5927	rs1774810	A	C	A	C	-0.1185	-0.00921	0.9287	0.918922	FALSE	FALSE	FALSE	veBhfS	0.025494	0.717938	486484	sepsis	TRUE	reported	textfile	0.0261	5.58E-06	528	M33391.m	TRUE	reported	Gfjy5a	2	TRUE	0.037574	20.53561
655	rs2846723	T	G	T	G	-0.0476	0.004764	0.5442	0.538577	FALSE	FALSE	FALSE	7BPGuQ	0.013708	0.728207	486484	sepsis	TRUE	reported	textfile	0.0105	5.76E-06	2639	M01123.m	TRUE	reported	WV2hVn	2	TRUE	0.007727	20.53554
8954	rs2707795	T	G	T	G	-0.3579	0.022335	0.972	0.984904	FALSE	FALSE	FALSE	Ywk2ve	0.058958	0.70482	486484	sepsis	TRUE	reported	textfile	0.0788	5.64E-06	437	M37112.m	TRUE	reported	EiAdDj	2	TRUE	0.045077	20.53425
1101	rs9353315	A	G	A	G	-0.0476	0.013586	0.8731	0.856115	FALSE	FALSE	FALSE	K3wtZo	0.019495	0.485859	486484	sepsis	TRUE	reported	textfile	0.0105	5.86E-06	2315	M01573.m	TRUE	reported	0D9CvA	2	TRUE	0.008799	20.53336
7605	rs4389303	T	G	T	G	-0.172	-0.03284	0.1027	0.105385	FALSE	FALSE	FALSE	PlYt5g	0.022471	0.143948	486484	sepsis	TRUE	reported	textfile	0.0378	5.35E-06	241	M34516.m	TRUE	reported	jX41tf	2	TRUE	0.079115	20.53308
8309	rs3813782	A	G	A	G	0.0535	0.002922	0.1154	0.132505	FALSE	FALSE	FALSE	xXnn6V	0.020224	0.885112	486484	sepsis	TRUE	reported	textfile	0.0118	5.81E-06	1766	M35626.m	TRUE	reported	WgWa24	2	TRUE	0.011506	20.53295
2807	rs2489774	T	C	T	C	0.0213	0.008501	0.0339	0.034611	FALSE	FALSE	FALSE	Sx5Uhq	0.03743	0.820326	486484	sepsis	TRUE	reported	textfile	0.0047	6.46E-06	7354	M19934.m	TRUE	reported	DijYTE	2	TRUE	0.002785	20.53267
1673	rs1740379	A	G	A	G	0.073	0.030846	0.1166	0.123785	FALSE	FALSE	FALSE	IIFBGY	0.020767	0.137455	486484	sepsis	TRUE	reported	textfile	0.0161	5.61E-06	1543	M12261.m	TRUE	reported	F5Vlan	2	TRUE	0.013149	20.53197
6593	rs6759985	T	G	T	G	0.2189	0.011018	0.0471	0.039921	FALSE	FALSE	FALSE	XO3rXq	0.035152	0.753956	486484	sepsis	TRUE	reported	textfile	0.0481	5.33E-06	228	M33877.m	TRUE	reported	p2sETg	2	TRUE	0.083273	20.52934
4272	rs2910745	C	G	C	G	-0.136	0.05764	0.0111	0.008671	FALSE	TRUE	FALSE	ylV4bp	0.073866	0.435196	486484	sepsis	TRUE	reported	textfile	0.03	5.70E-06	1762	M32632.m	TRUE	reported	hqHJoU	2	TRUE	0.011529	20.52778
3437	rs1003183	T	C	T	C	-0.034	-0.00411	0.6953	0.659746	FALSE	FALSE	FALSE	5dHWFk	0.014458	0.776102	486484	sepsis	TRUE	reported	textfile	0.0075	5.81E-06	1758	M27672.m	TRUE	reported	pEd8kY	2	TRUE	0.011555	20.52773
5118	rs7762480	A	T	A	T	0.0748	-0.02703	0.9273	0.921267	FALSE	TRUE	FALSE	O6ine1	0.025344	0.286109	486484	sepsis	TRUE	reported	textfile	0.0165	5.69E-06	1751	M33165.m	TRUE	reported	gGsqjW	2	TRUE	0.011601	20.52764
2294	rs4268384	A	G	A	G	0.029	0.024416	0.0624	0.065496	FALSE	FALSE	FALSE	43Yh3h	0.027762	0.379146	486484	sepsis	TRUE	reported	textfile	0.0064	5.01E-06	7055	M16818.m	TRUE	reported	beR4IT	2	TRUE	0.002902	20.52641
6730	rs1165542	A	T	A	T	0.0145	0.02286	0.1996	0.286081	FALSE	TRUE	FALSE	SpG9A1	0.015186	0.132236	486484	sepsis	TRUE	reported	textfile	0.0032	5.82E-06	6502	M33939.m	TRUE	reported	Tudxel	2	TRUE	0.003148	20.52591
8158	rs1781577	A	C	A	C	-0.1079	-0.04903	0.9885	0.987824	FALSE	FALSE	FALSE	sRlsb6	0.063071	0.436968	486484	sepsis	TRUE	reported	textfile	0.0238	5.94E-06	1375	M35331.m	TRUE	reported	GPijlC	2	TRUE	0.014728	20.52375
2314	rs976547	A	T	A	T	-0.0272	0.017288	0.5965	0.603248	FALSE	TRUE	FALSE	ZzUW8U	0.014021	0.21759	486484	sepsis	TRUE	reported	textfile	0.006	6.15E-06	1445	M16821.m	TRUE	reported	g2xs3i	2	TRUE	0.014023	20.52267
4660	rs7337711	T	C	T	C	0.0408	0.009567	0.1564	0.143108	FALSE	FALSE	FALSE	SyAFR2	0.019595	0.625392	486484	sepsis	TRUE	reported	textfile	0.009	5.44E-06	1435	M32755.m	TRUE	reported	prUo74	2	TRUE	0.014119	20.52247
4769	rs1742900	A	G	A	G	-0.0367	0.011049	0.91	0.874454	FALSE	FALSE	FALSE	mOrnK4	0.020699	0.593468	486484	sepsis	TRUE	reported	textfile	0.0081	6.65E-06	6169	M32795.m	TRUE	reported	7EGxuf	2	TRUE	0.003317	20.52207
4316	rs1075815	T	C	T	C	-0.0222	0.007302	0.1408	0.158161	FALSE	FALSE	FALSE	EOAssJ	0.018745	0.696854	486484	sepsis	TRUE	reported	textfile	0.0049	6.21E-06	7369	M32635.m	TRUE	reported	X1mTjy	2	TRUE	0.002778	20.52088
4629	rs649622	T	G	T	G	0.0444	0.009563	0.097	0.113891	FALSE	FALSE	FALSE	0oA38w	0.021507	0.656571	486484	sepsis	TRUE	reported	textfile	0.0098	5.89E-06	7356	M32753.m	TRUE	reported	dQUaUX	2	TRUE	0.002783	20.52087
6919	rs2663073	A	G	A	G	0.4837	0.029343	0.927	0.915528	FALSE	FALSE	FALSE	SvfSvH	0.024583	0.232611	486484	sepsis	TRUE	reported	textfile	0.1064	5.42E-06	282	M34109.m	TRUE	reported	7OSbiu	2	TRUE	0.068282	20.52002
7775	rs1782694	A	G	A	G	0.0897	0.05316	0.984	0.975904	FALSE	FALSE	FALSE	15BqPa	0.045768	0.245434	486484	sepsis	TRUE	reported	textfile	0.0198	5.56E-06	7364	M35126.m	TRUE	reported	zvDzP2	2	TRUE	0.002779	20.51807
7787	rs6972443	C	G	C	G	0.0299	-0.00795	0.8153	0.78993	FALSE	TRUE	FALSE	15BqPa	0.016763	0.635378	486484	sepsis	TRUE	reported	textfile	0.0066	5.78E-06	7364	M35126.m	TRUE	reported	zvDzP2	2	TRUE	0.002779	20.51807
5071	rs1156319	C	G	C	G	0.0299	-0.00637	0.0722	0.076105	FALSE	TRUE	FALSE	gqzGN4	0.025766	0.804649	486484	sepsis	TRUE	reported	textfile	0.0066	5.34E-06	7319	M33140.m	TRUE	reported	DKGBe9	2	TRUE	0.002796	20.51804
8964	rs1287987	T	C	T	C	-0.0299	0.008016	0.5575	0.556705	FALSE	FALSE	FALSE	2iAvlt	0.013815	0.561727	486484	sepsis	TRUE	reported	textfile	0.0066	5.17E-06	6934	M37190.m	TRUE	reported	hdOuHe	2	TRUE	0.002951	20.51773
8967	rs4372938	T	C	T	C	0.0299	0.018718	0.5433	0.541526	FALSE	FALSE	FALSE	2iAvlt	0.013828	0.175837	486484	sepsis	TRUE	reported	textfile	0.0066	6.09E-06	6934	M37190.m	TRUE	reported	hdOuHe	2	TRUE	0.002951	20.51773
852	rs35350	A	G	A	G	0.0376	0.024302	0.9692	0.971344	FALSE	FALSE	FALSE	1tFtaf	0.040705	0.550495	486484	sepsis	TRUE	reported	textfile	0.0083	5.44E-06	7323	M01359.m	TRUE	reported	qq3q6E	2	TRUE	0.002795	20.51639
2690	rs309873	C	G	C	G	0.0743	0.009761	0.065	0.069972	FALSE	TRUE	FALSE	ATsH6N	0.026803	0.715731	486484	sepsis	TRUE	reported	textfile	0.0164	5.63E-06	3783	M18497.m	TRUE	reported	IGuIRI	2	TRUE	0.005396	20.51447
4645	rs9543952	A	G	A	G	-0.053	-0.01411	0.8491	0.841923	FALSE	FALSE	FALSE	Dfaklt	0.018756	0.451822	486484	sepsis	TRUE	reported	textfile	0.0117	5.80E-06	6379	M32754.m	TRUE	reported	y21cDm	2	TRUE	0.003207	20.51369
6215	rs1802376	A	G	A	G	0.0875	-0.0784	0.0427	0.01931	FALSE	FALSE	FALSE	FQfT2o	0.049581	0.113805	486484	sepsis	TRUE	reported	textfile	0.0193	5.95E-06	972	M33508.m	TRUE	reported	JtbOy2	2	TRUE	0.020708	20.51195
3393	rs2130722	T	C	T	C	-0.0666	0.008068	0.0738	0.077968	FALSE	FALSE	FALSE	frOZiy	0.025507	0.751761	486484	sepsis	TRUE	reported	textfile	0.0147	6.28E-06	2759	M27447.m	TRUE	reported	2z9fSv	2	TRUE	0.007385	20.51157
1558	rs2725779	T	C	T	C	0.0077	-0.0257	0.4523	0.461841	FALSE	FALSE	FALSE	Ke0sNh	0.013775	0.062081	486484	sepsis	TRUE	reported	textfile	0.0017	3.27E-06	7799	M10642.m	TRUE	reported	ZINwVb	2	TRUE	0.002624	20.51031
1122	rs1049073	A	G	A	G	0.0077	0.001737	0.8314	0.832629	FALSE	FALSE	FALSE	nlqUuV	0.018324	0.924481	486484	sepsis	TRUE	reported	textfile	0.0017	7.94E-06	7371	M01604.m	TRUE	reported	Uro9nC	2	TRUE	0.002776	20.51
1128	rs1257914	T	C	T	C	-0.0077	-0.01034	0.1887	0.185828	FALSE	FALSE	FALSE	nlqUuV	0.017716	0.559532	486484	sepsis	TRUE	reported	textfile	0.0017	5.92E-06	7371	M01604.m	TRUE	reported	Uro9nC	2	TRUE	0.002776	20.51
1129	rs1286087	T	C	T	C	0.0077	0.019534	0.8367	0.846009	FALSE	FALSE	FALSE	nlqUuV	0.019003	0.303976	486484	sepsis	TRUE	reported	textfile	0.0017	8.08E-06	7371	M01604.m	TRUE	reported	Uro9nC	2	TRUE	0.002776	20.51
1782	rs1116275	A	G	A	G	-0.0077	-0.00487	0.5972	0.583273	FALSE	FALSE	FALSE	JEcCRZ	0.013866	0.725301	486484	sepsis	TRUE	reported	textfile	0.0017	8.08E-06	7368	M15140.m	TRUE	reported	xYCzKP	2	TRUE	0.002777	20.51
1350	rs7029664	T	C	T	C	0.0077	0.02521	0.5057	0.507327	FALSE	FALSE	FALSE	LDUJbh	0.013663	0.065028	486484	sepsis	TRUE	reported	textfile	0.0017	9.66E-06	7367	M01898.m	TRUE	reported	JRSLeH	2	TRUE	0.002777	20.51
739	rs4317453	A	G	A	G	-0.0077	0.008127	0.3363	0.371074	FALSE	FALSE	FALSE	9u0SC8	0.014743	0.581475	486484	sepsis	TRUE	reported	textfile	0.0017	9.12E-06	7363	M01301.m	TRUE	reported	H95uec	2	TRUE	0.002779	20.51
4143	rs1177928	A	T	A	T	0.0231	-0.01214	0.3845	0.352591	FALSE	TRUE	FALSE	CrVaiQ	0.014335	0.397055	486484	sepsis	TRUE	reported	textfile	0.0051	6.50E-06	7361	M32553.m	TRUE	reported	HsKdAI	2	TRUE	0.002779	20.51
714	rs7195332	T	C	T	C	0.0077	-0.04195	0.2152	0.20495	FALSE	FALSE	FALSE	H9l3s3	0.016973	0.013444	486484	sepsis	TRUE	reported	textfile	0.0017	4.05E-06	7358	M01299.m	TRUE	reported	kXWyUq	2	TRUE	0.00278	20.50999
98	rs33980	C	G	C	G	-0.0077	-0.0132	0.9071	0.901558	FALSE	TRUE	FALSE	xmGuol	0.022925	0.564802	486484	sepsis	TRUE	reported	textfile	0.0017	9.11E-06	7355	M00054.m	TRUE	reported	GW50Ri	2	TRUE	0.002782	20.50999
829	rs2280248	A	G	A	G	-0.0077	0.019601	0.4808	0.482707	FALSE	FALSE	FALSE	2qgDgB	0.013717	0.15302	486484	sepsis	TRUE	reported	textfile	0.0017	5.55E-06	7355	M01358.m	TRUE	reported	asmXq0	2	TRUE	0.002782	20.50999
835	rs4507103	A	G	A	G	0.0077	0.007771	0.397	0.39649	FALSE	FALSE	FALSE	2qgDgB	0.013972	0.5781	486484	sepsis	TRUE	reported	textfile	0.0017	6.89E-06	7355	M01358.m	TRUE	reported	asmXq0	2	TRUE	0.002782	20.50999
840	rs6883481	T	C	T	C	0.0077	0.000204	0.4737	0.475358	FALSE	FALSE	FALSE	2qgDgB	0.013772	0.988163	486484	sepsis	TRUE	reported	textfile	0.0017	6.17E-06	7355	M01358.m	TRUE	reported	asmXq0	2	TRUE	0.002782	20.50999
2601	rs2203995	A	G	A	G	0.0077	-0.00015	0.6055	0.612965	FALSE	FALSE	FALSE	ruvJh6	0.014152	0.991825	486484	sepsis	TRUE	reported	textfile	0.0017	7.26E-06	7354	M18369.m	TRUE	reported	GFmwGk	2	TRUE	0.002782	20.50999
2810	rs265487	A	G	A	G	-0.0077	-0.03634	0.4929	0.497497	FALSE	FALSE	FALSE	Sx5Uhq	0.013675	0.007883	486484	sepsis	TRUE	reported	textfile	0.0017	7.56E-06	7354	M19934.m	TRUE	reported	DijYTE	2	TRUE	0.002782	20.50999
782	rs1019978	T	C	T	C	0.0077	-0.00383	0.6173	0.61969	FALSE	FALSE	FALSE	dyT79I	0.014078	0.78576	486484	sepsis	TRUE	reported	textfile	0.0017	6.20E-06	7352	M01336.m	TRUE	reported	OemVJ2	2	TRUE	0.002783	20.50999
1968	rs2962321	T	C	T	C	-0.0077	0.035451	0.8968	0.903331	FALSE	FALSE	FALSE	v2KE89	0.023791	0.136198	486484	sepsis	TRUE	reported	textfile	0.0017	6.01E-06	7349	M15500.m	TRUE	reported	to2t7H	2	TRUE	0.002784	20.50999
1996	rs4811828	T	C	T	C	0.0077	-0.03418	0.8787	0.879505	FALSE	FALSE	FALSE	v2KE89	0.021004	0.10367	486484	sepsis	TRUE	reported	textfile	0.0017	5.89E-06	7349	M15500.m	TRUE	reported	to2t7H	2	TRUE	0.002784	20.50999
749	rs1742639	A	G	A	G	0.0077	-0.01555	0.8865	0.885404	FALSE	FALSE	FALSE	RHWptG	0.021572	0.470963	486484	sepsis	TRUE	reported	textfile	0.0017	7.94E-06	7347	M01302.m	TRUE	reported	z1OLfr	2	TRUE	0.002785	20.50999
756	rs6008767	C	G	C	G	-0.0077	0.010318	0.8182	0.808022	FALSE	TRUE	FALSE	RHWptG	0.017612	0.557982	486484	sepsis	TRUE	reported	textfile	0.0017	4.12E-06	7347	M01302.m	TRUE	reported	z1OLfr	2	TRUE	0.002785	20.50999
758	rs6533609	T	C	T	C	-0.0077	-0.00725	0.1429	0.149496	FALSE	FALSE	FALSE	RHWptG	0.019158	0.705001	486484	sepsis	TRUE	reported	textfile	0.0017	3.78E-06	7347	M01302.m	TRUE	reported	z1OLfr	2	TRUE	0.002785	20.50999
6706	rs2720162	T	G	T	G	-0.0154	0.004338	0.6076	0.597515	FALSE	FALSE	FALSE	bzT5yX	0.013969	0.756169	486484	sepsis	TRUE	reported	textfile	0.0034	7.31E-06	7345	M33936.m	TRUE	reported	W7BO8n	2	TRUE	0.002785	20.50998
8163	rs1215466	T	C	T	C	0.0077	0.015058	0.7624	0.742571	FALSE	FALSE	FALSE	8UhtPK	0.015925	0.344379	486484	sepsis	TRUE	reported	textfile	0.0017	4.65E-06	7345	M35397.m	TRUE	reported	rvVJba	2	TRUE	0.002785	20.50998
8180	rs4917204	A	G	A	G	-0.0077	-0.0111	0.7723	0.781879	FALSE	FALSE	FALSE	8UhtPK	0.01655	0.502392	486484	sepsis	TRUE	reported	textfile	0.0017	5.08E-06	7345	M35397.m	TRUE	reported	rvVJba	2	TRUE	0.002785	20.50998
3797	rs9367164	A	G	A	G	0.0077	-0.0094	0.6161	0.609244	FALSE	FALSE	FALSE	NW1aaR	0.01401	0.502044	486484	sepsis	TRUE	reported	textfile	0.0017	9.17E-06	7344	M32339.m	TRUE	reported	pBPkAB	2	TRUE	0.002786	20.50998
4964	rs4867926	T	G	T	G	0.0077	-0.00648	0.3914	0.368723	FALSE	FALSE	FALSE	bKmDMa	0.014256	0.64925	486484	sepsis	TRUE	reported	textfile	0.0017	8.29E-06	7344	M32910.m	TRUE	reported	nAuPzO	2	TRUE	0.002786	20.50998
3735	rs170734	A	G	A	G	-0.0154	0.004406	0.7263	0.723037	FALSE	FALSE	FALSE	k16olH	0.015305	0.773444	486484	sepsis	TRUE	reported	textfile	0.0034	6.79E-06	7340	M32328.m	TRUE	reported	TQA3sZ	2	TRUE	0.002787	20.50998
2131	rs1222036	A	G	A	G	-0.0077	0.008523	0.4107	0.413799	FALSE	FALSE	FALSE	TEupfd	0.013904	0.53988	486484	sepsis	TRUE	reported	textfile	0.0017	8.34E-06	7331	M15676.m	TRUE	reported	NXiaPC	2	TRUE	0.002791	20.50997
2146	rs7310929	T	C	T	C	-0.0077	-0.00295	0.6256	0.622411	FALSE	FALSE	FALSE	TEupfd	0.014086	0.834312	486484	sepsis	TRUE	reported	textfile	0.0017	9.38E-06	7331	M15676.m	TRUE	reported	NXiaPC	2	TRUE	0.002791	20.50997
2906	rs2011920	C	G	C	G	-0.0154	-0.01894	0.1375	0.151012	FALSE	TRUE	FALSE	FBDEQi	0.019213	0.324173	486484	sepsis	TRUE	reported	textfile	0.0034	5.13E-06	7327	M20699.m	TRUE	reported	nvNIlp	2	TRUE	0.002792	20.50997
1375	rs1511589	A	G	A	G	0.0077	0.010684	0.4559	0.447623	FALSE	FALSE	FALSE	KrWhws	0.013769	0.437774	486484	sepsis	TRUE	reported	textfile	0.0017	7.55E-06	7325	M02132.m	TRUE	reported	7pzpfK	2	TRUE	0.002793	20.50997
1383	rs229084	A	G	A	G	-0.0077	-0.00119	0.4727	0.457412	FALSE	FALSE	FALSE	KrWhws	0.013727	0.931064	486484	sepsis	TRUE	reported	textfile	0.0017	6.57E-06	7325	M02132.m	TRUE	reported	7pzpfK	2	TRUE	0.002793	20.50997
1391	rs6510448	T	G	T	G	0.0077	-0.00702	0.6691	0.663202	FALSE	FALSE	FALSE	KrWhws	0.014502	0.628129	486484	sepsis	TRUE	reported	textfile	0.0017	8.14E-06	7325	M02132.m	TRUE	reported	7pzpfK	2	TRUE	0.002793	20.50997
1395	rs758186	T	C	T	C	-0.0077	5.08E-05	0.6536	0.643687	FALSE	FALSE	FALSE	KrWhws	0.014434	0.997193	486484	sepsis	TRUE	reported	textfile	0.0017	8.69E-06	7325	M02132.m	TRUE	reported	7pzpfK	2	TRUE	0.002793	20.50997
1398	rs779463	T	G	T	G	-0.0077	-0.01349	0.3012	0.309315	FALSE	FALSE	FALSE	KrWhws	0.014789	0.36156	486484	sepsis	TRUE	reported	textfile	0.0017	9.46E-06	7325	M02132.m	TRUE	reported	7pzpfK	2	TRUE	0.002793	20.50997
2867	rs9915927	T	G	T	G	0.0077	0.00793	0.2687	0.282734	FALSE	FALSE	FALSE	Y5qWBm	0.015222	0.6024	486484	sepsis	TRUE	reported	textfile	0.0017	5.76E-06	7325	M20489.m	TRUE	reported	f2g9Su	2	TRUE	0.002793	20.50997
7623	rs6062634	A	C	A	C	-0.0154	-0.00157	0.4048	0.419147	FALSE	FALSE	FALSE	kVfKqT	0.013953	0.910223	486484	sepsis	TRUE	reported	textfile	0.0034	7.16E-06	7322	M34527.m	TRUE	reported	s4ti92	2	TRUE	0.002794	20.50997
4578	rs1271220	A	G	A	G	0.0077	-0.00915	0.4119	0.399996	FALSE	FALSE	FALSE	Pzplqu	0.013952	0.511745	486484	sepsis	TRUE	reported	textfile	0.0017	7.69E-06	7318	M32740.m	TRUE	reported	FjEBS5	2	TRUE	0.002796	20.50996
4585	rs1710109	C	G	C	G	-0.0077	0.004995	0.3183	0.323643	FALSE	TRUE	FALSE	Pzplqu	0.014662	0.733321	486484	sepsis	TRUE	reported	textfile	0.0017	9.76E-06	7318	M32740.m	TRUE	reported	FjEBS5	2	TRUE	0.002796	20.50996
4591	rs2147103	A	T	A	T	0.0077	0.001324	0.6044	0.58885	FALSE	TRUE	FALSE	Pzplqu	0.01392	0.92421	486484	sepsis	TRUE	reported	textfile	0.0017	8.51E-06	7318	M32740.m	TRUE	reported	FjEBS5	2	TRUE	0.002796	20.50996
4613	rs8005168	T	C	T	C	0.0077	-0.01196	0.3852	0.396733	FALSE	FALSE	FALSE	Pzplqu	0.013964	0.391778	486484	sepsis	TRUE	reported	textfile	0.0017	9.31E-06	7318	M32740.m	TRUE	reported	FjEBS5	2	TRUE	0.002796	20.50996
4617	rs9320187	A	G	A	G	-0.0154	-0.00045	0.065	0.062276	FALSE	FALSE	FALSE	Pzplqu	0.028237	0.987261	486484	sepsis	TRUE	reported	textfile	0.0034	4.90E-06	7318	M32740.m	TRUE	reported	FjEBS5	2	TRUE	0.002796	20.50996
1683	rs1093765	A	G	A	G	0.0077	0.016558	0.2676	0.270366	FALSE	FALSE	FALSE	f01en0	0.01548	0.284767	486484	sepsis	TRUE	reported	textfile	0.0017	3.50E-06	7314	M12593.m	TRUE	reported	EAsM6U	2	TRUE	0.002797	20.50996
1690	rs1556988	A	G	A	G	-0.0077	0.026406	0.7923	0.796392	FALSE	FALSE	FALSE	f01en0	0.0171	0.122532	486484	sepsis	TRUE	reported	textfile	0.0017	5.86E-06	7314	M12593.m	TRUE	reported	EAsM6U	2	TRUE	0.002797	20.50996
1697	rs6924754	A	G	A	G	0.0154	-0.01953	0.0606	0.097323	FALSE	FALSE	FALSE	f01en0	0.023425	0.404443	486484	sepsis	TRUE	reported	textfile	0.0034	6.43E-06	7314	M12593.m	TRUE	reported	EAsM6U	2	TRUE	0.002797	20.50996
6004	rs7901640	A	G	A	G	-0.0077	-0.02024	0.3935	0.394325	FALSE	FALSE	FALSE	3KsoXq	0.014005	0.148392	486484	sepsis	TRUE	reported	textfile	0.0017	9.06E-06	7306	M33422.m	TRUE	reported	aNr6Ug	2	TRUE	0.0028	20.50995
1102	rs1009006	T	C	T	C	0.0077	0.015615	0.8026	0.803588	FALSE	FALSE	FALSE	yahiGZ	0.017297	0.366639	486484	sepsis	TRUE	reported	textfile	0.0017	5.93E-06	7271	M01585.m	TRUE	reported	dwZ7vX	2	TRUE	0.002814	20.50993
3354	rs1342039	A	G	A	G	0.0154	-0.01788	0.0957	0.097678	FALSE	FALSE	FALSE	LY4Gus	0.023085	0.438716	486484	sepsis	TRUE	reported	textfile	0.0034	5.91E-06	7268	M27273.m	TRUE	reported	QKITBx	2	TRUE	0.002815	20.50993
3546	rs1317933	A	T	A	T	0.0154	-0.00224	0.2062	0.307902	FALSE	TRUE	FALSE	8luUON	0.015057	0.881856	486484	sepsis	TRUE	reported	textfile	0.0034	4.85E-06	7257	M31453.m	TRUE	reported	FBs6G4	2	TRUE	0.002819	20.50992
2737	rs7680308	A	G	A	G	0.0077	0.008002	0.4854	0.484058	FALSE	FALSE	FALSE	H4yqSK	0.013746	0.560475	486484	sepsis	TRUE	reported	textfile	0.0017	9.08E-06	7182	M19362.m	TRUE	reported	w2PDEO	2	TRUE	0.002848	20.50986
1295	rs1334235	C	G	C	G	0.0077	-0.01972	0.3644	0.371597	FALSE	TRUE	FALSE	WQdpmo	0.014118	0.162508	486484	sepsis	TRUE	reported	textfile	0.0017	9.97E-06	7136	M01769.m	TRUE	reported	BkptOK	2	TRUE	0.002867	20.50982
1297	rs2058473	A	G	A	G	-0.0077	-0.0052	0.6494	0.653926	FALSE	FALSE	FALSE	WQdpmo	0.014416	0.718343	486484	sepsis	TRUE	reported	textfile	0.0017	7.48E-06	7136	M01769.m	TRUE	reported	BkptOK	2	TRUE	0.002867	20.50982
1300	rs2272663	A	G	A	G	0.0077	-0.00507	0.5321	0.529431	FALSE	FALSE	FALSE	WQdpmo	0.013684	0.710974	486484	sepsis	TRUE	reported	textfile	0.0017	9.39E-06	7136	M01769.m	TRUE	reported	BkptOK	2	TRUE	0.002867	20.50982
1312	rs7562418	A	C	A	C	0.0077	-0.00435	0.5075	0.512047	FALSE	FALSE	FALSE	WQdpmo	0.013927	0.754607	486484	sepsis	TRUE	reported	textfile	0.0017	6.81E-06	7136	M01769.m	TRUE	reported	BkptOK	2	TRUE	0.002867	20.50982
1320	rs940511	A	G	A	G	-0.0077	0.004262	0.4652	0.456281	FALSE	FALSE	FALSE	WQdpmo	0.013803	0.75749	486484	sepsis	TRUE	reported	textfile	0.0017	6.71E-06	7136	M01769.m	TRUE	reported	BkptOK	2	TRUE	0.002867	20.50982
5827	rs28157	T	G	T	G	-0.0077	-0.0252	0.3075	0.315162	FALSE	FALSE	FALSE	R5joEf	0.014751	0.087567	486484	sepsis	TRUE	reported	textfile	0.0017	6.68E-06	7118	M33380.m	TRUE	reported	kRvbfZ	2	TRUE	0.002874	20.50981
5841	rs835800	T	C	T	C	0.0077	0.023768	0.2665	0.266846	FALSE	FALSE	FALSE	R5joEf	0.015512	0.125465	486484	sepsis	TRUE	reported	textfile	0.0017	9.61E-06	7118	M33380.m	TRUE	reported	kRvbfZ	2	TRUE	0.002874	20.50981
5844	rs941980	T	C	T	C	0.0077	-0.01499	0.5798	0.582025	FALSE	FALSE	FALSE	R5joEf	0.013894	0.280767	486484	sepsis	TRUE	reported	textfile	0.0017	4.23E-06	7118	M33380.m	TRUE	reported	kRvbfZ	2	TRUE	0.002874	20.50981
8691	rs1171158	A	G	A	G	-0.0077	-0.02666	0.4815	0.48126	FALSE	FALSE	FALSE	Bg4VnB	0.013752	0.052518	486484	sepsis	TRUE	reported	textfile	0.0017	5.19E-06	7092	M36552.m	TRUE	reported	6zdDpC	2	TRUE	0.002884	20.50979
8693	rs1279831	A	G	A	G	0.0077	-0.01375	0.5589	0.566797	FALSE	FALSE	FALSE	Bg4VnB	0.013785	0.318606	486484	sepsis	TRUE	reported	textfile	0.0017	4.74E-06	7092	M36552.m	TRUE	reported	6zdDpC	2	TRUE	0.002884	20.50979
1645	rs7279224	A	T	A	T	0.0077	0.002607	0.6952	0.634954	FALSE	TRUE	FALSE	n5rDuT	0.014271	0.855058	486484	sepsis	TRUE	reported	textfile	0.0017	6.87E-06	7081	M12067.m	TRUE	reported	ZQLTeR	2	TRUE	0.002889	20.50978
1460	rs1119917	A	C	A	C	0.0077	-0.03055	0.5221	0.535532	FALSE	FALSE	FALSE	pkb876	0.013716	0.025933	486484	sepsis	TRUE	reported	textfile	0.0017	9.33E-06	7037	M02734.m	TRUE	reported	0LCMVI	2	TRUE	0.002907	20.50974
1465	rs1254604	T	C	T	C	0.0077	-0.00088	0.4197	0.43722	FALSE	FALSE	FALSE	pkb876	0.013767	0.94914	486484	sepsis	TRUE	reported	textfile	0.0017	8.35E-06	7037	M02734.m	TRUE	reported	0LCMVI	2	TRUE	0.002907	20.50974
1480	rs353620	A	G	A	G	-0.0077	0.024948	0.6613	0.651501	FALSE	FALSE	FALSE	pkb876	0.01433	0.081703	486484	sepsis	TRUE	reported	textfile	0.0017	9.80E-06	7037	M02734.m	TRUE	reported	0LCMVI	2	TRUE	0.002907	20.50974
8844	rs1756740	A	G	A	G	0.0077	0.004725	0.2327	0.26747	FALSE	FALSE	FALSE	XIHeTV	0.015607	0.762065	486484	sepsis	TRUE	reported	textfile	0.0017	9.16E-06	6940	M36808.m	TRUE	reported	37Vi2D	2	TRUE	0.002947	20.50966
8015	rs8008572	T	C	T	C	-0.0154	0.012968	0.7565	0.752066	FALSE	FALSE	FALSE	Zj5bZH	0.015847	0.413177	486484	sepsis	TRUE	reported	textfile	0.0034	6.69E-06	6538	M35257.m	TRUE	reported	exssAk	2	TRUE	0.003128	20.5093
8115	rs2460405	A	G	A	G	-0.0077	0.001411	0.36	0.358425	FALSE	FALSE	FALSE	nNbXBS	0.014242	0.921076	486484	sepsis	TRUE	reported	textfile	0.0017	8.87E-06	5768	M35326.m	TRUE	reported	UNDorn	2	TRUE	0.003544	20.50846
2284	rs2473097	T	C	T	C	0.0154	0.002111	0.7894	0.782128	FALSE	FALSE	FALSE	mRULgB	0.016555	0.898519	486484	sepsis	TRUE	reported	textfile	0.0034	5.90E-06	4689	M16816.m	TRUE	reported	05IFsX	2	TRUE	0.004356	20.50682
8599	rs463106	T	C	T	C	0.0367	0.017308	0.6825	0.531414	FALSE	FALSE	FALSE	t5potg	0.013791	0.209476	486484	sepsis	TRUE	reported	textfile	0.0081	6.29E-06	1731	M36115.m	TRUE	reported	SSOHIA	2	TRUE	0.01172	20.50501
5622	rs2697339	T	C	T	C	0.0462	-0.0063	0.7684	0.767397	FALSE	FALSE	FALSE	8QFOKi	0.016158	0.696635	486484	sepsis	TRUE	reported	textfile	0.0102	6.39E-06	3740	M33188.m	TRUE	reported	DzRwMK	2	TRUE	0.005456	20.5046
6613	rs953628	T	G	T	G	-0.0607	0.017609	0.2013	0.203779	FALSE	FALSE	FALSE	VfOuo8	0.016966	0.299328	486484	sepsis	TRUE	reported	textfile	0.0134	5.74E-06	2725	M33883.m	TRUE	reported	BDe0Nk	2	TRUE	0.007474	20.50449
6402	rs8050423	A	G	A	G	0.1772	0.049824	0.0297	0.035422	FALSE	FALSE	FALSE	Z1h3mT	0.037202	0.18048	486484	sepsis	TRUE	reported	textfile	0.0391	5.72E-06	1192	M33652.m	TRUE	reported	nszuTQ	2	TRUE	0.016939	20.50428
4420	rs2512652	C	G	C	G	-0.0521	0.014425	0.704	0.700102	FALSE	TRUE	FALSE	NzBiVA	0.015305	0.345935	486484	sepsis	TRUE	reported	textfile	0.0115	5.76E-06	1764	M32672.m	TRUE	reported	YLlhHJ	2	TRUE	0.011502	20.50157
5706	rs9521845	A	G	A	G	0.0154	-0.00179	0.3291	0.292656	FALSE	FALSE	FALSE	1SbQTe	0.015004	0.904909	486484	sepsis	TRUE	reported	textfile	0.0034	7.19E-06	2683	M33204.m	TRUE	reported	x7MBDH	2	TRUE	0.007588	20.50028
8582	rs1358498	T	C	T	C	-0.024	0.005333	0.5824	0.580994	FALSE	FALSE	FALSE	iuJmRL	0.013855	0.700311	486484	sepsis	TRUE	reported	textfile	0.0053	5.01E-06	7311	M36103.m	TRUE	reported	jfJbVw	2	TRUE	0.002797	20.49991
2623	rs2564483	T	G	T	G	0.024	0.006455	0.6043	0.614624	FALSE	FALSE	FALSE	eygBBR	0.014064	0.646243	486484	sepsis	TRUE	reported	textfile	0.0053	6.42E-06	6970	M18394.m	TRUE	reported	hD79iE	2	TRUE	0.002933	20.49963
7930	rs1339098	T	C	T	C	0.024	0.003981	0.9232	0.921055	FALSE	FALSE	FALSE	kFTWXY	0.025303	0.874998	486484	sepsis	TRUE	reported	textfile	0.0053	6.56E-06	6543	M35193.m	TRUE	reported	cjJvJl	2	TRUE	0.003124	20.49925
8057	rs1041584	T	C	T	C	-0.024	-0.00724	0.1248	0.120627	FALSE	FALSE	FALSE	whAa18	0.020975	0.729985	486484	sepsis	TRUE	reported	textfile	0.0053	5.79E-06	5979	M35305.m	TRUE	reported	e6hvDm	2	TRUE	0.003418	20.49866
5053	rs6534104	C	G	C	G	-0.1087	-0.02578	0.0373	0.046072	FALSE	TRUE	FALSE	JLCSne	0.032719	0.430759	486484	sepsis	TRUE	reported	textfile	0.024	6.15E-06	2391	M33137.m	TRUE	reported	LGKYXK	2	TRUE	0.008506	20.49619
483	rs2711506	A	G	A	G	-0.0326	-0.00817	0.5343	0.526606	FALSE	FALSE	FALSE	ScJVI8	0.013673	0.549923	486484	sepsis	TRUE	reported	textfile	0.0072	6.35E-06	7371	M00542.m	TRUE	reported	Sa1L04	2	TRUE	0.002774	20.49521
4324	rs7210933	A	T	A	T	0.0163	-0.0086	0.7569	0.739625	FALSE	TRUE	FALSE	EOAssJ	0.015588	0.581321	486484	sepsis	TRUE	reported	textfile	0.0036	8.17E-06	7369	M32635.m	TRUE	reported	X1mTjy	2	TRUE	0.002774	20.49521
8973	rs1084749	A	G	A	G	0.0326	-0.01471	0.7519	0.734074	FALSE	FALSE	FALSE	snOepl	0.015544	0.344073	486484	sepsis	TRUE	reported	textfile	0.0072	5.89E-06	7355	M37202.m	TRUE	reported	PQx272	2	TRUE	0.00278	20.4952
7458	rs4505564	T	C	T	C	0.0163	0.009251	0.7913	0.790715	FALSE	FALSE	FALSE	7ubZow	0.016919	0.584515	486484	sepsis	TRUE	reported	textfile	0.0036	4.94E-06	7344	M34407.m	TRUE	reported	LRsXN2	2	TRUE	0.002784	20.49519
3188	rs1087792	A	G	A	G	-0.0163	-0.03457	0.0869	0.08926	FALSE	FALSE	FALSE	UfLkBq	0.023906	0.148206	486484	sepsis	TRUE	reported	textfile	0.0036	8.25E-06	7329	M22481.m	TRUE	reported	kWMdiT	2	TRUE	0.002789	20.49518
8311	rs1075972	A	G	A	G	0.0163	0.000775	0.4548	0.451195	FALSE	FALSE	FALSE	YZIMbv	0.013844	0.955363	486484	sepsis	TRUE	reported	textfile	0.0036	5.79E-06	7302	M35628.m	TRUE	reported	2aP0wZ	2	TRUE	0.0028	20.49516
8312	rs1291875	A	G	A	G	0.0163	-0.00658	0.5774	0.587881	FALSE	FALSE	FALSE	YZIMbv	0.013902	0.635984	486484	sepsis	TRUE	reported	textfile	0.0036	5.89E-06	7302	M35628.m	TRUE	reported	2aP0wZ	2	TRUE	0.0028	20.49516
3432	rs6997473	A	G	A	G	-0.0729	0.027648	0.9538	0.954241	FALSE	FALSE	FALSE	HHQf8w	0.032776	0.398921	486484	sepsis	TRUE	reported	textfile	0.0161	6.29E-06	5673	M27531.m	TRUE	reported	3vTluK	2	TRUE	0.003601	20.49511
9069	rs6779957	A	G	A	G	0.0163	-0.01136	0.6456	0.643163	FALSE	FALSE	FALSE	0l8IKx	0.014302	0.426883	486484	sepsis	TRUE	reported	textfile	0.0036	7.86E-06	7219	M38178.m	TRUE	reported	bWs4mL	2	TRUE	0.002832	20.49509
7564	rs1173242	T	C	T	C	-0.0163	-0.00293	0.7205	0.712613	FALSE	FALSE	FALSE	Ot2ZAn	0.015118	0.846351	486484	sepsis	TRUE	reported	textfile	0.0036	5.11E-06	7119	M34481.m	TRUE	reported	tpyFcK	2	TRUE	0.002871	20.49501
8215	rs9405175	A	G	A	G	-0.024	0.006583	0.178	0.191676	FALSE	FALSE	FALSE	aY1HfB	0.017541	0.70745	486484	sepsis	TRUE	reported	textfile	0.0053	5.83E-06	3784	M35433.m	TRUE	reported	H5O4i2	2	TRUE	0.00539	20.49468
3009	rs1759546	A	G	A	G	0.0163	-0.0295	0.864	0.864712	FALSE	FALSE	FALSE	gxupFu	0.01996	0.139424	486484	sepsis	TRUE	reported	textfile	0.0036	5.89E-06	6556	M21188.m	TRUE	reported	gwwXh2	2	TRUE	0.003117	20.49452
6909	rs1296969	A	T	A	T	0.447	0.03067	0.984	0.970567	FALSE	TRUE	FALSE	SvfSvH	0.040753	0.451698	486484	sepsis	TRUE	reported	textfile	0.098	5.09E-06	134	M34109.m	TRUE	reported	7OSbiu	2	TRUE	0.134394	20.49425
7742	rs2072583	A	G	A	G	0.0163	0.027558	0.185	0.243262	FALSE	FALSE	FALSE	HyMNYM	0.015911	0.083272	486484	sepsis	TRUE	reported	textfile	0.0036	6.42E-06	5885	M34912.m	TRUE	reported	Tl8Hcw	2	TRUE	0.003471	20.4938
4733	rs6850094	T	C	T	C	-0.0394	-0.00583	0.4631	0.449477	FALSE	FALSE	FALSE	fz63r4	0.013759	0.671633	486484	sepsis	TRUE	reported	textfile	0.0087	6.35E-06	2537	M32762.m	TRUE	reported	kgxlBo	2	TRUE	0.008019	20.49328
6926	rs7912030	A	G	A	G	0.821	0.095747	0.979	0.97493	FALSE	FALSE	FALSE	SvfSvH	0.044472	0.03132	486484	sepsis	TRUE	reported	textfile	0.18	5.09E-06	134	M34109.m	TRUE	reported	7OSbiu	2	TRUE	0.134388	20.49323
5810	rs6902872	T	C	T	C	0.0163	0.008398	0.6467	0.643461	FALSE	FALSE	FALSE	JE7L71	0.014307	0.557204	486484	sepsis	TRUE	reported	textfile	0.0036	4.89E-06	3890	M33364.m	TRUE	reported	FdLiEs	2	TRUE	0.005242	20.49023
7414	rs1700439	A	C	A	C	0.111	-0.00802	0.0314	0.024077	FALSE	FALSE	FALSE	7i5VUS	0.044706	0.857634	486484	sepsis	TRUE	reported	textfile	0.0245	5.73E-06	1104	M34395.m	TRUE	reported	64hEaL	2	TRUE	0.018253	20.48926
8517	rs7619989	C	G	C	G	-0.0308	0.003919	0.602	0.621309	FALSE	TRUE	FALSE	vHXktL	0.014112	0.78125	486484	sepsis	TRUE	reported	textfile	0.0068	5.82E-06	1517	M35978.m	TRUE	reported	ceap9w	2	TRUE	0.013343	20.48852
5193	rs1076600	A	G	A	G	0.1241	0.010319	0.42	0.440811	FALSE	FALSE	FALSE	q32d4E	0.013775	0.453762	486484	sepsis	TRUE	reported	textfile	0.0263	2.28E-06	25	M33178.m	TRUE	reported	cS3BwT	2	TRUE	0.471073	20.48424
2673	rs1695034	A	G	A	G	-0.0507	-0.02231	0.9243	0.924363	FALSE	FALSE	FALSE	s4UTyf	0.025943	0.389847	486484	sepsis	TRUE	reported	textfile	0.0112	6.36E-06	5376	M18494.m	TRUE	reported	ajPzhg	2	TRUE	0.003797	20.48417
5079	rs1014885	T	G	T	G	-0.0421	-0.00827	0.7171	0.695143	FALSE	FALSE	FALSE	VzfWsT	0.014993	0.5814	486484	sepsis	TRUE	reported	textfile	0.0093	6.13E-06	4817	M33144.m	TRUE	reported	ZtQP3u	2	TRUE	0.004236	20.48415
8294	rs9417900	T	C	T	C	0.0385	-0.02181	0.2502	0.25805	FALSE	FALSE	FALSE	vXjGkU	0.015816	0.167846	486484	sepsis	TRUE	reported	textfile	0.0085	6.25E-06	1291	M35527.m	TRUE	reported	oS5qaO	2	TRUE	0.015643	20.48379
4675	rs232668	A	G	A	G	-0.1354	-0.04216	0.0207	0.024462	FALSE	FALSE	FALSE	xaapK0	0.044787	0.346522	486484	sepsis	TRUE	reported	textfile	0.0299	5.97E-06	1761	M32757.m	TRUE	reported	64n1Z4	2	TRUE	0.011511	20.48337
2914	rs6128265	T	C	T	C	0.0797	0.029721	0.0155	0.0121	FALSE	FALSE	FALSE	FBDEQi	0.062773	0.635874	486484	sepsis	TRUE	reported	textfile	0.0176	6.01E-06	1748	M20699.m	TRUE	reported	nvNIlp	2	TRUE	0.011595	20.48303
3646	rs7776941	T	C	T	C	0.043	-0.00927	0.8002	0.789222	FALSE	FALSE	FALSE	Vq2efp	0.016778	0.580549	486484	sepsis	TRUE	reported	textfile	0.0095	6.33E-06	7363	M31787.m	TRUE	reported	krl4Tw	2	TRUE	0.002775	20.48197
8069	rs623248	A	G	A	G	0.0258	-0.01325	0.8882	0.885061	FALSE	FALSE	FALSE	7NkUCa	0.021462	0.536963	486484	sepsis	TRUE	reported	textfile	0.0057	7.08E-06	7359	M35320.m	TRUE	reported	GixRsK	2	TRUE	0.002776	20.48197
3719	rs1090691	A	C	A	C	0.0172	0.004659	0.7034	0.685649	FALSE	FALSE	FALSE	ZRfYkG	0.014762	0.752286	486484	sepsis	TRUE	reported	textfile	0.0038	4.81E-06	7356	M32319.m	TRUE	reported	phVUPX	2	TRUE	0.002777	20.48196
6775	rs6777718	T	G	T	G	-0.0086	0.001331	0.2783	0.278888	FALSE	FALSE	FALSE	Elf6MQ	0.015558	0.931826	486484	sepsis	TRUE	reported	textfile	0.0019	3.51E-06	7354	M33955.m	TRUE	reported	Lxn4RF	2	TRUE	0.002778	20.48196
6837	rs4288115	A	T	A	T	-0.0172	-0.01975	0.3119	0.357478	FALSE	TRUE	FALSE	qL0cJY	0.014428	0.171125	486484	sepsis	TRUE	reported	textfile	0.0038	7.20E-06	7349	M33971.m	TRUE	reported	wb1N61	2	TRUE	0.00278	20.48196
8368	rs1198896	T	C	T	C	-0.0086	-0.00889	0.7043	0.708574	FALSE	FALSE	FALSE	SCzh0A	0.015067	0.555141	486484	sepsis	TRUE	reported	textfile	0.0019	4.15E-06	7348	M35675.m	TRUE	reported	n1SREk	2	TRUE	0.00278	20.48196
8379	rs1699968	C	G	C	G	-0.0344	-0.0137	0.9821	0.976675	FALSE	TRUE	FALSE	SCzh0A	0.045219	0.761914	486484	sepsis	TRUE	reported	textfile	0.0076	6.03E-06	7348	M35675.m	TRUE	reported	n1SREk	2	TRUE	0.00278	20.48196
1848	rs1493749	A	G	A	G	0.0086	-0.03343	0.17	0.172451	FALSE	FALSE	FALSE	S1eWts	0.018831	0.075844	486484	sepsis	TRUE	reported	textfile	0.0019	3.73E-06	7345	M15488.m	TRUE	reported	511KI9	2	TRUE	0.002782	20.48196
8188	rs7812354	C	G	C	G	0.0172	0.045196	0.9508	0.935642	FALSE	TRUE	FALSE	8UhtPK	0.028016	0.106699	486484	sepsis	TRUE	reported	textfile	0.0038	6.29E-06	7345	M35397.m	TRUE	reported	rvVJba	2	TRUE	0.002782	20.48196
1397	rs7615965	T	C	T	C	0.0086	-0.00558	0.7121	0.703756	FALSE	FALSE	FALSE	KrWhws	0.014941	0.708758	486484	sepsis	TRUE	reported	textfile	0.0019	4.15E-06	7325	M02132.m	TRUE	reported	7pzpfK	2	TRUE	0.002789	20.48194
2505	rs1799977	A	G	A	G	0.0086	0.017217	0.6748	0.675771	FALSE	FALSE	FALSE	LwUYua	0.014616	0.238815	486484	sepsis	TRUE	reported	textfile	0.0019	3.91E-06	7315	M17945.m	TRUE	reported	SUN8dc	2	TRUE	0.002793	20.48193
5878	rs342170	T	C	T	C	-0.0086	0.005881	0.5871	0.600896	FALSE	FALSE	FALSE	tqW0ld	0.014347	0.681872	486484	sepsis	TRUE	reported	textfile	0.0019	4.82E-06	7315	M33389.m	TRUE	reported	Sv7EyV	2	TRUE	0.002793	20.48193
5883	rs433110	A	T	A	T	0.0086	0.005936	0.2962	0.304486	FALSE	TRUE	FALSE	tqW0ld	0.014849	0.68936	486484	sepsis	TRUE	reported	textfile	0.0019	3.52E-06	7315	M33389.m	TRUE	reported	Sv7EyV	2	TRUE	0.002793	20.48193
5893	rs709947	C	G	C	G	0.0086	0.004707	0.6413	0.649552	FALSE	TRUE	FALSE	tqW0ld	0.014541	0.746138	486484	sepsis	TRUE	reported	textfile	0.0019	3.41E-06	7315	M33389.m	TRUE	reported	Sv7EyV	2	TRUE	0.002793	20.48193
2191	rs1051564	A	G	A	G	-0.0086	0.010003	0.2493	0.25092	FALSE	FALSE	FALSE	Bf6uMK	0.015917	0.529712	486484	sepsis	TRUE	reported	textfile	0.0019	4.37E-06	7308	M15778.m	TRUE	reported	jowM2N	2	TRUE	0.002796	20.48193
2216	rs4672488	A	G	A	G	0.0086	-0.00014	0.2854	0.318654	FALSE	FALSE	FALSE	Bf6uMK	0.014741	0.992682	486484	sepsis	TRUE	reported	textfile	0.0019	4.08E-06	7308	M15778.m	TRUE	reported	jowM2N	2	TRUE	0.002796	20.48193
2218	rs4850051	A	G	A	G	-0.0086	-0.01083	0.2082	0.209455	FALSE	FALSE	FALSE	Bf6uMK	0.016962	0.522985	486484	sepsis	TRUE	reported	textfile	0.0019	3.84E-06	7308	M15778.m	TRUE	reported	jowM2N	2	TRUE	0.002796	20.48193
8028	rs1318832	T	C	T	C	-0.0086	-0.00346	0.1925	0.193549	FALSE	FALSE	FALSE	SwiqSi	0.017292	0.841194	486484	sepsis	TRUE	reported	textfile	0.0019	3.54E-06	7217	M35270.m	TRUE	reported	21ZlsQ	2	TRUE	0.002831	20.48186
8038	rs26764	C	G	C	G	0.0086	-0.04408	0.7599	0.742214	FALSE	TRUE	FALSE	SwiqSi	0.015597	0.00471	486484	sepsis	TRUE	reported	textfile	0.0019	3.71E-06	7217	M35270.m	TRUE	reported	21ZlsQ	2	TRUE	0.002831	20.48186
6271	rs402710	T	C	T	C	0.0086	0.007571	0.3378	0.345439	FALSE	FALSE	FALSE	MOonP9	0.014369	0.598272	486484	sepsis	TRUE	reported	textfile	0.0019	4.16E-06	7066	M33515.m	TRUE	reported	226GuP	2	TRUE	0.002891	20.48174

3527	rs1189338	T	C	T	C	0.0172	-0.00086	0.9023	0.90592	FALSE	FALSE	FALSE	rXzSlH	0.023427	0.970584	486484	sepsis	TRUE	reported	textfile	0.0038	6.62E-06	6795	M30805.m	TRUE	reported	7wg9QH	2	TRUE	0.003006	20.4815
8456	rs1869578	A	G	A	G	-0.0086	-0.00491	0.5791	0.571093	FALSE	FALSE	FALSE	uMajV6	0.01389	0.723936	486484	sepsis	TRUE	reported	textfile	0.0019	7.61E-06	6604	M35754.m	TRUE	reported	b8QB7S	2	TRUE	0.003093	20.48133
8480	rs939358	T	C	T	C	-0.0086	-0.02001	0.4477	0.433329	FALSE	FALSE	FALSE	uMajV6	0.013814	0.147471	486484	sepsis	TRUE	reported	textfile	0.0019	8.16E-06	6604	M35754.m	TRUE	reported	b8QB7S	2	TRUE	0.003093	20.48133
8906	rs242725	A	G	A	G	-0.0086	-0.01281	0.358	0.361342	FALSE	FALSE	FALSE	eLhsld	0.014258	0.36904	486484	sepsis	TRUE	reported	textfile	0.0019	3.51E-06	6552	M37058.m	TRUE	reported	RSeWE5	2	TRUE	0.003117	20.48128
3130	rs1339555	T	C	T	C	0.0086	-0.04919	0.4714	0.449646	FALSE	FALSE	FALSE	JiXR2t	0.013825	0.000374	486484	sepsis	TRUE	reported	textfile	0.0019	8.25E-06	6535	M22177.m	TRUE	reported	joi6p6	2	TRUE	0.003125	20.48126
3171	rs7776725	T	C	T	C	-0.0086	0.00737	0.7339	0.719829	FALSE	FALSE	FALSE	JiXR2t	0.015222	0.628236	486484	sepsis	TRUE	reported	textfile	0.0019	9.27E-06	6535	M22177.m	TRUE	reported	joi6p6	2	TRUE	0.003125	20.48126
4524	rs174892	T	G	T	G	0.0086	-0.02585	0.2779	0.280086	FALSE	FALSE	FALSE	r0qJaM	0.015234	0.089741	486484	sepsis	TRUE	reported	textfile	0.0019	6.63E-06	6497	M32729.m	TRUE	reported	vYv5FI	2	TRUE	0.003143	20.48123
4526	rs1864658	T	C	T	C	0.0086	0.042652	0.6948	0.696297	FALSE	FALSE	FALSE	r0qJaM	0.014857	0.004095	486484	sepsis	TRUE	reported	textfile	0.0019	7.67E-06	6497	M32729.m	TRUE	reported	vYv5FI	2	TRUE	0.003143	20.48123
4534	rs2934554	T	C	T	C	0.0086	0.002732	0.4337	0.452413	FALSE	FALSE	FALSE	r0qJaM	0.013789	0.842947	486484	sepsis	TRUE	reported	textfile	0.0019	4.53E-06	6497	M32729.m	TRUE	reported	vYv5FI	2	TRUE	0.003143	20.48123
4537	rs622997	T	C	T	C	-0.0086	-0.01097	0.4844	0.486738	FALSE	FALSE	FALSE	r0qJaM	0.013787	0.426223	486484	sepsis	TRUE	reported	textfile	0.0019	4.28E-06	6497	M32729.m	TRUE	reported	vYv5FI	2	TRUE	0.003143	20.48123
7718	rs1019437	A	C	A	C	-0.0086	-0.01056	0.3347	0.34032	FALSE	FALSE	FALSE	HyMNYM	0.014534	0.467293	486484	sepsis	TRUE	reported	textfile	0.0019	6.48E-06	5885	M34912.m	TRUE	reported	Tl8Hcw	2	TRUE	0.003469	20.48057
7730	rs1302363	T	G	T	G	0.0086	0.008455	0.4468	0.446416	FALSE	FALSE	FALSE	HyMNYM	0.013791	0.539819	486484	sepsis	TRUE	reported	textfile	0.0019	6.95E-06	5885	M34912.m	TRUE	reported	Tl8Hcw	2	TRUE	0.003469	20.48057
7741	rs202093	T	C	T	C	0.0086	-0.00343	0.3918	0.390831	FALSE	FALSE	FALSE	HyMNYM	0.014033	0.806718	486484	sepsis	TRUE	reported	textfile	0.0019	4.99E-06	5885	M34912.m	TRUE	reported	Tl8Hcw	2	TRUE	0.003469	20.48057
7745	rs4244568	T	G	T	G	-0.0086	0.037161	0.6271	0.611932	FALSE	FALSE	FALSE	HyMNYM	0.014212	0.008929	486484	sepsis	TRUE	reported	textfile	0.0019	5.41E-06	5885	M34912.m	TRUE	reported	Tl8Hcw	2	TRUE	0.003469	20.48057
6604	rs1226319	A	G	A	G	-0.0584	-0.01336	0.7306	0.721089	FALSE	FALSE	FALSE	VfOuo8	0.015247	0.380933	486484	sepsis	TRUE	reported	textfile	0.0129	6.34E-06	2725	M33883.m	TRUE	reported	BDe0Nk	2	TRUE	0.007465	20.47988
7837	rs4142154	A	G	A	G	0.0086	0.005953	0.3524	0.335959	FALSE	FALSE	FALSE	h5whIS	0.014475	0.680887	486484	sepsis	TRUE	reported	textfile	0.0019	7.38E-06	4900	M35137.m	TRUE	reported	xVITKF	2	TRUE	0.004164	20.47917
7847	rs6564173	A	G	A	G	0.0086	-0.00139	0.3642	0.35354	FALSE	FALSE	FALSE	h5whIS	0.014392	0.923199	486484	sepsis	TRUE	reported	textfile	0.0019	5.40E-06	4900	M35137.m	TRUE	reported	xVITKF	2	TRUE	0.004164	20.47917
6413	rs6442358	C	G	C	G	-0.0258	0.01227	0.3656	0.339744	FALSE	TRUE	FALSE	5CIPW7	0.014471	0.396492	486484	sepsis	TRUE	reported	textfile	0.0057	5.04E-06	4807	M33653.m	TRUE	reported	H4b2zx	2	TRUE	0.004244	20.47901
6054	rs8013127	A	G	A	G	-1.5304	-0.04327	0.0184	0.009253	FALSE	FALSE	FALSE	33ojbt	0.072827	0.552439	486484	sepsis	TRUE	reported	textfile	0.3325	4.16E-06	60	M33423.m	TRUE	reported	ivEtvl	2	TRUE	0.260946	20.47875
6717	rs1195382	T	C	T	C	0.0326	0.025852	0.3065	0.298629	FALSE	FALSE	FALSE	B6lZly	0.015012	0.085061	486484	sepsis	TRUE	reported	textfile	0.0072	6.54E-06	1759	M33937.m	TRUE	reported	qFPzjh	2	TRUE	0.011521	20.47746
5526	rs7015641	A	G	A	G	-0.3444	-0.05566	0.0227	0.01985	FALSE	FALSE	FALSE	q32d4E	0.049556	0.261345	486484	sepsis	TRUE	reported	textfile	0.073	2.38E-06	25	M33178.m	TRUE	reported	cS3BwT	2	TRUE	0.470986	20.4771
1817	rs1081165	A	G	A	G	-0.0611	0.005817	0.2599	0.296468	FALSE	FALSE	FALSE	Yub5KV	0.015111	0.700293	486484	sepsis	TRUE	reported	textfile	0.0135	6.22E-06	5606	M15335.m	TRUE	reported	mcnINa	2	TRUE	0.003641	20.4767
6727	rs7933681	A	G	A	G	0.0267	0.009519	0.094	0.099971	FALSE	FALSE	FALSE	B6lZly	0.022856	0.677063	486484	sepsis	TRUE	reported	textfile	0.0059	5.57E-06	7222	M33937.m	TRUE	reported	qFPzjh	2	TRUE	0.002828	20.47379
7264	rs1259233	T	C	T	C	-0.1292	0.021103	0.8495	0.843525	FALSE	FALSE	FALSE	P5Xm4S	0.018806	0.261787	486484	sepsis	TRUE	reported	textfile	0.0285	5.86E-06	525	M34350.m	TRUE	reported	YU4x0J	2	TRUE	0.03767	20.47282
5911	rs1176912	A	T	A	T	-0.1068	-0.00414	0.9758	0.956626	FALSE	TRUE	FALSE	iGV9aX	0.033497	0.901638	486484	sepsis	TRUE	reported	textfile	0.0236	5.83E-06	5185	M33390.m	TRUE	reported	sLbpnf	2	TRUE	0.003934	20.47156
6898	rs4627421	T	C	T	C	0.0267	0.011143	0.2337	0.253632	FALSE	FALSE	FALSE	MN2lbE	0.0157	0.477839	486484	sepsis	TRUE	reported	textfile	0.0059	7.19E-06	4961	M34106.m	TRUE	reported	q4zXEz	2	TRUE	0.004111	20.4712
8626	rs7812350	T	C	T	C	0.0344	0.04081	0.7854	0.770665	FALSE	FALSE	FALSE	MZ60v7	0.016277	0.012166	486484	sepsis	TRUE	reported	textfile	0.0076	6.29E-06	2432	M36134.m	TRUE	reported	dMQewN	2	TRUE	0.008354	20.47069
1828	rs1102058	A	G	A	G	-0.0362	-0.06368	0.9798	0.9792	FALSE	FALSE	FALSE	2TaDHm	0.048103	0.185534	486484	sepsis	TRUE	reported	textfile	0.008	7.02E-06	7337	M15365.m	TRUE	reported	LoLveg	2	TRUE	0.002783	20.47004
6658	rs3783687	A	C	A	C	-0.212	-0.13572	0.9851	0.988736	FALSE	FALSE	FALSE	REeQiq	0.064888	0.036475	486484	sepsis	TRUE	reported	textfile	0.0468	5.94E-06	819	M33892.m	TRUE	reported	cVOa3u	2	TRUE	0.024443	20.47002
7157	rs2925759	T	C	T	C	-0.067	-0.00989	0.1897	0.215795	FALSE	FALSE	FALSE	PnBT0M	0.016831	0.556915	486484	sepsis	TRUE	reported	textfile	0.0148	6.28E-06	1612	M34327.m	TRUE	reported	VUt4Q7	2	TRUE	0.012554	20.46855
4827	rs1099429	T	C	T	C	0.0738	-0.02611	0.0651	0.06304	FALSE	FALSE	FALSE	yLDcUj	0.028635	0.361937	486484	sepsis	TRUE	reported	textfile	0.0163	5.80E-06	1307	M32838.m	TRUE	reported	5dQgAN	2	TRUE	0.015442	20.46786
2282	rs1861007	C	G	C	G	-0.0181	-0.00644	0.1925	0.187499	FALSE	TRUE	FALSE	mRULgB	0.017569	0.713986	486484	sepsis	TRUE	reported	textfile	0.004	7.81E-06	4689	M16816.m	TRUE	reported	05lFsX	2	TRUE	0.004348	20.46689
6520	rs1708577	A	G	A	G	-0.0276	0.009932	0.0693	0.071052	FALSE	FALSE	FALSE	OKlXJ8	0.026511	0.707928	486484	sepsis	TRUE	reported	textfile	0.0061	6.88E-06	7362	M33821.m	TRUE	reported	XGkaXH	2	TRUE	0.002773	20.46635
4428	rs1162679	A	C	A	C	0.0276	0.030181	0.0117	0.017171	FALSE	FALSE	FALSE	WGPdpq	0.053376	0.571779	486484	sepsis	TRUE	reported	textfile	0.0061	6.35E-06	7338	M32675.m	TRUE	reported	u0eUAG	2	TRUE	0.002782	20.46634
4802	rs6540767	A	T	A	T	-0.0276	-0.01824	0.6977	0.681744	FALSE	TRUE	FALSE	9qWhFj	0.01465	0.213004	486484	sepsis	TRUE	reported	textfile	0.0061	5.05E-06	6200	M32808.m	TRUE	reported	MQcOSi	2	TRUE	0.003291	20.46531
5678	rs7916966	A	T	A	T	-0.3985	-0.04886	0.9755	0.985232	FALSE	TRUE	FALSE	AAOSvG	0.0578	0.397886	486484	sepsis	TRUE	reported	textfile	0.088	5.92E-06	996	M33197.m	TRUE	reported	aAmcal	2	TRUE	0.020173	20.46531
4200	rs751903	A	G	A	G	0.0647	-0.03676	0.0733	0.062565	FALSE	FALSE	FALSE	TxOCzG	0.028413	0.195801	486484	sepsis	TRUE	reported	textfile	0.0143	6.22E-06	6970	M32564.m	TRUE	reported	9JP6ND	2	TRUE	0.002928	20.465
3022	rs8094941	A	C	A	C	-0.0371	0.012004	0.9697	0.967711	FALSE	FALSE	FALSE	gxupFu	0.038674	0.756262	486484	sepsis	TRUE	reported	textfile	0.0082	5.42E-06	6556	M21188.m	TRUE	reported	gwwXh2	2	TRUE	0.003113	20.46386
2669	rs9301881	T	C	T	C	0.0593	0.013812	0.5692	0.577923	FALSE	FALSE	FALSE	cDEa3G	0.013867	0.319225	486484	sepsis	TRUE	reported	textfile	0.0131	5.79E-06	1457	M18477.m	TRUE	reported	kxZEfC	2	TRUE	0.013869	20.46304
5704	rs7953025	A	G	A	G	0.0172	-0.01047	0.5419	0.557444	FALSE	FALSE	FALSE	1SbQTe	0.013792	0.447946	486484	sepsis	TRUE	reported	textfile	0.0038	7.61E-06	1673	M33204.m	TRUE	reported	x7MBDH	2	TRUE	0.012098	20.46304
4785	rs7299188	C	G	C	G	-0.0371	0.033068	0.6604	0.625282	FALSE	TRUE	FALSE	MmZZoM	0.014221	0.020053	486484	sepsis	TRUE	reported	textfile	0.0082	6.53E-06	4274	M32800.m	TRUE	reported	70YSCO	2	TRUE	0.004767	20.46053
906	rs6497209	A	G	A	G	0.019	0.00611	0.2139	0.214945	FALSE	FALSE	FALSE	l6zQnf	0.016658	0.713766	486484	sepsis	TRUE	reported	textfile	0.0042	4.78E-06	7345	M01444.m	TRUE	reported	llzalm	2	TRUE	0.002778	20.45928
8931	rs4792110	T	G	T	G	0.114	0.025035	0.9378	0.915881	FALSE	FALSE	FALSE	ns6PV1	0.024784	0.312443	486484	sepsis	TRUE	reported	textfile	0.0252	6.31E-06	7014	M37097.m	TRUE	reported	ZYmYt4	2	TRUE	0.002909	20.45902
5649	rs4894432	A	G	A	G	-0.0475	0.033842	0.7331	0.745741	FALSE	FALSE	FALSE	BFsMtm	0.015721	0.031352	486484	sepsis	TRUE	reported	textfile	0.0105	6.85E-06	5716	M33192.m	TRUE	reported	NAJN3D	2	TRUE	0.003568	20.45769
6791	rs7727774	A	G	A	G	0.0267	0.019429	0.4534	0.48794	FALSE	FALSE	FALSE	kcZoFT	0.013713	0.156544	486484	sepsis	TRUE	reported	textfile	0.0059	6.38E-06	1730	M33957.m	TRUE	reported	aIW1w0	2	TRUE	0.011699	20.45578
5847	rs1124204	A	G	A	G	0.4771	0.034674	0.0216	0.012142	FALSE	FALSE	FALSE	Vkec6Y	0.062794	0.580815	486484	sepsis	TRUE	reported	textfile	0.1051	5.60E-06	272	M33384.m	TRUE	reported	eaxeyR	2	TRUE	0.070425	20.45541
4933	rs7828843	T	C	T	C	-0.1059	-0.02448	0.0202	0.012445	FALSE	FALSE	FALSE	clhN6v	0.062939	0.697371	486484	sepsis	TRUE	reported	textfile	0.0234	6.17E-06	1550	M32869.m	TRUE	reported	BzloWk	2	TRUE	0.013041	20.455
7227	rs6511851	C	G	C	G	-0.584	-0.04509	0.986	0.951907	FALSE	TRUE	FALSE	i1uJ7T	0.036789	0.220302	486484	sepsis	TRUE	reported	textfile	0.128	5.06E-06	115	M34338.m	TRUE	reported	pi3mKW	2	TRUE	0.153269	20.45438
6701	rs8085038	A	C	A	C	-0.0991	-0.03563	0.1003	0.090265	FALSE	FALSE	FALSE	AcRp2F	0.023959	0.136935	486484	sepsis	TRUE	reported	textfile	0.0219	6.32E-06	1738	M33935.m	TRUE	reported	FQCKJy	2	TRUE	0.011645	20.45309
6561	rs924931	T	C	T	C	-0.0353	0.028183	0.5401	0.54546	FALSE	FALSE	FALSE	58qeH5	0.013771	0.040697	486484	sepsis	TRUE	reported	textfile	0.0078	6.14E-06	1428	M33864.m	TRUE	reported	TBsy8r	2	TRUE	0.01414	20.45274
2460	rs9497904	A	G	A	G	-0.0543	-0.00955	0.4345	0.378868	FALSE	FALSE	FALSE	NBJ2Do	0.014119	0.498726	486484	sepsis	TRUE	reported	textfile	0.012	6.36E-06	1739	M17799.m	TRUE	reported	Uz0Wgl	2	TRUE	0.011637	20.45208
4562	rs7151027	A	T	A	T	-0.0294	0.023009	0.3207	0.323877	FALSE	TRUE	FALSE	KfoRxi	0.014603	0.115111	486484	sepsis	TRUE	reported	textfile	0.0065	6.92E-06	6000	M32735.m	TRUE	reported	UDRXAp	2	TRUE	0.003398	20.45141
2988	rs1162754	A	C	A	C	0.0294	-0.00387	0.1703	0.172767	FALSE	FALSE	FALSE	xmzJ5F	0.018081	0.830569	486484	sepsis	TRUE	reported	textfile	0.0065	6.87E-06	5436	M21184.m	TRUE	reported	xgwhDp	2	TRUE	0.003749	20.4507
3520	rs7132285	T	C	T	C	0.1371	0.05577	0.0108	0.006766	FALSE	FALSE	FALSE	hxuqls	0.084239	0.50794	486484	sepsis	TRUE	reported	textfile	0.0303	6.12E-06	1758	M27738.m	TRUE	reported	aueeZs	2	TRUE	0.011512	20.45009
4422	rs2551453	T	C	T	C	-0.0199	0.012848	0.2649	0.266499	FALSE	FALSE	FALSE	NzBiVA	0.01545	0.405648	486484	sepsis	TRUE	reported	textfile	0.0044	7.28E-06	7354	M32672.m	TRUE	reported	YLIhHJ	2	TRUE	0.002774	20.4495
7232	rs9826397	A	G	A	G	-0.2895	-0.02174	0.9782	0.969106	FALSE	FALSE	FALSE	i1uJ7T	0.040362	0.590196	486484	sepsis	TRUE	reported	textfile	0.0639	5.80E-06	535	M34338.m	TRUE	reported	pi3mKW	2	TRUE	0.036948	20.44885
4730	rs532	A	G	A	G	-0.0475	-6.28E-05	0.771	0.780813	FALSE	FALSE	FALSE	fz63r4	0.016522	0.996969	486484	sepsis	TRUE	reported	textfile	0.0105	6.06E-06	2537	M32762.m	TRUE	reported	kgxlBo	2	TRUE	0.008002	20.44872
8752	rs9399651	C	G	C	G	0.0389	0.001273	0.8405	0.816749	FALSE	TRUE	FALSE	FnYrPc	0.017746	0.942791	486484	sepsis	TRUE	reported	textfile	0.0086	5.64E-06	3346	M36585.m	TRUE	reported	amHtMH	2	TRUE	0.006078	20.44761
2364	rs1161243	A	G	A	G	-0.1389	0.111531	0.0367	0.030911	FALSE	FALSE	FALSE	NBJ2Do	0.039953	0.005245	486484	sepsis	TRUE	reported	textfile	0.0307	5.91E-06	1739	M17799.m	TRUE	reported	Uz0Wgl	2	TRUE	0.011634	20.44692
2786	rs818525	T	C	T	C	0.0199	0.027932	0.1436	0.157424	FALSE	FALSE	FALSE	yYuamw	0.018768	0.136675	486484	sepsis	TRUE	reported	textfile	0.0044	6.46E-06	4844	M19414.m	TRUE	reported	GxSb5G	2	TRUE	0.004205	20.44662
5077	rs798007	T	C	T	C	-0.0407	0.018203	0.9676	0.968167	FALSE	FALSE	FALSE	gqzGN4	0.039315	0.643357	486484	sepsis	TRUE	reported	textfile	0.009	6.32E-06	7319	M33140.m	TRUE	reported	DKGBe9	2	TRUE	0.002786	20.44491
8774	rs1878640	T	C	T	C	0.0303	-0.00812	0.911	0.915515	FALSE	FALSE	FALSE	7desNF	0.024556	0.740904	486484	sepsis	TRUE	reported	textfile	0.0067	5.41E-06	5292	M36673.m	TRUE	reported	RKbIT4	2	TRUE	0.00385	20.44426
4674	rs1719767	A	G	A	G	0.0751	0.010211	0.928	0.933453	FALSE	FALSE	FALSE	xaapK0	0.030635	0.738907	486484	sepsis	TRUE	reported	textfile	0.0166	6.41E-06	1761	M32757.m	TRUE	reported	64n1Z4	2	TRUE	0.011489	20.4442
5341	rs1709153	A	G	A	G	-0.3441	-0.04785	0.0227	0.067749	FALSE	FALSE	FALSE	q32d4E	0.027401	0.080751	486484	sepsis	TRUE	reported	textfile	0.073	2.46E-06	25	M33178.m	TRUE	reported	cS3BwT	2	TRUE	0.470552	20.44144
5554	rs7675814	A	C	A	C	0.3441	0.004758	0.9773	0.966419	FALSE	FALSE	FALSE	q32d4E	0.038159	0.900765	486484	sepsis	TRUE	reported	textfile	0.073	2.46E-06	25	M33178.m	TRUE	reported	cS3BwT	2	TRUE	0.470552	20.44144
5579	rs8192827	A	G	A	G	0.3441	0.047687	0.9773	0.968631	FALSE	FALSE	FALSE	q32d4E	0.039891	0.231917	486484	sepsis	TRUE	reported	textfile	0.073	2.46E-06	25	M33178.m	TRUE	reported	cS3BwT	2	TRUE	0.470552	20.44144
3700	rs197442	A	G	A	G	0.0104	0.010451	0.5962	0.600546	FALSE	FALSE	FALSE	agPgtF	0.013978	0.454668	486484	sepsis	TRUE	reported	textfile	0.0023	7.60E-06	7349	M32315.m	TRUE	reported	hMRjcV	2	TRUE	0.002774	20.44056
3703	rs342563	A	G	A	G	-0.0104	-0.01113	0.471	0.497427	FALSE	FALSE	FALSE	agPgtF	0.013718	0.417099	486484	sepsis	TRUE	reported	textfile	0.0023	7.86E-06	7349	M32315.m	TRUE	reported	hMRjcV	2	TRUE	0.002774	20.44056
2644	rs1185449	A	G	A	G	0.0869	0.028796	0.0972	0.101317	FALSE	FALSE	FALSE	9GoQdh	0.022811	0.206813	486484	sepsis	TRUE	reported	textfile	0.0192	6.16E-06	917	M18474.m	TRUE	reported	p6Dq8p	2	TRUE	0.021851	20.44037
8197	rs1714525	A	G	A	G	0.0312	0.002066	0.0292	0.030202	FALSE	FALSE	FALSE	iLlpho	0.040234	0.959047	486484	sepsis	TRUE	reported	textfile	0.0069	6.58E-06	7007	M35431.m	TRUE	reported	AAZ5ge	2	TRUE	0.002909	20.44029
3428	rs2708754	A	G	A	G	0.0312	0.021503	0.3968	0.39153	FALSE	FALSE	FALSE	HHQf8w	0.014065	0.126304	486484	sepsis	TRUE	reported	textfile	0.0069	6.52E-06	5673	M27531.m	TRUE	reported	3vTluK	2	TRUE	0.003591	20.43892
3562	rs1339805	A	G	A	G	0.038	0.002569	0.6148	0.610662	FALSE	FALSE	FALSE	lPkHXk	0.014101	0.855449	486484	sepsis	TRUE	reported	textfile	0.0084	5.76E-06	1558	M31522.m	TRUE	reported	jdWe3V	2	TRUE	0.012965	20.43858
597	rs1499094	T	G	T	G	0.0208	-0.01956	0.7686	0.77872	FALSE	FALSE	FALSE	KVK33e	0.016613	0.239148	486484	sepsis	TRUE	reported	textfile	0.0046	7.68E-06	5344	M01107.m	TRUE	reported	eAyR8w	2	TRUE	0.003811	20.43847
6382	rs262749	A	C	A	C	0.0208	0.002388	0.6982	0.699032	FALSE	FALSE	FALSE	pPr1v0	0.015012	0.873614	486484	sepsis	TRUE	reported	textfile	0.0046	5.33E-06	5080	M33637.m	TRUE	reported	Ide1A7	2	TRUE	0.004009	20.43808
6519	rs1701328	A	G	A	G	-0.0493	0.016966	0.0898	0.076409	FALSE	FALSE	FALSE	OKlXJ8	0.025974	0.513633	486484	sepsis	TRUE	reported	textfile	0.0109	5.90E-06	1764	M33821.m	TRUE	reported	XGkaXH	2	TRUE	0.011464	20.43375
8291	rs1551864	T	C	T	C	-0.038	0.011443	0.3052	0.329624	FALSE	FALSE	FALSE	vXjGkU	0.014573	0.432329	486484	sepsis	TRUE	reported	textfile	0.0084	5.90E-06	1291	M35527.m	TRUE	reported	oS5qaO	2	TRUE	0.015605	20.43315
4191	rs2051674	T	C	T	C	0.0434	0.011956	0.8325	0.820467	FALSE	FALSE	FALSE	TxOCzG	0.017889	0.50392	486484	sepsis	TRUE	reported	textfile	0.0096	5.89E-06	6970	M32564.m	TRUE	reported	9JP6ND	2	TRUE	0.002924	20.43207
4697	rs1074416	T	C	T	C	-0.0217	0.007165	0.3489	0.353722	FALSE	FALSE	FALSE	WlwKaF	0.014425	0.61937	486484	sepsis	TRUE	reported	textfile	0.0048	8.00E-06	6712	M32759.m	TRUE	reported	Ub4yxH	2	TRUE	0.003036	20.43184
1824	rs4917405	T	C	T	C	0.0972	-0.01493	0.0437	0.042927	FALSE	FALSE	FALSE	Yub5KV	0.033829	0.659024	486484	sepsis	TRUE	reported	textfile	0.0215	6.28E-06	5606	M15335.m	TRUE	reported	mcnINa	2	TRUE	0.003633	20.43152
7382	rs1763075	A	G	A	G	0.1768	0.042867	0.0279	0.030078	FALSE	FALSE	FALSE	iLnkG0	0.040023	0.284152	486484	sepsis	TRUE	reported	textfile	0.0391	5.99E-06	2779	M34384.m	TRUE	reported	JVsZvV	2	TRUE	0.007304	20.43141
4243	rs7942018	T	C	T	C	-0.0217	0.02473	0.4068	0.399143	FALSE	FALSE	FALSE	wWEMr1	0.01411	0.079647	486484	sepsis	TRUE	reported	textfile	0.0048	5.96E-06	5955	M32593.m	TRUE	reported	Mh2g4i	2	TRUE	0.00342	20.43107
8560	rs882014	A	G	A	G	-1.0082	-0.08183	0.0175	0.016057	FALSE	FALSE	FALSE	aQhCav	0.054178	0.130957	486484	sepsis	TRUE	reported	textfile	0.2226	5.94E-06	488	M36097.m	TRUE	reported	9DJS9j	2	TRUE	0.04034	20.42958
2954	rs1023229	A	G	A	G	0.0321	0.006719	0.3415	0.352556	FALSE	FALSE	FALSE	zndu0r	0.014374	0.640162	486484	sepsis	TRUE	reported	textfile	0.0071	6.03E-06	3422	M21049.m	TRUE	reported	2XpPDU	2	TRUE	0.005938	20.42864
6903	rs1204738	T	C	T	C	0.298	-0.00046	0.9679	0.931264	FALSE	FALSE	FALSE	SvfSvH	0.027488	0.98675	486484	sepsis	TRUE	reported	textfile	0.0657	5.80E-06	282	M34109.m	TRUE	reported	7OSbiu	2	TRUE	0.067994	20.42729
1149	rs2720445	T	C	T	C	0.033	-0.00273	0.536	0.542073	FALSE	FALSE	FALSE	QtY5Hc	0.013859	0.844079	486484	sepsis	TRUE	reported	textfile	0.0073	5.56E-06	4207	M01605.m	TRUE	reported	Q3iDQX	2	TRUE	0.004834	20.42564
1572	rs8128639	A	C	A	C	-0.0113	-0.02194	0.1698	0.230187	FALSE	FALSE	FALSE	Ke0sNh	0.016353	0.179695	486484	sepsis	TRUE	reported	textfile	0.0025	6.51E-06	7799	M10642.m	TRUE	reported	ZINwVb	2	TRUE	0.002613	20.42516
3849	rs7286668	A	C	A	C	-0.0113	-0.0118	0.827	0.834388	FALSE	FALSE	FALSE	tRX2u0	0.018395	0.52133	486484	sepsis	TRUE	reported	textfile	0.0025	7.86E-06	7365	M32348.m	TRUE	reported	ruBgwQ	2	TRUE	0.002766	20.42485
716	rs7834337	A	G	A	G	-0.0113	0.027133	0.0904	0.090286	FALSE	FALSE	FALSE	H9l3s3	0.023893	0.256104	486484	sepsis	TRUE	reported	textfile	0.0025	4.71E-06	7358	M01299.m	TRUE	reported	kXWyUq	2	TRUE	0.002769	20.42485
3759	rs491847	A	G	A	G	-0.0113	-0.02123	0.3457	0.353324	FALSE	FALSE	FALSE	OZLdls	0.014359	0.139222	486484	sepsis	TRUE	reported	textfile	0.0025	5.33E-06	7356	M32338.m	TRUE	reported	s2itmB	2	TRUE	0.00277	20.42485
823	rs1426441	A	G	A	G	0.0113	-0.00162	0.1423	0.163501	FALSE	FALSE	FALSE	2qgDgB	0.018624	0.930485	486484	sepsis	TRUE	reported	textfile	0.0025	4.87E-06	7355	M01358.m	TRUE	reported	asmXq0	2	TRUE	0.00277	20.42484
4036	rs1258857	A	G	A	G	0.0113	-0.05229	0.2168	0.224903	FALSE	FALSE	FALSE	SiYGYU	0.016387	0.001418	486484	sepsis	TRUE	reported	textfile	0.0025	6.12E-06	7355	M32492.m	TRUE	reported	GfQvpU	2	TRUE	0.00277	20.42484
585	rs6075489	A	G	A	G	0.0113	0.015433	0.7177	0.705583	FALSE	FALSE	FALSE	ZoqU2j	0.01511	0.307091	486484	sepsis	TRUE	reported	textfile	0.0025	9.51E-06	7333	M01105.m	TRUE	reported	2WxSOH	2	TRUE	0.002778	20.42483
1401	rs9354336	T	C	T	C	0.0113	-0.02469	0.8733	0.878191	FALSE	FALSE	FALSE	KrWhws	0.020984	0.239379	486484	sepsis	TRUE	reported	textfile	0.0025	8.57E-06	7325	M02132.m	TRUE	reported	7pzpfK	2	TRUE	0.002781	20.42482
3876	rs3780970	T	C	T	C	0.0113	-0.00754	0.2697	0.266793	FALSE	FALSE	FALSE	bwkKtK	0.015476	0.626297	486484	sepsis	TRUE	reported	textfile	0.0025	3.76E-06	7307	M32393.m	TRUE	reported	RvZzAy	2	TRUE	0.002788	20.42481
3041	rs7512200	A	G	A	G	0.0113	-0.00033	0.39	0.413034	FALSE	FALSE	FALSE	FJZEYz	0.013951	0.980862	486484	sepsis	TRUE	reported	textfile	0.0025	9.78E-06	7284	M21630.m	TRUE	reported	s6Pshg	2	TRUE	0.002797	20.42479
3353	rs1164238	A	G	A	G	0.0113	0.012109	0.753	0.7458	FALSE	FALSE	FALSE	LY4Gus	0.015837	0.444523	486484	sepsis	TRUE	reported	textfile	0.0025	7.79E-06	7268	M27273.m	TRUE	reported	QKlTBx	2	TRUE	0.002803	20.42478
7880	rs4703415	T	C	T	C	-0.0226	0.005216	0.1469	0.145355	FALSE	FALSE	FALSE	mpkL0o	0.019393	0.787965	486484	sepsis	TRUE	reported	textfile	0.005	6.55E-06	7263	M35160.m	TRUE	reported	MKkvYr	2	TRUE	0.002805	20.42477
3267	rs4811661	T	C	T	C	-0.0113	0.024425	0.3137	0.32793	FALSE	FALSE	FALSE	ZNPBqT	0.0146	0.094334	486484	sepsis	TRUE	reported	textfile	0.0025	6.59E-06	7256	M24074.m	TRUE	reported	8pPtOl	2	TRUE	0.002808	20.42477
2721	rs1259684	A	G	A	G	-0.0113	0.023593	0.1846	0.200242	FALSE	FALSE	FALSE	H4yqSK	0.017647	0.181233	486484	sepsis	TRUE	reported	textfile	0.0025	3.93E-06	7182	M19362.m	TRUE	reported	w2PDEO	2	TRUE	0.002837	20.42471
2731	rs3844066	T	C	T	C	0.0113	0.012614	0.8135	0.811193	FALSE	FALSE	FALSE	H4yqSK	0.017477	0.470452	486484	sepsis	TRUE	reported	textfile	0.0025	5.67E-06	7182	M19362.m	TRUE	reported	w2PDEO	2	TRUE	0.002837	20.42471
4806	rs1007599	A	G	A	G	-0.0113	0.024133	0.578	0.583832	FALSE	FALSE	FALSE	bJwLV5	0.013914	0.08283	486484	sepsis	TRUE	reported	textfile	0.0025	8.73E-06	7063	M32814.m	TRUE	reported	qte0qp	2	TRUE	0.002884	20.42461
6468	rs1156345	T	C	T	C	-0.0914	-0.02435	0.8849	0.873028	FALSE	FALSE	FALSE	ecwVg8	0.020614	0.237465	486484	sepsis	TRUE	reported	textfile	0.0202	5.78E-06	831	M33751.m	TRUE	reported	NnvblQ	2	TRUE	0.024045	20.42411
7560	rs4630827	T	C	T	C	0.0226	0.011117	0.201	0.216091	FALSE	FALSE	FALSE	APSWAU	0.0166	0.503064	486484	sepsis	TRUE	reported	textfile	0.005	7.34E-06	6144	M34469.m	TRUE	reported	frF3RX	2	TRUE	0.003314	20.42375
8208	rs1118028	A	G	A	G	0.123	-0.06343	0.9821	0.984936	FALSE	FALSE	FALSE	aY1HfB	0.05608	0.258031	486484	sepsis	TRUE	reported	textfile	0.0272	6.01E-06	1522	M35433.m	TRUE	reported	H5O4i2	2	TRUE	0.013257	20.42214
4841	rs1690279	C	G	C	G	0.0922	0.013225	0.0266	0.033451	FALSE	TRUE	FALSE	jvlpBt	0.038038	0.728075	486484	sepsis	TRUE	reported	textfile	0.0204	6.42E-06	6250	M32846.m	TRUE	reported	RAXcz5	2	TRUE	0.003258	20.42032
6172	rs4374940	T	C	T	C	0.1321	0.028604	0.0286	0.032598	FALSE	FALSE	FALSE	O4Ty2I	0.038966	0.462913	486484	sepsis	TRUE	reported	textfile	0.0292	5.89E-06	834	M33488.m	TRUE	reported	RGtF84	2	TRUE	0.023952	20.41725
2773	rs7359707	A	T	A	T	0.0235	0.007578	0.1937	0.224666	FALSE	TRUE	FALSE	JWylTj	0.016512	0.646261	486484	sepsis	TRUE	reported	textfile	0.0052	5.42E-06	6385	M19396.m	TRUE	reported	6S7kzt	2	TRUE	0.003188	20.41705
6844	rs6578042	T	C	T	C	0.047	0.008704	0.857	0.85016	FALSE	FALSE	FALSE	aWy2Uj	0.019206	0.650395	486484	sepsis	TRUE	reported	textfile	0.0104	5.93E-06	5561	M33973.m	TRUE	reported	SUSE3b	2	TRUE	0.003659	20.4161
7961	rs1745299	T	G	T	G	0.1406	0.019931	0.0125	0.02415	FALSE	FALSE	FALSE	zb8m4a	0.044589	0.654874	486484	sepsis	TRUE	reported	textfile	0.0311	6.09E-06	1761	M35253.m	TRUE	reported	tHnnnS	2	TRUE	0.011473	20.41533
6568	rs6034091	A	G	A	G	0.1302	0.04412	0.0124	0.012802	FALSE	FALSE	FALSE	aTDqRQ	0.061942	0.476289	486484	sepsis	TRUE	reported	textfile	0.0288	6.14E-06	1639	M33871.m	TRUE	reported	Lqimdw	2	TRUE	0.012316	20.41299
621	rs6535974	A	G	A	G	-0.0122	0.01444	0.8465	0.840693	FALSE	FALSE	FALSE	BYGzq4	0.018735	0.440853	486484	sepsis	TRUE	reported	textfile	0.0027	5.80E-06	7367	M01110.m	TRUE	reported	oZiXqP	2	TRUE	0.002764	20.41147
4495	rs269066	A	G	A	G	-0.0122	-0.02638	0.2794	0.279312	FALSE	FALSE	FALSE	1wNJ1E	0.015254	0.083709	486484	sepsis	TRUE	reported	textfile	0.0027	5.28E-06	7363	M32709.m	TRUE	reported	zihBHg	2	TRUE	0.002765	20.41146
6875	rs2301783	A	C	A	C	0.0122	0.037257	0.9151	0.882154	FALSE	FALSE	FALSE	kFWZjs	0.021475	0.082752	486484	sepsis	TRUE	reported	textfile	0.0027	4.90E-06	7362	M34062.m	TRUE	reported	eLONsg	2	TRUE	0.002766	20.41146
7462	rs8007846	A	G	A	G	0.0122	-0.00581	0.5509	0.546473	FALSE	FALSE	FALSE	7ubZow	0.0137	0.671463	486484	sepsis	TRUE	reported	textfile	0.0027	8.39E-06	7344	M34407.m	TRUE	reported	LRsXN2	2	TRUE	0.002772	20.41145
7463	rs891404	A	G	A	G	0.0122	0.012744	0.4842	0.488158	FALSE	FALSE	FALSE	7ubZow	0.013703	0.352351	486484	sepsis	TRUE	reported	textfile	0.0027	7.49E-06	7344	M34407.m	TRUE	reported	LRsXN2	2	TRUE	0.002772	20.41145
1832	rs1813982	T	C	T	C	-0.0122	-0.00059	0.2209	0.236849	FALSE	FALSE	FALSE	2TaDHm	0.016124	0.970824	486484	sepsis	TRUE	reported	textfile	0.0027	6.59E-06	7337	M15365.m	TRUE	reported	LoLveg	2	TRUE	0.002775	20.41144
4273	rs2979269	A	G	A	G	-0.0122	-0.02429	0.4706	0.473254	FALSE	FALSE	FALSE	ylV4bp	0.013686	0.075975	486484	sepsis	TRUE	reported	textfile	0.0027	4.59E-06	7337	M32632.m	TRUE	reported	hqHJoU	2	TRUE	0.002775	20.41144
2229	rs9514739	C	G	C	G	-0.0122	-0.00099	0.8962	0.882705	FALSE	TRUE	FALSE	Bf6uMK	0.021362	0.963012	486484	sepsis	TRUE	reported	textfile	0.0027	6.10E-06	7308	M15778.m	TRUE	reported	jowM2N	2	TRUE	0.002786	20.41142
2890	rs5027944	T	C	T	C	0.0122	0.008489	0.5795	0.585307	FALSE	FALSE	FALSE	mUzn1R	0.013864	0.540363	486484	sepsis	TRUE	reported	textfile	0.0027	3.93E-06	7301	M20675.m	TRUE	reported	XwY04K	2	TRUE	0.002789	20.41142
2891	rs5757568	A	G	A	G	0.0122	0.002616	0.388	0.370645	FALSE	FALSE	FALSE	mUzn1R	0.014145	0.853249	486484	sepsis	TRUE	reported	textfile	0.0027	5.10E-06	7301	M20675.m	TRUE	reported	XwY04K	2	TRUE	0.002789	20.41142
6239	rs248670	T	C	T	C	0.0122	0.001085	0.6181	0.614848	FALSE	FALSE	FALSE	vL52Yb	0.014061	0.938484	486484	sepsis	TRUE	reported	textfile	0.0027	4.78E-06	7269	M33510.m	TRUE	reported	JIO9VX	2	TRUE	0.002801	20.41139
7970	rs736396	T	C	T	C	-0.0122	0.023534	0.3554	0.344932	FALSE	FALSE	FALSE	zb8m4a	0.014502	0.104611	486484	sepsis	TRUE	reported	textfile	0.0027	4.68E-06	7261	M35253.m	TRUE	reported	tHnnnS	2	TRUE	0.002804	20.41139
8238	rs573812	T	C	T	C	0.0122	0.025769	0.8073	0.806894	FALSE	FALSE	FALSE	4YAWhA	0.017349	0.137451	486484	sepsis	TRUE	reported	textfile	0.0027	6.12E-06	7256	M35439.m	TRUE	reported	bSSC4Z	2	TRUE	0.002806	20.41138
546	rs1251583	T	G	T	G	0.0244	0.034436	0.8744	0.873307	FALSE	FALSE	FALSE	QUUN2g	0.020806	0.097904	486484	sepsis	TRUE	reported	textfile	0.0054	5.79E-06	7237	M00599.m	TRUE	reported	vxn2i7	2	TRUE	0.002813	20.41137
3407	rs1886152	A	G	A	G	0.0122	-0.01037	0.3789	0.376675	FALSE	FALSE	FALSE	VU62LG	0.014093	0.461636	486484	sepsis	TRUE	reported	textfile	0.0027	8.34E-06	7180	M27513.m	TRUE	reported	0didOx	2	TRUE	0.002836	20.41132
960	rs4746017	A	C	A	C	-0.0122	-0.00946	0.713	0.712843	FALSE	FALSE	FALSE	tWoDq4	0.015139	0.531901	486484	sepsis	TRUE	reported	textfile	0.0027	5.10E-06	7160	M01508.m	TRUE	reported	6ltDw0	2	TRUE	0.002843	20.41131
7983	rs3808756	A	G	A	G	0.0122	0.02141	0.6465	0.650798	FALSE	FALSE	FALSE	t02AUT	0.014406	0.137239	486484	sepsis	TRUE	reported	textfile	0.0027	5.65E-06	7100	M35254.m	TRUE	reported	uClJaT	2	TRUE	0.002867	20.41126
5726	rs1325431	T	C	T	C	0.0122	-0.00168	0.2929	0.293137	FALSE	FALSE	FALSE	YDQaWa	0.015075	0.911097	486484	sepsis	TRUE	reported	textfile	0.0027	7.58E-06	7063	M33228.m	TRUE	reported	BmYg8r	2	TRUE	0.002882	20.41123
6949	rs1158932	A	C	A	C	0.0122	-0.01885	0.5676	0.555781	FALSE	FALSE	FALSE	7Cw2XZ	0.013929	0.175984	486484	sepsis	TRUE	reported	textfile	0.0027	4.60E-06	7049	M34123.m	TRUE	reported	vrgMI3	2	TRUE	0.002888	20.41122
1724	rs2487411	T	C	T	C	-0.0122	-0.01757	0.3154	0.317192	FALSE	FALSE	FALSE	RmWDhQ	0.014669	0.231014	486484	sepsis	TRUE	reported	textfile	0.0027	4.68E-06	7034	M12768.m	TRUE	reported	f0vnEz	2	TRUE	0.002894	20.4112
1728	rs7127507	T	C	T	C	0.0122	-0.01779	0.6814	0.670179	FALSE	FALSE	FALSE	RmWDhQ	0.014547	0.22124	486484	sepsis	TRUE	reported	textfile	0.0027	4.95E-06	7034	M12768.m	TRUE	reported	f0vnEz	2	TRUE	0.002894	20.4112
8194	rs1247784	T	C	T	C	0.0122	0.004814	0.1863	0.191955	FALSE	FALSE	FALSE	iLlpho	0.017418	0.782262	486484	sepsis	TRUE	reported	textfile	0.0027	5.89E-06	7007	M35431.m	TRUE	reported	AAZ5ge	2	TRUE	0.002905	20.41118
2972	rs430508	A	G	A	G	-0.0122	-0.0048	0.4994	0.49963	FALSE	FALSE	FALSE	dOOXpo	0.013778	0.727455	486484	sepsis	TRUE	reported	textfile	0.0027	7.02E-06	6999	M21127.m	TRUE	reported	IdiP4j	2	TRUE	0.002909	20.41118
2975	rs6089908	T	C	T	C	-0.0122	0.006716	0.5596	0.564594	FALSE	FALSE	FALSE	dOOXpo	0.013952	0.630272	486484	sepsis	TRUE	reported	textfile	0.0027	7.19E-06	6999	M21127.m	TRUE	reported	IdiP4j	2	TRUE	0.002909	20.41118
7092	rs1389923	T	G	T	G	0.0122	-0.01185	0.2697	0.264552	FALSE	FALSE	FALSE	pxNICA	0.015509	0.444989	486484	sepsis	TRUE	reported	textfile	0.0027	7.18E-06	6994	M34306.m	TRUE	reported	xk5b9M	2	TRUE	0.002911	20.41117
7094	rs1735548	T	C	T	C	-0.0122	-0.02058	0.3071	0.348712	FALSE	FALSE	FALSE	pxNICA	0.01435	0.151494	486484	sepsis	TRUE	reported	textfile	0.0027	5.01E-06	6994	M34306.m	TRUE	reported	xk5b9M	2	TRUE	0.002911	20.41117
768	rs1271312	T	C	T	C	0.0122	0.03102	0.7564	0.758163	FALSE	FALSE	FALSE	tYvbNc	0.016109	0.054148	486484	sepsis	TRUE	reported	textfile	0.0027	6.74E-06	6946	M01303.m	TRUE	reported	hT3gsE	2	TRUE	0.002931	20.41113
2745	rs2151539	T	C	T	C	-0.0122	0.013225	0.7279	0.727574	FALSE	FALSE	FALSE	qFAbee	0.016192	0.414076	486484	sepsis	TRUE	reported	textfile	0.0027	4.57E-06	6929	M19364.m	TRUE	reported	tZgXNL	2	TRUE	0.002938	20.41112
1652	rs1232529	C	G	C	G	0.0122	-0.0031	0.6932	0.681039	FALSE	TRUE	FALSE	YzX5ZD	0.014736	0.833266	486484	sepsis	TRUE	reported	textfile	0.0027	6.94E-06	6864	M12129.m	TRUE	reported	6tMfaW	2	TRUE	0.002966	20.41106
7943	rs6973126	T	C	T	C	0.0122	0.005728	0.5122	0.526882	FALSE	FALSE	FALSE	kFTWXY	0.013685	0.675538	486484	sepsis	TRUE	reported	textfile	0.0027	7.06E-06	6543	M35193.m	TRUE	reported	cjJvJl	2	TRUE	0.003111	20.41077
6731	rs1169216	T	C	T	C	0.0122	-0.00515	0.2324	0.239747	FALSE	FALSE	FALSE	SpG9A1	0.016051	0.748461	486484	sepsis	TRUE	reported	textfile	0.0027	4.70E-06	6502	M33939.m	TRUE	reported	Tudxel	2	TRUE	0.00313	20.41073
5711	rs1713763	A	G	A	G	0.0845	-0.04332	0.0135	0.016045	FALSE	FALSE	FALSE	XDem1i	0.054829	0.429458	486484	sepsis	TRUE	reported	textfile	0.0187	6.37E-06	4665	M33221.m	TRUE	reported	KBV6FH	2	TRUE	0.004358	20.41005
7261	rs9971507	A	G	A	G	0.322	0.026846	0.0347	0.037047	FALSE	FALSE	FALSE	4t4W6t	0.036163	0.457867	486484	sepsis	TRUE	reported	textfile	0.0712	6.17E-06	955	M34344.m	TRUE	reported	Rz9mCt	2	TRUE	0.020967	20.40992
3488	rs2041992	T	C	T	C	-0.0122	-0.00592	0.3478	0.347974	FALSE	FALSE	FALSE	Awafb0	0.014423	0.68145	486484	sepsis	TRUE	reported	textfile	0.0027	8.55E-06	5562	M27728.m	TRUE	reported	Lfj8L0	2	TRUE	0.003657	20.40967
3490	rs2114588	A	G	A	G	-0.0122	0.024412	0.3114	0.308046	FALSE	FALSE	FALSE	Awafb0	0.014781	0.098637	486484	sepsis	TRUE	reported	textfile	0.0027	6.99E-06	5562	M27728.m	TRUE	reported	Lfj8L0	2	TRUE	0.003657	20.40967
4373	rs4752176	T	C	T	C	-0.0122	-0.00444	0.4565	0.454948	FALSE	FALSE	FALSE	SPhl52	0.013734	0.746224	486484	sepsis	TRUE	reported	textfile	0.0027	7.83E-06	5211	M32651.m	TRUE	reported	V0Bnpj	2	TRUE	0.003903	20.40917
7388	rs1166418	A	G	A	G	-0.0244	-0.03065	0.8302	0.837483	FALSE	FALSE	FALSE	AW6M5u	0.018559	0.098612	486484	sepsis	TRUE	reported	textfile	0.0054	6.66E-06	5195	M34389.m	TRUE	reported	6ZYYQK	2	TRUE	0.003915	20.40915
3965	rs4753364	A	C	A	C	-0.0244	-0.00344	0.2753	0.273946	FALSE	FALSE	FALSE	sKCkQs	0.015308	0.822112	486484	sepsis	TRUE	reported	textfile	0.0054	7.34E-06	5080	M32445.m	TRUE	reported	egPvJc	2	TRUE	0.004003	20.40897
2780	rs1742707	T	G	T	G	0.0122	-0.00024	0.4385	0.442021	FALSE	FALSE	FALSE	yYuamw	0.013797	0.986197	486484	sepsis	TRUE	reported	textfile	0.0027	4.88E-06	4844	M19414.m	TRUE	reported	GxSb5G	2	TRUE	0.004197	20.40858
2781	rs1959400	T	G	T	G	0.0122	0.013665	0.5551	0.559983	FALSE	FALSE	FALSE	yYuamw	0.013858	0.324113	486484	sepsis	TRUE	reported	textfile	0.0027	6.67E-06	4844	M19414.m	TRUE	reported	GxSb5G	2	TRUE	0.004197	20.40858
2279	rs1517397	T	C	T	C	0.0122	0.006994	0.3986	0.389553	FALSE	FALSE	FALSE	mRULgB	0.014043	0.61848	486484	sepsis	TRUE	reported	textfile	0.0027	4.44E-06	4689	M16816.m	TRUE	reported	05lFsX	2	TRUE	0.004335	20.4083
8518	rs9877943	A	G	A	G	0.0244	-0.01389	0.2282	0.223059	FALSE	FALSE	FALSE	vHXktL	0.016408	0.39735	486484	sepsis	TRUE	reported	textfile	0.0054	5.99E-06	4529	M35978.m	TRUE	reported	ceap9w	2	TRUE	0.004488	20.40799
8868	rs2053585	A	G	A	G	0.0556	-0.00267	0.6736	0.669016	FALSE	FALSE	FALSE	nWuK1Q	0.01459	0.854654	486484	sepsis	TRUE	reported	textfile	0.0123	6.58E-06	1583	M36850.m	TRUE	reported	2BA0SE	2	TRUE	0.012743	20.40752
6984	rs9347355	C	G	C	G	-0.0253	0.001254	0.8245	0.747491	FALSE	TRUE	FALSE	ZlvYs5	0.015979	0.937433	486484	sepsis	TRUE	reported	textfile	0.0056	7.17E-06	7351	M34214.m	TRUE	reported	qU57S1	2	TRUE	0.002769	20.40548
4181	rs1004405	A	G	A	G	0.0646	-0.03499	0.0711	0.080991	FALSE	FALSE	FALSE	TxOCzG	0.025156	0.164301	486484	sepsis	TRUE	reported	textfile	0.0143	6.59E-06	6970	M32564.m	TRUE	reported	9JP6ND	2	TRUE	0.002919	20.40179
7620	rs322906	T	G	T	G	-0.0235	-0.0095	0.4573	0.495991	FALSE	FALSE	FALSE	kVfKqT	0.01371	0.488292	486484	sepsis	TRUE	reported	textfile	0.0052	5.84E-06	1741	M34527.m	TRUE	reported	s4ti92	2	TRUE	0.011595	20.39999
58	rs1683628	A	C	A	C	0.0131	0.046825	0.044	0.046578	FALSE	FALSE	FALSE	xmGuol	0.032569	0.15052	486484	sepsis	TRUE	reported	textfile	0.0029	5.60E-06	7355	M00054.m	TRUE	reported	GW50Ri	2	TRUE	0.002767	20.39992
2074	rs1210500	A	G	A	G	-0.0131	0.008032	0.0487	0.054818	FALSE	FALSE	FALSE	NPVH5j	0.030032	0.789131	486484	sepsis	TRUE	reported	textfile	0.0029	7.55E-06	7310	M15506.m	TRUE	reported	r7r3vM	2	TRUE	0.002784	20.39989
2201	rs1289906	T	C	T	C	0.0131	-0.01071	0.7665	0.690583	FALSE	FALSE	FALSE	Bf6uMK	0.014813	0.469554	486484	sepsis	TRUE	reported	textfile	0.0029	8.58E-06	7308	M15778.m	TRUE	reported	jowM2N	2	TRUE	0.002784	20.39989
8720	rs594084	T	C	T	C	-0.0131	7.04E-05	0.6304	0.637114	FALSE	FALSE	FALSE	I0G0I1	0.014314	0.996073	486484	sepsis	TRUE	reported	textfile	0.0029	5.55E-06	6464	M36553.m	TRUE	reported	IBltb0	2	TRUE	0.003147	20.39916
6657	rs3007381	T	G	T	G	0.0131	0.013671	0.6033	0.600288	FALSE	FALSE	FALSE	REeQiq	0.01397	0.327775	486484	sepsis	TRUE	reported	textfile	0.0029	6.43E-06	6004	M33892.m	TRUE	reported	cVOa3u	2	TRUE	0.003387	20.39867
2339	rs1373765	T	C	T	C	-0.0637	0.001899	0.7973	0.73406	FALSE	FALSE	FALSE	13lycQ	0.015587	0.903056	486484	sepsis	TRUE	reported	textfile	0.0141	6.42E-06	3393	M16823.m	TRUE	reported	u2seZ2	2	TRUE	0.005979	20.39786
5212	rs1103854	T	C	T	C	-0.2283	-0.04035	0.9659	0.979626	FALSE	FALSE	FALSE	q32d4E	0.048884	0.40917	486484	sepsis	TRUE	reported	textfile	0.0505	6.23E-06	1017	M33178.m	TRUE	reported	cS3BwT	2	TRUE	0.0197	20.39737
6825	rs7164415	C	G	C	G	0.0958	0.038558	0.9751	0.981389	FALSE	TRUE	FALSE	wedECv	0.050856	0.448344	486484	sepsis	TRUE	reported	textfile	0.0212	5.95E-06	1751	M33968.m	TRUE	reported	GxGxRG	2	TRUE	0.011528	20.39684
7627	rs1088748	T	C	T	C	0.2627	-0.0175	0.0266	0.039954	FALSE	FALSE	FALSE	xGQKuv	0.035983	0.626728	486484	sepsis	TRUE	reported	textfile	0.0581	6.06E-06	829	M34530.m	TRUE	reported	wajzOs	2	TRUE	0.024068	20.39477
7981	rs1783249	T	C	T	C	0.0271	-0.02415	0.0521	0.057421	FALSE	FALSE	FALSE	t02AUT	0.029527	0.413445	486484	sepsis	TRUE	reported	textfile	0.006	6.16E-06	7100	M35254.m	TRUE	reported	uClJaT	2	TRUE	0.002865	20.39453
6300	rs7338260	T	C	T	C	0.0515	-0.04189	0.0857	0.10056	FALSE	FALSE	FALSE	VlzZVG	0.022972	0.068218	486484	sepsis	TRUE	reported	textfile	0.0114	5.94E-06	2859	M33531.m	TRUE	reported	XiYjn4	2	TRUE	0.007088	20.39393
6110	rs2733118	C	G	C	G	0.0271	-0.01244	0.118	0.11976	FALSE	TRUE	FALSE	a0osmD	0.021029	0.554227	486484	sepsis	TRUE	reported	textfile	0.006	5.49E-06	5717	M33453.m	TRUE	reported	CePFnK	2	TRUE	0.003556	20.39314
3875	rs1776854	T	C	T	C	-0.014	-0.00649	0.8282	0.834181	FALSE	FALSE	FALSE	bwkKtK	0.018361	0.723892	486484	sepsis	TRUE	reported	textfile	0.0031	8.33E-06	7307	M32393.m	TRUE	reported	RvZzAy	2	TRUE	0.002783	20.38984
1509	rs2900071	T	C	T	C	-0.014	-0.01415	0.2178	0.227675	FALSE	FALSE	FALSE	rzkxmh	0.016312	0.385776	486484	sepsis	TRUE	reported	textfile	0.0031	7.46E-06	6941	M03127.m	TRUE	reported	3lrzhA	2	TRUE	0.00293	20.38954
4726	rs2567308	A	G	A	G	-0.0524	-0.00508	0.8244	0.849818	FALSE	FALSE	FALSE	fz63r4	0.019209	0.791254	486484	sepsis	TRUE	reported	textfile	0.0116	5.86E-06	2537	M32762.m	TRUE	reported	kgxlBo	2	TRUE	0.007979	20.38938
2170	rs609236	C	G	C	G	0.028	-0.01088	0.1512	0.164176	FALSE	TRUE	FALSE	XCBNPg	0.018449	0.555325	486484	sepsis	TRUE	reported	textfile	0.0062	5.57E-06	5829	M15749.m	TRUE	reported	dKBewD	2	TRUE	0.003487	20.38842
7405	rs4805131	A	G	A	G	-0.028	-0.00208	0.7825	0.78303	FALSE	FALSE	FALSE	XfpKv0	0.016579	0.900265	486484	sepsis	TRUE	reported	textfile	0.0062	5.64E-06	5561	M34390.m	TRUE	reported	mmOsbK	2	TRUE	0.003654	20.38809
5381	rs1799453	C	G	C	G	0.2531	0.01035	0.9804	0.946435	FALSE	TRUE	FALSE	q32d4E	0.030608	0.73526	486484	sepsis	TRUE	reported	textfile	0.056	6.14E-06	1017	M33178.m	TRUE	reported	cS3BwT	2	TRUE	0.01969	20.387
7272	rs6793840	A	G	A	G	0.0411	-0.0086	0.5465	0.53109	FALSE	FALSE	FALSE	P5Xm4S	0.0138	0.533006	486484	sepsis	TRUE	reported	textfile	0.0091	5.72E-06	3491	M34350.m	TRUE	reported	YU4x0J	2	TRUE	0.005809	20.38694
6050	rs7520433	C	G	C	G	-0.8675	0.041578	0.0583	0.035786	FALSE	TRUE	FALSE	33ojbt	0.036964	0.260669	486484	sepsis	TRUE	reported	textfile	0.1889	4.38E-06	60	M33423.m	TRUE	reported	ivEtvl	2	TRUE	0.260081	20.38693
8298	rs1159296	T	C	T	C	-0.061	-0.00274	0.7444	0.728444	FALSE	FALSE	FALSE	HELgy7	0.01538	0.858572	486484	sepsis	TRUE	reported	textfile	0.0135	6.60E-06	1340	M35551.m	TRUE	reported	7x7uUn	2	TRUE	0.015008	20.38654
6374	rs6599251	T	G	T	G	-0.028	0.01158	0.553	0.550972	FALSE	FALSE	FALSE	PAgZZz	0.013786	0.400908	486484	sepsis	TRUE	reported	textfile	0.0062	5.41E-06	4323	M33633.m	TRUE	reported	I8R1KJ	2	TRUE	0.004696	20.38599
4990	rs1199948	A	G	A	G	0.0384	-0.00945	0.8305	0.830406	FALSE	FALSE	FALSE	MdUq0m	0.018203	0.603678	486484	sepsis	TRUE	reported	textfile	0.0085	6.47E-06	1618	M33009.m	TRUE	reported	icuhyC	2	TRUE	0.012457	20.38391
7022	rs1630427	C	G	C	G	-0.0289	0.002236	0.1047	0.114077	FALSE	TRUE	FALSE	zMIkYA	0.021553	0.917372	486484	sepsis	TRUE	reported	textfile	0.0064	7.48E-06	4908	M34245.m	TRUE	reported	itCtz8	2	TRUE	0.004137	20.38256
2933	rs729253	T	C	T	C	0.0262	0.001447	0.5812	0.555048	FALSE	FALSE	FALSE	fV4qul	0.013767	0.91631	486484	sepsis	TRUE	reported	textfile	0.0058	7.52E-06	1765	M21044.m	TRUE	reported	hbdBQJ	2	TRUE	0.011429	20.38235
725	rs1073838	A	G	A	G	-0.0131	-0.01238	0.6175	0.61147	FALSE	FALSE	FALSE	9u0SC8	0.014018	0.377221	486484	sepsis	TRUE	reported	textfile	0.0029	7.39E-06	1763	M01301.m	TRUE	reported	H95uec	2	TRUE	0.011442	20.38232
1744	rs2862982	A	G	A	G	-0.0149	-0.03114	0.8794	0.883496	FALSE	FALSE	FALSE	nFLpAP	0.021332	0.144404	486484	sepsis	TRUE	reported	textfile	0.0033	5.01E-06	7356	M12774.m	TRUE	reported	yOSQTj	2	TRUE	0.002764	20.38105
5074	rs3844297	T	C	T	C	-0.0149	-0.01309	0.5092	0.51511	FALSE	FALSE	FALSE	gqzGN4	0.013855	0.344834	486484	sepsis	TRUE	reported	textfile	0.0033	7.10E-06	7319	M33140.m	TRUE	reported	DKGBe9	2	TRUE	0.002778	20.38102
3265	rs4765356	A	G	A	G	0.0149	0.020774	0.8548	0.858109	FALSE	FALSE	FALSE	ZNPBqT	0.019855	0.295448	486484	sepsis	TRUE	reported	textfile	0.0033	7.08E-06	7256	M24074.m	TRUE	reported	8pPtOl	2	TRUE	0.002802	20.38097
871	rs6472299	A	G	A	G	-0.0149	0.013068	0.8381	0.8402	FALSE	FALSE	FALSE	Vg5OOR	0.01876	0.486051	486484	sepsis	TRUE	reported	textfile	0.0033	7.87E-06	7063	M01361.m	TRUE	reported	XnrS00	2	TRUE	0.002878	20.38082
8525	rs1836049	T	G	T	G	-0.0926	0.004061	0.9791	0.986471	FALSE	FALSE	FALSE	gM7v2m	0.059403	0.945497	486484	sepsis	TRUE	reported	textfile	0.0205	6.48E-06	1743	M36009.m	TRUE	reported	6Xcypd	2	TRUE	0.011571	20.38054
1580	rs1080062	A	G	A	G	-0.0262	-0.0079	0.2767	0.315363	FALSE	FALSE	FALSE	7QN2kA	0.01468	0.590402	486484	sepsis	TRUE	reported	textfile	0.0058	6.17E-06	1452	M12017.m	TRUE	reported	52YQ1d	2	TRUE	0.013859	20.37736
2337	rs1258463	A	G	A	G	-0.0945	0.007882	0.6398	0.657433	FALSE	FALSE	FALSE	13lycQ	0.014387	0.583795	486484	sepsis	TRUE	reported	textfile	0.0209	6.24E-06	604	M16823.m	TRUE	reported	u2seZ2	2	TRUE	0.03274	20.37655
4794	rs1042167	A	G	A	G	0.0307	-0.01211	0.2392	0.248539	FALSE	FALSE	FALSE	9qWhFj	0.015889	0.445876	486484	sepsis	TRUE	reported	textfile	0.0068	7.62E-06	6200	M32808.m	TRUE	reported	MQcOSi	2	TRUE	0.003277	20.37599
4511	rs1078462	T	C	T	C	0.0262	0.000969	0.7213	0.71414	FALSE	FALSE	FALSE	r0qJaM	0.015165	0.949049	486484	sepsis	TRUE	reported	textfile	0.0058	6.12E-06	1298	M32729.m	TRUE	reported	vYv5FI	2	TRUE	0.015477	20.37403
4318	rs1092491	T	C	T	C	-0.0158	0.027975	0.3733	0.394658	FALSE	FALSE	FALSE	EOAssJ	0.014049	0.046452	486484	sepsis	TRUE	reported	textfile	0.0035	5.48E-06	7369	M32635.m	TRUE	reported	X1mTjy	2	TRUE	0.002758	20.37324
2935	rs876927	T	G	T	G	0.0158	0.003854	0.2075	0.205772	FALSE	FALSE	FALSE	fV4qul	0.017679	0.827428	486484	sepsis	TRUE	reported	textfile	0.0035	5.42E-06	7366	M21044.m	TRUE	reported	hbdBQJ	2	TRUE	0.002759	20.37324
3727	rs4149121	C	G	C	G	0.0158	-0.00109	0.308	0.316904	FALSE	TRUE	FALSE	Z55Bsv	0.014694	0.940686	486484	sepsis	TRUE	reported	textfile	0.0035	7.69E-06	7357	M32322.m	TRUE	reported	6FDRcB	2	TRUE	0.002762	20.37324
6128	rs1248126	T	C	T	C	-0.0158	0.00338	0.9208	0.906363	FALSE	FALSE	FALSE	MiSJly	0.023426	0.885275	486484	sepsis	TRUE	reported	textfile	0.0035	8.56E-06	7307	M33477.m	TRUE	reported	WwL1hq	2	TRUE	0.002781	20.3732
7878	rs2458177	C	G	C	G	0.0158	-0.00839	0.3897	0.404083	FALSE	TRUE	FALSE	mpkL0o	0.013967	0.548244	486484	sepsis	TRUE	reported	textfile	0.0035	8.47E-06	7263	M35160.m	TRUE	reported	MKkvYr	2	TRUE	0.002798	20.37316
1706	rs1694034	A	G	A	G	0.0158	-0.03903	0.88	0.8505	FALSE	FALSE	FALSE	ZmI9HX	0.019185	0.041913	486484	sepsis	TRUE	reported	textfile	0.0035	5.36E-06	7258	M12626.m	TRUE	reported	9SUabZ	2	TRUE	0.0028	20.37316
2310	rs4510422	T	C	T	C	-0.0158	-0.00918	0.196	0.196183	FALSE	FALSE	FALSE	ZzUW8U	0.017268	0.594874	486484	sepsis	TRUE	reported	textfile	0.0035	7.04E-06	6733	M16821.m	TRUE	reported	g2xs3i	2	TRUE	0.003018	20.37272
4779	rs1173667	A	C	A	C	-0.1881	-0.03208	0.9635	0.961633	FALSE	FALSE	FALSE	MmZZoM	0.035689	0.36874	486484	sepsis	TRUE	reported	textfile	0.0416	6.11E-06	562	M32800.m	TRUE	reported	70YSCO	2	TRUE	0.035102	20.37242
8061	rs1554690	A	C	A	C	0.0158	0.014129	0.5949	0.593075	FALSE	FALSE	FALSE	whAa18	0.013967	0.311707	486484	sepsis	TRUE	reported	textfile	0.0035	8.17E-06	5979	M35305.m	TRUE	reported	e6hvDm	2	TRUE	0.003397	20.37196
3430	rs6450853	A	G	A	G	-0.0316	-0.03123	0.652	0.645321	FALSE	FALSE	FALSE	HHQf8w	0.014413	0.030245	486484	sepsis	TRUE	reported	textfile	0.007	5.73E-06	5673	M27531.m	TRUE	reported	3vTluK	2	TRUE	0.003579	20.37159
6403	rs9962038	T	C	T	C	0.0316	0.012562	0.2175	0.224938	FALSE	FALSE	FALSE	Z1h3mT	0.016654	0.450689	486484	sepsis	TRUE	reported	textfile	0.007	7.15E-06	5328	M33652.m	TRUE	reported	nszuTQ	2	TRUE	0.00381	20.37113
4683	rs1028885	A	G	A	G	0.042	0.00686	0.7832	0.755699	FALSE	FALSE	FALSE	MV108h	0.015912	0.66638	486484	sepsis	TRUE	reported	textfile	0.0093	6.06E-06	1612	M32758.m	TRUE	reported	btjNXQ	2	TRUE	0.012494	20.37012
3312	rs3751834	A	G	A	G	0.0167	-0.00831	0.8707	0.872157	FALSE	FALSE	FALSE	nqmBts	0.020498	0.685135	486484	sepsis	TRUE	reported	textfile	0.0037	8.17E-06	6388	M25599.m	TRUE	reported	VkwSh1	2	TRUE	0.003179	20.36543
4631	rs1051781	T	C	T	C	-0.0501	-0.01472	0.8138	0.806532	FALSE	FALSE	FALSE	Dfaklt	0.017599	0.402969	486484	sepsis	TRUE	reported	textfile	0.0111	6.62E-06	6379	M32754.m	TRUE	reported	y21cDm	2	TRUE	0.003183	20.36542
5489	rs588113	A	G	A	G	-0.1722	-0.02166	0.0476	0.02761	FALSE	FALSE	FALSE	q32d4E	0.041758	0.603957	486484	sepsis	TRUE	reported	textfile	0.0366	2.53E-06	25	M33178.m	TRUE	reported	cS3BwT	2	TRUE	0.469623	20.36535
7758	rs9357080	A	G	A	G	-0.0167	0.016101	0.9074	0.902333	FALSE	FALSE	FALSE	HyMNYM	0.022947	0.482874	486484	sepsis	TRUE	reported	textfile	0.0037	5.32E-06	5885	M34912.m	TRUE	reported	Tl8Hcw	2	TRUE	0.00345	20.36488

6965	rs6034944	C	G	C	G	0.0587	-0.01251	0.9395	0.960109	FALSE	TRUE	FALSE	7Cw2XZ	0.035413	0.723894	486484	sepsis	TRUE	reported	textfile	0.013	5.81E-06	1644	M34123.m	TRUE	reported	vrgMI3	2	TRUE	0.01225	20.36389
4929	rs4377848	A	G	A	G	0.0307	-0.00382	0.1394	0.133576	FALSE	FALSE	FALSE	clhN6v	0.020076	0.849045	486484	sepsis	TRUE	reported	textfile	0.0068	6.19E-06	2148	M32869.m	TRUE	reported	BzloWk	2	TRUE	0.0094	20.36359
3722	rs7342491	T	C	T	C	-0.1192	-0.04343	0.0167	0.012065	FALSE	FALSE	FALSE	ZRfYkG	0.063202	0.491936	486484	sepsis	TRUE	reported	textfile	0.0264	6.49E-06	1765	M32319.m	TRUE	reported	phVUPX	2	TRUE	0.011419	20.36349
3984	rs4458381	C	G	C	G	-0.0343	0.045786	0.967	0.9547	FALSE	TRUE	FALSE	XdJbEU	0.034356	0.182627	486484	sepsis	TRUE	reported	textfile	0.0076	6.25E-06	7364	M32452.m	TRUE	reported	V5xWKi	2	TRUE	0.002758	20.36306
6939	rs4455656	A	G	A	G	-0.0343	-0.03311	0.1597	0.162833	FALSE	FALSE	FALSE	oNv0vf	0.018459	0.072827	486484	sepsis	TRUE	reported	textfile	0.0076	6.88E-06	5581	M34112.m	TRUE	reported	l0pe0N	2	TRUE	0.003636	20.36129
2605	rs3846286	A	G	A	G	-0.0307	-0.00149	0.9275	0.927082	FALSE	FALSE	FALSE	ruvJh6	0.026395	0.95502	486484	sepsis	TRUE	reported	textfile	0.0068	6.64E-06	1762	M18369.m	TRUE	reported	GFmwGk	2	TRUE	0.011436	20.35943
7890	rs1984049	A	C	A	C	0.0307	0.000829	0.8108	0.807324	FALSE	FALSE	FALSE	pU8zps	0.017344	0.961895	486484	sepsis	TRUE	reported	textfile	0.0068	5.33E-06	1760	M35186.m	TRUE	reported	Oc2lOF	2	TRUE	0.011448	20.35941
1581	rs1096317	A	G	A	G	0.0352	-0.01189	0.9724	0.967145	FALSE	FALSE	FALSE	7QN2kA	0.038407	0.756932	486484	sepsis	TRUE	reported	textfile	0.0078	6.99E-06	5656	M12017.m	TRUE	reported	52YQ1d	2	TRUE	0.003588	20.35835
8769	rs1268042	T	C	T	C	0.0176	-0.00593	0.2899	0.300692	FALSE	FALSE	FALSE	7desNF	0.014938	0.691422	486484	sepsis	TRUE	reported	textfile	0.0039	7.77E-06	5292	M36673.m	TRUE	reported	RKbIT4	2	TRUE	0.003834	20.35785
3258	rs1726046	T	C	T	C	0.0361	0.009909	0.9112	0.85745	FALSE	FALSE	FALSE	ZNPBqT	0.019872	0.618052	486484	sepsis	TRUE	reported	textfile	0.008	5.59E-06	7256	M24074.m	TRUE	reported	8pPtOl	2	TRUE	0.002798	20.35704
2237	rs2345834	T	C	T	C	0.0176	0.010137	0.6652	0.672193	FALSE	FALSE	FALSE	cwj8fY	0.014592	0.487222	486484	sepsis	TRUE	reported	textfile	0.0039	5.45E-06	4538	M15958.m	TRUE	reported	dPwsBc	2	TRUE	0.004468	20.35657
1802	rs6575634	T	C	T	C	-0.0158	-0.00229	0.6184	0.623355	FALSE	FALSE	FALSE	JEcCRZ	0.014102	0.87099	486484	sepsis	TRUE	reported	textfile	0.0035	4.85E-06	1764	M15140.m	TRUE	reported	xYCzKP	2	TRUE	0.011421	20.35567
8654	rs7021014	T	G	T	G	0.0334	-0.01878	0.7224	0.70506	FALSE	FALSE	FALSE	J6i46i	0.014977	0.209788	486484	sepsis	TRUE	reported	textfile	0.0074	6.59E-06	2520	M36376.m	TRUE	reported	IdddPp	2	TRUE	0.008019	20.35564
3438	rs3847067	A	C	A	C	-0.0316	0.006518	0.2877	0.284322	FALSE	FALSE	FALSE	5dHWFk	0.015142	0.666854	486484	sepsis	TRUE	reported	textfile	0.007	6.69E-06	1758	M27672.m	TRUE	reported	pEd8kY	2	TRUE	0.011459	20.35559
647	rs1735132	A	G	A	G	-0.0185	-0.01078	0.8651	0.863581	FALSE	FALSE	FALSE	FfmsRf	0.020058	0.591085	486484	sepsis	TRUE	reported	textfile	0.0041	5.37E-06	7349	M01121.m	TRUE	reported	9R12A7	2	TRUE	0.002763	20.35436
3506	rs8124907	A	G	A	G	0.0307	-0.01046	0.7514	0.758131	FALSE	FALSE	FALSE	Awafb0	0.015947	0.511945	486484	sepsis	TRUE	reported	textfile	0.0068	5.85E-06	1227	M27728.m	TRUE	reported	Lfj8L0	2	TRUE	0.01634	20.34935
4807	rs1022480	T	C	T	C	0.0194	0.023755	0.8787	0.882804	FALSE	FALSE	FALSE	bJwLV5	0.021329	0.265402	486484	sepsis	TRUE	reported	textfile	0.0043	5.64E-06	7063	M32814.m	TRUE	reported	qte0qp	2	TRUE	0.002874	20.34902
641	rs7427777	A	C	A	C	0.1291	-0.06547	0.0483	0.036631	FALSE	FALSE	FALSE	F6Dn8H	0.036664	0.074148	486484	sepsis	TRUE	reported	textfile	0.0286	6.36E-06	1447	M01114.m	TRUE	reported	dVHatZ	2	TRUE	0.013886	20.34791
7642	rs2218275	A	T	A	T	0.0582	-0.06166	0.9535	0.954176	FALSE	TRUE	FALSE	wrTMYM	0.032825	0.060338	486484	sepsis	TRUE	reported	textfile	0.0129	6.60E-06	5856	M34533.m	TRUE	reported	qIRVku	2	TRUE	0.003464	20.34783
1579	rs1001217	T	C	T	C	0.0194	-0.00448	0.1065	0.113156	FALSE	FALSE	FALSE	7QN2kA	0.021626	0.835765	486484	sepsis	TRUE	reported	textfile	0.0043	5.78E-06	5656	M12017.m	TRUE	reported	52YQ1d	2	TRUE	0.003586	20.34759
6895	rs2798002	A	G	A	G	-0.1151	-0.01006	0.0628	0.019131	FALSE	FALSE	FALSE	MN2IbE	0.050036	0.840675	486484	sepsis	TRUE	reported	textfile	0.0255	6.48E-06	1481	M34106.m	TRUE	reported	q4zXEz	2	TRUE	0.01357	20.3462
7109	rs4454960	C	G	C	G	-0.0745	0.018623	0.8989	0.967057	FALSE	TRUE	FALSE	DfLd6z	0.038676	0.630152	486484	sepsis	TRUE	reported	textfile	0.0165	6.47E-06	979	M34314.m	TRUE	reported	OgloAQ	2	TRUE	0.020399	20.34495
3197	rs2268943	A	T	A	T	-0.0203	0.03443	0.0682	0.071423	FALSE	TRUE	FALSE	UfLkBq	0.026492	0.193713	486484	sepsis	TRUE	reported	textfile	0.0045	7.10E-06	7329	M22481.m	TRUE	reported	kWMdiT	2	TRUE	0.002769	20.34457
2231	rs9859467	A	G	A	G	-0.0203	0.003909	0.037	0.049221	FALSE	FALSE	FALSE	Bf6uMK	0.031571	0.901462	486484	sepsis	TRUE	reported	textfile	0.0045	5.75E-06	7308	M15778.m	TRUE	reported	jowM2N	2	TRUE	0.002777	20.34455
3414	rs426434	T	C	T	C	0.0203	-0.00567	0.1192	0.121264	FALSE	FALSE	FALSE	VU62LG	0.020949	0.786546	486484	sepsis	TRUE	reported	textfile	0.0045	6.99E-06	7180	M27513.m	TRUE	reported	0didOx	2	TRUE	0.002826	20.34445
2976	rs757717	T	C	T	C	0.0203	0.05909	0.1121	0.129457	FALSE	FALSE	FALSE	dOOXpo	0.020478	0.003908	486484	sepsis	TRUE	reported	textfile	0.0045	8.12E-06	6999	M21127.m	TRUE	reported	ldiP4j	2	TRUE	0.002899	20.34431
4853	rs4500414	A	G	A	G	-0.0203	-0.02839	0.6497	0.632079	FALSE	FALSE	FALSE	StMkBO	0.01424	0.046201	486484	sepsis	TRUE	reported	textfile	0.0045	7.44E-06	6967	M32847.m	TRUE	reported	uzQb8Q	2	TRUE	0.002912	20.34428
7253	rs1219492	T	C	T	C	0.5355	0.036493	0.0118	0.019156	FALSE	FALSE	FALSE	4t4W6t	0.049732	0.463069	486484	sepsis	TRUE	reported	textfile	0.1186	6.35E-06	955	M34344.m	TRUE	reported	Rz9mCt	2	TRUE	0.020901	20.34413
3032	rs2314600	A	C	A	C	-0.1047	0.041889	0.0144	0.009537	FALSE	FALSE	FALSE	FJZEYz	0.069826	0.548565	486484	sepsis	TRUE	reported	textfile	0.0232	6.67E-06	1755	M21630.m	TRUE	reported	s6Pshg	2	TRUE	0.011472	20.34334
6923	rs6812391	A	G	A	G	-0.7293	-0.03615	0.0441	0.056034	FALSE	FALSE	FALSE	SvfSvH	0.02978	0.224833	486484	sepsis	TRUE	reported	textfile	0.1606	5.61E-06	148	M34109.m	TRUE	reported	7OSbiu	2	TRUE	0.122295	20.34288
3356	rs1456374	T	C	T	C	-0.0176	-0.00037	0.6002	0.62574	FALSE	FALSE	FALSE	LY4Gus	0.014125	0.979328	486484	sepsis	TRUE	reported	textfile	0.0039	7.04E-06	1751	M27273.m	TRUE	reported	QKITBx	2	TRUE	0.011497	20.34229
1153	rs7463380	C	G	C	G	0.0763	-0.00263	0.8421	0.824261	FALSE	TRUE	FALSE	QtY5Hc	0.017923	0.883554	486484	sepsis	TRUE	reported	textfile	0.0169	6.40E-06	955	M01605.m	TRUE	reported	Q3iDQX	2	TRUE	0.020898	20.34067
6349	rs7222177	T	C	T	C	0.5557	0.103229	0.0108	0.01327	FALSE	FALSE	FALSE	qLUY00	0.063827	0.105808	486484	sepsis	TRUE	reported	textfile	0.122	5.20E-06	102	M33610.m	TRUE	reported	8cAJbB	2	TRUE	0.169024	20.34047
8785	rs1889603	A	G	A	G	-0.086	-0.00803	0.747	0.739259	FALSE	FALSE	FALSE	4bkfvN	0.015615	0.60708	486484	sepsis	TRUE	reported	textfile	0.019	6.00E-06	274	M36738.m	TRUE	reported	xcqyX2	2	TRUE	0.06957	20.33799
7205	rs1302821	C	G	C	G	-0.3375	-0.01101	0.9809	0.937945	FALSE	TRUE	FALSE	i1uJ7T	0.028957	0.703889	486484	sepsis	TRUE	reported	textfile	0.0747	6.30E-06	535	M34338.m	TRUE	reported	pi3mKW	2	TRUE	0.036753	20.33667
8958	rs4453613	T	C	T	C	0.06	0.015443	0.3355	0.33912	FALSE	FALSE	FALSE	Ywk2ve	0.014562	0.288917	486484	sepsis	TRUE	reported	textfile	0.0133	6.83E-06	2560	M37112.m	TRUE	reported	EiAdDj	2	TRUE	0.007887	20.33573
4178	rs7103589	T	G	T	G	0.0388	-0.02574	0.6053	0.599976	FALSE	FALSE	FALSE	Aq7H4p	0.01397	0.065358	486484	sepsis	TRUE	reported	textfile	0.0086	5.72E-06	2091	M32560.m	TRUE	reported	NIzbA7	2	TRUE	0.009641	20.33532
4196	rs3846714	A	G	A	G	0.0672	-0.03387	0.065	0.061507	FALSE	FALSE	FALSE	TxOCzG	0.029635	0.253095	486484	sepsis	TRUE	reported	textfile	0.0149	6.46E-06	6970	M32564.m	TRUE	reported	9JP6ND	2	TRUE	0.00291	20.33487
1821	rs2365522	A	C	A	C	0.0442	0.008587	0.7137	0.719471	FALSE	FALSE	FALSE	Yub5KV	0.015203	0.572202	486484	sepsis	TRUE	reported	textfile	0.0098	6.70E-06	5606	M15335.m	TRUE	reported	mcnINa	2	TRUE	0.003615	20.33468
1050	rs2239337	C	G	C	G	0.023	0.012047	0.0356	0.032574	FALSE	TRUE	FALSE	tRtQ2e	0.038712	0.755649	486484	sepsis	TRUE	reported	textfile	0.0051	7.51E-06	7364	M01564.m	TRUE	reported	Tbg0zM	2	TRUE	0.002754	20.33281
104	rs3780494	T	C	T	C	-0.023	-0.00711	0.9855	0.982705	FALSE	FALSE	FALSE	xmGuol	0.052459	0.892122	486484	sepsis	TRUE	reported	textfile	0.0051	7.02E-06	7355	M00054.m	TRUE	reported	GW50Ri	2	TRUE	0.002758	20.3328
624	rs7169869	A	G	A	G	-0.0194	-0.00469	0.5404	0.488779	FALSE	FALSE	FALSE	BYGzq4	0.013666	0.731499	486484	sepsis	TRUE	reported	textfile	0.0043	6.59E-06	1766	M01110.m	TRUE	reported	oZiXqP	2	TRUE	0.011395	20.33173
7039	rs1177874	T	C	T	C	-0.0194	0.000788	0.2583	0.255664	FALSE	FALSE	FALSE	uthruC	0.01574	0.960067	486484	sepsis	TRUE	reported	textfile	0.0043	5.25E-06	1755	M34283.m	TRUE	reported	ftTuRU	2	TRUE	0.011465	20.33159
5809	rs3791608	A	G	A	G	-0.0221	0.015735	0.1287	0.145877	FALSE	FALSE	FALSE	JE7L71	0.019453	0.418582	486484	sepsis	TRUE	reported	textfile	0.0049	7.00E-06	3890	M33364.m	TRUE	reported	FdLiEs	2	TRUE	0.005202	20.33148
2981	rs2717871	A	G	A	G	-0.0618	0.01087	0.8155	0.829237	FALSE	FALSE	FALSE	Tli4xl	0.018267	0.551786	486484	sepsis	TRUE	reported	textfile	0.0137	6.64E-06	2339	M21151.m	TRUE	reported	xaSytY	2	TRUE	0.008625	20.33126
7432	rs4398579	A	C	A	C	-0.023	0.00218	0.3581	0.385165	FALSE	FALSE	FALSE	NZPe5A	0.014117	0.877295	486484	sepsis	TRUE	reported	textfile	0.0051	7.53E-06	5689	M34400.m	TRUE	reported	OyPwYO	2	TRUE	0.003562	20.33118
651	rs1161665	A	G	A	G	-0.1069	0.009298	0.9444	0.939397	FALSE	FALSE	FALSE	7BPGuQ	0.028717	0.7461	486484	sepsis	TRUE	reported	textfile	0.0237	6.37E-06	2639	M01123.m	TRUE	reported	WV2hVn	2	TRUE	0.00765	20.32963
5650	rs6507387	T	C	T	C	-0.0469	0.003316	0.3592	0.367689	FALSE	FALSE	FALSE	BFsMtm	0.014217	0.815565	486484	sepsis	TRUE	reported	textfile	0.0104	7.06E-06	5716	M33192.m	TRUE	reported	NAJN3D	2	TRUE	0.003545	20.32952
2530	rs1777793	T	C	T	C	-0.0744	0.014951	0.0548	0.08705	FALSE	FALSE	FALSE	4HwTIG	0.024432	0.540563	486484	sepsis	TRUE	reported	textfile	0.0165	6.20E-06	7071	M18254.m	TRUE	reported	F9KrSC	2	TRUE	0.002867	20.32615
654	rs1774181	T	C	T	C	0.538	-0.00335	0.015	0.068153	FALSE	FALSE	FALSE	7BPGuQ	0.027396	0.902689	486484	sepsis	TRUE	reported	textfile	0.1192	6.39E-06	908	M01123.m	TRUE	reported	WV2hVn	2	TRUE	0.021943	20.32612
7497	rs1299693	A	G	A	G	0.1606	0.003632	0.0536	0.053489	FALSE	FALSE	FALSE	1aXQWs	0.030497	0.905193	486484	sepsis	TRUE	reported	textfile	0.0356	6.57E-06	1496	M34420.m	TRUE	reported	FOqhHW	2	TRUE	0.013421	20.32404
5086	rs1120715	C	G	C	G	0.3171	-0.00079	0.0118	0.077856	FALSE	TRUE	FALSE	Cira8M	0.025919	0.975542	486484	sepsis	TRUE	reported	textfile	0.0703	6.50E-06	1766	M33150.m	TRUE	reported	AofVV7	2	TRUE	0.01139	20.32309
7555	rs1235385	T	C	T	C	0.0257	0.006608	0.167	0.207699	FALSE	FALSE	FALSE	APSWAU	0.016809	0.694225	486484	sepsis	TRUE	reported	textfile	0.0057	5.66E-06	6144	M34469.m	TRUE	reported	frF3RX	2	TRUE	0.003298	20.32241
3611	rs4674858	T	C	T	C	0.1484	-0.06729	0.0101	0.011073	FALSE	FALSE	FALSE	2z5KhH	0.065519	0.304439	486484	sepsis	TRUE	reported	textfile	0.0329	6.61E-06	1713	M31555.m	TRUE	reported	5kcWr2	2	TRUE	0.011738	20.3221
2658	rs2718107	A	C	A	C	0.0257	0.010703	0.5408	0.545208	FALSE	FALSE	FALSE	UtYu7J	0.013733	0.435764	486484	sepsis	TRUE	reported	textfile	0.0057	5.85E-06	5624	M18476.m	TRUE	reported	wJx0A4	2	TRUE	0.003602	20.32179
6933	rs1087543	C	G	C	G	-0.0257	-0.00879	0.6582	0.644682	FALSE	TRUE	FALSE	oNv0vf	0.014283	0.538209	486484	sepsis	TRUE	reported	textfile	0.0057	7.69E-06	5581	M34112.m	TRUE	reported	l0pe0N	2	TRUE	0.003629	20.32174
2992	rs1889891	T	C	T	C	0.0257	0.009901	0.2641	0.269532	FALSE	FALSE	FALSE	xmzJ5F	0.015412	0.52062	486484	sepsis	TRUE	reported	textfile	0.0057	5.77E-06	5436	M21184.m	TRUE	reported	xgwhDp	2	TRUE	0.003726	20.32154
1186	rs9606290	A	G	A	G	0.159	0.020957	0.241	0.262057	FALSE	FALSE	FALSE	FgZx4a	0.015779	0.184111	486484	sepsis	TRUE	reported	textfile	0.0352	6.32E-06	496	M01640.m	TRUE	reported	hNPAJ8	2	TRUE	0.039511	20.32143
604	rs972709	A	C	A	C	0.0483	-0.00309	0.6548	0.660058	FALSE	FALSE	FALSE	KVK33e	0.014439	0.830626	486484	sepsis	TRUE	reported	textfile	0.0107	6.73E-06	736	M01107.m	TRUE	reported	eAyR8w	2	TRUE	0.026939	20.32099
5633	rs2932286	A	G	A	G	0.0717	0.030121	0.8764	0.885813	FALSE	FALSE	FALSE	U0d0ue	0.021485	0.160931	486484	sepsis	TRUE	reported	textfile	0.0159	6.83E-06	2616	M33190.m	TRUE	reported	FoODGF	2	TRUE	0.007713	20.31945
2268	rs1598817	T	G	T	G	0.0541	0.036963	0.9624	0.9621	FALSE	FALSE	FALSE	Mjgi2R	0.036067	0.305438	486484	sepsis	TRUE	reported	textfile	0.012	7.10E-06	7064	M16634.m	TRUE	reported	XO0GV7	2	TRUE	0.002869	20.31931
8880	rs195113	T	C	T	C	0.0221	0.017763	0.6742	0.679381	FALSE	FALSE	FALSE	Nv6gDO	0.014657	0.22554	486484	sepsis	TRUE	reported	textfile	0.0049	8.04E-06	1782	M37004.m	TRUE	reported	RqmQdg	2	TRUE	0.011286	20.31911
3082	rs2933405	A	G	A	G	0.0221	-0.01453	0.1507	0.167212	FALSE	FALSE	FALSE	esOP8m	0.018461	0.431355	486484	sepsis	TRUE	reported	textfile	0.0049	5.88E-06	1766	M22116.m	TRUE	reported	fDMh7Z	2	TRUE	0.011387	20.3189
3684	rs6810706	A	G	A	G	0.0221	0.006003	0.5523	0.519362	FALSE	FALSE	FALSE	rGHJcj	0.013684	0.660899	486484	sepsis	TRUE	reported	textfile	0.0049	5.44E-06	1760	M32198.m	TRUE	reported	6P1Nno	2	TRUE	0.011426	20.31883
7429	rs1395489	A	G	A	G	-0.0564	0.027495	0.1479	0.15499	FALSE	FALSE	FALSE	NZPe5A	0.018884	0.145389	486484	sepsis	TRUE	reported	textfile	0.0125	6.88E-06	1018	M34400.m	TRUE	reported	OyPwYO	2	TRUE	0.019606	20.31815
6449	rs1168135	A	G	A	G	0.0275	0.01698	0.318	0.335885	FALSE	FALSE	FALSE	OFlEwq	0.014591	0.244529	486484	sepsis	TRUE	reported	textfile	0.0061	7.56E-06	5694	M33675.m	TRUE	reported	T2BzXG	2	TRUE	0.003557	20.3167
7402	rs1820124	A	G	A	G	0.0275	-0.00821	0.2432	0.255096	FALSE	FALSE	FALSE	XfpKv0	0.015702	0.601088	486484	sepsis	TRUE	reported	textfile	0.0061	5.59E-06	5561	M34390.m	TRUE	reported	mmOsbK	2	TRUE	0.003641	20.31653
3827	rs1710131	T	C	T	C	-0.0284	-0.00015	0.973	0.966065	FALSE	FALSE	FALSE	tRX2u0	0.03791	0.996816	486484	sepsis	TRUE	reported	textfile	0.0063	5.95E-06	7365	M32348.m	TRUE	reported	ruBgwQ	2	TRUE	0.002752	20.31597
3519	rs576266	T	C	T	C	-0.0284	-0.00098	0.9071	0.910006	FALSE	FALSE	FALSE	hxuqls	0.023943	0.967219	486484	sepsis	TRUE	reported	textfile	0.0063	5.97E-06	7336	M27738.m	TRUE	reported	aueeZs	2	TRUE	0.002762	20.31595
8505	rs1043349	T	C	T	C	0.055	0.056341	0.9624	0.969331	FALSE	FALSE	FALSE	vHXktL	0.039881	0.157732	486484	sepsis	TRUE	reported	textfile	0.0122	6.67E-06	4529	M35978.m	TRUE	reported	ceap9w	2	TRUE	0.004467	20.31486
8509	rs1418811	T	G	T	G	0.0275	-0.01569	0.8452	0.842516	FALSE	FALSE	FALSE	vHXktL	0.018736	0.402325	486484	sepsis	TRUE	reported	textfile	0.0061	5.93E-06	4529	M35978.m	TRUE	reported	ceap9w	2	TRUE	0.004467	20.31486
7644	rs2326675	A	C	A	C	0.0284	0.030643	0.7231	0.729527	FALSE	FALSE	FALSE	wrTMYM	0.015412	0.046785	486484	sepsis	TRUE	reported	textfile	0.0063	6.39E-06	5856	M34533.m	TRUE	reported	qIRVku	2	TRUE	0.003458	20.31455
4084	rs838532	T	C	T	C	-0.0293	0.015001	0.9416	0.937393	FALSE	FALSE	FALSE	TyHtdh	0.028247	0.595367	486484	sepsis	TRUE	reported	textfile	0.0065	6.89E-06	7358	M32497.m	TRUE	reported	H3KmLR	2	TRUE	0.002754	20.31377
5583	rs890290	T	C	T	C	-0.3402	-0.0229	0.0266	0.012739	FALSE	FALSE	FALSE	q32d4E	0.060666	0.705768	486484	sepsis	TRUE	reported	textfile	0.0724	2.60E-06	25	M33178.m	TRUE	reported	cS3BwT	2	TRUE	0.468985	20.31325
4451	rs209338	A	T	A	T	0.078	-0.02103	0.9651	0.954187	FALSE	TRUE	FALSE	g2RNym	0.033109	0.525381	486484	sepsis	TRUE	reported	textfile	0.0173	6.39E-06	2575	M32691.m	TRUE	reported	35eo6p	2	TRUE	0.007833	20.31232
4783	rs4674860	T	C	T	C	-0.0284	-0.03819	0.4516	0.456382	FALSE	FALSE	FALSE	MmZZoM	0.013743	0.005459	486484	sepsis	TRUE	reported	textfile	0.0063	5.74E-06	4274	M32800.m	TRUE	reported	70YSCO	2	TRUE	0.004732	20.31198
2468	rs6557244	T	C	T	C	0.0302	0.057779	0.9101	0.899887	FALSE	FALSE	FALSE	C5wkoN	0.022791	0.01124	486484	sepsis	TRUE	reported	textfile	0.0067	7.32E-06	7353	M17805.m	TRUE	reported	F5VXY0	2	TRUE	0.002756	20.31169
2529	rs1338783	A	T	A	T	-0.0622	0.008308	0.9417	0.935693	FALSE	TRUE	FALSE	4HwTIG	0.028018	0.766828	486484	sepsis	TRUE	reported	textfile	0.0138	6.24E-06	7071	M18254.m	TRUE	reported	F9KrSC	2	TRUE	0.002865	20.30952
8564	rs1202158	A	G	A	G	0.0311	-0.00394	0.3846	0.365727	FALSE	FALSE	FALSE	Yjjldt	0.014201	0.781277	486484	sepsis	TRUE	reported	textfile	0.0069	6.00E-06	7054	M36098.m	TRUE	reported	U2ORsF	2	TRUE	0.002872	20.30951
1721	rs1671347	A	G	A	G	0.0311	-0.00519	0.0462	0.052477	FALSE	FALSE	FALSE	RmWDhQ	0.030884	0.86658	486484	sepsis	TRUE	reported	textfile	0.0069	7.26E-06	7034	M12768.m	TRUE	reported	f0vnEz	2	TRUE	0.00288	20.30949
8805	rs2081670	T	C	T	C	0.0284	-0.00467	0.1437	0.161291	FALSE	FALSE	FALSE	QWTooc	0.018618	0.801784	486484	sepsis	TRUE	reported	textfile	0.0063	6.77E-06	3248	M36756.m	TRUE	reported	OLsw6C	2	TRUE	0.006218	20.30898
8255	rs2209436	T	C	T	C	0.138	-0.00276	0.0297	0.029801	FALSE	FALSE	FALSE	G4plxe	0.040262	0.945251	486484	sepsis	TRUE	reported	textfile	0.0306	6.62E-06	1383	M35451.m	TRUE	reported	hNOVMI	2	TRUE	0.014493	20.30892
4493	rs2184568	A	G	A	G	-0.0248	0.011902	0.6762	0.665301	FALSE	FALSE	FALSE	1wNJ1E	0.014491	0.411475	486484	sepsis	TRUE	reported	textfile	0.0055	6.01E-06	1766	M32709.m	TRUE	reported	zihBHg	2	TRUE	0.011382	20.30887
5433	rs3780205	A	G	A	G	-0.1254	-0.01033	0.0519	0.072942	FALSE	FALSE	FALSE	q32d4E	0.026355	0.695056	486484	sepsis	TRUE	reported	textfile	0.0278	6.60E-06	1017	M33178.m	TRUE	reported	cS3BwT	2	TRUE	0.019615	20.30722
7475	rs1298120	A	C	A	C	0.0496	-0.00106	0.0814	0.088358	FALSE	FALSE	FALSE	SqfdcK	0.024069	0.964903	486484	sepsis	TRUE	reported	textfile	0.011	6.16E-06	1567	M34416.m	TRUE	reported	sRGRIK	2	TRUE	0.012809	20.30595
6396	rs1078328	A	G	A	G	0.032	-0.00341	0.2235	0.22824	FALSE	FALSE	FALSE	Z1h3mT	0.016266	0.834117	486484	sepsis	TRUE	reported	textfile	0.0071	5.86E-06	5328	M33652.m	TRUE	reported	nszuTQ	2	TRUE	0.003798	20.3058
508	rs1690297	T	G	T	G	-0.1325	0.065942	0.0232	0.033358	FALSE	FALSE	FALSE	ZOMIXT	0.038108	0.083556	486484	sepsis	TRUE	reported	textfile	0.0294	6.36E-06	7274	M00569.m	TRUE	reported	kZGouU	2	TRUE	0.002785	20.30569
5227	rs1162079	A	T	A	T	-0.0857	0.006015	0.705	0.682526	FALSE	TRUE	FALSE	q32d4E	0.014761	0.683644	486484	sepsis	TRUE	reported	textfile	0.019	6.64E-06	1017	M33178.m	TRUE	reported	cS3BwT	2	TRUE	0.019612	20.30484
5929	rs2008827	T	G	T	G	0.4093	0.040236	0.0302	0.028014	FALSE	FALSE	FALSE	veBhfS	0.041845	0.33628	486484	sepsis	TRUE	reported	textfile	0.0903	5.78E-06	171	M33391.m	TRUE	reported	Gfjy5a	2	TRUE	0.10726	20.30479
8629	rs1106806	A	C	A	C	-0.0284	0.014029	0.3883	0.393073	FALSE	FALSE	FALSE	4cZjpQ	0.014062	0.318453	486484	sepsis	TRUE	reported	textfile	0.0063	6.78E-06	2400	M36230.m	TRUE	reported	TPUeNs	2	TRUE	0.008396	20.30456
4986	rs6006250	T	C	T	C	-0.0532	0.018416	0.0613	0.047684	FALSE	FALSE	FALSE	AjJi2i	0.032206	0.567448	486484	sepsis	TRUE	reported	textfile	0.0118	6.49E-06	1767	M32980.m	TRUE	reported	gVSm9e	2	TRUE	0.011372	20.30334
5815	rs1157863	T	G	T	G	-0.0523	0.016509	0.0162	0.177028	FALSE	FALSE	FALSE	R5joEf	0.017895	0.356249	486484	sepsis	TRUE	reported	textfile	0.0116	6.24E-06	1582	M33380.m	TRUE	reported	kRvbfZ	2	TRUE	0.012686	20.30196
6042	rs4811103	T	C	T	C	-0.4766	-0.02467	0.3103	0.292493	FALSE	FALSE	FALSE	33ojbt	0.01881	0.189713	486484	sepsis	TRUE	reported	textfile	0.104	4.58E-06	60	M33423.m	TRUE	reported	ivEtvl	2	TRUE	0.259269	20.30103
5666	rs1755624	T	C	T	C	0.1361	0.00548	0.0315	0.049078	FALSE	FALSE	FALSE	wHu1Rk	0.031548	0.862108	486484	sepsis	TRUE	reported	textfile	0.0302	6.43E-06	4625	M33195.m	TRUE	reported	yvhnuQ	2	TRUE	0.004372	20.30086
1150	rs312306	C	G	C	G	-0.0654	-0.0185	0.2278	0.246996	FALSE	TRUE	FALSE	QtY5Hc	0.01587	0.243791	486484	sepsis	TRUE	reported	textfile	0.0145	6.23E-06	955	M01605.m	TRUE	reported	Q3iDQX	2	TRUE	0.020857	20.30061
1591	rs7908166	A	G	A	G	-0.0356	-0.06023	0.9724	0.959337	FALSE	FALSE	FALSE	7QN2kA	0.034475	0.080649	486484	sepsis	TRUE	reported	textfile	0.0079	7.68E-06	5656	M12017.m	TRUE	reported	52YQ1d	2	TRUE	0.003578	20.29982
8604	rs1225608	A	G	A	G	0.0559	-0.02993	0.9155	0.927122	FALSE	FALSE	FALSE	oHM5TD	0.02622	0.253693	486484	sepsis	TRUE	reported	textfile	0.0124	6.69E-06	1780	M36131.m	TRUE	reported	Xw6wmg	2	TRUE	0.011288	20.29981
7910	rs1175889	T	C	T	C	0.0766	0.059159	0.0242	0.033804	FALSE	FALSE	FALSE	8qXKkX	0.038317	0.1226	486484	sepsis	TRUE	reported	textfile	0.017	6.36E-06	6234	M35189.m	TRUE	reported	Nt6oaC	2	TRUE	0.003246	20.29646
7046	rs1317460	T	C	T	C	0.0604	0.004636	0.9576	0.929931	FALSE	FALSE	FALSE	uthruC	0.027497	0.866116	486484	sepsis	TRUE	reported	textfile	0.0134	6.15E-06	1755	M34283.m	TRUE	reported	ftTuRU	2	TRUE	0.011444	20.29407
479	rs1122400	A	G	A	G	0.0419	0.018985	0.177	0.223655	FALSE	FALSE	FALSE	ScJVI8	0.016453	0.248532	486484	sepsis	TRUE	reported	textfile	0.0093	7.07E-06	7371	M00542.m	TRUE	reported	Sa1L04	2	TRUE	0.002746	20.29291
6176	rs730596	A	G	A	G	0.0401	0.011228	0.9335	0.92704	FALSE	FALSE	FALSE	O4Ty2l	0.026458	0.671302	486484	sepsis	TRUE	reported	textfile	0.0089	6.43E-06	5107	M33488.m	TRUE	reported	RGtF84	2	TRUE	0.003959	20.29264
6700	rs768846	T	C	T	C	0.1284	-0.03037	0.9819	0.982424	FALSE	FALSE	FALSE	AcRp2F	0.052371	0.561987	486484	sepsis	TRUE	reported	textfile	0.0285	6.67E-06	6756	M33935.m	TRUE	reported	FQCKJy	2	TRUE	0.002995	20.29139
5641	rs9298802	T	C	T	C	-0.3291	0.000341	0.9866	0.995315	FALSE	FALSE	FALSE	U0d0ue	0.103027	0.997361	486484	sepsis	TRUE	reported	textfile	0.073	6.53E-06	1229	M33190.m	TRUE	reported	FoODGF	2	TRUE	0.016268	20.29097
7665	rs8180157	A	G	A	G	-0.041	0.002231	0.3926	0.378971	FALSE	FALSE	FALSE	z8S1DZ	0.014232	0.875436	486484	sepsis	TRUE	reported	textfile	0.0091	6.97E-06	4515	M34535.m	TRUE	reported	FNLxxh	2	TRUE	0.004476	20.29049
3667	rs7728541	A	G	A	G	0.0455	0.008764	0.0167	0.01854	FALSE	FALSE	FALSE	fnjup6	0.050913	0.863329	486484	sepsis	TRUE	reported	textfile	0.0101	7.09E-06	7346	M32197.m	TRUE	reported	oQzaMk	2	TRUE	0.002755	20.28905
3439	rs527140	A	G	A	G	-0.0329	0.011909	0.7481	0.77921	FALSE	FALSE	FALSE	5dHWFk	0.016507	0.470624	486484	sepsis	TRUE	reported	textfile	0.0073	6.77E-06	1758	M27672.m	TRUE	reported	pEd8kY	2	TRUE	0.011422	20.28858
3588	rs7638353	A	C	A	C	-0.1359	0.010601	0.5531	0.43051	FALSE	FALSE	FALSE	F1s6qg	0.01379	0.442066	486484	sepsis	TRUE	reported	textfile	0.0301	6.48E-06	423	M31536.m	TRUE	reported	Zm40GX	2	TRUE	0.045975	20.28839
983	rs400214	T	C	T	C	0.3258	0.006369	0.0185	0.02363	FALSE	FALSE	FALSE	xSRII4	0.045308	0.888212	486484	sepsis	TRUE	reported	textfile	0.0723	6.60E-06	2285	M01515.m	TRUE	reported	juZ5GE	2	TRUE	0.008808	20.28828
7680	rs7340748	A	G	A	G	0.0437	-0.02276	0.0539	0.096742	FALSE	FALSE	FALSE	iqkM9B	0.023503	0.332931	486484	sepsis	TRUE	reported	textfile	0.0097	5.92E-06	4800	M34539.m	TRUE	reported	5nFq6f	2	TRUE	0.004211	20.28796
8676	rs1783235	A	G	A	G	0.0347	-0.02719	0.7188	0.717846	FALSE	FALSE	FALSE	i4bSdJ	0.015275	0.075129	486484	sepsis	TRUE	reported	textfile	0.0077	6.59E-06	1908	M36468.m	TRUE	reported	qJLuZj	2	TRUE	0.010532	20.2872
4942	rs1540603	A	C	A	C	-0.0676	0.032468	0.0122	0.013332	FALSE	FALSE	FALSE	bKmDMa	0.059542	0.585554	486484	sepsis	TRUE	reported	textfile	0.015	7.00E-06	1760	M32910.m	TRUE	reported	nAuPzO	2	TRUE	0.011408	20.28696
7610	rs907609	T	C	T	C	0.1592	-0.01122	0.1354	0.151327	FALSE	FALSE	FALSE	PlYt5g	0.019034	0.555715	486484	sepsis	TRUE	reported	textfile	0.0352	6.18E-06	241	M34516.m	TRUE	reported	jX41tf	2	TRUE	0.078235	20.28531
6500	rs2623476	A	C	A	C	0.0446	0.042436	0.2405	0.262939	FALSE	FALSE	FALSE	HNc7C9	0.01555	0.006353	486484	sepsis	TRUE	reported	textfile	0.0099	6.56E-06	3962	M33782.m	TRUE	reported	SAGYGx	2	TRUE	0.005096	20.28523
4792	rs307944	A	G	A	G	-0.041	0.01157	0.205	0.206799	FALSE	FALSE	FALSE	dwreVz	0.018298	0.527187	486484	sepsis	TRUE	reported	textfile	0.0091	5.95E-06	2711	M32802.m	TRUE	reported	7YCayZ	2	TRUE	0.007432	20.28451
4672	rs1695866	A	G	A	G	-0.0509	-0.0112	0.0523	0.057565	FALSE	FALSE	FALSE	xaapK0	0.029366	0.702995	486484	sepsis	TRUE	reported	textfile	0.0113	6.26E-06	7250	M32757.m	TRUE	reported	64n1Z4	2	TRUE	0.002791	20.28425
986	rs9453415	A	G	A	G	0.374	-0.0667	0.011	0.014068	FALSE	FALSE	FALSE	xSRII4	0.058414	0.25352	486484	sepsis	TRUE	reported	textfile	0.083	6.61E-06	1803	M01515.m	TRUE	reported	juZ5GE	2	TRUE	0.011136	20.28173
7496	rs1208353	T	C	T	C	0.0527	-0.00783	0.2656	0.324062	FALSE	FALSE	FALSE	1aXQWs	0.014611	0.592101	486484	sepsis	TRUE	reported	textfile	0.0117	6.82E-06	4452	M34420.m	TRUE	reported	FOqhHW	2	TRUE	0.004536	20.27937
8516	rs6899809	T	C	T	C	-0.0554	0.003109	0.0436	0.050506	FALSE	FALSE	FALSE	vHXktL	0.031216	0.920653	486484	sepsis	TRUE	reported	textfile	0.0123	7.20E-06	4529	M35978.m	TRUE	reported	ceap9w	2	TRUE	0.004459	20.27764
5113	rs1094153	A	G	A	G	0.0401	-0.02611	0.6584	0.636336	FALSE	FALSE	FALSE	O6ine1	0.014187	0.065652	486484	sepsis	TRUE	reported	textfile	0.0089	6.29E-06	1751	M33165.m	TRUE	reported	gGsqjW	2	TRUE	0.011461	20.27741
8278	rs1248228	T	G	T	G	-0.1167	0.007241	0.9753	0.976122	FALSE	FALSE	FALSE	J6o4xC	0.045058	0.872321	486484	sepsis	TRUE	reported	textfile	0.0259	6.59E-06	1640	M35472.m	TRUE	reported	3XBcu9	2	TRUE	0.012228	20.2774
4920	rs1185269	T	C	T	C	-0.0446	0.001582	0.934	0.924858	FALSE	FALSE	FALSE	clhN6v	0.025948	0.951385	486484	sepsis	TRUE	reported	textfile	0.0099	6.41E-06	2148	M32869.m	TRUE	reported	BzloWk	2	TRUE	0.00936	20.27658
8261	rs7025875	A	T	A	T	-0.1843	-0.00678	0.9753	0.962896	FALSE	TRUE	FALSE	G4plxe	0.037596	0.856818	486484	sepsis	TRUE	reported	textfile	0.0409	6.54E-06	1383	M35451.m	TRUE	reported	hNOVMI	2	TRUE	0.014469	20.27569
6479	rs29766	A	G	A	G	0.1091	-0.01162	0.0862	0.089192	FALSE	FALSE	FALSE	ecwVg8	0.023982	0.628008	486484	sepsis	TRUE	reported	textfile	0.0242	6.39E-06	831	M33751.m	TRUE	reported	NnvbIQ	2	TRUE	0.023874	20.27553
1409	rs2349945	T	C	T	C	-0.0347	-0.00087	0.3863	0.362576	FALSE	FALSE	FALSE	33ptwk	0.014242	0.951022	486484	sepsis	TRUE	reported	textfile	0.0077	7.25E-06	1175	M02137.m	TRUE	reported	SQY1na	2	TRUE	0.01699	20.27392
1185	rs9419004	C	G	C	G	-0.254	-0.01818	0.186	0.273569	FALSE	TRUE	FALSE	FgZx4a	0.015327	0.235579	486484	sepsis	TRUE	reported	textfile	0.0563	6.53E-06	496	M01640.m	TRUE	reported	hNPAJ8	2	TRUE	0.039419	20.27197
2997	rs6005580	A	G	A	G	0.0446	-0.01736	0.7521	0.771127	FALSE	FALSE	FALSE	xmzJ5F	0.016302	0.28706	486484	sepsis	TRUE	reported	textfile	0.0099	6.38E-06	1690	M21184.m	TRUE	reported	xgwhDp	2	TRUE	0.011867	20.27146
2589	rs7697449	T	C	T	C	0.0383	0.024514	0.5636	0.556689	FALSE	FALSE	FALSE	JGJ9vH	0.013824	0.076173	486484	sepsis	TRUE	reported	textfile	0.0085	6.18E-06	1204	M18357.m	TRUE	reported	cOV2qw	2	TRUE	0.016583	20.26925
4501	rs7281501	T	G	T	G	0.0482	0.003381	0.9362	0.93514	FALSE	FALSE	FALSE	1wNJ1E	0.027691	0.902834	486484	sepsis	TRUE	reported	textfile	0.0107	7.18E-06	1766	M32709.m	TRUE	reported	zihBHg	2	TRUE	0.01136	20.2691
5630	rs2753980	A	G	A	G	-0.0793	-0.00217	0.6032	0.607363	FALSE	FALSE	FALSE	U0d0ue	0.014021	0.877119	486484	sepsis	TRUE	reported	textfile	0.0176	6.74E-06	1229	M33190.m	TRUE	reported	FoODGF	2	TRUE	0.01625	20.26813
7634	rs9277380	C	G	C	G	0.0631	0.018832	0.7148	0.700217	FALSE	TRUE	FALSE	xGQKuv	0.014901	0.206286	486484	sepsis	TRUE	reported	textfile	0.014	6.78E-06	829	M34530.m	TRUE	reported	wajzOs	2	TRUE	0.023919	20.26533
5689	rs1710456	A	G	A	G	0.1077	0.000271	0.292	0.287232	FALSE	FALSE	FALSE	KxtFnC	0.020952	0.989667	486484	sepsis	TRUE	reported	textfile	0.0239	6.56E-06	930	M33203.m	TRUE	reported	Uz0wKU	2	TRUE	0.021368	20.26285
5437	rs3811321	A	C	A	C	0.1266	-0.00736	0.9373	0.937807	FALSE	FALSE	FALSE	q32d4E	0.028345	0.795029	486484	sepsis	TRUE	reported	textfile	0.0281	6.78E-06	1017	M33178.m	TRUE	reported	cS3BwT	2	TRUE	0.019568	20.25815
5801	rs8011328	A	G	A	G	0.1081	0.045451	0.0129	0.021808	FALSE	FALSE	FALSE	ka9zG7	0.046846	0.33194	486484	sepsis	TRUE	reported	textfile	0.024	6.59E-06	1378	M33363.m	TRUE	reported	UFTVNc	2	TRUE	0.014509	20.25807
832	rs3748771	T	G	T	G	-0.0671	0.003066	0.9877	0.990927	FALSE	FALSE	FALSE	2qgDgB	0.072138	0.966096	486484	sepsis	TRUE	reported	textfile	0.0149	6.35E-06	1765	M01358.m	TRUE	reported	asmXq0	2	TRUE	0.01136	20.25723
8741	rs9922902	C	G	C	G	0.0635	-0.01244	0.0778	0.242168	FALSE	TRUE	FALSE	ERC6hD	0.016205	0.442712	486484	sepsis	TRUE	reported	textfile	0.0141	6.45E-06	1636	M36559.m	TRUE	reported	n8waJF	2	TRUE	0.012245	20.25713
4885	rs1117767	T	C	T	C	0.0743	0.001056	0.1109	0.125207	FALSE	FALSE	FALSE	ocaywK	0.020762	0.959436	486484	sepsis	TRUE	reported	textfile	0.0165	7.01E-06	1988	M32863.m	TRUE	reported	WH6KGr	2	TRUE	0.010097	20.25688
810	rs1706683	C	G	C	G	-0.0707	0.05921	0.0202	0.01836	FALSE	TRUE	FALSE	J58DI0	0.05123	0.247776	486484	sepsis	TRUE	reported	textfile	0.0157	6.24E-06	1755	M01356.m	TRUE	reported	zk9AMB	2	TRUE	0.011423	20.25556
3245	rs9477741	C	G	C	G	-0.0851	-0.03665	0.0113	0.013029	FALSE	TRUE	FALSE	COSeSD	0.06075	0.546319	486484	sepsis	TRUE	reported	textfile	0.0189	6.89E-06	1547	M22649.m	TRUE	reported	mnH8z2	2	TRUE	0.012936	20.24761
1562	rs4483514	T	G	T	G	0.009	-0.00283	0.357	0.3568	FALSE	FALSE	FALSE	Ke0sNh	0.014277	0.842968	486484	sepsis	TRUE	reported	textfile	0.002	6.80E-06	7799	M10642.m	TRUE	reported	ZINwVb	2	TRUE	0.00259	20.24481
1568	rs6828152	T	G	T	G	0.009	0.021818	0.359	0.366409	FALSE	FALSE	FALSE	Ke0sNh	0.014241	0.125506	486484	sepsis	TRUE	reported	textfile	0.002	6.80E-06	7799	M10642.m	TRUE	reported	ZINwVb	2	TRUE	0.00259	20.24481
1570	rs7859597	A	G	A	G	-0.009	0.000106	0.737	0.727082	FALSE	FALSE	FALSE	Ke0sNh	0.015371	0.99448	486484	sepsis	TRUE	reported	textfile	0.002	6.80E-06	7799	M10642.m	TRUE	reported	ZINwVb	2	TRUE	0.00259	20.24481
4088	rs1299971	A	T	A	T	-0.0153	0.016183	0.6299	0.630298	FALSE	TRUE	FALSE	j2JYxt	0.014173	0.253533	486484	sepsis	TRUE	reported	textfile	0.0034	8.38E-06	7373	M32504.m	TRUE	reported	poO7a5	2	TRUE	0.002739	20.24451
3	rs2199619	T	C	T	C	0.0054	-0.02073	0.2787	0.269636	FALSE	FALSE	FALSE	0cebDu	0.015411	0.178628	486484	sepsis	TRUE	reported	textfile	0.0012	5.23E-06	7372	M00053.m	TRUE	reported	i8iuxX	2	TRUE	0.002739	20.24451
1124	rs1113001	T	C	T	C	-0.0081	-0.00134	0.153	0.154942	FALSE	FALSE	FALSE	nIqUuV	0.018886	0.943569	486484	sepsis	TRUE	reported	textfile	0.0018	5.09E-06	7371	M01604.m	TRUE	reported	Uro9nC	2	TRUE	0.00274	20.24451
1143	rs9557545	A	T	A	T	0.018	-0.025	0.032	0.029878	FALSE	TRUE	FALSE	nIqUuV	0.040307	0.535073	486484	sepsis	TRUE	reported	textfile	0.004	7.83E-06	7371	M01604.m	TRUE	reported	Uro9nC	2	TRUE	0.00274	20.24451
4229	rs4447076	A	G	A	G	-0.0162	0.0092	0.6152	0.599272	FALSE	FALSE	FALSE	FDy32d	0.013921	0.508682	486484	sepsis	TRUE	reported	textfile	0.0036	8.58E-06	7369	M32587.m	TRUE	reported	S10k1h	2	TRUE	0.00274	20.2445
1797	rs3788706	A	G	A	G	-0.0117	0.005251	0.1302	0.13505	FALSE	FALSE	FALSE	JEcCRZ	0.020006	0.792976	486484	sepsis	TRUE	reported	textfile	0.0026	6.10E-06	7368	M15140.m	TRUE	reported	xYCzKP	2	TRUE	0.002741	20.2445
606	rs1010069	A	G	A	G	0.0081	0.021811	0.4623	0.456451	FALSE	FALSE	FALSE	BYGzq4	0.013725	0.112037	486484	sepsis	TRUE	reported	textfile	0.0018	8.58E-06	7367	M01110.m	TRUE	reported	oZiXqP	2	TRUE	0.002741	20.2445
615	rs180022	T	C	T	C	0.0117	0.00753	0.8328	0.838948	FALSE	FALSE	FALSE	BYGzq4	0.018582	0.685319	486484	sepsis	TRUE	reported	textfile	0.0026	9.43E-06	7367	M01110.m	TRUE	reported	oZiXqP	2	TRUE	0.002741	20.2445
1325	rs1051263	A	G	A	G	0.0117	0.035792	0.1405	0.138017	FALSE	FALSE	FALSE	LDUJbh	0.019785	0.070438	486484	sepsis	TRUE	reported	textfile	0.0026	8.59E-06	7367	M01898.m	TRUE	reported	JRSLeH	2	TRUE	0.002741	20.2445
9025	rs1271385	A	G	A	G	0.0081	0.0249	0.6774	0.671411	FALSE	FALSE	FALSE	df0MCM	0.014621	0.088558	486484	sepsis	TRUE	reported	textfile	0.0018	5.24E-06	7366	M37506.m	TRUE	reported	iUfr4u	2	TRUE	0.002742	20.2445
9037	rs2269282	T	C	T	C	0.0081	0.001242	0.6994	0.706092	FALSE	FALSE	FALSE	df0MCM	0.014985	0.933956	486484	sepsis	TRUE	reported	textfile	0.0018	4.53E-06	7366	M37506.m	TRUE	reported	iUfr4u	2	TRUE	0.002742	20.2445
9061	rs9947662	A	G	A	G	0.0081	0.032898	0.2582	0.264065	FALSE	FALSE	FALSE	df0MCM	0.015528	0.034115	486484	sepsis	TRUE	reported	textfile	0.0018	7.46E-06	7366	M37506.m	TRUE	reported	iUfr4u	2	TRUE	0.002742	20.2445
412	rs1479402	T	C	T	C	0.0306	-0.0394	0.0137	0.01229	FALSE	FALSE	FALSE	cTsNj6	0.06237	0.527593	486484	sepsis	TRUE	reported	textfile	0.0068	6.85E-06	7365	M00063.m	TRUE	reported	7KYTrQ	2	TRUE	0.002742	20.2445
416	rs1772892	A	C	A	C	0.0081	0.013298	0.1768	0.178997	FALSE	FALSE	FALSE	cTsNj6	0.017877	0.456947	486484	sepsis	TRUE	reported	textfile	0.0018	5.30E-06	7365	M00063.m	TRUE	reported	7KYTrQ	2	TRUE	0.002742	20.2445
441	rs886282	T	C	T	C	0.0081	-0.01372	0.1989	0.217944	FALSE	FALSE	FALSE	cTsNj6	0.016717	0.41183	486484	sepsis	TRUE	reported	textfile	0.0018	3.71E-06	7365	M00063.m	TRUE	reported	7KYTrQ	2	TRUE	0.002742	20.2445
3825	rs1420426	A	G	A	G	0.0081	0.002282	0.6202	0.618236	FALSE	FALSE	FALSE	tRX2u0	0.014089	0.871335	486484	sepsis	TRUE	reported	textfile	0.0018	4.16E-06	7365	M32348.m	TRUE	reported	ruBgwQ	2	TRUE	0.002742	20.2445
1039	rs1280330	A	T	A	T	-0.0081	-0.01963	0.6752	0.672856	FALSE	TRUE	FALSE	tRtQ2e	0.014611	0.179151	486484	sepsis	TRUE	reported	textfile	0.0018	5.97E-06	7364	M01564.m	TRUE	reported	Tbg0zM	2	TRUE	0.002742	20.2445
1047	rs1703468	T	G	T	G	0.0081	-0.01773	0.2881	0.288189	FALSE	FALSE	FALSE	tRtQ2e	0.015151	0.242023	486484	sepsis	TRUE	reported	textfile	0.0018	7.90E-06	7364	M01564.m	TRUE	reported	Tbg0zM	2	TRUE	0.002742	20.2445
1053	rs3193970	T	C	T	C	0.0081	-0.02028	0.5801	0.573165	FALSE	FALSE	FALSE	tRtQ2e	0.013867	0.143585	486484	sepsis	TRUE	reported	textfile	0.0018	6.07E-06	7364	M01564.m	TRUE	reported	Tbg0zM	2	TRUE	0.002742	20.2445
1062	rs7710027	T	C	T	C	0.0081	0.022414	0.2886	0.29254	FALSE	FALSE	FALSE	tRtQ2e	0.015033	0.135979	486484	sepsis	TRUE	reported	textfile	0.0018	9.16E-06	7364	M01564.m	TRUE	reported	Tbg0zM	2	TRUE	0.002742	20.2445
1064	rs7919076	T	C	T	C	-0.0081	-0.00889	0.3416	0.338936	FALSE	FALSE	FALSE	tRtQ2e	0.014564	0.541718	486484	sepsis	TRUE	reported	textfile	0.0018	7.79E-06	7364	M01564.m	TRUE	reported	Tbg0zM	2	TRUE	0.002742	20.2445
1071	rs9327706	A	C	A	C	0.0081	0.006404	0.5937	0.597954	FALSE	FALSE	FALSE	tRtQ2e	0.013928	0.645677	486484	sepsis	TRUE	reported	textfile	0.0018	5.61E-06	7364	M01564.m	TRUE	reported	Tbg0zM	2	TRUE	0.002742	20.2445
1072	rs9372398	T	C	T	C	0.0081	-0.00539	0.5453	0.448303	FALSE	FALSE	FALSE	tRtQ2e	0.013759	0.695255	486484	sepsis	TRUE	reported	textfile	0.0018	6.74E-06	7364	M01564.m	TRUE	reported	Tbg0zM	2	TRUE	0.002742	20.2445
3982	rs3806851	T	C	T	C	-0.0081	0.016066	0.4918	0.48303	FALSE	FALSE	FALSE	XdJbEU	0.013722	0.241681	486484	sepsis	TRUE	reported	textfile	0.0018	9.85E-06	7364	M32452.m	TRUE	reported	V5xWKi	2	TRUE	0.002742	20.2445
3989	rs7545139	A	G	A	G	0.0081	0.010803	0.5822	0.580253	FALSE	FALSE	FALSE	XdJbEU	0.013887	0.436634	486484	sepsis	TRUE	reported	textfile	0.0018	9.14E-06	7364	M32452.m	TRUE	reported	V5xWKi	2	TRUE	0.002742	20.2445
5745	rs1572948	A	G	A	G	-0.0126	0.011936	0.4296	0.421673	FALSE	FALSE	FALSE	bm6x2Z	0.01398	0.393227	486484	sepsis	TRUE	reported	textfile	0.0028	4.98E-06	7364	M33230.m	TRUE	reported	8Ccke3	2	TRUE	0.002742	20.2445
5750	rs7301800	A	G	A	G	0.0162	0.022655	0.2124	0.215043	FALSE	FALSE	FALSE	bm6x2Z	0.016836	0.178431	486484	sepsis	TRUE	reported	textfile	0.0036	8.73E-06	7364	M33230.m	TRUE	reported	8Ccke3	2	TRUE	0.002742	20.2445
6800	rs303610	A	T	A	T	-0.0117	-0.01367	0.2958	0.304449	FALSE	TRUE	FALSE	OZ0I1H	0.015057	0.363774	486484	sepsis	TRUE	reported	textfile	0.0026	8.88E-06	7364	M33960.m	TRUE	reported	GlPp4w	2	TRUE	0.002742	20.2445
6807	rs969948	T	C	T	C	-0.0117	0.01331	0.6023	0.590722	FALSE	FALSE	FALSE	OZ0I1H	0.013964	0.340488	486484	sepsis	TRUE	reported	textfile	0.0026	5.74E-06	7364	M33960.m	TRUE	reported	GlPp4w	2	TRUE	0.002742	20.2445
7779	rs3127434	A	G	A	G	0.0198	-0.01741	0.489	0.489578	FALSE	FALSE	FALSE	15BqPa	0.013654	0.202198	486484	sepsis	TRUE	reported	textfile	0.0044	7.05E-06	7364	M35126.m	TRUE	reported	zvDzP2	2	TRUE	0.002742	20.2445
734	rs1333926	A	G	A	G	-0.0081	0.03025	0.8587	0.857131	FALSE	FALSE	FALSE	9u0SC8	0.019615	0.123042	486484	sepsis	TRUE	reported	textfile	0.0018	6.52E-06	7363	M01301.m	TRUE	reported	H95uec	2	TRUE	0.002743	20.2445
3644	rs6904596	A	G	A	G	-0.0531	-0.01334	0.1218	0.125175	FALSE	FALSE	FALSE	Vq2efp	0.020611	0.517624	486484	sepsis	TRUE	reported	textfile	0.0118	7.36E-06	7363	M31787.m	TRUE	reported	krl4Tw	2	TRUE	0.002743	20.2445
4004	rs1293768	T	G	T	G	-0.0081	-0.00594	0.3221	0.325517	FALSE	FALSE	FALSE	V9AtId	0.01459	0.683743	486484	sepsis	TRUE	reported	textfile	0.0018	9.31E-06	7363	M32489.m	TRUE	reported	8B8S8a	2	TRUE	0.002743	20.2445
4012	rs2103340	T	C	T	C	-0.0081	0.001647	0.6181	0.621698	FALSE	FALSE	FALSE	V9AtId	0.014078	0.906846	486484	sepsis	TRUE	reported	textfile	0.0018	7.41E-06	7363	M32489.m	TRUE	reported	8B8S8a	2	TRUE	0.002743	20.2445
4016	rs2846444	A	G	A	G	-0.0081	-0.00513	0.5854	0.586717	FALSE	FALSE	FALSE	V9AtId	0.013901	0.711995	486484	sepsis	TRUE	reported	textfile	0.0018	6.34E-06	7363	M32489.m	TRUE	reported	8B8S8a	2	TRUE	0.002743	20.2445
4020	rs511319	C	G	C	G	0.0081	0.000597	0.3878	0.389314	FALSE	TRUE	FALSE	V9AtId	0.01409	0.966217	486484	sepsis	TRUE	reported	textfile	0.0018	7.27E-06	7363	M32489.m	TRUE	reported	8B8S8a	2	TRUE	0.002743	20.2445
4024	rs6886152	A	C	A	C	0.0081	-0.00861	0.5983	0.602336	FALSE	FALSE	FALSE	V9AtId	0.01401	0.538832	486484	sepsis	TRUE	reported	textfile	0.0018	6.12E-06	7363	M32489.m	TRUE	reported	8B8S8a	2	TRUE	0.002743	20.2445
880	rs1083149	A	G	A	G	0.0126	0.026408	0.2209	0.23537	FALSE	FALSE	FALSE	oi7KQI	0.016185	0.102759	486484	sepsis	TRUE	reported	textfile	0.0028	4.97E-06	7362	M01365.m	TRUE	reported	7NWGKA	2	TRUE	0.002743	20.2445
894	rs7016645	T	C	T	C	-0.0117	-0.01698	0.253	0.257847	FALSE	FALSE	FALSE	oi7KQI	0.015625	0.277236	486484	sepsis	TRUE	reported	textfile	0.0026	8.44E-06	7362	M01365.m	TRUE	reported	7NWGKA	2	TRUE	0.002743	20.2445
4281	rs1005075	A	G	A	G	-0.0081	-0.01632	0.4668	0.473665	FALSE	FALSE	FALSE	0tjjPC	0.013774	0.236118	486484	sepsis	TRUE	reported	textfile	0.0018	5.84E-06	7362	M32634.m	TRUE	reported	nJJFkX	2	TRUE	0.002743	20.2445
4285	rs1116185	T	C	T	C	0.0081	-0.00371	0.5897	0.597502	FALSE	FALSE	FALSE	0tjjPC	0.013933	0.790278	486484	sepsis	TRUE	reported	textfile	0.0018	5.41E-06	7362	M32634.m	TRUE	reported	nJJFkX	2	TRUE	0.002743	20.2445
4288	rs1412853	A	G	A	G	-0.0081	0.010546	0.2693	0.273898	FALSE	FALSE	FALSE	0tjjPC	0.015363	0.492449	486484	sepsis	TRUE	reported	textfile	0.0018	7.23E-06	7362	M32634.m	TRUE	reported	nJJFkX	2	TRUE	0.002743	20.2445
4289	rs1744401	A	G	A	G	0.0117	0.028281	0.8636	0.861713	FALSE	FALSE	FALSE	0tjjPC	0.020043	0.158246	486484	sepsis	TRUE	reported	textfile	0.0026	7.81E-06	7362	M32634.m	TRUE	reported	nJJFkX	2	TRUE	0.002743	20.2445
4290	rs174578	A	T	A	T	-0.0081	0.024753	0.3433	0.352673	FALSE	TRUE	FALSE	0tjjPC	0.014314	0.083768	486484	sepsis	TRUE	reported	textfile	0.0018	6.73E-06	7362	M32634.m	TRUE	reported	nJJFkX	2	TRUE	0.002743	20.2445
5042	rs9686531	T	C	T	C	0.0081	-0.00417	0.119	0.119666	FALSE	FALSE	FALSE	BNnOvt	0.021288	0.844598	486484	sepsis	TRUE	reported	textfile	0.0018	7.74E-06	7362	M33132.m	TRUE	reported	rHcCPG	2	TRUE	0.002743	20.2445
6518	rs1244947	T	C	T	C	-0.0162	0.004306	0.2067	0.209492	FALSE	FALSE	FALSE	OKIXJ8	0.016804	0.79778	486484	sepsis	TRUE	reported	textfile	0.0036	8.10E-06	7362	M33821.m	TRUE	reported	XGkaXH	2	TRUE	0.002743	20.2445
6862	rs1090664	A	G	A	G	0.0081	0.006058	0.8721	0.872366	FALSE	FALSE	FALSE	kFWZjs	0.020513	0.767752	486484	sepsis	TRUE	reported	textfile	0.0018	7.66E-06	7362	M34062.m	TRUE	reported	eLONsg	2	TRUE	0.002743	20.2445
6867	rs1288425	A	G	A	G	0.0081	0.008401	0.1395	0.160046	FALSE	FALSE	FALSE	kFWZjs	0.01867	0.652725	486484	sepsis	TRUE	reported	textfile	0.0018	8.02E-06	7362	M34062.m	TRUE	reported	eLONsg	2	TRUE	0.002743	20.2445
6883	rs5999780	T	C	T	C	0.0081	0.015677	0.1528	0.166175	FALSE	FALSE	FALSE	kFWZjs	0.018763	0.403409	486484	sepsis	TRUE	reported	textfile	0.0018	7.21E-06	7362	M34062.m	TRUE	reported	eLONsg	2	TRUE	0.002743	20.2445
456	rs1498350	A	C	A	C	0.0045	0.000705	0.4954	0.501727	FALSE	FALSE	FALSE	cl8ucN	0.013698	0.958976	486484	sepsis	TRUE	reported	textfile	0.001	1.88E-06	7361	M00513.m	TRUE	reported	Ian0Q8	2	TRUE	0.002743	20.2445
463	rs4460629	T	C	T	C	-0.0045	0.023595	0.5224	0.514288	FALSE	FALSE	FALSE	cl8ucN	0.013664	0.084192	486484	sepsis	TRUE	reported	textfile	0.001	2.21E-06	7361	M00513.m	TRUE	reported	Ian0Q8	2	TRUE	0.002743	20.2445
701	rs1512342	T	C	T	C	0.0072	-0.00147	0.3384	0.341357	FALSE	FALSE	FALSE	H9I3s3	0.014468	0.91918	486484	sepsis	TRUE	reported	textfile	0.0016	7.01E-06	7358	M01299.m	TRUE	reported	kXWyUq	2	TRUE	0.002745	20.2445
4070	rs1863824	A	G	A	G	0.0117	0.009667	0.613	0.61626	FALSE	FALSE	FALSE	TyHtdh	0.014088	0.492564	486484	sepsis	TRUE	reported	textfile	0.0026	9.64E-06	7358	M32497.m	TRUE	reported	H3KmLR	2	TRUE	0.002745	20.2445
1539	rs7896866	A	C	A	C	0.0108	0.009609	0.2982	0.278498	FALSE	FALSE	FALSE	YvgTeW	0.0153	0.529984	486484	sepsis	TRUE	reported	textfile	0.0024	8.34E-06	7357	M03141.m	TRUE	reported	srKjSc	2	TRUE	0.002745	20.2445
2190	rs7102716	A	T	A	T	0.027	-0.04132	0.7654	0.759444	FALSE	TRUE	FALSE	UokbxN	0.016052	0.010055	486484	sepsis	TRUE	reported	textfile	0.006	7.85E-06	7357	M15753.m	TRUE	reported	lUGKBi	2	TRUE	0.002745	20.2445
1606	rs2147618	A	C	A	C	-0.0081	0.008739	0.5036	0.493766	FALSE	FALSE	FALSE	zP3C2v	0.01367	0.522634	486484	sepsis	TRUE	reported	textfile	0.0018	5.31E-06	7356	M12035.m	TRUE	reported	1Pooe3	2	TRUE	0.002745	20.24449
1607	rs2286396	T	C	T	C	0.0117	0.026125	0.1505	0.157654	FALSE	FALSE	FALSE	zP3C2v	0.018751	0.163559	486484	sepsis	TRUE	reported	textfile	0.0026	5.60E-06	7356	M12035.m	TRUE	reported	1Pooe3	2	TRUE	0.002745	20.24449
1612	rs511545	T	C	T	C	0.0081	-0.00377	0.583	0.577107	FALSE	FALSE	FALSE	zP3C2v	0.013883	0.786012	486484	sepsis	TRUE	reported	textfile	0.0018	4.63E-06	7356	M12035.m	TRUE	reported	1Pooe3	2	TRUE	0.002745	20.24449
1616	rs7214330	T	C	T	C	0.0144	-0.02121	0.1256	0.142246	FALSE	FALSE	FALSE	zP3C2v	0.019525	0.277323	486484	sepsis	TRUE	reported	textfile	0.0032	9.76E-06	7356	M12035.m	TRUE	reported	1Pooe3	2	TRUE	0.002745	20.24449
1735	rs1089924	T	C	T	C	-0.0081	-0.00443	0.5316	0.52077	FALSE	FALSE	FALSE	nFLpAP	0.013748	0.747104	486484	sepsis	TRUE	reported	textfile	0.0018	7.02E-06	7356	M12774.m	TRUE	reported	yOSQTj	2	TRUE	0.002745	20.24449
1746	rs3219095	T	C	T	C	0.0117	0.028335	0.8471	0.839469	FALSE	FALSE	FALSE	nFLpAP	0.018629	0.12825	486484	sepsis	TRUE	reported	textfile	0.0026	9.79E-06	7356	M12774.m	TRUE	reported	yOSQTj	2	TRUE	0.002745	20.24449
1759	rs988143	T	C	T	C	0.0081	0.021566	0.4828	0.479797	FALSE	FALSE	FALSE	nFLpAP	0.013677	0.114844	486484	sepsis	TRUE	reported	textfile	0.0018	5.25E-06	7356	M12774.m	TRUE	reported	yOSQTj	2	TRUE	0.002745	20.24449
3766	rs8453	T	G	T	G	0.0144	-0.01139	0.158	0.156485	FALSE	FALSE	FALSE	OZLdls	0.018785	0.544134	486484	sepsis	TRUE	reported	textfile	0.0032	8.06E-06	7356	M32338.m	TRUE	reported	s2itmB	2	TRUE	0.002745	20.24449
33	rs1179636	A	C	A	C	-0.0045	0.007693	0.5356	0.535756	FALSE	FALSE	FALSE	xmGuol	0.013774	0.576486	486484	sepsis	TRUE	reported	textfile	0.001	1.83E-06	7355	M00054.m	TRUE	reported	GW50Ri	2	TRUE	0.002746	20.24449
822	rs1237044	T	C	T	C	0.0081	-0.0137	0.5836	0.578341	FALSE	FALSE	FALSE	2qgDgB	0.014077	0.330618	486484	sepsis	TRUE	reported	textfile	0.0018	5.10E-06	7355	M01358.m	TRUE	reported	asmXq0	2	TRUE	0.002746	20.24449
838	rs6453594	A	G	A	G	-0.0108	-0.00874	0.2196	0.242637	FALSE	FALSE	FALSE	2qgDgB	0.01596	0.584073	486484	sepsis	TRUE	reported	textfile	0.0024	7.01E-06	7355	M01358.m	TRUE	reported	asmXq0	2	TRUE	0.002746	20.24449

845	rs9568232	T	C	T	C	0.0126	0.025377	0.1152	0.064291	FALSE	FALSE	FALSE	2qgDgB	0.028041	0.365478	486484	sepsis	TRUE	reported	textfile	0.0028	5.06E-06	7355	M01358.m	TRUE	reported	asmXq0	2	TRUE	0.002746	20.24449
1200	rs1773910	A	G	A	G	-0.0198	0.018118	0.942	0.935981	FALSE	FALSE	FALSE	Ckdgh1	0.027923	0.516445	486484	sepsis	TRUE	reported	textfile	0.0044	8.09E-06	7355	M01644.m	TRUE	reported	nKxz7B	2	TRUE	0.002746	20.24449
1208	rs269194	A	G	A	G	-0.0081	-0.00385	0.4386	0.438229	FALSE	FALSE	FALSE	Ckdgh1	0.013806	0.780612	486484	sepsis	TRUE	reported	textfile	0.0018	9.01E-06	7355	M01644.m	TRUE	reported	nKxz7B	2	TRUE	0.002746	20.24449
4039	rs1373469	T	C	T	C	0.0081	0.031605	0.5235	0.520672	FALSE	FALSE	FALSE	SiYGYU	0.01366	0.020688	486484	sepsis	TRUE	reported	textfile	0.0018	4.85E-06	7355	M32492.m	TRUE	reported	GfQvpU	2	TRUE	0.002746	20.24449
4055	rs7712191	T	G	T	G	0.0081	-0.00072	0.6607	0.660843	FALSE	FALSE	FALSE	SiYGYU	0.014441	0.960409	486484	sepsis	TRUE	reported	textfile	0.0018	7.10E-06	7355	M32492.m	TRUE	reported	GfQvpU	2	TRUE	0.002746	20.24449
562	rs1482928	T	G	T	G	0.0081	-0.00194	0.7912	0.790194	FALSE	FALSE	FALSE	fNqkCW	0.016815	0.908197	486484	sepsis	TRUE	reported	textfile	0.0018	4.42E-06	7354	M00606.m	TRUE	reported	g9uwOn	2	TRUE	0.002746	20.24449
571	rs787496	A	G	A	G	0.0081	-0.01819	0.1702	0.193064	FALSE	FALSE	FALSE	fNqkCW	0.017388	0.295511	486484	sepsis	TRUE	reported	textfile	0.0018	5.98E-06	7354	M00606.m	TRUE	reported	g9uwOn	2	TRUE	0.002746	20.24449
939	rs7561255	A	G	A	G	-0.0072	-0.01154	0.7086	0.71287	FALSE	FALSE	FALSE	tv99UN	0.015126	0.445515	486484	sepsis	TRUE	reported	textfile	0.0016	4.72E-06	7354	M01494.m	TRUE	reported	osDclp	2	TRUE	0.002746	20.24449
2608	rs6768610	T	C	T	C	0.0144	-0.00165	0.914	0.921035	FALSE	FALSE	FALSE	ruvJh6	0.025411	0.948283	486484	sepsis	TRUE	reported	textfile	0.0032	7.08E-06	7354	M18369.m	TRUE	reported	GFmwGk	2	TRUE	0.002746	20.24449
2812	rs2807592	T	G	T	G	-0.0081	0.031267	0.6979	0.705466	FALSE	FALSE	FALSE	Sx5Uhq	0.015285	0.040795	486484	sepsis	TRUE	reported	textfile	0.0018	4.45E-06	7354	M19934.m	TRUE	reported	DijYTE	2	TRUE	0.002746	20.24449
6755	rs1091371	A	G	A	G	0.0081	-0.00035	0.212	0.21444	FALSE	FALSE	FALSE	Elf6MQ	0.01664	0.983328	486484	sepsis	TRUE	reported	textfile	0.0018	8.90E-06	7354	M33955.m	TRUE	reported	Lxn4RF	2	TRUE	0.002746	20.24449
6758	rs1165452	T	G	T	G	0.0081	-0.01213	0.1988	0.195036	FALSE	FALSE	FALSE	Elf6MQ	0.017294	0.482909	486484	sepsis	TRUE	reported	textfile	0.0018	8.24E-06	7354	M33955.m	TRUE	reported	Lxn4RF	2	TRUE	0.002746	20.24449
6762	rs1781486	A	G	A	G	-0.0081	-0.01141	0.7346	0.739811	FALSE	FALSE	FALSE	Elf6MQ	0.015624	0.465088	486484	sepsis	TRUE	reported	textfile	0.0018	8.40E-06	7354	M33955.m	TRUE	reported	Lxn4RF	2	TRUE	0.002746	20.24449
6774	rs6714201	A	G	A	G	0.0081	0.026104	0.483	0.496068	FALSE	FALSE	FALSE	Elf6MQ	0.013759	0.057786	486484	sepsis	TRUE	reported	textfile	0.0018	4.41E-06	7354	M33955.m	TRUE	reported	Lxn4RF	2	TRUE	0.002746	20.24449
6783	rs9880529	T	C	T	C	0.0117	-0.00985	0.8762	0.863252	FALSE	FALSE	FALSE	Elf6MQ	0.019982	0.621975	486484	sepsis	TRUE	reported	textfile	0.0026	7.47E-06	7354	M33955.m	TRUE	reported	Lxn4RF	2	TRUE	0.002746	20.24449
2470	rs9560579	A	G	A	G	-0.0153	-0.00473	0.5193	0.513367	FALSE	FALSE	FALSE	C5wkoN	0.013676	0.729568	486484	sepsis	TRUE	reported	textfile	0.0034	6.41E-06	7353	M17805.m	TRUE	reported	F5VXY0	2	TRUE	0.002746	20.24449
1769	rs2259038	A	G	A	G	-0.0216	-0.01379	0.203	0.042143	FALSE	FALSE	FALSE	wM5dkT	0.033878	0.684071	486484	sepsis	TRUE	reported	textfile	0.0048	7.51E-06	7352	M15122.m	TRUE	reported	CBpa4t	2	TRUE	0.002747	20.24449
1778	rs9878149	A	G	A	G	0.0117	0.01906	0.2674	0.269308	FALSE	FALSE	FALSE	wM5dkT	0.015513	0.219193	486484	sepsis	TRUE	reported	textfile	0.0026	5.48E-06	7352	M15122.m	TRUE	reported	CBpa4t	2	TRUE	0.002747	20.24449
674	rs503360	A	G	A	G	0.0081	-0.01992	0.8799	0.87707	FALSE	FALSE	FALSE	p4oCVP	0.020796	0.338039	486484	sepsis	TRUE	reported	textfile	0.0018	4.79E-06	7352	M01125.m	TRUE	reported	OwD5re	2	TRUE	0.002747	20.24449
6305	rs2838532	T	G	T	G	0.018	-0.01932	0.2993	0.297971	FALSE	FALSE	FALSE	ORVTMc	0.014945	0.196121	486484	sepsis	TRUE	reported	textfile	0.004	7.83E-06	7352	M33587.m	TRUE	reported	57AsXW	2	TRUE	0.002747	20.24449
266	rs1822834	C	G	C	G	0.0108	0.010741	0.0629	0.070214	FALSE	TRUE	FALSE	tbRL8J	0.026823	0.688836	486484	sepsis	TRUE	reported	textfile	0.0024	6.59E-06	7351	M00060.m	TRUE	reported	oOvRKT	2	TRUE	0.002747	20.24449
278	rs2285137	T	C	T	C	-0.0108	0.021144	0.9436	0.950358	FALSE	FALSE	FALSE	tbRL8J	0.031595	0.503357	486484	sepsis	TRUE	reported	textfile	0.0024	5.35E-06	7351	M00060.m	TRUE	reported	oOvRKT	2	TRUE	0.002747	20.24449
4116	rs3784934	A	C	A	C	0.0126	0.031361	0.0856	0.085484	FALSE	FALSE	FALSE	4rtLd0	0.024388	0.19848	486484	sepsis	TRUE	reported	textfile	0.0028	5.64E-06	7350	M32518.m	TRUE	reported	j7f1ER	2	TRUE	0.002748	20.24449
6534	rs2269543	T	C	T	C	-0.0162	0.008403	0.2719	0.25734	FALSE	FALSE	FALSE	DedZqr	0.015625	0.590731	486484	sepsis	TRUE	reported	textfile	0.0036	8.66E-06	7350	M33822.m	TRUE	reported	ciYOdn	2	TRUE	0.002748	20.24449
7897	rs4530945	T	C	T	C	-0.0117	-0.00783	0.732	0.735766	FALSE	FALSE	FALSE	pU8zps	0.015483	0.613025	486484	sepsis	TRUE	reported	textfile	0.0026	9.98E-06	7350	M35186.m	TRUE	reported	Oc2IOF	2	TRUE	0.002748	20.24449
2035	rs7799978	A	G	A	G	0.0072	-0.01677	0.8174	0.799909	FALSE	FALSE	FALSE	v2KE89	0.017022	0.324379	486484	sepsis	TRUE	reported	textfile	0.0016	4.54E-06	7349	M15500.m	TRUE	reported	to2t7H	2	TRUE	0.002748	20.24449
3714	rs841845	T	C	T	C	0.0108	0.000723	0.7848	0.788471	FALSE	FALSE	FALSE	agPgtF	0.016753	0.965559	486484	sepsis	TRUE	reported	textfile	0.0024	9.21E-06	7349	M32315.m	TRUE	reported	hMRjcV	2	TRUE	0.002748	20.24449
1268	rs6753621	A	G	A	G	-0.0117	0.016215	0.2183	0.224524	FALSE	FALSE	FALSE	gNG3wy	0.016415	0.323224	486484	sepsis	TRUE	reported	textfile	0.0026	7.02E-06	7348	M01670.m	TRUE	reported	t3lsMq	2	TRUE	0.002748	20.24449
8370	rs1235145	T	C	T	C	-0.0081	-0.01577	0.4209	0.43263	FALSE	FALSE	FALSE	SCzh0A	0.013948	0.258201	486484	sepsis	TRUE	reported	textfile	0.0018	9.08E-06	7348	M35675.m	TRUE	reported	n1SREk	2	TRUE	0.002748	20.24449
8394	rs4984992	C	G	C	G	0.0081	0.004093	0.6116	0.597358	FALSE	TRUE	FALSE	SCzh0A	0.014005	0.770077	486484	sepsis	TRUE	reported	textfile	0.0018	7.83E-06	7348	M35675.m	TRUE	reported	n1SREk	2	TRUE	0.002748	20.24449
8398	rs6851835	A	G	A	G	0.0081	-0.00397	0.5236	0.521827	FALSE	FALSE	FALSE	SCzh0A	0.01367	0.771349	486484	sepsis	TRUE	reported	textfile	0.0018	8.89E-06	7348	M35675.m	TRUE	reported	n1SREk	2	TRUE	0.002748	20.24449
1224	rs1173111	A	G	A	G	0.0081	0.007382	0.5958	0.590248	FALSE	FALSE	FALSE	V50rWC	0.013895	0.595228	486484	sepsis	TRUE	reported	textfile	0.0018	4.32E-06	7346	M01645.m	TRUE	reported	yN3gpQ	2	TRUE	0.002749	20.24449
3655	rs2817479	T	G	T	G	0.0126	-0.00431	0.5278	0.536646	FALSE	FALSE	FALSE	fnjup6	0.014917	0.772529	486484	sepsis	TRUE	reported	textfile	0.0028	6.92E-06	7346	M32197.m	TRUE	reported	oQzaMk	2	TRUE	0.002749	20.24449
897	rs1171840	T	C	T	C	0.0216	-0.00267	0.8358	0.832545	FALSE	FALSE	FALSE	l6zQnf	0.018273	0.883862	486484	sepsis	TRUE	reported	textfile	0.0048	8.43E-06	7345	M01444.m	TRUE	reported	llzalm	2	TRUE	0.002749	20.24449
6707	rs6907864	T	C	T	C	0.0261	-0.02603	0.1108	0.103934	FALSE	FALSE	FALSE	bzT5yX	0.022411	0.245469	486484	sepsis	TRUE	reported	textfile	0.0058	5.79E-06	7345	M33936.m	TRUE	reported	W7BO8n	2	TRUE	0.002749	20.24449
8165	rs1252875	A	G	A	G	0.0108	0.042948	0.1278	0.127527	FALSE	FALSE	FALSE	8UhtPK	0.020469	0.035882	486484	sepsis	TRUE	reported	textfile	0.0024	6.20E-06	7345	M35397.m	TRUE	reported	rvVJba	2	TRUE	0.002749	20.24449
3783	rs485878	A	G	A	G	-0.0081	-0.01361	0.4238	0.418215	FALSE	FALSE	FALSE	NW1aaR	0.013899	0.327378	486484	sepsis	TRUE	reported	textfile	0.0018	4.67E-06	7344	M32339.m	TRUE	reported	pBPkAB	2	TRUE	0.00275	20.24449
3800	rs9876174	T	C	T	C	0.0117	0.006225	0.8297	0.840456	FALSE	FALSE	FALSE	NW1aaR	0.018681	0.738965	486484	sepsis	TRUE	reported	textfile	0.0026	7.00E-06	7344	M32339.m	TRUE	reported	pBPkAB	2	TRUE	0.00275	20.24449
4949	rs2009217	T	C	T	C	-0.0081	0.008495	0.7257	0.72465	FALSE	FALSE	FALSE	bKmDMa	0.015289	0.578482	486484	sepsis	TRUE	reported	textfile	0.0018	4.91E-06	7344	M32910.m	TRUE	reported	nAuPzO	2	TRUE	0.00275	20.24449
4954	rs2382376	T	C	T	C	0.0081	0.016976	0.3053	0.317579	FALSE	FALSE	FALSE	bKmDMa	0.014776	0.250622	486484	sepsis	TRUE	reported	textfile	0.0018	6.50E-06	7344	M32910.m	TRUE	reported	nAuPzO	2	TRUE	0.00275	20.24449
4962	rs4084164	A	G	A	G	0.0081	0.014568	0.3942	0.394507	FALSE	FALSE	FALSE	bKmDMa	0.0142	0.304923	486484	sepsis	TRUE	reported	textfile	0.0018	4.04E-06	7344	M32910.m	TRUE	reported	nAuPzO	2	TRUE	0.00275	20.24449
3281	rs1760904	A	G	A	G	-0.0081	-0.01621	0.5414	0.540028	FALSE	FALSE	FALSE	vYWPe9	0.013718	0.237315	486484	sepsis	TRUE	reported	textfile	0.0018	7.90E-06	7340	M25459.m	TRUE	reported	VvJ6Vx	2	TRUE	0.002751	20.24448
3294	rs7467889	T	C	T	C	0.0081	-0.00086	0.4331	0.431267	FALSE	FALSE	FALSE	vYWPe9	0.013812	0.950294	486484	sepsis	TRUE	reported	textfile	0.0018	8.84E-06	7340	M25459.m	TRUE	reported	VvJ6Vx	2	TRUE	0.002751	20.24448
3744	rs883352	T	C	T	C	0.0189	0.017093	0.7693	0.731256	FALSE	FALSE	FALSE	k16olH	0.015476	0.269396	486484	sepsis	TRUE	reported	textfile	0.0042	7.55E-06	7340	M32328.m	TRUE	reported	TQA3sZ	2	TRUE	0.002751	20.24448
3622	rs349022	T	C	T	C	0.0297	-0.00572	0.6757	0.679418	FALSE	FALSE	FALSE	gq4ISJ	0.014633	0.695812	486484	sepsis	TRUE	reported	textfile	0.0066	7.81E-06	7338	M31591.m	TRUE	reported	wjwl4J	2	TRUE	0.002752	20.24448
4436	rs3737294	A	G	A	G	0.0081	-0.04632	0.857	0.859845	FALSE	FALSE	FALSE	WGPdpq	0.019705	0.018728	486484	sepsis	TRUE	reported	textfile	0.0018	4.83E-06	7338	M32675.m	TRUE	reported	u0eUAG	2	TRUE	0.002752	20.24448
4263	rs1723646	A	T	A	T	0.0189	-0.00364	0.1577	0.162698	FALSE	TRUE	FALSE	ylV4bp	0.018605	0.844697	486484	sepsis	TRUE	reported	textfile	0.0042	8.26E-06	7337	M32632.m	TRUE	reported	hqHJoU	2	TRUE	0.002752	20.24448
4266	rs1775098	A	G	A	G	-0.0162	-0.01735	0.2501	0.260936	FALSE	FALSE	FALSE	ylV4bp	0.015591	0.265681	486484	sepsis	TRUE	reported	textfile	0.0036	6.38E-06	7337	M32632.m	TRUE	reported	hqHJoU	2	TRUE	0.002752	20.24448
3516	rs2257061	T	C	T	C	-0.0162	-0.01329	0.6471	0.644061	FALSE	FALSE	FALSE	hxuqls	0.014318	0.353313	486484	sepsis	TRUE	reported	textfile	0.0036	6.33E-06	7336	M27738.m	TRUE	reported	aueeZs	2	TRUE	0.002753	20.24448
3523	rs8031609	T	G	T	G	0.0198	-0.0067	0.8038	0.797171	FALSE	FALSE	FALSE	hxuqls	0.016995	0.693415	486484	sepsis	TRUE	reported	textfile	0.0044	7.45E-06	7336	M27738.m	TRUE	reported	aueeZs	2	TRUE	0.002753	20.24448
3525	rs9572687	A	G	A	G	-0.0162	-0.00667	0.7063	0.699456	FALSE	FALSE	FALSE	hxuqls	0.01491	0.654763	486484	sepsis	TRUE	reported	textfile	0.0036	6.55E-06	7336	M27738.m	TRUE	reported	aueeZs	2	TRUE	0.002753	20.24448
6071	rs1161712	A	C	A	C	-0.0081	0.019177	0.8238	0.804257	FALSE	FALSE	FALSE	P8n0HP	0.017202	0.264949	486484	sepsis	TRUE	reported	textfile	0.0018	3.78E-06	7336	M33442.m	TRUE	reported	cljMTI	2	TRUE	0.002753	20.24448
805	rs1015633	A	G	A	G	-0.0153	-0.03172	0.8473	0.837495	FALSE	FALSE	FALSE	J58DI0	0.018721	0.090157	486484	sepsis	TRUE	reported	textfile	0.0034	7.17E-06	7335	M01356.m	TRUE	reported	zk9AMB	2	TRUE	0.002753	20.24448
816	rs9291034	T	C	T	C	-0.0117	-0.01071	0.4068	0.42227	FALSE	FALSE	FALSE	J58DI0	0.013822	0.438286	486484	sepsis	TRUE	reported	textfile	0.0026	5.42E-06	7335	M01356.m	TRUE	reported	zk9AMB	2	TRUE	0.002753	20.24448
6624	rs9654430	A	G	A	G	-0.0189	-0.00785	0.5156	0.495079	FALSE	FALSE	FALSE	2plv9p	0.013689	0.566586	486484	sepsis	TRUE	reported	textfile	0.0042	5.92E-06	7334	M33884.m	TRUE	reported	AoFcqC	2	TRUE	0.002754	20.24448
1083	rs1713197	A	G	A	G	-0.0216	0.010555	0.0607	0.034705	FALSE	FALSE	FALSE	JLtEiZ	0.037367	0.777578	486484	sepsis	TRUE	reported	textfile	0.0048	8.18E-06	7333	M01572.m	TRUE	reported	TSSe28	2	TRUE	0.002754	20.24448
4981	rs1253120	T	C	T	C	-0.0162	-0.03287	0.2025	0.21381	FALSE	FALSE	FALSE	AjJi2i	0.016723	0.049375	486484	sepsis	TRUE	reported	textfile	0.0036	7.24E-06	7330	M32980.m	TRUE	reported	gVSm9e	2	TRUE	0.002755	20.24447
4988	rs831471	A	G	A	G	-0.0162	-0.00243	0.4088	0.433709	FALSE	FALSE	FALSE	AjJi2i	0.013783	0.860083	486484	sepsis	TRUE	reported	textfile	0.0036	6.44E-06	7330	M32980.m	TRUE	reported	gVSm9e	2	TRUE	0.002755	20.24447
3201	rs2850874	A	G	A	G	0.0171	0.029778	0.0817	0.083674	FALSE	FALSE	FALSE	UfLkBq	0.024693	0.227854	486484	sepsis	TRUE	reported	textfile	0.0038	7.56E-06	7329	M22481.m	TRUE	reported	kWMdiT	2	TRUE	0.002755	20.24447
2899	rs1097375	A	G	A	G	-0.0117	-0.03207	0.8139	0.796251	FALSE	FALSE	FALSE	FBDEQi	0.017	0.059207	486484	sepsis	TRUE	reported	textfile	0.0026	8.25E-06	7327	M20699.m	TRUE	reported	nvNIlp	2	TRUE	0.002756	20.24447
2902	rs1726729	T	C	T	C	-0.0117	-0.02839	0.7884	0.752624	FALSE	FALSE	FALSE	FBDEQi	0.01584	0.073067	486484	sepsis	TRUE	reported	textfile	0.0026	6.68E-06	7327	M20699.m	TRUE	reported	nvNIlp	2	TRUE	0.002756	20.24447
1390	rs6040877	A	G	A	G	-0.0081	0.0205	0.2715	0.291188	FALSE	FALSE	FALSE	KrWhws	0.015059	0.173404	486484	sepsis	TRUE	reported	textfile	0.0018	4.01E-06	7325	M02132.m	TRUE	reported	7pzpfK	2	TRUE	0.002757	20.24447
1394	rs7012395	T	C	T	C	-0.0081	0.012286	0.3041	0.305262	FALSE	FALSE	FALSE	KrWhws	0.014903	0.409724	486484	sepsis	TRUE	reported	textfile	0.0018	4.96E-06	7325	M02132.m	TRUE	reported	7pzpfK	2	TRUE	0.002757	20.24447
4205	rs312994	A	G	A	G	-0.0198	-0.01337	0.5193	0.511267	FALSE	FALSE	FALSE	rdKSjG	0.013701	0.329058	486484	sepsis	TRUE	reported	textfile	0.0044	8.77E-06	7322	M32578.m	TRUE	reported	RAzSLK	2	TRUE	0.002758	20.24447
6817	rs1113348	T	G	T	G	-0.0189	-0.00356	0.2844	0.28919	FALSE	FALSE	FALSE	wedECv	0.015085	0.813242	486484	sepsis	TRUE	reported	textfile	0.0042	6.58E-06	7322	M33968.m	TRUE	reported	GxGxRG	2	TRUE	0.002758	20.24447
6818	rs1170940	A	G	A	G	0.0198	-0.01691	0.3576	0.3963	FALSE	FALSE	FALSE	wedECv	0.014038	0.228306	486484	sepsis	TRUE	reported	textfile	0.0044	5.63E-06	7322	M33968.m	TRUE	reported	GxGxRG	2	TRUE	0.002758	20.24447
7618	rs2076196	A	G	A	G	-0.0162	-0.00847	0.1888	0.199771	FALSE	FALSE	FALSE	kVfKqT	0.017117	0.620545	486484	sepsis	TRUE	reported	textfile	0.0036	6.46E-06	7322	M34527.m	TRUE	reported	s4ti92	2	TRUE	0.002758	20.24447
7624	rs659599	A	G	A	G	0.0117	0.000853	0.5251	0.533951	FALSE	FALSE	FALSE	kVfKqT	0.013699	0.950362	486484	sepsis	TRUE	reported	textfile	0.0026	9.44E-06	7322	M34527.m	TRUE	reported	s4ti92	2	TRUE	0.002758	20.24447
6752	rs9556	T	C	T	C	-0.0189	0.003509	0.3103	0.320593	FALSE	FALSE	FALSE	NZVg9N	0.014721	0.811591	486484	sepsis	TRUE	reported	textfile	0.0042	6.08E-06	7320	M33941.m	TRUE	reported	kRWNNK	2	TRUE	0.002759	20.24447
4595	rs3754909	A	G	A	G	-0.0081	-0.01938	0.6615	0.651012	FALSE	FALSE	FALSE	Pzplqu	0.014343	0.176732	486484	sepsis	TRUE	reported	textfile	0.0018	3.73E-06	7318	M32740.m	TRUE	reported	FjEBS5	2	TRUE	0.00276	20.24447
8317	rs1008945	A	C	A	C	-0.0126	-0.01099	0.5399	0.580007	FALSE	FALSE	FALSE	o0GzT1	0.01408	0.434932	486484	sepsis	TRUE	reported	textfile	0.0028	6.53E-06	7317	M35631.m	TRUE	reported	G1XFx0	2	TRUE	0.00276	20.24446
8323	rs2161134	A	G	A	G	0.0117	0.012169	0.6204	0.611039	FALSE	FALSE	FALSE	o0GzT1	0.014003	0.384811	486484	sepsis	TRUE	reported	textfile	0.0026	6.93E-06	7317	M35631.m	TRUE	reported	G1XFx0	2	TRUE	0.00276	20.24446
8335	rs7403602	T	C	T	C	-0.0117	-0.01554	0.5361	0.534877	FALSE	FALSE	FALSE	o0GzT1	0.013851	0.261859	486484	sepsis	TRUE	reported	textfile	0.0026	7.15E-06	7317	M35631.m	TRUE	reported	G1XFx0	2	TRUE	0.00276	20.24446
7060	rs313125	A	G	A	G	-0.0117	-0.021	0.1782	0.176454	FALSE	FALSE	FALSE	uthruC	0.017983	0.242818	486484	sepsis	TRUE	reported	textfile	0.0026	5.13E-06	7316	M34283.m	TRUE	reported	ftTuRU	2	TRUE	0.00276	20.24446
2502	rs1297537	A	G	A	G	-0.0081	-0.01656	0.5367	0.538666	FALSE	FALSE	FALSE	LwUYua	0.013732	0.227703	486484	sepsis	TRUE	reported	textfile	0.0018	9.15E-06	7315	M17945.m	TRUE	reported	SUN8dc	2	TRUE	0.002761	20.24446
2509	rs2745001	A	G	A	G	-0.0081	-0.01304	0.3976	0.403798	FALSE	FALSE	FALSE	LwUYua	0.013911	0.348618	486484	sepsis	TRUE	reported	textfile	0.0018	9.25E-06	7315	M17945.m	TRUE	reported	SUN8dc	2	TRUE	0.002761	20.24446
2090	rs9321388	A	G	A	G	-0.0072	0.027752	0.1772	0.196916	FALSE	FALSE	FALSE	NPVH5j	0.017216	0.106959	486484	sepsis	TRUE	reported	textfile	0.0016	7.58E-06	7310	M15506.m	TRUE	reported	r7r3vM	2	TRUE	0.002763	20.24446
2200	rs1278316	T	C	T	C	-0.0081	-0.03595	0.2909	0.336607	FALSE	FALSE	FALSE	Bf6uMK	0.014555	0.013505	486484	sepsis	TRUE	reported	textfile	0.0018	6.56E-06	7308	M15778.m	TRUE	reported	jowM2N	2	TRUE	0.002763	20.24446
4467	rs1687678	A	G	A	G	0.0144	0.045352	0.1222	0.128787	FALSE	FALSE	FALSE	g3iM2w	0.020449	0.026569	486484	sepsis	TRUE	reported	textfile	0.0032	8.18E-06	7308	M32698.m	TRUE	reported	ZQBn0h	2	TRUE	0.002763	20.24446
6118	rs1018947	A	C	A	C	-0.0081	-0.00275	0.4262	0.432452	FALSE	FALSE	FALSE	MiSJly	0.013825	0.842217	486484	sepsis	TRUE	reported	textfile	0.0018	5.91E-06	7307	M33477.m	TRUE	reported	WwL1hq	2	TRUE	0.002764	20.24446
6131	rs1338580	A	C	A	C	-0.0081	0.000981	0.7023	0.686203	FALSE	FALSE	FALSE	MiSJly	0.01475	0.946955	486484	sepsis	TRUE	reported	textfile	0.0018	9.39E-06	7307	M33477.m	TRUE	reported	WwL1hq	2	TRUE	0.002764	20.24446
6140	rs4141184	T	C	T	C	-0.0081	-0.01005	0.2907	0.287272	FALSE	FALSE	FALSE	MiSJly	0.015106	0.506023	486484	sepsis	TRUE	reported	textfile	0.0018	8.46E-06	7307	M33477.m	TRUE	reported	WwL1hq	2	TRUE	0.002764	20.24446
6148	rs6426492	T	C	T	C	-0.0081	0.00543	0.3336	0.337371	FALSE	FALSE	FALSE	MiSJly	0.014518	0.708388	486484	sepsis	TRUE	reported	textfile	0.0018	8.75E-06	7307	M33477.m	TRUE	reported	WwL1hq	2	TRUE	0.002764	20.24446
5997	rs4396797	T	C	T	C	0.0081	-0.00214	0.7483	0.750797	FALSE	FALSE	FALSE	3KsoXq	0.015799	0.892177	486484	sepsis	TRUE	reported	textfile	0.0018	5.39E-06	7306	M33422.m	TRUE	reported	aNr6Ug	2	TRUE	0.002764	20.24446
5989	rs2276228	A	C	A	C	0.0081	-0.00521	0.2783	0.280017	FALSE	FALSE	FALSE	3KsoXq	0.015192	0.731412	486484	sepsis	TRUE	reported	textfile	0.0018	5.35E-06	7306	M33422.m	TRUE	reported	aNr6Ug	2	TRUE	0.002764	20.24446
5996	rs40525	A	C	A	C	0.0081	0.019532	0.7741	0.770737	FALSE	FALSE	FALSE	3KsoXq	0.016286	0.230394	486484	sepsis	TRUE	reported	textfile	0.0018	5.94E-06	7306	M33422.m	TRUE	reported	aNr6Ug	2	TRUE	0.002764	20.24446
3335	rs278013	A	C	A	C	0.0081	-0.01575	0.8065	0.801466	FALSE	FALSE	FALSE	m66Eav	0.017154	0.358538	486484	sepsis	TRUE	reported	textfile	0.0018	5.51E-06	7303	M27256.m	TRUE	reported	pI2ECU	2	TRUE	0.002765	20.24445
3345	rs7956757	T	C	T	C	0.0081	0.012315	0.8055	0.801011	FALSE	FALSE	FALSE	m66Eav	0.01715	0.472692	486484	sepsis	TRUE	reported	textfile	0.0018	5.03E-06	7303	M27256.m	TRUE	reported	pI2ECU	2	TRUE	0.002765	20.24445
2885	rs4556997	A	C	A	C	-0.0189	0.045085	0.1388	0.135865	FALSE	FALSE	FALSE	mUzn1R	0.02003	0.024392	486484	sepsis	TRUE	reported	textfile	0.0042	6.40E-06	7301	M20675.m	TRUE	reported	XwY04K	2	TRUE	0.002766	20.24445
3026	rs1319093	A	G	A	G	-0.0117	0.011729	0.3445	0.362455	FALSE	FALSE	FALSE	FJZEYz	0.014214	0.409264	486484	sepsis	TRUE	reported	textfile	0.0026	6.58E-06	7284	M21630.m	TRUE	reported	s6Pshg	2	TRUE	0.002772	20.24444
1120	rs7250750	T	G	T	G	0.0081	-0.00536	0.8583	0.861084	FALSE	FALSE	FALSE	yahiGZ	0.019843	0.787184	486484	sepsis	TRUE	reported	textfile	0.0018	7.03E-06	7271	M01585.m	TRUE	reported	dwZ7vX	2	TRUE	0.002777	20.24443
7958	rs1258801	T	G	T	G	0.0117	-0.00661	0.3744	0.377784	FALSE	FALSE	FALSE	zb8m4a	0.014093	0.638927	486484	sepsis	TRUE	reported	textfile	0.0026	8.57E-06	7261	M35253.m	TRUE	reported	tHnnnS	2	TRUE	0.002781	20.24442
1707	rs1715056	T	C	T	C	0.0117	0.005914	0.7256	0.731727	FALSE	FALSE	FALSE	Zml9HX	0.015447	0.701846	486484	sepsis	TRUE	reported	textfile	0.0026	4.61E-06	7258	M12626.m	TRUE	reported	9SUabZ	2	TRUE	0.002782	20.24442
1708	rs4978089	A	G	A	G	0.0117	0.000813	0.2125	0.225491	FALSE	FALSE	FALSE	Zml9HX	0.016574	0.960895	486484	sepsis	TRUE	reported	textfile	0.0026	8.78E-06	7258	M12626.m	TRUE	reported	9SUabZ	2	TRUE	0.002782	20.24442
3543	rs1097693	A	G	A	G	-0.0117	-0.03422	0.7662	0.755514	FALSE	FALSE	FALSE	8luUON	0.015933	0.031754	486484	sepsis	TRUE	reported	textfile	0.0026	8.61E-06	7257	M31453.m	TRUE	reported	FBs6G4	2	TRUE	0.002783	20.24442
3556	rs6920798	A	G	A	G	-0.0117	-0.03278	0.787	0.793106	FALSE	FALSE	FALSE	8luUON	0.016868	0.051943	486484	sepsis	TRUE	reported	textfile	0.0026	9.51E-06	7257	M31453.m	TRUE	reported	FBs6G4	2	TRUE	0.002783	20.24442
8217	rs1024145	A	G	A	G	0.0117	0.001163	0.5409	0.562195	FALSE	FALSE	FALSE	4YAWhA	0.013912	0.933364	486484	sepsis	TRUE	reported	textfile	0.0026	7.64E-06	7256	M35439.m	TRUE	reported	bSSC4Z	2	TRUE	0.002783	20.24442
4666	rs1122836	A	G	A	G	-0.0225	-0.0037	0.4543	0.439578	FALSE	FALSE	FALSE	xaapK0	0.013833	0.788913	486484	sepsis	TRUE	reported	textfile	0.005	7.05E-06	7250	M32757.m	TRUE	reported	64n1Z4	2	TRUE	0.002785	20.24441
4328	rs1016295	T	C	T	C	0.0081	0.013119	0.6424	0.628631	FALSE	FALSE	FALSE	6VSoQQ	0.014192	0.355266	486484	sepsis	TRUE	reported	textfile	0.0018	9.14E-06	7229	M32644.m	TRUE	reported	oC2AV0	2	TRUE	0.002793	20.2444
4332	rs1102682	A	T	A	T	-0.0081	-0.01992	0.3792	0.379095	FALSE	TRUE	FALSE	6VSoQQ	0.014129	0.158634	486484	sepsis	TRUE	reported	textfile	0.0018	9.20E-06	7229	M32644.m	TRUE	reported	oC2AV0	2	TRUE	0.002793	20.2444
1445	rs3094600	A	G	A	G	-0.0099	0.038937	0.2637	0.271302	FALSE	FALSE	FALSE	1M4gqj	0.015353	0.011206	486484	sepsis	TRUE	reported	textfile	0.0022	6.21E-06	7226	M02730.m	TRUE	reported	RWyYEM	2	TRUE	0.002795	20.2444
1454	rs9436667	T	C	T	C	0.0135	-0.02858	0.1138	0.119654	FALSE	FALSE	FALSE	1M4gqj	0.021152	0.176671	486484	sepsis	TRUE	reported	textfile	0.003	7.30E-06	7226	M02730.m	TRUE	reported	RWyYEM	2	TRUE	0.002795	20.2444
6726	rs4328432	A	G	A	G	0.0207	0.020749	0.1812	0.202168	FALSE	FALSE	FALSE	B6lZly	0.017143	0.226143	486484	sepsis	TRUE	reported	textfile	0.0046	5.50E-06	7222	M33937.m	TRUE	reported	qFPzjh	2	TRUE	0.002796	20.24439
8020	rs1051986	A	G	A	G	0.0081	-0.00625	0.2313	0.225174	FALSE	FALSE	FALSE	SwiqSi	0.016372	0.702812	486484	sepsis	TRUE	reported	textfile	0.0018	7.04E-06	7217	M35270.m	TRUE	reported	21ZlsQ	2	TRUE	0.002798	20.24439
8037	rs2395911	T	G	T	G	0.0081	0.015885	0.7477	0.752855	FALSE	FALSE	FALSE	SwiqSi	0.015835	0.315761	486484	sepsis	TRUE	reported	textfile	0.0018	6.76E-06	7217	M35270.m	TRUE	reported	21ZlsQ	2	TRUE	0.002798	20.24439
2947	rs209755	C	G	C	G	0.0099	-0.00229	0.302	0.290763	FALSE	TRUE	FALSE	pn11BC	0.015314	0.880971	486484	sepsis	TRUE	reported	textfile	0.0022	8.51E-06	7203	M21047.m	TRUE	reported	KJF8U4	2	TRUE	0.002803	20.24438
3231	rs628238	A	C	A	C	-0.0144	0.000606	0.2548	0.261899	FALSE	FALSE	FALSE	I25GHB	0.015565	0.968932	486484	sepsis	TRUE	reported	textfile	0.0032	6.03E-06	7184	M22548.m	TRUE	reported	eBmTQD	2	TRUE	0.002811	20.24436
8648	rs4654649	T	C	T	C	0.0144	0.002003	0.5518	0.5493	FALSE	FALSE	FALSE	GgPG6k	0.013761	0.884279	486484	sepsis	TRUE	reported	textfile	0.0032	8.49E-06	7184	M36300.m	TRUE	reported	kT1yso	2	TRUE	0.002811	20.24436
5062	rs1768520	T	C	T	C	0.0333	0.040429	0.9177	0.895183	FALSE	FALSE	FALSE	nHZXjp	0.022899	0.077473	486484	sepsis	TRUE	reported	textfile	0.0074	6.83E-06	7166	M33138.m	TRUE	reported	ccoK1F	2	TRUE	0.002818	20.24435
968	rs9365106	A	G	A	G	-0.0117	-0.01047	0.6824	0.682847	FALSE	FALSE	FALSE	tWoDq4	0.014689	0.476163	486484	sepsis	TRUE	reported	textfile	0.0026	8.06E-06	7160	M01508.m	TRUE	reported	6ltDw0	2	TRUE	0.00282	20.24434
5830	rs4624748	A	G	A	G	-0.0081	0.006437	0.2344	0.228241	FALSE	FALSE	FALSE	R5joEf	0.016322	0.693287	486484	sepsis	TRUE	reported	textfile	0.0018	3.81E-06	7118	M33380.m	TRUE	reported	kRvbfZ	2	TRUE	0.002837	20.24431
1167	rs6920504	T	C	T	C	-0.0135	0.004506	0.7729	0.751289	FALSE	FALSE	FALSE	cSBFJ3	0.015889	0.776703	486484	sepsis	TRUE	reported	textfile	0.003	7.53E-06	7104	M01638.m	TRUE	reported	sUkjpE	2	TRUE	0.002842	20.2443
7984	rs486052	T	G	T	G	0.0117	-0.00543	0.6521	0.650258	FALSE	FALSE	FALSE	t02AUT	0.014499	0.708004	486484	sepsis	TRUE	reported	textfile	0.0026	9.46E-06	7100	M35254.m	TRUE	reported	uClJaT	2	TRUE	0.002844	20.2443
8699	rs2615697	T	C	T	C	-0.0108	-0.02977	0.8166	0.817584	FALSE	FALSE	FALSE	Bg4VnB	0.017729	0.093124	486484	sepsis	TRUE	reported	textfile	0.0024	7.72E-06	7092	M36552.m	TRUE	reported	6zdDpC	2	TRUE	0.002847	20.24429
8705	rs7037029	A	G	A	G	0.0108	0.016823	0.7501	0.730259	FALSE	FALSE	FALSE	Bg4VnB	0.015481	0.277189	486484	sepsis	TRUE	reported	textfile	0.0024	7.21E-06	7092	M36552.m	TRUE	reported	6zdDpC	2	TRUE	0.002847	20.24429
6278	rs4781872	C	G	C	G	-0.0081	-0.00543	0.58	0.585807	FALSE	TRUE	FALSE	MOonP9	0.013868	0.695271	486484	sepsis	TRUE	reported	textfile	0.0018	8.35E-06	7066	M33515.m	TRUE	reported	226GuP	2	TRUE	0.002858	20.24427
6287	rs7684565	T	C	T	C	0.0081	0.003825	0.4972	0.51185	FALSE	FALSE	FALSE	MOonP9	0.013729	0.780547	486484	sepsis	TRUE	reported	textfile	0.0018	8.67E-06	7066	M33515.m	TRUE	reported	226GuP	2	TRUE	0.002858	20.24427
861	rs1124520	T	C	T	C	0.018	0.003706	0.1119	0.108095	FALSE	FALSE	FALSE	Vg5OOR	0.022184	0.867311	486484	sepsis	TRUE	reported	textfile	0.004	8.94E-06	7063	M01361.m	TRUE	reported	XnrS00	2	TRUE	0.002859	20.24427
864	rs1705622	T	G	T	G	0.0153	0.018195	0.1416	0.14834	FALSE	FALSE	FALSE	Vg5OOR	0.019258	0.344767	486484	sepsis	TRUE	reported	textfile	0.0034	7.07E-06	7063	M01361.m	TRUE	reported	XnrS00	2	TRUE	0.002859	20.24427
874	rs7710912	A	G	A	G	-0.0189	0.019947	0.0728	0.072389	FALSE	FALSE	FALSE	Vg5OOR	0.026416	0.450184	486484	sepsis	TRUE	reported	textfile	0.0042	8.30E-06	7063	M01361.m	TRUE	reported	XnrS00	2	TRUE	0.002859	20.24427
4812	rs434943	A	G	A	G	0.0135	0.001712	0.3163	0.336217	FALSE	FALSE	FALSE	bJwLV5	0.014458	0.905727	486484	sepsis	TRUE	reported	textfile	0.003	8.57E-06	7063	M32814.m	TRUE	reported	qte0qp	2	TRUE	0.002859	20.24427
1470	rs1759345	C	G	C	G	0.0144	-0.01406	0.8978	0.877168	FALSE	TRUE	FALSE	pkb876	0.02105	0.504055	486484	sepsis	TRUE	reported	textfile	0.0032	7.09E-06	7037	M02734.m	TRUE	reported	0LCMVI	2	TRUE	0.002869	20.24424
1476	rs2396461	T	C	T	C	0.0117	0.003924	0.121	0.159091	FALSE	FALSE	FALSE	pkb876	0.018676	0.833576	486484	sepsis	TRUE	reported	textfile	0.0026	8.38E-06	7037	M02734.m	TRUE	reported	0LCMVI	2	TRUE	0.002869	20.24424
4855	rs7707678	A	G	A	G	0.0198	0.009593	0.4165	0.417712	FALSE	FALSE	FALSE	StMkBO	0.013896	0.489997	486484	sepsis	TRUE	reported	textfile	0.0044	8.10E-06	6967	M32847.m	TRUE	reported	uzQb8Q	2	TRUE	0.002898	20.24419
8494	rs917046	T	G	T	G	-0.0369	0.057412	0.9531	0.951252	FALSE	FALSE	FALSE	ABkZ7x	0.032039	0.073139	486484	sepsis	TRUE	reported	textfile	0.0082	6.71E-06	6960	M35854.m	TRUE	reported	wc0CeK	2	TRUE	0.002901	20.24418
1503	rs1728001	A	T	A	T	-0.0234	0.017858	0.2083	0.255033	FALSE	TRUE	FALSE	rzkxmh	0.017116	0.296789	486484	sepsis	TRUE	reported	textfile	0.0052	6.91E-06	6941	M03127.m	TRUE	reported	3lrzhA	2	TRUE	0.002909	20.24417
1518	rs7924591	T	C	T	C	0.0108	-0.01695	0.5985	0.601813	FALSE	FALSE	FALSE	rzkxmh	0.013976	0.225087	486484	sepsis	TRUE	reported	textfile	0.0024	9.52E-06	6941	M03127.m	TRUE	reported	3lrzhA	2	TRUE	0.002909	20.24417
8837	rs1163871	A	G	A	G	0.0081	-0.02441	0.7783	0.784005	FALSE	FALSE	FALSE	XIHeTV	0.016683	0.143435	486484	sepsis	TRUE	reported	textfile	0.0018	4.62E-06	6940	M36808.m	TRUE	reported	37Vi2D	2	TRUE	0.002909	20.24416
8838	rs1167905	T	C	T	C	0.0081	-0.00377	0.7521	0.757275	FALSE	FALSE	FALSE	XIHeTV	0.015979	0.813566	486484	sepsis	TRUE	reported	textfile	0.0018	4.14E-06	6940	M36808.m	TRUE	reported	37Vi2D	2	TRUE	0.002909	20.24416
8842	rs1302247	A	G	A	G	0.0081	0.015631	0.8262	0.823616	FALSE	FALSE	FALSE	XIHeTV	0.017904	0.382648	486484	sepsis	TRUE	reported	textfile	0.0018	6.50E-06	6940	M36808.m	TRUE	reported	37Vi2D	2	TRUE	0.002909	20.24416
8860	rs7129047	A	C	A	C	-0.0081	-0.00871	0.1751	0.179193	FALSE	FALSE	FALSE	XIHeTV	0.017826	0.625023	486484	sepsis	TRUE	reported	textfile	0.0018	7.21E-06	6940	M36808.m	TRUE	reported	37Vi2D	2	TRUE	0.002909	20.24416
2574	rs2511986	C	G	C	G	0.0153	-0.00981	0.7684	0.765939	FALSE	TRUE	FALSE	KHS91f	0.016262	0.546298	486484	sepsis	TRUE	reported	textfile	0.0034	7.63E-06	6939	M18349.m	TRUE	reported	PeVGil	2	TRUE	0.00291	20.24416
2757	rs947678	A	C	A	C	-0.0117	-0.02148	0.5736	0.616272	FALSE	FALSE	FALSE	qFAbee	0.014079	0.127103	486484	sepsis	TRUE	reported	textfile	0.0026	5.03E-06	6929	M19364.m	TRUE	reported	tZgXNL	2	TRUE	0.002914	20.24416
3478	rs934668	T	C	T	C	0.0252	0.003068	0.7739	0.705609	FALSE	FALSE	FALSE	w7juXi	0.014982	0.837746	486484	sepsis	TRUE	reported	textfile	0.0056	6.78E-06	6830	M27722.m	TRUE	reported	xzWmif	2	TRUE	0.002956	20.24407
3526	rs1049569	A	G	A	G	-0.0288	0.044986	0.9526	0.930456	FALSE	FALSE	FALSE	rXzSlH	0.02718	0.097902	486484	sepsis	TRUE	reported	textfile	0.0064	6.35E-06	6795	M30805.m	TRUE	reported	7wg9QH	2	TRUE	0.002971	20.24404
2254	rs7260033	T	C	T	C	-0.0117	-0.02393	0.4757	0.477556	FALSE	FALSE	FALSE	zxcPox	0.013689	0.080454	486484	sepsis	TRUE	reported	textfile	0.0026	8.79E-06	6782	M15990.m	TRUE	reported	34Vg8F	2	TRUE	0.002977	20.24403
6697	rs1741285	A	G	A	G	-0.0351	0.024042	0.4861	0.493088	FALSE	FALSE	FALSE	AcRp2F	0.013784	0.081132	486484	sepsis	TRUE	reported	textfile	0.0078	5.95E-06	6756	M33935.m	TRUE	reported	FQCKJy	2	TRUE	0.002988	20.24401
7281	rs1233196	A	C	A	C	0.0081	0.003035	0.4969	0.494669	FALSE	FALSE	FALSE	Vcz4EA	0.013661	0.824213	486484	sepsis	TRUE	reported	textfile	0.0018	4.00E-06	6755	M34359.m	TRUE	reported	aUSo2J	2	TRUE	0.002989	20.244
7287	rs136230	A	G	A	G	-0.0081	0.009891	0.5971	0.594612	FALSE	FALSE	FALSE	Vcz4EA	0.013897	0.476643	486484	sepsis	TRUE	reported	textfile	0.0018	5.83E-06	6755	M34359.m	TRUE	reported	aUSo2J	2	TRUE	0.002989	20.244
7296	rs2541164	A	G	A	G	-0.0081	0.003657	0.2961	0.302609	FALSE	FALSE	FALSE	Vcz4EA	0.014884	0.805895	486484	sepsis	TRUE	reported	textfile	0.0018	6.77E-06	6755	M34359.m	TRUE	reported	aUSo2J	2	TRUE	0.002989	20.244
3448	rs472847	T	C	T	C	0.0243	0.030057	0.3494	0.398518	FALSE	FALSE	FALSE	J1I92u	0.01404	0.03229	486484	sepsis	TRUE	reported	textfile	0.0054	7.14E-06	6715	M27710.m	TRUE	reported	9qjpIZ	2	TRUE	0.003007	20.24397
4710	rs9316990	A	G	A	G	0.0189	0.017112	0.433	0.441692	FALSE	FALSE	FALSE	WlwKaF	0.013753	0.213408	486484	sepsis	TRUE	reported	textfile	0.0042	7.24E-06	6712	M32759.m	TRUE	reported	Ub4yxH	2	TRUE	0.003008	20.24397
8280	rs1411737	T	C	T	C	0.0243	0.022494	0.7834	0.78052	FALSE	FALSE	FALSE	J6o4xC	0.016801	0.180618	486484	sepsis	TRUE	reported	textfile	0.0054	5.82E-06	6595	M35472.m	TRUE	reported	3XBcu9	2	TRUE	0.003061	20.24386
2328	rs6024831	A	G	A	G	-0.0189	0.03165	0.9232	0.926118	FALSE	FALSE	FALSE	lZXwpR	0.026209	0.227204	486484	sepsis	TRUE	reported	textfile	0.0042	7.09E-06	6549	M16822.m	TRUE	reported	Zx9k2V	2	TRUE	0.003083	20.24382
7931	rs1686598	A	G	A	G	-0.0162	0.019546	0.2149	0.219576	FALSE	FALSE	FALSE	kFTWXY	0.016513	0.236533	486484	sepsis	TRUE	reported	textfile	0.0036	6.32E-06	6543	M35193.m	TRUE	reported	cjJvJl	2	TRUE	0.003085	20.24381
8002	rs1115213	A	G	A	G	-0.0162	0.005834	0.8149	0.813518	FALSE	FALSE	FALSE	Zj5bZH	0.017575	0.73991	486484	sepsis	TRUE	reported	textfile	0.0036	6.23E-06	6538	M35257.m	TRUE	reported	exssAk	2	TRUE	0.003088	20.24381
993	rs1165061	C	G	C	G	0.0081	0.022957	0.6907	0.709931	FALSE	TRUE	FALSE	jfkZbM	0.015068	0.127599	486484	sepsis	TRUE	reported	textfile	0.0018	5.99E-06	6523	M01558.m	TRUE	reported	LFZvMG	2	TRUE	0.003095	20.24379
998	rs1556880	T	C	T	C	0.0081	0.00146	0.2976	0.304854	FALSE	FALSE	FALSE	jfkZbM	0.014929	0.922115	486484	sepsis	TRUE	reported	textfile	0.0018	6.04E-06	6523	M01558.m	TRUE	reported	LFZvMG	2	TRUE	0.003095	20.24379
1004	rs2252585	T	C	T	C	-0.0081	-0.03892	0.7349	0.730914	FALSE	FALSE	FALSE	jfkZbM	0.017265	0.024169	486484	sepsis	TRUE	reported	textfile	0.0018	5.97E-06	6523	M01558.m	TRUE	reported	LFZvMG	2	TRUE	0.003095	20.24379
1008	rs3813810	A	C	A	C	0.0117	-0.00678	0.1334	0.134458	FALSE	FALSE	FALSE	jfkZbM	0.02019	0.73685	486484	sepsis	TRUE	reported	textfile	0.0026	5.73E-06	6523	M01558.m	TRUE	reported	LFZvMG	2	TRUE	0.003095	20.24379
1011	rs4923082	T	C	T	C	0.0081	-0.00162	0.2518	0.256695	FALSE	FALSE	FALSE	jfkZbM	0.015652	0.917348	486484	sepsis	TRUE	reported	textfile	0.0018	6.95E-06	6523	M01558.m	TRUE	reported	LFZvMG	2	TRUE	0.003095	20.24379
1018	rs9354329	T	C	T	C	-0.0081	0.002521	0.6322	0.611809	FALSE	FALSE	FALSE	jfkZbM	0.014025	0.857377	486484	sepsis	TRUE	reported	textfile	0.0018	5.10E-06	6523	M01558.m	TRUE	reported	LFZvMG	2	TRUE	0.003095	20.24379
6570	rs8104825	A	G	A	G	0.0153	-0.00551	0.4384	0.438693	FALSE	FALSE	FALSE	aTDqRQ	0.013817	0.689878	486484	sepsis	TRUE	reported	textfile	0.0034	7.29E-06	6506	M33871.m	TRUE	reported	Lqimdw	2	TRUE	0.003103	20.24377
5101	rs239430	A	G	A	G	-0.0153	0.013515	0.7871	0.771989	FALSE	FALSE	FALSE	Uiz8VU	0.016292	0.406799	486484	sepsis	TRUE	reported	textfile	0.0034	8.78E-06	6404	M33163.m	TRUE	reported	ky0Bda	2	TRUE	0.003152	20.24368
5105	rs3886930	T	C	T	C	0.0117	-0.00036	0.6	0.60798	FALSE	FALSE	FALSE	Uiz8VU	0.013982	0.979635	486484	sepsis	TRUE	reported	textfile	0.0026	9.18E-06	6404	M33163.m	TRUE	reported	ky0Bda	2	TRUE	0.003152	20.24368
6433	rs1713003	T	C	T	C	-0.018	-0.03149	0.9127	0.91286	FALSE	FALSE	FALSE	QCw5dg	0.024334	0.195687	486484	sepsis	TRUE	reported	textfile	0.004	7.79E-06	6279	M33666.m	TRUE	reported	zYHmzg	2	TRUE	0.003215	20.24355
3054	rs4433392	T	C	T	C	0.0117	0.014508	0.3697	0.357152	FALSE	FALSE	FALSE	H6vcCG	0.014267	0.309203	486484	sepsis	TRUE	reported	textfile	0.0026	5.10E-06	6180	M22030.m	TRUE	reported	XetVX7	2	TRUE	0.003266	20.24345
7559	rs133565	A	G	A	G	-0.0189	0.002974	0.3318	0.339732	FALSE	FALSE	FALSE	APSWAU	0.014416	0.836551	486484	sepsis	TRUE	reported	textfile	0.0042	8.51E-06	6144	M34469.m	TRUE	reported	frF3RX	2	TRUE	0.003285	20.24341
3861	rs4694161	A	G	A	G	0.0513	-0.06606	0.0297	0.026234	FALSE	FALSE	FALSE	8gg1zS	0.043073	0.125109	486484	sepsis	TRUE	reported	textfile	0.0114	7.19E-06	6119	M32379.m	TRUE	reported	uZWtuS	2	TRUE	0.003298	20.24338
7743	rs2970366	A	G	A	G	-0.0126	0.008203	0.1664	0.17799	FALSE	FALSE	FALSE	HyMNYM	0.017903	0.646824	486484	sepsis	TRUE	reported	textfile	0.0028	4.95E-06	5885	M34912.m	TRUE	reported	Tl8Hcw	2	TRUE	0.003429	20.24312
7744	rs32487	A	G	A	G	-0.0126	0.016248	0.8291	0.826766	FALSE	FALSE	FALSE	HyMNYM	0.018051	0.368069	486484	sepsis	TRUE	reported	textfile	0.0028	6.25E-06	5885	M34912.m	TRUE	reported	Tl8Hcw	2	TRUE	0.003429	20.24312
7760	rs9807799	T	C	T	C	-0.0126	0.021972	0.1397	0.143387	FALSE	FALSE	FALSE	HyMNYM	0.019474	0.259186	486484	sepsis	TRUE	reported	textfile	0.0028	6.56E-06	5885	M34912.m	TRUE	reported	Tl8Hcw	2	TRUE	0.003429	20.24312
2162	rs1049327	A	T	A	T	0.0243	-0.00111	0.8124	0.817382	FALSE	TRUE	FALSE	XCBNPg	0.017723	0.95005	486484	sepsis	TRUE	reported	textfile	0.0054	5.83E-06	5829	M15749.m	TRUE	reported	dKBewD	2	TRUE	0.003462	20.24305
8114	rs2174753	A	T	A	T	0.0081	-0.00897	0.3905	0.397305	FALSE	TRUE	FALSE	nNbXBS	0.013993	0.521417	486484	sepsis	TRUE	reported	textfile	0.0018	3.58E-06	5768	M35326.m	TRUE	reported	UNDorn	2	TRUE	0.003498	20.24298
8119	rs4765845	A	G	A	G	0.0081	-0.00606	0.4785	0.482771	FALSE	FALSE	FALSE	nNbXBS	0.013667	0.657632	486484	sepsis	TRUE	reported	textfile	0.0018	5.57E-06	5768	M35326.m	TRUE	reported	UNDorn	2	TRUE	0.003498	20.24298
6454	rs6003815	T	C	T	C	0.0243	-0.01603	0.4411	0.444713	FALSE	FALSE	FALSE	OFlEwq	0.013752	0.243845	486484	sepsis	TRUE	reported	textfile	0.0054	6.58E-06	5694	M33675.m	TRUE	reported	T2BzXG	2	TRUE	0.003544	20.24289
3075	rs815198	T	C	T	C	-0.0126	-0.02098	0.4745	0.464593	FALSE	FALSE	FALSE	e3izea	0.013826	0.129144	486484	sepsis	TRUE	reported	textfile	0.0028	6.02E-06	5676	M22032.m	TRUE	reported	zODKFl	2	TRUE	0.003555	20.24286
8350	rs1696708	C	G	C	G	-0.0207	0.013007	0.3734	0.374359	FALSE	TRUE	FALSE	6VVCe4	0.014122	0.357028	486484	sepsis	TRUE	reported	textfile	0.0046	6.34E-06	5629	M35669.m	TRUE	reported	l0fk8X	2	TRUE	0.003585	20.24281
1136	rs510032	T	C	T	C	0.009	-0.01451	0.169	0.193497	FALSE	FALSE	FALSE	nlqUuV	0.017392	0.404088	486484	sepsis	TRUE	reported	textfile	0.002	6.80E-06	5604	M01604.m	TRUE	reported	Uro9nC	2	TRUE	0.0036	20.24277
1056	rs588973	A	G	A	G	0.009	0.00595	0.646	0.647252	FALSE	FALSE	FALSE	tRtQ2e	0.014284	0.676997	486484	sepsis	TRUE	reported	textfile	0.002	6.80E-06	5603	M01564.m	TRUE	reported	Tbg0zM	2	TRUE	0.003601	20.24277
620	rs532098	A	G	A	G	0.009	-0.02872	0.461	0.456883	FALSE	FALSE	FALSE	BYGzq4	0.013706	0.036163	486484	sepsis	TRUE	reported	textfile	0.002	6.80E-06	5601	M01110.m	TRUE	reported	oZiXqP	2	TRUE	0.003602	20.24277
1344	rs4978836	A	G	A	G	0.009	0.010741	0.391	0.38406	FALSE	FALSE	FALSE	LDUJbh	0.01409	0.445858	486484	sepsis	TRUE	reported	textfile	0.002	6.80E-06	5601	M01898.m	TRUE	reported	JRSLeH	2	TRUE	0.003602	20.24277
5028	rs1771851	T	C	T	C	0.009	0.010948	0.77	0.765534	FALSE	FALSE	FALSE	BNnOvt	0.016125	0.497185	486484	sepsis	TRUE	reported	textfile	0.002	6.80E-06	5600	M33132.m	TRUE	reported	rHcCPG	2	TRUE	0.003603	20.24277
5041	rs932748	T	G	T	G	0.009	-0.00539	0.878	0.881422	FALSE	FALSE	FALSE	BNnOvt	0.021328	0.800517	486484	sepsis	TRUE	reported	textfile	0.002	6.80E-06	5600	M33132.m	TRUE	reported	rHcCPG	2	TRUE	0.003603	20.24277
461	rs2521310	T	C	T	C	0.009	-0.00462	0.874	0.866911	FALSE	FALSE	FALSE	cl8ucN	0.020208	0.819261	486484	sepsis	TRUE	reported	textfile	0.002	6.80E-06	5598	M00513.m	TRUE	reported	lan0Q8	2	TRUE	0.003604	20.24277
464	rs6502247	A	G	A	G	-0.009	0.008777	0.119	0.119882	FALSE	FALSE	FALSE	cl8ucN	0.021998	0.689897	486484	sepsis	TRUE	reported	textfile	0.002	6.80E-06	5598	M00513.m	TRUE	reported	lan0Q8	2	TRUE	0.003604	20.24277
4286	rs1165162	A	G	A	G	0.009	0.000759	0.321	0.308426	FALSE	FALSE	FALSE	0tjjPC	0.016334	0.962942	486484	sepsis	TRUE	reported	textfile	0.002	6.80E-06	5598	M32634.m	TRUE	reported	nJJFkX	2	TRUE	0.003604	20.24277
4303	rs439401	T	C	T	C	-0.009	-0.01535	0.351	0.362706	FALSE	FALSE	FALSE	0tjjPC	0.014232	0.280762	486484	sepsis	TRUE	reported	textfile	0.002	6.80E-06	5598	M32634.m	TRUE	reported	nJJFkX	2	TRUE	0.003604	20.24277
717	rs8035304	T	G	T	G	0.009	0.009461	0.81	0.812215	FALSE	FALSE	FALSE	H9I3s3	0.017868	0.596469	486484	sepsis	TRUE	reported	textfile	0.002	6.80E-06	5597	M01299.m	TRUE	reported	kXWyUq	2	TRUE	0.003605	20.24276
4015	rs2467261	A	G	A	G	-0.009	-0.01771	0.735	0.731587	FALSE	FALSE	FALSE	V9AtId	0.015462	0.252112	486484	sepsis	TRUE	reported	textfile	0.002	6.80E-06	5596	M32489.m	TRUE	reported	8B8S8a	2	TRUE	0.003606	20.24276
1745	rs2980872	A	G	A	G	-0.009	-0.01103	0.461	0.473827	FALSE	FALSE	FALSE	nFLpAP	0.0138	0.424149	486484	sepsis	TRUE	reported	textfile	0.002	6.80E-06	5595	M12774.m	TRUE	reported	yOSQTj	2	TRUE	0.003606	20.24276
6872	rs1938299	A	G	A	G	0.009	-0.01683	0.889	0.892421	FALSE	FALSE	FALSE	kFWZjs	0.022044	0.445272	486484	sepsis	TRUE	reported	textfile	0.002	6.80E-06	5594	M34062.m	TRUE	reported	eLONsg	2	TRUE	0.003607	20.24276
131	rs6001129	T	C	T	C	-0.009	-0.0001	0.134	0.131565	FALSE	FALSE	FALSE	xmGuol	0.02023	0.995899	486484	sepsis	TRUE	reported	textfile	0.002	6.80E-06	5592	M00054.m	TRUE	reported	GW50Ri	2	TRUE	0.003608	20.24276
937	rs4646693	T	C	T	C	-0.009	0.015429	0.435	0.436411	FALSE	FALSE	FALSE	tv99UN	0.014009	0.270748	486484	sepsis	TRUE	reported	textfile	0.002	6.80E-06	5592	M01494.m	TRUE	reported	osDclp	2	TRUE	0.003608	20.24276
2598	rs200449	A	G	A	G	0.009	-0.01463	0.569	0.562024	FALSE	FALSE	FALSE	ruvJh6	0.013791	0.288678	486484	sepsis	TRUE	reported	textfile	0.002	6.80E-06	5592	M18369.m	TRUE	reported	GFmwGk	2	TRUE	0.003608	20.24276
2609	rs6797094	T	G	T	G	0.009	0.018546	0.611	0.596093	FALSE	FALSE	FALSE	ruvJh6	0.013957	0.183915	486484	sepsis	TRUE	reported	textfile	0.002	6.80E-06	5592	M18369.m	TRUE	reported	GFmwGk	2	TRUE	0.003608	20.24276
820	rs1075227	T	C	T	C	0.009	-0.01788	0.552	0.55074	FALSE	FALSE	FALSE	2qgDgB	0.013776	0.194203	486484	sepsis	TRUE	reported	textfile	0.002	6.80E-06	5590	M01358.m	TRUE	reported	asmXq0	2	TRUE	0.003609	20.24275
1985	rs4634560	T	C	T	C	-0.009	0.036771	0.906	0.912993	FALSE	FALSE	FALSE	v2KE89	0.024282	0.129938	486484	sepsis	TRUE	reported	textfile	0.002	6.80E-06	5590	M15500.m	TRUE	reported	to2t7H	2	TRUE	0.003609	20.24275
6763	rs1875897	A	G	A	G	0.009	0.013179	0.257	0.264624	FALSE	FALSE	FALSE	Elf6MQ	0.015612	0.39858	486484	sepsis	TRUE	reported	textfile	0.002	6.80E-06	5590	M33955.m	TRUE	reported	Lxn4RF	2	TRUE	0.003609	20.24275
1205	rs2073805	T	C	T	C	-0.009	0.045626	0.407	0.410223	FALSE	FALSE	FALSE	Ckdgh1	0.015395	0.003041	486484	sepsis	TRUE	reported	textfile	0.002	6.80E-06	5589	M01644.m	TRUE	reported	nKxz7B	2	TRUE	0.00361	20.24275
4054	rs7669416	A	G	A	G	-0.009	-0.02	0.549	0.543518	FALSE	FALSE	FALSE	SiYGYU	0.013716	0.144851	486484	sepsis	TRUE	reported	textfile	0.002	6.80E-06	5588	M32492.m	TRUE	reported	GfQvpU	2	TRUE	0.003611	20.24275
8176	rs3786099	T	C	T	C	-0.009	-0.00738	0.207	0.208212	FALSE	FALSE	FALSE	8UhtPK	0.016806	0.6605	486484	sepsis	TRUE	reported	textfile	0.002	6.80E-06	5587	M35397.m	TRUE	reported	rvVJba	2	TRUE	0.003611	20.24275
8177	rs4269090	T	C	T	C	0.009	0.027913	0.226	0.226696	FALSE	FALSE	FALSE	8UhtPK	0.0163	0.0868	486484	sepsis	TRUE	reported	textfile	0.002	6.80E-06	5587	M35397.m	TRUE	reported	rvVJba	2	TRUE	0.003611	20.24275
798	rs577266	A	C	A	C	-0.009	-0.02484	0.637	0.637893	FALSE	FALSE	FALSE	dyT79I	0.014227	0.08088	486484	sepsis	TRUE	reported	textfile	0.002	6.80E-06	5586	M01336.m	TRUE	reported	OemVJ2	2	TRUE	0.003612	20.24275
3942	rs1004569	T	C	T	C	-0.027	-0.00073	0.691	0.691406	FALSE	FALSE	FALSE	N6UQnQ	0.014842	0.960869	486484	sepsis	TRUE	reported	textfile	0.006	6.80E-06	5586	M32425.m	TRUE	reported	FEcGHY	2	TRUE	0.003612	20.24275
759	rs6766715	A	G	A	G	-0.009	-0.0042	0.128	0.131017	FALSE	FALSE	FALSE	RHWptG	0.020379	0.836736	486484	sepsis	TRUE	reported	textfile	0.002	6.80E-06	5585	M01302.m	TRUE	reported	z1OLfr	2	TRUE	0.003613	20.24275
1084	rs2643891	A	G	A	G	-0.018	0.033721	0.165	0.169686	FALSE	FALSE	FALSE	JLtEiZ	0.01823	0.064355	486484	sepsis	TRUE	reported	textfile	0.004	6.80E-06	5585	M01572.m	TRUE	reported	TSSe28	2	TRUE	0.003613	20.24275
4961	rs3887893	T	C	T	C	0.009	0.001733	0.617	0.606537	FALSE	FALSE	FALSE	bKmDMa	0.013998	0.901463	486484	sepsis	TRUE	reported	textfile	0.002	6.80E-06	5584	M32910.m	TRUE	reported	nAuPzO	2	TRUE	0.003613	20.24275
8395	rs6133587	T	G	T	G	0.009	-0.02973	0.719	0.893281	FALSE	FALSE	FALSE	SCzh0A	0.024216	0.219591	486484	sepsis	TRUE	reported	textfile	0.002	6.80E-06	5584	M35675.m	TRUE	reported	n1SREk	2	TRUE	0.003613	20.24275
3795	rs785896	A	G	A	G	0.009	0.009922	0.563	0.563712	FALSE	FALSE	FALSE	NW1aaR	0.013804	0.472307	486484	sepsis	TRUE	reported	textfile	0.002	6.80E-06	5583	M32339.m	TRUE	reported	pBPkAB	2	TRUE	0.003614	20.24275
2904	rs1755916	T	C	T	C	0.045	0.010404	0.027	0.159471	FALSE	FALSE	FALSE	FBDEQi	0.020293	0.608183	486484	sepsis	TRUE	reported	textfile	0.01	6.80E-06	5579	M20699.m	TRUE	reported	nvNIlp	2	TRUE	0.003617	20.24274
6121	rs1083268	A	G	A	G	-0.009	0.001289	0.547	0.556277	FALSE	FALSE	FALSE	MiSJly	0.013742	0.925285	486484	sepsis	TRUE	reported	textfile	0.002	6.80E-06	5573	M33477.m	TRUE	reported	WwL1hq	2	TRUE	0.00362	20.24273
6122	rs1093661	T	G	T	G	0.009	0.022024	0.675	0.672254	FALSE	FALSE	FALSE	MiSJly	0.014591	0.131183	486484	sepsis	TRUE	reported	textfile	0.002	6.80E-06	5573	M33477.m	TRUE	reported	WwL1hq	2	TRUE	0.00362	20.24273
8813	rs1250935	C	G	C	G	0.018	-0.0259	0.161	0.198462	FALSE	TRUE	FALSE	X2Rol2	0.017134	0.13062	486484	sepsis	TRUE	reported	textfile	0.004	6.80E-06	5568	M36776.m	TRUE	reported	nikHw1	2	TRUE	0.003624	20.24273
2139	rs2825115	T	C	T	C	0.009	-0.00984	0.568	0.565561	FALSE	FALSE	FALSE	TEupfd	0.013777	0.475242	486484	sepsis	TRUE	reported	textfile	0.002	6.80E-06	5566	M15676.m	TRUE	reported	NXiaPC	2	TRUE	0.003625	20.24272
2142	rs6596867	A	C	A	C	0.009	0.024537	0.543	0.546072	FALSE	FALSE	FALSE	TEupfd	0.013764	0.074629	486484	sepsis	TRUE	reported	textfile	0.002	6.80E-06	5566	M15676.m	TRUE	reported	NXiaPC	2	TRUE	0.003625	20.24272
7047	rs1324707	A	G	A	G	-0.009	-0.00277	0.506	0.508542	FALSE	FALSE	FALSE	uthruC	0.013667	0.839304	486484	sepsis	TRUE	reported	textfile	0.002	6.80E-06	5561	M34283.m	TRUE	reported	ftTuRU	2	TRUE	0.003628	20.24272

4605	rs6503078	T	G	T	G	-0.009	0.008666	0.283	0.280374	FALSE	FALSE	FALSE	Pzplqu	0.015604	0.578647	486484	sepsis	TRUE	reported	textfile	0.002	6.80E-06	5559	M32740.m	TRUE	reported	FjEBS5	2	TRUE	0.00363	20.24271
4615	rs9038	T	C	T	C	0.009	-0.01975	0.616	0.609475	FALSE	FALSE	FALSE	Pzplqu	0.01398	0.157639	486484	sepsis	TRUE	reported	textfile	0.002	6.80E-06	5559	M32740.m	TRUE	reported	FjEBS5	2	TRUE	0.00363	20.24271
4616	rs930551	A	G	A	G	-0.018	-0.00311	0.069	0.068236	FALSE	FALSE	FALSE	Pzplqu	0.027082	0.908627	486484	sepsis	TRUE	reported	textfile	0.004	6.80E-06	5559	M32740.m	TRUE	reported	FjEBS5	2	TRUE	0.00363	20.24271
4618	rs9322898	T	C	T	C	-0.009	-0.02228	0.318	0.318016	FALSE	FALSE	FALSE	Pzplqu	0.014701	0.129581	486484	sepsis	TRUE	reported	textfile	0.002	6.80E-06	5559	M32740.m	TRUE	reported	FjEBS5	2	TRUE	0.00363	20.24271
2524	rs9715724	T	C	T	C	0.009	0.024783	0.448	0.444344	FALSE	FALSE	FALSE	LwUYua	0.014689	0.091584	486484	sepsis	TRUE	reported	textfile	0.002	6.80E-06	5554	M17945.m	TRUE	reported	SUN8dc	2	TRUE	0.003633	20.24271
5825	rs208357	T	G	T	G	-0.009	-0.00619	0.25	0.247358	FALSE	FALSE	FALSE	R5joEf	0.015846	0.696182	486484	sepsis	TRUE	reported	textfile	0.002	6.80E-06	5536	M33380.m	TRUE	reported	kRvbfZ	2	TRUE	0.003645	20.24268
3132	rs1457192	A	G	A	G	-0.009	0.007325	0.272	0.27238	FALSE	FALSE	FALSE	JiXR2t	0.015351	0.633261	486484	sepsis	TRUE	reported	textfile	0.002	6.80E-06	5474	M22177.m	TRUE	reported	joi6p6	2	TRUE	0.003686	20.2426
4334	rs1173598	A	G	A	G	0.009	-0.00392	0.305	0.306144	FALSE	FALSE	FALSE	6VSoQQ	0.014832	0.791728	486484	sepsis	TRUE	reported	textfile	0.002	6.80E-06	5463	M32644.m	TRUE	reported	oC2AV0	2	TRUE	0.003693	20.24259
2738	rs7802565	C	G	C	G	-0.036	0.011283	0.024	0.026216	FALSE	TRUE	FALSE	H4yqSK	0.042832	0.792222	486484	sepsis	TRUE	reported	textfile	0.008	6.80E-06	5436	M19362.m	TRUE	reported	w2PDEO	2	TRUE	0.003711	20.24255
1288	rs1082430	A	G	A	G	0.009	-0.0253	0.383	0.390503	FALSE	FALSE	FALSE	WQdpmo	0.014049	0.071748	486484	sepsis	TRUE	reported	textfile	0.002	6.80E-06	5422	M01769.m	TRUE	reported	BkptOK	2	TRUE	0.003721	20.24253
1467	rs151743	T	C	T	C	-0.009	0.016295	0.426	0.422633	FALSE	FALSE	FALSE	pkb876	0.015178	0.282995	486484	sepsis	TRUE	reported	textfile	0.002	6.80E-06	5370	M02734.m	TRUE	reported	0LCMVI	2	TRUE	0.003757	20.24246
598	rs167277	A	C	A	C	-0.0207	0.010444	0.781	0.779773	FALSE	FALSE	FALSE	KVK33e	0.016502	0.526801	486484	sepsis	TRUE	reported	textfile	0.0046	7.92E-06	5344	M01107.m	TRUE	reported	eAyR8w	2	TRUE	0.003775	20.24242
8568	rs4141381	T	G	T	G	-0.144	0.001819	0.985	0.983478	FALSE	FALSE	FALSE	Yjjldt	0.055566	0.973889	486484	sepsis	TRUE	reported	textfile	0.032	6.80E-06	5329	M36098.m	TRUE	reported	U2ORsF	2	TRUE	0.003786	20.2424
2264	rs1041553	A	G	A	G	0.036	0.030006	0.167	0.178397	FALSE	FALSE	FALSE	Mjgi2R	0.017804	0.091928	486484	sepsis	TRUE	reported	textfile	0.008	6.80E-06	5315	M16634.m	TRUE	reported	XO0GV7	2	TRUE	0.003796	20.24238
4382	rs9626993	T	C	T	C	0.0126	0.001891	0.3485	0.339396	FALSE	FALSE	FALSE	SPhl52	0.014245	0.894414	486484	sepsis	TRUE	reported	textfile	0.0028	6.07E-06	5211	M32651.m	TRUE	reported	V0Bnpj	2	TRUE	0.003871	20.24223
4514	rs1177282	A	G	A	G	0.009	0.00116	0.323	0.329967	FALSE	FALSE	FALSE	r0qJaM	0.014567	0.936547	486484	sepsis	TRUE	reported	textfile	0.002	6.80E-06	5199	M32729.m	TRUE	reported	vYv5FI	2	TRUE	0.00388	20.24221
7392	rs3825895	T	C	T	C	0.0207	0.014134	0.4752	0.472209	FALSE	FALSE	FALSE	AW6M5u	0.013699	0.302183	486484	sepsis	TRUE	reported	textfile	0.0046	8.32E-06	5195	M34389.m	TRUE	reported	6ZYYQK	2	TRUE	0.003883	20.2422
7295	rs2281071	A	G	A	G	0.009	0.011501	0.346	0.342673	FALSE	FALSE	FALSE	Vcz4EA	0.014425	0.425275	486484	sepsis	TRUE	reported	textfile	0.002	6.80E-06	5179	M34359.m	TRUE	reported	aUSo2J	2	TRUE	0.003895	20.24218
8147	rs2498680	A	G	A	G	0.009	0.010201	0.81	0.808881	FALSE	FALSE	FALSE	lv3pAQ	0.017411	0.557962	486484	sepsis	TRUE	reported	textfile	0.002	6.80E-06	5160	M35327.m	TRUE	reported	3Vsglp	2	TRUE	0.003909	20.24215
7188	rs7795256	T	C	T	C	-0.0135	-0.02314	0.2434	0.240623	FALSE	FALSE	FALSE	2hlepp	0.016065	0.149738	486484	sepsis	TRUE	reported	textfile	0.003	5.33E-06	5081	M34336.m	TRUE	reported	XyK8mO	2	TRUE	0.00397	20.24203
3966	rs6517442	T	C	T	C	0.0252	-0.02482	0.6652	0.677811	FALSE	FALSE	FALSE	sKCkQs	0.01465	0.090219	486484	sepsis	TRUE	reported	textfile	0.0056	5.94E-06	5080	M32445.m	TRUE	reported	egPvJc	2	TRUE	0.00397	20.24203
2241	rs1076302	A	G	A	G	0.018	-0.00061	0.201	0.201447	FALSE	FALSE	FALSE	zxcPox	0.017041	0.97159	486484	sepsis	TRUE	reported	textfile	0.004	6.80E-06	5026	M15990.m	TRUE	reported	34Vg8F	2	TRUE	0.004013	20.24194
8883	rs1008284	T	C	T	C	0.009	0.003295	0.393	0.401746	FALSE	FALSE	FALSE	eLhsld	0.014038	0.814433	486484	sepsis	TRUE	reported	textfile	0.002	6.80E-06	5018	M37058.m	TRUE	reported	RSeWE5	2	TRUE	0.004019	20.24193
8886	rs1076230	A	G	A	G	0.009	-0.03061	0.532	0.53101	FALSE	FALSE	FALSE	eLhsld	0.013683	0.025291	486484	sepsis	TRUE	reported	textfile	0.002	6.80E-06	5018	M37058.m	TRUE	reported	RSeWE5	2	TRUE	0.004019	20.24193
8899	rs1727198	T	C	T	C	-0.009	0.031364	0.556	0.556969	FALSE	FALSE	FALSE	eLhsld	0.013753	0.022571	486484	sepsis	TRUE	reported	textfile	0.002	6.80E-06	5018	M37058.m	TRUE	reported	RSeWE5	2	TRUE	0.004019	20.24193
643	rs9851393	A	G	A	G	0.0297	0.019213	0.5199	0.521263	FALSE	FALSE	FALSE	F6Dn8H	0.0137	0.16079	486484	sepsis	TRUE	reported	textfile	0.0066	7.38E-06	4934	M01114.m	TRUE	reported	dVHatZ	2	TRUE	0.004087	20.24179
6112	rs4807122	T	C	T	C	0.018	0.009516	0.386	0.383945	FALSE	FALSE	FALSE	a0osmD	0.014208	0.503028	486484	sepsis	TRUE	reported	textfile	0.004	6.80E-06	4599	M33453.m	TRUE	reported	CePFnK	2	TRUE	0.004384	20.24119
3059	rs7377975	A	G	A	G	0.018	0.027112	0.189	0.199781	FALSE	FALSE	FALSE	H6vcCG	0.0171	0.112867	486484	sepsis	TRUE	reported	textfile	0.004	6.80E-06	4558	M22030.m	TRUE	reported	XetVX7	2	TRUE	0.004423	20.24111
5763	rs4767064	A	G	A	G	0.0216	-0.01278	0.3478	0.369283	FALSE	FALSE	FALSE	n16uO2	0.014162	0.36697	486484	sepsis	TRUE	reported	textfile	0.0048	8.17E-06	4409	M33250.m	TRUE	reported	9dhl67	2	TRUE	0.004572	20.24081
4790	rs1051101	T	C	T	C	0.0261	0.027936	0.2764	0.284335	FALSE	FALSE	FALSE	dwreVz	0.015197	0.066018	486484	sepsis	TRUE	reported	textfile	0.0058	6.31E-06	4311	M32802.m	TRUE	reported	7YCayZ	2	TRUE	0.004675	20.24061
7809	rs1006081	A	G	A	G	0.009	-0.03419	0.323	0.327922	FALSE	FALSE	FALSE	h5whlS	0.014746	0.020416	486484	sepsis	TRUE	reported	textfile	0.002	6.80E-06	4081	M35137.m	TRUE	reported	xVITKF	2	TRUE	0.004938	20.24008
6091	rs1243281	A	C	A	C	0.045	-0.00817	0.9717	0.969438	FALSE	FALSE	FALSE	xvzgym	0.03965	0.836792	486484	sepsis	TRUE	reported	textfile	0.01	7.17E-06	4004	M33443.m	TRUE	reported	KtBPyR	2	TRUE	0.005032	20.23989
8112	rs2149211	A	G	A	G	-0.009	0.020095	0.435	0.438903	FALSE	FALSE	FALSE	nNbXBS	0.01381	0.145653	486484	sepsis	TRUE	reported	textfile	0.002	6.80E-06	4004	M35326.m	TRUE	reported	UNDorn	2	TRUE	0.005032	20.23989
3116	rs2031504	T	G	T	G	0.0225	0.023026	0.4374	0.43152	FALSE	FALSE	FALSE	a9KeDe	0.013806	0.095348	486484	sepsis	TRUE	reported	textfile	0.005	8.14E-06	3838	M22175.m	TRUE	reported	shg5gu	2	TRUE	0.005248	20.23945
2963	rs2393374	T	G	T	G	0.0351	0.035234	0.3095	0.308408	FALSE	FALSE	FALSE	zndu0r	0.014848	0.017647	486484	sepsis	TRUE	reported	textfile	0.0078	6.39E-06	3422	M21049.m	TRUE	reported	2XpPDU	2	TRUE	0.005883	20.23816
5685	rs1181503	T	C	T	C	0.1134	-0.01049	0.0578	0.05341	FALSE	FALSE	FALSE	KxtFnC	0.03055	0.731208	486484	sepsis	TRUE	reported	textfile	0.0252	6.97E-06	3221	M33203.m	TRUE	reported	Uz0wKU	2	TRUE	0.006248	20.23743
6355	rs1029659	A	C	A	C	0.0198	-0.00681	0.1991	0.189893	FALSE	FALSE	FALSE	WWmDGM	0.017488	0.696805	486484	sepsis	TRUE	reported	textfile	0.0044	7.94E-06	2757	M33627.m	TRUE	reported	2kkEru	2	TRUE	0.007291	20.23531
6359	rs1460191	A	C	A	C	0.0207	-0.00259	0.1654	0.182987	FALSE	FALSE	FALSE	WWmDGM	0.017689	0.883542	486484	sepsis	TRUE	reported	textfile	0.0046	5.38E-06	2757	M33627.m	TRUE	reported	2kkEru	2	TRUE	0.007291	20.23531
5697	rs1113306	T	G	T	G	-0.0135	-0.0103	0.5241	0.540117	FALSE	FALSE	FALSE	1SbQTe	0.013748	0.453554	486484	sepsis	TRUE	reported	textfile	0.003	6.74E-06	2683	M33204.m	TRUE	reported	x7MBDH	2	TRUE	0.007491	20.2349
4883	rs7129619	C	G	C	G	0.0297	0.026508	0.6449	0.667583	FALSE	TRUE	FALSE	MDX5FX	0.014557	0.068614	486484	sepsis	TRUE	reported	textfile	0.0066	7.25E-06	2656	M32857.m	TRUE	reported	moAN2E	2	TRUE	0.007567	20.23475
8953	rs1718085	A	T	A	T	0.0837	0.005294	0.864	0.858796	FALSE	TRUE	FALSE	Ywk2ve	0.019727	0.788435	486484	sepsis	TRUE	reported	textfile	0.0186	6.76E-06	2560	M37112.m	TRUE	reported	EiAdDj	2	TRUE	0.007848	20.23418
6328	rs1083670	C	G	C	G	-0.1272	0.023448	0.326	0.299746	FALSE	TRUE	FALSE	qLUY00	0.014965	0.117129	486484	sepsis	TRUE	reported	textfile	0.028	5.53E-06	102	M33610.m	TRUE	reported	8cAJbB	2	TRUE	0.168281	20.23289
4928	rs3737137	T	C	T	C	-0.0306	0.010313	0.233	0.229496	FALSE	FALSE	FALSE	clhN6v	0.016234	0.525241	486484	sepsis	TRUE	reported	textfile	0.0068	7.99E-06	2148	M32869.m	TRUE	reported	BzloWk	2	TRUE	0.009339	20.23115
7130	rs1706698	T	C	T	C	0.1974	-0.01514	0.0473	0.065167	FALSE	FALSE	FALSE	de8tA6	0.027731	0.585042	486484	sepsis	TRUE	reported	textfile	0.0433	5.16E-06	75	M34322.m	TRUE	reported	sXPPBO	2	TRUE	0.216984	20.22926
7110	rs4953342	A	G	A	G	-0.0432	0.000452	0.7182	0.704533	FALSE	FALSE	FALSE	DfLd6z	0.015178	0.976236	486484	sepsis	TRUE	reported	textfile	0.0096	7.20E-06	1766	M34314.m	TRUE	reported	OgloAQ	2	TRUE	0.011337	20.22707
8130	rs7706802	A	G	A	G	-0.0189	-0.001	0.4621	0.536163	FALSE	FALSE	FALSE	nNbXBS	0.013721	0.942072	486484	sepsis	TRUE	reported	textfile	0.0042	7.06E-06	1764	M35326.m	TRUE	reported	UNDorn	2	TRUE	0.011349	20.22704
8385	rs2344567	T	G	T	G	0.0225	-0.00192	0.2843	0.290763	FALSE	FALSE	FALSE	SCzh0A	0.015095	0.898654	486484	sepsis	TRUE	reported	textfile	0.005	6.35E-06	1764	M35675.m	TRUE	reported	n1SREk	2	TRUE	0.011349	20.22704
449	rs1163838	T	C	T	C	-0.0171	0.010923	0.1875	0.185952	FALSE	FALSE	FALSE	cl8ucN	0.017909	0.541897	486484	sepsis	TRUE	reported	textfile	0.0038	5.06E-06	1763	M00513.m	TRUE	reported	lan0Q8	2	TRUE	0.011356	20.22703
532	rs1841535	T	G	T	G	0.0252	-0.00106	0.1482	0.254958	FALSE	FALSE	FALSE	wXMWHy	0.01568	0.94595	486484	sepsis	TRUE	reported	textfile	0.0056	7.00E-06	1754	M00584.m	TRUE	reported	7CKn2b	2	TRUE	0.011413	20.22691
2379	rs1382668	A	G	A	G	0.0927	0.022463	0.9273	0.95839	FALSE	FALSE	FALSE	NBJ2Do	0.034245	0.511852	486484	sepsis	TRUE	reported	textfile	0.0206	7.01E-06	1739	M17799.m	TRUE	reported	Uz0Wgl	2	TRUE	0.011511	20.22671
8228	rs1262524	T	G	T	G	-0.0297	0.014217	0.8274	0.872289	FALSE	FALSE	FALSE	4YAWhA	0.020582	0.489743	486484	sepsis	TRUE	reported	textfile	0.0066	7.16E-06	1718	M35439.m	TRUE	reported	bSSC4Z	2	TRUE	0.01165	20.22643
4639	rs335810	A	C	A	C	0.3722	0.020763	0.9883	0.989047	FALSE	FALSE	FALSE	Dfaklt	0.06674	0.75572	486484	sepsis	TRUE	reported	textfile	0.0827	6.80E-06	1368	M32754.m	TRUE	reported	y21cDm	2	TRUE	0.014591	20.22583
8619	rs1889157	A	G	A	G	-0.0342	-0.01393	0.4713	0.485584	FALSE	FALSE	FALSE	MZ60v7	0.014143	0.324529	486484	sepsis	TRUE	reported	textfile	0.0076	6.44E-06	1657	M36134.m	TRUE	reported	dMQewN	2	TRUE	0.012073	20.22556
4989	rs1019202	T	G	T	G	-0.0513	0.003527	0.0878	0.092083	FALSE	FALSE	FALSE	MdUq0m	0.024083	0.883558	486484	sepsis	TRUE	reported	textfile	0.0114	7.46E-06	1618	M33009.m	TRUE	reported	icuhyC	2	TRUE	0.012361	20.22497
8548	rs1689756	T	C	T	C	0.2875	-0.01483	0.8063	0.824987	FALSE	FALSE	FALSE	aQhCav	0.01821	0.415565	486484	sepsis	TRUE	reported	textfile	0.0638	6.70E-06	488	M36097.m	TRUE	reported	9DJS9j	2	TRUE	0.039949	20.22324
8260	rs6698967	A	G	A	G	-0.3177	0.037437	0.9723	0.972306	FALSE	FALSE	FALSE	G4plxe	0.041922	0.371842	486484	sepsis	TRUE	reported	textfile	0.0704	6.49E-06	284	M35451.m	TRUE	reported	hNOVMI	2	TRUE	0.06691	20.2218
6548	rs1402168	T	G	T	G	0.1077	0.03761	0.8834	0.863216	FALSE	FALSE	FALSE	zmDnD7	0.020073	0.060972	486484	sepsis	TRUE	reported	textfile	0.0239	6.49E-06	471	M33835.m	TRUE	reported	MLBiKM	2	TRUE	0.041332	20.2203
7905	rs1327695	A	C	A	C	0.0657	-0.02473	0.3037	0.300715	FALSE	FALSE	FALSE	AhsT9A	0.014954	0.098116	486484	sepsis	TRUE	reported	textfile	0.0146	6.50E-06	1254	M35187.m	TRUE	reported	uLU0Fy	2	TRUE	0.015892	20.2177
6573	rs1199373	T	C	T	C	-0.2732	-0.05077	0.9774	0.977566	FALSE	FALSE	FALSE	XO3rXq	0.046301	0.2729	486484	sepsis	TRUE	reported	textfile	0.0605	6.30E-06	228	M33877.m	TRUE	reported	p2sETg	2	TRUE	0.082094	20.2127
5511	rs6578873	T	G	T	G	-0.3042	0.003455	0.0535	0.542754	FALSE	FALSE	FALSE	q32d4E	0.013728	0.801275	486484	sepsis	TRUE	reported	textfile	0.0649	2.72E-06	25	M33178.m	TRUE	reported	cS3BwT	2	TRUE	0.467745	20.21235
6340	rs4291236	C	G	C	G	0.2588	-0.03409	0.0549	0.072201	FALSE	TRUE	FALSE	qLUY00	0.02663	0.200513	486484	sepsis	TRUE	reported	textfile	0.057	5.54E-06	102	M33610.m	TRUE	reported	8cAJbB	2	TRUE	0.168126	20.21057
5664	rs954483	A	G	A	G	0.0616	-0.03701	0.8406	0.843276	FALSE	FALSE	FALSE	mod1fD	0.018888	0.050029	486484	sepsis	TRUE	reported	textfile	0.0137	6.81E-06	5064	M33194.m	TRUE	reported	fSFVUj	2	TRUE	0.003976	20.20918
5724	rs1195499	A	T	A	T	0.121	0.086704	0.0131	0.01432	FALSE	TRUE	FALSE	YDQaWa	0.060152	0.149462	486484	sepsis	TRUE	reported	textfile	0.0269	6.85E-06	1612	M33228.m	TRUE	reported	BmYg8r	2	TRUE	0.012396	20.20817
7330	rs1177250	T	C	T	C	0.1134	-0.00278	0.1441	0.175124	FALSE	FALSE	FALSE	mm9ksf	0.018044	0.87735	486484	sepsis	TRUE	reported	textfile	0.0252	6.92E-06	954	M34365.m	TRUE	reported	AVwEqD	2	TRUE	0.020785	20.20755
5456	rs4379054	A	C	A	C	-0.2713	-0.0365	0.027	0.017872	FALSE	FALSE	FALSE	q32d4E	0.051507	0.478519	486484	sepsis	TRUE	reported	textfile	0.0603	6.89E-06	1017	M33178.m	TRUE	reported	cS3BwT	2	TRUE	0.019516	20.20273
499	rs6468437	T	C	T	C	0.1246	0.019158	0.8334	0.797878	FALSE	FALSE	FALSE	RtjZPn	0.017001	0.259807	486484	sepsis	TRUE	reported	textfile	0.0277	6.86E-06	1233	M00553.m	TRUE	reported	C3Ovsl	2	TRUE	0.016145	20.20094
5084	rs4364244	A	G	A	G	-0.0463	0.000768	0.7874	0.797232	FALSE	FALSE	FALSE	VzfWsT	0.017013	0.964014	486484	sepsis	TRUE	reported	textfile	0.0103	7.29E-06	4817	M33144.m	TRUE	reported	ZtQP3u	2	TRUE	0.004177	20.19794
8067	rs1698237	A	G	A	G	-0.2028	-0.07945	0.0111	0.011105	FALSE	FALSE	FALSE	7NkUCa	0.065468	0.224898	486484	sepsis	TRUE	reported	textfile	0.0451	7.02E-06	1765	M35320.m	TRUE	reported	GixRsK	2	TRUE	0.011326	20.19717
7595	rs1117179	T	C	T	C	0.3447	0.074146	0.957	0.950618	FALSE	FALSE	FALSE	PlYt5g	0.031573	0.018854	486484	sepsis	TRUE	reported	textfile	0.0761	5.86E-06	128	M34516.m	TRUE	reported	jX41tf	2	TRUE	0.138146	20.19639
5654	rs1048551	A	G	A	G	-0.0427	-0.01804	0.4042	0.401299	FALSE	FALSE	FALSE	mod1fD	0.013953	0.196108	486484	sepsis	TRUE	reported	textfile	0.0095	7.29E-06	5064	M33194.m	TRUE	reported	fSFVUj	2	TRUE	0.003974	20.19468
7214	rs1778496	T	G	T	G	-0.3165	0.023505	0.975	0.969724	FALSE	FALSE	FALSE	i1uJ7T	0.039811	0.554922	486484	sepsis	TRUE	reported	textfile	0.0703	6.76E-06	535	M34338.m	TRUE	reported	pi3mKW	2	TRUE	0.036503	20.19344
2365	rs1165210	T	C	T	C	-0.1214	-0.01947	0.04	0.042341	FALSE	FALSE	FALSE	NBJ2Do	0.034783	0.575729	486484	sepsis	TRUE	reported	textfile	0.027	6.71E-06	1739	M17799.m	TRUE	reported	Uz0Wgl	2	TRUE	0.011492	20.19343
3570	rs1254053	A	C	A	C	0.1022	0.003663	0.1129	0.11711	FALSE	FALSE	FALSE	F1s6qg	0.021257	0.863182	486484	sepsis	TRUE	reported	textfile	0.0227	6.74E-06	522	M31536.m	TRUE	reported	Zm40GX	2	TRUE	0.03738	20.19217
3866	rs9284892	T	C	T	C	-0.0382	0.028433	0.0588	0.058388	FALSE	FALSE	FALSE	8gg1zS	0.029315	0.332094	486484	sepsis	TRUE	reported	textfile	0.0085	7.03E-06	6119	M32379.m	TRUE	reported	uZWtuS	2	TRUE	0.00329	20.19049
3874	rs1688548	A	G	A	G	-0.0355	-0.00162	0.0207	0.028985	FALSE	FALSE	FALSE	bwkKtK	0.041144	0.968557	486484	sepsis	TRUE	reported	textfile	0.0079	7.48E-06	7307	M32393.m	TRUE	reported	RvZzAy	2	TRUE	0.002756	20.18755
1032	rs1051473	A	G	A	G	0.0346	-0.01405	0.985	0.983972	FALSE	FALSE	FALSE	tRtQ2e	0.054714	0.797298	486484	sepsis	TRUE	reported	textfile	0.0077	6.51E-06	7364	M01564.m	TRUE	reported	Tbg0zM	2	TRUE	0.002734	20.18612
7483	rs1244831	A	G	A	G	0.0346	0.025849	0.9776	0.960643	FALSE	FALSE	FALSE	jAhmnV	0.035124	0.461784	486484	sepsis	TRUE	reported	textfile	0.0077	6.99E-06	7348	M34419.m	TRUE	reported	F2Tg9L	2	TRUE	0.00274	20.1861
6505	rs756316	T	C	T	C	-0.0373	0.001788	0.6015	0.602377	FALSE	FALSE	FALSE	HNc7C9	0.014033	0.898611	486484	sepsis	TRUE	reported	textfile	0.0083	6.95E-06	3962	M33782.m	TRUE	reported	SAGYGx	2	TRUE	0.005072	20.18562
3955	rs1117727	A	T	A	T	0.0355	0.032626	0.8925	0.860009	FALSE	TRUE	FALSE	sKCkQs	0.019705	0.097778	486484	sepsis	TRUE	reported	textfile	0.0079	7.29E-06	5080	M32445.m	TRUE	reported	egPvJc	2	TRUE	0.003959	20.18513
582	rs2158555	C	G	C	G	-0.0337	0.012453	0.0331	0.022706	FALSE	TRUE	FALSE	ZoqU2j	0.046687	0.78968	486484	sepsis	TRUE	reported	textfile	0.0075	7.91E-06	7333	M01105.m	TRUE	reported	2WxSOH	2	TRUE	0.002746	20.18454
6816	rs1074283	T	C	T	C	-0.0337	0.041288	0.0697	0.0735	FALSE	FALSE	FALSE	wedECv	0.026146	0.114306	486484	sepsis	TRUE	reported	textfile	0.0075	7.82E-06	7322	M33968.m	TRUE	reported	GxGxRG	2	TRUE	0.00275	20.18453
8939	rs1317896	T	C	T	C	-0.0355	0.00853	0.8388	0.84671	FALSE	FALSE	FALSE	mXJP3h	0.018984	0.653197	486484	sepsis	TRUE	reported	textfile	0.0079	6.47E-06	4520	M37104.m	TRUE	reported	HRZBak	2	TRUE	0.004448	20.18414
3436	rs9549759	T	C	T	C	0.0337	-0.00996	0.4298	0.428865	FALSE	FALSE	FALSE	HHQf8w	0.013808	0.470887	486484	sepsis	TRUE	reported	textfile	0.0075	7.25E-06	5673	M27531.m	TRUE	reported	3vTluK	2	TRUE	0.003546	20.18293
2752	rs6579773	T	C	T	C	-0.0472	0.004563	0.933	0.955962	FALSE	FALSE	FALSE	qFAbee	0.033518	0.891713	486484	sepsis	TRUE	reported	textfile	0.0105	6.47E-06	1654	M19364.m	TRUE	reported	tZgXNL	2	TRUE	0.01207	20.18273
863	rs1683001	T	G	T	G	-0.0328	0.02851	0.9719	0.970156	FALSE	FALSE	FALSE	Vg5OOR	0.040365	0.479989	486484	sepsis	TRUE	reported	textfile	0.0073	7.41E-06	7063	M01361.m	TRUE	reported	XnrS00	2	TRUE	0.00285	20.18269
2676	rs392488	T	C	T	C	-0.0337	0.003788	0.1984	0.202924	FALSE	FALSE	FALSE	s4UTyf	0.017007	0.823725	486484	sepsis	TRUE	reported	textfile	0.0075	7.26E-06	5376	M18494.m	TRUE	reported	ajPzhg	2	TRUE	0.003742	20.18253
3446	rs1770991	A	G	A	G	0.0472	0.008614	0.7757	0.788889	FALSE	FALSE	FALSE	J1l92u	0.01674	0.606854	486484	sepsis	TRUE	reported	textfile	0.0105	6.41E-06	1510	M27710.m	TRUE	reported	9qjplZ	2	TRUE	0.013206	20.1804
7376	rs7771614	T	C	T	C	-0.162	0.005381	0.5	0.425589	FALSE	FALSE	FALSE	mm9ksf	0.013922	0.699125	486484	sepsis	TRUE	reported	textfile	0.034	1.85E-06	18	M34365.m	TRUE	reported	AVwEqD	2	TRUE	0.557766	20.17993
8571	rs791232	T	C	T	C	0.031	-0.01991	0.491	0.471429	FALSE	FALSE	FALSE	Yjjldt	0.013691	0.145887	486484	sepsis	TRUE	reported	textfile	0.0069	7.63E-06	7054	M36098.m	TRUE	reported	U2ORsF	2	TRUE	0.002853	20.17911
6459	rs1329128	T	C	T	C	0.1277	0.020542	0.0306	0.043665	FALSE	FALSE	FALSE	yLohrN	0.03439	0.55029	486484	sepsis	TRUE	reported	textfile	0.0284	6.77E-06	1027	M33683.m	TRUE	reported	mvhAZ4	2	TRUE	0.019307	20.17895
8630	rs1302519	C	G	C	G	-0.0364	0.027393	0.8014	0.797313	FALSE	TRUE	FALSE	4cZjpQ	0.017114	0.109453	486484	sepsis	TRUE	reported	textfile	0.0081	6.72E-06	2400	M36230.m	TRUE	reported	TPUeNs	2	TRUE	0.008344	20.17765
8966	rs3214031	A	C	A	C	-0.0301	0.009	0.7088	0.716581	FALSE	FALSE	FALSE	2iAvlt	0.015167	0.552926	486484	sepsis	TRUE	reported	textfile	0.0067	6.05E-06	6934	M37190.m	TRUE	reported	hdOuHe	2	TRUE	0.002902	20.17707
8830	rs4976017	T	C	T	C	0.031	-0.03493	0.0472	0.049562	FALSE	FALSE	FALSE	zuPm7L	0.031621	0.269265	486484	sepsis	TRUE	reported	textfile	0.0069	7.79E-06	4317	M36802.m	TRUE	reported	cn9d0n	2	TRUE	0.004654	20.17548
8201	rs4626843	A	G	A	G	0.0292	-0.00854	0.0341	0.040918	FALSE	FALSE	FALSE	iLlpho	0.034633	0.80525	486484	sepsis	TRUE	reported	textfile	0.0065	7.39E-06	7007	M35431.m	TRUE	reported	AAZ5ge	2	TRUE	0.002872	20.17507
6400	rs1692816	A	G	A	G	-0.0885	0.072977	0.974	0.970692	FALSE	FALSE	FALSE	Z1h3mT	0.04098	0.074948	486484	sepsis	TRUE	reported	textfile	0.0197	7.22E-06	5328	M33652.m	TRUE	reported	nszuTQ	2	TRUE	0.003774	20.17395
7254	rs1791914	A	G	A	G	-0.0566	0.023968	0.2847	0.387628	FALSE	FALSE	FALSE	4t4W6t	0.014238	0.092312	486484	sepsis	TRUE	reported	textfile	0.0126	6.63E-06	5270	M34344.m	TRUE	reported	Rz9mCt	2	TRUE	0.003814	20.17098
8541	rs1265023	T	G	T	G	0.5126	-0.0389	0.9469	0.947366	FALSE	FALSE	FALSE	aQhCav	0.030637	0.20424	486484	sepsis	TRUE	reported	textfile	0.1139	6.82E-06	488	M36097.m	TRUE	reported	9DJS9j	2	TRUE	0.03985	20.17094
3968	rs1100417	T	C	T	C	0.0274	-0.00914	0.9683	0.963752	FALSE	FALSE	FALSE	XdJbEU	0.036746	0.803553	486484	sepsis	TRUE	reported	textfile	0.0061	8.15E-06	7364	M32452.m	TRUE	reported	V5xWKi	2	TRUE	0.002732	20.17082
5153	rs4805580	T	C	T	C	0.964	-0.04773	0.9867	0.984138	FALSE	FALSE	FALSE	L2DJzK	0.054822	0.383974	486484	sepsis	TRUE	reported	textfile	0.2118	5.35E-06	76	M33173.m	TRUE	reported	hRG70D	2	TRUE	0.214193	20.17067
6590	rs4785153	A	G	A	G	0.3758	0.033274	0.0201	0.023652	FALSE	FALSE	FALSE	XO3rXq	0.04478	0.457443	486484	sepsis	TRUE	reported	textfile	0.0832	6.35E-06	176	M33877.m	TRUE	reported	p2sETg	2	TRUE	0.103878	20.16989
1097	rs4661299	T	G	T	G	0.0319	-0.00825	0.4551	0.455769	FALSE	FALSE	FALSE	K3wtZo	0.013748	0.548416	486484	sepsis	TRUE	reported	textfile	0.0071	7.63E-06	2315	M01573.m	TRUE	reported	0D9CvA	2	TRUE	0.008645	20.16923
4877	rs1176113	A	T	A	T	-0.0301	-0.00457	0.6277	0.616279	FALSE	TRUE	FALSE	MDX5FX	0.014074	0.7456	486484	sepsis	TRUE	reported	textfile	0.0067	6.27E-06	2656	M32857.m	TRUE	reported	moAN2E	2	TRUE	0.007542	20.16769
3721	rs6926138	T	C	T	C	-0.0337	0.011369	0.6583	0.62266	FALSE	FALSE	FALSE	ZRfYkG	0.014165	0.422171	486484	sepsis	TRUE	reported	textfile	0.0075	6.26E-06	1765	M32319.m	TRUE	reported	phVUPX	2	TRUE	0.01131	20.16717
3397	rs7804260	A	G	A	G	0.0292	-0.01781	0.4115	0.414243	FALSE	FALSE	FALSE	frOZiy	0.013876	0.199365	486484	sepsis	TRUE	reported	textfile	0.0065	7.01E-06	2759	M27447.m	TRUE	reported	2z9fSv	2	TRUE	0.007261	20.1662
6337	rs2803893	A	G	A	G	-0.0621	-0.00126	0.7876	0.786588	FALSE	FALSE	FALSE	qLUY00	0.016725	0.9399	486484	sepsis	TRUE	reported	textfile	0.0138	7.09E-06	470	M33610.m	TRUE	reported	8cAJbB	2	TRUE	0.041305	20.16383
6510	rs6893667	T	C	T	C	0.0319	0.003591	0.551	0.538011	FALSE	FALSE	FALSE	dluFOH	0.013698	0.79319	486484	sepsis	TRUE	reported	textfile	0.0071	7.19E-06	1761	M33801.m	TRUE	reported	JCXy06	2	TRUE	0.011333	20.16374
8767	rs877682	T	C	T	C	0.0795	0.013813	0.9622	0.963786	FALSE	FALSE	FALSE	KMisdy	0.036722	0.706793	486484	sepsis	TRUE	reported	textfile	0.0177	7.00E-06	3977	M36589.m	TRUE	reported	J1lSTP	2	TRUE	0.005047	20.16366
3767	rs952045	T	C	T	C	0.0247	0.023332	0.9494	0.943884	FALSE	FALSE	FALSE	OZLdls	0.02997	0.436257	486484	sepsis	TRUE	reported	textfile	0.0055	7.34E-06	7356	M32338.m	TRUE	reported	s2itmB	2	TRUE	0.002734	20.16278
8155	rs1098765	A	G	A	G	0.0346	-0.02147	0.1032	0.102227	FALSE	FALSE	FALSE	sRlsb6	0.022537	0.340783	486484	sepsis	TRUE	reported	textfile	0.0077	7.52E-06	1375	M35331.m	TRUE	reported	GPijlC	2	TRUE	0.014472	20.16223
903	rs4443239	T	G	T	G	0.031	-0.01488	0.7453	0.754159	FALSE	FALSE	FALSE	l6zQnf	0.015924	0.350118	486484	sepsis	TRUE	reported	textfile	0.0069	7.58E-06	1753	M01444.m	TRUE	reported	llzalm	2	TRUE	0.011383	20.16181
5008	rs1291642	A	G	A	G	-0.0274	-0.0154	0.2058	0.207993	FALSE	FALSE	FALSE	URCVGm	0.016883	0.361725	486484	sepsis	TRUE	reported	textfile	0.0061	8.06E-06	2773	M33131.m	TRUE	reported	4rQ2gs	2	TRUE	0.007223	20.16174
7129	rs159314	A	G	A	G	0.1866	0.023993	0.054	0.054387	FALSE	FALSE	FALSE	de8tA6	0.030278	0.428112	486484	sepsis	TRUE	reported	textfile	0.041	5.44E-06	75	M34322.m	TRUE	reported	sXPPBO	2	TRUE	0.216412	20.16124
1730	rs1008834	A	G	A	G	0.0238	0.020764	0.0749	0.093484	FALSE	FALSE	FALSE	nFLpAP	0.023606	0.37907	486484	sepsis	TRUE	reported	textfile	0.0053	6.75E-06	7356	M12774.m	TRUE	reported	yOSQTj	2	TRUE	0.002734	20.1597
7461	rs7668227	A	G	A	G	0.0238	0.008168	0.9017	0.885003	FALSE	FALSE	FALSE	7ubZow	0.021457	0.703467	486484	sepsis	TRUE	reported	textfile	0.0053	7.86E-06	7344	M34407.m	TRUE	reported	LRsXN2	2	TRUE	0.002738	20.15969
8602	rs9479657	T	C	T	C	-0.053	-0.01847	0.0711	0.072538	FALSE	FALSE	FALSE	t5potg	0.0265	0.485787	486484	sepsis	TRUE	reported	textfile	0.0118	7.75E-06	2709	M36115.m	TRUE	reported	SSOHIA	2	TRUE	0.007392	20.15891
6427	rs1182517	T	C	T	C	-0.0584	0.081302	0.0223	0.025436	FALSE	FALSE	FALSE	QCw5dg	0.043392	0.060976	486484	sepsis	TRUE	reported	textfile	0.013	6.84E-06	1764	M33666.m	TRUE	reported	zYHmzg	2	TRUE	0.011311	20.15795
7121	rs1165602	A	G	A	G	0.1074	0.015671	0.2014	0.229897	FALSE	FALSE	FALSE	de8tA6	0.016315	0.336787	486484	sepsis	TRUE	reported	textfile	0.0236	5.45E-06	75	M34322.m	TRUE	reported	sXPPBO	2	TRUE	0.216385	20.15794
5669	rs2490700	C	G	C	G	0.1208	-0.00626	0.0343	0.227877	FALSE	TRUE	FALSE	wHu1Rk	0.016275	0.700309	486484	sepsis	TRUE	reported	textfile	0.0269	7.35E-06	4625	M33195.m	TRUE	reported	yvhnuQ	2	TRUE	0.004341	20.15772
7410	rs1049251	T	C	T	C	-0.0238	0.031352	0.1563	0.158041	FALSE	FALSE	FALSE	7i5VUS	0.018753	0.094545	486484	sepsis	TRUE	reported	textfile	0.0053	7.65E-06	5208	M34395.m	TRUE	reported	64hEaL	2	TRUE	0.003857	20.15744
3613	rs7742715	A	G	A	G	0.0292	0.0056	0.356	0.364669	FALSE	FALSE	FALSE	2z5KhH	0.014181	0.69292	486484	sepsis	TRUE	reported	textfile	0.0065	6.59E-06	1713	M31555.m	TRUE	reported	5kcWr2	2	TRUE	0.011644	20.15727
6681	rs9864709	T	G	T	G	-0.0238	-0.00576	0.2158	0.222319	FALSE	FALSE	FALSE	6iZSOI	0.016465	0.726302	486484	sepsis	TRUE	reported	textfile	0.0053	5.74E-06	4533	M33901.m	TRUE	reported	ApKaGe	2	TRUE	0.004429	20.15629
6571	rs1083231	A	G	A	G	-0.1452	-0.0223	0.9183	0.917299	FALSE	FALSE	FALSE	XO3rXq	0.024872	0.370034	486484	sepsis	TRUE	reported	textfile	0.0322	6.67E-06	228	M33877.m	TRUE	reported	p2sETg	2	TRUE	0.081881	20.15557
971	rs1045872	A	G	A	G	-0.0786	-0.01755	0.609	0.602283	FALSE	FALSE	FALSE	xSRIl4	0.014008	0.210364	486484	sepsis	TRUE	reported	textfile	0.0175	7.23E-06	2285	M01515.m	TRUE	reported	juZ5GE	2	TRUE	0.008751	20.15527
4242	rs7783170	T	C	T	C	-0.0229	-0.01579	0.7251	0.736369	FALSE	FALSE	FALSE	wWEMr1	0.015562	0.310387	486484	sepsis	TRUE	reported	textfile	0.0051	7.96E-06	5955	M32593.m	TRUE	reported	Mh2g4i	2	TRUE	0.003374	20.15509
7343	rs157009	T	C	T	C	-0.2438	-0.01289	0.3529	0.394738	FALSE	FALSE	FALSE	mm9ksf	0.013997	0.35702	486484	sepsis	TRUE	reported	textfile	0.0512	1.92E-06	18	M34365.m	TRUE	reported	AVwEqD	2	TRUE	0.557456	20.15464
7608	rs7549636	A	G	A	G	0.1145	-0.01111	0.271	0.269568	FALSE	FALSE	FALSE	PlYt5g	0.015466	0.472407	486484	sepsis	TRUE	reported	textfile	0.0254	6.36E-06	241	M34516.m	TRUE	reported	jX41tf	2	TRUE	0.077762	20.15229
3857	rs1705804	A	G	A	G	-0.044	-0.02067	0.0554	0.060857	FALSE	FALSE	FALSE	8gg1zS	0.028618	0.470161	486484	sepsis	TRUE	reported	textfile	0.0098	7.33E-06	6119	M32379.m	TRUE	reported	uZWtuS	2	TRUE	0.003284	20.15168
8970	rs7831994	T	C	T	C	0.1316	0.000145	0.0356	0.028908	FALSE	FALSE	FALSE	2iAvlt	0.040681	0.997147	486484	sepsis	TRUE	reported	textfile	0.0293	6.91E-06	1745	M37190.m	TRUE	reported	hdOuHe	2	TRUE	0.011428	20.15016
7651	rs1385234	A	T	A	T	0.022	0.010928	0.3159	0.310356	FALSE	TRUE	FALSE	8W5cqf	0.014793	0.460057	486484	sepsis	TRUE	reported	textfile	0.0049	7.00E-06	4925	M34534.m	TRUE	reported	JV42Pw	2	TRUE	0.004076	20.15008
7140	rs4589435	T	C	T	C	-0.064	0.002981	0.7069	0.689659	FALSE	FALSE	FALSE	de8tA6	0.014872	0.841125	486484	sepsis	TRUE	reported	textfile	0.0142	6.20E-06	248	M34322.m	TRUE	reported	sXPPBO	2	TRUE	0.075708	20.14961
4722	rs798727	T	C	T	C	-0.0211	-0.00532	0.8227	0.809245	FALSE	FALSE	FALSE	L2WFM1	0.017456	0.760694	486484	sepsis	TRUE	reported	textfile	0.0047	8.00E-06	7313	M32761.m	TRUE	reported	5GHzph	2	TRUE	0.002748	20.14886
5115	rs1917331	A	T	A	T	0.0211	0.010617	0.8179	0.823386	FALSE	TRUE	FALSE	O6ine1	0.017937	0.553928	486484	sepsis	TRUE	reported	textfile	0.0047	5.58E-06	7263	M33165.m	TRUE	reported	gGsqjW	2	TRUE	0.002767	20.14882
3473	rs1221633	T	G	T	G	-0.0211	0.024572	0.1744	0.200417	FALSE	FALSE	FALSE	w7juXi	0.017137	0.151614	486484	sepsis	TRUE	reported	textfile	0.0047	5.99E-06	6830	M27722.m	TRUE	reported	xzWmif	2	TRUE	0.002942	20.14847
6170	rs3024981	A	G	A	G	-0.0862	0.057824	0.0147	0.026379	FALSE	FALSE	FALSE	O4Ty2l	0.042923	0.177928	486484	sepsis	TRUE	reported	textfile	0.0192	7.14E-06	5107	M33488.m	TRUE	reported	RGtF84	2	TRUE	0.003931	20.14846
4211	rs7866	T	C	T	C	-0.1033	-0.01572	0.0443	0.043286	FALSE	FALSE	FALSE	rdKSjG	0.033718	0.64097	486484	sepsis	TRUE	reported	textfile	0.023	6.95E-06	1726	M32578.m	TRUE	reported	RAzSLK	2	TRUE	0.011552	20.14844
2251	rs309293	T	C	T	C	0.0256	0.006594	0.4315	0.43233	FALSE	FALSE	FALSE	zxcPox	0.013805	0.632872	486484	sepsis	TRUE	reported	textfile	0.0057	6.90E-06	1756	M15990.m	TRUE	reported	34Vg8F	2	TRUE	0.011357	20.14816
8751	rs7220100	A	G	A	G	-0.044	-0.02075	0.8574	0.854555	FALSE	FALSE	FALSE	FnYrPc	0.019577	0.289117	486484	sepsis	TRUE	reported	textfile	0.0098	7.50E-06	3346	M36585.m	TRUE	reported	amHtMH	2	TRUE	0.005989	20.14622
1090	rs884870	C	G	C	G	-0.0202	-0.00297	0.0689	0.070805	FALSE	TRUE	FALSE	JLtEiZ	0.026675	0.911351	486484	sepsis	TRUE	reported	textfile	0.0045	7.40E-06	7333	M01572.m	TRUE	reported	TSSe28	2	TRUE	0.00274	20.14463
8782	rs1214340	T	G	T	G	-0.0328	0.019248	0.2971	0.301388	FALSE	FALSE	FALSE	4bkfvN	0.014935	0.197477	486484	sepsis	TRUE	reported	textfile	0.0073	6.05E-06	922	M36738.m	TRUE	reported	xcqyX2	2	TRUE	0.021427	20.14461
5287	rs1516626	C	G	C	G	-0.3444	0.003015	0.0239	0.016951	FALSE	TRUE	FALSE	q32d4E	0.05572	0.956846	486484	sepsis	TRUE	reported	textfile	0.0736	2.87E-06	25	M33178.m	TRUE	reported	cS3BwT	2	TRUE	0.466909	20.14459
2714	rs1187293	A	T	A	T	0.0202	-0.01045	0.1999	0.199488	FALSE	TRUE	FALSE	89XKxw	0.017135	0.541946	486484	sepsis	TRUE	reported	textfile	0.0045	6.61E-06	7251	M19324.m	TRUE	reported	gryJuD	2	TRUE	0.002771	20.14457
2153	rs1117428	T	C	T	C	-0.0615	-0.00544	0.0957	0.097258	FALSE	FALSE	FALSE	uuY1Mi	0.023072	0.813464	486484	sepsis	TRUE	reported	textfile	0.0137	6.89E-06	5502	M15677.m	TRUE	reported	nWMiPu	2	TRUE	0.003649	20.14425
2962	rs2118838	C	G	C	G	0.0422	0.001317	0.8319	0.840502	FALSE	TRUE	FALSE	zndu0r	0.018639	0.943664	486484	sepsis	TRUE	reported	textfile	0.0094	7.09E-06	3422	M21049.m	TRUE	reported	2XpPDU	2	TRUE	0.005855	20.14259
2827	rs744093	A	G	A	G	-0.0238	-0.02141	0.892	0.875145	FALSE	FALSE	FALSE	Sx5Uhq	0.020896	0.305662	486484	sepsis	TRUE	reported	textfile	0.0053	8.51E-06	1759	M19934.m	TRUE	reported	DijYTE	2	TRUE	0.011334	20.14226
6378	rs1079479	T	C	T	C	-0.0202	-0.02105	0.3454	0.339282	FALSE	FALSE	FALSE	pPr1v0	0.014437	0.14473	486484	sepsis	TRUE	reported	textfile	0.0045	6.82E-06	5080	M33637.m	TRUE	reported	Ide1A7	2	TRUE	0.003951	20.14219
6323	rs6978556	T	C	T	C	0.1712	0.053534	0.0175	0.018089	FALSE	FALSE	FALSE	5B5LTX	0.051513	0.298698	486484	sepsis	TRUE	reported	textfile	0.0381	7.08E-06	824	M33609.m	TRUE	reported	m0eM6N	2	TRUE	0.023918	20.14198
7028	rs317279	A	G	A	G	0.0202	0.006161	0.6525	0.626137	FALSE	FALSE	FALSE	zMIkYA	0.014128	0.662761	486484	sepsis	TRUE	reported	textfile	0.0045	7.62E-06	4908	M34245.m	TRUE	reported	itCtz8	2	TRUE	0.004089	20.14191
5709	rs1265373	C	G	C	G	0.0202	-0.02806	0.3139	0.324795	FALSE	TRUE	FALSE	XDem1i	0.014606	0.054705	486484	sepsis	TRUE	reported	textfile	0.0045	8.14E-06	4665	M33221.m	TRUE	reported	KBV6FH	2	TRUE	0.004301	20.14148
2928	rs2861960	T	C	T	C	-0.0193	-0.01578	0.8651	0.871601	FALSE	FALSE	FALSE	fV4quI	0.020626	0.444111	486484	sepsis	TRUE	reported	textfile	0.0043	7.98E-06	7366	M21044.m	TRUE	reported	hbdBQJ	2	TRUE	0.002727	20.14001
883	rs1296779	A	C	A	C	0.0193	-0.0252	0.0794	0.07669	FALSE	FALSE	FALSE	oi7KQI	0.025696	0.326691	486484	sepsis	TRUE	reported	textfile	0.0043	6.13E-06	7362	M01365.m	TRUE	reported	7NWGKA	2	TRUE	0.002729	20.14001
629	rs1713101	A	G	A	G	-0.0781	0.040974	0.0404	0.047738	FALSE	FALSE	FALSE	F6Dn8H	0.032206	0.203289	486484	sepsis	TRUE	reported	textfile	0.0174	6.88E-06	4934	M01114.m	TRUE	reported	dVHatZ	2	TRUE	0.004067	20.13852
4067	rs1280622	A	G	A	G	-0.1127	0.015015	0.024	0.258087	FALSE	FALSE	FALSE	TyHtdh	0.015668	0.337898	486484	sepsis	TRUE	reported	textfile	0.0251	7.05E-06	1767	M32497.m	TRUE	reported	H3KmLR	2	TRUE	0.011281	20.13764
3635	rs1018596	T	C	T	C	0.0754	-0.0257	0.0527	0.050925	FALSE	FALSE	FALSE	Vq2efp	0.031425	0.413557	486484	sepsis	TRUE	reported	textfile	0.0168	6.83E-06	7363	M31787.m	TRUE	reported	krl4Tw	2	TRUE	0.002728	20.13753
5934	rs904720	A	G	A	G	-0.1106	-0.00088	0.2414	0.340148	FALSE	FALSE	FALSE	veBhfS	0.014544	0.951759	486484	sepsis	TRUE	reported	textfile	0.0246	6.61E-06	528	M33391.m	TRUE	reported	Gfjy5a	2	TRUE	0.036871	20.13687
8156	rs1730139	T	C	T	C	-0.0202	0.001806	0.147	0.149463	FALSE	FALSE	FALSE	sRlsb6	0.019382	0.925775	486484	sepsis	TRUE	reported	textfile	0.0045	7.17E-06	3007	M35331.m	TRUE	reported	GPijlC	2	TRUE	0.006656	20.13672
5855	rs7145855	A	G	A	G	0.3181	-0.02122	0.9691	0.960962	FALSE	FALSE	FALSE	Vkec6Y	0.035742	0.552707	486484	sepsis	TRUE	reported	textfile	0.0708	7.07E-06	803	M33384.m	TRUE	reported	eaxeyR	2	TRUE	0.024522	20.13621
4347	rs4766159	A	C	A	C	-0.022	-0.02742	0.7548	0.778731	FALSE	FALSE	FALSE	6VSoQQ	0.016484	0.096163	486484	sepsis	TRUE	reported	textfile	0.0049	8.37E-06	1766	M32644.m	TRUE	reported	oC2AV0	2	TRUE	0.011286	20.13544
8991	rs1337985	T	C	T	C	-0.0184	0.000996	0.627	0.627506	FALSE	FALSE	FALSE	TfdxtP	0.014247	0.944256	486484	sepsis	TRUE	reported	textfile	0.0041	7.64E-06	7333	M37203.m	TRUE	reported	xicmWr	2	TRUE	0.002739	20.1349
3604	rs1175877	T	C	T	C	0.0184	-0.03632	0.7686	0.770494	FALSE	FALSE	FALSE	2z5KhH	0.016275	0.025648	486484	sepsis	TRUE	reported	textfile	0.0041	7.67E-06	7263	M31555.m	TRUE	reported	5kcWr2	2	TRUE	0.002765	20.13485
8865	rs1236525	T	C	T	C	-0.0359	0.030893	0.1707	0.205266	FALSE	FALSE	FALSE	nWuK1Q	0.016949	0.068344	486484	sepsis	TRUE	reported	textfile	0.008	7.78E-06	6511	M36850.m	TRUE	reported	2BA0SE	2	TRUE	0.003083	20.13147
2547	rs7663798	A	G	A	G	0.1966	-0.06504	0.0486	0.043527	FALSE	FALSE	FALSE	o0CFya	0.03357	0.052707	486484	sepsis	TRUE	reported	textfile	0.0438	7.23E-06	2433	M18281.m	TRUE	reported	pxbsnu	2	TRUE	0.008213	20.13083
7113	rs9345410	C	G	C	G	0.0835	-0.02663	0.9419	0.932731	FALSE	TRUE	FALSE	DfLd6z	0.027377	0.330742	486484	sepsis	TRUE	reported	textfile	0.0186	7.56E-06	1766	M34314.m	TRUE	reported	OgloAQ	2	TRUE	0.011283	20.13052
5944	rs679020	T	C	T	C	-0.0552	-0.00119	0.9156	0.919289	FALSE	FALSE	FALSE	yepzrL	0.025138	0.962188	486484	sepsis	TRUE	reported	textfile	0.0123	7.03E-06	3890	M33408.m	TRUE	reported	SY4CoS	2	TRUE	0.005151	20.13004
8075	rs9362985	T	C	T	C	-0.0175	-0.01065	0.3161	0.317624	FALSE	FALSE	FALSE	7NkUCa	0.014751	0.47009	486484	sepsis	TRUE	reported	textfile	0.0039	6.48E-06	7359	M35320.m	TRUE	reported	GixRsK	2	TRUE	0.002729	20.12931
8498	rs1254788	A	G	A	G	-0.0175	-0.03676	0.7663	0.766355	FALSE	FALSE	FALSE	4MCEPy	0.016221	0.023439	486484	sepsis	TRUE	reported	textfile	0.0039	6.32E-06	6901	M35977.m	TRUE	reported	B3kU6y	2	TRUE	0.002909	20.12894
9016	rs6082720	T	C	T	C	0.2245	0.036485	0.0112	0.014302	FALSE	FALSE	FALSE	uxRXvK	0.057685	0.527066	486484	sepsis	TRUE	reported	textfile	0.05	7.03E-06	1228	M37459.m	TRUE	reported	m4dgs3	2	TRUE	0.016152	20.12727
8776	rs4846407	T	C	T	C	-0.0175	0.008428	0.3452	0.353209	FALSE	FALSE	FALSE	7desNF	0.014311	0.555938	486484	sepsis	TRUE	reported	textfile	0.0039	5.60E-06	5292	M36673.m	TRUE	reported	RKbIT4	2	TRUE	0.00379	20.12717
806	rs1111889	T	C	T	C	0.0202	-0.00839	0.4675	0.418275	FALSE	FALSE	FALSE	J58DI0	0.013834	0.544148	486484	sepsis	TRUE	reported	textfile	0.0045	7.16E-06	1755	M01356.m	TRUE	reported	zk9AMB	2	TRUE	0.011351	20.12716
8879	rs1758367	T	C	T	C	0.0633	-0.04071	0.9631	0.963006	FALSE	FALSE	FALSE	Nv6gDO	0.036428	0.263721	486484	sepsis	TRUE	reported	textfile	0.0141	7.57E-06	1467	M37004.m	TRUE	reported	RqmQdg	2	TRUE	0.013552	20.12689
977	rs1164673	C	G	C	G	0.0754	0.023887	0.3743	0.382609	FALSE	TRUE	FALSE	xSRIl4	0.014082	0.089824	486484	sepsis	TRUE	reported	textfile	0.0168	7.27E-06	2285	M01515.m	TRUE	reported	juZ5GE	2	TRUE	0.008738	20.12537
4843	rs4721104	A	T	A	T	-0.0341	0.000403	0.2583	0.259458	FALSE	TRUE	FALSE	jvlpBt	0.015583	0.979348	486484	sepsis	TRUE	reported	textfile	0.0076	6.93E-06	6250	M32846.m	TRUE	reported	RAXcz5	2	TRUE	0.003211	20.12531
2871	rs1131718	A	G	A	G	-0.0166	0.004704	0.8565	0.852054	FALSE	FALSE	FALSE	mUzn1R	0.019485	0.809232	486484	sepsis	TRUE	reported	textfile	0.0037	5.71E-06	7301	M20675.m	TRUE	reported	XwY04K	2	TRUE	0.002749	20.12305
5119	rs7819602	C	G	C	G	-0.0166	0.037628	0.3909	0.386047	FALSE	TRUE	FALSE	O6ine1	0.014111	0.007661	486484	sepsis	TRUE	reported	textfile	0.0037	7.57E-06	7263	M33165.m	TRUE	reported	gGsqjW	2	TRUE	0.002764	20.12302
2475	rs1709156	T	C	T	C	0.0332	-0.01708	0.1249	0.12368	FALSE	FALSE	FALSE	SrfyMU	0.020776	0.411019	486484	sepsis	TRUE	reported	textfile	0.0074	7.53E-06	7226	M17807.m	TRUE	reported	0zrJsu	2	TRUE	0.002778	20.12299
4811	rs2576722	A	T	A	T	0.0166	-0.00516	0.8553	0.834613	FALSE	TRUE	FALSE	bJwLV5	0.018383	0.778951	486484	sepsis	TRUE	reported	textfile	0.0037	9.92E-06	7063	M32814.m	TRUE	reported	qte0qp	2	TRUE	0.002842	20.12286
2362	rs1138253	A	G	A	G	0.0579	-0.00461	0.4619	0.446156	FALSE	FALSE	FALSE	NBJ2Do	0.013731	0.736987	486484	sepsis	TRUE	reported	textfile	0.0129	7.88E-06	1739	M17799.m	TRUE	reported	Uz0Wgl	2	TRUE	0.011452	20.12232
6943	rs6596884	T	C	T	C	0.0332	-0.00045	0.4477	0.413343	FALSE	FALSE	FALSE	oNv0vf	0.0139	0.974137	486484	sepsis	TRUE	reported	textfile	0.0074	6.91E-06	5581	M34112.m	TRUE	reported	l0pe0N	2	TRUE	0.003594	20.12135
8250	rs1252270	A	G	A	G	0.2998	0.033907	0.0337	0.025686	FALSE	FALSE	FALSE	G4plxe	0.044331	0.444363	486484	sepsis	TRUE	reported	textfile	0.0666	6.84E-06	284	M35451.m	TRUE	reported	hNOVMI	2	TRUE	0.066599	20.12081
4387	rs1257343	A	C	A	C	0.0377	0.013311	0.087	0.092699	FALSE	FALSE	FALSE	UJygVd	0.023575	0.572335	486484	sepsis	TRUE	reported	textfile	0.0084	7.96E-06	1766	M32654.m	TRUE	reported	kv9R0W	2	TRUE	0.011277	20.12019
5752	rs9315938	T	C	T	C	0.0323	0.021696	0.0442	0.043119	FALSE	FALSE	FALSE	bm6x2Z	0.033716	0.5199	486484	sepsis	TRUE	reported	textfile	0.0072	6.92E-06	7364	M33230.m	TRUE	reported	8Ccke3	2	TRUE	0.002725	20.11973
6743	rs1220578	A	G	A	G	-0.0323	-0.0371	0.0778	0.085778	FALSE	FALSE	FALSE	NZVg9N	0.024724	0.133473	486484	sepsis	TRUE	reported	textfile	0.0072	6.89E-06	7320	M33941.m	TRUE	reported	kRWNNK	2	TRUE	0.002742	20.11969
4921	rs1213747	T	C	T	C	0.0359	-0.03243	0.1187	0.118747	FALSE	FALSE	FALSE	clhN6v	0.021411	0.129864	486484	sepsis	TRUE	reported	textfile	0.008	6.92E-06	2148	M32869.m	TRUE	reported	BzloWk	2	TRUE	0.009288	20.11891
7906	rs4084390	C	G	C	G	0.0323	0.018221	0.238	0.23261	FALSE	TRUE	FALSE	AhsT9A	0.016291	0.26336	486484	sepsis	TRUE	reported	textfile	0.0072	7.24E-06	5953	M35187.m	TRUE	reported	uLU0Fy	2	TRUE	0.003369	20.11843
8639	rs6533515	A	G	A	G	-0.035	0.001017	0.2112	0.202023	FALSE	FALSE	FALSE	4cZjpQ	0.017024	0.952341	486484	sepsis	TRUE	reported	textfile	0.0078	7.04E-06	2400	M36230.m	TRUE	reported	TPUeNs	2	TRUE	0.00832	20.118
4323	rs551488	T	C	T	C	0.0157	-0.00934	0.7412	0.740931	FALSE	FALSE	FALSE	EOAssJ	0.015598	0.549288	486484	sepsis	TRUE	reported	textfile	0.0035	7.41E-06	7369	M32635.m	TRUE	reported	X1mTjy	2	TRUE	0.002723	20.11617
4621	rs1329561	A	G	A	G	-0.0314	-0.0102	0.1636	0.164166	FALSE	FALSE	FALSE	0oA38w	0.018525	0.581894	486484	sepsis	TRUE	reported	textfile	0.007	6.86E-06	7356	M32753.m	TRUE	reported	dQUaUX	2	TRUE	0.002728	20.11616
1705	rs1042685	A	G	A	G	-0.0157	0.004746	0.8949	0.890721	FALSE	FALSE	FALSE	ZmI9HX	0.021916	0.828561	486484	sepsis	TRUE	reported	textfile	0.0035	8.06E-06	7258	M12626.m	TRUE	reported	9SUabZ	2	TRUE	0.002765	20.11609
2576	rs2932525	A	G	A	G	-0.0157	-0.005	0.8432	0.849777	FALSE	FALSE	FALSE	KHS91f	0.019232	0.794974	486484	sepsis	TRUE	reported	textfile	0.0035	7.55E-06	6939	M18349.m	TRUE	reported	PeVGil	2	TRUE	0.002891	20.11583
570	rs7593385	A	C	A	C	0.0359	-0.03137	0.9685	0.990492	FALSE	FALSE	FALSE	fNqkCW	0.071108	0.659051	486484	sepsis	TRUE	reported	textfile	0.008	6.87E-06	1766	M00606.m	TRUE	reported	g9uwOn	2	TRUE	0.011274	20.11485
5166	rs787878	T	C	T	C	0.2768	-0.00538	0.6233	0.61428	FALSE	FALSE	FALSE	L2DJzK	0.014054	0.702032	486484	sepsis	TRUE	reported	textfile	0.0609	5.51E-06	76	M33173.m	TRUE	reported	hRG70D	2	TRUE	0.213726	20.1148
6559	rs4947639	T	G	T	G	0.0561	-0.00842	0.1513	0.06749	FALSE	FALSE	FALSE	58qeH5	0.027442	0.759004	486484	sepsis	TRUE	reported	textfile	0.0125	6.69E-06	1428	M33864.m	TRUE	reported	TBsy8r	2	TRUE	0.013909	20.11393
8320	rs1710934	A	G	A	G	-0.0884	0.059819	0.9737	0.983317	FALSE	FALSE	FALSE	o0GzT1	0.05364	0.264766	486484	sepsis	TRUE	reported	textfile	0.0197	7.28E-06	1750	M35631.m	TRUE	reported	G1XFx0	2	TRUE	0.011375	20.11294
8079	rs1111974	C	G	C	G	-0.0949	-0.0256	0.9047	0.889025	FALSE	TRUE	FALSE	z65cF2	0.021985	0.244186	486484	sepsis	TRUE	reported	textfile	0.0211	6.69E-06	349	M35322.m	TRUE	reported	3GUSWk	2	TRUE	0.054786	20.11275
2585	rs1772858	A	G	A	G	-0.0341	0.02702	0.6211	0.640723	FALSE	FALSE	FALSE	JGJ9vH	0.014241	0.057779	486484	sepsis	TRUE	reported	textfile	0.0076	7.98E-06	2079	M18357.m	TRUE	reported	cOV2qw	2	TRUE	0.009591	20.11239
8581	rs1309827	A	G	A	G	-0.0305	0.012795	0.203	0.202825	FALSE	FALSE	FALSE	iuJmRL	0.017018	0.452141	486484	sepsis	TRUE	reported	textfile	0.0068	7.48E-06	7311	M36103.m	TRUE	reported	jfJbVw	2	TRUE	0.002744	20.11236
5967	rs1333677	A	C	A	C	0.0624	-0.02999	0.1736	0.151232	FALSE	FALSE	FALSE	YCswLG	0.019055	0.115549	486484	sepsis	TRUE	reported	textfile	0.0139	7.39E-06	977	M33420.m	TRUE	reported	WH7xfc	2	TRUE	0.020211	20.11174
693	rs9403794	A	C	A	C	0.0175	0.006148	0.5963	0.57521	FALSE	FALSE	FALSE	aT1Fw1	0.013958	0.659591	486484	sepsis	TRUE	reported	textfile	0.0039	8.09E-06	1679	M01284.m	TRUE	reported	mbTe8H	2	TRUE	0.01185	20.1108
7177	rs1696397	A	G	A	G	-0.0305	-0.02316	0.0772	0.164261	FALSE	FALSE	FALSE	2hlepp	0.018485	0.210158	486484	sepsis	TRUE	reported	textfile	0.0068	6.13E-06	5081	M34336.m	TRUE	reported	XyK8mO	2	TRUE	0.003944	20.10994
636	rs4244808	T	G	T	G	0.0305	0.005261	0.5307	0.540058	FALSE	FALSE	FALSE	F6Dn8H	0.013814	0.70332	486484	sepsis	TRUE	reported	textfile	0.0068	8.30E-06	4934	M01114.m	TRUE	reported	dVHatZ	2	TRUE	0.004061	20.10971
7687	rs2172784	T	C	T	C	0.0323	0.017052	0.8464	0.850434	FALSE	FALSE	FALSE	5FAxbZ	0.019176	0.373869	486484	sepsis	TRUE	reported	textfile	0.0072	7.50E-06	2545	M34674.m	TRUE	reported	TDQqa8	2	TRUE	0.007846	20.10938
586	rs7014120	A	G	A	G	0.0148	-0.01008	0.1341	0.147294	FALSE	FALSE	FALSE	ZoqU2j	0.019278	0.601136	486484	sepsis	TRUE	reported	textfile	0.0033	5.67E-06	7333	M01105.m	TRUE	reported	2WxSOH	2	TRUE	0.002735	20.10838
7612	rs1079737	A	G	A	G	-0.0148	-0.01219	0.6912	0.692803	FALSE	FALSE	FALSE	kVfKqT	0.014863	0.412171	486484	sepsis	TRUE	reported	textfile	0.0033	8.16E-06	7322	M34527.m	TRUE	reported	s4ti92	2	TRUE	0.00274	20.10837
1682	rs1027932	T	C	T	C	-0.0148	0.060178	0.9522	0.945662	FALSE	FALSE	FALSE	f01en0	0.030265	0.046767	486484	sepsis	TRUE	reported	textfile	0.0033	6.52E-06	7314	M12593.m	TRUE	reported	EAsM6U	2	TRUE	0.002743	20.10837
7247	rs749427	A	G	A	G	0.1032	0.010908	0.0625	0.075057	FALSE	FALSE	FALSE	RlcE3J	0.026245	0.677696	486484	sepsis	TRUE	reported	textfile	0.023	7.04E-06	1649	M34339.m	TRUE	reported	QQrwdm	2	TRUE	0.012062	20.10836
6018	rs1244356	A	C	A	C	-0.618	0.021821	0.1	0.225232	FALSE	FALSE	FALSE	33ojbt	0.01635	0.182002	486484	sepsis	TRUE	reported	textfile	0.1355	5.13E-06	60	M33423.m	TRUE	reported	ivEtvl	2	TRUE	0.257441	20.10829
4571	rs9440550	A	G	A	G	-0.0148	-0.00651	0.1936	0.206961	FALSE	FALSE	FALSE	ILOOFw	0.016867	0.699372	486484	sepsis	TRUE	reported	textfile	0.0033	6.70E-06	6042	M32739.m	TRUE	reported	TpvRBe	2	TRUE	0.003318	20.10721
4750	rs7870790	A	G	A	G	0.0287	-0.01331	0.309	0.302124	FALSE	FALSE	FALSE	oQPvO3	0.014877	0.371057	486484	sepsis	TRUE	reported	textfile	0.0064	6.97E-06	7330	M32786.m	TRUE	reported	Th0GcB	2	TRUE	0.002736	20.10413

6093	rs1882545	A	G	A	G	0.0148	-0.00784	0.6567	0.666769	FALSE	FALSE	FALSE	xvzgym	0.014502	0.588844	486484	sepsis	TRUE	reported	textfile	0.0033	9.36E-06	4004	M33443.m	TRUE	reported	KtBPyR	2	TRUE	0.004998	20.10382
6575	rs1308282:	T	G	T	G	-0.1452	-0.00492	0.8387	0.84672	FALSE	FALSE	FALSE	XO3rXq	0.019103	0.796741	486484	sepsis	TRUE	reported	textfile	0.0322	6.73E-06	176	M33877.m	TRUE	reported	p2sETg	2	TRUE	0.103568	20.10287
2993	rs2073030	A	T	A	T	0.0287	-0.0251	0.7836	0.785464	FALSE	TRUE	FALSE	xmzJ5F	0.016677	0.132329	486484	sepsis	TRUE	reported	textfile	0.0064	6.99E-06	5436	M21184.m	TRUE	reported	xgwhDp	2	TRUE	0.003686	20.10222
3469	rs3111102	A	G	A	G	-0.048	0.010779	0.0953	0.10949	FALSE	FALSE	FALSE	BQlbxe	0.021912	0.62277	486484	sepsis	TRUE	reported	textfile	0.0107	7.00E-06	1768	M27718.m	TRUE	reported	MnHHJ0	2	TRUE	0.011254	20.10126
5568	rs7944027	A	G	A	G	-0.3473	-0.0088	0.0253	0.022107	FALSE	FALSE	FALSE	q32d4E	0.047358	0.852529	486484	sepsis	TRUE	reported	textfile	0.0743	3.00E-06	25	M33178.m	TRUE	reported	cS3BwT	2	TRUE	0.466371	20.1011
1827	rs7240261	T	C	T	C	0.0565	-0.00863	0.562	0.555422	FALSE	FALSE	FALSE	Yub5KV	0.013964	0.536749	486484	sepsis	TRUE	reported	textfile	0.0126	7.52E-06	5606	M15335.m	TRUE	reported	mcnINa	2	TRUE	0.003574	20.10022
5271	rs1332905:	T	C	T	C	0.3543	-0.04491	0.9711	0.979414	FALSE	FALSE	FALSE	q32d4E	0.048369	0.353182	486484	sepsis	TRUE	reported	textfile	0.0758	2.91E-06	25	M33178.m	TRUE	reported	cS3BwT	2	TRUE	0.466355	20.0998
4752	rs994645	A	G	A	G	-0.0417	-0.01635	0.1146	0.119429	FALSE	FALSE	FALSE	oQPvO3	0.021096	0.438224	486484	sepsis	TRUE	reported	textfile	0.0093	8.18E-06	7330	M32786.m	TRUE	reported	Th0GcB	2	TRUE	0.002735	20.09961
1711	rs729761	T	G	T	G	-0.0139	-0.00263	0.2479	0.288123	FALSE	FALSE	FALSE	ZmI9HX	0.015236	0.863089	486484	sepsis	TRUE	reported	textfile	0.0031	8.52E-06	7258	M12626.m	TRUE	reported	9SUabZ	2	TRUE	0.002762	20.09956
1718	rs1120095	T	G	T	G	-0.0278	-0.07852	0.9527	0.951581	FALSE	FALSE	FALSE	RmWDhQ	0.032134	0.014549	486484	sepsis	TRUE	reported	textfile	0.0062	8.12E-06	7034	M12768.m	TRUE	reported	f0vnEz	2	TRUE	0.00285	20.09938
8871	rs4579381	T	C	T	C	-0.0278	-0.01833	0.6475	0.669335	FALSE	FALSE	FALSE	nWuK1Q	0.014552	0.207729	486484	sepsis	TRUE	reported	textfile	0.0062	7.00E-06	6511	M36850.m	TRUE	reported	2BA0SE	2	TRUE	0.003078	20.09892
1343	rs4580644	T	C	T	C	0.0157	-0.01614	0.4937	0.487688	FALSE	FALSE	FALSE	LDUJbh	0.013693	0.238568	486484	sepsis	TRUE	reported	textfile	0.0035	8.16E-06	1766	M01898.m	TRUE	reported	JRSLeH	2	TRUE	0.011266	20.09884
3086	rs6569976	A	C	A	C	0.0157	-0.00403	0.6201	0.605644	FALSE	FALSE	FALSE	esOP8m	0.014027	0.774127	486484	sepsis	TRUE	reported	textfile	0.0035	7.33E-06	1766	M22116.m	TRUE	reported	fDMh7Z	2	TRUE	0.011266	20.09884
5708	rs1166977(	T	C	T	C	0.0278	0.030972	0.138	0.165094	FALSE	FALSE	FALSE	XDem1i	0.018413	0.092547	486484	sepsis	TRUE	reported	textfile	0.0062	8.01E-06	4665	M33221.m	TRUE	reported	KBV6FH	2	TRUE	0.004291	20.09648
4201	rs1011177!	A	C	A	C	-0.0462	0.033906	0.7263	0.722766	FALSE	FALSE	FALSE	rdKSjG	0.015344	0.027121	486484	sepsis	TRUE	reported	textfile	0.0103	8.00E-06	1726	M32578.m	TRUE	reported	RAzSLK	2	TRUE	0.011522	20.09583
6373	rs2933259	T	C	T	C	-0.0278	0.008468	0.5499	0.535852	FALSE	FALSE	FALSE	PAgZZz	0.013768	0.538523	486484	sepsis	TRUE	reported	textfile	0.0062	6.53E-06	4323	M33633.m	TRUE	reported	I8R1KJ	2	TRUE	0.004629	20.0958
2348	rs1006884:	A	C	A	C	-0.1072	-0.00039	0.046	0.065454	FALSE	FALSE	FALSE	NBJ2Do	0.027838	0.988715	486484	sepsis	TRUE	reported	textfile	0.0239	7.43E-06	1739	M17799.m	TRUE	reported	Uz0WgI	2	TRUE	0.011437	20.09528
8116	rs268290	A	C	A	C	-0.061	0.017484	0.0174	0.014962	FALSE	FALSE	FALSE	nNbXBS	0.056613	0.757447	486484	sepsis	TRUE	reported	textfile	0.0136	7.58E-06	1764	M35326.m	TRUE	reported	UNDorn	2	TRUE	0.011276	20.09505
2487	rs878983	T	C	T	C	-0.0269	0.001703	0.2282	0.240721	FALSE	FALSE	FALSE	SrfyMU	0.015981	0.915117	486484	sepsis	TRUE	reported	textfile	0.006	8.15E-06	7226	M17807.m	TRUE	reported	0zrJsu	2	TRUE	0.002774	20.09471
7699	rs1244373(	T	G	T	G	0.0269	0.015032	0.1232	0.126822	FALSE	FALSE	FALSE	2eJIPM	0.020532	0.464077	486484	sepsis	TRUE	reported	textfile	0.006	8.29E-06	6381	M34761.m	TRUE	reported	druKtT	2	TRUE	0.00314	20.09398
5163	rs7082110	A	G	A	G	0.1386	-0.01362	0.8649	0.84412	FALSE	FALSE	FALSE	L2DJzK	0.019299	0.480242	486484	sepsis	TRUE	reported	textfile	0.0309	7.10E-06	1574	M33173.m	TRUE	reported	hRG70D	2	TRUE	0.012621	20.09358
3487	rs1735937:	T	C	T	C	-0.0323	0.003997	0.261	0.267137	FALSE	FALSE	FALSE	Awafb0	0.015654	0.798476	486484	sepsis	TRUE	reported	textfile	0.0072	7.85E-06	1227	M27728.m	TRUE	reported	Lfj8L0	2	TRUE	0.016137	20.09239
2457	rs886236	C	G	C	G	0.0749	-0.02685	0.7262	0.691638	FALSE	TRUE	FALSE	NBJ2Do	0.015358	0.080425	486484	sepsis	TRUE	reported	textfile	0.0167	7.71E-06	1739	M17799.m	TRUE	reported	Uz0WgI	2	TRUE	0.011435	20.09236
6216	rs42789	A	G	A	G	-0.0278	0.014915	0.426	0.425135	FALSE	FALSE	FALSE	FQfT2o	0.013877	0.282483	486484	sepsis	TRUE	reported	textfile	0.0062	7.33E-06	2759	M33508.m	TRUE	reported	JtbOy2	2	TRUE	0.007234	20.09052
1452	rs832404	T	C	T	C	-0.013	-0.01939	0.8684	0.875442	FALSE	FALSE	FALSE	1M4gqj	0.020698	0.348795	486484	sepsis	TRUE	reported	textfile	0.0029	7.53E-06	7226	M02730.m	TRUE	reported	RWyYEM	2	TRUE	0.002773	20.08956
8284	rs1763928(	T	C	T	C	0.026	-0.00017	0.2643	0.311773	FALSE	FALSE	FALSE	J6o4xC	0.014745	0.991066	486484	sepsis	TRUE	reported	textfile	0.0058	8.99E-06	6595	M35472.m	TRUE	reported	3XBcu9	2	TRUE	0.003038	20.08903
8709	rs1158161(	A	G	A	G	-0.013	-0.02445	0.3437	0.348514	FALSE	FALSE	FALSE	I0G0I1	0.01437	0.088904	486484	sepsis	TRUE	reported	textfile	0.0029	6.59E-06	6464	M36553.m	TRUE	reported	lBltb0	2	TRUE	0.003099	20.08891
6647	rs1544149	T	C	T	C	0.013	0.000368	0.6652	0.662876	FALSE	FALSE	FALSE	REeQiq	0.014576	0.979848	486484	sepsis	TRUE	reported	textfile	0.0029	7.22E-06	6004	M33892.m	TRUE	reported	cVOa3u	2	TRUE	0.003336	20.08843
6648	rs167675	C	G	C	G	0.013	-0.01983	0.371	0.369917	FALSE	TRUE	FALSE	REeQiq	0.014165	0.161521	486484	sepsis	TRUE	reported	textfile	0.0029	7.54E-06	6004	M33892.m	TRUE	reported	cVOa3u	2	TRUE	0.003336	20.08843
6944	rs7836759	A	C	A	C	-0.026	-0.00175	0.3393	0.341974	FALSE	FALSE	FALSE	oNv0vf	0.014472	0.903583	486484	sepsis	TRUE	reported	textfile	0.0058	6.61E-06	5581	M34112.m	TRUE	reported	l0pe0N	2	TRUE	0.003588	20.08792
2622	rs1767870(	A	G	A	G	0.0511	-0.02994	0.1871	0.213491	FALSE	FALSE	FALSE	eygBBR	0.01793	0.094942	486484	sepsis	TRUE	reported	textfile	0.0114	7.88E-06	6970	M18394.m	TRUE	reported	hD79iE	2	TRUE	0.002874	20.08665
5418	rs2804609	T	C	T	C	-0.2427	0.074276	0.0214	0.019012	FALSE	FALSE	FALSE	q32d4E	0.050245	0.139331	486484	sepsis	TRUE	reported	textfile	0.0541	7.13E-06	1017	M33178.m	TRUE	reported	cS3BwT	2	TRUE	0.019405	20.08585
6981	rs7903596	A	G	A	G	-0.1278	0.01346	0.988	0.981939	FALSE	FALSE	FALSE	ZlvYs5	0.052125	0.796228	486484	sepsis	TRUE	reported	textfile	0.0285	7.09E-06	1760	M34214.m	TRUE	reported	qU57S1	2	TRUE	0.011296	20.08529
3669	rs9973135	T	C	T	C	-0.0251	0.04681	0.0499	0.034305	FALSE	FALSE	FALSE	fnjup6	0.037684	0.214175	486484	sepsis	TRUE	reported	textfile	0.0056	8.32E-06	7346	M32197.m	TRUE	reported	oQzaMk	2	TRUE	0.002727	20.08414
8789	rs1157846(	C	G	C	G	-0.0251	0.005913	0.8688	0.86279	FALSE	TRUE	FALSE	0YKkSS	0.019865	0.765967	486484	sepsis	TRUE	reported	textfile	0.0056	8.40E-06	6876	M36754.m	TRUE	reported	cf9IBu	2	TRUE	0.002913	20.08376
8696	rs1698556'	T	C	T	C	-0.0139	0.015533	0.5111	0.524817	FALSE	FALSE	FALSE	Bg4VnB	0.013776	0.259526	486484	sepsis	TRUE	reported	textfile	0.0031	8.32E-06	1765	M36552.m	TRUE	reported	6zdDpC	2	TRUE	0.011263	20.08232
2411	rs2486578	A	G	A	G	0.1523	0.001294	0.9524	0.792409	FALSE	FALSE	FALSE	NBJ2Do	0.016858	0.938794	486484	sepsis	TRUE	reported	textfile	0.0326	2.99E-06	25	M17799.m	TRUE	reported	Uz0WgI	2	TRUE	0.466103	20.07948
6978	rs6973089	A	T	A	T	0.0408	0.029551	0.9182	0.910715	FALSE	TRUE	FALSE	ZlvYs5	0.024045	0.219087	486484	sepsis	TRUE	reported	textfile	0.0091	7.64E-06	1760	M34214.m	TRUE	reported	qU57S1	2	TRUE	0.011293	20.07908
4494	rs2256321	T	C	T	C	-0.0121	0.00413	0.6891	0.68813	FALSE	FALSE	FALSE	1wNJ1E	0.014827	0.780621	486484	sepsis	TRUE	reported	textfile	0.0027	5.53E-06	7363	M32709.m	TRUE	reported	zihBHg	2	TRUE	0.00272	20.07822
1731	rs1009692:	A	T	A	T	0.0121	0.034134	0.8346	0.823673	FALSE	TRUE	FALSE	nFLpAP	0.017962	0.057393	486484	sepsis	TRUE	reported	textfile	0.0027	5.42E-06	7356	M12774.m	TRUE	reported	yOSQTj	2	TRUE	0.002723	20.07822
1763	rs1083997:	T	G	T	G	0.0121	0.029384	0.1872	0.186172	FALSE	FALSE	FALSE	wM5dkT	0.017696	0.096819	486484	sepsis	TRUE	reported	textfile	0.0027	7.91E-06	7352	M15122.m	TRUE	reported	CBpa4t	2	TRUE	0.002724	20.07821
7895	rs381227	T	C	T	C	-0.0121	-0.02604	0.287	0.298372	FALSE	FALSE	FALSE	pU8zps	0.015007	0.082758	486484	sepsis	TRUE	reported	textfile	0.0027	6.30E-06	7350	M35186.m	TRUE	reported	Oc2lOF	2	TRUE	0.002725	20.07821
7003	rs1288686	A	G	A	G	-0.0121	-0.01542	0.3911	0.399548	FALSE	FALSE	FALSE	ePdfmg	0.013959	0.269233	486484	sepsis	TRUE	reported	textfile	0.0027	7.09E-06	7347	M34244.m	TRUE	reported	l9nzfX	2	TRUE	0.002726	20.07821
7008	rs1883995	A	G	A	G	0.0121	-0.01352	0.6271	0.619113	FALSE	FALSE	FALSE	ePdfmg	0.014086	0.337304	486484	sepsis	TRUE	reported	textfile	0.0027	6.57E-06	7347	M34244.m	TRUE	reported	l9nzfX	2	TRUE	0.002726	20.07821
7448	rs1105155:	A	G	A	G	-0.0121	-0.00189	0.5217	0.523959	FALSE	FALSE	FALSE	7ubZow	0.013697	0.890193	486484	sepsis	TRUE	reported	textfile	0.0027	8.47E-06	7344	M34407.m	TRUE	reported	LRsXN2	2	TRUE	0.002727	20.07821
8822	rs9954696	A	T	A	T	0.0121	0.002657	0.7848	0.781317	FALSE	TRUE	FALSE	X2RoI2	0.016736	0.873882	486484	sepsis	TRUE	reported	textfile	0.0027	5.36E-06	7335	M36776.m	TRUE	reported	nikHw1	2	TRUE	0.002731	20.0782
3214	rs8056954	T	G	T	G	-0.0121	0.009704	0.1734	0.166985	FALSE	FALSE	FALSE	UfLkBq	0.01833	0.596527	486484	sepsis	TRUE	reported	textfile	0.0027	8.27E-06	7329	M22481.m	TRUE	reported	kWMdiT	2	TRUE	0.002733	20.0782
5998	rs4524522	C	G	C	G	-0.0121	0.009648	0.0858	0.088962	FALSE	TRUE	FALSE	3KsoXq	0.023945	0.687005	486484	sepsis	TRUE	reported	textfile	0.0027	6.92E-06	7306	M33422.m	TRUE	reported	aNr6Ug	2	TRUE	0.002741	20.07818
6236	rs1683029:	T	G	T	G	-0.0121	-0.02018	0.3419	0.345266	FALSE	FALSE	FALSE	vL52Yb	0.014355	0.159815	486484	sepsis	TRUE	reported	textfile	0.0027	5.55E-06	7269	M33510.m	TRUE	reported	JIO9VX	2	TRUE	0.002755	20.07815
7964	rs2479415	T	C	T	C	0.0121	0.008972	0.5265	0.521503	FALSE	FALSE	FALSE	zb8m4a	0.013708	0.512781	486484	sepsis	TRUE	reported	textfile	0.0027	8.98E-06	7261	M35253.m	TRUE	reported	tHnnnS	2	TRUE	0.002758	20.07814
7965	rs329498	T	G	T	G	-0.0121	-0.00658	0.3106	0.307198	FALSE	FALSE	FALSE	zb8m4a	0.014834	0.657576	486484	sepsis	TRUE	reported	textfile	0.0027	5.67E-06	7261	M35253.m	TRUE	reported	tHnnnS	2	TRUE	0.002758	20.07814
4355	rs9570627	T	G	T	G	-0.0121	-0.03703	0.8432	0.811274	FALSE	FALSE	FALSE	6VSoQQ	0.017612	0.035503	486484	sepsis	TRUE	reported	textfile	0.0027	7.47E-06	7229	M32644.m	TRUE	reported	oC2AV0	2	TRUE	0.002771	20.07812
2937	rs1172360(	T	C	T	C	0.0121	0.02166	0.1817	0.239071	FALSE	FALSE	FALSE	pn11BC	0.016405	0.186711	486484	sepsis	TRUE	reported	textfile	0.0027	8.03E-06	7203	M21047.m	TRUE	reported	KJF8U4	2	TRUE	0.00278	20.0781
877	rs961959	A	C	A	C	-0.0121	0.036349	0.1925	0.198841	FALSE	FALSE	FALSE	Vg5OOR	0.017116	0.033699	486484	sepsis	TRUE	reported	textfile	0.0027	5.26E-06	7063	M01361.m	TRUE	reported	XnrS00	2	TRUE	0.002835	20.07799
5722	rs1023380:	T	C	T	C	0.0121	-0.00699	0.5881	0.587824	FALSE	FALSE	FALSE	YDQaWa	0.01388	0.614643	486484	sepsis	TRUE	reported	textfile	0.0027	7.69E-06	7063	M33228.m	TRUE	reported	BmYg8r	2	TRUE	0.002835	20.07799
5730	rs227769	T	C	T	C	-0.0121	-0.0016	0.467	0.470141	FALSE	FALSE	FALSE	YDQaWa	0.013679	0.906684	486484	sepsis	TRUE	reported	textfile	0.0027	9.32E-06	7063	M33228.m	TRUE	reported	BmYg8r	2	TRUE	0.002835	20.07799
1715	rs1051264!	A	G	A	G	-0.0121	0.005723	0.6525	0.649549	FALSE	FALSE	FALSE	RmWDhQ	0.014413	0.691323	486484	sepsis	TRUE	reported	textfile	0.0027	5.52E-06	7034	M12768.m	TRUE	reported	f0vnEz	2	TRUE	0.002847	20.07797
2977	rs954050	T	C	T	C	0.0121	-0.01996	0.5912	0.582956	FALSE	FALSE	FALSE	dOOXpo	0.013881	0.150557	486484	sepsis	TRUE	reported	textfile	0.0027	7.68E-06	6999	M21127.m	TRUE	reported	IdiP4j	2	TRUE	0.002861	20.07794
7097	rs2377324	T	C	T	C	-0.0121	-0.00722	0.7038	0.704467	FALSE	FALSE	FALSE	pxNICA	0.014967	0.629548	486484	sepsis	TRUE	reported	textfile	0.0027	7.39E-06	6994	M34306.m	TRUE	reported	xk5b9M	2	TRUE	0.002863	20.07793
7100	rs7080146	T	C	T	C	0.0121	0.023099	0.705	0.689482	FALSE	FALSE	FALSE	pxNICA	0.01478	0.118075	486484	sepsis	TRUE	reported	textfile	0.0027	8.00E-06	6994	M34306.m	TRUE	reported	xk5b9M	2	TRUE	0.002863	20.07793
765	rs1016648	T	C	T	C	-0.0121	-0.01194	0.3157	0.324	FALSE	FALSE	FALSE	tYvbNc	0.014602	0.413568	486484	sepsis	TRUE	reported	textfile	0.0027	5.29E-06	6946	M01303.m	TRUE	reported	hT3gsE	2	TRUE	0.002883	20.07789
775	rs690191	T	C	T	C	-0.0121	-0.02117	0.7821	0.779642	FALSE	FALSE	FALSE	tYvbNc	0.016467	0.198537	486484	sepsis	TRUE	reported	textfile	0.0027	9.23E-06	6946	M01303.m	TRUE	reported	hT3gsE	2	TRUE	0.002883	20.07789
2568	rs1162344:	T	C	T	C	0.0121	-0.01808	0.6919	0.686686	FALSE	FALSE	FALSE	KHS91f	0.014812	0.222335	486484	sepsis	TRUE	reported	textfile	0.0027	7.84E-06	6939	M18349.m	TRUE	reported	PeVGil	2	TRUE	0.002886	20.07789
1657	rs2130791	A	G	A	G	0.0121	-0.00912	0.7132	0.712725	FALSE	FALSE	FALSE	YzX5ZD	0.015109	0.546199	486484	sepsis	TRUE	reported	textfile	0.0027	7.96E-06	6864	M12129.m	TRUE	reported	6tMfaW	2	TRUE	0.002917	20.07782
6634	rs7655244	T	G	T	G	0.0735	0.00916	0.0616	0.059779	FALSE	FALSE	FALSE	IDG01N	0.028932	0.75155	486484	sepsis	TRUE	reported	textfile	0.0164	7.15E-06	4999	M33885.m	TRUE	reported	q3gy7x	2	TRUE	0.004002	20.07766
3015	rs4266604	T	C	T	C	-0.0121	-0.01403	0.7099	0.700127	FALSE	FALSE	FALSE	gxupFu	0.014905	0.346641	486484	sepsis	TRUE	reported	textfile	0.0027	9.19E-06	6556	M21188.m	TRUE	reported	gwwXh2	2	TRUE	0.003054	20.07755
7940	rs6107820	A	G	A	G	-0.0121	0.007383	0.5819	0.586682	FALSE	FALSE	FALSE	kFTWXY	0.013884	0.594914	486484	sepsis	TRUE	reported	textfile	0.0027	9.95E-06	6543	M35193.m	TRUE	reported	cjJvJl	2	TRUE	0.00306	20.07754
8004	rs1299717	T	C	T	C	0.0121	-0.00191	0.5646	0.565204	FALSE	FALSE	FALSE	Zj5bZH	0.013783	0.889754	486484	sepsis	TRUE	reported	textfile	0.0027	6.79E-06	6538	M35257.m	TRUE	reported	exssAk	2	TRUE	0.003062	20.07753
3236	rs1081081:	A	G	A	G	-0.0121	-0.0266	0.1837	0.187416	FALSE	FALSE	FALSE	COSeSD	0.017677	0.132397	486484	sepsis	TRUE	reported	textfile	0.0027	5.20E-06	6517	M22649.m	TRUE	reported	mnH8z2	2	TRUE	0.003072	20.07751
3238	rs1316352(	A	G	A	G	0.0121	0.041643	0.8339	0.826937	FALSE	FALSE	FALSE	COSeSD	0.018147	0.021745	486484	sepsis	TRUE	reported	textfile	0.0027	8.11E-06	6517	M22649.m	TRUE	reported	mnH8z2	2	TRUE	0.003072	20.07751
6740	rs817338	T	C	T	C	0.0121	0.021471	0.8271	0.825569	FALSE	FALSE	FALSE	SpG9A1	0.018063	0.234566	486484	sepsis	TRUE	reported	textfile	0.0027	7.06E-06	6502	M33939.m	TRUE	reported	Tudxel	2	TRUE	0.003079	20.0775
4532	rs261332	A	G	A	G	0.0121	-0.01278	0.1695	0.209873	FALSE	FALSE	FALSE	r0qJaM	0.016785	0.4465	486484	sepsis	TRUE	reported	textfile	0.0027	8.88E-06	6497	M32729.m	TRUE	reported	vYv5Fl	2	TRUE	0.003082	20.07749
5110	rs9472173	T	C	T	C	0.0121	-0.00809	0.4953	0.502064	FALSE	FALSE	FALSE	Uiz8VU	0.014375	0.573404	486484	sepsis	TRUE	reported	textfile	0.0027	8.68E-06	6404	M33163.m	TRUE	reported	ky0Bda	2	TRUE	0.003126	20.0774
3089	rs1235542'	A	G	A	G	-0.0121	-0.01751	0.277	0.274047	FALSE	FALSE	FALSE	mr0awv	0.015436	0.25655	486484	sepsis	TRUE	reported	textfile	0.0027	9.20E-06	5702	M22130.m	TRUE	reported	Y2TfWQ	2	TRUE	0.00351	20.07663
3505	rs7929077	A	G	A	G	0.0121	-0.01241	0.4899	0.490914	FALSE	FALSE	FALSE	Awafb0	0.013706	0.36506	486484	sepsis	TRUE	reported	textfile	0.0027	6.63E-06	5562	M27728.m	TRUE	reported	Lfj8L0	2	TRUE	0.003598	20.07645
4372	rs4316494	A	G	A	G	0.0121	-0.01448	0.4925	0.510585	FALSE	FALSE	FALSE	SPhl52	0.013744	0.292069	486484	sepsis	TRUE	reported	textfile	0.0027	8.33E-06	5211	M32651.m	TRUE	reported	V0Bnpj	2	TRUE	0.003839	20.07597
5663	rs8059937	T	G	T	G	-0.0605	-0.01151	0.8405	0.83077	FALSE	FALSE	FALSE	mod1fD	0.018305	0.52957	486484	sepsis	TRUE	reported	textfile	0.0135	7.88E-06	5064	M33194.m	TRUE	reported	fSFVUj	2	TRUE	0.00395	20.07574
4724	rs1223898!	C	G	C	G	0.1605	-0.04369	0.0233	0.013013	FALSE	TRUE	FALSE	fz63r4	0.06048	0.470093	486484	sepsis	TRUE	reported	textfile	0.0358	7.51E-06	1672	M32762.m	TRUE	reported	kgxlBo	2	TRUE	0.011878	20.0754
2285	rs4665701	T	C	T	C	-0.0121	0.013216	0.4689	0.465969	FALSE	FALSE	FALSE	mRULgB	0.013755	0.336644	486484	sepsis	TRUE	reported	textfile	0.0027	4.90E-06	4689	M16816.m	TRUE	reported	05lFsX	2	TRUE	0.004265	20.07511
5183	rs1027439'	T	G	T	G	0.348	-0.02411	0.9757	0.978042	FALSE	FALSE	FALSE	q32d4E	0.046822	0.606574	486484	sepsis	TRUE	reported	textfile	0.0745	2.99E-06	25	M33178.m	TRUE	reported	cS3BwT	2	TRUE	0.466035	20.07399
2471	rs1322254:	T	C	T	C	-0.0941	-0.08464	0.0194	0.019502	FALSE	FALSE	FALSE	SrfyMU	0.049349	0.086312	486484	sepsis	TRUE	reported	textfile	0.021	7.57E-06	7226	M17807.m	TRUE	reported	0zrJsu	2	TRUE	0.002771	20.07338
6317	rs1766502	A	G	A	G	-0.0628	0.010128	0.7991	0.789471	FALSE	FALSE	FALSE	5B5LTX	0.016736	0.545062	486484	sepsis	TRUE	reported	textfile	0.014	6.87E-06	824	M33609.m	TRUE	reported	m0eM6N	2	TRUE	0.023837	20.07279
4445	rs1082918(	A	G	A	G	0.0372	-0.01428	0.8267	0.816596	FALSE	FALSE	FALSE	g2RNym	0.017793	0.422176	486484	sepsis	TRUE	reported	textfile	0.0083	7.99E-06	2575	M32691.m	TRUE	reported	35eo6p	2	TRUE	0.007741	20.07207
4622	rs1690821:	T	C	T	C	0.0466	0.003465	0.924	0.915118	FALSE	FALSE	FALSE	0oA38w	0.024555	0.887787	486484	sepsis	TRUE	reported	textfile	0.0104	7.21E-06	7356	M32753.m	TRUE	reported	dQUaUX	2	TRUE	0.002722	20.07183
905	rs4717457	A	C	A	C	0.0233	0.046956	0.1392	0.143233	FALSE	FALSE	FALSE	I6zQnf	0.019583	0.016494	486484	sepsis	TRUE	reported	textfile	0.0052	6.80E-06	7345	M01444.m	TRUE	reported	llzalm	2	TRUE	0.002726	20.07183
2474	rs1693451:	A	C	A	C	-0.0233	-0.01436	0.5653	0.572023	FALSE	FALSE	FALSE	SrfyMU	0.013886	0.301219	486484	sepsis	TRUE	reported	textfile	0.0052	7.36E-06	7226	M17807.m	TRUE	reported	0zrJsu	2	TRUE	0.002771	20.07174
5950	rs1164492(	A	T	A	T	-0.0233	0.000797	0.6891	0.68728	FALSE	TRUE	FALSE	mDZrGw	0.014891	0.957335	486484	sepsis	TRUE	reported	textfile	0.0052	6.85E-06	6797	M33415.m	TRUE	reported	MzEP7R	2	TRUE	0.002945	20.07139
6366	rs1193482:	A	G	A	G	0.0354	0.005809	0.8122	0.81121	FALSE	FALSE	FALSE	PAgZZz	0.01753	0.740357	486484	sepsis	TRUE	reported	textfile	0.0079	7.80E-06	4323	M33633.m	TRUE	reported	I8R1KJ	2	TRUE	0.004623	20.07018
7649	rs1097929'	A	T	A	T	-0.0233	0.010435	0.6724	0.666906	FALSE	TRUE	FALSE	8W5cqf	0.014518	0.472287	486484	sepsis	TRUE	reported	textfile	0.0052	6.86E-06	4925	M34534.m	TRUE	reported	JV42Pw	2	TRUE	0.00406	20.06914
8286	rs6899136	A	G	A	G	0.0345	0.040941	0.0868	0.09057	FALSE	FALSE	FALSE	J6o4xC	0.023817	0.085617	486484	sepsis	TRUE	reported	textfile	0.0077	7.35E-06	6595	M35472.m	TRUE	reported	3XBcu9	2	TRUE	0.003035	20.06897
5787	rs1000011:	A	G	A	G	0.2505	0.025723	0.0351	0.038491	FALSE	FALSE	FALSE	lUkr5t	0.035656	0.470649	486484	sepsis	TRUE	reported	textfile	0.0558	7.12E-06	474	M33359.m	TRUE	reported	DjliwS	2	TRUE	0.040784	20.06831
8499	rs4567273	A	C	A	C	0.0251	-0.01758	0.2608	0.259802	FALSE	FALSE	FALSE	4MCEPy	0.015602	0.259829	486484	sepsis	TRUE	reported	textfile	0.0056	8.97E-06	1760	M35977.m	TRUE	reported	B3kU6y	2	TRUE	0.011286	20.06678
4897	rs7961980	C	G	C	G	-0.0493	-0.01564	0.6612	0.670334	FALSE	TRUE	FALSE	ocaywK	0.014538	0.281921	486484	sepsis	TRUE	reported	textfile	0.011	6.83E-06	1988	M32863.m	TRUE	reported	WH6KGr	2	TRUE	0.010003	20.06649
3688	rs1001972	C	G	C	G	0.0112	0.001734	0.8248	0.821058	FALSE	TRUE	FALSE	agPgtF	0.017924	0.922919	486484	sepsis	TRUE	reported	textfile	0.0025	8.36E-06	7349	M32315.m	TRUE	reported	hMRjcV	2	TRUE	0.002724	20.06494
1225	rs1185787!	T	G	T	G	0.0112	-0.00745	0.1952	0.20636	FALSE	FALSE	FALSE	V50rWC	0.016929	0.659971	486484	sepsis	TRUE	reported	textfile	0.0025	8.18E-06	7346	M01645.m	TRUE	reported	yN3gpQ	2	TRUE	0.002725	20.06494
3284	rs2895714	A	G	A	G	0.0112	0.016627	0.6755	0.675736	FALSE	FALSE	FALSE	vYWPe9	0.014707	0.258224	486484	sepsis	TRUE	reported	textfile	0.0025	9.64E-06	7340	M25459.m	TRUE	reported	VvJ6Vx	2	TRUE	0.002727	20.06493
4429	rs1175332(	T	C	T	C	0.0112	0.015539	0.9099	0.918325	FALSE	FALSE	FALSE	WGPdpq	0.024931	0.533105	486484	sepsis	TRUE	reported	textfile	0.0025	9.63E-06	7338	M32675.m	TRUE	reported	u0eUAG	2	TRUE	0.002728	20.06493
3873	rs1260464'	A	C	A	C	-0.0112	0.003478	0.2439	0.243967	FALSE	FALSE	FALSE	bwkKtK	0.016041	0.82836	486484	sepsis	TRUE	reported	textfile	0.0025	6.05E-06	7307	M32393.m	TRUE	reported	RvZzAy	2	TRUE	0.002739	20.06491
3355	rs1421165	T	C	T	C	0.0112	0.003899	0.7334	0.691837	FALSE	FALSE	FALSE	LY4Gus	0.014943	0.794142	486484	sepsis	TRUE	reported	textfile	0.0025	8.15E-06	7268	M27273.m	TRUE	reported	QKITBx	2	TRUE	0.002754	20.06488
3257	rs1534979	T	C	T	C	-0.0112	-0.02244	0.3253	0.33871	FALSE	FALSE	FALSE	ZNPBqT	0.014569	0.123517	486484	sepsis	TRUE	reported	textfile	0.0025	8.14E-06	7256	M24074.m	TRUE	reported	8pPtOl	2	TRUE	0.002758	20.06487
2736	rs7118352	A	G	A	G	-0.0112	-0.00763	0.8182	0.821829	FALSE	FALSE	FALSE	H4yqSK	0.017972	0.670996	486484	sepsis	TRUE	reported	textfile	0.0025	5.34E-06	7182	M19362.m	TRUE	reported	w2PDEO	2	TRUE	0.002787	20.06481
1471	rs1761655!	A	G	A	G	-0.0112	0.018882	0.1597	0.164096	FALSE	FALSE	FALSE	pkb876	0.018513	0.307757	486484	sepsis	TRUE	reported	textfile	0.0025	9.58E-06	7037	M02734.m	TRUE	reported	0LCMVl	2	TRUE	0.002844	20.0647
8734	rs1711226(	T	C	T	C	-0.1723	-0.09423	0.9839	0.993203	FALSE	FALSE	FALSE	ERC6hD	0.084098	0.262507	486484	sepsis	TRUE	reported	textfile	0.0384	7.20E-06	589	M36559.m	TRUE	reported	n8waJF	2	TRUE	0.033052	20.06462
1512	rs4147184	A	G	A	G	0.0112	0.004064	0.6082	0.614509	FALSE	FALSE	FALSE	rzkxmh	0.014121	0.773474	486484	sepsis	TRUE	reported	textfile	0.0025	6.73E-06	6941	M03127.m	TRUE	reported	3lrzhA	2	TRUE	0.002883	20.06462
8269	rs2633279	A	G	A	G	0.0112	0.015746	0.6235	0.643682	FALSE	FALSE	FALSE	TdsGCZ	0.014307	0.271055	486484	sepsis	TRUE	reported	textfile	0.0025	6.66E-06	6883	M35464.m	TRUE	reported	sOspot	2	TRUE	0.002907	20.06457
7598	rs1194736!	T	C	T	C	-0.1138	1.23E-05	0.727	0.728031	FALSE	FALSE	FALSE	PlYt5g	0.015519	0.99937	486484	sepsis	TRUE	reported	textfile	0.0253	7.07E-06	241	M34516.m	TRUE	reported	jX41tf	2	TRUE	0.077449	20.06432
2329	rs6748268	T	C	T	C	0.0112	-0.0054	0.6942	0.688211	FALSE	FALSE	FALSE	IZXwpR	0.014876	0.716737	486484	sepsis	TRUE	reported	textfile	0.0025	8.73E-06	6549	M16822.m	TRUE	reported	Zx9k2V	2	TRUE	0.003055	20.06427
6435	rs2218152	T	G	T	G	-0.0112	-0.01193	0.4105	0.426111	FALSE	FALSE	FALSE	QCw5dg	0.014066	0.396524	486484	sepsis	TRUE	reported	textfile	0.0025	5.78E-06	6279	M33666.m	TRUE	reported	zYHmzg	2	TRUE	0.003186	20.06401
512	rs6603123	T	G	T	G	0.0865	-0.01512	0.3939	0.415936	FALSE	FALSE	FALSE	ZOMIXT	0.013924	0.277459	486484	sepsis	TRUE	reported	textfile	0.0193	7.84E-06	1713	M00569.m	TRUE	reported	kZGouU	2	TRUE	0.01159	20.06366
6932	rs1072961	T	C	T	C	-0.0336	0.007012	0.2668	0.280393	FALSE	FALSE	FALSE	oNv0vf	0.015275	0.646194	486484	sepsis	TRUE	reported	textfile	0.0075	6.63E-06	5581	M34112.m	TRUE	reported	l0pe0N	2	TRUE	0.003583	20.06321
8974	rs1332550(	T	C	T	C	0.099	-0.00856	0.9736	0.976128	FALSE	FALSE	FALSE	snOepl	0.044766	0.848383	486484	sepsis	TRUE	reported	textfile	0.0221	7.28E-06	7355	M37202.m	TRUE	reported	PQx272	2	TRUE	0.002721	20.0617
5157	rs6014654	A	G	A	G	0.6332	-0.01535	0.9697	0.983125	FALSE	FALSE	FALSE	L2DJzK	0.055283	0.781334	486484	sepsis	TRUE	reported	textfile	0.1395	5.60E-06	76	M33173.m	TRUE	reported	hRG70D	2	TRUE	0.213276	20.06095
4254	rs8130944	A	G	A	G	-0.0439	0.008082	0.3688	0.392776	FALSE	FALSE	FALSE	KZ60wl	0.014023	0.564395	486484	sepsis	TRUE	reported	textfile	0.0098	7.84E-06	6904	M32616.m	TRUE	reported	SPDpZi	2	TRUE	0.002898	20.06093
8976	rs1825496	A	G	A	G	-0.0484	0.011003	0.5326	0.516049	FALSE	FALSE	FALSE	snOepl	0.013693	0.421658	486484	sepsis	TRUE	reported	textfile	0.0108	8.22E-06	1763	M37202.m	TRUE	reported	PQx272	2	TRUE	0.011263	20.06089
7451	rs1307513'	T	C	T	C	-0.0242	-0.01405	0.4448	0.482431	FALSE	FALSE	FALSE	7ubZow	0.013723	0.305816	486484	sepsis	TRUE	reported	textfile	0.0054	7.51E-06	1754	M34407.m	TRUE	reported	LRsXN2	2	TRUE	0.011321	20.06078
4146	rs1321150'	T	C	T	C	-0.0327	0.008032	0.8862	0.884537	FALSE	FALSE	FALSE	CrVaiQ	0.021373	0.707055	486484	sepsis	TRUE	reported	textfile	0.0073	6.87E-06	7361	M32553.m	TRUE	reported	HsKdAI	2	TRUE	0.002719	20.06004
8416	rs2286411	A	G	A	G	0.0327	-0.02787	0.1197	0.155023	FALSE	FALSE	FALSE	02RdNm	0.01907	0.143949	486484	sepsis	TRUE	reported	textfile	0.0073	8.31E-06	6447	M35678.m	TRUE	reported	BWN5JR	2	TRUE	0.003103	20.05927
5687	rs1205201!	C	G	C	G	-0.056	0.007299	0.6242	0.642522	FALSE	TRUE	FALSE	KxtFnC	0.014272	0.609025	486484	sepsis	TRUE	reported	textfile	0.0125	7.30E-06	3221	M33203.m	TRUE	reported	Uz0wKU	2	TRUE	0.006193	20.05794
4838	rs1158137!	T	C	T	C	-0.2174	-0.03977	0.9792	0.979368	FALSE	FALSE	FALSE	jvlpBt	0.049101	0.418016	486484	sepsis	TRUE	reported	textfile	0.0485	7.48E-06	1146	M32846.m	TRUE	reported	RAXcz5	2	TRUE	0.017231	20.05751
3895	rs900429	T	C	T	C	0.0215	-0.02436	0.8442	0.844906	FALSE	FALSE	FALSE	bmXxuz	0.019413	0.209587	486484	sepsis	TRUE	reported	textfile	0.0048	7.22E-06	7358	M32405.m	TRUE	reported	A9BFZF	2	TRUE	0.002719	20.05748
5712	rs1733033(	C	G	C	G	-0.0327	-0.00089	0.0771	0.076662	FALSE	TRUE	FALSE	XDem1i	0.02568	0.972464	486484	sepsis	TRUE	reported	textfile	0.0073	8.35E-06	4665	M33221.m	TRUE	reported	KBV6FH	2	TRUE	0.004283	20.05689
3148	rs3742766	A	G	A	G	-0.0215	-0.00677	0.0394	0.04679	FALSE	FALSE	FALSE	JiXR2t	0.032394	0.834545	486484	sepsis	TRUE	reported	textfile	0.0048	8.44E-06	6535	M22177.m	TRUE	reported	joi6p6	2	TRUE	0.003061	20.05679
3433	rs7084667	T	C	T	C	-0.0318	0.001634	0.3248	0.333433	FALSE	FALSE	FALSE	HHQf8w	0.014532	0.910451	486484	sepsis	TRUE	reported	textfile	0.0071	6.47E-06	5673	M27531.m	TRUE	reported	3vTluK	2	TRUE	0.003524	20.05323
7417	rs4244070	T	C	T	C	0.0502	0.0023	0.8314	0.838607	FALSE	FALSE	FALSE	7i5VUS	0.018622	0.901703	486484	sepsis	TRUE	reported	textfile	0.0112	7.77E-06	1104	M34395.m	TRUE	reported	64hEaL	2	TRUE	0.017872	20.05321
8738	rs4795411	A	G	A	G	-0.0466	0.019534	0.9339	0.926345	FALSE	FALSE	FALSE	ERC6hD	0.026089	0.454025	486484	sepsis	TRUE	reported	textfile	0.0104	6.90E-06	1636	M36559.m	TRUE	reported	n8waJF	2	TRUE	0.012123	20.05275
6371	rs1709014(	A	G	A	G	0.0636	-0.02157	0.0502	0.051258	FALSE	FALSE	FALSE	PAgZZz	0.030965	0.485976	486484	sepsis	TRUE	reported	textfile	0.0142	7.07E-06	4323	M33633.m	TRUE	reported	I8R1KJ	2	TRUE	0.004619	20.05102
1277	rs1198798:	T	C	T	C	-0.0457	-0.02341	0.099	0.074466	FALSE	FALSE	FALSE	nvWJJ3	0.026148	0.370729	486484	sepsis	TRUE	reported	textfile	0.0102	7.16E-06	1753	M01712.m	TRUE	reported	1x44lf	2	TRUE	0.011322	20.05101
2659	rs3807360	A	G	A	G	0.0735	-0.02588	0.2011	0.207533	FALSE	FALSE	FALSE	UtYu7J	0.016881	0.125235	486484	sepsis	TRUE	reported	textfile	0.0164	7.44E-06	1157	M18476.m	TRUE	reported	wJx0A4	2	TRUE	0.017064	20.05098
1781	rs1105769!	T	C	T	C	0.0206	-0.02779	0.0566	0.09141	FALSE	FALSE	FALSE	JEcCRZ	0.023874	0.244346	486484	sepsis	TRUE	reported	textfile	0.0046	8.03E-06	7368	M15140.m	TRUE	reported	xYCzKP	2	TRUE	0.002714	20.04938
729	rs1160068!	A	T	A	T	-0.0103	0.026032	0.1616	0.208065	FALSE	TRUE	FALSE	9u0SC8	0.016917	0.123846	486484	sepsis	TRUE	reported	textfile	0.0023	9.14E-06	7363	M01301.m	TRUE	reported	H95uec	2	TRUE	0.002716	20.04937
557	rs1170502(	A	G	A	G	-0.0206	0.011948	0.0859	0.110036	FALSE	FALSE	FALSE	fNqkCW	0.022381	0.593459	486484	sepsis	TRUE	reported	textfile	0.0046	8.85E-06	7354	M00606.m	TRUE	reported	g9uwOn	2	TRUE	0.00272	20.04937
923	rs1085353:	A	C	A	C	0.0103	-0.00689	0.8743	0.86945	FALSE	FALSE	FALSE	tv99UN	0.020344	0.734898	486484	sepsis	TRUE	reported	textfile	0.0023	6.09E-06	7354	M01494.m	TRUE	reported	osDclp	2	TRUE	0.00272	20.04937
7882	rs9600082	A	C	A	C	0.0206	0.010503	0.194	0.196231	FALSE	FALSE	FALSE	mpkL0o	0.017223	0.541985	486484	sepsis	TRUE	reported	textfile	0.0046	7.87E-06	7263	M35160.m	TRUE	reported	MKkvYr	2	TRUE	0.002754	20.0493
8926	rs2212465	A	T	A	T	-0.0927	0.010794	0.0432	0.049023	FALSE	TRUE	FALSE	ns6PV1	0.031769	0.734027	486484	sepsis	TRUE	reported	textfile	0.0207	7.30E-06	7014	M37097.m	TRUE	reported	ZYmYt4	2	TRUE	0.002851	20.0491
8195	rs1292932(	C	G	C	G	-0.0206	0.005819	0.8542	0.826007	FALSE	TRUE	FALSE	iLlpho	0.018314	0.75068	486484	sepsis	TRUE	reported	textfile	0.0046	8.89E-06	7007	M35431.m	TRUE	reported	AAZ5ge	2	TRUE	0.002854	20.0491
8539	rs1200587:	A	T	A	T	0.5499	-0.02308	0.8885	0.876102	FALSE	TRUE	FALSE	aQhCav	0.021165	0.275409	486484	sepsis	TRUE	reported	textfile	0.1218	6.39E-06	122	M36097.m	TRUE	reported	9DJS9j	2	TRUE	0.143157	20.04907
1500	rs1433818	T	C	T	C	0.0206	-0.00111	0.0808	0.077457	FALSE	FALSE	FALSE	rzkxmh	0.025892	0.965934	486484	sepsis	TRUE	reported	textfile	0.0046	6.99E-06	6941	M03127.m	TRUE	reported	3lrzhA	2	TRUE	0.002881	20.04904
4839	rs1253358!	T	C	T	C	0.0309	-0.00339	0.2893	0.294119	FALSE	FALSE	FALSE	jvlpBt	0.015087	0.82217	486484	sepsis	TRUE	reported	textfile	0.0069	7.82E-06	6250	M32846.m	TRUE	reported	RAXcz5	2	TRUE	0.003199	20.0484
5141	rs1706171(	C	G	C	G	0.3081	0.004308	0.8406	0.858291	FALSE	TRUE	FALSE	L2DJzK	0.019596	0.826004	486484	sepsis	TRUE	reported	textfile	0.0679	5.62E-06	76	M33173.m	TRUE	reported	hRG70D	2	TRUE	0.213164	20.04758
5692	rs2287358	T	C	T	C	0.1375	0.004508	0.0386	0.038724	FALSE	FALSE	FALSE	KxtFnC	0.035522	0.89901	486484	sepsis	TRUE	reported	textfile	0.0307	7.44E-06	3221	M33203.m	TRUE	reported	Uz0wKU	2	TRUE	0.006189	20.04744
601	rs2549842	T	C	T	C	-0.0206	-0.00982	0.786	0.773172	FALSE	FALSE	FALSE	KVK33e	0.016311	0.547129	486484	sepsis	TRUE	reported	textfile	0.0046	8.68E-06	5344	M01107.m	TRUE	reported	eAyR8w	2	TRUE	0.003739	20.04731
982	rs1768206(	A	G	A	G	-0.1299	-0.00867	0.9248	0.936139	FALSE	FALSE	FALSE	xSRIl4	0.028199	0.758583	486484	sepsis	TRUE	reported	textfile	0.029	7.45E-06	2285	M01515.m	TRUE	reported	juZ5GE	2	TRUE	0.008704	20.04666
7020	rs1288678:	A	G	A	G	-0.0206	0.001409	0.2265	0.226492	FALSE	FALSE	FALSE	zMIkYA	0.016337	0.931271	486484	sepsis	TRUE	reported	textfile	0.0046	8.11E-06	4908	M34245.m	TRUE	reported	itCtz8	2	TRUE	0.00407	20.04665
5422	rs2968779	T	C	T	C	-0.2532	0.02221	0.0267	0.038995	FALSE	FALSE	FALSE	q32d4E	0.035933	0.536515	486484	sepsis	TRUE	reported	textfile	0.0565	7.34E-06	1017	M33178.m	TRUE	reported	cS3BwT	2	TRUE	0.019365	20.04359
3940	rs9691574	T	C	T	C	0.03	-0.00372	0.0695	0.076544	FALSE	FALSE	FALSE	LlOaWH	0.025733	0.88503	486484	sepsis	TRUE	reported	textfile	0.0067	8.79E-06	7355	M32418.m	TRUE	reported	AtHOVw	2	TRUE	0.002718	20.04356
1425	rs1770893:	A	G	A	G	-0.0766	-0.03007	0.0376	0.053587	FALSE	FALSE	FALSE	ac6RGB	0.030821	0.329255	486484	sepsis	TRUE	reported	textfile	0.0171	7.22E-06	1741	M02342.m	TRUE	reported	dC6Rkp	2	TRUE	0.011394	20.04316
8538	rs1179292(	A	G	A	G	-0.4893	-0.02318	0.1274	0.134201	FALSE	FALSE	FALSE	aQhCav	0.02006	0.247904	486484	sepsis	TRUE	reported	textfile	0.1084	6.39E-06	122	M36097.m	TRUE	reported	9DJS9j	2	TRUE	0.143106	20.04072
1037	rs1212142(	T	C	T	C	-0.0197	0.009593	0.0535	0.057501	FALSE	FALSE	FALSE	tRtQ2e	0.030297	0.751512	486484	sepsis	TRUE	reported	textfile	0.0044	6.09E-06	7364	M01564.m	TRUE	reported	Tbg0zM	2	TRUE	0.002715	20.04053
8442	rs7784712	T	C	T	C	0.0197	-0.04293	0.0761	0.088738	FALSE	FALSE	FALSE	Nvt7pl	0.024128	0.075216	486484	sepsis	TRUE	reported	textfile	0.0044	7.00E-06	7358	M35718.m	TRUE	reported	WkA5Gq	2	TRUE	0.002717	20.04052
5977	rs1158851:	T	C	T	C	0.0197	0.038548	0.955	0.922912	FALSE	FALSE	FALSE	3KsoXq	0.026579	0.146968	486484	sepsis	TRUE	reported	textfile	0.0044	9.58E-06	7306	M33422.m	TRUE	reported	aNr6Ug	2	TRUE	0.002736	20.04048
5112	rs1076573'	A	G	A	G	-0.0197	0.022779	0.6388	0.652743	FALSE	FALSE	FALSE	O6ine1	0.01457	0.117947	486484	sepsis	TRUE	reported	textfile	0.0044	8.25E-06	7263	M33165.m	TRUE	reported	gGsqjW	2	TRUE	0.002752	20.04045
8082	rs1561164	A	G	A	G	0.1346	-0.02171	0.0405	0.030592	FALSE	FALSE	FALSE	z65cF2	0.039762	0.585043	486484	sepsis	TRUE	reported	textfile	0.03	7.45E-06	448	M35322.m	TRUE	reported	3GUSWk	2	TRUE	0.043001	20.04031
8077	rs1097258(	A	T	A	T	0.0839	-0.02132	0.0992	0.096299	FALSE	TRUE	FALSE	z65cF2	0.023354	0.361297	486484	sepsis	TRUE	reported	textfile	0.0187	7.22E-06	448	M35322.m	TRUE	reported	3GUSWk	2	TRUE	0.043001	20.03999
8421	rs749008	A	G	A	G	-0.0197	-0.0168	0.6386	0.631782	FALSE	FALSE	FALSE	02RdNm	0.014177	0.235908	486484	sepsis	TRUE	reported	textfile	0.0044	7.65E-06	6447	M35678.m	TRUE	reported	BWN5JR	2	TRUE	0.0031	20.03975
1410	rs2760277	A	G	A	G	0.0197	0.022555	0.7946	0.797446	FALSE	FALSE	FALSE	33ptwk	0.017002	0.18463	486484	sepsis	TRUE	reported	textfile	0.0044	8.26E-06	6252	M02137.m	TRUE	reported	SQY1na	2	TRUE	0.003196	20.03956
5047	rs1265906:	A	G	A	G	0.0618	-0.00398	0.1376	0.132945	FALSE	FALSE	FALSE	JLCSne	0.020123	0.843072	486484	sepsis	TRUE	reported	textfile	0.0138	7.46E-06	2391	M33137.m	TRUE	reported	LGKYXK	2	TRUE	0.008318	20.03805
6663	rs1017776(	A	G	A	G	-0.0197	-0.01403	0.6419	0.625747	FALSE	FALSE	FALSE	6iZSOI	0.014122	0.32061	486484	sepsis	TRUE	reported	textfile	0.0044	8.01E-06	4533	M33901.m	TRUE	reported	ApKaGe	2	TRUE	0.004403	20.03713
3856	rs1207935(	A	G	A	G	-0.0291	-0.00604	0.7102	0.686176	FALSE	FALSE	FALSE	8gg1zS	0.015495	0.696866	486484	sepsis	TRUE	reported	textfile	0.0065	8.42E-06	6119	M32379.m	TRUE	reported	uZWtuS	2	TRUE	0.003265	20.03629
6029	rs197623	A	C	A	C	-0.982	-0.04649	0.0417	0.028933	FALSE	FALSE	FALSE	33ojbt	0.040761	0.254031	486484	sepsis	TRUE	reported	textfile	0.2157	5.32E-06	60	M33423.m	TRUE	reported	ivEtvl	2	TRUE	0.256748	20.03547
7600	rs1396287	A	G	A	G	-0.1717	0.002744	0.8863	0.885727	FALSE	FALSE	FALSE	PlYt5g	0.021544	0.898668	486484	sepsis	TRUE	reported	textfile	0.0382	6.95E-06	241	M34516.m	TRUE	reported	jX41tf	2	TRUE	0.077346	20.03525
7493	rs1014444:	A	G	A	G	-0.0488	-0.00616	0.7294	0.729934	FALSE	FALSE	FALSE	1aXQWs	0.015476	0.690601	486484	sepsis	TRUE	reported	textfile	0.0109	7.92E-06	4452	M34420.m	TRUE	reported	FOqhHW	2	TRUE	0.004482	20.0351
4860	rs6763260	T	C	T	C	0.03	0.006853	0.6335	0.643512	FALSE	FALSE	FALSE	6UwyhO	0.014279	0.631257	486484	sepsis	TRUE	reported	textfile	0.0067	7.76E-06	2684	M32854.m	TRUE	reported	YFrJSY	2	TRUE	0.007414	20.03407
4872	rs3857224	T	C	T	C	0.0188	0.009432	0.3195	0.315409	FALSE	FALSE	FALSE	KBJVQO	0.014742	0.522276	486484	sepsis	TRUE	reported	textfile	0.0042	6.31E-06	7355	M32855.m	TRUE	reported	Xea7Fn	2	TRUE	0.002717	20.03083
1851	rs302121	A	T	A	T	0.0094	0.033818	0.819	0.792884	FALSE	TRUE	FALSE	S1eWts	0.017141	0.048504	486484	sepsis	TRUE	reported	textfile	0.0021	8.61E-06	7345	M15488.m	TRUE	reported	511Kl9	2	TRUE	0.00272	20.03083
6713	rs884760	T	C	T	C	0.0188	-0.00147	0.277	0.309987	FALSE	FALSE	FALSE	bzT5yX	0.014932	0.921357	486484	sepsis	TRUE	reported	textfile	0.0042	7.16E-06	7345	M33936.m	TRUE	reported	W7BO8n	2	TRUE	0.00272	20.03083
3513	rs1244795(	A	G	A	G	0.0188	0.010794	0.7028	0.689561	FALSE	FALSE	FALSE	hxuqls	0.01489	0.46848	486484	sepsis	TRUE	reported	textfile	0.0042	7.57E-06	7336	M27738.m	TRUE	reported	aueeZs	2	TRUE	0.002724	20.03082
2873	rs1158061:	T	C	T	C	0.0188	0.004912	0.1344	0.153996	FALSE	FALSE	FALSE	mUzn1R	0.018992	0.795914	486484	sepsis	TRUE	reported	textfile	0.0042	8.68E-06	7301	M20675.m	TRUE	reported	XwY04K	2	TRUE	0.002737	20.03079
2536	rs7558224	T	C	T	C	0.0376	-0.00261	0.7832	0.783886	FALSE	FALSE	FALSE	4HwTlG	0.016865	0.876953	486484	sepsis	TRUE	reported	textfile	0.0084	8.56E-06	7071	M18254.m	TRUE	reported	F9KrSC	2	TRUE	0.002826	20.03061
4700	rs1244695:	A	G	A	G	0.0282	-0.03469	0.1573	0.162937	FALSE	FALSE	FALSE	WlwKaF	0.018788	0.064872	486484	sepsis	TRUE	reported	textfile	0.0063	7.85E-06	6712	M32759.m	TRUE	reported	Ub4yxH	2	TRUE	0.002976	20.03031
4707	rs4711652	A	G	A	G	0.0188	-0.01174	0.5551	0.558979	FALSE	FALSE	FALSE	WlwKaF	0.013785	0.394422	486484	sepsis	TRUE	reported	textfile	0.0042	8.56E-06	6712	M32759.m	TRUE	reported	Ub4yxH	2	TRUE	0.002976	20.03031
7509	rs6943248	A	T	A	T	0.1334	-0.03533	0.0297	0.036336	FALSE	TRUE	FALSE	1aXQWs	0.037437	0.345344	486484	sepsis	TRUE	reported	textfile	0.0298	7.58E-06	4452	M34420.m	TRUE	reported	FOqhHW	2	TRUE	0.004481	20.03014
7554	rs1195892	T	C	T	C	-0.0188	0.001887	0.5662	0.549808	FALSE	FALSE	FALSE	APSWAU	0.013744	0.890778	486484	sepsis	TRUE	reported	textfile	0.0042	6.16E-06	6144	M34469.m	TRUE	reported	frF3RX	2	TRUE	0.003251	20.02976
7590	rs4901004	T	C	T	C	-0.0385	0.004668	0.8716	0.853115	FALSE	FALSE	FALSE	SbjFMF	0.019313	0.809015	486484	sepsis	TRUE	reported	textfile	0.0086	7.99E-06	3239	M34513.m	TRUE	reported	HVYiKM	2	TRUE	0.006149	20.02886
591	rs1114740	T	G	T	G	0.0282	0.005243	0.8872	0.886508	FALSE	FALSE	FALSE	KVK33e	0.021579	0.808049	486484	sepsis	TRUE	reported	textfile	0.0063	8.69E-06	5344	M01107.m	TRUE	reported	eAyR8w	2	TRUE	0.003735	20.02878
3100	rs4872094	T	C	T	C	-0.0327	-0.00345	0.8154	0.796549	FALSE	FALSE	FALSE	mr0awv	0.017002	0.839228	486484	sepsis	TRUE	reported	textfile	0.0073	8.22E-06	1074	M22130.m	TRUE	reported	Y2TfWQ	2	TRUE	0.01834	20.02812
8632	rs1909355	T	C	T	C	-0.0385	0.005297	0.0954	0.073261	FALSE	FALSE	FALSE	4cZjpQ	0.026467	0.841385	486484	sepsis	TRUE	reported	textfile	0.0086	8.54E-06	2400	M36230.m	TRUE	reported	TPUeNs	2	TRUE	0.008281	20.02454
776	rs7010256	C	G	C	G	0.1003	-0.06679	0.0158	0.01646	FALSE	TRUE	FALSE	tYvbNc	0.053762	0.214094	486484	sepsis	TRUE	reported	textfile	0.0224	7.58E-06	1578	M01303.m	TRUE	reported	hT3gsE	2	TRUE	0.012546	20.02419
7377	rs7900257	T	G	T	G	0.159	0.008661	0.3056	0.239888	FALSE	FALSE	FALSE	mm9ksf	0.016141	0.591536	486484	sepsis	TRUE	reported	textfile	0.0335	2.02E-06	18	M34365.m	TRUE	reported	AVwEqD	2	TRUE	0.555852	20.02406
3768	rs983498	C	G	C	G	-0.0197	0.004564	0.7106	0.729444	FALSE	TRUE	FALSE	OZLdls	0.015429	0.767384	486484	sepsis	TRUE	reported	textfile	0.0044	7.98E-06	1760	M32338.m	TRUE	reported	s2itmB	2	TRUE	0.011261	20.02319
6914	rs1652045	T	C	T	C	0.3852	0.032896	0.8547	0.893965	FALSE	FALSE	FALSE	SvfSvH	0.02217	0.137866	486484	sepsis	TRUE	reported	textfile	0.0855	6.59E-06	148	M34109.m	TRUE	reported	7OSbiu	2	TRUE	0.120604	20.02311
8425	rs9587762	T	C	T	C	0.0273	-0.01156	0.187	0.18522	FALSE	FALSE	FALSE	02RdNm	0.017689	0.513523	486484	sepsis	TRUE	reported	textfile	0.0061	7.77E-06	6447	M35678.m	TRUE	reported	BWN5JR	2	TRUE	0.003097	20.02308
6092	rs1371204	T	C	T	C	-0.03	0.025803	0.1619	0.170144	FALSE	FALSE	FALSE	xvzgym	0.018194	0.156135	486484	sepsis	TRUE	reported	textfile	0.0067	7.00E-06	1432	M33443.m	TRUE	reported	KtBPyR	2	TRUE	0.013807	20.02101
6899	rs4736435	A	C	A	C	0.0497	-0.01112	0.2466	0.255828	FALSE	FALSE	FALSE	MN2lbE	0.015666	0.477638	486484	sepsis	TRUE	reported	textfile	0.0111	8.00E-06	1481	M34106.m	TRUE	reported	q4zXEz	2	TRUE	0.013356	20.02073
487	rs7314742	T	G	T	G	-0.0358	0.037332	0.2662	0.268058	FALSE	FALSE	FALSE	ScJVl8	0.015461	0.015751	486484	sepsis	TRUE	reported	textfile	0.008	8.51E-06	7371	M00542.m	TRUE	reported	Sa1L04	2	TRUE	0.002709	20.02019
3595	rs1310507:	A	T	A	T	-0.1258	0.049143	0.9776	0.980746	FALSE	TRUE	FALSE	e4G8UU	0.049767	0.323419	486484	sepsis	TRUE	reported	textfile	0.0281	7.43E-06	1764	M31548.m	TRUE	reported	kWkkmH	2	TRUE	0.011234	20.01963
4566	rs1075071!	T	G	T	G	0.0179	-0.01801	0.877	0.87467	FALSE	FALSE	FALSE	ILOOFw	0.020637	0.382726	486484	sepsis	TRUE	reported	textfile	0.004	7.17E-06	6042	M32739.m	TRUE	reported	TpvRBe	2	TRUE	0.003303	20.019
3399	rs9827772	A	G	A	G	0.1061	-0.0384	0.9746	0.977832	FALSE	FALSE	FALSE	frOZiy	0.046777	0.411709	486484	sepsis	TRUE	reported	textfile	0.0237	7.70E-06	1758	M27447.m	TRUE	reported	2z9fSv	2	TRUE	0.011272	20.01888
6407	rs1106264(	T	G	T	G	-0.1164	-0.04321	0.0564	0.027385	FALSE	FALSE	FALSE	5ClPW7	0.042015	0.30379	486484	sepsis	TRUE	reported	textfile	0.026	7.36E-06	1603	M33653.m	TRUE	reported	H4b2zx	2	TRUE	0.012349	20.01783
4670	rs1368843	A	G	A	G	-0.0264	0.015938	0.4256	0.456513	FALSE	FALSE	FALSE	xaapK0	0.013694	0.244457	486484	sepsis	TRUE	reported	textfile	0.0059	6.34E-06	7250	M32757.m	TRUE	reported	64n1Z4	2	TRUE	0.002754	20.01631
6294	rs1096690:	A	G	A	G	0.0273	-0.00655	0.6275	0.610476	FALSE	FALSE	FALSE	VlzZVG	0.01403	0.640829	486484	sepsis	TRUE	reported	textfile	0.0061	8.07E-06	2859	M33531.m	TRUE	reported	XiYjn4	2	TRUE	0.006957	20.01528
6358	rs1276343'	A	G	A	G	0.0273	0.006503	0.0834	0.092106	FALSE	FALSE	FALSE	WWmDGM	0.023664	0.783451	486484	sepsis	TRUE	reported	textfile	0.0061	8.68E-06	2757	M33627.m	TRUE	reported	2kkEru	2	TRUE	0.007212	20.01476
3001	rs9313524	T	C	T	C	0.0264	-0.00856	0.4146	0.415442	FALSE	FALSE	FALSE	xmzJ5F	0.013849	0.536362	486484	sepsis	TRUE	reported	textfile	0.0059	8.91E-06	5436	M21184.m	TRUE	reported	xgwhDp	2	TRUE	0.00367	20.01447
2412	rs2623167	A	C	A	C	0.094	-0.00522	0.9047	0.899998	FALSE	FALSE	FALSE	NBJ2Do	0.023548	0.824536	486484	sepsis	TRUE	reported	textfile	0.021	7.57E-06	1739	M17799.m	TRUE	reported	Uz0WgI	2	TRUE	0.01139	20.01324
4550	rs1220290:	A	T	A	T	0.0349	-0.01426	0.2064	0.255701	FALSE	TRUE	FALSE	KfoRxi	0.015658	0.362468	486484	sepsis	TRUE	reported	textfile	0.0078	8.68E-06	6000	M32735.m	TRUE	reported	UDRXAp	2	TRUE	0.003326	20.01321
4655	rs3112734	T	C	T	C	-0.0604	0.050935	0.0221	0.023795	FALSE	FALSE	FALSE	SyAFR2	0.045458	0.262511	486484	sepsis	TRUE	reported	textfile	0.0135	7.79E-06	6658	M32755.m	TRUE	reported	prUo74	2	TRUE	0.002997	20.01133
2706	rs9500850	A	G	A	G	0.0555	0.013645	0.9644	0.944498	FALSE	FALSE	FALSE	XsQHZK	0.029895	0.648079	486484	sepsis	TRUE	reported	textfile	0.0124	8.12E-06	1748	M18929.m	TRUE	reported	k8R7hN	2	TRUE	0.011331	20.00992
618	rs4739402	T	C	T	C	-0.0085	-0.02454	0.5172	0.523774	FALSE	FALSE	FALSE	BYGzq4	0.01366	0.072359	486484	sepsis	TRUE	reported	textfile	0.0019	5.61E-06	7367	M01110.m	TRUE	reported	oZiXqP	2	TRUE	0.002709	20.00842
4302	rs435614	T	C	T	C	-0.0085	0.007287	0.2744	0.294884	FALSE	FALSE	FALSE	0tjjPC	0.015272	0.63325	486484	sepsis	TRUE	reported	textfile	0.0019	5.56E-06	7362	M32634.m	TRUE	reported	nJJFkX	2	TRUE	0.002711	20.00841
1756	rs8078118	A	C	A	C	0.0085	0.008582	0.4238	0.419789	FALSE	FALSE	FALSE	nFLpAP	0.01386	0.535823	486484	sepsis	TRUE	reported	textfile	0.0019	6.98E-06	7356	M12774.m	TRUE	reported	yOSQTj	2	TRUE	0.002713	20.00841
573	rs977712	A	C	A	C	-0.0085	0.033785	0.8633	0.865003	FALSE	FALSE	FALSE	fNqkCW	0.020106	0.092894	486484	sepsis	TRUE	reported	textfile	0.0019	6.32E-06	7354	M00606.m	TRUE	reported	g9uwOn	2	TRUE	0.002714	20.00841
8365	rs1018021(	A	T	A	T	-0.0085	0.002063	0.3776	0.388409	FALSE	TRUE	FALSE	SCzh0A	0.01417	0.884277	486484	sepsis	TRUE	reported	textfile	0.0019	4.78E-06	7348	M35675.m	TRUE	reported	n1SREk	2	TRUE	0.002716	20.0084
8373	rs1305917	T	C	T	C	0.0085	-0.00675	0.2947	0.285875	FALSE	FALSE	FALSE	SCzh0A	0.015094	0.654804	486484	sepsis	TRUE	reported	textfile	0.0019	5.02E-06	7348	M35675.m	TRUE	reported	n1SREk	2	TRUE	0.002716	20.0084
4435	rs2425463	T	G	T	G	0.0085	-0.00164	0.8519	0.855999	FALSE	FALSE	FALSE	WGPdpq	0.019821	0.934102	486484	sepsis	TRUE	reported	textfile	0.0019	4.04E-06	7338	M32675.m	TRUE	reported	u0eUAG	2	TRUE	0.00272	20.0084
3215	rs9375450	A	G	A	G	0.0085	-0.02093	0.5099	0.493499	FALSE	FALSE	FALSE	UfLkBq	0.013671	0.125696	486484	sepsis	TRUE	reported	textfile	0.0019	5.63E-06	7329	M22481.m	TRUE	reported	kWMdiT	2	TRUE	0.002723	20.00839
4908	rs2626332	A	C	A	C	0.0085	-0.00649	0.1274	0.136727	FALSE	FALSE	FALSE	VqcRGX	0.019846	0.743783	486484	sepsis	TRUE	reported	textfile	0.0019	4.88E-06	7329	M32867.m	TRUE	reported	psuFJy	2	TRUE	0.002723	20.00839
2497	rs1121053'	A	G	A	G	0.0085	-0.0049	0.3117	0.31481	FALSE	FALSE	FALSE	LwUYua	0.01473	0.739466	486484	sepsis	TRUE	reported	textfile	0.0019	4.96E-06	7315	M17945.m	TRUE	reported	SUN8dc	2	TRUE	0.002729	20.00838
2501	rs1263971!	T	C	T	C	0.0085	0.01898	0.6649	0.672136	FALSE	FALSE	FALSE	LwUYua	0.014629	0.194478	486484	sepsis	TRUE	reported	textfile	0.0019	4.18E-06	7315	M17945.m	TRUE	reported	SUN8dc	2	TRUE	0.002729	20.00838

5862	rs1157751:	A	G	A	G	0.0085	-0.01031	0.4077	0.418065	FALSE	FALSE	FALSE	tqW0ld	0.014076	0.463996	486484	sepsis	TRUE	reported	textfile	0.0019	5.28E-06	7315	M33389.m	TRUE	reported	Sv7EyV	2	TRUE	0.002729	20.00838
5890	rs6878354	C	G	C	G	0.0085	0.000771	0.3007	0.310634	FALSE	TRUE	FALSE	tqW0ld	0.014868	0.958647	486484	sepsis	TRUE	reported	textfile	0.0019	4.52E-06	7315	M33389.m	TRUE	reported	Sv7EyV	2	TRUE	0.002729	20.00838
5895	rs732787	A	G	A	G	0.0085	-0.0284	0.377	0.372523	FALSE	FALSE	FALSE	tqW0ld	0.014206	0.04555	486484	sepsis	TRUE	reported	textfile	0.0019	5.27E-06	7315	M33389.m	TRUE	reported	Sv7EyV	2	TRUE	0.002729	20.00838
7998	rs7733625	T	G	T	G	-0.017	0.04589	0.2771	0.279393	FALSE	FALSE	FALSE	Xrodum	0.015371	0.002831	486484	sepsis	TRUE	reported	textfile	0.0038	7.69E-06	7291	M35255.m	TRUE	reported	T8t1tl	2	TRUE	0.002737	20.00836
4351	rs7552457	A	C	A	C	-0.0085	0.010191	0.7283	0.725261	FALSE	FALSE	FALSE	6VSoQQ	0.015424	0.508803	486484	sepsis	TRUE	reported	textfile	0.0019	5.43E-06	7229	M32644.m	TRUE	reported	oC2AV0	2	TRUE	0.002761	20.00831
6853	rs4363421	A	G	A	G	0.017	-0.02862	0.7027	0.699221	FALSE	FALSE	FALSE	2pJy8f	0.014886	0.054552	486484	sepsis	TRUE	reported	textfile	0.0038	7.07E-06	7132	M34040.m	TRUE	reported	zuJC1r	2	TRUE	0.002798	20.00824
6291	rs8053839	T	G	T	G	0.0085	0.018481	0.5602	0.559077	FALSE	FALSE	FALSE	MOonP9	0.013865	0.18255	486484	sepsis	TRUE	reported	textfile	0.0019	5.14E-06	7066	M33515.m	TRUE	reported	226GuP	2	TRUE	0.002824	20.00819
8449	rs1256061	T	G	T	G	-0.0085	-0.02355	0.4673	0.475587	FALSE	FALSE	FALSE	uMajV6	0.013705	0.085777	486484	sepsis	TRUE	reported	textfile	0.0019	9.28E-06	6604	M35754.m	TRUE	reported	b8QB7S	2	TRUE	0.003021	20.00779
8451	rs1296988	A	C	A	C	0.0085	-0.0009	0.5532	0.538345	FALSE	FALSE	FALSE	uMajV6	0.013783	0.947986	486484	sepsis	TRUE	reported	textfile	0.0019	9.50E-06	6604	M35754.m	TRUE	reported	b8QB7S	2	TRUE	0.003021	20.00779
8469	rs6828013	T	C	T	C	0.017	-0.02613	0.0729	0.076316	FALSE	FALSE	FALSE	uMajV6	0.025722	0.309672	486484	sepsis	TRUE	reported	textfile	0.0038	8.79E-06	6604	M35754.m	TRUE	reported	b8QB7S	2	TRUE	0.003021	20.00779
8479	rs884957	A	G	A	G	-0.0085	0.011211	0.6492	0.650007	FALSE	FALSE	FALSE	uMajV6	0.014337	0.43425	486484	sepsis	TRUE	reported	textfile	0.0019	9.54E-06	6604	M35754.m	TRUE	reported	b8QB7S	2	TRUE	0.003021	20.00779
8890	rs1164883	A	G	A	G	-0.0085	-0.00252	0.5161	0.506617	FALSE	FALSE	FALSE	eLhsld	0.013694	0.853979	486484	sepsis	TRUE	reported	textfile	0.0019	6.38E-06	6552	M37058.m	TRUE	reported	RSeWE5	2	TRUE	0.003045	20.00774
8918	rs6958124	A	T	A	T	-0.0085	0.01074	0.3835	0.384454	FALSE	TRUE	FALSE	eLhsld	0.014106	0.446403	486484	sepsis	TRUE	reported	textfile	0.0019	4.65E-06	6552	M37058.m	TRUE	reported	RSeWE5	2	TRUE	0.003045	20.00774
4539	rs6732028	A	G	A	G	0.0085	-0.00911	0.5425	0.542775	FALSE	FALSE	FALSE	r0qJaM	0.013907	0.512322	486484	sepsis	TRUE	reported	textfile	0.0019	7.51E-06	6497	M32729.m	TRUE	reported	vYv5Fl	2	TRUE	0.003071	20.00769
4544	rs8008122	T	C	T	C	-0.0085	-0.02324	0.3667	0.380417	FALSE	FALSE	FALSE	r0qJaM	0.014148	0.10045	486484	sepsis	TRUE	reported	textfile	0.0019	7.87E-06	6497	M32729.m	TRUE	reported	vYv5Fl	2	TRUE	0.003071	20.00769
6639	rs1026686	T	C	T	C	0.017	0.006182	0.8064	0.795308	FALSE	FALSE	FALSE	REeQiq	0.016998	0.716066	486484	sepsis	TRUE	reported	textfile	0.0038	9.68E-06	6004	M33892.m	TRUE	reported	cVOa3u	2	TRUE	0.003322	20.00718
9004	rs1568101	T	C	T	C	-0.0255	-0.02067	0.912	0.914751	FALSE	FALSE	FALSE	EZQ1cW	0.024528	0.399452	486484	sepsis	TRUE	reported	textfile	0.0057	8.62E-06	5932	M37253.m	TRUE	reported	ddPpd8	2	TRUE	0.003363	20.0071
7720	rs1093497!	T	C	T	C	0.0085	5.02E-05	0.5406	0.53911	FALSE	FALSE	FALSE	HyMNYM	0.013832	0.997102	486484	sepsis	TRUE	reported	textfile	0.0019	6.32E-06	5885	M34912.m	TRUE	reported	Tl8Hcw	2	TRUE	0.003389	20.00705
7728	rs1221584(	T	C	T	C	0.0085	-0.01032	0.5911	0.586034	FALSE	FALSE	FALSE	HyMNYM	0.0139	0.457733	486484	sepsis	TRUE	reported	textfile	0.0019	6.09E-06	5885	M34912.m	TRUE	reported	Tl8Hcw	2	TRUE	0.003389	20.00705
7739	rs1887820	A	G	A	G	0.0085	-0.0121	0.6665	0.665207	FALSE	FALSE	FALSE	HyMNYM	0.014584	0.406791	486484	sepsis	TRUE	reported	textfile	0.0019	8.47E-06	5885	M34912.m	TRUE	reported	Tl8Hcw	2	TRUE	0.003389	20.00705
7747	rs4583642	T	C	T	C	-0.0085	0.000433	0.3636	0.361122	FALSE	FALSE	FALSE	HyMNYM	0.014344	0.975937	486484	sepsis	TRUE	reported	textfile	0.0019	6.57E-06	5885	M34912.m	TRUE	reported	Tl8Hcw	2	TRUE	0.003389	20.00705
7753	rs698015	T	C	T	C	0.0085	0.002682	0.6477	0.64674	FALSE	FALSE	FALSE	HyMNYM	0.014508	0.853345	486484	sepsis	TRUE	reported	textfile	0.0019	6.72E-06	5885	M34912.m	TRUE	reported	Tl8Hcw	2	TRUE	0.003389	20.00705
7755	rs762864	A	G	A	G	-0.0085	0.020408	0.6215	0.62109	FALSE	FALSE	FALSE	HyMNYM	0.014157	0.149436	486484	sepsis	TRUE	reported	textfile	0.0019	8.82E-06	5885	M34912.m	TRUE	reported	Tl8Hcw	2	TRUE	0.003389	20.00705
2168	rs4772056	C	G	C	G	-0.034	-0.02072	0.907	0.860895	FALSE	TRUE	FALSE	XCBNPg	0.019999	0.300287	486484	sepsis	TRUE	reported	textfile	0.0076	8.45E-06	5829	M15749.m	TRUE	reported	dKBewD	2	TRUE	0.003422	20.00698
6946	rs9297054	T	C	T	C	-0.0255	0.032553	0.5207	0.522636	FALSE	FALSE	FALSE	oNv0vf	0.013794	0.018276	486484	sepsis	TRUE	reported	textfile	0.0057	9.22E-06	5581	M34112.m	TRUE	reported	l0pe0N	2	TRUE	0.003573	20.00668
7817	rs1252076	A	C	A	C	0.0085	0.003323	0.3051	0.306779	FALSE	FALSE	FALSE	h5whIS	0.014914	0.823704	486484	sepsis	TRUE	reported	textfile	0.0019	8.10E-06	4900	M35137.m	TRUE	reported	xVITKF	2	TRUE	0.004068	20.00568
7818	rs1320236	A	G	A	G	0.0085	-0.01053	0.6956	0.701418	FALSE	FALSE	FALSE	h5whIS	0.015159	0.487233	486484	sepsis	TRUE	reported	textfile	0.0019	9.49E-06	4900	M35137.m	TRUE	reported	xVITKF	2	TRUE	0.004068	20.00568
7828	rs2351921	T	C	T	C	0.0085	0.039393	0.3571	0.396619	FALSE	FALSE	FALSE	h5whIS	0.01395	0.004746	486484	sepsis	TRUE	reported	textfile	0.0019	7.48E-06	4900	M35137.m	TRUE	reported	xVITKF	2	TRUE	0.004068	20.00568
7841	rs4908655	C	G	C	G	-0.0085	0.010248	0.2906	0.293265	FALSE	TRUE	FALSE	h5whIS	0.015088	0.497005	486484	sepsis	TRUE	reported	textfile	0.0019	8.42E-06	4900	M35137.m	TRUE	reported	xVITKF	2	TRUE	0.004068	20.00568
7842	rs4975842	C	G	C	G	0.0085	0.011685	0.4075	0.40142	FALSE	TRUE	FALSE	h5whIS	0.013977	0.403128	486484	sepsis	TRUE	reported	textfile	0.0019	7.49E-06	4900	M35137.m	TRUE	reported	xVITKF	2	TRUE	0.004068	20.00568
7849	rs6786214	A	G	A	G	-0.0085	-0.00337	0.3424	0.353584	FALSE	FALSE	FALSE	h5whIS	0.014403	0.815201	486484	sepsis	TRUE	reported	textfile	0.0019	8.88E-06	4900	M35137.m	TRUE	reported	xVITKF	2	TRUE	0.004068	20.00568
7851	rs6836081	T	C	T	C	-0.0085	-0.00592	0.3887	0.381299	FALSE	FALSE	FALSE	h5whIS	0.014154	0.675733	486484	sepsis	TRUE	reported	textfile	0.0019	6.85E-06	4900	M35137.m	TRUE	reported	xVITKF	2	TRUE	0.004068	20.00568
7860	rs764134	C	G	C	G	-0.0085	-0.00401	0.6366	0.645176	FALSE	TRUE	FALSE	h5whIS	0.014266	0.778757	486484	sepsis	TRUE	reported	textfile	0.0019	7.37E-06	4900	M35137.m	TRUE	reported	xVITKF	2	TRUE	0.004068	20.00568
7863	rs7927428	A	C	A	C	-0.0085	0.002008	0.6695	0.676448	FALSE	FALSE	FALSE	h5whIS	0.014611	0.890673	486484	sepsis	TRUE	reported	textfile	0.0019	7.58E-06	4900	M35137.m	TRUE	reported	xVITKF	2	TRUE	0.004068	20.00568
7864	rs8039730	T	C	T	C	-0.0085	-0.00018	0.3197	0.324949	FALSE	FALSE	FALSE	h5whIS	0.014609	0.990157	486484	sepsis	TRUE	reported	textfile	0.0019	8.56E-06	4900	M35137.m	TRUE	reported	xVITKF	2	TRUE	0.004068	20.00568
4625	rs2715026	C	G	C	G	-0.0452	-0.01559	0.4137	0.404121	FALSE	TRUE	FALSE	0oA38w	0.013951	0.263841	486484	sepsis	TRUE	reported	textfile	0.0101	7.35E-06	1763	M32753.m	TRUE	reported	dQUaUX	2	TRUE	0.011232	20.00512
2233	rs1688526	T	C	T	C	0.0255	-0.02432	0.1333	0.141174	FALSE	FALSE	FALSE	cwj8fY	0.019614	0.215085	486484	sepsis	TRUE	reported	textfile	0.0057	8.07E-06	4538	M15958.m	TRUE	reported	dPwsBc	2	TRUE	0.004391	20.00503
5238	rs1189331	A	G	A	G	0.2928	-0.00932	0.0187	0.060205	FALSE	FALSE	FALSE	q32d4E	0.029081	0.748705	486484	sepsis	TRUE	reported	textfile	0.0654	7.70E-06	1017	M33178.m	TRUE	reported	cS3BwT	2	TRUE	0.019328	20.00469
3264	rs4302786	A	T	A	T	-0.0501	-0.07049	0.0131	0.014239	FALSE	TRUE	FALSE	ZNPBqT	0.057843	0.222961	486484	sepsis	TRUE	reported	textfile	0.0112	7.43E-06	7256	M24074.m	TRUE	reported	8pPtOl	2	TRUE	0.00275	20.00413
8565	rs2399250	A	G	A	G	0.0416	-0.00539	0.8481	0.847595	FALSE	FALSE	FALSE	Yjjldt	0.019055	0.777385	486484	sepsis	TRUE	reported	textfile	0.0093	8.23E-06	7054	M36098.m	TRUE	reported	U2ORsF	2	TRUE	0.002828	20.00311
520	rs4663119	A	G	A	G	0.0331	0.015474	0.0773	0.078056	FALSE	FALSE	FALSE	AZraib	0.025487	0.543768	486484	sepsis	TRUE	reported	textfile	0.0074	7.32E-06	7333	M00577.m	TRUE	reported	2tucEZ	2	TRUE	0.002721	20.00203
7163	rs1107709	A	G	A	G	0.0694	0.00531	0.3208	0.285983	FALSE	FALSE	FALSE	VfvTtz	0.015101	0.72509	486484	sepsis	TRUE	reported	textfile	0.0155	7.51E-06	879	M34329.m	TRUE	reported	egac6r	2	TRUE	0.022298	20.00167
973	rs1074955	A	G	A	G	-0.0868	-0.0136	0.3074	0.337483	FALSE	FALSE	FALSE	xSRII4	0.014579	0.350909	486484	sepsis	TRUE	reported	textfile	0.0194	7.55E-06	2285	M01515.m	TRUE	reported	juZ5GE	2	TRUE	0.008685	20.00118
7196	rs1169433	A	G	A	G	0.2881	0.044314	0.0329	0.042364	FALSE	FALSE	FALSE	i1uJ7T	0.034296	0.196324	486484	sepsis	TRUE	reported	textfile	0.0643	7.33E-06	535	M34338.m	TRUE	reported	pi3mKW	2	TRUE	0.036167	20.00037
5299	rs1683709	A	G	A	G	0.3399	-0.04557	0.9693	0.987443	FALSE	FALSE	FALSE	q32d4E	0.063598	0.473647	486484	sepsis	TRUE	reported	textfile	0.0729	3.10E-06	25	M33178.m	TRUE	reported	cS3BwT	2	TRUE	0.465119	20.00024
8875	rs1009555	A	G	A	G	0.0264	0.007399	0.8043	0.812565	FALSE	FALSE	FALSE	Nv6gDO	0.017529	0.672958	486484	sepsis	TRUE	reported	textfile	0.0059	8.21E-06	1782	M37004.m	TRUE	reported	RqmQdg	2	TRUE	0.011111	19.99936
8458	rs2216327	C	G	C	G	-0.0246	-0.04055	0.9359	0.918672	FALSE	TRUE	FALSE	uMajV6	0.025332	0.109436	486484	sepsis	TRUE	reported	textfile	0.0055	8.26E-06	6604	M35754.m	TRUE	reported	b8QB7S	2	TRUE	0.00302	19.99923
2557	rs9565748	T	G	T	G	0.0246	-0.01447	0.6241	0.633272	FALSE	FALSE	FALSE	NxZhaQ	0.014193	0.30801	486484	sepsis	TRUE	reported	textfile	0.0055	6.97E-06	5984	M18283.m	TRUE	reported	5Pi7r3	2	TRUE	0.003332	19.9986
3249	rs2689327	A	G	A	G	0.0492	0.007785	0.7541	0.754049	FALSE	FALSE	FALSE	jxdGCf	0.015872	0.623769	486484	sepsis	TRUE	reported	textfile	0.011	7.74E-06	5599	M22842.m	TRUE	reported	f0L3pZ	2	TRUE	0.00356	19.99814
7652	rs1696667	T	C	T	C	0.115	-0.06783	0.0263	0.02374	FALSE	FALSE	FALSE	8W5cqf	0.045254	0.133912	486484	sepsis	TRUE	reported	textfile	0.0257	7.89E-06	1572	M34534.m	TRUE	reported	JV42Pw	2	TRUE	0.012577	19.99754
7848	rs6659436	T	C	T	C	0.0246	-0.01152	0.0333	0.036468	FALSE	FALSE	FALSE	h5whIS	0.036496	0.752335	486484	sepsis	TRUE	reported	textfile	0.0055	7.43E-06	4900	M35137.m	TRUE	reported	xVITKF	2	TRUE	0.004066	19.99712
8944	rs626747	T	C	T	C	0.0246	-0.00215	0.4816	0.478043	FALSE	FALSE	FALSE	mXJP3h	0.013808	0.876065	486484	sepsis	TRUE	reported	textfile	0.0055	6.95E-06	4520	M37104.m	TRUE	reported	HRZBak	2	TRUE	0.004406	19.99644
7070	rs1043464	A	G	A	G	-0.0255	-0.01619	0.8432	0.842397	FALSE	FALSE	FALSE	sg7nOB	0.018905	0.391765	486484	sepsis	TRUE	reported	textfile	0.0057	8.39E-06	2278	M34289.m	TRUE	reported	KemvlC	2	TRUE	0.008709	19.99628
6249	rs9825012	T	C	T	C	0.0434	0.003212	0.9575	0.979676	FALSE	FALSE	FALSE	vL52Yb	0.049106	0.947848	486484	sepsis	TRUE	reported	textfile	0.0097	7.70E-06	1718	M33510.m	TRUE	reported	JlO9VX	2	TRUE	0.011518	19.9954
1785	rs1280233	A	G	A	G	-0.0161	0.005474	0.0776	0.085831	FALSE	FALSE	FALSE	JEcCRZ	0.024367	0.822242	486484	sepsis	TRUE	reported	textfile	0.0036	5.95E-06	7368	M15140.m	TRUE	reported	xYCzKP	2	TRUE	0.002707	19.99534
2182	rs1878105	T	C	T	C	0.0966	0.080909	0.0183	0.024614	FALSE	FALSE	FALSE	UokbxN	0.044513	0.069115	486484	sepsis	TRUE	reported	textfile	0.0216	7.38E-06	7357	M15753.m	TRUE	reported	lUGKBi	2	TRUE	0.002711	19.99533
6770	rs4414129	T	C	T	C	-0.0161	0.000821	0.9342	0.930242	FALSE	FALSE	FALSE	Elf6MQ	0.026959	0.975691	486484	sepsis	TRUE	reported	textfile	0.0036	8.00E-06	7354	M33955.m	TRUE	reported	Lxn4RF	2	TRUE	0.002712	19.99533
3187	rs1045123	A	G	A	G	-0.0161	0.020792	0.8872	0.879386	FALSE	FALSE	FALSE	UfLkBq	0.021007	0.322302	486484	sepsis	TRUE	reported	textfile	0.0036	6.15E-06	7329	M22481.m	TRUE	reported	kWMdiT	2	TRUE	0.002722	19.99531
7877	rs1777774	A	C	A	C	0.0161	-0.00179	0.5951	0.583966	FALSE	FALSE	FALSE	mpkL0o	0.013951	0.898091	486484	sepsis	TRUE	reported	textfile	0.0036	9.97E-06	7263	M35160.m	TRUE	reported	MKkvYr	2	TRUE	0.002746	19.99526
551	rs4648464	A	G	A	G	-0.0161	0.01256	0.5522	0.556472	FALSE	FALSE	FALSE	QUUN2g	0.013849	0.36445	486484	sepsis	TRUE	reported	textfile	0.0036	9.37E-06	7237	M00599.m	TRUE	reported	vxn2i7	2	TRUE	0.002756	19.99524
3111	rs7989427	A	T	A	T	0.0492	0.026214	0.0528	0.06639	FALSE	TRUE	FALSE	ApMZhy	0.027723	0.344359	486484	sepsis	TRUE	reported	textfile	0.011	7.84E-06	3950	M22138.m	TRUE	reported	LiJliF	2	TRUE	0.005039	19.99516
7975	rs1104910	A	G	A	G	0.0161	-0.01597	0.1677	0.168827	FALSE	FALSE	FALSE	t02AUT	0.018209	0.380452	486484	sepsis	TRUE	reported	textfile	0.0036	6.38E-06	7100	M35254.m	TRUE	reported	uClJaT	2	TRUE	0.002809	19.99514
2297	rs4888946	A	T	A	T	-0.0161	0.017106	0.7754	0.776997	FALSE	TRUE	FALSE	43Yh3h	0.016409	0.297166	486484	sepsis	TRUE	reported	textfile	0.0036	8.78E-06	7055	M16818.m	TRUE	reported	beR4IT	2	TRUE	0.002827	19.9951
2312	rs7604529	A	G	A	G	-0.0161	0.001863	0.8443	0.846378	FALSE	FALSE	FALSE	ZzUW8U	0.019102	0.922294	486484	sepsis	TRUE	reported	textfile	0.0036	8.04E-06	6733	M16821.m	TRUE	reported	g2xs3i	2	TRUE	0.002962	19.99483
7946	rs9871214	T	G	T	G	-0.0161	0.01357	0.1789	0.17599	FALSE	FALSE	FALSE	kFTWXY	0.017941	0.449431	486484	sepsis	TRUE	reported	textfile	0.0036	8.39E-06	6543	M35193.m	TRUE	reported	cjJvJl	2	TRUE	0.003048	19.99466
8001	rs1058337	T	G	T	G	0.0161	-0.02224	0.7966	0.795248	FALSE	FALSE	FALSE	Zj5bZH	0.016967	0.189913	486484	sepsis	TRUE	reported	textfile	0.0036	5.58E-06	6538	M35257.m	TRUE	reported	exssAk	2	TRUE	0.00305	19.99465
8873	rs9904760	A	G	A	G	-0.0322	-0.00828	0.19	0.198975	FALSE	FALSE	FALSE	nWuK1Q	0.017124	0.628643	486484	sepsis	TRUE	reported	textfile	0.0072	8.12E-06	6511	M36850.m	TRUE	reported	2BA0SE	2	TRUE	0.003062	19.99463
6345	rs6803591	T	C	T	C	0.3527	-0.00195	0.0253	0.030425	FALSE	FALSE	FALSE	qLUY00	0.0403	0.961496	486484	sepsis	TRUE	reported	textfile	0.0781	6.25E-06	102	M33610.m	TRUE	reported	8cAJbB	2	TRUE	0.166628	19.99441
8064	rs8083552	A	G	A	G	-0.0161	-0.0117	0.3479	0.349889	FALSE	FALSE	FALSE	whAa18	0.01438	0.415943	486484	sepsis	TRUE	reported	textfile	0.0036	5.69E-06	5979	M35305.m	TRUE	reported	e6hvDm	2	TRUE	0.003334	19.99408
3480	rs1011704	T	G	T	G	0.0161	-0.01965	0.156	0.155095	FALSE	FALSE	FALSE	Awafb0	0.018912	0.298837	486484	sepsis	TRUE	reported	textfile	0.0036	6.13E-06	5562	M27728.m	TRUE	reported	Lfj8L0	2	TRUE	0.003583	19.99358
3507	rs908690	T	C	T	C	-0.0161	-0.00813	0.8554	0.861235	FALSE	FALSE	FALSE	Awafb0	0.019762	0.680884	486484	sepsis	TRUE	reported	textfile	0.0036	7.83E-06	5562	M27728.m	TRUE	reported	Lfj8L0	2	TRUE	0.003583	19.99358
2158	rs3783141	T	C	T	C	0.0559	-0.03806	0.8708	0.862446	FALSE	FALSE	FALSE	uuY1Mi	0.019912	0.055931	486484	sepsis	TRUE	reported	textfile	0.0125	7.75E-06	5502	M15677.m	TRUE	reported	nWMiPu	2	TRUE	0.003622	19.99151
5045	rs1246450	T	C	T	C	0.0658	0.009456	0.167	0.171987	FALSE	FALSE	FALSE	JLCSne	0.018097	0.601311	486484	sepsis	TRUE	reported	textfile	0.0147	7.10E-06	888	M33137.m	TRUE	reported	LGKYXK	2	TRUE	0.022066	19.99115
2854	rs6732398	T	C	T	C	-0.034	-0.00291	0.9627	0.962878	FALSE	FALSE	FALSE	Y5qWBm	0.036026	0.93555	486484	sepsis	TRUE	reported	textfile	0.0076	7.82E-06	1754	M20489.m	TRUE	reported	f2g9Su	2	TRUE	0.011282	19.99103
4154	rs7118058	T	G	T	G	0.0474	0.005472	0.0603	0.078908	FALSE	FALSE	FALSE	CrVaiQ	0.025712	0.831463	486484	sepsis	TRUE	reported	textfile	0.0106	8.13E-06	7361	M32553.m	TRUE	reported	HsKdAI	2	TRUE	0.002709	19.99065
3654	rs1763199	T	C	T	C	0.0237	-0.00408	0.0805	0.09827	FALSE	FALSE	FALSE	fnjup6	0.023182	0.860398	486484	sepsis	TRUE	reported	textfile	0.0053	6.81E-06	7346	M32197.m	TRUE	reported	oQzaMk	2	TRUE	0.002715	19.99064
5078	rs8058442	T	C	T	C	-0.0474	0.007015	0.9738	0.973346	FALSE	FALSE	FALSE	gqzGN4	0.043213	0.871032	486484	sepsis	TRUE	reported	textfile	0.0106	8.10E-06	7319	M33140.m	TRUE	reported	DKGBe9	2	TRUE	0.002725	19.99062
8199	rs2410833	T	C	T	C	-0.0237	0.018913	0.9447	0.913723	FALSE	FALSE	FALSE	iLlpho	0.024486	0.439899	486484	sepsis	TRUE	reported	textfile	0.0053	8.27E-06	7007	M35431.m	TRUE	reported	AAZ5ge	2	TRUE	0.002846	19.99038
4503	rs7982884	A	G	A	G	-0.0313	0.068777	0.0425	0.037213	FALSE	FALSE	FALSE	1wNJ1E	0.036251	0.057796	486484	sepsis	TRUE	reported	textfile	0.007	7.61E-06	7363	M32709.m	TRUE	reported	zihBHg	2	TRUE	0.002708	19.98824
656	rs4384764	A	G	A	G	-0.0568	-0.00079	0.2371	0.267792	FALSE	FALSE	FALSE	7BPGuQ	0.015453	0.959336	486484	sepsis	TRUE	reported	textfile	0.0127	7.90E-06	2639	M01123.m	TRUE	reported	WV2hVn	2	TRUE	0.007523	19.98757
7521	rs2903269	T	C	T	C	-0.0237	0.014289	0.2157	0.212481	FALSE	FALSE	FALSE	rp1LGA	0.016825	0.395735	486484	sepsis	TRUE	reported	textfile	0.0053	7.66E-06	4442	M34441.m	TRUE	reported	SU47tz	2	TRUE	0.004481	19.98708
6597	rs827283	A	G	A	G	-0.1151	0.018406	0.7613	0.782364	FALSE	FALSE	FALSE	XO3rXq	0.016602	0.267597	486484	sepsis	TRUE	reported	textfile	0.0256	6.95E-06	176	M33877.m	TRUE	reported	p2sETg	2	TRUE	0.103024	19.98514
6514	rs1003504	A	G	A	G	-0.0331	0.028681	0.2331	0.230246	FALSE	FALSE	FALSE	OKlXJ8	0.016306	0.078587	486484	sepsis	TRUE	reported	textfile	0.0074	8.39E-06	1764	M33821.m	TRUE	reported	XGkaXH	2	TRUE	0.011215	19.9848
5310	rs1689434	T	C	T	C	0.2045	0.063691	0.9687	0.962854	FALSE	FALSE	FALSE	q32d4E	0.036413	0.080268	486484	sepsis	TRUE	reported	textfile	0.0457	7.65E-06	1017	M33178.m	TRUE	reported	cS3BwT	2	TRUE	0.019309	19.98478
1553	rs1530919	A	G	A	G	0.0076	0.038104	0.3006	0.302902	FALSE	FALSE	FALSE	Ke0sNh	0.014856	0.010318	486484	sepsis	TRUE	reported	textfile	0.0017	5.92E-06	7799	M10642.m	TRUE	reported	ZlNwVb	2	TRUE	0.002556	19.98103
1555	rs1761689	A	T	A	T	-0.0076	-0.01975	0.6964	0.702196	FALSE	TRUE	FALSE	Ke0sNh	0.014973	0.187085	486484	sepsis	TRUE	reported	textfile	0.0017	4.65E-06	7799	M10642.m	TRUE	reported	ZlNwVb	2	TRUE	0.002556	19.98103
1567	rs6557106	T	C	T	C	0.0076	-0.00542	0.6715	0.669266	FALSE	FALSE	FALSE	Ke0sNh	0.014555	0.709519	486484	sepsis	TRUE	reported	textfile	0.0017	5.42E-06	7799	M10642.m	TRUE	reported	ZlNwVb	2	TRUE	0.002556	19.98103
623	rs712916	T	C	T	C	-0.0456	0.094807	0.9875	0.980185	FALSE	FALSE	FALSE	BYGzq4	0.049189	0.053931	486484	sepsis	TRUE	reported	textfile	0.0102	8.19E-06	7367	M01110.m	TRUE	reported	oZiXqP	2	TRUE	0.002706	19.98073
2632	rs1773650	C	G	C	G	-0.0684	0.018875	0.0125	0.017473	FALSE	TRUE	FALSE	UxuT5B	0.052419	0.718783	486484	sepsis	TRUE	reported	textfile	0.0153	7.68E-06	7367	M18467.m	TRUE	reported	eGQj6o	2	TRUE	0.002706	19.98073
406	rs1181314	A	G	A	G	0.0076	0.002818	0.3414	0.355677	FALSE	FALSE	FALSE	cTsNj6	0.014331	0.844123	486484	sepsis	TRUE	reported	textfile	0.0017	5.87E-06	7365	M00063.m	TRUE	reported	7KYTrQ	2	TRUE	0.002706	19.98073
454	rs1303139	T	G	T	G	-0.0076	-0.02636	0.2473	0.252591	FALSE	FALSE	FALSE	cl8ucN	0.015753	0.094321	486484	sepsis	TRUE	reported	textfile	0.0017	9.17E-06	7361	M00513.m	TRUE	reported	Ian0Q8	2	TRUE	0.002708	19.98073
697	rs1124392	T	C	T	C	-0.0076	-0.0056	0.7731	0.778771	FALSE	FALSE	FALSE	H9l3s3	0.016476	0.733929	486484	sepsis	TRUE	reported	textfile	0.0017	8.33E-06	7358	M01299.m	TRUE	reported	kXWyUq	2	TRUE	0.002709	19.98073
699	rs1323743	A	G	A	G	-0.0076	0.019621	0.193	0.199624	FALSE	FALSE	FALSE	H9l3s3	0.017084	0.250748	486484	sepsis	TRUE	reported	textfile	0.0017	7.26E-06	7358	M01299.m	TRUE	reported	kXWyUq	2	TRUE	0.002709	19.98073
719	rs906466	A	G	A	G	0.0076	-0.00869	0.1846	0.187322	FALSE	FALSE	FALSE	H9l3s3	0.017569	0.6207	486484	sepsis	TRUE	reported	textfile	0.0017	8.84E-06	7358	M01299.m	TRUE	reported	kXWyUq	2	TRUE	0.002709	19.98073
2602	rs2413113	T	C	T	C	-0.0076	-0.02396	0.4667	0.47063	FALSE	FALSE	FALSE	ruvJh6	0.013718	0.080644	486484	sepsis	TRUE	reported	textfile	0.0017	5.74E-06	7354	M18369.m	TRUE	reported	GFmwGk	2	TRUE	0.00271	19.98072
2604	rs3799105	A	C	A	C	0.0076	-0.00845	0.5951	0.606074	FALSE	FALSE	FALSE	ruvJh6	0.01399	0.545801	486484	sepsis	TRUE	reported	textfile	0.0017	6.08E-06	7354	M18369.m	TRUE	reported	GFmwGk	2	TRUE	0.00271	19.98072
2606	rs4926707	A	G	A	G	-0.0076	0.012683	0.4735	0.469573	FALSE	FALSE	FALSE	ruvJh6	0.013694	0.354346	486484	sepsis	TRUE	reported	textfile	0.0017	6.22E-06	7354	M18369.m	TRUE	reported	GFmwGk	2	TRUE	0.00271	19.98072
2612	rs7678928	T	C	T	C	0.0076	-0.01197	0.4678	0.464607	FALSE	FALSE	FALSE	ruvJh6	0.013728	0.383183	486484	sepsis	TRUE	reported	textfile	0.0017	5.49E-06	7354	M18369.m	TRUE	reported	GFmwGk	2	TRUE	0.00271	19.98072
793	rs2190223	A	G	A	G	0.0076	-0.00814	0.5733	0.575741	FALSE	FALSE	FALSE	dyT79I	0.013851	0.556834	486484	sepsis	TRUE	reported	textfile	0.0017	7.69E-06	7352	M01336.m	TRUE	reported	OemVJ2	2	TRUE	0.002711	19.98072
802	rs942888	A	G	A	G	-0.0076	-0.02358	0.4776	0.479027	FALSE	FALSE	FALSE	dyT79I	0.013718	0.085615	486484	sepsis	TRUE	reported	textfile	0.0017	6.29E-06	7352	M01336.m	TRUE	reported	OemVJ2	2	TRUE	0.002711	19.98072
377	rs8023420	A	C	A	C	-0.0076	-0.00741	0.0972	0.109692	FALSE	FALSE	FALSE	tbRL8J	0.022099	0.737238	486484	sepsis	TRUE	reported	textfile	0.0017	8.85E-06	7351	M00060.m	TRUE	reported	oOvRKT	2	TRUE	0.002711	19.98072
2029	rs7604464	A	G	A	G	0.0152	0.012968	0.9726	0.972201	FALSE	FALSE	FALSE	v2KE89	0.042115	0.758155	486484	sepsis	TRUE	reported	textfile	0.0034	5.87E-06	7349	M15500.m	TRUE	reported	to2t7H	2	TRUE	0.002712	19.98072
1262	rs1569823	T	C	T	C	-0.0152	0.013083	0.8757	0.866694	FALSE	FALSE	FALSE	gNG3wy	0.020131	0.515744	486484	sepsis	TRUE	reported	textfile	0.0034	8.25E-06	7348	M01670.m	TRUE	reported	t3lsMq	2	TRUE	0.002713	19.98072
1266	rs2424200	T	C	T	C	0.0152	0.038417	0.1163	0.105518	FALSE	FALSE	FALSE	gNG3wy	0.022222	0.083855	486484	sepsis	TRUE	reported	textfile	0.0034	6.43E-06	7348	M01670.m	TRUE	reported	t3lsMq	2	TRUE	0.002713	19.98072
533	rs2270451	A	G	A	G	0.0152	-0.02127	0.8941	0.89699	FALSE	FALSE	FALSE	wXMWHy	0.022629	0.347247	486484	sepsis	TRUE	reported	textfile	0.0034	7.53E-06	7345	M00584.m	TRUE	reported	7CKn2b	2	TRUE	0.002714	19.98072
6705	rs2133336	A	G	A	G	0.0152	0.035696	0.3986	0.401584	FALSE	FALSE	FALSE	bzT5yX	0.013999	0.010776	486484	sepsis	TRUE	reported	textfile	0.0034	9.56E-06	7345	M33936.m	TRUE	reported	W7BO8n	2	TRUE	0.002714	19.98072
6710	rs7334793	T	C	T	C	-0.0152	0.001471	0.4953	0.498299	FALSE	FALSE	FALSE	bzT5yX	0.013719	0.914585	486484	sepsis	TRUE	reported	textfile	0.0034	8.04E-06	7345	M33936.m	TRUE	reported	W7BO8n	2	TRUE	0.002714	19.98072
6839	rs703138	T	G	T	G	0.0152	-0.04027	0.3743	0.379909	FALSE	FALSE	FALSE	iVyBC0	0.014079	0.004233	486484	sepsis	TRUE	reported	textfile	0.0034	8.62E-06	7345	M33972.m	TRUE	reported	JyBTbA	2	TRUE	0.002714	19.98072
8173	rs2835977	A	G	A	G	-0.0076	0.016187	0.2484	0.254163	FALSE	FALSE	FALSE	8UhtPK	0.015819	0.306194	486484	sepsis	TRUE	reported	textfile	0.0017	5.23E-06	7345	M35397.m	TRUE	reported	rvVJba	2	TRUE	0.002714	19.98072
8184	rs7190694	C	G	C	G	-0.0076	-0.00035	0.2487	0.241161	FALSE	TRUE	FALSE	8UhtPK	0.015986	0.982726	486484	sepsis	TRUE	reported	textfile	0.0017	5.57E-06	7345	M35397.m	TRUE	reported	rvVJba	2	TRUE	0.002714	19.98072
8187	rs7674482	T	C	T	C	0.0076	0.017047	0.7627	0.758007	FALSE	FALSE	FALSE	8UhtPK	0.015969	0.285755	486484	sepsis	TRUE	reported	textfile	0.0017	5.51E-06	7345	M35397.m	TRUE	reported	rvVJba	2	TRUE	0.002714	19.98072
2136	rs1902781	T	G	T	G	0.0076	-0.00803	0.4272	0.425177	FALSE	FALSE	FALSE	TEupfd	0.013823	0.561135	486484	sepsis	TRUE	reported	textfile	0.0017	6.71E-06	7331	M15676.m	TRUE	reported	NXiaPC	2	TRUE	0.002719	19.98071
2137	rs219908	T	C	T	C	-0.0076	0.003268	0.407	0.39436	FALSE	FALSE	FALSE	TEupfd	0.013983	0.815197	486484	sepsis	TRUE	reported	textfile	0.0017	8.79E-06	7331	M15676.m	TRUE	reported	NXiaPC	2	TRUE	0.002719	19.98071
2152	rs963913	A	G	A	G	-0.0076	0.00093	0.4455	0.439087	FALSE	FALSE	FALSE	TEupfd	0.013836	0.946396	486484	sepsis	TRUE	reported	textfile	0.0017	9.04E-06	7331	M15676.m	TRUE	reported	NXiaPC	2	TRUE	0.002719	19.98071
1362	rs1048900	A	C	A	C	-0.0076	0.002458	0.4919	0.476664	FALSE	FALSE	FALSE	KrWhws	0.013858	0.859221	486484	sepsis	TRUE	reported	textfile	0.0017	7.37E-06	7325	M02132.m	TRUE	reported	7pzpfK	2	TRUE	0.002721	19.9807
1385	rs2506864	T	G	T	G	-0.0076	0.035845	0.4103	0.40074	FALSE	FALSE	FALSE	KrWhws	0.013953	0.0102	486484	sepsis	TRUE	reported	textfile	0.0017	8.81E-06	7325	M02132.m	TRUE	reported	7pzpfK	2	TRUE	0.002721	19.9807
2853	rs6604664	C	G	C	G	0.0076	0.021993	0.2321	0.236957	FALSE	TRUE	FALSE	Y5qWBm	0.016234	0.175504	486484	sepsis	TRUE	reported	textfile	0.0017	8.90E-06	7325	M20489.m	TRUE	reported	f2g9Su	2	TRUE	0.002721	19.9807
2865	rs9804190	T	C	T	C	0.0076	-0.00266	0.2232	0.216173	FALSE	FALSE	FALSE	Y5qWBm	0.016554	0.87224	486484	sepsis	TRUE	reported	textfile	0.0017	8.95E-06	7325	M20489.m	TRUE	reported	f2g9Su	2	TRUE	0.002721	19.9807
1698	rs699191	T	C	T	C	0.0076	-0.01944	0.1916	0.17793	FALSE	FALSE	FALSE	f01en0	0.018152	0.284065	486484	sepsis	TRUE	reported	textfile	0.0017	9.91E-06	7314	M12593.m	TRUE	reported	EAsM6U	2	TRUE	0.002725	19.98069
3342	rs708161	A	G	A	G	-0.0076	0.017866	0.6965	0.699067	FALSE	FALSE	FALSE	m66Eav	0.015091	0.236471	486484	sepsis	TRUE	reported	textfile	0.0017	8.75E-06	7303	M27256.m	TRUE	reported	pl2ECU	2	TRUE	0.002729	19.98069
3347	rs807531	T	C	T	C	-0.0152	-0.00989	0.9382	0.932298	FALSE	FALSE	FALSE	m66Eav	0.027395	0.718153	486484	sepsis	TRUE	reported	textfile	0.0034	8.06E-06	7303	M27256.m	TRUE	reported	pl2ECU	2	TRUE	0.002729	19.98069
949	rs1350858	A	C	A	C	0.0152	0.010186	0.1783	0.189778	FALSE	FALSE	FALSE	tWoDq4	0.017398	0.558244	486484	sepsis	TRUE	reported	textfile	0.0034	8.21E-06	7160	M01508.m	TRUE	reported	6ltDw0	2	TRUE	0.002784	19.98058
1302	rs2325727	T	C	T	C	-0.0076	-0.02398	0.4423	0.436315	FALSE	FALSE	FALSE	WQdpmo	0.013819	0.082745	486484	sepsis	TRUE	reported	textfile	0.0017	9.34E-06	7136	M01769.m	TRUE	reported	BkptOK	2	TRUE	0.002793	19.98056
1314	rs7670890	A	G	A	G	-0.0076	-0.01425	0.6344	0.633378	FALSE	FALSE	FALSE	WQdpmo	0.014177	0.314923	486484	sepsis	TRUE	reported	textfile	0.0017	9.64E-06	7136	M01769.m	TRUE	reported	BkptOK	2	TRUE	0.002793	19.98056
1318	rs9379820	T	C	T	C	-0.0076	-0.03465	0.5914	0.588162	FALSE	FALSE	FALSE	WQdpmo	0.013845	0.012332	486484	sepsis	TRUE	reported	textfile	0.0017	8.32E-06	7136	M01769.m	TRUE	reported	BkptOK	2	TRUE	0.002793	19.98056
5826	rs2492943	T	C	T	C	0.0076	0.022359	0.3964	0.394571	FALSE	FALSE	FALSE	R5joEf	0.013969	0.109456	486484	sepsis	TRUE	reported	textfile	0.0017	8.68E-06	7118	M33380.m	TRUE	reported	kRvbfZ	2	TRUE	0.0028	19.98054
8695	rs1537987	T	C	T	C	0.0076	-0.0079	0.5417	0.541963	FALSE	FALSE	FALSE	Bg4VnB	0.013781	0.566637	486484	sepsis	TRUE	reported	textfile	0.0017	5.79E-06	7092	M36552.m	TRUE	reported	6zdDpC	2	TRUE	0.00281	19.98052
2265	rs1174732	T	C	T	C	-0.0228	0.006919	0.2853	0.301446	FALSE	FALSE	FALSE	Mjgi2R	0.014965	0.643818	486484	sepsis	TRUE	reported	textfile	0.0051	6.45E-06	7064	M16634.m	TRUE	reported	XO0GV7	2	TRUE	0.002821	19.9805
3590	rs7797678	T	C	T	C	0.2817	-0.00656	0.0159	0.015372	FALSE	FALSE	FALSE	F1s6qg	0.055702	0.906228	486484	sepsis	TRUE	reported	textfile	0.0629	7.60E-06	522	M31536.m	TRUE	reported	Zm40GX	2	TRUE	0.037002	19.98045
9008	rs7702827	C	G	C	G	-0.0152	-0.01231	0.3005	0.282077	FALSE	TRUE	FALSE	EZQ1cW	0.015193	0.417647	486484	sepsis	TRUE	reported	textfile	0.0034	6.29E-06	5932	M37253.m	TRUE	reported	ddPpd8	2	TRUE	0.003358	19.97942
8575	rs1248821	T	C	T	C	-0.038	-0.02166	0.2512	0.255816	FALSE	FALSE	FALSE	3VcRoA	0.015669	0.166907	486484	sepsis	TRUE	reported	textfile	0.0085	7.10E-06	5696	M36099.m	TRUE	reported	zHE146	2	TRUE	0.003497	19.97914
3499	rs6699475	A	G	A	G	-0.0152	-0.01815	0.245	0.284767	FALSE	FALSE	FALSE	Awafb0	0.015212	0.232847	486484	sepsis	TRUE	reported	textfile	0.0034	8.91E-06	5562	M27728.m	TRUE	reported	Lfj8L0	2	TRUE	0.00358	19.97897
5759	rs2016979	T	C	T	C	0.0304	0.010831	0.7289	0.68719	FALSE	FALSE	FALSE	n16uO2	0.014968	0.469282	486484	sepsis	TRUE	reported	textfile	0.0068	7.48E-06	4409	M33250.m	TRUE	reported	9dhl67	2	TRUE	0.004513	19.97709
809	rs1182473	T	C	T	C	0.072	0.001861	0.0212	0.02282	FALSE	FALSE	FALSE	J58DI0	0.045709	0.967527	486484	sepsis	TRUE	reported	textfile	0.0161	7.64E-06	1755	M01356.m	TRUE	reported	zk9AMB	2	TRUE	0.011267	19.97644
2642	rs1051545	A	G	A	G	0.0868	-0.02074	0.1032	0.106203	FALSE	FALSE	FALSE	9GoQdh	0.022153	0.349169	486484	sepsis	TRUE	reported	textfile	0.0194	7.98E-06	917	M18474.m	TRUE	reported	p6Dq8p	2	TRUE	0.021364	19.97504
5792	rs6938176	A	G	A	G	0.0842	-0.00447	0.4494	0.457171	FALSE	FALSE	FALSE	lUkr5t	0.013745	0.745142	486484	sepsis	TRUE	reported	textfile	0.0188	7.45E-06	474	M33359.m	TRUE	reported	DjliwS	2	TRUE	0.0406	19.97433
2707	rs1006351	T	C	T	C	0.0474	-0.01053	0.0701	0.07435	FALSE	FALSE	FALSE	uNhql4	0.02614	0.687053	486484	sepsis	TRUE	reported	textfile	0.0106	8.19E-06	1766	M19323.m	TRUE	reported	h9HAwp	2	TRUE	0.011196	19.97344
8697	rs1751663	A	G	A	G	0.055	0.082986	0.017	0.018105	FALSE	FALSE	FALSE	Bg4VnB	0.051419	0.106545	486484	sepsis	TRUE	reported	textfile	0.0123	7.49E-06	1765	M36552.m	TRUE	reported	6zdDpC	2	TRUE	0.011202	19.97206
4768	rs1218709	A	G	A	G	0.0295	0.024269	0.1521	0.161016	FALSE	FALSE	FALSE	mOrnK4	0.018592	0.191786	486484	sepsis	TRUE	reported	textfile	0.0066	7.11E-06	6169	M32795.m	TRUE	reported	7EGxuf	2	TRUE	0.003228	19.97171
3944	rs1076471	T	G	T	G	0.0219	0.013964	0.4349	0.434921	FALSE	FALSE	FALSE	N6UQnQ	0.013866	0.313916	486484	sepsis	TRUE	reported	textfile	0.0049	9.62E-06	7346	M32425.m	TRUE	reported	FEcGHY	2	TRUE	0.002712	19.96999
3364	rs7811011	C	G	C	G	0.0219	-0.03854	0.0471	0.048022	FALSE	TRUE	FALSE	LY4Gus	0.032138	0.23041	486484	sepsis	TRUE	reported	textfile	0.0049	8.09E-06	7268	M27273.m	TRUE	reported	QKITBx	2	TRUE	0.002741	19.96993
5520	rs6798383	C	G	C	G	0.3429	-0.00238	0.9748	0.940742	FALSE	TRUE	FALSE	q32d4E	0.02937	0.935441	486484	sepsis	TRUE	reported	textfile	0.0736	3.21E-06	25	M33178.m	TRUE	reported	cS3BwT	2	TRUE	0.464737	19.9695
4705	rs2868035	A	G	A	G	0.0219	-0.01143	0.3422	0.356453	FALSE	FALSE	FALSE	WlwKaF	0.014272	0.423311	486484	sepsis	TRUE	reported	textfile	0.0049	7.52E-06	6712	M32759.m	TRUE	reported	Ub4yxH	2	TRUE	0.002967	19.96947
4696	rs7858984	A	G	A	G	-0.0219	0.007244	0.3026	0.311714	FALSE	FALSE	FALSE	MV108h	0.014882	0.626435	486484	sepsis	TRUE	reported	textfile	0.0049	8.88E-06	6642	M32758.m	TRUE	reported	btjNXQ	2	TRUE	0.002998	19.96941
8354	rs1988344	T	C	T	C	-0.0362	0.00043	0.9117	0.911777	FALSE	FALSE	FALSE	6VVCe4	0.024124	0.985782	486484	sepsis	TRUE	reported	textfile	0.0081	7.95E-06	5629	M35669.m	TRUE	reported	l0fk8X	2	TRUE	0.003536	19.96608
7325	rs1078714	T	C	T	C	-0.0993	-0.01327	0.7552	0.664712	FALSE	FALSE	FALSE	mm9ksf	0.014474	0.359263	486484	sepsis	TRUE	reported	textfile	0.0222	7.79E-06	954	M34365.m	TRUE	reported	AVwEqD	2	TRUE	0.020541	19.96554
5308	rs1687701	A	G	A	G	-0.3396	0.024217	0.0243	0.093582	FALSE	FALSE	FALSE	q32d4E	0.023453	0.301796	486484	sepsis	TRUE	reported	textfile	0.0729	3.18E-06	25	M33178.m	TRUE	reported	cS3BwT	2	TRUE	0.46468	19.96495
588	rs7898173	A	G	A	G	-0.0143	-0.02582	0.1419	0.136389	FALSE	FALSE	FALSE	ZoqU2j	0.020182	0.200751	486484	sepsis	TRUE	reported	textfile	0.0032	6.81E-06	7333	M01105.m	TRUE	reported	2WxSOH	2	TRUE	0.002716	19.96428
7111	rs6575039	T	C	T	C	0.0456	0.004476	0.7656	0.758617	FALSE	FALSE	FALSE	DfLd6z	0.015971	0.779274	486484	sepsis	TRUE	reported	textfile	0.0102	7.54E-06	1766	M34314.m	TRUE	reported	OgloAQ	2	TRUE	0.011191	19.96352
8254	rs1714270	T	C	T	C	-0.1807	0.02607	0.8725	0.867233	FALSE	FALSE	FALSE	G4plxe	0.020118	0.19502	486484	sepsis	TRUE	reported	textfile	0.0403	7.27E-06	284	M35451.m	TRUE	reported	hNOVMI	2	TRUE	0.066112	19.96351
4278	rs6916798	T	C	T	C	0.0608	0.065757	0.9454	0.953448	FALSE	FALSE	FALSE	ylV4bp	0.032576	0.043533	486484	sepsis	TRUE	reported	textfile	0.0136	7.41E-06	1762	M32632.m	TRUE	reported	hqHJoU	2	TRUE	0.011216	19.96347
6364	rs6432256	T	C	T	C	-0.0228	-0.00151	0.7655	0.767025	FALSE	FALSE	FALSE	WWmDGN	0.016291	0.925919	486484	sepsis	TRUE	reported	textfile	0.0051	7.48E-06	1745	M33627.m	TRUE	reported	2kkEru	2	TRUE	0.011324	19.96325
7768	rs677302	T	C	T	C	0.0228	-0.00109	0.4637	0.475972	FALSE	FALSE	FALSE	G4gUxB	0.013779	0.937105	486484	sepsis	TRUE	reported	textfile	0.0051	7.83E-06	1657	M35114.m	TRUE	reported	o79OO5	2	TRUE	0.011918	19.96204
6377	rs1022894	A	T	A	T	0.0711	0.025056	0.8711	0.836377	FALSE	TRUE	FALSE	pPr1v0	0.018474	0.175023	486484	sepsis	TRUE	reported	textfile	0.0159	7.75E-06	1155	M33637.m	TRUE	reported	Ide1A7	2	TRUE	0.017018	19.96146
2564	rs7088140	T	G	T	G	0.0353	0.019123	0.5864	0.574457	FALSE	FALSE	FALSE	WfyP8n	0.013852	0.167421	486484	sepsis	TRUE	reported	textfile	0.0079	7.26E-06	5899	M18335.m	TRUE	reported	mjljpe	2	TRUE	0.003373	19.95942
8315	rs7583683	A	G	A	G	0.021	-0.03386	0.8612	0.857855	FALSE	FALSE	FALSE	YZlMbv	0.019643	0.084758	486484	sepsis	TRUE	reported	textfile	0.0047	7.24E-06	7302	M35628.m	TRUE	reported	2aP0wZ	2	TRUE	0.002727	19.95832
4772	rs3750078	A	G	A	G	0.021	0.01983	0.5971	0.593595	FALSE	FALSE	FALSE	mOrnK4	0.01392	0.154289	486484	sepsis	TRUE	reported	textfile	0.0047	6.84E-06	6169	M32795.m	TRUE	reported	7EGxuf	2	TRUE	0.003226	19.95731
4163	rs1740074	C	G	C	G	-0.0697	0.016666	0.9615	0.928006	FALSE	TRUE	FALSE	stpu5O	0.027049	0.537799	486484	sepsis	TRUE	reported	textfile	0.0156	8.29E-06	7337	M32557.m	TRUE	reported	0oV8Ss	2	TRUE	0.002713	19.95712
1030	rs7930821	T	C	T	C	0.0666	0.033586	0.0202	0.019978	FALSE	FALSE	FALSE	haWxZg	0.048722	0.49062	486484	sepsis	TRUE	reported	textfile	0.0149	7.53E-06	1759	M01561.m	TRUE	reported	5wm9R3	2	TRUE	0.011231	19.95638
7252	rs1177887	T	C	T	C	-0.063	0.054003	0.9184	0.913246	FALSE	FALSE	FALSE	4t4W6t	0.024365	0.026665	486484	sepsis	TRUE	reported	textfile	0.0141	8.41E-06	5270	M34344.m	TRUE	reported	Rz9mCt	2	TRUE	0.003774	19.95621
8877	rs1708567	A	T	A	T	-0.0295	0.020189	0.1407	0.142614	FALSE	TRUE	FALSE	Nv6gDO	0.01953	0.301245	486484	sepsis	TRUE	reported	textfile	0.0066	7.95E-06	1782	M37004.m	TRUE	reported	RqmQdg	2	TRUE	0.011087	19.95577
2178	rs1153519	A	G	A	G	0.0277	-0.02588	0.8135	0.805275	FALSE	FALSE	FALSE	UokbxN	0.017291	0.134428	486484	sepsis	TRUE	reported	textfile	0.0062	7.48E-06	7357	M15753.m	TRUE	reported	lUGKBi	2	TRUE	0.002706	19.95529
6533	rs1272718	T	G	T	G	-0.0277	-0.03156	0.9165	0.911889	FALSE	FALSE	FALSE	DedZqr	0.024322	0.194373	486484	sepsis	TRUE	reported	textfile	0.0062	8.80E-06	7350	M33822.m	TRUE	reported	ciYOdn	2	TRUE	0.002708	19.95529
4759	rs2596574	A	G	A	G	0.0277	-0.05303	0.0789	0.087579	FALSE	FALSE	FALSE	RDfDIS	0.024179	0.028296	486484	sepsis	TRUE	reported	textfile	0.0062	7.49E-06	6857	M32787.m	TRUE	reported	Hq6nGt	2	TRUE	0.002903	19.9549
8655	rs1075106	A	C	A	C	0.0371	-0.00208	0.7777	0.762769	FALSE	FALSE	FALSE	BFEGyF	0.016082	0.896928	486484	sepsis	TRUE	reported	textfile	0.0083	8.52E-06	1582	M36399.m	TRUE	reported	nhT4FM	2	TRUE	0.012472	19.95456
2195	rs1151659	T	C	T	C	-0.0219	-0.02635	0.2122	0.204146	FALSE	FALSE	FALSE	Bf6uMK	0.017014	0.12145	486484	sepsis	TRUE	reported	textfile	0.0049	8.24E-06	1750	M15778.m	TRUE	reported	jowM2N	2	TRUE	0.011286	19.9526
5447	rs421380	T	C	T	C	0.353	-0.03009	0.9728	0.98392	FALSE	FALSE	FALSE	q32d4E	0.054239	0.579069	486484	sepsis	TRUE	reported	textfile	0.0758	3.24E-06	25	M33178.m	TRUE	reported	cS3BwT	2	TRUE	0.464526	19.95257
7379	rs820860	T	C	T	C	-0.1715	0.011596	0.6111	0.584296	FALSE	FALSE	FALSE	mm9ksf	0.013853	0.402536	486484	sepsis	TRUE	reported	textfile	0.0362	2.10E-06	18	M34365.m	TRUE	reported	AVwEqD	2	TRUE	0.554946	19.95072
5553	rs7674006	T	C	T	C	0.131	-0.01798	0.9415	0.942831	FALSE	FALSE	FALSE	q32d4E	0.029458	0.541639	486484	sepsis	TRUE	reported	textfile	0.0293	7.98E-06	1017	M33178.m	TRUE	reported	cS3BwT	2	TRUE	0.019277	19.95044
5420	rs288438	A	G	A	G	-0.1709	0.058301	0.0435	0.013691	FALSE	FALSE	FALSE	q32d4E	0.059368	0.326087	486484	sepsis	TRUE	reported	textfile	0.0367	3.21E-06	25	M33178.m	TRUE	reported	cS3BwT	2	TRUE	0.464492	19.94986
534	rs2272172	T	C	T	C	-0.0648	0.060487	0.0226	0.02112	FALSE	FALSE	FALSE	wXMWHy	0.048449	0.211856	486484	sepsis	TRUE	reported	textfile	0.0145	7.51E-06	1754	M00584.m	TRUE	reported	7CKn2b	2	TRUE	0.011258	19.94888
3248	rs1868259	A	G	A	G	0.0818	0.003085	0.5541	0.539531	FALSE	FALSE	FALSE	jxdGCf	0.013703	0.821896	486484	sepsis	TRUE	reported	textfile	0.0183	7.67E-06	1207	M22842.m	TRUE	reported	f0L3pZ	2	TRUE	0.016284	19.9473
2955	rs1084752	A	G	A	G	-0.0344	-0.0027	0.3904	0.368818	FALSE	FALSE	FALSE	zndu0r	0.014165	0.848785	486484	sepsis	TRUE	reported	textfile	0.0077	9.16E-06	3422	M21049.m	TRUE	reported	2XpPDU	2	TRUE	0.005799	19.94718
4142	rs1113336	T	C	T	C	-0.0715	-0.0248	0.9007	0.911761	FALSE	FALSE	FALSE	CrVaiQ	0.024177	0.304952	486484	sepsis	TRUE	reported	textfile	0.016	7.62E-06	1766	M32553.m	TRUE	reported	HsKdAI	2	TRUE	0.011181	19.94711
8685	rs1708708	T	C	T	C	0.0143	0.027153	0.975	0.988353	FALSE	FALSE	FALSE	X2e8qc	0.064955	0.675924	486484	sepsis	TRUE	reported	textfile	0.0032	8.47E-06	1763	M36515.m	TRUE	reported	OaWQSH	2	TRUE	0.0112	19.94707
837	rs4750059	C	G	C	G	-0.0134	-0.03357	0.7956	0.747728	FALSE	TRUE	FALSE	2qgDgB	0.015727	0.032784	486484	sepsis	TRUE	reported	textfile	0.003	6.49E-06	7355	M01358.m	TRUE	reported	asmXq0	2	TRUE	0.002705	19.94569
812	rs1801838	T	C	T	C	-0.0469	-0.00492	0.9655	0.886613	FALSE	FALSE	FALSE	J58DI0	0.021568	0.819716	486484	sepsis	TRUE	reported	textfile	0.0105	8.51E-06	7335	M01356.m	TRUE	reported	zk9AMB	2	TRUE	0.002713	19.94567
1516	rs6567408	C	G	C	G	0.0134	0.009432	0.3922	0.383605	FALSE	TRUE	FALSE	rzkxmh	0.01409	0.503234	486484	sepsis	TRUE	reported	textfile	0.003	8.07E-06	6941	M03127.m	TRUE	reported	3lrzhA	2	TRUE	0.002866	19.94536
8561	rs9787093	A	G	A	G	-1.323	0.101887	0.0167	0.012649	FALSE	FALSE	FALSE	aQhCav	0.061394	0.097004	486484	sepsis	TRUE	reported	textfile	0.2938	6.69E-06	122	M36097.m	TRUE	reported	9DJS9j	2	TRUE	0.142521	19.94516
5400	rs2235380	T	C	T	C	0.3385	0.014816	0.9753	0.947523	FALSE	FALSE	FALSE	q32d4E	0.030707	0.62946	486484	sepsis	TRUE	reported	textfile	0.0727	3.24E-06	25	M33178.m	TRUE	reported	cS3BwT	2	TRUE	0.464433	19.94511
1544	rs4694292	T	C	T	C	0.0201	-0.02898	0.1089	0.112002	FALSE	FALSE	FALSE	tApf9z	0.021766	0.183069	486484	sepsis	TRUE	reported	textfile	0.0045	6.44E-06	6515	M03147.m	TRUE	reported	tXdmtV	2	TRUE	0.003053	19.94499
2350	rs1042147	T	C	T	C	-0.0639	-0.0273	0.153	0.175906	FALSE	FALSE	FALSE	NBJ2Do	0.017956	0.128386	486484	sepsis	TRUE	reported	textfile	0.0143	7.68E-06	1739	M17799.m	TRUE	reported	Uz0Wgl	2	TRUE	0.011352	19.94481
[truncated: 514,729 more chars]
