# Supplementary material for: Mendelian randomization analysis reveals causal associations of serum metabolites with sepsis and 28-day mortality
Source: Sci Rep. 2024 May 21;14:11551. doi: 10.1038/s41598-024-58160-1 (PMC11109149; doi:10.1038/s41598-024-58160-1)
Supplement: Supplementary file 2 — Supplementary Table 2. [file 41598_2024_58160_MOESM2_ESM.pdf]

Supplementary table 2. The characteristics of SNPs and their genetic associations with known metabolites and sepsis mortality within 28 days

	SNP	effect_allel	other_allel	effect_allel	other_allel	beta.expos	beta.outco	eaf.exposu	eaf.outcon	remove	palindromi	ambiguous	id.outcome	chr	pos	se.outcom	samplesize	pval.outco	outcome	originalnar	outcome.d	mr_keep.o	data_sourc	se.exposur	pval.expos	samplesize	exposure	mr_keep.e	pval_origin	id.exposur	action	mr_keep	R2	F
1462	rs1119609	A	G	A	G	0.0111	0.024198	0.1128	0.108016	FALSE	FALSE	FALSE	ieu-b-508	10	1.15E+08	0.053776	486484	0.652734	Sepsis (28	Sepsis (28	Sepsis (28	TRUE	igd	0.0025	9.83E-06	7799	M10642.m	TRUE	reported	XKxUVi	2	TRUE	0.002521	19.70854
1463	rs1166419	T	C	T	C	-0.0082	-0.08625	0.592	0.606212	FALSE	FALSE	FALSE	ieu-b-508	18	74107920	0.034109	486484	0.011448	Sepsis (28	Sepsis (28	Sepsis (28	TRUE	igd	0.0016	6.47E-07	7799	M10642.m	TRUE	reported	XKxUVi	2	TRUE	0.003357	26.25889
1464	rs1200170	A	G	A	G	-0.0315	0.096399	0.052	0.036891	FALSE	FALSE	FALSE	ieu-b-508	9	79555787	0.088727	486484	0.277269	Sepsis (28	Sepsis (28	Sepsis (28	TRUE	igd	0.0066	1.89E-06	7799	M10642.m	TRUE	reported	XKxUVi	2	TRUE	0.002912	22.77308
1465	rs1202639	T	C	T	C	-0.014	-0.03449	0.8893	0.876115	FALSE	FALSE	FALSE	ieu-b-508	1	30866084	0.050743	486484	0.496734	Sepsis (28	Sepsis (28	Sepsis (28	TRUE	igd	0.0029	9.43E-07	7799	M10642.m	TRUE	reported	XKxUVi	2	TRUE	0.002979	23.29961
1466	rs1213605	T	C	T	C	-0.0731	0.099007	0.0129	0.0158	FALSE	FALSE	FALSE	ieu-b-508	1	33920374	0.137452	486484	0.471339	Sepsis (28	Sepsis (28	Sepsis (28	TRUE	igd	0.0153	1.75E-06	7799	M10642.m	TRUE	reported	XKxUVi	2	TRUE	0.002918	22.82131
1467	rs1258682	T	C	T	C	0.0129	-0.02625	0.1222	0.120333	FALSE	FALSE	FALSE	ieu-b-508	14	74869044	0.051716	486484	0.611773	Sepsis (28	Sepsis (28	Sepsis (28	TRUE	igd	0.0025	1.62E-07	7799	M10642.m	TRUE	reported	XKxUVi	2	TRUE	0.003402	26.61877
1468	rs1338027	T	C	T	C	-0.0077	0.045743	0.4149	0.41192	FALSE	FALSE	FALSE	ieu-b-508	15	88499185	0.033854	486484	0.176638	Sepsis (28	Sepsis (28	Sepsis (28	TRUE	igd	0.0016	2.90E-06	7799	M10642.m	TRUE	reported	XKxUVi	2	TRUE	0.002961	23.15422
1469	rs1391933	T	G	T	G	-0.0075	-0.01375	0.4399	0.446753	FALSE	FALSE	FALSE	ieu-b-508	3	3003351	0.033653	486484	0.682771	Sepsis (28	Sepsis (28	Sepsis (28	TRUE	igd	0.0016	5.79E-06	7799	M10642.m	TRUE	reported	XKxUVi	2	TRUE	0.002809	21.96702
1470	rs1530919	A	G	A	G	0.0076	0.025721	0.3006	0.302902	FALSE	FALSE	FALSE	ieu-b-508	11	1.2E+08	0.03618	486484	0.477141	Sepsis (28	Sepsis (28	Sepsis (28	TRUE	igd	0.0017	5.92E-06	7799	M10642.m	TRUE	reported	XKxUVi	2	TRUE	0.002556	19.98103
1471	rs1549556	T	C	T	C	-0.0075	0.060677	0.3719	0.383648	FALSE	FALSE	FALSE	ieu-b-508	1	80858494	0.034304	486484	0.076931	Sepsis (28	Sepsis (28	Sepsis (28	TRUE	igd	0.0017	7.95E-06	7799	M10642.m	TRUE	reported	XKxUVi	2	TRUE	0.002489	19.45868
1472	rs1761689	A	T	A	T	-0.0076	-0.01961	0.6964	0.702196	FALSE	TRUE	FALSE	ieu-b-508	14	41810300	0.036584	486484	0.591874	Sepsis (28	Sepsis (28	Sepsis (28	TRUE	igd	0.0017	4.65E-06	7799	M10642.m	TRUE	reported	XKxUVi	2	TRUE	0.002556	19.98103
1473	rs1769589	T	C	T	C	0.011	-0.08534	0.153	0.158668	FALSE	FALSE	FALSE	ieu-b-508	17	995866	0.045808	486484	0.062476	Sepsis (28	Sepsis (28	Sepsis (28	TRUE	igd	0.0025	8.03E-06	7799	M10642.m	TRUE	reported	XKxUVi	2	TRUE	0.002476	19.35504
1474	rs2720692	A	G	A	G	-0.0272	-0.02815	0.0648	0.080402	FALSE	FALSE	FALSE	ieu-b-508	8	1.29E+08	0.061423	486484	0.646726	Sepsis (28	Sepsis (28	Sepsis (28	TRUE	igd	0.006	5.63E-06	7799	M10642.m	TRUE	reported	XKxUVi	2	TRUE	0.002628	20.54584
1475	rs2725779	T	C	T	C	0.0077	-0.08774	0.4523	0.461841	FALSE	FALSE	FALSE	ieu-b-508	4	1.4E+08	0.033608	486484	0.009033	Sepsis (28	Sepsis (28	Sepsis (28	TRUE	igd	0.0017	3.27E-06	7799	M10642.m	TRUE	reported	XKxUVi	2	TRUE	0.002624	20.51031
1476	rs2938330	T	C	T	C	-0.0117	-0.05754	0.8105	0.856821	FALSE	FALSE	FALSE	ieu-b-508	1	1.15E+08	0.047506	486484	0.225813	Sepsis (28	Sepsis (28	Sepsis (28	TRUE	igd	0.0025	2.15E-06	7799	M10642.m	TRUE	reported	XKxUVi	2	TRUE	0.0028	21.89678
1477	rs3781273	A	G	A	G	0.0074	-0.01371	0.5139	0.516759	FALSE	FALSE	FALSE	ieu-b-508	10	95402973	0.033909	486484	0.686016	Sepsis (28	Sepsis (28	Sepsis (28	TRUE	igd	0.0017	8.52E-06	7799	M10642.m	TRUE	reported	XKxUVi	2	TRUE	0.002424	18.94324
1478	rs4436895	C	G	C	G	-0.01	-0.03856	0.252	0.278811	FALSE	TRUE	FALSE	ieu-b-508	2	1.4E+08	0.037368	486484	0.302137	Sepsis (28	Sepsis (28	Sepsis (28	TRUE	igd	0.0022	3.47E-06	7799	M10642.m	TRUE	reported	XKxUVi	2	TRUE	0.002642	20.65586
1479	rs4483514	T	G	T	G	0.009	0.010681	0.357	0.3568	FALSE	FALSE	FALSE	ieu-b-508	10	73710514	0.034832	486484	0.759109	Sepsis (28	Sepsis (28	Sepsis (28	TRUE	igd	0.002	6.80E-06	7799	M10642.m	TRUE	reported	XKxUVi	2	TRUE	0.00259	20.24481
1480	rs4812805	C	G	C	G	-0.0109	0.11024	0.8421	0.856269	FALSE	TRUE	FALSE	ieu-b-508	20	42853466	0.047836	486484	0.021192	Sepsis (28	Sepsis (28	Sepsis (28	TRUE	igd	0.0024	5.88E-06	7799	M10642.m	TRUE	reported	XKxUVi	2	TRUE	0.002638	20.62145
1481	rs4818244	T	C	T	C	0.0136	0.085015	0.0939	0.127076	FALSE	FALSE	FALSE	ieu-b-508	21	42894561	0.050229	486484	0.090544	Sepsis (28	Sepsis (28	Sepsis (28	TRUE	igd	0.003	6.61E-06	7799	M10642.m	TRUE	reported	XKxUVi	2	TRUE	0.002628	20.54584
1482	rs569114	A	G	A	G	-0.0079	0.006463	0.6199	0.626721	FALSE	FALSE	FALSE	ieu-b-508	9	14072370	0.034701	486484	0.852247	Sepsis (28	Sepsis (28	Sepsis (28	TRUE	igd	0.0017	1.99E-06	7799	M10642.m	TRUE	reported	XKxUVi	2	TRUE	0.002761	21.58962
1483	rs6540619	T	C	T	C	0.0076	-0.04236	0.434	0.42855	FALSE	FALSE	FALSE	ieu-b-508	1	2.11E+08	0.033738	486484	0.209321	Sepsis (28	Sepsis (28	Sepsis (28	TRUE	igd	0.0016	3.71E-06	7799	M10642.m	TRUE	reported	XKxUVi	2	TRUE	0.002885	22.55671
1484	rs6557106	T	C	T	C	0.0076	-0.0118	0.6715	0.669266	FALSE	FALSE	FALSE	ieu-b-508	6	1.51E+08	0.035509	486484	0.739561	Sepsis (28	Sepsis (28	Sepsis (28	TRUE	igd	0.0017	5.42E-06	7799	M10642.m	TRUE	reported	XKxUVi	2	TRUE	0.002556	19.98103
1485	rs6828152	T	G	T	G	0.009	0.013327	0.359	0.366409	FALSE	FALSE	FALSE	ieu-b-508	4	1.66E+08	0.034721	486484	0.701111	Sepsis (28	Sepsis (28	Sepsis (28	TRUE	igd	0.002	6.80E-06	7799	M10642.m	TRUE	reported	XKxUVi	2	TRUE	0.00259	20.24481
1486	rs7735310	T	G	T	G	-0.0078	-0.07194	0.7188	0.71342	FALSE	FALSE	FALSE	ieu-b-508	5	55706875	0.037012	486484	0.051921	Sepsis (28	Sepsis (28	Sepsis (28	TRUE	igd	0.0017	3.38E-06	7799	M10642.m	TRUE	reported	XKxUVi	2	TRUE	0.002692	21.0465
1487	rs7859597	A	G	A	G	-0.009	0.020967	0.737	0.727082	FALSE	FALSE	FALSE	ieu-b-508	9	15075185	0.03757	486484	0.576789	Sepsis (28	Sepsis (28	Sepsis (28	TRUE	igd	0.002	6.80E-06	7799	M10642.m	TRUE	reported	XKxUVi	2	TRUE	0.00259	20.24481
1488	rs8043217	A	G	A	G	0.009	0.020307	0.524	0.517314	FALSE	FALSE	FALSE	ieu-b-508	15	99100761	0.033628	486484	0.54594	Sepsis (28	Sepsis (28	Sepsis (28	TRUE	igd	0.0016	2.79E-08	7799	M10642.m	TRUE	reported	XKxUVi	2	TRUE	0.004041	31.63251
1489	rs8128639	A	C	A	C	-0.0113	-0.02275	0.1698	0.230187	FALSE	FALSE	FALSE	ieu-b-508	21	45175571	0.039931	486484	0.568812	Sepsis (28	Sepsis (28	Sepsis (28	TRUE	igd	0.0025	6.51E-06	7799	M10642.m	TRUE	reported	XKxUVi	2	TRUE	0.002613	20.42516
1490	rs947099	A	G	A	G	-0.008	-0.0166	0.3625	0.353475	FALSE	FALSE	FALSE	ieu-b-508	10	31129883	0.034804	486484	0.633467	Sepsis (28	Sepsis (28	Sepsis (28	TRUE	igd	0.0017	1.22E-06	7799	M10642.m	TRUE	reported	XKxUVi	2	TRUE	0.002831	22.13965
1491	rs9529827	T	C	T	C	-0.0397	0.068564	0.9843	0.979332	FALSE	FALSE	FALSE	ieu-b-508	13	71546251	0.120247	486484	0.568544	Sepsis (28	Sepsis (28	Sepsis (28	TRUE	igd	0.0087	5.14E-06	7799	M10642.m	TRUE	reported	XKxUVi	2	TRUE	0.002663	20.81762
1727	rs1073264	T	C	T	C	0.0369	-0.13739	0.0603	0.034601	FALSE	FALSE	FALSE	ieu-b-508	12	90157922	0.091441	486484	0.132958	Sepsis (28	Sepsis (28	Sepsis (28	TRUE	igd	0.0084	1.21E-05	7574	M15630.m	TRUE	reported	sqSK78	2	TRUE	0.002541	19.2921
1728	rs1088794	A	G	A	G	-0.0316	-0.08281	0.1172	0.187278	FALSE	FALSE	FALSE	ieu-b-508	10	91056866	0.043035	486484	0.054314	Sepsis (28	Sepsis (28	Sepsis (28	TRUE	igd	0.0073	1.51E-05	7574	M15630.m	TRUE	reported	sqSK78	2	TRUE	0.002468	18.73328
1729	rs1161741	A	G	A	G	0.0275	-0.0094	0.8761	0.820826	FALSE	FALSE	FALSE	ieu-b-508	13	1.08E+08	0.043574	486484	0.829248	Sepsis (28	Sepsis (28	Sepsis (28	TRUE	igd	0.0064	1.92E-05	7574	M15630.m	TRUE	reported	sqSK78	2	TRUE	0.002432	18.45826
1730	rs1264993	A	G	A	G	-0.0188	-0.00136	0.7072	0.710907	FALSE	FALSE	FALSE	ieu-b-508	4	18409874	0.036864	486484	0.970652	Sepsis (28	Sepsis (28	Sepsis (28	TRUE	igd	0.0043	1.12E-05	7574	M15630.m	TRUE	reported	sqSK78	2	TRUE	0.002517	19.11015
1731	rs1341501	T	C	T	C	0.034	-0.01877	0.69	0.715439	FALSE	FALSE	FALSE	ieu-b-508	2	74122223	0.042407	486484	0.657988	Sepsis (28	Sepsis (28	Sepsis (28	TRUE	igd	0.006	1.46E-08	7574	M15630.m	TRUE	reported	sqSK78	2	TRUE	0.004222	32.10263
1732	rs1342773	A	C	A	C	-0.0328	0.036022	0.0753	0.071542	FALSE	FALSE	FALSE	ieu-b-508	2	51881577	0.064869	486484	0.578691	Sepsis (28	Sepsis (28	Sepsis (28	TRUE	igd	0.0076	1.40E-05	7574	M15630.m	TRUE	reported	sqSK78	2	TRUE	0.002453	18.62112
1733	rs1472473	T	C	T	C	-0.0189	-0.04563	0.2543	0.260026	FALSE	FALSE	FALSE	ieu-b-508	3	3388486	0.038341	486484	0.234041	Sepsis (28	Sepsis (28	Sepsis (28	TRUE	igd	0.0043	1.41E-05	7574	M15630.m	TRUE	reported	sqSK78	2	TRUE	0.002544	19.31399
1734	rs15612	T	C	T	C	0.0187	0.03607	0.3004	0.291379	FALSE	FALSE	FALSE	ieu-b-508	5	33988535	0.036745	486484	0.326286	Sepsis (28	Sepsis (28	Sepsis (28	TRUE	igd	0.0044	1.67E-05	7574	M15630.m	TRUE	reported	sqSK78	2	TRUE	0.002379	18.05773
1735	rs1682385	A	G	A	G	0.0257	0.031842	0.8668	0.865753	FALSE	FALSE	FALSE	ieu-b-508	1	1.85E+08	0.04949	486484	0.519961	Sepsis (28	Sepsis (28	Sepsis (28	TRUE	igd	0.0059	1.48E-05	7574	M15630.m	TRUE	reported	sqSK78	2	TRUE	0.002499	18.96914
1736	rs1695062	A	C	A	C	-0.0385	0.031033	0.062	0.026401	FALSE	FALSE	FALSE	ieu-b-508	16	51995864	0.104066	486484	0.765545	Sepsis (28	Sepsis (28	Sepsis (28	TRUE	igd	0.0087	1.04E-05	7574	M15630.m	TRUE	reported	sqSK78	2	TRUE	0.002579	19.578
1737	rs2268365	T	C	T	C	0.0289	-0.022	0.8731	0.877716	FALSE	FALSE	FALSE	ieu-b-508	2	1.7E+08	0.051022	486484	0.666336	Sepsis (28	Sepsis (28	Sepsis (28	TRUE	igd	0.0059	1.11E-06	7574	M15630.m	TRUE	reported	sqSK78	2	TRUE	0.003158	23.98706
1738	rs4593503	T	C	T	C	0.0262	-0.01674	0.1143	0.11523	FALSE	FALSE	FALSE	ieu-b-508	8	1.29E+08	0.052204	486484	0.748418	Sepsis (28	Sepsis (28	Sepsis (28	TRUE	igd	0.006	1.25E-05	7574	M15630.m	TRUE	reported	sqSK78	2	TRUE	0.002511	19.06274
1739	rs4671125	A	C	A	C	0.0188	0.042024	0.5802	0.579808	FALSE	FALSE	FALSE	ieu-b-508	2	65542416	0.033873	486484	0.214741	Sepsis (28	Sepsis (28	Sepsis (28	TRUE	igd	0.0043	1.02E-05	7574	M15630.m	TRUE	reported	sqSK78	2	TRUE	0.002517	19.11015
1740	rs6732369	A	G	A	G	0.0219	0.006564	0.7778	0.774759	FALSE	FALSE	FALSE	ieu-b-508	2	73271874	0.040061	486484	0.869851	Sepsis (28	Sepsis (28	Sepsis (28	TRUE	igd	0.005	1.19E-05	7574	M15630.m	TRUE	reported	sqSK78	2	TRUE	0.002527	19.17933
1741	rs6817090	T	C	T	C	0.023	0.04882	0.8347	0.843593	FALSE	FALSE	FALSE	ieu-b-508	4	1.57E+08	0.046043	486484	0.288993	Sepsis (28	Sepsis (28	Sepsis (28	TRUE	igd	0.0052	1.01E-05	7574	M15630.m	TRUE	reported	sqSK78	2	TRUE	0.002576	19.55844
1742	rs6836758	A	G	A	G	-0.0184	0.014116	0.3079	0.29987	FALSE	FALSE	FALSE	ieu-b-508	4	1.25E+08	0.036359	486484	0.697839	Sepsis (28	Sepsis (28	Sepsis (28	TRUE	igd	0.0043	1.50E-05	7574	M15630.m	TRUE	reported	sqSK78	2	TRUE	0.002412	18.3056
1743	rs7309949	A	G	A	G	-0.132	-0.04352	0.0184	0.015071	FALSE	FALSE	FALSE	ieu-b-508	12	1.28E+08	0.140731	486484	0.757138	Sepsis (28	Sepsis (28	Sepsis (28	TRUE	igd	0.0277	1.93E-06	7574	M15630.m	TRUE	reported	sqSK78	2	TRUE	0.002989	22.7025
1744	rs7525711	A	G	A	G	-0.0186	0.023648	0.7081	0.710006	FALSE	FALSE	FALSE	ieu-b-508	1	2.01E+08	0.036795	486484	0.52042	Sepsis (28	Sepsis (28	Sepsis (28	TRUE	igd	0.0043	1.59E-05	7574	M15630.m	TRUE	reported	sqSK78	2	TRUE	0.002464	18.70571
1745	rs7557275	A	G	A	G	-0.0362	-0.06919	0.3196	0.300508	FALSE	FALSE	FALSE	ieu-b-508	2	1.18E+08	0.036335	486484	0.056883	Sepsis (28	Sepsis (28	Sepsis (28	TRUE	igd	0.0083	1.28E-05	7574	M15630.m	TRUE	reported	sqSK78	2	TRUE	0.002505	19.01719
1746	rs7585004	C	G	C	G	-0.1987	-0.03011	0.2303	0.235485	FALSE	TRUE	FALSE	ieu-b-508	2	73666645	0.039349	486484	0.4442	Sepsis (28	Sepsis (28	Sepsis (28	TRUE	igd	0.0048	1.00E-200	7574	M15630.m	TRUE	reported	sqSK78	2	TRUE	0.184505	1713.163
1747	rs9531784	T	C	T	C	-0.0216	-0.02501	0.7876	0.777481	FALSE	FALSE	FALSE	ieu-b-508	13	85982098	0.040551	486484	0.537347	Sepsis (28	Sepsis (28	Sepsis (28	TRUE	igd	0.005	1.49E-05	7574	M15630.m	TRUE	reported	sqSK78	2	TRUE	0.002458	18.65747
1748	rs9610113	A	G	A	G	-0.0208	0.023885	0.3153	0.314188	FALSE	FALSE	FALSE	ieu-b-508	22	35166990	0.035981	486484	0.506806	Sepsis (28	Sepsis (28	Sepsis (28	TRUE	igd	0.0043	1.25E-06	7574	M15630.m	TRUE	reported	sqSK78	2	TRUE	0.00308	23.39242
3338	rs1076210	T	G	T	G	-0.0153	0.033347	0.4391	0.442771	FALSE	FALSE	FALSE	ieu-b-508	10	68493081	0.033608	486484	0.321074	Sepsis (28	Sepsis (28	Sepsis (28	TRUE	igd	0.0035	1.65E-05	7796	M32412.m	TRUE	reported	mD0ZtL	2	TRUE	0.002445	19.10449
3339	rs1084814	A	G	A	G	-0.1354	0.023896	0.9825	0.992814	FALSE	FALSE	FALSE	ieu-b-508	12	1.23E+08	0.196482	486484	0.903201	Sepsis (28	Sepsis (28	Sepsis (28	TRUE	igd	0.0311	1.33E-05	7796	M32412.m	TRUE	reported	mD0ZtL	2	TRUE	0.002425	18.94982
3340	rs1084983	T	C	T	C	0.0499	0.064028	0.9124	0.907592	FALSE	FALSE	FALSE	ieu-b-508	12	1.21E+08	0.057638	486484	0.266624	Sepsis (28	Sepsis (28	Sepsis (28	TRUE	igd	0.0067	1.06E-13	7796	M32412.m	TRUE	reported	mD0ZtL	2	TRUE	0.007065	55.45492
3341	rs1109732	A	G	A	G	-0.0606	-0.14592	0.9753	0.982642	FALSE	FALSE	FALSE	ieu-b-508	12	1.03E+08	0.128174	486484	0.254948	Sepsis (28	Sepsis (28	Sepsis (28	TRUE	igd	0.014	1.47E-05	7796	M32412.m	TRUE	reported	mD0ZtL	2	TRUE	0.002398	18.73172
3342	rs1171617	T	G	T	G	0.0358	-0.02804	0.7771	0.767566	FALSE	FALSE	FALSE	ieu-b-508	10	61467182	0.039463	486484	0.477339	Sepsis (28	Sepsis (28	Sepsis (28	TRUE	igd	0.0047	1.61E-14	7796	M32412.m	TRUE	reported	mD0ZtL	2	TRUE	0.007387	58.00413
3343	rs1202591	T	C	T	C	0.0228	0.07551	0.8498	0.856363	FALSE	FALSE	FALSE	ieu-b-508	1	21002699	0.047812	486484	0.114267	Sepsis (28	Sepsis (28	Sepsis (28	TRUE	igd	0.0053	1.76E-05	7796	M32412.m	TRUE	reported	mD0ZtL	2	TRUE	0.002368	18.50148
3344	rs1225514	A	G	A	G	-0.0272	0.055538	0.907	0.900081	FALSE	FALSE	FALSE	ieu-b-508	10	1.14E+08	0.055988	486484	0.321213	Sepsis (28	Sepsis (28	Sepsis (28	TRUE	igd	0.0062	1.15E-05	7796	M32412.m	TRUE	reported	mD0ZtL	2	TRUE	0.002463	19.24168
3345	rs1225752	T	C	T	C	0.0248	-0.03476	0.8739	0.876221	FALSE	FALSE	FALSE	ieu-b-508	10	87928843	0.051132	486484	0.496659	Sepsis (28	Sepsis (28	Sepsis (28	TRUE	igd	0.0058	1.74E-05	7796	M32412.m	TRUE	reported	mD0ZtL	2	TRUE	0.00234	18.27831
3346	rs1256268	A	C	A	C	-0.036	0.019439	0.9454	0.940604	FALSE	FALSE	FALSE	ieu-b-508	1	2.21E+08	0.070896	486484	0.783942	Sepsis (28	Sepsis (28	Sepsis (28	TRUE	igd	0.0084	1.74E-05	7796	M32412.m	TRUE	reported	mD0ZtL	2	TRUE	0.00235	18.36263
3347	rs1336584	T	C	T	C	-0.018	0.016155	0.4071	0.416143	FALSE	FALSE	FALSE	ieu-b-508	1	88656688	0.033985	486484	0.634533	Sepsis (28	Sepsis (28	Sepsis (28	TRUE	igd	0.0042	1.91E-05	7796	M32412.m	TRUE	reported	mD0ZtL	2	TRUE	0.00235	18.36263
3348	rs1469231	A	G	A	G	-0.0188	0.02985	0.2917	0.288098	FALSE	FALSE	FALSE	ieu-b-508	7	37574668	0.037032	486484	0.420204	Sepsis (28	Sepsis (28	Sepsis (28	TRUE	igd	0.0043	1.00E-05	7796	M32412.m	TRUE	reported	mD0ZtL	2	TRUE	0.002446	19.11029
3349	rs1557852	A	G	A	G	-0.0181	-0.02048	0.3251	0.325302	FALSE	FALSE	FALSE	ieu-b-508	5	1.32E+08	0.035531	486484	0.564442	Sepsis (28	Sepsis (28	Sepsis (28	TRUE	igd	0.0042	1.53E-05	7796	M32412.m	TRUE	reported	mD0ZtL	2	TRUE	0.002377	18.56723
3350	rs1695005	A	G	A	G	0.0376	0.128462	0.9498	0.943482	FALSE	FALSE	FALSE	ieu-b-508	12	1.21E+08	0.071804	486484	0.073604	Sepsis (28	Sepsis (28	Sepsis (28	TRUE	igd	0.0087	1.50E-05	7796	M32412.m	TRUE	reported	mD0ZtL	2	TRUE	0.00239	18.6735
3351	rs1701010	C	G	C	G	-0.0382	0.011554	0.9521	0.941747	FALSE	TRUE	FALSE	ieu-b-508	1	2.22E+08	0.071583	486484	0.871775	Sepsis (28	Sepsis (28	Sepsis (28	TRUE	igd	0.0087	1.09E-05	7796	M32412.m	TRUE	reported	mD0ZtL	2	TRUE	0.002467	19.27422
3352	rs1705008	T	C	T	C	-0.0413	-0.06489	0.9582	0.959545	FALSE	FALSE	FALSE	ieu-b-508	2	59514159	0.085344	486484	0.447067	Sepsis (28	Sepsis (28	Sepsis (28	TRUE	igd	0.0096	1.90E-05	7796	M32412.m	TRUE	reported	mD0ZtL	2	TRUE	0.002368	18.50317
3353	rs1750767	T	C	T	C	-0.0512	0.021471	0.9538	0.946585	FALSE	FALSE	FALSE	ieu-b-508	12	1.21E+08	0.07415	486484	0.772152	Sepsis (28	Sepsis (28	Sepsis (28	TRUE	igd	0.0096	1.03E-07	7796	M32412.m	TRUE	reported	mD0ZtL	2	TRUE	0.003635	28.43715
3354	rs1768620	T	C	T	C	-0.0518	-0.2229	0.9707	0.971043	FALSE	FALSE	FALSE	ieu-b-508	10	28831564	0.099292	486484	0.024778	Sepsis (28	Sepsis (28	Sepsis (28	TRUE	igd	0.0118	1.06E-05	7796	M32412.m	TRUE	reported	mD0ZtL	2	TRUE	0.002466	19.26567
3355	rs1873745	A	G	A	G	-0.0195	0.074792	0.7904	0.789844	FALSE	FALSE	FALSE	ieu-b-508	8	96446999	0.041072	486484	0.068604	Sepsis (28	Sepsis (28	Sepsis (28	TRUE	igd	0.0044	1.01E-05	7796	M32412.m	TRUE	reported	mD0ZtL	2	TRUE	0.002513	19.63597
3356	rs1955919	A	G	A	G	-0.0467	0.067952	0.9663	0.958651	FALSE	FALSE	FALSE	ieu-b-508	1	1.16E+08	0.085187	486484	0.425055	Sepsis (28	Sepsis (28	Sepsis (28	TRUE	igd	0.0106	1.13E-05	7796	M32412.m	TRUE	reported	mD0ZtL	2	TRUE	0.002484	19.40486
3357	rs1957910	A	G	A	G	0.0376	-0.09271	0.9466	0.928051	FALSE	FALSE	FALSE	ieu-b-508	14	61861304	0.064564	486484	0.151029	Sepsis (28	Sepsis (28	Sepsis (28	TRUE	igd	0.0088	1.96E-05	7796	M32412.m	TRUE	reported	mD0ZtL	2	TRUE	0.002336	18.25151
3358	rs217272	T	C	T	C	0.0192	0.001891	0.7664	0.757901	FALSE	FALSE	FALSE	ieu-b-508	5	1.37E+08	0.038992	486484	0.961316	Sepsis (28	Sepsis (28	Sepsis (28	TRUE	igd	0.0044	1.02E-05	7796	M32412.m	TRUE	reported	mD0ZtL	2	TRUE	0.002436	19.03644
3359	rs2469211	A	T	A	T	-0.0342	-0.0558	0.8921	0.89144	FALSE	TRUE	FALSE	ieu-b-508	15	76599892	0.053703	486484	0.298755	Sepsis (28	Sepsis (28	Sepsis (28	TRUE	igd	0.006	1.06E-08	7796	M32412.m	TRUE	reported	mD0ZtL	2	TRUE	0.00415	32.48166
3360	rs2631693	C	G	C	G	-0.0233	-0.0285	0.8664	0.86573	FALSE	TRUE	FALSE	ieu-b-508	15	39989486	0.04892	486484	0.560199	Sepsis (28	Sepsis (28	Sepsis (28	TRUE	igd	0.0053	1.14E-05	7796	M32412.m	TRUE	reported	mD0ZtL	2	TRUE	0.002473	19.32185
3361	rs2708104	T	G	T	G	0.0464	0.021135	0.3881	0.38549	FALSE	FALSE	FALSE	ieu-b-508	12	1.21E+08	0.034477	486484	0.53986	Sepsis (28	Sepsis (28	Sepsis (28	TRUE	igd	0.0042	1.26E-28	7796	M32412.m	TRUE	reported	mD0ZtL	2	TRUE	0.015414	122.0186
3362	rs278136	T	C	T	C	-0.0259	0.026998	0.234	0.231581	FALSE	FALSE	FALSE	ieu-b-508	12	1.2E+08	0.039632	486484	0.495725	Sepsis (28	Sepsis (28	Sepsis (28	TRUE	igd	0.0043	1.96E-09	7796	M32412.m	TRUE	reported	mD0ZtL	2	TRUE	0.004632	36.2703
3363	rs3767512	A	G	A	G	0.0625	0.145331	0.0178	0.019585	FALSE	FALSE	FALSE	ieu-b-508	1	2.01E+08	0.120864	486484	0.229197	Sepsis (28	Sepsis (28	Sepsis (28	TRUE	igd	0.0146	1.91E-05	7796	M32412.m	TRUE	reported	mD0ZtL	2	TRUE	0.002345	18.32074
3364	rs4146382	T	C	T	C	-0.0186	-0.05287	0.6883	0.69113	FALSE	FALSE	FALSE	ieu-b-508	2	1.3E+08	0.036158	486484	0.143706	Sepsis (28	Sepsis (28	Sepsis (28	TRUE	igd	0.0043	1.25E-05	7796	M32412.m	TRUE	reported	mD0ZtL	2	TRUE	0.002394	18.70585
3365	rs4943508	T	C	T	C	0.0153	0.018084	0.5497	0.549579	FALSE	FALSE	FALSE	ieu-b-508	13	37984354	0.033575	486484	0.590152	Sepsis (28	Sepsis (28	Sepsis (28	TRUE	igd	0.0035	1.38E-05	7796	M32412.m	TRUE	reported	mD0ZtL	2	TRUE	0.002445	19.10449
3366	rs646454	T	C	T	C	0.0257	0.026264	0.8827	0.881315	FALSE	FALSE	FALSE	ieu-b-508	3	20527529	0.051608	486484	0.610814	Sepsis (28	Sepsis (28	Sepsis (28	TRUE	igd	0.0059	1.17E-05	7796	M32412.m	TRUE	reported	mD0ZtL	2	TRUE	0.002428	18.96928
3367	rs6468765	T	C	T	C	0.0156	0.045046	0.5426	0.540309	FALSE	FALSE	FALSE	ieu-b-508	8	1.02E+08	0.033769	486484	0.182228	Sepsis (28	Sepsis (28	Sepsis (28	TRUE	igd	0.0035	1.02E-05	7796	M32412.m	TRUE	reported	mD0ZtL	2	TRUE	0.002542	19.86103
3368	rs6496996	A	G	A	G	0.0197	0.051259	0.2007	0.203346	FALSE	FALSE	FALSE	ieu-b-508	15	93402496	0.041493	486484	0.216697	Sepsis (28	Sepsis (28	Sepsis (28	TRUE	igd	0.0045	1.05E-05	7796	M32412.m	TRUE	reported	mD0ZtL	2	TRUE	0.002452	19.16002
3369	rs696337	T	C	T	C	-0.1146	0.067098	0.5507	0.562886	FALSE	FALSE	FALSE	ieu-b-508	12	1.21E+08	0.033648	486484	0.046141	Sepsis (28	Sepsis (28	Sepsis (28	TRUE	igd	0.0034	1.00E-200	7796	M32412.m	TRUE	reported	mD0ZtL	2	TRUE	0.127192	1135.795
3370	rs7295193	T	C	T	C	-0.0288	0.053336	0.0835	0.083847	FALSE	FALSE	FALSE	ieu-b-508	12	44804533	0.060529	486484	0.378227	Sepsis (28	Sepsis (28	Sepsis (28	TRUE	igd	0.0067	1.58E-05	7796	M32412.m	TRUE	reported	mD0ZtL	2	TRUE	0.002364	18.47243
3371	rs7303401	A	T	A	T	-0.0692	-0.05123	0.1111	0.109882	FALSE	TRUE	FALSE	ieu-b-508	12	1.21E+08	0.053356	486484	0.336984	Sepsis (28	Sepsis (28	Sepsis (28	TRUE	igd	0.006	1.38E-30	7796	M32412.m	TRUE	reported	mD0ZtL	2	TRUE	0.016776	132.9837
3372	rs7312642	T	G	T	G	-0.0362	-0.01276	0.0717	0.074922	FALSE	FALSE	FALSE	ieu-b-508	12	1.22E+08	0.063403	486484	0.840512	Sepsis (28	Sepsis (28	Sepsis (28	TRUE	igd	0.0076	1.76E-06	7796	M32412.m	TRUE	reported	mD0ZtL	2	TRUE	0.002902	22.68185
3373	rs7954772	A	T	A	T	0.0182	0.005037	0.3805	0.373562	FALSE	TRUE	FALSE	ieu-b-508	12	47218144	0.03485	486484	0.885073	Sepsis (28	Sepsis (28	Sepsis (28	TRUE	igd	0.0042	1.22E-05	7796	M32412.m	TRUE	reported	mD0ZtL	2	TRUE	0.002403	18.77296
3374	rs9657933	A	C	A	C	0.0442	0.04608	0.9392	0.90581	FALSE	FALSE	FALSE	ieu-b-508	12	1.21E+08	0.058283	486484	0.429162	Sepsis (28	Sepsis (28	Sepsis (28	TRUE	igd	0.0091	1.05E-06	7796	M32412.m	TRUE	reported	mD0ZtL	2	TRUE	0.003017	23.58578
5725	rs1012820	A	G	A	G	-0.028	-0.01964	0.7856	0.774135	FALSE	FALSE	FALSE	ieu-b-508	10	64195873	0.039898	486484	0.622591	Sepsis (28	Sepsis (28	Sepsis (28	TRUE	igd	0.0065	1.85E-05	7796	M33507.m	TRUE	reported	4sCS0c	2	TRUE	0.002375	18.55145
5726	rs1077797	T	C	T	C	0.0225	0.004499	0.6647	0.660997	FALSE	FALSE	FALSE	ieu-b-508	12	99777018	0.035358	486484	0.898746	Sepsis (28	Sepsis (28	Sepsis (28	TRUE	igd	0.0051	1.19E-05	7500	M33507.m	TRUE	reported	4sCS0c	2	TRUE	0.002588	19.45848
5727	rs1087386	A	G	A	G	0.0261	-0.01616	0.1858	0.183552	FALSE	FALSE	FALSE	ieu-b-508	1	76487865	0.043172	486484	0.708096	Sepsis (28	Sepsis (28	Sepsis (28	TRUE	igd	0.0059	1.01E-05	7500	M33507.m	TRUE	reported	4sCS0c	2	TRUE	0.002602	19.56416
5728	rs1153987	A	G	A	G	-0.2222	0.038924	0.044	0.060305	FALSE	FALSE	FALSE	ieu-b-508	10	1E+08	0.070964	486484	0.58334	Sepsis (28	Sepsis (28	Sepsis (28	TRUE	igd	0.013	1.07E-65	7500	M33507.m	TRUE	reported	4sCS0c	2	TRUE	0.037492	292.0691
5729	rs1181637	T	C	T	C	0.1273	-0.31343	0.0299	0.015652	FALSE	FALSE	FALSE	ieu-b-508	10	1E+08	0.148338	486484	0.034607	Sepsis (28	Sepsis (28	Sepsis (28	TRUE	igd	0.0265	1.63E-06	7500	M33507.m	TRUE	reported	4sCS0c	2	TRUE	0.003067	23.07009
5730	rs1188211	A	T	A	T	-0.022	0.011475	0.669	0.649683	FALSE	TRUE	FALSE	ieu-b-508	14	56503838	0.035227	486484	0.74461	Sepsis (28	Sepsis (28	Sepsis (28	TRUE	igd	0.0051	1.63E-05	7500	M33507.m	TRUE	reported	4sCS0c	2	TRUE	0.002475	18.60327
5731	rs1205206	A	C	A	C	0.0223	-0.01915	0.6572	0.650589	FALSE	FALSE	FALSE	ieu-b-508	19	17241649	0.034945	486484	0.583708	Sepsis (28	Sepsis (28	Sepsis (28	TRUE	igd	0.0052	1.76E-05	7500	M33507.m	TRUE	reported	4sCS0c	2	TRUE	0.002446	18.386
5732	rs1221039	A	C	A	C	-0.0443	0.032885	0.0621	0.082745	FALSE	FALSE	FALSE	ieu-b-508	6	1.54E+08	0.061034	486484	0.590029	Sepsis (28	Sepsis (28	Sepsis (28	TRUE	igd	0.0103	1.63E-05	7500	M33507.m	TRUE	reported	4sCS0c	2	TRUE	0.00246	18.49342
5733	rs1246087	T	C	T	C	0.0263	-0.015	0.6114	0.605659	FALSE	FALSE	FALSE	ieu-b-508	19	33356891	0.034159	486484	0.660639	Sepsis (28	Sepsis (28	Sepsis (28	TRUE	igd	0.0051	1.85E-07	7500	M33507.m	TRUE	reported	4sCS0c	2	TRUE	0.003533	26.58614
5734	rs1325377	C	G	C	G	-0.0225	-0.01434	0.669	0.676902	FALSE	TRUE	FALSE	ieu-b-508	8	20071770	0.035737	486484	0.688313	Sepsis (28	Sepsis (28	Sepsis (28	TRUE	igd	0.0051	1.05E-05	7500	M33507.m	TRUE	reported	4sCS0c	2	TRUE	0.002588	19.45848
5735	rs1365	A	C	A	C	0.0752	0.005495	0.9458	0.942435	FALSE	FALSE	FALSE	ieu-b-508	10	1E+08	0.072355	486484	0.939468	Sepsis (28	Sepsis (28	Sepsis (28	TRUE	igd	0.0101	1.16E-13	7500	M33507.m	TRUE	reported	4sCS0c	2	TRUE	0.007337	55.42135
5736	rs1392989	T	G	T	G	0.035	0.065878	0.861	0.864978	FALSE	FALSE	FALSE	ieu-b-508	15	71921743	0.053636	486484	0.219355	Sepsis (28	Sepsis (28	Sepsis (28	TRUE	igd	0.008	1.21E-05	7500	M33507.m	TRUE	reported	4sCS0c	2	TRUE	0.002546	19.13552
5737	rs1491241	T	C	T	C	0.0255	0.068187	0.7825	0.78285	FALSE	FALSE	FALSE	ieu-b-508	4	1.01E+08	0.040528	486484	0.092476	Sepsis (28	Sepsis (28	Sepsis (28	TRUE	igd	0.0059	1.69E-05	7500	M33507.m	TRUE	reported	4sCS0c	2	TRUE	0.002484	18.675
5738	rs1713453	A	G	A	G	-0.0408	0.01425	0.1563	0.153546	FALSE	FALSE	FALSE	ieu-b-508	10	5246085	0.046305	486484	0.758289	Sepsis (28	Sepsis (28	Sepsis (28	TRUE	igd	0.0068	1.52E-09	7500	M33507.m	TRUE	reported	4sCS0c	2	TRUE	0.004777	35.9904
5739	rs1862262	T	G	T	G	0.0262	-0.00368	0.1903	0.196931	FALSE	FALSE	FALSE	ieu-b-508	19	13564788	0.042103	486484	0.930372	Sepsis (28	Sepsis (28	Sepsis (28	TRUE	igd	0.0061	1.53E-05	7500	M33507.m	TRUE	reported	4sCS0c	2	TRUE	0.002454	18.44281
5740	rs2274243	T	C	T	C	0.2313	-0.05506	0.7389	0.74274	FALSE	FALSE	FALSE	ieu-b-508	10	1E+08	0.038143	486484	0.148843	Sepsis (28	Sepsis (28	Sepsis (28	TRUE	igd	0.0055	1.00E-200	7500	M33507.m	TRUE	reported	4sCS0c	2	TRUE	0.190815	1768.113
5741	rs2754284	T	C	T	C	0.0218	-0.08906	0.4096	0.408408	FALSE	FALSE	FALSE	ieu-b-508	20	55567017	0.034413	486484	0.009656	Sepsis (28	Sepsis (28	Sepsis (28	TRUE	igd	0.0051	1.75E-05	7500	M33507.m	TRUE	reported	4sCS0c	2	TRUE	0.00243	18.26656
5742	rs2777878	T	C	T	C	0.0289	0.004994	0.1576	0.167524	FALSE	FALSE	FALSE	ieu-b-508	9	72015701	0.044577	486484	0.910803	Sepsis (28	Sepsis (28	Sepsis (28	TRUE	igd	0.0067	1.39E-05	7500	M33507.m	TRUE	reported	4sCS0c	2	TRUE	0.002475	18.60074
5743	rs3851647	A	G	A	G	-0.0217	-0.03604	0.4724	0.459601	FALSE	FALSE	FALSE	ieu-b-508	12	1.17E+08	0.03353	486484	0.282492	Sepsis (28	Sepsis (28	Sepsis (28	TRUE	igd	0.005	1.52E-05	7500	M33507.m	TRUE	reported	4sCS0c	2	TRUE	0.002505	18.83058
5744	rs4761	T	C	T	C	0.0228	-0.03503	0.6254	0.639199	FALSE	FALSE	FALSE	ieu-b-508	14	24641526	0.03524	486484	0.320214	Sepsis (28	Sepsis (28	Sepsis (28	TRUE	igd	0.0053	1.55E-05	7500	M33507.m	TRUE	reported	4sCS0c	2	TRUE	0.002461	18.50129
5745	rs4894502	A	C	A	C	0.0224	0.016096	0.6861	0.686872	FALSE	FALSE	FALSE	ieu-b-508	3	1.76E+08	0.036063	486484	0.655354	Sepsis (28	Sepsis (28	Sepsis (28	TRUE	igd	0.0051	1.14E-05	7500	M33507.m	TRUE	reported	4sCS0c	2	TRUE	0.002566	19.2859
5746	rs4919141	A	G	A	G	0.0282	-0.03022	0.7786	0.771427	FALSE	FALSE	FALSE	ieu-b-508	10	99561960	0.039749	486484	0.447072	Sepsis (28	Sepsis (28	Sepsis (28	TRUE	igd	0.0059	1.73E-06	7500	M33507.m	TRUE	reported	4sCS0c	2	TRUE	0.003037	22.83907
5747	rs4919234	T	G	T	G	0.0288	-0.00471	0.3296	0.31577	FALSE	FALSE	FALSE	ieu-b-508	10	1E+08	0.036041	486484	0.89605	Sepsis (28	Sepsis (28	Sepsis (28	TRUE	igd	0.0051	1.52E-08	7500	M33507.m	TRUE	reported	4sCS0c	2	TRUE	0.004234	31.88077
5748	rs6565153	A	C	A	C	-0.0223	-0.02229	0.3309	0.336337	FALSE	FALSE	FALSE	ieu-b-508	16	83232123	0.036101	486484	0.536933	Sepsis (28	Sepsis (28	Sepsis (28	TRUE	igd	0.0051	1.27E-05	7500	M33507.m	TRUE	reported	4sCS0c	2	TRUE	0.002543	19.11409
5749	rs6584214	T	C	T	C	-0.0435	0.012855	0.0633	0.060516	FALSE	FALSE	FALSE	ieu-b-508	10	1E+08	0.070166	486484	0.854634	Sepsis (28	Sepsis (28	Sepsis (28	TRUE	igd	0.01	1.35E-05	7500	M33507.m	TRUE	reported	4sCS0c	2	TRUE	0.002517	18.91745
5750	rs7095491	T	C	T	C	0.0218	-0.00261	0.4877	0.475986	FALSE	FALSE	FALSE	ieu-b-508	10	1.01E+08	0.033362	486484	0.937558	Sepsis (28	Sepsis (28	Sepsis (28	TRUE	igd	0.005	1.27E-05	7500	M33507.m	TRUE	reported	4sCS0c	2	TRUE	0.002528	19.00453
5751	rs7203786	A	G	A	G	-0.027	0.007699	0.358	0.375713	FALSE	FALSE	FALSE	ieu-b-508	16	84055898	0.034709	486484	0.824445	Sepsis (28	Sepsis (28	Sepsis (28	TRUE	igd	0.0062	1.48E-05	7500	M33507.m	TRUE	reported	4sCS0c	2	TRUE	0.002522	18.95956
5752	rs7505827	C	G	C	G	-0.0251	0.021296	0.2959	0.320402	FALSE	TRUE	FALSE	ieu-b-508	18	4092370	0.035762	486484	0.551518	Sepsis (28	Sepsis (28	Sepsis (28	TRUE	igd	0.0057	1.20E-05	7500	M33507.m	TRUE	reported	4sCS0c	2	TRUE	0.002579	19.38572
5753	rs942800	A	C	A	C	0.0362	0.021005	0.4265	0.440165	FALSE	FALSE	FALSE	ieu-b-508	10	1E+08	0.035273	486484	0.55151	Sepsis (28	Sepsis (28	Sepsis (28	TRUE	igd	0.0053	1.06E-11	7500	M33507.m	TRUE	reported	4sCS0c	2	TRUE	0.006182	46.63904
5754	rs9857669	T	C	T	C	0.0297	0.108133	0.145	0.161889	FALSE	FALSE	FALSE	ieu-b-508	3	45806066	0.045205	486484	0.016755	Sepsis (28	Sepsis (28	Sepsis (28	TRUE	igd	0.0068	1.25E-05	7500	M33507.m	TRUE	reported	4sCS0c	2	TRUE	0.002537	19.07125
4325	rs7969341	A	G	A	G	-0.2801	0.072243	0.8106	0.837659	FALSE	FALSE	FALSE	ieu-b-508	12	21385604	0.045255	486484	0.110408	Sepsis (28	Sepsis (28	Sepsis (28	TRUE	igd	0.0078	1.00E-200	6250	M32846.m	TRUE	reported	IwF2Yy	2	TRUE	0.171038	1289.134
1332	rs887829	T	C	T	C	0.1131	-0.01303	0.3411	0.316055	FALSE	FALSE	FALSE	ieu-b-508	2	2.35E+08	0.035821	486484	0.716143	Sepsis (28	Sepsis (28	Sepsis (28	TRUE	igd	0.0041	2.50E-168	6252	M02137.m	TRUE	reported	iRGqBE	2	TRUE	0.108507	760.709
6410	rs13538	A	G	A	G	0.1186	0.0227	0.7828	0.779077	FALSE	FALSE	FALSE	ieu-b-508	2	73871665	0.040209	486484	0.572373	Sepsis (28	Sepsis (28	Sepsis (28	TRUE	igd	0.0044	7.89E-157	7132	M34040.m	TRUE	reported	sJBy3i	2	TRUE	0.092453	726.3438
7447	rs3738934	T	C	T	C	-0.1056	-0.01438	0.6231	0.629287	FALSE	FALSE	FALSE	ieu-b-508	2	2.11E+08	0.034521	486484	0.676948	Sepsis (28	Sepsis (28	Sepsis (28	TRUE	igd	0.0043	1.21E-134	6234	M35189.m	TRUE	reported	54XZFj	2	TRUE	0.08821	602.9087
4492	rs1020689	T	C	T	C	0.0399	0.026254	0.78	0.779049	FALSE	FALSE	FALSE	ieu-b-508	2	73900900	0.040163	486484	0.513319	Sepsis (28	Sepsis (28	Sepsis (28	TRUE	igd	0.0017	7.74E-123	7362	M33132.m	TRUE	reported	dTXJa8	2	TRUE	0.069617	550.7189
4162	rs4148324	T	G	T	G	-0.1091	0.01033	0.6706	0.684664	FALSE	FALSE	FALSE	ieu-b-508	2	2.35E+08	0.035829	486484	0.773099	Sepsis (28	Sepsis (28	Sepsis (28	TRUE	igd	0.0048	1.52E-116	6642	M32758.m	TRUE	reported	AF0m66	2	TRUE	0.072167	516.4595
3041	rs1027804	A	G	A	G	-0.3609	0.053817	0.0419	0.059726	FALSE	FALSE	FALSE	ieu-b-508	7	99141373	0.070248	486484	0.443617	Sepsis (28	Sepsis (28	Sepsis (28	TRUE	igd	0.016	8.82E-113	7338	M31591.m	TRUE	reported	KltFxx	2	TRUE	0.06484	508.6457
5480	rs1024245	A	G	A	G	0.2212	-0.04688	0.9335	0.93212	FALSE	FALSE	FALSE	ieu-b-508	7	99240179	0.066448	486484	0.48047	Sepsis (28	Sepsis (28	Sepsis (28	TRUE	igd	0.0099	1.67E-109	6797	M33415.m	TRUE	reported	xHSphe	2	TRUE	0.068423	499.0822
906	rs1255072	T	C	T	C	0.0637	0.139977	0.9233	0.91425	FALSE	FALSE	FALSE	ieu-b-508	8	1.45E+08	0.059629	486484	0.018902	Sepsis (28	Sepsis (28	Sepsis (28	TRUE	igd	0.0029	5.97E-110	7354	M01494.m	TRUE	reported	7JWNKl	2	TRUE	0.061569	482.3527
3160	rs1116152	T	C	T	C	0.0703	0.021431	0.6983	0.69812	FALSE	FALSE	FALSE	ieu-b-508	1	76216330	0.03633	486484	0.555258	Sepsis (28	Sepsis (28	Sepsis (28	TRUE	igd	0.0033	6.42E-100	7340	M32328.m	TRUE	reported	YGsb6D	2	TRUE	0.058228	453.6954
1096	rs938554	C	G	C	G	-0.0348	0.036268	0.2256	0.218593	FALSE	TRUE	FALSE	ieu-b-508	4	9925692	0.040403	486484	0.369375	Sepsis (28	Sepsis (28	Sepsis (28	TRUE	igd	0.0017	5.85E-93	7371	M01604.m	TRUE	reported	hE3I2k	2	TRUE	0.053792	418.9313
8467	rs1472631	A	G	A	G	0.0366	0.003341	0.4919	0.487561	FALSE	FALSE	FALSE	ieu-b-508	15	63423739	0.033397	486484	0.920317	Sepsis (28	Sepsis (28	Sepsis (28	TRUE	igd	0.0018	1.85E-88	6552	M37058.m	TRUE	reported	32p8qV	2	TRUE	0.059357	413.3182
2448	rs7157785	T	G	T	G	0.0681	0.008458	0.1649	0.160732	FALSE	FALSE	FALSE	ieu-b-508	14	64235556	0.045509	486484	0.852554	Sepsis (28	Sepsis (28	Sepsis (28	TRUE	igd	0.0034	7.23E-87	7284	M21630.m	TRUE	reported	7eaO4H	2	TRUE	0.052201	401.0672
4350	rs1871395	A	G	A	G	-0.1101	0.071175	0.8385	0.847702	FALSE	FALSE	FALSE	ieu-b-508	12	21352315	0.046533	486484	0.126126	Sepsis (28	Sepsis (28	Sepsis (28	TRUE	igd	0.0055	1.06E-89	7355	M32855.m	TRUE	reported	AM8kCf	2	TRUE	0.051669	400.6186
591	rs174548	C	G	C	G	0.0488	-0.07283	0.7005	0.686724	FALSE	TRUE	FALSE	ieu-b-508	11	61571348	0.035919	486484	0.042613	Sepsis (28	Sepsis (28	Sepsis (28	TRUE	igd	0.0025	1.43E-84	7367	M01110.m	TRUE	reported	0A31Sl	2	TRUE	0.049178	380.927
6259	rs7552404	A	G	A	G	0.0746	0.013958	0.6984	0.694948	FALSE	FALSE	FALSE	ieu-b-508	1	76135946	0.03622	486484	0.699964	Sepsis (28	Sepsis (28	Sepsis (28	TRUE	igd	0.004	1.27E-77	7345	M33936.m	TRUE	reported	jwA4B2	2	TRUE	0.045214	347.7278
6398	rs1197470	A	G	A	G	0.1844	0.006122	0.9139	0.912907	FALSE	FALSE	FALSE	ieu-b-508	7	99166544	0.059312	486484	0.917791	Sepsis (28	Sepsis (28	Sepsis (28	TRUE	igd	0.01	2.80E-75	7326	M33973.m	TRUE	reported	rpygG6	2	TRUE	0.044356	339.9408
7431	rs1231726	A	G	A	G	-0.1377	0.071682	0.8337	0.847892	FALSE	FALSE	FALSE	ieu-b-508	12	21352541	0.046552	486484	0.123602	Sepsis (28	Sepsis (28	Sepsis (28	TRUE	igd	0.0079	2.09E-67	5953	M35187.m	TRUE	reported	Zx7JfE	2	TRUE	0.048558	303.7161
4506	rs1934963	T	C	T	C	0.0293	0.031312	0.8021	0.800605	FALSE	FALSE	FALSE	ieu-b-508	10	96734676	0.041729	486484	0.453035	Sepsis (28	Sepsis (28	Sepsis (28	TRUE	igd	0.0017	8.69E-65	7362	M33132.m	TRUE	reported	dTXJa8	2	TRUE	0.038785	296.9747
3546	rs9333029	A	G	A	G	-0.0695	-0.00815	0.8669	0.868114	FALSE	FALSE	FALSE	ieu-b-508	1	47396162	0.049286	486484	0.868688	Sepsis (28	Sepsis (28	Sepsis (28	TRUE	igd	0.0042	1.52E-61	7358	M32497.m	TRUE	reported	244bZN	2	TRUE	0.035879	273.7493
6913	rs4921914	T	C	T	C	0.0879	-0.02431	0.7848	0.777478	FALSE	FALSE	FALSE	ieu-b-508	8	18272438	0.040215	486484	0.545444	Sepsis (28	Sepsis (28	Sepsis (28	TRUE	igd	0.0054	1.09E-60	5195	M34389.m	TRUE	reported	suiRlN	2	TRUE	0.048529	264.864
977	rs721399	T	C	T	C	-0.0289	-0.0051	0.7176	0.71737	FALSE	FALSE	FALSE	ieu-b-508	8	18259366	0.037067	486484	0.890645	Sepsis (28	Sepsis (28	Sepsis (28	TRUE	igd	0.0018	3.95E-58	6523	M01558.m	TRUE	reported	AZK0p4	2	TRUE	0.038016	257.7018
4320	rs4149034	A	G	A	G	-0.1045	0.03342	0.325	0.338028	FALSE	FALSE	FALSE	ieu-b-508	12	21317922	0.035292	486484	0.343658	Sepsis (28	Sepsis (28	Sepsis (28	TRUE	igd	0.0069	2.74E-51	6250	M32846.m	TRUE	reported	IwF2Yy	2	TRUE	0.0354	229.2954
5755	rs1046996	A	G	A	G	0.1024	-0.0228	0.2526	0.252821	FALSE	FALSE	FALSE	ieu-b-508	2	73752368	0.038623	486484	0.554932	Sepsis (28	Sepsis (28	Sepsis (28	TRUE	igd	0.0068	1.12E-51	2759	M33508.m	TRUE	reported	UMXHcP	2	TRUE	0.07595	226.6038
5764	rs4488133	A	T	A	T	0.0985	-0.04295	0.7014	0.65566	FALSE	TRUE	FALSE	ieu-b-508	10	1E+08	0.035126	486484	0.221399	Sepsis (28	Sepsis (28	Sepsis (28	TRUE	igd	0.0067	1.26E-48	2759	M33508.m	TRUE	reported	UMXHcP	2	TRUE	0.072647	215.9772
7011	rs4253311	A	G	A	G	-0.1412	0.028665	0.4869	0.487409	FALSE	FALSE	FALSE	ieu-b-508	4	1.87E+08	0.03343	486484	0.391179	Sepsis (28	Sepsis (28	Sepsis (28	TRUE	igd	0.0097	3.56E-48	4452	M34420.m	TRUE	reported	20uofq	2	TRUE	0.045434	211.8024
7779	rs8012	A	G	A	G	0.0374	0.022147	0.4414	0.451946	FALSE	FALSE	FALSE	ieu-b-508	19	13010520	0.03304	486484	0.502653	Sepsis (28	Sepsis (28	Sepsis (28	TRUE	igd	0.0027	6.36E-45	7256	M35439.m	TRUE	reported	DnuAGZ	2	TRUE	0.025762	191.8209
2298	rs7570971	A	C	A	C	-0.0373	0.025425	0.3255	0.283862	FALSE	FALSE	FALSE	ieu-b-508	2	1.36E+08	0.037976	486484	0.50317	Sepsis (28	Sepsis (28	Sepsis (28	TRUE	igd	0.0027	7.86E-45	7301	M20675.m	TRUE	reported	CXe9rE	2	TRUE	0.025474	190.7968
6971	rs1195056	A	C	A	C	0.0362	0.007882	0.5231	0.524588	FALSE	FALSE	FALSE	ieu-b-508	5	1.32E+08	0.033371	486484	0.81329	Sepsis (28	Sepsis (28	Sepsis (28	TRUE	igd	0.0027	2.31E-41	7344	M34407.m	TRUE	reported	X7I83Y	2	TRUE	0.023892	179.7096
4561	rs3099557	A	G	A	G	-0.1484	0.014107	0.8504	0.852602	FALSE	FALSE	FALSE	ieu-b-508	4	22824021	0.047	486484	0.764059	Sepsis (28	Sepsis (28	Sepsis (28	TRUE	igd	0.0113	1.83E-39	4817	M33144.m	TRUE	reported	cgXZoM	2	TRUE	0.034567	172.3973
7473	rs9392556	T	C	T	C	-0.0358	0.033345	0.6581	0.652579	FALSE	FALSE	FALSE	ieu-b-508	6	4147657	0.035208	486484	0.343591	Sepsis (28	Sepsis (28	Sepsis (28	TRUE	igd	0.0028	4.03E-38	6543	M35193.m	TRUE	reported	BMMWXU	2	TRUE	0.024376	163.4245
2803	rs4902243	A	G	A	G	-0.0442	-0.00937	0.8624	0.839571	FALSE	FALSE	FALSE	ieu-b-508	14	64234243	0.045558	486484	0.836977	Sepsis (28	Sepsis (28	Sepsis (28	TRUE	igd	0.0035	3.41E-37	7347	M27278.m	TRUE	reported	kPgqm3	2	TRUE	0.021246	159.4374
3792	rs174535	T	C	T	C	-0.044	-0.06719	0.6677	0.649782	FALSE	FALSE	FALSE	ieu-b-508	11	61551356	0.034978	486484	0.054742	Sepsis (28	Sepsis (28	Sepsis (28	TRUE	igd	0.0035	2.82E-36	7369	M32635.m	TRUE	reported	jJI9P8	2	TRUE	0.020996	157.9979
6986	rs9635324	A	G	A	G	0.0337	-0.02751	0.6075	0.60205	FALSE	FALSE	FALSE	ieu-b-508	15	40721939	0.034031	486484	0.418929	Sepsis (28	Sepsis (28	Sepsis (28	TRUE	igd	0.0027	1.89E-35	7344	M34407.m	TRUE	reported	X7I83Y	2	TRUE	0.020772	155.745
3424	rs1235619	A	G	A	G	0.0328	-0.00299	0.836	0.832956	FALSE	FALSE	FALSE	ieu-b-508	10	61413353	0.044712	486484	0.946715	Sepsis (28	Sepsis (28	Sepsis (28	TRUE	igd	0.0027	1.96E-33	7364	M32452.m	TRUE	reported	8GtFpl	2	TRUE	0.019647	147.5374
6260	rs8396	T	C	T	C	0.0478	0.022544	0.6972	0.697287	FALSE	FALSE	FALSE	ieu-b-508	4	1.6E+08	0.036161	486484	0.532986	Sepsis (28	Sepsis (28	Sepsis (28	TRUE	igd	0.0041	1.38E-31	7345	M33936.m	TRUE	reported	jwA4B2	2	TRUE	0.018169	135.8845
7780	rs8056893	A	C	A	C	-0.0302	-0.01839	0.7336	0.732887	FALSE	FALSE	FALSE	ieu-b-508	16	68304392	0.037816	486484	0.626817	Sepsis (28	Sepsis (28	Sepsis (28	TRUE	igd	0.0026	2.23E-30	7256	M35439.m	TRUE	reported	DnuAGZ	2	TRUE	0.018254	134.88
2279	rs1246580	A	G	A	G	-0.0364	0.023569	0.31	0.276149	FALSE	FALSE	FALSE	ieu-b-508	2	1.36E+08	0.038777	486484	0.543317	Sepsis (28	Sepsis (28	Sepsis (28	TRUE	igd	0.0032	3.47E-29	7301	M20675.m	TRUE	reported	CXe9rE	2	TRUE	0.017414	129.3552
6269	rs2403254	T	C	T	C	-0.0409	-0.00942	0.5505	0.579221	FALSE	FALSE	FALSE	ieu-b-508	11	18325146	0.033878	486484	0.781043	Sepsis (28	Sepsis (28	Sepsis (28	TRUE	igd	0.0036	1.26E-30	7222	M33937.m	TRUE	reported	QanGCl	2	TRUE	0.017559	129.0391
8498	rs2405522	A	G	A	G	-0.1264	0.023909	0.1672	0.169924	FALSE	FALSE	FALSE	ieu-b-508	5	1.32E+08	0.044458	486484	0.590723	Sepsis (28	Sepsis (28	Sepsis (28	TRUE	igd	0.0113	4.49E-29	7014	M37097.m	TRUE	reported	wCbJj4	2	TRUE	0.017526	125.0874
3121	rs1163251	T	C	T	C	0.0188	-0.07102	0.6	0.594142	FALSE	FALSE	FALSE	ieu-b-508	1	1.2E+08	0.034357	486484	0.038723	Sepsis (28	Sepsis (28	Sepsis (28	TRUE	igd	0.0017	7.05E-27	7349	M32315.m	TRUE	reported	4igzkL	2	TRUE	0.016369	122.2643
7768	rs1337574	T	C	T	C	0.0297	-0.00427	0.7801	0.774476	FALSE	FALSE	FALSE	ieu-b-508	1	53695882	0.039904	486484	0.914855	Sepsis (28	Sepsis (28	Sepsis (28	TRUE	igd	0.0027	2.80E-28	7256	M35439.m	TRUE	reported	DnuAGZ	2	TRUE	0.016402	120.9666
6933	rs1495741	A	G	A	G	0.057	-0.02466	0.7841	0.779473	FALSE	FALSE	FALSE	ieu-b-508	8	18272881	0.040149	486484	0.539042	Sepsis (28	Sepsis (28	Sepsis (28	TRUE	igd	0.0052	1.04E-27	5208	M34395.m	TRUE	reported	QVOPA3	2	TRUE	0.022551	120.1092
3949	rs1260290	C	G	C	G	-0.0368	-0.03715	0.1717	0.180734	FALSE	TRUE	FALSE	ieu-b-508	17	4824625	0.043394	486484	0.391985	Sepsis (28	Sepsis (28	Sepsis (28	TRUE	igd	0.0034	1.15E-26	7363	M32709.m	TRUE	reported	YR8SB4	2	TRUE	0.015661	117.117
6160	rs6685187	A	G	A	G	-0.0432	0.046678	0.4088	0.389975	FALSE	FALSE	FALSE	ieu-b-508	1	1.52E+08	0.034215	486484	0.172481	Sepsis (28	Sepsis (28	Sepsis (28	TRUE	igd	0.0041	2.88E-26	7334	M33884.m	TRUE	reported	aAWgRQ	2	TRUE	0.014912	110.9894
3875	rs273913	T	C	T	C	0.0261	0.001322	0.3822	0.374367	FALSE	FALSE	FALSE	ieu-b-508	5	1.32E+08	0.034383	486484	0.969333	Sepsis (28	Sepsis (28	Sepsis (28	TRUE	igd	0.0025	1.08E-25	7361	M32654.m	TRUE	reported	6ISQOP	2	TRUE	0.014591	108.964
1657	rs8051149	A	G	A	G	0.0258	0.024137	0.2127	0.245418	FALSE	FALSE	FALSE	ieu-b-508	16	87878822	0.038945	486484	0.535401	Sepsis (28	Sepsis (28	Sepsis (28	TRUE	igd	0.0025	9.07E-26	7368	M15140.m	TRUE	reported	jEcqMJ	2	TRUE	0.014249	106.4735
3543	rs7556052	A	T	A	T	-0.0346	0.077747	0.6721	0.910181	FALSE	TRUE	FALSE	ieu-b-508	1	47414851	0.060602	486484	0.199526	Sepsis (28	Sepsis (28	Sepsis (28	TRUE	igd	0.0034	5.50E-24	7358	M32497.m	TRUE	reported	244bZN	2	TRUE	0.013879	103.5324
6150	rs2271316	C	G	C	G	0.1133	-0.03216	0.3822	0.40342	FALSE	TRUE	FALSE	ieu-b-508	17	6915401	0.034146	486484	0.346208	Sepsis (28	Sepsis (28	Sepsis (28	TRUE	igd	0.0113	1.79E-23	2725	M33883.m	TRUE	reported	RT7YNu	2	TRUE	0.03558	100.4579
5986	rs8002180	T	C	T	C	-0.0239	0.054915	0.7203	0.724916	FALSE	FALSE	FALSE	ieu-b-508	13	95922765	0.037326	486484	0.141229	Sepsis (28	Sepsis (28	Sepsis (28	TRUE	igd	0.0024	8.23E-23	6279	M33666.m	TRUE	reported	dvWEtk	2	TRUE	0.015548	99.13682
5600	rs2065396	C	G	C	G	0.035	-0.01524	0.3289	0.328774	FALSE	TRUE	FALSE	ieu-b-508	6	1.6E+08	0.035469	486484	0.667513	Sepsis (28	Sepsis (28	Sepsis (28	TRUE	igd	0.0036	2.77E-22	7365	M33441.m	TRUE	reported	CSdSpl	2	TRUE	0.012671	94.49594
7434	rs4149032	T	C	T	C	-0.0621	0.03665	0.3324	0.338222	FALSE	FALSE	FALSE	ieu-b-508	12	21317791	0.035283	486484	0.298928	Sepsis (28	Sepsis (28	Sepsis (28	TRUE	igd	0.0064	4.61E-22	5953	M35187.m	TRUE	reported	Zx7JfE	2	TRUE	0.015569	94.119
7773	rs1764197	T	C	T	C	-0.0252	-0.00303	0.6608	0.652367	FALSE	FALSE	FALSE	ieu-b-508	8	49884817	0.035192	486484	0.931377	Sepsis (28	Sepsis (28	Sepsis (28	TRUE	igd	0.0026	3.35E-22	7256	M35439.m	TRUE	reported	DnuAGZ	2	TRUE	0.012781	93.91494
4527	rs4253252	T	G	T	G	-0.0862	0.031428	0.4803	0.487908	FALSE	FALSE	FALSE	ieu-b-508	4	1.87E+08	0.033389	486484	0.346572	Sepsis (28	Sepsis (28	Sepsis (28	TRUE	igd	0.0089	2.83E-22	2391	M33137.m	TRUE	reported	ONVNk3	2	TRUE	0.037752	93.72838
2095	rs174556	T	C	T	C	-0.0356	0.076415	0.299	0.30722	FALSE	FALSE	FALSE	ieu-b-508	11	61580635	0.036131	486484	0.034437	Sepsis (28	Sepsis (28	Sepsis (28	TRUE	igd	0.0037	1.97E-22	7367	M18467.m	TRUE	reported	64PaNn	2	TRUE	0.01241	92.55047
8177	rs4362	T	C	T	C	0.0612	0.018466	0.5281	0.523127	FALSE	FALSE	FALSE	ieu-b-508	17	61574492	0.033628	486484	0.582915	Sepsis (28	Sepsis (28	Sepsis (28	TRUE	igd	0.0064	1.37E-21	2432	M36134.m	TRUE	reported	ekMsfG	2	TRUE	0.036237	91.36621
2509	rs4784054	A	G	A	G	0.0349	0.066059	0.1063	0.099208	FALSE	FALSE	FALSE	ieu-b-508	16	58776314	0.055796	486484	0.236434	Sepsis (28	Sepsis (28	Sepsis (28	TRUE	igd	0.0037	2.31E-21	5702	M22130.m	TRUE	reported	YDbJlz	2	TRUE	0.015364	88.93957
3296	rs4787294	A	T	A	T	0.075	-0.02643	0.9278	0.934916	FALSE	TRUE	FALSE	ieu-b-508	16	25002350	0.067621	486484	0.695873	Sepsis (28	Sepsis (28	Sepsis (28	TRUE	igd	0.008	9.64E-21	6119	M32379.m	TRUE	reported	MLimM6	2	TRUE	0.01416	87.8619
6528	rs4675874	A	G	A	G	-0.0243	-0.01224	0.7094	0.71145	FALSE	FALSE	FALSE	ieu-b-508	2	2.42E+08	0.036913	486484	0.740283	Sepsis (28	Sepsis (28	Sepsis (28	TRUE	igd	0.0026	3.13E-20	7049	M34123.m	TRUE	reported	9O5wo1	2	TRUE	0.01224	87.32581
8359	rs1282970	A	G	A	G	-0.042	0.026602	0.1927	0.184971	FALSE	FALSE	FALSE	ieu-b-508	12	21388621	0.04287	486484	0.534919	Sepsis (28	Sepsis (28	Sepsis (28	TRUE	igd	0.0045	9.49E-21	6876	M36754.m	TRUE	reported	f8bkFg	2	TRUE	0.01251	87.08577
3324	rs1394678	T	C	T	C	-0.0354	0.021814	0.2785	0.282022	FALSE	FALSE	FALSE	ieu-b-508	16	20491058	0.037435	486484	0.560098	Sepsis (28	Sepsis (28	Sepsis (28	TRUE	igd	0.0038	1.70E-20	7358	M32405.m	TRUE	reported	uzomIQ	2	TRUE	0.011657	86.76034
4313	rs1050587	A	G	A	G	0.0654	0.005531	0.2733	0.280358	FALSE	FALSE	FALSE	ieu-b-508	12	21171896	0.037296	486484	0.882105	Sepsis (28	Sepsis (28	Sepsis (28	TRUE	igd	0.0071	3.13E-20	6250	M32846.m	TRUE	reported	IwF2Yy	2	TRUE	0.013394	84.8203
5981	rs6804368	A	G	A	G	0.0221	-0.01992	0.5714	0.559719	FALSE	FALSE	FALSE	ieu-b-508	3	30753573	0.033671	486484	0.554159	Sepsis (28	Sepsis (28	Sepsis (28	TRUE	igd	0.0024	8.17E-21	6279	M33666.m	TRUE	reported	dvWEtk	2	TRUE	0.013324	84.76639
2528	rs4351	A	G	A	G	-0.0455	-0.03792	0.4754	0.479486	FALSE	FALSE	FALSE	ieu-b-508	17	61569732	0.033496	486484	0.257544	Sepsis (28	Sepsis (28	Sepsis (28	TRUE	igd	0.005	1.24E-19	3838	M22175.m	TRUE	reported	IlIqyJ	2	TRUE	0.021121	82.76685
252	rs1440581	T	C	T	C	-0.0081	0.000585	0.4592	0.467697	FALSE	FALSE	FALSE	ieu-b-508	4	89226422	0.033423	486484	0.986036	Sepsis (28	Sepsis (28	Sepsis (28	TRUE	igd	9.00E-04	1.44E-19	7351	M00060.m	TRUE	reported	kqwI4V	2	TRUE	0.010899	80.97796
3048	rs2572023	A	G	A	G	-0.0576	-0.01187	0.4687	0.476254	FALSE	FALSE	FALSE	ieu-b-508	7	99474427	0.033384	486484	0.722203	Sepsis (28	Sepsis (28	Sepsis (28	TRUE	igd	0.0064	4.18E-19	7338	M31591.m	TRUE	reported	KltFxx	2	TRUE	0.010918	80.97792
1444	rs1687639	T	C	T	C	0.0304	0.017214	0.8993	0.894471	FALSE	FALSE	FALSE	ieu-b-508	5	78346769	0.054214	486484	0.750846	Sepsis (28	Sepsis (28	Sepsis (28	TRUE	igd	0.0034	1.49E-19	7357	M03141.m	TRUE	reported	y4mG6m	2	TRUE	0.01075	79.9229
7438	rs1256623	A	C	A	C	-0.0446	-0.01664	0.7095	0.716119	FALSE	FALSE	FALSE	ieu-b-508	1	1.52E+08	0.037029	486484	0.653167	Sepsis (28	Sepsis (28	Sepsis (28	TRUE	igd	0.005	5.69E-19	6234	M35189.m	TRUE	reported	54XZFj	2	TRUE	0.012602	79.54087
6065	rs968567	T	C	T	C	0.04	0.004726	0.1798	0.187234	FALSE	FALSE	FALSE	ieu-b-508	11	61595564	0.042793	486484	0.912055	Sepsis (28	Sepsis (28	Sepsis (28	TRUE	igd	0.0045	2.84E-19	7362	M33821.m	TRUE	reported	eVYICL	2	TRUE	0.010618	78.99088
4191	rs7604035	T	C	T	C	0.031	0.002699	0.3261	0.33177	FALSE	FALSE	FALSE	ieu-b-508	2	32540334	0.035608	486484	0.939587	Sepsis (28	Sepsis (28	Sepsis (28	TRUE	igd	0.0035	9.39E-19	7313	M32761.m	TRUE	reported	QefVGc	2	TRUE	0.010613	78.42752
4	rs2657879	A	G	A	G	0.015	0.00847	0.8179	0.817272	FALSE	FALSE	FALSE	ieu-b-508	12	56865338	0.043121	486484	0.84427	Sepsis (28	Sepsis (28	Sepsis (28	TRUE	igd	0.0017	6.15E-18	7372	M00053.m	TRUE	reported	4Smluz	2	TRUE	0.01045	77.83355
1640	rs3184504	T	C	T	C	0.0149	-0.04238	0.4851	0.482729	FALSE	FALSE	FALSE	ieu-b-508	12	1.12E+08	0.033376	486484	0.204141	Sepsis (28	Sepsis (28	Sepsis (28	TRUE	igd	0.0017	6.05E-18	7368	M15140.m	TRUE	reported	jEcqMJ	2	TRUE	0.010319	76.79922
2467	rs493519	T	C	T	C	-0.0358	0.038542	0.1252	0.124372	FALSE	FALSE	FALSE	ieu-b-508	12	1.22E+08	0.050497	486484	0.445307	Sepsis (28	Sepsis (28	Sepsis (28	TRUE	igd	0.0041	1.32E-18	6180	M22030.m	TRUE	reported	ZNV0qR	2	TRUE	0.012187	76.21804
8458	rs1098821	A	G	A	G	-0.0164	0.044617	0.3883	0.396208	FALSE	FALSE	FALSE	ieu-b-508	9	1.32E+08	0.034385	486484	0.194437	Sepsis (28	Sepsis (28	Sepsis (28	TRUE	igd	0.0019	1.36E-18	6552	M37058.m	TRUE	reported	32p8qV	2	TRUE	0.011243	74.48141
4429	rs174675	T	C	T	C	0.0155	0.040174	0.2812	0.281659	FALSE	FALSE	FALSE	ieu-b-508	22	19934051	0.037154	486484	0.279569	Sepsis (28	Sepsis (28	Sepsis (28	TRUE	igd	0.0018	2.28E-18	7344	M32910.m	TRUE	reported	2OCBKh	2	TRUE	0.009996	74.13104
7130	rs2035647	A	G	A	G	-0.0292	0.01955	0.2052	0.207803	FALSE	FALSE	FALSE	ieu-b-508	7	1.38E+08	0.041226	486484	0.635351	Sepsis (28	Sepsis (28	Sepsis (28	TRUE	igd	0.0034	2.18E-17	7322	M34527.m	TRUE	reported	EWFGP8	2	TRUE	0.009973	73.73764
4071	rs6151429	T	C	T	C	-0.0289	0.04046	0.9291	0.921487	FALSE	FALSE	FALSE	ieu-b-508	22	51063477	0.061723	486484	0.512143	Sepsis (28	Sepsis (28	Sepsis (28	TRUE	igd	0.0034	6.51E-18	7318	M32740.m	TRUE	reported	YLshfP	2	TRUE	0.009776	72.23025
6400	rs474229	T	G	T	G	0.0492	0.037617	0.6017	0.590034	FALSE	FALSE	FALSE	ieu-b-508	7	99466558	0.033992	486484	0.268439	Sepsis (28	Sepsis (28	Sepsis (28	TRUE	igd	0.0058	3.51E-17	7326	M33973.m	TRUE	reported	rpygG6	2	TRUE	0.009727	71.93755
4190	rs6735065	C	G	C	G	0.0678	0.084691	0.953	0.93674	FALSE	TRUE	FALSE	ieu-b-508	2	31542938	0.06919	486484	0.220939	Sepsis (28	Sepsis (28	Sepsis (28	TRUE	igd	0.008	1.99E-17	7313	M32761.m	TRUE	reported	QefVGc	2	TRUE	0.009726	71.80598
724	rs2863979	A	G	A	G	0.0143	-0.0358	0.7241	0.710388	FALSE	FALSE	FALSE	ieu-b-508	16	68356187	0.03677	486484	0.330296	Sepsis (28	Sepsis (28	Sepsis (28	TRUE	igd	0.0017	1.44E-17	7363	M01301.m	TRUE	reported	w8TL71	2	TRUE	0.009518	70.73857
2523	rs8101881	T	C	T	C	0.0325	-0.01048	0.613	0.602566	FALSE	FALSE	FALSE	ieu-b-508	19	33364628	0.034123	486484	0.75885	Sepsis (28	Sepsis (28	Sepsis (28	TRUE	igd	0.0039	1.43E-16	3950	M22138.m	TRUE	reported	UbKBSS	2	TRUE	0.017277	69.40928
2446	rs4814176	T	C	T	C	0.0216	-0.05148	0.3938	0.392493	FALSE	FALSE	FALSE	ieu-b-508	20	12959398	0.034234	486484	0.132665	Sepsis (28	Sepsis (28	Sepsis (28	TRUE	igd	0.0026	2.97E-17	7284	M21630.m	TRUE	reported	7eaO4H	2	TRUE	0.009386	68.9988
7750	rs2270968	T	G	T	G	0.0307	-0.02759	0.2634	0.261096	FALSE	FALSE	FALSE	ieu-b-508	3	1.83E+08	0.037952	486484	0.467255	Sepsis (28	Sepsis (28	Sepsis (28	TRUE	igd	0.0037	1.31E-16	5306	M35433.m	TRUE	reported	XlYkLe	2	TRUE	0.012809	68.81919
3444	rs7727544	T	C	T	C	-0.0148	-0.01826	0.5553	0.558604	FALSE	FALSE	FALSE	ieu-b-508	5	1.32E+08	0.03357	486484	0.586412	Sepsis (28	Sepsis (28	Sepsis (28	TRUE	igd	0.0018	6.61E-16	7364	M32452.m	TRUE	reported	8GtFpl	2	TRUE	0.009097	67.58658
5486	rs1323055	T	C	T	C	-0.1701	0.184029	0.016	0.019966	FALSE	FALSE	FALSE	ieu-b-508	7	98730819	0.118869	486484	0.121583	Sepsis (28	Sepsis (28	Sepsis (28	TRUE	igd	0.0209	4.31E-16	6797	M33415.m	TRUE	reported	xHSphe	2	TRUE	0.009651	66.21986
3693	rs1934955	A	G	A	G	-0.0301	0.030157	0.713	0.7187	FALSE	FALSE	FALSE	ieu-b-508	10	96788561	0.037072	486484	0.415953	Sepsis (28	Sepsis (28	Sepsis (28	TRUE	igd	0.0037	3.33E-16	7369	M32587.m	TRUE	reported	uknv7G	2	TRUE	0.008901	66.16246
551	rs131794	A	C	A	C	0.0146	-0.01357	0.2074	0.209311	FALSE	FALSE	FALSE	ieu-b-508	22	50971752	0.040891	486484	0.739982	Sepsis (28	Sepsis (28	Sepsis (28	TRUE	igd	0.0018	4.37E-16	7354	M00606.m	TRUE	reported	r5smf7	2	TRUE	0.008867	65.77223
1432	rs6459467	A	G	A	G	0.0202	0.010339	0.3888	0.383205	FALSE	FALSE	FALSE	ieu-b-508	6	16288192	0.034383	486484	0.763646	Sepsis (28	Sepsis (28	Sepsis (28	TRUE	igd	0.0025	1.93E-16	6941	M03127.m	TRUE	reported	JscEZw	2	TRUE	0.009318	65.26759
6384	rs174601	T	C	T	C	-0.0336	0.0998	0.3398	0.374872	FALSE	FALSE	FALSE	ieu-b-508	11	61623140	0.03443	486484	0.003748	Sepsis (28	Sepsis (28	Sepsis (28	TRUE	igd	0.0042	7.93E-16	7330	M33969.m	TRUE	reported	0ujroW	2	TRUE	0.008656	63.98254
2288	rs3800993	T	C	T	C	0.0287	-0.03369	0.1697	0.175045	FALSE	FALSE	FALSE	ieu-b-508	7	1.42E+08	0.043855	486484	0.442377	Sepsis (28	Sepsis (28	Sepsis (28	TRUE	igd	0.0036	1.53E-15	7301	M20675.m	TRUE	reported	CXe9rE	2	TRUE	0.00863	63.53892
5984	rs7775554	A	C	A	C	0.019	0.06006	0.5279	0.524786	FALSE	FALSE	FALSE	ieu-b-508	6	89879168	0.033414	486484	0.072265	Sepsis (28	Sepsis (28	Sepsis (28	TRUE	igd	0.0024	1.35E-15	6279	M33666.m	TRUE	reported	dvWEtk	2	TRUE	0.009883	62.65365
8270	rs7822232	A	C	A	C	0.0299	0.129926	0.9203	0.914804	FALSE	FALSE	FALSE	ieu-b-508	8	1.45E+08	0.059802	486484	0.02981	Sepsis (28	Sepsis (28	Sepsis (28	TRUE	igd	0.0038	5.12E-15	7092	M36552.m	TRUE	reported	5CxMJz	2	TRUE	0.008654	61.89459
4643	rs6664483	T	G	T	G	1.1464	-0.01461	0.9803	0.951187	FALSE	FALSE	FALSE	ieu-b-508	1	2.01E+08	0.077081	486484	0.849658	Sepsis (28	Sepsis (28	Sepsis (28	TRUE	igd	0.1447	2.32E-15	76	M33173.m	TRUE	reported	04fkUQ	2	TRUE	0.452322	61.11579
1634	rs1763023	A	G	A	G	0.0131	-0.05625	0.4183	0.408736	FALSE	FALSE	FALSE	ieu-b-508	12	1.13E+08	0.033975	486484	0.097791	Sepsis (28	Sepsis (28	Sepsis (28	TRUE	igd	0.0017	3.41E-14	7368	M15140.m	TRUE	reported	jEcqMJ	2	TRUE	0.007995	59.3645
7902	rs6663731	A	T	A	T	0.0624	-0.013	0.8857	0.867839	FALSE	TRUE	FALSE	ieu-b-508	1	47347427	0.049321	486484	0.792076	Sepsis (28	Sepsis (28	Sepsis (28	TRUE	igd	0.0081	1.39E-14	5629	M35669.m	TRUE	reported	gSecuE	2	TRUE	0.010433	59.32596
3883	rs6897597	T	C	T	C	-0.02	-0.00306	0.2368	0.242198	FALSE	FALSE	FALSE	ieu-b-508	5	1.32E+08	0.039098	486484	0.937682	Sepsis (28	Sepsis (28	Sepsis (28	TRUE	igd	0.0026	1.10E-14	7361	M32654.m	TRUE	reported	6ISQOP	2	TRUE	0.007974	59.15552
626	rs603424	A	G	A	G	0.0292	-0.03229	0.1865	0.173202	FALSE	FALSE	FALSE	ieu-b-508	10	1.02E+08	0.044209	486484	0.465202	Sepsis (28	Sepsis (28	Sepsis (28	TRUE	igd	0.0038	1.56E-14	7349	M01121.m	TRUE	reported	HU0fhR	2	TRUE	0.007971	59.03102
3942	rs1005390	T	G	T	G	0.0261	0.030824	0.7998	0.792417	FALSE	FALSE	FALSE	ieu-b-508	7	1.51E+08	0.041453	486484	0.457118	Sepsis (28	Sepsis (28	Sepsis (28	TRUE	igd	0.0034	2.50E-14	7363	M32709.m	TRUE	reported	YR8SB4	2	TRUE	0.00794	58.91219
7774	rs246234	C	G	C	G	-0.0199	0.034371	0.2958	0.303591	FALSE	TRUE	FALSE	ieu-b-508	16	16128742	0.03642	486484	0.345303	Sepsis (28	Sepsis (28	Sepsis (28	TRUE	igd	0.0026	2.56E-14	7256	M35439.m	TRUE	reported	DnuAGZ	2	TRUE	0.008009	58.56521
3136	rs4947534	T	C	T	C	-0.0183	-0.01403	0.2479	0.248025	FALSE	FALSE	FALSE	ieu-b-508	7	56079094	0.038782	486484	0.717566	Sepsis (28	Sepsis (28	Sepsis (28	TRUE	igd	0.0024	1.96E-14	7349	M32315.m	TRUE	reported	4igzkL	2	TRUE	0.007849	58.1248
704	rs9400467	T	C	T	C	-0.0121	-0.04289	0.7059	0.709744	FALSE	FALSE	FALSE	ieu-b-508	6	1.11E+08	0.037408	486484	0.251513	Sepsis (28	Sepsis (28	Sepsis (28	TRUE	igd	0.0016	6.54E-14	7358	M01299.m	TRUE	reported	gfM0a3	2	TRUE	0.007713	57.17586
7440	rs1509821	T	C	T	C	0.0574	0.050357	0.1091	0.108902	FALSE	FALSE	FALSE	ieu-b-508	2	2.11E+08	0.053819	486484	0.349442	Sepsis (28	Sepsis (28	Sepsis (28	TRUE	igd	0.0076	5.29E-14	6234	M35189.m	TRUE	reported	54XZFj	2	TRUE	0.009067	57.02394
3552	rs174538	A	G	A	G	-0.0264	0.059194	0.3025	0.315646	FALSE	FALSE	FALSE	ieu-b-508	11	61560081	0.035873	486484	0.098924	Sepsis (28	Sepsis (28	Sepsis (28	TRUE	igd	0.0035	8.90E-14	7373	M32504.m	TRUE	reported	MN0yyR	2	TRUE	0.007658	56.87926
3391	rs1176152	T	C	T	C	-0.0675	-0.00675	0.0736	0.086688	FALSE	FALSE	FALSE	ieu-b-508	7	99118801	0.059294	486484	0.909371	Sepsis (28	Sepsis (28	Sepsis (28	TRUE	igd	0.009	6.70E-14	7346	M32425.m	TRUE	reported	u5RLGY	2	TRUE	0.007599	56.23469

3599	rs2071426	T	C	T	C	0.0547	0.027225	0.7107	0.718055	FALSE	FALSE	FALSE	ieu-b-508	10	96828323	0.037022	486484	0.462121	Sepsis (28	Sepsis (28	Sepsis (28	TRUE	igd	0.0073	6.87E-14	7256	M32549.m	TRUE	reported	W7Fgu2	2	TRUE	0.007679	56.13183
501	rs6968554	A	G	A	G	0.0644	0.028702	0.3756	0.366672	FALSE	FALSE	FALSE	ieu-b-508	7	17287106	0.034638	486484	0.407326	Sepsis (28	Sepsis (28	Sepsis (28	TRUE	igd	0.0086	8.68E-14	7274	M00569.m	TRUE	reported	1TjBaz	2	TRUE	0.00765	56.0603
199	rs1686824	C	G	C	G	0.0097	0.019585	0.2113	0.206694	FALSE	TRUE	FALSE	ieu-b-508	4	9978305	0.041226	486484	0.634748	Sepsis (28	Sepsis (28	Sepsis (28	TRUE	igd	0.0013	1.78E-14	7355	M00059.m	TRUE	reported	RX1zaI	2	TRUE	0.007513	55.65942
4491	rs7061710	C	G	C	G	0.0453	0.003824	0.2282	0.220637	FALSE	TRUE	FALSE	ieu-b-508	1	1.71E+08	0.040181	486484	0.92418	Sepsis (28	Sepsis (28	Sepsis (28	TRUE	igd	0.0061	7.52E-14	2773	M33131.m	TRUE	reported	uGKURO	2	TRUE	0.0195	55.10911
4027	rs4680	A	G	A	G	-0.0436	0.064336	0.5065	0.514871	FALSE	FALSE	FALSE	ieu-b-508	22	19951271	0.03338	486484	0.053932	Sepsis (28	Sepsis (28	Sepsis (28	TRUE	igd	0.0059	1.31E-13	6000	M32735.m	TRUE	reported	QE2vKQ	2	TRUE	0.00902	54.59139
5688	rs4687717	T	C	T	C	0.0133	-0.0091	0.4248	0.422068	FALSE	FALSE	FALSE	ieu-b-508	3	53282188	0.033919	486484	0.788521	Sepsis (28	Sepsis (28	Sepsis (28	TRUE	igd	0.0018	1.07E-13	7307	M33477.m	TRUE	reported	Aw8PBK	2	TRUE	0.007416	54.58074
1331	rs838705	A	G	A	G	0.0266	-0.00523	0.6134	0.606656	FALSE	FALSE	FALSE	ieu-b-508	2	2.34E+08	0.034118	486484	0.87819	Sepsis (28	Sepsis (28	Sepsis (28	TRUE	igd	0.0036	1.23E-13	6252	M02137.m	TRUE	reported	iRGqBE	2	TRUE	0.008657	54.57821
4471	rs2731672	T	C	T	C	-0.081	-0.08491	0.257	0.255166	FALSE	FALSE	FALSE	ieu-b-508	5	1.77E+08	0.038181	486484	0.026152	Sepsis (28	Sepsis (28	Sepsis (28	TRUE	igd	0.011	1.79E-13	3659	M33084.m	TRUE	reported	jrOsNK	2	TRUE	0.014603	54.1935
2263	rs4808136	A	G	A	G	0.0132	-0.0212	0.3392	0.351506	FALSE	FALSE	FALSE	ieu-b-508	19	18618867	0.035029	486484	0.544943	Sepsis (28	Sepsis (28	Sepsis (28	TRUE	igd	0.0018	5.42E-14	7354	M19934.m	TRUE	reported	HtcWcQ	2	TRUE	0.00726	53.76315
1009	rs170149	A	G	A	G	0.0256	-0.01295	0.074	0.073876	FALSE	FALSE	FALSE	ieu-b-508	17	6595930	0.063909	486484	0.839368	Sepsis (28	Sepsis (28	Sepsis (28	TRUE	igd	0.0035	1.42E-13	7364	M01564.m	TRUE	reported	IPUdyD	2	TRUE	0.007213	53.48425
3946	rs1110173	A	T	A	T	-0.0553	0.016414	0.9414	0.921699	FALSE	TRUE	FALSE	ieu-b-508	10	1.35E+08	0.062019	486484	0.791267	Sepsis (28	Sepsis (28	Sepsis (28	TRUE	igd	0.0076	3.28E-13	7363	M32709.m	TRUE	reported	YR8SB4	2	TRUE	0.007139	52.93039
5601	rs2404602	A	G	A	G	0.0259	-0.02338	0.4398	0.436121	FALSE	FALSE	FALSE	ieu-b-508	15	77030792	0.033619	486484	0.486827	Sepsis (28	Sepsis (28	Sepsis (28	TRUE	igd	0.0036	3.56E-13	7365	M33441.m	TRUE	reported	CSdSpl	2	TRUE	0.006979	51.74598
3085	rs4625783	T	C	T	C	-0.0187	0.03151	0.4268	0.408413	FALSE	FALSE	FALSE	ieu-b-508	17	80086395	0.033944	486484	0.353245	Sepsis (28	Sepsis (28	Sepsis (28	TRUE	igd	0.0026	3.90E-13	7346	M32197.m	TRUE	reported	VZjwa9	2	TRUE	0.006993	51.71521
4234	rs2734331	A	G	A	G	0.0726	0.021242	0.9651	0.968741	FALSE	FALSE	FALSE	ieu-b-508	6	31930351	0.095649	486484	0.824252	Sepsis (28	Sepsis (28	Sepsis (28	TRUE	igd	0.0101	6.56E-13	6857	M32787.m	TRUE	reported	fSRakm	2	TRUE	0.007479	51.65398
7014	rs5030062	A	C	A	C	-0.0718	0.013965	0.6147	0.628077	FALSE	FALSE	FALSE	ieu-b-508	3	1.86E+08	0.03445	486484	0.685215	Sepsis (28	Sepsis (28	Sepsis (28	TRUE	igd	0.01	5.94E-13	4452	M34420.m	TRUE	reported	20uofq	2	TRUE	0.011447	51.52924
3653	rs1853207	T	C	T	C	0.1085	-0.03639	0.0598	0.063202	FALSE	FALSE	FALSE	ieu-b-508	10	96665937	0.068832	486484	0.597044	Sepsis (28	Sepsis (28	Sepsis (28	TRUE	igd	0.0152	9.58E-13	6970	M32564.m	TRUE	reported	73MgOk	2	TRUE	0.007257	50.93868
2262	rs4788439	T	C	T	C	-0.0271	0.009324	0.0772	0.067708	FALSE	FALSE	FALSE	ieu-b-508	16	24931798	0.066619	486484	0.888696	Sepsis (28	Sepsis (28	Sepsis (28	TRUE	igd	0.0038	6.59E-13	7354	M19934.m	TRUE	reported	HtcWcQ	2	TRUE	0.006868	50.84559
3743	rs3761097	T	C	T	C	0.057	-0.09734	0.053	0.052456	FALSE	FALSE	FALSE	ieu-b-508	19	36290977	0.075914	486484	0.199753	Sepsis (28	Sepsis (28	Sepsis (28	TRUE	igd	0.008	1.04E-12	5575	M32632.m	TRUE	reported	pIHzPT	2	TRUE	0.009024	50.74741
7803	rs2041073	T	C	T	C	0.0178	0.055646	0.5366	0.535603	FALSE	FALSE	FALSE	ieu-b-508	14	73972535	0.033377	486484	0.095471	Sepsis (28	Sepsis (28	Sepsis (28	TRUE	igd	0.0025	3.41E-13	6883	M35464.m	TRUE	reported	Ir9dHp	2	TRUE	0.007311	50.67967
3397	rs296396	T	C	T	C	-0.0462	0.003997	0.1646	0.1616	FALSE	FALSE	FALSE	ieu-b-508	19	48365523	0.045353	486484	0.929776	Sepsis (28	Sepsis (28	Sepsis (28	TRUE	igd	0.0065	1.17E-12	7346	M32425.m	TRUE	reported	u5RLGY	2	TRUE	0.00683	50.50554
6411	rs1430346	T	C	T	C	0.0312	-0.03378	0.729	0.725572	FALSE	FALSE	FALSE	ieu-b-508	2	73372558	0.037418	486484	0.366675	Sepsis (28	Sepsis (28	Sepsis (28	TRUE	igd	0.0044	1.40E-12	7132	M34040.m	TRUE	reported	sJBy3i	2	TRUE	0.007001	50.26689
635	rs494562	A	G	A	G	-0.1182	0.04409	0.8988	0.904536	FALSE	FALSE	FALSE	ieu-b-508	6	86117129	0.056947	486484	0.438791	Sepsis (28	Sepsis (28	Sepsis (28	TRUE	igd	0.0167	1.34E-12	2639	M01123.m	TRUE	reported	NDTvfD	2	TRUE	0.018629	50.05791
2032	rs15676	A	G	A	G	0.0191	0.014213	0.7123	0.715932	FALSE	FALSE	FALSE	ieu-b-508	9	1.32E+08	0.037162	486484	0.702123	Sepsis (28	Sepsis (28	Sepsis (28	TRUE	igd	0.0027	1.33E-12	6939	M18349.m	TRUE	reported	M5qPs8	2	TRUE	0.00716	50.0281
5545	rs1122815	A	T	A	T	0.61	0.037725	0.9654	0.950015	FALSE	TRUE	FALSE	ieu-b-508	11	67968131	0.07692	486484	0.623815	Sepsis (28	Sepsis (28	Sepsis (28	TRUE	igd	0.0865	1.72E-12	1391	M33423.m	TRUE	reported	MoEnO7	2	TRUE	0.034518	49.65953
3744	rs4327428	A	C	A	C	-0.031	0.122928	0.1117	0.120156	FALSE	FALSE	FALSE	ieu-b-508	3	45809329	0.051257	486484	0.016472	Sepsis (28	Sepsis (28	Sepsis (28	TRUE	igd	0.0044	2.81E-12	7337	M32632.m	TRUE	reported	pIHzPT	2	TRUE	0.00672	49.6249
4299	rs2007084	A	G	A	G	-0.0683	0.052586	0.091	0.086702	FALSE	FALSE	FALSE	ieu-b-508	15	90345335	0.060316	486484	0.383294	Sepsis (28	Sepsis (28	Sepsis (28	TRUE	igd	0.0097	1.77E-12	7255	M32836.m	TRUE	reported	d4lVXe	2	TRUE	0.006787	49.56535
3107	rs274567	T	C	T	C	0.0176	0.005053	0.3709	0.366626	FALSE	FALSE	FALSE	ieu-b-508	5	1.32E+08	0.034539	486484	0.88369	Sepsis (28	Sepsis (28	Sepsis (28	TRUE	igd	0.0025	2.85E-12	7356	M32198.m	TRUE	reported	ndXmnp	2	TRUE	0.006692	49.54812
6612	rs7568884	T	C	T	C	-0.0183	0.02242	0.2496	0.241596	FALSE	FALSE	FALSE	ieu-b-508	2	1.37E+08	0.039517	486484	0.570483	Sepsis (28	Sepsis (28	Sepsis (28	TRUE	igd	0.0026	5.39E-12	6994	M34306.m	TRUE	reported	WPXPUN	2	TRUE	0.007033	49.52577
6266	rs1214104	T	C	T	C	-0.0253	0.003682	0.4692	0.469097	FALSE	FALSE	FALSE	ieu-b-508	1	1.2E+08	0.033546	486484	0.912607	Sepsis (28	Sepsis (28	Sepsis (28	TRUE	igd	0.0036	1.77E-12	7222	M33937.m	TRUE	reported	QanGCl	2	TRUE	0.006792	49.37598
7399	rs5760492	A	G	A	G	-0.0492	0.044502	0.333	0.344431	FALSE	FALSE	FALSE	ieu-b-508	22	24995202	0.035146	486484	0.205443	Sepsis (28	Sepsis (28	Sepsis (28	TRUE	igd	0.007	2.34E-12	1982	M35159.m	TRUE	reported	7EoJLB	2	TRUE	0.024319	49.35097
528	rs937813	T	C	T	C	0.0271	-0.04234	0.9025	0.889884	FALSE	FALSE	FALSE	ieu-b-508	2	28301540	0.053907	486484	0.432207	Sepsis (28	Sepsis (28	Sepsis (28	TRUE	igd	0.0039	2.65E-12	7345	M00584.m	TRUE	reported	oBt02X	2	TRUE	0.006531	48.27153
2323	rs7542172	A	G	A	G	-0.0236	0.007676	0.1535	0.196427	FALSE	FALSE	FALSE	ieu-b-508	1	46990681	0.042289	486484	0.855964	Sepsis (28	Sepsis (28	Sepsis (28	TRUE	igd	0.0034	1.98E-12	7327	M20699.m	TRUE	reported	Z3EKFa	2	TRUE	0.006533	48.16678
6659	rs707922	T	G	T	G	0.2854	0.143468	0.04	0.051157	FALSE	FALSE	FALSE	ieu-b-508	6	31625507	0.076457	486484	0.060593	Sepsis (28	Sepsis (28	Sepsis (28	TRUE	igd	0.0406	2.12E-12	75	M34322.m	TRUE	reported	16GdG9	2	TRUE	0.397177	48.09694
5489	rs2417971	A	G	A	G	-0.0401	0.022065	0.2843	0.291706	FALSE	FALSE	FALSE	ieu-b-508	12	21424661	0.036888	486484	0.549731	Sepsis (28	Sepsis (28	Sepsis (28	TRUE	igd	0.0058	3.83E-12	6797	M33415.m	TRUE	reported	xHSphe	2	TRUE	0.006983	47.78647
1926	rs1030420	T	C	T	C	-0.0221	0.025627	0.1884	0.184521	FALSE	FALSE	FALSE	ieu-b-508	8	67360725	0.043163	486484	0.552692	Sepsis (28	Sepsis (28	Sepsis (28	TRUE	igd	0.0032	1.02E-11	6549	M16822.m	TRUE	reported	UNI018	2	TRUE	0.00723	47.68172
8227	rs4986894	T	C	T	C	-0.0421	-0.06568	0.838	0.851041	FALSE	FALSE	FALSE	ieu-b-508	10	96522365	0.046705	486484	0.15964	Sepsis (28	Sepsis (28	Sepsis (28	TRUE	igd	0.0061	5.55E-12	6486	M36399.m	TRUE	reported	EdUpnt	2	TRUE	0.00729	47.61794
3047	rs182420	T	C	T	C	0.051	-0.00386	0.7553	0.757661	FALSE	FALSE	FALSE	ieu-b-508	19	48372195	0.039111	486484	0.921309	Sepsis (28	Sepsis (28	Sepsis (28	TRUE	igd	0.0074	6.24E-12	7338	M31591.m	TRUE	reported	KltFxx	2	TRUE	0.006431	47.48523
44	rs1312225	T	C	T	C	0.0062	-0.01725	0.5542	0.572215	FALSE	FALSE	FALSE	ieu-b-508	4	1.57E+08	0.03389	486484	0.610652	Sepsis (28	Sepsis (28	Sepsis (28	TRUE	igd	9.00E-04	8.95E-12	7355	M00054.m	TRUE	reported	QTDAnh	2	TRUE	0.006411	47.44389
5481	rs1121592	T	C	T	C	-0.0343	-0.01588	0.54	0.541704	FALSE	FALSE	FALSE	ieu-b-508	7	99523631	0.033466	486484	0.635063	Sepsis (28	Sepsis (28	Sepsis (28	TRUE	igd	0.005	6.38E-12	6797	M33415.m	TRUE	reported	xHSphe	2	TRUE	0.006876	47.04575
6522	rs2235649	T	C	T	C	-0.0184	-0.00375	0.2159	0.223707	FALSE	FALSE	FALSE	ieu-b-508	16	1837406	0.039971	486484	0.92531	Sepsis (28	Sepsis (28	Sepsis (28	TRUE	igd	0.0027	1.95E-11	7049	M34123.m	TRUE	reported	9O5wo1	2	TRUE	0.006545	46.42852
3169	rs272869	A	G	A	G	0.0224	0.003087	0.3706	0.367942	FALSE	FALSE	FALSE	ieu-b-508	5	1.32E+08	0.034515	486484	0.928725	Sepsis (28	Sepsis (28	Sepsis (28	TRUE	igd	0.0033	1.40E-11	7340	M32328.m	TRUE	reported	YGsb6D	2	TRUE	0.006238	46.06274
3867	rs1717158	T	G	T	G	0.0331	-0.0347	0.943	0.970317	FALSE	FALSE	FALSE	ieu-b-508	5	1.31E+08	0.098534	486484	0.724728	Sepsis (28	Sepsis (28	Sepsis (28	TRUE	igd	0.0049	2.06E-11	7361	M32654.m	TRUE	reported	6ISQOP	2	TRUE	0.006161	45.61901
1799	rs1164758	A	G	A	G	0.0303	-0.02618	0.7151	0.718851	FALSE	FALSE	FALSE	ieu-b-508	16	20449616	0.037393	486484	0.483767	Sepsis (28	Sepsis (28	Sepsis (28	TRUE	igd	0.0045	2.85E-11	5829	M15749.m	TRUE	reported	TR861N	2	TRUE	0.007718	45.32222
1982	rs9632722	A	G	A	G	0.0571	0.008727	0.9087	0.910834	FALSE	FALSE	FALSE	ieu-b-508	7	99115568	0.058806	486484	0.882029	Sepsis (28	Sepsis (28	Sepsis (28	TRUE	igd	0.0085	1.52E-11	7226	M17807.m	TRUE	reported	zd6sZl	2	TRUE	0.006206	45.11429
6217	rs2547231	A	C	A	C	0.0409	0.002245	0.833	0.833993	FALSE	FALSE	FALSE	ieu-b-508	19	48385057	0.044785	486484	0.960028	Sepsis (28	Sepsis (28	Sepsis (28	TRUE	igd	0.0061	2.82E-11	4533	M33901.m	TRUE	reported	mQkhrb	2	TRUE	0.00982	44.93609
2812	rs1182518	A	G	A	G	0.0761	-0.0269	0.0766	0.06734	FALSE	FALSE	FALSE	ieu-b-508	11	1.17E+08	0.066476	486484	0.685771	Sepsis (28	Sepsis (28	Sepsis (28	TRUE	igd	0.0114	2.93E-11	2759	M27447.m	TRUE	reported	s1NdyU	2	TRUE	0.015895	44.52918
5604	rs4886484	C	G	C	G	-0.024	-0.011	0.6217	0.62062	FALSE	TRUE	FALSE	ieu-b-508	15	76527955	0.034465	486484	0.74953	Sepsis (28	Sepsis (28	Sepsis (28	TRUE	igd	0.0036	2.69E-11	7365	M33441.m	TRUE	reported	CSdSpl	2	TRUE	0.005998	44.43238
5813	rs2160860	A	T	A	T	0.012	0.020008	0.6141	0.617882	FALSE	TRUE	FALSE	ieu-b-508	8	39823145	0.034601	486484	0.563097	Sepsis (28	Sepsis (28	Sepsis (28	TRUE	igd	0.0018	7.35E-11	7066	M33515.m	TRUE	reported	X9mul1	2	TRUE	0.006251	44.43186
3778	rs7499892	T	C	T	C	-0.02	0.048426	0.175	0.178341	FALSE	FALSE	FALSE	ieu-b-508	16	57006590	0.043677	486484	0.267543	Sepsis (28	Sepsis (28	Sepsis (28	TRUE	igd	0.003	2.62E-11	5598	M32634.m	TRUE	reported	7T2RUz	2	TRUE	0.007877	44.42857
6043	rs601338	A	G	A	G	0.0413	0.007387	0.4316	0.507835	FALSE	FALSE	FALSE	ieu-b-508	19	49206674	0.03347	486484	0.825313	Sepsis (28	Sepsis (28	Sepsis (28	TRUE	igd	0.0062	2.52E-11	3845	M33801.m	TRUE	reported	zhDCxK	2	TRUE	0.011409	44.34971
3162	rs1171615	T	C	T	C	0.0246	-0.0273	0.7778	0.768972	FALSE	FALSE	FALSE	ieu-b-508	10	61469090	0.039552	486484	0.490073	Sepsis (28	Sepsis (28	Sepsis (28	TRUE	igd	0.0037	4.05E-11	7340	M32328.m	TRUE	reported	YGsb6D	2	TRUE	0.005986	44.19248
7206	rs2727271	A	T	A	T	0.0551	-0.11767	0.8882	0.879856	FALSE	TRUE	FALSE	ieu-b-508	11	61604782	0.051127	486484	0.021361	Sepsis (28	Sepsis (28	Sepsis (28	TRUE	igd	0.0083	2.74E-11	2545	M34674.m	TRUE	reported	plxOKz	2	TRUE	0.017022	44.03577
2293	rs4954573	T	G	T	G	0.0219	-0.01928	0.7291	0.757222	FALSE	FALSE	FALSE	ieu-b-508	2	1.37E+08	0.039464	486484	0.625081	Sepsis (28	Sepsis (28	Sepsis (28	TRUE	igd	0.0033	2.53E-11	7301	M20675.m	TRUE	reported	CXe9rE	2	TRUE	0.005996	44.02926
7983	rs6498540	A	G	A	G	0.0172	-0.00969	0.7042	0.698603	FALSE	FALSE	FALSE	ieu-b-508	16	15130594	0.036461	486484	0.790393	Sepsis (28	Sepsis (28	Sepsis (28	TRUE	igd	0.0026	2.55E-11	7358	M35718.m	TRUE	reported	oUwc0a	2	TRUE	0.005913	43.75142
8470	rs1780688	T	C	T	C	-0.0224	0.004268	0.8806	0.882445	FALSE	FALSE	FALSE	ieu-b-508	3	67416322	0.051752	486484	0.934278	Sepsis (28	Sepsis (28	Sepsis (28	TRUE	igd	0.0034	7.15E-11	6552	M37058.m	TRUE	reported	32p8qV	2	TRUE	0.006581	43.39159
2302	rs1053941	T	G	T	G	-0.0171	-0.0654	0.2263	0.233145	FALSE	FALSE	FALSE	ieu-b-508	1	46084383	0.039456	486484	0.097407	Sepsis (28	Sepsis (28	Sepsis (28	TRUE	igd	0.0026	4.99E-11	7327	M20699.m	TRUE	reported	Z3EKFa	2	TRUE	0.005869	43.24411
2881	rs1167525	A	G	A	G	0.0697	-0.07396	0.0885	0.093019	FALSE	FALSE	FALSE	ieu-b-508	2	2.34E+08	0.057472	486484	0.198105	Sepsis (28	Sepsis (28	Sepsis (28	TRUE	igd	0.0106	5.81E-11	6393	M27716.m	TRUE	reported	N5hToq	2	TRUE	0.006718	43.2233
1292	rs1935815	A	T	A	T	0.0111	-0.00396	0.3513	0.349273	FALSE	TRUE	FALSE	ieu-b-508	10	97631492	0.035033	486484	0.909965	Sepsis (28	Sepsis (28	Sepsis (28	TRUE	igd	0.0017	1.55E-10	7325	M02132.m	TRUE	reported	uJFm2t	2	TRUE	0.005787	42.62158
1081	rs1173760	A	G	A	G	0.0104	0.026	0.6847	0.697225	FALSE	FALSE	FALSE	ieu-b-508	4	10488568	0.03647	486484	0.475895	Sepsis (28	Sepsis (28	Sepsis (28	TRUE	igd	0.0016	1.34E-10	7371	M01604.m	TRUE	reported	hE3I2k	2	TRUE	0.005699	42.23854
3247	rs1002246	T	C	T	C	-0.0117	0.028977	0.452	0.444355	FALSE	FALSE	FALSE	ieu-b-508	4	89243818	0.033629	486484	0.388862	Sepsis (28	Sepsis (28	Sepsis (28	TRUE	igd	0.0018	4.55E-11	7365	M32348.m	TRUE	reported	DJeLDx	2	TRUE	0.005704	42.23853
2542	rs1327884	A	G	A	G	-0.013	0.059347	0.73	0.736365	FALSE	FALSE	FALSE	ieu-b-508	8	26714874	0.038016	486484	0.118496	Sepsis (28	Sepsis (28	Sepsis (28	TRUE	igd	0.002	2.79E-11	6535	M22177.m	TRUE	reported	2s6Sly	2	TRUE	0.006424	42.23707
1451	rs567754	T	C	T	C	0.0162	0.047861	0.3247	0.340305	FALSE	FALSE	FALSE	ieu-b-508	5	78416416	0.035285	486484	0.174964	Sepsis (28	Sepsis (28	Sepsis (28	TRUE	igd	0.0025	4.96E-11	7357	M03141.m	TRUE	reported	y4mG6m	2	TRUE	0.005675	41.97898
5284	rs7962263	T	C	T	C	-0.0317	0.002542	0.2686	0.282798	FALSE	FALSE	FALSE	ieu-b-508	12	21466117	0.036981	486484	0.945208	Sepsis (28	Sepsis (28	Sepsis (28	TRUE	igd	0.0049	7.86E-11	4409	M33250.m	TRUE	reported	WIZI6U	2	TRUE	0.009403	41.83399
3873	rs2291428	C	G	C	G	-0.0168	-0.04144	0.2351	0.241829	FALSE	TRUE	FALSE	ieu-b-508	10	45958856	0.03897	486484	0.287647	Sepsis (28	Sepsis (28	Sepsis (28	TRUE	igd	0.0026	8.71E-11	7361	M32654.m	TRUE	reported	6ISQOP	2	TRUE	0.00564	41.74014
13	rs1016522	A	G	A	G	0.0058	-0.00132	0.5803	0.575669	FALSE	FALSE	FALSE	ieu-b-508	5	1.43E+08	0.033882	486484	0.968934	Sepsis (28	Sepsis (28	Sepsis (28	TRUE	igd	9.00E-04	1.59E-10	7355	M00054.m	TRUE	reported	QTDAnh	2	TRUE	0.005615	41.51957
498	rs2472297	T	C	T	C	-0.0668	0.004523	0.2362	0.261592	FALSE	FALSE	FALSE	ieu-b-508	15	75027880	0.037957	486484	0.905148	Sepsis (28	Sepsis (28	Sepsis (28	TRUE	igd	0.0104	1.50E-10	7274	M00569.m	TRUE	reported	1TjBaz	2	TRUE	0.00564	41.24457
7633	rs1224602	A	G	A	G	0.0115	-0.06988	0.2666	0.268367	FALSE	FALSE	FALSE	ieu-b-508	10	318076	0.03791	486484	0.065288	Sepsis (28	Sepsis (28	Sepsis (28	TRUE	igd	0.0018	1.05E-10	5768	M35326.m	TRUE	reported	jRytyJ	2	TRUE	0.007027	40.80375
1969	rs2199680	A	T	A	T	-0.0415	0.062865	0.8328	0.839848	FALSE	TRUE	FALSE	ieu-b-508	12	21415496	0.045979	486484	0.171543	Sepsis (28	Sepsis (28	Sepsis (28	TRUE	igd	0.0065	2.17E-10	7226	M17807.m	TRUE	reported	zd6sZl	2	TRUE	0.00561	40.75203
685	rs172650	A	C	A	C	0.0102	0.021603	0.6543	0.624275	FALSE	FALSE	FALSE	ieu-b-508	16	71612889	0.034471	486484	0.530866	Sepsis (28	Sepsis (28	Sepsis (28	TRUE	igd	0.0016	2.82E-10	7358	M01299.m	TRUE	reported	gfM0a3	2	TRUE	0.005493	40.62958
928	rs1395	A	G	A	G	0.0172	-0.03401	0.7034	0.695829	FALSE	FALSE	FALSE	ieu-b-508	2	27424636	0.036177	486484	0.347142	Sepsis (28	Sepsis (28	Sepsis (28	TRUE	igd	0.0027	8.14E-11	7160	M01508.m	TRUE	reported	go526k	2	TRUE	0.005636	40.57028
4938	rs3816015	A	T	A	T	-0.1041	-0.0401	0.3923	0.408994	FALSE	TRUE	FALSE	ieu-b-508	5	1.51E+08	0.033906	486484	0.236964	Sepsis (28	Sepsis (28	Sepsis (28	TRUE	igd	0.0164	2.20E-10	1017	M33178.m	TRUE	reported	g22WMS	2	TRUE	0.038108	40.21229
3064	rs1175428	A	G	A	G	0.0488	0.010064	0.4322	0.419466	FALSE	FALSE	FALSE	ieu-b-508	6	25776949	0.033744	486484	0.765514	Sepsis (28	Sepsis (28	Sepsis (28	TRUE	igd	0.0077	2.25E-10	7363	M31787.m	TRUE	reported	XMp8YI	2	TRUE	0.005426	40.15505
1032	rs835154	A	G	A	G	-0.0114	0.00318	0.5753	0.5557	FALSE	FALSE	FALSE	ieu-b-508	5	14876569	0.033842	486484	0.925131	Sepsis (28	Sepsis (28	Sepsis (28	TRUE	igd	0.0018	1.15E-10	7364	M01564.m	TRUE	reported	IPUdyD	2	TRUE	0.005417	40.10022
112	rs4306882	T	G	T	G	-0.0057	0.004642	0.6153	0.609648	FALSE	FALSE	FALSE	ieu-b-508	3	21062584	0.034311	486484	0.892391	Sepsis (28	Sepsis (28	Sepsis (28	TRUE	igd	9.00E-04	2.52E-10	7355	M00054.m	TRUE	reported	QTDAnh	2	TRUE	0.005424	40.1002
4074	rs651007	T	C	T	C	-0.0152	-0.03497	0.2007	0.207276	FALSE	FALSE	FALSE	ieu-b-508	9	1.36E+08	0.041051	486484	0.394312	Sepsis (28	Sepsis (28	Sepsis (28	TRUE	igd	0.0024	2.78E-10	7318	M32740.m	TRUE	reported	YLshfP	2	TRUE	0.005451	40.10015
2597	rs273914	A	T	A	T	0.0209	0.005997	0.3691	0.374818	FALSE	TRUE	FALSE	ieu-b-508	5	1.32E+08	0.034372	486484	0.861482	Sepsis (28	Sepsis (28	Sepsis (28	TRUE	igd	0.0033	4.02E-10	7259	M22189.m	TRUE	reported	YSD5fY	2	TRUE	0.005495	40.10006
2840	rs7809234	A	T	A	T	0.0285	0.01047	0.8755	0.882505	FALSE	TRUE	FALSE	ieu-b-508	7	50610379	0.051911	486484	0.840163	Sepsis (28	Sepsis (28	Sepsis (28	TRUE	igd	0.0045	1.60E-10	7180	M27513.m	TRUE	reported	o9Ff2u	2	TRUE	0.005555	40.09994
1450	rs495360	A	C	A	C	-0.019	0.050729	0.497	0.501258	FALSE	FALSE	FALSE	ieu-b-508	12	327421	0.033899	486484	0.134529	Sepsis (28	Sepsis (28	Sepsis (28	TRUE	igd	0.003	2.40E-10	5598	M03141.m	TRUE	reported	y4mG6m	2	TRUE	0.007114	40.09678
5544	rs1042327	T	G	T	G	1.0018	0.095336	0.9333	0.951285	FALSE	FALSE	FALSE	ieu-b-508	19	1310136	0.079301	486484	0.229287	Sepsis (28	Sepsis (28	Sepsis (28	TRUE	igd	0.1561	1.38E-10	60	M33423.m	TRUE	reported	MoEnO7	2	TRUE	0.407036	39.81374
6981	rs4706020	A	G	A	G	-0.0214	-0.05922	0.3834	0.378721	FALSE	FALSE	FALSE	ieu-b-508	5	1.31E+08	0.034554	486484	0.086581	Sepsis (28	Sepsis (28	Sepsis (28	TRUE	igd	0.0034	3.80E-10	7344	M34407.m	TRUE	reported	X7I83Y	2	TRUE	0.005365	39.60513
3861	rs1007003	A	G	A	G	-0.0409	0.0706	0.0297	0.028865	FALSE	FALSE	FALSE	ieu-b-508	5	1.3E+08	0.099478	486484	0.477887	Sepsis (28	Sepsis (28	Sepsis (28	TRUE	igd	0.0065	4.30E-10	7361	M32654.m	TRUE	reported	6ISQOP	2	TRUE	0.00535	39.58238
6409	rs1165209	A	G	A	G	-0.0226	-0.00871	0.5531	0.566604	FALSE	FALSE	FALSE	ieu-b-508	6	25801319	0.033638	486484	0.795711	Sepsis (28	Sepsis (28	Sepsis (28	TRUE	igd	0.0036	5.13E-10	7132	M34040.m	TRUE	reported	sJBy3i	2	TRUE	0.005496	39.39944
4638	rs4951117	A	T	A	T	-0.4779	-0.01217	0.0855	0.104557	FALSE	TRUE	FALSE	ieu-b-508	1	2.05E+08	0.054437	486484	0.823113	Sepsis (28	Sepsis (28	Sepsis (28	TRUE	igd	0.0752	2.05E-10	76	M33173.m	TRUE	reported	04fkUQ	2	TRUE	0.347004	39.32389
4096	rs556339	T	C	T	C	0.0382	-0.00863	0.2533	0.260572	FALSE	FALSE	FALSE	ieu-b-508	6	25870745	0.037999	486484	0.820378	Sepsis (28	Sepsis (28	Sepsis (28	TRUE	igd	0.0061	4.83E-10	7356	M32753.m	TRUE	reported	XlC6zq	2	TRUE	0.005303	39.20568
431	rs445925	A	G	A	G	-0.025	0.069979	0.079	0.108393	FALSE	FALSE	FALSE	ieu-b-508	19	45415640	0.05337	486484	0.189782	Sepsis (28	Sepsis (28	Sepsis (28	TRUE	igd	0.004	4.11E-10	5602	M00063.m	TRUE	reported	Hdsv1L	2	TRUE	0.006925	39.04855
4201	rs4149081	A	G	A	G	0.068	-0.0701	0.1794	0.162129	FALSE	FALSE	FALSE	ieu-b-508	12	21378021	0.045258	486484	0.12143	Sepsis (28	Sepsis (28	Sepsis (28	TRUE	igd	0.0109	5.22E-10	2537	M32762.m	TRUE	reported	Q5IKLH	2	TRUE	0.015109	38.8886
6872	rs2309461	T	C	T	C	-0.2016	0.035662	0.7222	0.667121	FALSE	FALSE	FALSE	ieu-b-508	4	1.82E+08	0.035602	486484	0.316484	Sepsis (28	Sepsis (28	Sepsis (28	TRUE	igd	0.0305	3.95E-11	18	M34365.m	TRUE	reported	8eexD0	2	TRUE	0.708218	38.8355
153	rs6935961	A	G	A	G	-0.0056	0.042025	0.5578	0.556347	FALSE	FALSE	FALSE	ieu-b-508	6	1.26E+08	0.033624	486484	0.211358	Sepsis (28	Sepsis (28	Sepsis (28	TRUE	igd	9.00E-04	3.75E-10	7355	M00054.m	TRUE	reported	QTDAnh	2	TRUE	0.005236	38.70552
3597	rs1165196	A	G	A	G	-0.0409	-0.00831	0.549	0.566584	FALSE	FALSE	FALSE	ieu-b-508	6	25813150	0.03363	486484	0.804813	Sepsis (28	Sepsis (28	Sepsis (28	TRUE	igd	0.0066	4.80E-10	7256	M32549.m	TRUE	reported	W7Fgu2	2	TRUE	0.005265	38.39185
3168	rs2070630	C	G	C	G	-0.021	-0.018	0.2948	0.302967	FALSE	TRUE	FALSE	ieu-b-508	4	1.6E+08	0.036382	486484	0.620862	Sepsis (28	Sepsis (28	Sepsis (28	TRUE	igd	0.0034	6.76E-10	7340	M32328.m	TRUE	reported	YGsb6D	2	TRUE	0.005171	38.13839
5492	rs6544713	T	C	T	C	-0.0314	0.005815	0.3211	0.32316	FALSE	FALSE	FALSE	ieu-b-508	2	44073881	0.035588	486484	0.870205	Sepsis (28	Sepsis (28	Sepsis (28	TRUE	igd	0.0051	8.97E-10	6797	M33415.m	TRUE	reported	xHSphe	2	TRUE	0.005546	37.8958
766	rs7503429	A	C	A	C	-0.016	0.033155	0.4255	0.418614	FALSE	FALSE	FALSE	ieu-b-508	17	80191425	0.034194	486484	0.332238	Sepsis (28	Sepsis (28	Sepsis (28	TRUE	igd	0.0026	1.64E-09	6946	M01303.m	TRUE	reported	3b8x0F	2	TRUE	0.005422	37.85892
6263	rs924135	A	T	A	T	-0.0209	0.024954	0.384	0.388339	FALSE	TRUE	FALSE	ieu-b-508	16	16123459	0.034258	486484	0.466351	Sepsis (28	Sepsis (28	Sepsis (28	TRUE	igd	0.0034	1.09E-09	7345	M33936.m	TRUE	reported	jwA4B2	2	TRUE	0.005118	37.77604
7170	rs4690909	C	G	C	G	0.0301	0.019474	0.6902	0.697724	FALSE	TRUE	FALSE	ieu-b-508	4	1.6E+08	0.036189	486484	0.590495	Sepsis (28	Sepsis (28	Sepsis (28	TRUE	igd	0.0049	1.06E-09	4925	M34534.m	TRUE	reported	iA78RM	2	TRUE	0.007604	37.71937
3793	rs2070895	A	G	A	G	0.0257	-0.03138	0.21	0.219377	FALSE	FALSE	FALSE	ieu-b-508	15	58723939	0.040342	486484	0.436649	Sepsis (28	Sepsis (28	Sepsis (28	TRUE	igd	0.0042	1.22E-09	7369	M32635.m	TRUE	reported	jJI9P8	2	TRUE	0.005055	37.43258
1031	rs807670	A	C	A	C	0.011	0.005632	0.421	0.432011	FALSE	FALSE	FALSE	ieu-b-508	22	19154608	0.033906	486484	0.868064	Sepsis (28	Sepsis (28	Sepsis (28	TRUE	igd	0.0018	6.84E-10	7364	M01564.m	TRUE	reported	IPUdyD	2	TRUE	0.005046	37.33554
5593	rs915282	A	G	A	G	-1.8595	-0.02144	0.0167	0.011635	FALSE	FALSE	FALSE	ieu-b-508	9	1.2E+08	0.156721	486484	0.891165	Sepsis (28	Sepsis (28	Sepsis (28	TRUE	igd	0.2997	5.49E-10	60	M33423.m	TRUE	reported	MoEnO7	2	TRUE	0.39084	37.21308
3736	rs1809148	A	G	A	G	-0.0262	-0.0478	0.136	0.130981	FALSE	FALSE	FALSE	ieu-b-508	8	1.45E+08	0.049429	486484	0.333555	Sepsis (28	Sepsis (28	Sepsis (28	TRUE	igd	0.0043	9.51E-10	7337	M32632.m	TRUE	reported	pIHzPT	2	TRUE	0.005034	37.11481
5562	rs1713299	T	G	T	G	-1.8613	0.17375	0.0177	0.038808	FALSE	FALSE	FALSE	ieu-b-508	6	177050	0.134413	486484	0.19613	Sepsis (28	Sepsis (28	Sepsis (28	TRUE	igd	0.3006	5.95E-10	60	M33423.m	TRUE	reported	MoEnO7	2	TRUE	0.389873	37.06223
5828	rs4843718	A	G	A	G	0.0158	0.024868	0.2413	0.245192	FALSE	FALSE	FALSE	ieu-b-508	16	87878476	0.03896	486484	0.523277	Sepsis (28	Sepsis (28	Sepsis (28	TRUE	igd	0.0026	1.71E-09	7066	M33515.m	TRUE	reported	X9mul1	2	TRUE	0.005199	36.91854
2411	rs964184	C	G	C	G	-0.0443	-0.04717	0.8615	0.86637	FALSE	TRUE	FALSE	ieu-b-508	11	1.17E+08	0.048981	486484	0.335505	Sepsis (28	Sepsis (28	Sepsis (28	TRUE	igd	0.0073	1.20E-09	5436	M21184.m	TRUE	reported	63ppuJ	2	TRUE	0.006729	36.81306
1621	rs1008593	T	C	T	C	-0.0103	-0.01627	0.3782	0.373392	FALSE	FALSE	FALSE	ieu-b-508	8	39806267	0.034603	486484	0.638194	Sepsis (28	Sepsis (28	Sepsis (28	TRUE	igd	0.0017	3.33E-09	7368	M15140.m	TRUE	reported	jEcqMJ	2	TRUE	0.004958	36.69938
7751	rs274570	T	C	T	C	0.0224	0.020129	0.2962	0.293331	FALSE	FALSE	FALSE	ieu-b-508	5	1.32E+08	0.036615	486484	0.582504	Sepsis (28	Sepsis (28	Sepsis (28	TRUE	igd	0.0037	1.78E-09	5306	M35433.m	TRUE	reported	XIYkLe	2	TRUE	0.00686	36.63776
7187	rs1213675	A	C	A	C	0.026	-0.02618	0.3343	0.305487	FALSE	FALSE	FALSE	ieu-b-508	1	76243438	0.036255	486484	0.470208	Sepsis (28	Sepsis (28	Sepsis (28	TRUE	igd	0.0043	1.24E-09	4800	M34539.m	TRUE	reported	puA8ni	2	TRUE	0.007559	36.54507
4213	rs1050967	C	G	C	G	-0.0471	-0.0487	0.1624	0.151929	FALSE	TRUE	FALSE	ieu-b-508	10	96236191	0.04653	486484	0.295301	Sepsis (28	Sepsis (28	Sepsis (28	TRUE	igd	0.0078	1.78E-09	7330	M32786.m	TRUE	reported	rwF0co	2	TRUE	0.00495	36.45307
5491	rs4917639	A	C	A	C	-0.0362	0.034924	0.8015	0.801852	FALSE	FALSE	FALSE	ieu-b-508	10	96725535	0.041818	486484	0.403646	Sepsis (28	Sepsis (28	Sepsis (28	TRUE	igd	0.006	1.39E-09	6797	M33415.m	TRUE	reported	xHSphe	2	TRUE	0.005327	36.3904
6297	rs4691536	A	G	A	G	0.0247	0.035819	0.3688	0.382599	FALSE	FALSE	FALSE	ieu-b-508	4	1.6E+08	0.034279	486484	0.296054	Sepsis (28	Sepsis (28	Sepsis (28	TRUE	igd	0.0041	1.62E-09	7320	M33941.m	TRUE	reported	culqiU	2	TRUE	0.004934	36.28336
4611	rs1242635	T	G	T	G	-1.192	0.107329	0.0132	0.008723	FALSE	FALSE	FALSE	ieu-b-508	12	1.07E+08	0.179523	486484	0.549938	Sepsis (28	Sepsis (28	Sepsis (28	TRUE	igd	0.1953	1.04E-09	76	M33173.m	TRUE	reported	04fkUQ	2	TRUE	0.328929	36.27155
434	rs602633	T	G	T	G	-0.0102	-0.10909	0.2163	0.217297	FALSE	FALSE	FALSE	ieu-b-508	1	1.1E+08	0.04034	486484	0.006848	Sepsis (28	Sepsis (28	Sepsis (28	TRUE	igd	0.0017	5.02E-09	7365	M00063.m	TRUE	reported	Hdsv1L	2	TRUE	0.004864	35.99022
3417	rs1012850	A	G	A	G	0.0108	-0.04056	0.5803	0.591661	FALSE	FALSE	FALSE	ieu-b-508	10	61521501	0.033938	486484	0.231986	Sepsis (28	Sepsis (28	Sepsis (28	TRUE	igd	0.0018	5.06E-09	7364	M32452.m	TRUE	reported	8GtFpl	2	TRUE	0.004865	35.99022
3556	rs1046801	T	C	T	C	0.0108	0.02023	0.289	0.295989	FALSE	FALSE	FALSE	ieu-b-508	15	58678512	0.036545	486484	0.579885	Sepsis (28	Sepsis (28	Sepsis (28	TRUE	igd	0.0018	3.45E-09	7350	M32518.m	TRUE	reported	V0ZCiU	2	TRUE	0.004874	35.9902
7764	rs1259788	T	G	T	G	0.0156	0.001799	0.3363	0.330182	FALSE	FALSE	FALSE	ieu-b-508	16	67170432	0.034855	486484	0.958836	Sepsis (28	Sepsis (28	Sepsis (28	TRUE	igd	0.0026	3.78E-09	7256	M35439.m	TRUE	reported	DnuAGZ	2	TRUE	0.004937	35.99008
93	rs284191	A	G	A	G	-0.006	0.04398	0.615	0.617284	FALSE	FALSE	FALSE	ieu-b-508	1	92236048	0.034455	486484	0.201802	Sepsis (28	Sepsis (28	Sepsis (28	TRUE	igd	0.001	1.97E-09	5592	M00054.m	TRUE	reported	QTDAnh	2	TRUE	0.006397	35.98712
292	rs2713737	A	G	A	G	-0.006	-0.02457	0.44	0.439028	FALSE	FALSE	FALSE	ieu-b-508	3	1.01E+08	0.033943	486484	0.469232	Sepsis (28	Sepsis (28	Sepsis (28	TRUE	igd	0.001	1.97E-09	5589	M00060.m	TRUE	reported	kqwl4V	2	TRUE	0.0064	35.98712
7380	rs7203065	T	G	T	G	0.0114	-0.03426	0.3934	0.389998	FALSE	FALSE	FALSE	ieu-b-508	16	84748515	0.035043	486484	0.328258	Sepsis (28	Sepsis (28	Sepsis (28	TRUE	igd	0.0019	2.00E-09	4900	M35137.m	TRUE	reported	Eq2siS	2	TRUE	0.007293	35.98531
6734	rs1912320	A	G	A	G	0.3974	-0.16185	0.0355	0.034393	FALSE	FALSE	FALSE	ieu-b-508	10	87209918	0.091903	486484	0.07822	Sepsis (28	Sepsis (28	Sepsis (28	TRUE	igd	0.0662	1.98E-09	535	M34338.m	TRUE	reported	h4FB9a	2	TRUE	0.063107	35.90155
4148	rs3732218	A	G	A	G	-0.0532	-3.69E-05	0.0805	0.082397	FALSE	FALSE	FALSE	ieu-b-508	2	2.35E+08	0.06092	486484	0.999516	Sepsis (28	Sepsis (28	Sepsis (28	TRUE	igd	0.0089	2.77E-09	7250	M32757.m	TRUE	reported	TJV9Xb	2	TRUE	0.004904	35.72099
6859	rs1269734	T	C	T	C	-0.2064	0.020245	0.8333	0.788994	FALSE	FALSE	FALSE	ieu-b-508	5	35712513	0.040764	486484	0.619448	Sepsis (28	Sepsis (28	Sepsis (28	TRUE	igd	0.0326	2.54E-10	18	M34365.m	TRUE	reported	8eexD0	2	TRUE	0.69011	35.6313
4000	rs3764261	A	C	A	C	0.0113	-0.06314	0.3354	0.323147	FALSE	FALSE	FALSE	ieu-b-508	16	56994528	0.035643	486484	0.076498	Sepsis (28	Sepsis (28	Sepsis (28	TRUE	igd	0.0019	3.39E-09	6497	M32729.m	TRUE	reported	XGRSN1	2	TRUE	0.005415	35.3603
3433	rs2715311	A	G	A	G	-0.0107	-0.01589	0.3884	0.385011	FALSE	FALSE	FALSE	ieu-b-508	18	63688253	0.034316	486484	0.643237	Sepsis (28	Sepsis (28	Sepsis (28	TRUE	igd	0.0018	6.57E-09	7364	M32452.m	TRUE	reported	8GtFpl	2	TRUE	0.004776	35.32682
6546	rs1077835	A	G	A	G	-0.0208	0.031263	0.7976	0.779568	FALSE	FALSE	FALSE	ieu-b-508	15	58723426	0.040315	486484	0.438065	Sepsis (28	Sepsis (28	Sepsis (28	TRUE	igd	0.0035	1.82E-09	7347	M34244.m	TRUE	reported	JyF0Sp	2	TRUE	0.004784	35.30794
6412	rs1466245	T	C	T	C	0.0481	0.001775	0.0511	0.052168	FALSE	FALSE	FALSE	ieu-b-508	3	15962611	0.075365	486484	0.981211	Sepsis (28	Sepsis (28	Sepsis (28	TRUE	igd	0.0081	2.78E-09	7132	M34040.m	TRUE	reported	sJBy3i	2	TRUE	0.00492	35.25318
6739	rs2830801	A	G	A	G	-1.072	0.020227	0.023	0.115068	FALSE	FALSE	FALSE	ieu-b-508	21	28620731	0.052483	486484	0.699935	Sepsis (28	Sepsis (28	Sepsis (28	TRUE	igd	0.179	2.11E-09	115	M34338.m	TRUE	reported	h4FB9a	2	TRUE	0.237734	35.24229
4239	rs6929324	T	G	T	G	-0.0896	0.000436	0.0166	0.012603	FALSE	FALSE	FALSE	ieu-b-508	6	30988886	0.149512	486484	0.997671	Sepsis (28	Sepsis (28	Sepsis (28	TRUE	igd	0.0151	3.25E-09	6857	M32787.m	TRUE	reported	fSRakm	2	TRUE	0.005109	35.19941
1316	rs1017016	T	C	T	C	0.0338	-0.01786	0.5675	0.625457	FALSE	FALSE	FALSE	ieu-b-508	2	2.35E+08	0.03445	486484	0.604227	Sepsis (28	Sepsis (28	Sepsis (28	TRUE	igd	0.0057	2.71E-09	6252	M02137.m	TRUE	reported	iRGqBE	2	TRUE	0.005593	35.15157
6618	rs1470777	T	C	T	C	-0.0515	0.058426	0.5972	0.603158	FALSE	FALSE	FALSE	ieu-b-508	8	1072775	0.034336	486484	0.088828	Sepsis (28	Sepsis (28	Sepsis (28	TRUE	igd	0.0087	3.73E-09	1766	M34314.m	TRUE	reported	wPKEWG	2	TRUE	0.019456	35.00127
3240	rs4870490	A	G	A	G	0.0857	-0.10175	0.066	0.072314	FALSE	FALSE	FALSE	ieu-b-508	6	1.57E+08	0.064299	486484	0.113564	Sepsis (28	Sepsis (28	Sepsis (28	TRUE	igd	0.0145	3.14E-09	6687	M32346.m	TRUE	reported	cnIFoV	2	TRUE	0.005197	34.92173
7160	rs279941	A	C	A	C	-0.0478	0.0304	0.1171	0.122154	FALSE	FALSE	FALSE	ieu-b-508	13	1.04E+08	0.050931	486484	0.550583	Sepsis (28	Sepsis (28	Sepsis (28	TRUE	igd	0.0081	4.31E-09	5856	M34533.m	TRUE	reported	SuKX4k	2	TRUE	0.005912	34.81268
162	rs7463805	T	C	T	C	-0.0053	-0.01977	0.5213	0.511221	FALSE	FALSE	FALSE	ieu-b-508	8	1.11E+08	0.0335	486484	0.555052	Sepsis (28	Sepsis (28	Sepsis (28	TRUE	igd	9.00E-04	4.60E-09	7355	M00054.m	TRUE	reported	QTDAnh	2	TRUE	0.004693	34.66958
220	rs1092301	A	G	A	G	0.0053	0.044187	0.6191	0.62302	FALSE	FALSE	FALSE	ieu-b-508	1	88516267	0.034472	486484	0.199904	Sepsis (28	Sepsis (28	Sepsis (28	TRUE	igd	9.00E-04	2.88E-09	7351	M00060.m	TRUE	reported	kqwl4V	2	TRUE	0.004695	34.66958
362	rs7647029	A	G	A	G	0.0053	-0.06176	0.6479	0.639897	FALSE	FALSE	FALSE	ieu-b-508	3	1.32E+08	0.034874	486484	0.076554	Sepsis (28	Sepsis (28	Sepsis (28	TRUE	igd	9.00E-04	1.02E-08	7351	M00060.m	TRUE	reported	kqwl4V	2	TRUE	0.004695	34.66958
4462	rs9318225	T	C	T	C	-0.0106	-0.00417	0.6829	0.684825	FALSE	FALSE	FALSE	ieu-b-508	13	74491265	0.035921	486484	0.907594	Sepsis (28	Sepsis (28	Sepsis (28	TRUE	igd	0.0018	1.95E-09	7344	M32910.m	TRUE	reported	2OCBKh	2	TRUE	0.0047	34.66957
5663	rs1026376	T	C	T	C	-0.0106	-0.0035	0.2442	0.246096	FALSE	FALSE	FALSE	ieu-b-508	7	55985288	0.038736	486484	0.928065	Sepsis (28	Sepsis (28	Sepsis (28	TRUE	igd	0.0018	7.47E-09	7307	M33477.m	TRUE	reported	Aw8PBK	2	TRUE	0.004724	34.66952
1535	rs1094564	A	T	A	T	0.0212	0.070104	0.1287	0.135836	FALSE	TRUE	FALSE	ieu-b-508	6	1.6E+08	0.048928	486484	0.151921	Sepsis (28	Sepsis (28	Sepsis (28	TRUE	igd	0.0036	4.29E-09	6864	M12129.m	TRUE	reported	iNzdgb	2	TRUE	0.005027	34.66891
1269	rs1016452	A	G	A	G	-0.01	0.03626	0.6775	0.669782	FALSE	FALSE	FALSE	ieu-b-508	2	2.12E+08	0.035586	486484	0.308229	Sepsis (28	Sepsis (28	Sepsis (28	TRUE	igd	0.0017	1.06E-08	7325	M02132.m	TRUE	reported	uJFm2t	2	TRUE	0.004702	34.59263
3050	rs4236541	T	G	T	G	0.0423	-0.01709	0.7287	0.735283	FALSE	FALSE	FALSE	ieu-b-508	7	98830439	0.037978	486484	0.652637	Sepsis (28	Sepsis (28	Sepsis (28	TRUE	igd	0.0072	5.09E-09	7338	M31591.m	TRUE	reported	KltFxx	2	TRUE	0.004682	34.50622
6660	rs726389	T	C	T	C	-0.31	-0.11641	0.9733	0.977712	FALSE	FALSE	FALSE	ieu-b-508	20	19615798	0.114725	486484	0.310249	Sepsis (28	Sepsis (28	Sepsis (28	TRUE	igd	0.0521	2.71E-09	75	M34322.m	TRUE	reported	16GdG9	2	TRUE	0.320675	34.45954
4271	rs1264510	A	G	A	G	0.0463	-0.01856	0.1473	0.152392	FALSE	FALSE	FALSE	ieu-b-508	4	69988999	0.046504	486484	0.689795	Sepsis (28	Sepsis (28	Sepsis (28	TRUE	igd	0.0079	4.61E-09	6200	M32808.m	TRUE	reported	vnr36k	2	TRUE	0.00551	34.33742
2301	rs9756306	A	G	A	G	-0.0158	-0.00181	0.3332	0.324404	FALSE	FALSE	FALSE	ieu-b-508	3	1.65E+08	0.035625	486484	0.959585	Sepsis (28	Sepsis (28	Sepsis (28	TRUE	igd	0.0027	3.64E-09	7301	M20675.m	TRUE	reported	CXe9rE	2	TRUE	0.004668	34.23479
4256	rs1705769	T	C	T	C	0.0831	-0.08874	0.0555	0.05084	FALSE	FALSE	FALSE	ieu-b-508	6	97779395	0.07576	486484	0.241456	Sepsis (28	Sepsis (28	Sepsis (28	TRUE	igd	0.0142	5.00E-09	4274	M32800.m	TRUE	reported	Q7MVsa	2	TRUE	0.007949	34.2312
4161	rs3820744	T	C	T	C	-0.0433	0.073001	0.9084	0.907302	FALSE	FALSE	FALSE	ieu-b-508	2	2.34E+08	0.057606	486484	0.205067	Sepsis (28	Sepsis (28	Sepsis (28	TRUE	igd	0.0074	4.28E-09	6642	M32758.m	TRUE	reported	AF0m66	2	TRUE	0.005128	34.228
7126	rs1166660	T	C	T	C	0.0234	0.00363	0.2406	0.236421	FALSE	FALSE	FALSE	ieu-b-508	19	12496934	0.039722	486484	0.927181	Sepsis (28	Sepsis (28	Sepsis (28	TRUE	igd	0.004	6.96E-09	7322	M34527.m	TRUE	reported	EWFGP8	2	TRUE	0.004652	34.21315
4644	rs690764	A	G	A	G	-0.6782	-0.1719	0.0574	0.071812	FALSE	FALSE	FALSE	ieu-b-508	16	84574353	0.064864	486484	0.008046	Sepsis (28	Sepsis (28	Sepsis (28	TRUE	igd	0.1147	3.36E-09	76	M33173.m	TRUE	reported	04fkUQ	2	TRUE	0.315077	34.04136
7947	rs9404466	T	C	T	C	0.0105	-0.02169	0.2671	0.273739	FALSE	FALSE	FALSE	ieu-b-508	6	1.04E+08	0.03746	486484	0.562648	Sepsis (28	Sepsis (28	Sepsis (28	TRUE	igd	0.0018	1.26E-08	7348	M35675.m	TRUE	reported	D7fdyZ	2	TRUE	0.00461	34.01852
5522	rs2263065	T	G	T	G	-0.0105	-0.03476	0.4417	0.519497	FALSE	FALSE	FALSE	ieu-b-508	12	1.02E+08	0.033559	486484	0.300285	Sepsis (28	Sepsis (28	Sepsis (28	TRUE	igd	0.0018	3.56E-09	7306	M33422.m	TRUE	reported	PLgjrT	2	TRUE	0.004636	34.01846
7129	rs1761493	A	G	A	G	0.0204	-0.00699	0.782	0.777585	FALSE	FALSE	FALSE	ieu-b-508	4	70360229	0.040557	486484	0.863147	Sepsis (28	Sepsis (28	Sepsis (28	TRUE	igd	0.0035	5.67E-09	7322	M34527.m	TRUE	reported	EWFGP8	2	TRUE	0.004618	33.96297
5774	rs6430553	T	C	T	C	-0.0204	0.039292	0.4007	0.371084	FALSE	FALSE	FALSE	ieu-b-508	2	1.36E+08	0.034696	486484	0.257439	Sepsis (28	Sepsis (28	Sepsis (28	TRUE	igd	0.0035	4.81E-09	7044	M33509.m	TRUE	reported	56SCCs	2	TRUE	0.0048	33.9626
1236	rs1093881	T	G	T	G	0.0099	0.051328	0.5932	0.587326	FALSE	FALSE	FALSE	ieu-b-508	4	20856353	0.033939	486484	0.130437	Sepsis (28	Sepsis (28	Sepsis (28	TRUE	igd	0.0017	8.38E-09	7136	M01769.m	TRUE	reported	rismUZ	2	TRUE	0.00473	33.90399
1971	rs296381	T	C	T	C	0.039	-0.00847	0.8282	0.835977	FALSE	FALSE	FALSE	ieu-b-508	19	48371102	0.045022	486484	0.850858	Sepsis (28	Sepsis (28	Sepsis (28	TRUE	igd	0.0067	4.83E-09	7226	M17807.m	TRUE	reported	zd6sZl	2	TRUE	0.004667	33.87345
3678	rs920812	T	C	T	C	0.0256	-0.01462	0.326	0.328569	FALSE	FALSE	FALSE	ieu-b-508	6	1.6E+08	0.035473	486484	0.680221	Sepsis (28	Sepsis (28	Sepsis (28	TRUE	igd	0.0044	8.19E-09	7322	M32578.m	TRUE	reported	LPkMFr	2	TRUE	0.004602	33.84199
2875	rs7948073	T	G	T	G	-0.0256	0.060901	0.5702	0.568527	FALSE	FALSE	FALSE	ieu-b-508	11	67375184	0.033718	486484	0.070894	Sepsis (28	Sepsis (28	Sepsis (28	TRUE	igd	0.0044	4.60E-09	6715	M27710.m	TRUE	reported	XeaFav	2	TRUE	0.005016	33.84116
4276	rs4149026	A	C	A	C	0.036	0.007067	0.6863	0.694115	FALSE	FALSE	FALSE	ieu-b-508	12	21315415	0.036535	486484	0.846623	Sepsis (28	Sepsis (28	Sepsis (28	TRUE	igd	0.0062	5.67E-09	6200	M32808.m	TRUE	reported	vnr36k	2	TRUE	0.005408	33.704
5246	rs3738544	T	C	T	C	0.0203	0.029256	0.8071	0.803494	FALSE	FALSE	FALSE	ieu-b-508	1	2.37E+08	0.042132	486484	0.487432	Sepsis (28	Sepsis (28	Sepsis (28	TRUE	igd	0.0035	8.05E-09	7063	M33228.m	TRUE	reported	JwOXbQ	2	TRUE	0.00474	33.63047
6103	rs1535	A	G	A	G	-0.0203	-0.08051	0.662	0.65351	FALSE	FALSE	FALSE	ieu-b-508	11	61597972	0.035048	486484	0.021611	Sepsis (28	Sepsis (28	Sepsis (28	TRUE	igd	0.0035	6.15E-09	6506	M33871.m	TRUE	reported	mQD6DP	2	TRUE	0.005144	33.62966
5558	rs1684815	A	G	A	G	-1.7999	-0.12686	0.0199	0.009966	FALSE	FALSE	FALSE	ieu-b-508	3	1.64E+08	0.167016	486484	0.4475	Sepsis (28	Sepsis (28	Sepsis (28	TRUE	igd	0.3054	3.79E-09	60	M33423.m	TRUE	reported	MoEnO7	2	TRUE	0.36665	33.5765
7080	rs3799344	T	C	T	C	0.0197	0.016006	0.4519	0.43657	FALSE	FALSE	FALSE	ieu-b-508	6	25786993	0.033568	486484	0.633493	Sepsis (28	Sepsis (28	Sepsis (28	TRUE	igd	0.0034	8.94E-09	7119	M34481.m	TRUE	reported	mXdL0W	2	TRUE	0.004694	33.56237
3309	rs1083801	A	G	A	G	-0.0139	-0.02077	0.7066	0.725819	FALSE	FALSE	FALSE	ieu-b-508	3	7523498	0.03863	486484	0.590764	Sepsis (28	Sepsis (28	Sepsis (28	TRUE	igd	0.0024	8.78E-09	7307	M32393.m	TRUE	reported	Pub0X0	2	TRUE	0.00457	33.53422
7254	rs1339995	A	C	A	C	0.011	-0.02489	0.4714	0.458232	FALSE	FALSE	FALSE	ieu-b-508	1	1.64E+08	0.033478	486484	0.457142	Sepsis (28	Sepsis (28	Sepsis (28	TRUE	igd	0.0019	1.63E-08	5885	M34912.m	TRUE	reported	W8lcT6	2	TRUE	0.005663	33.50661
5206	rs885479	A	G	A	G	0.1621	0.056927	0.0535	0.043909	FALSE	FALSE	FALSE	ieu-b-508	16	90118342	0.0824	486484	0.489651	Sepsis (28	Sepsis (28	Sepsis (28	TRUE	igd	0.028	7.00E-09	3221	M33203.m	TRUE	reported	Sto7pR	2	TRUE	0.010298	33.49502
6208	rs1084178	A	C	A	C	0.0301	-0.00491	0.7347	0.718232	FALSE	FALSE	FALSE	ieu-b-508	12	21467666	0.037018	486484	0.894384	Sepsis (28	Sepsis (28	Sepsis (28	TRUE	igd	0.0052	5.78E-09	4533	M33901.m	TRUE	reported	mQkhrb	2	TRUE	0.007337	33.4915
55	rs1559063	C	G	C	G	0.0052	0.001479	0.6209	0.618648	FALSE	TRUE	FALSE	ieu-b-508	5	1.72E+08	0.034352	486484	0.965654	Sepsis (28	Sepsis (28	Sepsis (28	TRUE	igd	9.00E-04	7.82E-09	7355	M00054.m	TRUE	reported	QTDAnh	2	TRUE	0.004518	33.37364
339	rs6735596	A	C	A	C	-0.0052	-0.04752	0.6267	0.62053	FALSE	FALSE	FALSE	ieu-b-508	2	2.28E+08	0.034351	486484	0.166516	Sepsis (28	Sepsis (28	Sepsis (28	TRUE	igd	9.00E-04	1.15E-08	7351	M00060.m	TRUE	reported	kqwl4V	2	TRUE	0.004521	33.37363
3590	rs8033940	A	G	A	G	0.0104	-0.00453	0.274	0.292807	FALSE	FALSE	FALSE	ieu-b-508	15	58725839	0.036841	486484	0.902226	Sepsis (28	Sepsis (28	Sepsis (28	TRUE	igd	0.0018	1.19E-08	7350	M32518.m	TRUE	reported	V0ZCiU	2	TRUE	0.004521	33.37363
3809	rs1219181	T	G	T	G	0.0104	0.021126	0.684	0.681355	FALSE	FALSE	FALSE	ieu-b-508	6	1.36E+08	0.035824	486484	0.555395	Sepsis (28	Sepsis (28	Sepsis (28	TRUE	igd	0.0018	1.66E-08	7229	M32644.m	TRUE	reported	JAa2WD	2	TRUE	0.004597	33.37348
7549	rs1006989	A	C	A	C	0.0104	-0.03599	0.1781	0.182629	FALSE	FALSE	FALSE	ieu-b-508	5	1.3E+08	0.043076	486484	0.403448	Sepsis (28	Sepsis (28	Sepsis (28	TRUE	igd	0.0018	1.42E-08	7217	M35270.m	TRUE	reported	Ct8V1n	2	TRUE	0.004604	33.37346
1411	rs9940990	A	T	A	T	-0.0104	0.008719	0.3545	0.358067	FALSE	TRUE	FALSE	ieu-b-508	16	71831343	0.034826	486484	0.802309	Sepsis (28	Sepsis (28	Sepsis (28	TRUE	igd	0.0018	4.75E-09	7037	M02734.m	TRUE	reported	0y0dDD	2	TRUE	0.004721	33.37323
4606	rs1084875	A	C	A	C	-0.9526	-0.05811	0.0197	0.020606	FALSE	FALSE	FALSE	ieu-b-508	12	3084576	0.119719	486484	0.627404	Sepsis (28	Sepsis (28	Sepsis (28	TRUE	igd	0.1628	4.85E-09	76	M33173.m	TRUE	reported	04fkUQ	2	TRUE	0.310585	33.3373
1007	rs1509123	T	C	T	C	-0.0248	0.011125	0.0476	0.062718	FALSE	FALSE	FALSE	ieu-b-508	17	6683878	0.069333	486484	0.87252	Sepsis (28	Sepsis (28	Sepsis (28	TRUE	igd	0.0043	5.20E-09	7364	M01564.m	TRUE	reported	IPUdyD	2	TRUE	0.004497	33.25435
1697	rs4970834	T	C	T	C	-0.0098	-0.08734	0.1821	0.186662	FALSE	FALSE	FALSE	ieu-b-508	1	1.1E+08	0.04303	486484	0.042371	Sepsis (28	Sepsis (28	Sepsis (28	TRUE	igd	0.0017	1.06E-08	7345	M15488.m	TRUE	reported	SPriBO	2	TRUE	0.004504	33.22279
7134	rs430200	T	G	T	G	0.0201	0.018232	0.7744	0.77917	FALSE	FALSE	FALSE	ieu-b-508	4	69597175	0.040293	486484	0.650927	Sepsis (28	Sepsis (28	Sepsis (28	TRUE	igd	0.0035	6.45E-09	7322	M34527.m	TRUE	reported	EWFGP8	2	TRUE	0.004484	32.9714
7385	rs7749940	A	C	A	C	0.0109	-0.04082	0.3736	0.36025	FALSE	FALSE	FALSE	ieu-b-508	6	1.56E+08	0.034705	486484	0.239474	Sepsis (28	Sepsis (28	Sepsis (28	TRUE	igd	0.0019	8.30E-09	4900	M35137.m	TRUE	reported	Eq2siS	2	TRUE	0.006672	32.89792
4532	rs698078	A	G	A	G	-0.0636	0.000814	0.5789	0.596094	FALSE	FALSE	FALSE	ieu-b-508	3	1.86E+08	0.033939	486484	0.980859	Sepsis (28	Sepsis (28	Sepsis (28	TRUE	igd	0.0111	1.02E-08	2391	M33137.m	TRUE	reported	ONVNk3	2	TRUE	0.013545	32.80234
4456	rs7707010	A	G	A	G	0.0252	0.025492	0.9582	0.95836	FALSE	FALSE	FALSE	ieu-b-508	5	1.38E+08	0.083302	486484	0.759591	Sepsis (28	Sepsis (28	Sepsis (28	TRUE	igd	0.0044	9.61E-09	7344	M32910.m	TRUE	reported	2OCBKh	2	TRUE	0.004447	32.79272
1134	rs1015340	A	G	A	G	-0.0103	0.034157	0.5342	0.538748	FALSE	FALSE	FALSE	ieu-b-508	18	41270672	0.03363	486484	0.309788	Sepsis (28	Sepsis (28	Sepsis (28	TRUE	igd	0.0018	9.27E-09	7355	M01644.m	TRUE	reported	IB0DTL	2	TRUE	0.004432	32.73492
6717	rs1232699	A	C	A	C	-1.038	-0.13962	0.011	0.036351	FALSE	FALSE	FALSE	ieu-b-508	18	32756493	0.09229	486484	0.13031	Sepsis (28	Sepsis (28	Sepsis (28	TRUE	igd	0.18	8.09E-09	115	M34338.m	TRUE	reported	h4FB9a	2	TRUE	0.224307	32.67611
6124	rs1711533	T	C	T	C	-0.6712	-0.05227	0.9866	0.973685	FALSE	FALSE	FALSE	ieu-b-508	14	45086814	0.107385	486484	0.6264	Sepsis (28	Sepsis (28	Sepsis (28	TRUE	igd	0.1168	9.04E-09	176	M33877.m	TRUE	reported	yDVi1f	2	TRUE	0.157988	32.64787
3306	rs3766198	A	C	A	C	-0.024	0.018508	0.274	0.268801	FALSE	FALSE	FALSE	ieu-b-508	1	47281780	0.037624	486484	0.622772	Sepsis (28	Sepsis (28	Sepsis (28	TRUE	igd	0.0042	1.46E-08	5130	M32388.m	TRUE	reported	yYczcA	2	TRUE	0.006325	32.64033
1080	rs1173032	T	C	T	C	0.0097	0.020894	0.6665	0.644844	FALSE	FALSE	FALSE	ieu-b-508	4	9719901	0.03622	486484	0.564035	Sepsis (28	Sepsis (28	Sepsis (28	TRUE	igd	0.0017	7.08E-09	7371	M01604.m	TRUE	reported	hE3I2k	2	TRUE	0.004397	32.54826
4439	rs2686184	A	G	A	G	-0.0097	0.005559	0.408	0.407878	FALSE	FALSE	FALSE	ieu-b-508	8	11649359	0.033983	486484	0.870048	Sepsis (28	Sepsis (28	Sepsis (28	TRUE	igd	0.0017	1.89E-08	7344	M32910.m	TRUE	reported	2OCBKh	2	TRUE	0.004414	32.54823
4619	rs1296083	T	C	T	C	-1.1492	-0.03713	0.0132	0.03284	FALSE	FALSE	FALSE	ieu-b-508	18	45481926	0.093516	486484	0.691296	Sepsis (28	Sepsis (28	Sepsis (28	TRUE	igd	0.1988	7.40E-09	76	M33173.m	TRUE	reported	04fkUQ	2	TRUE	0.305405	32.53693
7759	rs1108583	T	C	T	C	0.0154	-0.01223	0.2018	0.203646	FALSE	FALSE	FALSE	ieu-b-508	19	13332010	0.041651	486484	0.769081	Sepsis (28	Sepsis (28	Sepsis (28	TRUE	igd	0.0027	1.04E-08	7256	M35439.m	TRUE	reported	DnuAGZ	2	TRUE	0.004463	32.52327
5903	rs8082167	T	C	T	C	0.4457	0.054782	0.0288	0.022316	FALSE	FALSE	FALSE	ieu-b-508	17	52494156	0.116989	486484	0.639596	Sepsis (28	Sepsis (28	Sepsis (28	TRUE	igd	0.0774	8.49E-09	102	M33610.m	TRUE	reported	tmg43v	2	TRUE	0.245334	32.50897
6788	rs4001461	T	C	T	C	0.0513	-0.03102	0.4402	0.444794	FALSE	FALSE	FALSE	ieu-b-508	4	1.19E+08	0.033488	486484	0.354302	Sepsis (28	Sepsis (28	Sepsis (28	TRUE	igd	0.009	1.39E-08	3491	M34350.m	TRUE	reported	Cc3yW9	2	TRUE	0.009221	32.47139
2114	rs2912546	T	C	T	C	0.2679	-0.05691	0.0501	0.040697	FALSE	FALSE	FALSE	ieu-b-508	17	53822437	0.085793	486484	0.507145	Sepsis (28	Sepsis (28	Sepsis (28	TRUE	igd	0.0469	1.15E-08	257	M18474.m	TRUE	reported	B6Xd02	2	TRUE	0.112657	32.37478
2701	rs1800775	A	C	A	C	0.0108	-0.06425	0.4986	0.487001	FALSE	FALSE	FALSE	ieu-b-508	16	56995236	0.033324	486484	0.053859	Sepsis (28	Sepsis (28	Sepsis (28	TRUE	igd	0.0019	7.96E-09	7340	M25459.m	TRUE	reported	ACJY35	2	TRUE	0.004383	32.30145
8008	rs2708257	A	C	A	C	-0.0108	-0.00534	0.6508	0.647175	FALSE	FALSE	FALSE	ieu-b-508	7	1.48E+08	0.03503	486484	0.87883	Sepsis (28	Sepsis (28	Sepsis (28	TRUE	igd	0.0019	2.17E-08	6604	M35754.m	TRUE	reported	lIXOo1	2	TRUE	0.004869	32.30046
8486	rs6703518	T	G	T	G	-0.0108	0.012683	0.4273	0.425424	FALSE	FALSE	FALSE	ieu-b-508	1	1.86E+08	0.033739	486484	0.706983	Sepsis (28	Sepsis (28	Sepsis (28	TRUE	igd	0.0019	5.85E-09	6552	M37058.m	TRUE	reported	32p8qV	2	TRUE	0.004907	32.30039
2579	rs711995	T	C	T	C	0.0108	0.035205	0.4481	0.447732	FALSE	FALSE	FALSE	ieu-b-508	3	1.94E+08	0.033724	486484	0.296518	Sepsis (28	Sepsis (28	Sepsis (28	TRUE	igd	0.0019	2.70E-08	6535	M22177.m	TRUE	reported	2s6Sly	2	TRUE	0.00492	32.30036
7150	rs1101997	C	G	C	G	0.1331	-0.17132	0.0717	0.058637	FALSE	TRUE	FALSE	ieu-b-508	11	92225262	0.07101	486484	0.015838	Sepsis (28	Sepsis (28	Sepsis (28	TRUE	igd	0.0234	1.36E-08	1212	M34533.m	TRUE	reported	SuKX4k	2	TRUE	0.026	32.30034
1376	rs1219271	A	T	A	T	0.0142	0.074989	0.1412	0.145803	FALSE	TRUE	FALSE	ieu-b-508	6	1.12E+08	0.047414	486484	0.113742	Sepsis (28	Sepsis (28	Sepsis (28	TRUE	igd	0.0025	2.10E-08	7037	M02734.m	TRUE	reported	0y0dDD	2	TRUE	0.004564	32.25323
5596	rs9921315	A	G	A	G	0.7395	-0.01854	0.8729	0.86161	FALSE	FALSE	FALSE	ieu-b-508	16	88078224	0.048715	486484	0.703477	Sepsis (28	Sepsis (28	Sepsis (28	TRUE	igd	0.1281	7.82E-09	60	M33423.m	TRUE	reported	MoEnO7	2	TRUE	0.35709	32.21476
6059	rs3198697	T	C	T	C	0.0193	-0.03396	0.4174	0.407569	FALSE	FALSE	FALSE	ieu-b-508	16	15129940	0.033972	486484	0.317409	Sepsis (28	Sepsis (28	Sepsis (28	TRUE	igd	0.0034	1.65E-08	7362	M33821.m	TRUE	reported	eVYICL	2	TRUE	0.004358	32.21356
7227	rs8022857	T	C	T	C	0.0772	0.215423	0.9764	0.972759	FALSE	FALSE	FALSE	ieu-b-508	14	74093165	0.104302	486484	0.038888	Sepsis (28	Sepsis (28	Sepsis (28	TRUE	igd	0.0136	1.45E-08	6381	M34761.m	TRUE	reported	AMtp1w	2	TRUE	0.005024	32.21222
8197	rs649129	T	C	T	C	-0.0437	-0.03537	0.2141	0.207952	FALSE	FALSE	FALSE	ieu-b-508	9	1.36E+08	0.040853	486484	0.38666	Sepsis (28	Sepsis (28	Sepsis (28	TRUE	igd	0.0077	1.26E-08	2400	M36230.m	TRUE	reported	V5KJeR	2	TRUE	0.013243	32.18247
4149	rs4715354	A	G	A	G	0.0278	-0.01251	0.478	0.483689	FALSE	FALSE	FALSE	ieu-b-508	6	52708797	0.033368	486484	0.707773	Sepsis (28	Sepsis (28	Sepsis (28	TRUE	igd	0.0049	1.66E-08	7250	M32757.m	TRUE	reported	TJV9Xb	2	TRUE	0.00442	32.17938
6729	rs1689678	A	G	A	G	1.024	-0.1201	0.989	0.99122	FALSE	FALSE	FALSE	ieu-b-508	6	66247351	0.180735	486484	0.50635	Sepsis (28	Sepsis (28	Sepsis (28	TRUE	igd	0.179	1.06E-08	115	M34338.m	TRUE	reported	h4FB9a	2	TRUE	0.221532	32.15692
2229	rs3783200	A	C	A	C	-0.0414	0.043088	0.6859	0.679317	FALSE	FALSE	FALSE	ieu-b-508	13	46616462	0.035744	486484	0.228035	Sepsis (28	Sepsis (28	Sepsis (28	TRUE	igd	0.0073	1.51E-08	1643	M19396.m	TRUE	reported	Xa1QqA	2	TRUE	0.0192	32.12373
3185	rs2370998	A	G	A	G	-0.0136	0.03204	0.5725	0.559124	FALSE	FALSE	FALSE	ieu-b-508	2	2.11E+08	0.033742	486484	0.342338	Sepsis (28	Sepsis (28	Sepsis (28	TRUE	igd	0.0024	1.56E-08	7356	M32338.m	TRUE	reported	q5cuaP	2	TRUE	0.004346	32.10238
71	rs2111118	T	C	T	C	0.0051	-0.04219	0.6207	0.624241	FALSE	FALSE	FALSE	ieu-b-508	16	54049153	0.034397	486484	0.219973	Sepsis (28	Sepsis (28	Sepsis (28	TRUE	igd	9.00E-04	1.21E-08	7355	M00054.m	TRUE	reported	QTDAnh	2	TRUE	0.004347	32.10238
106	rs38271	A	G	A	G	-0.0051	-0.00832	0.5925	0.594601	FALSE	FALSE	FALSE	ieu-b-508	7	14307031	0.034006	486484	0.806757	Sepsis (28	Sepsis (28	Sepsis (28	TRUE	igd	9.00E-04	1.19E-08	7355	M00054.m	TRUE	reported	QTDAnh	2	TRUE	0.004347	32.10238
133	rs603446	T	C	T	C	0.0051	0.031937	0.4448	0.453206	FALSE	FALSE	FALSE	ieu-b-508	11	1.17E+08	0.033465	486484	0.339915	Sepsis (28	Sepsis (28	Sepsis (28	TRUE	igd	9.00E-04	1.38E-08	7355	M00054.m	TRUE	reported	QTDAnh	2	TRUE	0.004347	32.10238
340	rs6737109	T	C	T	C	0.0051	0.017323	0.5761	0.580037	FALSE	FALSE	FALSE	ieu-b-508	2	23179531	0.033773	486484	0.608003	Sepsis (28	Sepsis (28	Sepsis (28	TRUE	igd	9.00E-04	1.45E-08	7351	M00060.m	TRUE	reported	kqwl4V	2	TRUE	0.004349	32.10237
342	rs6820011	T	G	T	G	-0.0051	-0.03093	0.5152	0.506483	FALSE	FALSE	FALSE	ieu-b-508	4	1.67E+08	0.033465	486484	0.355411	Sepsis (28	Sepsis (28	Sepsis (28	TRUE	igd	9.00E-04	1.18E-08	7351	M00060.m	TRUE	reported	kqwl4V	2	TRUE	0.004349	32.10237
1407	rs875740	A	C	A	C	0.0102	-0.02144	0.6656	0.665835	FALSE	FALSE	FALSE	ieu-b-508	16	16123048	0.035394	486484	0.544664	Sepsis (28	Sepsis (28	Sepsis (28	TRUE	igd	0.0018	6.35E-09	7037	M02734.m	TRUE	reported	0y0dDD	2	TRUE	0.004542	32.10198
8492	rs8060756	A	G	A	G	0.0102	-0.02494	0.3703	0.371881	FALSE	FALSE	FALSE	ieu-b-508	16	63202385	0.034557	486484	0.470392	Sepsis (28	Sepsis (28	Sepsis (28	TRUE	igd	0.0018	4.07E-08	6552	M37058.m	TRUE	reported	32p8qV	2	TRUE	0.004877	32.10131
7783	rs1028074	T	C	T	C	-0.0838	-0.04772	0.8585	0.852839	FALSE	FALSE	FALSE	ieu-b-508	8	1.38E+08	0.047236	486484	0.312354	Sepsis (28	Sepsis (28	Sepsis (28	TRUE	igd	0.0148	1.52E-08	1383	M35451.m	TRUE	reported	GMRGAN	2	TRUE	0.022656	32.01372
5860	rs1233521	T	C	T	C	0.323	-0.18264	0.013	0.034581	FALSE	FALSE	FALSE	ieu-b-508	8	66023840	0.089379	486484	0.041008	Sepsis (28	Sepsis (28	Sepsis (28	TRUE	igd	0.057	1.46E-08	296	M33609.m	TRUE	reported	h7B9gN	2	TRUE	0.097867	31.89414
679	rs1272867	T	C	T	C	-0.0096	0.04921	0.2003	0.218978	FALSE	FALSE	FALSE	ieu-b-508	1	1.55E+08	0.040397	486484	0.22316	Sepsis (28	Sepsis (28	Sepsis (28	TRUE	igd	0.0017	2.19E-08	7358	M01299.m	TRUE	reported	gfM0a3	2	TRUE	0.004315	31.88061
1284	rs1509820	A	G	A	G	0.0096	-0.00058	0.5299	0.542114	FALSE	FALSE	FALSE	ieu-b-508	2	2.11E+08	0.033503	486484	0.986274	Sepsis (28	Sepsis (28	Sepsis (28	TRUE	igd	0.0017	1.75E-08	7325	M02132.m	TRUE	reported	uJFm2t	2	TRUE	0.004335	31.88057
7524	rs2121073	T	C	T	C	-0.0293	0.037818	0.1209	0.122112	FALSE	FALSE	FALSE	ieu-b-508	14	76665740	0.050911	486484	0.457588	Sepsis (28	Sepsis (28	Sepsis (28	TRUE	igd	0.0052	2.15E-08	7291	M35255.m	TRUE	reported	ZDjmUf	2	TRUE	0.004336	31.74018
7839	rs1218973	T	C	T	C	-0.0642	0.054493	0.8963	0.89088	FALSE	FALSE	FALSE	ieu-b-508	6	72293724	0.053622	486484	0.30952	Sepsis (28	Sepsis (28	Sepsis (28	TRUE	igd	0.0114	1.56E-08	5035	M35551.m	TRUE	reported	5Sxswl	2	TRUE	0.006259	31.70208
5253	rs748196	T	G	T	G	0.0152	0.033021	0.4596	0.465569	FALSE	FALSE	FALSE	ieu-b-508	17	17422768	0.033478	486484	0.323968	Sepsis (28	Sepsis (28	Sepsis (28	TRUE	igd	0.0027	1.70E-08	7063	M33228.m	TRUE	reported	JwOXbQ	2	TRUE	0.004467	31.68376
6294	rs2062541	A	G	A	G	0.0225	-0.02752	0.6144	0.612586	FALSE	FALSE	FALSE	ieu-b-508	16	16127235	0.034296	486484	0.42235	Sepsis (28	Sepsis (28	Sepsis (28	TRUE	igd	0.004	2.89E-08	7320	M33941.m	TRUE	reported	culqiU	2	TRUE	0.004304	31.63198
2458	rs1116011	T	C	T	C	-0.0146	0.008625	0.7128	0.694214	FALSE	FALSE	FALSE	ieu-b-508	14	93775073	0.036187	486484	0.811617	Sepsis (28	Sepsis (28	Sepsis (28	TRUE	igd	0.0026	2.34E-08	6180	M22030.m	TRUE	reported	ZNV0qR	2	TRUE	0.005076	31.52234
6722	rs1296746	T	C	T	C	1.008	-0.17563	0.987	0.962323	FALSE	FALSE	FALSE	ieu-b-508	18	19460437	0.092817	486484	0.058464	Sepsis (28	Sepsis (28	Sepsis (28	TRUE	igd	0.178	1.49E-08	115	M34338.m	TRUE	reported	h4FB9a	2	TRUE	0.218052	31.51096
1023	rs6507399	A	G	A	G	-0.0101	-0.08303	0.6713	0.664269	FALSE	FALSE	FALSE	ieu-b-508	18	38732045	0.035459	486484	0.019201	Sepsis (28	Sepsis (28	Sepsis (28	TRUE	igd	0.0018	1.70E-08	7364	M01564.m	TRUE	reported	IPUdyD	2	TRUE	0.004257	31.47602
1033	rs868395	T	G	T	G	0.0101	-0.01953	0.6653	0.660404	FALSE	FALSE	FALSE	ieu-b-508	9	85175793	0.035325	486484	0.580299	Sepsis (28	Sepsis (28	Sepsis (28	TRUE	igd	0.0018	2.03E-08	7364	M01564.m	TRUE	reported	IPUdyD	2	TRUE	0.004257	31.47602
1163	rs864428	T	C	T	C	0.0101	-0.01914	0.4058	0.412814	FALSE	FALSE	FALSE	ieu-b-508	7	1.37E+08	0.033972	486484	0.573135	Sepsis (28	Sepsis (28	Sepsis (28	TRUE	igd	0.0018	2.99E-08	7355	M01644.m	TRUE	reported	IB0DTL	2	TRUE	0.004262	31.47601
1409	rs956966	A	G	A	G	-0.0101	0.011473	0.4858	0.478082	FALSE	FALSE	FALSE	ieu-b-508	2	1.04E+08	0.033406	486484	0.731265	Sepsis (28	Sepsis (28	Sepsis (28	TRUE	igd	0.0018	1.48E-08	7037	M02734.m	TRUE	reported	0y0dDD	2	TRUE	0.004454	31.47562
687	rs174408	A	G	A	G	0.0129	0.062625	0.149	0.150129	FALSE	FALSE	FALSE	ieu-b-508	6	1.12E+08	0.046862	486484	0.181432	Sepsis (28	Sepsis (28	Sepsis (28	TRUE	igd	0.0023	1.41E-08	7358	M01299.m	TRUE	reported	gfM0a3	2	TRUE	0.004257	31.44892
4302	rs7535263	A	G	A	G	-0.0443	-0.02882	0.435	0.404243	FALSE	FALSE	FALSE	ieu-b-508	1	1.97E+08	0.033948	486484	0.395825	Sepsis (28	Sepsis (28	Sepsis (28	TRUE	igd	0.0079	2.20E-08	1711	M32836.m	TRUE	reported	d4lVXe	2	TRUE	0.018047	31.40836
4542	rs3923630	A	G	A	G	-0.028	-0.05137	0.333	0.331723	FALSE	FALSE	FALSE	ieu-b-508	12	71289175	0.035366	486484	0.146346	Sepsis (28	Sepsis (28	Sepsis (28	TRUE	igd	0.005	2.14E-08	5568	M33138.m	TRUE	reported	IUpLAI	2	TRUE	0.005601	31.34874
5877	rs1042548	A	G	A	G	0.5394	-0.13682	0.0157	0.010996	FALSE	FALSE	FALSE	ieu-b-508	19	41278020	0.161765	486484	0.397663	Sepsis (28	Sepsis (28	Sepsis (28	TRUE	igd	0.0954	1.59E-08	102	M33610.m	TRUE	reported	tmg43v	2	TRUE	0.238628	31.34187
558	rs532545	T	C	T	C	-0.0095	0.033786	0.3065	0.340111	FALSE	FALSE	FALSE	ieu-b-508	1	20915172	0.035221	486484	0.337426	Sepsis (28	Sepsis (28	Sepsis (28	TRUE	igd	0.0017	3.41E-08	7354	M00606.m	TRUE	reported	r5smf7	2	TRUE	0.004228	31.21988
3876	rs316019	A	C	A	C	-0.0229	0.062058	0.0986	0.10333	FALSE	FALSE	FALSE	ieu-b-508	6	1.61E+08	0.055015	486484	0.259312	Sepsis (28	Sepsis (28	Sepsis (28	TRUE	igd	0.0041	2.06E-08	7361	M32654.m	TRUE	reported	6ISQOP	2	TRUE	0.00422	31.18784
3859	rs9424148	A	C	A	C	-0.0201	-0.02505	0.7912	0.78401	FALSE	FALSE	FALSE	ieu-b-508	10	12146858	0.040697	486484	0.53814	Sepsis (28	Sepsis (28	Sepsis (28	TRUE	igd	0.0036	1.61E-08	5211	M32651.m	TRUE	reported	DeuPwv	2	TRUE	0.005947	31.16165

2604	rs1093830	T	C	T	C	-0.0106	-0.04733	0.4663	0.469713	FALSE	FALSE	FALSE	ieu-b-508	4	43781839	0.033451	486484	0.157068	Sepsis (28	Sepsis (28	Sepsis (28	TRUE	igd	0.0019	1.48E-08	7329	M22481.m	TRUE	reported	bXpf0k	2	TRUE	0.004229	31.11616
5453	rs1173635	C	G	C	G	0.516	0.111414	0.0263	0.012893	FALSE	TRUE	FALSE	ieu-b-508	4	81601668	0.148017	486484	0.451622	Sepsis (28	Sepsis (28	Sepsis (28	TRUE	igd	0.092	2.02E-08	171	M33391.m	TRUE	reported	EgMd1b	2	TRUE	0.155378	31.08954
1632	rs1692489	A	T	A	T	0.0814	0.009647	0.024	0.023955	FALSE	TRUE	FALSE	ieu-b-508	10	24845525	0.110263	486484	0.930284	Sepsis (28	Sepsis (28	Sepsis (28	TRUE	igd	0.0146	2.33E-08	1764	M15140.m	TRUE	reported	jEcqMJ	2	TRUE	0.017316	31.0492
6749	rs9566930	T	C	T	C	-0.989	0.056622	0.015	0.0173	FALSE	FALSE	FALSE	ieu-b-508	13	42717594	0.128242	486484	0.658832	Sepsis (28	Sepsis (28	Sepsis (28	TRUE	igd	0.176	1.92E-08	115	M34338.m	TRUE	reported	h4FB9a	2	TRUE	0.215428	31.02758
1694	rs2799635	T	C	T	C	0.0089	0.01533	0.3644	0.359149	FALSE	FALSE	FALSE	ieu-b-508	6	96378415	0.034786	486484	0.65944	Sepsis (28	Sepsis (28	Sepsis (28	TRUE	igd	0.0016	1.96E-08	7345	M15488.m	TRUE	reported	SPriBO	2	TRUE	0.004195	30.93298
1527	rs6140831	A	C	A	C	0.0089	-0.00206	0.5484	0.555286	FALSE	FALSE	FALSE	ieu-b-508	20	9008889	0.033598	486484	0.951208	Sepsis (28	Sepsis (28	Sepsis (28	TRUE	igd	0.0016	5.40E-08	7081	M12067.m	TRUE	reported	O5x7RD	2	TRUE	0.004351	30.93267
2359	rs1012025	A	G	A	G	-0.0484	-0.00725	0.8189	0.825398	FALSE	FALSE	FALSE	ieu-b-508	9	82645562	0.044183	486484	0.869674	Sepsis (28	Sepsis (28	Sepsis (28	TRUE	igd	0.0087	3.03E-08	3422	M21049.m	TRUE	reported	UDtLE4	2	TRUE	0.008963	30.93131
5307	rs1681529	A	G	A	G	0.4453	0.170672	0.0182	0.020203	FALSE	FALSE	FALSE	ieu-b-508	15	60504269	0.120409	486484	0.156356	Sepsis (28	Sepsis (28	Sepsis (28	TRUE	igd	0.0799	2.49E-08	474	M33359.m	TRUE	reported	dfJmmS	2	TRUE	0.061499	30.92968
3538	rs6077678	A	G	A	G	-0.0228	0.024816	0.848	0.832232	FALSE	FALSE	FALSE	ieu-b-508	20	1069103	0.044725	486484	0.578998	Sepsis (28	Sepsis (28	Sepsis (28	TRUE	igd	0.0041	3.38E-08	7358	M32497.m	TRUE	reported	244bZN	2	TRUE	0.004185	30.91604
3903	rs6867478	A	G	A	G	0.0695	0.018144	0.0139	0.011277	FALSE	FALSE	FALSE	ieu-b-508	5	92122345	0.161165	486484	0.910363	Sepsis (28	Sepsis (28	Sepsis (28	TRUE	igd	0.0125	2.97E-08	1756	M32675.m	TRUE	reported	9FmPh9	2	TRUE	0.0173	30.87839
8118	rs1165165	T	C	T	C	0.0439	-0.00248	0.2392	0.240954	FALSE	FALSE	FALSE	ieu-b-508	6	25862466	0.039031	486484	0.949421	Sepsis (28	Sepsis (28	Sepsis (28	TRUE	igd	0.0079	2.37E-08	7054	M36098.m	TRUE	reported	hs9SZI	2	TRUE	0.004359	30.87107
8622	rs633695	A	G	A	G	-0.01	0.004526	0.7183	0.707193	FALSE	FALSE	FALSE	ieu-b-508	15	58725839	0.036841	486484	0.902226	Sepsis (28	Sepsis (28	Sepsis (28	TRUE	igd	0.0018	2.47E-08	7366	M37506.m	TRUE	reported	m2kaRt	2	TRUE	0.004173	30.85582
47	rs1373962	T	C	T	C	0.005	-0.03063	0.5973	0.594481	FALSE	FALSE	FALSE	ieu-b-508	5	83527862	0.033995	486484	0.36765	Sepsis (28	Sepsis (28	Sepsis (28	TRUE	igd	9.00E-04	2.71E-08	7355	M00054.m	TRUE	reported	QTDAnh	2	TRUE	0.004179	30.8558
126	rs4958379	A	G	A	G	-0.005	0.011253	0.433	0.431617	FALSE	FALSE	FALSE	ieu-b-508	5	1.54E+08	0.033807	486484	0.739249	Sepsis (28	Sepsis (28	Sepsis (28	TRUE	igd	9.00E-04	2.18E-08	7355	M00054.m	TRUE	reported	QTDAnh	2	TRUE	0.004179	30.8558
157	rs710580	A	C	A	C	-0.005	-0.03023	0.3566	0.372877	FALSE	FALSE	FALSE	ieu-b-508	3	1.9E+08	0.034434	486484	0.379931	Sepsis (28	Sepsis (28	Sepsis (28	TRUE	igd	9.00E-04	3.57E-08	7355	M00054.m	TRUE	reported	QTDAnh	2	TRUE	0.004179	30.8558
164	rs7584842	T	C	T	C	-0.005	-0.08857	0.5377	0.544598	FALSE	FALSE	FALSE	ieu-b-508	2	11756260	0.033965	486484	0.00912	Sepsis (28	Sepsis (28	Sepsis (28	TRUE	igd	9.00E-04	4.15E-08	7355	M00054.m	TRUE	reported	QTDAnh	2	TRUE	0.004179	30.8558
186	rs9511152	A	G	A	G	-0.005	-0.01103	0.559	0.58113	FALSE	FALSE	FALSE	ieu-b-508	13	19596767	0.033835	486484	0.74443	Sepsis (28	Sepsis (28	Sepsis (28	TRUE	igd	9.00E-04	2.99E-08	7355	M00054.m	TRUE	reported	QTDAnh	2	TRUE	0.004179	30.8558
193	rs972459	T	C	T	C	0.005	0.012287	0.5787	0.584668	FALSE	FALSE	FALSE	ieu-b-508	3	1.92E+08	0.033931	486484	0.717254	Sepsis (28	Sepsis (28	Sepsis (28	TRUE	igd	9.00E-04	1.97E-08	7355	M00054.m	TRUE	reported	QTDAnh	2	TRUE	0.004179	30.8558
1203	rs9563502	A	G	A	G	-0.01	-0.01164	0.7115	0.713546	FALSE	FALSE	FALSE	ieu-b-508	13	57944356	0.036832	486484	0.751912	Sepsis (28	Sepsis (28	Sepsis (28	TRUE	igd	0.0018	2.56E-08	7346	M01645.m	TRUE	reported	d5nAn3	2	TRUE	0.004184	30.85579
5527	rs2817817	A	G	A	G	-0.01	-0.02298	0.2809	0.30403	FALSE	FALSE	FALSE	ieu-b-508	10	29576573	0.036586	486484	0.53	Sepsis (28	Sepsis (28	Sepsis (28	TRUE	igd	0.0018	2.14E-08	7306	M33422.m	TRUE	reported	PLgjrT	2	TRUE	0.004207	30.85575
3819	rs4541909	T	C	T	C	0.01	0.001429	0.674	0.675446	FALSE	FALSE	FALSE	ieu-b-508	8	74365745	0.035736	486484	0.968114	Sepsis (28	Sepsis (28	Sepsis (28	TRUE	igd	0.0018	4.65E-08	7229	M32644.m	TRUE	reported	JAa2WD	2	TRUE	0.004251	30.85566
7584	rs795213	A	G	A	G	0.01	0.069699	0.3339	0.367606	FALSE	FALSE	FALSE	ieu-b-508	6	4359705	0.034842	486484	0.045453	Sepsis (28	Sepsis (28	Sepsis (28	TRUE	igd	0.0018	2.95E-08	7217	M35270.m	TRUE	reported	Ct8V1n	2	TRUE	0.004258	30.85564
6924	rs2163783	T	C	T	C	-0.03	-0.03845	0.2984	0.295205	FALSE	FALSE	FALSE	ieu-b-508	5	1.68E+08	0.03812	486484	0.313166	Sepsis (28	Sepsis (28	Sepsis (28	TRUE	igd	0.0054	2.98E-08	5561	M34390.m	TRUE	reported	wruTbW	2	TRUE	0.005519	30.8531
4308	rs1683366	A	C	A	C	0.0211	0.029314	0.3149	0.309231	FALSE	FALSE	FALSE	ieu-b-508	1	1.52E+08	0.036179	486484	0.417796	Sepsis (28	Sepsis (28	Sepsis (28	TRUE	igd	0.0038	2.91E-08	6753	M32838.m	TRUE	reported	hyR7zA	2	TRUE	0.004545	30.82259
6721	rs1276971	T	C	T	C	-1.008	-0.13186	0.011	0.052588	FALSE	FALSE	FALSE	ieu-b-508	10	73160050	0.086192	486484	0.126058	Sepsis (28	Sepsis (28	Sepsis (28	TRUE	igd	0.18	2.14E-08	115	M34338.m	TRUE	reported	h4FB9a	2	TRUE	0.214266	30.81461
2839	rs6497490	T	G	T	G	-0.0305	-0.0537	0.8823	0.865078	FALSE	FALSE	FALSE	ieu-b-508	16	20477474	0.051575	486484	0.297815	Sepsis (28	Sepsis (28	Sepsis (28	TRUE	igd	0.0055	3.49E-08	7180	M27513.m	TRUE	reported	o9Ff2u	2	TRUE	0.004265	30.7435
2496	rs4941615	T	C	T	C	0.0133	0.014067	0.2119	0.208617	FALSE	FALSE	FALSE	ieu-b-508	13	48459901	0.041059	486484	0.731902	Sepsis (28	Sepsis (28	Sepsis (28	TRUE	igd	0.0024	4.54E-08	7329	M22116.m	TRUE	reported	bjUU6r	2	TRUE	0.004173	30.70169
1752	rs315988	T	C	T	C	0.0072	0.045645	0.2367	0.239914	FALSE	FALSE	FALSE	ieu-b-508	6	1.61E+08	0.039061	486484	0.242586	Sepsis (28	Sepsis (28	Sepsis (28	TRUE	igd	0.0013	1.08E-08	7365	M15650.m	TRUE	reported	NgezyT	2	TRUE	0.004148	30.66623
6975	rs2291449	A	G	A	G	-0.0288	-0.07486	0.9135	0.910424	FALSE	FALSE	FALSE	ieu-b-508	15	76579234	0.058454	486484	0.200336	Sepsis (28	Sepsis (28	Sepsis (28	TRUE	igd	0.0052	2.35E-08	7344	M34407.m	TRUE	reported	X7I83Y	2	TRUE	0.004159	30.6662
554	rs2686796	T	C	T	C	0.0094	0.040024	0.4524	0.439978	FALSE	FALSE	FALSE	ieu-b-508	7	48102911	0.033632	486484	0.234023	Sepsis (28	Sepsis (28	Sepsis (28	TRUE	igd	0.0017	1.89E-08	7354	M00606.m	TRUE	reported	r5smf7	2	TRUE	0.00414	30.56608
740	rs320485	T	C	T	C	0.0094	0.00158	0.1324	0.136228	FALSE	FALSE	FALSE	ieu-b-508	1	2.14E+08	0.048747	486484	0.974136	Sepsis (28	Sepsis (28	Sepsis (28	TRUE	igd	0.0017	4.97E-08	7347	M01302.m	TRUE	reported	sNp2lC	2	TRUE	0.004144	30.56607
1570	rs1013258	T	G	T	G	0.0094	-0.01122	0.1734	0.173495	FALSE	FALSE	FALSE	ieu-b-508	14	36118348	0.044129	486484	0.79937	Sepsis (28	Sepsis (28	Sepsis (28	TRUE	igd	0.0017	5.67E-08	7314	M12593.m	TRUE	reported	lBXgSU	2	TRUE	0.004163	30.56603
8495	rs1318497	A	T	A	T	-0.0763	0.087338	0.103	0.115561	FALSE	TRUE	FALSE	ieu-b-508	5	1.3E+08	0.052164	486484	0.094074	Sepsis (28	Sepsis (28	Sepsis (28	TRUE	igd	0.0138	3.29E-08	7014	M37097.m	TRUE	reported	wCbJj4	2	TRUE	0.004339	30.56096
2806	rs7632727	T	C	T	C	0.0199	0.081835	0.8935	0.888767	FALSE	FALSE	FALSE	ieu-b-508	3	98228007	0.052961	486484	0.1223	Sepsis (28	Sepsis (28	Sepsis (28	TRUE	igd	0.0036	2.68E-08	7347	M27278.m	TRUE	reported	kPgqm3	2	TRUE	0.004142	30.54801
8169	rs2881913	A	C	A	C	0.0315	-0.0168	0.449	0.443444	FALSE	FALSE	FALSE	ieu-b-508	4	1.11E+08	0.033532	486484	0.616342	Sepsis (28	Sepsis (28	Sepsis (28	TRUE	igd	0.0057	3.99E-08	1780	M36131.m	TRUE	reported	DbYm5t	2	TRUE	0.016868	30.50585
5680	rs2391217	A	C	A	C	0.0149	0.054964	0.1069	0.108711	FALSE	FALSE	FALSE	ieu-b-508	7	26048418	0.05374	486484	0.306411	Sepsis (28	Sepsis (28	Sepsis (28	TRUE	igd	0.0027	4.88E-08	7307	M33477.m	TRUE	reported	Aw8PBK	2	TRUE	0.00415	30.44571
7756	rs1059264	T	C	T	C	-0.0149	0.036766	0.3254	0.336355	FALSE	FALSE	FALSE	ieu-b-508	14	1.01E+08	0.035192	486484	0.29616	Sepsis (28	Sepsis (28	Sepsis (28	TRUE	igd	0.0027	5.57E-08	7256	M35439.m	TRUE	reported	DnuAGZ	2	TRUE	0.00418	30.44565
1354	rs1751956	T	G	T	G	0.016	0.018169	0.8647	0.857251	FALSE	FALSE	FALSE	ieu-b-508	10	29581998	0.047687	486484	0.703197	Sepsis (28	Sepsis (28	Sepsis (28	TRUE	igd	0.0029	2.35E-08	7226	M02730.m	TRUE	reported	XYa035	2	TRUE	0.004195	30.43153
5582	rs6818342	T	C	T	C	-0.849	0.008681	0.0593	0.042414	FALSE	FALSE	FALSE	ieu-b-508	4	1.85E+08	0.083144	486484	0.916839	Sepsis (28	Sepsis (28	Sepsis (28	TRUE	igd	0.1515	2.12E-08	60	M33423.m	TRUE	reported	MoEnO7	2	TRUE	0.343576	30.35756
3000	rs2277503	A	G	A	G	0.1667	0.003072	0.0502	0.045102	FALSE	FALSE	FALSE	ieu-b-508	14	65271780	0.079681	486484	0.969248	Sepsis (28	Sepsis (28	Sepsis (28	TRUE	igd	0.0302	3.43E-08	522	M31536.m	TRUE	reported	3nAuUI	2	TRUE	0.05515	30.3522
5868	rs1899124	T	C	T	C	0.304	0.01972	0.051	0.902431	FALSE	FALSE	FALSE	ieu-b-508	18	62488818	0.056009	486484	0.724778	Sepsis (28	Sepsis (28	Sepsis (28	TRUE	igd	0.055	3.25E-08	296	M33609.m	TRUE	reported	h7B9gN	2	TRUE	0.093556	30.34432
1194	rs6441206	A	G	A	G	0.0099	0.018225	0.4638	0.477931	FALSE	FALSE	FALSE	ieu-b-508	3	1.58E+08	0.033394	486484	0.585225	Sepsis (28	Sepsis (28	Sepsis (28	TRUE	igd	0.0018	2.09E-08	7346	M01645.m	TRUE	reported	d5nAn3	2	TRUE	0.004101	30.24176
6256	rs714462	T	C	T	C	0.0187	0.0356	0.3714	0.381552	FALSE	FALSE	FALSE	ieu-b-508	4	1.6E+08	0.034282	486484	0.299069	Sepsis (28	Sepsis (28	Sepsis (28	TRUE	igd	0.0034	5.54E-08	7345	M33936.m	TRUE	reported	jwA4B2	2	TRUE	0.004102	30.24176
3776	rs6593265	A	C	A	C	0.011	-0.01674	0.311	0.310218	FALSE	FALSE	FALSE	ieu-b-508	7	55656122	0.036186	486484	0.643661	Sepsis (28	Sepsis (28	Sepsis (28	TRUE	igd	0.002	3.80E-08	5598	M32634.m	TRUE	reported	7T2RUz	2	TRUE	0.005375	30.23919
6427	rs1107617	A	G	A	G	0.011	-0.0481	0.845	0.821907	FALSE	FALSE	FALSE	ieu-b-508	16	57006378	0.043749	486484	0.271527	Sepsis (28	Sepsis (28	Sepsis (28	TRUE	igd	0.002	3.80E-08	5594	M34062.m	TRUE	reported	RZyHKP	2	TRUE	0.005378	30.23918
3573	rs2935461	A	G	A	G	-0.011	-0.06514	0.81	0.809251	FALSE	FALSE	FALSE	ieu-b-508	13	56404249	0.042465	486484	0.12505	Sepsis (28	Sepsis (28	Sepsis (28	TRUE	igd	0.002	3.80E-08	5584	M32518.m	TRUE	reported	V0ZCiU	2	TRUE	0.005388	30.23917
1760	rs1108439	T	C	T	C	-0.011	0.018189	0.544	0.549258	FALSE	FALSE	FALSE	ieu-b-508	19	55865527	0.033417	486484	0.586222	Sepsis (28	Sepsis (28	Sepsis (28	TRUE	igd	0.002	3.80E-08	5566	M15676.m	TRUE	reported	s1NfKN	2	TRUE	0.005405	30.23913
2208	rs6053126	A	G	A	G	-0.022	-0.02121	0.253	0.344616	FALSE	FALSE	FALSE	ieu-b-508	20	564893	0.035281	486484	0.547753	Sepsis (28	Sepsis (28	Sepsis (28	TRUE	igd	0.004	3.80E-08	5275	M19364.m	TRUE	reported	XkjMEx	2	TRUE	0.005702	30.23853
8477	rs2686513	T	C	T	C	-0.011	-0.04123	0.552	0.553263	FALSE	FALSE	FALSE	ieu-b-508	7	22451353	0.033551	486484	0.2191	Sepsis (28	Sepsis (28	Sepsis (28	TRUE	igd	0.002	3.80E-08	5018	M37058.m	TRUE	reported	32p8qV	2	TRUE	0.005992	30.23794
5657	rs7036192	A	T	A	T	0.0835	0.014673	0.9301	0.9404	FALSE	TRUE	FALSE	ieu-b-508	9	12298496	0.070724	486484	0.835645	Sepsis (28	Sepsis (28	Sepsis (28	TRUE	igd	0.0152	3.50E-08	1118	M33453.m	TRUE	reported	OzEeLQ	2	TRUE	0.026283	30.12369
7304	rs4797105	C	G	C	G	0.0406	-0.03319	0.7968	0.766799	FALSE	TRUE	FALSE	ieu-b-508	18	3370562	0.039961	486484	0.406287	Sepsis (28	Sepsis (28	Sepsis (28	TRUE	igd	0.0074	3.59E-08	7364	M35126.m	TRUE	reported	1haOJA	2	TRUE	0.004071	30.09336
2427	rs6662425	C	G	C	G	0.0148	-0.01678	0.6385	0.613568	FALSE	TRUE	FALSE	ieu-b-508	1	4688033	0.034398	486484	0.625729	Sepsis (28	Sepsis (28	Sepsis (28	TRUE	igd	0.0027	3.51E-08	6556	M21188.m	TRUE	reported	kvdnjc	2	TRUE	0.004562	30.03747
7452	rs992029	T	C	T	C	-0.0422	-0.08424	0.0981	0.097047	FALSE	FALSE	FALSE	ieu-b-508	2	2.1E+08	0.056276	486484	0.13442	Sepsis (28	Sepsis (28	Sepsis (28	TRUE	igd	0.0077	3.93E-08	6234	M35189.m	TRUE	reported	54XZFj	2	TRUE	0.004795	30.02646
7331	rs9594738	T	C	T	C	0.0137	-0.03719	0.4782	0.486275	FALSE	FALSE	FALSE	ieu-b-508	13	42952145	0.033394	486484	0.265477	Sepsis (28	Sepsis (28	Sepsis (28	TRUE	igd	0.0025	4.53E-08	7344	M35127.m	TRUE	reported	RNsmjl	2	TRUE	0.004072	30.02222
2256	rs4131290	A	C	A	C	-0.0126	-0.06151	0.702	0.711383	FALSE	FALSE	FALSE	ieu-b-508	5	1.77E+08	0.037156	486484	0.097825	Sepsis (28	Sepsis (28	Sepsis (28	TRUE	igd	0.0023	7.47E-08	7354	M19934.m	TRUE	reported	HtcWcQ	2	TRUE	0.004064	30.00318
3411	rs2182325	A	G	A	G	-0.0378	-0.00889	0.2818	0.349675	FALSE	FALSE	FALSE	ieu-b-508	1	71371420	0.035014	486484	0.799551	Sepsis (28	Sepsis (28	Sepsis (28	TRUE	igd	0.0069	4.34E-08	5080	M32445.m	TRUE	reported	qcc6f3	2	TRUE	0.005873	29.99953
6276	rs893971	A	G	A	G	-0.0197	0.005652	0.5882	0.600922	FALSE	FALSE	FALSE	ieu-b-508	4	89203670	0.033972	486484	0.867872	Sepsis (28	Sepsis (28	Sepsis (28	TRUE	igd	0.0036	4.05E-08	7222	M33937.m	TRUE	reported	QanGCI	2	TRUE	0.004129	29.93692
3263	rs1864774	T	C	T	C	0.0093	0.050401	0.586	0.581461	FALSE	FALSE	FALSE	ieu-b-508	11	83199276	0.033842	486484	0.136405	Sepsis (28	Sepsis (28	Sepsis (28	TRUE	igd	0.0017	1.15E-07	7365	M32348.m	TRUE	reported	DJeLDx	2	TRUE	0.004047	29.91921
5454	rs1250831	A	T	A	T	-0.4363	0.047233	0.9577	0.926865	FALSE	TRUE	FALSE	ieu-b-508	4	34761270	0.064189	486484	0.461831	Sepsis (28	Sepsis (28	Sepsis (28	TRUE	igd	0.0793	3.71E-08	171	M33391.m	TRUE	reported	EgMd1b	2	TRUE	0.150398	29.91677
4212	rs9348689	T	G	T	G	0.0339	-0.04015	0.5165	0.524885	FALSE	FALSE	FALSE	ieu-b-508	6	25684653	0.034129	486484	0.239372	Sepsis (28	Sepsis (28	Sepsis (28	TRUE	igd	0.0062	4.41E-08	6150	M32769.m	TRUE	reported	W2ubRP	2	TRUE	0.004838	29.88648
8174	rs1226630	A	C	A	C	-0.1028	-0.08116	0.955	0.951661	FALSE	FALSE	FALSE	ieu-b-508	10	1.02E+08	0.078436	486484	0.300804	Sepsis (28	Sepsis (28	Sepsis (28	TRUE	igd	0.0188	4.71E-08	1657	M36134.m	TRUE	reported	ekMsfG	2	TRUE	0.017725	29.86387
867	rs6887589	A	G	A	G	-0.0142	-0.02311	0.7787	0.782892	FALSE	FALSE	FALSE	ieu-b-508	5	1.07E+08	0.040802	486484	0.571114	Sepsis (28	Sepsis (28	Sepsis (28	TRUE	igd	0.0026	4.33E-08	7063	M01361.m	TRUE	reported	NIAcQ3	2	TRUE	0.004205	29.81996
1499	rs339707	T	G	T	G	-0.0142	-0.07712	0.6768	0.66738	FALSE	FALSE	FALSE	ieu-b-508	3	42723827	0.035569	486484	0.030148	Sepsis (28	Sepsis (28	Sepsis (28	TRUE	igd	0.0026	6.35E-08	5656	M12017.m	TRUE	reported	bbGtsK	2	TRUE	0.005246	29.81785
6724	rs1309455	A	G	A	G	0.975	0.07002	0.985	0.948687	FALSE	FALSE	FALSE	ieu-b-508	3	1.57E+08	0.075591	486484	0.35429	Sepsis (28	Sepsis (28	Sepsis (28	TRUE	igd	0.177	3.62E-08	115	M34338.m	TRUE	reported	h4FB9a	2	TRUE	0.20877	29.81558
6484	rs2928923	A	G	A	G	0.4932	-0.20103	0.979	0.973313	FALSE	FALSE	FALSE	ieu-b-508	8	93313295	0.104697	486484	0.054839	Sepsis (28	Sepsis (28	Sepsis (28	TRUE	igd	0.0901	4.38E-08	282	M34109.m	TRUE	reported	piJuC7	2	TRUE	0.096049	29.75127
3913	rs1277892	A	C	A	C	0.0371	0.02584	0.2745	0.27816	FALSE	FALSE	FALSE	ieu-b-508	10	92054100	0.037313	486484	0.48861	Sepsis (28	Sepsis (28	Sepsis (28	TRUE	igd	0.0068	6.05E-08	2575	M32691.m	TRUE	reported	SiuD6F	2	TRUE	0.011428	29.74353
3163	rs1175399	A	G	A	G	-0.0229	0.004717	0.1568	0.17093	FALSE	FALSE	FALSE	ieu-b-508	6	1.61E+08	0.04443	486484	0.915457	Sepsis (28	Sepsis (28	Sepsis (28	TRUE	igd	0.0042	7.24E-08	7340	M32328.m	TRUE	reported	YGsb6D	2	TRUE	0.004034	29.72036
4735	rs1212279	A	G	A	G	-0.1811	-0.05887	0.038	0.027773	FALSE	FALSE	FALSE	ieu-b-508	1	44989685	0.101845	486484	0.563215	Sepsis (28	Sepsis (28	Sepsis (28	TRUE	igd	0.0332	4.87E-08	1017	M33178.m	TRUE	reported	g22WMS	2	TRUE	0.028426	29.69654
619	rs8192870	T	G	T	G	-0.0365	-0.04875	0.3498	0.342035	FALSE	FALSE	FALSE	ieu-b-508	8	59412066	0.035092	486484	0.164787	Sepsis (28	Sepsis (28	Sepsis (28	TRUE	igd	0.0067	3.99E-08	4934	M01114.m	TRUE	reported	fqHdM2	2	TRUE	0.005979	29.66607
5598	rs1146579	T	C	T	C	-0.0196	0.02803	0.6674	0.662041	FALSE	FALSE	FALSE	ieu-b-508	1	76209792	0.035295	486484	0.4271	Sepsis (28	Sepsis (28	Sepsis (28	TRUE	igd	0.0036	5.78E-08	7365	M33441.m	TRUE	reported	CSdSpl	2	TRUE	0.004009	29.63393
3425	rs1389939	C	G	C	G	0.0098	0.05184	0.4085	0.417072	FALSE	TRUE	FALSE	ieu-b-508	1	79992759	0.03389	486484	0.126103	Sepsis (28	Sepsis (28	Sepsis (28	TRUE	igd	0.0018	1.13E-07	7364	M32452.m	TRUE	reported	8GtFpl	2	TRUE	0.004009	29.63392
53	rs154246	T	C	T	C	-0.0049	0.016981	0.6103	0.620378	FALSE	FALSE	FALSE	ieu-b-508	5	16602451	0.034391	486484	0.621478	Sepsis (28	Sepsis (28	Sepsis (28	TRUE	igd	9.00E-04	6.70E-08	7355	M00054.m	TRUE	reported	QTDAnh	2	TRUE	0.004014	29.63391
59	rs1697901	A	G	A	G	0.0049	-0.04273	0.3501	0.357057	FALSE	FALSE	FALSE	ieu-b-508	5	11019405	0.035219	486484	0.225057	Sepsis (28	Sepsis (28	Sepsis (28	TRUE	igd	9.00E-04	6.31E-08	7355	M00054.m	TRUE	reported	QTDAnh	2	TRUE	0.004014	29.63391
90	rs2798184	T	C	T	C	-0.0049	0.02044	0.4314	0.428102	FALSE	FALSE	FALSE	ieu-b-508	1	1.09E+08	0.034026	486484	0.548023	Sepsis (28	Sepsis (28	Sepsis (28	TRUE	igd	9.00E-04	6.37E-08	7355	M00054.m	TRUE	reported	QTDAnh	2	TRUE	0.004014	29.63391
116	rs4615256	A	G	A	G	0.0049	0.023385	0.5327	0.52837	FALSE	FALSE	FALSE	ieu-b-508	5	62700867	0.033439	486484	0.484332	Sepsis (28	Sepsis (28	Sepsis (28	TRUE	igd	9.00E-04	4.99E-08	7355	M00054.m	TRUE	reported	QTDAnh	2	TRUE	0.004014	29.63391
139	rs6480970	A	G	A	G	-0.0049	-0.0063	0.6159	0.606311	FALSE	FALSE	FALSE	ieu-b-508	10	54568664	0.034261	486484	0.854041	Sepsis (28	Sepsis (28	Sepsis (28	TRUE	igd	9.00E-04	4.29E-08	7355	M00054.m	TRUE	reported	QTDAnh	2	TRUE	0.004014	29.63391
170	rs8021242	T	C	T	C	0.0049	-0.00497	0.5899	0.583544	FALSE	FALSE	FALSE	ieu-b-508	14	22398968	0.033868	486484	0.883245	Sepsis (28	Sepsis (28	Sepsis (28	TRUE	igd	9.00E-04	5.18E-08	7355	M00054.m	TRUE	reported	QTDAnh	2	TRUE	0.004014	29.63391
1138	rs1084819	A	G	A	G	-0.0098	0.003102	0.5584	0.555714	FALSE	FALSE	FALSE	ieu-b-508	12	1.31E+08	0.033555	486484	0.926356	Sepsis (28	Sepsis (28	Sepsis (28	TRUE	igd	0.0018	5.25E-08	7355	M01644.m	TRUE	reported	IB0DTL	2	TRUE	0.004014	29.63391
253	rs1463301	T	C	T	C	-0.0049	0.020639	0.5742	0.579952	FALSE	FALSE	FALSE	ieu-b-508	12	61929312	0.03382	486484	0.54168	Sepsis (28	Sepsis (28	Sepsis (28	TRUE	igd	9.00E-04	7.49E-08	7351	M00060.m	TRUE	reported	kqwl4V	2	TRUE	0.004016	29.63391
263	rs1733198	A	G	A	G	0.0049	-0.00925	0.6308	0.622084	FALSE	FALSE	FALSE	ieu-b-508	3	1.04E+08	0.034423	486484	0.788045	Sepsis (28	Sepsis (28	Sepsis (28	TRUE	igd	9.00E-04	7.62E-08	7351	M00060.m	TRUE	reported	kqwl4V	2	TRUE	0.004016	29.63391
343	rs6820313	T	G	T	G	0.0049	0.014533	0.5169	0.517975	FALSE	FALSE	FALSE	ieu-b-508	4	1.4E+08	0.033571	486484	0.665083	Sepsis (28	Sepsis (28	Sepsis (28	TRUE	igd	9.00E-04	4.65E-08	7351	M00060.m	TRUE	reported	kqwl4V	2	TRUE	0.004016	29.63391
368	rs7740614	A	G	A	G	-0.0049	0.007786	0.4838	0.486488	FALSE	FALSE	FALSE	ieu-b-508	6	45545851	0.033459	486484	0.815979	Sepsis (28	Sepsis (28	Sepsis (28	TRUE	igd	9.00E-04	4.75E-08	7351	M00060.m	TRUE	reported	kqwl4V	2	TRUE	0.004016	29.63391
373	rs7915	A	C	A	C	-0.0049	-0.04385	0.6374	0.627082	FALSE	FALSE	FALSE	ieu-b-508	15	41106485	0.034425	486484	0.202774	Sepsis (28	Sepsis (28	Sepsis (28	TRUE	igd	9.00E-04	5.82E-08	7351	M00060.m	TRUE	reported	kqwl4V	2	TRUE	0.004016	29.63391
7932	rs4132537	A	G	A	G	-0.0098	0.027885	0.4952	0.514623	FALSE	FALSE	FALSE	ieu-b-508	3	1E+08	0.033506	486484	0.405274	Sepsis (28	Sepsis (28	Sepsis (28	TRUE	igd	0.0018	8.29E-08	7348	M35675.m	TRUE	reported	D7fdyZ	2	TRUE	0.004018	29.63391
1962	rs1390947	A	C	A	C	-0.0272	0.017461	0.4471	0.450785	FALSE	FALSE	FALSE	ieu-b-508	9	76125591	0.033646	486484	0.603783	Sepsis (28	Sepsis (28	Sepsis (28	TRUE	igd	0.005	5.72E-08	7226	M17807.m	TRUE	reported	zd6sZI	2	TRUE	0.004079	29.58541
3261	rs1766896	A	C	A	C	0.0326	-0.09785	0.0383	0.049554	FALSE	FALSE	FALSE	ieu-b-508	6	84811129	0.077147	486484	0.204684	Sepsis (28	Sepsis (28	Sepsis (28	TRUE	igd	0.006	5.33E-08	7365	M32348.m	TRUE	reported	DJeLDx	2	TRUE	0.003992	29.51309
2460	rs1163502	A	G	A	G	-0.0152	-0.02243	0.508	0.514561	FALSE	FALSE	FALSE	ieu-b-508	15	25158051	0.03355	486484	0.503729	Sepsis (28	Sepsis (28	Sepsis (28	TRUE	igd	0.0028	4.25E-08	6180	M22030.m	TRUE	reported	ZNV0qR	2	TRUE	0.004746	29.45985
2304	rs1247033	A	T	A	T	-0.038	0.024771	0.97	0.9683	FALSE	TRUE	FALSE	ieu-b-508	2	2.01E+08	0.095503	486484	0.795345	Sepsis (28	Sepsis (28	Sepsis (28	TRUE	igd	0.007	5.68E-08	5579	M20699.m	TRUE	reported	Z3EKFa	2	TRUE	0.005254	29.45882
3918	rs457273	A	G	A	G	-0.0597	0.058935	0.8172	0.846916	FALSE	FALSE	FALSE	ieu-b-508	5	4264392	0.046881	486484	0.208713	Sepsis (28	Sepsis (28	Sepsis (28	TRUE	igd	0.011	5.34E-08	1675	M32691.m	TRUE	reported	SiuD6F	2	TRUE	0.017281	29.42012
6963	rs1944577	T	C	T	C	-0.0733	0.023506	0.2625	0.253079	FALSE	FALSE	FALSE	ieu-b-508	18	45730103	0.038442	486484	0.540894	Sepsis (28	Sepsis (28	Sepsis (28	TRUE	igd	0.0135	5.42E-08	777	M34404.m	TRUE	reported	8FMHfo	2	TRUE	0.036555	29.40499
1439	rs1215182	T	C	T	C	-0.0141	-0.05269	0.8159	0.809735	FALSE	FALSE	FALSE	ieu-b-508	2	1.36E+08	0.042495	486484	0.214976	Sepsis (28	Sepsis (28	Sepsis (28	TRUE	igd	0.0026	6.35E-08	7357	M03141.m	TRUE	reported	y4mG6m	2	TRUE	0.003982	29.40177
7657	rs622034	T	C	T	C	0.0141	-0.01166	0.128	0.121834	FALSE	FALSE	FALSE	ieu-b-508	10	11185749	0.051241	486484	0.820022	Sepsis (28	Sepsis (28	Sepsis (28	TRUE	igd	0.0026	4.20E-08	5768	M35326.m	TRUE	reported	jRytyJ	2	TRUE	0.005073	29.39957
2540	rs1299626	C	G	C	G	-0.0103	-0.04141	0.6148	0.594706	FALSE	TRUE	FALSE	ieu-b-508	2	23296529	0.034181	486484	0.225755	Sepsis (28	Sepsis (28	Sepsis (28	TRUE	igd	0.0019	1.23E-07	6535	M22177.m	TRUE	reported	2s6Sly	2	TRUE	0.004477	29.37882
2582	rs7541235	T	G	T	G	-0.0103	-0.00981	0.6521	0.644471	FALSE	FALSE	FALSE	ieu-b-508	1	81765344	0.035101	486484	0.779913	Sepsis (28	Sepsis (28	Sepsis (28	TRUE	igd	0.0019	1.15E-07	6535	M22177.m	TRUE	reported	2s6Sly	2	TRUE	0.004477	29.37882
4514	rs739626	A	C	A	C	0.013	0.066176	0.0935	0.117727	FALSE	FALSE	FALSE	ieu-b-508	7	24852791	0.051801	486484	0.201419	Sepsis (28	Sepsis (28	Sepsis (28	TRUE	igd	0.0024	7.96E-08	7362	M33132.m	TRUE	reported	dTXJa8	2	TRUE	0.00397	29.33231
5977	rs2980782	C	G	C	G	-0.013	-0.01574	0.3307	0.34671	FALSE	TRUE	FALSE	ieu-b-508	8	3803786	0.035053	486484	0.653427	Sepsis (28	Sepsis (28	Sepsis (28	TRUE	igd	0.0024	6.71E-08	6279	M33666.m	TRUE	reported	dvWEtk	2	TRUE	0.004651	29.33093
4226	rs1079865	A	C	A	C	0.0184	-0.00435	0.4816	0.480583	FALSE	FALSE	FALSE	ieu-b-508	1	1.79E+08	0.033363	486484	0.896169	Sepsis (28	Sepsis (28	Sepsis (28	TRUE	igd	0.0034	7.63E-08	6857	M32787.m	TRUE	reported	fSRakm	2	TRUE	0.004253	29.27865
8423	rs6107895	A	G	A	G	-0.0249	0.005177	0.1405	0.148499	FALSE	FALSE	FALSE	ieu-b-508	20	6922676	0.046991	486484	0.912282	Sepsis (28	Sepsis (28	Sepsis (28	TRUE	igd	0.0046	6.18E-08	1764	M36808.m	TRUE	reported	u7lqaj	2	TRUE	0.016339	29.26782
2639	rs1186547	A	G	A	G	-0.0146	0.025087	0.506	0.493652	FALSE	FALSE	FALSE	ieu-b-508	16	1184967	0.033321	486484	0.451514	Sepsis (28	Sepsis (28	Sepsis (28	TRUE	igd	0.0027	6.71E-08	7184	M22548.m	TRUE	reported	mZtwX2	2	TRUE	0.004054	29.23191
7119	rs6541298	A	G	A	G	0.8157	-0.22636	0.0117	0.015629	FALSE	FALSE	FALSE	ieu-b-508	1	2.3E+08	0.134455	486484	0.092278	Sepsis (28	Sepsis (28	Sepsis (28	TRUE	igd	0.1497	5.09E-08	128	M34516.m	TRUE	reported	7Ah2wy	2	TRUE	0.188283	29.22657
6603	rs1452253	A	C	A	C	0.0173	-0.00208	0.6045	0.549642	FALSE	FALSE	FALSE	ieu-b-508	12	85235569	0.03397	486484	0.951226	Sepsis (28	Sepsis (28	Sepsis (28	TRUE	igd	0.0032	6.60E-08	6994	M34306.m	TRUE	reported	WPXPUN	2	TRUE	0.004162	29.21918
8143	rs2819047	A	G	A	G	-0.0373	0.01697	0.2125	0.199108	FALSE	FALSE	FALSE	ieu-b-508	10	10214666	0.041912	486484	0.685551	Sepsis (28	Sepsis (28	Sepsis (28	TRUE	igd	0.0069	5.82E-08	7311	M36103.m	TRUE	reported	sjeaPC	2	TRUE	0.003981	29.21465
5578	rs4516063	T	G	T	G	0.8886	-0.00791	0.9154	0.903789	FALSE	FALSE	FALSE	ieu-b-508	13	21680070	0.056822	486484	0.889246	Sepsis (28	Sepsis (28	Sepsis (28	TRUE	igd	0.1617	3.92E-08	60	M33423.m	TRUE	reported	MoEnO7	2	TRUE	0.334804	29.19237
6121	rs1699435	T	G	T	G	-0.4144	-0.11225	0.9773	0.978857	FALSE	FALSE	FALSE	ieu-b-508	21	38005642	0.116149	486484	0.333829	Sepsis (28	Sepsis (28	Sepsis (28	TRUE	igd	0.0763	5.60E-08	176	M33877.m	TRUE	reported	yDVi1f	2	TRUE	0.143543	29.16265
2331	rs1710134	A	G	A	G	-0.0189	0.000859	0.7777	0.764104	FALSE	FALSE	FALSE	ieu-b-508	14	33970832	0.039476	486484	0.98263	Sepsis (28	Sepsis (28	Sepsis (28	TRUE	igd	0.0035	5.64E-08	7366	M21044.m	TRUE	reported	Ym5rae	2	TRUE	0.003943	29.15208
1719	rs4487482	A	G	A	G	0.0081	-0.086	0.8039	0.803596	FALSE	FALSE	FALSE	ieu-b-508	5	1.35E+08	0.042134	486484	0.041231	Sepsis (28	Sepsis (28	Sepsis (28	TRUE	igd	0.0015	1.82E-07	7310	M15506.m	TRUE	reported	mVRmZO	2	TRUE	0.003973	29.15202
5758	rs1229587	A	G	A	G	0.2913	0.025469	0.018	0.015039	FALSE	FALSE	FALSE	ieu-b-508	11	82897094	0.138144	486484	0.853725	Sepsis (28	Sepsis (28	Sepsis (28	TRUE	igd	0.0539	6.67E-08	972	M33508.m	TRUE	reported	UMXHcP	2	TRUE	0.029173	29.14801
4219	rs7495382	T	G	T	G	-0.0448	0.072363	0.2056	0.22372	FALSE	FALSE	FALSE	ieu-b-508	15	23824746	0.04013	486484	0.071356	Sepsis (28	Sepsis (28	Sepsis (28	TRUE	igd	0.0083	7.19E-08	7330	M32786.m	TRUE	reported	rwF0co	2	TRUE	0.003959	29.12603
5724	rs7942396	T	C	T	C	-0.048	0.027998	0.0664	0.073275	FALSE	FALSE	FALSE	ieu-b-508	11	1.21E+08	0.064212	486484	0.662821	Sepsis (28	Sepsis (28	Sepsis (28	TRUE	igd	0.0089	7.68E-08	5107	M33488.m	TRUE	reported	mZmDY6	2	TRUE	0.005663	29.07585
8554	rs4398451	T	C	T	C	-0.0345	-0.03594	0.5546	0.54838	FALSE	FALSE	FALSE	ieu-b-508	3	20010886	0.033523	486484	0.283703	Sepsis (28	Sepsis (28	Sepsis (28	TRUE	igd	0.0064	7.26E-08	7355	M37202.m	TRUE	reported	BtslDx	2	TRUE	0.003935	29.05094
6378	rs2279501	C	G	C	G	0.0221	-0.04871	0.5933	0.603268	FALSE	TRUE	FALSE	ieu-b-508	1	1.52E+08	0.034182	486484	0.154118	Sepsis (28	Sepsis (28	Sepsis (28	TRUE	igd	0.0041	5.60E-08	7322	M33968.m	TRUE	reported	sgVOvT	2	TRUE	0.003952	29.04679
3001	rs2838057	A	C	A	C	-0.1987	0.251356	0.9626	0.967143	FALSE	FALSE	FALSE	ieu-b-508	21	42922817	0.093708	486484	0.007311	Sepsis (28	Sepsis (28	Sepsis (28	TRUE	igd	0.0368	6.91E-08	522	M31536.m	TRUE	reported	3nAuUI	2	TRUE	0.052897	29.04243
3768	rs2098462	A	G	A	G	0.0097	0.008056	0.7432	0.746835	FALSE	FALSE	FALSE	ieu-b-508	2	2.06E+08	0.038457	486484	0.834069	Sepsis (28	Sepsis (28	Sepsis (28	TRUE	igd	0.0018	1.04E-07	7362	M32634.m	TRUE	reported	7T2RUz	2	TRUE	0.003929	29.03223
4510	rs4900819	T	C	T	C	-0.0097	0.006697	0.8744	0.871601	FALSE	FALSE	FALSE	ieu-b-508	14	48369678	0.049897	486484	0.893228	Sepsis (28	Sepsis (28	Sepsis (28	TRUE	igd	0.0018	6.88E-08	7362	M33132.m	TRUE	reported	dTXJa8	2	TRUE	0.003929	29.03223
3515	rs8059670	T	C	T	C	-0.0097	-0.00869	0.4437	0.456107	FALSE	FALSE	FALSE	ieu-b-508	16	61624701	0.033513	486484	0.795315	Sepsis (28	Sepsis (28	Sepsis (28	TRUE	igd	0.0018	3.72E-08	7355	M32492.m	TRUE	reported	rZZFcE	2	TRUE	0.003933	29.03223
4419	rs1041280	T	C	T	C	0.0097	0.018238	0.3119	0.316942	FALSE	FALSE	FALSE	ieu-b-508	19	46877975	0.035761	486484	0.610048	Sepsis (28	Sepsis (28	Sepsis (28	TRUE	igd	0.0018	4.42E-08	7344	M32910.m	TRUE	reported	2OCBKh	2	TRUE	0.003939	29.03221
1303	rs682103	T	C	T	C	-0.0097	0.085946	0.7055	0.703762	FALSE	FALSE	FALSE	ieu-b-508	5	53394938	0.036819	486484	0.019579	Sepsis (28	Sepsis (28	Sepsis (28	TRUE	igd	0.0018	4.26E-08	7325	M02132.m	TRUE	reported	uJFm2t	2	TRUE	0.003949	29.03219
5703	rs9555127	A	G	A	G	-0.0097	-0.01561	0.3316	0.342323	FALSE	FALSE	FALSE	ieu-b-508	13	88791291	0.035287	486484	0.658137	Sepsis (28	Sepsis (28	Sepsis (28	TRUE	igd	0.0018	7.00E-08	7307	M33477.m	TRUE	reported	Aw8PBK	2	TRUE	0.003959	29.03217
1061	rs1114413	T	G	T	G	0.0097	0.028078	0.1375	0.141422	FALSE	FALSE	FALSE	ieu-b-508	9	71502351	0.047878	486484	0.557573	Sepsis (28	Sepsis (28	Sepsis (28	TRUE	igd	0.0018	4.86E-08	7271	M01585.m	TRUE	reported	M7QiSM	2	TRUE	0.003978	29.03214
978	rs7228099	T	C	T	C	-0.0097	-0.06559	0.4364	0.432448	FALSE	FALSE	FALSE	ieu-b-508	18	22786478	0.033758	486484	0.052036	Sepsis (28	Sepsis (28	Sepsis (28	TRUE	igd	0.0018	3.61E-08	6523	M01558.m	TRUE	reported	AZK0p4	2	TRUE	0.004432	29.03122
6728	rs1683176	A	G	A	G	0.44	0.037505	0.988	0.991793	FALSE	FALSE	FALSE	ieu-b-508	3	1.21E+08	0.188498	486484	0.842288	Sepsis (28	Sepsis (28	Sepsis (28	TRUE	igd	0.081	5.57E-08	115	M34338.m	TRUE	reported	h4FB9a	2	TRUE	0.204195	28.99452
7859	rs1533302	A	G	A	G	-0.014	-0.03894	0.4472	0.445389	FALSE	FALSE	FALSE	ieu-b-508	2	1.78E+08	0.033872	486484	0.250334	Sepsis (28	Sepsis (28	Sepsis (28	TRUE	igd	0.0026	1.09E-07	7317	M35631.m	TRUE	reported	002wka	2	TRUE	0.003947	28.98616
7506	rs1314948	A	G	A	G	0.014	-0.0747	0.5433	0.538521	FALSE	FALSE	FALSE	ieu-b-508	4	40450756	0.033561	486484	0.026024	Sepsis (28	Sepsis (28	Sepsis (28	TRUE	igd	0.0026	1.24E-07	7100	M35254.m	TRUE	reported	HfVgPn	2	TRUE	0.004067	28.98592
760	rs26724	A	G	A	G	0.014	0.07729	0.3507	0.354682	FALSE	FALSE	FALSE	ieu-b-508	5	1.07E+08	0.034853	486484	0.026579	Sepsis (28	Sepsis (28	Sepsis (28	TRUE	igd	0.0026	1.26E-07	6946	M01303.m	TRUE	reported	3b8x0F	2	TRUE	0.004157	28.98573
2196	rs1021152	A	G	A	G	-0.014	-0.03833	0.4195	0.423896	FALSE	FALSE	FALSE	ieu-b-508	2	65208074	0.033899	486484	0.258202	Sepsis (28	Sepsis (28	Sepsis (28	TRUE	igd	0.0026	5.39E-08	6929	M19364.m	TRUE	reported	XkjMEx	2	TRUE	0.004167	28.98571
2197	rs1021452	T	C	T	C	0.014	0.077184	0.642	0.627483	FALSE	FALSE	FALSE	ieu-b-508	11	1.3E+08	0.034582	486484	0.02562	Sepsis (28	Sepsis (28	Sepsis (28	TRUE	igd	0.0026	7.83E-08	6929	M19364.m	TRUE	reported	XkjMEx	2	TRUE	0.004167	28.98571
5199	rs155146	T	C	T	C	-0.4768	-0.1406	0.9834	0.987092	FALSE	FALSE	FALSE	ieu-b-508	2	1.82E+08	0.148574	486484	0.343999	Sepsis (28	Sepsis (28	Sepsis (28	TRUE	igd	0.0885	7.24E-08	930	M33203.m	TRUE	reported	Sto7pR	2	TRUE	0.030266	28.9635
8009	rs3110907	C	G	C	G	-0.0199	0.055614	0.8988	0.86727	FALSE	TRUE	FALSE	ieu-b-508	10	60634371	0.049372	486484	0.259981	Sepsis (28	Sepsis (28	Sepsis (28	TRUE	igd	0.0037	1.04E-07	6604	M35754.m	TRUE	reported	IlXOo1	2	TRUE	0.004361	28.91819
3155	rs2037458	A	G	A	G	0.0188	-0.00867	0.6676	0.666818	FALSE	FALSE	FALSE	ieu-b-508	4	59321537	0.035344	486484	0.806264	Sepsis (28	Sepsis (28	Sepsis (28	TRUE	igd	0.0035	8.78E-08	7357	M32322.m	TRUE	reported	p4H9pm	2	TRUE	0.003906	28.8444
4580	rs1246710	A	G	A	G	0.0188	-0.09402	0.1697	0.169787	FALSE	FALSE	FALSE	ieu-b-508	2	79447701	0.044413	486484	0.034271	Sepsis (28	Sepsis (28	Sepsis (28	TRUE	igd	0.0035	8.38E-08	6404	M33163.m	TRUE	reported	YzGXkR	2	TRUE	0.004485	28.84323
6370	rs1037422	A	G	A	G	-0.0446	0.005037	0.2223	0.264813	FALSE	FALSE	FALSE	ieu-b-508	15	70194334	0.037931	486484	0.894366	Sepsis (28	Sepsis (28	Sepsis (28	TRUE	igd	0.0083	7.41E-08	1751	M33968.m	TRUE	reported	sgVOvT	2	TRUE	0.016223	28.84146
4102	rs1405215	A	G	A	G	0.0623	-0.01672	0.165	0.17387	FALSE	FALSE	FALSE	ieu-b-508	2	1.51E+08	0.044078	486484	0.704474	Sepsis (28	Sepsis (28	Sepsis (28	TRUE	igd	0.0116	8.00E-08	6379	M32754.m	TRUE	reported	n0okFy	2	TRUE	0.004501	28.83526
1556	rs9841498	T	C	T	C	0.0145	-0.02659	0.2663	0.261969	FALSE	FALSE	FALSE	ieu-b-508	3	1.83E+08	0.03792	486484	0.483164	Sepsis (28	Sepsis (28	Sepsis (28	TRUE	igd	0.0027	7.12E-08	6864	M12129.m	TRUE	reported	iNzdgb	2	TRUE	0.004184	28.83247
2160	rs7607746	T	C	T	C	-0.0333	0.087742	0.1582	0.183926	FALSE	FALSE	FALSE	ieu-b-508	2	1.88E+08	0.04312	486484	0.041868	Sepsis (28	Sepsis (28	Sepsis (28	TRUE	igd	0.0062	8.35E-08	1748	M18929.m	TRUE	reported	2vYFb2	2	TRUE	0.016235	28.81429
3412	rs3525171	A	C	A	C	-0.0865	-0.00435	0.1112	0.10398	FALSE	FALSE	FALSE	ieu-b-508	4	1.32E+08	0.054726	486484	0.936662	Sepsis (28	Sepsis (28	Sepsis (28	TRUE	igd	0.0161	8.30E-08	1120	M32445.m	TRUE	reported	qcc6f3	2	TRUE	0.025125	28.81405
2616	rs3104893	T	C	T	C	-0.0102	0.075808	0.5566	0.560877	FALSE	FALSE	FALSE	ieu-b-508	8	96523611	0.033608	486484	0.02409	Sepsis (28	Sepsis (28	Sepsis (28	TRUE	igd	0.0019	6.03E-08	7329	M22481.m	TRUE	reported	bXpf0k	2	TRUE	0.003917	28.81208
7307	rs629187	A	G	A	G	-0.0327	0.031087	0.8438	0.847052	FALSE	FALSE	FALSE	ieu-b-508	3	1.05E+08	0.046378	486484	0.502674	Sepsis (28	Sepsis (28	Sepsis (28	TRUE	igd	0.0061	1.03E-07	7364	M35126.m	TRUE	reported	1haOJA	2	TRUE	0.003887	28.72883
7062	rs1075621	T	C	T	C	0.0268	0.009213	0.2358	0.231857	FALSE	FALSE	FALSE	ieu-b-508	9	11262339	0.039531	486484	0.815724	Sepsis (28	Sepsis (28	Sepsis (28	TRUE	igd	0.005	6.63E-08	6144	M34469.m	TRUE	reported	WMOqbm	2	TRUE	0.004654	28.72025
5240	rs1246367	A	G	A	G	0.015	-0.03102	0.6888	0.686852	FALSE	FALSE	FALSE	ieu-b-508	2	1.79E+08	0.03598	486484	0.388639	Sepsis (28	Sepsis (28	Sepsis (28	TRUE	igd	0.0028	5.14E-08	7063	M33228.m	TRUE	reported	JwOXbQ	2	TRUE	0.004047	28.69085
3842	rs1703546	T	C	T	C	-0.015	-0.0185	0.3092	0.33235	FALSE	FALSE	FALSE	ieu-b-508	4	1.58E+08	0.035306	486484	0.600257	Sepsis (28	Sepsis (28	Sepsis (28	TRUE	igd	0.0028	1.20E-07	5211	M32651.m	TRUE	reported	DeuPwv	2	TRUE	0.005477	28.68796
4098	rs939885	A	G	A	G	0.0316	-0.02408	0.4911	0.484186	FALSE	FALSE	FALSE	ieu-b-508	3	1.96E+08	0.033297	486484	0.469512	Sepsis (28	Sepsis (28	Sepsis (28	TRUE	igd	0.0059	6.88E-08	7356	M32753.m	TRUE	reported	XlC6zq	2	TRUE	0.003885	28.67821
2847	rs1095432	T	C	T	C	0.2208	0.060753	0.026	0.019491	FALSE	FALSE	FALSE	ieu-b-508	7	1.31E+08	0.122811	486484	0.620822	Sepsis (28	Sepsis (28	Sepsis (28	TRUE	igd	0.0412	8.46E-08	1309	M27531.m	TRUE	reported	v01zDr	2	TRUE	0.02147	28.67739
2242	rs1265238	A	G	A	G	0.0091	-0.01069	0.6121	0.619256	FALSE	FALSE	FALSE	ieu-b-508	5	1.7E+08	0.034787	486484	0.758583	Sepsis (28	Sepsis (28	Sepsis (28	TRUE	igd	0.0017	1.57E-07	7354	M19934.m	TRUE	reported	HtcWcQ	2	TRUE	0.003881	28.64619
7699	rs1251276	T	C	T	C	0.0091	0.049522	0.7813	0.80825	FALSE	FALSE	FALSE	ieu-b-508	4	1.15E+08	0.0428	486484	0.247249	Sepsis (28	Sepsis (28	Sepsis (28	TRUE	igd	0.0017	9.07E-08	7345	M35397.m	TRUE	reported	Y56mZr	2	TRUE	0.003886	28.64618
4453	rs6824211	A	G	A	G	-0.0091	-0.00794	0.3182	0.312687	FALSE	FALSE	FALSE	ieu-b-508	4	1.86E+08	0.036271	486484	0.826641	Sepsis (28	Sepsis (28	Sepsis (28	TRUE	igd	0.0017	2.16E-07	7344	M32910.m	TRUE	reported	2OCBKh	2	TRUE	0.003887	28.64618
1273	rs1085807	T	C	T	C	-0.0091	-0.04276	0.4061	0.403767	FALSE	FALSE	FALSE	ieu-b-508	1	1.16E+08	0.034103	486484	0.209889	Sepsis (28	Sepsis (28	Sepsis (28	TRUE	igd	0.0017	1.12E-07	7325	M02132.m	TRUE	reported	uJFm2t	2	TRUE	0.003897	28.64616
4077	rs6928245	A	G	A	G	0.0091	-0.00061	0.3637	0.363118	FALSE	FALSE	FALSE	ieu-b-508	6	1.01E+08	0.034708	486484	0.986056	Sepsis (28	Sepsis (28	Sepsis (28	TRUE	igd	0.0017	1.79E-07	7318	M32740.m	TRUE	reported	YLshfP	2	TRUE	0.0039	28.64615
5369	rs944243	A	G	A	G	-0.0091	-0.0136	0.3604	0.363317	FALSE	FALSE	FALSE	ieu-b-508	20	60172462	0.034918	486484	0.696875	Sepsis (28	Sepsis (28	Sepsis (28	TRUE	igd	0.0017	1.48E-07	7118	M33380.m	TRUE	reported	OHRfl5	2	TRUE	0.004009	28.64593
1526	rs4519482	A	T	A	T	-0.0091	3.21E-05	0.291	0.335168	FALSE	TRUE	FALSE	ieu-b-508	2	1.73E+08	0.035457	486484	0.999278	Sepsis (28	Sepsis (28	Sepsis (28	TRUE	igd	0.0017	8.79E-08	7081	M12067.m	TRUE	reported	O5x7RD	2	TRUE	0.00403	28.64589
3694	rs2241480	T	C	T	C	0.0198	-0.00435	0.2872	0.287173	FALSE	FALSE	FALSE	ieu-b-508	4	10089763	0.036858	486484	0.905987	Sepsis (28	Sepsis (28	Sepsis (28	TRUE	igd	0.0037	7.72E-08	7369	M32587.m	TRUE	reported	uknv7G	2	TRUE	0.003871	28.62919
8037	rs1961334	A	G	A	G	-0.0198	-0.02295	0.4773	0.468908	FALSE	FALSE	FALSE	ieu-b-508	10	45612074	0.033448	486484	0.492545	Sepsis (28	Sepsis (28	Sepsis (28	TRUE	igd	0.0037	6.03E-08	6960	M35854.m	TRUE	reported	EujB0p	2	TRUE	0.004098	28.62873
3817	rs334698	C	G	C	G	-0.0246	0.108737	0.0445	0.045693	FALSE	TRUE	FALSE	ieu-b-508	1	61620993	0.08089	486484	0.178862	Sepsis (28	Sepsis (28	Sepsis (28	TRUE	igd	0.0046	7.72E-08	7229	M32644.m	TRUE	reported	JAa2WD	2	TRUE	0.003941	28.59133
2308	rs1731817	A	G	A	G	0.0139	-0.06292	0.1785	0.185684	FALSE	FALSE	FALSE	ieu-b-508	10	6954242	0.043401	486484	0.147115	Sepsis (28	Sepsis (28	Sepsis (28	TRUE	igd	0.0026	1.28E-07	7327	M20699.m	TRUE	reported	Z3EKFa	2	TRUE	0.003886	28.57356
2647	rs710719	C	G	C	G	-0.0139	-0.05435	0.3209	0.317661	FALSE	TRUE	FALSE	ieu-b-508	12	70069619	0.035907	486484	0.13015	Sepsis (28	Sepsis (28	Sepsis (28	TRUE	igd	0.0026	5.58E-08	7184	M22548.m	TRUE	reported	mZtwX2	2	TRUE	0.003963	28.5734
754	rs1089938	A	G	A	G	0.0139	0.014338	0.3819	0.377903	FALSE	FALSE	FALSE	ieu-b-508	11	77218468	0.034766	486484	0.680041	Sepsis (28	Sepsis (28	Sepsis (28	TRUE	igd	0.0026	1.27E-07	6946	M01303.m	TRUE	reported	3b8x0F	2	TRUE	0.004098	28.57313
1349	rs1043257	A	G	A	G	0.0738	0.002023	0.0145	0.021814	FALSE	FALSE	FALSE	ieu-b-508	2	2.42E+08	0.116087	486484	0.986094	Sepsis (28	Sepsis (28	Sepsis (28	TRUE	igd	0.0138	9.50E-08	1766	M02730.m	TRUE	reported	XYa035	2	TRUE	0.015936	28.56686
6199	rs2116833	A	G	A	G	-0.0155	0.023053	0.35	0.354154	FALSE	FALSE	FALSE	ieu-b-508	10	78712361	0.03535	486484	0.514321	Sepsis (28	Sepsis (28	Sepsis (28	TRUE	igd	0.0029	1.05E-07	6004	M33892.m	TRUE	reported	w7HgU7	2	TRUE	0.004735	28.55767
8099	rs1479400	T	C	T	C	0.5062	-0.01183	0.8279	0.855413	FALSE	FALSE	FALSE	ieu-b-508	3	61934466	0.047331	486484	0.802622	Sepsis (28	Sepsis (28	Sepsis (28	TRUE	igd	0.094	7.19E-08	122	M36097.m	TRUE	reported	RHr18S	2	TRUE	0.19205	28.52397
2337	rs4670465	T	G	T	G	-0.0144	0.086815	0.5498	0.541709	FALSE	FALSE	FALSE	ieu-b-508	2	35621644	0.033498	486484	0.009553	Sepsis (28	Sepsis (28	Sepsis (28	TRUE	igd	0.0027	6.37E-08	7366	M21044.m	TRUE	reported	Ym5rae	2	TRUE	0.003847	28.43672
419	rs1776525	A	G	A	G	0.0096	0.008075	0.1845	0.18921	FALSE	FALSE	FALSE	ieu-b-508	12	54099369	0.042903	486484	0.8507	Sepsis (28	Sepsis (28	Sepsis (28	TRUE	igd	0.0018	8.27E-08	7365	M00063.m	TRUE	reported	Hdsv1L	2	TRUE	0.003847	28.43672
3753	rs1020066	T	G	T	G	0.0096	-0.00125	0.7374	0.735476	FALSE	FALSE	FALSE	ieu-b-508	15	60025177	0.037945	486484	0.973723	Sepsis (28	Sepsis (28	Sepsis (28	TRUE	igd	0.0018	1.44E-07	7362	M32634.m	TRUE	reported	7T2RUz	2	TRUE	0.003849	28.43672
449	rs1217510	A	C	A	C	0.0048	-0.01284	0.4153	0.429966	FALSE	FALSE	FALSE	ieu-b-508	6	1.01E+08	0.033739	486484	0.703433	Sepsis (28	Sepsis (28	Sepsis (28	TRUE	igd	9.00E-04	4.86E-07	7361	M00513.m	TRUE	reported	ohacnP	2	TRUE	0.003849	28.43672
63	rs1790398	T	C	T	C	0.0048	-0.02122	0.5227	0.531419	FALSE	FALSE	FALSE	ieu-b-508	11	73882260	0.033439	486484	0.525619	Sepsis (28	Sepsis (28	Sepsis (28	TRUE	igd	9.00E-04	5.79E-08	7355	M00054.m	TRUE	reported	QTDAnh	2	TRUE	0.003852	28.43671
84	rs2658761	T	C	T	C	-0.0048	-0.00504	0.4343	0.436236	FALSE	FALSE	FALSE	ieu-b-508	18	54059039	0.033705	486484	0.881024	Sepsis (28	Sepsis (28	Sepsis (28	TRUE	igd	9.00E-04	6.75E-08	7355	M00054.m	TRUE	reported	QTDAnh	2	TRUE	0.003852	28.43671
95	rs295142	A	G	A	G	-0.0048	-0.00326	0.5909	0.600658	FALSE	FALSE	FALSE	ieu-b-508	2	2.01E+08	0.034041	486484	0.923745	Sepsis (28	Sepsis (28	Sepsis (28	TRUE	igd	9.00E-04	1.54E-07	7355	M00054.m	TRUE	reported	QTDAnh	2	TRUE	0.003852	28.43671
125	rs4926571	T	C	T	C	-0.0048	0.017502	0.5231	0.533092	FALSE	FALSE	FALSE	ieu-b-508	1	53096614	0.033737	486484	0.603919	Sepsis (28	Sepsis (28	Sepsis (28	TRUE	igd	9.00E-04	9.03E-08	7355	M00054.m	TRUE	reported	QTDAnh	2	TRUE	0.003852	28.43671
150	rs6826247	T	C	T	C	0.0048	0.018647	0.5071	0.509922	FALSE	FALSE	FALSE	ieu-b-508	4	1.62E+08	0.033459	486484	0.577326	Sepsis (28	Sepsis (28	Sepsis (28	TRUE	igd	9.00E-04	8.80E-08	7355	M00054.m	TRUE	reported	QTDAnh	2	TRUE	0.003852	28.43671
181	rs9315440	A	G	A	G	0.0048	0.008199	0.5843	0.57781	FALSE	FALSE	FALSE	ieu-b-508	13	37293989	0.033732	486484	0.807961	Sepsis (28	Sepsis (28	Sepsis (28	TRUE	igd	9.00E-04	6.71E-08	7355	M00054.m	TRUE	reported	QTDAnh	2	TRUE	0.003852	28.43671
6316	rs2490289	T	C	T	C	0.0096	-0.05009	0.2222	0.229001	FALSE	FALSE	FALSE	ieu-b-508	10	1.01E+08	0.040201	486484	0.212808	Sepsis (28	Sepsis (28	Sepsis (28	TRUE	igd	0.0018	1.53E-07	7354	M33955.m	TRUE	reported	mhHFnT	2	TRUE	0.003853	28.43671
6332	rs8052052	T	G	T	G	-0.0096	-0.03793	0.2037	0.199206	FALSE	FALSE	FALSE	ieu-b-508	16	54276243	0.04177	486484	0.363849	Sepsis (28	Sepsis (28	Sepsis (28	TRUE	igd	0.0018	1.86E-07	7354	M33955.m	TRUE	reported	mhHFnT	2	TRUE	0.003853	28.43671
210	rs1043991	T	C	T	C	0.0048	0.033094	0.6375	0.625513	FALSE	FALSE	FALSE	ieu-b-508	9	1.12E+08	0.03446	486484	0.336865	Sepsis (28	Sepsis (28	Sepsis (28	TRUE	igd	9.00E-04	8.33E-08	7351	M00060.m	TRUE	reported	kqwl4V	2	TRUE	0.003855	28.43671
215	rs1076394	A	G	A	G	0.0048	0.017178	0.3654	0.373453	FALSE	FALSE	FALSE	ieu-b-508	10	33751360	0.034591	486484	0.619468	Sepsis (28	Sepsis (28	Sepsis (28	TRUE	igd	9.00E-04	1.17E-07	7351	M00060.m	TRUE	reported	kqwl4V	2	TRUE	0.003855	28.43671
254	rs1541245	T	C	T	C	-0.0048	-0.02585	0.5318	0.52296	FALSE	FALSE	FALSE	ieu-b-508	1	1.65E+08	0.033478	486484	0.43999	Sepsis (28	Sepsis (28	Sepsis (28	TRUE	igd	9.00E-04	7.99E-08	7351	M00060.m	TRUE	reported	kqwl4V	2	TRUE	0.003855	28.43671
257	rs1654495	A	C	A	C	0.0048	-0.02241	0.5709	0.55807	FALSE	FALSE	FALSE	ieu-b-508	19	55501579	0.033745	486484	0.506565	Sepsis (28	Sepsis (28	Sepsis (28	TRUE	igd	9.00E-04	1.04E-07	7351	M00060.m	TRUE	reported	kqwl4V	2	TRUE	0.003855	28.43671
271	rs1952935	A	C	A	C	-0.0048	0.051023	0.5806	0.585009	FALSE	FALSE	FALSE	ieu-b-508	14	40859517	0.034032	486484	0.133806	Sepsis (28	Sepsis (28	Sepsis (28	TRUE	igd	9.00E-04	1.07E-07	7351	M00060.m	TRUE	reported	kqwl4V	2	TRUE	0.003855	28.43671
283	rs242082	T	C	T	C	0.0048	-0.03815	0.3867	0.389483	FALSE	FALSE	FALSE	ieu-b-508	22	33224439	0.03442	486484	0.267655	Sepsis (28	Sepsis (28	Sepsis (28	TRUE	igd	9.00E-04	1.38E-07	7351	M00060.m	TRUE	reported	kqwl4V	2	TRUE	0.003855	28.43671
285	rs2465250	A	C	A	C	0.0048	0.037418	0.5503	0.540415	FALSE	FALSE	FALSE	ieu-b-508	4	1.69E+08	0.033539	486484	0.264575	Sepsis (28	Sepsis (28	Sepsis (28	TRUE	igd	9.00E-04	7.76E-08	7351	M00060.m	TRUE	reported	kqwl4V	2	TRUE	0.003855	28.43671
286	rs2471650	T	C	T	C	-0.0048	-0.01149	0.5252	0.524594	FALSE	FALSE	FALSE	ieu-b-508	12	75578076	0.033509	486484	0.73167	Sepsis (28	Sepsis (28	Sepsis (28	TRUE	igd	9.00E-04	1.16E-07	7351	M00060.m	TRUE	reported	kqwl4V	2	TRUE	0.003855	28.43671
288	rs248065	A	G	A	G	-0.0048	-0.04391	0.5081	0.504241	FALSE	FALSE	FALSE	ieu-b-508	5	1.27E+08	0.033434	486484	0.189044	Sepsis (28	Sepsis (28	Sepsis (28	TRUE	igd	9.00E-04	1.13E-07	7351	M00060.m	TRUE	reported	kqwl4V	2	TRUE	0.003855	28.43671
293	rs2722276	A	G	A	G	0.0048	0.029157	0.523	0.526219	FALSE	FALSE	FALSE	ieu-b-508	7	37989178	0.033516	486484	0.384325	Sepsis (28	Sepsis (28	Sepsis (28	TRUE	igd	9.00E-04	8.70E-08	7351	M00060.m	TRUE	reported	kqwl4V	2	TRUE	0.003855	28.43671
299	rs3102519	A	G	A	G	0.0048	-0.01668	0.375	0.379059	FALSE	FALSE	FALSE	ieu-b-508	8	96791410	0.034365	486484	0.627506	Sepsis (28	Sepsis (28	Sepsis (28	TRUE	igd	9.00E-04	7.83E-08	7351	M00060.m	TRUE	reported	kqwl4V	2	TRUE	0.003855	28.43671
316	rs4758270	A	G	A	G	-0.0048	-0.05232	0.6483	0.648976	FALSE	FALSE	FALSE	ieu-b-508	11	8042616	0.035039	486484	0.135392	Sepsis (28	Sepsis (28	Sepsis (28	TRUE	igd	9.00E-04	2.01E-07	7351	M00060.m	TRUE	reported	kqwl4V	2	TRUE	0.003855	28.43671
358	rs7584322	A	C	A	C	-0.0048	0.042278	0.4742	0.47369	FALSE	FALSE	FALSE	ieu-b-508	2	71140960	0.03358	486484	0.208025	Sepsis (28	Sepsis (28	Sepsis (28	TRUE	igd	9.00E-04	7.03E-08	7351	M00060.m	TRUE	reported	kqwl4V	2	TRUE	0.003855	28.43671
7946	rs7930371	T	C	T	C	-0.0096	0.069586	0.3408	0.352849	FALSE	FALSE	FALSE	ieu-b-508	11	1.04E+08	0.034927	486484	0.046336	Sepsis (28	Sepsis (28	Sepsis (28	TRUE	igd	0.0018	1.82E-07	7348	M35675.m	TRUE	reported	D7fdyZ	2	TRUE	0.003856	28.4367
1175	rs1294060	A	C	A	C	-0.0096	-0.03051	0.6059	0.610077	FALSE	FALSE	FALSE	ieu-b-508	17	12655432	0.034378	486484	0.374861	Sepsis (28	Sepsis (28	Sepsis (28	TRUE	igd	0.0018	1.82E-07	7346	M01645.m	TRUE	reported	d5nAn3	2	TRUE	0.003857	28.4367
3822	rs6466580	T	C	T	C	-0.0096	-0.02089	0.6573	0.651137	FALSE	FALSE	FALSE	ieu-b-508	7	1.16E+08	0.035002	486484	0.550558	Sepsis (28	Sepsis (28	Sepsis (28	TRUE	igd	0.0018	1.41E-07	7229	M32644.m	TRUE	reported	JAa2WD	2	TRUE	0.003919	28.43657
6803	rs1329833	T	C	T	C	0.0096	-0.01452	0.6276	0.636652	FALSE	FALSE	FALSE	ieu-b-508	9	77520324	0.034621	486484	0.674979	Sepsis (28	Sepsis (28	Sepsis (28	TRUE	igd	0.0018	1.05E-07	6755	M34359.m	TRUE	reported	0UotaJ	2	TRUE	0.004193	28.43602
6825	rs4905134	A	G	A	G	-0.0096	0.006477	0.5097	0.505696	FALSE	FALSE	FALSE	ieu-b-508	14	94459845	0.03357	486484	0.846997	Sepsis (28	Sepsis (28	Sepsis (28	TRUE	igd	0.0018	6.15E-08	6755	M34359.m	TRUE	reported	0UotaJ	2	TRUE	0.004193	28.43602
7148	rs1050586	T	G	T	G	-0.0288	0.04994	0.665	0.660561	FALSE	FALSE	FALSE	ieu-b-508	12	20911587	0.035227	486484	0.156287	Sepsis (28	Sepsis (28	Sepsis (28	TRUE	igd	0.0054	9.92E-08	5856	M34533.m	TRUE	reported	SuKX4k	2	TRUE	0.004834	28.43473
1425	rs275721	A	C	A	C	-0.016	0.032971	0.509	0.503189	FALSE	FALSE	FALSE	ieu-b-508	15	40115920	0.033375	486484	0.323202	Sepsis (28	Sepsis (28	Sepsis (28	TRUE	igd	0.003	9.64E-08	5196	M03127.m	TRUE	reported	JscEZw	2	TRUE	0.005444	28.4335
6707	rs894127	A	G	A	G	0.0208	-0.09485	0.1321	0.145218	FALSE	FALSE	FALSE	ieu-b-508	8	70452311	0.047573	486484	0.046182	Sepsis (28	Sepsis (28	Sepsis (28	TRUE	igd	0.0039	1.23E-07	5081	M34336.m	TRUE	reported	jrSSxL	2	TRUE	0.005567	28.43325
5649	rs1748431	C	G	C	G	0.0618	0.10086	0.0326	0.045311	FALSE	TRUE	FALSE	ieu-b-508	8	16010732	0.079914	486484	0.206913	Sepsis (28	Sepsis (28	Sepsis (28	TRUE	igd	0.0116	1.07E-07	5717	M33453.m	TRUE	reported	OzEeLQ	2	TRUE	0.00494	28.37325
7013	rs4899048	A	G	A	G	-0.0959	0.022074	0.0941	0.084575	FALSE	FALSE	FALSE	ieu-b-508	14	61909343	0.060044	486484	0.71315	Sepsis (28	Sepsis (28	Sepsis (28	TRUE	igd	0.018	9.37E-08	4452	M34420.m	TRUE	reported	20uofq	2	TRUE	0.006335	28.37246
6787	rs3917538	A	G	A	G	-0.0586	0.028257	0.2133	0.229851	FALSE	FALSE	FALSE	ieu-b-508	7	94937893	0.039687	486484	0.476461	Sepsis (28	Sepsis (28	Sepsis (28	TRUE	igd	0.011	9.75E-08	3491	M34350.m	TRUE	reported	Cc3yW9	2	TRUE	0.008064	28.36358
7414	rs1703172	C	G	C	G	-0.0181	0.092999	0.8162	0.822412	FALSE	TRUE	FALSE	ieu-b-508	4	1.55E+08	0.043651	486484	0.033128	Sepsis (28	Sepsis (28	Sepsis (28	TRUE	igd	0.0034	7.68E-08	7350	M35186.m	TRUE	reported	UZfu6Z	2	TRUE	0.003841	28.33225
6011	rs2292787	A	G	A	G	-0.6793	-0.0754	0.0326	0.017509	FALSE	FALSE	FALSE	ieu-b-508	2	1.69E+08	0.128341	486484	0.556883	Sepsis (28	Sepsis (28	Sepsis (28	TRUE	igd	0.126	6.93E-08	79	M33751.m	TRUE	reported	Cxdkr4	2	TRUE	0.268964	28.32995
2960	rs281499	A	G	A	G	0.0149	0.022759	0.5873	0.571516	FALSE	FALSE	FALSE	ieu-b-508	2	46418606	0.033856	486484	0.501433	Sepsis (28	Sepsis (28	Sepsis (28	TRUE	igd	0.0028	7.82E-08	6795	M30805.m	TRUE	reported	YHV70c	2	TRUE	0.00415	28.30927
7898	rs2554887	A	T	A	T	0.0282	-0.02645	0.683	0.680261	FALSE	TRUE	FALSE	ieu-b-508	15	50488859	0.035868	486484	0.460813	Sepsis (28	Sepsis (28	Sepsis (28	TRUE	igd	0.0053	1.29E-07	5629	M35669.m	TRUE	reported	gSecuE	2	TRUE	0.005004	28.30037
848	rs6453591	A	T	A	T	0.0133	-0.00187	0.771	0.757363	FALSE	TRUE	FALSE	ieu-b-508	6	73335108	0.038929	486484	0.961775	Sepsis (28	Sepsis (28	Sepsis (28	TRUE	igd	0.0025	8.55E-08	7323	M01359.m	TRUE	reported	7L9x1s	2	TRUE	0.00385	28.29467
1261	rs7814638	A	G	A	G	-0.0133	0.012263	0.141	0.142969	FALSE	FALSE	FALSE	ieu-b-508	8	40213193	0.047685	486484	0.797053	Sepsis (28	Sepsis (28	Sepsis (28	TRUE	igd	0.0025	1.23E-07	7136	M01769.m	TRUE	reported	rismUZ	2	TRUE	0.00395	28.29447
2305	rs1262913	T	G	T	G	0.0234	0.031157	0.0631	0.066448	FALSE	FALSE	FALSE	ieu-b-508	3	53373238	0.0667	486484	0.640417	Sepsis (28	Sepsis (28	Sepsis (28	TRUE	igd	0.0044	1.29E-07	7327	M20699.m	TRUE	reported	Z3EKFa	2	TRUE	0.003845	28.27534
2722	rs1294679	A	G	A	G	0.0234	0.027309	0.8939	0.885946	FALSE	FALSE	FALSE	ieu-b-508	17	55316065	0.052691	486484	0.604259	Sepsis (28	Sepsis (28	Sepsis (28	TRUE	igd	0.0044	8.83E-08	6388	M25599.m	TRUE	reported	fRZxwP	2	TRUE	0.004408	28.2742
6402	rs7078211	A	G	A	G	-0.0404	-0.01371	0.8445	0.846308	FALSE	FALSE	FALSE	ieu-b-508	10	5253638	0.046257	486484	0.766911	Sepsis (28	Sepsis (28	Sepsis (28	TRUE	igd	0.0076	1.26E-07	7326	M33973.m	TRUE	reported	rpygG6	2	TRUE	0.003842	28.2499
2546	rs1630999	C	G	C	G	-0.0101	0.000265	0.4049	0.390765	FALSE	TRUE	FALSE	ieu-b-508	3	10305551	0.034246	486484	0.993831	Sepsis (28	Sepsis (28	Sepsis (28	TRUE	igd	0.0019	1.93E-07	6535	M22177.m	TRUE	reported	2s6Sly	2	TRUE	0.004305	28.24897
7247	rs1188026	T	C	T	C	-0.0335	-0.00653	0.2898	0.296224	FALSE	FALSE	FALSE	ieu-b-508	19	40788641	0.036573	486484	0.85837	Sepsis (28	Sepsis (28	Sepsis (28	TRUE	igd	0.0063	1.30E-07	1060	M34912.m	TRUE	reported	W8lcT6	2	TRUE	0.025982	28.22203
639	rs1002471	A	G	A	G	0.0085	-0.00852	0.3772	0.367194	FALSE	FALSE	FALSE	ieu-b-508	4	89219645	0.034638	486484	0.805737	Sepsis (28	Sepsis (28	Sepsis (28	TRUE	igd	0.0016	7.50E-08	7352	M01125.m	TRUE	reported	yw0clB	2	TRUE	0.003824	28.21498
6770	rs1050970	C	G	C	G	-0.1004	-0.14435	0.9525	0.95136	FALSE	TRUE	FALSE	ieu-b-508	10	98164357	0.077568	486484	0.062748	Sepsis (28	Sepsis (28	Sepsis (28	TRUE	igd	0.0189	1.09E-07	5270	M34344.m	TRUE	reported	enFm6p	2	TRUE	0.005326	28.20843
3253	rs1187926	A	G	A	G	-0.0138	-0.09629	0.17	0.16875	FALSE	FALSE	FALSE	ieu-b-508	19	3763118	0.044736	486484	0.031367	Sepsis (28	Sepsis (28	Sepsis (28	TRUE	igd	0.0026	8.70E-08	7365	M32348.m	TRUE	reported	DJeLDx	2	TRUE	0.00381	28.16395
4574	rs1076223	A	G	A	G	-0.0138	-0.0098	0.3787	0.377605	FALSE	FALSE	FALSE	ieu-b-508	10	70323114	0.034627	486484	0.777073	Sepsis (28	Sepsis (28	Sepsis (28	TRUE	igd	0.0026	1.68E-07	6404	M33163.m	TRUE	reported	YzGXkR	2	TRUE	0.00438	28.1628
6733	rs1779696	T	C	T	C	0.202	0.024685	0.9439	0.930536	FALSE	FALSE	FALSE	ieu-b-508	6	1.3E+08	0.06574	486484	0.707292	Sepsis (28	Sepsis (28	Sepsis (28	TRUE	igd	0.038	1.06E-07	535	M34338.m	TRUE	reported	h4FB9a	2	TRUE	0.050168	28.15198
3423	rs1220235	T	C	T	C	0.0191	-0.01128	0.9123	0.902127	FALSE	FALSE	FALSE	ieu-b-508	6	1.6E+08	0.05607	486484	0.840575	Sepsis (28	Sepsis (28	Sepsis (28	TRUE	igd	0.0036	1.14E-07	7364	M32452.m	TRUE	reported	8GtFpl	2	TRUE	0.003808	28.14127
1537	rs1185260	A	G	A	G	-0.0191	0.034074	0.1225	0.123442	FALSE	FALSE	FALSE	ieu-b-508	12	1.22E+08	0.050651	486484	0.50113	Sepsis (28	Sepsis (28	Sepsis (28	TRUE	igd	0.0036	1.14E-07	6864	M12129.m	TRUE	reported	iNzdgb	2	TRUE	0.004084	28.14072
4229	rs176400	A	G	A	G	0.0191	0.006353	0.331	0.320254	FALSE	FALSE	FALSE	ieu-b-508	2	32633569	0.035918	486484	0.859609	Sepsis (28	Sepsis (28	Sepsis (28	TRUE	igd	0.0036	1.20E-07	6857	M32787.m	TRUE	reported	fSRakm	2	TRUE	0.004088	28.14071
5441	rs1258966	T	G	T	G	-0.0382	-0.04042	0.3791	0.380883	FALSE	FALSE	FALSE	ieu-b-508	14	24995175	0.034422	486484	0.240283	Sepsis (28	Sepsis (28	Sepsis (28	TRUE	igd	0.0072	1.26E-07	5185	M33390.m	TRUE	reported	t0EYdH	2	TRUE	0.0054	28.13806
2185	rs1709612	A	G	A	G	0.0435	-0.05846	0.9614	0.958921	FALSE	FALSE	FALSE	ieu-b-508	14	30366612	0.084094	486484	0.486907	Sepsis (28	Sepsis (28	Sepsis (28	TRUE	igd	0.0082	1.06E-07	1746	M19362.m	TRUE	reported	SkNEKw	2	TRUE	0.015862	28.1095
5871	rs542663	T	C	T	C	-0.0674	0.023751	0.8073	0.815146	FALSE	FALSE	FALSE	ieu-b-508	9	1.35E+08	0.042939	486484	0.580167	Sepsis (28	Sepsis (28	Sepsis (28	TRUE	igd	0.0127	1.12E-07	824	M33609.m	TRUE	reported	h7B9gN	2	TRUE	0.033051	28.09681
1784	rs7761964	T	C	T	C	0.0175	-0.04053	0.6305	0.623564	FALSE	FALSE	FALSE	ieu-b-508	6	1.35E+08	0.034417	486484	0.238997	Sepsis (28	Sepsis (28	Sepsis (28	TRUE	igd	0.0033	1.71E-07	1765	M15676.m	TRUE	reported	s1NfKN	2	TRUE	0.015683	28.09026
2567	rs4878628	T	C	T	C	0.0106	-0.01355	0.2753	0.271546	FALSE	FALSE	FALSE	ieu-b-508	9	35766561	0.037555	486484	0.718294	Sepsis (28	Sepsis (28	Sepsis (28	TRUE	igd	0.002	6.18E-08	6535	M22177.m	TRUE	reported	2s6Sly	2	TRUE	0.00428	28.0814
7180	rs705795	T	C	T	C	0.0551	-0.022	0.2579	0.266021	FALSE	FALSE	FALSE	ieu-b-508	6	1.66E+08	0.038251	486484	0.565162	Sepsis (28	Sepsis (28	Sepsis (28	TRUE	igd	0.0104	1.13E-07	4515	M34535.m	TRUE	reported	z9BiJF	2	TRUE	0.006179	28.05719
3467	rs1748974	A	T	A	T	0.0143	0.008985	0.1166	0.117618	FALSE	TRUE	FALSE	ieu-b-508	3	1.11E+08	0.051739	486484	0.862133	Sepsis (28	Sepsis (28	Sepsis (28	TRUE	igd	0.0027	1.53E-07	7363	M32489.m	TRUE	reported	hnljaD	2	TRUE	0.003795	28.04314
458	rs2289804	A	G	A	G	-0.0143	0.035854	0.9343	0.93512	FALSE	FALSE	FALSE	ieu-b-508	10	69993032	0.067991	486484	0.59796	Sepsis (28	Sepsis (28	Sepsis (28	TRUE	igd	0.0027	1.25E-07	7361	M00513.m	TRUE	reported	ohacnP	2	TRUE	0.003796	28.04313
545	rs1103350	A	G	A	G	0.0143	-0.07159	0.9307	0.92809	FALSE	FALSE	FALSE	ieu-b-508	11	36246782	0.065065	486484	0.271203	Sepsis (28	Sepsis (28	Sepsis (28	TRUE	igd	0.0027	9.46E-08	7354	M00606.m	TRUE	reported	r5smf7	2	TRUE	0.0038	28.04313
8497	rs2255908	A	G	A	G	0.0466	0.021762	0.6525	0.646732	FALSE	FALSE	FALSE	ieu-b-508	15	93146609	0.035038	486484	0.534546	Sepsis (28	Sepsis (28	Sepsis (28	TRUE	igd	0.0088	1.04E-07	7014	M37097.m	TRUE	reported	wCbJj4	2	TRUE	0.003982	28.03384
401	rs1049354	A	G	A	G	-0.009	-0.00745	0.7989	0.801287	FALSE	FALSE	FALSE	ieu-b-508	1	75311145	0.041798	486484	0.858598	Sepsis (28	Sepsis (28	Sepsis (28	TRUE	igd	0.0017	2.27E-07	7365	M00063.m	TRUE	reported	Hdsv1L	2	TRUE	0.003791	28.02007
625	rs1822906	A	G	A	G	0.018	0.042882	0.2048	0.800281	FALSE	FALSE	FALSE	ieu-b-508	3	1.45E+08	0.042114	486484	0.308572	Sepsis (28	Sepsis (28	Sepsis (28	TRUE	igd	0.0034	9.61E-08	7349	M01121.m	TRUE	reported	HU0fhR	2	TRUE	0.003799	28.02005
1762	rs1161586	A	G	A	G	-0.009	0.006566	0.3917	0.376308	FALSE	FALSE	FALSE	ieu-b-508	12	30175212	0.034443	486484	0.848805	Sepsis (28	Sepsis (28	Sepsis (28	TRUE	igd	0.0017	1.34E-07	7331	M15676.m	TRUE	reported	s1NfKN	2	TRUE	0.003809	28.02004

4058	rs2038410	T	C	T	C	-0.009	-0.01951	0.4395	0.444539	FALSE	FALSE	FALSE	ieu-b-508	20	5249302	0.033606	486484	0.561587	Sepsis (28	Sepsis (28	Sepsis (28	TRUE	igd	0.0017	1.65E-07	7318	M32740.m	TRUE	reported	YLshfP	2	TRUE	0.003815	28.02002
2183	rs1655155	T	C	T	C	-0.009	0.017895	0.5977	0.60913	FALSE	FALSE	FALSE	ieu-b-508	10	1.33E+08	0.034809	486484	0.607185	Sepsis (28	Sepsis (28	Sepsis (28	TRUE	igd	0.0017	1.51E-07	7182	M19362.m	TRUE	reported	SkNEKw	2	TRUE	0.003887	28.01988
6777	rs7524781	A	G	A	G	0.1192	-0.02497	0.1738	0.170513	FALSE	FALSE	FALSE	ieu-b-508	1	1.07E+08	0.044291	486484	0.572974	Sepsis (28	Sepsis (28	Sepsis (28	TRUE	igd	0.0225	1.20E-07	955	M34344.m	TRUE	reported	enFm6p	2	TRUE	0.02855	28.00767
5947	rs1014242	A	C	A	C	-0.0328	0.046955	0.6825	0.67167	FALSE	FALSE	FALSE	ieu-b-508	14	99673324	0.035853	486484	0.190314	Sepsis (28	Sepsis (28	Sepsis (28	TRUE	igd	0.0062	1.33E-07	5328	M33652.m	TRUE	reported	vYkdTv	2	TRUE	0.005225	27.97701
2529	rs495828	T	G	T	G	-0.0312	-0.03537	0.217	0.207952	FALSE	FALSE	FALSE	ieu-b-508	9	1.36E+08	0.040853	486484	0.38666	Sepsis (28	Sepsis (28	Sepsis (28	TRUE	igd	0.0059	1.43E-07	3838	M22175.m	TRUE	reported	IIlqyJ	2	TRUE	0.007233	27.94981
4368	rs1289375	T	C	T	C	0.1566	-0.13292	0.9005	0.842101	FALSE	FALSE	FALSE	ieu-b-508	14	20233840	0.058603	486484	0.023323	Sepsis (28	Sepsis (28	Sepsis (28	TRUE	igd	0.0296	1.26E-07	1162	M32863.m	TRUE	reported	cLOFYE	2	TRUE	0.023521	27.94164
1608	rs1321675	A	G	A	G	0.0555	0.050138	0.0117	0.015076	FALSE	FALSE	FALSE	ieu-b-508	6	44653210	0.137242	486484	0.714872	Sepsis (28	Sepsis (28	Sepsis (28	TRUE	igd	0.0105	1.31E-07	7352	M15122.m	TRUE	reported	VlnI8T	2	TRUE	0.003786	27.93118
2925	rs7095787	A	T	A	T	0.0185	0.058658	0.788	0.775657	FALSE	TRUE	FALSE	ieu-b-508	10	85679116	0.040269	486484	0.145213	Sepsis (28	Sepsis (28	Sepsis (28	TRUE	igd	0.0035	1.58E-07	5562	M27728.m	TRUE	reported	WOp1XE	2	TRUE	0.004998	27.92873
8500	rs3828671	A	G	A	G	-0.1357	0.056422	0.0276	0.028874	FALSE	FALSE	FALSE	ieu-b-508	5	1.32E+08	0.100122	486484	0.573069	Sepsis (28	Sepsis (28	Sepsis (28	TRUE	igd	0.0257	1.24E-07	7014	M37097.m	TRUE	reported	wCbJj4	2	TRUE	0.003959	27.87209
921	rs9964014	T	C	T	C	-0.0132	0.042266	0.1115	0.139808	FALSE	FALSE	FALSE	ieu-b-508	18	3794759	0.049334	486484	0.391592	Sepsis (28	Sepsis (28	Sepsis (28	TRUE	igd	0.0025	1.80E-07	7354	M01494.m	TRUE	reported	7JWNKI	2	TRUE	0.003777	27.87082
1401	rs5010651	T	C	T	C	-0.0132	0.006569	0.8332	0.680879	FALSE	FALSE	FALSE	ieu-b-508	3	30904511	0.035815	486484	0.854469	Sepsis (28	Sepsis (28	Sepsis (28	TRUE	igd	0.0025	1.77E-07	7037	M02734.m	TRUE	reported	0y0dDD	2	TRUE	0.003946	27.87048
7632	rs1204979	A	G	A	G	0.0132	0.010351	0.1285	0.121903	FALSE	FALSE	FALSE	ieu-b-508	11	97388680	0.051292	486484	0.840062	Sepsis (28	Sepsis (28	Sepsis (28	TRUE	igd	0.0025	1.99E-07	5768	M35326.m	TRUE	reported	jRytyJ	2	TRUE	0.00481	27.86873
4263	rs9953591	A	T	A	T	0.0681	-0.05482	0.0705	0.079449	FALSE	TRUE	FALSE	ieu-b-508	18	70122049	0.062092	486484	0.377297	Sepsis (28	Sepsis (28	Sepsis (28	TRUE	igd	0.0129	1.40E-07	4274	M32800.m	TRUE	reported	Q7MVsa	2	TRUE	0.006478	27.85554
1635	rs21327	A	G	A	G	0.0095	-0.01765	0.286	0.28648	FALSE	FALSE	FALSE	ieu-b-508	1	1.47E+08	0.036928	486484	0.632759	Sepsis (28	Sepsis (28	Sepsis (28	TRUE	igd	0.0018	1.25E-07	7368	M15140.m	TRUE	reported	jEcqMJ	2	TRUE	0.003766	27.84738
4496	rs1118796	A	G	A	G	0.0095	-0.00434	0.1222	0.130403	FALSE	FALSE	FALSE	ieu-b-508	10	96231169	0.0497	486484	0.93039	Sepsis (28	Sepsis (28	Sepsis (28	TRUE	igd	0.0018	2.05E-07	7362	M33132.m	TRUE	reported	dTXJa8	2	TRUE	0.003769	27.84737
7950	rs9543004	T	C	T	C	-0.0095	0.013621	0.6185	0.627053	FALSE	FALSE	FALSE	ieu-b-508	13	73136378	0.034701	486484	0.694663	Sepsis (28	Sepsis (28	Sepsis (28	TRUE	igd	0.0018	2.16E-07	7348	M35675.m	TRUE	reported	D7fdyZ	2	TRUE	0.003777	27.84736
1690	rs1211923	T	C	T	C	0.0095	-0.03832	0.2374	0.234153	FALSE	FALSE	FALSE	ieu-b-508	1	72297766	0.040325	486484	0.342025	Sepsis (28	Sepsis (28	Sepsis (28	TRUE	igd	0.0018	7.11E-08	7345	M15488.m	TRUE	reported	SPriBO	2	TRUE	0.003778	27.84735
4443	rs350729	T	G	T	G	-0.0095	-0.03974	0.313	0.305088	FALSE	FALSE	FALSE	ieu-b-508	2	52983773	0.036315	486484	0.273756	Sepsis (28	Sepsis (28	Sepsis (28	TRUE	igd	0.0018	6.90E-08	7344	M32910.m	TRUE	reported	2OCBKh	2	TRUE	0.003779	27.84735
4041	rs1091668	A	T	A	T	0.0095	0.026598	0.6493	0.632213	FALSE	TRUE	FALSE	ieu-b-508	1	20309565	0.034914	486484	0.446168	Sepsis (28	Sepsis (28	Sepsis (28	TRUE	igd	0.0018	8.65E-08	7318	M32740.m	TRUE	reported	YLshfP	2	TRUE	0.003792	27.84733
5670	rs1124111	A	G	A	G	0.0095	0.054943	0.7231	0.716373	FALSE	FALSE	FALSE	ieu-b-508	5	1.11E+08	0.03706	486484	0.138192	Sepsis (28	Sepsis (28	Sepsis (28	TRUE	igd	0.0018	2.15E-07	7307	M33477.m	TRUE	reported	Aw8PBK	2	TRUE	0.003798	27.84731
5537	rs7111143	A	G	A	G	-0.0095	0.004799	0.2847	0.332102	FALSE	FALSE	FALSE	ieu-b-508	11	1.31E+08	0.035918	486484	0.893705	Sepsis (28	Sepsis (28	Sepsis (28	TRUE	igd	0.0018	5.35E-08	7306	M33422.m	TRUE	reported	PLgjrT	2	TRUE	0.003798	27.84731
5805	rs1166040	T	C	T	C	0.0095	-0.01277	0.604	0.597895	FALSE	FALSE	FALSE	ieu-b-508	18	71472768	0.034091	486484	0.707947	Sepsis (28	Sepsis (28	Sepsis (28	TRUE	igd	0.0018	2.21E-07	7066	M33515.m	TRUE	reported	X9mul1	2	TRUE	0.003927	27.84705
5830	rs4902201	A	G	A	G	0.0095	0.012164	0.3616	0.370225	FALSE	FALSE	FALSE	ieu-b-508	14	63566635	0.034786	486484	0.726568	Sepsis (28	Sepsis (28	Sepsis (28	TRUE	igd	0.0018	2.21E-07	7066	M33515.m	TRUE	reported	X9mul1	2	TRUE	0.003927	27.84705
982	rs9940567	T	C	T	C	0.0095	-0.0133	0.7319	0.734741	FALSE	FALSE	FALSE	ieu-b-508	16	62285678	0.037775	486484	0.724696	Sepsis (28	Sepsis (28	Sepsis (28	TRUE	igd	0.0018	1.57E-07	6523	M01558.m	TRUE	reported	AZK0p4	2	TRUE	0.004252	27.8464
7844	rs7171302	C	G	C	G	-0.0438	-0.0329	0.2838	0.295231	FALSE	TRUE	FALSE	ieu-b-508	15	61767705	0.036886	486484	0.372477	Sepsis (28	Sepsis (28	Sepsis (28	TRUE	igd	0.0083	1.54E-07	5035	M35551.m	TRUE	reported	5Sxswl	2	TRUE	0.0055	27.83681
2735	rs6688960	A	G	A	G	-0.1911	-0.29567	0.9856	0.984296	FALSE	FALSE	FALSE	ieu-b-508	1	2.14E+08	0.139027	486484	0.033447	Sepsis (28	Sepsis (28	Sepsis (28	TRUE	igd	0.0362	1.34E-07	1358	M25599.m	TRUE	reported	fRZxwP	2	TRUE	0.020109	27.82686
8	rs780093	T	C	T	C	-0.0058	0.005915	0.3995	0.38476	FALSE	FALSE	FALSE	ieu-b-508	2	27742603	0.034302	486484	0.863098	Sepsis (28	Sepsis (28	Sepsis (28	TRUE	igd	0.0011	1.93E-07	7372	M00053.m	TRUE	reported	4Smluz	2	TRUE	0.003757	27.79411
7316	rs1192055	A	G	A	G	0.0174	0.040244	0.218	0.218949	FALSE	FALSE	FALSE	ieu-b-508	3	57128211	0.040526	486484	0.320684	Sepsis (28	Sepsis (28	Sepsis (28	TRUE	igd	0.0033	1.63E-07	7344	M35127.m	TRUE	reported	RNsmjl	2	TRUE	0.003771	27.79408
4624	rs1702200	A	G	A	G	-0.7015	-0.08922	0.0339	0.014367	FALSE	FALSE	FALSE	ieu-b-508	2	38331037	0.140387	486484	0.525076	Sepsis (28	Sepsis (28	Sepsis (28	TRUE	igd	0.1313	9.16E-08	76	M33173.m	TRUE	reported	04fkUQ	2	TRUE	0.273038	27.79355
2846	rs1086501	A	G	A	G	0.0453	0.023704	0.1816	0.188581	FALSE	FALSE	FALSE	ieu-b-508	2	98989304	0.042773	486484	0.57945	Sepsis (28	Sepsis (28	Sepsis (28	TRUE	igd	0.0086	1.36E-07	5673	M27531.m	TRUE	reported	v01zDr	2	TRUE	0.004867	27.73616
1706	rs1165621	T	C	T	C	-0.0079	-0.04378	0.498	0.525293	FALSE	FALSE	FALSE	ieu-b-508	17	17453067	0.033362	486484	0.189413	Sepsis (28	Sepsis (28	Sepsis (28	TRUE	igd	0.0015	7.48E-08	7310	M15506.m	TRUE	reported	mVRmZO	2	TRUE	0.00378	27.73019
6693	rs1341995	T	C	T	C	-0.0158	-0.00536	0.7317	0.720031	FALSE	FALSE	FALSE	ieu-b-508	2	2.09E+08	0.037131	486484	0.885212	Sepsis (28	Sepsis (28	Sepsis (28	TRUE	igd	0.003	9.57E-08	5081	M34336.m	TRUE	reported	jrSSxL	2	TRUE	0.005429	27.72686
4137	rs1203824	A	G	A	G	0.0258	-0.00479	0.4301	0.437906	FALSE	FALSE	FALSE	ieu-b-508	1	1.2E+08	0.033727	486484	0.887167	Sepsis (28	Sepsis (28	Sepsis (28	TRUE	igd	0.0049	1.84E-07	7250	M32757.m	TRUE	reported	TJV9Xb	2	TRUE	0.003809	27.7158
4114	rs1000491	A	G	A	G	0.0179	0.024204	0.5631	0.564363	FALSE	FALSE	FALSE	ieu-b-508	4	57968444	0.033679	486484	0.472357	Sepsis (28	Sepsis (28	Sepsis (28	TRUE	igd	0.0034	1.62E-07	6658	M32755.m	TRUE	reported	WDrNdi	2	TRUE	0.004146	27.7088
4384	rs1245254	A	T	A	T	-0.01	0.054539	0.8568	0.844489	FALSE	TRUE	FALSE	ieu-b-508	17	70073175	0.046218	486484	0.23799	Sepsis (28	Sepsis (28	Sepsis (28	TRUE	igd	0.0019	7.64E-08	7329	M32867.m	TRUE	reported	Jcsyzm	2	TRUE	0.003765	27.69327
8022	rs7147975	A	G	A	G	-0.01	0.01232	0.4965	0.502254	FALSE	FALSE	FALSE	ieu-b-508	14	22491372	0.033456	486484	0.712692	Sepsis (28	Sepsis (28	Sepsis (28	TRUE	igd	0.0019	2.44E-07	6604	M35754.m	TRUE	reported	IlXOo1	2	TRUE	0.004177	27.69244
2556	rs2514587	A	G	A	G	0.01	-0.03576	0.6223	0.627679	FALSE	FALSE	FALSE	ieu-b-508	8	1.2E+08	0.03493	486484	0.305888	Sepsis (28	Sepsis (28	Sepsis (28	TRUE	igd	0.0019	3.00E-07	6535	M22177.m	TRUE	reported	2s6Sly	2	TRUE	0.004221	27.69235
7382	rs7446715	A	G	A	G	-0.01	0.031098	0.6732	0.657081	FALSE	FALSE	FALSE	ieu-b-508	5	84069439	0.035172	486484	0.376617	Sepsis (28	Sepsis (28	Sepsis (28	TRUE	igd	0.0019	1.81E-07	4900	M35137.m	TRUE	reported	Eq2siS	2	TRUE	0.005621	27.68952
2000	rs1768206	A	G	A	G	-0.1836	-0.14893	0.9239	0.936139	FALSE	FALSE	FALSE	ieu-b-508	8	1.19E+08	0.068924	486484	0.030713	Sepsis (28	Sepsis (28	Sepsis (28	TRUE	igd	0.0349	1.45E-07	2433	M18281.m	TRUE	reported	3mLp7z	2	TRUE	0.011247	27.65269
3178	rs11578	T	C	T	C	0.0142	-0.05211	0.2919	0.290093	FALSE	FALSE	FALSE	ieu-b-508	20	57253275	0.036629	486484	0.154816	Sepsis (28	Sepsis (28	Sepsis (28	TRUE	igd	0.0027	1.93E-07	7356	M32338.m	TRUE	reported	q5cuaP	2	TRUE	0.003746	27.65229
2420	rs2014576	A	G	A	G	-0.0142	-0.02194	0.4568	0.449484	FALSE	FALSE	FALSE	ieu-b-508	19	46269076	0.033539	486484	0.512998	Sepsis (28	Sepsis (28	Sepsis (28	TRUE	igd	0.0027	1.15E-07	6556	M21188.m	TRUE	reported	kvdnjc	2	TRUE	0.004201	27.65137
6768	rs9667032	T	G	T	G	0.3286	0.033506	0.0244	0.031179	FALSE	FALSE	FALSE	ieu-b-508	11	71344441	0.096053	486484	0.727215	Sepsis (28	Sepsis (28	Sepsis (28	TRUE	igd	0.0624	1.38E-07	633	M34339.m	TRUE	reported	RwGZ7c	2	TRUE	0.04197	27.64341
894	rs1192521	A	T	A	T	-0.0163	0.004476	0.7755	0.730302	FALSE	TRUE	FALSE	ieu-b-508	20	56679345	0.037731	486484	0.905572	Sepsis (28	Sepsis (28	Sepsis (28	TRUE	igd	0.0031	1.40E-07	7307	M01493.m	TRUE	reported	eLLRt7	2	TRUE	0.003769	27.63968
6087	rs542320	A	T	A	T	0.0853	-0.01923	0.3812	0.398427	FALSE	TRUE	FALSE	ieu-b-508	15	29536945	0.034415	486484	0.576371	Sepsis (28	Sepsis (28	Sepsis (28	TRUE	igd	0.0162	1.52E-07	471	M33835.m	TRUE	reported	crhBhm	2	TRUE	0.055591	27.60705
3133	rs3828090	A	G	A	G	0.0126	-0.01396	0.2682	0.277079	FALSE	FALSE	FALSE	ieu-b-508	1	1.2E+08	0.037719	486484	0.711366	Sepsis (28	Sepsis (28	Sepsis (28	TRUE	igd	0.0024	9.97E-08	7349	M32315.m	TRUE	reported	4igzkL	2	TRUE	0.003736	27.555
4160	rs3133577	T	C	T	C	-0.0252	-0.02042	0.6707	0.667203	FALSE	FALSE	FALSE	ieu-b-508	8	1.02E+08	0.035692	486484	0.567255	Sepsis (28	Sepsis (28	Sepsis (28	TRUE	igd	0.0048	1.98E-07	6642	M32758.m	TRUE	reported	AF0m66	2	TRUE	0.004133	27.5542
1327	rs6823453	A	G	A	G	0.0231	-0.03891	0.2233	0.229804	FALSE	FALSE	FALSE	ieu-b-508	4	85470969	0.039622	486484	0.326138	Sepsis (28	Sepsis (28	Sepsis (28	TRUE	igd	0.0044	1.78E-07	6252	M02137.m	TRUE	reported	iRGqBE	2	TRUE	0.004389	27.55368
3598	rs1224843	T	C	T	C	0.0467	0.052397	0.8404	0.847722	FALSE	FALSE	FALSE	ieu-b-508	10	96306509	0.046474	486484	0.259557	Sepsis (28	Sepsis (28	Sepsis (28	TRUE	igd	0.0089	1.53E-07	7256	M32549.m	TRUE	reported	W7Fgu2	2	TRUE	0.00378	27.52542
2153	rs1799405	A	T	A	T	0.032	-0.03252	0.9643	0.952511	FALSE	TRUE	FALSE	ieu-b-508	18	52944025	0.078447	486484	0.678456	Sepsis (28	Sepsis (28	Sepsis (28	TRUE	igd	0.0061	1.81E-07	7286	M18929.m	TRUE	reported	2vYFb2	2	TRUE	0.003763	27.51193
7106	rs1046301	A	G	A	G	-0.796	-0.04767	0.9883	0.98664	FALSE	FALSE	FALSE	ieu-b-508	5	1.71E+08	0.143959	486484	0.740553	Sepsis (28	Sepsis (28	Sepsis (28	TRUE	igd	0.1506	1.24E-07	128	M34516.m	TRUE	reported	7Ah2wy	2	TRUE	0.179154	27.50026
4974	rs4805151	A	G	A	G	-0.2498	0.0923	0.0347	0.054451	FALSE	FALSE	FALSE	ieu-b-508	19	28805168	0.073442	486484	0.208831	Sepsis (28	Sepsis (28	Sepsis (28	TRUE	igd	0.0476	1.50E-07	1017	M33178.m	TRUE	reported	g22WMS	2	TRUE	0.026366	27.48629
3120	rs1092389	T	C	T	C	-0.0388	-0.0504	0.9802	0.979721	FALSE	FALSE	FALSE	ieu-b-508	1	1.2E+08	0.120071	486484	0.674655	Sepsis (28	Sepsis (28	Sepsis (28	TRUE	igd	0.0074	1.82E-07	7349	M32315.m	TRUE	reported	4igzkL	2	TRUE	0.003727	27.48412
6967	rs9945857	A	G	A	G	0.1688	0.020552	0.0947	0.112796	FALSE	FALSE	FALSE	ieu-b-508	18	64412293	0.052752	486484	0.69684	Sepsis (28	Sepsis (28	Sepsis (28	TRUE	igd	0.0321	1.51E-07	314	M34404.m	TRUE	reported	8FMHfo	2	TRUE	0.080938	27.4764
7609	rs1051423	A	G	A	G	0.1745	-0.00926	0.04	0.041181	FALSE	FALSE	FALSE	ieu-b-508	18	74910478	0.083919	486484	0.912152	Sepsis (28	Sepsis (28	Sepsis (28	TRUE	igd	0.0332	1.49E-07	349	M35322.m	TRUE	reported	om3h9x	2	TRUE	0.073351	27.46748
5297	rs2320173	T	C	T	C	-0.0493	-0.00608	0.3067	0.332196	FALSE	FALSE	FALSE	ieu-b-508	5	71111127	0.035984	486484	0.865721	Sepsis (28	Sepsis (28	Sepsis (28	TRUE	igd	0.0094	1.35E-07	1273	M33353.m	TRUE	reported	ZIN4gk	2	TRUE	0.021151	27.46346
6873	rs2402511	A	G	A	G	0.2034	0.02836	0.3786	0.398857	FALSE	FALSE	FALSE	ieu-b-508	14	95285264	0.034168	486484	0.406529	Sepsis (28	Sepsis (28	Sepsis (28	TRUE	igd	0.0366	2.82E-08	18	M34365.m	TRUE	reported	8eexD0	2	TRUE	0.631785	27.45284
435	rs6438950	C	G	C	G	0.0131	-0.03558	0.9036	0.905934	FALSE	TRUE	FALSE	ieu-b-508	3	96806729	0.057548	486484	0.536352	Sepsis (28	Sepsis (28	Sepsis (28	TRUE	igd	0.0025	1.54E-07	7365	M00063.m	TRUE	reported	Hdsv1L	2	TRUE	0.003714	27.45014
5979	rs6047134	A	G	A	G	-0.0131	0.022613	0.2672	0.277795	FALSE	FALSE	FALSE	ieu-b-508	20	2141054	0.03748	486484	0.546295	Sepsis (28	Sepsis (28	Sepsis (28	TRUE	igd	0.0025	8.93E-08	6279	M33666.m	TRUE	reported	dvWEtk	2	TRUE	0.004354	27.44885
638	rs9359674	A	T	A	T	0.1142	0.057389	0.0565	0.058117	FALSE	TRUE	FALSE	ieu-b-508	6	86705909	0.071833	486484	0.424331	Sepsis (28	Sepsis (28	Sepsis (28	TRUE	igd	0.0218	1.57E-07	2639	M01123.m	TRUE	reported	NDTvfD	2	TRUE	0.010292	27.42142
8591	rs1018051	T	C	T	C	0.0089	-0.0146	0.4607	0.445759	FALSE	FALSE	FALSE	ieu-b-508	2	1.27E+08	0.033721	486484	0.665125	Sepsis (28	Sepsis (28	Sepsis (28	TRUE	igd	0.0017	3.75E-07	7366	M37506.m	TRUE	reported	m2kaRt	2	TRUE	0.003707	27.40086
703	rs936632	A	C	A	C	-0.0089	-0.06132	0.1787	0.177243	FALSE	FALSE	FALSE	ieu-b-508	1	53114103	0.043625	486484	0.159835	Sepsis (28	Sepsis (28	Sepsis (28	TRUE	igd	0.0017	2.69E-07	7358	M01299.m	TRUE	reported	gfM0a3	2	TRUE	0.003711	27.40085
2269	rs7544659	A	G	A	G	0.0089	-0.01915	0.4048	0.387307	FALSE	FALSE	FALSE	ieu-b-508	1	1.76E+08	0.034279	486484	0.576408	Sepsis (28	Sepsis (28	Sepsis (28	TRUE	igd	0.0017	3.13E-07	7354	M19934.m	TRUE	reported	HtcWcQ	2	TRUE	0.003713	27.40085
773	rs1113389	A	G	A	G	0.0089	-0.05769	0.4427	0.437868	FALSE	FALSE	FALSE	ieu-b-508	5	17255026	0.034075	486484	0.09043	Sepsis (28	Sepsis (28	Sepsis (28	TRUE	igd	0.0017	1.56E-07	7352	M01336.m	TRUE	reported	FAIrya	2	TRUE	0.003714	27.40085
1242	rs1724833	T	C	T	C	-0.0089	0.043709	0.5848	0.578463	FALSE	FALSE	FALSE	ieu-b-508	8	1.08E+08	0.033849	486484	0.196604	Sepsis (28	Sepsis (28	Sepsis (28	TRUE	igd	0.0017	1.70E-07	7136	M01769.m	TRUE	reported	rismUZ	2	TRUE	0.003826	27.40062
1373	rs1116545	T	C	T	C	0.0089	0.025587	0.5583	0.569018	FALSE	FALSE	FALSE	ieu-b-508	1	96104296	0.033764	486484	0.44855	Sepsis (28	Sepsis (28	Sepsis (28	TRUE	igd	0.0017	2.79E-07	7037	M02734.m	TRUE	reported	0y0dDD	2	TRUE	0.00388	27.40051
1387	rs1864151	A	C	A	C	0.0089	-0.05776	0.5575	0.558514	FALSE	FALSE	FALSE	ieu-b-508	16	87641993	0.033576	486484	0.085391	Sepsis (28	Sepsis (28	Sepsis (28	TRUE	igd	0.0017	2.40E-07	7037	M02734.m	TRUE	reported	0y0dDD	2	TRUE	0.00388	27.40051
7660	rs6912426	A	G	A	G	-0.0089	0.011296	0.3535	0.35276	FALSE	FALSE	FALSE	ieu-b-508	6	25409785	0.035176	486484	0.74812	Sepsis (28	Sepsis (28	Sepsis (28	TRUE	igd	0.0017	3.19E-07	5768	M35326.m	TRUE	reported	jRytyJ	2	TRUE	0.004729	27.3988
2167	rs1049107	T	C	T	C	0.122	0.014764	0.0175	0.021814	FALSE	FALSE	FALSE	ieu-b-508	10	65817857	0.115172	486484	0.897998	Sepsis (28	Sepsis (28	Sepsis (28	TRUE	igd	0.0233	1.66E-07	1738	M19324.m	TRUE	reported	1pj7ZT	2	TRUE	0.01553	27.38469
1612	rs2501242	T	G	T	G	-0.0136	-0.06365	0.223	0.230583	FALSE	FALSE	FALSE	ieu-b-508	13	70576428	0.039944	486484	0.111047	Sepsis (28	Sepsis (28	Sepsis (28	TRUE	igd	0.0026	1.84E-07	7352	M15122.m	TRUE	reported	VlnI8T	2	TRUE	0.003708	27.3535
4397	rs862226	T	G	T	G	0.0068	0.020619	0.2555	0.262859	FALSE	FALSE	FALSE	ieu-b-508	13	77468236	0.037821	486484	0.58564	Sepsis (28	Sepsis (28	Sepsis (28	TRUE	igd	0.0013	4.34E-07	7329	M32867.m	TRUE	reported	Jcsyzm	2	TRUE	0.003719	27.35348
7310	rs879092	A	T	A	T	0.0434	-0.07804	0.9154	0.90644	FALSE	TRUE	FALSE	ieu-b-508	1	4820966	0.057321	486484	0.173375	Sepsis (28	Sepsis (28	Sepsis (28	TRUE	igd	0.0083	1.56E-07	7364	M35126.m	TRUE	reported	1haOJA	2	TRUE	0.003699	27.33413
7077	rs1320667	T	C	T	C	-0.0737	-0.02778	0.0161	0.016682	FALSE	FALSE	FALSE	ieu-b-508	6	1.6E+08	0.132795	486484	0.83427	Sepsis (28	Sepsis (28	Sepsis (28	TRUE	igd	0.0141	1.67E-07	7119	M34481.m	TRUE	reported	mXdL0W	2	TRUE	0.003823	27.31333
5963	rs6502323	A	T	A	T	0.0434	-0.00664	0.2996	0.335103	FALSE	TRUE	FALSE	ieu-b-508	17	13900090	0.035405	486484	0.851171	Sepsis (28	Sepsis (28	Sepsis (28	TRUE	igd	0.0083	1.56E-07	1860	M33658.m	TRUE	reported	9QszGO	2	TRUE	0.014487	27.31216
3281	rs6972344	T	C	T	C	0.0094	-0.0064	0.3962	0.416189	FALSE	FALSE	FALSE	ieu-b-508	7	1.36E+08	0.033898	486484	0.850286	Sepsis (28	Sepsis (28	Sepsis (28	TRUE	igd	0.0018	1.11E-07	7365	M32348.m	TRUE	reported	DJeLDx	2	TRUE	0.003689	27.2642
3436	rs3829681	C	G	C	G	-0.0141	0.046702	0.8259	0.806366	FALSE	TRUE	FALSE	ieu-b-508	19	14951511	0.042254	486484	0.269045	Sepsis (28	Sepsis (28	Sepsis (28	TRUE	igd	0.0027	1.68E-07	7364	M32452.m	TRUE	reported	8GtFpl	2	TRUE	0.00369	27.2642
3445	rs8181126	T	C	T	C	0.0094	0.023919	0.4188	0.419959	FALSE	FALSE	FALSE	ieu-b-508	9	27918516	0.033837	486484	0.479641	Sepsis (28	Sepsis (28	Sepsis (28	TRUE	igd	0.0018	2.73E-07	7364	M32452.m	TRUE	reported	8GtFpl	2	TRUE	0.00369	27.2642
4512	rs666276	T	C	T	C	-0.0094	0.015392	0.1213	0.11658	FALSE	FALSE	FALSE	ieu-b-508	8	2832449	0.051909	486484	0.766839	Sepsis (28	Sepsis (28	Sepsis (28	TRUE	igd	0.0018	2.19E-07	7362	M33132.m	TRUE	reported	dTXJa8	2	TRUE	0.003691	27.2642
6448	rs6749150	A	G	A	G	0.0094	-0.06341	0.8699	0.831073	FALSE	FALSE	FALSE	ieu-b-508	2	2.4E+08	0.044462	486484	0.153801	Sepsis (28	Sepsis (28	Sepsis (28	TRUE	igd	0.0018	3.52E-07	7362	M34062.m	TRUE	reported	RZyHKP	2	TRUE	0.003691	27.2642
56	rs1663501	A	G	A	G	-0.0047	0.034535	0.4634	0.454681	FALSE	FALSE	FALSE	ieu-b-508	21	28219307	0.033498	486484	0.302562	Sepsis (28	Sepsis (28	Sepsis (28	TRUE	igd	9.00E-04	2.44E-07	7355	M00054.m	TRUE	reported	QTDAnh	2	TRUE	0.003694	27.26419
65	rs1995960	A	G	A	G	-0.0047	-0.02717	0.5949	0.601923	FALSE	FALSE	FALSE	ieu-b-508	4	1.43E+08	0.03413	486484	0.426062	Sepsis (28	Sepsis (28	Sepsis (28	TRUE	igd	9.00E-04	1.63E-07	7355	M00054.m	TRUE	reported	QTDAnh	2	TRUE	0.003694	27.26419
67	rs201262	T	G	T	G	0.0047	0.039703	0.5563	0.570191	FALSE	FALSE	FALSE	ieu-b-508	6	9987615	0.033753	486484	0.239483	Sepsis (28	Sepsis (28	Sepsis (28	TRUE	igd	9.00E-04	1.36E-07	7355	M00054.m	TRUE	reported	QTDAnh	2	TRUE	0.003694	27.26419
158	rs7109477	A	G	A	G	-0.0047	-0.01753	0.4588	0.449037	FALSE	FALSE	FALSE	ieu-b-508	11	21413780	0.033478	486484	0.600443	Sepsis (28	Sepsis (28	Sepsis (28	TRUE	igd	9.00E-04	1.89E-07	7355	M00054.m	TRUE	reported	QTDAnh	2	TRUE	0.003694	27.26419
166	rs7638383	A	G	A	G	0.0047	-0.01172	0.3984	0.401311	FALSE	FALSE	FALSE	ieu-b-508	3	4994341	0.033979	486484	0.730242	Sepsis (28	Sepsis (28	Sepsis (28	TRUE	igd	9.00E-04	1.64E-07	7355	M00054.m	TRUE	reported	QTDAnh	2	TRUE	0.003694	27.26419
174	rs860916	A	G	A	G	-0.0047	0.012457	0.4232	0.417302	FALSE	FALSE	FALSE	ieu-b-508	18	60665539	0.033838	486484	0.712764	Sepsis (28	Sepsis (28	Sepsis (28	TRUE	igd	9.00E-04	1.51E-07	7355	M00054.m	TRUE	reported	QTDAnh	2	TRUE	0.003694	27.26419
559	rs6988242	T	G	T	G	0.0094	0.013375	0.769	0.718969	FALSE	FALSE	FALSE	ieu-b-508	8	25792789	0.038067	486484	0.725317	Sepsis (28	Sepsis (28	Sepsis (28	TRUE	igd	0.0018	1.31E-07	7354	M00606.m	TRUE	reported	r5smf7	2	TRUE	0.003695	27.26419
1619	rs9374302	A	G	A	G	-0.0141	-0.00662	0.1744	0.175583	FALSE	FALSE	FALSE	ieu-b-508	6	1.12E+08	0.043898	486484	0.880158	Sepsis (28	Sepsis (28	Sepsis (28	TRUE	igd	0.0027	1.72E-07	7352	M15122.m	TRUE	reported	VlnI8T	2	TRUE	0.003696	27.26419
246	rs1314757	A	G	A	G	-0.0047	-0.00354	0.5809	0.58404	FALSE	FALSE	FALSE	ieu-b-508	4	18293144	0.033915	486484	0.91684	Sepsis (28	Sepsis (28	Sepsis (28	TRUE	igd	9.00E-04	1.99E-07	7351	M00060.m	TRUE	reported	kqwl4V	2	TRUE	0.003696	27.26419
268	rs1862710	A	G	A	G	0.0047	-0.02347	0.5489	0.554919	FALSE	FALSE	FALSE	ieu-b-508	1	15844057	0.033525	486484	0.483796	Sepsis (28	Sepsis (28	Sepsis (28	TRUE	igd	9.00E-04	1.17E-07	7351	M00060.m	TRUE	reported	kqwl4V	2	TRUE	0.003696	27.26419
284	rs2454043	A	C	A	C	-0.0047	0.017159	0.588	0.586279	FALSE	FALSE	FALSE	ieu-b-508	8	1.04E+08	0.034144	486484	0.61527	Sepsis (28	Sepsis (28	Sepsis (28	TRUE	igd	9.00E-04	1.85E-07	7351	M00060.m	TRUE	reported	kqwl4V	2	TRUE	0.003696	27.26419
315	rs4741887	A	G	A	G	-0.0047	-0.00146	0.4584	0.46471	FALSE	FALSE	FALSE	ieu-b-508	9	4079470	0.033463	486484	0.965252	Sepsis (28	Sepsis (28	Sepsis (28	TRUE	igd	9.00E-04	1.68E-07	7351	M00060.m	TRUE	reported	kqwl4V	2	TRUE	0.003696	27.26419
317	rs4767788	A	G	A	G	0.0047	0.051287	0.4189	0.419645	FALSE	FALSE	FALSE	ieu-b-508	12	1.2E+08	0.033745	486484	0.128554	Sepsis (28	Sepsis (28	Sepsis (28	TRUE	igd	9.00E-04	5.74E-07	7351	M00060.m	TRUE	reported	kqwl4V	2	TRUE	0.003696	27.26419
318	rs477992	A	G	A	G	-0.0047	0.074745	0.3228	0.320749	FALSE	FALSE	FALSE	ieu-b-508	1	1.2E+08	0.035684	486484	0.036206	Sepsis (28	Sepsis (28	Sepsis (28	TRUE	igd	9.00E-04	2.00E-07	7351	M00060.m	TRUE	reported	kqwl4V	2	TRUE	0.003696	27.26419
319	rs4795552	A	G	A	G	-0.0047	0.066261	0.3894	0.389303	FALSE	FALSE	FALSE	ieu-b-508	17	25613381	0.034352	486484	0.053743	Sepsis (28	Sepsis (28	Sepsis (28	TRUE	igd	9.00E-04	1.55E-07	7351	M00060.m	TRUE	reported	kqwl4V	2	TRUE	0.003696	27.26419
370	rs7825809	T	C	T	C	-0.0047	0.015007	0.4319	0.422111	FALSE	FALSE	FALSE	ieu-b-508	8	93190822	0.033789	486484	0.65695	Sepsis (28	Sepsis (28	Sepsis (28	TRUE	igd	9.00E-04	1.55E-07	7351	M00060.m	TRUE	reported	kqwl4V	2	TRUE	0.003696	27.26419
386	rs911981	A	G	A	G	-0.0047	0.016266	0.5283	0.533397	FALSE	FALSE	FALSE	ieu-b-508	6	44508113	0.033541	486484	0.627716	Sepsis (28	Sepsis (28	Sepsis (28	TRUE	igd	9.00E-04	2.40E-07	7351	M00060.m	TRUE	reported	kqwl4V	2	TRUE	0.003696	27.26419
3900	rs3800014	A	C	A	C	-0.0094	0.006959	0.1334	0.138381	FALSE	FALSE	FALSE	ieu-b-508	6	57214742	0.048239	486484	0.885302	Sepsis (28	Sepsis (28	Sepsis (28	TRUE	igd	0.0018	2.68E-07	7338	M32675.m	TRUE	reported	9FmPh9	2	TRUE	0.003703	27.26417
5616	rs1730463	A	T	A	T	-0.0094	0.056939	0.8349	0.826106	FALSE	TRUE	FALSE	ieu-b-508	19	35948797	0.044212	486484	0.197798	Sepsis (28	Sepsis (28	Sepsis (28	TRUE	igd	0.0018	8.64E-08	7336	M33442.m	TRUE	reported	B1Peb3	2	TRUE	0.003704	27.26417
2609	rs1769965	T	C	T	C	0.0141	0.031503	0.2136	0.219274	FALSE	FALSE	FALSE	ieu-b-508	3	71256309	0.040588	486484	0.437644	Sepsis (28	Sepsis (28	Sepsis (28	TRUE	igd	0.0027	2.28E-07	7329	M22481.m	TRUE	reported	bXpf0k	2	TRUE	0.003707	27.26416
5414	rs4700085	T	C	T	C	0.0094	-0.04838	0.3754	0.354544	FALSE	FALSE	FALSE	ieu-b-508	5	64837105	0.034892	486484	0.165592	Sepsis (28	Sepsis (28	Sepsis (28	TRUE	igd	0.0018	2.98E-07	7315	M33389.m	TRUE	reported	Hf6XVM	2	TRUE	0.003714	27.26415
1855	rs4852620	A	G	A	G	0.0141	-0.01093	0.1096	0.118917	FALSE	FALSE	FALSE	ieu-b-508	2	70722946	0.051587	486484	0.832138	Sepsis (28	Sepsis (28	Sepsis (28	TRUE	igd	0.0027	1.73E-07	7308	M15778.m	TRUE	reported	941o3I	2	TRUE	0.003718	27.26414
5674	rs1302272	A	G	A	G	0.0094	-0.02489	0.3471	0.360071	FALSE	FALSE	FALSE	ieu-b-508	2	19770970	0.0348	486484	0.474453	Sepsis (28	Sepsis (28	Sepsis (28	TRUE	igd	0.0018	2.46E-07	7307	M33477.m	TRUE	reported	Aw8PBK	2	TRUE	0.003718	27.26414
1509	rs1122530	A	G	A	G	-0.0094	-0.01967	0.8519	0.842435	FALSE	FALSE	FALSE	ieu-b-508	9	87464352	0.045985	486484	0.668858	Sepsis (28	Sepsis (28	Sepsis (28	TRUE	igd	0.0018	1.49E-07	7081	M12067.m	TRUE	reported	O5x7RD	2	TRUE	0.003837	27.2639
5255	rs823554	A	T	A	T	-0.0188	0.027322	0.8205	0.801881	FALSE	TRUE	FALSE	ieu-b-508	2	2468365	0.04226	486484	0.517945	Sepsis (28	Sepsis (28	Sepsis (28	TRUE	igd	0.0036	2.45E-07	7063	M33228.m	TRUE	reported	JwOXbQ	2	TRUE	0.003846	27.26388
6818	rs2861609	A	G	A	G	-0.0094	-0.00947	0.3031	0.298569	FALSE	FALSE	FALSE	ieu-b-508	2	78633880	0.036521	486484	0.795454	Sepsis (28	Sepsis (28	Sepsis (28	TRUE	igd	0.0018	1.63E-07	6755	M34359.m	TRUE	reported	0UotaJ	2	TRUE	0.004021	27.26353
6819	rs2881130	A	G	A	G	-0.0094	0.090824	0.3385	0.339239	FALSE	FALSE	FALSE	ieu-b-508	11	6728220	0.035253	486484	0.009986	Sepsis (28	Sepsis (28	Sepsis (28	TRUE	igd	0.0018	1.25E-07	6755	M34359.m	TRUE	reported	0UotaJ	2	TRUE	0.004021	27.26353
953	rs1113643	T	C	T	C	0.0094	0.010112	0.2769	0.278633	FALSE	FALSE	FALSE	ieu-b-508	2	29539536	0.037346	486484	0.786562	Sepsis (28	Sepsis (28	Sepsis (28	TRUE	igd	0.0018	2.24E-07	6523	M01558.m	TRUE	reported	AZK0p4	2	TRUE	0.004163	27.26324
956	rs1280717	A	G	A	G	0.0094	-0.00706	0.2512	0.241103	FALSE	FALSE	FALSE	ieu-b-508	6	54497728	0.038871	486484	0.855839	Sepsis (28	Sepsis (28	Sepsis (28	TRUE	igd	0.0018	2.01E-07	6523	M01558.m	TRUE	reported	AZK0p4	2	TRUE	0.004163	27.26324
8333	rs1004581	A	G	A	G	0.0235	-0.06537	0.2081	0.216104	FALSE	FALSE	FALSE	ieu-b-508	5	1.65E+08	0.040637	486484	0.107721	Sepsis (28	Sepsis (28	Sepsis (28	TRUE	igd	0.0045	2.27E-07	5292	M36673.m	TRUE	reported	CD5lvR	2	TRUE	0.005127	27.2613
984	rs1024570	T	C	T	C	-0.0663	-0.08916	0.0181	0.019778	FALSE	FALSE	FALSE	ieu-b-508	7	26129672	0.122474	486484	0.466622	Sepsis (28	Sepsis (28	Sepsis (28	TRUE	igd	0.0127	1.95E-07	7276	M01561.m	TRUE	reported	Zd9SUU	2	TRUE	0.003732	27.24584
4236	rs3740393	C	G	C	G	-0.0261	0.104514	0.1428	0.13742	FALSE	TRUE	FALSE	ieu-b-508	10	1.05E+08	0.048657	486484	0.031715	Sepsis (28	Sepsis (28	Sepsis (28	TRUE	igd	0.005	1.59E-07	6857	M32787.m	TRUE	reported	fSRakm	2	TRUE	0.003958	27.24045
2857	rs7276515	T	C	T	C	-0.0454	-0.01961	0.8161	0.807473	FALSE	FALSE	FALSE	ieu-b-508	21	38703319	0.042403	486484	0.643668	Sepsis (28	Sepsis (28	Sepsis (28	TRUE	igd	0.0087	2.00E-07	5673	M27531.m	TRUE	reported	v01zDr	2	TRUE	0.004777	27.222
1352	rs1273784	T	C	T	C	0.012	0.015952	0.7976	0.796649	FALSE	FALSE	FALSE	ieu-b-508	1	1.67E+08	0.041475	486484	0.700518	Sepsis (28	Sepsis (28	Sepsis (28	TRUE	igd	0.0023	1.96E-07	7226	M02730.m	TRUE	reported	XYa035	2	TRUE	0.003753	27.21364
5999	rs8085059	A	G	A	G	-0.1427	0.006101	0.4278	0.433575	FALSE	FALSE	FALSE	ieu-b-508	18	13492909	0.034011	486484	0.857637	Sepsis (28	Sepsis (28	Sepsis (28	TRUE	igd	0.0273	1.69E-07	477	M33683.m	TRUE	reported	wSozpU	2	TRUE	0.054177	27.20808
3886	rs9382940	T	C	T	C	0.0172	0.04722	0.1616	0.173184	FALSE	FALSE	FALSE	ieu-b-508	6	14133573	0.044339	486484	0.286886	Sepsis (28	Sepsis (28	Sepsis (28	TRUE	igd	0.0033	2.31E-07	7361	M32654.m	TRUE	reported	6lSQOP	2	TRUE	0.003677	27.15883
2671	rs1123692	C	G	C	G	-0.0172	-0.07603	0.1592	0.158164	FALSE	TRUE	FALSE	ieu-b-508	11	70468387	0.045717	486484	0.096299	Sepsis (28	Sepsis (28	Sepsis (28	TRUE	igd	0.0033	1.50E-07	7256	M24074.m	TRUE	reported	Lk6D2A	2	TRUE	0.00373	27.15872
5434	rs9822180	T	C	T	C	0.0099	0.03218	0.7155	0.718795	FALSE	FALSE	FALSE	ieu-b-508	3	1.05E+08	0.037113	486484	0.385898	Sepsis (28	Sepsis (28	Sepsis (28	TRUE	igd	0.0019	2.58E-07	7315	M33389.m	TRUE	reported	Hf6XVM	2	TRUE	0.003698	27.14216
3245	rs9297619	A	G	A	G	0.1272	-0.04187	0.0979	0.089761	FALSE	FALSE	FALSE	ieu-b-508	8	1.21E+08	0.058362	486484	0.47314	Sepsis (28	Sepsis (28	Sepsis (28	TRUE	igd	0.0244	1.93E-07	1565	M32346.m	TRUE	reported	cnIFoV	2	TRUE	0.017069	27.14184
7994	rs1214542	A	G	A	G	0.0099	0.007042	0.3757	0.368595	FALSE	FALSE	FALSE	ieu-b-508	1	73769279	0.034591	486484	0.838679	Sepsis (28	Sepsis (28	Sepsis (28	TRUE	igd	0.0019	2.22E-07	6604	M35754.m	TRUE	reported	IlXOo1	2	TRUE	0.004094	27.14136
3992	rs2170489	A	G	A	G	-0.0099	-0.02906	0.4041	0.405736	FALSE	FALSE	FALSE	ieu-b-508	11	98303416	0.033991	486484	0.392649	Sepsis (28	Sepsis (28	Sepsis (28	TRUE	igd	0.0019	1.57E-07	6497	M32729.m	TRUE	reported	XGRSN1	2	TRUE	0.004161	27.14123
4009	rs7819333	C	G	C	G	-0.0099	0.007117	0.3311	0.327824	FALSE	TRUE	FALSE	ieu-b-508	8	32397721	0.035674	486484	0.841874	Sepsis (28	Sepsis (28	Sepsis (28	TRUE	igd	0.0019	1.45E-07	6497	M32729.m	TRUE	reported	XGRSN1	2	TRUE	0.004161	27.14123
7367	rs550328	A	G	A	G	0.0099	-0.0309	0.2687	0.27544	FALSE	FALSE	FALSE	ieu-b-508	11	95965054	0.037457	486484	0.409347	Sepsis (28	Sepsis (28	Sepsis (28	TRUE	igd	0.0019	2.54E-07	4900	M35137.m	TRUE	reported	Eq2siS	2	TRUE	0.00551	27.1385
7517	rs1046315	T	C	T	C	-0.0224	-0.01785	0.2297	0.268815	FALSE	FALSE	FALSE	ieu-b-508	5	1.65E+08	0.037639	486484	0.635237	Sepsis (28	Sepsis (28	Sepsis (28	TRUE	igd	0.0043	2.39E-07	7291	M35255.m	TRUE	reported	ZDjmUf	2	TRUE	0.003708	27.12939
3448	rs9450092	A	G	A	G	0.037	0.022931	0.886	0.899433	FALSE	FALSE	FALSE	ieu-b-508	6	85644418	0.055162	486484	0.677631	Sepsis (28	Sepsis (28	Sepsis (28	TRUE	igd	0.0071	2.11E-07	1766	M32452.m	TRUE	reported	8GtFpl	2	TRUE	0.015145	27.12655
1040	rs1106141	A	G	A	G	-0.0125	0.060464	0.6428	0.648699	FALSE	FALSE	FALSE	ieu-b-508	12	1.32E+08	0.034755	486484	0.081911	Sepsis (28	Sepsis (28	Sepsis (28	TRUE	igd	0.0024	1.76E-07	7333	M01572.m	TRUE	reported	EOUAWy	2	TRUE	0.003686	27.11934
5900	rs7870108	T	G	T	G	0.6206	-0.05313	0.0102	0.015185	FALSE	FALSE	FALSE	ieu-b-508	9	87813359	0.148905	486484	0.721239	Sepsis (28	Sepsis (28	Sepsis (28	TRUE	igd	0.118	1.46E-07	102	M33610.m	TRUE	reported	tmg43v	2	TRUE	0.21333	27.11811
4125	rs4866873	T	C	T	C	0.0521	-0.08435	0.8725	0.863898	FALSE	FALSE	FALSE	ieu-b-508	5	44161075	0.048928	486484	0.084699	Sepsis (28	Sepsis (28	Sepsis (28	TRUE	igd	0.01	1.71E-07	1435	M32755.m	TRUE	reported	WDrNdi	2	TRUE	0.018565	27.10627
8228	rs967942	T	C	T	C	0.0255	0.004953	0.2883	0.302116	FALSE	FALSE	FALSE	ieu-b-508	4	20663866	0.036386	486484	0.891725	Sepsis (28	Sepsis (28	Sepsis (28	TRUE	igd	0.0049	1.64E-07	6486	M36399.m	TRUE	reported	EdUpnt	2	TRUE	0.004158	27.07411
4210	rs4633	T	C	T	C	0.0307	0.060648	0.5168	0.515271	FALSE	FALSE	FALSE	ieu-b-508	22	19950235	0.033499	486484	0.070231	Sepsis (28	Sepsis (28	Sepsis (28	TRUE	igd	0.0059	1.84E-07	6150	M32769.m	TRUE	reported	W2ubRP	2	TRUE	0.004383	27.06646
2530	rs4968748	A	G	A	G	-0.0307	-0.00944	0.232	0.223073	FALSE	FALSE	FALSE	ieu-b-508	17	61069500	0.040101	486484	0.81392	Sepsis (28	Sepsis (28	Sepsis (28	TRUE	igd	0.0059	1.83E-07	3838	M22175.m	TRUE	reported	IIlqyJ	2	TRUE	0.007005	27.06116
2013	rs1686540	A	T	A	T	-0.0385	0.023144	0.7722	0.787862	FALSE	TRUE	FALSE	ieu-b-508	3	1.9E+08	0.040998	486484	0.57241	Sepsis (28	Sepsis (28	Sepsis (28	TRUE	igd	0.0074	1.78E-07	5984	M18283.m	TRUE	reported	oyZdTE	2	TRUE	0.004503	27.05907
2020	rs2726698	C	G	C	G	-0.0791	0.075486	0.6936	0.847632	FALSE	TRUE	FALSE	ieu-b-508	8	50050384	0.046534	486484	0.10477	Sepsis (28	Sepsis (28	Sepsis (28	TRUE	igd	0.0152	2.12E-07	1452	M18335.m	TRUE	reported	IdUJrL	2	TRUE	0.018309	27.04377
584	rs1024196	A	G	A	G	0.0182	-0.07398	0.0952	0.099684	FALSE	FALSE	FALSE	ieu-b-508	7	19042114	0.05574	486484	0.184434	Sepsis (28	Sepsis (28	Sepsis (28	TRUE	igd	0.0035	2.25E-07	7367	M01110.m	TRUE	reported	0A31SI	2	TRUE	0.003657	27.03266
6333	rs9318232	T	C	T	C	-0.0104	0.04192	0.2312	0.237039	FALSE	FALSE	FALSE	ieu-b-508	13	74530279	0.039303	486484	0.28616	Sepsis (28	Sepsis (28	Sepsis (28	TRUE	igd	0.002	1.05E-07	7354	M33955.m	TRUE	reported	mhHFnT	2	TRUE	0.003663	27.03265
8246	rs169291	C	G	C	G	0.0052	-0.0706	0.7729	0.749342	FALSE	TRUE	FALSE	ieu-b-508	6	1.41E+08	0.038461	486484	0.066431	Sepsis (28	Sepsis (28	Sepsis (28	TRUE	igd	0.001	3.10E-07	7330	M36515.m	TRUE	reported	yZaYzr	2	TRUE	0.003675	27.03262
2487	rs1074150	A	T	A	T	-0.026	-0.0705	0.041	0.043222	FALSE	TRUE	FALSE	ieu-b-508	11	88436209	0.082036	486484	0.390134	Sepsis (28	Sepsis (28	Sepsis (28	TRUE	igd	0.005	2.57E-07	7329	M22116.m	TRUE	reported	bjUU6r	2	TRUE	0.003676	27.03262
2643	rs4073054	A	C	A	C	0.013	-0.03992	0.6227	0.627017	FALSE	FALSE	FALSE	ieu-b-508	1	1.61E+08	0.034425	486484	0.246205	Sepsis (28	Sepsis (28	Sepsis (28	TRUE	igd	0.0025	2.74E-07	7184	M22548.m	TRUE	reported	mZtwX2	2	TRUE	0.00375	27.03247
853	rs1103689	T	C	T	C	-0.013	-0.03027	0.3592	0.366326	FALSE	FALSE	FALSE	ieu-b-508	11	42466106	0.03467	486484	0.382582	Sepsis (28	Sepsis (28	Sepsis (28	TRUE	igd	0.0025	3.01E-07	7063	M01361.m	TRUE	reported	NIAcQ3	2	TRUE	0.003814	27.03234
6767	rs862029	A	G	A	G	0.2654	-0.04498	0.0214	0.011835	FALSE	FALSE	FALSE	ieu-b-508	14	74994201	0.161648	486484	0.780804	Sepsis (28	Sepsis (28	Sepsis (28	TRUE	igd	0.051	1.92E-07	633	M34339.m	TRUE	reported	RwGZ7c	2	TRUE	0.041026	26.99524
4554	rs7589832	A	C	A	C	-0.0213	0.014308	0.8207	0.845898	FALSE	FALSE	FALSE	ieu-b-508	2	1.37E+08	0.04679	486484	0.759765	Sepsis (28	Sepsis (28	Sepsis (28	TRUE	igd	0.0041	2.08E-07	7319	M33140.m	TRUE	reported	eBOUtb	2	TRUE	0.003674	26.98192
4286	rs2433679	A	G	A	G	0.0213	-0.02648	0.8868	0.901555	FALSE	FALSE	FALSE	ieu-b-508	2	1.7E+08	0.056181	486484	0.637385	Sepsis (28	Sepsis (28	Sepsis (28	TRUE	igd	0.0041	1.65E-07	7063	M32814.m	TRUE	reported	z0OeTx	2	TRUE	0.003807	26.98165
7066	rs1258042	A	G	A	G	0.0431	0.056798	0.9345	0.932383	FALSE	FALSE	FALSE	ieu-b-508	12	1.09E+08	0.066471	486484	0.392845	Sepsis (28	Sepsis (28	Sepsis (28	TRUE	igd	0.0083	2.20E-07	6144	M34469.m	TRUE	reported	WMOqbm	2	TRUE	0.00437	26.95609
5518	rs1693723	A	G	A	G	0.0405	0.261014	0.9882	0.979464	FALSE	FALSE	FALSE	ieu-b-508	8	71884673	0.118405	486484	0.027494	Sepsis (28	Sepsis (28	Sepsis (28	TRUE	igd	0.0078	1.80E-07	7306	M33422.m	TRUE	reported	PLgjrT	2	TRUE	0.003677	26.95268
2203	rs2846556	T	G	T	G	-0.0135	0.054277	0.3337	0.330295	FALSE	FALSE	FALSE	ieu-b-508	6	1.63E+08	0.035684	486484	0.128247	Sepsis (28	Sepsis (28	Sepsis (28	TRUE	igd	0.0026	2.03E-07	6929	M19364.m	TRUE	reported	XkjMEx	2	TRUE	0.003876	26.95228
4581	rs1336659	A	G	A	G	0.0135	0.01112	0.5079	0.521129	FALSE	FALSE	FALSE	ieu-b-508	13	1.03E+08	0.033728	486484	0.74163	Sepsis (28	Sepsis (28	Sepsis (28	TRUE	igd	0.0026	3.01E-07	6404	M33163.m	TRUE	reported	YzGXkR	2	TRUE	0.004192	26.95164
8085	rs7697282	T	C	T	C	0.0997	0.139897	0.9785	0.979081	FALSE	FALSE	FALSE	ieu-b-508	4	1.11E+08	0.118085	486484	0.236129	Sepsis (28	Sepsis (28	Sepsis (28	TRUE	igd	0.0192	1.95E-07	2651	M36009.m	TRUE	reported	2EUZ1U	2	TRUE	0.010069	26.94388
6629	rs1048576	T	C	T	C	-0.2299	0.093736	0.9733	0.956035	FALSE	FALSE	FALSE	ieu-b-508	20	12649066	0.081245	486484	0.248606	Sepsis (28	Sepsis (28	Sepsis (28	TRUE	igd	0.0437	1.48E-07	75	M34322.m	TRUE	reported	16GdG9	2	TRUE	0.269552	26.9387
481	rs1683077	C	G	C	G	-0.5244	-0.17764	0.0197	0.024296	FALSE	TRUE	FALSE	ieu-b-508	2	1.35E+08	0.109269	486484	0.104003	Sepsis (28	Sepsis (28	Sepsis (28	TRUE	igd	0.1007	1.92E-07	283	M00553.m	TRUE	reported	IFjXiL	2	TRUE	0.087446	26.9269
3308	rs7499321	T	C	T	C	0.0192	-0.06747	0.3347	0.355779	FALSE	FALSE	FALSE	ieu-b-508	16	10270314	0.034885	486484	0.053113	Sepsis (28	Sepsis (28	Sepsis (28	TRUE	igd	0.0037	2.14E-07	6066	M32388.m	TRUE	reported	yYczcA	2	TRUE	0.004419	26.91881
5472	rs2252553	T	G	T	G	-0.0358	-0.00282	0.4533	0.465713	FALSE	FALSE	FALSE	ieu-b-508	8	1.34E+08	0.033446	486484	0.9327	Sepsis (28	Sepsis (28	Sepsis (28	TRUE	igd	0.0069	1.82E-07	3890	M33408.m	TRUE	reported	tWZWla	2	TRUE	0.006873	26.90571
1699	rs7288834	T	G	T	G	-0.0083	0.050923	0.3986	0.461114	FALSE	FALSE	FALSE	ieu-b-508	22	17832142	0.033511	486484	0.128623	Sepsis (28	Sepsis (28	Sepsis (28	TRUE	igd	0.0016	1.51E-07	7345	M15488.m	TRUE	reported	SPriBO	2	TRUE	0.00365	26.90283
1725	rs8033100	T	C	T	C	-0.0083	-0.00742	0.8565	0.855227	FALSE	FALSE	FALSE	ieu-b-508	15	71977446	0.04742	486484	0.875671	Sepsis (28	Sepsis (28	Sepsis (28	TRUE	igd	0.0016	4.94E-07	7310	M15506.m	TRUE	reported	mVRmZO	2	TRUE	0.003668	26.90279
3682	rs1173658	T	C	T	C	-0.0332	0.01952	0.7281	0.725707	FALSE	FALSE	FALSE	ieu-b-508	4	99820360	0.037314	486484	0.600879	Sepsis (28	Sepsis (28	Sepsis (28	TRUE	igd	0.0064	2.19E-07	7303	M32586.m	TRUE	reported	f6n0gX	2	TRUE	0.003671	26.90279
1106	rs2246012	T	C	T	C	-0.0166	-0.00074	0.8388	0.833936	FALSE	FALSE	FALSE	ieu-b-508	6	1.32E+08	0.044887	486484	0.986926	Sepsis (28	Sepsis (28	Sepsis (28	TRUE	igd	0.0032	2.23E-07	7104	M01638.m	TRUE	reported	8dp0Ac	2	TRUE	0.003774	26.90258
3608	rs1319847	A	G	A	G	0.042	0.051893	0.0945	0.094248	FALSE	FALSE	FALSE	ieu-b-508	6	25874423	0.057251	486484	0.364712	Sepsis (28	Sepsis (28	Sepsis (28	TRUE	igd	0.0081	2.24E-07	7361	M32553.m	TRUE	reported	vHSHqn	2	TRUE	0.003639	26.87884
5803	rs1102290	A	G	A	G	-0.014	-0.0213	0.154	0.14822	FALSE	FALSE	FALSE	ieu-b-508	11	13632483	0.046969	486484	0.650187	Sepsis (28	Sepsis (28	Sepsis (28	TRUE	igd	0.0027	1.86E-07	7066	M33515.m	TRUE	reported	X9mul1	2	TRUE	0.003791	26.87854
5251	rs6784447	A	G	A	G	0.014	0.014676	0.3405	0.341233	FALSE	FALSE	FALSE	ieu-b-508	3	1.06E+08	0.035311	486484	0.67769	Sepsis (28	Sepsis (28	Sepsis (28	TRUE	igd	0.0027	2.91E-07	7063	M33228.m	TRUE	reported	JwOXbQ	2	TRUE	0.003792	26.87853
1757	rs1008258	A	T	A	T	-0.0259	-0.0698	0.0409	0.043192	FALSE	TRUE	FALSE	ieu-b-508	11	88433862	0.082092	486484	0.395196	Sepsis (28	Sepsis (28	Sepsis (28	TRUE	igd	0.005	1.88E-07	7331	M15676.m	TRUE	reported	s1NfKN	2	TRUE	0.003647	26.82508
5580	rs4663518	C	G	C	G	-1.0035	-0.08113	0.0508	0.089085	FALSE	TRUE	FALSE	ieu-b-508	2	2.36E+08	0.058667	486484	0.166703	Sepsis (28	Sepsis (28	Sepsis (28	TRUE	igd	0.1905	1.39E-07	60	M33423.m	TRUE	reported	MoEnO7	2	TRUE	0.31623	26.82388
4608	rs1113719	A	G	A	G	-0.7694	0.087895	0.0263	0.021429	FALSE	FALSE	FALSE	ieu-b-508	9	1.41E+08	0.115335	486484	0.446011	Sepsis (28	Sepsis (28	Sepsis (28	TRUE	igd	0.1466	1.53E-07	76	M33173.m	TRUE	reported	04fkUQ	2	TRUE	0.266017	26.81974
5397	rs1868852	A	C	A	C	0.0145	-0.06148	0.8553	0.811083	FALSE	FALSE	FALSE	ieu-b-508	2	2692598	0.04274	486484	0.150299	Sepsis (28	Sepsis (28	Sepsis (28	TRUE	igd	0.0028	1.57E-07	7315	M33389.m	TRUE	reported	Hf6XVM	2	TRUE	0.003653	26.81027
2955	rs1311508	A	G	A	G	0.0145	0.016533	0.792	0.779277	FALSE	FALSE	FALSE	ieu-b-508	4	4546923	0.040401	486484	0.682375	Sepsis (28	Sepsis (28	Sepsis (28	TRUE	igd	0.0028	3.10E-07	6795	M30805.m	TRUE	reported	YHV70c	2	TRUE	0.003931	26.80971
1809	rs9355342	A	G	A	G	0.1456	-0.1545	0.9692	0.963615	FALSE	FALSE	FALSE	ieu-b-508	6	1.62E+08	0.093491	486484	0.098424	Sepsis (28	Sepsis (28	Sepsis (28	TRUE	igd	0.0281	2.19E-07	1259	M15749.m	TRUE	reported	TR861N	2	TRUE	0.02088	26.80524
5499	rs1060467	A	G	A	G	0.0233	-0.01066	0.5903	0.578805	FALSE	FALSE	FALSE	ieu-b-508	19	16024538	0.033836	486484	0.752702	Sepsis (28	Sepsis (28	Sepsis (28	TRUE	igd	0.0045	2.61E-07	5822	M33420.m	TRUE	reported	eTToDb	2	TRUE	0.004584	26.80017
5717	rs328833	A	G	A	G	0.0233	-0.00171	0.6759	0.678505	FALSE	FALSE	FALSE	ieu-b-508	5	1.34E+08	0.03581	486484	0.961964	Sepsis (28	Sepsis (28	Sepsis (28	TRUE	igd	0.0045	2.72E-07	5107	M33488.m	TRUE	reported	mZmDY6	2	TRUE	0.005222	26.79888
3283	rs7310323	A	C	A	C	0.0088	0.009246	0.5455	0.501812	FALSE	FALSE	FALSE	ieu-b-508	12	60498560	0.033359	486484	0.781649	Sepsis (28	Sepsis (28	Sepsis (28	TRUE	igd	0.0017	4.07E-07	7365	M32348.m	TRUE	reported	DJeLDx	2	TRUE	0.003625	26.78857
5619	rs3853497	T	C	T	C	-0.0088	-0.10471	0.2686	0.251972	FALSE	FALSE	FALSE	ieu-b-508	1	1.09E+08	0.03859	486484	0.00666	Sepsis (28	Sepsis (28	Sepsis (28	TRUE	igd	0.0017	2.91E-07	7336	M33442.m	TRUE	reported	B1Peb3	2	TRUE	0.003639	26.78854
1708	rs1203911	T	C	T	C	-0.0088	0.107351	0.1289	0.132598	FALSE	FALSE	FALSE	ieu-b-508	1	62995263	0.04909	486484	0.028755	Sepsis (28	Sepsis (28	Sepsis (28	TRUE	igd	0.0017	1.93E-07	7310	M15506.m	TRUE	reported	mVRmZO	2	TRUE	0.003652	26.78852
1529	rs6967282	A	G	A	G	0.0088	0.03497	0.797	0.779882	FALSE	FALSE	FALSE	ieu-b-508	7	1.51E+08	0.040401	486484	0.386728	Sepsis (28	Sepsis (28	Sepsis (28	TRUE	igd	0.0017	3.96E-07	7081	M12067.m	TRUE	reported	O5x7RD	2	TRUE	0.00377	26.78828
1530	rs7069652	A	C	A	C	-0.0088	-0.02471	0.1869	0.190612	FALSE	FALSE	FALSE	ieu-b-508	10	1.22E+08	0.042582	486484	0.561694	Sepsis (28	Sepsis (28	Sepsis (28	TRUE	igd	0.0017	4.03E-07	7081	M12067.m	TRUE	reported	O5x7RD	2	TRUE	0.00377	26.78828
1370	rs1049827	A	G	A	G	0.0088	0.015797	0.6222	0.619128	FALSE	FALSE	FALSE	ieu-b-508	14	21106527	0.034378	486484	0.645876	Sepsis (28	Sepsis (28	Sepsis (28	TRUE	igd	0.0017	4.73E-07	7037	M02734.m	TRUE	reported	0y0dDD	2	TRUE	0.003793	26.78823
1399	rs4572660	T	G	T	G	-0.0088	-0.01458	0.6118	0.613978	FALSE	FALSE	FALSE	ieu-b-508	20	12928988	0.034365	486484	0.671347	Sepsis (28	Sepsis (28	Sepsis (28	TRUE	igd	0.0017	4.69E-07	7037	M02734.m	TRUE	reported	0y0dDD	2	TRUE	0.003793	26.78823
6117	rs1322565	A	T	A	T	-0.1616	0.005512	0.8762	0.855042	FALSE	TRUE	FALSE	ieu-b-508	7	20793432	0.047919	486484	0.908421	Sepsis (28	Sepsis (28	Sepsis (28	TRUE	igd	0.0311	2.08E-07	228	M33877.m	TRUE	reported	yDVi1f	2	TRUE	0.105882	26.76305
4917	rs2891717	A	G	A	G	0.3226	-0.13707	0.981	0.971516	FALSE	FALSE	FALSE	ieu-b-508	8	1.24E+08	0.100964	486484	0.174576	Sepsis (28	Sepsis (28	Sepsis (28	TRUE	igd	0.0623	2.26E-07	1017	M33178.m	TRUE	reported	g22WMS	2	TRUE	0.025688	26.76072
5550	rs1177309	C	G	C	G	0.9721	0.15542	0.9667	0.984336	FALSE	TRUE	FALSE	ieu-b-508	7	14100880	0.133503	486484	0.244353	Sepsis (28	Sepsis (28	Sepsis (28	TRUE	igd	0.1848	1.45E-07	60	M33423.m	TRUE	reported	MoEnO7	2	TRUE	0.31562	26.74821
8348	rs1209853	T	C	T	C	0.0642	0.047616	0.0626	0.056889	FALSE	FALSE	FALSE	ieu-b-508	10	78422157	0.072132	486484	0.50917	Sepsis (28	Sepsis (28	Sepsis (28	TRUE	igd	0.0124	2.21E-07	922	M36738.m	TRUE	reported	mVQQcD	2	TRUE	0.028252	26.74752
2873	rs4774192	T	C	T	C	0.0709	0.048229	0.1306	0.134538	FALSE	FALSE	FALSE	ieu-b-508	15	55102387	0.048751	486484	0.322519	Sepsis (28	Sepsis (28	Sepsis (28	TRUE	igd	0.0137	2.06E-07	1510	M27710.m	TRUE	reported	XeaFav	2	TRUE	0.017428	26.74704
8054	rs9831949	T	G	T	G	-0.0693	0.093368	0.0277	0.024425	FALSE	FALSE	FALSE	ieu-b-508	3	1.43E+08	0.107866	486484	0.386711	Sepsis (28	Sepsis (28	Sepsis (28	TRUE	igd	0.0134	2.38E-07	6901	M35977.m	TRUE	reported	NHnUo7	2	TRUE	0.003861	26.73813
3154	rs1087977	T	C	T	C	-0.0181	0.042842	0.3441	0.347641	FALSE	FALSE	FALSE	ieu-b-508	12	74832190	0.03514	486484	0.222781	Sepsis (28	Sepsis (28	Sepsis (28	TRUE	igd	0.0035	2.59E-07	7357	M32322.m	TRUE	reported	p4H9pm	2	TRUE	0.003622	26.7364
3834	rs9936831	A	T	A	T	0.0212	0.092273	0.9266	0.889986	FALSE	TRUE	FALSE	ieu-b-508	16	31287148	0.053754	486484	0.086058	Sepsis (28	Sepsis (28	Sepsis (28	TRUE	igd	0.0041	2.88E-07	7229	M32644.m	TRUE	reported	JAa2WD	2	TRUE	0.003685	26.72907
1941	rs8112502	A	G	A	G	-0.0212	-0.04919	0.0918	0.087086	FALSE	FALSE	FALSE	ieu-b-508	19	52623322	0.059313	486484	0.406937	Sepsis (28	Sepsis (28	Sepsis (28	TRUE	igd	0.0041	2.64E-07	6549	M16822.m	TRUE	reported	UNI018	2	TRUE	0.004066	26.7283
1035	rs910616	T	C	T	C	-0.0093	0.026136	0.4991	0.500328	FALSE	FALSE	FALSE	ieu-b-508	6	44074976	0.033385	486484	0.433713	Sepsis (28	Sepsis (28	Sepsis (28	TRUE	igd	0.0018	1.97E-07	7364	M01564.m	TRUE	reported	IPUdyD	2	TRUE	0.003612	26.68719
728	rs6587731	T	G	T	G	-0.0093	0.005941	0.1626	0.166798	FALSE	FALSE	FALSE	ieu-b-508	1	1.53E+08	0.045809	486484	0.896811	Sepsis (28	Sepsis (28	Sepsis (28	TRUE	igd	0.0018	1.96E-07	7363	M01301.m	TRUE	reported	w8TL71	2	TRUE	0.003612	26.68719
3771	rs3740779	A	G	A	G	0.0093	0.075578	0.3688	0.383381	FALSE	FALSE	FALSE	ieu-b-508	11	76372052	0.034876	486484	0.03023	Sepsis (28	Sepsis (28	Sepsis (28	TRUE	igd	0.0018	2.97E-07	7362	M32634.m	TRUE	reported	7T2RUz	2	TRUE	0.003613	26.68719
6451	rs9973286	C	G	C	G	-0.0093	0.013072	0.8771	0.864646	FALSE	TRUE	FALSE	ieu-b-508	19	45215711	0.049102	486484	0.790069	Sepsis (28	Sepsis (28	Sepsis (28	TRUE	igd	0.0018	3.23E-07	7362	M34062.m	TRUE	reported	RZyHKP	2	TRUE	0.003613	26.68719
1457	rs9462856	T	C	T	C	-0.0093	0.021695	0.4711	0.460895	FALSE	FALSE	FALSE	ieu-b-508	6	42926024	0.033416	486484	0.516183	Sepsis (28	Sepsis (28	Sepsis (28	TRUE	igd	0.0018	2.00E-07	7357	M03141.m	TRUE	reported	y4mG6m	2	TRUE	0.003615	26.68719
1145	rs1752058	A	G	A	G	0.0093	0.039607	0.332	0.331505	FALSE	FALSE	FALSE	ieu-b-508	10	92247237	0.035487	486484	0.264376	Sepsis (28	Sepsis (28	Sepsis (28	TRUE	igd	0.0018	3.60E-07	7355	M01644.m	TRUE	reported	IB0DTL	2	TRUE	0.003616	26.68719
1158	rs630782	T	C	T	C	0.0093	0.016814	0.4713	0.459751	FALSE	FALSE	FALSE	ieu-b-508	11	1.07E+08	0.033497	486484	0.615703	Sepsis (28	Sepsis (28	Sepsis (28	TRUE	igd	0.0018	2.32E-07	7355	M01644.m	TRUE	reported	IB0DTL	2	TRUE	0.003616	26.68719
3486	rs1004733	A	G	A	G	-0.0093	-0.04581	0.6773	0.673037	FALSE	FALSE	FALSE	ieu-b-508	10	92342511	0.035626	486484	0.198449	Sepsis (28	Sepsis (28	Sepsis (28	TRUE	igd	0.0018	2.43E-07	7355	M32492.m	TRUE	reported	rZZFcE	2	TRUE	0.003616	26.68719
1188	rs4327548	A	G	A	G	-0.0093	-0.01337	0.2904	0.282351	FALSE	FALSE	FALSE	ieu-b-508	4	57999268	0.037132	486484	0.718898	Sepsis (28	Sepsis (28	Sepsis (28	TRUE	igd	0.0018	2.16E-07	7346	M01645.m	TRUE	reported	d5nAn3	2	TRUE	0.003621	26.68718
3223	rs703462	C	G	C	G	0.0093	-0.01748	0.3569	0.352398	FALSE	TRUE	FALSE	ieu-b-508	10	1.07E+08	0.034946	486484	0.616886	Sepsis (28	Sepsis (28	Sepsis (28	TRUE	igd	0.0018	1.06E-07	7344	M32339.m	TRUE	reported	SVp504	2	TRUE	0.003622	26.68717
2715	rs7523745	T	C	T	C	-0.0093	-0.01357	0.4559	0.461125	FALSE	FALSE	FALSE	ieu-b-508	1	1.81E+08	0.033518	486484	0.685556	Sepsis (28	Sepsis (28	Sepsis (28	TRUE	igd	0.0018	3.42E-07	7340	M25459.m	TRUE	reported	ACJY35	2	TRUE	0.003624	26.68717
4060	rs2371276	T	C	T	C	-0.0093	-0.02131	0.4783	0.475598	FALSE	FALSE	FALSE	ieu-b-508	2	2.13E+08	0.033793	486484	0.528271	Sepsis (28	Sepsis (28	Sepsis (28	TRUE	igd	0.0018	1.91E-07	7318	M32740.m	TRUE	reported	YLshfP	2	TRUE	0.003635	26.68715
4069	rs529833	A	G	A	G	-0.0093	0.046896	0.6913	0.695234	FALSE	FALSE	FALSE	ieu-b-508	6	83006636	0.03627	486484	0.196023	Sepsis (28	Sepsis (28	Sepsis (28	TRUE	igd	0.0018	1.32E-07	7318	M32740.m	TRUE	reported	YLshfP	2	TRUE	0.003635	26.68715
5823	rs4634107	T	G	T	G	-0.0093	0.013362	0.5163	0.527821	FALSE	FALSE	FALSE	ieu-b-508	3	1.86E+08	0.033481	486484	0.689825	Sepsis (28	Sepsis (28	Sepsis (28	TRUE	igd	0.0018	3.25E-07	7066	M33515.m	TRUE	reported	X9mul1	2	TRUE	0.003764	26.68689
5832	rs6536380	T	C	T	C	0.0093	-0.06506	0.6346	0.630786	FALSE	FALSE	FALSE	ieu-b-508	4	1.6E+08	0.034733	486484	0.061053	Sepsis (28	Sepsis (28	Sepsis (28	TRUE	igd	0.0018	4.49E-07	7066	M33515.m	TRUE	reported	X9mul1	2	TRUE	0.003764	26.68689
6520	rs2187448	T	C	T	C	-0.0186	0.049603	0.1296	0.126111	FALSE	FALSE	FALSE	ieu-b-508	11	79333777	0.050218	486484	0.323274	Sepsis (28	Sepsis (28	Sepsis (28	TRUE	igd	0.0036	1.79E-07	7049	M34123.m	TRUE	reported	9O5wo1	2	TRUE	0.003773	26.68687
768	rs912170	A	G	A	G	-0.0186	-0.02123	0.8773	0.879174	FALSE	FALSE	FALSE	ieu-b-508	9	656736	0.051115	486484	0.677844	Sepsis (28	Sepsis (28	Sepsis (28	TRUE	igd	0.0036	2.09E-07	6946	M01303.m	TRUE	reported	3b8x0F	2	TRUE	0.003828	26.68676
6811	rs1905351	T	C	T	C	0.0093	0.043721	0.3924	0.40245	FALSE	FALSE	FALSE	ieu-b-508	3	1.09E+08	0.034179	486484	0.200836	Sepsis (28	Sepsis (28	Sepsis (28	TRUE	igd	0.0018	1.38E-07	6755	M34359.m	TRUE	reported	0UotaJ	2	TRUE	0.003936	26.68654
7226	rs633290	A	G	A	G	-0.0217	0.029911	0.6791	0.67558	FALSE	FALSE	FALSE	ieu-b-508	6	4114788	0.035657	486484	0.401557	Sepsis (28	Sepsis (28	Sepsis (28	TRUE	igd	0.0042	2.13E-07	6381	M34761.m	TRUE	reported	AMtp1w	2	TRUE	0.004166	26.68608
6699	rs4533654	T	G	T	G	-0.0155	0.003257	0.5883	0.601491	FALSE	FALSE	FALSE	ieu-b-508	3	1.33E+08	0.034398	486484	0.924567	Sepsis (28	Sepsis (28	Sepsis (28	TRUE	igd	0.003	1.61E-07	5081	M34336.m	TRUE	reported	jrSSxL	2	TRUE	0.005226	26.68394
4351	rs1899256	C	G	C	G	0.0377	0.067971	0.9149	0.915157	FALSE	TRUE	FALSE	ieu-b-508	14	30502105	0.059587	486484	0.253993	Sepsis (28	Sepsis (28	Sepsis (28	TRUE	igd	0.0073	2.61E-07	7355	M32855.m	TRUE	reported	AM8kCf	2	TRUE	0.003613	26.66361
8214	rs2874702	T	C	T	C	-0.0496	-0.01186	0.4189	0.406372	FALSE	FALSE	FALSE	ieu-b-508	4	1.66E+08	0.033981	486484	0.727108	Sepsis (28	Sepsis (28	Sepsis (28	TRUE	igd	0.0096	2.47E-07	1529	M36376.m	TRUE	reported	k6uC1I	2	TRUE	0.017159	26.65953
1975	rs6603803	A	G	A	G	-0.0253	-0.00536	0.4879	0.492489	FALSE	FALSE	FALSE	ieu-b-508	1	1812688	0.033352	486484	0.872333	Sepsis (28	Sepsis (28	Sepsis (28	TRUE	igd	0.0049	2.93E-07	7226	M17807.m	TRUE	reported	zd6sZI	2	TRUE	0.003676	26.65193
8493	rs1007983	T	G	T	G	-0.1332	0.053462	0.0301	0.02847	FALSE	FALSE	FALSE	ieu-b-508	5	1.31E+08	0.100425	486484	0.594474	Sepsis (28	Sepsis (28	Sepsis (28	TRUE	igd	0.0258	2.33E-07	7014	M37097.m	TRUE	reported	wCbJj4	2	TRUE	0.003786	26.64681
7463	rs2326537	A	T	A	T	-0.1064	-0.00966	0.0294	0.041108	FALSE	TRUE	FALSE	ieu-b-508	20	4563033	0.084048	486484	0.908498	Sepsis (28	Sepsis (28	Sepsis (28	TRUE	igd	0.0206	2.43E-07	1525	M35193.m	TRUE	reported	BMMWXU	2	TRUE	0.017193	26.64274
916	rs5764925	A	G	A	G	-0.016	-0.01519	0.0772	0.086068	FALSE	FALSE	FALSE	ieu-b-508	22	44763799	0.060399	486484	0.80145	Sepsis (28	Sepsis (28	Sepsis (28	TRUE	igd	0.0031	1.91E-07	7354	M01494.m	TRUE	reported	7JWNKI	2	TRUE	0.003609	26.63167
1768	rs1687022	A	G	A	G	0.0129	0.102513	0.1456	0.14317	FALSE	FALSE	FALSE	ieu-b-508	8	1.04E+08	0.047655	486484	0.031464	Sepsis (28	Sepsis (28	Sepsis (28	TRUE	igd	0.0025	3.23E-07	7331	M15676.m	TRUE	reported	s1NfKN	2	TRUE	0.003619	26.61834
1434	rs7644390	A	T	A	T	0.0129	0.017581	0.3478	0.354162	FALSE	TRUE	FALSE	ieu-b-508	3	1.22E+08	0.034968	486484	0.615123	Sepsis (28	Sepsis (28	Sepsis (28	TRUE	igd	0.0025	1.76E-07	6941	M03127.m	TRUE	reported	JscEZw	2	TRUE	0.003821	26.61793
4615	rs1263280	T	C	T	C	-0.783	-0.04869	0.0407	0.031519	FALSE	FALSE	FALSE	ieu-b-508	5	15204361	0.094831	486484	0.607634	Sepsis (28	Sepsis (28	Sepsis (28	TRUE	igd	0.1498	1.73E-07	76	M33173.m	TRUE	reported	04fkUQ	2	TRUE	0.26443	26.60223
1843	rs247616	T	C	T	C	0.0098	-0.06872	0.322	0.32435	FALSE	FALSE	FALSE	ieu-b-508	16	56989590	0.035617	486484	0.05369	Sepsis (28	Sepsis (28	Sepsis (28	TRUE	igd	0.0019	1.32E-07	7308	M15778.m	TRUE	reported	941o3I	2	TRUE	0.003627	26.5966
3807	rs1189652	A	G	A	G	-0.0098	0.03303	0.3492	0.340575	FALSE	FALSE	FALSE	ieu-b-508	2	1.25E+08	0.035278	486484	0.349121	Sepsis (28	Sepsis (28	Sepsis (28	TRUE	igd	0.0019	1.28E-07	7229	M32644.m	TRUE	reported	JAa2WD	2	TRUE	0.003667	26.59652
5804	rs1110277	A	G	A	G	-0.0098	0.021037	0.675	0.661621	FALSE	FALSE	FALSE	ieu-b-508	20	4854682	0.035317	486484	0.551402	Sepsis (28	Sepsis (28	Sepsis (28	TRUE	igd	0.0019	1.47E-07	7066	M33515.m	TRUE	reported	X9mul1	2	TRUE	0.003751	26.59635
8018	rs6832838	T	C	T	C	0.0098	0.026527	0.5177	0.525589	FALSE	FALSE	FALSE	ieu-b-508	4	1.85E+08	0.03338	486484	0.426796	Sepsis (28	Sepsis (28	Sepsis (28	TRUE	igd	0.0019	3.36E-07	6604	M35754.m	TRUE	reported	IlXOo1	2	TRUE	0.004012	26.59582
8480	rs327737	T	C	T	C	0.0098	-0.04363	0.577	0.577215	FALSE	FALSE	FALSE	ieu-b-508	7	80858234	0.034615	486484	0.207502	Sepsis (28	Sepsis (28	Sepsis (28	TRUE	igd	0.0019	1.18E-07	6552	M37058.m	TRUE	reported	32p8qV	2	TRUE	0.004044	26.59576
2560	rs328370	T	G	T	G	0.0098	-0.04139	0.722	0.706978	FALSE	FALSE	FALSE	ieu-b-508	16	73797008	0.036746	486484	0.26006	Sepsis (28	Sepsis (28	Sepsis (28	TRUE	igd	0.0019	5.30E-07	6535	M22177.m	TRUE	reported	2s6Sly	2	TRUE	0.004054	26.59574
4622	rs1689278	C	G	C	G	-0.6355	0.100465	0.04	0.019054	FALSE	TRUE	FALSE	ieu-b-508	6	39937820	0.122981	486484	0.413976	Sepsis (28	Sepsis (28	Sepsis (28	TRUE	igd	0.1216	1.73E-07	76	M33173.m	TRUE	reported	04fkUQ	2	TRUE	0.264369	26.5939
7379	rs7179088	T	C	T	C	0.0098	0.024539	0.3056	0.302467	FALSE	FALSE	FALSE	ieu-b-508	15	31791957	0.036411	486484	0.500356	Sepsis (28	Sepsis (28	Sepsis (28	TRUE	igd	0.0019	2.86E-07	4900	M35137.m	TRUE	reported	Eq2siS	2	TRUE	0.0054	26.59302
422	rs2130712	A	C	A	C	0.0165	-0.09555	0.92	0.918891	FALSE	FALSE	FALSE	ieu-b-508	4	1.55E+08	0.06113	486484	0.118027	Sepsis (28	Sepsis (28	Sepsis (28	TRUE	igd	0.0032	2.43E-07	7365	M00063.m	TRUE	reported	Hdsv1L	2	TRUE	0.003597	26.57969
6852	rs1196522	A	C	A	C	0.0965	-0.06937	0.3865	0.381115	FALSE	FALSE	FALSE	ieu-b-508	6	1.32E+08	0.034445	486484	0.044003	Sepsis (28	Sepsis (28	Sepsis (28	TRUE	igd	0.0187	2.53E-07	954	M34365.m	TRUE	reported	8eexD0	2	TRUE	0.027156	26.57419
7605	rs7249061	T	C	T	C	0.0201	-0.02989	0.5464	0.538413	FALSE	FALSE	FALSE	ieu-b-508	19	51236834	0.033527	486484	0.372674	Sepsis (28	Sepsis (28	Sepsis (28	TRUE	igd	0.0039	2.62E-07	7359	M35320.m	TRUE	reported	ijCk2X	2	TRUE	0.003596	26.55491
7212	rs9806159	T	G	T	G	-0.05	-0.05307	0.1395	0.141805	FALSE	FALSE	FALSE	ieu-b-508	15	31209654	0.047935	486484	0.268243	Sepsis (28	Sepsis (28	Sepsis (28	TRUE	igd	0.0097	2.56E-07	1560	M34674.m	TRUE	reported	plxOKz	2	TRUE	0.016747	26.53624
7820	rs1704783	T	G	T	G	0.0768	0.035449	0.9243	0.928201	FALSE	FALSE	FALSE	ieu-b-508	3	68609856	0.064523	486484	0.582734	Sepsis (28	Sepsis (28	Sepsis (28	TRUE	igd	0.0149	2.51E-07	1640	M35472.m	TRUE	reported	oZU10n	2	TRUE	0.015941	26.53505
566	rs1077122	A	G	A	G	0.0237	-0.03363	0.78	0.773514	FALSE	FALSE	FALSE	ieu-b-508	12	25651899	0.039633	486484	0.396176	Sepsis (28	Sepsis (28	Sepsis (28	TRUE	igd	0.0046	2.87E-07	5344	M01107.m	TRUE	reported	EbPV1D	2	TRUE	0.004943	26.53496
2404	rs2529488	T	C	T	C	-0.0376	-0.01144	0.8627	0.861338	FALSE	FALSE	FALSE	ieu-b-508	7	1.11E+08	0.048381	486484	0.813037	Sepsis (28	Sepsis (28	Sepsis (28	TRUE	igd	0.0073	2.32E-07	5436	M21184.m	TRUE	reported	63ppuJ	2	TRUE	0.004857	26.51979
3112	rs662138	C	G	C	G	0.0139	0.001614	0.8297	0.814014	FALSE	TRUE	FALSE	ieu-b-508	6	1.61E+08	0.042865	486484	0.969958	Sepsis (28	Sepsis (28	Sepsis (28	TRUE	igd	0.0027	2.95E-07	7356	M32198.m	TRUE	reported	ndXmnp	2	TRUE	0.00359	26.49622
2933	rs9471691	T	C	T	C	0.0139	0.005424	0.6461	0.648412	FALSE	FALSE	FALSE	ieu-b-508	6	12411155	0.03499	486484	0.876805	Sepsis (28	Sepsis (28	Sepsis (28	TRUE	igd	0.0027	2.77E-07	5562	M27728.m	TRUE	reported	WOp1XE	2	TRUE	0.004742	26.4939

1521	rs1723332	A	G	A	G	-0.0175	0.082292	0.9519	0.962019	FALSE	FALSE	FALSE	ieu-b-508	5	81017141	0.087192	486484	0.345271	Sepsis (28	Sepsis (28	Sepsis (28	TRUE	igd	0.0034	3.74E-07	7081	M12067.m	TRUE	reported	O5x7RD	2	TRUE	0.003727	26.48473
665	rs1709295	T	G	T	G	-0.0175	-0.17794	0.1024	0.109991	FALSE	FALSE	FALSE	ieu-b-508	14	96081498	0.053807	486484	0.000943	Sepsis (28	Sepsis (28	Sepsis (28	TRUE	igd	0.0034	3.39E-07	5687	M01284.m	TRUE	reported	E4hc9c	2	TRUE	0.004637	26.4829
4656	rs1004469	A	G	A	G	0.3085	0.081558	0.9784	0.98343	FALSE	FALSE	FALSE	ieu-b-508	5	1.72E+08	0.130321	486484	0.53143	Sepsis (28	Sepsis (28	Sepsis (28	TRUE	igd	0.0599	2.58E-07	1017	M33178.m	TRUE	reported	g22WMS	2	TRUE	0.025419	26.47292
4526	rs2937754	T	C	T	C	-0.0597	0.055261	0.8251	0.838226	FALSE	FALSE	FALSE	ieu-b-508	5	17255803	0.045532	486484	0.224866	Sepsis (28	Sepsis (28	Sepsis (28	TRUE	igd	0.0116	2.91E-07	2391	M33137.m	TRUE	reported	ONVNk3	2	TRUE	0.010956	26.46484
203	rs7014133	A	G	A	G	-0.0561	-0.07555	0.0119	0.009312	FALSE	FALSE	FALSE	ieu-b-508	8	1.32E+08	0.179263	486484	0.673435	Sepsis (28	Sepsis (28	Sepsis (28	TRUE	igd	0.0109	2.74E-07	1762	M00059.m	TRUE	reported	RX1zal	2	TRUE	0.014811	26.45937
7026	rs1703308	T	C	T	C	-0.0484	0.018719	0.3485	0.317396	FALSE	FALSE	FALSE	ieu-b-508	3	35404592	0.035848	486484	0.601542	Sepsis (28	Sepsis (28	Sepsis (28	TRUE	igd	0.0094	3.02E-07	997	M34441.m	TRUE	reported	UpgxQm	2	TRUE	0.025903	26.45836
6987	rs9775485	T	C	T	C	-0.0144	-0.02845	0.5852	0.570899	FALSE	FALSE	FALSE	ieu-b-508	9	95611913	0.033678	486484	0.39826	Sepsis (28	Sepsis (28	Sepsis (28	TRUE	igd	0.0028	2.01E-07	7344	M34407.m	TRUE	reported	X7I83Y	2	TRUE	0.003589	26.44178
7557	rs1240394	A	C	A	C	-0.0144	0.086399	0.0874	0.095911	FALSE	FALSE	FALSE	ieu-b-508	1	2.16E+08	0.056586	486484	0.126791	Sepsis (28	Sepsis (28	Sepsis (28	TRUE	igd	0.0028	1.59E-07	7217	M35270.m	TRUE	reported	Ct8V1n	2	TRUE	0.003651	26.44165
2834	rs289999	T	C	T	C	-0.018	0.000979	0.6577	0.667709	FALSE	FALSE	FALSE	ieu-b-508	7	25564438	0.035916	486484	0.978263	Sepsis (28	Sepsis (28	Sepsis (28	TRUE	igd	0.0035	1.91E-07	7180	M27513.m	TRUE	reported	o9Ff2u	2	TRUE	0.00367	26.44161
3611	rs1690178	A	C	A	C	0.0365	0.043231	0.1157	0.136691	FALSE	FALSE	FALSE	ieu-b-508	6	26555433	0.048443	486484	0.372172	Sepsis (28	Sepsis (28	Sepsis (28	TRUE	igd	0.0071	2.64E-07	7361	M32553.m	TRUE	reported	vHSHqn	2	TRUE	0.003577	26.42111
4126	rs4963728	T	C	T	C	-0.0257	0.040417	0.1436	0.140814	FALSE	FALSE	FALSE	ieu-b-508	12	24036855	0.048194	486484	0.401679	Sepsis (28	Sepsis (28	Sepsis (28	TRUE	igd	0.005	2.59E-07	6658	M32755.m	TRUE	reported	WDrNdi	2	TRUE	0.003952	26.41166
7798	rs6542859	T	C	T	C	0.1378	-0.00491	0.0339	0.047932	FALSE	FALSE	FALSE	ieu-b-508	2	99668835	0.07974	486484	0.950927	Sepsis (28	Sepsis (28	Sepsis (28	TRUE	igd	0.0268	2.68E-07	1383	M35451.m	TRUE	reported	GMRGAN	2	TRUE	0.018758	26.39978
4301	rs4253236	T	C	T	C	-0.037	-0.00261	0.362	0.367998	FALSE	FALSE	FALSE	ieu-b-508	4	1.87E+08	0.034528	486484	0.939828	Sepsis (28	Sepsis (28	Sepsis (28	TRUE	igd	0.0072	2.61E-07	5544	M32836.m	TRUE	reported	d4IVXe	2	TRUE	0.004741	26.39865
3701	rs1448663	A	G	A	G	0.0334	-0.02751	0.1756	0.195988	FALSE	FALSE	FALSE	ieu-b-508	7	17513190	0.042671	486484	0.519166	Sepsis (28	Sepsis (28	Sepsis (28	TRUE	igd	0.0065	3.47E-07	5955	M32593.m	TRUE	reported	6zVI1i	2	TRUE	0.004414	26.39492
6757	rs1270117	T	G	T	G	-0.11	-0.00629	0.9455	0.951157	FALSE	FALSE	FALSE	ieu-b-508	7	3844849	0.077493	486484	0.93532	Sepsis (28	Sepsis (28	Sepsis (28	TRUE	igd	0.0214	2.59E-07	1649	M34339.m	TRUE	reported	RwGZ7c	2	TRUE	0.01577	26.38948
8510	rs1322179	T	C	T	C	0.0411	0.066649	0.1489	0.148882	FALSE	FALSE	FALSE	ieu-b-508	10	96575242	0.046783	486484	0.154264	Sepsis (28	Sepsis (28	Sepsis (28	TRUE	igd	0.008	3.09E-07	4520	M37104.m	TRUE	reported	bR3g7d	2	TRUE	0.005805	26.38223
3043	rs1474102	T	C	T	C	-0.0339	0.007085	0.337	0.344112	FALSE	FALSE	FALSE	ieu-b-508	2	1.41E+08	0.035288	486484	0.840874	Sepsis (28	Sepsis (28	Sepsis (28	TRUE	igd	0.0066	3.32E-07	7338	M31591.m	TRUE	reported	KltFxx	2	TRUE	0.003582	26.37504
6419	rs6689147	T	C	T	C	0.0226	-0.02922	0.2377	0.238077	FALSE	FALSE	FALSE	ieu-b-508	1	1.12E+08	0.039371	486484	0.457967	Sepsis (28	Sepsis (28	Sepsis (28	TRUE	igd	0.0044	3.86E-07	7132	M34040.m	TRUE	reported	sJBy3i	2	TRUE	0.003686	26.37483
7184	rs1104291	T	C	T	C	0.0226	-0.0349	0.2479	0.245292	FALSE	FALSE	FALSE	ieu-b-508	11	10705161	0.039027	486484	0.37119	Sepsis (28	Sepsis (28	Sepsis (28	TRUE	igd	0.0044	2.80E-07	4800	M34539.m	TRUE	reported	puA8ni	2	TRUE	0.005466	26.37124
6315	rs2273534	T	C	T	C	0.019	0.063332	0.947	0.945415	FALSE	FALSE	FALSE	ieu-b-508	20	56285540	0.07345	486484	0.388553	Sepsis (28	Sepsis (28	Sepsis (28	TRUE	igd	0.0037	4.20E-07	7354	M33955.m	TRUE	reported	mhHFnT	2	TRUE	0.003573	26.36244
8642	rs193538	T	G	T	G	0.019	-0.03433	0.7047	0.6974	FALSE	FALSE	FALSE	ieu-b-508	16	16127916	0.036434	486484	0.346076	Sepsis (28	Sepsis (28	Sepsis (28	TRUE	igd	0.0037	2.27E-07	7219	M38178.m	TRUE	reported	GgCXMN	2	TRUE	0.00364	26.36231
3413	rs4668464	A	G	A	G	0.0272	0.010033	0.3316	0.340981	FALSE	FALSE	FALSE	ieu-b-508	2	15900788	0.03519	486484	0.775561	Sepsis (28	Sepsis (28	Sepsis (28	TRUE	igd	0.0053	2.93E-07	5080	M32445.m	TRUE	reported	qcc6f3	2	TRUE	0.005158	26.32783
904	rs1214358	A	G	A	G	0.0118	0.035648	0.1428	0.177098	FALSE	FALSE	FALSE	ieu-b-508	1	2.39E+08	0.044389	486484	0.421924	Sepsis (28	Sepsis (28	Sepsis (28	TRUE	igd	0.0023	3.38E-07	7354	M01494.m	TRUE	reported	7JWNKI	2	TRUE	0.003566	26.3142
5469	rs1684440	A	G	A	G	-0.1401	0.027608	0.9409	0.93862	FALSE	FALSE	FALSE	ieu-b-508	2	1.41E+08	0.069733	486484	0.692177	Sepsis (28	Sepsis (28	Sepsis (28	TRUE	igd	0.0273	2.97E-07	1094	M33408.m	TRUE	reported	tWZWIa	2	TRUE	0.023507	26.28792
1067	rs266742	T	G	T	G	0.0082	-0.00035	0.7307	0.675196	FALSE	FALSE	FALSE	ieu-b-508	3	1.86E+08	0.035675	486484	0.992179	Sepsis (28	Sepsis (28	Sepsis (28	TRUE	igd	0.0016	5.68E-07	7271	M01585.m	TRUE	reported	M7QiSM	2	TRUE	0.003599	26.2584
8261	rs2028012	T	C	T	C	-0.0123	-0.02454	0.1636	0.164743	FALSE	FALSE	FALSE	ieu-b-508	7	18820482	0.045008	486484	0.585661	Sepsis (28	Sepsis (28	Sepsis (28	TRUE	igd	0.0024	4.78E-07	7092	M36552.m	TRUE	reported	5CxMJz	2	TRUE	0.00369	26.25822
7836	rs1008297	T	C	T	C	-0.0451	-0.05277	0.1881	0.181407	FALSE	FALSE	FALSE	ieu-b-508	12	1.32E+08	0.043385	486484	0.223864	Sepsis (28	Sepsis (28	Sepsis (28	TRUE	igd	0.0088	2.99E-07	5035	M35551.m	TRUE	reported	5Sxswl	2	TRUE	0.00519	26.25519
4110	rs6415698	A	C	A	C	0.0548	-0.02408	0.7774	0.778634	FALSE	FALSE	FALSE	ieu-b-508	8	39687113	0.040367	486484	0.5508	Sepsis (28	Sepsis (28	Sepsis (28	TRUE	igd	0.0107	3.10E-07	6379	M32754.m	TRUE	reported	n0okFy	2	TRUE	0.004095	26.22149
2405	rs4937147	A	G	A	G	0.0379	0.007163	0.1341	0.146782	FALSE	FALSE	FALSE	ieu-b-508	11	1.26E+08	0.047318	486484	0.879678	Sepsis (28	Sepsis (28	Sepsis (28	TRUE	igd	0.0074	3.11E-07	5436	M21184.m	TRUE	reported	63ppuJ	2	TRUE	0.004802	26.22136
5319	rs7083972	A	T	A	T	-0.0292	0.039461	0.2206	0.20133	FALSE	TRUE	FALSE	ieu-b-508	10	68608284	0.04157	486484	0.342485	Sepsis (28	Sepsis (28	Sepsis (28	TRUE	igd	0.0057	3.63E-07	1378	M33363.m	TRUE	reported	7K0MM0	2	TRUE	0.018688	26.20506
1082	rs1174118	T	C	T	C	-0.0087	0.045908	0.177	0.169875	FALSE	FALSE	FALSE	ieu-b-508	5	1.8E+08	0.044683	486484	0.304229	Sepsis (28	Sepsis (28	Sepsis (28	TRUE	igd	0.0017	3.78E-07	7371	M01604.m	TRUE	reported	hE3I2k	2	TRUE	0.003541	26.18321
433	rs4941743	T	C	T	C	0.0087	0.003524	0.2179	0.227874	FALSE	FALSE	FALSE	ieu-b-508	13	31664439	0.039983	486484	0.929769	Sepsis (28	Sepsis (28	Sepsis (28	TRUE	igd	0.0017	3.28E-07	7365	M00063.m	TRUE	reported	Hdsv1L	2	TRUE	0.003543	26.1832
814	rs1477025	T	C	T	C	0.0087	0.066616	0.6547	0.657068	FALSE	FALSE	FALSE	ieu-b-508	16	49811433	0.035349	486484	0.059498	Sepsis (28	Sepsis (28	Sepsis (28	TRUE	igd	0.0017	4.83E-07	7355	M01358.m	TRUE	reported	kSPQgd	2	TRUE	0.003548	26.18319
2247	rs2160717	T	C	T	C	-0.0087	-0.06622	0.4042	0.41851	FALSE	FALSE	FALSE	ieu-b-508	19	29822729	0.033788	486484	0.050007	Sepsis (28	Sepsis (28	Sepsis (28	TRUE	igd	0.0017	4.64E-07	7354	M19934.m	TRUE	reported	HtcWcQ	2	TRUE	0.003549	26.18319
779	rs1293126	T	C	T	C	-0.0087	-0.03336	0.6329	0.631948	FALSE	FALSE	FALSE	ieu-b-508	16	49804502	0.034559	486484	0.334397	Sepsis (28	Sepsis (28	Sepsis (28	TRUE	igd	0.0017	2.91E-07	7352	M01336.m	TRUE	reported	FAIrya	2	TRUE	0.00355	26.18319
235	rs1199926	A	T	A	T	-0.0087	0.072596	0.0943	0.092775	FALSE	TRUE	FALSE	ieu-b-508	9	1.08E+08	0.057747	486484	0.208709	Sepsis (28	Sepsis (28	Sepsis (28	TRUE	igd	0.0017	6.56E-07	7351	M00060.m	TRUE	reported	kqwl4V	2	TRUE	0.00355	26.18319
7723	rs7551359	A	C	A	C	0.0087	0.018355	0.7125	0.6941	FALSE	FALSE	FALSE	ieu-b-508	1	2.05E+08	0.03643	486484	0.614371	Sepsis (28	Sepsis (28	Sepsis (28	TRUE	igd	0.0017	2.43E-07	7345	M35397.m	TRUE	reported	Y56mZr	2	TRUE	0.003553	26.18318
3214	rs465152	A	G	A	G	-0.0087	-0.02789	0.5429	0.541074	FALSE	FALSE	FALSE	ieu-b-508	3	6919079	0.033582	486484	0.406189	Sepsis (28	Sepsis (28	Sepsis (28	TRUE	igd	0.0017	6.45E-07	7344	M32339.m	TRUE	reported	SVp504	2	TRUE	0.003554	26.18318
1275	rs1090132	A	G	A	G	0.0087	0.001153	0.4313	0.430189	FALSE	FALSE	FALSE	ieu-b-508	9	1.33E+08	0.033753	486484	0.972744	Sepsis (28	Sepsis (28	Sepsis (28	TRUE	igd	0.0017	4.34E-07	7325	M02132.m	TRUE	reported	uJFm2t	2	TRUE	0.003563	26.18316
3073	rs6947392	T	C	T	C	0.0486	-0.01504	0.2858	0.252835	FALSE	FALSE	FALSE	ieu-b-508	7	7988212	0.038405	486484	0.695287	Sepsis (28	Sepsis (28	Sepsis (28	TRUE	igd	0.0095	2.81E-07	7363	M31787.m	TRUE	reported	XMp8YI	2	TRUE	0.003542	26.16419
7728	rs1086670	A	C	A	C	-0.0133	-0.06423	0.7567	0.755639	FALSE	FALSE	FALSE	ieu-b-508	5	1.77E+08	0.039154	486484	0.100923	Sepsis (28	Sepsis (28	Sepsis (28	TRUE	igd	0.0026	3.47E-07	7007	M35431.m	TRUE	reported	tLfE4I	2	TRUE	0.003721	26.15969
2207	rs505125	C	G	C	G	0.0133	0.055764	0.7328	0.732954	FALSE	TRUE	FALSE	ieu-b-508	8	93512199	0.038	486484	0.142254	Sepsis (28	Sepsis (28	Sepsis (28	TRUE	igd	0.0026	5.22E-07	6929	M19364.m	TRUE	reported	XkjMEx	2	TRUE	0.003762	26.15961
2226	rs764428	T	C	T	C	-0.0133	-0.00844	0.6245	0.619529	FALSE	FALSE	FALSE	ieu-b-508	17	14178470	0.034391	486484	0.806063	Sepsis (28	Sepsis (28	Sepsis (28	TRUE	igd	0.0026	2.46E-07	6678	M19368.m	TRUE	reported	QaKPad	2	TRUE	0.003903	26.15932
3408	rs1707120	A	G	A	G	-0.0312	-0.01402	0.7844	0.795559	FALSE	FALSE	FALSE	ieu-b-508	4	1.82E+08	0.041643	486484	0.736397	Sepsis (28	Sepsis (28	Sepsis (28	TRUE	igd	0.0061	3.92E-07	5080	M32445.m	TRUE	reported	qcc6f3	2	TRUE	0.005123	26.15041
1020	rs4999864	C	G	C	G	0.0179	-0.02573	0.9329	0.934841	FALSE	TRUE	FALSE	ieu-b-508	17	66790174	0.068201	486484	0.705987	Sepsis (28	Sepsis (28	Sepsis (28	TRUE	igd	0.0035	4.77E-07	7364	M01564.m	TRUE	reported	IPUdyD	2	TRUE	0.003539	26.14881
2913	rs210331	A	G	A	G	-0.0179	-0.06419	0.8206	0.828912	FALSE	FALSE	FALSE	ieu-b-508	14	54058327	0.044561	486484	0.149742	Sepsis (28	Sepsis (28	Sepsis (28	TRUE	igd	0.0035	3.53E-07	5562	M27728.m	TRUE	reported	WOp1XE	2	TRUE	0.004681	26.14651
7526	rs7277840	A	G	A	G	0.0266	-0.04112	0.5377	0.531157	FALSE	FALSE	FALSE	ieu-b-508	21	40365504	0.033885	486484	0.224974	Sepsis (28	Sepsis (28	Sepsis (28	TRUE	igd	0.0052	3.76E-07	1767	M35255.m	TRUE	reported	ZDjmUf	2	TRUE	0.014593	26.13754
1219	rs1010874	A	G	A	G	-0.0491	-0.06628	0.0629	0.046417	FALSE	FALSE	FALSE	ieu-b-508	10	84501091	0.079894	486484	0.406732	Sepsis (28	Sepsis (28	Sepsis (28	TRUE	igd	0.0096	3.17E-07	1753	M01712.m	TRUE	reported	JfdAWN	2	TRUE	0.014703	26.12912
603	rs9679675	A	C	A	C	0.0092	-0.00185	0.4732	0.470515	FALSE	FALSE	FALSE	ieu-b-508	2	1.75E+08	0.033447	486484	0.955879	Sepsis (28	Sepsis (28	Sepsis (28	TRUE	igd	0.0018	4.17E-07	7367	M01110.m	TRUE	reported	0A31SI	2	TRUE	0.003533	26.11636
8609	rs1789679	A	G	A	G	0.0092	0.006199	0.689	0.689894	FALSE	FALSE	FALSE	ieu-b-508	11	74935046	0.03612	486484	0.863746	Sepsis (28	Sepsis (28	Sepsis (28	TRUE	igd	0.0018	2.92E-07	7366	M37506.m	TRUE	reported	m2kaRt	2	TRUE	0.003534	26.11636
8624	rs6708367	A	G	A	G	0.0092	-0.04609	0.7078	0.704035	FALSE	FALSE	FALSE	ieu-b-508	2	1.4E+08	0.03656	486484	0.207445	Sepsis (28	Sepsis (28	Sepsis (28	TRUE	igd	0.0018	3.09E-07	7366	M37506.m	TRUE	reported	m2kaRt	2	TRUE	0.003534	26.11636
3252	rs1152592	A	G	A	G	-0.0092	-0.03368	0.6183	0.627005	FALSE	FALSE	FALSE	ieu-b-508	14	64676098	0.034492	486484	0.328899	Sepsis (28	Sepsis (28	Sepsis (28	TRUE	igd	0.0018	1.57E-07	7365	M32348.m	TRUE	reported	DJeLDx	2	TRUE	0.003534	26.11636
3272	rs4315324	A	G	A	G	0.0092	-0.04137	0.5036	0.508199	FALSE	FALSE	FALSE	ieu-b-508	16	48665975	0.033387	486484	0.215341	Sepsis (28	Sepsis (28	Sepsis (28	TRUE	igd	0.0018	1.39E-07	7365	M32348.m	TRUE	reported	DJeLDx	2	TRUE	0.003534	26.11636
3770	rs288500	A	G	A	G	-0.0092	0.039044	0.5823	0.594735	FALSE	FALSE	FALSE	ieu-b-508	3	69630735	0.033975	486484	0.250468	Sepsis (28	Sepsis (28	Sepsis (28	TRUE	igd	0.0018	2.36E-07	7362	M32634.m	TRUE	reported	7T2RUz	2	TRUE	0.003536	26.11636
3775	rs626787	C	G	C	G	0.0092	-0.04361	0.6639	0.647179	FALSE	TRUE	FALSE	ieu-b-508	1	62901243	0.03496	486484	0.212278	Sepsis (28	Sepsis (28	Sepsis (28	TRUE	igd	0.0018	3.80E-07	7362	M32634.m	TRUE	reported	7T2RUz	2	TRUE	0.003536	26.11636
4495	rs1107347	A	G	A	G	-0.0092	0.041984	0.1492	0.179622	FALSE	FALSE	FALSE	ieu-b-508	15	96806278	0.043805	486484	0.337851	Sepsis (28	Sepsis (28	Sepsis (28	TRUE	igd	0.0018	5.55E-07	7362	M33132.m	TRUE	reported	dTXJa8	2	TRUE	0.003536	26.11636
4507	rs2009767	T	C	T	C	0.0092	0.033991	0.8814	0.878218	FALSE	FALSE	FALSE	ieu-b-508	14	43980703	0.051281	486484	0.507427	Sepsis (28	Sepsis (28	Sepsis (28	TRUE	igd	0.0018	4.14E-07	7362	M33132.m	TRUE	reported	dTXJa8	2	TRUE	0.003536	26.11636
6049	rs1018724	A	T	A	T	-0.0184	-0.00694	0.7692	0.757521	FALSE	TRUE	FALSE	ieu-b-508	2	98816010	0.039215	486484	0.859478	Sepsis (28	Sepsis (28	Sepsis (28	TRUE	igd	0.0036	2.96E-07	7362	M33821.m	TRUE	reported	eVYICL	2	TRUE	0.003536	26.11636
26	rs1092710	A	C	A	C	-0.0046	-0.01024	0.3738	0.372671	FALSE	FALSE	FALSE	ieu-b-508	1	2.44E+08	0.034413	486484	0.76603	Sepsis (28	Sepsis (28	Sepsis (28	TRUE	igd	9.00E-04	2.72E-07	7355	M00054.m	TRUE	reported	QTDAnh	2	TRUE	0.003539	26.11635
31	rs1172086	T	G	T	G	-0.0046	-0.03071	0.6034	0.60546	FALSE	FALSE	FALSE	ieu-b-508	3	1838974	0.034294	486484	0.37047	Sepsis (28	Sepsis (28	Sepsis (28	TRUE	igd	9.00E-04	3.58E-07	7355	M00054.m	TRUE	reported	QTDAnh	2	TRUE	0.003539	26.11635
36	rs1220605	A	G	A	G	-0.0046	-0.00394	0.6312	0.626949	FALSE	FALSE	FALSE	ieu-b-508	13	24789809	0.03442	486484	0.908913	Sepsis (28	Sepsis (28	Sepsis (28	TRUE	igd	9.00E-04	9.29E-07	7355	M00054.m	TRUE	reported	QTDAnh	2	TRUE	0.003539	26.11635
124	rs4880888	A	G	A	G	-0.0046	0.036248	0.4785	0.487946	FALSE	FALSE	FALSE	ieu-b-508	10	1661627	0.033411	486484	0.277961	Sepsis (28	Sepsis (28	Sepsis (28	TRUE	igd	9.00E-04	3.24E-07	7355	M00054.m	TRUE	reported	QTDAnh	2	TRUE	0.003539	26.11635
127	rs4972588	A	G	A	G	-0.0046	0.03475	0.5138	0.512925	FALSE	FALSE	FALSE	ieu-b-508	2	1.74E+08	0.033366	486484	0.297656	Sepsis (28	Sepsis (28	Sepsis (28	TRUE	igd	9.00E-04	3.74E-07	7355	M00054.m	TRUE	reported	QTDAnh	2	TRUE	0.003539	26.11635
129	rs502654	T	C	T	C	-0.0046	0.063045	0.559	0.552772	FALSE	FALSE	FALSE	ieu-b-508	1	31061501	0.033796	486484	0.062117	Sepsis (28	Sepsis (28	Sepsis (28	TRUE	igd	9.00E-04	2.60E-07	7355	M00054.m	TRUE	reported	QTDAnh	2	TRUE	0.003539	26.11635
169	rs8006663	A	G	A	G	-0.0046	0.033966	0.4556	0.468163	FALSE	FALSE	FALSE	ieu-b-508	14	69171003	0.033557	486484	0.311443	Sepsis (28	Sepsis (28	Sepsis (28	TRUE	igd	9.00E-04	3.02E-07	7355	M00054.m	TRUE	reported	QTDAnh	2	TRUE	0.003539	26.11635
183	rs937382	T	C	T	C	-0.0046	0.022985	0.4466	0.447111	FALSE	FALSE	FALSE	ieu-b-508	4	1.9E+08	0.033512	486484	0.492797	Sepsis (28	Sepsis (28	Sepsis (28	TRUE	igd	9.00E-04	2.31E-07	7355	M00054.m	TRUE	reported	QTDAnh	2	TRUE	0.003539	26.11635
187	rs9517869	T	C	T	C	-0.0046	0.018525	0.3661	0.366053	FALSE	FALSE	FALSE	ieu-b-508	13	1E+08	0.034741	486484	0.593867	Sepsis (28	Sepsis (28	Sepsis (28	TRUE	igd	9.00E-04	3.46E-07	7355	M00054.m	TRUE	reported	QTDAnh	2	TRUE	0.003539	26.11635
544	rs1007797	T	C	T	C	-0.0092	0.059558	0.879	0.87685	FALSE	FALSE	FALSE	ieu-b-508	11	70734035	0.050891	486484	0.24188	Sepsis (28	Sepsis (28	Sepsis (28	TRUE	igd	0.0018	6.49E-07	7354	M00606.m	TRUE	reported	r5smf7	2	TRUE	0.00354	26.11635
1609	rs1614303	T	G	T	G	0.0138	-0.00675	0.8159	0.816176	FALSE	FALSE	FALSE	ieu-b-508	10	1.23E+08	0.043115	486484	0.87558	Sepsis (28	Sepsis (28	Sepsis (28	TRUE	igd	0.0027	2.40E-07	7352	M15122.m	TRUE	reported	VlnI8T	2	TRUE	0.003541	26.11635
211	rs1048737	A	G	A	G	0.0046	0.012395	0.5764	0.599071	FALSE	FALSE	FALSE	ieu-b-508	7	1.17E+08	0.033956	486484	0.715086	Sepsis (28	Sepsis (28	Sepsis (28	TRUE	igd	9.00E-04	4.09E-07	7351	M00060.m	TRUE	reported	kqwl4V	2	TRUE	0.003541	26.11635
217	rs1081058	T	C	T	C	0.0046	-0.02355	0.5583	0.550117	FALSE	FALSE	FALSE	ieu-b-508	9	16629923	0.033405	486484	0.480915	Sepsis (28	Sepsis (28	Sepsis (28	TRUE	igd	9.00E-04	3.28E-07	7351	M00060.m	TRUE	reported	kqwl4V	2	TRUE	0.003541	26.11635
234	rs1198809	T	C	T	C	-0.0046	0.025374	0.6185	0.623239	FALSE	FALSE	FALSE	ieu-b-508	8	85654878	0.034554	486484	0.462735	Sepsis (28	Sepsis (28	Sepsis (28	TRUE	igd	9.00E-04	3.29E-07	7351	M00060.m	TRUE	reported	kqwl4V	2	TRUE	0.003541	26.11635
236	rs1204761	T	G	T	G	0.0046	0.062019	0.3271	0.320841	FALSE	FALSE	FALSE	ieu-b-508	1	2.03E+08	0.03595	486484	0.084505	Sepsis (28	Sepsis (28	Sepsis (28	TRUE	igd	9.00E-04	4.25E-07	7351	M00060.m	TRUE	reported	kqwl4V	2	TRUE	0.003541	26.11635
309	rs417144	T	C	T	C	0.0046	-0.01266	0.3507	0.340128	FALSE	FALSE	FALSE	ieu-b-508	2	79251847	0.035183	486484	0.718919	Sepsis (28	Sepsis (28	Sepsis (28	TRUE	igd	9.00E-04	2.57E-07	7351	M00060.m	TRUE	reported	kqwl4V	2	TRUE	0.003541	26.11635
335	rs6475359	A	G	A	G	-0.0046	0.021141	0.4127	0.424088	FALSE	FALSE	FALSE	ieu-b-508	9	1975279	0.034675	486484	0.542066	Sepsis (28	Sepsis (28	Sepsis (28	TRUE	igd	9.00E-04	3.57E-07	7351	M00060.m	TRUE	reported	kqwl4V	2	TRUE	0.003541	26.11635
345	rs6833720	A	G	A	G	-0.0046	-0.02	0.4766	0.476424	FALSE	FALSE	FALSE	ieu-b-508	4	19924591	0.033688	486484	0.55264	Sepsis (28	Sepsis (28	Sepsis (28	TRUE	igd	9.00E-04	2.22E-07	7351	M00060.m	TRUE	reported	kqwl4V	2	TRUE	0.003541	26.11635
365	rs7689825	T	C	T	C	0.0046	0.04606	0.643	0.634374	FALSE	FALSE	FALSE	ieu-b-508	4	1.01E+08	0.034652	486484	0.183781	Sepsis (28	Sepsis (28	Sepsis (28	TRUE	igd	9.00E-04	3.97E-07	7351	M00060.m	TRUE	reported	kqwl4V	2	TRUE	0.003541	26.11635
3566	rs1771823	A	G	A	G	0.0092	-0.01838	0.706	0.708818	FALSE	FALSE	FALSE	ieu-b-508	6	1.51E+08	0.036665	486484	0.616186	Sepsis (28	Sepsis (28	Sepsis (28	TRUE	igd	0.0018	4.94E-07	7350	M32518.m	TRUE	reported	V0ZCiU	2	TRUE	0.003542	26.11635
7909	rs1084140	A	G	A	G	-0.0092	-3.83E-05	0.3964	0.384954	FALSE	FALSE	FALSE	ieu-b-508	12	20073094	0.034332	486484	0.999111	Sepsis (28	Sepsis (28	Sepsis (28	TRUE	igd	0.0018	4.94E-07	7348	M35675.m	TRUE	reported	D7fdyZ	2	TRUE	0.003543	26.11635
7934	rs4643071	T	C	T	C	-0.0092	0.034051	0.6782	0.666169	FALSE	FALSE	FALSE	ieu-b-508	11	7662732	0.035512	486484	0.337636	Sepsis (28	Sepsis (28	Sepsis (28	TRUE	igd	0.0018	5.07E-07	7348	M35675.m	TRUE	reported	D7fdyZ	2	TRUE	0.003543	26.11635
1193	rs6112257	A	G	A	G	-0.0092	-0.00835	0.6843	0.68039	FALSE	FALSE	FALSE	ieu-b-508	20	19195319	0.035767	486484	0.815466	Sepsis (28	Sepsis (28	Sepsis (28	TRUE	igd	0.0018	3.39E-07	7346	M01645.m	TRUE	reported	d5nAn3	2	TRUE	0.003544	26.11634
4423	rs1424092	T	G	T	G	-0.0092	-0.03062	0.3648	0.364041	FALSE	FALSE	FALSE	ieu-b-508	16	75810140	0.034908	486484	0.380336	Sepsis (28	Sepsis (28	Sepsis (28	TRUE	igd	0.0018	1.78E-07	7344	M32910.m	TRUE	reported	2OCBKh	2	TRUE	0.003545	26.11634
5383	rs1011090	A	G	A	G	-0.0092	-0.00106	0.468	0.460597	FALSE	FALSE	FALSE	ieu-b-508	8	1.29E+08	0.0335	486484	0.974781	Sepsis (28	Sepsis (28	Sepsis (28	TRUE	igd	0.0018	4.83E-07	7315	M33389.m	TRUE	reported	Hf6XVM	2	TRUE	0.003559	26.11631
1835	rs1263785	A	T	A	T	-0.0138	-0.08037	0.8926	0.870426	FALSE	TRUE	FALSE	ieu-b-508	3	22426059	0.04993	486484	0.10748	Sepsis (28	Sepsis (28	Sepsis (28	TRUE	igd	0.0027	4.33E-07	7308	M15778.m	TRUE	reported	941o3I	2	TRUE	0.003562	26.11631
1848	rs3765035	A	G	A	G	0.0092	-0.02287	0.3438	0.354991	FALSE	FALSE	FALSE	ieu-b-508	2	1.2E+08	0.035043	486484	0.513926	Sepsis (28	Sepsis (28	Sepsis (28	TRUE	igd	0.0018	3.76E-07	7308	M15778.m	TRUE	reported	941o3I	2	TRUE	0.003562	26.11631
5690	rs4853388	A	G	A	G	-0.0092	0.007761	0.2394	0.237822	FALSE	FALSE	FALSE	ieu-b-508	2	78649422	0.03918	486484	0.842984	Sepsis (28	Sepsis (28	Sepsis (28	TRUE	igd	0.0018	5.38E-07	7307	M33477.m	TRUE	reported	Aw8PBK	2	TRUE	0.003562	26.11631
5799	rs1008717	T	C	T	C	-0.0092	0.010905	0.6135	0.596977	FALSE	FALSE	FALSE	ieu-b-508	8	75184592	0.034009	486484	0.74849	Sepsis (28	Sepsis (28	Sepsis (28	TRUE	igd	0.0018	5.13E-07	7066	M33515.m	TRUE	reported	X9mul1	2	TRUE	0.003683	26.11606
5256	rs9462177	A	G	A	G	0.0138	0.000552	0.6032	0.599518	FALSE	FALSE	FALSE	ieu-b-508	6	36312907	0.034075	486484	0.987079	Sepsis (28	Sepsis (28	Sepsis (28	TRUE	igd	0.0027	4.82E-07	7063	M33228.m	TRUE	reported	JwOXbQ	2	TRUE	0.003685	26.11606
2381	rs4774615	A	G	A	G	-0.0184	0.117168	0.8406	0.831705	FALSE	FALSE	FALSE	ieu-b-508	15	52533360	0.044656	486484	0.008695	Sepsis (28	Sepsis (28	Sepsis (28	TRUE	igd	0.0036	4.07E-07	6999	M21127.m	TRUE	reported	WCjhS3	2	TRUE	0.003719	26.11599
7456	rs1235299	T	C	T	C	0.0184	0.007938	0.8104	0.812198	FALSE	FALSE	FALSE	ieu-b-508	9	11019846	0.042922	486484	0.853267	Sepsis (28	Sepsis (28	Sepsis (28	TRUE	igd	0.0036	2.54E-07	6543	M35193.m	TRUE	reported	BMMWXU	2	TRUE	0.003977	26.11547
7464	rs2378668	A	C	A	C	0.0138	0.004093	0.3623	0.360193	FALSE	FALSE	FALSE	ieu-b-508	9	87197225	0.034781	486484	0.906314	Sepsis (28	Sepsis (28	Sepsis (28	TRUE	igd	0.0027	5.04E-07	6543	M35193.m	TRUE	reported	BMMWXU	2	TRUE	0.003977	26.11547
2929	rs757704	A	G	A	G	0.0138	-0.06247	0.5037	0.494834	FALSE	FALSE	FALSE	ieu-b-508	7	8540236	0.033754	486484	0.064186	Sepsis (28	Sepsis (28	Sepsis (28	TRUE	igd	0.0027	2.22E-07	5562	M27728.m	TRUE	reported	WOp1XE	2	TRUE	0.004675	26.11406
5712	rs2197399	A	G	A	G	-0.023	-0.01948	0.5961	0.578521	FALSE	FALSE	FALSE	ieu-b-508	12	46531876	0.033757	486484	0.563854	Sepsis (28	Sepsis (28	Sepsis (28	TRUE	igd	0.0045	2.68E-07	5107	M33488.m	TRUE	reported	mZmDY6	2	TRUE	0.005089	26.11323
4562	rs3916092	A	G	A	G	0.0511	0.063161	0.2029	0.208186	FALSE	FALSE	FALSE	ieu-b-508	3	19946057	0.041904	486484	0.131737	Sepsis (28	Sepsis (28	Sepsis (28	TRUE	igd	0.01	3.22E-07	4817	M33144.m	TRUE	reported	cgXZoM	2	TRUE	0.005392	26.10126
1124	rs6713914	T	C	T	C	0.0593	-0.06785	0.5741	0.53945	FALSE	FALSE	FALSE	ieu-b-508	2	59523041	0.033457	486484	0.042548	Sepsis (28	Sepsis (28	Sepsis (28	TRUE	igd	0.0116	3.22E-07	1567	M01640.m	TRUE	reported	o3qxzl	2	TRUE	0.016404	26.09989
1340	rs2742351	C	G	C	G	-0.0235	-0.01575	0.2209	0.279648	FALSE	TRUE	FALSE	ieu-b-508	2	1.8E+08	0.037451	486484	0.674058	Sepsis (28	Sepsis (28	Sepsis (28	TRUE	igd	0.0046	3.23E-07	5791	M02342.m	TRUE	reported	EhZr3g	2	TRUE	0.004487	26.08976
6579	rs923673	A	C	A	C	0.0235	0.008745	0.7212	0.712673	FALSE	FALSE	FALSE	ieu-b-508	4	20791198	0.036994	486484	0.81312	Sepsis (28	Sepsis (28	Sepsis (28	TRUE	igd	0.0046	2.98E-07	4908	M34245.m	TRUE	reported	pRwBKr	2	TRUE	0.005289	26.08814
513	rs1167691	A	G	A	G	-0.0429	0.051941	0.0216	0.03425	FALSE	FALSE	FALSE	ieu-b-508	2	1.29E+08	0.095685	486484	0.587246	Sepsis (28	Sepsis (28	Sepsis (28	TRUE	igd	0.0084	3.66E-07	7345	M00584.m	TRUE	reported	oBt02X	2	TRUE	0.003539	26.07581
4231	rs2174474	T	C	T	C	0.0327	-0.02205	0.7012	0.739073	FALSE	FALSE	FALSE	ieu-b-508	10	76958658	0.038021	486484	0.562022	Sepsis (28	Sepsis (28	Sepsis (28	TRUE	igd	0.0064	3.67E-07	1593	M32787.m	TRUE	reported	fSRakm	2	TRUE	0.016124	26.07294
8598	rs1247804	A	G	A	G	0.0097	-0.00565	0.2527	0.253903	FALSE	FALSE	FALSE	ieu-b-508	2	2.31E+08	0.038489	486484	0.883206	Sepsis (28	Sepsis (28	Sepsis (28	TRUE	igd	0.0019	1.49E-07	7366	M37506.m	TRUE	reported	m2kaRt	2	TRUE	0.003526	26.05664
7927	rs2285688	A	G	A	G	0.0097	0.023381	0.705	0.699786	FALSE	FALSE	FALSE	ieu-b-508	7	24874459	0.036539	486484	0.522248	Sepsis (28	Sepsis (28	Sepsis (28	TRUE	igd	0.0019	1.82E-07	7348	M35675.m	TRUE	reported	D7fdyZ	2	TRUE	0.003535	26.05662
2600	rs1012455	A	G	A	G	-0.0097	0.007623	0.5085	0.504163	FALSE	FALSE	FALSE	ieu-b-508	1	80739543	0.03341	486484	0.819509	Sepsis (28	Sepsis (28	Sepsis (28	TRUE	igd	0.0019	2.16E-07	7329	M22481.m	TRUE	reported	bXpf0k	2	TRUE	0.003544	26.0566
1222	rs1185513	A	G	A	G	-0.0618	0.072144	0.0406	0.037466	FALSE	FALSE	FALSE	ieu-b-508	15	57770091	0.090343	486484	0.424548	Sepsis (28	Sepsis (28	Sepsis (28	TRUE	igd	0.0121	2.99E-07	1753	M01712.m	TRUE	reported	JfdAWN	2	TRUE	0.014663	26.05616
2553	rs227375	T	C	T	C	0.0097	-0.02945	0.4802	0.489404	FALSE	FALSE	FALSE	ieu-b-508	4	1.04E+08	0.033391	486484	0.377809	Sepsis (28	Sepsis (28	Sepsis (28	TRUE	igd	0.0019	5.76E-07	6535	M22177.m	TRUE	reported	2s6Sly	2	TRUE	0.003972	26.05574
7626	rs1048875	T	C	T	C	0.0097	-0.0438	0.675	0.679875	FALSE	FALSE	FALSE	ieu-b-508	11	1.03E+08	0.035736	486484	0.220319	Sepsis (28	Sepsis (28	Sepsis (28	TRUE	igd	0.0019	2.13E-07	5768	M35326.m	TRUE	reported	jRytyJ	2	TRUE	0.004498	26.05467
7337	rs1113199	T	G	T	G	-0.0097	-0.02816	0.6998	0.698125	FALSE	FALSE	FALSE	ieu-b-508	4	1.81E+08	0.036346	486484	0.43854	Sepsis (28	Sepsis (28	Sepsis (28	TRUE	igd	0.0019	4.20E-07	4900	M35137.m	TRUE	reported	Eq2siS	2	TRUE	0.005291	26.05307
7352	rs2468526	T	C	T	C	0.0097	-0.02239	0.6827	0.684035	FALSE	FALSE	FALSE	ieu-b-508	5	36515990	0.035923	486484	0.533048	Sepsis (28	Sepsis (28	Sepsis (28	TRUE	igd	0.0019	3.48E-07	4900	M35137.m	TRUE	reported	Eq2siS	2	TRUE	0.005291	26.05307
7391	rs9649625	A	C	A	C	-0.0097	0.047228	0.2883	0.285441	FALSE	FALSE	FALSE	ieu-b-508	7	75387937	0.037193	486484	0.204155	Sepsis (28	Sepsis (28	Sepsis (28	TRUE	igd	0.0019	5.81E-07	4900	M35137.m	TRUE	reported	Eq2siS	2	TRUE	0.005291	26.05307
7775	rs3809197	T	C	T	C	0.1072	0.034267	0.0135	0.01151	FALSE	FALSE	FALSE	ieu-b-508	12	96883418	0.16053	486484	0.830968	Sepsis (28	Sepsis (28	Sepsis (28	TRUE	igd	0.021	3.25E-07	1718	M35439.m	TRUE	reported	DnuAGZ	2	TRUE	0.014941	26.02826
7451	rs7741438	A	G	A	G	0.0255	0.021022	0.4744	0.478613	FALSE	FALSE	FALSE	ieu-b-508	6	5723118	0.033809	486484	0.534075	Sepsis (28	Sepsis (28	Sepsis (28	TRUE	igd	0.005	3.38E-07	6234	M35189.m	TRUE	reported	54XZFj	2	TRUE	0.004155	26.00166
3641	rs4687396	A	C	A	C	-0.1489	0.075482	0.9784	0.988674	FALSE	FALSE	FALSE	ieu-b-508	3	1.93E+08	0.167653	486484	0.652547	Sepsis (28	Sepsis (28	Sepsis (28	TRUE	igd	0.0292	3.34E-07	2091	M32560.m	TRUE	reported	sLwsEw	2	TRUE	0.012283	25.97814
8323	rs1710294	A	G	A	G	0.0836	0.03255	0.9519	0.954724	FALSE	FALSE	FALSE	ieu-b-508	14	72239765	0.080321	486484	0.685294	Sepsis (28	Sepsis (28	Sepsis (28	TRUE	igd	0.0164	3.51E-07	3977	M36589.m	TRUE	reported	27GADo	2	TRUE	0.006491	25.97206
7695	rs9885127	T	C	T	C	-0.1433	-0.13366	0.9868	0.978075	FALSE	FALSE	FALSE	ieu-b-508	5	68321834	0.121143	486484	0.269882	Sepsis (28	Sepsis (28	Sepsis (28	TRUE	igd	0.0281	3.32E-07	1375	M35331.m	TRUE	reported	GBnxhN	2	TRUE	0.018563	25.96854
2736	rs6767775	A	G	A	G	-0.0311	-0.04439	0.4648	0.476571	FALSE	FALSE	FALSE	ieu-b-508	3	84157105	0.033588	486484	0.186332	Sepsis (28	Sepsis (28	Sepsis (28	TRUE	igd	0.0061	4.06E-07	1358	M25599.m	TRUE	reported	fRZxwP	2	TRUE	0.018781	25.955
2807	rs1023792	A	G	A	G	0.0382	-0.01198	0.749	0.731091	FALSE	FALSE	FALSE	ieu-b-508	7	88675915	0.037578	486484	0.749915	Sepsis (28	Sepsis (28	Sepsis (28	TRUE	igd	0.0075	3.11E-07	2759	M27447.m	TRUE	reported	s1NdyU	2	TRUE	0.009315	25.92324
2	rs1697704	T	C	T	C	-0.0056	-0.01493	0.7034	0.708292	FALSE	FALSE	FALSE	ieu-b-508	16	27924612	0.036541	486484	0.682854	Sepsis (28	Sepsis (28	Sepsis (28	TRUE	igd	0.0011	1.08E-06	7372	M00053.m	TRUE	reported	4Smluz	2	TRUE	0.003503	25.91032
3523	rs1168060	C	G	C	G	0.0168	0.026248	0.7095	0.703265	FALSE	TRUE	FALSE	ieu-b-508	2	48928533	0.036609	486484	0.473388	Sepsis (28	Sepsis (28	Sepsis (28	TRUE	igd	0.0033	3.48E-07	7358	M32497.m	TRUE	reported	244bZN	2	TRUE	0.00351	25.91031
1459	rs1125012	A	G	A	G	-0.0494	0.099067	0.043	0.058544	FALSE	FALSE	FALSE	ieu-b-508	8	11093364	0.071153	486484	0.163831	Sepsis (28	Sepsis (28	Sepsis (28	TRUE	igd	0.0097	3.83E-07	1748	M03147.m	TRUE	reported	D9eDjf	2	TRUE	0.014621	25.90677
6031	rs1267078	A	G	A	G	-0.1499	-0.02782	0.8761	0.885846	FALSE	FALSE	FALSE	ieu-b-508	7	26019953	0.052661	486484	0.597288	Sepsis (28	Sepsis (28	Sepsis (28	TRUE	igd	0.0294	3.30E-07	581	M33782.m	TRUE	reported	875Pal	2	TRUE	0.042827	25.90664
5293	rs1343863	A	G	A	G	-0.0601	-0.03818	0.7954	0.761733	FALSE	FALSE	FALSE	ieu-b-508	4	1.81E+08	0.039742	486484	0.336669	Sepsis (28	Sepsis (28	Sepsis (28	TRUE	igd	0.0118	3.80E-07	1273	M33353.m	TRUE	reported	ZIN4gk	2	TRUE	0.019971	25.90014
1892	rs7711255	A	G	A	G	-0.0173	-0.00324	0.7968	0.768625	FALSE	FALSE	FALSE	ieu-b-508	5	1.41E+08	0.03973	486484	0.934904	Sepsis (28	Sepsis (28	Sepsis (28	TRUE	igd	0.0034	4.92E-07	6782	M15990.m	TRUE	reported	15fe4N	2	TRUE	0.003803	25.8825
6810	rs1763887	T	C	T	C	0.0173	0.111549	0.9074	0.860692	FALSE	FALSE	FALSE	ieu-b-508	17	61894076	0.047977	486484	0.020068	Sepsis (28	Sepsis (28	Sepsis (28	TRUE	igd	0.0034	2.44E-07	6755	M34359.m	TRUE	reported	0UotaJ	2	TRUE	0.003818	25.88247
7439	rs1300312	T	C	T	C	0.0346	-0.07167	0.1214	0.120968	FALSE	FALSE	FALSE	ieu-b-508	2	2.12E+08	0.051213	486484	0.161651	Sepsis (28	Sepsis (28	Sepsis (28	TRUE	igd	0.0068	3.63E-07	6234	M35189.m	TRUE	reported	54XZFj	2	TRUE	0.004136	25.88183
2147	rs7809441	A	G	A	G	0.0748	-0.01552	0.0807	0.085485	FALSE	FALSE	FALSE	ieu-b-508	7	31155802	0.060331	486484	0.796997	Sepsis (28	Sepsis (28	Sepsis (28	TRUE	igd	0.0147	3.80E-07	3783	M18497.m	TRUE	reported	90JFPc	2	TRUE	0.006798	25.87849
6351	rs1708956	T	C	T	C	0.0468	-0.0179	0.0209	0.03532	FALSE	FALSE	FALSE	ieu-b-508	4	60176254	0.091095	486484	0.844215	Sepsis (28	Sepsis (28	Sepsis (28	TRUE	igd	0.0092	3.39E-07	7364	M33960.m	TRUE	reported	o8RcxU	2	TRUE	0.003502	25.8701
1766	rs1245558	T	C	T	C	0.0117	-0.05011	0.3104	0.379655	FALSE	FALSE	FALSE	ieu-b-508	18	75638023	0.034683	486484	0.148512	Sepsis (28	Sepsis (28	Sepsis (28	TRUE	igd	0.0023	4.39E-07	7331	M15676.m	TRUE	reported	s1NfKN	2	TRUE	0.003517	25.87007
7043	rs165582	A	G	A	G	0.0234	0.053207	0.1156	0.133224	FALSE	FALSE	FALSE	ieu-b-508	4	1.53E+08	0.049339	486484	0.280852	Sepsis (28	Sepsis (28	Sepsis (28	TRUE	igd	0.0046	3.31E-07	5207	M34456.m	TRUE	reported	Nh457q	2	TRUE	0.004945	25.86719
8195	rs4949966	T	C	T	C	0.0468	-0.02965	0.874	0.86653	FALSE	FALSE	FALSE	ieu-b-508	1	95969035	0.049075	486484	0.54572	Sepsis (28	Sepsis (28	Sepsis (28	TRUE	igd	0.0092	3.56E-07	2400	M36230.m	TRUE	reported	V5KJeR	2	TRUE	0.010667	25.85556
2368	rs1785007	C	G	C	G	0.1233	0.002562	0.1024	0.09151	FALSE	TRUE	FALSE	ieu-b-508	11	1.16E+08	0.058142	486484	0.964851	Sepsis (28	Sepsis (28	Sepsis (28	TRUE	igd	0.0242	3.35E-07	413	M21049.m	TRUE	reported	UDtLE4	2	TRUE	0.059139	25.83373
6205	rs6891011	A	G	A	G	-0.0244	-0.0665	0.8862	0.860106	FALSE	FALSE	FALSE	ieu-b-508	5	1.03E+08	0.04832	486484	0.168738	Sepsis (28	Sepsis (28	Sepsis (28	TRUE	igd	0.0048	3.64E-07	6004	M33892.m	TRUE	reported	w7HgU7	2	TRUE	0.004285	25.83167
1550	rs7446718	A	G	A	G	-0.0417	-0.05946	0.8591	0.834337	FALSE	FALSE	FALSE	ieu-b-508	5	12077683	0.044849	486484	0.184898	Sepsis (28	Sepsis (28	Sepsis (28	TRUE	igd	0.0082	3.83E-07	1586	M12129.m	TRUE	reported	iNzdgb	2	TRUE	0.016044	25.82833
4621	rs162882	C	G	C	G	0.4501	0.066482	0.9145	0.900227	FALSE	TRUE	FALSE	ieu-b-508	5	1.32E+08	0.055903	486484	0.23434	Sepsis (28	Sepsis (28	Sepsis (28	TRUE	igd	0.0874	2.57E-07	76	M33173.m	TRUE	reported	04fkUQ	2	TRUE	0.258691	25.82339
7785	rs1076659	A	G	A	G	-0.1244	-0.00927	0.2938	0.327509	FALSE	FALSE	FALSE	ieu-b-508	11	19834989	0.035603	486484	0.794537	Sepsis (28	Sepsis (28	Sepsis (28	TRUE	igd	0.0244	3.26E-07	284	M35451.m	TRUE	reported	GMRGAN	2	TRUE	0.083851	25.81023
6886	rs6464160	T	G	T	G	0.1498	-0.01528	0.2552	0.305163	FALSE	FALSE	FALSE	ieu-b-508	7	1.51E+08	0.036296	486484	0.673741	Sepsis (28	Sepsis (28	Sepsis (28	TRUE	igd	0.0278	6.94E-08	18	M34365.m	TRUE	reported	8eexD0	2	TRUE	0.617313	25.80961
3426	rs1417985	A	G	A	G	0.0315	0.081423	0.9726	0.971005	FALSE	FALSE	FALSE	ieu-b-508	13	47296625	0.100539	486484	0.418021	Sepsis (28	Sepsis (28	Sepsis (28	TRUE	igd	0.0062	4.65E-07	7364	M32452.m	TRUE	reported	8GtFpl	2	TRUE	0.003493	25.80594
3188	rs329319	A	G	A	G	0.0127	-0.03043	0.4276	0.42825	FALSE	FALSE	FALSE	ieu-b-508	5	1.34E+08	0.034986	486484	0.384354	Sepsis (28	Sepsis (28	Sepsis (28	TRUE	igd	0.0025	6.50E-07	7356	M32338.m	TRUE	reported	q5cuaP	2	TRUE	0.003496	25.79938
8368	rs1167464	C	G	C	G	-0.0381	-0.02313	0.1049	0.122975	FALSE	TRUE	FALSE	ieu-b-508	2	1.18E+08	0.05074	486484	0.648505	Sepsis (28	Sepsis (28	Sepsis (28	TRUE	igd	0.0075	3.46E-07	3248	M36756.m	TRUE	reported	QsW4oR	2	TRUE	0.007883	25.79051
6242	rs1713747	T	C	T	C	-0.0386	-0.03323	0.5085	0.500405	FALSE	FALSE	FALSE	ieu-b-508	7	17296072	0.033415	486484	0.320035	Sepsis (28	Sepsis (28	Sepsis (28	TRUE	igd	0.0076	3.86E-07	6756	M33935.m	TRUE	reported	ELBTDc	2	TRUE	0.003804	25.78807
3843	rs1714248	T	G	T	G	0.0391	-0.03307	0.0349	0.048895	FALSE	FALSE	FALSE	ieu-b-508	10	7145539	0.07696	486484	0.667369	Sepsis (28	Sepsis (28	Sepsis (28	TRUE	igd	0.0077	4.07E-07	5211	M32651.m	TRUE	reported	DeuPwv	2	TRUE	0.004924	25.7754
3420	rs1123954	A	T	A	T	-0.0132	-0.04833	0.2416	0.246501	FALSE	TRUE	FALSE	ieu-b-508	10	46012245	0.038669	486484	0.211324	Sepsis (28	Sepsis (28	Sepsis (28	TRUE	igd	0.0026	5.22E-07	7364	M32452.m	TRUE	reported	8GtFpl	2	TRUE	0.003488	25.76815
3945	rs1077177	A	G	A	G	0.0132	0.017416	0.5978	0.596348	FALSE	FALSE	FALSE	ieu-b-508	12	31008385	0.034042	486484	0.60892	Sepsis (28	Sepsis (28	Sepsis (28	TRUE	igd	0.0026	2.91E-07	7363	M32709.m	TRUE	reported	YR8SB4	2	TRUE	0.003488	25.76815
3907	rs9566165	T	C	T	C	0.0132	-0.0039	0.0679	0.065771	FALSE	FALSE	FALSE	ieu-b-508	13	37403981	0.067416	486484	0.95384	Sepsis (28	Sepsis (28	Sepsis (28	TRUE	igd	0.0026	4.47E-07	7338	M32675.m	TRUE	reported	9FmPh9	2	TRUE	0.0035	25.76812
5626	rs7090877	A	C	A	C	0.0132	-0.10485	0.0784	0.073146	FALSE	FALSE	FALSE	ieu-b-508	10	1.31E+08	0.064259	486484	0.102733	Sepsis (28	Sepsis (28	Sepsis (28	TRUE	igd	0.0026	2.56E-07	7336	M33442.m	TRUE	reported	B1Peb3	2	TRUE	0.003501	25.76812
1928	rs1333292	A	C	A	C	0.0132	-0.09296	0.2301	0.224186	FALSE	FALSE	FALSE	ieu-b-508	16	80396241	0.04023	486484	0.020844	Sepsis (28	Sepsis (28	Sepsis (28	TRUE	igd	0.0026	4.76E-07	6549	M16822.m	TRUE	reported	UNI018	2	TRUE	0.00392	25.76728
6845	rs1089202	A	G	A	G	0.1609	-0.04343	0.4724	0.458288	FALSE	FALSE	FALSE	ieu-b-508	11	98471532	0.033673	486484	0.197172	Sepsis (28	Sepsis (28	Sepsis (28	TRUE	igd	0.0299	7.01E-08	18	M34365.m	TRUE	reported	8eexD0	2	TRUE	0.616679	25.74051
8596	rs1178238	T	C	T	C	-0.0137	0.03962	0.1092	0.112483	FALSE	FALSE	FALSE	ieu-b-508	8	9201787	0.052531	486484	0.45071	Sepsis (28	Sepsis (28	Sepsis (28	TRUE	igd	0.0027	3.09E-07	7366	M37506.m	TRUE	reported	m2kaRt	2	TRUE	0.003483	25.73924
6558	rs2444830	T	C	T	C	0.0137	0.003581	0.6646	0.663869	FALSE	FALSE	FALSE	ieu-b-508	6	81341175	0.035198	486484	0.918963	Sepsis (28	Sepsis (28	Sepsis (28	TRUE	igd	0.0027	4.52E-07	7347	M34244.m	TRUE	reported	JyF0Sp	2	TRUE	0.003492	25.73922
3731	rs1511782	A	C	A	C	-0.0137	-0.00182	0.543	0.533714	FALSE	FALSE	FALSE	ieu-b-508	4	1.65E+08	0.033424	486484	0.956649	Sepsis (28	Sepsis (28	Sepsis (28	TRUE	igd	0.0027	3.74E-07	7337	M32632.m	TRUE	reported	plHzPT	2	TRUE	0.003497	25.73921
5416	rs588136	T	C	T	C	-0.0137	0.037664	0.7976	0.786715	FALSE	FALSE	FALSE	ieu-b-508	15	58730498	0.040887	486484	0.356949	Sepsis (28	Sepsis (28	Sepsis (28	TRUE	igd	0.0027	2.79E-07	7315	M33389.m	TRUE	reported	Hf6XVM	2	TRUE	0.003507	25.73919
7762	rs1220202	T	C	T	C	-0.0137	0.029604	0.7878	0.78863	FALSE	FALSE	FALSE	ieu-b-508	6	19351564	0.041237	486484	0.472819	Sepsis (28	Sepsis (28	Sepsis (28	TRUE	igd	0.0027	3.91E-07	7256	M35439.m	TRUE	reported	DnuAGZ	2	TRUE	0.003536	25.73913
2659	rs774211	T	C	T	C	0.0137	-0.02341	0.8303	0.833525	FALSE	FALSE	FALSE	ieu-b-508	12	56920939	0.044759	486484	0.600951	Sepsis (28	Sepsis (28	Sepsis (28	TRUE	igd	0.0027	5.19E-07	6517	M22649.m	TRUE	reported	NAzwRF	2	TRUE	0.003935	25.73833
5926	rs7231703	A	T	A	T	0.035	0.055386	0.3913	0.394624	FALSE	TRUE	FALSE	ieu-b-508	18	3329846	0.034236	486484	0.105714	Sepsis (28	Sepsis (28	Sepsis (28	TRUE	igd	0.0069	3.67E-07	4323	M33633.m	TRUE	reported	e36XGE	2	TRUE	0.005917	25.71798
4339	rs6723705	A	G	A	G	0.0411	0.044281	0.5871	0.616205	FALSE	FALSE	FALSE	ieu-b-508	2	2.13E+08	0.03436	486484	0.197488	Sepsis (28	Sepsis (28	Sepsis (28	TRUE	igd	0.0081	3.46E-07	1756	M32854.m	TRUE	reported	QgJDAK	2	TRUE	0.01445	25.7169
4159	rs1786376	A	G	A	G	-0.0705	0.064668	0.0292	0.029083	FALSE	FALSE	FALSE	ieu-b-508	2	2.35E+08	0.101288	486484	0.523176	Sepsis (28	Sepsis (28	Sepsis (28	TRUE	igd	0.0139	3.80E-07	6642	M32758.m	TRUE	reported	AF0m66	2	TRUE	0.003858	25.71685
1329	rs741382	T	C	T	C	-0.0472	0.039047	0.2234	0.197862	FALSE	FALSE	FALSE	ieu-b-508	2	2.18E+08	0.042013	486484	0.352679	Sepsis (28	Sepsis (28	Sepsis (28	TRUE	igd	0.0093	3.74E-07	1175	M02137.m	TRUE	reported	iRGqBE	2	TRUE	0.021452	25.71451
2959	rs2016976	T	C	T	C	-0.0142	0.039466	0.2607	0.264548	FALSE	FALSE	FALSE	ieu-b-508	1	2.15E+08	0.037868	486484	0.297311	Sepsis (28	Sepsis (28	Sepsis (28	TRUE	igd	0.0028	4.10E-07	6795	M30805.m	TRUE	reported	YHV70c	2	TRUE	0.003771	25.71182
6631	rs1105943	T	C	T	C	0.3901	0.02448	0.0133	0.012389	FALSE	FALSE	FALSE	ieu-b-508	12	1.29E+08	0.160949	486484	0.87911	Sepsis (28	Sepsis (28	Sepsis (28	TRUE	igd	0.0759	2.74E-07	75	M34322.m	TRUE	reported	16GdG9	2	TRUE	0.260472	25.71165
8636	rs4656083	A	G	A	G	0.0345	-0.01751	0.4386	0.436457	FALSE	FALSE	FALSE	ieu-b-508	1	89510106	0.033563	486484	0.601808	Sepsis (28	Sepsis (28	Sepsis (28	TRUE	igd	0.0068	4.09E-07	1765	M38150.m	TRUE	reported	u3Ckmr	2	TRUE	0.014374	25.71153
2719	rs1015126	T	C	T	C	0.0142	0.004202	0.3406	0.343971	FALSE	FALSE	FALSE	ieu-b-508	14	63061920	0.035225	486484	0.905056	Sepsis (28	Sepsis (28	Sepsis (28	TRUE	igd	0.0028	5.77E-07	6388	M25599.m	TRUE	reported	fRZxwP	2	TRUE	0.00401	25.71134
4992	rs6066168	A	G	A	G	-0.3223	-0.02149	0.0218	0.028326	FALSE	FALSE	FALSE	ieu-b-508	20	45655123	0.099689	486484	0.829299	Sepsis (28	Sepsis (28	Sepsis (28	TRUE	igd	0.0635	3.82E-07	1017	M33178.m	TRUE	reported	g22WMS	2	TRUE	0.024705	25.71096
7059	rs918174	T	C	T	C	-0.0142	0.036956	0.687	0.690958	FALSE	FALSE	FALSE	ieu-b-508	18	3736629	0.036826	486484	0.315606	Sepsis (28	Sepsis (28	Sepsis (28	TRUE	igd	0.0028	5.61E-07	5207	M34456.m	TRUE	reported	Nh457q	2	TRUE	0.004915	25.70951
4577	rs1110732	C	G	C	G	0.036	0.021069	0.9536	0.9576	FALSE	TRUE	FALSE	ieu-b-508	12	94405399	0.082966	486484	0.799535	Sepsis (28	Sepsis (28	Sepsis (28	TRUE	igd	0.0071	3.70E-07	6404	M33163.m	TRUE	reported	YzGXkR	2	TRUE	0.003998	25.70116
6184	rs1093836	A	C	A	C	0.0147	-0.02756	0.4014	0.388517	FALSE	FALSE	FALSE	ieu-b-508	4	44794259	0.034375	486484	0.422695	Sepsis (28	Sepsis (28	Sepsis (28	TRUE	igd	0.0029	4.12E-07	6004	M33892.m	TRUE	reported	w7HgU7	2	TRUE	0.004261	25.68585
5098	rs9401158	A	G	A	G	-0.2648	0.134242	0.0266	0.08823	FALSE	FALSE	FALSE	ieu-b-508	6	1.2E+08	0.059498	486484	0.024055	Sepsis (28	Sepsis (28	Sepsis (28	TRUE	igd	0.0522	3.91E-07	1017	M33178.m	TRUE	reported	g22WMS	2	TRUE	0.024679	25.68266
6706	rs893712	T	C	T	C	-0.0152	0.02101	0.7669	0.758968	FALSE	FALSE	FALSE	ieu-b-508	5	1.25E+08	0.038961	486484	0.589708	Sepsis (28	Sepsis (28	Sepsis (28	TRUE	igd	0.003	2.99E-07	5081	M34336.m	TRUE	reported	jrSSxL	2	TRUE	0.005027	25.66101
3189	rs4761007	T	C	T	C	0.0157	-0.098	0.7354	0.727683	FALSE	FALSE	FALSE	ieu-b-508	12	1.28E+08	0.037469	486484	0.008909	Sepsis (28	Sepsis (28	Sepsis (28	TRUE	igd	0.0031	4.13E-07	7356	M32338.m	TRUE	reported	q5cuaP	2	TRUE	0.003475	25.64235
8641	rs1339080	A	G	A	G	0.0395	0.109007	0.0557	0.06942	FALSE	FALSE	FALSE	ieu-b-508	2	1.25E+08	0.065944	486484	0.098326	Sepsis (28	Sepsis (28	Sepsis (28	TRUE	igd	0.0078	4.12E-07	7219	M38178.m	TRUE	reported	GgCXMN	2	TRUE	0.00354	25.63803
930	rs1708260	T	C	T	C	0.0476	-0.07064	0.0193	0.017644	FALSE	FALSE	FALSE	ieu-b-508	13	26228314	0.127602	486484	0.57984	Sepsis (28	Sepsis (28	Sepsis (28	TRUE	igd	0.0094	4.08E-07	7160	M01508.m	TRUE	reported	go526k	2	TRUE	0.003569	25.63521
5874	rs7239639	T	C	T	C	-0.254	0.033413	0.938	0.723124	FALSE	FALSE	FALSE	ieu-b-508	18	70498620	0.037241	486484	0.369596	Sepsis (28	Sepsis (28	Sepsis (28	TRUE	igd	0.05	3.77E-07	296	M33609.m	TRUE	reported	h7B9gN	2	TRUE	0.080192	25.63203
7791	rs1293588	A	G	A	G	0.381	-0.01671	0.0392	0.039667	FALSE	FALSE	FALSE	ieu-b-508	17	77317764	0.085976	486484	0.845863	Sepsis (28	Sepsis (28	Sepsis (28	TRUE	igd	0.075	3.72E-07	284	M35451.m	TRUE	reported	GMRGAN	2	TRUE	0.083298	25.62466
6736	rs207703	T	C	T	C	0.2845	-0.07911	0.9769	0.958898	FALSE	FALSE	FALSE	ieu-b-508	4	16828794	0.085017	486484	0.352078	Sepsis (28	Sepsis (28	Sepsis (28	TRUE	igd	0.0561	3.94E-07	535	M34338.m	TRUE	reported	h4FB9a	2	TRUE	0.045866	25.62195
7795	rs2663644	A	G	A	G	0.5247	0.001743	0.0123	0.012985	FALSE	FALSE	FALSE	ieu-b-508	2	2.1E+08	0.149887	486484	0.990723	Sepsis (28	Sepsis (28	Sepsis (28	TRUE	igd	0.1033	3.81E-07	284	M35451.m	TRUE	reported	GMRGAN	2	TRUE	0.08328	25.61841
5370	rs1052118	T	C	T	C	-0.3993	-0.08689	0.9682	0.964675	FALSE	FALSE	FALSE	ieu-b-508	17	11249920	0.090736	486484	0.338233	Sepsis (28	Sepsis (28	Sepsis (28	TRUE	igd	0.0786	3.71E-07	272	M33384.m	TRUE	reported	IOffrZ	2	TRUE	0.08666	25.61819
4337	rs1042830	T	C	T	C	0.0324	-0.04633	0.6101	0.620065	FALSE	FALSE	FALSE	ieu-b-508	4	1.43E+08	0.034524	486484	0.179645	Sepsis (28	Sepsis (28	Sepsis (28	TRUE	igd	0.0064	4.64E-07	2684	M32854.m	TRUE	reported	QgJDAK	2	TRUE	0.009458	25.60981
5883	rs1572704	A	G	A	G	0.3904	0.060944	0.0272	0.028939	FALSE	FALSE	FALSE	ieu-b-508	1	2.38E+08	0.099889	486484	0.541784	Sepsis (28	Sepsis (28	Sepsis (28	TRUE	igd	0.0764	3.29E-07	102	M33610.m	TRUE	reported	tmg43v	2	TRUE	0.203819	25.59957
1565	rs4632709	T	C	T	C	-0.1504	-0.08587	0.95	0.943017	FALSE	FALSE	FALSE	ieu-b-508	4	1.59E+08	0.072399	486484	0.23561	Sepsis (28	Sepsis (28	Sepsis (28	TRUE	igd	0.0297	4.00E-07	963	M12261.m	TRUE	reported	taUUcM	2	TRUE	0.025938	25.59056
1622	rs1085731	T	C	T	C	-0.0086	0.045863	0.3481	0.351072	FALSE	FALSE	FALSE	ieu-b-508	4	1.6E+08	0.034967	486484	0.189657	Sepsis (28	Sepsis (28	Sepsis (28	TRUE	igd	0.0017	9.45E-07	7368	M15140.m	TRUE	reported	jEcqMJ	2	TRUE	0.003461	25.58475
1626	rs1164684	A	G	A	G	-0.0086	0.033099	0.5005	0.501563	FALSE	FALSE	FALSE	ieu-b-508	16	82817251	0.033376	486484	0.321347	Sepsis (28	Sepsis (28	Sepsis (28	TRUE	igd	0.0017	6.40E-07	7368	M15140.m	TRUE	reported	jEcqMJ	2	TRUE	0.003461	25.58475
8621	rs6043498	T	G	T	G	-0.0172	0.007772	0.0688	0.078511	FALSE	FALSE	FALSE	ieu-b-508	20	15716517	0.062112	486484	0.900419	Sepsis (28	Sepsis (28	Sepsis (28	TRUE	igd	0.0034	4.81E-07	7366	M37506.m	TRUE	reported	m2kaRt	2	TRUE	0.003462	25.58475
7409	rs1004436	T	C	T	C	0.0172	0.008864	0.763	0.761149	FALSE	FALSE	FALSE	ieu-b-508	5	52171635	0.03918	486484	0.821012	Sepsis (28	Sepsis (28	Sepsis (28	TRUE	igd	0.0034	3.26E-07	7350	M35186.m	TRUE	reported	UZfu6Z	2	TRUE	0.00347	25.58473
3141	rs7022371	T	C	T	C	0.0086	-0.01316	0.5008	0.497788	FALSE	FALSE	FALSE	ieu-b-508	9	1.15E+08	0.03333	486484	0.692988	Sepsis (28	Sepsis (28	Sepsis (28	TRUE	igd	0.0017	6.54E-07	7349	M32315.m	TRUE	reported	4igzkL	2	TRUE	0.00347	25.58473
7707	rs2090103	A	G	A	G	-0.0086	-0.01524	0.2395	0.224078	FALSE	FALSE	FALSE	ieu-b-508	4	1.28E+08	0.039917	486484	0.702567	Sepsis (28	Sepsis (28	Sepsis (28	TRUE	igd	0.0017	3.37E-07	7345	M35397.m	TRUE	reported	Y56mZr	2	TRUE	0.003472	25.58473
4452	rs549114	A	G	A	G	0.0086	0.038354	0.3334	0.333023	FALSE	FALSE	FALSE	ieu-b-508	1	94534354	0.035441	486484	0.279174	Sepsis (28	Sepsis (28	Sepsis (28	TRUE	igd	0.0017	7.57E-07	7344	M32910.m	TRUE	reported	2OCBKh	2	TRUE	0.003473	25.58473
1279	rs1270126	A	G	A	G	0.0086	0.033621	0.3919	0.394675	FALSE	FALSE	FALSE	ieu-b-508	7	32971236	0.034088	486484	0.323984	Sepsis (28	Sepsis (28	Sepsis (28	TRUE	igd	0.0017	7.37E-07	7325	M02132.m	TRUE	reported	uJFm2t	2	TRUE	0.003482	25.58471
1313	rs879126	T	G	T	G	-0.0086	-0.00547	0.3286	0.334996	FALSE	FALSE	FALSE	ieu-b-508	2	2.08E+08	0.035457	486484	0.877375	Sepsis (28	Sepsis (28	Sepsis (28	TRUE	igd	0.0017	7.47E-07	7325	M02132.m	TRUE	reported	uJFm2t	2	TRUE	0.003482	25.58471
4188	rs2954021	A	G	A	G	0.0172	0.022264	0.5064	0.495425	FALSE	FALSE	FALSE	ieu-b-508	8	1.26E+08	0.033354	486484	0.50445	Sepsis (28	Sepsis (28	Sepsis (28	TRUE	igd	0.0034	3.45E-07	7313	M32761.m	TRUE	reported	QefVGc	2	TRUE	0.003487	25.5847
1063	rs1554074	A	G	A	G	0.0086	0.036216	0.1925	0.191246	FALSE	FALSE	FALSE	ieu-b-508	12	1.01E+08	0.042189	486484	0.390651	Sepsis (28	Sepsis (28	Sepsis (28	TRUE	igd	0.0017	6.39E-07	7271	M01585.m	TRUE	reported	M7QiSM	2	TRUE	0.003507	25.58466
1254	rs4717222	A	C	A	C	-0.0086	-0.06772	0.4686	0.463741	FALSE	FALSE	FALSE	ieu-b-508	7	64340294	0.033394	486484	0.042573	Sepsis (28	Sepsis (28	Sepsis (28	TRUE	igd	0.0017	4.34E-07	7136	M01769.m	TRUE	reported	rismUZ	2	TRUE	0.003573	25.58452
7797	rs2850065	T	C	T	C	0.5411	-0.00052	0.0146	0.462603	FALSE	FALSE	FALSE	ieu-b-508	21	37337880	0.033436	486484	0.987523	Sepsis (28	Sepsis (28	Sepsis (28	TRUE	igd	0.1066	3.87E-07	284	M35451.m	TRUE	reported	GMRGAN	2	TRUE	0.083178	25.58418
658	rs1043011	T	G	T	G	0.0172	-0.01013	0.1722	0.182598	FALSE	FALSE	FALSE	ieu-b-508	12	56865040	0.043138	486484	0.8143	Sepsis (28	Sepsis (28	Sepsis (28	TRUE	igd	0.0034	5.21E-07	5687	M01284.m	TRUE	reported	E4hc9c	2	TRUE	0.00448	25.5827
5202	rs1866724	T	C	T	C	-0.0688	0.033297	0.7129	0.70999	FALSE	FALSE	FALSE	ieu-b-508	8	2138234	0.036854	486484	0.366268	Sepsis (28	Sepsis (28	Sepsis (28	TRUE	igd	0.0136	4.25E-07	3221	M33203.m	TRUE	reported	Sto7pR	2	TRUE	0.007883	25.5758
2782	rs6012418	A	G	A	G	-0.0177	0.14071	0.0853	0.08379	FALSE	FALSE	FALSE	ieu-b-508	20	47036502	0.060335	486484	0.019693	Sepsis (28	Sepsis (28	Sepsis (28	TRUE	igd	0.0035	3.82E-07	7268	M27273.m	TRUE	reported	EvU2od	2	TRUE	0.003506	25.56766
6936	rs202534	A	G	A	G	-0.042	-0.00404	0.5576	0.495962	FALSE	FALSE	FALSE	ieu-b-508	20	1667860	0.033364	486484	0.903682	Sepsis (28	Sepsis (28	Sepsis (28	TRUE	igd	0.0083	3.65E-07	1104	M34395.m	TRUE	reported	QVOPA3	2	TRUE	0.022668	25.55965
1631	rs1496635	A	C	A	C	-0.0091	-0.03508	0.3407	0.328944	FALSE	FALSE	FALSE	ieu-b-508	19	31692461	0.035469	486484	0.322667	Sepsis (28	Sepsis (28	Sepsis (28	TRUE	igd	0.0018	2.12E-07	7368	M15140.m	TRUE	reported	jEcqMJ	2	TRUE	0.003457	25.5517
1025	rs7105815	A	G	A	G	0.0091	-0.02991	0.2968	0.29715	FALSE	FALSE	FALSE	ieu-b-508	11	12309693	0.036447	486484	0.41191	Sepsis (28	Sepsis (28	Sepsis (28	TRUE	igd	0.0018	4.93E-07	7364	M01564.m	TRUE	reported	IPUdyD	2	TRUE	0.003459	25.5517
3465	rs1683282	T	C	T	C	-0.0091	0.02967	0.6632	0.655572	FALSE	FALSE	FALSE	ieu-b-508	1	30197407	0.035474	486484	0.402929	Sepsis (28	Sepsis (28	Sepsis (28	TRUE	igd	0.0018	5.52E-07	7363	M32489.m	TRUE	reported	hnljaD	2	TRUE	0.003459	25.5517
3787	rs981735	T	C	T	C	0.0091	0.021773	0.759	0.758285	FALSE	FALSE	FALSE	ieu-b-508	8	1.1E+08	0.038986	486484	0.576505	Sepsis (28	Sepsis (28	Sepsis (28	TRUE	igd	0.0018	6.08E-07	7362	M32634.m	TRUE	reported	7T2RUz	2	TRUE	0.00346	25.5517
3504	rs3795579	T	G	T	G	0.0091	0.002049	0.3479	0.344558	FALSE	FALSE	FALSE	ieu-b-508	1	2.04E+08	0.035093	486484	0.953444	Sepsis (28	Sepsis (28	Sepsis (28	TRUE	igd	0.0018	3.69E-07	7355	M32492.m	TRUE	reported	rZZFcE	2	TRUE	0.003463	25.55169
644	rs1618996	A	G	A	G	0.0091	-0.02433	0.1298	0.133278	FALSE	FALSE	FALSE	ieu-b-508	8	13185926	0.049545	486484	0.623356	Sepsis (28	Sepsis (28	Sepsis (28	TRUE	igd	0.0018	3.78E-07	7352	M01125.m	TRUE	reported	yw0clB	2	TRUE	0.003464	25.55169
2696	rs1312792	A	G	A	G	-0.0091	0.024056	0.5059	0.501377	FALSE	FALSE	FALSE	ieu-b-508	4	1.18E+08	0.033706	486484	0.475413	Sepsis (28	Sepsis (28	Sepsis (28	TRUE	igd	0.0018	4.90E-07	7340	M25459.m	TRUE	reported	ACJY35	2	TRUE	0.00347	25.55168
2704	rs4697525	T	C	T	C	-0.0091	0.045483	0.546	0.543121	FALSE	FALSE	FALSE	ieu-b-508	4	25018625	0.033564	486484	0.175379	Sepsis (28	Sepsis (28	Sepsis (28	TRUE	igd	0.0018	5.60E-07	7340	M25459.m	TRUE	reported	ACJY35	2	TRUE	0.00347	25.55168
5512	rs119194	A	C	A	C	-0.0091	0.038894	0.2105	0.216209	FALSE	FALSE	FALSE	ieu-b-508	6	4569275	0.040661	486484	0.338805	Sepsis (28	Sepsis (28	Sepsis (28	TRUE	igd	0.0018	4.33E-07	7306	M33422.m	TRUE	reported	PLgjrT	2	TRUE	0.003486	25.55165
5524	rs2298381	C	G	C	G	-0.0091	-0.05504	0.2267	0.209519	FALSE	TRUE	FALSE	ieu-b-508	22	24726752	0.0409	486484	0.17843	Sepsis (28	Sepsis (28	Sepsis (28	TRUE	igd	0.0018	5.23E-07	7306	M33422.m	TRUE	reported	PLgjrT	2	TRUE	0.003486	25.55165
2754	rs2531899	A	G	A	G	-0.0091	0.090741	0.1941	0.196227	FALSE	FALSE	FALSE	ieu-b-508	17	58007193	0.041832	486484	0.030068	Sepsis (28	Sepsis (28	Sepsis (28	TRUE	igd	0.0018	4.86E-07	7303	M27256.m	TRUE	reported	mJwzsi	2	TRUE	0.003488	25.55164
7555	rs1197907	A	G	A	G	-0.0091	0.005289	0.7762	0.76344	FALSE	FALSE	FALSE	ieu-b-508	7	1.57E+08	0.039231	486484	0.892749	Sepsis (28	Sepsis (28	Sepsis (28	TRUE	igd	0.0018	5.12E-07	7217	M35270.m	TRUE	reported	Ct8V1n	2	TRUE	0.003529	25.55156
7560	rs1462120	T	C	T	C	0.0182	0.013523	0.0656	0.05154	FALSE	FALSE	FALSE	ieu-b-508	5	1.53E+08	0.075426	486484	0.857711	Sepsis (28	Sepsis (28	Sepsis (28	TRUE	igd	0.0036	3.28E-07	7217	M35270.m	TRUE	reported	Ct8V1n	2	TRUE	0.003529	25.55156
7575	rs484080	T	C	T	C	-0.0182	0.05082	0.0506	0.083159	FALSE	FALSE	FALSE	ieu-b-508	6	1.04E+08	0.060363	486484	0.399835	Sepsis (28	Sepsis (28	Sepsis (28	TRUE	igd	0.0036	5.87E-07	7217	M35270.m	TRUE	reported	Ct8V1n	2	TRUE	0.003529	25.55156
7586	rs852030	A	G	A	G	0.0091	-0.02816	0.1953	0.199167	FALSE	FALSE	FALSE	ieu-b-508	20	17084107	0.042019	486484	0.502719	Sepsis (28	Sepsis (28	Sepsis (28	TRUE	igd	0.0018	6.32E-07	7217	M35270.m	TRUE	reported	Ct8V1n	2	TRUE	0.003529	25.55156
1513	rs1282625	T	C	T	C	0.0091	0.038536	0.1364	0.144722	FALSE	FALSE	FALSE	ieu-b-508	12	1.31E+08	0.047657	486484	0.418741	Sepsis (28	Sepsis (28	Sepsis (28	TRUE	igd	0.0018	7.05E-07	7081	M12067.m	TRUE	reported	O5x7RD	2	TRUE	0.003596	25.55142
1520	rs1714533	T	C	T	C	-0.0091	0.03148	0.8525	0.851319	FALSE	FALSE	FALSE	ieu-b-508	7	21808198	0.046852	486484	0.501646	Sepsis (28	Sepsis (28	Sepsis (28	TRUE	igd	0.0018	3.58E-07	7081	M12067.m	TRUE	reported	O5x7RD	2	TRUE	0.003596	25.55142
1522	rs1864609	A	C	A	C	-0.0091	0.081327	0.8606	0.860305	FALSE	FALSE	FALSE	ieu-b-508	7	1.37E+08	0.048278	486484	0.092077	Sepsis (28	Sepsis (28	Sepsis (28	TRUE	igd	0.0018	3.27E-07	7081	M12067.m	TRUE	reported	O5x7RD	2	TRUE	0.003596	25.55142
5815	rs2280821	T	G	T	G	0.0091	0.020358	0.508	0.527502	FALSE	FALSE	FALSE	ieu-b-508	8	1897900	0.033477	486484	0.54312	Sepsis (28	Sepsis (28	Sepsis (28	TRUE	igd	0.0018	6.54E-07	7066	M33515.m	TRUE	reported	X9mul1	2	TRUE	0.003604	25.55141
1404	rs8013373	A	G	A	G	-0.0091	0.030316	0.3428	0.358602	FALSE	FALSE	FALSE	ieu-b-508	14	63072399	0.034878	486484	0.384741	Sepsis (28	Sepsis (28	Sepsis (28	TRUE	igd	0.0018	4.99E-07	7037	M02734.m	TRUE	reported	0y0dDD	2	TRUE	0.003619	25.55138
8425	rs6538186	T	C	T	C	0.0091	0.06384	0.2552	0.310201	FALSE	FALSE	FALSE	ieu-b-508	12	77848715	0.036101	486484	0.077003	Sepsis (28	Sepsis (28	Sepsis (28	TRUE	igd	0.0018	1.95E-07	6940	M36808.m	TRUE	reported	u7lqaj	2	TRUE	0.003669	25.55128
6808	rs1757545	A	C	A	C	-0.0091	0.03031	0.3094	0.305519	FALSE	FALSE	FALSE	ieu-b-508	2	76624220	0.03646	486484	0.405788	Sepsis (28	Sepsis (28	Sepsis (28	TRUE	igd	0.0018	3.57E-07	6755	M34359.m	TRUE	reported	0UotaJ	2	TRUE	0.003769	25.55107
5288	rs1205044	A	T	A	T	-0.044	-0.01226	0.656	0.637036	FALSE	TRUE	FALSE	ieu-b-508	14	42955506	0.034699	486484	0.723899	Sepsis (28	Sepsis (28	Sepsis (28	TRUE	igd	0.0087	4.10E-07	1273	M33353.m	TRUE	reported	ZIN4gk	2	TRUE	0.019697	25.53783
2890	rs1145077	T	G	T	G	0.0187	-0.02343	0.3769	0.373291	FALSE	FALSE	FALSE	ieu-b-508	15	45683795	0.03445	486484	0.496366	Sepsis (28	Sepsis (28	Sepsis (28	TRUE	igd	0.0037	3.99E-07	7373	M27718.m	TRUE	reported	PHL57z	2	TRUE	0.003452	25.53653
1864	rs9394526	A	G	A	G	-0.0187	-0.06768	0.0529	0.057822	FALSE	FALSE	FALSE	ieu-b-508	6	12090563	0.071793	486484	0.345801	Sepsis (28	Sepsis (28	Sepsis (28	TRUE	igd	0.0037	4.28E-07	7308	M15778.m	TRUE	reported	941o3I	2	TRUE	0.003483	25.53647
4249	rs7520193	A	C	A	C	0.0283	-0.01894	0.1862	0.187765	FALSE	FALSE	FALSE	ieu-b-508	1	68393522	0.04285	486484	0.658455	Sepsis (28	Sepsis (28	Sepsis (28	TRUE	igd	0.0056	4.55E-07	6169	M32795.m	TRUE	reported	0YBWkh	2	TRUE	0.004123	25.5303
8001	rs1696215	T	C	T	C	-0.0192	-0.02457	0.9219	0.916287	FALSE	FALSE	FALSE	ieu-b-508	16	13592059	0.060438	486484	0.684313	Sepsis (28	Sepsis (28	Sepsis (28	TRUE	igd	0.0038	4.57E-07	6604	M35754.m	TRUE	reported	IlXOo1	2	TRUE	0.003851	25.52135
2565	rs4434479	A	G	A	G	0.0096	0.01898	0.2901	0.287868	FALSE	FALSE	FALSE	ieu-b-508	6	96289803	0.037056	486484	0.608517	Sepsis (28	Sepsis (28	Sepsis (28	TRUE	igd	0.0019	8.84E-07	6535	M22177.m	TRUE	reported	2s6Sly	2	TRUE	0.003891	25.52127
3994	rs2243498	A	G	A	G	0.0096	0.009983	0.4479	0.444079	FALSE	FALSE	FALSE	ieu-b-508	21	34653666	0.033776	486484	0.76757	Sepsis (28	Sepsis (28	Sepsis (28	TRUE	igd	0.0019	2.95E-07	6497	M22729.m	TRUE	reported	XGRSN1	2	TRUE	0.003914	25.52123
5547	rs1158943	T	C	T	C	-1.6514	0.13818	0.025	0.037741	FALSE	FALSE	FALSE	ieu-b-508	1	1.46E+08	0.087033	486484	0.11236	Sepsis (28	Sepsis (28	Sepsis (28	TRUE	igd	0.3214	2.77E-07	60	M33423.m	TRUE	reported	MoEnO7	2	TRUE	0.30556	25.52052
7369	rs6026567	A	G	A	G	-0.0096	-0.01243	0.5792	0.575419	FALSE	FALSE	FALSE	ieu-b-508	20	57444915	0.033995	486484	0.714582	Sepsis (28	Sepsis (28	Sepsis (28	TRUE	igd	0.0019	5.87E-07	4900	M35137.m	TRUE	reported	Eq2siS	2	TRUE	0.005183	25.51867
4245	rs1766086	A	G	A	G	0.175	-0.09628	0.93	0.967172	FALSE	FALSE	FALSE	ieu-b-508	9	4292848	0.093437	486484	0.302818	Sepsis (28	Sepsis (28	Sepsis (28	TRUE	igd	0.0346	4.37E-07	798	M32795.m	TRUE	reported	0YBWkh	2	TRUE	0.031061	25.51726
5996	rs6823047	A	C	A	C	-0.0982	0.039179	0.7299	0.710925	FALSE	FALSE	FALSE	ieu-b-508	4	14326193	0.036949	486484	0.288981	Sepsis (28	Sepsis (28	Sepsis (28	TRUE	igd	0.0194	3.91E-07	477	M33683.m	TRUE	reported	wSozpU	2	TRUE	0.050977	25.51495

5872	rs548027	A	G	A	G	0.2591	-0.10821	0.0208	0.015455	FALSE	FALSE	FALSE	ieu-b-508	15	53390144	0.135559	486484	0.424711	Sepsis (28	Sepsis (28	Sepsis (28	TRUE	igd	0.0512	4.17E-07	528	M33609.m	TRUE	reported	h7B9gN	2	TRUE	0.046259	25.51213
8012	rs4852787	A	C	A	C	-0.0101	-0.00766	0.4642	0.470365	FALSE	FALSE	FALSE	ieu-b-508	2	71706697	0.033399	486484	0.818541	Sepsis (28	Sepsis (28	Sepsis (28	TRUE	igd	0.002	2.86E-07	6604	M35754.m	TRUE	reported	IlXOo1	2	TRUE	0.003847	25.49478
3872	rs2269340	T	C	T	C	-0.0222	0.050927	0.9246	0.920577	FALSE	FALSE	FALSE	ieu-b-508	1	1.1E+08	0.061689	486484	0.409059	Sepsis (28	Sepsis (28	Sepsis (28	TRUE	igd	0.0044	3.58E-07	7361	M32654.m	TRUE	reported	6lSQOP	2	TRUE	0.003446	25.44969
1365	rs7068278	T	G	T	G	0.0116	-0.01587	0.1968	0.199327	FALSE	FALSE	FALSE	ieu-b-508	10	99357142	0.041874	486484	0.704617	Sepsis (28	Sepsis (28	Sepsis (28	TRUE	igd	0.0023	6.55E-07	7226	M02730.m	TRUE	reported	XYa035	2	TRUE	0.003508	25.42963
4593	rs1012601	C	G	C	G	0.056	-0.00807	0.8372	0.848971	FALSE	TRUE	FALSE	ieu-b-508	4	1.06E+08	0.046811	486484	0.863136	Sepsis (28	Sepsis (28	Sepsis (28	TRUE	igd	0.0111	4.87E-07	1751	M33165.m	TRUE	reported	RZt3uS	2	TRUE	0.014328	25.42341
6173	rs335952	T	C	T	C	-0.0494	-0.02979	0.1902	0.191294	FALSE	FALSE	FALSE	ieu-b-508	5	16391881	0.042429	486484	0.482604	Sepsis (28	Sepsis (28	Sepsis (28	TRUE	igd	0.0098	4.05E-07	4999	M33885.m	TRUE	reported	uCOy6o	2	TRUE	0.005057	25.39966
8543	rs7794170	T	G	T	G	0.0378	-0.00531	0.2421	0.243484	FALSE	FALSE	FALSE	ieu-b-508	7	99840461	0.038795	486484	0.891105	Sepsis (28	Sepsis (28	Sepsis (28	TRUE	igd	0.0075	4.34E-07	6934	M37190.m	TRUE	reported	tiNuK9	2	TRUE	0.00365	25.39427
7810	rs398172	A	G	A	G	-0.0126	0.026927	0.3361	0.339449	FALSE	FALSE	FALSE	ieu-b-508	5	1.29E+08	0.03519	486484	0.444158	Sepsis (28	Sepsis (28	Sepsis (28	TRUE	igd	0.0025	4.04E-07	6883	M35464.m	TRUE	reported	lr9dHp	2	TRUE	0.003677	25.39422
6127	rs2826321	A	G	A	G	-0.5286	0.095151	0.9847	0.983491	FALSE	FALSE	FALSE	ieu-b-508	21	21859776	0.131287	486484	0.4686	Sepsis (28	Sepsis (28	Sepsis (28	TRUE	igd	0.1043	4.00E-07	176	M33877.m	TRUE	reported	yDVi1f	2	TRUE	0.127354	25.39348
2789	rs1189070	T	C	T	C	-0.0257	0.033922	0.9322	0.929784	FALSE	FALSE	FALSE	ieu-b-508	2	2.35E+08	0.065625	486484	0.605215	Sepsis (28	Sepsis (28	Sepsis (28	TRUE	igd	0.0051	4.83E-07	7347	M27278.m	TRUE	reported	kPgqm3	2	TRUE	0.003444	25.38678
8144	rs478259	A	G	A	G	-0.0393	0.041258	0.858	0.859624	FALSE	FALSE	FALSE	ieu-b-508	3	1.05E+08	0.048125	486484	0.391266	Sepsis (28	Sepsis (28	Sepsis (28	TRUE	igd	0.0078	5.27E-07	7311	M36103.m	TRUE	reported	sjeaPC	2	TRUE	0.00346	25.37915
4648	rs7863822	T	C	T	C	-1.0516	-0.03208	0.0132	0.025949	FALSE	FALSE	FALSE	ieu-b-508	9	1.2E+08	0.104678	486484	0.759256	Sepsis (28	Sepsis (28	Sepsis (28	TRUE	igd	0.206	3.32E-07	76	M33173.m	TRUE	reported	04fkUQ	2	TRUE	0.255337	25.37376
7313	rs1095462	T	C	T	C	-0.0252	0.0159	0.3553	0.380898	FALSE	FALSE	FALSE	ieu-b-508	2	1.85E+08	0.034346	486484	0.643416	Sepsis (28	Sepsis (28	Sepsis (28	TRUE	igd	0.005	4.13E-07	1762	M35127.m	TRUE	reported	RNsmjl	2	TRUE	0.014211	25.37277
3447	rs9399110	T	C	T	C	0.0267	-0.05077	0.0497	0.049602	FALSE	FALSE	FALSE	ieu-b-508	6	99724096	0.076851	486484	0.508862	Sepsis (28	Sepsis (28	Sepsis (28	TRUE	igd	0.0053	4.29E-07	7364	M32452.m	TRUE	reported	8GtFpl	2	TRUE	0.003434	25.37189
436	rs6511720	T	G	T	G	-0.0136	-0.02197	0.1197	0.118817	FALSE	FALSE	FALSE	ieu-b-508	19	11202306	0.051583	486484	0.670114	Sepsis (28	Sepsis (28	Sepsis (28	TRUE	igd	0.0027	3.69E-07	7365	M00063.m	TRUE	reported	Hdsv1L	2	TRUE	0.003433	25.36485
893	rs1170186	A	C	A	C	-0.0136	-0.04989	0.1761	0.192476	FALSE	FALSE	FALSE	ieu-b-508	21	43556732	0.042334	486484	0.238575	Sepsis (28	Sepsis (28	Sepsis (28	TRUE	igd	0.0027	2.75E-07	7307	M01493.m	TRUE	reported	eLLRt7	2	TRUE	0.00346	25.3648
2376	rs1544542	A	C	A	C	0.0136	-0.00044	0.4642	0.466273	FALSE	FALSE	FALSE	ieu-b-508	12	1.27E+08	0.033547	486484	0.989595	Sepsis (28	Sepsis (28	Sepsis (28	TRUE	igd	0.0027	5.40E-07	6999	M21127.m	TRUE	reported	WCjhS3	2	TRUE	0.003612	25.36449
5456	rs1336286	T	C	T	C	-0.1665	0.184871	0.9493	0.944971	FALSE	FALSE	FALSE	ieu-b-508	10	83952605	0.073716	486484	0.012146	Sepsis (28	Sepsis (28	Sepsis (28	TRUE	igd	0.033	4.54E-07	528	M33391.m	TRUE	reported	EgMd1b	2	TRUE	0.045996	25.36019
8080	rs2243168	A	T	A	T	0.0524	-0.00858	0.9071	0.915943	FALSE	TRUE	FALSE	ieu-b-508	1	2.07E+08	0.060329	486484	0.886884	Sepsis (28	Sepsis (28	Sepsis (28	TRUE	igd	0.0104	4.88E-07	1743	M36009.m	TRUE	reported	2EUZ1U	2	TRUE	0.014356	25.35697
2953	rs1252264	T	C	T	C	-0.0141	-0.05275	0.7914	0.723036	FALSE	FALSE	FALSE	ieu-b-508	5	55741171	0.037512	486484	0.15964	Sepsis (28	Sepsis (28	Sepsis (28	TRUE	igd	0.0028	7.20E-07	6795	M30805.m	TRUE	reported	YHV70c	2	TRUE	0.003718	25.35095
2962	rs559135	T	C	T	C	-0.0141	-0.00054	0.2066	0.200674	FALSE	FALSE	FALSE	ieu-b-508	9	1.2E+08	0.041844	486484	0.989693	Sepsis (28	Sepsis (28	Sepsis (28	TRUE	igd	0.0028	6.72E-07	6795	M30805.m	TRUE	reported	YHV70c	2	TRUE	0.003718	25.35095
2734	rs6681770	A	G	A	G	-0.0141	-0.01271	0.762	0.755445	FALSE	FALSE	FALSE	ieu-b-508	1	2.43E+08	0.038978	486484	0.744434	Sepsis (28	Sepsis (28	Sepsis (28	TRUE	igd	0.0028	3.91E-07	6388	M25599.m	TRUE	reported	fRZxwP	2	TRUE	0.003954	25.35048
7051	rs6049700	T	G	T	G	-0.0141	0.017286	0.2893	0.291826	FALSE	FALSE	FALSE	ieu-b-508	20	24430014	0.036913	486484	0.639579	Sepsis (28	Sepsis (28	Sepsis (28	TRUE	igd	0.0028	4.95E-07	5207	M34456.m	TRUE	reported	Nh457q	2	TRUE	0.004846	25.34868
4457	rs7748892	A	G	A	G	0.0287	-0.03213	0.9721	0.965703	FALSE	FALSE	FALSE	ieu-b-508	6	1.37E+08	0.09351	486484	0.731144	Sepsis (28	Sepsis (28	Sepsis (28	TRUE	igd	0.0057	4.30E-07	7344	M32910.m	TRUE	reported	2OCBKh	2	TRUE	0.00344	25.3452
6974	rs1687416	A	G	A	G	0.137	0.125268	0.9858	0.976643	FALSE	FALSE	FALSE	ieu-b-508	8	1.07E+08	0.110699	486484	0.257801	Sepsis (28	Sepsis (28	Sepsis (28	TRUE	igd	0.0272	4.49E-07	1754	M34407.m	TRUE	reported	X7I83Y	2	TRUE	0.014257	25.34007
3402	rs794614	A	G	A	G	0.0292	0.029608	0.7931	0.74537	FALSE	FALSE	FALSE	ieu-b-508	6	1.19E+08	0.038426	486484	0.440993	Sepsis (28	Sepsis (28	Sepsis (28	TRUE	igd	0.0058	5.44E-07	7346	M32425.m	TRUE	reported	u5RLGY	2	TRUE	0.003438	25.33912
3647	rs1018352	C	G	C	G	0.0896	0.020677	0.9442	0.927894	FALSE	TRUE	FALSE	ieu-b-508	2	2.12E+08	0.064676	486484	0.749199	Sepsis (28	Sepsis (28	Sepsis (28	TRUE	igd	0.0178	5.09E-07	6970	M32564.m	TRUE	reported	73MgOk	2	TRUE	0.003622	25.33094
3098	rs1003746	T	G	T	G	0.0302	-0.07393	0.968	0.968863	FALSE	FALSE	FALSE	ieu-b-508	5	1.3E+08	0.096133	486484	0.441879	Sepsis (28	Sepsis (28	Sepsis (28	TRUE	igd	0.006	5.97E-07	7356	M32198.m	TRUE	reported	ndXmnp	2	TRUE	0.003432	25.32756
6858	rs1249809	A	G	A	G	-0.1601	0.010519	0.5278	0.576092	FALSE	FALSE	FALSE	ieu-b-508	3	73275538	0.033779	486484	0.755484	Sepsis (28	Sepsis (28	Sepsis (28	TRUE	igd	0.03	9.17E-08	18	M34365.m	TRUE	reported	8eexD0	2	TRUE	0.612737	25.31557
8439	rs4148987	A	G	A	G	-0.0317	0.014308	0.3217	0.322065	FALSE	FALSE	FALSE	ieu-b-508	12	21482481	0.035658	486484	0.688234	Sepsis (28	Sepsis (28	Sepsis (28	TRUE	igd	0.0063	4.09E-07	6511	M36850.m	TRUE	reported	j6lLja	2	TRUE	0.003874	25.31069
1225	rs1288349	T	C	T	C	0.0166	-0.00059	0.6656	0.658126	FALSE	FALSE	FALSE	ieu-b-508	14	91195372	0.03532	486484	0.986754	Sepsis (28	Sepsis (28	Sepsis (28	TRUE	igd	0.0033	3.76E-07	7346	M01712.m	TRUE	reported	JfdAWN	2	TRUE	0.003433	25.29706
5760	rs1450233	A	T	A	T	-0.0584	0.035531	0.3174	0.318865	FALSE	TRUE	FALSE	ieu-b-508	4	1.45E+08	0.035786	486484	0.320775	Sepsis (28	Sepsis (28	Sepsis (28	TRUE	igd	0.0116	4.32E-07	972	M33508.m	TRUE	reported	UMXHcP	2	TRUE	0.025413	25.29386
5866	rs1743324	A	C	A	C	0.328	-0.03769	0.023	0.063803	FALSE	FALSE	FALSE	ieu-b-508	22	27557349	0.06927	486484	0.586396	Sepsis (28	Sepsis (28	Sepsis (28	TRUE	igd	0.065	4.51E-07	296	M33609.m	TRUE	reported	h7B9gN	2	TRUE	0.079212	25.29162
7644	rs2061282	A	G	A	G	-0.0171	-0.05231	0.0735	0.079327	FALSE	FALSE	FALSE	ieu-b-508	5	58533719	0.061813	486484	0.397428	Sepsis (28	Sepsis (28	Sepsis (28	TRUE	igd	0.0034	5.26E-07	5768	M35326.m	TRUE	reported	jRytyJ	2	TRUE	0.004366	25.28621
923	rs1091904	A	C	A	C	0.0176	-0.07941	0.1408	0.142042	FALSE	FALSE	FALSE	ieu-b-508	1	1.69E+08	0.04797	486484	0.097825	Sepsis (28	Sepsis (28	Sepsis (28	TRUE	igd	0.0035	6.75E-07	7160	M01508.m	TRUE	reported	go526k	2	TRUE	0.003519	25.27947
8069	rs6751559	T	G	T	G	-0.0357	-0.02296	0.8962	0.898578	FALSE	FALSE	FALSE	ieu-b-508	2	28367761	0.055566	486484	0.67946	Sepsis (28	Sepsis (28	Sepsis (28	TRUE	igd	0.0071	5.48E-07	4529	M35978.m	TRUE	reported	CN0wRR	2	TRUE	0.005551	25.27132
1239	rs1173830	T	C	T	C	-0.0171	-0.02145	0.6542	0.625807	FALSE	FALSE	FALSE	ieu-b-508	1	2.46E+08	0.034488	486484	0.533948	Sepsis (28	Sepsis (28	Sepsis (28	TRUE	igd	0.0034	6.60E-07	1714	M01769.m	TRUE	reported	rismUZ	2	TRUE	0.014543	25.26547
2274	rs1074643	A	G	A	G	0.0558	-0.21206	0.9808	0.979019	FALSE	FALSE	FALSE	ieu-b-508	1	2.11E+08	0.117793	486484	0.071821	Sepsis (28	Sepsis (28	Sepsis (28	TRUE	igd	0.0111	5.10E-07	7301	M20675.m	TRUE	reported	CXe9rE	2	TRUE	0.003449	25.26408
2048	rs4905425	A	C	A	C	0.0377	0.045295	0.5066	0.498649	FALSE	FALSE	FALSE	ieu-b-508	14	96537280	0.033385	486484	0.174863	Sepsis (28	Sepsis (28	Sepsis (28	TRUE	igd	0.0075	5.72E-07	2079	M18357.m	TRUE	reported	o7AvpG	2	TRUE	0.012008	25.24307
6616	rs1003095	T	C	T	C	-0.0704	-0.02786	0.3662	0.389216	FALSE	FALSE	FALSE	ieu-b-508	4	94938117	0.034296	486484	0.416663	Sepsis (28	Sepsis (28	Sepsis (28	TRUE	igd	0.014	4.70E-07	979	M34314.m	TRUE	reported	wPKEWG	2	TRUE	0.025179	25.23487
6829	rs6507247	A	G	A	G	-0.0191	0.01326	0.4643	0.495373	FALSE	FALSE	FALSE	ieu-b-508	18	35830154	0.033421	486484	0.691551	Sepsis (28	Sepsis (28	Sepsis (28	TRUE	igd	0.0038	4.43E-07	1576	M34359.m	TRUE	reported	0UotaJ	2	TRUE	0.015777	25.23179
4235	rs3135377	A	G	A	G	0.0216	-0.04742	0.2078	0.233314	FALSE	FALSE	FALSE	ieu-b-508	6	32385399	0.039564	486484	0.230727	Sepsis (28	Sepsis (28	Sepsis (28	TRUE	igd	0.0043	4.57E-07	6857	M32787.m	TRUE	reported	fSRakm	2	TRUE	0.003666	25.22574
2200	rs1333626	A	C	A	C	-0.0226	0.008677	0.828	0.797032	FALSE	FALSE	FALSE	ieu-b-508	16	757459	0.041672	486484	0.835052	Sepsis (28	Sepsis (28	Sepsis (28	TRUE	igd	0.0045	5.91E-07	6929	M19364.m	TRUE	reported	XkjMEx	2	TRUE	0.003627	25.21544
6866	rs1735789	C	G	C	G	-0.1895	0.044028	0.901	0.831342	FALSE	TRUE	FALSE	ieu-b-508	1	1.89E+08	0.044561	486484	0.323138	Sepsis (28	Sepsis (28	Sepsis (28	TRUE	igd	0.0377	4.94E-07	954	M34365.m	TRUE	reported	8eexD0	2	TRUE	0.025801	25.21299
6569	rs1461941	C	G	C	G	0.0226	-0.00901	0.6294	0.60971	FALSE	TRUE	FALSE	ieu-b-508	16	51788611	0.034416	486484	0.793367	Sepsis (28	Sepsis (28	Sepsis (28	TRUE	igd	0.0045	5.01E-07	4908	M34245.m	TRUE	reported	pRwBKr	2	TRUE	0.005113	25.21244
7960	rs1764453	C	G	C	G	-0.2588	-0.37758	0.9834	0.99386	FALSE	TRUE	FALSE	ieu-b-508	16	79056445	0.220346	486484	0.086608	Sepsis (28	Sepsis (28	Sepsis (28	TRUE	igd	0.0515	4.99E-07	1020	M35678.m	TRUE	reported	56yR01	2	TRUE	0.02416	25.20355
6148	rs1494950	A	G	A	G	0.1843	-0.16245	0.9762	0.984098	FALSE	FALSE	FALSE	ieu-b-508	4	14967169	0.134225	486484	0.226168	Sepsis (28	Sepsis (28	Sepsis (28	TRUE	igd	0.0367	5.31E-07	2725	M33883.m	TRUE	reported	RT7YNu	2	TRUE	0.00917	25.19995
1967	rs1864468	A	G	A	G	-0.0251	-0.00489	0.6161	0.618397	FALSE	FALSE	FALSE	ieu-b-508	2	1.25E+08	0.034305	486484	0.886659	Sepsis (28	Sepsis (28	Sepsis (28	TRUE	igd	0.005	5.94E-07	7226	M17807.m	TRUE	reported	zd6sZI	2	TRUE	0.003475	25.19343
1973	rs450940	T	C	T	C	-0.0251	-0.04535	0.5116	0.524582	FALSE	FALSE	FALSE	ieu-b-508	20	19907398	0.033326	486484	0.173603	Sepsis (28	Sepsis (28	Sepsis (28	TRUE	igd	0.005	5.04E-07	7226	M17807.m	TRUE	reported	zd6sZI	2	TRUE	0.003475	25.19343
6237	rs2246344	T	G	T	G	-0.0261	-0.00555	0.7708	0.766034	FALSE	FALSE	FALSE	ieu-b-508	13	39196090	0.039276	486484	0.887541	Sepsis (28	Sepsis (28	Sepsis (28	TRUE	igd	0.0052	4.60E-07	5534	M33910.m	TRUE	reported	YwFZg1	2	TRUE	0.004532	25.18357
5146	rs6904241	T	G	T	G	0.0527	-0.01337	0.5433	0.543106	FALSE	FALSE	FALSE	ieu-b-508	6	1.12E+08	0.033438	486484	0.689357	Sepsis (28	Sepsis (28	Sepsis (28	TRUE	igd	0.0105	5.36E-07	2616	M33190.m	TRUE	reported	UkhAw5	2	TRUE	0.009538	25.17158
7868	rs2762662	A	G	A	G	-0.0577	0.076305	0.0601	0.059476	FALSE	FALSE	FALSE	ieu-b-508	10	10176338	0.070734	486484	0.280695	Sepsis (28	Sepsis (28	Sepsis (28	TRUE	igd	0.0115	5.19E-07	1750	M35631.m	TRUE	reported	002wka	2	TRUE	0.014181	25.14544
4415	rs6912503	A	G	A	G	-0.0311	0.050838	0.8251	0.848382	FALSE	FALSE	FALSE	ieu-b-508	6	27209642	0.04647	486484	0.273949	Sepsis (28	Sepsis (28	Sepsis (28	TRUE	igd	0.0062	6.16E-07	2148	M32869.m	TRUE	reported	Rvim1n	2	TRUE	0.011578	25.13812
7083	rs7761031	A	G	A	G	-0.0406	0.11169	0.96	0.956179	FALSE	FALSE	FALSE	ieu-b-508	6	1.61E+08	0.081852	486484	0.172399	Sepsis (28	Sepsis (28	Sepsis (28	TRUE	igd	0.0081	5.87E-07	7119	M34481.m	TRUE	reported	mXdL0W	2	TRUE	0.003517	25.11655
7035	rs1048312	A	G	A	G	0.0346	-0.27161	0.0205	0.020788	FALSE	FALSE	FALSE	ieu-b-508	22	27258789	0.115985	486484	0.019192	Sepsis (28	Sepsis (28	Sepsis (28	TRUE	igd	0.0069	5.61E-07	1755	M34453.m	TRUE	reported	3EAMbN	2	TRUE	0.014125	25.11648
8105	rs2956731	T	G	T	G	0.4689	0.019312	0.931	0.923304	FALSE	FALSE	FALSE	ieu-b-508	8	38190769	0.062894	486484	0.758799	Sepsis (28	Sepsis (28	Sepsis (28	TRUE	igd	0.0934	5.18E-07	488	M36097.m	TRUE	reported	RHr18S	2	TRUE	0.049111	25.10055
4203	rs625532	A	G	A	G	-0.0561	0.02351	0.1697	0.160403	FALSE	FALSE	FALSE	ieu-b-508	11	74097186	0.045472	486484	0.605144	Sepsis (28	Sepsis (28	Sepsis (28	TRUE	igd	0.0112	5.31E-07	2537	M32762.m	TRUE	reported	Q5lKLH	2	TRUE	0.009793	25.06959
4477	rs7309973	T	C	T	C	-0.0701	-0.04641	0.0502	0.028617	FALSE	FALSE	FALSE	ieu-b-508	12	1.05E+08	0.101045	486484	0.646023	Sepsis (28	Sepsis (28	Sepsis (28	TRUE	igd	0.014	5.85E-07	5425	M33084.m	TRUE	reported	jrOsNK	2	TRUE	0.0046	25.06224
6479	rs2002691	A	C	A	C	0.5054	0.036526	0.9844	0.975291	FALSE	FALSE	FALSE	ieu-b-508	14	65899385	0.107229	486484	0.733378	Sepsis (28	Sepsis (28	Sepsis (28	TRUE	igd	0.1006	5.00E-07	282	M34109.m	TRUE	reported	piJuC7	2	TRUE	0.082148	25.06014
6488	rs6944023	A	C	A	C	-0.9465	0.100877	0.0219	0.031974	FALSE	FALSE	FALSE	ieu-b-508	7	5477406	0.095161	486484	0.289112	Sepsis (28	Sepsis (28	Sepsis (28	TRUE	igd	0.1878	4.67E-07	148	M34109.m	TRUE	reported	piJuC7	2	TRUE	0.146487	25.0577
8117	rs9961807	T	C	T	C	-0.32	0.08823	0.2187	0.193119	FALSE	FALSE	FALSE	ieu-b-508	18	53819904	0.042135	486484	0.036261	Sepsis (28	Sepsis (28	Sepsis (28	TRUE	igd	0.0638	5.35E-07	488	M36097.m	TRUE	reported	RHr18S	2	TRUE	0.049024	25.05388
1790	rs2126638	A	G	A	G	0.0816	0.006665	0.9266	0.919716	FALSE	FALSE	FALSE	ieu-b-508	9	22962118	0.061852	486484	0.914194	Sepsis (28	Sepsis (28	Sepsis (28	TRUE	igd	0.0163	5.78E-07	5502	M15677.m	TRUE	reported	1CuWi9	2	TRUE	0.004534	25.05228
1087	rs1825043	A	G	A	G	0.019	-0.01762	0.9571	0.946326	FALSE	FALSE	FALSE	ieu-b-508	4	9573740	0.07511	486484	0.814538	Sepsis (28	Sepsis (28	Sepsis (28	TRUE	igd	0.0038	5.26E-07	7371	M01604.m	TRUE	reported	hE3I2k	2	TRUE	0.00338	24.99322
1094	rs7906282	T	G	T	G	0.008	0.000636	0.3075	0.309156	FALSE	FALSE	FALSE	ieu-b-508	10	19083596	0.036044	486484	0.985924	Sepsis (28	Sepsis (28	Sepsis (28	TRUE	igd	0.0016	1.11E-06	7371	M01604.m	TRUE	reported	hE3I2k	2	TRUE	0.00338	24.99322
1644	rs4820242	A	G	A	G	-0.0085	-0.08218	0.6201	0.615143	FALSE	FALSE	FALSE	ieu-b-508	22	36982675	0.03433	486484	0.016676	Sepsis (28	Sepsis (28	Sepsis (28	TRUE	igd	0.0017	9.03E-07	7368	M15140.m	TRUE	reported	jEcqMJ	2	TRUE	0.003382	24.99321
1649	rs6815057	A	G	A	G	0.0085	0.005128	0.5786	0.576571	FALSE	FALSE	FALSE	ieu-b-508	4	97782257	0.034161	486484	0.880677	Sepsis (28	Sepsis (28	Sepsis (28	TRUE	igd	0.0017	1.12E-06	7368	M15140.m	TRUE	reported	jEcqMJ	2	TRUE	0.003382	24.99321
2340	rs872745	A	C	A	C	-0.0135	-0.01801	0.4114	0.405906	FALSE	FALSE	FALSE	ieu-b-508	16	82578355	0.03405	486484	0.596814	Sepsis (28	Sepsis (28	Sepsis (28	TRUE	igd	0.0027	4.93E-07	7366	M21044.m	TRUE	reported	Ym5rae	2	TRUE	0.003382	24.99321
8613	rs2979471	A	T	A	T	-0.009	-0.08331	0.2882	0.300699	FALSE	TRUE	FALSE	ieu-b-508	8	30289667	0.036559	486484	0.022674	Sepsis (28	Sepsis (28	Sepsis (28	TRUE	igd	0.0018	5.34E-07	7366	M37506.m	TRUE	reported	m2kaRt	2	TRUE	0.003382	24.99321
8616	rs420422	T	C	T	C	-0.009	0.010367	0.542	0.560988	FALSE	FALSE	FALSE	ieu-b-508	5	1436408	0.033821	486484	0.7592	Sepsis (28	Sepsis (28	Sepsis (28	TRUE	igd	0.0018	3.01E-07	7366	M37506.m	TRUE	reported	m2kaRt	2	TRUE	0.003382	24.99321
3250	rs1079007	T	C	T	C	-0.009	0.033982	0.611	0.611867	FALSE	FALSE	FALSE	ieu-b-508	11	1.15E+08	0.034645	486484	0.326664	Sepsis (28	Sepsis (28	Sepsis (28	TRUE	igd	0.0018	3.15E-07	7365	M32348.m	TRUE	reported	DJeLDx	2	TRUE	0.003383	24.99321
996	rs1078730	A	G	A	G	-0.009	0.012638	0.2701	0.272417	FALSE	FALSE	FALSE	ieu-b-508	10	1.13E+08	0.037473	486484	0.735917	Sepsis (28	Sepsis (28	Sepsis (28	TRUE	igd	0.0018	6.04E-07	7364	M01564.m	TRUE	reported	IPUdyD	2	TRUE	0.003383	24.99321
1028	rs7851531	A	G	A	G	-0.009	0.063062	0.6594	0.633781	FALSE	FALSE	FALSE	ieu-b-508	9	85126285	0.034833	486484	0.070228	Sepsis (28	Sepsis (28	Sepsis (28	TRUE	igd	0.0018	6.47E-07	7364	M01564.m	TRUE	reported	IPUdyD	2	TRUE	0.003383	24.99321
3446	rs842636	A	G	A	G	-0.009	0.036304	0.4429	0.435417	FALSE	FALSE	FALSE	ieu-b-508	2	61091950	0.033574	486484	0.279555	Sepsis (28	Sepsis (28	Sepsis (28	TRUE	igd	0.0018	9.72E-07	7364	M32452.m	TRUE	reported	8GtFpl	2	TRUE	0.003383	24.99321
6350	rs1696871	A	G	A	G	0.0205	-0.01058	0.8679	0.87042	FALSE	FALSE	FALSE	ieu-b-508	15	39593159	0.049996	486484	0.832383	Sepsis (28	Sepsis (28	Sepsis (28	TRUE	igd	0.0041	4.65E-07	7364	M33960.m	TRUE	reported	o8RcxU	2	TRUE	0.003383	24.99321
3482	rs6925339	A	G	A	G	0.009	-0.04246	0.3097	0.304697	FALSE	FALSE	FALSE	ieu-b-508	6	1.1E+08	0.036279	486484	0.241885	Sepsis (28	Sepsis (28	Sepsis (28	TRUE	igd	0.0018	6.67E-07	7363	M32489.m	TRUE	reported	hnljaD	2	TRUE	0.003384	24.99321
3784	rs7952698	A	G	A	G	0.009	0.045173	0.2345	0.238165	FALSE	FALSE	FALSE	ieu-b-508	11	79170555	0.039084	486484	0.247766	Sepsis (28	Sepsis (28	Sepsis (28	TRUE	igd	0.0018	8.69E-07	7362	M32634.m	TRUE	reported	7T2RUz	2	TRUE	0.003384	24.99321
6050	rs1089911	A	G	A	G	0.026	0.097799	0.8976	0.905195	FALSE	FALSE	FALSE	ieu-b-508	11	75470588	0.056936	486484	0.08585	Sepsis (28	Sepsis (28	Sepsis (28	TRUE	igd	0.0052	4.58E-07	7362	M33821.m	TRUE	reported	eVYlCL	2	TRUE	0.003384	24.99321
6440	rs3729908	T	C	T	C	0.009	-0.0282	0.1234	0.116483	FALSE	FALSE	FALSE	ieu-b-508	16	24231248	0.052737	486484	0.59279	Sepsis (28	Sepsis (28	Sepsis (28	TRUE	igd	0.0018	9.91E-07	7362	M34062.m	TRUE	reported	RZyHKP	2	TRUE	0.003384	24.99321
445	rs1051944	A	G	A	G	-0.0045	-0.03244	0.5338	0.536125	FALSE	FALSE	FALSE	ieu-b-508	15	24106808	0.033602	486484	0.334413	Sepsis (28	Sepsis (28	Sepsis (28	TRUE	igd	9.00E-04	1.85E-06	7361	M00513.m	TRUE	reported	ohacnP	2	TRUE	0.003385	24.99321
1442	rs1535039	T	C	T	C	0.0175	0.024175	0.912	0.878248	FALSE	FALSE	FALSE	ieu-b-508	6	29411432	0.050974	486484	0.635321	Sepsis (28	Sepsis (28	Sepsis (28	TRUE	igd	0.0035	4.49E-07	7357	M03141.m	TRUE	reported	y4mG6m	2	TRUE	0.003387	24.9932
18	rs1078449	A	G	A	G	0.0045	-0.03481	0.5375	0.531303	FALSE	FALSE	FALSE	ieu-b-508	12	66160971	0.033425	486484	0.2977	Sepsis (28	Sepsis (28	Sepsis (28	TRUE	igd	9.00E-04	5.09E-07	7355	M00054.m	TRUE	reported	QTDAnh	2	TRUE	0.003388	24.9932
23	rs1084666	T	C	T	C	0.0045	0.004006	0.4861	0.483873	FALSE	FALSE	FALSE	ieu-b-508	12	1.25E+08	0.033628	486484	0.905174	Sepsis (28	Sepsis (28	Sepsis (28	TRUE	igd	9.00E-04	1.65E-06	7355	M00054.m	TRUE	reported	QTDAnh	2	TRUE	0.003388	24.9932
39	rs1253393	A	T	A	T	0.0045	-0.04363	0.3887	0.382543	FALSE	TRUE	FALSE	ieu-b-508	7	1.58E+08	0.034304	486484	0.203451	Sepsis (28	Sepsis (28	Sepsis (28	TRUE	igd	9.00E-04	6.52E-07	7355	M00054.m	TRUE	reported	QTDAnh	2	TRUE	0.003388	24.9932
54	rs1549225	T	C	T	C	0.0045	0.001538	0.607	0.601646	FALSE	FALSE	FALSE	ieu-b-508	5	1.75E+08	0.033994	486484	0.963915	Sepsis (28	Sepsis (28	Sepsis (28	TRUE	igd	9.00E-04	6.06E-07	7355	M00054.m	TRUE	reported	QTDAnh	2	TRUE	0.003388	24.9932
66	rs1997238	A	G	A	G	0.0045	-0.04486	0.5117	0.50945	FALSE	FALSE	FALSE	ieu-b-508	7	48538770	0.033347	486484	0.178571	Sepsis (28	Sepsis (28	Sepsis (28	TRUE	igd	9.00E-04	6.07E-07	7355	M00054.m	TRUE	reported	QTDAnh	2	TRUE	0.003388	24.9932
80	rs2510595	A	G	A	G	-0.0045	-0.02715	0.6113	0.599706	FALSE	FALSE	FALSE	ieu-b-508	11	1.07E+08	0.034197	486484	0.427257	Sepsis (28	Sepsis (28	Sepsis (28	TRUE	igd	9.00E-04	4.99E-07	7355	M00054.m	TRUE	reported	QTDAnh	2	TRUE	0.003388	24.9932
81	rs2520117	A	G	A	G	0.0045	0.044891	0.4545	0.455817	FALSE	FALSE	FALSE	ieu-b-508	16	26576299	0.033571	486484	0.181164	Sepsis (28	Sepsis (28	Sepsis (28	TRUE	igd	9.00E-04	5.06E-07	7355	M00054.m	TRUE	reported	QTDAnh	2	TRUE	0.003388	24.9932
85	rs2662269	A	C	A	C	0.0045	0.000784	0.417	0.40956	FALSE	FALSE	FALSE	ieu-b-508	5	98076517	0.033986	486484	0.981601	Sepsis (28	Sepsis (28	Sepsis (28	TRUE	igd	9.00E-04	7.12E-07	7355	M00054.m	TRUE	reported	QTDAnh	2	TRUE	0.003388	24.9932
92	rs2834164	A	C	A	C	-0.0045	-0.04067	0.5041	0.496224	FALSE	FALSE	FALSE	ieu-b-508	21	34621948	0.033361	486484	0.222855	Sepsis (28	Sepsis (28	Sepsis (28	TRUE	igd	9.00E-04	4.76E-07	7355	M00054.m	TRUE	reported	QTDAnh	2	TRUE	0.003388	24.9932
111	rs428185	T	C	T	C	-0.0045	0.068995	0.5851	0.577921	FALSE	FALSE	FALSE	ieu-b-508	14	48321936	0.033802	486484	0.041236	Sepsis (28	Sepsis (28	Sepsis (28	TRUE	igd	9.00E-04	6.25E-07	7355	M00054.m	TRUE	reported	QTDAnh	2	TRUE	0.003388	24.9932
118	rs4695289	A	G	A	G	-0.0045	0.025887	0.3489	0.343522	FALSE	FALSE	FALSE	ieu-b-508	4	43965710	0.035958	486484	0.471575	Sepsis (28	Sepsis (28	Sepsis (28	TRUE	igd	9.00E-04	7.95E-07	7355	M00054.m	TRUE	reported	QTDAnh	2	TRUE	0.003388	24.9932
120	rs4751282	T	C	T	C	0.0045	0.021603	0.5421	0.552864	FALSE	FALSE	FALSE	ieu-b-508	10	1.29E+08	0.033905	486484	0.524027	Sepsis (28	Sepsis (28	Sepsis (28	TRUE	igd	9.00E-04	7.41E-07	7355	M00054.m	TRUE	reported	QTDAnh	2	TRUE	0.003388	24.9932
128	rs4989308	A	G	A	G	-0.0045	-0.01933	0.3746	0.37913	FALSE	FALSE	FALSE	ieu-b-508	4	1.15E+08	0.034455	486484	0.574728	Sepsis (28	Sepsis (28	Sepsis (28	TRUE	igd	9.00E-04	8.43E-07	7355	M00054.m	TRUE	reported	QTDAnh	2	TRUE	0.003388	24.9932
155	rs7006068	T	C	T	C	0.0045	-0.01615	0.6264	0.621111	FALSE	FALSE	FALSE	ieu-b-508	8	1.43E+08	0.034409	486484	0.638799	Sepsis (28	Sepsis (28	Sepsis (28	TRUE	igd	9.00E-04	4.80E-07	7355	M00054.m	TRUE	reported	QTDAnh	2	TRUE	0.003388	24.9932
167	rs7810390	A	G	A	G	-0.0045	0.029574	0.5928	0.591016	FALSE	FALSE	FALSE	ieu-b-508	7	82662097	0.033981	486484	0.384136	Sepsis (28	Sepsis (28	Sepsis (28	TRUE	igd	9.00E-04	5.61E-07	7355	M00054.m	TRUE	reported	QTDAnh	2	TRUE	0.003388	24.9932
173	rs8110890	T	G	T	G	0.0045	0.05965	0.4741	0.474728	FALSE	FALSE	FALSE	ieu-b-508	19	19848485	0.033651	486484	0.076294	Sepsis (28	Sepsis (28	Sepsis (28	TRUE	igd	9.00E-04	7.51E-07	7355	M00054.m	TRUE	reported	QTDAnh	2	TRUE	0.003388	24.9932
827	rs4710948	A	G	A	G	-0.0085	-0.02224	0.3382	0.347428	FALSE	FALSE	FALSE	ieu-b-508	6	10305925	0.035176	486484	0.527261	Sepsis (28	Sepsis (28	Sepsis (28	TRUE	igd	0.0017	6.94E-07	7355	M01358.m	TRUE	reported	kSPQgd	2	TRUE	0.003388	24.9932
3377	rs1172879	T	C	T	C	-0.021	-0.00899	0.7845	0.783605	FALSE	FALSE	FALSE	ieu-b-508	4	83491535	0.040737	486484	0.825333	Sepsis (28	Sepsis (28	Sepsis (28	TRUE	igd	0.0042	7.15E-07	7355	M32418.m	TRUE	reported	y8cN4Y	2	TRUE	0.003388	24.9932
562	rs7948201	T	C	T	C	-0.023	-0.01543	0.9623	0.923357	FALSE	FALSE	FALSE	ieu-b-508	11	1.3E+08	0.062737	486484	0.805775	Sepsis (28	Sepsis (28	Sepsis (28	TRUE	igd	0.0046	6.77E-07	7354	M00606.m	TRUE	reported	r5smf7	2	TRUE	0.003388	24.9932
6330	rs8028440	T	C	T	C	0.0095	0.066327	0.2136	0.215091	FALSE	FALSE	FALSE	ieu-b-508	15	22920718	0.040481	486484	0.101326	Sepsis (28	Sepsis (28	Sepsis (28	TRUE	igd	0.0019	3.15E-07	7354	M33955.m	TRUE	reported	mhHFnT	2	TRUE	0.003388	24.9932
244	rs1292666	T	C	T	C	0.0045	0.010984	0.4667	0.463271	FALSE	FALSE	FALSE	ieu-b-508	1	10908412	0.033632	486484	0.743971	Sepsis (28	Sepsis (28	Sepsis (28	TRUE	igd	9.00E-04	6.44E-07	7351	M00060.m	TRUE	reported	kqwl4V	2	TRUE	0.003389	24.9932
251	rs1428203	T	G	T	G	0.0045	-0.01728	0.4117	0.411322	FALSE	FALSE	FALSE	ieu-b-508	5	24164613	0.033925	486484	0.610513	Sepsis (28	Sepsis (28	Sepsis (28	TRUE	igd	9.00E-04	5.51E-07	7351	M00060.m	TRUE	reported	kqwl4V	2	TRUE	0.003389	24.9932
255	rs1558789	A	C	A	C	0.0045	0.010289	0.51	0.507691	FALSE	FALSE	FALSE	ieu-b-508	12	15168791	0.033504	486484	0.75877	Sepsis (28	Sepsis (28	Sepsis (28	TRUE	igd	9.00E-04	3.52E-07	7351	M00060.m	TRUE	reported	kqwl4V	2	TRUE	0.003389	24.9932
256	rs1564480	A	G	A	G	0.0045	0.000169	0.4765	0.471641	FALSE	FALSE	FALSE	ieu-b-508	8	21402658	0.033383	486484	0.995972	Sepsis (28	Sepsis (28	Sepsis (28	TRUE	igd	9.00E-04	6.68E-07	7351	M00060.m	TRUE	reported	kqwl4V	2	TRUE	0.003389	24.9932
295	rs2834578	T	C	T	C	0.0045	-0.01131	0.3986	0.398924	FALSE	FALSE	FALSE	ieu-b-508	21	36059609	0.034221	486484	0.741082	Sepsis (28	Sepsis (28	Sepsis (28	TRUE	igd	9.00E-04	7.24E-07	7351	M00060.m	TRUE	reported	kqwl4V	2	TRUE	0.003389	24.9932
303	rs3802681	T	C	T	C	0.0045	0.042182	0.6144	0.627027	FALSE	FALSE	FALSE	ieu-b-508	10	1.04E+08	0.034428	486484	0.220496	Sepsis (28	Sepsis (28	Sepsis (28	TRUE	igd	9.00E-04	5.44E-07	7351	M00060.m	TRUE	reported	kqwl4V	2	TRUE	0.003389	24.9932
304	rs390836	A	T	A	T	0.0045	0.012645	0.415	0.417154	FALSE	TRUE	FALSE	ieu-b-508	20	15057002	0.033902	486484	0.709163	Sepsis (28	Sepsis (28	Sepsis (28	TRUE	igd	9.00E-04	8.06E-07	7351	M00060.m	TRUE	reported	kqwl4V	2	TRUE	0.003389	24.9932
307	rs4077470	T	C	T	C	-0.0045	0.025621	0.5403	0.542297	FALSE	FALSE	FALSE	ieu-b-508	12	5596812	0.033533	486484	0.444824	Sepsis (28	Sepsis (28	Sepsis (28	TRUE	igd	9.00E-04	4.65E-07	7351	M00060.m	TRUE	reported	kqwl4V	2	TRUE	0.003389	24.9932
325	rs4935115	A	G	A	G	0.0045	0.038252	0.529	0.539587	FALSE	FALSE	FALSE	ieu-b-508	10	56396781	0.033499	486484	0.253505	Sepsis (28	Sepsis (28	Sepsis (28	TRUE	igd	9.00E-04	5.75E-07	7351	M00060.m	TRUE	reported	kqwl4V	2	TRUE	0.003389	24.9932
327	rs5018486	A	C	A	C	0.005	0.027721	0.4559	0.462503	FALSE	FALSE	FALSE	ieu-b-508	2	6087070	0.033542	486484	0.408553	Sepsis (28	Sepsis (28	Sepsis (28	TRUE	igd	0.001	2.70E-07	7351	M00060.m	TRUE	reported	kqwl4V	2	TRUE	0.003389	24.9932
341	rs679563	A	G	A	G	-0.0045	0.037691	0.4837	0.491278	FALSE	FALSE	FALSE	ieu-b-508	1	7971078	0.033348	486484	0.258377	Sepsis (28	Sepsis (28	Sepsis (28	TRUE	igd	9.00E-04	7.13E-07	7351	M00060.m	TRUE	reported	kqwl4V	2	TRUE	0.003389	24.9932
364	rs7685009	A	G	A	G	-0.0045	0.033262	0.6582	0.652168	FALSE	FALSE	FALSE	ieu-b-508	4	90480831	0.034962	486484	0.341407	Sepsis (28	Sepsis (28	Sepsis (28	TRUE	igd	9.00E-04	6.44E-07	7351	M00060.m	TRUE	reported	kqwl4V	2	TRUE	0.003389	24.9932
391	rs9403527	T	C	T	C	0.0045	0.008754	0.3456	0.345078	FALSE	FALSE	FALSE	ieu-b-508	6	1.44E+08	0.03546	486484	0.805019	Sepsis (28	Sepsis (28	Sepsis (28	TRUE	igd	9.00E-04	9.91E-07	7351	M00060.m	TRUE	reported	kqwl4V	2	TRUE	0.003389	24.9932
3571	rs2434532	A	G	A	G	-0.0095	-0.0553	0.1919	0.184627	FALSE	FALSE	FALSE	ieu-b-508	5	1.54E+08	0.042973	486484	0.198124	Sepsis (28	Sepsis (28	Sepsis (28	TRUE	igd	0.0019	2.84E-07	7350	M32518.m	TRUE	reported	V0ZCiU	2	TRUE	0.00339	24.9932
3572	rs2831549	T	C	T	C	-0.009	-0.01683	0.7077	0.67212	FALSE	FALSE	FALSE	ieu-b-508	21	29527720	0.035485	486484	0.635226	Sepsis (28	Sepsis (28	Sepsis (28	TRUE	igd	0.0018	4.90E-07	7350	M32518.m	TRUE	reported	V0ZCiU	2	TRUE	0.00339	24.9932
3118	rs1042725	T	C	T	C	-0.0085	0.014388	0.4977	0.494168	FALSE	FALSE	FALSE	ieu-b-508	12	66358347	0.033382	486484	0.666464	Sepsis (28	Sepsis (28	Sepsis (28	TRUE	igd	0.0017	7.98E-07	7349	M32315.m	TRUE	reported	4igzkL	2	TRUE	0.00339	24.9932
733	rs1123210	A	C	A	C	0.0085	-0.01903	0.1151	0.112197	FALSE	FALSE	FALSE	ieu-b-508	11	80092415	0.05381	486484	0.723624	Sepsis (28	Sepsis (28	Sepsis (28	TRUE	igd	0.0017	3.70E-07	7347	M01302.m	TRUE	reported	sNp2lC	2	TRUE	0.003391	24.99319
1177	rs1530439	T	G	T	G	0.009	0.020353	0.2946	0.307643	FALSE	FALSE	FALSE	ieu-b-508	10	63645959	0.036366	486484	0.575698	Sepsis (28	Sepsis (28	Sepsis (28	TRUE	igd	0.0018	6.08E-07	7346	M01645.m	TRUE	reported	d5nAn3	2	TRUE	0.003392	24.99319
1180	rs2069151	A	G	A	G	0.009	0.080041	0.7083	0.72055	FALSE	FALSE	FALSE	ieu-b-508	1	2.11E+08	0.037182	486484	0.031345	Sepsis (28	Sepsis (28	Sepsis (28	TRUE	igd	0.0018	4.68E-07	7346	M01645.m	TRUE	reported	d5nAn3	2	TRUE	0.003392	24.99319
1198	rs6973824	A	G	A	G	0.009	0.038361	0.3374	0.344941	FALSE	FALSE	FALSE	ieu-b-508	7	1.34E+08	0.035164	486484	0.275309	Sepsis (28	Sepsis (28	Sepsis (28	TRUE	igd	0.0018	4.02E-07	7346	M01645.m	TRUE	reported	d5nAn3	2	TRUE	0.003392	24.99319
7711	rs3781638	T	G	T	G	0.008	0.022537	0.5337	0.54374	FALSE	FALSE	FALSE	ieu-b-508	11	92713507	0.033581	486484	0.502149	Sepsis (28	Sepsis (28	Sepsis (28	TRUE	igd	0.0016	4.32E-07	7345	M35397.m	TRUE	reported	Y56mZr	2	TRUE	0.003392	24.99319
3206	rs1376492	A	T	A	T	0.009	-0.00906	0.6524	0.657391	FALSE	TRUE	FALSE	ieu-b-508	11	96192116	0.035163	486484	0.796642	Sepsis (28	Sepsis (28	Sepsis (28	TRUE	igd	0.0018	3.59E-07	7344	M32339.m	TRUE	reported	SVp504	2	TRUE	0.003393	24.99319
4431	rs1921984	T	C	T	C	-0.0085	0.041557	0.5754	0.556995	FALSE	FALSE	FALSE	ieu-b-508	2	2.18E+08	0.03388	486484	0.219972	Sepsis (28	Sepsis (28	Sepsis (28	TRUE	igd	0.0017	9.00E-07	7344	M32910.m	TRUE	reported	2OCBKh	2	TRUE	0.003393	24.99319
6969	rs1088902	A	G	A	G	-0.0135	-0.02433	0.4193	0.42616	FALSE	FALSE	FALSE	ieu-b-508	1	57625649	0.033707	486484	0.470426	Sepsis (28	Sepsis (28	Sepsis (28	TRUE	igd	0.0027	7.12E-07	7344	M34407.m	TRUE	reported	X7I83Y	2	TRUE	0.003393	24.99319
2946	rs7216075	A	G	A	G	-0.0215	-0.04556	0.2494	0.27307	FALSE	FALSE	FALSE	ieu-b-508	17	32026379	0.037403	486484	0.223162	Sepsis (28	Sepsis (28	Sepsis (28	TRUE	igd	0.0043	4.39E-07	7336	M27738.m	TRUE	reported	UtPBd9	2	TRUE	0.003396	24.99318
5618	rs2465657	A	G	A	G	-0.0085	0.044656	0.1764	0.170886	FALSE	FALSE	FALSE	ieu-b-508	2	2.01E+08	0.044351	486484	0.313994	Sepsis (28	Sepsis (28	Sepsis (28	TRUE	igd	0.0017	9.90E-07	7336	M33442.m	TRUE	reported	B1Peb3	2	TRUE	0.003396	24.99318
8378	rs1162721	A	T	A	T	0.013	0.0073	0.7766	0.7639	FALSE	TRUE	FALSE	ieu-b-508	14	59219801	0.039253	486484	0.852462	Sepsis (28	Sepsis (28	Sepsis (28	TRUE	igd	0.0026	6.46E-07	7335	M36776.m	TRUE	reported	VnYglx	2	TRUE	0.003397	24.99318
1270	rs1023418	T	C	T	C	0.0085	0.055253	0.3185	0.325101	FALSE	FALSE	FALSE	ieu-b-508	7	11216782	0.035563	486484	0.120264	Sepsis (28	Sepsis (28	Sepsis (28	TRUE	igd	0.0017	1.07E-06	7325	M02132.m	TRUE	reported	uJFm2t	2	TRUE	0.003401	24.99317
1274	rs1090104	T	C	T	C	-0.009	0.058659	0.5877	0.596618	FALSE	FALSE	FALSE	ieu-b-508	9	1.33E+08	0.03414	486484	0.085763	Sepsis (28	Sepsis (28	Sepsis (28	TRUE	igd	0.0018	2.96E-07	7325	M02132.m	TRUE	reported	uJFm2t	2	TRUE	0.003401	24.99317
1289	rs1768168	A	G	A	G	0.0085	-0.03111	0.3158	0.329235	FALSE	FALSE	FALSE	ieu-b-508	17	9792768	0.035604	486484	0.382268	Sepsis (28	Sepsis (28	Sepsis (28	TRUE	igd	0.0017	1.04E-06	7325	M02132.m	TRUE	reported	uJFm2t	2	TRUE	0.003401	24.99317
4043	rs1169187	T	C	T	C	-0.009	-0.00803	0.3084	0.301319	FALSE	FALSE	FALSE	ieu-b-508	2	78654537	0.036434	486484	0.825624	Sepsis (28	Sepsis (28	Sepsis (28	TRUE	igd	0.0018	2.83E-07	7318	M32740.m	TRUE	reported	YLshfP	2	TRUE	0.003405	24.99317
5384	rs1045744	T	C	T	C	-0.018	-0.06348	0.0849	0.13225	FALSE	FALSE	FALSE	ieu-b-508	6	1.23E+08	0.049299	486484	0.197845	Sepsis (28	Sepsis (28	Sepsis (28	TRUE	igd	0.0036	4.77E-07	7315	M33389.m	TRUE	reported	Hf6XVM	2	TRUE	0.003406	24.99316
5427	rs928177	T	C	T	C	-0.0135	-0.00104	0.146	0.169899	FALSE	FALSE	FALSE	ieu-b-508	9	1.21E+08	0.0446	486484	0.981367	Sepsis (28	Sepsis (28	Sepsis (28	TRUE	igd	0.0027	7.14E-07	7315	M33389.m	TRUE	reported	Hf6XVM	2	TRUE	0.003406	24.99316
1592	rs9396681	T	C	T	C	-0.012	-0.04709	0.9072	0.905996	FALSE	FALSE	FALSE	ieu-b-508	6	16493089	0.057666	486484	0.414139	Sepsis (28	Sepsis (28	Sepsis (28	TRUE	igd	0.0024	6.25E-07	7314	M12593.m	TRUE	reported	IBXgSU	2	TRUE	0.003406	24.99316
1860	rs7561849	A	G	A	G	0.009	-0.00622	0.2168	0.230545	FALSE	FALSE	FALSE	ieu-b-508	2	1.48E+08	0.040023	486484	0.876589	Sepsis (28	Sepsis (28	Sepsis (28	TRUE	igd	0.0018	1.01E-06	7308	M15778.m	TRUE	reported	941o3I	2	TRUE	0.003409	24.99316
5676	rs191149	A	C	A	C	0.009	-0.01874	0.7214	0.735668	FALSE	FALSE	FALSE	ieu-b-508	6	1.61E+08	0.037847	486484	0.620556	Sepsis (28	Sepsis (28	Sepsis (28	TRUE	igd	0.0018	9.14E-07	7307	M33477.m	TRUE	reported	Aw8PBK	2	TRUE	0.00341	24.99316
5699	rs741301	T	C	T	C	0.009	-0.00037	0.6849	0.682178	FALSE	FALSE	FALSE	ieu-b-508	7	36917995	0.035825	486484	0.991767	Sepsis (28	Sepsis (28	Sepsis (28	TRUE	igd	0.0018	8.12E-07	7307	M33477.m	TRUE	reported	Aw8PBK	2	TRUE	0.00341	24.99316
2753	rs2287011	A	G	A	G	-0.009	-0.0295	0.2273	0.235032	FALSE	FALSE	FALSE	ieu-b-508	2	2.02E+08	0.040074	486484	0.461576	Sepsis (28	Sepsis (28	Sepsis (28	TRUE	igd	0.0018	4.84E-07	7303	M27256.m	TRUE	reported	mJwzsi	2	TRUE	0.003412	24.99315
2300	rs9556974	C	G	C	G	0.0135	-0.01985	0.3426	0.346155	FALSE	TRUE	FALSE	ieu-b-508	13	99216302	0.035256	486484	0.573502	Sepsis (28	Sepsis (28	Sepsis (28	TRUE	igd	0.0027	5.36E-07	7301	M20675.m	TRUE	reported	CXe9rE	2	TRUE	0.003413	24.99315
2152	rs1682870	A	G	A	G	0.0135	0.056167	0.7407	0.737338	FALSE	FALSE	FALSE	ieu-b-508	2	1.49E+08	0.037832	486484	0.137639	Sepsis (28	Sepsis (28	Sepsis (28	TRUE	igd	0.0027	9.08E-07	7286	M18929.m	TRUE	reported	2vYFb2	2	TRUE	0.00342	24.99314
2157	rs2806302	T	G	T	G	0.0125	0.000464	0.688	0.673705	FALSE	FALSE	FALSE	ieu-b-508	13	1E+08	0.036029	486484	0.989723	Sepsis (28	Sepsis (28	Sepsis (28	TRUE	igd	0.0025	6.71E-07	7286	M18929.m	TRUE	reported	2vYFb2	2	TRUE	0.00342	24.99314
2443	rs307128	A	G	A	G	-0.013	-0.00554	0.681	0.68835	FALSE	FALSE	FALSE	ieu-b-508	18	37504251	0.036112	486484	0.878112	Sepsis (28	Sepsis (28	Sepsis (28	TRUE	igd	0.0026	4.33E-07	7284	M21630.m	TRUE	reported	7eaO4H	2	TRUE	0.00342	24.99314
1057	rs1012520	C	G	C	G	0.009	0.091814	0.8698	0.86373	FALSE	TRUE	FALSE	ieu-b-508	9	83708171	0.04868	486484	0.059284	Sepsis (28	Sepsis (28	Sepsis (28	TRUE	igd	0.0018	4.11E-07	7271	M01585.m	TRUE	reported	M7QiSM	2	TRUE	0.003427	24.99312
1073	rs6690829	T	C	T	C	-0.008	0.058813	0.24	0.25443	FALSE	FALSE	FALSE	ieu-b-508	1	2.46E+08	0.038433	486484	0.125951	Sepsis (28	Sepsis (28	Sepsis (28	TRUE	igd	0.0016	1.24E-06	7271	M01585.m	TRUE	reported	M7QiSM	2	TRUE	0.003427	24.99312
5795	rs935270	A	G	A	G	-0.0135	0.017594	0.4556	0.448462	FALSE	FALSE	FALSE	ieu-b-508	10	49757251	0.034016	486484	0.605008	Sepsis (28	Sepsis (28	Sepsis (28	TRUE	igd	0.0027	4.66E-07	7269	M33510.m	TRUE	reported	WxGKnn	2	TRUE	0.003427	24.99312
7496	rs485868	A	G	A	G	-0.0135	-0.01277	0.7155	0.713335	FALSE	FALSE	FALSE	ieu-b-508	11	1.28E+08	0.037053	486484	0.730279	Sepsis (28	Sepsis (28	Sepsis (28	TRUE	igd	0.0027	4.36E-07	7261	M35253.m	TRUE	reported	VboRmj	2	TRUE	0.003431	24.99311
2969	rs1171316	A	G	A	G	-0.017	-0.01108	0.8575	0.841307	FALSE	FALSE	FALSE	ieu-b-508	3	30953169	0.045998	486484	0.809731	Sepsis (28	Sepsis (28	Sepsis (28	TRUE	igd	0.0034	5.63E-07	7257	M31453.m	TRUE	reported	vdTd9M	2	TRUE	0.003433	24.99311
536	rs1319586	A	C	A	C	0.022	0.032376	0.2043	0.204844	FALSE	FALSE	FALSE	ieu-b-508	6	1E+08	0.041406	486484	0.434264	Sepsis (28	Sepsis (28	Sepsis (28	TRUE	igd	0.0044	4.13E-07	7237	M00599.m	TRUE	reported	nRJY3s	2	TRUE	0.003443	24.99309
3805	rs1165406	T	C	T	C	0.009	0.012238	0.5837	0.590778	FALSE	FALSE	FALSE	ieu-b-508	17	63875004	0.034076	486484	0.71948	Sepsis (28	Sepsis (28	Sepsis (28	TRUE	igd	0.0018	8.68E-07	7229	M32644.m	TRUE	reported	JAa2WD	2	TRUE	0.003446	24.99308
3825	rs723967	T	C	T	C	0.0095	-0.04496	0.4929	0.487863	FALSE	FALSE	FALSE	ieu-b-508	15	58696300	0.033551	486484	0.180241	Sepsis (28	Sepsis (28	Sepsis (28	TRUE	igd	0.0019	3.27E-07	7229	M32644.m	TRUE	reported	JAa2WD	2	TRUE	0.003446	24.99308
1362	rs4880550	T	C	T	C	0.015	-0.03536	0.1292	0.158607	FALSE	FALSE	FALSE	ieu-b-508	10	2101863	0.045669	486484	0.438716	Sepsis (28	Sepsis (28	Sepsis (28	TRUE	igd	0.003	6.32E-07	7226	M02730.m	TRUE	reported	XYa035	2	TRUE	0.003448	24.99308
7562	rs1776453	A	G	A	G	0.009	-0.02302	0.1973	0.198056	FALSE	FALSE	FALSE	ieu-b-508	16	49040995	0.042348	486484	0.586728	Sepsis (28	Sepsis (28	Sepsis (28	TRUE	igd	0.0018	9.31E-07	7217	M35270.m	TRUE	reported	Ct8V1n	2	TRUE	0.003452	24.99307
2641	rs1472923	T	C	T	C	-0.0165	0.044452	0.1656	0.15969	FALSE	FALSE	FALSE	ieu-b-508	4	1.27E+08	0.045617	486484	0.329825	Sepsis (28	Sepsis (28	Sepsis (28	TRUE	igd	0.0033	8.09E-07	7184	M22548.m	TRUE	reported	mZtwX2	2	TRUE	0.003468	24.99304
1990	rs2059238	A	C	A	C	-0.0435	-0.02728	0.1958	0.214002	FALSE	FALSE	FALSE	ieu-b-508	16	78258810	0.040699	486484	0.502734	Sepsis (28	Sepsis (28	Sepsis (28	TRUE	igd	0.0087	6.60E-07	7071	M18254.m	TRUE	reported	clvu6V	2	TRUE	0.003523	24.99293
5827	rs4823474	T	C	T	C	0.009	0.039083	0.5551	0.550714	FALSE	FALSE	FALSE	ieu-b-508	22	47560778	0.033649	486484	0.245434	Sepsis (28	Sepsis (28	Sepsis (28	TRUE	igd	0.0018	8.28E-07	7066	M33515.m	TRUE	reported	X9mul1	2	TRUE	0.003526	24.99292
5768	rs1295340	T	C	T	C	0.022	-0.02487	0.1905	0.187168	FALSE	FALSE	FALSE	ieu-b-508	18	45852017	0.042779	486484	0.560965	Sepsis (28	Sepsis (28	Sepsis (28	TRUE	igd	0.0044	7.33E-07	7044	M33509.m	TRUE	reported	56SCCs	2	TRUE	0.003537	24.9929
1382	rs1560792	A	G	A	G	0.009	0.002851	0.3354	0.345131	FALSE	FALSE	FALSE	ieu-b-508	7	48436309	0.035386	486484	0.935778	Sepsis (28	Sepsis (28	Sepsis (28	TRUE	igd	0.0018	3.33E-07	7037	M02734.m	TRUE	reported	0y0dDD	2	TRUE	0.00354	24.99289
8422	rs510573	T	G	T	G	-0.0085	-0.02558	0.6641	0.674735	FALSE	FALSE	FALSE	ieu-b-508	18	4330954	0.036047	486484	0.477959	Sepsis (28	Sepsis (28	Sepsis (28	TRUE	igd	0.0017	9.11E-07	6940	M36808.m	TRUE	reported	u7Iqaj	2	TRUE	0.003589	24.9928
2026	rs1055689	A	G	A	G	-0.0135	0.059695	0.2969	0.306843	FALSE	FALSE	FALSE	ieu-b-508	20	42805719	0.036236	486484	0.099474	Sepsis (28	Sepsis (28	Sepsis (28	TRUE	igd	0.0027	4.26E-07	6939	M18349.m	TRUE	reported	M5qPs8	2	TRUE	0.00359	24.99279
7673	rs1110027	A	G	A	G	-0.009	0.010426	0.8163	0.789707	FALSE	FALSE	FALSE	ieu-b-508	4	1.61E+08	0.040827	486484	0.798446	Sepsis (28	Sepsis (28	Sepsis (28	TRUE	igd	0.0018	7.74E-07	6920	M35327.m	TRUE	reported	SquFiY	2	TRUE	0.0036	24.99277
7678	rs1798613	A	G	A	G	0.009	-0.05724	0.2257	0.270146	FALSE	FALSE	FALSE	ieu-b-508	12	32277522	0.037551	486484	0.127434	Sepsis (28	Sepsis (28	Sepsis (28	TRUE	igd	0.0018	3.74E-07	6920	M35327.m	TRUE	reported	SquFiY	2	TRUE	0.0036	24.99277
6239	rs1189639	T	C	T	C	0.122	0.136978	0.0255	0.02999	FALSE	FALSE	FALSE	ieu-b-508	2	1.96E+08	0.09735	486484	0.159407	Sepsis (28	Sepsis (28	Sepsis (28	TRUE	igd	0.0244	5.47E-07	6756	M33935.m	TRUE	reported	ELBTDc	2	TRUE	0.003687	24.9926
6794	rs1075206	A	G	A	G	-0.009	-0.01783	0.669	0.665661	FALSE	FALSE	FALSE	ieu-b-508	10	16853900	0.035463	486484	0.615184	Sepsis (28	Sepsis (28	Sepsis (28	TRUE	igd	0.0018	4.61E-07	6755	M34359.m	TRUE	reported	0UotaJ	2	TRUE	0.003687	24.9926
6832	rs749514	A	G	A	G	-0.009	-0.01507	0.3055	0.307016	FALSE	FALSE	FALSE	ieu-b-508	11	1.27E+08	0.036221	486484	0.677312	Sepsis (28	Sepsis (28	Sepsis (28	TRUE	igd	0.0018	4.71E-07	6755	M34359.m	TRUE	reported	0UotaJ	2	TRUE	0.003687	24.9926
7995	rs1225903	A	G	A	G	0.014	-0.08765	0.1453	0.149786	FALSE	FALSE	FALSE	ieu-b-508	10	64412618	0.046806	486484	0.061108	Sepsis (28	Sepsis (28	Sepsis (28	TRUE	igd	0.0028	9.07E-07	6604	M35754.m	TRUE	reported	IlXOo1	2	TRUE	0.003771	24.99243
2412	rs1073964	A	G	A	G	-0.0135	-0.00256	0.4429	0.439096	FALSE	FALSE	FALSE	ieu-b-508	9	1.27E+08	0.033765	486484	0.939508	Sepsis (28	Sepsis (28	Sepsis (28	TRUE	igd	0.0027	4.06E-07	6556	M21188.m	TRUE	reported	kvdnjc	2	TRUE	0.003799	24.99237
2413	rs1078376	T	G	T	G	0.0135	-0.038	0.7251	0.725855	FALSE	FALSE	FALSE	ieu-b-508	12	56039025	0.037427	486484	0.309985	Sepsis (28	Sepsis (28	Sepsis (28	TRUE	igd	0.0027	6.63E-07	6556	M21188.m	TRUE	reported	kvdnjc	2	TRUE	0.003799	24.99237
8462	rs1211447	A	G	A	G	-0.009	0.041446	0.4738	0.474264	FALSE	FALSE	FALSE	ieu-b-508	8	66383566	0.033425	486484	0.214991	Sepsis (28	Sepsis (28	Sepsis (28	TRUE	igd	0.0018	1.22E-06	6552	M37058.m	TRUE	reported	32p8qV	2	TRUE	0.003801	24.99237
3972	rs1050482	A	G	A	G	-0.0095	0.022054	0.7046	0.701518	FALSE	FALSE	FALSE	ieu-b-508	8	87167184	0.036484	486484	0.54553	Sepsis (28	Sepsis (28	Sepsis (28	TRUE	igd	0.0019	5.81E-07	6497	M32729.m	TRUE	reported	XGRSN1	2	TRUE	0.003833	24.9923
3987	rs1735765	T	C	T	C	-0.0095	0.077694	0.6988	0.698555	FALSE	FALSE	FALSE	ieu-b-508	2	1.9E+08	0.036405	486484	0.03283	Sepsis (28	Sepsis (28	Sepsis (28	TRUE	igd	0.0019	5.48E-07	6497	M32729.m	TRUE	reported	XGRSN1	2	TRUE	0.003833	24.9923
4006	rs6791299	A	G	A	G	-0.0095	0.011323	0.534	0.535495	FALSE	FALSE	FALSE	ieu-b-508	3	6634839	0.033577	486484	0.735951	Sepsis (28	Sepsis (28	Sepsis (28	TRUE	igd	0.0019	3.71E-07	6497	M32729.m	TRUE	reported	XGRSN1	2	TRUE	0.003833	24.9923
2233	rs789736	T	G	T	G	0.019	0.03771	0.2464	0.238968	FALSE	FALSE	FALSE	ieu-b-508	12	64538324	0.038998	486484	0.333554	Sepsis (28	Sepsis (28	Sepsis (28	TRUE	igd	0.0038	4.03E-07	6385	M19396.m	TRUE	reported	Xa1QqA	2	TRUE	0.0039	24.99217
2471	rs7934036	T	G	T	G	0.0125	0.014071	0.3475	0.340771	FALSE	FALSE	FALSE	ieu-b-508	11	65451020	0.03516	486484	0.689003	Sepsis (28	Sepsis (28	Sepsis (28	TRUE	igd	0.0025	9.76E-07	6180	M22030.m	TRUE	reported	ZNV0qR	2	TRUE	0.004029	24.99191
4251	rs930609	A	G	A	G	-0.028	-0.1065	0.7886	0.783111	FALSE	FALSE	FALSE	ieu-b-508	12	1.18E+08	0.040451	486484	0.008469	Sepsis (28	Sepsis (28	Sepsis (28	TRUE	igd	0.0056	4.98E-07	6169	M32795.m	TRUE	reported	0YBWkh	2	TRUE	0.004036	24.99189
7257	rs1422587	T	C	T	C	0.0095	-0.02146	0.3521	0.353302	FALSE	FALSE	FALSE	ieu-b-508	5	1.47E+08	0.034872	486484	0.538235	Sepsis (28	Sepsis (28	Sepsis (28	TRUE	igd	0.0019	4.87E-07	5885	M34912.m	TRUE	reported	W8lcT6	2	TRUE	0.00423	24.9915
7650	rs4480029	A	C	A	C	0.009	0.021448	0.5598	0.547359	FALSE	FALSE	FALSE	ieu-b-508	7	1.52E+08	0.033721	486484	0.52476	Sepsis (28	Sepsis (28	Sepsis (28	TRUE	igd	0.0018	9.94E-07	5768	M35326.m	TRUE	reported	jRytyJ	2	TRUE	0.004316	24.99133
7666	rs924321	T	C	T	C	0.0115	0.008738	0.305	0.318463	FALSE	FALSE	FALSE	ieu-b-508	3	1.93E+08	0.035847	486484	0.807423	Sepsis (28	Sepsis (28	Sepsis (28	TRUE	igd	0.0023	5.74E-07	5768	M35326.m	TRUE	reported	jRytyJ	2	TRUE	0.004316	24.99133
7667	rs9290704	T	G	T	G	-0.0085	-0.00614	0.5814	0.585315	FALSE	FALSE	FALSE	ieu-b-508	3	1.02E+08	0.034063	486484	0.856923	Sepsis (28	Sepsis (28	Sepsis (28	TRUE	igd	0.0017	8.98E-07	5768	M35326.m	TRUE	reported	jRytyJ	2	TRUE	0.004316	24.99133
1638	rs2491294	A	G	A	G	-0.01	0.020736	0.31	0.311805	FALSE	FALSE	FALSE	ieu-b-508	1	88302604	0.036044	486484	0.565094	Sepsis (28	Sepsis (28	Sepsis (28	TRUE	igd	0.002	5.73E-07	5604	M15140.m	TRUE	reported	jEcqMJ	2	TRUE	0.004441	24.99108
8615	rs39578	A	G	A	G	-0.01	-0.02619	0.729	0.736659	FALSE	FALSE	FALSE	ieu-b-508	19	21386126	0.037887	486484	0.489409	Sepsis (28	Sepsis (28	Sepsis (28	TRUE	igd	0.002	5.73E-07	5604	M37506.m	TRUE	reported	m2kaRt	2	TRUE	0.004441	24.99108
3783	rs7912716	T	C	T	C	0.015	0.023242	0.104	0.108705	FALSE	FALSE	FALSE	ieu-b-508	10	16985317	0.053502	486484	0.663983	Sepsis (28	Sepsis (28	Sepsis (28	TRUE	igd	0.003	5.73E-07	5598	M32634.m	TRUE	reported	7T2RUz	2	TRUE	0.004446	24.99107
3961	rs4838726	C	G	C	G	0.045	0.041358	0.032	0.031054	FALSE	TRUE	FALSE	ieu-b-508	10	1.35E+08	0.096449	486484	0.668067	Sepsis (28	Sepsis (28	Sepsis (28	TRUE	igd	0.009	5.73E-07	5597	M32709.m	TRUE	reported	YR8SB4	2	TRUE	0.004447	24.99107
9	rs1000137	T	C	T	C	-0.005	0.044258	0.475	0.477687	FALSE	FALSE	FALSE	ieu-b-508	7	1.58E+08	0.033435	486484	0.185603	Sepsis (28	Sepsis (28	Sepsis (28	TRUE	igd	0.001	5.73E-07	5592	M00054.m	TRUE	reported	QTDAnh	2	TRUE	0.004451	24.99106
22	rs1083636	T	C	T	C	0.005	0.016745	0.607	0.610918	FALSE	FALSE	FALSE	ieu-b-508	11	35306345	0.034271	486484	0.625128	Sepsis (28	Sepsis (28	Sepsis (28	TRUE	igd	0.001	5.73E-07	5592	M00054.m	TRUE	reported	QTDAnh	2	TRUE	0.004451	24.99106
75	rs2230532	T	C	T	C	0.005	-0.04264	0.349	0.345677	FALSE	FALSE	FALSE	ieu-b-508	14	97321689	0.035048	486484	0.223779	Sepsis (28	Sepsis (28	Sepsis (28	TRUE	igd	0.001	5.73E-07	5592	M00054.m	TRUE	reported	QTDAnh	2	TRUE	0.004451	24.99106
79	rs2505644	T	C	T	C	0.005	-0.03614	0.558	0.556744	FALSE	FALSE	FALSE	ieu-b-508	10	44175098	0.033626	486484	0.282534	Sepsis (28	Sepsis (28	Sepsis (28	TRUE	igd	0.001	5.73E-07	5592	M00054.m	TRUE	reported	QTDAnh	2	TRUE	0.004451	24.99106
98	rs2977272	T	C	T	C	0.005	-0.03643	0.341	0.346977	FALSE	FALSE	FALSE	ieu-b-508	1	17536952	0.034944	486484	0.29716	Sepsis (28	Sepsis (28	Sepsis (28	TRUE	igd	0.001	5.73E-07	5592	M00054.m	TRUE	reported	QTDAnh	2	TRUE	0.004451	24.99106
100	rs341973	T	C	T	C	0.005	0.018831	0.493	0.494256	FALSE	FALSE	FALSE	ieu-b-508	3	5268162	0.033543	486484	0.574537	Sepsis (28	Sepsis (28	Sepsis (28	TRUE	igd	0.001	5.73E-07	5592	M00054.m	TRUE	reported	QTDAnh	2	TRUE	0.004451	24.99106
114	rs4407399	T	C	T	C	-0.005	0.048512	0.603	0.606617	FALSE	FALSE	FALSE	ieu-b-508	3	1.88E+08	0.034158	486484	0.155551	Sepsis (28	Sepsis (28	Sepsis (28	TRUE	igd	0.001	5.73E-07	5592	M00054.m	TRUE	reported	QTDAnh	2	TRUE	0.004451	24.99106
131	rs5997110	T	C	T	C	-0.005	-0.07729	0.523	0.51616	FALSE	FALSE	FALSE	ieu-b-508	22	27036505	0.033525	486484	0.021141	Sepsis (28	Sepsis (28	Sepsis (28	TRUE	igd	0.001	5.73E-07	5592	M00054.m	TRUE	reported	QTDAnh	2	TRUE	0.004451	24.99106
204	rs1000428	A	G	A	G	0.005	0.037256	0.374	0.377414	FALSE	FALSE	FALSE	ieu-b-508	4	59559715	0.03446	486484	0.279629	Sepsis (28	Sepsis (28	Sepsis (28	TRUE	igd	0.001	5.73E-07	5589	M00060.m	TRUE	reported	kqwl4V	2	TRUE	0.004453	24.99105
205	rs1003887	T	C	T	C	-0.005	-0.00246	0.651	0.645013	FALSE	FALSE	FALSE	ieu-b-508	19	17927400	0.034787	486484	0.943547	Sepsis (28	Sepsis (28	Sepsis (28	TRUE	igd	0.001	5.73E-07	5589	M00060.m	TRUE	reported	kqwl4V	2	TRUE	0.004453	24.99105
206	rs1019018	T	C	T	C	0.005	-0.01565	0.662	0.657368	FALSE	FALSE	FALSE	ieu-b-508	2	49894154	0.035229	486484	0.656839	Sepsis (28	Sepsis (28	Sepsis (28	TRUE	igd	0.001	5.73E-07	5589	M00060.m	TRUE	reported	kqwl4V	2	TRUE	0.004453	24.99105
266	rs1818106	A	C	A	C	0.005	-0.05934	0.501	0.501212	FALSE	FALSE	FALSE	ieu-b-508	11	1.04E+08	0.033577	486484	0.077195	Sepsis (28	Sepsis (28	Sepsis (28	TRUE	igd	0.001	5.73E-07	5589	M00060.m	TRUE	reported	kqwl4V	2	TRUE	0.004453	24.99105
297	rs2898645	T	C	T	C	-0.005	0.010307	0.545	0.550173	FALSE	FALSE	FALSE	ieu-b-508	17	75332699	0.033573	486484	0.758831	Sepsis (28	Sepsis (28	Sepsis (28	TRUE	igd	0.001	5.73E-07	5589	M00060.m	TRUE	reported	kqwl4V	2	TRUE	0.004453	24.99105
308	rs4148593	A	G	A	G	0.005	-0.004	0.624	0.617911	FALSE	FALSE	FALSE	ieu-b-508	3	1.84E+08	0.034322	486484	0.907303	Sepsis (28	Sepsis (28	Sepsis (28	TRUE	igd	0.001	5.73E-07	5589	M00060.m	TRUE	reported	kqwl4V	2	TRUE	0.004453	24.99105
334	rs6470194	A	G	A	G	0.005	-0.04164	0.406	0.410037	FALSE	FALSE	FALSE	ieu-b-508	8	1.25E+08	0.035821	486484	0.245017	Sepsis (28	Sepsis (28	Sepsis (28	TRUE	igd	0.001	5.73E-07	5589	M00060.m	TRUE	reported	kqwl4V	2	TRUE	0.004453	24.99105
650	rs4347830	A	G	A	G	0.01	0.037294	0.337	0.343257	FALSE	FALSE	FALSE	ieu-b-508	2	2.28E+08	0.035263	486484	0.290237	Sepsis (28	Sepsis (28	Sepsis (28	TRUE	igd	0.002	5.73E-07	5588	M01125.m	TRUE	reported	yw0clB	2	TRUE	0.004454	24.99105
785	rs4420815	A	G	A	G	-0.015	-0.09085	0.143	0.15028	FALSE	FALSE	FALSE	ieu-b-508	3	33794769	0.046574	486484	0.051107	Sepsis (28	Sepsis (28	Sepsis (28	TRUE	igd	0.003	5.73E-07	5586	M01336.m	TRUE	reported	FAlrya	2	TRUE	0.004456	24.99105
2692	rs1022713	T	C	T	C	0.01	-0.001	0.435	0.437786	FALSE	FALSE	FALSE	ieu-b-508	7	1.57E+08	0.03357	486484	0.976272	Sepsis (28	Sepsis (28	Sepsis (28	TRUE	igd	0.002	5.73E-07	5584	M25459.m	TRUE	reported	ACJY35	2	TRUE	0.004457	24.99105
2693	rs1050457	A	G	A	G	-0.01	0.049517	0.531	0.548035	FALSE	FALSE	FALSE	ieu-b-508	19	731144	0.033583	486484	0.140356	Sepsis (28	Sepsis (28	Sepsis (28	TRUE	igd	0.002	5.73E-07	5584	M25459.m	TRUE	reported	ACJY35	2	TRUE	0.004457	24.99105
1701	rs9811522	A	G	A	G	0.01	0.034766	0.174	0.185035	FALSE	FALSE	FALSE	ieu-b-508	3	5879366	0.043777	486484	0.427098	Sepsis (28	Sepsis (28	Sepsis (28	TRUE	igd	0.002	5.73E-07	5583	M15488.m	TRUE	reported	SPriBO	2	TRUE	0.004458	24.99104
2906	rs1105769	A	C	A	C	-0.0135	-0.02406	0.3077	0.305056	FALSE	FALSE	FALSE	ieu-b-508	5	1.19E+08	0.036171	486484	0.505903	Sepsis (28	Sepsis (28	Sepsis (28	TRUE	igd	0.0027	7.19E-07	5562	M27728.m	TRUE	reported	WOp1XE	2	TRUE	0.004475	24.99101
2739	rs1012431	T	C	T	C	-0.01	0.093437	0.235	0.232678	FALSE	FALSE	FALSE	ieu-b-508	9	1.25E+08	0.039531	486484	0.018096	Sepsis (28	Sepsis (28	Sepsis (28	TRUE	igd	0.002	5.73E-07	5542	M27256.m	TRUE	reported	mJwzsi	2	TRUE	0.004491	24.99098
7754	rs1000347	T	C	T	C	0.02	0.026181	0.112	0.116445	FALSE	FALSE	FALSE	ieu-b-508	4	92200866	0.051988	486484	0.614538	Sepsis (28	Sepsis (28	Sepsis (28	TRUE	igd	0.004	5.73E-07	5538	M35439.m	TRUE	reported	DnuAGZ	2	TRUE	0.004494	24.99097
5364	rs8130833	A	G	A	G	-0.01	-0.04294	0.707	0.716733	FALSE	FALSE	FALSE	ieu-b-508	21	42556885	0.036914	486484	0.244746	Sepsis (28	Sepsis (28	Sepsis (28	TRUE	igd	0.002	5.73E-07	5536	M33380.m	TRUE	reported	OHRfl5	2	TRUE	0.004496	24.99097
3823	rs6593266	A	G	A	G	0.01	0.006528	0.306	0.31247	FALSE	FALSE	FALSE	ieu-b-508	7	55658709	0.036269	486484	0.857156	Sepsis (28	Sepsis (28	Sepsis (28	TRUE	igd	0.002	5.73E-07	5463	M32644.m	TRUE	reported	JAa2WD	2	TRUE	0.004555	24.99085
5822	rs4626675	T	C	T	C	0.01	0.055973	0.466	0.471528	FALSE	FALSE	FALSE	ieu-b-508	9	77860670	0.034684	486484	0.106573	Sepsis (28	Sepsis (28	Sepsis (28	TRUE	igd	0.002	5.73E-07	5382	M33515.m	TRUE	reported	X9mul1	2	TRUE	0.004624	24.99071
6611	rs7120732	T	C	T	C	-0.055	-0.10301	0.018	0.019763	FALSE	FALSE	FALSE	ieu-b-508	11	24838844	0.119512	486484	0.38873	Sepsis (28	Sepsis (28	Sepsis (28	TRUE	igd	0.011	5.73E-07	5321	M34306.m	TRUE	reported	WPXPUN	2	TRUE	0.004676	24.9906
8335	rs1169860	A	G	A	G	-0.0235	-0.02242	0.828	0.81107	FALSE	FALSE	FALSE	ieu-b-508	20	17351160	0.042746	486484	0.599895	Sepsis (28	Sepsis (28	Sepsis (28	TRUE	igd	0.0047	6.70E-07	5292	M36673.m	TRUE	reported	CD5lvR	2	TRUE	0.004702	24.99055
2961	rs4148088	C	G	C	G	0.025	0.073564	0.926	0.918884	FALSE	TRUE	FALSE	ieu-b-508	21	43628935	0.061395	486484	0.230834	Sepsis (28	Sepsis (28	Sepsis (28	TRUE	igd	0.005	5.73E-07	5286	M30805.m	TRUE	reported	YHV70c	2	TRUE	0.004707	24.99054
7368	rs595286	A	G	A	G	-0.0095	-0.01956	0.5466	0.554473	FALSE	FALSE	FALSE	ieu-b-508	18	60280097	0.03388	486484	0.563636	Sepsis (28	Sepsis (28	Sepsis (28	TRUE	igd	0.0019	8.62E-07	4900	M35137.m	TRUE	reported	Eq2siS	2	TRUE	0.005076	24.9898
506	rs526744	T	C	T	C	-0.021	0.025949	0.243	0.244488	FALSE	FALSE	FALSE	ieu-b-508	19	49300605	0.039424	486484	0.510414	Sepsis (28	Sepsis (28	Sepsis (28	TRUE	igd	0.0042	7.14E-07	4500	M00575.m	TRUE	reported	Mv0t8k	2	TRUE	0.005525	24.98889
4489	rs4363506	T	C	T	C	0.0245	0.024451	0.5678	0.575479	FALSE	FALSE	FALSE	ieu-b-508	10	1.29E+08	0.033985	486484	0.47186	Sepsis (28	Sepsis (28	Sepsis (28	TRUE	igd	0.0049	6.01E-07	2773	M33131.m	TRUE	reported	uGKURO	2	TRUE	0.008935	24.98197
6588	rs1690878	A	G	A	G	-0.0255	-0.00154	0.2086	0.211799	FALSE	FALSE	FALSE	ieu-b-508	12	13375486	0.040968	486484	0.97005	Sepsis (28	Sepsis (28	Sepsis (28	TRUE	igd	0.0051	5.52E-07	2278	M34289.m	TRUE	reported	Knp8ry	2	TRUE	0.010855	24.97805
4398	rs894282	A	T	A	T	0.013	-0.08616	0.0899	0.09346	FALSE	TRUE	FALSE	ieu-b-508	4	1.74E+08	0.057692	486484	0.13532	Sepsis (28	Sepsis (28	Sepsis (28	TRUE	igd	0.0026	9.04E-07	1763	M32867.m	TRUE	reported	Jcsyzm	2	TRUE	0.013982	24.97164
8248	rs1849217	T	C	T	C	0.007	-0.05372	0.8178	0.828082	FALSE	FALSE	FALSE	ieu-b-508	4	77259248	0.044299	486484	0.225289	Sepsis (28	Sepsis (28	Sepsis (28	TRUE	igd	0.0014	8.98E-07	1763	M36515.m	TRUE	reported	yZaYzr	2	TRUE	0.013982	24.97164
694	rs703014	A	G	A	G	0.0135	0.031018	0.326	0.329627	FALSE	FALSE	FALSE	ieu-b-508	10	28641805	0.035972	486484	0.388532	Sepsis (28	Sepsis (28	Sepsis (28	TRUE	igd	0.0027	7.19E-07	1761	M01299.m	TRUE	reported	gfM0a3	2	TRUE	0.013998	24.97161
6556	rs1822378	T	C	T	C	0.0315	0.062362	0.6721	0.699001	FALSE	FALSE	FALSE	ieu-b-508	18	11913615	0.036708	486484	0.089347	Sepsis (28	Sepsis (28	Sepsis (28	TRUE	igd	0.0063	6.37E-07	1761	M34244.m	TRUE	reported	JyF0Sp	2	TRUE	0.013998	24.97161
2864	rs9489956	T	C	T	C	0.0395	-0.0261	0.782	0.783185	FALSE	FALSE	FALSE	ieu-b-508	6	1.21E+08	0.040656	486484	0.520872	Sepsis (28	Sepsis (28	Sepsis (28	TRUE	igd	0.0079	5.28E-07	1758	M27672.m	TRUE	reported	5cTg55	2	TRUE	0.014021	24.97156
7826	rs8031307	A	G	A	G	-0.052	0.015171	0.8267	0.810365	FALSE	FALSE	FALSE	ieu-b-508	15	96583154	0.043538	486484	0.727497	Sepsis (28	Sepsis (28	Sepsis (28	TRUE	igd	0.0104	5.14E-07	1640	M35472.m	TRUE	reported	oZU10n	2	TRUE	0.015015	24.96951
6672	rs2826581	T	G	T	G	-0.0695	0.022023	0.789	0.775199	FALSE	FALSE	FALSE	ieu-b-508	21	22249511	0.040041	486484	0.582316	Sepsis (28	Sepsis (28	Sepsis (28	TRUE	igd	0.0139	5.58E-07	1612	M34327.m	TRUE	reported	Il4Lrr	2	TRUE	0.015272	24.96898
8298	rs1715247	T	C	T	C	0.2502	-0.07793	0.0133	0.039841	FALSE	FALSE	FALSE	ieu-b-508	10	12960211	0.085628	486484	0.362788	Sepsis (28	Sepsis (28	Sepsis (28	TRUE	igd	0.05	5.58E-07	589	M36559.m	TRUE	reported	g6l9hg	2	TRUE	0.040779	24.95499
8350	rs1491633	A	G	A	G	0.059	0.12615	0.0765	0.073929	FALSE	FALSE	FALSE	ieu-b-508	15	68957142	0.063748	486484	0.047829	Sepsis (28	Sepsis (28	Sepsis (28	TRUE	igd	0.0118	5.48E-07	922	M36738.m	TRUE	reported	mVQQcD	2	TRUE	0.026399	24.94577
4279	rs6686996	T	C	T	C	0.0919	0.063723	0.0256	0.024698	FALSE	FALSE	FALSE	ieu-b-508	1	57065295	0.108714	486484	0.55777	Sepsis (28	Sepsis (28	Sepsis (28	TRUE	igd	0.0184	5.81E-07	6200	M32808.m	TRUE	reported	vnr36k	2	TRUE	0.004007	24.93763
4298	rs1715081	A	C	A	C	-0.0714	-0.02956	0.0377	0.053668	FALSE	FALSE	FALSE	ieu-b-508	8	9790737	0.075742	486484	0.696325	Sepsis (28	Sepsis (28	Sepsis (28	TRUE	igd	0.0143	6.11E-07	7255	M32836.m	TRUE	reported	d4IVXe	2	TRUE	0.003425	24.92325

8527	rs328637	A	G	A	G	-0.0864	0.025526	0.1638	0.16458	FALSE	FALSE	FALSE	ieu-b-508	2	9243530	0.04504	486484	0.570897	Sepsis (28	Sepsis (28	Sepsis (28	TRUE	igd	0.0173	6.13E-07	2560	M37112.m	TRUE	reported	P3GiAg	2	TRUE	0.009649	24.92274
6454	rs1258813	C	G	C	G	0.0694	0.003089	0.0374	0.040532	FALSE	TRUE	FALSE	ieu-b-508	14	98698325	0.085104	486484	0.971042	Sepsis (28	Sepsis (28	Sepsis (28	TRUE	igd	0.0139	6.33E-07	4961	M34106.m	TRUE	reported	8DuoNM	2	TRUE	0.005	24.91806
2002	rs219633	A	G	A	G	0.1483	0.005206	0.121	0.114932	FALSE	FALSE	FALSE	ieu-b-508	21	27868059	0.052479	486484	0.920975	Sepsis (28	Sepsis (28	Sepsis (28	TRUE	igd	0.0297	5.96E-07	2433	M18281.m	TRUE	reported	3mLp7z	2	TRUE	0.010144	24.91221
6146	rs138832	A	G	A	G	0.0779	0.062105	0.7832	0.783963	FALSE	FALSE	FALSE	ieu-b-508	22	50178082	0.040611	486484	0.126199	Sepsis (28	Sepsis (28	Sepsis (28	TRUE	igd	0.0156	6.18E-07	1742	M33883.m	TRUE	reported	RT7YNu	2	TRUE	0.014113	24.90731
944	rs7458704	T	G	T	G	-0.0544	-0.10826	0.984	0.97943	FALSE	FALSE	FALSE	ieu-b-508	7	67422093	0.117575	486484	0.357163	Sepsis (28	Sepsis (28	Sepsis (28	TRUE	igd	0.0109	5.60E-07	7160	M01508.m	TRUE	reported	go526k	2	TRUE	0.003467	24.90138
6455	rs1552059	A	G	A	G	0.0519	-0.09313	0.0942	0.113926	FALSE	FALSE	FALSE	ieu-b-508	4	5732227	0.052571	486484	0.076472	Sepsis (28	Sepsis (28	Sepsis (28	TRUE	igd	0.0104	6.38E-07	4961	M34106.m	TRUE	reported	8DuoNM	2	TRUE	0.004995	24.8939
4642	rs61130478	A	G	A	G	0.8585	-0.18331	0.9803	0.980055	FALSE	FALSE	FALSE	ieu-b-508	20	42533655	0.119418	486484	0.124778	Sepsis (28	Sepsis (28	Sepsis (28	TRUE	igd	0.1698	4.28E-07	76	M33173.m	TRUE	reported	04fkUQ	2	TRUE	0.251693	24.88991
5150	rs1168794	T	G	T	G	-0.0484	0.052285	0.5576	0.55444	FALSE	FALSE	FALSE	ieu-b-508	2	31032243	0.033772	486484	0.121574	Sepsis (28	Sepsis (28	Sepsis (28	TRUE	igd	0.0097	6.03E-07	5716	M33192.m	TRUE	reported	CyfjKE	2	TRUE	0.004337	24.8883
8545	rs9602130	A	C	A	C	0.0579	0.008177	0.3642	0.368688	FALSE	FALSE	FALSE	ieu-b-508	13	83793707	0.034656	486484	0.813474	Sepsis (28	Sepsis (28	Sepsis (28	TRUE	igd	0.0116	5.72E-07	1745	M37190.m	TRUE	reported	tiNuK9	2	TRUE	0.014076	24.88531
4369	rs1748316	A	G	A	G	-0.0544	-0.00779	0.6475	0.643341	FALSE	FALSE	FALSE	ieu-b-508	8	15872655	0.03497	486484	0.823612	Sepsis (28	Sepsis (28	Sepsis (28	TRUE	igd	0.0109	6.58E-07	1988	M32863.m	TRUE	reported	cLOFYE	2	TRUE	0.012374	24.88328
8078	rs1695174	A	G	A	G	0.0449	0.010099	0.1023	0.110509	FALSE	FALSE	FALSE	ieu-b-508	18	7442604	0.053328	486484	0.849803	Sepsis (28	Sepsis (28	Sepsis (28	TRUE	igd	0.009	6.09E-07	2651	M36009.m	TRUE	reported	2EUZ1U	2	TRUE	0.009301	24.87024
5188	rs7808781	A	G	A	G	0.1777	-0.10101	0.1358	0.129259	FALSE	FALSE	FALSE	ieu-b-508	7	52233497	0.050789	486484	0.046728	Sepsis (28	Sepsis (28	Sepsis (28	TRUE	igd	0.0356	5.98E-07	996	M33197.m	TRUE	reported	D6xaBg	2	TRUE	0.024405	24.86577
5308	rs2207775	A	G	A	G	0.0454	0.036833	0.5486	0.543728	FALSE	FALSE	FALSE	ieu-b-508	10	83776091	0.033664	486484	0.273892	Sepsis (28	Sepsis (28	Sepsis (28	TRUE	igd	0.0091	6.13E-07	1863	M33359.m	TRUE	reported	dfJmmS	2	TRUE	0.013184	24.86351
8086	rs1076503	T	C	T	C	-0.0798	-0.05944	0.1892	0.193596	FALSE	FALSE	FALSE	ieu-b-508	10	1.33E+08	0.042422	486484	0.161169	Sepsis (28	Sepsis (28	Sepsis (28	TRUE	igd	0.016	6.34E-07	3939	M36095.m	TRUE	reported	9OLvcB	2	TRUE	0.006275	24.86253
5956	rs1007401	A	G	A	G	0.0424	-0.02512	0.2515	0.26135	FALSE	FALSE	FALSE	ieu-b-508	5	1.24E+08	0.038057	486484	0.509168	Sepsis (28	Sepsis (28	Sepsis (28	TRUE	igd	0.0085	5.48E-07	1860	M33658.m	TRUE	reported	9QszGO	2	TRUE	0.013201	24.85574
5708	rs1114993	A	G	A	G	-0.0349	0.064575	0.1083	0.108141	FALSE	FALSE	FALSE	ieu-b-508	16	73653046	0.054145	486484	0.233019	Sepsis (28	Sepsis (28	Sepsis (28	TRUE	igd	0.007	6.24E-07	5107	M33488.m	TRUE	reported	mZmDY6	2	TRUE	0.004844	24.84761
8314	rs6761802	A	G	A	G	-0.0334	-0.01477	0.3637	0.365481	FALSE	FALSE	FALSE	ieu-b-508	2	31223514	0.034827	486484	0.671399	Sepsis (28	Sepsis (28	Sepsis (28	TRUE	igd	0.0067	6.53E-07	3346	M36585.m	TRUE	reported	pXZg13	2	TRUE	0.007372	24.83611
2169	rs1184450	C	G	C	G	-0.1381	-0.11104	0.9853	0.981734	FALSE	TRUE	FALSE	ieu-b-508	14	30813871	0.126445	486484	0.379857	Sepsis (28	Sepsis (28	Sepsis (28	TRUE	igd	0.0277	6.28E-07	1738	M19324.m	TRUE	reported	1pj7ZT	2	TRUE	0.0141	24.8272
3392	rs1212158	A	G	A	G	-0.0289	-0.02307	0.2068	0.229944	FALSE	FALSE	FALSE	ieu-b-508	1	2.28E+08	0.039739	486484	0.56153	Sepsis (28	Sepsis (28	Sepsis (28	TRUE	igd	0.0058	5.43E-07	7346	M32425.m	TRUE	reported	u5RLGY	2	TRUE	0.003368	24.82112
7401	rs1028374	A	G	A	G	0.0264	0.06943	0.1409	0.152626	FALSE	FALSE	FALSE	ieu-b-508	1	1.68E+08	0.047796	486484	0.146328	Sepsis (28	Sepsis (28	Sepsis (28	TRUE	igd	0.0053	5.88E-07	7263	M35160.m	TRUE	reported	eRox3e	2	TRUE	0.003405	24.80484
6344	rs8081778	T	C	T	C	0.0259	0.03082	0.1587	0.162731	FALSE	FALSE	FALSE	ieu-b-508	17	73312272	0.04523	486484	0.495611	Sepsis (28	Sepsis (28	Sepsis (28	TRUE	igd	0.0052	7.06E-07	6998	M33957.m	TRUE	reported	mrKpMv	2	TRUE	0.003532	24.80097
5556	rs1515977	A	G	A	G	-0.9112	-0.06362	0.0594	0.039977	FALSE	FALSE	FALSE	ieu-b-508	2	17291222	0.085439	486484	0.456525	Sepsis (28	Sepsis (28	Sepsis (28	TRUE	igd	0.1799	4.06E-07	60	M33423.m	TRUE	reported	MoEnO7	2	TRUE	0.299512	24.79944
8067	rs4665383	C	G	C	G	-0.0254	-0.04371	0.7261	0.743643	FALSE	TRUE	FALSE	ieu-b-508	2	27791555	0.038287	486484	0.253562	Sepsis (28	Sepsis (28	Sepsis (28	TRUE	igd	0.0051	5.49E-07	4529	M35978.m	TRUE	reported	CN0wRR	2	TRUE	0.005447	24.79335
3707	rs2977258	A	G	A	G	0.0244	-0.00302	0.3036	0.309074	FALSE	FALSE	FALSE	ieu-b-508	1	17501898	0.036316	486484	0.933787	Sepsis (28	Sepsis (28	Sepsis (28	TRUE	igd	0.0049	5.39E-07	5955	M32593.m	TRUE	reported	6zVl1i	2	TRUE	0.004147	24.78801
5117	rs974135	T	C	T	C	-0.0912	0.053196	0.2826	0.327035	FALSE	FALSE	FALSE	ieu-b-508	2	60333030	0.035675	486484	0.135925	Sepsis (28	Sepsis (28	Sepsis (28	TRUE	igd	0.0183	5.84E-07	1017	M33178.m	TRUE	reported	g22WMS	2	TRUE	0.023839	24.78749
6376	rs1771181	T	C	T	C	0.0214	0.013084	0.2564	0.258373	FALSE	FALSE	FALSE	ieu-b-508	7	22268760	0.03835	486484	0.732976	Sepsis (28	Sepsis (28	Sepsis (28	TRUE	igd	0.0043	5.09E-07	7322	M33968.m	TRUE	reported	sgVOvT	2	TRUE	0.003371	24.76122
7183	rs1001046	T	G	T	G	0.0214	-0.03021	0.6845	0.69096	FALSE	FALSE	FALSE	ieu-b-508	4	20601311	0.036216	486484	0.404181	Sepsis (28	Sepsis (28	Sepsis (28	TRUE	igd	0.0043	6.53E-07	4800	M34539.m	TRUE	reported	puA8ni	2	TRUE	0.005134	24.75766
3640	rs4650516	T	C	T	C	-0.0448	-0.0174	0.5865	0.588182	FALSE	FALSE	FALSE	ieu-b-508	1	81683122	0.033911	486484	0.607848	Sepsis (28	Sepsis (28	Sepsis (28	TRUE	igd	0.009	5.75E-07	2091	M32560.m	TRUE	reported	sLwsEw	2	TRUE	0.011711	24.75457
6982	rs7106005	T	C	T	C	0.0204	0.002861	0.8158	0.797753	FALSE	FALSE	FALSE	ieu-b-508	11	11598372	0.041689	486484	0.945291	Sepsis (28	Sepsis (28	Sepsis (28	TRUE	igd	0.0041	7.66E-07	7344	M34407.m	TRUE	reported	X7I83Y	2	TRUE	0.00336	24.74995
2999	rs2073779	T	C	T	C	-0.2562	0.040622	0.9759	0.970613	FALSE	FALSE	FALSE	ieu-b-508	22	20077863	0.098663	486484	0.680539	Sepsis (28	Sepsis (28	Sepsis (28	TRUE	igd	0.0514	6.09E-07	522	M31536.m	TRUE	reported	3nAuUI	2	TRUE	0.045433	24.74941
6240	rs1248539	A	G	A	G	0.0403	-0.01381	0.3273	0.34007	FALSE	FALSE	FALSE	ieu-b-508	3	1.92E+08	0.035306	486484	0.695671	Sepsis (28	Sepsis (28	Sepsis (28	TRUE	igd	0.0081	6.08E-07	6756	M33935.m	TRUE	reported	ELBTDc	2	TRUE	0.003651	24.74637
5448	rs4148622	A	G	A	G	0.0398	0.012368	0.2618	0.266694	FALSE	FALSE	FALSE	ieu-b-508	11	17449002	0.038136	486484	0.745703	Sepsis (28	Sepsis (28	Sepsis (28	TRUE	igd	0.008	7.66E-07	5185	M33390.m	TRUE	reported	t0EYdH	2	TRUE	0.004751	24.74108
7296	rs1268073	A	G	A	G	0.206	0.020827	0.9872	0.987822	FALSE	FALSE	FALSE	ieu-b-508	8	77608979	0.153292	486484	0.89193	Sepsis (28	Sepsis (28	Sepsis (28	TRUE	igd	0.0414	6.44E-07	1764	M35126.m	TRUE	reported	1haOJA	2	TRUE	0.013841	24.73097
4306	rs1171660	A	G	A	G	-0.0189	0.003456	0.6827	0.679364	FALSE	FALSE	FALSE	ieu-b-508	10	61443808	0.035767	486484	0.923024	Sepsis (28	Sepsis (28	Sepsis (28	TRUE	igd	0.0038	6.89E-07	6753	M32838.m	TRUE	reported	hyR7zA	2	TRUE	0.00365	24.73021
6731	rs1741548	A	G	A	G	-0.898	-0.06013	0.989	0.892071	FALSE	FALSE	FALSE	ieu-b-508	10	22597174	0.054904	486484	0.273446	Sepsis (28	Sepsis (28	Sepsis (28	TRUE	igd	0.179	5.26E-07	115	M34338.m	TRUE	reported	h4FB9a	2	TRUE	0.179555	24.73018
5301	rs6760934	T	C	T	C	-0.1921	-0.14353	0.9725	0.978203	FALSE	FALSE	FALSE	ieu-b-508	2	1.39E+08	0.114867	486484	0.211473	Sepsis (28	Sepsis (28	Sepsis (28	TRUE	igd	0.0385	5.91E-07	292	M33353.m	TRUE	reported	ZIN4gk	2	TRUE	0.078563	24.72569
2280	rs1260353	T	C	T	C	-0.0368	0.157475	0.0394	0.059281	FALSE	FALSE	FALSE	ieu-b-508	17	19242017	0.070613	486484	0.02574	Sepsis (28	Sepsis (28	Sepsis (28	TRUE	igd	0.0074	6.62E-07	7301	M20675.m	TRUE	reported	CXe9rE	2	TRUE	0.003376	24.72369
486	rs2234233	A	G	A	G	-0.1234	-0.10735	0.135	0.16795	FALSE	FALSE	FALSE	ieu-b-508	5	9629529	0.044655	486484	0.016213	Sepsis (28	Sepsis (28	Sepsis (28	TRUE	igd	0.0248	6.38E-07	1233	M00553.m	TRUE	reported	IFjXiL	2	TRUE	0.019685	24.71849
7847	rs9849918	A	C	A	C	0.0363	-0.06444	0.6538	0.665364	FALSE	FALSE	FALSE	ieu-b-508	3	1.87E+08	0.035566	486484	0.070016	Sepsis (28	Sepsis (28	Sepsis (28	TRUE	igd	0.0073	7.74E-07	5035	M35551.m	TRUE	reported	5Sxswl	2	TRUE	0.004887	24.71696
2101	rs6468384	A	C	A	C	0.0179	-0.01904	0.5691	0.552655	FALSE	FALSE	FALSE	ieu-b-508	8	36772358	0.033477	486484	0.569557	Sepsis (28	Sepsis (28	Sepsis (28	TRUE	igd	0.0036	5.63E-07	7367	M18467.m	TRUE	reported	64PaNn	2	TRUE	0.003345	24.71628
5714	rs2466085	A	G	A	G	0.1762	-0.03141	0.0186	0.012352	FALSE	FALSE	FALSE	ieu-b-508	8	32519332	0.154851	486484	0.839241	Sepsis (28	Sepsis (28	Sepsis (28	TRUE	igd	0.0354	6.70E-07	834	M33488.m	TRUE	reported	mZmDY6	2	TRUE	0.028849	24.71511
1123	rs577596	A	G	A	G	-0.0567	0.014093	0.3267	0.347183	FALSE	FALSE	FALSE	ieu-b-508	22	25931606	0.035048	486484	0.687613	Sepsis (28	Sepsis (28	Sepsis (28	TRUE	igd	0.0114	6.68E-07	2063	M01640.m	TRUE	reported	o3qxzl	2	TRUE	0.011849	24.71355
2283	rs1499686	A	G	A	G	-0.0174	0.055738	0.8079	0.81472	FALSE	FALSE	FALSE	ieu-b-508	8	3322528	0.043387	486484	0.1989	Sepsis (28	Sepsis (28	Sepsis (28	TRUE	igd	0.0035	7.49E-07	7301	M20675.m	TRUE	reported	CXe9rE	2	TRUE	0.003374	24.70833
4482	rs1249168	C	G	C	G	-0.0368	0.014614	0.7659	0.752445	FALSE	TRUE	FALSE	ieu-b-508	3	1.76E+08	0.038683	486484	0.705581	Sepsis (28	Sepsis (28	Sepsis (28	TRUE	igd	0.0074	7.13E-07	1767	M33131.m	TRUE	reported	uGKURO	2	TRUE	0.013803	24.70247
4616	rs1278449	A	T	A	T	0.9912	0.032565	0.9821	0.666449	FALSE	TRUE	FALSE	ieu-b-508	10	14463299	0.035438	486484	0.358133	Sepsis (28	Sepsis (28	Sepsis (28	TRUE	igd	0.1968	4.76E-07	76	M33173.m	TRUE	reported	04fkUQ	2	TRUE	0.250251	24.69963
491	rs7701941	C	G	C	G	-0.5561	0.047651	0.0194	0.019614	FALSE	TRUE	FALSE	ieu-b-508	5	1.35E+08	0.120637	486484	0.692848	Sepsis (28	Sepsis (28	Sepsis (28	TRUE	igd	0.1115	6.10E-07	283	M00553.m	TRUE	reported	IFjXiL	2	TRUE	0.080795	24.6988
5314	rs1716546	T	C	T	C	0.0358	0.04603	0.1252	0.224122	FALSE	FALSE	FALSE	ieu-b-508	7	12034187	0.040065	486484	0.250596	Sepsis (28	Sepsis (28	Sepsis (28	TRUE	igd	0.0072	7.83E-07	1378	M33363.m	TRUE	reported	7K0MM0	2	TRUE	0.017625	24.68711
5463	rs739484	T	C	T	C	0.1354	-0.02399	0.0864	0.118721	FALSE	FALSE	FALSE	ieu-b-508	12	1.12E+08	0.052035	486484	0.644834	Sepsis (28	Sepsis (28	Sepsis (28	TRUE	igd	0.0272	6.56E-07	528	M33391.m	TRUE	reported	EgMd1b	2	TRUE	0.044828	24.68604
2141	rs1948095	A	C	A	C	-0.1423	-0.09678	0.8599	0.865565	FALSE	FALSE	FALSE	ieu-b-508	11	98747735	0.04909	486484	0.048667	Sepsis (28	Sepsis (28	Sepsis (28	TRUE	igd	0.0286	6.39E-07	698	M18497.m	TRUE	reported	90JFPc	2	TRUE	0.034252	24.68491
8552	rs2762353	A	G	A	G	0.0318	0.008605	0.4552	0.433382	FALSE	FALSE	FALSE	ieu-b-508	6	25794431	0.033638	486484	0.798103	Sepsis (28	Sepsis (28	Sepsis (28	TRUE	igd	0.0064	6.70E-07	7355	M37202.m	TRUE	reported	BtsIDx	2	TRUE	0.003345	24.68176
3939	rs9457843	T	C	T	C	0.0159	0.089871	0.1218	0.118684	FALSE	FALSE	FALSE	ieu-b-508	6	1.61E+08	0.051836	486484	0.08296	Sepsis (28	Sepsis (28	Sepsis (28	TRUE	igd	0.0032	9.53E-07	7308	M32698.m	TRUE	reported	srqncz	2	TRUE	0.003367	24.68172
3067	rs1752156	A	G	A	G	-0.0472	0.037786	0.803	0.797268	FALSE	FALSE	FALSE	ieu-b-508	10	96634310	0.041482	486484	0.362354	Sepsis (28	Sepsis (28	Sepsis (28	TRUE	igd	0.0095	6.44E-07	7363	M31787.m	TRUE	reported	XMp8YI	2	TRUE	0.003341	24.6785
4037	rs1261781	T	G	T	G	0.0477	-0.08419	0.0449	0.042326	FALSE	FALSE	FALSE	ieu-b-508	2	6980931	0.08308	486484	0.310887	Sepsis (28	Sepsis (28	Sepsis (28	TRUE	igd	0.0096	7.54E-07	1748	M32739.m	TRUE	reported	dYSC7n	2	TRUE	0.013927	24.66023
4836	rs1710604	T	C	T	C	-0.255	-0.12959	0.0221	0.022656	FALSE	FALSE	FALSE	ieu-b-508	14	69087864	0.113896	486484	0.255208	Sepsis (28	Sepsis (28	Sepsis (28	TRUE	igd	0.0513	6.61E-07	1017	M33178.m	TRUE	reported	g22WMS	2	TRUE	0.023719	24.65987
2578	rs6919834	T	C	T	C	0.0144	0.005038	0.1237	0.128479	FALSE	FALSE	FALSE	ieu-b-508	6	1.06E+08	0.050014	486484	0.91976	Sepsis (28	Sepsis (28	Sepsis (28	TRUE	igd	0.0029	8.27E-07	6535	M22177.m	TRUE	reported	2s6Sly	2	TRUE	0.003759	24.64882
7443	rs1786534	A	G	A	G	-0.0412	0.059782	0.089	0.092975	FALSE	FALSE	FALSE	ieu-b-508	4	1.18E+08	0.057946	486484	0.302216	Sepsis (28	Sepsis (28	Sepsis (28	TRUE	igd	0.0083	6.16E-07	6234	M35189.m	TRUE	reported	54XZFj	2	TRUE	0.003937	24.63196
4022	rs1689751	A	C	A	C	-0.0412	0.068964	0.1641	0.190447	FALSE	FALSE	FALSE	ieu-b-508	6	27278020	0.0425	486484	0.104656	Sepsis (28	Sepsis (28	Sepsis (28	TRUE	igd	0.0083	7.25E-07	6000	M32735.m	TRUE	reported	QE2vKQ	2	TRUE	0.00409	24.63165
587	rs1235282	A	G	A	G	-0.0134	-0.01857	0.1506	0.151043	FALSE	FALSE	FALSE	ieu-b-508	9	74006667	0.046397	486484	0.68902	Sepsis (28	Sepsis (28	Sepsis (28	TRUE	igd	0.0027	5.84E-07	7367	M01110.m	TRUE	reported	0A31SI	2	TRUE	0.003332	24.62431
6428	rs1158384	A	C	A	C	0.0134	0.04363	0.9439	0.939713	FALSE	FALSE	FALSE	ieu-b-508	1	2.31E+08	0.070266	486484	0.534646	Sepsis (28	Sepsis (28	Sepsis (28	TRUE	igd	0.0027	6.34E-07	7362	M34062.m	TRUE	reported	RZyHKP	2	TRUE	0.003335	24.62431
1215	rs7739842	T	G	T	G	0.0134	-0.00669	0.8202	0.810187	FALSE	FALSE	FALSE	ieu-b-508	6	1.32E+08	0.042801	486484	0.875763	Sepsis (28	Sepsis (28	Sepsis (28	TRUE	igd	0.0027	8.00E-07	7348	M01670.m	TRUE	reported	kjghbc	2	TRUE	0.003341	24.6243
7772	rs1760863	A	G	A	G	-0.0134	-0.02613	0.2319	0.247986	FALSE	FALSE	FALSE	ieu-b-508	4	45079676	0.038731	486484	0.499887	Sepsis (28	Sepsis (28	Sepsis (28	TRUE	igd	0.0027	5.01E-07	7256	M35439.m	TRUE	reported	DnuAGZ	2	TRUE	0.003383	24.62421
1544	rs1738658	A	C	A	C	-0.0134	0.052096	0.2495	0.257242	FALSE	FALSE	FALSE	ieu-b-508	13	1.09E+08	0.038429	486484	0.175213	Sepsis (28	Sepsis (28	Sepsis (28	TRUE	igd	0.0027	9.74E-07	6864	M12129.m	TRUE	reported	iNzdgb	2	TRUE	0.003576	24.62382
2430	rs7504246	A	G	A	G	-0.0134	0.008677	0.2945	0.293604	FALSE	FALSE	FALSE	ieu-b-508	18	37850005	0.036749	486484	0.813347	Sepsis (28	Sepsis (28	Sepsis (28	TRUE	igd	0.0027	7.62E-07	6556	M21188.m	TRUE	reported	kvdnjc	2	TRUE	0.003743	24.62349
5980	rs6060270	T	G	T	G	0.0134	-0.05886	0.2057	0.211375	FALSE	FALSE	FALSE	ieu-b-508	20	33736814	0.040761	486484	0.148742	Sepsis (28	Sepsis (28	Sepsis (28	TRUE	igd	0.0027	8.58E-07	6279	M33666.m	TRUE	reported	dvWEtk	2	TRUE	0.003907	24.62316
2927	rs734567	T	C	T	C	0.0134	0.04685	0.5906	0.596781	FALSE	FALSE	FALSE	ieu-b-508	19	17015607	0.034167	486484	0.170312	Sepsis (28	Sepsis (28	Sepsis (28	TRUE	igd	0.0027	7.83E-07	5562	M27728.m	TRUE	reported	WOp1XE	2	TRUE	0.004409	24.62214
4525	rs2926419	T	C	T	C	-0.0551	-0.04948	0.2092	0.203479	FALSE	FALSE	FALSE	ieu-b-508	4	1.31E+08	0.041804	486484	0.236587	Sepsis (28	Sepsis (28	Sepsis (28	TRUE	igd	0.0111	7.12E-07	2391	M33137.m	TRUE	reported	ONVNk3	2	TRUE	0.010201	24.62033
2105	rs1026775	T	C	T	C	-0.0591	-0.01506	0.4495	0.448265	FALSE	FALSE	FALSE	ieu-b-508	3	1.4E+08	0.033618	486484	0.654255	Sepsis (28	Sepsis (28	Sepsis (28	TRUE	igd	0.0119	6.40E-07	917	M18474.m	TRUE	reported	B6Xd02	2	TRUE	0.026193	24.6112
7984	rs7142493	T	C	T	C	-0.0129	-0.00256	0.7195	0.715861	FALSE	FALSE	FALSE	ieu-b-508	14	62276237	0.037052	486484	0.944841	Sepsis (28	Sepsis (28	Sepsis (28	TRUE	igd	0.0026	1.11E-06	7358	M35718.m	TRUE	reported	oUwc0a	2	TRUE	0.003334	24.61017
5631	rs9633478	A	G	A	G	-0.0129	0.061261	0.0763	0.071816	FALSE	FALSE	FALSE	ieu-b-508	1	77885282	0.064583	486484	0.34284	Sepsis (28	Sepsis (28	Sepsis (28	TRUE	igd	0.0026	5.31E-07	7336	M33442.m	TRUE	reported	B1Peb3	2	TRUE	0.003344	24.61015
8532	rs6050798	T	C	T	C	-0.0655	0.003898	0.4924	0.487549	FALSE	FALSE	FALSE	ieu-b-508	20	2621998	0.033534	486484	0.907458	Sepsis (28	Sepsis (28	Sepsis (28	TRUE	igd	0.0132	7.03E-07	2560	M37112.m	TRUE	reported	P3GiAg	2	TRUE	0.009527	24.60341
2593	rs1250154	A	C	A	C	-0.0253	-0.00781	0.8955	0.890469	FALSE	FALSE	FALSE	ieu-b-508	4	1.18E+08	0.053561	486484	0.88411	Sepsis (28	Sepsis (28	Sepsis (28	TRUE	igd	0.0051	7.87E-07	7259	M22189.m	TRUE	reported	YSD5fY	2	TRUE	0.003379	24.6026
7320	rs1896198	A	G	A	G	-0.0124	0.051645	0.416	0.422529	FALSE	FALSE	FALSE	ieu-b-508	8	18822249	0.033927	486484	0.127946	Sepsis (28	Sepsis (28	Sepsis (28	TRUE	igd	0.0025	8.67E-07	7344	M35127.m	TRUE	reported	RNsmjl	2	TRUE	0.003339	24.5949
3314	rs2164397	C	G	C	G	-0.0124	0.024119	0.2279	0.200291	FALSE	TRUE	FALSE	ieu-b-508	2	2.38E+08	0.041767	486484	0.563623	Sepsis (28	Sepsis (28	Sepsis (28	TRUE	igd	0.0025	8.73E-07	7307	M32393.m	TRUE	reported	Pub0X0	2	TRUE	0.003356	24.59487
1424	rs2247330	T	C	T	C	0.0124	0.028051	0.6524	0.648995	FALSE	FALSE	FALSE	ieu-b-508	12	96424122	0.035103	486484	0.424238	Sepsis (28	Sepsis (28	Sepsis (28	TRUE	igd	0.0025	4.56E-07	6941	M03127.m	TRUE	reported	JscEZw	2	TRUE	0.003532	24.59451
4154	rs1082359	T	C	T	C	-0.0248	0.023222	0.7417	0.739711	FALSE	FALSE	FALSE	ieu-b-508	10	72442042	0.038142	486484	0.542636	Sepsis (28	Sepsis (28	Sepsis (28	TRUE	igd	0.005	8.42E-07	6642	M32758.m	TRUE	reported	AF0m66	2	TRUE	0.00369	24.59419
5968	rs1311144	T	C	T	C	0.0124	0.045823	0.7906	0.793845	FALSE	FALSE	FALSE	ieu-b-508	4	1.77E+08	0.041281	486484	0.266982	Sepsis (28	Sepsis (28	Sepsis (28	TRUE	igd	0.0025	1.04E-06	6279	M33666.m	TRUE	reported	dvWEtk	2	TRUE	0.003903	24.59376
3394	rs1307289	A	G	A	G	0.0243	0.043331	0.628	0.633655	FALSE	FALSE	FALSE	ieu-b-508	3	20028570	0.034629	486484	0.210826	Sepsis (28	Sepsis (28	Sepsis (28	TRUE	igd	0.0049	7.95E-07	7346	M32425.m	TRUE	reported	u5RLGY	2	TRUE	0.003337	24.58681
4740	rs1224636	A	C	A	C	0.2665	-0.20173	0.9792	0.978619	FALSE	FALSE	FALSE	ieu-b-508	10	72007149	0.115782	486484	0.081457	Sepsis (28	Sepsis (28	Sepsis (28	TRUE	igd	0.0537	6.93E-07	1017	M33178.m	TRUE	reported	g22WMS	2	TRUE	0.023645	24.58051
2997	rs1418200	T	C	T	C	0.2117	0.055743	0.0408	0.03039	FALSE	FALSE	FALSE	ieu-b-508	1	2.34E+08	0.100277	486484	0.578288	Sepsis (28	Sepsis (28	Sepsis (28	TRUE	igd	0.0426	6.63E-07	423	M31536.m	TRUE	reported	3nAuUI	2	TRUE	0.055162	24.579
1088	rs2231142	T	G	T	G	0.0119	-0.03336	0.1106	0.113225	FALSE	FALSE	FALSE	ieu-b-508	4	89052323	0.052486	486484	0.525079	Sepsis (28	Sepsis (28	Sepsis (28	TRUE	igd	0.0024	8.43E-07	7371	M01604.m	TRUE	reported	hE3I2k	2	TRUE	0.003324	24.5784
1414	rs1126072	C	G	C	G	-0.0119	-0.04011	0.4106	0.382912	FALSE	TRUE	FALSE	ieu-b-508	1	16703448	0.034239	486484	0.241399	Sepsis (28	Sepsis (28	Sepsis (28	TRUE	igd	0.0024	8.41E-07	6941	M03127.m	TRUE	reported	JscEZw	2	TRUE	0.00353	24.57799
7814	rs887733	T	C	T	C	0.0119	0.007592	0.5524	0.565009	FALSE	FALSE	FALSE	ieu-b-508	4	10183108	0.033701	486484	0.82176	Sepsis (28	Sepsis (28	Sepsis (28	TRUE	igd	0.0024	1.02E-06	6883	M35464.m	TRUE	reported	Ir9dHp	2	TRUE	0.003559	24.57793
8571	rs7183977	T	C	T	C	-0.0233	0.014324	0.6755	0.652885	FALSE	FALSE	FALSE	ieu-b-508	15	40377092	0.03526	486484	0.684573	Sepsis (28	Sepsis (28	Sepsis (28	TRUE	igd	0.0047	7.19E-07	7333	M37203.m	TRUE	reported	3glIlv	2	TRUE	0.00334	24.56958
6743	rs3750318	T	C	T	C	0.895	0.109508	0.012	0.030845	FALSE	FALSE	FALSE	ieu-b-508	9	1.32E+08	0.096501	486484	0.256467	Sepsis (28	Sepsis (28	Sepsis (28	TRUE	igd	0.179	5.73E-07	115	M34338.m	TRUE	reported	h4FB9a	2	TRUE	0.178571	24.56522
6744	rs4976704	T	C	T	C	-0.905	0.034688	0.989	0.817213	FALSE	FALSE	FALSE	ieu-b-508	5	1.77E+08	0.04316	486484	0.421562	Sepsis (28	Sepsis (28	Sepsis (28	TRUE	igd	0.181	5.73E-07	115	M34338.m	TRUE	reported	h4FB9a	2	TRUE	0.178571	24.56522
7104	rs1017988	T	C	T	C	0.2058	-0.07371	0.168	0.183841	FALSE	FALSE	FALSE	ieu-b-508	2	1.27E+08	0.043316	486484	0.08882	Sepsis (28	Sepsis (28	Sepsis (28	TRUE	igd	0.0412	5.90E-07	128	M34516.m	TRUE	reported	7Ah2wy	2	TRUE	0.163133	24.56161
2854	rs6957252	T	C	T	C	0.0739	0.003596	0.6329	0.622183	FALSE	FALSE	FALSE	ieu-b-508	7	89437129	0.034473	486484	0.916913	Sepsis (28	Sepsis (28	Sepsis (28	TRUE	igd	0.0149	6.84E-07	1309	M27531.m	TRUE	reported	v01zDr	2	TRUE	0.018446	24.56135
1187	rs369895	A	G	A	G	-0.0114	-0.03679	0.574	0.551415	FALSE	FALSE	FALSE	ieu-b-508	8	1.44E+08	0.033496	486484	0.272029	Sepsis (28	Sepsis (28	Sepsis (28	TRUE	igd	0.0023	1.08E-06	7346	M01645.m	TRUE	reported	d5nAn3	2	TRUE	0.003333	24.56042
1782	rs7405977	T	C	T	C	-0.0114	0.040083	0.3918	0.418063	FALSE	FALSE	FALSE	ieu-b-508	17	39278305	0.033871	486484	0.236654	Sepsis (28	Sepsis (28	Sepsis (28	TRUE	igd	0.0023	1.17E-06	7331	M15676.m	TRUE	reported	s1NfKN	2	TRUE	0.00334	24.56041
5589	rs7628540	T	G	T	G	-0.6303	-0.02273	0.1273	0.179158	FALSE	FALSE	FALSE	ieu-b-508	3	1375734	0.043535	486484	0.601531	Sepsis (28	Sepsis (28	Sepsis (28	TRUE	igd	0.1251	4.67E-07	60	M33423.m	TRUE	reported	MoEnO7	2	TRUE	0.297302	24.53899
2333	rs2067997	T	C	T	C	-0.0213	-0.05358	0.8694	0.862937	FALSE	FALSE	FALSE	ieu-b-508	6	35471246	0.048601	486484	0.270234	Sepsis (28	Sepsis (28	Sepsis (28	TRUE	igd	0.0043	9.40E-07	7366	M21044.m	TRUE	reported	Ym5rae	2	TRUE	0.00332	24.53038
2466	rs4631845	A	G	A	G	0.0213	0.057151	0.0851	0.092342	FALSE	FALSE	FALSE	ieu-b-508	11	99225548	0.057791	486484	0.322704	Sepsis (28	Sepsis (28	Sepsis (28	TRUE	igd	0.0043	9.87E-07	6180	M22030.m	TRUE	reported	ZNV0qR	2	TRUE	0.003955	24.52911
6219	rs4402594	T	C	T	C	-0.0451	-0.04367	0.4067	0.404384	FALSE	FALSE	FALSE	ieu-b-508	16	74701360	0.034026	486484	0.199318	Sepsis (28	Sepsis (28	Sepsis (28	TRUE	igd	0.0091	7.99E-07	1294	M33901.m	TRUE	reported	mQkhrb	2	TRUE	0.018628	24.52441
6949	rs1250572	T	C	T	C	-0.0917	0.003515	0.1409	0.219091	FALSE	FALSE	FALSE	ieu-b-508	4	1.83E+08	0.040366	486484	0.930605	Sepsis (28	Sepsis (28	Sepsis (28	TRUE	igd	0.0185	7.38E-07	1018	M34400.m	TRUE	reported	QXmzfR	2	TRUE	0.023566	24.52117
2452	rs7522439	A	G	A	G	0.0208	0.116487	0.8977	0.900891	FALSE	FALSE	FALSE	ieu-b-508	1	17577394	0.055863	486484	0.037048	Sepsis (28	Sepsis (28	Sepsis (28	TRUE	igd	0.0042	7.34E-07	7284	M21630.m	TRUE	reported	7eaO4H	2	TRUE	0.003356	24.51934
4175	rs1764950	A	G	A	G	-0.0416	0.017726	0.9227	0.896823	FALSE	FALSE	FALSE	ieu-b-508	13	51480318	0.054916	486484	0.746856	Sepsis (28	Sepsis (28	Sepsis (28	TRUE	igd	0.0084	8.11E-07	6712	M32759.m	TRUE	reported	9eIHr7	2	TRUE	0.003641	24.51877
6641	rs1335444	A	G	A	G	-0.087	0.047288	0.8928	0.963679	FALSE	FALSE	FALSE	ieu-b-508	5	40189846	0.089197	486484	0.596005	Sepsis (28	Sepsis (28	Sepsis (28	TRUE	igd	0.0175	6.33E-07	248	M34322.m	TRUE	reported	16GdG9	2	TRUE	0.090626	24.51579
8104	rs1865661	A	T	A	T	1.4423	0.02894	0.9836	0.979012	FALSE	TRUE	FALSE	ieu-b-508	2	52075051	0.118412	486484	0.806919	Sepsis (28	Sepsis (28	Sepsis (28	TRUE	igd	0.2889	5.95E-07	122	M36097.m	TRUE	reported	RHr18S	2	TRUE	0.169638	24.51532
6414	rs2469215	T	C	T	C	-0.0307	0.057837	0.1051	0.110336	FALSE	FALSE	FALSE	ieu-b-508	15	76611933	0.0534	486484	0.278765	Sepsis (28	Sepsis (28	Sepsis (28	TRUE	igd	0.0062	6.70E-07	7132	M34040.m	TRUE	reported	sJBy3i	2	TRUE	0.003426	24.51159
5503	rs5994305	A	G	A	G	0.0307	0.022263	0.8318	0.831409	FALSE	FALSE	FALSE	ieu-b-508	22	30820707	0.044688	486484	0.618347	Sepsis (28	Sepsis (28	Sepsis (28	TRUE	igd	0.0062	7.15E-07	5822	M33420.m	TRUE	reported	eTToDb	2	TRUE	0.004194	24.51005
5974	rs1742392	T	C	T	C	0.0203	-0.00801	0.91	0.909823	FALSE	FALSE	FALSE	ieu-b-508	5	1.23E+08	0.058749	486484	0.89149	Sepsis (28	Sepsis (28	Sepsis (28	TRUE	igd	0.0041	5.47E-07	6279	M33666.m	TRUE	reported	dvWEtk	2	TRUE	0.003889	24.50677
4116	rs1365505	T	C	T	C	0.0198	-0.02083	0.681	0.682068	FALSE	FALSE	FALSE	ieu-b-508	15	50488041	0.035915	486484	0.561994	Sepsis (28	Sepsis (28	Sepsis (28	TRUE	igd	0.004	1.06E-06	6658	M32755.m	TRUE	reported	WDrNdi	2	TRUE	0.003667	24.49514
7457	rs1319751	T	C	T	C	-0.0396	0.010537	0.0369	0.041499	FALSE	FALSE	FALSE	ieu-b-508	6	4171143	0.083704	486484	0.89982	Sepsis (28	Sepsis (28	Sepsis (28	TRUE	igd	0.008	6.63E-07	6543	M35193.m	TRUE	reported	BMMWXU	2	TRUE	0.003731	24.49501
2537	rs1261620	A	G	A	G	0.0099	0.011545	0.2818	0.279487	FALSE	FALSE	FALSE	ieu-b-508	2	28474423	0.037263	486484	0.756695	Sepsis (28	Sepsis (28	Sepsis (28	TRUE	igd	0.002	4.16E-07	6535	M22177.m	TRUE	reported	2s6Sly	2	TRUE	0.003735	24.495
7956	rs1246410	A	G	A	G	0.0297	-0.00455	0.1572	0.16152	FALSE	FALSE	FALSE	ieu-b-508	2	4974780	0.045475	486484	0.920339	Sepsis (28	Sepsis (28	Sepsis (28	TRUE	igd	0.006	8.24E-07	6447	M35678.m	TRUE	reported	56yR01	2	TRUE	0.003786	24.4949
6338	rs1220119	A	T	A	T	-0.0391	-0.00892	0.9375	0.929466	FALSE	TRUE	FALSE	ieu-b-508	6	18139802	0.065622	486484	0.891853	Sepsis (28	Sepsis (28	Sepsis (28	TRUE	igd	0.0079	7.65E-07	6998	M33957.m	TRUE	reported	mrKpMv	2	TRUE	0.003488	24.48923
3079	rs1701017	C	G	C	G	-0.0292	0.014768	0.9497	0.941798	FALSE	TRUE	FALSE	ieu-b-508	1	2.22E+08	0.071672	486484	0.836754	Sepsis (28	Sepsis (28	Sepsis (28	TRUE	igd	0.0059	6.61E-07	7346	M32197.m	TRUE	reported	VZjwa9	2	TRUE	0.003323	24.48744
6759	rs1721615	A	T	A	T	-0.1713	-0.0466	0.9738	0.938603	FALSE	TRUE	FALSE	ieu-b-508	4	1.83E+08	0.071771	486484	0.516152	Sepsis (28	Sepsis (28	Sepsis (28	TRUE	igd	0.0346	7.13E-07	1649	M34339.m	TRUE	reported	RwGZ7c	2	TRUE	0.014647	24.48136
7796	rs2728817	A	T	A	T	0.1986	0.117518	0.0954	0.090694	FALSE	TRUE	FALSE	ieu-b-508	12	22456347	0.058431	486484	0.0443	Sepsis (28	Sepsis (28	Sepsis (28	TRUE	igd	0.04	6.95E-07	284	M35451.m	TRUE	reported	GMRGAN	2	TRUE	0.079868	24.47762
1167	rs1003973	A	G	A	G	0.0198	0.033113	0.5787	0.612863	FALSE	FALSE	FALSE	ieu-b-508	5	1.11E+08	0.034443	486484	0.336346	Sepsis (28	Sepsis (28	Sepsis (28	TRUE	igd	0.004	8.47E-07	1767	M01645.m	TRUE	reported	d5nAn3	2	TRUE	0.013677	24.47477
6474	rs1304928	A	T	A	T	-0.957	0.057898	0.011	0.032751	FALSE	TRUE	FALSE	ieu-b-508	21	22817279	0.098711	486484	0.55751	Sepsis (28	Sepsis (28	Sepsis (28	TRUE	igd	0.192	6.22E-07	134	M34109.m	TRUE	reported	piJuC7	2	TRUE	0.156405	24.47319
6894	rs7721259	T	C	T	C	0.2104	-0.00664	0.1111	0.118719	FALSE	FALSE	FALSE	ieu-b-508	5	1.36E+08	0.051508	486484	0.897495	Sepsis (28	Sepsis (28	Sepsis (28	TRUE	igd	0.0401	1.53E-07	18	M34365.m	TRUE	reported	8eexD0	2	TRUE	0.604654	24.47091
3209	rs1710664	T	C	T	C	0.0282	-0.07872	0.0296	0.03032	FALSE	FALSE	FALSE	ieu-b-508	1	81998027	0.098388	486484	0.423662	Sepsis (28	Sepsis (28	Sepsis (28	TRUE	igd	0.0057	8.85E-07	7344	M32339.m	TRUE	reported	SVp504	2	TRUE	0.003322	24.46979
5389	rs1178671	C	G	C	G	-0.0094	0.039264	0.6543	0.633383	FALSE	TRUE	FALSE	ieu-b-508	8	10838413	0.034971	486484	0.261531	Sepsis (28	Sepsis (28	Sepsis (28	TRUE	igd	0.0019	4.26E-07	7315	M33389.m	TRUE	reported	Hf6XVM	2	TRUE	0.003335	24.46976
5529	rs3810252	A	G	A	G	-0.0094	-0.07199	0.8089	0.805003	FALSE	FALSE	FALSE	ieu-b-508	19	14608314	0.041971	486484	0.086282	Sepsis (28	Sepsis (28	Sepsis (28	TRUE	igd	0.0019	5.21E-07	7306	M33422.m	TRUE	reported	PLgjrT	2	TRUE	0.003339	24.46975
3828	rs759287	A	G	A	G	0.0094	-0.00603	0.3082	0.322382	FALSE	FALSE	FALSE	ieu-b-508	2	49197684	0.035756	486484	0.86599	Sepsis (28	Sepsis (28	Sepsis (28	TRUE	igd	0.0019	4.48E-07	7229	M32644.m	TRUE	reported	JAa2WD	2	TRUE	0.003374	24.46968
5807	rs1218924	A	G	A	G	0.0094	-0.03413	0.6201	0.620118	FALSE	FALSE	FALSE	ieu-b-508	11	1.01E+08	0.034398	486484	0.321122	Sepsis (28	Sepsis (28	Sepsis (28	TRUE	igd	0.0019	3.39E-07	7066	M33515.m	TRUE	reported	X9mul1	2	TRUE	0.003452	24.46953
7997	rs1257009	T	C	T	C	-0.0094	-0.00448	0.5847	0.58231	FALSE	FALSE	FALSE	ieu-b-508	10	9710675	0.033861	486484	0.894755	Sepsis (28	Sepsis (28	Sepsis (28	TRUE	igd	0.0019	9.18E-07	6604	M35754.m	TRUE	reported	IlXOo1	2	TRUE	0.003693	24.46904
8010	rs4641528	T	C	T	C	0.0094	0.020677	0.5683	0.569383	FALSE	FALSE	FALSE	ieu-b-508	12	72387342	0.033676	486484	0.539221	Sepsis (28	Sepsis (28	Sepsis (28	TRUE	igd	0.0019	9.44E-07	6604	M35754.m	TRUE	reported	IlXOo1	2	TRUE	0.003693	24.46904
8011	rs4792953	T	C	T	C	0.0094	-0.03996	0.3951	0.395911	FALSE	FALSE	FALSE	ieu-b-508	17	40873303	0.034128	486484	0.241634	Sepsis (28	Sepsis (28	Sepsis (28	TRUE	igd	0.0019	8.92E-07	6604	M35754.m	TRUE	reported	IlXOo1	2	TRUE	0.003693	24.46904
8479	rs300177	C	G	C	G	-0.0094	-0.03871	0.367	0.366881	FALSE	TRUE	FALSE	ieu-b-508	2	17954421	0.034577	486484	0.262932	Sepsis (28	Sepsis (28	Sepsis (28	TRUE	igd	0.0019	4.30E-07	6552	M37058.m	TRUE	reported	32p8qV	2	TRUE	0.003722	24.46898
7246	rs1180210	A	G	A	G	-0.0094	0.030665	0.5682	0.556047	FALSE	FALSE	FALSE	ieu-b-508	1	2.22E+08	0.033622	486484	0.361731	Sepsis (28	Sepsis (28	Sepsis (28	TRUE	igd	0.0019	6.80E-07	5885	M34912.m	TRUE	reported	W8lcT6	2	TRUE	0.004142	24.46814
7335	rs1096239	T	C	T	C	-0.0094	-0.01046	0.5964	0.583356	FALSE	FALSE	FALSE	ieu-b-508	9	16363553	0.034466	486484	0.761479	Sepsis (28	Sepsis (28	Sepsis (28	TRUE	igd	0.0019	9.52E-07	4900	M35137.m	TRUE	reported	Eq2siS	2	TRUE	0.00497	24.46646
7359	rs3843430	T	C	T	C	-0.0094	-0.03946	0.371	0.375114	FALSE	FALSE	FALSE	ieu-b-508	4	14101273	0.034689	486484	0.25526	Sepsis (28	Sepsis (28	Sepsis (28	TRUE	igd	0.0019	5.81E-07	4900	M35137.m	TRUE	reported	Eq2siS	2	TRUE	0.00497	24.46646
7381	rs744432	A	C	A	C	-0.0094	0.046837	0.4475	0.449258	FALSE	FALSE	FALSE	ieu-b-508	8	74255500	0.03375	486484	0.165211	Sepsis (28	Sepsis (28	Sepsis (28	TRUE	igd	0.0019	6.34E-07	4900	M35137.m	TRUE	reported	Eq2siS	2	TRUE	0.00497	24.46646
7390	rs9297758	A	G	A	G	-0.0094	-0.05778	0.3801	0.380943	FALSE	FALSE	FALSE	ieu-b-508	8	1.28E+08	0.034322	486484	0.092266	Sepsis (28	Sepsis (28	Sepsis (28	TRUE	igd	0.0019	8.02E-07	4900	M35137.m	TRUE	reported	Eq2siS	2	TRUE	0.00497	24.46646
1986	rs1234181	T	C	T	C	-0.093	-0.02556	0.9564	0.935492	FALSE	FALSE	FALSE	ieu-b-508	9	17107467	0.067885	486484	0.706479	Sepsis (28	Sepsis (28	Sepsis (28	TRUE	igd	0.0188	7.44E-07	7071	M18254.m	TRUE	reported	clvu6V	2	TRUE	0.003449	24.46399
7461	rs1991108	T	C	T	C	-0.0183	-0.05325	0.1597	0.163236	FALSE	FALSE	FALSE	ieu-b-508	2	2.21E+08	0.045088	486484	0.237589	Sepsis (28	Sepsis (28	Sepsis (28	TRUE	igd	0.0037	5.77E-07	6543	M35193.m	TRUE	reported	BMMWXU	2	TRUE	0.003725	24.4549
2516	rs9467632	A	G	A	G	-0.0183	-0.01923	0.8773	0.880799	FALSE	FALSE	FALSE	ieu-b-508	6	25889706	0.051499	486484	0.708841	Sepsis (28	Sepsis (28	Sepsis (28	TRUE	igd	0.0037	8.50E-07	5702	M22130.m	TRUE	reported	YDbJlz	2	TRUE	0.004272	24.4538
8626	rs6911080	T	C	T	C	-0.0089	0.082547	0.7145	0.698068	FALSE	FALSE	FALSE	ieu-b-508	6	58337439	0.036268	486484	0.022843	Sepsis (28	Sepsis (28	Sepsis (28	TRUE	igd	0.0018	5.23E-07	7366	M37506.m	TRUE	reported	m2kaRt	2	TRUE	0.003308	24.44089
1003	rs1313002	T	C	T	C	-0.0089	0.030062	0.4312	0.437398	FALSE	FALSE	FALSE	ieu-b-508	4	2277763	0.033688	486484	0.372204	Sepsis (28	Sepsis (28	Sepsis (28	TRUE	igd	0.0018	1.41E-06	7364	M01564.m	TRUE	reported	IPUdyD	2	TRUE	0.003309	24.44089
3434	rs3766553	A	G	A	G	0.0089	-0.04267	0.5153	0.519722	FALSE	FALSE	FALSE	ieu-b-508	1	2.03E+08	0.033512	486484	0.202911	Sepsis (28	Sepsis (28	Sepsis (28	TRUE	igd	0.0018	1.05E-06	7364	M32452.m	TRUE	reported	8GtFpl	2	TRUE	0.003309	24.44089
717	rs1292795	A	G	A	G	0.0089	-0.00921	0.7248	0.699561	FALSE	FALSE	FALSE	ieu-b-508	16	67727069	0.036331	486484	0.79998	Sepsis (28	Sepsis (28	Sepsis (28	TRUE	igd	0.0018	3.76E-07	7363	M01301.m	TRUE	reported	w8TL71	2	TRUE	0.003309	24.44089
3767	rs1997111	A	C	A	C	-0.0089	0.044613	0.3558	0.357673	FALSE	FALSE	FALSE	ieu-b-508	12	1.19E+08	0.034712	486484	0.19872	Sepsis (28	Sepsis (28	Sepsis (28	TRUE	igd	0.0018	8.72E-07	7362	M32634.m	TRUE	reported	7T2RUz	2	TRUE	0.00331	24.44089
3782	rs7837038	A	G	A	G	0.0089	-0.06029	0.4808	0.493655	FALSE	FALSE	FALSE	ieu-b-508	8	10783074	0.033403	486484	0.07109	Sepsis (28	Sepsis (28	Sepsis (28	TRUE	igd	0.0018	5.54E-07	7362	M32634.m	TRUE	reported	7T2RUz	2	TRUE	0.00331	24.44089
4515	rs7794651	T	C	T	C	0.0089	0.017985	0.1191	0.120977	FALSE	FALSE	FALSE	ieu-b-508	7	1.09E+08	0.05145	486484	0.72667	Sepsis (28	Sepsis (28	Sepsis (28	TRUE	igd	0.0018	7.66E-07	7362	M33132.m	TRUE	reported	dTXJa8	2	TRUE	0.00331	24.44089
1160	rs6860349	A	G	A	G	-0.0089	0.006811	0.4108	0.405994	FALSE	FALSE	FALSE	ieu-b-508	5	2133412	0.033986	486484	0.841171	Sepsis (28	Sepsis (28	Sepsis (28	TRUE	igd	0.0018	8.67E-07	7355	M01644.m	TRUE	reported	IB0DTL	2	TRUE	0.003313	24.44088
3505	rs4834846	A	G	A	G	0.0089	0.038166	0.5503	0.55681	FALSE	FALSE	FALSE	ieu-b-508	4	12615274	0.033644	486484	0.256622	Sepsis (28	Sepsis (28	Sepsis (28	TRUE	igd	0.0018	4.70E-07	7355	M32492.m	TRUE	reported	rZZFcE	2	TRUE	0.003313	24.44088
555	rs2904665	A	G	A	G	-0.0089	-0.07871	0.861	0.861051	FALSE	FALSE	FALSE	ieu-b-508	8	17035927	0.048538	486484	0.104873	Sepsis (28	Sepsis (28	Sepsis (28	TRUE	igd	0.0018	9.19E-07	7354	M00606.m	TRUE	reported	r5smf7	2	TRUE	0.003313	24.44088
2234	rs1008667	T	C	T	C	0.0089	0.022087	0.2938	0.289078	FALSE	FALSE	FALSE	ieu-b-508	8	10305317	0.036723	486484	0.54755	Sepsis (28	Sepsis (28	Sepsis (28	TRUE	igd	0.0018	5.26E-07	7354	M19934.m	TRUE	reported	HtcWcQ	2	TRUE	0.003313	24.44088
2270	rs7837995	T	C	T	C	0.0089	0.005941	0.4485	0.442392	FALSE	FALSE	FALSE	ieu-b-508	8	11485820	0.033993	486484	0.861252	Sepsis (28	Sepsis (28	Sepsis (28	TRUE	igd	0.0018	4.28E-07	7354	M19934.m	TRUE	reported	HtcWcQ	2	TRUE	0.003313	24.44088
1172	rs1197872	A	T	A	T	-0.0089	0.033644	0.6876	0.683833	FALSE	TRUE	FALSE	ieu-b-508	7	38750371	0.035838	486484	0.347855	Sepsis (28	Sepsis (28	Sepsis (28	TRUE	igd	0.0018	5.18E-07	7346	M01645.m	TRUE	reported	d5nAn3	2	TRUE	0.003317	24.44087
3204	rs1189399	A	G	A	G	0.0089	0.041757	0.4228	0.426329	FALSE	FALSE	FALSE	ieu-b-508	2	44894142	0.034358	486484	0.224233	Sepsis (28	Sepsis (28	Sepsis (28	TRUE	igd	0.0018	8.89E-07	7344	M32339.m	TRUE	reported	SVp504	2	TRUE	0.003318	24.44087
4426	rs1558688	T	C	T	C	-0.0089	0.005403	0.2928	0.30135	FALSE	FALSE	FALSE	ieu-b-508	7	36915185	0.036441	486484	0.882123	Sepsis (28	Sepsis (28	Sepsis (28	TRUE	igd	0.0018	4.34E-07	7344	M32910.m	TRUE	reported	2OCBKh	2	TRUE	0.003318	24.44087
2705	rs4937602	A	G	A	G	0.0089	-0.003	0.5448	0.539456	FALSE	FALSE	FALSE	ieu-b-508	11	1.31E+08	0.033631	486484	0.928815	Sepsis (28	Sepsis (28	Sepsis (28	TRUE	igd	0.0018	9.63E-07	7340	M25459.m	TRUE	reported	ACJY35	2	TRUE	0.00332	24.44087
2711	rs7144509	A	G	A	G	-0.0089	-0.03924	0.4147	0.413599	FALSE	FALSE	FALSE	ieu-b-508	14	53041549	0.033842	486484	0.246308	Sepsis (28	Sepsis (28	Sepsis (28	TRUE	igd	0.0018	1.37E-06	7340	M25459.m	TRUE	reported	ACJY35	2	TRUE	0.00332	24.44087
1829	rs1098145	A	G	A	G	-0.0089	-0.024	0.3039	0.324587	FALSE	FALSE	FALSE	ieu-b-508	9	1.15E+08	0.03563	486484	0.500638	Sepsis (28	Sepsis (28	Sepsis (28	TRUE	igd	0.0018	1.08E-06	7308	M15778.m	TRUE	reported	941o3I	2	TRUE	0.003334	24.44084
5679	rs2101874	T	C	T	C	0.0089	0.031702	0.7429	0.763661	FALSE	FALSE	FALSE	ieu-b-508	6	19401418	0.039404	486484	0.421088	Sepsis (28	Sepsis (28	Sepsis (28	TRUE	igd	0.0018	1.10E-06	7307	M33477.m	TRUE	reported	Aw8PBK	2	TRUE	0.003335	24.44084
5695	rs6948214	A	T	A	T	0.0089	0.005356	0.2357	0.236652	FALSE	TRUE	FALSE	ieu-b-508	7	89941068	0.039259	486484	0.891476	Sepsis (28	Sepsis (28	Sepsis (28	TRUE	igd	0.0018	1.36E-06	7307	M33477.m	TRUE	reported	Aw8PBK	2	TRUE	0.003335	24.44084
5514	rs1231258	A	T	A	T	0.0089	-0.01498	0.8015	0.794115	FALSE	TRUE	FALSE	ieu-b-508	12	1.03E+08	0.041324	486484	0.716985	Sepsis (28	Sepsis (28	Sepsis (28	TRUE	igd	0.0018	9.62E-07	7306	M33422.m	TRUE	reported	PLgjrT	2	TRUE	0.003335	24.44084
5515	rs1250088	C	G	C	G	-0.0089	-0.01558	0.7842	0.782691	FALSE	TRUE	FALSE	ieu-b-508	4	1.4E+08	0.040546	486484	0.700847	Sepsis (28	Sepsis (28	Sepsis (28	TRUE	igd	0.0018	7.04E-07	7306	M33422.m	TRUE	reported	PLgjrT	2	TRUE	0.003335	24.44084
5538	rs738873	T	C	T	C	0.0089	0.002729	0.6892	0.670425	FALSE	FALSE	FALSE	ieu-b-508	22	22563154	0.035607	486484	0.938917	Sepsis (28	Sepsis (28	Sepsis (28	TRUE	igd	0.0018	5.02E-07	7306	M33422.m	TRUE	reported	PLgjrT	2	TRUE	0.003335	24.44084
3802	rs1086997	A	G	A	G	-0.0089	-0.03697	0.4381	0.436643	FALSE	FALSE	FALSE	ieu-b-508	9	80457276	0.033698	486484	0.272621	Sepsis (28	Sepsis (28	Sepsis (28	TRUE	igd	0.0018	9.69E-07	7229	M32644.m	TRUE	reported	JAa2WD	2	TRUE	0.00337	24.44077
1533	rs7666900	T	C	T	C	0.0089	-0.0038	0.1418	0.148169	FALSE	FALSE	FALSE	ieu-b-508	4	1.4E+08	0.047042	486484	0.935685	Sepsis (28	Sepsis (28	Sepsis (28	TRUE	igd	0.0018	6.93E-07	7081	M12067.m	TRUE	reported	O5x7RD	2	TRUE	0.003441	24.44063
5831	rs4980965	T	C	T	C	-0.0089	0.013717	0.5402	0.539117	FALSE	FALSE	FALSE	ieu-b-508	12	833418	0.033686	486484	0.683855	Sepsis (28	Sepsis (28	Sepsis (28	TRUE	igd	0.0018	1.28E-06	7066	M33515.m	TRUE	reported	X9mul1	2	TRUE	0.003448	24.44061
7671	rs1029481	A	T	A	T	-0.0089	-0.0577	0.7306	0.689503	FALSE	TRUE	FALSE	ieu-b-508	7	43254559	0.036334	486484	0.112263	Sepsis (28	Sepsis (28	Sepsis (28	TRUE	igd	0.0018	8.81E-07	6920	M35327.m	TRUE	reported	SquFiY	2	TRUE	0.00352	24.44047
6657	rs6961104	T	C	T	C	-0.247	-0.01094	0.9667	0.92463	FALSE	FALSE	FALSE	ieu-b-508	7	25697638	0.063757	486484	0.863733	Sepsis (28	Sepsis (28	Sepsis (28	TRUE	igd	0.0493	5.56E-07	75	M34322.m	TRUE	reported	16GdG9	2	TRUE	0.250761	24.43215
6957	rs739163	T	C	T	C	0.0262	-0.01671	0.3656	0.379384	FALSE	FALSE	FALSE	ieu-b-508	22	44833580	0.03455	486484	0.628686	Sepsis (28	Sepsis (28	Sepsis (28	TRUE	igd	0.0053	6.09E-07	5689	M34400.m	TRUE	reported	QXmzfR	2	TRUE	0.004277	24.42858
8376	rs1050167	A	C	A	C	-0.0346	-0.02027	0.0254	0.028803	FALSE	FALSE	FALSE	ieu-b-508	11	88406300	0.099754	486484	0.838976	Sepsis (28	Sepsis (28	Sepsis (28	TRUE	igd	0.007	7.05E-07	7335	M36776.m	TRUE	reported	VnYglx	2	TRUE	0.00332	24.42518
1939	rs6959584	T	C	T	C	0.0173	-0.0487	0.1278	0.129255	FALSE	FALSE	FALSE	ieu-b-508	7	1.06E+08	0.049694	486484	0.32711	Sepsis (28	Sepsis (28	Sepsis (28	TRUE	igd	0.0035	6.10E-07	6549	M16822.m	TRUE	reported	UNI018	2	TRUE	0.003717	24.42438
6119	rs1687578	A	G	A	G	0.5363	-0.01337	0.0114	0.014928	FALSE	FALSE	FALSE	ieu-b-508	5	5579439	0.139453	486484	0.923633	Sepsis (28	Sepsis (28	Sepsis (28	TRUE	igd	0.1079	6.74E-07	176	M33877.m	TRUE	reported	yDVi1f	2	TRUE	0.123088	24.42358
3600	rs2187662	A	G	A	G	0.0939	0.014247	0.0332	0.028951	FALSE	FALSE	FALSE	ieu-b-508	16	14270121	0.100611	486484	0.88739	Sepsis (28	Sepsis (28	Sepsis (28	TRUE	igd	0.019	7.61E-07	7256	M32549.m	TRUE	reported	W7Fgu2	2	TRUE	0.003355	24.41767
4626	rs1709537	A	G	A	G	0.5603	-0.18578	0.9671	0.994299	FALSE	FALSE	FALSE	ieu-b-508	12	45403273	0.224215	486484	0.407349	Sepsis (28	Sepsis (28	Sepsis (28	TRUE	igd	0.1119	5.45E-07	76	M33173.m	TRUE	reported	04fkUQ	2	TRUE	0.248057	24.41177
4268	rs853725	T	C	T	C	0.044	-0.03647	0.5592	0.553934	FALSE	FALSE	FALSE	ieu-b-508	4	1.5E+08	0.033554	486484	0.277033	Sepsis (28	Sepsis (28	Sepsis (28	TRUE	igd	0.0089	8.62E-07	1600	M32802.m	TRUE	reported	m8vmvZ	2	TRUE	0.015046	24.41081
1637	rs2375475	T	C	T	C	-0.0084	0.020773	0.3236	0.337946	FALSE	FALSE	FALSE	ieu-b-508	1	65235535	0.035386	486484	0.557179	Sepsis (28	Sepsis (28	Sepsis (28	TRUE	igd	0.0017	1.41E-06	7368	M15140.m	TRUE	reported	jEcqMJ	2	TRUE	0.003303	24.4086
8625	rs6745464	T	C	T	C	-0.0084	-0.05361	0.5494	0.542552	FALSE	FALSE	FALSE	ieu-b-508	2	1.1E+08	0.03352	486484	0.109717	Sepsis (28	Sepsis (28	Sepsis (28	TRUE	igd	0.0017	1.39E-06	7366	M37506.m	TRUE	reported	m2kaRt	2	TRUE	0.003304	24.4086
429	rs3897960	A	T	A	T	-0.0084	-0.03899	0.2083	0.205881	FALSE	TRUE	FALSE	ieu-b-508	4	83701791	0.041343	486484	0.345668	Sepsis (28	Sepsis (28	Sepsis (28	TRUE	igd	0.0017	1.29E-06	7365	M00063.m	TRUE	reported	Hdsv1L	2	TRUE	0.003304	24.40859
3269	rs37379	A	G	A	G	0.0168	-0.05402	0.0868	0.100332	FALSE	FALSE	FALSE	ieu-b-508	5	35045826	0.05586	486484	0.333489	Sepsis (28	Sepsis (28	Sepsis (28	TRUE	igd	0.0034	7.94E-07	7365	M32348.m	TRUE	reported	DJeLDx	2	TRUE	0.003304	24.40859
700	rs8111703	A	T	A	T	-0.0084	0.00579	0.2142	0.246211	FALSE	TRUE	FALSE	ieu-b-508	19	3685054	0.038824	486484	0.881449	Sepsis (28	Sepsis (28	Sepsis (28	TRUE	igd	0.0017	1.47E-06	7358	M01299.m	TRUE	reported	gfM0a3	2	TRUE	0.003307	24.40859
3530	rs17685	A	G	A	G	-0.0168	-0.0574	0.2781	0.277402	FALSE	FALSE	FALSE	ieu-b-508	7	75616105	0.037156	486484	0.122401	Sepsis (28	Sepsis (28	Sepsis (28	TRUE	igd	0.0034	6.44E-07	7358	M32497.m	TRUE	reported	244bZN	2	TRUE	0.003307	24.40859
4088	rs1076131	T	C	T	C	-0.0336	0.041052	0.2803	0.30376	FALSE	FALSE	FALSE	ieu-b-508	9	96895320	0.036535	486484	0.261158	Sepsis (28	Sepsis (28	Sepsis (28	TRUE	igd	0.0068	7.22E-07	7356	M32753.m	TRUE	reported	XIC6zq	2	TRUE	0.003308	24.40859
817	rs2100884	T	C	T	C	-0.0084	-0.03353	0.4375	0.446949	FALSE	FALSE	FALSE	ieu-b-508	11	6159019	0.033631	486484	0.318729	Sepsis (28	Sepsis (28	Sepsis (28	TRUE	igd	0.0017	8.60E-07	7355	M01358.m	TRUE	reported	kSPQgd	2	TRUE	0.003309	24.40859
3125	rs1299292	A	G	A	G	0.0084	0.082666	0.5789	0.570298	FALSE	FALSE	FALSE	ieu-b-508	2	1.8E+08	0.033805	486484	0.014468	Sepsis (28	Sepsis (28	Sepsis (28	TRUE	igd	0.0017	1.31E-06	7349	M32315.m	TRUE	reported	4igzkL	2	TRUE	0.003311	24.40858
3147	rs9874508	A	G	A	G	-0.0084	-0.01004	0.4625	0.454359	FALSE	FALSE	FALSE	ieu-b-508	3	1.26E+08	0.033585	486484	0.764901	Sepsis (28	Sepsis (28	Sepsis (28	TRUE	igd	0.0017	1.42E-06	7349	M32315.m	TRUE	reported	4igzkL	2	TRUE	0.003311	24.40858
7710	rs2841549	A	G	A	G	-0.0084	0.01253	0.762	0.761076	FALSE	FALSE	FALSE	ieu-b-508	6	13489554	0.039116	486484	0.748714	Sepsis (28	Sepsis (28	Sepsis (28	TRUE	igd	0.0017	4.47E-07	7345	M35397.m	TRUE	reported	Y56mZr	2	TRUE	0.003313	24.40858
7722	rs7546483	C	G	C	G	0.0084	0.047314	0.7027	0.699938	FALSE	TRUE	FALSE	ieu-b-508	1	2.01E+08	0.036425	486484	0.193956	Sepsis (28	Sepsis (28	Sepsis (28	TRUE	igd	0.0017	6.20E-07	7345	M35397.m	TRUE	reported	Y56mZr	2	TRUE	0.003313	24.40858
4050	rs1289948	A	G	A	G	0.0084	0.001803	0.7034	0.709386	FALSE	FALSE	FALSE	ieu-b-508	15	96241595	0.036778	486484	0.960898	Sepsis (28	Sepsis (28	Sepsis (28	TRUE	igd	0.0017	1.59E-06	7318	M32740.m	TRUE	reported	YLshfP	2	TRUE	0.003325	24.40855
1575	rs1204294	A	G	A	G	-0.0084	0.000786	0.8045	0.795447	FALSE	FALSE	FALSE	ieu-b-508	1	84849664	0.041379	486484	0.984839	Sepsis (28	Sepsis (28	Sepsis (28	TRUE	igd	0.0017	1.40E-06	7314	M12593.m	TRUE	reported	IBXgSU	2	TRUE	0.003327	24.40855
4184	rs1340306	C	G	C	G	0.0168	-0.03566	0.3866	0.405571	FALSE	TRUE	FALSE	ieu-b-508	2	33555255	0.034137	486484	0.296228	Sepsis (28	Sepsis (28	Sepsis (28	TRUE	igd	0.0034	9.92E-07	7313	M32761.m	TRUE	reported	QefVGc	2	TRUE	0.003327	24.40855
495	rs1397829	A	G	A	G	0.042	0.083994	0.5328	0.541572	FALSE	FALSE	FALSE	ieu-b-508	4	23587996	0.03365	486484	0.012557	Sepsis (28	Sepsis (28	Sepsis (28	TRUE	igd	0.0085	7.08E-07	7274	M00569.m	TRUE	reported	1TjBaz	2	TRUE	0.003345	24.40851
2177	rs1247391	A	C	A	C	-0.0084	0.011068	0.4341	0.433494	FALSE	FALSE	FALSE	ieu-b-508	2	67417246	0.033668	486484	0.742356	Sepsis (28	Sepsis (28	Sepsis (28	TRUE	igd	0.0017	1.13E-06	7182	M19362.m	TRUE	reported	SkNEKw	2	TRUE	0.003388	24.40843
2189	rs4337809	T	C	T	C	-0.0084	-0.00994	0.45	0.445857	FALSE	FALSE	FALSE	ieu-b-508	5	61318693	0.033677	486484	0.767816	Sepsis (28	Sepsis (28	Sepsis (28	TRUE	igd	0.0017	1.22E-06	7182	M19362.m	TRUE	reported	SkNEKw	2	TRUE	0.003388	24.40843
937	rs4269521	A	G	A	G	0.0168	0.0343	0.8431	0.848928	FALSE	FALSE	FALSE	ieu-b-508	8	1.32E+08	0.046558	486484	0.461305	Sepsis (28	Sepsis (28	Sepsis (28	TRUE	igd	0.0034	1.03E-06	7160	M01508.m	TRUE	reported	go526k	2	TRUE	0.003398	24.40841
1244	rs2118096	A	C	A	C	0.0084	-0.03296	0.6068	0.60519	FALSE	FALSE	FALSE	ieu-b-508	3	1.62E+08	0.034206	486484	0.335259	Sepsis (28	Sepsis (28	Sepsis (28	TRUE	igd	0.0017	1.13E-06	7136	M01769.m	TRUE	reported	rismUZ	2	TRUE	0.00341	24.40838
1253	rs4653629	T	C	T	C	-0.0084	0.012231	0.5584	0.5539	FALSE	FALSE	FALSE	ieu-b-508	1	2.26E+08	0.03359	486484	0.715766	Sepsis (28	Sepsis (28	Sepsis (28	TRUE	igd	0.0017	1.25E-06	7136	M01769.m	TRUE	reported	rismUZ	2	TRUE	0.00341	24.40838
1256	rs6017596	T	C	T	C	-0.0084	0.029224	0.5825	0.578685	FALSE	FALSE	FALSE	ieu-b-508	20	35868219	0.033901	486484	0.388666	Sepsis (28	Sepsis (28	Sepsis (28	TRUE	igd	0.0017	8.31E-07	7136	M01769.m	TRUE	reported	rismUZ	2	TRUE	0.00341	24.40838
1515	rs1688339	A	C	A	C	-0.0084	-0.02152	0.1921	0.222493	FALSE	FALSE	FALSE	ieu-b-508	5	9697886	0.040254	486484	0.592994	Sepsis (28	Sepsis (28	Sepsis (28	TRUE	igd	0.0017	1.20E-06	7081	M12067.m	TRUE	reported	O5x7RD	2	TRUE	0.003436	24.40833
1377	rs1227610	T	C	T	C	-0.0084	-0.00301	0.4352	0.442024	FALSE	FALSE	FALSE	ieu-b-508	11	33088587	0.033653	486484	0.928742	Sepsis (28	Sepsis (28	Sepsis (28	TRUE	igd	0.0017	1.56E-06	7037	M02734.m	TRUE	reported	0y0dDD	2	TRUE	0.003458	24.40829
1393	rs322614	A	G	A	G	0.0084	0.038656	0.3892	0.382842	FALSE	FALSE	FALSE	ieu-b-508	11	29755547	0.034228	486484	0.258745	Sepsis (28	Sepsis (28	Sepsis (28	TRUE	igd	0.0017	1.70E-06	7037	M02734.m	TRUE	reported	0y0dDD	2	TRUE	0.003458	24.40829
8417	rs300585	A	G	A	G	0.0084	-0.02962	0.3107	0.337785	FALSE	FALSE	FALSE	ieu-b-508	20	4395778	0.035697	486484	0.406703	Sepsis (28	Sepsis (28	Sepsis (28	TRUE	igd	0.0017	1.19E-06	6940	M36808.m	TRUE	reported	u7lqaj	2	TRUE	0.003506	24.40819
7682	rs4307594	T	C	T	C	0.0084	-0.02648	0.6911	0.702166	FALSE	FALSE	FALSE	ieu-b-508	1	1.08E+08	0.036385	486484	0.466835	Sepsis (28	Sepsis (28	Sepsis (28	TRUE	igd	0.0017	9.86E-07	6920	M35327.m	TRUE	reported	SquFiY	2	TRUE	0.003516	24.40817
6280	rs1285732	T	C	T	C	0.0168	-0.1171	0.8752	0.878885	FALSE	FALSE	FALSE	ieu-b-508	13	20838486	0.051017	486484	0.021722	Sepsis (28	Sepsis (28	Sepsis (28	TRUE	igd	0.0034	9.75E-07	6502	M33939.m	TRUE	reported	PeKlgz	2	TRUE	0.003741	24.40771
6720	rs1270261	A	G	A	G	0.892	0.023063	0.011	0.030852	FALSE	FALSE	FALSE	ieu-b-508	7	7556557	0.097116	486484	0.812289	Sepsis (28	Sepsis (28	Sepsis (28	TRUE	igd	0.179	6.25E-07	115	M34338.m	TRUE	reported	h4FB9a	2	TRUE	0.177589	24.40081
6891	rs7463317	A	G	A	G	0.2216	0.041625	0.108	0.166398	FALSE	FALSE	FALSE	ieu-b-508	8	66402871	0.04476	486484	0.352387	Sepsis (28	Sepsis (28	Sepsis (28	TRUE	igd	0.0423	1.60E-07	18	M34365.m	TRUE	reported	8eexD0	2	TRUE	0.603914	24.39531
6251	rs1763853	A	G	A	G	-0.0652	-0.10474	0.0208	0.019216	FALSE	FALSE	FALSE	ieu-b-508	6	51934092	0.122037	486484	0.390765	Sepsis (28	Sepsis (28	Sepsis (28	TRUE	igd	0.0132	8.75E-07	7345	M33936.m	TRUE	reported	jwA4B2	2	TRUE	0.003311	24.39097
4189	rs458582	T	G	T	G	0.0163	-0.0636	0.4996	0.492335	FALSE	FALSE	FALSE	ieu-b-508	21	42804506	0.033437	486484	0.057154	Sepsis (28	Sepsis (28	Sepsis (28	TRUE	igd	0.0033	1.04E-06	7313	M32761.m	TRUE	reported	QefVGc	2	TRUE	0.003325	24.39094
7085	rs1244308	A	G	A	G	-0.0726	-0.06677	0.0904	0.088991	FALSE	FALSE	FALSE	ieu-b-508	15	83627652	0.058839	486484	0.256475	Sepsis (28	Sepsis (28	Sepsis (28	TRUE	igd	0.0147	7.92E-07	4296	M34499.m	TRUE	reported	JqFBiM	2	TRUE	0.005646	24.38015
424	rs2407893	A	G	A	G	0.0079	0.016712	0.427	0.418435	FALSE	FALSE	FALSE	ieu-b-508	16	61623478	0.033798	486484	0.620971	Sepsis (28	Sepsis (28	Sepsis (28	TRUE	igd	0.0016	1.29E-06	7365	M00063.m	TRUE	reported	Hdsv1L	2	TRUE	0.003299	24.37229
695	rs7151615	T	G	T	G	-0.0079	0.023586	0.273	0.2747	FALSE	FALSE	FALSE	ieu-b-508	14	20731947	0.037277	486484	0.526926	Sepsis (28	Sepsis (28	Sepsis (28	TRUE	igd	0.0016	1.47E-06	7358	M01299.m	TRUE	reported	gfM0a3	2	TRUE	0.003302	24.37228
1722	rs7215931	A	G	A	G	0.0079	-0.00308	0.2013	0.215733	FALSE	FALSE	FALSE	ieu-b-508	17	30099125	0.040556	486484	0.939516	Sepsis (28	Sepsis (28	Sepsis (28	TRUE	igd	0.0016	1.29E-06	7310	M15506.m	TRUE	reported	mVRmZO	2	TRUE	0.003324	24.37224
5005	rs6534458	T	C	T	C	-0.3044	0.193141	0.0185	0.01584	FALSE	FALSE	FALSE	ieu-b-508	4	1.26E+08	0.134825	486484	0.151991	Sepsis (28	Sepsis (28	Sepsis (28	TRUE	igd	0.0616	7.65E-07	1017	M33178.m	TRUE	reported	g22WMS	2	TRUE	0.023448	24.37098
460	rs394282	A	T	A	T	-0.0163	-0.09436	0.1112	0.06855	FALSE	TRUE	FALSE	ieu-b-508	1	8284657	0.066047	486484	0.153099	Sepsis (28	Sepsis (28	Sepsis (28	TRUE	igd	0.0033	1.02E-06	1763	M00513.m	TRUE	reported	ohacnP	2	TRUE	0.01365	24.36994
8562	rs1250835	T	G	T	G	-0.0232	-0.03706	0.328	0.324276	FALSE	FALSE	FALSE	ieu-b-508	4	1.35E+08	0.035757	486484	0.299967	Sepsis (28	Sepsis (28	Sepsis (28	TRUE	igd	0.0047	8.24E-07	7333	M37203.m	TRUE	reported	3glIlv	2	TRUE	0.003312	24.35913
579	rs8108144	T	G	T	G	-0.0232	-0.00775	0.788	0.797373	FALSE	FALSE	FALSE	ieu-b-508	19	23373200	0.041726	486484	0.85256	Sepsis (28	Sepsis (28	Sepsis (28	TRUE	igd	0.0047	6.44E-07	5344	M01107.m	TRUE	reported	EbPV1D	2	TRUE	0.004539	24.35666
3065	rs1245864	T	C	T	C	0.1377	-0.07677	0.0419	0.067228	FALSE	FALSE	FALSE	ieu-b-508	18	14921927	0.070552	486484	0.276523	Sepsis (28	Sepsis (28	Sepsis (28	TRUE	igd	0.0279	7.92E-07	7363	M31787.m	TRUE	reported	XMp8YI	2	TRUE	0.003297	24.35238
3197	rs8082170	T	C	T	C	0.0153	0.011922	0.1804	0.180396	FALSE	FALSE	FALSE	ieu-b-508	17	62395382	0.043382	486484	0.783462	Sepsis (28	Sepsis (28	Sepsis (28	TRUE	igd	0.0031	1.07E-06	7356	M32338.m	TRUE	reported	q5cuaP	2	TRUE	0.003301	24.35238
2850	rs2484454	A	G	A	G	-0.1096	0.015455	0.8988	0.912658	FALSE	FALSE	FALSE	ieu-b-508	10	84465884	0.059338	486484	0.794516	Sepsis (28	Sepsis (28	Sepsis (28	TRUE	igd	0.0222	7.71E-07	1309	M27531.m	TRUE	reported	v01zDr	2	TRUE	0.018279	24.33611
5579	rs4527784	A	G	A	G	1.0631	-0.01534	0.9569	0.94136	FALSE	FALSE	FALSE	ieu-b-508	7	17052493	0.071488	486484	0.830076	Sepsis (28	Sepsis (28	Sepsis (28	TRUE	igd	0.2119	5.25E-07	60	M33423.m	TRUE	reported	MoEnO7	2	TRUE	0.295528	24.33117
796	rs1167446	T	C	T	C	0.0148	-0.00709	0.4181	0.425306	FALSE	FALSE	FALSE	ieu-b-508	2	38621456	0.033635	486484	0.832961	Sepsis (28	Sepsis (28	Sepsis (28	TRUE	igd	0.003	7.01E-07	7335	M01356.m	TRUE	reported	ah1g5W	2	TRUE	0.003307	24.33114
1333	rs1051605	T	C	T	C	-0.0222	-0.03003	0.1978	0.207372	FALSE	FALSE	FALSE	ieu-b-508	5	1.68E+08	0.043236	486484	0.487352	Sepsis (28	Sepsis (28	Sepsis (28	TRUE	igd	0.0045	6.99E-07	5791	M02342.m	TRUE	reported	EhZr3g	2	TRUE	0.004185	24.32937
1321	rs1891053	A	G	A	G	0.1831	0.080101	0.0145	0.012604	FALSE	FALSE	FALSE	ieu-b-508	10	15725671	0.151008	486484	0.595808	Sepsis (28	Sepsis (28	Sepsis (28	TRUE	igd	0.0371	8.03E-07	1175	M02137.m	TRUE	reported	iRGqBE	2	TRUE	0.020309	24.31583
2876	rs797072	A	G	A	G	0.0217	-0.01528	0.5347	0.538018	FALSE	FALSE	FALSE	ieu-b-508	2	2.12E+08	0.033774	486484	0.651073	Sepsis (28	Sepsis (28	Sepsis (28	TRUE	igd	0.0044	6.28E-07	6715	M27710.m	TRUE	reported	XeaFav	2	TRUE	0.003609	24.31559
7346	rs1734031	C	G	C	G	0.0217	-0.05518	0.9347	0.882968	FALSE	TRUE	FALSE	ieu-b-508	8	1.07E+08	0.052135	486484	0.289893	Sepsis (28	Sepsis (28	Sepsis (28	TRUE	igd	0.0044	8.78E-07	4900	M35137.m	TRUE	reported	Eq2siS	2	TRUE	0.004939	24.3129
8369	rs1306018	T	C	T	C	-0.0217	-0.07484	0.5699	0.565259	FALSE	FALSE	FALSE	ieu-b-508	3	1.12E+08	0.033726	486484	0.026489	Sepsis (28	Sepsis (28	Sepsis (28	TRUE	igd	0.0044	8.17E-07	3248	M36756.m	TRUE	reported	QsW4oR	2	TRUE	0.007433	24.30785
7987	rs7748665	A	G	A	G	-0.0212	0.130205	0.9206	0.91506	FALSE	FALSE	FALSE	ieu-b-508	6	1.63E+08	0.060582	486484	0.031614	Sepsis (28	Sepsis (28	Sepsis (28	TRUE	igd	0.0043	6.71E-07	7358	M35718.m	TRUE	reported	oUwc0a	2	TRUE	0.003293	24.30059
992	rs7238006	T	C	T	C	0.0281	0.068378	0.9262	0.930523	FALSE	FALSE	FALSE	ieu-b-508	18	31927898	0.066276	486484	0.302209	Sepsis (28	Sepsis (28	Sepsis (28	TRUE	igd	0.0057	6.77E-07	7276	M01561.m	TRUE	reported	Zd9SUU	2	TRUE	0.003329	24.29649
6498	rs1412535	T	C	T	C	-0.0281	0.017006	0.5882	0.590211	FALSE	FALSE	FALSE	ieu-b-508	9	16244120	0.034244	486484	0.619471	Sepsis (28	Sepsis (28	Sepsis (28	TRUE	igd	0.0057	8.90E-07	5581	M34112.m	TRUE	reported	97JqFb	2	TRUE	0.004336	24.29446
7081	rs3912161	T	C	T	C	-0.035	0.017336	0.9396	0.941188	FALSE	FALSE	FALSE	ieu-b-508	6	1.61E+08	0.073016	486484	0.812323	Sepsis (28	Sepsis (28	Sepsis (28	TRUE	igd	0.0071	9.26E-07	7119	M34481.m	TRUE	reported	mXdL0W	2	TRUE	0.003402	24.29391
8355	rs690111	T	G	T	G	-0.037	-0.03158	0.2596	0.253988	FALSE	FALSE	FALSE	ieu-b-508	9	93377290	0.038597	486484	0.4133	Sepsis (28	Sepsis (28	Sepsis (28	TRUE	igd	0.0075	8.30E-07	922	M36738.m	TRUE	reported	mVQQcD	2	TRUE	0.025718	24.28498
8551	rs2130589	A	G	A	G	0.0345	-0.00517	0.3137	0.327087	FALSE	FALSE	FALSE	ieu-b-508	6	1.24E+08	0.035621	486484	0.884503	Sepsis (28	Sepsis (28	Sepsis (28	TRUE	igd	0.007	9.18E-07	7355	M37202.m	TRUE	reported	BtsIDx	2	TRUE	0.003292	24.28421
6284	rs4666270	T	G	T	G	-0.0138	-0.01181	0.8392	0.846313	FALSE	FALSE	FALSE	ieu-b-508	2	29989203	0.048249	486484	0.806677	Sepsis (28	Sepsis (28	Sepsis (28	TRUE	igd	0.0028	1.02E-06	6502	M33939.m	TRUE	reported	PeKlgz	2	TRUE	0.003722	24.28334
4031	rs9308696	A	G	A	G	-0.1558	0.080926	0.9751	0.973929	FALSE	FALSE	FALSE	ieu-b-508	2	1.15E+08	0.104763	486484	0.439837	Sepsis (28	Sepsis (28	Sepsis (28	TRUE	igd	0.0316	8.19E-07	1677	M32735.m	TRUE	reported	QE2vKQ	2	TRUE	0.014288	24.27965
2130	rs6585252	C	G	C	G	-0.1538	0.010461	0.9571	0.954365	FALSE	TRUE	FALSE	ieu-b-508	10	1.16E+08	0.080073	486484	0.896054	Sepsis (28	Sepsis (28	Sepsis (28	TRUE	igd	0.0312	8.55E-07	1457	M18477.m	TRUE	reported	p0vcCU	2	TRUE	0.016404	24.26649
4483	rs1256546	A	G	A	G	0.034	0.002307	0.8419	0.828233	FALSE	FALSE	FALSE	ieu-b-508	1	1.77E+08	0.044323	486484	0.958482	Sepsis (28	Sepsis (28	Sepsis (28	TRUE	igd	0.0069	8.34E-07	2773	M33131.m	TRUE	reported	uGKURO	2	TRUE	0.00868	24.2631
6271	rs2906973	A	T	A	T	-0.0468	-0.01056	0.9593	0.95534	FALSE	TRUE	FALSE	ieu-b-508	17	15369693	0.081319	486484	0.896684	Sepsis (28	Sepsis (28	Sepsis (28	TRUE	igd	0.0095	8.26E-07	7222	M33937.m	TRUE	reported	QanGCI	2	TRUE	0.003349	24.26187
1828	rs1074573	A	G	A	G	0.0133	-0.08725	0.907	0.905153	FALSE	FALSE	FALSE	ieu-b-508	12	96279745	0.057315	486484	0.127952	Sepsis (28	Sepsis (28	Sepsis (28	TRUE	igd	0.0027	1.15E-06	7308	M15778.m	TRUE	reported	941o3I	2	TRUE	0.003309	24.25811

3799	rs1002820	T	C	T	C	-0.0133	-0.01336	0.1218	0.125677	FALSE	FALSE	FALSE	ieu-b-508	4	37716343	0.050326	486484	0.79069	Sepsis (28	Sepsis (28	Sepsis (28	TRUE	igd	0.0027	8.97E-07	7229	M32644.m	TRUE	reported	JAa2WD	2	TRUE	0.003345	24.25803
7507	rs1440201	C	G	C	G	0.0133	0.021803	0.3764	0.383324	FALSE	TRUE	FALSE	ieu-b-508	14	33779953	0.034516	486484	0.527595	Sepsis (28	Sepsis (28	Sepsis (28	TRUE	igd	0.0027	6.13E-07	7100	M35254.m	TRUE	reported	HfVgPn	2	TRUE	0.003406	24.25791
1516	rs1703163	A	G	A	G	0.0133	0.036364	0.9284	0.922687	FALSE	FALSE	FALSE	ieu-b-508	2	1.06E+08	0.062788	486484	0.562485	Sepsis (28	Sepsis (28	Sepsis (28	TRUE	igd	0.0027	5.69E-07	7081	M12067.m	TRUE	reported	O5x7RD	2	TRUE	0.003415	24.25789
6529	rs4738400	T	C	T	C	0.0133	0.012189	0.2947	0.29871	FALSE	FALSE	FALSE	ieu-b-508	8	74857165	0.036435	486484	0.737965	Sepsis (28	Sepsis (28	Sepsis (28	TRUE	igd	0.0027	6.33E-07	7049	M34123.m	TRUE	reported	9O5wo1	2	TRUE	0.00343	24.25786
1541	rs1295097	T	C	T	C	-0.0133	-0.00936	0.6497	0.644111	FALSE	FALSE	FALSE	ieu-b-508	17	74519300	0.035008	486484	0.789242	Sepsis (28	Sepsis (28	Sepsis (28	TRUE	igd	0.0027	9.84E-07	6864	M12129.m	TRUE	reported	iNzdgb	2	TRUE	0.003523	24.25768
2417	rs1726015	T	C	T	C	-0.0133	0.051663	0.7525	0.751819	FALSE	FALSE	FALSE	ieu-b-508	7	87981432	0.038762	486484	0.182593	Sepsis (28	Sepsis (28	Sepsis (28	TRUE	igd	0.0027	1.08E-06	6556	M21188.m	TRUE	reported	kvdnjc	2	TRUE	0.003688	24.25734
3836	rs1074468	A	G	A	G	0.0133	0.028824	0.3319	0.336793	FALSE	FALSE	FALSE	ieu-b-508	12	5555040	0.03545	486484	0.416161	Sepsis (28	Sepsis (28	Sepsis (28	TRUE	igd	0.0027	1.25E-06	5211	M32651.m	TRUE	reported	DeuPwv	2	TRUE	0.004635	24.25543
8274	rs1714555	T	C	T	C	-0.033	0.022172	0.951	0.944097	FALSE	FALSE	FALSE	ieu-b-508	11	56968560	0.072814	486484	0.760745	Sepsis (28	Sepsis (28	Sepsis (28	TRUE	igd	0.0067	7.79E-07	6464	M36553.m	TRUE	reported	2Rk7nd	2	TRUE	0.003739	24.25179
2296	rs5998272	A	C	A	C	0.0261	0.023936	0.9257	0.919833	FALSE	FALSE	FALSE	ieu-b-508	22	32560045	0.061238	486484	0.695892	Sepsis (28	Sepsis (28	Sepsis (28	TRUE	igd	0.0053	1.08E-06	7301	M20675.m	TRUE	reported	CXe9rE	2	TRUE	0.003311	24.24434
5870	rs2801848	T	C	T	C	0.0764	-0.04049	0.1289	0.123522	FALSE	FALSE	FALSE	ieu-b-508	10	28382756	0.050363	486484	0.421396	Sepsis (28	Sepsis (28	Sepsis (28	TRUE	igd	0.0155	8.58E-07	824	M33609.m	TRUE	reported	h7B9gN	2	TRUE	0.02864	24.23639
6502	rs2705534	T	G	T	G	0.0517	-0.13055	0.9192	0.916772	FALSE	FALSE	FALSE	ieu-b-508	3	1.12E+08	0.060517	486484	0.030992	Sepsis (28	Sepsis (28	Sepsis (28	TRUE	igd	0.0105	7.90E-07	5581	M34112.m	TRUE	reported	97JqFb	2	TRUE	0.004325	24.23521
715	rs1179979	A	G	A	G	-0.0128	-0.02883	0.9214	0.920391	FALSE	FALSE	FALSE	ieu-b-508	14	37346974	0.061752	486484	0.640568	Sepsis (28	Sepsis (28	Sepsis (28	TRUE	igd	0.0026	9.49E-07	7363	M01301.m	TRUE	reported	w8TL71	2	TRUE	0.003281	24.2301
7990	rs9373682	T	C	T	C	-0.0128	0.068875	0.2404	0.258705	FALSE	FALSE	FALSE	ieu-b-508	6	1.04E+08	0.038253	486484	0.071779	Sepsis (28	Sepsis (28	Sepsis (28	TRUE	igd	0.0026	1.15E-06	7358	M35718.m	TRUE	reported	oUwc0a	2	TRUE	0.003283	24.2301
7765	rs1262114	A	G	A	G	-0.0128	0.001492	0.4155	0.419211	FALSE	FALSE	FALSE	ieu-b-508	2	99927112	0.033807	486484	0.964797	Sepsis (28	Sepsis (28	Sepsis (28	TRUE	igd	0.0026	9.23E-07	7256	M35439.m	TRUE	reported	DnuAGZ	2	TRUE	0.003329	24.23001
6862	rs1327041	T	C	T	C	-0.1222	-0.03686	0.8578	0.828859	FALSE	FALSE	FALSE	ieu-b-508	8	21334882	0.044573	486484	0.408301	Sepsis (28	Sepsis (28	Sepsis (28	TRUE	igd	0.0248	8.00E-07	954	M34365.m	TRUE	reported	8eexD0	2	TRUE	0.024819	24.22856
7631	rs1187601	T	C	T	C	-0.0128	0.055041	0.1123	0.115567	FALSE	FALSE	FALSE	ieu-b-508	14	95683892	0.053625	486484	0.304698	Sepsis (28	Sepsis (28	Sepsis (28	TRUE	igd	0.0026	1.15E-06	5768	M35326.m	TRUE	reported	jRytyJ	2	TRUE	0.004184	24.22828
1914	rs1262950	A	G	A	G	0.0128	0.006944	0.3983	0.399278	FALSE	FALSE	FALSE	ieu-b-508	3	99904783	0.034222	486484	0.839207	Sepsis (28	Sepsis (28	Sepsis (28	TRUE	igd	0.0026	1.10E-06	4689	M16816.m	TRUE	reported	J1abbO	2	TRUE	0.005142	24.22635
4272	rs1293988	A	G	A	G	-0.0527	0.07133	0.4973	0.464622	FALSE	FALSE	FALSE	ieu-b-508	1	1.86E+08	0.033432	486484	0.032877	Sepsis (28	Sepsis (28	Sepsis (28	TRUE	igd	0.0107	9.39E-07	1298	M32808.m	TRUE	reported	vnr36k	2	TRUE	0.018346	24.22055
5455	rs1278214	A	G	A	G	0.2983	0.067777	0.0137	0.020669	FALSE	FALSE	FALSE	ieu-b-508	10	86505933	0.121265	486484	0.576221	Sepsis (28	Sepsis (28	Sepsis (28	TRUE	igd	0.0605	8.10E-07	528	M33391.m	TRUE	reported	EgMd1b	2	TRUE	0.044016	24.21852
6874	rs2408190	A	G	A	G	-0.0867	-0.05778	0.5271	0.53485	FALSE	FALSE	FALSE	ieu-b-508	16	63042304	0.033681	486484	0.08627	Sepsis (28	Sepsis (28	Sepsis (28	TRUE	igd	0.0176	7.87E-07	954	M34365.m	TRUE	reported	8eexD0	2	TRUE	0.024806	24.21595
6503	rs2821373	T	C	T	C	0.0561	-0.01252	0.9257	0.899272	FALSE	FALSE	FALSE	ieu-b-508	1	2E+08	0.056362	486484	0.824214	Sepsis (28	Sepsis (28	Sepsis (28	TRUE	igd	0.0114	8.61E-07	5581	M34112.m	TRUE	reported	97JqFb	2	TRUE	0.00432	24.20808
6585	rs16215	T	C	T	C	0.0694	-0.14974	0.9711	0.969358	FALSE	FALSE	FALSE	ieu-b-508	7	24258793	0.097081	486484	0.122972	Sepsis (28	Sepsis (28	Sepsis (28	TRUE	igd	0.0141	8.05E-07	2278	M34289.m	TRUE	reported	Knp8ry	2	TRUE	0.010523	24.20467
8575	rs1019600	T	C	T	C	-0.032	0.017629	0.8157	0.803946	FALSE	FALSE	FALSE	ieu-b-508	2	1.45E+08	0.042018	486484	0.674805	Sepsis (28	Sepsis (28	Sepsis (28	TRUE	igd	0.0065	7.63E-07	1411	M37253.m	TRUE	reported	PYMqzt	2	TRUE	0.016887	24.20233
1642	rs3789978	A	G	A	G	0.0123	0.035529	0.8469	0.842616	FALSE	FALSE	FALSE	ieu-b-508	12	32947193	0.045902	486484	0.438918	Sepsis (28	Sepsis (28	Sepsis (28	TRUE	igd	0.0025	1.32E-06	7368	M15140.m	TRUE	reported	jEcqMJ	2	TRUE	0.003275	24.19983
8377	rs1159305	T	G	T	G	0.0123	-0.01175	0.2479	0.27495	FALSE	FALSE	FALSE	ieu-b-508	10	1.26E+08	0.037446	486484	0.753758	Sepsis (28	Sepsis (28	Sepsis (28	TRUE	igd	0.0025	1.12E-06	7335	M36776.m	TRUE	reported	VnYglx	2	TRUE	0.003289	24.1998
4550	rs1712741	A	G	A	G	0.0246	-0.03757	0.8728	0.872002	FALSE	FALSE	FALSE	ieu-b-508	8	18749120	0.049958	486484	0.452023	Sepsis (28	Sepsis (28	Sepsis (28	TRUE	igd	0.005	7.54E-07	7319	M33140.m	TRUE	reported	eBOUtb	2	TRUE	0.003296	24.19979
3824	rs7025315	C	G	C	G	0.0123	0.020977	0.3317	0.354876	FALSE	TRUE	FALSE	ieu-b-508	9	9010336	0.035082	486484	0.549865	Sepsis (28	Sepsis (28	Sepsis (28	TRUE	igd	0.0025	6.54E-07	7229	M32644.m	TRUE	reported	JAa2WD	2	TRUE	0.003337	24.1997
1265	rs9391679	A	C	A	C	0.0123	-0.03797	0.157	0.146203	FALSE	FALSE	FALSE	ieu-b-508	6	30228652	0.047133	486484	0.420433	Sepsis (28	Sepsis (28	Sepsis (28	TRUE	igd	0.0025	5.82E-07	7136	M01769.m	TRUE	reported	rismUZ	2	TRUE	0.003381	24.19962
7805	rs2088148	T	G	T	G	0.0123	0.043502	0.341	0.33289	FALSE	FALSE	FALSE	ieu-b-508	5	96646957	0.035347	486484	0.218438	Sepsis (28	Sepsis (28	Sepsis (28	TRUE	igd	0.0025	1.08E-06	6883	M35464.m	TRUE	reported	lr9dHp	2	TRUE	0.003505	24.19937
4414	rs6769112	T	C	T	C	0.0251	0.049013	0.2973	0.303755	FALSE	FALSE	FALSE	ieu-b-508	3	15981306	0.036586	486484	0.180354	Sepsis (28	Sepsis (28	Sepsis (28	TRUE	igd	0.0051	7.28E-07	2148	M32869.m	TRUE	reported	Rvim1n	2	TRUE	0.011151	24.19928
1925	rs1009184	T	G	T	G	-0.0123	-0.05205	0.5453	0.550294	FALSE	FALSE	FALSE	ieu-b-508	10	1.09E+08	0.033633	486484	0.121743	Sepsis (28	Sepsis (28	Sepsis (28	TRUE	igd	0.0025	6.00E-07	6549	M16822.m	TRUE	reported	UNI018	2	TRUE	0.003683	24.19901
3971	rs1048586	T	C	T	C	0.0123	0.042328	0.8306	0.853616	FALSE	FALSE	FALSE	ieu-b-508	7	4090283	0.04735	486484	0.371351	Sepsis (28	Sepsis (28	Sepsis (28	TRUE	igd	0.0025	1.07E-06	6497	M32729.m	TRUE	reported	XGRSN1	2	TRUE	0.003712	24.19895
8288	rs1104551	T	C	T	C	-0.0379	0.01428	0.1391	0.15243	FALSE	FALSE	FALSE	ieu-b-508	12	20956268	0.046708	486484	0.75981	Sepsis (28	Sepsis (28	Sepsis (28	TRUE	igd	0.0077	8.66E-07	1636	M36559.m	TRUE	reported	g6I9hg	2	TRUE	0.014592	24.19723
6781	rs1083956	C	G	C	G	-0.0797	-0.02214	0.8384	0.698091	FALSE	TRUE	FALSE	ieu-b-508	11	6433265	0.03817	486484	0.561838	Sepsis (28	Sepsis (28	Sepsis (28	TRUE	igd	0.0162	9.03E-07	3491	M34350.m	TRUE	reported	Cc3yW9	2	TRUE	0.006886	24.1901
5858	rs9906465	A	G	A	G	-0.0364	0.087175	0.9319	0.908078	FALSE	FALSE	FALSE	ieu-b-508	17	59343880	0.057815	486484	0.131599	Sepsis (28	Sepsis (28	Sepsis (28	TRUE	igd	0.0074	8.97E-07	7352	M33587.m	TRUE	reported	ssG6bF	2	TRUE	0.00328	24.18918
2765	rs723078	C	G	C	G	-0.0182	0.094983	0.9499	0.931307	FALSE	TRUE	FALSE	ieu-b-508	11	1.13E+08	0.066032	486484	0.150311	Sepsis (28	Sepsis (28	Sepsis (28	TRUE	igd	0.0037	6.84E-07	7303	M27256.m	TRUE	reported	mJwzsi	2	TRUE	0.003302	24.18914
1897	rs4690523	T	C	T	C	0.0182	0.04284	0.5243	0.512978	FALSE	FALSE	FALSE	ieu-b-508	4	1.78E+08	0.033403	486484	0.199658	Sepsis (28	Sepsis (28	Sepsis (28	TRUE	igd	0.0037	1.04E-06	7281	M15996.m	TRUE	reported	nXFvB9	2	TRUE	0.003312	24.18912
6413	rs1933041	A	G	A	G	-0.0182	-0.04213	0.6651	0.661217	FALSE	FALSE	FALSE	ieu-b-508	9	1196586	0.035284	486484	0.232433	Sepsis (28	Sepsis (28	Sepsis (28	TRUE	igd	0.0037	7.23E-07	7132	M34040.m	TRUE	reported	sJBy3i	2	TRUE	0.003381	24.18898
5920	rs1434384	A	G	A	G	-0.0305	-0.03043	0.5567	0.557951	FALSE	FALSE	FALSE	ieu-b-508	4	1.58E+08	0.03372	486484	0.366801	Sepsis (28	Sepsis (28	Sepsis (28	TRUE	igd	0.0062	7.15E-07	4323	M33633.m	TRUE	reported	e36XGE	2	TRUE	0.005567	24.18886
6539	rs2267575	A	T	A	T	-0.0423	0.036547	0.8736	0.835942	FALSE	TRUE	FALSE	ieu-b-508	19	35825205	0.045785	486484	0.424738	Sepsis (28	Sepsis (28	Sepsis (28	TRUE	igd	0.0086	8.73E-07	6987	M34221.m	TRUE	reported	nUe3TL	2	TRUE	0.003451	24.18575
5443	rs1695882	T	C	T	C	-0.0605	0.144582	0.9132	0.912292	FALSE	FALSE	FALSE	ieu-b-508	16	82924952	0.059139	486484	0.014493	Sepsis (28	Sepsis (28	Sepsis (28	TRUE	igd	0.0123	9.48E-07	5185	M33390.m	TRUE	reported	t0EYdH	2	TRUE	0.004644	24.18427
1511	rs1211644	T	C	T	C	-0.0679	0.136659	0.0144	0.015792	FALSE	FALSE	FALSE	ieu-b-508	1	33478877	0.133651	486484	0.306543	Sepsis (28	Sepsis (28	Sepsis (28	TRUE	igd	0.0138	9.28E-07	1747	M12067.m	TRUE	reported	O5x7RD	2	TRUE	0.013668	24.18154
7298	rs2025277	A	G	A	G	-0.03	0.025099	0.1872	0.184533	FALSE	FALSE	FALSE	ieu-b-508	10	10239143	0.043239	486484	0.561599	Sepsis (28	Sepsis (28	Sepsis (28	TRUE	igd	0.0061	7.93E-07	7364	M35126.m	TRUE	reported	1haOJA	2	TRUE	0.003274	24.18048
3536	rs590582	C	G	C	G	0.03	-0.05505	0.9437	0.939579	FALSE	TRUE	FALSE	ieu-b-508	1	47266629	0.070271	486484	0.433361	Sepsis (28	Sepsis (28	Sepsis (28	TRUE	igd	0.0061	7.95E-07	7358	M32497.m	TRUE	reported	244bZN	2	TRUE	0.003276	24.18047
2825	rs1165019	A	G	A	G	0.03	0.006554	0.0782	0.108759	FALSE	FALSE	FALSE	ieu-b-508	17	11667132	0.054039	486484	0.903471	Sepsis (28	Sepsis (28	Sepsis (28	TRUE	igd	0.0061	7.59E-07	7180	M27513.m	TRUE	reported	o9Ff2u	2	TRUE	0.003357	24.18031
7538	rs1730076	A	G	A	G	0.03	0.000299	0.9399	0.939413	FALSE	FALSE	FALSE	ieu-b-508	21	47894724	0.069778	486484	0.996583	Sepsis (28	Sepsis (28	Sepsis (28	TRUE	igd	0.0061	7.48E-07	6538	M35257.m	TRUE	reported	iphouU	2	TRUE	0.003686	24.17965
2365	rs1582961	T	C	T	C	0.0423	0.061596	0.2291	0.24245	FALSE	FALSE	FALSE	ieu-b-508	19	34061975	0.040547	486484	0.128729	Sepsis (28	Sepsis (28	Sepsis (28	TRUE	igd	0.0086	8.08E-07	3422	M21049.m	TRUE	reported	UDtLE4	2	TRUE	0.00702	24.17853
200	rs2176664	T	C	T	C	-0.0059	-0.00841	0.2846	0.284541	FALSE	FALSE	FALSE	ieu-b-508	17	12519871	0.037355	486484	0.821975	Sepsis (28	Sepsis (28	Sepsis (28	TRUE	igd	0.0012	2.03E-06	7355	M00059.m	TRUE	reported	RX1zal	2	TRUE	0.003276	24.16704
6391	rs6986249	T	C	T	C	-0.0177	0.018692	0.4413	0.447188	FALSE	FALSE	FALSE	ieu-b-508	8	36788693	0.03346	486484	0.576413	Sepsis (28	Sepsis (28	Sepsis (28	TRUE	igd	0.0036	6.30E-07	7330	M33969.m	TRUE	reported	0ujroW	2	TRUE	0.003287	24.16702
2672	rs1251677	A	G	A	G	0.0118	-0.04291	0.5206	0.506137	FALSE	FALSE	FALSE	ieu-b-508	5	57034653	0.03356	486484	0.201052	Sepsis (28	Sepsis (28	Sepsis (28	TRUE	igd	0.0024	1.26E-06	7256	M24074.m	TRUE	reported	Lk6D2A	2	TRUE	0.00332	24.16695
8083	rs6124466	T	C	T	C	-0.0359	-0.03831	0.8283	0.8347	FALSE	FALSE	FALSE	ieu-b-508	20	41122106	0.045442	486484	0.399158	Sepsis (28	Sepsis (28	Sepsis (28	TRUE	igd	0.0073	7.67E-07	2651	M36009.m	TRUE	reported	2EUZ1U	2	TRUE	0.00904	24.16659
5652	rs2471644	A	G	A	G	0.0177	-0.0141	0.6142	0.61605	FALSE	FALSE	FALSE	ieu-b-508	12	75593188	0.034313	486484	0.681152	Sepsis (28	Sepsis (28	Sepsis (28	TRUE	igd	0.0036	6.50E-07	5717	M33453.m	TRUE	reported	OzEeLQ	2	TRUE	0.004211	24.16515
6083	rs1310356	T	C	T	C	-0.0418	-0.0886	0.7102	0.700002	FALSE	FALSE	FALSE	ieu-b-508	4	73528682	0.03702	486484	0.016702	Sepsis (28	Sepsis (28	Sepsis (28	TRUE	igd	0.0085	9.87E-07	2660	M33835.m	TRUE	reported	crhBhm	2	TRUE	0.00901	24.16507
5561	rs1703360	A	G	A	G	-1.5889	-0.13787	0.0249	0.0377	FALSE	FALSE	FALSE	ieu-b-508	3	162346	0.099379	486484	0.165335	Sepsis (28	Sepsis (28	Sepsis (28	TRUE	igd	0.3178	5.75E-07	60	M33423.m	TRUE	reported	MoEnO7	2	TRUE	0.294092	24.16363
5897	rs7147680	A	G	A	G	0.5941	-0.03462	0.0114	0.014506	FALSE	FALSE	FALSE	ieu-b-508	14	90027391	0.141796	486484	0.807134	Sepsis (28	Sepsis (28	Sepsis (28	TRUE	igd	0.1197	6.94E-07	102	M33610.m	TRUE	reported	tmg43v	2	TRUE	0.194528	24.15075
2255	rs3872668	C	G	C	G	-0.0177	0.036664	0.3688	0.352272	FALSE	TRUE	FALSE	ieu-b-508	3	314429	0.034996	486484	0.294792	Sepsis (28	Sepsis (28	Sepsis (28	TRUE	igd	0.0036	7.34E-07	1759	M19934.m	TRUE	reported	HtcWcQ	2	TRUE	0.013557	24.14613
6250	rs1146594	A	G	A	G	0.0516	-0.07372	0.0294	0.032227	FALSE	FALSE	FALSE	ieu-b-508	1	76235229	0.094548	486484	0.435564	Sepsis (28	Sepsis (28	Sepsis (28	TRUE	igd	0.0105	8.65E-07	7345	M33936.m	TRUE	reported	jwA4B2	2	TRUE	0.003277	24.14363
2823	rs1104328	T	C	T	C	0.057	-0.07407	0.0188	0.020272	FALSE	FALSE	FALSE	ieu-b-508	12	1.22E+08	0.118807	486484	0.532981	Sepsis (28	Sepsis (28	Sepsis (28	TRUE	igd	0.0116	8.02E-07	7180	M27513.m	TRUE	reported	o9Ff2u	2	TRUE	0.003352	24.13864
630	rs1707758	A	G	A	G	0.1032	0.006897	0.9187	0.922638	FALSE	FALSE	FALSE	ieu-b-508	18	65292921	0.063145	486484	0.913019	Sepsis (28	Sepsis (28	Sepsis (28	TRUE	igd	0.021	9.20E-07	2639	M01123.m	TRUE	reported	NDTvfD	2	TRUE	0.009068	24.1319
402	rs1077834	T	C	T	C	-0.0113	0.031478	0.7912	0.780636	FALSE	FALSE	FALSE	ieu-b-508	15	58723479	0.040382	486484	0.435677	Sepsis (28	Sepsis (28	Sepsis (28	TRUE	igd	0.0023	6.87E-07	7365	M00063.m	TRUE	reported	Hdsv1L	2	TRUE	0.003267	24.13144
908	rs1301322	C	G	C	G	0.0113	-0.00274	0.1794	0.194448	FALSE	TRUE	FALSE	ieu-b-508	2	53430486	0.042306	486484	0.9484	Sepsis (28	Sepsis (28	Sepsis (28	TRUE	igd	0.0023	7.04E-07	7354	M01494.m	TRUE	reported	7JWNKI	2	TRUE	0.003272	24.13143
2186	rs2041343	T	C	T	C	0.0113	-0.05516	0.4795	0.503259	FALSE	FALSE	FALSE	ieu-b-508	7	2729743	0.03389	486484	0.103581	Sepsis (28	Sepsis (28	Sepsis (28	TRUE	igd	0.0023	6.67E-07	7182	M19362.m	TRUE	reported	SkNEKw	2	TRUE	0.00335	24.13127
7897	rs2468240	A	G	A	G	-0.0226	-0.01984	0.5882	0.582486	FALSE	FALSE	FALSE	ieu-b-508	12	88444592	0.033773	486484	0.556963	Sepsis (28	Sepsis (28	Sepsis (28	TRUE	igd	0.0046	1.01E-06	5629	M35669.m	TRUE	reported	gSecuE	2	TRUE	0.00427	24.12942
8307	rs1314234	T	G	T	G	0.0339	-0.04532	0.2825	0.279416	FALSE	FALSE	FALSE	ieu-b-508	4	1.21E+08	0.037223	486484	0.223374	Sepsis (28	Sepsis (28	Sepsis (28	TRUE	igd	0.0069	8.46E-07	3346	M36585.m	TRUE	reported	pXZg13	2	TRUE	0.007162	24.12357
5879	rs1174740	A	T	A	T	0.2269	0.04636	0.0366	0.04627	FALSE	TRUE	FALSE	ieu-b-508	5	1.56E+08	0.079256	486484	0.558587	Sepsis (28	Sepsis (28	Sepsis (28	TRUE	igd	0.0461	8.78E-07	470	M33610.m	TRUE	reported	tmg43v	2	TRUE	0.049016	24.1221
3262	rs1776347	A	C	A	C	0.0167	0.054057	0.0905	0.097925	FALSE	FALSE	FALSE	ieu-b-508	10	77103188	0.056606	486484	0.339597	Sepsis (28	Sepsis (28	Sepsis (28	TRUE	igd	0.0034	9.81E-07	7365	M32348.m	TRUE	reported	DJeLDx	2	TRUE	0.003265	24.11888
3226	rs7791060	A	G	A	G	-0.0167	0.005942	0.9237	0.924867	FALSE	FALSE	FALSE	ieu-b-508	7	32397269	0.063598	486484	0.925566	Sepsis (28	Sepsis (28	Sepsis (28	TRUE	igd	0.0034	6.96E-07	7344	M32339.m	TRUE	reported	SVp504	2	TRUE	0.003274	24.11886
6968	rs1052007	A	G	A	G	-0.0167	-0.04356	0.3469	0.329982	FALSE	FALSE	FALSE	ieu-b-508	5	1.3E+08	0.035499	486484	0.219739	Sepsis (28	Sepsis (28	Sepsis (28	TRUE	igd	0.0034	1.36E-06	7344	M34407.m	TRUE	reported	X7I83Y	2	TRUE	0.003274	24.11886
2462	rs1457613	A	C	A	C	0.0167	0.06604	0.8556	0.870668	FALSE	FALSE	FALSE	ieu-b-508	12	13645414	0.049823	486484	0.185008	Sepsis (28	Sepsis (28	Sepsis (28	TRUE	igd	0.0034	1.23E-06	6180	M22030.m	TRUE	reported	ZNV0qR	2	TRUE	0.003889	24.11762
6468	rs1246268	A	T	A	T	0.95	-0.04571	0.988	0.963544	FALSE	TRUE	FALSE	ieu-b-508	19	23378438	0.095828	486484	0.633393	Sepsis (28	Sepsis (28	Sepsis (28	TRUE	igd	0.192	7.50E-07	134	M34109.m	TRUE	reported	piJuC7	2	TRUE	0.154477	24.11648
2272	rs9947181	C	G	C	G	-0.0275	-0.03534	0.0304	0.028902	FALSE	TRUE	FALSE	ieu-b-508	18	45580229	0.100543	486484	0.725217	Sepsis (28	Sepsis (28	Sepsis (28	TRUE	igd	0.0056	9.24E-07	7354	M19934.m	TRUE	reported	HtcWcQ	2	TRUE	0.003268	24.10856
2397	rs1707103	A	G	A	G	-0.0329	0.038005	0.8334	0.834023	FALSE	FALSE	FALSE	ieu-b-508	4	1.82E+08	0.045042	486484	0.398793	Sepsis (28	Sepsis (28	Sepsis (28	TRUE	igd	0.0067	8.57E-07	5436	M21184.m	TRUE	reported	63ppuJ	2	TRUE	0.004416	24.10363
6851	rs1192474	A	G	A	G	-0.1786	0.02838	0.6176	0.57223	FALSE	FALSE	FALSE	ieu-b-508	3	64459732	0.033726	486484	0.400076	Sepsis (28	Sepsis (28	Sepsis (28	TRUE	igd	0.0343	1.89E-07	18	M34365.m	TRUE	reported	8eexD0	2	TRUE	0.601	24.10028
883	rs4109037	A	T	A	T	0.0162	0.018589	0.146	0.143559	FALSE	TRUE	FALSE	ieu-b-508	4	57775609	0.047987	486484	0.698474	Sepsis (28	Sepsis (28	Sepsis (28	TRUE	igd	0.0033	9.49E-07	7362	M01365.m	TRUE	reported	N1cZTs	2	TRUE	0.003263	24.09263
4356	rs7687485	A	T	A	T	-0.0324	-0.02251	0.0947	0.092258	FALSE	TRUE	FALSE	ieu-b-508	4	29335928	0.057869	486484	0.697291	Sepsis (28	Sepsis (28	Sepsis (28	TRUE	igd	0.0066	8.28E-07	7355	M32855.m	TRUE	reported	AM8kCf	2	TRUE	0.003266	24.09262
6530	rs523932	T	C	T	C	0.054	-0.15374	0.9868	0.983533	FALSE	FALSE	FALSE	ieu-b-508	11	59468550	0.13068	486484	0.239413	Sepsis (28	Sepsis (28	Sepsis (28	TRUE	igd	0.011	8.30E-07	7049	M34123.m	TRUE	reported	9O5wo1	2	TRUE	0.003407	24.09234
6988	rs1042933	T	C	T	C	0.0329	-0.02559	0.2704	0.26442	FALSE	FALSE	FALSE	ieu-b-508	8	68453664	0.038275	486484	0.503833	Sepsis (28	Sepsis (28	Sepsis (28	TRUE	igd	0.0067	9.14E-07	1567	M34416.m	TRUE	reported	q9OtaZ	2	TRUE	0.015154	24.08172
8531	rs4761693	T	C	T	C	0.0643	-0.01521	0.6193	0.614294	FALSE	FALSE	FALSE	ieu-b-508	12	95258824	0.0342	486484	0.656446	Sepsis (28	Sepsis (28	Sepsis (28	TRUE	igd	0.0131	9.65E-07	2560	M37112.m	TRUE	reported	P3GiAg	2	TRUE	0.009323	24.07354
1751	rs1490672	T	C	T	C	-0.0108	-0.00706	0.2361	0.235553	FALSE	FALSE	FALSE	ieu-b-508	4	1.55E+08	0.039293	486484	0.857453	Sepsis (28	Sepsis (28	Sepsis (28	TRUE	igd	0.0022	1.13E-06	1763	M15650.m	TRUE	reported	NgezyT	2	TRUE	0.013485	24.07183
7489	rs1717637	A	G	A	G	0.0211	0.034044	0.1078	0.106161	FALSE	FALSE	FALSE	ieu-b-508	7	39036411	0.054141	486484	0.529472	Sepsis (28	Sepsis (28	Sepsis (28	TRUE	igd	0.0043	8.44E-07	7261	M35253.m	TRUE	reported	VboRmj	2	TRUE	0.003305	24.07179
7010	rs2938675	A	C	A	C	0.0476	0.031934	0.3923	0.382829	FALSE	FALSE	FALSE	ieu-b-508	18	23537365	0.034377	486484	0.35291	Sepsis (28	Sepsis (28	Sepsis (28	TRUE	igd	0.0097	9.79E-07	4452	M34420.m	TRUE	reported	20uofq	2	TRUE	0.00538	24.06996
6139	rs9317012	T	C	T	C	-0.1562	0.086024	0.8991	0.909299	FALSE	FALSE	FALSE	ieu-b-508	13	59334465	0.058373	486484	0.140565	Sepsis (28	Sepsis (28	Sepsis (28	TRUE	igd	0.0317	8.49E-07	228	M33877.m	TRUE	reported	yDVi1f	2	TRUE	0.096241	24.06673
839	rs1301953	C	G	C	G	0.0157	0.019564	0.841	0.841827	FALSE	TRUE	FALSE	ieu-b-508	2	48976577	0.045998	486484	0.67061	Sepsis (28	Sepsis (28	Sepsis (28	TRUE	igd	0.0032	7.52E-07	7323	M01359.m	TRUE	reported	7L9x1s	2	TRUE	0.003276	24.06471
1268	rs9674799	A	G	A	G	0.0157	0.029321	0.8784	0.879419	FALSE	FALSE	FALSE	ieu-b-508	17	1984540	0.051171	486484	0.566644	Sepsis (28	Sepsis (28	Sepsis (28	TRUE	igd	0.0032	7.47E-07	7136	M01769.m	TRUE	reported	rismUZ	2	TRUE	0.003362	24.06454
4413	rs671307	A	G	A	G	0.0319	-0.02809	0.1553	0.155625	FALSE	FALSE	FALSE	ieu-b-508	11	78524245	0.046264	486484	0.543712	Sepsis (28	Sepsis (28	Sepsis (28	TRUE	igd	0.0065	7.95E-07	2148	M32869.m	TRUE	reported	Rvim1n	2	TRUE	0.011089	24.06302
7830	rs1439737	T	C	T	C	0.0368	0.024445	0.5832	0.596457	FALSE	FALSE	FALSE	ieu-b-508	2	2.1E+08	0.03394	486484	0.471378	Sepsis (28	Sepsis (28	Sepsis (28	TRUE	igd	0.0075	1.08E-06	1291	M35527.m	TRUE	reported	UAMhnu	2	TRUE	0.018307	24.03808
2211	rs7415896	A	G	A	G	0.0304	0.062162	0.0488	0.055302	FALSE	FALSE	FALSE	ieu-b-508	1	5534441	0.073682	486484	0.398867	Sepsis (28	Sepsis (28	Sepsis (28	TRUE	igd	0.0062	8.77E-07	6929	M19364.m	TRUE	reported	XkjMEx	2	TRUE	0.003458	24.03468
7202	rs1687949	A	C	A	C	0.052	0.064844	0.9479	0.979543	FALSE	FALSE	FALSE	ieu-b-508	4	27576781	0.118012	486484	0.582684	Sepsis (28	Sepsis (28	Sepsis (28	TRUE	igd	0.0106	9.81E-07	1560	M34674.m	TRUE	reported	plxOKz	2	TRUE	0.015192	24.03465
502	rs946712	A	C	A	C	0.1039	0.154172	0.9559	0.954463	FALSE	FALSE	FALSE	ieu-b-508	9	1.34E+08	0.080242	486484	0.054689	Sepsis (28	Sepsis (28	Sepsis (28	TRUE	igd	0.0212	9.59E-07	7274	M00569.m	TRUE	reported	1TjBaz	2	TRUE	0.003291	24.01264
1119	rs1116790	T	C	T	C	0.0804	0.058169	0.8545	0.842405	FALSE	FALSE	FALSE	ieu-b-508	5	1.45E+08	0.046257	486484	0.208572	Sepsis (28	Sepsis (28	Sepsis (28	TRUE	igd	0.0164	9.83E-07	2063	M01640.m	TRUE	reported	o3qxzl	2	TRUE	0.011516	24.01061
2849	rs1765149	A	G	A	G	0.1034	0.132252	0.9423	0.935551	FALSE	FALSE	FALSE	ieu-b-508	4	31641899	0.067645	486484	0.050572	Sepsis (28	Sepsis (28	Sepsis (28	TRUE	igd	0.0211	9.13E-07	5673	M27531.m	TRUE	reported	v01zDr	2	TRUE	0.004215	24.00618
4180	rs7215713	T	C	T	C	0.0245	0.012858	0.2296	0.225648	FALSE	FALSE	FALSE	ieu-b-508	17	5576634	0.040339	486484	0.749908	Sepsis (28	Sepsis (28	Sepsis (28	TRUE	igd	0.005	1.18E-06	6712	M32759.m	TRUE	reported	9elHr7	2	TRUE	0.003564	24.00285
4036	rs1235776	T	C	T	C	0.0343	0.101886	0.0454	0.049561	FALSE	FALSE	FALSE	ieu-b-508	10	67512471	0.076997	486484	0.185752	Sepsis (28	Sepsis (28	Sepsis (28	TRUE	igd	0.007	8.35E-07	6042	M32739.m	TRUE	reported	dYSC7n	2	TRUE	0.003958	24.00205
5061	rs7755459	A	C	A	C	0.3099	-0.04355	0.9804	0.977346	FALSE	FALSE	FALSE	ieu-b-508	6	97545709	0.112824	486484	0.699489	Sepsis (28	Sepsis (28	Sepsis (28	TRUE	igd	0.0632	9.49E-07	1017	M33178.m	TRUE	reported	g22WMS	2	TRUE	0.023096	23.99684
5291	rs1305496	A	G	A	G	0.05	-0.00919	0.2239	0.224469	FALSE	FALSE	FALSE	ieu-b-508	22	27692054	0.040189	486484	0.819177	Sepsis (28	Sepsis (28	Sepsis (28	TRUE	igd	0.0102	9.07E-07	1273	M33353.m	TRUE	reported	ZIN4gk	2	TRUE	0.018526	23.99147
6690	rs1103935	T	C	T	C	0.0338	-0.16192	0.0406	0.048353	FALSE	FALSE	FALSE	ieu-b-508	11	5885846	0.07855	486484	0.039266	Sepsis (28	Sepsis (28	Sepsis (28	TRUE	igd	0.0069	8.47E-07	5081	M34336.m	TRUE	reported	jrSSxL	2	TRUE	0.0047	23.98635
2874	rs780245	T	C	T	C	0.0289	0.007007	0.1757	0.168224	FALSE	FALSE	FALSE	ieu-b-508	1	2.34E+08	0.044655	486484	0.875319	Sepsis (28	Sepsis (28	Sepsis (28	TRUE	igd	0.0059	1.15E-06	6715	M27710.m	TRUE	reported	XeaFav	2	TRUE	0.00356	23.98625
6125	rs1716908	A	G	A	G	0.5339	0.050787	0.0134	0.054508	FALSE	FALSE	FALSE	ieu-b-508	7	97402027	0.074974	486484	0.49815	Sepsis (28	Sepsis (28	Sepsis (28	TRUE	igd	0.1084	8.32E-07	176	M33877.m	TRUE	reported	yDVi1f	2	TRUE	0.121135	23.98269
4360	rs498275	A	G	A	G	0.0338	0.009245	0.3246	0.338148	FALSE	FALSE	FALSE	ieu-b-508	9	1.08E+08	0.035384	486484	0.793889	Sepsis (28	Sepsis (28	Sepsis (28	TRUE	igd	0.0069	9.93E-07	2656	M32857.m	TRUE	reported	MVAWzT	2	TRUE	0.008954	23.97773
6719	rs1262616	C	G	C	G	0.2276	0.072569	0.0363	0.046055	FALSE	TRUE	FALSE	ieu-b-508	20	49769883	0.080313	486484	0.366215	Sepsis (28	Sepsis (28	Sepsis (28	TRUE	igd	0.0464	9.37E-07	535	M34338.m	TRUE	reported	h4FB9a	2	TRUE	0.043038	23.97077
3196	rs804895	T	C	T	C	-0.0235	0.035072	0.0781	0.085133	FALSE	FALSE	FALSE	ieu-b-508	16	81099713	0.059815	486484	0.557647	Sepsis (28	Sepsis (28	Sepsis (28	TRUE	igd	0.0048	8.44E-07	7356	M32338.m	TRUE	reported	q5cuaP	2	TRUE	0.003248	23.96267
6298	rs6475272	T	C	T	C	-0.0235	0.014237	0.2277	0.223626	FALSE	FALSE	FALSE	ieu-b-508	9	18951598	0.039978	486484	0.72175	Sepsis (28	Sepsis (28	Sepsis (28	TRUE	igd	0.0048	9.07E-07	7320	M33941.m	TRUE	reported	culqiU	2	TRUE	0.003264	23.96264
1110	rs511304	A	C	A	C	0.0191	0.024186	0.4368	0.43912	FALSE	FALSE	FALSE	ieu-b-508	3	1.74E+08	0.03413	486484	0.478533	Sepsis (28	Sepsis (28	Sepsis (28	TRUE	igd	0.0039	1.01E-06	1735	M01638.m	TRUE	reported	8dp0Ac	2	TRUE	0.013636	23.95723
6006	rs1684379	T	C	T	C	-0.7079	0.009473	0.974	0.978435	FALSE	FALSE	FALSE	ieu-b-508	2	1.34E+08	0.116814	486484	0.935368	Sepsis (28	Sepsis (28	Sepsis (28	TRUE	igd	0.1428	7.10E-07	79	M33751.m	TRUE	reported	Cxdkr4	2	TRUE	0.237265	23.95251
3870	rs1948709	T	C	T	C	0.0186	-0.01898	0.8952	0.894734	FALSE	FALSE	FALSE	ieu-b-508	4	53450096	0.054468	486484	0.727438	Sepsis (28	Sepsis (28	Sepsis (28	TRUE	igd	0.0038	7.72E-07	7361	M32654.m	TRUE	reported	6ISQOP	2	TRUE	0.003244	23.95194
546	rs1160770	T	C	T	C	0.0093	0.03831	0.8783	0.871592	FALSE	FALSE	FALSE	ieu-b-508	11	1.14E+08	0.049833	486484	0.442031	Sepsis (28	Sepsis (28	Sepsis (28	TRUE	igd	0.0019	6.94E-07	7354	M00606.m	TRUE	reported	r5smf7	2	TRUE	0.003247	23.95193
2605	rs1269420	T	C	T	C	0.0093	-0.0327	0.4344	0.441797	FALSE	FALSE	FALSE	ieu-b-508	2	2.11E+08	0.033693	486484	0.331765	Sepsis (28	Sepsis (28	Sepsis (28	TRUE	igd	0.0019	6.54E-07	7329	M22481.m	TRUE	reported	bXpf0k	2	TRUE	0.003258	23.95191
5399	rs236403	T	C	T	C	-0.0093	0.012157	0.624	0.614409	FALSE	FALSE	FALSE	ieu-b-508	6	36771587	0.034317	486484	0.723136	Sepsis (28	Sepsis (28	Sepsis (28	TRUE	igd	0.0019	5.87E-07	7315	M33389.m	TRUE	reported	Hf6XVM	2	TRUE	0.003265	23.9519
1899	rs7150776	T	C	T	C	0.0279	0.031412	0.1105	0.106147	FALSE	FALSE	FALSE	ieu-b-508	14	93113821	0.054326	486484	0.563116	Sepsis (28	Sepsis (28	Sepsis (28	TRUE	igd	0.0057	9.22E-07	7281	M15996.m	TRUE	reported	nXFvB9	2	TRUE	0.00328	23.95187
7581	rs7508059	A	G	A	G	0.0093	0.045072	0.1808	0.815766	FALSE	FALSE	FALSE	ieu-b-508	19	21841076	0.04293	486484	0.293766	Sepsis (28	Sepsis (28	Sepsis (28	TRUE	igd	0.0019	6.27E-07	7217	M35270.m	TRUE	reported	Ct8V1n	2	TRUE	0.003309	23.95181
8025	rs7862610	A	C	A	C	0.0093	0.013796	0.4276	0.436185	FALSE	FALSE	FALSE	ieu-b-508	9	8160552	0.033662	486484	0.681922	Sepsis (28	Sepsis (28	Sepsis (28	TRUE	igd	0.0019	1.36E-06	6604	M35754.m	TRUE	reported	IlXOo1	2	TRUE	0.003615	23.95119
2539	rs1287183	A	C	A	C	-0.0093	-0.04309	0.4654	0.481307	FALSE	FALSE	FALSE	ieu-b-508	13	98748212	0.03406	486484	0.205823	Sepsis (28	Sepsis (28	Sepsis (28	TRUE	igd	0.0019	1.53E-06	6535	M22177.m	TRUE	reported	2s6Sly	2	TRUE	0.003653	23.95112
2569	rs571139	A	G	A	G	0.0093	-0.00457	0.3354	0.348692	FALSE	FALSE	FALSE	ieu-b-508	11	1.16E+08	0.035059	486484	0.896362	Sepsis (28	Sepsis (28	Sepsis (28	TRUE	igd	0.0019	1.62E-06	6535	M22177.m	TRUE	reported	2s6Sly	2	TRUE	0.003653	23.95112
7350	rs2267290	C	G	C	G	-0.0093	0.009578	0.6914	0.691852	FALSE	TRUE	FALSE	ieu-b-508	22	34198628	0.036129	486484	0.790924	Sepsis (28	Sepsis (28	Sepsis (28	TRUE	igd	0.0019	8.92E-07	4900	M35137.m	TRUE	reported	Eq2siS	2	TRUE	0.004866	23.94867
4252	rs1025573	T	C	T	C	0.0558	-0.00926	0.0859	0.092574	FALSE	FALSE	FALSE	ieu-b-508	9	1.11E+08	0.05789	486484	0.872957	Sepsis (28	Sepsis (28	Sepsis (28	TRUE	igd	0.0114	9.52E-07	4274	M32800.m	TRUE	reported	Q7MVsa	2	TRUE	0.005574	23.94724
5180	rs2493950	T	C	T	C	-0.2434	-0.05049	0.9465	0.94568	FALSE	FALSE	FALSE	ieu-b-508	6	95352036	0.073766	486484	0.493683	Sepsis (28	Sepsis (28	Sepsis (28	TRUE	igd	0.0497	9.90E-07	1247	M33195.m	TRUE	reported	SI4STh	2	TRUE	0.018871	23.94591
470	rs1340344	A	T	A	T	-0.0235	0.024078	0.8588	0.874969	FALSE	TRUE	FALSE	ieu-b-508	2	83225802	0.050562	486484	0.633924	Sepsis (28	Sepsis (28	Sepsis (28	TRUE	igd	0.0048	1.02E-06	1761	M00527.m	TRUE	reported	UYN7vF	2	TRUE	0.013428	23.94196
2395	rs822406	A	G	A	G	0.1625	-0.06853	0.063	0.091749	FALSE	FALSE	FALSE	ieu-b-508	5	1.55E+08	0.059116	486484	0.246355	Sepsis (28	Sepsis (28	Sepsis (28	TRUE	igd	0.0332	1.03E-06	2339	M21151.m	TRUE	reported	LfwdLx	2	TRUE	0.010139	23.93641
5782	rs1674726	A	G	A	G	-0.0137	0.022181	0.3501	0.366891	FALSE	FALSE	FALSE	ieu-b-508	6	1.54E+08	0.034771	486484	0.523524	Sepsis (28	Sepsis (28	Sepsis (28	TRUE	igd	0.0028	8.82E-07	7269	M33510.m	TRUE	reported	WxGKnn	2	TRUE	0.003283	23.93346
6605	rs21806	T	C	T	C	-0.0137	0.05533	0.3173	0.339219	FALSE	FALSE	FALSE	ieu-b-508	4	1.11E+08	0.035299	486484	0.117007	Sepsis (28	Sepsis (28	Sepsis (28	TRUE	igd	0.0028	1.17E-06	6994	M34306.m	TRUE	reported	WPXPUN	2	TRUE	0.003411	23.93321
3858	rs9423399	A	G	A	G	0.0137	-0.00708	0.3176	0.32543	FALSE	FALSE	FALSE	ieu-b-508	10	5311075	0.0356	486484	0.842306	Sepsis (28	Sepsis (28	Sepsis (28	TRUE	igd	0.0028	5.89E-07	5211	M32651.m	TRUE	reported	DeuPwv	2	TRUE	0.004573	23.93086
1912	rs1098032	T	C	T	C	0.0411	0.073704	0.9677	0.962168	FALSE	FALSE	FALSE	ieu-b-508	9	1.13E+08	0.08698	486484	0.39679	Sepsis (28	Sepsis (28	Sepsis (28	TRUE	igd	0.0084	9.21E-07	4689	M16816.m	TRUE	reported	J1abbO	2	TRUE	0.00508	23.92984
6262	rs888860	A	C	A	C	-0.0181	-0.01261	0.3513	0.354471	FALSE	FALSE	FALSE	ieu-b-508	19	35465187	0.034949	486484	0.718213	Sepsis (28	Sepsis (28	Sepsis (28	TRUE	igd	0.0037	9.39E-07	7345	M33936.m	TRUE	reported	jwA4B2	2	TRUE	0.003248	23.92409
3856	rs728661	T	C	T	C	-0.0181	0.030206	0.8438	0.84937	FALSE	FALSE	FALSE	ieu-b-508	12	96071320	0.046695	486484	0.517708	Sepsis (28	Sepsis (28	Sepsis (28	TRUE	igd	0.0037	1.28E-06	5211	M32651.m	TRUE	reported	DeuPwv	2	TRUE	0.004571	23.92142
2081	rs4410790	T	C	T	C	0.0313	0.025506	0.3695	0.36919	FALSE	FALSE	FALSE	ieu-b-508	7	17284577	0.034594	486484	0.460936	Sepsis (28	Sepsis (28	Sepsis (28	TRUE	igd	0.0064	8.96E-07	7322	M18392.m	TRUE	reported	fBRMfe	2	TRUE	0.003256	23.91168
7032	rs7923671	A	G	A	G	-0.0538	0.009002	0.0466	0.066829	FALSE	FALSE	FALSE	ieu-b-508	10	48414570	0.067146	486484	0.893346	Sepsis (28	Sepsis (28	Sepsis (28	TRUE	igd	0.011	9.82E-07	4442	M34441.m	TRUE	reported	UpgxQm	2	TRUE	0.005356	23.91022
4911	rs275362	A	G	A	G	-0.1249	0.005436	0.4565	0.490616	FALSE	FALSE	FALSE	ieu-b-508	3	81428950	0.034224	486484	0.87381	Sepsis (28	Sepsis (28	Sepsis (28	TRUE	igd	0.0245	3.39E-07	25	M33178.m	TRUE	reported	g22WMS	2	TRUE	0.5097	23.91005
4479	rs1159324	A	G	A	G	-0.0494	-0.03268	0.937	0.928581	FALSE	FALSE	FALSE	ieu-b-508	10	1.19E+08	0.064729	486484	0.613667	Sepsis (28	Sepsis (28	Sepsis (28	TRUE	igd	0.0101	1.07E-06	2773	M33131.m	TRUE	reported	uGKURO	2	TRUE	0.008553	23.9055
6741	rs3735210	T	C	T	C	-0.873	0.02499	0.985	0.983562	FALSE	FALSE	FALSE	ieu-b-508	7	2069588	0.130861	486484	0.848551	Sepsis (28	Sepsis (28	Sepsis (28	TRUE	igd	0.177	8.13E-07	115	M34338.m	TRUE	reported	h4FB9a	2	TRUE	0.174601	23.90356
5919	rs1337683	A	G	A	G	-0.0401	-0.00426	0.8266	0.83126	FALSE	FALSE	FALSE	ieu-b-508	10	95352173	0.044584	486484	0.923946	Sepsis (28	Sepsis (28	Sepsis (28	TRUE	igd	0.0082	1.13E-06	4323	M33633.m	TRUE	reported	e36XGE	2	TRUE	0.005501	23.90342
1126	rs6834631	T	G	T	G	0.1306	0.090716	0.9596	0.956031	FALSE	FALSE	FALSE	ieu-b-508	4	1.22E+08	0.08144	486484	0.265326	Sepsis (28	Sepsis (28	Sepsis (28	TRUE	igd	0.0267	1.03E-06	2063	M01640.m	TRUE	reported	o3qxzl	2	TRUE	0.011465	23.90246
8629	rs9266813	C	G	C	G	-0.0088	0.004858	0.7349	0.742286	FALSE	TRUE	FALSE	ieu-b-508	6	31381321	0.038163	486484	0.898703	Sepsis (28	Sepsis (28	Sepsis (28	TRUE	igd	0.0018	1.02E-06	7366	M37506.m	TRUE	reported	m2kaRt	2	TRUE	0.003234	23.89474
3265	rs2018507	A	G	A	G	-0.0088	0.041617	0.3629	0.358739	FALSE	FALSE	FALSE	ieu-b-508	10	88168024	0.034832	486484	0.232175	Sepsis (28	Sepsis (28	Sepsis (28	TRUE	igd	0.0018	7.06E-07	7365	M32348.m	TRUE	reported	DJeLDx	2	TRUE	0.003235	23.89474
3266	rs2635351	A	C	A	C	-0.0088	-0.04202	0.5749	0.580481	FALSE	FALSE	FALSE	ieu-b-508	4	1.18E+08	0.034081	486484	0.217583	Sepsis (28	Sepsis (28	Sepsis (28	TRUE	igd	0.0018	5.95E-07	7365	M32348.m	TRUE	reported	DJeLDx	2	TRUE	0.003235	23.89474
3428	rs1500394	T	C	T	C	-0.0088	0.016853	0.5727	0.575934	FALSE	FALSE	FALSE	ieu-b-508	4	1.56E+08	0.035136	486484	0.631481	Sepsis (28	Sepsis (28	Sepsis (28	TRUE	igd	0.0018	1.45E-06	7364	M32452.m	TRUE	reported	8GtFpl	2	TRUE	0.003235	23.89474
729	rs7572149	T	C	T	C	0.0088	0.042111	0.133	0.132792	FALSE	FALSE	FALSE	ieu-b-508	2	1.05E+08	0.049201	486484	0.392046	Sepsis (28	Sepsis (28	Sepsis (28	TRUE	igd	0.0018	1.00E-06	7363	M01301.m	TRUE	reported	w8TL71	2	TRUE	0.003236	23.89474
3457	rs1235857	T	C	T	C	-0.0088	0.001234	0.7176	0.723339	FALSE	FALSE	FALSE	ieu-b-508	10	1.23E+08	0.037377	486484	0.973658	Sepsis (28	Sepsis (28	Sepsis (28	TRUE	igd	0.0018	1.53E-06	7363	M32489.m	TRUE	reported	hnljaD	2	TRUE	0.003236	23.89474
446	rs1105432	T	C	T	C	0.0088	-0.05224	0.1072	0.101597	FALSE	FALSE	FALSE	ieu-b-508	12	11663755	0.055333	486484	0.345084	Sepsis (28	Sepsis (28	Sepsis (28	TRUE	igd	0.0018	1.46E-06	7361	M00513.m	TRUE	reported	ohacnP	2	TRUE	0.003237	23.89474
451	rs1280155	T	C	T	C	0.0088	-0.05405	0.8705	0.867245	FALSE	FALSE	FALSE	ieu-b-508	11	13993754	0.049179	486484	0.271725	Sepsis (28	Sepsis (28	Sepsis (28	TRUE	igd	0.0018	9.64E-07	7361	M00513.m	TRUE	reported	ohacnP	2	TRUE	0.003237	23.89474
10	rs1000770	T	C	T	C	-0.0044	0.001815	0.6418	0.640743	FALSE	FALSE	FALSE	ieu-b-508	4	44276751	0.034849	486484	0.958464	Sepsis (28	Sepsis (28	Sepsis (28	TRUE	igd	9.00E-04	1.24E-06	7355	M00054.m	TRUE	reported	QTDAnh	2	TRUE	0.003239	23.89474
25	rs1090078	A	C	A	C	-0.0044	0.024282	0.4158	0.421535	FALSE	FALSE	FALSE	ieu-b-508	5	1.26E+08	0.033725	486484	0.471512	Sepsis (28	Sepsis (28	Sepsis (28	TRUE	igd	9.00E-04	9.74E-07	7355	M00054.m	TRUE	reported	QTDAnh	2	TRUE	0.003239	23.89474
38	rs1246808	T	C	T	C	-0.0044	-0.03777	0.5436	0.535194	FALSE	FALSE	FALSE	ieu-b-508	2	2.31E+08	0.033701	486484	0.26234	Sepsis (28	Sepsis (28	Sepsis (28	TRUE	igd	9.00E-04	1.25E-06	7355	M00054.m	TRUE	reported	QTDAnh	2	TRUE	0.003239	23.89474
46	rs1349704	A	G	A	G	0.0044	-0.01725	0.4611	0.45823	FALSE	FALSE	FALSE	ieu-b-508	11	96777666	0.033491	486484	0.606423	Sepsis (28	Sepsis (28	Sepsis (28	TRUE	igd	9.00E-04	8.31E-07	7355	M00054.m	TRUE	reported	QTDAnh	2	TRUE	0.003239	23.89474
52	rs1519309	T	C	T	C	-0.0044	-0.02949	0.6544	0.647523	FALSE	FALSE	FALSE	ieu-b-508	4	1.87E+08	0.034945	486484	0.398677	Sepsis (28	Sepsis (28	Sepsis (28	TRUE	igd	9.00E-04	1.21E-06	7355	M00054.m	TRUE	reported	QTDAnh	2	TRUE	0.003239	23.89474
64	rs1871053	A	G	A	G	-0.0044	-0.04459	0.4514	0.449437	FALSE	FALSE	FALSE	ieu-b-508	11	88476191	0.033691	486484	0.185638	Sepsis (28	Sepsis (28	Sepsis (28	TRUE	igd	9.00E-04	7.68E-07	7355	M00054.m	TRUE	reported	QTDAnh	2	TRUE	0.003239	23.89474
82	rs2580344	T	C	T	C	0.0044	-0.00177	0.6238	0.632002	FALSE	FALSE	FALSE	ieu-b-508	2	1.22E+08	0.034726	486484	0.959347	Sepsis (28	Sepsis (28	Sepsis (28	TRUE	igd	9.00E-04	7.98E-07	7355	M00054.m	TRUE	reported	QTDAnh	2	TRUE	0.003239	23.89474
83	rs2604365	T	G	T	G	0.0044	0.007799	0.6371	0.633293	FALSE	FALSE	FALSE	ieu-b-508	8	15614315	0.034769	486484	0.822514	Sepsis (28	Sepsis (28	Sepsis (28	TRUE	igd	9.00E-04	1.45E-06	7355	M00054.m	TRUE	reported	QTDAnh	2	TRUE	0.003239	23.89474
91	rs2813760	A	G	A	G	0.0044	-0.03414	0.5775	0.587314	FALSE	FALSE	FALSE	ieu-b-508	1	90569361	0.034101	486484	0.316762	Sepsis (28	Sepsis (28	Sepsis (28	TRUE	igd	9.00E-04	1.19E-06	7355	M00054.m	TRUE	reported	QTDAnh	2	TRUE	0.003239	23.89474
97	rs2970335	A	C	A	C	0.0044	0.075931	0.4039	0.415305	FALSE	FALSE	FALSE	ieu-b-508	11	14325401	0.033913	486484	0.025158	Sepsis (28	Sepsis (28	Sepsis (28	TRUE	igd	9.00E-04	1.02E-06	7355	M00054.m	TRUE	reported	QTDAnh	2	TRUE	0.003239	23.89474
101	rs3742929	T	C	T	C	-0.0044	0.02729	0.512	0.515793	FALSE	FALSE	FALSE	ieu-b-508	14	33165182	0.033412	486484	0.414047	Sepsis (28	Sepsis (28	Sepsis (28	TRUE	igd	9.00E-04	1.01E-06	7355	M00054.m	TRUE	reported	QTDAnh	2	TRUE	0.003239	23.89474
115	rs4611118	A	G	A	G	-0.0044	-0.00203	0.609	0.612037	FALSE	FALSE	FALSE	ieu-b-508	10	1.22E+08	0.034262	486484	0.952807	Sepsis (28	Sepsis (28	Sepsis (28	TRUE	igd	9.00E-04	9.41E-07	7355	M00054.m	TRUE	reported	QTDAnh	2	TRUE	0.003239	23.89474
149	rs682359	T	G	T	G	0.0044	0.021548	0.5812	0.578534	FALSE	FALSE	FALSE	ieu-b-508	15	42426833	0.033907	486484	0.525098	Sepsis (28	Sepsis (28	Sepsis (28	TRUE	igd	9.00E-04	6.54E-07	7355	M00054.m	TRUE	reported	QTDAnh	2	TRUE	0.003239	23.89474
159	rs719564	A	G	A	G	0.0044	0.000426	0.6101	0.625714	FALSE	FALSE	FALSE	ieu-b-508	2	2.42E+08	0.034484	486484	0.990133	Sepsis (28	Sepsis (28	Sepsis (28	TRUE	igd	9.00E-04	1.28E-06	7355	M00054.m	TRUE	reported	QTDAnh	2	TRUE	0.003239	23.89474
160	rs7230740	A	C	A	C	0.0044	-0.01683	0.6655	0.672118	FALSE	FALSE	FALSE	ieu-b-508	18	3636288	0.035402	486484	0.634508	Sepsis (28	Sepsis (28	Sepsis (28	TRUE	igd	9.00E-04	1.56E-06	7355	M00054.m	TRUE	reported	QTDAnh	2	TRUE	0.003239	23.89474
172	rs8088963	A	G	A	G	0.0044	-0.03453	0.6387	0.636583	FALSE	FALSE	FALSE	ieu-b-508	18	59047314	0.034652	486484	0.319032	Sepsis (28	Sepsis (28	Sepsis (28	TRUE	igd	9.00E-04	8.29E-07	7355	M00054.m	TRUE	reported	QTDAnh	2	TRUE	0.003239	23.89474
177	rs881562	T	G	T	G	0.0044	-0.03314	0.4978	0.506632	FALSE	FALSE	FALSE	ieu-b-508	16	7760884	0.033943	486484	0.328828	Sepsis (28	Sepsis (28	Sepsis (28	TRUE	igd	9.00E-04	1.45E-06	7355	M00054.m	TRUE	reported	QTDAnh	2	TRUE	0.003239	23.89474
3512	rs7200919	A	G	A	G	-0.0088	-0.00455	0.4101	0.401328	FALSE	FALSE	FALSE	ieu-b-508	16	67316600	0.034003	486484	0.893452	Sepsis (28	Sepsis (28	Sepsis (28	TRUE	igd	0.0018	7.75E-07	7355	M32492.m	TRUE	reported	rZZFcE	2	TRUE	0.003239	23.89474
3517	rs9403095	A	C	A	C	-0.0088	0.020818	0.3305	0.331258	FALSE	FALSE	FALSE	ieu-b-508	6	1.4E+08	0.035481	486484	0.557381	Sepsis (28	Sepsis (28	Sepsis (28	TRUE	igd	0.0018	9.32E-07	7355	M32492.m	TRUE	reported	rZZFcE	2	TRUE	0.003239	23.89474
556	rs2982907	T	G	T	G	-0.0088	-0.02096	0.1465	0.160726	FALSE	FALSE	FALSE	ieu-b-508	6	1.64E+08	0.045337	486484	0.643804	Sepsis (28	Sepsis (28	Sepsis (28	TRUE	igd	0.0018	1.28E-06	7354	M00606.m	TRUE	reported	r5smf7	2	TRUE	0.00324	23.89473
208	rs1025860	A	G	A	G	0.0044	-0.02547	0.4382	0.453791	FALSE	FALSE	FALSE	ieu-b-508	7	51371109	0.033626	486484	0.448786	Sepsis (28	Sepsis (28	Sepsis (28	TRUE	igd	9.00E-04	6.73E-07	7351	M00060.m	TRUE	reported	kqwl4V	2	TRUE	0.003241	23.89473
221	rs1093776	A	G	A	G	0.0044	-0.01341	0.5674	0.559169	FALSE	FALSE	FALSE	ieu-b-508	4	6918679	0.033791	486484	0.691516	Sepsis (28	Sepsis (28	Sepsis (28	TRUE	igd	9.00E-04	1.53E-06	7351	M00060.m	TRUE	reported	kqwl4V	2	TRUE	0.003241	23.89473
242	rs1261200	A	G	A	G	0.0044	-0.00146	0.3461	0.338549	FALSE	FALSE	FALSE	ieu-b-508	2	1.08E+08	0.035267	486484	0.96706	Sepsis (28	Sepsis (28	Sepsis (28	TRUE	igd	9.00E-04	1.20E-06	7351	M00060.m	TRUE	reported	kqwl4V	2	TRUE	0.003241	23.89473
248	rs1334318	A	G	A	G	0.0044	-0.035	0.3572	0.363527	FALSE	FALSE	FALSE	ieu-b-508	6	97079610	0.03468	486484	0.312921	Sepsis (28	Sepsis (28	Sepsis (28	TRUE	igd	9.00E-04	1.01E-06	7351	M00060.m	TRUE	reported	kqwl4V	2	TRUE	0.003241	23.89473
259	rs1677193	A	C	A	C	-0.0044	-0.01537	0.6122	0.607768	FALSE	FALSE	FALSE	ieu-b-508	12	31756155	0.034421	486484	0.655156	Sepsis (28	Sepsis (28	Sepsis (28	TRUE	igd	9.00E-04	1.25E-06	7351	M00060.m	TRUE	reported	kqwl4V	2	TRUE	0.003241	23.89473
262	rs1717319	T	C	T	C	-0.0044	0.026636	0.3872	0.384533	FALSE	FALSE	FALSE	ieu-b-508	7	1.51E+08	0.034449	486484	0.439417	Sepsis (28	Sepsis (28	Sepsis (28	TRUE	igd	9.00E-04	2.46E-06	7351	M00060.m	TRUE	reported	kqwl4V	2	TRUE	0.003241	23.89473
265	rs1795964	A	G	A	G	-0.0044	0.008037	0.4091	0.418577	FALSE	FALSE	FALSE	ieu-b-508	12	1.22E+08	0.035803	486484	0.822377	Sepsis (28	Sepsis (28	Sepsis (28	TRUE	igd	9.00E-04	1.98E-06	7351	M00060.m	TRUE	reported	kqwl4V	2	TRUE	0.003241	23.89473
276	rs2060490	T	C	T	C	-0.0044	-0.0229	0.474	0.473842	FALSE	FALSE	FALSE	ieu-b-508	2	1.79E+08	0.033401	486484	0.493039	Sepsis (28	Sepsis (28	Sepsis (28	TRUE	igd	9.00E-04	9.10E-07	7351	M00060.m	TRUE	reported	kqwl4V	2	TRUE	0.003241	23.89473
300	rs3775574	A	G	A	G	-0.0044	-0.00898	0.6047	0.60579	FALSE	FALSE	FALSE	ieu-b-508	4	1.85E+08	0.034295	486484	0.793442	Sepsis (28	Sepsis (28	Sepsis (28	TRUE	igd	9.00E-04	3.13E-06	7351	M00060.m	TRUE	reported	kqwl4V	2	TRUE	0.003241	23.89473
301	rs37959	T	C	T	C	0.0044	-0.00666	0.3912	0.38572	FALSE	FALSE	FALSE	ieu-b-508	7	8002389	0.034312	486484	0.846081	Sepsis (28	Sepsis (28	Sepsis (28	TRUE	igd	9.00E-04	1.71E-06	7351	M00060.m	TRUE	reported	kqwl4V	2	TRUE	0.003241	23.89473
320	rs481781	T	C	T	C	0.0044	-0.00981	0.5409	0.537912	FALSE	FALSE	FALSE	ieu-b-508	3	1.7E+08	0.033597	486484	0.770345	Sepsis (28	Sepsis (28	Sepsis (28	TRUE	igd	9.00E-04	9.14E-07	7351	M00060.m	TRUE	reported	kqwl4V	2	TRUE	0.003241	23.89473
367	rs7738860	T	C	T	C	-0.0044	-0.01237	0.4518	0.440564	FALSE	FALSE	FALSE	ieu-b-508	6	1.56E+08	0.033619	486484	0.712899	Sepsis (28	Sepsis (28	Sepsis (28	TRUE	igd	9.00E-04	1.31E-06	7351	M00060.m	TRUE	reported	kqwl4V	2	TRUE	0.003241	23.89473
369	rs7795093	A	G	A	G	-0.0044	-0.0127	0.3813	0.374036	FALSE	FALSE	FALSE	ieu-b-508	7	1.31E+08	0.034765	486484	0.71499	Sepsis (28	Sepsis (28	Sepsis (28	TRUE	igd	9.00E-04	1.47E-06	7351	M00060.m	TRUE	reported	kqwl4V	2	TRUE	0.003241	23.89473
374	rs7923885	A	G	A	G	-0.0044	-0.02318	0.5257	0.521805	FALSE	FALSE	FALSE	ieu-b-508	10	18654871	0.033421	486484	0.487941	Sepsis (28	Sepsis (28	Sepsis (28	TRUE	igd	9.00E-04	8.08E-07	7351	M00060.m	TRUE	reported	kqwl4V	2	TRUE	0.003241	23.89473
385	rs900532	T	C	T	C	-0.0044	-0.05606	0.4524	0.443278	FALSE	FALSE	FALSE	ieu-b-508	5	10636077	0.033585	486484	0.095074	Sepsis (28	Sepsis (28	Sepsis (28	TRUE	igd	9.00E-04	8.99E-07	7351	M00060.m	TRUE	reported	kqwl4V	2	TRUE	0.003241	23.89473
393	rs982790	T	C	T	C	-0.0044	-0.01396	0.4106	0.411129	FALSE	FALSE	FALSE	ieu-b-508	13	91231773	0.034117	486484	0.68232	Sepsis (28	Sepsis (28	Sepsis (28	TRUE	igd	9.00E-04	3.74E-06	7351	M00060.m	TRUE	reported	kqwl4V	2	TRUE	0.003241	23.89473
3582	rs4841120	T	G	T	G	0.0088	-0.06025	0.714	0.702261	FALSE	FALSE	FALSE	ieu-b-508	8	9139055	0.036411	486484	0.097978	Sepsis (28	Sepsis (28	Sepsis (28	TRUE	igd	0.0018	1.47E-06	7350	M32518.m	TRUE	reported	V0ZCiU	2	TRUE	0.003241	23.89473
3586	rs6946189	A	G	A	G	-0.0088	0.042834	0.2499	0.254868	FALSE	FALSE	FALSE	ieu-b-508	7	26000137	0.038542	486484	0.266416	Sepsis (28	Sepsis (28	Sepsis (28	TRUE	igd	0.0018	1.52E-06	7350	M32518.m	TRUE	reported	V0ZCiU	2	TRUE	0.003241	23.89473
7935	rs4670599	A	G	A	G	-0.0088	-0.02399	0.5558	0.53382	FALSE	FALSE	FALSE	ieu-b-508	2	36963955	0.033679	486484	0.476275	Sepsis (28	Sepsis (28	Sepsis (28	TRUE	igd	0.0018	1.29E-06	7348	M35675.m	TRUE	reported	D7fdyZ	2	TRUE	0.003242	23.89473
7951	rs9602121	T	C	T	C	-0.0088	-0.01306	0.6481	0.623559	FALSE	FALSE	FALSE	ieu-b-508	13	83758824	0.034491	486484	0.704974	Sepsis (28	Sepsis (28	Sepsis (28	TRUE	igd	0.0018	1.86E-06	7348	M35675.m	TRUE	reported	D7fdyZ	2	TRUE	0.003242	23.89473
3210	rs1752013	T	C	T	C	-0.0088	0.055349	0.5973	0.596069	FALSE	FALSE	FALSE	ieu-b-508	4	21324191	0.034057	486484	0.104125	Sepsis (28	Sepsis (28	Sepsis (28	TRUE	igd	0.0018	6.85E-07	7344	M32339.m	TRUE	reported	SVp504	2	TRUE	0.003244	23.89473
3896	rs1950305	T	C	T	C	0.0088	0.000178	0.1208	0.117921	FALSE	FALSE	FALSE	ieu-b-508	14	63648805	0.05212	486484	0.997276	Sepsis (28	Sepsis (28	Sepsis (28	TRUE	igd	0.0018	1.72E-06	7338	M32675.m	TRUE	reported	9FmPh9	2	TRUE	0.003247	23.89472
5607	rs1051331	A	G	A	G	-0.0088	0.053884	0.8435	0.840307	FALSE	FALSE	FALSE	ieu-b-508	21	46226786	0.045929	486484	0.240714	Sepsis (28	Sepsis (28	Sepsis (28	TRUE	igd	0.0018	6.47E-07	7336	M33442.m	TRUE	reported	B1Peb3	2	TRUE	0.003247	23.89472
1845	rs310597	A	G	A	G	-0.0088	0.063752	0.6532	0.632415	FALSE	FALSE	FALSE	ieu-b-508	7	1.51E+08	0.03466	486484	0.065863	Sepsis (28	Sepsis (28	Sepsis (28	TRUE	igd	0.0018	1.30E-06	7308	M15778.m	TRUE	reported	941o3l	2	TRUE	0.00326	23.89469
1851	rs4515826	T	G	T	G	-0.0132	0.016875	0.1021	0.129927	FALSE	FALSE	FALSE	ieu-b-508	1	2.49E+08	0.049773	486484	0.734571	Sepsis (28	Sepsis (28	Sepsis (28	TRUE	igd	0.0027	1.31E-06	7308	M15778.m	TRUE	reported	941o3l	2	TRUE	0.00326	23.89469
1856	rs6841005	T	C	T	C	0.0088	-0.06133	0.3264	0.341543	FALSE	FALSE	FALSE	ieu-b-508	4	25067377	0.035075	486484	0.080356	Sepsis (28	Sepsis (28	Sepsis (28	TRUE	igd	0.0018	1.15E-06	7308	M15778.m	TRUE	reported	941o3l	2	TRUE	0.00326	23.89469
5668	rs1106598	A	G	A	G	-0.0088	0.066757	0.5821	0.583631	FALSE	FALSE	FALSE	ieu-b-508	12	1.12E+08	0.033868	486484	0.048716	Sepsis (28	Sepsis (28	Sepsis (28	TRUE	igd	0.0018	8.30E-07	7307	M33477.m	TRUE	reported	Aw8PBK	2	TRUE	0.00326	23.89469
5669	rs1116054	A	G	A	G	-0.0088	-0.01019	0.2939	0.288941	FALSE	FALSE	FALSE	ieu-b-508	14	1E+08	0.036763	486484	0.781561	Sepsis (28	Sepsis (28	Sepsis (28	TRUE	igd	0.0018	1.54E-06	7307	M33477.m	TRUE	reported	Aw8PBK	2	TRUE	0.00326	23.89469
2746	rs1185528	T	C	T	C	-0.0088	-0.00737	0.8068	0.801746	FALSE	FALSE	FALSE	ieu-b-508	15	58689187	0.041764	486484	0.859852	Sepsis (28	Sepsis (28	Sepsis (28	TRUE	igd	0.0018	1.93E-06	7303	M27256.m	TRUE	reported	mJwzsi	2	TRUE	0.003262	23.89469
2978	rs2710833	T	C	T	C	-0.0132	-0.01455	0.1979	0.193305	FALSE	FALSE	FALSE	ieu-b-508	4	1.69E+08	0.042169	486484	0.730103	Sepsis (28	Sepsis (28	Sepsis (28	TRUE	igd	0.0027	1.20E-06	7257	M31453.m	TRUE	reported	vdTd9M	2	TRUE	0.003283	23.89465
3813	rs2581354	T	C	T	C	0.0088	-0.0128	0.5938	0.5788	FALSE	FALSE	FALSE	ieu-b-508	15	1E+08	0.033933	486484	0.706111	Sepsis (28	Sepsis (28	Sepsis (28	TRUE	igd	0.0018	1.37E-06	7229	M32644.m	TRUE	reported	JAa2WD	2	TRUE	0.003295	23.89462
7567	rs2267292	T	C	T	C	-0.0088	0.037789	0.2242	0.217006	FALSE	FALSE	FALSE	ieu-b-508	22	34210998	0.040521	486484	0.351035	Sepsis (28	Sepsis (28	Sepsis (28	TRUE	igd	0.0018	1.03E-06	7217	M35270.m	TRUE	reported	Ct8V1n	2	TRUE	0.003301	23.89461
7570	rs2727985	C	G	C	G	-0.0088	-0.05592	0.7599	0.738407	FALSE	TRUE	FALSE	ieu-b-508	3	1117218	0.037986	486484	0.141001	Sepsis (28	Sepsis (28	Sepsis (28	TRUE	igd	0.0018	2.03E-06	7217	M35270.m	TRUE	reported	Ct8V1n	2	TRUE	0.003301	23.89461
1252	rs4470135	A	C	A	C	0.0088	0.019037	0.5203	0.526134	FALSE	FALSE	FALSE	ieu-b-508	16	59190194	0.033501	486484	0.569868	Sepsis (28	Sepsis (28	Sepsis (28	TRUE	igd	0.0018	5.17E-07	7136	M01769.m	TRUE	reported	rismUZ	2	TRUE	0.003338	23.89454
5333	rs1121287	C	G	C	G	-0.0088	0.046118	0.7979	0.797042	FALSE	TRUE	FALSE	ieu-b-508	11	1.09E+08	0.041604	486484	0.267649	Sepsis (28	Sepsis (28	Sepsis (28	TRUE	igd	0.0018	7.82E-07	7118	M33380.m	TRUE	reported	OHRfI5	2	TRUE	0.003347	23.89452
1524	rs2471075	A	G	A	G	-0.0176	0.021113	0.9318	0.881594	FALSE	FALSE	FALSE	ieu-b-508	5	38123664	0.052078	486484	0.685174	Sepsis (28	Sepsis (28	Sepsis (28	TRUE	igd	0.0036	8.39E-07	7081	M12067.m	TRUE	reported	O5x7RD	2	TRUE	0.003364	23.89448
5800	rs1039386	A	G	A	G	-0.0088	0.004227	0.5733	0.591112	FALSE	FALSE	FALSE	ieu-b-508	4	1.7E+08	0.03424	486484	0.901741	Sepsis (28	Sepsis (28	Sepsis (28	TRUE	igd	0.0018	1.36E-06	7066	M33515.m	TRUE	reported	X9mul1	2	TRUE	0.003371	23.89447
5801	rs1086802	A	G	A	G	0.0088	0.00056	0.6116	0.623163	FALSE	FALSE	FALSE	ieu-b-508	9	86164176	0.034403	486484	0.987007	Sepsis (28	Sepsis (28	Sepsis (28	TRUE	igd	0.0018	1.52E-06	7066	M33515.m	TRUE	reported	X9mul1	2	TRUE	0.003371	23.89447
759	rs2288867	T	C	T	C	-0.0132	-0.03943	0.2334	0.247087	FALSE	FALSE	FALSE	ieu-b-508	19	19765568	0.038725	486484	0.308569	Sepsis (28	Sepsis (28	Sepsis (28	TRUE	igd	0.0027	1.02E-06	6946	M01303.m	TRUE	reported	3b8x0F	2	TRUE	0.003429	23.89435
8402	rs1079174	A	C	A	C	0.0132	-0.00273	0.0747	0.078476	FALSE	FALSE	FALSE	ieu-b-508	11	1.05E+08	0.061588	486484	0.964614	Sepsis (28	Sepsis (28	Sepsis (28	TRUE	igd	0.0027	9.72E-07	6940	M36808.m	TRUE	reported	u7lqaj	2	TRUE	0.003432	23.89435
8403	rs1119957	A	G	A	G	0.0088	-0.01009	0.2001	0.209427	FALSE	FALSE	FALSE	ieu-b-508	10	1.23E+08	0.041084	486484	0.806038	Sepsis (28	Sepsis (28	Sepsis (28	TRUE	igd	0.0018	8.70E-07	6940	M36808.m	TRUE	reported	u7lqaj	2	TRUE	0.003432	23.89435
1551	rs7544738	A	G	A	G	0.0132	0.010075	0.4114	0.420511	FALSE	FALSE	FALSE	ieu-b-508	1	37727248	0.033885	486484	0.766216	Sepsis (28	Sepsis (28	Sepsis (28	TRUE	igd	0.0027	7.15E-07	6864	M12129.m	TRUE	reported	iNzdgb	2	TRUE	0.00347	23.89427
6809	rs1761768	A	G	A	G	0.0088	0.003525	0.3771	0.39299	FALSE	FALSE	FALSE	ieu-b-508	6	43680462	0.034408	486484	0.918409	Sepsis (28	Sepsis (28	Sepsis (28	TRUE	igd	0.0018	6.07E-07	6755	M34359.m	TRUE	reported	0UotaJ	2	TRUE	0.003526	23.89416
952	rs1102338	A	G	A	G	0.0088	0.054312	0.4591	0.543107	FALSE	FALSE	FALSE	ieu-b-508	11	14930058	0.033489	486484	0.10485	Sepsis (28	Sepsis (28	Sepsis (28	TRUE	igd	0.0018	5.37E-07	6523	M01558.m	TRUE	reported	AZK0p4	2	TRUE	0.003651	23.89391
967	rs2290156	C	G	C	G	0.0088	-0.02022	0.2806	0.279115	FALSE	TRUE	FALSE	ieu-b-508	2	11333208	0.037139	486484	0.586177	Sepsis (28	Sepsis (28	Sepsis (28	TRUE	igd	0.0018	1.05E-06	6523	M01558.m	TRUE	reported	AZK0p4	2	TRUE	0.003651	23.89391
2606	rs1284408	T	G	T	G	-0.0269	-0.00061	0.5194	0.525184	FALSE	FALSE	FALSE	ieu-b-508	18	13548430	0.033595	486484	0.985616	Sepsis (28	Sepsis (28	Sepsis (28	TRUE	igd	0.0055	7.95E-07	1756	M22481.m	TRUE	reported	bXpf0k	2	TRUE	0.013439	23.89375
4326	rs1240164	A	G	A	G	0.1345	-0.0207	0.9861	0.986015	FALSE	FALSE	FALSE	ieu-b-508	1	59357800	0.141874	486484	0.883986	Sepsis (28	Sepsis (28	Sepsis (28	TRUE	igd	0.0275	1.03E-06	1727	M32847.m	TRUE	reported	eYme29	2	TRUE	0.013662	23.89329
7655	rs565666	T	C	T	C	-0.0088	-0.03585	0.3033	0.311026	FALSE	FALSE	FALSE	ieu-b-508	5	39936705	0.035985	486484	0.319116	Sepsis (28	Sepsis (28	Sepsis (28	TRUE	igd	0.0018	5.12E-07	5768	M35326.m	TRUE	reported	jRytyJ	2	TRUE	0.004127	23.89295
2507	rs2032995	T	G	T	G	0.0132	-0.04812	0.7853	0.763338	FALSE	FALSE	FALSE	ieu-b-508	3	1.89E+08	0.039405	486484	0.222005	Sepsis (28	Sepsis (28	Sepsis (28	TRUE	igd	0.0027	1.35E-06	5702	M22130.m	TRUE	reported	YDbJlz	2	TRUE	0.004174	23.89285
659	rs1043367	T	C	T	C	0.0264	0.150325	0.9584	0.95825	FALSE	FALSE	FALSE	ieu-b-508	4	71761726	0.083689	486484	0.072459	Sepsis (28	Sepsis (28	Sepsis (28	TRUE	igd	0.0054	1.24E-06	5687	M01284.m	TRUE	reported	E4hc9c	2	TRUE	0.004185	23.89283
1821	rs4338651	A	G	A	G	-0.088	0.087399	0.026	0.026015	FALSE	FALSE	FALSE	ieu-b-508	13	1.09E+08	0.105212	486484	0.406149	Sepsis (28	Sepsis (28	Sepsis (28	TRUE	igd	0.018	1.01E-06	5599	M15753.m	TRUE	reported	VDJYDI	2	TRUE	0.004251	23.8927
3853	rs6782877	A	G	A	G	0.0132	0.034825	0.349	0.344118	FALSE	FALSE	FALSE	ieu-b-508	3	1.45E+08	0.03514	486484	0.321673	Sepsis (28	Sepsis (28	Sepsis (28	TRUE	igd	0.0027	1.08E-06	5211	M32651.m	TRUE	reported	DeuPwv	2	TRUE	0.004566	23.89206
8519	rs8180176	T	C	T	C	0.0264	-0.00973	0.4591	0.453323	FALSE	FALSE	FALSE	ieu-b-508	4	55808036	0.033581	486484	0.771935	Sepsis (28	Sepsis (28	Sepsis (28	TRUE	igd	0.0054	8.50E-07	4520	M37104.m	TRUE	reported	bR3g7d	2	TRUE	0.00526	23.89066
7025	rs1702447	A	G	A	G	0.022	-0.00364	0.2904	0.293666	FALSE	FALSE	FALSE	ieu-b-508	18	73813586	0.036664	486484	0.920921	Sepsis (28	Sepsis (28	Sepsis (28	TRUE	igd	0.0045	8.34E-07	4442	M34441.m	TRUE	reported	UpgxQm	2	TRUE	0.005352	23.89047
5959	rs1314675	T	C	T	C	0.1467	-0.01905	0.0199	0.02423	FALSE	FALSE	FALSE	ieu-b-508	4	91121751	0.108097	486484	0.86014	Sepsis (28	Sepsis (28	Sepsis (28	TRUE	igd	0.03	1.02E-06	1860	M33658.m	TRUE	reported	9QszGO	2	TRUE	0.012693	23.88639
6761	rs1764606	T	C	T	C	-0.2932	-0.06377	0.9899	0.970988	FALSE	FALSE	FALSE	ieu-b-508	13	42803263	0.100077	486484	0.524015	Sepsis (28	Sepsis (28	Sepsis (28	TRUE	igd	0.0599	1.01E-06	633	M34339.m	TRUE	reported	RwGZ7c	2	TRUE	0.03647	23.88361
6218	rs2867882	A	G	A	G	0.0215	0.051766	0.4696	0.464106	FALSE	FALSE	FALSE	ieu-b-508	19	28651702	0.033464	486484	0.121889	Sepsis (28	Sepsis (28	Sepsis (28	TRUE	igd	0.0044	9.66E-07	4533	M33901.m	TRUE	reported	mQkhrb	2	TRUE	0.00524	23.86602
6548	rs1095672	T	C	T	C	-0.0342	0.035635	0.9414	0.958174	FALSE	FALSE	FALSE	ieu-b-508	8	1.35E+08	0.082973	486484	0.667578	Sepsis (28	Sepsis (28	Sepsis (28	TRUE	igd	0.007	1.19E-06	7347	M34244.m	TRUE	reported	JyF0Sp	2	TRUE	0.003238	23.86371
3172	rs6553778	T	C	T	C	-0.0171	-0.04003	0.2295	0.237627	FALSE	FALSE	FALSE	ieu-b-508	4	1.68E+08	0.039246	486484	0.307778	Sepsis (28	Sepsis (28	Sepsis (28	TRUE	igd	0.0035	1.18E-06	7340	M32328.m	TRUE	reported	YGsb6D	2	TRUE	0.003242	23.8637
1677	rs2517897	A	C	A	C	0.0171	0.051728	0.1141	0.152648	FALSE	FALSE	FALSE	ieu-b-508	6	29800101	0.046448	486484	0.265421	Sepsis (28	Sepsis (28	Sepsis (28	TRUE	igd	0.0035	1.23E-06	7337	M15365.m	TRUE	reported	TReTCM	2	TRUE	0.003243	23.8637
5615	rs1315759	T	C	T	C	0.0171	0.179449	0.0447	0.048817	FALSE	FALSE	FALSE	ieu-b-508	5	1.23E+08	0.078029	486484	0.021462	Sepsis (28	Sepsis (28	Sepsis (28	TRUE	igd	0.0035	1.17E-06	7336	M33442.m	TRUE	reported	B1Peb3	2	TRUE	0.003243	23.8637
6492	rs7970370	T	C	T	C	-0.5726	0.005144	0.0206	0.013654	FALSE	FALSE	FALSE	ieu-b-508	12	1.04E+08	0.146253	486484	0.971941	Sepsis (28	Sepsis (28	Sepsis (28	TRUE	igd	0.1168	9.37E-07	282	M34109.m	TRUE	reported	piJuC7	2	TRUE	0.078532	23.86305
6762	rs3810529	T	C	T	C	0.1173	-0.10223	0.0437	0.043544	FALSE	FALSE	FALSE	ieu-b-508	20	46488862	0.08192	486484	0.212081	Sepsis (28	Sepsis (28	Sepsis (28	TRUE	igd	0.024	1.04E-06	1649	M34339.m	TRUE	reported	RwGZ7c	2	TRUE	0.014279	23.85868
2797	rs1753103	C	G	C	G	0.0127	-0.00108	0.2505	0.257931	FALSE	TRUE	FALSE	ieu-b-508	4	1.14E+08	0.038144	486484	0.977319	Sepsis (28	Sepsis (28	Sepsis (28	TRUE	igd	0.0026	1.32E-06	7347	M27278.m	TRUE	reported	kPgqm3	2	TRUE	0.003237	23.85297
5625	rs6543592	A	G	A	G	-0.0127	-0.07372	0.9265	0.921589	FALSE	FALSE	FALSE	ieu-b-508	2	31240246	0.062172	486484	0.235743	Sepsis (28	Sepsis (28	Sepsis (28	TRUE	igd	0.0026	8.84E-07	7336	M33442.m	TRUE	reported	B1Peb3	2	TRUE	0.003242	23.85296
797	rs1178070	T	C	T	C	-0.0127	-0.06799	0.7557	0.756377	FALSE	FALSE	FALSE	ieu-b-508	8	1.4E+08	0.038728	486484	0.079141	Sepsis (28	Sepsis (28	Sepsis (28	TRUE	igd	0.0026	9.89E-07	7335	M01356.m	TRUE	reported	ah1g5W	2	TRUE	0.003242	23.85296
1846	rs3112496	T	C	T	C	0.0127	-0.0372	0.1331	0.115906	FALSE	FALSE	FALSE	ieu-b-508	2	1.37E+08	0.052845	486484	0.481455	Sepsis (28	Sepsis (28	Sepsis (28	TRUE	igd	0.0026	1.13E-06	7308	M15778.m	TRUE	reported	941o3l	2	TRUE	0.003254	23.85294

2438	rs1520218	A	C	A	C	0.0127	-0.01262	0.2558	0.276477	FALSE	FALSE	FALSE	ieu-b-508	12	1.03E+08	0.03731	486484	0.735135	Sepsis (28	Sepsis (28	Sepsis (28	TRUE	igd	0.0026	1.39E-06	7284	M21630.m	TRUE	reported	7eaO4H	2	TRUE	0.003265	23.85292
7663	rs762941	A	G	A	G	0.0127	0.040597	0.1129	0.113498	FALSE	FALSE	FALSE	ieu-b-508	22	49636451	0.053347	486484	0.44665	Sepsis (28	Sepsis (28	Sepsis (28	TRUE	igd	0.0026	1.06E-06	5768	M35326.m	TRUE	reported	jRytyJ	2	TRUE	0.004119	23.85119
7460	rs1853936	T	C	T	C	0.021	0.005777	0.1762	0.189932	FALSE	FALSE	FALSE	ieu-b-508	6	76728046	0.042564	486484	0.892047	Sepsis (28	Sepsis (28	Sepsis (28	TRUE	igd	0.0043	8.96E-07	6543	M35193.m	TRUE	reported	BMMWXU	2	TRUE	0.003632	23.84344
7223	rs2878733	T	C	T	C	0.021	-0.01363	0.703	0.703463	FALSE	FALSE	FALSE	ieu-b-508	14	74218968	0.037014	486484	0.712759	Sepsis (28	Sepsis (28	Sepsis (28	TRUE	igd	0.0043	1.18E-06	6381	M34761.m	TRUE	reported	AMtp1w	2	TRUE	0.003724	23.84325
3292	rs2574985	A	G	A	G	-0.021	0.041215	0.2764	0.29434	FALSE	FALSE	FALSE	ieu-b-508	10	52129486	0.036646	486484	0.260716	Sepsis (28	Sepsis (28	Sepsis (28	TRUE	igd	0.0043	1.27E-06	6119	M32379.m	TRUE	reported	MLimM6	2	TRUE	0.003883	23.84293
8185	rs7980973	T	G	T	G	-0.0381	-0.05164	0.7914	0.779461	FALSE	FALSE	FALSE	ieu-b-508	12	32092117	0.040748	486484	0.205064	Sepsis (28	Sepsis (28	Sepsis (28	TRUE	igd	0.0078	1.10E-06	2432	M36134.m	TRUE	reported	ekMsfG	2	TRUE	0.009715	23.83985
8206	rs1374273	A	G	A	G	0.0254	0.010543	0.6234	0.628609	FALSE	FALSE	FALSE	ieu-b-508	2	47051551	0.034825	486484	0.762079	Sepsis (28	Sepsis (28	Sepsis (28	TRUE	igd	0.0052	1.05E-06	1732	M36300.m	TRUE	reported	ZjeC9m	2	TRUE	0.013588	23.83192
722	rs1894947	T	C	T	C	0.0083	-0.00779	0.1801	0.181927	FALSE	FALSE	FALSE	ieu-b-508	16	56006578	0.043497	486484	0.857837	Sepsis (28	Sepsis (28	Sepsis (28	TRUE	igd	0.0017	1.85E-06	7363	M01301.m	TRUE	reported	w8TL71	2	TRUE	0.003227	23.8309
878	rs1339301	A	G	A	G	0.0415	-0.05849	0.0218	0.028508	FALSE	FALSE	FALSE	ieu-b-508	2	1.96E+08	0.101529	486484	0.564562	Sepsis (28	Sepsis (28	Sepsis (28	TRUE	igd	0.0085	1.08E-06	7362	M01365.m	TRUE	reported	N1cZTs	2	TRUE	0.003227	23.83089
4516	rs7992587	T	C	T	C	-0.0083	-0.01577	0.2605	0.24964	FALSE	FALSE	FALSE	ieu-b-508	13	1.01E+08	0.038905	486484	0.685144	Sepsis (28	Sepsis (28	Sepsis (28	TRUE	igd	0.0017	7.72E-07	7362	M33132.m	TRUE	reported	dTXJa8	2	TRUE	0.003227	23.83089
832	rs727892	A	G	A	G	0.0083	0.016036	0.5021	0.510813	FALSE	FALSE	FALSE	ieu-b-508	22	29443709	0.033638	486484	0.633569	Sepsis (28	Sepsis (28	Sepsis (28	TRUE	igd	0.0017	8.15E-07	7355	M01358.m	TRUE	reported	kSPQgd	2	TRUE	0.003231	23.83089
557	rs4286846	C	G	C	G	0.0083	0.0391	0.7232	0.709235	FALSE	TRUE	FALSE	ieu-b-508	7	33054812	0.036879	486484	0.289045	Sepsis (28	Sepsis (28	Sepsis (28	TRUE	igd	0.0017	1.26E-06	7354	M00606.m	TRUE	reported	r5smf7	2	TRUE	0.003231	23.83089
2252	rs2667755	T	C	T	C	-0.0083	-0.02939	0.6304	0.630655	FALSE	FALSE	FALSE	ieu-b-508	16	24893768	0.034701	486484	0.396955	Sepsis (28	Sepsis (28	Sepsis (28	TRUE	igd	0.0017	1.84E-06	7354	M19934.m	TRUE	reported	HtcWcQ	2	TRUE	0.003231	23.83089
2261	rs4759824	T	C	T	C	0.0083	-0.02619	0.6793	0.678223	FALSE	FALSE	FALSE	ieu-b-508	12	1.31E+08	0.035766	486484	0.463993	Sepsis (28	Sepsis (28	Sepsis (28	TRUE	igd	0.0017	1.85E-06	7354	M19934.m	TRUE	reported	HtcWcQ	2	TRUE	0.003231	23.83089
3131	rs2762343	A	G	A	G	0.0083	-0.00792	0.5133	0.527108	FALSE	FALSE	FALSE	ieu-b-508	6	35066091	0.03346	486484	0.812887	Sepsis (28	Sepsis (28	Sepsis (28	TRUE	igd	0.0017	1.22E-06	7349	M32315.m	TRUE	reported	4igzkL	2	TRUE	0.003233	23.83088
3902	rs6434188	T	C	T	C	0.0083	0.002064	0.2169	0.226204	FALSE	FALSE	FALSE	ieu-b-508	2	1.87E+08	0.039895	486484	0.958737	Sepsis (28	Sepsis (28	Sepsis (28	TRUE	igd	0.0017	9.92E-07	7338	M32675.m	TRUE	reported	9FmPh9	2	TRUE	0.003238	23.83087
5613	rs1287262	C	G	C	G	0.0083	0.057742	0.1938	0.192229	FALSE	TRUE	FALSE	ieu-b-508	5	36058174	0.042456	486484	0.173816	Sepsis (28	Sepsis (28	Sepsis (28	TRUE	igd	0.0017	1.16E-06	7336	M33442.m	TRUE	reported	B1Peb3	2	TRUE	0.003239	23.83087
5617	rs2209196	T	G	T	G	-0.0083	-0.01462	0.1924	0.191638	FALSE	FALSE	FALSE	ieu-b-508	1	2.09E+08	0.043046	486484	0.734047	Sepsis (28	Sepsis (28	Sepsis (28	TRUE	igd	0.0017	2.21E-06	7336	M33442.m	TRUE	reported	B1Peb3	2	TRUE	0.003239	23.83087
1783	rs7648992	T	C	T	C	0.0083	0.062725	0.3947	0.403075	FALSE	FALSE	FALSE	ieu-b-508	3	40537016	0.034181	486484	0.066495	Sepsis (28	Sepsis (28	Sepsis (28	TRUE	igd	0.0017	1.53E-06	7331	M15676.m	TRUE	reported	s1NfKN	2	TRUE	0.003241	23.83087
2772	rs984326	A	G	A	G	0.0083	-0.00222	0.2537	0.2981	FALSE	FALSE	FALSE	ieu-b-508	16	7772234	0.036499	486484	0.951511	Sepsis (28	Sepsis (28	Sepsis (28	TRUE	igd	0.0017	1.50E-06	7303	M27256.m	TRUE	reported	mJwzsi	2	TRUE	0.003253	23.83084
1233	rs1045465	T	C	T	C	0.0083	-0.01711	0.5849	0.583292	FALSE	FALSE	FALSE	ieu-b-508	13	75591823	0.033874	486484	0.613396	Sepsis (28	Sepsis (28	Sepsis (28	TRUE	igd	0.0017	1.64E-06	7136	M01769.m	TRUE	reported	rismUZ	2	TRUE	0.003329	23.83069
1389	rs2250515	T	G	T	G	-0.0083	-0.04954	0.5346	0.536097	FALSE	FALSE	FALSE	ieu-b-508	10	88408892	0.033465	486484	0.138734	Sepsis (28	Sepsis (28	Sepsis (28	TRUE	igd	0.0017	1.60E-06	7037	M02734.m	TRUE	reported	0y0dDD	2	TRUE	0.003376	23.8306
1398	rs4487134	T	G	T	G	-0.0083	-0.01383	0.355	0.357351	FALSE	FALSE	FALSE	ieu-b-508	2	43107129	0.034887	486484	0.691899	Sepsis (28	Sepsis (28	Sepsis (28	TRUE	igd	0.0017	1.99E-06	7037	M02734.m	TRUE	reported	0y0dDD	2	TRUE	0.003376	23.8306
2029	rs1164780	A	C	A	C	0.0249	0.052631	0.0735	0.084232	FALSE	FALSE	FALSE	ieu-b-508	16	58770881	0.059959	486484	0.380062	Sepsis (28	Sepsis (28	Sepsis (28	TRUE	igd	0.0051	8.57E-07	6939	M18349.m	TRUE	reported	M5qPs8	2	TRUE	0.003424	23.8305
8296	rs1439977	T	C	T	C	-0.0254	0.005587	0.3281	0.327651	FALSE	FALSE	FALSE	ieu-b-508	2	1.74E+08	0.035524	486484	0.875036	Sepsis (28	Sepsis (28	Sepsis (28	TRUE	igd	0.0052	1.10E-06	1636	M36559.m	TRUE	reported	g6I9hg	2	TRUE	0.014374	23.8303
1929	rs1525549	A	G	A	G	0.0166	0.016161	0.1421	0.14437	FALSE	FALSE	FALSE	ieu-b-508	7	3213851	0.047587	486484	0.734157	Sepsis (28	Sepsis (28	Sepsis (28	TRUE	igd	0.0034	1.11E-06	6549	M16822.m	TRUE	reported	UNI018	2	TRUE	0.003627	23.83009
7639	rs1571011	A	C	A	C	-0.0083	0.014204	0.5825	0.585107	FALSE	FALSE	FALSE	ieu-b-508	10	90757787	0.033914	486484	0.675342	Sepsis (28	Sepsis (28	Sepsis (28	TRUE	igd	0.0017	2.28E-06	5768	M35326.m	TRUE	reported	jRytyJ	2	TRUE	0.004116	23.8291
8239	rs327759	T	C	T	C	0.042	-0.03517	0.2319	0.228196	FALSE	FALSE	FALSE	ieu-b-508	9	7805650	0.040766	486484	0.38827	Sepsis (28	Sepsis (28	Sepsis (28	TRUE	igd	0.0086	1.02E-06	1908	M36468.m	TRUE	reported	vycAFr	2	TRUE	0.012346	23.82573
8234	rs1297404	T	C	T	C	-0.0503	0.04199	0.2168	0.221676	FALSE	FALSE	FALSE	ieu-b-508	19	18061639	0.040934	486484	0.30498	Sepsis (28	Sepsis (28	Sepsis (28	TRUE	igd	0.0103	1.14E-06	1908	M36468.m	TRUE	reported	vycAFr	2	TRUE	0.012345	23.82353
2120	rs1710702	T	C	T	C	-0.0781	-0.07494	0.9608	0.964696	FALSE	FALSE	FALSE	ieu-b-508	14	69961833	0.090937	486484	0.409868	Sepsis (28	Sepsis (28	Sepsis (28	TRUE	igd	0.016	1.08E-06	5624	M18476.m	TRUE	reported	6GnBtw	2	TRUE	0.004219	23.81813
2041	rs7727858	A	G	A	G	-0.0205	0.000228	0.8891	0.890052	FALSE	FALSE	FALSE	ieu-b-508	5	32924873	0.053449	486484	0.996594	Sepsis (28	Sepsis (28	Sepsis (28	TRUE	igd	0.0042	1.08E-06	6939	M18349.m	TRUE	reported	M5qPs8	2	TRUE	0.003422	23.81683
4018	rs1252109	T	C	T	C	0.0327	0.015089	0.2679	0.264373	FALSE	FALSE	FALSE	ieu-b-508	1	2.37E+08	0.037812	486484	0.689863	Sepsis (28	Sepsis (28	Sepsis (28	TRUE	igd	0.0067	8.88E-07	6000	M32735.m	TRUE	reported	QE2vKQ	2	TRUE	0.003954	23.81229
2057	rs1718587	A	G	A	G	-0.0122	-0.04034	0.8515	0.844406	FALSE	FALSE	FALSE	ieu-b-508	8	13025438	0.046254	486484	0.383096	Sepsis (28	Sepsis (28	Sepsis (28	TRUE	igd	0.0025	8.68E-07	7354	M18369.m	TRUE	reported	ykBjIT	2	TRUE	0.003228	23.80792
8564	rs1252510	A	G	A	G	0.0244	-0.01178	0.771	0.756507	FALSE	FALSE	FALSE	ieu-b-508	6	1.41E+08	0.039173	486484	0.763674	Sepsis (28	Sepsis (28	Sepsis (28	TRUE	igd	0.005	8.91E-07	7333	M37203.m	TRUE	reported	3glllv	2	TRUE	0.003237	23.8079
2677	rs1775647	C	G	C	G	0.0122	-0.01164	0.7131	0.705775	FALSE	TRUE	FALSE	ieu-b-508	4	7323248	0.036761	486484	0.751439	Sepsis (28	Sepsis (28	Sepsis (28	TRUE	igd	0.0025	1.20E-06	7256	M24074.m	TRUE	reported	Lk6D2A	2	TRUE	0.003271	23.80784
5366	rs870031	A	G	A	G	0.0122	-0.08844	0.1164	0.113641	FALSE	FALSE	FALSE	ieu-b-508	1	1.55E+08	0.052661	486484	0.093055	Sepsis (28	Sepsis (28	Sepsis (28	TRUE	igd	0.0025	1.27E-06	7118	M33380.m	TRUE	reported	OHRfl5	2	TRUE	0.003335	23.80771
858	rs1738584	A	G	A	G	0.0122	-0.03611	0.6443	0.6612	FALSE	FALSE	FALSE	ieu-b-508	2	17277900	0.035499	486484	0.309003	Sepsis (28	Sepsis (28	Sepsis (28	TRUE	igd	0.0025	1.16E-06	7063	M01361.m	TRUE	reported	NIAcQ3	2	TRUE	0.00336	23.80766
2868	rs1266123	A	G	A	G	0.0244	0.021279	0.61	0.599749	FALSE	FALSE	FALSE	ieu-b-508	6	69099509	0.034064	486484	0.532168	Sepsis (28	Sepsis (28	Sepsis (28	TRUE	igd	0.005	1.26E-06	6715	M27710.m	TRUE	reported	XeaFav	2	TRUE	0.003534	23.80731
6383	rs1123651	T	C	T	C	0.0283	0.104713	0.897	0.906281	FALSE	FALSE	FALSE	ieu-b-508	11	75451668	0.057062	486484	0.066494	Sepsis (28	Sepsis (28	Sepsis (28	TRUE	igd	0.0058	1.24E-06	7330	M33969.m	TRUE	reported	0ujroW	2	TRUE	0.003237	23.80117
7086	rs1719882	A	G	A	G	-0.3408	0.414154	0.9879	0.982673	FALSE	FALSE	FALSE	ieu-b-508	20	49790340	0.128119	486484	0.001227	Sepsis (28	Sepsis (28	Sepsis (28	TRUE	igd	0.0698	1.06E-06	1235	M34499.m	TRUE	reported	JqFBiM	2	TRUE	0.018937	23.80041
8091	rs1167079	T	C	T	C	0.5691	0.031634	0.8964	0.858254	FALSE	FALSE	FALSE	ieu-b-508	19	35795416	0.048112	486484	0.510855	Sepsis (28	Sepsis (28	Sepsis (28	TRUE	igd	0.1157	8.77E-07	122	M36097.m	TRUE	reported	RHr18S	2	TRUE	0.165493	23.79753
3535	rs3828128	A	G	A	G	-0.0161	-0.02388	0.2536	0.259589	FALSE	FALSE	FALSE	ieu-b-508	1	2.31E+08	0.038158	486484	0.531459	Sepsis (28	Sepsis (28	Sepsis (28	TRUE	igd	0.0033	1.35E-06	7358	M32497.m	TRUE	reported	244bZN	2	TRUE	0.003224	23.7961
2972	rs1329362	T	C	T	C	0.1054	0.147088	0.0166	0.025995	FALSE	FALSE	FALSE	ieu-b-508	9	30193931	0.105588	486484	0.16361	Sepsis (28	Sepsis (28	Sepsis (28	TRUE	igd	0.0216	1.07E-06	1750	M31453.m	TRUE	reported	vdTd9M	2	TRUE	0.013424	23.78357
1558	rs1124193	A	T	A	T	-0.061	0.026908	0.2705	0.283581	FALSE	TRUE	FALSE	ieu-b-508	5	1.27E+08	0.037128	486484	0.46861	Sepsis (28	Sepsis (28	Sepsis (28	TRUE	igd	0.0125	1.05E-06	1543	M12261.m	TRUE	reported	taUUcM	2	TRUE	0.015199	23.78353
510	rs661454	A	G	A	G	0.0317	-0.07698	0.0848	0.087879	FALSE	FALSE	FALSE	ieu-b-508	6	50620678	0.05886	486484	0.190917	Sepsis (28	Sepsis (28	Sepsis (28	TRUE	igd	0.0065	1.31E-06	7333	M00577.m	TRUE	reported	YzFebE	2	TRUE	0.003233	23.77789
4677	rs1049511	A	C	A	C	0.3075	0.061459	0.9818	0.984095	FALSE	FALSE	FALSE	ieu-b-508	1	2.19E+08	0.133896	486484	0.646231	Sepsis (28	Sepsis (28	Sepsis (28	TRUE	igd	0.063	1.06E-06	1017	M33178.m	TRUE	reported	g22WMS	2	TRUE	0.022889	23.77685
1326	rs6503066	T	C	T	C	0.0317	-0.00383	0.0984	0.104747	FALSE	FALSE	FALSE	ieu-b-508	17	7766228	0.054744	486484	0.94419	Sepsis (28	Sepsis (28	Sepsis (28	TRUE	igd	0.0065	9.49E-07	6252	M02137.m	TRUE	reported	iRGqBE	2	TRUE	0.00379	23.77677
5116	rs9689446	T	C	T	C	0.1635	0.045802	0.962	0.959808	FALSE	FALSE	FALSE	ieu-b-508	6	1.34E+08	0.085577	486484	0.5925	Sepsis (28	Sepsis (28	Sepsis (28	TRUE	igd	0.0335	1.04E-06	1017	M33178.m	TRUE	reported	g22WMS	2	TRUE	0.022886	23.77338
3230	rs9582849	A	G	A	G	0.02	0.046871	0.7575	0.885689	FALSE	FALSE	FALSE	ieu-b-508	13	1.05E+08	0.06237	486484	0.45235	Sepsis (28	Sepsis (28	Sepsis (28	TRUE	igd	0.0041	1.34E-06	1761	M32339.m	TRUE	reported	SVp504	2	TRUE	0.013332	23.76834
7592	rs1386540	T	C	T	C	-0.0361	-0.016	0.3484	0.35494	FALSE	FALSE	FALSE	ieu-b-508	2	1.88E+08	0.034858	486484	0.646141	Sepsis (28	Sepsis (28	Sepsis (28	TRUE	igd	0.0074	1.14E-06	1505	M35305.m	TRUE	reported	6ybfdq	2	TRUE	0.015567	23.76695
1627	rs1208239	T	C	T	C	0.0117	-0.03212	0.5855	0.580852	FALSE	FALSE	FALSE	ieu-b-508	1	25238631	0.033776	486484	0.341573	Sepsis (28	Sepsis (28	Sepsis (28	TRUE	igd	0.0024	9.33E-07	7368	M15140.m	TRUE	reported	jEcqMJ	2	TRUE	0.003215	23.75917
1633	rs1697485	T	C	T	C	-0.0234	0.100157	0.038	0.035906	FALSE	FALSE	FALSE	ieu-b-508	17	68029032	0.089902	486484	0.265252	Sepsis (28	Sepsis (28	Sepsis (28	TRUE	igd	0.0048	1.15E-06	7368	M15140.m	TRUE	reported	jEcqMJ	2	TRUE	0.003215	23.75917
469	rs1330523	A	G	A	G	-0.0117	0.024877	0.2326	0.2237	FALSE	FALSE	FALSE	ieu-b-508	13	1.05E+08	0.040091	486484	0.534914	Sepsis (28	Sepsis (28	Sepsis (28	TRUE	igd	0.0024	1.56E-06	7365	M00527.m	TRUE	reported	UYN7vF	2	TRUE	0.003216	23.75917
781	rs1348656	T	C	T	C	-0.0117	0.041128	0.798	0.798722	FALSE	FALSE	FALSE	ieu-b-508	3	1.45E+08	0.041981	486484	0.327249	Sepsis (28	Sepsis (28	Sepsis (28	TRUE	igd	0.0024	9.07E-07	7352	M01336.m	TRUE	reported	FAlrya	2	TRUE	0.003222	23.75916
3140	rs6723481	T	C	T	C	0.0117	-0.00241	0.2248	0.222691	FALSE	FALSE	FALSE	ieu-b-508	2	1.15E+08	0.04006	486484	0.952031	Sepsis (28	Sepsis (28	Sepsis (28	TRUE	igd	0.0024	1.21E-06	7349	M32315.m	TRUE	reported	4igzkL	2	TRUE	0.003223	23.75916
1772	rs2404519	A	G	A	G	0.0117	-0.00388	0.1587	0.174997	FALSE	FALSE	FALSE	ieu-b-508	5	26766426	0.044195	486484	0.930007	Sepsis (28	Sepsis (28	Sepsis (28	TRUE	igd	0.0024	1.60E-06	7331	M15676.m	TRUE	reported	s1NfKN	2	TRUE	0.003231	23.75914
1576	rs1244477	T	C	T	C	-0.0156	-0.07002	0.0607	0.071423	FALSE	FALSE	FALSE	ieu-b-508	16	81773227	0.066271	486484	0.290705	Sepsis (28	Sepsis (28	Sepsis (28	TRUE	igd	0.0032	1.61E-06	7314	M12593.m	TRUE	reported	IBXgSU	2	TRUE	0.003239	23.75913
3036	rs400120	A	G	A	G	0.0195	0.039223	0.6474	0.627096	FALSE	FALSE	FALSE	ieu-b-508	12	52831340	0.034488	486484	0.255417	Sepsis (28	Sepsis (28	Sepsis (28	TRUE	igd	0.004	1.30E-06	7263	M31555.m	TRUE	reported	iQ5E3v	2	TRUE	0.003261	23.75908
6151	rs6580981	A	G	A	G	-0.0551	-0.0363	0.4501	0.459406	FALSE	FALSE	FALSE	ieu-b-508	12	54723028	0.033582	486484	0.279731	Sepsis (28	Sepsis (28	Sepsis (28	TRUE	igd	0.0113	1.12E-06	2725	M33883.m	TRUE	reported	RT7YNu	2	TRUE	0.00865	23.75896
5342	rs1363574	A	G	A	G	-0.0195	0.030427	0.9545	0.953255	FALSE	FALSE	FALSE	ieu-b-508	5	1.65E+08	0.08004	486484	0.703837	Sepsis (28	Sepsis (28	Sepsis (28	TRUE	igd	0.004	1.29E-06	7118	M33380.m	TRUE	reported	OHRfl5	2	TRUE	0.003328	23.75895
7726	rs1013947	T	G	T	G	-0.0156	-0.02192	0.2538	0.282926	FALSE	FALSE	FALSE	ieu-b-508	14	92150089	0.037766	486484	0.561688	Sepsis (28	Sepsis (28	Sepsis (28	TRUE	igd	0.0032	1.47E-06	7007	M35431.m	TRUE	reported	tLfE4I	2	TRUE	0.00338	23.75884
2878	rs805936	A	G	A	G	-0.0897	0.072271	0.9841	0.981144	FALSE	FALSE	FALSE	ieu-b-508	20	38258767	0.124023	486484	0.560081	Sepsis (28	Sepsis (28	Sepsis (28	TRUE	igd	0.0184	1.16E-06	6715	M27710.m	TRUE	reported	XeaFav	2	TRUE	0.003527	23.75855
2266	rs6948277	A	G	A	G	0.0951	-0.06904	0.0135	0.020759	FALSE	FALSE	FALSE	ieu-b-508	7	10871979	0.116823	486484	0.554532	Sepsis (28	Sepsis (28	Sepsis (28	TRUE	igd	0.0195	1.01E-06	1759	M19934.m	TRUE	reported	HtcWcQ	2	TRUE	0.013341	23.75734
1823	rs475322	A	C	A	C	0.117	-0.05757	0.016	0.035952	FALSE	FALSE	FALSE	ieu-b-508	1	53975436	0.092211	486484	0.532438	Sepsis (28	Sepsis (28	Sepsis (28	TRUE	igd	0.024	1.09E-06	5599	M15753.m	TRUE	reported	VDJYDI	2	TRUE	0.004227	23.75714
2518	rs2215015	T	C	T	C	-0.0195	0.052421	0.3426	0.35554	FALSE	FALSE	FALSE	ieu-b-508	14	72597951	0.034957	486484	0.133725	Sepsis (28	Sepsis (28	Sepsis (28	TRUE	igd	0.004	1.25E-06	3950	M22138.m	TRUE	reported	UbKBSS	2	TRUE	0.005981	23.75359
4375	rs465818	T	C	T	C	-0.2195	-0.04605	0.963	0.968701	FALSE	FALSE	FALSE	ieu-b-508	5	1.28E+08	0.095886	486484	0.631075	Sepsis (28	Sepsis (28	Sepsis (28	TRUE	igd	0.045	1.07E-06	1162	M32863.m	TRUE	reported	cLOFYE	2	TRUE	0.020065	23.75176
8351	rs1724952	T	G	T	G	0.0322	-0.03475	0.3177	0.303825	FALSE	FALSE	FALSE	ieu-b-508	8	34797403	0.036325	486484	0.338709	Sepsis (28	Sepsis (28	Sepsis (28	TRUE	igd	0.0066	1.22E-06	922	M36738.m	TRUE	reported	mVQQcD	2	TRUE	0.025167	23.75094
6737	rs2212841	T	C	T	C	0.87	0.120943	0.015	0.018214	FALSE	FALSE	FALSE	ieu-b-508	18	73360511	0.123744	486484	0.328392	Sepsis (28	Sepsis (28	Sepsis (28	TRUE	igd	0.177	8.87E-07	115	M34338.m	TRUE	reported	h4FB9a	2	TRUE	0.173611	23.73956
4261	rs7305915	A	G	A	G	-0.0307	-0.00458	0.3824	0.383082	FALSE	FALSE	FALSE	ieu-b-508	12	1.31E+08	0.034369	486484	0.893909	Sepsis (28	Sepsis (28	Sepsis (28	TRUE	igd	0.0063	9.85E-07	4274	M32800.m	TRUE	reported	Q7MVsa	2	TRUE	0.005525	23.73517
2934	rs9608300	C	G	C	G	-0.137	0.156293	0.0264	0.01433	FALSE	TRUE	FALSE	ieu-b-508	22	24980306	0.147739	486484	0.290102	Sepsis (28	Sepsis (28	Sepsis (28	TRUE	igd	0.0281	1.06E-06	1227	M27728.m	TRUE	reported	WOp1XE	2	TRUE	0.019004	23.73122
5321	rs1188168	A	G	A	G	-0.0273	0.028838	0.779	0.809845	FALSE	FALSE	FALSE	ieu-b-508	19	7610952	0.042482	486484	0.497251	Sepsis (28	Sepsis (28	Sepsis (28	TRUE	igd	0.0056	1.06E-06	1338	M33364.m	TRUE	reported	9Zrr7Y	2	TRUE	0.017452	23.7301
515	rs1221854	C	G	C	G	-0.019	0.002655	0.0836	0.11212	FALSE	TRUE	FALSE	ieu-b-508	10	1.25E+08	0.05283	486484	0.959919	Sepsis (28	Sepsis (28	Sepsis (28	TRUE	igd	0.0039	1.28E-06	7345	M00584.m	TRUE	reported	oBt02X	2	TRUE	0.003221	23.72792
1689	rs1184372	T	C	T	C	0.019	0.021385	0.9644	0.959595	FALSE	FALSE	FALSE	ieu-b-508	13	95326452	0.085294	486484	0.802028	Sepsis (28	Sepsis (28	Sepsis (28	TRUE	igd	0.0039	9.47E-07	7345	M15488.m	TRUE	reported	SPriBO	2	TRUE	0.003221	23.72792
1955	rs7679137	T	C	T	C	-0.2217	-0.22352	0.9857	0.984946	FALSE	FALSE	FALSE	ieu-b-508	4	72909736	0.1379	486484	0.10504	Sepsis (28	Sepsis (28	Sepsis (28	TRUE	igd	0.0455	1.11E-06	3393	M16823.m	TRUE	reported	fNy2PD	2	TRUE	0.006949	23.72753
3242	rs7793797	A	T	A	T	0.038	-0.06078	0.7018	0.696611	FALSE	TRUE	FALSE	ieu-b-508	7	25234926	0.036387	486484	0.094866	Sepsis (28	Sepsis (28	Sepsis (28	TRUE	igd	0.0078	1.26E-06	6687	M32346.m	TRUE	reported	cnIFoV	2	TRUE	0.003537	23.72729
1498	rs1281570	A	G	A	G	0.019	0.007134	0.2806	0.320689	FALSE	FALSE	FALSE	ieu-b-508	12	47715378	0.037003	486484	0.84713	Sepsis (28	Sepsis (28	Sepsis (28	TRUE	igd	0.0039	9.10E-07	5656	M12017.m	TRUE	reported	bbGtsK	2	TRUE	0.004179	23.72599
8221	rs1469456	T	C	T	C	-0.0263	-0.00742	0.7562	0.752219	FALSE	FALSE	FALSE	ieu-b-508	5	1.03E+08	0.038828	486484	0.848421	Sepsis (28	Sepsis (28	Sepsis (28	TRUE	igd	0.0054	1.36E-06	6486	M36399.m	TRUE	reported	EdUpnt	2	TRUE	0.003644	23.71319
4134	rs944846	T	C	T	C	0.0575	0.069656	0.0802	0.083371	FALSE	FALSE	FALSE	ieu-b-508	20	56688965	0.060351	486484	0.248425	Sepsis (28	Sepsis (28	Sepsis (28	TRUE	igd	0.0118	1.19E-06	1435	M32755.m	TRUE	reported	WDrNdi	2	TRUE	0.016278	23.71188
8508	rs1246726	T	C	T	C	0.0263	0.012508	0.4786	0.482727	FALSE	FALSE	FALSE	ieu-b-508	1	1.91E+08	0.03335	486484	0.707618	Sepsis (28	Sepsis (28	Sepsis (28	TRUE	igd	0.0054	1.09E-06	4520	M37104.m	TRUE	reported	bR3g7d	2	TRUE	0.005221	23.71001
2107	rs1173669	A	C	A	C	-0.24	0.024903	0.9602	0.94654	FALSE	FALSE	FALSE	ieu-b-508	4	1.55E+08	0.075195	486484	0.740508	Sepsis (28	Sepsis (28	Sepsis (28	TRUE	igd	0.0491	1.04E-06	257	M18474.m	TRUE	reported	B6Xd02	2	TRUE	0.085059	23.70645
903	rs1158214	C	G	C	G	-0.0112	0.025334	0.1443	0.157731	FALSE	TRUE	FALSE	ieu-b-508	1	2.12E+08	0.045777	486484	0.57998	Sepsis (28	Sepsis (28	Sepsis (28	TRUE	igd	0.0023	9.32E-07	7354	M01494.m	TRUE	reported	7JWNKI	2	TRUE	0.003214	23.70622
6072	rs7151939	T	G	T	G	-0.0224	0.032754	0.1357	0.137462	FALSE	FALSE	FALSE	ieu-b-508	14	76614949	0.048442	486484	0.498947	Sepsis (28	Sepsis (28	Sepsis (28	TRUE	igd	0.0046	1.22E-06	7350	M33822.m	TRUE	reported	Wic4uS	2	TRUE	0.003216	23.70621
845	rs4353846	T	C	T	C	-0.0112	0.024273	0.5643	0.545597	FALSE	FALSE	FALSE	ieu-b-508	3	1.77E+08	0.033678	486484	0.471078	Sepsis (28	Sepsis (28	Sepsis (28	TRUE	igd	0.0023	1.70E-06	7323	M01359.m	TRUE	reported	7L9x1s	2	TRUE	0.003228	23.70619
8226	rs4542627	A	T	A	T	-0.0224	-0.00382	0.3909	0.418173	FALSE	TRUE	FALSE	ieu-b-508	15	78070743	0.033992	486484	0.910611	Sepsis (28	Sepsis (28	Sepsis (28	TRUE	igd	0.0046	1.50E-06	6486	M36399.m	TRUE	reported	EdUpnt	2	TRUE	0.003643	23.70535
7394	rs1289566	T	C	T	C	-0.0302	0.032946	0.6305	0.637242	FALSE	FALSE	FALSE	ieu-b-508	14	33208717	0.034882	486484	0.344908	Sepsis (28	Sepsis (28	Sepsis (28	TRUE	igd	0.0062	1.24E-06	1982	M35159.m	TRUE	reported	7EoJLB	2	TRUE	0.011829	23.70238
8068	rs4854073	T	C	T	C	-0.0224	0.013922	0.4114	0.427419	FALSE	FALSE	FALSE	ieu-b-508	2	2.41E+08	0.034074	486484	0.682848	Sepsis (28	Sepsis (28	Sepsis (28	TRUE	igd	0.0046	1.25E-06	4529	M35978.m	TRUE	reported	CN0wRR	2	TRUE	0.005208	23.70219
4270	rs1171135	A	T	A	T	0.0297	0.004412	0.582	0.597319	FALSE	TRUE	FALSE	ieu-b-508	3	1.12E+08	0.034429	486484	0.898041	Sepsis (28	Sepsis (28	Sepsis (28	TRUE	igd	0.0061	9.67E-07	6200	M32808.m	TRUE	reported	vnr36k	2	TRUE	0.003809	23.69808
7958	rs1329347	C	G	C	G	-0.037	-0.02296	0.9111	0.908379	FALSE	TRUE	FALSE	ieu-b-508	9	18621725	0.058171	486484	0.693089	Sepsis (28	Sepsis (28	Sepsis (28	TRUE	igd	0.0076	1.30E-06	6447	M35678.m	TRUE	reported	56yR01	2	TRUE	0.003663	23.69417
6495	rs9929954	T	G	T	G	0.1231	0.034216	0.7364	0.729135	FALSE	FALSE	FALSE	ieu-b-508	16	5022314	0.038104	486484	0.369216	Sepsis (28	Sepsis (28	Sepsis (28	TRUE	igd	0.0252	9.87E-07	282	M34109.m	TRUE	reported	piJuC7	2	TRUE	0.078017	23.69321
582	rs9834988	T	C	T	C	0.0185	0.000301	0.5297	0.530723	FALSE	FALSE	FALSE	ieu-b-508	3	79630364	0.033484	486484	0.992828	Sepsis (28	Sepsis (28	Sepsis (28	TRUE	igd	0.0038	7.98E-07	5344	M01107.m	TRUE	reported	EbPV1D	2	TRUE	0.004416	23.69265
3625	rs1695726	C	G	C	G	-0.0443	0.013552	0.9068	0.910201	FALSE	TRUE	FALSE	ieu-b-508	13	88302780	0.058585	486484	0.817065	Sepsis (28	Sepsis (28	Sepsis (28	TRUE	igd	0.0091	1.09E-06	7337	M32557.m	TRUE	reported	qR7rZX	2	TRUE	0.00322	23.69225
2697	rs1322212	A	G	A	G	-0.1154	0.070308	0.9863	0.931973	FALSE	FALSE	FALSE	ieu-b-508	7	53070386	0.066544	486484	0.290709	Sepsis (28	Sepsis (28	Sepsis (28	TRUE	igd	0.0237	1.09E-06	1756	M25459.m	TRUE	reported	ACJY35	2	TRUE	0.013322	23.68209
4322	rs4762836	T	C	T	C	0.0584	-0.04816	0.0748	0.072437	FALSE	FALSE	FALSE	ieu-b-508	12	21664879	0.064405	486484	0.454604	Sepsis (28	Sepsis (28	Sepsis (28	TRUE	igd	0.012	1.01E-06	6250	M32846.m	TRUE	reported	lwF2Yy	2	TRUE	0.003775	23.67687
6663	rs8014529	A	G	A	G	-0.3141	-0.02207	0.9782	0.405576	FALSE	FALSE	FALSE	ieu-b-508	14	1.07E+08	0.033979	486484	0.515946	Sepsis (28	Sepsis (28	Sepsis (28	TRUE	igd	0.0637	8.21E-07	75	M34322.m	TRUE	reported	16GdG9	2	TRUE	0.24482	23.66566
3690	rs1711003	T	C	T	C	-0.0433	0.001283	0.961	0.961597	FALSE	FALSE	FALSE	ieu-b-508	10	96206045	0.086863	486484	0.988214	Sepsis (28	Sepsis (28	Sepsis (28	TRUE	igd	0.0089	1.18E-06	7369	M32587.m	TRUE	reported	uknv7G	2	TRUE	0.003202	23.66344
4600	rs7648714	A	G	A	G	0.018	0.04152	0.4717	0.474138	FALSE	FALSE	FALSE	ieu-b-508	3	71934042	0.033633	486484	0.217023	Sepsis (28	Sepsis (28	Sepsis (28	TRUE	igd	0.0037	1.06E-06	7263	M33165.m	TRUE	reported	RZt3uS	2	TRUE	0.003248	23.66039
1405	rs8132248	T	C	T	C	0.018	0.004657	0.0986	0.136558	FALSE	FALSE	FALSE	ieu-b-508	21	18678620	0.049194	486484	0.924581	Sepsis (28	Sepsis (28	Sepsis (28	TRUE	igd	0.0037	1.43E-06	7037	M02734.m	TRUE	reported	0y0dDD	2	TRUE	0.003352	23.66018
6807	rs1478747	A	G	A	G	0.018	-0.01345	0.214	0.236462	FALSE	FALSE	FALSE	ieu-b-508	11	1.35E+08	0.043846	486484	0.759081	Sepsis (28	Sepsis (28	Sepsis (28	TRUE	igd	0.0037	1.27E-06	6755	M34359.m	TRUE	reported	0UotaJ	2	TRUE	0.003491	23.6599
570	rs1271859	T	C	T	C	0.018	-0.03377	0.5178	0.512092	FALSE	FALSE	FALSE	ieu-b-508	7	50428445	0.033351	486484	0.311308	Sepsis (28	Sepsis (28	Sepsis (28	TRUE	igd	0.0037	1.49E-06	5344	M01107.m	TRUE	reported	EbPV1D	2	TRUE	0.004409	23.65805
637	rs7795118	T	C	T	C	0.0905	-0.02914	0.2305	0.269166	FALSE	FALSE	FALSE	ieu-b-508	7	1.06E+08	0.039571	486484	0.461496	Sepsis (28	Sepsis (28	Sepsis (28	TRUE	igd	0.0186	1.21E-06	2639	M01123.m	TRUE	reported	NDTvfD	2	TRUE	0.008891	23.65604
5964	rs7956425	A	G	A	G	-0.093	0.029086	0.8522	0.854194	FALSE	FALSE	FALSE	ieu-b-508	12	42754176	0.047115	486484	0.537	Sepsis (28	Sepsis (28	Sepsis (28	TRUE	igd	0.0191	1.21E-06	860	M33658.m	TRUE	reported	9QszGO	2	TRUE	0.026828	23.6531
3081	rs1738925	T	C	T	C	-0.0214	-0.03944	0.9163	0.917168	FALSE	FALSE	FALSE	ieu-b-508	10	78794407	0.060759	486484	0.516246	Sepsis (28	Sepsis (28	Sepsis (28	TRUE	igd	0.0044	1.03E-06	7346	M32197.m	TRUE	reported	VZjwa9	2	TRUE	0.00321	23.64852
1360	rs35592	T	C	T	C	0.0107	-1.11E-05	0.7618	0.765565	FALSE	FALSE	FALSE	ieu-b-508	16	16141823	0.039323	486484	0.999774	Sepsis (28	Sepsis (28	Sepsis (28	TRUE	igd	0.0022	1.24E-06	7226	M02730.m	TRUE	reported	XYa035	2	TRUE	0.003263	23.64841
8159	rs1076417	A	T	A	T	-0.0579	-0.07272	0.9265	0.930832	FALSE	TRUE	FALSE	ieu-b-508	10	20219268	0.066163	486484	0.271713	Sepsis (28	Sepsis (28	Sepsis (28	TRUE	igd	0.0119	1.22E-06	1780	M36131.m	TRUE	reported	DbYm5t	2	TRUE	0.013125	23.64694
7121	rs7655071	C	G	C	G	0.1543	-0.02963	0.1519	0.183575	FALSE	TRUE	FALSE	ieu-b-508	4	81710326	0.043258	486484	0.493408	Sepsis (28	Sepsis (28	Sepsis (28	TRUE	igd	0.0316	1.04E-06	241	M34516.m	TRUE	reported	7Ah2wy	2	TRUE	0.090026	23.64496
2011	rs1321208	T	C	T	C	-0.0355	0.017288	0.1913	0.227301	FALSE	FALSE	FALSE	ieu-b-508	6	1.1E+08	0.039714	486484	0.663334	Sepsis (28	Sepsis (28	Sepsis (28	TRUE	igd	0.0073	1.05E-06	5984	M18283.m	TRUE	reported	oyZdTE	2	TRUE	0.003936	23.641
7904	rs704622	T	C	T	C	-0.3467	0.020374	0.9863	0.987964	FALSE	FALSE	FALSE	ieu-b-508	11	29633417	0.152726	486484	0.893874	Sepsis (28	Sepsis (28	Sepsis (28	TRUE	igd	0.0712	1.13E-06	659	M35669.m	TRUE	reported	gSecuE	2	TRUE	0.03473	23.63892
6582	rs1153203	A	G	A	G	-0.0535	-0.1457	0.9564	0.947882	FALSE	FALSE	FALSE	ieu-b-508	11	1.3E+08	0.074847	486484	0.051573	Sepsis (28	Sepsis (28	Sepsis (28	TRUE	igd	0.011	1.17E-06	2278	M34289.m	TRUE	reported	Knp8ry	2	TRUE	0.010277	23.63419
4292	rs7095335	A	T	A	T	-0.0141	0.026445	0.6604	0.653823	FALSE	TRUE	FALSE	ieu-b-508	10	1.22E+08	0.035051	486484	0.450563	Sepsis (28	Sepsis (28	Sepsis (28	TRUE	igd	0.0029	1.11E-06	7063	M32814.m	TRUE	reported	z0OeTx	2	TRUE	0.003336	23.63302
2996	rs1275921	A	G	A	G	-0.1242	0.009469	0.9097	0.899592	FALSE	FALSE	FALSE	ieu-b-508	1	1.64E+08	0.055588	486484	0.864739	Sepsis (28	Sepsis (28	Sepsis (28	TRUE	igd	0.0255	1.05E-06	522	M31536.m	TRUE	reported	3nAuUI	2	TRUE	0.04347	23.63174
6977	rs2963485	T	C	T	C	-0.0175	0.038065	0.1869	0.189321	FALSE	FALSE	FALSE	ieu-b-508	5	1.48E+08	0.042663	486484	0.372276	Sepsis (28	Sepsis (28	Sepsis (28	TRUE	igd	0.0036	1.06E-06	7344	M34407.m	TRUE	reported	X7I83Y	2	TRUE	0.003207	23.62397
3334	rs8093526	T	C	T	C	0.0355	0.031485	0.4585	0.457352	FALSE	FALSE	FALSE	ieu-b-508	18	4442517	0.033832	486484	0.352042	Sepsis (28	Sepsis (28	Sepsis (28	TRUE	igd	0.0073	1.02E-06	1766	M32405.m	TRUE	reported	uzomIQ	2	TRUE	0.013214	23.62212
8328	rs2824447	A	G	A	G	-0.035	-0.03633	0.6792	0.673913	FALSE	FALSE	FALSE	ieu-b-508	21	19066534	0.035725	486484	0.309192	Sepsis (28	Sepsis (28	Sepsis (28	TRUE	igd	0.0072	1.21E-06	3977	M36589.m	TRUE	reported	27GADo	2	TRUE	0.005907	23.61852
5325	rs2062528	C	G	C	G	0.0175	0.034888	0.6616	0.668041	FALSE	TRUE	FALSE	ieu-b-508	12	1.17E+08	0.035528	486484	0.326099	Sepsis (28	Sepsis (28	Sepsis (28	TRUE	igd	0.0036	9.19E-07	3890	M33364.m	TRUE	reported	9Zrr7Y	2	TRUE	0.006038	23.61825
2990	rs1007243	T	C	T	C	0.3673	0.09017	0.0175	0.042562	FALSE	FALSE	FALSE	ieu-b-508	5	1.63E+08	0.082891	486484	0.27668	Sepsis (28	Sepsis (28	Sepsis (28	TRUE	igd	0.0754	1.10E-06	423	M31536.m	TRUE	reported	3nAuUI	2	TRUE	0.053119	23.61788
882	rs3759526	T	C	T	C	-0.0209	0.038095	0.0806	0.076923	FALSE	FALSE	FALSE	ieu-b-508	14	51292535	0.063287	486484	0.547216	Sepsis (28	Sepsis (28	Sepsis (28	TRUE	igd	0.0043	8.93E-07	7362	M01365.m	TRUE	reported	N1cZTs	2	TRUE	0.003199	23.6177
1603	rs1003652	A	G	A	G	-0.0209	0.066333	0.0757	0.075813	FALSE	FALSE	FALSE	ieu-b-508	5	1.1E+08	0.063305	486484	0.294716	Sepsis (28	Sepsis (28	Sepsis (28	TRUE	igd	0.0043	9.58E-07	7352	M15122.m	TRUE	reported	VlnI8T	2	TRUE	0.003203	23.61769
3300	rs780241	A	G	A	G	0.0209	0.03188	0.2584	0.249315	FALSE	FALSE	FALSE	ieu-b-508	1	2.34E+08	0.038645	486484	0.409412	Sepsis (28	Sepsis (28	Sepsis (28	TRUE	igd	0.0043	1.24E-06	6119	M32379.m	TRUE	reported	MLimM6	2	TRUE	0.003846	23.6164
3624	rs1447149	A	G	A	G	-0.0311	0.032514	0.2486	0.234974	FALSE	FALSE	FALSE	ieu-b-508	11	1.2E+08	0.039511	486484	0.410557	Sepsis (28	Sepsis (28	Sepsis (28	TRUE	igd	0.0064	1.08E-06	7337	M32557.m	TRUE	reported	qR7rZX	2	TRUE	0.003208	23.60709
1127	rs7112460	T	C	T	C	0.1084	-0.11012	0.0658	0.063011	FALSE	FALSE	FALSE	ieu-b-508	11	1.26E+08	0.068534	486484	0.108101	Sepsis (28	Sepsis (28	Sepsis (28	TRUE	igd	0.0223	1.14E-06	2063	M01640.m	TRUE	reported	o3qxzl	2	TRUE	0.011324	23.60628
4323	rs7150683	A	G	A	G	-0.0379	0.014529	0.2063	0.212069	FALSE	FALSE	FALSE	ieu-b-508	14	61711788	0.040859	486484	0.722159	Sepsis (28	Sepsis (28	Sepsis (28	TRUE	igd	0.0078	1.13E-06	6250	M32846.m	TRUE	reported	lwF2Yy	2	TRUE	0.003763	23.60208
6839	rs1014637	C	G	C	G	0.1099	-0.00699	0.247	0.18747	FALSE	TRUE	FALSE	ieu-b-508	14	63790366	0.042761	486484	0.870159	Sepsis (28	Sepsis (28	Sepsis (28	TRUE	igd	0.0226	1.22E-06	954	M34365.m	TRUE	reported	8eexD0	2	TRUE	0.024188	23.59756
8302	rs6938391	A	G	A	G	0.0802	0.1992	0.0262	0.03458	FALSE	FALSE	FALSE	ieu-b-508	6	1.45E+08	0.092503	486484	0.031283	Sepsis (28	Sepsis (28	Sepsis (28	TRUE	igd	0.0165	1.19E-06	1636	M36559.m	TRUE	reported	g6I9hg	2	TRUE	0.014235	23.59661
5898	rs7185611	T	C	T	C	-0.4842	0.351508	0.98	0.989967	FALSE	FALSE	FALSE	ieu-b-508	16	20730690	0.169117	486484	0.037664	Sepsis (28	Sepsis (28	Sepsis (28	TRUE	igd	0.0987	9.41E-07	102	M33610.m	TRUE	reported	tmg43v	2	TRUE	0.190904	23.59473
5894	rs6679176	T	C	T	C	-0.3414	-0.0012	0.9608	0.829539	FALSE	FALSE	FALSE	ieu-b-508	1	2.46E+08	0.044425	486484	0.978489	Sepsis (28	Sepsis (28	Sepsis (28	TRUE	igd	0.0696	9.20E-07	102	M33610.m	TRUE	reported	tmg43v	2	TRUE	0.190866	23.58894
7143	rs1196213	A	G	A	G	0.0753	0.010703	0.037	0.037447	FALSE	FALSE	FALSE	ieu-b-508	6	66116731	0.088063	486484	0.903265	Sepsis (28	Sepsis (28	Sepsis (28	TRUE	igd	0.0155	1.12E-06	3813	M34530.m	TRUE	reported	8plvGA	2	TRUE	0.006151	23.58841
3887	rs9551761	A	G	A	G	0.017	-0.00609	0.8809	0.928703	FALSE	FALSE	FALSE	ieu-b-508	13	30452085	0.0648	486484	0.925108	Sepsis (28	Sepsis (28	Sepsis (28	TRUE	igd	0.0035	1.51E-06	7361	M32654.m	TRUE	reported	6lSQOP	2	TRUE	0.003195	23.58543
4381	rs1109583	A	G	A	G	-0.0068	0.098403	0.235	0.240825	FALSE	FALSE	FALSE	ieu-b-508	6	47677886	0.039145	486484	0.011944	Sepsis (28	Sepsis (28	Sepsis (28	TRUE	igd	0.0014	8.70E-07	7329	M32867.m	TRUE	reported	Jcsyzm	2	TRUE	0.003209	23.5854
5360	rs7526119	A	G	A	G	-0.0136	0.03762	0.1692	0.21776	FALSE	FALSE	FALSE	ieu-b-508	1	14850351	0.040823	486484	0.356775	Sepsis (28	Sepsis (28	Sepsis (28	TRUE	igd	0.0028	1.77E-06	7118	M33380.m	TRUE	reported	OHRfl5	2	TRUE	0.003303	23.58521
1895	rs7795076	T	C	T	C	-0.017	-0.0025	0.8166	0.830944	FALSE	FALSE	FALSE	ieu-b-508	7	49145008	0.044564	486484	0.955325	Sepsis (28	Sepsis (28	Sepsis (28	TRUE	igd	0.0035	1.62E-06	6782	M15990.m	TRUE	reported	15fe4N	2	TRUE	0.003467	23.58488
4130	rs760955	T	C	T	C	0.0238	-0.00558	0.281	0.344649	FALSE	FALSE	FALSE	ieu-b-508	6	1.44E+08	0.036032	486484	0.87693	Sepsis (28	Sepsis (28	Sepsis (28	TRUE	igd	0.0049	1.09E-06	6658	M32755.m	TRUE	reported	WDrNdi	2	TRUE	0.003531	23.58475
4587	rs4130215	A	C	A	C	0.0238	-0.02713	0.1106	0.130813	FALSE	FALSE	FALSE	ieu-b-508	7	48196475	0.049333	486484	0.582412	Sepsis (28	Sepsis (28	Sepsis (28	TRUE	igd	0.0049	1.32E-06	6404	M33163.m	TRUE	reported	YzGXkR	2	TRUE	0.00367	23.58447
7067	rs1264047	C	G	C	G	-0.0204	0.041872	0.413	0.414637	FALSE	TRUE	FALSE	ieu-b-508	4	1.45E+08	0.033997	486484	0.218082	Sepsis (28	Sepsis (28	Sepsis (28	TRUE	igd	0.0042	1.46E-06	6144	M34469.m	TRUE	reported	WMOqbm	2	TRUE	0.003825	23.58416
2504	rs1471834	A	G	A	G	0.0136	0.013714	0.7493	0.75275	FALSE	FALSE	FALSE	ieu-b-508	15	65208227	0.039367	486484	0.72757	Sepsis (28	Sepsis (28	Sepsis (28	TRUE	igd	0.0028	1.45E-06	5702	M22130.m	TRUE	reported	YDbJlz	2	TRUE	0.00412	23.58356
7040	rs1333111	A	G	A	G	-0.0136	-0.01679	0.3146	0.319802	FALSE	FALSE	FALSE	ieu-b-508	9	9416962	0.035918	486484	0.640187	Sepsis (28	Sepsis (28	Sepsis (28	TRUE	igd	0.0028	9.84E-07	5207	M34456.m	TRUE	reported	Nh457q	2	TRUE	0.00451	23.58278
8047	rs1105315	T	C	T	C	0.102	0.212508	0.012	0.023626	FALSE	FALSE	FALSE	ieu-b-508	12	34342352	0.121204	486484	0.07955	Sepsis (28	Sepsis (28	Sepsis (28	TRUE	igd	0.021	1.19E-06	5141	M35977.m	TRUE	reported	NHnUo7	2	TRUE	0.004568	23.58266
7174	rs943087	T	C	T	C	0.0306	0.041288	0.1704	0.182645	FALSE	FALSE	FALSE	ieu-b-508	6	76754976	0.043659	486484	0.344309	Sepsis (28	Sepsis (28	Sepsis (28	TRUE	igd	0.0063	1.40E-06	4925	M34534.m	TRUE	reported	iA78RM	2	TRUE	0.004767	23.58226
8107	rs4387456	A	G	A	G	1.6505	0.03976	0.9863	0.984879	FALSE	FALSE	FALSE	ieu-b-508	13	19287779	0.138044	486484	0.773332	Sepsis (28	Sepsis (28	Sepsis (28	TRUE	igd	0.3371	9.78E-07	122	M36097.m	TRUE	reported	RHr18S	2	TRUE	0.164226	23.57952
5324	rs1713335	A	G	A	G	-0.0403	0.072886	0.9531	0.948531	FALSE	FALSE	FALSE	ieu-b-508	5	1.11E+08	0.0757	486484	0.335631	Sepsis (28	Sepsis (28	Sepsis (28	TRUE	igd	0.0083	1.07E-06	3890	M33364.m	TRUE	reported	9Zrr7Y	2	TRUE	0.006024	23.563
5767	rs6781351	A	C	A	C	-0.0792	0.099383	0.8686	0.870734	FALSE	FALSE	FALSE	ieu-b-508	3	6559180	0.049873	486484	0.04629	Sepsis (28	Sepsis (28	Sepsis (28	TRUE	igd	0.0163	1.14E-06	972	M33508.m	TRUE	reported	UMXHcP	2	TRUE	0.023713	23.56029
6513	rs9319761	T	G	T	G	-0.0869	-0.02131	0.9697	0.949131	FALSE	FALSE	FALSE	ieu-b-508	18	66098859	0.076119	486484	0.779473	Sepsis (28	Sepsis (28	Sepsis (28	TRUE	igd	0.0179	1.21E-06	5581	M34112.m	TRUE	reported	97JqFb	2	TRUE	0.004205	23.56014
1954	rs6008912	A	G	A	G	0.1439	0.027917	0.159	0.192496	FALSE	FALSE	FALSE	ieu-b-508	22	46962477	0.042605	486484	0.512315	Sepsis (28	Sepsis (28	Sepsis (28	TRUE	igd	0.0296	1.19E-06	604	M16823.m	TRUE	reported	fNy2PD	2	TRUE	0.037656	23.55579
8276	rs1783280	C	G	C	G	0.0233	-0.00177	0.1092	0.142088	FALSE	TRUE	FALSE	ieu-b-508	14	57818966	0.048267	486484	0.970815	Sepsis (28	Sepsis (28	Sepsis (28	TRUE	igd	0.0048	1.55E-06	6464	M36553.m	TRUE	reported	2Rk7nd	2	TRUE	0.003632	23.55564
3912	rs1262149	A	C	A	C	-0.1005	0.016219	0.0275	0.040778	FALSE	FALSE	FALSE	ieu-b-508	2	1.98E+08	0.084217	486484	0.847288	Sepsis (28	Sepsis (28	Sepsis (28	TRUE	igd	0.0207	1.27E-06	2575	M32691.m	TRUE	reported	SiuD6F	2	TRUE	0.009071	23.55342
7168	rs274559	A	G	A	G	-0.0233	-0.00338	0.6171	0.626867	FALSE	FALSE	FALSE	ieu-b-508	5	1.32E+08	0.034396	486484	0.921767	Sepsis (28	Sepsis (28	Sepsis (28	TRUE	igd	0.0048	1.50E-06	4925	M34534.m	TRUE	reported	iA78RM	2	TRUE	0.004762	23.55337
5271	rs1262932	T	C	T	C	-0.0233	0.005333	0.7183	0.715208	FALSE	FALSE	FALSE	ieu-b-508	3	33991854	0.037092	486484	0.885686	Sepsis (28	Sepsis (28	Sepsis (28	TRUE	igd	0.0048	1.44E-06	4409	M33250.m	TRUE	reported	WIZI6U	2	TRUE	0.005316	23.55225
6290	rs1003620	T	C	T	C	0.0199	0.052296	0.3421	0.329329	FALSE	FALSE	FALSE	ieu-b-508	5	1.32E+08	0.035675	486484	0.142669	Sepsis (28	Sepsis (28	Sepsis (28	TRUE	igd	0.0041	1.49E-06	7320	M33941.m	TRUE	reported	culqiU	2	TRUE	0.003208	23.55156
8535	rs1118074	A	G	A	G	0.0398	-0.06768	0.1661	0.165012	FALSE	FALSE	FALSE	ieu-b-508	12	76303759	0.04647	486484	0.145272	Sepsis (28	Sepsis (28	Sepsis (28	TRUE	igd	0.0082	1.33E-06	6934	M37190.m	TRUE	reported	tiNuK9	2	TRUE	0.003386	23.55121
2329	rs1247056	T	C	T	C	0.0165	0.003868	0.5681	0.592685	FALSE	FALSE	FALSE	ieu-b-508	2	2.41E+08	0.03431	486484	0.910232	Sepsis (28	Sepsis (28	Sepsis (28	TRUE	igd	0.0034	1.11E-06	7366	M21044.m	TRUE	reported	Ym5rae	2	TRUE	0.003187	23.54464
2264	rs5994710	A	G	A	G	-0.0165	0.074121	0.0773	0.078451	FALSE	FALSE	FALSE	ieu-b-508	22	33682273	0.064341	486484	0.249314	Sepsis (28	Sepsis (28	Sepsis (28	TRUE	igd	0.0034	1.16E-06	7354	M19934.m	TRUE	reported	HtcWcQ	2	TRUE	0.003192	23.54463
3713	rs1823803	T	C	T	C	-0.0427	-0.01897	0.4614	0.449794	FALSE	FALSE	FALSE	ieu-b-508	2	2.35E+08	0.033503	486484	0.571248	Sepsis (28	Sepsis (28	Sepsis (28	TRUE	igd	0.0088	1.22E-06	6904	M32616.m	TRUE	reported	vrkjBE	2	TRUE	0.003399	23.53773
2791	rs1276852	T	C	T	C	-0.0131	-0.01805	0.2137	0.232981	FALSE	FALSE	FALSE	ieu-b-508	10	1.23E+08	0.039542	486484	0.648076	Sepsis (28	Sepsis (28	Sepsis (28	TRUE	igd	0.0027	1.06E-06	7347	M27278.m	TRUE	reported	kPgqm3	2	TRUE	0.003194	23.53406
1673	rs1106213	A	G	A	G	0.0131	-0.03231	0.1724	0.175921	FALSE	FALSE	FALSE	ieu-b-508	12	2280942	0.043887	486484	0.461654	Sepsis (28	Sepsis (28	Sepsis (28	TRUE	igd	0.0027	1.46E-06	7337	M15365.m	TRUE	reported	TReTCM	2	TRUE	0.003198	23.53405
1547	rs6477873	T	C	T	C	0.0131	-0.07118	0.6394	0.64593	FALSE	FALSE	FALSE	ieu-b-508	9	1.15E+08	0.03489	486484	0.041346	Sepsis (28	Sepsis (28	Sepsis (28	TRUE	igd	0.0027	9.51E-07	6864	M12129.m	TRUE	reported	iNzdgb	2	TRUE	0.003418	23.53361
1883	rs1923140	A	G	A	G	0.0131	0.037734	0.5322	0.542375	FALSE	FALSE	FALSE	ieu-b-508	1	34337704	0.033458	486484	0.259395	Sepsis (28	Sepsis (28	Sepsis (28	TRUE	igd	0.0027	8.57E-07	6782	M15990.m	TRUE	reported	15fe4N	2	TRUE	0.003459	23.53352
2656	rs2280289	A	G	A	G	-0.0131	-0.02708	0.8397	0.839601	FALSE	FALSE	FALSE	ieu-b-508	2	2.38E+08	0.045295	486484	0.549983	Sepsis (28	Sepsis (28	Sepsis (28	TRUE	igd	0.0027	1.38E-06	6517	M22649.m	TRUE	reported	NAzwRF	2	TRUE	0.003599	23.53324
6286	rs508482	T	G	T	G	0.0131	0.080178	0.8237	0.824343	FALSE	FALSE	FALSE	ieu-b-508	15	55410507	0.043955	486484	0.06814	Sepsis (28	Sepsis (28	Sepsis (28	TRUE	igd	0.0027	1.12E-06	6502	M33939.m	TRUE	reported	PeKlgz	2	TRUE	0.003607	23.53323
6126	rs244904	A	G	A	G	-0.0916	-0.03525	0.5145	0.4779	FALSE	FALSE	FALSE	ieu-b-508	5	1.68E+08	0.033529	486484	0.293126	Sepsis (28	Sepsis (28	Sepsis (28	TRUE	igd	0.0188	1.15E-06	228	M33877.m	TRUE	reported	yDVi1f	2	TRUE	0.094303	23.53146
3845	rs1991401	A	G	A	G	0.0131	0.011652	0.6758	0.687167	FALSE	FALSE	FALSE	ieu-b-508	17	62502435	0.036102	486484	0.74688	Sepsis (28	Sepsis (28	Sepsis (28	TRUE	igd	0.0027	1.91E-06	5211	M32651.m	TRUE	reported	DeuPwv	2	TRUE	0.004497	23.53143
3685	rs7855272	A	G	A	G	-0.0359	-0.01324	0.2071	0.219424	FALSE	FALSE	FALSE	ieu-b-508	9	1.29E+08	0.040528	486484	0.743959	Sepsis (28	Sepsis (28	Sepsis (28	TRUE	igd	0.0074	1.14E-06	7303	M32586.m	TRUE	reported	f6n0gX	2	TRUE	0.003212	23.52916
7926	rs2177207	T	C	T	C	-0.0927	-0.27095	0.9801	0.97257	FALSE	FALSE	FALSE	ieu-b-508	11	80771015	0.104104	486484	0.00925	Sepsis (28	Sepsis (28	Sepsis (28	TRUE	igd	0.0191	1.21E-06	1764	M35675.m	TRUE	reported	D7fdyZ	2	TRUE	0.013178	23.52882
7230	rs1175299	T	C	T	C	0.0927	0.06972	0.0666	0.030856	FALSE	FALSE	FALSE	ieu-b-508	6	25268520	0.09761	486484	0.475062	Sepsis (28	Sepsis (28	Sepsis (28	TRUE	igd	0.0191	1.26E-06	1763	M34878.m	TRUE	reported	Cpq9yU	2	TRUE	0.013185	23.5288
7235	rs32281	T	C	T	C	-0.1422	-0.29619	0.9844	0.987741	FALSE	FALSE	FALSE	ieu-b-508	5	11183411	0.152796	486484	0.052567	Sepsis (28	Sepsis (28	Sepsis (28	TRUE	igd	0.0293	1.22E-06	1763	M34878.m	TRUE	reported	Cpq9yU	2	TRUE	0.013184	23.52724
8224	rs2311717	A	T	A	T	0.0364	-0.0208	0.6829	0.702579	FALSE	TRUE	FALSE	ieu-b-508	18	25876887	0.036572	486484	0.569503	Sepsis (28	Sepsis (28	Sepsis (28	TRUE	igd	0.0075	1.30E-06	1582	M36399.m	TRUE	reported	EdUpnt	2	TRUE	0.014671	23.52507
5187	rs7732078	A	G	A	G	0.0524	0.034934	0.6742	0.666281	FALSE	FALSE	FALSE	ieu-b-508	5	1.75E+08	0.03541	486484	0.323858	Sepsis (28	Sepsis (28	Sepsis (28	TRUE	igd	0.0108	1.23E-06	2860	M33197.m	TRUE	reported	D6xaBg	2	TRUE	0.008164	23.524
5881	rs1249801	T	C	T	C	0.3027	0.204708	0.0395	0.025204	FALSE	FALSE	FALSE	ieu-b-508	3	66668599	0.105919	486484	0.053275	Sepsis (28	Sepsis (28	Sepsis (28	TRUE	igd	0.0618	9.79E-07	102	M33610.m	TRUE	reported	tmg43v	2	TRUE	0.190418	23.52056
381	rs825885	A	G	A	G	-0.0097	-0.00358	0.1469	0.200866	FALSE	FALSE	FALSE	ieu-b-508	12	1.3E+08	0.04182	486484	0.931849	Sepsis (28	Sepsis (28	Sepsis (28	TRUE	igd	0.002	1.56E-06	7351	M00060.m	TRUE	reported	kqwl4V	2	TRUE	0.00319	23.5161
1342	rs5764174	T	C	T	C	-0.0194	-0.056	0.2445	0.240135	FALSE	FALSE	FALSE	ieu-b-508	22	44033934	0.038944	486484	0.150473	Sepsis (28	Sepsis (28	Sepsis (28	TRUE	igd	0.004	9.61E-07	5791	M02342.m	TRUE	reported	EhZr3g	2	TRUE	0.004045	23.51438
3615	rs4324798	A	G	A	G	0.0354	0.000362	0.1136	0.117102	FALSE	FALSE	FALSE	ieu-b-508	6	28776117	0.051851	486484	0.99443	Sepsis (28	Sepsis (28	Sepsis (28	TRUE	igd	0.0073	1.08E-06	7361	M32553.m	TRUE	reported	vHSHqn	2	TRUE	0.003184	23.50947
2118	rs1073826	T	C	T	C	0.0354	-0.00815	0.1746	0.181542	FALSE	FALSE	FALSE	ieu-b-508	9	1223322	0.043146	486484	0.850112	Sepsis (28	Sepsis (28	Sepsis (28	TRUE	igd	0.0073	1.13E-06	5624	M18476.m	TRUE	reported	6GnBtw	2	TRUE	0.004164	23.50749
3672	rs4365292	A	C	A	C	0.0257	-0.03984	0.1924	0.194235	FALSE	FALSE	FALSE	ieu-b-508	16	26982655	0.042163	486484	0.344748	Sepsis (28	Sepsis (28	Sepsis (28	TRUE	igd	0.0053	1.51E-06	7322	M32578.m	TRUE	reported	LPkMFr	2	TRUE	0.003201	23.50693
6925	rs4331333	T	C	T	C	-0.0257	-0.01294	0.6194	0.615552	FALSE	FALSE	FALSE	ieu-b-508	16	51108719	0.03425	486484	0.705467	Sepsis (28	Sepsis (28	Sepsis (28	TRUE	igd	0.0053	1.16E-06	5561	M34390.m	TRUE	reported	wruTbW	2	TRUE	0.00421	23.50489
8162	rs1411176	T	C	T	C	-0.0359	0.067734	0.3811	0.381654	FALSE	FALSE	FALSE	ieu-b-508	8	1.32E+08	0.034386	486484	0.04886	Sepsis (28	Sepsis (28	Sepsis (28	TRUE	igd	0.0074	1.28E-06	1489	M36131.m	TRUE	reported	DbYm5t	2	TRUE	0.01556	23.504
1882	rs1731292	A	G	A	G	0.016	-0.01537	0.3493	0.348719	FALSE	FALSE	FALSE	ieu-b-508	12	12647902	0.035061	486484	0.661124	Sepsis (28	Sepsis (28	Sepsis (28	TRUE	igd	0.0033	1.42E-06	6782	M15990.m	TRUE	reported	15fe4N	2	TRUE	0.003454	23.50087
7824	rs397175	A	G	A	G	0.032	0.02666	0.1373	0.138466	FALSE	FALSE	FALSE	ieu-b-508	18	5159004	0.049432	486484	0.589665	Sepsis (28	Sepsis (28	Sepsis (28	TRUE	igd	0.0066	1.36E-06	6595	M35472.m	TRUE	reported	oZU10n	2	TRUE	0.003552	23.50068
1345	rs8113773	A	G	A	G	0.016	0.048834	0.5616	0.552873	FALSE	FALSE	FALSE	ieu-b-508	19	30350139	0.03381	486484	0.148631	Sepsis (28	Sepsis (28	Sepsis (28	TRUE	igd	0.0033	1.12E-06	5791	M02342.m	TRUE	reported	EhZr3g	2	TRUE	0.004043	23.49969
2424	rs3741298	T	C	T	C	-0.0325	0.025792	0.782	0.808348	FALSE	FALSE	FALSE	ieu-b-508	11	1.17E+08	0.042337	486484	0.542388	Sepsis (28	Sepsis (28	Sepsis (28	TRUE	igd	0.0067	1.34E-06	1408	M21188.m	TRUE	reported	kvdnjc	2	TRUE	0.016437	23.49632
48	rs1412972	T	G	T	G	0.0097	0.00367	0.5006	0.51539	FALSE	FALSE	FALSE	ieu-b-508	1	1.9E+08	0.033368	486484	0.91243	Sepsis (28	Sepsis (28	Sepsis (28	TRUE	igd	0.002	1.63E-06	1763	M00054.m	TRUE	reported	QTDAnh	2	TRUE	0.013167	23.49582
2761	rs6126354	T	C	T	C	-0.0194	0.012827	0.7911	0.651168	FALSE	FALSE	FALSE	ieu-b-508	20	50470585	0.035079	486484	0.714625	Sepsis (28	Sepsis (28	Sepsis (28	TRUE	igd	0.004	9.75E-07	1761	M27256.m	TRUE	reported	mJwzsi	2	TRUE	0.013181	23.49579
6179	rs1002384	T	G	T	G	0.0606	-0.02331	0.9859	0.975927	FALSE	FALSE	FALSE	ieu-b-508	4	32446683	0.10823	486484	0.829507	Sepsis (28	Sepsis (28	Sepsis (28	TRUE	igd	0.0125	1.27E-06	6004	M33892.m	TRUE	reported	w7HgU7	2	TRUE	0.003899	23.49527
6742	rs3749229	T	C	T	C	0.88	0.083147	0.014	0.096528	FALSE	FALSE	FALSE	ieu-b-508	3	1.84E+08	0.05665	486484	0.14218	Sepsis (28	Sepsis (28	Sepsis (28	TRUE	igd	0.18	1.01E-06	115	M34338.m	TRUE	reported	h4FB9a	2	TRUE	0.172074	23.48556
6962	rs1199720	C	G	C	G	-0.0807	-0.01207	0.3709	0.376795	FALSE	TRUE	FALSE	ieu-b-508	8	1.28E+08	0.034405	486484	0.725621	Sepsis (28	Sepsis (28	Sepsis (28	TRUE	igd	0.0166	1.13E-06	314	M34404.m	TRUE	reported	8FMHfo	2	TRUE	0.069998	23.48312
3256	rs1325625	A	G	A	G	-0.0126	-0.00349	0.1668	0.159727	FALSE	FALSE	FALSE	ieu-b-508	8	68797014	0.045789	486484	0.939329	Sepsis (28	Sepsis (28	Sepsis (28	TRUE	igd	0.0026	8.30E-07	7365	M32348.m	TRUE	reported	DJeLDx	2	TRUE	0.003179	23.47883
1024	rs6678076	A	G	A	G	-0.0252	-0.17148	0.0339	0.03688	FALSE	FALSE	FALSE	ieu-b-508	1	1.99E+08	0.089363	486484	0.054997	Sepsis (28	Sepsis (28	Sepsis (28	TRUE	igd	0.0052	1.08E-06	7364	M01564.m	TRUE	reported	IPUdyD	2	TRUE	0.003179	23.47883
1139	rs1295337	A	T	A	T	0.0126	-0.00468	0.8162	0.771846	FALSE	TRUE	FALSE	ieu-b-508	18	75916505	0.039932	486484	0.90662	Sepsis (28	Sepsis (28	Sepsis (28	TRUE	igd	0.0026	1.34E-06	7355	M01644.m	TRUE	reported	IB0DTL	2	TRUE	0.003183	23.47882
3093	rs718377	A	G	A	G	-0.0126	0.005193	0.3343	0.333537	FALSE	FALSE	FALSE	ieu-b-508	5	1.03E+08	0.035432	486484	0.883489	Sepsis (28	Sepsis (28	Sepsis (28	TRUE	igd	0.0026	1.29E-06	7346	M32197.m	TRUE	reported	VZjwa9	2	TRUE	0.003187	23.47881
3096	rs8024	A	C	A	C	0.0126	0.014329	0.2975	0.336218	FALSE	FALSE	FALSE	ieu-b-508	1	2.02E+08	0.035303	486484	0.684832	Sepsis (28	Sepsis (28	Sepsis (28	TRUE	igd	0.0026	1.58E-06	7346	M32197.m	TRUE	reported	VZjwa9	2	TRUE	0.003187	23.47881
4395	rs7829432	T	C	T	C	0.0063	-0.05756	0.346	0.349934	FALSE	FALSE	FALSE	ieu-b-508	8	9135117	0.035144	486484	0.101483	Sepsis (28	Sepsis (28	Sepsis (28	TRUE	igd	0.0013	1.14E-06	7329	M32867.m	TRUE	reported	Jcsyzm	2	TRUE	0.003194	23.4788
3933	rs295893	T	C	T	C	-0.0126	-0.02208	0.8293	0.818439	FALSE	FALSE	FALSE	ieu-b-508	5	1.52E+08	0.043505	486484	0.611767	Sepsis (28	Sepsis (28	Sepsis (28	TRUE	igd	0.0026	1.50E-06	7308	M32698.m	TRUE	reported	srqncz	2	TRUE	0.003203	23.47878
2740	rs1046720	A	G	A	G	-0.0126	-0.03781	0.9094	0.903476	FALSE	FALSE	FALSE	ieu-b-508	12	45547736	0.057045	486484	0.507445	Sepsis (28	Sepsis (28	Sepsis (28	TRUE	igd	0.0026	1.77E-06	7303	M27256.m	TRUE	reported	mJwzsi	2	TRUE	0.003206	23.47878
2292	rs4791715	T	G	T	G	-0.0126	-0.04647	0.594	0.57943	FALSE	FALSE	FALSE	ieu-b-508	17	8428148	0.033831	486484	0.169575	Sepsis (28	Sepsis (28	Sepsis (28	TRUE	igd	0.0026	1.66E-06	7301	M20675.m	TRUE	reported	CXe9rE	2	TRUE	0.003206	23.47877
869	rs7791503	T	C	T	C	0.0126	-0.05044	0.269	0.26897	FALSE	FALSE	FALSE	ieu-b-508	7	46007683	0.037839	486484	0.182558	Sepsis (28	Sepsis (28	Sepsis (28	TRUE	igd	0.0026	9.65E-07	7063	M01361.m	TRUE	reported	NIAcQ3	2	TRUE	0.003314	23.47856
8649	rs3851552	T	C	T	C	0.0126	-0.06137	0.6232	0.620089	FALSE	FALSE	FALSE	ieu-b-508	10	49799453	0.035048	486484	0.079948	Sepsis (28	Sepsis (28	Sepsis (28	TRUE	igd	0.0026	1.47E-06	6942	M38768.m	TRUE	reported	a2b8mH	2	TRUE	0.003372	23.47844
4598	rs2298423	T	G	T	G	0.0761	-0.02297	0.9286	0.924511	FALSE	FALSE	FALSE	ieu-b-508	8	37834312	0.063494	486484	0.71753	Sepsis (28	Sepsis (28	Sepsis (28	TRUE	igd	0.0157	1.24E-06	1751	M33165.m	TRUE	reported	RZt3uS	2	TRUE	0.01324	23.46787
6854	rs1205151	A	G	A	G	0.1668	-0.07058	0.0791	0.091212	FALSE	FALSE	FALSE	ieu-b-508	16	80942877	0.058596	486484	0.22836	Sepsis (28	Sepsis (28	Sepsis (28	TRUE	igd	0.0344	1.24E-06	954	M34365.m	TRUE	reported	8eexD0	2	TRUE	0.024052	23.46193
6573	rs2912035	T	G	T	G	0.0218	0.028829	0.6198	0.606475	FALSE	FALSE	FALSE	ieu-b-508	8	6632149	0.034498	486484	0.403345	Sepsis (28	Sepsis (28	Sepsis (28	TRUE	igd	0.0045	1.30E-06	4908	M34245.m	TRUE	reported	pRwBKr	2	TRUE	0.004759	23.45908
675	rs1003362	T	C	T	C	0.0126	0.000909	0.6115	0.648733	FALSE	FALSE	FALSE	ieu-b-508	4	1.87E+08	0.035314	486484	0.979454	Sepsis (28	Sepsis (28	Sepsis (28	TRUE	igd	0.0026	1.35E-06	1761	M01299.m	TRUE	reported	gfM0a3	2	TRUE	0.013161	23.45853
3888	rs1051173	C	G	C	G	-0.0189	-0.01807	0.8576	0.885417	FALSE	TRUE	FALSE	ieu-b-508	9	23624261	0.052335	486484	0.729948	Sepsis (28	Sepsis (28	Sepsis (28	TRUE	igd	0.0039	1.66E-06	1756	M32675.m	TRUE	reported	9FmPh9	2	TRUE	0.013198	23.45846
8505	rs9651696	T	C	T	C	-0.0756	0.008814	0.556	0.538	FALSE	FALSE	FALSE	ieu-b-508	11	1.21E+08	0.033664	486484	0.793467	Sepsis (28	Sepsis (28	Sepsis (28	TRUE	igd	0.0156	1.20E-06	1672	M37097.m	TRUE	reported	wCbJj4	2	TRUE	0.013852	23.45711
5446	rs1746325	T	C	T	C	0.1105	-0.00515	0.0671	0.06123	FALSE	FALSE	FALSE	ieu-b-508	2	3924349	0.069833	486484	0.941164	Sepsis (28	Sepsis (28	Sepsis (28	TRUE	igd	0.0228	1.31E-06	1464	M33390.m	TRUE	reported	t0EYdH	2	TRUE	0.015791	23.45639
5628	rs8026775	A	G	A	G	-0.0155	0.038046	0.068	0.063096	FALSE	FALSE	FALSE	ieu-b-508	15	85511647	0.068406	486484	0.578092	Sepsis (28	Sepsis (28	Sepsis (28	TRUE	igd	0.0032	1.16E-06	7336	M33442.m	TRUE	reported	B1Peb3	2	TRUE	0.003188	23.45552
6486	rs6745020	A	G	A	G	-0.932	-5.34E-05	0.011	0.015581	FALSE	FALSE	FALSE	ieu-b-508	2	1.08E+08	0.136406	486484	0.999688	Sepsis (28	Sepsis (28	Sepsis (28	TRUE	igd	0.191	1.06E-06	134	M34109.m	TRUE	reported	piJuC7	2	TRUE	0.150879	23.45493
5997	rs6930522	A	G	A	G	-0.0509	-0.03844	0.6305	0.635032	FALSE	FALSE	FALSE	ieu-b-508	6	1.02E+08	0.034737	486484	0.268474	Sepsis (28	Sepsis (28	Sepsis (28	TRUE	igd	0.0105	1.18E-06	1027	M33683.m	TRUE	reported	wSozpU	2	TRUE	0.02237	23.45365
7466	rs4536931	C	G	C	G	-0.0494	-0.00178	0.0288	0.057238	FALSE	TRUE	FALSE	ieu-b-508	4	1.07E+08	0.071947	486484	0.980248	Sepsis (28	Sepsis (28	Sepsis (28	TRUE	igd	0.0102	1.37E-06	6543	M35193.m	TRUE	reported	BMMWXU	2	TRUE	0.003572	23.44881
5946	rs9375094	T	C	T	C	-0.0678	0.134345	0.0286	0.031033	FALSE	FALSE	FALSE	ieu-b-508	6	1.22E+08	0.096466	486484	0.163723	Sepsis (28	Sepsis (28	Sepsis (28	TRUE	igd	0.014	1.28E-06	6109	M33638.m	TRUE	reported	hVuSGd	2	TRUE	0.003824	23.44559
7942	rs7152842	A	G	A	G	0.0092	-0.01028	0.7508	0.747111	FALSE	FALSE	FALSE	ieu-b-508	14	71849740	0.038389	486484	0.78884	Sepsis (28	Sepsis (28	Sepsis (28	TRUE	igd	0.0019	6.13E-07	7348	M35675.m	TRUE	reported	D7fdyZ	2	TRUE	0.003181	23.4396
5391	rs1253906	T	C	T	C	0.0092	0.025734	0.3111	0.314766	FALSE	FALSE	FALSE	ieu-b-508	7	82553949	0.035895	486484	0.473417	Sepsis (28	Sepsis (28	Sepsis (28	TRUE	igd	0.0019	9.03E-07	7315	M33389.m	TRUE	reported	Hf6XVM	2	TRUE	0.003195	23.43957
7992	rs1100886	A	G	A	G	-0.0092	0.004746	0.4749	0.471499	FALSE	FALSE	FALSE	ieu-b-508	10	19478056	0.033447	486484	0.887157	Sepsis (28	Sepsis (28	Sepsis (28	TRUE	igd	0.0019	1.53E-06	6604	M35754.m	TRUE	reported	lIXOo1	2	TRUE	0.003538	23.43888
8024	rs7814471	A	G	A	G	-0.0092	0.028381	0.4642	0.452787	FALSE	FALSE	FALSE	ieu-b-508	8	58700518	0.034113	486484	0.405419	Sepsis (28	Sepsis (28	Sepsis (28	TRUE	igd	0.0019	1.78E-06	6604	M35754.m	TRUE	reported	lIXOo1	2	TRUE	0.003538	23.43888
8027	rs8103504	T	C	T	C	0.0092	0.064865	0.5809	0.577907	FALSE	FALSE	FALSE	ieu-b-508	19	29808196	0.033791	486484	0.054911	Sepsis (28	Sepsis (28	Sepsis (28	TRUE	igd	0.0019	1.62E-06	6604	M35754.m	TRUE	reported	lIXOo1	2	TRUE	0.003538	23.43888
8453	rs1016145	T	C	T	C	0.0092	-0.01813	0.5665	0.571995	FALSE	FALSE	FALSE	ieu-b-508	12	20526181	0.03367	486484	0.590334	Sepsis (28	Sepsis (28	Sepsis (28	TRUE	igd	0.0019	2.03E-06	6552	M37058.m	TRUE	reported	32p8qV	2	TRUE	0.003566	23.43883
2570	rs598236	T	C	T	C	-0.0092	-0.05531	0.7138	0.711145	FALSE	FALSE	FALSE	ieu-b-508	19	6009445	0.037037	486484	0.135318	Sepsis (28	Sepsis (28	Sepsis (28	TRUE	igd	0.0019	2.28E-06	6535	M22177.m	TRUE	reported	2s6Sly	2	TRUE	0.003575	23.43881
2586	rs8180794	A	G	A	G	0.0092	-0.02436	0.4133	0.395009	FALSE	FALSE	FALSE	ieu-b-508	7	1.26E+08	0.034164	486484	0.475744	Sepsis (28	Sepsis (28	Sepsis (28	TRUE	igd	0.0019	2.02E-06	6535	M22177.m	TRUE	reported	2s6Sly	2	TRUE	0.003575	23.43881
3970	rs1012798	T	C	T	C	0.0092	-0.04189	0.4763	0.47959	FALSE	FALSE	FALSE	ieu-b-508	1	1.94E+08	0.03375	486484	0.214529	Sepsis (28	Sepsis (28	Sepsis (28	TRUE	igd	0.0019	1.13E-06	6497	M32729.m	TRUE	reported	XGRSN1	2	TRUE	0.003596	23.43877
4002	rs4542981	A	C	A	C	-0.0092	-0.03662	0.6707	0.673525	FALSE	FALSE	FALSE	ieu-b-508	3	1.55E+08	0.035505	486484	0.302308	Sepsis (28	Sepsis (28	Sepsis (28	TRUE	igd	0.0019	1.24E-06	6497	M32729.m	TRUE	reported	XGRSN1	2	TRUE	0.003596	23.43877
7242	rs1113071	C	G	C	G	-0.0092	-0.00372	0.4027	0.41224	FALSE	TRUE	FALSE	ieu-b-508	3	59492701	0.033938	486484	0.912789	Sepsis (28	Sepsis (28	Sepsis (28	TRUE	igd	0.0019	1.55E-06	5885	M34912.m	TRUE	reported	W8lcT6	2	TRUE	0.003968	23.43802
7244	rs1160659	A	C	A	C	-0.0092	0.010496	0.3632	0.374925	FALSE	FALSE	FALSE	ieu-b-508	11	56969436	0.034466	486484	0.760727	Sepsis (28	Sepsis (28	Sepsis (28	TRUE	igd	0.0019	1.11E-06	5885	M34912.m	TRUE	reported	W8lcT6	2	TRUE	0.003968	23.43802
7251	rs1270885	A	C	A	C	-0.0092	0.014003	0.5148	0.54359	FALSE	FALSE	FALSE	ieu-b-508	16	63699511	0.033486	486484	0.675817	Sepsis (28	Sepsis (28	Sepsis (28	TRUE	igd	0.0019	1.17E-06	5885	M34912.m	TRUE	reported	W8lcT6	2	TRUE	0.003968	23.43802
7273	rs6796650	A	G	A	G	-0.0092	0.001861	0.5569	0.54814	FALSE	FALSE	FALSE	ieu-b-508	3	88377822	0.033585	486484	0.955814	Sepsis (28	Sepsis (28	Sepsis (28	TRUE	igd	0.0019	1.17E-06	5885	M34912.m	TRUE	reported	W8lcT6	2	TRUE	0.003968	23.43802
7274	rs6834482	T	C	T	C	-0.0092	0.040704	0.6503	0.648276	FALSE	FALSE	FALSE	ieu-b-508	4	41359965	0.03524	486484	0.248061	Sepsis (28	Sepsis (28	Sepsis (28	TRUE	igd	0.0019	1.45E-06	5885	M34912.m	TRUE	reported	W8lcT6	2	TRUE	0.003968	23.43802
7353	rs2928949	T	C	T	C	0.0092	-0.04867	0.6858	0.667928	FALSE	FALSE	FALSE	ieu-b-508	18	49469609	0.035537	486484	0.170862	Sepsis (28	Sepsis (28	Sepsis (28	TRUE	igd	0.0019	1.52E-06	4900	M35137.m	TRUE	reported	Eq2siS	2	TRUE	0.004762	23.43641
6536	rs1196656	C	G	C	G	0.0305	-0.00809	0.8182	0.776378	FALSE	TRUE	FALSE	ieu-b-508	6	72451617	0.04039	486484	0.84133	Sepsis (28	Sepsis (28	Sepsis (28	TRUE	igd	0.0063	1.31E-06	6987	M34221.m	TRUE	reported	nUe3TL	2	TRUE	0.003343	23.43118
1538	rs1185806	T	C	T	C	0.0213	0.032025	0.9142	0.882621	FALSE	FALSE	FALSE	ieu-b-508	15	98662139	0.051886	486484	0.537082	Sepsis (28	Sepsis (28	Sepsis (28	TRUE	igd	0.0044	1.65E-06	6864	M12129.m	TRUE	reported	iNzdgb	2	TRUE	0.003402	23.42757
7967	rs7523522	T	C	T	C	-0.0213	-0.01819	0.6548	0.647295	FALSE	FALSE	FALSE	ieu-b-508	1	89101978	0.035042	486484	0.603671	Sepsis (28	Sepsis (28	Sepsis (28	TRUE	igd	0.0044	1.32E-06	6447	M35678.m	TRUE	reported	56yR01	2	TRUE	0.003622	23.42713
7840	rs1716788	T	C	T	C	-0.0852	0.020586	0.9594	0.955801	FALSE	FALSE	FALSE	ieu-b-508	7	96516313	0.08182	486484	0.801354	Sepsis (28	Sepsis (28	Sepsis (28	TRUE	igd	0.0176	1.30E-06	5035	M35551.m	TRUE	reported	5Sxswl	2	TRUE	0.004633	23.42509
6215	rs1854521	A	G	A	G	0.0213	-0.02698	0.4968	0.493425	FALSE	FALSE	FALSE	ieu-b-508	13	1.04E+08	0.033365	486484	0.418693	Sepsis (28	Sepsis (28	Sepsis (28	TRUE	igd	0.0044	1.17E-06	4533	M33901.m	TRUE	reported	mQkhrb	2	TRUE	0.005143	23.42406

407	rs1161093	T	C	T	C	-0.0121	-0.05752	0.0989	0.097501	FALSE	FALSE	FALSE	ieu-b-508	12	95285404	0.056085	486484	0.305129	Sepsis (28	Sepsis (28	Sepsis (28	TRUE	igd	0.0025	1.34E-06	7365	M00063.m	TRUE	reported	Hdsv1L	2	TRUE	0.003171	23.41924
6356	rs6977605	A	C	A	C	-0.0242	0.023834	0.9311	0.918515	FALSE	FALSE	FALSE	ieu-b-508	7	44685667	0.060974	486484	0.695883	Sepsis (28	Sepsis (28	Sepsis (28	TRUE	igd	0.005	1.67E-06	7364	M33960.m	TRUE	reported	o8RcxU	2	TRUE	0.003171	23.41924
1054	rs6896132	C	G	C	G	0.0121	0.019413	0.219	0.223159	FALSE	TRUE	FALSE	ieu-b-508	5	1.31E+08	0.040137	486484	0.628614	Sepsis (28	Sepsis (28	Sepsis (28	TRUE	igd	0.0025	1.80E-06	7333	M01572.m	TRUE	reported	EOUAWy	2	TRUE	0.003184	23.41921
5536	rs6417039	A	G	A	G	-0.0121	-0.08948	0.1602	0.198813	FALSE	FALSE	FALSE	ieu-b-508	18	6568416	0.043113	486484	0.037954	Sepsis (28	Sepsis (28	Sepsis (28	TRUE	igd	0.0025	1.11E-06	7306	M33422.m	TRUE	reported	PLgjrT	2	TRUE	0.003196	23.41919
1240	rs1328020	A	C	A	C	0.0121	0.024232	0.805	0.725999	FALSE	FALSE	FALSE	ieu-b-508	8	5857112	0.038019	486484	0.523883	Sepsis (28	Sepsis (28	Sepsis (28	TRUE	igd	0.0025	2.02E-06	7136	M01769.m	TRUE	reported	rismUZ	2	TRUE	0.003272	23.41903
7821	rs1728046	T	C	T	C	-0.0242	0.065809	0.2358	0.231247	FALSE	FALSE	FALSE	ieu-b-508	7	17099868	0.039713	486484	0.097495	Sepsis (28	Sepsis (28	Sepsis (28	TRUE	igd	0.005	1.31E-06	6595	M35472.m	TRUE	reported	oZU10n	2	TRUE	0.003539	23.4185
508	rs2456584	A	G	A	G	-0.0179	-0.01447	0.5393	0.536706	FALSE	FALSE	FALSE	ieu-b-508	19	51313145	0.033398	486484	0.664935	Sepsis (28	Sepsis (28	Sepsis (28	TRUE	igd	0.0037	1.64E-06	7333	M00577.m	TRUE	reported	YzFebE	2	TRUE	0.003182	23.39829
5162	rs1241361	T	C	T	C	-0.2541	0.098846	0.9528	0.955385	FALSE	FALSE	FALSE	ieu-b-508	10	2738192	0.086473	486484	0.252999	Sepsis (28	Sepsis (28	Sepsis (28	TRUE	igd	0.0525	1.29E-06	1586	M33194.m	TRUE	reported	xRUveq	2	TRUE	0.014555	23.39606
5330	rs7432894	T	C	T	C	-0.0179	0.005501	0.6903	0.690831	FALSE	FALSE	FALSE	ieu-b-508	3	1.96E+08	0.036125	486484	0.878978	Sepsis (28	Sepsis (28	Sepsis (28	TRUE	igd	0.0037	1.82E-06	3890	M33364.m	TRUE	reported	9Zrr7Y	2	TRUE	0.005981	23.39264
5462	rs427619	T	C	T	C	0.3498	0.060463	0.0263	0.022933	FALSE	FALSE	FALSE	ieu-b-508	7	1.59E+08	0.112716	486484	0.591667	Sepsis (28	Sepsis (28	Sepsis (28	TRUE	igd	0.0719	1.15E-06	171	M33391.m	TRUE	reported	EgMd1b	2	TRUE	0.121586	23.39227
6155	rs1188325	C	G	C	G	0.0208	-0.04117	0.3339	0.341896	FALSE	TRUE	FALSE	ieu-b-508	19	31790369	0.035331	486484	0.243856	Sepsis (28	Sepsis (28	Sepsis (28	TRUE	igd	0.0043	1.05E-06	7334	M33884.m	TRUE	reported	aAWgRQ	2	TRUE	0.00318	23.39221
3034	rs1927938	T	C	T	C	-0.0237	-0.04328	0.154	0.163131	FALSE	FALSE	FALSE	ieu-b-508	9	73921879	0.045248	486484	0.338843	Sepsis (28	Sepsis (28	Sepsis (28	TRUE	igd	0.0049	1.39E-06	7263	M31555.m	TRUE	reported	iQ5E3v	2	TRUE	0.003211	23.38756
8242	rs7211140	A	G	A	G	0.0358	-0.01558	0.4598	0.459005	FALSE	FALSE	FALSE	ieu-b-508	17	72173910	0.033766	486484	0.64457	Sepsis (28	Sepsis (28	Sepsis (28	TRUE	igd	0.0074	1.41E-06	1908	M36468.m	TRUE	reported	vycAFr	2	TRUE	0.012118	23.38014
1100	rs1111498	T	C	T	C	-0.0324	0.008769	0.0439	0.044885	FALSE	FALSE	FALSE	ieu-b-508	12	82102912	0.080644	486484	0.913415	Sepsis (28	Sepsis (28	Sepsis (28	TRUE	igd	0.0067	1.41E-06	7104	M01638.m	TRUE	reported	8dp0Ac	2	TRUE	0.003281	23.37858
1517	rs170631	C	G	C	G	-0.0208	-0.02209	0.8913	0.902549	FALSE	TRUE	FALSE	ieu-b-508	1	7852117	0.055907	486484	0.692815	Sepsis (28	Sepsis (28	Sepsis (28	TRUE	igd	0.0043	1.78E-06	1747	M12067.m	TRUE	reported	O5x7RD	2	TRUE	0.013217	23.37181
490	rs7482087	T	C	T	C	-0.5162	-0.04628	0.0254	0.032948	FALSE	FALSE	FALSE	ieu-b-508	11	11655316	0.092919	486484	0.618448	Sepsis (28	Sepsis (28	Sepsis (28	TRUE	igd	0.1064	1.24E-06	283	M00553.m	TRUE	reported	IFjXiL	2	TRUE	0.076784	23.37075
5181	rs1011894	T	C	T	C	0.1001	-0.08889	0.0815	0.109156	FALSE	FALSE	FALSE	ieu-b-508	9	86769142	0.055587	486484	0.1098	Sepsis (28	Sepsis (28	Sepsis (28	TRUE	igd	0.0207	1.31E-06	2860	M33197.m	TRUE	reported	D6xaBg	2	TRUE	0.00811	23.36811
7006	rs1734645	T	C	T	C	-0.1344	0.167698	0.9542	0.948623	FALSE	FALSE	FALSE	ieu-b-508	8	4560547	0.075521	486484	0.026381	Sepsis (28	Sepsis (28	Sepsis (28	TRUE	igd	0.0278	1.30E-06	4452	M34420.m	TRUE	reported	20uofq	2	TRUE	0.005223	23.3622
3104	rs1655902	A	G	A	G	-0.0295	0.040677	0.8197	0.82953	FALSE	FALSE	FALSE	ieu-b-508	6	29916855	0.043916	486484	0.354318	Sepsis (28	Sepsis (28	Sepsis (28	TRUE	igd	0.0061	1.29E-06	1760	M32198.m	TRUE	reported	ndXmnp	2	TRUE	0.013114	23.36095
5272	rs1343232	T	C	T	C	-0.0933	0.120204	0.016	0.016692	FALSE	FALSE	FALSE	ieu-b-508	2	18627062	0.130136	486484	0.355655	Sepsis (28	Sepsis (28	Sepsis (28	TRUE	igd	0.0193	1.27E-06	4409	M33250.m	TRUE	reported	WIZI6U	2	TRUE	0.005272	23.35886
3009	rs7165202	T	C	T	C	-0.1564	-0.14329	0.9469	0.952018	FALSE	FALSE	FALSE	ieu-b-508	15	50146582	0.077876	486484	0.065782	Sepsis (28	Sepsis (28	Sepsis (28	TRUE	igd	0.0323	1.29E-06	522	M31536.m	TRUE	reported	3nAuUI	2	TRUE	0.042985	23.35615
6	rs7078003	T	C	T	C	0.0087	-0.02565	0.1654	0.178694	FALSE	FALSE	FALSE	ieu-b-508	10	99359412	0.043653	486484	0.556766	Sepsis (28	Sepsis (28	Sepsis (28	TRUE	igd	0.0018	1.84E-06	7372	M00053.m	TRUE	reported	4Smluz	2	TRUE	0.003159	23.35477
3276	rs597467	T	C	T	C	0.0087	-0.00492	0.3784	0.385738	FALSE	FALSE	FALSE	ieu-b-508	7	51951935	0.034282	486484	0.885844	Sepsis (28	Sepsis (28	Sepsis (28	TRUE	igd	0.0018	8.06E-07	7365	M32348.m	TRUE	reported	DJeLDx	2	TRUE	0.003162	23.35477
3284	rs755403	T	C	T	C	0.0087	0.011403	0.5969	0.596543	FALSE	FALSE	FALSE	ieu-b-508	4	6389642	0.034464	486484	0.740757	Sepsis (28	Sepsis (28	Sepsis (28	TRUE	igd	0.0018	9.52E-07	7365	M32348.m	TRUE	reported	DJeLDx	2	TRUE	0.003162	23.35477
1012	rs2236299	A	G	A	G	0.0087	0.077377	0.3065	0.295546	FALSE	FALSE	FALSE	ieu-b-508	14	55255335	0.036723	486484	0.035116	Sepsis (28	Sepsis (28	Sepsis (28	TRUE	igd	0.0018	1.69E-06	7364	M01564.m	TRUE	reported	IPUdyD	2	TRUE	0.003162	23.35477
3418	rs1081892	T	C	T	C	-0.0087	-0.02548	0.6238	0.616463	FALSE	FALSE	FALSE	ieu-b-508	9	1.27E+08	0.034316	486484	0.457815	Sepsis (28	Sepsis (28	Sepsis (28	TRUE	igd	0.0018	2.35E-06	7364	M32452.m	TRUE	reported	8GtFpl	2	TRUE	0.003162	23.35477
721	rs1573544	T	C	T	C	0.0087	-0.00591	0.1484	0.158108	FALSE	FALSE	FALSE	ieu-b-508	11	15195648	0.045582	486484	0.89688	Sepsis (28	Sepsis (28	Sepsis (28	TRUE	igd	0.0018	9.64E-07	7363	M01301.m	TRUE	reported	w8TL71	2	TRUE	0.003163	23.35477
3473	rs331721	T	C	T	C	0.0087	-0.05763	0.7235	0.725919	FALSE	FALSE	FALSE	ieu-b-508	5	1.25E+08	0.037429	486484	0.123613	Sepsis (28	Sepsis (28	Sepsis (28	TRUE	igd	0.0018	1.85E-06	7363	M32489.m	TRUE	reported	hnljaD	2	TRUE	0.003163	23.35477
3477	rs6494904	A	G	A	G	0.0087	0.006597	0.7315	0.732832	FALSE	FALSE	FALSE	ieu-b-508	15	71610835	0.03762	486484	0.860805	Sepsis (28	Sepsis (28	Sepsis (28	TRUE	igd	0.0018	1.91E-06	7363	M32489.m	TRUE	reported	hnljaD	2	TRUE	0.003163	23.35477
3772	rs382543	T	C	T	C	0.0087	-0.03975	0.2625	0.289315	FALSE	FALSE	FALSE	ieu-b-508	5	76323206	0.036977	486484	0.282342	Sepsis (28	Sepsis (28	Sepsis (28	TRUE	igd	0.0018	1.94E-06	7362	M32634.m	TRUE	reported	7T2RUz	2	TRUE	0.003163	23.35476
6441	rs3764387	A	G	A	G	-0.0087	0.032724	0.1754	0.210662	FALSE	FALSE	FALSE	ieu-b-508	17	53920169	0.040862	486484	0.423228	Sepsis (28	Sepsis (28	Sepsis (28	TRUE	igd	0.0018	1.61E-06	7362	M34062.m	TRUE	reported	RZyHKP	2	TRUE	0.003163	23.35476
3187	rs3012739	A	G	A	G	0.0116	-0.00292	0.4834	0.480064	FALSE	FALSE	FALSE	ieu-b-508	9	1.35E+08	0.033537	486484	0.930682	Sepsis (28	Sepsis (28	Sepsis (28	TRUE	igd	0.0024	2.13E-06	7356	M32338.m	TRUE	reported	q5cuaP	2	TRUE	0.003166	23.35476
1133	rs1009287	T	C	T	C	0.0087	0.01942	0.4173	0.411429	FALSE	FALSE	FALSE	ieu-b-508	8	89823675	0.034008	486484	0.567969	Sepsis (28	Sepsis (28	Sepsis (28	TRUE	igd	0.0018	1.25E-06	7355	M01644.m	TRUE	reported	IB0DTL	2	TRUE	0.003166	23.35476
1166	rs990054	T	C	T	C	-0.0087	-0.03864	0.5955	0.597836	FALSE	FALSE	FALSE	ieu-b-508	21	25434305	0.034314	486484	0.260116	Sepsis (28	Sepsis (28	Sepsis (28	TRUE	igd	0.0018	1.33E-06	7355	M01644.m	TRUE	reported	IB0DTL	2	TRUE	0.003166	23.35476
3501	rs2704668	T	C	T	C	-0.0087	-0.02446	0.6202	0.614874	FALSE	FALSE	FALSE	ieu-b-508	7	16520193	0.034464	486484	0.477793	Sepsis (28	Sepsis (28	Sepsis (28	TRUE	igd	0.0018	8.77E-07	7355	M32492.m	TRUE	reported	rZZFcE	2	TRUE	0.003166	23.35476
3510	rs7136574	T	C	T	C	0.0087	-0.0085	0.4342	0.422651	FALSE	FALSE	FALSE	ieu-b-508	12	1.2E+08	0.033729	486484	0.800951	Sepsis (28	Sepsis (28	Sepsis (28	TRUE	igd	0.0018	8.27E-07	7355	M32492.m	TRUE	reported	rZZFcE	2	TRUE	0.003166	23.35476
6320	rs3851441	C	G	C	G	-0.0087	-0.03642	0.2195	0.218305	FALSE	TRUE	FALSE	ieu-b-508	5	41517506	0.040387	486484	0.367178	Sepsis (28	Sepsis (28	Sepsis (28	TRUE	igd	0.0018	2.30E-06	7354	M33955.m	TRUE	reported	mhHFnT	2	TRUE	0.003167	23.35476
774	rs1119363	T	C	T	C	0.0087	0.00559	0.6724	0.673596	FALSE	FALSE	FALSE	ieu-b-508	10	1.09E+08	0.035893	486484	0.876247	Sepsis (28	Sepsis (28	Sepsis (28	TRUE	igd	0.0018	7.34E-07	7352	M01336.m	TRUE	reported	FAIrya	2	TRUE	0.003167	23.35476
328	rs6003916	T	C	T	C	0.0087	-0.02228	0.8966	0.888786	FALSE	FALSE	FALSE	ieu-b-508	22	24186823	0.053146	486484	0.675016	Sepsis (28	Sepsis (28	Sepsis (28	TRUE	igd	0.0018	1.06E-06	7351	M00060.m	TRUE	reported	kqwl4V	2	TRUE	0.003168	23.35476
7925	rs2167730	T	C	T	C	-0.0087	0.049554	0.6541	0.666266	FALSE	FALSE	FALSE	ieu-b-508	8	78103417	0.035396	486484	0.161517	Sepsis (28	Sepsis (28	Sepsis (28	TRUE	igd	0.0018	2.32E-06	7348	M35675.m	TRUE	reported	D7fdyZ	2	TRUE	0.003169	23.35475
7939	rs6564157	T	C	T	C	-0.0087	-0.01583	0.4074	0.440392	FALSE	FALSE	FALSE	ieu-b-508	16	74710254	0.03371	486484	0.638691	Sepsis (28	Sepsis (28	Sepsis (28	TRUE	igd	0.0018	2.12E-06	7348	M35675.m	TRUE	reported	D7fdyZ	2	TRUE	0.003169	23.35475
1173	rs1246605	A	G	A	G	0.0087	-0.04648	0.402	0.422036	FALSE	FALSE	FALSE	ieu-b-508	2	35935217	0.033838	486484	0.169583	Sepsis (28	Sepsis (28	Sepsis (28	TRUE	igd	0.0018	8.44E-07	7346	M01645.m	TRUE	reported	d5nAn3	2	TRUE	0.00317	23.35475
3202	rs1165255	T	C	T	C	0.0087	0.040418	0.3265	0.327568	FALSE	FALSE	FALSE	ieu-b-508	17	4338964	0.035528	486484	0.255264	Sepsis (28	Sepsis (28	Sepsis (28	TRUE	igd	0.0018	1.52E-06	7344	M32339.m	TRUE	reported	SVp504	2	TRUE	0.003171	23.35475
3219	rs651158	A	G	A	G	0.0087	0.036396	0.6393	0.652698	FALSE	FALSE	FALSE	ieu-b-508	6	35906389	0.03504	486484	0.298946	Sepsis (28	Sepsis (28	Sepsis (28	TRUE	igd	0.0018	8.12E-07	7344	M32339.m	TRUE	reported	SVp504	2	TRUE	0.003171	23.35475
4440	rs282145	T	C	T	C	0.0087	0.020311	0.6353	0.649234	FALSE	FALSE	FALSE	ieu-b-508	20	551921	0.035185	486484	0.563767	Sepsis (28	Sepsis (28	Sepsis (28	TRUE	igd	0.0018	7.18E-07	7344	M32910.m	TRUE	reported	2OCBKh	2	TRUE	0.003171	23.35475
4450	rs4983559	A	G	A	G	-0.0087	-0.00816	0.6109	0.613581	FALSE	FALSE	FALSE	ieu-b-508	14	1.05E+08	0.034215	486484	0.811468	Sepsis (28	Sepsis (28	Sepsis (28	TRUE	igd	0.0018	1.35E-06	7344	M32910.m	TRUE	reported	2OCBKh	2	TRUE	0.003171	23.35475
2708	rs6475805	T	C	T	C	-0.0087	-0.01205	0.514	0.524556	FALSE	FALSE	FALSE	ieu-b-508	9	24658725	0.033457	486484	0.718651	Sepsis (28	Sepsis (28	Sepsis (28	TRUE	igd	0.0018	1.81E-06	7340	M25459.m	TRUE	reported	ACJY35	2	TRUE	0.003173	23.35475
3730	rs1474920	T	G	T	G	-0.0174	-0.00843	0.1861	0.199175	FALSE	FALSE	FALSE	ieu-b-508	14	23587555	0.042108	486484	0.841253	Sepsis (28	Sepsis (28	Sepsis (28	TRUE	igd	0.0036	1.46E-06	7337	M32632.m	TRUE	reported	plHzPT	2	TRUE	0.003174	23.35474
5620	rs4129608	A	G	A	G	0.0087	-0.03434	0.8509	0.852255	FALSE	FALSE	FALSE	ieu-b-508	9	18327215	0.047072	486484	0.465677	Sepsis (28	Sepsis (28	Sepsis (28	TRUE	igd	0.0018	7.16E-07	7336	M33442.m	TRUE	reported	B1Peb3	2	TRUE	0.003174	23.35474
8244	rs1262611	T	C	T	C	-0.0058	0.025606	0.2385	0.241767	FALSE	FALSE	FALSE	ieu-b-508	20	54318925	0.039267	486484	0.514338	Sepsis (28	Sepsis (28	Sepsis (28	TRUE	igd	0.0012	9.78E-07	7330	M36515.m	TRUE	reported	yZaYzr	2	TRUE	0.003177	23.35474
2498	rs791623	T	G	T	G	-0.0116	-0.06149	0.2041	0.198185	FALSE	FALSE	FALSE	ieu-b-508	5	1.73E+08	0.041844	486484	0.141675	Sepsis (28	Sepsis (28	Sepsis (28	TRUE	igd	0.0024	1.68E-06	7329	M22116.m	TRUE	reported	bjUU6r	2	TRUE	0.003177	23.35474
1858	rs7283154	T	C	T	C	-0.0087	-0.02153	0.2516	0.248387	FALSE	FALSE	FALSE	ieu-b-508	21	42282735	0.038526	486484	0.576278	Sepsis (28	Sepsis (28	Sepsis (28	TRUE	igd	0.0018	2.32E-06	7308	M15778.m	TRUE	reported	941o3l	2	TRUE	0.003186	23.35472
1861	rs8008040	T	C	T	C	-0.0087	-0.05428	0.7276	0.710754	FALSE	FALSE	FALSE	ieu-b-508	14	22392807	0.036699	486484	0.139108	Sepsis (28	Sepsis (28	Sepsis (28	TRUE	igd	0.0018	2.06E-06	7308	M15778.m	TRUE	reported	941o3l	2	TRUE	0.003186	23.35472
5684	rs398462	T	C	T	C	0.0087	0.01122	0.5177	0.547671	FALSE	FALSE	FALSE	ieu-b-508	19	5996439	0.033665	486484	0.738919	Sepsis (28	Sepsis (28	Sepsis (28	TRUE	igd	0.0018	1.39E-06	7307	M33477.m	TRUE	reported	Aw8PBK	2	TRUE	0.003187	23.35472
5526	rs2758886	A	G	A	G	0.0087	-0.02939	0.2863	0.29755	FALSE	FALSE	FALSE	ieu-b-508	6	39250837	0.036412	486484	0.419606	Sepsis (28	Sepsis (28	Sepsis (28	TRUE	igd	0.0018	7.46E-07	7306	M33422.m	TRUE	reported	PLgjrT	2	TRUE	0.003187	23.35472
5528	rs2874279	A	G	A	G	-0.0087	0.013629	0.7386	0.73899	FALSE	FALSE	FALSE	ieu-b-508	4	18197469	0.038163	486484	0.721006	Sepsis (28	Sepsis (28	Sepsis (28	TRUE	igd	0.0018	1.02E-06	7306	M33422.m	TRUE	reported	PLgjrT	2	TRUE	0.003187	23.35472
2748	rs1323688	T	C	T	C	0.0087	-0.03629	0.2268	0.223705	FALSE	FALSE	FALSE	ieu-b-508	10	95040595	0.040081	486484	0.365242	Sepsis (28	Sepsis (28	Sepsis (28	TRUE	igd	0.0018	6.90E-07	7303	M27256.m	TRUE	reported	mJwzsi	2	TRUE	0.003189	23.35471
2756	rs2876814	T	G	T	G	-0.0087	0.019805	0.185	0.18583	FALSE	FALSE	FALSE	ieu-b-508	13	84916858	0.04351	486484	0.648984	Sepsis (28	Sepsis (28	Sepsis (28	TRUE	igd	0.0018	1.08E-06	7303	M27256.m	TRUE	reported	mJwzsi	2	TRUE	0.003189	23.35471
2669	rs1081787	T	C	T	C	0.0116	-0.00499	0.5666	0.55299	FALSE	FALSE	FALSE	ieu-b-508	9	1.19E+08	0.033696	486484	0.882363	Sepsis (28	Sepsis (28	Sepsis (28	TRUE	igd	0.0024	1.46E-06	7256	M24074.m	TRUE	reported	Lk6D2A	2	TRUE	0.003209	23.35467
3826	rs738715	A	C	A	C	0.0087	-0.02433	0.5394	0.537822	FALSE	FALSE	FALSE	ieu-b-508	22	48020172	0.033566	486484	0.468637	Sepsis (28	Sepsis (28	Sepsis (28	TRUE	igd	0.0018	1.83E-06	7229	M32644.m	TRUE	reported	JAa2WD	2	TRUE	0.003221	23.35465
5814	rs2278908	T	C	T	C	0.0087	0.050962	0.359	0.374158	FALSE	FALSE	FALSE	ieu-b-508	11	68523910	0.034845	486484	0.14359	Sepsis (28	Sepsis (28	Sepsis (28	TRUE	igd	0.0018	2.55E-06	7066	M33515.m	TRUE	reported	X9mul1	2	TRUE	0.003295	23.3545
5824	rs4669966	A	G	A	G	0.0087	0.016525	0.5646	0.565276	FALSE	FALSE	FALSE	ieu-b-508	2	6075682	0.033879	486484	0.625716	Sepsis (28	Sepsis (28	Sepsis (28	TRUE	igd	0.0018	1.86E-06	7066	M33515.m	TRUE	reported	X9mul1	2	TRUE	0.003295	23.3545
5840	rs9972361	A	G	A	G	-0.0087	-0.02216	0.3351	0.340635	FALSE	FALSE	FALSE	ieu-b-508	15	37486228	0.035258	486484	0.529598	Sepsis (28	Sepsis (28	Sepsis (28	TRUE	igd	0.0018	2.32E-06	7066	M33515.m	TRUE	reported	X9mul1	2	TRUE	0.003295	23.3545
1369	rs1001373	A	G	A	G	0.0087	-0.01007	0.6839	0.670447	FALSE	FALSE	FALSE	ieu-b-508	4	37391031	0.035602	486484	0.777229	Sepsis (28	Sepsis (28	Sepsis (28	TRUE	igd	0.0018	7.90E-07	7037	M02734.m	TRUE	reported	0y0dDD	2	TRUE	0.003309	23.35447
5095	rs9389954	T	C	T	C	0.119	-0.05152	0.8466	0.824599	FALSE	FALSE	FALSE	ieu-b-508	6	1.42E+08	0.043944	486484	0.241029	Sepsis (28	Sepsis (28	Sepsis (28	TRUE	igd	0.0246	1.30E-06	1017	M33178.m	TRUE	reported	g22WMS	2	TRUE	0.022492	23.3544
8401	rs1049465	T	G	T	G	-0.0087	0.019482	0.8351	0.834707	FALSE	FALSE	FALSE	ieu-b-508	1	1.92E+08	0.044964	486484	0.664812	Sepsis (28	Sepsis (28	Sepsis (28	TRUE	igd	0.0018	2.04E-06	6940	M36808.m	TRUE	reported	u7Iqaj	2	TRUE	0.003355	23.35438
7684	rs6477329	A	T	A	T	0.0087	-0.1138	0.6832	0.653466	FALSE	TRUE	FALSE	ieu-b-508	9	8736878	0.035157	486484	0.001208	Sepsis (28	Sepsis (28	Sepsis (28	TRUE	igd	0.0018	9.49E-07	6920	M35327.m	TRUE	reported	SquFiY	2	TRUE	0.003365	23.35436
6836	rs973634	T	C	T	C	0.0087	-0.03334	0.505	0.523651	FALSE	FALSE	FALSE	ieu-b-508	10	83871324	0.033468	486484	0.319231	Sepsis (28	Sepsis (28	Sepsis (28	TRUE	igd	0.0018	1.00E-06	6755	M34359.m	TRUE	reported	0UotaJ	2	TRUE	0.003446	23.35419
8465	rs1254802	C	G	C	G	-0.0087	0.037193	0.4	0.400794	FALSE	TRUE	FALSE	ieu-b-508	8	25630547	0.034039	486484	0.274541	Sepsis (28	Sepsis (28	Sepsis (28	TRUE	igd	0.0018	2.68E-06	6552	M37058.m	TRUE	reported	32p8qV	2	TRUE	0.003553	23.35398
1936	rs504899	T	C	T	C	-0.0232	0.026945	0.0734	0.082263	FALSE	FALSE	FALSE	ieu-b-508	7	80041938	0.060725	486484	0.657251	Sepsis (28	Sepsis (28	Sepsis (28	TRUE	igd	0.0048	1.42E-06	6549	M16822.m	TRUE	reported	UNI018	2	TRUE	0.003554	23.35398
2721	rs1266549	A	G	A	G	0.029	-0.05209	0.9441	0.935015	FALSE	FALSE	FALSE	ieu-b-508	6	1.3E+08	0.06836	486484	0.446102	Sepsis (28	Sepsis (28	Sepsis (28	TRUE	igd	0.006	1.28E-06	6388	M25599.m	TRUE	reported	fRZxwP	2	TRUE	0.003644	23.3538
7630	rs1165327	T	G	T	G	-0.0087	0.065448	0.721	0.703574	FALSE	FALSE	FALSE	ieu-b-508	17	78639760	0.036535	486484	0.073232	Sepsis (28	Sepsis (28	Sepsis (28	TRUE	igd	0.0018	1.05E-06	5768	M35326.m	TRUE	reported	jRytyJ	2	TRUE	0.004034	23.35301
7669	rs9951062	A	G	A	G	0.0087	-0.02882	0.7122	0.712471	FALSE	FALSE	FALSE	ieu-b-508	18	37586676	0.036885	486484	0.434543	Sepsis (28	Sepsis (28	Sepsis (28	TRUE	igd	0.0018	1.05E-06	5768	M35326.m	TRUE	reported	jRytyJ	2	TRUE	0.004034	23.35301
2481	rs333063	T	C	T	C	0.029	-0.01341	0.0879	0.118535	FALSE	FALSE	FALSE	ieu-b-508	8	19034022	0.051475	486484	0.79453	Sepsis (28	Sepsis (28	Sepsis (28	TRUE	igd	0.006	1.49E-06	5676	M22032.m	TRUE	reported	xpe96K	2	TRUE	0.004099	23.35288
6564	rs1051273	C	G	C	G	0.0261	-0.05354	0.8047	0.788791	FALSE	TRUE	FALSE	ieu-b-508	5	40308790	0.04118	486484	0.193588	Sepsis (28	Sepsis (28	Sepsis (28	TRUE	igd	0.0054	1.15E-06	4908	M34245.m	TRUE	reported	pRwBKr	2	TRUE	0.004737	23.35159
8400	rs8031716	T	C	T	C	-0.0145	0.048969	0.555	0.549138	FALSE	FALSE	FALSE	ieu-b-508	15	52998283	0.034077	486484	0.150708	Sepsis (28	Sepsis (28	Sepsis (28	TRUE	igd	0.003	1.45E-06	4317	M36802.m	TRUE	reported	njeQfy	2	TRUE	0.005382	23.35029
6471	rs1270253	A	G	A	G	0.925	-0.0221	0.986	0.910412	FALSE	FALSE	FALSE	ieu-b-508	7	1321193	0.058379	486484	0.705055	Sepsis (28	Sepsis (28	Sepsis (28	TRUE	igd	0.19	1.13E-06	134	M34109.m	TRUE	reported	piJuC7	2	TRUE	0.150294	23.34777
7091	rs1002490	A	C	A	C	0.0377	-0.02249	0.1598	0.149506	FALSE	FALSE	FALSE	ieu-b-508	4	24358657	0.046744	486484	0.63039	Sepsis (28	Sepsis (28	Sepsis (28	TRUE	igd	0.0078	1.30E-06	3239	M34513.m	TRUE	reported	1hoso6	2	TRUE	0.007161	23.34669
2712	rs7160179	C	G	C	G	-0.0701	-0.01296	0.9807	0.849335	FALSE	TRUE	FALSE	ieu-b-508	14	80984413	0.046631	486484	0.78102	Sepsis (28	Sepsis (28	Sepsis (28	TRUE	igd	0.0145	1.28E-06	1756	M25459.m	TRUE	reported	ACJY35	2	TRUE	0.013135	23.3456
1568	rs7961079	A	G	A	G	0.0527	-0.10264	0.6241	0.620876	FALSE	FALSE	FALSE	ieu-b-508	12	1.09E+08	0.034344	486484	0.002802	Sepsis (28	Sepsis (28	Sepsis (28	TRUE	igd	0.0109	1.46E-06	1543	M12261.m	TRUE	reported	taUUcM	2	TRUE	0.014924	23.3456
6713	rs1157359	A	G	A	G	-0.3896	0.055793	0.0204	0.020513	FALSE	FALSE	FALSE	ieu-b-508	1	45292173	0.119342	486484	0.64014	Sepsis (28	Sepsis (28	Sepsis (28	TRUE	igd	0.0805	1.32E-06	535	M34338.m	TRUE	reported	h4FB9a	2	TRUE	0.041945	23.33563
4019	rs1278781	T	C	T	C	-0.0372	-0.04908	0.814	0.804802	FALSE	FALSE	FALSE	ieu-b-508	11	20545926	0.042188	486484	0.244649	Sepsis (28	Sepsis (28	Sepsis (28	TRUE	igd	0.0077	1.23E-06	6000	M32735.m	TRUE	reported	QE2vKQ	2	TRUE	0.003875	23.33241
2778	rs1714188	C	G	C	G	-0.0314	0.024289	0.9716	0.970256	FALSE	TRUE	FALSE	ieu-b-508	10	6857690	0.098738	486484	0.805684	Sepsis (28	Sepsis (28	Sepsis (28	TRUE	igd	0.0065	1.13E-06	7268	M27273.m	TRUE	reported	EvU2od	2	TRUE	0.003201	23.32991
6481	rs2582067	A	G	A	G	0.91	0.022278	0.977	0.845912	FALSE	FALSE	FALSE	ieu-b-508	1	2.14E+08	0.049307	486484	0.651394	Sepsis (28	Sepsis (28	Sepsis (28	TRUE	igd	0.187	1.14E-06	134	M34109.m	TRUE	reported	piJuC7	2	TRUE	0.150183	23.32753
3670	rs1884917	C	G	C	G	-0.0256	-0.01878	0.2335	0.22644	FALSE	TRUE	FALSE	ieu-b-508	20	4576069	0.040017	486484	0.63886	Sepsis (28	Sepsis (28	Sepsis (28	TRUE	igd	0.0053	1.51E-06	7322	M32578.m	TRUE	reported	LPkMFr	2	TRUE	0.003176	23.32435
1670	rs7184992	T	C	T	C	0.0807	0.021884	0.3003	0.305354	FALSE	FALSE	FALSE	ieu-b-508	16	27432010	0.036268	486484	0.546254	Sepsis (28	Sepsis (28	Sepsis (28	TRUE	igd	0.0167	1.31E-06	1522	M15335.m	TRUE	reported	a7RjFn	2	TRUE	0.015111	23.32078
7620	rs385748	T	C	T	C	-0.3443	-0.16358	0.9894	0.98673	FALSE	FALSE	FALSE	ieu-b-508	13	1.04E+08	0.146902	486484	0.265471	Sepsis (28	Sepsis (28	Sepsis (28	TRUE	igd	0.0711	1.28E-06	349	M35322.m	TRUE	reported	om3h9x	2	TRUE	0.06296	23.31519
899	rs6975921	A	G	A	G	0.0343	0.051754	0.9095	0.919455	FALSE	FALSE	FALSE	ieu-b-508	7	15152813	0.061398	486484	0.399272	Sepsis (28	Sepsis (28	Sepsis (28	TRUE	igd	0.0071	1.25E-06	1766	M01493.m	TRUE	reported	eLLRt7	2	TRUE	0.013043	23.31199
7910	rs1173142	C	G	C	G	0.0169	0.141982	0.9058	0.901716	FALSE	TRUE	FALSE	ieu-b-508	4	1.71E+08	0.055879	486484	0.011058	Sepsis (28	Sepsis (28	Sepsis (28	TRUE	igd	0.0035	1.69E-06	7348	M35675.m	TRUE	reported	D7fdyZ	2	TRUE	0.003163	23.30876
905	rs1229418	T	C	T	C	0.014	-0.05265	0.0987	0.131913	FALSE	FALSE	FALSE	ieu-b-508	11	12240720	0.050305	486484	0.295238	Sepsis (28	Sepsis (28	Sepsis (28	TRUE	igd	0.0029	1.07E-06	7354	M01494.m	TRUE	reported	7JWNKI	2	TRUE	0.003159	23.29925
8287	rs7499206	T	C	T	C	0.014	0.048804	0.29	0.290819	FALSE	FALSE	FALSE	ieu-b-508	16	53046134	0.036696	486484	0.183532	Sepsis (28	Sepsis (28	Sepsis (28	TRUE	igd	0.0029	1.40E-06	6464	M36553.m	TRUE	reported	2Rk7nd	2	TRUE	0.003592	23.29838
7224	rs512251	A	T	A	T	0.028	0.055675	0.8615	0.856318	FALSE	TRUE	FALSE	ieu-b-508	1	1.12E+08	0.047543	486484	0.241577	Sepsis (28	Sepsis (28	Sepsis (28	TRUE	igd	0.0058	1.44E-06	6381	M34761.m	TRUE	reported	AMtp1w	2	TRUE	0.003639	23.29828
6195	rs1746802	T	C	T	C	0.014	-0.0122	0.4198	0.429349	FALSE	FALSE	FALSE	ieu-b-508	4	14393864	0.034016	486484	0.71991	Sepsis (28	Sepsis (28	Sepsis (28	TRUE	igd	0.0029	1.49E-06	6004	M33892.m	TRUE	reported	w7HgU7	2	TRUE	0.003867	23.29783
6197	rs1995777	T	C	T	C	-0.014	0.063478	0.5594	0.564228	FALSE	FALSE	FALSE	ieu-b-508	5	32987956	0.03421	486484	0.063519	Sepsis (28	Sepsis (28	Sepsis (28	TRUE	igd	0.0029	1.37E-06	6004	M33892.m	TRUE	reported	w7HgU7	2	TRUE	0.003867	23.29783
492	rs9614985	A	G	A	G	-0.0855	0.003447	0.4177	0.41821	FALSE	FALSE	FALSE	ieu-b-508	22	45320631	0.033996	486484	0.919244	Sepsis (28	Sepsis (28	Sepsis (28	TRUE	igd	0.0177	1.28E-06	1233	M00553.m	TRUE	reported	IFjXiL	2	TRUE	0.018573	23.29596
1812	rs1040090	A	G	A	G	-0.0251	0.053427	0.3089	0.31521	FALSE	FALSE	FALSE	ieu-b-508	15	79231616	0.035976	486484	0.137523	Sepsis (28	Sepsis (28	Sepsis (28	TRUE	igd	0.0052	1.62E-06	7357	M15753.m	TRUE	reported	VDJYDI	2	TRUE	0.003157	23.29285
8189	rs1712143	A	G	A	G	0.1236	0.146499	0.9807	0.980218	FALSE	FALSE	FALSE	ieu-b-508	8	15274318	0.125531	486484	0.243197	Sepsis (28	Sepsis (28	Sepsis (28	TRUE	igd	0.0256	1.36E-06	2400	M36230.m	TRUE	reported	V5KJeR	2	TRUE	0.009619	23.29137
6464	rs6911308	T	G	T	G	-0.0251	-0.0009	0.5796	0.611689	FALSE	FALSE	FALSE	ieu-b-508	6	3375697	0.034318	486484	0.979014	Sepsis (28	Sepsis (28	Sepsis (28	TRUE	igd	0.0052	1.34E-06	4961	M34106.m	TRUE	reported	8DuoNM	2	TRUE	0.004675	23.28979
6493	rs9465476	T	C	T	C	-0.43	-0.02223	0.14	0.141731	FALSE	FALSE	FALSE	ieu-b-508	6	19672138	0.050281	486484	0.658426	Sepsis (28	Sepsis (28	Sepsis (28	TRUE	igd	0.0885	1.19E-06	148	M34109.m	TRUE	reported	piJuC7	2	TRUE	0.137567	23.2885
1629	rs1293763	T	C	T	C	-0.0082	-0.0292	0.5397	0.538974	FALSE	FALSE	FALSE	ieu-b-508	17	47444442	0.03351	486484	0.383554	Sepsis (28	Sepsis (28	Sepsis (28	TRUE	igd	0.0017	2.28E-06	7368	M15140.m	TRUE	reported	jEcqMJ	2	TRUE	0.003148	23.26012
1651	rs739744	T	C	T	C	-0.0082	0.018522	0.5529	0.557867	FALSE	FALSE	FALSE	ieu-b-508	12	1.13E+08	0.033739	486484	0.583021	Sepsis (28	Sepsis (28	Sepsis (28	TRUE	igd	0.0017	1.88E-06	7368	M15140.m	TRUE	reported	jEcqMJ	2	TRUE	0.003148	23.26012
1658	rs886125	A	G	A	G	0.0082	-0.02508	0.463	0.458778	FALSE	FALSE	FALSE	ieu-b-508	12	1.11E+08	0.033512	486484	0.454254	Sepsis (28	Sepsis (28	Sepsis (28	TRUE	igd	0.0017	1.86E-06	7368	M15140.m	TRUE	reported	jEcqMJ	2	TRUE	0.003148	23.26012
415	rs1718278	T	C	T	C	-0.0082	-0.0205	0.253	0.265386	FALSE	FALSE	FALSE	ieu-b-508	2	1.66E+08	0.037621	486484	0.585833	Sepsis (28	Sepsis (28	Sepsis (28	TRUE	igd	0.0017	2.10E-06	7365	M00063.m	TRUE	reported	Hdsv1L	2	TRUE	0.003149	23.26012
714	rs1165980	C	G	C	G	-0.0082	0.034145	0.1964	0.230471	FALSE	TRUE	FALSE	ieu-b-508	18	33101254	0.040057	486484	0.393987	Sepsis (28	Sepsis (28	Sepsis (28	TRUE	igd	0.0017	2.16E-06	7363	M01301.m	TRUE	reported	w8TL71	2	TRUE	0.00315	23.26012
730	rs7583413	T	C	T	C	0.0082	-0.0611	0.8245	0.806094	FALSE	FALSE	FALSE	ieu-b-508	2	2.28E+08	0.042466	486484	0.150229	Sepsis (28	Sepsis (28	Sepsis (28	TRUE	igd	0.0017	2.62E-06	7363	M01301.m	TRUE	reported	w8TL71	2	TRUE	0.00315	23.26012
7985	rs732647	A	G	A	G	0.0246	0.053258	0.0598	0.054856	FALSE	FALSE	FALSE	ieu-b-508	6	53122053	0.073304	486484	0.467506	Sepsis (28	Sepsis (28	Sepsis (28	TRUE	igd	0.0051	1.49E-06	7358	M35718.m	TRUE	reported	oUwc0a	2	TRUE	0.003152	23.26011
830	rs6733405	A	G	A	G	-0.0082	0.084823	0.3867	0.377833	FALSE	FALSE	FALSE	ieu-b-508	2	22973119	0.0346	486484	0.014225	Sepsis (28	Sepsis (28	Sepsis (28	TRUE	igd	0.0017	2.42E-06	7355	M01358.m	TRUE	reported	kSPQgd	2	TRUE	0.003153	23.26011
2062	rs2201453	A	G	A	G	0.0082	0.054346	0.438	0.451009	FALSE	FALSE	FALSE	ieu-b-508	1	80040353	0.033562	486484	0.105394	Sepsis (28	Sepsis (28	Sepsis (28	TRUE	igd	0.0017	1.14E-06	7354	M18369.m	TRUE	reported	ykBjIT	2	TRUE	0.003154	23.26011
2066	rs2573124	A	C	A	C	-0.0082	0.059379	0.4579	0.455777	FALSE	FALSE	FALSE	ieu-b-508	16	87643917	0.033669	486484	0.077798	Sepsis (28	Sepsis (28	Sepsis (28	TRUE	igd	0.0017	1.13E-06	7354	M18369.m	TRUE	reported	ykBjIT	2	TRUE	0.003154	23.26011
775	rs1122247	T	G	T	G	-0.0082	-0.06095	0.6036	0.607528	FALSE	FALSE	FALSE	ieu-b-508	11	1.31E+08	0.034264	486484	0.075258	Sepsis (28	Sepsis (28	Sepsis (28	TRUE	igd	0.0017	1.42E-06	7352	M01336.m	TRUE	reported	FAIrya	2	TRUE	0.003155	23.26011
261	rs1710797	T	C	T	C	-0.0082	-0.03747	0.1068	0.124341	FALSE	FALSE	FALSE	ieu-b-508	14	22055136	0.05087	486484	0.461422	Sepsis (28	Sepsis (28	Sepsis (28	TRUE	igd	0.0017	1.50E-06	7351	M00060.m	TRUE	reported	kqwl4V	2	TRUE	0.003155	23.26011
3126	rs1303342	T	C	T	C	0.0082	0.030099	0.4434	0.430508	FALSE	FALSE	FALSE	ieu-b-508	2	98758323	0.033702	486484	0.371812	Sepsis (28	Sepsis (28	Sepsis (28	TRUE	igd	0.0017	1.92E-06	7349	M32315.m	TRUE	reported	4igzkL	2	TRUE	0.003156	23.2601
3135	rs4241779	A	G	A	G	0.0082	0.018421	0.5776	0.564816	FALSE	FALSE	FALSE	ieu-b-508	4	1.85E+08	0.033614	486484	0.583687	Sepsis (28	Sepsis (28	Sepsis (28	TRUE	igd	0.0017	2.83E-06	7349	M32315.m	TRUE	reported	4igzkL	2	TRUE	0.003156	23.2601
3137	rs580333	A	G	A	G	0.0082	0.02392	0.4934	0.498021	FALSE	FALSE	FALSE	ieu-b-508	10	84643651	0.033414	486484	0.474071	Sepsis (28	Sepsis (28	Sepsis (28	TRUE	igd	0.0017	1.81E-06	7349	M32315.m	TRUE	reported	4igzkL	2	TRUE	0.003156	23.2601
3216	rs4980554	A	G	A	G	0.0082	-0.08045	0.4561	0.455962	FALSE	FALSE	FALSE	ieu-b-508	11	69281227	0.033597	486484	0.016643	Sepsis (28	Sepsis (28	Sepsis (28	TRUE	igd	0.0017	2.87E-06	7344	M32339.m	TRUE	reported	SVp504	2	TRUE	0.003158	23.2601
1290	rs1782177	T	G	T	G	-0.0082	-0.00599	0.3485	0.35014	FALSE	FALSE	FALSE	ieu-b-508	7	33973249	0.034975	486484	0.863969	Sepsis (28	Sepsis (28	Sepsis (28	TRUE	igd	0.0017	1.92E-06	7325	M02132.m	TRUE	reported	uJFm2t	2	TRUE	0.003166	23.26008
4048	rs1274287	C	G	C	G	-0.0082	-0.00929	0.3923	0.398566	FALSE	TRUE	FALSE	ieu-b-508	1	76908156	0.034463	486484	0.787497	Sepsis (28	Sepsis (28	Sepsis (28	TRUE	igd	0.0017	2.70E-06	7318	M32740.m	TRUE	reported	YLshfP	2	TRUE	0.003169	23.26008
4078	rs7151149	T	C	T	C	-0.0082	-0.00578	0.2898	0.298182	FALSE	FALSE	FALSE	ieu-b-508	14	34969433	0.03644	486484	0.873917	Sepsis (28	Sepsis (28	Sepsis (28	TRUE	igd	0.0017	3.10E-06	7318	M32740.m	TRUE	reported	YLshfP	2	TRUE	0.003169	23.26008
1589	rs7914384	T	C	T	C	-0.0082	-0.03804	0.1806	0.182776	FALSE	FALSE	FALSE	ieu-b-508	10	53639321	0.043318	486484	0.379833	Sepsis (28	Sepsis (28	Sepsis (28	TRUE	igd	0.0017	1.85E-06	7314	M12593.m	TRUE	reported	IBXgSU	2	TRUE	0.003171	23.26007
5535	rs5751928	A	G	A	G	-0.0082	0.004217	0.4847	0.503127	FALSE	FALSE	FALSE	ieu-b-508	22	25075402	0.033411	486484	0.899551	Sepsis (28	Sepsis (28	Sepsis (28	TRUE	igd	0.0017	2.31E-06	7306	M33422.m	TRUE	reported	PLgjrT	2	TRUE	0.003174	23.26007
2175	rs1211978	A	G	A	G	-0.0082	0.013352	0.4048	0.405187	FALSE	FALSE	FALSE	ieu-b-508	1	4823492	0.034054	486484	0.694994	Sepsis (28	Sepsis (28	Sepsis (28	TRUE	igd	0.0017	2.38E-06	7182	M19362.m	TRUE	reported	SkNEKw	2	TRUE	0.003229	23.25996
1259	rs7661472	A	C	A	C	-0.0082	-0.03554	0.4922	0.496622	FALSE	FALSE	FALSE	ieu-b-508	4	1.42E+08	0.033518	486484	0.289049	Sepsis (28	Sepsis (28	Sepsis (28	TRUE	igd	0.0017	1.38E-06	7136	M01769.m	TRUE	reported	rismUZ	2	TRUE	0.00325	23.25992
5340	rs1292357	A	G	A	G	0.0082	-0.0069	0.2397	0.238062	FALSE	FALSE	FALSE	ieu-b-508	16	57756643	0.039094	486484	0.859836	Sepsis (28	Sepsis (28	Sepsis (28	TRUE	igd	0.0017	2.92E-06	7118	M33380.m	TRUE	reported	OHRfl5	2	TRUE	0.003258	23.2599
1528	rs686321	T	C	T	C	0.0082	-0.00258	0.8232	0.811404	FALSE	FALSE	FALSE	ieu-b-508	13	52426096	0.042731	486484	0.951868	Sepsis (28	Sepsis (28	Sepsis (28	TRUE	igd	0.0017	2.14E-06	7081	M12067.m	TRUE	reported	O5x7RD	2	TRUE	0.003275	23.25986
7674	rs1212622	A	G	A	G	0.0082	0.008575	0.2807	0.305799	FALSE	FALSE	FALSE	ieu-b-508	1	81774822	0.036392	486484	0.813716	Sepsis (28	Sepsis (28	Sepsis (28	TRUE	igd	0.0017	3.01E-06	6920	M35327.m	TRUE	reported	SquFiY	2	TRUE	0.003351	23.25971
7683	rs450852	A	G	A	G	0.0082	0.008318	0.3243	0.330242	FALSE	FALSE	FALSE	ieu-b-508	11	2756314	0.035705	486484	0.815782	Sepsis (28	Sepsis (28	Sepsis (28	TRUE	igd	0.0017	1.91E-06	6920	M35327.m	TRUE	reported	SquFiY	2	TRUE	0.003351	23.25971
2225	rs606114	T	C	T	C	-0.0164	-0.01503	0.1601	0.158333	FALSE	FALSE	FALSE	ieu-b-508	1	2.02E+08	0.045716	486484	0.742306	Sepsis (28	Sepsis (28	Sepsis (28	TRUE	igd	0.0034	1.48E-06	6678	M19368.m	TRUE	reported	QaKPad	2	TRUE	0.003472	23.25947
4132	rs780094	T	C	T	C	-0.0164	0.005915	0.3929	0.38476	FALSE	FALSE	FALSE	ieu-b-508	2	27742603	0.034302	486484	0.863098	Sepsis (28	Sepsis (28	Sepsis (28	TRUE	igd	0.0034	1.61E-06	6658	M32755.m	TRUE	reported	WDrNdi	2	TRUE	0.003482	23.25945
7532	rs1171256	T	C	T	C	0.0164	-0.04038	0.311	0.318836	FALSE	FALSE	FALSE	ieu-b-508	3	15147989	0.035873	486484	0.260311	Sepsis (28	Sepsis (28	Sepsis (28	TRUE	igd	0.0034	1.50E-06	6538	M35257.m	TRUE	reported	iphouU	2	TRUE	0.003546	23.25932
6109	rs6593732	T	C	T	C	-0.0164	-0.06453	0.4672	0.474633	FALSE	FALSE	FALSE	ieu-b-508	1	1.47E+08	0.033815	486484	0.056343	Sepsis (28	Sepsis (28	Sepsis (28	TRUE	igd	0.0034	1.63E-06	6506	M33871.m	TRUE	reported	mQD6DP	2	TRUE	0.003563	23.25928
7628	rs1118890	A	G	A	G	0.0082	-0.05481	0.6306	0.625393	FALSE	FALSE	FALSE	ieu-b-508	10	98508282	0.034447	486484	0.111571	Sepsis (28	Sepsis (28	Sepsis (28	TRUE	igd	0.0017	2.77E-06	5768	M35326.m	TRUE	reported	jRytyJ	2	TRUE	0.004018	23.25837
7662	rs7327332	T	G	T	G	-0.0082	0.020906	0.6628	0.665672	FALSE	FALSE	FALSE	ieu-b-508	13	43472671	0.035379	486484	0.554581	Sepsis (28	Sepsis (28	Sepsis (28	TRUE	igd	0.0017	2.73E-06	5768	M35326.m	TRUE	reported	jRytyJ	2	TRUE	0.004018	23.25837
504	rs1242613	A	G	A	G	0.0246	0.079241	0.1446	0.165478	FALSE	FALSE	FALSE	ieu-b-508	12	46567909	0.045718	486484	0.083046	Sepsis (28	Sepsis (28	Sepsis (28	TRUE	igd	0.0051	1.68E-06	5502	M00575.m	TRUE	reported	Mv0t8k	2	TRUE	0.004211	23.25798
6157	rs2123517	T	C	T	C	0.1158	-0.14017	0.023	0.016419	FALSE	FALSE	FALSE	ieu-b-508	2	1.3E+08	0.133751	486484	0.294634	Sepsis (28	Sepsis (28	Sepsis (28	TRUE	igd	0.024	1.46E-06	1766	M33884.m	TRUE	reported	aAWgRQ	2	TRUE	0.013011	23.25426
2080	rs3846406	A	C	A	C	0.0299	0.100264	0.4896	0.497589	FALSE	FALSE	FALSE	ieu-b-508	4	14131479	0.033364	486484	0.002654	Sepsis (28	Sepsis (28	Sepsis (28	TRUE	igd	0.0062	1.46E-06	7322	M18392.m	TRUE	reported	fBRMfe	2	TRUE	0.003166	23.25093
1800	rs1184794	T	C	T	C	-0.0217	0.041648	0.3031	0.31906	FALSE	FALSE	FALSE	ieu-b-508	14	99093183	0.035976	486484	0.247006	Sepsis (28	Sepsis (28	Sepsis (28	TRUE	igd	0.0045	1.54E-06	5829	M15749.m	TRUE	reported	TR861N	2	TRUE	0.003973	23.24585
7099	rs2816252	T	C	T	C	0.082	-0.05178	0.066	0.223581	FALSE	FALSE	FALSE	ieu-b-508	6	1454972	0.040913	486484	0.205636	Sepsis (28	Sepsis (28	Sepsis (28	TRUE	igd	0.017	1.41E-06	2168	M34513.m	TRUE	reported	1hoso6	2	TRUE	0.010618	23.24497
5709	rs1168780	T	C	T	C	-0.0217	-0.02053	0.3033	0.308677	FALSE	FALSE	FALSE	ieu-b-508	2	78978011	0.036137	486484	0.569868	Sepsis (28	Sepsis (28	Sepsis (28	TRUE	igd	0.0045	1.38E-06	5107	M33488.m	TRUE	reported	mZmDY6	2	TRUE	0.004533	23.24472
1419	rs1694829	A	T	A	T	-0.041	0.052937	0.0694	0.067807	FALSE	TRUE	FALSE	ieu-b-508	15	94164895	0.067033	486484	0.429692	Sepsis (28	Sepsis (28	Sepsis (28	TRUE	igd	0.0085	1.35E-06	1745	M03127.m	TRUE	reported	JscEZw	2	TRUE	0.013158	23.23977
1367	rs849039	T	G	T	G	-0.0135	0.010721	0.779	0.74431	FALSE	FALSE	FALSE	ieu-b-508	8	5378100	0.038486	486484	0.780581	Sepsis (28	Sepsis (28	Sepsis (28	TRUE	igd	0.0028	1.41E-06	7226	M02730.m	TRUE	reported	XYa035	2	TRUE	0.003207	23.23974
7734	rs1709857	A	C	A	C	-0.027	0.043377	0.9522	0.939871	FALSE	FALSE	FALSE	ieu-b-508	12	63314504	0.070038	486484	0.535698	Sepsis (28	Sepsis (28	Sepsis (28	TRUE	igd	0.0056	1.54E-06	7007	M35431.m	TRUE	reported	tLfE4l	2	TRUE	0.003307	23.23954
7018	rs961928	T	C	T	C	0.0487	0.036587	0.3628	0.365674	FALSE	FALSE	FALSE	ieu-b-508	7	78473591	0.035299	486484	0.299973	Sepsis (28	Sepsis (28	Sepsis (28	TRUE	igd	0.0101	1.58E-06	4452	M34420.m	TRUE	reported	20uofq	2	TRUE	0.005195	23.23914
2728	rs1752600	A	G	A	G	0.0135	-0.0603	0.2579	0.257554	FALSE	FALSE	FALSE	ieu-b-508	15	84232366	0.038146	486484	0.113921	Sepsis (28	Sepsis (28	Sepsis (28	TRUE	igd	0.0028	1.04E-06	6388	M25599.m	TRUE	reported	fRZxwP	2	TRUE	0.003626	23.2389
6785	rs3093135	A	T	A	T	0.0569	0.027182	0.8288	0.836231	FALSE	TRUE	FALSE	ieu-b-508	19	16004371	0.045109	486484	0.546774	Sepsis (28	Sepsis (28	Sepsis (28	TRUE	igd	0.0118	1.29E-06	3491	M34350.m	TRUE	reported	Cc3yW9	2	TRUE	0.006616	23.23869
2485	rs7397059	T	C	T	C	-0.0135	0.033661	0.5732	0.576755	FALSE	FALSE	FALSE	ieu-b-508	12	1.1E+08	0.033823	486484	0.31963	Sepsis (28	Sepsis (28	Sepsis (28	TRUE	igd	0.0028	1.22E-06	5676	M22032.m	TRUE	reported	xpe96K	2	TRUE	0.004079	23.23798
3109	rs3843502	A	G	A	G	-0.0323	0.02287	0.0286	0.029436	FALSE	FALSE	FALSE	ieu-b-508	5	1.31E+08	0.098959	486484	0.817235	Sepsis (28	Sepsis (28	Sepsis (28	TRUE	igd	0.0067	1.63E-06	7356	M32198.m	TRUE	reported	ndXmnp	2	TRUE	0.00315	23.23471
6511	rs7971484	T	G	T	G	-0.0323	-0.00087	0.1909	0.182891	FALSE	FALSE	FALSE	ieu-b-508	12	68487789	0.043223	486484	0.984024	Sepsis (28	Sepsis (28	Sepsis (28	TRUE	igd	0.0067	1.54E-06	5581	M34112.m	TRUE	reported	97JqFb	2	TRUE	0.004147	23.23271
1344	rs7868774	A	G	A	G	-0.0188	0.018568	0.2709	0.266021	FALSE	FALSE	FALSE	ieu-b-508	9	1.38E+08	0.037881	486484	0.624022	Sepsis (28	Sepsis (28	Sepsis (28	TRUE	igd	0.0039	1.78E-06	5791	M02342.m	TRUE	reported	EhZr3g	2	TRUE	0.003997	23.22932
7111	rs1244181	A	T	A	T	0.2952	-0.03136	0.0435	0.040301	FALSE	TRUE	FALSE	ieu-b-508	15	50210933	0.086017	486484	0.715425	Sepsis (28	Sepsis (28	Sepsis (28	TRUE	igd	0.061	1.31E-06	241	M34516.m	TRUE	reported	7Ah2wy	2	TRUE	0.088569	23.2249
5154	rs1705421	A	C	A	C	0.0829	-0.01086	0.3934	0.465315	FALSE	FALSE	FALSE	ieu-b-508	13	21171438	0.034627	486484	0.753793	Sepsis (28	Sepsis (28	Sepsis (28	TRUE	igd	0.0172	1.54E-06	5716	M33192.m	TRUE	reported	CyfjKE	2	TRUE	0.004048	23.22203
2939	rs1277997	A	G	A	G	0.0347	0.036044	0.9388	0.942136	FALSE	FALSE	FALSE	ieu-b-508	10	46042932	0.072641	486484	0.619761	Sepsis (28	Sepsis (28	Sepsis (28	TRUE	igd	0.0072	1.26E-06	7336	M27738.m	TRUE	reported	UtPBd9	2	TRUE	0.003156	23.22071
3897	rs2101330	A	G	A	G	0.0135	0.006952	0.5266	0.546421	FALSE	FALSE	FALSE	ieu-b-508	2	1.35E+08	0.033673	486484	0.836426	Sepsis (28	Sepsis (28	Sepsis (28	TRUE	igd	0.0028	1.08E-06	1756	M32675.m	TRUE	reported	9FmPh9	2	TRUE	0.013065	23.2197
2223	rs4778640	A	G	A	G	0.04	0.055194	0.9704	0.966054	FALSE	FALSE	FALSE	ieu-b-508	15	81604131	0.092006	486484	0.548574	Sepsis (28	Sepsis (28	Sepsis (28	TRUE	igd	0.0083	1.39E-06	6678	M19368.m	TRUE	reported	QaKPad	2	TRUE	0.003466	23.21848
8236	rs151316	T	C	T	C	0.082	0.003576	0.183	0.198388	FALSE	FALSE	FALSE	ieu-b-508	11	2819620	0.04579	486484	0.937756	Sepsis (28	Sepsis (28	Sepsis (28	TRUE	igd	0.017	1.41E-06	932	M36468.m	TRUE	reported	vycAFr	2	TRUE	0.024356	23.21651
8138	rs1698451	A	G	A	G	0.202	-0.06156	0.9848	0.982134	FALSE	FALSE	FALSE	ieu-b-508	2	18420872	0.125837	486484	0.624714	Sepsis (28	Sepsis (28	Sepsis (28	TRUE	igd	0.0419	1.41E-06	1750	M36103.m	TRUE	reported	sjeaPC	2	TRUE	0.013107	23.2155
7742	rs6764615	C	G	C	G	0.0159	-0.00897	0.8298	0.838613	FALSE	TRUE	FALSE	ieu-b-508	3	1.75E+08	0.045609	486484	0.844123	Sepsis (28	Sepsis (28	Sepsis (28	TRUE	igd	0.0033	2.17E-06	7007	M35431.m	TRUE	reported	tLfE4l	2	TRUE	0.003302	23.20825
4171	rs1156411	A	G	A	G	0.0395	0.016734	0.9236	0.916566	FALSE	FALSE	FALSE	ieu-b-508	12	40507773	0.060187	486484	0.780986	Sepsis (28	Sepsis (28	Sepsis (28	TRUE	igd	0.0082	1.58E-06	6712	M32759.m	TRUE	reported	9eIHr7	2	TRUE	0.003445	23.19728
4016	rs1019890	A	G	A	G	-0.0342	-0.01251	0.295	0.29926	FALSE	FALSE	FALSE	ieu-b-508	2	71907425	0.036626	486484	0.732647	Sepsis (28	Sepsis (28	Sepsis (28	TRUE	igd	0.0071	1.61E-06	6000	M32735.m	TRUE	reported	QE2vKQ	2	TRUE	0.003852	23.19481
5781	rs1421106	T	G	T	G	-0.0236	0.060022	0.9228	0.923042	FALSE	FALSE	FALSE	ieu-b-508	12	14125734	0.063924	486484	0.347748	Sepsis (28	Sepsis (28	Sepsis (28	TRUE	igd	0.0049	1.79E-06	7269	M33510.m	TRUE	reported	WxGKnn	2	TRUE	0.003181	23.19062
6245	rs6074527	T	C	T	C	0.0419	-0.02363	0.7046	0.694152	FALSE	FALSE	FALSE	ieu-b-508	20	12929239	0.036179	486484	0.513599	Sepsis (28	Sepsis (28	Sepsis (28	TRUE	igd	0.0087	1.43E-06	6756	M33935.m	TRUE	reported	ELBTDc	2	TRUE	0.003421	23.18788
2896	rs1189197	A	G	A	G	-0.0183	-0.08674	0.3109	0.312081	FALSE	FALSE	FALSE	ieu-b-508	2	1.77E+08	0.036246	486484	0.016702	Sepsis (28	Sepsis (28	Sepsis (28	TRUE	igd	0.0038	1.35E-06	6830	M27722.m	TRUE	reported	rzVgUy	2	TRUE	0.003384	23.18504
3385	rs8088457	A	G	A	G	0.0496	0.035551	0.0323	0.029937	FALSE	FALSE	FALSE	ieu-b-508	18	13929778	0.098147	486484	0.717183	Sepsis (28	Sepsis (28	Sepsis (28	TRUE	igd	0.0103	1.59E-06	7355	M32418.m	TRUE	reported	y8cN4Y	2	TRUE	0.003143	23.18306
5298	rs3751035	A	G	A	G	0.0802	-0.03613	0.1812	0.199306	FALSE	FALSE	FALSE	ieu-b-508	11	1.31E+08	0.041797	486484	0.387349	Sepsis (28	Sepsis (28	Sepsis (28	TRUE	igd	0.0166	1.40E-06	292	M33353.m	TRUE	reported	ZIN4gk	2	TRUE	0.07402	23.18183
6578	rs807741	T	G	T	G	-0.0805	-0.02408	0.0918	0.085035	FALSE	FALSE	FALSE	ieu-b-508	22	19103298	0.059632	486484	0.686377	Sepsis (28	Sepsis (28	Sepsis (28	TRUE	igd	0.0167	1.38E-06	788	M34245.m	TRUE	reported	pRwBKr	2	TRUE	0.028643	23.17689
6309	rs1235119	A	T	A	T	-0.013	0.005707	0.8949	0.875258	FALSE	TRUE	FALSE	ieu-b-508	20	12826073	0.052016	486484	0.912637	Sepsis (28	Sepsis (28	Sepsis (28	TRUE	igd	0.0027	1.08E-06	7354	M33955.m	TRUE	reported	mhHFnT	2	TRUE	0.003142	23.17614
6547	rs1095669	T	C	T	C	0.013	-0.01796	0.4838	0.490025	FALSE	FALSE	FALSE	ieu-b-508	8	1.34E+08	0.034548	486484	0.603102	Sepsis (28	Sepsis (28	Sepsis (28	TRUE	igd	0.0027	1.43E-06	7347	M34244.m	TRUE	reported	JyF0Sp	2	TRUE	0.003145	23.17613
3089	rs6691915	A	G	A	G	0.013	0.029771	0.2814	0.289688	FALSE	FALSE	FALSE	ieu-b-508	1	37716718	0.036964	486484	0.420581	Sepsis (28	Sepsis (28	Sepsis (28	TRUE	igd	0.0027	8.95E-07	7346	M32197.m	TRUE	reported	VZjwa9	2	TRUE	0.003146	23.17613
6980	rs4609066	T	C	T	C	0.013	-0.03155	0.5741	0.568577	FALSE	FALSE	FALSE	ieu-b-508	6	1.23E+08	0.033748	486484	0.349889	Sepsis (28	Sepsis (28	Sepsis (28	TRUE	igd	0.0027	1.60E-06	7344	M34407.m	TRUE	reported	X7I83Y	2	TRUE	0.003147	23.17613
3737	rs2416366	A	C	A	C	0.013	0.000425	0.4649	0.461764	FALSE	FALSE	FALSE	ieu-b-508	5	1.14E+08	0.033659	486484	0.989915	Sepsis (28	Sepsis (28	Sepsis (28	TRUE	igd	0.0027	1.17E-06	7337	M32632.m	TRUE	reported	plHzPT	2	TRUE	0.00315	23.17612
7867	rs2606621	A	G	A	G	-0.013	-0.02556	0.2991	0.289492	FALSE	FALSE	FALSE	ieu-b-508	6	1.22E+08	0.036894	486484	0.48847	Sepsis (28	Sepsis (28	Sepsis (28	TRUE	igd	0.0027	9.59E-07	7317	M35631.m	TRUE	reported	002wka	2	TRUE	0.003158	23.17611
2278	rs1172236	T	C	T	C	0.013	-0.00944	0.3602	0.347574	FALSE	FALSE	FALSE	ieu-b-508	4	14934107	0.035014	486484	0.78741	Sepsis (28	Sepsis (28	Sepsis (28	TRUE	igd	0.0027	1.03E-06	7301	M20675.m	TRUE	reported	CXe9rE	2	TRUE	0.003165	23.17609
7483	rs1095161	C	G	C	G	-0.013	-0.03518	0.3777	0.384899	FALSE	TRUE	FALSE	ieu-b-508	7	39851066	0.0344	486484	0.306399	Sepsis (28	Sepsis (28	Sepsis (28	TRUE	igd	0.0027	1.02E-06	7261	M35253.m	TRUE	reported	VboRmj	2	TRUE	0.003183	23.17606
2824	rs1124295	A	G	A	G	-0.013	-0.05309	0.5528	0.560065	FALSE	FALSE	FALSE	ieu-b-508	6	4515551	0.033629	486484	0.114385	Sepsis (28	Sepsis (28	Sepsis (28	TRUE	igd	0.0027	1.67E-06	7180	M27513.m	TRUE	reported	o9Ff2u	2	TRUE	0.003218	23.17598
2838	rs4324578	C	G	C	G	0.013	0.07692	0.6112	0.605783	FALSE	TRUE	FALSE	ieu-b-508	4	44363889	0.034263	486484	0.024768	Sepsis (28	Sepsis (28	Sepsis (28	TRUE	igd	0.0027	1.99E-06	7180	M27513.m	TRUE	reported	o9Ff2u	2	TRUE	0.003218	23.17598
5244	rs203846	T	C	T	C	-0.013	0.040309	0.6027	0.603101	FALSE	FALSE	FALSE	ieu-b-508	1	1.68E+08	0.034227	486484	0.23892	Sepsis (28	Sepsis (28	Sepsis (28	TRUE	igd	0.0027	1.63E-06	7063	M33228.m	TRUE	reported	JwOXbQ	2	TRUE	0.003271	23.17588
6596	rs1168101	A	G	A	G	-0.013	-0.06081	0.3403	0.358014	FALSE	FALSE	FALSE	ieu-b-508	2	1.35E+08	0.035128	486484	0.083457	Sepsis (28	Sepsis (28	Sepsis (28	TRUE	igd	0.0027	1.27E-06	6994	M34306.m	TRUE	reported	WPXPUN	2	TRUE	0.003304	23.17581
6601	rs1299655	C	G	C	G	-0.013	0.020285	0.7649	0.744053	FALSE	TRUE	FALSE	ieu-b-508	2	1.38E+08	0.038313	486484	0.596497	Sepsis (28	Sepsis (28	Sepsis (28	TRUE	igd	0.0027	2.15E-06	6994	M34306.m	TRUE	reported	WPXPUN	2	TRUE	0.003304	23.17581
2416	rs1456959	T	C	T	C	0.013	0.002432	0.3972	0.389449	FALSE	FALSE	FALSE	ieu-b-508	9	20196466	0.034402	486484	0.943649	Sepsis (28	Sepsis (28	Sepsis (28	TRUE	igd	0.0027	1.30E-06	6556	M21188.m	TRUE	reported	kvdnjc	2	TRUE	0.003524	23.17537
2654	rs1929842	C	G	C	G	0.013	0.046345	0.2049	0.221426	FALSE	TRUE	FALSE	ieu-b-508	9	1.08E+08	0.040243	486484	0.249474	Sepsis (28	Sepsis (28	Sepsis (28	TRUE	igd	0.0027	1.94E-06	6517	M22649.m	TRUE	reported	NAzwRF	2	TRUE	0.003545	23.17533
2726	rs1309556	T	C	T	C	0.013	0.064606	0.7452	0.747687	FALSE	FALSE	FALSE	ieu-b-508	3	1.26E+08	0.038522	486484	0.093519	Sepsis (28	Sepsis (28	Sepsis (28	TRUE	igd	0.0027	2.07E-06	6388	M25599.m	TRUE	reported	fRZxwP	2	TRUE	0.003616	23.17518
2506	rs1821685	T	C	T	C	0.013	-0.0031	0.3252	0.354864	FALSE	FALSE	FALSE	ieu-b-508	4	29311144	0.034901	486484	0.929309	Sepsis (28	Sepsis (28	Sepsis (28	TRUE	igd	0.0027	1.71E-06	5702	M22130.m	TRUE	reported	YDbJlz	2	TRUE	0.004049	23.17431
2285	rs1687134	T	C	T	C	0.0289	0.055918	0.7206	0.722755	FALSE	FALSE	FALSE	ieu-b-508	6	47100710	0.037645	486484	0.137438	Sepsis (28	Sepsis (28	Sepsis (28	TRUE	igd	0.006	1.58E-06	1747	M20675.m	TRUE	reported	CXe9rE	2	TRUE	0.013106	23.17372
3835	rs1003737	T	C	T	C	0.013	0.024537	0.5465	0.551035	FALSE	FALSE	FALSE	ieu-b-508	5	1.45E+08	0.033818	486484	0.468097	Sepsis (28	Sepsis (28	Sepsis (28	TRUE	igd	0.0027	1.77E-06	5211	M32651.m	TRUE	reported	DeuPwv	2	TRUE	0.004429	23.17354
2622	rs5768188	A	G	A	G	0.0467	-0.21417	0.9868	0.985395	FALSE	FALSE	FALSE	ieu-b-508	22	48328070	0.142127	486484	0.131845	Sepsis (28	Sepsis (28	Sepsis (28	TRUE	igd	0.0097	1.44E-06	7329	M22481.m	TRUE	reported	bXpf0k	2	TRUE	0.003153	23.17244
7199	rs9348403	T	C	T	C	-0.0207	0.042429	0.6666	0.67038	FALSE	FALSE	FALSE	ieu-b-508	6	19959784	0.035691	486484	0.234523	Sepsis (28	Sepsis (28	Sepsis (28	TRUE	igd	0.0043	1.61E-06	4800	M34539.m	TRUE	reported	puA8ni	2	TRUE	0.004805	23.16449
1976	rs6900835	T	C	T	C	-0.0284	-0.0042	0.2163	0.220472	FALSE	FALSE	FALSE	ieu-b-508	6	1.34E+08	0.040229	486484	0.916875	Sepsis (28	Sepsis (28	Sepsis (28	TRUE	igd	0.0059	1.44E-06	7226	M17807.m	TRUE	reported	zd6sZI	2	TRUE	0.003196	23.16394
6480	rs2255960	C	G	C	G	-0.931	-0.10846	0.01	0.020363	FALSE	TRUE	FALSE	ieu-b-508	10	1.2E+08	0.120127	486484	0.366593	Sepsis (28	Sepsis (28	Sepsis (28	TRUE	igd	0.192	1.24E-06	134	M34109.m	TRUE	reported	piJuC7	2	TRUE	0.149273	23.16147
5282	rs7238314	T	G	T	G	-0.0775	-0.16713	0.9792	0.980493	FALSE	FALSE	FALSE	ieu-b-508	18	68929159	0.119538	486484	0.162074	Sepsis (28	Sepsis (28	Sepsis (28	TRUE	igd	0.0161	1.49E-06	4409	M33250.m	TRUE	reported	WIZI6U	2	TRUE	0.005228	23.16086
3060	rs9824080	T	C	T	C	0.0438	-0.02787	0.1602	0.168163	FALSE	FALSE	FALSE	ieu-b-508	3	13297174	0.044742	486484	0.533293	Sepsis (28	Sepsis (28	Sepsis (28	TRUE	igd	0.0091	1.62E-06	7338	M31591.m	TRUE	reported	KltFxx	2	TRUE	0.003147	23.16045
2519	rs2243617	A	G	A	G	-0.0284	-0.03266	0.8747	0.864067	FALSE	FALSE	FALSE	ieu-b-508	10	17685727	0.049125	486484	0.506202	Sepsis (28	Sepsis (28	Sepsis (28	TRUE	igd	0.0059	1.62E-06	3950	M22138.m	TRUE	reported	UbKBSS	2	TRUE	0.005832	23.15862
697	rs7641634	A	G	A	G	-0.0077	-0.06732	0.266	0.277966	FALSE	FALSE	FALSE	ieu-b-508	3	1.44E+08	0.037179	486484	0.070171	Sepsis (28	Sepsis (28	Sepsis (28	TRUE	igd	0.0016	2.56E-06	7358	M01299.m	TRUE	reported	gfM0a3	2	TRUE	0.003138	23.15386
1716	rs3786284	A	G	A	G	0.0077	-0.08983	0.1481	0.135998	FALSE	FALSE	FALSE	ieu-b-508	18	12010646	0.048873	486484	0.066068	Sepsis (28	Sepsis (28	Sepsis (28	TRUE	igd	0.0016	2.04E-06	7310	M15506.m	TRUE	reported	mVRmZO	2	TRUE	0.003158	23.15382
1717	rs4242454	A	T	A	T	0.0077	0.017692	0.1377	0.140139	FALSE	TRUE	FALSE	ieu-b-508	8	50675489	0.048115	486484	0.713096	Sepsis (28	Sepsis (28	Sepsis (28	TRUE	igd	0.0016	3.25E-06	7310	M15506.m	TRUE	reported	mVRmZO	2	TRUE	0.003158	23.15382
4289	rs550214	A	G	A	G	-0.0154	-0.06388	0.2053	0.213764	FALSE	FALSE	FALSE	ieu-b-508	11	1.28E+08	0.040867	486484	0.118042	Sepsis (28	Sepsis (28	Sepsis (28	TRUE	igd	0.0032	1.78E-06	7063	M32814.m	TRUE	reported	z0OeTx	2	TRUE	0.003268	23.1536
5144	rs6426944	A	G	A	G	-0.0568	0.04489	0.2621	0.26855	FALSE	FALSE	FALSE	ieu-b-508	1	1.55E+08	0.037795	486484	0.234942	Sepsis (28	Sepsis (28	Sepsis (28	TRUE	igd	0.0118	1.55E-06	2616	M33190.m	TRUE	reported	UkhAw5	2	TRUE	0.008779	23.15264
4373	rs2714775	T	C	T	C	-0.1526	-0.17944	0.9695	0.962144	FALSE	FALSE	FALSE	ieu-b-508	15	25436505	0.087019	486484	0.039199	Sepsis (28	Sepsis (28	Sepsis (28	TRUE	igd	0.0317	1.52E-06	1988	M32863.m	TRUE	reported	cLOFYE	2	TRUE	0.011522	23.15013
5477	rs7512146	T	G	T	G	0.0332	0.07843	0.5108	0.507569	FALSE	FALSE	FALSE	ieu-b-508	1	34283008	0.033324	486484	0.018596	Sepsis (28	Sepsis (28	Sepsis (28	TRUE	igd	0.0069	1.38E-06	3890	M33408.m	TRUE	reported	tWZWIa	2	TRUE	0.005916	23.13954
5606	rs959046	C	G	C	G	0.0178	-0.02652	0.4	0.396603	FALSE	TRUE	FALSE	ieu-b-508	11	1.34E+08	0.034154	486484	0.437513	Sepsis (28	Sepsis (28	Sepsis (28	TRUE	igd	0.0037	1.12E-06	7365	M33441.m	TRUE	reported	CSdSpl	2	TRUE	0.003133	23.13762
3993	rs2237231	T	C	T	C	0.0178	0.110755	0.068	0.054579	FALSE	FALSE	FALSE	ieu-b-508	6	26057050	0.073543	486484	0.132066	Sepsis (28	Sepsis (28	Sepsis (28	TRUE	igd	0.0037	1.85E-06	6497	M32729.m	TRUE	reported	XGRSN1	2	TRUE	0.00355	23.13678
4264	rs1017275	T	C	T	C	0.0433	-0.16488	0.0898	0.089705	FALSE	FALSE	FALSE	ieu-b-508	11	10627711	0.059377	486484	0.00549	Sepsis (28	Sepsis (28	Sepsis (28	TRUE	igd	0.009	1.50E-06	4311	M32802.m	TRUE	reported	m8vmvZ	2	TRUE	0.005341	23.13605
586	rs1183742	A	G	A	G	0.0385	0.006324	0.088	0.085967	FALSE	FALSE	FALSE	ieu-b-508	12	1.25E+08	0.059725	486484	0.915676	Sepsis (28	Sepsis (28	Sepsis (28	TRUE	igd	0.008	1.52E-06	1766	M01110.m	TRUE	reported	0A31Sl	2	TRUE	0.012945	23.13393
5270	rs1121140	A	C	A	C	0.0279	0.020436	0.7964	0.791664	FALSE	FALSE	FALSE	ieu-b-508	1	47413786	0.041246	486484	0.620272	Sepsis (28	Sepsis (28	Sepsis (28	TRUE	igd	0.0058	1.45E-06	4409	M33250.m	TRUE	reported	WIZI6U	2	TRUE	0.005221	23.12892
4195	rs1117449	A	G	A	G	0.0433	-0.02069	0.3526	0.365142	FALSE	FALSE	FALSE	ieu-b-508	12	62858342	0.034682	486484	0.550772	Sepsis (28	Sepsis (28	Sepsis (28	TRUE	igd	0.009	1.42E-06	2537	M32762.m	TRUE	reported	Q5lKLH	2	TRUE	0.009041	23.12854
5447	rs1773598	T	C	T	C	-0.1949	0.119574	0.9801	0.984327	FALSE	FALSE	FALSE	ieu-b-508	8	95300879	0.13773	486484	0.385298	Sepsis (28	Sepsis (28	Sepsis (28	TRUE	igd	0.0405	1.51E-06	1464	M33390.m	TRUE	reported	t0EYdH	2	TRUE	0.015572	23.12703
2844	rs1051897	T	C	T	C	0.0505	-0.02161	0.11	0.120119	FALSE	FALSE	FALSE	ieu-b-508	15	58650355	0.051523	486484	0.674871	Sepsis (28	Sepsis (28	Sepsis (28	TRUE	igd	0.0105	1.35E-06	5673	M27531.m	TRUE	reported	v01zDr	2	TRUE	0.004061	23.12336
6675	rs898219	T	G	T	G	-0.0664	0.018128	0.6202	0.627375	FALSE	FALSE	FALSE	ieu-b-508	5	71240138	0.034535	486484	0.599643	Sepsis (28	Sepsis (28	Sepsis (28	TRUE	igd	0.0138	1.44E-06	1612	M34327.m	TRUE	reported	Il4Lrr	2	TRUE	0.014159	23.12271
5311	rs7799018	T	C	T	C	-0.0433	0.009135	0.427	0.435208	FALSE	FALSE	FALSE	ieu-b-508	7	1.45E+08	0.033693	486484	0.786301	Sepsis (28	Sepsis (28	Sepsis (28	TRUE	igd	0.009	1.62E-06	1863	M33359.m	TRUE	reported	dfJmmS	2	TRUE	0.012272	23.12194
8347	rs1104436	C	G	C	G	0.0982	0.11748	0.9662	0.961071	FALSE	TRUE	FALSE	ieu-b-508	12	19086146	0.087957	486484	0.181663	Sepsis (28	Sepsis (28	Sepsis (28	TRUE	igd	0.0204	1.41E-06	922	M36738.m	TRUE	reported	mVQQcD	2	TRUE	0.024516	23.12169
5715	rs3020376	A	G	A	G	0.0544	0.058138	0.7234	0.740932	FALSE	FALSE	FALSE	ieu-b-508	6	1.52E+08	0.03875	486484	0.133528	Sepsis (28	Sepsis (28	Sepsis (28	TRUE	igd	0.0113	1.33E-06	834	M33488.m	TRUE	reported	mZmDY6	2	TRUE	0.027038	23.12055
5164	rs1683963	A	G	A	G	0.0957	-0.0733	0.9359	0.928494	FALSE	FALSE	FALSE	ieu-b-508	1	2.4E+08	0.064864	486484	0.25847	Sepsis (28	Sepsis (28	Sepsis (28	TRUE	igd	0.0199	1.51E-06	5064	M33194.m	TRUE	reported	xRUveq	2	TRUE	0.004546	23.11778
1089	rs233340	A	G	A	G	-0.0125	-0.08798	0.0707	0.072744	FALSE	FALSE	FALSE	ieu-b-508	5	13592425	0.064184	486484	0.170472	Sepsis (28	Sepsis (28	Sepsis (28	TRUE	igd	0.0026	2.54E-06	7371	M01604.m	TRUE	reported	hE3I2k	2	TRUE	0.003126	23.10763
8602	rs1432288	T	G	T	G	0.0125	0.00295	0.1421	0.13885	FALSE	FALSE	FALSE	ieu-b-508	2	1.34E+08	0.048627	486484	0.951624	Sepsis (28	Sepsis (28	Sepsis (28	TRUE	igd	0.0026	1.18E-06	7366	M37506.m	TRUE	reported	m2kaRt	2	TRUE	0.003128	23.10763
3522	rs1126502	A	G	A	G	0.0125	0.006739	0.4733	0.462971	FALSE	FALSE	FALSE	ieu-b-508	1	1.59E+08	0.033395	486484	0.840078	Sepsis (28	Sepsis (28	Sepsis (28	TRUE	igd	0.0026	1.85E-06	7358	M32497.m	TRUE	reported	244bZN	2	TRUE	0.003131	23.10762
7979	rs1752812	A	G	A	G	-0.0125	0.022343	0.755	0.742849	FALSE	FALSE	FALSE	ieu-b-508	13	62868719	0.038352	486484	0.560178	Sepsis (28	Sepsis (28	Sepsis (28	TRUE	igd	0.0026	2.11E-06	7358	M35718.m	TRUE	reported	oUwc0a	2	TRUE	0.003131	23.10762
2318	rs6106307	T	C	T	C	0.0125	-0.01932	0.7398	0.734684	FALSE	FALSE	FALSE	ieu-b-508	20	2142755	0.037996	486484	0.611211	Sepsis (28	Sepsis (28	Sepsis (28	TRUE	igd	0.0026	8.85E-07	7327	M20699.m	TRUE	reported	Z3EKFa	2	TRUE	0.003145	23.1076
4042	rs1105470	A	G	A	G	-0.0125	0.059018	0.1397	0.151055	FALSE	FALSE	FALSE	ieu-b-508	12	12299769	0.046813	486484	0.207411	Sepsis (28	Sepsis (28	Sepsis (28	TRUE	igd	0.0026	1.32E-06	7318	M32740.m	TRUE	reported	YLshfP	2	TRUE	0.003149	23.10759
7879	rs9854668	A	C	A	C	0.0125	-0.05171	0.702	0.690944	FALSE	FALSE	FALSE	ieu-b-508	3	1.53E+08	0.036087	486484	0.151872	Sepsis (28	Sepsis (28	Sepsis (28	TRUE	igd	0.0026	2.52E-06	7317	M35631.m	TRUE	reported	002wka	2	TRUE	0.003149	23.10759
3941	rs9809318	T	C	T	C	0.0125	-0.05949	0.7743	0.77694	FALSE	FALSE	FALSE	ieu-b-508	3	31713353	0.040277	486484	0.13969	Sepsis (28	Sepsis (28	Sepsis (28	TRUE	igd	0.0026	9.84E-07	7308	M32698.m	TRUE	reported	srqncz	2	TRUE	0.003153	23.10758
896	rs1342408	A	G	A	G	-0.0125	0.022077	0.1735	0.184515	FALSE	FALSE	FALSE	ieu-b-508	2	1.8E+08	0.043125	486484	0.608693	Sepsis (28	Sepsis (28	Sepsis (28	TRUE	igd	0.0026	1.80E-06	7307	M01493.m	TRUE	reported	eLLRt7	2	TRUE	0.003153	23.10758
2741	rs1048952	A	G	A	G	0.0125	-0.02693	0.0955	0.108434	FALSE	FALSE	FALSE	ieu-b-508	1	37164087	0.053929	486484	0.617497	Sepsis (28	Sepsis (28	Sepsis (28	TRUE	igd	0.0026	1.47E-06	7303	M27256.m	TRUE	reported	mJwzsi	2	TRUE	0.003155	23.10758
2437	rs1325526	T	C	T	C	-0.0125	0.071042	0.2518	0.243204	FALSE	FALSE	FALSE	ieu-b-508	8	79313373	0.038945	486484	0.068126	Sepsis (28	Sepsis (28	Sepsis (28	TRUE	igd	0.0026	1.96E-06	7284	M21630.m	TRUE	reported	7eaO4H	2	TRUE	0.003163	23.10756
5787	rs303031	A	G	A	G	-0.0125	-0.01057	0.5388	0.543967	FALSE	FALSE	FALSE	ieu-b-508	6	25036980	0.033792	486484	0.754343	Sepsis (28	Sepsis (28	Sepsis (28	TRUE	igd	0.0026	1.72E-06	7269	M33510.m	TRUE	reported	WxGKnn	2	TRUE	0.00317	23.10755
5791	rs6055874	T	C	T	C	0.0125	-0.04733	0.4882	0.487597	FALSE	FALSE	FALSE	ieu-b-508	20	144382	0.033538	486484	0.158127	Sepsis (28	Sepsis (28	Sepsis (28	TRUE	igd	0.0026	2.15E-06	7269	M33510.m	TRUE	reported	WxGKnn	2	TRUE	0.00317	23.10755
5793	rs7327971	A	G	A	G	-0.0125	0.061106	0.3546	0.372212	FALSE	FALSE	FALSE	ieu-b-508	13	1.02E+08	0.035429	486484	0.084573	Sepsis (28	Sepsis (28	Sepsis (28	TRUE	igd	0.0026	2.25E-06	7269	M33510.m	TRUE	reported	WxGKnn	2	TRUE	0.00317	23.10755
7778	rs7633111	T	C	T	C	-0.0125	-0.05995	0.5766	0.55449	FALSE	FALSE	FALSE	ieu-b-508	3	30840808	0.033755	486484	0.075739	Sepsis (28	Sepsis (28	Sepsis (28	TRUE	igd	0.0026	1.93E-06	7256	M35439.m	TRUE	reported	DnuAGZ	2	TRUE	0.003175	23.10753
933	rs2173615	T	G	T	G	0.0125	0.004022	0.3153	0.30454	FALSE	FALSE	FALSE	ieu-b-508	3	1.92E+08	0.036215	486484	0.911571	Sepsis (28	Sepsis (28	Sepsis (28	TRUE	igd	0.0026	1.69E-06	7160	M01508.m	TRUE	reported	go526k	2	TRUE	0.003218	23.10745

1518	rs1709456	A	T	A	T	-0.0125	0.088191	0.9357	0.935824	FALSE	TRUE	FALSE	ieu-b-508	1	74348796	0.06865	486484	0.198917	Sepsis (28	Sepsis (28	Sepsis (28	TRUE	igd	0.0026	1.88E-06	7081	M12067.m	TRUE	reported	O5x7RD	2	TRUE	0.003254	23.10738
2039	rs6910570	A	G	A	G	0.0125	0.017187	0.5538	0.57289	FALSE	FALSE	FALSE	ieu-b-508	6	1.06E+08	0.033781	486484	0.610908	Sepsis (28	Sepsis (28	Sepsis (28	TRUE	igd	0.0026	1.84E-06	6939	M18349.m	TRUE	reported	M5qPs8	2	TRUE	0.00332	23.10724
5327	rs310104	T	C	T	C	-0.0558	0.006626	0.0428	0.053585	FALSE	FALSE	FALSE	ieu-b-508	1	1.53E+08	0.075245	486484	0.929832	Sepsis (28	Sepsis (28	Sepsis (28	TRUE	igd	0.0116	1.52E-06	1338	M33364.m	TRUE	reported	9Zrr7Y	2	TRUE	0.017	23.10483
1922	rs6810964	A	G	A	G	0.0125	-0.00568	0.5495	0.527871	FALSE	FALSE	FALSE	ieu-b-508	4	1.66E+08	0.033385	486484	0.864797	Sepsis (28	Sepsis (28	Sepsis (28	TRUE	igd	0.0026	2.08E-06	4689	M16816.m	TRUE	reported	J1abbO	2	TRUE	0.004905	23.10405
8243	rs802524	T	C	T	C	0.0875	0.028739	0.9577	0.954122	FALSE	FALSE	FALSE	ieu-b-508	7	1.46E+08	0.080706	486484	0.72177	Sepsis (28	Sepsis (28	Sepsis (28	TRUE	igd	0.0182	1.47E-06	1908	M36468.m	TRUE	reported	vycAFr	2	TRUE	0.011969	23.08968
1663	rs1696775	T	C	T	C	0.0495	-0.04358	0.3037	0.297349	FALSE	FALSE	FALSE	ieu-b-508	16	64636513	0.036504	486484	0.232501	Sepsis (28	Sepsis (28	Sepsis (28	TRUE	igd	0.0103	1.43E-06	5606	M15335.m	TRUE	reported	a7RjFn	2	TRUE	0.004103	23.08772
4502	rs1749046	A	G	A	G	-0.0173	-0.09074	0.0364	0.039015	FALSE	FALSE	FALSE	ieu-b-508	7	1.04E+08	0.086873	486484	0.296225	Sepsis (28	Sepsis (28	Sepsis (28	TRUE	igd	0.0036	1.22E-06	7362	M33132.m	TRUE	reported	dTXJa8	2	TRUE	0.003127	23.08709
7845	rs7326215	T	C	T	C	0.0495	0.016684	0.8718	0.876758	FALSE	FALSE	FALSE	ieu-b-508	13	59965281	0.050827	486484	0.742716	Sepsis (28	Sepsis (28	Sepsis (28	TRUE	igd	0.0103	1.49E-06	5035	M35551.m	TRUE	reported	5Sxswl	2	TRUE	0.004566	23.08678
765	rs726900	T	G	T	G	0.0173	-0.06629	0.8497	0.844698	FALSE	FALSE	FALSE	ieu-b-508	9	76638581	0.045974	486484	0.149314	Sepsis (28	Sepsis (28	Sepsis (28	TRUE	igd	0.0036	1.59E-06	6946	M01303.m	TRUE	reported	3b8x0F	2	TRUE	0.003314	23.08671
6996	rs6466344	C	G	C	G	-0.0173	-0.01858	0.2679	0.269322	FALSE	TRUE	FALSE	ieu-b-508	7	1.1E+08	0.037888	486484	0.623798	Sepsis (28	Sepsis (28	Sepsis (28	TRUE	igd	0.0036	1.22E-06	6929	M34416.m	TRUE	reported	q9OtaZ	2	TRUE	0.003322	23.0867
8329	rs4490450	A	G	A	G	0.0346	0.050705	0.6949	0.687523	FALSE	FALSE	FALSE	ieu-b-508	4	85545644	0.035976	486484	0.158721	Sepsis (28	Sepsis (28	Sepsis (28	TRUE	igd	0.0072	1.47E-06	3977	M36589.m	TRUE	reported	27GADo	2	TRUE	0.005773	23.08175
3854	rs6898211	A	C	A	C	0.037	0.004966	0.0348	0.037738	FALSE	FALSE	FALSE	ieu-b-508	5	1.41E+08	0.087118	486484	0.954543	Sepsis (28	Sepsis (28	Sepsis (28	TRUE	igd	0.0077	1.62E-06	5211	M32651.m	TRUE	reported	DeuPwv	2	TRUE	0.004411	23.08104
6051	rs1206213	T	C	T	C	-0.0639	0.187475	0.9849	0.980161	FALSE	FALSE	FALSE	ieu-b-508	1	55906839	0.119862	486484	0.117796	Sepsis (28	Sepsis (28	Sepsis (28	TRUE	igd	0.0133	1.49E-06	7362	M33821.m	TRUE	reported	eVYICL	2	TRUE	0.003126	23.07706
2238	rs1178708	A	G	A	G	0.0221	0.029943	0.0475	0.055705	FALSE	FALSE	FALSE	ieu-b-508	8	49665502	0.072896	486484	0.681246	Sepsis (28	Sepsis (28	Sepsis (28	TRUE	igd	0.0046	1.63E-06	7354	M19934.m	TRUE	reported	HtcWcQ	2	TRUE	0.003129	23.07548
8648	rs3751196	A	G	A	G	0.0221	0.031746	0.0984	0.104603	FALSE	FALSE	FALSE	ieu-b-508	12	1.04E+08	0.054578	486484	0.560794	Sepsis (28	Sepsis (28	Sepsis (28	TRUE	igd	0.0046	1.99E-06	6942	M38768.m	TRUE	reported	a2b8mH	2	TRUE	0.003314	23.07511
2392	rs7133595	A	G	A	G	0.0519	-0.0023	0.3868	0.383755	FALSE	FALSE	FALSE	ieu-b-508	12	1.3E+08	0.034324	486484	0.946595	Sepsis (28	Sepsis (28	Sepsis (28	TRUE	igd	0.0108	1.66E-06	2339	M21151.m	TRUE	reported	LfwdLx	2	TRUE	0.009777	23.07362
3080	rs1710140	A	G	A	G	0.0245	0.054451	0.0774	0.043533	FALSE	FALSE	FALSE	ieu-b-508	14	1.04E+08	0.082532	486484	0.509409	Sepsis (28	Sepsis (28	Sepsis (28	TRUE	igd	0.0051	1.85E-06	7346	M32197.m	TRUE	reported	VZjwa9	2	TRUE	0.003132	23.07138
5907	rs1252105	A	C	A	C	-0.0197	0.039217	0.402	0.3816	FALSE	FALSE	FALSE	ieu-b-508	5	76426987	0.034244	486484	0.252108	Sepsis (28	Sepsis (28	Sepsis (28	TRUE	igd	0.0041	1.50E-06	2757	M33627.m	TRUE	reported	4RHaUX	2	TRUE	0.008304	23.07011
5572	rs2494014	A	G	A	G	-0.1788	0.07571	0.8251	0.827297	FALSE	FALSE	FALSE	ieu-b-508	1	1.12E+08	0.044201	486484	0.086738	Sepsis (28	Sepsis (28	Sepsis (28	TRUE	igd	0.0372	1.56E-06	1391	M33423.m	TRUE	reported	MoEnO7	2	TRUE	0.016337	23.06876
2132	rs806784	T	C	T	C	-0.0952	-0.03864	0.7156	0.714978	FALSE	FALSE	FALSE	ieu-b-508	12	32438189	0.036959	486484	0.295794	Sepsis (28	Sepsis (28	Sepsis (28	TRUE	igd	0.0198	1.48E-06	868	M18477.m	TRUE	reported	p0vcCU	2	TRUE	0.025942	23.06437
6211	rs1305484	A	T	A	T	0.0293	0.011476	0.8357	0.833654	FALSE	TRUE	FALSE	ieu-b-508	22	48304985	0.044932	486484	0.798404	Sepsis (28	Sepsis (28	Sepsis (28	TRUE	igd	0.0061	1.27E-06	4533	M33901.m	TRUE	reported	mQkhrb	2	TRUE	0.005064	23.06131
4467	rs1263030	T	C	T	C	0.0711	0.071049	0.0603	0.063362	FALSE	FALSE	FALSE	ieu-b-508	3	6924680	0.068441	486484	0.299218	Sepsis (28	Sepsis (28	Sepsis (28	TRUE	igd	0.0148	1.65E-06	1462	M33009.m	TRUE	reported	8RTEdM	2	TRUE	0.015541	23.04736
7611	rs1101192	T	C	T	C	-0.1477	0.143005	0.0435	0.037732	FALSE	FALSE	FALSE	ieu-b-508	10	18279756	0.087634	486484	0.102711	Sepsis (28	Sepsis (28	Sepsis (28	TRUE	igd	0.0307	1.52E-06	448	M35322.m	TRUE	reported	om3h9x	2	TRUE	0.049128	23.04311
1125	rs6826474	T	C	T	C	-0.1383	-0.03582	0.0393	0.028048	FALSE	FALSE	FALSE	ieu-b-508	4	96650120	0.101179	486484	0.723311	Sepsis (28	Sepsis (28	Sepsis (28	TRUE	igd	0.0288	1.56E-06	2063	M01640.m	TRUE	reported	o3qxzl	2	TRUE	0.011054	23.03765
468	rs1193345	A	G	A	G	-0.0216	2.40E-05	0.9357	0.934468	FALSE	FALSE	FALSE	ieu-b-508	1	17913714	0.067319	486484	0.999716	Sepsis (28	Sepsis (28	Sepsis (28	TRUE	igd	0.0045	1.43E-06	7365	M00527.m	TRUE	reported	UYN7vF	2	TRUE	0.003119	23.03374
3766	rs1992232	A	G	A	G	0.0216	0.020379	0.958	0.953078	FALSE	FALSE	FALSE	ieu-b-508	4	1.5E+08	0.078769	486484	0.795857	Sepsis (28	Sepsis (28	Sepsis (28	TRUE	igd	0.0045	1.41E-06	7362	M32634.m	TRUE	reported	7T2RUz	2	TRUE	0.00312	23.03374
3618	rs7714057	T	C	T	C	0.0456	-0.05113	0.0685	0.080125	FALSE	FALSE	FALSE	ieu-b-508	5	1.68E+08	0.0618	486484	0.408063	Sepsis (28	Sepsis (28	Sepsis (28	TRUE	igd	0.0095	1.75E-06	7361	M32553.m	TRUE	reported	vHSHqn	2	TRUE	0.00312	23.03374
1443	rs164372	T	C	T	C	0.012	-0.05658	0.8036	0.80088	FALSE	FALSE	FALSE	ieu-b-508	5	1.61E+08	0.041809	486484	0.175949	Sepsis (28	Sepsis (28	Sepsis (28	TRUE	igd	0.0025	2.64E-06	7357	M03141.m	TRUE	reported	y4mG6m	2	TRUE	0.003122	23.03374
3387	rs9888349	T	C	T	C	-0.0192	-0.02668	0.7742	0.7727	FALSE	FALSE	FALSE	ieu-b-508	12	2153818	0.039845	486484	0.503053	Sepsis (28	Sepsis (28	Sepsis (28	TRUE	igd	0.004	2.02E-06	7355	M32418.m	TRUE	reported	y8cN4Y	2	TRUE	0.003123	23.03373
2804	rs4949008	A	G	A	G	0.012	-0.05551	0.6154	0.613763	FALSE	FALSE	FALSE	ieu-b-508	10	42884169	0.034315	486484	0.105733	Sepsis (28	Sepsis (28	Sepsis (28	TRUE	igd	0.0025	2.48E-06	7347	M27278.m	TRUE	reported	kPgqm3	2	TRUE	0.003126	23.03373
4461	rs9315310	T	C	T	C	0.012	-0.08344	0.1535	0.216885	FALSE	FALSE	FALSE	ieu-b-508	13	35351431	0.040657	486484	0.040144	Sepsis (28	Sepsis (28	Sepsis (28	TRUE	igd	0.0025	1.84E-06	7344	M32910.m	TRUE	reported	2OCBKh	2	TRUE	0.003127	23.03373
3159	rs1087387	T	G	T	G	-0.0216	-0.00292	0.8609	0.860112	FALSE	FALSE	FALSE	ieu-b-508	1	76779877	0.047973	486484	0.951491	Sepsis (28	Sepsis (28	Sepsis (28	TRUE	igd	0.0045	1.40E-06	7340	M32328.m	TRUE	reported	YGsb6D	2	TRUE	0.003129	23.03372
1043	rs1250778	A	G	A	G	-0.012	-0.04463	0.2395	0.247653	FALSE	FALSE	FALSE	ieu-b-508	4	1.38E+08	0.038656	486484	0.248229	Sepsis (28	Sepsis (28	Sepsis (28	TRUE	igd	0.0025	1.78E-06	7333	M01572.m	TRUE	reported	EOUAWy	2	TRUE	0.003132	23.03372
2488	rs1094780	T	C	T	C	-0.012	-0.00065	0.8118	0.809992	FALSE	FALSE	FALSE	ieu-b-508	6	39323615	0.042608	486484	0.987842	Sepsis (28	Sepsis (28	Sepsis (28	TRUE	igd	0.0025	1.39E-06	7329	M22116.m	TRUE	reported	bjUU6r	2	TRUE	0.003134	23.03371
3938	rs6996022	C	G	C	G	-0.012	0.09983	0.1711	0.169658	FALSE	TRUE	FALSE	ieu-b-508	8	15755815	0.044541	486484	0.025005	Sepsis (28	Sepsis (28	Sepsis (28	TRUE	igd	0.0025	2.35E-06	7308	M32698.m	TRUE	reported	srqncz	2	TRUE	0.003143	23.03369
532	rs1121626	T	C	T	C	0.0168	0.017368	0.6435	0.655398	FALSE	FALSE	FALSE	ieu-b-508	11	1.17E+08	0.035137	486484	0.621092	Sepsis (28	Sepsis (28	Sepsis (28	TRUE	igd	0.0035	1.56E-06	7237	M00599.m	TRUE	reported	nRJY3s	2	TRUE	0.003174	23.03363
542	rs7941871	T	G	T	G	-0.024	-0.05635	0.8353	0.826955	FALSE	FALSE	FALSE	ieu-b-508	11	13228063	0.044138	486484	0.201735	Sepsis (28	Sepsis (28	Sepsis (28	TRUE	igd	0.005	1.56E-06	7237	M00599.m	TRUE	reported	nRJY3s	2	TRUE	0.003174	23.03363
8266	rs4684437	T	C	T	C	-0.012	-0.01334	0.158	0.155491	FALSE	FALSE	FALSE	ieu-b-508	3	4722784	0.046102	486484	0.772284	Sepsis (28	Sepsis (28	Sepsis (28	TRUE	igd	0.0025	1.34E-06	7092	M36552.m	TRUE	reported	5CxMJz	2	TRUE	0.003238	23.0335
5836	rs7922903	T	G	T	G	-0.0168	0.009863	0.0945	0.093034	FALSE	FALSE	FALSE	ieu-b-508	10	98764694	0.057691	486484	0.864256	Sepsis (28	Sepsis (28	Sepsis (28	TRUE	igd	0.0035	2.10E-06	7066	M33515.m	TRUE	reported	X9mul1	2	TRUE	0.00325	23.03348
1402	rs5751902	T	C	T	C	0.012	-0.03885	0.3762	0.380665	FALSE	FALSE	FALSE	ieu-b-508	22	24996630	0.035796	486484	0.277781	Sepsis (28	Sepsis (28	Sepsis (28	TRUE	igd	0.0025	2.38E-06	7037	M02734.m	TRUE	reported	0y0dDD	2	TRUE	0.003263	23.03345
5485	rs1317415	T	G	T	G	-0.024	-0.05212	0.4549	0.458015	FALSE	FALSE	FALSE	ieu-b-508	5	1.26E+08	0.033509	486484	0.119854	Sepsis (28	Sepsis (28	Sepsis (28	TRUE	igd	0.005	1.82E-06	6797	M33415.m	TRUE	reported	xHSphe	2	TRUE	0.003378	23.03322
2431	rs7746531	T	C	T	C	0.0216	-0.01949	0.9206	0.919571	FALSE	FALSE	FALSE	ieu-b-508	6	44540935	0.061255	486484	0.750385	Sepsis (28	Sepsis (28	Sepsis (28	TRUE	igd	0.0045	2.00E-06	6556	M21188.m	TRUE	reported	kvdnjc	2	TRUE	0.003502	23.03297
3998	rs2828004	T	G	T	G	0.0216	-0.06182	0.9477	0.945571	FALSE	FALSE	FALSE	ieu-b-508	21	24646748	0.072952	486484	0.396803	Sepsis (28	Sepsis (28	Sepsis (28	TRUE	igd	0.0045	1.87E-06	6497	M32729.m	TRUE	reported	XGRSN1	2	TRUE	0.003534	23.03291
4250	rs9277015	A	G	A	G	0.024	0.002651	0.358	0.346857	FALSE	FALSE	FALSE	ieu-b-508	6	32993266	0.034931	486484	0.939494	Sepsis (28	Sepsis (28	Sepsis (28	TRUE	igd	0.005	1.59E-06	5371	M32795.m	TRUE	reported	0YBWkh	2	TRUE	0.004271	23.03142
6915	rs6803606	T	C	T	C	-0.0216	0.036528	0.7239	0.718809	FALSE	FALSE	FALSE	ieu-b-508	3	1.97E+08	0.0371	486484	0.32483	Sepsis (28	Sepsis (28	Sepsis (28	TRUE	igd	0.0045	2.06E-06	5195	M34389.m	TRUE	reported	suiRlN	2	TRUE	0.004415	23.03113
5278	rs3093198	A	G	A	G	0.024	0.043715	0.279	0.288233	FALSE	FALSE	FALSE	ieu-b-508	19	15990048	0.036929	486484	0.236506	Sepsis (28	Sepsis (28	Sepsis (28	TRUE	igd	0.005	1.59E-06	4227	M33250.m	TRUE	reported	WlZI6U	2	TRUE	0.005421	23.0291
3950	rs1275547	A	G	A	G	-0.0773	0.011816	0.031	0.042161	FALSE	FALSE	FALSE	ieu-b-508	1	38041482	0.083238	486484	0.887121	Sepsis (28	Sepsis (28	Sepsis (28	TRUE	igd	0.0161	1.59E-06	1766	M32709.m	TRUE	reported	YR8SB4	2	TRUE	0.012885	23.02582
7676	rs1712268	A	G	A	G	-0.0216	0.101381	0.1379	0.140935	FALSE	FALSE	FALSE	ieu-b-508	14	51080422	0.048021	486484	0.034758	Sepsis (28	Sepsis (28	Sepsis (28	TRUE	igd	0.0045	1.47E-06	1760	M35327.m	TRUE	reported	SquFiY	2	TRUE	0.012922	23.01382
571	rs1320934	A	T	A	T	0.1215	-0.04119	0.9599	0.938297	FALSE	TRUE	FALSE	ieu-b-508	3	61884650	0.069376	486484	0.552691	Sepsis (28	Sepsis (28	Sepsis (28	TRUE	igd	0.0253	1.51E-06	736	M01107.m	TRUE	reported	EbPV1D	2	TRUE	0.030383	23.0001
4028	rs591058	T	C	T	C	0.0283	-0.01072	0.5062	0.514694	FALSE	FALSE	FALSE	ieu-b-508	11	1.03E+08	0.033404	486484	0.748325	Sepsis (28	Sepsis (28	Sepsis (28	TRUE	igd	0.0059	1.74E-06	6000	M32735.m	TRUE	reported	QE2vKQ	2	TRUE	0.00382	22.9998
2016	rs6725791	T	C	T	C	0.0259	-0.02469	0.4058	0.412207	FALSE	FALSE	FALSE	ieu-b-508	2	69728152	0.033964	486484	0.467297	Sepsis (28	Sepsis (28	Sepsis (28	TRUE	igd	0.0054	1.81E-06	5984	M18283.m	TRUE	reported	oyZdTE	2	TRUE	0.00383	22.99677
7033	rs802424	C	G	C	G	-0.048	-0.09653	0.3093	0.305604	FALSE	TRUE	FALSE	ieu-b-508	7	86294694	0.036146	486484	0.007572	Sepsis (28	Sepsis (28	Sepsis (28	TRUE	igd	0.01	1.52E-06	997	M34441.m	TRUE	reported	UpgxQm	2	TRUE	0.022587	22.99378
7430	rs1216076	A	G	A	G	0.0681	-0.0307	0.9499	0.949174	FALSE	FALSE	FALSE	ieu-b-508	22	31794754	0.076284	486484	0.687312	Sepsis (28	Sepsis (28	Sepsis (28	TRUE	igd	0.0142	1.78E-06	5953	M35187.m	TRUE	reported	Zx7JfE	2	TRUE	0.003849	22.99173
3548	rs1201686	C	G	C	G	-0.0422	0.109905	0.0467	0.053295	FALSE	TRUE	FALSE	ieu-b-508	20	45610923	0.076584	486484	0.151258	Sepsis (28	Sepsis (28	Sepsis (28	TRUE	igd	0.0088	1.60E-06	7373	M32504.m	TRUE	reported	MN0yyR	2	TRUE	0.003109	22.99015
7953	rs1086964	A	G	A	G	-0.0211	-0.00693	0.4086	0.409526	FALSE	FALSE	FALSE	ieu-b-508	9	78426135	0.033956	486484	0.838232	Sepsis (28	Sepsis (28	Sepsis (28	TRUE	igd	0.0044	1.37E-06	6447	M35678.m	TRUE	reported	56yR01	2	TRUE	0.003554	22.98925
5937	rs1016651	A	G	A	G	-0.0211	-0.0801	0.6837	0.683177	FALSE	FALSE	FALSE	ieu-b-508	2	82035216	0.036004	486484	0.026104	Sepsis (28	Sepsis (28	Sepsis (28	TRUE	igd	0.0044	2.06E-06	6109	M33638.m	TRUE	reported	hVuSGd	2	TRUE	0.00375	22.98886
4218	rs4918797	T	C	T	C	0.0374	-0.03526	0.1997	0.199663	FALSE	FALSE	FALSE	ieu-b-508	10	96750251	0.041705	486484	0.397885	Sepsis (28	Sepsis (28	Sepsis (28	TRUE	igd	0.0078	1.56E-06	7330	M32786.m	TRUE	reported	rwF0co	2	TRUE	0.003127	22.98452
1560	rs1194903	A	G	A	G	0.0662	0.013125	0.1629	0.164485	FALSE	FALSE	FALSE	ieu-b-508	5	2370336	0.044956	486484	0.770325	Sepsis (28	Sepsis (28	Sepsis (28	TRUE	igd	0.0138	1.52E-06	1543	M12261.m	TRUE	reported	taUUcM	2	TRUE	0.014695	22.98235
6752	rs1017123	T	C	T	C	0.1402	0.175724	0.0634	0.055727	FALSE	FALSE	FALSE	ieu-b-508	2	72480536	0.072575	486484	0.015466	Sepsis (28	Sepsis (28	Sepsis (28	TRUE	igd	0.0292	1.51E-06	633	M34339.m	TRUE	reported	RwGZ7c	2	TRUE	0.035139	22.98031
2328	rs1178804	A	G	A	G	0.0163	0.013016	0.2425	0.252392	FALSE	FALSE	FALSE	ieu-b-508	9	81650381	0.038543	486484	0.735592	Sepsis (28	Sepsis (28	Sepsis (28	TRUE	igd	0.0034	1.94E-06	7366	M21044.m	TRUE	reported	Ym5rae	2	TRUE	0.003111	22.97732
6534	rs878013	C	G	C	G	0.0163	-0.06807	0.1689	0.177975	FALSE	TRUE	FALSE	ieu-b-508	16	6531618	0.043858	486484	0.120672	Sepsis (28	Sepsis (28	Sepsis (28	TRUE	igd	0.0034	1.97E-06	7049	M34123.m	TRUE	reported	9O5wo1	2	TRUE	0.00325	22.97704
4120	rs1860538	T	G	T	G	0.0163	0.016842	0.5561	0.549815	FALSE	FALSE	FALSE	ieu-b-508	7	77969293	0.033629	486484	0.616508	Sepsis (28	Sepsis (28	Sepsis (28	TRUE	igd	0.0034	1.67E-06	6658	M32755.m	TRUE	reported	WDrNdi	2	TRUE	0.00344	22.97666
7096	rs1252111	T	G	T	G	0.0374	-0.00971	0.1521	0.152272	FALSE	FALSE	FALSE	ieu-b-508	5	66936418	0.046548	486484	0.834751	Sepsis (28	Sepsis (28	Sepsis (28	TRUE	igd	0.0078	1.46E-06	3239	M34513.m	TRUE	reported	1hoso6	2	TRUE	0.007048	22.9766
8275	rs1765184	A	G	A	G	0.0441	-0.03383	0.031	0.032874	FALSE	FALSE	FALSE	ieu-b-508	15	52882872	0.093746	486484	0.71817	Sepsis (28	Sepsis (28	Sepsis (28	TRUE	igd	0.0092	1.52E-06	6464	M36553.m	TRUE	reported	2Rk7nd	2	TRUE	0.003542	22.97032
2555	rs2434210	A	T	A	T	-0.0139	-0.06952	0.8346	0.82668	FALSE	TRUE	FALSE	ieu-b-508	16	10299190	0.044111	486484	0.115002	Sepsis (28	Sepsis (28	Sepsis (28	TRUE	igd	0.0029	1.30E-06	6535	M22177.m	TRUE	reported	2s6Sly	2	TRUE	0.003503	22.96681
2573	rs6441104	A	G	A	G	0.0139	0.015834	0.1267	0.122513	FALSE	FALSE	FALSE	ieu-b-508	3	1.57E+08	0.050995	486484	0.756187	Sepsis (28	Sepsis (28	Sepsis (28	TRUE	igd	0.0029	1.73E-06	6535	M22177.m	TRUE	reported	2s6Sly	2	TRUE	0.003503	22.96681
8100	rs1687071	A	C	A	C	-0.5095	0.02583	0.0674	0.061052	FALSE	FALSE	FALSE	ieu-b-508	6	93806878	0.06976	486484	0.711181	Sepsis (28	Sepsis (28	Sepsis (28	TRUE	igd	0.1061	1.55E-06	488	M36097.m	TRUE	reported	RHr18S	2	TRUE	0.045122	22.9654
3044	rs1695865	T	C	T	C	0.0647	-0.10649	0.9423	0.942194	FALSE	FALSE	FALSE	ieu-b-508	16	11873158	0.071478	486484	0.136257	Sepsis (28	Sepsis (28	Sepsis (28	TRUE	igd	0.0135	1.68E-06	7338	M31591.m	TRUE	reported	KltFxx	2	TRUE	0.00312	22.96268
5290	rs1254491	C	G	C	G	-0.1079	-0.00711	0.0372	0.052975	FALSE	TRUE	FALSE	ieu-b-508	8	13475151	0.074883	486484	0.9244	Sepsis (28	Sepsis (28	Sepsis (28	TRUE	igd	0.0225	1.72E-06	1273	M33353.m	TRUE	reported	ZIN4gk	2	TRUE	0.017745	22.96122
5941	rs1165189	A	C	A	C	-0.0254	0.002493	0.7496	0.743675	FALSE	FALSE	FALSE	ieu-b-508	6	25849779	0.03824	486484	0.948011	Sepsis (28	Sepsis (28	Sepsis (28	TRUE	igd	0.0053	1.46E-06	6109	M33638.m	TRUE	reported	hVuSGd	2	TRUE	0.003746	22.96008
6598	rs1247510	A	C	A	C	-0.0369	-0.12889	0.0269	0.028183	FALSE	FALSE	FALSE	ieu-b-508	2	66882225	0.101503	486484	0.20417	Sepsis (28	Sepsis (28	Sepsis (28	TRUE	igd	0.0077	1.56E-06	6994	M34306.m	TRUE	reported	WPXPUN	2	TRUE	0.003273	22.95869
5178	rs2298126	A	C	A	C	-0.0484	-0.01776	0.3406	0.346166	FALSE	FALSE	FALSE	ieu-b-508	10	16558985	0.035229	486484	0.614161	Sepsis (28	Sepsis (28	Sepsis (28	TRUE	igd	0.0101	1.57E-06	4625	M33195.m	TRUE	reported	SI4STh	2	TRUE	0.004941	22.95409
3184	rs229914	A	G	A	G	-0.0115	0.03538	0.5846	0.566774	FALSE	FALSE	FALSE	ieu-b-508	6	1.34E+08	0.034004	486484	0.298125	Sepsis (28	Sepsis (28	Sepsis (28	TRUE	igd	0.0024	2.40E-06	7356	M32338.m	TRUE	reported	q5cuaP	2	TRUE	0.003112	22.95383
8263	rs2997247	T	C	T	C	0.0115	-0.02633	0.8295	0.839561	FALSE	FALSE	FALSE	ieu-b-508	10	1.23E+08	0.045463	486484	0.562529	Sepsis (28	Sepsis (28	Sepsis (28	TRUE	igd	0.0024	1.77E-06	7092	M36552.m	TRUE	reported	5CxMJz	2	TRUE	0.003227	22.95359
5971	rs1568846	A	G	A	G	-0.0115	0.021899	0.5288	0.528552	FALSE	FALSE	FALSE	ieu-b-508	7	14737999	0.033578	486484	0.514282	Sepsis (28	Sepsis (28	Sepsis (28	TRUE	igd	0.0024	1.74E-06	6279	M33666.m	TRUE	reported	dvWEtk	2	TRUE	0.003643	22.95276
5170	rs6574474	A	G	A	G	0.046	-0.0108	0.5481	0.544129	FALSE	FALSE	FALSE	ieu-b-508	14	20672016	0.033607	486484	0.747982	Sepsis (28	Sepsis (28	Sepsis (28	TRUE	igd	0.0096	1.52E-06	5064	M33194.m	TRUE	reported	xRUveq	2	TRUE	0.004514	22.951
4343	rs1099585	A	G	A	G	0.0321	-0.05543	0.8946	0.891404	FALSE	FALSE	FALSE	ieu-b-508	10	66026415	0.053557	486484	0.300646	Sepsis (28	Sepsis (28	Sepsis (28	TRUE	igd	0.0067	1.95E-06	7355	M32855.m	TRUE	reported	AM8kCf	2	TRUE	0.003111	22.94787
7624	rs905176	A	G	A	G	-0.0576	-0.06132	0.3698	0.359771	FALSE	FALSE	FALSE	ieu-b-508	5	18795048	0.03494	486484	0.079248	Sepsis (28	Sepsis (28	Sepsis (28	TRUE	igd	0.012	1.50E-06	448	M35322.m	TRUE	reported	om3h9x	2	TRUE	0.048913	22.93714
4607	rs1108271	A	G	A	G	0.3479	-0.10036	0.9531	0.934225	FALSE	FALSE	FALSE	ieu-b-508	18	46389383	0.067689	486484	0.138156	Sepsis (28	Sepsis (28	Sepsis (28	TRUE	igd	0.0726	1.68E-06	1574	M33173.m	TRUE	reported	04fkUQ	2	TRUE	0.014379	22.93419
3461	rs1333190	T	C	T	C	0.0091	0.082178	0.5895	0.590739	FALSE	FALSE	FALSE	ieu-b-508	1	3289729	0.034186	486484	0.016224	Sepsis (28	Sepsis (28	Sepsis (28	TRUE	igd	0.0019	8.90E-07	7363	M32489.m	TRUE	reported	hnljaD	2	TRUE	0.003106	22.93283
3869	rs1776847	T	C	T	C	-0.0273	0.019295	0.0454	0.060797	FALSE	FALSE	FALSE	ieu-b-508	5	1.29E+08	0.069688	486484	0.781878	Sepsis (28	Sepsis (28	Sepsis (28	TRUE	igd	0.0057	1.50E-06	7361	M32654.m	TRUE	reported	6lSQOP	2	TRUE	0.003107	22.93283
2254	rs2834059	A	G	A	G	-0.0091	-0.0238	0.4831	0.491617	FALSE	FALSE	FALSE	ieu-b-508	21	34375461	0.033785	486484	0.481136	Sepsis (28	Sepsis (28	Sepsis (28	TRUE	igd	0.0019	9.96E-07	7354	M19934.m	TRUE	reported	HtcWcQ	2	TRUE	0.00311	22.93282
3581	rs4814243	A	G	A	G	-0.0091	0.044788	0.8087	0.808367	FALSE	FALSE	FALSE	ieu-b-508	20	1409435	0.042565	486484	0.292696	Sepsis (28	Sepsis (28	Sepsis (28	TRUE	igd	0.0019	1.00E-06	7350	M32518.m	TRUE	reported	V0ZCiU	2	TRUE	0.003111	22.93282
1183	rs2304767	A	G	A	G	0.0091	-0.00312	0.3402	0.347635	FALSE	FALSE	FALSE	ieu-b-508	13	1.11E+08	0.035172	486484	0.929212	Sepsis (28	Sepsis (28	Sepsis (28	TRUE	igd	0.0019	1.22E-06	7346	M01645.m	TRUE	reported	d5nAn3	2	TRUE	0.003113	22.93281
3400	rs4470864	A	G	A	G	-0.0273	-0.00331	0.2175	0.220804	FALSE	FALSE	FALSE	ieu-b-508	6	1.34E+08	0.040216	486484	0.934404	Sepsis (28	Sepsis (28	Sepsis (28	TRUE	igd	0.0057	2.02E-06	7346	M32425.m	TRUE	reported	u5RLGY	2	TRUE	0.003113	22.93281
2615	rs2863821	A	G	A	G	-0.0091	-0.04684	0.4839	0.483188	FALSE	FALSE	FALSE	ieu-b-508	5	1.29E+08	0.033707	486484	0.164628	Sepsis (28	Sepsis (28	Sepsis (28	TRUE	igd	0.0019	1.41E-06	7329	M22481.m	TRUE	reported	bXpf0k	2	TRUE	0.00312	22.9328
2617	rs3213827	A	G	A	G	-0.0091	0.005157	0.5701	0.574664	FALSE	FALSE	FALSE	ieu-b-508	2	16137237	0.033728	486484	0.878468	Sepsis (28	Sepsis (28	Sepsis (28	TRUE	igd	0.0019	1.10E-06	7329	M22481.m	TRUE	reported	bXpf0k	2	TRUE	0.00312	22.9328
5390	rs1189802	T	C	T	C	0.0091	-0.0589	0.7118	0.713419	FALSE	FALSE	FALSE	ieu-b-508	2	1.25E+08	0.036991	486484	0.111315	Sepsis (28	Sepsis (28	Sepsis (28	TRUE	igd	0.0019	1.06E-06	7315	M33389.m	TRUE	reported	Hf6XVM	2	TRUE	0.003126	22.93279
5422	rs7311763	A	C	A	C	-0.0091	0.029269	0.3591	0.360949	FALSE	FALSE	FALSE	ieu-b-508	12	74296773	0.034673	486484	0.398587	Sepsis (28	Sepsis (28	Sepsis (28	TRUE	igd	0.0019	9.26E-07	7315	M33389.m	TRUE	reported	Hf6XVM	2	TRUE	0.003126	22.93279
5426	rs7760528	T	C	T	C	-0.0091	0.020976	0.68	0.679385	FALSE	FALSE	FALSE	ieu-b-508	6	20442354	0.036192	486484	0.562196	Sepsis (28	Sepsis (28	Sepsis (28	TRUE	igd	0.0019	9.17E-07	7315	M33389.m	TRUE	reported	Hf6XVM	2	TRUE	0.003126	22.93279
8000	rs165558	T	C	T	C	-0.0091	-0.03832	0.5657	0.565689	FALSE	FALSE	FALSE	ieu-b-508	20	12899896	0.033771	486484	0.256475	Sepsis (28	Sepsis (28	Sepsis (28	TRUE	igd	0.0019	2.00E-06	6604	M35754.m	TRUE	reported	lIXOo1	2	TRUE	0.003461	22.93211
8002	rs1722784	A	G	A	G	-0.0091	-0.03792	0.5093	0.495566	FALSE	FALSE	FALSE	ieu-b-508	1	1.51E+08	0.033388	486484	0.256126	Sepsis (28	Sepsis (28	Sepsis (28	TRUE	igd	0.0019	2.40E-06	6604	M35754.m	TRUE	reported	lIXOo1	2	TRUE	0.003461	22.93211
8023	rs7520333	A	G	A	G	-0.0091	0.000429	0.554	0.556576	FALSE	FALSE	FALSE	ieu-b-508	1	41090250	0.033657	486484	0.989825	Sepsis (28	Sepsis (28	Sepsis (28	TRUE	igd	0.0019	2.30E-06	6604	M35754.m	TRUE	reported	lIXOo1	2	TRUE	0.003461	22.93211
2534	rs1177690	A	G	A	G	-0.0091	-0.02515	0.6567	0.647935	FALSE	FALSE	FALSE	ieu-b-508	8	4170978	0.034882	486484	0.470847	Sepsis (28	Sepsis (28	Sepsis (28	TRUE	igd	0.0019	3.21E-06	6535	M22177.m	TRUE	reported	2s6Sly	2	TRUE	0.003498	22.93204
2577	rs6874723	T	C	T	C	0.0091	-0.01515	0.3661	0.364997	FALSE	FALSE	FALSE	ieu-b-508	5	1.75E+08	0.034848	486484	0.663748	Sepsis (28	Sepsis (28	Sepsis (28	TRUE	igd	0.0019	3.17E-06	6535	M22177.m	TRUE	reported	2s6Sly	2	TRUE	0.003498	22.93204
2587	rs892857	T	C	T	C	-0.0091	0.006071	0.6572	0.643722	FALSE	FALSE	FALSE	ieu-b-508	11	78707366	0.035193	486484	0.863032	Sepsis (28	Sepsis (28	Sepsis (28	TRUE	igd	0.0019	2.83E-06	6535	M22177.m	TRUE	reported	2s6Sly	2	TRUE	0.003498	22.93204
4004	rs6706970	T	C	T	C	-0.0091	-0.0175	0.5982	0.6082	FALSE	FALSE	FALSE	ieu-b-508	2	1.28E+08	0.034254	486484	0.60952	Sepsis (28	Sepsis (28	Sepsis (28	TRUE	igd	0.0019	1.25E-06	6497	M32729.m	TRUE	reported	XGRSN1	2	TRUE	0.003518	22.932
7270	rs4674025	T	C	T	C	-0.0091	-0.00306	0.4561	0.452513	FALSE	FALSE	FALSE	ieu-b-508	2	2.17E+08	0.033858	486484	0.927869	Sepsis (28	Sepsis (28	Sepsis (28	TRUE	igd	0.0019	2.03E-06	5885	M34912.m	TRUE	reported	W8lcT6	2	TRUE	0.003883	22.93126
7276	rs7022352	A	G	A	G	0.0091	-0.01636	0.5336	0.547817	FALSE	FALSE	FALSE	ieu-b-508	9	1.04E+08	0.033991	486484	0.630279	Sepsis (28	Sepsis (28	Sepsis (28	TRUE	igd	0.0019	1.48E-06	5885	M34912.m	TRUE	reported	W8lcT6	2	TRUE	0.003883	22.93126
7396	rs2688	T	G	T	G	0.0321	0.015272	0.6039	0.606529	FALSE	FALSE	FALSE	ieu-b-508	17	36046931	0.034198	486484	0.655185	Sepsis (28	Sepsis (28	Sepsis (28	TRUE	igd	0.0067	1.46E-06	1982	M35159.m	TRUE	reported	7EoJLB	2	TRUE	0.011449	22.93095
7338	rs1165700	T	G	T	G	-0.0091	-0.02008	0.7079	0.699585	FALSE	FALSE	FALSE	ieu-b-508	17	52454579	0.036395	486484	0.581085	Sepsis (28	Sepsis (28	Sepsis (28	TRUE	igd	0.0019	2.23E-06	4900	M35137.m	TRUE	reported	Eq2siS	2	TRUE	0.00466	22.9297
7357	rs3121910	T	G	T	G	-0.0091	0.020204	0.7221	0.714461	FALSE	FALSE	FALSE	ieu-b-508	1	2.32E+08	0.036888	486484	0.583895	Sepsis (28	Sepsis (28	Sepsis (28	TRUE	igd	0.0019	1.99E-06	4900	M35137.m	TRUE	reported	Eq2siS	2	TRUE	0.00466	22.9297
7363	rs4398300	A	G	A	G	0.0091	-0.01372	0.6235	0.622029	FALSE	FALSE	FALSE	ieu-b-508	2	79158022	0.034338	486484	0.68943	Sepsis (28	Sepsis (28	Sepsis (28	TRUE	igd	0.0019	1.84E-06	4900	M35137.m	TRUE	reported	Eq2siS	2	TRUE	0.00466	22.9297
7376	rs6871718	T	C	T	C	-0.0091	-0.01246	0.665	0.666504	FALSE	FALSE	FALSE	ieu-b-508	5	1.6E+08	0.035385	486484	0.72467	Sepsis (28	Sepsis (28	Sepsis (28	TRUE	igd	0.0019	1.75E-06	4900	M35137.m	TRUE	reported	Eq2siS	2	TRUE	0.00466	22.9297
6738	rs2390633	A	C	A	C	-0.2461	-0.00699	0.0402	0.045378	FALSE	FALSE	FALSE	ieu-b-508	7	22390445	0.079932	486484	0.930335	Sepsis (28	Sepsis (28	Sepsis (28	TRUE	igd	0.0513	1.58E-06	535	M34338.m	TRUE	reported	h4FB9a	2	TRUE	0.041242	22.92778
8134	rs1051174	A	T	A	T	0.068	0.065092	0.9606	0.956638	FALSE	TRUE	FALSE	ieu-b-508	9	24219684	0.082041	486484	0.427538	Sepsis (28	Sepsis (28	Sepsis (28	TRUE	igd	0.0142	1.73E-06	7311	M36103.m	TRUE	reported	sjeaPC	2	TRUE	0.003127	22.92568
6571	rs168190	T	C	T	C	0.0465	0.018956	0.4522	0.462654	FALSE	FALSE	FALSE	ieu-b-508	5	77936965	0.033885	486484	0.575881	Sepsis (28	Sepsis (28	Sepsis (28	TRUE	igd	0.0097	1.81E-06	788	M34245.m	TRUE	reported	pRwBKr	2	TRUE	0.028337	22.92233
4220	rs7772658	A	G	A	G	0.0565	0.005038	0.0782	0.0961	FALSE	FALSE	FALSE	ieu-b-508	6	1.28E+08	0.056584	486484	0.929059	Sepsis (28	Sepsis (28	Sepsis (28	TRUE	igd	0.0118	1.68E-06	7330	M32786.m	TRUE	reported	rwF0co	2	TRUE	0.003118	22.91999
7031	rs7074531	T	C	T	C	0.0249	0.004761	0.6964	0.704396	FALSE	FALSE	FALSE	ieu-b-508	10	1.32E+08	0.036642	486484	0.896622	Sepsis (28	Sepsis (28	Sepsis (28	TRUE	igd	0.0052	1.43E-06	4442	M34441.m	TRUE	reported	UpgxQm	2	TRUE	0.005135	22.91904
482	rs1780051	T	G	T	G	-0.1308	-0.07399	0.1085	0.100505	FALSE	FALSE	FALSE	ieu-b-508	19	51314692	0.055414	486484	0.181796	Sepsis (28	Sepsis (28	Sepsis (28	TRUE	igd	0.0273	1.58E-06	1233	M00553.m	TRUE	reported	IFjXiL	2	TRUE	0.018277	22.91845
770	rs1011200	A	G	A	G	0.0158	-0.00202	0.0782	0.077044	FALSE	FALSE	FALSE	ieu-b-508	8	34360493	0.062678	486484	0.974254	Sepsis (28	Sepsis (28	Sepsis (28	TRUE	igd	0.0033	1.73E-06	7352	M01336.m	TRUE	reported	FAlrya	2	TRUE	0.003108	22.91755
8211	rs1192458	A	G	A	G	0.034	-0.01025	0.6223	0.606006	FALSE	FALSE	FALSE	ieu-b-508	6	62672201	0.034076	486484	0.763551	Sepsis (28	Sepsis (28	Sepsis (28	TRUE	igd	0.0071	1.89E-06	2520	M36376.m	TRUE	reported	k6uC1l	2	TRUE	0.009018	22.91376
7285	rs1204948	T	G	T	G	0.0273	-0.01963	0.744	0.759402	FALSE	FALSE	FALSE	ieu-b-508	1	2.2E+08	0.038962	486484	0.614403	Sepsis (28	Sepsis (28	Sepsis (28	TRUE	igd	0.0057	1.81E-06	1657	M35114.m	TRUE	reported	a6NQjV	2	TRUE	0.013655	22.91137
6380	rs6544157	A	G	A	G	-0.0292	-0.01931	0.9013	0.895243	FALSE	FALSE	FALSE	ieu-b-508	2	38546918	0.055273	486484	0.72682	Sepsis (28	Sepsis (28	Sepsis (28	TRUE	igd	0.0061	2.01E-06	7322	M33968.m	TRUE	reported	sgVOvT	2	TRUE	0.00312	22.90801
4646	rs7071224	C	G	C	G	0.3778	0.073368	0.9005	0.8323	FALSE	TRUE	FALSE	ieu-b-508	10	98318820	0.044618	486484	0.100105	Sepsis (28	Sepsis (28	Sepsis (28	TRUE	igd	0.0779	1.22E-06	76	M33173.m	TRUE	reported	04fkUQ	2	TRUE	0.236339	22.90167
6735	rs1954396	A	G	A	G	-0.2752	0.08696	0.9704	0.96422	FALSE	FALSE	FALSE	ieu-b-508	14	43601836	0.089661	486484	0.332105	Sepsis (28	Sepsis (28	Sepsis (28	TRUE	igd	0.0574	1.65E-06	535	M34338.m	TRUE	reported	h4FB9a	2	TRUE	0.041195	22.90058
5267	rs7603154	A	G	A	G	0.0134	-0.03708	0.5252	0.5312	FALSE	FALSE	FALSE	ieu-b-508	2	1.07E+08	0.033428	486484	0.267298	Sepsis (28	Sepsis (28	Sepsis (28	TRUE	igd	0.0028	1.18E-06	7364	M33230.m	TRUE	reported	nzcTOn	2	TRUE	0.0031	22.89684
1684	rs874758	A	G	A	G	-0.0134	-0.03906	0.1855	0.180374	FALSE	FALSE	FALSE	ieu-b-508	10	88100089	0.043702	486484	0.371427	Sepsis (28	Sepsis (28	Sepsis (28	TRUE	igd	0.0028	1.43E-06	7337	M15365.m	TRUE	reported	TReTCM	2	TRUE	0.003112	22.89682
3684	rs4149000	T	C	T	C	0.0402	-0.05971	0.139	0.129189	FALSE	FALSE	FALSE	ieu-b-508	12	21447997	0.050613	486484	0.238143	Sepsis (28	Sepsis (28	Sepsis (28	TRUE	igd	0.0084	1.72E-06	7303	M32586.m	TRUE	reported	f6n0gX	2	TRUE	0.003126	22.89679
5299	rs4325604	T	C	T	C	0.0771	0.015279	0.0727	0.078004	FALSE	FALSE	FALSE	ieu-b-508	17	3119008	0.062147	486484	0.805801	Sepsis (28	Sepsis (28	Sepsis (28	TRUE	igd	0.0161	1.63E-06	1273	M33353.m	TRUE	reported	ZIN4gk	2	TRUE	0.017696	22.89677
7220	rs1419916	T	C	T	C	0.0201	-0.00788	0.3141	0.297675	FALSE	FALSE	FALSE	ieu-b-508	7	33507361	0.03644	486484	0.828842	Sepsis (28	Sepsis (28	Sepsis (28	TRUE	igd	0.0042	2.27E-06	6381	M34761.m	TRUE	reported	AMtp1w	2	TRUE	0.003576	22.89588
4324	rs7309696	T	C	T	C	0.0335	-0.05933	0.3022	0.339441	FALSE	FALSE	FALSE	ieu-b-508	12	20951307	0.035192	486484	0.091842	Sepsis (28	Sepsis (28	Sepsis (28	TRUE	igd	0.007	1.95E-06	6250	M32846.m	TRUE	reported	lwF2Yy	2	TRUE	0.003651	22.89573
2921	rs4901357	A	G	A	G	-0.0134	0.026583	0.3396	0.332157	FALSE	FALSE	FALSE	ieu-b-508	14	53632171	0.035405	486484	0.452762	Sepsis (28	Sepsis (28	Sepsis (28	TRUE	igd	0.0028	1.25E-06	5562	M27728.m	TRUE	reported	WOp1XE	2	TRUE	0.004101	22.89483
3910	rs1178091	C	G	C	G	0.1939	0.013842	0.9873	0.959252	FALSE	TRUE	FALSE	ieu-b-508	8	2381251	0.084383	486484	0.869706	Sepsis (28	Sepsis (28	Sepsis (28	TRUE	igd	0.0405	1.68E-06	1675	M32691.m	TRUE	reported	SiuD6F	2	TRUE	0.0135	22.89426
2889	rs1090012	A	G	A	G	-0.0383	-0.07316	0.7361	0.734744	FALSE	FALSE	FALSE	ieu-b-508	10	45365561	0.037822	486484	0.053085	Sepsis (28	Sepsis (28	Sepsis (28	TRUE	igd	0.008	1.74E-06	1768	M27718.m	TRUE	reported	PHL57z	2	TRUE	0.012798	22.89423
1996	rs1088687	T	C	T	C	-0.1704	-0.03202	0.9072	0.853497	FALSE	FALSE	FALSE	ieu-b-508	10	85425691	0.047617	486484	0.501366	Sepsis (28	Sepsis (28	Sepsis (28	TRUE	igd	0.0356	1.75E-06	2433	M18281.m	TRUE	reported	3mLp7z	2	TRUE	0.009329	22.89191
4629	rs1923325	T	C	T	C	0.5416	-0.02797	0.9478	0.938875	FALSE	FALSE	FALSE	ieu-b-508	10	1.08E+08	0.069603	486484	0.687817	Sepsis (28	Sepsis (28	Sepsis (28	TRUE	igd	0.1117	1.26E-06	76	M33173.m	TRUE	reported	04fkUQ	2	TRUE	0.236257	22.89123
5543	rs1011820	C	G	C	G	-0.7381	0.009191	0.0667	0.100583	FALSE	TRUE	FALSE	ieu-b-508	9	18825134	0.055792	486484	0.869145	Sepsis (28	Sepsis (28	Sepsis (28	TRUE	igd	0.1517	1.14E-06	60	M33423.m	TRUE	reported	MoEnO7	2	TRUE	0.282926	22.88421
5645	rs1043909	T	C	T	C	-0.0244	-0.00392	0.1912	0.207687	FALSE	FALSE	FALSE	ieu-b-508	1	2.3E+08	0.04131	486484	0.924486	Sepsis (28	Sepsis (28	Sepsis (28	TRUE	igd	0.0051	2.19E-06	5717	M33453.m	TRUE	reported	OzEeLQ	2	TRUE	0.003988	22.88165
5138	rs2822577	T	C	T	C	0.0512	0.006648	0.3412	0.323379	FALSE	FALSE	FALSE	ieu-b-508	21	15705554	0.035741	486484	0.852439	Sepsis (28	Sepsis (28	Sepsis (28	TRUE	igd	0.0107	1.56E-06	2616	M33190.m	TRUE	reported	UkhAw5	2	TRUE	0.008677	22.87917
7525	rs2490288	T	C	T	C	0.0177	-0.03464	0.3319	0.329849	FALSE	FALSE	FALSE	ieu-b-508	10	1.01E+08	0.035457	486484	0.328561	Sepsis (28	Sepsis (28	Sepsis (28	TRUE	igd	0.0037	1.54E-06	7291	M35255.m	TRUE	reported	ZDjmUf	2	TRUE	0.003129	22.87831
2686	rs5995601	A	G	A	G	0.0177	-0.01799	0.8544	0.831256	FALSE	FALSE	FALSE	ieu-b-508	22	22524871	0.044551	486484	0.686272	Sepsis (28	Sepsis (28	Sepsis (28	TRUE	igd	0.0037	1.70E-06	7256	M24074.m	TRUE	reported	Lk6D2A	2	TRUE	0.003144	22.87828
8042	rs6730977	A	G	A	G	0.0177	0.023941	0.6251	0.612379	FALSE	FALSE	FALSE	ieu-b-508	2	51181831	0.034356	486484	0.485905	Sepsis (28	Sepsis (28	Sepsis (28	TRUE	igd	0.0037	1.38E-06	6960	M35854.m	TRUE	reported	EujB0p	2	TRUE	0.003277	22.87801
3596	rs1084414	A	C	A	C	-0.0464	-0.0573	0.8691	0.878229	FALSE	FALSE	FALSE	ieu-b-508	12	32319818	0.051029	486484	0.261466	Sepsis (28	Sepsis (28	Sepsis (28	TRUE	igd	0.0097	1.84E-06	7256	M32549.m	TRUE	reported	W7Fgu2	2	TRUE	0.003144	22.87561
6296	rs2156436	T	C	T	C	0.1445	0.086858	0.0307	0.024616	FALSE	FALSE	FALSE	ieu-b-508	18	57457267	0.112431	486484	0.439792	Sepsis (28	Sepsis (28	Sepsis (28	TRUE	igd	0.0302	1.73E-06	1765	M33941.m	TRUE	reported	culqiU	2	TRUE	0.012805	22.86806
8523	rs1560661	T	C	T	C	0.1129	-0.01028	0.6472	0.574315	FALSE	FALSE	FALSE	ieu-b-508	5	1.5E+08	0.034708	486484	0.767141	Sepsis (28	Sepsis (28	Sepsis (28	TRUE	igd	0.0236	1.77E-06	2560	M37112.m	TRUE	reported	P3GiAg	2	TRUE	0.008861	22.8678
8555	rs6588480	A	G	A	G	-0.044	-0.02991	0.1493	0.151946	FALSE	FALSE	FALSE	ieu-b-508	1	53978119	0.046451	486484	0.519578	Sepsis (28	Sepsis (28	Sepsis (28	TRUE	igd	0.0092	1.82E-06	7355	M37202.m	TRUE	reported	BtslDx	2	TRUE	0.0031	22.86713
6077	rs7096907	T	C	T	C	-0.022	-0.03125	0.181	0.181586	FALSE	FALSE	FALSE	ieu-b-508	10	19026841	0.043484	486484	0.472411	Sepsis (28	Sepsis (28	Sepsis (28	TRUE	igd	0.0046	2.15E-06	6327	M33833.m	TRUE	reported	LMlPLH	2	TRUE	0.003602	22.86612
8204	rs1089991	T	C	T	C	0.0244	-0.01622	0.3837	0.404498	FALSE	FALSE	FALSE	ieu-b-508	10	44302862	0.033928	486484	0.632508	Sepsis (28	Sepsis (28	Sepsis (28	TRUE	igd	0.0051	2.07E-06	1732	M36300.m	TRUE	reported	ZjeC9m	2	TRUE	0.013043	22.86323
6091	rs1013631	A	G	A	G	-0.0378	-0.02434	0.5986	0.600781	FALSE	FALSE	FALSE	ieu-b-508	14	69063564	0.034099	486484	0.475328	Sepsis (28	Sepsis (28	Sepsis (28	TRUE	igd	0.0079	1.86E-06	1428	M33864.m	TRUE	reported	yr5h52	2	TRUE	0.01578	22.86234
8139	rs1712534	T	C	T	C	-0.0526	0.083844	0.0709	0.040426	FALSE	FALSE	FALSE	ieu-b-508	11	1.21E+08	0.084754	486484	0.322532	Sepsis (28	Sepsis (28	Sepsis (28	TRUE	igd	0.011	1.61E-06	7311	M36103.m	TRUE	reported	sjeaPC	2	TRUE	0.003118	22.85953
2110	rs1252615	A	C	A	C	0.4507	-0.00717	0.0139	0.028093	FALSE	FALSE	FALSE	ieu-b-508	6	38199488	0.101361	486484	0.94358	Sepsis (28	Sepsis (28	Sepsis (28	TRUE	igd	0.0939	1.61E-06	257	M18474.m	TRUE	reported	B6Xd02	2	TRUE	0.082267	22.85867
4627	rs1806077	A	G	A	G	-0.4253	0.086292	0.0205	0.034363	FALSE	FALSE	FALSE	ieu-b-508	5	9203274	0.095678	486484	0.367109	Sepsis (28	Sepsis (28	Sepsis (28	TRUE	igd	0.0889	1.73E-06	1574	M33173.m	TRUE	reported	04fkUQ	2	TRUE	0.014332	22.85783
1184	rs2423542	A	G	A	G	0.0153	0.007492	0.8036	0.739855	FALSE	FALSE	FALSE	ieu-b-508	20	11094509	0.042301	486484	0.859413	Sepsis (28	Sepsis (28	Sepsis (28	TRUE	igd	0.0032	1.91E-06	7346	M01645.m	TRUE	reported	d5nAn3	2	TRUE	0.003102	22.85413
4370	rs1770028	C	G	C	G	0.0617	0.061319	0.5903	0.598794	FALSE	TRUE	FALSE	ieu-b-508	15	36627882	0.034588	486484	0.076254	Sepsis (28	Sepsis (28	Sepsis (28	TRUE	igd	0.0129	1.77E-06	1988	M32863.m	TRUE	reported	cLOFYE	2	TRUE	0.011376	22.85356
959	rs1538330	T	G	T	G	-0.0306	-0.1054	0.0213	0.023436	FALSE	FALSE	FALSE	ieu-b-508	6	715684	0.110269	486484	0.339153	Sepsis (28	Sepsis (28	Sepsis (28	TRUE	igd	0.0064	1.73E-06	6523	M01558.m	TRUE	reported	AZK0p4	2	TRUE	0.003492	22.85334
5437	rs1101935	T	C	T	C	-0.0459	-0.08009	0.8389	0.837139	FALSE	FALSE	FALSE	ieu-b-508	11	90990885	0.045194	486484	0.076375	Sepsis (28	Sepsis (28	Sepsis (28	TRUE	igd	0.0096	1.77E-06	5185	M33390.m	TRUE	reported	t0EYdH	2	TRUE	0.00439	22.85153
5978	rs3124018	A	G	A	G	-0.0196	-0.02696	0.9208	0.917452	FALSE	FALSE	FALSE	ieu-b-508	9	1.08E+08	0.061028	486484	0.658611	Sepsis (28	Sepsis (28	Sepsis (28	TRUE	igd	0.0041	1.78E-06	6279	M33666.m	TRUE	reported	dvWEtk	2	TRUE	0.003626	22.84578
7178	rs1174382	T	C	T	C	0.0545	-0.03953	0.2041	0.220603	FALSE	FALSE	FALSE	ieu-b-508	5	1.68E+08	0.040479	486484	0.328771	Sepsis (28	Sepsis (28	Sepsis (28	TRUE	igd	0.0114	1.64E-06	4515	M34535.m	TRUE	reported	z9BiJF	2	TRUE	0.005037	22.84499
3259	rs1682600	T	C	T	C	-0.0239	-0.0187	0.9608	0.960345	FALSE	FALSE	FALSE	ieu-b-508	2	1.34E+08	0.085244	486484	0.826384	Sepsis (28	Sepsis (28	Sepsis (28	TRUE	igd	0.005	1.72E-06	7365	M32348.m	TRUE	reported	DJeLDx	2	TRUE	0.003093	22.8422
4141	rs1543433	A	G	A	G	0.0239	0.055733	0.366	0.36445	FALSE	FALSE	FALSE	ieu-b-508	20	20772930	0.034754	486484	0.108791	Sepsis (28	Sepsis (28	Sepsis (28	TRUE	igd	0.005	1.99E-06	7250	M32757.m	TRUE	reported	TJV9Xb	2	TRUE	0.003142	22.8421
4543	rs4363657	T	C	T	C	-0.0239	0.068014	0.8302	0.836244	FALSE	FALSE	FALSE	ieu-b-508	12	21368722	0.045162	486484	0.132067	Sepsis (28	Sepsis (28	Sepsis (28	TRUE	igd	0.005	1.48E-06	7166	M33138.m	TRUE	reported	IUpLAI	2	TRUE	0.003178	22.84202
4522	rs1263245	T	C	T	C	0.0459	0.023381	0.6341	0.623732	FALSE	FALSE	FALSE	ieu-b-508	3	28020552	0.034841	486484	0.502168	Sepsis (28	Sepsis (28	Sepsis (28	TRUE	igd	0.0096	1.93E-06	2391	M33137.m	TRUE	reported	ONVNk3	2	TRUE	0.00947	22.84123
7176	rs1107021	T	C	T	C	0.0435	0.013815	0.4394	0.440795	FALSE	FALSE	FALSE	ieu-b-508	15	39821867	0.033579	486484	0.680769	Sepsis (28	Sepsis (28	Sepsis (28	TRUE	igd	0.0091	1.55E-06	4515	M34535.m	TRUE	reported	z9BiJF	2	TRUE	0.005036	22.84038
2012	rs1392786	T	C	T	C	-0.0282	0.046052	0.4017	0.434235	FALSE	FALSE	FALSE	ieu-b-508	3	1.13E+08	0.033658	486484	0.171239	Sepsis (28	Sepsis (28	Sepsis (28	TRUE	igd	0.0059	1.93E-06	5984	M18283.m	TRUE	reported	oyZdTE	2	TRUE	0.003803	22.83752
8066	rs3789107	A	C	A	C	0.0636	-0.026	0.8931	0.884175	FALSE	FALSE	FALSE	ieu-b-508	2	1.12E+08	0.054542	486484	0.633598	Sepsis (28	Sepsis (28	Sepsis (28	TRUE	igd	0.0133	1.90E-06	1517	M35978.m	TRUE	reported	CN0wRR	2	TRUE	0.01485	22.83694
6695	rs1714408	A	T	A	T	-0.0282	0.08754	0.057	0.063095	FALSE	TRUE	FALSE	ieu-b-508	16	7597659	0.06868	486484	0.202448	Sepsis (28	Sepsis (28	Sepsis (28	TRUE	igd	0.0059	1.89E-06	5081	M34336.m	TRUE	reported	jrSSxL	2	TRUE	0.004476	22.83617
5440	rs1209223	T	G	T	G	0.1535	-0.07025	0.0315	0.029979	FALSE	FALSE	FALSE	ieu-b-508	1	14289509	0.097875	486484	0.472901	Sepsis (28	Sepsis (28	Sepsis (28	TRUE	igd	0.0321	1.69E-06	1464	M33390.m	TRUE	reported	t0EYdH	2	TRUE	0.015379	22.83563
5011	rs6670038	T	C	T	C	-0.2511	-0.11366	0.0235	0.024996	FALSE	FALSE	FALSE	ieu-b-508	1	1.03E+08	0.107304	486484	0.289483	Sepsis (28	Sepsis (28	Sepsis (28	TRUE	igd	0.0525	1.72E-06	1017	M33178.m	TRUE	reported	g22WMS	2	TRUE	0.021999	22.83074
1337	rs1264612	T	C	T	C	-0.0497	0.117813	0.0308	0.019073	FALSE	FALSE	FALSE	ieu-b-508	4	1.56E+08	0.121964	486484	0.33406	Sepsis (28	Sepsis (28	Sepsis (28	TRUE	igd	0.0104	1.80E-06	5791	M02342.m	TRUE	reported	EhZr3g	2	TRUE	0.003928	22.82948
1078	rs1084431	T	G	T	G	-0.0086	-0.05925	0.1637	0.141524	FALSE	FALSE	FALSE	ieu-b-508	12	32874640	0.04774	486484	0.214585	Sepsis (28	Sepsis (28	Sepsis (28	TRUE	igd	0.0018	1.12E-06	7371	M01604.m	TRUE	reported	hE3I2k	2	TRUE	0.003087	22.82097
589	rs1430209	T	C	T	C	-0.0086	0.001692	0.5313	0.523317	FALSE	FALSE	FALSE	ieu-b-508	2	2.22E+08	0.033358	486484	0.959548	Sepsis (28	Sepsis (28	Sepsis (28	TRUE	igd	0.0018	1.87E-06	7367	M01110.m	TRUE	reported	0A31Sl	2	TRUE	0.003089	22.82096
8619	rs534863	T	C	T	C	-0.0086	4.99E-05	0.3246	0.306619	FALSE	FALSE	FALSE	ieu-b-508	11	79109590	0.036135	486484	0.998899	Sepsis (28	Sepsis (28	Sepsis (28	TRUE	igd	0.0018	1.48E-06	7366	M37506.m	TRUE	reported	m2kaRt	2	TRUE	0.003089	22.82096
398	rs1001109	T	C	T	C	0.0086	0.014572	0.8159	0.811894	FALSE	FALSE	FALSE	ieu-b-508	4	1.61E+08	0.042712	486484	0.732987	Sepsis (28	Sepsis (28	Sepsis (28	TRUE	igd	0.0018	2.15E-06	7365	M00063.m	TRUE	reported	Hdsv1L	2	TRUE	0.00309	22.82096
428	rs3802195	T	G	T	G	0.0086	0.01829	0.1886	0.18295	FALSE	FALSE	FALSE	ieu-b-508	8	99140208	0.0434	486484	0.673449	Sepsis (28	Sepsis (28	Sepsis (28	TRUE	igd	0.0018	1.13E-06	7365	M00063.m	TRUE	reported	Hdsv1L	2	TRUE	0.00309	22.82096
1001	rs1262582	T	G	T	G	0.0086	-0.03029	0.3366	0.345432	FALSE	FALSE	FALSE	ieu-b-508	20	48844585	0.03512	486484	0.388444	Sepsis (28	Sepsis (28	Sepsis (28	TRUE	igd	0.0018	2.06E-06	7364	M01564.m	TRUE	reported	IPUdyD	2	TRUE	0.00309	22.82096
1014	rs2370136	T	C	T	C	-0.0086	0.034206	0.4875	0.466404	FALSE	FALSE	FALSE	ieu-b-508	19	57619241	0.033477	486484	0.306887	Sepsis (28	Sepsis (28	Sepsis (28	TRUE	igd	0.0018	1.25E-06	7364	M01564.m	TRUE	reported	IPUdyD	2	TRUE	0.00309	22.82096
707	rs1009541	A	G	A	G	0.0086	-0.06677	0.8619	0.852448	FALSE	FALSE	FALSE	ieu-b-508	8	20583511	0.046888	486484	0.154414	Sepsis (28	Sepsis (28	Sepsis (28	TRUE	igd	0.0018	2.29E-06	7363	M01301.m	TRUE	reported	w8TL71	2	TRUE	0.003091	22.82096
711	rs1091791	A	C	A	C	-0.0086	-0.01359	0.8597	0.860563	FALSE	FALSE	FALSE	ieu-b-508	1	1.64E+08	0.048158	486484	0.77785	Sepsis (28	Sepsis (28	Sepsis (28	TRUE	igd	0.0018	1.65E-06	7363	M01301.m	TRUE	reported	w8TL71	2	TRUE	0.003091	22.82096
731	rs9642758	A	G	A	G	0.0086	-0.09917	0.1578	0.151763	FALSE	FALSE	FALSE	ieu-b-508	8	35721159	0.047162	486484	0.035478	Sepsis (28	Sepsis (28	Sepsis (28	TRUE	igd	0.0018	1.28E-06	7363	M01301.m	TRUE	reported	w8TL71	2	TRUE	0.003091	22.82096
3484	rs8087127	A	G	A	G	0.0086	0.036959	0.5695	0.573277	FALSE	FALSE	FALSE	ieu-b-508	18	9680186	0.03374	486484	0.273334	Sepsis (28	Sepsis (28	Sepsis (28	TRUE	igd	0.0018	1.62E-06	7363	M32489.m	TRUE	reported	hnljaD	2	TRUE	0.003091	22.82096
3777	rs709189	A	G	A	G	-0.0086	-0.00652	0.7218	0.713828	FALSE	FALSE	FALSE	ieu-b-508	1	5748792	0.037032	486484	0.860291	Sepsis (28	Sepsis (28	Sepsis (28	TRUE	igd	0.0018	1.60E-06	7362	M32634.m	TRUE	reported	7T2RUz	2	TRUE	0.003091	22.82096
4501	rs1742383	A	G	A	G	0.0086	-0.00782	0.8836	0.882321	FALSE	FALSE	FALSE	ieu-b-508	3	1.11E+08	0.051697	486484	0.87979	Sepsis (28	Sepsis (28	Sepsis (28	TRUE	igd	0.0018	3.06E-06	7362	M33132.m	TRUE	reported	dTXJa8	2	TRUE	0.003091	22.82096
4508	rs2924762	T	C	T	C	0.0086	0.03643	0.8777	0.870635	FALSE	FALSE	FALSE	ieu-b-508	8	4923507	0.049642	486484	0.463034	Sepsis (28	Sepsis (28	Sepsis (28	TRUE	igd	0.0018	2.25E-06	7362	M33132.m	TRUE	reported	dTXJa8	2	TRUE	0.003091	22.82096
464	rs9312777	T	C	T	C	0.0043	-0.00568	0.4788	0.480363	FALSE	FALSE	FALSE	ieu-b-508	5	11556754	0.033644	486484	0.86602	Sepsis (28	Sepsis (28	Sepsis (28	TRUE	igd	9.00E-04	4.68E-06	7361	M00513.m	TRUE	reported	ohacnP	2	TRUE	0.003092	22.82096
12	rs1015795	A	G	A	G	0.0043	0.028324	0.5122	0.512713	FALSE	FALSE	FALSE	ieu-b-508	13	1.03E+08	0.033328	486484	0.395404	Sepsis (28	Sepsis (28	Sepsis (28	TRUE	igd	9.00E-04	2.26E-06	7355	M00054.m	TRUE	reported	QTDAnh	2	TRUE	0.003094	22.82095
16	rs1049826	T	G	T	G	0.0043	0.032879	0.4674	0.485478	FALSE	FALSE	FALSE	ieu-b-508	14	20691610	0.033403	486484	0.324968	Sepsis (28	Sepsis (28	Sepsis (28	TRUE	igd	9.00E-04	2.35E-06	7355	M00054.m	TRUE	reported	QTDAnh	2	TRUE	0.003094	22.82095
19	rs1080550	T	C	T	C	0.0043	0.003919	0.6272	0.622354	FALSE	FALSE	FALSE	ieu-b-508	5	57390158	0.034481	486484	0.909503	Sepsis (28	Sepsis (28	Sepsis (28	TRUE	igd	9.00E-04	2.00E-06	7355	M00054.m	TRUE	reported	QTDAnh	2	TRUE	0.003094	22.82095
20	rs1080732	A	T	A	T	0.0043	-0.02413	0.5866	0.591895	FALSE	TRUE	FALSE	ieu-b-508	6	45542617	0.033946	486484	0.477169	Sepsis (28	Sepsis (28	Sepsis (28	TRUE	igd	9.00E-04	1.97E-06	7355	M00054.m	TRUE	reported	QTDAnh	2	TRUE	0.003094	22.82095
28	rs1120103	A	T	A	T	0.0043	0.037577	0.6064	0.605256	FALSE	TRUE	FALSE	ieu-b-508	10	81745367	0.034252	486484	0.272611	Sepsis (28	Sepsis (28	Sepsis (28	TRUE	igd	9.00E-04	1.89E-06	7355	M00054.m	TRUE	reported	QTDAnh	2	TRUE	0.003094	22.82095
37	rs1227876	T	C	T	C	0.0043	-0.00749	0.5229	0.512784	FALSE	FALSE	FALSE	ieu-b-508	11	98781993	0.033476	486484	0.822977	Sepsis (28	Sepsis (28	Sepsis (28	TRUE	igd	9.00E-04	1.56E-06	7355	M00054.m	TRUE	reported	QTDAnh	2	TRUE	0.003094	22.82095
43	rs1305696	T	C	T	C	0.0043	0.012588	0.4376	0.418294	FALSE	FALSE	FALSE	ieu-b-508	22	33622667	0.033812	486484	0.709673	Sepsis (28	Sepsis (28	Sepsis (28	TRUE	igd	9.00E-04	5.58E-06	7355	M00054.m	TRUE	reported	QTDAnh	2	TRUE	0.003094	22.82095
69	rs207938	A	G	A	G	-0.0043	-0.03131	0.3963	0.40186	FALSE	FALSE	FALSE	ieu-b-508	2	2.17E+08	0.034079	486484	0.358238	Sepsis (28	Sepsis (28	Sepsis (28	TRUE	igd	9.00E-04	1.32E-06	7355	M00054.m	TRUE	reported	QTDAnh	2	TRUE	0.003094	22.82095
70	rs2091289	A	G	A	G	0.0043	0.042135	0.5943	0.595894	FALSE	FALSE	FALSE	ieu-b-508	11	1.12E+08	0.03393	486484	0.214313	Sepsis (28	Sepsis (28	Sepsis (28	TRUE	igd	9.00E-04	1.50E-06	7355	M00054.m	TRUE	reported	QTDAnh	2	TRUE	0.003094	22.82095
74	rs2216061	A	C	A	C	0.0043	-0.00274	0.512	0.509191	FALSE	FALSE	FALSE	ieu-b-508	16	52914547	0.03339	486484	0.934525	Sepsis (28	Sepsis (28	Sepsis (28	TRUE	igd	9.00E-04	2.21E-06	7355	M00054.m	TRUE	reported	QTDAnh	2	TRUE	0.003094	22.82095
77	rs2383131	T	C	T	C	-0.0043	0.018993	0.4403	0.439499	FALSE	FALSE	FALSE	ieu-b-508	9	19902822	0.033663	486484	0.572602	Sepsis (28	Sepsis (28	Sepsis (28	TRUE	igd	9.00E-04	3.05E-06	7355	M00054.m	TRUE	reported	QTDAnh	2	TRUE	0.003094	22.82095
78	rs2484178	T	C	T	C	0.0043	-0.01622	0.6063	0.605478	FALSE	FALSE	FALSE	ieu-b-508	10	27691799	0.034	486484	0.633414	Sepsis (28	Sepsis (28	Sepsis (28	TRUE	igd	9.00E-04	1.75E-06	7355	M00054.m	TRUE	reported	QTDAnh	2	TRUE	0.003094	22.82095
86	rs2688858	T	C	T	C	0.0043	0.034647	0.5742	0.582663	FALSE	FALSE	FALSE	ieu-b-508	10	14764993	0.034014	486484	0.308376	Sepsis (28	Sepsis (28	Sepsis (28	TRUE	igd	9.00E-04	2.84E-06	7355	M00054.m	TRUE	reported	QTDAnh	2	TRUE	0.003094	22.82095
87	rs2717586	A	G	A	G	0.0043	-0.01002	0.3962	0.399925	FALSE	FALSE	FALSE	ieu-b-508	8	1.44E+08	0.034274	486484	0.769956	Sepsis (28	Sepsis (28	Sepsis (28	TRUE	igd	9.00E-04	2.31E-06	7355	M00054.m	TRUE	reported	QTDAnh	2	TRUE	0.003094	22.82095
108	rs4063769	T	C	T	C	-0.0043	0.014259	0.5301	0.533444	FALSE	FALSE	FALSE	ieu-b-508	2	1.33E+08	0.033488	486484	0.670247	Sepsis (28	Sepsis (28	Sepsis (28	TRUE	igd	9.00E-04	1.30E-06	7355	M00054.m	TRUE	reported	QTDAnh	2	TRUE	0.003094	22.82095
141	rs6534667	C	G	C	G	-0.0043	0.036307	0.4186	0.408912	FALSE	TRUE	FALSE	ieu-b-508	4	1.29E+08	0.033867	486484	0.283693	Sepsis (28	Sepsis (28	Sepsis (28	TRUE	igd	9.00E-04	1.49E-06	7355	M00054.m	TRUE	reported	QTDAnh	2	TRUE	0.003094	22.82095
143	rs6586235	A	C	A	C	0.0043	0.022507	0.4598	0.463526	FALSE	FALSE	FALSE	ieu-b-508	21	43174436	0.033585	486484	0.502764	Sepsis (28	Sepsis (28	Sepsis (28	TRUE	igd	9.00E-04	1.62E-06	7355	M00054.m	TRUE	reported	QTDAnh	2	TRUE	0.003094	22.82095
148	rs6819555	A	G	A	G	-0.0043	0.021054	0.5862	0.586771	FALSE	FALSE	FALSE	ieu-b-508	4	27342170	0.03389	486484	0.534434	Sepsis (28	Sepsis (28	Sepsis (28	TRUE	igd	9.00E-04	3.69E-06	7355	M00054.m	TRUE	reported	QTDAnh	2	TRUE	0.003094	22.82095
161	rs7246393	A	G	A	G	0.0043	-0.03323	0.5222	0.517799	FALSE	FALSE	FALSE	ieu-b-508	19	23388805	0.033453	486484	0.320573	Sepsis (28	Sepsis (28	Sepsis (28	TRUE	igd	9.00E-04	1.55E-06	7355	M00054.m	TRUE	reported	QTDAnh	2	TRUE	0.003094	22.82095
163	rs7505177	T	G	T	G	-0.0043	-0.01582	0.4132	0.418399	FALSE	FALSE	FALSE	ieu-b-508	18	75220892	0.033818	486484	0.639981	Sepsis (28	Sepsis (28	Sepsis (28	TRUE	igd	9.00E-04	5.86E-06	7355	M00054.m	TRUE	reported	QTDAnh	2	TRUE	0.003094	22.82095
176	rs875283	A	G	A	G	-0.0043	0.046088	0.4896	0.490423	FALSE	FALSE	FALSE	ieu-b-508	19	51767485	0.03363	486484	0.17055	Sepsis (28	Sepsis (28	Sepsis (28	TRUE	igd	9.00E-04	2.34E-06	7355	M00054.m	TRUE	reported	QTDAnh	2	TRUE	0.003094	22.82095
188	rs9523030	T	G	T	G	0.0043	0.017315	0.6269	0.626803	FALSE	FALSE	FALSE	ieu-b-508	13	91262300	0.03529	486484	0.623676	Sepsis (28	Sepsis (28	Sepsis (28	TRUE	igd	9.00E-04	5.65E-06	7355	M00054.m	TRUE	reported	QTDAnh	2	TRUE	0.003094	22.82095
191	rs9610322	A	G	A	G	-0.0043	-0.0003	0.5195	0.528354	FALSE	FALSE	FALSE	ieu-b-508	22	35980173	0.033526	486484	0.992775	Sepsis (28	Sepsis (28	Sepsis (28	TRUE	igd	9.00E-04	1.12E-06	7355	M00054.m	TRUE	reported	QTDAnh	2	TRUE	0.003094	22.82095
1150	rs203591	T	C	T	C	-0.0086	0.051551	0.3697	0.364985	FALSE	FALSE	FALSE	ieu-b-508	14	27774944	0.03466	486484	0.136933	Sepsis (28	Sepsis (28	Sepsis (28	TRUE	igd	0.0018	2.01E-06	7355	M01644.m	TRUE	reported	IB0DTL	2	TRUE	0.003094	22.82095
3492	rs1247803	T	C	T	C	-0.0086	0.031173	0.6526	0.657424	FALSE	FALSE	FALSE	ieu-b-508	2	11248242	0.035214	486484	0.376029	Sepsis (28	Sepsis (28	Sepsis (28	TRUE	igd	0.0018	2.79E-06	7355	M32492.m	TRUE	reported	rZZFcE	2	TRUE	0.003094	22.82095
3502	rs2706095	A	G	A	G	-0.0086	-0.07649	0.3359	0.355974	FALSE	FALSE	FALSE	ieu-b-508	4	1.68E+08	0.034987	486484	0.028791	Sepsis (28	Sepsis (28	Sepsis (28	TRUE	igd	0.0018	2.38E-06	7355	M32492.m	TRUE	reported	rZZFcE	2	TRUE	0.003094	22.82095
553	rs163425	A	G	A	G	0.0086	-0.02005	0.8734	0.870653	FALSE	FALSE	FALSE	ieu-b-508	5	1.57E+08	0.049872	486484	0.6877	Sepsis (28	Sepsis (28	Sepsis (28	TRUE	igd	0.0018	2.62E-06	7354	M00606.m	TRUE	reported	r5smf7	2	TRUE	0.003094	22.82095
2246	rs1944518	T	C	T	C	-0.0086	0.008809	0.3068	0.324905	FALSE	FALSE	FALSE	ieu-b-508	11	1.21E+08	0.035717	486484	0.805188	Sepsis (28	Sepsis (28	Sepsis (28	TRUE	igd	0.0018	1.28E-06	7354	M19934.m	TRUE	reported	HtcWcQ	2	TRUE	0.003094	22.82095
6304	rs1081458	A	G	A	G	0.0086	-0.01093	0.3122	0.317622	FALSE	FALSE	FALSE	ieu-b-508	9	3772674	0.036271	486484	0.763203	Sepsis (28	Sepsis (28	Sepsis (28	TRUE	igd	0.0018	1.53E-06	7354	M33955.m	TRUE	reported	mhHFnT	2	TRUE	0.003094	22.82095
6328	rs7547153	A	G	A	G	-0.0086	-0.04828	0.5893	0.58799	FALSE	FALSE	FALSE	ieu-b-508	1	62130419	0.034139	486484	0.157294	Sepsis (28	Sepsis (28	Sepsis (28	TRUE	igd	0.0018	1.13E-06	7354	M33955.m	TRUE	reported	mhHFnT	2	TRUE	0.003094	22.82095
222	rs1095441	A	T	A	T	0.0043	0.061652	0.3772	0.359756	FALSE	TRUE	FALSE	ieu-b-508	7	1.33E+08	0.034767	486484	0.07618	Sepsis (28	Sepsis (28	Sepsis (28	TRUE	igd	9.00E-04	1.81E-06	7351	M00060.m	TRUE	reported	kqwl4V	2	TRUE	0.003096	22.82095
224	rs1100708	T	G	T	G	0.0043	0.015023	0.3779	0.379086	FALSE	FALSE	FALSE	ieu-b-508	10	19090565	0.034323	486484	0.661604	Sepsis (28	Sepsis (28	Sepsis (28	TRUE	igd	9.00E-04	1.84E-06	7351	M00060.m	TRUE	reported	kqwl4V	2	TRUE	0.003096	22.82095
227	rs1112474	A	G	A	G	0.0043	0.046738	0.6354	0.62961	FALSE	FALSE	FALSE	ieu-b-508	2	40725639	0.034704	486484	0.178057	Sepsis (28	Sepsis (28	Sepsis (28	TRUE	igd	9.00E-04	1.91E-06	7351	M00060.m	TRUE	reported	kqwl4V	2	TRUE	0.003096	22.82095
230	rs1120304	A	G	A	G	-0.0043	-0.04879	0.4458	0.447084	FALSE	FALSE	FALSE	ieu-b-508	10	90988886	0.033569	486484	0.146114	Sepsis (28	Sepsis (28	Sepsis (28	TRUE	igd	9.00E-04	1.95E-06	7351	M00060.m	TRUE	reported	kqwl4V	2	TRUE	0.003096	22.82095
260	rs1705832	T	C	T	C	0.0043	0.005016	0.395	0.39505	FALSE	FALSE	FALSE	ieu-b-508	3	3201432	0.034107	486484	0.883077	Sepsis (28	Sepsis (28	Sepsis (28	TRUE	igd	9.00E-04	1.90E-06	7351	M00060.m	TRUE	reported	kqwl4V	2	TRUE	0.003096	22.82095
270	rs1931001	T	G	T	G	-0.0043	0.013857	0.3503	0.345403	FALSE	FALSE	FALSE	ieu-b-508	6	1.32E+08	0.035128	486484	0.693221	Sepsis (28	Sepsis (28	Sepsis (28	TRUE	igd	9.00E-04	1.62E-06	7351	M00060.m	TRUE	reported	kqwl4V	2	TRUE	0.003096	22.82095
278	rs2273875	C	G	C	G	0.0043	0.076251	0.37	0.363352	FALSE	TRUE	FALSE	ieu-b-508	1	2.35E+08	0.034721	486484	0.028083	Sepsis (28	Sepsis (28	Sepsis (28	TRUE	igd	9.00E-04	1.82E-06	7351	M00060.m	TRUE	reported	kqwl4V	2	TRUE	0.003096	22.82095
294	rs2819322	T	C	T	C	-0.0043	-0.03988	0.4589	0.458099	FALSE	FALSE	FALSE	ieu-b-508	1	1.62E+08	0.03353	486484	0.234289	Sepsis (28	Sepsis (28	Sepsis (28	TRUE	igd	9.00E-04	1.79E-06	7351	M00060.m	TRUE	reported	kqwl4V	2	TRUE	0.003096	22.82095
326	rs4968791	A	G	A	G	-0.0043	0.00252	0.5344	0.53217	FALSE	FALSE	FALSE	ieu-b-508	17	67402795	0.033605	486484	0.94023	Sepsis (28	Sepsis (28	Sepsis (28	TRUE	igd	9.00E-04	1.48E-06	7351	M00060.m	TRUE	reported	kqwl4V	2	TRUE	0.003096	22.82095
330	rs6103894	T	G	T	G	-0.0043	-0.0343	0.5939	0.605357	FALSE	FALSE	FALSE	ieu-b-508	20	43480679	0.034131	486484	0.314929	Sepsis (28	Sepsis (28	Sepsis (28	TRUE	igd	9.00E-04	2.71E-06	7351	M00060.m	TRUE	reported	kqwl4V	2	TRUE	0.003096	22.82095
336	rs6518309	A	C	A	C	0.0043	-0.00809	0.6195	0.607629	FALSE	FALSE	FALSE	ieu-b-508	21	45047992	0.034204	486484	0.813009	Sepsis (28	Sepsis (28	Sepsis (28	TRUE	igd	9.00E-04	1.78E-06	7351	M00060.m	TRUE	reported	kqwl4V	2	TRUE	0.003096	22.82095
337	rs6562776	A	G	A	G	-0.0043	0.038892	0.6116	0.60265	FALSE	FALSE	FALSE	ieu-b-508	13	74196887	0.034158	486484	0.254874	Sepsis (28	Sepsis (28	Sepsis (28	TRUE	igd	9.00E-04	1.65E-06	7351	M00060.m	TRUE	reported	kqwl4V	2	TRUE	0.003096	22.82095
350	rs720293	A	G	A	G	0.0043	-0.01602	0.4312	0.423747	FALSE	FALSE	FALSE	ieu-b-508	11	43036313	0.033781	486484	0.635265	Sepsis (28	Sepsis (28	Sepsis (28	TRUE	igd	9.00E-04	2.53E-06	7351	M00060.m	TRUE	reported	kqwl4V	2	TRUE	0.003096	22.82095
352	rs7301176	T	C	T	C	0.0043	-0.02106	0.4603	0.453092	FALSE	FALSE	FALSE	ieu-b-508	12	48790991	0.033739	486484	0.532551	Sepsis (28	Sepsis (28	Sepsis (28	TRUE	igd	9.00E-04	1.86E-06	7351	M00060.m	TRUE	reported	kqwl4V	2	TRUE	0.003096	22.82095
371	rs7911876	T	C	T	C	0.0043	0.026758	0.4276	0.428668	FALSE	FALSE	FALSE	ieu-b-508	10	14494126	0.034497	486484	0.437951	Sepsis (28	Sepsis (28	Sepsis (28	TRUE	igd	9.00E-04	2.76E-06	7351	M00060.m	TRUE	reported	kqwl4V	2	TRUE	0.003096	22.82095
390	rs9369741	T	G	T	G	0.0043	0.050703	0.5822	0.581551	FALSE	FALSE	FALSE	ieu-b-508	6	47702934	0.033889	486484	0.134621	Sepsis (28	Sepsis (28	Sepsis (28	TRUE	igd	9.00E-04	2.42E-06	7351	M00060.m	TRUE	reported	kqwl4V	2	TRUE	0.003096	22.82095
392	rs979242	T	C	T	C	-0.0043	0.006786	0.3692	0.373345	FALSE	FALSE	FALSE	ieu-b-508	20	18201837	0.034575	486484	0.844397	Sepsis (28	Sepsis (28	Sepsis (28	TRUE	igd	9.00E-04	2.45E-06	7351	M00060.m	TRUE	reported	kqwl4V	2	TRUE	0.003096	22.82095
394	rs9837954	T	C	T	C	-0.0043	0.024547	0.6418	0.636952	FALSE	FALSE	FALSE	ieu-b-508	3	1.57E+08	0.034931	486484	0.482223	Sepsis (28	Sepsis (28	Sepsis (28	TRUE	igd	9.00E-04	2.64E-06	7351	M00060.m	TRUE	reported	kqwl4V	2	TRUE	0.003096	22.82095
3567	rs2053595	A	G	A	G	0.0086	0.024121	0.312	0.342847	FALSE	FALSE	FALSE	ieu-b-508	17	7029549	0.035205	486484	0.49324	Sepsis (28	Sepsis (28	Sepsis (28	TRUE	igd	0.0018	2.98E-06	7350	M32518.m	TRUE	reported	V0ZCiU	2	TRUE	0.003096	22.82095
7917	rs1307330	A	G	A	G	0.0086	-0.00484	0.5896	0.602437	FALSE	FALSE	FALSE	ieu-b-508	3	8384412	0.034149	486484	0.887194	Sepsis (28	Sepsis (28	Sepsis (28	TRUE	igd	0.0018	2.70E-06	7348	M35675.m	TRUE	reported	D7fdyZ	2	TRUE	0.003097	22.82095
7936	rs4935623	A	G	A	G	-0.0086	-0.00909	0.4782	0.481908	FALSE	FALSE	FALSE	ieu-b-508	10	52098379	0.033431	486484	0.785614	Sepsis (28	Sepsis (28	Sepsis (28	TRUE	igd	0.0018	2.28E-06	7348	M35675.m	TRUE	reported	D7fdyZ	2	TRUE	0.003097	22.82095

3228	rs890230	T	C	T	C	0.0086	0.010817	0.4592	0.452164	FALSE	FALSE	FALSE	ieu-b-508	8	6660502	0.033479	486484	0.746628	Sepsis (28	Sepsis (28	Sepsis (28	TRUE	igd	0.0018	1.06E-06	7344	M32339.m	TRUE	reported	SVp504	2	TRUE	0.003099	22.82094
4432	rs1981409	A	T	A	T	-0.0086	-0.02268	0.6036	0.601302	FALSE	TRUE	FALSE	ieu-b-508	11	21426436	0.035465	486484	0.522562	Sepsis (28	Sepsis (28	Sepsis (28	TRUE	igd	0.0018	9.93E-07	7344	M32910.m	TRUE	reported	2OCBKh	2	TRUE	0.003099	22.82094
3058	rs9676475	T	C	T	C	0.0387	0.007473	0.2123	0.19004	FALSE	FALSE	FALSE	ieu-b-508	19	9177777	0.042711	486484	0.861099	Sepsis (28	Sepsis (28	Sepsis (28	TRUE	igd	0.0081	1.72E-06	7338	M31591.m	TRUE	reported	KltFxx	2	TRUE	0.003101	22.82094
8249	rs2839579	A	G	A	G	0.0043	-0.02193	0.312	0.301576	FALSE	FALSE	FALSE	ieu-b-508	21	44155445	0.036643	486484	0.54944	Sepsis (28	Sepsis (28	Sepsis (28	TRUE	igd	9.00E-04	4.45E-06	7330	M36515.m	TRUE	reported	yZaYzr	2	TRUE	0.003105	22.82093
8253	rs8184882	T	C	T	C	0.0043	0.002556	0.6224	0.615242	FALSE	FALSE	FALSE	ieu-b-508	20	58611406	0.034196	486484	0.940407	Sepsis (28	Sepsis (28	Sepsis (28	TRUE	igd	9.00E-04	7.27E-06	7330	M36515.m	TRUE	reported	yZaYzr	2	TRUE	0.003105	22.82093
2621	rs4702556	T	C	T	C	0.0129	-0.04105	0.3237	0.446039	FALSE	FALSE	FALSE	ieu-b-508	5	8507041	0.033633	486484	0.22224	Sepsis (28	Sepsis (28	Sepsis (28	TRUE	igd	0.0027	1.99E-06	7329	M22481.m	TRUE	reported	bXpf0k	2	TRUE	0.003105	22.82093
1299	rs539904	A	G	A	G	-0.0086	0.040153	0.7056	0.700386	FALSE	FALSE	FALSE	ieu-b-508	18	22085351	0.036737	486484	0.274397	Sepsis (28	Sepsis (28	Sepsis (28	TRUE	igd	0.0018	1.51E-06	7325	M02132.m	TRUE	reported	uJFm2t	2	TRUE	0.003107	22.82093
1311	rs8021336	A	G	A	G	-0.0086	0.036402	0.313	0.319689	FALSE	FALSE	FALSE	ieu-b-508	14	32733465	0.035867	486484	0.310139	Sepsis (28	Sepsis (28	Sepsis (28	TRUE	igd	0.0018	9.89E-07	7325	M02132.m	TRUE	reported	uJFm2t	2	TRUE	0.003107	22.82093
5382	rs1008970	T	G	T	G	-0.0086	-0.00401	0.4652	0.461048	FALSE	FALSE	FALSE	ieu-b-508	8	21276398	0.033516	486484	0.904862	Sepsis (28	Sepsis (28	Sepsis (28	TRUE	igd	0.0018	3.15E-06	7315	M33389.m	TRUE	reported	Hf6XVM	2	TRUE	0.003111	22.82092
1842	rs2317834	T	G	T	G	0.0129	0.023894	0.9041	0.885435	FALSE	FALSE	FALSE	ieu-b-508	16	82321726	0.052591	486484	0.649591	Sepsis (28	Sepsis (28	Sepsis (28	TRUE	igd	0.0027	2.31E-06	7308	M15778.m	TRUE	reported	941o3I	2	TRUE	0.003114	22.82091
1844	rs310528	A	G	A	G	0.0086	0.030896	0.2233	0.228205	FALSE	FALSE	FALSE	ieu-b-508	5	82853209	0.039985	486484	0.439699	Sepsis (28	Sepsis (28	Sepsis (28	TRUE	igd	0.0018	2.59E-06	7308	M15778.m	TRUE	reported	941o3I	2	TRUE	0.003114	22.82091
1859	rs7513165	T	C	T	C	0.0086	0.055499	0.7526	0.751663	FALSE	FALSE	FALSE	ieu-b-508	1	2.04E+08	0.038813	486484	0.152747	Sepsis (28	Sepsis (28	Sepsis (28	TRUE	igd	0.0018	2.83E-06	7308	M15778.m	TRUE	reported	941o3I	2	TRUE	0.003114	22.82091
1866	rs969075	T	C	T	C	-0.0086	0.01264	0.3346	0.335177	FALSE	FALSE	FALSE	ieu-b-508	20	17792323	0.035423	486484	0.721215	Sepsis (28	Sepsis (28	Sepsis (28	TRUE	igd	0.0018	2.63E-06	7308	M15778.m	TRUE	reported	941o3I	2	TRUE	0.003114	22.82091
5667	rs1105484	A	G	A	G	-0.0086	-0.03023	0.3623	0.366155	FALSE	FALSE	FALSE	ieu-b-508	12	12515329	0.03475	486484	0.384291	Sepsis (28	Sepsis (28	Sepsis (28	TRUE	igd	0.0018	2.85E-06	7307	M33477.m	TRUE	reported	Aw8PBK	2	TRUE	0.003114	22.82091
5677	rs201252	T	C	T	C	0.0086	0.056211	0.5963	0.597853	FALSE	FALSE	FALSE	ieu-b-508	6	10011084	0.034094	486484	0.099209	Sepsis (28	Sepsis (28	Sepsis (28	TRUE	igd	0.0018	1.61E-06	7307	M33477.m	TRUE	reported	Aw8PBK	2	TRUE	0.003114	22.82091
5691	rs612149	A	G	A	G	0.0129	0.055516	0.1538	0.164236	FALSE	FALSE	FALSE	ieu-b-508	1	2.45E+08	0.045143	486484	0.21878	Sepsis (28	Sepsis (28	Sepsis (28	TRUE	igd	0.0027	2.04E-06	7307	M33477.m	TRUE	reported	Aw8PBK	2	TRUE	0.003114	22.82091
5521	rs2241217	T	C	T	C	0.0086	-0.02958	0.7557	0.741043	FALSE	FALSE	FALSE	ieu-b-508	16	49888469	0.038623	486484	0.443802	Sepsis (28	Sepsis (28	Sepsis (28	TRUE	igd	0.0018	2.73E-06	7306	M33422.m	TRUE	reported	PLgjrT	2	TRUE	0.003115	22.82091
2759	rs4832148	T	C	T	C	0.0086	-0.06514	0.8088	0.805246	FALSE	FALSE	FALSE	ieu-b-508	2	85434540	0.042943	486484	0.129267	Sepsis (28	Sepsis (28	Sepsis (28	TRUE	igd	0.0018	2.65E-06	7303	M27256.m	TRUE	reported	mJwzsi	2	TRUE	0.003116	22.82091
1060	rs1104542	A	G	A	G	0.0086	-0.00846	0.1266	0.131372	FALSE	FALSE	FALSE	ieu-b-508	12	20893980	0.049861	486484	0.865203	Sepsis (28	Sepsis (28	Sepsis (28	TRUE	igd	0.0018	1.83E-06	7271	M01585.m	TRUE	reported	M7QiSM	2	TRUE	0.00313	22.82088
3815	rs267583	A	G	A	G	0.0086	-0.00214	0.2882	0.293685	FALSE	FALSE	FALSE	ieu-b-508	9	82503489	0.036783	486484	0.953525	Sepsis (28	Sepsis (28	Sepsis (28	TRUE	igd	0.0018	2.88E-06	7229	M32644.m	TRUE	reported	JAa2WD	2	TRUE	0.003148	22.82085
1355	rs1760243	T	C	T	C	0.0172	-0.06897	0.079	0.068106	FALSE	FALSE	FALSE	ieu-b-508	12	47185518	0.066245	486484	0.297828	Sepsis (28	Sepsis (28	Sepsis (28	TRUE	igd	0.0036	1.57E-06	7226	M02730.m	TRUE	reported	XYa035	2	TRUE	0.003149	22.82084
7576	rs4886189	A	G	A	G	0.0086	0.006033	0.4854	0.503288	FALSE	FALSE	FALSE	ieu-b-508	13	60229350	0.033661	486484	0.857749	Sepsis (28	Sepsis (28	Sepsis (28	TRUE	igd	0.0018	1.77E-06	7217	M35270.m	TRUE	reported	Ct8V1n	2	TRUE	0.003153	22.82083
7587	rs9351602	T	C	T	C	-0.0086	0.014976	0.2627	0.272883	FALSE	FALSE	FALSE	ieu-b-508	6	67555226	0.037617	486484	0.690545	Sepsis (28	Sepsis (28	Sepsis (28	TRUE	igd	0.0018	1.36E-06	7217	M35270.m	TRUE	reported	Ct8V1n	2	TRUE	0.003153	22.82083
6421	rs7718044	T	C	T	C	0.0172	0.013205	0.4678	0.460891	FALSE	FALSE	FALSE	ieu-b-508	5	1.01E+08	0.033686	486484	0.695064	Sepsis (28	Sepsis (28	Sepsis (28	TRUE	igd	0.0036	2.30E-06	7132	M34040.m	TRUE	reported	sJBy3i	2	TRUE	0.00319	22.82076
5355	rs4748632	A	G	A	G	0.0086	-0.02949	0.8015	0.788892	FALSE	FALSE	FALSE	ieu-b-508	10	20316072	0.041078	486484	0.472882	Sepsis (28	Sepsis (28	Sepsis (28	TRUE	igd	0.0018	1.45E-06	7118	M33380.m	TRUE	reported	OHRfl5	2	TRUE	0.003197	22.82075
1523	rs196885	T	C	T	C	-0.0086	-0.01582	0.8501	0.845262	FALSE	FALSE	FALSE	ieu-b-508	8	24736887	0.046256	486484	0.732411	Sepsis (28	Sepsis (28	Sepsis (28	TRUE	igd	0.0018	1.22E-06	7081	M12067.m	TRUE	reported	O5x7RD	2	TRUE	0.003213	22.82071
5802	rs1097688	A	G	A	G	0.0086	-0.0343	0.6202	0.620204	FALSE	FALSE	FALSE	ieu-b-508	9	8257948	0.034702	486484	0.322887	Sepsis (28	Sepsis (28	Sepsis (28	TRUE	igd	0.0018	2.79E-06	7066	M33515.m	TRUE	reported	X9mul1	2	TRUE	0.00322	22.8207
5825	rs4766046	A	C	A	C	-0.0086	0.042605	0.5752	0.564554	FALSE	FALSE	FALSE	ieu-b-508	12	3222714	0.033728	486484	0.206518	Sepsis (28	Sepsis (28	Sepsis (28	TRUE	igd	0.0018	2.69E-06	7066	M33515.m	TRUE	reported	X9mul1	2	TRUE	0.00322	22.8207
5829	rs4890333	A	G	A	G	-0.0086	0.021338	0.5104	0.503935	FALSE	FALSE	FALSE	ieu-b-508	18	44134563	0.033351	486484	0.522302	Sepsis (28	Sepsis (28	Sepsis (28	TRUE	igd	0.0018	2.06E-06	7066	M33515.m	TRUE	reported	X9mul1	2	TRUE	0.00322	22.8207
5834	rs6764891	A	G	A	G	0.0086	0.005593	0.3475	0.354699	FALSE	FALSE	FALSE	ieu-b-508	3	1.53E+08	0.035083	486484	0.873344	Sepsis (28	Sepsis (28	Sepsis (28	TRUE	igd	0.0018	2.89E-06	7066	M33515.m	TRUE	reported	X9mul1	2	TRUE	0.00322	22.8207
1378	rs1242920	A	G	A	G	0.0086	-0.01514	0.3376	0.343571	FALSE	FALSE	FALSE	ieu-b-508	13	51446114	0.035125	486484	0.666443	Sepsis (28	Sepsis (28	Sepsis (28	TRUE	igd	0.0018	1.85E-06	7037	M02734.m	TRUE	reported	0y0dDD	2	TRUE	0.003233	22.82067
7730	rs1100625	T	C	T	C	0.0129	-0.00826	0.201	0.206746	FALSE	FALSE	FALSE	ieu-b-508	10	60514349	0.04135	486484	0.841584	Sepsis (28	Sepsis (28	Sepsis (28	TRUE	igd	0.0027	1.55E-06	7007	M35431.m	TRUE	reported	tLfE4l	2	TRUE	0.003247	22.82064
7670	rs1002567	T	G	T	G	-0.0086	0.037737	0.1997	0.197513	FALSE	FALSE	FALSE	ieu-b-508	4	1.19E+08	0.042223	486484	0.371451	Sepsis (28	Sepsis (28	Sepsis (28	TRUE	igd	0.0018	1.27E-06	6920	M35327.m	TRUE	reported	SquFiY	2	TRUE	0.003288	22.82056
7687	rs9787087	A	G	A	G	0.0086	0.04178	0.1893	0.189787	FALSE	FALSE	FALSE	ieu-b-508	1	84206260	0.042615	486484	0.326883	Sepsis (28	Sepsis (28	Sepsis (28	TRUE	igd	0.0018	1.47E-06	6920	M35327.m	TRUE	reported	SquFiY	2	TRUE	0.003288	22.82056
631	rs1722427	A	G	A	G	0.0951	0.127552	0.0778	0.097738	FALSE	FALSE	FALSE	ieu-b-508	2	1.82E+08	0.056398	486484	0.023719	Sepsis (28	Sepsis (28	Sepsis (28	TRUE	igd	0.0199	1.83E-06	2639	M01123.m	TRUE	reported	NDTvfD	2	TRUE	0.00858	22.82052
6804	rs1338182	C	G	C	G	0.0086	0.013189	0.6082	0.606574	FALSE	TRUE	FALSE	ieu-b-508	18	56878511	0.034204	486484	0.699787	Sepsis (28	Sepsis (28	Sepsis (28	TRUE	igd	0.0018	1.51E-06	6755	M34359.m	TRUE	reported	0UotaJ	2	TRUE	0.003368	22.8204
2423	rs2865986	A	G	A	G	-0.0129	0.032197	0.6566	0.65923	FALSE	FALSE	FALSE	ieu-b-508	4	99067907	0.035118	486484	0.359231	Sepsis (28	Sepsis (28	Sepsis (28	TRUE	igd	0.0027	1.63E-06	6556	M21188.m	TRUE	reported	kvdnjc	2	TRUE	0.00347	22.8202
968	rs2604548	T	C	T	C	-0.0086	0.019548	0.6463	0.643628	FALSE	FALSE	FALSE	ieu-b-508	4	15090453	0.035049	486484	0.577031	Sepsis (28	Sepsis (28	Sepsis (28	TRUE	igd	0.0018	1.29E-06	6523	M01558.m	TRUE	reported	AZK0p4	2	TRUE	0.003487	22.82016
975	rs604388	T	C	T	C	0.0086	-0.03705	0.6845	0.67443	FALSE	FALSE	FALSE	ieu-b-508	1	1.78E+08	0.035608	486484	0.29815	Sepsis (28	Sepsis (28	Sepsis (28	TRUE	igd	0.0018	1.56E-06	6523	M01558.m	TRUE	reported	AZK0p4	2	TRUE	0.003487	22.82016
5190	rs9656510	A	G	A	G	0.252	-0.06135	0.0464	0.025865	FALSE	FALSE	FALSE	ieu-b-508	7	1.39E+08	0.105429	486484	0.560657	Sepsis (28	Sepsis (28	Sepsis (28	TRUE	igd	0.0527	1.71E-06	996	M33197.m	TRUE	reported	D6xaBg	2	TRUE	0.022442	22.81954
7629	rs1162399	A	G	A	G	-0.0086	0.021056	0.3453	0.338577	FALSE	FALSE	FALSE	ieu-b-508	14	22905725	0.035238	486484	0.550142	Sepsis (28	Sepsis (28	Sepsis (28	TRUE	igd	0.0018	9.23E-07	5768	M35326.m	TRUE	reported	jRytyJ	2	TRUE	0.003942	22.81925
2503	rs1341513	T	C	T	C	-0.0129	0.001566	0.2763	0.283863	FALSE	FALSE	FALSE	ieu-b-508	2	47081551	0.037133	486484	0.966364	Sepsis (28	Sepsis (28	Sepsis (28	TRUE	igd	0.0027	2.90E-06	5702	M22130.m	TRUE	reported	YDbJlz	2	TRUE	0.003987	22.81915
2513	rs747902	A	G	A	G	-0.0129	0.000705	0.6122	0.648765	FALSE	FALSE	FALSE	ieu-b-508	12	1.09E+08	0.035316	486484	0.984081	Sepsis (28	Sepsis (28	Sepsis (28	TRUE	igd	0.0027	2.13E-06	5702	M22130.m	TRUE	reported	YDbJlz	2	TRUE	0.003987	22.81915
7038	rs1079399	T	G	T	G	-0.0129	-0.02013	0.6284	0.623726	FALSE	FALSE	FALSE	ieu-b-508	10	1.27E+08	0.034498	486484	0.559479	Sepsis (28	Sepsis (28	Sepsis (28	TRUE	igd	0.0027	2.55E-06	5207	M34456.m	TRUE	reported	Nh457q	2	TRUE	0.004365	22.81839
6919	rs9910557	A	G	A	G	-0.0215	0.022715	0.4874	0.482973	FALSE	FALSE	FALSE	ieu-b-508	17	13662685	0.033493	486484	0.497633	Sepsis (28	Sepsis (28	Sepsis (28	TRUE	igd	0.0045	1.62E-06	5195	M34389.m	TRUE	reported	suiRIN	2	TRUE	0.004375	22.81837
3652	rs1341146	T	G	T	G	-0.0406	-0.04536	0.734	0.735647	FALSE	FALSE	FALSE	ieu-b-508	1	2.14E+08	0.038232	486484	0.235433	Sepsis (28	Sepsis (28	Sepsis (28	TRUE	igd	0.0085	1.66E-06	6970	M32564.m	TRUE	reported	73MgOk	2	TRUE	0.003263	22.80812
5471	rs1769838	T	C	T	C	0.0406	-0.02808	0.7946	0.783193	FALSE	FALSE	FALSE	ieu-b-508	14	98542862	0.040383	486484	0.486914	Sepsis (28	Sepsis (28	Sepsis (28	TRUE	igd	0.0085	1.88E-06	3890	M33408.m	TRUE	reported	tWZWla	2	TRUE	0.005831	22.80294
4393	rs7037165	A	G	A	G	0.0277	0.047734	0.0124	0.01137	FALSE	FALSE	FALSE	ieu-b-508	9	1.14E+08	0.155731	486484	0.75921	Sepsis (28	Sepsis (28	Sepsis (28	TRUE	igd	0.0058	2.03E-06	7329	M32867.m	TRUE	reported	Jcsyzm	2	TRUE	0.003102	22.80263
604	rs1115989	T	C	T	C	0.032	-0.01254	0.3295	0.320157	FALSE	FALSE	FALSE	ieu-b-508	14	89721819	0.035754	486484	0.72586	Sepsis (28	Sepsis (28	Sepsis (28	TRUE	igd	0.0067	1.77E-06	4934	M01114.m	TRUE	reported	fqHdM2	2	TRUE	0.004602	22.80207
2646	rs6890028	T	C	T	C	0.0258	-0.00062	0.2604	0.287631	FALSE	FALSE	FALSE	ieu-b-508	5	3094727	0.036955	486484	0.986541	Sepsis (28	Sepsis (28	Sepsis (28	TRUE	igd	0.0054	1.73E-06	1749	M22548.m	TRUE	reported	mZtwX2	2	TRUE	0.012883	22.80106
8087	rs2163880	A	G	A	G	0.1237	-0.05506	0.9321	0.890901	FALSE	FALSE	FALSE	ieu-b-508	12	73067540	0.053508	486484	0.303462	Sepsis (28	Sepsis (28	Sepsis (28	TRUE	igd	0.0259	1.78E-06	3939	M36095.m	TRUE	reported	9OLvcB	2	TRUE	0.005758	22.79918
2148	rs9815713	A	G	A	G	-0.0917	0.091938	0.9315	0.879743	FALSE	FALSE	FALSE	ieu-b-508	3	1.54E+08	0.052022	486484	0.077179	Sepsis (28	Sepsis (28	Sepsis (28	TRUE	igd	0.0192	1.79E-06	3783	M18497.m	TRUE	reported	90JFPc	2	TRUE	0.005994	22.79851
4618	rs1279781	T	C	T	C	-0.7626	-0.16705	0.0291	0.011793	FALSE	FALSE	FALSE	ieu-b-508	11	74235336	0.156723	486484	0.286459	Sepsis (28	Sepsis (28	Sepsis (28	TRUE	igd	0.1576	1.31E-06	76	M33173.m	TRUE	reported	04fkUQ	2	TRUE	0.235522	22.79813
6168	rs1167951	A	G	A	G	-0.0702	-0.09321	0.0743	0.070895	FALSE	FALSE	FALSE	ieu-b-508	2	85388186	0.067032	486484	0.164352	Sepsis (28	Sepsis (28	Sepsis (28	TRUE	igd	0.0147	1.89E-06	4999	M33885.m	TRUE	reported	uCOy6o	2	TRUE	0.004541	22.79637
6857	rs1222151	T	G	T	G	-0.1711	0.082255	0.9281	0.945729	FALSE	FALSE	FALSE	ieu-b-508	11	23561654	0.073889	486484	0.265607	Sepsis (28	Sepsis (28	Sepsis (28	TRUE	igd	0.0358	1.81E-06	954	M34365.m	TRUE	reported	8eexD0	2	TRUE	0.023384	22.7941
3697	rs6490461	A	C	A	C	0.0401	-0.09491	0.0491	0.067744	FALSE	FALSE	FALSE	ieu-b-508	13	30976845	0.068923	486484	0.168499	Sepsis (28	Sepsis (28	Sepsis (28	TRUE	igd	0.0084	1.60E-06	7369	M32587.m	TRUE	reported	uknv7G	2	TRUE	0.003083	22.78307
5843	rs1162343	T	C	T	C	-0.0296	-0.01485	0.4589	0.460039	FALSE	FALSE	FALSE	ieu-b-508	14	37045492	0.033495	486484	0.657505	Sepsis (28	Sepsis (28	Sepsis (28	TRUE	igd	0.0062	1.48E-06	2859	M33531.m	TRUE	reported	kNM0s1	2	TRUE	0.007909	22.77698
6856	rs1219155	T	C	T	C	-0.1094	-0.05861	0.2583	0.247235	FALSE	FALSE	FALSE	ieu-b-508	6	99537716	0.038821	486484	0.131137	Sepsis (28	Sepsis (28	Sepsis (28	TRUE	igd	0.0229	1.79E-06	954	M34365.m	TRUE	reported	8eexD0	2	TRUE	0.023364	22.77468
5262	rs2657880	C	G	C	G	0.021	-0.00847	0.136	0.182728	FALSE	TRUE	FALSE	ieu-b-508	12	56865338	0.043121	486484	0.84427	Sepsis (28	Sepsis (28	Sepsis (28	TRUE	igd	0.0044	2.09E-06	7364	M33230.m	TRUE	reported	nzcTOn	2	TRUE	0.003084	22.77274
8331	rs7666614	A	G	A	G	-0.0463	0.035543	0.1545	0.142787	FALSE	FALSE	FALSE	ieu-b-508	4	1.15E+08	0.047694	486484	0.456129	Sepsis (28	Sepsis (28	Sepsis (28	TRUE	igd	0.0097	1.81E-06	3977	M36589.m	TRUE	reported	27GADo	2	TRUE	0.005696	22.77194
1460	rs4673167	A	G	A	G	-0.021	-0.0443	0.889	0.901788	FALSE	FALSE	FALSE	ieu-b-508	2	2.28E+08	0.056499	486484	0.433039	Sepsis (28	Sepsis (28	Sepsis (28	TRUE	igd	0.0044	1.70E-06	6515	M03147.m	TRUE	reported	D9eDjf	2	TRUE	0.003484	22.77193
5201	rs1712218	T	C	T	C	-0.1494	-0.01198	0.9553	0.937707	FALSE	FALSE	FALSE	ieu-b-508	12	59554040	0.069181	486484	0.862478	Sepsis (28	Sepsis (28	Sepsis (28	TRUE	igd	0.0313	1.82E-06	3221	M33203.m	TRUE	reported	Sto7pR	2	TRUE	0.007024	22.76894
5882	rs1408509	A	G	A	G	0.0526	0.059171	0.3568	0.340281	FALSE	FALSE	FALSE	ieu-b-508	9	29447596	0.035179	486484	0.092568	Sepsis (28	Sepsis (28	Sepsis (28	TRUE	igd	0.011	1.78E-06	470	M33610.m	TRUE	reported	tmg43v	2	TRUE	0.046394	22.76848
8146	rs727276	T	C	T	C	0.1628	-0.00038	0.9671	0.963656	FALSE	FALSE	FALSE	ieu-b-508	1	80943475	0.089112	486484	0.996582	Sepsis (28	Sepsis (28	Sepsis (28	TRUE	igd	0.0341	1.73E-06	1750	M36103.m	TRUE	reported	sjeaPC	2	TRUE	0.012857	22.76687
3683	rs1996490	A	G	A	G	-0.0272	-0.04818	0.4179	0.423771	FALSE	FALSE	FALSE	ieu-b-508	3	1.16E+08	0.033753	486484	0.153492	Sepsis (28	Sepsis (28	Sepsis (28	TRUE	igd	0.0057	2.08E-06	7303	M32586.m	TRUE	reported	f6n0gX	2	TRUE	0.003108	22.76508
7017	rs9394200	T	C	T	C	0.1613	0.043813	0.0244	0.030922	FALSE	FALSE	FALSE	ieu-b-508	6	34224546	0.097271	486484	0.652405	Sepsis (28	Sepsis (28	Sepsis (28	TRUE	igd	0.0338	1.88E-06	4452	M34420.m	TRUE	reported	20uofq	2	TRUE	0.005089	22.76356
2399	rs182119	C	G	C	G	-0.0272	-0.0337	0.7302	0.726627	FALSE	TRUE	FALSE	ieu-b-508	5	6485363	0.037601	486484	0.37007	Sepsis (28	Sepsis (28	Sepsis (28	TRUE	igd	0.0057	1.73E-06	5436	M21184.m	TRUE	reported	63ppuJ	2	TRUE	0.004172	22.76294
7986	rs7615671	T	G	T	G	0.0253	0.013661	0.6922	0.7141	FALSE	FALSE	FALSE	ieu-b-508	3	1.11E+08	0.037014	486484	0.712066	Sepsis (28	Sepsis (28	Sepsis (28	TRUE	igd	0.0053	1.52E-06	1766	M35718.m	TRUE	reported	oUwc0a	2	TRUE	0.012739	22.76131
2787	rs1103006	T	C	T	C	-0.0167	0.023825	0.1273	0.141964	FALSE	FALSE	FALSE	ieu-b-508	11	27618490	0.047584	486484	0.616585	Sepsis (28	Sepsis (28	Sepsis (28	TRUE	igd	0.0035	2.13E-06	7347	M27278.m	TRUE	reported	kPgqm3	2	TRUE	0.003089	22.76033
3167	rs1998881	T	C	T	C	-0.0167	-0.00252	0.2091	0.218659	FALSE	FALSE	FALSE	ieu-b-508	10	8863208	0.040373	486484	0.95021	Sepsis (28	Sepsis (28	Sepsis (28	TRUE	igd	0.0035	1.33E-06	7340	M32328.m	TRUE	reported	YGsb6D	2	TRUE	0.003092	22.76033
7853	rs1470121	A	G	A	G	0.0167	0.001581	0.3619	0.360887	FALSE	FALSE	FALSE	ieu-b-508	3	1.43E+08	0.034803	486484	0.963762	Sepsis (28	Sepsis (28	Sepsis (28	TRUE	igd	0.0035	2.36E-06	7302	M35628.m	TRUE	reported	qFJY9w	2	TRUE	0.003108	22.76029
2640	rs1316091	A	G	A	G	0.0167	0.054948	0.8729	0.869492	FALSE	FALSE	FALSE	ieu-b-508	5	1.29E+08	0.049488	486484	0.266857	Sepsis (28	Sepsis (28	Sepsis (28	TRUE	igd	0.0035	2.35E-06	7184	M22548.m	TRUE	reported	mZtwX2	2	TRUE	0.003159	22.76019
5468	rs1609276	C	G	C	G	0.1088	0.073297	0.9727	0.980555	FALSE	TRUE	FALSE	ieu-b-508	7	78929161	0.12067	486484	0.543576	Sepsis (28	Sepsis (28	Sepsis (28	TRUE	igd	0.0228	1.83E-06	3890	M33408.m	TRUE	reported	tWZWla	2	TRUE	0.00582	22.75961
1501	rs6592988	T	C	T	C	0.0167	-0.04587	0.1439	0.156867	FALSE	FALSE	FALSE	ieu-b-508	7	52281433	0.046098	486484	0.319712	Sepsis (28	Sepsis (28	Sepsis (28	TRUE	igd	0.0035	2.40E-06	5656	M12017.m	TRUE	reported	bbGtsK	2	TRUE	0.004009	22.75848
1999	rs1702178	A	G	A	G	-0.3999	0.120059	0.9857	0.977182	FALSE	FALSE	FALSE	ieu-b-508	3	28757739	0.11183	486484	0.283011	Sepsis (28	Sepsis (28	Sepsis (28	TRUE	igd	0.0838	1.84E-06	2433	M18281.m	TRUE	reported	3mLp7z	2	TRUE	0.009273	22.75399
6965	rs7042159	A	C	A	C	0.0683	-0.01738	0.2478	0.261316	FALSE	FALSE	FALSE	ieu-b-508	9	18545209	0.038187	486484	0.649069	Sepsis (28	Sepsis (28	Sepsis (28	TRUE	igd	0.0143	1.71E-06	777	M34404.m	TRUE	reported	8FMHfo	2	TRUE	0.028522	22.75359
6283	rs3891367	T	C	T	C	0.0358	-0.14094	0.8915	0.911794	FALSE	FALSE	FALSE	ieu-b-508	4	59478444	0.058886	486484	0.01669	Sepsis (28	Sepsis (28	Sepsis (28	TRUE	igd	0.0075	1.73E-06	1404	M33939.m	TRUE	reported	PeKlgz	2	TRUE	0.015969	22.75225
4103	rs1692695	T	G	T	G	-0.052	-0.00532	0.1948	0.202665	FALSE	FALSE	FALSE	ieu-b-508	9	8021065	0.04157	486484	0.898243	Sepsis (28	Sepsis (28	Sepsis (28	TRUE	igd	0.0109	1.77E-06	6379	M32754.m	TRUE	reported	n0okFy	2	TRUE	0.003555	22.75189
6921	rs1277798	T	C	T	C	-0.0291	0.04781	0.2212	0.21544	FALSE	FALSE	FALSE	ieu-b-508	10	1.29E+08	0.040913	486484	0.242578	Sepsis (28	Sepsis (28	Sepsis (28	TRUE	igd	0.0061	2.14E-06	5561	M34390.m	TRUE	reported	wruTbW	2	TRUE	0.004076	22.74941
7344	rs1698618	C	G	C	G	-0.1275	-0.01228	0.9857	0.98002	FALSE	TRUE	FALSE	ieu-b-508	20	35146984	0.119976	486484	0.918487	Sepsis (28	Sepsis (28	Sepsis (28	TRUE	igd	0.0267	1.81E-06	819	M35137.m	TRUE	reported	Eq2siS	2	TRUE	0.027089	22.74762
4948	rs42441	T	G	T	G	0.2726	-0.02785	0.9799	0.978935	FALSE	FALSE	FALSE	ieu-b-508	5	96045137	0.116167	486484	0.81054	Sepsis (28	Sepsis (28	Sepsis (28	TRUE	igd	0.0571	1.80E-06	1017	M33178.m	TRUE	reported	g22WMS	2	TRUE	0.02192	22.74702
6023	rs7925072	T	C	T	C	-0.0826	0.025707	0.822	0.823318	FALSE	FALSE	FALSE	ieu-b-508	11	20646145	0.04372	486484	0.556548	Sepsis (28	Sepsis (28	Sepsis (28	TRUE	igd	0.0173	1.78E-06	831	M33751.m	TRUE	reported	Cxdkr4	2	TRUE	0.0267	22.74162
3863	rs1158981	A	G	A	G	0.0124	-0.00471	0.7885	0.793514	FALSE	FALSE	FALSE	ieu-b-508	1	2.12E+08	0.041194	486484	0.909049	Sepsis (28	Sepsis (28	Sepsis (28	TRUE	igd	0.0026	2.46E-06	7361	M32654.m	TRUE	reported	6ISQOP	2	TRUE	0.00308	22.73938
3491	rs1181567	A	G	A	G	-0.0124	-0.06691	0.1429	0.162331	FALSE	FALSE	FALSE	ieu-b-508	10	1.22E+08	0.046512	486484	0.150289	Sepsis (28	Sepsis (28	Sepsis (28	TRUE	igd	0.0026	1.93E-06	7355	M32492.m	TRUE	reported	rZZFcE	2	TRUE	0.003083	22.73938
2611	rs2322487	A	T	A	T	-0.0124	0.002992	0.6629	0.628232	FALSE	TRUE	FALSE	ieu-b-508	6	1.65E+08	0.034545	486484	0.930978	Sepsis (28	Sepsis (28	Sepsis (28	TRUE	igd	0.0026	2.00E-06	7329	M22481.m	TRUE	reported	bXpf0k	2	TRUE	0.003094	22.73936
2574	rs6476317	T	C	T	C	0.0186	-0.07741	0.0729	0.080002	FALSE	FALSE	FALSE	ieu-b-508	9	31990046	0.061496	486484	0.208097	Sepsis (28	Sepsis (28	Sepsis (28	TRUE	igd	0.0039	1.68E-06	6535	M22177.m	TRUE	reported	2s6Sly	2	TRUE	0.003469	22.7386
4025	rs2814818	A	G	A	G	0.031	0.01322	0.6985	0.689067	FALSE	FALSE	FALSE	ieu-b-508	6	1682567	0.036108	486484	0.714268	Sepsis (28	Sepsis (28	Sepsis (28	TRUE	igd	0.0065	2.15E-06	6000	M32735.m	TRUE	reported	QE2vKQ	2	TRUE	0.003777	22.73798
1950	rs1757966	T	G	T	G	-0.0601	0.006532	0.2583	0.274607	FALSE	FALSE	FALSE	ieu-b-508	7	70128708	0.037425	486484	0.861449	Sepsis (28	Sepsis (28	Sepsis (28	TRUE	igd	0.0126	1.84E-06	3393	M16823.m	TRUE	reported	fNy2PD	2	TRUE	0.006661	22.73797
1497	rs1253851	C	G	C	G	0.0124	0.008799	0.3714	0.390647	FALSE	TRUE	FALSE	ieu-b-508	7	1.51E+08	0.034127	486484	0.796538	Sepsis (28	Sepsis (28	Sepsis (28	TRUE	igd	0.0026	2.43E-06	5656	M12017.m	TRUE	reported	bbGtsK	2	TRUE	0.004005	22.73752
1503	rs7751499	A	G	A	G	0.0124	-0.0184	0.684	0.67414	FALSE	FALSE	FALSE	ieu-b-508	6	1.55E+08	0.035718	486484	0.606418	Sepsis (28	Sepsis (28	Sepsis (28	TRUE	igd	0.0026	2.57E-06	5656	M12017.m	TRUE	reported	bbGtsK	2	TRUE	0.004005	22.73752
7118	rs6111973	T	C	T	C	0.1379	-0.07302	0.2105	0.208831	FALSE	FALSE	FALSE	ieu-b-508	20	18445266	0.041012	486484	0.075015	Sepsis (28	Sepsis (28	Sepsis (28	TRUE	igd	0.0288	1.65E-06	241	M34516.m	TRUE	reported	7Ah2wy	2	TRUE	0.086868	22.73654
2391	rs6016809	T	C	T	C	-0.1345	0.001441	0.9615	0.961387	FALSE	FALSE	FALSE	ieu-b-508	20	41122834	0.087291	486484	0.986828	Sepsis (28	Sepsis (28	Sepsis (28	TRUE	igd	0.0282	1.82E-06	2339	M21151.m	TRUE	reported	LfwdLx	2	TRUE	0.009632	22.72871
6589	rs1821897	C	G	C	G	0.0248	-0.0248	0.7792	0.755734	FALSE	TRUE	FALSE	ieu-b-508	2	12283275	0.039255	486484	0.527595	Sepsis (28	Sepsis (28	Sepsis (28	TRUE	igd	0.0052	1.49E-06	2278	M34289.m	TRUE	reported	Knp8ry	2	TRUE	0.009886	22.72559
6494	rs9558761	C	G	C	G	-0.5296	-0.10005	0.0177	0.026179	FALSE	TRUE	FALSE	ieu-b-508	13	1.07E+08	0.105049	486484	0.34091	Sepsis (28	Sepsis (28	Sepsis (28	TRUE	igd	0.1107	1.71E-06	282	M34109.m	TRUE	reported	piJuC7	2	TRUE	0.075069	22.7253
2134	rs1051447	A	G	A	G	0.0391	-0.02557	0.4619	0.453228	FALSE	FALSE	FALSE	ieu-b-508	16	80023841	0.033897	486484	0.450685	Sepsis (28	Sepsis (28	Sepsis (28	TRUE	igd	0.0082	1.80E-06	3783	M18497.m	TRUE	reported	90JFPc	2	TRUE	0.005974	22.72459
6363	rs1489866	T	G	T	G	0.0205	0.046922	0.8208	0.824916	FALSE	FALSE	FALSE	ieu-b-508	14	62998256	0.043773	486484	0.283753	Sepsis (28	Sepsis (28	Sepsis (28	TRUE	igd	0.0043	1.72E-06	7368	M33961.m	TRUE	reported	10AWw0	2	TRUE	0.003075	22.72233
802	rs281216	T	G	T	G	0.0205	-0.05378	0.9184	0.913609	FALSE	FALSE	FALSE	ieu-b-508	15	47708579	0.059322	486484	0.36461	Sepsis (28	Sepsis (28	Sepsis (28	TRUE	igd	0.0043	1.70E-06	7335	M01356.m	TRUE	reported	ah1g5W	2	TRUE	0.003089	22.7223
6212	rs1685374	T	C	T	C	0.0739	0.091423	0.9739	0.955396	FALSE	FALSE	FALSE	ieu-b-508	3	1.69E+08	0.080812	486484	0.257926	Sepsis (28	Sepsis (28	Sepsis (28	TRUE	igd	0.0155	1.98E-06	4533	M33901.m	TRUE	reported	mQkhrb	2	TRUE	0.00499	22.72133
1318	rs1237160	T	C	T	C	-0.0205	0.052922	0.7709	0.779881	FALSE	FALSE	FALSE	ieu-b-508	12	21391336	0.040267	486484	0.18875	Sepsis (28	Sepsis (28	Sepsis (28	TRUE	igd	0.0043	2.18E-06	6252	M02137.m	TRUE	reported	iRGqBE	2	TRUE	0.003622	22.72123
5211	rs1694433	A	T	A	T	-0.0248	0.033735	0.1526	0.168953	FALSE	TRUE	FALSE	ieu-b-508	16	81016014	0.044624	486484	0.449665	Sepsis (28	Sepsis (28	Sepsis (28	TRUE	igd	0.0052	1.99E-06	1673	M33204.m	TRUE	reported	ROvPWK	2	TRUE	0.013413	22.71837
8232	rs1296296	T	C	T	C	0.0453	0.010228	0.17	0.150733	FALSE	FALSE	FALSE	ieu-b-508	18	4077811	0.047032	486484	0.82785	Sepsis (28	Sepsis (28	Sepsis (28	TRUE	igd	0.0095	1.83E-06	1908	M36468.m	TRUE	reported	vycAFr	2	TRUE	0.011777	22.71401
5273	rs1720779	T	C	T	C	0.0429	0.039507	0.1399	0.270346	FALSE	FALSE	FALSE	ieu-b-508	16	85569338	0.037622	486484	0.29367	Sepsis (28	Sepsis (28	Sepsis (28	TRUE	igd	0.009	1.91E-06	4409	M33250.m	TRUE	reported	WIZI6U	2	TRUE	0.005127	22.7108
1630	rs1426134	C	G	C	G	-0.0081	-0.02602	0.3715	0.368216	FALSE	TRUE	FALSE	ieu-b-508	4	14179729	0.034665	486484	0.45284	Sepsis (28	Sepsis (28	Sepsis (28	TRUE	igd	0.0017	3.84E-06	7368	M15140.m	TRUE	reported	jEcqMJ	2	TRUE	0.003072	22.69626
1636	rs2320536	T	C	T	C	0.0081	-0.00208	0.4131	0.417406	FALSE	FALSE	FALSE	ieu-b-508	4	1.41E+08	0.034001	486484	0.951118	Sepsis (28	Sepsis (28	Sepsis (28	TRUE	igd	0.0017	3.32E-06	7368	M15140.m	TRUE	reported	jEcqMJ	2	TRUE	0.003072	22.69626
8618	rs484061	A	G	A	G	-0.0081	0.034627	0.5129	0.524635	FALSE	FALSE	FALSE	ieu-b-508	3	1.27E+08	0.033671	486484	0.303754	Sepsis (28	Sepsis (28	Sepsis (28	TRUE	igd	0.0017	3.62E-06	7366	M37506.m	TRUE	reported	m2kaRt	2	TRUE	0.003073	22.69626
438	rs6603141	A	G	A	G	-0.0081	0.009957	0.258	0.263523	FALSE	FALSE	FALSE	ieu-b-508	19	8158074	0.037905	486484	0.792795	Sepsis (28	Sepsis (28	Sepsis (28	TRUE	igd	0.0017	2.61E-06	7365	M00063.m	TRUE	reported	Hdsv1L	2	TRUE	0.003073	22.69626
683	rs1532775	A	G	A	G	0.0081	0.002105	0.7796	0.771092	FALSE	FALSE	FALSE	ieu-b-508	1	1.15E+08	0.039679	486484	0.957687	Sepsis (28	Sepsis (28	Sepsis (28	TRUE	igd	0.0017	1.04E-06	7358	M01299.m	TRUE	reported	gfM0a3	2	TRUE	0.003076	22.69625
686	rs1729382	T	G	T	G	0.0081	0.038324	0.8016	0.801443	FALSE	FALSE	FALSE	ieu-b-508	8	14111623	0.041787	486484	0.359069	Sepsis (28	Sepsis (28	Sepsis (28	TRUE	igd	0.0017	2.01E-06	7358	M01299.m	TRUE	reported	gfM0a3	2	TRUE	0.003076	22.69625
689	rs2879560	T	C	T	C	-0.0081	0.024661	0.2226	0.203576	FALSE	FALSE	FALSE	ieu-b-508	3	1.82E+08	0.044901	486484	0.582848	Sepsis (28	Sepsis (28	Sepsis (28	TRUE	igd	0.0017	3.48E-06	7358	M01299.m	TRUE	reported	gfM0a3	2	TRUE	0.003076	22.69625
821	rs2489020	T	C	T	C	-0.0081	-0.07815	0.5262	0.52191	FALSE	FALSE	FALSE	ieu-b-508	10	1.02E+08	0.033626	486484	0.020122	Sepsis (28	Sepsis (28	Sepsis (28	TRUE	igd	0.0017	2.30E-06	7355	M01358.m	TRUE	reported	kSPQgd	2	TRUE	0.003077	22.69625
2054	rs1318821	A	C	A	C	0.0081	-0.01098	0.4184	0.422765	FALSE	FALSE	FALSE	ieu-b-508	5	1.7E+08	0.033862	486484	0.745727	Sepsis (28	Sepsis (28	Sepsis (28	TRUE	igd	0.0017	1.77E-06	7354	M18369.m	TRUE	reported	ykBjIT	2	TRUE	0.003078	22.69625
786	rs4957697	A	G	A	G	-0.0081	-0.03065	0.4208	0.432088	FALSE	FALSE	FALSE	ieu-b-508	5	1.07E+08	0.033736	486484	0.36362	Sepsis (28	Sepsis (28	Sepsis (28	TRUE	igd	0.0017	1.73E-06	7352	M01336.m	TRUE	reported	FAlrya	2	TRUE	0.003078	22.69625
1698	rs6455128	A	C	A	C	0.0081	0.013289	0.1757	0.193425	FALSE	FALSE	FALSE	ieu-b-508	6	62703955	0.042254	486484	0.753137	Sepsis (28	Sepsis (28	Sepsis (28	TRUE	igd	0.0017	2.21E-06	7345	M15488.m	TRUE	reported	SPriBO	2	TRUE	0.003081	22.69624
7702	rs1877564	T	C	T	C	0.0081	0.013761	0.7887	0.794634	FALSE	FALSE	FALSE	ieu-b-508	3	39160399	0.041561	486484	0.740568	Sepsis (28	Sepsis (28	Sepsis (28	TRUE	igd	0.0017	2.20E-06	7345	M35397.m	TRUE	reported	Y56mZr	2	TRUE	0.003081	22.69624
1769	rs1732932	T	C	T	C	0.0081	-0.00174	0.4292	0.43082	FALSE	FALSE	FALSE	ieu-b-508	2	36004210	0.033689	486484	0.9587	Sepsis (28	Sepsis (28	Sepsis (28	TRUE	igd	0.0017	3.49E-06	7331	M15676.m	TRUE	reported	s1NfKN	2	TRUE	0.003087	22.69623
1775	rs4570993	T	C	T	C	0.0081	-0.0204	0.5322	0.53424	FALSE	FALSE	FALSE	ieu-b-508	19	3656901	0.03358	486484	0.543487	Sepsis (28	Sepsis (28	Sepsis (28	TRUE	igd	0.0017	2.43E-06	7331	M15676.m	TRUE	reported	s1NfKN	2	TRUE	0.003087	22.69623
1779	rs703147	T	C	T	C	0.0081	-0.04169	0.3801	0.382694	FALSE	FALSE	FALSE	ieu-b-508	1	1.59E+08	0.03437	486484	0.225125	Sepsis (28	Sepsis (28	Sepsis (28	TRUE	igd	0.0017	2.41E-06	7331	M15676.m	TRUE	reported	s1NfKN	2	TRUE	0.003087	22.69623
1282	rs1326718	A	G	A	G	0.0081	-0.03959	0.4794	0.490879	FALSE	FALSE	FALSE	ieu-b-508	8	10466482	0.033352	486484	0.235204	Sepsis (28	Sepsis (28	Sepsis (28	TRUE	igd	0.0017	3.78E-06	7325	M02132.m	TRUE	reported	uJFm2t	2	TRUE	0.00309	22.69622
1291	rs1920325	T	C	T	C	-0.0081	-0.07276	0.6425	0.640313	FALSE	FALSE	FALSE	ieu-b-508	3	1.04E+08	0.034757	486484	0.03631	Sepsis (28	Sepsis (28	Sepsis (28	TRUE	igd	0.0017	3.66E-06	7325	M02132.m	TRUE	reported	uJFm2t	2	TRUE	0.00309	22.69622
1295	rs2487410	T	C	T	C	-0.0081	-0.01445	0.4696	0.523784	FALSE	FALSE	FALSE	ieu-b-508	6	70562615	0.033361	486484	0.665002	Sepsis (28	Sepsis (28	Sepsis (28	TRUE	igd	0.0017	1.84E-06	7325	M02132.m	TRUE	reported	uJFm2t	2	TRUE	0.00309	22.69622
4557	rs8089236	T	C	T	C	-0.0162	0.018008	0.7336	0.738863	FALSE	FALSE	FALSE	ieu-b-508	18	40730509	0.037974	486484	0.635344	Sepsis (28	Sepsis (28	Sepsis (28	TRUE	igd	0.0034	2.74E-06	7319	M33140.m	TRUE	reported	eBOUtb	2	TRUE	0.003092	22.69622
4057	rs2029209	A	G	A	G	0.0081	-0.02203	0.5491	0.533184	FALSE	FALSE	FALSE	ieu-b-508	9	11861841	0.03346	486484	0.510256	Sepsis (28	Sepsis (28	Sepsis (28	TRUE	igd	0.0017	2.08E-06	7318	M32740.m	TRUE	reported	YLshfP	2	TRUE	0.003093	22.69622
2154	rs2028973	T	C	T	C	0.0162	-0.11936	0.1086	0.108187	FALSE	FALSE	FALSE	ieu-b-508	6	25138627	0.053701	486484	0.026231	Sepsis (28	Sepsis (28	Sepsis (28	TRUE	igd	0.0034	2.66E-06	7286	M18929.m	TRUE	reported	2vYFb2	2	TRUE	0.003106	22.69619
1074	rs7178551	T	C	T	C	0.0081	0.032066	0.1545	0.196213	FALSE	FALSE	FALSE	ieu-b-508	15	81263637	0.042231	486484	0.447672	Sepsis (28	Sepsis (28	Sepsis (28	TRUE	igd	0.0017	2.66E-06	7271	M01585.m	TRUE	reported	M7QiSM	2	TRUE	0.003113	22.69618
1249	rs2351174	T	G	T	G	-0.0081	-0.00177	0.6063	0.621704	FALSE	FALSE	FALSE	ieu-b-508	14	61013237	0.034453	486484	0.958964	Sepsis (28	Sepsis (28	Sepsis (28	TRUE	igd	0.0017	2.63E-06	7136	M01769.m	TRUE	reported	rismUZ	2	TRUE	0.003171	22.69606
1262	rs7837792	T	C	T	C	-0.0081	-0.00834	0.5936	0.591734	FALSE	FALSE	FALSE	ieu-b-508	8	1.24E+08	0.034215	486484	0.807478	Sepsis (28	Sepsis (28	Sepsis (28	TRUE	igd	0.0017	2.78E-06	7136	M01769.m	TRUE	reported	rismUZ	2	TRUE	0.003171	22.69606
5363	rs8058227	T	C	T	C	0.0081	0.006249	0.4024	0.401779	FALSE	FALSE	FALSE	ieu-b-508	16	9298257	0.034202	486484	0.855026	Sepsis (28	Sepsis (28	Sepsis (28	TRUE	igd	0.0017	1.35E-06	7118	M33380.m	TRUE	reported	OHRfl5	2	TRUE	0.003179	22.69604
4127	rs4977264	T	C	T	C	-0.0162	0.002272	0.5535	0.549788	FALSE	FALSE	FALSE	ieu-b-508	9	18616787	0.033681	486484	0.946211	Sepsis (28	Sepsis (28	Sepsis (28	TRUE	igd	0.0034	2.47E-06	6658	M32755.m	TRUE	reported	WDrNdi	2	TRUE	0.003398	22.6956
7627	rs1077862	A	C	A	C	0.0081	0.039005	0.3766	0.379695	FALSE	FALSE	FALSE	ieu-b-508	12	1.09E+08	0.034507	486484	0.258331	Sepsis (28	Sepsis (28	Sepsis (28	TRUE	igd	0.0017	3.82E-06	5768	M35326.m	TRUE	reported	jRytyJ	2	TRUE	0.00392	22.69455
2128	rs2420201	A	G	A	G	0.1473	0.150659	0.0494	0.056618	FALSE	FALSE	FALSE	ieu-b-508	2	72356370	0.072577	486484	0.037908	Sepsis (28	Sepsis (28	Sepsis (28	TRUE	igd	0.0309	1.90E-06	1457	M18477.m	TRUE	reported	p0vcCU	2	TRUE	0.015357	22.693
2348	rs1694853	A	G	A	G	-0.0224	-0.03996	0.8805	0.882211	FALSE	FALSE	FALSE	ieu-b-508	17	13699102	0.05167	486484	0.439278	Sepsis (28	Sepsis (28	Sepsis (28	TRUE	igd	0.0047	2.23E-06	1764	M21047.m	TRUE	reported	lSF1Cr	2	TRUE	0.012713	22.6886
2121	rs1780783	A	G	A	G	0.0343	0.012824	0.8027	0.793742	FALSE	FALSE	FALSE	ieu-b-508	12	83960889	0.041415	486484	0.756838	Sepsis (28	Sepsis (28	Sepsis (28	TRUE	igd	0.0072	1.71E-06	5624	M18476.m	TRUE	reported	6GnBtw	2	TRUE	0.004019	22.68657
4151	rs9362410	T	C	T	C	0.0262	-0.00642	0.3749	0.390701	FALSE	FALSE	FALSE	ieu-b-508	6	64753874	0.034363	486484	0.851886	Sepsis (28	Sepsis (28	Sepsis (28	TRUE	igd	0.0055	1.83E-06	7250	M32757.m	TRUE	reported	TJV9Xb	2	TRUE	0.00312	22.68597
1987	rs1289589	A	G	A	G	-0.0443	-0.0333	0.8278	0.804617	FALSE	FALSE	FALSE	ieu-b-508	14	26708629	0.042054	486484	0.428458	Sepsis (28	Sepsis (28	Sepsis (28	TRUE	igd	0.0093	1.96E-06	7071	M18254.m	TRUE	reported	clvu6V	2	TRUE	0.003199	22.68395
576	rs1799412	C	G	C	G	-0.0262	-0.03101	0.8394	0.815135	FALSE	TRUE	FALSE	ieu-b-508	3	40210125	0.042917	486484	0.469996	Sepsis (28	Sepsis (28	Sepsis (28	TRUE	igd	0.0055	2.28E-06	5344	M01107.m	TRUE	reported	EbPV1D	2	TRUE	0.004228	22.68374
2619	rs3798343	C	G	C	G	-0.0362	-0.12142	0.9771	0.977054	FALSE	TRUE	FALSE	ieu-b-508	6	35357693	0.111043	486484	0.274197	Sepsis (28	Sepsis (28	Sepsis (28	TRUE	igd	0.0076	2.07E-06	7329	M22481.m	TRUE	reported	bXpf0k	2	TRUE	0.003086	22.68148
3940	rs9544223	A	C	A	C	-0.0181	0.023768	0.8771	0.807479	FALSE	FALSE	FALSE	ieu-b-508	13	76809200	0.042744	486484	0.578164	Sepsis (28	Sepsis (28	Sepsis (28	TRUE	igd	0.0038	2.07E-06	7308	M32698.m	TRUE	reported	srqncz	2	TRUE	0.003095	22.68146
5235	rs9615728	A	G	A	G	0.0543	-0.01967	0.0399	0.06672	FALSE	FALSE	FALSE	ieu-b-508	22	48397623	0.06699	486484	0.769091	Sepsis (28	Sepsis (28	Sepsis (28	TRUE	igd	0.0114	2.01E-06	4665	M33221.m	TRUE	reported	8ZrV1r	2	TRUE	0.00484	22.67795
4342	rs1051644	A	G	A	G	0.0281	-0.03325	0.8742	0.871428	FALSE	FALSE	FALSE	ieu-b-508	4	1E+08	0.050774	486484	0.512523	Sepsis (28	Sepsis (28	Sepsis (28	TRUE	igd	0.0059	2.13E-06	7355	M32855.m	TRUE	reported	AM8kCf	2	TRUE	0.003075	22.67726
986	rs1114533	A	C	A	C	0.0324	-0.01388	0.8908	0.887075	FALSE	FALSE	FALSE	ieu-b-508	9	71733603	0.052407	486484	0.791199	Sepsis (28	Sepsis (28	Sepsis (28	TRUE	igd	0.0068	1.95E-06	1759	M01561.m	TRUE	reported	Zd9SUU	2	TRUE	0.012742	22.67661
485	rs2211372	T	C	T	C	0.3317	-0.17994	0.985	0.984942	FALSE	FALSE	FALSE	ieu-b-508	6	22587398	0.138783	486484	0.194778	Sepsis (28	Sepsis (28	Sepsis (28	TRUE	igd	0.0696	1.87E-06	1233	M00553.m	TRUE	reported	IFjXiL	2	TRUE	0.018088	22.67605
3540	rs7013605	T	C	T	C	0.02	-0.00837	0.1199	0.144539	FALSE	FALSE	FALSE	ieu-b-508	8	1.4E+08	0.047719	486484	0.860841	Sepsis (28	Sepsis (28	Sepsis (28	TRUE	igd	0.0042	2.42E-06	7358	M32497.m	TRUE	reported	244bZN	2	TRUE	0.003072	22.66957
3547	rs998531	A	G	A	G	0.02	0.028764	0.8796	0.870793	FALSE	FALSE	FALSE	ieu-b-508	12	77542760	0.049813	486484	0.563645	Sepsis (28	Sepsis (28	Sepsis (28	TRUE	igd	0.0042	1.89E-06	7358	M32497.m	TRUE	reported	244bZN	2	TRUE	0.003072	22.66957
2477	rs1237556	A	G	A	G	-0.0386	0.006233	0.2606	0.235121	FALSE	FALSE	FALSE	ieu-b-508	9	16805155	0.039431	486484	0.874395	Sepsis (28	Sepsis (28	Sepsis (28	TRUE	igd	0.0081	2.16E-06	1087	M22032.m	TRUE	reported	xpe96K	2	TRUE	0.020464	22.66756
3648	rs1050824	A	G	A	G	0.0519	0.007278	0.1246	0.106916	FALSE	FALSE	FALSE	ieu-b-508	10	2944715	0.054585	486484	0.893925	Sepsis (28	Sepsis (28	Sepsis (28	TRUE	igd	0.0109	1.92E-06	6970	M32564.m	TRUE	reported	73MgOk	2	TRUE	0.003242	22.66507
7294	rs9513567	C	G	C	G	0.0262	0.00466	0.2905	0.285561	FALSE	TRUE	FALSE	ieu-b-508	13	99798641	0.037001	486484	0.899785	Sepsis (28	Sepsis (28	Sepsis (28	TRUE	igd	0.0055	2.08E-06	1657	M35114.m	TRUE	reported	a6NQjV	2	TRUE	0.01351	22.66484
2112	rs1599081	T	G	T	G	-0.0691	0.053266	0.788	0.793984	FALSE	FALSE	FALSE	ieu-b-508	15	31740923	0.041562	486484	0.199977	Sepsis (28	Sepsis (28	Sepsis (28	TRUE	igd	0.0145	1.90E-06	917	M18474.m	TRUE	reported	B6Xd02	2	TRUE	0.024167	22.66062
7523	rs2076060	C	G	C	G	0.0219	0.011005	0.2579	0.2832	FALSE	TRUE	FALSE	ieu-b-508	6	1.07E+08	0.037049	486484	0.766438	Sepsis (28	Sepsis (28	Sepsis (28	TRUE	igd	0.0046	1.55E-06	7291	M35255.m	TRUE	reported	ZDjmUf	2	TRUE	0.003099	22.65966
6485	rs4448374	T	C	T	C	-0.2957	-0.18067	0.0283	0.044799	FALSE	FALSE	FALSE	ieu-b-508	9	20563365	0.080412	486484	0.024651	Sepsis (28	Sepsis (28	Sepsis (28	TRUE	igd	0.0619	1.80E-06	282	M34109.m	TRUE	reported	piJuC7	2	TRUE	0.074865	22.65845
5796	rs9451877	A	G	A	G	-0.0943	0.029045	0.9703	0.975787	FALSE	FALSE	FALSE	ieu-b-508	6	92701566	0.108269	486484	0.788491	Sepsis (28	Sepsis (28	Sepsis (28	TRUE	igd	0.0198	1.92E-06	1718	M33510.m	TRUE	reported	WxGKnn	2	TRUE	0.013031	22.6562
7315	rs1136613	A	G	A	G	-0.0781	0.023865	0.0194	0.015697	FALSE	FALSE	FALSE	ieu-b-508	17	45735975	0.13514	486484	0.85983	Sepsis (28	Sepsis (28	Sepsis (28	TRUE	igd	0.0164	2.01E-06	1762	M35127.m	TRUE	reported	RNsmjl	2	TRUE	0.012707	22.65276
8385	rs6755317	A	G	A	G	0.0357	0.069908	0.9729	0.97571	FALSE	FALSE	FALSE	ieu-b-508	2	98939253	0.113048	486484	0.536314	Sepsis (28	Sepsis (28	Sepsis (28	TRUE	igd	0.0075	2.03E-06	7335	M36776.m	TRUE	reported	VnYglx	2	TRUE	0.003079	22.65142
3320	rs9894902	A	G	A	G	0.0119	0.037203	0.2086	0.216886	FALSE	FALSE	FALSE	ieu-b-508	17	51881281	0.040536	486484	0.358736	Sepsis (28	Sepsis (28	Sepsis (28	TRUE	igd	0.0025	2.35E-06	7307	M32393.m	TRUE	reported	Pub0X0	2	TRUE	0.003091	22.6514
7812	rs4789522	A	C	A	C	0.0119	-0.00263	0.666	0.661128	FALSE	FALSE	FALSE	ieu-b-508	17	75959825	0.035316	486484	0.94054	Sepsis (28	Sepsis (28	Sepsis (28	TRUE	igd	0.0025	2.08E-06	6883	M35464.m	TRUE	reported	Ir9dHp	2	TRUE	0.003281	22.65102
2216	rs2263487	T	C	T	C	-0.0119	0.004998	0.5184	0.515352	FALSE	FALSE	FALSE	ieu-b-508	12	47250827	0.033318	486484	0.880746	Sepsis (28	Sepsis (28	Sepsis (28	TRUE	igd	0.0025	3.05E-06	6678	M19368.m	TRUE	reported	QaKPad	2	TRUE	0.003381	22.65081
971	rs4443239	T	G	T	G	0.02	0.020627	0.7446	0.754159	FALSE	FALSE	FALSE	ieu-b-508	4	1.7E+08	0.038894	486484	0.595888	Sepsis (28	Sepsis (28	Sepsis (28	TRUE	igd	0.0042	2.27E-06	1608	M01558.m	TRUE	reported	AZK0p4	2	TRUE	0.013906	22.64753
6725	rs1312171	A	C	A	C	-0.3919	0.127921	0.9794	0.971629	FALSE	FALSE	FALSE	ieu-b-508	4	5233536	0.100464	486484	0.202912	Sepsis (28	Sepsis (28	Sepsis (28	TRUE	igd	0.0822	1.86E-06	535	M34338.m	TRUE	reported	h4FB9a	2	TRUE	0.040755	22.64542
8170	rs4403944	T	G	T	G	-0.0638	-0.02173	0.0458	0.055212	FALSE	FALSE	FALSE	ieu-b-508	13	62624271	0.073354	486484	0.767044	Sepsis (28	Sepsis (28	Sepsis (28	TRUE	igd	0.0134	2.04E-06	1780	M36131.m	TRUE	reported	DbYm5t	2	TRUE	0.012575	22.6435
6566	rs1106869	A	G	A	G	-0.0395	-0.00588	0.0667	0.068637	FALSE	FALSE	FALSE	ieu-b-508	12	1.18E+08	0.066143	486484	0.929186	Sepsis (28	Sepsis (28	Sepsis (28	TRUE	igd	0.0083	2.06E-06	4908	M34245.m	TRUE	reported	pRwBKr	2	TRUE	0.004593	22.6392
5493	rs6719679	A	G	A	G	0.0276	0.022352	0.7751	0.772601	FALSE	FALSE	FALSE	ieu-b-508	2	2.39E+08	0.039918	486484	0.575526	Sepsis (28	Sepsis (28	Sepsis (28	TRUE	igd	0.0058	2.20E-06	6797	M33415.m	TRUE	reported	xHSphe	2	TRUE	0.00332	22.63781
4209	rs300250	A	G	A	G	0.0276	-0.02796	0.635	0.642625	FALSE	FALSE	FALSE	ieu-b-508	5	73883887	0.034798	486484	0.421663	Sepsis (28	Sepsis (28	Sepsis (28	TRUE	igd	0.0058	2.25E-06	6150	M32769.m	TRUE	reported	W2ubRP	2	TRUE	0.003669	22.63711
4712	rs1160873	T	G	T	G	0.1062	-0.01646	0.8494	0.876813	FALSE	FALSE	FALSE	ieu-b-508	12	1.2E+08	0.050835	486484	0.74604	Sepsis (28	Sepsis (28	Sepsis (28	TRUE	igd	0.0223	1.82E-06	1017	M33178.m	TRUE	reported	g22WMS	2	TRUE	0.021814	22.6352
5205	rs7693835	A	G	A	G	-0.1204	-0.09874	0.9106	0.888548	FALSE	FALSE	FALSE	ieu-b-508	4	1.78E+08	0.053152	486484	0.063208	Sepsis (28	Sepsis (28	Sepsis (28	TRUE	igd	0.0253	2.01E-06	3221	M33203.m	TRUE	reported	Sto7pR	2	TRUE	0.006982	22.633
6021	rs7334694	T	C	T	C	-0.0643	0.001973	0.4609	0.458427	FALSE	FALSE	FALSE	ieu-b-508	13	55546160	0.033477	486484	0.953004	Sepsis (28	Sepsis (28	Sepsis (28	TRUE	igd	0.0135	1.86E-06	831	M33751.m	TRUE	reported	Cxdkr4	2	TRUE	0.026574	22.63122
662	rs1099316	A	G	A	G	0.0295	-0.05508	0.9587	0.969822	FALSE	FALSE	FALSE	ieu-b-508	9	97068926	0.097892	486484	0.573655	Sepsis (28	Sepsis (28	Sepsis (28	TRUE	igd	0.0062	1.68E-06	5687	M01284.m	TRUE	reported	E4hc9c	2	TRUE	0.003965	22.63122
8339	rs1554057	A	G	A	G	0.0295	-0.05367	0.9059	0.914618	FALSE	FALSE	FALSE	ieu-b-508	8	66289587	0.059745	486484	0.36901	Sepsis (28	Sepsis (28	Sepsis (28	TRUE	igd	0.0062	1.78E-06	5292	M36673.m	TRUE	reported	CD5lvR	2	TRUE	0.00426	22.63062
3066	rs1265532	A	G	A	G	0.0733	-0.06882	0.5227	0.520363	FALSE	FALSE	FALSE	ieu-b-508	5	1.76E+08	0.033383	486484	0.039254	Sepsis (28	Sepsis (28	Sepsis (28	TRUE	igd	0.0154	2.02E-06	1760	M31787.m	TRUE	reported	XMp8YI	2	TRUE	0.012709	22.62938
599	rs6568389	T	C	T	C	0.0157	0.053713	0.8061	0.699348	FALSE	FALSE	FALSE	ieu-b-508	6	1.06E+08	0.036373	486484	0.139745	Sepsis (28	Sepsis (28	Sepsis (28	TRUE	igd	0.0033	2.16E-06	7367	M01110.m	TRUE	reported	0A31Sl	2	TRUE	0.003063	22.62838
4407	rs2324594	T	C	T	C	-0.0357	0.012183	0.1825	0.170587	FALSE	FALSE	FALSE	ieu-b-508	13	40892121	0.04546	486484	0.788706	Sepsis (28	Sepsis (28	Sepsis (28	TRUE	igd	0.0075	2.16E-06	1550	M32869.m	TRUE	reported	Rvim1n	2	TRUE	0.014407	22.62836
5609	rs1104776	T	C	T	C	-0.0157	-0.03352	0.0528	0.060776	FALSE	FALSE	FALSE	ieu-b-508	12	25172939	0.070285	486484	0.633375	Sepsis (28	Sepsis (28	Sepsis (28	TRUE	igd	0.0033	1.49E-06	7336	M33442.m	TRUE	reported	B1Peb3	2	TRUE	0.003076	22.62836
5565	rs1937053	A	T	A	T	0.9018	-0.03851	0.9239	0.867573	FALSE	TRUE	FALSE	ieu-b-508	20	26093132	0.049872	486484	0.439969	Sepsis (28	Sepsis (28	Sepsis (28	TRUE	igd	0.1864	1.31E-06	60	M33423.m	TRUE	reported	MoEnO7	2	TRUE	0.280628	22.62588
2883	rs249619	T	C	T	C	-0.0533	0.030091	0.5601	0.569229	FALSE	FALSE	FALSE	ieu-b-508	12	98170673	0.033835	486484	0.373822	Sepsis (28	Sepsis (28	Sepsis (28	TRUE	igd	0.0112	1.79E-06	1720	M27716.m	TRUE	reported	N5hToq	2	TRUE	0.012996	22.62107
3195	rs7709785	T	C	T	C	-0.0176	0.009648	0.148	0.198644	FALSE	FALSE	FALSE	ieu-b-508	5	1.15E+08	0.042276	486484	0.819481	Sepsis (28	Sepsis (28	Sepsis (28	TRUE	igd	0.0037	1.99E-06	7356	M32338.m	TRUE	reported	q5cuaP	2	TRUE	0.003067	22.62058
4230	rs1939769	A	G	A	G	0.0352	0.027453	0.0647	0.066517	FALSE	FALSE	FALSE	ieu-b-508	11	62914202	0.067255	486484	0.683134	Sepsis (28	Sepsis (28	Sepsis (28	TRUE	igd	0.0074	1.77E-06	6857	M32787.m	TRUE	reported	fSRakm	2	TRUE	0.003289	22.62014
7546	rs847391	A	G	A	G	0.039	0.043434	0.9514	0.933734	FALSE	FALSE	FALSE	ieu-b-508	7	16970675	0.069261	486484	0.530589	Sepsis (28	Sepsis (28	Sepsis (28	TRUE	igd	0.0082	2.16E-06	6538	M35257.m	TRUE	reported	iphouU	2	TRUE	0.003448	22.61354
8499	rs3821142	T	C	T	C	0.0747	-0.00022	0.5584	0.527518	FALSE	FALSE	FALSE	ieu-b-508	2	37575287	0.03341	486484	0.994761	Sepsis (28	Sepsis (28	Sepsis (28	TRUE	igd	0.0157	2.09E-06	1672	M37097.m	TRUE	reported	wCbJj4	2	TRUE	0.013359	22.61112
6643	rs1447600	T	C	T	C	0.11	0.019607	0.81	0.788793	FALSE	FALSE	FALSE	ieu-b-508	3	1.35E+08	0.040834	486484	0.631123	Sepsis (28	Sepsis (28	Sepsis (28	TRUE	igd	0.023	1.73E-06	173	M34322.m	TRUE	reported	16GdG9	2	TRUE	0.116776	22.60891
5249	rs540613	T	C	T	C	0.0214	-0.02177	0.1462	0.193676	FALSE	FALSE	FALSE	ieu-b-508	11	61680870	0.042855	486484	0.611447	Sepsis (28	Sepsis (28	Sepsis (28	TRUE	igd	0.0045	1.92E-06	7063	M33228.m	TRUE	reported	JwOXbQ	2	TRUE	0.003192	22.6089
6609	rs7073702	T	C	T	C	-0.0214	0.039242	0.9282	0.927939	FALSE	FALSE	FALSE	ieu-b-508	10	28216528	0.064327	486484	0.54183	Sepsis (28	Sepsis (28	Sepsis (28	TRUE	igd	0.0045	2.66E-06	6994	M34306.m	TRUE	reported	WPXPUN	2	TRUE	0.003223	22.60884
1416	rs1159507	T	G	T	G	-0.0214	-0.0756	0.0951	0.090256	FALSE	FALSE	FALSE	ieu-b-508	10	87819082	0.058646	486484	0.197374	Sepsis (28	Sepsis (28	Sepsis (28	TRUE	igd	0.0045	1.89E-06	6941	M03127.m	TRUE	reported	JscEZw	2	TRUE	0.003248	22.60879
5938	rs1086138	A	G	A	G	-0.0214	-0.05319	0.7004	0.697383	FALSE	FALSE	FALSE	ieu-b-508	12	1.06E+08	0.036457	486484	0.144557	Sepsis (28	Sepsis (28	Sepsis (28	TRUE	igd	0.0045	1.59E-06	6109	M33638.m	TRUE	reported	hVuSGd	2	TRUE	0.003688	22.6079
7055	rs7215587	T	G	T	G	0.0214	0.061049	0.1312	0.130758	FALSE	FALSE	FALSE	ieu-b-508	17	2348817	0.049705	486484	0.21936	Sepsis (28	Sepsis (28	Sepsis (28	TRUE	igd	0.0045	1.55E-06	5207	M34456.m	TRUE	reported	Nh457q	2	TRUE	0.004324	22.60662
7004	rs1708887	A	G	A	G	0.0856	-0.00424	0.9182	0.916764	FALSE	FALSE	FALSE	ieu-b-508	18	71823296	0.060226	486484	0.943908	Sepsis (28	Sepsis (28	Sepsis (28	TRUE	igd	0.018	2.06E-06	4452	M34420.m	TRUE	reported	20uofq	2	TRUE	0.005054	22.60515
5770	rs1693991	A	T	A	T	-0.0333	0.107221	0.2762	0.288427	FALSE	TRUE	FALSE	ieu-b-508	18	12837993	0.036888	486484	0.003653	Sepsis (28	Sepsis (28	Sepsis (28	TRUE	igd	0.007	1.92E-06	1733	M33509.m	TRUE	reported	56SCCs	2	TRUE	0.01289	22.60429
5571	rs2365913	T	C	T	C	1.0329	0.007191	0.9519	0.698885	FALSE	FALSE	FALSE	ieu-b-508	12	74831961	0.036394	486484	0.843376	Sepsis (28	Sepsis (28	Sepsis (28	TRUE	igd	0.2136	1.34E-06	60	M33423.m	TRUE	reported	MoEnO7	2	TRUE	0.280435	22.60429
2172	rs2241216	C	G	C	G	0.0252	0.062253	0.199	0.239909	FALSE	TRUE	FALSE	ieu-b-508	16	49888310	0.040066	486484	0.120241	Sepsis (28	Sepsis (28	Sepsis (28	TRUE	igd	0.0053	2.32E-06	7251	M19324.m	TRUE	reported	1pj7ZT	2	TRUE	0.003108	22.6011
8404	rs1123296	T	G	T	G	0.0176	0.004917	0.2475	0.270607	FALSE	FALSE	FALSE	ieu-b-508	11	81717389	0.037459	486484	0.895572	Sepsis (28	Sepsis (28	Sepsis (28	TRUE	igd	0.0037	1.60E-06	1764	M36808.m	TRUE	reported	u7lqaj	2	TRUE	0.012665	22.60108
4331	rs2831156	A	G	A	G	-0.0252	0.036598	0.1791	0.180074	FALSE	FALSE	FALSE	ieu-b-508	21	29143094	0.04344	486484	0.399515	Sepsis (28	Sepsis (28	Sepsis (28	TRUE	igd	0.0053	2.38E-06	6967	M32847.m	TRUE	reported	eYme29	2	TRUE	0.003234	22.60084
3416	rs6834891	T	C	T	C	0.0252	0.033892	0.6263	0.620761	FALSE	FALSE	FALSE	ieu-b-508	4	77587257	0.034542	486484	0.326502	Sepsis (28	Sepsis (28	Sepsis (28	TRUE	igd	0.0053	2.20E-06	5080	M32445.m	TRUE	reported	qcc6f3	2	TRUE	0.004431	22.59843
705	rs9497139	A	G	A	G	-0.029	0.299748	0.0155	0.016868	FALSE	FALSE	FALSE	ieu-b-508	6	1.45E+08	0.130436	486484	0.021559	Sepsis (28	Sepsis (28	Sepsis (28	TRUE	igd	0.0061	1.97E-06	7358	M01299.m	TRUE	reported	gfM0a3	2	TRUE	0.003062	22.59531
6617	rs1082686	T	C	T	C	-0.039	-0.03349	0.6363	0.635329	FALSE	FALSE	FALSE	ieu-b-508	10	31023578	0.034742	486484	0.33513	Sepsis (28	Sepsis (28	Sepsis (28	TRUE	igd	0.0082	1.74E-06	1766	M34314.m	TRUE	reported	wPKEWG	2	TRUE	0.012647	22.59485
6793	rs9538657	A	C	A	C	0.4491	-0.04534	0.0228	0.016664	FALSE	FALSE	FALSE	ieu-b-508	13	60928244	0.139327	486484	0.74485	Sepsis (28	Sepsis (28	Sepsis (28	TRUE	igd	0.0943	1.93E-06	525	M34350.m	TRUE	reported	Cc3yW9	2	TRUE	0.041413	22.59462
6698	rs2380875	T	G	T	G	0.2035	-0.04327	0.0551	0.066806	FALSE	FALSE	FALSE	ieu-b-508	9	3671063	0.067945	486484	0.524229	Sepsis (28	Sepsis (28	Sepsis (28	TRUE	igd	0.0427	1.86E-06	369	M34336.m	TRUE	reported	jrSSxL	2	TRUE	0.057984	22.58982
5542	rs1007469	C	G	C	G	0.5873	0.019671	0.8839	0.850323	FALSE	TRUE	FALSE	ieu-b-508	5	1.35E+08	0.046818	486484	0.674362	Sepsis (28	Sepsis (28	Sepsis (28	TRUE	igd	0.1215	1.33E-06	60	M33423.m	TRUE	reported	MoEnO7	2	TRUE	0.280274	22.58625
1346	rs825278	T	C	T	C	-0.0913	-0.00642	0.0201	0.014292	FALSE	FALSE	FALSE	ieu-b-508	2	2.23E+08	0.142003	486484	0.963935	Sepsis (28	Sepsis (28	Sepsis (28	TRUE	igd	0.0192	2.06E-06	1741	M02342.m	TRUE	reported	EhZr3g	2	TRUE	0.012821	22.58603
4346	rs11982	A	G	A	G	0.0385	-0.0493	0.0691	0.075095	FALSE	FALSE	FALSE	ieu-b-508	14	77935677	0.063734	486484	0.439184	Sepsis (28	Sepsis (28	Sepsis (28	TRUE	igd	0.0081	2.26E-06	7355	M32855.m	TRUE	reported	AM8kCf	2	TRUE	0.003062	22.58569
2033	rs1707366	A	G	A	G	0.0856	0.000613	0.023	0.018755	FALSE	FALSE	FALSE	ieu-b-508	6	1.13E+08	0.123755	486484	0.996049	Sepsis (28	Sepsis (28	Sepsis (28	TRUE	igd	0.018	1.84E-06	1492	M18349.m	TRUE	reported	M5qPs8	2	TRUE	0.014931	22.58499
5888	rs321188	A	G	A	G	-0.536	-0.13638	0.9806	0.983844	FALSE	FALSE	FALSE	ieu-b-508	7	1.37E+08	0.130813	486484	0.297138	Sepsis (28	Sepsis (28	Sepsis (28	TRUE	igd	0.1117	1.59E-06	102	M33610.m	TRUE	reported	tmg43v	2	TRUE	0.184171	22.57476
8516	rs631791	A	G	A	G	0.0628	0.022673	0.3017	0.315805	FALSE	FALSE	FALSE	ieu-b-508	11	57675410	0.035925	486484	0.527965	Sepsis (28	Sepsis (28	Sepsis (28	TRUE	igd	0.0132	2.06E-06	751	M37104.m	TRUE	reported	bR3g7d	2	TRUE	0.029257	22.57425
4359	rs1578963	C	G	C	G	0.0846	0.020441	0.0342	0.037851	FALSE	TRUE	FALSE	ieu-b-508	12	82068639	0.087658	486484	0.815615	Sepsis (28	Sepsis (28	Sepsis (28	TRUE	igd	0.0178	1.91E-06	2656	M32857.m	TRUE	reported	MVAWzT	2	TRUE	0.008433	22.57218
5859	rs1183014	T	G	T	G	0.088	0.026559	0.0928	0.102431	FALSE	FALSE	FALSE	ieu-b-508	12	49709867	0.055301	486484	0.631043	Sepsis (28	Sepsis (28	Sepsis (28	TRUE	igd	0.0185	2.00E-06	824	M33609.m	TRUE	reported	h7B9gN	2	TRUE	0.026726	22.57182
6716	rs1207101	T	C	T	C	0.1843	-0.02932	0.2889	0.323926	FALSE	FALSE	FALSE	ieu-b-508	1	1.58E+08	0.035805	486484	0.412835	Sepsis (28	Sepsis (28	Sepsis (28	TRUE	igd	0.0387	1.96E-06	420	M34338.m	TRUE	reported	h4FB9a	2	TRUE	0.051232	22.57126
3711	rs1083610	C	G	C	G	0.086	0.123804	0.0682	0.079915	FALSE	TRUE	FALSE	ieu-b-508	11	4491308	0.061975	486484	0.045756	Sepsis (28	Sepsis (28	Sepsis (28	TRUE	igd	0.0181	1.94E-06	6904	M32616.m	TRUE	reported	vrkjBE	2	TRUE	0.003259	22.56908
4674	rs1047116	T	C	T	C	-0.1783	-0.00153	0.0667	0.0815	FALSE	FALSE	FALSE	ieu-b-508	4	1.69E+08	0.061078	486484	0.979977	Sepsis (28	Sepsis (28	Sepsis (28	TRUE	igd	0.036	7.43E-07	25	M33178.m	TRUE	reported	g22WMS	2	TRUE	0.495255	22.56761
8353	rs1959363	T	C	T	C	-0.0447	0.039095	0.1197	0.124569	FALSE	FALSE	FALSE	ieu-b-508	14	45035016	0.050457	486484	0.438445	Sepsis (28	Sepsis (28	Sepsis (28	TRUE	igd	0.0094	2.06E-06	922	M36738.m	TRUE	reported	mVQQcD	2	TRUE	0.023939	22.56401
8533	rs7797103	T	C	T	C	-0.159	0.001144	0.7905	0.773562	FALSE	FALSE	FALSE	ieu-b-508	7	1.55E+08	0.039917	486484	0.977145	Sepsis (28	Sepsis (28	Sepsis (28	TRUE	igd	0.0334	1.94E-06	437	M37112.m	TRUE	reported	P3GiAg	2	TRUE	0.049302	22.55844
3794	rs2828209	T	C	T	C	-0.0361	0.106528	0.051	0.051372	FALSE	FALSE	FALSE	ieu-b-508	21	24878381	0.075879	486484	0.160346	Sepsis (28	Sepsis (28	Sepsis (28	TRUE	igd	0.0076	1.78E-06	7369	M32635.m	TRUE	reported	jJI9P8	2	TRUE	0.003052	22.55638
5259	rs1314028	A	G	A	G	0.0133	-0.00684	0.5842	0.589147	FALSE	FALSE	FALSE	ieu-b-508	4	1.77E+08	0.033985	486484	0.840429	Sepsis (28	Sepsis (28	Sepsis (28	TRUE	igd	0.0028	1.59E-06	7364	M33230.m	TRUE	reported	nzcTOn	2	TRUE	0.003055	22.55637
6439	rs325614	A	G	A	G	0.0171	0.136224	0.9673	0.962703	FALSE	FALSE	FALSE	ieu-b-508	11	6217152	0.08846	486484	0.123572	Sepsis (28	Sepsis (28	Sepsis (28	TRUE	igd	0.0036	2.63E-06	7362	M34062.m	TRUE	reported	RZyHKP	2	TRUE	0.003055	22.55637
693	rs6493	A	G	A	G	0.0114	-0.0816	0.1195	0.162663	FALSE	FALSE	FALSE	ieu-b-508	14	78140329	0.045134	486484	0.07062	Sepsis (28	Sepsis (28	Sepsis (28	TRUE	igd	0.0024	2.35E-06	7358	M01299.m	TRUE	reported	gfM0a3	2	TRUE	0.003057	22.55637
702	rs9327433	T	G	T	G	-0.0076	-0.04995	0.5134	0.495704	FALSE	FALSE	FALSE	ieu-b-508	5	1.27E+08	0.033699	486484	0.138296	Sepsis (28	Sepsis (28	Sepsis (28	TRUE	igd	0.0016	2.53E-06	7358	M01299.m	TRUE	reported	gfM0a3	2	TRUE	0.003057	22.55637
147	rs6759991	T	G	T	G	0.0076	-0.05922	0.8623	0.842839	FALSE	FALSE	FALSE	ieu-b-508	2	75696816	0.045863	486484	0.196601	Sepsis (28	Sepsis (28	Sepsis (28	TRUE	igd	0.0016	2.52E-06	7355	M00054.m	TRUE	reported	QTDAnh	2	TRUE	0.003058	22.55636
621	rs1111812	A	C	A	C	-0.019	-0.05666	0.8501	0.854411	FALSE	FALSE	FALSE	ieu-b-508	1	2.19E+08	0.048098	486484	0.238809	Sepsis (28	Sepsis (28	Sepsis (28	TRUE	igd	0.004	1.97E-06	7349	M01121.m	TRUE	reported	HU0fhR	2	TRUE	0.003061	22.55636
1703	rs9998852	A	G	A	G	0.0076	-0.00305	0.6611	0.641767	FALSE	FALSE	FALSE	ieu-b-508	4	1.26E+08	0.034977	486484	0.930515	Sepsis (28	Sepsis (28	Sepsis (28	TRUE	igd	0.0016	2.77E-06	7345	M15488.m	TRUE	reported	SPriBO	2	TRUE	0.003062	22.55636
7697	rs1168162	T	G	T	G	-0.0076	0.025218	0.4034	0.413321	FALSE	FALSE	FALSE	ieu-b-508	2	71974813	0.03403	486484	0.458659	Sepsis (28	Sepsis (28	Sepsis (28	TRUE	igd	0.0016	2.99E-06	7345	M35397.m	TRUE	reported	Y56mZr	2	TRUE	0.003062	22.55636
7706	rs2075158	T	C	T	C	-0.0076	0.053108	0.3188	0.331652	FALSE	FALSE	FALSE	ieu-b-508	16	11945231	0.035454	486484	0.134141	Sepsis (28	Sepsis (28	Sepsis (28	TRUE	igd	0.0016	3.23E-06	7345	M35397.m	TRUE	reported	Y56mZr	2	TRUE	0.003062	22.55636
3623	rs1344215	A	G	A	G	0.0418	-0.01044	0.4834	0.521405	FALSE	FALSE	FALSE	ieu-b-508	3	1.87E+08	0.034609	486484	0.763015	Sepsis (28	Sepsis (28	Sepsis (28	TRUE	igd	0.0088	1.76E-06	7337	M32557.m	TRUE	reported	qR7rZX	2	TRUE	0.003066	22.55635
4385	rs1316154	T	C	T	C	-0.0057	0.027278	0.5812	0.59337	FALSE	FALSE	FALSE	ieu-b-508	5	1.5E+08	0.034106	486484	0.423819	Sepsis (28	Sepsis (28	Sepsis (28	TRUE	igd	0.0012	4.84E-06	7329	M32867.m	TRUE	reported	Jcsyzm	2	TRUE	0.003069	22.55634
1573	rs1171591	T	C	T	C	-0.0076	-0.00078	0.2789	0.306207	FALSE	FALSE	FALSE	ieu-b-508	3	49455330	0.036211	486484	0.982894	Sepsis (28	Sepsis (28	Sepsis (28	TRUE	igd	0.0016	3.77E-06	7314	M12593.m	TRUE	reported	IBXgSU	2	TRUE	0.003075	22.55633
1580	rs1615157	T	C	T	C	0.0076	0.026339	0.5764	0.592249	FALSE	FALSE	FALSE	ieu-b-508	3	13164783	0.034177	486484	0.440905	Sepsis (28	Sepsis (28	Sepsis (28	TRUE	igd	0.0016	2.22E-06	7314	M12593.m	TRUE	reported	IBXgSU	2	TRUE	0.003075	22.55633
1707	rs1193049	A	G	A	G	-0.0076	-0.03205	0.803	0.802222	FALSE	FALSE	FALSE	ieu-b-508	4	1.32E+08	0.04199	486484	0.445363	Sepsis (28	Sepsis (28	Sepsis (28	TRUE	igd	0.0016	1.52E-06	7310	M15506.m	TRUE	reported	mVRmZO	2	TRUE	0.003077	22.55633
1076	rs9663814	A	G	A	G	0.0076	-0.01255	0.6175	0.59571	FALSE	FALSE	FALSE	ieu-b-508	10	77738382	0.034235	486484	0.713977	Sepsis (28	Sepsis (28	Sepsis (28	TRUE	igd	0.0016	1.95E-06	7271	M01585.m	TRUE	reported	M7QiSM	2	TRUE	0.003093	22.55629
6755	rs1187167	T	C	T	C	0.0537	-0.02538	0.2673	0.282176	FALSE	FALSE	FALSE	ieu-b-508	17	71409026	0.037042	486484	0.49324	Sepsis (28	Sepsis (28	Sepsis (28	TRUE	igd	0.0113	2.18E-06	1649	M34339.m	TRUE	reported	RwGZ7c	2	TRUE	0.01351	22.55613
2954	rs1272885	A	G	A	G	0.0133	-0.02072	0.5552	0.551328	FALSE	FALSE	FALSE	ieu-b-508	1	45111360	0.033855	486484	0.5406	Sepsis (28	Sepsis (28	Sepsis (28	TRUE	igd	0.0028	2.04E-06	6795	M30805.m	TRUE	reported	YHV70c	2	TRUE	0.003309	22.55586
6101	rs1221004	A	G	A	G	-0.0171	0.054802	0.4239	0.420784	FALSE	FALSE	FALSE	ieu-b-508	6	69433777	0.033786	486484	0.104792	Sepsis (28	Sepsis (28	Sepsis (28	TRUE	igd	0.0036	2.13E-06	6506	M33871.m	TRUE	reported	mQD6DP	2	TRUE	0.003456	22.55556
4280	rs6977159	A	G	A	G	-0.0285	0.037561	0.5935	0.600297	FALSE	FALSE	FALSE	ieu-b-508	7	24930069	0.034262	486484	0.272958	Sepsis (28	Sepsis (28	Sepsis (28	TRUE	igd	0.006	2.00E-06	6200	M32808.m	TRUE	reported	vnr36k	2	TRUE	0.003626	22.55522

2018	rs9854875	A	G	A	G	0.0266	0.014399	0.3185	0.32466	FALSE	FALSE	FALSE	ieu-b-508	3	1.11E+08	0.035849	486484	0.687941	Sepsis (28	Sepsis (28	Sepsis (28	TRUE	igd	0.0056	2.05E-06	5984	M18283.m	TRUE	reported	oyZdTE	2	TRUE	0.003756	22.55496
5501	rs261301	T	C	T	C	0.0323	0.004705	0.1314	0.139747	FALSE	FALSE	FALSE	ieu-b-508	15	58686939	0.04813	486484	0.922124	Sepsis (28	Sepsis (28	Sepsis (28	TRUE	igd	0.0068	2.06E-06	5822	M33420.m	TRUE	reported	eTToDb	2	TRUE	0.00386	22.55475
5658	rs7075080	A	G	A	G	-0.0171	0.026092	0.6326	0.629904	FALSE	FALSE	FALSE	ieu-b-508	10	1E+08	0.034534	486484	0.449932	Sepsis (28	Sepsis (28	Sepsis (28	TRUE	igd	0.0036	2.61E-06	5717	M33453.m	TRUE	reported	OzEeLQ	2	TRUE	0.003931	22.55461
2512	rs6966840	A	G	A	G	0.0133	0.034366	0.7671	0.748275	FALSE	FALSE	FALSE	ieu-b-508	7	1.51E+08	0.038609	486484	0.373412	Sepsis (28	Sepsis (28	Sepsis (28	TRUE	igd	0.0028	2.01E-06	5702	M22130.m	TRUE	reported	YDbJlz	2	TRUE	0.003941	22.55459
3274	rs4717210	A	G	A	G	0.019	-0.05066	0.082	0.085793	FALSE	FALSE	FALSE	ieu-b-508	7	64238697	0.059274	486484	0.392759	Sepsis (28	Sepsis (28	Sepsis (28	TRUE	igd	0.004	2.03E-06	5600	M32348.m	TRUE	reported	DJeLDx	2	TRUE	0.004013	22.55444
2071	rs6470195	A	T	A	T	0.019	0.03531	0.89	0.832862	FALSE	TRUE	FALSE	ieu-b-508	8	1.25E+08	0.048494	486484	0.466533	Sepsis (28	Sepsis (28	Sepsis (28	TRUE	igd	0.004	2.03E-06	5592	M18369.m	TRUE	reported	ykBjIT	2	TRUE	0.004019	22.55443
3173	rs658591	T	G	T	G	-0.019	-0.00448	0.798	0.799785	FALSE	FALSE	FALSE	ieu-b-508	17	26610769	0.041668	486484	0.914322	Sepsis (28	Sepsis (28	Sepsis (28	TRUE	igd	0.004	2.03E-06	5575	M32328.m	TRUE	reported	YGsb6D	2	TRUE	0.004031	22.55441
2187	rs2389336	A	T	A	T	-0.038	0.026405	0.023	0.027863	FALSE	TRUE	FALSE	ieu-b-508	12	34254175	0.101035	486484	0.793827	Sepsis (28	Sepsis (28	Sepsis (28	TRUE	igd	0.008	2.03E-06	5436	M19362.m	TRUE	reported	SkNEKw	2	TRUE	0.004133	22.5542
8225	rs2842707	C	G	C	G	0.057	0.017958	0.399	0.414038	FALSE	TRUE	FALSE	ieu-b-508	1	2.07E+08	0.036185	486484	0.619694	Sepsis (28	Sepsis (28	Sepsis (28	TRUE	igd	0.012	2.03E-06	4904	M36399.m	TRUE	reported	EdUpnt	2	TRUE	0.00458	22.5533
8019	rs6895134	C	G	C	G	0.057	-0.02162	0.011	0.017463	FALSE	TRUE	FALSE	ieu-b-508	5	1.01E+08	0.128931	486484	0.866819	Sepsis (28	Sepsis (28	Sepsis (28	TRUE	igd	0.012	2.03E-06	4895	M35754.m	TRUE	reported	IlXOo1	2	TRUE	0.004588	22.55328
6104	rs247197	T	C	T	C	-0.019	-0.0688	0.382	0.387947	FALSE	FALSE	FALSE	ieu-b-508	5	1.28E+08	0.034221	486484	0.04438	Sepsis (28	Sepsis (28	Sepsis (28	TRUE	igd	0.004	2.03E-06	4867	M33871.m	TRUE	reported	mQD6DP	2	TRUE	0.004614	22.55323
6028	rs1106641	A	C	A	C	0.0893	0.138848	0.0516	0.050563	FALSE	FALSE	FALSE	ieu-b-508	12	1.13E+08	0.07656	486484	0.069743	Sepsis (28	Sepsis (28	Sepsis (28	TRUE	igd	0.0188	2.11E-06	3962	M33782.m	TRUE	reported	875Pal	2	TRUE	0.005662	22.55111
2988	rs241323	A	G	A	G	-0.0794	-0.07574	0.1166	0.105247	FALSE	FALSE	FALSE	ieu-b-508	1	2.29E+08	0.054243	486484	0.162642	Sepsis (28	Sepsis (28	Sepsis (28	TRUE	igd	0.0167	1.88E-06	789	M31522.m	TRUE	reported	gM9gAj	2	TRUE	0.027852	22.54788
8546	rs1002131	T	C	T	C	-0.2423	0.114258	0.9892	0.992716	FALSE	FALSE	FALSE	ieu-b-508	4	76957243	0.198022	486484	0.563943	Sepsis (28	Sepsis (28	Sepsis (28	TRUE	igd	0.051	2.01E-06	1763	M37202.m	TRUE	reported	BtslDx	2	TRUE	0.012641	22.54621
3006	rs626720	A	G	A	G	0.1018	0.025581	0.1195	0.13094	FALSE	FALSE	FALSE	ieu-b-508	4	90112443	0.049855	486484	0.60787	Sepsis (28	Sepsis (28	Sepsis (28	TRUE	igd	0.0214	1.86E-06	522	M31536.m	TRUE	reported	3nAuUI	2	TRUE	0.04155	22.54244
7618	rs2819083	A	G	A	G	0.059	0.007014	0.3744	0.379886	FALSE	FALSE	FALSE	ieu-b-508	6	1.57E+08	0.03463	486484	0.839504	Sepsis (28	Sepsis (28	Sepsis (28	TRUE	igd	0.0124	1.96E-06	448	M35322.m	TRUE	reported	om3h9x	2	TRUE	0.048103	22.53811
8168	rs2854152	A	G	A	G	-0.0304	0.000451	0.3025	0.321746	FALSE	FALSE	FALSE	ieu-b-508	17	61986027	0.035879	486484	0.989964	Sepsis (28	Sepsis (28	Sepsis (28	TRUE	igd	0.0064	2.09E-06	1780	M36131.m	TRUE	reported	DbYm5t	2	TRUE	0.012517	22.53715
2450	rs7499814	A	C	A	C	-0.0285	0.070407	0.8177	0.824058	FALSE	FALSE	FALSE	ieu-b-508	16	88052161	0.044049	486484	0.109962	Sepsis (28	Sepsis (28	Sepsis (28	TRUE	igd	0.006	2.05E-06	1755	M21630.m	TRUE	reported	7eaO4H	2	TRUE	0.012693	22.53679
2168	rs1050108	T	C	T	C	0.0836	-0.09844	0.0309	0.038901	FALSE	FALSE	FALSE	ieu-b-508	11	27788859	0.086324	486484	0.25416	Sepsis (28	Sepsis (28	Sepsis (28	TRUE	igd	0.0176	2.10E-06	1738	M19324.m	TRUE	reported	1pj7ZT	2	TRUE	0.012816	22.53654
7828	rs1193247	A	G	A	G	0.0361	-0.01803	0.4287	0.437714	FALSE	FALSE	FALSE	ieu-b-508	4	77468196	0.033774	486484	0.593469	Sepsis (28	Sepsis (28	Sepsis (28	TRUE	igd	0.0076	2.42E-06	1291	M35527.m	TRUE	reported	UAMhnu	2	TRUE	0.017177	22.52755
5886	rs1759425	A	T	A	T	-0.0742	-0.02559	0.1399	0.172715	FALSE	TRUE	FALSE	ieu-b-508	7	77743726	0.044177	486484	0.562372	Sepsis (28	Sepsis (28	Sepsis (28	TRUE	igd	0.0156	2.10E-06	470	M33610.m	TRUE	reported	tmg43v	2	TRUE	0.045924	22.52717
6140	rs1053871	T	C	T	C	0.0527	0.061304	0.615	0.597139	FALSE	FALSE	FALSE	ieu-b-508	16	3181963	0.033972	486484	0.071146	Sepsis (28	Sepsis (28	Sepsis (28	TRUE	igd	0.0111	2.14E-06	2725	M33883.m	TRUE	reported	RT7YNu	2	TRUE	0.008204	22.52456
8179	rs4954715	A	G	A	G	-0.0527	0.029014	0.0853	0.096429	FALSE	FALSE	FALSE	ieu-b-508	2	1.39E+08	0.056529	486484	0.607763	Sepsis (28	Sepsis (28	Sepsis (28	TRUE	igd	0.0111	2.28E-06	2432	M36134.m	TRUE	reported	ekMsfG	2	TRUE	0.009183	22.52257
2093	rs1258300	T	C	T	C	-0.0318	0.041741	0.0723	0.083657	FALSE	FALSE	FALSE	ieu-b-508	13	1.04E+08	0.060587	486484	0.490852	Sepsis (28	Sepsis (28	Sepsis (28	TRUE	igd	0.0067	1.75E-06	7367	M18467.m	TRUE	reported	64PaNn	2	TRUE	0.003049	22.52095
3714	rs2104340	C	G	C	G	0.0617	-0.00755	0.2526	0.260376	FALSE	TRUE	FALSE	ieu-b-508	9	2196447	0.038082	486484	0.842876	Sepsis (28	Sepsis (28	Sepsis (28	TRUE	igd	0.013	2.16E-06	6904	M32616.m	TRUE	reported	vrkjBE	2	TRUE	0.003252	22.51945
1245	rs219918	C	G	C	G	-0.0299	-0.01072	0.0224	0.021951	FALSE	TRUE	FALSE	ieu-b-508	12	14100129	0.114307	486484	0.925266	Sepsis (28	Sepsis (28	Sepsis (28	TRUE	igd	0.0063	2.48E-06	7136	M01769.m	TRUE	reported	rismUZ	2	TRUE	0.003147	22.5185
4973	rs4787041	T	C	T	C	0.1672	0.22335	0.9626	0.984596	FALSE	FALSE	FALSE	ieu-b-508	16	7581318	0.137224	486484	0.103603	Sepsis (28	Sepsis (28	Sepsis (28	TRUE	igd	0.0352	2.05E-06	1017	M33178.m	TRUE	reported	g22WMS	2	TRUE	0.021704	22.51813
922	rs9987070	C	G	C	G	-0.028	-0.08482	0.9808	0.974646	FALSE	TRUE	FALSE	ieu-b-508	7	19197941	0.106994	486484	0.427914	Sepsis (28	Sepsis (28	Sepsis (28	TRUE	igd	0.0059	2.43E-06	7354	M01494.m	TRUE	reported	7JWNKI	2	TRUE	0.003053	22.51614
8101	rs1687893	A	T	A	T	-0.444	-0.01438	0.0909	0.036027	FALSE	TRUE	FALSE	ieu-b-508	5	1498340	0.090302	486484	0.873494	Sepsis (28	Sepsis (28	Sepsis (28	TRUE	igd	0.0928	1.74E-06	122	M36097.m	TRUE	reported	RHr18S	2	TRUE	0.157989	22.51601
2863	rs9379897	T	C	T	C	-0.0261	-0.04515	0.8707	0.868785	FALSE	FALSE	FALSE	ieu-b-508	6	26601526	0.049308	486484	0.359821	Sepsis (28	Sepsis (28	Sepsis (28	TRUE	igd	0.0055	2.04E-06	7339	M27672.m	TRUE	reported	5cTg55	2	TRUE	0.003059	22.5132
5129	rs3780277	A	G	A	G	0.1767	-0.06643	0.0847	0.08781	FALSE	FALSE	FALSE	ieu-b-508	9	1.34E+08	0.060792	486484	0.274514	Sepsis (28	Sepsis (28	Sepsis (28	TRUE	igd	0.0372	2.07E-06	868	M33188.m	TRUE	reported	xmgbws	2	TRUE	0.025335	22.51051
6665	rs9375892	T	G	T	G	-0.0867	-0.00305	0.8819	0.942365	FALSE	FALSE	FALSE	ieu-b-508	6	99523104	0.071202	486484	0.965884	Sepsis (28	Sepsis (28	Sepsis (28	TRUE	igd	0.0182	2.02E-06	248	M34322.m	TRUE	reported	16GdG9	2	TRUE	0.083834	22.51017
1983	rs9972424	A	G	A	G	-0.0242	0.024524	0.6602	0.653553	FALSE	FALSE	FALSE	ieu-b-508	15	40365851	0.035025	486484	0.483824	Sepsis (28	Sepsis (28	Sepsis (28	TRUE	igd	0.0051	2.04E-06	7226	M17807.m	TRUE	reported	zd6sZl	2	TRUE	0.003106	22.50972
6786	rs35620	C	G	C	G	0.056	0.018132	0.8372	0.836859	FALSE	TRUE	FALSE	ieu-b-508	16	16168340	0.045497	486484	0.690245	Sepsis (28	Sepsis (28	Sepsis (28	TRUE	igd	0.0118	2.23E-06	3491	M34350.m	TRUE	reported	Cc3yW9	2	TRUE	0.00641	22.50936
7392	rs1115007	A	G	A	G	0.0394	0.000137	0.7148	0.648529	FALSE	FALSE	FALSE	ieu-b-508	16	78419522	0.035049	486484	0.996872	Sepsis (28	Sepsis (28	Sepsis (28	TRUE	igd	0.0083	2.38E-06	1630	M35159.m	TRUE	reported	7EoJLB	2	TRUE	0.013636	22.50625
6135	rs7908275	A	C	A	C	-0.3564	0.233365	0.979	0.98635	FALSE	FALSE	FALSE	ieu-b-508	10	1.33E+08	0.146033	486484	0.110035	Sepsis (28	Sepsis (28	Sepsis (28	TRUE	igd	0.0747	1.81E-06	176	M33877.m	TRUE	reported	yDVi1f	2	TRUE	0.114524	22.50457
4473	rs6029632	A	G	A	G	0.0223	0.003861	0.3782	0.397661	FALSE	FALSE	FALSE	ieu-b-508	20	39960342	0.034073	486484	0.909792	Sepsis (28	Sepsis (28	Sepsis (28	TRUE	igd	0.0047	2.60E-06	5425	M33084.m	TRUE	reported	jrOsNK	2	TRUE	0.004133	22.5037
489	rs7359970	C	G	C	G	0.2147	0.067405	0.8975	0.911599	FALSE	TRUE	FALSE	ieu-b-508	19	48696935	0.059102	486484	0.254088	Sepsis (28	Sepsis (28	Sepsis (28	TRUE	igd	0.0451	1.93E-06	283	M00553.m	TRUE	reported	IFjXiL	2	TRUE	0.074143	22.50251
8371	rs1942651	T	C	T	C	-0.0242	-0.04247	0.6705	0.635164	FALSE	FALSE	FALSE	ieu-b-508	7	15770629	0.034622	486484	0.219901	Sepsis (28	Sepsis (28	Sepsis (28	TRUE	igd	0.0051	2.14E-06	3248	M36756.m	TRUE	reported	QsW4oR	2	TRUE	0.006885	22.50209
1870	rs1768319	A	G	A	G	-0.0446	-0.04622	0.0385	0.043757	FALSE	FALSE	FALSE	ieu-b-508	18	35238850	0.081628	486484	0.571204	Sepsis (28	Sepsis (28	Sepsis (28	TRUE	igd	0.0094	1.88E-06	4538	M15958.m	TRUE	reported	3HF6lf	2	TRUE	0.004936	22.50207
6407	rs6125916	T	G	T	G	-0.0204	-0.04113	0.1936	0.205119	FALSE	FALSE	FALSE	ieu-b-508	20	36328382	0.041363	486484	0.320007	Sepsis (28	Sepsis (28	Sepsis (28	TRUE	igd	0.0043	1.80E-06	7338	M34035.m	TRUE	reported	W4G5qR	2	TRUE	0.003058	22.50117
5785	rs2328991	C	G	C	G	0.0204	0.03438	0.1132	0.122048	FALSE	TRUE	FALSE	ieu-b-508	13	77397101	0.051002	486484	0.500259	Sepsis (28	Sepsis (28	Sepsis (28	TRUE	igd	0.0043	1.88E-06	7269	M33510.m	TRUE	reported	WxGKnn	2	TRUE	0.003087	22.50111
6223	rs6584331	T	C	T	C	0.0204	0.03902	0.4873	0.475342	FALSE	FALSE	FALSE	ieu-b-508	10	1.02E+08	0.033435	486484	0.243194	Sepsis (28	Sepsis (28	Sepsis (28	TRUE	igd	0.0043	2.55E-06	4533	M33901.m	TRUE	reported	mQkhrb	2	TRUE	0.004941	22.49737
607	rs1717523	A	G	A	G	0.0593	0.014643	0.9348	0.953695	FALSE	FALSE	FALSE	ieu-b-508	13	94965048	0.080378	486484	0.855441	Sepsis (28	Sepsis (28	Sepsis (28	TRUE	igd	0.0125	1.99E-06	4934	M01114.m	TRUE	reported	fqHdM2	2	TRUE	0.004541	22.49641
2135	rs1076656	T	C	T	C	0.0389	-0.00261	0.4367	0.449415	FALSE	FALSE	FALSE	ieu-b-508	11	19505217	0.033529	486484	0.93803	Sepsis (28	Sepsis (28	Sepsis (28	TRUE	igd	0.0082	1.97E-06	3783	M18497.m	TRUE	reported	90JFPc	2	TRUE	0.005914	22.49271
2144	rs7108724	A	G	A	G	-0.0389	0.01328	0.6246	0.627702	FALSE	FALSE	FALSE	ieu-b-508	11	1.29E+08	0.034506	486484	0.700351	Sepsis (28	Sepsis (28	Sepsis (28	TRUE	igd	0.0082	2.13E-06	3783	M18497.m	TRUE	reported	90JFPc	2	TRUE	0.005914	22.49271
8076	rs1185800	T	C	T	C	-0.0408	-0.08151	0.8867	0.886708	FALSE	FALSE	FALSE	ieu-b-508	13	39979645	0.052765	486484	0.122386	Sepsis (28	Sepsis (28	Sepsis (28	TRUE	igd	0.0086	2.28E-06	2651	M36009.m	TRUE	reported	2EUZ1U	2	TRUE	0.008419	22.49032
2315	rs254840	A	G	A	G	-0.0166	0.005456	0.0939	0.105694	FALSE	FALSE	FALSE	ieu-b-508	5	1.59E+08	0.054335	486484	0.92001	Sepsis (28	Sepsis (28	Sepsis (28	TRUE	igd	0.0035	3.03E-06	7327	M20699.m	TRUE	reported	Z3EKFa	2	TRUE	0.003061	22.48855
4115	rs1190591	A	G	A	G	-0.0166	-0.03027	0.5878	0.588043	FALSE	FALSE	FALSE	ieu-b-508	20	36097077	0.034234	486484	0.376529	Sepsis (28	Sepsis (28	Sepsis (28	TRUE	igd	0.0035	1.92E-06	6658	M32755.m	TRUE	reported	WDrNdi	2	TRUE	0.003367	22.48794
8580	rs4732543	A	G	A	G	0.0166	-0.01817	0.2404	0.245937	FALSE	FALSE	FALSE	ieu-b-508	7	83745910	0.038708	486484	0.638837	Sepsis (28	Sepsis (28	Sepsis (28	TRUE	igd	0.0035	1.56E-06	5932	M37253.m	TRUE	reported	PYMqzt	2	TRUE	0.003778	22.48711
5313	rs1246985	A	T	A	T	-0.0204	0.062443	0.707	0.730617	FALSE	TRUE	FALSE	ieu-b-508	2	1.5E+08	0.037697	486484	0.097636	Sepsis (28	Sepsis (28	Sepsis (28	TRUE	igd	0.0043	2.12E-06	2212	M33363.m	TRUE	reported	7K0MM0	2	TRUE	0.010073	22.48695
738	rs2418886	A	G	A	G	0.0223	-0.02473	0.0489	0.050101	FALSE	FALSE	FALSE	ieu-b-508	1	1.9E+08	0.076653	486484	0.74697	Sepsis (28	Sepsis (28	Sepsis (28	TRUE	igd	0.0047	1.63E-06	1762	M01302.m	TRUE	reported	sNp2lC	2	TRUE	0.012615	22.48644
7237	rs9344588	T	C	T	C	0.0389	-0.03582	0.1831	0.159185	FALSE	FALSE	FALSE	ieu-b-508	6	86956248	0.045909	486484	0.435204	Sepsis (28	Sepsis (28	Sepsis (28	TRUE	igd	0.0082	2.31E-06	2474	M34878.m	TRUE	reported	Cpq9yU	2	TRUE	0.009014	22.48642
4377	rs7856961	A	G	A	G	-0.0778	0.041281	0.1517	0.174901	FALSE	FALSE	FALSE	ieu-b-508	9	1927121	0.04423	486484	0.350647	Sepsis (28	Sepsis (28	Sepsis (28	TRUE	igd	0.0164	2.15E-06	1988	M32863.m	TRUE	reported	cLOFYE	2	TRUE	0.011194	22.48197
538	rs2964063	T	C	T	C	-0.0735	-0.12594	0.9853	0.983483	FALSE	FALSE	FALSE	ieu-b-508	5	1.74E+08	0.130396	486484	0.33413	Sepsis (28	Sepsis (28	Sepsis (28	TRUE	igd	0.0155	2.25E-06	7237	M00599.m	TRUE	reported	nRJY3s	2	TRUE	0.003097	22.47974
1453	rs7126003	T	C	T	C	0.0185	0.013371	0.6259	0.628538	FALSE	FALSE	FALSE	ieu-b-508	11	10888433	0.034615	486484	0.699299	Sepsis (28	Sepsis (28	Sepsis (28	TRUE	igd	0.0039	2.58E-06	1759	M03141.m	TRUE	reported	y4mG6m	2	TRUE	0.012631	22.47606
6030	rs1240513	A	G	A	G	0.0697	-0.09117	0.0929	0.090521	FALSE	FALSE	FALSE	ieu-b-508	1	2.43E+08	0.058611	486484	0.11982	Sepsis (28	Sepsis (28	Sepsis (28	TRUE	igd	0.0147	2.06E-06	3962	M33782.m	TRUE	reported	875Pal	2	TRUE	0.005642	22.47044
1136	rs1049222	T	C	T	C	-0.0128	0.002151	0.8498	0.852195	FALSE	FALSE	FALSE	ieu-b-508	12	96461306	0.046924	486484	0.963438	Sepsis (28	Sepsis (28	Sepsis (28	TRUE	igd	0.0027	1.60E-06	7355	M01644.m	TRUE	reported	IB0DTL	2	TRUE	0.003046	22.46851
3589	rs7959422	T	C	T	C	0.0128	-0.01057	0.888	0.889257	FALSE	FALSE	FALSE	ieu-b-508	12	91692508	0.05316	486484	0.84243	Sepsis (28	Sepsis (28	Sepsis (28	TRUE	igd	0.0027	2.04E-06	7350	M32518.m	TRUE	reported	V0ZCiU	2	TRUE	0.003048	22.46851
7411	rs1104858	A	G	A	G	-0.0128	0.016085	0.7449	0.754122	FALSE	FALSE	FALSE	ieu-b-508	12	26726859	0.039026	486484	0.680224	Sepsis (28	Sepsis (28	Sepsis (28	TRUE	igd	0.0027	1.85E-06	7350	M35186.m	TRUE	reported	UZfu6Z	2	TRUE	0.003048	22.46851
7923	rs1739166	A	G	A	G	0.0128	-0.00377	0.1367	0.132969	FALSE	FALSE	FALSE	ieu-b-508	13	1.09E+08	0.04912	486484	0.938882	Sepsis (28	Sepsis (28	Sepsis (28	TRUE	igd	0.0027	2.26E-06	7348	M35675.m	TRUE	reported	D7fdyZ	2	TRUE	0.003049	22.46851
2702	rs2223810	A	C	A	C	-0.0128	0.025714	0.8498	0.844972	FALSE	FALSE	FALSE	ieu-b-508	6	1.04E+08	0.046035	486484	0.576449	Sepsis (28	Sepsis (28	Sepsis (28	TRUE	igd	0.0027	2.18E-06	7340	M25459.m	TRUE	reported	ACJY35	2	TRUE	0.003053	22.4685
3750	rs9322744	T	C	T	C	0.0128	-0.03385	0.3958	0.40069	FALSE	FALSE	FALSE	ieu-b-508	6	1.04E+08	0.033993	486484	0.31937	Sepsis (28	Sepsis (28	Sepsis (28	TRUE	igd	0.0027	1.91E-06	7337	M32632.m	TRUE	reported	pIHzPT	2	TRUE	0.003054	22.4685
2627	rs7990504	A	C	A	C	0.0128	-0.02127	0.8066	0.804934	FALSE	FALSE	FALSE	ieu-b-508	13	42600003	0.042147	486484	0.613872	Sepsis (28	Sepsis (28	Sepsis (28	TRUE	igd	0.0027	3.13E-06	7329	M22481.m	TRUE	reported	bXpf0k	2	TRUE	0.003057	22.46849
3012	rs749429	T	G	T	G	-0.142	-0.05882	0.9406	0.933053	FALSE	FALSE	FALSE	ieu-b-508	11	20585803	0.067027	486484	0.38021	Sepsis (28	Sepsis (28	Sepsis (28	TRUE	igd	0.0299	2.07E-06	522	M31536.m	TRUE	reported	3nAuUI	2	TRUE	0.041418	22.46814
1553	rs789195	A	G	A	G	-0.0128	0.031353	0.7657	0.767274	FALSE	FALSE	FALSE	ieu-b-508	12	70455012	0.039474	486484	0.427036	Sepsis (28	Sepsis (28	Sepsis (28	TRUE	igd	0.0027	2.53E-06	6864	M12129.m	TRUE	reported	iNzdgb	2	TRUE	0.003264	22.46807
7453	rs1021115	T	G	T	G	-0.0128	0.048367	0.4131	0.403778	FALSE	FALSE	FALSE	ieu-b-508	2	76815174	0.033907	486484	0.153732	Sepsis (28	Sepsis (28	Sepsis (28	TRUE	igd	0.0027	2.90E-06	6543	M35193.m	TRUE	reported	BMMWXU	2	TRUE	0.003423	22.46775
2718	rs1007865	T	C	T	C	0.0128	-0.03091	0.7163	0.719132	FALSE	FALSE	FALSE	ieu-b-508	5	1.04E+08	0.037359	486484	0.408014	Sepsis (28	Sepsis (28	Sepsis (28	TRUE	igd	0.0027	2.85E-06	6388	M25599.m	TRUE	reported	fRZxwP	2	TRUE	0.003506	22.46759
2725	rs1307318	C	G	C	G	0.0128	-0.02531	0.3048	0.310882	FALSE	TRUE	FALSE	ieu-b-508	3	56572454	0.036224	486484	0.484795	Sepsis (28	Sepsis (28	Sepsis (28	TRUE	igd	0.0027	3.02E-06	6388	M25599.m	TRUE	reported	fRZxwP	2	TRUE	0.003506	22.46759
4651	rs899311	A	T	A	T	0.4035	-0.02738	0.8971	0.865472	FALSE	TRUE	FALSE	ieu-b-508	16	7092265	0.049026	486484	0.576513	Sepsis (28	Sepsis (28	Sepsis (28	TRUE	igd	0.084	1.55E-06	76	M33173.m	TRUE	reported	04fkUQ	2	TRUE	0.232899	22.46708
3691	rs1745093	A	G	A	G	-0.0365	0.049816	0.0875	0.126446	FALSE	FALSE	FALSE	ieu-b-508	8	1.28E+08	0.0519	486484	0.337132	Sepsis (28	Sepsis (28	Sepsis (28	TRUE	igd	0.0077	2.21E-06	7369	M32587.m	TRUE	reported	uknv7G	2	TRUE	0.00304	22.46396
4552	rs429020	A	G	A	G	-0.0365	-0.02711	0.0511	0.050987	FALSE	FALSE	FALSE	ieu-b-508	8	26433269	0.07658	486484	0.723284	Sepsis (28	Sepsis (28	Sepsis (28	TRUE	igd	0.0077	2.01E-06	7319	M33140.m	TRUE	reported	eBOUtb	2	TRUE	0.003061	22.46392
1985	rs1191565	A	G	A	G	-0.0474	-0.00244	0.1557	0.145862	FALSE	FALSE	FALSE	ieu-b-508	3	1.75E+08	0.04903	486484	0.960266	Sepsis (28	Sepsis (28	Sepsis (28	TRUE	igd	0.01	2.22E-06	7071	M18254.m	TRUE	reported	clvu6V	2	TRUE	0.003167	22.46125
426	rs2870664	T	C	T	C	0.0147	-0.00457	0.4767	0.463005	FALSE	FALSE	FALSE	ieu-b-508	20	54283260	0.033531	486484	0.891494	Sepsis (28	Sepsis (28	Sepsis (28	TRUE	igd	0.0031	1.60E-06	1763	M00063.m	TRUE	reported	Hdsv1L	2	TRUE	0.012594	22.46044
8340	rs1757342	A	G	A	G	0.0237	0.010371	0.1497	0.159681	FALSE	FALSE	FALSE	ieu-b-508	4	1.36E+08	0.046124	486484	0.822099	Sepsis (28	Sepsis (28	Sepsis (28	TRUE	igd	0.005	1.70E-06	5292	M36673.m	TRUE	reported	CD5lvR	2	TRUE	0.004228	22.45911
1105	rs2180566	T	C	T	C	-0.0109	-0.00052	0.6433	0.643133	FALSE	FALSE	FALSE	ieu-b-508	20	30042033	0.03516	486484	0.988196	Sepsis (28	Sepsis (28	Sepsis (28	TRUE	igd	0.0023	2.11E-06	7104	M01638.m	TRUE	reported	8dp0Ac	2	TRUE	0.003152	22.45303
5228	rs2711484	A	G	A	G	-0.0218	0.020088	0.3034	0.30078	FALSE	FALSE	FALSE	ieu-b-508	7	68210911	0.036433	486484	0.581384	Sepsis (28	Sepsis (28	Sepsis (28	TRUE	igd	0.0046	1.89E-06	4665	M33221.m	TRUE	reported	8ZrV1r	2	TRUE	0.004791	22.44973
2920	rs4860118	T	C	T	C	0.0716	0.015392	0.9631	0.960554	FALSE	FALSE	FALSE	ieu-b-508	4	63535037	0.085755	486484	0.857555	Sepsis (28	Sepsis (28	Sepsis (28	TRUE	igd	0.0151	2.04E-06	1227	M27728.m	TRUE	reported	WOp1XE	2	TRUE	0.017995	22.44728
7599	rs1188998	T	C	T	C	0.0493	-0.01087	0.7004	0.649997	FALSE	FALSE	FALSE	ieu-b-508	2	1.71E+08	0.035112	486484	0.756817	Sepsis (28	Sepsis (28	Sepsis (28	TRUE	igd	0.0104	2.12E-06	1765	M35320.m	TRUE	reported	ijCk2X	2	TRUE	0.012572	22.44578
3645	rs9901192	A	G	A	G	0.1267	0.065024	0.0959	0.110511	FALSE	FALSE	FALSE	ieu-b-508	17	6095435	0.053144	486484	0.221127	Sepsis (28	Sepsis (28	Sepsis (28	TRUE	igd	0.0267	2.00E-06	604	M32560.m	TRUE	reported	sLwsEw	2	TRUE	0.035942	22.44348
3170	rs2915420	T	C	T	C	-0.0199	0.013652	0.1595	0.149259	FALSE	FALSE	FALSE	ieu-b-508	9	1.04E+08	0.047046	486484	0.771681	Sepsis (28	Sepsis (28	Sepsis (28	TRUE	igd	0.0042	2.10E-06	7340	M32328.m	TRUE	reported	YGsb6D	2	TRUE	0.003049	22.44343
8502	rs4898658	A	C	A	C	-0.0777	-0.02543	0.0682	0.06816	FALSE	FALSE	FALSE	ieu-b-508	14	50939208	0.066349	486484	0.701465	Sepsis (28	Sepsis (28	Sepsis (28	TRUE	igd	0.0164	2.05E-06	7014	M37097.m	TRUE	reported	wCbJj4	2	TRUE	0.00319	22.44039
3791	rs1157445	A	C	A	C	-0.0379	0.051842	0.0424	0.045401	FALSE	FALSE	FALSE	ieu-b-508	4	74846661	0.081543	486484	0.524934	Sepsis (28	Sepsis (28	Sepsis (28	TRUE	igd	0.008	1.96E-06	7369	M32635.m	TRUE	reported	jJI9P8	2	TRUE	0.003036	22.43781
4589	rs7146383	T	C	T	C	0.1237	0.173395	0.0124	0.009175	FALSE	FALSE	FALSE	ieu-b-508	14	32312297	0.179666	486484	0.334495	Sepsis (28	Sepsis (28	Sepsis (28	TRUE	igd	0.0261	2.15E-06	1531	M33163.m	TRUE	reported	YzGXkR	2	TRUE	0.01446	22.43317
1872	rs2133896	T	G	T	G	0.0469	-0.11748	0.0416	0.04235	FALSE	FALSE	FALSE	ieu-b-508	12	99848900	0.083994	486484	0.161908	Sepsis (28	Sepsis (28	Sepsis (28	TRUE	igd	0.0099	2.36E-06	4538	M15958.m	TRUE	reported	3HF6lf	2	TRUE	0.004921	22.43282
7601	rs4741433	T	G	T	G	0.018	-0.00104	0.438	0.412846	FALSE	FALSE	FALSE	ieu-b-508	9	1497818	0.034125	486484	0.975579	Sepsis (28	Sepsis (28	Sepsis (28	TRUE	igd	0.0038	2.64E-06	7359	M35320.m	TRUE	reported	ijCk2X	2	TRUE	0.00304	22.43158
3329	rs377483	T	C	T	C	-0.018	0.033427	0.6051	0.60226	FALSE	FALSE	FALSE	ieu-b-508	21	37920943	0.034294	486484	0.329708	Sepsis (28	Sepsis (28	Sepsis (28	TRUE	igd	0.0038	1.60E-06	7358	M32405.m	TRUE	reported	uzomIQ	2	TRUE	0.00304	22.43157
3331	rs6011040	A	G	A	G	-0.018	-0.04332	0.3141	0.332641	FALSE	FALSE	FALSE	ieu-b-508	20	62337406	0.035364	486484	0.220571	Sepsis (28	Sepsis (28	Sepsis (28	TRUE	igd	0.0038	2.72E-06	7358	M32405.m	TRUE	reported	uzomIQ	2	TRUE	0.00304	22.43157
3336	rs9636231	A	G	A	G	0.018	-0.07323	0.2952	0.289951	FALSE	FALSE	FALSE	ieu-b-508	2	1.4E+08	0.036694	486484	0.045972	Sepsis (28	Sepsis (28	Sepsis (28	TRUE	igd	0.0038	2.13E-06	7358	M32405.m	TRUE	reported	uzomIQ	2	TRUE	0.00304	22.43157
3570	rs2431704	A	C	A	C	-0.009	0.049706	0.8029	0.801818	FALSE	FALSE	FALSE	ieu-b-508	5	1.28E+08	0.041977	486484	0.236361	Sepsis (28	Sepsis (28	Sepsis (28	TRUE	igd	0.0019	1.04E-06	7350	M32518.m	TRUE	reported	V0ZCiU	2	TRUE	0.003043	22.43157
7929	rs25846	T	C	T	C	-0.009	0.008628	0.3109	0.310492	FALSE	FALSE	FALSE	ieu-b-508	5	1.49E+08	0.036587	486484	0.813565	Sepsis (28	Sepsis (28	Sepsis (28	TRUE	igd	0.0019	1.62E-06	7348	M35675.m	TRUE	reported	D7fdyZ	2	TRUE	0.003044	22.43157
2607	rs1299549	A	G	A	G	0.009	0.014009	0.5359	0.535896	FALSE	FALSE	FALSE	ieu-b-508	2	28651505	0.033553	486484	0.676303	Sepsis (28	Sepsis (28	Sepsis (28	TRUE	igd	0.0019	1.84E-06	7329	M22481.m	TRUE	reported	bXpf0k	2	TRUE	0.003052	22.43155
2620	rs4279521	A	C	A	C	0.009	-0.03168	0.475	0.47755	FALSE	FALSE	FALSE	ieu-b-508	7	75452584	0.033361	486484	0.342369	Sepsis (28	Sepsis (28	Sepsis (28	TRUE	igd	0.0019	1.42E-06	7329	M22481.m	TRUE	reported	bXpf0k	2	TRUE	0.003052	22.43155
2632	rs9668088	T	C	T	C	-0.009	0.080806	0.4178	0.425179	FALSE	FALSE	FALSE	ieu-b-508	12	95281404	0.033788	486484	0.016778	Sepsis (28	Sepsis (28	Sepsis (28	TRUE	igd	0.0019	1.28E-06	7329	M22481.m	TRUE	reported	bXpf0k	2	TRUE	0.003052	22.43155
3830	rs8082649	A	G	A	G	0.009	-0.01069	0.3275	0.330064	FALSE	FALSE	FALSE	ieu-b-508	17	72117069	0.03561	486484	0.764114	Sepsis (28	Sepsis (28	Sepsis (28	TRUE	igd	0.0019	1.37E-06	7229	M32644.m	TRUE	reported	JAa2WD	2	TRUE	0.003094	22.43147
7561	rs1762563	T	C	T	C	-0.009	-0.00316	0.1889	0.196118	FALSE	FALSE	FALSE	ieu-b-508	1	1.78E+08	0.04191	486484	0.939896	Sepsis (28	Sepsis (28	Sepsis (28	TRUE	igd	0.0019	1.32E-06	7217	M35270.m	TRUE	reported	Ct8V1n	2	TRUE	0.003099	22.43146
3649	rs1125853	C	G	C	G	0.045	-0.02702	0.1705	0.178737	FALSE	TRUE	FALSE	ieu-b-508	8	30568635	0.04362	486484	0.535598	Sepsis (28	Sepsis (28	Sepsis (28	TRUE	igd	0.0095	2.08E-06	6970	M32564.m	TRUE	reported	73MgOk	2	TRUE	0.003209	22.43123
6822	rs3875143	A	C	A	C	0.009	0.049749	0.6316	0.632641	FALSE	FALSE	FALSE	ieu-b-508	19	51401807	0.034738	486484	0.152105	Sepsis (28	Sepsis (28	Sepsis (28	TRUE	igd	0.0019	1.76E-06	6755	M34359.m	TRUE	reported	0UotaJ	2	TRUE	0.003311	22.43103
8007	rs2683822	T	C	T	C	-0.009	0.068407	0.6125	0.62328	FALSE	FALSE	FALSE	ieu-b-508	2	1.42E+08	0.034411	486484	0.04682	Sepsis (28	Sepsis (28	Sepsis (28	TRUE	igd	0.0019	3.46E-06	6604	M35754.m	TRUE	reported	IlXOo1	2	TRUE	0.003386	22.43088
8013	rs4968679	T	C	T	C	-0.009	-0.02883	0.4992	0.492202	FALSE	FALSE	FALSE	ieu-b-508	17	62046741	0.033555	486484	0.390269	Sepsis (28	Sepsis (28	Sepsis (28	TRUE	igd	0.0019	3.09E-06	6604	M35754.m	TRUE	reported	IlXOo1	2	TRUE	0.003386	22.43088
8020	rs7036147	T	C	T	C	-0.009	0.00488	0.4125	0.41125	FALSE	FALSE	FALSE	ieu-b-508	9	1.23E+08	0.033982	486484	0.885809	Sepsis (28	Sepsis (28	Sepsis (28	TRUE	igd	0.0019	2.86E-06	6604	M35754.m	TRUE	reported	IlXOo1	2	TRUE	0.003386	22.43088
8026	rs791320	T	C	T	C	-0.009	0.00501	0.5496	0.558812	FALSE	FALSE	FALSE	ieu-b-508	9	88951838	0.033596	486484	0.881446	Sepsis (28	Sepsis (28	Sepsis (28	TRUE	igd	0.0019	2.57E-06	6604	M35754.m	TRUE	reported	IlXOo1	2	TRUE	0.003386	22.43088
8461	rs1171378	A	G	A	G	-0.009	0.015825	0.6291	0.634827	FALSE	FALSE	FALSE	ieu-b-508	3	74432477	0.034881	486484	0.650064	Sepsis (28	Sepsis (28	Sepsis (28	TRUE	igd	0.0019	1.70E-06	6552	M37058.m	TRUE	reported	32p8qV	2	TRUE	0.003413	22.43082
8463	rs1228241	T	C	T	C	0.009	-0.0325	0.475	0.469394	FALSE	FALSE	FALSE	ieu-b-508	11	13441917	0.033425	486484	0.330823	Sepsis (28	Sepsis (28	Sepsis (28	TRUE	igd	0.0019	1.24E-06	6552	M37058.m	TRUE	reported	32p8qV	2	TRUE	0.003413	22.43082
1944	rs9604751	A	G	A	G	0.018	-0.01152	0.1344	0.207142	FALSE	FALSE	FALSE	ieu-b-508	22	17986344	0.04287	486484	0.788194	Sepsis (28	Sepsis (28	Sepsis (28	TRUE	igd	0.0038	2.04E-06	6549	M16822.m	TRUE	reported	UNI018	2	TRUE	0.003414	22.43082
2572	rs625676	T	C	T	C	-0.009	0.041269	0.6649	0.660966	FALSE	FALSE	FALSE	ieu-b-508	11	1.32E+08	0.035277	486484	0.242056	Sepsis (28	Sepsis (28	Sepsis (28	TRUE	igd	0.0019	3.67E-06	6535	M22177.m	TRUE	reported	2s6Sly	2	TRUE	0.003422	22.43081
2588	rs922048	A	T	A	T	-0.009	-0.01983	0.4049	0.400581	FALSE	TRUE	FALSE	ieu-b-508	18	56876386	0.034074	486484	0.560604	Sepsis (28	Sepsis (28	Sepsis (28	TRUE	igd	0.0019	3.33E-06	6535	M22177.m	TRUE	reported	2s6Sly	2	TRUE	0.003422	22.43081
3979	rs1225182	A	G	A	G	-0.009	0.003806	0.6878	0.695103	FALSE	FALSE	FALSE	ieu-b-508	10	2377838	0.036191	486484	0.916253	Sepsis (28	Sepsis (28	Sepsis (28	TRUE	igd	0.0019	2.08E-06	6497	M32729.m	TRUE	reported	XGRSN1	2	TRUE	0.003442	22.43077
3985	rs1424532	A	G	A	G	-0.009	-0.05149	0.6678	0.670436	FALSE	FALSE	FALSE	ieu-b-508	2	1.86E+08	0.035723	486484	0.14947	Sepsis (28	Sepsis (28	Sepsis (28	TRUE	igd	0.0019	1.65E-06	6497	M32729.m	TRUE	reported	XGRSN1	2	TRUE	0.003442	22.43077
3991	rs2000813	T	C	T	C	0.009	0.035926	0.2942	0.297929	FALSE	FALSE	FALSE	ieu-b-508	18	47093864	0.036383	486484	0.32343	Sepsis (28	Sepsis (28	Sepsis (28	TRUE	igd	0.0019	2.31E-06	6497	M32729.m	TRUE	reported	XGRSN1	2	TRUE	0.003442	22.43077
2131	rs7856830	T	C	T	C	-0.0891	0.042538	0.1533	0.151616	FALSE	FALSE	FALSE	ieu-b-508	9	1.36E+08	0.046692	486484	0.362273	Sepsis (28	Sepsis (28	Sepsis (28	TRUE	igd	0.0188	2.27E-06	1457	M18477.m	TRUE	reported	p0vcCU	2	TRUE	0.015182	22.43072
8318	rs1045987	C	G	C	G	-0.0559	-0.02782	0.9167	0.911903	FALSE	TRUE	FALSE	ieu-b-508	16	81093067	0.058932	486484	0.636882	Sepsis (28	Sepsis (28	Sepsis (28	TRUE	igd	0.0118	2.28E-06	3977	M36589.m	TRUE	reported	27GADo	2	TRUE	0.005611	22.43061
7245	rs1160752	T	C	T	C	0.009	0.00872	0.5424	0.537961	FALSE	FALSE	FALSE	ieu-b-508	11	13261390	0.033595	486484	0.795205	Sepsis (28	Sepsis (28	Sepsis (28	TRUE	igd	0.0019	1.82E-06	5885	M34912.m	TRUE	reported	W8lcT6	2	TRUE	0.003798	22.43005
7253	rs1326186	A	G	A	G	-0.009	0.048835	0.4078	0.40073	FALSE	FALSE	FALSE	ieu-b-508	8	77920007	0.034029	486484	0.151264	Sepsis (28	Sepsis (28	Sepsis (28	TRUE	igd	0.0019	1.92E-06	5885	M34912.m	TRUE	reported	W8lcT6	2	TRUE	0.003798	22.43005
7255	rs1347222	T	C	T	C	0.009	-0.00907	0.3688	0.371406	FALSE	FALSE	FALSE	ieu-b-508	12	82394154	0.034629	486484	0.793323	Sepsis (28	Sepsis (28	Sepsis (28	TRUE	igd	0.0019	2.01E-06	5885	M34912.m	TRUE	reported	W8lcT6	2	TRUE	0.003798	22.43005
7262	rs1933830	T	C	T	C	-0.009	-0.0226	0.4953	0.491568	FALSE	FALSE	FALSE	ieu-b-508	6	1.21E+08	0.033289	486484	0.49717	Sepsis (28	Sepsis (28	Sepsis (28	TRUE	igd	0.0019	1.87E-06	5885	M34912.m	TRUE	reported	W8lcT6	2	TRUE	0.003798	22.43005
6678	rs1097120	T	C	T	C	-0.0436	0.014425	0.4075	0.419228	FALSE	FALSE	FALSE	ieu-b-508	9	32899739	0.033986	486484	0.671245	Sepsis (28	Sepsis (28	Sepsis (28	TRUE	igd	0.0092	2.09E-06	1492	M34329.m	TRUE	reported	mgPKP5	2	TRUE	0.01483	22.42925
7334	rs1049244	T	G	T	G	0.009	0.026477	0.6924	0.690003	FALSE	FALSE	FALSE	ieu-b-508	13	46755880	0.036394	486484	0.466906	Sepsis (28	Sepsis (28	Sepsis (28	TRUE	igd	0.0019	2.31E-06	4900	M35137.m	TRUE	reported	Eq2siS	2	TRUE	0.004558	22.42851
7374	rs6788007	T	C	T	C	0.009	0.079662	0.4017	0.394246	FALSE	FALSE	FALSE	ieu-b-508	3	1.17E+08	0.034207	486484	0.019868	Sepsis (28	Sepsis (28	Sepsis (28	TRUE	igd	0.0019	2.17E-06	4900	M35137.m	TRUE	reported	Eq2siS	2	TRUE	0.004558	22.42851
6131	rs4897530	T	C	T	C	0.4842	0.155942	0.0228	0.031009	FALSE	FALSE	FALSE	ieu-b-508	6	1.32E+08	0.096374	486484	0.105641	Sepsis (28	Sepsis (28	Sepsis (28	TRUE	igd	0.1018	1.97E-06	228	M33877.m	TRUE	reported	yDVi1f	2	TRUE	0.090268	22.42475
6360	rs1110089	A	G	A	G	0.0161	-0.01265	0.4863	0.474502	FALSE	FALSE	FALSE	ieu-b-508	4	1.47E+08	0.033771	486484	0.708026	Sepsis (28	Sepsis (28	Sepsis (28	TRUE	igd	0.0034	2.61E-06	7368	M33961.m	TRUE	reported	10AWw0	2	TRUE	0.003034	22.41692
2800	rs3849097	T	G	T	G	-0.0161	0.034096	0.8494	0.800015	FALSE	FALSE	FALSE	ieu-b-508	5	16367855	0.041828	486484	0.414997	Sepsis (28	Sepsis (28	Sepsis (28	TRUE	igd	0.0034	1.73E-06	7347	M27278.m	TRUE	reported	kPgqm3	2	TRUE	0.003043	22.41691
7715	rs4898845	A	G	A	G	-0.0161	-0.07251	0.0654	0.0629	FALSE	FALSE	FALSE	ieu-b-508	14	55130718	0.069024	486484	0.293505	Sepsis (28	Sepsis (28	Sepsis (28	TRUE	igd	0.0034	2.03E-06	7345	M35397.m	TRUE	reported	Y56mZr	2	TRUE	0.003044	22.4169
2314	rs2404571	A	G	A	G	0.0161	0.030625	0.8739	0.862223	FALSE	FALSE	FALSE	ieu-b-508	3	1.61E+08	0.04946	486484	0.535794	Sepsis (28	Sepsis (28	Sepsis (28	TRUE	igd	0.0034	2.92E-06	7327	M20699.m	TRUE	reported	Z3EKFa	2	TRUE	0.003051	22.41689
5784	rs1775463	A	G	A	G	-0.0161	-0.03057	0.7461	0.737217	FALSE	FALSE	FALSE	ieu-b-508	3	23806758	0.038295	486484	0.4247	Sepsis (28	Sepsis (28	Sepsis (28	TRUE	igd	0.0034	2.25E-06	7269	M33510.m	TRUE	reported	WxGKnn	2	TRUE	0.003075	22.41684
8608	rs1773401	A	C	A	C	0.0232	0.062029	0.9604	0.927064	FALSE	FALSE	FALSE	ieu-b-508	2	24690025	0.064383	486484	0.335322	Sepsis (28	Sepsis (28	Sepsis (28	TRUE	igd	0.0049	2.03E-06	7366	M37506.m	TRUE	reported	m2kaRt	2	TRUE	0.003034	22.41124
3379	rs1508782	A	C	A	C	0.0232	0.023391	0.1332	0.151905	FALSE	FALSE	FALSE	ieu-b-508	5	1.08E+08	0.046586	486484	0.615601	Sepsis (28	Sepsis (28	Sepsis (28	TRUE	igd	0.0049	2.09E-06	7355	M32418.m	TRUE	reported	y8cN4Y	2	TRUE	0.003039	22.41123
4832	rs1709322	T	C	T	C	-0.1649	-0.07958	0.0368	0.036424	FALSE	FALSE	FALSE	ieu-b-508	14	96141149	0.089177	486484	0.372186	Sepsis (28	Sepsis (28	Sepsis (28	TRUE	igd	0.0348	2.16E-06	1017	M33178.m	TRUE	reported	g22WMS	2	TRUE	0.021601	22.40928
5549	rs1169745	A	G	A	G	1.2995	-0.10462	0.974	0.95231	FALSE	FALSE	FALSE	ieu-b-508	20	5335724	0.078716	486484	0.183831	Sepsis (28	Sepsis (28	Sepsis (28	TRUE	igd	0.2699	1.47E-06	60	M33423.m	TRUE	reported	MoEnO7	2	TRUE	0.278688	22.40905
3246	rs9551641	T	C	T	C	-0.1241	0.072449	0.0894	0.075677	FALSE	FALSE	FALSE	ieu-b-508	13	29876339	0.063367	486484	0.25291	Sepsis (28	Sepsis (28	Sepsis (28	TRUE	igd	0.0262	2.24E-06	1565	M32346.m	TRUE	reported	cnIFoV	2	TRUE	0.014133	22.4071
3304	rs1684691	T	C	T	C	-0.0398	-0.03848	0.7553	0.734097	FALSE	FALSE	FALSE	ieu-b-508	1	2.27E+08	0.037773	486484	0.308326	Sepsis (28	Sepsis (28	Sepsis (28	TRUE	igd	0.0084	2.45E-06	936	M32388.m	TRUE	reported	yYczcA	2	TRUE	0.023423	22.40158
4744	rs1241335	A	T	A	T	-0.2563	0.230111	0.0177	0.014706	FALSE	TRUE	FALSE	ieu-b-508	10	37922293	0.13885	486484	0.097465	Sepsis (28	Sepsis (28	Sepsis (28	TRUE	igd	0.0541	2.13E-06	1017	M33178.m	TRUE	reported	g22WMS	2	TRUE	0.021592	22.39999
2001	rs1951656	T	C	T	C	-0.42	-0.1016	0.986	0.965931	FALSE	FALSE	FALSE	ieu-b-508	14	54530629	0.092009	486484	0.269478	Sepsis (28	Sepsis (28	Sepsis (28	TRUE	igd	0.0887	2.20E-06	2078	M18281.m	TRUE	reported	3mLp7z	2	TRUE	0.010674	22.39923
3881	rs6817256	T	C	T	C	0.0213	-0.05769	0.0773	0.081099	FALSE	FALSE	FALSE	ieu-b-508	4	1.9E+08	0.06116	486484	0.345533	Sepsis (28	Sepsis (28	Sepsis (28	TRUE	igd	0.0045	1.99E-06	7361	M32654.m	TRUE	reported	6lSQOP	2	TRUE	0.003034	22.39836
918	rs764206	A	G	A	G	0.0071	0.015145	0.6546	0.657489	FALSE	FALSE	FALSE	ieu-b-508	1	1.72E+08	0.03516	486484	0.666653	Sepsis (28	Sepsis (28	Sepsis (28	TRUE	igd	0.0015	4.58E-06	7354	M01494.m	TRUE	reported	7JWNKI	2	TRUE	0.003037	22.39835
4603	rs7943834	C	G	C	G	0.0639	0.071818	0.9792	0.9791	FALSE	TRUE	FALSE	ieu-b-508	11	7163584	0.117338	486484	0.5405	Sepsis (28	Sepsis (28	Sepsis (28	TRUE	igd	0.0135	2.14E-06	7263	M33165.m	TRUE	reported	RZt3uS	2	TRUE	0.003075	22.39827
2595	rs1715787	A	T	A	T	-0.0284	0.056266	0.9213	0.904718	FALSE	TRUE	FALSE	ieu-b-508	10	2263125	0.056987	486484	0.323471	Sepsis (28	Sepsis (28	Sepsis (28	TRUE	igd	0.006	2.32E-06	7259	M22189.m	TRUE	reported	YSD5fY	2	TRUE	0.003077	22.39827
4273	rs1383432	T	G	T	G	-0.0284	0.053579	0.6573	0.661995	FALSE	FALSE	FALSE	ieu-b-508	4	71229662	0.03527	486484	0.128736	Sepsis (28	Sepsis (28	Sepsis (28	TRUE	igd	0.006	2.36E-06	6200	M32808.m	TRUE	reported	vnr36k	2	TRUE	0.003601	22.39722
661	rs1079857	T	G	T	G	-0.0142	-0.013	0.2212	0.324571	FALSE	FALSE	FALSE	ieu-b-508	1	1.78E+08	0.035663	486484	0.715548	Sepsis (28	Sepsis (28	Sepsis (28	TRUE	igd	0.003	2.03E-06	5687	M01284.m	TRUE	reported	E4hc9c	2	TRUE	0.003924	22.39657
6701	rs7029730	A	C	A	C	-0.0142	-0.03696	0.2493	0.257582	FALSE	FALSE	FALSE	ieu-b-508	9	16178816	0.038254	486484	0.333934	Sepsis (28	Sepsis (28	Sepsis (28	TRUE	igd	0.003	1.57E-06	5081	M34336.m	TRUE	reported	jrSSxL	2	TRUE	0.00439	22.39563
5033	rs7089115	A	G	A	G	-0.2733	0.008105	0.0178	0.023172	FALSE	FALSE	FALSE	ieu-b-508	10	7805801	0.112248	486484	0.942438	Sepsis (28	Sepsis (28	Sepsis (28	TRUE	igd	0.0577	2.19E-06	1017	M33178.m	TRUE	reported	g22WMS	2	TRUE	0.021584	22.39096
1237	rs1115509	A	C	A	C	-0.0999	0.135717	0.0138	0.019105	FALSE	FALSE	FALSE	ieu-b-508	17	20928954	0.120676	486484	0.260739	Sepsis (28	Sepsis (28	Sepsis (28	TRUE	igd	0.0211	2.13E-06	1714	M01769.m	TRUE	reported	rismUZ	2	TRUE	0.01291	22.39025
6906	rs7975440	T	C	T	C	-0.0885	0.04122	0.1425	0.161025	FALSE	FALSE	FALSE	ieu-b-508	12	14262889	0.045746	486484	0.367561	Sepsis (28	Sepsis (28	Sepsis (28	TRUE	igd	0.0187	2.31E-06	2779	M34384.m	TRUE	reported	fl3hiS	2	TRUE	0.007995	22.38158
866	rs6783294	A	G	A	G	-0.0213	0.046338	0.4281	0.384437	FALSE	FALSE	FALSE	ieu-b-508	3	1.34E+08	0.034318	486484	0.176933	Sepsis (28	Sepsis (28	Sepsis (28	TRUE	igd	0.0045	2.41E-06	1700	M01361.m	TRUE	reported	NIAcQ3	2	TRUE	0.013008	22.37809
3033	rs1431844	T	C	T	C	0.0563	0.234544	0.0256	0.023344	FALSE	FALSE	FALSE	ieu-b-508	18	40071148	0.110343	486484	0.033537	Sepsis (28	Sepsis (28	Sepsis (28	TRUE	igd	0.0119	2.30E-06	7263	M31555.m	TRUE	reported	iQ5E3v	2	TRUE	0.003072	22.37707
3659	rs2807872	T	C	T	C	-0.0833	0.033326	0.1863	0.18354	FALSE	FALSE	FALSE	ieu-b-508	1	2.21E+08	0.043367	486484	0.442212	Sepsis (28	Sepsis (28	Sepsis (28	TRUE	igd	0.0176	2.11E-06	1718	M32564.m	TRUE	reported	73MgOk	2	TRUE	0.012871	22.37478
3798	rs9570044	A	C	A	C	0.0246	0.085647	0.1057	0.116807	FALSE	FALSE	FALSE	ieu-b-508	13	59667807	0.051922	486484	0.09904	Sepsis (28	Sepsis (28	Sepsis (28	TRUE	igd	0.0052	2.38E-06	7369	M32635.m	TRUE	reported	jJI9P8	2	TRUE	0.003028	22.3741
8623	rs6589371	T	C	T	C	0.0123	-0.03308	0.1212	0.135406	FALSE	FALSE	FALSE	ieu-b-508	11	1.13E+08	0.048693	486484	0.496876	Sepsis (28	Sepsis (28	Sepsis (28	TRUE	igd	0.0026	3.24E-06	7366	M37506.m	TRUE	reported	m2kaRt	2	TRUE	0.003029	22.3741
3954	rs1781045	A	G	A	G	-0.0123	0.034539	0.6796	0.671221	FALSE	FALSE	FALSE	ieu-b-508	17	9789466	0.035627	486484	0.332325	Sepsis (28	Sepsis (28	Sepsis (28	TRUE	igd	0.0026	3.16E-06	7363	M32709.m	TRUE	reported	YR8SB4	2	TRUE	0.00303	22.3741
3962	rs4961335	A	G	A	G	0.0123	-0.00099	0.5531	0.552324	FALSE	FALSE	FALSE	ieu-b-508	8	1.42E+08	0.033717	486484	0.976473	Sepsis (28	Sepsis (28	Sepsis (28	TRUE	igd	0.0026	1.89E-06	7363	M32709.m	TRUE	reported	YR8SB4	2	TRUE	0.00303	22.3741
3964	rs7165678	A	G	A	G	-0.0123	0.00254	0.6088	0.61947	FALSE	FALSE	FALSE	ieu-b-508	15	47568706	0.034328	486484	0.941028	Sepsis (28	Sepsis (28	Sepsis (28	TRUE	igd	0.0026	2.63E-06	7363	M32709.m	TRUE	reported	YR8SB4	2	TRUE	0.00303	22.3741
3884	rs7819084	A	G	A	G	0.0123	-0.0352	0.2474	0.243832	FALSE	FALSE	FALSE	ieu-b-508	8	1.28E+08	0.039264	486484	0.37	Sepsis (28	Sepsis (28	Sepsis (28	TRUE	igd	0.0026	2.45E-06	7361	M32654.m	TRUE	reported	6lSQOP	2	TRUE	0.003031	22.3741
1618	rs806214	A	G	A	G	0.0123	-0.07228	0.2175	0.209465	FALSE	FALSE	FALSE	ieu-b-508	7	1.27E+08	0.040963	486484	0.077641	Sepsis (28	Sepsis (28	Sepsis (28	TRUE	igd	0.0026	2.94E-06	7352	M15122.m	TRUE	reported	Vlnl8T	2	TRUE	0.003035	22.37409
1210	rs2049664	C	G	C	G	0.0123	0.062418	0.1916	0.197035	FALSE	TRUE	FALSE	ieu-b-508	6	1.08E+08	0.042115	486484	0.138314	Sepsis (28	Sepsis (28	Sepsis (28	TRUE	igd	0.0026	3.01E-06	7348	M01670.m	TRUE	reported	kjghbc	2	TRUE	0.003037	22.37409
2796	rs1730499	A	G	A	G	0.0123	-0.03211	0.7653	0.765073	FALSE	FALSE	FALSE	ieu-b-508	3	53070755	0.039508	486484	0.416314	Sepsis (28	Sepsis (28	Sepsis (28	TRUE	igd	0.0026	3.10E-06	7347	M27278.m	TRUE	reported	kPgqm3	2	TRUE	0.003037	22.37409
3077	rs1552425	A	T	A	T	-0.0123	-0.04498	0.5806	0.584197	FALSE	TRUE	FALSE	ieu-b-508	16	55231196	0.033701	486484	0.181982	Sepsis (28	Sepsis (28	Sepsis (28	TRUE	igd	0.0026	1.54E-06	7346	M32197.m	TRUE	reported	VZjwa9	2	TRUE	0.003037	22.37408
8384	rs2625530	A	G	A	G	0.0123	0.000102	0.2039	0.209194	FALSE	FALSE	FALSE	ieu-b-508	15	72136243	0.041082	486484	0.998014	Sepsis (28	Sepsis (28	Sepsis (28	TRUE	igd	0.0026	2.41E-06	7335	M36776.m	TRUE	reported	VnYglx	2	TRUE	0.003042	22.37408
1053	rs6743210	T	C	T	C	0.0123	-0.00719	0.6199	0.60749	FALSE	FALSE	FALSE	ieu-b-508	2	2.36E+08	0.037377	486484	0.847401	Sepsis (28	Sepsis (28	Sepsis (28	TRUE	igd	0.0026	2.21E-06	7333	M01572.m	TRUE	reported	EOUAWy	2	TRUE	0.003043	22.37407
7873	rs6799366	T	C	T	C	0.0123	0.022538	0.3135	0.326621	FALSE	FALSE	FALSE	ieu-b-508	3	3670883	0.035756	486484	0.528468	Sepsis (28	Sepsis (28	Sepsis (28	TRUE	igd	0.0026	3.50E-06	7317	M35631.m	TRUE	reported	002wka	2	TRUE	0.003049	22.37406
2444	rs3755750	T	C	T	C	-0.0123	-0.00296	0.3156	0.322533	FALSE	FALSE	FALSE	ieu-b-508	3	1.23E+08	0.03581	486484	0.93419	Sepsis (28	Sepsis (28	Sepsis (28	TRUE	igd	0.0026	1.81E-06	7284	M21630.m	TRUE	reported	7eaO4H	2	TRUE	0.003063	22.37403
2781	rs4287862	A	G	A	G	0.0123	-0.02398	0.1739	0.1793	FALSE	FALSE	FALSE	ieu-b-508	22	22486735	0.043617	486484	0.582523	Sepsis (28	Sepsis (28	Sepsis (28	TRUE	igd	0.0026	2.16E-06	7268	M27273.m	TRUE	reported	EvU2od	2	TRUE	0.00307	22.37402
2784	rs7184060	A	G	A	G	0.0123	-0.0169	0.1847	0.181215	FALSE	FALSE	FALSE	ieu-b-508	16	54521649	0.043563	486484	0.698007	Sepsis (28	Sepsis (28	Sepsis (28	TRUE	igd	0.0026	1.80E-06	7268	M27273.m	TRUE	reported	EvU2od	2	TRUE	0.00307	22.37402
1602	rs8105499	A	C	A	C	0.0123	0.040313	0.2909	0.312526	FALSE	FALSE	FALSE	ieu-b-508	19	32023957	0.036399	486484	0.268066	Sepsis (28	Sepsis (28	Sepsis (28	TRUE	igd	0.0026	1.57E-06	7258	M12626.m	TRUE	reported	17lClc	2	TRUE	0.003074	22.37401
859	rs1777339	A	G	A	G	0.0123	0.041586	0.2915	0.313367	FALSE	FALSE	FALSE	ieu-b-508	4	6173521	0.035794	486484	0.245306	Sepsis (28	Sepsis (28	Sepsis (28	TRUE	igd	0.0026	3.25E-06	7063	M01361.m	TRUE	reported	NIAcQ3	2	TRUE	0.003159	22.37384
7743	rs7946997	T	C	T	C	-0.0123	-0.01582	0.2974	0.302657	FALSE	FALSE	FALSE	ieu-b-508	11	11069843	0.036283	486484	0.662839	Sepsis (28	Sepsis (28	Sepsis (28	TRUE	igd	0.0026	2.97E-06	7007	M35431.m	TRUE	reported	tLfE4l	2	TRUE	0.003184	22.37379
2030	rs1252652	T	C	T	C	-0.0369	0.132361	0.0349	0.054212	FALSE	FALSE	FALSE	ieu-b-508	6	1.18E+08	0.073619	486484	0.072191	Sepsis (28	Sepsis (28	Sepsis (28	TRUE	igd	0.0078	2.55E-06	6939	M18349.m	TRUE	reported	M5qPs8	2	TRUE	0.003215	22.37373
2227	rs9494155	A	C	A	C	0.0123	-0.06166	0.5988	0.586152	FALSE	FALSE	FALSE	ieu-b-508	6	1.35E+08	0.033978	486484	0.069583	Sepsis (28	Sepsis (28	Sepsis (28	TRUE	igd	0.0026	1.99E-06	6678	M19368.m	TRUE	reported	QaKPad	2	TRUE	0.00334	22.37347
2649	rs1020807	A	G	A	G	0.0123	0.006754	0.689	0.66301	FALSE	FALSE	FALSE	ieu-b-508	2	1.93E+08	0.035257	486484	0.848073	Sepsis (28	Sepsis (28	Sepsis (28	TRUE	igd	0.0026	1.51E-06	6517	M22649.m	TRUE	reported	NAzwRF	2	TRUE	0.003422	22.37331
2514	rs7689395	T	C	T	C	-0.0246	-0.06227	0.0682	0.094223	FALSE	FALSE	FALSE	ieu-b-508	4	1.16E+08	0.057139	486484	0.275823	Sepsis (28	Sepsis (28	Sepsis (28	TRUE	igd	0.0052	2.34E-06	5702	M22130.m	TRUE	reported	YDbJlz	2	TRUE	0.00391	22.37233
568	rs1213279	C	G	C	G	0.0615	0.137467	0.9749	0.969259	FALSE	TRUE	FALSE	ieu-b-508	1	2.16E+08	0.096108	486484	0.152617	Sepsis (28	Sepsis (28	Sepsis (28	TRUE	igd	0.013	2.09E-06	5344	M01107.m	TRUE	reported	EbPV1D	2	TRUE	0.00417	22.3718
3734	rs1738284	A	G	A	G	0.1202	-0.19093	0.9864	0.985357	FALSE	FALSE	FALSE	ieu-b-508	18	5521132	0.14004	486484	0.172765	Sepsis (28	Sepsis (28	Sepsis (28	TRUE	igd	0.0254	2.28E-06	1762	M32632.m	TRUE	reported	pIHzPT	2	TRUE	0.01255	22.36909
2231	rs4998531	T	C	T	C	0.0175	-0.0405	0.7039	0.70652	FALSE	FALSE	FALSE	ieu-b-508	3	1.34E+08	0.036677	486484	0.269546	Sepsis (28	Sepsis (28	Sepsis (28	TRUE	igd	0.0037	2.32E-06	6385	M19396.m	TRUE	reported	Xa1QqA	2	TRUE	0.003491	22.36334
478	rs1244001	C	G	C	G	0.1008	0.025459	0.7673	0.761996	FALSE	TRUE	FALSE	ieu-b-508	15	78926726	0.039383	486484	0.517986	Sepsis (28	Sepsis (28	Sepsis (28	TRUE	igd	0.0213	2.18E-06	1233	M00553.m	TRUE	reported	IFjXiL	2	TRUE	0.017839	22.35923
5638	rs4325575	T	C	T	C	-0.0175	-0.03324	0.7167	0.712854	FALSE	FALSE	FALSE	ieu-b-508	16	79533286	0.036842	486484	0.36699	Sepsis (28	Sepsis (28	Sepsis (28	TRUE	igd	0.0037	2.75E-06	4004	M33443.m	TRUE	reported	G1JrRV	2	TRUE	0.005556	22.35917
6112	rs1171478	T	C	T	C	-0.3581	-0.01339	0.9731	0.988465	FALSE	FALSE	FALSE	ieu-b-508	3	71186010	0.158039	486484	0.932498	Sepsis (28	Sepsis (28	Sepsis (28	TRUE	igd	0.0753	1.96E-06	176	M33877.m	TRUE	reported	yDVi1f	2	TRUE	0.113869	22.35915
4379	rs982503	T	C	T	C	0.0667	0.010728	0.8001	0.794298	FALSE	FALSE	FALSE	ieu-b-508	8	98168814	0.041476	486484	0.795903	Sepsis (28	Sepsis (28	Sepsis (28	TRUE	igd	0.0141	2.21E-06	1988	M32863.m	TRUE	reported	cLOFYE	2	TRUE	0.011131	22.35508
4155	rs1093989	T	C	T	C	0.0279	0.057019	0.1641	0.161027	FALSE	FALSE	FALSE	ieu-b-508	5	60556773	0.045776	486484	0.212905	Sepsis (28	Sepsis (28	Sepsis (28	TRUE	igd	0.0059	2.18E-06	6642	M32758.m	TRUE	reported	AF0m66	2	TRUE	0.003355	22.35494
6375	rs1248441	A	G	A	G	0.0492	0.00765	0.8721	0.864268	FALSE	FALSE	FALSE	ieu-b-508	22	21301336	0.048917	486484	0.875721	Sepsis (28	Sepsis (28	Sepsis (28	TRUE	igd	0.0104	2.04E-06	1751	M33968.m	TRUE	reported	sgVOvT	2	TRUE	0.01262	22.35461
5915	rs9521789	T	C	T	C	0.0175	0.016305	0.6021	0.603892	FALSE	FALSE	FALSE	ieu-b-508	13	1.11E+08	0.034154	486484	0.633081	Sepsis (28	Sepsis (28	Sepsis (28	TRUE	igd	0.0037	2.29E-06	2757	M33627.m	TRUE	reported	4RHaUX	2	TRUE	0.008049	22.35412
3239	rs2078391	T	G	T	G	-0.0331	0.006431	0.4558	0.452756	FALSE	FALSE	FALSE	ieu-b-508	9	72726597	0.033522	486484	0.84786	Sepsis (28	Sepsis (28	Sepsis (28	TRUE	igd	0.007	2.56E-06	6687	M32346.m	TRUE	reported	cnIFoV	2	TRUE	0.003333	22.3527
7191	rs1711005	A	C	A	C	0.0331	-0.01504	0.1356	0.144373	FALSE	FALSE	FALSE	ieu-b-508	5	1.49E+08	0.047918	486484	0.75355	Sepsis (28	Sepsis (28	Sepsis (28	TRUE	igd	0.0071	2.08E-06	4800	M34935.m	TRUE	reported	puA8ni	2	TRUE	0.004637	22.35236
6222	rs6565488	A	C	A	C	0.0331	-0.04636	0.1172	0.138482	FALSE	FALSE	FALSE	ieu-b-508	17	78882972	0.048258	486484	0.336715	Sepsis (28	Sepsis (28	Sepsis (28	TRUE	igd	0.007	1.98E-06	4533	M33901.m	TRUE	reported	mQkhrb	2	TRUE	0.004908	22.34952
2942	rs2303010	T	C	T	C	-0.1471	0.287218	0.0102	0.009392	FALSE	FALSE	FALSE	ieu-b-508	5	1.8E+08	0.177614	486484	0.105859	Sepsis (28	Sepsis (28	Sepsis (28	TRUE	igd	0.0311	2.32E-06	1758	M27769.m	TRUE	reported	UtPBd9	2	TRUE	0.017692	22.34651
7564	rs197926	A	G	A	G	-0.0175	0.046109	0.5181	0.499169	FALSE	FALSE	FALSE	ieu-b-508	17	45012087	0.033557	486484	0.166883	Sepsis (28	Sepsis (28	Sepsis (28	TRUE	igd	0.0037	2.19E-06	6701	M35270.m	TRUE	reported	Ct8V1n	2	TRUE	0.003326	22.34396
1095	rs872114	A	G	A	G	-0.0208	-0.01351	0.9074	0.970876	FALSE	FALSE	FALSE	ieu-b-508	4	68560123	0.099396	486484	0.891872	Sepsis (28	Sepsis (28	Sepsis (28	TRUE	igd	0.0044	2.20E-06	7371	M01604.m	TRUE	reported	hE3l2k	2	TRUE	0.003007	22.34104
463	rs6663614	A	G	A	G	-0.0104	0.067436	0.2085	0.284023	FALSE	FALSE	FALSE	ieu-b-508	1	2.13E+08	0.037045	486484	0.068707	Sepsis (28	Sepsis (28	Sepsis (28	TRUE	igd	0.0022	2.08E-06	7361	M00511.m	TRUE	reported	ohacnP	2	TRUE	0.003027	22.34104
3142	rs7908297	T	C	T	C	0.0156	0.013774	0.1133	0.153704	FALSE	FALSE	FALSE	ieu-b-508	10	52517002	0.046261	486484	0.765924	Sepsis (28	Sepsis (28	Sepsis (28	TRUE	igd	0.0033	1.56E-06	7349	M31935.m	TRUE	reported	4igzkL	2	TRUE	0.003032	22.34103
8205	rs1167037	A	G	A	G	-0.0208	-0.03719	0.8641	0.871494	FALSE	FALSE	FALSE	ieu-b-508	19	46148237	0.050039	486484	0.457394	Sepsis (28	Sepsis (28	Sepsis (28	TRUE	igd	0.0044	2.21E-06	7184	M36048.m	TRUE	reported	ZjeC9m	2	TRUE	0.003101	22.34089
7727	rs1048483	T	C	T	C	0.0208	0.112697	0.0654	0.065775	FALSE	FALSE	FALSE	ieu-b-508	6	4553691	0.068543	486484	0.100139	Sepsis (28	Sepsis (28	Sepsis (28	TRUE	igd	0.0044	2.17E-06	7007	M35431.m	TRUE	reported	tLfE4l	2	TRUE	0.003179	22.34075
958	rs1493031	T	C	T	C	0.0208	0.108185	0.0434	0.04327	FALSE	FALSE	FALSE	ieu-b-508	8	18148612	0.082024	486484	0.187188	Sepsis (28	Sepsis (28	Sepsis (28	TRUE	igd	0.0044	1.96E-06	6523	M01504.m	TRUE	reported	AZK0p4	2	TRUE	0.003416	22.34025
6637	rs1207086	T	C	T	C	-0.1619	-0.11953	0.9322	0.926344	FALSE	FALSE	FALSE	ieu-b-508	15	93240812	0.064798	486484	0.065119	Sepsis (28	Sepsis (28	Sepsis (28	TRUE	igd	0.0344	2.07E-06	[illegible]	M34263.m	TRUE	reported	16GdG9	2	TRUE	0.023453	22.3317
4157	rs1246135	C	G	C	G	0.0449	0.033996	0.9375	0.914394	FALSE	TRUE	FALSE	ieu-b-508	19	46696941	0.059195	486484	0.565753	Sepsis (28	Sepsis (28	Sepsis (28	TRUE	igd	0.0095	2.29E-06	6642	M32758.m	TRUE	reported	AF0m66	2	TRUE	0.003352	22.33133
5135	rs2392757	A	G	A	G	-0.0572	0.077072	0.2245	0.23077	FALSE	FALSE	FALSE	ieu-b-508	8	2.22E+08	0.039748	486484	0.052484	Sepsis (28	Sepsis (28	Sepsis (28	TRUE	igd	0.0121	2.12E-06	3460	M33190.m	TRUE	reported	UkhAw5	2	TRUE	0.00847	22.33002
6035	rs4107301	A	G	A	G	-0.0605	-0.00748	0.1135	0.132322	FALSE	FALSE	FALSE	ieu-b-508	13	1.09E+08	0.049336	486484	0.879433	Sepsis (28	Sepsis (28	Sepsis (28	TRUE	igd	0.0128	2.34E-06	3972	M33782.m	TRUE	reported	875Pal	2	TRUE	0.005605	22.32975
6132	rs6109922	A	T	A	T	-0.4676	0.060392	0.9822	0.979643	FALSE	TRUE	FALSE	ieu-b-508	20	15044427	0.122609	486484	0.622328	Sepsis (28	Sepsis (28	Sepsis (28	TRUE	igd	0.0984	2.01E-06	176	M33877.m	TRUE	reported	yDVi1f	2	TRUE	0.113715	22.3252
2150	rs1106154	A	G	A	G	-0.0241	-0.07855	0.95	0.950579	FALSE	FALSE	FALSE	ieu-b-508	12	1.32E+08	0.077415	486484	0.310253	Sepsis (28	Sepsis (28	Sepsis (28	TRUE	igd	0.0051	2.11E-06	7286	M18497.m	TRUE	reported	2vYFb2	2	TRUE	0.003355	22.32446
5133	rs1247824	A	G	A	G	-0.0501	0.058776	0.9345	0.414801	FALSE	FALSE	FALSE	ieu-b-508	5	6389216	0.033932	486484	0.083243	Sepsis (28	Sepsis (28	Sepsis (28	TRUE	igd	0.0106	[illegible]	3460	M33190.m	TRUE	reported	UkhAw5	2	TRUE	0.008467	22.32192
8196	rs6070840	A	C	A	C	0.0397	0.010874	0.2034	0.204427	FALSE	FALSE	FALSE	ieu-b-508	20	58117030	0.041536	486484	0.793487	Sepsis (28	Sepsis (28	Sepsis (28	TRUE	igd	0.0084	2.48E-06	2400	M36230.m	TRUE	reported	V5KleR	2	TRUE	0.009121	22.31826
1500	rs6442530	T	C	T	C	-0.026	-0.02521	0.3251	0.334889	FALSE	FALSE	FALSE	ieu-b-508	3	15555422	0.035618	486484	0.479071	Sepsis (28	Sepsis (28	Sepsis (28	TRUE	igd	0.0055	2.56E-06	1452	M10275.m	TRUE	reported	bbGIsK	2	TRUE	0.015157	22.31633
997	rs1099272	A	C	A	C	0.0326	0.078833	0.0163	0.018717	FALSE	FALSE	FALSE	ieu-b-508	9	96163761	0.122402	486484	0.519541	Sepsis (28	Sepsis (28	Sepsis (28	TRUE	igd	0.0069	2.44E-06	7364	M01564.m	TRUE	reported	IPUdyD	2	TRUE	0.003022	22.31614
1761	rs1156366	A	G	A	G	0.0657	-0.20114	0.0136	0.009673	FALSE	FALSE	FALSE	ieu-b-508	7	1.27E+08	0.173076	486484	0.245179	Sepsis (28	Sepsis (28	Sepsis (28	TRUE	igd	0.0139	2.28E-06	1765	M15155.m	TRUE	reported	s1NfKN	2	TRUE	0.0125	22.31561
6789	rs4631721	A	G	A	G	0.043	-0.04354	0.6423	0.630288	FALSE	FALSE	FALSE	ieu-b-508	1	27468019	0.035366	486484	0.218312	Sepsis (28	Sepsis (28	Sepsis (28	TRUE	igd	0.0091	2.49E-06	3491	M34350.m	TRUE	reported	Cc3yW9	2	TRUE	0.006386	22.31557
7752	rs6025080	T	C	T	C	-0.1125	-0.1281	0.9782	0.983443	FALSE	FALSE	FALSE	ieu-b-508	20	52271025	0.132281	486484	0.332833	Sepsis (28	Sepsis (28	Sepsis (28	TRUE	igd	0.0238	2.25E-06	1760	M35431.m	TRUE	reported	XfYkLe	2	TRUE	0.014468	22.31414
2533	rs1122208	C	G	C	G	-0.0137	0.085227	0.2851	0.881689	FALSE	TRUE	FALSE	ieu-b-508	11	19917838	0.050791	486484	0.093458	Sepsis (28	Sepsis (28	Sepsis (28	TRUE	igd	0.0029	2.56E-06	6503	M22177.m	TRUE	reported	2s6Sly	2	TRUE	0.003422	22.31065
8585	rs8176623	A	G	A	G	-0.0411	0.045589	0.9284	0.917609	FALSE	FALSE	FALSE	ieu-b-508	9	1.36E+08	0.062634	486484	0.466702	Sepsis (28	Sepsis (28	Sepsis (28	TRUE	igd	0.0087	2.06E-06	5932	M37253.m	TRUE	reported	PYMqzt	2	TRUE	0.003748	22.30995
3046	rs1763809	T	C	T	C	-0.0359	-0.00346	0.2983	0.286182	FALSE	FALSE	FALSE	ieu-b-508	1	77372677	0.035047	486484	0.921463	Sepsis (28	Sepsis (28	Sepsis (28	TRUE	igd	0.0076	2.12E-06	7365	M32055.m	TRUE	reported	KltFxx	2	TRUE	0.003032	22.30711
3911	rs1245020	T	C	T	C	-0.0841	0.019315	0.9812	0.952219	FALSE	FALSE	FALSE	ieu-b-508	10	50927702	0.116544	486484	0.868428	Sepsis (28	Sepsis (28	Sepsis (28	TRUE	igd	0.0178	2.33E-06	2575	M32698.m	TRUE	reported	SiuD6F	2	TRUE	0.008595	22.30563
6489	rs7088815	A	G	A	G	0.885	-0.03121	0.984	0.985485	FALSE	FALSE	FALSE	ieu-b-508	10	1.09E+08	0.14009	486484	0.823699	Sepsis (28	Sepsis (28	Sepsis (28	TRUE	igd	0.186	1.96E-06	134	M34109.m	TRUE	reported	piJuC7	2	TRUE	0.144531	22.30219
8140	rs2072901	A	G	A	G	0.0392	-0.05622	0.8764	0.879089	FALSE	FALSE	FALSE	ieu-b-508	3	48290835	0.051334	486484	0.273406	Sepsis (28	Sepsis (28	Sepsis (28	TRUE	igd	0.0083	2.47E-06	7311	M36103.m	TRUE	reported	sjeAPC	2	TRUE	0.003042	22.2996
8229	rs1075399	A	C	A	C	-0.0326	-0.0175	0.3849	0.383609	FALSE	FALSE	FALSE	ieu-b-508	1	1.92E+08	0.03443	486484	0.611264	Sepsis (28	Sepsis (28	Sepsis (28	TRUE	igd	0.0069	2.50E-06	1908	M36468.m	TRUE	reported	vycAFr	2	TRUE	0.011564	22.2988
1211	rs2170700	A	G	A	G	0.0326	0.055538	0.8663	0.896183	FALSE	FALSE	FALSE	ieu-b-508	15	93855208	0.055632	486484	0.318126	Sepsis (28	Sepsis (28	Sepsis (28	TRUE	igd	0.0069	2.45E-06	7348	M01670.m	TRUE	reported	kjghbc	2	TRUE	0.012517	22.29685
8357	rs1173371	T	C	T	C	-0.0704	-0.02851	0.0461	0.057828	FALSE	FALSE	FALSE	ieu-b-508	9	94824773	0.071135	486484	0.688585	Sepsis (28	Sepsis (28	Sepsis (28	TRUE	igd	0.0149	2.44E-06	1506	M36754.m	TRUE	reported	fBbKFg	2	TRUE	0.014607	22.29439
8601	rs1323664	A	G	A	G	-0.0085	0.015836	0.7326	0.718667	FALSE	FALSE	FALSE	ieu-b-508	7	22806718	0.037291	486484	0.671028	Sepsis (28	Sepsis (28	Sepsis (28	TRUE	igd	0.0018	2.28E-06	7366	M37506.m	TRUE	reported	m2kaRt	2	TRUE	0.00303	22.29335
8627	rs7185774	T	C	T	C	0.0085	-0.0465	0.3408	0.326129	FALSE	FALSE	FALSE	ieu-b-508	16	81249927	0.035439	486484	0.189445	Sepsis (28	Sepsis (28	Sepsis (28	TRUE	igd	0.0018	2.16E-06	7366	M37506.m	TRUE	reported	m2kaRt	2	TRUE	0.00303	22.29333
8630	rs9572325	T	C	T	C	0.0085	0.050453	0.4286	0.419367	FALSE	FALSE	FALSE	ieu-b-508	13	70553135	0.034316	486484	0.141486	Sepsis (28	Sepsis (28	Sepsis (28	TRUE	igd	0.0018	2.19E-06	7366	M37506.m	TRUE	reported	m2kaRt	2	TRUE	0.00303	22.29333
437	rs6573766	T	C	T	C	-0.0085	0.014141	0.801	0.79868	FALSE	FALSE	FALSE	ieu-b-508	14	24852046	0.041624	486484	0.734062	Sepsis (28	Sepsis (28	Sepsis (28	TRUE	igd	0.0018	1.55E-06	7365	M00063.m	TRUE	reported	Hdsv1L	2	TRUE	0.003018	22.29333
3270	rs3961919	A	G	A	G	-0.0085	-0.00655	0.4308	0.421181	FALSE	FALSE	FALSE	ieu-b-508	2	1.13E+08	0.034098	486484	0.847698	Sepsis (28	Sepsis (28	Sepsis (28	TRUE	igd	0.0018	2.35E-06	7365	M32348.m	TRUE	reported	DJeLDx	2	TRUE	0.003018	22.29333
3275	rs4815285	A	G	A	G	-0.0085	0.031037	0.4616	0.460779	FALSE	FALSE	FALSE	ieu-b-508	20	24594269	0.033524	486484	0.354531	Sepsis (28	Sepsis (28	Sepsis (28	TRUE	igd	0.0018	2.57E-06	7365	M32348.m	TRUE	reported	DJeLDx	2	TRUE	0.003018	22.29333
3289	rs9821422	T	C	T	C	-0.0085	0.0057	0.5535	0.558024	FALSE	FALSE	FALSE	ieu-b-508	3	88419120	0.033611	486484	0.865513	Sepsis (28	Sepsis (28	Sepsis (28	TRUE	igd	0.0018	2.56E-06	7365	M32348.m	TRUE	reported	DJeLDx	2	TRUE	0.003018	22.29333
5605	rs7820633	A	G	A	G	-0.017	0.015073	0.6205	0.628712	FALSE	FALSE	FALSE	ieu-b-508	8	26683792	0.035657	486484	0.762974	Sepsis (28	Sepsis (28	Sepsis (28	TRUE	igd	0.0036	2.94E-06	7365	M33441.m	TRUE	reported	CSdSpl	2	TRUE	0.003019	22.29333
999	rs1125471	A	G	A	G	-0.0085	-0.01485	0.2928	0.294132	FALSE	FALSE	FALSE	ieu-b-508	10	25094926	0.036723	486484	0.685586	Sepsis (28	Sepsis (28	Sepsis (28	TRUE	igd	0.0018	2.29E-06	7364	M01564.m	TRUE	reported	IPUdyD	2	TRUE	0.003019	22.29333
1022	rs6077792	T	C	T	C	-0.0085	-0.03953	0.3	0.304062	FALSE	FALSE	FALSE	ieu-b-508	20	16173967	0.036244	486484	0.275401	Sepsis (28	Sepsis (28	Sepsis (28	TRUE	igd	0.0018	2.42E-06	7364	M01564.m	TRUE	reported	IPUdyD	2	TRUE	0.003019	22.29333
3421	rs1187389	A	G	A	G	-0.0085	-0.04697	0.6166	0.614276	FALSE	FALSE	FALSE	ieu-b-508	12	22886718	0.034393	486484	0.172094	Sepsis (28	Sepsis (28	Sepsis (28	TRUE	igd	0.0018	2.49E-06	7364	M32436.m	TRUE	reported	8GtFpl	2	TRUE	0.003019	22.29333
5265	rs569688	T	C	T	C	0.017	-0.0168	0.2186	0.219909	FALSE	FALSE	FALSE	ieu-b-508	8	60961821	0.040323	486484	0.67688	Sepsis (28	Sepsis (28	Sepsis (28	TRUE	igd	0.0036	2.49E-06	7364	M33302.m	TRUE	reported	nzcTOn	2	TRUE	0.003019	22.29333
710	rs1085090	T	C	T	C	0.0085	-0.05715	0.127	0.125837	FALSE	FALSE	FALSE	ieu-b-508	18	73281398	0.049838	486484	0.251728	Sepsis (28	Sepsis (28	Sepsis (28	TRUE	igd	0.0018	2.38E-06	7363	M01301.m	TRUE	reported	w8TL71	2	TRUE	0.003019	22.29333
712	rs1115113	A	G	A	G	0.0085	-0.08453	0.1379	0.137202	FALSE	FALSE	FALSE	ieu-b-508	18	73323599	0.048776	486484	0.083106	Sepsis (28	Sepsis (28	Sepsis (28	TRUE	igd	0.0018	1.79E-06	7363	M01301.m	TRUE	reported	w8TL71	2	TRUE	0.003019	22.29333

725	rs3024536	T	C	T	C	-0.0085	0.048829	0.1443	0.144204	FALSE	FALSE	FALSE	ieu-b-508	16	27352713	0.047509	486484	0.304056	Sepsis (28	Sepsis (28	Sepsis (28	TRUE	igd	0.0018	1.69E-06	7363	M01301.m	TRUE	reported	w8TL71	2	TRUE	0.003019	22.29333
3452	rs1050506	T	C	T	C	-0.0085	-0.04762	0.4005	0.388187	FALSE	FALSE	FALSE	ieu-b-508	8	1.06E+08	0.034212	486484	0.163903	Sepsis (28	Sepsis (28	Sepsis (28	TRUE	igd	0.0018	1.85E-06	7363	M32489.m	TRUE	reported	hnljaD	2	TRUE	0.003019	22.29333
3483	rs7617041	A	G	A	G	0.0085	0.041067	0.6691	0.672545	FALSE	FALSE	FALSE	ieu-b-508	3	12709167	0.035488	486484	0.247183	Sepsis (28	Sepsis (28	Sepsis (28	TRUE	igd	0.0018	2.90E-06	7363	M32489.m	TRUE	reported	hnljaD	2	TRUE	0.003019	22.29333
3779	rs750658	A	G	A	G	0.0085	-0.0083	0.2567	0.258784	FALSE	FALSE	FALSE	ieu-b-508	14	69289901	0.03821	486484	0.828033	Sepsis (28	Sepsis (28	Sepsis (28	TRUE	igd	0.0018	2.87E-06	7362	M32634.m	TRUE	reported	7T2RUz	2	TRUE	0.00302	22.29332
4509	rs4521827	A	T	A	T	0.0085	0.035446	0.1344	0.140934	FALSE	TRUE	FALSE	ieu-b-508	9	74124229	0.048283	486484	0.46287	Sepsis (28	Sepsis (28	Sepsis (28	TRUE	igd	0.0018	2.17E-06	7362	M33132.m	TRUE	reported	dTXJa8	2	TRUE	0.00302	22.29332
3878	rs4781555	A	G	A	G	0.017	0.009476	0.8048	0.762425	FALSE	FALSE	FALSE	ieu-b-508	16	13894030	0.039308	486484	0.80951	Sepsis (28	Sepsis (28	Sepsis (28	TRUE	igd	0.0036	2.03E-06	7361	M32654.m	TRUE	reported	6lSQOP	2	TRUE	0.00302	22.29332
1437	rs1015306	T	G	T	G	0.0085	-0.02425	0.4356	0.444455	FALSE	FALSE	FALSE	ieu-b-508	16	7257971	0.033644	486484	0.471098	Sepsis (28	Sepsis (28	Sepsis (28	TRUE	igd	0.0018	2.12E-06	7357	M03141.m	TRUE	reported	y4mG6m	2	TRUE	0.003022	22.29332
1142	rs1310909	A	C	A	C	0.0085	-0.06316	0.614	0.608694	FALSE	FALSE	FALSE	ieu-b-508	4	1.49E+08	0.034187	486484	0.064677	Sepsis (28	Sepsis (28	Sepsis (28	TRUE	igd	0.0018	4.25E-06	7355	M01644.m	TRUE	reported	IB0DTL	2	TRUE	0.003023	22.29332
1155	rs4618485	A	G	A	G	-0.0085	0.037868	0.595	0.602764	FALSE	FALSE	FALSE	ieu-b-508	6	73555917	0.034838	486484	0.277059	Sepsis (28	Sepsis (28	Sepsis (28	TRUE	igd	0.0018	3.04E-06	7355	M01644.m	TRUE	reported	IB0DTL	2	TRUE	0.003023	22.29332
1165	rs989248	A	G	A	G	-0.0085	0.025434	0.6475	0.646773	FALSE	FALSE	FALSE	ieu-b-508	5	39526442	0.034968	486484	0.467006	Sepsis (28	Sepsis (28	Sepsis (28	TRUE	igd	0.0018	2.96E-06	7355	M01644.m	TRUE	reported	IB0DTL	2	TRUE	0.003023	22.29332
550	rs1258070	T	C	T	C	0.0085	0.005844	0.1328	0.134355	FALSE	FALSE	FALSE	ieu-b-508	12	98248254	0.048758	486484	0.9046	Sepsis (28	Sepsis (28	Sepsis (28	TRUE	igd	0.0018	4.55E-06	7354	M00606.m	TRUE	reported	r5smf7	2	TRUE	0.003023	22.29332
6307	rs1096601	A	G	A	G	-0.0085	-0.05371	0.2684	0.296246	FALSE	FALSE	FALSE	ieu-b-508	9	23681867	0.036872	486484	0.145192	Sepsis (28	Sepsis (28	Sepsis (28	TRUE	igd	0.0018	3.23E-06	7354	M33955.m	TRUE	reported	mhHFnT	2	TRUE	0.003023	22.29332
6310	rs1288091	T	C	T	C	-0.0085	0.028529	0.7389	0.735328	FALSE	FALSE	FALSE	ieu-b-508	14	62467133	0.037884	486484	0.451423	Sepsis (28	Sepsis (28	Sepsis (28	TRUE	igd	0.0018	2.45E-06	7354	M33955.m	TRUE	reported	mhHFnT	2	TRUE	0.003023	22.29332
6312	rs1442583	A	G	A	G	-0.017	-0.08583	0.0594	0.062196	FALSE	FALSE	FALSE	ieu-b-508	3	1.38E+08	0.0691	486484	0.214192	Sepsis (28	Sepsis (28	Sepsis (28	TRUE	igd	0.0036	1.94E-06	7354	M33955.m	TRUE	reported	mhHFnT	2	TRUE	0.003023	22.29332
6317	rs2748805	T	C	T	C	0.0085	0.019753	0.3004	0.314287	FALSE	FALSE	FALSE	ieu-b-508	14	99757151	0.036007	486484	0.583277	Sepsis (28	Sepsis (28	Sepsis (28	TRUE	igd	0.0018	2.31E-06	7354	M33955.m	TRUE	reported	mhHFnT	2	TRUE	0.003023	22.29332
6318	rs353694	A	G	A	G	-0.0085	-0.02774	0.4518	0.455738	FALSE	FALSE	FALSE	ieu-b-508	19	4243792	0.033624	486484	0.409419	Sepsis (28	Sepsis (28	Sepsis (28	TRUE	igd	0.0018	4.09E-06	7354	M33955.m	TRUE	reported	mhHFnT	2	TRUE	0.003023	22.29332
656	rs7530270	A	C	A	C	-0.0085	-0.02198	0.2	0.273442	FALSE	FALSE	FALSE	ieu-b-508	1	2.42E+08	0.037465	486484	0.557444	Sepsis (28	Sepsis (28	Sepsis (28	TRUE	igd	0.0018	1.51E-06	7352	M01125.m	TRUE	reported	yw0clB	2	TRUE	0.003024	22.29332
3574	rs2991721	A	G	A	G	-0.0085	-0.00666	0.3833	0.419368	FALSE	FALSE	FALSE	ieu-b-508	1	88430943	0.033811	486484	0.843865	Sepsis (28	Sepsis (28	Sepsis (28	TRUE	igd	0.0018	3.43E-06	7350	M32518.m	TRUE	reported	V0ZCiU	2	TRUE	0.003025	22.29331
3576	rs35441	T	C	T	C	0.0085	0.020486	0.3871	0.381768	FALSE	FALSE	FALSE	ieu-b-508	12	1.16E+08	0.034289	486484	0.550207	Sepsis (28	Sepsis (28	Sepsis (28	TRUE	igd	0.0018	2.12E-06	7350	M32518.m	TRUE	reported	V0ZCiU	2	TRUE	0.003025	22.29331
7915	rs1269811	A	G	A	G	0.0085	-0.00702	0.3013	0.312614	FALSE	FALSE	FALSE	ieu-b-508	7	1.58E+08	0.035843	486484	0.844777	Sepsis (28	Sepsis (28	Sepsis (28	TRUE	igd	0.0018	4.15E-06	7348	M35675.m	TRUE	reported	D7fdyZ	2	TRUE	0.003026	22.29331
7931	rs2901027	A	T	A	T	-0.0085	0.007426	0.341	0.361409	FALSE	TRUE	FALSE	ieu-b-508	10	92230423	0.034873	486484	0.83138	Sepsis (28	Sepsis (28	Sepsis (28	TRUE	igd	0.0018	4.10E-06	7348	M35675.m	TRUE	reported	D7fdyZ	2	TRUE	0.003026	22.29331
1205	rs9852606	C	G	C	G	-0.0085	-0.05095	0.3771	0.359949	FALSE	TRUE	FALSE	ieu-b-508	3	68466154	0.034895	486484	0.144242	Sepsis (28	Sepsis (28	Sepsis (28	TRUE	igd	0.0018	1.48E-06	7346	M01645.m	TRUE	reported	d5nAn3	2	TRUE	0.003026	22.29331
4451	rs5019028	T	G	T	G	0.0085	-0.02252	0.7021	0.699026	FALSE	FALSE	FALSE	ieu-b-508	4	94222825	0.036384	486484	0.536027	Sepsis (28	Sepsis (28	Sepsis (28	TRUE	igd	0.0018	2.27E-06	7344	M32910.m	TRUE	reported	2OCBKh	2	TRUE	0.003027	22.29331
3893	rs1345255	A	G	A	G	-0.0085	0.030764	0.8677	0.863478	FALSE	FALSE	FALSE	ieu-b-508	7	22106466	0.048815	486484	0.528558	Sepsis (28	Sepsis (28	Sepsis (28	TRUE	igd	0.0018	2.12E-06	7338	M32675.m	TRUE	reported	9FmPh9	2	TRUE	0.00303	22.2933
5621	rs4697477	T	C	T	C	-0.0085	-0.08755	0.8334	0.827407	FALSE	FALSE	FALSE	ieu-b-508	4	24723251	0.044405	486484	0.048653	Sepsis (28	Sepsis (28	Sepsis (28	TRUE	igd	0.0018	2.19E-06	7336	M33442.m	TRUE	reported	B1Peb3	2	TRUE	0.003031	22.2933
4064	rs3915879	T	C	T	C	-0.0085	-0.03311	0.3166	0.305554	FALSE	FALSE	FALSE	ieu-b-508	4	7154743	0.037649	486484	0.379097	Sepsis (28	Sepsis (28	Sepsis (28	TRUE	igd	0.0018	3.91E-06	7318	M32740.m	TRUE	reported	YLshfP	2	TRUE	0.003038	22.29329
5402	rs288514	T	C	T	C	-0.0085	0.040823	0.5866	0.596295	FALSE	FALSE	FALSE	ieu-b-508	3	69620621	0.034003	486484	0.229912	Sepsis (28	Sepsis (28	Sepsis (28	TRUE	igd	0.0018	4.14E-06	7315	M33389.m	TRUE	reported	Hf6XVM	2	TRUE	0.003039	22.29329
5417	rs686189	A	G	A	G	-0.0085	0.050022	0.5854	0.56919	FALSE	FALSE	FALSE	ieu-b-508	8	8623637	0.033765	486484	0.138475	Sepsis (28	Sepsis (28	Sepsis (28	TRUE	igd	0.0018	3.86E-06	7315	M33389.m	TRUE	reported	Hf6XVM	2	TRUE	0.003039	22.29329
1838	rs1315168	T	C	T	C	0.0085	-0.05278	0.3407	0.370789	FALSE	FALSE	FALSE	ieu-b-508	4	21312957	0.034656	486484	0.127778	Sepsis (28	Sepsis (28	Sepsis (28	TRUE	igd	0.0018	3.81E-06	7308	M15778.m	TRUE	reported	941o3l	2	TRUE	0.003042	22.29328
1853	rs4741625	A	G	A	G	0.0085	-0.01175	0.2777	0.294579	FALSE	FALSE	FALSE	ieu-b-508	9	1894517	0.036905	486484	0.750233	Sepsis (28	Sepsis (28	Sepsis (28	TRUE	igd	0.0018	3.50E-06	7308	M15778.m	TRUE	reported	941o3l	2	TRUE	0.003042	22.29328
5700	rs7767693	A	G	A	G	0.0085	0.026414	0.2941	0.296707	FALSE	FALSE	FALSE	ieu-b-508	6	98137914	0.036611	486484	0.470617	Sepsis (28	Sepsis (28	Sepsis (28	TRUE	igd	0.0018	2.73E-06	7307	M33477.m	TRUE	reported	Aw8PBK	2	TRUE	0.003042	22.29328
2750	rs1379560	T	C	T	C	-0.0085	0.04271	0.1931	0.197169	FALSE	FALSE	FALSE	ieu-b-508	5	1.14E+08	0.041987	486484	0.309048	Sepsis (28	Sepsis (28	Sepsis (28	TRUE	igd	0.0018	2.27E-06	7303	M27256.m	TRUE	reported	mJwzsi	2	TRUE	0.003044	22.29328
2751	rs2159022	T	C	T	C	0.0085	0.057061	0.8241	0.827648	FALSE	FALSE	FALSE	ieu-b-508	7	1.03E+08	0.044135	486484	0.196056	Sepsis (28	Sepsis (28	Sepsis (28	TRUE	igd	0.0018	1.96E-06	7303	M27256.m	TRUE	reported	mJwzsi	2	TRUE	0.003044	22.29328
2769	rs9518163	A	G	A	G	-0.0085	-0.04112	0.7373	0.723407	FALSE	FALSE	FALSE	ieu-b-508	13	88362635	0.037319	486484	0.270548	Sepsis (28	Sepsis (28	Sepsis (28	TRUE	igd	0.0018	2.37E-06	7303	M27256.m	TRUE	reported	mJwzsi	2	TRUE	0.003044	22.29328
1071	rs6462723	A	G	A	G	-0.0085	0.006702	0.121	0.123393	FALSE	FALSE	FALSE	ieu-b-508	7	36957350	0.05089	486484	0.895228	Sepsis (28	Sepsis (28	Sepsis (28	TRUE	igd	0.0018	1.92E-06	7271	M01585.m	TRUE	reported	M7QiSM	2	TRUE	0.003058	22.29325
3803	rs1092735	C	G	C	G	0.0085	-0.02051	0.3072	0.311132	FALSE	TRUE	FALSE	ieu-b-508	1	2.45E+08	0.036066	486484	0.56959	Sepsis (28	Sepsis (28	Sepsis (28	TRUE	igd	0.0018	4.41E-06	7229	M32644.m	TRUE	reported	JAa2WD	2	TRUE	0.003075	22.29321
7553	rs1118482	A	G	A	G	-0.0255	0.231431	0.0247	0.03166	FALSE	FALSE	FALSE	ieu-b-508	1	1.07E+08	0.094875	486484	0.014715	Sepsis (28	Sepsis (28	Sepsis (28	TRUE	igd	0.0054	2.61E-06	7217	M35270.m	TRUE	reported	Ct8V1n	2	TRUE	0.00308	22.2932
7559	rs1331927	C	G	C	G	-0.0085	0.013364	0.7984	0.792825	FALSE	TRUE	FALSE	ieu-b-508	13	84318449	0.041445	486484	0.747104	Sepsis (28	Sepsis (28	Sepsis (28	TRUE	igd	0.0018	3.82E-06	7217	M35270.m	TRUE	reported	Ct8V1n	2	TRUE	0.00308	22.2932
7572	rs3770124	T	C	T	C	-0.0085	-0.00827	0.802	0.800278	FALSE	FALSE	FALSE	ieu-b-508	2	1.82E+08	0.041642	486484	0.842565	Sepsis (28	Sepsis (28	Sepsis (28	TRUE	igd	0.0018	3.23E-06	7217	M35270.m	TRUE	reported	Ct8V1n	2	TRUE	0.00308	22.2932
5798	rs1001659	A	C	A	C	-0.0085	0.059969	0.5815	0.578911	FALSE	FALSE	FALSE	ieu-b-508	4	77901449	0.033782	486484	0.075872	Sepsis (28	Sepsis (28	Sepsis (28	TRUE	igd	0.0018	3.12E-06	7066	M33515.m	TRUE	reported	X9mul1	2	TRUE	0.003146	22.29307
8419	rs3797510	C	G	C	G	-0.0085	-0.04825	0.8426	0.843686	FALSE	TRUE	FALSE	ieu-b-508	5	1.07E+08	0.046307	486484	0.297401	Sepsis (28	Sepsis (28	Sepsis (28	TRUE	igd	0.0018	2.71E-06	6940	M36808.m	TRUE	reported	u7lqaj	2	TRUE	0.003203	22.29296
7685	rs731908	A	C	A	C	-0.0085	0.068831	0.193	0.198544	FALSE	FALSE	FALSE	ieu-b-508	13	1.04E+08	0.042156	486484	0.102515	Sepsis (28	Sepsis (28	Sepsis (28	TRUE	igd	0.0018	2.73E-06	6920	M35327.m	TRUE	reported	SquFiY	2	TRUE	0.003212	22.29294
8364	rs631871	A	G	A	G	-0.017	-0.00031	0.5197	0.522219	FALSE	FALSE	FALSE	ieu-b-508	2	1.65E+08	0.033568	486484	0.992691	Sepsis (28	Sepsis (28	Sepsis (28	TRUE	igd	0.0036	1.76E-06	6876	M36754.m	TRUE	reported	f8bkFg	2	TRUE	0.003233	22.2929
7089	rs34170	T	C	T	C	0.1342	0.009682	0.9477	0.943781	FALSE	FALSE	FALSE	ieu-b-508	7	31732461	0.075563	486484	0.898047	Sepsis (28	Sepsis (28	Sepsis (28	TRUE	igd	0.0284	2.32E-06	1235	M34499.m	TRUE	reported	JqFBiM	2	TRUE	0.017759	22.29279
8466	rs1339703	T	C	T	C	-0.0085	-0.01047	0.4316	0.431239	FALSE	FALSE	FALSE	ieu-b-508	2	1.87E+08	0.033708	486484	0.756209	Sepsis (28	Sepsis (28	Sepsis (28	TRUE	igd	0.0018	4.27E-06	6552	M37058.m	TRUE	reported	32p8qV	2	TRUE	0.003392	22.29258
950	rs1041603	A	G	A	G	0.0085	-0.05604	0.3202	0.31937	FALSE	FALSE	FALSE	ieu-b-508	19	1095151	0.036152	486484	0.121119	Sepsis (28	Sepsis (28	Sepsis (28	TRUE	igd	0.0018	3.84E-06	6523	M01558.m	TRUE	reported	AZK0p4	2	TRUE	0.003407	22.29255
963	rs1812125	T	G	T	G	-0.0085	0.003589	0.7578	0.75771	FALSE	FALSE	FALSE	ieu-b-508	18	44138761	0.039079	486484	0.926819	Sepsis (28	Sepsis (28	Sepsis (28	TRUE	igd	0.0018	3.48E-06	6523	M01558.m	TRUE	reported	AZK0p4	2	TRUE	0.003407	22.29255
3299	rs6950034	A	G	A	G	0.1663	-0.15151	0.0206	0.032558	FALSE	FALSE	FALSE	ieu-b-508	7	6760229	0.09389	486484	0.106591	Sepsis (28	Sepsis (28	Sepsis (28	TRUE	igd	0.0352	2.25E-06	1502	M32379.m	TRUE	reported	MLimM6	2	TRUE	0.014643	22.29054
4372	rs213190	T	G	T	G	0.0529	-0.04201	0.5931	0.576362	FALSE	FALSE	FALSE	ieu-b-508	7	52653869	0.033824	486484	0.214203	Sepsis (28	Sepsis (28	Sepsis (28	TRUE	igd	0.0112	2.36E-06	1988	M32863.m	TRUE	reported	cLOFYE	2	TRUE	0.011097	22.28631
3880	rs6691848	A	G	A	G	0.0288	-0.02032	0.9639	0.96973	FALSE	FALSE	FALSE	ieu-b-508	1	35474188	0.097709	486484	0.83526	Sepsis (28	Sepsis (28	Sepsis (28	TRUE	igd	0.0061	2.12E-06	7361	M32654.m	TRUE	reported	6lSQOP	2	TRUE	0.003019	22.28473
2798	rs1994851	A	G	A	G	-0.0203	0.077459	0.0758	0.097728	FALSE	FALSE	FALSE	ieu-b-508	8	62377604	0.05633	486484	0.1691	Sepsis (28	Sepsis (28	Sepsis (28	TRUE	igd	0.0043	2.69E-06	7347	M27278.m	TRUE	reported	kPgqm3	2	TRUE	0.003024	22.28112
531	rs1103873	A	T	A	T	-0.0203	-0.05577	0.7937	0.789415	FALSE	TRUE	FALSE	ieu-b-508	11	46017895	0.040851	486484	0.172155	Sepsis (28	Sepsis (28	Sepsis (28	TRUE	igd	0.0043	3.01E-06	7237	M00599.m	TRUE	reported	nRJY3s	2	TRUE	0.00307	22.28102
6923	rs1892651	A	G	A	G	0.0496	-0.02464	0.4236	0.38488	FALSE	FALSE	FALSE	ieu-b-508	21	33541098	0.034762	486484	0.478521	Sepsis (28	Sepsis (28	Sepsis (28	TRUE	igd	0.0105	2.40E-06	1312	M34390.m	TRUE	reported	wruTbW	2	TRUE	0.016723	22.28036
3298	rs6829935	T	C	T	C	-0.0203	0.035232	0.2937	0.313367	FALSE	FALSE	FALSE	ieu-b-508	4	16861512	0.036134	486484	0.329541	Sepsis (28	Sepsis (28	Sepsis (28	TRUE	igd	0.0043	2.57E-06	6119	M32379.m	TRUE	reported	MLimM6	2	TRUE	0.003629	22.2799
3927	rs1110654	T	C	T	C	-0.0321	0.086147	0.9498	0.952664	FALSE	FALSE	FALSE	ieu-b-508	12	92818102	0.078675	486484	0.273531	Sepsis (28	Sepsis (28	Sepsis (28	TRUE	igd	0.0068	2.31E-06	7308	M32698.m	TRUE	reported	srqncz	2	TRUE	0.00304	22.27785
6632	rs1107458	T	C	T	C	-0.3698	-0.08104	0.9867	0.984775	FALSE	FALSE	FALSE	ieu-b-508	20	18986728	0.135502	486484	0.54979	Sepsis (28	Sepsis (28	Sepsis (28	TRUE	igd	0.0773	1.70E-06	75	M34322.m	TRUE	reported	16GdG9	2	TRUE	0.233805	22.27596
1207	rs1265481	A	G	A	G	0.0118	0.031591	0.2913	0.344149	FALSE	FALSE	FALSE	ieu-b-508	5	1.77E+08	0.035071	486484	0.367719	Sepsis (28	Sepsis (28	Sepsis (28	TRUE	igd	0.0025	3.36E-06	7348	M01670.m	TRUE	reported	kjghbc	2	TRUE	0.003023	22.27234
3094	rs7204324	A	T	A	T	-0.0236	-0.03508	0.9189	0.915782	FALSE	TRUE	FALSE	ieu-b-508	16	58750869	0.059965	486484	0.558492	Sepsis (28	Sepsis (28	Sepsis (28	TRUE	igd	0.005	2.12E-06	7346	M32197.m	TRUE	reported	VZjwa9	2	TRUE	0.003024	22.27233
1758	rs1050886	A	G	A	G	0.0118	-0.02915	0.8446	0.851789	FALSE	FALSE	FALSE	ieu-b-508	10	43224701	0.048324	486484	0.546365	Sepsis (28	Sepsis (28	Sepsis (28	TRUE	igd	0.0025	1.64E-06	7331	M15676.m	TRUE	reported	s1NfKN	2	TRUE	0.00303	22.27232
7804	rs2067776	T	C	T	C	0.0118	0.044535	0.5198	0.515677	FALSE	FALSE	FALSE	ieu-b-508	8	56207293	0.033381	486484	0.182161	Sepsis (28	Sepsis (28	Sepsis (28	TRUE	igd	0.0025	3.41E-06	6883	M35464.m	TRUE	reported	Ir9dHp	2	TRUE	0.003226	22.27193
1940	rs7699551	T	C	T	C	-0.0118	0.0606	0.5871	0.58128	FALSE	FALSE	FALSE	ieu-b-508	4	1.18E+08	0.034073	486484	0.075313	Sepsis (28	Sepsis (28	Sepsis (28	TRUE	igd	0.0025	3.31E-06	6549	M16822.m	TRUE	reported	UNI018	2	TRUE	0.00339	22.2716
4243	rs1726043	A	G	A	G	-0.0269	0.02546	0.1739	0.176874	FALSE	FALSE	FALSE	ieu-b-508	1	2.11E+08	0.04373	486484	0.560423	Sepsis (28	Sepsis (28	Sepsis (28	TRUE	igd	0.0057	2.15E-06	6169	M32795.m	TRUE	reported	0YBWkh	2	TRUE	0.003597	22.26456
4531	rs6835379	A	G	A	G	0.0439	-0.03838	0.5036	0.507054	FALSE	FALSE	FALSE	ieu-b-508	4	14807194	0.033711	486484	0.25485	Sepsis (28	Sepsis (28	Sepsis (28	TRUE	igd	0.0093	2.16E-06	2391	M33137.m	TRUE	reported	ONVNk3	2	TRUE	0.009233	22.26382
6506	rs4750514	A	G	A	G	0.0269	0.035358	0.5822	0.589186	FALSE	FALSE	FALSE	ieu-b-508	10	5645895	0.03403	486484	0.29879	Sepsis (28	Sepsis (28	Sepsis (28	TRUE	igd	0.0057	2.60E-06	5581	M34112.m	TRUE	reported	97JqFb	2	TRUE	0.003975	22.26379
3628	rs2834622	T	C	T	C	0.0302	0.016723	0.2733	0.292487	FALSE	FALSE	FALSE	ieu-b-508	21	36120774	0.036652	486484	0.648206	Sepsis (28	Sepsis (28	Sepsis (28	TRUE	igd	0.0064	2.34E-06	7337	M32557.m	TRUE	reported	qR7rZX	2	TRUE	0.003026	22.26053
4628	rs1911951	A	C	A	C	0.203	0.035813	0.934	0.939075	FALSE	FALSE	FALSE	ieu-b-508	19	31257696	0.069898	486484	0.608403	Sepsis (28	Sepsis (28	Sepsis (28	TRUE	igd	0.043	2.35E-06	1498	M33173.m	TRUE	reported	04fkUQ	2	TRUE	0.01466	22.25743
2137	rs1207017	T	C	T	C	-0.2041	-0.06517	0.951	0.941058	FALSE	FALSE	FALSE	ieu-b-508	1	1.54E+08	0.070883	486484	0.357852	Sepsis (28	Sepsis (28	Sepsis (28	TRUE	igd	0.0432	2.31E-06	698	M18497.m	TRUE	reported	90JFPc	2	TRUE	0.030988	22.25729
7537	rs1716085	T	G	T	G	0.0335	-0.01838	0.9515	0.966683	FALSE	FALSE	FALSE	ieu-b-508	7	32103632	0.09408	486484	0.845097	Sepsis (28	Sepsis (28	Sepsis (28	TRUE	igd	0.0071	2.05E-06	6538	M35257.m	TRUE	reported	iphouU	2	TRUE	0.003394	22.25564
990	rs2074731	A	C	A	C	-0.0184	-0.01957	0.1654	0.162066	FALSE	FALSE	FALSE	ieu-b-508	22	30733914	0.045309	486484	0.665777	Sepsis (28	Sepsis (28	Sepsis (28	TRUE	igd	0.0039	2.31E-06	7276	M01561.m	TRUE	reported	Zd9SUU	2	TRUE	0.00305	22.25292
4634	rs4299028	A	G	A	G	-0.9695	-0.11542	0.0171	0.007656	FALSE	FALSE	FALSE	ieu-b-508	13	80596188	0.193661	486484	0.551174	Sepsis (28	Sepsis (28	Sepsis (28	TRUE	igd	0.2028	1.75E-06	76	M33173.m	TRUE	reported	04fkUQ	2	TRUE	0.231188	22.25245
7139	rs1082940	A	G	A	G	0.0434	0.055344	0.1231	0.129946	FALSE	FALSE	FALSE	ieu-b-508	10	1.3E+08	0.049698	486484	0.265445	Sepsis (28	Sepsis (28	Sepsis (28	TRUE	igd	0.0092	2.32E-06	3813	M34530.m	TRUE	reported	8plvGA	2	TRUE	0.005802	22.24211
645	rs1895999	A	G	A	G	-0.0151	0.04671	0.1699	0.165552	FALSE	FALSE	FALSE	ieu-b-508	12	48952204	0.044921	486484	0.298417	Sepsis (28	Sepsis (28	Sepsis (28	TRUE	igd	0.0032	3.43E-06	1764	M01125.m	TRUE	reported	yw0clB	2	TRUE	0.012465	22.24136
2977	rs2314026	A	G	A	G	0.0283	0.11766	0.9644	0.961446	FALSE	FALSE	FALSE	ieu-b-508	5	1.55E+08	0.087572	486484	0.179082	Sepsis (28	Sepsis (28	Sepsis (28	TRUE	igd	0.006	2.53E-06	7257	M31453.m	TRUE	reported	vdTd9M	2	TRUE	0.003056	22.24081
2664	rs1772351	A	G	A	G	-0.0481	-0.02754	0.6672	0.653061	FALSE	FALSE	FALSE	ieu-b-508	18	63554245	0.035123	486484	0.432912	Sepsis (28	Sepsis (28	Sepsis (28	TRUE	igd	0.0102	2.30E-06	5599	M22842.m	TRUE	reported	0t12oo	2	TRUE	0.003956	22.22975
5987	rs842456	A	G	A	G	-0.0217	0.049708	0.7803	0.780763	FALSE	FALSE	FALSE	ieu-b-508	7	1.59E+08	0.040153	486484	0.215725	Sepsis (28	Sepsis (28	Sepsis (28	TRUE	igd	0.0046	1.76E-06	1764	M33666.m	TRUE	reported	dvWEtk	2	TRUE	0.012458	22.22855
2866	rs1103039	A	G	A	G	-0.0434	-0.02317	0.3845	0.368726	FALSE	FALSE	FALSE	ieu-b-508	11	28653727	0.034626	486484	0.503356	Sepsis (28	Sepsis (28	Sepsis (28	TRUE	igd	0.0092	2.61E-06	1510	M27710.m	TRUE	reported	XeaFav	2	TRUE	0.014524	22.22431
7770	rs1524307	T	C	T	C	0.025	0.000886	0.4846	0.498185	FALSE	FALSE	FALSE	ieu-b-508	3	22673600	0.033582	486484	0.978951	Sepsis (28	Sepsis (28	Sepsis (28	TRUE	igd	0.0053	1.96E-06	1718	M35439.m	TRUE	reported	DnuAGZ	2	TRUE	0.012785	22.22401
8346	rs1014551	A	G	A	G	-0.0373	-0.02606	0.7599	0.731208	FALSE	FALSE	FALSE	ieu-b-508	14	39808311	0.037588	486484	0.488063	Sepsis (28	Sepsis (28	Sepsis (28	TRUE	igd	0.0079	2.46E-06	648	M36738.m	TRUE	reported	mVQQcD	2	TRUE	0.033258	22.22394
5960	rs1735821	T	C	T	C	0.1099	-0.0474	0.0313	0.054657	FALSE	FALSE	FALSE	ieu-b-508	5	71014850	0.076371	486484	0.534796	Sepsis (28	Sepsis (28	Sepsis (28	TRUE	igd	0.0233	2.36E-06	1860	M33658.m	TRUE	reported	9QszGO	2	TRUE	0.01182	22.2237
1130	rs8105491	T	G	T	G	-0.0698	-0.02873	0.154	0.151561	FALSE	FALSE	FALSE	ieu-b-508	19	20403988	0.046361	486484	0.535475	Sepsis (28	Sepsis (28	Sepsis (28	TRUE	igd	0.0148	2.30E-06	2063	M01640.m	TRUE	reported	o3qxzl	2	TRUE	0.010667	22.22113
3422	rs1202925	A	C	A	C	0.0132	0.045989	0.1421	0.142278	FALSE	FALSE	FALSE	ieu-b-508	1	22686493	0.047698	486484	0.334962	Sepsis (28	Sepsis (28	Sepsis (28	TRUE	igd	0.0028	1.57E-06	7364	M32452.m	TRUE	reported	8GtFpl	2	TRUE	0.003009	22.21845
732	rs1006645	C	G	C	G	0.0165	-0.15403	0.0387	0.027272	FALSE	TRUE	FALSE	ieu-b-508	5	91387973	0.104658	486484	0.141104	Sepsis (28	Sepsis (28	Sepsis (28	TRUE	igd	0.0035	1.93E-06	7347	M01302.m	TRUE	reported	sNp2lC	2	TRUE	0.003016	22.21844
3086	rs4641779	A	C	A	C	0.0165	0.032024	0.1417	0.152256	FALSE	FALSE	FALSE	ieu-b-508	17	64283326	0.046444	486484	0.4905	Sepsis (28	Sepsis (28	Sepsis (28	TRUE	igd	0.0035	2.37E-06	7346	M32197.m	TRUE	reported	VZjwa9	2	TRUE	0.003016	22.21844
6249	rs1086845	A	T	A	T	-0.0165	0.03496	0.3461	0.354504	FALSE	TRUE	FALSE	ieu-b-508	9	89277187	0.034833	486484	0.315537	Sepsis (28	Sepsis (28	Sepsis (28	TRUE	igd	0.0035	1.92E-06	7345	M33936.m	TRUE	reported	jwA4B2	2	TRUE	0.003017	22.21844
3203	rs1170495	A	C	A	C	-0.0231	-0.04614	0.0362	0.036267	FALSE	FALSE	FALSE	ieu-b-508	22	26665804	0.090045	486484	0.608328	Sepsis (28	Sepsis (28	Sepsis (28	TRUE	igd	0.0049	2.85E-06	7344	M32339.m	TRUE	reported	SVp504	2	TRUE	0.003017	22.21844
2630	rs942358	T	C	T	C	-0.0132	0.050175	0.1543	0.152758	FALSE	FALSE	FALSE	ieu-b-508	13	1.08E+08	0.046356	486484	0.279083	Sepsis (28	Sepsis (28	Sepsis (28	TRUE	igd	0.0028	1.94E-06	7329	M22481.m	TRUE	reported	bXpf0k	2	TRUE	0.003023	22.21842
1712	rs1764274	T	G	T	G	-0.0165	-0.13093	0.9518	0.925002	FALSE	FALSE	FALSE	ieu-b-508	10	1.2E+08	0.063205	486484	0.038312	Sepsis (28	Sepsis (28	Sepsis (28	TRUE	igd	0.0035	2.68E-06	7310	M15506.m	TRUE	reported	mVRmZO	2	TRUE	0.003031	22.21841
5356	rs4919062	A	C	A	C	0.0198	0.143635	0.9652	0.966948	FALSE	FALSE	FALSE	ieu-b-508	10	98786856	0.094446	486484	0.128304	Sepsis (28	Sepsis (28	Sepsis (28	TRUE	igd	0.0042	3.17E-06	7118	M33380.m	TRUE	reported	OHRfl5	2	TRUE	0.003113	22.21825
5250	rs6517062	A	G	A	G	0.0132	-0.06582	0.3577	0.359497	FALSE	FALSE	FALSE	ieu-b-508	21	33243423	0.034944	486484	0.059607	Sepsis (28	Sepsis (28	Sepsis (28	TRUE	igd	0.0028	1.57E-06	7063	M33228.m	TRUE	reported	JwOXbQ	2	TRUE	0.003137	22.2182
2390	rs3112989	A	G	A	G	-0.0514	0.023161	0.5555	0.542551	FALSE	FALSE	FALSE	ieu-b-508	2	95745120	0.033653	486484	0.491318	Sepsis (28	Sepsis (28	Sepsis (28	TRUE	igd	0.0109	2.23E-06	2339	M21151.m	TRUE	reported	LfwdLx	2	TRUE	0.009417	22.21783
8021	rs7039918	T	C	T	C	0.0132	0.018219	0.8007	0.788391	FALSE	FALSE	FALSE	ieu-b-508	9	929611	0.04166	486484	0.661872	Sepsis (28	Sepsis (28	Sepsis (28	TRUE	igd	0.0028	2.76E-06	6604	M35754.m	TRUE	reported	IlXOo1	2	TRUE	0.003354	22.21776
2414	rs1264104	A	G	A	G	-0.0165	0.031056	0.8189	0.822505	FALSE	FALSE	FALSE	ieu-b-508	4	5532985	0.043694	486484	0.477238	Sepsis (28	Sepsis (28	Sepsis (28	TRUE	igd	0.0035	2.38E-06	6556	M21188.m	TRUE	reported	kvdnjc	2	TRUE	0.003378	22.21771
1109	rs3011362	A	G	A	G	-0.0349	0.073349	0.0334	0.043032	FALSE	FALSE	FALSE	ieu-b-508	10	1.23E+08	0.082312	486484	0.372871	Sepsis (28	Sepsis (28	Sepsis (28	TRUE	igd	0.0074	2.37E-06	1735	M01638.m	TRUE	reported	8dp0Ac	2	TRUE	0.012658	22.21706
7243	rs1126376	A	G	A	G	0.0132	-0.00802	0.7777	0.762317	FALSE	FALSE	FALSE	ieu-b-508	17	36182400	0.039312	486484	0.838407	Sepsis (28	Sepsis (28	Sepsis (28	TRUE	igd	0.0028	2.01E-06	5885	M34912.m	TRUE	reported	W8lcT6	2	TRUE	0.003762	22.21694
7155	rs1473500	T	C	T	C	-0.0891	-0.24896	0.0202	0.02296	FALSE	FALSE	FALSE	ieu-b-508	6	1.69E+08	0.111426	486484	0.025463	Sepsis (28	Sepsis (28	Sepsis (28	TRUE	igd	0.0189	2.36E-06	5856	M34533.m	TRUE	reported	SuKX4k	2	TRUE	0.003781	22.2169
4374	rs4447447	A	G	A	G	0.2288	-0.02849	0.0449	0.061338	FALSE	FALSE	FALSE	ieu-b-508	16	11482993	0.070037	486484	0.6842	Sepsis (28	Sepsis (28	Sepsis (28	TRUE	igd	0.0485	2.36E-06	1162	M32863.m	TRUE	reported	cLOFYE	2	TRUE	0.018792	22.21674
5646	rs1272575	A	T	A	T	-0.0198	0.045021	0.6521	0.647361	FALSE	TRUE	FALSE	ieu-b-508	1	88641534	0.034956	486484	0.197771	Sepsis (28	Sepsis (28	Sepsis (28	TRUE	igd	0.0042	2.96E-06	5717	M33453.m	TRUE	reported	OzEeLQ	2	TRUE	0.003872	22.21671
2483	rs4147977	T	C	T	C	-0.0132	0.000511	0.4483	0.457757	FALSE	FALSE	FALSE	ieu-b-508	17	66925910	0.03425	486484	0.988091	Sepsis (28	Sepsis (28	Sepsis (28	TRUE	igd	0.0028	1.82E-06	5676	M22032.m	TRUE	reported	xpe96K	2	TRUE	0.0039	22.21666
2918	rs2982376	T	C	T	C	-0.0132	0.077585	0.4212	0.420025	FALSE	FALSE	FALSE	ieu-b-508	1	11390336	0.034044	486484	0.02267	Sepsis (28	Sepsis (28	Sepsis (28	TRUE	igd	0.0028	2.93E-06	5562	M27728.m	TRUE	reported	WOp1XE	2	TRUE	0.00398	22.2165
5721	rs7227090	A	G	A	G	0.0297	0.103729	0.8661	0.867184	FALSE	FALSE	FALSE	ieu-b-508	18	50467744	0.049218	486484	0.035072	Sepsis (28	Sepsis (28	Sepsis (28	TRUE	igd	0.0063	2.28E-06	5107	M33488.m	TRUE	reported	mZmDY6	2	TRUE	0.004333	22.21579
7378	rs6979908	A	G	A	G	-0.0132	0.041161	0.1274	0.145843	FALSE	FALSE	FALSE	ieu-b-508	7	10971055	0.04753	486484	0.386492	Sepsis (28	Sepsis (28	Sepsis (28	TRUE	igd	0.0028	3.05E-06	4900	M35137.m	TRUE	reported	Eq2siS	2	TRUE	0.004515	22.21542
1919	rs1861371	A	G	A	G	0.0165	0.041969	0.1774	0.182111	FALSE	FALSE	FALSE	ieu-b-508	12	97774934	0.043348	486484	0.332958	Sepsis (28	Sepsis (28	Sepsis (28	TRUE	igd	0.0035	2.35E-06	4689	M16816.m	TRUE	reported	J1abbO	2	TRUE	0.004717	22.21501
5931	rs1993342	A	C	A	C	-0.0533	-0.035	0.2758	0.284077	FALSE	FALSE	FALSE	ieu-b-508	8	15701883	0.037126	486484	0.345789	Sepsis (28	Sepsis (28	Sepsis (28	TRUE	igd	0.0113	2.45E-06	1155	M33637.m	TRUE	reported	tfLznu	2	TRUE	0.018899	22.20981
8186	rs1106666	A	C	A	C	-0.0297	0.06693	0.4127	0.426412	FALSE	FALSE	FALSE	ieu-b-508	12	1.14E+08	0.033729	486484	0.047219	Sepsis (28	Sepsis (28	Sepsis (28	TRUE	igd	0.0063	2.18E-06	2400	M36230.m	TRUE	reported	V5KJeR	2	TRUE	0.009175	22.20597
4685	rs1084192	C	G	C	G	0.1066	0.029576	0.6919	0.624522	FALSE	TRUE	FALSE	ieu-b-508	12	22195202	0.034452	486484	0.390637	Sepsis (28	Sepsis (28	Sepsis (28	TRUE	igd	0.0226	2.38E-06	1017	M33178.m	TRUE	reported	g22WMS	2	TRUE	0.021408	22.20458
3438	rs6102466	T	C	T	C	-0.0264	-0.05893	0.7812	0.782022	FALSE	FALSE	FALSE	ieu-b-508	20	40188916	0.040421	486484	0.144851	Sepsis (28	Sepsis (28	Sepsis (28	TRUE	igd	0.0056	2.68E-06	1766	M32452.m	TRUE	reported	8GtFpl	2	TRUE	0.012428	22.19932
7397	rs4340260	T	C	T	C	-0.0363	0.052239	0.4537	0.442275	FALSE	FALSE	FALSE	ieu-b-508	14	96547541	0.033701	486484	0.12113	Sepsis (28	Sepsis (28	Sepsis (28	TRUE	igd	0.0077	2.67E-06	1630	M35159.m	TRUE	reported	7EoJLB	2	TRUE	0.013451	22.19722
6185	rs1190205	A	C	A	C	0.0622	0.127583	0.986	0.981215	FALSE	FALSE	FALSE	ieu-b-508	2	1.9E+08	0.123833	486484	0.30288	Sepsis (28	Sepsis (28	Sepsis (28	TRUE	igd	0.0132	2.27E-06	6004	M33892.m	TRUE	reported	w7HgU7	2	TRUE	0.003685	22.19669
6945	rs9907459	T	C	T	C	-0.2136	-0.12825	0.9803	0.949358	FALSE	FALSE	FALSE	ieu-b-508	17	55685579	0.075998	486484	0.091491	Sepsis (28	Sepsis (28	Sepsis (28	TRUE	igd	0.0453	2.38E-06	1104	M34395.m	TRUE	reported	QVOPA3	2	TRUE	0.019741	22.19313
6081	rs1026162	T	G	T	G	-0.041	-0.04069	0.6829	0.66554	FALSE	FALSE	FALSE	ieu-b-508	7	1.5E+08	0.03549	486484	0.251629	Sepsis (28	Sepsis (28	Sepsis (28	TRUE	igd	0.0087	2.27E-06	2660	M33835.m	TRUE	reported	crhBhm	2	TRUE	0.00828	22.19231
6537	rs1717000	C	G	C	G	0.0245	-0.04208	0.8052	0.78027	FALSE	TRUE	FALSE	ieu-b-508	7	1.46E+08	0.040499	486484	0.298756	Sepsis (28	Sepsis (28	Sepsis (28	TRUE	igd	0.0052	2.00E-06	6987	M34221.m	TRUE	reported	nUe3TL	2	TRUE	0.003167	22.19224
6389	rs4811800	A	G	A	G	-0.0212	-0.03026	0.484	0.48757	FALSE	FALSE	FALSE	ieu-b-508	20	55663870	0.033377	486484	0.364578	Sepsis (28	Sepsis (28	Sepsis (28	TRUE	igd	0.0045	2.52E-06	7330	M33969.m	TRUE	reported	0ujroW	2	TRUE	0.003019	22.18851
5765	rs4899659	C	G	C	G	-0.0311	0.008941	0.3679	0.369859	FALSE	TRUE	FALSE	ieu-b-508	14	78038642	0.034605	486484	0.796119	Sepsis (28	Sepsis (28	Sepsis (28	TRUE	igd	0.0066	2.42E-06	2759	M33508.m	TRUE	reported	UMXHcP	2	TRUE	0.007984	22.18799
6272	rs3125596	A	G	A	G	-0.0542	0.045602	0.9148	0.901076	FALSE	FALSE	FALSE	ieu-b-508	10	1.33E+08	0.055938	486484	0.414952	Sepsis (28	Sepsis (28	Sepsis (28	TRUE	igd	0.0115	2.39E-06	1759	M33937.m	TRUE	reported	QanGCI	2	TRUE	0.012471	22.18752
4224	rs9299150	T	C	T	C	0.0542	-0.00419	0.3005	0.363476	FALSE	FALSE	FALSE	ieu-b-508	9	1.11E+08	0.034703	486484	0.903931	Sepsis (28	Sepsis (28	Sepsis (28	TRUE	igd	0.0115	2.34E-06	1751	M32786.m	TRUE	reported	rwF0co	2	TRUE	0.012527	22.18741
5227	rs1865099	A	G	A	G	0.0212	-0.03119	0.6476	0.626699	FALSE	FALSE	FALSE	ieu-b-508	19	29512573	0.034548	486484	0.366575	Sepsis (28	Sepsis (28	Sepsis (28	TRUE	igd	0.0045	2.60E-06	4665	M33221.m	TRUE	reported	8ZrV1r	2	TRUE	0.004735	22.18505
7289	rs4737772	A	C	A	C	0.0952	0.043327	0.0139	0.012163	FALSE	FALSE	FALSE	ieu-b-508	8	67192398	0.15691	486484	0.782453	Sepsis (28	Sepsis (28	Sepsis (28	TRUE	igd	0.0202	2.55E-06	1657	M35114.m	TRUE	reported	a6NQjV	2	TRUE	0.013227	22.18435
3330	rs4954419	C	G	C	G	-0.0179	-0.06122	0.7089	0.723279	FALSE	TRUE	FALSE	ieu-b-508	2	1.37E+08	0.037551	486484	0.103052	Sepsis (28	Sepsis (28	Sepsis (28	TRUE	igd	0.0038	1.89E-06	7358	M32405.m	TRUE	reported	uzomlQ	2	TRUE	0.003007	22.18303
4330	rs1746184	T	C	T	C	-0.0358	0.106736	0.9096	0.909786	FALSE	FALSE	FALSE	ieu-b-508	5	1.49E+08	0.059313	486484	0.071935	Sepsis (28	Sepsis (28	Sepsis (28	TRUE	igd	0.0076	2.86E-06	6967	M32847.m	TRUE	reported	eYme29	2	TRUE	0.003175	22.18269
8004	rs2041264	T	C	T	C	-0.0179	-0.00804	0.9069	0.900802	FALSE	FALSE	FALSE	ieu-b-508	17	68656936	0.055619	486484	0.885029	Sepsis (28	Sepsis (28	Sepsis (28	TRUE	igd	0.0038	2.53E-06	6604	M35754.m	TRUE	reported	IlXOo1	2	TRUE	0.003349	22.18234
6879	rs4413146	T	G	T	G	-0.1504	0.032155	0.8985	0.891938	FALSE	FALSE	FALSE	ieu-b-508	2	59605724	0.053658	486484	0.549002	Sepsis (28	Sepsis (28	Sepsis (28	TRUE	igd	0.0319	2.39E-06	954	M34365.m	TRUE	reported	8eexD0	2	TRUE	0.02277	22.18211
8588	rs1272771	T	C	T	C	-0.1065	-0.03379	0.0307	0.039775	FALSE	FALSE	FALSE	ieu-b-508	1	18393640	0.085939	486484	0.694156	Sepsis (28	Sepsis (28	Sepsis (28	TRUE	igd	0.0226	2.37E-06	1805	M37459.m	TRUE	reported	55F8jy	2	TRUE	0.012153	22.18201
8141	rs2280042	A	G	A	G	-0.2177	-0.03857	0.0129	0.008421	FALSE	FALSE	FALSE	ieu-b-508	2	2.17E+08	0.186839	486484	0.836454	Sepsis (28	Sepsis (28	Sepsis (28	TRUE	igd	0.0462	2.48E-06	1750	M36103.m	TRUE	reported	sjeaPC	2	TRUE	0.012529	22.17871
8362	rs359428	A	G	A	G	-0.0471	0.024891	0.0344	0.04431	FALSE	FALSE	FALSE	ieu-b-508	5	1.73E+08	0.081733	486484	0.760715	Sepsis (28	Sepsis (28	Sepsis (28	TRUE	igd	0.01	2.44E-06	6876	M36754.m	TRUE	reported	f8bkFg	2	TRUE	0.003216	22.17765
6027	rs9466463	C	G	C	G	0.1372	0.018004	0.0541	0.061027	FALSE	TRUE	FALSE	ieu-b-508	6	10504532	0.069916	486484	0.79678	Sepsis (28	Sepsis (28	Sepsis (28	TRUE	igd	0.0291	2.37E-06	831	M33751.m	TRUE	reported	Cxdkr4	2	TRUE	0.026053	22.17562
7330	rs9528023	T	C	T	C	-0.0146	-0.01802	0.2466	0.274778	FALSE	FALSE	FALSE	ieu-b-508	13	60226991	0.037402	486484	0.629957	Sepsis (28	Sepsis (28	Sepsis (28	TRUE	igd	0.0031	3.36E-06	7344	M35127.m	TRUE	reported	RNsmjl	2	TRUE	0.003011	22.17502
3004	rs3794836	A	G	A	G	0.1656	-0.13874	0.0439	0.03675	FALSE	FALSE	FALSE	ieu-b-508	18	74719729	0.088881	486484	0.118524	Sepsis (28	Sepsis (28	Sepsis (28	TRUE	igd	0.0351	2.42E-06	522	M31536.m	TRUE	reported	3nAuUl	2	TRUE	0.040898	22.17376
477	rs1176413	T	C	T	C	-0.1032	0.024821	0.1873	0.184636	FALSE	FALSE	FALSE	ieu-b-508	10	1.33E+08	0.043083	486484	0.56454	Sepsis (28	Sepsis (28	Sepsis (28	TRUE	igd	0.0219	2.55E-06	1233	M00553.m	TRUE	reported	IFjXiL	2	TRUE	0.017691	22.17002
5949	rs1114802	A	G	A	G	-0.0405	-0.04721	0.8748	0.87254	FALSE	FALSE	FALSE	ieu-b-508	13	47645689	0.050203	486484	0.347	Sepsis (28	Sepsis (28	Sepsis (28	TRUE	igd	0.0086	2.74E-06	5328	M33652.m	TRUE	reported	vYkdTv	2	TRUE	0.004145	22.1692
8213	rs2174099	C	G	C	G	0.0471	-0.05193	0.1469	0.204329	FALSE	TRUE	FALSE	ieu-b-508	8	946165	0.041514	486484	0.210964	Sepsis (28	Sepsis (28	Sepsis (28	TRUE	igd	0.01	2.60E-06	2520	M36376.m	TRUE	reported	k6uC1l	2	TRUE	0.008726	22.16649
4111	rs7321884	T	C	T	C	0.1488	-0.02935	0.0207	0.016759	FALSE	FALSE	FALSE	ieu-b-508	13	1.02E+08	0.13132	486484	0.823149	Sepsis (28	Sepsis (28	Sepsis (28	TRUE	igd	0.0316	2.43E-06	6379	M32754.m	TRUE	reported	n0okFy	2	TRUE	0.003464	22.16642
5564	rs1761630	A	T	A	T	-1.4131	0.043776	0.0236	0.01986	FALSE	TRUE	FALSE	ieu-b-508	12	1.17E+08	0.121319	486484	0.718226	Sepsis (28	Sepsis (28	Sepsis (28	TRUE	igd	0.2951	1.68E-06	60	M33423.m	TRUE	reported	MoEnO7	2	TRUE	0.2765	22.16584
6171	rs1538388	A	T	A	T	0.0372	0.004833	0.3972	0.391043	FALSE	TRUE	FALSE	ieu-b-508	1	1.12E+08	0.034119	486484	0.887359	Sepsis (28	Sepsis (28	Sepsis (28	TRUE	igd	0.0079	2.60E-06	4999	M33885.m	TRUE	reported	uCOy6o	2	TRUE	0.004416	22.1645
8074	rs1160377	A	G	A	G	-0.0292	-0.0072	0.269	0.269775	FALSE	FALSE	FALSE	ieu-b-508	11	36890993	0.037814	486484	0.848972	Sepsis (28	Sepsis (28	Sepsis (28	TRUE	igd	0.0062	2.32E-06	2651	M36009.m	TRUE	reported	2EUZ1U	2	TRUE	0.008298	22.16433
3193	rs7582822	A	G	A	G	0.0113	0.054834	0.388	0.385761	FALSE	FALSE	FALSE	ieu-b-508	2	1.72E+08	0.034463	486484	0.111586	Sepsis (28	Sepsis (28	Sepsis (28	TRUE	igd	0.0024	3.84E-06	7356	M32338.m	TRUE	reported	q5cuaP	2	TRUE	0.003005	22.16238
1202	rs891531	T	G	T	G	0.0113	-0.03895	0.1846	0.226642	FALSE	FALSE	FALSE	ieu-b-508	2	67987821	0.039951	486484	0.32957	Sepsis (28	Sepsis (28	Sepsis (28	TRUE	igd	0.0024	3.81E-06	7346	M01645.m	TRUE	reported	d5nAn3	2	TRUE	0.003009	22.16237
7704	rs1943505	T	C	T	C	0.0113	-0.02628	0.1067	0.104425	FALSE	FALSE	FALSE	ieu-b-508	11	95212878	0.055058	486484	0.63311	Sepsis (28	Sepsis (28	Sepsis (28	TRUE	igd	0.0024	3.20E-06	7345	M35397.m	TRUE	reported	Y56mZr	2	TRUE	0.003009	22.16237
8574	rs9938618	A	G	A	G	-0.0452	0.236867	0.943	0.945264	FALSE	FALSE	FALSE	ieu-b-508	16	56626769	0.073115	486484	0.001197	Sepsis (28	Sepsis (28	Sepsis (28	TRUE	igd	0.0096	2.74E-06	7333	M37203.m	TRUE	reported	3gIIIv	2	TRUE	0.003014	22.16236
7580	rs7153218	A	G	A	G	0.0113	-0.00088	0.2736	0.310946	FALSE	FALSE	FALSE	ieu-b-508	14	95901182	0.036136	486484	0.980679	Sepsis (28	Sepsis (28	Sepsis (28	TRUE	igd	0.0024	3.27E-06	7217	M35270.m	TRUE	reported	Ct8V1n	2	TRUE	0.003062	22.16226
5339	rs1282122	T	C	T	C	0.0226	-0.00305	0.0348	0.039387	FALSE	FALSE	FALSE	ieu-b-508	12	13588748	0.085359	486484	0.971506	Sepsis (28	Sepsis (28	Sepsis (28	TRUE	igd	0.0048	2.11E-06	7118	M33380.m	TRUE	reported	OHRfl5	2	TRUE	0.003105	22.16217
7179	rs2941589	A	G	A	G	0.0598	0.023708	0.8527	0.846687	FALSE	FALSE	FALSE	ieu-b-508	2	54905778	0.047003	486484	0.613977	Sepsis (28	Sepsis (28	Sepsis (28	TRUE	igd	0.0127	2.58E-06	4515	M34535.m	TRUE	reported	z9BiJF	2	TRUE	0.004887	22.16167
6662	rs745932	A	G	A	G	-0.0846	0.090368	0.1263	0.123949	FALSE	FALSE	FALSE	ieu-b-508	18	60495520	0.050504	486484	0.073567	Sepsis (28	Sepsis (28	Sepsis (28	TRUE	igd	0.0179	2.25E-06	248	M34322.m	TRUE	reported	16GdG9	2	TRUE	0.082628	22.15736
1981	rs9552597	A	G	A	G	0.0306	-0.05128	0.1845	0.190696	FALSE	FALSE	FALSE	ieu-b-508	13	22690127	0.042494	486484	0.227527	Sepsis (28	Sepsis (28	Sepsis (28	TRUE	igd	0.0065	2.15E-06	7226	M17807.m	TRUE	reported	zd6sZl	2	TRUE	0.003058	22.15623
7356	rs2982694	T	G	T	G	-0.041	-0.03761	0.8716	0.842559	FALSE	FALSE	FALSE	ieu-b-508	6	1.52E+08	0.045873	486484	0.412245	Sepsis (28	Sepsis (28	Sepsis (28	TRUE	igd	0.0087	2.49E-06	819	M35137.m	TRUE	reported	Eq2siS	2	TRUE	0.026401	22.15478
5028	rs6987404	T	C	T	C	-0.163	-0.05055	0.0588	0.059019	FALSE	FALSE	FALSE	ieu-b-508	8	41548312	0.070777	486484	0.475069	Sepsis (28	Sepsis (28	Sepsis (28	TRUE	igd	0.0346	2.53E-06	1017	M33178.m	TRUE	reported	g22WMS	2	TRUE	0.021356	22.14971
6654	rs418889	A	G	A	G	0.3821	-0.05993	0.0143	0.01816	FALSE	FALSE	FALSE	ieu-b-508	19	18149975	0.126416	486484	0.635463	Sepsis (28	Sepsis (28	Sepsis (28	TRUE	igd	0.0801	1.86E-06	75	M34322.m	TRUE	reported	16GdG9	2	TRUE	0.232781	22.14882
6293	rs1715749	A	G	A	G	0.0353	-0.07745	0.074	0.076041	FALSE	FALSE	FALSE	ieu-b-508	5	1.04E+08	0.062473	486484	0.215087	Sepsis (28	Sepsis (28	Sepsis (28	TRUE	igd	0.0075	2.21E-06	7320	M33941.m	TRUE	reported	culqiU	2	TRUE	0.003017	22.14666
5862	rs1391470	T	C	T	C	0.2838	0.10549	0.0161	0.019416	FALSE	FALSE	FALSE	ieu-b-508	15	76429785	0.12051	486484	0.381375	Sepsis (28	Sepsis (28	Sepsis (28	TRUE	igd	0.0602	2.45E-06	528	M33609.m	TRUE	reported	h7B9gN	2	TRUE	0.040392	22.14031
1639	rs2651516	T	C	T	C	0.008	0.098081	0.6585	0.655452	FALSE	FALSE	FALSE	ieu-b-508	15	53302309	0.035122	486484	0.005229	Sepsis (28	Sepsis (28	Sepsis (28	TRUE	igd	0.0017	4.30E-06	7368	M15140.m	TRUE	reported	jEcqMJ	2	TRUE	0.002997	22.13932
1648	rs6770323	T	C	T	C	-0.008	-0.00668	0.4299	0.428736	FALSE	FALSE	FALSE	ieu-b-508	3	1.62E+08	0.033833	486484	0.843545	Sepsis (28	Sepsis (28	Sepsis (28	TRUE	igd	0.0017	3.33E-06	7368	M15140.m	TRUE	reported	jEcqMJ	2	TRUE	0.002997	22.13932
1654	rs7619096	A	C	A	C	0.008	-0.06252	0.6886	0.687962	FALSE	FALSE	FALSE	ieu-b-508	3	84199075	0.036096	486484	0.083288	Sepsis (28	Sepsis (28	Sepsis (28	TRUE	igd	0.0017	4.92E-06	7368	M15140.m	TRUE	reported	jEcqMJ	2	TRUE	0.002997	22.13932
1660	rs9857268	A	G	A	G	0.008	-0.00556	0.411	0.418605	FALSE	FALSE	FALSE	ieu-b-508	3	83398721	0.033804	486484	0.869298	Sepsis (28	Sepsis (28	Sepsis (28	TRUE	igd	0.0017	3.07E-06	7368	M15140.m	TRUE	reported	jEcqMJ	2	TRUE	0.002997	22.13932
8614	rs3733593	A	G	A	G	0.008	0.0482	0.4373	0.439084	FALSE	FALSE	FALSE	ieu-b-508	4	15854725	0.03364	486484	0.151912	Sepsis (28	Sepsis (28	Sepsis (28	TRUE	igd	0.0017	4.66E-06	7366	M37506.m	TRUE	reported	m2kaRt	2	TRUE	0.002997	22.13932
420	rs2026590	T	G	T	G	0.008	-0.00959	0.2176	0.207226	FALSE	FALSE	FALSE	ieu-b-508	9	24833789	0.041181	486484	0.815806	Sepsis (28	Sepsis (28	Sepsis (28	TRUE	igd	0.0017	3.76E-06	7365	M00063.m	TRUE	reported	Hdsv1L	2	TRUE	0.002998	22.13932
441	rs810551	C	G	C	G	0.008	0.032728	0.7904	0.780879	FALSE	TRUE	FALSE	ieu-b-508	7	73492093	0.040269	486484	0.416379	Sepsis (28	Sepsis (28	Sepsis (28	TRUE	igd	0.0017	4.66E-06	7365	M00063.m	TRUE	reported	Hdsv1L	2	TRUE	0.002998	22.13932
4503	rs1759502	T	C	T	C	-0.008	0.019178	0.1176	0.114773	FALSE	FALSE	FALSE	ieu-b-508	15	91790302	0.052466	486484	0.714717	Sepsis (28	Sepsis (28	Sepsis (28	TRUE	igd	0.0017	4.76E-06	7362	M33132.m	TRUE	reported	dTXJa8	2	TRUE	0.002999	22.13931
688	rs1851740	A	C	A	C	-0.008	0.02177	0.185	0.184666	FALSE	FALSE	FALSE	ieu-b-508	3	77739202	0.043219	486484	0.614466	Sepsis (28	Sepsis (28	Sepsis (28	TRUE	igd	0.0017	2.74E-06	7358	M01299.m	TRUE	reported	gfM0a3	2	TRUE	0.003001	22.13931
819	rs216283	A	C	A	C	0.008	-0.02739	0.4171	0.427589	FALSE	FALSE	FALSE	ieu-b-508	19	3007698	0.034129	486484	0.422261	Sepsis (28	Sepsis (28	Sepsis (28	TRUE	igd	0.0017	2.63E-06	7355	M01358.m	TRUE	reported	kSPQgd	2	TRUE	0.003002	22.13931
825	rs3863373	A	C	A	C	-0.008	-0.04942	0.3335	0.332215	FALSE	FALSE	FALSE	ieu-b-508	12	71288396	0.035369	486484	0.162315	Sepsis (28	Sepsis (28	Sepsis (28	TRUE	igd	0.0017	4.21E-06	7355	M01358.m	TRUE	reported	kSPQgd	2	TRUE	0.003002	22.13931
2074	rs6951006	A	G	A	G	-0.008	0.064273	0.4884	0.488455	FALSE	FALSE	FALSE	ieu-b-508	7	1.56E+08	0.033925	486484	0.058154	Sepsis (28	Sepsis (28	Sepsis (28	TRUE	igd	0.0017	3.53E-06	7354	M18369.m	TRUE	reported	ykBjIT	2	TRUE	0.003002	22.13931
2078	rs9827101	A	C	A	C	0.008	-0.04803	0.5393	0.517279	FALSE	FALSE	FALSE	ieu-b-508	3	1.32E+08	0.033518	486484	0.151846	Sepsis (28	Sepsis (28	Sepsis (28	TRUE	igd	0.0017	2.99E-06	7354	M18369.m	TRUE	reported	ykBjIT	2	TRUE	0.003002	22.13931
2245	rs1764963	T	C	T	C	-0.008	-0.01886	0.654	0.663023	FALSE	FALSE	FALSE	ieu-b-508	13	86259295	0.035372	486484	0.593939	Sepsis (28	Sepsis (28	Sepsis (28	TRUE	igd	0.0017	4.40E-06	7354	M19934.m	TRUE	reported	HtcWcQ	2	TRUE	0.003002	22.13931
2267	rs722419	A	C	A	C	0.008	-0.00164	0.4468	0.472521	FALSE	FALSE	FALSE	ieu-b-508	19	13360233	0.033577	486484	0.961104	Sepsis (28	Sepsis (28	Sepsis (28	TRUE	igd	0.0017	4.03E-06	7354	M19934.m	TRUE	reported	HtcWcQ	2	TRUE	0.003002	22.13931
2271	rs9317855	T	C	T	C	-0.008	-0.02822	0.3513	0.358256	FALSE	FALSE	FALSE	ieu-b-508	13	70543601	0.034825	486484	0.417794	Sepsis (28	Sepsis (28	Sepsis (28	TRUE	igd	0.0017	4.35E-06	7354	M19934.m	TRUE	reported	HtcWcQ	2	TRUE	0.003002	22.13931
640	rs1006320	T	C	T	C	0.008	0.013079	0.1559	0.189025	FALSE	FALSE	FALSE	ieu-b-508	5	1.59E+08	0.045274	486484	0.772668	Sepsis (28	Sepsis (28	Sepsis (28	TRUE	igd	0.0017	4.06E-06	7352	M01125.m	TRUE	reported	yw0clB	2	TRUE	0.003003	22.1393
788	rs6540294	A	C	A	C	-0.008	0.067298	0.451	0.450836	FALSE	FALSE	FALSE	ieu-b-508	16	86479404	0.03363	486484	0.045381	Sepsis (28	Sepsis (28	Sepsis (28	TRUE	igd	0.0017	2.87E-06	7352	M01336.m	TRUE	reported	FAlrya	2	TRUE	0.003003	22.1393
792	rs9809122	A	G	A	G	-0.008	-0.02743	0.5296	0.524047	FALSE	FALSE	FALSE	ieu-b-508	3	70084253	0.033438	486484	0.412101	Sepsis (28	Sepsis (28	Sepsis (28	TRUE	igd	0.0017	2.45E-06	7352	M01336.m	TRUE	reported	FAlrya	2	TRUE	0.003003	22.1393
7701	rs1261383	T	C	T	C	-0.008	0.013472	0.7597	0.772893	FALSE	FALSE	FALSE	ieu-b-508	2	2.4E+08	0.039826	486484	0.735153	Sepsis (28	Sepsis (28	Sepsis (28	TRUE	igd	0.0017	1.93E-06	7345	M35397.m	TRUE	reported	Y56mZr	2	TRUE	0.003006	22.1393
4460	rs866142	A	G	A	G	0.008	0.022919	0.605	0.57677	FALSE	FALSE	FALSE	ieu-b-508	6	72048250	0.033739	486484	0.49695	Sepsis (28	Sepsis (28	Sepsis (28	TRUE	igd	0.0017	3.79E-06	7344	M32910.m	TRUE	reported	2OCBKh	2	TRUE	0.003006	22.1393
1778	rs699530	T	G	T	G	-0.008	0.020141	0.5636	0.55929	FALSE	FALSE	FALSE	ieu-b-508	1	90947926	0.033829	486484	0.551601	Sepsis (28	Sepsis (28	Sepsis (28	TRUE	igd	0.0017	2.78E-06	7331	M15676.m	TRUE	reported	s1NfKN	2	TRUE	0.003012	22.13929
1785	rs7986369	A	C	A	C	-0.008	0.065942	0.3795	0.381947	FALSE	FALSE	FALSE	ieu-b-508	13	48463123	0.034253	486484	0.054209	Sepsis (28	Sepsis (28	Sepsis (28	TRUE	igd	0.0017	2.97E-06	7331	M15676.m	TRUE	reported	s1NfKN	2	TRUE	0.003012	22.13929
1312	rs8033140	T	C	T	C	0.008	0.04685	0.3547	0.364727	FALSE	FALSE	FALSE	ieu-b-508	15	68212656	0.034935	486484	0.179902	Sepsis (28	Sepsis (28	Sepsis (28	TRUE	igd	0.0017	4.32E-06	7325	M02132.m	TRUE	reported	uJFm2t	2	TRUE	0.003014	22.13928
4044	rs1177828	A	G	A	G	0.008	0.046293	0.3156	0.322107	FALSE	FALSE	FALSE	ieu-b-508	8	63735445	0.035815	486484	0.196159	Sepsis (28	Sepsis (28	Sepsis (28	TRUE	igd	0.0017	5.03E-06	7318	M32740.m	TRUE	reported	YLshfP	2	TRUE	0.003017	22.13928
4066	rs4562058	T	C	T	C	-0.008	0.014214	0.3093	0.309825	FALSE	FALSE	FALSE	ieu-b-508	5	1.72E+08	0.036067	486484	0.69351	Sepsis (28	Sepsis (28	Sepsis (28	TRUE	igd	0.0017	3.88E-06	7318	M32740.m	TRUE	reported	YLshfP	2	TRUE	0.003017	22.13928
1588	rs7068714	A	C	A	C	0.008	-0.00052	0.1774	0.178368	FALSE	FALSE	FALSE	ieu-b-508	10	87401397	0.043553	486484	0.990446	Sepsis (28	Sepsis (28	Sepsis (28	TRUE	igd	0.0017	2.97E-06	7314	M12593.m	TRUE	reported	lBXgSU	2	TRUE	0.003019	22.13927
4187	rs2279399	T	C	T	C	0.016	-0.01443	0.4592	0.44372	FALSE	FALSE	FALSE	ieu-b-508	12	54581483	0.033902	486484	0.67031	Sepsis (28	Sepsis (28	Sepsis (28	TRUE	igd	0.0034	3.29E-06	7313	M32761.m	TRUE	reported	QefVGc	2	TRUE	0.003019	22.13927
2757	rs453766	C	G	C	G	0.008	0.003627	0.7548	0.748061	FALSE	TRUE	FALSE	ieu-b-508	19	11406839	0.038454	486484	0.924845	Sepsis (28	Sepsis (28	Sepsis (28	TRUE	igd	0.0017	4.23E-06	7303	M27256.m	TRUE	reported	mJwzsi	2	TRUE	0.003023	22.13926
2439	rs2035247	T	G	T	G	-0.016	-0.02831	0.8304	0.823354	FALSE	FALSE	FALSE	ieu-b-508	16	7069056	0.043975	486484	0.51973	Sepsis (28	Sepsis (28	Sepsis (28	TRUE	igd	0.0034	1.94E-06	7284	M21630.m	TRUE	reported	7eaO4H	2	TRUE	0.003031	22.13925
2687	rs6832163	A	G	A	G	-0.016	0.056344	0.2711	0.29616	FALSE	FALSE	FALSE	ieu-b-508	4	1.15E+08	0.037312	486484	0.131021	Sepsis (28	Sepsis (28	Sepsis (28	TRUE	igd	0.0034	3.48E-06	7256	M24074.m	TRUE	reported	Lk6D2A	2	TRUE	0.003043	22.13922
2634	rs1054634	A	G	A	G	-0.016	0.02035	0.8558	0.85715	FALSE	FALSE	FALSE	ieu-b-508	11	1756225	0.047674	486484	0.669484	Sepsis (28	Sepsis (28	Sepsis (28	TRUE	igd	0.0034	2.60E-06	7184	M22548.m	TRUE	reported	mZtwX2	2	TRUE	0.003073	22.13916
2180	rs1382606	A	G	A	G	0.008	0.024264	0.5044	0.507521	FALSE	FALSE	FALSE	ieu-b-508	8	98582216	0.033568	486484	0.469785	Sepsis (28	Sepsis (28	Sepsis (28	TRUE	igd	0.0017	3.11E-06	7182	M19362.m	TRUE	reported	SkNEKw	2	TRUE	0.003074	22.13916
1267	rs960381	A	C	A	C	0.008	0.054617	0.5958	0.588819	FALSE	FALSE	FALSE	ieu-b-508	7	49558713	0.033858	486484	0.106718	Sepsis (28	Sepsis (28	Sepsis (28	TRUE	igd	0.0017	3.56E-06	7136	M01769.m	TRUE	reported	rismUZ	2	TRUE	0.003094	22.13912
5357	rs6080978	T	C	T	C	-0.008	-0.0168	0.299	0.298197	FALSE	FALSE	FALSE	ieu-b-508	20	1862420	0.037307	486484	0.652427	Sepsis (28	Sepsis (28	Sepsis (28	TRUE	igd	0.0017	3.18E-06	7118	M33380.m	TRUE	reported	OHRfl5	2	TRUE	0.003102	22.13911
5358	rs6605235	T	C	T	C	-0.008	0.019547	0.7072	0.711759	FALSE	FALSE	FALSE	ieu-b-508	1	82055488	0.036914	486484	0.596429	Sepsis (28	Sepsis (28	Sepsis (28	TRUE	igd	0.0017	3.54E-06	7118	M33380.m	TRUE	reported	OHRfl5	2	TRUE	0.003102	22.13911
1380	rs1340971	T	C	T	C	-0.008	0.059885	0.6654	0.670345	FALSE	FALSE	FALSE	ieu-b-508	6	70536658	0.035575	486484	0.092306	Sepsis (28	Sepsis (28	Sepsis (28	TRUE	igd	0.0017	4.66E-06	7037	M02734.m	TRUE	reported	0y0dDD	2	TRUE	0.003137	22.13903
4841	rs1714055	A	G	A	G	0.3375	-0.17366	0.9707	0.943083	FALSE	FALSE	FALSE	ieu-b-508	5	1.16E+08	0.072053	486484	0.015943	Sepsis (28	Sepsis (28	Sepsis (28	TRUE	igd	0.0688	9.24E-07	25	M33178.m	TRUE	reported	g22WMS	2	TRUE	0.490463	22.13903
3294	rs2761735	T	C	T	C	-0.024	-0.05857	0.8145	0.808048	FALSE	FALSE	FALSE	ieu-b-508	9	9796116	0.04278	486484	0.170959	Sepsis (28	Sepsis (28	Sepsis (28	TRUE	igd	0.0051	2.67E-06	6119	M32379.m	TRUE	reported	MLimM6	2	TRUE	0.003606	22.13809
6220	rs4953276	A	C	A	C	-0.032	-0.03278	0.1272	0.13585	FALSE	FALSE	FALSE	ieu-b-508	2	46083842	0.048801	486484	0.501795	Sepsis (28	Sepsis (28	Sepsis (28	TRUE	igd	0.0068	2.42E-06	4533	M33901.m	TRUE	reported	mQkhrb	2	TRUE	0.004862	22.13556
4072	rs6429032	T	C	T	C	-0.0273	0.038921	0.0905	0.112042	FALSE	FALSE	FALSE	ieu-b-508	1	2.38E+08	0.052966	486484	0.462447	Sepsis (28	Sepsis (28	Sepsis (28	TRUE	igd	0.0058	2.12E-06	1759	M32740.m	TRUE	reported	YLshfP	2	TRUE	0.012438	22.12968
6069	rs1312182	A	G	A	G	0.0207	-0.02339	0.2121	0.28444	FALSE	FALSE	FALSE	ieu-b-508	4	25097862	0.03744	486484	0.532138	Sepsis (28	Sepsis (28	Sepsis (28	TRUE	igd	0.0044	2.23E-06	7350	M33822.m	TRUE	reported	Wic4uS	2	TRUE	0.003002	22.12673
6542	rs4955937	A	G	A	G	-0.0207	-0.02555	0.5451	0.541054	FALSE	FALSE	FALSE	ieu-b-508	3	55194254	0.03371	486484	0.448422	Sepsis (28	Sepsis (28	Sepsis (28	TRUE	igd	0.0044	2.17E-06	6987	M34221.m	TRUE	reported	nUe3TL	2	TRUE	0.003158	22.12641
7152	rs1175738	T	C	T	C	0.0998	0.044043	0.1781	0.142635	FALSE	FALSE	FALSE	ieu-b-508	6	298700	0.065231	486484	0.499562	Sepsis (28	Sepsis (28	Sepsis (28	TRUE	igd	0.0212	2.45E-06	1212	M34533.m	TRUE	reported	SuKX4k	2	TRUE	0.017956	22.12443
8050	rs1262368	A	G	A	G	0.0334	0.003003	0.0629	0.087388	FALSE	FALSE	FALSE	ieu-b-508	2	28383413	0.059315	486484	0.959616	Sepsis (28	Sepsis (28	Sepsis (28	TRUE	igd	0.0071	2.59E-06	6901	M35977.m	TRUE	reported	NHnUo7	2	TRUE	0.003196	22.12332
3440	rs6881997	A	G	A	G	-0.0127	0.106601	0.8282	0.824724	FALSE	FALSE	FALSE	ieu-b-508	5	1.35E+08	0.044041	486484	0.015499	Sepsis (28	Sepsis (28	Sepsis (28	TRUE	igd	0.0027	2.06E-06	7364	M32452.m	TRUE	reported	8GtFpl	2	TRUE	0.002995	22.11882
7978	rs1491024	A	G	A	G	-0.0127	-0.01615	0.7762	0.7763	FALSE	FALSE	FALSE	ieu-b-508	13	90715598	0.040125	486484	0.687405	Sepsis (28	Sepsis (28	Sepsis (28	TRUE	igd	0.0027	2.89E-06	7358	M35718.m	TRUE	reported	oUwc0a	2	TRUE	0.002998	22.11881
1607	rs1310635	A	T	A	T	-0.0127	-0.02999	0.2123	0.219655	FALSE	TRUE	FALSE	ieu-b-508	4	1.68E+08	0.04052	486484	0.45927	Sepsis (28	Sepsis (28	Sepsis (28	TRUE	igd	0.0027	2.31E-06	7352	M15122.m	TRUE	reported	Vlnl8T	2	TRUE	0.003	22.11881
1617	rs641939	A	G	A	G	0.0127	0.052837	0.7987	0.790855	FALSE	FALSE	FALSE	ieu-b-508	2	72611175	0.041141	486484	0.199041	Sepsis (28	Sepsis (28	Sepsis (28	TRUE	igd	0.0027	2.13E-06	7352	M15122.m	TRUE	reported	Vlnl8T	2	TRUE	0.003	22.11881
3560	rs1257796	A	G	A	G	-0.0127	0.010696	0.1103	0.107686	FALSE	FALSE	FALSE	ieu-b-508	11	46820788	0.053799	486484	0.84241	Sepsis (28	Sepsis (28	Sepsis (28	TRUE	igd	0.0027	2.85E-06	7350	M32518.m	TRUE	reported	V0ZCiU	2	TRUE	0.003001	22.11881
3588	rs7349641	T	C	T	C	0.0127	0.039132	0.8878	0.889003	FALSE	FALSE	FALSE	ieu-b-508	4	41281031	0.053266	486484	0.462547	Sepsis (28	Sepsis (28	Sepsis (28	TRUE	igd	0.0027	2.72E-06	7350	M32518.m	TRUE	reported	V0ZCiU	2	TRUE	0.003001	22.11881
6553	rs1341296	T	C	T	C	0.0127	-0.01631	0.4596	0.462422	FALSE	FALSE	FALSE	ieu-b-508	11	29363716	0.033557	486484	0.626878	Sepsis (28	Sepsis (28	Sepsis (28	TRUE	igd	0.0027	2.79E-06	7347	M34244.m	TRUE	reported	JyF0Sp	2	TRUE	0.003002	22.11881
2691	rs1020973	A	G	A	G	-0.0127	-0.06502	0.7536	0.74961	FALSE	FALSE	FALSE	ieu-b-508	2	1.82E+08	0.038553	486484	0.091702	Sepsis (28	Sepsis (28	Sepsis (28	TRUE	igd	0.0027	2.21E-06	7340	M25459.m	TRUE	reported	ACJY35	2	TRUE	0.003005	22.1188
1678	rs333383	A	G	A	G	-0.0127	0.057251	0.8384	0.838968	FALSE	FALSE	FALSE	ieu-b-508	4	1.73E+08	0.045426	486484	0.207549	Sepsis (28	Sepsis (28	Sepsis (28	TRUE	igd	0.0027	3.38E-06	7337	M15365.m	TRUE	reported	TReTCM	2	TRUE	0.003006	22.1188
3738	rs2449529	A	G	A	G	-0.0127	-0.02992	0.5572	0.555595	FALSE	FALSE	FALSE	ieu-b-508	8	98785523	0.033578	486484	0.372862	Sepsis (28	Sepsis (28	Sepsis (28	TRUE	igd	0.0027	2.76E-06	7337	M32632.m	TRUE	reported	plHzPT	2	TRUE	0.003006	22.1188
2015	rs4708792	A	G	A	G	-0.0673	0.048326	0.1015	0.105946	FALSE	FALSE	FALSE	ieu-b-508	6	1.59E+08	0.054495	486484	0.375197	Sepsis (28	Sepsis (28	Sepsis (28	TRUE	igd	0.0143	2.61E-06	1448	M18283.m	TRUE	reported	oyZdTE	2	TRUE	0.015066	22.11861
7515	rs9470238	T	G	T	G	-0.0127	0.039932	0.3575	0.358651	FALSE	FALSE	FALSE	ieu-b-508	6	36228600	0.035363	486484	0.258811	Sepsis (28	Sepsis (28	Sepsis (28	TRUE	igd	0.0027	3.89E-06	7100	M35254.m	TRUE	reported	HfVgPn	2	TRUE	0.003106	22.1186
2377	rs2378442	A	T	A	T	0.0127	-0.02376	0.3445	0.333919	FALSE	TRUE	FALSE	ieu-b-508	3	1.88E+08	0.035502	486484	0.503271	Sepsis (28	Sepsis (28	Sepsis (28	TRUE	igd	0.0027	3.98E-06	6999	M21127.m	TRUE	reported	WCjhS3	2	TRUE	0.003151	22.11851
6599	rs1259892	T	G	T	G	-0.0127	0.001805	0.2613	0.265048	FALSE	FALSE	FALSE	ieu-b-508	16	3985081	0.037732	486484	0.961853	Sepsis (28	Sepsis (28	Sepsis (28	TRUE	igd	0.0027	2.82E-06	6994	M34306.m	TRUE	reported	WPXPUN	2	TRUE	0.003153	22.1185
7154	rs1351284	A	C	A	C	0.0254	-0.02974	0.6287	0.618017	FALSE	FALSE	FALSE	ieu-b-508	5	76544051	0.03429	486484	0.385789	Sepsis (28	Sepsis (28	Sepsis (28	TRUE	igd	0.0054	2.63E-06	5856	M34533.m	TRUE	reported	SuKX4k	2	TRUE	0.003764	22.11727
2473	rs1003138	A	G	A	G	0.0254	-0.05163	0.9233	0.922318	FALSE	FALSE	FALSE	ieu-b-508	4	13301722	0.062684	486484	0.410092	Sepsis (28	Sepsis (28	Sepsis (28	TRUE	igd	0.0054	3.02E-06	5676	M22032.m	TRUE	reported	xpe96K	2	TRUE	0.003883	22.11703
3848	rs4305648	A	G	A	G	0.0127	-0.0306	0.3639	0.366169	FALSE	FALSE	FALSE	ieu-b-508	5	28172825	0.034741	486484	0.378498	Sepsis (28	Sepsis (28	Sepsis (28	TRUE	igd	0.0027	2.96E-06	5211	M32651.m	TRUE	reported	DeuPwv	2	TRUE	0.004228	22.11634
6940	rs7705239	A	G	A	G	0.0254	-0.00341	0.8521	0.848872	FALSE	FALSE	FALSE	ieu-b-508	5	38049522	0.04669	486484	0.941756	Sepsis (28	Sepsis (28	Sepsis (28	TRUE	igd	0.0054	2.30E-06	5208	M34395.m	TRUE	reported	QVOPA3	2	TRUE	0.00423	22.11633
3381	rs4612338	A	G	A	G	-0.0367	0.014874	0.1596	0.157475	FALSE	FALSE	FALSE	ieu-b-508	8	84511490	0.045714	486484	0.744897	Sepsis (28	Sepsis (28	Sepsis (28	TRUE	igd	0.0078	2.44E-06	1767	M32418.m	TRUE	reported	y8cN4Y	2	TRUE	0.012374	22.11317
6016	rs5998862	A	C	A	C	-0.6716	-0.0596	0.9487	0.947988	FALSE	FALSE	FALSE	ieu-b-508	22	33697548	0.078032	486484	0.444958	Sepsis (28	Sepsis (28	Sepsis (28	TRUE	igd	0.141	1.91E-06	79	M33751.m	TRUE	reported	Cxdkr4	2	TRUE	0.223109	22.11296
2679	rs2034360	T	C	T	C	0.0174	0.006658	0.8625	0.84349	FALSE	FALSE	FALSE	ieu-b-508	2	46386298	0.045903	486484	0.884673	Sepsis (28	Sepsis (28	Sepsis (28	TRUE	igd	0.0037	2.73E-06	7256	M24074.m	TRUE	reported	Lk6D2A	2	TRUE	0.003039	22.10932
4257	rs4437633	T	G	T	G	-0.065	-0.04733	0.468	0.498471	FALSE	FALSE	FALSE	ieu-b-508	8	69504822	0.033465	486484	0.157268	Sepsis (28	Sepsis (28	Sepsis (28	TRUE	igd	0.0138	2.44E-06	562	M32800.m	TRUE	reported	Q7MVsa	2	TRUE	0.037977	22.10651
7787	rs1181484	T	C	T	C	-0.1595	0.050113	0.9778	0.934884	FALSE	FALSE	FALSE	ieu-b-508	10	56606071	0.067882	486484	0.460366	Sepsis (28	Sepsis (28	Sepsis (28	TRUE	igd	0.0339	2.48E-06	1383	M35451.m	TRUE	reported	GMRGAN	2	TRUE	0.015754	22.10515
8569	rs3733475	T	G	T	G	-0.0221	-0.02607	0.3611	0.401898	FALSE	FALSE	FALSE	ieu-b-508	4	1.85E+08	0.034263	486484	0.446662	Sepsis (28	Sepsis (28	Sepsis (28	TRUE	igd	0.0047	2.78E-06	7333	M37203.m	TRUE	reported	3gIIIv	2	TRUE	0.003006	22.10397
3919	rs6563540	T	G	T	G	0.0428	0.008083	0.1256	0.12783	FALSE	FALSE	FALSE	ieu-b-508	13	38008009	0.050073	486484	0.871761	Sepsis (28	Sepsis (28	Sepsis (28	TRUE	igd	0.0091	2.72E-06	2575	M32691.m	TRUE	reported	SiuD6F	2	TRUE	0.008518	22.10382
8257	rs1282331	T	C	T	C	-0.0221	0.125777	0.0408	0.040118	FALSE	FALSE	FALSE	ieu-b-508	12	95008004	0.085831	486484	0.142811	Sepsis (28	Sepsis (28	Sepsis (28	TRUE	igd	0.0047	2.28E-06	7092	M36552.m	TRUE	reported	5CxMJz	2	TRUE	0.003108	22.10377
2963	rs6890263	T	C	T	C	0.0221	-0.02432	0.9379	0.938288	FALSE	FALSE	FALSE	ieu-b-508	5	1.15E+08	0.069595	486484	0.726798	Sepsis (28	Sepsis (28	Sepsis (28	TRUE	igd	0.0047	3.18E-06	6795	M30805.m	TRUE	reported	YHV70c	2	TRUE	0.003243	22.1035
7449	rs4687148	T	G	T	G	0.0489	-0.05026	0.9265	0.896295	FALSE	FALSE	FALSE	ieu-b-508	3	1.9E+08	0.054428	486484	0.355773	Sepsis (28	Sepsis (28	Sepsis (28	TRUE	igd	0.0104	2.65E-06	6234	M35189.m	TRUE	reported	54XZFj	2	TRUE	0.003534	22.10099
2811	rs1123667	T	C	T	C	0.0348	-0.04158	0.3167	0.348128	FALSE	FALSE	FALSE	ieu-b-508	11	75983754	0.035009	486484	0.234968	Sepsis (28	Sepsis (28	Sepsis (28	TRUE	igd	0.0074	2.58E-06	2759	M27447.m	TRUE	reported	s1NdyU	2	TRUE	0.007952	22.09938
6178	rs9839976	A	G	A	G	-0.1468	0.039993	0.0718	0.077312	FALSE	FALSE	FALSE	ieu-b-508	3	31527862	0.062685	486484	0.523475	Sepsis (28	Sepsis (28	Sepsis (28	TRUE	igd	0.0312	2.53E-06	1100	M33885.m	TRUE	reported	uCOy6o	2	TRUE	0.019729	22.09798
2111	rs1290508	A	G	A	G	-0.0574	-0.00968	0.4166	0.443855	FALSE	FALSE	FALSE	ieu-b-508	15	59250000	0.033735	486484	0.774066	Sepsis (28	Sepsis (28	Sepsis (28	TRUE	igd	0.0122	2.58E-06	917	M18474.m	TRUE	reported	B6Xd02	2	TRUE	0.023571	22.08797
7063	rs1190579	A	G	A	G	-0.0997	-0.02885	0.0405	0.059503	FALSE	FALSE	FALSE	ieu-b-508	20	62471198	0.070942	486484	0.684255	Sepsis (28	Sepsis (28	Sepsis (28	TRUE	igd	0.0212	2.50E-06	1462	M34469.m	TRUE	reported	WMOqbm	2	TRUE	0.014902	22.08636
1615	rs5022234	A	C	A	C	0.0221	0.003941	0.6303	0.674151	FALSE	FALSE	FALSE	ieu-b-508	13	82053416	0.035622	486484	0.911906	Sepsis (28	Sepsis (28	Sepsis (28	TRUE	igd	0.0047	2.15E-06	1757	M15122.m	TRUE	reported	Vlnl8T	2	TRUE	0.012428	22.08484

6064	rs9321466	C	G	C	G	0.0376	-0.05606	0.9628	0.961639	FALSE	TRUE	FALSE	ieu-b-508	6	1.35E+08	0.087724	486484	0.522807	Sepsis (28	Sepsis (28	Sepsis (28	TRUE	igd	0.008	2.74E-06	7362	M33821.m	TRUE	reported	eVYICL	2	TRUE	0.002992	22.084
1050	rs2907828	C	G	C	G	-0.0188	-0.03903	0.1375	0.175395	FALSE	TRUE	FALSE	ieu-b-508	5	56902155	0.044179	486484	0.377007	Sepsis (28	Sepsis (28	Sepsis (28	TRUE	igd	0.004	2.74E-06	7333	M01572.m	TRUE	reported	EOUAWy	2	TRUE	0.003003	22.08398
1780	rs7222769	T	C	T	C	0.0141	0.034451	0.8765	0.872642	FALSE	FALSE	FALSE	ieu-b-508	17	53962112	0.051495	486484	0.503484	Sepsis (28	Sepsis (28	Sepsis (28	TRUE	igd	0.003	2.97E-06	7331	M15676.m	TRUE	reported	s1NfKN	2	TRUE	0.003004	22.08397
8250	rs2850161	T	G	T	G	0.0047	0.038341	0.3664	0.369139	FALSE	FALSE	FALSE	ieu-b-508	21	45698562	0.034507	486484	0.266516	Sepsis (28	Sepsis (28	Sepsis (28	TRUE	igd	0.001	4.84E-06	7330	M36515.m	TRUE	reported	yZaYzr	2	TRUE	0.003005	22.08397
2571	rs624947	A	G	A	G	-0.0094	0.050883	0.6784	0.686726	FALSE	FALSE	FALSE	ieu-b-508	11	68140782	0.03677	486484	0.166418	Sepsis (28	Sepsis (28	Sepsis (28	TRUE	igd	0.002	1.90E-06	6535	M22177.m	TRUE	reported	2s6Sly	2	TRUE	0.003369	22.08324
4275	rs1686888	T	C	T	C	-0.1646	-0.14074	0.9702	0.976855	FALSE	FALSE	FALSE	ieu-b-508	5	39439942	0.114605	486484	0.219441	Sepsis (28	Sepsis (28	Sepsis (28	TRUE	igd	0.035	2.55E-06	1298	M32808.m	TRUE	reported	vnr36k	2	TRUE	0.016754	22.08279
3499	rs2239855	T	C	T	C	-0.047	0.023963	0.986	0.976535	FALSE	FALSE	FALSE	ieu-b-508	22	26891923	0.111065	486484	0.82918	Sepsis (28	Sepsis (28	Sepsis (28	TRUE	igd	0.01	2.60E-06	5588	M32492.m	TRUE	reported	rZZFcE	2	TRUE	0.003938	22.08209
8338	rs1390370	A	G	A	G	0.0235	-0.0613	0.8174	0.79732	FALSE	FALSE	FALSE	ieu-b-508	13	44245602	0.042336	486484	0.147645	Sepsis (28	Sepsis (28	Sepsis (28	TRUE	igd	0.005	2.62E-06	5292	M36673.m	TRUE	reported	CD5lvR	2	TRUE	0.004157	22.08165
6688	rs1005464	A	G	A	G	0.0141	0.044005	0.229	0.226225	FALSE	FALSE	FALSE	ieu-b-508	20	6756148	0.039783	486484	0.26868	Sepsis (28	Sepsis (28	Sepsis (28	TRUE	igd	0.003	2.20E-06	5081	M34336.m	TRUE	reported	jrSSxL	2	TRUE	0.004329	22.0813
4765	rs1338583	A	G	A	G	-0.1364	-0.11378	0.1003	0.123664	FALSE	FALSE	FALSE	ieu-b-508	2	95868247	0.051026	486484	0.025764	Sepsis (28	Sepsis (28	Sepsis (28	TRUE	igd	0.029	2.61E-06	1017	M33178.m	TRUE	reported	g22WMS	2	TRUE	0.02129	22.07892
6128	rs3752559	T	G	T	G	0.5132	-0.01446	0.0114	0.019688	FALSE	FALSE	FALSE	ieu-b-508	20	42197904	0.120589	486484	0.904542	Sepsis (28	Sepsis (28	Sepsis (28	TRUE	igd	0.1086	2.29E-06	176	M33877.m	TRUE	reported	yDVi1f	2	TRUE	0.112596	22.07752
5159	rs6878813	T	C	T	C	0.0672	0.045217	0.1376	0.151943	FALSE	FALSE	FALSE	ieu-b-508	5	61463949	0.046554	486484	0.331406	Sepsis (28	Sepsis (28	Sepsis (28	TRUE	igd	0.0143	2.62E-06	5716	M33192.m	TRUE	reported	CyfjKE	2	TRUE	0.003849	22.0757
7480	rs1702260	A	G	A	G	-0.0771	0.086154	0.9828	0.98771	FALSE	FALSE	FALSE	ieu-b-508	4	1.48E+08	0.151392	486484	0.569301	Sepsis (28	Sepsis (28	Sepsis (28	TRUE	igd	0.0164	2.46E-06	1673	M35240.m	TRUE	reported	55Kiv4	2	TRUE	0.013038	22.07504
7880	rs1246187	A	C	A	C	0.0569	0.0573	0.2704	0.278979	FALSE	FALSE	FALSE	ieu-b-508	19	17180358	0.03728	486484	0.124287	Sepsis (28	Sepsis (28	Sepsis (28	TRUE	igd	0.0121	2.70E-06	1098	M35635.m	TRUE	reported	qymryH	2	TRUE	0.019742	22.07303
5989	rs1121845	A	G	A	G	-0.0442	0.006221	0.5504	0.546519	FALSE	FALSE	FALSE	ieu-b-508	11	1.22E+08	0.03355	486484	0.852901	Sepsis (28	Sepsis (28	Sepsis (28	TRUE	igd	0.0094	2.49E-06	1027	M33683.m	TRUE	reported	wSozpU	2	TRUE	0.021075	22.06695
5194	rs1173719	T	C	T	C	-0.3889	-0.07933	0.9824	0.977612	FALSE	FALSE	FALSE	ieu-b-508	4	1.05E+08	0.112626	486484	0.481187	Sepsis (28	Sepsis (28	Sepsis (28	TRUE	igd	0.0827	2.59E-06	930	M33203.m	TRUE	reported	Sto7pR	2	TRUE	0.023226	22.06632
6939	rs4356633	A	C	A	C	-0.0249	0.039248	0.1592	0.154138	FALSE	FALSE	FALSE	ieu-b-508	2	1.76E+08	0.046112	486484	0.394686	Sepsis (28	Sepsis (28	Sepsis (28	TRUE	igd	0.0053	2.73E-06	5208	M34395.m	TRUE	reported	QVOPA3	2	TRUE	0.00422	22.06379
2088	rs27667	T	G	T	G	-0.0564	0.028955	0.6338	0.628009	FALSE	FALSE	FALSE	ieu-b-508	3	10377312	0.034473	486484	0.400946	Sepsis (28	Sepsis (28	Sepsis (28	TRUE	igd	0.012	2.65E-06	1640	M18394.m	TRUE	reported	Lt9awu	2	TRUE	0.01329	22.06306
3061	rs1015592	T	C	T	C	-0.0404	0.103998	0.7352	0.733574	FALSE	FALSE	FALSE	ieu-b-508	2	59700640	0.037896	486484	0.006064	Sepsis (28	Sepsis (28	Sepsis (28	TRUE	igd	0.0086	3.01E-06	7363	M31787.m	TRUE	reported	XMp8YI	2	TRUE	0.002988	22.06215
517	rs1247409	A	G	A	G	-0.0202	-0.09318	0.0649	0.075822	FALSE	FALSE	FALSE	ieu-b-508	2	26832666	0.062808	486484	0.137916	Sepsis (28	Sepsis (28	Sepsis (28	TRUE	igd	0.0043	1.97E-06	7345	M00584.m	TRUE	reported	oBt02X	2	TRUE	0.002996	22.06214
6374	rs1194194	A	G	A	G	-0.0202	-0.0383	0.2281	0.237716	FALSE	FALSE	FALSE	ieu-b-508	4	1.68E+08	0.039215	486484	0.328705	Sepsis (28	Sepsis (28	Sepsis (28	TRUE	igd	0.0043	2.82E-06	7322	M33968.m	TRUE	reported	sgVOvT	2	TRUE	0.003005	22.06212
1536	rs1160505	T	C	T	C	-0.0202	0.015365	0.8787	0.876499	FALSE	FALSE	FALSE	ieu-b-508	11	86704096	0.050943	486484	0.762952	Sepsis (28	Sepsis (28	Sepsis (28	TRUE	igd	0.0043	2.24E-06	6864	M12129.m	TRUE	reported	iNzdgb	2	TRUE	0.003205	22.06171
7882	rs1253725	A	G	A	G	0.0912	-0.0081	0.1088	0.184174	FALSE	FALSE	FALSE	ieu-b-508	7	2668045	0.043305	486484	0.851554	Sepsis (28	Sepsis (28	Sepsis (28	TRUE	igd	0.0194	2.48E-06	1098	M35635.m	TRUE	reported	qymryH	2	TRUE	0.01973	22.05944
4480	rs1163364	A	G	A	G	-0.0296	-0.00267	0.3194	0.345806	FALSE	FALSE	FALSE	ieu-b-508	15	80720155	0.035067	486484	0.939392	Sepsis (28	Sepsis (28	Sepsis (28	TRUE	igd	0.0063	2.35E-06	2773	M33131.m	TRUE	reported	uGKURO	2	TRUE	0.007898	22.05916
6393	rs220992	A	G	A	G	0.0155	0.025349	0.4501	0.466376	FALSE	FALSE	FALSE	ieu-b-508	17	7049788	0.033394	486484	0.447801	Sepsis (28	Sepsis (28	Sepsis (28	TRUE	igd	0.0033	2.69E-06	7349	M33971.m	TRUE	reported	dHZ6ha	2	TRUE	0.002993	22.05552
5627	rs7939695	T	C	T	C	-0.0155	0.049326	0.0489	0.072433	FALSE	FALSE	FALSE	ieu-b-508	11	2728090	0.064506	486484	0.444466	Sepsis (28	Sepsis (28	Sepsis (28	TRUE	igd	0.0033	2.76E-06	7336	M33442.m	TRUE	reported	B1Peb3	2	TRUE	0.002998	22.05551
4355	rs4141475	A	C	A	C	0.039	-0.05781	0.2691	0.339662	FALSE	FALSE	FALSE	ieu-b-508	12	20951861	0.035192	486484	0.100466	Sepsis (28	Sepsis (28	Sepsis (28	TRUE	igd	0.0083	2.65E-06	1763	M32855.m	TRUE	reported	AM8kCf	2	TRUE	0.012368	22.05363
2393	rs7628676	A	G	A	G	-0.0653	-0.01317	0.8283	0.896926	FALSE	FALSE	FALSE	ieu-b-508	3	1.75E+08	0.055545	486484	0.812635	Sepsis (28	Sepsis (28	Sepsis (28	TRUE	igd	0.0139	2.50E-06	2339	M21151.m	TRUE	reported	LfwdLx	2	TRUE	0.009347	22.05085
8088	rs5765217	A	G	A	G	-0.0573	0.029514	0.2914	0.288538	FALSE	FALSE	FALSE	ieu-b-508	22	45595721	0.037016	486484	0.425257	Sepsis (28	Sepsis (28	Sepsis (28	TRUE	igd	0.0122	2.73E-06	3939	M36095.m	TRUE	reported	9OLvcB	2	TRUE	0.005569	22.04799
3084	rs3749572	T	C	T	C	0.0808	0.204596	0.0283	0.027579	FALSE	FALSE	FALSE	ieu-b-508	4	1.7E+08	0.102363	486484	0.045638	Sepsis (28	Sepsis (28	Sepsis (28	TRUE	igd	0.0172	2.62E-06	1761	M32197.m	TRUE	reported	VZjwa9	2	TRUE	0.012377	22.04308
7500	rs7767262	T	C	T	C	0.0324	-0.0625	0.9628	0.96134	FALSE	FALSE	FALSE	ieu-b-508	6	1.35E+08	0.087458	486484	0.474813	Sepsis (28	Sepsis (28	Sepsis (28	TRUE	igd	0.0069	2.64E-06	7261	M35253.m	TRUE	reported	VboRmj	2	TRUE	0.003027	22.04308
2354	rs243180	A	G	A	G	0.0108	-0.02952	0.7695	0.767286	FALSE	FALSE	FALSE	ieu-b-508	14	90054689	0.039588	486484	0.455918	Sepsis (28	Sepsis (28	Sepsis (28	TRUE	igd	0.0023	3.16E-06	7203	M21047.m	TRUE	reported	ISF1Cr	2	TRUE	0.003052	22.04303
6416	rs4434099	T	C	T	C	-0.0216	-0.00318	0.8282	0.826625	FALSE	FALSE	FALSE	ieu-b-508	3	1.95E+08	0.044275	486484	0.942665	Sepsis (28	Sepsis (28	Sepsis (28	TRUE	igd	0.0046	2.82E-06	7132	M34040.m	TRUE	reported	sJBy3i	2	TRUE	0.003082	22.04297
572	rs1431793	A	C	A	C	-0.0216	0.009048	0.7526	0.7497	FALSE	FALSE	FALSE	ieu-b-508	2	2.36E+08	0.038793	486484	0.815573	Sepsis (28	Sepsis (28	Sepsis (28	TRUE	igd	0.0046	2.90E-06	5344	M01107.m	TRUE	reported	EbPV1D	2	TRUE	0.004109	22.0409
4211	rs824764	T	C	T	C	0.0709	0.024476	0.9563	0.953647	FALSE	FALSE	FALSE	ieu-b-508	13	64440625	0.078837	486484	0.756209	Sepsis (28	Sepsis (28	Sepsis (28	TRUE	igd	0.0151	2.73E-06	6150	M32769.m	TRUE	reported	W2ubRP	2	TRUE	0.003572	22.03928
6038	rs7544128	C	G	C	G	0.0432	0.042436	0.7224	0.712901	FALSE	TRUE	FALSE	ieu-b-508	1	97871760	0.037096	486484	0.252654	Sepsis (28	Sepsis (28	Sepsis (28	TRUE	igd	0.0092	2.80E-06	3962	M33782.m	TRUE	reported	875Pal	2	TRUE	0.005534	22.03802
6026	rs922025	A	G	A	G	-0.0846	0.115136	0.1942	0.227783	FALSE	FALSE	FALSE	ieu-b-508	5	2302513	0.040228	486484	0.004208	Sepsis (28	Sepsis (28	Sepsis (28	TRUE	igd	0.018	2.51E-06	831	M33751.m	TRUE	reported	Cxdkr4	2	TRUE	0.025894	22.03684
3915	rs2244987	A	T	A	T	-0.0479	-0.04629	0.8885	0.873654	FALSE	TRUE	FALSE	ieu-b-508	3	1.94E+08	0.050519	486484	0.359513	Sepsis (28	Sepsis (28	Sepsis (28	TRUE	igd	0.0102	2.73E-06	2575	M32691.m	TRUE	reported	SiuD6F	2	TRUE	0.008492	22.03602
3620	rs1050346	C	G	C	G	-0.0338	0.031146	0.8052	0.806948	FALSE	TRUE	FALSE	ieu-b-508	8	13525311	0.042521	486484	0.463865	Sepsis (28	Sepsis (28	Sepsis (28	TRUE	igd	0.0072	3.14E-06	7337	M32557.m	TRUE	reported	qR7rZX	2	TRUE	0.002995	22.0318
7214	rs4680092	T	G	T	G	-0.0169	0.0052	0.2203	0.217919	FALSE	FALSE	FALSE	ieu-b-508	3	1.54E+08	0.040545	486484	0.897945	Sepsis (28	Sepsis (28	Sepsis (28	TRUE	igd	0.0036	2.19E-06	6735	M34732.m	TRUE	reported	IRNSa4	2	TRUE	0.003261	22.03126
7596	rs7800039	A	G	A	G	-0.0169	-0.00711	0.3424	0.343284	FALSE	FALSE	FALSE	ieu-b-508	7	87977805	0.035442	486484	0.840986	Sepsis (28	Sepsis (28	Sepsis (28	TRUE	igd	0.0036	2.13E-06	5979	M35305.m	TRUE	reported	6ybfdq	2	TRUE	0.003672	22.03044
7656	rs6054468	T	G	T	G	-0.0169	-0.00931	0.0819	0.1239	FALSE	FALSE	FALSE	ieu-b-508	20	6674573	0.050688	486484	0.854242	Sepsis (28	Sepsis (28	Sepsis (28	TRUE	igd	0.0036	2.50E-06	5768	M35326.m	TRUE	reported	jRytyJ	2	TRUE	0.003806	22.03017
6575	rs311496	T	C	T	C	-0.0338	-0.04881	0.0866	0.152241	FALSE	FALSE	FALSE	ieu-b-508	20	62221610	0.046488	486484	0.293718	Sepsis (28	Sepsis (28	Sepsis (28	TRUE	igd	0.0072	3.05E-06	4908	M34245.m	TRUE	reported	pRwBKr	2	TRUE	0.00447	22.02883
8103	rs1712697	A	G	A	G	0.4543	-0.00416	0.8598	0.779661	FALSE	FALSE	FALSE	ieu-b-508	14	91185867	0.040312	486484	0.917752	Sepsis (28	Sepsis (28	Sepsis (28	TRUE	igd	0.0966	2.59E-06	488	M36097.m	TRUE	reported	RHr18S	2	TRUE	0.043357	22.02661
2347	rs1391874	A	G	A	G	0.023	0.070527	0.9599	0.956778	FALSE	FALSE	FALSE	ieu-b-508	11	88435739	0.082035	486484	0.389945	Sepsis (28	Sepsis (28	Sepsis (28	TRUE	igd	0.0049	2.57E-06	7203	M21047.m	TRUE	reported	ISF1Cr	2	TRUE	0.003049	22.02637
7234	rs1859570	T	C	T	C	0.1451	0.215224	0.0272	0.015583	FALSE	FALSE	FALSE	ieu-b-508	7	1.41E+08	0.134513	486484	0.109594	Sepsis (28	Sepsis (28	Sepsis (28	TRUE	igd	0.0309	2.65E-06	1763	M34878.m	TRUE	reported	Cpq9yU	2	TRUE	0.012353	22.02546
6747	rs7954487	C	G	C	G	0.4415	0.296044	0.9869	0.987825	FALSE	TRUE	FALSE	ieu-b-508	12	383837	0.153888	486484	0.054384	Sepsis (28	Sepsis (28	Sepsis (28	TRUE	igd	0.0939	2.57E-06	535	M34338.m	TRUE	reported	h4FB9a	2	TRUE	0.039682	22.02438
5568	rs2077726	T	G	T	G	-0.1686	-0.01644	0.7831	0.765793	FALSE	FALSE	FALSE	ieu-b-508	5	1.2E+08	0.040969	486484	0.688289	Sepsis (28	Sepsis (28	Sepsis (28	TRUE	igd	0.0359	2.71E-06	1391	M33423.m	TRUE	reported	MoEnO7	2	TRUE	0.015609	22.02426
4469	rs9635387	A	G	A	G	0.0324	-0.01556	0.5273	0.51123	FALSE	FALSE	FALSE	ieu-b-508	15	36928867	0.033375	486484	0.640988	Sepsis (28	Sepsis (28	Sepsis (28	TRUE	igd	0.0069	2.72E-06	1462	M33009.m	TRUE	reported	8RTEdM	2	TRUE	0.014857	22.01899
5318	rs6903432	C	G	C	G	-0.069	0.136058	0.0183	0.033955	FALSE	TRUE	FALSE	ieu-b-508	6	1.36E+08	0.092458	486484	0.141137	Sepsis (28	Sepsis (28	Sepsis (28	TRUE	igd	0.0147	2.77E-06	2212	M33363.m	TRUE	reported	7K0MM0	2	TRUE	0.009862	22.01257
1018	rs4589188	A	G	A	G	0.0122	0.059456	0.2827	0.307226	FALSE	FALSE	FALSE	ieu-b-508	10	5567914	0.036675	486484	0.104988	Sepsis (28	Sepsis (28	Sepsis (28	TRUE	igd	0.0026	3.34E-06	7364	M01564.m	TRUE	reported	IPUdyD	2	TRUE	0.002981	22.01177
3966	rs7806249	T	C	T	C	0.0122	-0.01332	0.6546	0.656704	FALSE	FALSE	FALSE	ieu-b-508	7	94119	0.035175	486484	0.704891	Sepsis (28	Sepsis (28	Sepsis (28	TRUE	igd	0.0026	2.62E-06	7363	M32709.m	TRUE	reported	YR8SB4	2	TRUE	0.002981	22.01177
875	rs1225060	T	C	T	C	0.0122	0.09123	0.751	0.747461	FALSE	FALSE	FALSE	ieu-b-508	10	1.2E+08	0.038429	486484	0.017598	Sepsis (28	Sepsis (28	Sepsis (28	TRUE	igd	0.0026	2.02E-06	7362	M01365.m	TRUE	reported	N1cZTs	2	TRUE	0.002982	22.01177
3520	rs1083788	T	G	T	G	-0.0122	-0.01403	0.6036	0.5958	FALSE	FALSE	FALSE	ieu-b-508	11	42609917	0.033948	486484	0.679423	Sepsis (28	Sepsis (28	Sepsis (28	TRUE	igd	0.0026	4.08E-06	7358	M32497.m	TRUE	reported	244bZN	2	TRUE	0.002983	22.01177
3537	rs6062362	A	G	A	G	0.0122	-0.01218	0.5705	0.562217	FALSE	FALSE	FALSE	ieu-b-508	20	62911229	0.03383	486484	0.718917	Sepsis (28	Sepsis (28	Sepsis (28	TRUE	igd	0.0026	3.38E-06	7358	M32497.m	TRUE	reported	244bZN	2	TRUE	0.002983	22.01177
3539	rs6801666	A	G	A	G	-0.0122	-0.05926	0.4209	0.424049	FALSE	FALSE	FALSE	ieu-b-508	3	20345376	0.033774	486484	0.079303	Sepsis (28	Sepsis (28	Sepsis (28	TRUE	igd	0.0026	2.89E-06	7358	M32497.m	TRUE	reported	244bZN	2	TRUE	0.002983	22.01177
198	rs1527683	T	C	T	C	0.0061	-0.02802	0.8	0.781567	FALSE	FALSE	FALSE	ieu-b-508	1	74822937	0.040509	486484	0.489126	Sepsis (28	Sepsis (28	Sepsis (28	TRUE	igd	0.0013	2.17E-06	7355	M00059.m	TRUE	reported	RX1zal	2	TRUE	0.002985	22.01176
8556	rs7172197	A	G	A	G	-0.0305	-0.03795	0.5376	0.520489	FALSE	FALSE	FALSE	ieu-b-508	15	68763881	0.033488	486484	0.257105	Sepsis (28	Sepsis (28	Sepsis (28	TRUE	igd	0.0065	3.16E-06	7355	M37202.m	TRUE	reported	BtslDx	2	TRUE	0.002985	22.01176
7427	rs7307046	A	G	A	G	0.0122	-0.06226	0.4072	0.416195	FALSE	FALSE	FALSE	ieu-b-508	12	48178194	0.033828	486484	0.065675	Sepsis (28	Sepsis (28	Sepsis (28	TRUE	igd	0.0026	2.33E-06	7350	M35186.m	TRUE	reported	UZfu6Z	2	TRUE	0.002987	22.01176
1216	rs7990832	A	G	A	G	0.0122	0.012465	0.1792	0.177391	FALSE	FALSE	FALSE	ieu-b-508	13	43743933	0.043849	486484	0.776211	Sepsis (28	Sepsis (28	Sepsis (28	TRUE	igd	0.0026	3.87E-06	7348	M01670.m	TRUE	reported	kjghbc	2	TRUE	0.002987	22.01176
527	rs7747345	A	T	A	T	0.0122	0.032691	0.8153	0.801616	FALSE	TRUE	FALSE	ieu-b-508	6	20512373	0.041764	486484	0.43377	Sepsis (28	Sepsis (28	Sepsis (28	TRUE	igd	0.0026	2.52E-06	7345	M00584.m	TRUE	reported	oBt02X	2	TRUE	0.002989	22.01176
7871	rs4687841	A	G	A	G	0.0122	-0.02289	0.6302	0.625282	FALSE	FALSE	FALSE	ieu-b-508	3	1.19E+08	0.03443	486484	0.506211	Sepsis (28	Sepsis (28	Sepsis (28	TRUE	igd	0.0026	3.34E-06	7317	M35631.m	TRUE	reported	002wka	2	TRUE	0.003	22.01173
2441	rs230943	A	C	A	C	-0.0122	-0.04056	0.5083	0.490608	FALSE	FALSE	FALSE	ieu-b-508	17	15135272	0.033438	486484	0.225163	Sepsis (28	Sepsis (28	Sepsis (28	TRUE	igd	0.0026	1.64E-06	7284	M21630.m	TRUE	reported	7eaO4H	2	TRUE	0.003014	22.01171
5346	rs1764225	T	G	T	G	0.0122	0.010563	0.0886	0.087597	FALSE	FALSE	FALSE	ieu-b-508	16	64487715	0.05895	486484	0.857794	Sepsis (28	Sepsis (28	Sepsis (28	TRUE	igd	0.0026	4.06E-06	7118	M33380.m	TRUE	reported	OHRfl5	2	TRUE	0.003084	22.01156
8114	rs873413	T	C	T	C	0.3898	-0.0514	0.5579	0.605855	FALSE	FALSE	FALSE	ieu-b-508	6	89283640	0.034113	486484	0.131844	Sepsis (28	Sepsis (28	Sepsis (28	TRUE	igd	0.0824	2.27E-06	122	M36097.m	TRUE	reported	RHr18S	2	TRUE	0.154998	22.01155
6805	rs1352907	T	C	T	C	0.0122	-0.06437	0.1679	0.182768	FALSE	FALSE	FALSE	ieu-b-508	12	20014904	0.043247	486484	0.136633	Sepsis (28	Sepsis (28	Sepsis (28	TRUE	igd	0.0026	4.02E-06	6755	M34359.m	TRUE	reported	0UotaJ	2	TRUE	0.003249	22.01123
2221	rs3112726	T	C	T	C	0.0122	0.020699	0.455	0.465622	FALSE	FALSE	FALSE	ieu-b-508	16	2748699	0.033669	486484	0.538698	Sepsis (28	Sepsis (28	Sepsis (28	TRUE	igd	0.0026	2.93E-06	6678	M19368.m	TRUE	reported	QaKPad	2	TRUE	0.003286	22.01116
2657	rs249462	A	T	A	T	-0.0122	0.022855	0.3223	0.318246	FALSE	TRUE	FALSE	ieu-b-508	5	4996414	0.036423	486484	0.530331	Sepsis (28	Sepsis (28	Sepsis (28	TRUE	igd	0.0026	2.45E-06	6517	M22649.m	TRUE	reported	NAzwRF	2	TRUE	0.003367	22.01099
4575	rs1091042	T	C	T	C	-0.0122	0.011466	0.4474	0.444302	FALSE	FALSE	FALSE	ieu-b-508	1	2.35E+08	0.033633	486484	0.733165	Sepsis (28	Sepsis (28	Sepsis (28	TRUE	igd	0.0026	3.92E-06	6404	M33163.m	TRUE	reported	YzGXkR	2	TRUE	0.003426	22.01088
7222	rs2162518	A	C	A	C	-0.0305	0.054235	0.8896	0.894644	FALSE	FALSE	FALSE	ieu-b-508	2	49837515	0.054599	486484	0.320548	Sepsis (28	Sepsis (28	Sepsis (28	TRUE	igd	0.0065	3.05E-06	6381	M34761.m	TRUE	reported	AMtp1w	2	TRUE	0.003439	22.01085
671	rs4822129	A	G	A	G	0.0122	0.003033	0.194	0.196979	FALSE	FALSE	FALSE	ieu-b-508	22	42823916	0.042177	486484	0.942668	Sepsis (28	Sepsis (28	Sepsis (28	TRUE	igd	0.0026	3.76E-06	5687	M01284.m	TRUE	reported	E4hc9c	2	TRUE	0.003857	22.01001
6236	rs1952103	T	G	T	G	-0.0244	0.066979	0.7585	0.774623	FALSE	FALSE	FALSE	ieu-b-508	10	91809374	0.039838	486484	0.092702	Sepsis (28	Sepsis (28	Sepsis (28	TRUE	igd	0.0052	3.07E-06	5534	M33910.m	TRUE	reported	YwFZg1	2	TRUE	0.003963	22.00979
2077	rs7830772	A	C	A	C	-0.0399	0.087199	0.9671	0.973708	FALSE	FALSE	FALSE	ieu-b-508	8	60338131	0.10441	486484	0.403624	Sepsis (28	Sepsis (28	Sepsis (28	TRUE	igd	0.0085	2.95E-06	1762	M18369.m	TRUE	reported	ykBjIT	2	TRUE	0.012351	22.00973
862	rs3900774	T	G	T	G	0.061	0.134242	0.988	0.977508	FALSE	FALSE	FALSE	ieu-b-508	6	1.69E+08	0.113805	486484	0.238169	Sepsis (28	Sepsis (28	Sepsis (28	TRUE	igd	0.013	2.70E-06	5363	M01361.m	TRUE	reported	NIAcQ3	2	TRUE	0.004089	22.00954
6170	rs1511938	A	G	A	G	-0.0366	-0.00407	0.5175	0.506419	FALSE	FALSE	FALSE	ieu-b-508	18	35967920	0.0334	486484	0.903005	Sepsis (28	Sepsis (28	Sepsis (28	TRUE	igd	0.0078	2.82E-06	4999	M33885.m	TRUE	reported	uCOy6o	2	TRUE	0.004385	22.00894
3916	rs2932198	A	G	A	G	0.0305	0.002348	0.3908	0.405693	FALSE	FALSE	FALSE	ieu-b-508	15	57790391	0.034021	486484	0.944985	Sepsis (28	Sepsis (28	Sepsis (28	TRUE	igd	0.0065	2.50E-06	2575	M32691.m	TRUE	reported	SiuD6F	2	TRUE	0.008478	22.00065
5296	rs1836845	T	G	T	G	-0.0399	0.02576	0.6005	0.596993	FALSE	FALSE	FALSE	ieu-b-508	13	1.02E+08	0.033984	486484	0.448449	Sepsis (28	Sepsis (28	Sepsis (28	TRUE	igd	0.0085	2.73E-06	1273	M33353.m	TRUE	reported	ZIN4gk	2	TRUE	0.017015	22.00012
7007	rs2390196	A	G	A	G	-0.0699	0.037123	0.1233	0.12287	FALSE	FALSE	FALSE	ieu-b-508	7	19864026	0.05084	486484	0.465271	Sepsis (28	Sepsis (28	Sepsis (28	TRUE	igd	0.0149	2.94E-06	4452	M34420.m	TRUE	reported	20uofq	2	TRUE	0.004919	21.99818
7217	rs1083330	A	G	A	G	-0.0258	-0.0131	0.1893	0.239223	FALSE	FALSE	FALSE	ieu-b-508	11	20378045	0.03934	486484	0.739203	Sepsis (28	Sepsis (28	Sepsis (28	TRUE	igd	0.0055	2.43E-06	6381	M34761.m	TRUE	reported	AMtp1w	2	TRUE	0.003437	21.99773
5167	rs4126458	A	G	A	G	0.0887	0.038988	0.5107	0.478773	FALSE	FALSE	FALSE	ieu-b-508	14	36812308	0.033351	486484	0.242393	Sepsis (28	Sepsis (28	Sepsis (28	TRUE	igd	0.0189	2.83E-06	1586	M33194.m	TRUE	reported	xRUveq	2	TRUE	0.013697	21.99762
2044	rs1709236	A	G	A	G	-0.0671	-0.02212	0.9096	0.891078	FALSE	FALSE	FALSE	ieu-b-508	14	57338592	0.053427	486484	0.678899	Sepsis (28	Sepsis (28	Sepsis (28	TRUE	igd	0.0143	2.61E-06	2079	M18357.m	TRUE	reported	o7AvpG	2	TRUE	0.01048	21.99657
7208	rs6436677	T	C	T	C	0.1131	0.210084	0.0189	0.010743	FALSE	FALSE	FALSE	ieu-b-508	2	2.28E+08	0.167559	486484	0.209917	Sepsis (28	Sepsis (28	Sepsis (28	TRUE	igd	0.0241	2.66E-06	1560	M34674.m	TRUE	reported	plxOKz	2	TRUE	0.013921	21.99551
5553	rs1241718	A	T	A	T	-0.2877	-0.05917	0.0796	0.137556	FALSE	TRUE	FALSE	ieu-b-508	11	59907222	0.048552	486484	0.222982	Sepsis (28	Sepsis (28	Sepsis (28	TRUE	igd	0.0613	2.71E-06	1391	M33423.m	TRUE	reported	MoEnO7	2	TRUE	0.015589	21.9955
4753	rs1267960	A	G	A	G	-0.1582	-0.10878	0.0561	0.035318	FALSE	FALSE	FALSE	ieu-b-508	8	1.26E+08	0.090558	486484	0.229671	Sepsis (28	Sepsis (28	Sepsis (28	TRUE	igd	0.0337	2.72E-06	1017	M33178.m	TRUE	reported	g22WMS	2	TRUE	0.021209	21.9937
2257	rs4399682	A	T	A	T	0.0183	-0.03575	0.7244	0.712577	FALSE	TRUE	FALSE	ieu-b-508	2	1.85E+08	0.036941	486484	0.333187	Sepsis (28	Sepsis (28	Sepsis (28	TRUE	igd	0.0039	2.06E-06	1759	M19934.m	TRUE	reported	HtcWcQ	2	TRUE	0.012362	21.99272
3023	rs2274629	A	G	A	G	0.0333	0.014287	0.2873	0.268319	FALSE	FALSE	FALSE	ieu-b-508	10	97106465	0.037589	486484	0.70389	Sepsis (28	Sepsis (28	Sepsis (28	TRUE	igd	0.0071	3.01E-06	5229	M31548.m	TRUE	reported	bgRTBS	2	TRUE	0.004189	21.98901
7016	rs754739	A	C	A	C	-0.053	0.038249	0.2707	0.267527	FALSE	FALSE	FALSE	ieu-b-508	4	8331883	0.037867	486484	0.31245	Sepsis (28	Sepsis (28	Sepsis (28	TRUE	igd	0.0113	2.79E-06	4452	M34420.m	TRUE	reported	20uofq	2	TRUE	0.004917	21.98871
8281	rs4657463	C	G	C	G	0.0136	-0.01581	0.6945	0.701313	FALSE	TRUE	FALSE	ieu-b-508	1	1.66E+08	0.036494	486484	0.664808	Sepsis (28	Sepsis (28	Sepsis (28	TRUE	igd	0.0029	2.80E-06	6464	M36553.m	TRUE	reported	2Rk7nd	2	TRUE	0.003391	21.98606
8284	rs6563831	T	C	T	C	0.0136	-0.06239	0.652	0.647593	FALSE	FALSE	FALSE	ieu-b-508	13	40975937	0.035147	486484	0.07586	Sepsis (28	Sepsis (28	Sepsis (28	TRUE	igd	0.0029	2.33E-06	6464	M36553.m	TRUE	reported	2Rk7nd	2	TRUE	0.003391	21.98606
6200	rs2889961	A	G	A	G	0.0136	0.003107	0.3641	0.361023	FALSE	FALSE	FALSE	ieu-b-508	9	87193435	0.034792	486484	0.928833	Sepsis (28	Sepsis (28	Sepsis (28	TRUE	igd	0.0029	2.70E-06	6004	M33892.m	TRUE	reported	w7HgU7	2	TRUE	0.00365	21.98554
2662	rs1523437	A	G	A	G	-0.0619	0.024278	0.8196	0.824475	FALSE	FALSE	FALSE	ieu-b-508	3	63302571	0.043992	486484	0.581039	Sepsis (28	Sepsis (28	Sepsis (28	TRUE	igd	0.0132	2.75E-06	5599	M22842.m	TRUE	reported	0t12oo	2	TRUE	0.003912	21.98256
7816	rs1125923	A	G	A	G	-0.0347	-0.08154	0.8943	0.894546	FALSE	FALSE	FALSE	ieu-b-508	10	14667316	0.054676	486484	0.135887	Sepsis (28	Sepsis (28	Sepsis (28	TRUE	igd	0.0074	2.39E-06	6595	M35472.m	TRUE	reported	oZU10n	2	TRUE	0.003323	21.98183
8175	rs1240400	A	G	A	G	0.2036	0.028202	0.9894	0.938651	FALSE	FALSE	FALSE	ieu-b-508	1	2.38E+08	0.07253	486484	0.697405	Sepsis (28	Sepsis (28	Sepsis (28	TRUE	igd	0.0434	2.74E-06	1657	M36134.m	TRUE	reported	ekMsfG	2	TRUE	0.013108	21.98121
3674	rs520906	A	G	A	G	0.0211	-0.00335	0.6353	0.633931	FALSE	FALSE	FALSE	ieu-b-508	11	1.08E+08	0.034584	486484	0.922891	Sepsis (28	Sepsis (28	Sepsis (28	TRUE	igd	0.0045	2.23E-06	7322	M32578.m	TRUE	reported	LPkMFr	2	TRUE	0.002994	21.97967
2230	rs4884317	A	G	A	G	-0.0211	0.051519	0.211	0.215826	FALSE	FALSE	FALSE	ieu-b-508	13	53591426	0.04155	486484	0.214994	Sepsis (28	Sepsis (28	Sepsis (28	TRUE	igd	0.0045	2.44E-06	6385	M19396.m	TRUE	reported	Xa1QqA	2	TRUE	0.003432	21.97879
2908	rs1234223	C	G	C	G	0.0211	-0.04516	0.0908	0.093902	FALSE	TRUE	FALSE	ieu-b-508	10	89675590	0.056976	486484	0.42801	Sepsis (28	Sepsis (28	Sepsis (28	TRUE	igd	0.0045	3.02E-06	5562	M27728.m	TRUE	reported	WOp1XE	2	TRUE	0.003937	21.97777
4485	rs2197598	T	C	T	C	-0.0408	0.038289	0.1051	0.10538	FALSE	FALSE	FALSE	ieu-b-508	11	18669600	0.054599	486484	0.48313	Sepsis (28	Sepsis (28	Sepsis (28	TRUE	igd	0.0087	2.76E-06	2773	M33131.m	TRUE	reported	uGKURO	2	TRUE	0.007869	21.977
6386	rs2427612	T	C	T	C	-0.0286	-0.0079	0.364	0.380043	FALSE	FALSE	FALSE	ieu-b-508	20	62818891	0.034823	486484	0.820429	Sepsis (28	Sepsis (28	Sepsis (28	TRUE	igd	0.0061	3.01E-06	7330	M33969.m	TRUE	reported	0ujroW	2	TRUE	0.00299	21.97626
1794	rs7151608	A	G	A	G	0.0497	-0.01703	0.8127	0.811498	FALSE	FALSE	FALSE	ieu-b-508	14	39673861	0.042713	486484	0.690176	Sepsis (28	Sepsis (28	Sepsis (28	TRUE	igd	0.0106	2.86E-06	5502	M15677.m	TRUE	reported	1CuWi9	2	TRUE	0.00398	21.97572
4192	rs7841945	A	T	A	T	0.0394	-0.05838	0.8698	0.880862	FALSE	TRUE	FALSE	ieu-b-508	8	69733257	0.051389	486484	0.255962	Sepsis (28	Sepsis (28	Sepsis (28	TRUE	igd	0.0084	2.49E-06	1734	M32761.m	TRUE	reported	QefVGc	2	TRUE	0.012529	21.97519
5575	rs4240294	T	C	T	C	-0.3218	0.07897	0.0413	0.056025	FALSE	FALSE	FALSE	ieu-b-508	4	1.15E+08	0.072643	486484	0.276991	Sepsis (28	Sepsis (28	Sepsis (28	TRUE	igd	0.0686	2.73E-06	1391	M33423.m	TRUE	reported	MoEnO7	2	TRUE	0.015573	21.97349
3702	rs1714898	C	G	C	G	0.0361	-0.03153	0.0869	0.099533	FALSE	TRUE	FALSE	ieu-b-508	7	75649821	0.055704	486484	0.571408	Sepsis (28	Sepsis (28	Sepsis (28	TRUE	igd	0.0077	2.52E-06	5955	M32593.m	TRUE	reported	6zVI1i	2	TRUE	0.003677	21.97288
3016	rs1049505	A	G	A	G	-0.0872	0.047393	0.9683	0.970429	FALSE	FALSE	FALSE	ieu-b-508	1	2.17E+08	0.098752	486484	0.631284	Sepsis (28	Sepsis (28	Sepsis (28	TRUE	igd	0.0186	2.77E-06	5229	M31548.m	TRUE	reported	bgRTBS	2	TRUE	0.004186	21.97055
7593	rs1507362	T	C	T	C	0.0333	0.044535	0.3901	0.394409	FALSE	FALSE	FALSE	ieu-b-508	4	1.35E+08	0.034068	486484	0.191127	Sepsis (28	Sepsis (28	Sepsis (28	TRUE	igd	0.0071	3.02E-06	1505	M35305.m	TRUE	reported	6ybfdq	2	TRUE	0.014406	21.96819
706	rs9594421	A	T	A	T	0.0075	0.049475	0.2757	0.294598	FALSE	TRUE	FALSE	ieu-b-508	13	41091759	0.036753	486484	0.178251	Sepsis (28	Sepsis (28	Sepsis (28	TRUE	igd	0.0016	4.59E-06	7358	M01299.m	TRUE	reported	gfM0a3	2	TRUE	0.002977	21.96668
910	rs1578743	A	C	A	C	0.0075	-0.0734	0.6068	0.60373	FALSE	FALSE	FALSE	ieu-b-508	10	5722784	0.034347	486484	0.032605	Sepsis (28	Sepsis (28	Sepsis (28	TRUE	igd	0.0016	1.70E-06	7354	M01494.m	TRUE	reported	7JWNKI	2	TRUE	0.002979	21.96668
745	rs6071991	T	C	T	C	-0.0075	0.000918	0.1495	0.157929	FALSE	FALSE	FALSE	ieu-b-508	20	38889983	0.045833	486484	0.984012	Sepsis (28	Sepsis (28	Sepsis (28	TRUE	igd	0.0016	3.81E-06	7347	M01302.m	TRUE	reported	sNp2IC	2	TRUE	0.002982	21.96667
1691	rs1248263	T	C	T	C	0.0075	-0.02831	0.2693	0.293695	FALSE	FALSE	FALSE	ieu-b-508	21	38419782	0.036748	486484	0.441115	Sepsis (28	Sepsis (28	Sepsis (28	TRUE	igd	0.0016	5.40E-06	7345	M15488.m	TRUE	reported	SPriBO	2	TRUE	0.002983	21.96667
2176	rs1229866	C	G	C	G	-0.015	0.02435	0.9	0.895497	FALSE	TRUE	FALSE	ieu-b-508	12	76902090	0.054729	486484	0.656378	Sepsis (28	Sepsis (28	Sepsis (28	TRUE	igd	0.0032	2.94E-06	7182	M19362.m	TRUE	reported	SkNEKw	2	TRUE	0.00305	21.96654
6538	rs1767986	T	C	T	C	-0.0525	0.011905	0.0844	0.11858	FALSE	FALSE	FALSE	ieu-b-508	17	14140080	0.05141	486484	0.816874	Sepsis (28	Sepsis (28	Sepsis (28	TRUE	igd	0.0112	2.85E-06	6987	M34221.m	TRUE	reported	nUe3TL	2	TRUE	0.003135	21.96637
1334	rs1088142	T	G	T	G	-0.015	0.017181	0.5558	0.556435	FALSE	FALSE	FALSE	ieu-b-508	1	1.07E+08	0.033556	486484	0.608646	Sepsis (28	Sepsis (28	Sepsis (28	TRUE	igd	0.0032	3.69E-06	5791	M02342.m	TRUE	reported	EhZr3g	2	TRUE	0.00378	21.96507
1341	rs3753556	T	C	T	C	0.015	-0.00984	0.4325	0.427329	FALSE	FALSE	FALSE	ieu-b-508	1	1.75E+08	0.034036	486484	0.772474	Sepsis (28	Sepsis (28	Sepsis (28	TRUE	igd	0.0032	3.66E-06	5791	M02342.m	TRUE	reported	EhZr3g	2	TRUE	0.00378	21.96507
7171	rs4836309	T	C	T	C	-0.0225	0.050365	0.5412	0.541793	FALSE	FALSE	FALSE	ieu-b-508	5	1.26E+08	0.033504	486484	0.132776	Sepsis (28	Sepsis (28	Sepsis (28	TRUE	igd	0.0048	2.38E-06	4925	M34534.m	TRUE	reported	iA78RM	2	TRUE	0.004442	21.96373
4112	rs768328	T	C	T	C	-0.0464	-0.0374	0.228	0.225767	FALSE	FALSE	FALSE	ieu-b-508	1	49573780	0.039834	486484	0.347798	Sepsis (28	Sepsis (28	Sepsis (28	TRUE	igd	0.0099	3.09E-06	6379	M32754.m	TRUE	reported	n0okFy	2	TRUE	0.003432	21.95985
2179	rs1270339	T	C	T	C	0.0314	0.083291	0.9778	0.974206	FALSE	FALSE	FALSE	ieu-b-508	7	1.41E+08	0.105018	486484	0.427713	Sepsis (28	Sepsis (28	Sepsis (28	TRUE	igd	0.0067	3.00E-06	7182	M19362.m	TRUE	reported	SkNEKw	2	TRUE	0.003049	21.9578
3407	rs1260374	T	C	T	C	-0.0389	0.026714	0.88	0.860267	FALSE	FALSE	FALSE	ieu-b-508	17	77262120	0.048463	486484	0.581483	Sepsis (28	Sepsis (28	Sepsis (28	TRUE	igd	0.0083	2.95E-06	5080	M32445.m	TRUE	reported	qcc6f3	2	TRUE	0.004305	21.95695
537	rs2833657	A	G	A	G	0.0239	-0.04001	0.8254	0.825775	FALSE	FALSE	FALSE	ieu-b-508	21	33478113	0.043933	486484	0.362444	Sepsis (28	Sepsis (28	Sepsis (28	TRUE	igd	0.0051	3.02E-06	7237	M00599.m	TRUE	reported	nRJY3s	2	TRUE	0.003025	21.9551
5475	rs6717460	A	C	A	C	-0.1646	0.11623	0.962	0.969405	FALSE	FALSE	FALSE	ieu-b-508	2	60127231	0.096962	486484	0.23064	Sepsis (28	Sepsis (28	Sepsis (28	TRUE	igd	0.0351	2.77E-06	1094	M33408.m	TRUE	reported	tWZWla	2	TRUE	0.019705	21.95082
2164	rs1808751	A	G	A	G	0.0164	0.021164	0.7375	0.725391	FALSE	FALSE	FALSE	ieu-b-508	3	1.52E+08	0.0374	486484	0.571476	Sepsis (28	Sepsis (28	Sepsis (28	TRUE	igd	0.0035	3.58E-06	7369	M19323.m	TRUE	reported	yzKFKX	2	TRUE	0.002971	21.94996
3947	rs1180318	T	C	T	C	0.0164	0.003244	0.8545	0.829442	FALSE	FALSE	FALSE	ieu-b-508	1	30729133	0.044615	486484	0.942032	Sepsis (28	Sepsis (28	Sepsis (28	TRUE	igd	0.0035	3.96E-06	7363	M32709.m	TRUE	reported	YR8SB4	2	TRUE	0.002973	21.94995
6061	rs6817243	A	G	A	G	0.0164	-0.049	0.2809	0.275764	FALSE	FALSE	FALSE	ieu-b-508	4	1.21E+08	0.037467	486484	0.190964	Sepsis (28	Sepsis (28	Sepsis (28	TRUE	igd	0.0035	3.29E-06	7362	M33821.m	TRUE	reported	eVYICL	2	TRUE	0.002973	21.94995
3862	rs1079209	T	G	T	G	0.0164	-0.01011	0.1084	0.114399	FALSE	FALSE	FALSE	ieu-b-508	11	57153948	0.052339	486484	0.846901	Sepsis (28	Sepsis (28	Sepsis (28	TRUE	igd	0.0035	3.15E-06	7361	M32654.m	TRUE	reported	6ISQOP	2	TRUE	0.002974	21.94995
3165	rs1706419	A	G	A	G	-0.0164	0.004716	0.7977	0.804349	FALSE	FALSE	FALSE	ieu-b-508	13	43689407	0.042047	486484	0.910704	Sepsis (28	Sepsis (28	Sepsis (28	TRUE	igd	0.0035	2.71E-06	7340	M32328.m	TRUE	reported	YGsb6D	2	TRUE	0.002982	21.94994
4549	rs1289336	A	G	A	G	-0.0164	-0.0274	0.7444	0.74546	FALSE	FALSE	FALSE	ieu-b-508	14	41789550	0.038388	486484	0.475372	Sepsis (28	Sepsis (28	Sepsis (28	TRUE	igd	0.0035	2.28E-06	7319	M33140.m	TRUE	reported	eBOUtb	2	TRUE	0.002991	21.94992
539	rs3925033	T	G	T	G	-0.0164	-0.00191	0.4591	0.44852	FALSE	FALSE	FALSE	ieu-b-508	12	18456713	0.033579	486484	0.954598	Sepsis (28	Sepsis (28	Sepsis (28	TRUE	igd	0.0035	2.59E-06	7237	M00599.m	TRUE	reported	nRJY3s	2	TRUE	0.003025	21.94985
925	rs1254456	A	G	A	G	0.0164	-0.05341	0.148	0.140807	FALSE	FALSE	FALSE	ieu-b-508	8	21368451	0.0479	486484	0.264809	Sepsis (28	Sepsis (28	Sepsis (28	TRUE	igd	0.0035	3.32E-06	7160	M01508.m	TRUE	reported	go526k	2	TRUE	0.003057	21.94979
1531	rs7134822	A	G	A	G	-0.0164	-0.14557	0.9597	0.96034	FALSE	FALSE	FALSE	ieu-b-508	12	16198974	0.08673	486484	0.09327	Sepsis (28	Sepsis (28	Sepsis (28	TRUE	igd	0.0035	3.87E-06	7081	M12067.m	TRUE	reported	O5x7RD	2	TRUE	0.003091	21.94972
7534	rs1316848	T	C	T	C	0.0164	-0.00507	0.2029	0.199134	FALSE	FALSE	FALSE	ieu-b-508	5	84852951	0.041787	486484	0.903432	Sepsis (28	Sepsis (28	Sepsis (28	TRUE	igd	0.0035	2.68E-06	6538	M35257.m	TRUE	reported	iphouU	2	TRUE	0.003347	21.9492
7539	rs1770008	T	C	T	C	0.0164	0.068781	0.2073	0.218161	FALSE	FALSE	FALSE	ieu-b-508	18	44263237	0.040687	486484	0.090935	Sepsis (28	Sepsis (28	Sepsis (28	TRUE	igd	0.0035	2.83E-06	6538	M35257.m	TRUE	reported	iphouU	2	TRUE	0.003347	21.9492
202	rs4768741	C	G	C	G	-0.015	0.056582	0.9153	0.913671	FALSE	TRUE	FALSE	ieu-b-508	12	47070105	0.059355	486484	0.34045	Sepsis (28	Sepsis (28	Sepsis (28	TRUE	igd	0.0032	3.52E-06	1762	M00059.m	TRUE	reported	RX1zal	2	TRUE	0.012317	21.94772
8077	rs1262265	A	G	A	G	-0.0314	0.034222	0.7765	0.769357	FALSE	FALSE	FALSE	ieu-b-508	2	99546355	0.040022	486484	0.392508	Sepsis (28	Sepsis (28	Sepsis (28	TRUE	igd	0.0067	2.89E-06	2651	M36009.m	TRUE	reported	2EUZ1U	2	TRUE	0.008217	21.94734
3238	rs2044048	A	G	A	G	-0.0417	0.018484	0.2224	0.217251	FALSE	FALSE	FALSE	ieu-b-508	8	98176043	0.04052	486484	0.648272	Sepsis (28	Sepsis (28	Sepsis (28	TRUE	igd	0.0089	2.63E-06	6687	M32346.m	TRUE	reported	cnIFoV	2	TRUE	0.003272	21.94634
5918	rs1246172	A	G	A	G	0.0328	-0.05976	0.2836	0.300551	FALSE	FALSE	FALSE	ieu-b-508	6	1.57E+08	0.036508	486484	0.101672	Sepsis (28	Sepsis (28	Sepsis (28	TRUE	igd	0.007	2.52E-06	4323	M33633.m	TRUE	reported	e36XGE	2	TRUE	0.005053	21.94576
3375	rs1009343	A	C	A	C	0.1003	0.045415	0.9622	0.962186	FALSE	FALSE	FALSE	ieu-b-508	12	5001593	0.089377	486484	0.611366	Sepsis (28	Sepsis (28	Sepsis (28	TRUE	igd	0.0214	2.69E-06	1767	M32418.m	TRUE	reported	y8cN4Y	2	TRUE	0.012279	21.94232
6076	rs685747	C	G	C	G	-0.0431	0.007629	0.9581	0.951113	FALSE	TRUE	FALSE	ieu-b-508	11	78699340	0.078057	486484	0.922141	Sepsis (28	Sepsis (28	Sepsis (28	TRUE	igd	0.0092	2.96E-06	6327	M33833.m	TRUE	reported	LMIPLH	2	TRUE	0.003457	21.94025
7842	rs4841110	A	G	A	G	0.0342	0.103558	0.5869	0.585984	FALSE	FALSE	FALSE	ieu-b-508	8	9086023	0.033974	486484	0.002303	Sepsis (28	Sepsis (28	Sepsis (28	TRUE	igd	0.0073	2.60E-06	5035	M35551.m	TRUE	reported	5Sxswl	2	TRUE	0.00434	21.93986
5444	rs1709762	T	C	T	C	-0.104	0.083883	0.9727	0.968957	FALSE	FALSE	FALSE	ieu-b-508	12	47521738	0.096807	486484	0.386214	Sepsis (28	Sepsis (28	Sepsis (28	TRUE	igd	0.0222	2.72E-06	5185	M33390.m	TRUE	reported	t0EYdH	2	TRUE	0.004215	21.93781
404	rs1110613	T	G	T	G	-0.0089	-0.02726	0.2379	0.244839	FALSE	FALSE	FALSE	ieu-b-508	12	78045649	0.038966	486484	0.484185	Sepsis (28	Sepsis (28	Sepsis (28	TRUE	igd	0.0019	1.81E-06	7365	M00063.m	TRUE	reported	Hdsv1L	2	TRUE	0.00297	21.93587
3114	rs7332276	T	G	T	G	-0.0178	0.021216	0.4699	0.526337	FALSE	FALSE	FALSE	ieu-b-508	13	1.08E+08	0.034574	486484	0.539442	Sepsis (28	Sepsis (28	Sepsis (28	TRUE	igd	0.0038	3.14E-06	7356	M32198.m	TRUE	reported	ndXmnp	2	TRUE	0.002974	21.93586
1149	rs1889463	T	C	T	C	0.0089	-0.01052	0.628	0.639032	FALSE	FALSE	FALSE	ieu-b-508	1	1.07E+08	0.036122	486484	0.770816	Sepsis (28	Sepsis (28	Sepsis (28	TRUE	igd	0.0019	3.36E-06	7355	M01644.m	TRUE	reported	IB0DTL	2	TRUE	0.002974	21.93586
3564	rs1691607	T	G	T	G	0.0089	-0.02951	0.806	0.797762	FALSE	FALSE	FALSE	ieu-b-508	8	94600938	0.04163	486484	0.478408	Sepsis (28	Sepsis (28	Sepsis (28	TRUE	igd	0.0019	2.69E-06	7350	M32518.m	TRUE	reported	V0ZCiU	2	TRUE	0.002976	21.93586
7920	rs158896	T	C	T	C	0.0089	-0.00706	0.2658	0.261117	FALSE	FALSE	FALSE	ieu-b-508	5	1.68E+08	0.038198	486484	0.853326	Sepsis (28	Sepsis (28	Sepsis (28	TRUE	igd	0.0019	1.38E-06	7348	M35675.m	TRUE	reported	D7fdyZ	2	TRUE	0.002977	21.93586
7948	rs9410293	A	T	A	T	0.0089	-0.03115	0.7111	0.709543	FALSE	TRUE	FALSE	ieu-b-508	9	91631896	0.037671	486484	0.408309	Sepsis (28	Sepsis (28	Sepsis (28	TRUE	igd	0.0019	4.67E-06	7348	M35675.m	TRUE	reported	D7fdyZ	2	TRUE	0.002977	21.93586
2623	rs625842	A	C	A	C	0.0089	0.019513	0.5072	0.514271	FALSE	FALSE	FALSE	ieu-b-508	2	12828973	0.033397	486484	0.559038	Sepsis (28	Sepsis (28	Sepsis (28	TRUE	igd	0.0019	1.85E-06	7329	M22481.m	TRUE	reported	bXpf0k	2	TRUE	0.002985	21.93584
5392	rs1288410	A	C	A	C	-0.0089	0.012914	0.4607	0.567194	FALSE	FALSE	FALSE	ieu-b-508	14	88529291	0.03375	486484	0.701987	Sepsis (28	Sepsis (28	Sepsis (28	TRUE	igd	0.0019	1.84E-06	7315	M33389.m	TRUE	reported	Hf6XVM	2	TRUE	0.002991	21.93583
5407	rs348661	A	T	A	T	0.0089	0.044887	0.3458	0.350188	FALSE	TRUE	FALSE	ieu-b-508	12	62455504	0.035048	486484	0.200289	Sepsis (28	Sepsis (28	Sepsis (28	TRUE	igd	0.0019	1.78E-06	7315	M33389.m	TRUE	reported	Hf6XVM	2	TRUE	0.002991	21.93583
5429	rs939291	T	C	T	C	0.0089	0.044443	0.711	0.70015	FALSE	FALSE	FALSE	ieu-b-508	3	14659008	0.036594	486484	0.224558	Sepsis (28	Sepsis (28	Sepsis (28	TRUE	igd	0.0019	2.63E-06	7315	M33389.m	TRUE	reported	Hf6XVM	2	TRUE	0.002991	21.93583
1840	rs1687615	T	G	T	G	-0.0089	0.077587	0.2054	0.207148	FALSE	FALSE	FALSE	ieu-b-508	6	47628260	0.041273	486484	0.060127	Sepsis (28	Sepsis (28	Sepsis (28	TRUE	igd	0.0019	1.65E-06	7308	M15778.m	TRUE	reported	941o3l	2	TRUE	0.002993	21.93582
5686	rs4505759	T	C	T	C	-0.0089	0.031459	0.3074	0.306477	FALSE	FALSE	FALSE	ieu-b-508	4	1003022	0.036309	486484	0.386257	Sepsis (28	Sepsis (28	Sepsis (28	TRUE	igd	0.0019	2.36E-06	7307	M33477.m	TRUE	reported	Aw8PBK	2	TRUE	0.002994	21.93582
5838	rs8045299	A	G	A	G	0.0089	-0.03278	0.3403	0.333528	FALSE	FALSE	FALSE	ieu-b-508	16	1316225	0.035422	486484	0.354747	Sepsis (28	Sepsis (28	Sepsis (28	TRUE	igd	0.0019	1.81E-06	7066	M33515.m	TRUE	reported	X9mul1	2	TRUE	0.003096	21.93562
8046	rs9960323	T	C	T	C	0.0178	-0.00257	0.2721	0.283489	FALSE	FALSE	FALSE	ieu-b-508	18	74352728	0.037034	486484	0.944596	Sepsis (28	Sepsis (28	Sepsis (28	TRUE	igd	0.0038	2.37E-06	6960	M35854.m	TRUE	reported	EujB0p	2	TRUE	0.003143	21.93552
4311	rs981831	T	C	T	C	0.0267	0.030117	0.123	0.121851	FALSE	FALSE	FALSE	ieu-b-508	4	1.28E+08	0.050898	486484	0.554045	Sepsis (28	Sepsis (28	Sepsis (28	TRUE	igd	0.0057	2.48E-06	6753	M32838.m	TRUE	reported	hyR7zA	2	TRUE	0.003239	21.93533
7999	rs1322878	A	C	A	C	-0.0089	-0.03106	0.4235	0.43118	FALSE	FALSE	FALSE	ieu-b-508	7	1.1E+08	0.03376	486484	0.357552	Sepsis (28	Sepsis (28	Sepsis (28	TRUE	igd	0.0019	3.32E-06	6604	M35754.m	TRUE	reported	IlXOo1	2	TRUE	0.003312	21.93518
8016	rs6827956	A	G	A	G	0.0089	-0.01806	0.4111	0.423174	FALSE	FALSE	FALSE	ieu-b-508	4	11910466	0.033816	486484	0.593291	Sepsis (28	Sepsis (28	Sepsis (28	TRUE	igd	0.0019	3.66E-06	6604	M35754.m	TRUE	reported	IlXOo1	2	TRUE	0.003312	21.93518
8456	rs1080903	A	C	A	C	-0.0089	-0.06056	0.6121	0.610671	FALSE	FALSE	FALSE	ieu-b-508	9	10230321	0.034309	486484	0.077539	Sepsis (28	Sepsis (28	Sepsis (28	TRUE	igd	0.0019	1.75E-06	6552	M37058.m	TRUE	reported	32p8qV	2	TRUE	0.003338	21.93513
8460	rs1168556	A	G	A	G	0.0089	-0.01208	0.6358	0.630969	FALSE	FALSE	FALSE	ieu-b-508	14	38873594	0.034763	486484	0.728221	Sepsis (28	Sepsis (28	Sepsis (28	TRUE	igd	0.0019	1.47E-06	6552	M37058.m	TRUE	reported	32p8qV	2	TRUE	0.003338	21.93513
8483	rs4837828	A	G	A	G	-0.0089	-0.02042	0.6656	0.66502	FALSE	FALSE	FALSE	ieu-b-508	9	1.24E+08	0.035327	486484	0.563229	Sepsis (28	Sepsis (28	Sepsis (28	TRUE	igd	0.0019	1.54E-06	6552	M37058.m	TRUE	reported	32p8qV	2	TRUE	0.003338	21.93513
2576	rs6738212	A	G	A	G	-0.0089	-0.00551	0.3037	0.328941	FALSE	FALSE	FALSE	ieu-b-508	2	2.24E+08	0.035526	486484	0.876672	Sepsis (28	Sepsis (28	Sepsis (28	TRUE	igd	0.0019	4.29E-06	6535	M22177.m	TRUE	reported	2s6Sly	2	TRUE	0.003346	21.93511
2589	rs9539356	A	C	A	C	0.0089	0.003071	0.7252	0.745016	FALSE	FALSE	FALSE	ieu-b-508	13	62463331	0.038352	486484	0.936177	Sepsis (28	Sepsis (28	Sepsis (28	TRUE	igd	0.0019	4.75E-06	6535	M22177.m	TRUE	reported	2s6Sly	2	TRUE	0.003346	21.93511
7248	rs1219563	T	C	T	C	-0.0089	0.009661	0.6339	0.62588	FALSE	FALSE	FALSE	ieu-b-508	6	23316593	0.034439	486484	0.779083	Sepsis (28	Sepsis (28	Sepsis (28	TRUE	igd	0.0019	2.49E-06	5885	M34912.m	TRUE	reported	W8lcT6	2	TRUE	0.003715	21.93437
7278	rs7755681	T	C	T	C	0.0089	-0.04038	0.5943	0.574046	FALSE	FALSE	FALSE	ieu-b-508	6	1.62E+08	0.033834	486484	0.232727	Sepsis (28	Sepsis (28	Sepsis (28	TRUE	igd	0.0019	2.58E-06	5885	M34912.m	TRUE	reported	W8lcT6	2	TRUE	0.003715	21.93437
7283	rs982702	T	C	T	C	-0.0089	0.016136	0.3591	0.353544	FALSE	FALSE	FALSE	ieu-b-508	1	1.68E+08	0.035039	486484	0.645145	Sepsis (28	Sepsis (28	Sepsis (28	TRUE	igd	0.0019	2.49E-06	5885	M34912.m	TRUE	reported	W8lcT6	2	TRUE	0.003715	21.93437
6686	rs7655049	T	C	T	C	-0.0553	0.066396	0.1616	0.159886	FALSE	FALSE	FALSE	ieu-b-508	4	1.06E+08	0.045632	486484	0.145663	Sepsis (28	Sepsis (28	Sepsis (28	TRUE	igd	0.0118	2.91E-06	1492	M34329.m	TRUE	reported	mgPKP5	2	TRUE	0.014507	21.93329
7355	rs2969990	A	T	A	T	0.0089	-0.01318	0.3814	0.389454	FALSE	TRUE	FALSE	ieu-b-508	18	47961018	0.034288	486484	0.700698	Sepsis (28	Sepsis (28	Sepsis (28	TRUE	igd	0.0019	2.82E-06	4900	M35137.m	TRUE	reported	Eq2siS	2	TRUE	0.004458	21.93287
7377	rs696958	A	G	A	G	-0.0089	-0.03858	0.3585	0.346586	FALSE	FALSE	FALSE	ieu-b-508	1	2.09E+08	0.039104	486484	0.323844	Sepsis (28	Sepsis (28	Sepsis (28	TRUE	igd	0.0019	3.05E-06	4900	M35137.m	TRUE	reported	Eq2siS	2	TRUE	0.004458	21.93287
2258	rs4431081	T	C	T	C	0.0164	0.049168	0.4481	0.452746	FALSE	FALSE	FALSE	ieu-b-508	3	1.11E+08	0.033497	486484	0.142154	Sepsis (28	Sepsis (28	Sepsis (28	TRUE	igd	0.0035	2.18E-06	1759	M19934.m	TRUE	reported	HtcWcQ	2	TRUE	0.012328	21.93095
5479	rs7767760	T	C	T	C	0.0356	-0.01204	0.2932	0.297773	FALSE	FALSE	FALSE	ieu-b-508	6	1.6E+08	0.036572	486484	0.742038	Sepsis (28	Sepsis (28	Sepsis (28	TRUE	igd	0.0076	2.97E-06	3890	M33408.m	TRUE	reported	tWZWla	2	TRUE	0.005609	21.93055
3924	rs913080	T	C	T	C	-0.0417	-0.04606	0.3661	0.342971	FALSE	FALSE	FALSE	ieu-b-508	9	1.17E+08	0.035183	486484	0.190525	Sepsis (28	Sepsis (28	Sepsis (28	TRUE	igd	0.0089	2.64E-06	1675	M32691.m	TRUE	reported	SiuD6F	2	TRUE	0.012937	21.9267
5720	rs6862546	T	C	T	C	-0.0281	0.02812	0.1752	0.16818	FALSE	FALSE	FALSE	ieu-b-508	5	21826564	0.044685	486484	0.529151	Sepsis (28	Sepsis (28	Sepsis (28	TRUE	igd	0.006	3.31E-06	5107	M33488.m	TRUE	reported	mZmDY6	2	TRUE	0.004276	21.92502
6869	rs1806807	A	G	A	G	-0.1364	0.0573	0.772	0.754608	FALSE	FALSE	FALSE	ieu-b-508	9	1.38E+08	0.038948	486484	0.141245	Sepsis (28	Sepsis (28	Sepsis (28	TRUE	igd	0.0291	2.69E-06	954	M34365.m	TRUE	reported	8eexD0	2	TRUE	0.022512	21.92458
5958	rs1104626	T	C	T	C	-0.0431	0.043476	0.2995	0.313228	FALSE	FALSE	FALSE	ieu-b-508	12	22189630	0.036694	486484	0.236078	Sepsis (28	Sepsis (28	Sepsis (28	TRUE	igd	0.0092	2.53E-06	1860	M33658.m	TRUE	reported	9QszGO	2	TRUE	0.011662	21.92359
1906	rs2216140	A	G	A	G	-0.1026	-0.05369	0.0341	0.027843	FALSE	FALSE	FALSE	ieu-b-508	13	72282407	0.10281	486484	0.601484	Sepsis (28	Sepsis (28	Sepsis (28	TRUE	igd	0.0219	2.73E-06	1749	M16634.m	TRUE	reported	P9BdXh	2	TRUE	0.012394	21.92348
4402	rs1163690	A	C	A	C	0.0623	-0.07347	0.0306	0.037784	FALSE	FALSE	FALSE	ieu-b-508	15	46994087	0.088624	486484	0.407114	Sepsis (28	Sepsis (28	Sepsis (28	TRUE	igd	0.0133	2.84E-06	2148	M32869.m	TRUE	reported	Rvim1n	2	TRUE	0.010112	21.9214
4376	rs7539884	T	C	T	C	0.0623	-0.03348	0.749	0.773435	FALSE	FALSE	FALSE	ieu-b-508	1	16055931	0.039951	486484	0.402009	Sepsis (28	Sepsis (28	Sepsis (28	TRUE	igd	0.0133	2.67E-06	1988	M32863.m	TRUE	reported	cLOFYE	2	TRUE	0.010917	21.91975
503	rs1209117	A	G	A	G	0.127	0.013653	0.0264	0.224278	FALSE	FALSE	FALSE	ieu-b-508	1	2.16E+08	0.040058	486484	0.73323	Sepsis (28	Sepsis (28	Sepsis (28	TRUE	igd	0.0271	2.81E-06	1002	M00575.m	TRUE	reported	Mv0t8k	2	TRUE	0.021448	21.91801
5864	rs1706515	A	G	A	G	0.1289	-0.08469	0.0388	0.03887	FALSE	FALSE	FALSE	ieu-b-508	8	549716	0.08708	486484	0.330768	Sepsis (28	Sepsis (28	Sepsis (28	TRUE	igd	0.0275	2.81E-06	824	M33609.m	TRUE	reported	h7B9gN	2	TRUE	0.025971	21.9172
4494	rs1092071	A	G	A	G	-0.0178	0.060734	0.8234	0.821921	FALSE	FALSE	FALSE	ieu-b-508	1	1.9E+08	0.043985	486484	0.16734	Sepsis (28	Sepsis (28	Sepsis (28	TRUE	igd	0.0038	2.27E-06	1762	M33132.m	TRUE	reported	dTXJa8	2	TRUE	0.0123	21.91692
3179	rs1194262	T	G	T	G	0.0712	0.229542	0.9829	0.987334	FALSE	FALSE	FALSE	ieu-b-508	4	12360318	0.148464	486484	0.122077	Sepsis (28	Sepsis (28	Sepsis (28	TRUE	igd	0.0152	2.68E-06	1760	M32338.m	TRUE	reported	q5cuaP	2	TRUE	0.012313	21.91689
8132	rs3935503	T	G	T	G	-0.0398	-0.01578	0.7581	0.761371	FALSE	FALSE	FALSE	ieu-b-508	18	27584537	0.039339	486484	0.688369	Sepsis (28	Sepsis (28	Sepsis (28	TRUE	igd	0.0085	2.66E-06	5696	M36099.m	TRUE	reported	bFHsTx	2	TRUE	0.003834	21.91673
8182	rs7187550	T	C	T	C	0.0473	-0.02952	0.1217	0.116841	FALSE	FALSE	FALSE	ieu-b-508	16	54673195	0.051866	486484	0.569205	Sepsis (28	Sepsis (28	Sepsis (28	TRUE	igd	0.0101	2.67E-06	2432	M36134.m	TRUE	reported	ekMsfG	2	TRUE	0.008938	21.91403
912	rs2115151	A	T	A	T	0.0103	-0.02927	0.8627	0.864621	FALSE	TRUE	FALSE	ieu-b-508	4	1.24E+08	0.048565	486484	0.546665	Sepsis (28	Sepsis (28	Sepsis (28	TRUE	igd	0.0022	3.75E-06	7354	M01494.m	TRUE	reported	7JWNKI	2	TRUE	0.002972	21.91346
5608	rs1080985	A	G	A	G	-0.0103	0.041902	0.8089	0.791088	FALSE	FALSE	FALSE	ieu-b-508	9	12908303	0.041028	486484	0.307109	Sepsis (28	Sepsis (28	Sepsis (28	TRUE	igd	0.0022	4.16E-06	7336	M33442.m	TRUE	reported	B1Peb3	2	TRUE	0.002979	21.91345
2596	rs2485904	A	C	A	C	0.0206	0.012295	0.1564	0.148556	FALSE	FALSE	FALSE	ieu-b-508	6	76794103	0.0481	486484	0.798247	Sepsis (28	Sepsis (28	Sepsis (28	TRUE	igd	0.0044	2.88E-06	7259	M22189.m	TRUE	reported	YSD5fY	2	TRUE	0.003011	21.91338
1350	rs1049717	T	C	T	C	0.0103	-0.03392	0.2732	0.266111	FALSE	FALSE	FALSE	ieu-b-508	2	1.57E+08	0.038238	486484	0.375066	Sepsis (28	Sepsis (28	Sepsis (28	TRUE	igd	0.0022	4.16E-06	7226	M02730.m	TRUE	reported	XYa035	2	TRUE	0.003024	21.91335
1357	rs192598	A	C	A	C	0.0103	-0.09836	0.3185	0.315379	FALSE	FALSE	FALSE	ieu-b-508	16	82886133	0.035888	486484	0.006128	Sepsis (28	Sepsis (28	Sepsis (28	TRUE	igd	0.0022	1.99E-06	7226	M02730.m	TRUE	reported	XYa035	2	TRUE	0.003024	21.91335
5300	rs6565887	T	C	T	C	-0.0773	-0.05161	0.927	0.92813	FALSE	FALSE	FALSE	ieu-b-508	18	74182975	0.06488	486484	0.426381	Sepsis (28	Sepsis (28	Sepsis (28	TRUE	igd	0.0165	2.90E-06	1273	M33353.m	TRUE	reported	ZIN4gk	2	TRUE	0.016949	21.91332
924	rs1171963	A	G	A	G	-0.0206	0.022308	0.0945	0.098411	FALSE	FALSE	FALSE	ieu-b-508	3	20402881	0.056476	486484	0.692838	Sepsis (28	Sepsis (28	Sepsis (28	TRUE	igd	0.0044	3.01E-06	7160	M01508.m	TRUE	reported	go526k	2	TRUE	0.003052	21.9133
7547	rs9405761	A	T	A	T	0.0206	-0.04633	0.8788	0.865015	FALSE	TRUE	FALSE	ieu-b-508	6	4687048	0.048849	486484	0.342949	Sepsis (28	Sepsis (28	Sepsis (28	TRUE	igd	0.0044	2.95E-06	6538	M35257.m	TRUE	reported	iphouU	2	TRUE	0.003341	21.91272
7968	rs9304337	A	G	A	G	0.0206	-0.02706	0.5538	0.555102	FALSE	FALSE	FALSE	ieu-b-508	18	44493094	0.033653	486484	0.421301	Sepsis (28	Sepsis (28	Sepsis (28	TRUE	igd	0.0044	2.26E-06	6447	M35678.m	TRUE	reported	56yR01	2	TRUE	0.003388	21.91262
615	rs696155	A	G	A	G	-0.0412	0.054613	0.1888	0.192488	FALSE	FALSE	FALSE	ieu-b-508	5	33552040	0.042405	486484	0.197783	Sepsis (28	Sepsis (28	Sepsis (28	TRUE	igd	0.0088	2.92E-06	4934	M01114.m	TRUE	reported	fqHdM2	2	TRUE	0.004423	21.91054
8153	rs2357258	A	C	A	C	-0.037	0.021654	0.6387	0.647154	FALSE	FALSE	FALSE	ieu-b-508	2	9806527	0.034993	486484	0.536039	Sepsis (28	Sepsis (28	Sepsis (28	TRUE	igd	0.0079	2.54E-06	1731	M36115.m	TRUE	reported	ZV1jWp	2	TRUE	0.012514	21.91024
6377	rs2194101	A	G	A	G	0.0323	-0.0477	0.8777	0.8569	FALSE	FALSE	FALSE	ieu-b-508	4	1.82E+08	0.047808	486484	0.318409	Sepsis (28	Sepsis (28	Sepsis (28	TRUE	igd	0.0069	3.41E-06	7322	M33968.m	TRUE	reported	sgVOvT	2	TRUE	0.002984	21.90727
6177	rs7718486	T	C	T	C	0.0426	-0.03513	0.7269	0.734051	FALSE	FALSE	FALSE	ieu-b-508	5	1.79E+08	0.037819	486484	0.352941	Sepsis (28	Sepsis (28	Sepsis (28	TRUE	igd	0.0091	2.68E-06	4999	M33885.m	TRUE	reported	uCOy6o	2	TRUE	0.004365	21.90598
5216	rs4807944	T	C	T	C	0.0206	-0.11761	0.1282	0.147262	FALSE	FALSE	FALSE	ieu-b-508	19	1642781	0.046993	486484	0.012322	Sepsis (28	Sepsis (28	Sepsis (28	TRUE	igd	0.0044	3.32E-06	2683	M33204.m	TRUE	reported	ROvPWK	2	TRUE	0.008104	21.90308
3613	rs1764259	C	G	C	G	-0.0337	-0.09521	0.8943	0.909613	FALSE	TRUE	FALSE	ieu-b-508	10	1.2E+08	0.058419	486484	0.103151	Sepsis (28	Sepsis (28	Sepsis (28	TRUE	igd	0.0072	2.67E-06	7361	M32553.m	TRUE	reported	vHSHqn	2	TRUE	0.002967	21.90165
4152	rs9445291	T	C	T	C	-0.0337	-0.01472	0.1411	0.139155	FALSE	FALSE	FALSE	ieu-b-508	6	95197882	0.048627	486484	0.762107	Sepsis (28	Sepsis (28	Sepsis (28	TRUE	igd	0.0072	3.28E-06	7250	M32757.m	TRUE	reported	TJV9Xb	2	TRUE	0.003013	21.90156
6583	rs1163471	A	G	A	G	-0.0206	-0.00923	0.3458	0.351945	FALSE	FALSE	FALSE	ieu-b-508	15	25306646	0.035106	486484	0.792589	Sepsis (28	Sepsis (28	Sepsis (28	TRUE	igd	0.0044	2.84E-06	2278	M34289.m	TRUE	reported	Knp8ry	2	TRUE	0.009531	21.90018
4038	rs7946041	A	G	A	G	0.0454	0.032058	0.0211	0.024901	FALSE	FALSE	FALSE	ieu-b-508	11	1.15E+08	0.107292	486484	0.765098	Sepsis (28	Sepsis (28	Sepsis (28	TRUE	igd	0.0097	2.75E-06	6042	M32739.m	TRUE	reported	dYSC7n	2	TRUE	0.003613	21.89901
6036	rs4695275	A	T	A	T	0.1561	-0.0111	0.1015	0.102301	FALSE	TRUE	FALSE	ieu-b-508	4	47775045	0.055259	486484	0.84079	Sepsis (28	Sepsis (28	Sepsis (28	TRUE	igd	0.0333	2.73E-06	581	M33782.m	TRUE	reported	875Pal	2	TRUE	0.036443	21.89877
3273	rs4671635	A	G	A	G	-0.0117	0.014422	0.2317	0.217848	FALSE	FALSE	FALSE	ieu-b-508	2	65180817	0.040469	486484	0.721569	Sepsis (28	Sepsis (28	Sepsis (28	TRUE	igd	0.0025	2.35E-06	7365	M32348.m	TRUE	reported	DJeLDx	2	TRUE	0.002965	21.89645
3111	rs6583751	C	G	C	G	-0.0117	0.085972	0.5964	0.591266	FALSE	TRUE	FALSE	ieu-b-508	10	92838776	0.034061	486484	0.011601	Sepsis (28	Sepsis (28	Sepsis (28	TRUE	igd	0.0025	3.60E-06	7356	M32198.m	TRUE	reported	ndXmnp	2	TRUE	0.002969	21.89645
3123	rs1237812	T	C	T	C	0.0117	0.038234	0.8196	0.822232	FALSE	FALSE	FALSE	ieu-b-508	9	34317588	0.043587	486484	0.380386	Sepsis (28	Sepsis (28	Sepsis (28	TRUE	igd	0.0025	3.91E-06	7349	M32315.m	TRUE	reported	4igzkL	2	TRUE	0.002971	21.89644
3389	rs1023682	T	C	T	C	-0.0585	-0.04901	0.9564	0.938653	FALSE	FALSE	FALSE	ieu-b-508	7	41664903	0.069854	486484	0.482949	Sepsis (28	Sepsis (28	Sepsis (28	TRUE	igd	0.0125	2.68E-06	7346	M32425.m	TRUE	reported	u5RLGY	2	TRUE	0.002973	21.89644
4458	rs8038921	A	C	A	C	0.0117	0.027783	0.1345	0.136059	FALSE	FALSE	FALSE	ieu-b-508	15	37057906	0.048966	486484	0.570449	Sepsis (28	Sepsis (28	Sepsis (28	TRUE	igd	0.0025	4.67E-06	7344	M32910.m	TRUE	reported	2OCBKh	2	TRUE	0.002973	21.89644
4146	rs2828489	T	C	T	C	0.0234	0.010661	0.6364	0.641205	FALSE	FALSE	FALSE	ieu-b-508	21	25112425	0.034761	486484	0.759077	Sepsis (28	Sepsis (28	Sepsis (28	TRUE	igd	0.005	2.31E-06	7250	M32757.m	TRUE	reported	TJV9Xb	2	TRUE	0.003012	21.89636
2355	rs703654	A	G	A	G	-0.0117	0.020899	0.8216	0.815419	FALSE	FALSE	FALSE	ieu-b-508	12	1.04E+08	0.043192	486484	0.628478	Sepsis (28	Sepsis (28	Sepsis (28	TRUE	igd	0.0025	2.89E-06	7203	M21047.m	TRUE	reported	ISF1Cr	2	TRUE	0.003032	21.89632
2637	rs1115739	T	C	T	C	0.0117	-0.0043	0.5826	0.575961	FALSE	FALSE	FALSE	ieu-b-508	14	22549886	0.033711	486484	0.898602	Sepsis (28	Sepsis (28	Sepsis (28	TRUE	igd	0.0025	3.69E-06	7184	M22548.m	TRUE	reported	mZtwX2	2	TRUE	0.00304	21.8963
2181	rs1460542	A	C	A	C	0.0117	-0.07334	0.1671	0.169732	FALSE	FALSE	FALSE	ieu-b-508	10	44471109	0.044395	486484	0.09853	Sepsis (28	Sepsis (28	Sepsis (28	TRUE	igd	0.0025	2.93E-06	7182	M19362.m	TRUE	reported	SkNEKw	2	TRUE	0.00304	21.8963
5367	rs9370455	T	C	T	C	-0.0117	-0.015	0.8882	0.86007	FALSE	FALSE	FALSE	ieu-b-508	6	55594431	0.048104	486484	0.755151	Sepsis (28	Sepsis (28	Sepsis (28	TRUE	igd	0.0025	2.35E-06	7118	M33380.m	TRUE	reported	OHRfl5	2	TRUE	0.003068	21.89625
861	rs3135095	A	G	A	G	-0.0117	-0.02653	0.2993	0.31118	FALSE	FALSE	FALSE	ieu-b-508	4	2389800	0.036078	486484	0.462079	Sepsis (28	Sepsis (28	Sepsis (28	TRUE	igd	0.0025	3.92E-06	7063	M01361.m	TRUE	reported	NIAcQ3	2	TRUE	0.003091	21.8962
4295	rs8012350	A	G	A	G	-0.0117	0.060066	0.4879	0.485254	FALSE	FALSE	FALSE	ieu-b-508	14	1.01E+08	0.033584	486484	0.073689	Sepsis (28	Sepsis (28	Sepsis (28	TRUE	igd	0.0025	3.79E-06	7063	M32814.m	TRUE	reported	z0OeTx	2	TRUE	0.003091	21.8962
7651	rs4693661	A	G	A	G	0.0117	0.024323	0.1547	0.156974	FALSE	FALSE	FALSE	ieu-b-508	4	84857956	0.046092	486484	0.5977	Sepsis (28	Sepsis (28	Sepsis (28	TRUE	igd	0.0025	3.24E-06	5768	M35326.m	TRUE	reported	jRytyJ	2	TRUE	0.003783	21.89481
2403	rs2143069	A	G	A	G	-0.0234	-0.07993	0.4261	0.424167	FALSE	FALSE	FALSE	ieu-b-508	6	1.64E+08	0.034239	486484	0.019572	Sepsis (28	Sepsis (28	Sepsis (28	TRUE	igd	0.005	2.64E-06	5436	M21184.m	TRUE	reported	63ppuJ	2	TRUE	0.004013	21.89434
8441	rs7500975	A	G	A	G	-0.0833	-0.16853	0.0376	0.046253	FALSE	FALSE	FALSE	ieu-b-508	16	7641721	0.081013	486484	0.037501	Sepsis (28	Sepsis (28	Sepsis (28	TRUE	igd	0.0178	2.87E-06	6511	M36850.m	TRUE	reported	j6lLja	2	TRUE	0.003352	21.89357
60	rs1711435	A	G	A	G	0.0248	-0.31142	0.9874	0.985399	FALSE	FALSE	FALSE	ieu-b-508	12	75291946	0.137783	486484	0.023807	Sepsis (28	Sepsis (28	Sepsis (28	TRUE	igd	0.0053	3.52E-06	7355	M00054.m	TRUE	reported	QTDAnh	2	TRUE	0.002968	21.88938
1	rs1159660	A	G	A	G	0.0323	-0.11809	0.0236	0.047958	FALSE	FALSE	FALSE	ieu-b-508	10	87141944	0.078156	486484	0.130798	Sepsis (28	Sepsis (28	Sepsis (28	TRUE	igd	0.0069	2.52E-06	1768	M00053.m	TRUE	reported	4Smluz	2	TRUE	0.012243	21.88846
1662	rs1148709	A	G	A	G	0.248	-0.10469	0.012	0.010444	FALSE	FALSE	FALSE	ieu-b-508	7	1.08E+08	0.167372	486484	0.531643	Sepsis (28	Sepsis (28	Sepsis (28	TRUE	igd	0.053	2.88E-06	4084	M15335.m	TRUE	reported	a7RjFn	2	TRUE	0.005333	21.88461
2117	rs7546527	A	G	A	G	0.4654	-0.11725	0.012	0.017741	FALSE	FALSE	FALSE	ieu-b-508	1	65846564	0.126726	486484	0.35487	Sepsis (28	Sepsis (28	Sepsis (28	TRUE	igd	0.0991	2.64E-06	257	M18474.m	TRUE	reported	B6Xd02	2	TRUE	0.079034	21.88328
2612	rs2671471	A	G	A	G	-0.0131	0.036966	0.1641	0.158395	FALSE	FALSE	FALSE	ieu-b-508	10	65752909	0.045821	486484	0.419814	Sepsis (28	Sepsis (28	Sepsis (28	TRUE	igd	0.0028	2.67E-06	7329	M22481.m	TRUE	reported	bXpf0k	2	TRUE	0.002978	21.88306
2042	rs7805747	A	G	A	G	0.0131	-0.04137	0.2679	0.276613	FALSE	FALSE	FALSE	ieu-b-508	7	1.51E+08	0.037242	486484	0.266624	Sepsis (28	Sepsis (28	Sepsis (28	TRUE	igd	0.0028	3.77E-06	6939	M18349.m	TRUE	reported	M5qPs8	2	TRUE	0.003145	21.88272
1552	rs7660017	A	G	A	G	-0.0131	0.042555	0.3331	0.334387	FALSE	FALSE	FALSE	ieu-b-508	4	38300473	0.035415	486484	0.229514	Sepsis (28	Sepsis (28	Sepsis (28	TRUE	igd	0.0028	2.34E-06	6864	M12129.m	TRUE	reported	iNzdgb	2	TRUE	0.003179	21.88265
7881	rs1248083	A	G	A	G	-0.081	-0.07617	0.0804	0.08789	FALSE	FALSE	FALSE	ieu-b-508	20	30508331	0.058894	486484	0.19591	Sepsis (28	Sepsis (28	Sepsis (28	TRUE	igd	0.0173	2.89E-06	1098	M35635.m	TRUE	reported	qymryH	2	TRUE	0.019574	21.88195
4253	rs1169078	C	G	C	G	0.051	-0.05129	0.1054	0.126538	FALSE	TRUE	FALSE	ieu-b-508	2	66421703	0.050994	486484	0.31448	Sepsis (28	Sepsis (28	Sepsis (28	TRUE	igd	0.0109	3.10E-06	4274	M32800.m	TRUE	reported	Q7MVsa	2	TRUE	0.005096	21.88185
7899	rs2566347	A	C	A	C	-0.0262	-0.04565	0.2063	0.206069	FALSE	FALSE	FALSE	ieu-b-508	3	1.58E+08	0.041357	486484	0.269639	Sepsis (28	Sepsis (28	Sepsis (28	TRUE	igd	0.0056	2.44E-06	5629	M35669.m	TRUE	reported	gSecuE	2	TRUE	0.003874	21.88125
8584	rs7725624	A	C	A	C	-0.044	-0.07359	0.0865	0.103989	FALSE	FALSE	FALSE	ieu-b-508	5	63010725	0.054409	486484	0.176224	Sepsis (28	Sepsis (28	Sepsis (28	TRUE	igd	0.0094	3.02E-06	1411	M37253.m	TRUE	reported	PYMqzt	2	TRUE	0.015291	21.87931
6992	rs1221918	T	C	T	C	0.0407	0.023883	0.9516	0.781985	FALSE	FALSE	FALSE	ieu-b-508	10	68270163	0.040554	486484	0.555912	Sepsis (28	Sepsis (28	Sepsis (28	TRUE	igd	0.0087	2.67E-06	6929	M34416.m	TRUE	reported	q9OtaZ	2	TRUE	0.003149	21.87887

6748	rs8187736	T	C	T	C	0.2254	-0.04288	0.0534	0.049328	FALSE	FALSE	FALSE	ieu-b-508	15	85430954	0.077556	486484	0.580311	Sepsis (28	Sepsis (28	Sepsis (28	TRUE	igd	0.0481	2.73E-06	535	M34338.m	TRUE	reported	h4FB9a	2	TRUE	0.039427	21.87717
7229	rs1110278	A	G	A	G	0.0379	-0.00275	0.1984	0.202729	FALSE	FALSE	FALSE	ieu-b-508	1	1.15E+08	0.041821	486484	0.947534	Sepsis (28	Sepsis (28	Sepsis (28	TRUE	igd	0.0081	3.16E-06	2474	M34878.m	TRUE	reported	Cpq9yU	2	TRUE	0.008772	21.87546
4632	rs3850788	A	G	A	G	-0.3028	0.055516	0.0225	0.027847	FALSE	FALSE	FALSE	ieu-b-508	18	4413203	0.102646	486484	0.588613	Sepsis (28	Sepsis (28	Sepsis (28	TRUE	igd	0.0647	2.84E-06	1574	M33173.m	TRUE	reported	04fkUQ	2	TRUE	0.013725	21.87515
1421	rs1824583	A	G	A	G	-0.0145	0.072013	0.7906	0.781202	FALSE	FALSE	FALSE	ieu-b-508	11	99064051	0.040535	486484	0.075642	Sepsis (28	Sepsis (28	Sepsis (28	TRUE	igd	0.0031	3.38E-06	6941	M03127.m	TRUE	reported	JscEZw	2	TRUE	0.003142	21.87195
2865	rs1089683	T	C	T	C	0.087	0.126614	0.9822	0.981026	FALSE	FALSE	FALSE	ieu-b-508	11	58575572	0.123041	486484	0.303459	Sepsis (28	Sepsis (28	Sepsis (28	TRUE	igd	0.0186	3.04E-06	6715	M27710.m	TRUE	reported	XeaFav	2	TRUE	0.003248	21.87174
7536	rs1715617	T	C	T	C	0.029	-0.05399	0.9129	0.877099	FALSE	FALSE	FALSE	ieu-b-508	7	1.08E+08	0.05074	486484	0.287262	Sepsis (28	Sepsis (28	Sepsis (28	TRUE	igd	0.0062	3.30E-06	6538	M35257.m	TRUE	reported	iphouU	2	TRUE	0.003335	21.87156
5976	rs2567470	T	C	T	C	-0.0248	-0.04166	0.1528	0.139505	FALSE	FALSE	FALSE	ieu-b-508	17	70739396	0.048184	486484	0.387255	Sepsis (28	Sepsis (28	Sepsis (28	TRUE	igd	0.0053	2.72E-06	1764	M33666.m	TRUE	reported	dvWEtk	2	TRUE	0.01226	21.87051
1953	rs343836	T	C	T	C	-0.1801	-0.04185	0.9796	0.972192	FALSE	FALSE	FALSE	ieu-b-508	5	1.21E+08	0.101775	486484	0.680915	Sepsis (28	Sepsis (28	Sepsis (28	TRUE	igd	0.0385	2.83E-06	3393	M16823.m	TRUE	reported	fNy2PD	2	TRUE	0.006408	21.87006
5972	rs1696535	A	G	A	G	-0.0304	-0.08341	0.035	0.061796	FALSE	FALSE	FALSE	ieu-b-508	13	1.05E+08	0.069724	486484	0.231601	Sepsis (28	Sepsis (28	Sepsis (28	TRUE	igd	0.0065	2.78E-06	6279	M33666.m	TRUE	reported	dvWEtk	2	TRUE	0.003472	21.86664
8435	rs1261199	T	C	T	C	-0.0627	0.043104	0.245	0.224297	FALSE	FALSE	FALSE	ieu-b-508	2	2.37E+08	0.039962	486484	0.280756	Sepsis (28	Sepsis (28	Sepsis (28	TRUE	igd	0.0134	2.87E-06	1583	M36850.m	TRUE	reported	j6lLja	2	TRUE	0.013642	21.86636
8245	rs1263025	T	C	T	C	-0.0262	-0.21357	0.0162	0.01692	FALSE	FALSE	FALSE	ieu-b-508	3	3509168	0.130138	486484	0.100778	Sepsis (28	Sepsis (28	Sepsis (28	TRUE	igd	0.0056	3.09E-06	1763	M36515.m	TRUE	reported	yZaYzr	2	TRUE	0.012264	21.8642
3090	rs6699507	T	C	T	C	-0.0159	0.006462	0.818	0.803372	FALSE	FALSE	FALSE	ieu-b-508	1	2.1E+08	0.04194	486484	0.877557	Sepsis (28	Sepsis (28	Sepsis (28	TRUE	igd	0.0034	3.25E-06	7346	M32197.m	TRUE	reported	VZjwa9	2	TRUE	0.002968	21.86342
7321	rs1930131	T	C	T	C	-0.0159	-0.0052	0.821	0.816858	FALSE	FALSE	FALSE	ieu-b-508	9	1.01E+08	0.043259	486484	0.904392	Sepsis (28	Sepsis (28	Sepsis (28	TRUE	igd	0.0034	2.35E-06	7344	M35127.m	TRUE	reported	RNsmjl	2	TRUE	0.002969	21.86342
1042	rs1172419	A	G	A	G	0.0159	-0.01228	0.8734	0.845608	FALSE	FALSE	FALSE	ieu-b-508	4	1.69E+08	0.046162	486484	0.790249	Sepsis (28	Sepsis (28	Sepsis (28	TRUE	igd	0.0034	2.29E-06	7333	M01572.m	TRUE	reported	EOUAWy	2	TRUE	0.002973	21.86341
7125	rs1104079	A	T	A	T	0.0159	-0.00069	0.7347	0.735226	FALSE	TRUE	FALSE	ieu-b-508	11	6221214	0.037688	486484	0.98543	Sepsis (28	Sepsis (28	Sepsis (28	TRUE	igd	0.0034	2.73E-06	7322	M34527.m	TRUE	reported	EWFGP8	2	TRUE	0.002978	21.8634
6525	rs3773977	T	C	T	C	-0.0159	-0.00051	0.7484	0.727965	FALSE	FALSE	FALSE	ieu-b-508	3	1.9E+08	0.038478	486484	0.989504	Sepsis (28	Sepsis (28	Sepsis (28	TRUE	igd	0.0034	2.63E-06	7049	M34123.m	TRUE	reported	9O5wo1	2	TRUE	0.003093	21.86317
5487	rs1883349	A	G	A	G	-0.0318	-0.03925	0.1609	0.16332	FALSE	FALSE	FALSE	ieu-b-508	22	44331943	0.045093	486484	0.384079	Sepsis (28	Sepsis (28	Sepsis (28	TRUE	igd	0.0068	2.48E-06	6797	M33415.m	TRUE	reported	xHSphe	2	TRUE	0.003207	21.86294
8503	rs719672	A	C	A	C	0.0917	0.058168	0.8137	0.809574	FALSE	FALSE	FALSE	ieu-b-508	6	51165634	0.042547	486484	0.171574	Sepsis (28	Sepsis (28	Sepsis (28	TRUE	igd	0.0196	2.91E-06	1672	M37097.m	TRUE	reported	wCbJj4	2	TRUE	0.012922	21.86285
7544	rs7588373	A	C	A	C	0.0159	0.002859	0.7215	0.722744	FALSE	FALSE	FALSE	ieu-b-508	2	1.34E+08	0.037478	486484	0.939183	Sepsis (28	Sepsis (28	Sepsis (28	TRUE	igd	0.0034	2.97E-06	6538	M35257.m	TRUE	reported	iphouU	2	TRUE	0.003334	21.86269
8303	rs7574665	A	G	A	G	-0.2023	0.28466	0.9858	0.990157	FALSE	FALSE	FALSE	ieu-b-508	2	1.04E+08	0.171697	486484	0.097333	Sepsis (28	Sepsis (28	Sepsis (28	TRUE	igd	0.0432	2.79E-06	589	M36559.m	TRUE	reported	g6l9hg	2	TRUE	0.035895	21.85481
4650	rs7934811	A	G	A	G	-0.6419	-0.10757	0.0329	0.015972	FALSE	FALSE	FALSE	ieu-b-508	11	1.13E+08	0.138621	486484	0.43776	Sepsis (28	Sepsis (28	Sepsis (28	TRUE	igd	0.1355	2.17E-06	76	M33173.m	TRUE	reported	04fkUQ	2	TRUE	0.22797	21.85115
6756	rs1188240	A	C	A	C	0.0721	0.011095	0.5129	0.509374	FALSE	FALSE	FALSE	ieu-b-508	9	1.11E+08	0.033335	486484	0.739275	Sepsis (28	Sepsis (28	Sepsis (28	TRUE	igd	0.0154	2.93E-06	633	M34339.m	TRUE	reported	RwGZ7c	2	TRUE	0.033469	21.85017
3619	rs9682960	T	C	T	C	-0.0388	-0.09621	0.8843	0.878798	FALSE	FALSE	FALSE	ieu-b-508	3	11089345	0.051202	486484	0.060238	Sepsis (28	Sepsis (28	Sepsis (28	TRUE	igd	0.0083	3.36E-06	7361	M32553.m	TRUE	reported	vHSHqn	2	TRUE	0.00296	21.84687
3727	rs1162913	T	C	T	C	-0.0201	-0.03872	0.1656	0.172492	FALSE	FALSE	FALSE	ieu-b-508	14	34511741	0.044224	486484	0.381334	Sepsis (28	Sepsis (28	Sepsis (28	TRUE	igd	0.0043	3.09E-06	7337	M32632.m	TRUE	reported	pIHzPT	2	TRUE	0.002969	21.84423
3749	rs7541494	T	C	T	C	-0.0201	-0.00144	0.8786	0.880918	FALSE	FALSE	FALSE	ieu-b-508	1	2.33E+08	0.051578	486484	0.977796	Sepsis (28	Sepsis (28	Sepsis (28	TRUE	igd	0.0043	3.45E-06	7337	M32632.m	TRUE	reported	pIHzPT	2	TRUE	0.002969	21.84423
1903	rs1294991	T	C	T	C	-0.0201	0.009745	0.584	0.575774	FALSE	FALSE	FALSE	ieu-b-508	17	40526273	0.03384	486484	0.773369	Sepsis (28	Sepsis (28	Sepsis (28	TRUE	igd	0.0043	2.36E-06	7064	M16634.m	TRUE	reported	P9BdXh	2	TRUE	0.003084	21.844
6952	rs4358841	A	G	A	G	0.079	0.289546	0.9839	0.983919	FALSE	FALSE	FALSE	ieu-b-508	8	89716685	0.136161	486484	0.033462	Sepsis (28	Sepsis (28	Sepsis (28	TRUE	igd	0.0169	3.00E-06	5689	M34400.m	TRUE	reported	QXmzfR	2	TRUE	0.003826	21.84379
4158	rs1326601	A	C	A	C	-0.0201	0.008734	0.5953	0.579064	FALSE	FALSE	FALSE	ieu-b-508	8	18474596	0.033834	486484	0.796303	Sepsis (28	Sepsis (28	Sepsis (28	TRUE	igd	0.0043	2.35E-06	6642	M32758.m	TRUE	reported	AF0m66	2	TRUE	0.003279	21.84361
2228	rs1773930	A	G	A	G	-0.0215	-0.06198	0.845	0.803066	FALSE	FALSE	FALSE	ieu-b-508	2	1.27E+08	0.041834	486484	0.138432	Sepsis (28	Sepsis (28	Sepsis (28	TRUE	igd	0.0046	2.29E-06	6385	M19396.m	TRUE	reported	Xa1QqA	2	TRUE	0.00341	21.83862
5961	rs1892513	A	G	A	G	-0.0346	0.005572	0.5107	0.503285	FALSE	FALSE	FALSE	ieu-b-508	6	6979273	0.033578	486484	0.868194	Sepsis (28	Sepsis (28	Sepsis (28	TRUE	igd	0.0074	3.18E-06	1860	M33658.m	TRUE	reported	9QszGO	2	TRUE	0.011617	21.83844
3055	rs7859457	T	C	T	C	-0.0519	0.0353	0.3974	0.417204	FALSE	FALSE	FALSE	ieu-b-508	9	1.3E+08	0.033884	486484	0.297506	Sepsis (28	Sepsis (28	Sepsis (28	TRUE	igd	0.0111	2.93E-06	1760	M31591.m	TRUE	reported	KltFxx	2	TRUE	0.012269	21.8371
6911	rs332176	A	G	A	G	-0.0215	-0.09071	0.7408	0.743758	FALSE	FALSE	FALSE	ieu-b-508	10	28905079	0.038146	486484	0.017413	Sepsis (28	Sepsis (28	Sepsis (28	TRUE	igd	0.0046	2.52E-06	5195	M34389.m	TRUE	reported	suiRIN	2	TRUE	0.004187	21.83705
778	rs1235345	A	G	A	G	-0.0229	0.078571	0.0373	0.046925	FALSE	FALSE	FALSE	ieu-b-508	9	1.38E+08	0.078835	486484	0.318933	Sepsis (28	Sepsis (28	Sepsis (28	TRUE	igd	0.0049	3.12E-06	7352	M01336.m	TRUE	reported	FAlrya	2	TRUE	0.002962	21.83537
5092	rs9363575	T	C	T	C	-0.2507	0.042629	0.0374	0.074012	FALSE	FALSE	FALSE	ieu-b-508	6	67037367	0.069778	486484	0.541249	Sepsis (28	Sepsis (28	Sepsis (28	TRUE	igd	0.0536	2.85E-06	1017	M33178.m	TRUE	reported	g22WMS	2	TRUE	0.021058	21.83354
7982	rs4978408	A	G	A	G	-0.0243	-0.04359	0.066	0.069424	FALSE	FALSE	FALSE	ieu-b-508	9	1.13E+08	0.065483	486484	0.505585	Sepsis (28	Sepsis (28	Sepsis (28	TRUE	igd	0.0052	3.11E-06	7358	M35718.m	TRUE	reported	oUwc0a	2	TRUE	0.002959	21.83171
6122	rs1700632	A	G	A	G	-0.1619	0.041535	0.9636	0.962626	FALSE	FALSE	FALSE	ieu-b-508	3	69713930	0.088707	486484	0.63962	Sepsis (28	Sepsis (28	Sepsis (28	TRUE	igd	0.0345	2.62E-06	228	M33877.m	TRUE	reported	yDVi1f	2	TRUE	0.08808	21.82876
2986	rs1160305	A	T	A	T	0.1122	0.067269	0.0414	0.036855	FALSE	TRUE	FALSE	ieu-b-508	11	1.1E+08	0.089126	486484	0.450394	Sepsis (28	Sepsis (28	Sepsis (28	TRUE	igd	0.024	2.84E-06	1558	M31522.m	TRUE	reported	gM9gAj	2	TRUE	0.013834	21.82757
8124	rs4322806	C	G	C	G	0.0813	0.020096	0.037	0.039902	FALSE	TRUE	FALSE	ieu-b-508	2	1.85E+08	0.085271	486484	0.813688	Sepsis (28	Sepsis (28	Sepsis (28	TRUE	igd	0.0174	2.94E-06	7054	M36098.m	TRUE	reported	hs9SZl	2	TRUE	0.003085	21.82526
6460	rs2992439	A	G	A	G	-0.0271	0.013651	0.7289	0.724873	FALSE	FALSE	FALSE	ieu-b-508	1	39010828	0.038156	486484	0.72052	Sepsis (28	Sepsis (28	Sepsis (28	TRUE	igd	0.0058	3.01E-06	4961	M34106.m	TRUE	reported	8DuoNM	2	TRUE	0.004381	21.82265
6726	rs1314751	T	C	T	C	0.787	-0.07747	0.028	0.098721	FALSE	FALSE	FALSE	ieu-b-508	4	8645076	0.055973	486484	0.166327	Sepsis (28	Sepsis (28	Sepsis (28	TRUE	igd	0.167	2.45E-06	115	M34338.m	TRUE	reported	h4FB9a	2	TRUE	0.161859	21.82213
2082	rs884648	C	G	C	G	-0.0327	0.045218	0.4032	0.414189	FALSE	TRUE	FALSE	ieu-b-508	12	1.28E+08	0.034037	486484	0.184019	Sepsis (28	Sepsis (28	Sepsis (28	TRUE	igd	0.007	2.91E-06	7322	M18392.m	TRUE	reported	fBRMfe	2	TRUE	0.002972	21.81628
5599	rs1326231	A	G	A	G	0.0668	-0.08575	0.9831	0.976795	FALSE	FALSE	FALSE	ieu-b-508	8	61798817	0.111488	486484	0.441789	Sepsis (28	Sepsis (28	Sepsis (28	TRUE	igd	0.0143	3.03E-06	7365	M33441.m	TRUE	reported	CSdSpl	2	TRUE	0.002954	21.81539
2098	rs2068592	A	G	A	G	0.071	0.104532	0.0144	0.017564	FALSE	FALSE	FALSE	ieu-b-508	7	70856133	0.127388	486484	0.411888	Sepsis (28	Sepsis (28	Sepsis (28	TRUE	igd	0.0152	2.96E-06	7367	M18467.m	TRUE	reported	64PaNn	2	TRUE	0.002953	21.81281
2852	rs6024969	T	C	T	C	-0.3856	0.01462	0.9884	0.988969	FALSE	FALSE	FALSE	ieu-b-508	20	55155548	0.158769	486484	0.92663	Sepsis (28	Sepsis (28	Sepsis (28	TRUE	igd	0.0825	2.97E-06	1309	M27531.m	TRUE	reported	v01zDr	2	TRUE	0.016415	21.81233
611	rs2391088	A	G	A	G	-0.0341	-0.02885	0.6873	0.679926	FALSE	FALSE	FALSE	ieu-b-508	7	25183508	0.035751	486484	0.419724	Sepsis (28	Sepsis (28	Sepsis (28	TRUE	igd	0.0073	2.92E-06	4934	M01114.m	TRUE	reported	fqHdM2	2	TRUE	0.004403	21.81157
806	rs9543361	T	C	T	C	-0.0514	-0.04317	0.0422	0.043294	FALSE	FALSE	FALSE	ieu-b-508	13	73976863	0.082259	486484	0.599697	Sepsis (28	Sepsis (28	Sepsis (28	TRUE	igd	0.011	3.18E-06	1755	M01356.m	TRUE	reported	ah1g5W	2	TRUE	0.012288	21.8095
3057	rs807019	T	C	T	C	0.0425	-0.06382	0.1496	0.156286	FALSE	FALSE	FALSE	ieu-b-508	10	1.03E+08	0.045939	486484	0.164779	Sepsis (28	Sepsis (28	Sepsis (28	TRUE	igd	0.0091	3.18E-06	7338	M31591.m	TRUE	reported	KltFxx	2	TRUE	0.002964	21.80603
4470	rs2324913	T	C	T	C	-0.0411	0.0049	0.12	0.1009	FALSE	FALSE	FALSE	ieu-b-508	13	67074875	0.056664	486484	0.931085	Sepsis (28	Sepsis (28	Sepsis (28	TRUE	igd	0.0088	3.31E-06	5425	M33084.m	TRUE	reported	jrOsNK	2	TRUE	0.004005	21.8051
6008	rs1709163	A	G	A	G	0.173	-0.02968	0.027	0.031628	FALSE	FALSE	FALSE	ieu-b-508	14	95385621	0.09549	486484	0.755932	Sepsis (28	Sepsis (28	Sepsis (28	TRUE	igd	0.037	2.93E-06	752	M33751.m	TRUE	reported	Cxdkr4	2	TRUE	0.02825	21.8038
8192	rs2278161	T	C	T	C	-0.0341	-0.04287	0.7661	0.764711	FALSE	FALSE	FALSE	ieu-b-508	18	72176083	0.039327	486484	0.275616	Sepsis (28	Sepsis (28	Sepsis (28	TRUE	igd	0.0073	3.25E-06	2400	M36230.m	TRUE	reported	V5KJeR	2	TRUE	0.00901	21.80223
8295	rs1340847	A	G	A	G	0.0594	0.029035	0.1647	0.182001	FALSE	FALSE	FALSE	ieu-b-508	2	2.12E+08	0.043372	486484	0.503219	Sepsis (28	Sepsis (28	Sepsis (28	TRUE	igd	0.0127	2.98E-06	589	M36559.m	TRUE	reported	g6l9hg	2	TRUE	0.035811	21.80159
1129	rs808686	A	G	A	G	0.0598	-0.05523	0.6086	0.510496	FALSE	FALSE	FALSE	ieu-b-508	20	19717135	0.03345	486484	0.098714	Sepsis (28	Sepsis (28	Sepsis (28	TRUE	igd	0.0128	3.01E-06	1567	M01640.m	TRUE	reported	o3qxzl	2	TRUE	0.013737	21.79856
4408	rs2501628	A	C	A	C	0.0299	-0.00751	0.3167	0.309589	FALSE	FALSE	FALSE	ieu-b-508	6	92496405	0.036108	486484	0.835268	Sepsis (28	Sepsis (28	Sepsis (28	TRUE	igd	0.0064	2.85E-06	1550	M32869.m	TRUE	reported	Rvim1n	2	TRUE	0.013886	21.79825
4199	rs2592288	T	C	T	C	-0.092	0.153937	0.9423	0.954738	FALSE	FALSE	FALSE	ieu-b-508	12	1.18E+08	0.080867	486484	0.056964	Sepsis (28	Sepsis (28	Sepsis (28	TRUE	igd	0.0197	3.15E-06	2537	M32762.m	TRUE	reported	Q5lKLH	2	TRUE	0.008523	21.79218
5902	rs8036131	T	G	T	G	0.0608	-0.06533	0.7378	0.758441	FALSE	FALSE	FALSE	ieu-b-508	15	96785496	0.038984	486484	0.093761	Sepsis (28	Sepsis (28	Sepsis (28	TRUE	igd	0.013	3.15E-06	470	M33610.m	TRUE	reported	tmg43v	2	TRUE	0.04447	21.78053
6674	rs7036822	A	G	A	G	-0.0663	-0.03144	0.807	0.796753	FALSE	FALSE	FALSE	ieu-b-508	9	1450428	0.041636	486484	0.450125	Sepsis (28	Sepsis (28	Sepsis (28	TRUE	igd	0.0142	3.12E-06	1612	M34327.m	TRUE	reported	Il4Lrr	2	TRUE	0.013343	21.77265
596	rs4982495	T	C	T	C	-0.0084	0.014358	0.4275	0.426609	FALSE	FALSE	FALSE	ieu-b-508	14	22220569	0.033774	486484	0.670755	Sepsis (28	Sepsis (28	Sepsis (28	TRUE	igd	0.0018	3.27E-06	7367	M01110.m	TRUE	reported	0A31Sl	2	TRUE	0.002947	21.77187
8597	rs1193885	T	C	T	C	-0.0084	-0.04085	0.3657	0.368493	FALSE	FALSE	FALSE	ieu-b-508	4	64671240	0.034616	486484	0.237955	Sepsis (28	Sepsis (28	Sepsis (28	TRUE	igd	0.0018	1.59E-06	7366	M37506.m	TRUE	reported	m2kaRt	2	TRUE	0.002948	21.77186
1750	rs1266253	A	T	A	T	-0.0056	-0.0437	0.2919	0.301235	FALSE	TRUE	FALSE	ieu-b-508	6	33635407	0.036432	486484	0.230321	Sepsis (28	Sepsis (28	Sepsis (28	TRUE	igd	0.0012	6.66E-06	7365	M15650.m	TRUE	reported	NgezyT	2	TRUE	0.002948	21.77186
1756	rs6108469	A	G	A	G	0.0056	0.02742	0.7569	0.751776	FALSE	FALSE	FALSE	ieu-b-508	20	10304374	0.038976	486484	0.481744	Sepsis (28	Sepsis (28	Sepsis (28	TRUE	igd	0.0012	6.36E-06	7365	M15650.m	TRUE	reported	NgezyT	2	TRUE	0.002948	21.77186
3254	rs1188565	T	C	T	C	-0.0084	-0.04155	0.485	0.476291	FALSE	FALSE	FALSE	ieu-b-508	2	34200798	0.033444	486484	0.214049	Sepsis (28	Sepsis (28	Sepsis (28	TRUE	igd	0.0018	1.76E-06	7365	M32348.m	TRUE	reported	DJeLDx	2	TRUE	0.002948	21.77186
1006	rs1404373	T	C	T	C	-0.0084	0.045892	0.6525	0.648553	FALSE	FALSE	FALSE	ieu-b-508	4	58204024	0.034995	486484	0.189723	Sepsis (28	Sepsis (28	Sepsis (28	TRUE	igd	0.0018	2.92E-06	7364	M01564.m	TRUE	reported	IPUdyD	2	TRUE	0.002949	21.77186
3439	rs6560147	T	C	T	C	0.0126	0.011544	0.1449	0.139525	FALSE	FALSE	FALSE	ieu-b-508	9	73250899	0.048067	486484	0.810197	Sepsis (28	Sepsis (28	Sepsis (28	TRUE	igd	0.0027	4.20E-06	7364	M32452.m	TRUE	reported	8GtFpl	2	TRUE	0.002949	21.77186
3443	rs7587616	A	G	A	G	-0.0084	0.05625	0.4743	0.479246	FALSE	FALSE	FALSE	ieu-b-508	2	1.35E+08	0.033521	486484	0.093334	Sepsis (28	Sepsis (28	Sepsis (28	TRUE	igd	0.0018	4.71E-06	7364	M32452.m	TRUE	reported	8GtFpl	2	TRUE	0.002949	21.77186
3063	rs1049383	T	G	T	G	-0.0406	-0.02334	0.7644	0.764681	FALSE	FALSE	FALSE	ieu-b-508	1	90191203	0.039395	486484	0.553517	Sepsis (28	Sepsis (28	Sepsis (28	TRUE	igd	0.0087	3.20E-06	7363	M31787.m	TRUE	reported	XMp8YI	2	TRUE	0.002949	21.77186
3451	rs1023115	A	G	A	G	-0.0084	-0.00513	0.5301	0.53845	FALSE	FALSE	FALSE	ieu-b-508	1	1.61E+08	0.033505	486484	0.878344	Sepsis (28	Sepsis (28	Sepsis (28	TRUE	igd	0.0018	2.68E-06	7363	M32489.m	TRUE	reported	hnljaD	2	TRUE	0.002949	21.77186
3462	rs1360501	T	C	T	C	-0.0084	-0.01284	0.6909	0.682399	FALSE	FALSE	FALSE	ieu-b-508	9	2359459	0.035868	486484	0.720377	Sepsis (28	Sepsis (28	Sepsis (28	TRUE	igd	0.0018	3.49E-06	7363	M32489.m	TRUE	reported	hnljaD	2	TRUE	0.002949	21.77186
3754	rs1022407	T	C	T	C	-0.0084	-0.01072	0.2481	0.250547	FALSE	FALSE	FALSE	ieu-b-508	7	10318768	0.038702	486484	0.781757	Sepsis (28	Sepsis (28	Sepsis (28	TRUE	igd	0.0018	4.69E-06	7362	M32634.m	TRUE	reported	7T2RUz	2	TRUE	0.002949	21.77186
3763	rs1888108	A	G	A	G	0.0084	0.061623	0.6614	0.645644	FALSE	FALSE	FALSE	ieu-b-508	9	24572490	0.035022	486484	0.078487	Sepsis (28	Sepsis (28	Sepsis (28	TRUE	igd	0.0018	3.11E-06	7362	M32634.m	TRUE	reported	7T2RUz	2	TRUE	0.002949	21.77186
3785	rs928563	T	C	T	C	0.0084	0.035274	0.737	0.741224	FALSE	FALSE	FALSE	ieu-b-508	9	72065331	0.038041	486484	0.353797	Sepsis (28	Sepsis (28	Sepsis (28	TRUE	igd	0.0018	3.74E-06	7362	M32634.m	TRUE	reported	7T2RUz	2	TRUE	0.002949	21.77186
4500	rs1716881	T	G	T	G	-0.0084	0.064841	0.134	0.152734	FALSE	FALSE	FALSE	ieu-b-508	7	1.37E+08	0.046575	486484	0.163862	Sepsis (28	Sepsis (28	Sepsis (28	TRUE	igd	0.0018	3.68E-06	7362	M33132.m	TRUE	reported	dTXJa8	2	TRUE	0.002949	21.77186
6434	rs1926815	A	C	A	C	-0.0084	-0.0116	0.1804	0.220991	FALSE	FALSE	FALSE	ieu-b-508	10	4379965	0.040078	486484	0.772241	Sepsis (28	Sepsis (28	Sepsis (28	TRUE	igd	0.0018	1.85E-06	7362	M34062.m	TRUE	reported	RZyHKP	2	TRUE	0.002949	21.77186
450	rs1232423	T	C	T	C	0.0042	0.015932	0.5209	0.517075	FALSE	FALSE	FALSE	ieu-b-508	15	75806911	0.033354	486484	0.632881	Sepsis (28	Sepsis (28	Sepsis (28	TRUE	igd	9.00E-04	8.68E-06	7361	M00513.m	TRUE	reported	ohacnP	2	TRUE	0.00295	21.77186
3327	rs2324600	T	C	T	C	0.0266	0.116505	0.1179	0.119639	FALSE	FALSE	FALSE	ieu-b-508	13	40943836	0.051446	486484	0.023536	Sepsis (28	Sepsis (28	Sepsis (28	TRUE	igd	0.0057	3.47E-06	7358	M32405.m	TRUE	reported	uzomIQ	2	TRUE	0.002951	21.77186
17	rs1075041	T	G	T	G	0.0042	-0.00315	0.3959	0.417226	FALSE	FALSE	FALSE	ieu-b-508	11	99750593	0.033955	486484	0.926177	Sepsis (28	Sepsis (28	Sepsis (28	TRUE	igd	9.00E-04	2.77E-06	7355	M00054.m	TRUE	reported	QTDAnh	2	TRUE	0.002952	21.77186
21	rs1082205	T	C	T	C	0.0042	-0.04113	0.5141	0.530739	FALSE	FALSE	FALSE	ieu-b-508	10	64514111	0.033421	486484	0.218473	Sepsis (28	Sepsis (28	Sepsis (28	TRUE	igd	9.00E-04	2.65E-06	7355	M00054.m	TRUE	reported	QTDAnh	2	TRUE	0.002952	21.77186
27	rs1093104	T	C	T	C	-0.0042	0.018306	0.5756	0.575961	FALSE	FALSE	FALSE	ieu-b-508	2	1.84E+08	0.033974	486484	0.590005	Sepsis (28	Sepsis (28	Sepsis (28	TRUE	igd	9.00E-04	3.36E-06	7355	M00054.m	TRUE	reported	QTDAnh	2	TRUE	0.002952	21.77186
40	rs1260326	T	C	T	C	0.0042	-0.02078	0.404	0.395705	FALSE	FALSE	FALSE	ieu-b-508	2	27730940	0.034143	486484	0.542863	Sepsis (28	Sepsis (28	Sepsis (28	TRUE	igd	9.00E-04	2.74E-06	7355	M00054.m	TRUE	reported	QTDAnh	2	TRUE	0.002952	21.77186
42	rs1300809	A	C	A	C	-0.0042	0.076313	0.4038	0.411536	FALSE	FALSE	FALSE	ieu-b-508	2	62792689	0.034058	486484	0.025048	Sepsis (28	Sepsis (28	Sepsis (28	TRUE	igd	9.00E-04	3.32E-06	7355	M00054.m	TRUE	reported	QTDAnh	2	TRUE	0.002952	21.77186
45	rs1330918	A	C	A	C	0.0042	0.003137	0.6132	0.607339	FALSE	FALSE	FALSE	ieu-b-508	13	23373129	0.034143	486484	0.926797	Sepsis (28	Sepsis (28	Sepsis (28	TRUE	igd	9.00E-04	2.21E-06	7355	M00054.m	TRUE	reported	QTDAnh	2	TRUE	0.002952	21.77186
50	rs1501803	T	C	T	C	0.0042	-0.00128	0.4152	0.409709	FALSE	FALSE	FALSE	ieu-b-508	5	1.66E+08	0.035009	486484	0.970905	Sepsis (28	Sepsis (28	Sepsis (28	TRUE	igd	9.00E-04	4.57E-06	7355	M00054.m	TRUE	reported	QTDAnh	2	TRUE	0.002952	21.77186
51	rs1508513	C	G	C	G	0.0042	0.043877	0.6354	0.638855	FALSE	TRUE	FALSE	ieu-b-508	4	1.72E+08	0.034838	486484	0.207868	Sepsis (28	Sepsis (28	Sepsis (28	TRUE	igd	9.00E-04	2.36E-06	7355	M00054.m	TRUE	reported	QTDAnh	2	TRUE	0.002952	21.77186
72	rs2126498	T	G	T	G	-0.0042	-0.05085	0.6176	0.601139	FALSE	FALSE	FALSE	ieu-b-508	6	1.1E+08	0.03419	486484	0.136907	Sepsis (28	Sepsis (28	Sepsis (28	TRUE	igd	9.00E-04	2.84E-06	7355	M00054.m	TRUE	reported	QTDAnh	2	TRUE	0.002952	21.77186
73	rs2178260	T	C	T	C	0.0042	0.049667	0.4251	0.424139	FALSE	FALSE	FALSE	ieu-b-508	11	62069365	0.033836	486484	0.142141	Sepsis (28	Sepsis (28	Sepsis (28	TRUE	igd	9.00E-04	3.59E-06	7355	M00054.m	TRUE	reported	QTDAnh	2	TRUE	0.002952	21.77186
96	rs2966079	T	C	T	C	-0.0042	-0.00454	0.5425	0.549892	FALSE	FALSE	FALSE	ieu-b-508	16	81511806	0.03352	486484	0.892287	Sepsis (28	Sepsis (28	Sepsis (28	TRUE	igd	9.00E-04	2.53E-06	7355	M00054.m	TRUE	reported	QTDAnh	2	TRUE	0.002952	21.77186
107	rs3927971	T	C	T	C	0.0042	0.009615	0.5178	0.512394	FALSE	FALSE	FALSE	ieu-b-508	2	8776102	0.033471	486484	0.773915	Sepsis (28	Sepsis (28	Sepsis (28	TRUE	igd	9.00E-04	8.98E-06	7355	M00054.m	TRUE	reported	QTDAnh	2	TRUE	0.002952	21.77186
117	rs4684599	T	C	T	C	-0.0042	-0.03073	0.6319	0.628567	FALSE	FALSE	FALSE	ieu-b-508	3	8626572	0.034508	486484	0.373246	Sepsis (28	Sepsis (28	Sepsis (28	TRUE	igd	9.00E-04	2.89E-06	7355	M00054.m	TRUE	reported	QTDAnh	2	TRUE	0.002952	21.77186
130	rs575257	A	G	A	G	-0.0042	-0.00262	0.6005	0.605691	FALSE	FALSE	FALSE	ieu-b-508	11	78869070	0.034146	486484	0.93891	Sepsis (28	Sepsis (28	Sepsis (28	TRUE	igd	9.00E-04	3.51E-06	7355	M00054.m	TRUE	reported	QTDAnh	2	TRUE	0.002952	21.77186
140	rs6517254	T	C	T	C	-0.0042	0.000966	0.3971	0.397614	FALSE	FALSE	FALSE	ieu-b-508	21	36080290	0.034168	486484	0.977443	Sepsis (28	Sepsis (28	Sepsis (28	TRUE	igd	9.00E-04	3.12E-06	7355	M00054.m	TRUE	reported	QTDAnh	2	TRUE	0.002952	21.77186
144	rs6685230	T	C	T	C	-0.0042	-0.02058	0.3976	0.411012	FALSE	FALSE	FALSE	ieu-b-508	1	1.68E+08	0.035601	486484	0.56328	Sepsis (28	Sepsis (28	Sepsis (28	TRUE	igd	9.00E-04	7.78E-06	7355	M00054.m	TRUE	reported	QTDAnh	2	TRUE	0.002952	21.77186
151	rs688627	T	C	T	C	0.0042	-0.01572	0.5878	0.59415	FALSE	FALSE	FALSE	ieu-b-508	1	44048283	0.03396	486484	0.643366	Sepsis (28	Sepsis (28	Sepsis (28	TRUE	igd	9.00E-04	3.62E-06	7355	M00054.m	TRUE	reported	QTDAnh	2	TRUE	0.002952	21.77186
178	rs893922	A	G	A	G	0.0042	-0.01345	0.366	0.368748	FALSE	FALSE	FALSE	ieu-b-508	9	1.12E+08	0.034676	486484	0.698168	Sepsis (28	Sepsis (28	Sepsis (28	TRUE	igd	9.00E-04	3.95E-06	7355	M00054.m	TRUE	reported	QTDAnh	2	TRUE	0.002952	21.77186
180	rs928963	A	C	A	C	0.0042	0.013808	0.3985	0.399748	FALSE	FALSE	FALSE	ieu-b-508	16	13616231	0.034259	486484	0.686917	Sepsis (28	Sepsis (28	Sepsis (28	TRUE	igd	9.00E-04	3.62E-06	7355	M00054.m	TRUE	reported	QTDAnh	2	TRUE	0.002952	21.77186
189	rs9541214	T	C	T	C	-0.0042	0.017036	0.653	0.652646	FALSE	FALSE	FALSE	ieu-b-508	13	35112824	0.035132	486484	0.627742	Sepsis (28	Sepsis (28	Sepsis (28	TRUE	igd	9.00E-04	3.80E-06	7355	M00054.m	TRUE	reported	QTDAnh	2	TRUE	0.002952	21.77186
194	rs993927	T	G	T	G	0.0042	0.042155	0.3773	0.378752	FALSE	FALSE	FALSE	ieu-b-508	18	50101294	0.034382	486484	0.220168	Sepsis (28	Sepsis (28	Sepsis (28	TRUE	igd	9.00E-04	2.64E-06	7355	M00054.m	TRUE	reported	QTDAnh	2	TRUE	0.002952	21.77186
197	rs9989564	A	G	A	G	-0.0042	-0.04181	0.5142	0.535082	FALSE	FALSE	FALSE	ieu-b-508	18	41305359	0.033385	486484	0.210424	Sepsis (28	Sepsis (28	Sepsis (28	TRUE	igd	9.00E-04	3.10E-06	7355	M00054.m	TRUE	reported	QTDAnh	2	TRUE	0.002952	21.77186
808	rs1016016	A	G	A	G	0.0084	-0.10159	0.6329	0.605957	FALSE	FALSE	FALSE	ieu-b-508	10	91856824	0.034117	486484	0.002904	Sepsis (28	Sepsis (28	Sepsis (28	TRUE	igd	0.0018	3.78E-06	7355	M01358.m	TRUE	reported	kSPQgd	2	TRUE	0.002952	21.77186
1137	rs1074032	T	C	T	C	0.0084	0.029004	0.5935	0.590704	FALSE	FALSE	FALSE	ieu-b-508	10	71502192	0.033858	486484	0.391635	Sepsis (28	Sepsis (28	Sepsis (28	TRUE	igd	0.0018	4.10E-06	7355	M01644.m	TRUE	reported	IB0DTL	2	TRUE	0.002952	21.77186
3488	rs1076249	A	C	A	C	0.0084	0.010429	0.3563	0.356868	FALSE	FALSE	FALSE	ieu-b-508	10	73709990	0.034838	486484	0.76467	Sepsis (28	Sepsis (28	Sepsis (28	TRUE	igd	0.0018	2.25E-06	7355	M32492.m	TRUE	reported	rZZFcE	2	TRUE	0.002952	21.77186
3500	rs2414622	A	G	A	G	-0.0084	-0.05047	0.6434	0.643726	FALSE	FALSE	FALSE	ieu-b-508	15	59533696	0.034859	486484	0.14765	Sepsis (28	Sepsis (28	Sepsis (28	TRUE	igd	0.0018	2.93E-06	7355	M32492.m	TRUE	reported	rZZFcE	2	TRUE	0.002952	21.77186
3506	rs4858696	T	C	T	C	-0.0084	-0.00021	0.5733	0.562125	FALSE	FALSE	FALSE	ieu-b-508	3	25101270	0.033739	486484	0.99505	Sepsis (28	Sepsis (28	Sepsis (28	TRUE	igd	0.0018	2.15E-06	7355	M32492.m	TRUE	reported	rZZFcE	2	TRUE	0.002952	21.77186
3518	rs940597	T	C	T	C	-0.0084	-0.02091	0.3479	0.350514	FALSE	FALSE	FALSE	ieu-b-508	7	67775112	0.035169	486484	0.552086	Sepsis (28	Sepsis (28	Sepsis (28	TRUE	igd	0.0018	3.00E-06	7355	M32492.m	TRUE	reported	rZZFcE	2	TRUE	0.002952	21.77186
549	rs1254473	C	G	C	G	-0.0084	0.052611	0.8378	0.834536	FALSE	TRUE	FALSE	ieu-b-508	8	53929703	0.04499	486484	0.242244	Sepsis (28	Sepsis (28	Sepsis (28	TRUE	igd	0.0018	2.74E-06	7354	M00606.m	TRUE	reported	r5smf7	2	TRUE	0.002953	21.77186
907	rs1261178	T	C	T	C	-0.007	-0.01441	0.6267	0.646614	FALSE	FALSE	FALSE	ieu-b-508	2	31342175	0.034956	486484	0.680257	Sepsis (28	Sepsis (28	Sepsis (28	TRUE	igd	0.0015	5.51E-06	7354	M01494.m	TRUE	reported	7JWNKI	2	TRUE	0.002953	21.77186
914	rs306676	A	G	A	G	0.0112	-0.02148	0.1304	0.137508	FALSE	FALSE	FALSE	ieu-b-508	13	93606814	0.048889	486484	0.660356	Sepsis (28	Sepsis (28	Sepsis (28	TRUE	igd	0.0024	2.48E-06	7354	M01494.m	TRUE	reported	7JWNKI	2	TRUE	0.002953	21.77186
2059	rs1768345	A	T	A	T	0.0112	-0.03656	0.8525	0.855375	FALSE	TRUE	FALSE	ieu-b-508	4	28363171	0.0477	486484	0.443377	Sepsis (28	Sepsis (28	Sepsis (28	TRUE	igd	0.0024	4.14E-06	7354	M18369.m	TRUE	reported	ykBjIT	2	TRUE	0.002953	21.77186
6322	rs4459845	A	C	A	C	0.0084	-0.01065	0.8009	0.802465	FALSE	FALSE	FALSE	ieu-b-508	3	1.26E+08	0.041908	486484	0.799306	Sepsis (28	Sepsis (28	Sepsis (28	TRUE	igd	0.0018	5.08E-06	7354	M33955.m	TRUE	reported	mhHFnT	2	TRUE	0.002953	21.77186
1959	rs6738713	T	C	T	C	-0.0308	-0.01679	0.9295	0.923169	FALSE	FALSE	FALSE	ieu-b-508	2	49727334	0.062858	486484	0.789405	Sepsis (28	Sepsis (28	Sepsis (28	TRUE	igd	0.0066	3.38E-06	7353	M17805.m	TRUE	reported	ZDRoA2	2	TRUE	0.002953	21.77185
212	rs1050090	A	G	A	G	0.0042	-0.01103	0.3597	0.362908	FALSE	FALSE	FALSE	ieu-b-508	11	21415127	0.034696	486484	0.750478	Sepsis (28	Sepsis (28	Sepsis (28	TRUE	igd	9.00E-04	3.55E-06	7351	M00060.m	TRUE	reported	kqwl4V	2	TRUE	0.002954	21.77185
214	rs1076384	A	G	A	G	-0.0042	0.018449	0.4283	0.422174	FALSE	FALSE	FALSE	ieu-b-508	10	31158591	0.03372	486484	0.584297	Sepsis (28	Sepsis (28	Sepsis (28	TRUE	igd	9.00E-04	3.45E-06	7351	M00060.m	TRUE	reported	kqwl4V	2	TRUE	0.002954	21.77185
216	rs1079858	A	G	A	G	-0.0042	-0.05619	0.4216	0.422428	FALSE	FALSE	FALSE	ieu-b-508	1	1.78E+08	0.034013	486484	0.09856	Sepsis (28	Sepsis (28	Sepsis (28	TRUE	igd	9.00E-04	3.64E-06	7351	M00060.m	TRUE	reported	kqwl4V	2	TRUE	0.002954	21.77185
228	rs1114451	T	C	T	C	-0.0042	0.035049	0.4497	0.464968	FALSE	FALSE	FALSE	ieu-b-508	9	78218231	0.035714	486484	0.326398	Sepsis (28	Sepsis (28	Sepsis (28	TRUE	igd	9.00E-04	8.42E-06	7351	M00060.m	TRUE	reported	kqwl4V	2	TRUE	0.002954	21.77185
233	rs1186894	A	G	A	G	-0.0042	-0.00885	0.6435	0.638807	FALSE	FALSE	FALSE	ieu-b-508	17	10214042	0.034789	486484	0.79928	Sepsis (28	Sepsis (28	Sepsis (28	TRUE	igd	9.00E-04	4.63E-06	7351	M00060.m	TRUE	reported	kqwl4V	2	TRUE	0.002954	21.77185
240	rs1249870	A	G	A	G	-0.0042	0.008644	0.5998	0.586073	FALSE	FALSE	FALSE	ieu-b-508	4	1.56E+08	0.034077	486484	0.799749	Sepsis (28	Sepsis (28	Sepsis (28	TRUE	igd	9.00E-04	2.86E-06	7351	M00060.m	TRUE	reported	kqwl4V	2	TRUE	0.002954	21.77185
243	rs1278312	T	C	T	C	-0.0042	-0.03586	0.4008	0.409264	FALSE	FALSE	FALSE	ieu-b-508	10	1.28E+08	0.033959	486484	0.291005	Sepsis (28	Sepsis (28	Sepsis (28	TRUE	igd	9.00E-04	2.87E-06	7351	M00060.m	TRUE	reported	kqwl4V	2	TRUE	0.002954	21.77185
249	rs1360820	A	G	A	G	-0.0042	-0.03206	0.4963	0.511484	FALSE	FALSE	FALSE	ieu-b-508	13	93554207	0.033487	486484	0.338348	Sepsis (28	Sepsis (28	Sepsis (28	TRUE	igd	9.00E-04	2.15E-06	7351	M00060.m	TRUE	reported	kqwl4V	2	TRUE	0.002954	21.77185
264	rs174476	T	C	T	C	-0.0042	-0.04968	0.4174	0.419611	FALSE	FALSE	FALSE	ieu-b-508	11	61674118	0.03376	486484	0.141162	Sepsis (28	Sepsis (28	Sepsis (28	TRUE	igd	9.00E-04	5.70E-06	7351	M00060.m	TRUE	reported	kqwl4V	2	TRUE	0.002954	21.77185
280	rs2302423	A	G	A	G	0.0042	0.002993	0.55	0.549172	FALSE	FALSE	FALSE	ieu-b-508	11	22868937	0.033519	486484	0.928841	Sepsis (28	Sepsis (28	Sepsis (28	TRUE	igd	9.00E-04	2.82E-06	7351	M00060.m	TRUE	reported	kqwl4V	2	TRUE	0.002954	21.77185
281	rs2357713	T	G	T	G	-0.0042	-0.03439	0.447	0.449775	FALSE	FALSE	FALSE	ieu-b-508	7	13657472	0.033862	486484	0.30978	Sepsis (28	Sepsis (28	Sepsis (28	TRUE	igd	9.00E-04	3.00E-06	7351	M00060.m	TRUE	reported	kqwl4V	2	TRUE	0.002954	21.77185
298	rs2933284	T	C	T	C	0.0042	0.094341	0.3898	0.390073	FALSE	FALSE	FALSE	ieu-b-508	3	11021945	0.03452	486484	0.006278	Sepsis (28	Sepsis (28	Sepsis (28	TRUE	igd	9.00E-04	2.86E-06	7351	M00060.m	TRUE	reported	kqwl4V	2	TRUE	0.002954	21.77185
302	rs3802644	A	T	A	T	-0.0042	-0.00383	0.4006	0.407724	FALSE	TRUE	FALSE	ieu-b-508	10	80782924	0.033918	486484	0.909983	Sepsis (28	Sepsis (28	Sepsis (28	TRUE	igd	9.00E-04	2.94E-06	7351	M00060.m	TRUE	reported	kqwl4V	2	TRUE	0.002954	21.77185
313	rs4681618	A	G	A	G	-0.0042	-0.00516	0.5793	0.581743	FALSE	FALSE	FALSE	ieu-b-508	3	1.5E+08	0.033842	486484	0.878851	Sepsis (28	Sepsis (28	Sepsis (28	TRUE	igd	9.00E-04	3.15E-06	7351	M00060.m	TRUE	reported	kqwl4V	2	TRUE	0.002954	21.77185
314	rs4726308	T	G	T	G	-0.0042	-0.02971	0.3944	0.392993	FALSE	FALSE	FALSE	ieu-b-508	7	1.53E+08	0.034119	486484	0.3838	Sepsis (28	Sepsis (28	Sepsis (28	TRUE	igd	9.00E-04	3.35E-06	7351	M00060.m	TRUE	reported	kqwl4V	2	TRUE	0.002954	21.77185
321	rs4875397	A	G	A	G	-0.0042	0.014227	0.5599	0.564212	FALSE	FALSE	FALSE	ieu-b-508	8	4700011	0.033724	486484	0.673119	Sepsis (28	Sepsis (28	Sepsis (28	TRUE	igd	9.00E-04	3.04E-06	7351	M00060.m	TRUE	reported	kqwl4V	2	TRUE	0.002954	21.77185
324	rs4921412	C	G	C	G	-0.0042	0.025445	0.3864	0.390391	FALSE	TRUE	FALSE	ieu-b-508	5	1.61E+08	0.03414	486484	0.456094	Sepsis (28	Sepsis (28	Sepsis (28	TRUE	igd	9.00E-04	2.78E-06	7351	M00060.m	TRUE	reported	kqwl4V	2	TRUE	0.002954	21.77185
347	rs6982408	T	G	T	G	-0.0042	0.03236	0.4435	0.440361	FALSE	FALSE	FALSE	ieu-b-508	8	60673486	0.0336	486484	0.335494	Sepsis (28	Sepsis (28	Sepsis (28	TRUE	igd	9.00E-04	2.32E-06	7351	M00060.m	TRUE	reported	kqwl4V	2	TRUE	0.002954	21.77185
353	rs7329192	T	C	T	C	-0.0042	0.02449	0.5277	0.520854	FALSE	FALSE	FALSE	ieu-b-508	13	85068774	0.033434	486484	0.463875	Sepsis (28	Sepsis (28	Sepsis (28	TRUE	igd	9.00E-04	3.17E-06	7351	M00060.m	TRUE	reported	kqwl4V	2	TRUE	0.002954	21.77185
356	rs757378	A	T	A	T	-0.0042	0.032895	0.6095	0.608136	FALSE	TRUE	FALSE	ieu-b-508	17	29722619	0.034273	486484	0.337169	Sepsis (28	Sepsis (28	Sepsis (28	TRUE	igd	9.00E-04	3.57E-06	7351	M00060.m	TRUE	reported	kqwl4V	2	TRUE	0.002954	21.77185
359	rs759456	T	G	T	G	0.0042	-0.03512	0.6263	0.636335	FALSE	FALSE	FALSE	ieu-b-508	2	65194302	0.03486	486484	0.313735	Sepsis (28	Sepsis (28	Sepsis (28	TRUE	igd	9.00E-04	3.73E-06	7351	M00060.m	TRUE	reported	kqwl4V	2	TRUE	0.002954	21.77185
361	rs7625991	A	C	A	C	-0.0042	-0.06877	0.345	0.356789	FALSE	FALSE	FALSE	ieu-b-508	3	44136424	0.034988	486484	0.049338	Sepsis (28	Sepsis (28	Sepsis (28	TRUE	igd	9.00E-04	5.90E-06	7351	M00060.m	TRUE	reported	kqwl4V	2	TRUE	0.002954	21.77185
377	rs8003702	T	G	T	G	0.0042	-0.00158	0.536	0.536253	FALSE	FALSE	FALSE	ieu-b-508	14	25787602	0.033524	486484	0.962525	Sepsis (28	Sepsis (28	Sepsis (28	TRUE	igd	9.00E-04	2.45E-06	7351	M00060.m	TRUE	reported	kqwl4V	2	TRUE	0.002954	21.77185
378	rs8012205	A	G	A	G	-0.0042	0.000537	0.4966	0.491627	FALSE	FALSE	FALSE	ieu-b-508	14	25426899	0.033455	486484	0.987184	Sepsis (28	Sepsis (28	Sepsis (28	TRUE	igd	9.00E-04	2.56E-06	7351	M00060.m	TRUE	reported	kqwl4V	2	TRUE	0.002954	21.77185
382	rs849327	A	G	A	G	0.0042	-0.04968	0.3492	0.342693	FALSE	FALSE	FALSE	ieu-b-508	7	28232457	0.035154	486484	0.157593	Sepsis (28	Sepsis (28	Sepsis (28	TRUE	igd	9.00E-04	3.45E-06	7351	M00060.m	TRUE	reported	kqwl4V	2	TRUE	0.002954	21.77185
384	rs899273	T	C	T	C	0.0042	-0.07019	0.5202	0.523539	FALSE	FALSE	FALSE	ieu-b-508	4	1.9E+08	0.033477	486484	0.036032	Sepsis (28	Sepsis (28	Sepsis (28	TRUE	igd	9.00E-04	3.33E-06	7351	M00060.m	TRUE	reported	kqwl4V	2	TRUE	0.002954	21.77185
3559	rs1188542	A	T	A	T	0.0084	0.058763	0.2326	0.245114	FALSE	TRUE	FALSE	ieu-b-508	2	30590548	0.038897	486484	0.130851	Sepsis (28	Sepsis (28	Sepsis (28	TRUE	igd	0.0018	4.34E-06	7350	M32518.m	TRUE	reported	V0ZCiU	2	TRUE	0.002954	21.77185
3562	rs1342020	A	C	A	C	-0.0084	-0.03108	0.7652	0.744007	FALSE	FALSE	FALSE	ieu-b-508	2	28594035	0.038487	486484	0.419371	Sepsis (28	Sepsis (28	Sepsis (28	TRUE	igd	0.0018	5.55E-06	7350	M32518.m	TRUE	reported	V0ZCiU	2	TRUE	0.002954	21.77185
3575	rs345513	A	G	A	G	-0.0084	0.002261	0.6132	0.613463	FALSE	FALSE	FALSE	ieu-b-508	4	79240063	0.03419	486484	0.94728	Sepsis (28	Sepsis (28	Sepsis (28	TRUE	igd	0.0018	2.86E-06	7350	M32518.m	TRUE	reported	V0ZCiU	2	TRUE	0.002954	21.77185
3580	rs4724070	T	C	T	C	0.0084	-0.08987	0.2039	0.19916	FALSE	FALSE	FALSE	ieu-b-508	7	41783359	0.041707	486484	0.031171	Sepsis (28	Sepsis (28	Sepsis (28	TRUE	igd	0.0018	4.96E-06	7350	M32518.m	TRUE	reported	V0ZCiU	2	TRUE	0.002954	21.77185
3591	rs906771	C	G	C	G	-0.0084	0.038798	0.3769	0.404024	FALSE	TRUE	FALSE	ieu-b-508	9	1053497	0.034126	486484	0.255572	Sepsis (28	Sepsis (28	Sepsis (28	TRUE	igd	0.0018	2.90E-06	7350	M32518.m	TRUE	reported	V0ZCiU	2	TRUE	0.002954	21.77185
7933	rs4482740	A	G	A	G	0.0084	-0.1011	0.3507	0.349014	FALSE	FALSE	FALSE	ieu-b-508	4	1.55E+08	0.035325	486484	0.00421	Sepsis (28	Sepsis (28	Sepsis (28	TRUE	igd	0.0018	4.53E-06	7348	M35675.m	TRUE	reported	D7fdyZ	2	TRUE	0.002955	21.77185
1169	rs1158985	T	C	T	C	-0.0084	0.01047	0.3809	0.377178	FALSE	FALSE	FALSE	ieu-b-508	1	1.55E+08	0.034623	486484	0.762355	Sepsis (28	Sepsis (28	Sepsis (28	TRUE	igd	0.0018	2.22E-06	7346	M01645.m	TRUE	reported	d5nAn3	2	TRUE	0.002956	21.77185
1186	rs287276	A	G	A	G	0.0084	0.096908	0.3317	0.344404	FALSE	FALSE	FALSE	ieu-b-508	5	55726631	0.0352	486484	0.005905	Sepsis (28	Sepsis (28	Sepsis (28	TRUE	igd	0.0018	2.65E-06	7346	M01645.m	TRUE	reported	d5nAn3	2	TRUE	0.002956	21.77185
1197	rs696979	T	C	T	C	0.0084	0.018178	0.6026	0.581469	FALSE	FALSE	FALSE	ieu-b-508	1	2.09E+08	0.033858	486484	0.59134	Sepsis (28	Sepsis (28	Sepsis (28	TRUE	igd	0.0018	2.05E-06	7346	M01645.m	TRUE	reported	d5nAn3	2	TRUE	0.002956	21.77185
518	rs1311647	A	G	A	G	-0.0448	0.100983	0.9853	0.983076	FALSE	FALSE	FALSE	ieu-b-508	4	39184712	0.131277	486484	0.44175	Sepsis (28	Sepsis (28	Sepsis (28	TRUE	igd	0.0096	2.93E-06	7345	M00584.m	TRUE	reported	oBt02X	2	TRUE	0.002956	21.77185
3205	rs1289671	A	G	A	G	-0.0084	0.003096	0.6005	0.594908	FALSE	FALSE	FALSE	ieu-b-508	1	1.18E+08	0.034055	486484	0.927555	Sepsis (28	Sepsis (28	Sepsis (28	TRUE	igd	0.0018	1.56E-06	7344	M32339.m	TRUE	reported	SVp504	2	TRUE	0.002957	21.77185
3224	rs7191435	T	C	T	C	0.0084	0.019762	0.6525	0.650007	FALSE	FALSE	FALSE	ieu-b-508	16	12363035	0.035116	486484	0.573601	Sepsis (28	Sepsis (28	Sepsis (28	TRUE	igd	0.0018	2.53E-06	7344	M32339.m	TRUE	reported	SVp504	2	TRUE	0.002957	21.77185
3225	rs7712111	A	G	A	G	0.0084	0.031423	0.6383	0.639604	FALSE	FALSE	FALSE	ieu-b-508	5	1.69E+08	0.034888	486484	0.36776	Sepsis (28	Sepsis (28	Sepsis (28	TRUE	igd	0.0018	1.63E-06	7344	M32339.m	TRUE	reported	SVp504	2	TRUE	0.002957	21.77185
4424	rs1480792	A	G	A	G	0.0084	0.006674	0.3268	0.327543	FALSE	FALSE	FALSE	ieu-b-508	8	1.36E+08	0.035635	486484	0.851441	Sepsis (28	Sepsis (28	Sepsis (28	TRUE	igd	0.0018	1.84E-06	7344	M32910.m	TRUE	reported	2OCBKh	2	TRUE	0.002957	21.77185
6976	rs2852371	A	G	A	G	0.0126	-0.04666	0.5831	0.583634	FALSE	FALSE	FALSE	ieu-b-508	11	1.06E+08	0.033828	486484	0.167767	Sepsis (28	Sepsis (28	Sepsis (28	TRUE	igd	0.0027	3.97E-06	7344	M34407.m	TRUE	reported	X7I83Y	2	TRUE	0.002957	21.77185
6978	rs383911	A	G	A	G	-0.0126	0.061342	0.5551	0.555345	FALSE	FALSE	FALSE	ieu-b-508	3	41122345	0.033478	486484	0.066902	Sepsis (28	Sepsis (28	Sepsis (28	TRUE	igd	0.0027	2.98E-06	7344	M34407.m	TRUE	reported	X7I83Y	2	TRUE	0.002957	21.77185
2699	rs1746357	A	G	A	G	0.0084	0.036905	0.4831	0.482094	FALSE	FALSE	FALSE	ieu-b-508	4	44887793	0.033518	486484	0.27088	Sepsis (28	Sepsis (28	Sepsis (28	TRUE	igd	0.0018	3.37E-06	7340	M25459.m	TRUE	reported	ACJY35	2	TRUE	0.002958	21.77184
3725	rs1088231	A	G	A	G	-0.0126	-0.0022	0.5268	0.515508	FALSE	FALSE	FALSE	ieu-b-508	10	95458897	0.033323	486484	0.947268	Sepsis (28	Sepsis (28	Sepsis (28	TRUE	igd	0.0027	2.77E-06	7337	M32632.m	TRUE	reported	pIHzPT	2	TRUE	0.002959	21.77184
2631	rs957671	A	G	A	G	0.0168	0.057038	0.9045	0.907312	FALSE	FALSE	FALSE	ieu-b-508	20	41443492	0.05819	486484	0.326984	Sepsis (28	Sepsis (28	Sepsis (28	TRUE	igd	0.0036	3.02E-06	7329	M22481.m	TRUE	reported	bXpf0k	2	TRUE	0.002963	21.77183
2324	rs7632086	C	G	C	G	-0.0126	0.059526	0.8243	0.812491	FALSE	TRUE	FALSE	ieu-b-508	3	1.77E+08	0.042889	486484	0.16517	Sepsis (28	Sepsis (28	Sepsis (28	TRUE	igd	0.0027	2.47E-06	7327	M20699.m	TRUE	reported	Z3EKFa	2	TRUE	0.002963	21.77183
1300	rs554710	T	C	T	C	-0.0084	0.033066	0.7074	0.709528	FALSE	FALSE	FALSE	ieu-b-508	9	1.36E+08	0.036659	486484	0.367058	Sepsis (28	Sepsis (28	Sepsis (28	TRUE	igd	0.0018	1.59E-06	7325	M02132.m	TRUE	reported	uJFm2t	2	TRUE	0.002964	21.77183
4553	rs6948511	T	C	T	C	-0.028	0.034621	0.9206	0.914988	FALSE	FALSE	FALSE	ieu-b-508	7	27978715	0.060183	486484	0.565118	Sepsis (28	Sepsis (28	Sepsis (28	TRUE	igd	0.006	3.30E-06	7319	M33140.m	TRUE	reported	eBOUtb	2	TRUE	0.002967	21.77183
4051	rs1309883	A	G	A	G	-0.0084	-0.02366	0.7208	0.721739	FALSE	FALSE	FALSE	ieu-b-508	3	20428568	0.037251	486484	0.525399	Sepsis (28	Sepsis (28	Sepsis (28	TRUE	igd	0.0018	1.93E-06	7318	M32740.m	TRUE	reported	YLshfP	2	TRUE	0.002967	21.77183
4068	rs4981021	T	C	T	C	-0.0084	-0.0499	0.282	0.273712	FALSE	FALSE	FALSE	ieu-b-508	12	1.04E+08	0.037586	486484	0.184281	Sepsis (28	Sepsis (28	Sepsis (28	TRUE	igd	0.0018	1.92E-06	7318	M32740.m	TRUE	reported	YLshfP	2	TRUE	0.002967	21.77183
4080	rs7560697	T	C	T	C	0.0084	0.020592	0.2811	0.270952	FALSE	FALSE	FALSE	ieu-b-508	2	1.87E+08	0.037712	486484	0.585046	Sepsis (28	Sepsis (28	Sepsis (28	TRUE	igd	0.0018	1.79E-06	7318	M32740.m	TRUE	reported	YLshfP	2	TRUE	0.002967	21.77183
7865	rs2305779	A	G	A	G	0.0196	-0.05637	0.1237	0.123644	FALSE	FALSE	FALSE	ieu-b-508	19	14045033	0.051164	486484	0.270601	Sepsis (28	Sepsis (28	Sepsis (28	TRUE	igd	0.0042	2.79E-06	7317	M35631.m	TRUE	reported	002wka	2	TRUE	0.002967	21.77183
5394	rs1337483	T	C	T	C	0.0168	0.009261	0.9021	0.898947	FALSE	FALSE	FALSE	ieu-b-508	1	22140348	0.056011	486484	0.86867	Sepsis (28	Sepsis (28	Sepsis (28	TRUE	igd	0.0036	3.30E-06	7315	M33389.m	TRUE	reported	Hf6XVM	2	TRUE	0.002968	21.77182
1582	rs2422370	A	G	A	G	-0.0084	-0.01407	0.8094	0.821362	FALSE	FALSE	FALSE	ieu-b-508	11	36868287	0.047656	486484	0.767741	Sepsis (28	Sepsis (28	Sepsis (28	TRUE	igd	0.0018	5.96E-06	7314	M12593.m	TRUE	reported	IBXgSU	2	TRUE	0.002969	21.77182
1704	rs1019407	A	C	A	C	-0.007	0.002806	0.7918	0.790362	FALSE	FALSE	FALSE	ieu-b-508	2	38117987	0.040947	486484	0.94536	Sepsis (28	Sepsis (28	Sepsis (28	TRUE	igd	0.0015	6.33E-06	7310	M15506.m	TRUE	reported	mVRmZO	2	TRUE	0.00297	21.77182
1713	rs2080224	A	G	A	G	-0.0112	-0.01041	0.9304	0.925914	FALSE	FALSE	FALSE	ieu-b-508	7	19528604	0.064023	486484	0.870787	Sepsis (28	Sepsis (28	Sepsis (28	TRUE	igd	0.0024	1.97E-06	7310	M15506.m	TRUE	reported	mVRmZO	2	TRUE	0.00297	21.77182
1862	rs9314172	C	G	C	G	-0.0084	-0.01052	0.6969	0.685175	FALSE	TRUE	FALSE	ieu-b-508	5	92107935	0.036359	486484	0.772259	Sepsis (28	Sepsis (28	Sepsis (28	TRUE	igd	0.0018	4.50E-06	7308	M15778.m	TRUE	reported	941o3l	2	TRUE	0.002971	21.77182
5682	rs2487521	T	C	T	C	0.0084	0.015911	0.6858	0.680754	FALSE	FALSE	FALSE	ieu-b-508	1	2.24E+08	0.035694	486484	0.655781	Sepsis (28	Sepsis (28	Sepsis (28	TRUE	igd	0.0018	3.54E-06	7307	M33477.m	TRUE	reported	Aw8PBK	2	TRUE	0.002972	21.77182
5689	rs476093	A	C	A	C	-0.0084	-0.05894	0.7566	0.751256	FALSE	FALSE	FALSE	ieu-b-508	1	1.82E+08	0.03857	486484	0.12647	Sepsis (28	Sepsis (28	Sepsis (28	TRUE	igd	0.0018	4.98E-06	7307	M33477.m	TRUE	reported	Aw8PBK	2	TRUE	0.002972	21.77182
5705	rs9888676	A	G	A	G	0.0084	-0.04904	0.2454	0.249815	FALSE	FALSE	FALSE	ieu-b-508	15	56116501	0.038698	486484	0.205115	Sepsis (28	Sepsis (28	Sepsis (28	TRUE	igd	0.0018	5.44E-06	7307	M33477.m	TRUE	reported	Aw8PBK	2	TRUE	0.002972	21.77182
5517	rs1669715	T	C	T	C	-0.0126	0.042947	0.0915	0.090276	FALSE	FALSE	FALSE	ieu-b-508	8	593641	0.058065	486484	0.459522	Sepsis (28	Sepsis (28	Sepsis (28	TRUE	igd	0.0027	2.30E-06	7306	M33422.m	TRUE	reported	PLgjrT	2	TRUE	0.002972	21.77182
2282	rs1472578	A	T	A	T	-0.0126	-0.05124	0.6894	0.687349	FALSE	TRUE	FALSE	ieu-b-508	3	1.59E+08	0.036004	486484	0.154709	Sepsis (28	Sepsis (28	Sepsis (28	TRUE	igd	0.0027	2.44E-06	7301	M20675.m	TRUE	reported	CXe9rE	2	TRUE	0.002974	21.77181
2290	rs4671111	T	C	T	C	-0.0126	0.001456	0.5592	0.546257	FALSE	FALSE	FALSE	ieu-b-508	2	65130811	0.033751	486484	0.965589	Sepsis (28	Sepsis (28	Sepsis (28	TRUE	igd	0.0027	2.00E-06	7301	M20675.m	TRUE	reported	CXe9rE	2	TRUE	0.002974	21.77181
1059	rs1098835	C	G	C	G	0.0084	0.124866	0.1244	0.134187	FALSE	TRUE	FALSE	ieu-b-508	9	1.32E+08	0.049743	486484	0.012065	Sepsis (28	Sepsis (28	Sepsis (28	TRUE	igd	0.0018	3.09E-06	7271	M01585.m	TRUE	reported	M7QiSM	2	TRUE	0.002986	21.77179
7404	rs360020	A	G	A	G	0.0196	0.078124	0.6799	0.699029	FALSE	FALSE	FALSE	ieu-b-508	5	1.73E+08	0.036554	486484	0.032578	Sepsis (28	Sepsis (28	Sepsis (28	TRUE	igd	0.0042	2.48E-06	7263	M35160.m	TRUE	reported	eRox3e	2	TRUE	0.002989	21.77178
1601	rs7844733	T	C	T	C	-0.0126	-0.0145	0.1881	0.18937	FALSE	FALSE	FALSE	ieu-b-508	8	3540955	0.042611	486484	0.733699	Sepsis (28	Sepsis (28	Sepsis (28	TRUE	igd	0.0027	2.68E-06	7258	M12626.m	TRUE	reported	17lClc	2	TRUE	0.002992	21.77178
3801	rs1075833	T	C	T	C	-0.0084	0.019364	0.6855	0.669378	FALSE	FALSE	FALSE	ieu-b-508	9	35966277	0.035533	486484	0.585794	Sepsis (28	Sepsis (28	Sepsis (28	TRUE	igd	0.0018	4.46E-06	7229	M32644.m	TRUE	reported	JAa2WD	2	TRUE	0.003004	21.77175
3812	rs1313204	T	C	T	C	-0.0084	0.104081	0.2928	0.300308	FALSE	FALSE	FALSE	ieu-b-508	4	1.42E+08	0.036431	486484	0.004277	Sepsis (28	Sepsis (28	Sepsis (28	TRUE	igd	0.0018	4.58E-06	7229	M32644.m	TRUE	reported	JAa2WD	2	TRUE	0.003004	21.77175
3814	rs2657761	T	C	T	C	-0.0084	0.019802	0.7123	0.704297	FALSE	FALSE	FALSE	ieu-b-508	18	69512950	0.036738	486484	0.589883	Sepsis (28	Sepsis (28	Sepsis (28	TRUE	igd	0.0018	4.99E-06	7229	M32644.m	TRUE	reported	JAa2WD	2	TRUE	0.003004	21.77175
3829	rs769108	T	C	T	C	-0.0084	0.00162	0.6089	0.668052	FALSE	FALSE	FALSE	ieu-b-508	6	25257966	0.035801	486484	0.963904	Sepsis (28	Sepsis (28	Sepsis (28	TRUE	igd	0.0018	3.73E-06	7229	M32644.m	TRUE	reported	JAa2WD	2	TRUE	0.003004	21.77175
1353	rs1704186	T	C	T	C	0.0196	-0.05617	0.939	0.935871	FALSE	FALSE	FALSE	ieu-b-508	2	1.12E+08	0.068136	486484	0.409744	Sepsis (28	Sepsis (28	Sepsis (28	TRUE	igd	0.0042	4.02E-06	7226	M02730.m	TRUE	reported	XYa035	2	TRUE	0.003005	21.77175
7554	rs1182282	A	G	A	G	0.0084	0.0092	0.7848	0.786894	FALSE	FALSE	FALSE	ieu-b-508	11	1.23E+08	0.04078	486484	0.821521	Sepsis (28	Sepsis (28	Sepsis (28	TRUE	igd	0.0018	4.30E-06	7217	M35270.m	TRUE	reported	Ct8V1n	2	TRUE	0.003008	21.77174
7563	rs1883261	A	G	A	G	0.0084	-0.02459	0.4801	0.471898	FALSE	FALSE	FALSE	ieu-b-508	6	25601365	0.033418	486484	0.461877	Sepsis (28	Sepsis (28	Sepsis (28	TRUE	igd	0.0018	1.92E-06	7217	M35270.m	TRUE	reported	Ct8V1n	2	TRUE	0.003008	21.77174
7577	rs6559771	C	G	C	G	0.0084	0.00378	0.775	0.773097	FALSE	TRUE	FALSE	ieu-b-508	9	86792561	0.039897	486484	0.924522	Sepsis (28	Sepsis (28	Sepsis (28	TRUE	igd	0.0018	4.92E-06	7217	M35270.m	TRUE	reported	Ct8V1n	2	TRUE	0.003008	21.77174
7582	rs7670227	A	C	A	C	-0.0084	-0.05164	0.2569	0.254113	FALSE	FALSE	FALSE	ieu-b-508	4	44904253	0.038519	486484	0.180013	Sepsis (28	Sepsis (28	Sepsis (28	TRUE	igd	0.0018	3.66E-06	7217	M35270.m	TRUE	reported	Ct8V1n	2	TRUE	0.003008	21.77174
2357	rs791630	A	G	A	G	-0.0112	-0.07656	0.1945	0.189962	FALSE	FALSE	FALSE	ieu-b-508	5	1.73E+08	0.042639	486484	0.072577	Sepsis (28	Sepsis (28	Sepsis (28	TRUE	igd	0.0024	2.80E-06	7203	M21047.m	TRUE	reported	ISF1Cr	2	TRUE	0.003014	21.77173
4541	rs3779259	T	G	T	G	0.0308	-0.09508	0.0881	0.0895	FALSE	FALSE	FALSE	ieu-b-508	7	1.23E+08	0.058479	486484	0.103992	Sepsis (28	Sepsis (28	Sepsis (28	TRUE	igd	0.0066	2.98E-06	7166	M33138.m	TRUE	reported	IUpLAl	2	TRUE	0.00303	21.7717
935	rs2967437	A	G	A	G	-0.0126	-0.04177	0.6902	0.686352	FALSE	FALSE	FALSE	ieu-b-508	19	37086645	0.035924	486484	0.244962	Sepsis (28	Sepsis (28	Sepsis (28	TRUE	igd	0.0027	1.88E-06	7160	M01508.m	TRUE	reported	go526k	2	TRUE	0.003032	21.77169
5818	rs2715313	A	G	A	G	-0.0084	-0.01296	0.3788	0.383906	FALSE	FALSE	FALSE	ieu-b-508	18	63687612	0.034317	486484	0.705609	Sepsis (28	Sepsis (28	Sepsis (28	TRUE	igd	0.0018	4.60E-06	7066	M33515.m	TRUE	reported	X9mul1	2	TRUE	0.003073	21.77161
5820	rs403018	A	G	A	G	-0.0084	0.019425	0.6712	0.665009	FALSE	FALSE	FALSE	ieu-b-508	16	24151620	0.035441	486484	0.583626	Sepsis (28	Sepsis (28	Sepsis (28	TRUE	igd	0.0018	5.36E-06	7066	M33515.m	TRUE	reported	X9mul1	2	TRUE	0.003073	21.77161
5243	rs1845546	T	C	T	C	-0.0126	-0.04084	0.3674	0.372329	FALSE	FALSE	FALSE	ieu-b-508	4	70314998	0.034512	486484	0.236614	Sepsis (28	Sepsis (28	Sepsis (28	TRUE	igd	0.0027	3.25E-06	7063	M33228.m	TRUE	reported	JwOXbQ	2	TRUE	0.003074	21.77161
5247	rs418874	A	G	A	G	0.0126	-0.014	0.6931	0.692075	FALSE	FALSE	FALSE	ieu-b-508	3	83345924	0.036097	486484	0.698059	Sepsis (28	Sepsis (28	Sepsis (28	TRUE	igd	0.0027	3.64E-06	7063	M33228.m	TRUE	reported	JwOXbQ	2	TRUE	0.003074	21.77161
6524	rs27419	T	C	T	C	-0.0126	-0.03473	0.6409	0.634157	FALSE	FALSE	FALSE	ieu-b-508	5	52420938	0.034562	486484	0.314922	Sepsis (28	Sepsis (28	Sepsis (28	TRUE	igd	0.0027	2.60E-06	7049	M34123.m	TRUE	reported	9O5wo1	2	TRUE	0.00308	21.7716
4336	rs792222	C	G	C	G	-0.0224	0.063245	0.7494	0.734169	FALSE	TRUE	FALSE	ieu-b-508	10	90108268	0.037857	486484	0.094794	Sepsis (28	Sepsis (28	Sepsis (28	TRUE	igd	0.0048	3.59E-06	6967	M32847.m	TRUE	reported	eYme29	2	TRUE	0.003116	21.77153
8650	rs4765789	T	C	T	C	-0.0112	0.033774	0.4153	0.415979	FALSE	FALSE	FALSE	ieu-b-508	12	4950048	0.033964	486484	0.320032	Sepsis (28	Sepsis (28	Sepsis (28	TRUE	igd	0.0024	3.88E-06	6942	M38768.m	TRUE	reported	a2b8mH	2	TRUE	0.003127	21.7715
8415	rs2305929	A	G	A	G	0.0084	-0.02077	0.819	0.823263	FALSE	FALSE	FALSE	ieu-b-508	2	28113911	0.044033	486484	0.637115	Sepsis (28	Sepsis (28	Sepsis (28	TRUE	igd	0.0018	2.62E-06	6940	M36808.m	TRUE	reported	u7lqaj	2	TRUE	0.003128	21.7715
6991	rs1125006	A	C	A	C	-0.0168	0.066466	0.3131	0.32124	FALSE	FALSE	FALSE	ieu-b-508	8	10581882	0.035955	486484	0.064515	Sepsis (28	Sepsis (28	Sepsis (28	TRUE	igd	0.0036	2.47E-06	6929	M34416.m	TRUE	reported	q9OtaZ	2	TRUE	0.003133	21.77149
1539	rs1186971	A	G	A	G	-0.0126	0.046357	0.7762	0.774418	FALSE	FALSE	FALSE	ieu-b-508	17	48587307	0.040029	486484	0.246833	Sepsis (28	Sepsis (28	Sepsis (28	TRUE	igd	0.0027	4.25E-06	6864	M12129.m	TRUE	reported	iNzdgb	2	TRUE	0.003163	21.77143
8122	rs2674357	C	G	C	G	-0.0775	-0.00736	0.8039	0.806486	FALSE	TRUE	FALSE	ieu-b-508	6	93469885	0.042263	486484	0.861681	Sepsis (28	Sepsis (28	Sepsis (28	TRUE	igd	0.0166	2.96E-06	1725	M36098.m	TRUE	reported	hs9SZl	2	TRUE	0.012478	21.77125
8473	rs2206426	A	G	A	G	-0.0084	-0.03578	0.4177	0.418486	FALSE	FALSE	FALSE	ieu-b-508	20	41286043	0.033966	486484	0.292097	Sepsis (28	Sepsis (28	Sepsis (28	TRUE	igd	0.0018	4.92E-06	6552	M37058.m	TRUE	reported	32p8qV	2	TRUE	0.003313	21.77113
8481	rs367213	A	G	A	G	-0.0084	-0.04054	0.5133	0.51645	FALSE	FALSE	FALSE	ieu-b-508	6	1.61E+08	0.033275	486484	0.223082	Sepsis (28	Sepsis (28	Sepsis (28	TRUE	igd	0.0018	4.74E-06	6552	M37058.m	TRUE	reported	32p8qV	2	TRUE	0.003313	21.77113
7542	rs416324	A	G	A	G	-0.0126	0.003354	0.5573	0.552943	FALSE	FALSE	FALSE	ieu-b-508	8	90815235	0.03359	486484	0.920463	Sepsis (28	Sepsis (28	Sepsis (28	TRUE	igd	0.0027	2.10E-06	6538	M35257.m	TRUE	reported	iphouU	2	TRUE	0.00332	21.77112
7543	rs4270639	A	G	A	G	-0.0168	-0.03654	0.7232	0.716522	FALSE	FALSE	FALSE	ieu-b-508	4	6391735	0.036919	486484	0.322282	Sepsis (28	Sepsis (28	Sepsis (28	TRUE	igd	0.0036	3.78E-06	6538	M35257.m	TRUE	reported	iphouU	2	TRUE	0.00332	21.77112
969	rs304171	T	C	T	C	-0.0168	-0.02711	0.0624	0.061865	FALSE	FALSE	FALSE	ieu-b-508	3	62780809	0.069215	486484	0.695333	Sepsis (28	Sepsis (28	Sepsis (28	TRUE	igd	0.0036	2.17E-06	6523	M01558.m	TRUE	reported	AZK0p4	2	TRUE	0.003328	21.7711
8438	rs2609982	T	C	T	C	-0.0294	-0.01525	0.2865	0.283071	FALSE	FALSE	FALSE	ieu-b-508	8	57496477	0.036993	486484	0.680229	Sepsis (28	Sepsis (28	Sepsis (28	TRUE	igd	0.0063	3.10E-06	6511	M36850.m	TRUE	reported	j6lLja	2	TRUE	0.003334	21.77109
2730	rs2211843	T	G	T	G	-0.0126	0.077652	0.2444	0.236203	FALSE	FALSE	FALSE	ieu-b-508	21	39184185	0.039514	486484	0.04939	Sepsis (28	Sepsis (28	Sepsis (28	TRUE	igd	0.0027	4.23E-06	6388	M25599.m	TRUE	reported	fRZxwP	2	TRUE	0.003398	21.77096
3303	rs1539549	T	C	T	C	-0.0369	0.021487	0.305	0.311654	FALSE	FALSE	FALSE	ieu-b-508	13	36451881	0.038024	486484	0.572017	Sepsis (28	Sepsis (28	Sepsis (28	TRUE	igd	0.0079	2.72E-06	936	M32388.m	TRUE	reported	yYczcA	2	TRUE	0.022778	21.77056
6947	rs1108665	T	G	T	G	0.0252	-0.02243	0.2207	0.250122	FALSE	FALSE	FALSE	ieu-b-508	20	57198838	0.040291	486484	0.577755	Sepsis (28	Sepsis (28	Sepsis (28	TRUE	igd	0.0054	3.24E-06	5689	M34400.m	TRUE	reported	QXmzfR	2	TRUE	0.003813	21.77012
1496	rs1213178	A	G	A	G	0.0126	0.037827	0.7091	0.695999	FALSE	FALSE	FALSE	ieu-b-508	1	1.67E+08	0.036631	486484	0.301778	Sepsis (28	Sepsis (28	Sepsis (28	TRUE	igd	0.0027	2.59E-06	5656	M12017.m	TRUE	reported	bbGtsK	2	TRUE	0.003836	21.77008
7900	rs4551320	T	G	T	G	0.0434	0.066439	0.0759	0.10482	FALSE	FALSE	FALSE	ieu-b-508	8	98886485	0.055146	486484	0.228291	Sepsis (28	Sepsis (28	Sepsis (28	TRUE	igd	0.0093	2.88E-06	5629	M35669.m	TRUE	reported	gSecuE	2	TRUE	0.003854	21.77004
5	rs2748991	T	C	T	C	-0.007	-0.02555	0.446	0.452764	FALSE	FALSE	FALSE	ieu-b-508	6	52596516	0.033447	486484	0.444875	Sepsis (28	Sepsis (28	Sepsis (28	TRUE	igd	0.0015	2.34E-06	5604	M00053.m	TRUE	reported	4Smluz	2	TRUE	0.003871	21.77001
588	rs1253211	T	C	T	C	-0.028	-0.03912	0.961	0.964547	FALSE	FALSE	FALSE	ieu-b-508	7	90484736	0.090449	486484	0.665376	Sepsis (28	Sepsis (28	Sepsis (28	TRUE	igd	0.006	3.06E-06	5601	M01110.m	TRUE	reported	0A31Sl	2	TRUE	0.003873	21.77
4499	rs1704400	T	G	T	G	-0.014	-0.0096	0.074	0.079005	FALSE	FALSE	FALSE	ieu-b-508	4	1.65E+08	0.061887	486484	0.876683	Sepsis (28	Sepsis (28	Sepsis (28	TRUE	igd	0.003	3.06E-06	5600	M33132.m	TRUE	reported	dTXJa8	2	TRUE	0.003874	21.77
6355	rs6061764	A	G	A	G	-0.014	0.031732	0.314	0.314952	FALSE	FALSE	FALSE	ieu-b-508	20	59979354	0.035968	486484	0.377648	Sepsis (28	Sepsis (28	Sepsis (28	TRUE	igd	0.003	3.06E-06	5597	M33960.m	TRUE	reported	o8RcxU	2	TRUE	0.003876	21.77
3866	rs1578905	T	G	T	G	0.042	0.042088	0.977	0.971129	FALSE	FALSE	FALSE	ieu-b-508	14	57640614	0.101801	486484	0.67929	Sepsis (28	Sepsis (28	Sepsis (28	TRUE	igd	0.009	3.06E-06	5595	M32654.m	TRUE	reported	6ISQOP	2	TRUE	0.003877	21.76999
103	rs3772343	A	C	A	C	0.028	0.137667	0.987	0.972126	FALSE	FALSE	FALSE	ieu-b-508	3	1273716	0.102614	486484	0.179724	Sepsis (28	Sepsis (28	Sepsis (28	TRUE	igd	0.006	3.06E-06	5592	M00054.m	TRUE	reported	QTDAnh	2	TRUE	0.003879	21.76999
789	rs7565567	A	C	A	C	-0.042	-0.03175	0.982	0.97778	FALSE	FALSE	FALSE	ieu-b-508	2	11711222	0.114052	486484	0.780692	Sepsis (28	Sepsis (28	Sepsis (28	TRUE	igd	0.009	3.06E-06	5586	M01336.m	TRUE	reported	FAlrya	2	TRUE	0.003883	21.76998
3563	rs1388331	A	G	A	G	0.014	0.089163	0.913	0.916689	FALSE	FALSE	FALSE	ieu-b-508	4	23778350	0.060903	486484	0.143189	Sepsis (28	Sepsis (28	Sepsis (28	TRUE	igd	0.003	3.06E-06	5584	M32518.m	TRUE	reported	V0ZCiU	2	TRUE	0.003885	21.76998
4421	rs1154147	A	G	A	G	-0.014	-0.01545	0.152	0.146399	FALSE	FALSE	FALSE	ieu-b-508	22	19906511	0.047205	486484	0.743449	Sepsis (28	Sepsis (28	Sepsis (28	TRUE	igd	0.003	3.06E-06	5584	M32910.m	TRUE	reported	2OCBKh	2	TRUE	0.003885	21.76998
2490	rs1156408	T	C	T	C	-0.028	-0.05204	0.053	0.075465	FALSE	FALSE	FALSE	ieu-b-508	9	1.32E+08	0.066772	486484	0.435743	Sepsis (28	Sepsis (28	Sepsis (28	TRUE	igd	0.006	3.06E-06	5563	M22116.m	TRUE	reported	bjUU6r	2	TRUE	0.003899	21.76995
5248	rs4392177	A	G	A	G	-0.014	-0.02576	0.547	0.566858	FALSE	FALSE	FALSE	ieu-b-508	19	1684137	0.033619	486484	0.443562	Sepsis (28	Sepsis (28	Sepsis (28	TRUE	igd	0.003	3.06E-06	5451	M33228.m	TRUE	reported	JwOXbQ	2	TRUE	0.003979	21.76979
2833	rs2494523	T	C	T	C	-0.014	0.004877	0.492	0.490814	FALSE	FALSE	FALSE	ieu-b-508	1	1.6E+08	0.033339	486484	0.883699	Sepsis (28	Sepsis (28	Sepsis (28	TRUE	igd	0.003	3.06E-06	5442	M27513.m	TRUE	reported	o9Ff2u	2	TRUE	0.003986	21.76977
2351	rs1895981	T	C	T	C	0.014	-0.02324	0.841	0.841493	FALSE	FALSE	FALSE	ieu-b-508	12	1.14E+08	0.045518	486484	0.609631	Sepsis (28	Sepsis (28	Sepsis (28	TRUE	igd	0.003	3.06E-06	5439	M21047.m	TRUE	reported	ISF1Cr	2	TRUE	0.003988	21.76977
1250	rs2364598	A	C	A	C	-0.014	0.00553	0.149	0.158604	FALSE	FALSE	FALSE	ieu-b-508	12	53795365	0.045711	486484	0.903715	Sepsis (28	Sepsis (28	Sepsis (28	TRUE	igd	0.003	3.06E-06	5422	M01769.m	TRUE	reported	rismUZ	2	TRUE	0.004	21.76974
7740	rs4719207	T	C	T	C	-0.014	-0.04269	0.211	0.216005	FALSE	FALSE	FALSE	ieu-b-508	7	71550079	0.040511	486484	0.291951	Sepsis (28	Sepsis (28	Sepsis (28	TRUE	igd	0.003	3.06E-06	5312	M35431.m	TRUE	reported	tLfE4l	2	TRUE	0.004083	21.76958
3017	rs1079970	A	G	A	G	0.0308	-0.00158	0.435	0.460382	FALSE	FALSE	FALSE	ieu-b-508	1	21820990	0.033467	486484	0.96243	Sepsis (28	Sepsis (28	Sepsis (28	TRUE	igd	0.0066	2.69E-06	5229	M31548.m	TRUE	reported	bgRTBS	2	TRUE	0.004148	21.76945
3024	rs2327511	A	T	A	T	0.035	-0.03458	0.2333	0.244886	FALSE	TRUE	FALSE	ieu-b-508	6	12090331	0.038988	486484	0.3751	Sepsis (28	Sepsis (28	Sepsis (28	TRUE	igd	0.0075	3.44E-06	5229	M31548.m	TRUE	reported	bgRTBS	2	TRUE	0.004148	21.76945
7053	rs6785727	A	G	A	G	0.0126	0.04469	0.5467	0.59428	FALSE	FALSE	FALSE	ieu-b-508	3	1.19E+08	0.033938	486484	0.187902	Sepsis (28	Sepsis (28	Sepsis (28	TRUE	igd	0.0027	4.52E-06	5207	M34456.m	TRUE	reported	Nh457q	2	TRUE	0.004165	21.76941

8418	rs3014614	T	G	T	G	-0.014	0.010529	0.922	0.913859	FALSE	FALSE	FALSE	ieu-b-508	1	2.04E+08	0.060255	486484	0.861283	Sepsis (28	Sepsis (28	Sepsis (28	TRUE	igd	0.003	3.06E-06	5176	M36808.m	TRUE	reported	u7Iqaj	2	TRUE	0.00419	21.76936
2421	rs2049974	A	G	A	G	-0.014	0.044797	0.296	0.291343	FALSE	FALSE	FALSE	ieu-b-508	22	36942323	0.036771	486484	0.223117	Sepsis (28	Sepsis (28	Sepsis (28	TRUE	igd	0.003	3.06E-06	5148	M21188.m	TRUE	reported	kvdnjc	2	TRUE	0.004213	21.76932
1934	rs4325745	T	C	T	C	-0.014	-0.0465	0.596	0.60095	FALSE	FALSE	FALSE	ieu-b-508	2	2.28E+08	0.034416	486484	0.176696	Sepsis (28	Sepsis (28	Sepsis (28	TRUE	igd	0.003	3.06E-06	4962	M16822.m	TRUE	reported	UNI018	2	TRUE	0.00437	21.769
7259	rs1740655	T	C	T	C	0.028	0.025056	0.959	0.949227	FALSE	FALSE	FALSE	ieu-b-508	8	4107011	0.075779	486484	0.740918	Sepsis (28	Sepsis (28	Sepsis (28	TRUE	igd	0.006	3.06E-06	4825	M34912.m	TRUE	reported	W8lcT6	2	TRUE	0.004493	21.76875
5474	rs6685925	A	G	A	G	0.0364	0.008987	0.2447	0.25598	FALSE	FALSE	FALSE	ieu-b-508	1	2.34E+08	0.03822	486484	0.814093	Sepsis (28	Sepsis (28	Sepsis (28	TRUE	igd	0.0078	2.82E-06	3890	M33408.m	TRUE	reported	tWZWIa	2	TRUE	0.005567	21.76658
6885	rs606242	T	C	T	C	-0.0934	-0.03183	0.6853	0.721699	FALSE	FALSE	FALSE	ieu-b-508	6	1.17E+08	0.037241	486484	0.392751	Sepsis (28	Sepsis (28	Sepsis (28	TRUE	igd	0.02	3.00E-06	954	M34365.m	TRUE	reported	8eexD0	2	TRUE	0.02235	21.76318
816	rs1922419	A	G	A	G	-0.0182	-0.02948	0.7807	0.768332	FALSE	FALSE	FALSE	ieu-b-508	7	1.33E+08	0.039561	486484	0.456202	Sepsis (28	Sepsis (28	Sepsis (28	TRUE	igd	0.0039	3.37E-06	1765	M01358.m	TRUE	reported	kSPQgd	2	TRUE	0.012188	21.7531
3149	rs1357743	T	G	T	G	0.0322	-0.00193	0.5585	0.562263	FALSE	FALSE	FALSE	ieu-b-508	7	1.13E+08	0.0336	486484	0.954149	Sepsis (28	Sepsis (28	Sepsis (28	TRUE	igd	0.0069	2.79E-06	1765	M32319.m	TRUE	reported	9YFmUx	2	TRUE	0.012188	21.7531
4387	rs2039906	C	G	C	G	0.0084	0.002772	0.2696	0.261364	FALSE	TRUE	FALSE	ieu-b-508	6	4930812	0.038041	486484	0.941906	Sepsis (28	Sepsis (28	Sepsis (28	TRUE	igd	0.0018	1.94E-06	1763	M32867.m	TRUE	reported	Jcsyzm	2	TRUE	0.012202	21.75307
6972	rs1249425	A	G	A	G	0.0308	0.003006	0.7684	0.792931	FALSE	FALSE	FALSE	ieu-b-508	3	99003734	0.041395	486484	0.942115	Sepsis (28	Sepsis (28	Sepsis (28	TRUE	igd	0.0066	2.72E-06	1754	M34407.m	TRUE	reported	X7I83Y	2	TRUE	0.012264	21.75295
2678	rs2007443	A	T	A	T	0.028	0.056511	0.1321	0.103662	FALSE	TRUE	FALSE	ieu-b-508	15	49857177	0.054913	486484	0.303434	Sepsis (28	Sepsis (28	Sepsis (28	TRUE	igd	0.006	2.87E-06	1751	M24074.m	TRUE	reported	Lk6D2A	2	TRUE	0.012285	21.7529
7757	rs1089551	T	C	T	C	-0.0532	-0.16062	0.9515	0.958926	FALSE	FALSE	FALSE	ieu-b-508	11	1.04E+08	0.084957	486484	0.058671	Sepsis (28	Sepsis (28	Sepsis (28	TRUE	igd	0.0114	2.90E-06	1718	M35439.m	TRUE	reported	DnuAGZ	2	TRUE	0.012518	21.75243
1984	rs1079172	A	T	A	T	-0.0658	-0.00302	0.3242	0.346562	FALSE	TRUE	FALSE	ieu-b-508	11	1.05E+08	0.035028	486484	0.931199	Sepsis (28	Sepsis (28	Sepsis (28	TRUE	igd	0.0141	2.96E-06	1659	M18254.m	TRUE	reported	clvu6V	2	TRUE	0.012957	21.75152
6093	rs1360605	C	G	C	G	0.1092	-0.08514	0.9704	0.970949	FALSE	TRUE	FALSE	ieu-b-508	6	1.38E+08	0.100768	486484	0.398186	Sepsis (28	Sepsis (28	Sepsis (28	TRUE	igd	0.0234	2.96E-06	1428	M33864.m	TRUE	reported	yr5h52	2	TRUE	0.015021	21.74728
4640	rs575723	A	G	A	G	-0.9929	0.116935	0.0135	0.015572	FALSE	FALSE	FALSE	ieu-b-508	6	1.12E+08	0.133275	486484	0.38027	Sepsis (28	Sepsis (28	Sepsis (28	TRUE	igd	0.2101	2.29E-06	76	M33173.m	TRUE	reported	04fkUQ	2	TRUE	0.227121	21.74588
6082	rs1041323	T	C	T	C	-0.0729	0.061639	0.3811	0.379869	FALSE	FALSE	FALSE	ieu-b-508	9	90065039	0.034472	486484	0.073765	Sepsis (28	Sepsis (28	Sepsis (28	TRUE	igd	0.0156	3.00E-06	471	M33835.m	TRUE	reported	crhBhm	2	TRUE	0.04431	21.74492
4633	rs4279445	A	T	A	T	-0.9923	0.119369	0.0132	0.024988	FALSE	TRUE	FALSE	ieu-b-508	6	97698269	0.107549	486484	0.267042	Sepsis (28	Sepsis (28	Sepsis (28	TRUE	igd	0.21	2.30E-06	76	M33173.m	TRUE	reported	04fkUQ	2	TRUE	0.227076	21.7403
2484	rs739167	A	G	A	G	-0.0476	-0.0111	0.8667	0.881826	FALSE	FALSE	FALSE	ieu-b-508	22	37810777	0.051897	486484	0.830662	Sepsis (28	Sepsis (28	Sepsis (28	TRUE	igd	0.0102	3.25E-06	1087	M22032.m	TRUE	reported	xpe96K	2	TRUE	0.019641	21.73771
8538	rs1685083	A	G	A	G	0.0429	-0.01037	0.1266	0.128495	FALSE	FALSE	FALSE	ieu-b-508	3	1.62E+08	0.050006	486484	0.835694	Sepsis (28	Sepsis (28	Sepsis (28	TRUE	igd	0.0092	3.12E-06	6934	M37190.m	TRUE	reported	tiNuK9	2	TRUE	0.003126	21.7377
7616	rs1715158	A	C	A	C	0.0514	-0.0741	0.6597	0.67895	FALSE	FALSE	FALSE	ieu-b-508	8	10149212	0.035866	486484	0.038835	Sepsis (28	Sepsis (28	Sepsis (28	TRUE	igd	0.011	3.11E-06	448	M35322.m	TRUE	reported	om3h9x	2	TRUE	0.046473	21.73691
3607	rs1319504	A	G	A	G	-0.0373	0.002418	0.9137	0.88928	FALSE	FALSE	FALSE	ieu-b-508	6	27413924	0.053113	486484	0.963693	Sepsis (28	Sepsis (28	Sepsis (28	TRUE	igd	0.008	3.59E-06	7361	M32553.m	TRUE	reported	vHSHqn	2	TRUE	0.002945	21.733
6469	rs1248391	T	C	T	C	0.4285	0.030068	0.9829	0.978141	FALSE	FALSE	FALSE	ieu-b-508	22	35359743	0.115346	486484	0.794344	Sepsis (28	Sepsis (28	Sepsis (28	TRUE	igd	0.0916	2.90E-06	282	M34109.m	TRUE	reported	piJuC7	2	TRUE	0.072012	21.72799
2670	rs1110651	A	G	A	G	-0.0331	0.027903	0.9495	0.94027	FALSE	FALSE	FALSE	ieu-b-508	12	92729243	0.071192	486484	0.695103	Sepsis (28	Sepsis (28	Sepsis (28	TRUE	igd	0.0071	3.52E-06	7256	M24074.m	TRUE	reported	Lk6D2A	2	TRUE	0.002986	21.72799
6638	rs1263903	T	C	T	C	-0.1844	-0.14946	0.975	0.963987	FALSE	FALSE	FALSE	ieu-b-508	3	1.54E+08	0.090208	486484	0.097557	Sepsis (28	Sepsis (28	Sepsis (28	TRUE	igd	0.0394	2.84E-06	248	M34322.m	TRUE	reported	16GdG9	2	TRUE	0.081156	21.72765
6775	rs2513248	T	C	T	C	0.0662	-0.05465	0.083	0.088481	FALSE	FALSE	FALSE	ieu-b-508	11	1.05E+08	0.058963	486484	0.353992	Sepsis (28	Sepsis (28	Sepsis (28	TRUE	igd	0.0142	3.01E-06	5270	M34344.m	TRUE	reported	enFm6p	2	TRUE	0.004107	21.72573
3877	rs3822311	T	C	T	C	0.0303	-0.01882	0.9654	0.962683	FALSE	FALSE	FALSE	ieu-b-508	5	1.31E+08	0.088301	486484	0.831255	Sepsis (28	Sepsis (28	Sepsis (28	TRUE	igd	0.0065	3.43E-06	7361	M32654.m	TRUE	reported	6lSQOP	2	TRUE	0.002943	21.72404
543	rs924483	A	G	A	G	-0.0289	0.046282	0.0916	0.095901	FALSE	FALSE	FALSE	ieu-b-508	13	27061003	0.056781	486484	0.415022	Sepsis (28	Sepsis (28	Sepsis (28	TRUE	igd	0.0062	3.25E-06	7237	M00599.m	TRUE	reported	nRJY3s	2	TRUE	0.002993	21.72162
5942	rs1383950	T	C	T	C	0.1548	-0.23557	0.9813	0.985329	FALSE	FALSE	FALSE	ieu-b-508	8	3587622	0.139464	486484	0.091203	Sepsis (28	Sepsis (28	Sepsis (28	TRUE	igd	0.0332	3.13E-06	1660	M33638.m	TRUE	reported	hVuSGd	2	TRUE	0.012927	21.71412
7225	rs5997515	T	C	T	C	-0.0508	-0.06756	0.0469	0.044027	FALSE	FALSE	FALSE	ieu-b-508	22	30140278	0.081362	486484	0.406327	Sepsis (28	Sepsis (28	Sepsis (28	TRUE	igd	0.0109	3.27E-06	6381	M34761.m	TRUE	reported	AMtp1w	2	TRUE	0.003392	21.71392
2299	rs7915784	A	G	A	G	0.0247	0.00964	0.0662	0.070291	FALSE	FALSE	FALSE	ieu-b-508	10	28286740	0.065595	486484	0.883158	Sepsis (28	Sepsis (28	Sepsis (28	TRUE	igd	0.0053	2.65E-06	7301	M20675.m	TRUE	reported	CXe9rE	2	TRUE	0.002966	21.71317
8644	rs6971411	A	G	A	G	0.0247	-0.03171	0.1381	0.148043	FALSE	FALSE	FALSE	ieu-b-508	7	1.55E+08	0.046893	486484	0.498899	Sepsis (28	Sepsis (28	Sepsis (28	TRUE	igd	0.0053	2.92E-06	7219	M38178.m	TRUE	reported	GgCXMN	2	TRUE	0.003	21.7131
2457	rs1079516	T	C	T	C	-0.0233	-0.00609	0.9261	0.924433	FALSE	FALSE	FALSE	ieu-b-508	10	4458818	0.063183	486484	0.92319	Sepsis (28	Sepsis (28	Sepsis (28	TRUE	igd	0.005	3.51E-06	6180	M22030.m	TRUE	reported	ZNV0qR	2	TRUE	0.003502	21.70857
872	rs1000529	A	G	A	G	-0.0634	-0.00547	0.0267	0.044906	FALSE	FALSE	FALSE	ieu-b-508	4	91882724	0.081256	486484	0.946336	Sepsis (28	Sepsis (28	Sepsis (28	TRUE	igd	0.0136	3.40E-06	1768	M01365.m	TRUE	reported	N1cZTs	2	TRUE	0.012143	21.70747
6405	rs1313219	A	T	A	T	0.0317	0.068669	0.2154	0.193555	FALSE	TRUE	FALSE	ieu-b-508	4	1.03E+08	0.042478	486484	0.10597	Sepsis (28	Sepsis (28	Sepsis (28	TRUE	igd	0.0068	3.54E-06	1768	M34035.m	TRUE	reported	W4G5qR	2	TRUE	0.012143	21.70747
524	rs2304684	T	C	T	C	-0.0438	-0.01971	0.0182	0.019486	FALSE	FALSE	FALSE	ieu-b-508	2	27466368	0.124474	486484	0.874167	Sepsis (28	Sepsis (28	Sepsis (28	TRUE	igd	0.0094	3.02E-06	7345	M00584.m	TRUE	reported	oBt02X	2	TRUE	0.002947	21.70572
7552	rs1089230	A	T	A	T	-0.0219	-0.01017	0.0417	0.062443	FALSE	TRUE	FALSE	ieu-b-508	11	1.19E+08	0.069224	486484	0.883207	Sepsis (28	Sepsis (28	Sepsis (28	TRUE	igd	0.0047	3.23E-06	7217	M35270.m	TRUE	reported	Ct8V1n	2	TRUE	0.002999	21.70562
8300	rs2954636	T	C	T	C	0.0317	0.01944	0.1715	0.180838	FALSE	FALSE	FALSE	ieu-b-508	8	3740896	0.043349	486484	0.653823	Sepsis (28	Sepsis (28	Sepsis (28	TRUE	igd	0.0068	3.18E-06	1636	M36559.m	TRUE	reported	g6I9hg	2	TRUE	0.01311	21.70548
3720	rs7847689	T	C	T	C	0.0438	0.042691	0.7003	0.709555	FALSE	FALSE	FALSE	ieu-b-508	9	4434601	0.036899	486484	0.247288	Sepsis (28	Sepsis (28	Sepsis (28	TRUE	igd	0.0094	3.44E-06	6904	M32616.m	TRUE	reported	vrkjBE	2	TRUE	0.003135	21.70534
8219	rs1310060	T	G	T	G	-0.0219	0.041166	0.3761	0.397863	FALSE	FALSE	FALSE	ieu-b-508	3	1.37E+08	0.034069	486484	0.226934	Sepsis (28	Sepsis (28	Sepsis (28	TRUE	igd	0.0047	3.14E-06	6486	M36399.m	TRUE	reported	EdUpnt	2	TRUE	0.003336	21.70494
5302	rs7425298	A	T	A	T	-0.2286	0.082896	0.9846	0.975184	FALSE	TRUE	FALSE	ieu-b-508	2	51510144	0.107946	486484	0.442523	Sepsis (28	Sepsis (28	Sepsis (28	TRUE	igd	0.0489	2.88E-06	292	M33353.m	TRUE	reported	ZIN4gk	2	TRUE	0.069632	21.7045
577	rs2293319	T	C	T	C	-0.0219	0.010004	0.7852	0.781983	FALSE	FALSE	FALSE	ieu-b-508	15	31235357	0.040452	486484	0.804675	Sepsis (28	Sepsis (28	Sepsis (28	TRUE	igd	0.0047	3.59E-06	5344	M01107.m	TRUE	reported	EbPV1D	2	TRUE	0.004046	21.70351
4410	rs2899743	C	G	C	G	-0.0261	-0.01244	0.6897	0.672583	FALSE	TRUE	FALSE	ieu-b-508	15	68921524	0.035843	486484	0.728519	Sepsis (28	Sepsis (28	Sepsis (28	TRUE	igd	0.0056	3.79E-06	2148	M32869.m	TRUE	reported	Rvim1n	2	TRUE	0.010012	21.70203
7518	rs1167190	A	C	A	C	0.0205	0.0241	0.7672	0.76176	FALSE	FALSE	FALSE	ieu-b-508	19	4246693	0.039181	486484	0.538495	Sepsis (28	Sepsis (28	Sepsis (28	TRUE	igd	0.0044	3.63E-06	7291	M35255.m	TRUE	reported	ZDjmUf	2	TRUE	0.002968	21.70117
4363	rs6747014	T	C	T	C	0.048	-0.09594	0.8967	0.903007	FALSE	FALSE	FALSE	ieu-b-508	2	1.51E+08	0.05628	486484	0.088265	Sepsis (28	Sepsis (28	Sepsis (28	TRUE	igd	0.0103	3.12E-06	2656	M32857.m	TRUE	reported	MVAWzT	2	TRUE	0.00811	21.70106
7952	rs1077012	A	G	A	G	0.0205	0.054068	0.5547	0.568123	FALSE	FALSE	FALSE	ieu-b-508	11	10616209	0.033617	486484	0.10776	Sepsis (28	Sepsis (28	Sepsis (28	TRUE	igd	0.0044	2.68E-06	6447	M35678.m	TRUE	reported	56yR01	2	TRUE	0.003356	21.70039
3302	rs1112615	A	G	A	G	-0.0205	0.053301	0.7711	0.750002	FALSE	FALSE	FALSE	ieu-b-508	2	68002545	0.038467	486484	0.165862	Sepsis (28	Sepsis (28	Sepsis (28	TRUE	igd	0.0044	3.22E-06	6066	M32388.m	TRUE	reported	yYczcA	2	TRUE	0.003566	21.69997
4300	rs3133745	T	C	T	C	-0.0396	-0.01531	0.1259	0.130506	FALSE	FALSE	FALSE	ieu-b-508	8	96534806	0.052242	486484	0.769428	Sepsis (28	Sepsis (28	Sepsis (28	TRUE	igd	0.0085	2.71E-06	7255	M32836.m	TRUE	reported	d4lVXe	2	TRUE	0.002983	21.69865
6129	rs3791160	T	C	T	C	0.4005	0.013739	0.0205	0.029603	FALSE	FALSE	FALSE	ieu-b-508	17	80764771	0.099003	486484	0.889627	Sepsis (28	Sepsis (28	Sepsis (28	TRUE	igd	0.0856	2.87E-06	228	M33877.m	TRUE	reported	yDVi1f	2	TRUE	0.087601	21.69857
7036	rs6429539	A	G	A	G	-0.0261	-0.18596	0.0308	0.020237	FALSE	FALSE	FALSE	ieu-b-508	1	44860224	0.118531	486484	0.116685	Sepsis (28	Sepsis (28	Sepsis (28	TRUE	igd	0.0056	3.54E-06	1755	M34453.m	TRUE	reported	3EAMbN	2	TRUE	0.012226	21.6975
3882	rs6862720	T	C	T	C	0.0191	-0.06088	0.0785	0.082896	FALSE	FALSE	FALSE	ieu-b-508	5	21261450	0.060271	486484	0.312452	Sepsis (28	Sepsis (28	Sepsis (28	TRUE	igd	0.0041	4.28E-06	7361	M32654.m	TRUE	reported	6lSQOP	2	TRUE	0.00294	21.69607
3497	rs1684443	A	G	A	G	-0.0191	-0.00616	0.9276	0.924316	FALSE	FALSE	FALSE	ieu-b-508	3	1.37E+08	0.063845	486484	0.923163	Sepsis (28	Sepsis (28	Sepsis (28	TRUE	igd	0.0041	2.72E-06	7355	M32492.m	TRUE	reported	rZZFcE	2	TRUE	0.002942	21.69606
8570	rs6755484	T	C	T	C	-0.0191	0.002767	0.4847	0.488378	FALSE	FALSE	FALSE	ieu-b-508	2	1.12E+08	0.033416	486484	0.934	Sepsis (28	Sepsis (28	Sepsis (28	TRUE	igd	0.0041	3.11E-06	7333	M37203.m	TRUE	reported	3glllv	2	TRUE	0.002951	21.69604
3005	rs4384785	A	G	A	G	0.1372	0.023492	0.1018	0.111287	FALSE	FALSE	FALSE	ieu-b-508	2	1.07E+08	0.053382	486484	0.659886	Sepsis (28	Sepsis (28	Sepsis (28	TRUE	igd	0.0294	3.16E-06	522	M31536.m	TRUE	reported	3nAuUl	2	TRUE	0.040049	21.69434
5234	rs8796	T	G	T	G	0.0634	-0.02139	0.1633	0.203821	FALSE	FALSE	FALSE	ieu-b-508	8	98864102	0.041641	486484	0.607466	Sepsis (28	Sepsis (28	Sepsis (28	TRUE	igd	0.0136	3.21E-06	1142	M33221.m	TRUE	reported	8ZrV1r	2	TRUE	0.018674	21.69399
5292	rs130798	A	T	A	T	0.0578	0.002267	0.1603	0.182531	FALSE	TRUE	FALSE	ieu-b-508	22	48800080	0.043232	486484	0.95818	Sepsis (28	Sepsis (28	Sepsis (28	TRUE	igd	0.0124	2.99E-06	1273	M33353.m	TRUE	reported	ZIN4gk	2	TRUE	0.016782	21.69349
4367	rs1247994	T	G	T	G	-0.0918	-0.029	0.9236	0.924344	FALSE	FALSE	FALSE	ieu-b-508	20	45373828	0.063315	486484	0.646941	Sepsis (28	Sepsis (28	Sepsis (28	TRUE	igd	0.0197	3.39E-06	1988	M32863.m	TRUE	reported	cLOFYE	2	TRUE	0.010805	21.69281
6433	rs17352	T	G	T	G	0.0233	0.022838	0.8945	0.888189	FALSE	FALSE	FALSE	ieu-b-508	20	5097976	0.053014	486484	0.666623	Sepsis (28	Sepsis (28	Sepsis (28	TRUE	igd	0.005	3.13E-06	1768	M34062.m	TRUE	reported	RZyHKP	2	TRUE	0.012134	21.69103
4305	rs1113499	T	C	T	C	0.0177	0.046633	0.3569	0.368408	FALSE	FALSE	FALSE	ieu-b-508	5	1.76E+08	0.034987	486484	0.182576	Sepsis (28	Sepsis (28	Sepsis (28	TRUE	igd	0.0038	2.96E-06	6753	M32838.m	TRUE	reported	hyR7zA	2	TRUE	0.003203	21.68956
8279	rs3790310	A	T	A	T	0.0177	-0.03168	0.8295	0.828755	FALSE	TRUE	FALSE	ieu-b-508	20	17632823	0.044311	486484	0.47469	Sepsis (28	Sepsis (28	Sepsis (28	TRUE	igd	0.0038	3.46E-06	6464	M36553.m	TRUE	reported	2Rk7nd	2	TRUE	0.003345	21.68927
6183	rs1076483	T	C	T	C	-0.0177	-0.05569	0.788	0.788982	FALSE	FALSE	FALSE	ieu-b-508	10	1.31E+08	0.041299	486484	0.177535	Sepsis (28	Sepsis (28	Sepsis (28	TRUE	igd	0.0038	3.78E-06	6004	M33892.m	TRUE	reported	w7HgU7	2	TRUE	0.003601	21.68876
8334	rs1158895	C	G	C	G	-0.0177	-0.01039	0.6174	0.678363	FALSE	TRUE	FALSE	ieu-b-508	1	45119965	0.035687	486484	0.770985	Sepsis (28	Sepsis (28	Sepsis (28	TRUE	igd	0.0038	3.57E-06	5292	M36673.m	TRUE	reported	CD5lvR	2	TRUE	0.004083	21.68778
7883	rs1705941	A	G	A	G	0.1061	0.106162	0.3922	0.371775	FALSE	FALSE	FALSE	ieu-b-508	18	74177219	0.034666	486484	0.002195	Sepsis (28	Sepsis (28	Sepsis (28	TRUE	igd	0.0227	2.86E-06	274	M35635.m	TRUE	reported	qymryH	2	TRUE	0.073844	21.6869
5852	rs1341788	A	G	A	G	0.034	-0.04816	0.9333	0.936055	FALSE	FALSE	FALSE	ieu-b-508	2	1.67E+08	0.067796	486484	0.47752	Sepsis (28	Sepsis (28	Sepsis (28	TRUE	igd	0.0073	3.41E-06	7352	M33587.m	TRUE	reported	ssG6bF	2	TRUE	0.002942	21.68672
5893	rs601208	T	C	T	C	0.0518	-0.01841	0.6648	0.655522	FALSE	FALSE	FALSE	ieu-b-508	11	79624615	0.035106	486484	0.600018	Sepsis (28	Sepsis (28	Sepsis (28	TRUE	igd	0.0111	3.04E-06	470	M33610.m	TRUE	reported	tmg43v	2	TRUE	0.044284	21.68511
2373	rs9521664	T	C	T	C	-0.0708	-0.10592	0.9351	0.93545	FALSE	FALSE	FALSE	ieu-b-508	13	1.11E+08	0.069878	486484	0.129569	Sepsis (28	Sepsis (28	Sepsis (28	TRUE	igd	0.0152	3.24E-06	3422	M21049.m	TRUE	reported	UDtLE4	2	TRUE	0.0063	21.6833
941	rs6774965	A	G	A	G	-0.0163	-0.00118	0.8582	0.868491	FALSE	FALSE	FALSE	ieu-b-508	3	27549021	0.049462	486484	0.980908	Sepsis (28	Sepsis (28	Sepsis (28	TRUE	igd	0.0035	3.16E-06	7160	M01508.m	TRUE	reported	go526k	2	TRUE	0.00302	21.68292
2085	rs1166005	T	C	T	C	-0.0326	0.059555	0.8153	0.790465	FALSE	FALSE	FALSE	ieu-b-508	18	49151252	0.041035	486484	0.146691	Sepsis (28	Sepsis (28	Sepsis (28	TRUE	igd	0.007	3.68E-06	6970	M18394.m	TRUE	reported	Lt9awu	2	TRUE	0.003102	21.68276
4238	rs6762129	T	C	T	C	0.0163	0.032983	0.3537	0.35818	FALSE	FALSE	FALSE	ieu-b-508	3	9074865	0.034951	486484	0.345327	Sepsis (28	Sepsis (28	Sepsis (28	TRUE	igd	0.0035	3.06E-06	6857	M32787.m	TRUE	reported	fSRakm	2	TRUE	0.003153	21.68265
2321	rs6591040	T	G	T	G	-0.0205	-0.0562	0.5899	0.59247	FALSE	FALSE	FALSE	ieu-b-508	11	1.04E+08	0.03389	486484	0.097281	Sepsis (28	Sepsis (28	Sepsis (28	TRUE	igd	0.0044	3.34E-06	1748	M20699.m	TRUE	reported	Z3EKFa	2	TRUE	0.012266	21.68229
2456	rs1022117	T	C	T	C	0.0163	0.13304	0.1464	0.156776	FALSE	FALSE	FALSE	ieu-b-508	16	80924740	0.045924	486484	0.003768	Sepsis (28	Sepsis (28	Sepsis (28	TRUE	igd	0.0035	2.32E-06	6180	M22030.m	TRUE	reported	ZNV0qR	2	TRUE	0.003497	21.68196
7622	rs724467	T	C	T	C	0.0602	0.048204	0.2576	0.242604	FALSE	FALSE	FALSE	ieu-b-508	11	1.24E+08	0.03912	486484	0.217868	Sepsis (28	Sepsis (28	Sepsis (28	TRUE	igd	0.0129	2.92E-06	448	M35322.m	TRUE	reported	om3h9x	2	TRUE	0.046358	21.68056
2935	rs1009794	A	G	A	G	0.0475	-0.08518	0.9488	0.923218	FALSE	FALSE	FALSE	ieu-b-508	8	1.41E+08	0.063343	486484	0.178717	Sepsis (28	Sepsis (28	Sepsis (28	TRUE	igd	0.0102	3.41E-06	7336	M27738.m	TRUE	reported	UtPBd9	2	TRUE	0.002947	21.68046
1832	rs1183853	A	G	A	G	0.0284	-0.17565	0.0205	0.021107	FALSE	FALSE	FALSE	ieu-b-508	13	48668018	0.115658	486484	0.128839	Sepsis (28	Sepsis (28	Sepsis (28	TRUE	igd	0.0061	3.38E-06	7308	M15778.m	TRUE	reported	941o3l	2	TRUE	0.002957	21.66996
5142	rs4542650	T	C	T	C	0.0624	-0.08106	0.1769	0.176582	FALSE	FALSE	FALSE	ieu-b-508	16	12965048	0.043826	486484	0.064384	Sepsis (28	Sepsis (28	Sepsis (28	TRUE	igd	0.0134	3.19E-06	2616	M33190.m	TRUE	reported	UkhAw5	2	TRUE	0.008221	21.66843
4297	rs1192325	A	G	A	G	-0.0419	0.09905	0.9158	0.914495	FALSE	FALSE	FALSE	ieu-b-508	2	84959080	0.059677	486484	0.096964	Sepsis (28	Sepsis (28	Sepsis (28	TRUE	igd	0.009	3.30E-06	7255	M32836.m	TRUE	reported	d4lVXe	2	TRUE	0.002979	21.66822
7896	rs2127434	A	G	A	G	0.0284	-0.00917	0.7956	0.779386	FALSE	FALSE	FALSE	ieu-b-508	17	71737392	0.04074	486484	0.821846	Sepsis (28	Sepsis (28	Sepsis (28	TRUE	igd	0.0061	3.23E-06	5629	M35669.m	TRUE	reported	gSecuE	2	TRUE	0.003836	21.66819
6937	rs3852445	C	G	C	G	0.0284	0.050313	0.1284	0.136613	FALSE	TRUE	FALSE	ieu-b-508	10	71592174	0.049284	486484	0.307309	Sepsis (28	Sepsis (28	Sepsis (28	TRUE	igd	0.0061	2.95E-06	5208	M34395.m	TRUE	reported	QVOPA3	2	TRUE	0.004145	21.66757
6958	rs9333273	A	G	A	G	0.0554	-0.10957	0.0869	0.018331	FALSE	FALSE	FALSE	ieu-b-508	10	15618890	0.123039	486484	0.373199	Sepsis (28	Sepsis (28	Sepsis (28	TRUE	igd	0.0119	3.54E-06	5689	M34400.m	TRUE	reported	QXmzfR	2	TRUE	0.003795	21.66571
3403	rs9316766	A	G	A	G	-0.027	0.034127	0.7962	0.797514	FALSE	FALSE	FALSE	ieu-b-508	13	22692292	0.041749	486484	0.413678	Sepsis (28	Sepsis (28	Sepsis (28	TRUE	igd	0.0058	2.82E-06	7346	M32425.m	TRUE	reported	u5RLGY	2	TRUE	0.002941	21.66473
6594	rs1083586	A	G	A	G	0.1318	-0.01162	0.9826	0.587851	FALSE	FALSE	FALSE	ieu-b-508	11	32230877	0.033808	486484	0.731126	Sepsis (28	Sepsis (28	Sepsis (28	TRUE	igd	0.0283	3.31E-06	1673	M34306.m	TRUE	reported	WPXPUN	2	TRUE	0.012799	21.66399
6193	rs1738009	T	C	T	C	-0.0135	-0.01441	0.5345	0.550007	FALSE	FALSE	FALSE	ieu-b-508	2	83637055	0.034103	486484	0.672662	Sepsis (28	Sepsis (28	Sepsis (28	TRUE	igd	0.0029	2.75E-06	6004	M33892.m	TRUE	reported	w7HgU7	2	TRUE	0.003596	21.66341
6198	rs20301	A	G	A	G	0.0135	-0.00372	0.5617	0.557914	FALSE	FALSE	FALSE	ieu-b-508	22	33983594	0.033603	486484	0.91179	Sepsis (28	Sepsis (28	Sepsis (28	TRUE	igd	0.0029	2.98E-06	6004	M33892.m	TRUE	reported	w7HgU7	2	TRUE	0.003596	21.66341
6029	rs1160835	T	C	T	C	0.2555	0.217679	0.0342	0.032267	FALSE	FALSE	FALSE	ieu-b-508	12	44701141	0.094857	486484	0.021744	Sepsis (28	Sepsis (28	Sepsis (28	TRUE	igd	0.0548	3.07E-06	581	M33782.m	TRUE	reported	875Pal	2	TRUE	0.036066	21.66323
4186	rs2132551	A	C	A	C	0.054	0.119532	0.964	0.958601	FALSE	FALSE	FALSE	ieu-b-508	20	24282913	0.083885	486484	0.154172	Sepsis (28	Sepsis (28	Sepsis (28	TRUE	igd	0.0116	3.56E-06	5579	M32761.m	TRUE	reported	QefVGc	2	TRUE	0.003869	21.66286
1666	rs4404993	T	G	T	G	0.0964	0.027977	0.1848	0.20702	FALSE	FALSE	FALSE	ieu-b-508	9	19367826	0.041269	486484	0.497831	Sepsis (28	Sepsis (28	Sepsis (28	TRUE	igd	0.0207	3.22E-06	1522	M15335.m	TRUE	reported	a7RjFn	2	TRUE	0.014049	21.6592
2306	rs1269917	A	G	A	G	-0.0461	0.029278	0.9499	0.957632	FALSE	FALSE	FALSE	ieu-b-508	7	1746349	0.082911	486484	0.723996	Sepsis (28	Sepsis (28	Sepsis (28	TRUE	igd	0.0099	3.03E-06	1748	M20699.m	TRUE	reported	Z3EKFa	2	TRUE	0.012253	21.65879
692	rs4863214	T	C	T	C	-0.0149	-0.03534	0.7398	0.688595	FALSE	FALSE	FALSE	ieu-b-508	4	1.91E+08	0.03654	486484	0.333512	Sepsis (28	Sepsis (28	Sepsis (28	TRUE	igd	0.0032	3.98E-06	1761	M01299.m	TRUE	reported	gfM0a3	2	TRUE	0.012162	21.65604
8512	rs1748359	T	C	T	C	0.1024	0.121784	0.9827	0.982206	FALSE	FALSE	FALSE	ieu-b-508	2	61632918	0.126247	486484	0.334723	Sepsis (28	Sepsis (28	Sepsis (28	TRUE	igd	0.022	3.16E-06	4520	M37104.m	TRUE	reported	bR3g7d	2	TRUE	0.00477	21.65521
6345	rs1046508	A	T	A	T	-0.0121	-0.00252	0.2818	0.280587	FALSE	TRUE	FALSE	ieu-b-508	9	34012222	0.037125	486484	0.945805	Sepsis (28	Sepsis (28	Sepsis (28	TRUE	igd	0.0026	4.69E-06	7364	M33960.m	TRUE	reported	o8RcxU	2	TRUE	0.002932	21.6524
3955	rs1801394	A	G	A	G	0.0121	-0.07558	0.4675	0.436014	FALSE	FALSE	FALSE	ieu-b-508	5	7870973	0.033663	486484	0.024761	Sepsis (28	Sepsis (28	Sepsis (28	TRUE	igd	0.0026	3.33E-06	7363	M32709.m	TRUE	reported	YR8SB4	2	TRUE	0.002933	21.6524
3116	rs9348257	A	T	A	T	0.0121	-0.00854	0.2629	0.251625	FALSE	TRUE	FALSE	ieu-b-508	6	1.7E+08	0.038671	486484	0.825191	Sepsis (28	Sepsis (28	Sepsis (28	TRUE	igd	0.0026	2.74E-06	7356	M32198.m	TRUE	reported	ndXmnp	2	TRUE	0.002936	21.6524
772	rs1024217	A	G	A	G	-0.0121	-0.00753	0.1355	0.139437	FALSE	FALSE	FALSE	ieu-b-508	7	28944145	0.048196	486484	0.875899	Sepsis (28	Sepsis (28	Sepsis (28	TRUE	igd	0.0026	3.73E-06	7352	M01336.m	TRUE	reported	FAlrya	2	TRUE	0.002937	21.65239
7412	rs1108167	T	C	T	C	0.0121	0.045955	0.3179	0.340467	FALSE	FALSE	FALSE	ieu-b-508	18	76708276	0.03541	486484	0.194358	Sepsis (28	Sepsis (28	Sepsis (28	TRUE	igd	0.0026	3.70E-06	7350	M35186.m	TRUE	reported	UZfu6Z	2	TRUE	0.002938	21.65239
519	rs1693280	T	G	T	G	-0.0121	0.088684	0.1673	0.168115	FALSE	FALSE	FALSE	ieu-b-508	9	35961087	0.04488	486484	0.048152	Sepsis (28	Sepsis (28	Sepsis (28	TRUE	igd	0.0026	5.01E-06	7345	M00584.m	TRUE	reported	oBt02X	2	TRUE	0.00294	21.65239
1305	rs6994363	A	G	A	G	-0.0121	-0.01667	0.8662	0.84654	FALSE	FALSE	FALSE	ieu-b-508	8	11125062	0.046916	486484	0.722296	Sepsis (28	Sepsis (28	Sepsis (28	TRUE	igd	0.0026	2.50E-06	7325	M02132.m	TRUE	reported	uJFm2t	2	TRUE	0.002948	21.65237
2151	rs1280328	A	G	A	G	-0.0121	0.023593	0.2119	0.229516	FALSE	FALSE	FALSE	ieu-b-508	11	1.21E+08	0.039626	486484	0.551583	Sepsis (28	Sepsis (28	Sepsis (28	TRUE	igd	0.0026	4.31E-06	7286	M18929.m	TRUE	reported	2vYFb2	2	TRUE	0.002964	21.65234
2158	rs4904262	T	C	T	C	-0.0121	0.018968	0.1727	0.173911	FALSE	FALSE	FALSE	ieu-b-508	14	86064367	0.044011	486484	0.666481	Sepsis (28	Sepsis (28	Sepsis (28	TRUE	igd	0.0026	4.91E-06	7286	M18929.m	TRUE	reported	2vYFb2	2	TRUE	0.002964	21.65234
7502	rs9293713	A	G	A	G	-0.0242	0.091577	0.0653	0.068287	FALSE	FALSE	FALSE	ieu-b-508	5	76705255	0.066724	486484	0.16992	Sepsis (28	Sepsis (28	Sepsis (28	TRUE	igd	0.0052	3.39E-06	7261	M35253.m	TRUE	reported	VboRmj	2	TRUE	0.002974	21.65232
7761	rs1219213	A	G	A	G	-0.0121	0.01211	0.262	0.276038	FALSE	FALSE	FALSE	ieu-b-508	6	82001561	0.037272	486484	0.745257	Sepsis (28	Sepsis (28	Sepsis (28	TRUE	igd	0.0026	4.75E-06	7256	M35439.m	TRUE	reported	DnuAGZ	2	TRUE	0.002976	21.65231
2648	rs805780	A	C	A	C	0.0121	-0.03641	0.7634	0.774561	FALSE	FALSE	FALSE	ieu-b-508	7	1.23E+08	0.039982	486484	0.362523	Sepsis (28	Sepsis (28	Sepsis (28	TRUE	igd	0.0026	3.47E-06	7184	M22548.m	TRUE	reported	mZtwX2	2	TRUE	0.003006	21.65225
5833	rs6694467	A	G	A	G	0.0121	0.020579	0.8153	0.825973	FALSE	FALSE	FALSE	ieu-b-508	1	71892420	0.044026	486484	0.640198	Sepsis (28	Sepsis (28	Sepsis (28	TRUE	igd	0.0026	4.44E-06	7066	M33515.m	TRUE	reported	X9mul1	2	TRUE	0.003056	21.65215
4285	rs1581762	A	G	A	G	-0.0121	0.020379	0.4121	0.422327	FALSE	FALSE	FALSE	ieu-b-508	1	58251060	0.033932	486484	0.54812	Sepsis (28	Sepsis (28	Sepsis (28	TRUE	igd	0.0026	3.28E-06	7063	M32814.m	TRUE	reported	z0OeTx	2	TRUE	0.003057	21.65215
7741	rs4730501	A	G	A	G	-0.0121	0.055852	0.239	0.243408	FALSE	FALSE	FALSE	ieu-b-508	7	1.11E+08	0.038888	486484	0.150938	Sepsis (28	Sepsis (28	Sepsis (28	TRUE	igd	0.0026	4.18E-06	7007	M35431.m	TRUE	reported	tLfE4l	2	TRUE	0.003081	21.6521
6833	rs7763535	A	C	A	C	0.0121	-0.05447	0.7642	0.707286	FALSE	FALSE	FALSE	ieu-b-508	6	35662106	0.036694	486484	0.137697	Sepsis (28	Sepsis (28	Sepsis (28	TRUE	igd	0.0026	2.06E-06	6755	M34359.m	TRUE	reported	0UotaJ	2	TRUE	0.003196	21.65187
6281	rs1869289	A	C	A	C	-0.0121	-0.00252	0.2164	0.221508	FALSE	FALSE	FALSE	ieu-b-508	8	1.14E+08	0.040189	486484	0.950101	Sepsis (28	Sepsis (28	Sepsis (28	TRUE	igd	0.0026	4.31E-06	6502	M33939.m	TRUE	reported	PeKlgz	2	TRUE	0.00332	21.65162
4576	rs1093283	A	G	A	G	0.0121	-0.04	0.5337	0.542255	FALSE	FALSE	FALSE	ieu-b-508	2	2.21E+08	0.033549	486484	0.233172	Sepsis (28	Sepsis (28	Sepsis (28	TRUE	igd	0.0026	3.86E-06	6404	M33163.m	TRUE	reported	YzGXkR	2	TRUE	0.003371	21.65152
6877	rs3759903	T	G	T	G	0.1253	-0.03555	0.1612	0.074933	FALSE	FALSE	FALSE	ieu-b-508	15	99768642	0.06369	486484	0.576735	Sepsis (28	Sepsis (28	Sepsis (28	TRUE	igd	0.0269	3.09E-06	954	M34365.m	TRUE	reported	8eexD0	2	TRUE	0.022237	21.65141
5373	rs1690511	A	T	A	T	0.3196	0.006739	0.0413	0.048982	FALSE	TRUE	FALSE	ieu-b-508	8	1.35E+08	0.078133	486484	0.931264	Sepsis (28	Sepsis (28	Sepsis (28	TRUE	igd	0.0686	3.17E-06	803	M33384.m	TRUE	reported	lOffrZ	2	TRUE	0.026319	21.65122
5994	rs2074116	A	G	A	G	-0.0503	0.0011	0.7665	0.779447	FALSE	FALSE	FALSE	ieu-b-508	7	1.11E+08	0.040426	486484	0.978287	Sepsis (28	Sepsis (28	Sepsis (28	TRUE	igd	0.0108	3.61E-06	1027	M33683.m	TRUE	reported	wSozpU	2	TRUE	0.020684	21.6492
7311	rs929005	A	T	A	T	-0.0349	0.033299	0.1602	0.221982	FALSE	TRUE	FALSE	ieu-b-508	4	3709627	0.04092	486484	0.415779	Sepsis (28	Sepsis (28	Sepsis (28	TRUE	igd	0.0075	3.18E-06	7364	M35126.m	TRUE	reported	1haOJA	2	TRUE	0.002932	21.64763
7236	rs7942717	A	G	A	G	-0.1094	-0.01888	0.9543	0.919133	FALSE	FALSE	FALSE	ieu-b-508	11	61647288	0.061893	486484	0.760325	Sepsis (28	Sepsis (28	Sepsis (28	TRUE	igd	0.0235	3.18E-06	1763	M34878.m	TRUE	reported	Cpq9yU	2	TRUE	0.012143	21.64741
1097	rs9549021	T	C	T	C	-0.0135	0.048935	0.6112	0.626747	FALSE	FALSE	FALSE	ieu-b-508	13	40543493	0.03465	486484	0.157877	Sepsis (28	Sepsis (28	Sepsis (28	TRUE	igd	0.0029	2.94E-06	1767	M01604.m	TRUE	reported	hE3l2k	2	TRUE	0.012115	21.6461
804	rs6486528	C	G	C	G	0.0228	-0.01505	0.9236	0.921633	FALSE	TRUE	FALSE	ieu-b-508	12	1.31E+08	0.063271	486484	0.811958	Sepsis (28	Sepsis (28	Sepsis (28	TRUE	igd	0.0049	3.30E-06	7335	M01356.m	TRUE	reported	ah1g5W	2	TRUE	0.002943	21.64508
4478	rs786272	A	G	A	G	0.0228	0.038593	0.6761	0.662684	FALSE	FALSE	FALSE	ieu-b-508	2	1.46E+08	0.035363	486484	0.275124	Sepsis (28	Sepsis (28	Sepsis (28	TRUE	igd	0.0049	3.02E-06	5425	M33084.m	TRUE	reported	jrOsNK	2	TRUE	0.003975	21.643
3762	rs1783633	C	G	C	G	0.0335	-0.20681	0.9808	0.986661	FALSE	TRUE	FALSE	ieu-b-508	9	23619254	0.145304	486484	0.15465	Sepsis (28	Sepsis (28	Sepsis (28	TRUE	igd	0.0072	2.99E-06	7362	M32634.m	TRUE	reported	7T2RUz	2	TRUE	0.002932	21.64246
5276	rs2526978	T	C	T	C	-0.0228	0.028333	0.2984	0.309899	FALSE	FALSE	FALSE	ieu-b-508	7	1.06E+08	0.036005	486484	0.43132	Sepsis (28	Sepsis (28	Sepsis (28	TRUE	igd	0.0049	2.63E-06	4409	M33250.m	TRUE	reported	WIZl6U	2	TRUE	0.004887	21.64116
6763	rs666676	C	G	C	G	-0.298	0.138871	0.989	0.983447	FALSE	TRUE	FALSE	ieu-b-508	18	3001658	0.131457	486484	0.290786	Sepsis (28	Sepsis (28	Sepsis (28	TRUE	igd	0.064	3.22E-06	1016	M34339.m	TRUE	reported	RwGZ7c	2	TRUE	0.020893	21.63799
5557	rs1683482	T	C	T	C	-1.557	-0.11496	0.0179	0.02456	FALSE	FALSE	FALSE	ieu-b-508	1	2.37E+08	0.109335	486484	0.293036	Sepsis (28	Sepsis (28	Sepsis (28	TRUE	igd	0.3291	2.23E-06	60	M33423.m	TRUE	reported	MoEnO7	2	TRUE	0.271696	21.63706
1102	rs1271426	A	G	A	G	-0.0107	-0.03713	0.5733	0.570769	FALSE	FALSE	FALSE	ieu-b-508	2	29318077	0.03391	486484	0.273505	Sepsis (28	Sepsis (28	Sepsis (28	TRUE	igd	0.0023	3.98E-06	7104	M01638.m	TRUE	reported	8dp0Ac	2	TRUE	0.003037	21.63663
2124	rs7837471	A	G	A	G	0.3403	0.196509	0.015	0.027966	FALSE	FALSE	FALSE	ieu-b-508	8	1.27E+08	0.100861	486484	0.051377	Sepsis (28	Sepsis (28	Sepsis (28	TRUE	igd	0.0731	3.27E-06	1157	M18476.m	TRUE	reported	6GnBtw	2	TRUE	0.018386	21.63405
7068	rs1281014	T	C	T	C	0.0754	-0.03043	0.079	0.114251	FALSE	FALSE	FALSE	ieu-b-508	12	71891734	0.052338	486484	0.560985	Sepsis (28	Sepsis (28	Sepsis (28	TRUE	igd	0.0162	3.35E-06	1462	M34469.m	TRUE	reported	WMOqbm	2	TRUE	0.014601	21.63307
7955	rs1207084	A	G	A	G	0.0414	-0.1261	0.0702	0.085161	FALSE	FALSE	FALSE	ieu-b-508	1	1.09E+08	0.059552	486484	0.034222	Sepsis (28	Sepsis (28	Sepsis (28	TRUE	igd	0.0089	3.16E-06	6447	M35678.m	TRUE	reported	56yR01	2	TRUE	0.003345	21.63146
8557	rs7968170	T	C	T	C	-0.04	0.024985	0.4284	0.458576	FALSE	FALSE	FALSE	ieu-b-508	12	16149335	0.033695	486484	0.45839	Sepsis (28	Sepsis (28	Sepsis (28	TRUE	igd	0.0086	3.16E-06	7355	M37202.m	TRUE	reported	BtslDx	2	TRUE	0.002933	21.62743
7419	rs2504453	A	T	A	T	0.02	0.03891	0.8969	0.886885	FALSE	TRUE	FALSE	ieu-b-508	1	1.08E+08	0.052855	486484	0.461624	Sepsis (28	Sepsis (28	Sepsis (28	TRUE	igd	0.0043	3.35E-06	7350	M35186.m	TRUE	reported	UZfu6Z	2	TRUE	0.002935	21.62743
3663	rs603677	A	C	A	C	0.12	0.038431	0.9724	0.982439	FALSE	FALSE	FALSE	ieu-b-508	9	1.38E+08	0.128391	486484	0.764689	Sepsis (28	Sepsis (28	Sepsis (28	TRUE	igd	0.0258	3.35E-06	6970	M32564.m	TRUE	reported	73MgOk	2	TRUE	0.003094	21.62711
3293	rs2575043	A	T	A	T	-0.02	0.050374	0.2748	0.281522	FALSE	TRUE	FALSE	ieu-b-508	8	31284500	0.037138	486484	0.174971	Sepsis (28	Sepsis (28	Sepsis (28	TRUE	igd	0.0043	3.63E-06	6119	M32379.m	TRUE	reported	MLimM6	2	TRUE	0.003523	21.62624
2022	rs6955908	T	C	T	C	0.0386	0.021784	0.4325	0.427344	FALSE	FALSE	FALSE	ieu-b-508	7	1.57E+08	0.033914	486484	0.520663	Sepsis (28	Sepsis (28	Sepsis (28	TRUE	igd	0.0083	3.50E-06	5899	M18335.m	TRUE	reported	IdUJrL	2	TRUE	0.003653	21.62077
68	rs2063601	A	C	A	C	-0.0107	0.059509	0.2487	0.236742	FALSE	FALSE	FALSE	ieu-b-508	6	78578306	0.039278	486484	0.129757	Sepsis (28	Sepsis (28	Sepsis (28	TRUE	igd	0.0023	5.24E-06	1763	M00054.m	TRUE	reported	QTDAnh	2	TRUE	0.012127	21.61817
5377	rs281083	A	G	A	G	-0.1634	0.04291	0.1001	0.129836	FALSE	FALSE	FALSE	ieu-b-508	13	1.04E+08	0.049854	486484	0.389404	Sepsis (28	Sepsis (28	Sepsis (28	TRUE	igd	0.0351	3.24E-06	803	M33384.m	TRUE	reported	lOffrZ	2	TRUE	0.026279	21.61757
1815	rs1246642	A	G	A	G	0.0279	0.021943	0.6945	0.694189	FALSE	FALSE	FALSE	ieu-b-508	2	10332057	0.036018	486484	0.54237	Sepsis (28	Sepsis (28	Sepsis (28	TRUE	igd	0.006	3.96E-06	7357	M15753.m	TRUE	reported	VDJYDI	2	TRUE	0.00293	21.61662
7325	rs4839333	A	T	A	T	0.0186	-0.07998	0.8828	0.886905	FALSE	TRUE	FALSE	ieu-b-508	1	1.14E+08	0.052625	486484	0.128574	Sepsis (28	Sepsis (28	Sepsis (28	TRUE	igd	0.004	2.91E-06	7344	M35127.m	TRUE	reported	RNsmjl	2	TRUE	0.002936	21.61661
3056	rs8020226	T	C	T	C	0.0465	0.029949	0.8887	0.879989	FALSE	FALSE	FALSE	ieu-b-508	14	50603129	0.051359	486484	0.559807	Sepsis (28	Sepsis (28	Sepsis (28	TRUE	igd	0.01	3.40E-06	7338	M31591.m	TRUE	reported	KltFxx	2	TRUE	0.002938	21.61661
6533	rs7483614	T	C	T	C	0.0186	0.056566	0.8665	0.837766	FALSE	FALSE	FALSE	ieu-b-508	11	32729647	0.045352	486484	0.2123	Sepsis (28	Sepsis (28	Sepsis (28	TRUE	igd	0.004	3.97E-06	7049	M34123.m	TRUE	reported	9O5wo1	2	TRUE	0.003058	21.61637
3011	rs737853	A	G	A	G	-0.0736	0.0089	0.617	0.530644	FALSE	FALSE	FALSE	ieu-b-508	22	19861157	0.033787	486484	0.792222	Sepsis (28	Sepsis (28	Sepsis (28	TRUE	igd	0.0158	3.05E-06	522	M31536.m	TRUE	reported	3nAuUl	2	TRUE	0.03991	21.61595
2538	rs1278732	T	G	T	G	0.0093	-0.10648	0.6649	0.650066	FALSE	FALSE	FALSE	ieu-b-508	11	1.26E+08	0.035174	486484	0.002469	Sepsis (28	Sepsis (28	Sepsis (28	TRUE	igd	0.002	1.65E-06	6535	M22177.m	TRUE	reported	2s6Sly	2	TRUE	0.003298	21.61588
1343	rs6466265	A	G	A	G	0.0186	0.00897	0.7977	0.802858	FALSE	FALSE	FALSE	ieu-b-508	7	78160248	0.042241	486484	0.831829	Sepsis (28	Sepsis (28	Sepsis (28	TRUE	igd	0.004	4.15E-06	5791	M02342.m	TRUE	reported	EhZr3g	2	TRUE	0.00372	21.61503
931	rs1775180	A	G	A	G	0.0628	0.148401	0.9645	0.96565	FALSE	FALSE	FALSE	ieu-b-508	14	65034363	0.092692	486484	0.109373	Sepsis (28	Sepsis (28	Sepsis (28	TRUE	igd	0.0135	3.40E-06	1650	M01508.m	TRUE	reported	go526k	2	TRUE	0.012945	21.6135
8506	rs1046376	T	C	T	C	-0.0372	-0.02336	0.1434	0.145405	FALSE	FALSE	FALSE	ieu-b-508	5	1.23E+08	0.047482	486484	0.622798	Sepsis (28	Sepsis (28	Sepsis (28	TRUE	igd	0.008	3.37E-06	4520	M37104.m	TRUE	reported	bR3g7d	2	TRUE	0.004761	21.61293
4726	rs1187664	T	C	T	C	-0.348	0.258972	0.0271	0.005743	FALSE	FALSE	FALSE	ieu-b-508	18	73010900	0.222395	486484	0.244234	Sepsis (28	Sepsis (28	Sepsis (28	TRUE	igd	0.0718	1.23E-06	25	M33178.m	TRUE	reported	g22WMS	2	TRUE	0.484445	21.61212
2937	rs1191458	T	C	T	C	-0.0307	0.015228	0.3531	0.348842	FALSE	FALSE	FALSE	ieu-b-508	3	1.89E+08	0.035001	486484	0.663506	Sepsis (28	Sepsis (28	Sepsis (28	TRUE	igd	0.0066	2.89E-06	1758	M27738.m	TRUE	reported	UtPBd9	2	TRUE	0.012158	21.61198
3635	rs1195197	A	G	A	G	-0.0493	0.008703	0.352	0.424367	FALSE	FALSE	FALSE	ieu-b-508	5	1.08E+08	0.034148	486484	0.798831	Sepsis (28	Sepsis (28	Sepsis (28	TRUE	igd	0.0106	3.15E-06	2091	M32560.m	TRUE	reported	sLwsEw	2	TRUE	0.010239	21.61058
8289	rs1172292	T	C	T	C	-0.0307	0.028033	0.8291	0.819389	FALSE	FALSE	FALSE	ieu-b-508	4	82083445	0.043531	486484	0.51959	Sepsis (28	Sepsis (28	Sepsis (28	TRUE	igd	0.0066	3.74E-06	1636	M36559.m	TRUE	reported	g6I9hg	2	TRUE	0.013053	21.61014
7100	rs2974445	T	C	T	C	0.0279	-0.03082	0.5529	0.550707	FALSE	FALSE	FALSE	ieu-b-508	5	82481380	0.033438	486484	0.356608	Sepsis (28	Sepsis (28	Sepsis (28	TRUE	igd	0.006	3.11E-06	3239	M34513.m	TRUE	reported	1hoso6	2	TRUE	0.006631	21.60915
6216	rs2432026	A	G	A	G	-0.0451	0.086164	0.0587	0.058453	FALSE	FALSE	FALSE	ieu-b-508	5	97406001	0.071147	486484	0.225865	Sepsis (28	Sepsis (28	Sepsis (28	TRUE	igd	0.0097	3.49E-06	4533	M33901.m	TRUE	reported	mQkhrb	2	TRUE	0.004746	21.60817
5110	rs9574766	A	G	A	G	-0.3499	-0.10214	0.025	0.046651	FALSE	FALSE	FALSE	ieu-b-508	13	81723607	0.088984	486484	0.251015	Sepsis (28	Sepsis (28	Sepsis (28	TRUE	igd	0.0722	1.25E-06	25	M33178.m	TRUE	reported	g22WMS	2	TRUE	0.48439	21.60734
7128	rs1326499	A	G	A	G	-0.0293	0.058157	0.2032	0.19159	FALSE	FALSE	FALSE	ieu-b-508	8	1.4E+08	0.042477	486484	0.170959	Sepsis (28	Sepsis (28	Sepsis (28	TRUE	igd	0.0063	3.46E-06	1741	M34527.m	TRUE	reported	EWFGP8	2	TRUE	0.012271	21.60503
7216	rs6907935	T	C	T	C	-0.0172	-0.05339	0.6945	0.688897	FALSE	FALSE	FALSE	ieu-b-508	6	41717855	0.036048	486484	0.13855	Sepsis (28	Sepsis (28	Sepsis (28	TRUE	igd	0.0037	3.81E-06	6735	M34732.m	TRUE	reported	IRNSa4	2	TRUE	0.003198	21.60352
8282	rs4762693	A	G	A	G	0.0172	-0.00465	0.2859	0.292848	FALSE	FALSE	FALSE	ieu-b-508	12	21162243	0.036938	486484	0.899774	Sepsis (28	Sepsis (28	Sepsis (28	TRUE	igd	0.0037	4.07E-06	6464	M36553.m	TRUE	reported	2Rk7nd	2	TRUE	0.003332	21.60325
2482	rs354468	T	C	T	C	0.0172	0.05537	0.1829	0.184453	FALSE	FALSE	FALSE	ieu-b-508	13	1.07E+08	0.043096	486484	0.198858	Sepsis (28	Sepsis (28	Sepsis (28	TRUE	igd	0.0037	4.59E-06	5676	M22032.m	TRUE	reported	xpe96K	2	TRUE	0.003793	21.60232
3642	rs4742370	C	G	C	G	-0.0465	0.104358	0.3392	0.342557	FALSE	TRUE	FALSE	ieu-b-508	9	7540384	0.035479	486484	0.003268	Sepsis (28	Sepsis (28	Sepsis (28	TRUE	igd	0.01	3.24E-06	2091	M32560.m	TRUE	reported	sLwsEw	2	TRUE	0.010235	21.60182
7048	rs4505228	T	C	T	C	-0.0172	-0.12689	0.8488	0.859324	FALSE	FALSE	FALSE	ieu-b-508	14	47676287	0.048864	486484	0.009411	Sepsis (28	Sepsis (28	Sepsis (28	TRUE	igd	0.0037	3.82E-06	5207	M34456.m	TRUE	reported	Nh457q	2	TRUE	0.004133	21.60163
5294	rs1681855	T	C	T	C	0.0507	-0.04602	0.2032	0.285022	FALSE	FALSE	FALSE	ieu-b-508	1	43357600	0.036984	486484	0.213335	Sepsis (28	Sepsis (28	Sepsis (28	TRUE	igd	0.0109	3.43E-06	1273	M33353.m	TRUE	reported	ZIN4gk	2	TRUE	0.016712	21.60131
8536	rs1118657	A	G	A	G	-0.1609	0.046051	0.9729	0.966351	FALSE	FALSE	FALSE	ieu-b-508	10	93217601	0.092052	486484	0.616879	Sepsis (28	Sepsis (28	Sepsis (28	TRUE	igd	0.0346	3.40E-06	1745	M37190.m	TRUE	reported	tiNuK9	2	TRUE	0.012241	21.6004
6037	rs7132897	A	T	A	T	0.1811	0.02598	0.0742	0.085068	FALSE	TRUE	FALSE	ieu-b-508	12	10495118	0.059666	486484	0.663254	Sepsis (28	Sepsis (28	Sepsis (28	TRUE	igd	0.0389	3.20E-06	581	M33782.m	TRUE	reported	875Pal	2	TRUE	0.035963	21.59932
480	rs1605457	T	C	T	C	0.3731	0.064761	0.9643	0.950834	FALSE	FALSE	FALSE	ieu-b-508	18	68662821	0.077082	486484	0.400821	Sepsis (28	Sepsis (28	Sepsis (28	TRUE	igd	0.08	3.10E-06	283	M00553.m	TRUE	reported	IFjXiL	2	TRUE	0.071372	21.59685
7448	rs3742758	A	G	A	G	0.0739	0.000377	0.0191	0.020926	FALSE	FALSE	FALSE	ieu-b-508	14	76670036	0.116549	486484	0.99742	Sepsis (28	Sepsis (28	Sepsis (28	TRUE	igd	0.0159	3.45E-06	6234	M35189.m	TRUE	reported	54XZFj	2	TRUE	0.003453	21.5951
7747	rs1194525	T	C	T	C	-0.033	0.08873	0.0592	0.061666	FALSE	FALSE	FALSE	ieu-b-508	4	65511966	0.069834	486484	0.203873	Sepsis (28	Sepsis (28	Sepsis (28	TRUE	igd	0.0071	3.82E-06	5306	M35433.m	TRUE	reported	XlYkLe	2	TRUE	0.004055	21.59471
3237	rs1602290	A	G	A	G	0.0488	0.116928	0.1276	0.126831	FALSE	FALSE	FALSE	ieu-b-508	4	44292501	0.050041	486484	0.019457	Sepsis (28	Sepsis (28	Sepsis (28	TRUE	igd	0.0105	3.25E-06	6687	M32346.m	TRUE	reported	cnIFoV	2	TRUE	0.00322	21.5939
1090	rs235320	A	G	A	G	-0.0079	0.034778	0.8241	0.80565	FALSE	FALSE	FALSE	ieu-b-508	21	46277214	0.042337	486484	0.411383	Sepsis (28	Sepsis (28	Sepsis (28	TRUE	igd	0.0017	3.68E-06	7371	M01604.m	TRUE	reported	hE3l2k	2	TRUE	0.002921	21.5893
8620	rs559346	T	C	T	C	0.0079	0.040158	0.4193	0.421261	FALSE	FALSE	FALSE	ieu-b-508	1	28393814	0.03378	486484	0.23451	Sepsis (28	Sepsis (28	Sepsis (28	TRUE	igd	0.0017	6.62E-06	7366	M37506.m	TRUE	reported	m2kaRt	2	TRUE	0.002923	21.58929
439	rs6970076	T	C	T	C	-0.0079	0.04672	0.7679	0.766622	FALSE	FALSE	FALSE	ieu-b-508	7	1.06E+08	0.039485	486484	0.23672	Sepsis (28	Sepsis (28	Sepsis (28	TRUE	igd	0.0017	4.37E-06	7365	M00063.m	TRUE	reported	Hdsv1L	2	TRUE	0.002924	21.58929
440	rs7595385	T	C	T	C	0.0237	-0.05007	0.9759	0.957312	FALSE	FALSE	FALSE	ieu-b-508	2	1.67E+08	0.084267	486484	0.5524	Sepsis (28	Sepsis (28	Sepsis (28	TRUE	igd	0.0051	3.83E-06	7365	M00063.m	TRUE	reported	Hdsv1L	2	TRUE	0.002924	21.58929
444	rs927961	T	G	T	G	-0.0079	-0.05906	0.7597	0.759188	FALSE	FALSE	FALSE	ieu-b-508	1	41246301	0.039027	486484	0.130215	Sepsis (28	Sepsis (28	Sepsis (28	TRUE	igd	0.0017	5.06E-06	7365	M00063.m	TRUE	reported	Hdsv1L	2	TRUE	0.002924	21.58929
727	rs4426541	A	G	A	G	-0.0079	-0.03243	0.2229	0.226659	FALSE	FALSE	FALSE	ieu-b-508	2	1.67E+08	0.039884	486484	0.416115	Sepsis (28	Sepsis (28	Sepsis (28	TRUE	igd	0.0017	5.40E-06	7363	M01301.m	TRUE	reported	w8TL71	2	TRUE	0.002924	21.58929
681	rs1475943	T	C	T	C	0.0079	0.02584	0.2215	0.239415	FALSE	FALSE	FALSE	ieu-b-508	22	47305436	0.039213	486484	0.509925	Sepsis (28	Sepsis (28	Sepsis (28	TRUE	igd	0.0017	3.69E-06	7358	M01299.m	TRUE	reported	gfM0a3	2	TRUE	0.002926	21.58929
810	rs1075783	T	C	T	C	0.0079	-0.0764	0.6521	0.670488	FALSE	FALSE	FALSE	ieu-b-508	9	2954622	0.035594	486484	0.031834	Sepsis (28	Sepsis (28	Sepsis (28	TRUE	igd	0.0017	4.74E-06	7355	M01358.m	TRUE	reported	kSPQgd	2	TRUE	0.002928	21.58928
815	rs1662999	T	C	T	C	0.0079	0.067978	0.6325	0.621997	FALSE	FALSE	FALSE	ieu-b-508	2	26886802	0.034427	486484	0.048322	Sepsis (28	Sepsis (28	Sepsis (28	TRUE	igd	0.0017	4.21E-06	7355	M01358.m	TRUE	reported	kSPQgd	2	TRUE	0.002928	21.58928
818	rs2115366	T	C	T	C	-0.0079	-0.04851	0.4418	0.441553	FALSE	FALSE	FALSE	ieu-b-508	2	8464031	0.033679	486484	0.149796	Sepsis (28	Sepsis (28	Sepsis (28	TRUE	igd	0.0017	3.93E-06	7355	M01358.m	TRUE	reported	kSPQgd	2	TRUE	0.002928	21.58928
834	rs7996008	A	G	A	G	0.0079	-0.04766	0.2935	0.294869	FALSE	FALSE	FALSE	ieu-b-508	13	58365712	0.036564	486484	0.19237	Sepsis (28	Sepsis (28	Sepsis (28	TRUE	igd	0.0017	5.69E-06	7355	M01358.m	TRUE	reported	kSPQgd	2	TRUE	0.002928	21.58928
2067	rs3747115	T	C	T	C	0.0079	0.003751	0.4954	0.501869	FALSE	FALSE	FALSE	ieu-b-508	22	24815288	0.033343	486484	0.910431	Sepsis (28	Sepsis (28	Sepsis (28	TRUE	igd	0.0017	2.32E-06	7354	M18369.m	TRUE	reported	ykBjIT	2	TRUE	0.002928	21.58928
7420	rs35544	A	G	A	G	0.0158	-0.08103	0.8264	0.83526	FALSE	FALSE	FALSE	ieu-b-508	5	1.07E+08	0.045019	486484	0.071867	Sepsis (28	Sepsis (28	Sepsis (28	TRUE	igd	0.0034	4.26E-06	7350	M35186.m	TRUE	reported	UZfu6Z	2	TRUE	0.00293	21.58928
1206	rs1007518	T	G	T	G	0.0316	0.111225	0.9687	0.967152	FALSE	FALSE	FALSE	ieu-b-508	5	4145329	0.094096	486484	0.237189	Sepsis (28	Sepsis (28	Sepsis (28	TRUE	igd	0.0068	3.44E-06	7348	M01670.m	TRUE	reported	kjghbc	2	TRUE	0.00293	21.58928
752	rs9598459	A	G	A	G	0.0079	-0.03489	0.896	0.892532	FALSE	FALSE	FALSE	ieu-b-508	13	63035349	0.053663	486484	0.515568	Sepsis (28	Sepsis (28	Sepsis (28	TRUE	igd	0.0017	5.17E-06	7347	M01302.m	TRUE	reported	sNp2IC	2	TRUE	0.002931	21.58928
7714	rs4690099	T	C	T	C	0.0079	0.003856	0.2323	0.227227	FALSE	FALSE	FALSE	ieu-b-508	4	3454454	0.03984	486484	0.922886	Sepsis (28	Sepsis (28	Sepsis (28	TRUE	igd	0.0017	2.72E-06	7345	M35397.m	TRUE	reported	Y56mZr	2	TRUE	0.002931	21.58928
7719	rs6963604	A	G	A	G	0.0079	-0.04143	0.2522	0.251183	FALSE	FALSE	FALSE	ieu-b-508	7	21168716	0.038542	486484	0.282411	Sepsis (28	Sepsis (28	Sepsis (28	TRUE	igd	0.0017	2.20E-06	7345	M35397.m	TRUE	reported	Y56mZr	2	TRUE	0.002931	21.58928
4422	rs1194940	T	C	T	C	-0.0079	-0.04639	0.686	0.689441	FALSE	FALSE	FALSE	ieu-b-508	5	1.77E+08	0.036182	486484	0.199805	Sepsis (28	Sepsis (28	Sepsis (28	TRUE	igd	0.0017	6.79E-06	7344	M32910.m	TRUE	reported	2OCBKh	2	TRUE	0.002932	21.58927
3890	rs1110233	A	G	A	G	-0.0079	0.003798	0.1938	0.201014	FALSE	FALSE	FALSE	ieu-b-508	1	1.12E+08	0.041577	486484	0.927224	Sepsis (28	Sepsis (28	Sepsis (28	TRUE	igd	0.0017	5.37E-06	7338	M32675.m	TRUE	reported	9FmPh9	2	TRUE	0.002934	21.58927
3895	rs1867017	T	G	T	G	-0.0079	-0.00609	0.221	0.254222	FALSE	FALSE	FALSE	ieu-b-508	7	52762340	0.038569	486484	0.8746	Sepsis (28	Sepsis (28	Sepsis (28	TRUE	igd	0.0017	2.75E-06	7338	M32675.m	TRUE	reported	9FmPh9	2	TRUE	0.002934	21.58927
1767	rs1261661	A	G	A	G	0.0079	-0.02652	0.5527	0.542412	FALSE	FALSE	FALSE	ieu-b-508	2	1.54E+08	0.033758	486484	0.432154	Sepsis (28	Sepsis (28	Sepsis (28	TRUE	igd	0.0017	3.83E-06	7331	M15676.m	TRUE	reported	s1NfKN	2	TRUE	0.002937	21.58926
4052	rs1620675	T	G	T	G	0.0079	0.02009	0.3434	0.346705	FALSE	FALSE	FALSE	ieu-b-508	3	3192927	0.035023	486484	0.566224	Sepsis (28	Sepsis (28	Sepsis (28	TRUE	igd	0.0017	6.02E-06	7318	M32740.m	TRUE	reported	YLshfP	2	TRUE	0.002942	21.58925
4062	rs2875878	C	G	C	G	-0.0079	-0.00665	0.7045	0.698163	FALSE	TRUE	FALSE	ieu-b-508	6	1.26E+08	0.036314	486484	0.854593	Sepsis (28	Sepsis (28	Sepsis (28	TRUE	igd	0.0017	6.17E-06	7318	M32740.m	TRUE	reported	YLshfP	2	TRUE	0.002942	21.58925
4070	rs569137	A	G	A	G	-0.0079	0.033412	0.3585	0.35685	FALSE	FALSE	FALSE	ieu-b-508	8	23696290	0.035069	486484	0.340727	Sepsis (28	Sepsis (28	Sepsis (28	TRUE	igd	0.0017	5.13E-06	7318	M32740.m	TRUE	reported	YLshfP	2	TRUE	0.002942	21.58925
4076	rs6832662	A	G	A	G	-0.0079	0.00781	0.4257	0.427474	FALSE	FALSE	FALSE	ieu-b-508	4	1.37E+08	0.033666	486484	0.816545	Sepsis (28	Sepsis (28	Sepsis (28	TRUE	igd	0.0017	6.07E-06	7318	M32740.m	TRUE	reported	YLshfP	2	TRUE	0.002942	21.58925
1578	rs1379459	T	C	T	C	0.0079	0.044699	0.8083	0.782633	FALSE	FALSE	FALSE	ieu-b-508	2	6720877	0.040399	486484	0.268537	Sepsis (28	Sepsis (28	Sepsis (28	TRUE	igd	0.0017	3.14E-06	7314	M12593.m	TRUE	reported	IBXgSU	2	TRUE	0.002944	21.58925
1591	rs905508	T	C	T	C	0.0079	0.048659	0.1655	0.162788	FALSE	FALSE	FALSE	ieu-b-508	15	69571668	0.045404	486484	0.283856	Sepsis (28	Sepsis (28	Sepsis (28	TRUE	igd	0.0017	5.94E-06	7314	M12593.m	TRUE	reported	IBXgSU	2	TRUE	0.002944	21.58925
2743	rs1107270	T	C	T	C	0.0079	0.022303	0.71	0.715048	FALSE	FALSE	FALSE	ieu-b-508	6	22852032	0.036891	486484	0.545479	Sepsis (28	Sepsis (28	Sepsis (28	TRUE	igd	0.0017	4.44E-06	7303	M27256.m	TRUE	reported	mJwzsi	2	TRUE	0.002948	21.58924
2770	rs9520114	A	G	A	G	0.0079	-0.05379	0.2732	0.311048	FALSE	FALSE	FALSE	ieu-b-508	13	1.07E+08	0.036253	486484	0.137859	Sepsis (28	Sepsis (28	Sepsis (28	TRUE	igd	0.0017	3.70E-06	7303	M27256.m	TRUE	reported	mJwzsi	2	TRUE	0.002948	21.58924
2773	rs1157606	A	G	A	G	-0.0158	-0.06726	0.1125	0.127611	FALSE	FALSE	FALSE	ieu-b-508	1	1.53E+08	0.051243	486484	0.189316	Sepsis (28	Sepsis (28	Sepsis (28	TRUE	igd	0.0034	3.19E-06	7268	M27273.m	TRUE	reported	EvU2od	2	TRUE	0.002962	21.58921
2173	rs1111986	T	C	T	C	-0.0079	-0.03242	0.4341	0.43298	FALSE	FALSE	FALSE	ieu-b-508	1	2.12E+08	0.033591	486484	0.334544	Sepsis (28	Sepsis (28	Sepsis (28	TRUE	igd	0.0017	5.45E-06	7182	M19362.m	TRUE	reported	SkNEKw	2	TRUE	0.002998	21.58914
2174	rs1185470	A	G	A	G	-0.0079	-0.01405	0.4635	0.468052	FALSE	FALSE	FALSE	ieu-b-508	15	54460325	0.033664	486484	0.676505	Sepsis (28	Sepsis (28	Sepsis (28	TRUE	igd	0.0017	4.57E-06	7182	M19362.m	TRUE	reported	SkNEKw	2	TRUE	0.002998	21.58914
2182	rs1602068	T	C	T	C	-0.0079	0.056347	0.5655	0.559933	FALSE	FALSE	FALSE	ieu-b-508	4	1.19E+08	0.033748	486484	0.094991	Sepsis (28	Sepsis (28	Sepsis (28	TRUE	igd	0.0017	5.67E-06	7182	M19362.m	TRUE	reported	SkNEKw	2	TRUE	0.002998	21.58914
1255	rs4930767	T	C	T	C	-0.0079	0.039894	0.4862	0.47833	FALSE	FALSE	FALSE	ieu-b-508	12	5597381	0.033533	486484	0.234166	Sepsis (28	Sepsis (28	Sepsis (28	TRUE	igd	0.0017	3.69E-06	7136	M01769.m	TRUE	reported	rismUZ	2	TRUE	0.003017	21.5891
1395	rs3819535	T	C	T	C	-0.0079	-0.04679	0.618	0.627889	FALSE	FALSE	FALSE	ieu-b-508	12	2438810	0.034503	486484	0.175057	Sepsis (28	Sepsis (28	Sepsis (28	TRUE	igd	0.0017	5.02E-06	7037	M02734.m	TRUE	reported	0y0dDD	2	TRUE	0.003059	21.58902
8426	rs6688105	T	C	T	C	-0.0079	0.113841	0.703	0.694376	FALSE	FALSE	FALSE	ieu-b-508	1	1.66E+08	0.036349	486484	0.001737	Sepsis (28	Sepsis (28	Sepsis (28	TRUE	igd	0.0017	4.86E-06	6940	M36808.m	TRUE	reported	u7Iqaj	2	TRUE	0.003102	21.58893
7672	rs1106275	A	G	A	G	-0.0079	0.014759	0.7472	0.743279	FALSE	FALSE	FALSE	ieu-b-508	12	3773857	0.038517	486484	0.701578	Sepsis (28	Sepsis (28	Sepsis (28	TRUE	igd	0.0017	5.07E-06	6920	M35327.m	TRUE	reported	SquFiY	2	TRUE	0.003111	21.58891
4131	rs7702466	A	G	A	G	0.0316	0.107446	0.1021	0.127283	FALSE	FALSE	FALSE	ieu-b-508	5	81009238	0.050369	486484	0.032909	Sepsis (28	Sepsis (28	Sepsis (28	TRUE	igd	0.0068	3.01E-06	6658	M32755.m	TRUE	reported	WDrNdi	2	TRUE	0.003233	21.58867
7454	rs1115099	T	C	T	C	-0.0158	-0.01213	0.6181	0.588614	FALSE	FALSE	FALSE	ieu-b-508	18	74763117	0.034156	486484	0.722445	Sepsis (28	Sepsis (28	Sepsis (28	TRUE	igd	0.0034	3.54E-06	6543	M35193.m	TRUE	reported	BMMWXU	2	TRUE	0.00329	21.58855
4579	rs1221117	A	G	A	G	0.0158	0.039207	0.2158	0.208332	FALSE	FALSE	FALSE	ieu-b-508	6	1.57E+08	0.041255	486484	0.341938	Sepsis (28	Sepsis (28	Sepsis (28	TRUE	igd	0.0034	4.57E-06	6404	M33163.m	TRUE	reported	YzGXkR	2	TRUE	0.003361	21.58841
4105	rs1782572	C	G	C	G	-0.0711	-0.06025	0.0842	0.091687	FALSE	TRUE	FALSE	ieu-b-508	2	1.19E+08	0.05788	486484	0.297888	Sepsis (28	Sepsis (28	Sepsis (28	TRUE	igd	0.0153	3.36E-06	6379	M32754.m	TRUE	reported	n0okFy	2	TRUE	0.003374	21.58839
7636	rs1300462	A	C	A	C	0.0079	0.009085	0.5442	0.543156	FALSE	FALSE	FALSE	ieu-b-508	2	1.15E+08	0.033447	486484	0.785905	Sepsis (28	Sepsis (28	Sepsis (28	TRUE	igd	0.0017	4.07E-06	5768	M35326.m	TRUE	reported	jRytyJ	2	TRUE	0.00373	21.58767
7637	rs1313763	T	C	T	C	0.0079	-0.01423	0.3423	0.33915	FALSE	FALSE	FALSE	ieu-b-508	4	1.68E+08	0.035269	486484	0.686657	Sepsis (28	Sepsis (28	Sepsis (28	TRUE	igd	0.0017	6.26E-06	5768	M35326.m	TRUE	reported	jRytyJ	2	TRUE	0.00373	21.58767
7642	rs1838137	A	G	A	G	0.0079	0.004218	0.6843	0.683111	FALSE	FALSE	FALSE	ieu-b-508	3	1.57E+08	0.035849	486484	0.906344	Sepsis (28	Sepsis (28	Sepsis (28	TRUE	igd	0.0017	6.70E-06	5768	M35326.m	TRUE	reported	jRytyJ	2	TRUE	0.00373	21.58767
5374	rs1701102	A	G	A	G	-0.1149	0.005579	0.7728	0.786132	FALSE	FALSE	FALSE	ieu-b-508	4	86751384	0.040876	486484	0.891443	Sepsis (28	Sepsis (28	Sepsis (28	TRUE	igd	0.0247	3.30E-06	803	M33384.m	TRUE	reported	lOffrZ	2	TRUE	0.026241	21.58555
4138	rs1251271	C	G	C	G	-0.0423	-0.0393	0.6294	0.629752	FALSE	TRUE	FALSE	ieu-b-508	4	1.56E+08	0.03474	486484	0.258003	Sepsis (28	Sepsis (28	Sepsis (28	TRUE	igd	0.0091	3.63E-06	1761	M32757.m	TRUE	reported	TJV9Xb	2	TRUE	0.012121	21.58263
4560	rs294095	A	G	A	G	-0.086	0.082468	0.1424	0.131781	FALSE	FALSE	FALSE	ieu-b-508	5	1.03E+08	0.049484	486484	0.095607	Sepsis (28	Sepsis (28	Sepsis (28	TRUE	igd	0.0185	3.18E-06	1570	M33144.m	TRUE	reported	cgXZoM	2	TRUE	0.013577	21.58241
4487	rs2501556	A	G	A	G	-0.0237	0.060898	0.522	0.510799	FALSE	FALSE	FALSE	ieu-b-508	9	1.41E+08	0.033437	486484	0.068563	Sepsis (28	Sepsis (28	Sepsis (28	TRUE	igd	0.0051	3.48E-06	2773	M33131.m	TRUE	reported	uGKURO	2	TRUE	0.007727	21.57958
4178	rs3108908	T	C	T	C	0.0223	0.023276	0.3316	0.332797	FALSE	FALSE	FALSE	ieu-b-508	8	1.02E+08	0.035671	486484	0.51407	Sepsis (28	Sepsis (28	Sepsis (28	TRUE	igd	0.0048	3.03E-06	6712	M32759.m	TRUE	reported	9elHr7	2	TRUE	0.003205	21.57734
2043	rs940008	A	G	A	G	0.0251	0.045929	0.4416	0.460649	FALSE	FALSE	FALSE	ieu-b-508	17	25920891	0.033394	486484	0.169015	Sepsis (28	Sepsis (28	Sepsis (28	TRUE	igd	0.0054	2.64E-06	1492	M18349.m	TRUE	reported	M5qPs8	2	TRUE	0.014274	21.57632
8517	rs7754899	A	G	A	G	0.059	0.013367	0.0573	0.037773	FALSE	FALSE	FALSE	ieu-b-508	6	7840255	0.08787	486484	0.879094	Sepsis (28	Sepsis (28	Sepsis (28	TRUE	igd	0.0127	3.33E-06	4520	M37104.m	TRUE	reported	bR3g7d	2	TRUE	0.004752	21.57269
891	rs1090004	T	C	T	C	-0.0144	-0.04514	0.8351	0.78607	FALSE	FALSE	FALSE	ieu-b-508	10	44953571	0.040614	486484	0.266398	Sepsis (28	Sepsis (28	Sepsis (28	TRUE	igd	0.0031	3.40E-06	7307	M01493.m	TRUE	reported	eLLRt7	2	TRUE	0.002944	21.57162
8433	rs1231385	T	C	T	C	-0.0288	0.004186	0.513	0.526491	FALSE	FALSE	FALSE	ieu-b-508	9	25765893	0.033471	486484	0.900473	Sepsis (28	Sepsis (28	Sepsis (28	TRUE	igd	0.0062	3.38E-06	6511	M36850.m	TRUE	reported	j6lLja	2	TRUE	0.003303	21.5709
8238	rs2404867	A	C	A	C	0.053	0.037955	0.7977	0.797654	FALSE	FALSE	FALSE	ieu-b-508	4	1.37E+08	0.041513	486484	0.360559	Sepsis (28	Sepsis (28	Sepsis (28	TRUE	igd	0.0114	3.14E-06	976	M36468.m	TRUE	reported	vycAFr	2	TRUE	0.021666	21.57005
7195	rs3751563	A	G	A	G	0.0288	0.040058	0.1086	0.10938	FALSE	FALSE	FALSE	ieu-b-508	15	29415698	0.053349	486484	0.452731	Sepsis (28	Sepsis (28	Sepsis (28	TRUE	igd	0.0062	3.03E-06	4800	M34539.m	TRUE	reported	puA8ni	2	TRUE	0.004475	21.56853
5378	rs308097	A	G	A	G	0.3482	0.06281	0.0377	0.045844	FALSE	FALSE	FALSE	ieu-b-508	7	5880377	0.079256	486484	0.428073	Sepsis (28	Sepsis (28	Sepsis (28	TRUE	igd	0.0747	3.16E-06	272	M33384.m	TRUE	reported	lOffrZ	2	TRUE	0.073973	21.56807
4121	rs2032205	T	C	T	C	-0.0706	-0.07718	0.0171	0.019117	FALSE	FALSE	FALSE	ieu-b-508	21	24493528	0.12463	486484	0.535731	Sepsis (28	Sepsis (28	Sepsis (28	TRUE	igd	0.0152	3.19E-06	6658	M32755.m	TRUE	reported	WDrNdi	2	TRUE	0.00323	21.5671
3610	rs1452040	A	C	A	C	-0.0539	0.011064	0.7973	0.77348	FALSE	FALSE	FALSE	ieu-b-508	5	4795354	0.039916	486484	0.781635	Sepsis (28	Sepsis (28	Sepsis (28	TRUE	igd	0.0116	3.68E-06	1766	M32553.m	TRUE	reported	vHSHqn	2	TRUE	0.012078	21.56599
6086	rs4530331	A	G	A	G	-0.0367	-0.00691	0.3391	0.332104	FALSE	FALSE	FALSE	ieu-b-508	2	1.87E+08	0.035405	486484	0.845162	Sepsis (28	Sepsis (28	Sepsis (28	TRUE	igd	0.0079	3.39E-06	2660	M33835.m	TRUE	reported	crhBhm	2	TRUE	0.008048	21.56509
3568	rs2295308	A	C	A	C	0.0209	-0.14775	0.9608	0.950334	FALSE	FALSE	FALSE	ieu-b-508	14	24761805	0.076702	486484	0.054062	Sepsis (28	Sepsis (28	Sepsis (28	TRUE	igd	0.0045	3.07E-06	7350	M32518.m	TRUE	reported	V0ZCiU	2	TRUE	0.002926	21.56499
6595	rs1124386	T	C	T	C	-0.0209	0.03561	0.0768	0.077701	FALSE	FALSE	FALSE	ieu-b-508	6	91803337	0.062112	486484	0.566427	Sepsis (28	Sepsis (28	Sepsis (28	TRUE	igd	0.0045	3.22E-06	6994	M34306.m	TRUE	reported	WPXPUN	2	TRUE	0.003075	21.5647
3633	rs1103498	A	G	A	G	-0.1561	0.006956	0.9783	0.980978	FALSE	FALSE	FALSE	ieu-b-508	11	38908483	0.122576	486484	0.954746	Sepsis (28	Sepsis (28	Sepsis (28	TRUE	igd	0.0336	3.51E-06	2091	M32560.m	TRUE	reported	sLwsEw	2	TRUE	0.010217	21.56312
5875	rs756247	A	T	A	T	-0.1619	-0.04854	0.9396	0.95692	FALSE	TRUE	FALSE	ieu-b-508	12	81306677	0.084121	486484	0.563891	Sepsis (28	Sepsis (28	Sepsis (28	TRUE	igd	0.0348	3.24E-06	528	M33609.m	TRUE	reported	h7B9gN	2	TRUE	0.039378	21.5619
2116	rs7279572	T	C	T	C	-0.2727	-0.04597	0.9644	0.973031	FALSE	FALSE	FALSE	ieu-b-508	21	21210594	0.10439	486484	0.659661	Sepsis (28	Sepsis (28	Sepsis (28	TRUE	igd	0.0585	3.09E-06	257	M18474.m	TRUE	reported	B6Xd02	2	TRUE	0.077961	21.56084
4100	rs1243532	C	G	C	G	-0.4219	-0.0555	0.0126	0.010382	FALSE	TRUE	FALSE	ieu-b-508	14	70570480	0.171358	486484	0.746017	Sepsis (28	Sepsis (28	Sepsis (28	TRUE	igd	0.0908	3.37E-06	1368	M32754.m	TRUE	reported	n0okFy	2	TRUE	0.015537	21.55817
1297	rs310101	A	G	A	G	0.0367	-0.00659	0.9579	0.946415	FALSE	FALSE	FALSE	ieu-b-508	1	1.53E+08	0.075245	486484	0.930199	Sepsis (28	Sepsis (28	Sepsis (28	TRUE	igd	0.0079	3.86E-06	1753	M02132.m	TRUE	reported	uJFm2t	2	TRUE	0.012161	21.55669
5847	rs6129197	A	G	A	G	0.0418	0.085	0.8768	0.847381	FALSE	FALSE	FALSE	ieu-b-508	20	37771045	0.046478	486484	0.067428	Sepsis (28	Sepsis (28	Sepsis (28	TRUE	igd	0.009	3.79E-06	2859	M33531.m	TRUE	reported	kNM0s1	2	TRUE	0.007488	21.55577
3644	rs899236	A	G	A	G	-0.0497	0.051922	0.2189	0.212034	FALSE	FALSE	FALSE	ieu-b-508	16	56352905	0.041146	486484	0.206985	Sepsis (28	Sepsis (28	Sepsis (28	TRUE	igd	0.0107	3.60E-06	2091	M32560.m	TRUE	reported	sLwsEw	2	TRUE	0.010213	21.55409

4380	rs1049215	T	C	T	C	0.0065	0.042676	0.7656	0.766846	FALSE	FALSE	FALSE	ieu-b-508	12	1.08E+08	0.039444	486484	0.279277	Sepsis (28	Sepsis (28	Sepsis (28	TRUE	igd	0.0014	1.92E-06	7329	M32867.m	TRUE	reported	Jcsyzm	2	TRUE	0.002933	21.55024
7583	rs7815445	A	G	A	G	-0.013	0.003095	0.9218	0.922859	FALSE	FALSE	FALSE	ieu-b-508	8	34352349	0.06263	486484	0.960585	Sepsis (28	Sepsis (28	Sepsis (28	TRUE	igd	0.0028	2.42E-06	7217	M35270.m	TRUE	reported	Ct8V1n	2	TRUE	0.002978	21.55015
7516	rs9483731	T	C	T	C	-0.0325	0.04841	0.0382	0.038364	FALSE	FALSE	FALSE	ieu-b-508	6	1.35E+08	0.0877	486484	0.580952	Sepsis (28	Sepsis (28	Sepsis (28	TRUE	igd	0.007	3.17E-06	7100	M35254.m	TRUE	reported	HfVgPn	2	TRUE	0.003027	21.55005
767	rs8073225	T	C	T	C	-0.013	0.042429	0.7657	0.778617	FALSE	FALSE	FALSE	ieu-b-508	17	77092159	0.040156	486484	0.290699	Sepsis (28	Sepsis (28	Sepsis (28	TRUE	igd	0.0028	3.94E-06	6946	M01303.m	TRUE	reported	3b8x0F	2	TRUE	0.003094	21.54992
4118	rs174605	T	G	T	G	0.0195	0.070739	0.284	0.296105	FALSE	FALSE	FALSE	ieu-b-508	11	61626921	0.036553	486484	0.052963	Sepsis (28	Sepsis (28	Sepsis (28	TRUE	igd	0.0042	3.07E-06	6658	M32755.m	TRUE	reported	WDrNdi	2	TRUE	0.003227	21.54965
7268	rs4520895	A	C	A	C	0.013	-0.02596	0.8582	0.854718	FALSE	FALSE	FALSE	ieu-b-508	17	45169760	0.047246	486484	0.582729	Sepsis (28	Sepsis (28	Sepsis (28	TRUE	igd	0.0028	4.28E-06	5885	M34912.m	TRUE	reported	W8lcT6	2	TRUE	0.00365	21.5488
7056	rs7924532	A	T	A	T	-0.013	0.044848	0.6705	0.665973	FALSE	TRUE	FALSE	ieu-b-508	11	5016330	0.03556	486484	0.207241	Sepsis (28	Sepsis (28	Sepsis (28	TRUE	igd	0.0028	2.49E-06	5207	M34456.m	TRUE	reported	Nh457q	2	TRUE	0.004123	21.54784
5583	rs6940545	A	C	A	C	1.6279	-0.07922	0.9822	0.845954	FALSE	FALSE	FALSE	ieu-b-508	6	1.31E+08	0.046322	486484	0.087231	Sepsis (28	Sepsis (28	Sepsis (28	TRUE	igd	0.3448	2.35E-06	60	M33423.m	TRUE	reported	MoEnO7	2	TRUE	0.270876	21.54754
7333	rs1015380	T	C	T	C	0.013	0.045006	0.878	0.884787	FALSE	FALSE	FALSE	ieu-b-508	8	20927542	0.053019	486484	0.39596	Sepsis (28	Sepsis (28	Sepsis (28	TRUE	igd	0.0028	2.93E-06	4900	M35137.m	TRUE	reported	Eq2siS	2	TRUE	0.00438	21.54732
4524	rs1307730	T	C	T	C	-0.0553	0.033413	0.6692	0.696172	FALSE	FALSE	FALSE	ieu-b-508	3	1.21E+08	0.036255	486484	0.356731	Sepsis (28	Sepsis (28	Sepsis (28	TRUE	igd	0.0119	3.55E-06	888	M33137.m	TRUE	reported	ONVNk3	2	TRUE	0.023742	21.54652
2947	rs7332927	A	G	A	G	0.1384	0.03425	0.9802	0.987499	FALSE	FALSE	FALSE	ieu-b-508	13	1.01E+08	0.150549	486484	0.820033	Sepsis (28	Sepsis (28	Sepsis (28	TRUE	igd	0.0298	3.37E-06	1758	M27738.m	TRUE	reported	UtPBd9	2	TRUE	0.012121	21.54494
1157	rs6051643	A	G	A	G	0.0441	0.023667	0.986	0.975482	FALSE	FALSE	FALSE	ieu-b-508	20	3185946	0.111366	486484	0.831708	Sepsis (28	Sepsis (28	Sepsis (28	TRUE	igd	0.0095	3.76E-06	7355	M01644.m	TRUE	reported	IB0DTL	2	TRUE	0.002921	21.54328
6730	rs1692861	T	C	T	C	-0.3934	-0.08062	0.0213	0.017161	FALSE	FALSE	FALSE	ieu-b-508	10	28509296	0.129664	486484	0.534093	Sepsis (28	Sepsis (28	Sepsis (28	TRUE	igd	0.0846	3.28E-06	535	M34338.m	TRUE	reported	h4FB9a	2	TRUE	0.038848	21.54276
5279	rs4676262	T	C	T	C	0.0798	0.072555	0.4367	0.466759	FALSE	FALSE	FALSE	ieu-b-508	2	1.07E+08	0.033481	486484	0.030233	Sepsis (28	Sepsis (28	Sepsis (28	TRUE	igd	0.0171	2.91E-06	182	M33250.m	TRUE	reported	WIZI6U	2	TRUE	0.10687	21.53846
5303	rs911685	T	G	T	G	-0.0771	-0.06694	0.9276	0.929728	FALSE	FALSE	FALSE	ieu-b-508	14	96047547	0.065185	486484	0.304451	Sepsis (28	Sepsis (28	Sepsis (28	TRUE	igd	0.0166	3.20E-06	1273	M33353.m	TRUE	reported	ZIN4gk	2	TRUE	0.016664	21.53822
2350	rs1752858	A	G	A	G	-0.0246	-0.13692	0.0514	0.072904	FALSE	FALSE	FALSE	ieu-b-508	9	7294013	0.063965	486484	0.032317	Sepsis (28	Sepsis (28	Sepsis (28	TRUE	igd	0.0053	3.10E-06	7203	M21047.m	TRUE	reported	ISF1Cr	2	TRUE	0.002982	21.53763
6463	rs6848587	T	C	T	C	-0.0311	-0.04874	0.1655	0.164303	FALSE	FALSE	FALSE	ieu-b-508	4	1.4E+08	0.045595	486484	0.285081	Sepsis (28	Sepsis (28	Sepsis (28	TRUE	igd	0.0067	3.70E-06	4961	M34106.m	TRUE	reported	8DuoNM	2	TRUE	0.004324	21.53754
2378	rs2584543	A	G	A	G	0.0246	-0.13565	0.0698	0.070326	FALSE	FALSE	FALSE	ieu-b-508	9	17466395	0.06468	486484	0.035971	Sepsis (28	Sepsis (28	Sepsis (28	TRUE	igd	0.0053	3.24E-06	6999	M21127.m	TRUE	reported	WCjhS3	2	TRUE	0.003069	21.53745
1546	rs3778889	T	C	T	C	0.0246	-0.00264	0.0616	0.063331	FALSE	FALSE	FALSE	ieu-b-508	7	38432283	0.069058	486484	0.969483	Sepsis (28	Sepsis (28	Sepsis (28	TRUE	igd	0.0053	3.42E-06	6864	M12129.m	TRUE	reported	iNzdgb	2	TRUE	0.003129	21.53733
7395	rs1762464	C	G	C	G	-0.0325	0.004511	0.2139	0.224812	FALSE	TRUE	FALSE	ieu-b-508	5	1.44E+08	0.039903	486484	0.909997	Sepsis (28	Sepsis (28	Sepsis (28	TRUE	igd	0.007	3.81E-06	1982	M35159.m	TRUE	reported	7EoJLB	2	TRUE	0.010759	21.53437
7623	rs748276	A	G	A	G	0.0879	0.096772	0.0832	0.080521	FALSE	FALSE	FALSE	ieu-b-508	10	71244351	0.061437	486484	0.115223	Sepsis (28	Sepsis (28	Sepsis (28	TRUE	igd	0.0189	3.35E-06	448	M35322.m	TRUE	reported	om3h9x	2	TRUE	0.046057	21.53332
5844	rs1236848	T	C	T	C	0.0376	-0.01858	0.135	0.141814	FALSE	FALSE	FALSE	ieu-b-508	12	1.07E+08	0.047976	486484	0.698506	Sepsis (28	Sepsis (28	Sepsis (28	TRUE	igd	0.0081	3.21E-06	2859	M33531.m	TRUE	reported	kNM0s1	2	TRUE	0.00748	21.53286
7108	rs1168639	T	C	T	C	-0.1328	-0.02955	0.8106	0.795609	FALSE	FALSE	FALSE	ieu-b-508	2	84623163	0.041505	486484	0.476477	Sepsis (28	Sepsis (28	Sepsis (28	TRUE	igd	0.0285	3.27E-06	241	M34516.m	TRUE	reported	7Ah2wy	2	TRUE	0.082647	21.53214
5719	rs555096	T	C	T	C	-0.0543	-0.03133	0.039	0.034698	FALSE	FALSE	FALSE	ieu-b-508	1	1.95E+08	0.091988	486484	0.733427	Sepsis (28	Sepsis (28	Sepsis (28	TRUE	igd	0.0117	3.86E-06	5107	M33488.m	TRUE	reported	mZmDY6	2	TRUE	0.0042	21.53068
5993	rs1711529	T	C	T	C	-0.0966	-0.05354	0.9457	0.933197	FALSE	FALSE	FALSE	ieu-b-508	15	25388614	0.066934	486484	0.423759	Sepsis (28	Sepsis (28	Sepsis (28	TRUE	igd	0.0208	3.31E-06	1027	M33683.m	TRUE	reported	wSozpU	2	TRUE	0.02057	21.52688
3864	rs1213875	A	G	A	G	0.0116	-0.04386	0.3354	0.358201	FALSE	FALSE	FALSE	ieu-b-508	1	2.27E+08	0.03521	486484	0.212932	Sepsis (28	Sepsis (28	Sepsis (28	TRUE	igd	0.0025	3.60E-06	7361	M32654.m	TRUE	reported	6lSQOP	2	TRUE	0.002916	21.52375
6555	rs1744827	A	G	A	G	0.0232	-0.01827	0.9163	0.892786	FALSE	FALSE	FALSE	ieu-b-508	4	78609860	0.054215	486484	0.736131	Sepsis (28	Sepsis (28	Sepsis (28	TRUE	igd	0.005	3.92E-06	7347	M34244.m	TRUE	reported	JyF0Sp	2	TRUE	0.002922	21.52374
838	rs1245144	A	G	A	G	-0.0116	-0.00237	0.2677	0.271325	FALSE	FALSE	FALSE	ieu-b-508	17	51587882	0.037657	486484	0.94985	Sepsis (28	Sepsis (28	Sepsis (28	TRUE	igd	0.0025	2.30E-06	7323	M01359.m	TRUE	reported	7L9x1s	2	TRUE	0.002931	21.52372
1247	rs227421	T	C	T	C	0.0116	-0.00699	0.1489	0.155053	FALSE	FALSE	FALSE	ieu-b-508	14	70458035	0.046121	486484	0.879615	Sepsis (28	Sepsis (28	Sepsis (28	TRUE	igd	0.0025	4.04E-06	7136	M01769.m	TRUE	reported	rismUZ	2	TRUE	0.003008	21.52357
7813	rs806044	T	G	T	G	-0.0116	-0.00438	0.6844	0.667736	FALSE	FALSE	FALSE	ieu-b-508	9	4091648	0.035411	486484	0.901488	Sepsis (28	Sepsis (28	Sepsis (28	TRUE	igd	0.0025	4.07E-06	6883	M35464.m	TRUE	reported	Ir9dHp	2	TRUE	0.003118	21.52334
8294	rs1325513	T	C	T	C	0.0441	0.028261	0.0791	0.078464	FALSE	FALSE	FALSE	ieu-b-508	8	63409288	0.061992	486484	0.648473	Sepsis (28	Sepsis (28	Sepsis (28	TRUE	igd	0.0095	3.24E-06	1636	M36559.m	TRUE	reported	g6I9hg	2	TRUE	0.013001	21.5228
7640	rs1744710	A	T	A	T	0.0116	-0.05083	0.8434	0.835359	FALSE	TRUE	FALSE	ieu-b-508	13	1.07E+08	0.044919	486484	0.257819	Sepsis (28	Sepsis (28	Sepsis (28	TRUE	igd	0.0025	4.80E-06	5768	M35326.m	TRUE	reported	jRytyJ	2	TRUE	0.003719	21.52213
8313	rs6138327	C	G	C	G	-0.1123	-0.08684	0.98	0.972651	FALSE	TRUE	FALSE	ieu-b-508	20	24549637	0.10312	486484	0.399733	Sepsis (28	Sepsis (28	Sepsis (28	TRUE	igd	0.0242	3.52E-06	3346	M36585.m	TRUE	reported	pXZg13	2	TRUE	0.006395	21.52133
6475	rs1319041	T	G	T	G	0.169	0.031648	0.1061	0.140173	FALSE	FALSE	FALSE	ieu-b-508	5	61961975	0.04797	486484	0.509409	Sepsis (28	Sepsis (28	Sepsis (28	TRUE	igd	0.0363	3.14E-06	282	M34109.m	TRUE	reported	piJuC7	2	TRUE	0.071376	21.52133
3629	rs6692597	A	C	A	C	-0.0515	-0.01052	0.0597	0.056721	FALSE	FALSE	FALSE	ieu-b-508	1	2.39E+08	0.071761	486484	0.88348	Sepsis (28	Sepsis (28	Sepsis (28	TRUE	igd	0.0111	3.24E-06	7337	M32557.m	TRUE	reported	qR7rZX	2	TRUE	0.002925	21.52039
998	rs1112072	A	G	A	G	-0.0181	-0.03067	0.4596	0.4403	FALSE	FALSE	FALSE	ieu-b-508	1	2.16E+08	0.033914	486484	0.365839	Sepsis (28	Sepsis (28	Sepsis (28	TRUE	igd	0.0039	4.61E-06	1761	M01564.m	TRUE	reported	IPUdyD	2	TRUE	0.012083	21.51466
7893	rs1756490	A	G	A	G	-0.045	0.008354	0.9315	0.907277	FALSE	FALSE	FALSE	ieu-b-508	5	26972016	0.057323	486484	0.884124	Sepsis (28	Sepsis (28	Sepsis (28	TRUE	igd	0.0097	3.97E-06	5629	M35669.m	TRUE	reported	gSecuE	2	TRUE	0.003809	21.5143
5757	rs1167627	A	G	A	G	-0.0348	-0.05933	0.2042	0.204526	FALSE	FALSE	FALSE	ieu-b-508	2	1.06E+08	0.041294	486484	0.15082	Sepsis (28	Sepsis (28	Sepsis (28	TRUE	igd	0.0075	3.86E-06	2759	M33508.m	TRUE	reported	UMXHcP	2	TRUE	0.007743	21.51399
7619	rs3212446	T	C	T	C	0.0516	-0.08603	0.6284	0.676597	FALSE	FALSE	FALSE	ieu-b-508	5	52339114	0.035634	486484	0.015766	Sepsis (28	Sepsis (28	Sepsis (28	TRUE	igd	0.0111	3.41E-06	448	M35322.m	TRUE	reported	om3h9x	2	TRUE	0.046017	21.51346
5603	rs3798189	T	G	T	G	0.0167	0.079411	0.4912	0.496962	FALSE	FALSE	FALSE	ieu-b-508	6	1.6E+08	0.033671	486484	0.018351	Sepsis (28	Sepsis (28	Sepsis (28	TRUE	igd	0.0036	3.42E-06	7365	M33441.m	TRUE	reported	CSdSpl	2	TRUE	0.002913	21.51345
6062	rs7181157	C	G	C	G	0.0167	0.062601	0.2136	0.215381	FALSE	TRUE	FALSE	ieu-b-508	15	22918379	0.040473	486484	0.121931	Sepsis (28	Sepsis (28	Sepsis (28	TRUE	igd	0.0036	4.83E-06	7362	M33821.m	TRUE	reported	eVYlCL	2	TRUE	0.002915	21.51344
1685	rs9695287	A	G	A	G	-0.0167	0.144704	0.0949	0.096653	FALSE	FALSE	FALSE	ieu-b-508	9	82117642	0.056643	486484	0.010629	Sepsis (28	Sepsis (28	Sepsis (28	TRUE	igd	0.0036	4.16E-06	7337	M15365.m	TRUE	reported	TReTCM	2	TRUE	0.002924	21.51342
5393	rs1299698	A	G	A	G	-0.0167	0.06178	0.0786	0.084398	FALSE	FALSE	FALSE	ieu-b-508	2	1.45E+08	0.059925	486484	0.302564	Sepsis (28	Sepsis (28	Sepsis (28	TRUE	igd	0.0036	3.78E-06	7315	M33389.m	TRUE	reported	Hf6XVM	2	TRUE	0.002933	21.51341
5546	rs1156903	T	C	T	C	-0.9284	0.1066	0.05	0.022595	FALSE	FALSE	FALSE	ieu-b-508	4	1.11E+08	0.111723	486484	0.340009	Sepsis (28	Sepsis (28	Sepsis (28	TRUE	igd	0.1968	2.39E-06	60	M33423.m	TRUE	reported	MoEnO7	2	TRUE	0.270558	21.51279
8218	rs1161322	C	G	C	G	0.0334	-0.05732	0.8706	0.871978	FALSE	TRUE	FALSE	ieu-b-508	12	94532532	0.049855	486484	0.250246	Sepsis (28	Sepsis (28	Sepsis (28	TRUE	igd	0.0072	4.05E-06	6486	M36399.m	TRUE	reported	EdUpnt	2	TRUE	0.003307	21.51265
7591	rs1221057	T	C	T	C	-0.0167	-0.04577	0.4836	0.46947	FALSE	FALSE	FALSE	ieu-b-508	6	1.03E+08	0.033443	486484	0.171104	Sepsis (28	Sepsis (28	Sepsis (28	TRUE	igd	0.0036	3.06E-06	5979	M35305.m	TRUE	reported	6ybfdq	2	TRUE	0.003586	21.51209
5660	rs883044	C	G	C	G	0.0167	0.02704	0.6316	0.65197	FALSE	TRUE	FALSE	ieu-b-508	5	1.25E+08	0.034967	486484	0.439346	Sepsis (28	Sepsis (28	Sepsis (28	TRUE	igd	0.0036	3.23E-06	5717	M33453.m	TRUE	reported	OzEeLQ	2	TRUE	0.00375	21.51176
2480	rs2059405	A	G	A	G	0.0167	-0.00738	0.798	0.797149	FALSE	FALSE	FALSE	ieu-b-508	12	46175469	0.041524	486484	0.858855	Sepsis (28	Sepsis (28	Sepsis (28	TRUE	igd	0.0036	4.73E-06	5676	M22032.m	TRUE	reported	xpe96K	2	TRUE	0.003777	21.51171
6790	rs516834	T	C	T	C	0.045	-0.02049	0.6097	0.598143	FALSE	FALSE	FALSE	ieu-b-508	13	49504810	0.033985	486484	0.546648	Sepsis (28	Sepsis (28	Sepsis (28	TRUE	igd	0.0097	3.86E-06	3491	M34350.m	TRUE	reported	Cc3yW9	2	TRUE	0.006127	21.50962
8395	rs190756	A	G	A	G	-0.0167	0.046462	0.2755	0.277562	FALSE	FALSE	FALSE	ieu-b-508	6	1.19E+08	0.037386	486484	0.213947	Sepsis (28	Sepsis (28	Sepsis (28	TRUE	igd	0.0036	3.37E-06	4317	M36802.m	TRUE	reported	njeQfy	2	TRUE	0.00496	21.50932
627	rs7583745	T	C	T	C	-0.0348	0.076626	0.884	0.902762	FALSE	FALSE	FALSE	ieu-b-508	2	79538898	0.056249	486484	0.173111	Sepsis (28	Sepsis (28	Sepsis (28	TRUE	igd	0.0075	3.06E-06	1768	M01121.m	TRUE	reported	HU0fhR	2	TRUE	0.012031	21.50525
8635	rs2672893	A	G	A	G	-0.0218	-0.00193	0.1652	0.164581	FALSE	FALSE	FALSE	ieu-b-508	17	78827863	0.04534	486484	0.966085	Sepsis (28	Sepsis (28	Sepsis (28	TRUE	igd	0.0047	4.35E-06	4754	M38150.m	TRUE	reported	u3Ckmr	2	TRUE	0.004505	21.50476
5912	rs4696727	T	C	T	C	0.0167	-0.01763	0.649	0.638129	FALSE	FALSE	FALSE	ieu-b-508	4	7992786	0.035053	486484	0.614945	Sepsis (28	Sepsis (28	Sepsis (28	TRUE	igd	0.0036	2.88E-06	2757	M33627.m	TRUE	reported	4RHaUX	2	TRUE	0.007745	21.50368
7442	rs1774357	T	C	T	C	-0.0269	0.016218	0.8014	0.816545	FALSE	FALSE	FALSE	ieu-b-508	14	47883942	0.043271	486484	0.707815	Sepsis (28	Sepsis (28	Sepsis (28	TRUE	igd	0.0058	3.74E-06	6234	M35189.m	TRUE	reported	54XZFj	2	TRUE	0.003439	21.5035
5145	rs6765892	T	C	T	C	0.1002	0.059197	0.0793	0.098202	FALSE	FALSE	FALSE	ieu-b-508	3	74263840	0.056977	486484	0.298826	Sepsis (28	Sepsis (28	Sepsis (28	TRUE	igd	0.0216	3.63E-06	2616	M33190.m	TRUE	reported	UkhAw5	2	TRUE	0.008159	21.50284
6900	rs9815685	A	G	A	G	0.1928	-0.0223	0.1775	0.233764	FALSE	FALSE	FALSE	ieu-b-508	3	1.9E+08	0.039383	486484	0.571251	Sepsis (28	Sepsis (28	Sepsis (28	TRUE	igd	0.0392	8.95E-07	18	M34365.m	TRUE	reported	8eexD0	2	TRUE	0.573362	21.50252
2100	rs2364754	A	G	A	G	-0.032	-0.01253	0.0751	0.109802	FALSE	FALSE	FALSE	ieu-b-508	3	26007868	0.053544	486484	0.81495	Sepsis (28	Sepsis (28	Sepsis (28	TRUE	igd	0.0069	3.69E-06	7367	M18467.m	TRUE	reported	64PaNn	2	TRUE	0.002911	21.50225
534	rs1133034	T	C	T	C	0.032	0.090994	0.0757	0.071518	FALSE	FALSE	FALSE	ieu-b-508	3	1.83E+08	0.065054	486484	0.161886	Sepsis (28	Sepsis (28	Sepsis (28	TRUE	igd	0.0069	3.91E-06	7237	M00599.m	TRUE	reported	nRJY3s	2	TRUE	0.002963	21.50214
4314	rs1052032	A	G	A	G	-0.032	0.068938	0.6558	0.661213	FALSE	FALSE	FALSE	ieu-b-508	2	81840224	0.035266	486484	0.050609	Sepsis (28	Sepsis (28	Sepsis (28	TRUE	igd	0.0069	3.27E-06	6250	M32846.m	TRUE	reported	IwF2Yy	2	TRUE	0.003429	21.5012
1952	rs269263	T	C	T	C	-0.0487	-0.03475	0.3981	0.402661	FALSE	FALSE	FALSE	ieu-b-508	7	1.4E+08	0.034037	486484	0.30722	Sepsis (28	Sepsis (28	Sepsis (28	TRUE	igd	0.0105	3.32E-06	3393	M16823.m	TRUE	reported	fNy2PD	2	TRUE	0.0063	21.49925
5584	rs7123491	A	C	A	C	-1.7152	-0.02666	0.016	0.016021	FALSE	FALSE	FALSE	ieu-b-508	11	10391044	0.133802	486484	0.842086	Sepsis (28	Sepsis (28	Sepsis (28	TRUE	igd	0.3637	2.40E-06	60	M33423.m	TRUE	reported	MoEnO7	2	TRUE	0.270432	21.49907
4772	rs1444113	A	C	A	C	-0.297	0.062395	0.0191	0.022188	FALSE	FALSE	FALSE	ieu-b-508	18	65248701	0.114404	486484	0.585485	Sepsis (28	Sepsis (28	Sepsis (28	TRUE	igd	0.064	3.45E-06	1017	M33178.m	TRUE	reported	g22WMS	2	TRUE	0.020736	21.49305
6477	rs1565932	A	T	A	T	0.2624	-0.13291	0.9122	0.892736	FALSE	TRUE	FALSE	ieu-b-508	4	11541107	0.05634	486484	0.018325	Sepsis (28	Sepsis (28	Sepsis (28	TRUE	igd	0.0564	3.34E-06	282	M34109.m	TRUE	reported	piJuC7	2	TRUE	0.071286	21.49208
6476	rs1339453	A	G	A	G	-0.3573	0.083403	0.0248	0.02859	FALSE	FALSE	FALSE	ieu-b-508	2	1.69E+08	0.09937	486484	0.401295	Sepsis (28	Sepsis (28	Sepsis (28	TRUE	igd	0.0768	3.27E-06	282	M34109.m	TRUE	reported	piJuC7	2	TRUE	0.071282	21.4908
7312	rs973579	A	G	A	G	-0.0204	0.018488	0.5112	0.500545	FALSE	FALSE	FALSE	ieu-b-508	19	49241976	0.033406	486484	0.579961	Sepsis (28	Sepsis (28	Sepsis (28	TRUE	igd	0.0044	4.64E-06	7364	M35126.m	TRUE	reported	1haOJA	2	TRUE	0.002911	21.49003
4092	rs2488751	A	G	A	G	-0.0306	-0.01569	0.4539	0.475588	FALSE	FALSE	FALSE	ieu-b-508	10	37112220	0.033889	486484	0.64329	Sepsis (28	Sepsis (28	Sepsis (28	TRUE	igd	0.0066	3.22E-06	7356	M32753.m	TRUE	reported	XlC6zq	2	TRUE	0.002914	21.49002
6073	rs7737048	T	C	T	C	0.0306	0.02024	0.08	0.094852	FALSE	FALSE	FALSE	ieu-b-508	5	78867249	0.056826	486484	0.721705	Sepsis (28	Sepsis (28	Sepsis (28	TRUE	igd	0.0066	3.92E-06	7350	M33822.m	TRUE	reported	Wic4uS	2	TRUE	0.002916	21.49002
3739	rs2595191	T	C	T	C	0.0204	-0.08004	0.8884	0.888917	FALSE	FALSE	FALSE	ieu-b-508	2	46166361	0.053011	486484	0.131087	Sepsis (28	Sepsis (28	Sepsis (28	TRUE	igd	0.0044	2.76E-06	7337	M32632.m	TRUE	reported	pIHzPT	2	TRUE	0.002921	21.49001
2673	rs1258375	A	C	A	C	-0.0255	-0.02988	0.0954	0.161661	FALSE	FALSE	FALSE	ieu-b-508	13	1.11E+08	0.047403	486484	0.528462	Sepsis (28	Sepsis (28	Sepsis (28	TRUE	igd	0.0055	3.65E-06	7256	M24074.m	TRUE	reported	Lk6D2A	2	TRUE	0.002954	21.48994
2684	rs4798481	A	G	A	G	-0.0153	0.061163	0.1447	0.13349	FALSE	FALSE	FALSE	ieu-b-508	18	6693107	0.049206	486484	0.213869	Sepsis (28	Sepsis (28	Sepsis (28	TRUE	igd	0.0033	2.96E-06	7256	M24074.m	TRUE	reported	Lk6D2A	2	TRUE	0.002954	21.48994
4573	rs7325308	T	C	T	C	0.0422	0.002825	0.3593	0.380207	FALSE	FALSE	FALSE	ieu-b-508	13	46517275	0.034464	486484	0.934667	Sepsis (28	Sepsis (28	Sepsis (28	TRUE	igd	0.0091	3.46E-06	2777	M33150.m	TRUE	reported	H9SSMD	2	TRUE	0.007685	21.48964
5376	rs2722578	T	G	T	G	0.1411	0.022746	0.1259	0.123526	FALSE	FALSE	FALSE	ieu-b-508	2	5867165	0.051124	486484	0.656379	Sepsis (28	Sepsis (28	Sepsis (28	TRUE	igd	0.0304	3.38E-06	803	M33384.m	TRUE	reported	IOffrZ	2	TRUE	0.026127	21.48938
7963	rs4972811	T	C	T	C	0.0204	0.043192	0.428	0.427762	FALSE	FALSE	FALSE	ieu-b-508	2	1.73E+08	0.034014	486484	0.204142	Sepsis (28	Sepsis (28	Sepsis (28	TRUE	igd	0.0044	4.22E-06	6447	M35678.m	TRUE	reported	56yR01	2	TRUE	0.003323	21.4892
2928	rs7428286	A	G	A	G	-0.0561	-0.03115	0.0142	0.020754	FALSE	FALSE	FALSE	ieu-b-508	3	95398058	0.118101	486484	0.791938	Sepsis (28	Sepsis (28	Sepsis (28	TRUE	igd	0.0121	3.88E-06	5562	M27728.m	TRUE	reported	WOp1XE	2	TRUE	0.00385	21.48814
3070	rs2813524	A	G	A	G	0.0705	-0.06829	0.6401	0.630318	FALSE	FALSE	FALSE	ieu-b-508	6	1.53E+08	0.034493	486484	0.047723	Sepsis (28	Sepsis (28	Sepsis (28	TRUE	igd	0.0152	3.31E-06	1760	M31787.m	TRUE	reported	XMp8YI	2	TRUE	0.012075	21.48806
6930	rs1024417	A	G	A	G	0.0204	0.03583	0.6552	0.650346	FALSE	FALSE	FALSE	ieu-b-508	7	29557072	0.035002	486484	0.306005	Sepsis (28	Sepsis (28	Sepsis (28	TRUE	igd	0.0044	3.73E-06	5208	M34395.m	TRUE	reported	QVOPA3	2	TRUE	0.004111	21.48761
5269	rs1104963	A	G	A	G	0.0816	0.137222	0.9833	0.983792	FALSE	FALSE	FALSE	ieu-b-508	12	28614181	0.132608	486484	0.300765	Sepsis (28	Sepsis (28	Sepsis (28	TRUE	igd	0.0176	3.41E-06	4409	M33250.m	TRUE	reported	WIZI6U	2	TRUE	0.004852	21.48612
8310	rs2130048	T	G	T	G	-0.051	0.062383	0.9023	0.895863	FALSE	FALSE	FALSE	ieu-b-508	11	1.04E+08	0.054734	486484	0.254385	Sepsis (28	Sepsis (28	Sepsis (28	TRUE	igd	0.011	3.45E-06	3346	M36585.m	TRUE	reported	pXZg13	2	TRUE	0.006383	21.48302
4255	rs1200042	A	G	A	G	0.3394	-0.05007	0.0143	0.04352	FALSE	FALSE	FALSE	ieu-b-508	9	97586149	0.082975	486484	0.546221	Sepsis (28	Sepsis (28	Sepsis (28	TRUE	igd	0.0731	3.49E-06	562	M32800.m	TRUE	reported	Q7MVsa	2	TRUE	0.036941	21.48031
5210	rs1327266	T	C	T	C	0.0357	0.025014	0.9338	0.943974	FALSE	FALSE	FALSE	ieu-b-508	6	51171829	0.072664	486484	0.730664	Sepsis (28	Sepsis (28	Sepsis (28	TRUE	igd	0.0077	3.88E-06	2683	M33204.m	TRUE	reported	ROvPWK	2	TRUE	0.007948	21.47984
5106	rs9559615	T	G	T	G	-0.2969	0.089245	0.0188	0.032921	FALSE	FALSE	FALSE	ieu-b-508	13	1.1E+08	0.093752	486484	0.341129	Sepsis (28	Sepsis (28	Sepsis (28	TRUE	igd	0.064	3.43E-06	1017	M33178.m	TRUE	reported	g22WMS	2	TRUE	0.020723	21.47858
3565	rs1757974	A	G	A	G	0.0292	-0.0352	0.9713	0.957279	FALSE	FALSE	FALSE	ieu-b-508	14	76657203	0.08361	486484	0.673794	Sepsis (28	Sepsis (28	Sepsis (28	TRUE	igd	0.0063	3.87E-06	7350	M32518.m	TRUE	reported	V0ZCiU	2	TRUE	0.002914	21.47664
2994	rs1122713	A	G	A	G	-0.4069	-0.22265	0.9845	0.978467	FALSE	FALSE	FALSE	ieu-b-508	11	64923055	0.116967	486484	0.056974	Sepsis (28	Sepsis (28	Sepsis (28	TRUE	igd	0.0876	3.40E-06	423	M31536.m	TRUE	reported	3nAuUI	2	TRUE	0.048531	21.4738
2982	rs7127674	A	G	A	G	-0.0482	0.091574	0.0147	0.017555	FALSE	FALSE	FALSE	ieu-b-508	11	6517553	0.12738	486484	0.472202	Sepsis (28	Sepsis (28	Sepsis (28	TRUE	igd	0.0104	3.33E-06	7257	M31453.m	TRUE	reported	vdTd9M	2	TRUE	0.002951	21.47374
926	rs1321284	A	C	A	C	-0.0241	-0.04034	0.9252	0.906697	FALSE	FALSE	FALSE	ieu-b-508	6	50301100	0.057364	486484	0.481905	Sepsis (28	Sepsis (28	Sepsis (28	TRUE	igd	0.0052	2.88E-06	7160	M01508.m	TRUE	reported	go526k	2	TRUE	0.002991	21.47366
4569	rs1709334	A	G	A	G	-0.1043	-0.0789	0.0344	0.029378	FALSE	FALSE	FALSE	ieu-b-508	14	60674490	0.0996	486484	0.428266	Sepsis (28	Sepsis (28	Sepsis (28	TRUE	igd	0.0225	3.47E-06	2777	M33150.m	TRUE	reported	H9SSMD	2	TRUE	0.007679	21.4729
6459	rs28384	T	C	T	C	0.0241	-0.01095	0.2933	0.285373	FALSE	FALSE	FALSE	ieu-b-508	22	20156415	0.037324	486484	0.769244	Sepsis (28	Sepsis (28	Sepsis (28	TRUE	igd	0.0052	4.46E-06	4961	M34106.m	TRUE	reported	8DuoNM	2	TRUE	0.004311	21.471
6842	rs1024457	A	G	A	G	-0.2133	0.016962	0.8206	0.871014	FALSE	FALSE	FALSE	ieu-b-508	7	20157230	0.050206	486484	0.735479	Sepsis (28	Sepsis (28	Sepsis (28	TRUE	igd	0.0434	8.89E-07	18	M34365.m	TRUE	reported	8eexD0	2	TRUE	0.573002	21.47087
7196	rs6802120	T	C	T	C	0.0241	0.043395	0.1586	0.154964	FALSE	FALSE	FALSE	ieu-b-508	3	1.78E+08	0.046332	486484	0.34896	Sepsis (28	Sepsis (28	Sepsis (28	TRUE	igd	0.0052	3.99E-06	4800	M34539.m	TRUE	reported	puA8ni	2	TRUE	0.004455	21.47071
4357	rs910923	A	G	A	G	0.019	-0.00011	0.5988	0.603938	FALSE	FALSE	FALSE	ieu-b-508	22	46487902	0.034421	486484	0.997362	Sepsis (28	Sepsis (28	Sepsis (28	TRUE	igd	0.0041	3.90E-06	7355	M32855.m	TRUE	reported	AM8kCf	2	TRUE	0.002911	21.46947
622	rs1194003	T	C	T	C	0.019	0.04686	0.842	0.820378	FALSE	FALSE	FALSE	ieu-b-508	4	58835528	0.043327	486484	0.279455	Sepsis (28	Sepsis (28	Sepsis (28	TRUE	igd	0.0041	2.82E-06	7349	M01121.m	TRUE	reported	HU0fhR	2	TRUE	0.002914	21.46947
2312	rs2165127	A	G	A	G	-0.0751	-0.02202	0.0444	0.192337	FALSE	FALSE	FALSE	ieu-b-508	18	1843583	0.043376	486484	0.611715	Sepsis (28	Sepsis (28	Sepsis (28	TRUE	igd	0.0162	3.37E-06	1748	M20699.m	TRUE	reported	Z3EKFa	2	TRUE	0.012145	21.46608
7303	rs4628823	T	C	T	C	0.0468	0.195941	0.9434	0.947315	FALSE	FALSE	FALSE	ieu-b-508	13	94419836	0.075267	486484	0.009234	Sepsis (28	Sepsis (28	Sepsis (28	TRUE	igd	0.0101	3.68E-06	7364	M35126.m	TRUE	reported	1haOJA	2	TRUE	0.002907	21.465
7076	rs1252712	A	C	A	C	0.0607	-0.02331	0.9801	0.982227	FALSE	FALSE	FALSE	ieu-b-508	6	1.61E+08	0.125561	486484	0.852752	Sepsis (28	Sepsis (28	Sepsis (28	TRUE	igd	0.0131	3.83E-06	7119	M34481.m	TRUE	reported	mXdL0W	2	TRUE	0.003007	21.4641
5773	rs537721	T	C	T	C	-0.0417	0.116773	0.9625	0.953047	FALSE	FALSE	FALSE	ieu-b-508	6	1.63E+08	0.078966	486484	0.139198	Sepsis (28	Sepsis (28	Sepsis (28	TRUE	igd	0.009	3.89E-06	7044	M33509.m	TRUE	reported	56SCCs	2	TRUE	0.003038	21.46168
6535	rs1092700	T	C	T	C	0.0278	0.052536	0.8659	0.856332	FALSE	FALSE	FALSE	ieu-b-508	1	2.44E+08	0.047596	486484	0.26968	Sepsis (28	Sepsis (28	Sepsis (28	TRUE	igd	0.006	3.24E-06	6987	M34221.m	TRUE	reported	nUe3TL	2	TRUE	0.003063	21.46163
6544	rs9569679	T	G	T	G	0.0278	-0.04097	0.149	0.176006	FALSE	FALSE	FALSE	ieu-b-508	13	57964877	0.043807	486484	0.349695	Sepsis (28	Sepsis (28	Sepsis (28	TRUE	igd	0.006	3.62E-06	6987	M34221.m	TRUE	reported	nUe3TL	2	TRUE	0.003063	21.46163
8391	rs1087019	A	G	A	G	-0.019	0.034684	0.613	0.616567	FALSE	FALSE	FALSE	ieu-b-508	9	1.4E+08	0.034252	486484	0.311244	Sepsis (28	Sepsis (28	Sepsis (28	TRUE	igd	0.0041	3.70E-06	2952	M36802.m	TRUE	reported	njeQfy	2	TRUE	0.007222	21.46076
6012	rs2859567	A	C	A	C	-0.1461	-0.04226	0.947	0.9265	FALSE	FALSE	FALSE	ieu-b-508	16	81421590	0.06391	486484	0.50845	Sepsis (28	Sepsis (28	Sepsis (28	TRUE	igd	0.0315	3.52E-06	831	M33751.m	TRUE	reported	Cxdkr4	2	TRUE	0.025234	21.46015
5442	rs1693699	A	G	A	G	0.1488	0.008073	0.0319	0.029731	FALSE	FALSE	FALSE	ieu-b-508	10	80924955	0.099218	486484	0.935154	Sepsis (28	Sepsis (28	Sepsis (28	TRUE	igd	0.0321	3.46E-06	1464	M33390.m	TRUE	reported	t0EYdH	2	TRUE	0.014465	21.45864
3068	rs2125644	A	G	A	G	-0.1928	0.178355	0.0317	0.031852	FALSE	FALSE	FALSE	ieu-b-508	12	46622346	0.095976	486484	0.063122	Sepsis (28	Sepsis (28	Sepsis (28	TRUE	igd	0.0416	3.66E-06	1760	M31787.m	TRUE	reported	XMp8YI	2	TRUE	0.012057	21.45525
8374	rs7048966	C	G	C	G	0.0278	-0.02459	0.1755	0.174812	FALSE	TRUE	FALSE	ieu-b-508	9	80675646	0.043838	486484	0.574848	Sepsis (28	Sepsis (28	Sepsis (28	TRUE	igd	0.006	3.71E-06	3248	M36756.m	TRUE	reported	QsW4oR	2	TRUE	0.006566	21.45456
4353	rs2226274	T	C	T	C	-0.0403	-0.04932	0.9342	0.934299	FALSE	FALSE	FALSE	ieu-b-508	13	99237856	0.06795	486484	0.467914	Sepsis (28	Sepsis (28	Sepsis (28	TRUE	igd	0.0087	3.33E-06	7355	M32855.m	TRUE	reported	AM8kCf	2	TRUE	0.002909	21.45129
8230	rs1178617	A	C	A	C	-0.0519	-0.01187	0.1501	0.141375	FALSE	FALSE	FALSE	ieu-b-508	8	28038789	0.048657	486484	0.807345	Sepsis (28	Sepsis (28	Sepsis (28	TRUE	igd	0.0112	3.61E-06	1908	M36468.m	TRUE	reported	vycAFr	2	TRUE	0.011129	21.45079
493	rs1039293	A	G	A	G	0.0709	0.026688	0.4948	0.470613	FALSE	FALSE	FALSE	ieu-b-508	3	25155294	0.033544	486484	0.426265	Sepsis (28	Sepsis (28	Sepsis (28	TRUE	igd	0.0153	3.39E-06	1713	M00569.m	TRUE	reported	1TjBaz	2	TRUE	0.012381	21.44876
1564	rs2032297	C	G	C	G	0.057	0.021641	0.7857	0.780249	FALSE	TRUE	FALSE	ieu-b-508	21	22455320	0.04026	486484	0.590895	Sepsis (28	Sepsis (28	Sepsis (28	TRUE	igd	0.0123	3.85E-06	1543	M12261.m	TRUE	reported	taUUcM	2	TRUE	0.013727	21.44748
6417	rs4865204	T	C	T	C	-0.0329	-0.00406	0.481	0.484756	FALSE	FALSE	FALSE	ieu-b-508	4	58103527	0.033395	486484	0.903168	Sepsis (28	Sepsis (28	Sepsis (28	TRUE	igd	0.0071	3.77E-06	1714	M34040.m	TRUE	reported	sJBy3i	2	TRUE	0.012373	21.44707
2891	rs1230164	C	G	C	G	0.0176	-0.01077	0.721	0.718775	FALSE	TRUE	FALSE	ieu-b-508	4	99986023	0.037444	486484	0.773528	Sepsis (28	Sepsis (28	Sepsis (28	TRUE	igd	0.0038	3.08E-06	7373	M27718.m	TRUE	reported	PHL57z	2	TRUE	0.002901	21.4457
1192	rs4930046	A	G	A	G	0.0088	-0.04963	0.4652	0.476961	FALSE	FALSE	FALSE	ieu-b-508	11	2197148	0.033407	486484	0.137417	Sepsis (28	Sepsis (28	Sepsis (28	TRUE	igd	0.0019	2.72E-06	7346	M01645.m	TRUE	reported	d5nAn3	2	TRUE	0.002912	21.44568
2633	rs9789183	T	C	T	C	0.0088	0.011484	0.517	0.522354	FALSE	FALSE	FALSE	ieu-b-508	18	5877869	0.033536	486484	0.732027	Sepsis (28	Sepsis (28	Sepsis (28	TRUE	igd	0.0019	3.27E-06	7329	M22481.m	TRUE	reported	bXpf0k	2	TRUE	0.002918	21.44567
5412	rs4602028	A	C	A	C	-0.0088	0.008142	0.3766	0.37664	FALSE	FALSE	FALSE	ieu-b-508	16	83575572	0.034591	486484	0.81391	Sepsis (28	Sepsis (28	Sepsis (28	TRUE	igd	0.0019	2.07E-06	7315	M33389.m	TRUE	reported	Hf6XVM	2	TRUE	0.002924	21.44566
5661	rs1011985	A	G	A	G	0.0088	-0.03414	0.5446	0.541683	FALSE	FALSE	FALSE	ieu-b-508	9	1.11E+08	0.034164	486484	0.317626	Sepsis (28	Sepsis (28	Sepsis (28	TRUE	igd	0.0019	2.60E-06	7307	M33477.m	TRUE	reported	Aw8PBK	2	TRUE	0.002927	21.44565
2352	rs2075383	T	C	T	C	-0.0176	0.028814	0.9371	0.934286	FALSE	FALSE	FALSE	ieu-b-508	12	1.07E+08	0.067259	486484	0.668355	Sepsis (28	Sepsis (28	Sepsis (28	TRUE	igd	0.0038	3.86E-06	7203	M21047.m	TRUE	reported	ISF1Cr	2	TRUE	0.002969	21.44557
8028	rs819909	A	G	A	G	0.0088	-0.01344	0.5236	0.514866	FALSE	FALSE	FALSE	ieu-b-508	1	99762785	0.033378	486484	0.687119	Sepsis (28	Sepsis (28	Sepsis (28	TRUE	igd	0.0019	4.24E-06	6604	M35754.m	TRUE	reported	IlXOo1	2	TRUE	0.003238	21.44503
8490	rs7177	A	C	A	C	-0.0088	-0.00987	0.5419	0.526944	FALSE	FALSE	FALSE	ieu-b-508	11	69466115	0.033545	486484	0.768497	Sepsis (28	Sepsis (28	Sepsis (28	TRUE	igd	0.0019	2.74E-06	6552	M37058.m	TRUE	reported	32p8qV	2	TRUE	0.003263	21.44498
2541	rs1326822	T	C	T	C	-0.0088	-0.0093	0.5305	0.526119	FALSE	FALSE	FALSE	ieu-b-508	8	27877350	0.033434	486484	0.780793	Sepsis (28	Sepsis (28	Sepsis (28	TRUE	igd	0.0019	6.22E-06	6535	M22177.m	TRUE	reported	2s6Sly	2	TRUE	0.003272	21.44496
2550	rs1876088	A	C	A	C	-0.0088	0.061365	0.6596	0.654134	FALSE	FALSE	FALSE	ieu-b-508	2	51342409	0.035123	486484	0.080608	Sepsis (28	Sepsis (28	Sepsis (28	TRUE	igd	0.0019	5.77E-06	6535	M22177.m	TRUE	reported	2s6Sly	2	TRUE	0.003272	21.44496
2551	rs1998125	T	G	T	G	-0.0088	-0.02531	0.5805	0.550475	FALSE	FALSE	FALSE	ieu-b-508	14	38081005	0.03366	486484	0.452009	Sepsis (28	Sepsis (28	Sepsis (28	TRUE	igd	0.0019	5.75E-06	6535	M22177.m	TRUE	reported	2s6Sly	2	TRUE	0.003272	21.44496
2558	rs2870925	C	G	C	G	-0.0088	0.005488	0.6398	0.636872	FALSE	TRUE	FALSE	ieu-b-508	12	67313182	0.034803	486484	0.874696	Sepsis (28	Sepsis (28	Sepsis (28	TRUE	igd	0.0019	5.84E-06	6535	M22177.m	TRUE	reported	2s6Sly	2	TRUE	0.003272	21.44496
2568	rs534408	T	G	T	G	0.0088	0.003626	0.7263	0.718286	FALSE	FALSE	FALSE	ieu-b-508	11	79363044	0.037369	486484	0.922691	Sepsis (28	Sepsis (28	Sepsis (28	TRUE	igd	0.0019	6.07E-06	6535	M22177.m	TRUE	reported	2s6Sly	2	TRUE	0.003272	21.44496
2575	rs6513545	A	G	A	G	0.0088	-0.03688	0.6085	0.620885	FALSE	FALSE	FALSE	ieu-b-508	20	59472167	0.034509	486484	0.28525	Sepsis (28	Sepsis (28	Sepsis (28	TRUE	igd	0.0019	5.25E-06	6535	M22177.m	TRUE	reported	2s6Sly	2	TRUE	0.003272	21.44496
2585	rs7800932	T	G	T	G	0.0088	0.042648	0.4133	0.396588	FALSE	FALSE	FALSE	ieu-b-508	7	1.54E+08	0.034102	486484	0.211089	Sepsis (28	Sepsis (28	Sepsis (28	TRUE	igd	0.0019	5.52E-06	6535	M22177.m	TRUE	reported	2s6Sly	2	TRUE	0.003272	21.44496
3974	rs1075383	A	G	A	G	0.0088	-0.0034	0.4657	0.476899	FALSE	FALSE	FALSE	ieu-b-508	1	1.99E+08	0.033483	486484	0.919219	Sepsis (28	Sepsis (28	Sepsis (28	TRUE	igd	0.0019	2.49E-06	6497	M32729.m	TRUE	reported	XGRSN1	2	TRUE	0.003291	21.44492
3976	rs1102407	T	C	T	C	-0.0088	-0.0492	0.7057	0.695327	FALSE	FALSE	FALSE	ieu-b-508	11	16917219	0.036273	486484	0.174974	Sepsis (28	Sepsis (28	Sepsis (28	TRUE	igd	0.0019	3.28E-06	6497	M32729.m	TRUE	reported	XGRSN1	2	TRUE	0.003291	21.44492
3983	rs1311150	T	C	T	C	0.0088	-0.01252	0.6934	0.703405	FALSE	FALSE	FALSE	ieu-b-508	4	1.71E+08	0.037486	486484	0.738436	Sepsis (28	Sepsis (28	Sepsis (28	TRUE	igd	0.0019	4.16E-06	6497	M32729.m	TRUE	reported	XGRSN1	2	TRUE	0.003291	21.44492
4012	rs9322929	T	C	T	C	-0.0088	-0.02814	0.6994	0.693931	FALSE	FALSE	FALSE	ieu-b-508	14	34307720	0.036267	486484	0.43778	Sepsis (28	Sepsis (28	Sepsis (28	TRUE	igd	0.0019	3.57E-06	6497	M32729.m	TRUE	reported	XGRSN1	2	TRUE	0.003291	21.44492
4014	rs990482	T	G	T	G	0.0088	-0.00724	0.2786	0.273536	FALSE	FALSE	FALSE	ieu-b-508	6	90806835	0.03873	486484	0.851646	Sepsis (28	Sepsis (28	Sepsis (28	TRUE	igd	0.0019	4.44E-06	6497	M32729.m	TRUE	reported	XGRSN1	2	TRUE	0.003291	21.44492
7250	rs1228679	A	C	A	C	-0.0088	0.040381	0.5493	0.516808	FALSE	FALSE	FALSE	ieu-b-508	11	79122209	0.0335	486484	0.228041	Sepsis (28	Sepsis (28	Sepsis (28	TRUE	igd	0.0019	3.71E-06	5885	M34912.m	TRUE	reported	W8lcT6	2	TRUE	0.003632	21.44423
7260	rs1771292	A	G	A	G	0.0088	0.058029	0.3432	0.348026	FALSE	FALSE	FALSE	ieu-b-508	18	47130342	0.034971	486484	0.097042	Sepsis (28	Sepsis (28	Sepsis (28	TRUE	igd	0.0019	3.59E-06	5885	M34912.m	TRUE	reported	W8lcT6	2	TRUE	0.003632	21.44423
7284	rs9845510	T	C	T	C	-0.0088	-0.00779	0.5512	0.557771	FALSE	FALSE	FALSE	ieu-b-508	3	1.86E+08	0.033625	486484	0.816703	Sepsis (28	Sepsis (28	Sepsis (28	TRUE	igd	0.0019	2.92E-06	5885	M34912.m	TRUE	reported	W8lcT6	2	TRUE	0.003632	21.44423
5149	rs1153452	T	C	T	C	-0.0616	-0.0356	0.8392	0.843304	FALSE	FALSE	FALSE	ieu-b-508	3	3311720	0.046804	486484	0.44689	Sepsis (28	Sepsis (28	Sepsis (28	TRUE	igd	0.0133	3.62E-06	5716	M33192.m	TRUE	reported	CyfjKE	2	TRUE	0.003739	21.44402
7339	rs1249967	T	C	T	C	-0.0088	0.001367	0.3638	0.394479	FALSE	FALSE	FALSE	ieu-b-508	4	1.75E+08	0.034533	486484	0.968414	Sepsis (28	Sepsis (28	Sepsis (28	TRUE	igd	0.0019	3.70E-06	4900	M35137.m	TRUE	reported	Eq2siS	2	TRUE	0.004359	21.44277
7386	rs7770207	T	C	T	C	-0.0088	-0.01061	0.689	0.69587	FALSE	FALSE	FALSE	ieu-b-508	6	40699218	0.036344	486484	0.770266	Sepsis (28	Sepsis (28	Sepsis (28	TRUE	igd	0.0019	3.94E-06	4900	M35137.m	TRUE	reported	Eq2siS	2	TRUE	0.004359	21.44277
5461	rs2927446	A	C	A	C	-0.4392	-0.02055	0.9737	0.977493	FALSE	FALSE	FALSE	ieu-b-508	19	45318564	0.112547	486484	0.855127	Sepsis (28	Sepsis (28	Sepsis (28	TRUE	igd	0.0943	3.24E-06	171	M33391.m	TRUE	reported	EgMd1b	2	TRUE	0.112574	21.43838
8210	rs6913176	T	C	T	C	0.082	0.165475	0.9804	0.987472	FALSE	FALSE	FALSE	ieu-b-508	6	24957511	0.150676	486484	0.272108	Sepsis (28	Sepsis (28	Sepsis (28	TRUE	igd	0.0177	3.80E-06	1732	M36300.m	TRUE	reported	ZjeC9m	2	TRUE	0.01224	21.43776
5097	rs9398363	T	C	T	C	-0.2359	0.021961	0.0232	0.031015	FALSE	FALSE	FALSE	ieu-b-508	6	97658270	0.095729	486484	0.818555	Sepsis (28	Sepsis (28	Sepsis (28	TRUE	igd	0.0509	3.58E-06	1017	M33178.m	TRUE	reported	g22WMS	2	TRUE	0.020683	21.43707
6264	rs1115159	A	C	A	C	0.0213	0.044477	0.7667	0.757298	FALSE	FALSE	FALSE	ieu-b-508	18	68152416	0.039571	486484	0.261021	Sepsis (28	Sepsis (28	Sepsis (28	TRUE	igd	0.0046	4.11E-06	7222	M33937.m	TRUE	reported	QanGCl	2	TRUE	0.00296	21.43499
7901	rs4799429	T	C	T	C	-0.0213	-0.02513	0.48	0.487102	FALSE	FALSE	FALSE	ieu-b-508	18	34302276	0.033436	486484	0.452243	Sepsis (28	Sepsis (28	Sepsis (28	TRUE	igd	0.0046	3.27E-06	5629	M35669.m	TRUE	reported	gSecuE	2	TRUE	0.003795	21.43331
7993	rs1202287	A	G	A	G	-0.0403	0.054545	0.8244	0.85318	FALSE	FALSE	FALSE	ieu-b-508	1	1.63E+08	0.047214	486484	0.247973	Sepsis (28	Sepsis (28	Sepsis (28	TRUE	igd	0.0087	4.17E-06	1709	M35754.m	TRUE	reported	IlXOo1	2	TRUE	0.0124	21.43202
5230	rs2816826	T	C	T	C	0.0213	0.019766	0.2779	0.279325	FALSE	FALSE	FALSE	ieu-b-508	10	52743962	0.037277	486484	0.595931	Sepsis (28	Sepsis (28	Sepsis (28	TRUE	igd	0.0046	2.82E-06	4665	M33221.m	TRUE	reported	8ZrV1r	2	TRUE	0.004575	21.43173
5233	rs7254306	A	G	A	G	0.0213	-0.05017	0.3907	0.405739	FALSE	FALSE	FALSE	ieu-b-508	19	29368109	0.034144	486484	0.141736	Sepsis (28	Sepsis (28	Sepsis (28	TRUE	igd	0.0046	3.25E-06	4665	M33221.m	TRUE	reported	8ZrV1r	2	TRUE	0.004575	21.43173
2119	rs1259011	T	G	T	G	-0.0463	-0.0711	0.9201	0.920205	FALSE	FALSE	FALSE	ieu-b-508	14	30857912	0.061574	486484	0.248186	Sepsis (28	Sepsis (28	Sepsis (28	TRUE	igd	0.01	3.84E-06	5624	M18476.m	TRUE	reported	6GnBtw	2	TRUE	0.003797	21.42928
3427	rs1420139	C	G	C	G	-0.0125	-0.08027	0.1735	0.211411	FALSE	TRUE	FALSE	ieu-b-508	7	29547240	0.041237	486484	0.051579	Sepsis (28	Sepsis (28	Sepsis (28	TRUE	igd	0.0027	3.28E-06	7364	M32452.m	TRUE	reported	8GtFpl	2	TRUE	0.002902	21.42765
3466	rs1743536	T	C	T	C	-0.0125	0.017279	0.1195	0.111206	FALSE	FALSE	FALSE	ieu-b-508	8	1.03E+08	0.053059	486484	0.744679	Sepsis (28	Sepsis (28	Sepsis (28	TRUE	igd	0.0027	3.20E-06	7363	M32489.m	TRUE	reported	hnljaD	2	TRUE	0.002903	21.42765
3100	rs1098941	T	C	T	C	0.0125	0.011541	0.1927	0.191152	FALSE	FALSE	FALSE	ieu-b-508	9	1.04E+08	0.042415	486484	0.78555	Sepsis (28	Sepsis (28	Sepsis (28	TRUE	igd	0.0027	3.82E-06	7356	M32198.m	TRUE	reported	ndXmnp	2	TRUE	0.002905	21.42764
7429	rs9624896	T	C	T	C	-0.0125	0.02893	0.2909	0.316477	FALSE	FALSE	FALSE	ieu-b-508	22	26173184	0.036085	486484	0.422715	Sepsis (28	Sepsis (28	Sepsis (28	TRUE	igd	0.0027	2.40E-06	7350	M35186.m	TRUE	reported	UZfu6Z	2	TRUE	0.002908	21.42764
2792	rs1296569	T	C	T	C	-0.0125	-0.00083	0.7726	0.770612	FALSE	FALSE	FALSE	ieu-b-508	18	14121617	0.039694	486484	0.983251	Sepsis (28	Sepsis (28	Sepsis (28	TRUE	igd	0.0027	2.76E-06	7347	M27278.m	TRUE	reported	kPgqm3	2	TRUE	0.002909	21.42764
2793	rs1311709	A	G	A	G	0.0125	0.02849	0.1954	0.194846	FALSE	FALSE	FALSE	ieu-b-508	4	77726588	0.042665	486484	0.504288	Sepsis (28	Sepsis (28	Sepsis (28	TRUE	igd	0.0027	4.89E-06	7347	M27278.m	TRUE	reported	kPgqm3	2	TRUE	0.002909	21.42764
7875	rs7290374	A	G	A	G	0.0125	-0.03552	0.4047	0.402103	FALSE	FALSE	FALSE	ieu-b-508	22	47566744	0.035224	486484	0.31323	Sepsis (28	Sepsis (28	Sepsis (28	TRUE	igd	0.0027	3.64E-06	7317	M35631.m	TRUE	reported	002wka	2	TRUE	0.002921	21.42761
2291	rs4742351	T	C	T	C	-0.0125	0.005596	0.393	0.393506	FALSE	FALSE	FALSE	ieu-b-508	9	7489720	0.034309	486484	0.870423	Sepsis (28	Sepsis (28	Sepsis (28	TRUE	igd	0.0027	2.81E-06	7301	M20675.m	TRUE	reported	CXe9rE	2	TRUE	0.002927	21.4276
2967	rs1092697	T	C	T	C	-0.0125	0.020092	0.2524	0.257289	FALSE	FALSE	FALSE	ieu-b-508	1	2.43E+08	0.038036	486484	0.597334	Sepsis (28	Sepsis (28	Sepsis (28	TRUE	igd	0.0027	2.66E-06	7257	M31453.m	TRUE	reported	vdTd9M	2	TRUE	0.002945	21.42756
2822	rs1013612	A	G	A	G	-0.0125	0.00651	0.4965	0.498024	FALSE	FALSE	FALSE	ieu-b-508	7	1.24E+08	0.033388	486484	0.845408	Sepsis (28	Sepsis (28	Sepsis (28	TRUE	igd	0.0027	4.48E-06	7180	M27513.m	TRUE	reported	o9Ff2u	2	TRUE	0.002976	21.4275
2836	rs4144140	T	C	T	C	0.0125	-0.01346	0.4524	0.459354	FALSE	FALSE	FALSE	ieu-b-508	13	38443040	0.033603	486484	0.688747	Sepsis (28	Sepsis (28	Sepsis (28	TRUE	igd	0.0027	4.75E-06	7180	M27513.m	TRUE	reported	o9Ff2u	2	TRUE	0.002976	21.4275
7511	rs2319973	A	C	A	C	0.0125	0.036684	0.3507	0.351381	FALSE	FALSE	FALSE	ieu-b-508	18	56465318	0.034886	486484	0.293019	Sepsis (28	Sepsis (28	Sepsis (28	TRUE	igd	0.0027	2.82E-06	7100	M35254.m	TRUE	reported	HfVgPn	2	TRUE	0.00301	21.42743
5252	rs706041	T	C	T	C	-0.0125	-0.0433	0.3351	0.334347	FALSE	FALSE	FALSE	ieu-b-508	7	16735586	0.037642	486484	0.249961	Sepsis (28	Sepsis (28	Sepsis (28	TRUE	igd	0.0027	4.45E-06	7063	M33228.m	TRUE	reported	JwOXbQ	2	TRUE	0.003025	21.4274
6532	rs6431170	A	C	A	C	0.0125	0.038108	0.2833	0.302301	FALSE	FALSE	FALSE	ieu-b-508	2	1.27E+08	0.036447	486484	0.295756	Sepsis (28	Sepsis (28	Sepsis (28	TRUE	igd	0.0027	3.75E-06	7049	M34123.m	TRUE	reported	9O5wo1	2	TRUE	0.003031	21.42739
2379	rs400511	T	C	T	C	0.0125	0.073219	0.6391	0.631063	FALSE	FALSE	FALSE	ieu-b-508	4	31490392	0.034635	486484	0.034516	Sepsis (28	Sepsis (28	Sepsis (28	TRUE	igd	0.0027	4.17E-06	6999	M21127.m	TRUE	reported	WCjhS3	2	TRUE	0.003053	21.42735
2382	rs4821424	A	G	A	G	-0.0125	-0.002	0.5199	0.513086	FALSE	FALSE	FALSE	ieu-b-508	22	36001860	0.033365	486484	0.952213	Sepsis (28	Sepsis (28	Sepsis (28	TRUE	igd	0.0027	3.97E-06	6999	M21127.m	TRUE	reported	WCjhS3	2	TRUE	0.003053	21.42735
6608	rs4622179	T	C	T	C	-0.0125	-0.04035	0.7749	0.770552	FALSE	FALSE	FALSE	ieu-b-508	10	58457011	0.039596	486484	0.308203	Sepsis (28	Sepsis (28	Sepsis (28	TRUE	igd	0.0027	4.73E-06	6994	M34306.m	TRUE	reported	WPXPUN	2	TRUE	0.003055	21.42734
761	rs2869990	T	C	T	C	-0.0125	-0.09973	0.7192	0.714466	FALSE	FALSE	FALSE	ieu-b-508	4	89991614	0.036935	486484	0.006933	Sepsis (28	Sepsis (28	Sepsis (28	TRUE	igd	0.0027	2.83E-06	6946	M01303.m	TRUE	reported	3b8x0F	2	TRUE	0.003076	21.4273
8478	rs2705088	T	G	T	G	-0.0125	0.038577	0.7887	0.777455	FALSE	FALSE	FALSE	ieu-b-508	8	17313916	0.040268	486484	0.338048	Sepsis (28	Sepsis (28	Sepsis (28	TRUE	igd	0.0027	4.17E-06	6552	M37058.m	TRUE	reported	32p8qV	2	TRUE	0.003261	21.42693
7467	rs4960438	T	C	T	C	-0.0125	-0.01714	0.6378	0.638595	FALSE	FALSE	FALSE	ieu-b-508	6	8548630	0.034677	486484	0.621058	Sepsis (28	Sepsis (28	Sepsis (28	TRUE	igd	0.0027	4.75E-06	6543	M35193.m	TRUE	reported	BMMWXU	2	TRUE	0.003265	21.42692
2652	rs1177601	T	C	T	C	0.0125	0.014601	0.8309	0.826374	FALSE	FALSE	FALSE	ieu-b-508	8	13208668	0.044355	486484	0.742019	Sepsis (28	Sepsis (28	Sepsis (28	TRUE	igd	0.0027	3.52E-06	6517	M22649.m	TRUE	reported	NAzwRF	2	TRUE	0.003278	21.42689
2724	rs1304928	C	G	C	G	0.0125	0.039283	0.6544	0.633085	FALSE	TRUE	FALSE	ieu-b-508	21	40594656	0.034608	486484	0.256338	Sepsis (28	Sepsis (28	Sepsis (28	TRUE	igd	0.0027	4.13E-06	6388	M25599.m	TRUE	reported	fRZxwP	2	TRUE	0.003344	21.42676
3692	rs1811763	A	G	A	G	-0.025	-0.03061	0.144	0.141642	FALSE	FALSE	FALSE	ieu-b-508	3	1.73E+08	0.047749	486484	0.521507	Sepsis (28	Sepsis (28	Sepsis (28	TRUE	igd	0.0054	3.53E-06	5605	M32587.m	TRUE	reported	uknv7G	2	TRUE	0.003809	21.42582
2907	rs1107824	T	C	T	C	-0.0125	0.004008	0.6743	0.668694	FALSE	FALSE	FALSE	ieu-b-508	17	14386357	0.035762	486484	0.910754	Sepsis (28	Sepsis (28	Sepsis (28	TRUE	igd	0.0027	3.67E-06	5562	M27728.m	TRUE	reported	WOp1XE	2	TRUE	0.003839	21.42576
2909	rs1317833	A	G	A	G	-0.0125	0.031737	0.6631	0.652678	FALSE	FALSE	FALSE	ieu-b-508	10	87939804	0.035806	486484	0.375429	Sepsis (28	Sepsis (28	Sepsis (28	TRUE	igd	0.0027	4.38E-06	5562	M27728.m	TRUE	reported	WOp1XE	2	TRUE	0.003839	21.42576
2910	rs1401635	C	G	C	G	-0.0125	0.011729	0.3132	0.319872	FALSE	TRUE	FALSE	ieu-b-508	11	27693991	0.03569	486484	0.742441	Sepsis (28	Sepsis (28	Sepsis (28	TRUE	igd	0.0027	4.91E-06	5562	M27728.m	TRUE	reported	WOp1XE	2	TRUE	0.003839	21.42576
2916	rs2253834	T	C	T	C	-0.0125	-0.02008	0.5521	0.553999	FALSE	FALSE	FALSE	ieu-b-508	20	23387816	0.033517	486484	0.54918	Sepsis (28	Sepsis (28	Sepsis (28	TRUE	igd	0.0027	4.55E-06	5562	M27728.m	TRUE	reported	WOp1XE	2	TRUE	0.003839	21.42576
3850	rs452767	T	C	T	C	0.0125	0.01122	0.5251	0.547671	FALSE	FALSE	FALSE	ieu-b-508	19	5996439	0.033665	486484	0.738919	Sepsis (28	Sepsis (28	Sepsis (28	TRUE	igd	0.0027	5.24E-06	5211	M32651.m	TRUE	reported	DeuPwv	2	TRUE	0.004096	21.42524
3716	rs4149056	T	C	T	C	-0.0537	0.069603	0.8373	0.84838	FALSE	FALSE	FALSE	ieu-b-508	12	21331549	0.046537	486484	0.134746	Sepsis (28	Sepsis (28	Sepsis (28	TRUE	igd	0.0116	4.00E-06	6904	M32616.m	TRUE	reported	vrkjBE	2	TRUE	0.003094	21.42431
5317	rs4416818	A	G	A	G	0.0213	0.011203	0.2365	0.250979	FALSE	FALSE	FALSE	ieu-b-508	8	84587462	0.03843	486484	0.770653	Sepsis (28	Sepsis (28	Sepsis (28	TRUE	igd	0.0046	2.79E-06	2212	M33363.m	TRUE	reported	7K0MM0	2	TRUE	0.0096	21.42154
5505	rs7038957	T	C	T	C	-0.0287	0.025933	0.8312	0.831529	FALSE	FALSE	FALSE	ieu-b-508	9	1.07E+08	0.044618	486484	0.561096	Sepsis (28	Sepsis (28	Sepsis (28	TRUE	igd	0.0062	3.86E-06	5822	M33420.m	TRUE	reported	eTToDb	2	TRUE	0.003667	21.42058
6002	rs1265852	T	C	T	C	0.1682	-2.47E-05	0.0382	0.038162	FALSE	FALSE	FALSE	ieu-b-508	5	57231934	0.087155	486484	0.999774	Sepsis (28	Sepsis (28	Sepsis (28	TRUE	igd	0.0363	3.59E-06	831	M33751.m	TRUE	reported	Cxdkr4	2	TRUE	0.025186	21.41866
5182	rs1292729	A	T	A	T	-0.05	0.064261	0.7061	0.715314	FALSE	TRUE	FALSE	ieu-b-508	16	58634475	0.036984	486484	0.082293	Sepsis (28	Sepsis (28	Sepsis (28	TRUE	igd	0.0108	3.99E-06	2860	M33197.m	TRUE	reported	D6xaBg	2	TRUE	0.007438	21.41848
7021	rs1231157	T	C	T	C	-0.0287	0.06132	0.8478	0.837092	FALSE	FALSE	FALSE	ieu-b-508	12	1.01E+08	0.045493	486484	0.177692	Sepsis (28	Sepsis (28	Sepsis (28	TRUE	igd	0.0062	3.26E-06	4442	M34441.m	TRUE	reported	UpgxQm	2	TRUE	0.004801	21.41829
2332	rs1712256	T	C	T	C	-0.0162	-0.00833	0.2523	0.266371	FALSE	FALSE	FALSE	ieu-b-508	12	48412410	0.037772	486484	0.825507	Sepsis (28	Sepsis (28	Sepsis (28	TRUE	igd	0.0035	2.75E-06	7366	M21044.m	TRUE	reported	Ym5rae	2	TRUE	0.0029	21.41786
4094	rs3746024	T	C	T	C	-0.0324	-0.03524	0.8273	0.824452	FALSE	FALSE	FALSE	ieu-b-508	19	33317690	0.043961	486484	0.422817	Sepsis (28	Sepsis (28	Sepsis (28	TRUE	igd	0.007	3.46E-06	7356	M32753.m	TRUE	reported	XlC6zq	2	TRUE	0.002904	21.41785
6559	rs3807839	T	C	T	C	0.0162	-0.01543	0.2604	0.268577	FALSE	FALSE	FALSE	ieu-b-508	7	8191966	0.037809	486484	0.683142	Sepsis (28	Sepsis (28	Sepsis (28	TRUE	igd	0.0035	4.40E-06	7347	M34244.m	TRUE	reported	JyF0Sp	2	TRUE	0.002907	21.41784
3158	rs1051850	T	C	T	C	0.0162	-0.01601	0.7866	0.774069	FALSE	FALSE	FALSE	ieu-b-508	1	75926899	0.040067	486484	0.689407	Sepsis (28	Sepsis (28	Sepsis (28	TRUE	igd	0.0035	3.29E-06	7340	M32328.m	TRUE	reported	YGsb6D	2	TRUE	0.00291	21.41784
6406	rs2242415	A	C	A	C	0.0162	0.040243	0.6232	0.616528	FALSE	FALSE	FALSE	ieu-b-508	5	17225333	0.034657	486484	0.245566	Sepsis (28	Sepsis (28	Sepsis (28	TRUE	igd	0.0035	4.36E-06	7338	M34035.m	TRUE	reported	W4G5qR	2	TRUE	0.002911	21.41783
8386	rs7225312	A	T	A	T	-0.0162	-0.01285	0.8749	0.843382	FALSE	TRUE	FALSE	ieu-b-508	17	55862854	0.045819	486484	0.779067	Sepsis (28	Sepsis (28	Sepsis (28	TRUE	igd	0.0035	3.16E-06	7335	M36776.m	TRUE	reported	VnYglx	2	TRUE	0.002912	21.41783
1116	rs7717247	A	C	A	C	0.0162	0.061943	0.8061	0.836482	FALSE	FALSE	FALSE	ieu-b-508	5	27839258	0.045453	486484	0.172947	Sepsis (28	Sepsis (28	Sepsis (28	TRUE	igd	0.0035	3.35E-06	7104	M01638.m	TRUE	reported	8dp0Ac	2	TRUE	0.003007	21.41764
3662	rs4750519	T	G	T	G	-0.0324	-0.03531	0.4178	0.410805	FALSE	FALSE	FALSE	ieu-b-508	10	5645947	0.03403	486484	0.299429	Sepsis (28	Sepsis (28	Sepsis (28	TRUE	igd	0.007	4.10E-06	6970	M32564.m	TRUE	reported	73MgOk	2	TRUE	0.003064	21.41753
8360	rs1761839	A	G	A	G	0.0162	0.023627	0.5998	0.586552	FALSE	FALSE	FALSE	ieu-b-508	7	38839591	0.033957	486484	0.486557	Sepsis (28	Sepsis (28	Sepsis (28	TRUE	igd	0.0035	5.10E-06	6876	M36754.m	TRUE	reported	f8bkFg	2	TRUE	0.003106	21.41744
4228	rs1475961	A	G	A	G	0.0162	0.013942	0.6271	0.630235	FALSE	FALSE	FALSE	ieu-b-508	6	32194609	0.034571	486484	0.68673	Sepsis (28	Sepsis (28	Sepsis (28	TRUE	igd	0.0035	2.69E-06	6857	M32787.m	TRUE	reported	fSRakm	2	TRUE	0.003115	21.41742
1927	rs1185635	T	C	T	C	-0.0162	0.000441	0.8796	0.880604	FALSE	FALSE	FALSE	ieu-b-508	15	36971349	0.052371	486484	0.993284	Sepsis (28	Sepsis (28	Sepsis (28	TRUE	igd	0.0035	2.87E-06	6549	M16822.m	TRUE	reported	UNI018	2	TRUE	0.003261	21.41713
4591	rs896401	T	C	T	C	-0.0162	0.030939	0.7318	0.74569	FALSE	FALSE	FALSE	ieu-b-508	16	8454655	0.040268	486484	0.442288	Sepsis (28	Sepsis (28	Sepsis (28	TRUE	igd	0.0035	3.43E-06	6404	M33163.m	TRUE	reported	YzGXkR	2	TRUE	0.003334	21.41698
3931	rs1707062	T	C	T	C	-0.0426	-0.13252	0.0388	0.026573	FALSE	FALSE	FALSE	ieu-b-508	18	60721642	0.104949	486484	0.206695	Sepsis (28	Sepsis (28	Sepsis (28	TRUE	igd	0.0092	3.88E-06	1757	M32698.m	TRUE	reported	srqncz	2	TRUE	0.012056	21.41652
1559	rs1153699	T	G	T	G	0.0602	0.078009	0.2385	0.242129	FALSE	FALSE	FALSE	ieu-b-508	2	1.76E+08	0.039521	486484	0.048396	Sepsis (28	Sepsis (28	Sepsis (28	TRUE	igd	0.013	3.89E-06	1543	M12261.m	TRUE	reported	taUUcM	2	TRUE	0.013707	21.41623
623	rs1466228	T	C	T	C	-0.0361	0.043352	0.0348	0.044601	FALSE	FALSE	FALSE	ieu-b-508	4	32428311	0.080152	486484	0.5886	Sepsis (28	Sepsis (28	Sepsis (28	TRUE	igd	0.0078	3.21E-06	7349	M01121.m	TRUE	reported	HU0fhR	2	TRUE	0.002906	21.41445
2126	rs9914239	A	G	A	G	-0.1056	-0.11683	0.9123	0.922167	FALSE	FALSE	FALSE	ieu-b-508	17	26709913	0.06244	486484	0.061334	Sepsis (28	Sepsis (28	Sepsis (28	TRUE	igd	0.0228	3.64E-06	1157	M18476.m	TRUE	reported	6GnBtw	2	TRUE	0.018203	21.41444
7109	rs1189335	A	G	A	G	0.5065	0.253905	0.0161	0.020515	FALSE	FALSE	FALSE	ieu-b-508	2	2.29E+08	0.116836	486484	0.029767	Sepsis (28	Sepsis (28	Sepsis (28	TRUE	igd	0.1086	3.08E-06	128	M34516.m	TRUE	reported	7Ah2wy	2	TRUE	0.145254	21.41213
7422	rs39741	A	G	A	G	-0.0199	-0.05046	0.0945	0.103851	FALSE	FALSE	FALSE	ieu-b-508	7	81889209	0.055098	486484	0.359715	Sepsis (28	Sepsis (28	Sepsis (28	TRUE	igd	0.0043	4.51E-06	7350	M35186.m	TRUE	reported	UZfu6Z	2	TRUE	0.002905	21.4117
3724	rs1026341	A	T	A	T	0.0199	0.070218	0.8572	0.851164	FALSE	TRUE	FALSE	ieu-b-508	7	41293296	0.046848	486484	0.133908	Sepsis (28	Sepsis (28	Sepsis (28	TRUE	igd	0.0043	3.58E-06	7337	M32632.m	TRUE	reported	pIHzPT	2	TRUE	0.002911	21.41168
2345	rs1249813	A	G	A	G	-0.0199	0.004233	0.0744	0.087204	FALSE	FALSE	FALSE	ieu-b-508	3	1.21E+08	0.059146	486484	0.942946	Sepsis (28	Sepsis (28	Sepsis (28	TRUE	igd	0.0043	4.35E-06	7203	M21047.m	TRUE	reported	ISF1Cr	2	TRUE	0.002965	21.41158
7677	rs1765427	A	G	A	G	-0.0199	-0.07639	0.9626	0.963783	FALSE	FALSE	FALSE	ieu-b-508	3	1.16E+08	0.08955	486484	0.393633	Sepsis (28	Sepsis (28	Sepsis (28	TRUE	igd	0.0043	4.01E-06	6920	M35327.m	TRUE	reported	SquFiY	2	TRUE	0.003085	21.41133
4122	rs2395859	A	G	A	G	0.0199	0.002424	0.8015	0.773625	FALSE	FALSE	FALSE	ieu-b-508	7	1.07E+08	0.039787	486484	0.951417	Sepsis (28	Sepsis (28	Sepsis (28	TRUE	igd	0.0043	3.88E-06	6658	M32755.m	TRUE	reported	WDrNdi	2	TRUE	0.003206	21.41109
6229	rs1048781	A	C	A	C	-0.0199	-0.0097	0.4438	0.441563	FALSE	FALSE	FALSE	ieu-b-508	7	1.24E+08	0.033686	486484	0.773314	Sepsis (28	Sepsis (28	Sepsis (28	TRUE	igd	0.0043	3.94E-06	5534	M33910.m	TRUE	reported	YwFZg1	2	TRUE	0.003855	21.40978
2394	rs8078549	A	G	A	G	0.1884	-0.03945	0.0266	0.02219	FALSE	FALSE	FALSE	ieu-b-508	17	62206367	0.11512	486484	0.731865	Sepsis (28	Sepsis (28	Sepsis (28	TRUE	igd	0.0407	3.60E-06	2339	M21151.m	TRUE	reported	LfwdLx	2	TRUE	0.009078	21.40925
7300	rs2217851	A	G	A	G	0.0236	0.035821	0.5917	0.618379	FALSE	FALSE	FALSE	ieu-b-508	4	1.75E+08	0.034555	486484	0.299912	Sepsis (28	Sepsis (28	Sepsis (28	TRUE	igd	0.0051	3.01E-06	7364	M35126.m	TRUE	reported	1haOJA	2	TRUE	0.002899	21.40749
6158	rs3770914	T	C	T	C	0.0537	0.07121	0.8905	0.882648	FALSE	FALSE	FALSE	ieu-b-508	2	36636830	0.051921	486484	0.170219	Sepsis (28	Sepsis (28	Sepsis (28	TRUE	igd	0.0116	3.94E-06	1766	M33884.m	TRUE	reported	aAWgRQ	2	TRUE	0.01199	21.40624
7784	rs1050412	A	G	A	G	-0.0588	-0.01074	0.817	0.806477	FALSE	FALSE	FALSE	ieu-b-508	8	52528272	0.042206	486484	0.799118	Sepsis (28	Sepsis (28	Sepsis (28	TRUE	igd	0.0127	3.76E-06	1383	M35451.m	TRUE	reported	GMRGAN	2	TRUE	0.015263	21.40517
2049	rs7349940	A	T	A	T	-0.0611	-0.00933	0.1269	0.115254	FALSE	TRUE	FALSE	ieu-b-508	6	1.51E+08	0.053327	486484	0.861085	Sepsis (28	Sepsis (28	Sepsis (28	TRUE	igd	0.0132	3.59E-06	2079	M18357.m	TRUE	reported	o7AvpG	2	TRUE	0.010201	21.40507
3051	rs4439540	A	G	A	G	0.0574	-0.03976	0.7407	0.73219	FALSE	FALSE	FALSE	ieu-b-508	11	5372147	0.037681	486484	0.291289	Sepsis (28	Sepsis (28	Sepsis (28	TRUE	igd	0.0124	3.80E-06	1760	M31591.m	TRUE	reported	KltFxx	2	TRUE	0.012029	21.40359
793	rs1002106	T	C	T	C	0.031	-0.04232	0.9664	0.957797	FALSE	FALSE	FALSE	ieu-b-508	4	32355847	0.082266	486484	0.606988	Sepsis (28	Sepsis (28	Sepsis (28	TRUE	igd	0.0067	4.07E-06	7335	M01356.m	TRUE	reported	ah1g5W	2	TRUE	0.00291	21.40205
3603	rs4726900	T	G	T	G	0.031	0.00576	0.6601	0.658094	FALSE	FALSE	FALSE	ieu-b-508	7	1.48E+08	0.035252	486484	0.870208	Sepsis (28	Sepsis (28	Sepsis (28	TRUE	igd	0.0067	3.89E-06	7256	M32549.m	TRUE	reported	W7Fgu2	2	TRUE	0.002942	21.40199
8354	rs4930211	T	G	T	G	0.0477	0.079921	0.9025	0.894495	FALSE	FALSE	FALSE	ieu-b-508	11	67423892	0.054355	486484	0.141464	Sepsis (28	Sepsis (28	Sepsis (28	TRUE	igd	0.0103	3.30E-06	922	M36738.m	TRUE	reported	mVQQcD	2	TRUE	0.022732	21.40027
3054	rs6963509	C	G	C	G	-0.0731	0.003837	0.0423	0.045051	FALSE	TRUE	FALSE	ieu-b-508	7	98356872	0.080048	486484	0.961768	Sepsis (28	Sepsis (28	Sepsis (28	TRUE	igd	0.0158	3.46E-06	7338	M31591.m	TRUE	reported	KltFxx	2	TRUE	0.002909	21.39943
3038	rs6752425	T	C	T	C	0.0972	-0.07571	0.0314	0.927117	FALSE	FALSE	FALSE	ieu-b-508	2	1.59E+08	0.064474	486484	0.240312	Sepsis (28	Sepsis (28	Sepsis (28	TRUE	igd	0.021	3.79E-06	1713	M31555.m	TRUE	reported	iQ5E3v	2	TRUE	0.012352	21.39866
5892	rs5022652	A	G	A	G	0.0649	0.016581	0.1582	0.15676	FALSE	FALSE	FALSE	ieu-b-508	9	4766355	0.045857	486484	0.717669	Sepsis (28	Sepsis (28	Sepsis (28	TRUE	igd	0.014	3.84E-06	470	M33610.m	TRUE	reported	tmg43v	2	TRUE	0.043724	21.3984
3923	rs7997650	A	G	A	G	-0.0509	-0.08867	0.0812	0.088421	FALSE	FALSE	FALSE	ieu-b-508	13	34996421	0.058888	486484	0.132142	Sepsis (28	Sepsis (28	Sepsis (28	TRUE	igd	0.011	3.78E-06	2575	M32691.m	TRUE	reported	SiuD6F	2	TRUE	0.008247	21.39502
6636	rs1187858	C	G	C	G	-0.3746	-0.08624	0.986	0.986615	FALSE	TRUE	FALSE	ieu-b-508	19	20059509	0.144196	486484	0.549795	Sepsis (28	Sepsis (28	Sepsis (28	TRUE	igd	0.0799	2.79E-06	75	M34322.m	TRUE	reported	16GdG9	2	TRUE	0.22665	21.39457
8330	rs749275	A	C	A	C	0.0495	-0.01915	0.1139	0.113536	FALSE	FALSE	FALSE	ieu-b-508	3	70151307	0.05262	486484	0.715888	Sepsis (28	Sepsis (28	Sepsis (28	TRUE	igd	0.0107	3.54E-06	3977	M36589.m	TRUE	reported	27GADo	2	TRUE	0.005352	21.39067
3654	rs1884646	C	G	C	G	-0.087	0.004913	0.1687	0.164954	FALSE	TRUE	FALSE	ieu-b-508	20	6107933	0.044948	486484	0.91296	Sepsis (28	Sepsis (28	Sepsis (28	TRUE	igd	0.0188	3.81E-06	1718	M32564.m	TRUE	reported	73MgOk	2	TRUE	0.012312	21.3903
6867	rs1740198	A	G	A	G	0.2037	0.104066	0.0527	0.055417	FALSE	FALSE	FALSE	ieu-b-508	20	20258183	0.072916	486484	0.153523	Sepsis (28	Sepsis (28	Sepsis (28	TRUE	igd	0.044	3.66E-06	954	M34365.m	TRUE	reported	8eexD0	2	TRUE	0.021972	21.38776
6784	rs1781901	T	G	T	G	0.0717	-0.02599	0.0876	0.088065	FALSE	FALSE	FALSE	ieu-b-508	15	55513086	0.059367	486484	0.661595	Sepsis (28	Sepsis (28	Sepsis (28	TRUE	igd	0.0155	3.90E-06	3491	M34350.m	TRUE	reported	Cc3yW9	2	TRUE	0.006092	21.38583
467	rs1078823	T	C	T	C	-0.0111	0.009933	0.6825	0.658631	FALSE	FALSE	FALSE	ieu-b-508	10	1.24E+08	0.035468	486484	0.779435	Sepsis (28	Sepsis (28	Sepsis (28	TRUE	igd	0.0024	2.46E-06	7365	M00527.m	TRUE	reported	UYN7vF	2	TRUE	0.002896	21.38482
473	rs2242578	C	G	C	G	-0.0111	0.020874	0.6704	0.656826	FALSE	TRUE	FALSE	ieu-b-508	12	57853153	0.035594	486484	0.557584	Sepsis (28	Sepsis (28	Sepsis (28	TRUE	igd	0.0024	5.08E-06	7365	M00527.m	TRUE	reported	UYN7vF	2	TRUE	0.002896	21.38482
3192	rs6951820	A	G	A	G	0.0111	-0.02105	0.4338	0.449111	FALSE	FALSE	FALSE	ieu-b-508	7	1.54E+08	0.033581	486484	0.5307	Sepsis (28	Sepsis (28	Sepsis (28	TRUE	igd	0.0024	4.77E-06	7356	M32338.m	TRUE	reported	q5cuaP	2	TRUE	0.002899	21.38481
909	rs1315940	T	G	T	G	-0.0148	-0.00621	0.0652	0.071319	FALSE	FALSE	FALSE	ieu-b-508	5	55128472	0.064545	486484	0.923307	Sepsis (28	Sepsis (28	Sepsis (28	TRUE	igd	0.0032	2.74E-06	7354	M01494.m	TRUE	reported	7JWNKI	2	TRUE	0.0029	21.38481
395	rs9889080	A	G	A	G	0.0074	0.000758	0.1641	0.163474	FALSE	FALSE	FALSE	ieu-b-508	16	55926873	0.045157	486484	0.986614	Sepsis (28	Sepsis (28	Sepsis (28	TRUE	igd	0.0016	4.21E-06	7351	M00060.m	TRUE	reported	kqwl4V	2	TRUE	0.002901	21.38481
743	rs487946	T	G	T	G	-0.0074	0.043258	0.8292	0.810715	FALSE	FALSE	FALSE	ieu-b-508	12	63212116	0.042619	486484	0.310113	Sepsis (28	Sepsis (28	Sepsis (28	TRUE	igd	0.0016	3.10E-06	7347	M01302.m	TRUE	reported	sNp2IC	2	TRUE	0.002903	21.3848
1178	rs1694244	A	G	A	G	-0.0259	0.235484	0.0314	0.033194	FALSE	FALSE	FALSE	ieu-b-508	15	89441356	0.093792	486484	0.012049	Sepsis (28	Sepsis (28	Sepsis (28	TRUE	igd	0.0056	3.32E-06	7346	M01645.m	TRUE	reported	d5nAn3	2	TRUE	0.002903	21.3848
1693	rs2044899	A	C	A	C	0.0074	-0.02317	0.3787	0.371841	FALSE	FALSE	FALSE	ieu-b-508	8	96084835	0.034675	486484	0.504081	Sepsis (28	Sepsis (28	Sepsis (28	TRUE	igd	0.0016	3.54E-06	7345	M15488.m	TRUE	reported	SPriBO	2	TRUE	0.002904	21.3848
7703	rs1878022	T	C	T	C	-0.0074	0.042915	0.6634	0.665407	FALSE	FALSE	FALSE	ieu-b-508	12	1.09E+08	0.035246	486484	0.223387	Sepsis (28	Sepsis (28	Sepsis (28	TRUE	igd	0.0016	6.26E-06	7345	M35397.m	TRUE	reported	Y56mZr	2	TRUE	0.002904	21.3848

7705	rs1995087	A	G	A	G	-0.0074	0.02103	0.3435	0.355432	FALSE	FALSE	FALSE	ieu-b-508	8	19962013	0.03506	486484	0.548627	Sepsis (28	Sepsis (28	Sepsis (28	TRUE	igd	0.0016	6.10E-06	7345	M35397.m	TRUE	reported	Y56mZr	2	TRUE	0.002904	21.3848
3627	rs1757898	T	C	T	C	0.0259	0.066562	0.6347	0.636149	FALSE	FALSE	FALSE	ieu-b-508	1	2.35E+08	0.034827	486484	0.055973	Sepsis (28	Sepsis (28	Sepsis (28	TRUE	igd	0.0056	3.10E-06	7337	M32557.m	TRUE	reported	qR7rZX	2	TRUE	0.002907	21.38479
1051	rs338526	T	C	T	C	-0.0111	0.01004	0.7181	0.716926	FALSE	FALSE	FALSE	ieu-b-508	15	86261840	0.037037	486484	0.786325	Sepsis (28	Sepsis (28	Sepsis (28	TRUE	igd	0.0024	5.61E-06	7333	M01572.m	TRUE	reported	EOUAWy	2	TRUE	0.002909	21.38479
1593	rs9861011	A	G	A	G	-0.0074	0.050145	0.6558	0.657406	FALSE	FALSE	FALSE	ieu-b-508	3	1.31E+08	0.035117	486484	0.153312	Sepsis (28	Sepsis (28	Sepsis (28	TRUE	igd	0.0016	3.80E-06	7314	M12593.m	TRUE	reported	IBXgSU	2	TRUE	0.002916	21.38478
1715	rs2824226	A	G	A	G	0.0074	-0.09121	0.8186	0.81956	FALSE	FALSE	FALSE	ieu-b-508	21	18457828	0.044036	486484	0.038328	Sepsis (28	Sepsis (28	Sepsis (28	TRUE	igd	0.0016	3.19E-06	7310	M15506.m	TRUE	reported	mVRmZO	2	TRUE	0.002918	21.38477
2344	rs1190577	A	C	A	C	0.0111	-0.04521	0.1708	0.161829	FALSE	FALSE	FALSE	ieu-b-508	20	17331246	0.045693	486484	0.322508	Sepsis (28	Sepsis (28	Sepsis (28	TRUE	igd	0.0024	3.96E-06	7203	M21047.m	TRUE	reported	ISF1Cr	2	TRUE	0.002961	21.38469
2346	rs1251548	T	C	T	C	-0.0148	0.055816	0.0969	0.102586	FALSE	FALSE	FALSE	ieu-b-508	5	25951104	0.055207	486484	0.312	Sepsis (28	Sepsis (28	Sepsis (28	TRUE	igd	0.0032	4.11E-06	7203	M21047.m	TRUE	reported	ISF1Cr	2	TRUE	0.002961	21.38469
2190	rs4569257	A	G	A	G	-0.0111	-0.04431	0.4136	0.411984	FALSE	FALSE	FALSE	ieu-b-508	16	8611633	0.0342	486484	0.19511	Sepsis (28	Sepsis (28	Sepsis (28	TRUE	igd	0.0024	2.62E-06	7182	M19362.m	TRUE	reported	SkNEKw	2	TRUE	0.00297	21.38467
2885	rs4738080	A	G	A	G	0.0555	-0.04226	0.0687	0.068863	FALSE	FALSE	FALSE	ieu-b-508	8	71129813	0.066263	486484	0.523631	Sepsis (28	Sepsis (28	Sepsis (28	TRUE	igd	0.012	3.44E-06	6393	M27716.m	TRUE	reported	N5hToq	2	TRUE	0.003335	21.38393
8191	rs205208	T	C	T	C	-0.0495	0.021888	0.096	0.094524	FALSE	FALSE	FALSE	ieu-b-508	6	89726410	0.057508	486484	0.703495	Sepsis (28	Sepsis (28	Sepsis (28	TRUE	igd	0.0107	4.11E-06	2400	M36230.m	TRUE	reported	V5KJeR	2	TRUE	0.008838	21.3836
663	rs1324247	A	G	A	G	0.0111	-0.01851	0.7395	0.73544	FALSE	FALSE	FALSE	ieu-b-508	7	20441065	0.037983	486484	0.626031	Sepsis (28	Sepsis (28	Sepsis (28	TRUE	igd	0.0024	5.47E-06	5687	M01284.m	TRUE	reported	E4hc9c	2	TRUE	0.003747	21.3831
667	rs202490	T	C	T	C	-0.0111	-0.03343	0.3533	0.353535	FALSE	FALSE	FALSE	ieu-b-508	20	1748126	0.035002	486484	0.339481	Sepsis (28	Sepsis (28	Sepsis (28	TRUE	igd	0.0024	4.45E-06	5687	M01284.m	TRUE	reported	E4hc9c	2	TRUE	0.003747	21.3831
5541	rs9584195	T	C	T	C	0.037	-0.00788	0.014	0.018831	FALSE	FALSE	FALSE	ieu-b-508	13	94818360	0.123194	486484	0.949025	Sepsis (28	Sepsis (28	Sepsis (28	TRUE	igd	0.008	3.75E-06	5558	M33422.m	TRUE	reported	PLgjrT	2	TRUE	0.003834	21.38293
7888	rs7110135	C	G	C	G	0.0523	0.029162	0.6932	0.696613	FALSE	TRUE	FALSE	ieu-b-508	11	99733936	0.036361	486484	0.42255	Sepsis (28	Sepsis (28	Sepsis (28	TRUE	igd	0.0113	4.00E-06	1098	M35635.m	TRUE	reported	qymryH	2	TRUE	0.019136	21.38231
8293	rs1315965	T	C	T	C	0.031	-0.00063	0.2052	0.207962	FALSE	FALSE	FALSE	ieu-b-508	5	86130997	0.041169	486484	0.987821	Sepsis (28	Sepsis (28	Sepsis (28	TRUE	igd	0.0067	3.34E-06	1636	M36559.m	TRUE	reported	g6I9hg	2	TRUE	0.012916	21.38171
5229	rs277311	T	C	T	C	0.0259	0.044798	0.1536	0.154088	FALSE	FALSE	FALSE	ieu-b-508	5	1.54E+08	0.046253	486484	0.332771	Sepsis (28	Sepsis (28	Sepsis (28	TRUE	igd	0.0056	4.06E-06	4665	M33221.m	TRUE	reported	8ZrV1r	2	TRUE	0.004564	21.38145
3110	rs4976950	A	C	A	C	-0.068	0.070305	0.0296	0.02113	FALSE	FALSE	FALSE	ieu-b-508	8	1.43E+08	0.116752	486484	0.547058	Sepsis (28	Sepsis (28	Sepsis (28	TRUE	igd	0.0147	3.66E-06	1760	M32198.m	TRUE	reported	ndXmnp	2	TRUE	0.012012	21.37417
8521	rs1258740	A	C	A	C	-0.0888	0.047361	0.1352	0.142795	FALSE	FALSE	FALSE	ieu-b-508	14	68207889	0.047844	486484	0.322216	Sepsis (28	Sepsis (28	Sepsis (28	TRUE	igd	0.0192	3.86E-06	2560	M37112.m	TRUE	reported	P3GiAg	2	TRUE	0.008286	21.37391
6792	rs7551986	A	T	A	T	-0.08	0.132191	0.0797	0.073967	FALSE	TRUE	FALSE	ieu-b-508	1	1.12E+08	0.063797	486484	0.038261	Sepsis (28	Sepsis (28	Sepsis (28	TRUE	igd	0.0173	3.80E-06	3491	M34350.m	TRUE	reported	Cc3yW9	2	TRUE	0.006088	21.37169
7094	rs1234318	A	G	A	G	0.037	0.072483	0.645	0.641082	FALSE	FALSE	FALSE	ieu-b-508	1	1.73E+08	0.034765	486484	0.037075	Sepsis (28	Sepsis (28	Sepsis (28	TRUE	igd	0.008	3.75E-06	2168	M34513.m	TRUE	reported	1hoso6	2	TRUE	0.00977	21.37089
5889	rs357118	A	G	A	G	0.1438	-0.01524	0.8019	0.752422	FALSE	FALSE	FALSE	ieu-b-508	3	13232489	0.038933	486484	0.695378	Sepsis (28	Sepsis (28	Sepsis (28	TRUE	igd	0.0308	3.03E-06	102	M33610.m	TRUE	reported	tmg43v	2	TRUE	0.176077	21.37057
5445	rs1716669	A	C	A	C	-0.0897	-0.06018	0.9655	0.95456	FALSE	FALSE	FALSE	ieu-b-508	7	94820252	0.080349	486484	0.453868	Sepsis (28	Sepsis (28	Sepsis (28	TRUE	igd	0.0194	3.96E-06	5185	M33390.m	TRUE	reported	t0EYdH	2	TRUE	0.004106	21.37046
7841	rs215934	T	C	T	C	0.043	-0.0206	0.1585	0.154061	FALSE	FALSE	FALSE	ieu-b-508	6	85487246	0.046091	486484	0.654948	Sepsis (28	Sepsis (28	Sepsis (28	TRUE	igd	0.0093	3.58E-06	5035	M35551.m	TRUE	reported	5Sxswl	2	TRUE	0.004228	21.3697
6709	rs1002099	A	G	A	G	-0.1357	0.018127	0.1116	0.108042	FALSE	FALSE	FALSE	ieu-b-508	4	1.06E+08	0.053847	486484	0.736397	Sepsis (28	Sepsis (28	Sepsis (28	TRUE	igd	0.0293	3.69E-06	535	M34338.m	TRUE	reported	h4FB9a	2	TRUE	0.038548	21.36967
5457	rs1431419	T	C	T	C	-0.3534	-0.01188	0.9649	0.951484	FALSE	FALSE	FALSE	ieu-b-508	18	66738929	0.078117	486484	0.879147	Sepsis (28	Sepsis (28	Sepsis (28	TRUE	igd	0.076	3.27E-06	171	M33391.m	TRUE	reported	EgMd1b	2	TRUE	0.112253	21.36961
1991	rs29456	T	C	T	C	-0.0356	-0.013	0.2189	0.21518	FALSE	FALSE	FALSE	ieu-b-508	5	65539847	0.040753	486484	0.749808	Sepsis (28	Sepsis (28	Sepsis (28	TRUE	igd	0.0077	4.10E-06	7071	M18254.m	TRUE	reported	clvu6V	2	TRUE	0.003014	21.36957
5143	rs593634	C	G	C	G	0.0763	-0.06543	0.1084	0.11126	FALSE	TRUE	FALSE	ieu-b-508	1	41133265	0.053579	486484	0.222049	Sepsis (28	Sepsis (28	Sepsis (28	TRUE	igd	0.0165	3.50E-06	2616	M33190.m	TRUE	reported	UkhAw5	2	TRUE	0.008108	21.36727
3406	rs1244366	C	G	C	G	-0.0356	-0.07466	0.1372	0.134438	FALSE	TRUE	FALSE	ieu-b-508	16	9633308	0.049098	486484	0.128354	Sepsis (28	Sepsis (28	Sepsis (28	TRUE	igd	0.0077	4.40E-06	5080	M32445.m	TRUE	reported	qcc6f3	2	TRUE	0.00419	21.3672
3723	rs1018900	T	C	T	C	-0.0703	0.184851	0.0531	0.075849	FALSE	FALSE	FALSE	ieu-b-508	2	68530681	0.062868	486484	0.003279	Sepsis (28	Sepsis (28	Sepsis (28	TRUE	igd	0.0152	3.66E-06	1762	M32632.m	TRUE	reported	pIHzPT	2	TRUE	0.011994	21.36635
5473	rs296429	A	G	A	G	-0.0356	0.015602	0.728	0.736746	FALSE	FALSE	FALSE	ieu-b-508	6	1.34E+08	0.037994	486484	0.681326	Sepsis (28	Sepsis (28	Sepsis (28	TRUE	igd	0.0077	3.52E-06	3890	M33408.m	TRUE	reported	tWZWIa	2	TRUE	0.005465	21.36462
4468	rs8008553	A	G	A	G	0.0333	0.01708	0.7132	0.733567	FALSE	FALSE	FALSE	ieu-b-508	14	30298091	0.037835	486484	0.651667	Sepsis (28	Sepsis (28	Sepsis (28	TRUE	igd	0.0072	3.98E-06	1618	M33009.m	TRUE	reported	8RTEdM	2	TRUE	0.013048	21.36418
7092	rs1083665	A	G	A	G	0.0319	-0.02329	0.2061	0.206185	FALSE	FALSE	FALSE	ieu-b-508	11	4814246	0.041341	486484	0.573193	Sepsis (28	Sepsis (28	Sepsis (28	TRUE	igd	0.0069	4.41E-06	3239	M34513.m	TRUE	reported	1hoso6	2	TRUE	0.006556	21.36067
3007	rs6994108	A	G	A	G	0.2358	-0.01217	0.0236	0.038018	FALSE	FALSE	FALSE	ieu-b-508	8	28313280	0.086928	486484	0.888654	Sepsis (28	Sepsis (28	Sepsis (28	TRUE	igd	0.0509	3.68E-06	423	M31536.m	TRUE	reported	3nAuUI	2	TRUE	0.048286	21.35963
5567	rs2028039	A	C	A	C	-1.5512	0.068515	0.0177	0.037251	FALSE	FALSE	FALSE	ieu-b-508	7	1.08E+08	0.088039	486484	0.436436	Sepsis (28	Sepsis (28	Sepsis (28	TRUE	igd	0.33	2.58E-06	60	M33423.m	TRUE	reported	MoEnO7	2	TRUE	0.269146	21.35917
6147	rs1397793	A	G	A	G	-0.0564	-0.05661	0.6623	0.661071	FALSE	FALSE	FALSE	ieu-b-508	5	90471451	0.036613	486484	0.122081	Sepsis (28	Sepsis (28	Sepsis (28	TRUE	igd	0.0122	3.65E-06	2725	M33883.m	TRUE	reported	RT7YNu	2	TRUE	0.007782	21.35599
7886	rs4796899	T	G	T	G	0.093	-0.02209	0.098	0.10004	FALSE	FALSE	FALSE	ieu-b-508	18	1086189	0.057494	486484	0.700808	Sepsis (28	Sepsis (28	Sepsis (28	TRUE	igd	0.0201	3.55E-06	824	M35635.m	TRUE	reported	qymryH	2	TRUE	0.025323	21.35593
3687	rs1225805	A	G	A	G	0.0171	-0.0258	0.3022	0.299218	FALSE	FALSE	FALSE	ieu-b-508	10	96036696	0.036478	486484	0.47934	Sepsis (28	Sepsis (28	Sepsis (28	TRUE	igd	0.0037	3.14E-06	7369	M32587.m	TRUE	reported	uknv7G	2	TRUE	0.00289	21.35359
3688	rs1284443	T	C	T	C	-0.0171	0.062466	0.2702	0.266069	FALSE	FALSE	FALSE	ieu-b-508	18	13595438	0.03786	486484	0.098958	Sepsis (28	Sepsis (28	Sepsis (28	TRUE	igd	0.0037	4.97E-06	7369	M32587.m	TRUE	reported	uknv7G	2	TRUE	0.00289	21.35359
3601	rs2272861	A	G	A	G	-0.0342	-0.01469	0.7054	0.692795	FALSE	FALSE	FALSE	ieu-b-508	22	35937568	0.036296	486484	0.685589	Sepsis (28	Sepsis (28	Sepsis (28	TRUE	igd	0.0074	3.45E-06	7256	M32549.m	TRUE	reported	W7Fgu2	2	TRUE	0.002935	21.3535
5659	rs8102864	A	C	A	C	0.0171	0.013575	0.5284	0.528479	FALSE	FALSE	FALSE	ieu-b-508	19	15611753	0.033544	486484	0.685701	Sepsis (28	Sepsis (28	Sepsis (28	TRUE	igd	0.0037	4.56E-06	5717	M33453.m	TRUE	reported	OzEeLQ	2	TRUE	0.003722	21.35191
4529	rs4581716	A	G	A	G	0.049	-0.0311	0.6695	0.615659	FALSE	FALSE	FALSE	ieu-b-508	16	7458135	0.034284	486484	0.364305	Sepsis (28	Sepsis (28	Sepsis (28	TRUE	igd	0.0106	3.69E-06	2391	M33137.m	TRUE	reported	ONVNk3	2	TRUE	0.008858	21.35094
5436	rs1087332	T	G	T	G	0.0476	0.011346	0.1828	0.212343	FALSE	FALSE	FALSE	ieu-b-508	14	26133060	0.040903	486484	0.781484	Sepsis (28	Sepsis (28	Sepsis (28	TRUE	igd	0.0103	3.68E-06	5185	M33390.m	TRUE	reported	t0EYdH	2	TRUE	0.004102	21.34872
409	rs1264876	A	G	A	G	0.0564	-0.05863	0.9861	0.979926	FALSE	FALSE	FALSE	ieu-b-508	4	60544511	0.118865	486484	0.62182	Sepsis (28	Sepsis (28	Sepsis (28	TRUE	igd	0.0122	3.57E-06	1763	M00063.m	TRUE	reported	Hdsv1L	2	TRUE	0.011977	21.34743
5560	rs1696258	T	C	T	C	-1.2674	0.055353	0.0271	0.047589	FALSE	FALSE	FALSE	ieu-b-508	15	36418198	0.078914	486484	0.483034	Sepsis (28	Sepsis (28	Sepsis (28	TRUE	igd	0.2697	2.62E-06	60	M33423.m	TRUE	reported	MoEnO7	2	TRUE	0.269036	21.34727
7892	rs1724409	A	C	A	C	-0.0439	-0.04635	0.1095	0.166744	FALSE	FALSE	FALSE	ieu-b-508	9	19941355	0.044587	486484	0.298538	Sepsis (28	Sepsis (28	Sepsis (28	TRUE	igd	0.0095	4.00E-06	5629	M35669.m	TRUE	reported	gSecuE	2	TRUE	0.003779	21.34654
5208	rs1095221	T	C	T	C	0.0282	0.050936	0.1046	0.10709	FALSE	FALSE	FALSE	ieu-b-508	7	10472275	0.054149	486484	0.346883	Sepsis (28	Sepsis (28	Sepsis (28	TRUE	igd	0.0061	4.38E-06	1673	M33204.m	TRUE	reported	ROvPWK	2	TRUE	0.012613	21.34613
2408	rs7998013	A	G	A	G	-0.0573	0.042918	0.9344	0.939771	FALSE	FALSE	FALSE	ieu-b-508	13	32171381	0.071981	486484	0.551011	Sepsis (28	Sepsis (28	Sepsis (28	TRUE	igd	0.0124	4.08E-06	5436	M21184.m	TRUE	reported	63ppuJ	2	TRUE	0.003913	21.34549
1422	rs2113305	T	G	T	G	-0.0134	0.055092	0.2651	0.28931	FALSE	FALSE	FALSE	ieu-b-508	16	79051997	0.03695	486484	0.135968	Sepsis (28	Sepsis (28	Sepsis (28	TRUE	igd	0.0029	4.95E-06	6941	M03127.m	TRUE	reported	JscEZw	2	TRUE	0.003067	21.34462
2956	rs1545442	T	C	T	C	0.0134	0.030636	0.8127	0.814758	FALSE	FALSE	FALSE	ieu-b-508	7	37988291	0.043091	486484	0.4771	Sepsis (28	Sepsis (28	Sepsis (28	TRUE	igd	0.0029	2.87E-06	6795	M30805.m	TRUE	reported	YHV70c	2	TRUE	0.003132	21.34449
8286	rs6859143	A	G	A	G	0.0134	-0.0217	0.7032	0.691555	FALSE	FALSE	FALSE	ieu-b-508	5	1.03E+08	0.036293	486484	0.54986	Sepsis (28	Sepsis (28	Sepsis (28	TRUE	igd	0.0029	3.55E-06	6464	M36553.m	TRUE	reported	2Rk7nd	2	TRUE	0.003292	21.34417
6196	rs1861512	T	C	T	C	-0.0134	-0.01551	0.3349	0.337923	FALSE	FALSE	FALSE	ieu-b-508	2	2.06E+08	0.035383	486484	0.661078	Sepsis (28	Sepsis (28	Sepsis (28	TRUE	igd	0.0029	3.81E-06	6004	M33892.m	TRUE	reported	w7HgU7	2	TRUE	0.003543	21.34366
6203	rs4467622	C	G	C	G	-0.0134	-0.00527	0.5956	0.591067	FALSE	TRUE	FALSE	ieu-b-508	4	1.85E+08	0.03401	486484	0.876847	Sepsis (28	Sepsis (28	Sepsis (28	TRUE	igd	0.0029	3.53E-06	6004	M33892.m	TRUE	reported	w7HgU7	2	TRUE	0.003543	21.34366
5494	rs1007793	T	C	T	C	-0.0402	-0.06413	0.1376	0.185438	FALSE	FALSE	FALSE	ieu-b-508	5	2450679	0.04308	486484	0.136593	Sepsis (28	Sepsis (28	Sepsis (28	TRUE	igd	0.0087	4.08E-06	5822	M33420.m	TRUE	reported	eTToDb	2	TRUE	0.003654	21.34344
2407	rs7525018	T	G	T	G	-0.0268	0.042076	0.7223	0.695618	FALSE	FALSE	FALSE	ieu-b-508	1	97583471	0.03635	486484	0.247053	Sepsis (28	Sepsis (28	Sepsis (28	TRUE	igd	0.0058	3.64E-06	5436	M21184.m	TRUE	reported	63ppuJ	2	TRUE	0.003912	21.34292
7634	rs1258789	T	C	T	C	0.0416	0.028079	0.9619	0.964239	FALSE	FALSE	FALSE	ieu-b-508	14	87606646	0.08993	486484	0.754859	Sepsis (28	Sepsis (28	Sepsis (28	TRUE	igd	0.009	3.91E-06	1764	M35326.m	TRUE	reported	jRytyJ	2	TRUE	0.011967	21.34071
4362	rs671321	T	G	T	G	0.0305	0.049964	0.5978	0.578613	FALSE	FALSE	FALSE	ieu-b-508	11	83292373	0.033841	486484	0.139824	Sepsis (28	Sepsis (28	Sepsis (28	TRUE	igd	0.0066	4.01E-06	2656	M32857.m	TRUE	reported	MVAWzT	2	TRUE	0.007976	21.33952
4547	rs1039444	T	C	T	C	0.0231	0.00031	0.8895	0.880522	FALSE	FALSE	FALSE	ieu-b-508	15	63483202	0.051681	486484	0.995209	Sepsis (28	Sepsis (28	Sepsis (28	TRUE	igd	0.005	4.76E-06	7319	M33140.m	TRUE	reported	eBOUtb	2	TRUE	0.002908	21.33857
6658	rs7009700	C	G	C	G	-0.1461	0.082961	0.9503	0.946472	FALSE	TRUE	FALSE	ieu-b-508	8	69301426	0.074163	486484	0.263296	Sepsis (28	Sepsis (28	Sepsis (28	TRUE	igd	0.0315	3.54E-06	248	M34322.m	TRUE	reported	16GdG9	2	TRUE	0.079818	21.33844
4352	rs2016052	C	G	C	G	0.0194	-0.01805	0.6469	0.643262	FALSE	TRUE	FALSE	ieu-b-508	6	1775463	0.034896	486484	0.604984	Sepsis (28	Sepsis (28	Sepsis (28	TRUE	igd	0.0042	2.92E-06	7355	M32855.m	TRUE	reported	AM8kCf	2	TRUE	0.002892	21.3298
6399	rs1419352	C	G	C	G	0.0291	0.029097	0.3905	0.400966	FALSE	TRUE	FALSE	ieu-b-508	10	92440959	0.034278	486484	0.395963	Sepsis (28	Sepsis (28	Sepsis (28	TRUE	igd	0.0063	3.72E-06	7326	M33973.m	TRUE	reported	rpygG6	2	TRUE	0.002904	21.32978
4163	rs4430526	A	G	A	G	0.0194	0.023156	0.5227	0.523445	FALSE	FALSE	FALSE	ieu-b-508	11	1.26E+08	0.033475	486484	0.489112	Sepsis (28	Sepsis (28	Sepsis (28	TRUE	igd	0.0042	3.72E-06	6642	M32758.m	TRUE	reported	AF0m66	2	TRUE	0.003202	21.32918
3384	rs7100861	T	C	T	C	-0.0268	-0.01253	0.5232	0.517687	FALSE	FALSE	FALSE	ieu-b-508	10	31270278	0.033355	486484	0.707146	Sepsis (28	Sepsis (28	Sepsis (28	TRUE	igd	0.0058	3.53E-06	1767	M32418.m	TRUE	reported	y8cN4Y	2	TRUE	0.011939	21.32661
6591	rs4714588	T	C	T	C	0.0231	0.031139	0.7866	0.808384	FALSE	FALSE	FALSE	ieu-b-508	6	12446932	0.043117	486484	0.47017	Sepsis (28	Sepsis (28	Sepsis (28	TRUE	igd	0.005	4.86E-06	2278	M34289.m	TRUE	reported	Knp8ry	2	TRUE	0.009283	21.32566
3008	rs7039553	T	C	T	C	-0.0694	-0.00739	0.6695	0.681638	FALSE	FALSE	FALSE	ieu-b-508	9	22471832	0.035988	486484	0.837218	Sepsis (28	Sepsis (28	Sepsis (28	TRUE	igd	0.015	3.56E-06	522	M31536.m	TRUE	reported	3nAuUI	2	TRUE	0.039392	21.32403
2127	rs1774654	A	G	A	G	-0.0883	0.006368	0.5073	0.505763	FALSE	FALSE	FALSE	ieu-b-508	4	55227440	0.033378	486484	0.848696	Sepsis (28	Sepsis (28	Sepsis (28	TRUE	igd	0.0191	3.67E-06	868	M18477.m	TRUE	reported	p0vcCU	2	TRUE	0.024031	21.32322
7042	rs1506682	A	C	A	C	0.0416	-0.01647	0.1331	0.144291	FALSE	FALSE	FALSE	ieu-b-508	11	60549966	0.047686	486484	0.729753	Sepsis (28	Sepsis (28	Sepsis (28	TRUE	igd	0.009	3.87E-06	1019	M34456.m	TRUE	reported	Nh457q	2	TRUE	0.020536	21.32301
2880	rs1021551	A	G	A	G	0.0499	0.017856	0.5372	0.519537	FALSE	FALSE	FALSE	ieu-b-508	7	66258080	0.033403	486484	0.592957	Sepsis (28	Sepsis (28	Sepsis (28	TRUE	igd	0.0108	3.85E-06	1720	M27716.m	TRUE	reported	N5hToq	2	TRUE	0.012259	21.323
3025	rs388600	A	C	A	C	0.0448	0.020882	0.868	0.864776	FALSE	FALSE	FALSE	ieu-b-508	3	10202211	0.048981	486484	0.669872	Sepsis (28	Sepsis (28	Sepsis (28	TRUE	igd	0.0097	4.00E-06	5229	M31548.m	TRUE	reported	bgRTBS	2	TRUE	0.004063	21.32291
3632	rs883118	A	G	A	G	0.0254	0.008477	0.6702	0.666781	FALSE	FALSE	FALSE	ieu-b-508	7	55307104	0.035439	486484	0.810942	Sepsis (28	Sepsis (28	Sepsis (28	TRUE	igd	0.0055	4.22E-06	7337	M32557.m	TRUE	reported	qR7rZX	2	TRUE	0.002898	21.32179
7193	rs2026781	C	G	C	G	0.0351	-0.09383	0.7702	0.734783	FALSE	TRUE	FALSE	ieu-b-508	10	1.34E+08	0.037863	486484	0.013209	Sepsis (28	Sepsis (28	Sepsis (28	TRUE	igd	0.0076	3.83E-06	4800	M34539.m	TRUE	reported	puA8ni	2	TRUE	0.004424	21.32093
3257	rs1328846	T	C	T	C	-0.0157	-0.02227	0.9192	0.918788	FALSE	FALSE	FALSE	ieu-b-508	9	18326806	0.061487	486484	0.717178	Sepsis (28	Sepsis (28	Sepsis (28	TRUE	igd	0.0034	5.26E-06	7365	M32348.m	TRUE	reported	DJeLDx	2	TRUE	0.002887	21.31687
3045	rs1752362	A	T	A	T	0.0471	0.040569	0.8853	0.876695	FALSE	TRUE	FALSE	ieu-b-508	10	96905714	0.050689	486484	0.423515	Sepsis (28	Sepsis (28	Sepsis (28	TRUE	igd	0.0102	3.57E-06	7338	M31591.m	TRUE	reported	KltFxx	2	TRUE	0.002897	21.31685
1711	rs1749738	T	G	T	G	0.0157	-0.10368	0.0722	0.054754	FALSE	FALSE	FALSE	ieu-b-508	1	83181649	0.075896	486484	0.171893	Sepsis (28	Sepsis (28	Sepsis (28	TRUE	igd	0.0034	5.34E-06	7310	M15506.m	TRUE	reported	mVRmZO	2	TRUE	0.002908	21.31683
3934	rs2980973	T	C	T	C	-0.0157	0.028983	0.9084	0.903457	FALSE	FALSE	FALSE	ieu-b-508	18	60077764	0.056405	486484	0.607365	Sepsis (28	Sepsis (28	Sepsis (28	TRUE	igd	0.0034	4.96E-06	7308	M32698.m	TRUE	reported	srqncz	2	TRUE	0.002909	21.31683
7760	rs1178021	A	G	A	G	0.0157	-0.04265	0.8469	0.816002	FALSE	FALSE	FALSE	ieu-b-508	8	1.18E+08	0.043046	486484	0.321786	Sepsis (28	Sepsis (28	Sepsis (28	TRUE	igd	0.0034	3.67E-06	7256	M35439.m	TRUE	reported	DnuAGZ	2	TRUE	0.00293	21.31679
2025	rs1015947	A	G	A	G	0.0157	0.018703	0.8276	0.806934	FALSE	FALSE	FALSE	ieu-b-508	10	16303374	0.042204	486484	0.657656	Sepsis (28	Sepsis (28	Sepsis (28	TRUE	igd	0.0034	4.09E-06	6939	M18349.m	TRUE	reported	M5qPs8	2	TRUE	0.003063	21.31652
3718	rs6453786	T	G	T	G	-0.0471	-0.04776	0.2993	0.320214	FALSE	FALSE	FALSE	ieu-b-508	6	75532250	0.035951	486484	0.184009	Sepsis (28	Sepsis (28	Sepsis (28	TRUE	igd	0.0102	3.87E-06	6904	M32616.m	TRUE	reported	vrkjBE	2	TRUE	0.003079	21.31649
7540	rs2073234	A	G	A	G	0.0157	0.021867	0.6908	0.686469	FALSE	FALSE	FALSE	ieu-b-508	20	12891577	0.036669	486484	0.550959	Sepsis (28	Sepsis (28	Sepsis (28	TRUE	igd	0.0034	4.46E-06	6538	M35257.m	TRUE	reported	iphouU	2	TRUE	0.003251	21.31614
6118	rs1329418	A	G	A	G	-0.6236	-0.49961	0.9887	0.994806	FALSE	FALSE	FALSE	ieu-b-508	9	37372437	0.240074	486484	0.037427	Sepsis (28	Sepsis (28	Sepsis (28	TRUE	igd	0.1343	3.43E-06	176	M33877.m	TRUE	reported	yDVi1f	2	TRUE	0.109134	21.31556
6865	rs1614887	A	G	A	G	0.1758	-0.00204	0.4688	0.548205	FALSE	FALSE	FALSE	ieu-b-508	6	26393021	0.033508	486484	0.951553	Sepsis (28	Sepsis (28	Sepsis (28	TRUE	igd	0.0359	1.01E-06	18	M34365.m	TRUE	reported	8eexD0	2	TRUE	0.571224	21.31554
7295	rs9992384	T	G	T	G	0.0157	0.027187	0.2562	0.251451	FALSE	FALSE	FALSE	ieu-b-508	4	14811260	0.038526	486484	0.480387	Sepsis (28	Sepsis (28	Sepsis (28	TRUE	igd	0.0034	2.81E-06	5804	M35114.m	TRUE	reported	a6NQjV	2	TRUE	0.00366	21.31532
1910	rs1025417	T	C	T	C	-0.0157	-0.018	0.2329	0.23465	FALSE	FALSE	FALSE	ieu-b-508	7	1.53E+08	0.039411	486484	0.647873	Sepsis (28	Sepsis (28	Sepsis (28	TRUE	igd	0.0034	3.93E-06	4689	M16816.m	TRUE	reported	J1abbO	2	TRUE	0.004527	21.31357
5136	rs2486001	T	C	T	C	0.0702	0.033261	0.1433	0.135114	FALSE	FALSE	FALSE	ieu-b-508	1	44475987	0.049219	486484	0.499188	Sepsis (28	Sepsis (28	Sepsis (28	TRUE	igd	0.0152	3.91E-06	2616	M33190.m	TRUE	reported	UkhAw5	2	TRUE	0.008088	21.31351
5183	rs1457182	A	G	A	G	-0.194	-0.13463	0.982	0.980151	FALSE	FALSE	FALSE	ieu-b-508	8	4649142	0.119308	486484	0.259131	Sepsis (28	Sepsis (28	Sepsis (28	TRUE	igd	0.042	3.86E-06	1864	M33197.m	TRUE	reported	D6xaBg	2	TRUE	0.011317	21.31271
1141	rs1308371	T	C	T	C	0.0194	-0.02898	0.5762	0.549935	FALSE	FALSE	FALSE	ieu-b-508	3	6109274	0.03369	486484	0.389635	Sepsis (28	Sepsis (28	Sepsis (28	TRUE	igd	0.0042	4.84E-06	1766	M01644.m	TRUE	reported	IB0DTL	2	TRUE	0.011937	21.31144
1705	rs1105833	A	G	A	G	-0.0217	0.033525	0.0228	0.020874	FALSE	FALSE	FALSE	ieu-b-508	12	1.26E+08	0.117429	486484	0.77527	Sepsis (28	Sepsis (28	Sepsis (28	TRUE	igd	0.0047	4.26E-06	7310	M15506.m	TRUE	reported	mVRmZO	2	TRUE	0.002908	21.31105
4307	rs1303767	A	G	A	G	-0.0217	-0.0347	0.7782	0.769411	FALSE	FALSE	FALSE	ieu-b-508	20	8324256	0.039754	486484	0.382788	Sepsis (28	Sepsis (28	Sepsis (28	TRUE	igd	0.0047	5.01E-06	6753	M32838.m	TRUE	reported	hyR7zA	2	TRUE	0.003147	21.31057
5896	rs7105058	A	G	A	G	0.0532	0.010426	0.4597	0.474682	FALSE	FALSE	FALSE	ieu-b-508	11	1.08E+08	0.033376	486484	0.754758	Sepsis (28	Sepsis (28	Sepsis (28	TRUE	igd	0.0115	3.36E-06	470	M33610.m	TRUE	reported	tmg43v	2	TRUE	0.04355	21.30961
1813	rs1084778	A	G	A	G	0.0277	0.009618	0.2367	0.24897	FALSE	FALSE	FALSE	ieu-b-508	12	1.3E+08	0.038612	486484	0.803281	Sepsis (28	Sepsis (28	Sepsis (28	TRUE	igd	0.006	4.28E-06	7357	M15753.m	TRUE	reported	VDJYDI	2	TRUE	0.002889	21.30782
5821	rs4509324	A	G	A	G	-0.0277	0.053381	0.9671	0.965915	FALSE	FALSE	FALSE	ieu-b-508	8	12684459	0.091961	486484	0.561595	Sepsis (28	Sepsis (28	Sepsis (28	TRUE	igd	0.006	3.27E-06	7066	M33515.m	TRUE	reported	X9mul1	2	TRUE	0.003007	21.30758
6727	rs1618850	T	G	T	G	-0.0911	0.037246	0.6732	0.664776	FALSE	FALSE	FALSE	ieu-b-508	10	8172573	0.035296	486484	0.291309	Sepsis (28	Sepsis (28	Sepsis (28	TRUE	igd	0.0197	3.77E-06	535	M34338.m	TRUE	reported	h4FB9a	2	TRUE	0.038435	21.30481
6003	rs1322668	C	G	C	G	-0.0975	-0.0194	0.136	0.160674	FALSE	TRUE	FALSE	ieu-b-508	6	44473677	0.045363	486484	0.668889	Sepsis (28	Sepsis (28	Sepsis (28	TRUE	igd	0.0211	3.92E-06	831	M33751.m	TRUE	reported	Cxdkr4	2	TRUE	0.025051	21.30089
4645	rs6912629	C	G	C	G	-0.1016	0.018352	0.3944	0.409847	FALSE	TRUE	FALSE	ieu-b-508	6	1.49E+08	0.033943	486484	0.588747	Sepsis (28	Sepsis (28	Sepsis (28	TRUE	igd	0.022	3.97E-06	1574	M33173.m	TRUE	reported	04fkUQ	2	TRUE	0.013369	21.3005
7095	rs1241943	T	C	T	C	-0.0277	-0.00279	0.3944	0.385926	FALSE	FALSE	FALSE	ieu-b-508	11	75969520	0.034258	486484	0.935004	Sepsis (28	Sepsis (28	Sepsis (28	TRUE	igd	0.006	4.05E-06	3239	M34513.m	TRUE	reported	1hoso6	2	TRUE	0.006537	21.30045
7009	rs2803054	A	C	A	C	-0.0457	0.011454	0.5973	0.594113	FALSE	FALSE	FALSE	ieu-b-508	6	1.63E+08	0.033965	486484	0.735938	Sepsis (28	Sepsis (28	Sepsis (28	TRUE	igd	0.0099	4.23E-06	4452	M34420.m	TRUE	reported	20uofq	2	TRUE	0.004764	21.29938
5139	rs2906297	A	G	A	G	0.0554	-0.02844	0.2427	0.239524	FALSE	FALSE	FALSE	ieu-b-508	7	1.48E+08	0.039106	486484	0.467057	Sepsis (28	Sepsis (28	Sepsis (28	TRUE	igd	0.012	4.13E-06	2616	M33190.m	TRUE	reported	UkhAw5	2	TRUE	0.008082	21.29732
1645	rs511797	A	C	A	C	0.012	0.14217	0.1261	0.120106	FALSE	FALSE	FALSE	ieu-b-508	11	95895139	0.051423	486484	0.005697	Sepsis (28	Sepsis (28	Sepsis (28	TRUE	igd	0.0026	3.77E-06	7368	M15140.m	TRUE	reported	jEcqMJ	2	TRUE	0.002883	21.29599
585	rs1051449	T	C	T	C	0.012	0.016178	0.6782	0.673642	FALSE	FALSE	FALSE	ieu-b-508	16	80315263	0.035768	486484	0.651055	Sepsis (28	Sepsis (28	Sepsis (28	TRUE	igd	0.0026	2.86E-06	7367	M01110.m	TRUE	reported	0A31Sl	2	TRUE	0.002883	21.29599
3255	rs1195490	T	C	T	C	0.012	0.043761	0.8579	0.849236	FALSE	FALSE	FALSE	ieu-b-508	5	1.33E+08	0.046923	486484	0.351021	Sepsis (28	Sepsis (28	Sepsis (28	TRUE	igd	0.0026	3.60E-06	7365	M32348.m	TRUE	reported	DJeLDx	2	TRUE	0.002884	21.29599
1038	rs9389071	A	G	A	G	-0.012	-0.02705	0.1579	0.157894	FALSE	FALSE	FALSE	ieu-b-508	6	1.34E+08	0.045865	486484	0.555353	Sepsis (28	Sepsis (28	Sepsis (28	TRUE	igd	0.0026	5.64E-06	7364	M01564.m	TRUE	reported	IPUdyD	2	TRUE	0.002884	21.29599
6347	rs1260565	T	C	T	C	0.012	0.064234	0.3031	0.32036	FALSE	FALSE	FALSE	ieu-b-508	18	65214082	0.035917	486484	0.073709	Sepsis (28	Sepsis (28	Sepsis (28	TRUE	igd	0.0026	5.13E-06	7364	M33960.m	TRUE	reported	o8RcxU	2	TRUE	0.002884	21.29599
6353	rs4736583	A	C	A	C	-0.012	-0.01521	0.6256	0.62355	FALSE	FALSE	FALSE	ieu-b-508	8	1.33E+08	0.034471	486484	0.658939	Sepsis (28	Sepsis (28	Sepsis (28	TRUE	igd	0.0026	4.89E-06	7364	M33960.m	TRUE	reported	o8RcxU	2	TRUE	0.002884	21.29599
3459	rs1258298	A	G	A	G	-0.012	-0.00966	0.1673	0.207193	FALSE	FALSE	FALSE	ieu-b-508	12	69477770	0.041208	486484	0.814714	Sepsis (28	Sepsis (28	Sepsis (28	TRUE	igd	0.0026	4.13E-06	7363	M32489.m	TRUE	reported	hnljaD	2	TRUE	0.002885	21.29599
3479	rs6711493	A	G	A	G	-0.012	0.047149	0.8695	0.875405	FALSE	FALSE	FALSE	ieu-b-508	2	1.37E+08	0.051401	486484	0.359002	Sepsis (28	Sepsis (28	Sepsis (28	TRUE	igd	0.0026	3.76E-06	7363	M32489.m	TRUE	reported	hnljaD	2	TRUE	0.002885	21.29599
3948	rs1184512	C	G	C	G	-0.012	-0.06562	0.4016	0.411006	FALSE	TRUE	FALSE	ieu-b-508	14	54372739	0.033927	486484	0.053085	Sepsis (28	Sepsis (28	Sepsis (28	TRUE	igd	0.0026	4.24E-06	7363	M32709.m	TRUE	reported	YR8SB4	2	TRUE	0.002885	21.29599
3956	rs2070554	T	G	T	G	0.012	-0.02634	0.3446	0.338269	FALSE	FALSE	FALSE	ieu-b-508	21	45640139	0.035286	486484	0.455323	Sepsis (28	Sepsis (28	Sepsis (28	TRUE	igd	0.0026	4.92E-06	7363	M32709.m	TRUE	reported	YR8SB4	2	TRUE	0.002885	21.29599
3616	rs7047770	A	G	A	G	-0.024	0.007544	0.6563	0.641912	FALSE	FALSE	FALSE	ieu-b-508	9	1.4E+08	0.034804	486484	0.82839	Sepsis (28	Sepsis (28	Sepsis (28	TRUE	igd	0.0052	4.49E-06	7361	M32553.m	TRUE	reported	vHSHqn	2	TRUE	0.002886	21.29599
1447	rs3797248	A	G	A	G	0.012	-0.08768	0.1651	0.17072	FALSE	FALSE	FALSE	ieu-b-508	5	53509186	0.044761	486484	0.050122	Sepsis (28	Sepsis (28	Sepsis (28	TRUE	igd	0.0026	4.62E-06	7357	M03141.m	TRUE	reported	y4mG6m	2	TRUE	0.002887	21.29598
646	rs2397972	A	G	A	G	0.012	-0.03337	0.054	0.059562	FALSE	FALSE	FALSE	ieu-b-508	15	96358652	0.070269	486484	0.634892	Sepsis (28	Sepsis (28	Sepsis (28	TRUE	igd	0.0026	4.05E-06	7352	M01125.m	TRUE	reported	yw0clB	2	TRUE	0.002889	21.29598
5853	rs2527641	A	G	A	G	-0.018	-0.03127	0.5656	0.554702	FALSE	FALSE	FALSE	ieu-b-508	7	68260664	0.033533	486484	0.351017	Sepsis (28	Sepsis (28	Sepsis (28	TRUE	igd	0.0039	4.94E-06	7352	M33587.m	TRUE	reported	ssG6bF	2	TRUE	0.002889	21.29598
7423	rs4257232	A	G	A	G	0.012	-0.04514	0.5675	0.591213	FALSE	FALSE	FALSE	ieu-b-508	16	76480663	0.034118	486484	0.185822	Sepsis (28	Sepsis (28	Sepsis (28	TRUE	igd	0.0026	4.17E-06	7350	M35186.m	TRUE	reported	UZfu6Z	2	TRUE	0.00289	21.29598
7428	rs7599943	T	C	T	C	-0.012	-0.02674	0.3473	0.353786	FALSE	FALSE	FALSE	ieu-b-508	2	2.42E+08	0.0349	486484	0.443611	Sepsis (28	Sepsis (28	Sepsis (28	TRUE	igd	0.0026	5.17E-06	7350	M35186.m	TRUE	reported	UZfu6Z	2	TRUE	0.00289	21.29598
1218	rs980922	C	G	C	G	-0.012	0.000301	0.8056	0.78819	FALSE	TRUE	FALSE	ieu-b-508	1	95853985	0.040929	486484	0.994132	Sepsis (28	Sepsis (28	Sepsis (28	TRUE	igd	0.0026	3.57E-06	7348	M01670.m	TRUE	reported	kjghbc	2	TRUE	0.002891	21.29598
7327	rs737842	A	G	A	G	-0.012	-0.01997	0.5825	0.579934	FALSE	FALSE	FALSE	ieu-b-508	22	22517205	0.033811	486484	0.554751	Sepsis (28	Sepsis (28	Sepsis (28	TRUE	igd	0.0026	3.05E-06	7344	M35127.m	TRUE	reported	RNsmjl	2	TRUE	0.002892	21.29597
1777	rs6846111	T	C	T	C	-0.012	-0.02343	0.1508	0.153926	FALSE	FALSE	FALSE	ieu-b-508	4	1.3E+08	0.046117	486484	0.611433	Sepsis (28	Sepsis (28	Sepsis (28	TRUE	igd	0.0026	5.46E-06	7331	M15676.m	TRUE	reported	s1NfKN	2	TRUE	0.002897	21.29596
2326	rs9814648	A	G	A	G	0.012	-0.01781	0.2592	0.275632	FALSE	FALSE	FALSE	ieu-b-508	3	3155229	0.037585	486484	0.635647	Sepsis (28	Sepsis (28	Sepsis (28	TRUE	igd	0.0026	4.60E-06	7327	M20699.m	TRUE	reported	Z3EKFa	2	TRUE	0.002899	21.29596
890	rs1049742	T	C	T	C	0.012	-0.09269	0.8064	0.809973	FALSE	FALSE	FALSE	ieu-b-508	2	1.76E+08	0.042809	486484	0.030368	Sepsis (28	Sepsis (28	Sepsis (28	TRUE	igd	0.0026	4.47E-06	7307	M01493.m	TRUE	reported	eLLRt7	2	TRUE	0.002907	21.29594
2976	rs1948930	A	G	A	G	0.012	0.039584	0.297	0.310101	FALSE	FALSE	FALSE	ieu-b-508	8	19053212	0.036203	486484	0.274226	Sepsis (28	Sepsis (28	Sepsis (28	TRUE	igd	0.0026	4.21E-06	7257	M31453.m	TRUE	reported	vdTd9M	2	TRUE	0.002927	21.2959
7579	rs708496	T	C	T	C	-0.012	-0.023	0.5445	0.615886	FALSE	FALSE	FALSE	ieu-b-508	14	52772695	0.034989	486484	0.511045	Sepsis (28	Sepsis (28	Sepsis (28	TRUE	igd	0.0026	4.39E-06	7217	M35270.m	TRUE	reported	Ct8V1n	2	TRUE	0.002943	21.29587
934	rs2526477	A	G	A	G	-0.012	0.031277	0.609	0.610565	FALSE	FALSE	FALSE	ieu-b-508	17	20131193	0.0342	486484	0.36044	Sepsis (28	Sepsis (28	Sepsis (28	TRUE	igd	0.0026	2.99E-06	7160	M01508.m	TRUE	reported	go526k	2	TRUE	0.002966	21.29582
6342	rs6833678	T	C	T	C	-0.018	-0.02555	0.3165	0.305667	FALSE	FALSE	FALSE	ieu-b-508	4	88307986	0.036505	486484	0.484069	Sepsis (28	Sepsis (28	Sepsis (28	TRUE	igd	0.0039	3.50E-06	6998	M33957.m	TRUE	reported	mrKpMv	2	TRUE	0.003035	21.29569
1884	rs1955742	A	T	A	T	-0.012	0.016064	0.599	0.596561	FALSE	TRUE	FALSE	ieu-b-508	14	37067827	0.033993	486484	0.636518	Sepsis (28	Sepsis (28	Sepsis (28	TRUE	igd	0.0026	6.24E-06	6782	M15990.m	TRUE	reported	15fe4N	2	TRUE	0.003131	21.29549
4588	rs4453360	A	G	A	G	-0.012	-0.01851	0.4648	0.449473	FALSE	FALSE	FALSE	ieu-b-508	13	82125852	0.033891	486484	0.58503	Sepsis (28	Sepsis (28	Sepsis (28	TRUE	igd	0.0026	4.28E-06	6404	M33163.m	TRUE	reported	YzGXkR	2	TRUE	0.003315	21.29512
7445	rs2048073	A	G	A	G	-0.03	-0.07156	0.1579	0.189123	FALSE	FALSE	FALSE	ieu-b-508	4	1.78E+08	0.042968	486484	0.095847	Sepsis (28	Sepsis (28	Sepsis (28	TRUE	igd	0.0065	4.13E-06	6234	M35189.m	TRUE	reported	54XZFj	2	TRUE	0.003405	21.29494
5168	rs432097	A	G	A	G	-0.048	0.020895	0.2928	0.29804	FALSE	FALSE	FALSE	ieu-b-508	5	1.06E+08	0.036479	486484	0.566786	Sepsis (28	Sepsis (28	Sepsis (28	TRUE	igd	0.0104	4.08E-06	5064	M33194.m	TRUE	reported	xRUveq	2	TRUE	0.004189	21.29336
7317	rs1270382	A	T	A	T	-0.0217	-0.01511	0.6236	0.633849	FALSE	TRUE	FALSE	ieu-b-508	7	1.46E+08	0.034614	486484	0.662488	Sepsis (28	Sepsis (28	Sepsis (28	TRUE	igd	0.0047	4.64E-06	1762	M35127.m	TRUE	reported	RNsmjl	2	TRUE	0.011954	21.29269
1643	rs3809198	A	C	A	C	0.0554	0.019465	0.0199	0.011387	FALSE	FALSE	FALSE	ieu-b-508	12	96883306	0.161387	486484	0.904002	Sepsis (28	Sepsis (28	Sepsis (28	TRUE	igd	0.012	4.28E-06	1764	M15140.m	TRUE	reported	jEcqMJ	2	TRUE	0.011938	21.28945
2713	rs7242260	A	C	A	C	0.0277	0.007182	0.8606	0.870494	FALSE	FALSE	FALSE	ieu-b-508	18	897563	0.04988	486484	0.885505	Sepsis (28	Sepsis (28	Sepsis (28	TRUE	igd	0.006	3.52E-06	1756	M25459.m	TRUE	reported	ACJY35	2	TRUE	0.011992	21.28934
5166	rs2959857	A	T	A	T	0.0988	-0.01955	0.2731	0.2729	FALSE	TRUE	FALSE	ieu-b-508	15	76461848	0.037607	486484	0.60325	Sepsis (28	Sepsis (28	Sepsis (28	TRUE	igd	0.0214	3.81E-06	1586	M33194.m	TRUE	reported	xRUveq	2	TRUE	0.013261	21.28817
6635	rs1181731	T	C	T	C	-0.17	-0.09094	0.9575	0.966745	FALSE	FALSE	FALSE	ieu-b-508	10	43283026	0.093435	486484	0.330383	Sepsis (28	Sepsis (28	Sepsis (28	TRUE	igd	0.0367	3.61E-06	248	M34322.m	TRUE	reported	16GdG9	2	TRUE	0.07963	21.2838
1817	rs1343188	T	C	T	C	-0.0263	-0.03288	0.5646	0.537753	FALSE	FALSE	FALSE	ieu-b-508	13	36467079	0.033586	486484	0.32759	Sepsis (28	Sepsis (28	Sepsis (28	TRUE	igd	0.0057	4.17E-06	7357	M15753.m	TRUE	reported	VDJYDI	2	TRUE	0.002885	21.28353
8651	rs7187181	T	C	T	C	0.0263	0.00425	0.0635	0.070927	FALSE	FALSE	FALSE	ieu-b-508	16	82274265	0.066095	486484	0.948728	Sepsis (28	Sepsis (28	Sepsis (28	TRUE	igd	0.0057	3.34E-06	6942	M38768.m	TRUE	reported	a2b8mH	2	TRUE	0.003057	21.28319
1806	rs6851666	A	T	A	T	-0.0526	-0.02166	0.9632	0.957476	FALSE	TRUE	FALSE	ieu-b-508	4	1.7E+08	0.082737	486484	0.793504	Sepsis (28	Sepsis (28	Sepsis (28	TRUE	igd	0.0114	4.06E-06	5829	M15749.m	TRUE	reported	TR861N	2	TRUE	0.003639	21.28202
5151	rs1233996	C	G	C	G	0.0789	-0.02378	0.0852	0.090283	FALSE	TRUE	FALSE	ieu-b-508	9	80745500	0.058743	486484	0.685629	Sepsis (28	Sepsis (28	Sepsis (28	TRUE	igd	0.0171	3.71E-06	5716	M33192.m	TRUE	reported	CyfjKE	2	TRUE	0.003711	21.28187
6507	rs4850779	A	G	A	G	-0.0263	0.041246	0.415	0.40632	FALSE	FALSE	FALSE	ieu-b-508	2	1.98E+08	0.033896	486484	0.223663	Sepsis (28	Sepsis (28	Sepsis (28	TRUE	igd	0.0057	4.40E-06	5581	M34112.m	TRUE	reported	97JqFb	2	TRUE	0.0038	21.28169
4905	rs2361394	A	G	A	G	0.1413	-0.04658	0.9584	0.956838	FALSE	FALSE	FALSE	ieu-b-508	5	35800547	0.083369	486484	0.576346	Sepsis (28	Sepsis (28	Sepsis (28	TRUE	igd	0.0306	3.75E-06	1017	M33178.m	TRUE	reported	g22WMS	2	TRUE	0.020536	21.28073
5372	rs163589	T	G	T	G	0.6297	-0.04069	0.011	0.017198	FALSE	FALSE	FALSE	ieu-b-508	5	34978489	0.1288	486484	0.752096	Sepsis (28	Sepsis (28	Sepsis (28	TRUE	igd	0.136	3.63E-06	272	M33384.m	TRUE	reported	lOffrZ	2	TRUE	0.073059	21.28063
7305	rs4938200	T	C	T	C	-0.0203	-0.04154	0.4388	0.439258	FALSE	FALSE	FALSE	ieu-b-508	11	1.15E+08	0.033612	486484	0.21652	Sepsis (28	Sepsis (28	Sepsis (28	TRUE	igd	0.0044	4.31E-06	7364	M35126.m	TRUE	reported	1haOJA	2	TRUE	0.002882	21.27986
5779	rs1279565	A	T	A	T	-0.0203	-0.01516	0.8826	0.891025	FALSE	TRUE	FALSE	ieu-b-508	11	79185332	0.053474	486484	0.776791	Sepsis (28	Sepsis (28	Sepsis (28	TRUE	igd	0.0044	3.34E-06	7269	M33510.m	TRUE	reported	WxGKnn	2	TRUE	0.00292	21.27978
2644	rs6078040	T	C	T	C	-0.0203	-0.05124	0.9256	0.914642	FALSE	FALSE	FALSE	ieu-b-508	20	1159395	0.059874	486484	0.392101	Sepsis (28	Sepsis (28	Sepsis (28	TRUE	igd	0.0044	4.82E-06	7184	M22548.m	TRUE	reported	mZtwX2	2	TRUE	0.002954	21.27971
6746	rs693833	A	C	A	C	0.591	-0.00124	0.983	0.978415	FALSE	FALSE	FALSE	ieu-b-508	1	1.94E+08	0.115955	486484	0.991448	Sepsis (28	Sepsis (28	Sepsis (28	TRUE	igd	0.127	3.26E-06	115	M34338.m	TRUE	reported	h4FB9a	2	TRUE	0.158468	21.27885
6450	rs7167675	C	G	C	G	-0.06	0.172494	0.9875	0.990575	FALSE	TRUE	FALSE	ieu-b-508	15	93270322	0.174116	486484	0.321839	Sepsis (28	Sepsis (28	Sepsis (28	TRUE	igd	0.013	3.68E-06	1768	M34062.m	TRUE	reported	RZyHKP	2	TRUE	0.011905	21.27768
8409	rs1258238	T	C	T	C	0.036	0.095835	0.0454	0.046476	FALSE	FALSE	FALSE	ieu-b-508	12	82517212	0.080641	486484	0.23467	Sepsis (28	Sepsis (28	Sepsis (28	TRUE	igd	0.0078	3.64E-06	1764	M36808.m	TRUE	reported	u7lqaj	2	TRUE	0.011932	21.27762
3904	rs7010049	A	G	A	G	-0.024	0.078036	0.9352	0.935228	FALSE	FALSE	FALSE	ieu-b-508	8	2539444	0.067993	486484	0.251093	Sepsis (28	Sepsis (28	Sepsis (28	TRUE	igd	0.0052	3.53E-06	1756	M32675.m	TRUE	reported	9FmPh9	2	TRUE	0.011985	21.27751
533	rs1125992	A	G	A	G	0.042	-0.02338	0.8139	0.791596	FALSE	FALSE	FALSE	ieu-b-508	15	84535704	0.041002	486484	0.568558	Sepsis (28	Sepsis (28	Sepsis (28	TRUE	igd	0.0091	3.92E-06	1723	M00599.m	TRUE	reported	nRJY3s	2	TRUE	0.012212	21.27705
7200	rs9832461	A	G	A	G	-0.0203	-0.05151	0.7731	0.776976	FALSE	FALSE	FALSE	ieu-b-508	3	39749593	0.040288	486484	0.201026	Sepsis (28	Sepsis (28	Sepsis (28	TRUE	igd	0.0044	4.10E-06	4800	M34539.m	TRUE	reported	puA8ni	2	TRUE	0.004415	21.27677
7745	rs9642829	A	G	A	G	-0.0346	-0.05901	0.9744	0.978297	FALSE	FALSE	FALSE	ieu-b-508	8	1.16E+08	0.114864	486484	0.607441	Sepsis (28	Sepsis (28	Sepsis (28	TRUE	igd	0.0075	3.61E-06	7007	M35431.m	TRUE	reported	tLfE4l	2	TRUE	0.003028	21.27677
4927	rs33469	A	G	A	G	0.1639	0.152732	0.9553	0.97976	FALSE	FALSE	FALSE	ieu-b-508	3	42408066	0.119962	486484	0.202957	Sepsis (28	Sepsis (28	Sepsis (28	TRUE	igd	0.0355	4.01E-06	1017	M33178.m	TRUE	reported	g22WMS	2	TRUE	0.020529	21.27386
3305	rs2903593	C	G	C	G	-0.0374	-0.003	0.6229	0.587902	FALSE	TRUE	FALSE	ieu-b-508	19	31318358	0.033835	486484	0.929351	Sepsis (28	Sepsis (28	Sepsis (28	TRUE	igd	0.0081	4.52E-06	936	M32388.m	TRUE	reported	yYczcA	2	TRUE	0.02227	21.27376
6624	rs6597533	A	G	A	G	0.1306	-0.0049	0.9771	0.972775	FALSE	FALSE	FALSE	ieu-b-508	9	1.35E+08	0.103403	486484	0.962229	Sepsis (28	Sepsis (28	Sepsis (28	TRUE	igd	0.0283	4.04E-06	1766	M34314.m	TRUE	reported	wPKEWG	2	TRUE	0.011916	21.27264
8366	rs1054812	A	G	A	G	-0.0203	-0.07825	0.6283	0.621511	FALSE	FALSE	FALSE	ieu-b-508	13	43537189	0.034551	486484	0.023521	Sepsis (28	Sepsis (28	Sepsis (28	TRUE	igd	0.0044	4.28E-06	3248	M36756.m	TRUE	reported	QsW4oR	2	TRUE	0.006511	21.27253
8373	rs518385	A	G	A	G	-0.0203	0.001683	0.5033	0.51385	FALSE	FALSE	FALSE	ieu-b-508	11	56544745	0.033425	486484	0.959839	Sepsis (28	Sepsis (28	Sepsis (28	TRUE	igd	0.0044	3.29E-06	3248	M36756.m	TRUE	reported	QsW4oR	2	TRUE	0.006511	21.27253
3838	rs1099586	A	C	A	C	0.0286	-0.02081	0.0589	0.060439	FALSE	FALSE	FALSE	ieu-b-508	10	52914898	0.070782	486484	0.768785	Sepsis (28	Sepsis (28	Sepsis (28	TRUE	igd	0.0062	3.92E-06	5211	M32651.m	TRUE	reported	DeuPwv	2	TRUE	0.004067	21.27071
1675	rs1312063	T	G	T	G	0.0443	0.021754	0.9213	0.928398	FALSE	FALSE	FALSE	ieu-b-508	4	1.87E+08	0.065151	486484	0.738455	Sepsis (28	Sepsis (28	Sepsis (28	TRUE	igd	0.0096	3.96E-06	1760	M15365.m	TRUE	reported	TReTCM	2	TRUE	0.011954	21.27018
7233	rs1696425	T	C	T	C	-0.0669	-0.02839	0.9549	0.957044	FALSE	FALSE	FALSE	ieu-b-508	13	1.05E+08	0.082295	486484	0.730066	Sepsis (28	Sepsis (28	Sepsis (28	TRUE	igd	0.0145	4.02E-06	2474	M34878.m	TRUE	reported	Cpq9yU	2	TRUE	0.008531	21.26988
3031	rs1244517	A	C	A	C	-0.0226	-0.05201	0.8312	0.818862	FALSE	FALSE	FALSE	ieu-b-508	16	8251747	0.043355	486484	0.230251	Sepsis (28	Sepsis (28	Sepsis (28	TRUE	igd	0.0049	3.61E-06	7263	M31555.m	TRUE	reported	iQ5E3v	2	TRUE	0.00292	21.26695
4139	rs1366688	C	G	C	G	0.0226	-0.03624	0.6094	0.602271	FALSE	TRUE	FALSE	ieu-b-508	4	1.48E+08	0.034677	486484	0.296025	Sepsis (28	Sepsis (28	Sepsis (28	TRUE	igd	0.0049	5.15E-06	7250	M32757.m	TRUE	reported	TJV9Xb	2	TRUE	0.002926	21.26693
7116	rs1708348	T	C	T	C	-0.1292	-0.0025	0.2175	0.222184	FALSE	FALSE	FALSE	ieu-b-508	4	54656948	0.040066	486484	0.950257	Sepsis (28	Sepsis (28	Sepsis (28	TRUE	igd	0.0279	3.63E-06	241	M34516.m	TRUE	reported	7Ah2wy	2	TRUE	0.081711	21.26657
1011	rs2143327	T	C	T	C	0.0392	0.144174	0.0129	0.018485	FALSE	FALSE	FALSE	ieu-b-508	6	1.47E+08	0.125158	486484	0.24935	Sepsis (28	Sepsis (28	Sepsis (28	TRUE	igd	0.0085	4.28E-06	7364	M01564.m	TRUE	reported	IPUdyD	2	TRUE	0.00288	21.2626
594	rs3798211	A	C	A	C	-0.0083	0.001391	0.4247	0.425821	FALSE	FALSE	FALSE	ieu-b-508	6	1.6E+08	0.03371	486484	0.967076	Sepsis (28	Sepsis (28	Sepsis (28	TRUE	igd	0.0018	4.58E-06	7367	M01110.m	TRUE	reported	0A31Sl	2	TRUE	0.002878	21.25657
8603	rs1445447	A	G	A	G	-0.0083	-0.00029	0.7448	0.743124	FALSE	FALSE	FALSE	ieu-b-508	16	59813971	0.038132	486484	0.993831	Sepsis (28	Sepsis (28	Sepsis (28	TRUE	igd	0.0018	3.91E-06	7366	M37506.m	TRUE	reported	m2kaRt	2	TRUE	0.002878	21.25657
8612	rs2294070	A	T	A	T	-0.0083	0.018444	0.7404	0.736916	FALSE	TRUE	FALSE	ieu-b-508	8	2050383	0.03788	486484	0.626334	Sepsis (28	Sepsis (28	Sepsis (28	TRUE	igd	0.0018	3.87E-06	7366	M37506.m	TRUE	reported	m2kaRt	2	TRUE	0.002878	21.25657
410	rs1288179	T	G	T	G	-0.0083	-0.03903	0.7603	0.745516	FALSE	FALSE	FALSE	ieu-b-508	14	63023507	0.038593	486484	0.311878	Sepsis (28	Sepsis (28	Sepsis (28	TRUE	igd	0.0018	5.91E-06	7365	M00063.m	TRUE	reported	Hdsv1L	2	TRUE	0.002879	21.25657
427	rs3747924	T	C	T	C	0.0083	-0.06681	0.1781	0.178878	FALSE	FALSE	FALSE	ieu-b-508	20	6182755	0.044032	486484	0.129168	Sepsis (28	Sepsis (28	Sepsis (28	TRUE	igd	0.0018	4.24E-06	7365	M00063.m	TRUE	reported	Hdsv1L	2	TRUE	0.002879	21.25657
3251	rs1089800	T	C	T	C	0.0083	0.025431	0.5314	0.53667	FALSE	FALSE	FALSE	ieu-b-508	11	82328339	0.03367	486484	0.450061	Sepsis (28	Sepsis (28	Sepsis (28	TRUE	igd	0.0018	2.30E-06	7365	M32348.m	TRUE	reported	DJeLDx	2	TRUE	0.002879	21.25657
3264	rs192812	T	C	T	C	0.0083	0.034866	0.4356	0.438499	FALSE	FALSE	FALSE	ieu-b-508	3	1.61E+08	0.033666	486484	0.30037	Sepsis (28	Sepsis (28	Sepsis (28	TRUE	igd	0.0018	2.89E-06	7365	M32348.m	TRUE	reported	DJeLDx	2	TRUE	0.002879	21.25657
3288	rs9323955	A	G	A	G	0.0083	-0.02734	0.4396	0.440146	FALSE	FALSE	FALSE	ieu-b-508	14	97294935	0.033603	486484	0.415874	Sepsis (28	Sepsis (28	Sepsis (28	TRUE	igd	0.0018	2.70E-06	7365	M32348.m	TRUE	reported	DJeLDx	2	TRUE	0.002879	21.25657
1004	rs1380634	A	G	A	G	-0.0083	-0.00243	0.7425	0.740988	FALSE	FALSE	FALSE	ieu-b-508	8	80237893	0.038274	486484	0.949353	Sepsis (28	Sepsis (28	Sepsis (28	TRUE	igd	0.0018	5.17E-06	7364	M01564.m	TRUE	reported	IPUdyD	2	TRUE	0.002879	21.25657
1015	rs2534835	T	G	T	G	-0.0083	-0.00728	0.2407	0.245481	FALSE	FALSE	FALSE	ieu-b-508	5	56948443	0.038947	486484	0.851657	Sepsis (28	Sepsis (28	Sepsis (28	TRUE	igd	0.0018	6.23E-06	7364	M01564.m	TRUE	reported	IPUdyD	2	TRUE	0.002879	21.25657
1017	rs4235093	A	G	A	G	-0.0083	-0.00205	0.2874	0.292324	FALSE	FALSE	FALSE	ieu-b-508	4	72346252	0.036835	486484	0.955536	Sepsis (28	Sepsis (28	Sepsis (28	TRUE	igd	0.0018	5.04E-06	7364	M01564.m	TRUE	reported	IPUdyD	2	TRUE	0.002879	21.25657
1026	rs7584253	A	C	A	C	0.0083	0.024559	0.7047	0.702211	FALSE	FALSE	FALSE	ieu-b-508	2	48927246	0.036575	486484	0.501929	Sepsis (28	Sepsis (28	Sepsis (28	TRUE	igd	0.0018	5.04E-06	7364	M01564.m	TRUE	reported	IPUdyD	2	TRUE	0.002879	21.25657
1030	rs8017368	A	C	A	C	-0.0083	-0.01571	0.6452	0.630534	FALSE	FALSE	FALSE	ieu-b-508	14	63527183	0.034559	486484	0.649491	Sepsis (28	Sepsis (28	Sepsis (28	TRUE	igd	0.0018	3.85E-06	7364	M01564.m	TRUE	reported	IPUdyD	2	TRUE	0.002879	21.25657
3453	rs1054053	A	G	A	G	0.0083	0.009501	0.4028	0.405125	FALSE	FALSE	FALSE	ieu-b-508	10	81688670	0.034031	486484	0.780112	Sepsis (28	Sepsis (28	Sepsis (28	TRUE	igd	0.0018	3.82E-06	7363	M32489.m	TRUE	reported	hnljaD	2	TRUE	0.002879	21.25657
3756	rs1162300	A	C	A	C	0.0083	-0.02713	0.5662	0.565635	FALSE	FALSE	FALSE	ieu-b-508	14	87292755	0.03369	486484	0.420595	Sepsis (28	Sepsis (28	Sepsis (28	TRUE	igd	0.0018	3.25E-06	7362	M32634.m	TRUE	reported	7T2RUz	2	TRUE	0.00288	21.25657
3758	rs1264950	T	G	T	G	0.0083	0.003649	0.2978	0.302562	FALSE	FALSE	FALSE	ieu-b-508	4	76463089	0.036408	486484	0.920175	Sepsis (28	Sepsis (28	Sepsis (28	TRUE	igd	0.0018	5.14E-06	7362	M32634.m	TRUE	reported	7T2RUz	2	TRUE	0.00288	21.25657
4513	rs6969691	A	G	A	G	-0.0083	0.033651	0.8775	0.88018	FALSE	FALSE	FALSE	ieu-b-508	7	92734983	0.051335	486484	0.512137	Sepsis (28	Sepsis (28	Sepsis (28	TRUE	igd	0.0018	5.29E-06	7362	M33132.m	TRUE	reported	dTXJa8	2	TRUE	0.00288	21.25657
6429	rs1265800	T	G	T	G	0.0083	0.045263	0.2091	0.232222	FALSE	FALSE	FALSE	ieu-b-508	5	1.21E+08	0.03955	486484	0.252442	Sepsis (28	Sepsis (28	Sepsis (28	TRUE	igd	0.0018	2.66E-06	7362	M34062.m	TRUE	reported	RZyHKP	2	TRUE	0.00288	21.25657
6432	rs1715036	A	T	A	T	0.0083	0.027005	0.8838	0.881384	FALSE	TRUE	FALSE	ieu-b-508	7	21312125	0.051848	486484	0.602476	Sepsis (28	Sepsis (28	Sepsis (28	TRUE	igd	0.0018	6.17E-06	7362	M34062.m	TRUE	reported	RZyHKP	2	TRUE	0.00288	21.25657
6437	rs2162481	T	C	T	C	-0.0083	-0.03159	0.1224	0.120591	FALSE	FALSE	FALSE	ieu-b-508	2	2.31E+08	0.051141	486484	0.536819	Sepsis (28	Sepsis (28	Sepsis (28	TRUE	igd	0.0018	6.89E-06	7362	M34062.m	TRUE	reported	RZyHKP	2	TRUE	0.00288	21.25657
6443	rs4748538	T	C	T	C	-0.0083	0.003343	0.88	0.859842	FALSE	FALSE	FALSE	ieu-b-508	10	6966044	0.048396	486484	0.944935	Sepsis (28	Sepsis (28	Sepsis (28	TRUE	igd	0.0018	7.41E-06	7362	M34062.m	TRUE	reported	RZyHKP	2	TRUE	0.00288	21.25657
691	rs4808739	T	C	T	C	0.0083	-0.05437	0.7578	0.763828	FALSE	FALSE	FALSE	ieu-b-508	19	18159516	0.039373	486484	0.167309	Sepsis (28	Sepsis (28	Sepsis (28	TRUE	igd	0.0018	2.42E-06	7358	M01299.m	TRUE	reported	gfM0a3	2	TRUE	0.002881	21.25657
1449	rs4646359	T	C	T	C	-0.0083	-0.00886	0.5297	0.526301	FALSE	FALSE	FALSE	ieu-b-508	17	17470609	0.033409	486484	0.790802	Sepsis (28	Sepsis (28	Sepsis (28	TRUE	igd	0.0018	3.20E-06	7357	M03141.m	TRUE	reported	y4mG6m	2	TRUE	0.002882	21.25657
57	rs1682395	T	G	T	G	-0.0083	0.086312	0.9069	0.902679	FALSE	FALSE	FALSE	ieu-b-508	1	1.85E+08	0.056228	486484	0.124775	Sepsis (28	Sepsis (28	Sepsis (28	TRUE	igd	0.0018	5.08E-06	7355	M00054.m	TRUE	reported	QTDAnh	2	TRUE	0.002883	21.25656
835	rs954820	A	G	A	G	0.0083	0.02271	0.5619	0.552677	FALSE	FALSE	FALSE	ieu-b-508	10	1.34E+08	0.033668	486484	0.499976	Sepsis (28	Sepsis (28	Sepsis (28	TRUE	igd	0.0018	2.57E-06	7355	M01358.m	TRUE	reported	kSPQgd	2	TRUE	0.002883	21.25656
1135	rs1026672	T	C	T	C	-0.0083	-0.09344	0.4261	0.406072	FALSE	FALSE	FALSE	ieu-b-508	7	3146081	0.033953	486484	0.005924	Sepsis (28	Sepsis (28	Sepsis (28	TRUE	igd	0.0018	4.00E-06	7355	M01644.m	TRUE	reported	IB0DTL	2	TRUE	0.002883	21.25656
1144	rs1728793	T	C	T	C	0.0083	0.027429	0.4687	0.464841	FALSE	FALSE	FALSE	ieu-b-508	13	22423925	0.033361	486484	0.410968	Sepsis (28	Sepsis (28	Sepsis (28	TRUE	igd	0.0018	5.24E-06	7355	M01644.m	TRUE	reported	IB0DTL	2	TRUE	0.002883	21.25656
1148	rs1866411	A	C	A	C	-0.0083	0.101765	0.3473	0.34778	FALSE	FALSE	FALSE	ieu-b-508	2	1.48E+08	0.035038	486484	0.003679	Sepsis (28	Sepsis (28	Sepsis (28	TRUE	igd	0.0018	5.23E-06	7355	M01644.m	TRUE	reported	IB0DTL	2	TRUE	0.002883	21.25656
1153	rs2291199	A	G	A	G	0.0083	0.069364	0.5373	0.539105	FALSE	FALSE	FALSE	ieu-b-508	4	1244267	0.03341	486484	0.03788	Sepsis (28	Sepsis (28	Sepsis (28	TRUE	igd	0.0018	5.18E-06	7355	M01644.m	TRUE	reported	IB0DTL	2	TRUE	0.002883	21.25656
3489	rs1110290	A	G	A	G	-0.0083	-0.01148	0.4032	0.399855	FALSE	FALSE	FALSE	ieu-b-508	1	1.16E+08	0.034132	486484	0.736607	Sepsis (28	Sepsis (28	Sepsis (28	TRUE	igd	0.0018	2.68E-06	7355	M32492.m	TRUE	reported	rZZFcE	2	TRUE	0.002883	21.25656
3516	rs9384386	T	C	T	C	0.0083	0.001076	0.3966	0.397958	FALSE	FALSE	FALSE	ieu-b-508	6	1.56E+08	0.034251	486484	0.97494	Sepsis (28	Sepsis (28	Sepsis (28	TRUE	igd	0.0018	3.26E-06	7355	M32492.m	TRUE	reported	rZZFcE	2	TRUE	0.002883	21.25656
563	rs9601054	T	C	T	C	0.0083	-0.04763	0.162	0.164292	FALSE	FALSE	FALSE	ieu-b-508	13	79082602	0.045086	486484	0.29078	Sepsis (28	Sepsis (28	Sepsis (28	TRUE	igd	0.0018	4.24E-06	7354	M00606.m	TRUE	reported	r5smf7	2	TRUE	0.002883	21.25656
2240	rs1219123	C	G	C	G	0.0083	-0.02122	0.6904	0.673831	FALSE	TRUE	FALSE	ieu-b-508	6	1.57E+08	0.035598	486484	0.551075	Sepsis (28	Sepsis (28	Sepsis (28	TRUE	igd	0.0018	2.27E-06	7354	M19934.m	TRUE	reported	HtcWcQ	2	TRUE	0.002883	21.25656
2241	rs1258922	T	C	T	C	0.0083	-0.02823	0.3252	0.330859	FALSE	FALSE	FALSE	ieu-b-508	14	91820675	0.03556	486484	0.427325	Sepsis (28	Sepsis (28	Sepsis (28	TRUE	igd	0.0018	2.41E-06	7354	M19934.m	TRUE	reported	HtcWcQ	2	TRUE	0.002883	21.25656
2244	rs1699639	A	T	A	T	-0.0083	-0.00296	0.6946	0.699096	FALSE	TRUE	FALSE	ieu-b-508	20	9753835	0.036436	486484	0.935276	Sepsis (28	Sepsis (28	Sepsis (28	TRUE	igd	0.0018	3.18E-06	7354	M19934.m	TRUE	reported	HtcWcQ	2	TRUE	0.002883	21.25656
6302	rs1005989	T	C	T	C	-0.0083	0.01865	0.786	0.789666	FALSE	FALSE	FALSE	ieu-b-508	5	23241847	0.041752	486484	0.655105	Sepsis (28	Sepsis (28	Sepsis (28	TRUE	igd	0.0018	5.63E-06	7354	M33955.m	TRUE	reported	mhHFnT	2	TRUE	0.002883	21.25656
6324	rs476092	A	C	A	C	0.0083	-0.01623	0.2852	0.285782	FALSE	FALSE	FALSE	ieu-b-508	11	1.28E+08	0.037529	486484	0.665468	Sepsis (28	Sepsis (28	Sepsis (28	TRUE	igd	0.0018	3.25E-06	7354	M33955.m	TRUE	reported	mhHFnT	2	TRUE	0.002883	21.25656
647	rs2470439	A	G	A	G	0.0083	-0.04126	0.11	0.112618	FALSE	FALSE	FALSE	ieu-b-508	12	2015160	0.052762	486484	0.434171	Sepsis (28	Sepsis (28	Sepsis (28	TRUE	igd	0.0018	5.69E-06	7352	M01125.m	TRUE	reported	yw0clB	2	TRUE	0.002884	21.25656
649	rs4086666	T	G	T	G	0.0083	-0.01102	0.8804	0.873957	FALSE	FALSE	FALSE	ieu-b-508	11	13875937	0.05038	486484	0.826786	Sepsis (28	Sepsis (28	Sepsis (28	TRUE	igd	0.0018	2.42E-06	7352	M01125.m	TRUE	reported	yw0clB	2	TRUE	0.002884	21.25656
1610	rs2076799	C	G	C	G	0.0166	0.020327	0.0853	0.069815	FALSE	TRUE	FALSE	ieu-b-508	13	36721182	0.065716	486484	0.757084	Sepsis (28	Sepsis (28	Sepsis (28	TRUE	igd	0.0036	3.89E-06	7352	M15122.m	TRUE	reported	VlnI8T	2	TRUE	0.002884	21.25656
3558	rs1161461	A	C	A	C	-0.0083	0.017792	0.7392	0.713895	FALSE	FALSE	FALSE	ieu-b-508	12	12997687	0.037077	486484	0.631324	Sepsis (28	Sepsis (28	Sepsis (28	TRUE	igd	0.0018	5.84E-06	7350	M32518.m	TRUE	reported	V0ZCiU	2	TRUE	0.002884	21.25656
3584	rs4936882	T	G	T	G	0.0083	-0.03152	0.2446	0.234689	FALSE	FALSE	FALSE	ieu-b-508	11	1.24E+08	0.039284	486484	0.422412	Sepsis (28	Sepsis (28	Sepsis (28	TRUE	igd	0.0018	5.44E-06	7350	M32518.m	TRUE	reported	V0ZCiU	2	TRUE	0.002884	21.25656
3119	rs1081205	A	G	A	G	-0.0083	0.023122	0.5108	0.505998	FALSE	FALSE	FALSE	ieu-b-508	9	24495241	0.033364	486484	0.488289	Sepsis (28	Sepsis (28	Sepsis (28	TRUE	igd	0.0018	2.56E-06	7349	M32315.m	TRUE	reported	4igzkL	2	TRUE	0.002885	21.25656
3128	rs1874795	A	G	A	G	-0.0083	0.025253	0.4303	0.441108	FALSE	FALSE	FALSE	ieu-b-508	11	80729659	0.033669	486484	0.453233	Sepsis (28	Sepsis (28	Sepsis (28	TRUE	igd	0.0018	2.26E-06	7349	M32315.m	TRUE	reported	4igzkL	2	TRUE	0.002885	21.25656
7945	rs742914	A	C	A	C	-0.0083	0.001653	0.7235	0.711074	FALSE	FALSE	FALSE	ieu-b-508	20	39432249	0.036944	486484	0.964315	Sepsis (28	Sepsis (28	Sepsis (28	TRUE	igd	0.0018	7.46E-06	7348	M35675.m	TRUE	reported	D7fdyZ	2	TRUE	0.002885	21.25656
739	rs2780242	T	C	T	C	-0.0083	0.013045	0.1138	0.129229	FALSE	FALSE	FALSE	ieu-b-508	9	1.21E+08	0.050347	486484	0.795558	Sepsis (28	Sepsis (28	Sepsis (28	TRUE	igd	0.0018	3.85E-06	7347	M01302.m	TRUE	reported	sNp2IC	2	TRUE	0.002886	21.25656
1204	rs9815886	A	C	A	C	-0.0083	0.002884	0.4756	0.453201	FALSE	FALSE	FALSE	ieu-b-508	3	72396241	0.033679	486484	0.931757	Sepsis (28	Sepsis (28	Sepsis (28	TRUE	igd	0.0018	2.55E-06	7346	M01645.m	TRUE	reported	d5nAn3	2	TRUE	0.002886	21.25656
2706	rs575528	A	G	A	G	0.0083	0.03514	0.5409	0.544812	FALSE	FALSE	FALSE	ieu-b-508	1	81563249	0.03346	486484	0.293627	Sepsis (28	Sepsis (28	Sepsis (28	TRUE	igd	0.0018	4.60E-06	7340	M25459.m	TRUE	reported	ACJY35	2	TRUE	0.002888	21.25655
2707	rs5758064	T	C	T	C	-0.0083	-0.02273	0.5194	0.523401	FALSE	FALSE	FALSE	ieu-b-508	22	41153879	0.033557	486484	0.49817	Sepsis (28	Sepsis (28	Sepsis (28	TRUE	igd	0.0018	3.91E-06	7340	M25459.m	TRUE	reported	ACJY35	2	TRUE	0.002888	21.25655
2936	rs1051218	T	C	T	C	0.0166	-0.03519	0.6502	0.643846	FALSE	FALSE	FALSE	ieu-b-508	9	90051391	0.035054	486484	0.315445	Sepsis (28	Sepsis (28	Sepsis (28	TRUE	igd	0.0036	3.32E-06	7336	M27738.m	TRUE	reported	UtPBd9	2	TRUE	0.00289	21.25655
5630	rs9540840	T	C	T	C	0.0083	-0.10625	0.8471	0.848718	FALSE	FALSE	FALSE	ieu-b-508	13	67184560	0.047344	486484	0.024818	Sepsis (28	Sepsis (28	Sepsis (28	TRUE	igd	0.0018	3.82E-06	7336	M33442.m	TRUE	reported	B1Peb3	2	TRUE	0.00289	21.25655
4396	rs804854	A	T	A	T	0.0083	-0.089	0.1417	0.140765	FALSE	TRUE	FALSE	ieu-b-508	6	37578883	0.048225	486484	0.064977	Sepsis (28	Sepsis (28	Sepsis (28	TRUE	igd	0.0018	2.68E-06	7329	M32867.m	TRUE	reported	Jcsyzm	2	TRUE	0.002893	21.25654
4087	rs9397197	A	G	A	G	0.0083	-0.00338	0.2948	0.290721	FALSE	FALSE	FALSE	ieu-b-508	6	1.55E+08	0.036776	486484	0.926738	Sepsis (28	Sepsis (28	Sepsis (28	TRUE	igd	0.0018	2.21E-06	7318	M32740.m	TRUE	reported	YLshfP	2	TRUE	0.002897	21.25653
5420	rs707	T	C	T	C	-0.0083	-0.03934	0.4558	0.413091	FALSE	FALSE	FALSE	ieu-b-508	7	1.43E+08	0.034149	486484	0.249275	Sepsis (28	Sepsis (28	Sepsis (28	TRUE	igd	0.0018	5.95E-06	7315	M33389.m	TRUE	reported	Hf6XVM	2	TRUE	0.002898	21.25653
5425	rs7661478	T	C	T	C	0.0083	0.032532	0.6299	0.637706	FALSE	FALSE	FALSE	ieu-b-508	4	21603519	0.0347	486484	0.348486	Sepsis (28	Sepsis (28	Sepsis (28	TRUE	igd	0.0018	6.17E-06	7315	M33389.m	TRUE	reported	Hf6XVM	2	TRUE	0.002898	21.25653
5687	rs4687367	T	C	T	C	0.0083	-0.01252	0.4659	0.470629	FALSE	FALSE	FALSE	ieu-b-508	3	1.93E+08	0.033591	486484	0.709346	Sepsis (28	Sepsis (28	Sepsis (28	TRUE	igd	0.0018	4.21E-06	7307	M33477.m	TRUE	reported	Aw8PBK	2	TRUE	0.002901	21.25653
5701	rs8182062	A	G	A	G	-0.0083	0.011997	0.7075	0.713017	FALSE	FALSE	FALSE	ieu-b-508	15	51254502	0.036852	486484	0.744775	Sepsis (28	Sepsis (28	Sepsis (28	TRUE	igd	0.0018	4.75E-06	7307	M33477.m	TRUE	reported	Aw8PBK	2	TRUE	0.002901	21.25653
5702	rs951062	T	C	T	C	0.0083	0.000375	0.703	0.697015	FALSE	FALSE	FALSE	ieu-b-508	12	19988377	0.036426	486484	0.991791	Sepsis (28	Sepsis (28	Sepsis (28	TRUE	igd	0.0018	5.68E-06	7307	M33477.m	TRUE	reported	Aw8PBK	2	TRUE	0.002901	21.25653
5508	rs1050074	T	C	T	C	0.0083	0.002493	0.7541	0.748203	FALSE	FALSE	FALSE	ieu-b-508	11	11771889	0.038672	486484	0.9486	Sepsis (28	Sepsis (28	Sepsis (28	TRUE	igd	0.0018	4.57E-06	7306	M33422.m	TRUE	reported	PLgjrT	2	TRUE	0.002902	21.25653
5516	rs1503575	A	G	A	G	0.0083	0.050896	0.1815	0.181318	FALSE	FALSE	FALSE	ieu-b-508	12	61043705	0.04339	486484	0.240802	Sepsis (28	Sepsis (28	Sepsis (28	TRUE	igd	0.0018	6.18E-06	7306	M33422.m	TRUE	reported	PLgjrT	2	TRUE	0.002902	21.25653
5525	rs2731506	A	G	A	G	-0.0083	0.028509	0.2438	0.244749	FALSE	FALSE	FALSE	ieu-b-508	3	1888860	0.038772	486484	0.462156	Sepsis (28	Sepsis (28	Sepsis (28	TRUE	igd	0.0018	3.78E-06	7306	M33422.m	TRUE	reported	PLgjrT	2	TRUE	0.002902	21.25653
2760	rs4899091	A	G	A	G	-0.0083	-0.00171	0.7678	0.759115	FALSE	FALSE	FALSE	ieu-b-508	14	63141952	0.039017	486484	0.965048	Sepsis (28	Sepsis (28	Sepsis (28	TRUE	igd	0.0018	2.47E-06	7303	M27256.m	TRUE	reported	mJwzsi	2	TRUE	0.002903	21.25652
1066	rs2596194	A	T	A	T	-0.0083	0.03238	0.1255	0.136855	FALSE	TRUE	FALSE	ieu-b-508	15	33686777	0.049208	486484	0.510518	Sepsis (28	Sepsis (28	Sepsis (28	TRUE	igd	0.0018	2.59E-06	7271	M01585.m	TRUE	reported	M7QiSM	2	TRUE	0.002916	21.2565
3820	rs4711082	T	C	T	C	-0.0083	-0.0238	0.6337	0.636961	FALSE	FALSE	FALSE	ieu-b-508	6	25379926	0.034657	486484	0.492231	Sepsis (28	Sepsis (28	Sepsis (28	TRUE	igd	0.0018	5.92E-06	7229	M32644.m	TRUE	reported	JAa2WD	2	TRUE	0.002933	21.25646
7556	rs1229658	A	G	A	G	-0.0083	0.057686	0.2306	0.23981	FALSE	FALSE	FALSE	ieu-b-508	7	26435397	0.039119	486484	0.140311	Sepsis (28	Sepsis (28	Sepsis (28	TRUE	igd	0.0018	6.13E-06	7217	M35270.m	TRUE	reported	Ct8V1n	2	TRUE	0.002937	21.25645
7566	rs2078875	T	C	T	C	-0.0083	-0.02164	0.2436	0.233451	FALSE	FALSE	FALSE	ieu-b-508	15	91791639	0.039461	486484	0.583346	Sepsis (28	Sepsis (28	Sepsis (28	TRUE	igd	0.0018	4.46E-06	7217	M35270.m	TRUE	reported	Ct8V1n	2	TRUE	0.002937	21.25645
6267	rs1689205	C	G	C	G	0.0775	0.000623	0.0265	0.010409	FALSE	TRUE	FALSE	ieu-b-508	4	15496020	0.165553	486484	0.996997	Sepsis (28	Sepsis (28	Sepsis (28	TRUE	igd	0.0168	3.81E-06	1759	M33937.m	TRUE	reported	QanGCI	2	TRUE	0.011954	21.25645

2191	rs5746800	A	G	A	G	0.0083	-0.07595	0.5003	0.516737	FALSE	FALSE	FALSE	ieu-b-508	22	19594436	0.033719	486484	0.024296	Sepsis (28	Sepsis (28	Sepsis (28	TRUE	igd	0.0018	2.44E-06	7182	M19362.m	TRUE	reported	SkNEKw	2	TRUE	0.002952	21.25642
5347	rs1874617	A	G	A	G	-0.0083	0.055178	0.7831	0.772772	FALSE	FALSE	FALSE	ieu-b-508	4	1.4E+08	0.039854	486484	0.166204	Sepsis (28	Sepsis (28	Sepsis (28	TRUE	igd	0.0018	4.23E-06	7118	M33380.m	TRUE	reported	OHRfl5	2	TRUE	0.002978	21.25637
5359	rs688608	A	G	A	G	-0.0083	0.019865	0.778	0.767221	FALSE	FALSE	FALSE	ieu-b-508	10	38331624	0.039456	486484	0.614637	Sepsis (28	Sepsis (28	Sepsis (28	TRUE	igd	0.0018	2.61E-06	7118	M33380.m	TRUE	reported	OHRfl5	2	TRUE	0.002978	21.25637
5806	rs1180647	T	C	T	C	0.0083	0.013109	0.3749	0.372522	FALSE	FALSE	FALSE	ieu-b-508	1	1.51E+08	0.034571	486484	0.70455	Sepsis (28	Sepsis (28	Sepsis (28	TRUE	igd	0.0018	6.10E-06	7066	M33515.m	TRUE	reported	X9mul1	2	TRUE	0.003	21.25633
5812	rs2067434	T	C	T	C	-0.0083	-0.05519	0.4351	0.427055	FALSE	FALSE	FALSE	ieu-b-508	2	1.91E+08	0.033633	486484	0.100788	Sepsis (28	Sepsis (28	Sepsis (28	TRUE	igd	0.0018	5.24E-06	7066	M33515.m	TRUE	reported	X9mul1	2	TRUE	0.003	21.25633
5816	rs2494461	A	C	A	C	0.0083	-0.02378	0.6292	0.623608	FALSE	FALSE	FALSE	ieu-b-508	1	1.82E+08	0.034481	486484	0.490429	Sepsis (28	Sepsis (28	Sepsis (28	TRUE	igd	0.0018	6.27E-06	7066	M33515.m	TRUE	reported	X9mul1	2	TRUE	0.003	21.25633
5837	rs792369	A	C	A	C	-0.0083	0.002272	0.659	0.663447	FALSE	FALSE	FALSE	ieu-b-508	17	55446071	0.035405	486484	0.94883	Sepsis (28	Sepsis (28	Sepsis (28	TRUE	igd	0.0018	6.65E-06	7066	M33515.m	TRUE	reported	X9mul1	2	TRUE	0.003	21.25633
1397	rs4465961	T	C	T	C	0.0083	-0.02962	0.3479	0.353439	FALSE	FALSE	FALSE	ieu-b-508	3	21063058	0.035074	486484	0.398342	Sepsis (28	Sepsis (28	Sepsis (28	TRUE	igd	0.0018	3.12E-06	7037	M02734.m	TRUE	reported	0y0dDD	2	TRUE	0.003012	21.2563
8411	rs1309903	A	C	A	C	0.0083	-0.00755	0.181	0.187838	FALSE	FALSE	FALSE	ieu-b-508	3	21771225	0.043222	486484	0.861353	Sepsis (28	Sepsis (28	Sepsis (28	TRUE	igd	0.0018	4.09E-06	6940	M36808.m	TRUE	reported	u7lqaj	2	TRUE	0.003054	21.25622
8416	rs27852	A	C	A	C	-0.0083	-0.00147	0.2959	0.296255	FALSE	FALSE	FALSE	ieu-b-508	5	96046039	0.036552	486484	0.967926	Sepsis (28	Sepsis (28	Sepsis (28	TRUE	igd	0.0018	2.18E-06	6940	M36808.m	TRUE	reported	u7lqaj	2	TRUE	0.003054	21.25622
8420	rs4518383	A	G	A	G	-0.0083	-0.0066	0.8068	0.802654	FALSE	FALSE	FALSE	ieu-b-508	5	1.13E+08	0.04191	486484	0.87483	Sepsis (28	Sepsis (28	Sepsis (28	TRUE	igd	0.0018	3.89E-06	6940	M36808.m	TRUE	reported	u7lqaj	2	TRUE	0.003054	21.25622
8431	rs7580094	A	G	A	G	0.0083	0.053037	0.7814	0.765073	FALSE	FALSE	FALSE	ieu-b-508	2	1.72E+08	0.039428	486484	0.178571	Sepsis (28	Sepsis (28	Sepsis (28	TRUE	igd	0.0018	3.46E-06	6940	M36808.m	TRUE	reported	u7lqaj	2	TRUE	0.003054	21.25622
6795	rs1088075	A	G	A	G	-0.0083	0.055418	0.488	0.487885	FALSE	FALSE	FALSE	ieu-b-508	12	38627485	0.033457	486484	0.097645	Sepsis (28	Sepsis (28	Sepsis (28	TRUE	igd	0.0018	2.51E-06	6755	M34359.m	TRUE	reported	0UotaJ	2	TRUE	0.003138	21.25605
6802	rs1321291	A	G	A	G	-0.0083	-0.00215	0.6812	0.682645	FALSE	FALSE	FALSE	ieu-b-508	6	22450262	0.03605	486484	0.952514	Sepsis (28	Sepsis (28	Sepsis (28	TRUE	igd	0.0018	2.92E-06	6755	M34359.m	TRUE	reported	0UotaJ	2	TRUE	0.003138	21.25605
6816	rs2712549	A	G	A	G	0.0083	0.026735	0.472	0.478293	FALSE	FALSE	FALSE	ieu-b-508	12	1.28E+08	0.033371	486484	0.42305	Sepsis (28	Sepsis (28	Sepsis (28	TRUE	igd	0.0018	3.22E-06	6755	M34359.m	TRUE	reported	0UotaJ	2	TRUE	0.003138	21.25605
6821	rs310325	T	C	T	C	-0.0083	0.058219	0.4417	0.447502	FALSE	FALSE	FALSE	ieu-b-508	8	23685567	0.033609	486484	0.083232	Sepsis (28	Sepsis (28	Sepsis (28	TRUE	igd	0.0018	2.68E-06	6755	M34359.m	TRUE	reported	0UotaJ	2	TRUE	0.003138	21.25605
6827	rs501438	C	G	C	G	0.0083	0.00625	0.3909	0.394851	FALSE	TRUE	FALSE	ieu-b-508	2	1.74E+08	0.034116	486484	0.854652	Sepsis (28	Sepsis (28	Sepsis (28	TRUE	igd	0.0018	2.35E-06	6755	M34359.m	TRUE	reported	0UotaJ	2	TRUE	0.003138	21.25605
976	rs6500864	A	G	A	G	-0.0083	-0.02615	0.2762	0.279675	FALSE	FALSE	FALSE	ieu-b-508	16	6938132	0.037345	486484	0.483714	Sepsis (28	Sepsis (28	Sepsis (28	TRUE	igd	0.0018	4.34E-06	6523	M01558.m	TRUE	reported	AZK0p4	2	TRUE	0.003249	21.25583
5213	rs1741879	T	C	T	C	-0.0535	0.021956	0.9675	0.922376	FALSE	FALSE	FALSE	ieu-b-508	6	15834753	0.062258	486484	0.724339	Sepsis (28	Sepsis (28	Sepsis (28	TRUE	igd	0.0116	4.24E-06	2683	M33204.m	TRUE	reported	ROvPWK	2	TRUE	0.007866	21.25532
7668	rs952108	A	G	A	G	0.0083	-0.04886	0.4705	0.449626	FALSE	FALSE	FALSE	ieu-b-508	19	51511930	0.033839	486484	0.148751	Sepsis (28	Sepsis (28	Sepsis (28	TRUE	igd	0.0018	2.14E-06	5768	M35326.m	TRUE	reported	jRytyJ	2	TRUE	0.003673	21.25497
7164	rs1290262	C	G	C	G	0.0332	-0.09094	0.8789	0.87867	FALSE	TRUE	FALSE	ieu-b-508	15	82533236	0.051101	486484	0.075136	Sepsis (28	Sepsis (28	Sepsis (28	TRUE	igd	0.0072	4.27E-06	4925	M34534.m	TRUE	reported	iA78RM	2	TRUE	0.004299	21.25371
5185	rs2163086	A	G	A	G	0.0498	0.011345	0.2974	0.303398	FALSE	FALSE	FALSE	ieu-b-508	7	90936457	0.036405	486484	0.755308	Sepsis (28	Sepsis (28	Sepsis (28	TRUE	igd	0.0108	3.93E-06	2860	M33197.m	TRUE	reported	D6xaBg	2	TRUE	0.00738	21.24748
6853	rs1200575	A	G	A	G	0.0909	0.006957	0.4099	0.406428	FALSE	FALSE	FALSE	ieu-b-508	9	33522045	0.034036	486484	0.838049	Sepsis (28	Sepsis (28	Sepsis (28	TRUE	igd	0.0197	4.02E-06	954	M34365.m	TRUE	reported	8eexD0	2	TRUE	0.02183	21.24633
4858	rs1747065	A	T	A	T	0.3263	0.020894	0.944	0.969079	FALSE	TRUE	FALSE	ieu-b-508	12	18267613	0.099768	486484	0.83412	Sepsis (28	Sepsis (28	Sepsis (28	TRUE	igd	0.0679	1.54E-06	25	M33178.m	TRUE	reported	g22WMS	2	TRUE	0.480182	21.24626
7203	rs1742313	A	G	A	G	0.0498	0.007999	0.0656	0.072099	FALSE	FALSE	FALSE	ieu-b-508	3	1.61E+08	0.065009	486484	0.902068	Sepsis (28	Sepsis (28	Sepsis (28	TRUE	igd	0.0108	3.75E-06	2545	M34674.m	TRUE	reported	plxOKz	2	TRUE	0.008285	21.24564
7135	rs4953640	T	C	T	C	0.0189	0.012855	0.1512	0.168642	FALSE	FALSE	FALSE	ieu-b-508	2	49126802	0.044699	486484	0.773665	Sepsis (28	Sepsis (28	Sepsis (28	TRUE	igd	0.0041	4.16E-06	7322	M34527.m	TRUE	reported	EWFGP8	2	TRUE	0.002894	21.24405
2336	rs4388837	T	G	T	G	0.1102	0.05195	0.9828	0.98128	FALSE	FALSE	FALSE	ieu-b-508	11	26722592	0.123633	486484	0.674343	Sepsis (28	Sepsis (28	Sepsis (28	TRUE	igd	0.0239	4.15E-06	1765	M21044.m	TRUE	reported	Ym5rae	2	TRUE	0.011902	21.23611
8317	rs1013196	A	G	A	G	-0.0295	0.03639	0.6038	0.591269	FALSE	FALSE	FALSE	ieu-b-508	14	89328429	0.033957	486484	0.283878	Sepsis (28	Sepsis (28	Sepsis (28	TRUE	igd	0.0064	4.05E-06	3977	M36589.m	TRUE	reported	27GADo	2	TRUE	0.005314	21.23565
900	rs1001210	T	C	T	C	-0.0106	0.012754	0.8353	0.84115	FALSE	FALSE	FALSE	ieu-b-508	6	16728780	0.045694	486484	0.78015	Sepsis (28	Sepsis (28	Sepsis (28	TRUE	igd	0.0023	3.80E-06	7354	M01494.m	TRUE	reported	7JWNKI	2	TRUE	0.00288	21.2343
1710	rs1417497	C	G	C	G	-0.0106	0.05775	0.072	0.066991	FALSE	TRUE	FALSE	ieu-b-508	13	88128461	0.06655	486484	0.385525	Sepsis (28	Sepsis (28	Sepsis (28	TRUE	igd	0.0023	5.35E-06	7310	M15506.m	TRUE	reported	mVRmZO	2	TRUE	0.002897	21.23426
1101	rs1153903	A	G	A	G	-0.0106	-0.03564	0.3856	0.401436	FALSE	FALSE	FALSE	ieu-b-508	7	54741212	0.03401	486484	0.294606	Sepsis (28	Sepsis (28	Sepsis (28	TRUE	igd	0.0023	4.19E-06	7104	M01638.m	TRUE	reported	8dp0Ac	2	TRUE	0.002981	21.2341
3658	rs2722639	T	C	T	C	-0.0318	0.023862	0.5475	0.556687	FALSE	FALSE	FALSE	ieu-b-508	3	1.64E+08	0.033573	486484	0.477236	Sepsis (28	Sepsis (28	Sepsis (28	TRUE	igd	0.0069	4.43E-06	6970	M32564.m	TRUE	reported	73MgOk	2	TRUE	0.003038	21.23398
2453	rs897656	A	T	A	T	-0.0521	0.021875	0.0443	0.042174	FALSE	TRUE	FALSE	ieu-b-508	12	1.32E+08	0.083307	486484	0.792874	Sepsis (28	Sepsis (28	Sepsis (28	TRUE	igd	0.0113	4.06E-06	1755	M21630.m	TRUE	reported	7eaO4H	2	TRUE	0.011968	21.23359
8306	rs1091613	T	C	T	C	-0.0401	0.024683	0.8277	0.824023	FALSE	FALSE	FALSE	ieu-b-508	1	2.28E+08	0.044042	486484	0.57518	Sepsis (28	Sepsis (28	Sepsis (28	TRUE	igd	0.0087	3.86E-06	3346	M36585.m	TRUE	reported	pXZg13	2	TRUE	0.006309	21.23198
7078	rs1641387	T	C	T	C	0.0355	0.020471	0.7786	0.637549	FALSE	FALSE	FALSE	ieu-b-508	2	1.51E+08	0.03471	486484	0.555343	Sepsis (28	Sepsis (28	Sepsis (28	TRUE	igd	0.0077	4.23E-06	1651	M34481.m	TRUE	reported	mXdL0W	2	TRUE	0.012711	21.22994
4617	rs1279200	A	G	A	G	-0.2024	0.138986	0.0653	0.041162	FALSE	FALSE	FALSE	ieu-b-508	11	1630177	0.084615	486484	0.100473	Sepsis (28	Sepsis (28	Sepsis (28	TRUE	igd	0.0439	4.01E-06	1574	M33173.m	TRUE	reported	04fkUQ	2	TRUE	0.013325	21.2295
5943	rs2776982	A	C	A	C	0.0447	0.024606	0.053	0.056683	FALSE	FALSE	FALSE	ieu-b-508	13	30081384	0.072479	486484	0.734238	Sepsis (28	Sepsis (28	Sepsis (28	TRUE	igd	0.0097	4.52E-06	6109	M33638.m	TRUE	reported	hVuSGd	2	TRUE	0.003464	21.22899
6597	rs1192598	T	C	T	C	-0.0341	-0.02384	0.0325	0.053438	FALSE	FALSE	FALSE	ieu-b-508	3	99162278	0.07492	486484	0.7503	Sepsis (28	Sepsis (28	Sepsis (28	TRUE	igd	0.0074	4.08E-06	6994	M34306.m	TRUE	reported	WPXPUN	2	TRUE	0.003027	21.22859
6244	rs506506	A	G	A	G	0.0341	-0.01457	0.5029	0.498032	FALSE	FALSE	FALSE	ieu-b-508	11	90365458	0.033472	486484	0.663263	Sepsis (28	Sepsis (28	Sepsis (28	TRUE	igd	0.0074	4.16E-06	6756	M33935.m	TRUE	reported	ELBTDc	2	TRUE	0.003133	21.22837
1974	rs480891	A	G	A	G	-0.0235	0.022253	0.6432	0.6297	FALSE	FALSE	FALSE	ieu-b-508	18	7468961	0.034772	486484	0.522189	Sepsis (28	Sepsis (28	Sepsis (28	TRUE	igd	0.0051	4.15E-06	7226	M17807.m	TRUE	reported	zd6sZI	2	TRUE	0.00293	21.22634
7444	rs1947110	A	G	A	G	0.0235	-0.03085	0.284	0.281593	FALSE	FALSE	FALSE	ieu-b-508	2	47066282	0.037134	486484	0.406177	Sepsis (28	Sepsis (28	Sepsis (28	TRUE	igd	0.0051	3.34E-06	6234	M35189.m	TRUE	reported	54XZFj	2	TRUE	0.003394	21.22541
8345	rs659974	A	G	A	G	0.0235	0.038961	0.1642	0.185505	FALSE	FALSE	FALSE	ieu-b-508	16	89663072	0.043	486484	0.364903	Sepsis (28	Sepsis (28	Sepsis (28	TRUE	igd	0.0051	4.61E-06	5292	M36673.m	TRUE	reported	CD5lvR	2	TRUE	0.003996	21.22419
8121	rs2581383	C	G	C	G	0.0364	-0.02358	0.6655	0.653899	FALSE	TRUE	FALSE	ieu-b-508	15	1E+08	0.035404	486484	0.505393	Sepsis (28	Sepsis (28	Sepsis (28	TRUE	igd	0.0079	3.72E-06	7054	M36098.m	TRUE	reported	hs9SZI	2	TRUE	0.003001	21.22391
6586	rs1684155	A	C	A	C	-0.0212	-0.01361	0.6765	0.670678	FALSE	FALSE	FALSE	ieu-b-508	1	2.31E+08	0.035758	486484	0.703442	Sepsis (28	Sepsis (28	Sepsis (28	TRUE	igd	0.0046	4.48E-06	2278	M34289.m	TRUE	reported	Knp8ry	2	TRUE	0.009238	21.22143
2790	rs1233749	T	C	T	C	-0.0129	-0.03224	0.2157	0.232594	FALSE	FALSE	FALSE	ieu-b-508	9	1.16E+08	0.039486	486484	0.41417	Sepsis (28	Sepsis (28	Sepsis (28	TRUE	igd	0.0028	3.81E-06	7347	M27278.m	TRUE	reported	kPgqm3	2	TRUE	0.002881	21.21999
1361	rs4587207	A	G	A	G	-0.0129	-0.01428	0.8566	0.855717	FALSE	FALSE	FALSE	ieu-b-508	6	30766945	0.047668	486484	0.764568	Sepsis (28	Sepsis (28	Sepsis (28	TRUE	igd	0.0028	3.19E-06	7226	M02730.m	TRUE	reported	XYa035	2	TRUE	0.002929	21.21989
755	rs1099030	T	C	T	C	-0.0129	-0.02331	0.2309	0.229237	FALSE	FALSE	FALSE	ieu-b-508	9	98410405	0.040558	486484	0.565474	Sepsis (28	Sepsis (28	Sepsis (28	TRUE	igd	0.0028	3.94E-06	6946	M01303.m	TRUE	reported	3b8x0F	2	TRUE	0.003047	21.21965
2040	rs7503709	T	C	T	C	-0.0129	0.098912	0.4574	0.457622	FALSE	FALSE	FALSE	ieu-b-508	17	21428752	0.035529	486484	0.00537	Sepsis (28	Sepsis (28	Sepsis (28	TRUE	igd	0.0028	3.30E-06	6939	M18349.m	TRUE	reported	M5qPs8	2	TRUE	0.00305	21.21965
3981	rs1274280	T	C	T	C	0.0129	0.020782	0.1181	0.117132	FALSE	FALSE	FALSE	ieu-b-508	18	369148	0.05423	486484	0.701559	Sepsis (28	Sepsis (28	Sepsis (28	TRUE	igd	0.0028	4.95E-06	6497	M32729.m	TRUE	reported	XGRSN1	2	TRUE	0.003256	21.21923
7281	rs9459207	A	G	A	G	-0.0258	0.105902	0.0331	0.034509	FALSE	FALSE	FALSE	ieu-b-508	6	1.65E+08	0.091805	486484	0.248682	Sepsis (28	Sepsis (28	Sepsis (28	TRUE	igd	0.0056	3.36E-06	5885	M34912.m	TRUE	reported	W8lcT6	2	TRUE	0.003594	21.21855
2922	rs498494	C	G	C	G	0.0258	-0.09313	0.9184	0.901991	FALSE	TRUE	FALSE	ieu-b-508	3	1.9E+08	0.055927	486484	0.095856	Sepsis (28	Sepsis (28	Sepsis (28	TRUE	igd	0.0056	4.27E-06	5562	M27728.m	TRUE	reported	WOp1XE	2	TRUE	0.003802	21.21813
2923	rs552279	A	G	A	G	-0.0129	0.011015	0.6732	0.676227	FALSE	FALSE	FALSE	ieu-b-508	1	1.16E+08	0.036035	486484	0.759857	Sepsis (28	Sepsis (28	Sepsis (28	TRUE	igd	0.0028	4.90E-06	5562	M27728.m	TRUE	reported	WOp1XE	2	TRUE	0.003802	21.21813
1972	rs4393440	T	C	T	C	-0.129	0.033132	0.987	0.951565	FALSE	FALSE	FALSE	ieu-b-508	13	64343702	0.077345	486484	0.668381	Sepsis (28	Sepsis (28	Sepsis (28	TRUE	igd	0.028	4.08E-06	5482	M17807.m	TRUE	reported	zd6sZI	2	TRUE	0.003857	21.21802
2400	rs188595	A	C	A	C	0.0258	-0.02183	0.653	0.666201	FALSE	FALSE	FALSE	ieu-b-508	3	30757592	0.035711	486484	0.541068	Sepsis (28	Sepsis (28	Sepsis (28	TRUE	igd	0.0056	3.49E-06	5436	M21184.m	TRUE	reported	63ppuJ	2	TRUE	0.003889	21.21796
3837	rs1095733	A	G	A	G	0.0129	0.007961	0.2929	0.295035	FALSE	FALSE	FALSE	ieu-b-508	8	65783695	0.036694	486484	0.828243	Sepsis (28	Sepsis (28	Sepsis (28	TRUE	igd	0.0028	4.29E-06	5211	M32651.m	TRUE	reported	DeuPwv	2	TRUE	0.004057	21.21762
3847	rs3762920	A	G	A	G	0.0129	0.030622	0.6758	0.683243	FALSE	FALSE	FALSE	ieu-b-508	4	1.86E+08	0.035954	486484	0.394382	Sepsis (28	Sepsis (28	Sepsis (28	TRUE	igd	0.0028	3.15E-06	5211	M32651.m	TRUE	reported	DeuPwv	2	TRUE	0.004057	21.21762
6060	rs4504394	T	C	T	C	0.0318	0.0401	0.2788	0.298642	FALSE	FALSE	FALSE	ieu-b-508	5	31189907	0.036571	486484	0.272865	Sepsis (28	Sepsis (28	Sepsis (28	TRUE	igd	0.0069	3.97E-06	1764	M33821.m	TRUE	reported	eVYICL	2	TRUE	0.011898	21.21599
3706	rs2698512	A	G	A	G	-0.0539	-0.04609	0.0391	0.04088	FALSE	FALSE	FALSE	ieu-b-508	7	1.55E+08	0.084637	486484	0.586019	Sepsis (28	Sepsis (28	Sepsis (28	TRUE	igd	0.0117	4.09E-06	5955	M32593.m	TRUE	reported	6zVl1i	2	TRUE	0.003551	21.21582
4135	rs9513800	A	G	A	G	-0.041	-0.03295	0.0449	0.042822	FALSE	FALSE	FALSE	ieu-b-508	13	1.01E+08	0.082351	486484	0.689065	Sepsis (28	Sepsis (28	Sepsis (28	TRUE	igd	0.0089	3.85E-06	6658	M32755.m	TRUE	reported	WDrNdi	2	TRUE	0.003177	21.21569
5212	rs1698499	T	C	T	C	0.0364	0.070684	0.0398	0.046265	FALSE	FALSE	FALSE	ieu-b-508	21	31165317	0.079964	486484	0.376723	Sepsis (28	Sepsis (28	Sepsis (28	TRUE	igd	0.0079	4.43E-06	2683	M33204.m	TRUE	reported	ROvPWK	2	TRUE	0.007851	21.21411
1563	rs1898906	A	G	A	G	0.1378	0.029565	0.0296	0.030023	FALSE	FALSE	FALSE	ieu-b-508	2	1.89E+08	0.097832	486484	0.762495	Sepsis (28	Sepsis (28	Sepsis (28	TRUE	igd	0.0299	4.09E-06	1543	M12261.m	TRUE	reported	taUUcM	2	TRUE	0.013579	21.21254
4897	rs2249531	A	G	A	G	-0.0816	0.002357	0.5482	0.57593	FALSE	FALSE	FALSE	ieu-b-508	13	98406102	0.034185	486484	0.945038	Sepsis (28	Sepsis (28	Sepsis (28	TRUE	igd	0.0177	4.19E-06	1017	M33178.m	TRUE	reported	g22WMS	2	TRUE	0.020471	21.21187
2783	rs6941976	T	G	T	G	0.0447	-0.0762	0.0447	0.035202	FALSE	FALSE	FALSE	ieu-b-508	6	1.57E+08	0.090555	486484	0.400088	Sepsis (28	Sepsis (28	Sepsis (28	TRUE	igd	0.0097	3.90E-06	1751	M27273.m	TRUE	reported	EvU2od	2	TRUE	0.011983	21.21169
8507	rs1081350	T	C	T	C	-0.0281	0.043731	0.3812	0.4168	FALSE	FALSE	FALSE	ieu-b-508	9	31183273	0.034016	486484	0.19858	Sepsis (28	Sepsis (28	Sepsis (28	TRUE	igd	0.0061	4.23E-06	4520	M37104.m	TRUE	reported	bR3g7d	2	TRUE	0.004673	21.21098
8607	rs1753984	A	G	A	G	-0.0152	0.106432	0.0938	0.100621	FALSE	FALSE	FALSE	ieu-b-508	18	51652728	0.055354	486484	0.054512	Sepsis (28	Sepsis (28	Sepsis (28	TRUE	igd	0.0033	4.80E-06	7366	M37506.m	TRUE	reported	m2kaRt	2	TRUE	0.002872	21.21003
3382	rs4755163	A	G	A	G	0.0152	-0.00152	0.5331	0.544987	FALSE	FALSE	FALSE	ieu-b-508	11	42641136	0.033481	486484	0.963767	Sepsis (28	Sepsis (28	Sepsis (28	TRUE	igd	0.0033	3.51E-06	7355	M32418.m	TRUE	reported	y8cN4Y	2	TRUE	0.002876	21.21003
800	rs1751156	T	C	T	C	0.0152	0.006324	0.8423	0.823397	FALSE	FALSE	FALSE	ieu-b-508	6	4461144	0.044311	486484	0.886506	Sepsis (28	Sepsis (28	Sepsis (28	TRUE	igd	0.0033	5.26E-06	7335	M01356.m	TRUE	reported	ah1g5W	2	TRUE	0.002884	21.21001
1045	rs1310284	C	G	C	G	-0.0152	0.038809	0.8686	0.876891	FALSE	TRUE	FALSE	ieu-b-508	4	1.08E+08	0.050792	486484	0.44482	Sepsis (28	Sepsis (28	Sepsis (28	TRUE	igd	0.0033	3.13E-06	7333	M01572.m	TRUE	reported	EOUAWy	2	TRUE	0.002885	21.21001
3316	rs4661470	T	G	T	G	-0.0152	-0.03886	0.1974	0.20563	FALSE	FALSE	FALSE	ieu-b-508	1	14407802	0.041126	486484	0.34466	Sepsis (28	Sepsis (28	Sepsis (28	TRUE	igd	0.0033	5.44E-06	7307	M32393.m	TRUE	reported	Pub0X0	2	TRUE	0.002895	21.20999
3317	rs708891	T	C	T	C	-0.0152	0.039143	0.8739	0.872577	FALSE	FALSE	FALSE	ieu-b-508	12	1.2E+08	0.05008	486484	0.434447	Sepsis (28	Sepsis (28	Sepsis (28	TRUE	igd	0.0033	3.80E-06	7307	M32393.m	TRUE	reported	Pub0X0	2	TRUE	0.002895	21.20999
3602	rs3736083	T	C	T	C	0.0304	-0.06377	0.5544	0.555137	FALSE	FALSE	FALSE	ieu-b-508	4	877709	0.033905	486484	0.059978	Sepsis (28	Sepsis (28	Sepsis (28	TRUE	igd	0.0066	3.98E-06	7256	M32549.m	TRUE	reported	W7Fgu2	2	TRUE	0.002915	21.20995
1423	rs2145985	T	C	T	C	-0.0152	0.035446	0.16	0.168021	FALSE	FALSE	FALSE	ieu-b-508	9	99174531	0.044514	486484	0.425859	Sepsis (28	Sepsis (28	Sepsis (28	TRUE	igd	0.0033	3.91E-06	6941	M03127.m	TRUE	reported	JscEZw	2	TRUE	0.003047	21.20968
1889	rs633114	T	C	T	C	-0.0152	0.003264	0.3884	0.395526	FALSE	FALSE	FALSE	ieu-b-508	10	1.3E+08	0.03419	486484	0.923943	Sepsis (28	Sepsis (28	Sepsis (28	TRUE	igd	0.0033	3.91E-06	6782	M15990.m	TRUE	reported	15fe4N	2	TRUE	0.003118	21.20954
4614	rs1254457	C	G	C	G	-0.3743	-0.13687	0.0921	0.07022	FALSE	TRUE	FALSE	ieu-b-508	8	18671542	0.074024	486484	0.064459	Sepsis (28	Sepsis (28	Sepsis (28	TRUE	igd	0.0802	3.07E-06	76	M33173.m	TRUE	reported	04fkUQ	2	TRUE	0.222758	21.20846
668	rs3857856	A	G	A	G	0.0152	-0.03317	0.8885	0.892992	FALSE	FALSE	FALSE	ieu-b-508	7	1.3E+08	0.054142	486484	0.540061	Sepsis (28	Sepsis (28	Sepsis (28	TRUE	igd	0.0033	4.93E-06	5687	M01284.m	TRUE	reported	E4hc9c	2	TRUE	0.003717	21.20833
3511	rs7172565	T	C	T	C	0.0705	0.033049	0.0289	0.025812	FALSE	FALSE	FALSE	ieu-b-508	15	75278170	0.105973	486484	0.755146	Sepsis (28	Sepsis (28	Sepsis (28	TRUE	igd	0.0153	3.93E-06	1767	M32492.m	TRUE	reported	rZZFcE	2	TRUE	0.011873	21.20819
6893	rs7693137	T	C	T	C	-0.148	0.08846	0.5556	0.614121	FALSE	FALSE	FALSE	ieu-b-508	4	57214772	0.034241	486484	0.009782	Sepsis (28	Sepsis (28	Sepsis (28	TRUE	igd	0.0303	1.02E-06	18	M34365.m	TRUE	reported	8eexD0	2	TRUE	0.569977	21.20731
6172	rs1810195	A	C	A	C	-0.0608	-0.00185	0.9065	0.908545	FALSE	FALSE	FALSE	ieu-b-508	8	23481910	0.057612	486484	0.974351	Sepsis (28	Sepsis (28	Sepsis (28	TRUE	igd	0.0132	3.97E-06	4999	M33885.m	TRUE	reported	uCOy6o	2	TRUE	0.004226	21.20731
7101	rs317894	A	G	A	G	-0.0281	-0.02844	0.3357	0.342998	FALSE	FALSE	FALSE	ieu-b-508	4	16132175	0.035293	486484	0.420364	Sepsis (28	Sepsis (28	Sepsis (28	TRUE	igd	0.0061	3.45E-06	3239	M34513.m	TRUE	reported	1hoso6	2	TRUE	0.006509	21.20727
2958	rs1769434	A	C	A	C	0.0479	-0.0719	0.9783	0.969937	FALSE	FALSE	FALSE	ieu-b-508	2	1.88E+08	0.099846	486484	0.471485	Sepsis (28	Sepsis (28	Sepsis (28	TRUE	igd	0.0104	3.96E-06	6795	M30805.m	TRUE	reported	YHV70c	2	TRUE	0.003112	21.20687
3614	rs3010305	T	C	T	C	0.0327	-0.07762	0.1318	0.13561	FALSE	FALSE	FALSE	ieu-b-508	6	1.4E+08	0.049187	486484	0.114535	Sepsis (28	Sepsis (28	Sepsis (28	TRUE	igd	0.0071	4.44E-06	7361	M32553.m	TRUE	reported	vHSHqn	2	TRUE	0.002873	21.2061
3818	rs3757514	A	T	A	T	-0.0364	-0.06471	0.9211	0.896136	FALSE	TRUE	FALSE	ieu-b-508	7	8246150	0.054605	486484	0.235965	Sepsis (28	Sepsis (28	Sepsis (28	TRUE	igd	0.0079	3.71E-06	1766	M32644.m	TRUE	reported	JAa2WD	2	TRUE	0.011879	21.20589
2815	rs4797120	C	G	C	G	-0.0364	-0.01374	0.3947	0.396746	FALSE	TRUE	FALSE	ieu-b-508	18	3725189	0.034046	486484	0.686439	Sepsis (28	Sepsis (28	Sepsis (28	TRUE	igd	0.0079	4.05E-06	1758	M27447.m	TRUE	reported	s1NdyU	2	TRUE	0.011932	21.20578
3664	rs644396	T	C	T	C	-0.0327	-0.01145	0.5465	0.544002	FALSE	FALSE	FALSE	ieu-b-508	11	70120501	0.033561	486484	0.7329	Sepsis (28	Sepsis (28	Sepsis (28	TRUE	igd	0.0071	4.45E-06	6970	M32564.m	TRUE	reported	73MgOk	2	TRUE	0.003034	21.20578
3325	rs1561277	A	C	A	C	0.0175	0.015051	0.2892	0.254654	FALSE	FALSE	FALSE	ieu-b-508	2	1.36E+08	0.039589	486484	0.703821	Sepsis (28	Sepsis (28	Sepsis (28	TRUE	igd	0.0038	4.39E-06	7358	M32405.m	TRUE	reported	uzomIQ	2	TRUE	0.002874	21.20268
6190	rs1271618	A	T	A	T	0.0175	0.006856	0.2885	0.325948	FALSE	TRUE	FALSE	ieu-b-508	5	7038531	0.035966	486484	0.848812	Sepsis (28	Sepsis (28	Sepsis (28	TRUE	igd	0.0038	3.90E-06	6004	M33892.m	TRUE	reported	w7HgU7	2	TRUE	0.00352	21.20138
6681	rs1204632	A	C	A	C	-0.0493	0.00905	0.7637	0.760261	FALSE	FALSE	FALSE	ieu-b-508	1	79506410	0.039652	486484	0.819471	Sepsis (28	Sepsis (28	Sepsis (28	TRUE	igd	0.0107	4.14E-06	1492	M34329.m	TRUE	reported	mgPKP5	2	TRUE	0.014029	21.20038
2019	rs1720931	C	G	C	G	0.0723	0.037287	0.1068	0.143062	FALSE	TRUE	FALSE	ieu-b-508	13	39950120	0.049058	486484	0.447219	Sepsis (28	Sepsis (28	Sepsis (28	TRUE	igd	0.0157	4.17E-06	5899	M18335.m	TRUE	reported	IdUJrL	2	TRUE	0.003582	21.19971
4583	rs2448148	T	C	T	C	-0.0258	-0.03178	0.6465	0.681517	FALSE	FALSE	FALSE	ieu-b-508	8	98626459	0.035955	486484	0.376793	Sepsis (28	Sepsis (28	Sepsis (28	TRUE	igd	0.0056	4.39E-06	1531	M33163.m	TRUE	reported	YzGXkR	2	TRUE	0.013674	21.19804
2097	rs179976	T	C	T	C	-0.0198	0.01793	0.7581	0.744712	FALSE	FALSE	FALSE	ieu-b-508	6	16359308	0.038493	486484	0.641365	Sepsis (28	Sepsis (28	Sepsis (28	TRUE	igd	0.0043	3.92E-06	7367	M18467.m	TRUE	reported	64PaNn	2	TRUE	0.00287	21.19706
3703	rs1978915	A	C	A	C	0.0198	0.005253	0.506	0.512099	FALSE	FALSE	FALSE	ieu-b-508	15	39615145	0.033387	486484	0.874989	Sepsis (28	Sepsis (28	Sepsis (28	TRUE	igd	0.0043	5.48E-06	5955	M32593.m	TRUE	reported	6zVl1i	2	TRUE	0.003548	21.19569
2899	rs4692675	T	C	T	C	0.0861	-0.12695	0.0135	0.023003	FALSE	FALSE	FALSE	ieu-b-508	4	1.7E+08	0.115595	486484	0.272088	Sepsis (28	Sepsis (28	Sepsis (28	TRUE	igd	0.0187	4.36E-06	6830	M27722.m	TRUE	reported	rzVgUy	2	TRUE	0.003094	21.19317
1613	rs2941840	A	G	A	G	0.0221	-0.01899	0.0691	0.091184	FALSE	FALSE	FALSE	ieu-b-508	18	76526710	0.058148	486484	0.744005	Sepsis (28	Sepsis (28	Sepsis (28	TRUE	igd	0.0048	3.71E-06	7352	M15122.m	TRUE	reported	VlnI8T	2	TRUE	0.002875	21.19258
6778	rs9561023	A	G	A	G	0.064	-0.01321	0.0941	0.095587	FALSE	FALSE	FALSE	ieu-b-508	13	93022401	0.056627	486484	0.815535	Sepsis (28	Sepsis (28	Sepsis (28	TRUE	igd	0.0139	4.19E-06	5270	M34344.m	TRUE	reported	enFm6p	2	TRUE	0.004007	21.19169
1436	rs9379061	C	G	C	G	-0.0456	-0.00184	0.9562	0.963929	FALSE	TRUE	FALSE	ieu-b-508	6	6735912	0.091311	486484	0.983948	Sepsis (28	Sepsis (28	Sepsis (28	TRUE	igd	0.0099	3.92E-06	1745	M03127.m	TRUE	reported	JscEZw	2	TRUE	0.012012	21.19148
5281	rs4775058	A	G	A	G	0.0663	-0.03605	0.0236	0.033469	FALSE	FALSE	FALSE	ieu-b-508	15	58777278	0.093388	486484	0.699513	Sepsis (28	Sepsis (28	Sepsis (28	TRUE	igd	0.0144	3.85E-06	4409	M33250.m	TRUE	reported	WIZI6U	2	TRUE	0.004785	21.18873
6540	rs2275353	A	G	A	G	0.0244	-0.02645	0.1625	0.154335	FALSE	FALSE	FALSE	ieu-b-508	1	2.03E+08	0.046056	486484	0.565783	Sepsis (28	Sepsis (28	Sepsis (28	TRUE	igd	0.0053	4.51E-06	6987	M34221.m	TRUE	reported	nUe3TL	2	TRUE	0.003024	21.18866
5990	rs1247058	A	G	A	G	0.405	-0.01033	0.0118	0.019325	FALSE	FALSE	FALSE	ieu-b-508	2	1.23E+08	0.122909	486484	0.933037	Sepsis (28	Sepsis (28	Sepsis (28	TRUE	igd	0.0878	3.99E-06	477	M33683.m	TRUE	reported	wSozpU	2	TRUE	0.042702	21.18831
2146	rs7171216	T	G	T	G	-0.0442	-0.00793	0.4139	0.421929	FALSE	FALSE	FALSE	ieu-b-508	15	98893001	0.03373	486484	0.814235	Sepsis (28	Sepsis (28	Sepsis (28	TRUE	igd	0.0096	4.41E-06	3783	M18497.m	TRUE	reported	90JFPc	2	TRUE	0.005572	21.18714
4176	rs2268181	T	C	T	C	-0.0267	-0.01141	0.8405	0.8301	FALSE	FALSE	FALSE	ieu-b-508	1	54361288	0.044527	486484	0.797828	Sepsis (28	Sepsis (28	Sepsis (28	TRUE	igd	0.0058	4.78E-06	6712	M32759.m	TRUE	reported	9elHr7	2	TRUE	0.003147	21.18542
6508	rs6502243	T	G	T	G	-0.0267	-0.05042	0.5849	0.574092	FALSE	FALSE	FALSE	ieu-b-508	17	13134967	0.0337	486484	0.134654	Sepsis (28	Sepsis (28	Sepsis (28	TRUE	igd	0.0058	3.48E-06	5581	M34112.m	TRUE	reported	97JqFb	2	TRUE	0.003783	21.18414
2409	rs9296444	T	C	T	C	0.0267	0.042181	0.7828	0.788838	FALSE	FALSE	FALSE	ieu-b-508	6	44620507	0.041307	486484	0.307183	Sepsis (28	Sepsis (28	Sepsis (28	TRUE	igd	0.0058	4.79E-06	5436	M21184.m	TRUE	reported	63ppuJ	2	TRUE	0.003883	21.18394
6079	rs7575509	T	C	T	C	0.035	-0.01071	0.4488	0.454736	FALSE	FALSE	FALSE	ieu-b-508	2	1.43E+08	0.033513	486484	0.749219	Sepsis (28	Sepsis (28	Sepsis (28	TRUE	igd	0.0076	4.64E-06	1631	M33833.m	TRUE	reported	LMIPLH	2	TRUE	0.012836	21.18244
2900	rs6486548	C	G	C	G	-0.0502	0.028557	0.8143	0.874198	FALSE	TRUE	FALSE	ieu-b-508	12	1.31E+08	0.050865	486484	0.574511	Sepsis (28	Sepsis (28	Sepsis (28	TRUE	igd	0.0109	4.35E-06	1460	M27722.m	TRUE	reported	rzVgUy	2	TRUE	0.01432	21.18162
6395	rs530942	A	G	A	G	-0.0373	-0.05146	0.86	0.867548	FALSE	FALSE	FALSE	ieu-b-508	19	6004105	0.049132	486484	0.294887	Sepsis (28	Sepsis (28	Sepsis (28	TRUE	igd	0.0081	4.69E-06	1766	M33972.m	TRUE	reported	Fu46Yl	2	TRUE	0.011865	21.18144
2237	rs1050371	A	G	A	G	0.0313	-0.06014	0.9815	0.88636	FALSE	FALSE	FALSE	ieu-b-508	8	21850656	0.052549	486484	0.252477	Sepsis (28	Sepsis (28	Sepsis (28	TRUE	igd	0.0068	4.85E-06	7354	M19934.m	TRUE	reported	HtcWcQ	2	TRUE	0.002873	21.18131
4032	rs1022039	A	T	A	T	0.0198	-0.0128	0.2991	0.285948	FALSE	TRUE	FALSE	ieu-b-508	5	1.75E+08	0.03767	486484	0.734083	Sepsis (28	Sepsis (28	Sepsis (28	TRUE	igd	0.0043	5.47E-06	1748	M32739.m	TRUE	reported	dYSC7n	2	TRUE	0.011984	21.17855
6010	rs2173524	T	G	T	G	0.0622	-0.03979	0.3588	0.344125	FALSE	FALSE	FALSE	ieu-b-508	10	1687799	0.035107	486484	0.257057	Sepsis (28	Sepsis (28	Sepsis (28	TRUE	igd	0.0135	4.02E-06	831	M33751.m	TRUE	reported	Cxdkr4	2	TRUE	0.024909	21.17711
8450	rs9561537	A	G	A	G	0.0221	-0.0486	0.5764	0.55729	FALSE	FALSE	FALSE	ieu-b-508	13	94975867	0.033672	486484	0.148955	Sepsis (28	Sepsis (28	Sepsis (28	TRUE	igd	0.0048	4.04E-06	1782	M37004.m	TRUE	reported	EKCQye	2	TRUE	0.011756	21.17456
6403	rs7152358	A	G	A	G	0.0428	0.029814	0.889	0.879995	FALSE	FALSE	FALSE	ieu-b-508	14	50602875	0.051353	486484	0.561529	Sepsis (28	Sepsis (28	Sepsis (28	TRUE	igd	0.0093	3.83E-06	7326	M33973.m	TRUE	reported	rpygG6	2	TRUE	0.002883	21.17401
6642	rs1342906	T	C	T	C	0.2346	0.322574	0.0343	0.025543	FALSE	FALSE	FALSE	ieu-b-508	2	52621687	0.10671	486484	0.002504	Sepsis (28	Sepsis (28	Sepsis (28	TRUE	igd	0.0503	3.08E-06	75	M34322.m	TRUE	reported	16GdG9	2	TRUE	0.224831	21.17296
3550	rs1693690	A	G	A	G	-0.0709	-0.01135	0.954	0.961647	FALSE	FALSE	FALSE	ieu-b-508	10	80898551	0.08703	486484	0.896277	Sepsis (28	Sepsis (28	Sepsis (28	TRUE	igd	0.0154	4.09E-06	1768	M32504.m	TRUE	reported	MN0yyR	2	TRUE	0.011847	21.17188
5173	rs1119231	A	G	A	G	0.0428	0.017829	0.4433	0.453626	FALSE	FALSE	FALSE	ieu-b-508	10	83817243	0.03365	486484	0.596218	Sepsis (28	Sepsis (28	Sepsis (28	TRUE	igd	0.0093	3.69E-06	4625	M33195.m	TRUE	reported	Sl4STh	2	TRUE	0.004559	21.17063
7238	rs9924628	A	C	A	C	-0.0313	-0.02316	0.3336	0.329772	FALSE	FALSE	FALSE	ieu-b-508	16	82306551	0.035718	486484	0.516699	Sepsis (28	Sepsis (28	Sepsis (28	TRUE	igd	0.0068	3.56E-06	2474	M34878.m	TRUE	reported	Cpq9yU	2	TRUE	0.008491	21.16994
2808	rs1050107	A	G	A	G	0.0359	-0.00089	0.7994	0.803517	FALSE	FALSE	FALSE	ieu-b-508	11	27025520	0.042121	486484	0.983196	Sepsis (28	Sepsis (28	Sepsis (28	TRUE	igd	0.0078	4.42E-06	2759	M27447.m	TRUE	reported	s1NdyU	2	TRUE	0.007619	21.16824
4020	rs1316249	A	G	A	G	-0.0566	0.017417	0.0653	0.072295	FALSE	FALSE	FALSE	ieu-b-508	5	1.15E+08	0.064564	486484	0.787338	Sepsis (28	Sepsis (28	Sepsis (28	TRUE	igd	0.0123	4.35E-06	6000	M32735.m	TRUE	reported	QE2vKQ	2	TRUE	0.003517	21.1679
8098	rs1323558	T	C	T	C	0.3204	-0.00109	0.227	0.224977	FALSE	FALSE	FALSE	ieu-b-508	7	1.01E+08	0.039826	486484	0.978225	Sepsis (28	Sepsis (28	Sepsis (28	TRUE	igd	0.0695	4.04E-06	488	M36097.m	TRUE	reported	RHr18S	2	TRUE	0.041733	21.16566
8113	rs8101059	A	G	A	G	0.2683	0.006763	0.4901	0.523184	FALSE	FALSE	FALSE	ieu-b-508	19	57445734	0.034445	486484	0.844335	Sepsis (28	Sepsis (28	Sepsis (28	TRUE	igd	0.0582	4.05E-06	488	M36097.m	TRUE	reported	RHr18S	2	TRUE	0.041731	21.16469
5285	rs1008904	A	C	A	C	-0.2008	-0.01687	0.9474	0.708886	FALSE	FALSE	FALSE	ieu-b-508	8	84133048	0.036701	486484	0.645687	Sepsis (28	Sepsis (28	Sepsis (28	TRUE	igd	0.0435	3.84E-06	292	M33353.m	TRUE	reported	ZIN4gk	2	TRUE	0.068011	21.16236
4520	rs1123372	T	C	T	C	-0.0902	-0.01509	0.0578	0.054807	FALSE	FALSE	FALSE	ieu-b-508	11	83424094	0.073892	486484	0.838237	Sepsis (28	Sepsis (28	Sepsis (28	TRUE	igd	0.0196	4.06E-06	2391	M33137.m	TRUE	reported	ONVNk3	2	TRUE	0.00878	21.16106
5904	rs9833839	T	C	T	C	0.2858	0.15792	0.0127	0.011394	FALSE	FALSE	FALSE	ieu-b-508	3	1.74E+08	0.159262	486484	0.321407	Sepsis (28	Sepsis (28	Sepsis (28	TRUE	igd	0.062	3.96E-06	470	M33610.m	TRUE	reported	tmg43v	2	TRUE	0.043255	21.1587
6499	rs1706449	A	T	A	T	0.2812	0.045743	0.0225	0.018382	FALSE	TRUE	FALSE	ieu-b-508	8	1953485	0.124546	486484	0.713413	Sepsis (28	Sepsis (28	Sepsis (28	TRUE	igd	0.0611	4.20E-06	1654	M34112.m	TRUE	reported	97JqFb	2	TRUE	0.012644	21.15547
6750	rs9812186	A	G	A	G	-0.3327	-0.04435	0.9771	0.972097	FALSE	FALSE	FALSE	ieu-b-508	3	1.73E+08	0.101259	486484	0.661423	Sepsis (28	Sepsis (28	Sepsis (28	TRUE	igd	0.0722	4.04E-06	535	M34338.m	TRUE	reported	h4FB9a	2	TRUE	0.038175	21.15459
7887	rs6500859	A	T	A	T	-0.0511	-0.03972	0.699	0.713912	FALSE	TRUE	FALSE	ieu-b-508	16	6914358	0.036839	486484	0.280969	Sepsis (28	Sepsis (28	Sepsis (28	TRUE	igd	0.0111	4.56E-06	1098	M35635.m	TRUE	reported	qymryH	2	TRUE	0.018936	21.15456
399	rs1005596	A	G	A	G	0.0115	0.034807	0.1014	0.107995	FALSE	FALSE	FALSE	ieu-b-508	17	33142245	0.054091	486484	0.519904	Sepsis (28	Sepsis (28	Sepsis (28	TRUE	igd	0.0025	4.20E-06	7365	M00063.m	TRUE	reported	Hdsv1L	2	TRUE	0.002865	21.15425
4498	rs1290297	T	C	T	C	-0.0115	0.004677	0.9093	0.903009	FALSE	FALSE	FALSE	ieu-b-508	15	45267670	0.056517	486484	0.934047	Sepsis (28	Sepsis (28	Sepsis (28	TRUE	igd	0.0025	4.19E-06	7362	M33132.m	TRUE	reported	dTXJa8	2	TRUE	0.002866	21.15425
6063	rs7659812	A	G	A	G	-0.0161	0.017779	0.5527	0.544393	FALSE	FALSE	FALSE	ieu-b-508	4	1.78E+08	0.03359	486484	0.596597	Sepsis (28	Sepsis (28	Sepsis (28	TRUE	igd	0.0035	3.55E-06	7362	M33821.m	TRUE	reported	eVYICL	2	TRUE	0.002866	21.15425
6066	rs980142	A	G	A	G	-0.023	0.034398	0.1644	0.215017	FALSE	FALSE	FALSE	ieu-b-508	21	18731623	0.040936	486484	0.400748	Sepsis (28	Sepsis (28	Sepsis (28	TRUE	igd	0.005	4.44E-06	7362	M33821.m	TRUE	reported	eVYICL	2	TRUE	0.002866	21.15425
3495	rs1325436	T	C	T	C	0.0115	0.029552	0.1852	0.20939	FALSE	FALSE	FALSE	ieu-b-508	8	30165168	0.041448	486484	0.475847	Sepsis (28	Sepsis (28	Sepsis (28	TRUE	igd	0.0025	5.57E-06	7355	M32492.m	TRUE	reported	rZZFcE	2	TRUE	0.002869	21.15425
3498	rs2049791	A	G	A	G	-0.0115	-0.0207	0.7537	0.755922	FALSE	FALSE	FALSE	ieu-b-508	14	22420469	0.03873	486484	0.5931	Sepsis (28	Sepsis (28	Sepsis (28	TRUE	igd	0.0025	3.30E-06	7355	M32492.m	TRUE	reported	rZZFcE	2	TRUE	0.002869	21.15425
2795	rs1704674	A	G	A	G	0.0161	-0.01764	0.1133	0.113832	FALSE	FALSE	FALSE	ieu-b-508	2	25165825	0.052507	486484	0.736935	Sepsis (28	Sepsis (28	Sepsis (28	TRUE	igd	0.0035	4.58E-06	7347	M27278.m	TRUE	reported	kPgqm3	2	TRUE	0.002872	21.15424
6554	rs1391971	A	G	A	G	-0.0161	-0.09499	0.1795	0.177132	FALSE	FALSE	FALSE	ieu-b-508	5	39747893	0.043652	486484	0.029546	Sepsis (28	Sepsis (28	Sepsis (28	TRUE	igd	0.0035	3.90E-06	7347	M34244.m	TRUE	reported	JyF0Sp	2	TRUE	0.002872	21.15424
1179	rs1699045	T	C	T	C	0.0161	0.11442	0.0722	0.083522	FALSE	FALSE	FALSE	ieu-b-508	19	417714	0.06029	486484	0.057718	Sepsis (28	Sepsis (28	Sepsis (28	TRUE	igd	0.0035	4.18E-06	7346	M01645.m	TRUE	reported	d5nAn3	2	TRUE	0.002872	21.15424
2943	rs3766606	T	G	T	G	0.0207	-0.00661	0.1659	0.170781	FALSE	FALSE	FALSE	ieu-b-508	1	8022197	0.044258	486484	0.881257	Sepsis (28	Sepsis (28	Sepsis (28	TRUE	igd	0.0045	4.93E-06	7336	M27738.m	TRUE	reported	UtPBd9	2	TRUE	0.002876	21.15423
6392	rs7142028	A	C	A	C	-0.0161	0.055643	0.5693	0.562377	FALSE	FALSE	FALSE	ieu-b-508	14	89389073	0.03371	486484	0.098812	Sepsis (28	Sepsis (28	Sepsis (28	TRUE	igd	0.0035	4.06E-06	7330	M33969.m	TRUE	reported	0ujroW	2	TRUE	0.002878	21.15423
1590	rs8046494	A	G	A	G	0.0138	0.018714	0.9288	0.920075	FALSE	FALSE	FALSE	ieu-b-508	16	83219456	0.061281	486484	0.760085	Sepsis (28	Sepsis (28	Sepsis (28	TRUE	igd	0.003	4.08E-06	7314	M12593.m	TRUE	reported	IBXgSU	2	TRUE	0.002885	21.15421
3928	rs1246593	A	G	A	G	0.0115	0.054102	0.3006	0.303228	FALSE	FALSE	FALSE	ieu-b-508	2	2.11E+08	0.036293	486484	0.136044	Sepsis (28	Sepsis (28	Sepsis (28	TRUE	igd	0.0025	5.13E-06	7308	M32698.m	TRUE	reported	srqncz	2	TRUE	0.002887	21.15421
3319	rs878947	A	G	A	G	-0.0115	0.037851	0.7845	0.782355	FALSE	FALSE	FALSE	ieu-b-508	1	2.13E+08	0.040527	486484	0.350309	Sepsis (28	Sepsis (28	Sepsis (28	TRUE	igd	0.0025	5.34E-06	7307	M32393.m	TRUE	reported	Pub0X0	2	TRUE	0.002887	21.15421
2752	rs2213261	T	C	T	C	0.0115	-0.00112	0.9061	0.908547	FALSE	FALSE	FALSE	ieu-b-508	3	96763148	0.058258	486484	0.984689	Sepsis (28	Sepsis (28	Sepsis (28	TRUE	igd	0.0025	5.93E-06	7303	M27256.m	TRUE	reported	mJwzsi	2	TRUE	0.002889	21.15421
2445	rs4719411	T	G	T	G	-0.0115	-0.02319	0.5112	0.523446	FALSE	FALSE	FALSE	ieu-b-508	7	14598424	0.03334	486484	0.486697	Sepsis (28	Sepsis (28	Sepsis (28	TRUE	igd	0.0025	5.69E-06	7284	M21630.m	TRUE	reported	7eaO4H	2	TRUE	0.002897	21.15419
494	rs1093589	C	G	C	G	-0.0506	-0.05084	0.8197	0.816205	FALSE	TRUE	FALSE	ieu-b-508	3	1.52E+08	0.042862	486484	0.235523	Sepsis (28	Sepsis (28	Sepsis (28	TRUE	igd	0.011	4.54E-06	7274	M00569.m	TRUE	reported	1TjBaz	2	TRUE	0.002901	21.15418
2780	rs2694777	A	G	A	G	0.0138	-0.08243	0.8261	0.791095	FALSE	FALSE	FALSE	ieu-b-508	10	1.18E+08	0.042334	486484	0.051511	Sepsis (28	Sepsis (28	Sepsis (28	TRUE	igd	0.003	5.60E-06	7268	M27273.m	TRUE	reported	EvU2od	2	TRUE	0.002903	21.15418
3035	rs2197296	A	G	A	G	0.0207	-0.03781	0.2529	0.285355	FALSE	FALSE	FALSE	ieu-b-508	6	1.61E+08	0.037282	486484	0.310484	Sepsis (28	Sepsis (28	Sepsis (28	TRUE	igd	0.0045	4.75E-06	7263	M31555.m	TRUE	reported	iQ5E3v	2	TRUE	0.002905	21.15417
7495	rs4507280	A	T	A	T	0.0161	-0.02846	0.1488	0.154767	FALSE	TRUE	FALSE	ieu-b-508	3	88185509	0.046197	486484	0.537813	Sepsis (28	Sepsis (28	Sepsis (28	TRUE	igd	0.0035	3.49E-06	7261	M35253.m	TRUE	reported	VboRmj	2	TRUE	0.002906	21.15417
2676	rs1775517	A	T	A	T	-0.0115	0.015796	0.6786	0.645048	FALSE	TRUE	FALSE	ieu-b-508	2	1.88E+08	0.034859	486484	0.65044	Sepsis (28	Sepsis (28	Sepsis (28	TRUE	igd	0.0025	3.44E-06	7256	M24074.m	TRUE	reported	Lk6D2A	2	TRUE	0.002908	21.15417
2681	rs4075958	A	G	A	G	0.0115	0.046099	0.262	0.26302	FALSE	FALSE	FALSE	ieu-b-508	5	1.77E+08	0.037814	486484	0.222802	Sepsis (28	Sepsis (28	Sepsis (28	TRUE	igd	0.0025	5.28E-06	7256	M24074.m	TRUE	reported	Lk6D2A	2	TRUE	0.002908	21.15417
1363	rs4981468	A	G	A	G	0.0207	-0.03114	0.0509	0.051928	FALSE	FALSE	FALSE	ieu-b-508	14	23797519	0.075751	486484	0.681056	Sepsis (28	Sepsis (28	Sepsis (28	TRUE	igd	0.0045	4.76E-06	7226	M02730.m	TRUE	reported	XYa035	2	TRUE	0.00292	21.15414
8203	rs1089426	A	C	A	C	0.0161	-0.02491	0.5535	0.567466	FALSE	FALSE	FALSE	ieu-b-508	11	1.31E+08	0.033971	486484	0.463309	Sepsis (28	Sepsis (28	Sepsis (28	TRUE	igd	0.0035	4.84E-06	7184	M36300.m	TRUE	reported	ZjeC9m	2	TRUE	0.002937	21.15411
6420	rs7607175	T	C	T	C	0.0345	0.049782	0.9247	0.915203	FALSE	FALSE	FALSE	ieu-b-508	2	2.24E+08	0.059721	486484	0.404516	Sepsis (28	Sepsis (28	Sepsis (28	TRUE	igd	0.0075	4.55E-06	7132	M34040.m	TRUE	reported	sJBy3i	2	TRUE	0.002958	21.15407
8271	rs7910605	A	G	A	G	-0.0138	0.067478	0.876	0.876248	FALSE	FALSE	FALSE	ieu-b-508	10	94239793	0.05064	486484	0.182693	Sepsis (28	Sepsis (28	Sepsis (28	TRUE	igd	0.003	5.45E-06	7092	M36552.m	TRUE	reported	5CxMJz	2	TRUE	0.002975	21.15403
864	rs6471261	T	G	T	G	0.0115	0.035383	0.2806	0.291259	FALSE	FALSE	FALSE	ieu-b-508	8	91890170	0.036734	486484	0.335439	Sepsis (28	Sepsis (28	Sepsis (28	TRUE	igd	0.0025	6.16E-06	7063	M01361.m	TRUE	reported	NIAcQ3	2	TRUE	0.002987	21.15401
6518	rs1725671	T	C	T	C	0.0207	0.014789	0.0762	0.09011	FALSE	FALSE	FALSE	ieu-b-508	14	24639699	0.058036	486484	0.798853	Sepsis (28	Sepsis (28	Sepsis (28	TRUE	igd	0.0045	3.70E-06	7049	M34123.m	TRUE	reported	9O5wo1	2	TRUE	0.002993	21.154
5776	rs9648853	T	C	T	C	-0.0161	-0.00988	0.6009	0.602196	FALSE	FALSE	FALSE	ieu-b-508	7	1.48E+08	0.034285	486484	0.773284	Sepsis (28	Sepsis (28	Sepsis (28	TRUE	igd	0.0035	3.67E-06	7044	M33509.m	TRUE	reported	56SCCs	2	TRUE	0.002995	21.15399
8504	rs7582926	T	C	T	C	-0.0437	-0.01175	0.7138	0.711477	FALSE	FALSE	FALSE	ieu-b-508	2	2.18E+08	0.037015	486484	0.75095	Sepsis (28	Sepsis (28	Sepsis (28	TRUE	igd	0.0095	3.89E-06	7014	M37097.m	TRUE	reported	wCbJj4	2	TRUE	0.003008	21.15397
3651	rs1312965	C	G	C	G	0.0437	0.013146	0.8162	0.801758	FALSE	TRUE	FALSE	ieu-b-508	4	1.25E+08	0.041869	486484	0.753533	Sepsis (28	Sepsis (28	Sepsis (28	TRUE	igd	0.0095	4.02E-06	6970	M32564.m	TRUE	reported	73MgOk	2	TRUE	0.003027	21.15393
7801	rs1011502	A	G	A	G	0.0115	-0.02896	0.6952	0.708747	FALSE	FALSE	FALSE	ieu-b-508	9	1.29E+08	0.036706	486484	0.430084	Sepsis (28	Sepsis (28	Sepsis (28	TRUE	igd	0.0025	4.66E-06	6883	M35464.m	TRUE	reported	lr9dHp	2	TRUE	0.003065	21.15385
6830	rs6687408	C	G	C	G	0.0115	0.102869	0.828	0.837636	FALSE	TRUE	FALSE	ieu-b-508	1	77238418	0.04536	486484	0.02334	Sepsis (28	Sepsis (28	Sepsis (28	TRUE	igd	0.0025	5.18E-06	6755	M34359.m	TRUE	reported	0UotaJ	2	TRUE	0.003123	21.15374
1930	rs2333040	A	G	A	G	0.0115	0.005296	0.4575	0.485999	FALSE	FALSE	FALSE	ieu-b-508	3	1.25E+08	0.033484	486484	0.874325	Sepsis (28	Sepsis (28	Sepsis (28	TRUE	igd	0.0025	4.16E-06	6549	M16822.m	TRUE	reported	UNI018	2	TRUE	0.003221	21.15354
4578	rs1184878	A	T	A	T	-0.0161	-0.04357	0.2102	0.198405	FALSE	TRUE	FALSE	ieu-b-508	14	54931610	0.041906	486484	0.298452	Sepsis (28	Sepsis (28	Sepsis (28	TRUE	igd	0.0035	3.16E-06	6404	M33163.m	TRUE	reported	YzGXkR	2	TRUE	0.003293	21.15339
2464	rs2278441	T	G	T	G	0.0115	0.002271	0.4291	0.413612	FALSE	FALSE	FALSE	ieu-b-508	19	29585250	0.034065	486484	0.94684	Sepsis (28	Sepsis (28	Sepsis (28	TRUE	igd	0.0025	5.68E-06	6180	M22030.m	TRUE	reported	ZNV0qR	2	TRUE	0.003412	21.15315
2010	rs1276483	A	G	A	G	0.0253	-0.03155	0.6005	0.583586	FALSE	FALSE	FALSE	ieu-b-508	10	96997167	0.033959	486484	0.352783	Sepsis (28	Sepsis (28	Sepsis (28	TRUE	igd	0.0055	4.46E-06	5984	M18283.m	TRUE	reported	oyZdTE	2	TRUE	0.003524	21.15293
5289	rs1244143	A	T	A	T	-0.1298	-0.05264	0.0273	0.022091	FALSE	TRUE	FALSE	ieu-b-508	15	62139993	0.113841	486484	0.643769	Sepsis (28	Sepsis (28	Sepsis (28	TRUE	igd	0.0282	4.31E-06	1273	M33353.m	TRUE	reported	ZIN4gk	2	TRUE	0.01637	21.15282
5495	rs1013104	T	C	T	C	-0.0207	-0.04962	0.4354	0.441583	FALSE	FALSE	FALSE	ieu-b-508	17	12187236	0.03366	486484	0.140464	Sepsis (28	Sepsis (28	Sepsis (28	TRUE	igd	0.0045	3.83E-06	5822	M33420.m	TRUE	reported	eTToDb	2	TRUE	0.003621	21.15273
5655	rs2746997	A	G	A	G	0.0161	-0.01806	0.4486	0.44934	FALSE	FALSE	FALSE	ieu-b-508	14	86067774	0.033616	486484	0.591134	Sepsis (28	Sepsis (28	Sepsis (28	TRUE	igd	0.0035	6.00E-06	5717	M33453.m	TRUE	reported	OzEeLQ	2	TRUE	0.003688	21.1526
7308	rs6429216	A	G	A	G	-0.023	0.025275	0.464	0.46139	FALSE	FALSE	FALSE	ieu-b-508	1	2.41E+08	0.033338	486484	0.44836	Sepsis (28	Sepsis (28	Sepsis (28	TRUE	igd	0.005	4.23E-06	5600	M35126.m	TRUE	reported	1haOJA	2	TRUE	0.003764	21.15244
3943	rs1006553	A	G	A	G	0.046	-0.01314	0.025	0.035923	FALSE	FALSE	FALSE	ieu-b-508	5	1.7E+08	0.092297	486484	0.886825	Sepsis (28	Sepsis (28	Sepsis (28	TRUE	igd	0.01	4.23E-06	5597	M32709.m	TRUE	reported	YR8SB4	2	TRUE	0.003766	21.15244
3675	rs697387	T	C	T	C	-0.023	-0.02782	0.299	0.299485	FALSE	FALSE	FALSE	ieu-b-508	1	1.11E+08	0.041313	486484	0.500628	Sepsis (28	Sepsis (28	Sepsis (28	TRUE	igd	0.005	4.23E-06	5596	M32578.m	TRUE	reported	LPkMFr	2	TRUE	0.003767	21.15244
1616	rs5759809	T	C	T	C	0.069	0.024159	0.011	0.059664	FALSE	FALSE	FALSE	ieu-b-508	22	23786770	0.070532	486484	0.731963	Sepsis (28	Sepsis (28	Sepsis (28	TRUE	igd	0.015	4.23E-06	5595	M15122.m	TRUE	reported	VlnI8T	2	TRUE	0.003768	21.15244
1047	rs1708674	T	C	T	C	-0.046	-0.22811	0.02	0.021141	FALSE	FALSE	FALSE	ieu-b-508	4	57259999	0.11689	486484	0.050998	Sepsis (28	Sepsis (28	Sepsis (28	TRUE	igd	0.01	4.23E-06	5585	M01572.m	TRUE	reported	EOUAWy	2	TRUE	0.003774	21.15242
7815	rs1098273	A	G	A	G	0.023	0.014832	0.627	0.628119	FALSE	FALSE	FALSE	ieu-b-508	9	1.18E+08	0.034561	486484	0.667807	Sepsis (28	Sepsis (28	Sepsis (28	TRUE	igd	0.005	4.23E-06	4955	M35472.m	TRUE	reported	oZU10n	2	TRUE	0.004252	21.15146
4704	rs1112171	T	C	T	C	0.2845	-0.04323	0.9816	0.959867	FALSE	FALSE	FALSE	ieu-b-508	1	11345339	0.085585	486484	0.613455	Sepsis (28	Sepsis (28	Sepsis (28	TRUE	igd	0.0618	4.12E-06	1017	M33178.m	TRUE	reported	g22WMS	2	TRUE	0.020413	21.15109
5650	rs1786356	A	G	A	G	0.023	0.011704	0.606	0.596289	FALSE	FALSE	FALSE	ieu-b-508	21	21806268	0.035298	486484	0.74021	Sepsis (28	Sepsis (28	Sepsis (28	TRUE	igd	0.005	4.23E-06	4599	M33453.m	TRUE	reported	OzEeLQ	2	TRUE	0.00458	21.1508
8397	rs4729705	T	C	T	C	0.0138	0.015423	0.539	0.540746	FALSE	FALSE	FALSE	ieu-b-508	7	1.01E+08	0.033496	486484	0.645191	Sepsis (28	Sepsis (28	Sepsis (28	TRUE	igd	0.003	5.06E-06	4317	M36802.m	TRUE	reported	njeQfy	2	TRUE	0.004878	21.1502
6916	rs704852	A	C	A	C	-0.023	0.000917	0.758	0.769505	FALSE	FALSE	FALSE	ieu-b-508	1	1.67E+08	0.045458	486484	0.983903	Sepsis (28	Sepsis (28	Sepsis (28	TRUE	igd	0.005	4.23E-06	4020	M34389.m	TRUE	reported	suiRIN	2	TRUE	0.005236	21.14947
5587	rs7279663	T	G	T	G	1.5318	-0.05814	0.9746	0.971825	FALSE	FALSE	FALSE	ieu-b-508	21	45056212	0.10298	486484	0.572343	Sepsis (28	Sepsis (28	Sepsis (28	TRUE	igd	0.3275	2.90E-06	60	M33423.m	TRUE	reported	MoEnO7	2	TRUE	0.267191	21.14746
1120	rs1306999	T	C	T	C	-0.0506	0.00699	0.3781	0.390328	FALSE	FALSE	FALSE	ieu-b-508	3	1.16E+08	0.034219	486484	0.838139	Sepsis (28	Sepsis (28	Sepsis (28	TRUE	igd	0.011	4.44E-06	2063	M01640.m	TRUE	reported	o3qxzl	2	TRUE	0.010153	21.13949
7192	rs1718724	T	C	T	C	-0.0607	0.068872	0.9712	0.974079	FALSE	FALSE	FALSE	ieu-b-508	1	2.08E+08	0.105797	486484	0.515061	Sepsis (28	Sepsis (28	Sepsis (28	TRUE	igd	0.0132	3.91E-06	4800	M34539.m	TRUE	reported	puA8ni	2	TRUE	0.004386	21.13725
1881	rs170555	T	C	T	C	-0.0253	-0.00188	0.5607	0.57791	FALSE	FALSE	FALSE	ieu-b-508	1	2.1E+08	0.033781	486484	0.955585	Sepsis (28	Sepsis (28	Sepsis (28	TRUE	igd	0.0055	3.84E-06	1756	M15990.m	TRUE	reported	15fe4N	2	TRUE	0.011907	21.1359
6966	rs9415646	T	G	T	G	-0.088	0.039224	0.691	0.683177	FALSE	FALSE	FALSE	ieu-b-508	10	63882016	0.035959	486484	0.275353	Sepsis (28	Sepsis (28	Sepsis (28	TRUE	igd	0.0191	4.00E-06	463	M34404.m	TRUE	reported	8FMHfo	2	TRUE	0.043838	21.13579
5174	rs1256685	T	C	T	C	0.0515	0.009095	0.7395	0.739576	FALSE	FALSE	FALSE	ieu-b-508	1	2.36E+08	0.038033	486484	0.810997	Sepsis (28	Sepsis (28	Sepsis (28	TRUE	igd	0.0112	4.70E-06	4625	M33195.m	TRUE	reported	Sl4STh	2	TRUE	0.004551	21.13443
6466	rs1184283	A	G	A	G	0.2985	-0.00987	0.9424	0.875603	FALSE	FALSE	FALSE	ieu-b-508	13	41910692	0.050589	486484	0.845262	Sepsis (28	Sepsis (28	Sepsis (28	TRUE	igd	0.0647	3.97E-06	282	M34109.m	TRUE	reported	piJuC7	2	TRUE	0.070182	21.13436
6671	rs1373562	T	C	T	C	0.0713	0.030031	0.1574	0.163639	FALSE	FALSE	FALSE	ieu-b-508	15	26061504	0.045173	486484	0.50618	Sepsis (28	Sepsis (28	Sepsis (28	TRUE	igd	0.0155	3.89E-06	1612	M34327.m	TRUE	reported	lI4Lrr	2	TRUE	0.012956	21.13375
6998	rs1007455	A	G	A	G	-0.0492	-0.05567	0.4717	0.481072	FALSE	FALSE	FALSE	ieu-b-508	5	1.29E+08	0.03337	486484	0.095288	Sepsis (28	Sepsis (28	Sepsis (28	TRUE	igd	0.0107	4.54E-06	4452	M34420.m	TRUE	reported	20uofq	2	TRUE	0.004727	21.13331
2145	rs7139744	T	G	T	G	0.0423	-0.09324	0.3014	0.306841	FALSE	FALSE	FALSE	ieu-b-508	13	41039186	0.036186	486484	0.009977	Sepsis (28	Sepsis (28	Sepsis (28	TRUE	igd	0.0092	3.87E-06	3783	M18497.m	TRUE	reported	90JFPc	2	TRUE	0.005557	21.12883
3622	rs1292053	A	G	A	G	0.0308	0.049521	0.7386	0.729798	FALSE	FALSE	FALSE	ieu-b-508	16	20421556	0.040297	486484	0.219106	Sepsis (28	Sepsis (28	Sepsis (28	TRUE	igd	0.0067	4.37E-06	7337	M32557.m	TRUE	reported	qR7rZX	2	TRUE	0.002872	21.12679
4168	rs1013521	T	C	T	C	-0.0262	0.102797	0.8095	0.811254	FALSE	FALSE	FALSE	ieu-b-508	14	76158998	0.042675	486484	0.016003	Sepsis (28	Sepsis (28	Sepsis (28	TRUE	igd	0.0057	4.13E-06	6712	M32759.m	TRUE	reported	9elHr7	2	TRUE	0.003138	21.12144
5478	rs7708182	C	G	C	G	-0.1012	0.010604	0.8862	0.867704	FALSE	TRUE	FALSE	ieu-b-508	5	235608	0.049171	486484	0.829261	Sepsis (28	Sepsis (28	Sepsis (28	TRUE	igd	0.022	4.41E-06	1094	M33408.m	TRUE	reported	tWZWla	2	TRUE	0.018975	21.12132
3010	rs7315595	T	G	T	G	-0.0838	-0.00325	0.7851	0.77018	FALSE	FALSE	FALSE	ieu-b-508	12	80944011	0.039596	486484	0.934564	Sepsis (28	Sepsis (28	Sepsis (28	TRUE	igd	0.0182	3.95E-06	522	M31536.m	TRUE	reported	3nAuUI	2	TRUE	0.039029	21.11923
8148	rs9348951	A	C	A	C	0.0239	-0.02586	0.4014	0.414758	FALSE	FALSE	FALSE	ieu-b-508	6	11692419	0.033833	486484	0.444714	Sepsis (28	Sepsis (28	Sepsis (28	TRUE	igd	0.0052	5.05E-06	7311	M36103.m	TRUE	reported	sjeaPC	2	TRUE	0.002881	21.11885
6225	rs8024954	A	G	A	G	0.1812	0.024285	0.0304	0.03813	FALSE	FALSE	FALSE	ieu-b-508	15	23054608	0.091975	486484	0.791754	Sepsis (28	Sepsis (28	Sepsis (28	TRUE	igd	0.0394	4.33E-06	1294	M33901.m	TRUE	reported	mQkhrb	2	TRUE	0.016082	21.11797
8212	rs1893285	A	G	A	G	-0.0331	-0.0171	0.5927	0.617468	FALSE	FALSE	FALSE	ieu-b-508	18	6668781	0.034721	486484	0.622405	Sepsis (28	Sepsis (28	Sepsis (28	TRUE	igd	0.0072	4.81E-06	2520	M36376.m	TRUE	reported	k6uC1I	2	TRUE	0.008317	21.11768
5506	rs7350776	C	G	C	G	0.0239	0.00174	0.697	0.697156	FALSE	TRUE	FALSE	ieu-b-508	15	33689075	0.036393	486484	0.961866	Sepsis (28	Sepsis (28	Sepsis (28	TRUE	igd	0.0052	3.86E-06	5822	M33420.m	TRUE	reported	eTToDb	2	TRUE	0.003615	21.11737
8156	rs7992673	T	C	T	C	0.161	-0.01468	0.922	0.787807	FALSE	FALSE	FALSE	ieu-b-508	13	41776362	0.040796	486484	0.718962	Sepsis (28	Sepsis (28	Sepsis (28	TRUE	igd	0.035	4.23E-06	978	M36115.m	TRUE	reported	ZV1jWp	2	TRUE	0.021178	21.11673
6846	rs1101222	T	C	T	C	-0.1173	-0.0431	0.8856	0.861664	FALSE	FALSE	FALSE	ieu-b-508	10	20939445	0.048306	486484	0.372267	Sepsis (28	Sepsis (28	Sepsis (28	TRUE	igd	0.0255	4.16E-06	954	M34365.m	TRUE	reported	8eexD0	2	TRUE	0.021699	21.11564
840	rs1749522	A	G	A	G	0.0216	-0.09786	0.0566	0.055445	FALSE	FALSE	FALSE	ieu-b-508	4	37642746	0.073067	486484	0.180483	Sepsis (28	Sepsis (28	Sepsis (28	TRUE	igd	0.0047	5.67E-06	7323	M01359.m	TRUE	reported	7L9x1s	2	TRUE	0.002876	21.1151
7115	rs1695521	A	G	A	G	-0.5039	0.030514	0.9818	0.993971	FALSE	FALSE	FALSE	ieu-b-508	16	81411495	0.217675	486484	0.888515	Sepsis (28	Sepsis (28	Sepsis (28	TRUE	igd	0.1088	3.66E-06	128	M34516.m	TRUE	reported	7Ah2wy	2	TRUE	0.143527	21.11502
8217	rs1088971	T	C	T	C	0.0216	-0.017	0.3474	0.363142	FALSE	FALSE	FALSE	ieu-b-508	1	41371638	0.034752	486484	0.624612	Sepsis (28	Sepsis (28	Sepsis (28	TRUE	igd	0.0047	4.74E-06	6486	M36399.m	TRUE	reported	EdUpnt	2	TRUE	0.003246	21.11436
6230	rs1049161	T	G	T	G	-0.0841	0.010744	0.9743	0.956853	FALSE	FALSE	FALSE	ieu-b-508	9	33514843	0.084291	486484	0.898572	Sepsis (28	Sepsis (28	Sepsis (28	TRUE	igd	0.0183	4.32E-06	5534	M33910.m	TRUE	reported	YwFZg1	2	TRUE	0.003802	21.11217
2090	rs755677	T	G	T	G	0.0754	0.030049	0.87	0.888265	FALSE	FALSE	FALSE	ieu-b-508	17	70768773	0.053636	486484	0.575314	Sepsis (28	Sepsis (28	Sepsis (28	TRUE	igd	0.0164	4.51E-06	1640	M18394.m	TRUE	reported	Lt9awu	2	TRUE	0.012725	21.11179
4657	rs1004644	T	C	T	C	-0.1182	0.001411	0.0771	0.08049	FALSE	FALSE	FALSE	ieu-b-508	6	39339013	0.061713	486484	0.981761	Sepsis (28	Sepsis (28	Sepsis (28	TRUE	igd	0.0257	4.20E-06	1017	M33178.m	TRUE	reported	g22WMS	2	TRUE	0.020375	21.11124
4544	rs7916009	A	G	A	G	0.0193	0.02091	0.6156	0.60067	FALSE	FALSE	FALSE	ieu-b-508	10	55241540	0.034155	486484	0.540397	Sepsis (28	Sepsis (28	Sepsis (28	TRUE	igd	0.0042	3.92E-06	7166	M33138.m	TRUE	reported	IUpLAI	2	TRUE	0.002938	21.11032
4167	rs7906674	T	G	T	G	-0.0193	-0.06053	0.479	0.471361	FALSE	FALSE	FALSE	ieu-b-508	10	4829053	0.03336	486484	0.069592	Sepsis (28	Sepsis (28	Sepsis (28	TRUE	igd	0.0042	4.81E-06	6642	M32758.m	TRUE	reported	AF0m66	2	TRUE	0.003169	21.10985
8549	rs1724350	T	C	T	C	-0.0363	0.022688	0.1932	0.187778	FALSE	FALSE	FALSE	ieu-b-508	5	81255228	0.042556	486484	0.593936	Sepsis (28	Sepsis (28	Sepsis (28	TRUE	igd	0.0079	4.54E-06	7355	M37202.m	TRUE	reported	BtsIDx	2	TRUE	0.002862	21.1077
2103	rs703811	T	C	T	C	0.017	0.030325	0.7585	0.759279	FALSE	FALSE	FALSE	ieu-b-508	8	1.02E+08	0.03901	486484	0.436944	Sepsis (28	Sepsis (28	Sepsis (28	TRUE	igd	0.0037	4.20E-06	7367	M18467.m	TRUE	reported	64PaNn	2	TRUE	0.002857	21.10457
8039	rs422249	T	C	T	C	-0.017	0.086756	0.309	0.346819	FALSE	FALSE	FALSE	ieu-b-508	11	61639488	0.035206	486484	0.01373	Sepsis (28	Sepsis (28	Sepsis (28	TRUE	igd	0.0037	5.44E-06	6960	M35854.m	TRUE	reported	EujB0p	2	TRUE	0.003024	21.10423
7231	rs1384609	T	C	T	C	-0.046	-0.06102	0.474	0.47328	FALSE	FALSE	FALSE	ieu-b-508	10	1.27E+08	0.033389	486484	0.067613	Sepsis (28	Sepsis (28	Sepsis (28	TRUE	igd	0.01	4.23E-06	711	M34878.m	TRUE	reported	Cpq9yU	2	TRUE	0.028901	21.10048
5901	rs8003714	T	G	T	G	0.4616	0.218627	0.017	0.012003	FALSE	FALSE	FALSE	ieu-b-508	14	86595036	0.154698	486484	0.157582	Sepsis (28	Sepsis (28	Sepsis (28	TRUE	igd	0.0995	3.46E-06	102	M33610.m	TRUE	reported	tmg43v	2	TRUE	0.174237	21.10014
7012	rs4743627	A	T	A	T	0.0804	-0.01827	0.896	0.906246	FALSE	TRUE	FALSE	ieu-b-508	9	98949350	0.057934	486484	0.752435	Sepsis (28	Sepsis (28	Sepsis (28	TRUE	igd	0.0175	4.60E-06	4452	M34420.m	TRUE	reported	20uofq	2	TRUE	0.004719	21.09798
3631	rs8071320	T	G	T	G	-0.0294	-0.01162	0.2306	0.230133	FALSE	FALSE	FALSE	ieu-b-508	17	13875301	0.039522	486484	0.768805	Sepsis (28	Sepsis (28	Sepsis (28	TRUE	igd	0.0064	4.16E-06	7337	M32557.m	TRUE	reported	qR7rZX	2	TRUE	0.002868	21.09679
7890	rs1322106	A	G	A	G	-0.0294	-0.03534	0.8289	0.82814	FALSE	FALSE	FALSE	ieu-b-508	7	1.05E+08	0.04456	486484	0.427716	Sepsis (28	Sepsis (28	Sepsis (28	TRUE	igd	0.0064	4.78E-06	5629	M35669.m	TRUE	reported	gSecuE	2	TRUE	0.003735	21.09504
1977	rs7610725	A	G	A	G	-0.1714	-0.17698	0.9698	0.985054	FALSE	FALSE	FALSE	ieu-b-508	3	19976454	0.14323	486484	0.216606	Sepsis (28	Sepsis (28	Sepsis (28	TRUE	igd	0.0373	4.42E-06	1744	M17807.m	TRUE	reported	zd6sZI	2	TRUE	0.011963	21.09141
4536	rs1250078	A	T	A	T	-0.0395	-0.18728	0.9481	0.944405	FALSE	TRUE	FALSE	ieu-b-508	4	1.53E+08	0.072969	486484	0.010271	Sepsis (28	Sepsis (28	Sepsis (28	TRUE	igd	0.0086	4.76E-06	7166	M33138.m	TRUE	reported	IUpLAI	2	TRUE	0.002935	21.08997
3236	rs1245609	A	G	A	G	-0.0395	0.011506	0.7235	0.664904	FALSE	FALSE	FALSE	ieu-b-508	18	42880242	0.03538	486484	0.745015	Sepsis (28	Sepsis (28	Sepsis (28	TRUE	igd	0.0086	4.75E-06	6687	M32346.m	TRUE	reported	cnIFoV	2	TRUE	0.003145	21.08955
2092	rs915529	A	G	A	G	-0.1861	-0.16617	0.0187	0.018334	FALSE	FALSE	FALSE	ieu-b-508	21	33726326	0.125266	486484	0.184666	Sepsis (28	Sepsis (28	Sepsis (28	TRUE	igd	0.0405	4.23E-06	1640	M18394.m	TRUE	reported	Lt9awu	2	TRUE	0.012711	21.08884
6123	rs1707339	T	C	T	C	0.4078	-0.01673	0.0276	0.017441	FALSE	FALSE	FALSE	ieu-b-508	18	62314823	0.127917	486484	0.895953	Sepsis (28	Sepsis (28	Sepsis (28	TRUE	igd	0.0883	3.87E-06	176	M33877.m	TRUE	reported	yDVi1f	2	TRUE	0.108089	21.08675
3769	rs284431	A	C	A	C	0.0124	0.081901	0.1069	0.106696	FALSE	FALSE	FALSE	ieu-b-508	6	1.57E+08	0.054197	486484	0.130744	Sepsis (28	Sepsis (28	Sepsis (28	TRUE	igd	0.0027	4.58E-06	7362	M32634.m	TRUE	reported	7T2RUz	2	TRUE	0.002857	21.08618
3871	rs2186677	A	G	A	G	-0.0124	-0.01993	0.2785	0.297355	FALSE	FALSE	FALSE	ieu-b-508	11	76339194	0.037553	486484	0.595614	Sepsis (28	Sepsis (28	Sepsis (28	TRUE	igd	0.0027	5.86E-06	7361	M32654.m	TRUE	reported	6lSQOP	2	TRUE	0.002857	21.08618
3102	rs1318437	T	C	T	C	-0.0124	0.017077	0.1519	0.14694	FALSE	FALSE	FALSE	ieu-b-508	5	30721169	0.0472	486484	0.717508	Sepsis (28	Sepsis (28	Sepsis (28	TRUE	igd	0.0027	6.38E-06	7356	M32198.m	TRUE	reported	ndXmnp	2	TRUE	0.002859	21.08617
7426	rs6722402	T	G	T	G	-0.0124	0.008839	0.7106	0.712146	FALSE	FALSE	FALSE	ieu-b-508	2	2.24E+08	0.036952	486484	0.81095	Sepsis (28	Sepsis (28	Sepsis (28	TRUE	igd	0.0027	2.99E-06	7350	M35186.m	TRUE	reported	UZfu6Z	2	TRUE	0.002861	21.08617
7328	rs748651	T	C	T	C	-0.0124	-0.07841	0.5135	0.525451	FALSE	FALSE	FALSE	ieu-b-508	4	1011115	0.033665	486484	0.019845	Sepsis (28	Sepsis (28	Sepsis (28	TRUE	igd	0.0027	4.06E-06	7344	M35127.m	TRUE	reported	RNsmjl	2	TRUE	0.002864	21.08616
1681	rs6130362	C	G	C	G	0.0124	0.030165	0.2071	0.221344	FALSE	TRUE	FALSE	ieu-b-508	20	42016265	0.040541	486484	0.456836	Sepsis (28	Sepsis (28	Sepsis (28	TRUE	igd	0.0027	5.62E-06	7337	M15365.m	TRUE	reported	TReTCM	2	TRUE	0.002866	21.08616
3740	rs2776859	T	C	T	C	0.0124	-0.03864	0.5308	0.529042	FALSE	FALSE	FALSE	ieu-b-508	9	80946291	0.033855	486484	0.253763	Sepsis (28	Sepsis (28	Sepsis (28	TRUE	igd	0.0027	5.68E-06	7337	M32632.m	TRUE	reported	pIHzPT	2	TRUE	0.002866	21.08616
5419	rs6977786	A	G	A	G	-0.0124	0.012413	0.1753	0.212496	FALSE	FALSE	FALSE	ieu-b-508	7	67590626	0.040811	486484	0.761015	Sepsis (28	Sepsis (28	Sepsis (28	TRUE	igd	0.0027	3.23E-06	7315	M33389.m	TRUE	reported	Hf6XVM	2	TRUE	0.002875	21.08614
2273	rs1050843	A	G	A	G	-0.0124	-0.06974	0.7069	0.708486	FALSE	FALSE	FALSE	ieu-b-508	10	11825696	0.036701	486484	0.05741	Sepsis (28	Sepsis (28	Sepsis (28	TRUE	igd	0.0027	4.07E-06	7301	M20675.m	TRUE	reported	CXe9rE	2	TRUE	0.002881	21.08613
5788	rs3783853	A	G	A	G	0.0124	-0.03141	0.4188	0.41167	FALSE	FALSE	FALSE	ieu-b-508	14	89803269	0.034071	486484	0.356633	Sepsis (28	Sepsis (28	Sepsis (28	TRUE	igd	0.0027	3.27E-06	7269	M33510.m	TRUE	reported	WxGKnn	2	TRUE	0.002893	21.0861
7484	rs1107825	A	G	A	G	0.0124	-0.03058	0.3384	0.345874	FALSE	FALSE	FALSE	ieu-b-508	17	14464463	0.035376	486484	0.387338	Sepsis (28	Sepsis (28	Sepsis (28	TRUE	igd	0.0027	3.09E-06	7261	M35253.m	TRUE	reported	VboRmj	2	TRUE	0.002896	21.0861

7486	rs1215584	T	C	T	C	0.0124	-0.03649	0.6503	0.654831	FALSE	FALSE	FALSE	ieu-b-508	8	58702529	0.035757	486484	0.307503	Sepsis (28	Sepsis (28	Sepsis (28	TRUE	igd	0.0027	3.64E-06	7261	M35253.m	TRUE	reported	VboRmj	2	TRUE	0.002896	21.0861
7492	rs2236434	C	G	C	G	-0.0124	0.060442	0.6878	0.680194	FALSE	TRUE	FALSE	ieu-b-508	21	38176332	0.035843	486484	0.09174	Sepsis (28	Sepsis (28	Sepsis (28	TRUE	igd	0.0027	3.71E-06	7261	M35253.m	TRUE	reported	VboRmj	2	TRUE	0.002896	21.0861
7497	rs4905	A	G	A	G	0.0124	0.004644	0.7122	0.716094	FALSE	FALSE	FALSE	ieu-b-508	19	4237067	0.037022	486484	0.900182	Sepsis (28	Sepsis (28	Sepsis (28	TRUE	igd	0.0027	2.92E-06	7261	M35253.m	TRUE	reported	VboRmj	2	TRUE	0.002896	21.0861
3810	rs1227200	A	T	A	T	-0.0124	-0.00232	0.8447	0.850229	FALSE	TRUE	FALSE	ieu-b-508	11	1.24E+08	0.047349	486484	0.960943	Sepsis (28	Sepsis (28	Sepsis (28	TRUE	igd	0.0027	3.68E-06	7229	M32644.m	TRUE	reported	JAa2WD	2	TRUE	0.002909	21.08607
6408	rs1020972	A	G	A	G	0.0248	0.057789	0.1471	0.151774	FALSE	FALSE	FALSE	ieu-b-508	2	1.22E+08	0.046797	486484	0.216872	Sepsis (28	Sepsis (28	Sepsis (28	TRUE	igd	0.0054	5.04E-06	7132	M34040.m	TRUE	reported	sJBy3i	2	TRUE	0.002949	21.08599
2375	rs1283035	T	G	T	G	0.0124	0.042926	0.3375	0.331616	FALSE	FALSE	FALSE	ieu-b-508	12	28911388	0.035422	486484	0.225577	Sepsis (28	Sepsis (28	Sepsis (28	TRUE	igd	0.0027	5.88E-06	6999	M21127.m	TRUE	reported	WCjhS3	2	TRUE	0.003005	21.08588
6615	rs9972792	A	C	A	C	0.0124	0.010744	0.6339	0.645893	FALSE	FALSE	FALSE	ieu-b-508	16	66452197	0.035006	486484	0.758904	Sepsis (28	Sepsis (28	Sepsis (28	TRUE	igd	0.0027	3.18E-06	6994	M34306.m	TRUE	reported	WPXPUN	2	TRUE	0.003007	21.08588
1542	rs1337278	A	G	A	G	0.0124	0.024555	0.6107	0.616143	FALSE	FALSE	FALSE	ieu-b-508	13	86316853	0.034384	486484	0.475146	Sepsis (28	Sepsis (28	Sepsis (28	TRUE	igd	0.0027	3.68E-06	6864	M12129.m	TRUE	reported	iNzdgb	2	TRUE	0.003063	21.08576
1543	rs1448817	A	G	A	G	0.0124	-0.08686	0.7393	0.752245	FALSE	FALSE	FALSE	ieu-b-508	4	1.12E+08	0.038696	486484	0.024797	Sepsis (28	Sepsis (28	Sepsis (28	TRUE	igd	0.0027	4.56E-06	6864	M12129.m	TRUE	reported	iNzdgb	2	TRUE	0.003063	21.08576
1886	rs2834399	T	G	T	G	0.0124	-0.00593	0.4704	0.466002	FALSE	FALSE	FALSE	ieu-b-508	21	35549478	0.033733	486484	0.860559	Sepsis (28	Sepsis (28	Sepsis (28	TRUE	igd	0.0027	3.23E-06	6782	M15990.m	TRUE	reported	15fe4N	2	TRUE	0.0031	21.08569
2426	rs4711151	A	G	A	G	-0.0124	0.010475	0.3776	0.380961	FALSE	FALSE	FALSE	ieu-b-508	6	27380101	0.034329	486484	0.760262	Sepsis (28	Sepsis (28	Sepsis (28	TRUE	igd	0.0027	3.50E-06	6556	M21188.m	TRUE	reported	kvdnjc	2	TRUE	0.003207	21.08547
2429	rs7152341	A	G	A	G	0.0124	0.015465	0.2566	0.250972	FALSE	FALSE	FALSE	ieu-b-508	14	39884605	0.038492	486484	0.687849	Sepsis (28	Sepsis (28	Sepsis (28	TRUE	igd	0.0027	5.26E-06	6556	M21188.m	TRUE	reported	kvdnjc	2	TRUE	0.003207	21.08547
7465	rs3752192	A	G	A	G	0.0124	-0.01271	0.5526	0.555625	FALSE	FALSE	FALSE	ieu-b-508	19	14951605	0.033696	486484	0.706077	Sepsis (28	Sepsis (28	Sepsis (28	TRUE	igd	0.0027	5.05E-06	6543	M35193.m	TRUE	reported	BMMWXU	2	TRUE	0.003213	21.08546
7472	rs7942744	T	C	T	C	0.0124	-0.0658	0.556	0.559875	FALSE	FALSE	FALSE	ieu-b-508	11	6743890	0.03359	486484	0.050123	Sepsis (28	Sepsis (28	Sepsis (28	TRUE	igd	0.0027	6.42E-06	6543	M35193.m	TRUE	reported	BMMWXU	2	TRUE	0.003213	21.08546
7228	rs9788090	C	G	C	G	0.0248	0.05525	0.7338	0.74612	FALSE	TRUE	FALSE	ieu-b-508	12	67889960	0.039196	486484	0.158665	Sepsis (28	Sepsis (28	Sepsis (28	TRUE	igd	0.0054	5.22E-06	6381	M34761.m	TRUE	reported	AMtp1w	2	TRUE	0.003295	21.0853
8180	rs6089829	A	G	A	G	0.0294	0.058928	0.3531	0.354137	FALSE	FALSE	FALSE	ieu-b-508	20	61669036	0.034927	486484	0.091565	Sepsis (28	Sepsis (28	Sepsis (28	TRUE	igd	0.0064	4.75E-06	2432	M36134.m	TRUE	reported	ekMsfG	2	TRUE	0.008602	21.08519
4241	rs1107326	T	C	T	C	0.0248	-0.01774	0.5956	0.575324	FALSE	FALSE	FALSE	ieu-b-508	15	37951344	0.033999	486484	0.601822	Sepsis (28	Sepsis (28	Sepsis (28	TRUE	igd	0.0054	5.26E-06	6169	M32795.m	TRUE	reported	0YBWkh	2	TRUE	0.003407	21.08507
6647	rs1761927	A	G	A	G	0.071	0.038601	0.1542	0.054523	FALSE	FALSE	FALSE	ieu-b-508	13	68233727	0.072996	486484	0.596936	Sepsis (28	Sepsis (28	Sepsis (28	TRUE	igd	0.0154	4.02E-06	248	M34322.m	TRUE	reported	16GdG9	2	TRUE	0.078942	21.08428
1792	rs2969612	T	C	T	C	-0.0496	0.066292	0.1583	0.159536	FALSE	FALSE	FALSE	ieu-b-508	4	1.28E+08	0.04583	486484	0.14804	Sepsis (28	Sepsis (28	Sepsis (28	TRUE	igd	0.0108	4.75E-06	5502	M15677.m	TRUE	reported	1CuWi9	2	TRUE	0.003819	21.08424
3839	rs1116690	A	G	A	G	0.0124	0.034285	0.3594	0.372264	FALSE	FALSE	FALSE	ieu-b-508	8	1.36E+08	0.034613	486484	0.321921	Sepsis (28	Sepsis (28	Sepsis (28	TRUE	igd	0.0027	5.60E-06	5211	M32651.m	TRUE	reported	DeuPwv	2	TRUE	0.004031	21.08381
3840	rs1271934	A	G	A	G	0.0124	0.091901	0.3179	0.317123	FALSE	FALSE	FALSE	ieu-b-508	5	1.25E+08	0.035864	486484	0.010392	Sepsis (28	Sepsis (28	Sepsis (28	TRUE	igd	0.0027	6.09E-06	5211	M32651.m	TRUE	reported	DeuPwv	2	TRUE	0.004031	21.08381
3857	rs7924340	T	C	T	C	0.0124	-0.04315	0.4691	0.469048	FALSE	FALSE	FALSE	ieu-b-508	11	46164157	0.033582	486484	0.198861	Sepsis (28	Sepsis (28	Sepsis (28	TRUE	igd	0.0027	4.63E-06	5211	M32651.m	TRUE	reported	DeuPwv	2	TRUE	0.004031	21.08381
8110	rs6956438	A	G	A	G	-0.4219	0.014229	0.8991	0.890132	FALSE	FALSE	FALSE	ieu-b-508	7	7856669	0.054142	486484	0.792702	Sepsis (28	Sepsis (28	Sepsis (28	TRUE	igd	0.0917	4.20E-06	488	M36097.m	TRUE	reported	RHr18S	2	TRUE	0.041574	21.08127
5438	rs1160269	T	C	T	C	-0.0326	-0.01456	0.4442	0.449055	FALSE	FALSE	FALSE	ieu-b-508	8	5516034	0.03352	486484	0.664122	Sepsis (28	Sepsis (28	Sepsis (28	TRUE	igd	0.0071	3.70E-06	5185	M33390.m	TRUE	reported	t0EYdH	2	TRUE	0.00405	21.07419
5152	rs1252900	A	G	A	G	0.146	-0.05397	0.9751	0.971914	FALSE	FALSE	FALSE	ieu-b-508	6	18980752	0.101725	486484	0.595703	Sepsis (28	Sepsis (28	Sepsis (28	TRUE	igd	0.0318	4.39E-06	5716	M33192.m	TRUE	reported	CyfjKE	2	TRUE	0.003674	21.0717
5261	rs1692364	T	C	T	C	0.0202	-0.05488	0.1285	0.125212	FALSE	FALSE	FALSE	ieu-b-508	12	21451395	0.051284	486484	0.284581	Sepsis (28	Sepsis (28	Sepsis (28	TRUE	igd	0.0044	4.57E-06	7364	M33230.m	TRUE	reported	nzcTOn	2	TRUE	0.002854	21.07072
7522	rs1703298	T	G	T	G	0.0303	0.011926	0.103	0.112001	FALSE	FALSE	FALSE	ieu-b-508	3	10529069	0.054266	486484	0.826054	Sepsis (28	Sepsis (28	Sepsis (28	TRUE	igd	0.0066	4.70E-06	7291	M35255.m	TRUE	reported	ZDjmUf	2	TRUE	0.002882	21.07066
2835	rs404632	T	C	T	C	-0.0202	-0.02538	0.1335	0.139767	FALSE	FALSE	FALSE	ieu-b-508	19	3114545	0.048088	486484	0.597632	Sepsis (28	Sepsis (28	Sepsis (28	TRUE	igd	0.0044	5.36E-06	7180	M27513.m	TRUE	reported	o9Ff2u	2	TRUE	0.002927	21.07058
7079	rs2453573	T	C	T	C	-0.0202	-0.04879	0.8177	0.807584	FALSE	FALSE	FALSE	ieu-b-508	17	19452656	0.042254	486484	0.248208	Sepsis (28	Sepsis (28	Sepsis (28	TRUE	igd	0.0044	3.68E-06	7119	M34481.m	TRUE	reported	mXdL0W	2	TRUE	0.002952	21.07053
4405	rs1326510	T	C	T	C	0.0271	-0.05793	0.6244	0.62366	FALSE	FALSE	FALSE	ieu-b-508	8	1.07E+08	0.034462	486484	0.092756	Sepsis (28	Sepsis (28	Sepsis (28	TRUE	igd	0.0059	4.73E-06	1550	M32869.m	TRUE	reported	Rvim1n	2	TRUE	0.013429	21.07045
8305	rs9931063	A	G	A	G	0.0395	-0.03603	0.8768	0.831544	FALSE	FALSE	FALSE	ieu-b-508	16	81037883	0.044697	486484	0.420191	Sepsis (28	Sepsis (28	Sepsis (28	TRUE	igd	0.0086	4.32E-06	1636	M36559.m	TRUE	reported	g6l9hg	2	TRUE	0.012731	21.07007
1319	rs1249696	A	G	A	G	0.0202	0.037537	0.7844	0.778484	FALSE	FALSE	FALSE	ieu-b-508	3	24791310	0.040251	486484	0.351047	Sepsis (28	Sepsis (28	Sepsis (28	TRUE	igd	0.0044	4.28E-06	6252	M02137.m	TRUE	reported	iRGqBE	2	TRUE	0.00336	21.0697
3018	rs1156455	A	G	A	G	0.0303	0.0719	0.4808	0.474306	FALSE	FALSE	FALSE	ieu-b-508	19	48592183	0.033437	486484	0.031528	Sepsis (28	Sepsis (28	Sepsis (28	TRUE	igd	0.0066	3.87E-06	5229	M31548.m	TRUE	reported	bgRTBS	2	TRUE	0.004015	21.06838
1876	rs7243187	A	C	A	C	-0.0455	0.016515	0.2661	0.271123	FALSE	FALSE	FALSE	ieu-b-508	18	38803713	0.037512	486484	0.659748	Sepsis (28	Sepsis (28	Sepsis (28	TRUE	igd	0.0099	3.87E-06	773	M15958.m	TRUE	reported	3HF6If	2	TRUE	0.026599	21.06819
6379	rs4668061	A	G	A	G	-0.0583	-0.1654	0.0379	0.051471	FALSE	FALSE	FALSE	ieu-b-508	2	1.69E+08	0.075416	486484	0.028299	Sepsis (28	Sepsis (28	Sepsis (28	TRUE	igd	0.0127	4.67E-06	7322	M33968.m	TRUE	reported	sgVOvT	2	TRUE	0.00287	21.0674
7020	rs1168366	A	G	A	G	0.0202	-0.01507	0.6195	0.612699	FALSE	FALSE	FALSE	ieu-b-508	2	2.28E+08	0.034531	486484	0.662598	Sepsis (28	Sepsis (28	Sepsis (28	TRUE	igd	0.0044	4.38E-06	4442	M34441.m	TRUE	reported	UpgxQm	2	TRUE	0.004722	21.06696
6227	rs1027200	T	C	T	C	0.101	-0.06468	0.022	0.097657	FALSE	FALSE	FALSE	ieu-b-508	7	9018968	0.067433	486484	0.33748	Sepsis (28	Sepsis (28	Sepsis (28	TRUE	igd	0.022	4.41E-06	4351	M33910.m	TRUE	reported	YwFZg1	2	TRUE	0.004821	21.06676
1796	rs9915627	A	C	A	C	0.1117	0.016569	0.1829	0.188411	FALSE	FALSE	FALSE	ieu-b-508	17	13675618	0.052154	486484	0.750717	Sepsis (28	Sepsis (28	Sepsis (28	TRUE	igd	0.0243	4.29E-06	658	M15677.m	TRUE	reported	1CuWi9	2	TRUE	0.031113	21.0655
3027	rs4957657	T	C	T	C	0.0482	0.022882	0.1104	0.103087	FALSE	FALSE	FALSE	ieu-b-508	5	1.12E+08	0.055542	486484	0.680353	Sepsis (28	Sepsis (28	Sepsis (28	TRUE	igd	0.0105	4.53E-06	5229	M31548.m	TRUE	reported	bgRTBS	2	TRUE	0.004014	21.06441
657	rs1023493	A	G	A	G	0.028	0.049828	0.035	0.056353	FALSE	FALSE	FALSE	ieu-b-508	7	19607850	0.072468	486484	0.491716	Sepsis (28	Sepsis (28	Sepsis (28	TRUE	igd	0.0061	4.88E-06	5687	M01284.m	TRUE	reported	E4hc9c	2	TRUE	0.003691	21.0622
8178	rs4897622	A	G	A	G	-0.0574	-0.041	0.1147	0.118783	FALSE	FALSE	FALSE	ieu-b-508	6	1.33E+08	0.051602	486484	0.426889	Sepsis (28	Sepsis (28	Sepsis (28	TRUE	igd	0.0125	4.10E-06	1657	M36134.m	TRUE	reported	ekMsfG	2	TRUE	0.012566	21.06101
3937	rs4857879	A	C	A	C	0.0225	0.062038	0.8382	0.84814	FALSE	FALSE	FALSE	ieu-b-508	3	1.28E+08	0.046558	486484	0.182704	Sepsis (28	Sepsis (28	Sepsis (28	TRUE	igd	0.0049	3.56E-06	1757	M32698.m	TRUE	reported	srqncz	2	TRUE	0.011858	21.06096
6964	rs6911611	A	T	A	T	0.1861	0.032532	0.9734	0.970406	FALSE	TRUE	FALSE	ieu-b-508	6	1.36E+08	0.098528	486484	0.741268	Sepsis (28	Sepsis (28	Sepsis (28	TRUE	igd	0.0405	4.25E-06	777	M34404.m	TRUE	reported	8FMHfo	2	TRUE	0.026456	21.06024
3069	rs2553679	A	T	A	T	0.0537	-0.0579	0.8642	0.860246	FALSE	TRUE	FALSE	ieu-b-508	8	68645159	0.048525	486484	0.232829	Sepsis (28	Sepsis (28	Sepsis (28	TRUE	igd	0.0117	4.25E-06	7363	M31787.m	TRUE	reported	XMp8YI	2	TRUE	0.002853	21.06002
3333	rs6957675	T	C	T	C	-0.0179	-0.03803	0.2559	0.256918	FALSE	FALSE	FALSE	ieu-b-508	7	1.1E+08	0.038504	486484	0.323353	Sepsis (28	Sepsis (28	Sepsis (28	TRUE	igd	0.0039	3.51E-06	7358	M32405.m	TRUE	reported	uzomIQ	2	TRUE	0.002855	21.06002
1406	rs848767	T	C	T	C	-0.0179	-0.00966	0.0696	0.076785	FALSE	FALSE	FALSE	ieu-b-508	22	49718065	0.062519	486484	0.877251	Sepsis (28	Sepsis (28	Sepsis (28	TRUE	igd	0.0039	3.46E-06	7037	M02734.m	TRUE	reported	0y0dDD	2	TRUE	0.002985	21.05976
1430	rs4676313	T	C	T	C	0.0179	-0.04207	0.8778	0.892913	FALSE	FALSE	FALSE	ieu-b-508	2	1.08E+08	0.053738	486484	0.433683	Sepsis (28	Sepsis (28	Sepsis (28	TRUE	igd	0.0039	3.54E-06	6941	M03127.m	TRUE	reported	JscEZw	2	TRUE	0.003026	21.05968
5957	rs1077957	A	G	A	G	0.0427	0.029191	0.209	0.214016	FALSE	FALSE	FALSE	ieu-b-508	1	2.12E+08	0.040685	486484	0.473064	Sepsis (28	Sepsis (28	Sepsis (28	TRUE	igd	0.0093	4.07E-06	1860	M33658.m	TRUE	reported	9QszGO	2	TRUE	0.011207	21.05827
6340	rs6530980	T	C	T	C	0.0491	-0.23223	0.0364	0.034876	FALSE	FALSE	FALSE	ieu-b-508	8	12633235	0.091826	486484	0.011439	Sepsis (28	Sepsis (28	Sepsis (28	TRUE	igd	0.0107	4.02E-06	6998	M33957.m	TRUE	reported	mrKpMv	2	TRUE	0.003	21.05093
8290	rs1192152	A	G	A	G	-0.0303	0.030125	0.1844	0.185124	FALSE	FALSE	FALSE	ieu-b-508	3	14568071	0.043142	486484	0.485009	Sepsis (28	Sepsis (28	Sepsis (28	TRUE	igd	0.0066	4.99E-06	1636	M36559.m	TRUE	reported	g6l9hg	2	TRUE	0.012719	21.05068
2848	rs1252704	T	C	T	C	0.1239	0.006727	0.0167	0.036437	FALSE	FALSE	FALSE	ieu-b-508	6	50666968	0.089242	486484	0.939917	Sepsis (28	Sepsis (28	Sepsis (28	TRUE	igd	0.027	4.61E-06	5673	M27531.m	TRUE	reported	v01zDr	2	TRUE	0.003698	21.05048
7029	rs640631	T	C	T	C	0.0335	-0.00912	0.904	0.945066	FALSE	FALSE	FALSE	ieu-b-508	17	42086180	0.073123	486484	0.900733	Sepsis (28	Sepsis (28	Sepsis (28	TRUE	igd	0.0073	4.42E-06	4442	M34441.m	TRUE	reported	UpgxQm	2	TRUE	0.004719	21.04982
6092	rs1298686	T	C	T	C	0.0404	0.005255	0.2858	0.295209	FALSE	FALSE	FALSE	ieu-b-508	2	43132547	0.036641	486484	0.885965	Sepsis (28	Sepsis (28	Sepsis (28	TRUE	igd	0.0088	5.01E-06	1428	M33864.m	TRUE	reported	yr5h52	2	TRUE	0.014545	21.04693
2138	rs1298445	T	C	T	C	-0.0413	-0.0432	0.3058	0.314267	FALSE	FALSE	FALSE	ieu-b-508	19	54021597	0.03597	486484	0.229753	Sepsis (28	Sepsis (28	Sepsis (28	TRUE	igd	0.009	4.71E-06	3783	M18497.m	TRUE	reported	90JFPc	2	TRUE	0.005536	21.04677
1085	rs1331801	T	C	T	C	-0.0078	0.014417	0.8378	0.809101	FALSE	FALSE	FALSE	ieu-b-508	3	1.07E+08	0.042676	486484	0.735495	Sepsis (28	Sepsis (28	Sepsis (28	TRUE	igd	0.0017	8.36E-06	7371	M01604.m	TRUE	reported	hE3l2k	2	TRUE	0.002848	21.04619
400	rs1020731	T	G	T	G	0.0078	-0.02288	0.2432	0.247887	FALSE	FALSE	FALSE	ieu-b-508	2	21196346	0.038725	486484	0.554654	Sepsis (28	Sepsis (28	Sepsis (28	TRUE	igd	0.0017	4.79E-06	7365	M00063.m	TRUE	reported	Hdsv1L	2	TRUE	0.00285	21.04619
406	rs1124515	A	G	A	G	-0.0078	-0.07185	0.2539	0.24816	FALSE	FALSE	FALSE	ieu-b-508	11	1.26E+08	0.038589	486484	0.062622	Sepsis (28	Sepsis (28	Sepsis (28	TRUE	igd	0.0017	6.01E-06	7365	M00063.m	TRUE	reported	Hdsv1L	2	TRUE	0.00285	21.04619
416	rs1729344	T	C	T	C	0.0078	0.018144	0.7718	0.771873	FALSE	FALSE	FALSE	ieu-b-508	15	67437863	0.039688	486484	0.647548	Sepsis (28	Sepsis (28	Sepsis (28	TRUE	igd	0.0017	8.09E-06	7365	M00063.m	TRUE	reported	Hdsv1L	2	TRUE	0.00285	21.04619
417	rs1768356	T	C	T	C	0.0078	0.008729	0.2146	0.216779	FALSE	FALSE	FALSE	ieu-b-508	8	1.34E+08	0.040483	486484	0.829285	Sepsis (28	Sepsis (28	Sepsis (28	TRUE	igd	0.0017	7.57E-06	7365	M00063.m	TRUE	reported	Hdsv1L	2	TRUE	0.00285	21.04619
421	rs2050223	A	G	A	G	-0.0078	-0.02335	0.2016	0.200176	FALSE	FALSE	FALSE	ieu-b-508	20	15701814	0.041684	486484	0.575421	Sepsis (28	Sepsis (28	Sepsis (28	TRUE	igd	0.0017	7.22E-06	7365	M00063.m	TRUE	reported	Hdsv1L	2	TRUE	0.00285	21.04619
423	rs2259571	T	G	T	G	-0.0078	0.045066	0.6893	0.652881	FALSE	FALSE	FALSE	ieu-b-508	6	31583827	0.035039	486484	0.198392	Sepsis (28	Sepsis (28	Sepsis (28	TRUE	igd	0.0017	2.86E-06	7365	M00063.m	TRUE	reported	Hdsv1L	2	TRUE	0.00285	21.04619
425	rs2869804	A	G	A	G	0.0078	-0.01929	0.2489	0.25263	FALSE	FALSE	FALSE	ieu-b-508	18	14141133	0.038642	486484	0.617662	Sepsis (28	Sepsis (28	Sepsis (28	TRUE	igd	0.0017	6.50E-06	7365	M00063.m	TRUE	reported	Hdsv1L	2	TRUE	0.00285	21.04619
723	rs2383208	A	G	A	G	0.0078	-0.03181	0.8223	0.821932	FALSE	FALSE	FALSE	ieu-b-508	9	22132076	0.043533	486484	0.464986	Sepsis (28	Sepsis (28	Sepsis (28	TRUE	igd	0.0017	8.79E-06	7363	M01301.m	TRUE	reported	w8TL71	2	TRUE	0.002851	21.04618
822	rs3087751	A	C	A	C	0.0078	0.003815	0.423	0.44744	FALSE	FALSE	FALSE	ieu-b-508	20	1147410	0.033487	486484	0.909289	Sepsis (28	Sepsis (28	Sepsis (28	TRUE	igd	0.0017	5.23E-06	7355	M01358.m	TRUE	reported	kSPQgd	2	TRUE	0.002854	21.04618
824	rs3793900	A	C	A	C	-0.0078	-0.04324	0.6337	0.6407	FALSE	FALSE	FALSE	ieu-b-508	10	88718833	0.034924	486484	0.215686	Sepsis (28	Sepsis (28	Sepsis (28	TRUE	igd	0.0017	5.73E-06	7355	M01358.m	TRUE	reported	kSPQgd	2	TRUE	0.002854	21.04618
833	rs7681360	A	G	A	G	0.0078	0.031609	0.5825	0.584412	FALSE	FALSE	FALSE	ieu-b-508	4	57973165	0.033813	486484	0.349886	Sepsis (28	Sepsis (28	Sepsis (28	TRUE	igd	0.0017	4.19E-06	7355	M01358.m	TRUE	reported	kSPQgd	2	TRUE	0.002854	21.04618
2058	rs1765261	A	G	A	G	-0.0078	-0.02503	0.5436	0.541141	FALSE	FALSE	FALSE	ieu-b-508	3	7371919	0.033813	486484	0.459173	Sepsis (28	Sepsis (28	Sepsis (28	TRUE	igd	0.0017	7.29E-06	7354	M18369.m	TRUE	reported	ykBjIT	2	TRUE	0.002854	21.04618
2259	rs4541491	A	C	A	C	-0.0078	-0.0175	0.6185	0.61753	FALSE	FALSE	FALSE	ieu-b-508	4	82604912	0.034329	486484	0.61028	Sepsis (28	Sepsis (28	Sepsis (28	TRUE	igd	0.0017	6.60E-06	7354	M19934.m	TRUE	reported	HtcWcQ	2	TRUE	0.002854	21.04618
776	rs1214752	A	G	A	G	0.0078	-0.04668	0.4485	0.456497	FALSE	FALSE	FALSE	ieu-b-508	14	33554293	0.033527	486484	0.163834	Sepsis (28	Sepsis (28	Sepsis (28	TRUE	igd	0.0017	5.92E-06	7352	M01336.m	TRUE	reported	FAlrya	2	TRUE	0.002855	21.04618
780	rs1335597	A	G	A	G	-0.0078	0.048981	0.5663	0.562125	FALSE	FALSE	FALSE	ieu-b-508	5	32984015	0.033634	486484	0.145313	Sepsis (28	Sepsis (28	Sepsis (28	TRUE	igd	0.0017	3.94E-06	7352	M01336.m	TRUE	reported	FAlrya	2	TRUE	0.002855	21.04618
783	rs2931383	T	G	T	G	0.0078	-0.02514	0.5816	0.574748	FALSE	FALSE	FALSE	ieu-b-508	3	19939707	0.033731	486484	0.456139	Sepsis (28	Sepsis (28	Sepsis (28	TRUE	igd	0.0017	4.68E-06	7352	M01336.m	TRUE	reported	FAlrya	2	TRUE	0.002855	21.04618
735	rs1683931	T	C	T	C	-0.0078	0.091352	0.8726	0.867981	FALSE	FALSE	FALSE	ieu-b-508	3	1.32E+08	0.049256	486484	0.063649	Sepsis (28	Sepsis (28	Sepsis (28	TRUE	igd	0.0017	2.75E-06	7347	M01302.m	TRUE	reported	sNp2lC	2	TRUE	0.002857	21.04617
741	rs3740996	A	G	A	G	0.0078	-0.06042	0.1208	0.127081	FALSE	FALSE	FALSE	ieu-b-508	11	5701281	0.049941	486484	0.226372	Sepsis (28	Sepsis (28	Sepsis (28	TRUE	igd	0.0017	4.33E-06	7347	M01302.m	TRUE	reported	sNp2lC	2	TRUE	0.002857	21.04617
748	rs6960830	T	C	T	C	0.0078	-0.06909	0.8851	0.875592	FALSE	FALSE	FALSE	ieu-b-508	7	1.56E+08	0.050725	486484	0.17317	Sepsis (28	Sepsis (28	Sepsis (28	TRUE	igd	0.0017	4.16E-06	7347	M01302.m	TRUE	reported	sNp2lC	2	TRUE	0.002857	21.04617
1686	rs1049024	A	G	A	G	0.0078	0.019126	0.8318	0.826663	FALSE	FALSE	FALSE	ieu-b-508	2	50361463	0.044128	486484	0.664704	Sepsis (28	Sepsis (28	Sepsis (28	TRUE	igd	0.0017	5.17E-06	7345	M15488.m	TRUE	reported	SPriBO	2	TRUE	0.002858	21.04617
1696	rs3904667	A	T	A	T	-0.0078	-0.02172	0.2059	0.204351	FALSE	TRUE	FALSE	ieu-b-508	13	63486561	0.041298	486484	0.598913	Sepsis (28	Sepsis (28	Sepsis (28	TRUE	igd	0.0017	3.20E-06	7345	M15488.m	TRUE	reported	SPriBO	2	TRUE	0.002858	21.04617
7696	rs1095516	A	G	A	G	0.0078	-0.03194	0.7713	0.784825	FALSE	FALSE	FALSE	ieu-b-508	8	99377659	0.04069	486484	0.43244	Sepsis (28	Sepsis (28	Sepsis (28	TRUE	igd	0.0017	4.70E-06	7345	M35397.m	TRUE	reported	Y56mZr	2	TRUE	0.002858	21.04617
7720	rs6998531	T	C	T	C	0.0078	-0.0171	0.2087	0.203556	FALSE	FALSE	FALSE	ieu-b-508	8	31237833	0.041378	486484	0.679426	Sepsis (28	Sepsis (28	Sepsis (28	TRUE	igd	0.0017	4.34E-06	7345	M35397.m	TRUE	reported	Y56mZr	2	TRUE	0.002858	21.04617
4417	rs1005824	T	C	T	C	-0.0078	-0.01513	0.4208	0.42116	FALSE	FALSE	FALSE	ieu-b-508	5	65909196	0.034312	486484	0.659301	Sepsis (28	Sepsis (28	Sepsis (28	TRUE	igd	0.0017	5.47E-06	7344	M32910.m	TRUE	reported	2OCBKh	2	TRUE	0.002858	21.04617
4444	rs3856584	T	C	T	C	0.0078	0.044444	0.3934	0.386015	FALSE	FALSE	FALSE	ieu-b-508	3	73806887	0.035043	486484	0.204705	Sepsis (28	Sepsis (28	Sepsis (28	TRUE	igd	0.0017	6.62E-06	7344	M32910.m	TRUE	reported	2OCBKh	2	TRUE	0.002858	21.04617
4454	rs7255053	A	C	A	C	0.0078	-0.07536	0.6266	0.635295	FALSE	FALSE	FALSE	ieu-b-508	19	48508641	0.036436	486484	0.038615	Sepsis (28	Sepsis (28	Sepsis (28	TRUE	igd	0.0017	6.36E-06	7344	M32910.m	TRUE	reported	2OCBKh	2	TRUE	0.002858	21.04617
4463	rs9371494	A	G	A	G	0.0078	0.053717	0.6805	0.684679	FALSE	FALSE	FALSE	ieu-b-508	6	1.51E+08	0.036035	486484	0.136044	Sepsis (28	Sepsis (28	Sepsis (28	TRUE	igd	0.0017	7.38E-06	7344	M32910.m	TRUE	reported	2OCBKh	2	TRUE	0.002858	21.04617
1764	rs1196687	T	C	T	C	0.0078	-0.01769	0.6211	0.614948	FALSE	FALSE	FALSE	ieu-b-508	2	1.5E+08	0.034321	486484	0.606235	Sepsis (28	Sepsis (28	Sepsis (28	TRUE	igd	0.0017	5.82E-06	7331	M15676.m	TRUE	reported	s1NfKN	2	TRUE	0.002863	21.04616
4216	rs2870709	T	C	T	C	0.039	-0.03204	0.1734	0.192198	FALSE	FALSE	FALSE	ieu-b-508	4	94410573	0.042395	486484	0.449835	Sepsis (28	Sepsis (28	Sepsis (28	TRUE	igd	0.0085	4.20E-06	7330	M32786.m	TRUE	reported	rwF0co	2	TRUE	0.002864	21.04616
1298	rs4768769	T	C	T	C	-0.0078	-0.00034	0.6012	0.592335	FALSE	FALSE	FALSE	ieu-b-508	12	47423032	0.033937	486484	0.992098	Sepsis (28	Sepsis (28	Sepsis (28	TRUE	igd	0.0017	6.64E-06	7325	M02132.m	TRUE	reported	uJFm2t	2	TRUE	0.002866	21.04616
4047	rs1273066	A	G	A	G	0.0078	-0.09782	0.6775	0.681433	FALSE	FALSE	FALSE	ieu-b-508	1	96683642	0.035966	486484	0.006535	Sepsis (28	Sepsis (28	Sepsis (28	TRUE	igd	0.0017	7.23E-06	7318	M32740.m	TRUE	reported	YLshfP	2	TRUE	0.002868	21.04615
4056	rs1992235	A	G	A	G	-0.0078	0.025809	0.3191	0.33093	FALSE	FALSE	FALSE	ieu-b-508	2	2.16E+08	0.035588	486484	0.468313	Sepsis (28	Sepsis (28	Sepsis (28	TRUE	igd	0.0017	7.66E-06	7318	M32740.m	TRUE	reported	YLshfP	2	TRUE	0.002868	21.04615
4075	rs6545816	A	C	A	C	-0.0078	-0.01155	0.4566	0.456945	FALSE	FALSE	FALSE	ieu-b-508	2	60714861	0.033403	486484	0.729552	Sepsis (28	Sepsis (28	Sepsis (28	TRUE	igd	0.0017	6.06E-06	7318	M32740.m	TRUE	reported	YLshfP	2	TRUE	0.002868	21.04615
4079	rs743760	C	G	C	G	-0.0078	-0.0352	0.3375	0.348797	FALSE	TRUE	FALSE	ieu-b-508	13	33071127	0.035198	486484	0.317258	Sepsis (28	Sepsis (28	Sepsis (28	TRUE	igd	0.0017	7.18E-06	7318	M32740.m	TRUE	reported	YLshfP	2	TRUE	0.002868	21.04615
4082	rs890498	C	G	C	G	-0.0078	-0.04754	0.6478	0.662217	FALSE	TRUE	FALSE	ieu-b-508	15	42111298	0.035356	486484	0.178791	Sepsis (28	Sepsis (28	Sepsis (28	TRUE	igd	0.0017	7.23E-06	7318	M32740.m	TRUE	reported	YLshfP	2	TRUE	0.002868	21.04615
1583	rs2705962	A	G	A	G	0.0078	0.067504	0.8068	0.823108	FALSE	FALSE	FALSE	ieu-b-508	4	1.79E+08	0.043674	486484	0.122191	Sepsis (28	Sepsis (28	Sepsis (28	TRUE	igd	0.0017	4.63E-06	7314	M12593.m	TRUE	reported	IBXgSU	2	TRUE	0.00287	21.04615
1584	rs6129758	T	G	T	G	0.0078	0.035296	0.843	0.839804	FALSE	FALSE	FALSE	ieu-b-508	20	39743921	0.045469	486484	0.437602	Sepsis (28	Sepsis (28	Sepsis (28	TRUE	igd	0.0017	7.88E-06	7314	M12593.m	TRUE	reported	IBXgSU	2	TRUE	0.00287	21.04615
2771	rs9836183	T	C	T	C	-0.0078	-0.02457	0.2976	0.318458	FALSE	FALSE	FALSE	ieu-b-508	3	1.11E+08	0.035985	486484	0.49483	Sepsis (28	Sepsis (28	Sepsis (28	TRUE	igd	0.0017	6.82E-06	7303	M27256.m	TRUE	reported	mJwzsi	2	TRUE	0.002874	21.04614
1064	rs2019107	T	C	T	C	-0.0078	0.114549	0.6257	0.584142	FALSE	FALSE	FALSE	ieu-b-508	19	1181717	0.034023	486484	0.000761	Sepsis (28	Sepsis (28	Sepsis (28	TRUE	igd	0.0017	6.66E-06	7271	M01585.m	TRUE	reported	M7QiSM	2	TRUE	0.002887	21.04611
8207	rs1449969	A	G	A	G	-0.0156	0.05176	0.7512	0.715033	FALSE	FALSE	FALSE	ieu-b-508	2	23379606	0.037127	486484	0.163278	Sepsis (28	Sepsis (28	Sepsis (28	TRUE	igd	0.0034	5.28E-06	7184	M36300.m	TRUE	reported	ZjeC9m	2	TRUE	0.002922	21.04604
1235	rs1085173	A	G	A	G	0.0078	-0.07531	0.6345	0.645826	FALSE	FALSE	FALSE	ieu-b-508	15	64216247	0.035024	486484	0.031545	Sepsis (28	Sepsis (28	Sepsis (28	TRUE	igd	0.0017	6.05E-06	7136	M01769.m	TRUE	reported	rismUZ	2	TRUE	0.002941	21.046
1251	rs3095571	A	G	A	G	0.0078	0.009915	0.4465	0.439448	FALSE	FALSE	FALSE	ieu-b-508	16	53544993	0.033778	486484	0.769106	Sepsis (28	Sepsis (28	Sepsis (28	TRUE	igd	0.0017	6.74E-06	7136	M01769.m	TRUE	reported	rismUZ	2	TRUE	0.002941	21.046
1257	rs6539409	T	G	T	G	-0.0078	0.038586	0.5663	0.564176	FALSE	FALSE	FALSE	ieu-b-508	12	1.08E+08	0.033662	486484	0.251673	Sepsis (28	Sepsis (28	Sepsis (28	TRUE	igd	0.0017	4.95E-06	7136	M01769.m	TRUE	reported	rismUZ	2	TRUE	0.002941	21.046
1263	rs7915877	T	C	T	C	-0.0078	0.010458	0.5509	0.567624	FALSE	FALSE	FALSE	ieu-b-508	10	78727967	0.033701	486484	0.756312	Sepsis (28	Sepsis (28	Sepsis (28	TRUE	igd	0.0017	4.42E-06	7136	M01769.m	TRUE	reported	rismUZ	2	TRUE	0.002941	21.046
5351	rs2830527	T	C	T	C	0.0078	0.016717	0.2444	0.246403	FALSE	FALSE	FALSE	ieu-b-508	21	28166355	0.039099	486484	0.668975	Sepsis (28	Sepsis (28	Sepsis (28	TRUE	igd	0.0017	5.51E-06	7118	M33380.m	TRUE	reported	OHRfl5	2	TRUE	0.002949	21.04599
8255	rs1173021	T	C	T	C	-0.0078	-0.01832	0.495	0.505273	FALSE	FALSE	FALSE	ieu-b-508	4	1.09E+08	0.033468	486484	0.584207	Sepsis (28	Sepsis (28	Sepsis (28	TRUE	igd	0.0017	3.42E-06	7092	M36552.m	TRUE	reported	5CxMJz	2	TRUE	0.00296	21.04597
8267	rs6535384	A	G	A	G	0.0078	0.005014	0.5194	0.52253	FALSE	FALSE	FALSE	ieu-b-508	4	83639493	0.033426	486484	0.880768	Sepsis (28	Sepsis (28	Sepsis (28	TRUE	igd	0.0017	4.14E-06	7092	M36552.m	TRUE	reported	5CxMJz	2	TRUE	0.00296	21.04597
1386	rs1772037	T	C	T	C	-0.0078	-0.04384	0.4737	0.472843	FALSE	FALSE	FALSE	ieu-b-508	7	22391763	0.033375	486484	0.188958	Sepsis (28	Sepsis (28	Sepsis (28	TRUE	igd	0.0017	6.33E-06	7037	M02734.m	TRUE	reported	0y0dDD	2	TRUE	0.002983	21.04592
7625	rs1015501	A	G	A	G	-0.0078	-0.00333	0.3436	0.350826	FALSE	FALSE	FALSE	ieu-b-508	3	1.13E+08	0.035033	486484	0.924195	Sepsis (28	Sepsis (28	Sepsis (28	TRUE	igd	0.0017	7.23E-06	5768	M35326.m	TRUE	reported	jRytyJ	2	TRUE	0.003637	21.0446
8133	rs951918	A	G	A	G	0.0546	0.02093	0.8704	0.850105	FALSE	FALSE	FALSE	ieu-b-508	5	1.23E+08	0.04677	486484	0.654511	Sepsis (28	Sepsis (28	Sepsis (28	TRUE	igd	0.0119	4.67E-06	5696	M36099.m	TRUE	reported	bFHsTx	2	TRUE	0.003682	21.04451
7211	rs8178281	A	G	A	G	-0.0849	-0.0055	0.9728	0.988135	FALSE	FALSE	FALSE	ieu-b-508	17	56343394	0.155099	486484	0.971695	Sepsis (28	Sepsis (28	Sepsis (28	TRUE	igd	0.0185	4.30E-06	2545	M34674.m	TRUE	reported	plxOKz	2	TRUE	0.008207	21.04411
1446	rs2396726	T	C	T	C	-0.0179	0.023794	0.5395	0.512755	FALSE	FALSE	FALSE	ieu-b-508	7	1.14E+08	0.033423	486484	0.476525	Sepsis (28	Sepsis (28	Sepsis (28	TRUE	igd	0.0039	4.52E-06	1759	M03141.m	TRUE	reported	y4mG6m	2	TRUE	0.011834	21.04179
8542	rs666518	T	C	T	C	-0.0523	0.003861	0.0891	0.098842	FALSE	FALSE	FALSE	ieu-b-508	6	5075864	0.055885	486484	0.944914	Sepsis (28	Sepsis (28	Sepsis (28	TRUE	igd	0.0114	4.24E-06	6934	M37190.m	TRUE	reported	tiNuK9	2	TRUE	0.003026	21.0411
4636	rs4784988	T	C	T	C	-0.8149	0.109188	0.0216	0.014575	FALSE	FALSE	FALSE	ieu-b-508	16	58859968	0.140166	486484	0.435985	Sepsis (28	Sepsis (28	Sepsis (28	TRUE	igd	0.1753	3.35E-06	76	M33173.m	TRUE	reported	04fkUQ	2	TRUE	0.221387	21.04083
7501	rs7987737	T	C	T	C	-0.0578	-0.07282	0.986	0.987547	FALSE	FALSE	FALSE	ieu-b-508	13	26867314	0.152167	486484	0.632247	Sepsis (28	Sepsis (28	Sepsis (28	TRUE	igd	0.0126	4.48E-06	7261	M35253.m	TRUE	reported	VboRmj	2	TRUE	0.00289	21.03754
8563	rs1252199	A	G	A	G	0.05	0.201813	0.9559	0.955883	FALSE	FALSE	FALSE	ieu-b-508	5	3833240	0.081015	486484	0.012736	Sepsis (28	Sepsis (28	Sepsis (28	TRUE	igd	0.0109	4.46E-06	7333	M37203.m	TRUE	reported	3gIIlv	2	TRUE	0.002861	21.03626
1723	rs7687295	A	G	A	G	0.0211	-0.04222	0.0249	0.023914	FALSE	FALSE	FALSE	ieu-b-508	4	1.82E+08	0.110864	486484	0.703325	Sepsis (28	Sepsis (28	Sepsis (28	TRUE	igd	0.0046	4.32E-06	7310	M15506.m	TRUE	reported	mVRmZO	2	TRUE	0.00287	21.03441
939	rs6034318	A	G	A	G	0.1083	-0.12271	0.013	0.00889	FALSE	FALSE	FALSE	ieu-b-508	20	15863999	0.177243	486484	0.488738	Sepsis (28	Sepsis (28	Sepsis (28	TRUE	igd	0.0236	4.52E-06	1650	M01508.m	TRUE	reported	go526k	2	TRUE	0.012602	21.03324
2474	rs1078505	T	G	T	G	0.0211	-0.01676	0.1146	0.124114	FALSE	FALSE	FALSE	ieu-b-508	12	74006401	0.05063	486484	0.740563	Sepsis (28	Sepsis (28	Sepsis (28	TRUE	igd	0.0046	4.81E-06	5676	M22032.m	TRUE	reported	xpe96K	2	TRUE	0.003693	21.03276
7905	rs7233515	A	G	A	G	-0.0211	-0.03019	0.3974	0.399321	FALSE	FALSE	FALSE	ieu-b-508	18	44587654	0.034122	486484	0.376297	Sepsis (28	Sepsis (28	Sepsis (28	TRUE	igd	0.0046	4.74E-06	5629	M35669.m	TRUE	reported	gSecuE	2	TRUE	0.003724	21.03269
573	rs1447307	T	G	T	G	-0.0211	0.014634	0.227	0.21696	FALSE	FALSE	FALSE	ieu-b-508	8	94286040	0.040985	486484	0.721044	Sepsis (28	Sepsis (28	Sepsis (28	TRUE	igd	0.0046	5.69E-06	5344	M01107.m	TRUE	reported	EbPV1D	2	TRUE	0.003922	21.0323
5221	rs1000643	T	C	T	C	-0.0211	0.061662	0.2626	0.262391	FALSE	FALSE	FALSE	ieu-b-508	4	4326463	0.037947	486484	0.104177	Sepsis (28	Sepsis (28	Sepsis (28	TRUE	igd	0.0046	4.02E-06	4665	M33221.m	TRUE	reported	8ZrV1r	2	TRUE	0.00449	21.03115
6166	rs1028224	C	G	C	G	0.1019	0.006344	0.1667	0.179898	FALSE	TRUE	FALSE	ieu-b-508	7	57258950	0.043566	486484	0.884216	Sepsis (28	Sepsis (28	Sepsis (28	TRUE	igd	0.0222	4.45E-06	1100	M33885.m	TRUE	reported	uCOy6o	2	TRUE	0.018794	21.03062
7509	rs1775140	T	G	T	G	-0.0477	-0.08275	0.0263	0.024871	FALSE	FALSE	FALSE	ieu-b-508	7	3601075	0.107928	486484	0.443261	Sepsis (28	Sepsis (28	Sepsis (28	TRUE	igd	0.0104	4.52E-06	7100	M35254.m	TRUE	reported	HfVgPn	2	TRUE	0.002954	21.03041
6424	rs1080101	T	C	T	C	0.0156	-0.06343	0.2695	0.303932	FALSE	FALSE	FALSE	ieu-b-508	11	44104222	0.036232	486484	0.079987	Sepsis (28	Sepsis (28	Sepsis (28	TRUE	igd	0.0034	3.72E-06	1768	M34062.m	TRUE	reported	RZyHKP	2	TRUE	0.011767	21.02809
3332	rs6957421	A	G	A	G	-0.0468	0.034865	0.1573	0.172709	FALSE	FALSE	FALSE	ieu-b-508	7	1.24E+08	0.044218	486484	0.430418	Sepsis (28	Sepsis (28	Sepsis (28	TRUE	igd	0.0102	4.57E-06	1766	M32405.m	TRUE	reported	uzomIQ	2	TRUE	0.01178	21.02806
8264	rs329033	A	C	A	C	-0.0133	0.03696	0.8429	0.844522	FALSE	FALSE	FALSE	ieu-b-508	18	9584171	0.046062	486484	0.422331	Sepsis (28	Sepsis (28	Sepsis (28	TRUE	igd	0.0029	4.22E-06	7092	M36552.m	TRUE	reported	5CxMJz	2	TRUE	0.002957	21.02736
8311	rs2499690	A	G	A	G	-0.0766	-0.14575	0.9593	0.97253	FALSE	FALSE	FALSE	ieu-b-508	6	34041418	0.102929	486484	0.156761	Sepsis (28	Sepsis (28	Sepsis (28	TRUE	igd	0.0167	4.37E-06	3346	M36585.m	TRUE	reported	pXZg13	2	TRUE	0.006249	21.0264
6189	rs1252168	A	G	A	G	-0.0133	0.014495	0.6013	0.599064	FALSE	FALSE	FALSE	ieu-b-508	5	97861982	0.033998	486484	0.669846	Sepsis (28	Sepsis (28	Sepsis (28	TRUE	igd	0.0029	5.05E-06	6004	M33892.m	TRUE	reported	w7HgU7	2	TRUE	0.003491	21.02629
3700	rs1341748	T	C	T	C	-0.0266	0.069406	0.8097	0.798796	FALSE	FALSE	FALSE	ieu-b-508	2	2.06E+08	0.041631	486484	0.09548	Sepsis (28	Sepsis (28	Sepsis (28	TRUE	igd	0.0058	5.43E-06	5955	M32593.m	TRUE	reported	6zVl1i	2	TRUE	0.00352	21.02623
4240	rs1043189	A	G	A	G	-0.0454	-0.04571	0.0582	0.082788	FALSE	FALSE	FALSE	ieu-b-508	16	20618902	0.060333	486484	0.448703	Sepsis (28	Sepsis (28	Sepsis (28	TRUE	igd	0.0099	4.92E-06	6169	M32795.m	TRUE	reported	0YBWkh	2	TRUE	0.003397	21.02328
4143	rs1706104	T	C	T	C	-0.0642	-0.01752	0.9677	0.966165	FALSE	FALSE	FALSE	ieu-b-508	13	40833360	0.092695	486484	0.850099	Sepsis (28	Sepsis (28	Sepsis (28	TRUE	igd	0.014	4.71E-06	7250	M32757.m	TRUE	reported	TJV9Xb	2	TRUE	0.002892	21.02297
6903	rs2688892	T	C	T	C	0.0688	0.014308	0.2396	0.243128	FALSE	FALSE	FALSE	ieu-b-508	2	50381744	0.038963	486484	0.713447	Sepsis (28	Sepsis (28	Sepsis (28	TRUE	igd	0.015	4.47E-06	2779	M34384.m	TRUE	reported	fI3hiS	2	TRUE	0.007513	21.02237
8436	rs1688884	T	C	T	C	0.2982	-0.14333	0.0145	0.01478	FALSE	FALSE	FALSE	ieu-b-508	8	39979995	0.138563	486484	0.300959	Sepsis (28	Sepsis (28	Sepsis (28	TRUE	igd	0.065	4.43E-06	1583	M36850.m	TRUE	reported	j6lLja	2	TRUE	0.013121	21.02033
2882	rs1328713	A	G	A	G	0.0656	0.01536	0.8214	0.809836	FALSE	FALSE	FALSE	ieu-b-508	9	1.13E+08	0.042633	486484	0.718646	Sepsis (28	Sepsis (28	Sepsis (28	TRUE	igd	0.0143	4.80E-06	1720	M27716.m	TRUE	reported	N5hToq	2	TRUE	0.012087	21.01988
3621	rs1280416	C	G	C	G	0.0376	-0.02755	0.1235	0.140923	FALSE	TRUE	FALSE	ieu-b-508	11	44899772	0.048229	486484	0.56789	Sepsis (28	Sepsis (28	Sepsis (28	TRUE	igd	0.0082	5.13E-06	7337	M32557.m	TRUE	reported	qR7rZX	2	TRUE	0.002858	21.01985
6299	rs6537038	A	G	A	G	-0.0188	-0.02402	0.4014	0.386346	FALSE	FALSE	FALSE	ieu-b-508	4	1.42E+08	0.035041	486484	0.493011	Sepsis (28	Sepsis (28	Sepsis (28	TRUE	igd	0.0041	4.93E-06	7320	M33941.m	TRUE	reported	cuIqiU	2	TRUE	0.002864	21.01984
6959	rs1091611	T	C	T	C	-0.1021	0.032767	0.8253	0.819601	FALSE	FALSE	FALSE	ieu-b-508	1	2.27E+08	0.043246	486484	0.448642	Sepsis (28	Sepsis (28	Sepsis (28	TRUE	igd	0.0222	4.24E-06	314	M34404.m	TRUE	reported	8FMHfo	2	TRUE	0.063111	21.01699
2492	rs276166	T	G	T	G	-0.0243	-0.04857	0.1052	0.147483	FALSE	FALSE	FALSE	ieu-b-508	4	1.56E+08	0.048664	486484	0.318201	Sepsis (28	Sepsis (28	Sepsis (28	TRUE	igd	0.0053	5.33E-06	7329	M22116.m	TRUE	reported	bjUU6r	2	TRUE	0.00286	21.01562
628	rs10489	A	C	A	C	0.1743	0.040409	0.0243	0.031444	FALSE	FALSE	FALSE	ieu-b-508	3	69151152	0.096627	486484	0.675804	Sepsis (28	Sepsis (28	Sepsis (28	TRUE	igd	0.038	4.60E-06	1731	M01123.m	TRUE	reported	NDTvfD	2	TRUE	0.012008	21.01481
5944	rs3024298	A	C	A	C	0.0729	0.043115	0.9784	0.972733	FALSE	FALSE	FALSE	ieu-b-508	17	43037917	0.103135	486484	0.675912	Sepsis (28	Sepsis (28	Sepsis (28	TRUE	igd	0.0159	4.32E-06	6109	M33638.m	TRUE	reported	hVuSGd	2	TRUE	0.003429	21.01448
7877	rs7706198	A	G	A	G	-0.0344	-0.01651	0.1782	0.195899	FALSE	FALSE	FALSE	ieu-b-508	5	1.12E+08	0.042209	486484	0.695756	Sepsis (28	Sepsis (28	Sepsis (28	TRUE	igd	0.0075	4.55E-06	1750	M35631.m	TRUE	reported	002wka	2	TRUE	0.011879	21.01347
2831	rs2002614	A	G	A	G	0.0651	0.092914	0.0114	0.013168	FALSE	FALSE	FALSE	ieu-b-508	17	60788803	0.144985	486484	0.521617	Sepsis (28	Sepsis (28	Sepsis (28	TRUE	igd	0.0142	4.55E-06	7180	M27513.m	TRUE	reported	o9Ff2u	2	TRUE	0.002919	21.01185
1566	rs4896370	A	G	A	G	0.0555	-0.04719	0.3542	0.407802	FALSE	FALSE	FALSE	ieu-b-508	6	1.39E+08	0.034287	486484	0.168699	Sepsis (28	Sepsis (28	Sepsis (28	TRUE	igd	0.0121	4.19E-06	1543	M12261.m	TRUE	reported	taUUcM	2	TRUE	0.013451	21.01125
7589	rs1001966	A	G	A	G	-0.0344	0.009738	0.441	0.437302	FALSE	FALSE	FALSE	ieu-b-508	4	1.37E+08	0.03374	486484	0.77288	Sepsis (28	Sepsis (28	Sepsis (28	TRUE	igd	0.0075	5.21E-06	1505	M35305.m	TRUE	reported	6ybfdq	2	TRUE	0.013786	21.00955
2006	rs735507	A	G	A	G	-0.1128	0.018719	0.8134	0.788652	FALSE	FALSE	FALSE	ieu-b-508	19	58720360	0.040804	486484	0.646408	Sepsis (28	Sepsis (28	Sepsis (28	TRUE	igd	0.0246	4.60E-06	2433	M18281.m	TRUE	reported	3mLp7z	2	TRUE	0.008568	21.0083
8127	rs9616173	T	C	T	C	-0.0518	-0.01001	0.0924	0.087192	FALSE	FALSE	FALSE	ieu-b-508	22	47345487	0.05927	486484	0.865931	Sepsis (28	Sepsis (28	Sepsis (28	TRUE	igd	0.0113	4.33E-06	7054	M36098.m	TRUE	reported	hs9SZl	2	TRUE	0.00297	21.00775
7030	rs6824135	T	C	T	C	-0.0601	0.017583	0.1356	0.120854	FALSE	FALSE	FALSE	ieu-b-508	4	61290584	0.05123	486484	0.731435	Sepsis (28	Sepsis (28	Sepsis (28	TRUE	igd	0.0131	4.80E-06	997	M34441.m	TRUE	reported	UpgxQm	2	TRUE	0.020675	21.00556
2817	rs5997236	T	C	T	C	0.0298	0.035875	0.4	0.399769	FALSE	FALSE	FALSE	ieu-b-508	22	27760086	0.034077	486484	0.292457	Sepsis (28	Sepsis (28	Sepsis (28	TRUE	igd	0.0065	5.28E-06	2759	M27447.m	TRUE	reported	s1NdyU	2	TRUE	0.007561	21.00346
7	rs7394051	T	C	T	C	0.0055	0.043903	0.7014	0.711452	FALSE	FALSE	FALSE	ieu-b-508	10	1.22E+08	0.036921	486484	0.2344	Sepsis (28	Sepsis (28	Sepsis (28	TRUE	igd	0.0012	2.18E-06	7372	M00053.m	TRUE	reported	4Smluz	2	TRUE	0.002841	21.00125
2099	rs2290140	A	G	A	G	-0.0165	0.048714	0.3486	0.343712	FALSE	FALSE	FALSE	ieu-b-508	2	1.43E+08	0.035251	486484	0.166989	Sepsis (28	Sepsis (28	Sepsis (28	TRUE	igd	0.0036	5.80E-06	7367	M18467.m	TRUE	reported	64PaNn	2	TRUE	0.002843	21.00124
1749	rs1051386	A	G	A	G	0.0055	0.073941	0.2661	0.261722	FALSE	FALSE	FALSE	ieu-b-508	3	1.91E+08	0.037987	486484	0.051594	Sepsis (28	Sepsis (28	Sepsis (28	TRUE	igd	0.0012	7.33E-06	7365	M15650.m	TRUE	reported	NgezyT	2	TRUE	0.002844	21.00124
1753	rs369402	A	G	A	G	-0.0055	-0.01292	0.3892	0.391335	FALSE	FALSE	FALSE	ieu-b-508	5	31696941	0.034167	486484	0.705227	Sepsis (28	Sepsis (28	Sepsis (28	TRUE	igd	0.0012	5.86E-06	7365	M15650.m	TRUE	reported	NgezyT	2	TRUE	0.002844	21.00124
6319	rs3735474	A	G	A	G	-0.0165	-0.06678	0.9341	0.931895	FALSE	FALSE	FALSE	ieu-b-508	7	44713264	0.066282	486484	0.313669	Sepsis (28	Sepsis (28	Sepsis (28	TRUE	igd	0.0036	3.97E-06	7354	M33955.m	TRUE	reported	mhHFnT	2	TRUE	0.002848	21.00123
3127	rs1715449	A	C	A	C	-0.011	-0.1184	0.1953	0.210964	FALSE	FALSE	FALSE	ieu-b-508	7	1.07E+08	0.04148	486484	0.00431	Sepsis (28	Sepsis (28	Sepsis (28	TRUE	igd	0.0024	6.29E-06	7349	M32315.m	TRUE	reported	4igzkL	2	TRUE	0.00285	21.00123
1044	rs1286613	A	C	A	C	0.011	0.058061	0.2805	0.287668	FALSE	FALSE	FALSE	ieu-b-508	13	71781111	0.036902	486484	0.115628	Sepsis (28	Sepsis (28	Sepsis (28	TRUE	igd	0.0024	5.62E-06	7333	M01572.m	TRUE	reported	EOUAWy	2	TRUE	0.002857	21.00122
8561	rs1008223	T	C	T	C	-0.0385	0.167142	0.0735	0.071632	FALSE	FALSE	FALSE	ieu-b-508	1	1.54E+08	0.064946	486484	0.010067	Sepsis (28	Sepsis (28	Sepsis (28	TRUE	igd	0.0084	4.09E-06	7333	M37203.m	TRUE	reported	3gIIlv	2	TRUE	0.002857	21.00122
4055	rs1776158	A	G	A	G	0.011	-0.04642	0.8209	0.80895	FALSE	FALSE	FALSE	ieu-b-508	10	60766127	0.042429	486484	0.273925	Sepsis (28	Sepsis (28	Sepsis (28	TRUE	igd	0.0024	6.91E-06	7318	M32740.m	TRUE	reported	YLshfP	2	TRUE	0.002862	21.0012
898	rs4764034	A	G	A	G	-0.011	-0.04912	0.6869	0.698305	FALSE	FALSE	FALSE	ieu-b-508	12	13960034	0.036377	486484	0.176894	Sepsis (28	Sepsis (28	Sepsis (28	TRUE	igd	0.0024	7.67E-06	7307	M01493.m	TRUE	reported	eLLRt7	2	TRUE	0.002867	21.00119
2744	rs1120115	T	C	T	C	0.022	0.047025	0.0306	0.035472	FALSE	FALSE	FALSE	ieu-b-508	10	86425061	0.091267	486484	0.606382	Sepsis (28	Sepsis (28	Sepsis (28	TRUE	igd	0.0048	5.42E-06	7303	M27256.m	TRUE	reported	mJwzsi	2	TRUE	0.002868	21.00119
6995	rs2043422	A	G	A	G	0.0165	-0.02955	0.2928	0.290757	FALSE	FALSE	FALSE	ieu-b-508	8	1.42E+08	0.036856	486484	0.42276	Sepsis (28	Sepsis (28	Sepsis (28	TRUE	igd	0.0036	4.06E-06	6929	M34416.m	TRUE	reported	q9OtaZ	2	TRUE	0.003023	21.00088
8363	rs3794989	T	C	T	C	0.0165	0.008211	0.3285	0.333407	FALSE	FALSE	FALSE	ieu-b-508	19	15723528	0.035332	486484	0.816221	Sepsis (28	Sepsis (28	Sepsis (28	TRUE	igd	0.0036	4.95E-06	6876	M36754.m	TRUE	reported	f8bkFg	2	TRUE	0.003046	21.00083
2727	rs1429219	A	G	A	G	0.033	-0.00302	0.0353	0.033392	FALSE	FALSE	FALSE	ieu-b-508	2	5937233	0.094763	486484	0.974617	Sepsis (28	Sepsis (28	Sepsis (28	TRUE	igd	0.0072	4.53E-06	6388	M25599.m	TRUE	reported	fRZxwP	2	TRUE	0.003278	21.00037
6587	rs1685327	T	G	T	G	0.0298	0.015205	0.8831	0.876707	FALSE	FALSE	FALSE	ieu-b-508	1	14284642	0.051312	486484	0.766983	Sepsis (28	Sepsis (28	Sepsis (28	TRUE	igd	0.0065	5.04E-06	2278	M34289.m	TRUE	reported	Knp8ry	2	TRUE	0.009142	21.00024
2468	rs674635	T	C	T	C	0.0275	-0.01129	0.0415	0.042184	FALSE	FALSE	FALSE	ieu-b-508	5	39848889	0.083051	486484	0.891864	Sepsis (28	Sepsis (28	Sepsis (28	TRUE	igd	0.006	3.96E-06	6180	M22030.m	TRUE	reported	ZNV0qR	2	TRUE	0.003388	21.00015
1335	rs1095303	T	C	T	C	0.022	0.116407	0.8497	0.844183	FALSE	FALSE	FALSE	ieu-b-508	7	90857574	0.046202	486484	0.011751	Sepsis (28	Sepsis (28	Sepsis (28	TRUE	igd	0.0048	4.12E-06	5791	M02342.m	TRUE	reported	EhZr3g	2	TRUE	0.003614	20.99969
5651	rs1978273	A	G	A	G	-0.0165	-0.02333	0.4362	0.452449	FALSE	FALSE	FALSE	ieu-b-508	2	1.92E+08	0.033453	486484	0.485559	Sepsis (28	Sepsis (28	Sepsis (28	TRUE	igd	0.0036	3.51E-06	5717	M33453.m	TRUE	reported	OzEeLQ	2	TRUE	0.003661	20.9996
7843	rs6942053	C	G	C	G	0.033	-0.08044	0.4176	0.410875	FALSE	TRUE	FALSE	ieu-b-508	6	71077023	0.034054	486484	0.018162	Sepsis (28	Sepsis (28	Sepsis (28	TRUE	igd	0.0072	4.77E-06	5035	M35551.m	TRUE	reported	5Sxswl	2	TRUE	0.004155	20.9986
8637	rs6089828	T	C	T	C	-0.0165	-0.05834	0.6506	0.645915	FALSE	FALSE	FALSE	ieu-b-508	20	61668998	0.034934	486484	0.094923	Sepsis (28	Sepsis (28	Sepsis (28	TRUE	igd	0.0036	5.86E-06	4754	M38150.m	TRUE	reported	u3Ckmr	2	TRUE	0.004399	20.99811
4731	rs1195793	A	G	A	G	-0.2362	0.0539	0.0459	0.063176	FALSE	FALSE	FALSE	ieu-b-508	5	1.24E+08	0.069002	486484	0.434726	Sepsis (28	Sepsis (28	Sepsis (28	TRUE	igd	0.0515	4.58E-06	1017	M33178.m	TRUE	reported	g22WMS	2	TRUE	0.020264	20.99377
2102	rs7005406	T	C	T	C	0.0353	-0.00369	0.8036	0.814562	FALSE	FALSE	FALSE	ieu-b-508	8	1.35E+08	0.043139	486484	0.931854	Sepsis (28	Sepsis (28	Sepsis (28	TRUE	igd	0.0077	5.29E-06	1767	M18467.m	TRUE	reported	64PaNn	2	TRUE	0.011754	20.99308
8052	rs4719788	T	C	T	C	-0.0926	-0.01504	0.0317	0.022708	FALSE	FALSE	FALSE	ieu-b-508	7	25025257	0.111597	486484	0.892804	Sepsis (28	Sepsis (28	Sepsis (28	TRUE	igd	0.0202	4.59E-06	1760	M35977.m	TRUE	reported	NHnUo7	2	TRUE	0.011799	20.99063
6927	rs8069788	T	C	T	C	-0.0486	0.02609	0.4918	0.481973	FALSE	FALSE	FALSE	ieu-b-508	17	2758657	0.033867	486484	0.441083	Sepsis (28	Sepsis (28	Sepsis (28	TRUE	igd	0.0106	4.25E-06	1312	M34390.m	TRUE	reported	wruTbW	2	TRUE	0.01577	20.98932
2842	rs9319010	A	G	A	G	0.1201	0.114429	0.0156	0.012734	FALSE	FALSE	FALSE	ieu-b-508	13	84591199	0.149327	486484	0.443499	Sepsis (28	Sepsis (28	Sepsis (28	TRUE	igd	0.0262	4.44E-06	1738	M27513.m	TRUE	reported	o9Ff2u	2	TRUE	0.011946	20.9886
6194	rs1742061	C	G	C	G	0.0307	0.004139	0.0545	0.063345	FALSE	TRUE	FALSE	ieu-b-508	8	20452094	0.068609	486484	0.951892	Sepsis (28	Sepsis (28	Sepsis (28	TRUE	igd	0.0067	4.16E-06	6004	M33892.m	TRUE	reported	w7HgU7	2	TRUE	0.003485	20.98855
7190	rs1490956	A	G	A	G	0.0362	0.040998	0.8464	0.814513	FALSE	FALSE	FALSE	ieu-b-508	17	27340606	0.046976	486484	0.382805	Sepsis (28	Sepsis (28	Sepsis (28	TRUE	igd	0.0079	4.02E-06	4800	M34539.m	TRUE	reported	puA8ni	2	TRUE	0.004355	20.98853
5114	rs9672326	T	G	T	G	0.155	-0.01374	0.9622	0.978902	FALSE	FALSE	FALSE	ieu-b-508	15	49157606	0.115766	486484	0.905506	Sepsis (28	Sepsis (28	Sepsis (28	TRUE	igd	0.0338	4.44E-06	1017	M33178.m	TRUE	reported	g22WMS	2	TRUE	0.020259	20.98819
2091	rs7927424	A	G	A	G	-0.115	-0.05024	0.0147	0.021186	FALSE	FALSE	FALSE	ieu-b-508	11	1.34E+08	0.117125	486484	0.667969	Sepsis (28	Sepsis (28	Sepsis (28	TRUE	igd	0.0251	4.79E-06	6970	M18394.m	TRUE	reported	Lt9awu	2	TRUE	0.003003	20.98571
2510	rs4799200	A	G	A	G	0.0252	-0.0641	0.0528	0.056035	FALSE	FALSE	FALSE	ieu-b-508	18	76549058	0.072688	486484	0.377883	Sepsis (28	Sepsis (28	Sepsis (28	TRUE	igd	0.0055	3.79E-06	5702	M22130.m	TRUE	reported	YDbJlz	2	TRUE	0.003668	20.98569
8518	rs7829309	A	G	A	G	0.0252	-0.03517	0.424	0.423589	FALSE	FALSE	FALSE	ieu-b-508	8	26129295	0.034162	486484	0.303302	Sepsis (28	Sepsis (28	Sepsis (28	TRUE	igd	0.0055	4.07E-06	4520	M37104.m	TRUE	reported	bR3g7d	2	TRUE	0.004623	20.98377
902	rs1089051	T	C	T	C	-0.0197	-0.0865	0.0433	0.045729	FALSE	FALSE	FALSE	ieu-b-508	2	1408443	0.081326	486484	0.287508	Sepsis (28	Sepsis (28	Sepsis (28	TRUE	igd	0.0043	3.45E-06	7354	M01494.m	TRUE	reported	7JWNKl	2	TRUE	0.002846	20.98348
8443	rs1004587	T	G	T	G	-0.0935	0.000188	0.0213	0.028907	FALSE	FALSE	FALSE	ieu-b-508	5	27307727	0.099176	486484	0.998491	Sepsis (28	Sepsis (28	Sepsis (28	TRUE	igd	0.0204	4.78E-06	1782	M37004.m	TRUE	reported	EKCQye	2	TRUE	0.011651	20.98337
3430	rs1701542	C	G	C	G	-0.022	0.024393	0.5942	0.600493	FALSE	TRUE	FALSE	ieu-b-508	2	1.28E+08	0.034183	486484	0.475463	Sepsis (28	Sepsis (28	Sepsis (28	TRUE	igd	0.0048	4.10E-06	1766	M32452.m	TRUE	reported	8GtFpl	2	TRUE	0.011755	20.98315
8208	rs1557339	A	C	A	C	0.0275	0.004978	0.7675	0.750841	FALSE	FALSE	FALSE	ieu-b-508	18	35129076	0.038902	486484	0.898172	Sepsis (28	Sepsis (28	Sepsis (28	TRUE	igd	0.006	4.42E-06	1732	M36300.m	TRUE	reported	ZjeC9m	2	TRUE	0.011983	20.98269
6584	rs1322876	C	G	C	G	-0.0582	0.034516	0.0663	0.217435	FALSE	TRUE	FALSE	ieu-b-508	7	1004248	0.04046	486484	0.393611	Sepsis (28	Sepsis (28	Sepsis (28	TRUE	igd	0.0127	4.71E-06	2278	M34289.m	TRUE	reported	Knp8ry	2	TRUE	0.009135	20.98249
1324	rs280194	T	C	T	C	-0.0197	-0.01514	0.7311	0.734181	FALSE	FALSE	FALSE	ieu-b-508	2	1.22E+08	0.037847	486484	0.689044	Sepsis (28	Sepsis (28	Sepsis (28	TRUE	igd	0.0043	5.77E-06	6252	M02137.m	TRUE	reported	iRGqBE	2	TRUE	0.003346	20.98247
7441	rs1693551	T	C	T	C	-0.0197	-0.0051	0.5342	0.537808	FALSE	FALSE	FALSE	ieu-b-508	8	1.02E+08	0.033399	486484	0.87857	Sepsis (28	Sepsis (28	Sepsis (28	TRUE	igd	0.0043	4.79E-06	6234	M35189.m	TRUE	reported	54XZFj	2	TRUE	0.003356	20.98245
5160	rs9892714	T	C	T	C	0.1324	-0.06692	0.031	0.031981	FALSE	FALSE	FALSE	ieu-b-508	17	1687560	0.09446	486484	0.478657	Sepsis (28	Sepsis (28	Sepsis (28	TRUE	igd	0.0289	4.67E-06	5716	M33192.m	TRUE	reported	CyfjKE	2	TRUE	0.003658	20.9811
5071	rs7942841	A	G	A	G	-0.0917	0.044844	0.8086	0.800411	FALSE	FALSE	FALSE	ieu-b-508	11	35546361	0.04299	486484	0.296894	Sepsis (28	Sepsis (28	Sepsis (28	TRUE	igd	0.02	4.42E-06	1017	M33178.m	TRUE	reported	g22WMS	2	TRUE	0.020252	20.98088
8393	rs1728624	A	G	A	G	0.0197	0.02453	0.7403	0.735932	FALSE	FALSE	FALSE	ieu-b-508	12	96088546	0.0378	486484	0.51637	Sepsis (28	Sepsis (28	Sepsis (28	TRUE	igd	0.0043	4.25E-06	4317	M36802.m	TRUE	reported	njeQfy	2	TRUE	0.004838	20.97946
6231	rs1159426	A	G	A	G	0.0339	-0.05767	0.0971	0.089891	FALSE	FALSE	FALSE	ieu-b-508	10	1.18E+08	0.058926	486484	0.327715	Sepsis (28	Sepsis (28	Sepsis (28	TRUE	igd	0.0074	5.22E-06	5534	M33910.m	TRUE	reported	YwFZg1	2	TRUE	0.003778	20.97872
842	rs2017823	A	G	A	G	0.0142	0.005751	0.841	0.832639	FALSE	FALSE	FALSE	ieu-b-508	18	2383523	0.044663	486484	0.897545	Sepsis (28	Sepsis (28	Sepsis (28	TRUE	igd	0.0031	4.85E-06	7323	M01359.m	TRUE	reported	7L9x1s	2	TRUE	0.002857	20.97658
8063	rs1692839	T	C	T	C	0.0623	-0.00578	0.9707	0.97468	FALSE	FALSE	FALSE	ieu-b-508	12	1490829	0.105684	486484	0.956391	Sepsis (28	Sepsis (28	Sepsis (28	TRUE	igd	0.0136	4.57E-06	4529	M35978.m	TRUE	reported	CN0wRR	2	TRUE	0.004612	20.97522
8152	rs1699291	A	G	A	G	0.0646	-0.01192	0.0528	0.057414	FALSE	FALSE	FALSE	ieu-b-508	22	34127504	0.072268	486484	0.868983	Sepsis (28	Sepsis (28	Sepsis (28	TRUE	igd	0.0141	4.79E-06	2709	M36115.m	TRUE	reported	ZV1jWp	2	TRUE	0.007689	20.9752
1898	rs6854832	T	C	T	C	0.0472	-0.06918	0.1513	0.138124	FALSE	FALSE	FALSE	ieu-b-508	4	1.45E+08	0.048326	486484	0.152288	Sepsis (28	Sepsis (28	Sepsis (28	TRUE	igd	0.0103	4.98E-06	1721	M15996.m	TRUE	reported	nXFvB9	2	TRUE	0.012055	20.97512
4630	rs265795	C	G	C	G	-0.2845	0.021528	0.1061	0.044722	FALSE	TRUE	FALSE	ieu-b-508	3	1364796	0.081318	486484	0.791212	Sepsis (28	Sepsis (28	Sepsis (28	TRUE	igd	0.0613	3.51E-06	76	M33173.m	TRUE	reported	04fkUQ	2	TRUE	0.220832	20.97306
8396	rs327837	A	G	A	G	0.0142	-0.03067	0.376	0.38559	FALSE	FALSE	FALSE	ieu-b-508	5	1.25E+08	0.034285	486484	0.371059	Sepsis (28	Sepsis (28	Sepsis (28	TRUE	igd	0.0031	4.52E-06	4317	M36802.m	TRUE	reported	njeQfy	2	TRUE	0.004837	20.97259
873	rs1016415	A	G	A	G	0.0229	-0.01873	0.0577	0.059369	FALSE	FALSE	FALSE	ieu-b-508	18	66766874	0.071009	486484	0.791916	Sepsis (28	Sepsis (28	Sepsis (28	TRUE	igd	0.005	4.18E-06	7362	M01365.m	TRUE	reported	N1cZTs	2	TRUE	0.002841	20.9707
6685	rs472038	C	G	C	G	0.111	-0.07493	0.077	0.082054	FALSE	TRUE	FALSE	ieu-b-508	11	74527143	0.060913	486484	0.218659	Sepsis (28	Sepsis (28	Sepsis (28	TRUE	igd	0.0242	4.49E-06	613	M34329.m	TRUE	reported	mgPKP5	2	TRUE	0.033182	20.96988
7688	rs1010813	T	C	T	C	0.0142	-0.05415	0.6059	0.595083	FALSE	FALSE	FALSE	ieu-b-508	8	1.35E+08	0.034056	486484	0.111857	Sepsis (28	Sepsis (28	Sepsis (28	TRUE	igd	0.0031	6.45E-06	3007	M35331.m	TRUE	reported	GBnxhN	2	TRUE	0.006929	20.96835
3612	rs1762801	A	G	A	G	0.0316	0.054169	0.1561	0.168712	FALSE	FALSE	FALSE	ieu-b-508	4	1.71E+08	0.044707	486484	0.225653	Sepsis (28	Sepsis (28	Sepsis (28	TRUE	igd	0.0069	4.12E-06	7361	M32553.m	TRUE	reported	vHSHqn	2	TRUE	0.002841	20.96805
5217	rs7151750	C	G	C	G	-0.0142	-0.02536	0.5979	0.60979	FALSE	TRUE	FALSE	ieu-b-508	14	22394510	0.034132	486484	0.457491	Sepsis (28	Sepsis (28	Sepsis (28	TRUE	igd	0.0031	4.12E-06	2683	M33204.m	TRUE	reported	ROvPWK	2	TRUE	0.00776	20.96667
4174	rs1689958	C	G	C	G	0.0719	-0.0104	0.0382	0.07979	FALSE	TRUE	FALSE	ieu-b-508	8	40422353	0.063704	486484	0.870375	Sepsis (28	Sepsis (28	Sepsis (28	TRUE	igd	0.0157	5.00E-06	6712	M32759.m	TRUE	reported	9elHr7	2	TRUE	0.003115	20.96665
5855	rs6465164	A	G	A	G	-0.0394	0.042748	0.1492	0.156421	FALSE	FALSE	FALSE	ieu-b-508	7	88467385	0.046008	486484	0.352813	Sepsis (28	Sepsis (28	Sepsis (28	TRUE	igd	0.0086	5.06E-06	1768	M33587.m	TRUE	reported	ssG6bF	2	TRUE	0.011732	20.96544
282	rs2373642	A	G	A	G	-0.0536	-0.01583	0.0138	0.036064	FALSE	FALSE	FALSE	ieu-b-508	7	1.5E+08	0.09062	486484	0.861337	Sepsis (28	Sepsis (28	Sepsis (28	TRUE	igd	0.0117	4.72E-06	1762	M00060.m	TRUE	reported	kqwl4V	2	TRUE	0.011771	20.96354
5925	rs6917152	A	C	A	C	0.0893	0.014905	0.0265	0.027926	FALSE	FALSE	FALSE	ieu-b-508	6	6412535	0.101031	486484	0.882716	Sepsis (28	Sepsis (28	Sepsis (28	TRUE	igd	0.0195	4.92E-06	4323	M33633.m	TRUE	reported	e36XGE	2	TRUE	0.004828	20.962
6431	rs1710548	A	G	A	G	0.0087	0.062168	0.8711	0.868576	FALSE	FALSE	FALSE	ieu-b-508	12	68822840	0.049535	486484	0.209466	Sepsis (28	Sepsis (28	Sepsis (28	TRUE	igd	0.0019	3.10E-06	7362	M34062.m	TRUE	reported	RZyHKP	2	TRUE	0.00284	20.96106
3321	rs1172181	T	C	T	C	0.0174	-0.00331	0.6543	0.667438	FALSE	FALSE	FALSE	ieu-b-508	4	5560266	0.035415	486484	0.925431	Sepsis (28	Sepsis (28	Sepsis (28	TRUE	igd	0.0038	3.42E-06	7358	M32405.m	TRUE	reported	uzomIQ	2	TRUE	0.002841	20.96106
7912	rs1215192	T	C	T	C	-0.0087	-0.04269	0.7465	0.746343	FALSE	FALSE	FALSE	ieu-b-508	20	56044837	0.0385	486484	0.267474	Sepsis (28	Sepsis (28	Sepsis (28	TRUE	igd	0.0019	3.53E-06	7348	M35675.m	TRUE	reported	D7fdyZ	2	TRUE	0.002845	20.96105
7940	rs679959	A	G	A	G	0.0087	0.042197	0.3159	0.31671	FALSE	FALSE	FALSE	ieu-b-508	4	1.38E+08	0.036017	486484	0.241367	Sepsis (28	Sepsis (28	Sepsis (28	TRUE	igd	0.0019	2.79E-06	7348	M35675.m	TRUE	reported	D7fdyZ	2	TRUE	0.002845	20.96105
7943	rs7223436	A	G	A	G	-0.0087	-0.0237	0.2539	0.276345	FALSE	FALSE	FALSE	ieu-b-508	17	25564116	0.037278	486484	0.524949	Sepsis (28	Sepsis (28	Sepsis (28	TRUE	igd	0.0019	3.37E-06	7348	M35675.m	TRUE	reported	D7fdyZ	2	TRUE	0.002845	20.96105
2717	rs884518	A	G	A	G	-0.0087	0.015083	0.5041	0.523873	FALSE	FALSE	FALSE	ieu-b-508	1	1.64E+08	0.033406	486484	0.651625	Sepsis (28	Sepsis (28	Sepsis (28	TRUE	igd	0.0019	4.78E-06	7340	M25459.m	TRUE	reported	ACJY35	2	TRUE	0.002848	20.96105
5385	rs1094924	T	C	T	C	0.0087	0.020709	0.5212	0.509458	FALSE	FALSE	FALSE	ieu-b-508	6	14371336	0.033524	486484	0.53675	Sepsis (28	Sepsis (28	Sepsis (28	TRUE	igd	0.0019	3.69E-06	7315	M33389.m	TRUE	reported	Hf6XVM	2	TRUE	0.002858	20.96103
5388	rs1171706	T	C	T	C	-0.0087	0.010488	0.6324	0.617024	FALSE	FALSE	FALSE	ieu-b-508	3	1.42E+08	0.034302	486484	0.759796	Sepsis (28	Sepsis (28	Sepsis (28	TRUE	igd	0.0019	2.94E-06	7315	M33389.m	TRUE	reported	Hf6XVM	2	TRUE	0.002858	20.96103
5400	rs2373262	T	C	T	C	-0.0087	0.001299	0.705	0.70492	FALSE	FALSE	FALSE	ieu-b-508	14	87214383	0.036703	486484	0.97176	Sepsis (28	Sepsis (28	Sepsis (28	TRUE	igd	0.0019	2.80E-06	7315	M33389.m	TRUE	reported	Hf6XVM	2	TRUE	0.002858	20.96103
5405	rs33131	A	G	A	G	-0.0087	0.010011	0.669	0.660696	FALSE	FALSE	FALSE	ieu-b-508	16	83837529	0.035509	486484	0.777992	Sepsis (28	Sepsis (28	Sepsis (28	TRUE	igd	0.0019	3.12E-06	7315	M33389.m	TRUE	reported	Hf6XVM	2	TRUE	0.002858	20.96103
5415	rs52701	T	C	T	C	0.0087	-0.05857	0.3727	0.368437	FALSE	FALSE	FALSE	ieu-b-508	14	69214958	0.034693	486484	0.09134	Sepsis (28	Sepsis (28	Sepsis (28	TRUE	igd	0.0019	2.69E-06	7315	M33389.m	TRUE	reported	Hf6XVM	2	TRUE	0.002858	20.96103
5424	rs7435399	T	C	T	C	0.0087	-0.00656	0.66	0.658797	FALSE	FALSE	FALSE	ieu-b-508	4	24765819	0.035326	486484	0.852635	Sepsis (28	Sepsis (28	Sepsis (28	TRUE	igd	0.0019	2.48E-06	7315	M33389.m	TRUE	reported	Hf6XVM	2	TRUE	0.002858	20.96103
5428	rs9375723	T	C	T	C	0.0087	0.053031	0.2664	0.258632	FALSE	FALSE	FALSE	ieu-b-508	6	1.31E+08	0.038354	486484	0.166769	Sepsis (28	Sepsis (28	Sepsis (28	TRUE	igd	0.0019	3.39E-06	7315	M33389.m	TRUE	reported	Hf6XVM	2	TRUE	0.002858	20.96103
5432	rs962423	A	C	A	C	-0.0087	0.022335	0.4645	0.469667	FALSE	FALSE	FALSE	ieu-b-508	2	64696502	0.033411	486484	0.503823	Sepsis (28	Sepsis (28	Sepsis (28	TRUE	igd	0.0019	4.27E-06	7315	M33389.m	TRUE	reported	Hf6XVM	2	TRUE	0.002858	20.96103
1841	rs1791600	T	C	T	C	-0.0087	-0.01088	0.2072	0.201672	FALSE	FALSE	FALSE	ieu-b-508	11	1.06E+08	0.041578	486484	0.793546	Sepsis (28	Sepsis (28	Sepsis (28	TRUE	igd	0.0019	4.09E-06	7308	M15778.m	TRUE	reported	941o3l	2	TRUE	0.002861	20.96102
5673	rs1274976	A	G	A	G	-0.0087	0.029921	0.271	0.280009	FALSE	FALSE	FALSE	ieu-b-508	1	4111187	0.037126	486484	0.420279	Sepsis (28	Sepsis (28	Sepsis (28	TRUE	igd	0.0019	5.49E-06	7307	M33477.m	TRUE	reported	Aw8PBK	2	TRUE	0.002861	20.96102
5692	rs6135736	A	G	A	G	-0.0087	-0.00672	0.248	0.247043	FALSE	FALSE	FALSE	ieu-b-508	20	1687450	0.039159	486484	0.863678	Sepsis (28	Sepsis (28	Sepsis (28	TRUE	igd	0.0019	2.78E-06	7307	M33477.m	TRUE	reported	Aw8PBK	2	TRUE	0.002861	20.96102
2599	rs6738800	A	G	A	G	0.0261	0.010899	0.1176	0.163401	FALSE	FALSE	FALSE	ieu-b-508	2	1.28E+08	0.045654	486484	0.811317	Sepsis (28	Sepsis (28	Sepsis (28	TRUE	igd	0.0057	5.20E-06	7259	M22189.m	TRUE	reported	YSD5fY	2	TRUE	0.00288	20.96098
5361	rs7624812	T	C	T	C	-0.0087	0.028612	0.1734	0.182286	FALSE	FALSE	FALSE	ieu-b-508	3	1.11E+08	0.043171	486484	0.507475	Sepsis (28	Sepsis (28	Sepsis (28	TRUE	igd	0.0019	2.77E-06	7118	M33380.m	TRUE	reported	OHRfl5	2	TRUE	0.002937	20.96087
2965	rs8081052	A	G	A	G	0.0174	0.00545	0.1073	0.109809	FALSE	FALSE	FALSE	ieu-b-508	17	8891595	0.053529	486484	0.918898	Sepsis (28	Sepsis (28	Sepsis (28	TRUE	igd	0.0038	4.64E-06	6795	M30805.m	TRUE	reported	YHV70c	2	TRUE	0.003076	20.96059
8031	rs9563725	T	C	T	C	-0.0087	-0.04877	0.4437	0.45296	FALSE	FALSE	FALSE	ieu-b-508	13	60010888	0.033566	486484	0.146252	Sepsis (28	Sepsis (28	Sepsis (28	TRUE	igd	0.0019	6.02E-06	6604	M35754.m	TRUE	reported	IlXOo1	2	TRUE	0.003165	20.96041
8033	rs970719	T	C	T	C	0.0087	-0.00332	0.4248	0.425079	FALSE	FALSE	FALSE	ieu-b-508	11	29254490	0.033839	486484	0.921806	Sepsis (28	Sepsis (28	Sepsis (28	TRUE	igd	0.0019	6.44E-06	6604	M35754.m	TRUE	reported	IlXOo1	2	TRUE	0.003165	20.96041
8476	rs267939	T	C	T	C	-0.0087	-0.03002	0.6348	0.6463	FALSE	FALSE	FALSE	ieu-b-508	5	10752315	0.034878	486484	0.389469	Sepsis (28	Sepsis (28	Sepsis (28	TRUE	igd	0.0019	2.59E-06	6552	M37058.m	TRUE	reported	32p8qV	2	TRUE	0.00319	20.96036
8485	rs6470127	A	G	A	G	0.0087	-0.00958	0.6245	0.613502	FALSE	FALSE	FALSE	ieu-b-508	8	1.24E+08	0.034332	486484	0.780168	Sepsis (28	Sepsis (28	Sepsis (28	TRUE	igd	0.0019	2.87E-06	6552	M37058.m	TRUE	reported	32p8qV	2	TRUE	0.00319	20.96036
2531	rs1016245	T	C	T	C	0.0087	-0.00934	0.7178	0.717951	FALSE	FALSE	FALSE	ieu-b-508	14	78105188	0.037099	486484	0.801141	Sepsis (28	Sepsis (28	Sepsis (28	TRUE	igd	0.0019	8.50E-06	6535	M22177.m	TRUE	reported	2s6Sly	2	TRUE	0.003198	20.96034

2532	rs109843	A	T	A	T	-0.0087	0.019789	0.5886	0.584995	FALSE	TRUE	FALSE	ieu-b-508	5	98341053	0.03402	486484	0.56077	Sepsis (28	Sepsis (28	Sepsis (28	TRUE	igd	0.0019	7.15E-06	6535	M22177.m	TRUE	reported	2s6Sly	2	TRUE	0.003198	20.96034
2552	rs2256490	A	T	A	T	0.0087	0.045429	0.2844	0.29189	FALSE	TRUE	FALSE	ieu-b-508	21	46087077	0.036594	486484	0.214444	Sepsis (28	Sepsis (28	Sepsis (28	TRUE	igd	0.0019	7.73E-06	6535	M22177.m	TRUE	reported	2s6Sly	2	TRUE	0.003198	20.96034
2557	rs2781537	T	C	T	C	0.0087	0.000722	0.5547	0.574356	FALSE	FALSE	FALSE	ieu-b-508	9	72078593	0.034064	486484	0.983097	Sepsis (28	Sepsis (28	Sepsis (28	TRUE	igd	0.0019	7.72E-06	6535	M22177.m	TRUE	reported	2s6Sly	2	TRUE	0.003198	20.96034
2562	rs3845671	C	G	C	G	-0.0087	-0.01563	0.4044	0.408374	FALSE	TRUE	FALSE	ieu-b-508	2	1.3E+08	0.034017	486484	0.645987	Sepsis (28	Sepsis (28	Sepsis (28	TRUE	igd	0.0019	7.34E-06	6535	M22177.m	TRUE	reported	2s6Sly	2	TRUE	0.003198	20.96034
2563	rs4147331	A	G	A	G	0.0087	0.0048	0.393	0.396645	FALSE	FALSE	FALSE	ieu-b-508	8	1.15E+08	0.034146	486484	0.888205	Sepsis (28	Sepsis (28	Sepsis (28	TRUE	igd	0.0019	7.47E-06	6535	M22177.m	TRUE	reported	2s6Sly	2	TRUE	0.003198	20.96034
2564	rs4298477	T	C	T	C	0.0087	-0.01278	0.534	0.546501	FALSE	FALSE	FALSE	ieu-b-508	8	40647884	0.033588	486484	0.703605	Sepsis (28	Sepsis (28	Sepsis (28	TRUE	igd	0.0019	8.40E-06	6535	M22177.m	TRUE	reported	2s6Sly	2	TRUE	0.003198	20.96034
2580	rs7194277	C	G	C	G	-0.0087	-0.05677	0.6775	0.676108	FALSE	TRUE	FALSE	ieu-b-508	16	8571350	0.03579	486484	0.112717	Sepsis (28	Sepsis (28	Sepsis (28	TRUE	igd	0.0019	8.33E-06	6535	M22177.m	TRUE	reported	2s6Sly	2	TRUE	0.003198	20.96034
2583	rs7677906	T	C	T	C	0.0087	-0.02872	0.5094	0.506111	FALSE	FALSE	FALSE	ieu-b-508	4	1.79E+08	0.033443	486484	0.390384	Sepsis (28	Sepsis (28	Sepsis (28	TRUE	igd	0.0019	7.07E-06	6535	M22177.m	TRUE	reported	2s6Sly	2	TRUE	0.003198	20.96034
949	rs1012282	T	G	T	G	0.0087	-0.01887	0.3503	0.334612	FALSE	FALSE	FALSE	ieu-b-508	9	1.39E+08	0.035377	486484	0.593796	Sepsis (28	Sepsis (28	Sepsis (28	TRUE	igd	0.0019	2.79E-06	6523	M01558.m	TRUE	reported	AZK0p4	2	TRUE	0.003204	20.96033
3973	rs1074115	T	C	T	C	-0.0087	0.025008	0.3902	0.386214	FALSE	FALSE	FALSE	ieu-b-508	10	1.29E+08	0.034249	486484	0.465285	Sepsis (28	Sepsis (28	Sepsis (28	TRUE	igd	0.0019	4.02E-06	6497	M32729.m	TRUE	reported	XGRSN1	2	TRUE	0.003217	20.9603
3980	rs1243588	A	G	A	G	-0.0087	-0.02147	0.4175	0.411269	FALSE	FALSE	FALSE	ieu-b-508	14	53468249	0.034027	486484	0.528125	Sepsis (28	Sepsis (28	Sepsis (28	TRUE	igd	0.0019	4.04E-06	6497	M32729.m	TRUE	reported	XGRSN1	2	TRUE	0.003217	20.9603
7279	rs922331	T	C	T	C	0.0087	0.059025	0.3698	0.370985	FALSE	FALSE	FALSE	ieu-b-508	3	1.93E+08	0.035409	486484	0.095524	Sepsis (28	Sepsis (28	Sepsis (28	TRUE	igd	0.0019	6.14E-06	5885	M34912.m	TRUE	reported	W8lcT6	2	TRUE	0.00355	20.95963
7336	rs1106609	T	C	T	C	0.0087	-0.03251	0.6756	0.67066	FALSE	FALSE	FALSE	ieu-b-508	2	2.21E+08	0.035653	486484	0.361837	Sepsis (28	Sepsis (28	Sepsis (28	TRUE	igd	0.0019	4.81E-06	4900	M35137.m	TRUE	reported	Eq2siS	2	TRUE	0.004261	20.9582
7342	rs1464136	A	C	A	C	0.0087	0.042002	0.7294	0.7336	FALSE	FALSE	FALSE	ieu-b-508	2	1.9E+08	0.037645	486484	0.264535	Sepsis (28	Sepsis (28	Sepsis (28	TRUE	igd	0.0019	5.32E-06	4900	M35137.m	TRUE	reported	Eq2siS	2	TRUE	0.004261	20.9582
7348	rs2064443	A	G	A	G	-0.0087	-0.07061	0.5417	0.540037	FALSE	FALSE	FALSE	ieu-b-508	20	19358824	0.033684	486484	0.036057	Sepsis (28	Sepsis (28	Sepsis (28	TRUE	igd	0.0019	4.22E-06	4900	M35137.m	TRUE	reported	Eq2siS	2	TRUE	0.004261	20.9582
7349	rs2214626	C	G	C	G	-0.0087	-0.00523	0.6223	0.607942	FALSE	TRUE	FALSE	ieu-b-508	7	16315652	0.034385	486484	0.879079	Sepsis (28	Sepsis (28	Sepsis (28	TRUE	igd	0.0019	4.28E-06	4900	M35137.m	TRUE	reported	Eq2siS	2	TRUE	0.004261	20.9582
7354	rs2966713	A	G	A	G	-0.0087	0.066991	0.3387	0.356814	FALSE	FALSE	FALSE	ieu-b-508	7	1.43E+08	0.03479	486484	0.054158	Sepsis (28	Sepsis (28	Sepsis (28	TRUE	igd	0.0019	5.90E-06	4900	M35137.m	TRUE	reported	Eq2siS	2	TRUE	0.004261	20.9582
7358	rs3801969	T	G	T	G	-0.0087	0.010738	0.4459	0.436985	FALSE	FALSE	FALSE	ieu-b-508	7	1.07E+08	0.033736	486484	0.750259	Sepsis (28	Sepsis (28	Sepsis (28	TRUE	igd	0.0019	4.72E-06	4900	M35137.m	TRUE	reported	Eq2siS	2	TRUE	0.004261	20.9582
7362	rs4326611	T	C	T	C	-0.0087	-0.00468	0.4285	0.456629	FALSE	FALSE	FALSE	ieu-b-508	1	2.45E+08	0.033675	486484	0.889414	Sepsis (28	Sepsis (28	Sepsis (28	TRUE	igd	0.0019	6.06E-06	4900	M35137.m	TRUE	reported	Eq2siS	2	TRUE	0.004261	20.9582
7370	rs618791	T	C	T	C	-0.0087	0.015767	0.6833	0.680107	FALSE	FALSE	FALSE	ieu-b-508	17	26899502	0.035863	486484	0.660208	Sepsis (28	Sepsis (28	Sepsis (28	TRUE	igd	0.0019	4.42E-06	4900	M35137.m	TRUE	reported	Eq2siS	2	TRUE	0.004261	20.9582
7383	rs7505882	T	C	T	C	-0.0087	0.052639	0.4807	0.491426	FALSE	FALSE	FALSE	ieu-b-508	18	69260510	0.03359	486484	0.117093	Sepsis (28	Sepsis (28	Sepsis (28	TRUE	igd	0.0019	5.43E-06	4900	M35137.m	TRUE	reported	Eq2siS	2	TRUE	0.004261	20.9582
7389	rs820140	T	C	T	C	-0.0087	-0.0209	0.4898	0.48135	FALSE	FALSE	FALSE	ieu-b-508	17	73608203	0.033763	486484	0.535902	Sepsis (28	Sepsis (28	Sepsis (28	TRUE	igd	0.0019	4.65E-06	4900	M35137.m	TRUE	reported	Eq2siS	2	TRUE	0.004261	20.9582
4147	rs2852424	A	G	A	G	0.0554	0.060913	0.9371	0.92026	FALSE	FALSE	FALSE	ieu-b-508	11	1.14E+08	0.061312	486484	0.320471	Sepsis (28	Sepsis (28	Sepsis (28	TRUE	igd	0.0121	4.71E-06	7250	M32757.m	TRUE	reported	TJV9Xb	2	TRUE	0.002883	20.95699
8365	rs1042565	A	G	A	G	-0.0522	0.179386	0.9597	0.961852	FALSE	FALSE	FALSE	ieu-b-508	19	13589472	0.087803	486484	0.041048	Sepsis (28	Sepsis (28	Sepsis (28	TRUE	igd	0.0114	4.96E-06	3248	M36756.m	TRUE	reported	QsW4oR	2	TRUE	0.006414	20.95385
2794	rs1314945	T	C	T	C	-0.0293	0.048233	0.0539	0.066042	FALSE	FALSE	FALSE	ieu-b-508	4	97865943	0.067934	486484	0.4777	Sepsis (28	Sepsis (28	Sepsis (28	TRUE	igd	0.0064	4.34E-06	7347	M27278.m	TRUE	reported	kPgqm3	2	TRUE	0.002845	20.95352
8151	rs1261709	T	C	T	C	-0.0348	-0.07107	0.4512	0.396502	FALSE	FALSE	FALSE	ieu-b-508	2	64700471	0.035263	486484	0.043859	Sepsis (28	Sepsis (28	Sepsis (28	TRUE	igd	0.0076	4.81E-06	2709	M36115.m	TRUE	reported	ZV1jWp	2	TRUE	0.00768	20.95128
8167	rs2254145	A	G	A	G	-0.0316	0.016985	0.2138	0.21433	FALSE	FALSE	FALSE	ieu-b-508	16	85821586	0.040772	486484	0.676983	Sepsis (28	Sepsis (28	Sepsis (28	TRUE	igd	0.0069	4.29E-06	1780	M36131.m	TRUE	reported	DbYm5t	2	TRUE	0.011646	20.95018
7435	rs8063217	T	C	T	C	0.1278	-0.0049	0.0594	0.052702	FALSE	FALSE	FALSE	ieu-b-508	16	20422369	0.074603	486484	0.947652	Sepsis (28	Sepsis (28	Sepsis (28	TRUE	igd	0.0279	4.70E-06	1254	M35187.m	TRUE	reported	Zx7JfE	2	TRUE	0.016457	20.94885
5641	rs7692407	T	C	T	C	-0.0293	0.033528	0.9336	0.926015	FALSE	FALSE	FALSE	ieu-b-508	4	1.55E+08	0.064179	486484	0.601387	Sepsis (28	Sepsis (28	Sepsis (28	TRUE	igd	0.0064	5.36E-06	4004	M33443.m	TRUE	reported	G1JrRV	2	TRUE	0.005207	20.94876
4486	rs2408393	T	C	T	C	-0.0403	0.056837	0.866	0.864225	FALSE	FALSE	FALSE	ieu-b-508	11	1.02E+08	0.048681	486484	0.242995	Sepsis (28	Sepsis (28	Sepsis (28	TRUE	igd	0.0088	5.01E-06	1767	M33131.m	TRUE	reported	uGKURO	2	TRUE	0.01173	20.9485
6918	rs9837363	T	C	T	C	-0.0206	0.038329	0.4628	0.475676	FALSE	FALSE	FALSE	ieu-b-508	3	68336119	0.036518	486484	0.293904	Sepsis (28	Sepsis (28	Sepsis (28	TRUE	igd	0.0045	3.60E-06	5195	M34389.m	TRUE	reported	suiRIN	2	TRUE	0.004018	20.94798
3922	rs7843564	T	G	T	G	0.0554	0.022759	0.9307	0.920301	FALSE	FALSE	FALSE	ieu-b-508	8	20461123	0.06176	486484	0.7125	Sepsis (28	Sepsis (28	Sepsis (28	TRUE	igd	0.0121	4.64E-06	2575	M32691.m	TRUE	reported	SiuD6F	2	TRUE	0.008075	20.94649
2964	rs7397233	A	G	A	G	0.098	-0.0278	0.0335	0.137881	FALSE	FALSE	FALSE	ieu-b-508	12	84470694	0.048634	486484	0.56756	Sepsis (28	Sepsis (28	Sepsis (28	TRUE	igd	0.0214	4.47E-06	1509	M30805.m	TRUE	reported	YHV70c	2	TRUE	0.013707	20.94347
2260	rs4659612	A	G	A	G	0.0174	-0.02562	0.7371	0.744127	FALSE	FALSE	FALSE	ieu-b-508	1	2.36E+08	0.038246	486484	0.502896	Sepsis (28	Sepsis (28	Sepsis (28	TRUE	igd	0.0038	5.92E-06	1759	M19934.m	TRUE	reported	HtcWcQ	2	TRUE	0.011779	20.94292
3944	rs1008342	T	C	T	C	0.0119	-0.00851	0.3043	0.301652	FALSE	FALSE	FALSE	ieu-b-508	14	77161621	0.036345	486484	0.814846	Sepsis (28	Sepsis (28	Sepsis (28	TRUE	igd	0.0026	6.74E-06	7363	M32709.m	TRUE	reported	YR8SB4	2	TRUE	0.002837	20.94253
3953	rs1721799	T	C	T	C	0.0119	-0.07252	0.6869	0.644522	FALSE	FALSE	FALSE	ieu-b-508	9	13791428	0.035169	486484	0.039215	Sepsis (28	Sepsis (28	Sepsis (28	TRUE	igd	0.0026	7.26E-06	7363	M32709.m	TRUE	reported	YR8SB4	2	TRUE	0.002837	20.94253
3764	rs1990385	T	C	T	C	0.0119	-0.01359	0.8651	0.845845	FALSE	FALSE	FALSE	ieu-b-508	12	6394635	0.046322	486484	0.769265	Sepsis (28	Sepsis (28	Sepsis (28	TRUE	igd	0.0026	4.95E-06	7362	M32634.m	TRUE	reported	7T2RUz	2	TRUE	0.002837	20.94253
6442	rs4394864	T	C	T	C	-0.0119	0.045069	0.0645	0.075732	FALSE	FALSE	FALSE	ieu-b-508	11	1.12E+08	0.063486	486484	0.477767	Sepsis (28	Sepsis (28	Sepsis (28	TRUE	igd	0.0026	5.52E-06	7362	M34062.m	TRUE	reported	RZyHKP	2	TRUE	0.002837	20.94253
6445	rs559422	A	C	A	C	0.0119	-0.09053	0.9115	0.916768	FALSE	FALSE	FALSE	ieu-b-508	11	248181	0.061314	486484	0.139813	Sepsis (28	Sepsis (28	Sepsis (28	TRUE	igd	0.0026	5.77E-06	7362	M34062.m	TRUE	reported	RZyHKP	2	TRUE	0.002837	20.94253
7410	rs1010377	T	C	T	C	0.0119	0.006353	0.3033	0.288678	FALSE	FALSE	FALSE	ieu-b-508	20	40696227	0.036949	486484	0.86349	Sepsis (28	Sepsis (28	Sepsis (28	TRUE	igd	0.0026	6.76E-06	7350	M35186.m	TRUE	reported	UZfu6Z	2	TRUE	0.002842	20.94252
7415	rs1715284	T	C	T	C	-0.0119	-0.01021	0.7203	0.710468	FALSE	FALSE	FALSE	ieu-b-508	8	10942929	0.036777	486484	0.781228	Sepsis (28	Sepsis (28	Sepsis (28	TRUE	igd	0.0026	5.93E-06	7350	M35186.m	TRUE	reported	UZfu6Z	2	TRUE	0.002842	20.94252
749	rs7570141	A	G	A	G	-0.0119	0.030784	0.104	0.128492	FALSE	FALSE	FALSE	ieu-b-508	2	18275380	0.051513	486484	0.550103	Sepsis (28	Sepsis (28	Sepsis (28	TRUE	igd	0.0026	6.21E-06	7347	M01302.m	TRUE	reported	sNp2lC	2	TRUE	0.002843	20.94252
3078	rs1574228	A	G	A	G	0.0119	-0.01445	0.7061	0.712747	FALSE	FALSE	FALSE	ieu-b-508	10	5312310	0.03687	486484	0.695109	Sepsis (28	Sepsis (28	Sepsis (28	TRUE	igd	0.0026	6.71E-06	7346	M32197.m	TRUE	reported	VZjwa9	2	TRUE	0.002844	20.94252
4420	rs1148526	A	G	A	G	-0.0119	-0.08616	0.8872	0.88587	FALSE	FALSE	FALSE	ieu-b-508	1	75197533	0.05258	486484	0.101268	Sepsis (28	Sepsis (28	Sepsis (28	TRUE	igd	0.0026	6.74E-06	7344	M32910.m	TRUE	reported	2OCBKh	2	TRUE	0.002844	20.94252
3157	rs1009567	T	C	T	C	-0.0238	0.051593	0.9066	0.906815	FALSE	FALSE	FALSE	ieu-b-508	8	1.33E+08	0.057472	486484	0.36934	Sepsis (28	Sepsis (28	Sepsis (28	TRUE	igd	0.0052	4.02E-06	7340	M32328.m	TRUE	reported	YGsb6D	2	TRUE	0.002846	20.94252
1682	rs7160223	A	T	A	T	-0.0119	0.031853	0.419	0.413414	FALSE	TRUE	FALSE	ieu-b-508	14	72899529	0.034163	486484	0.351138	Sepsis (28	Sepsis (28	Sepsis (28	TRUE	igd	0.0026	5.52E-06	7337	M15365.m	TRUE	reported	TReTCM	2	TRUE	0.002847	20.94251
8375	rs1006415	A	C	A	C	-0.0119	0.033494	0.7322	0.722383	FALSE	FALSE	FALSE	ieu-b-508	5	17802524	0.037236	486484	0.368384	Sepsis (28	Sepsis (28	Sepsis (28	TRUE	igd	0.0026	3.78E-06	7335	M36776.m	TRUE	reported	VnYglx	2	TRUE	0.002848	20.94251
2489	rs1113667	A	G	A	G	-0.0119	-0.00183	0.715	0.715256	FALSE	FALSE	FALSE	ieu-b-508	8	3778035	0.037217	486484	0.960846	Sepsis (28	Sepsis (28	Sepsis (28	TRUE	igd	0.0026	7.49E-06	7329	M22116.m	TRUE	reported	bjUU6r	2	TRUE	0.00285	20.94251
2610	rs2202185	A	G	A	G	-0.0119	-0.00442	0.3139	0.366584	FALSE	FALSE	FALSE	ieu-b-508	14	79463780	0.034652	486484	0.898535	Sepsis (28	Sepsis (28	Sepsis (28	TRUE	igd	0.0026	6.16E-06	7329	M22481.m	TRUE	reported	bXpf0k	2	TRUE	0.00285	20.94251
1276	rs1111995	T	C	T	C	0.0119	0.062871	0.1175	0.110685	FALSE	FALSE	FALSE	ieu-b-508	1	2.13E+08	0.053069	486484	0.236136	Sepsis (28	Sepsis (28	Sepsis (28	TRUE	igd	0.0026	4.36E-06	7325	M02132.m	TRUE	reported	uJFm2t	2	TRUE	0.002852	20.94251
7862	rs184943	A	T	A	T	-0.0119	-0.02162	0.7036	0.701873	FALSE	TRUE	FALSE	ieu-b-508	5	50913447	0.036534	486484	0.55392	Sepsis (28	Sepsis (28	Sepsis (28	TRUE	igd	0.0026	7.23E-06	7317	M35631.m	TRUE	reported	002wka	2	TRUE	0.002855	20.9425
7864	rs2278476	T	C	T	C	0.0119	-0.01779	0.4871	0.485654	FALSE	FALSE	FALSE	ieu-b-508	3	1.49E+08	0.033312	486484	0.593353	Sepsis (28	Sepsis (28	Sepsis (28	TRUE	igd	0.0026	5.00E-06	7317	M35631.m	TRUE	reported	002wka	2	TRUE	0.002855	20.9425
897	rs2351725	A	T	A	T	-0.0119	0.00338	0.7947	0.784403	FALSE	TRUE	FALSE	ieu-b-508	6	66524413	0.040693	486484	0.933807	Sepsis (28	Sepsis (28	Sepsis (28	TRUE	igd	0.0026	5.84E-06	7307	M01493.m	TRUE	reported	eLLRt7	2	TRUE	0.002859	20.94249
5706	rs9990565	T	C	T	C	-0.0119	-0.0131	0.1528	0.144658	FALSE	FALSE	FALSE	ieu-b-508	4	4405881	0.04763	486484	0.783336	Sepsis (28	Sepsis (28	Sepsis (28	TRUE	igd	0.0026	5.48E-06	7307	M33477.m	TRUE	reported	Aw8PBK	2	TRUE	0.002859	20.94249
5789	rs4073619	T	G	T	G	-0.0119	0.011036	0.5405	0.530167	FALSE	FALSE	FALSE	ieu-b-508	11	7490548	0.033488	486484	0.741729	Sepsis (28	Sepsis (28	Sepsis (28	TRUE	igd	0.0026	6.83E-06	7269	M33510.m	TRUE	reported	WxGKnn	2	TRUE	0.002874	20.94246
4604	rs9650602	T	C	T	C	-0.0238	-0.08119	0.197	0.253952	FALSE	FALSE	FALSE	ieu-b-508	8	20441920	0.038901	486484	0.036877	Sepsis (28	Sepsis (28	Sepsis (28	TRUE	igd	0.0052	4.43E-06	7263	M33165.m	TRUE	reported	RZt3uS	2	TRUE	0.002876	20.94246
2983	rs7932167	T	G	T	G	0.0119	-0.00991	0.7711	0.784934	FALSE	FALSE	FALSE	ieu-b-508	11	620599	0.040719	486484	0.80768	Sepsis (28	Sepsis (28	Sepsis (28	TRUE	igd	0.0026	6.66E-06	7257	M31453.m	TRUE	reported	vdTd9M	2	TRUE	0.002878	20.94245
7777	rs6745507	C	G	C	G	-0.0119	0.034193	0.6155	0.585488	FALSE	TRUE	FALSE	ieu-b-508	2	2.14E+08	0.034202	486484	0.317434	Sepsis (28	Sepsis (28	Sepsis (28	TRUE	igd	0.0026	3.65E-06	7256	M35439.m	TRUE	reported	DnuAGZ	2	TRUE	0.002879	20.94245
2635	rs1111778	T	C	T	C	-0.0119	-0.01434	0.6373	0.625209	FALSE	FALSE	FALSE	ieu-b-508	1	2.17E+08	0.034613	486484	0.678717	Sepsis (28	Sepsis (28	Sepsis (28	TRUE	igd	0.0026	3.10E-06	7184	M22548.m	TRUE	reported	mZtwX2	2	TRUE	0.002907	20.94239
2638	rs1167586	T	C	T	C	0.0119	-0.03829	0.7367	0.746813	FALSE	FALSE	FALSE	ieu-b-508	2	2.09E+08	0.038353	486484	0.318103	Sepsis (28	Sepsis (28	Sepsis (28	TRUE	igd	0.0026	5.84E-06	7184	M22548.m	TRUE	reported	mZtwX2	2	TRUE	0.002907	20.94239
5337	rs1228381	A	G	A	G	-0.0119	-0.07909	0.1003	0.099521	FALSE	FALSE	FALSE	ieu-b-508	11	1.23E+08	0.056025	486484	0.15803	Sepsis (28	Sepsis (28	Sepsis (28	TRUE	igd	0.0026	4.01E-06	7118	M33380.m	TRUE	reported	OHRfl5	2	TRUE	0.002934	20.94234
7505	rs1263632	T	C	T	C	-0.0119	0.009509	0.4945	0.495685	FALSE	FALSE	FALSE	ieu-b-508	2	2.08E+08	0.033475	486484	0.776369	Sepsis (28	Sepsis (28	Sepsis (28	TRUE	igd	0.0026	6.16E-06	7100	M35254.m	TRUE	reported	HfVgPn	2	TRUE	0.002942	20.94232
852	rs1050984	A	G	A	G	-0.0119	-0.03057	0.6879	0.743825	FALSE	FALSE	FALSE	ieu-b-508	10	1.09E+08	0.038683	486484	0.429304	Sepsis (28	Sepsis (28	Sepsis (28	TRUE	igd	0.0026	4.23E-06	7063	M01361.m	TRUE	reported	NIAcQ3	2	TRUE	0.002957	20.94229
870	rs8098317	T	G	T	G	-0.0119	-0.00418	0.7857	0.781102	FALSE	FALSE	FALSE	ieu-b-508	18	22207719	0.040484	486484	0.917846	Sepsis (28	Sepsis (28	Sepsis (28	TRUE	igd	0.0026	6.64E-06	7063	M01361.m	TRUE	reported	NIAcQ3	2	TRUE	0.002957	20.94229
2199	rs1261411	T	C	T	C	0.0119	0.042873	0.4052	0.4202	FALSE	FALSE	FALSE	ieu-b-508	1	57017739	0.034057	486484	0.208082	Sepsis (28	Sepsis (28	Sepsis (28	TRUE	igd	0.0026	3.29E-06	6929	M19364.m	TRUE	reported	XkjMEx	2	TRUE	0.003014	20.94218
2212	rs7864694	A	G	A	G	0.0119	-0.01513	0.3364	0.346526	FALSE	FALSE	FALSE	ieu-b-508	9	12414681	0.035047	486484	0.665905	Sepsis (28	Sepsis (28	Sepsis (28	TRUE	igd	0.0026	4.04E-06	6929	M19364.m	TRUE	reported	XkjMEx	2	TRUE	0.003014	20.94218
2213	rs8071524	A	G	A	G	-0.0119	-0.02255	0.3003	0.307023	FALSE	FALSE	FALSE	ieu-b-508	17	71429776	0.0362	486484	0.533357	Sepsis (28	Sepsis (28	Sepsis (28	TRUE	igd	0.0026	4.55E-06	6929	M19364.m	TRUE	reported	XkjMEx	2	TRUE	0.003014	20.94218
2222	rs4344410	A	C	A	C	0.0119	-0.04992	0.503	0.490723	FALSE	FALSE	FALSE	ieu-b-508	10	1.34E+08	0.03339	486484	0.134901	Sepsis (28	Sepsis (28	Sepsis (28	TRUE	igd	0.0026	3.52E-06	6678	M19368.m	TRUE	reported	QaKPad	2	TRUE	0.003127	20.94195
7964	rs6870033	A	T	A	T	-0.0238	-0.04467	0.7179	0.688355	FALSE	TRUE	FALSE	ieu-b-508	5	81625648	0.036148	486484	0.216551	Sepsis (28	Sepsis (28	Sepsis (28	TRUE	igd	0.0052	4.14E-06	6447	M35678.m	TRUE	reported	56yR01	2	TRUE	0.003239	20.94173
2461	rs1277442	A	C	A	C	0.0119	-0.02056	0.3106	0.316018	FALSE	FALSE	FALSE	ieu-b-508	12	1.19E+08	0.035838	486484	0.566166	Sepsis (28	Sepsis (28	Sepsis (28	TRUE	igd	0.0026	3.21E-06	6180	M22030.m	TRUE	reported	ZNV0qR	2	TRUE	0.003378	20.94145
2984	rs9572772	T	G	T	G	-0.0902	-0.15265	0.0129	0.009357	FALSE	FALSE	FALSE	ieu-b-508	13	72299073	0.173157	486484	0.378005	Sepsis (28	Sepsis (28	Sepsis (28	TRUE	igd	0.0197	4.65E-06	1750	M31453.m	TRUE	reported	vdTd9M	2	TRUE	0.011838	20.94035
4998	rs633810	T	C	T	C	-0.1081	-0.08148	0.094	0.116988	FALSE	FALSE	FALSE	ieu-b-508	11	1.25E+08	0.05236	486484	0.119675	Sepsis (28	Sepsis (28	Sepsis (28	TRUE	igd	0.0236	4.79E-06	1017	M33178.m	TRUE	reported	g22WMS	2	TRUE	0.020213	20.9398
1916	rs1552422	A	G	A	G	-0.0119	-0.01377	0.5052	0.496895	FALSE	FALSE	FALSE	ieu-b-508	12	1.3E+08	0.033431	486484	0.680384	Sepsis (28	Sepsis (28	Sepsis (28	TRUE	igd	0.0026	6.00E-06	4689	M16816.m	TRUE	reported	J1abbO	2	TRUE	0.004448	20.93929
6470	rs1266106	T	C	T	C	-0.1644	-0.04056	0.1122	0.123043	FALSE	FALSE	FALSE	ieu-b-508	6	33896120	0.051747	486484	0.43314	Sepsis (28	Sepsis (28	Sepsis (28	TRUE	igd	0.0358	4.40E-06	282	M34109.m	TRUE	reported	piJuC7	2	TRUE	0.069577	20.93854
2970	rs1205212	T	C	T	C	0.0389	-0.01481	0.1205	0.122873	FALSE	FALSE	FALSE	ieu-b-508	19	53187079	0.050727	486484	0.770391	Sepsis (28	Sepsis (28	Sepsis (28	TRUE	igd	0.0085	4.18E-06	7257	M31453.m	TRUE	reported	vdTd9M	2	TRUE	0.002878	20.93831
6697	rs1752155	T	C	T	C	0.3198	-0.23206	0.0166	0.015487	FALSE	FALSE	FALSE	ieu-b-508	18	25478278	0.137465	486484	0.09139	Sepsis (28	Sepsis (28	Sepsis (28	TRUE	igd	0.0697	4.46E-06	369	M34336.m	TRUE	reported	jrSSxL	2	TRUE	0.053972	20.9378
3952	rs1704780	T	C	T	C	-0.038	-0.08	0.8891	0.878063	FALSE	FALSE	FALSE	ieu-b-508	2	56553005	0.050994	486484	0.116678	Sepsis (28	Sepsis (28	Sepsis (28	TRUE	igd	0.0083	4.12E-06	1766	M32709.m	TRUE	reported	YR8SB4	2	TRUE	0.01173	20.93721
2219	rs2823491	T	C	T	C	0.038	0.16352	0.9047	0.910891	FALSE	FALSE	FALSE	ieu-b-508	21	17161984	0.058678	486484	0.005324	Sepsis (28	Sepsis (28	Sepsis (28	TRUE	igd	0.0083	5.27E-06	1678	M19368.m	TRUE	reported	QaKPad	2	TRUE	0.012338	20.93597
6691	rs1105889	T	C	T	C	-0.027	0.069779	0.0551	0.058264	FALSE	FALSE	FALSE	ieu-b-508	12	1.27E+08	0.071656	486484	0.330153	Sepsis (28	Sepsis (28	Sepsis (28	TRUE	igd	0.0059	4.76E-06	5081	M34336.m	TRUE	reported	jrSSxL	2	TRUE	0.004105	20.93401
5846	rs1708109	A	G	A	G	0.0357	0.022149	0.8144	0.788167	FALSE	FALSE	FALSE	ieu-b-508	13	25322453	0.04106	486484	0.589588	Sepsis (28	Sepsis (28	Sepsis (28	TRUE	igd	0.0078	4.93E-06	2859	M33531.m	TRUE	reported	kNM0s1	2	TRUE	0.007274	20.93357
4759	rs1295001	T	C	T	C	0.0861	0.02588	0.5363	0.522417	FALSE	FALSE	FALSE	ieu-b-508	17	49737304	0.033483	486484	0.439558	Sepsis (28	Sepsis (28	Sepsis (28	TRUE	igd	0.0188	4.66E-06	1017	M33178.m	TRUE	reported	g22WMS	2	TRUE	0.020207	20.9332
1625	rs1159304	A	T	A	T	0.0151	-0.02683	0.0919	0.092269	FALSE	TRUE	FALSE	ieu-b-508	10	6933467	0.057983	486484	0.643539	Sepsis (28	Sepsis (28	Sepsis (28	TRUE	igd	0.0033	4.55E-06	7368	M15140.m	TRUE	reported	jEcqMJ	2	TRUE	0.002834	20.93187
1229	rs4439706	T	C	T	C	0.0151	-0.0036	0.7156	0.714137	FALSE	FALSE	FALSE	ieu-b-508	15	47129544	0.037152	486484	0.92275	Sepsis (28	Sepsis (28	Sepsis (28	TRUE	igd	0.0033	3.78E-06	7346	M01712.m	TRUE	reported	JfdAWN	2	TRUE	0.002842	20.93186
4045	rs1256901	A	T	A	T	-0.0151	-0.04191	0.0734	0.076832	FALSE	TRUE	FALSE	ieu-b-508	1	1.11E+08	0.062677	486484	0.503671	Sepsis (28	Sepsis (28	Sepsis (28	TRUE	igd	0.0033	6.39E-06	7318	M32740.m	TRUE	reported	YLshfP	2	TRUE	0.002853	20.93184
5792	rs7133996	T	C	T	C	-0.0151	-0.0164	0.2736	0.273421	FALSE	FALSE	FALSE	ieu-b-508	12	32931925	0.037557	486484	0.662337	Sepsis (28	Sepsis (28	Sepsis (28	TRUE	igd	0.0033	5.83E-06	7269	M33510.m	TRUE	reported	WxGKnn	2	TRUE	0.002872	20.9318
5343	rs1509091	T	C	T	C	0.0151	-0.04547	0.9357	0.918906	FALSE	FALSE	FALSE	ieu-b-508	13	1.08E+08	0.061966	486484	0.463044	Sepsis (28	Sepsis (28	Sepsis (28	TRUE	igd	0.0033	6.30E-06	7118	M33380.m	TRUE	reported	OHRfl5	2	TRUE	0.002933	20.93167
6045	rs7579137	A	G	A	G	-0.027	0.009615	0.5493	0.564845	FALSE	FALSE	FALSE	ieu-b-508	2	1.12E+08	0.033653	486484	0.775106	Sepsis (28	Sepsis (28	Sepsis (28	TRUE	igd	0.0059	4.77E-06	3845	M33801.m	TRUE	reported	zhDCxK	2	TRUE	0.005417	20.93136
7221	rs1759720	A	G	A	G	-0.0302	0.109499	0.8973	0.892699	FALSE	FALSE	FALSE	ieu-b-508	4	1E+08	0.053888	486484	0.042159	Sepsis (28	Sepsis (28	Sepsis (28	TRUE	igd	0.0066	5.01E-06	6381	M34761.m	TRUE	reported	AMtp1w	2	TRUE	0.003271	20.93099
7835	rs9580319	T	C	T	C	-0.0641	-0.00904	0.9296	0.91618	FALSE	FALSE	FALSE	ieu-b-508	13	22763034	0.059993	486484	0.880173	Sepsis (28	Sepsis (28	Sepsis (28	TRUE	igd	0.014	4.44E-06	1291	M35527.m	TRUE	reported	UAMhnu	2	TRUE	0.015979	20.93084
3921	rs7513605	T	C	T	C	0.0389	0.11573	0.1575	0.161011	FALSE	FALSE	FALSE	ieu-b-508	1	1.75E+08	0.045287	486484	0.010604	Sepsis (28	Sepsis (28	Sepsis (28	TRUE	igd	0.0085	4.10E-06	2575	M32691.m	TRUE	reported	SiuD6F	2	TRUE	0.008068	20.92782
5555	rs1462054	A	G	A	G	1.266	-0.0281	0.9746	0.987922	FALSE	FALSE	FALSE	ieu-b-508	2	6591865	0.154512	486484	0.855702	Sepsis (28	Sepsis (28	Sepsis (28	TRUE	igd	0.2721	3.27E-06	60	M33423.m	TRUE	reported	MoEnO7	2	TRUE	0.265135	20.92604
5334	rs1154486	T	C	T	C	0.0183	-0.00408	0.943	0.927103	FALSE	FALSE	FALSE	ieu-b-508	4	1E+08	0.064312	486484	0.949466	Sepsis (28	Sepsis (28	Sepsis (28	TRUE	igd	0.004	3.71E-06	7118	M33380.m	TRUE	reported	OHRfl5	2	TRUE	0.002932	20.92474
2322	rs6821567	C	G	C	G	-0.0476	-0.08635	0.9423	0.829764	FALSE	TRUE	FALSE	ieu-b-508	4	1.12E+08	0.044302	486484	0.051287	Sepsis (28	Sepsis (28	Sepsis (28	TRUE	igd	0.0104	4.38E-06	1748	M20699.m	TRUE	reported	Z3EKFa	2	TRUE	0.011842	20.92426
4206	rs9751295	A	G	A	G	-0.1428	-0.08178	0.9778	0.984689	FALSE	FALSE	FALSE	ieu-b-508	2	2.3E+08	0.13557	486484	0.546356	Sepsis (28	Sepsis (28	Sepsis (28	TRUE	igd	0.0312	4.68E-06	1672	M32762.m	TRUE	reported	Q5lKLH	2	TRUE	0.012374	20.92317
8202	rs9574536	T	C	T	C	-0.0517	-0.01602	0.3565	0.406997	FALSE	FALSE	FALSE	ieu-b-508	13	80585091	0.034041	486484	0.637859	Sepsis (28	Sepsis (28	Sepsis (28	TRUE	igd	0.0113	5.06E-06	2400	M36230.m	TRUE	reported	V5KJeR	2	TRUE	0.008647	20.91521
7323	rs270771	T	C	T	C	-0.0279	-0.09814	0.0551	0.053251	FALSE	FALSE	FALSE	ieu-b-508	19	55220626	0.074222	486484	0.186086	Sepsis (28	Sepsis (28	Sepsis (28	TRUE	igd	0.0061	4.02E-06	7344	M35127.m	TRUE	reported	RNsmjl	2	TRUE	0.00284	20.91368
8109	rs6829571	A	G	A	G	-0.4173	0.080525	0.7541	0.749295	FALSE	FALSE	FALSE	ieu-b-508	4	1.41E+08	0.038601	486484	0.036969	Sepsis (28	Sepsis (28	Sepsis (28	TRUE	igd	0.0905	3.95E-06	122	M36097.m	TRUE	reported	RHr18S	2	TRUE	0.148412	20.91323
4281	rs9881563	T	C	T	C	0.0279	0.035238	0.6543	0.667567	FALSE	FALSE	FALSE	ieu-b-508	3	1.13E+08	0.035412	486484	0.319702	Sepsis (28	Sepsis (28	Sepsis (28	TRUE	igd	0.0061	4.60E-06	6200	M32808.m	TRUE	reported	vnr36k	2	TRUE	0.003363	20.91263
5096	rs9395772	A	G	A	G	-0.2838	-0.15947	0.0176	0.016087	FALSE	FALSE	FALSE	ieu-b-508	6	52122014	0.133376	486484	0.231823	Sepsis (28	Sepsis (28	Sepsis (28	TRUE	igd	0.062	4.67E-06	1017	M33178.m	TRUE	reported	g22WMS	2	TRUE	0.020187	20.91156
1555	rs9460599	T	C	T	C	0.0302	0.054073	0.2443	0.248708	FALSE	FALSE	FALSE	ieu-b-508	6	21139758	0.039014	486484	0.165756	Sepsis (28	Sepsis (28	Sepsis (28	TRUE	igd	0.0066	4.86E-06	1586	M12129.m	TRUE	reported	iNzdgb	2	TRUE	0.013029	20.91115
6870	rs1941075	T	C	T	C	-0.1094	-0.01055	0.7538	0.747536	FALSE	FALSE	FALSE	ieu-b-508	18	12774848	0.038505	486484	0.784141	Sepsis (28	Sepsis (28	Sepsis (28	TRUE	igd	0.0239	4.71E-06	954	M34365.m	TRUE	reported	8eexD0	2	TRUE	0.021491	20.90872
642	rs1270743	A	G	A	G	-0.0366	0.30639	0.9639	0.976885	FALSE	FALSE	FALSE	ieu-b-508	7	1.39E+08	0.110893	486484	0.005729	Sepsis (28	Sepsis (28	Sepsis (28	TRUE	igd	0.008	4.65E-06	1764	M01125.m	TRUE	reported	yw0clB	2	TRUE	0.011726	20.90689
4246	rs3729621	A	G	A	G	0.0503	-0.12492	0.9564	0.953146	FALSE	FALSE	FALSE	ieu-b-508	7	29521433	0.079114	486484	0.114342	Sepsis (28	Sepsis (28	Sepsis (28	TRUE	igd	0.011	4.92E-06	6169	M32795.m	TRUE	reported	0YBWkh	2	TRUE	0.003378	20.90306
6961	rs1188573	A	T	A	T	0.0824	-0.01246	0.6877	0.664462	FALSE	TRUE	FALSE	ieu-b-508	2	17224592	0.035437	486484	0.725232	Sepsis (28	Sepsis (28	Sepsis (28	TRUE	igd	0.018	4.69E-06	777	M34404.m	TRUE	reported	8FMHfo	2	TRUE	0.026262	20.90211
1514	rs1408276	A	C	A	C	-0.043	0.066951	0.955	0.951881	FALSE	FALSE	FALSE	ieu-b-508	6	1.22E+08	0.078202	486484	0.391925	Sepsis (28	Sepsis (28	Sepsis (28	TRUE	igd	0.0094	4.59E-06	1747	M12067.m	TRUE	reported	O5x7RD	2	TRUE	0.011836	20.9018
5379	rs3784457	A	G	A	G	0.2165	-0.02944	0.0539	0.063398	FALSE	FALSE	FALSE	ieu-b-508	15	1.02E+08	0.068188	486484	0.665905	Sepsis (28	Sepsis (28	Sepsis (28	TRUE	igd	0.0473	4.66E-06	803	M33384.m	TRUE	reported	lOffrZ	2	TRUE	0.025427	20.89828
7973	rs1163654	T	G	T	G	0.0192	0.029682	0.1055	0.151903	FALSE	FALSE	FALSE	ieu-b-508	15	37089269	0.047432	486484	0.531457	Sepsis (28	Sepsis (28	Sepsis (28	TRUE	igd	0.0042	3.99E-06	7358	M35718.m	TRUE	reported	oUwc0a	2	TRUE	0.002832	20.89228
807	rs1015277	T	C	T	C	-0.0224	0.080108	0.0425	0.047425	FALSE	FALSE	FALSE	ieu-b-508	15	80674262	0.078797	486484	0.309322	Sepsis (28	Sepsis (28	Sepsis (28	TRUE	igd	0.0049	4.73E-06	7355	M01358.m	TRUE	reported	kSPQgd	2	TRUE	0.002833	20.89228
3164	rs1685253	A	G	A	G	0.016	0.049643	0.3193	0.373051	FALSE	FALSE	FALSE	ieu-b-508	1	2.31E+08	0.034809	486484	0.15383	Sepsis (28	Sepsis (28	Sepsis (28	TRUE	igd	0.0035	3.83E-06	7340	M32328.m	TRUE	reported	YGsb6D	2	TRUE	0.002839	20.89226
8381	rs1428365	T	C	T	C	-0.0192	0.091126	0.9228	0.92151	FALSE	FALSE	FALSE	ieu-b-508	5	24482475	0.062258	486484	0.143279	Sepsis (28	Sepsis (28	Sepsis (28	TRUE	igd	0.0042	4.52E-06	7335	M36776.m	TRUE	reported	VnYglx	2	TRUE	0.002841	20.89226
4399	rs931761	C	G	C	G	0.0064	-0.02559	0.755	0.747582	FALSE	TRUE	FALSE	ieu-b-508	3	59528623	0.038348	486484	0.504553	Sepsis (28	Sepsis (28	Sepsis (28	TRUE	igd	0.0014	2.80E-06	7329	M32867.m	TRUE	reported	Jcsyzm	2	TRUE	0.002843	20.89226
8147	rs7332616	T	C	T	C	-0.032	0.01233	0.7928	0.7942	FALSE	FALSE	FALSE	ieu-b-508	13	21677331	0.04141	486484	0.765886	Sepsis (28	Sepsis (28	Sepsis (28	TRUE	igd	0.007	5.18E-06	7311	M36103.m	TRUE	reported	sjeaPC	2	TRUE	0.00285	20.89224
3935	rs3770063	A	T	A	T	-0.0256	-0.02422	0.0352	0.034809	FALSE	TRUE	FALSE	ieu-b-508	2	86403499	0.090642	486484	0.789291	Sepsis (28	Sepsis (28	Sepsis (28	TRUE	igd	0.0056	5.88E-06	7308	M32698.m	TRUE	reported	srqncz	2	TRUE	0.002851	20.89224
5511	rs1162336	T	C	T	C	0.016	0.106338	0.0594	0.063509	FALSE	FALSE	FALSE	ieu-b-508	14	89924833	0.069532	486484	0.126182	Sepsis (28	Sepsis (28	Sepsis (28	TRUE	igd	0.0035	5.60E-06	7306	M33422.m	TRUE	reported	PLgjrT	2	TRUE	0.002852	20.89224
2286	rs1721249	A	G	A	G	0.016	0.013628	0.1645	0.169008	FALSE	FALSE	FALSE	ieu-b-508	14	44198202	0.044594	486484	0.759913	Sepsis (28	Sepsis (28	Sepsis (28	TRUE	igd	0.0035	5.93E-06	7301	M20675.m	TRUE	reported	CXe9rE	2	TRUE	0.002854	20.89223
2297	rs618900	C	G	C	G	-0.016	-0.05323	0.8136	0.81069	FALSE	TRUE	FALSE	ieu-b-508	11	1.25E+08	0.042451	486484	0.20988	Sepsis (28	Sepsis (28	Sepsis (28	TRUE	igd	0.0035	6.00E-06	7301	M20675.m	TRUE	reported	CXe9rE	2	TRUE	0.002854	20.89223
2979	rs4602889	A	G	A	G	0.016	-0.03975	0.8859	0.861876	FALSE	FALSE	FALSE	ieu-b-508	8	15155912	0.049014	486484	0.417385	Sepsis (28	Sepsis (28	Sepsis (28	TRUE	igd	0.0035	3.57E-06	7257	M31453.m	TRUE	reported	vdTd9M	2	TRUE	0.002871	20.8922
1364	rs6488270	A	G	A	G	0.0096	0.013068	0.4076	0.392371	FALSE	FALSE	FALSE	ieu-b-508	12	10350587	0.034124	486484	0.701746	Sepsis (28	Sepsis (28	Sepsis (28	TRUE	igd	0.0021	4.95E-06	7226	M02730.m	TRUE	reported	XYa035	2	TRUE	0.002884	20.89218
6273	rs3821477	T	C	T	C	-0.0288	0.04411	0.927	0.933021	FALSE	FALSE	FALSE	ieu-b-508	3	59946930	0.066648	486484	0.508079	Sepsis (28	Sepsis (28	Sepsis (28	TRUE	igd	0.0063	5.35E-06	7222	M33937.m	TRUE	reported	QanGCI	2	TRUE	0.002885	20.89217
4545	rs894137	A	G	A	G	-0.0192	0.009922	0.2902	0.290273	FALSE	FALSE	FALSE	ieu-b-508	11	13130196	0.036728	486484	0.787055	Sepsis (28	Sepsis (28	Sepsis (28	TRUE	igd	0.0042	4.99E-06	7166	M33138.m	TRUE	reported	lUpLAI	2	TRUE	0.002908	20.89213
946	rs8065435	A	G	A	G	-0.0128	-0.04394	0.327	0.331339	FALSE	FALSE	FALSE	ieu-b-508	17	56157617	0.036959	486484	0.234537	Sepsis (28	Sepsis (28	Sepsis (28	TRUE	igd	0.0028	5.03E-06	7160	M01508.m	TRUE	reported	go526k	2	TRUE	0.00291	20.89212
2089	rs6779362	T	C	T	C	-0.0288	-0.03346	0.7633	0.769276	FALSE	FALSE	FALSE	ieu-b-508	3	1.97E+08	0.039502	486484	0.396919	Sepsis (28	Sepsis (28	Sepsis (28	TRUE	igd	0.0063	5.74E-06	6970	M18394.m	TRUE	reported	Lt9awu	2	TRUE	0.002989	20.89196
6997	rs6990912	A	C	A	C	-0.016	0.059056	0.3602	0.356322	FALSE	FALSE	FALSE	ieu-b-508	8	9200472	0.034962	486484	0.091189	Sepsis (28	Sepsis (28	Sepsis (28	TRUE	igd	0.0035	4.81E-06	6929	M34416.m	TRUE	reported	q9OtaZ	2	TRUE	0.003007	20.89193
4232	rs2236525	T	C	T	C	0.016	0.033481	0.5285	0.531039	FALSE	FALSE	FALSE	ieu-b-508	20	60848835	0.033393	486484	0.316033	Sepsis (28	Sepsis (28	Sepsis (28	TRUE	igd	0.0035	4.02E-06	6857	M32787.m	TRUE	reported	fSRakm	2	TRUE	0.003038	20.89186
4303	rs1002322	T	C	T	C	-0.0256	0.023313	0.8808	0.88182	FALSE	FALSE	FALSE	ieu-b-508	4	1.71E+08	0.051779	486484	0.652541	Sepsis (28	Sepsis (28	Sepsis (28	TRUE	igd	0.0056	5.58E-06	6753	M32838.m	TRUE	reported	hyR7zA	2	TRUE	0.003085	20.89177
3234	rs1105842	A	C	A	C	-0.048	0.002681	0.1435	0.155539	FALSE	FALSE	FALSE	ieu-b-508	7	1.51E+08	0.04639	486484	0.953919	Sepsis (28	Sepsis (28	Sepsis (28	TRUE	igd	0.0105	4.44E-06	6687	M32346.m	TRUE	reported	cnIFoV	2	TRUE	0.003115	20.89171
7818	rs1308427	A	G	A	G	-0.0192	0.021922	0.4129	0.406978	FALSE	FALSE	FALSE	ieu-b-508	3	1.06E+08	0.034178	486484	0.521252	Sepsis (28	Sepsis (28	Sepsis (28	TRUE	igd	0.0042	5.54E-06	6595	M35472.m	TRUE	reported	oZU10n	2	TRUE	0.003159	20.89162
2731	rs2934216	A	G	A	G	-0.0128	0.080184	0.7472	0.737857	FALSE	FALSE	FALSE	ieu-b-508	5	52222508	0.038108	486484	0.035368	Sepsis (28	Sepsis (28	Sepsis (28	TRUE	igd	0.0028	3.10E-06	6388	M25599.m	TRUE	reported	fRZxwP	2	TRUE	0.003261	20.89142
2459	rs1159893	A	T	A	T	0.016	0.05639	0.1347	0.135494	FALSE	TRUE	FALSE	ieu-b-508	15	55111076	0.048602	486484	0.245951	Sepsis (28	Sepsis (28	Sepsis (28	TRUE	igd	0.0035	3.55E-06	6180	M22030.m	TRUE	reported	ZNV0qR	2	TRUE	0.00337	20.8912
7071	rs8086010	A	C	A	C	-0.0192	0.006304	0.6663	0.649268	FALSE	FALSE	FALSE	ieu-b-508	18	74626997	0.034898	486484	0.856655	Sepsis (28	Sepsis (28	Sepsis (28	TRUE	igd	0.0042	5.48E-06	6144	M34469.m	TRUE	reported	WMOqbm	2	TRUE	0.00339	20.89116
8014	rs6475283	T	C	T	C	0.0343	-0.0023	0.2824	0.274524	FALSE	FALSE	FALSE	ieu-b-508	9	18962486	0.037558	486484	0.951262	Sepsis (28	Sepsis (28	Sepsis (28	TRUE	igd	0.0075	5.12E-06	1709	M35754.m	TRUE	reported	lIXOo1	2	TRUE	0.01209	20.8909
2515	rs8061077	T	C	T	C	-0.0128	0.058763	0.2169	0.214822	FALSE	FALSE	FALSE	ieu-b-508	16	58697786	0.040628	486484	0.148077	Sepsis (28	Sepsis (28	Sepsis (28	TRUE	igd	0.0028	4.06E-06	5702	M22130.m	TRUE	reported	YDbJlz	2	TRUE	0.003652	20.89063
7045	rs1800600	A	G	A	G	0.0128	-0.04546	0.6929	0.685766	FALSE	FALSE	FALSE	ieu-b-508	1	1.57E+08	0.036119	486484	0.208142	Sepsis (28	Sepsis (28	Sepsis (28	TRUE	igd	0.0028	4.06E-06	5207	M34456.m	TRUE	reported	Nh457q	2	TRUE	0.003997	20.88993
7047	rs3800552	A	T	A	T	-0.0128	-0.02952	0.3513	0.353845	FALSE	TRUE	FALSE	ieu-b-508	6	47234245	0.03502	486484	0.399291	Sepsis (28	Sepsis (28	Sepsis (28	TRUE	igd	0.0028	3.30E-06	5207	M34456.m	TRUE	reported	Nh457q	2	TRUE	0.003997	20.88993
7052	rs6430457	A	C	A	C	-0.0128	-0.0156	0.6797	0.67971	FALSE	FALSE	FALSE	ieu-b-508	2	1.35E+08	0.036035	486484	0.664987	Sepsis (28	Sepsis (28	Sepsis (28	TRUE	igd	0.0028	3.82E-06	5207	M34456.m	TRUE	reported	Nh457q	2	TRUE	0.003997	20.88993
5707	rs1018657	A	G	A	G	0.0288	-0.092	0.1328	0.13952	FALSE	FALSE	FALSE	ieu-b-508	2	1.98E+08	0.048104	486484	0.055801	Sepsis (28	Sepsis (28	Sepsis (28	TRUE	igd	0.0063	5.10E-06	5107	M33488.m	TRUE	reported	mZmDY6	2	TRUE	0.004075	20.88978
7364	rs4659909	A	G	A	G	-0.0128	0.006113	0.1755	0.24269	FALSE	FALSE	FALSE	ieu-b-508	1	2.39E+08	0.038962	486484	0.875328	Sepsis (28	Sepsis (28	Sepsis (28	TRUE	igd	0.0028	5.06E-06	4900	M35137.m	TRUE	reported	Eq2siS	2	TRUE	0.004247	20.88943
1923	rs8105530	T	C	T	C	0.0128	-0.01632	0.5286	0.520753	FALSE	FALSE	FALSE	ieu-b-508	19	55839272	0.033401	486484	0.62503	Sepsis (28	Sepsis (28	Sepsis (28	TRUE	igd	0.0028	4.85E-06	4689	M16816.m	TRUE	reported	J1abbO	2	TRUE	0.004437	20.88905
8319	rs1095163	A	C	A	C	0.0288	0.039103	0.4616	0.459866	FALSE	FALSE	FALSE	ieu-b-508	7	40386835	0.033499	486484	0.24309	Sepsis (28	Sepsis (28	Sepsis (28	TRUE	igd	0.0063	5.68E-06	3977	M36589.m	TRUE	reported	27GADo	2	TRUE	0.005227	20.88745
5322	rs1211769	T	C	T	C	0.0192	-0.01392	0.7484	0.718906	FALSE	FALSE	FALSE	ieu-b-508	1	2.3E+08	0.03704	486484	0.706994	Sepsis (28	Sepsis (28	Sepsis (28	TRUE	igd	0.0042	5.24E-06	3890	M33364.m	TRUE	reported	9Zrr7Y	2	TRUE	0.005344	20.88721
2142	rs226212	A	G	A	G	-0.0384	0.009219	0.5696	0.57125	FALSE	FALSE	FALSE	ieu-b-508	17	37036554	0.033807	486484	0.785093	Sepsis (28	Sepsis (28	Sepsis (28	TRUE	igd	0.0084	4.84E-06	3783	M18497.m	TRUE	reported	90JFPc	2	TRUE	0.005494	20.88691
6905	rs4708385	A	G	A	G	-0.0608	0.02182	0.3704	0.406441	FALSE	FALSE	FALSE	ieu-b-508	6	1.69E+08	0.034058	486484	0.521742	Sepsis (28	Sepsis (28	Sepsis (28	TRUE	igd	0.0133	5.20E-06	2779	M34384.m	TRUE	reported	fl3hiS	2	TRUE	0.007464	20.88292
5910	rs1852749	A	T	A	T	-0.0352	-0.11502	0.942	0.946627	FALSE	TRUE	FALSE	ieu-b-508	4	17447699	0.074763	486484	0.123943	Sepsis (28	Sepsis (28	Sepsis (28	TRUE	igd	0.0077	4.80E-06	2757	M33627.m	TRUE	reported	4RHaUX	2	TRUE	0.007523	20.8828
4631	rs2840962	A	G	A	G	-0.4719	0.027934	0.0467	0.033521	FALSE	FALSE	FALSE	ieu-b-508	8	37309527	0.092604	486484	0.762918	Sepsis (28	Sepsis (28	Sepsis (28	TRUE	igd	0.1019	3.65E-06	76	M33173.m	TRUE	reported	04fkUQ	2	TRUE	0.220083	20.88189
3002	rs3123713	A	G	A	G	0.114	-0.00036	0.0862	0.10475	FALSE	FALSE	FALSE	ieu-b-508	10	43524522	0.054696	486484	0.99478	Sepsis (28	Sepsis (28	Sepsis (28	TRUE	igd	0.0249	4.88E-06	522	M31536.m	TRUE	reported	3nAuUl	2	TRUE	0.038605	20.88064
6667	rs1091677	A	T	A	T	0.0837	-0.06529	0.8056	0.813887	FALSE	TRUE	FALSE	ieu-b-508	1	20668375	0.042876	486484	0.127795	Sepsis (28	Sepsis (28	Sepsis (28	TRUE	igd	0.0183	5.05E-06	1009	M34327.m	TRUE	reported	ll4Lrr	2	TRUE	0.020312	20.87791
1122	rs2070006	T	C	T	C	0.0512	0.008323	0.3713	0.375133	FALSE	FALSE	FALSE	ieu-b-508	4	1.56E+08	0.034457	486484	0.80912	Sepsis (28	Sepsis (28	Sepsis (28	TRUE	igd	0.0112	4.76E-06	2063	M01640.m	TRUE	reported	o3qxzl	2	TRUE	0.010028	20.8777
6022	rs7665221	T	C	T	C	-0.0796	0.017784	0.8107	0.813022	FALSE	FALSE	FALSE	ieu-b-508	4	1.61E+08	0.042854	486484	0.678146	Sepsis (28	Sepsis (28	Sepsis (28	TRUE	igd	0.0174	4.71E-06	831	M33751.m	TRUE	reported	Cxdkr4	2	TRUE	0.024565	20.87763
8231	rs1192742	A	G	A	G	0.08	0.11935	0.9586	0.96256	FALSE	FALSE	FALSE	ieu-b-508	3	1785254	0.08893	486484	0.179575	Sepsis (28	Sepsis (28	Sepsis (28	TRUE	igd	0.0175	4.77E-06	1908	M36468.m	TRUE	reported	vycAFr	2	TRUE	0.010834	20.87605
2886	rs4918207	T	C	T	C	0.0361	-0.01547	0.4099	0.435895	FALSE	FALSE	FALSE	ieu-b-508	10	1.08E+08	0.033598	486484	0.645149	Sepsis (28	Sepsis (28	Sepsis (28	TRUE	igd	0.0079	4.36E-06	6393	M27716.m	TRUE	reported	N5hToq	2	TRUE	0.003256	20.8749
1652	rs7548008	A	G	A	G	0.016	-0.0276	0.504	0.522682	FALSE	FALSE	FALSE	ieu-b-508	1	43202095	0.033357	486484	0.407944	Sepsis (28	Sepsis (28	Sepsis (28	TRUE	igd	0.0035	3.56E-06	1764	M15140.m	TRUE	reported	jEcqMJ	2	TRUE	0.011708	20.87427
476	rs4718532	T	C	T	C	-0.016	0.06492	0.5052	0.532127	FALSE	FALSE	FALSE	ieu-b-508	7	66805000	0.033396	486484	0.051902	Sepsis (28	Sepsis (28	Sepsis (28	TRUE	igd	0.0035	6.61E-06	1761	M00527.m	TRUE	reported	UYN7vF	2	TRUE	0.011728	20.87422
1339	rs2036136	T	C	T	C	-0.0256	0.017308	0.4587	0.432072	FALSE	FALSE	FALSE	ieu-b-508	4	1.47E+08	0.033779	486484	0.608383	Sepsis (28	Sepsis (28	Sepsis (28	TRUE	igd	0.0056	5.11E-06	1741	M02342.m	TRUE	reported	EhZr3g	2	TRUE	0.011861	20.87395
6500	rs1774532	T	C	T	C	0.0722	-0.01558	0.9628	0.944667	FALSE	FALSE	FALSE	ieu-b-508	12	11704746	0.072744	486484	0.830446	Sepsis (28	Sepsis (28	Sepsis (28	TRUE	igd	0.0158	4.97E-06	5581	M34112.m	TRUE	reported	97JqFb	2	TRUE	0.003728	20.87395
7614	rs1243683	A	G	A	G	0.0522	0.005185	0.5918	0.597566	FALSE	FALSE	FALSE	ieu-b-508	14	21652837	0.034066	486484	0.879026	Sepsis (28	Sepsis (28	Sepsis (28	TRUE	igd	0.0114	5.04E-06	448	M35322.m	TRUE	reported	om3h9x	2	TRUE	0.044708	20.87316
4197	rs1940528	A	G	A	G	-0.0649	0.028194	0.8479	0.800841	FALSE	FALSE	FALSE	ieu-b-508	11	1.12E+08	0.042375	486484	0.505829	Sepsis (28	Sepsis (28	Sepsis (28	TRUE	igd	0.0142	4.71E-06	2537	M32762.m	TRUE	reported	Q5lKLH	2	TRUE	0.008166	20.87229
1185	rs2491274	A	G	A	G	-0.0297	-0.03781	0.9732	0.958325	FALSE	FALSE	FALSE	ieu-b-508	1	88253549	0.08377	486484	0.651729	Sepsis (28	Sepsis (28	Sepsis (28	TRUE	igd	0.0065	4.63E-06	7346	M01645.m	TRUE	reported	d5nAn3	2	TRUE	0.002834	20.87219
7508	rs1708452	A	G	A	G	0.0233	-0.02606	0.0965	0.090089	FALSE	FALSE	FALSE	ieu-b-508	13	69620171	0.05922	486484	0.659929	Sepsis (28	Sepsis (28	Sepsis (28	TRUE	igd	0.0051	4.74E-06	7100	M35254.m	TRUE	reported	HfVgPn	2	TRUE	0.002931	20.86648
7954	rs1172061	T	C	T	C	0.0233	0.039522	0.2508	0.24451	FALSE	FALSE	FALSE	ieu-b-508	3	1.26E+08	0.038796	486484	0.308343	Sepsis (28	Sepsis (28	Sepsis (28	TRUE	igd	0.0051	4.86E-06	6447	M35678.m	TRUE	reported	56yR01	2	TRUE	0.003227	20.86588
6708	rs9462736	T	C	T	C	0.0742	0.010266	0.3451	0.344891	FALSE	FALSE	FALSE	ieu-b-508	6	41655340	0.035108	486484	0.769981	Sepsis (28	Sepsis (28	Sepsis (28	TRUE	igd	0.0162	4.91E-06	369	M34336.m	TRUE	reported	jrSSxL	2	TRUE	0.053794	20.86496
6056	rs2482705	A	G	A	G	-0.0201	0.070358	0.1531	0.148678	FALSE	FALSE	FALSE	ieu-b-508	9	94182502	0.046988	486484	0.133391	Sepsis (28	Sepsis (28	Sepsis (28	TRUE	igd	0.0044	4.06E-06	7362	M33821.m	TRUE	reported	eVYlCL	2	TRUE	0.002827	20.86262
6154	rs1171725	A	G	A	G	-0.0201	0.029531	0.6186	0.602984	FALSE	FALSE	FALSE	ieu-b-508	3	22106222	0.034278	486484	0.38896	Sepsis (28	Sepsis (28	Sepsis (28	TRUE	igd	0.0044	4.33E-06	7334	M33884.m	TRUE	reported	aAWgRQ	2	TRUE	0.002837	20.86259
7866	rs2477833	T	C	T	C	-0.0201	0.074858	0.1056	0.12802	FALSE	FALSE	FALSE	ieu-b-508	6	41446021	0.050009	486484	0.134416	Sepsis (28	Sepsis (28	Sepsis (28	TRUE	igd	0.0044	4.81E-06	7317	M35631.m	TRUE	reported	002wka	2	TRUE	0.002844	20.86258
2680	rs2049216	C	G	C	G	0.0201	0.030069	0.1233	0.170872	FALSE	TRUE	FALSE	ieu-b-508	3	1.05E+08	0.044855	486484	0.502624	Sepsis (28	Sepsis (28	Sepsis (28	TRUE	igd	0.0044	5.43E-06	7256	M24074.m	TRUE	reported	Lk6D2A	2	TRUE	0.002868	20.86253
6514	rs1041099	A	C	A	C	0.0201	0.055762	0.9182	0.908509	FALSE	FALSE	FALSE	ieu-b-508	19	54005308	0.057811	486484	0.334765	Sepsis (28	Sepsis (28	Sepsis (28	TRUE	igd	0.0044	6.06E-06	7049	M34123.m	TRUE	reported	9O5wo1	2	TRUE	0.002952	20.86236
3180	rs1234961	T	C	T	C	0.0338	-0.25366	0.0551	0.019253	FALSE	FALSE	FALSE	ieu-b-508	9	1.18E+08	0.122612	486484	0.038564	Sepsis (28	Sepsis (28	Sepsis (28	TRUE	igd	0.0074	5.62E-06	7356	M32338.m	TRUE	reported	q5cuaP	2	TRUE	0.002828	20.857
6067	rs1177340	T	C	T	C	-0.0169	0.014586	0.7497	0.752326	FALSE	FALSE	FALSE	ieu-b-508	7	16149832	0.038697	486484	0.706235	Sepsis (28	Sepsis (28	Sepsis (28	TRUE	igd	0.0037	5.30E-06	7350	M33822.m	TRUE	reported	Wic4uS	2	TRUE	0.00283	20.857
991	rs261342	C	G	C	G	-0.0169	0.03888	0.794	0.779179	FALSE	TRUE	FALSE	ieu-b-508	15	58731153	0.040408	486484	0.335955	Sepsis (28	Sepsis (28	Sepsis (28	TRUE	igd	0.0037	5.41E-06	7276	M01561.m	TRUE	reported	Zd9SUU	2	TRUE	0.002859	20.85694
4596	rs163027	A	G	A	G	0.0169	-0.03472	0.5505	0.54942	FALSE	FALSE	FALSE	ieu-b-508	5	76782241	0.03355	486484	0.300758	Sepsis (28	Sepsis (28	Sepsis (28	TRUE	igd	0.0037	4.45E-06	7263	M33165.m	TRUE	reported	RZt3uS	2	TRUE	0.002864	20.85693
4605	rs986611	C	G	C	G	-0.0169	0.006962	0.6362	0.641909	FALSE	TRUE	FALSE	ieu-b-508	3	62015294	0.034831	486484	0.841563	Sepsis (28	Sepsis (28	Sepsis (28	TRUE	igd	0.0037	5.62E-06	7263	M33165.m	TRUE	reported	RZt3uS	2	TRUE	0.002864	20.85693
6270	rs2679896	T	C	T	C	0.0169	-0.00358	0.2902	0.312183	FALSE	FALSE	FALSE	ieu-b-508	2	1.06E+08	0.036408	486484	0.921761	Sepsis (28	Sepsis (28	Sepsis (28	TRUE	igd	0.0037	3.96E-06	7222	M33937.m	TRUE	reported	QanGCI	2	TRUE	0.00288	20.8569
1328	rs7045137	A	G	A	G	-0.0169	-0.00066	0.3947	0.393878	FALSE	FALSE	FALSE	ieu-b-508	9	1.17E+08	0.034299	486484	0.984592	Sepsis (28	Sepsis (28	Sepsis (28	TRUE	igd	0.0037	4.00E-06	6252	M02137.m	TRUE	reported	iRGqBE	2	TRUE	0.003326	20.856
7046	rs358374	A	G	A	G	0.0169	-0.01967	0.1773	0.175585	FALSE	FALSE	FALSE	ieu-b-508	3	13186109	0.043893	486484	0.654124	Sepsis (28	Sepsis (28	Sepsis (28	TRUE	igd	0.0037	4.96E-06	5207	M34456.m	TRUE	reported	Nh457q	2	TRUE	0.003991	20.85466
1763	rs1195460	T	C	T	C	0.0306	0.112845	0.9767	0.977114	FALSE	FALSE	FALSE	ieu-b-508	5	1.36E+08	0.112181	486484	0.314455	Sepsis (28	Sepsis (28	Sepsis (28	TRUE	igd	0.0067	4.83E-06	7331	M15676.m	TRUE	reported	s1NfKN	2	TRUE	0.002837	20.8533
6812	rs1910775	A	G	A	G	0.0297	-0.02837	0.0927	0.09242	FALSE	FALSE	FALSE	ieu-b-508	12	1.28E+08	0.057798	486484	0.623515	Sepsis (28	Sepsis (28	Sepsis (28	TRUE	igd	0.0065	4.34E-06	1576	M34359.m	TRUE	reported	0UotaJ	2	TRUE	0.013074	20.85138
4472	rs3793499	C	G	C	G	-0.0306	0.062474	0.8598	0.870463	FALSE	TRUE	FALSE	ieu-b-508	9	2131990	0.049608	486484	0.207905	Sepsis (28	Sepsis (28	Sepsis (28	TRUE	igd	0.0067	5.06E-06	5425	M33084.m	TRUE	reported	jrOsNK	2	TRUE	0.00383	20.8513
989	rs1532701	A	G	A	G	0.0137	-0.02721	0.5456	0.538158	FALSE	FALSE	FALSE	ieu-b-508	16	55698027	0.033463	486484	0.41609	Sepsis (28	Sepsis (28	Sepsis (28	TRUE	igd	0.003	5.07E-06	7276	M01561.m	TRUE	reported	Zd9SUU	2	TRUE	0.002858	20.84871
8233	rs1296620	A	T	A	T	0.0434	-0.04377	0.8443	0.841359	FALSE	TRUE	FALSE	ieu-b-508	18	39401020	0.045588	486484	0.340164	Sepsis (28	Sepsis (28	Sepsis (28	TRUE	igd	0.0095	5.30E-06	1908	M36468.m	TRUE	reported	vycAFr	2	TRUE	0.01082	20.84859
1413	rs1087807	A	G	A	G	0.0137	0.051878	0.6887	0.683537	FALSE	FALSE	FALSE	ieu-b-508	12	64056815	0.035787	486484	0.14716	Sepsis (28	Sepsis (28	Sepsis (28	TRUE	igd	0.003	7.14E-06	6941	M03127.m	TRUE	reported	JscEZw	2	TRUE	0.002996	20.84844
5913	rs562210	A	C	A	C	0.0169	0.062745	0.6864	0.70955	FALSE	FALSE	FALSE	ieu-b-508	8	81407796	0.036747	486484	0.087734	Sepsis (28	Sepsis (28	Sepsis (28	TRUE	igd	0.0037	4.72E-06	2757	M33627.m	TRUE	reported	4RHaUX	2	TRUE	0.00751	20.84754
1667	rs488225	A	G	A	G	-0.0411	0.000415	0.3902	0.394685	FALSE	FALSE	FALSE	ieu-b-508	12	3229827	0.034135	486484	0.99029	Sepsis (28	Sepsis (28	Sepsis (28	TRUE	igd	0.009	4.87E-06	5606	M15335.m	TRUE	reported	a7RjFn	2	TRUE	0.003706	20.847
3917	rs3133571	T	C	T	C	-0.0338	-0.0316	0.2559	0.268401	FALSE	FALSE	FALSE	ieu-b-508	8	1.02E+08	0.03782	486484	0.403464	Sepsis (28	Sepsis (28	Sepsis (28	TRUE	igd	0.0074	5.05E-06	2575	M32691.m	TRUE	reported	SiuD6F	2	TRUE	0.008037	20.84647
5023	rs6873660	A	G	A	G	0.3413	-0.05472	0.9683	0.94915	FALSE	FALSE	FALSE	ieu-b-508	5	45732184	0.075871	486484	0.470744	Sepsis (28	Sepsis (28	Sepsis (28	TRUE	igd	0.0717	1.93E-06	25	M33178.m	TRUE	reported	g22WMS	2	TRUE	0.475436	20.84597
7186	rs1208393	T	C	T	C	0.0274	0.097027	0.1261	0.134774	FALSE	FALSE	FALSE	ieu-b-508	1	1.84E+08	0.049277	486484	0.048953	Sepsis (28	Sepsis (28	Sepsis (28	TRUE	igd	0.006	4.50E-06	4800	M34539.m	TRUE	reported	puA8ni	2	TRUE	0.004326	20.84576
5459	rs194546	A	G	A	G	-0.4891	-0.03915	0.9795	0.98208	FALSE	FALSE	FALSE	ieu-b-508	16	21605967	0.125378	486484	0.754863	Sepsis (28	Sepsis (28	Sepsis (28	TRUE	igd	0.1065	4.37E-06	171	M33391.m	TRUE	reported	EgMd1b	2	TRUE	0.109797	20.84427
8280	rs4610008	T	C	T	C	-0.0562	-0.0043	0.6607	0.568481	FALSE	FALSE	FALSE	ieu-b-508	2	1.09E+08	0.033835	486484	0.89891	Sepsis (28	Sepsis (28	Sepsis (28	TRUE	igd	0.0123	5.20E-06	1260	M36553.m	TRUE	reported	2Rk7nd	2	TRUE	0.016299	20.84359
7093	rs1104422	C	G	C	G	-0.0274	0.049712	0.6326	0.623335	FALSE	TRUE	FALSE	ieu-b-508	12	18777451	0.034578	486484	0.150526	Sepsis (28	Sepsis (28	Sepsis (28	TRUE	igd	0.006	5.49E-06	3239	M34513.m	TRUE	reported	1hoso6	2	TRUE	0.006397	20.84157
6864	rs1606973	A	C	A	C	-0.184	0.027032	0.6944	0.806974	FALSE	FALSE	FALSE	ieu-b-508	2	51874700	0.042239	486484	0.52218	Sepsis (28	Sepsis (28	Sepsis (28	TRUE	igd	0.038	1.32E-06	18	M34365.m	TRUE	reported	8eexD0	2	TRUE	0.5657	20.84087
5570	rs2287296	T	C	T	C	-1.2634	-0.00549	0.025	0.02668	FALSE	FALSE	FALSE	ieu-b-508	9	1.32E+08	0.10545	486484	0.958509	Sepsis (28	Sepsis (28	Sepsis (28	TRUE	igd	0.2721	3.44E-06	60	M33423.m	TRUE	reported	MoEnO7	2	TRUE	0.264334	20.84018
6368	rs7305796	A	G	A	G	0.0338	0.059978	0.8905	0.89469	FALSE	FALSE	FALSE	ieu-b-508	12	10292859	0.054433	486484	0.270522	Sepsis (28	Sepsis (28	Sepsis (28	TRUE	igd	0.0074	4.30E-06	1765	M33961.m	TRUE	reported	10AWw0	2	TRUE	0.011682	20.83903
507	rs7790791	T	C	T	C	0.0594	-0.00103	0.0873	0.092717	FALSE	FALSE	FALSE	ieu-b-508	7	1.4E+08	0.057863	486484	0.985807	Sepsis (28	Sepsis (28	Sepsis (28	TRUE	igd	0.013	4.67E-06	1002	M00575.m	TRUE	reported	Mv0t8k	2	TRUE	0.020411	20.8362
2327	rs1016131	T	G	T	G	0.0315	0.016072	0.9568	0.955503	FALSE	FALSE	FALSE	ieu-b-508	12	1.08E+08	0.081545	486484	0.843752	Sepsis (28	Sepsis (28	Sepsis (28	TRUE	igd	0.0069	5.10E-06	7366	M21044.m	TRUE	reported	Ym5rae	2	TRUE	0.002821	20.83555
1104	rs1770653	A	G	A	G	0.0105	0.015635	0.6331	0.631833	FALSE	FALSE	FALSE	ieu-b-508	19	13015530	0.03456	486484	0.650987	Sepsis (28	Sepsis (28	Sepsis (28	TRUE	igd	0.0023	6.97E-06	7104	M01638.m	TRUE	reported	8dp0Ac	2	TRUE	0.002925	20.83534
1114	rs720118	T	C	T	C	-0.0105	0.012059	0.6152	0.621928	FALSE	FALSE	FALSE	ieu-b-508	2	6037363	0.034499	486484	0.72667	Sepsis (28	Sepsis (28	Sepsis (28	TRUE	igd	0.0023	4.55E-06	7104	M01638.m	TRUE	reported	8dp0Ac	2	TRUE	0.002925	20.83534
1118	rs901272	A	C	A	C	-0.0105	-0.02296	0.6207	0.613949	FALSE	FALSE	FALSE	ieu-b-508	15	63755235	0.034279	486484	0.503065	Sepsis (28	Sepsis (28	Sepsis (28	TRUE	igd	0.0023	6.49E-06	7104	M01638.m	TRUE	reported	8dp0Ac	2	TRUE	0.002925	20.83534
4310	rs9318132	A	C	A	C	-0.021	0.001378	0.2664	0.270616	FALSE	FALSE	FALSE	ieu-b-508	13	73571073	0.037604	486484	0.970767	Sepsis (28	Sepsis (28	Sepsis (28	TRUE	igd	0.0046	5.27E-06	6753	M32838.m	TRUE	reported	hyR7zA	2	TRUE	0.003077	20.83504
1885	rs1978450	A	C	A	C	0.1329	0.027532	0.017	0.151139	FALSE	FALSE	FALSE	ieu-b-508	5	1.56E+08	0.04656	486484	0.554301	Sepsis (28	Sepsis (28	Sepsis (28	TRUE	igd	0.0291	4.75E-06	1756	M15990.m	TRUE	reported	15fe4N	2	TRUE	0.011738	20.83383
569	rs1218955	A	T	A	T	0.021	-0.03318	0.7684	0.781983	FALSE	TRUE	FALSE	ieu-b-508	6	91104402	0.040407	486484	0.411609	Sepsis (28	Sepsis (28	Sepsis (28	TRUE	igd	0.0046	5.06E-06	5344	M01107.m	TRUE	reported	EbPV1D	2	TRUE	0.003885	20.83341
6754	rs1054363	T	G	T	G	0.2377	-0.08339	0.019	0.028887	FALSE	FALSE	FALSE	ieu-b-508	16	572855	0.10015	486484	0.405062	Sepsis (28	Sepsis (28	Sepsis (28	TRUE	igd	0.052	4.84E-06	633	M34339.m	TRUE	reported	RwGZ7c	2	TRUE	0.031955	20.82943
1431	rs4776752	A	G	A	G	0.0178	0.060134	0.1512	0.15223	FALSE	FALSE	FALSE	ieu-b-508	15	66451799	0.046608	486484	0.196976	Sepsis (28	Sepsis (28	Sepsis (28	TRUE	igd	0.0039	4.23E-06	6941	M03127.m	TRUE	reported	JscEZw	2	TRUE	0.002992	20.82503
5176	rs1764758	A	G	A	G	0.0744	0.075528	0.1044	0.154588	FALSE	FALSE	FALSE	ieu-b-508	13	69265591	0.047959	486484	0.115291	Sepsis (28	Sepsis (28	Sepsis (28	TRUE	igd	0.0163	4.76E-06	4625	M33195.m	TRUE	reported	Sl4STh	2	TRUE	0.004484	20.82489
8032	rs9605002	A	G	A	G	-0.0178	0.013326	0.1	0.105969	FALSE	FALSE	FALSE	ieu-b-508	22	19665415	0.055082	486484	0.808838	Sepsis (28	Sepsis (28	Sepsis (28	TRUE	igd	0.0039	4.24E-06	6604	M35754.m	TRUE	reported	lIXOo1	2	TRUE	0.003144	20.82472
7477	rs1151428	A	G	A	G	-0.0178	-0.02644	0.1545	0.16048	FALSE	FALSE	FALSE	ieu-b-508	13	1.12E+08	0.045397	486484	0.56027	Sepsis (28	Sepsis (28	Sepsis (28	TRUE	igd	0.0039	4.77E-06	5694	M35240.m	TRUE	reported	55Kiv4	2	TRUE	0.003645	20.82372
6504	rs2839011	T	C	T	C	0.0356	0.003303	0.2016	0.223059	FALSE	FALSE	FALSE	ieu-b-508	21	47243074	0.040076	486484	0.93431	Sepsis (28	Sepsis (28	Sepsis (28	TRUE	igd	0.0078	5.33E-06	5581	M34112.m	TRUE	reported	97JqFb	2	TRUE	0.003719	20.82357
8164	rs1695973	T	C	T	C	-0.0484	0.031297	0.1032	0.127591	FALSE	FALSE	FALSE	ieu-b-508	15	47891232	0.050094	486484	0.532125	Sepsis (28	Sepsis (28	Sepsis (28	TRUE	igd	0.0106	5.56E-06	1489	M36131.m	TRUE	reported	DbYm5t	2	TRUE	0.013808	20.8207
5295	rs1727996	A	G	A	G	-0.1137	-0.18891	0.9618	0.931111	FALSE	FALSE	FALSE	ieu-b-508	5	36204338	0.065731	486484	0.004053	Sepsis (28	Sepsis (28	Sepsis (28	TRUE	igd	0.0249	4.96E-06	1273	M33353.m	TRUE	reported	ZIN4gk	2	TRUE	0.016115	20.81802
4658	rs1006053	C	G	C	G	0.153	-0.01995	0.9646	0.984502	FALSE	TRUE	FALSE	ieu-b-508	5	1.17E+08	0.138541	486484	0.885521	Sepsis (28	Sepsis (28	Sepsis (28	TRUE	igd	0.0335	4.88E-06	1017	M33178.m	TRUE	reported	g22WMS	2	TRUE	0.020098	20.81797
5323	rs1221631	A	T	A	T	-0.0429	0.077466	0.0351	0.03213	FALSE	TRUE	FALSE	ieu-b-508	6	93244623	0.094377	486484	0.411754	Sepsis (28	Sepsis (28	Sepsis (28	TRUE	igd	0.0094	5.49E-06	3890	M33364.m	TRUE	reported	9Zrr7Y	2	TRUE	0.005326	20.81783
6680	rs1109726	T	C	T	C	0.0557	-0.01257	0.1593	0.144973	FALSE	FALSE	FALSE	ieu-b-508	4	77114936	0.047118	486484	0.789662	Sepsis (28	Sepsis (28	Sepsis (28	TRUE	igd	0.0122	5.11E-06	1492	M34329.m	TRUE	reported	mgPKP5	2	TRUE	0.013778	20.81652
2845	rs1077449	A	G	A	G	0.0397	-0.03472	0.1908	0.20131	FALSE	FALSE	FALSE	ieu-b-508	12	1.2E+08	0.04171	486484	0.405186	Sepsis (28	Sepsis (28	Sepsis (28	TRUE	igd	0.0087	5.39E-06	5673	M27531.m	TRUE	reported	v01zDr	2	TRUE	0.003657	20.81562
2858	rs7539563	T	G	T	G	0.0397	-0.03754	0.2058	0.208187	FALSE	FALSE	FALSE	ieu-b-508	1	20049085	0.041167	486484	0.361808	Sepsis (28	Sepsis (28	Sepsis (28	TRUE	igd	0.0087	5.47E-06	5673	M27531.m	TRUE	reported	v01zDr	2	TRUE	0.003657	20.81562
3634	rs1105306	T	C	T	C	0.1636	0.014282	0.0474	0.042844	FALSE	FALSE	FALSE	ieu-b-508	12	34176861	0.082965	486484	0.863323	Sepsis (28	Sepsis (28	Sepsis (28	TRUE	igd	0.0358	4.84E-06	604	M32560.m	TRUE	reported	sLwsEw	2	TRUE	0.03342	20.81422
8150	rs1249475	A	C	A	C	0.0429	-0.02265	0.1264	0.124665	FALSE	FALSE	FALSE	ieu-b-508	3	23769897	0.051029	486484	0.657198	Sepsis (28	Sepsis (28	Sepsis (28	TRUE	igd	0.0094	4.94E-06	2709	M36115.m	TRUE	reported	ZV1jWp	2	TRUE	0.00763	20.81317
5316	rs2642911	T	G	T	G	0.0178	0.035453	0.6151	0.61833	FALSE	FALSE	FALSE	ieu-b-508	3	27677178	0.03439	486484	0.302586	Sepsis (28	Sepsis (28	Sepsis (28	TRUE	igd	0.0039	4.53E-06	2212	M33363.m	TRUE	reported	7K0MM0	2	TRUE	0.009329	20.8122
8172	rs890554	C	G	C	G	-0.0283	0.06833	0.6895	0.687925	FALSE	TRUE	FALSE	ieu-b-508	8	82233910	0.035993	486484	0.05764	Sepsis (28	Sepsis (28	Sepsis (28	TRUE	igd	0.0062	5.75E-06	1780	M36131.m	TRUE	reported	DbYm5t	2	TRUE	0.01157	20.8114
2895	rs7319920	A	G	A	G	-0.0219	0.041422	0.1522	0.153957	FALSE	FALSE	FALSE	ieu-b-508	13	35867044	0.046241	486484	0.370374	Sepsis (28	Sepsis (28	Sepsis (28	TRUE	igd	0.0048	4.39E-06	7373	M27718.m	TRUE	reported	PHL57z	2	TRUE	0.002815	20.81076
7848	rs1493534	A	C	A	C	0.0219	0.035158	0.8645	0.856696	FALSE	FALSE	FALSE	ieu-b-508	11	1.29E+08	0.048392	486484	0.467515	Sepsis (28	Sepsis (28	Sepsis (28	TRUE	igd	0.0048	4.97E-06	7364	M35626.m	TRUE	reported	0cF47S	2	TRUE	0.002819	20.81075
919	rs7973508	A	G	A	G	-0.0073	0.00926	0.2831	0.27595	FALSE	FALSE	FALSE	ieu-b-508	12	1.15E+08	0.03729	486484	0.80389	Sepsis (28	Sepsis (28	Sepsis (28	TRUE	igd	0.0016	5.40E-06	7354	M01494.m	TRUE	reported	7JWNKI	2	TRUE	0.002823	20.81075
1226	rs1381274	T	C	T	C	-0.0146	0.028362	0.4672	0.463429	FALSE	FALSE	FALSE	ieu-b-508	14	98655131	0.033395	486484	0.395735	Sepsis (28	Sepsis (28	Sepsis (28	TRUE	igd	0.0032	3.63E-06	7346	M01712.m	TRUE	reported	JfdAWN	2	TRUE	0.002826	20.81074
6826	rs4925536	A	G	A	G	0.0146	-0.01143	0.8988	0.890866	FALSE	FALSE	FALSE	ieu-b-508	1	2.48E+08	0.053892	486484	0.83206	Sepsis (28	Sepsis (28	Sepsis (28	TRUE	igd	0.0032	7.17E-06	6755	M34359.m	TRUE	reported	0UotaJ	2	TRUE	0.003072	20.81024
7173	rs7162018	A	G	A	G	-0.0219	0.067176	0.4943	0.501233	FALSE	FALSE	FALSE	ieu-b-508	15	39365195	0.033532	486484	0.045141	Sepsis (28	Sepsis (28	Sepsis (28	TRUE	igd	0.0048	4.54E-06	4925	M34534.m	TRUE	reported	iA78RM	2	TRUE	0.004209	20.80795
1868	rs1052038	T	G	T	G	0.0219	-0.05996	0.1733	0.167632	FALSE	FALSE	FALSE	ieu-b-508	4	1.79E+08	0.04465	486484	0.179324	Sepsis (28	Sepsis (28	Sepsis (28	TRUE	igd	0.0048	4.92E-06	4538	M15958.m	TRUE	reported	3HF6lf	2	TRUE	0.004566	20.80723
5772	rs281796	A	C	A	C	-0.0356	-0.00682	0.8247	0.827763	FALSE	FALSE	FALSE	ieu-b-508	3	1.93E+08	0.044259	486484	0.877491	Sepsis (28	Sepsis (28	Sepsis (28	TRUE	igd	0.0078	5.62E-06	1733	M33509.m	TRUE	reported	56SCCs	2	TRUE	0.011877	20.80699
8309	rs1736651	A	G	A	G	-0.0584	0.054527	0.9343	0.933186	FALSE	FALSE	FALSE	ieu-b-508	2	11320668	0.0672	486484	0.41713	Sepsis (28	Sepsis (28	Sepsis (28	TRUE	igd	0.0128	4.75E-06	3346	M36585.m	TRUE	reported	pXZg13	2	TRUE	0.006183	20.80396
4537	rs1321237	T	G	T	G	-0.0333	-0.09069	0.0741	0.06783	FALSE	FALSE	FALSE	ieu-b-508	6	87780670	0.067158	486484	0.176868	Sepsis (28	Sepsis (28	Sepsis (28	TRUE	igd	0.0073	4.61E-06	7166	M33138.m	TRUE	reported	lUpLAI	2	TRUE	0.002895	20.80279
6650	rs197712	T	C	T	C	0.098	0.009806	0.8636	0.863625	FALSE	FALSE	FALSE	ieu-b-508	15	67308988	0.048531	486484	0.839876	Sepsis (28	Sepsis (28	Sepsis (28	TRUE	igd	0.0214	4.67E-06	248	M34322.m	TRUE	reported	16GdG9	2	TRUE	0.077968	20.80214

1963	rs1398802	T	G	T	G	-0.026	0.040455	0.6877	0.673165	FALSE	FALSE	FALSE	ieu-b-508	6	67197675	0.035644	486484	0.256389	Sepsis (28	Sepsis (28	Sepsis (28	TRUE	igd	0.0057	6.12E-06	7226	M17807.m	TRUE	reported	zd6sZl	2	TRUE	0.002871	20.80064
8043	rs7586148	T	C	T	C	-0.0324	-0.02064	0.5142	0.484812	FALSE	FALSE	FALSE	ieu-b-508	2	31342086	0.033391	486484	0.536413	Sepsis (28	Sepsis (28	Sepsis (28	TRUE	igd	0.0071	5.53E-06	1632	M35854.m	TRUE	reported	EujB0p	2	TRUE	0.012599	20.79892
8633	rs9922615	T	C	T	C	0.0187	-0.10699	0.9445	0.941627	FALSE	FALSE	FALSE	ieu-b-508	16	67398369	0.07184	486484	0.136409	Sepsis (28	Sepsis (28	Sepsis (28	TRUE	igd	0.0041	6.28E-06	7366	M37506.m	TRUE	reported	m2kaRt	2	TRUE	0.002816	20.79685
5857	rs7314742	T	G	T	G	-0.0187	0.052561	0.2658	0.268058	FALSE	FALSE	FALSE	ieu-b-508	12	1.22E+08	0.037766	486484	0.163997	Sepsis (28	Sepsis (28	Sepsis (28	TRUE	igd	0.0041	4.53E-06	7352	M33587.m	TRUE	reported	ssG6bF	2	TRUE	0.002822	20.79684
8566	rs1341951	A	G	A	G	-0.0187	-0.06654	0.5995	0.5974	FALSE	FALSE	FALSE	ieu-b-508	2	1.06E+08	0.034051	486484	0.050683	Sepsis (28	Sepsis (28	Sepsis (28	TRUE	igd	0.0041	5.78E-06	7333	M37203.m	TRUE	reported	3gIIlv	2	TRUE	0.002829	20.79682
8567	rs1476117	A	G	A	G	0.0187	0.013245	0.5513	0.539168	FALSE	FALSE	FALSE	ieu-b-508	16	12442286	0.033595	486484	0.693387	Sepsis (28	Sepsis (28	Sepsis (28	TRUE	igd	0.0041	5.86E-06	7333	M37203.m	TRUE	reported	3gIIlv	2	TRUE	0.002829	20.79682
1271	rs1047745	T	C	T	C	-0.0374	0.198637	0.0169	0.019821	FALSE	FALSE	FALSE	ieu-b-508	5	1.1E+08	0.120385	486484	0.098942	Sepsis (28	Sepsis (28	Sepsis (28	TRUE	igd	0.0082	4.65E-06	7325	M02132.m	TRUE	reported	uJFm2t	2	TRUE	0.002832	20.79682
3680	rs1079699	T	C	T	C	0.0374	-0.01291	0.865	0.855785	FALSE	FALSE	FALSE	ieu-b-508	1	1.58E+08	0.047581	486484	0.786131	Sepsis (28	Sepsis (28	Sepsis (28	TRUE	igd	0.0082	4.81E-06	7303	M32586.m	TRUE	reported	f6n0gX	2	TRUE	0.00284	20.7968
2526	rs1697378	A	G	A	G	-0.073	-0.29566	0.036	0.051506	FALSE	FALSE	FALSE	ieu-b-508	18	3445826	0.076184	486484	0.000104	Sepsis (28	Sepsis (28	Sepsis (28	TRUE	igd	0.016	5.06E-06	2088	M22175.m	TRUE	reported	IIlqyJ	2	TRUE	0.009871	20.79647
4128	rs7228173	T	C	T	C	0.0187	-0.05127	0.2694	0.290607	FALSE	FALSE	FALSE	ieu-b-508	18	35278788	0.036816	486484	0.163714	Sepsis (28	Sepsis (28	Sepsis (28	TRUE	igd	0.0041	6.11E-06	6658	M32755.m	TRUE	reported	WDrNdi	2	TRUE	0.003115	20.79625
6088	rs9317917	A	C	A	C	-0.0479	0.059511	0.1628	0.19051	FALSE	FALSE	FALSE	ieu-b-508	13	70872455	0.042645	486484	0.162866	Sepsis (28	Sepsis (28	Sepsis (28	TRUE	igd	0.0105	5.30E-06	2660	M33835.m	TRUE	reported	crhBhm	2	TRUE	0.007763	20.79533
6835	rs8071165	T	G	T	G	-0.1013	0.096111	0.0115	0.02667	FALSE	FALSE	FALSE	ieu-b-508	17	14038916	0.104029	486484	0.355549	Sepsis (28	Sepsis (28	Sepsis (28	TRUE	igd	0.0222	5.09E-06	1576	M34359.m	TRUE	reported	0UotaJ	2	TRUE	0.013039	20.79512
4389	rs2370933	A	G	A	G	0.0073	-0.03857	0.3578	0.352476	FALSE	FALSE	FALSE	ieu-b-508	14	79655012	0.034858	486484	0.268491	Sepsis (28	Sepsis (28	Sepsis (28	TRUE	igd	0.0016	2.85E-06	1763	M32867.m	TRUE	reported	Jcsyzm	2	TRUE	0.01167	20.79279
6033	rs1986163	T	C	T	C	0.264	-0.1086	0.0335	0.023189	FALSE	FALSE	FALSE	ieu-b-508	4	82344387	0.111418	486484	0.329695	Sepsis (28	Sepsis (28	Sepsis (28	TRUE	igd	0.0578	4.88E-06	581	M33782.m	TRUE	reported	875PaI	2	TRUE	0.034662	20.79001
879	rs1715594	T	C	T	C	0.0698	0.015718	0.9746	0.986758	FALSE	FALSE	FALSE	ieu-b-508	5	1.03E+08	0.147465	486484	0.915115	Sepsis (28	Sepsis (28	Sepsis (28	TRUE	igd	0.0153	4.75E-06	1768	M01365.m	TRUE	reported	N1cZTs	2	TRUE	0.011635	20.78914
7694	rs764419	T	G	T	G	-0.0187	0.058065	0.1973	0.208673	FALSE	FALSE	FALSE	ieu-b-508	6	1.55E+08	0.041188	486484	0.158611	Sepsis (28	Sepsis (28	Sepsis (28	TRUE	igd	0.0041	4.99E-06	3007	M35331.m	TRUE	reported	GBnxhN	2	TRUE	0.00687	20.78866
881	rs314241	T	C	T	C	-0.0114	0.001638	0.4625	0.445918	FALSE	FALSE	FALSE	ieu-b-508	17	7045661	0.033761	486484	0.961301	Sepsis (28	Sepsis (28	Sepsis (28	TRUE	igd	0.0025	5.25E-06	7362	M01365.m	TRUE	reported	N1cZTs	2	TRUE	0.002816	20.78795
884	rs453612	T	C	T	C	0.0114	-0.01926	0.3549	0.350134	FALSE	FALSE	FALSE	ieu-b-508	20	15402178	0.035071	486484	0.582954	Sepsis (28	Sepsis (28	Sepsis (28	TRUE	igd	0.0025	5.85E-06	7362	M01365.m	TRUE	reported	N1cZTs	2	TRUE	0.002816	20.78795
885	rs6467872	A	C	A	C	0.0114	-0.03709	0.6087	0.583851	FALSE	FALSE	FALSE	ieu-b-508	7	81404882	0.034097	486484	0.276736	Sepsis (28	Sepsis (28	Sepsis (28	TRUE	igd	0.0025	5.64E-06	7362	M01365.m	TRUE	reported	N1cZTs	2	TRUE	0.002816	20.78795
676	rs1051646	T	C	T	C	-0.0114	0.049571	0.1	0.10414	FALSE	FALSE	FALSE	ieu-b-508	4	1.02E+08	0.0555	486484	0.371764	Sepsis (28	Sepsis (28	Sepsis (28	TRUE	igd	0.0025	5.89E-06	7358	M01299.m	TRUE	reported	gfM0a3	2	TRUE	0.002818	20.78795
3076	rs1285355	A	G	A	G	0.0228	0.009718	0.088	0.094176	FALSE	FALSE	FALSE	ieu-b-508	13	71748180	0.057385	486484	0.865519	Sepsis (28	Sepsis (28	Sepsis (28	TRUE	igd	0.005	4.63E-06	7346	M32197.m	TRUE	reported	VZjwa9	2	TRUE	0.002823	20.78794
3393	rs1260148	T	C	T	C	-0.0342	-0.01155	0.8888	0.887366	FALSE	FALSE	FALSE	ieu-b-508	17	54309754	0.053109	486484	0.827819	Sepsis (28	Sepsis (28	Sepsis (28	TRUE	igd	0.0075	4.43E-06	7346	M32425.m	TRUE	reported	u5RLGY	2	TRUE	0.002823	20.78794
7318	rs177387	A	G	A	G	-0.0114	0.051864	0.5143	0.524282	FALSE	FALSE	FALSE	ieu-b-508	14	73728957	0.033736	486484	0.124201	Sepsis (28	Sepsis (28	Sepsis (28	TRUE	igd	0.0025	6.19E-06	7344	M35127.m	TRUE	reported	RNsmjl	2	TRUE	0.002823	20.78794
7319	rs1812081	T	G	T	G	0.0114	-0.03068	0.3963	0.398214	FALSE	FALSE	FALSE	ieu-b-508	2	1.34E+08	0.034068	486484	0.367904	Sepsis (28	Sepsis (28	Sepsis (28	TRUE	igd	0.0025	5.48E-06	7344	M35127.m	TRUE	reported	RNsmjl	2	TRUE	0.002823	20.78794
8568	rs2295709	T	C	T	C	0.0228	0.01962	0.7937	0.77923	FALSE	FALSE	FALSE	ieu-b-508	6	1.19E+08	0.040144	486484	0.625022	Sepsis (28	Sepsis (28	Sepsis (28	TRUE	igd	0.005	4.81E-06	7333	M37203.m	TRUE	reported	3gIIlv	2	TRUE	0.002828	20.78793
2491	rs1741183	A	C	A	C	-0.0114	0.073618	0.7981	0.795221	FALSE	FALSE	FALSE	ieu-b-508	5	1.07E+08	0.041502	486484	0.076092	Sepsis (28	Sepsis (28	Sepsis (28	TRUE	igd	0.0025	3.21E-06	7329	M22116.m	TRUE	reported	bjUU6r	2	TRUE	0.002829	20.78793
837	rs1074661	A	G	A	G	-0.0114	0.005598	0.2333	0.237609	FALSE	FALSE	FALSE	ieu-b-508	9	82616777	0.039337	486484	0.886828	Sepsis (28	Sepsis (28	Sepsis (28	TRUE	igd	0.0025	4.05E-06	7323	M01359.m	TRUE	reported	7L9x1s	2	TRUE	0.002831	20.78792
5569	rs2111849	A	C	A	C	-1.5433	-0.0946	0.0175	0.017228	FALSE	FALSE	FALSE	ieu-b-508	2	1.63E+08	0.128417	486484	0.461321	Sepsis (28	Sepsis (28	Sepsis (28	TRUE	igd	0.3328	3.54E-06	60	M33423.m	TRUE	reported	MoEnO7	2	TRUE	0.263846	20.78791
3926	rs1028578	T	C	T	C	0.0114	0.017528	0.183	0.185788	FALSE	FALSE	FALSE	ieu-b-508	14	86196704	0.042922	486484	0.683007	Sepsis (28	Sepsis (28	Sepsis (28	TRUE	igd	0.0025	7.52E-06	7308	M32698.m	TRUE	reported	srqncz	2	TRUE	0.002837	20.78791
3936	rs4537895	A	G	A	G	0.0114	-0.01061	0.7962	0.79409	FALSE	FALSE	FALSE	ieu-b-508	13	65376034	0.041504	486484	0.79815	Sepsis (28	Sepsis (28	Sepsis (28	TRUE	igd	0.0025	6.62E-06	7308	M32698.m	TRUE	reported	srqncz	2	TRUE	0.002837	20.78791
1117	rs837763	T	C	T	C	0.0114	-0.04332	0.5528	0.556074	FALSE	FALSE	FALSE	ieu-b-508	16	88853729	0.033518	486484	0.196183	Sepsis (28	Sepsis (28	Sepsis (28	TRUE	igd	0.0025	6.03E-06	7104	M01638.m	TRUE	reported	8dp0Ac	2	TRUE	0.002918	20.78775
7807	rs2592126	A	G	A	G	-0.0114	-0.00491	0.4137	0.434845	FALSE	FALSE	FALSE	ieu-b-508	2	2.34E+08	0.033699	486484	0.884161	Sepsis (28	Sepsis (28	Sepsis (28	TRUE	igd	0.0025	4.75E-06	6883	M35464.m	TRUE	reported	Ir9dHp	2	TRUE	0.003012	20.78756
2867	rs1247053	T	C	T	C	0.0228	0.041143	0.6097	0.604076	FALSE	FALSE	FALSE	ieu-b-508	2	46738846	0.034166	486484	0.228517	Sepsis (28	Sepsis (28	Sepsis (28	TRUE	igd	0.005	5.45E-06	6715	M27710.m	TRUE	reported	XeaFav	2	TRUE	0.003087	20.78741
1942	rs9393768	A	G	A	G	0.0114	-0.00927	0.3579	0.357625	FALSE	FALSE	FALSE	ieu-b-508	6	26885619	0.034802	486484	0.789911	Sepsis (28	Sepsis (28	Sepsis (28	TRUE	igd	0.0025	6.24E-06	6549	M16822.m	TRUE	reported	UNI018	2	TRUE	0.003165	20.78725
5970	rs1443751	T	C	T	C	0.0114	0.020699	0.7823	0.779769	FALSE	FALSE	FALSE	ieu-b-508	7	1.22E+08	0.040241	486484	0.606986	Sepsis (28	Sepsis (28	Sepsis (28	TRUE	igd	0.0025	5.36E-06	6279	M33666.m	TRUE	reported	dvWEtk	2	TRUE	0.003301	20.78698
2455	rs1015339	T	G	T	G	-0.0114	0.028158	0.4862	0.483223	FALSE	FALSE	FALSE	ieu-b-508	18	49960237	0.033544	486484	0.401236	Sepsis (28	Sepsis (28	Sepsis (28	TRUE	igd	0.0025	6.59E-06	6180	M22030.m	TRUE	reported	ZNV0qR	2	TRUE	0.003353	20.78687
2463	rs2054299	A	G	A	G	-0.0114	0.040795	0.4546	0.478667	FALSE	FALSE	FALSE	ieu-b-508	18	65475074	0.033529	486484	0.223709	Sepsis (28	Sepsis (28	Sepsis (28	TRUE	igd	0.0025	6.30E-06	6180	M22030.m	TRUE	reported	ZNV0qR	2	TRUE	0.003353	20.78687
3704	rs2093237	T	C	T	C	0.0228	-0.01964	0.2815	0.297315	FALSE	FALSE	FALSE	ieu-b-508	6	90139533	0.036652	486484	0.592126	Sepsis (28	Sepsis (28	Sepsis (28	TRUE	igd	0.005	5.93E-06	5955	M32593.m	TRUE	reported	6zVl1i	2	TRUE	0.00348	20.78662
660	rs1079536	C	G	C	G	0.0114	0.090248	0.6957	0.731785	FALSE	TRUE	FALSE	ieu-b-508	10	6701714	0.03781	486484	0.016991	Sepsis (28	Sepsis (28	Sepsis (28	TRUE	igd	0.0025	3.76E-06	5687	M01284.m	TRUE	reported	E4hc9c	2	TRUE	0.003643	20.78629
8220	rs1312517	C	G	C	G	-0.0406	-0.02329	0.1863	0.195476	FALSE	TRUE	FALSE	ieu-b-508	4	48241460	0.04216	486484	0.580636	Sepsis (28	Sepsis (28	Sepsis (28	TRUE	igd	0.0089	5.47E-06	1582	M36399.m	TRUE	reported	EdUpnt	2	TRUE	0.012983	20.78369
2205	rs4280872	A	G	A	G	-0.0269	0.003747	0.0491	0.055617	FALSE	FALSE	FALSE	ieu-b-508	5	3057392	0.07332	486484	0.959239	Sepsis (28	Sepsis (28	Sepsis (28	TRUE	igd	0.0059	5.48E-06	6929	M19364.m	TRUE	reported	XkjMEx	2	TRUE	0.002991	20.78142
5131	rs1087844	A	G	A	G	0.0944	0.03097	0.0829	0.081391	FALSE	FALSE	FALSE	ieu-b-508	12	66940670	0.061927	486484	0.617003	Sepsis (28	Sepsis (28	Sepsis (28	TRUE	igd	0.0207	5.21E-06	2616	M33190.m	TRUE	reported	UkhAw5	2	TRUE	0.007887	20.78122
8166	rs2068007	T	C	T	C	0.0374	0.002847	0.9124	0.953881	FALSE	FALSE	FALSE	ieu-b-508	12	71589616	0.08066	486484	0.971848	Sepsis (28	Sepsis (28	Sepsis (28	TRUE	igd	0.0082	5.49E-06	1780	M36131.m	TRUE	reported	DbYm5t	2	TRUE	0.011552	20.77912
4315	rs1102970	T	C	T	C	-0.0424	-0.00874	0.1475	0.169757	FALSE	FALSE	FALSE	ieu-b-508	11	26791046	0.04484	486484	0.845518	Sepsis (28	Sepsis (28	Sepsis (28	TRUE	igd	0.0093	5.34E-06	6250	M32846.m	TRUE	reported	IwF2Yy	2	TRUE	0.003315	20.7791
8183	rs7474722	T	C	T	C	-0.0757	0.011097	0.9636	0.965685	FALSE	FALSE	FALSE	ieu-b-508	10	59493227	0.091409	486484	0.903375	Sepsis (28	Sepsis (28	Sepsis (28	TRUE	igd	0.0166	5.29E-06	2432	M36134.m	TRUE	reported	ekMsfG	2	TRUE	0.008478	20.7787
1874	rs2579931	A	G	A	G	0.0269	-0.03543	0.1044	0.10035	FALSE	FALSE	FALSE	ieu-b-508	6	1.02E+08	0.055831	486484	0.525641	Sepsis (28	Sepsis (28	Sepsis (28	TRUE	igd	0.0059	4.23E-06	4538	M15958.m	TRUE	reported	3HF6lf	2	TRUE	0.00456	20.77826
7972	rs1084231	T	G	T	G	0.0155	0.064081	0.1288	0.123422	FALSE	FALSE	FALSE	ieu-b-508	12	24443270	0.051028	486484	0.209188	Sepsis (28	Sepsis (28	Sepsis (28	TRUE	igd	0.0034	5.84E-06	7358	M35718.m	TRUE	reported	oUwc0a	2	TRUE	0.002817	20.77722
1209	rs1792544	A	G	A	G	-0.0155	-0.00621	0.8963	0.906793	FALSE	FALSE	FALSE	ieu-b-508	11	15180533	0.057356	486484	0.913766	Sepsis (28	Sepsis (28	Sepsis (28	TRUE	igd	0.0034	5.83E-06	7348	M01670.m	TRUE	reported	kjghbc	2	TRUE	0.00282	20.77722
1308	rs7597477	A	G	A	G	-0.0155	0.098237	0.9327	0.932347	FALSE	FALSE	FALSE	ieu-b-508	2	1.05E+08	0.066356	486484	0.138753	Sepsis (28	Sepsis (28	Sepsis (28	TRUE	igd	0.0034	6.80E-06	7325	M02132.m	TRUE	reported	uJFm2t	2	TRUE	0.002829	20.7772
2276	rs1156470	C	G	C	G	0.0155	0.031579	0.8162	0.799568	FALSE	TRUE	FALSE	ieu-b-508	11	2206326	0.041801	486484	0.449976	Sepsis (28	Sepsis (28	Sepsis (28	TRUE	igd	0.0034	6.40E-06	7301	M20675.m	TRUE	reported	CXe9rE	2	TRUE	0.002838	20.77718
2592	rs1188835	T	C	T	C	-0.0155	-0.00187	0.5436	0.538076	FALSE	FALSE	FALSE	ieu-b-508	6	1.39E+08	0.033491	486484	0.955436	Sepsis (28	Sepsis (28	Sepsis (28	TRUE	igd	0.0034	4.09E-06	7259	M22189.m	TRUE	reported	YSD5fY	2	TRUE	0.002855	20.77715
6801	rs1251697	C	G	C	G	0.0155	0.049813	0.9076	0.878339	FALSE	TRUE	FALSE	ieu-b-508	5	89715768	0.051739	486484	0.335654	Sepsis (28	Sepsis (28	Sepsis (28	TRUE	igd	0.0034	6.47E-06	6755	M34359.m	TRUE	reported	0UotaJ	2	TRUE	0.003067	20.77672
8581	rs6064820	T	C	T	C	0.0155	0.031102	0.7229	0.723601	FALSE	FALSE	FALSE	ieu-b-508	20	58206004	0.037352	486484	0.405033	Sepsis (28	Sepsis (28	Sepsis (28	TRUE	igd	0.0034	4.01E-06	5932	M37253.m	TRUE	reported	PYMqzt	2	TRUE	0.003491	20.77586
1351	rs1115140	A	T	A	T	0.0301	0.140175	0.9444	0.950712	FALSE	TRUE	FALSE	ieu-b-508	18	65658146	0.077146	486484	0.069218	Sepsis (28	Sepsis (28	Sepsis (28	TRUE	igd	0.0066	5.61E-06	1766	M02730.m	TRUE	reported	XYa035	2	TRUE	0.01164	20.77557
1913	rs1193567	A	G	A	G	-0.0155	-0.04421	0.202	0.200887	FALSE	FALSE	FALSE	ieu-b-508	4	1.86E+08	0.041713	486484	0.289174	Sepsis (28	Sepsis (28	Sepsis (28	TRUE	igd	0.0034	6.80E-06	4689	M16816.m	TRUE	reported	J1abbO	2	TRUE	0.004413	20.77401
7884	rs2459791	T	C	T	C	0.0552	0.075058	0.4055	0.40006	FALSE	FALSE	FALSE	ieu-b-508	5	17434249	0.034757	486484	0.030809	Sepsis (28	Sepsis (28	Sepsis (28	TRUE	igd	0.0121	5.24E-06	1098	M35635.m	TRUE	reported	qymryH	2	TRUE	0.018602	20.77378
855	rs1179116	T	G	T	G	0.0196	-0.00509	0.0803	0.102444	FALSE	FALSE	FALSE	ieu-b-508	9	38285271	0.055312	486484	0.926703	Sepsis (28	Sepsis (28	Sepsis (28	TRUE	igd	0.0043	4.35E-06	7063	M01361.m	TRUE	reported	NIAcQ3	2	TRUE	0.002933	20.77075
6668	rs1220077	A	G	A	G	0.0871	-0.06635	0.0859	0.084185	FALSE	FALSE	FALSE	ieu-b-508	6	96714195	0.060793	486484	0.275082	Sepsis (28	Sepsis (28	Sepsis (28	TRUE	igd	0.0191	5.10E-06	1612	M34327.m	TRUE	reported	Il4Lrr	2	TRUE	0.012736	20.76971
3669	rs186053	T	G	T	G	-0.0237	-0.03197	0.704	0.752406	FALSE	FALSE	FALSE	ieu-b-508	4	69601023	0.038827	486484	0.410279	Sepsis (28	Sepsis (28	Sepsis (28	TRUE	igd	0.0052	6.01E-06	7322	M32578.m	TRUE	reported	LPkMFr	2	TRUE	0.002829	20.76689
7758	rs1091873	A	T	A	T	0.0237	0.060065	0.9478	0.93515	FALSE	TRUE	FALSE	ieu-b-508	1	1.68E+08	0.068127	486484	0.377955	Sepsis (28	Sepsis (28	Sepsis (28	TRUE	igd	0.0052	6.02E-06	7256	M35439.m	TRUE	reported	DnuAGZ	2	TRUE	0.002855	20.76683
6106	rs432642	A	T	A	T	-0.0237	-0.02076	0.1441	0.164084	FALSE	TRUE	FALSE	ieu-b-508	5	1.17E+08	0.045095	486484	0.645254	Sepsis (28	Sepsis (28	Sepsis (28	TRUE	igd	0.0052	4.09E-06	6506	M33871.m	TRUE	reported	mQD6DP	2	TRUE	0.003183	20.76617
6285	rs4831776	T	C	T	C	0.0237	-0.0117	0.9508	0.952315	FALSE	FALSE	FALSE	ieu-b-508	8	15753366	0.079357	486484	0.882755	Sepsis (28	Sepsis (28	Sepsis (28	TRUE	igd	0.0052	6.28E-06	6502	M33939.m	TRUE	reported	PeKlgz	2	TRUE	0.003185	20.76617
6684	rs2876587	T	C	T	C	0.0684	0.049148	0.1046	0.114792	FALSE	FALSE	FALSE	ieu-b-508	6	21258254	0.052445	486484	0.348686	Sepsis (28	Sepsis (28	Sepsis (28	TRUE	igd	0.015	5.01E-06	1492	M34329.m	TRUE	reported	mgPKP5	2	TRUE	0.013745	20.76573
8193	rs297192	T	G	T	G	0.031	0.008151	0.4024	0.411186	FALSE	FALSE	FALSE	ieu-b-508	5	17193033	0.034002	486484	0.810535	Sepsis (28	Sepsis (28	Sepsis (28	TRUE	igd	0.0068	4.54E-06	2400	M36230.m	TRUE	reported	V5KJeR	2	TRUE	0.008585	20.76555
6224	rs6987078	A	G	A	G	0.0237	-0.01375	0.2224	0.223972	FALSE	FALSE	FALSE	ieu-b-508	8	1.03E+08	0.040459	486484	0.733946	Sepsis (28	Sepsis (28	Sepsis (28	TRUE	igd	0.0052	5.33E-06	4533	M33901.m	TRUE	reported	mQkhrb	2	TRUE	0.004562	20.76339
3639	rs4630914	T	C	T	C	0.062	0.123809	0.1001	0.109445	FALSE	FALSE	FALSE	ieu-b-508	3	1.11E+08	0.053867	486484	0.021538	Sepsis (28	Sepsis (28	Sepsis (28	TRUE	igd	0.0136	4.90E-06	2091	M32560.m	TRUE	reported	sLwsEw	2	TRUE	0.009841	20.76299
4207	rs1259878	C	G	C	G	0.0278	0.01083	0.599	0.600043	FALSE	TRUE	FALSE	ieu-b-508	16	52164830	0.034077	486484	0.750622	Sepsis (28	Sepsis (28	Sepsis (28	TRUE	igd	0.0061	4.89E-06	6150	M32769.m	TRUE	reported	W2ubRP	2	TRUE	0.003366	20.76293
608	rs1764134	C	G	C	G	0.0725	-0.00393	0.1639	0.175347	FALSE	TRUE	FALSE	ieu-b-508	16	76318584	0.044021	486484	0.928849	Sepsis (28	Sepsis (28	Sepsis (28	TRUE	igd	0.0159	5.26E-06	1447	M01114.m	TRUE	reported	fqHdM2	2	TRUE	0.014165	20.76257
7075	rs1223875	T	C	T	C	0.036	-0.1786	0.0429	0.043609	FALSE	FALSE	FALSE	ieu-b-508	9	1.03E+08	0.081642	486484	0.028699	Sepsis (28	Sepsis (28	Sepsis (28	TRUE	igd	0.0079	5.12E-06	7119	M34481.m	TRUE	reported	mXdL0W	2	TRUE	0.002908	20.76007
5951	rs1374486	A	G	A	G	-0.0319	0.028817	0.2098	0.211876	FALSE	FALSE	FALSE	ieu-b-508	1	1.6E+08	0.04077	486484	0.479675	Sepsis (28	Sepsis (28	Sepsis (28	TRUE	igd	0.007	5.52E-06	5328	M33652.m	TRUE	reported	vYkdTv	2	TRUE	0.003883	20.75976
6277	rs1049243	T	C	T	C	-0.036	-0.13081	0.0203	0.018923	FALSE	FALSE	FALSE	ieu-b-508	13	26254210	0.123409	486484	0.289175	Sepsis (28	Sepsis (28	Sepsis (28	TRUE	igd	0.0079	5.15E-06	6502	M33939.m	TRUE	reported	PeKlgz	2	TRUE	0.003184	20.75952
5602	rs3134034	T	C	T	C	0.031	-0.01416	0.5354	0.531267	FALSE	FALSE	FALSE	ieu-b-508	8	1.2E+08	0.033578	486484	0.673152	Sepsis (28	Sepsis (28	Sepsis (28	TRUE	igd	0.0068	4.51E-06	1767	M33441.m	TRUE	reported	CSdSpl	2	TRUE	0.011625	20.75935
6014	rs4691942	A	G	A	G	-0.2972	0.10483	0.8916	0.855824	FALSE	FALSE	FALSE	ieu-b-508	4	1.65E+08	0.047424	486484	0.027072	Sepsis (28	Sepsis (28	Sepsis (28	TRUE	igd	0.0644	3.91E-06	79	M33751.m	TRUE	reported	Cxdkr4	2	TRUE	0.212342	20.75819
5195	rs1178367	C	G	C	G	-0.0916	0.025213	0.8364	0.813746	FALSE	TRUE	FALSE	ieu-b-508	8	1.32E+08	0.043601	486484	0.56308	Sepsis (28	Sepsis (28	Sepsis (28	TRUE	igd	0.0201	5.45E-06	3221	M33203.m	TRUE	reported	Sto7pR	2	TRUE	0.006406	20.7553
5927	rs1018664	A	G	A	G	-0.1988	0.154842	0.0229	0.027518	FALSE	FALSE	FALSE	ieu-b-508	2	1.69E+08	0.101691	486484	0.127841	Sepsis (28	Sepsis (28	Sepsis (28	TRUE	igd	0.0436	5.12E-06	1155	M33637.m	TRUE	reported	tfLznu	2	TRUE	0.017682	20.75425
6090	rs1008810	A	G	A	G	-0.0465	-0.02661	0.809	0.80722	FALSE	FALSE	FALSE	ieu-b-508	8	20062812	0.042637	486484	0.532605	Sepsis (28	Sepsis (28	Sepsis (28	TRUE	igd	0.0102	5.18E-06	1428	M33864.m	TRUE	reported	yr5h52	2	TRUE	0.014345	20.75376
8181	rs7099968	T	C	T	C	-0.0556	0.004828	0.0466	0.036129	FALSE	FALSE	FALSE	ieu-b-508	10	1.32E+08	0.089529	486484	0.95699	Sepsis (28	Sepsis (28	Sepsis (28	TRUE	igd	0.0122	4.66E-06	2432	M36134.m	TRUE	reported	ekMsfG	2	TRUE	0.008468	20.75261
1121	rs1310369	T	G	T	G	-0.0474	-0.01867	0.5359	0.541852	FALSE	FALSE	FALSE	ieu-b-508	4	9972778	0.033475	486484	0.576927	Sepsis (28	Sepsis (28	Sepsis (28	TRUE	igd	0.0104	5.20E-06	2063	M01640.m	TRUE	reported	o3qxzl	2	TRUE	0.009969	20.75242
8084	rs743745	T	G	T	G	0.0319	-0.05754	0.1734	0.166632	FALSE	FALSE	FALSE	ieu-b-508	22	33116779	0.044789	486484	0.198901	Sepsis (28	Sepsis (28	Sepsis (28	TRUE	igd	0.007	6.00E-06	2651	M36009.m	TRUE	reported	2EUZ1U	2	TRUE	0.007773	20.75188
3532	rs2042807	T	C	T	C	-0.0237	0.020438	0.573	0.564926	FALSE	FALSE	FALSE	ieu-b-508	8	53333501	0.033931	486484	0.546953	Sepsis (28	Sepsis (28	Sepsis (28	TRUE	igd	0.0052	5.40E-06	1767	M32497.m	TRUE	reported	244bZN	2	TRUE	0.011619	20.74905
2973	rs1337857	A	C	A	C	-0.0711	-0.01823	0.0303	0.040213	FALSE	FALSE	FALSE	ieu-b-508	13	65748169	0.085898	486484	0.831927	Sepsis (28	Sepsis (28	Sepsis (28	TRUE	igd	0.0156	4.81E-06	1750	M31453.m	TRUE	reported	vdTd9M	2	TRUE	0.011731	20.74882
5756	rs1049259	A	C	A	C	0.1605	0.013861	0.0227	0.014126	FALSE	FALSE	FALSE	ieu-b-508	13	67637548	0.142251	486484	0.922379	Sepsis (28	Sepsis (28	Sepsis (28	TRUE	igd	0.0352	5.08E-06	972	M33508.m	TRUE	reported	UMXHcP	2	TRUE	0.020941	20.74771
6592	rs574773	T	C	T	C	-0.036	0.018337	0.0733	0.080763	FALSE	FALSE	FALSE	ieu-b-508	1	2.07E+08	0.061323	486484	0.764918	Sepsis (28	Sepsis (28	Sepsis (28	TRUE	igd	0.0079	5.97E-06	2278	M34289.m	TRUE	reported	Knp8ry	2	TRUE	0.009034	20.74767
3689	rs1624064	T	C	T	C	-0.0164	0.017612	0.5678	0.579779	FALSE	FALSE	FALSE	ieu-b-508	6	26378681	0.033843	486484	0.602787	Sepsis (28	Sepsis (28	Sepsis (28	TRUE	igd	0.0036	6.36E-06	7369	M32587.m	TRUE	reported	uknv7G	2	TRUE	0.002808	20.74745
3797	rs7529462	C	G	C	G	0.0164	-0.01274	0.7318	0.714618	FALSE	TRUE	FALSE	ieu-b-508	1	2.3E+08	0.037091	486484	0.731318	Sepsis (28	Sepsis (28	Sepsis (28	TRUE	igd	0.0036	4.93E-06	7369	M32635.m	TRUE	reported	jJl9P8	2	TRUE	0.002808	20.74745
6364	rs1595416	A	G	A	G	0.0164	0.015774	0.2639	0.253379	FALSE	FALSE	FALSE	ieu-b-508	2	1.34E+08	0.03851	486484	0.682089	Sepsis (28	Sepsis (28	Sepsis (28	TRUE	igd	0.0036	6.93E-06	7368	M33961.m	TRUE	reported	10AWw0	2	TRUE	0.002809	20.74745
8594	rs1168264	A	G	A	G	0.0082	0.071264	0.5871	0.570835	FALSE	FALSE	FALSE	ieu-b-508	2	16767106	0.033843	486484	0.035227	Sepsis (28	Sepsis (28	Sepsis (28	TRUE	igd	0.0018	2.66E-06	7366	M37506.m	TRUE	reported	m2kaRt	2	TRUE	0.00281	20.74745
8617	rs4749219	A	G	A	G	0.0082	-0.01049	0.2784	0.285084	FALSE	FALSE	FALSE	ieu-b-508	10	27343232	0.036953	486484	0.776603	Sepsis (28	Sepsis (28	Sepsis (28	TRUE	igd	0.0018	7.02E-06	7366	M37506.m	TRUE	reported	m2kaRt	2	TRUE	0.00281	20.74745
411	rs1289681	T	C	T	C	-0.0082	-0.01931	0.1853	0.18393	FALSE	FALSE	FALSE	ieu-b-508	14	43029500	0.043135	486484	0.654404	Sepsis (28	Sepsis (28	Sepsis (28	TRUE	igd	0.0018	3.52E-06	7365	M00063.m	TRUE	reported	Hdsv1L	2	TRUE	0.00281	20.74745
430	rs4401492	T	G	T	G	0.0082	-0.10036	0.1795	0.186892	FALSE	FALSE	FALSE	ieu-b-508	4	1.85E+08	0.042811	486484	0.019061	Sepsis (28	Sepsis (28	Sepsis (28	TRUE	igd	0.0018	4.52E-06	7365	M00063.m	TRUE	reported	Hdsv1L	2	TRUE	0.00281	20.74745
442	rs871748	T	C	T	C	-0.0082	0.00688	0.182	0.186196	FALSE	FALSE	FALSE	ieu-b-508	10	5701795	0.043323	486484	0.873824	Sepsis (28	Sepsis (28	Sepsis (28	TRUE	igd	0.0018	8.56E-06	7365	M00063.m	TRUE	reported	Hdsv1L	2	TRUE	0.00281	20.74745
3249	rs1049128	A	G	A	G	-0.0082	-0.0763	0.658	0.647076	FALSE	FALSE	FALSE	ieu-b-508	5	1.26E+08	0.034993	486484	0.029226	Sepsis (28	Sepsis (28	Sepsis (28	TRUE	igd	0.0018	3.73E-06	7365	M32348.m	TRUE	reported	DJeLDx	2	TRUE	0.00281	20.74745
3271	rs405945	T	C	T	C	-0.0082	-0.04494	0.4396	0.432693	FALSE	FALSE	FALSE	ieu-b-508	7	1.08E+08	0.033695	486484	0.182255	Sepsis (28	Sepsis (28	Sepsis (28	TRUE	igd	0.0018	3.15E-06	7365	M32348.m	TRUE	reported	DJeLDx	2	TRUE	0.00281	20.74745
3278	rs6486771	T	C	T	C	-0.0082	0.043895	0.3983	0.389625	FALSE	FALSE	FALSE	ieu-b-508	12	1.3E+08	0.034214	486484	0.199508	Sepsis (28	Sepsis (28	Sepsis (28	TRUE	igd	0.0018	3.45E-06	7365	M32348.m	TRUE	reported	DJeLDx	2	TRUE	0.00281	20.74745
994	rs1004301	T	C	T	C	0.0082	-0.01282	0.7113	0.708727	FALSE	FALSE	FALSE	ieu-b-508	5	1.69E+08	0.036948	486484	0.7287	Sepsis (28	Sepsis (28	Sepsis (28	TRUE	igd	0.0018	5.58E-06	7364	M01564.m	TRUE	reported	IPUdyD	2	TRUE	0.00281	20.74745
1034	rs889128	T	G	T	G	0.0082	-0.01658	0.6665	0.658174	FALSE	FALSE	FALSE	ieu-b-508	19	10131693	0.035159	486484	0.63714	Sepsis (28	Sepsis (28	Sepsis (28	TRUE	igd	0.0018	6.75E-06	7364	M01564.m	TRUE	reported	IPUdyD	2	TRUE	0.00281	20.74745
1039	rs940697	A	G	A	G	0.0082	-0.04793	0.2701	0.286492	FALSE	FALSE	FALSE	ieu-b-508	7	1.43E+08	0.036958	486484	0.194631	Sepsis (28	Sepsis (28	Sepsis (28	TRUE	igd	0.0018	5.71E-06	7364	M01564.m	TRUE	reported	IPUdyD	2	TRUE	0.00281	20.74745
3429	rs1613813	A	G	A	G	-0.0082	0.008892	0.5082	0.503663	FALSE	FALSE	FALSE	ieu-b-508	12	1.16E+08	0.033443	486484	0.790329	Sepsis (28	Sepsis (28	Sepsis (28	TRUE	igd	0.0018	8.30E-06	7364	M32452.m	TRUE	reported	8GtFpl	2	TRUE	0.00281	20.74745
3431	rs2164187	T	G	T	G	0.0082	0.015473	0.4788	0.447442	FALSE	FALSE	FALSE	ieu-b-508	9	83113481	0.033674	486484	0.645879	Sepsis (28	Sepsis (28	Sepsis (28	TRUE	igd	0.0018	9.33E-06	7364	M32452.m	TRUE	reported	8GtFpl	2	TRUE	0.00281	20.74745
3441	rs7028146	A	G	A	G	0.0082	0.019551	0.6217	0.616742	FALSE	FALSE	FALSE	ieu-b-508	9	35150228	0.034404	486484	0.56984	Sepsis (28	Sepsis (28	Sepsis (28	TRUE	igd	0.0018	7.77E-06	7364	M32452.m	TRUE	reported	8GtFpl	2	TRUE	0.00281	20.74745
3449	rs9503551	A	G	A	G	-0.0164	-0.04858	0.0954	0.111387	FALSE	FALSE	FALSE	ieu-b-508	6	3340717	0.054031	486484	0.368567	Sepsis (28	Sepsis (28	Sepsis (28	TRUE	igd	0.0036	3.75E-06	7364	M32452.m	TRUE	reported	8GtFpl	2	TRUE	0.00281	20.74745
708	rs1049143	A	C	A	C	0.0082	0.021802	0.1435	0.142278	FALSE	FALSE	FALSE	ieu-b-508	5	35968000	0.047874	486484	0.648817	Sepsis (28	Sepsis (28	Sepsis (28	TRUE	igd	0.0018	3.80E-06	7363	M01301.m	TRUE	reported	w8TL71	2	TRUE	0.002811	20.74745
716	rs1226825	T	C	T	C	-0.0082	-0.02902	0.1339	0.132538	FALSE	FALSE	FALSE	ieu-b-508	10	25864681	0.049437	486484	0.557211	Sepsis (28	Sepsis (28	Sepsis (28	TRUE	igd	0.0018	5.59E-06	7363	M01301.m	TRUE	reported	w8TL71	2	TRUE	0.002811	20.74745
3456	rs1193347	T	G	T	G	-0.0082	0.015494	0.3025	0.314956	FALSE	FALSE	FALSE	ieu-b-508	4	1.68E+08	0.035884	486484	0.665909	Sepsis (28	Sepsis (28	Sepsis (28	TRUE	igd	0.0018	6.54E-06	7363	M32489.m	TRUE	reported	hnljaD	2	TRUE	0.002811	20.74745
3458	rs1250171	T	C	T	C	0.0082	0.008032	0.3105	0.310801	FALSE	FALSE	FALSE	ieu-b-508	18	12027028	0.036998	486484	0.828131	Sepsis (28	Sepsis (28	Sepsis (28	TRUE	igd	0.0018	6.05E-06	7363	M32489.m	TRUE	reported	hnljaD	2	TRUE	0.002811	20.74745
3469	rs2325358	T	C	T	C	0.0082	-0.05309	0.7049	0.702553	FALSE	FALSE	FALSE	ieu-b-508	13	71873267	0.036686	486484	0.147856	Sepsis (28	Sepsis (28	Sepsis (28	TRUE	igd	0.0018	5.59E-06	7363	M32489.m	TRUE	reported	hnljaD	2	TRUE	0.002811	20.74745
3470	rs2467041	T	C	T	C	-0.0082	0.0106	0.6501	0.646401	FALSE	FALSE	FALSE	ieu-b-508	2	1.99E+08	0.034873	486484	0.761172	Sepsis (28	Sepsis (28	Sepsis (28	TRUE	igd	0.0018	5.71E-06	7363	M32489.m	TRUE	reported	hnljaD	2	TRUE	0.002811	20.74745
3475	rs4704495	T	C	T	C	0.0082	-0.02755	0.6216	0.627254	FALSE	FALSE	FALSE	ieu-b-508	5	77624910	0.034778	486484	0.428197	Sepsis (28	Sepsis (28	Sepsis (28	TRUE	igd	0.0018	4.89E-06	7363	M32489.m	TRUE	reported	hnljaD	2	TRUE	0.002811	20.74745
3478	rs6498908	T	C	T	C	-0.0082	0.007182	0.3408	0.327739	FALSE	FALSE	FALSE	ieu-b-508	16	63633828	0.035785	486484	0.840928	Sepsis (28	Sepsis (28	Sepsis (28	TRUE	igd	0.0018	5.76E-06	7363	M32489.m	TRUE	reported	hnljaD	2	TRUE	0.002811	20.74745
3752	rs1012972	A	G	A	G	-0.0082	0.005271	0.3124	0.300255	FALSE	FALSE	FALSE	ieu-b-508	14	26714192	0.036396	486484	0.884853	Sepsis (28	Sepsis (28	Sepsis (28	TRUE	igd	0.0018	5.95E-06	7362	M32634.m	TRUE	reported	7T2RUz	2	TRUE	0.002811	20.74745
3786	rs9570619	A	G	A	G	-0.0082	-0.01863	0.7337	0.723547	FALSE	FALSE	FALSE	ieu-b-508	13	62321073	0.037945	486484	0.623534	Sepsis (28	Sepsis (28	Sepsis (28	TRUE	igd	0.0018	6.39E-06	7362	M32634.m	TRUE	reported	7T2RUz	2	TRUE	0.002811	20.74745
6423	rs1049768	A	G	A	G	0.0082	-0.04661	0.8809	0.884375	FALSE	FALSE	FALSE	ieu-b-508	2	1.89E+08	0.052134	486484	0.371287	Sepsis (28	Sepsis (28	Sepsis (28	TRUE	igd	0.0018	9.20E-06	7362	M34062.m	TRUE	reported	RZyHKP	2	TRUE	0.002811	20.74745
6436	rs2029490	A	G	A	G	0.0082	-0.0373	0.2811	0.288918	FALSE	FALSE	FALSE	ieu-b-508	1	4355593	0.037096	486484	0.314594	Sepsis (28	Sepsis (28	Sepsis (28	TRUE	igd	0.0018	5.60E-06	7362	M34062.m	TRUE	reported	RZyHKP	2	TRUE	0.002811	20.74745
6449	rs6898299	T	G	T	G	0.0082	-0.06259	0.1197	0.120921	FALSE	FALSE	FALSE	ieu-b-508	5	1.6E+08	0.051423	486484	0.223572	Sepsis (28	Sepsis (28	Sepsis (28	TRUE	igd	0.0018	7.50E-06	7362	M34062.m	TRUE	reported	RZyHKP	2	TRUE	0.002811	20.74745
455	rs1753352	T	C	T	C	0.0082	0.059822	0.1534	0.158573	FALSE	FALSE	FALSE	ieu-b-508	5	1.07E+08	0.045937	486484	0.192824	Sepsis (28	Sepsis (28	Sepsis (28	TRUE	igd	0.0018	5.04E-06	7361	M00513.m	TRUE	reported	ohacnP	2	TRUE	0.002811	20.74745
465	rs9456538	A	G	A	G	-0.0082	0.026254	0.1493	0.143328	FALSE	FALSE	FALSE	ieu-b-508	6	1.61E+08	0.047801	486484	0.582845	Sepsis (28	Sepsis (28	Sepsis (28	TRUE	igd	0.0018	4.20E-06	7361	M00513.m	TRUE	reported	ohacnP	2	TRUE	0.002811	20.74745
7981	rs1899398	T	C	T	C	0.0123	-0.03579	0.799	0.794528	FALSE	FALSE	FALSE	ieu-b-508	3	3770669	0.041463	486484	0.388091	Sepsis (28	Sepsis (28	Sepsis (28	TRUE	igd	0.0027	4.94E-06	7358	M35718.m	TRUE	reported	oUwc0a	2	TRUE	0.002813	20.74745
11	rs1013667	A	G	A	G	-0.0041	-0.05421	0.6087	0.610687	FALSE	FALSE	FALSE	ieu-b-508	3	1.91E+08	0.034204	486484	0.11297	Sepsis (28	Sepsis (28	Sepsis (28	TRUE	igd	9.00E-04	5.67E-06	7355	M00054.m	TRUE	reported	QTDAnh	2	TRUE	0.002814	20.74744
24	rs1088100	T	C	T	C	0.0041	-0.00534	0.4824	0.484641	FALSE	FALSE	FALSE	ieu-b-508	12	47236571	0.033326	486484	0.872654	Sepsis (28	Sepsis (28	Sepsis (28	TRUE	igd	9.00E-04	3.39E-06	7355	M00054.m	TRUE	reported	QTDAnh	2	TRUE	0.002814	20.74744
29	rs1167885	T	C	T	C	0.0041	-0.01453	0.4138	0.418686	FALSE	FALSE	FALSE	ieu-b-508	2	2.34E+08	0.033994	486484	0.669049	Sepsis (28	Sepsis (28	Sepsis (28	TRUE	igd	9.00E-04	5.94E-06	7355	M00054.m	TRUE	reported	QTDAnh	2	TRUE	0.002814	20.74744
30	rs1171967	A	T	A	T	-0.0041	-0.00754	0.3965	0.402494	FALSE	TRUE	FALSE	ieu-b-508	3	1.68E+08	0.034156	486484	0.825206	Sepsis (28	Sepsis (28	Sepsis (28	TRUE	igd	9.00E-04	5.12E-06	7355	M00054.m	TRUE	reported	QTDAnh	2	TRUE	0.002814	20.74744
34	rs1180241	T	C	T	C	-0.0041	0.013214	0.5816	0.573372	FALSE	FALSE	FALSE	ieu-b-508	1	84276046	0.033836	486484	0.696139	Sepsis (28	Sepsis (28	Sepsis (28	TRUE	igd	9.00E-04	4.47E-06	7355	M00054.m	TRUE	reported	QTDAnh	2	TRUE	0.002814	20.74744
35	rs1180414	T	C	T	C	0.0041	0.008905	0.4678	0.468027	FALSE	FALSE	FALSE	ieu-b-508	1	77457748	0.03342	486484	0.789894	Sepsis (28	Sepsis (28	Sepsis (28	TRUE	igd	9.00E-04	4.61E-06	7355	M00054.m	TRUE	reported	QTDAnh	2	TRUE	0.002814	20.74744
61	rs1769824	A	C	A	C	-0.0041	-0.04319	0.5682	0.560304	FALSE	FALSE	FALSE	ieu-b-508	16	87319938	0.033755	486484	0.200706	Sepsis (28	Sepsis (28	Sepsis (28	TRUE	igd	9.00E-04	5.50E-06	7355	M00054.m	TRUE	reported	QTDAnh	2	TRUE	0.002814	20.74744
62	rs1774450	T	C	T	C	-0.0041	0.003009	0.4582	0.460403	FALSE	FALSE	FALSE	ieu-b-508	15	1.02E+08	0.033565	486484	0.928578	Sepsis (28	Sepsis (28	Sepsis (28	TRUE	igd	9.00E-04	4.16E-06	7355	M00054.m	TRUE	reported	QTDAnh	2	TRUE	0.002814	20.74744
76	rs2349729	A	G	A	G	-0.0041	-0.00197	0.5141	0.511894	FALSE	FALSE	FALSE	ieu-b-508	2	2.03E+08	0.033451	486484	0.953033	Sepsis (28	Sepsis (28	Sepsis (28	TRUE	igd	9.00E-04	6.45E-06	7355	M00054.m	TRUE	reported	QTDAnh	2	TRUE	0.002814	20.74744
88	rs2722407	A	G	A	G	0.0041	0.003266	0.587	0.591968	FALSE	FALSE	FALSE	ieu-b-508	7	24371586	0.033913	486484	0.923276	Sepsis (28	Sepsis (28	Sepsis (28	TRUE	igd	9.00E-04	6.47E-06	7355	M00054.m	TRUE	reported	QTDAnh	2	TRUE	0.002814	20.74744
102	rs3751181	A	G	A	G	0.0041	0.029752	0.6085	0.606098	FALSE	FALSE	FALSE	ieu-b-508	12	1.26E+08	0.034252	486484	0.385061	Sepsis (28	Sepsis (28	Sepsis (28	TRUE	igd	9.00E-04	5.19E-06	7355	M00054.m	TRUE	reported	QTDAnh	2	TRUE	0.002814	20.74744
113	rs430727	T	C	T	C	0.0041	-0.05593	0.4628	0.440979	FALSE	FALSE	FALSE	ieu-b-508	3	41128564	0.033502	486484	0.095001	Sepsis (28	Sepsis (28	Sepsis (28	TRUE	igd	9.00E-04	5.13E-06	7355	M00054.m	TRUE	reported	QTDAnh	2	TRUE	0.002814	20.74744
119	rs4702007	A	G	A	G	-0.0041	0.01349	0.3929	0.390897	FALSE	FALSE	FALSE	ieu-b-508	5	14006306	0.03429	486484	0.694015	Sepsis (28	Sepsis (28	Sepsis (28	TRUE	igd	9.00E-04	6.11E-06	7355	M00054.m	TRUE	reported	QTDAnh	2	TRUE	0.002814	20.74744
121	rs4755291	A	G	A	G	-0.0041	0.002949	0.4644	0.459976	FALSE	FALSE	FALSE	ieu-b-508	11	44821921	0.033539	486484	0.929928	Sepsis (28	Sepsis (28	Sepsis (28	TRUE	igd	9.00E-04	5.25E-06	7355	M00054.m	TRUE	reported	QTDAnh	2	TRUE	0.002814	20.74744
122	rs4849291	C	G	C	G	0.0041	0.006072	0.6092	0.61142	FALSE	TRUE	FALSE	ieu-b-508	2	1.15E+08	0.034296	486484	0.85947	Sepsis (28	Sepsis (28	Sepsis (28	TRUE	igd	9.00E-04	5.93E-06	7355	M00054.m	TRUE	reported	QTDAnh	2	TRUE	0.002814	20.74744
123	rs4870304	A	G	A	G	0.0041	0.033687	0.5823	0.574412	FALSE	FALSE	FALSE	ieu-b-508	6	1.55E+08	0.033892	486484	0.320252	Sepsis (28	Sepsis (28	Sepsis (28	TRUE	igd	9.00E-04	8.31E-06	7355	M00054.m	TRUE	reported	QTDAnh	2	TRUE	0.002814	20.74744
134	rs6077611	T	G	T	G	-0.0041	0.030426	0.4794	0.46483	FALSE	FALSE	FALSE	ieu-b-508	20	9893691	0.033579	486484	0.364873	Sepsis (28	Sepsis (28	Sepsis (28	TRUE	igd	9.00E-04	5.32E-06	7355	M00054.m	TRUE	reported	QTDAnh	2	TRUE	0.002814	20.74744
146	rs6747650	A	G	A	G	-0.0041	-0.02687	0.5825	0.584689	FALSE	FALSE	FALSE	ieu-b-508	2	3451507	0.033811	486484	0.426751	Sepsis (28	Sepsis (28	Sepsis (28	TRUE	igd	9.00E-04	4.36E-06	7355	M00054.m	TRUE	reported	QTDAnh	2	TRUE	0.002814	20.74744
152	rs6910731	A	G	A	G	0.0041	-0.1026	0.4497	0.439244	FALSE	FALSE	FALSE	ieu-b-508	6	23415462	0.033731	486484	0.002352	Sepsis (28	Sepsis (28	Sepsis (28	TRUE	igd	9.00E-04	6.68E-06	7355	M00054.m	TRUE	reported	QTDAnh	2	TRUE	0.002814	20.74744
156	rs7009696	T	C	T	C	0.0041	0.001252	0.6243	0.620712	FALSE	FALSE	FALSE	ieu-b-508	8	72731717	0.034411	486484	0.970987	Sepsis (28	Sepsis (28	Sepsis (28	TRUE	igd	9.00E-04	6.17E-06	7355	M00054.m	TRUE	reported	QTDAnh	2	TRUE	0.002814	20.74744
168	rs7843149	T	C	T	C	-0.0041	-0.00285	0.3896	0.389678	FALSE	FALSE	FALSE	ieu-b-508	8	91474961	0.034204	486484	0.933691	Sepsis (28	Sepsis (28	Sepsis (28	TRUE	igd	9.00E-04	6.55E-06	7355	M00054.m	TRUE	reported	QTDAnh	2	TRUE	0.002814	20.74744
175	rs875184	T	C	T	C	-0.0041	-0.02935	0.403	0.410268	FALSE	FALSE	FALSE	ieu-b-508	5	1.7E+08	0.03428	486484	0.391943	Sepsis (28	Sepsis (28	Sepsis (28	TRUE	igd	9.00E-04	5.69E-06	7355	M00054.m	TRUE	reported	QTDAnh	2	TRUE	0.002814	20.74744
179	rs915281	T	C	T	C	-0.0041	-0.00293	0.5076	0.505111	FALSE	FALSE	FALSE	ieu-b-508	9	1.2E+08	0.033327	486484	0.929829	Sepsis (28	Sepsis (28	Sepsis (28	TRUE	igd	9.00E-04	4.42E-06	7355	M00054.m	TRUE	reported	QTDAnh	2	TRUE	0.002814	20.74744
185	rs9464137	A	C	A	C	0.0041	0.032361	0.5592	0.566808	FALSE	FALSE	FALSE	ieu-b-508	6	54669826	0.033602	486484	0.335524	Sepsis (28	Sepsis (28	Sepsis (28	TRUE	igd	9.00E-04	4.45E-06	7355	M00054.m	TRUE	reported	QTDAnh	2	TRUE	0.002814	20.74744
190	rs9543391	T	G	T	G	0.0041	-0.03966	0.3993	0.414817	FALSE	FALSE	FALSE	ieu-b-508	13	74115140	0.034099	486484	0.244825	Sepsis (28	Sepsis (28	Sepsis (28	TRUE	igd	9.00E-04	6.39E-06	7355	M00054.m	TRUE	reported	QTDAnh	2	TRUE	0.002814	20.74744
192	rs9649963	T	C	T	C	0.0041	-0.0596	0.634	0.634741	FALSE	FALSE	FALSE	ieu-b-508	8	1.32E+08	0.03484	486484	0.08712	Sepsis (28	Sepsis (28	Sepsis (28	TRUE	igd	9.00E-04	5.84E-06	7355	M00054.m	TRUE	reported	QTDAnh	2	TRUE	0.002814	20.74744
1152	rs2091331	A	G	A	G	0.0082	0.010161	0.4127	0.40564	FALSE	FALSE	FALSE	ieu-b-508	10	73741706	0.03396	486484	0.764779	Sepsis (28	Sepsis (28	Sepsis (28	TRUE	igd	0.0018	5.71E-06	7355	M01644.m	TRUE	reported	IB0DTL	2	TRUE	0.002814	20.74744
1156	rs498266	A	G	A	G	0.0082	-0.0188	0.5852	0.577111	FALSE	FALSE	FALSE	ieu-b-508	9	79306919	0.033774	486484	0.577678	Sepsis (28	Sepsis (28	Sepsis (28	TRUE	igd	0.0018	6.15E-06	7355	M01644.m	TRUE	reported	IB0DTL	2	TRUE	0.002814	20.74744
3503	rs351755	A	G	A	G	-0.0082	0.030194	0.5614	0.562027	FALSE	FALSE	FALSE	ieu-b-508	6	1.1E+08	0.033701	486484	0.370286	Sepsis (28	Sepsis (28	Sepsis (28	TRUE	igd	0.0018	3.53E-06	7355	M32492.m	TRUE	reported	rZZFcE	2	TRUE	0.002814	20.74744
3507	rs4898707	T	C	T	C	-0.0082	0.013986	0.4396	0.439196	FALSE	FALSE	FALSE	ieu-b-508	14	52205481	0.033657	486484	0.677751	Sepsis (28	Sepsis (28	Sepsis (28	TRUE	igd	0.0018	4.35E-06	7355	M32492.m	TRUE	reported	rZZFcE	2	TRUE	0.002814	20.74744
3519	rs9924102	A	G	A	G	-0.0082	-0.00952	0.6426	0.653288	FALSE	FALSE	FALSE	ieu-b-508	16	11511856	0.035078	486484	0.786153	Sepsis (28	Sepsis (28	Sepsis (28	TRUE	igd	0.0018	4.25E-06	7355	M32492.m	TRUE	reported	rZZFcE	2	TRUE	0.002814	20.74744
913	rs2592916	T	C	T	C	-0.0123	0.10776	0.1013	0.095169	FALSE	FALSE	FALSE	ieu-b-508	11	1.27E+08	0.056872	486484	0.058123	Sepsis (28	Sepsis (28	Sepsis (28	TRUE	igd	0.0027	6.55E-06	7354	M01494.m	TRUE	reported	7JWNKI	2	TRUE	0.002814	20.74744
2239	rs1193409	A	G	A	G	-0.0082	0.039193	0.7176	0.71614	FALSE	FALSE	FALSE	ieu-b-508	4	11884262	0.036976	486484	0.289155	Sepsis (28	Sepsis (28	Sepsis (28	TRUE	igd	0.0018	3.07E-06	7354	M19934.m	TRUE	reported	HtcWcQ	2	TRUE	0.002814	20.74744
6329	rs7759176	T	G	T	G	0.0082	0.028705	0.2976	0.28987	FALSE	FALSE	FALSE	ieu-b-508	6	2915664	0.037038	486484	0.438335	Sepsis (28	Sepsis (28	Sepsis (28	TRUE	igd	0.0018	4.35E-06	7354	M33955.m	TRUE	reported	mhHFnT	2	TRUE	0.002814	20.74744
653	rs6084839	A	C	A	C	-0.0123	-0.12634	0.9446	0.937331	FALSE	FALSE	FALSE	ieu-b-508	20	4685707	0.069327	486484	0.068391	Sepsis (28	Sepsis (28	Sepsis (28	TRUE	igd	0.0027	3.99E-06	7352	M01125.m	TRUE	reported	yw0clB	2	TRUE	0.002815	20.74744
1606	rs1233747	T	C	T	C	-0.0123	0.010581	0.8324	0.825317	FALSE	FALSE	FALSE	ieu-b-508	9	8097415	0.044087	486484	0.810325	Sepsis (28	Sepsis (28	Sepsis (28	TRUE	igd	0.0027	5.90E-06	7352	M15122.m	TRUE	reported	VlnI8T	2	TRUE	0.002815	20.74744
207	rs1025466	T	C	T	C	-0.0041	-0.05362	0.4062	0.406834	FALSE	FALSE	FALSE	ieu-b-508	5	1.59E+08	0.034464	486484	0.119748	Sepsis (28	Sepsis (28	Sepsis (28	TRUE	igd	9.00E-04	6.25E-06	7351	M00060.m	TRUE	reported	kqwl4V	2	TRUE	0.002815	20.74744
223	rs1097368	A	C	A	C	-0.0041	0.019107	0.4002	0.407894	FALSE	FALSE	FALSE	ieu-b-508	9	38151268	0.03394	486484	0.573472	Sepsis (28	Sepsis (28	Sepsis (28	TRUE	igd	9.00E-04	6.54E-06	7351	M00060.m	TRUE	reported	kqwl4V	2	TRUE	0.002815	20.74744
225	rs1104999	T	C	T	C	0.0041	0.002783	0.4822	0.482453	FALSE	FALSE	FALSE	ieu-b-508	9	1.2E+08	0.033253	486484	0.933314	Sepsis (28	Sepsis (28	Sepsis (28	TRUE	igd	9.00E-04	3.51E-06	7351	M00060.m	TRUE	reported	kqwl4V	2	TRUE	0.002815	20.74744
229	rs1118362	A	G	A	G	0.0041	0.000589	0.4676	0.466525	FALSE	FALSE	FALSE	ieu-b-508	12	47212370	0.033713	486484	0.986067	Sepsis (28	Sepsis (28	Sepsis (28	TRUE	igd	9.00E-04	4.68E-06	7351	M00060.m	TRUE	reported	kqwl4V	2	TRUE	0.002815	20.74744
231	rs1157892	A	C	A	C	0.0041	0.047998	0.4246	0.427091	FALSE	FALSE	FALSE	ieu-b-508	1	84926750	0.034194	486484	0.160412	Sepsis (28	Sepsis (28	Sepsis (28	TRUE	igd	9.00E-04	4.75E-06	7351	M00060.m	TRUE	reported	kqwl4V	2	TRUE	0.002815	20.74744
232	rs1165712	T	C	T	C	0.0041	-0.02029	0.5604	0.560837	FALSE	FALSE	FALSE	ieu-b-508	17	66767551	0.033515	486484	0.544959	Sepsis (28	Sepsis (28	Sepsis (28	TRUE	igd	9.00E-04	5.71E-06	7351	M00060.m	TRUE	reported	kqwl4V	2	TRUE	0.002815	20.74744
239	rs1246854	A	G	A	G	0.0041	0.01917	0.5563	0.551759	FALSE	FALSE	FALSE	ieu-b-508	2	35448317	0.033514	486484	0.567325	Sepsis (28	Sepsis (28	Sepsis (28	TRUE	igd	9.00E-04	5.76E-06	7351	M00060.m	TRUE	reported	kqwl4V	2	TRUE	0.002815	20.74744
241	rs1251709	A	G	A	G	0.0041	-0.00636	0.422	0.412452	FALSE	FALSE	FALSE	ieu-b-508	5	1.2E+08	0.033969	486484	0.851418	Sepsis (28	Sepsis (28	Sepsis (28	TRUE	igd	9.00E-04	8.41E-06	7351	M00060.m	TRUE	reported	kqwl4V	2	TRUE	0.002815	20.74744
247	rs1316507	A	G	A	G	0.0041	-0.01239	0.3821	0.370763	FALSE	FALSE	FALSE	ieu-b-508	5	1.21E+08	0.034732	486484	0.721199	Sepsis (28	Sepsis (28	Sepsis (28	TRUE	igd	9.00E-04	8.98E-06	7351	M00060.m	TRUE	reported	kqwl4V	2	TRUE	0.002815	20.74744
258	rs167648	A	C	A	C	-0.0041	-0.01955	0.4439	0.454091	FALSE	FALSE	FALSE	ieu-b-508	2	2.12E+08	0.033723	486484	0.562082	Sepsis (28	Sepsis (28	Sepsis (28	TRUE	igd	9.00E-04	4.52E-06	7351	M00060.m	TRUE	reported	kqwl4V	2	TRUE	0.002815	20.74744
272	rs1971105	T	G	T	G	-0.0041	0.017315	0.4305	0.424021	FALSE	FALSE	FALSE	ieu-b-508	2	55055307	0.033862	486484	0.609105	Sepsis (28	Sepsis (28	Sepsis (28	TRUE	igd	9.00E-04	6.27E-06	7351	M00060.m	TRUE	reported	kqwl4V	2	TRUE	0.002815	20.74744
274	rs1999120	A	G	A	G	-0.0041	0.036934	0.5692	0.568411	FALSE	FALSE	FALSE	ieu-b-508	6	1.59E+08	0.033747	486484	0.273754	Sepsis (28	Sepsis (28	Sepsis (28	TRUE	igd	9.00E-04	4.04E-06	7351	M00060.m	TRUE	reported	kqwl4V	2	TRUE	0.002815	20.74744
275	rs2057435	A	G	A	G	0.0041	0.00928	0.535	0.547756	FALSE	FALSE	FALSE	ieu-b-508	10	77674635	0.033628	486484	0.782586	Sepsis (28	Sepsis (28	Sepsis (28	TRUE	igd	9.00E-04	7.95E-06	7351	M00060.m	TRUE	reported	kqwl4V	2	TRUE	0.002815	20.74744
287	rs2478832	T	C	T	C	-0.0041	0.0286	0.3996	0.396873	FALSE	FALSE	FALSE	ieu-b-508	10	30346833	0.034396	486484	0.405703	Sepsis (28	Sepsis (28	Sepsis (28	TRUE	igd	9.00E-04	8.05E-06	7351	M00060.m	TRUE	reported	kqwl4V	2	TRUE	0.002815	20.74744
291	rs2667698	A	C	A	C	0.0041	0.01592	0.3681	0.366604	FALSE	FALSE	FALSE	ieu-b-508	3	1.9E+08	0.03455	486484	0.644954	Sepsis (28	Sepsis (28	Sepsis (28	TRUE	igd	9.00E-04	7.07E-06	7351	M00060.m	TRUE	reported	kqwl4V	2	TRUE	0.002815	20.74744
305	rs3924223	A	G	A	G	-0.0041	0.00978	0.549	0.544258	FALSE	FALSE	FALSE	ieu-b-508	1	2.22E+08	0.033563	486484	0.770753	Sepsis (28	Sepsis (28	Sepsis (28	TRUE	igd	9.00E-04	6.18E-06	7351	M00060.m	TRUE	reported	kqwl4V	2	TRUE	0.002815	20.74744
310	rs4276770	A	G	A	G	-0.0041	0.02686	0.3999	0.399316	FALSE	FALSE	FALSE	ieu-b-508	9	25256654	0.034315	486484	0.433775	Sepsis (28	Sepsis (28	Sepsis (28	TRUE	igd	9.00E-04	5.11E-06	7351	M00060.m	TRUE	reported	kqwl4V	2	TRUE	0.002815	20.74744
312	rs4531478	A	G	A	G	0.0041	0.053307	0.4713	0.474882	FALSE	FALSE	FALSE	ieu-b-508	11	68482489	0.033442	486484	0.110937	Sepsis (28	Sepsis (28	Sepsis (28	TRUE	igd	9.00E-04	4.45E-06	7351	M00060.m	TRUE	reported	kqwl4V	2	TRUE	0.002815	20.74744
322	rs4889294	T	C	T	C	-0.0041	0.061797	0.5449	0.551547	FALSE	FALSE	FALSE	ieu-b-508	16	81303759	0.033497	486484	0.065061	Sepsis (28	Sepsis (28	Sepsis (28	TRUE	igd	9.00E-04	3.65E-06	7351	M00060.m	TRUE	reported	kqwl4V	2	TRUE	0.002815	20.74744
331	rs618001	T	C	T	C	0.0041	-0.04632	0.5857	0.585799	FALSE	FALSE	FALSE	ieu-b-508	11	85756362	0.033832	486484	0.170987	Sepsis (28	Sepsis (28	Sepsis (28	TRUE	igd	9.00E-04	4.18E-06	7351	M00060.m	TRUE	reported	kqwl4V	2	TRUE	0.002815	20.74744
332	rs631750	T	C	T	C	0.0041	-0.00775	0.3544	0.365826	FALSE	FALSE	FALSE	ieu-b-508	12	4075855	0.034669	486484	0.823048	Sepsis (28	Sepsis (28	Sepsis (28	TRUE	igd	9.00E-04	7.51E-06	7351	M00060.m	TRUE	reported	kqwl4V	2	TRUE	0.002815	20.74744
333	rs637828	A	C	A	C	0.0041	0.020425	0.5424	0.534658	FALSE	FALSE	FALSE	ieu-b-508	18	60153289	0.033561	486484	0.542795	Sepsis (28	Sepsis (28	Sepsis (28	TRUE	igd	9.00E-04	5.81E-06	7351	M00060.m	TRUE	reported	kqwl4V	2	TRUE	0.002815	20.74744
344	rs6822279	A	C	A	C	-0.0041	-0.02171	0.3969	0.395468	FALSE	FALSE	FALSE	ieu-b-508	4	99745705	0.034432	486484	0.528324	Sepsis (28	Sepsis (28	Sepsis (28	TRUE	igd	9.00E-04	4.79E-06	7351	M00060.m	TRUE	reported	kqwl4V	2	TRUE	0.002815	20.74744
346	rs697311	A	G	A	G	0.0041	0.036807	0.3792	0.373905	FALSE	FALSE	FALSE	ieu-b-508	11	29675343	0.034513	486484	0.286205	Sepsis (28	Sepsis (28	Sepsis (28	TRUE	igd	9.00E-04	4.58E-06	7351	M00060.m	TRUE	reported	kqwl4V	2	TRUE	0.002815	20.74744
357	rs7574695	T	C	T	C	-0.0041	0.025878	0.4769	0.48425	FALSE	FALSE	FALSE	ieu-b-508	2	33953186	0.03342	486484	0.438733	Sepsis (28	Sepsis (28	Sepsis (28	TRUE	igd	9.00E-04	5.97E-06	7351	M00060.m	TRUE	reported	kqwl4V	2	TRUE	0.002815	20.74744
360	rs7622687	A	G	A	G	-0.0041	0.005954	0.4032	0.413949	FALSE	FALSE	FALSE	ieu-b-508	3	58168406	0.034031	486484	0.861105	Sepsis (28	Sepsis (28	Sepsis (28	TRUE	igd	9.00E-04	5.21E-06	7351	M00060.m	TRUE	reported	kqwl4V	2	TRUE	0.002815	20.74744
366	rs769630	T	C	T	C	0.0041	-0.01562	0.6016	0.605598	FALSE	FALSE	FALSE	ieu-b-508	3	3822559	0.034281	486484	0.648543	Sepsis (28	Sepsis (28	Sepsis (28	TRUE	igd	9.00E-04	5.06E-06	7351	M00060.m	TRUE	reported	kqwl4V	2	TRUE	0.002815	20.74744
372	rs7914851	C	G	C	G	0.0041	-0.05525	0.3443	0.343221	FALSE	TRUE	FALSE	ieu-b-508	10	92211107	0.035268	486484	0.117221	Sepsis (28	Sepsis (28	Sepsis (28	TRUE	igd	9.00E-04	6.79E-06	7351	M00060.m	TRUE	reported	kqwl4V	2	TRUE	0.002815	20.74744
380	rs8131355	A	G	A	G	-0.0041	-0.04884	0.5488	0.535777	FALSE	FALSE	FALSE	ieu-b-508	21	40867016	0.033361	486484	0.143232	Sepsis (28	Sepsis (28	Sepsis (28	TRUE	igd	9.00E-04	5.68E-06	7351	M00060.m	TRUE	reported	kqwl4V	2	TRUE	0.002815	20.74744
383	rs891878	T	G	T	G	-0.0041	0.067701	0.4844	0.491143	FALSE	FALSE	FALSE	ieu-b-508	2	1992624	0.03336	486484	0.042418	Sepsis (28	Sepsis (28	Sepsis (28	TRUE	igd	9.00E-04	6.03E-06	7351	M00060.m	TRUE	reported	kqwl4V	2	TRUE	0.002815	20.74744
387	rs923818	A	G	A	G	0.0041	0.000649	0.5072	0.510598	FALSE	FALSE	FALSE	ieu-b-508	3	1.92E+08	0.033461	486484	0.984525	Sepsis (28	Sepsis (28	Sepsis (28	TRUE	igd	9.00E-04	6.23E-06	7351	M00060.m	TRUE	reported	kqwl4V	2	TRUE	0.002815	20.74744
388	rs9292230	T	C	T	C	0.0041	0.03718	0.6218	0.624157	FALSE	FALSE	FALSE	ieu-b-508	5	27906291	0.034562	486484	0.282043	Sepsis (28	Sepsis (28	Sepsis (28	TRUE	igd	9.00E-04	4.63E-06	7351	M00060.m	TRUE	reported	kqwl4V	2	TRUE	0.002815	20.74744
396	rs989960	T	C	T	C	0.0041	-0.03012	0.4175	0.422877	FALSE	FALSE	FALSE	ieu-b-508	7	1.07E+08	0.033798	486484	0.372798	Sepsis (28	Sepsis (28	Sepsis (28	TRUE	igd	9.00E-04	4.66E-06	7351	M00060.m	TRUE	reported	kqwl4V	2	TRUE	0.002815	20.74744
397	rs9903421	T	C	T	C	-0.0041	-0.02799	0.5633	0.563554	FALSE	FALSE	FALSE	ieu-b-508	17	39067912	0.033625	486484	0.405214	Sepsis (28	Sepsis (28	Sepsis (28	TRUE	igd	9.00E-04	7.39E-06	7351	M00060.m	TRUE	reported	kqwl4V	2	TRUE	0.002815	20.74744
3579	rs4516432	T	C	T	C	0.0082	0.021636	0.7795	0.775061	FALSE	FALSE	FALSE	ieu-b-508	2	1.16E+08	0.040094	486484	0.589451	Sepsis (28	Sepsis (28	Sepsis (28	TRUE	igd	0.0018	7.61E-06	7350	M32518.m	TRUE	reported	V0ZCiU	2	TRUE	0.002816	20.74744
3583	rs4904578	T	C	T	C	0.0082	-0.06261	0.3808	0.404733	FALSE	FALSE	FALSE	ieu-b-508	14	89901260	0.033948	486484	0.065157	Sepsis (28	Sepsis (28	Sepsis (28	TRUE	igd	0.0018	6.29E-06	7350	M32518.m	TRUE	reported	V0ZCiU	2	TRUE	0.002816	20.74744
7413	rs1161762	T	C	T	C	0.0123	0.048736	0.7232	0.71816	FALSE	FALSE	FALSE	ieu-b-508	6	1167568	0.037432	486484	0.19293	Sepsis (28	Sepsis (28	Sepsis (28	TRUE	igd	0.0027	4.02E-06	7350	M35186.m	TRUE	reported	UZfu6Z	2	TRUE	0.002816	20.74744
7418	rs2392820	A	G	A	G	0.0123	0.062044	0.28	0.279729	FALSE	FALSE	FALSE	ieu-b-508	2	2.22E+08	0.037407	486484	0.09719	Sepsis (28	Sepsis (28	Sepsis (28	TRUE	igd	0.0027	3.86E-06	7350	M35186.m	TRUE	reported	UZfu6Z	2	TRUE	0.002816	20.74744
7924	rs1880030	A	G	A	G	-0.0082	0.009825	0.4317	0.447209	FALSE	FALSE	FALSE	ieu-b-508	12	1.22E+08	0.033518	486484	0.769423	Sepsis (28	Sepsis (28	Sepsis (28	TRUE	igd	0.0018	5.81E-06	7348	M35675.m	TRUE	reported	D7fdyZ	2	TRUE	0.002816	20.74744
7949	rs9541642	A	G	A	G	-0.0082	-0.02995	0.6955	0.685795	FALSE	FALSE	FALSE	ieu-b-508	13	69524989	0.036145	486484	0.40735	Sepsis (28	Sepsis (28	Sepsis (28	TRUE	igd	0.0018	8.36E-06	7348	M35675.m	TRUE	reported	D7fdyZ	2	TRUE	0.002816	20.74744
6550	rs1122871	A	G	A	G	-0.0123	0.013563	0.6124	0.608963	FALSE	FALSE	FALSE	ieu-b-508	11	56439065	0.03416	486484	0.691334	Sepsis (28	Sepsis (28	Sepsis (28	TRUE	igd	0.0027	4.14E-06	7347	M34244.m	TRUE	reported	JyF0Sp	2	TRUE	0.002817	20.74744
1196	rs6765113	T	C	T	C	0.0082	0.010774	0.5408	0.550112	FALSE	FALSE	FALSE	ieu-b-508	3	1.96E+08	0.033756	486484	0.749609	Sepsis (28	Sepsis (28	Sepsis (28	TRUE	igd	0.0018	8.16E-06	7346	M01645.m	TRUE	reported	d5nAn3	2	TRUE	0.002817	20.74744
1200	rs7011528	A	C	A	C	-0.0082	-0.05709	0.4515	0.441362	FALSE	FALSE	FALSE	ieu-b-508	8	1.25E+08	0.033582	486484	0.089148	Sepsis (28	Sepsis (28	Sepsis (28	TRUE	igd	0.0018	4.01E-06	7346	M01645.m	TRUE	reported	d5nAn3	2	TRUE	0.002817	20.74744
3207	rs1440327	T	C	T	C	-0.0082	0.071496	0.4308	0.432675	FALSE	FALSE	FALSE	ieu-b-508	18	68050456	0.034346	486484	0.037372	Sepsis (28	Sepsis (28	Sepsis (28	TRUE	igd	0.0018	6.00E-06	7344	M32339.m	TRUE	reported	SVp504	2	TRUE	0.002818	20.74743
3208	rs1566951	T	C	T	C	-0.0082	-0.03544	0.3943	0.391263	FALSE	FALSE	FALSE	ieu-b-508	9	76315155	0.034142	486484	0.299244	Sepsis (28	Sepsis (28	Sepsis (28	TRUE	igd	0.0018	4.52E-06	7344	M32339.m	TRUE	reported	SVp504	2	TRUE	0.002818	20.74743
3212	rs2064272	A	G	A	G	-0.0082	-0.00721	0.346	0.344505	FALSE	FALSE	FALSE	ieu-b-508	20	8512967	0.035047	486484	0.837029	Sepsis (28	Sepsis (28	Sepsis (28	TRUE	igd	0.0018	4.49E-06	7344	M32339.m	TRUE	reported	SVp504	2	TRUE	0.002818	20.74743
3221	rs6839120	A	G	A	G	0.0082	0.002635	0.6771	0.671188	FALSE	FALSE	FALSE	ieu-b-508	4	82365496	0.035563	486484	0.94093	Sepsis (28	Sepsis (28	Sepsis (28	TRUE	igd	0.0018	4.03E-06	7344	M32339.m	TRUE	reported	SVp504	2	TRUE	0.002818	20.74743
4436	rs222836	A	G	A	G	-0.0082	-0.04073	0.5336	0.515715	FALSE	FALSE	FALSE	ieu-b-508	17	7133162	0.033375	486484	0.22229	Sepsis (28	Sepsis (28	Sepsis (28	TRUE	igd	0.0018	3.90E-06	7344	M32910.m	TRUE	reported	2OCBKh	2	TRUE	0.002818	20.74743
4455	rs7589568	T	C	T	C	-0.0123	-0.07656	0.8796	0.875916	FALSE	FALSE	FALSE	ieu-b-508	2	1.29E+08	0.050681	486484	0.130865	Sepsis (28	Sepsis (28	Sepsis (28	TRUE	igd	0.0027	5.65E-06	7344	M32910.m	TRUE	reported	2OCBKh	2	TRUE	0.002818	20.74743
2694	rs1080288	A	G	A	G	-0.0082	0.009868	0.3775	0.365349	FALSE	FALSE	FALSE	ieu-b-508	1	2.41E+08	0.034705	486484	0.776147	Sepsis (28	Sepsis (28	Sepsis (28	TRUE	igd	0.0018	6.65E-06	7340	M25459.m	TRUE	reported	ACJY35	2	TRUE	0.002819	20.74743
2710	rs6986204	A	C	A	C	-0.0082	-0.00273	0.4755	0.478597	FALSE	FALSE	FALSE	ieu-b-508	8	77245331	0.033443	486484	0.935055	Sepsis (28	Sepsis (28	Sepsis (28	TRUE	igd	0.0018	5.63E-06	7340	M25459.m	TRUE	reported	ACJY35	2	TRUE	0.002819	20.74743
3894	rs1511592	A	C	A	C	-0.0082	-0.00912	0.8679	0.865924	FALSE	FALSE	FALSE	ieu-b-508	3	1.69E+08	0.04881	486484	0.851733	Sepsis (28	Sepsis (28	Sepsis (28	TRUE	igd	0.0018	8.34E-06	7338	M32675.m	TRUE	reported	9FmPh9	2	TRUE	0.00282	20.74743
3901	rs41070	T	C	T	C	0.0082	-0.09345	0.8499	0.85599	FALSE	FALSE	FALSE	ieu-b-508	16	59668949	0.047524	486484	0.049253	Sepsis (28	Sepsis (28	Sepsis (28	TRUE	igd	0.0018	4.35E-06	7338	M32675.m	TRUE	reported	9FmPh9	2	TRUE	0.00282	20.74743
3905	rs8007741	A	G	A	G	0.0082	-0.03373	0.8554	0.857602	FALSE	FALSE	FALSE	ieu-b-508	14	87071714	0.048327	486484	0.4852	Sepsis (28	Sepsis (28	Sepsis (28	TRUE	igd	0.0018	5.06E-06	7338	M32675.m	TRUE	reported	9FmPh9	2	TRUE	0.00282	20.74743
1680	rs4816048	C	G	C	G	0.0123	0.01147	0.1794	0.188808	FALSE	TRUE	FALSE	ieu-b-508	20	8150561	0.042762	486484	0.788513	Sepsis (28	Sepsis (28	Sepsis (28	TRUE	igd	0.0027	7.13E-06	7337	M15365.m	TRUE	reported	TReTCM	2	TRUE	0.002821	20.74743
3746	rs4834604	T	C	T	C	-0.0123	-0.04488	0.5456	0.538807	FALSE	FALSE	FALSE	ieu-b-508	4	1.18E+08	0.03353	486484	0.180756	Sepsis (28	Sepsis (28	Sepsis (28	TRUE	igd	0.0027	4.92E-06	7337	M32632.m	TRUE	reported	pIHzPT	2	TRUE	0.002821	20.74743
3668	rs1205497	A	G	A	G	-0.0205	-0.00655	0.6371	0.643029	FALSE	FALSE	FALSE	ieu-b-508	6	22830924	0.034995	486484	0.851421	Sepsis (28	Sepsis (28	Sepsis (28	TRUE	igd	0.0045	6.09E-06	7322	M32578.m	TRUE	reported	LPkMFr	2	TRUE	0.002826	20.74742
3676	rs759400	T	C	T	C	0.0246	-0.10994	0.8126	0.821862	FALSE	FALSE	FALSE	ieu-b-508	2	10709816	0.043752	486484	0.011982	Sepsis (28	Sepsis (28	Sepsis (28	TRUE	igd	0.0054	5.26E-06	7322	M32578.m	TRUE	reported	LPkMFr	2	TRUE	0.002826	20.74742
5409	rs394811	A	G	A	G	-0.0082	-0.0523	0.399	0.414783	FALSE	FALSE	FALSE	ieu-b-508	17	8792514	0.033902	486484	0.122927	Sepsis (28	Sepsis (28	Sepsis (28	TRUE	igd	0.0018	9.89E-06	7315	M33389.m	TRUE	reported	Hf6XVM	2	TRUE	0.002829	20.74741
5430	rs9471904	T	C	T	C	-0.0082	0.020953	0.4795	0.492836	FALSE	FALSE	FALSE	ieu-b-508	6	42671099	0.033298	486484	0.529165	Sepsis (28	Sepsis (28	Sepsis (28	TRUE	igd	0.0018	7.40E-06	7315	M33389.m	TRUE	reported	Hf6XVM	2	TRUE	0.002829	20.74741
1833	rs1213278	T	C	T	C	0.0082	0.084445	0.3331	0.318436	FALSE	FALSE	FALSE	ieu-b-508	1	2.08E+08	0.035821	486484	0.018401	Sepsis (28	Sepsis (28	Sepsis (28	TRUE	igd	0.0018	6.82E-06	7308	M15778.m	TRUE	reported	941o3l	2	TRUE	0.002832	20.74741
1847	rs3131535	A	G	A	G	-0.0205	-0.01538	0.9652	0.958339	FALSE	FALSE	FALSE	ieu-b-508	1	2.22E+08	0.083329	486484	0.853557	Sepsis (28	Sepsis (28	Sepsis (28	TRUE	igd	0.0045	6.30E-06	7308	M15778.m	TRUE	reported	941o3l	2	TRUE	0.002832	20.74741
1849	rs4353947	A	G	A	G	-0.0082	-0.01673	0.2304	0.234326	FALSE	FALSE	FALSE	ieu-b-508	4	32729725	0.0394	486484	0.671177	Sepsis (28	Sepsis (28	Sepsis (28	TRUE	igd	0.0018	8.70E-06	7308	M15778.m	TRUE	reported	941o3l	2	TRUE	0.002832	20.74741
5664	rs1076599	A	C	A	C	0.0082	0.034337	0.6859	0.681651	FALSE	FALSE	FALSE	ieu-b-508	11	12843878	0.035946	486484	0.339464	Sepsis (28	Sepsis (28	Sepsis (28	TRUE	igd	0.0018	5.13E-06	7307	M33477.m	TRUE	reported	Aw8PBK	2	TRUE	0.002832	20.74741
5671	rs1209617	A	G	A	G	0.0082	0.029966	0.4016	0.416968	FALSE	FALSE	FALSE	ieu-b-508	1	1.92E+08	0.033799	486484	0.375308	Sepsis (28	Sepsis (28	Sepsis (28	TRUE	igd	0.0018	4.97E-06	7307	M33477.m	TRUE	reported	Aw8PBK	2	TRUE	0.002832	20.74741
5683	rs270144	T	C	T	C	-0.0082	-0.01389	0.6901	0.648266	FALSE	FALSE	FALSE	ieu-b-508	3	1.87E+08	0.035077	486484	0.692064	Sepsis (28	Sepsis (28	Sepsis (28	TRUE	igd	0.0018	6.90E-06	7307	M33477.m	TRUE	reported	Aw8PBK	2	TRUE	0.002832	20.74741
5694	rs6434958	C	G	C	G	-0.0123	0.035345	0.1118	0.113448	FALSE	TRUE	FALSE	ieu-b-508	2	1.99E+08	0.052571	486484	0.501378	Sepsis (28	Sepsis (28	Sepsis (28	TRUE	igd	0.0027	7.10E-06	7307	M33477.m	TRUE	reported	Aw8PBK	2	TRUE	0.002832	20.74741
5697	rs7316944	C	G	C	G	-0.0082	0.027953	0.2987	0.293875	FALSE	TRUE	FALSE	ieu-b-508	13	1E+08	0.036806	486484	0.447566	Sepsis (28	Sepsis (28	Sepsis (28	TRUE	igd	0.0018	6.25E-06	7307	M33477.m	TRUE	reported	Aw8PBK	2	TRUE	0.002832	20.74741
5704	rs9677367	A	G	A	G	0.0082	-0.06499	0.6892	0.668331	FALSE	FALSE	FALSE	ieu-b-508	2	1.29E+08	0.035477	486484	0.06697	Sepsis (28	Sepsis (28	Sepsis (28	TRUE	igd	0.0018	5.39E-06	7307	M33477.m	TRUE	reported	Aw8PBK	2	TRUE	0.002832	20.74741
5507	rs1011654	A	G	A	G	0.0123	0.07862	0.0816	0.078972	FALSE	FALSE	FALSE	ieu-b-508	9	1.17E+08	0.061611	486484	0.201933	Sepsis (28	Sepsis (28	Sepsis (28	TRUE	igd	0.0027	6.81E-06	7306	M33422.m	TRUE	reported	PLgjrT	2	TRUE	0.002833	20.74741
5519	rs1764109	A	G	A	G	0.0082	0.022273	0.2241	0.218469	FALSE	FALSE	FALSE	ieu-b-508	6	938900	0.040527	486484	0.582605	Sepsis (28	Sepsis (28	Sepsis (28	TRUE	igd	0.0018	7.20E-06	7306	M33422.m	TRUE	reported	PLgjrT	2	TRUE	0.002833	20.74741
5533	rs4870483	T	G	T	G	0.0082	0.04012	0.7193	0.718352	FALSE	FALSE	FALSE	ieu-b-508	6	1.57E+08	0.037118	486484	0.279746	Sepsis (28	Sepsis (28	Sepsis (28	TRUE	igd	0.0018	3.85E-06	7306	M33422.m	TRUE	reported	PLgjrT	2	TRUE	0.002833	20.74741
2742	rs1106851	A	C	A	C	-0.0082	0.05034	0.1958	0.190076	FALSE	FALSE	FALSE	ieu-b-508	12	1.18E+08	0.04281	486484	0.23963	Sepsis (28	Sepsis (28	Sepsis (28	TRUE	igd	0.0018	3.71E-06	7303	M27256.m	TRUE	reported	mJwzsi	2	TRUE	0.002834	20.7474

2749	rs1330292	T	G	T	G	-0.0082	0.021039	0.8039	0.811309	FALSE	FALSE	FALSE	ieu-b-508	9	1.36E+08	0.042605	486484	0.621444	Sepsis (28	Sepsis (28	Sepsis (28	TRUE	igd	0.0018	4.26E-06	7303	M27256.m	TRUE	reported	mJwzsi	2	TRUE	0.002834	20.7474
2762	rs660240	T	C	T	C	-0.0082	-0.09815	0.2096	0.215159	FALSE	FALSE	FALSE	ieu-b-508	1	1.1E+08	0.040557	486484	0.015523	Sepsis (28	Sepsis (28	Sepsis (28	TRUE	igd	0.0018	3.61E-06	7303	M27256.m	TRUE	reported	mJwzsi	2	TRUE	0.002834	20.7474
2440	rs2239068	T	C	T	C	0.0123	0.043725	0.7471	0.758181	FALSE	FALSE	FALSE	ieu-b-508	12	2534466	0.039029	486484	0.262572	Sepsis (28	Sepsis (28	Sepsis (28	TRUE	igd	0.0027	3.78E-06	7284	M21630.m	TRUE	reported	7eaO4H	2	TRUE	0.002841	20.74739
1065	rs2486925	T	G	T	G	-0.0082	0.033416	0.1462	0.145446	FALSE	FALSE	FALSE	ieu-b-508	1	2.03E+08	0.047492	486484	0.481674	Sepsis (28	Sepsis (28	Sepsis (28	TRUE	igd	0.0018	3.47E-06	7271	M01585.m	TRUE	reported	M7QiSM	2	TRUE	0.002846	20.74738
1069	rs513209	A	C	A	C	-0.0082	-0.13946	0.8824	0.882192	FALSE	FALSE	FALSE	ieu-b-508	11	1.25E+08	0.051564	486484	0.006838	Sepsis (28	Sepsis (28	Sepsis (28	TRUE	igd	0.0018	6.60E-06	7271	M01585.m	TRUE	reported	M7QiSM	2	TRUE	0.002846	20.74738
5790	rs4878060	T	G	T	G	-0.0123	-0.00944	0.6539	0.654398	FALSE	FALSE	FALSE	ieu-b-508	9	89955458	0.035842	486484	0.792157	Sepsis (28	Sepsis (28	Sepsis (28	TRUE	igd	0.0027	6.17E-06	7269	M33510.m	TRUE	reported	WxGKnn	2	TRUE	0.002847	20.74738
2690	rs8137744	A	G	A	G	0.0123	0.033776	0.3814	0.406592	FALSE	FALSE	FALSE	ieu-b-508	22	44697280	0.034556	486484	0.328361	Sepsis (28	Sepsis (28	Sepsis (28	TRUE	igd	0.0027	5.55E-06	7256	M24074.m	TRUE	reported	Lk6D2A	2	TRUE	0.002852	20.74737
3808	rs1208624	A	G	A	G	-0.0123	-0.01774	0.1209	0.123139	FALSE	FALSE	FALSE	ieu-b-508	1	1.62E+08	0.050646	486484	0.726104	Sepsis (28	Sepsis (28	Sepsis (28	TRUE	igd	0.0027	5.77E-06	7229	M32644.m	TRUE	reported	JAa2WD	2	TRUE	0.002863	20.74734
3816	rs2897863	T	G	T	G	0.0082	0.023942	0.697	0.691447	FALSE	FALSE	FALSE	ieu-b-508	11	1.02E+08	0.036295	486484	0.509486	Sepsis (28	Sepsis (28	Sepsis (28	TRUE	igd	0.0018	7.50E-06	7229	M32644.m	TRUE	reported	JAa2WD	2	TRUE	0.002863	20.74734
3833	rs992837	C	G	C	G	-0.0082	0.002942	0.6787	0.6752	FALSE	TRUE	FALSE	ieu-b-508	15	92599074	0.035678	486484	0.934272	Sepsis (28	Sepsis (28	Sepsis (28	TRUE	igd	0.0018	8.92E-06	7229	M32644.m	TRUE	reported	JAa2WD	2	TRUE	0.002863	20.74734
8639	rs1091015	T	C	T	C	-0.0164	-0.02105	0.4179	0.422412	FALSE	FALSE	FALSE	ieu-b-508	1	2.34E+08	0.033692	486484	0.532054	Sepsis (28	Sepsis (28	Sepsis (28	TRUE	igd	0.0036	6.24E-06	7219	M38178.m	TRUE	reported	GgCXMN	2	TRUE	0.002867	20.74734
7565	rs1994003	A	C	A	C	0.0082	0.008333	0.1798	0.188078	FALSE	FALSE	FALSE	ieu-b-508	10	45176945	0.042585	486484	0.844863	Sepsis (28	Sepsis (28	Sepsis (28	TRUE	igd	0.0018	9.74E-06	7217	M35270.m	TRUE	reported	Ct8V1n	2	TRUE	0.002867	20.74734
7571	rs2834543	T	C	T	C	-0.0082	-0.01619	0.7707	0.76899	FALSE	FALSE	FALSE	ieu-b-508	21	35935371	0.039582	486484	0.682534	Sepsis (28	Sepsis (28	Sepsis (28	TRUE	igd	0.0018	7.42E-06	7217	M35270.m	TRUE	reported	Ct8V1n	2	TRUE	0.002867	20.74734
7578	rs6784156	C	G	C	G	-0.0082	0.021034	0.7475	0.753307	FALSE	TRUE	FALSE	ieu-b-508	3	1.21E+08	0.038689	486484	0.586665	Sepsis (28	Sepsis (28	Sepsis (28	TRUE	igd	0.0018	5.61E-06	7217	M35270.m	TRUE	reported	Ct8V1n	2	TRUE	0.002867	20.74734
7585	rs8069296	T	C	T	C	0.0082	-0.01442	0.7988	0.799004	FALSE	FALSE	FALSE	ieu-b-508	17	42935059	0.041567	486484	0.728638	Sepsis (28	Sepsis (28	Sepsis (28	TRUE	igd	0.0018	9.97E-06	7217	M35270.m	TRUE	reported	Ct8V1n	2	TRUE	0.002867	20.74734
5345	rs1694532	A	T	A	T	-0.0082	-0.05043	0.2334	0.230434	FALSE	TRUE	FALSE	ieu-b-508	12	1.16E+08	0.039647	486484	0.203375	Sepsis (28	Sepsis (28	Sepsis (28	TRUE	igd	0.0018	2.72E-06	7118	M33380.m	TRUE	reported	OHRfl5	2	TRUE	0.002907	20.74726
5353	rs4517836	T	C	T	C	0.0082	-0.00435	0.7357	0.743296	FALSE	FALSE	FALSE	ieu-b-508	17	16851908	0.038377	486484	0.909745	Sepsis (28	Sepsis (28	Sepsis (28	TRUE	igd	0.0018	3.00E-06	7118	M33380.m	TRUE	reported	OHRfl5	2	TRUE	0.002907	20.74726
5808	rs1223104	A	G	A	G	0.0164	-0.01512	0.922	0.91291	FALSE	FALSE	FALSE	ieu-b-508	12	1.11E+08	0.058992	486484	0.797697	Sepsis (28	Sepsis (28	Sepsis (28	TRUE	igd	0.0036	4.60E-06	7066	M33515.m	TRUE	reported	X9mul1	2	TRUE	0.002928	20.74721
5810	rs1373219	T	C	T	C	0.0082	-0.03844	0.5411	0.503245	FALSE	FALSE	FALSE	ieu-b-508	2	31429832	0.033447	486484	0.250462	Sepsis (28	Sepsis (28	Sepsis (28	TRUE	igd	0.0018	8.22E-06	7066	M33515.m	TRUE	reported	X9mul1	2	TRUE	0.002928	20.74721
5811	rs1924466	A	C	A	C	0.0082	0.012231	0.4544	0.460008	FALSE	FALSE	FALSE	ieu-b-508	6	17772764	0.033532	486484	0.715288	Sepsis (28	Sepsis (28	Sepsis (28	TRUE	igd	0.0018	8.17E-06	7066	M33515.m	TRUE	reported	X9mul1	2	TRUE	0.002928	20.74721
6519	rs202815	A	G	A	G	-0.0123	-0.01051	0.3799	0.383195	FALSE	FALSE	FALSE	ieu-b-508	5	1.58E+08	0.034514	486484	0.760726	Sepsis (28	Sepsis (28	Sepsis (28	TRUE	igd	0.0027	3.49E-06	7049	M34123.m	TRUE	reported	9O5wo1	2	TRUE	0.002935	20.7472
5775	rs7600589	C	G	C	G	0.0164	-0.04037	0.7048	0.699553	FALSE	TRUE	FALSE	ieu-b-508	2	1.19E+08	0.036391	486484	0.267277	Sepsis (28	Sepsis (28	Sepsis (28	TRUE	igd	0.0036	5.59E-06	7044	M33509.m	TRUE	reported	56SCCs	2	TRUE	0.002938	20.74719
1408	rs9310707	A	G	A	G	-0.0082	-0.02451	0.3473	0.357692	FALSE	FALSE	FALSE	ieu-b-508	3	2332104	0.034734	486484	0.480326	Sepsis (28	Sepsis (28	Sepsis (28	TRUE	igd	0.0018	6.38E-06	7037	M02734.m	TRUE	reported	0y0dDD	2	TRUE	0.00294	20.74719
757	rs1939471	A	G	A	G	-0.0123	-0.08938	0.7209	0.71159	FALSE	FALSE	FALSE	ieu-b-508	11	95837084	0.036849	486484	0.015288	Sepsis (28	Sepsis (28	Sepsis (28	TRUE	igd	0.0027	4.06E-06	6946	M01303.m	TRUE	reported	3b8x0F	2	TRUE	0.002979	20.74711
8427	rs6774259	A	G	A	G	-0.0082	-0.00414	0.829	0.829674	FALSE	FALSE	FALSE	ieu-b-508	3	1.93E+08	0.044563	486484	0.925905	Sepsis (28	Sepsis (28	Sepsis (28	TRUE	igd	0.0018	5.13E-06	6940	M36808.m	TRUE	reported	u7lqaj	2	TRUE	0.002981	20.74711
8430	rs7519669	A	G	A	G	0.0082	0.022324	0.1935	0.190482	FALSE	FALSE	FALSE	ieu-b-508	1	1.07E+08	0.04239	486484	0.598445	Sepsis (28	Sepsis (28	Sepsis (28	TRUE	igd	0.0018	4.37E-06	6940	M36808.m	TRUE	reported	u7lqaj	2	TRUE	0.002981	20.74711
2038	rs4719964	A	G	A	G	-0.0123	-0.04167	0.2554	0.261188	FALSE	FALSE	FALSE	ieu-b-508	7	3539520	0.038015	486484	0.272966	Sepsis (28	Sepsis (28	Sepsis (28	TRUE	igd	0.0027	4.46E-06	6939	M18349.m	TRUE	reported	M5qPs8	2	TRUE	0.002982	20.7471
6798	rs1184360	A	G	A	G	-0.0082	-0.04532	0.5105	0.509378	FALSE	FALSE	FALSE	ieu-b-508	13	45417450	0.033388	486484	0.174692	Sepsis (28	Sepsis (28	Sepsis (28	TRUE	igd	0.0018	2.85E-06	6755	M34359.m	TRUE	reported	0UotaJ	2	TRUE	0.003063	20.74694
6823	rs4281086	A	C	A	C	0.0082	0.017514	0.667	0.665593	FALSE	FALSE	FALSE	ieu-b-508	8	10352308	0.035406	486484	0.620832	Sepsis (28	Sepsis (28	Sepsis (28	TRUE	igd	0.0018	4.77E-06	6755	M34359.m	TRUE	reported	0UotaJ	2	TRUE	0.003063	20.74694
6824	rs4664490	C	G	C	G	0.0082	-0.06154	0.3876	0.390021	FALSE	TRUE	FALSE	ieu-b-508	2	1.52E+08	0.034334	486484	0.073067	Sepsis (28	Sepsis (28	Sepsis (28	TRUE	igd	0.0018	4.47E-06	6755	M34359.m	TRUE	reported	0UotaJ	2	TRUE	0.003063	20.74694
2415	rs1271338	A	G	A	G	0.0123	-0.02153	0.7335	0.736468	FALSE	FALSE	FALSE	ieu-b-508	2	58623301	0.037982	486484	0.570828	Sepsis (28	Sepsis (28	Sepsis (28	TRUE	igd	0.0027	7.86E-06	6556	M21188.m	TRUE	reported	kvdnjc	2	TRUE	0.003156	20.74676
2428	rs7032741	T	C	T	C	-0.0205	-0.04122	0.0891	0.09988	FALSE	FALSE	FALSE	ieu-b-508	9	1.38E+08	0.055829	486484	0.460265	Sepsis (28	Sepsis (28	Sepsis (28	TRUE	igd	0.0045	5.12E-06	6556	M21188.m	TRUE	reported	kvdnjc	2	TRUE	0.003156	20.74676
8454	rs1026192	A	C	A	C	-0.0123	0.056175	0.1617	0.166572	FALSE	FALSE	FALSE	ieu-b-508	7	1.32E+08	0.04474	486484	0.209264	Sepsis (28	Sepsis (28	Sepsis (28	TRUE	igd	0.0027	6.54E-06	6552	M37058.m	TRUE	reported	32p8qV	2	TRUE	0.003157	20.74675
8482	rs4548847	A	G	A	G	0.0082	0.056815	0.5209	0.533629	FALSE	FALSE	FALSE	ieu-b-508	16	60405936	0.033495	486484	0.089842	Sepsis (28	Sepsis (28	Sepsis (28	TRUE	igd	0.0018	9.56E-06	6552	M37058.m	TRUE	reported	32p8qV	2	TRUE	0.003157	20.74675
7470	rs6817210	A	C	A	C	-0.0123	-0.03502	0.5169	0.52401	FALSE	FALSE	FALSE	ieu-b-508	4	70004438	0.033412	486484	0.294524	Sepsis (28	Sepsis (28	Sepsis (28	TRUE	igd	0.0027	6.58E-06	6543	M35193.m	TRUE	reported	BMMWXU	2	TRUE	0.003162	20.74674
7529	rs1023143	T	C	T	C	-0.0246	0.04016	0.0747	0.075241	FALSE	FALSE	FALSE	ieu-b-508	7	1.05E+08	0.064	486484	0.530329	Sepsis (28	Sepsis (28	Sepsis (28	TRUE	igd	0.0054	6.26E-06	6538	M35257.m	TRUE	reported	iphouU	2	TRUE	0.003164	20.74674
957	rs1316441	T	C	T	C	0.0082	0.010331	0.7246	0.716074	FALSE	FALSE	FALSE	ieu-b-508	5	1.05E+08	0.037085	486484	0.780568	Sepsis (28	Sepsis (28	Sepsis (28	TRUE	igd	0.0018	5.33E-06	6523	M01558.m	TRUE	reported	AZK0p4	2	TRUE	0.003171	20.74672
973	rs4692012	A	G	A	G	-0.0082	-0.01741	0.5389	0.524496	FALSE	FALSE	FALSE	ieu-b-508	4	1.66E+08	0.033635	486484	0.604628	Sepsis (28	Sepsis (28	Sepsis (28	TRUE	igd	0.0018	3.35E-06	6523	M01558.m	TRUE	reported	AZK0p4	2	TRUE	0.003171	20.74672
7161	rs4764937	T	G	T	G	-0.0246	-0.04614	0.6365	0.636159	FALSE	FALSE	FALSE	ieu-b-508	12	1.03E+08	0.034627	486484	0.182688	Sepsis (28	Sepsis (28	Sepsis (28	TRUE	igd	0.0054	5.87E-06	5856	M34533.m	TRUE	reported	SuKX4k	2	TRUE	0.003531	20.746
7638	rs1398096	T	C	T	C	0.0082	-0.02531	0.7377	0.736204	FALSE	FALSE	FALSE	ieu-b-508	3	62661009	0.038291	486484	0.50866	Sepsis (28	Sepsis (28	Sepsis (28	TRUE	igd	0.0018	4.41E-06	5768	M35326.m	TRUE	reported	jRytyJ	2	TRUE	0.003585	20.74589
7653	rs4803892	T	C	T	C	-0.0082	0.041021	0.7313	0.732591	FALSE	FALSE	FALSE	ieu-b-508	19	46547023	0.038065	486484	0.281189	Sepsis (28	Sepsis (28	Sepsis (28	TRUE	igd	0.0018	7.13E-06	5768	M35326.m	TRUE	reported	jRytyJ	2	TRUE	0.003585	20.74589
7661	rs7117598	T	C	T	C	0.0082	0.037218	0.6692	0.663599	FALSE	FALSE	FALSE	ieu-b-508	11	23515684	0.035673	486484	0.296805	Sepsis (28	Sepsis (28	Sepsis (28	TRUE	igd	0.0018	3.74E-06	5768	M35326.m	TRUE	reported	jRytyJ	2	TRUE	0.003585	20.74589
5653	rs2649049	T	C	T	C	-0.0164	-0.01519	0.5773	0.567861	FALSE	FALSE	FALSE	ieu-b-508	11	9873283	0.033644	486484	0.651648	Sepsis (28	Sepsis (28	Sepsis (28	TRUE	igd	0.0036	4.63E-06	5717	M33453.m	TRUE	reported	OzEeLQ	2	TRUE	0.003617	20.74583
670	rs4516857	C	G	C	G	0.0205	-0.03564	0.0991	0.091584	FALSE	TRUE	FALSE	ieu-b-508	5	43937133	0.058241	486484	0.540625	Sepsis (28	Sepsis (28	Sepsis (28	TRUE	igd	0.0045	4.93E-06	5687	M01284.m	TRUE	reported	E4hc9c	2	TRUE	0.003636	20.74579
2667	rs310271	A	G	A	G	-0.0533	0.058331	0.2583	0.27735	FALSE	FALSE	FALSE	ieu-b-508	8	23644666	0.037338	486484	0.118235	Sepsis (28	Sepsis (28	Sepsis (28	TRUE	igd	0.0117	5.53E-06	5599	M22842.m	TRUE	reported	0t12oo	2	TRUE	0.003693	20.74567
2919	rs4236964	T	C	T	C	-0.0123	0.012263	0.3256	0.317501	FALSE	FALSE	FALSE	ieu-b-508	8	70239113	0.035877	486484	0.732491	Sepsis (28	Sepsis (28	Sepsis (28	TRUE	igd	0.0027	5.74E-06	5562	M27728.m	TRUE	reported	WOp1XE	2	TRUE	0.003717	20.74562
2398	rs1772999	A	G	A	G	-0.0492	0.059685	0.0612	0.065082	FALSE	FALSE	FALSE	ieu-b-508	8	96154908	0.067644	486484	0.377597	Sepsis (28	Sepsis (28	Sepsis (28	TRUE	igd	0.0108	5.23E-06	5436	M21184.m	TRUE	reported	63ppuJ	2	TRUE	0.003803	20.74545
7748	rs1289637	A	T	A	T	0.0205	0.029708	0.1961	0.209979	FALSE	TRUE	FALSE	ieu-b-508	14	30235469	0.041113	486484	0.469925	Sepsis (28	Sepsis (28	Sepsis (28	TRUE	igd	0.0045	5.72E-06	5306	M35433.m	TRUE	reported	XlYkLe	2	TRUE	0.003896	20.74526
3844	rs1948260	T	C	T	C	0.0123	0.009655	0.5206	0.519035	FALSE	FALSE	FALSE	ieu-b-508	8	82604242	0.033422	486484	0.772679	Sepsis (28	Sepsis (28	Sepsis (28	TRUE	igd	0.0027	7.10E-06	5211	M32651.m	TRUE	reported	DeuPwv	2	TRUE	0.003967	20.74512
3846	rs2555100	C	G	C	G	0.0123	0.043827	0.6638	0.670032	FALSE	TRUE	FALSE	ieu-b-508	2	17835064	0.035571	486484	0.217906	Sepsis (28	Sepsis (28	Sepsis (28	TRUE	igd	0.0027	6.86E-06	5211	M32651.m	TRUE	reported	DeuPwv	2	TRUE	0.003967	20.74512
3855	rs6953983	A	G	A	G	0.0123	-0.05254	0.3217	0.3239	FALSE	FALSE	FALSE	ieu-b-508	7	96407773	0.035792	486484	0.142151	Sepsis (28	Sepsis (28	Sepsis (28	TRUE	igd	0.0027	6.79E-06	5211	M32651.m	TRUE	reported	DeuPwv	2	TRUE	0.003967	20.74512
6944	rs9846511	A	C	A	C	-0.0205	-0.08357	0.2559	0.250097	FALSE	FALSE	FALSE	ieu-b-508	3	1.79E+08	0.03865	486484	0.030604	Sepsis (28	Sepsis (28	Sepsis (28	TRUE	igd	0.0045	4.25E-06	5208	M34395.m	TRUE	reported	QVOPA3	2	TRUE	0.003969	20.74512
612	rs3752241	C	G	C	G	0.0574	-0.00517	0.8684	0.831084	FALSE	TRUE	FALSE	ieu-b-508	19	1053524	0.044858	486484	0.908316	Sepsis (28	Sepsis (28	Sepsis (28	TRUE	igd	0.0126	5.52E-06	4934	M01114.m	TRUE	reported	fqHdM2	2	TRUE	0.004189	20.74467
488	rs6816298	A	G	A	G	0.1842	0.070496	0.8254	0.815118	FALSE	FALSE	FALSE	ieu-b-508	4	65144051	0.043146	486484	0.102281	Sepsis (28	Sepsis (28	Sepsis (28	TRUE	igd	0.0403	4.76E-06	283	M00553.m	TRUE	reported	IFjXiL	2	TRUE	0.068747	20.74383
5636	rs2129376	A	G	A	G	0.0164	-0.03638	0.7795	0.745029	FALSE	FALSE	FALSE	ieu-b-508	4	30646641	0.038444	486484	0.343956	Sepsis (28	Sepsis (28	Sepsis (28	TRUE	igd	0.0036	5.67E-06	4004	M33443.m	TRUE	reported	G1JrRV	2	TRUE	0.005156	20.74272
8324	rs1965169	T	C	T	C	0.0848	0.003745	0.1956	0.180967	FALSE	FALSE	FALSE	ieu-b-508	10	49794610	0.043555	486484	0.931471	Sepsis (28	Sepsis (28	Sepsis (28	TRUE	igd	0.0186	4.89E-06	952	M36589.m	TRUE	reported	27GADo	2	TRUE	0.021367	20.74209
2366	rs1684921	C	G	C	G	-0.0369	0.017795	0.7736	0.762489	FALSE	TRUE	FALSE	ieu-b-508	2	1.65E+08	0.03924	486484	0.650199	Sepsis (28	Sepsis (28	Sepsis (28	TRUE	igd	0.0081	5.57E-06	3422	M21049.m	TRUE	reported	UDtLE4	2	TRUE	0.006028	20.74096
413	rs1433904	T	C	T	C	0.0442	-0.01567	0.0228	0.028788	FALSE	FALSE	FALSE	ieu-b-508	11	4819790	0.100819	486484	0.876487	Sepsis (28	Sepsis (28	Sepsis (28	TRUE	igd	0.0097	5.06E-06	1763	M00063.m	TRUE	reported	Hdsv1L	2	TRUE	0.01164	20.73997
3026	rs4699296	A	G	A	G	0.0483	-0.00312	0.1693	0.188353	FALSE	FALSE	FALSE	ieu-b-508	4	97700960	0.042734	486484	0.941801	Sepsis (28	Sepsis (28	Sepsis (28	TRUE	igd	0.0106	4.78E-06	1764	M31548.m	TRUE	reported	bgRTBS	2	TRUE	0.011633	20.7391
6649	rs1824189	T	C	T	C	-0.0631	-0.07277	0.6979	0.708338	FALSE	FALSE	FALSE	ieu-b-508	15	37753443	0.036688	486484	0.047303	Sepsis (28	Sepsis (28	Sepsis (28	TRUE	igd	0.0138	5.25E-06	248	M34322.m	TRUE	reported	16GdG9	2	TRUE	0.07775	20.73882
1875	rs4296931	A	G	A	G	0.1523	0.122022	0.0244	0.046319	FALSE	FALSE	FALSE	ieu-b-508	6	3211615	0.080524	486484	0.129687	Sepsis (28	Sepsis (28	Sepsis (28	TRUE	igd	0.0334	5.10E-06	773	M15958.m	TRUE	reported	3HF6lf	2	TRUE	0.026194	20.73871
5186	rs7595056	T	C	T	C	0.0533	-0.03806	0.7661	0.774762	FALSE	FALSE	FALSE	ieu-b-508	2	58826238	0.039966	486484	0.340927	Sepsis (28	Sepsis (28	Sepsis (28	TRUE	igd	0.0117	5.38E-06	2860	M33197.m	TRUE	reported	D6xaBg	2	TRUE	0.007204	20.73857
8082	rs6060489	A	C	A	C	0.0524	-0.03233	0.0753	0.074011	FALSE	FALSE	FALSE	ieu-b-508	20	30237905	0.063721	486484	0.611898	Sepsis (28	Sepsis (28	Sepsis (28	TRUE	igd	0.0115	4.89E-06	1743	M36009.m	TRUE	reported	2EUZ1U	2	TRUE	0.011771	20.73807
8155	rs7229244	A	C	A	C	-0.0328	-0.01239	0.7276	0.726196	FALSE	FALSE	FALSE	ieu-b-508	18	52838987	0.03744	486484	0.740703	Sepsis (28	Sepsis (28	Sepsis (28	TRUE	igd	0.0072	5.67E-06	2709	M36115.m	TRUE	reported	ZV1jWp	2	TRUE	0.007603	20.73776
5498	rs1052084	A	C	A	C	0.191	-0.16185	0.0165	0.012398	FALSE	FALSE	FALSE	ieu-b-508	5	16879933	0.150781	486484	0.283083	Sepsis (28	Sepsis (28	Sepsis (28	TRUE	igd	0.0419	5.27E-06	977	M33420.m	TRUE	reported	eTToDb	2	TRUE	0.020826	20.73713
6009	rs2087937	C	G	C	G	-0.934	-0.03854	0.9873	0.990699	FALSE	TRUE	FALSE	ieu-b-508	6	92815392	0.175232	486484	0.825917	Sepsis (28	Sepsis (28	Sepsis (28	TRUE	igd	0.2025	3.96E-06	79	M33751.m	TRUE	reported	Cxdkr4	2	TRUE	0.212157	20.73516
6015	rs4822679	A	G	A	G	-0.934	-0.29716	0.9873	0.997265	FALSE	FALSE	FALSE	ieu-b-508	22	26392172	0.330306	486484	0.368304	Sepsis (28	Sepsis (28	Sepsis (28	TRUE	igd	0.2025	3.96E-06	79	M33751.m	TRUE	reported	Cxdkr4	2	TRUE	0.212157	20.73516
6019	rs7150140	T	C	T	C	-0.934	-0.10516	0.9873	0.98458	FALSE	FALSE	FALSE	ieu-b-508	14	89656376	0.138164	486484	0.44658	Sepsis (28	Sepsis (28	Sepsis (28	TRUE	igd	0.2025	3.96E-06	79	M33751.m	TRUE	reported	Cxdkr4	2	TRUE	0.212157	20.73516
6024	rs7934379	T	C	T	C	0.934	0.15367	0.0127	0.010742	FALSE	FALSE	FALSE	ieu-b-508	11	83661168	0.167002	486484	0.357483	Sepsis (28	Sepsis (28	Sepsis (28	TRUE	igd	0.2025	3.96E-06	79	M33751.m	TRUE	reported	Cxdkr4	2	TRUE	0.212157	20.73516
4717	rs1167212	A	G	A	G	-0.2949	-0.04166	0.0233	0.039156	FALSE	FALSE	FALSE	ieu-b-508	19	11194823	0.089572	486484	0.641852	Sepsis (28	Sepsis (28	Sepsis (28	TRUE	igd	0.0647	5.18E-06	1017	M33178.m	TRUE	reported	g22WMS	2	TRUE	0.020019	20.73414
8558	rs933775	A	G	A	G	0.0337	0.036712	0.7146	0.712307	FALSE	FALSE	FALSE	ieu-b-508	5	51748671	0.036947	486484	0.320406	Sepsis (28	Sepsis (28	Sepsis (28	TRUE	igd	0.0074	4.81E-06	7355	M37202.m	TRUE	reported	BtslDx	2	TRUE	0.002812	20.73377
6472	rs1293728	T	C	T	C	-0.568	-0.23451	0.0146	0.031438	FALSE	FALSE	FALSE	ieu-b-508	17	14385160	0.100097	486484	0.019136	Sepsis (28	Sepsis (28	Sepsis (28	TRUE	igd	0.1243	4.89E-06	282	M34109.m	TRUE	reported	piJuC7	2	TRUE	0.068942	20.73306
2888	rs7659567	A	G	A	G	0.0337	0.07909	0.2037	0.206658	FALSE	FALSE	FALSE	ieu-b-508	4	1.01E+08	0.041332	486484	0.055678	Sepsis (28	Sepsis (28	Sepsis (28	TRUE	igd	0.0074	5.01E-06	6393	M27716.m	TRUE	reported	N5hToq	2	TRUE	0.003234	20.73292
7272	rs4976671	C	G	C	G	0.0989	-0.04656	0.9854	0.981261	FALSE	TRUE	FALSE	ieu-b-508	5	1.76E+08	0.130836	486484	0.721927	Sepsis (28	Sepsis (28	Sepsis (28	TRUE	igd	0.0217	5.01E-06	1060	M34912.m	TRUE	reported	W8lcT6	2	TRUE	0.019219	20.73256
2364	rs1261667	T	G	T	G	0.1084	0.135673	0.9778	0.979255	FALSE	FALSE	FALSE	ieu-b-508	2	1.4E+08	0.117994	486484	0.250214	Sepsis (28	Sepsis (28	Sepsis (28	TRUE	igd	0.0238	5.05E-06	3422	M21049.m	TRUE	reported	UDtLE4	2	TRUE	0.006026	20.73246
5309	rs2792707	T	C	T	C	0.0984	-0.02327	0.0427	0.042324	FALSE	FALSE	FALSE	ieu-b-508	10	1.14E+08	0.083236	486484	0.779778	Sepsis (28	Sepsis (28	Sepsis (28	TRUE	igd	0.0216	5.10E-06	1863	M33359.m	TRUE	reported	dfJmmS	2	TRUE	0.011017	20.73081
4981	rs4964992	T	C	T	C	0.0752	-0.00699	0.5326	0.486337	FALSE	FALSE	FALSE	ieu-b-508	12	1.32E+08	0.033394	486484	0.834095	Sepsis (28	Sepsis (28	Sepsis (28	TRUE	igd	0.0165	5.01E-06	1017	M33178.m	TRUE	reported	g22WMS	2	TRUE	0.020015	20.73065
8514	rs4923384	T	C	T	C	-0.0337	-0.0085	0.7967	0.785609	FALSE	FALSE	FALSE	ieu-b-508	11	26756556	0.040619	486484	0.83416	Sepsis (28	Sepsis (28	Sepsis (28	TRUE	igd	0.0074	4.90E-06	4520	M37104.m	TRUE	reported	bR3g7d	2	TRUE	0.004567	20.73023
2163	rs1078882	T	G	T	G	0.0369	-0.0187	0.1884	0.155394	FALSE	FALSE	FALSE	ieu-b-508	1	1.52E+08	0.04595	486484	0.68408	Sepsis (28	Sepsis (28	Sepsis (28	TRUE	igd	0.0081	5.96E-06	1766	M19323.m	TRUE	reported	yzKFKX	2	TRUE	0.011615	20.72958
6071	rs6727350	A	T	A	T	-0.041	-0.0128	0.1525	0.149832	FALSE	TRUE	FALSE	ieu-b-508	2	22615230	0.046888	486484	0.784847	Sepsis (28	Sepsis (28	Sepsis (28	TRUE	igd	0.009	5.00E-06	1765	M33822.m	TRUE	reported	Wic4uS	2	TRUE	0.011621	20.72957
456	rs1990178	T	C	T	C	-0.0164	-0.00118	0.1943	0.236461	FALSE	FALSE	FALSE	ieu-b-508	7	21300265	0.039332	486484	0.975989	Sepsis (28	Sepsis (28	Sepsis (28	TRUE	igd	0.0036	6.27E-06	1763	M00513.m	TRUE	reported	ohacnP	2	TRUE	0.011635	20.72954
4040	rs1076285	A	G	A	G	-0.0164	0.028542	0.6966	0.732667	FALSE	FALSE	FALSE	ieu-b-508	10	80996513	0.038085	486484	0.453594	Sepsis (28	Sepsis (28	Sepsis (28	TRUE	igd	0.0036	6.86E-06	1759	M32740.m	TRUE	reported	YLshfP	2	TRUE	0.011661	20.72949
3715	rs2402228	A	T	A	T	-0.051	0.052236	0.1901	0.197248	FALSE	TRUE	FALSE	ieu-b-508	7	1.17E+08	0.042269	486484	0.216538	Sepsis (28	Sepsis (28	Sepsis (28	TRUE	igd	0.0112	5.27E-06	6904	M32616.m	TRUE	reported	vrkjBE	2	TRUE	0.002994	20.72901
4475	rs6480159	A	G	A	G	0.0255	-0.02463	0.2212	0.2205	FALSE	FALSE	FALSE	ieu-b-508	10	68079348	0.04034	486484	0.541492	Sepsis (28	Sepsis (28	Sepsis (28	TRUE	igd	0.0056	5.33E-06	5425	M33084.m	TRUE	reported	jrOsNK	2	TRUE	0.003808	20.72737
2374	rs1214593	T	C	T	C	0.0287	-0.03204	0.5511	0.58926	FALSE	FALSE	FALSE	ieu-b-508	1	80298833	0.034003	486484	0.346113	Sepsis (28	Sepsis (28	Sepsis (28	TRUE	igd	0.0063	6.21E-06	1608	M21127.m	TRUE	reported	WCjhS3	2	TRUE	0.012742	20.72727
1664	rs1998053	A	G	A	G	0.0724	0.066201	0.0855	0.082717	FALSE	FALSE	FALSE	ieu-b-508	14	1.01E+08	0.060837	486484	0.276525	Sepsis (28	Sepsis (28	Sepsis (28	TRUE	igd	0.0159	5.60E-06	5606	M15335.m	TRUE	reported	a7RjFn	2	TRUE	0.003685	20.72659
3176	rs1111150	T	C	T	C	-0.0214	-0.02234	0.0757	0.083019	FALSE	FALSE	FALSE	ieu-b-508	12	1.04E+08	0.060876	486484	0.713577	Sepsis (28	Sepsis (28	Sepsis (28	TRUE	igd	0.0047	5.92E-06	7356	M32338.m	TRUE	reported	q5cuaP	2	TRUE	0.00281	20.72592
6268	rs2378733	A	G	A	G	0.0214	0.106479	0.1266	0.127537	FALSE	FALSE	FALSE	ieu-b-508	9	89985014	0.050197	486484	0.033901	Sepsis (28	Sepsis (28	Sepsis (28	TRUE	igd	0.0047	5.07E-06	7222	M33937.m	TRUE	reported	QanGCl	2	TRUE	0.002862	20.72581
8277	rs2581320	T	G	T	G	0.0214	0.028411	0.8846	0.87866	FALSE	FALSE	FALSE	ieu-b-508	16	86304383	0.050995	486484	0.57744	Sepsis (28	Sepsis (28	Sepsis (28	TRUE	igd	0.0047	6.20E-06	6464	M36553.m	TRUE	reported	2Rk7nd	2	TRUE	0.003197	20.72514
6075	rs1271578	A	G	A	G	0.0214	-0.0046	0.8197	0.82706	FALSE	FALSE	FALSE	ieu-b-508	4	68694085	0.044088	486484	0.916851	Sepsis (28	Sepsis (28	Sepsis (28	TRUE	igd	0.0047	5.16E-06	6327	M33833.m	TRUE	reported	LMIPLH	2	TRUE	0.003266	20.725
4248	rs3791660	T	G	T	G	-0.0214	-0.04084	0.463	0.461206	FALSE	FALSE	FALSE	ieu-b-508	2	56129880	0.03348	486484	0.222488	Sepsis (28	Sepsis (28	Sepsis (28	TRUE	igd	0.0047	4.49E-06	6169	M32795.m	TRUE	reported	0YBWkh	2	TRUE	0.003349	20.72483
4612	rs1251651	A	T	A	T	0.1608	-0.01822	0.0973	0.107061	FALSE	TRUE	FALSE	ieu-b-508	5	1.54E+08	0.053976	486484	0.735714	Sepsis (28	Sepsis (28	Sepsis (28	TRUE	igd	0.0353	5.33E-06	1574	M33173.m	TRUE	reported	04fkUQ	2	TRUE	0.013012	20.72385
6960	rs1095510	A	C	A	C	-0.0717	0.036131	0.4839	0.490079	FALSE	FALSE	FALSE	ieu-b-508	8	98230545	0.033471	486484	0.28037	Sepsis (28	Sepsis (28	Sepsis (28	TRUE	igd	0.0157	5.14E-06	314	M34404.m	TRUE	reported	8FMHfo	2	TRUE	0.062285	20.72354
1948	rs1333695	T	C	T	C	0.1061	0.009521	0.0541	0.069347	FALSE	FALSE	FALSE	ieu-b-508	16	17366562	0.065879	486484	0.885087	Sepsis (28	Sepsis (28	Sepsis (28	TRUE	igd	0.0233	5.48E-06	3393	M16823.m	TRUE	reported	fNy2PD	2	TRUE	0.006074	20.72349
2104	rs8107558	T	C	T	C	0.0665	-0.00921	0.9541	0.937451	FALSE	FALSE	FALSE	ieu-b-508	19	17884120	0.069258	486484	0.894191	Sepsis (28	Sepsis (28	Sepsis (28	TRUE	igd	0.0146	5.52E-06	1767	M18467.m	TRUE	reported	64PaNn	2	TRUE	0.011605	20.72267
7607	rs841225	T	C	T	C	-0.0173	0.028625	0.5927	0.591365	FALSE	FALSE	FALSE	ieu-b-508	16	4736316	0.03392	486484	0.398731	Sepsis (28	Sepsis (28	Sepsis (28	TRUE	igd	0.0038	5.37E-06	7359	M35320.m	TRUE	reported	ijCk2X	2	TRUE	0.002809	20.72082
8388	rs9830523	T	C	T	C	-0.0346	-0.16007	0.9753	0.972917	FALSE	FALSE	FALSE	ieu-b-508	3	1.17E+08	0.103992	486484	0.123733	Sepsis (28	Sepsis (28	Sepsis (28	TRUE	igd	0.0076	5.70E-06	7335	M36776.m	TRUE	reported	VnYglx	2	TRUE	0.002818	20.7208
8038	rs3785236	A	C	A	C	-0.0173	-0.00103	0.2425	0.245841	FALSE	FALSE	FALSE	ieu-b-508	16	7667199	0.038976	486484	0.978848	Sepsis (28	Sepsis (28	Sepsis (28	TRUE	igd	0.0038	5.25E-06	6960	M35854.m	TRUE	reported	EujB0p	2	TRUE	0.002969	20.7205
4204	rs6464572	C	G	C	G	-0.0551	-0.05899	0.8341	0.841698	FALSE	TRUE	FALSE	ieu-b-508	7	1.44E+08	0.04583	486484	0.19804	Sepsis (28	Sepsis (28	Sepsis (28	TRUE	igd	0.0121	5.65E-06	2537	M32762.m	TRUE	reported	Q5IKLH	2	TRUE	0.008107	20.72001
5911	rs3923017	T	C	T	C	-0.0469	-0.07276	0.0439	0.077625	FALSE	FALSE	FALSE	ieu-b-508	17	86633	0.062385	486484	0.24352	Sepsis (28	Sepsis (28	Sepsis (28	TRUE	igd	0.0103	5.31E-06	2757	M33627.m	TRUE	reported	4RHaUX	2	TRUE	0.007464	20.71839
7400	rs7218918	A	G	A	G	-0.0296	0.022222	0.4081	0.40718	FALSE	FALSE	FALSE	ieu-b-508	17	77100721	0.033928	486484	0.512482	Sepsis (28	Sepsis (28	Sepsis (28	TRUE	igd	0.0065	4.63E-06	1982	M35159.m	TRUE	reported	7EoJLB	2	TRUE	0.010355	20.71659
5107	rs9562411	T	G	T	G	0.1795	-0.03103	0.9478	0.95856	FALSE	FALSE	FALSE	ieu-b-508	13	32595109	0.08374	486484	0.710997	Sepsis (28	Sepsis (28	Sepsis (28	TRUE	igd	0.0394	5.20E-06	1017	M33178.m	TRUE	reported	g22WMS	2	TRUE	0.020001	20.71484
8056	rs1026725	T	C	T	C	-0.0305	-0.00763	0.8786	0.866632	FALSE	FALSE	FALSE	ieu-b-508	7	46168740	0.049094	486484	0.876512	Sepsis (28	Sepsis (28	Sepsis (28	TRUE	igd	0.0067	4.71E-06	4529	M35978.m	TRUE	reported	CN0wRR	2	TRUE	0.004555	20.71373
5891	rs4458182	A	T	A	T	-0.0862	0.020852	0.0854	0.078129	FALSE	TRUE	FALSE	ieu-b-508	2	1.5E+08	0.061936	486484	0.736372	Sepsis (28	Sepsis (28	Sepsis (28	TRUE	igd	0.0189	5.30E-06	470	M33610.m	TRUE	reported	tmg43v	2	TRUE	0.042382	20.71281
6210	rs1287739	A	C	A	C	-0.0437	-0.06064	0.8993	0.85975	FALSE	FALSE	FALSE	ieu-b-508	13	90101790	0.051177	486484	0.236061	Sepsis (28	Sepsis (28	Sepsis (28	TRUE	igd	0.0096	5.85E-06	4533	M33901.m	TRUE	reported	mQkhrb	2	TRUE	0.00455	20.71232
1418	rs1587235	A	G	A	G	-0.0132	-0.01629	0.3312	0.334382	FALSE	FALSE	FALSE	ieu-b-508	12	1.31E+08	0.035542	486484	0.646778	Sepsis (28	Sepsis (28	Sepsis (28	TRUE	igd	0.0029	6.47E-06	6941	M03127.m	TRUE	reported	JscEZw	2	TRUE	0.002976	20.71222
7675	rs1257125	T	C	T	C	-0.0264	0.03364	0.0192	0.021215	FALSE	FALSE	FALSE	ieu-b-508	10	81223950	0.114776	486484	0.769451	Sepsis (28	Sepsis (28	Sepsis (28	TRUE	igd	0.0058	5.39E-06	6920	M35327.m	TRUE	reported	SquFiY	2	TRUE	0.002985	20.7122
4568	rs1302548	A	G	A	G	0.205	0.091478	0.977	0.958783	FALSE	FALSE	FALSE	ieu-b-508	2	1.44E+08	0.083989	486484	0.276081	Sepsis (28	Sepsis (28	Sepsis (28	TRUE	igd	0.045	5.23E-06	1011	M33150.m	TRUE	reported	H9SSMD	2	TRUE	0.020114	20.71203
8285	rs6771638	T	G	T	G	-0.0132	0.007095	0.2911	0.294336	FALSE	FALSE	FALSE	ieu-b-508	3	1.2E+08	0.036568	486484	0.846156	Sepsis (28	Sepsis (28	Sepsis (28	TRUE	igd	0.0029	5.38E-06	6464	M36553.m	TRUE	reported	2Rk7nd	2	TRUE	0.003195	20.71178
2003	rs37058	A	T	A	T	0.1894	-0.05866	0.0686	0.12364	FALSE	TRUE	FALSE	ieu-b-508	16	58563199	0.051044	486484	0.250472	Sepsis (28	Sepsis (28	Sepsis (28	TRUE	igd	0.0416	5.25E-06	2433	M18281.m	TRUE	reported	3mLp7z	2	TRUE	0.008448	20.71172
8200	rs7840544	A	G	A	G	0.0947	0.126405	0.9725	0.975126	FALSE	FALSE	FALSE	ieu-b-508	8	51657742	0.107523	486484	0.239752	Sepsis (28	Sepsis (28	Sepsis (28	TRUE	igd	0.0208	5.39E-06	2400	M36230.m	TRUE	reported	V5KJeR	2	TRUE	0.008563	20.71148
6180	rs1015298	A	G	A	G	-0.0132	-0.06487	0.6622	0.658625	FALSE	FALSE	FALSE	ieu-b-508	15	27508143	0.035383	486484	0.066764	Sepsis (28	Sepsis (28	Sepsis (28	TRUE	igd	0.0029	5.99E-06	6004	M33892.m	TRUE	reported	w7HgU7	2	TRUE	0.003439	20.71129
6204	rs6882710	A	G	A	G	-0.0132	-0.03918	0.5077	0.507138	FALSE	FALSE	FALSE	ieu-b-508	5	1.08E+08	0.033321	486484	0.239725	Sepsis (28	Sepsis (28	Sepsis (28	TRUE	igd	0.0029	5.26E-06	6004	M33892.m	TRUE	reported	w7HgU7	2	TRUE	0.003439	20.71129
2843	rs1028060	A	C	A	C	-0.0396	-0.01888	0.8277	0.818808	FALSE	FALSE	FALSE	ieu-b-508	7	95554916	0.043549	486484	0.66459	Sepsis (28	Sepsis (28	Sepsis (28	TRUE	igd	0.0087	4.96E-06	5673	M27531.m	TRUE	reported	v01zDr	2	TRUE	0.003639	20.71089
5466	rs1026362	T	C	T	C	-0.0569	-0.01673	0.9157	0.895475	FALSE	FALSE	FALSE	ieu-b-508	7	64894137	0.054886	486484	0.760451	Sepsis (28	Sepsis (28	Sepsis (28	TRUE	igd	0.0125	5.34E-06	3890	M33408.m	TRUE	reported	tWZWla	2	TRUE	0.005298	20.71005
617	rs7269193	A	G	A	G	-0.0528	0.103437	0.9092	0.893958	FALSE	FALSE	FALSE	ieu-b-508	20	46858654	0.054398	486484	0.057237	Sepsis (28	Sepsis (28	Sepsis (28	TRUE	igd	0.0116	5.03E-06	4934	M01114.m	TRUE	reported	fqHdM2	2	TRUE	0.004182	20.70979
7345	rs1711352	T	G	T	G	-0.0132	-0.00615	0.8744	0.869556	FALSE	FALSE	FALSE	ieu-b-508	14	43277268	0.049593	486484	0.901377	Sepsis (28	Sepsis (28	Sepsis (28	TRUE	igd	0.0029	3.68E-06	4900	M35137.m	TRUE	reported	Eq2siS	2	TRUE	0.00421	20.70974
8577	rs1179333	T	C	T	C	0.0619	-0.09999	0.0242	0.052628	FALSE	FALSE	FALSE	ieu-b-508	9	1.33E+08	0.076624	486484	0.191929	Sepsis (28	Sepsis (28	Sepsis (28	TRUE	igd	0.0136	5.09E-06	5932	M37253.m	TRUE	reported	PYMqzt	2	TRUE	0.00348	20.7089
1604	rs1021654	A	G	A	G	-0.0214	-0.01087	0.4694	0.482719	FALSE	FALSE	FALSE	ieu-b-508	8	68776627	0.033589	486484	0.746191	Sepsis (28	Sepsis (28	Sepsis (28	TRUE	igd	0.0047	5.19E-06	1757	M15122.m	TRUE	reported	VlnI8T	2	TRUE	0.011662	20.70795
516	rs1241436	C	G	C	G	0.0214	-0.00434	0.7696	0.79707	FALSE	TRUE	FALSE	ieu-b-508	10	4436442	0.041687	486484	0.917098	Sepsis (28	Sepsis (28	Sepsis (28	TRUE	igd	0.0047	4.89E-06	1754	M00584.m	TRUE	reported	oBt02X	2	TRUE	0.011682	20.70791
474	rs3785024	A	G	A	G	-0.0223	-0.02289	0.9469	0.947041	FALSE	FALSE	FALSE	ieu-b-508	16	84136233	0.075179	486484	0.760726	Sepsis (28	Sepsis (28	Sepsis (28	TRUE	igd	0.0049	5.56E-06	7365	M00527.m	TRUE	reported	UYN7vF	2	TRUE	0.002804	20.70616
5254	rs8127620	C	G	C	G	-0.0223	0.010887	0.1786	0.224237	FALSE	TRUE	FALSE	ieu-b-508	21	18732635	0.040369	486484	0.787403	Sepsis (28	Sepsis (28	Sepsis (28	TRUE	igd	0.0049	5.93E-06	7063	M33228.m	TRUE	reported	JwOXbQ	2	TRUE	0.002924	20.70592
8094	rs1219969	A	T	A	T	0.4221	0.033563	0.7393	0.746147	FALSE	TRUE	FALSE	ieu-b-508	6	89717261	0.03828	486484	0.380606	Sepsis (28	Sepsis (28	Sepsis (28	TRUE	igd	0.092	4.50E-06	122	M36097.m	TRUE	reported	RHr18S	2	TRUE	0.147152	20.70506
2810	rs1118384	A	G	A	G	-0.0965	0.083665	0.9518	0.948756	FALSE	FALSE	FALSE	ieu-b-508	12	47716293	0.07723	486484	0.278665	Sepsis (28	Sepsis (28	Sepsis (28	TRUE	igd	0.0212	5.49E-06	2759	M27447.m	TRUE	reported	s1NdyU	2	TRUE	0.007454	20.70465
6017	rs6758453	T	C	T	C	0.0656	-0.04422	0.4234	0.412	FALSE	FALSE	FALSE	ieu-b-508	2	39980322	0.033938	486484	0.192635	Sepsis (28	Sepsis (28	Sepsis (28	TRUE	igd	0.0144	4.99E-06	831	M33751.m	TRUE	reported	Cxdkr4	2	TRUE	0.024365	20.70314
6753	rs1049605	T	C	T	C	-0.0906	0.056556	0.9228	0.910171	FALSE	FALSE	FALSE	ieu-b-508	2	56010255	0.058726	486484	0.33552	Sepsis (28	Sepsis (28	Sepsis (28	TRUE	igd	0.0199	5.42E-06	1649	M34339.m	TRUE	reported	RwGZ7c	2	TRUE	0.012414	20.70252
3657	rs2679891	T	G	T	G	0.0719	-0.01404	0.0672	0.076162	FALSE	FALSE	FALSE	ieu-b-508	2	1.06E+08	0.062879	486484	0.823264	Sepsis (28	Sepsis (28	Sepsis (28	TRUE	igd	0.0158	5.05E-06	6970	M32564.m	TRUE	reported	73MgOk	2	TRUE	0.002962	20.70232
1816	rs1266097	T	G	T	G	0.0405	0.035071	0.1536	0.171573	FALSE	FALSE	FALSE	ieu-b-508	6	1.66E+08	0.04595	486484	0.445322	Sepsis (28	Sepsis (28	Sepsis (28	TRUE	igd	0.0089	5.67E-06	7357	M15753.m	TRUE	reported	VDJYDI	2	TRUE	0.002807	20.70198
2204	rs4128864	T	C	T	C	-0.0405	0.053606	0.0303	0.041666	FALSE	FALSE	FALSE	ieu-b-508	1	2.41E+08	0.083748	486484	0.52212	Sepsis (28	Sepsis (28	Sepsis (28	TRUE	igd	0.0089	5.00E-06	6929	M19364.m	TRUE	reported	XkjMEx	2	TRUE	0.00298	20.70164
7057	rs7961249	T	C	T	C	-0.0419	0.001578	0.1505	0.178628	FALSE	FALSE	FALSE	ieu-b-508	12	61876035	0.043683	486484	0.97118	Sepsis (28	Sepsis (28	Sepsis (28	TRUE	igd	0.0092	5.18E-06	1019	M34456.m	TRUE	reported	Nh457q	2	TRUE	0.019949	20.70137
3977	rs1175552	T	C	T	C	-0.0405	0.003837	0.0152	0.020678	FALSE	FALSE	FALSE	ieu-b-508	6	37569093	0.116085	486484	0.97363	Sepsis (28	Sepsis (28	Sepsis (28	TRUE	igd	0.0089	5.70E-06	6497	M32729.m	TRUE	reported	XGRSN1	2	TRUE	0.003177	20.70124
475	rs4635489	A	G	A	G	0.0364	0.003282	0.0177	0.019796	FALSE	FALSE	FALSE	ieu-b-508	2	30126856	0.119846	486484	0.978152	Sepsis (28	Sepsis (28	Sepsis (28	TRUE	igd	0.008	5.66E-06	7365	M00527.m	TRUE	reported	UYN7vF	2	TRUE	0.002803	20.69688
5594	rs986842	T	C	T	C	-1.5302	-0.01541	0.0167	0.047737	FALSE	FALSE	FALSE	ieu-b-508	16	24443107	0.081456	486484	0.849972	Sepsis (28	Sepsis (28	Sepsis (28	TRUE	igd	0.3307	3.71E-06	60	M33423.m	TRUE	reported	MoEnO7	2	TRUE	0.262995	20.69687
1820	rs3892034	A	T	A	T	-0.0273	-0.001	0.7703	0.756861	FALSE	TRUE	FALSE	ieu-b-508	16	87572899	0.03903	486484	0.979501	Sepsis (28	Sepsis (28	Sepsis (28	TRUE	igd	0.006	4.75E-06	7357	M15753.m	TRUE	reported	VDJYDI	2	TRUE	0.002806	20.69687
3380	rs4129516	T	C	T	C	-0.0182	-0.04709	0.7269	0.268267	FALSE	FALSE	FALSE	ieu-b-508	7	49097903	0.037629	486484	0.21081	Sepsis (28	Sepsis (28	Sepsis (28	TRUE	igd	0.004	5.51E-06	7355	M32418.m	TRUE	reported	y8cN4Y	2	TRUE	0.002807	20.69687
1228	rs1491606	A	T	A	T	0.0182	-0.04522	0.2992	0.351995	FALSE	TRUE	FALSE	ieu-b-508	3	70586528	0.035097	486484	0.19761	Sepsis (28	Sepsis (28	Sepsis (28	TRUE	igd	0.004	5.80E-06	7346	M01712.m	TRUE	reported	JfdAWN	2	TRUE	0.00281	20.69686
1041	rs1161822	T	C	T	C	-0.0182	0.022596	0.1008	0.170021	FALSE	FALSE	FALSE	ieu-b-508	13	26299649	0.044432	486484	0.611069	Sepsis (28	Sepsis (28	Sepsis (28	TRUE	igd	0.004	6.00E-06	7333	M01572.m	TRUE	reported	EOUAWy	2	TRUE	0.002815	20.69685
1721	rs4784175	A	T	A	T	-0.0091	-0.02336	0.8586	0.846128	FALSE	TRUE	FALSE	ieu-b-508	16	62213038	0.046628	486484	0.616452	Sepsis (28	Sepsis (28	Sepsis (28	TRUE	igd	0.002	8.64E-06	7310	M15506.m	TRUE	reported	mVRmZO	2	TRUE	0.002824	20.69684
6989	rs1099578	T	C	T	C	-0.0915	-0.01248	0.9786	0.977433	FALSE	FALSE	FALSE	ieu-b-508	10	65814632	0.113528	486484	0.912467	Sepsis (28	Sepsis (28	Sepsis (28	TRUE	igd	0.0201	5.21E-06	1567	M34416.m	TRUE	reported	q9OtaZ	2	TRUE	0.013052	20.69643
8520	rs9833886	C	G	C	G	0.2264	0.151793	0.0137	0.020225	FALSE	TRUE	FALSE	ieu-b-508	3	65358504	0.119891	486484	0.205482	Sepsis (28	Sepsis (28	Sepsis (28	TRUE	igd	0.0497	5.21E-06	751	M37104.m	TRUE	reported	bR3g7d	2	TRUE	0.026888	20.69579
2009	rs1234003	T	G	T	G	-0.0273	0.006004	0.4693	0.468914	FALSE	FALSE	FALSE	ieu-b-508	9	38282346	0.033663	486484	0.858445	Sepsis (28	Sepsis (28	Sepsis (28	TRUE	igd	0.006	5.59E-06	5984	M18283.m	TRUE	reported	oyZdTE	2	TRUE	0.003448	20.69558
7980	rs1816995	A	G	A	G	0.0264	-0.02223	0.7712	0.779863	FALSE	FALSE	FALSE	ieu-b-508	3	30007227	0.040215	486484	0.580432	Sepsis (28	Sepsis (28	Sepsis (28	TRUE	igd	0.0058	5.86E-06	1766	M35718.m	TRUE	reported	oUwc0a	2	TRUE	0.011596	20.69473
6047	rs9684934	T	G	T	G	0.0951	0.117627	0.0187	0.017887	FALSE	FALSE	FALSE	ieu-b-508	4	27434730	0.127476	486484	0.356144	Sepsis (28	Sepsis (28	Sepsis (28	TRUE	igd	0.0209	5.17E-06	3845	M33801.m	TRUE	reported	zhDCxK	2	TRUE	0.005356	20.69391
6581	rs1048908	T	C	T	C	0.0223	0.033597	0.7357	0.744667	FALSE	FALSE	FALSE	ieu-b-508	4	13716029	0.038272	486484	0.38003	Sepsis (28	Sepsis (28	Sepsis (28	TRUE	igd	0.0049	4.20E-06	2278	M34289.m	TRUE	reported	Knp8ry	2	TRUE	0.00901	20.6936
4104	rs1704035	A	G	A	G	0.3091	-0.12653	0.9825	0.984463	FALSE	FALSE	FALSE	ieu-b-508	12	1.09E+08	0.134263	486484	0.345986	Sepsis (28	Sepsis (28	Sepsis (28	TRUE	igd	0.0679	5.38E-06	1368	M32754.m	TRUE	reported	n0okFy	2	TRUE	0.014923	20.69298
8370	rs1433673	A	G	A	G	0.0428	0.009738	0.3426	0.378342	FALSE	FALSE	FALSE	ieu-b-508	4	94201395	0.034425	486484	0.777263	Sepsis (28	Sepsis (28	Sepsis (28	TRUE	igd	0.0094	5.33E-06	1054	M36756.m	TRUE	reported	QsW4oR	2	TRUE	0.01929	20.69221
2520	rs4452760	A	G	A	G	-0.0182	0.016605	0.569	0.575352	FALSE	FALSE	FALSE	ieu-b-508	8	92681583	0.033793	486484	0.623158	Sepsis (28	Sepsis (28	Sepsis (28	TRUE	igd	0.004	4.33E-06	3950	M22138.m	TRUE	reported	UbKBSS	2	TRUE	0.005214	20.69202
2517	rs1261555	A	G	A	G	-0.0569	0.001412	0.9347	0.936045	FALSE	FALSE	FALSE	ieu-b-508	2	1.19E+08	0.067723	486484	0.983363	Sepsis (28	Sepsis (28	Sepsis (28	TRUE	igd	0.0125	5.47E-06	1397	M22138.m	TRUE	reported	UbKBSS	2	TRUE	0.014616	20.69104
614	rs4579412	A	C	A	C	-0.0414	0.059776	0.1469	0.138636	FALSE	FALSE	FALSE	ieu-b-508	7	66945831	0.048285	486484	0.215728	Sepsis (28	Sepsis (28	Sepsis (28	TRUE	igd	0.0091	4.77E-06	4934	M01114.m	TRUE	reported	fqHdM2	2	TRUE	0.004177	20.68911
2992	rs1099571	T	G	T	G	0.1194	-0.07001	0.0683	0.064373	FALSE	FALSE	FALSE	ieu-b-508	10	65712395	0.068305	486484	0.305398	Sepsis (28	Sepsis (28	Sepsis (28	TRUE	igd	0.0262	5.23E-06	522	M31536.m	TRUE	reported	3nAuUI	2	TRUE	0.038264	20.68897
3450	rs1001159	T	C	T	C	0.0223	0.021602	0.8351	0.833627	FALSE	FALSE	FALSE	ieu-b-508	4	1.24E+08	0.044884	486484	0.630316	Sepsis (28	Sepsis (28	Sepsis (28	TRUE	igd	0.0049	5.43E-06	1767	M32489.m	TRUE	reported	hnljaD	2	TRUE	0.011586	20.68834
8149	rs1107967	T	C	T	C	-0.0819	-0.0532	0.9555	0.953139	FALSE	FALSE	FALSE	ieu-b-508	17	64925975	0.079099	486484	0.501235	Sepsis (28	Sepsis (28	Sepsis (28	TRUE	igd	0.018	5.27E-06	2709	M36115.m	TRUE	reported	ZV1jWp	2	TRUE	0.007584	20.68722
5155	rs1706826	T	C	T	C	-0.1633	-0.07501	0.9803	0.97569	FALSE	FALSE	FALSE	ieu-b-508	13	33414639	0.109249	486484	0.492313	Sepsis (28	Sepsis (28	Sepsis (28	TRUE	igd	0.0359	5.33E-06	5716	M33192.m	TRUE	reported	CyfjKE	2	TRUE	0.003607	20.68386
6928	rs9305620	A	G	A	G	-0.0487	0.016705	0.3537	0.377452	FALSE	FALSE	FALSE	ieu-b-508	21	38926300	0.034689	486484	0.630118	Sepsis (28	Sepsis (28	Sepsis (28	TRUE	igd	0.0107	5.52E-06	1312	M34390.m	TRUE	reported	wruTbW	2	TRUE	0.015544	20.68368
2524	rs1214310	T	C	T	C	0.0232	0.038415	0.5922	0.591332	FALSE	FALSE	FALSE	ieu-b-508	1	2.42E+08	0.033881	486484	0.256872	Sepsis (28	Sepsis (28	Sepsis (28	TRUE	igd	0.0051	4.77E-06	3838	M22175.m	TRUE	reported	IllqyJ	2	TRUE	0.005363	20.6828
7326	rs645288	C	G	C	G	-0.0141	-0.03458	0.2747	0.266184	FALSE	TRUE	FALSE	ieu-b-508	9	2547822	0.037887	486484	0.361418	Sepsis (28	Sepsis (28	Sepsis (28	TRUE	igd	0.0031	6.45E-06	7344	M35127.m	TRUE	reported	RNsmjl	2	TRUE	0.002809	20.68219
8240	rs3846241	A	G	A	G	-0.0478	-0.02251	0.404	0.462385	FALSE	FALSE	FALSE	ieu-b-508	4	1.61E+08	0.033626	486484	0.503137	Sepsis (28	Sepsis (28	Sepsis (28	TRUE	igd	0.0105	5.75E-06	976	M36468.m	TRUE	reported	vycAFr	2	TRUE	0.020792	20.6817
7138	rs1007405	A	G	A	G	-0.0373	-0.02389	0.8297	0.832997	FALSE	FALSE	FALSE	ieu-b-508	5	1.24E+08	0.045007	486484	0.595588	Sepsis (28	Sepsis (28	Sepsis (28	TRUE	igd	0.0082	5.24E-06	3813	M34530.m	TRUE	reported	8plvGA	2	TRUE	0.005397	20.68055
2136	rs1081335	A	G	A	G	0.0373	0.010876	0.4043	0.414599	FALSE	FALSE	FALSE	ieu-b-508	9	30755626	0.033912	486484	0.748428	Sepsis (28	Sepsis (28	Sepsis (28	TRUE	igd	0.0082	5.62E-06	3783	M18497.m	TRUE	reported	90JFPc	2	TRUE	0.00544	20.68046
7393	rs1226089	T	C	T	C	-0.0596	0.087589	0.9341	0.931989	FALSE	FALSE	FALSE	ieu-b-508	10	53889409	0.066188	486484	0.185719	Sepsis (28	Sepsis (28	Sepsis (28	TRUE	igd	0.0131	5.63E-06	1982	M35159.m	TRUE	reported	7EoJLB	2	TRUE	0.010336	20.67814
8299	rs1716328	A	C	A	C	-0.0273	0.021708	0.7217	0.727807	FALSE	FALSE	FALSE	ieu-b-508	7	7136077	0.037583	486484	0.563543	Sepsis (28	Sepsis (28	Sepsis (28	TRUE	igd	0.006	4.61E-06	1636	M36559.m	TRUE	reported	g6l9hg	2	TRUE	0.012496	20.67719
2004	rs3800052	T	C	T	C	0.1715	-0.00537	0.0846	0.096376	FALSE	FALSE	FALSE	ieu-b-508	6	1773297	0.05775	486484	0.925946	Sepsis (28	Sepsis (28	Sepsis (28	TRUE	igd	0.0377	5.46E-06	2433	M18281.m	TRUE	reported	3mLp7z	2	TRUE	0.008434	20.67704
5305	rs1159425	A	T	A	T	-0.1101	0.12395	0.0335	0.031999	FALSE	TRUE	FALSE	ieu-b-508	10	32499740	0.094332	486484	0.188853	Sepsis (28	Sepsis (28	Sepsis (28	TRUE	igd	0.0242	5.34E-06	1863	M33359.m	TRUE	reported	dfJmmS	2	TRUE	0.010988	20.67652
4345	rs1166860	A	G	A	G	-0.0191	0.004298	0.4922	0.523286	FALSE	FALSE	FALSE	ieu-b-508	19	16069643	0.033481	486484	0.897846	Sepsis (28	Sepsis (28	Sepsis (28	TRUE	igd	0.0042	5.15E-06	7355	M32855.m	TRUE	reported	AM8kCf	2	TRUE	0.002804	20.67522
5771	rs1742123	C	G	C	G	-0.0191	-0.00277	0.2887	0.304082	FALSE	TRUE	FALSE	ieu-b-508	7	1.25E+08	0.036582	486484	0.939534	Sepsis (28	Sepsis (28	Sepsis (28	TRUE	igd	0.0042	6.18E-06	7044	M33509.m	TRUE	reported	56SCCs	2	TRUE	0.002927	20.67497
1391	rs2504282	A	G	A	G	0.0191	-0.0665	0.9338	0.920344	FALSE	FALSE	FALSE	ieu-b-508	6	78355433	0.062257	486484	0.285441	Sepsis (28	Sepsis (28	Sepsis (28	TRUE	igd	0.0042	6.70E-06	7037	M02734.m	TRUE	reported	0y0dDD	2	TRUE	0.00293	20.67496
4108	rs4919957	A	G	A	G	0.1629	-0.0398	0.0653	0.053169	FALSE	FALSE	FALSE	ieu-b-508	21	43370291	0.074672	486484	0.593994	Sepsis (28	Sepsis (28	Sepsis (28	TRUE	igd	0.0358	5.40E-06	1368	M32754.m	TRUE	reported	n0okFy	2	TRUE	0.01491	20.67477
7061	rs1027574	T	C	T	C	0.0191	0.026581	0.4335	0.449905	FALSE	FALSE	FALSE	ieu-b-508	7	36116702	0.033533	486484	0.427965	Sepsis (28	Sepsis (28	Sepsis (28	TRUE	igd	0.0042	4.95E-06	6144	M34469.m	TRUE	reported	WMOqbm	2	TRUE	0.003355	20.67411
4035	rs1196661	A	G	A	G	-0.0191	-0.05038	0.8665	0.85956	FALSE	FALSE	FALSE	ieu-b-508	2	1.5E+08	0.048132	486484	0.295243	Sepsis (28	Sepsis (28	Sepsis (28	TRUE	igd	0.0042	5.89E-06	6042	M32739.m	TRUE	reported	dYSC7n	2	TRUE	0.003411	20.67399
7889	rs1248584	T	C	T	C	0.102	0.075773	0.0771	0.056937	FALSE	FALSE	FALSE	ieu-b-508	3	16233240	0.073438	486484	0.302169	Sepsis (28	Sepsis (28	Sepsis (28	TRUE	igd	0.0224	5.40E-06	659	M35669.m	TRUE	reported	gSecuE	2	TRUE	0.030505	20.67208
5637	rs4076939	A	C	A	C	0.0191	0.014424	0.2178	0.221201	FALSE	FALSE	FALSE	ieu-b-508	4	1.71E+08	0.040189	486484	0.719669	Sepsis (28	Sepsis (28	Sepsis (28	TRUE	igd	0.0042	5.49E-06	4004	M33443.m	TRUE	reported	G1JrRV	2	TRUE	0.005139	20.67051
1325	rs4944238	A	G	A	G	-0.0241	-0.05155	0.8712	0.872998	FALSE	FALSE	FALSE	ieu-b-508	11	79335591	0.050039	486484	0.302963	Sepsis (28	Sepsis (28	Sepsis (28	TRUE	igd	0.0053	4.88E-06	6252	M02137.m	TRUE	reported	iRGqBE	2	TRUE	0.003296	20.67014
5141	rs4233480	A	G	A	G	0.0473	0.026307	0.5412	0.53596	FALSE	FALSE	FALSE	ieu-b-508	1	2.4E+08	0.03354	486484	0.43283	Sepsis (28	Sepsis (28	Sepsis (28	TRUE	igd	0.0104	5.43E-06	2616	M33190.m	TRUE	reported	UkhAw5	2	TRUE	0.007845	20.66919
8553	rs4334280	A	G	A	G	-0.0291	-0.01181	0.3817	0.386269	FALSE	FALSE	FALSE	ieu-b-508	15	98913205	0.034249	486484	0.730148	Sepsis (28	Sepsis (28	Sepsis (28	TRUE	igd	0.0064	6.10E-06	7355	M37202.m	TRUE	reported	BtslDx	2	TRUE	0.002803	20.66845
2594	rs155394	A	G	A	G	-0.0291	-0.07401	0.9262	0.914103	FALSE	FALSE	FALSE	ieu-b-508	3	1423634	0.060192	486484	0.218858	Sepsis (28	Sepsis (28	Sepsis (28	TRUE	igd	0.0064	5.40E-06	7259	M22189.m	TRUE	reported	YSD5fY	2	TRUE	0.00284	20.66838
6943	rs8043508	A	G	A	G	-0.0773	-0.21203	0.9848	0.983029	FALSE	FALSE	FALSE	ieu-b-508	15	66347331	0.13083	486484	0.105099	Sepsis (28	Sepsis (28	Sepsis (28	TRUE	igd	0.017	5.38E-06	5208	M34395.m	TRUE	reported	QVOPA3	2	TRUE	0.003954	20.6678
5759	rs1280976	T	C	T	C	-0.0523	0.011725	0.9163	0.88955	FALSE	FALSE	FALSE	ieu-b-508	1	10975582	0.05341	486484	0.826243	Sepsis (28	Sepsis (28	Sepsis (28	TRUE	igd	0.0115	5.70E-06	2759	M33508.m	TRUE	reported	UMXHcP	2	TRUE	0.007441	20.66773
4925	rs3169950	A	G	A	G	0.0901	-0.00711	0.409	0.45075	FALSE	FALSE	FALSE	ieu-b-508	17	4699552	0.033454	486484	0.831643	Sepsis (28	Sepsis (28	Sepsis (28	TRUE	igd	0.0198	5.32E-06	1017	M33178.m	TRUE	reported	g22WMS	2	TRUE	0.019955	20.66637
6042	rs1206664	T	C	T	C	-0.0482	-0.02407	0.0891	0.103782	FALSE	FALSE	FALSE	ieu-b-508	1	56240278	0.054698	486484	0.659883	Sepsis (28	Sepsis (28	Sepsis (28	TRUE	igd	0.0106	4.94E-06	3845	M33801.m	TRUE	reported	zhDCxK	2	TRUE	0.005349	20.666
3019	rs1193324	A	G	A	G	-0.0341	-0.00097	0.3129	0.310051	FALSE	FALSE	FALSE	ieu-b-508	4	69132978	0.036695	486484	0.978912	Sepsis (28	Sepsis (28	Sepsis (28	TRUE	igd	0.0075	5.31E-06	5229	M31548.m	TRUE	reported	bgRTBS	2	TRUE	0.003938	20.66427
3398	rs356296	A	G	A	G	-0.0882	0.176106	0.016	0.022665	FALSE	FALSE	FALSE	ieu-b-508	1	80993467	0.112802	486484	0.118479	Sepsis (28	Sepsis (28	Sepsis (28	TRUE	igd	0.0194	5.57E-06	7346	M32425.m	TRUE	reported	u5RLGY	2	TRUE	0.002806	20.66405
7894	rs1766164	C	G	C	G	-0.0441	0.038002	0.9412	0.940831	FALSE	TRUE	FALSE	ieu-b-508	4	4901671	0.070663	486484	0.590718	Sepsis (28	Sepsis (28	Sepsis (28	TRUE	igd	0.0097	5.57E-06	5629	M35669.m	TRUE	reported	gSecuE	2	TRUE	0.003659	20.66233
1574	rs1187499	T	C	T	C	-0.0523	-0.04951	0.0175	0.025031	FALSE	FALSE	FALSE	ieu-b-508	18	73526221	0.106885	486484	0.643185	Sepsis (28	Sepsis (28	Sepsis (28	TRUE	igd	0.0115	5.62E-06	1750	M12593.m	TRUE	reported	IBXgSU	2	TRUE	0.011681	20.65908
1159	rs676094	A	G	A	G	0.0191	-0.06543	0.4178	0.407379	FALSE	FALSE	FALSE	ieu-b-508	1	29084689	0.033998	486484	0.0543	Sepsis (28	Sepsis (28	Sepsis (28	TRUE	igd	0.0042	6.61E-06	1766	M01644.m	TRUE	reported	IB0DTL	2	TRUE	0.011575	20.65742
4686	rs1084253	T	C	T	C	-0.1888	-0.09021	0.04	0.048744	FALSE	FALSE	FALSE	ieu-b-508	12	25499342	0.07799	486484	0.247382	Sepsis (28	Sepsis (28	Sepsis (28	TRUE	igd	0.0415	5.27E-06	1017	M33178.m	TRUE	reported	g22WMS	2	TRUE	0.019945	20.65632
3533	rs2373319	A	T	A	T	-0.015	0.009314	0.7383	0.725452	FALSE	TRUE	FALSE	ieu-b-508	7	1.48E+08	0.037562	486484	0.804158	Sepsis (28	Sepsis (28	Sepsis (28	TRUE	igd	0.0033	5.87E-06	7358	M32497.m	TRUE	reported	244bZN	2	TRUE	0.0028	20.65554
844	rs4287543	A	G	A	G	0.015	0.047613	0.8771	0.873709	FALSE	FALSE	FALSE	ieu-b-508	15	92113505	0.051047	486484	0.350961	Sepsis (28	Sepsis (28	Sepsis (28	TRUE	igd	0.0033	4.54E-06	7323	M01359.m	TRUE	reported	7L9x1s	2	TRUE	0.002813	20.65551
2281	rs1267706	A	G	A	G	-0.015	-0.02343	0.29	0.335335	FALSE	FALSE	FALSE	ieu-b-508	8	60383409	0.035276	486484	0.506562	Sepsis (28	Sepsis (28	Sepsis (28	TRUE	igd	0.0033	5.51E-06	7301	M20675.m	TRUE	reported	CXe9rE	2	TRUE	0.002822	20.6555
8541	rs650849	T	G	T	G	0.03	-0.00271	0.6496	0.6558	FALSE	FALSE	FALSE	ieu-b-508	6	93101639	0.035061	486484	0.938384	Sepsis (28	Sepsis (28	Sepsis (28	TRUE	igd	0.0066	5.64E-06	6934	M37190.m	TRUE	reported	tiNuK9	2	TRUE	0.002971	20.6552
5482	rs1159134	A	T	A	T	-0.045	0.051066	0.0643	0.0622	FALSE	TRUE	FALSE	ieu-b-508	10	1.1E+08	0.069045	486484	0.459542	Sepsis (28	Sepsis (28	Sepsis (28	TRUE	igd	0.0099	5.05E-06	6797	M33415.m	TRUE	reported	xHSphe	2	TRUE	0.003031	20.65508
5484	rs1176828	A	G	A	G	0.05	0.021774	0.0589	0.06752	FALSE	FALSE	FALSE	ieu-b-508	7	18436488	0.067255	486484	0.746122	Sepsis (28	Sepsis (28	Sepsis (28	TRUE	igd	0.011	5.90E-06	6797	M33415.m	TRUE	reported	xHSphe	2	TRUE	0.003031	20.65508
7957	rs1271423	T	C	T	C	-0.02	-0.02791	0.5232	0.513595	FALSE	FALSE	FALSE	ieu-b-508	2	28597305	0.033342	486484	0.402514	Sepsis (28	Sepsis (28	Sepsis (28	TRUE	igd	0.0044	7.07E-06	6447	M35678.m	TRUE	reported	56yR01	2	TRUE	0.003195	20.65475
8587	rs9544785	A	C	A	C	-0.015	0.048535	0.3614	0.357783	FALSE	FALSE	FALSE	ieu-b-508	13	78971070	0.035339	486484	0.169621	Sepsis (28	Sepsis (28	Sepsis (28	TRUE	igd	0.0033	6.63E-06	5932	M37253.m	TRUE	reported	PYMqzt	2	TRUE	0.003471	20.65419
7159	rs2429426	A	G	A	G	0.025	-0.03899	0.7093	0.716143	FALSE	FALSE	FALSE	ieu-b-508	17	61393045	0.037053	486484	0.292658	Sepsis (28	Sepsis (28	Sepsis (28	TRUE	igd	0.0055	4.70E-06	5856	M34533.m	TRUE	reported	SuKX4k	2	TRUE	0.003516	20.6541
7481	rs1981524	T	C	T	C	0.015	0.040897	0.2032	0.188143	FALSE	FALSE	FALSE	ieu-b-508	5	1.32E+08	0.042751	486484	0.338753	Sepsis (28	Sepsis (28	Sepsis (28	TRUE	igd	0.0033	4.71E-06	5694	M35240.m	TRUE	reported	55Kiv4	2	TRUE	0.003615	20.6539
2125	rs795543	T	C	T	C	-0.025	-0.0093	0.4091	0.41155	FALSE	FALSE	FALSE	ieu-b-508	5	13798819	0.0339	486484	0.78372	Sepsis (28	Sepsis (28	Sepsis (28	TRUE	igd	0.0055	5.19E-06	5624	M18476.m	TRUE	reported	6GnBtw	2	TRUE	0.00366	20.65381
7027	rs2850377	T	C	T	C	0.02	0.030882	0.41	0.431942	FALSE	FALSE	FALSE	ieu-b-508	4	1.03E+08	0.033675	486484	0.359105	Sepsis (28	Sepsis (28	Sepsis (28	TRUE	igd	0.0044	5.52E-06	4442	M34441.m	TRUE	reported	UpgxQm	2	TRUE	0.00463	20.65185
5640	rs7690478	T	C	T	C	0.015	0.024846	0.304	0.287803	FALSE	FALSE	FALSE	ieu-b-508	4	40928213	0.03711	486484	0.503159	Sepsis (28	Sepsis (28	Sepsis (28	TRUE	igd	0.0033	6.43E-06	4004	M33443.m	TRUE	reported	G1JrRV	2	TRUE	0.005134	20.65084
8064	rs1693619	C	G	C	G	-0.1014	0.02931	0.0247	0.027253	FALSE	TRUE	FALSE	ieu-b-508	9	1979400	0.10239	486484	0.774683	Sepsis (28	Sepsis (28	Sepsis (28	TRUE	igd	0.0223	5.41E-06	1517	M35978.m	TRUE	reported	CN0wRR	2	TRUE	0.013446	20.64872
7786	rs1115525	A	G	A	G	-0.0914	0.065405	0.066	0.063585	FALSE	FALSE	FALSE	ieu-b-508	6	96301493	0.068752	486484	0.341448	Sepsis (28	Sepsis (28	Sepsis (28	TRUE	igd	0.0201	5.74E-06	1383	M35451.m	TRUE	reported	GMRGAN	2	TRUE	0.014731	20.6477
8171	rs4486099	A	C	A	C	-0.0391	0.007324	0.1884	0.201612	FALSE	FALSE	FALSE	ieu-b-508	7	5373370	0.041801	486484	0.860904	Sepsis (28	Sepsis (28	Sepsis (28	TRUE	igd	0.0086	5.79E-06	1780	M36131.m	TRUE	reported	DbYm5t	2	TRUE	0.011479	20.64754
4972	rs4786186	A	C	A	C	0.2679	-0.20125	0.9792	0.968557	FALSE	FALSE	FALSE	ieu-b-508	16	7764496	0.09671	486484	0.03744	Sepsis (28	Sepsis (28	Sepsis (28	TRUE	igd	0.0589	5.34E-06	1017	M33178.m	TRUE	reported	g22WMS	2	TRUE	0.019936	20.64714
8320	rs1168837	T	C	T	C	-0.0655	0.037155	0.4786	0.493246	FALSE	FALSE	FALSE	ieu-b-508	2	2.21E+08	0.033408	486484	0.266065	Sepsis (28	Sepsis (28	Sepsis (28	TRUE	igd	0.0144	5.31E-06	952	M36589.m	TRUE	reported	27GADo	2	TRUE	0.021271	20.6464
4566	rs1264047	T	C	T	C	-0.04	0.003766	0.3034	0.289257	FALSE	FALSE	FALSE	ieu-b-508	4	19955070	0.037008	486484	0.918948	Sepsis (28	Sepsis (28	Sepsis (28	TRUE	igd	0.0088	5.88E-06	2777	M33150.m	TRUE	reported	H9SSMD	2	TRUE	0.007385	20.64628
2201	rs1758386	A	G	A	G	-0.0782	0.088516	0.0199	0.026152	FALSE	FALSE	FALSE	ieu-b-508	9	38994130	0.107386	486484	0.409782	Sepsis (28	Sepsis (28	Sepsis (28	TRUE	igd	0.0172	5.72E-06	1654	M19364.m	TRUE	reported	XkjMEx	2	TRUE	0.012343	20.64577
7209	rs6947774	T	C	T	C	0.025	-0.05538	0.3078	0.300328	FALSE	FALSE	FALSE	ieu-b-508	7	76914373	0.036313	486484	0.127257	Sepsis (28	Sepsis (28	Sepsis (28	TRUE	igd	0.0055	6.65E-06	2545	M34674.m	TRUE	reported	plxOKz	2	TRUE	0.008053	20.64492
5315	rs2416328	T	C	T	C	0.02	-0.08016	0.7592	0.758498	FALSE	FALSE	FALSE	ieu-b-508	5	1.13E+08	0.039127	486484	0.040489	Sepsis (28	Sepsis (28	Sepsis (28	TRUE	igd	0.0044	6.35E-06	2212	M33363.m	TRUE	reported	7K0MM0	2	TRUE	0.009254	20.64248
6241	rs1688032	T	G	T	G	0.1136	-0.26532	0.978	0.974829	FALSE	FALSE	FALSE	ieu-b-508	5	8302587	0.107574	486484	0.013649	Sepsis (28	Sepsis (28	Sepsis (28	TRUE	igd	0.025	5.76E-06	6756	M33935.m	TRUE	reported	ELBTDc	2	TRUE	0.003047	20.64182
8235	rs1479073	T	C	T	C	0.035	0.00311	0.7131	0.687222	FALSE	FALSE	FALSE	ieu-b-508	5	1.01E+08	0.036138	486484	0.931411	Sepsis (28	Sepsis (28	Sepsis (28	TRUE	igd	0.0077	5.97E-06	1908	M36468.m	TRUE	reported	vycAFr	2	TRUE	0.010713	20.6395
6235	rs1750335	A	G	A	G	0.0777	-0.09733	0.0288	0.056802	FALSE	FALSE	FALSE	ieu-b-508	2	51022513	0.07248	486484	0.179326	Sepsis (28	Sepsis (28	Sepsis (28	TRUE	igd	0.0171	5.41E-06	5534	M33910.m	TRUE	reported	YwFZg1	2	TRUE	0.003717	20.6392
5232	rs6921033	A	G	A	G	0.1937	0.070566	0.0116	0.013663	FALSE	FALSE	FALSE	ieu-b-508	6	10184123	0.145341	486484	0.627307	Sepsis (28	Sepsis (28	Sepsis (28	TRUE	igd	0.0426	5.34E-06	1142	M33221.m	TRUE	reported	8ZrV1r	2	TRUE	0.017782	20.63853
6018	rs6938476	T	C	T	C	0.0705	-0.01042	0.224	0.240797	FALSE	FALSE	FALSE	ieu-b-508	6	1.63E+08	0.03912	486484	0.789951	Sepsis (28	Sepsis (28	Sepsis (28	TRUE	igd	0.0155	5.38E-06	831	M33751.m	TRUE	reported	Cxdkr4	2	TRUE	0.02429	20.63803
2668	rs9510021	A	C	A	C	-0.0468	0.033851	0.5753	0.573272	FALSE	FALSE	FALSE	ieu-b-508	13	22757355	0.033769	486484	0.316138	Sepsis (28	Sepsis (28	Sepsis (28	TRUE	igd	0.0103	5.77E-06	5599	M22842.m	TRUE	reported	0t12oo	2	TRUE	0.003674	20.63774
7717	rs572132	A	T	A	T	-0.025	0.191236	0.9365	0.926901	FALSE	TRUE	FALSE	ieu-b-508	15	48168748	0.064306	486484	0.002941	Sepsis (28	Sepsis (28	Sepsis (28	TRUE	igd	0.0055	5.17E-06	1758	M35397.m	TRUE	reported	Y56mZr	2	TRUE	0.011616	20.63765
6426	rs1102974	T	G	T	G	0.0209	-0.19232	0.0206	0.021473	FALSE	FALSE	FALSE	ieu-b-508	11	26952457	0.115234	486484	0.09512	Sepsis (28	Sepsis (28	Sepsis (28	TRUE	igd	0.0046	5.55E-06	7362	M34062.m	TRUE	reported	RZyHKP	2	TRUE	0.002796	20.63759
5681	rs244523	A	T	A	T	-0.02	0.013339	0.2872	0.296087	FALSE	TRUE	FALSE	ieu-b-508	5	1.44E+08	0.036606	486484	0.715563	Sepsis (28	Sepsis (28	Sepsis (28	TRUE	igd	0.0044	6.53E-06	1734	M33477.m	TRUE	reported	Aw8PBK	2	TRUE	0.011775	20.63733
6232	rs1167149	A	G	A	G	0.1123	-0.03195	0.0411	0.051747	FALSE	FALSE	FALSE	ieu-b-508	19	11176201	0.075547	486484	0.672404	Sepsis (28	Sepsis (28	Sepsis (28	TRUE	igd	0.0247	5.23E-06	1183	M33910.m	TRUE	reported	YwFZg1	2	TRUE	0.017173	20.63625
1994	rs4661452	T	C	T	C	-0.0577	-0.04969	0.9194	0.886299	FALSE	FALSE	FALSE	ieu-b-508	1	14272107	0.053099	486484	0.349421	Sepsis (28	Sepsis (28	Sepsis (28	TRUE	igd	0.0127	5.18E-06	7071	M18254.m	TRUE	reported	clvu6V	2	TRUE	0.002911	20.6358
4528	rs4540481	T	C	T	C	-0.0459	0.021423	0.2868	0.292064	FALSE	FALSE	FALSE	ieu-b-508	9	29992643	0.036743	486484	0.559852	Sepsis (28	Sepsis (28	Sepsis (28	TRUE	igd	0.0101	5.04E-06	2391	M33137.m	TRUE	reported	ONVNk3	2	TRUE	0.008564	20.6357
1454	rs7668550	T	G	T	G	-0.0368	-0.13678	0.0157	0.016804	FALSE	FALSE	FALSE	ieu-b-508	4	1.04E+08	0.128398	486484	0.286756	Sepsis (28	Sepsis (28	Sepsis (28	TRUE	igd	0.0081	5.54E-06	7357	M03141.m	TRUE	reported	y4mG6m	2	TRUE	0.002798	20.63514
3960	rs4422759	T	C	T	C	0.0159	0.072575	0.1519	0.154779	FALSE	FALSE	FALSE	ieu-b-508	8	1.33E+08	0.046089	486484	0.115335	Sepsis (28	Sepsis (28	Sepsis (28	TRUE	igd	0.0035	6.73E-06	7363	M32709.m	TRUE	reported	YR8SB4	2	TRUE	0.002795	20.63195
6058	rs272461	A	G	A	G	0.0159	0.03287	0.2709	0.272864	FALSE	FALSE	FALSE	ieu-b-508	5	6488117	0.037656	486484	0.382704	Sepsis (28	Sepsis (28	Sepsis (28	TRUE	igd	0.0035	6.26E-06	7362	M33821.m	TRUE	reported	eVYICL	2	TRUE	0.002795	20.63194
4441	rs2904562	A	T	A	T	0.0159	0.032724	0.9187	0.90635	FALSE	TRUE	FALSE	ieu-b-508	22	19849021	0.057717	486484	0.570738	Sepsis (28	Sepsis (28	Sepsis (28	TRUE	igd	0.0035	4.67E-06	7344	M32910.m	TRUE	reported	2OCBKh	2	TRUE	0.002802	20.63193
2447	rs6508364	A	G	A	G	-0.0159	-0.03032	0.1404	0.135578	FALSE	FALSE	FALSE	ieu-b-508	18	22924491	0.049048	486484	0.536476	Sepsis (28	Sepsis (28	Sepsis (28	TRUE	igd	0.0035	6.52E-06	7284	M21630.m	TRUE	reported	7eaO4H	2	TRUE	0.002825	20.63188
7485	rs1194999	C	G	C	G	0.0159	-0.0493	0.8327	0.834838	FALSE	TRUE	FALSE	ieu-b-508	5	25880530	0.04495	486484	0.272749	Sepsis (28	Sepsis (28	Sepsis (28	TRUE	igd	0.0035	5.61E-06	7261	M35253.m	TRUE	reported	VboRmj	2	TRUE	0.002834	20.63187
7488	rs1272691	T	G	T	G	-0.0159	-0.0145	0.1774	0.179856	FALSE	FALSE	FALSE	ieu-b-508	1	1.57E+08	0.043951	486484	0.741426	Sepsis (28	Sepsis (28	Sepsis (28	TRUE	igd	0.0035	4.04E-06	7261	M35253.m	TRUE	reported	VboRmj	2	TRUE	0.002834	20.63187

6523	rs251456	A	G	A	G	0.0159	-0.0204	0.1523	0.147404	FALSE	FALSE	FALSE	ieu-b-508	3	1.19E+08	0.047332	486484	0.666414	Sepsis (28	Sepsis (28	Sepsis (28	TRUE	igd	0.0035	6.63E-06	7049	M34123.m	TRUE	reported	9O5wo1	2	TRUE	0.002919	20.6317
2037	rs314707	A	G	A	G	-0.0159	0.042192	0.8305	0.835612	FALSE	FALSE	FALSE	ieu-b-508	2	1.26E+08	0.045122	486484	0.349756	Sepsis (28	Sepsis (28	Sepsis (28	TRUE	igd	0.0035	4.43E-06	6939	M18349.m	TRUE	reported	M5qPs8	2	TRUE	0.002965	20.6316
7462	rs2278350	A	C	A	C	-0.0159	0.014568	0.3247	0.347694	FALSE	FALSE	FALSE	ieu-b-508	18	73836420	0.035163	486484	0.67866	Sepsis (28	Sepsis (28	Sepsis (28	TRUE	igd	0.0035	4.62E-06	6543	M35193.m	TRUE	reported	BMMWXU	2	TRUE	0.003144	20.63124
1804	rs584630	A	C	A	C	-0.0318	-0.03276	0.1052	0.14689	FALSE	FALSE	FALSE	ieu-b-508	10	38338699	0.047073	486484	0.486514	Sepsis (28	Sepsis (28	Sepsis (28	TRUE	igd	0.007	6.27E-06	5829	M15749.m	TRUE	reported	TR861N	2	TRUE	0.003528	20.63047
666	rs1726825	T	C	T	C	-0.0159	-0.09009	0.1349	0.163023	FALSE	FALSE	FALSE	ieu-b-508	4	1.4E+08	0.045293	486484	0.046701	Sepsis (28	Sepsis (28	Sepsis (28	TRUE	igd	0.0035	7.31E-06	5687	M01284.m	TRUE	reported	E4hc9c	2	TRUE	0.003616	20.63029
4217	rs4885169	A	G	A	G	-0.0427	-0.03043	0.1078	0.096835	FALSE	FALSE	FALSE	ieu-b-508	13	74900793	0.056347	486484	0.589226	Sepsis (28	Sepsis (28	Sepsis (28	TRUE	igd	0.0094	5.17E-06	7330	M32786.m	TRUE	reported	rwF0co	2	TRUE	0.002807	20.62916
5464	rs8094976	T	C	T	C	0.6419	0.002118	0.0131	0.016557	FALSE	FALSE	FALSE	ieu-b-508	18	7803579	0.131489	486484	0.987151	Sepsis (28	Sepsis (28	Sepsis (28	TRUE	igd	0.1405	4.93E-06	171	M33391.m	TRUE	reported	EgMd1b	2	TRUE	0.108785	20.62874
3161	rs1122413	T	C	T	C	-0.0409	0.010884	0.1324	0.165072	FALSE	FALSE	FALSE	ieu-b-508	11	1.35E+08	0.045092	486484	0.809274	Sepsis (28	Sepsis (28	Sepsis (28	TRUE	igd	0.009	5.31E-06	1765	M32328.m	TRUE	reported	YGsb6D	2	TRUE	0.011566	20.62857
7084	rs1182294	T	C	T	C	0.0477	0.001702	0.8079	0.789276	FALSE	FALSE	FALSE	ieu-b-508	11	69873933	0.041069	486484	0.966946	Sepsis (28	Sepsis (28	Sepsis (28	TRUE	igd	0.0105	5.69E-06	4296	M34499.m	TRUE	reported	JqFBiM	2	TRUE	0.004781	20.62794
6683	rs1685588	T	G	T	G	0.1068	0.050039	0.0407	0.042215	FALSE	FALSE	FALSE	ieu-b-508	2	1.7E+08	0.084369	486484	0.553114	Sepsis (28	Sepsis (28	Sepsis (28	TRUE	igd	0.0235	5.32E-06	1492	M34329.m	TRUE	reported	mgPKP5	2	TRUE	0.013654	20.62644
6711	rs1040865	T	C	T	C	0.2407	-0.02842	0.0449	0.071187	FALSE	FALSE	FALSE	ieu-b-508	19	42531185	0.064649	486484	0.660278	Sepsis (28	Sepsis (28	Sepsis (28	TRUE	igd	0.0529	5.25E-06	535	M34338.m	TRUE	reported	h4FB9a	2	TRUE	0.037256	20.62596
6942	rs7973154	C	G	C	G	-0.0595	0.127133	0.0265	0.030435	FALSE	TRUE	FALSE	ieu-b-508	12	1.29E+08	0.097896	486484	0.194063	Sepsis (28	Sepsis (28	Sepsis (28	TRUE	igd	0.0131	5.31E-06	5208	M34395.m	TRUE	reported	QVOPA3	2	TRUE	0.003946	20.6217
677	rs1087315	T	C	T	C	-0.0109	0.015479	0.0933	0.090697	FALSE	FALSE	FALSE	ieu-b-508	14	63100706	0.058129	486484	0.790013	Sepsis (28	Sepsis (28	Sepsis (28	TRUE	igd	0.0024	8.62E-06	7358	M01299.m	TRUE	reported	gfM0a3	2	TRUE	0.002795	20.62113
690	rs2891733	T	C	T	C	-0.0109	-0.09409	0.8941	0.893981	FALSE	FALSE	FALSE	ieu-b-508	2	2.2E+08	0.054488	486484	0.084198	Sepsis (28	Sepsis (28	Sepsis (28	TRUE	igd	0.0024	8.47E-06	7358	M01299.m	TRUE	reported	gfM0a3	2	TRUE	0.002795	20.62113
1052	rs4836200	A	G	A	G	-0.0109	-0.05929	0.6785	0.660664	FALSE	FALSE	FALSE	ieu-b-508	5	1.25E+08	0.035313	486484	0.093154	Sepsis (28	Sepsis (28	Sepsis (28	TRUE	igd	0.0024	5.91E-06	7333	M01572.m	TRUE	reported	EOUAWy	2	TRUE	0.002805	20.62111
2495	rs3798221	T	G	T	G	0.0109	-0.04892	0.1914	0.191035	FALSE	FALSE	FALSE	ieu-b-508	6	1.61E+08	0.042404	486484	0.248663	Sepsis (28	Sepsis (28	Sepsis (28	TRUE	igd	0.0024	8.31E-06	7329	M22116.m	TRUE	reported	bjUU6r	2	TRUE	0.002807	20.62111
2079	rs2081811	A	G	A	G	0.0327	0.125783	0.7543	0.749757	FALSE	FALSE	FALSE	ieu-b-508	3	42933507	0.038706	486484	0.001155	Sepsis (28	Sepsis (28	Sepsis (28	TRUE	igd	0.0072	5.81E-06	7322	M18392.m	TRUE	reported	fBRMfe	2	TRUE	0.002809	20.6211
1577	rs1289648	T	C	T	C	-0.0109	-0.02606	0.8919	0.878071	FALSE	FALSE	FALSE	ieu-b-508	14	44527268	0.051322	486484	0.611657	Sepsis (28	Sepsis (28	Sepsis (28	TRUE	igd	0.0024	4.17E-06	7314	M12593.m	TRUE	reported	IBXgSU	2	TRUE	0.002812	20.6211
987	rs1199243	A	G	A	G	0.0327	0.01843	0.9488	0.94184	FALSE	FALSE	FALSE	ieu-b-508	8	81526083	0.072282	486484	0.79874	Sepsis (28	Sepsis (28	Sepsis (28	TRUE	igd	0.0072	6.38E-06	7276	M01561.m	TRUE	reported	Zd9SUU	2	TRUE	0.002827	20.62107
6341	rs6573597	A	G	A	G	-0.0327	-0.10257	0.0874	0.094406	FALSE	FALSE	FALSE	ieu-b-508	14	65720883	0.056926	486484	0.071575	Sepsis (28	Sepsis (28	Sepsis (28	TRUE	igd	0.0072	6.33E-06	6998	M33957.m	TRUE	reported	mrKpMv	2	TRUE	0.002939	20.62084
1415	rs113515	C	G	C	G	-0.0109	-0.00059	0.5982	0.605419	FALSE	TRUE	FALSE	ieu-b-508	22	43556922	0.034118	486484	0.986221	Sepsis (28	Sepsis (28	Sepsis (28	TRUE	igd	0.0024	8.09E-06	6941	M03127.m	TRUE	reported	JscEZw	2	TRUE	0.002963	20.62079
1427	rs2939707	A	C	A	C	0.0109	0.008515	0.5698	0.563397	FALSE	FALSE	FALSE	ieu-b-508	4	37528330	0.033585	486484	0.799864	Sepsis (28	Sepsis (28	Sepsis (28	TRUE	igd	0.0024	7.39E-06	6941	M03127.m	TRUE	reported	JscEZw	2	TRUE	0.002963	20.62079
4227	rs1091624	A	T	A	T	-0.0218	-0.04473	0.8305	0.803325	FALSE	TRUE	FALSE	ieu-b-508	1	2.24E+08	0.042049	486484	0.287425	Sepsis (28	Sepsis (28	Sepsis (28	TRUE	igd	0.0048	5.10E-06	6857	M32787.m	TRUE	reported	fSRakm	2	TRUE	0.002999	20.62072
6099	rs1007251	C	G	C	G	0.0218	0.015738	0.2388	0.159035	FALSE	TRUE	FALSE	ieu-b-508	5	1.47E+08	0.045666	486484	0.730373	Sepsis (28	Sepsis (28	Sepsis (28	TRUE	igd	0.0048	5.04E-06	6506	M33871.m	TRUE	reported	mQD6DP	2	TRUE	0.00316	20.6204
5969	rs1316002	C	G	C	G	0.0109	0.000359	0.6331	0.629325	FALSE	TRUE	FALSE	ieu-b-508	5	6327639	0.034573	486484	0.991713	Sepsis (28	Sepsis (28	Sepsis (28	TRUE	igd	0.0024	4.67E-06	6279	M33666.m	TRUE	reported	dvWEtk	2	TRUE	0.003274	20.62017
3699	rs1116197	C	G	C	G	0.0218	0.016013	0.3812	0.389032	FALSE	TRUE	FALSE	ieu-b-508	1	87753920	0.034217	486484	0.639794	Sepsis (28	Sepsis (28	Sepsis (28	TRUE	igd	0.0048	4.81E-06	5955	M32593.m	TRUE	reported	6zVI1i	2	TRUE	0.003452	20.61981
6676	rs1000702	T	G	T	G	-0.0418	0.000762	0.4182	0.425886	FALSE	FALSE	FALSE	ieu-b-508	12	95256296	0.033675	486484	0.981941	Sepsis (28	Sepsis (28	Sepsis (28	TRUE	igd	0.0092	5.39E-06	1492	M34329.m	TRUE	reported	mgPKP5	2	TRUE	0.013647	20.61552
3728	rs1274801	A	G	A	G	-0.0277	-0.11216	0.0816	0.100788	FALSE	FALSE	FALSE	ieu-b-508	1	2.18E+08	0.055331	486484	0.042664	Sepsis (28	Sepsis (28	Sepsis (28	TRUE	igd	0.0061	5.62E-06	7337	M32632.m	TRUE	reported	pIHzPT	2	TRUE	0.002803	20.61491
497	rs2446427	T	C	T	C	-0.0554	0.074254	0.8371	0.833308	FALSE	FALSE	FALSE	ieu-b-508	15	51672513	0.045115	486484	0.099791	Sepsis (28	Sepsis (28	Sepsis (28	TRUE	igd	0.0122	5.21E-06	7274	M00569.m	TRUE	reported	1TjBaz	2	TRUE	0.002827	20.61486
5184	rs1782668	A	G	A	G	-0.0813	-0.0541	0.8905	0.838633	FALSE	FALSE	FALSE	ieu-b-508	3	1.82E+08	0.045259	486484	0.231945	Sepsis (28	Sepsis (28	Sepsis (28	TRUE	igd	0.0179	5.29E-06	2860	M33197.m	TRUE	reported	D6xaBg	2	TRUE	0.007161	20.61442
483	rs1818902	T	C	T	C	0.5718	-0.03792	0.9701	0.981133	FALSE	FALSE	FALSE	ieu-b-508	3	81947741	0.122844	486484	0.757541	Sepsis (28	Sepsis (28	Sepsis (28	TRUE	igd	0.1255	5.21E-06	283	M00553.m	TRUE	reported	IFjXiL	2	TRUE	0.06834	20.61203
2820	rs804441	T	C	T	C	-0.0327	-0.00942	0.29	0.292079	FALSE	FALSE	FALSE	ieu-b-508	12	1.2E+08	0.036712	486484	0.797463	Sepsis (28	Sepsis (28	Sepsis (28	TRUE	igd	0.0072	5.87E-06	2759	M27447.m	TRUE	reported	s1NdyU	2	TRUE	0.007421	20.61178
5778	rs1242993	T	C	T	C	-0.0586	-0.08438	0.9468	0.920611	FALSE	FALSE	FALSE	ieu-b-508	13	91507351	0.061892	486484	0.172755	Sepsis (28	Sepsis (28	Sepsis (28	TRUE	igd	0.0129	5.31E-06	1718	M33510.m	TRUE	reported	WxGKnn	2	TRUE	0.011869	20.61152
2892	rs1282074	A	G	A	G	-0.0168	-0.00486	0.5528	0.564957	FALSE	FALSE	FALSE	ieu-b-508	12	1.06E+08	0.033702	486484	0.885279	Sepsis (28	Sepsis (28	Sepsis (28	TRUE	igd	0.0037	5.06E-06	7373	M27718.m	TRUE	reported	PHL57z	2	TRUE	0.002788	20.61092
3322	rs1317320	A	G	A	G	0.0168	-0.05103	0.5657	0.559348	FALSE	FALSE	FALSE	ieu-b-508	5	7569871	0.033694	486484	0.129854	Sepsis (28	Sepsis (28	Sepsis (28	TRUE	igd	0.0037	7.35E-06	7358	M32405.m	TRUE	reported	uzomIQ	2	TRUE	0.002794	20.6109
7528	rs9857115	A	C	A	C	0.0168	-0.02311	0.538	0.565103	FALSE	FALSE	FALSE	ieu-b-508	3	1.9E+08	0.033768	486484	0.493728	Sepsis (28	Sepsis (28	Sepsis (28	TRUE	igd	0.0037	4.24E-06	7291	M35255.m	TRUE	reported	ZDjmUf	2	TRUE	0.00282	20.61085
541	rs6895278	T	C	T	C	0.0168	-0.00082	0.3354	0.33938	FALSE	FALSE	FALSE	ieu-b-508	5	1.47E+08	0.035463	486484	0.981477	Sepsis (28	Sepsis (28	Sepsis (28	TRUE	igd	0.0037	4.13E-06	7237	M00599.m	TRUE	reported	nRJY3s	2	TRUE	0.002841	20.61081
7073	rs1112383	A	G	A	G	-0.0168	-0.03034	0.24	0.246856	FALSE	FALSE	FALSE	ieu-b-508	2	1.01E+08	0.039064	486484	0.437377	Sepsis (28	Sepsis (28	Sepsis (28	TRUE	igd	0.0037	4.24E-06	7119	M34481.m	TRUE	reported	mXdL0W	2	TRUE	0.002888	20.61072
5236	rs980833	T	C	T	C	0.0445	0.036753	0.9438	0.91941	FALSE	FALSE	FALSE	ieu-b-508	10	78852031	0.061468	486484	0.549898	Sepsis (28	Sepsis (28	Sepsis (28	TRUE	igd	0.0098	5.99E-06	4665	M33221.m	TRUE	reported	8ZrV1r	2	TRUE	0.0044	20.61017
8163	rs1498100	A	T	A	T	0.0318	0.017949	0.3919	0.371279	FALSE	TRUE	FALSE	ieu-b-508	5	19854620	0.034595	486484	0.603883	Sepsis (28	Sepsis (28	Sepsis (28	TRUE	igd	0.007	5.06E-06	1489	M36131.m	TRUE	reported	DbYm5t	2	TRUE	0.013671	20.60983
6456	rs1692488	A	G	A	G	-0.0636	0.006369	0.0992	0.098204	FALSE	FALSE	FALSE	ieu-b-508	10	24843786	0.056278	486484	0.909893	Sepsis (28	Sepsis (28	Sepsis (28	TRUE	igd	0.014	5.95E-06	1481	M34106.m	TRUE	reported	8DuoNM	2	TRUE	0.013743	20.60968
2476	rs1224851	T	C	T	C	0.0168	-0.02561	0.186	0.187982	FALSE	FALSE	FALSE	ieu-b-508	10	57736009	0.042833	486484	0.549917	Sepsis (28	Sepsis (28	Sepsis (28	TRUE	igd	0.0037	5.15E-06	5676	M22032.m	TRUE	reported	xpe96K	2	TRUE	0.003619	20.60924
6453	rs1159459	C	G	C	G	0.0336	-0.0394	0.1287	0.13429	FALSE	TRUE	FALSE	ieu-b-508	10	30130884	0.049429	486484	0.425398	Sepsis (28	Sepsis (28	Sepsis (28	TRUE	igd	0.0074	6.10E-06	4961	M34106.m	TRUE	reported	8DuoNM	2	TRUE	0.004139	20.6082
1907	rs7857624	T	C	T	C	0.0395	0.014751	0.9289	0.899311	FALSE	FALSE	FALSE	ieu-b-508	9	78279942	0.055379	486484	0.789963	Sepsis (28	Sepsis (28	Sepsis (28	TRUE	igd	0.0087	6.22E-06	7064	M16634.m	TRUE	reported	P9BdXh	2	TRUE	0.00291	20.60785
8638	rs749902	T	C	T	C	0.0168	0.011321	0.3362	0.336695	FALSE	FALSE	FALSE	ieu-b-508	1	1.18E+08	0.035246	486484	0.748052	Sepsis (28	Sepsis (28	Sepsis (28	TRUE	igd	0.0037	4.61E-06	4754	M38150.m	TRUE	reported	u3Ckmr	2	TRUE	0.004318	20.60784
2434	rs1221724	A	T	A	T	-0.0227	0.050088	0.9351	0.936758	FALSE	TRUE	FALSE	ieu-b-508	10	4025644	0.068331	486484	0.463545	Sepsis (28	Sepsis (28	Sepsis (28	TRUE	igd	0.005	6.31E-06	7284	M21630.m	TRUE	reported	7eaO4H	2	TRUE	0.002822	20.60594
1980	rs932412	A	C	A	C	-0.0227	0.048059	0.3987	0.389739	FALSE	FALSE	FALSE	ieu-b-508	6	16425403	0.034209	486484	0.160064	Sepsis (28	Sepsis (28	Sepsis (28	TRUE	igd	0.005	5.84E-06	7226	M17807.m	TRUE	reported	zd6sZI	2	TRUE	0.002844	20.6059
4534	rs1084960	A	T	A	T	-0.0227	0.072686	0.171	0.182782	FALSE	TRUE	FALSE	ieu-b-508	12	1063419	0.043167	486484	0.092213	Sepsis (28	Sepsis (28	Sepsis (28	TRUE	igd	0.005	6.51E-06	7166	M33138.m	TRUE	reported	lUpLAl	2	TRUE	0.002868	20.60585
7450	rs7740539	T	C	T	C	-0.0227	0.098988	0.7104	0.716195	FALSE	FALSE	FALSE	ieu-b-508	6	1.07E+08	0.037086	486484	0.007604	Sepsis (28	Sepsis (28	Sepsis (28	TRUE	igd	0.005	6.73E-06	6234	M35189.m	TRUE	reported	54XZFj	2	TRUE	0.003295	20.60499
7072	rs9635567	T	C	T	C	0.0227	0.047019	0.7787	0.789028	FALSE	FALSE	FALSE	ieu-b-508	16	76137258	0.040881	486484	0.250083	Sepsis (28	Sepsis (28	Sepsis (28	TRUE	igd	0.005	5.73E-06	6144	M34469.m	TRUE	reported	WMOqbm	2	TRUE	0.003344	20.60489
2613	rs278815	T	C	T	C	-0.1249	0.213602	0.989	0.959083	FALSE	FALSE	FALSE	ieu-b-508	18	76266696	0.084781	486484	0.011753	Sepsis (28	Sepsis (28	Sepsis (28	TRUE	igd	0.0275	5.73E-06	1756	M22481.m	TRUE	reported	bXpf0k	2	TRUE	0.011611	20.60462
5264	rs4240475	A	C	A	C	0.0286	-0.04076	0.0592	0.059376	FALSE	FALSE	FALSE	ieu-b-508	9	1.25E+08	0.07075	486484	0.564513	Sepsis (28	Sepsis (28	Sepsis (28	TRUE	igd	0.0063	6.21E-06	7364	M33230.m	TRUE	reported	nzcTOn	2	TRUE	0.002791	20.60312
8065	rs2921448	A	G	A	G	0.0227	0.012188	0.2771	0.31232	FALSE	FALSE	FALSE	ieu-b-508	10	74497403	0.035967	486484	0.734705	Sepsis (28	Sepsis (28	Sepsis (28	TRUE	igd	0.005	5.25E-06	4529	M35978.m	TRUE	reported	CN0wRR	2	TRUE	0.00453	20.6025
6097	rs6925144	T	C	T	C	0.0527	0.001628	0.5429	0.489997	FALSE	FALSE	FALSE	ieu-b-508	6	1.23E+08	0.033453	486484	0.961176	Sepsis (28	Sepsis (28	Sepsis (28	TRUE	igd	0.0116	5.76E-06	1069	M33864.m	TRUE	reported	yr5h52	2	TRUE	0.018942	20.60117
1317	rs1019595	T	C	T	C	-0.0345	-0.04482	0.9326	0.940053	FALSE	FALSE	FALSE	ieu-b-508	10	82585950	0.070687	486484	0.526083	Sepsis (28	Sepsis (28	Sepsis (28	TRUE	igd	0.0076	5.46E-06	6252	M02137.m	TRUE	reported	iRGqBE	2	TRUE	0.003285	20.60023
4030	rs831607	T	C	T	C	-0.0404	0.065512	0.5112	0.51957	FALSE	FALSE	FALSE	ieu-b-508	11	33718074	0.034169	486484	0.0552	Sepsis (28	Sepsis (28	Sepsis (28	TRUE	igd	0.0089	6.13E-06	6000	M32735.m	TRUE	reported	QE2vKQ	2	TRUE	0.003422	20.59861
4165	rs504026	A	G	A	G	0.1099	-0.14543	0.9734	0.970394	FALSE	FALSE	FALSE	ieu-b-508	19	21470726	0.097488	486484	0.135764	Sepsis (28	Sepsis (28	Sepsis (28	TRUE	igd	0.0242	5.53E-06	1612	M32758.m	TRUE	reported	AF0m66	2	TRUE	0.012632	20.59802
1789	rs1311223	C	G	C	G	0.0404	0.049974	0.3279	0.324784	FALSE	TRUE	FALSE	ieu-b-508	4	96728564	0.035615	486484	0.160561	Sepsis (28	Sepsis (28	Sepsis (28	TRUE	igd	0.0089	5.90E-06	5502	M15677.m	TRUE	reported	1CuWi9	2	TRUE	0.003731	20.59799
5880	rs1193867	T	G	T	G	0.2083	0.091054	0.0205	0.035525	FALSE	FALSE	FALSE	ieu-b-508	4	36532015	0.090815	486484	0.316041	Sepsis (28	Sepsis (28	Sepsis (28	TRUE	igd	0.0458	5.38E-06	470	M33610.m	TRUE	reported	tmg43v	2	TRUE	0.042155	20.5966
7906	rs8192287	T	G	T	G	0.0522	-0.01947	0.0436	0.05978	FALSE	FALSE	FALSE	ieu-b-508	4	24796568	0.070316	486484	0.781867	Sepsis (28	Sepsis (28	Sepsis (28	TRUE	igd	0.0115	5.84E-06	5629	M35669.m	TRUE	reported	gSecuE	2	TRUE	0.003647	20.59638
7858	rs1280552	T	C	T	C	0.0999	0.142752	0.985	0.986753	FALSE	FALSE	FALSE	ieu-b-508	11	38705379	0.146645	486484	0.330331	Sepsis (28	Sepsis (28	Sepsis (28	TRUE	igd	0.022	5.86E-06	1750	M35631.m	TRUE	reported	002wka	2	TRUE	0.011646	20.59629
4262	rs9897258	T	C	T	C	-0.0463	0.020937	0.8542	0.781447	FALSE	FALSE	FALSE	ieu-b-508	17	79389251	0.04033	486484	0.603659	Sepsis (28	Sepsis (28	Sepsis (28	TRUE	igd	0.0102	6.07E-06	4274	M32800.m	TRUE	reported	Q7MVsa	2	TRUE	0.004798	20.59484
1755	rs6094988	C	G	C	G	0.0059	0.011652	0.234	0.243301	FALSE	TRUE	FALSE	ieu-b-508	20	46776312	0.038916	486484	0.764629	Sepsis (28	Sepsis (28	Sepsis (28	TRUE	igd	0.0013	3.14E-06	7365	M15650.m	TRUE	reported	NgezyT	2	TRUE	0.002789	20.59204
6346	rs1220235	T	C	T	C	0.0118	0.017509	0.7073	0.705439	FALSE	FALSE	FALSE	ieu-b-508	6	1.14E+08	0.03674	486484	0.633671	Sepsis (28	Sepsis (28	Sepsis (28	TRUE	igd	0.0026	7.14E-06	7364	M33960.m	TRUE	reported	o8RcxU	2	TRUE	0.002789	20.59204
6348	rs1366823	A	G	A	G	0.0118	-0.03883	0.6447	0.653199	FALSE	FALSE	FALSE	ieu-b-508	2	83659578	0.035152	486484	0.269322	Sepsis (28	Sepsis (28	Sepsis (28	TRUE	igd	0.0026	6.94E-06	7364	M33960.m	TRUE	reported	o8RcxU	2	TRUE	0.002789	20.59204
7306	rs551938	A	G	A	G	-0.0236	-0.0124	0.2537	0.253607	FALSE	FALSE	FALSE	ieu-b-508	9	1.28E+08	0.039802	486484	0.755478	Sepsis (28	Sepsis (28	Sepsis (28	TRUE	igd	0.0052	5.63E-06	7364	M35126.m	TRUE	reported	1haOJA	2	TRUE	0.002789	20.59204
718	rs1330221	A	G	A	G	-0.0118	0.048473	0.932	0.937178	FALSE	FALSE	FALSE	ieu-b-508	9	1.13E+08	0.068812	486484	0.481172	Sepsis (28	Sepsis (28	Sepsis (28	TRUE	igd	0.0026	6.80E-06	7363	M01301.m	TRUE	reported	w8TL71	2	TRUE	0.00279	20.59204
3951	rs1352815	A	C	A	C	-0.0118	0.027052	0.6865	0.664601	FALSE	FALSE	FALSE	ieu-b-508	14	80566731	0.035633	486484	0.447742	Sepsis (28	Sepsis (28	Sepsis (28	TRUE	igd	0.0026	8.16E-06	7363	M32709.m	TRUE	reported	YR8SB4	2	TRUE	0.00279	20.59204
880	rs1936396	A	G	A	G	-0.0118	0.030199	0.2253	0.215495	FALSE	FALSE	FALSE	ieu-b-508	10	45548068	0.040676	486484	0.457816	Sepsis (28	Sepsis (28	Sepsis (28	TRUE	igd	0.0026	5.67E-06	7362	M01365.m	TRUE	reported	N1cZTs	2	TRUE	0.00279	20.59204
3525	rs1236046	A	G	A	G	0.0118	-0.06334	0.5969	0.601817	FALSE	FALSE	FALSE	ieu-b-508	10	1.02E+08	0.034069	486484	0.062996	Sepsis (28	Sepsis (28	Sepsis (28	TRUE	igd	0.0026	5.69E-06	7358	M32497.m	TRUE	reported	244bZN	2	TRUE	0.002792	20.59203
3534	rs2968858	T	C	T	C	-0.0118	0.012215	0.5283	0.514711	FALSE	FALSE	FALSE	ieu-b-508	7	1.51E+08	0.033629	486484	0.716447	Sepsis (28	Sepsis (28	Sepsis (28	TRUE	igd	0.0026	7.25E-06	7358	M32497.m	TRUE	reported	244bZN	2	TRUE	0.002792	20.59203
3544	rs8054389	T	C	T	C	-0.0118	0.015663	0.493	0.49251	FALSE	FALSE	FALSE	ieu-b-508	16	48541394	0.033446	486484	0.639568	Sepsis (28	Sepsis (28	Sepsis (28	TRUE	igd	0.0026	6.42E-06	7358	M32497.m	TRUE	reported	244bZN	2	TRUE	0.002792	20.59203
1441	rs1256921	A	C	A	C	-0.0118	0.06558	0.1612	0.158935	FALSE	FALSE	FALSE	ieu-b-508	1	2.04E+08	0.045558	486484	0.150014	Sepsis (28	Sepsis (28	Sepsis (28	TRUE	igd	0.0026	5.82E-06	7357	M03141.m	TRUE	reported	y4mG6m	2	TRUE	0.002792	20.59203
1162	rs8079173	A	G	A	G	0.0118	-0.05636	0.1402	0.138747	FALSE	FALSE	FALSE	ieu-b-508	17	70075796	0.04829	486484	0.243171	Sepsis (28	Sepsis (28	Sepsis (28	TRUE	igd	0.0026	8.93E-06	7355	M01644.m	TRUE	reported	IB0DTL	2	TRUE	0.002793	20.59203
2075	rs7574847	A	G	A	G	-0.0118	0.01148	0.1481	0.154332	FALSE	FALSE	FALSE	ieu-b-508	2	65251696	0.046238	486484	0.803926	Sepsis (28	Sepsis (28	Sepsis (28	TRUE	igd	0.0026	3.98E-06	7354	M18369.m	TRUE	reported	ykBjIT	2	TRUE	0.002793	20.59203
3592	rs9322458	A	T	A	T	-0.0118	-0.04488	0.8754	0.854948	FALSE	TRUE	FALSE	ieu-b-508	6	1.55E+08	0.047573	486484	0.345455	Sepsis (28	Sepsis (28	Sepsis (28	TRUE	igd	0.0026	7.60E-06	7350	M32518.m	TRUE	reported	V0ZCiU	2	TRUE	0.002795	20.59203
2805	rs6498634	T	G	T	G	0.0118	0.017114	0.7656	0.766541	FALSE	FALSE	FALSE	ieu-b-508	16	16988283	0.03929	486484	0.663141	Sepsis (28	Sepsis (28	Sepsis (28	TRUE	igd	0.0026	7.87E-06	7347	M27278.m	TRUE	reported	kPgqm3	2	TRUE	0.002796	20.59203
511	rs10736	T	C	T	C	-0.0118	0.047515	0.2011	0.203294	FALSE	FALSE	FALSE	ieu-b-508	7	32526687	0.041467	486484	0.251858	Sepsis (28	Sepsis (28	Sepsis (28	TRUE	igd	0.0026	4.73E-06	7345	M00584.m	TRUE	reported	oBt02X	2	TRUE	0.002796	20.59202
4437	rs2295465	A	C	A	C	-0.0118	0.000913	0.8798	0.87845	FALSE	FALSE	FALSE	ieu-b-508	1	46726057	0.051008	486484	0.985716	Sepsis (28	Sepsis (28	Sepsis (28	TRUE	igd	0.0026	6.65E-06	7344	M32910.m	TRUE	reported	2OCBKh	2	TRUE	0.002797	20.59202
5624	rs5761606	T	G	T	G	0.0118	0.013748	0.07	0.075848	FALSE	FALSE	FALSE	ieu-b-508	22	26963743	0.063409	486484	0.828352	Sepsis (28	Sepsis (28	Sepsis (28	TRUE	igd	0.0026	7.86E-06	7336	M33442.m	TRUE	reported	B1Peb3	2	TRUE	0.0028	20.59202
803	rs4472784	T	C	T	C	-0.0118	0.015426	0.3048	0.319807	FALSE	FALSE	FALSE	ieu-b-508	1	1.73E+08	0.035896	486484	0.667389	Sepsis (28	Sepsis (28	Sepsis (28	TRUE	igd	0.0026	4.78E-06	7335	M01356.m	TRUE	reported	ah1g5W	2	TRUE	0.0028	20.59202
8379	rs1221784	A	G	A	G	0.0118	0.00171	0.287	0.286633	FALSE	FALSE	FALSE	ieu-b-508	10	79958794	0.04063	486484	0.966436	Sepsis (28	Sepsis (28	Sepsis (28	TRUE	igd	0.0026	5.74E-06	7335	M36776.m	TRUE	reported	VnYglx	2	TRUE	0.0028	20.59202
8387	rs9369302	T	C	T	C	0.0118	0.016114	0.7183	0.709983	FALSE	FALSE	FALSE	ieu-b-508	6	41664121	0.036841	486484	0.661828	Sepsis (28	Sepsis (28	Sepsis (28	TRUE	igd	0.0026	4.15E-06	7335	M36776.m	TRUE	reported	VnYglx	2	TRUE	0.0028	20.59202
8389	rs9953686	T	C	T	C	0.0118	0.001292	0.8128	0.812105	FALSE	FALSE	FALSE	ieu-b-508	18	43360005	0.042688	486484	0.975847	Sepsis (28	Sepsis (28	Sepsis (28	TRUE	igd	0.0026	6.93E-06	7335	M36776.m	TRUE	reported	VnYglx	2	TRUE	0.0028	20.59202
4388	rs2251468	A	C	A	C	-0.0059	-0.03494	0.6513	0.661203	FALSE	FALSE	FALSE	ieu-b-508	12	1.21E+08	0.035262	486484	0.321733	Sepsis (28	Sepsis (28	Sepsis (28	TRUE	igd	0.0013	3.43E-06	7329	M32867.m	TRUE	reported	Jcsyzm	2	TRUE	0.002803	20.59201
1278	rs1214717	C	G	C	G	-0.0118	-0.12073	0.1169	0.112193	FALSE	TRUE	FALSE	ieu-b-508	14	20529449	0.052742	486484	0.022073	Sepsis (28	Sepsis (28	Sepsis (28	TRUE	igd	0.0026	4.76E-06	7325	M02132.m	TRUE	reported	uJFm2t	2	TRUE	0.002804	20.59201
1581	rs1756283	T	C	T	C	0.0118	0.076008	0.0769	0.077966	FALSE	FALSE	FALSE	ieu-b-508	18	33564002	0.062031	486484	0.220456	Sepsis (28	Sepsis (28	Sepsis (28	TRUE	igd	0.0026	4.17E-06	7314	M12593.m	TRUE	reported	IBXgSU	2	TRUE	0.002808	20.592
2156	rs2363698	A	T	A	T	0.0118	-0.04174	0.7857	0.784143	FALSE	TRUE	FALSE	ieu-b-508	12	73970265	0.040489	486484	0.302643	Sepsis (28	Sepsis (28	Sepsis (28	TRUE	igd	0.0026	5.19E-06	7286	M18929.m	TRUE	reported	2vYFb2	2	TRUE	0.002819	20.59198
5794	rs733017	T	C	T	C	-0.0118	0.013164	0.6092	0.613134	FALSE	FALSE	FALSE	ieu-b-508	16	54517482	0.034689	486484	0.704333	Sepsis (28	Sepsis (28	Sepsis (28	TRUE	igd	0.0026	6.94E-06	7269	M33510.m	TRUE	reported	WxGKnn	2	TRUE	0.002826	20.59197
7491	rs2122506	A	G	A	G	0.0118	0.016705	0.3253	0.330125	FALSE	FALSE	FALSE	ieu-b-508	4	1.4E+08	0.035592	486484	0.638819	Sepsis (28	Sepsis (28	Sepsis (28	TRUE	igd	0.0026	9.05E-06	7261	M35253.m	TRUE	reported	VboRmj	2	TRUE	0.002829	20.59196
7498	rs6903630	C	G	C	G	-0.0118	-0.04136	0.6277	0.624738	FALSE	TRUE	FALSE	ieu-b-508	6	1.35E+08	0.034496	486484	0.230531	Sepsis (28	Sepsis (28	Sepsis (28	TRUE	igd	0.0026	6.98E-06	7261	M35253.m	TRUE	reported	VboRmj	2	TRUE	0.002829	20.59196
940	rs6078500	T	C	T	C	-0.0118	0.053395	0.5117	0.493675	FALSE	FALSE	FALSE	ieu-b-508	20	182013	0.033403	486484	0.109927	Sepsis (28	Sepsis (28	Sepsis (28	TRUE	igd	0.0026	4.47E-06	7160	M01508.m	TRUE	reported	go526k	2	TRUE	0.002869	20.59188
945	rs7574257	T	C	T	C	0.0118	-0.05897	0.325	0.33282	FALSE	FALSE	FALSE	ieu-b-508	2	1.06E+08	0.035554	486484	0.097187	Sepsis (28	Sepsis (28	Sepsis (28	TRUE	igd	0.0026	7.76E-06	7160	M01508.m	TRUE	reported	go526k	2	TRUE	0.002869	20.59188
1508	rs1083956	C	G	C	G	-0.0177	0.031449	0.9453	0.931726	FALSE	TRUE	FALSE	ieu-b-508	11	6401598	0.066468	486484	0.63611	Sepsis (28	Sepsis (28	Sepsis (28	TRUE	igd	0.0039	6.46E-06	7081	M12067.m	TRUE	reported	O5x7RD	2	TRUE	0.0029	20.59182
860	rs1830241	A	G	A	G	0.0118	-0.03913	0.3431	0.42797	FALSE	FALSE	FALSE	ieu-b-508	3	22116524	0.033855	486484	0.247745	Sepsis (28	Sepsis (28	Sepsis (28	TRUE	igd	0.0026	7.50E-06	7063	M01361.m	TRUE	reported	NIAcQ3	2	TRUE	0.002908	20.5918
6600	rs1290317	T	C	T	C	0.0118	-0.05975	0.6383	0.629432	FALSE	FALSE	FALSE	ieu-b-508	15	61369210	0.034765	486484	0.085684	Sepsis (28	Sepsis (28	Sepsis (28	TRUE	igd	0.0026	7.87E-06	6994	M34306.m	TRUE	reported	WPXPUN	2	TRUE	0.002936	20.59174
2198	rs1210012	A	G	A	G	-0.0295	0.011887	0.0425	0.056033	FALSE	FALSE	FALSE	ieu-b-508	13	1.05E+08	0.072214	486484	0.869256	Sepsis (28	Sepsis (28	Sepsis (28	TRUE	igd	0.0065	5.23E-06	6929	M19364.m	TRUE	reported	XkjMEx	2	TRUE	0.002964	20.59169
1878	rs1102543	A	G	A	G	-0.0118	0.019403	0.4231	0.428445	FALSE	FALSE	FALSE	ieu-b-508	11	20213955	0.033723	486484	0.565041	Sepsis (28	Sepsis (28	Sepsis (28	TRUE	igd	0.0026	8.33E-06	6782	M15990.m	TRUE	reported	15fe4N	2	TRUE	0.003028	20.59156
1943	rs9525877	A	G	A	G	-0.0118	0.085647	0.7816	0.779838	FALSE	FALSE	FALSE	ieu-b-508	13	44519331	0.040298	486484	0.033558	Sepsis (28	Sepsis (28	Sepsis (28	TRUE	igd	0.0026	6.50E-06	6549	M16822.m	TRUE	reported	UNI018	2	TRUE	0.003135	20.59134
4585	rs2463492	T	G	T	G	0.0118	-0.05546	0.4249	0.428673	FALSE	FALSE	FALSE	ieu-b-508	2	2.01E+08	0.03367	486484	0.099552	Sepsis (28	Sepsis (28	Sepsis (28	TRUE	igd	0.0026	6.95E-06	6404	M33163.m	TRUE	reported	YzGXkR	2	TRUE	0.003206	20.5912
7177	rs1107861	C	G	C	G	0.3903	-0.40198	0.0126	0.013322	FALSE	TRUE	FALSE	ieu-b-508	17	6154473	0.146807	486484	0.006179	Sepsis (28	Sepsis (28	Sepsis (28	TRUE	igd	0.0859	5.58E-06	754	M34535.m	TRUE	reported	z9BiJF	2	TRUE	0.026651	20.59004
6700	rs606567	A	G	A	G	0.0177	0.003931	0.8204	0.820695	FALSE	FALSE	FALSE	ieu-b-508	11	1.25E+08	0.043653	486484	0.928255	Sepsis (28	Sepsis (28	Sepsis (28	TRUE	igd	0.0039	6.60E-06	5081	M34336.m	TRUE	reported	jrSSxL	2	TRUE	0.004037	20.58953
2005	rs7016707	T	G	T	G	0.1439	-0.05422	0.099	0.10391	FALSE	FALSE	FALSE	ieu-b-508	8	41586822	0.054776	486484	0.322262	Sepsis (28	Sepsis (28	Sepsis (28	TRUE	igd	0.0317	5.81E-06	2433	M18281.m	TRUE	reported	3mLp7z	2	TRUE	0.008398	20.58951
5224	rs1442060	A	G	A	G	-0.0236	0.010395	0.5118	0.511285	FALSE	FALSE	FALSE	ieu-b-508	4	46366067	0.03385	486484	0.758768	Sepsis (28	Sepsis (28	Sepsis (28	TRUE	igd	0.0052	6.81E-06	4665	M33221.m	TRUE	reported	8ZrV1r	2	TRUE	0.004396	20.5888
6209	rs1286736	A	C	A	C	-0.0236	0.029495	0.7789	0.77522	FALSE	FALSE	FALSE	ieu-b-508	13	77365977	0.039948	486484	0.460317	Sepsis (28	Sepsis (28	Sepsis (28	TRUE	igd	0.0052	5.44E-06	4533	M33901.m	TRUE	reported	mQkhrb	2	TRUE	0.004523	20.58855
6712	rs1099516	A	C	A	C	0.3473	0.026211	0.9644	0.960324	FALSE	FALSE	FALSE	ieu-b-508	10	64212306	0.085202	486484	0.758359	Sepsis (28	Sepsis (28	Sepsis (28	TRUE	igd	0.0764	5.44E-06	535	M34338.m	TRUE	reported	h4FB9a	2	TRUE	0.037189	20.58715
1388	rs2111718	A	G	A	G	-0.0227	-0.11265	0.8615	0.849813	FALSE	FALSE	FALSE	ieu-b-508	2	1.72E+08	0.046873	486484	0.016243	Sepsis (28	Sepsis (28	Sepsis (28	TRUE	igd	0.005	5.00E-06	1667	M02734.m	TRUE	reported	0y0dDD	2	TRUE	0.012213	20.58687
7846	rs9784679	T	C	T	C	0.0363	0.004359	0.2789	0.276236	FALSE	FALSE	FALSE	ieu-b-508	5	1.07E+08	0.037662	486484	0.907854	Sepsis (28	Sepsis (28	Sepsis (28	TRUE	igd	0.008	6.20E-06	5035	M35551.m	TRUE	reported	5Sxswl	2	TRUE	0.004073	20.58073
1894	rs7788421	A	G	A	G	-0.0245	0.102883	0.9134	0.908112	FALSE	FALSE	FALSE	ieu-b-508	7	1.57E+08	0.057868	486484	0.075419	Sepsis (28	Sepsis (28	Sepsis (28	TRUE	igd	0.0054	5.18E-06	6782	M15990.m	TRUE	reported	15fe4N	2	TRUE	0.003026	20.57863
6955	rs4780314	C	G	C	G	-0.0245	0.00237	0.7909	0.787158	FALSE	TRUE	FALSE	ieu-b-508	16	10700879	0.041081	486484	0.953995	Sepsis (28	Sepsis (28	Sepsis (28	TRUE	igd	0.0054	5.05E-06	5689	M34400.m	TRUE	reported	QXmzfR	2	TRUE	0.003605	20.57747
7398	rs4758441	A	G	A	G	-0.0295	0.014086	0.2648	0.256892	FALSE	FALSE	FALSE	ieu-b-508	11	6627092	0.038226	486484	0.712508	Sepsis (28	Sepsis (28	Sepsis (28	TRUE	igd	0.0065	5.31E-06	1982	M35159.m	TRUE	reported	7EoJLB	2	TRUE	0.010285	20.57685
5710	rs1299909	T	G	T	G	0.0245	0.134203	0.8061	0.800896	FALSE	FALSE	FALSE	ieu-b-508	2	1.89E+08	0.041748	486484	0.001306	Sepsis (28	Sepsis (28	Sepsis (28	TRUE	igd	0.0054	5.92E-06	5107	M33488.m	TRUE	reported	mZmDY6	2	TRUE	0.004015	20.57664
8529	rs4331456	T	C	T	C	-0.1664	0.032416	0.2107	0.245674	FALSE	FALSE	FALSE	ieu-b-508	2	2.39E+08	0.039156	486484	0.407747	Sepsis (28	Sepsis (28	Sepsis (28	TRUE	igd	0.0366	5.52E-06	437	M37112.m	TRUE	reported	P3GiAg	2	TRUE	0.045164	20.57559
602	rs8123073	A	G	A	G	-0.0372	-0.07046	0.983	0.979596	FALSE	FALSE	FALSE	ieu-b-508	20	31446858	0.117451	486484	0.548567	Sepsis (28	Sepsis (28	Sepsis (28	TRUE	igd	0.0082	5.94E-06	7367	M01110.m	TRUE	reported	0A31Sl	2	TRUE	0.002786	20.57502
6292	rs1293243	T	G	T	G	0.0186	-0.02225	0.7139	0.71847	FALSE	FALSE	FALSE	ieu-b-508	16	7114043	0.037063	486484	0.548206	Sepsis (28	Sepsis (28	Sepsis (28	TRUE	igd	0.0041	6.34E-06	7320	M33941.m	TRUE	reported	cuIqiU	2	TRUE	0.002804	20.57498
4054	rs1719079	A	G	A	G	0.0186	-0.01324	0.9476	0.928146	FALSE	FALSE	FALSE	ieu-b-508	2	1.6E+08	0.064744	486484	0.837989	Sepsis (28	Sepsis (28	Sepsis (28	TRUE	igd	0.0041	4.92E-06	7318	M32740.m	TRUE	reported	YLshfP	2	TRUE	0.002804	20.57498
1507	rs1052019	A	G	A	G	0.0186	-0.03307	0.0296	0.03276	FALSE	FALSE	FALSE	ieu-b-508	4	1.72E+08	0.093963	486484	0.724845	Sepsis (28	Sepsis (28	Sepsis (28	TRUE	igd	0.0041	5.64E-06	7081	M12067.m	TRUE	reported	O5x7RD	2	TRUE	0.002898	20.57479
988	rs1404410	C	G	C	G	-0.0236	-0.0151	0.7853	0.80147	FALSE	TRUE	FALSE	ieu-b-508	7	1.24E+08	0.041947	486484	0.718927	Sepsis (28	Sepsis (28	Sepsis (28	TRUE	igd	0.0052	4.57E-06	1759	M01561.m	TRUE	reported	Zd9SUU	2	TRUE	0.011574	20.57421
2159	rs746764	T	C	T	C	-0.0236	-0.09489	0.1676	0.156851	FALSE	FALSE	FALSE	ieu-b-508	20	21476637	0.046316	486484	0.040478	Sepsis (28	Sepsis (28	Sepsis (28	TRUE	igd	0.0052	6.63E-06	1748	M18929.m	TRUE	reported	2vYFb2	2	TRUE	0.011646	20.57407
2472	rs967725	A	T	A	T	-0.0372	0.118936	0.0226	0.019916	FALSE	TRUE	FALSE	ieu-b-508	2	2.01E+08	0.121392	486484	0.327202	Sepsis (28	Sepsis (28	Sepsis (28	TRUE	igd	0.0082	6.15E-06	6180	M22030.m	TRUE	reported	ZNV0qR	2	TRUE	0.003319	20.57395
3297	rs4931472	A	G	A	G	-0.0186	-0.01448	0.3954	0.386742	FALSE	FALSE	FALSE	ieu-b-508	12	31385675	0.034935	486484	0.678571	Sepsis (28	Sepsis (28	Sepsis (28	TRUE	igd	0.0041	7.30E-06	6119	M32379.m	TRUE	reported	MLimM6	2	TRUE	0.003352	20.57388
6108	rs6426630	A	G	A	G	-0.0885	0.046575	0.0286	0.055647	FALSE	FALSE	FALSE	ieu-b-508	1	19339070	0.072999	486484	0.523461	Sepsis (28	Sepsis (28	Sepsis (28	TRUE	igd	0.0195	5.37E-06	1639	M33871.m	TRUE	reported	mQD6DP	2	TRUE	0.012411	20.5725
2139	rs1695509	T	C	T	C	0.3552	0.197127	0.015	0.017698	FALSE	FALSE	FALSE	ieu-b-508	17	5692999	0.12591	486484	0.117438	Sepsis (28	Sepsis (28	Sepsis (28	TRUE	igd	0.0782	5.61E-06	698	M18497.m	TRUE	reported	90JFPc	2	TRUE	0.02871	20.57246
8036	rs1245110	T	C	T	C	-0.059	0.020649	0.0806	0.075822	FALSE	FALSE	FALSE	ieu-b-508	17	133779	0.063536	486484	0.745185	Sepsis (28	Sepsis (28	Sepsis (28	TRUE	igd	0.013	5.77E-06	1632	M35854.m	TRUE	reported	EujB0p	2	TRUE	0.012464	20.57239
6715	rs1172553	A	G	A	G	-0.2122	-0.02124	0.9605	0.955488	FALSE	FALSE	FALSE	ieu-b-508	4	1.86E+08	0.080915	486484	0.79297	Sepsis (28	Sepsis (28	Sepsis (28	TRUE	igd	0.0467	5.43E-06	535	M34338.m	TRUE	reported	h4FB9a	2	TRUE	0.037158	20.56982
5623	rs5749010	A	G	A	G	-0.0127	-0.0405	0.0828	0.08095	FALSE	FALSE	FALSE	ieu-b-508	22	17765505	0.062207	486484	0.515063	Sepsis (28	Sepsis (28	Sepsis (28	TRUE	igd	0.0028	4.42E-06	7336	M33442.m	TRUE	reported	B1Peb3	2	TRUE	0.002797	20.5671
2618	rs3734747	A	G	A	G	-0.0127	-0.00414	0.164	0.15821	FALSE	FALSE	FALSE	ieu-b-508	6	1.08E+08	0.04582	486484	0.927943	Sepsis (28	Sepsis (28	Sepsis (28	TRUE	igd	0.0028	4.31E-06	7329	M22481.m	TRUE	reported	bXpf0k	2	TRUE	0.002799	20.56709
1277	rs1199300	A	G	A	G	-0.0127	-0.02557	0.3167	0.362275	FALSE	FALSE	FALSE	ieu-b-508	8	28307911	0.034675	486484	0.460928	Sepsis (28	Sepsis (28	Sepsis (28	TRUE	igd	0.0028	5.24E-06	7325	M02132.m	TRUE	reported	uJFm2t	2	TRUE	0.002801	20.56709
6614	rs9393409	A	G	A	G	-0.0127	-0.01632	0.3342	0.328834	FALSE	FALSE	FALSE	ieu-b-508	6	23383429	0.035621	486484	0.646873	Sepsis (28	Sepsis (28	Sepsis (28	TRUE	igd	0.0028	4.11E-06	6994	M34306.m	TRUE	reported	WPXPUN	2	TRUE	0.002933	20.56682
3989	rs1757091	A	G	A	G	0.0127	0.011556	0.1402	0.163756	FALSE	FALSE	FALSE	ieu-b-508	14	26750711	0.045238	486484	0.798369	Sepsis (28	Sepsis (28	Sepsis (28	TRUE	igd	0.0028	4.93E-06	6497	M32729.m	TRUE	reported	XGRSN1	2	TRUE	0.003156	20.56637
2014	rs2035344	A	G	A	G	0.0254	0.037509	0.6893	0.69033	FALSE	FALSE	FALSE	ieu-b-508	15	1.01E+08	0.036357	486484	0.302218	Sepsis (28	Sepsis (28	Sepsis (28	TRUE	igd	0.0056	6.74E-06	5984	M18283.m	TRUE	reported	oyZdTE	2	TRUE	0.003426	20.56583
2505	rs1704258	T	G	T	G	0.0127	-0.04701	0.7446	0.729981	FALSE	FALSE	FALSE	ieu-b-508	3	5493089	0.03756	486484	0.210684	Sepsis (28	Sepsis (28	Sepsis (28	TRUE	igd	0.0028	4.75E-06	5702	M22130.m	TRUE	reported	YDbJlz	2	TRUE	0.003595	20.56549
2475	rs1083164	T	C	T	C	0.0127	-0.00416	0.4756	0.473288	FALSE	FALSE	FALSE	ieu-b-508	11	11596860	0.033618	486484	0.901574	Sepsis (28	Sepsis (28	Sepsis (28	TRUE	igd	0.0028	4.73E-06	5676	M22032.m	TRUE	reported	xpe96K	2	TRUE	0.003611	20.56546
7060	rs9696823	A	G	A	G	-0.0127	-0.00787	0.6502	0.651002	FALSE	FALSE	FALSE	ieu-b-508	9	26754888	0.035088	486484	0.822545	Sepsis (28	Sepsis (28	Sepsis (28	TRUE	igd	0.0028	4.13E-06	5207	M34456.m	TRUE	reported	Nh457q	2	TRUE	0.003935	20.5648
8131	rs1686411	T	C	T	C	0.1474	0.019109	0.0154	0.034013	FALSE	FALSE	FALSE	ieu-b-508	3	1.89E+08	0.091821	486484	0.835143	Sepsis (28	Sepsis (28	Sepsis (28	TRUE	igd	0.0325	5.88E-06	5696	M36099.m	TRUE	reported	bFHsTx	2	TRUE	0.003598	20.56249
3241	rs7645550	T	C	T	C	-0.0322	0.037163	0.4017	0.400174	FALSE	FALSE	FALSE	ieu-b-508	3	1.79E+08	0.034034	486484	0.274859	Sepsis (28	Sepsis (28	Sepsis (28	TRUE	igd	0.0071	5.84E-06	6687	M32346.m	TRUE	reported	cnIFoV	2	TRUE	0.003066	20.56199
8125	rs6552978	A	G	A	G	-0.0735	-0.04775	0.808	0.810042	FALSE	FALSE	FALSE	ieu-b-508	4	1.7E+08	0.042451	486484	0.260692	Sepsis (28	Sepsis (28	Sepsis (28	TRUE	igd	0.0162	5.70E-06	1725	M36098.m	TRUE	reported	hs9SZl	2	TRUE	0.011792	20.56084
3874	rs2610815	T	C	T	C	-0.0195	0.097727	0.9355	0.935763	FALSE	FALSE	FALSE	ieu-b-508	10	13962655	0.068191	486484	0.151817	Sepsis (28	Sepsis (28	Sepsis (28	TRUE	igd	0.0043	5.84E-06	7361	M32654.m	TRUE	reported	6ISQOP	2	TRUE	0.002786	20.55958
762	rs4768795	T	G	T	G	0.0195	-0.00898	0.3587	0.432011	FALSE	FALSE	FALSE	ieu-b-508	12	47712974	0.033686	486484	0.789792	Sepsis (28	Sepsis (28	Sepsis (28	TRUE	igd	0.0043	6.19E-06	6946	M01303.m	TRUE	reported	3b8x0F	2	TRUE	0.002952	20.55925
7541	rs2627272	T	C	T	C	-0.0195	0.049059	0.8848	0.883097	FALSE	FALSE	FALSE	ieu-b-508	8	2755070	0.052315	486484	0.348372	Sepsis (28	Sepsis (28	Sepsis (28	TRUE	igd	0.0043	6.49E-06	6538	M35257.m	TRUE	reported	iphouU	2	TRUE	0.003136	20.55888
7218	rs1191963	A	G	A	G	0.0195	0.024407	0.7317	0.732627	FALSE	FALSE	FALSE	ieu-b-508	3	1.26E+08	0.037654	486484	0.516859	Sepsis (28	Sepsis (28	Sepsis (28	TRUE	igd	0.0043	5.96E-06	6381	M34761.m	TRUE	reported	AMtp1w	2	TRUE	0.003213	20.55872
6233	rs1336198	A	G	A	G	0.0195	-0.03203	0.4413	0.433724	FALSE	FALSE	FALSE	ieu-b-508	5	41017006	0.03362	486484	0.340813	Sepsis (28	Sepsis (28	Sepsis (28	TRUE	igd	0.0043	6.53E-06	5534	M33910.m	TRUE	reported	YwFZg1	2	TRUE	0.003702	20.55774
6143	rs1116363	A	C	A	C	0.0508	0.003641	0.4597	0.448599	FALSE	FALSE	FALSE	ieu-b-508	1	83477356	0.033637	486484	0.913799	Sepsis (28	Sepsis (28	Sepsis (28	TRUE	igd	0.0112	5.60E-06	2725	M33883.m	TRUE	reported	RT7YNu	2	TRUE	0.007493	20.5576
6331	rs804572	T	G	T	G	-0.0372	0.025222	0.0607	0.064367	FALSE	FALSE	FALSE	ieu-b-508	20	22152171	0.068207	486484	0.711536	Sepsis (28	Sepsis (28	Sepsis (28	TRUE	igd	0.0082	5.72E-06	1764	M33955.m	TRUE	reported	mhHFnT	2	TRUE	0.011532	20.55727
5998	rs723124	T	C	T	C	0.1622	0.038528	0.0634	0.089937	FALSE	FALSE	FALSE	ieu-b-508	10	58518676	0.059238	486484	0.515433	Sepsis (28	Sepsis (28	Sepsis (28	TRUE	igd	0.0357	5.40E-06	477	M33683.m	TRUE	reported	wSozpU	2	TRUE	0.041481	20.55609
8241	rs6999153	A	G	A	G	0.0313	-0.05213	0.5597	0.554521	FALSE	FALSE	FALSE	ieu-b-508	8	9193501	0.033753	486484	0.122468	Sepsis (28	Sepsis (28	Sepsis (28	TRUE	igd	0.0069	5.97E-06	1908	M36468.m	TRUE	reported	vycAFr	2	TRUE	0.01067	20.55583
7175	rs1080168	T	G	T	G	-0.4744	0.018843	0.0209	0.511458	FALSE	FALSE	FALSE	ieu-b-508	1	88235193	0.034562	486484	0.585613	Sepsis (28	Sepsis (28	Sepsis (28	TRUE	igd	0.1045	5.67E-06	754	M34535.m	TRUE	reported	z9BiJF	2	TRUE	0.026606	20.55433
6025	rs843172	T	G	T	G	-0.7724	0.1318	0.9755	0.979764	FALSE	FALSE	FALSE	ieu-b-508	9	1.06E+08	0.119174	486484	0.268749	Sepsis (28	Sepsis (28	Sepsis (28	TRUE	igd	0.1682	4.37E-06	79	M33751.m	TRUE	reported	Cxdkr4	2	TRUE	0.210693	20.55399
8447	rs1710313	A	G	A	G	0.044	0.065025	0.0712	0.077164	FALSE	FALSE	FALSE	ieu-b-508	14	75774346	0.063069	486484	0.302534	Sepsis (28	Sepsis (28	Sepsis (28	TRUE	igd	0.0097	5.98E-06	1782	M37004.m	TRUE	reported	EKCQye	2	TRUE	0.011415	20.55295
6169	rs1205060	C	G	C	G	-0.0662	-0.00263	0.0791	0.07331	FALSE	TRUE	FALSE	ieu-b-508	15	42396187	0.064364	486484	0.967434	Sepsis (28	Sepsis (28	Sepsis (28	TRUE	igd	0.0146	5.73E-06	4999	M33885.m	TRUE	reported	uCOy6o	2	TRUE	0.004096	20.55117
5513	rs1214427	A	C	A	C	-0.0635	0.002946	0.0297	0.054909	FALSE	FALSE	FALSE	ieu-b-508	1	94277582	0.0737	486484	0.968111	Sepsis (28	Sepsis (28	Sepsis (28	TRUE	igd	0.014	5.46E-06	1748	M33422.m	TRUE	reported	PLgjrT	2	TRUE	0.011632	20.54917
5917	rs1207117	A	G	A	G	0.0934	-0.05596	0.0265	0.032755	FALSE	FALSE	FALSE	ieu-b-508	1	1.18E+08	0.094592	486484	0.554142	Sepsis (28	Sepsis (28	Sepsis (28	TRUE	igd	0.0206	5.58E-06	4323	M33633.m	TRUE	reported	e36XGE	2	TRUE	0.004733	20.54747
4519	rs994713	T	C	T	C	-0.0204	0.01872	0.0245	0.025141	FALSE	FALSE	FALSE	ieu-b-508	12	32989025	0.106907	486484	0.861	Sepsis (28	Sepsis (28	Sepsis (28	TRUE	igd	0.0045	6.37E-06	7362	M33132.m	TRUE	reported	dTXJa8	2	TRUE	0.002784	20.54553
751	rs9410970	A	C	A	C	-0.0068	-0.01684	0.2201	0.215502	FALSE	FALSE	FALSE	ieu-b-508	9	90415220	0.040858	486484	0.680147	Sepsis (28	Sepsis (28	Sepsis (28	TRUE	igd	0.0015	5.49E-06	7347	M01302.m	TRUE	reported	sNp2IC	2	TRUE	0.002789	20.54552
6159	rs393228	T	G	T	G	0.034	-0.03818	0.0697	0.066851	FALSE	FALSE	FALSE	ieu-b-508	6	24962428	0.066582	486484	0.566389	Sepsis (28	Sepsis (28	Sepsis (28	TRUE	igd	0.0075	5.62E-06	7334	M33884.m	TRUE	reported	aAWgRQ	2	TRUE	0.002794	20.54551
2779	rs2269713	A	G	A	G	0.034	-0.13894	0.9761	0.971097	FALSE	FALSE	FALSE	ieu-b-508	6	31141127	0.10082	486484	0.168172	Sepsis (28	Sepsis (28	Sepsis (28	TRUE	igd	0.0075	5.65E-06	7268	M27273.m	TRUE	reported	EvU2od	2	TRUE	0.00282	20.54546
2184	rs1686471	A	T	A	T	0.034	0.116374	0.0172	0.02096	FALSE	TRUE	FALSE	ieu-b-508	2	6246620	0.116128	486484	0.316286	Sepsis (28	Sepsis (28	Sepsis (28	TRUE	igd	0.0075	6.76E-06	7182	M19362.m	TRUE	reported	SkNEKw	2	TRUE	0.002853	20.54539
7731	rs1163950	A	G	A	G	-0.034	0.023042	0.9736	0.947764	FALSE	FALSE	FALSE	ieu-b-508	16	80444668	0.075788	486484	0.761106	Sepsis (28	Sepsis (28	Sepsis (28	TRUE	igd	0.0075	5.69E-06	7007	M35431.m	TRUE	reported	tLfE4l	2	TRUE	0.002924	20.54525
1320	rs1786837	A	C	A	C	-0.034	-0.00856	0.932	0.932479	FALSE	FALSE	FALSE	ieu-b-508	2	2.35E+08	0.066704	486484	0.897937	Sepsis (28	Sepsis (28	Sepsis (28	TRUE	igd	0.0075	6.49E-06	6252	M02137.m	TRUE	reported	iRGqBE	2	TRUE	0.003276	20.54454
4274	rs1473993	T	C	T	C	0.0272	0.049365	0.6241	0.595022	FALSE	FALSE	FALSE	ieu-b-508	12	20859650	0.034006	486484	0.146597	Sepsis (28	Sepsis (28	Sepsis (28	TRUE	igd	0.006	5.85E-06	6200	M32808.m	TRUE	reported	vnr36k	2	TRUE	0.003304	20.54448
4277	rs514659	A	C	A	C	0.0272	0.006825	0.6737	0.682658	FALSE	FALSE	FALSE	ieu-b-508	9	1.36E+08	0.035652	486484	0.84818	Sepsis (28	Sepsis (28	Sepsis (28	TRUE	igd	0.006	6.61E-06	6200	M32808.m	TRUE	reported	vnr36k	2	TRUE	0.003304	20.54448
7476	rs1027416	T	G	T	G	-0.0136	-0.01655	0.4586	0.455978	FALSE	FALSE	FALSE	ieu-b-508	7	1.44E+08	0.033951	486484	0.625972	Sepsis (28	Sepsis (28	Sepsis (28	TRUE	igd	0.003	5.25E-06	5694	M35240.m	TRUE	reported	55Kiv4	2	TRUE	0.003596	20.54389
2129	rs4338010	T	C	T	C	-0.1084	-0.05531	0.0788	0.088855	FALSE	FALSE	FALSE	ieu-b-508	7	1.53E+08	0.058797	486484	0.346846	Sepsis (28	Sepsis (28	Sepsis (28	TRUE	igd	0.0239	5.73E-06	1457	M18477.m	TRUE	reported	p0vcCU	2	TRUE	0.013922	20.54311
5932	rs2171596	A	C	A	C	-0.0204	0.064357	0.6593	0.655627	FALSE	FALSE	FALSE	ieu-b-508	3	2016318	0.035132	486484	0.066973	Sepsis (28	Sepsis (28	Sepsis (28	TRUE	igd	0.0045	5.38E-06	5080	M33637.m	TRUE	reported	tfLznu	2	TRUE	0.004029	20.54302
7286	rs1243184	T	C	T	C	0.0975	0.292097	0.014	0.010412	FALSE	FALSE	FALSE	ieu-b-508	14	40136316	0.165993	486484	0.07846	Sepsis (28	Sepsis (28	Sepsis (28	TRUE	igd	0.0215	6.12E-06	1657	M35114.m	TRUE	reported	a6NQjV	2	TRUE	0.012259	20.54035
5995	rs6450591	A	G	A	G	-0.0871	0.041133	0.0819	0.085605	FALSE	FALSE	FALSE	ieu-b-508	5	28024827	0.059492	486484	0.489309	Sepsis (28	Sepsis (28	Sepsis (28	TRUE	igd	0.0192	5.77E-06	1027	M33683.m	TRUE	reported	wSozpU	2	TRUE	0.019645	20.53938
4119	rs1752555	T	C	T	C	-0.0417	0.062158	0.956	0.951822	FALSE	FALSE	FALSE	ieu-b-508	6	91601565	0.078216	486484	0.426787	Sepsis (28	Sepsis (28	Sepsis (28	TRUE	igd	0.0092	6.07E-06	6658	M32755.m	TRUE	reported	WDrNdi	2	TRUE	0.003076	20.53837
3660	rs2884692	T	C	T	C	-0.0349	-0.01173	0.3823	0.377268	FALSE	FALSE	FALSE	ieu-b-508	1	1.98E+08	0.034501	486484	0.73387	Sepsis (28	Sepsis (28	Sepsis (28	TRUE	igd	0.0077	6.34E-06	6970	M32564.m	TRUE	reported	73MgOk	2	TRUE	0.002939	20.53737
6032	rs1722092	T	C	T	C	0.0757	-0.04284	0.0707	0.076223	FALSE	FALSE	FALSE	ieu-b-508	10	1.09E+08	0.063299	486484	0.498502	Sepsis (28	Sepsis (28	Sepsis (28	TRUE	igd	0.0167	5.72E-06	3962	M33782.m	TRUE	reported	875Pal	2	TRUE	0.005159	20.53712
7022	rs1323365	A	G	A	G	0.0485	-0.0579	0.9528	0.933439	FALSE	FALSE	FALSE	ieu-b-508	7	70039026	0.067232	486484	0.389163	Sepsis (28	Sepsis (28	Sepsis (28	TRUE	igd	0.0107	5.67E-06	4442	M34441.m	TRUE	reported	UpgxQm	2	TRUE	0.004604	20.53621
5263	rs3811273	A	G	A	G	-0.0281	-0.02973	0.9383	0.926186	FALSE	FALSE	FALSE	ieu-b-508	14	22715158	0.063993	486484	0.642245	Sepsis (28	Sepsis (28	Sepsis (28	TRUE	igd	0.0062	5.13E-06	7364	M33230.m	TRUE	reported	nzcTOn	2	TRUE	0.002782	20.53578
5458	rs1774810	A	C	A	C	-0.1185	0.020411	0.9287	0.918922	FALSE	FALSE	FALSE	ieu-b-508	3	80099394	0.062249	486484	0.742988	Sepsis (28	Sepsis (28	Sepsis (28	TRUE	igd	0.0261	5.58E-06	528	M33391.m	TRUE	reported	EgMd1b	2	TRUE	0.037574	20.53561
633	rs2846723	T	G	T	G	-0.0476	0.001511	0.5442	0.538577	FALSE	FALSE	FALSE	ieu-b-508	11	1.03E+08	0.033452	486484	0.96397	Sepsis (28	Sepsis (28	Sepsis (28	TRUE	igd	0.0105	5.76E-06	2639	M01123.m	TRUE	reported	NDTvfD	2	TRUE	0.007727	20.53554
8526	rs2707795	T	G	T	G	-0.3579	-0.26263	0.972	0.984904	FALSE	FALSE	FALSE	ieu-b-508	2	1.45E+08	0.144004	486484	0.068192	Sepsis (28	Sepsis (28	Sepsis (28	TRUE	igd	0.0788	5.64E-06	437	M37112.m	TRUE	reported	P3GiAg	2	TRUE	0.045077	20.53425
7117	rs4389303	T	G	T	G	-0.172	-0.02227	0.1027	0.105385	FALSE	FALSE	FALSE	ieu-b-508	2	1.72E+08	0.054791	486484	0.684387	Sepsis (28	Sepsis (28	Sepsis (28	TRUE	igd	0.0378	5.35E-06	241	M34516.m	TRUE	reported	7Ah2wy	2	TRUE	0.079115	20.53308
7849	rs3813782	A	G	A	G	0.0535	0.050639	0.1154	0.132505	FALSE	FALSE	FALSE	ieu-b-508	19	8154350	0.049288	486484	0.304227	Sepsis (28	Sepsis (28	Sepsis (28	TRUE	igd	0.0118	5.81E-06	1766	M35626.m	TRUE	reported	0cF47S	2	TRUE	0.011506	20.53295
2248	rs2489774	T	C	T	C	0.0213	-0.10379	0.0339	0.034611	FALSE	FALSE	FALSE	ieu-b-508	1	2.08E+08	0.091317	486484	0.255694	Sepsis (28	Sepsis (28	Sepsis (28	TRUE	igd	0.0047	6.46E-06	7354	M19934.m	TRUE	reported	HtcWcQ	2	TRUE	0.002785	20.53267
1562	rs1740379	A	G	A	G	0.073	0.12984	0.1166	0.123785	FALSE	FALSE	FALSE	ieu-b-508	11	10581217	0.050745	486484	0.010507	Sepsis (28	Sepsis (28	Sepsis (28	TRUE	igd	0.0161	5.61E-06	1543	M12261.m	TRUE	reported	taUUcM	2	TRUE	0.013149	20.53197
6133	rs6759985	T	G	T	G	0.2189	-0.06185	0.0471	0.039921	FALSE	FALSE	FALSE	ieu-b-508	2	2.12E+08	0.085567	486484	0.469783	Sepsis (28	Sepsis (28	Sepsis (28	TRUE	igd	0.0481	5.33E-06	228	M33877.m	TRUE	reported	yDVi1f	2	TRUE	0.083273	20.52934
3741	rs2910745	C	G	C	G	-0.136	0.116693	0.0111	0.008671	FALSE	TRUE	FALSE	ieu-b-508	5	37885330	0.180318	486484	0.517535	Sepsis (28	Sepsis (28	Sepsis (28	TRUE	igd	0.03	5.70E-06	1762	M32632.m	TRUE	reported	pIHzPT	2	TRUE	0.011529	20.52778
2860	rs1003183	T	C	T	C	-0.034	0.052874	0.6953	0.659746	FALSE	FALSE	FALSE	ieu-b-508	4	1.36E+08	0.035199	486484	0.133062	Sepsis (28	Sepsis (28	Sepsis (28	TRUE	igd	0.0075	5.81E-06	1758	M27672.m	TRUE	reported	5cTg55	2	TRUE	0.011555	20.52773
4601	rs7762480	A	T	A	T	0.0748	0.081284	0.9273	0.921267	FALSE	TRUE	FALSE	ieu-b-508	6	1.47E+08	0.061806	486484	0.18846	Sepsis (28	Sepsis (28	Sepsis (28	TRUE	igd	0.0165	5.69E-06	1751	M33165.m	TRUE	reported	RZt3uS	2	TRUE	0.011601	20.52764
6278	rs1165542	A	T	A	T	0.0145	0.031251	0.1996	0.286081	FALSE	TRUE	FALSE	ieu-b-508	17	76251518	0.03708	486484	0.39934	Sepsis (28	Sepsis (28	Sepsis (28	TRUE	igd	0.0032	5.82E-06	6502	M33939.m	TRUE	reported	PeKlgz	2	TRUE	0.003148	20.52591
7692	rs1781577	A	C	A	C	-0.1079	-0.0489	0.9885	0.987824	FALSE	FALSE	FALSE	ieu-b-508	8	1.19E+08	0.153956	486484	0.750765	Sepsis (28	Sepsis (28	Sepsis (28	TRUE	igd	0.0238	5.94E-06	1375	M35331.m	TRUE	reported	GBnxhN	2	TRUE	0.014728	20.52375
4129	rs7337711	T	C	T	C	0.0408	0.00518	0.1564	0.143108	FALSE	FALSE	FALSE	ieu-b-508	13	34836113	0.047787	486484	0.913683	Sepsis (28	Sepsis (28	Sepsis (28	TRUE	igd	0.009	5.44E-06	1435	M32755.m	TRUE	reported	WDrNdi	2	TRUE	0.014119	20.52247
4244	rs1742900	A	G	A	G	-0.0367	0.064745	0.91	0.874454	FALSE	FALSE	FALSE	ieu-b-508	1	48823047	0.050532	486484	0.200106	Sepsis (28	Sepsis (28	Sepsis (28	TRUE	igd	0.0081	6.65E-06	6169	M32795.m	TRUE	reported	0YBWkh	2	TRUE	0.003317	20.52207
3788	rs1075815	T	C	T	C	-0.0222	0.04582	0.1408	0.158161	FALSE	FALSE	FALSE	ieu-b-508	9	32722876	0.045738	486484	0.31645	Sepsis (28	Sepsis (28	Sepsis (28	TRUE	igd	0.0049	6.21E-06	7369	M32635.m	TRUE	reported	jJI9P8	2	TRUE	0.002778	20.52088
4097	rs649622	T	G	T	G	0.0444	-0.00899	0.097	0.113891	FALSE	FALSE	FALSE	ieu-b-508	12	1.21E+08	0.052507	486484	0.864078	Sepsis (28	Sepsis (28	Sepsis (28	TRUE	igd	0.0098	5.89E-06	7356	M32753.m	TRUE	reported	XlC6zq	2	TRUE	0.002783	20.52087
6483	rs2663073	A	G	A	G	0.4837	0.194724	0.927	0.915528	FALSE	FALSE	FALSE	ieu-b-508	5	72018529	0.059963	486484	0.001165	Sepsis (28	Sepsis (28	Sepsis (28	TRUE	igd	0.1064	5.42E-06	282	M34109.m	TRUE	reported	piJuC7	2	TRUE	0.068282	20.52002
7297	rs1782694	A	G	A	G	0.0897	0.045052	0.984	0.975904	FALSE	FALSE	FALSE	ieu-b-508	14	95175027	0.110728	486484	0.684105	Sepsis (28	Sepsis (28	Sepsis (28	TRUE	igd	0.0198	5.56E-06	7364	M35126.m	TRUE	reported	1haOJA	2	TRUE	0.002779	20.51807
7309	rs6972443	C	G	C	G	0.0299	0.03075	0.8153	0.78993	FALSE	TRUE	FALSE	ieu-b-508	7	96453085	0.040823	486484	0.451295	Sepsis (28	Sepsis (28	Sepsis (28	TRUE	igd	0.0066	5.78E-06	7364	M35126.m	TRUE	reported	1haOJA	2	TRUE	0.002779	20.51807
4548	rs1156319	C	G	C	G	0.0299	-0.02354	0.0722	0.076105	FALSE	TRUE	FALSE	ieu-b-508	2	2.35E+08	0.062839	486484	0.707938	Sepsis (28	Sepsis (28	Sepsis (28	TRUE	igd	0.0066	5.34E-06	7319	M33140.m	TRUE	reported	eBOUtb	2	TRUE	0.002796	20.51804
8537	rs1287987	T	C	T	C	-0.0299	0.006294	0.5575	0.556705	FALSE	FALSE	FALSE	ieu-b-508	14	95106408	0.033727	486484	0.851953	Sepsis (28	Sepsis (28	Sepsis (28	TRUE	igd	0.0066	5.17E-06	6934	M37190.m	TRUE	reported	tiNuK9	2	TRUE	0.002951	20.51773
8540	rs4372938	T	C	T	C	0.0299	0.043177	0.5433	0.541526	FALSE	FALSE	FALSE	ieu-b-508	2	716503	0.033758	486484	0.2009	Sepsis (28	Sepsis (28	Sepsis (28	TRUE	igd	0.0066	6.09E-06	6934	M37190.m	TRUE	reported	tiNuK9	2	TRUE	0.002951	20.51773
843	rs35350	A	G	A	G	0.0376	0.011023	0.9692	0.971344	FALSE	FALSE	FALSE	ieu-b-508	12	1.16E+08	0.099585	486484	0.911861	Sepsis (28	Sepsis (28	Sepsis (28	TRUE	igd	0.0083	5.44E-06	7323	M01359.m	TRUE	reported	7L9x1s	2	TRUE	0.002795	20.51639
2143	rs309873	C	G	C	G	0.0743	-0.08137	0.065	0.069972	FALSE	TRUE	FALSE	ieu-b-508	4	48336820	0.065294	486484	0.212676	Sepsis (28	Sepsis (28	Sepsis (28	TRUE	igd	0.0164	5.63E-06	3783	M18497.m	TRUE	reported	90JFPc	2	TRUE	0.005396	20.51447
4113	rs9543952	A	G	A	G	-0.053	0.012445	0.8491	0.841923	FALSE	FALSE	FALSE	ieu-b-508	13	76084690	0.0457	486484	0.785373	Sepsis (28	Sepsis (28	Sepsis (28	TRUE	igd	0.0117	5.80E-06	6379	M32754.m	TRUE	reported	n0okFy	2	TRUE	0.003207	20.51369
5762	rs1802376	A	G	A	G	0.0875	-0.33022	0.0427	0.01931	FALSE	FALSE	FALSE	ieu-b-508	15	64428559	0.120799	486484	0.006264	Sepsis (28	Sepsis (28	Sepsis (28	TRUE	igd	0.0193	5.95E-06	972	M33508.m	TRUE	reported	UMXHcP	2	TRUE	0.020708	20.51195
2814	rs2130722	T	C	T	C	-0.0666	0.03717	0.0738	0.077968	FALSE	FALSE	FALSE	ieu-b-508	9	1.17E+08	0.062211	486484	0.550184	Sepsis (28	Sepsis (28	Sepsis (28	TRUE	igd	0.0147	6.28E-06	2759	M27447.m	TRUE	reported	s1NdyU	2	TRUE	0.007385	20.51157
1077	rs1049073	A	G	A	G	0.0077	-0.01118	0.8314	0.832629	FALSE	FALSE	FALSE	ieu-b-508	2	1.38E+08	0.04461	486484	0.802038	Sepsis (28	Sepsis (28	Sepsis (28	TRUE	igd	0.0017	7.94E-06	7371	M01604.m	TRUE	reported	hE3I2k	2	TRUE	0.002776	20.51
1083	rs1257914	T	C	T	C	-0.0077	-0.008	0.1887	0.185828	FALSE	FALSE	FALSE	ieu-b-508	12	26803493	0.043318	486484	0.853477	Sepsis (28	Sepsis (28	Sepsis (28	TRUE	igd	0.0017	5.92E-06	7371	M01604.m	TRUE	reported	hE3I2k	2	TRUE	0.002776	20.51
1084	rs1286087	T	C	T	C	0.0077	-0.03028	0.8367	0.846009	FALSE	FALSE	FALSE	ieu-b-508	14	91437370	0.04642	486484	0.514167	Sepsis (28	Sepsis (28	Sepsis (28	TRUE	igd	0.0017	8.08E-06	7371	M01604.m	TRUE	reported	hE3I2k	2	TRUE	0.002776	20.51
1624	rs1116275	A	G	A	G	-0.0077	-0.04994	0.5972	0.583273	FALSE	FALSE	FALSE	ieu-b-508	1	80060337	0.033866	486484	0.140335	Sepsis (28	Sepsis (28	Sepsis (28	TRUE	igd	0.0017	8.08E-06	7368	M15140.m	TRUE	reported	jEcqMJ	2	TRUE	0.002777	20.51
726	rs4317453	A	G	A	G	-0.0077	0.018871	0.3363	0.371074	FALSE	FALSE	FALSE	ieu-b-508	6	1424362	0.035998	486484	0.600113	Sepsis (28	Sepsis (28	Sepsis (28	TRUE	igd	0.0017	9.12E-06	7363	M01301.m	TRUE	reported	w8TL71	2	TRUE	0.002779	20.51
3606	rs1177928	A	T	A	T	0.0231	-0.02165	0.3845	0.352591	FALSE	TRUE	FALSE	ieu-b-508	8	1.27E+08	0.035045	486484	0.536676	Sepsis (28	Sepsis (28	Sepsis (28	TRUE	igd	0.0051	6.50E-06	7361	M32553.m	TRUE	reported	vHSHqn	2	TRUE	0.002779	20.51
696	rs7195332	T	C	T	C	0.0077	-0.03184	0.2152	0.20495	FALSE	FALSE	FALSE	ieu-b-508	16	19925963	0.041344	486484	0.44123	Sepsis (28	Sepsis (28	Sepsis (28	TRUE	igd	0.0017	4.05E-06	7358	M01299.m	TRUE	reported	gfM0a3	2	TRUE	0.00278	20.50999
3528	rs1558852	A	G	A	G	0.0154	-0.03852	0.7951	0.820191	FALSE	FALSE	FALSE	ieu-b-508	2	51288569	0.043618	486484	0.377204	Sepsis (28	Sepsis (28	Sepsis (28	TRUE	igd	0.0034	7.03E-06	7358	M32497.m	TRUE	reported	244bZN	2	TRUE	0.00278	20.50999
99	rs33980	C	G	C	G	-0.0077	0.021916	0.9071	0.901558	FALSE	TRUE	FALSE	ieu-b-508	5	1.42E+08	0.055983	486484	0.69545	Sepsis (28	Sepsis (28	Sepsis (28	TRUE	igd	0.0017	9.11E-06	7355	M00054.m	TRUE	reported	QTDAnh	2	TRUE	0.002782	20.50999
820	rs2280248	A	G	A	G	-0.0077	0.037698	0.4808	0.482707	FALSE	FALSE	FALSE	ieu-b-508	15	42433802	0.033453	486484	0.259795	Sepsis (28	Sepsis (28	Sepsis (28	TRUE	igd	0.0017	5.55E-06	7355	M01358.m	TRUE	reported	kSPQgd	2	TRUE	0.002782	20.50999
826	rs4507103	A	G	A	G	0.0077	-0.00553	0.397	0.39649	FALSE	FALSE	FALSE	ieu-b-508	2	2.15E+08	0.034143	486484	0.871426	Sepsis (28	Sepsis (28	Sepsis (28	TRUE	igd	0.0017	6.89E-06	7355	M01358.m	TRUE	reported	kSPQgd	2	TRUE	0.002782	20.50999
831	rs6883481	T	C	T	C	0.0077	-0.07842	0.4737	0.475358	FALSE	FALSE	FALSE	ieu-b-508	5	1.66E+08	0.03361	486484	0.01964	Sepsis (28	Sepsis (28	Sepsis (28	TRUE	igd	0.0017	6.17E-06	7355	M01358.m	TRUE	reported	kSPQgd	2	TRUE	0.002782	20.50999
2063	rs2203995	A	G	A	G	0.0077	0.033117	0.6055	0.612965	FALSE	FALSE	FALSE	ieu-b-508	12	77866800	0.034554	486484	0.337846	Sepsis (28	Sepsis (28	Sepsis (28	TRUE	igd	0.0017	7.26E-06	7354	M18369.m	TRUE	reported	ykBjIT	2	TRUE	0.002782	20.50999
2251	rs265487	A	G	A	G	-0.0077	0.0259	0.4929	0.497497	FALSE	FALSE	FALSE	ieu-b-508	10	83117262	0.033373	486484	0.437708	Sepsis (28	Sepsis (28	Sepsis (28	TRUE	igd	0.0017	7.56E-06	7354	M19934.m	TRUE	reported	HtcWcQ	2	TRUE	0.002782	20.50999
771	rs1019978	T	C	T	C	0.0077	-0.04132	0.6173	0.61969	FALSE	FALSE	FALSE	ieu-b-508	2	11092613	0.034337	486484	0.228817	Sepsis (28	Sepsis (28	Sepsis (28	TRUE	igd	0.0017	6.20E-06	7352	M01336.m	TRUE	reported	FAIrya	2	TRUE	0.002783	20.50999
736	rs1742639	A	G	A	G	0.0077	-0.02857	0.8865	0.885404	FALSE	FALSE	FALSE	ieu-b-508	14	46562320	0.052632	486484	0.587241	Sepsis (28	Sepsis (28	Sepsis (28	TRUE	igd	0.0017	7.94E-06	7347	M01302.m	TRUE	reported	sNp2IC	2	TRUE	0.002785	20.50999
744	rs6008767	C	G	C	G	-0.0077	0.026249	0.8182	0.808022	FALSE	TRUE	FALSE	ieu-b-508	22	46741974	0.042956	486484	0.541157	Sepsis (28	Sepsis (28	Sepsis (28	TRUE	igd	0.0017	4.12E-06	7347	M01302.m	TRUE	reported	sNp2IC	2	TRUE	0.002785	20.50999
746	rs6533609	T	C	T	C	-0.0077	0.034564	0.1429	0.149496	FALSE	FALSE	FALSE	ieu-b-508	4	1.13E+08	0.046723	486484	0.459445	Sepsis (28	Sepsis (28	Sepsis (28	TRUE	igd	0.0017	3.78E-06	7347	M01302.m	TRUE	reported	sNp2IC	2	TRUE	0.002785	20.50999
6253	rs2720162	T	G	T	G	-0.0154	0.017722	0.6076	0.597515	FALSE	FALSE	FALSE	ieu-b-508	2	2.32E+08	0.034079	486484	0.603052	Sepsis (28	Sepsis (28	Sepsis (28	TRUE	igd	0.0034	7.31E-06	7345	M33936.m	TRUE	reported	jwA4B2	2	TRUE	0.002785	20.50998
7698	rs1215466	T	C	T	C	0.0077	-0.00706	0.7624	0.742571	FALSE	FALSE	FALSE	ieu-b-508	7	96039063	0.038797	486484	0.855608	Sepsis (28	Sepsis (28	Sepsis (28	TRUE	igd	0.0017	4.65E-06	7345	M35397.m	TRUE	reported	Y56mZr	2	TRUE	0.002785	20.50998
7716	rs4917204	A	G	A	G	-0.0077	0.074171	0.7723	0.781879	FALSE	FALSE	FALSE	ieu-b-508	7	49195695	0.04035	486484	0.066037	Sepsis (28	Sepsis (28	Sepsis (28	TRUE	igd	0.0017	5.08E-06	7345	M35397.m	TRUE	reported	Y56mZr	2	TRUE	0.002785	20.50998
3229	rs9367164	A	G	A	G	0.0077	0.008157	0.6161	0.609244	FALSE	FALSE	FALSE	ieu-b-508	6	42765884	0.034214	486484	0.811559	Sepsis (28	Sepsis (28	Sepsis (28	TRUE	igd	0.0017	9.17E-06	7344	M32339.m	TRUE	reported	SVp504	2	TRUE	0.002786	20.50998
4449	rs4867926	T	G	T	G	0.0077	0.008416	0.3914	0.368723	FALSE	FALSE	FALSE	ieu-b-508	5	1.7E+08	0.034818	486484	0.80899	Sepsis (28	Sepsis (28	Sepsis (28	TRUE	igd	0.0017	8.29E-06	7344	M32910.m	TRUE	reported	2OCBKh	2	TRUE	0.002786	20.50998
3166	rs170734	A	G	A	G	-0.0154	0.00661	0.7263	0.723037	FALSE	FALSE	FALSE	ieu-b-508	5	98147041	0.037375	486484	0.859624	Sepsis (28	Sepsis (28	Sepsis (28	TRUE	igd	0.0034	6.79E-06	7340	M32328.m	TRUE	reported	YGsb6D	2	TRUE	0.002787	20.50998
1765	rs1222036	A	G	A	G	-0.0077	0.01168	0.4107	0.413799	FALSE	FALSE	FALSE	ieu-b-508	10	67234827	0.03393	486484	0.730675	Sepsis (28	Sepsis (28	Sepsis (28	TRUE	igd	0.0017	8.34E-06	7331	M15676.m	TRUE	reported	s1NfKN	2	TRUE	0.002791	20.50997
1781	rs7310929	T	C	T	C	-0.0077	-0.02561	0.6256	0.622411	FALSE	FALSE	FALSE	ieu-b-508	12	14593902	0.034399	486484	0.456584	Sepsis (28	Sepsis (28	Sepsis (28	TRUE	igd	0.0017	9.38E-06	7331	M15676.m	TRUE	reported	s1NfKN	2	TRUE	0.002791	20.50997
2311	rs2011920	C	G	C	G	-0.0154	0.000403	0.1375	0.151012	FALSE	TRUE	FALSE	ieu-b-508	10	71492635	0.046828	486484	0.993129	Sepsis (28	Sepsis (28	Sepsis (28	TRUE	igd	0.0034	5.13E-06	7327	M20699.m	TRUE	reported	Z3EKFa	2	TRUE	0.002792	20.50997

1285	rs1511589	A	G	A	G	0.0077	-0.02177	0.4559	0.447623	FALSE	FALSE	FALSE	ieu-b-508	12	84061431	0.033652	486484	0.517706	Sepsis (28	Sepsis (28	Sepsis (28	TRUE	igd	0.0017	7.55E-06	7325	M02132.m	TRUE	reported	uJFm2t	2	TRUE	0.002793	20.50997
1293	rs229084	A	G	A	G	-0.0077	0.028719	0.4727	0.457412	FALSE	FALSE	FALSE	ieu-b-508	21	28251329	0.033548	486484	0.391968	Sepsis (28	Sepsis (28	Sepsis (28	TRUE	igd	0.0017	6.57E-06	7325	M02132.m	TRUE	reported	uJFm2t	2	TRUE	0.002793	20.50997
1302	rs6510448	T	G	T	G	0.0077	-0.0192	0.6691	0.663202	FALSE	FALSE	FALSE	ieu-b-508	19	35240154	0.035366	486484	0.587228	Sepsis (28	Sepsis (28	Sepsis (28	TRUE	igd	0.0017	8.14E-06	7325	M02132.m	TRUE	reported	uJFm2t	2	TRUE	0.002793	20.50997
1307	rs758186	T	C	T	C	-0.0077	0.008471	0.6536	0.643687	FALSE	FALSE	FALSE	ieu-b-508	17	12500658	0.035215	486484	0.809912	Sepsis (28	Sepsis (28	Sepsis (28	TRUE	igd	0.0017	8.69E-06	7325	M02132.m	TRUE	reported	uJFm2t	2	TRUE	0.002793	20.50997
1310	rs779463	T	G	T	G	-0.0077	0.00372	0.3012	0.309315	FALSE	FALSE	FALSE	ieu-b-508	6	69942066	0.036067	486484	0.917853	Sepsis (28	Sepsis (28	Sepsis (28	TRUE	igd	0.0017	9.46E-06	7325	M02132.m	TRUE	reported	uJFm2t	2	TRUE	0.002793	20.50997
7136	rs6062634	A	C	A	C	-0.0154	0.00789	0.4048	0.419147	FALSE	FALSE	FALSE	ieu-b-508	20	62751812	0.034073	486484	0.816876	Sepsis (28	Sepsis (28	Sepsis (28	TRUE	igd	0.0034	7.16E-06	7322	M34527.m	TRUE	reported	EWFGP8	2	TRUE	0.002794	20.50997
4046	rs1271220	A	G	A	G	0.0077	-0.00142	0.4119	0.399996	FALSE	FALSE	FALSE	ieu-b-508	2	1.06E+08	0.034032	486484	0.96663	Sepsis (28	Sepsis (28	Sepsis (28	TRUE	igd	0.0017	7.69E-06	7318	M32740.m	TRUE	reported	YLshfP	2	TRUE	0.002796	20.50996
4053	rs1710109	C	G	C	G	-0.0077	-0.02978	0.3183	0.323643	FALSE	TRUE	FALSE	ieu-b-508	14	33898496	0.035751	486484	0.404826	Sepsis (28	Sepsis (28	Sepsis (28	TRUE	igd	0.0017	9.76E-06	7318	M32740.m	TRUE	reported	YLshfP	2	TRUE	0.002796	20.50996
4059	rs2147103	A	T	A	T	0.0077	0.064607	0.6044	0.58885	FALSE	TRUE	FALSE	ieu-b-508	14	54374023	0.033944	486484	0.056997	Sepsis (28	Sepsis (28	Sepsis (28	TRUE	igd	0.0017	8.51E-06	7318	M32740.m	TRUE	reported	YLshfP	2	TRUE	0.002796	20.50996
4081	rs8005168	T	C	T	C	0.0077	-0.08025	0.3852	0.396733	FALSE	FALSE	FALSE	ieu-b-508	14	23028364	0.034062	486484	0.018479	Sepsis (28	Sepsis (28	Sepsis (28	TRUE	igd	0.0017	9.31E-06	7318	M32740.m	TRUE	reported	YLshfP	2	TRUE	0.002796	20.50996
4085	rs9320187	A	G	A	G	-0.0154	-0.1441	0.065	0.062276	FALSE	FALSE	FALSE	ieu-b-508	6	1.07E+08	0.069077	486484	0.036975	Sepsis (28	Sepsis (28	Sepsis (28	TRUE	igd	0.0034	4.90E-06	7318	M32740.m	TRUE	reported	YLshfP	2	TRUE	0.002796	20.50996
1572	rs1093765	A	G	A	G	0.0077	0.078617	0.2676	0.270366	FALSE	FALSE	FALSE	ieu-b-508	4	5682811	0.03775	486484	0.03729	Sepsis (28	Sepsis (28	Sepsis (28	TRUE	igd	0.0017	3.50E-06	7314	M12593.m	TRUE	reported	IBXgSU	2	TRUE	0.002797	20.50996
1579	rs1556988	A	G	A	G	-0.0077	0.042042	0.7923	0.796392	FALSE	FALSE	FALSE	ieu-b-508	9	4049357	0.041682	486484	0.313157	Sepsis (28	Sepsis (28	Sepsis (28	TRUE	igd	0.0017	5.86E-06	7314	M12593.m	TRUE	reported	IBXgSU	2	TRUE	0.002797	20.50996
1586	rs6924754	A	G	A	G	0.0154	0.050052	0.0606	0.097323	FALSE	FALSE	FALSE	ieu-b-508	6	1.12E+08	0.057189	486484	0.381471	Sepsis (28	Sepsis (28	Sepsis (28	TRUE	igd	0.0034	6.43E-06	7314	M12593.m	TRUE	reported	IBXgSU	2	TRUE	0.002797	20.50996
5539	rs7901640	A	G	A	G	-0.0077	-0.06373	0.3935	0.394325	FALSE	FALSE	FALSE	ieu-b-508	10	18656953	0.034182	486484	0.062256	Sepsis (28	Sepsis (28	Sepsis (28	TRUE	igd	0.0017	9.06E-06	7306	M33422.m	TRUE	reported	PLgjrT	2	TRUE	0.0028	20.50995
1056	rs1009006	T	C	T	C	0.0077	-0.04226	0.8026	0.803588	FALSE	FALSE	FALSE	ieu-b-508	8	92896022	0.04207	486484	0.315121	Sepsis (28	Sepsis (28	Sepsis (28	TRUE	igd	0.0017	5.93E-06	7271	M01585.m	TRUE	reported	M7QiSM	2	TRUE	0.002814	20.50993
2775	rs1342039	A	G	A	G	0.0154	-0.076	0.0957	0.097678	FALSE	FALSE	FALSE	ieu-b-508	2	55058720	0.056456	486484	0.178241	Sepsis (28	Sepsis (28	Sepsis (28	TRUE	igd	0.0034	5.91E-06	7268	M27273.m	TRUE	reported	EvU2od	2	TRUE	0.002815	20.50993
2971	rs1317933	A	T	A	T	0.0154	0.056618	0.2062	0.307902	FALSE	TRUE	FALSE	ieu-b-508	5	27225776	0.036708	486484	0.122985	Sepsis (28	Sepsis (28	Sepsis (28	TRUE	igd	0.0034	4.85E-06	7257	M31453.m	TRUE	reported	vdTd9M	2	TRUE	0.002819	20.50992
2194	rs7680308	A	G	A	G	0.0077	0.022674	0.4854	0.484058	FALSE	FALSE	FALSE	ieu-b-508	4	46953347	0.033528	486484	0.49888	Sepsis (28	Sepsis (28	Sepsis (28	TRUE	igd	0.0017	9.08E-06	7182	M19362.m	TRUE	reported	SkNEKw	2	TRUE	0.002848	20.50986
1241	rs1334235	C	G	C	G	0.0077	-0.00704	0.3644	0.371597	FALSE	TRUE	FALSE	ieu-b-508	13	68013111	0.034485	486484	0.838333	Sepsis (28	Sepsis (28	Sepsis (28	TRUE	igd	0.0017	9.97E-06	7136	M01769.m	TRUE	reported	rismUZ	2	TRUE	0.002867	20.50982
1243	rs2058473	A	G	A	G	-0.0077	0.01218	0.6494	0.653926	FALSE	FALSE	FALSE	ieu-b-508	12	6093926	0.035207	486484	0.729382	Sepsis (28	Sepsis (28	Sepsis (28	TRUE	igd	0.0017	7.48E-06	7136	M01769.m	TRUE	reported	rismUZ	2	TRUE	0.002867	20.50982
1246	rs2272663	A	G	A	G	0.0077	0.014852	0.5321	0.529431	FALSE	FALSE	FALSE	ieu-b-508	8	1.46E+08	0.033403	486484	0.656588	Sepsis (28	Sepsis (28	Sepsis (28	TRUE	igd	0.0017	9.39E-06	7136	M01769.m	TRUE	reported	rismUZ	2	TRUE	0.002867	20.50982
1258	rs7562418	A	C	A	C	0.0077	0.000344	0.5075	0.512047	FALSE	FALSE	FALSE	ieu-b-508	2	7343159	0.033964	486484	0.991917	Sepsis (28	Sepsis (28	Sepsis (28	TRUE	igd	0.0017	6.81E-06	7136	M01769.m	TRUE	reported	rismUZ	2	TRUE	0.002867	20.50982
1266	rs940511	A	G	A	G	-0.0077	0.029162	0.4652	0.456281	FALSE	FALSE	FALSE	ieu-b-508	6	82346606	0.033694	486484	0.386776	Sepsis (28	Sepsis (28	Sepsis (28	TRUE	igd	0.0017	6.71E-06	7136	M01769.m	TRUE	reported	rismUZ	2	TRUE	0.002867	20.50982
5350	rs28157	T	G	T	G	-0.0077	-0.00809	0.3075	0.315162	FALSE	FALSE	FALSE	ieu-b-508	5	1.03E+08	0.035978	486484	0.822163	Sepsis (28	Sepsis (28	Sepsis (28	TRUE	igd	0.0017	6.68E-06	7118	M33380.m	TRUE	reported	OHRfl5	2	TRUE	0.002874	20.50981
5365	rs835800	T	C	T	C	0.0077	-0.01092	0.2665	0.266846	FALSE	FALSE	FALSE	ieu-b-508	11	44872419	0.037876	486484	0.773054	Sepsis (28	Sepsis (28	Sepsis (28	TRUE	igd	0.0017	9.61E-06	7118	M33380.m	TRUE	reported	OHRfl5	2	TRUE	0.002874	20.50981
5368	rs941980	T	C	T	C	0.0077	-0.00389	0.5798	0.582025	FALSE	FALSE	FALSE	ieu-b-508	1	90270846	0.033869	486484	0.908466	Sepsis (28	Sepsis (28	Sepsis (28	TRUE	igd	0.0017	4.23E-06	7118	M33380.m	TRUE	reported	OHRfl5	2	TRUE	0.002874	20.50981
8254	rs1171158	A	G	A	G	-0.0077	-0.03192	0.4815	0.48126	FALSE	FALSE	FALSE	ieu-b-508	3	1.11E+08	0.033564	486484	0.341617	Sepsis (28	Sepsis (28	Sepsis (28	TRUE	igd	0.0017	5.19E-06	7092	M36552.m	TRUE	reported	5CxMJz	2	TRUE	0.002884	20.50979
8256	rs1279831	A	G	A	G	0.0077	-0.00075	0.5589	0.566797	FALSE	FALSE	FALSE	ieu-b-508	3	1.36E+08	0.033648	486484	0.982227	Sepsis (28	Sepsis (28	Sepsis (28	TRUE	igd	0.0017	4.74E-06	7092	M36552.m	TRUE	reported	5CxMJz	2	TRUE	0.002884	20.50979
1532	rs7279224	A	T	A	T	0.0077	0.044965	0.6952	0.634954	FALSE	TRUE	FALSE	ieu-b-508	21	41095241	0.034821	486484	0.196593	Sepsis (28	Sepsis (28	Sepsis (28	TRUE	igd	0.0017	6.87E-06	7081	M12067.m	TRUE	reported	O5x7RD	2	TRUE	0.002889	20.50978
1374	rs1119917	A	C	A	C	0.0077	-0.0258	0.5221	0.535532	FALSE	FALSE	FALSE	ieu-b-508	10	1.22E+08	0.03346	486484	0.440734	Sepsis (28	Sepsis (28	Sepsis (28	TRUE	igd	0.0017	9.33E-06	7037	M02734.m	TRUE	reported	0y0dDD	2	TRUE	0.002907	20.50974
1379	rs1254604	T	C	T	C	0.0077	0.001113	0.4197	0.43722	FALSE	FALSE	FALSE	ieu-b-508	8	1.46E+08	0.033622	486484	0.973596	Sepsis (28	Sepsis (28	Sepsis (28	TRUE	igd	0.0017	8.35E-06	7037	M02734.m	TRUE	reported	0y0dDD	2	TRUE	0.002907	20.50974
1394	rs353620	A	G	A	G	-0.0077	0.045906	0.6613	0.651501	FALSE	FALSE	FALSE	ieu-b-508	11	35174136	0.034925	486484	0.188709	Sepsis (28	Sepsis (28	Sepsis (28	TRUE	igd	0.0017	9.80E-06	7037	M02734.m	TRUE	reported	0y0dDD	2	TRUE	0.002907	20.50974
8412	rs1756740	A	G	A	G	0.0077	-0.01587	0.2327	0.26747	FALSE	FALSE	FALSE	ieu-b-508	10	30204072	0.038062	486484	0.676711	Sepsis (28	Sepsis (28	Sepsis (28	TRUE	igd	0.0017	9.16E-06	6940	M36808.m	TRUE	reported	u7lqaj	2	TRUE	0.002947	20.50966
7545	rs8008572	T	C	T	C	-0.0154	0.042954	0.7565	0.752066	FALSE	FALSE	FALSE	ieu-b-508	14	76351840	0.038698	486484	0.267004	Sepsis (28	Sepsis (28	Sepsis (28	TRUE	igd	0.0034	6.69E-06	6538	M35257.m	TRUE	reported	iphouU	2	TRUE	0.003128	20.5093
7647	rs2460405	A	G	A	G	-0.0077	0.007475	0.36	0.358425	FALSE	FALSE	FALSE	ieu-b-508	2	7149509	0.034785	486484	0.829845	Sepsis (28	Sepsis (28	Sepsis (28	TRUE	igd	0.0017	8.87E-06	5768	M35326.m	TRUE	reported	jRytyJ	2	TRUE	0.003544	20.50846
1920	rs2473097	T	C	T	C	0.0154	0.021159	0.7894	0.782128	FALSE	FALSE	FALSE	ieu-b-508	6	1.38E+08	0.040361	486484	0.600113	Sepsis (28	Sepsis (28	Sepsis (28	TRUE	igd	0.0034	5.90E-06	4689	M16816.m	TRUE	reported	J1abbO	2	TRUE	0.004356	20.50682
8154	rs463106	T	C	T	C	0.0367	0.034232	0.6825	0.531414	FALSE	FALSE	FALSE	ieu-b-508	5	1.23E+08	0.033614	486484	0.308506	Sepsis (28	Sepsis (28	Sepsis (28	TRUE	igd	0.0081	6.29E-06	1731	M36115.m	TRUE	reported	ZV1jWp	2	TRUE	0.01172	20.50501
5128	rs2697339	T	C	T	C	0.0462	-0.01704	0.7684	0.767397	FALSE	FALSE	FALSE	ieu-b-508	2	1.51E+08	0.039411	486484	0.665531	Sepsis (28	Sepsis (28	Sepsis (28	TRUE	igd	0.0102	6.39E-06	3740	M33188.m	TRUE	reported	xmgbws	2	TRUE	0.005456	20.5046
6153	rs953628	T	G	T	G	-0.0607	-0.00811	0.2013	0.203779	FALSE	FALSE	FALSE	ieu-b-508	3	98838914	0.041397	486484	0.84477	Sepsis (28	Sepsis (28	Sepsis (28	TRUE	igd	0.0134	5.74E-06	2725	M33883.m	TRUE	reported	RT7YNu	2	TRUE	0.007474	20.50449
5954	rs8050423	A	G	A	G	0.1772	0.055475	0.0297	0.035422	FALSE	FALSE	FALSE	ieu-b-508	16	79188279	0.090747	486484	0.540995	Sepsis (28	Sepsis (28	Sepsis (28	TRUE	igd	0.0391	5.72E-06	1192	M33652.m	TRUE	reported	vYkdTv	2	TRUE	0.016939	20.50428
5220	rs9521845	A	G	A	G	0.0154	-0.00242	0.3291	0.292656	FALSE	FALSE	FALSE	ieu-b-508	13	1.11E+08	0.036619	486484	0.947339	Sepsis (28	Sepsis (28	Sepsis (28	TRUE	igd	0.0034	7.19E-06	2683	M33204.m	TRUE	reported	ROvPWK	2	TRUE	0.007588	20.50028
8137	rs1358498	T	C	T	C	-0.024	0.027353	0.5824	0.580994	FALSE	FALSE	FALSE	ieu-b-508	7	1.21E+08	0.033784	486484	0.418143	Sepsis (28	Sepsis (28	Sepsis (28	TRUE	igd	0.0053	5.01E-06	7311	M36103.m	TRUE	reported	sjeaPC	2	TRUE	0.002797	20.49991
2087	rs2564483	T	G	T	G	0.024	-0.05077	0.6043	0.614624	FALSE	FALSE	FALSE	ieu-b-508	18	57208826	0.034353	486484	0.139425	Sepsis (28	Sepsis (28	Sepsis (28	TRUE	igd	0.0053	6.42E-06	6970	M18394.m	TRUE	reported	Lt9awu	2	TRUE	0.002933	20.49963
7458	rs1339098	T	C	T	C	0.024	-0.00474	0.9232	0.921055	FALSE	FALSE	FALSE	ieu-b-508	2	19072345	0.061958	486484	0.939029	Sepsis (28	Sepsis (28	Sepsis (28	TRUE	igd	0.0053	6.56E-06	6543	M35193.m	TRUE	reported	BMMWXU	2	TRUE	0.003124	20.49925
7590	rs1041584	T	C	T	C	-0.024	-0.01446	0.1248	0.120627	FALSE	FALSE	FALSE	ieu-b-508	19	19505087	0.051184	486484	0.777567	Sepsis (28	Sepsis (28	Sepsis (28	TRUE	igd	0.0053	5.79E-06	5979	M35305.m	TRUE	reported	6ybfdq	2	TRUE	0.003418	20.49866
4530	rs6534104	C	G	C	G	-0.1087	0.014826	0.0373	0.046072	FALSE	TRUE	FALSE	ieu-b-508	4	79915309	0.079937	486484	0.85286	Sepsis (28	Sepsis (28	Sepsis (28	TRUE	igd	0.024	6.15E-06	2391	M33137.m	TRUE	reported	ONVNk3	2	TRUE	0.008506	20.49619
3796	rs7210933	A	T	A	T	0.0163	-0.01903	0.7569	0.739625	FALSE	TRUE	FALSE	ieu-b-508	17	46812838	0.037986	486484	0.616372	Sepsis (28	Sepsis (28	Sepsis (28	TRUE	igd	0.0036	8.17E-06	7369	M32635.m	TRUE	reported	jJI9P8	2	TRUE	0.002774	20.49521
8547	rs1084749	A	G	A	G	0.0326	-0.06781	0.7519	0.734074	FALSE	FALSE	FALSE	ieu-b-508	12	1.28E+08	0.037905	486484	0.073621	Sepsis (28	Sepsis (28	Sepsis (28	TRUE	igd	0.0072	5.89E-06	7355	M37202.m	TRUE	reported	BtsIDx	2	TRUE	0.00278	20.4952
6979	rs4505564	T	C	T	C	0.0163	-0.002	0.7913	0.790715	FALSE	FALSE	FALSE	ieu-b-508	2	2.41E+08	0.041229	486484	0.961356	Sepsis (28	Sepsis (28	Sepsis (28	TRUE	igd	0.0036	4.94E-06	7344	M34407.m	TRUE	reported	X7I83Y	2	TRUE	0.002784	20.49519
2602	rs1087792	A	G	A	G	-0.0163	-0.15868	0.0869	0.08926	FALSE	FALSE	FALSE	ieu-b-508	12	63240672	0.058347	486484	0.006536	Sepsis (28	Sepsis (28	Sepsis (28	TRUE	igd	0.0036	8.25E-06	7329	M22481.m	TRUE	reported	bXpf0k	2	TRUE	0.002789	20.49518
7851	rs1075972	A	G	A	G	0.0163	-0.0144	0.4548	0.451195	FALSE	FALSE	FALSE	ieu-b-508	9	1.17E+08	0.033835	486484	0.670511	Sepsis (28	Sepsis (28	Sepsis (28	TRUE	igd	0.0036	5.79E-06	7302	M35628.m	TRUE	reported	qFJY9w	2	TRUE	0.0028	20.49516
7852	rs1291875	A	G	A	G	0.0163	-0.03282	0.5774	0.587881	FALSE	FALSE	FALSE	ieu-b-508	16	84967089	0.033944	486484	0.333616	Sepsis (28	Sepsis (28	Sepsis (28	TRUE	igd	0.0036	5.89E-06	7302	M35628.m	TRUE	reported	qFJY9w	2	TRUE	0.0028	20.49516
2855	rs6997473	A	G	A	G	-0.0729	0.060396	0.9538	0.954241	FALSE	FALSE	FALSE	ieu-b-508	8	59406223	0.079609	486484	0.44806	Sepsis (28	Sepsis (28	Sepsis (28	TRUE	igd	0.0161	6.29E-06	5673	M27531.m	TRUE	reported	v01zDr	2	TRUE	0.003601	20.49511
8643	rs6779957	A	G	A	G	0.0163	-0.01755	0.6456	0.643163	FALSE	FALSE	FALSE	ieu-b-508	3	65433434	0.034901	486484	0.615048	Sepsis (28	Sepsis (28	Sepsis (28	TRUE	igd	0.0036	7.86E-06	7219	M38178.m	TRUE	reported	GgCXMN	2	TRUE	0.002832	20.49509
7074	rs1173242	T	C	T	C	-0.0163	0.009553	0.7205	0.712613	FALSE	FALSE	FALSE	ieu-b-508	5	4794725	0.036906	486484	0.795752	Sepsis (28	Sepsis (28	Sepsis (28	TRUE	igd	0.0036	5.11E-06	7119	M34481.m	TRUE	reported	mXdL0W	2	TRUE	0.002871	20.49501
7753	rs9405175	A	G	A	G	-0.024	0.033394	0.178	0.191676	FALSE	FALSE	FALSE	ieu-b-508	6	2584919	0.042773	486484	0.434965	Sepsis (28	Sepsis (28	Sepsis (28	TRUE	igd	0.0053	5.83E-06	3784	M35433.m	TRUE	reported	XlYkLe	2	TRUE	0.00539	20.49468
2419	rs1759546	A	G	A	G	0.0163	0.001933	0.864	0.864712	FALSE	FALSE	FALSE	ieu-b-508	4	1.2E+08	0.048747	486484	0.968373	Sepsis (28	Sepsis (28	Sepsis (28	TRUE	igd	0.0036	5.89E-06	6556	M21188.m	TRUE	reported	kvdnjc	2	TRUE	0.003117	20.49452
6473	rs1296969	A	T	A	T	0.447	-0.00612	0.984	0.970567	FALSE	TRUE	FALSE	ieu-b-508	18	19782500	0.09951	486484	0.950969	Sepsis (28	Sepsis (28	Sepsis (28	TRUE	igd	0.098	5.09E-06	134	M34109.m	TRUE	reported	piJuC7	2	TRUE	0.134394	20.49425
7264	rs2072583	A	G	A	G	0.0163	0.013138	0.185	0.243262	FALSE	FALSE	FALSE	ieu-b-508	3	8775457	0.038873	486484	0.735379	Sepsis (28	Sepsis (28	Sepsis (28	TRUE	igd	0.0036	6.42E-06	5885	M34912.m	TRUE	reported	W8lcT6	2	TRUE	0.003471	20.4938
4205	rs6850094	T	C	T	C	-0.0394	-0.0062	0.4631	0.449477	FALSE	FALSE	FALSE	ieu-b-508	4	1.86E+08	0.033589	486484	0.853463	Sepsis (28	Sepsis (28	Sepsis (28	TRUE	igd	0.0087	6.35E-06	2537	M32762.m	TRUE	reported	Q5lKLH	2	TRUE	0.008019	20.49328
6490	rs7912030	A	G	A	G	0.821	0.166775	0.979	0.97493	FALSE	FALSE	FALSE	ieu-b-508	10	58797872	0.10938	486484	0.127325	Sepsis (28	Sepsis (28	Sepsis (28	TRUE	igd	0.18	5.09E-06	134	M34109.m	TRUE	reported	piJuC7	2	TRUE	0.134388	20.49323
5329	rs6902872	T	C	T	C	0.0163	0.065184	0.6467	0.643461	FALSE	FALSE	FALSE	ieu-b-508	6	1.35E+08	0.034959	486484	0.062236	Sepsis (28	Sepsis (28	Sepsis (28	TRUE	igd	0.0036	4.89E-06	3890	M33364.m	TRUE	reported	9Zrr7Y	2	TRUE	0.005242	20.49023
6935	rs1700439	A	C	A	C	0.111	-0.112	0.0314	0.024077	FALSE	FALSE	FALSE	ieu-b-508	21	24887373	0.109825	486484	0.307839	Sepsis (28	Sepsis (28	Sepsis (28	TRUE	igd	0.0245	5.73E-06	1104	M34395.m	TRUE	reported	QVOPA3	2	TRUE	0.018253	20.48926
8071	rs7619989	C	G	C	G	-0.0308	-0.02979	0.602	0.621309	FALSE	TRUE	FALSE	ieu-b-508	3	1.87E+08	0.034411	486484	0.386719	Sepsis (28	Sepsis (28	Sepsis (28	TRUE	igd	0.0068	5.82E-06	1517	M35978.m	TRUE	reported	CN0wRR	2	TRUE	0.013343	20.48852
4679	rs1076600	A	G	A	G	0.1241	0.001906	0.42	0.440811	FALSE	FALSE	FALSE	ieu-b-508	11	12902966	0.03369	486484	0.954879	Sepsis (28	Sepsis (28	Sepsis (28	TRUE	igd	0.0263	2.28E-06	25	M33178.m	TRUE	reported	g22WMS	2	TRUE	0.471073	20.48424
4558	rs1014885	T	G	T	G	-0.0421	-0.02504	0.7171	0.695143	FALSE	FALSE	FALSE	ieu-b-508	14	33176678	0.036569	486484	0.493428	Sepsis (28	Sepsis (28	Sepsis (28	TRUE	igd	0.0093	6.13E-06	4817	M33144.m	TRUE	reported	cgXZoM	2	TRUE	0.004236	20.48415
7834	rs9417900	T	C	T	C	0.0385	-0.06547	0.2502	0.25805	FALSE	FALSE	FALSE	ieu-b-508	10	19444182	0.038675	486484	0.0905	Sepsis (28	Sepsis (28	Sepsis (28	TRUE	igd	0.0085	6.25E-06	1291	M35527.m	TRUE	reported	UAMhnu	2	TRUE	0.015643	20.48379
4145	rs232668	A	G	A	G	-0.1354	0.051687	0.0207	0.024462	FALSE	FALSE	FALSE	ieu-b-508	20	7087736	0.10925	486484	0.636139	Sepsis (28	Sepsis (28	Sepsis (28	TRUE	igd	0.0299	5.97E-06	1761	M32757.m	TRUE	reported	TJV9Xb	2	TRUE	0.011511	20.48337
2319	rs6128265	T	C	T	C	0.0797	0.03251	0.0155	0.0121	FALSE	FALSE	FALSE	ieu-b-508	20	56645434	0.153131	486484	0.831873	Sepsis (28	Sepsis (28	Sepsis (28	TRUE	igd	0.0176	6.01E-06	1748	M20699.m	TRUE	reported	Z3EKFa	2	TRUE	0.011595	20.48303
3074	rs7776941	T	C	T	C	0.043	-0.04617	0.8002	0.789222	FALSE	FALSE	FALSE	ieu-b-508	7	26519717	0.04095	486484	0.259549	Sepsis (28	Sepsis (28	Sepsis (28	TRUE	igd	0.0095	6.33E-06	7363	M31787.m	TRUE	reported	XMp8YI	2	TRUE	0.002775	20.48197
7602	rs623248	A	G	A	G	0.0258	-0.1051	0.8882	0.885061	FALSE	FALSE	FALSE	ieu-b-508	18	60152275	0.052315	486484	0.044536	Sepsis (28	Sepsis (28	Sepsis (28	TRUE	igd	0.0057	7.08E-06	7359	M35320.m	TRUE	reported	ijCk2X	2	TRUE	0.002776	20.48197
3148	rs1090691	A	C	A	C	0.0172	0.008848	0.7034	0.685649	FALSE	FALSE	FALSE	ieu-b-508	10	15549612	0.036019	486484	0.805948	Sepsis (28	Sepsis (28	Sepsis (28	TRUE	igd	0.0038	4.81E-06	7356	M32319.m	TRUE	reported	9YFmUx	2	TRUE	0.002777	20.48196
6326	rs6777718	T	G	T	G	-0.0086	0.011145	0.2783	0.278888	FALSE	FALSE	FALSE	ieu-b-508	3	1.25E+08	0.037985	486484	0.769221	Sepsis (28	Sepsis (28	Sepsis (28	TRUE	igd	0.0019	3.51E-06	7354	M33955.m	TRUE	reported	mhHFnT	2	TRUE	0.002778	20.48196
6394	rs4288115	A	T	A	T	-0.0172	-0.02177	0.3119	0.357478	FALSE	TRUE	FALSE	ieu-b-508	5	17249656	0.035185	486484	0.536069	Sepsis (28	Sepsis (28	Sepsis (28	TRUE	igd	0.0038	7.20E-06	7349	M33971.m	TRUE	reported	dHZ6ha	2	TRUE	0.00278	20.48196
7911	rs1198896	T	C	T	C	-0.0086	-0.0264	0.7043	0.708574	FALSE	FALSE	FALSE	ieu-b-508	8	30178879	0.036802	486484	0.473161	Sepsis (28	Sepsis (28	Sepsis (28	TRUE	igd	0.0019	4.15E-06	7348	M35675.m	TRUE	reported	D7fdyZ	2	TRUE	0.00278	20.48196
7922	rs1699968	C	G	C	G	-0.0344	-0.27119	0.9821	0.976675	FALSE	TRUE	FALSE	ieu-b-508	22	49065908	0.111027	486484	0.014585	Sepsis (28	Sepsis (28	Sepsis (28	TRUE	igd	0.0076	6.03E-06	7348	M35675.m	TRUE	reported	D7fdyZ	2	TRUE	0.00278	20.48196
1692	rs1493749	A	G	A	G	0.0086	0.003533	0.17	0.172451	FALSE	FALSE	FALSE	ieu-b-508	12	44854404	0.046009	486484	0.938798	Sepsis (28	Sepsis (28	Sepsis (28	TRUE	igd	0.0019	3.73E-06	7345	M15488.m	TRUE	reported	SPriBO	2	TRUE	0.002782	20.48196
7725	rs7812354	C	G	C	G	0.0172	-0.04469	0.9508	0.935642	FALSE	TRUE	FALSE	ieu-b-508	8	5995366	0.06826	486484	0.5127	Sepsis (28	Sepsis (28	Sepsis (28	TRUE	igd	0.0038	6.29E-06	7345	M35397.m	TRUE	reported	Y56mZr	2	TRUE	0.002782	20.48196
1309	rs7615965	T	C	T	C	0.0086	0.02893	0.7121	0.703756	FALSE	FALSE	FALSE	ieu-b-508	3	1.71E+08	0.036451	486484	0.427383	Sepsis (28	Sepsis (28	Sepsis (28	TRUE	igd	0.0019	4.15E-06	7325	M02132.m	TRUE	reported	uJFm2t	2	TRUE	0.002789	20.48194
5406	rs342170	T	C	T	C	-0.0086	-0.06464	0.5871	0.600896	FALSE	FALSE	FALSE	ieu-b-508	12	63113828	0.035044	486484	0.065107	Sepsis (28	Sepsis (28	Sepsis (28	TRUE	igd	0.0019	4.82E-06	7315	M33389.m	TRUE	reported	Hf6XVM	2	TRUE	0.002793	20.48193
5411	rs433110	A	T	A	T	0.0086	0.052712	0.2962	0.304486	FALSE	TRUE	FALSE	ieu-b-508	5	2596368	0.036251	486484	0.145931	Sepsis (28	Sepsis (28	Sepsis (28	TRUE	igd	0.0019	3.52E-06	7315	M33389.m	TRUE	reported	Hf6XVM	2	TRUE	0.002793	20.48193
5421	rs709947	C	G	C	G	0.0086	-0.05008	0.6413	0.649552	FALSE	TRUE	FALSE	ieu-b-508	14	57270155	0.035445	486484	0.157659	Sepsis (28	Sepsis (28	Sepsis (28	TRUE	igd	0.0019	3.41E-06	7315	M33389.m	TRUE	reported	Hf6XVM	2	TRUE	0.002793	20.48193
1827	rs1051564	A	G	A	G	-0.0086	0.024536	0.2493	0.25092	FALSE	FALSE	FALSE	ieu-b-508	5	1.5E+08	0.038955	486484	0.528796	Sepsis (28	Sepsis (28	Sepsis (28	TRUE	igd	0.0019	4.37E-06	7308	M15778.m	TRUE	reported	941o3l	2	TRUE	0.002796	20.48193
1852	rs4672488	A	G	A	G	0.0086	-0.02553	0.2854	0.318654	FALSE	FALSE	FALSE	ieu-b-508	2	62500181	0.035995	486484	0.478125	Sepsis (28	Sepsis (28	Sepsis (28	TRUE	igd	0.0019	4.08E-06	7308	M15778.m	TRUE	reported	941o3l	2	TRUE	0.002796	20.48193
1854	rs4850051	A	G	A	G	-0.0086	-0.07509	0.2082	0.209455	FALSE	FALSE	FALSE	ieu-b-508	2	4141080	0.041354	486484	0.069407	Sepsis (28	Sepsis (28	Sepsis (28	TRUE	igd	0.0019	3.84E-06	7308	M15778.m	TRUE	reported	941o3l	2	TRUE	0.002796	20.48193
7558	rs1318832	T	C	T	C	-0.0086	-0.03868	0.1925	0.193549	FALSE	FALSE	FALSE	ieu-b-508	5	1.12E+08	0.042218	486484	0.359609	Sepsis (28	Sepsis (28	Sepsis (28	TRUE	igd	0.0019	3.54E-06	7217	M35270.m	TRUE	reported	Ct8V1n	2	TRUE	0.002831	20.48186
7569	rs26764	C	G	C	G	0.0086	-0.01092	0.7599	0.742214	FALSE	TRUE	FALSE	ieu-b-508	16	23711925	0.03803	486484	0.77392	Sepsis (28	Sepsis (28	Sepsis (28	TRUE	igd	0.0019	3.71E-06	7217	M35270.m	TRUE	reported	Ct8V1n	2	TRUE	0.002831	20.48186
5819	rs402710	T	C	T	C	0.0086	-0.00844	0.3378	0.345439	FALSE	FALSE	FALSE	ieu-b-508	5	1320722	0.035067	486484	0.809731	Sepsis (28	Sepsis (28	Sepsis (28	TRUE	igd	0.0019	4.16E-06	7066	M33515.m	TRUE	reported	X9mul1	2	TRUE	0.002891	20.48174
2952	rs1189338	T	C	T	C	0.0172	0.067753	0.9023	0.90592	FALSE	FALSE	FALSE	ieu-b-508	2	1.14E+08	0.057277	486484	0.236845	Sepsis (28	Sepsis (28	Sepsis (28	TRUE	igd	0.0038	6.62E-06	6795	M30805.m	TRUE	reported	YHV70c	2	TRUE	0.003006	20.4815
8003	rs1869578	A	G	A	G	-0.0086	-0.01038	0.5791	0.571093	FALSE	FALSE	FALSE	ieu-b-508	8	14909909	0.033887	486484	0.75932	Sepsis (28	Sepsis (28	Sepsis (28	TRUE	igd	0.0019	7.61E-06	6604	M35754.m	TRUE	reported	IlXOo1	2	TRUE	0.003093	20.48133
8030	rs939358	T	C	T	C	-0.0086	-0.01499	0.4477	0.433329	FALSE	FALSE	FALSE	ieu-b-508	17	41666423	0.033655	486484	0.656109	Sepsis (28	Sepsis (28	Sepsis (28	TRUE	igd	0.0019	8.16E-06	6604	M35754.m	TRUE	reported	IlXOo1	2	TRUE	0.003093	20.48133
8475	rs242725	A	G	A	G	-0.0086	-3.99E-05	0.358	0.361342	FALSE	FALSE	FALSE	ieu-b-508	3	10285868	0.034784	486484	0.999086	Sepsis (28	Sepsis (28	Sepsis (28	TRUE	igd	0.0019	3.51E-06	6552	M37058.m	TRUE	reported	32p8qV	2	TRUE	0.003117	20.48128
2543	rs1339555	T	C	T	C	0.0086	-0.05779	0.4714	0.449646	FALSE	FALSE	FALSE	ieu-b-508	2	85976236	0.033716	486484	0.086507	Sepsis (28	Sepsis (28	Sepsis (28	TRUE	igd	0.0019	8.25E-06	6535	M22177.m	TRUE	reported	2s6Sly	2	TRUE	0.003125	20.48126
2584	rs7776725	T	C	T	C	-0.0086	0.04641	0.7339	0.719829	FALSE	FALSE	FALSE	ieu-b-508	7	1.21E+08	0.037163	486484	0.211727	Sepsis (28	Sepsis (28	Sepsis (28	TRUE	igd	0.0019	9.27E-06	6535	M22177.m	TRUE	reported	2s6Sly	2	TRUE	0.003125	20.48126
3988	rs174892	T	G	T	G	0.0086	-0.00968	0.2779	0.280086	FALSE	FALSE	FALSE	ieu-b-508	1	1.13E+08	0.03716	486484	0.794485	Sepsis (28	Sepsis (28	Sepsis (28	TRUE	igd	0.0019	6.63E-06	6497	M32729.m	TRUE	reported	XGRSN1	2	TRUE	0.003143	20.48123
3990	rs1864658	T	C	T	C	0.0086	0.084149	0.6948	0.696297	FALSE	FALSE	FALSE	ieu-b-508	11	14256444	0.036264	486484	0.020315	Sepsis (28	Sepsis (28	Sepsis (28	TRUE	igd	0.0019	7.67E-06	6497	M32729.m	TRUE	reported	XGRSN1	2	TRUE	0.003143	20.48123
3999	rs2934554	T	C	T	C	0.0086	-0.01558	0.4337	0.452413	FALSE	FALSE	FALSE	ieu-b-508	5	2960058	0.033634	486484	0.64325	Sepsis (28	Sepsis (28	Sepsis (28	TRUE	igd	0.0019	4.53E-06	6497	M32729.m	TRUE	reported	XGRSN1	2	TRUE	0.003143	20.48123
4003	rs622997	T	C	T	C	-0.0086	-0.00337	0.4844	0.486738	FALSE	FALSE	FALSE	ieu-b-508	2	2.35E+08	0.03367	486484	0.920317	Sepsis (28	Sepsis (28	Sepsis (28	TRUE	igd	0.0019	4.28E-06	6497	M32729.m	TRUE	reported	XGRSN1	2	TRUE	0.003143	20.48123
7239	rs1019437	A	C	A	C	-0.0086	-0.01466	0.3347	0.34032	FALSE	FALSE	FALSE	ieu-b-508	2	1.28E+08	0.035448	486484	0.679227	Sepsis (28	Sepsis (28	Sepsis (28	TRUE	igd	0.0019	6.48E-06	5885	M34912.m	TRUE	reported	W8lcT6	2	TRUE	0.003469	20.48057
7252	rs1302363	T	G	T	G	0.0086	0.062511	0.4468	0.446416	FALSE	FALSE	FALSE	ieu-b-508	2	1.17E+08	0.033599	486484	0.062815	Sepsis (28	Sepsis (28	Sepsis (28	TRUE	igd	0.0019	6.95E-06	5885	M34912.m	TRUE	reported	W8lcT6	2	TRUE	0.003469	20.48057
7263	rs202093	T	C	T	C	0.0086	0.037998	0.3918	0.390831	FALSE	FALSE	FALSE	ieu-b-508	13	30814918	0.034263	486484	0.267417	Sepsis (28	Sepsis (28	Sepsis (28	TRUE	igd	0.0019	4.99E-06	5885	M34912.m	TRUE	reported	W8lcT6	2	TRUE	0.003469	20.48057
7267	rs4244568	T	G	T	G	-0.0086	0.065858	0.6271	0.611932	FALSE	FALSE	FALSE	ieu-b-508	15	39319915	0.034697	486484	0.057682	Sepsis (28	Sepsis (28	Sepsis (28	TRUE	igd	0.0019	5.41E-06	5885	M34912.m	TRUE	reported	W8lcT6	2	TRUE	0.003469	20.48057
6144	rs1226319	A	G	A	G	-0.0584	0.000572	0.7306	0.721089	FALSE	FALSE	FALSE	ieu-b-508	10	73641906	0.037254	486484	0.987744	Sepsis (28	Sepsis (28	Sepsis (28	TRUE	igd	0.0129	6.34E-06	2725	M33883.m	TRUE	reported	RT7YNu	2	TRUE	0.007465	20.47988
7361	rs4142154	A	G	A	G	0.0086	-0.02448	0.3524	0.335959	FALSE	FALSE	FALSE	ieu-b-508	2	2.12E+08	0.035393	486484	0.489158	Sepsis (28	Sepsis (28	Sepsis (28	TRUE	igd	0.0019	7.38E-06	4900	M35137.m	TRUE	reported	Eq2siS	2	TRUE	0.004164	20.47917
7371	rs6564173	A	G	A	G	0.0086	0.045123	0.3642	0.35354	FALSE	FALSE	FALSE	ieu-b-508	16	74861095	0.03511	486484	0.19873	Sepsis (28	Sepsis (28	Sepsis (28	TRUE	igd	0.0019	5.40E-06	4900	M35137.m	TRUE	reported	Eq2siS	2	TRUE	0.004164	20.47917
5592	rs8013127	A	G	A	G	-1.5304	-0.14691	0.0184	0.009253	FALSE	FALSE	FALSE	ieu-b-508	14	56434779	0.180975	486484	0.416923	Sepsis (28	Sepsis (28	Sepsis (28	TRUE	igd	0.3325	4.16E-06	60	M33423.m	TRUE	reported	MoEnO7	2	TRUE	0.260946	20.47875
6265	rs1195382	T	C	T	C	0.0326	0.052092	0.3065	0.298629	FALSE	FALSE	FALSE	ieu-b-508	5	1.07E+08	0.036624	486484	0.154926	Sepsis (28	Sepsis (28	Sepsis (28	TRUE	igd	0.0072	6.54E-06	1759	M33937.m	TRUE	reported	QanGCI	2	TRUE	0.011521	20.47746
5029	rs7015641	A	G	A	G	-0.3444	0.214825	0.0227	0.01985	FALSE	FALSE	FALSE	ieu-b-508	8	1.06E+08	0.120827	486484	0.075412	Sepsis (28	Sepsis (28	Sepsis (28	TRUE	igd	0.073	2.38E-06	25	M33178.m	TRUE	reported	g22WMS	2	TRUE	0.470986	20.4771
1661	rs1081165	A	G	A	G	-0.0611	0.01919	0.2599	0.296468	FALSE	FALSE	FALSE	ieu-b-508	9	22128600	0.036863	486484	0.602655	Sepsis (28	Sepsis (28	Sepsis (28	TRUE	igd	0.0135	6.22E-06	5606	M15335.m	TRUE	reported	a7RjFn	2	TRUE	0.003641	20.4767
6275	rs7933681	A	G	A	G	0.0267	-0.0328	0.094	0.099971	FALSE	FALSE	FALSE	ieu-b-508	11	82270844	0.05585	486484	0.556955	Sepsis (28	Sepsis (28	Sepsis (28	TRUE	igd	0.0059	5.57E-06	7222	M33937.m	TRUE	reported	QanGCI	2	TRUE	0.002828	20.47379
6783	rs1259233	T	C	T	C	-0.1292	0.008267	0.8495	0.843525	FALSE	FALSE	FALSE	ieu-b-508	15	48645280	0.045862	486484	0.856948	Sepsis (28	Sepsis (28	Sepsis (28	TRUE	igd	0.0285	5.86E-06	525	M34350.m	TRUE	reported	Cc3yW9	2	TRUE	0.03767	20.47282
5439	rs1176912	A	T	A	T	-0.1068	-0.14981	0.9758	0.956626	FALSE	TRUE	FALSE	ieu-b-508	7	1.57E+08	0.081835	486484	0.067152	Sepsis (28	Sepsis (28	Sepsis (28	TRUE	igd	0.0236	5.83E-06	5185	M33390.m	TRUE	reported	t0EYdH	2	TRUE	0.003934	20.47156
6461	rs4627421	T	C	T	C	0.0267	0.027924	0.2337	0.253632	FALSE	FALSE	FALSE	ieu-b-508	17	7394281	0.038349	486484	0.466525	Sepsis (28	Sepsis (28	Sepsis (28	TRUE	igd	0.0059	7.19E-06	4961	M34106.m	TRUE	reported	8DuoNM	2	TRUE	0.004111	20.4712
8158	rs1073847	T	C	T	C	0.0335	-0.03195	0.1772	0.157457	FALSE	FALSE	FALSE	ieu-b-508	9	16939751	0.04568	486484	0.484342	Sepsis (28	Sepsis (28	Sepsis (28	TRUE	igd	0.0074	6.34E-06	1780	M36131.m	TRUE	reported	DbYm5t	2	TRUE	0.011382	20.47095
8184	rs7812350	T	C	T	C	0.0344	0.085415	0.7854	0.770665	FALSE	FALSE	FALSE	ieu-b-508	8	15672534	0.039644	486484	0.031198	Sepsis (28	Sepsis (28	Sepsis (28	TRUE	igd	0.0076	6.29E-06	2432	M36134.m	TRUE	reported	ekMsfG	2	TRUE	0.008354	20.47069
1672	rs1102058	A	G	A	G	-0.0362	-0.01369	0.9798	0.9792	FALSE	FALSE	FALSE	ieu-b-508	11	93643089	0.117264	486484	0.90708	Sepsis (28	Sepsis (28	Sepsis (28	TRUE	igd	0.008	7.02E-06	7337	M15365.m	TRUE	reported	TReTCM	2	TRUE	0.002783	20.47004
6202	rs3783687	A	C	A	C	-0.212	-0.08681	0.9851	0.988736	FALSE	FALSE	FALSE	ieu-b-508	14	58478941	0.159107	486484	0.585318	Sepsis (28	Sepsis (28	Sepsis (28	TRUE	igd	0.0468	5.94E-06	819	M33892.m	TRUE	reported	w7HgU7	2	TRUE	0.024443	20.47002
6673	rs2925759	T	C	T	C	-0.067	-0.02444	0.1897	0.215795	FALSE	FALSE	FALSE	ieu-b-508	2	1.61E+08	0.041001	486484	0.551088	Sepsis (28	Sepsis (28	Sepsis (28	TRUE	igd	0.0148	6.28E-06	1612	M34327.m	TRUE	reported	Il4Lrr	2	TRUE	0.012554	20.46855
4304	rs1099429	T	C	T	C	0.0738	-0.02715	0.0651	0.06304	FALSE	FALSE	FALSE	ieu-b-508	10	51554821	0.069622	486484	0.696564	Sepsis (28	Sepsis (28	Sepsis (28	TRUE	igd	0.0163	5.80E-06	1307	M32838.m	TRUE	reported	hyR7zA	2	TRUE	0.015442	20.46786
1918	rs1861007	C	G	C	G	-0.0181	0.028107	0.1925	0.187499	FALSE	TRUE	FALSE	ieu-b-508	7	55139751	0.042861	486484	0.511971	Sepsis (28	Sepsis (28	Sepsis (28	TRUE	igd	0.004	7.81E-06	4689	M16816.m	TRUE	reported	J1abbO	2	TRUE	0.004348	20.46689
6055	rs1708577	A	G	A	G	-0.0276	-0.13911	0.0693	0.071052	FALSE	FALSE	FALSE	ieu-b-508	6	1.55E+08	0.064511	486484	0.031051	Sepsis (28	Sepsis (28	Sepsis (28	TRUE	igd	0.0061	6.88E-06	7362	M33821.m	TRUE	reported	eVYICL	2	TRUE	0.002773	20.46635
3891	rs1162679	A	C	A	C	0.0276	0.066685	0.0117	0.017171	FALSE	FALSE	FALSE	ieu-b-508	14	65596148	0.130476	486484	0.609284	Sepsis (28	Sepsis (28	Sepsis (28	TRUE	igd	0.0061	6.35E-06	7338	M32675.m	TRUE	reported	9FmPh9	2	TRUE	0.002782	20.46634
4278	rs6540767	A	T	A	T	-0.0276	-0.04448	0.6977	0.681744	FALSE	TRUE	FALSE	ieu-b-508	1	2.13E+08	0.035779	486484	0.213818	Sepsis (28	Sepsis (28	Sepsis (28	TRUE	igd	0.0061	5.05E-06	6200	M32808.m	TRUE	reported	vnr36k	2	TRUE	0.003291	20.46531
5189	rs7916966	A	T	A	T	-0.3985	0.038986	0.9755	0.985232	FALSE	TRUE	FALSE	ieu-b-508	10	78552627	0.142095	486484	0.783803	Sepsis (28	Sepsis (28	Sepsis (28	TRUE	igd	0.088	5.92E-06	996	M33197.m	TRUE	reported	D6xaBg	2	TRUE	0.020173	20.46531
3665	rs751903	A	G	A	G	0.0647	0.039987	0.0733	0.062565	FALSE	FALSE	FALSE	ieu-b-508	2	1.05E+08	0.069214	486484	0.563452	Sepsis (28	Sepsis (28	Sepsis (28	TRUE	igd	0.0143	6.22E-06	6970	M32564.m	TRUE	reported	73MgOk	2	TRUE	0.002928	20.465
8015	rs6486785	A	G	A	G	0.0371	-0.03589	0.0193	0.023686	FALSE	FALSE	FALSE	ieu-b-508	12	1.22E+08	0.110694	486484	0.745794	Sepsis (28	Sepsis (28	Sepsis (28	TRUE	igd	0.0082	5.99E-06	6604	M35754.m	TRUE	reported	IlXOo1	2	TRUE	0.00309	20.46391
2432	rs8094941	A	C	A	C	-0.0371	0.044656	0.9697	0.967711	FALSE	FALSE	FALSE	ieu-b-508	18	71808546	0.094828	486484	0.637704	Sepsis (28	Sepsis (28	Sepsis (28	TRUE	igd	0.0082	5.42E-06	6556	M21188.m	TRUE	reported	kvdnjc	2	TRUE	0.003113	20.46386
2133	rs9301881	T	C	T	C	0.0593	0.006675	0.5692	0.577923	FALSE	FALSE	FALSE	ieu-b-508	13	94105286	0.033862	486484	0.84372	Sepsis (28	Sepsis (28	Sepsis (28	TRUE	igd	0.0131	5.79E-06	1457	M18477.m	TRUE	reported	p0vcCU	2	TRUE	0.013869	20.46304
5218	rs7953025	A	G	A	G	0.0172	-0.01543	0.5419	0.557444	FALSE	FALSE	FALSE	ieu-b-508	12	12557507	0.033692	486484	0.647026	Sepsis (28	Sepsis (28	Sepsis (28	TRUE	igd	0.0038	7.61E-06	1673	M33204.m	TRUE	reported	ROvPWK	2	TRUE	0.012098	20.46304
4260	rs7299188	C	G	C	G	-0.0371	0.040056	0.6604	0.625282	FALSE	TRUE	FALSE	ieu-b-508	12	1.3E+08	0.034651	486484	0.247685	Sepsis (28	Sepsis (28	Sepsis (28	TRUE	igd	0.0082	6.53E-06	4274	M32800.m	TRUE	reported	Q7MVsa	2	TRUE	0.004767	20.46053
8501	rs4792110	T	G	T	G	0.114	0.013195	0.9378	0.915881	FALSE	FALSE	FALSE	ieu-b-508	17	11148440	0.060542	486484	0.827464	Sepsis (28	Sepsis (28	Sepsis (28	TRUE	igd	0.0252	6.31E-06	7014	M37097.m	TRUE	reported	wCbJj4	2	TRUE	0.002909	20.45902
5156	rs4894432	A	G	A	G	-0.0475	0.058162	0.7331	0.745741	FALSE	FALSE	FALSE	ieu-b-508	3	1.71E+08	0.038412	486484	0.129988	Sepsis (28	Sepsis (28	Sepsis (28	TRUE	igd	0.0105	6.85E-06	5716	M33192.m	TRUE	reported	CyfjKE	2	TRUE	0.003568	20.45769
6343	rs7727774	A	G	A	G	0.0267	0.064466	0.4534	0.48794	FALSE	FALSE	FALSE	ieu-b-508	5	53164235	0.033424	486484	0.053765	Sepsis (28	Sepsis (28	Sepsis (28	TRUE	igd	0.0059	6.38E-06	1730	M33957.m	TRUE	reported	mrKpMv	2	TRUE	0.011699	20.45578
5371	rs1124204	A	G	A	G	0.4771	0.353974	0.0216	0.012142	FALSE	FALSE	FALSE	ieu-b-508	5	1.3E+08	0.15334	486484	0.020976	Sepsis (28	Sepsis (28	Sepsis (28	TRUE	igd	0.1051	5.60E-06	272	M33384.m	TRUE	reported	IOffrZ	2	TRUE	0.070425	20.45541
4416	rs7828843	T	C	T	C	-0.1059	0.034175	0.0202	0.012445	FALSE	FALSE	FALSE	ieu-b-508	8	11089499	0.153562	486484	0.82389	Sepsis (28	Sepsis (28	Sepsis (28	TRUE	igd	0.0234	6.17E-06	1550	M32869.m	TRUE	reported	Rvim1n	2	TRUE	0.013041	20.455
6745	rs6511851	C	G	C	G	-0.584	0.008514	0.986	0.951907	FALSE	TRUE	FALSE	ieu-b-508	19	13283675	0.089444	486484	0.924168	Sepsis (28	Sepsis (28	Sepsis (28	TRUE	igd	0.128	5.06E-06	115	M34338.m	TRUE	reported	h4FB9a	2	TRUE	0.153269	20.45438
6248	rs8085038	A	C	A	C	-0.0991	-0.06108	0.1003	0.090265	FALSE	FALSE	FALSE	ieu-b-508	18	27798556	0.058328	486484	0.295001	Sepsis (28	Sepsis (28	Sepsis (28	TRUE	igd	0.0219	6.32E-06	1738	M33935.m	TRUE	reported	ELBTDc	2	TRUE	0.011645	20.45309
6098	rs924931	T	C	T	C	-0.0353	0.028926	0.5401	0.54546	FALSE	FALSE	FALSE	ieu-b-508	3	16235042	0.033658	486484	0.390115	Sepsis (28	Sepsis (28	Sepsis (28	TRUE	igd	0.0078	6.14E-06	1428	M33864.m	TRUE	reported	yr5h52	2	TRUE	0.01414	20.45274
4029	rs7151027	A	T	A	T	-0.0294	0.013631	0.3207	0.323877	FALSE	TRUE	FALSE	ieu-b-508	14	92588917	0.035588	486484	0.701701	Sepsis (28	Sepsis (28	Sepsis (28	TRUE	igd	0.0065	6.92E-06	6000	M32735.m	TRUE	reported	QE2vKQ	2	TRUE	0.003398	20.45141
2396	rs1162754	A	C	A	C	0.0294	0.02859	0.1703	0.172767	FALSE	FALSE	FALSE	ieu-b-508	14	70365924	0.04414	486484	0.51718	Sepsis (28	Sepsis (28	Sepsis (28	TRUE	igd	0.0065	6.87E-06	5436	M21184.m	TRUE	reported	63ppuJ	2	TRUE	0.003749	20.4507
2945	rs7132285	T	C	T	C	0.1371	0.006291	0.0108	0.006766	FALSE	FALSE	FALSE	ieu-b-508	12	1776217	0.206657	486484	0.975714	Sepsis (28	Sepsis (28	Sepsis (28	TRUE	igd	0.0303	6.12E-06	1758	M27738.m	TRUE	reported	UtPBd9	2	TRUE	0.011512	20.45009
6751	rs9826397	A	G	A	G	-0.2895	0.112053	0.9782	0.969106	FALSE	FALSE	FALSE	ieu-b-508	3	1.55E+08	0.097826	486484	0.252026	Sepsis (28	Sepsis (28	Sepsis (28	TRUE	igd	0.0639	5.80E-06	535	M34338.m	TRUE	reported	h4FB9a	2	TRUE	0.036948	20.44885
4202	rs532	A	G	A	G	-0.0475	-0.0368	0.771	0.780813	FALSE	FALSE	FALSE	ieu-b-508	14	39651193	0.040237	486484	0.360451	Sepsis (28	Sepsis (28	Sepsis (28	TRUE	igd	0.0105	6.06E-06	2537	M32762.m	TRUE	reported	Q5lKLH	2	TRUE	0.008002	20.44872
8316	rs9399651	C	G	C	G	0.0389	0.005474	0.8405	0.816749	FALSE	TRUE	FALSE	ieu-b-508	6	1.49E+08	0.043342	486484	0.899491	Sepsis (28	Sepsis (28	Sepsis (28	TRUE	igd	0.0086	5.64E-06	3346	M36585.m	TRUE	reported	pXZg13	2	TRUE	0.006078	20.44761
4555	rs798007	T	C	T	C	-0.0407	0.040014	0.9676	0.968167	FALSE	FALSE	FALSE	ieu-b-508	1	1.17E+08	0.096036	486484	0.67693	Sepsis (28	Sepsis (28	Sepsis (28	TRUE	igd	0.009	6.32E-06	7319	M33140.m	TRUE	reported	eBOUtb	2	TRUE	0.002786	20.44491
8341	rs1878640	T	C	T	C	0.0303	-0.02261	0.911	0.915515	FALSE	FALSE	FALSE	ieu-b-508	2	1.32E+08	0.060152	486484	0.706952	Sepsis (28	Sepsis (28	Sepsis (28	TRUE	igd	0.0067	5.41E-06	5292	M36673.m	TRUE	reported	CD5lvR	2	TRUE	0.00385	20.44426
4144	rs1719767	A	G	A	G	0.0751	0.103143	0.928	0.933453	FALSE	FALSE	FALSE	ieu-b-508	14	22481451	0.075308	486484	0.170806	Sepsis (28	Sepsis (28	Sepsis (28	TRUE	igd	0.0166	6.41E-06	1761	M32757.m	TRUE	reported	TJV9Xb	2	TRUE	0.011489	20.4442
4571	rs2667615	C	G	C	G	-0.1185	0.048372	0.0264	0.111062	FALSE	TRUE	FALSE	ieu-b-508	16	78561592	0.053289	486484	0.364018	Sepsis (28	Sepsis (28	Sepsis (28	TRUE	igd	0.0262	5.89E-06	2777	M33150.m	TRUE	reported	H9SSMD	2	TRUE	0.007313	20.4419
4831	rs1709153	A	G	A	G	-0.3441	-0.16499	0.0227	0.067749	FALSE	FALSE	FALSE	ieu-b-508	8	19667559	0.066753	486484	0.01345	Sepsis (28	Sepsis (28	Sepsis (28	TRUE	igd	0.073	2.46E-06	25	M33178.m	TRUE	reported	g22WMS	2	TRUE	0.470552	20.44144
5056	rs7675814	A	C	A	C	0.3441	0.106174	0.9773	0.966419	FALSE	FALSE	FALSE	ieu-b-508	4	37684465	0.09323	486484	0.254773	Sepsis (28	Sepsis (28	Sepsis (28	TRUE	igd	0.073	2.46E-06	25	M33178.m	TRUE	reported	g22WMS	2	TRUE	0.470552	20.44144
5083	rs8192827	A	G	A	G	0.3441	-0.04378	0.9773	0.968631	FALSE	FALSE	FALSE	ieu-b-508	7	1.4E+08	0.097082	486484	0.65199	Sepsis (28	Sepsis (28	Sepsis (28	TRUE	igd	0.073	2.46E-06	25	M33178.m	TRUE	reported	g22WMS	2	TRUE	0.470552	20.44144
3129	rs197442	A	G	A	G	0.0104	-0.02392	0.5962	0.600546	FALSE	FALSE	FALSE	ieu-b-508	5	1.17E+08	0.034113	486484	0.483251	Sepsis (28	Sepsis (28	Sepsis (28	TRUE	igd	0.0023	7.60E-06	7349	M32315.m	TRUE	reported	4igzkL	2	TRUE	0.002774	20.44056
3132	rs342563	A	G	A	G	-0.0104	0.010928	0.471	0.497427	FALSE	FALSE	FALSE	ieu-b-508	5	16187424	0.033503	486484	0.744297	Sepsis (28	Sepsis (28	Sepsis (28	TRUE	igd	0.0023	7.86E-06	7349	M32315.m	TRUE	reported	4igzkL	2	TRUE	0.002774	20.44056
2108	rs1185449	A	G	A	G	0.0869	0.039178	0.0972	0.101317	FALSE	FALSE	FALSE	ieu-b-508	15	62041433	0.05568	486484	0.481667	Sepsis (28	Sepsis (28	Sepsis (28	TRUE	igd	0.0192	6.16E-06	917	M18474.m	TRUE	reported	B6Xd02	2	TRUE	0.021851	20.44037
7735	rs1714525	A	G	A	G	0.0312	0.092289	0.0292	0.030202	FALSE	FALSE	FALSE	ieu-b-508	10	9151229	0.098115	486484	0.346899	Sepsis (28	Sepsis (28	Sepsis (28	TRUE	igd	0.0069	6.58E-06	7007	M35431.m	TRUE	reported	tLfE4l	2	TRUE	0.002909	20.44029
2851	rs2708754	A	G	A	G	0.0312	0.033904	0.3968	0.39153	FALSE	FALSE	FALSE	ieu-b-508	19	54025972	0.034337	486484	0.323463	Sepsis (28	Sepsis (28	Sepsis (28	TRUE	igd	0.0069	6.52E-06	5673	M27531.m	TRUE	reported	v01zDr	2	TRUE	0.003591	20.43892
2987	rs1339805	A	G	A	G	0.038	-0.02891	0.6148	0.610662	FALSE	FALSE	FALSE	ieu-b-508	2	2.27E+08	0.034446	486484	0.401267	Sepsis (28	Sepsis (28	Sepsis (28	TRUE	igd	0.0084	5.76E-06	1558	M31522.m	TRUE	reported	gM9gAj	2	TRUE	0.012965	20.43858
574	rs1499094	T	G	T	G	0.0208	0.005886	0.7686	0.77872	FALSE	FALSE	FALSE	ieu-b-508	3	1244434	0.04055	486484	0.88458	Sepsis (28	Sepsis (28	Sepsis (28	TRUE	igd	0.0046	7.68E-06	5344	M01107.m	TRUE	reported	EbPV1D	2	TRUE	0.003811	20.43847
5933	rs262749	A	C	A	C	0.0208	-0.01572	0.6982	0.699032	FALSE	FALSE	FALSE	ieu-b-508	5	1.65E+08	0.036636	486484	0.667941	Sepsis (28	Sepsis (28	Sepsis (28	TRUE	igd	0.0046	5.33E-06	5080	M33637.m	TRUE	reported	tfLznu	2	TRUE	0.004009	20.43808
6054	rs1701328	A	G	A	G	-0.0493	-0.01561	0.0898	0.076409	FALSE	FALSE	FALSE	ieu-b-508	4	88762296	0.063507	486484	0.805873	Sepsis (28	Sepsis (28	Sepsis (28	TRUE	igd	0.0109	5.90E-06	1764	M33821.m	TRUE	reported	eVYICL	2	TRUE	0.011464	20.43375
7831	rs1551864	T	C	T	C	-0.038	0.071535	0.3052	0.329624	FALSE	FALSE	FALSE	ieu-b-508	15	27845275	0.035509	486484	0.043949	Sepsis (28	Sepsis (28	Sepsis (28	TRUE	igd	0.0084	5.90E-06	1291	M35527.m	TRUE	reported	UAMhnu	2	TRUE	0.015605	20.43315
3656	rs2051674	T	C	T	C	0.0434	-0.06463	0.8325	0.820467	FALSE	FALSE	FALSE	ieu-b-508	17	5901652	0.043659	486484	0.13879	Sepsis (28	Sepsis (28	Sepsis (28	TRUE	igd	0.0096	5.89E-06	6970	M32564.m	TRUE	reported	73MgOk	2	TRUE	0.002924	20.43207
4169	rs1074416	T	C	T	C	-0.0217	0.021857	0.3489	0.353722	FALSE	FALSE	FALSE	ieu-b-508	12	1.24E+08	0.035233	486484	0.535022	Sepsis (28	Sepsis (28	Sepsis (28	TRUE	igd	0.0048	8.00E-06	6712	M32759.m	TRUE	reported	9elHr7	2	TRUE	0.003036	20.43184
1668	rs4917405	T	C	T	C	0.0972	0.027111	0.0437	0.042927	FALSE	FALSE	FALSE	ieu-b-508	10	1.06E+08	0.082982	486484	0.743885	Sepsis (28	Sepsis (28	Sepsis (28	TRUE	igd	0.0215	6.28E-06	5606	M15335.m	TRUE	reported	a7RjFn	2	TRUE	0.003633	20.43152
6902	rs1763075	A	G	A	G	0.1768	0.054057	0.0279	0.030078	FALSE	FALSE	FALSE	ieu-b-508	18	37460638	0.098326	486484	0.582473	Sepsis (28	Sepsis (28	Sepsis (28	TRUE	igd	0.0391	5.99E-06	2779	M34384.m	TRUE	reported	fl3hiS	2	TRUE	0.007304	20.43141
3710	rs7942018	T	C	T	C	-0.0217	0.117268	0.4068	0.399433	FALSE	FALSE	FALSE	ieu-b-508	11	13005461	0.034433	486484	0.00066	Sepsis (28	Sepsis (28	Sepsis (28	TRUE	igd	0.0048	5.96E-06	5955	M32593.m	TRUE	reported	6zVI1i	2	TRUE	0.00342	20.43107
8115	rs882014	A	G	A	G	-1.0082	0.007358	0.0175	0.016057	FALSE	FALSE	FALSE	ieu-b-508	13	77396207	0.132044	486484	0.955563	Sepsis (28	Sepsis (28	Sepsis (28	TRUE	igd	0.2226	5.94E-06	488	M36097.m	TRUE	reported	RHr18S	2	TRUE	0.04034	20.42958
2360	rs1023229	A	G	A	G	0.0321	0.036048	0.3415	0.352556	FALSE	FALSE	FALSE	ieu-b-508	7	1.27E+08	0.035207	486484	0.305893	Sepsis (28	Sepsis (28	Sepsis (28	TRUE	igd	0.0071	6.03E-06	3422	M21049.m	TRUE	reported	UDtLE4	2	TRUE	0.005938	20.42864
6467	rs1204738	T	C	T	C	0.298	-0.01125	0.9679	0.931264	FALSE	FALSE	FALSE	ieu-b-508	1	2.31E+08	0.067255	486484	0.867125	Sepsis (28	Sepsis (28	Sepsis (28	TRUE	igd	0.0657	5.80E-06	282	M34109.m	TRUE	reported	piJuC7	2	TRUE	0.067994	20.42729
3282	rs7286668	A	C	A	C	-0.0113	-0.02875	0.827	0.834388	FALSE	FALSE	FALSE	ieu-b-508	22	35442750	0.044936	486484	0.522307	Sepsis (28	Sepsis (28	Sepsis (28	TRUE	igd	0.0025	7.86E-06	7365	M32348.m	TRUE	reported	DJeLDx	2	TRUE	0.002766	20.42485
698	rs7834337	A	G	A	G	-0.0113	0.063085	0.0904	0.090286	FALSE	FALSE	FALSE	ieu-b-508	8	655828	0.058091	486484	0.277486	Sepsis (28	Sepsis (28	Sepsis (28	TRUE	igd	0.0025	4.71E-06	7358	M01299.m	TRUE	reported	gfM0a3	2	TRUE	0.002769	20.42485
3190	rs491847	A	G	A	G	-0.0113	0.046948	0.3457	0.353324	FALSE	FALSE	FALSE	ieu-b-508	12	1.01E+08	0.035079	486484	0.180774	Sepsis (28	Sepsis (28	Sepsis (28	TRUE	igd	0.0025	5.33E-06	7356	M32338.m	TRUE	reported	q5cuaP	2	TRUE	0.00277	20.42485
813	rs1426441	A	G	A	G	0.0113	-0.03555	0.1423	0.163501	FALSE	FALSE	FALSE	ieu-b-508	12	1.14E+08	0.045605	486484	0.435636	Sepsis (28	Sepsis (28	Sepsis (28	TRUE	igd	0.0025	4.87E-06	7355	M01358.m	TRUE	reported	kSPQgd	2	TRUE	0.00277	20.42484
3493	rs1258857	A	G	A	G	0.0113	-0.07525	0.2168	0.224903	FALSE	FALSE	FALSE	ieu-b-508	14	75005309	0.039955	486484	0.059636	Sepsis (28	Sepsis (28	Sepsis (28	TRUE	igd	0.0025	6.12E-06	7355	M32492.m	TRUE	reported	rZZFcE	2	TRUE	0.00277	20.42484
4428	rs1684483	T	C	T	C	-0.0113	0.043395	0.1846	0.18657	FALSE	FALSE	FALSE	ieu-b-508	2	2.12E+08	0.042746	486484	0.310021	Sepsis (28	Sepsis (28	Sepsis (28	TRUE	igd	0.0025	5.00E-06	7344	M32910.m	TRUE	reported	2OCBKh	2	TRUE	0.002774	20.42484
1314	rs9354336	T	C	T	C	0.0113	0.007416	0.8733	0.878191	FALSE	FALSE	FALSE	ieu-b-508	6	66609594	0.051198	486484	0.884838	Sepsis (28	Sepsis (28	Sepsis (28	TRUE	igd	0.0025	8.57E-06	7325	M02132.m	TRUE	reported	uJFm2t	2	TRUE	0.002781	20.42482
3315	rs3780970	T	C	T	C	0.0113	-0.04352	0.2697	0.266793	FALSE	FALSE	FALSE	ieu-b-508	10	16823493	0.037835	486484	0.25004	Sepsis (28	Sepsis (28	Sepsis (28	TRUE	igd	0.0025	3.76E-06	7307	M32393.m	TRUE	reported	Pub0X0	2	TRUE	0.002788	20.42481
2451	rs7512200	A	G	A	G	0.0113	0.036703	0.39	0.413034	FALSE	FALSE	FALSE	ieu-b-508	1	61978694	0.034054	486484	0.281131	Sepsis (28	Sepsis (28	Sepsis (28	TRUE	igd	0.0025	9.78E-06	7284	M21630.m	TRUE	reported	7eaO4H	2	TRUE	0.002797	20.42479
2774	rs1164238	A	G	A	G	0.0113	0.043356	0.753	0.7458	FALSE	FALSE	FALSE	ieu-b-508	16	81488358	0.038704	486484	0.262624	Sepsis (28	Sepsis (28	Sepsis (28	TRUE	igd	0.0025	7.79E-06	7268	M27273.m	TRUE	reported	EvU2od	2	TRUE	0.002803	20.42478
7405	rs4703415	T	C	T	C	-0.0226	0.030389	0.1469	0.145355	FALSE	FALSE	FALSE	ieu-b-508	5	35589089	0.047292	486484	0.520492	Sepsis (28	Sepsis (28	Sepsis (28	TRUE	igd	0.005	6.55E-06	7263	M35160.m	TRUE	reported	eRox3e	2	TRUE	0.002805	20.42477
2685	rs4811661	T	C	T	C	-0.0113	0.002463	0.3137	0.32793	FALSE	FALSE	FALSE	ieu-b-508	20	54760515	0.035628	486484	0.944893	Sepsis (28	Sepsis (28	Sepsis (28	TRUE	igd	0.0025	6.59E-06	7256	M24074.m	TRUE	reported	Lk6D2A	2	TRUE	0.002808	20.42477
2178	rs1259684	A	G	A	G	-0.0113	-0.01081	0.1846	0.200242	FALSE	FALSE	FALSE	ieu-b-508	16	14206749	0.043091	486484	0.801925	Sepsis (28	Sepsis (28	Sepsis (28	TRUE	igd	0.0025	3.93E-06	7182	M19362.m	TRUE	reported	SkNEKw	2	TRUE	0.002837	20.42471
2188	rs3844066	T	C	T	C	0.0113	0.043875	0.8135	0.811193	FALSE	FALSE	FALSE	ieu-b-508	21	43155068	0.042615	486484	0.303217	Sepsis (28	Sepsis (28	Sepsis (28	TRUE	igd	0.0025	5.67E-06	7182	M19362.m	TRUE	reported	SkNEKw	2	TRUE	0.002837	20.42471
4282	rs1007599	A	G	A	G	-0.0113	0.014339	0.578	0.583832	FALSE	FALSE	FALSE	ieu-b-508	5	1.48E+08	0.034048	486484	0.673654	Sepsis (28	Sepsis (28	Sepsis (28	TRUE	igd	0.0025	8.73E-06	7063	M32814.m	TRUE	reported	z0OeTx	2	TRUE	0.002884	20.42461
2651	rs1106530	T	C	T	C	0.0113	-0.00672	0.3445	0.391652	FALSE	FALSE	FALSE	ieu-b-508	12	1.21E+08	0.034342	486484	0.844787	Sepsis (28	Sepsis (28	Sepsis (28	TRUE	igd	0.0025	8.59E-06	6517	M22649.m	TRUE	reported	NAzwRF	2	TRUE	0.003125	20.42413
6001	rs1156345	T	C	T	C	-0.0914	-0.03724	0.8849	0.873028	FALSE	FALSE	FALSE	ieu-b-508	7	1.27E+08	0.050343	486484	0.459449	Sepsis (28	Sepsis (28	Sepsis (28	TRUE	igd	0.0202	5.78E-06	831	M33751.m	TRUE	reported	Cxdkr4	2	TRUE	0.024045	20.42411
7070	rs4630827	T	C	T	C	0.0226	0.004576	0.201	0.216091	FALSE	FALSE	FALSE	ieu-b-508	20	14436938	0.040491	486484	0.910014	Sepsis (28	Sepsis (28	Sepsis (28	TRUE	igd	0.005	7.34E-06	6144	M34469.m	TRUE	reported	WMOqbm	2	TRUE	0.003314	20.42375
7746	rs1118028	A	G	A	G	0.123	-0.32799	0.9821	0.984936	FALSE	FALSE	FALSE	ieu-b-508	12	75307993	0.136036	486484	0.015907	Sepsis (28	Sepsis (28	Sepsis (28	TRUE	igd	0.0272	6.01E-06	1522	M35433.m	TRUE	reported	XlYkLe	2	TRUE	0.013257	20.42214
6388	rs4529031	T	C	T	C	-0.0841	-0.1228	0.9555	0.03326	FALSE	FALSE	FALSE	ieu-b-508	4	1.52E+08	0.093267	486484	0.187943	Sepsis (28	Sepsis (28	Sepsis (28	TRUE	igd	0.0186	6.41E-06	1760	M33969.m	TRUE	reported	0ujroW	2	TRUE	0.011483	20.42078
4319	rs1690279	C	G	C	G	0.0922	0.010356	0.0266	0.033451	FALSE	TRUE	FALSE	ieu-b-508	5	87073972	0.093409	486484	0.911721	Sepsis (28	Sepsis (28	Sepsis (28	TRUE	igd	0.0204	6.42E-06	6250	M32846.m	TRUE	reported	lwF2Yy	2	TRUE	0.003258	20.42032
5718	rs4374940	T	C	T	C	0.1321	-0.07593	0.0286	0.032598	FALSE	FALSE	FALSE	ieu-b-508	7	52266800	0.095283	486484	0.42552	Sepsis (28	Sepsis (28	Sepsis (28	TRUE	igd	0.0292	5.89E-06	834	M33488.m	TRUE	reported	mZmDY6	2	TRUE	0.023952	20.41725
2232	rs7359707	A	T	A	T	0.0235	0.016522	0.1937	0.224666	FALSE	TRUE	FALSE	ieu-b-508	18	22903341	0.040311	486484	0.681908	Sepsis (28	Sepsis (28	Sepsis (28	TRUE	igd	0.0052	5.42E-06	6385	M19396.m	TRUE	reported	Xa1QqA	2	TRUE	0.003188	20.41705
6401	rs6578042	T	C	T	C	0.047	0.013688	0.857	0.85016	FALSE	FALSE	FALSE	ieu-b-508	8	1.41E+08	0.046887	486484	0.770334	Sepsis (28	Sepsis (28	Sepsis (28	TRUE	igd	0.0104	5.93E-06	5561	M33973.m	TRUE	reported	rpygG6	2	TRUE	0.003659	20.4161
7490	rs1745299	T	G	T	G	0.1406	0.078392	0.0125	0.02415	FALSE	FALSE	FALSE	ieu-b-508	4	74447109	0.108955	486484	0.471836	Sepsis (28	Sepsis (28	Sepsis (28	TRUE	igd	0.0311	6.09E-06	1761	M35253.m	TRUE	reported	VboRmj	2	TRUE	0.011473	20.41533
6107	rs6034091	A	G	A	G	0.1302	-0.00594	0.0124	0.012802	FALSE	FALSE	FALSE	ieu-b-508	20	15020018	0.151746	486484	0.968779	Sepsis (28	Sepsis (28	Sepsis (28	TRUE	igd	0.0288	6.14E-06	1639	M33871.m	TRUE	reported	mQD6DP	2	TRUE	0.012316	20.41299
598	rs6535974	A	G	A	G	-0.0122	0.017725	0.8465	0.840693	FALSE	FALSE	FALSE	ieu-b-508	4	1.4E+08	0.045646	486484	0.697784	Sepsis (28	Sepsis (28	Sepsis (28	TRUE	igd	0.0027	5.80E-06	7367	M01110.m	TRUE	reported	0A31Sl	2	TRUE	0.002764	20.41147
1019	rs4779800	A	G	A	G	-0.0122	-0.0146	0.8831	0.892096	FALSE	FALSE	FALSE	ieu-b-508	15	29412169	0.054083	486484	0.787162	Sepsis (28	Sepsis (28	Sepsis (28	TRUE	igd	0.0027	6.06E-06	7364	M01564.m	TRUE	reported	IPUdyD	2	TRUE	0.002765	20.41146
3959	rs269066	A	G	A	G	-0.0122	-0.01522	0.2794	0.279312	FALSE	FALSE	FALSE	ieu-b-508	1	1.13E+08	0.037206	486484	0.682406	Sepsis (28	Sepsis (28	Sepsis (28	TRUE	igd	0.0027	5.28E-06	7363	M32709.m	TRUE	reported	YR8SB4	2	TRUE	0.002765	20.41146
6438	rs2301783	A	C	A	C	0.0122	-0.03954	0.9151	0.882154	FALSE	FALSE	FALSE	ieu-b-508	12	48182241	0.052492	486484	0.451333	Sepsis (28	Sepsis (28	Sepsis (28	TRUE	igd	0.0027	4.90E-06	7362	M34062.m	TRUE	reported	RZyHKP	2	TRUE	0.002766	20.41146
3101	rs1261587	A	T	A	T	-0.0122	0.074801	0.172	0.163233	FALSE	TRUE	FALSE	ieu-b-508	2	73082263	0.044982	486484	0.09633	Sepsis (28	Sepsis (28	Sepsis (28	TRUE	igd	0.0027	6.20E-06	7356	M32198.m	TRUE	reported	ndXmnp	2	TRUE	0.002768	20.41146
6984	rs8007846	A	G	A	G	0.0122	0.039725	0.5509	0.546473	FALSE	FALSE	FALSE	ieu-b-508	14	66262963	0.033437	486484	0.23481	Sepsis (28	Sepsis (28	Sepsis (28	TRUE	igd	0.0027	8.39E-06	7344	M34407.m	TRUE	reported	X7I83Y	2	TRUE	0.002772	20.41145
6985	rs891404	A	G	A	G	0.0122	-0.02171	0.4842	0.488158	FALSE	FALSE	FALSE	ieu-b-508	8	40276391	0.033413	486484	0.515822	Sepsis (28	Sepsis (28	Sepsis (28	TRUE	igd	0.0027	7.49E-06	7344	M34407.m	TRUE	reported	X7I83Y	2	TRUE	0.002772	20.41145
1676	rs1813982	T	C	T	C	-0.0122	-0.00142	0.2209	0.236849	FALSE	FALSE	FALSE	ieu-b-508	15	37794229	0.039334	486484	0.971141	Sepsis (28	Sepsis (28	Sepsis (28	TRUE	igd	0.0027	6.59E-06	7337	M15365.m	TRUE	reported	TReTCM	2	TRUE	0.002775	20.41144
3742	rs2979269	A	G	A	G	-0.0122	-0.04667	0.4706	0.473254	FALSE	FALSE	FALSE	ieu-b-508	8	8901140	0.033378	486484	0.162017	Sepsis (28	Sepsis (28	Sepsis (28	TRUE	igd	0.0027	4.59E-06	7337	M32632.m	TRUE	reported	pIHzPT	2	TRUE	0.002775	20.41144
1865	rs9514739	C	G	C	G	-0.0122	0.018221	0.8962	0.882705	FALSE	TRUE	FALSE	ieu-b-508	13	1.08E+08	0.052309	486484	0.72759	Sepsis (28	Sepsis (28	Sepsis (28	TRUE	igd	0.0027	6.10E-06	7308	M15778.m	TRUE	reported	941o3l	2	TRUE	0.002786	20.41142
2294	rs5027944	T	C	T	C	0.0122	0.029288	0.5795	0.585307	FALSE	FALSE	FALSE	ieu-b-508	5	1.17E+08	0.033829	486484	0.386631	Sepsis (28	Sepsis (28	Sepsis (28	TRUE	igd	0.0027	3.93E-06	7301	M20675.m	TRUE	reported	CXe9rE	2	TRUE	0.002789	20.41142
2295	rs5757568	A	G	A	G	0.0122	-0.02068	0.388	0.370645	FALSE	FALSE	FALSE	ieu-b-508	22	22583571	0.034466	486484	0.54857	Sepsis (28	Sepsis (28	Sepsis (28	TRUE	igd	0.0027	5.10E-06	7301	M20675.m	TRUE	reported	CXe9rE	2	TRUE	0.002789	20.41142
5786	rs248670	T	C	T	C	0.0122	-0.00837	0.6181	0.614848	FALSE	FALSE	FALSE	ieu-b-508	5	6055696	0.034361	486484	0.807526	Sepsis (28	Sepsis (28	Sepsis (28	TRUE	igd	0.0027	4.78E-06	7269	M33510.m	TRUE	reported	WxGKnn	2	TRUE	0.002801	20.41139
7499	rs736396	T	C	T	C	-0.0122	-0.02303	0.3554	0.344932	FALSE	FALSE	FALSE	ieu-b-508	14	94355461	0.035398	486484	0.515305	Sepsis (28	Sepsis (28	Sepsis (28	TRUE	igd	0.0027	4.68E-06	7261	M35253.m	TRUE	reported	VboRmj	2	TRUE	0.002804	20.41139
7776	rs573812	T	C	T	C	0.0122	0.059085	0.8073	0.806894	FALSE	FALSE	FALSE	ieu-b-508	10	25631649	0.042473	486484	0.164189	Sepsis (28	Sepsis (28	Sepsis (28	TRUE	igd	0.0027	6.12E-06	7256	M35439.m	TRUE	reported	DnuAGZ	2	TRUE	0.002806	20.41138
535	rs1251583	T	G	T	G	0.0244	0.086757	0.8744	0.873307	FALSE	FALSE	FALSE	ieu-b-508	5	1.01E+08	0.050724	486484	0.087195	Sepsis (28	Sepsis (28	Sepsis (28	TRUE	igd	0.0054	5.79E-06	7237	M00599.m	TRUE	reported	nRJY3s	2	TRUE	0.002813	20.41137
2830	rs1886152	A	G	A	G	0.0122	0.077878	0.3789	0.376675	FALSE	FALSE	FALSE	ieu-b-508	13	43550021	0.034412	486484	0.023629	Sepsis (28	Sepsis (28	Sepsis (28	TRUE	igd	0.0027	8.34E-06	7180	M27513.m	TRUE	reported	o9Ff2u	2	TRUE	0.002836	20.41132
938	rs4746017	A	C	A	C	-0.0122	-0.00484	0.713	0.712843	FALSE	FALSE	FALSE	ieu-b-508	10	71774872	0.036946	486484	0.895684	Sepsis (28	Sepsis (28	Sepsis (28	TRUE	igd	0.0027	5.10E-06	7160	M01508.m	TRUE	reported	go526k	2	TRUE	0.002843	20.41131
7512	rs3808756	A	G	A	G	0.0122	0.029069	0.6465	0.650798	FALSE	FALSE	FALSE	ieu-b-508	9	17613834	0.035176	486484	0.408596	Sepsis (28	Sepsis (28	Sepsis (28	TRUE	igd	0.0027	5.65E-06	7100	M35254.m	TRUE	reported	HfVgPn	2	TRUE	0.002867	20.41126
5241	rs1325431	T	C	T	C	0.0122	0.010875	0.2929	0.293137	FALSE	FALSE	FALSE	ieu-b-508	8	52385606	0.036833	486484	0.767791	Sepsis (28	Sepsis (28	Sepsis (28	TRUE	igd	0.0027	7.58E-06	7063	M33228.m	TRUE	reported	JwOXbQ	2	TRUE	0.002882	20.41123
6515	rs1158932	A	C	A	C	0.0122	-0.09249	0.5676	0.555781	FALSE	FALSE	FALSE	ieu-b-508	21	28657200	0.034009	486484	0.006538	Sepsis (28	Sepsis (28	Sepsis (28	TRUE	igd	0.0027	4.60E-06	7049	M34123.m	TRUE	reported	9O5wo1	2	TRUE	0.002888	20.41122
7732	rs1247784	T	C	T	C	0.0122	0.001799	0.1863	0.191955	FALSE	FALSE	FALSE	ieu-b-508	2	1.45E+08	0.04253	486484	0.966255	Sepsis (28	Sepsis (28	Sepsis (28	TRUE	igd	0.0027	5.89E-06	7007	M35431.m	TRUE	reported	tLfE4l	2	TRUE	0.002905	20.41118
2380	rs430508	A	G	A	G	-0.0122	0.03442	0.4994	0.49963	FALSE	FALSE	FALSE	ieu-b-508	5	73265685	0.03363	486484	0.306073	Sepsis (28	Sepsis (28	Sepsis (28	TRUE	igd	0.0027	7.02E-06	6999	M21127.m	TRUE	reported	WCjhS3	2	TRUE	0.002909	20.41118
2383	rs6089908	T	C	T	C	-0.0122	0.00872	0.5596	0.564594	FALSE	FALSE	FALSE	ieu-b-508	20	62048654	0.034068	486484	0.797995	Sepsis (28	Sepsis (28	Sepsis (28	TRUE	igd	0.0027	7.19E-06	6999	M21127.m	TRUE	reported	WCjhS3	2	TRUE	0.002909	20.41118
6602	rs1389923	T	G	T	G	0.0122	0.034247	0.2697	0.264552	FALSE	FALSE	FALSE	ieu-b-508	6	1.66E+08	0.037896	486484	0.366147	Sepsis (28	Sepsis (28	Sepsis (28	TRUE	igd	0.0027	7.18E-06	6994	M34306.m	TRUE	reported	WPXPUN	2	TRUE	0.002911	20.41117
6604	rs1735548	T	C	T	C	-0.0122	0.018028	0.3071	0.348712	FALSE	FALSE	FALSE	ieu-b-508	2	5475784	0.035077	486484	0.60729	Sepsis (28	Sepsis (28	Sepsis (28	TRUE	igd	0.0027	5.01E-06	6994	M34306.m	TRUE	reported	WPXPUN	2	TRUE	0.002911	20.41117
756	rs1271312	T	C	T	C	0.0122	0.028259	0.7564	0.758163	FALSE	FALSE	FALSE	ieu-b-508	2	50876684	0.039329	486484	0.472434	Sepsis (28	Sepsis (28	Sepsis (28	TRUE	igd	0.0027	6.74E-06	6946	M01303.m	TRUE	reported	3b8x0F	2	TRUE	0.002931	20.41113
2202	rs2151539	T	C	T	C	-0.0122	0.022363	0.7279	0.727574	FALSE	FALSE	FALSE	ieu-b-508	13	93235051	0.039648	486484	0.572729	Sepsis (28	Sepsis (28	Sepsis (28	TRUE	igd	0.0027	4.57E-06	6929	M19364.m	TRUE	reported	XkjMEx	2	TRUE	0.002938	20.41112
1540	rs1232529	C	G	C	G	0.0122	-0.00109	0.6932	0.681039	FALSE	TRUE	FALSE	ieu-b-508	16	86663253	0.035903	486484	0.975689	Sepsis (28	Sepsis (28	Sepsis (28	TRUE	igd	0.0027	6.94E-06	6864	M12129.m	TRUE	reported	iNzdgb	2	TRUE	0.002966	20.41106
7471	rs6973126	T	C	T	C	0.0122	-0.05352	0.5122	0.526882	FALSE	FALSE	FALSE	ieu-b-508	7	95131217	0.03341	486484	0.109178	Sepsis (28	Sepsis (28	Sepsis (28	TRUE	igd	0.0027	7.06E-06	6543	M35193.m	TRUE	reported	BMMWXU	2	TRUE	0.003111	20.41077

6279	rs1169216	T	C	T	C	0.0122	-0.03548	0.2324	0.239747	FALSE	FALSE	FALSE	ieu-b-508	2	2.12E+08	0.039232	486484	0.365735	Sepsis (28	Sepsis (28	Sepsis (28	TRUE	igd	0.0027	4.70E-06	6502	M33939.m	TRUE	reported	PeKlgz	2	TRUE	0.00313	20.41073
5225	rs1713763	A	G	A	G	0.0845	-0.02396	0.0135	0.016045	FALSE	FALSE	FALSE	ieu-b-508	5	1.01E+08	0.134213	486484	0.858284	Sepsis (28	Sepsis (28	Sepsis (28	TRUE	igd	0.0187	6.37E-06	4665	M33221.m	TRUE	reported	8ZrV1r	2	TRUE	0.004358	20.41005
6780	rs9971507	A	G	A	G	0.322	0.038082	0.0347	0.037047	FALSE	FALSE	FALSE	ieu-b-508	11	10169895	0.087654	486484	0.663952	Sepsis (28	Sepsis (28	Sepsis (28	TRUE	igd	0.0712	6.17E-06	955	M34344.m	TRUE	reported	enFm6p	2	TRUE	0.020967	20.40992
2912	rs2041992	T	C	T	C	-0.0122	-0.02965	0.3478	0.347974	FALSE	FALSE	FALSE	ieu-b-508	19	46524471	0.035191	486484	0.399489	Sepsis (28	Sepsis (28	Sepsis (28	TRUE	igd	0.0027	8.55E-06	5562	M27728.m	TRUE	reported	WOp1XE	2	TRUE	0.003657	20.40967
2914	rs2114588	A	G	A	G	-0.0122	0.004327	0.3114	0.308046	FALSE	FALSE	FALSE	ieu-b-508	2	2.31E+08	0.036072	486484	0.904516	Sepsis (28	Sepsis (28	Sepsis (28	TRUE	igd	0.0027	6.99E-06	5562	M27728.m	TRUE	reported	WOp1XE	2	TRUE	0.003657	20.40967
3851	rs4752176	T	C	T	C	-0.0122	0.003541	0.4565	0.454948	FALSE	FALSE	FALSE	ieu-b-508	10	1.2E+08	0.033496	486484	0.915801	Sepsis (28	Sepsis (28	Sepsis (28	TRUE	igd	0.0027	7.83E-06	5211	M32651.m	TRUE	reported	DeuPwv	2	TRUE	0.003903	20.40917
6908	rs1166418	A	G	A	G	-0.0244	-0.01311	0.8302	0.837483	FALSE	FALSE	FALSE	ieu-b-508	18	57599723	0.045299	486484	0.772264	Sepsis (28	Sepsis (28	Sepsis (28	TRUE	igd	0.0054	6.66E-06	5195	M34389.m	TRUE	reported	suiRlN	2	TRUE	0.003915	20.40915
3414	rs4753364	A	C	A	C	-0.0244	-0.04632	0.2753	0.273946	FALSE	FALSE	FALSE	ieu-b-508	11	91758752	0.037373	486484	0.215192	Sepsis (28	Sepsis (28	Sepsis (28	TRUE	igd	0.0054	7.34E-06	5080	M32445.m	TRUE	reported	qcc6f3	2	TRUE	0.004003	20.40897
1915	rs1517397	T	C	T	C	0.0122	0.045496	0.3986	0.389553	FALSE	FALSE	FALSE	ieu-b-508	2	72189250	0.034322	486484	0.184986	Sepsis (28	Sepsis (28	Sepsis (28	TRUE	igd	0.0027	4.44E-06	4689	M16816.m	TRUE	reported	J1abbO	2	TRUE	0.004335	20.4083
8072	rs9877943	A	G	A	G	0.0244	0.02195	0.2282	0.223059	FALSE	FALSE	FALSE	ieu-b-508	3	37914922	0.039989	486484	0.583072	Sepsis (28	Sepsis (28	Sepsis (28	TRUE	igd	0.0054	5.99E-06	4529	M35978.m	TRUE	reported	CN0wRR	2	TRUE	0.004488	20.40799
8437	rs2053585	A	G	A	G	0.0556	-0.00686	0.6736	0.669016	FALSE	FALSE	FALSE	ieu-b-508	17	66804173	0.035557	486484	0.846973	Sepsis (28	Sepsis (28	Sepsis (28	TRUE	igd	0.0123	6.58E-06	1583	M36850.m	TRUE	reported	j6lLja	2	TRUE	0.012743	20.40752
3646	rs1004405	A	G	A	G	0.0646	0.014112	0.0711	0.080991	FALSE	FALSE	FALSE	ieu-b-508	5	1.03E+08	0.061511	486484	0.818541	Sepsis (28	Sepsis (28	Sepsis (28	TRUE	igd	0.0143	6.59E-06	6970	M32564.m	TRUE	reported	73MgOk	2	TRUE	0.002919	20.40179
7133	rs322906	T	G	T	G	-0.0235	0.000754	0.4573	0.495991	FALSE	FALSE	FALSE	ieu-b-508	1	1.99E+08	0.033524	486484	0.982048	Sepsis (28	Sepsis (28	Sepsis (28	TRUE	igd	0.0052	5.84E-06	1741	M34527.m	TRUE	reported	EWFGP8	2	TRUE	0.011595	20.39999
58	rs1683628	A	C	A	C	0.0131	0.057995	0.044	0.046578	FALSE	FALSE	FALSE	ieu-b-508	4	4807203	0.079473	486484	0.465546	Sepsis (28	Sepsis (28	Sepsis (28	TRUE	igd	0.0029	5.60E-06	7355	M00054.m	TRUE	reported	QTDAnh	2	TRUE	0.002767	20.39992
1709	rs1210500	A	G	A	G	-0.0131	0.046922	0.0487	0.054818	FALSE	FALSE	FALSE	ieu-b-508	2	44382375	0.073913	486484	0.525548	Sepsis (28	Sepsis (28	Sepsis (28	TRUE	igd	0.0029	7.55E-06	7310	M15506.m	TRUE	reported	mVRmZO	2	TRUE	0.002784	20.39989
1837	rs1289906	T	C	T	C	0.0131	-0.02833	0.7665	0.690583	FALSE	FALSE	FALSE	ieu-b-508	15	71016383	0.036136	486484	0.433075	Sepsis (28	Sepsis (28	Sepsis (28	TRUE	igd	0.0029	8.58E-06	7308	M15778.m	TRUE	reported	941o3l	2	TRUE	0.002784	20.39989
8283	rs594084	T	C	T	C	-0.0131	0.056954	0.6304	0.637114	FALSE	FALSE	FALSE	ieu-b-508	9	3925954	0.03496	486484	0.10329	Sepsis (28	Sepsis (28	Sepsis (28	TRUE	igd	0.0029	5.55E-06	6464	M36553.m	TRUE	reported	2Rk7nd	2	TRUE	0.003147	20.39916
6201	rs3007381	T	G	T	G	0.0131	0.017413	0.6033	0.600288	FALSE	FALSE	FALSE	ieu-b-508	1	1.69E+08	0.034092	486484	0.609523	Sepsis (28	Sepsis (28	Sepsis (28	TRUE	igd	0.0029	6.43E-06	6004	M33892.m	TRUE	reported	w7HgU7	2	TRUE	0.003387	20.39867
1949	rs1373765	T	C	T	C	-0.0637	0.07527	0.7973	0.73406	FALSE	FALSE	FALSE	ieu-b-508	18	49746600	0.038056	486484	0.047942	Sepsis (28	Sepsis (28	Sepsis (28	TRUE	igd	0.0141	6.42E-06	3393	M16823.m	TRUE	reported	fNy2PD	2	TRUE	0.005979	20.39786
4699	rs1103854	T	C	T	C	-0.2283	-0.11191	0.9659	0.979626	FALSE	FALSE	FALSE	ieu-b-508	11	5670332	0.119096	486484	0.347385	Sepsis (28	Sepsis (28	Sepsis (28	TRUE	igd	0.0505	6.23E-06	1017	M33178.m	TRUE	reported	g22WMS	2	TRUE	0.0197	20.39737
6381	rs7164415	C	G	C	G	0.0958	0.132494	0.9751	0.981389	FALSE	TRUE	FALSE	ieu-b-508	15	60106610	0.125078	486484	0.28947	Sepsis (28	Sepsis (28	Sepsis (28	TRUE	igd	0.0212	5.95E-06	1751	M33968.m	TRUE	reported	sgVOvT	2	TRUE	0.011528	20.39684
7140	rs1088748	T	C	T	C	0.2627	-0.06775	0.0266	0.039954	FALSE	FALSE	FALSE	ieu-b-508	10	87220042	0.08772	486484	0.439913	Sepsis (28	Sepsis (28	Sepsis (28	TRUE	igd	0.0581	6.06E-06	829	M34530.m	TRUE	reported	8plvGA	2	TRUE	0.024068	20.39477
7510	rs1783249	T	C	T	C	0.0271	0.017093	0.0521	0.057421	FALSE	FALSE	FALSE	ieu-b-508	14	56341221	0.072031	486484	0.812418	Sepsis (28	Sepsis (28	Sepsis (28	TRUE	igd	0.006	6.16E-06	7100	M35254.m	TRUE	reported	HfVgPn	2	TRUE	0.002865	20.39453
5848	rs7338260	T	C	T	C	0.0515	-0.01474	0.0857	0.10056	FALSE	FALSE	FALSE	ieu-b-508	13	46152169	0.056051	486484	0.792609	Sepsis (28	Sepsis (28	Sepsis (28	TRUE	igd	0.0114	5.94E-06	2859	M33531.m	TRUE	reported	kNM0s1	2	TRUE	0.007088	20.39393
5654	rs2733118	C	G	C	G	0.0271	-0.01879	0.118	0.11976	FALSE	TRUE	FALSE	ieu-b-508	18	28268836	0.05136	486484	0.714509	Sepsis (28	Sepsis (28	Sepsis (28	TRUE	igd	0.006	5.49E-06	5717	M33453.m	TRUE	reported	OzEeLQ	2	TRUE	0.003556	20.39314
3313	rs1776854	T	C	T	C	-0.014	0.01218	0.8282	0.834181	FALSE	FALSE	FALSE	ieu-b-508	10	46052917	0.044749	486484	0.785476	Sepsis (28	Sepsis (28	Sepsis (28	TRUE	igd	0.0031	8.33E-06	7307	M32393.m	TRUE	reported	Pub0X0	2	TRUE	0.002783	20.38984
1426	rs2900071	T	C	T	C	-0.014	-0.02643	0.2178	0.227675	FALSE	FALSE	FALSE	ieu-b-508	5	1.13E+08	0.0398	486484	0.506648	Sepsis (28	Sepsis (28	Sepsis (28	TRUE	igd	0.0031	7.46E-06	6941	M03127.m	TRUE	reported	JscEZw	2	TRUE	0.00293	20.38954
4198	rs2567308	A	G	A	G	-0.0524	-0.01536	0.8244	0.849818	FALSE	FALSE	FALSE	ieu-b-508	9	1.04E+08	0.046823	486484	0.742805	Sepsis (28	Sepsis (28	Sepsis (28	TRUE	igd	0.0116	5.86E-06	2537	M32762.m	TRUE	reported	Q5lKLH	2	TRUE	0.007979	20.38938
1805	rs609236	C	G	C	G	0.028	-0.00314	0.1512	0.164176	FALSE	TRUE	FALSE	ieu-b-508	11	94845775	0.044944	486484	0.944327	Sepsis (28	Sepsis (28	Sepsis (28	TRUE	igd	0.0062	5.57E-06	5829	M15749.m	TRUE	reported	TR861N	2	TRUE	0.003487	20.38842
6926	rs4805131	A	G	A	G	-0.028	-0.07843	0.7825	0.78303	FALSE	FALSE	FALSE	ieu-b-508	19	36097297	0.040432	486484	0.052399	Sepsis (28	Sepsis (28	Sepsis (28	TRUE	igd	0.0062	5.64E-06	5561	M34390.m	TRUE	reported	wruTbW	2	TRUE	0.003654	20.38809
4872	rs1799453	C	G	C	G	0.2531	-0.01998	0.9804	0.946435	FALSE	TRUE	FALSE	ieu-b-508	12	16322243	0.075071	486484	0.790142	Sepsis (28	Sepsis (28	Sepsis (28	TRUE	igd	0.056	6.14E-06	1017	M33178.m	TRUE	reported	g22WMS	2	TRUE	0.01969	20.387
6791	rs6793840	A	G	A	G	0.0411	-0.05761	0.5465	0.53109	FALSE	FALSE	FALSE	ieu-b-508	3	1.18E+08	0.033687	486484	0.087215	Sepsis (28	Sepsis (28	Sepsis (28	TRUE	igd	0.0091	5.72E-06	3491	M34350.m	TRUE	reported	Cc3yW9	2	TRUE	0.005809	20.38694
5588	rs7520433	C	G	C	G	-0.8675	0.094414	0.0583	0.035786	FALSE	TRUE	FALSE	ieu-b-508	1	2.35E+08	0.090768	486484	0.298261	Sepsis (28	Sepsis (28	Sepsis (28	TRUE	igd	0.1889	4.38E-06	60	M33423.m	TRUE	reported	MoEnO7	2	TRUE	0.260081	20.38693
7838	rs1159296	T	C	T	C	-0.061	-0.03787	0.7444	0.728444	FALSE	FALSE	FALSE	ieu-b-508	18	10864553	0.037608	486484	0.313896	Sepsis (28	Sepsis (28	Sepsis (28	TRUE	igd	0.0135	6.60E-06	1340	M35551.m	TRUE	reported	5Sxswl	2	TRUE	0.015008	20.38654
5924	rs6599251	T	G	T	G	-0.028	0.048624	0.553	0.550972	FALSE	FALSE	FALSE	ieu-b-508	3	38785809	0.033644	486484	0.148396	Sepsis (28	Sepsis (28	Sepsis (28	TRUE	igd	0.0062	5.41E-06	4323	M33633.m	TRUE	reported	e36XGE	2	TRUE	0.004696	20.38599
4466	rs1199948	A	G	A	G	0.0384	-0.01466	0.8305	0.830406	FALSE	FALSE	FALSE	ieu-b-508	9	25667662	0.044338	486484	0.740947	Sepsis (28	Sepsis (28	Sepsis (28	TRUE	igd	0.0085	6.47E-06	1618	M33009.m	TRUE	reported	8RTEdM	2	TRUE	0.012457	20.38391
6570	rs1630427	C	G	C	G	-0.0289	-0.00371	0.1047	0.114077	FALSE	TRUE	FALSE	ieu-b-508	11	82330204	0.052651	486484	0.943836	Sepsis (28	Sepsis (28	Sepsis (28	TRUE	igd	0.0064	7.48E-06	4908	M34245.m	TRUE	reported	pRwBKr	2	TRUE	0.004137	20.38256
2339	rs729253	T	C	T	C	0.0262	0.02426	0.5812	0.555048	FALSE	FALSE	FALSE	ieu-b-508	2	1.18E+08	0.033619	486484	0.470526	Sepsis (28	Sepsis (28	Sepsis (28	TRUE	igd	0.0058	7.52E-06	1765	M21044.m	TRUE	reported	Ym5rae	2	TRUE	0.011429	20.38235
709	rs1073838	A	G	A	G	-0.0131	-0.05817	0.6175	0.61147	FALSE	FALSE	FALSE	ieu-b-508	9	15016042	0.034205	486484	0.088992	Sepsis (28	Sepsis (28	Sepsis (28	TRUE	igd	0.0029	7.39E-06	1763	M01301.m	TRUE	reported	w8TL71	2	TRUE	0.011442	20.38232
4551	rs3844297	T	C	T	C	-0.0149	-0.05079	0.5092	0.51511	FALSE	FALSE	FALSE	ieu-b-508	4	71406944	0.033819	486484	0.133154	Sepsis (28	Sepsis (28	Sepsis (28	TRUE	igd	0.0033	7.10E-06	7319	M33140.m	TRUE	reported	eBOUtb	2	TRUE	0.002778	20.38102
2683	rs4765356	A	G	A	G	0.0149	0.035924	0.8548	0.858109	FALSE	FALSE	FALSE	ieu-b-508	12	1.25E+08	0.048458	486484	0.458484	Sepsis (28	Sepsis (28	Sepsis (28	TRUE	igd	0.0033	7.08E-06	7256	M24074.m	TRUE	reported	Lk6D2A	2	TRUE	0.002802	20.38097
865	rs6472299	A	G	A	G	-0.0149	0.054596	0.8381	0.8402	FALSE	FALSE	FALSE	ieu-b-508	8	68365314	0.045626	486484	0.23147	Sepsis (28	Sepsis (28	Sepsis (28	TRUE	igd	0.0033	7.87E-06	7063	M01361.m	TRUE	reported	NIAcQ3	2	TRUE	0.002878	20.38082
8079	rs1836049	T	G	T	G	-0.0926	0.090861	0.9791	0.986471	FALSE	FALSE	FALSE	ieu-b-508	8	16540239	0.144319	486484	0.528969	Sepsis (28	Sepsis (28	Sepsis (28	TRUE	igd	0.0205	6.48E-06	1743	M36009.m	TRUE	reported	2EUZ1U	2	TRUE	0.011571	20.38054
1493	rs1080062	A	G	A	G	-0.0262	0.029329	0.2767	0.315363	FALSE	FALSE	FALSE	ieu-b-508	1	1.99E+08	0.035841	486484	0.413176	Sepsis (28	Sepsis (28	Sepsis (28	TRUE	igd	0.0058	6.17E-06	1452	M12017.m	TRUE	reported	bbGtsK	2	TRUE	0.013859	20.37736
1947	rs1258463	A	G	A	G	-0.0945	0.003593	0.6398	0.657433	FALSE	FALSE	FALSE	ieu-b-508	13	1.11E+08	0.035102	486484	0.91846	Sepsis (28	Sepsis (28	Sepsis (28	TRUE	igd	0.0209	6.24E-06	604	M16823.m	TRUE	reported	fNy2PD	2	TRUE	0.03274	20.37655
4269	rs1042167	A	G	A	G	0.0307	0.03098	0.2392	0.248539	FALSE	FALSE	FALSE	ieu-b-508	19	58112844	0.038804	486484	0.424648	Sepsis (28	Sepsis (28	Sepsis (28	TRUE	igd	0.0068	7.62E-06	6200	M32808.m	TRUE	reported	vnr36k	2	TRUE	0.003277	20.37599
3975	rs1078462	T	C	T	C	0.0262	0.017527	0.7213	0.71414	FALSE	FALSE	FALSE	ieu-b-508	12	67769982	0.037015	486484	0.635853	Sepsis (28	Sepsis (28	Sepsis (28	TRUE	igd	0.0058	6.12E-06	1298	M32729.m	TRUE	reported	XGRSN1	2	TRUE	0.015477	20.37403
3790	rs1092491	T	C	T	C	-0.0158	0.012474	0.3733	0.394658	FALSE	FALSE	FALSE	ieu-b-508	1	2.47E+08	0.034248	486484	0.715679	Sepsis (28	Sepsis (28	Sepsis (28	TRUE	igd	0.0035	5.48E-06	7369	M32635.m	TRUE	reported	jJl9P8	2	TRUE	0.002758	20.37324
2341	rs876927	T	G	T	G	0.0158	-0.00111	0.2075	0.205772	FALSE	FALSE	FALSE	ieu-b-508	9	88117073	0.043231	486484	0.979447	Sepsis (28	Sepsis (28	Sepsis (28	TRUE	igd	0.0035	5.42E-06	7366	M21044.m	TRUE	reported	Ym5rae	2	TRUE	0.002759	20.37324
3156	rs4149121	C	G	C	G	0.0158	-0.02792	0.308	0.316904	FALSE	TRUE	FALSE	ieu-b-508	12	21015046	0.035918	486484	0.436933	Sepsis (28	Sepsis (28	Sepsis (28	TRUE	igd	0.0035	7.69E-06	7357	M32322.m	TRUE	reported	p4H9pm	2	TRUE	0.002762	20.37324
5672	rs1248126	T	C	T	C	-0.0158	-0.04988	0.9208	0.906363	FALSE	FALSE	FALSE	ieu-b-508	20	42079562	0.056891	486484	0.380591	Sepsis (28	Sepsis (28	Sepsis (28	TRUE	igd	0.0035	8.56E-06	7307	M33477.m	TRUE	reported	Aw8PBK	2	TRUE	0.002781	20.3732
7403	rs2458177	C	G	C	G	0.0158	0.001208	0.3897	0.404083	FALSE	TRUE	FALSE	ieu-b-508	15	70605521	0.034149	486484	0.97177	Sepsis (28	Sepsis (28	Sepsis (28	TRUE	igd	0.0035	8.47E-06	7263	M35160.m	TRUE	reported	eRox3e	2	TRUE	0.002798	20.37316
1595	rs1694034	A	G	A	G	0.0158	-0.06552	0.88	0.8505	FALSE	FALSE	FALSE	ieu-b-508	16	23644586	0.046663	486484	0.160309	Sepsis (28	Sepsis (28	Sepsis (28	TRUE	igd	0.0035	5.36E-06	7258	M12626.m	TRUE	reported	17lClc	2	TRUE	0.0028	20.37316
4254	rs1173667	A	C	A	C	-0.1881	0.147745	0.9635	0.961633	FALSE	FALSE	FALSE	ieu-b-508	4	14852818	0.087185	486484	0.090149	Sepsis (28	Sepsis (28	Sepsis (28	TRUE	igd	0.0416	6.11E-06	562	M32800.m	TRUE	reported	Q7MVsa	2	TRUE	0.035102	20.37242
7708	rs2421332	T	C	T	C	-0.014	-0.03808	0.7667	0.763596	FALSE	FALSE	FALSE	ieu-b-508	2	61878318	0.039662	486484	0.337019	Sepsis (28	Sepsis (28	Sepsis (28	TRUE	igd	0.0031	5.92E-06	1758	M35397.m	TRUE	reported	Y56mZr	2	TRUE	0.011468	20.37222
7594	rs1554690	A	C	A	C	0.0158	0.06344	0.5949	0.593075	FALSE	FALSE	FALSE	ieu-b-508	7	1.46E+08	0.034083	486484	0.062698	Sepsis (28	Sepsis (28	Sepsis (28	TRUE	igd	0.0035	8.17E-06	5979	M35305.m	TRUE	reported	6ybfdq	2	TRUE	0.003397	20.37196
2853	rs6450853	A	G	A	G	-0.0316	0.015724	0.652	0.645321	FALSE	FALSE	FALSE	ieu-b-508	5	31678239	0.035189	486484	0.654997	Sepsis (28	Sepsis (28	Sepsis (28	TRUE	igd	0.007	5.73E-06	5673	M27531.m	TRUE	reported	v01zDr	2	TRUE	0.003579	20.37159
5955	rs9962038	T	C	T	C	0.0316	0.052376	0.2175	0.224938	FALSE	FALSE	FALSE	ieu-b-508	18	74760015	0.04068	486484	0.197922	Sepsis (28	Sepsis (28	Sepsis (28	TRUE	igd	0.007	7.15E-06	5328	M33652.m	TRUE	reported	vYkdTv	2	TRUE	0.00381	20.37113
4153	rs1028885	A	G	A	G	0.042	-0.04853	0.7832	0.755699	FALSE	FALSE	FALSE	ieu-b-508	6	25434518	0.038916	486484	0.212389	Sepsis (28	Sepsis (28	Sepsis (28	TRUE	igd	0.0093	6.06E-06	1612	M32758.m	TRUE	reported	AF0m66	2	TRUE	0.012494	20.37012
2732	rs3751834	A	G	A	G	0.0167	-0.06565	0.8707	0.872157	FALSE	FALSE	FALSE	ieu-b-508	16	79016408	0.050131	486484	0.190355	Sepsis (28	Sepsis (28	Sepsis (28	TRUE	igd	0.0037	8.17E-06	6388	M25599.m	TRUE	reported	fRZxwP	2	TRUE	0.003179	20.36543
4099	rs1051781	T	C	T	C	-0.0501	-0.05719	0.8138	0.806532	FALSE	FALSE	FALSE	ieu-b-508	4	1.66E+08	0.042946	486484	0.18297	Sepsis (28	Sepsis (28	Sepsis (28	TRUE	igd	0.0111	6.62E-06	6379	M32754.m	TRUE	reported	n0okFy	2	TRUE	0.003183	20.36542
4987	rs588113	A	G	A	G	-0.1722	-0.09999	0.0476	0.02761	FALSE	FALSE	FALSE	ieu-b-508	1	1.07E+08	0.101682	486484	0.325447	Sepsis (28	Sepsis (28	Sepsis (28	TRUE	igd	0.0366	2.53E-06	25	M33178.m	TRUE	reported	g22WMS	2	TRUE	0.469623	20.36535
7280	rs9357080	A	G	A	G	-0.0167	-0.01351	0.9074	0.902333	FALSE	FALSE	FALSE	ieu-b-508	6	29322866	0.056287	486484	0.810364	Sepsis (28	Sepsis (28	Sepsis (28	TRUE	igd	0.0037	5.32E-06	5885	M34912.m	TRUE	reported	W8lcT6	2	TRUE	0.00345	20.36488
6531	rs6034944	C	G	C	G	0.0587	-0.16686	0.9395	0.960109	FALSE	TRUE	FALSE	ieu-b-508	20	18024553	0.086512	486484	0.053759	Sepsis (28	Sepsis (28	Sepsis (28	TRUE	igd	0.013	5.81E-06	1644	M34123.m	TRUE	reported	9O5wo1	2	TRUE	0.01225	20.36389
4412	rs4377848	A	G	A	G	0.0307	0.046304	0.1394	0.133576	FALSE	FALSE	FALSE	ieu-b-508	7	39341026	0.049133	486484	0.345978	Sepsis (28	Sepsis (28	Sepsis (28	TRUE	igd	0.0068	6.19E-06	2148	M32869.m	TRUE	reported	Rvim1n	2	TRUE	0.0094	20.36359
3151	rs7342491	T	C	T	C	-0.1192	-0.13372	0.0167	0.012065	FALSE	FALSE	FALSE	ieu-b-508	14	84926611	0.155433	486484	0.389624	Sepsis (28	Sepsis (28	Sepsis (28	TRUE	igd	0.0264	6.49E-06	1765	M32319.m	TRUE	reported	9YFmUx	2	TRUE	0.011419	20.36349
3437	rs4458381	C	G	C	G	-0.0343	0.176832	0.967	0.9547	FALSE	TRUE	FALSE	ieu-b-508	3	1.75E+08	0.08377	486484	0.03478	Sepsis (28	Sepsis (28	Sepsis (28	TRUE	igd	0.0076	6.25E-06	7364	M32452.m	TRUE	reported	8GtFpl	2	TRUE	0.002758	20.36306
6505	rs4455656	A	G	A	G	-0.0343	-0.05361	0.1597	0.162833	FALSE	FALSE	FALSE	ieu-b-508	6	18429920	0.045137	486484	0.234971	Sepsis (28	Sepsis (28	Sepsis (28	TRUE	igd	0.0076	6.88E-06	5581	M34112.m	TRUE	reported	97JqFb	2	TRUE	0.003636	20.36129
2069	rs3846286	A	G	A	G	-0.0307	-0.02792	0.9275	0.927082	FALSE	FALSE	FALSE	ieu-b-508	4	89501246	0.06442	486484	0.66472	Sepsis (28	Sepsis (28	Sepsis (28	TRUE	igd	0.0068	6.64E-06	1762	M18369.m	TRUE	reported	ykBjlT	2	TRUE	0.011436	20.35943
7416	rs1984049	A	C	A	C	0.0307	0.024561	0.8108	0.807324	FALSE	FALSE	FALSE	ieu-b-508	9	12916710	0.042354	486484	0.561983	Sepsis (28	Sepsis (28	Sepsis (28	TRUE	igd	0.0068	5.33E-06	1760	M35186.m	TRUE	reported	UZfu6Z	2	TRUE	0.011448	20.35941
1494	rs1096317	A	G	A	G	0.0352	-0.05459	0.9724	0.967145	FALSE	FALSE	FALSE	ieu-b-508	9	17636441	0.093623	486484	0.559804	Sepsis (28	Sepsis (28	Sepsis (28	TRUE	igd	0.0078	6.99E-06	5656	M12017.m	TRUE	reported	bbGtsK	2	TRUE	0.003588	20.35835
8336	rs1268042	T	C	T	C	0.0176	-0.0055	0.2899	0.300692	FALSE	FALSE	FALSE	ieu-b-508	8	1.32E+08	0.036525	486484	0.880315	Sepsis (28	Sepsis (28	Sepsis (28	TRUE	igd	0.0039	7.77E-06	5292	M36673.m	TRUE	reported	CD5lvR	2	TRUE	0.003834	20.35785
2675	rs1726046	T	C	T	C	0.0361	0.109053	0.9112	0.85745	FALSE	FALSE	FALSE	ieu-b-508	9	20209701	0.048448	486484	0.02439	Sepsis (28	Sepsis (28	Sepsis (28	TRUE	igd	0.008	5.59E-06	7256	M24074.m	TRUE	reported	Lk6D2A	2	TRUE	0.002798	20.35704
1873	rs2345834	T	C	T	C	0.0176	-0.00883	0.6652	0.672193	FALSE	FALSE	FALSE	ieu-b-508	2	18565167	0.035617	486484	0.804213	Sepsis (28	Sepsis (28	Sepsis (28	TRUE	igd	0.0039	5.45E-06	4538	M15958.m	TRUE	reported	3HF6lf	2	TRUE	0.004468	20.35657
1646	rs6575634	T	C	T	C	-0.0158	0.025461	0.6184	0.623355	FALSE	FALSE	FALSE	ieu-b-508	14	98248954	0.034401	486484	0.459223	Sepsis (28	Sepsis (28	Sepsis (28	TRUE	igd	0.0035	4.85E-06	1764	M15140.m	TRUE	reported	jEcqMJ	2	TRUE	0.011421	20.35567
8215	rs7021014	T	G	T	G	0.0334	-0.00759	0.7224	0.70506	FALSE	FALSE	FALSE	ieu-b-508	9	82517423	0.036522	486484	0.835402	Sepsis (28	Sepsis (28	Sepsis (28	TRUE	igd	0.0074	6.59E-06	2520	M36376.m	TRUE	reported	k6uC1l	2	TRUE	0.008019	20.35564
2861	rs3847067	A	C	A	C	-0.0316	0.04124	0.2877	0.284322	FALSE	FALSE	FALSE	ieu-b-508	7	1.01E+08	0.036971	486484	0.264644	Sepsis (28	Sepsis (28	Sepsis (28	TRUE	igd	0.007	6.69E-06	1758	M27672.m	TRUE	reported	5cTg55	2	TRUE	0.011459	20.35559
3383	rs6075489	A	G	A	G	0.0185	0.051193	0.7177	0.705583	FALSE	FALSE	FALSE	ieu-b-508	20	19208970	0.036899	486484	0.165321	Sepsis (28	Sepsis (28	Sepsis (28	TRUE	igd	0.0041	5.76E-06	7355	M32418.m	TRUE	reported	y8cN4Y	2	TRUE	0.002761	20.35437
624	rs1735132	A	G	A	G	-0.0185	-0.06728	0.8651	0.863581	FALSE	FALSE	FALSE	ieu-b-508	5	1.07E+08	0.049047	486484	0.170168	Sepsis (28	Sepsis (28	Sepsis (28	TRUE	igd	0.0041	5.37E-06	7349	M01121.m	TRUE	reported	HU0fhR	2	TRUE	0.002763	20.35436
2931	rs8124907	A	G	A	G	0.0307	-0.06328	0.7514	0.758131	FALSE	FALSE	FALSE	ieu-b-508	20	60913127	0.038943	486484	0.104199	Sepsis (28	Sepsis (28	Sepsis (28	TRUE	igd	0.0068	5.85E-06	1227	M27728.m	TRUE	reported	WOp1XE	2	TRUE	0.01634	20.34935
4283	rs1022480	T	C	T	C	0.0194	0.048298	0.8787	0.882804	FALSE	FALSE	FALSE	ieu-b-508	7	8336923	0.052028	486484	0.353246	Sepsis (28	Sepsis (28	Sepsis (28	TRUE	igd	0.0043	5.64E-06	7063	M32814.m	TRUE	reported	z0OeTx	2	TRUE	0.002874	20.34902
618	rs7427777	A	C	A	C	0.1291	-0.13466	0.0483	0.036631	FALSE	FALSE	FALSE	ieu-b-508	3	1.82E+08	0.090152	486484	0.135256	Sepsis (28	Sepsis (28	Sepsis (28	TRUE	igd	0.0286	6.36E-06	1447	M01114.m	TRUE	reported	fqHdM2	2	TRUE	0.013886	20.34791
7156	rs2218275	A	T	A	T	0.0582	-0.01646	0.9535	0.954176	FALSE	TRUE	FALSE	ieu-b-508	12	71014525	0.079968	486484	0.836965	Sepsis (28	Sepsis (28	Sepsis (28	TRUE	igd	0.0129	6.60E-06	5856	M34533.m	TRUE	reported	SuKX4k	2	TRUE	0.003464	20.34783
1492	rs1001217	T	C	T	C	0.0194	0.027406	0.1065	0.113156	FALSE	FALSE	FALSE	ieu-b-508	4	90332273	0.052647	486484	0.602679	Sepsis (28	Sepsis (28	Sepsis (28	TRUE	igd	0.0043	5.78E-06	5656	M12017.m	TRUE	reported	bbGtsK	2	TRUE	0.003586	20.34759
6458	rs2798002	A	G	A	G	-0.1151	-0.31497	0.0628	0.019131	FALSE	FALSE	FALSE	ieu-b-508	10	95968214	0.122544	486484	0.010163	Sepsis (28	Sepsis (28	Sepsis (28	TRUE	igd	0.0255	6.48E-06	1481	M34106.m	TRUE	reported	8DuoNM	2	TRUE	0.01357	20.3462
6620	rs4454960	C	G	C	G	-0.0745	0.113679	0.8989	0.967057	FALSE	TRUE	FALSE	ieu-b-508	16	83242605	0.094564	486484	0.229306	Sepsis (28	Sepsis (28	Sepsis (28	TRUE	igd	0.0165	6.47E-06	979	M34314.m	TRUE	reported	wPKEWG	2	TRUE	0.020399	20.34495
2625	rs6683669	A	G	A	G	-0.0203	0.057325	0.0682	0.07142	FALSE	FALSE	FALSE	ieu-b-508	1	59041092	0.064604	486484	0.374899	Sepsis (28	Sepsis (28	Sepsis (28	TRUE	igd	0.0045	7.10E-06	7329	M22481.m	TRUE	reported	bXpf0k	2	TRUE	0.002769	20.34457
1867	rs9859467	A	G	A	G	-0.0203	-0.04127	0.037	0.049221	FALSE	FALSE	FALSE	ieu-b-508	3	1.18E+08	0.077207	486484	0.592925	Sepsis (28	Sepsis (28	Sepsis (28	TRUE	igd	0.0045	5.75E-06	7308	M15778.m	TRUE	reported	941o3l	2	TRUE	0.002777	20.34455
2837	rs426434	T	C	T	C	0.0203	-0.01372	0.1192	0.121264	FALSE	FALSE	FALSE	ieu-b-508	17	28925353	0.050926	486484	0.787562	Sepsis (28	Sepsis (28	Sepsis (28	TRUE	igd	0.0045	6.99E-06	7180	M27513.m	TRUE	reported	o9Ff2u	2	TRUE	0.002826	20.34445
2384	rs757717	T	C	T	C	0.0203	0.117516	0.1121	0.129457	FALSE	FALSE	FALSE	ieu-b-508	7	1.01E+08	0.049975	486484	0.018697	Sepsis (28	Sepsis (28	Sepsis (28	TRUE	igd	0.0045	8.12E-06	6999	M21127.m	TRUE	reported	WCjhS3	2	TRUE	0.002899	20.34431
4333	rs4500414	A	G	A	G	-0.0203	-0.01009	0.6497	0.632079	FALSE	FALSE	FALSE	ieu-b-508	10	95973328	0.034755	486484	0.771488	Sepsis (28	Sepsis (28	Sepsis (28	TRUE	igd	0.0045	7.44E-06	6967	M32847.m	TRUE	reported	eYme29	2	TRUE	0.002912	20.34428
6772	rs1219492	T	C	T	C	0.5355	-0.06837	0.0118	0.019156	FALSE	FALSE	FALSE	ieu-b-508	6	96496813	0.121641	486484	0.574072	Sepsis (28	Sepsis (28	Sepsis (28	TRUE	igd	0.1186	6.35E-06	955	M34344.m	TRUE	reported	enFm6p	2	TRUE	0.020901	20.34413
2442	rs2314600	A	C	A	C	-0.1047	-0.09978	0.0144	0.009537	FALSE	FALSE	FALSE	ieu-b-508	4	17139699	0.169612	486484	0.556351	Sepsis (28	Sepsis (28	Sepsis (28	TRUE	igd	0.0232	6.67E-06	1755	M21630.m	TRUE	reported	7eaO4H	2	TRUE	0.011472	20.34334
6487	rs6812391	A	G	A	G	-0.7293	-0.10244	0.0441	0.056034	FALSE	FALSE	FALSE	ieu-b-508	4	6963893	0.072519	486484	0.157792	Sepsis (28	Sepsis (28	Sepsis (28	TRUE	igd	0.1606	5.61E-06	148	M34109.m	TRUE	reported	piJuC7	2	TRUE	0.122295	20.34288
2777	rs1456374	T	C	T	C	-0.0176	0.005085	0.6002	0.62574	FALSE	FALSE	FALSE	ieu-b-508	4	1.17E+08	0.034498	486484	0.882825	Sepsis (28	Sepsis (28	Sepsis (28	TRUE	igd	0.0039	7.04E-06	1751	M27273.m	TRUE	reported	EvU2od	2	TRUE	0.011497	20.34229
5899	rs7222177	T	C	T	C	0.5557	-0.37816	0.0108	0.01327	FALSE	FALSE	FALSE	ieu-b-508	17	32344384	0.156384	486484	0.0156	Sepsis (28	Sepsis (28	Sepsis (28	TRUE	igd	0.122	5.20E-06	102	M33610.m	TRUE	reported	tmg43v	2	TRUE	0.169024	20.34047
8352	rs1889603	A	G	A	G	-0.086	-0.05375	0.747	0.739259	FALSE	FALSE	FALSE	ieu-b-508	1	71154774	0.038051	486484	0.15782	Sepsis (28	Sepsis (28	Sepsis (28	TRUE	igd	0.019	6.00E-06	274	M36738.m	TRUE	reported	mVQQcD	2	TRUE	0.06957	20.33799
6723	rs1302821	C	G	C	G	-0.3375	0.076345	0.9809	0.937945	FALSE	TRUE	FALSE	ieu-b-508	2	83975589	0.070518	486484	0.278971	Sepsis (28	Sepsis (28	Sepsis (28	TRUE	igd	0.0747	6.30E-06	535	M34338.m	TRUE	reported	h4FB9a	2	TRUE	0.036753	20.33667
3401	rs628137	A	C	A	C	-0.0221	-0.01689	0.3719	0.365755	FALSE	FALSE	FALSE	ieu-b-508	13	22318853	0.034724	486484	0.626588	Sepsis (28	Sepsis (28	Sepsis (28	TRUE	igd	0.0049	6.69E-06	7346	M32425.m	TRUE	reported	u5RLGY	2	TRUE	0.002761	20.3364
8530	rs4453613	T	C	T	C	0.06	-0.00826	0.3355	0.33912	FALSE	FALSE	FALSE	ieu-b-508	18	64921663	0.035519	486484	0.81617	Sepsis (28	Sepsis (28	Sepsis (28	TRUE	igd	0.0133	6.83E-06	2560	M37112.m	TRUE	reported	P3GiAg	2	TRUE	0.007887	20.33573
3643	rs7103589	T	G	T	G	0.0388	0.011638	0.6053	0.599976	FALSE	FALSE	FALSE	ieu-b-508	11	11601537	0.034081	486484	0.73275	Sepsis (28	Sepsis (28	Sepsis (28	TRUE	igd	0.0086	5.72E-06	2091	M32560.m	TRUE	reported	sLwsEw	2	TRUE	0.009641	20.33532
3661	rs3846714	A	G	A	G	0.0672	-0.08494	0.065	0.061507	FALSE	FALSE	FALSE	ieu-b-508	5	1.51E+08	0.072637	486484	0.242228	Sepsis (28	Sepsis (28	Sepsis (28	TRUE	igd	0.0149	6.46E-06	6970	M32564.m	TRUE	reported	73MgOk	2	TRUE	0.00291	20.33487
1665	rs2365522	A	C	A	C	0.0442	-0.0421	0.7137	0.719471	FALSE	FALSE	FALSE	ieu-b-508	4	72776163	0.037059	486484	0.255993	Sepsis (28	Sepsis (28	Sepsis (28	TRUE	igd	0.0098	6.70E-06	5606	M15335.m	TRUE	reported	a7RjFn	2	TRUE	0.003615	20.33468
6362	rs1397262	T	C	T	C	0.023	-0.02664	0.1269	0.137144	FALSE	FALSE	FALSE	ieu-b-508	15	36322534	0.048528	486484	0.583057	Sepsis (28	Sepsis (28	Sepsis (28	TRUE	igd	0.0051	5.66E-06	7368	M33961.m	TRUE	reported	10AWw0	2	TRUE	0.002753	20.33281
1013	rs2239337	C	G	C	G	0.023	0.078361	0.0356	0.032574	FALSE	TRUE	FALSE	ieu-b-508	16	22911553	0.094181	486484	0.40539	Sepsis (28	Sepsis (28	Sepsis (28	TRUE	igd	0.0051	7.51E-06	7364	M01564.m	TRUE	reported	IPUdyD	2	TRUE	0.002754	20.33281
105	rs3780494	T	C	T	C	-0.023	0.019375	0.9855	0.982705	FALSE	FALSE	FALSE	ieu-b-508	9	33126224	0.127744	486484	0.87945	Sepsis (28	Sepsis (28	Sepsis (28	TRUE	igd	0.0051	7.02E-06	7355	M00054.m	TRUE	reported	QTDAnh	2	TRUE	0.002758	20.3328
601	rs7169869	A	G	A	G	-0.0194	-0.02836	0.5404	0.488779	FALSE	FALSE	FALSE	ieu-b-508	15	85478918	0.033348	486484	0.39512	Sepsis (28	Sepsis (28	Sepsis (28	TRUE	igd	0.0043	6.59E-06	1766	M01110.m	TRUE	reported	0A31Sl	2	TRUE	0.011395	20.33173
5328	rs3791608	A	G	A	G	-0.0221	-0.04638	0.1287	0.145877	FALSE	FALSE	FALSE	ieu-b-508	2	2.4E+08	0.047469	486484	0.32855	Sepsis (28	Sepsis (28	Sepsis (28	TRUE	igd	0.0049	7.00E-06	3890	M33364.m	TRUE	reported	9Zrr7Y	2	TRUE	0.005202	20.33148
2389	rs2717871	A	G	A	G	-0.0618	0.06489	0.8155	0.829237	FALSE	FALSE	FALSE	ieu-b-508	7	25316325	0.044621	486484	0.145879	Sepsis (28	Sepsis (28	Sepsis (28	TRUE	igd	0.0137	6.64E-06	2339	M21151.m	TRUE	reported	LfwdLx	2	TRUE	0.008625	20.33126
6953	rs4398579	A	C	A	C	-0.023	0.042402	0.3581	0.385165	FALSE	FALSE	FALSE	ieu-b-508	4	1.38E+08	0.034481	486484	0.218796	Sepsis (28	Sepsis (28	Sepsis (28	TRUE	igd	0.0051	7.53E-06	5689	M34400.m	TRUE	reported	QXmzfR	2	TRUE	0.003562	20.33118
629	rs1161665	A	G	A	G	-0.1069	-0.06028	0.9444	0.939397	FALSE	FALSE	FALSE	ieu-b-508	13	23683941	0.070237	486484	0.390783	Sepsis (28	Sepsis (28	Sepsis (28	TRUE	igd	0.0237	6.37E-06	2639	M01123.m	TRUE	reported	NDTvfD	2	TRUE	0.00765	20.32963
5157	rs6507387	T	C	T	C	-0.0469	-0.01483	0.3592	0.367689	FALSE	FALSE	FALSE	ieu-b-508	18	38593162	0.034638	486484	0.668502	Sepsis (28	Sepsis (28	Sepsis (28	TRUE	igd	0.0104	7.06E-06	5716	M33192.m	TRUE	reported	CyfjKE	2	TRUE	0.003545	20.32952
5344	rs1564511	T	C	T	C	-0.0194	0.033861	0.8539	0.133009	FALSE	FALSE	FALSE	ieu-b-508	4	12658525	0.049203	486484	0.491339	Sepsis (28	Sepsis (28	Sepsis (28	TRUE	igd	0.0043	6.12E-06	1582	M33380.m	TRUE	reported	OHRfl5	2	TRUE	0.012703	20.32905
1989	rs1777793	T	C	T	C	-0.0744	-0.03643	0.0548	0.08705	FALSE	FALSE	FALSE	ieu-b-508	8	84937766	0.059637	486484	0.541285	Sepsis (28	Sepsis (28	Sepsis (28	TRUE	igd	0.0165	6.20E-06	7071	M18254.m	TRUE	reported	clvu6V	2	TRUE	0.002867	20.32615
632	rs1774181	T	C	T	C	0.538	0.015876	0.015	0.068153	FALSE	FALSE	FALSE	ieu-b-508	21	35669770	0.066871	486484	0.812338	Sepsis (28	Sepsis (28	Sepsis (28	TRUE	igd	0.1192	6.39E-06	908	M01123.m	TRUE	reported	NDTvfD	2	TRUE	0.021943	20.32612
2661	rs1099948	T	G	T	G	-0.0496	-0.02943	0.7102	0.699077	FALSE	FALSE	FALSE	ieu-b-508	10	72478945	0.036552	486484	0.420759	Sepsis (28	Sepsis (28	Sepsis (28	TRUE	igd	0.011	7.28E-06	5599	M22842.m	TRUE	reported	0t12oo	2	TRUE	0.003618	20.32464
7003	rs1299693	A	G	A	G	0.1606	-0.07979	0.0536	0.053489	FALSE	FALSE	FALSE	ieu-b-508	2	18898271	0.074327	486484	0.283032	Sepsis (28	Sepsis (28	Sepsis (28	TRUE	igd	0.0356	6.57E-06	1496	M34420.m	TRUE	reported	20uofq	2	TRUE	0.013421	20.32404
8524	rs1582016	T	C	T	C	0.0681	-0.00131	0.2424	0.239035	FALSE	FALSE	FALSE	ieu-b-508	9	18632108	0.039198	486484	0.973321	Sepsis (28	Sepsis (28	Sepsis (28	TRUE	igd	0.0151	6.09E-06	2560	M37112.m	TRUE	reported	P3GiAg	2	TRUE	0.007882	20.32361
4565	rs1120715	C	G	C	G	0.3171	-0.03311	0.0118	0.077856	FALSE	TRUE	FALSE	ieu-b-508	1	58485447	0.063165	486484	0.600149	Sepsis (28	Sepsis (28	Sepsis (28	TRUE	igd	0.0703	6.50E-06	1766	M33150.m	TRUE	reported	H9SSMD	2	TRUE	0.01139	20.32309
7065	rs1235385	T	C	T	C	0.0257	0.037461	0.167	0.207699	FALSE	FALSE	FALSE	ieu-b-508	8	91212707	0.04109	486484	0.361939	Sepsis (28	Sepsis (28	Sepsis (28	TRUE	igd	0.0057	5.66E-06	6144	M34469.m	TRUE	reported	WMOqbm	2	TRUE	0.003298	20.32241
3037	rs4674858	T	C	T	C	0.1484	-0.12861	0.0101	0.011073	FALSE	FALSE	FALSE	ieu-b-508	2	2.25E+08	0.159476	486484	0.419979	Sepsis (28	Sepsis (28	Sepsis (28	TRUE	igd	0.0329	6.61E-06	1713	M31555.m	TRUE	reported	iQ5E3v	2	TRUE	0.011738	20.3221
2122	rs2718107	A	C	A	C	0.0257	-0.01055	0.5408	0.545208	FALSE	FALSE	FALSE	ieu-b-508	7	1.29E+08	0.033524	486484	0.753	Sepsis (28	Sepsis (28	Sepsis (28	TRUE	igd	0.0057	5.85E-06	5624	M18476.m	TRUE	reported	6GnBtw	2	TRUE	0.003602	20.32179
6497	rs1087543	C	G	C	G	-0.0257	-0.02318	0.6582	0.644682	FALSE	TRUE	FALSE	ieu-b-508	8	1.36E+08	0.034834	486484	0.505832	Sepsis (28	Sepsis (28	Sepsis (28	TRUE	igd	0.0057	7.69E-06	5581	M34112.m	TRUE	reported	97JqFb	2	TRUE	0.003629	20.32174
2401	rs1889891	T	C	T	C	0.0257	-0.00294	0.2641	0.269532	FALSE	FALSE	FALSE	ieu-b-508	6	1.3E+08	0.037625	486484	0.937781	Sepsis (28	Sepsis (28	Sepsis (28	TRUE	igd	0.0057	5.77E-06	5436	M21184.m	TRUE	reported	63ppuJ	2	TRUE	0.003726	20.32154
1132	rs9606290	A	G	A	G	0.159	0.087632	0.241	0.262057	FALSE	FALSE	FALSE	ieu-b-508	22	20218237	0.038409	486484	0.022517	Sepsis (28	Sepsis (28	Sepsis (28	TRUE	igd	0.0352	6.32E-06	496	M01640.m	TRUE	reported	o3qxzl	2	TRUE	0.039511	20.32143
581	rs972709	A	C	A	C	0.0483	0.018655	0.6548	0.660058	FALSE	FALSE	FALSE	ieu-b-508	6	66255788	0.035237	486484	0.596517	Sepsis (28	Sepsis (28	Sepsis (28	TRUE	igd	0.0107	6.73E-06	736	M01107.m	TRUE	reported	EbPV1D	2	TRUE	0.026939	20.32099
5140	rs2932286	A	G	A	G	0.0717	0.045942	0.8764	0.885813	FALSE	FALSE	FALSE	ieu-b-508	3	87910931	0.052354	486484	0.380202	Sepsis (28	Sepsis (28	Sepsis (28	TRUE	igd	0.0159	6.83E-06	2616	M33190.m	TRUE	reported	UkhAw5	2	TRUE	0.007713	20.31945
1904	rs1598817	T	G	T	G	0.0541	0.014375	0.9624	0.9621	FALSE	FALSE	FALSE	ieu-b-508	5	1.25E+08	0.088055	486484	0.870323	Sepsis (28	Sepsis (28	Sepsis (28	TRUE	igd	0.012	7.10E-06	7064	M16634.m	TRUE	reported	P9BdXh	2	TRUE	0.002869	20.31931
8449	rs195113	T	C	T	C	0.0221	-0.0318	0.6742	0.679381	FALSE	FALSE	FALSE	ieu-b-508	4	5413525	0.035768	486484	0.373911	Sepsis (28	Sepsis (28	Sepsis (28	TRUE	igd	0.0049	8.04E-06	1782	M37004.m	TRUE	reported	EKCQye	2	TRUE	0.011286	20.31911
2493	rs2933405	A	G	A	G	0.0221	0.090281	0.1507	0.167212	FALSE	FALSE	FALSE	ieu-b-508	12	31285101	0.045059	486484	0.045113	Sepsis (28	Sepsis (28	Sepsis (28	TRUE	igd	0.0049	5.88E-06	1766	M22116.m	TRUE	reported	bjUU6r	2	TRUE	0.011387	20.3189
3113	rs6810706	A	G	A	G	0.0221	-0.02917	0.5523	0.519362	FALSE	FALSE	FALSE	ieu-b-508	4	18750217	0.033398	486484	0.382489	Sepsis (28	Sepsis (28	Sepsis (28	TRUE	igd	0.0049	5.44E-06	1760	M32198.m	TRUE	reported	ndXmnp	2	TRUE	0.011426	20.31883
6950	rs1395489	A	G	A	G	-0.0564	0.008591	0.1479	0.15499	FALSE	FALSE	FALSE	ieu-b-508	12	62048478	0.046126	486484	0.852242	Sepsis (28	Sepsis (28	Sepsis (28	TRUE	igd	0.0125	6.88E-06	1018	M34400.m	TRUE	reported	QXmzfR	2	TRUE	0.019606	20.31815
6922	rs1820124	A	G	A	G	0.0275	-0.01164	0.2432	0.255096	FALSE	FALSE	FALSE	ieu-b-508	5	56817523	0.038308	486484	0.761155	Sepsis (28	Sepsis (28	Sepsis (28	TRUE	igd	0.0061	5.59E-06	5561	M34390.m	TRUE	reported	wruTbW	2	TRUE	0.003641	20.31653
3260	rs1710131	T	C	T	C	-0.0284	0.097607	0.973	0.966065	FALSE	FALSE	FALSE	ieu-b-508	14	64068193	0.092849	486484	0.293144	Sepsis (28	Sepsis (28	Sepsis (28	TRUE	igd	0.0063	5.95E-06	7365	M32348.m	TRUE	reported	DJeLDx	2	TRUE	0.002752	20.31597
2944	rs576266	T	C	T	C	-0.0284	0.044208	0.9071	0.910006	FALSE	FALSE	FALSE	ieu-b-508	11	1.16E+08	0.058406	486484	0.449112	Sepsis (28	Sepsis (28	Sepsis (28	TRUE	igd	0.0063	5.97E-06	7336	M27738.m	TRUE	reported	UtPBd9	2	TRUE	0.002762	20.31595
8057	rs1043349	T	C	T	C	0.055	0.029099	0.9624	0.969331	FALSE	FALSE	FALSE	ieu-b-508	3	1.91E+08	0.097152	486484	0.764543	Sepsis (28	Sepsis (28	Sepsis (28	TRUE	igd	0.0122	6.67E-06	4529	M35978.m	TRUE	reported	CN0wRR	2	TRUE	0.004467	20.31486
8061	rs1418811	T	G	T	G	0.0275	-0.08251	0.8452	0.842516	FALSE	FALSE	FALSE	ieu-b-508	1	1.9E+08	0.045707	486484	0.071049	Sepsis (28	Sepsis (28	Sepsis (28	TRUE	igd	0.0061	5.93E-06	4529	M35978.m	TRUE	reported	CN0wRR	2	TRUE	0.004467	20.31486
7158	rs2326675	A	C	A	C	0.0284	-0.00108	0.7231	0.729527	FALSE	FALSE	FALSE	ieu-b-508	6	5862758	0.037623	486484	0.97709	Sepsis (28	Sepsis (28	Sepsis (28	TRUE	igd	0.0063	6.39E-06	5856	M34533.m	TRUE	reported	SuKX4k	2	TRUE	0.003458	20.31455
3545	rs838532	T	C	T	C	-0.0293	-0.07646	0.9416	0.937393	FALSE	FALSE	FALSE	ieu-b-508	1	1.2E+08	0.068929	486484	0.267331	Sepsis (28	Sepsis (28	Sepsis (28	TRUE	igd	0.0065	6.89E-06	7358	M32497.m	TRUE	reported	244bZN	2	TRUE	0.002754	20.31377
5086	rs890290	T	C	T	C	-0.3402	-0.06641	0.0266	0.012739	FALSE	FALSE	FALSE	ieu-b-508	15	69922693	0.147913	486484	0.653469	Sepsis (28	Sepsis (28	Sepsis (28	TRUE	igd	0.0724	2.60E-06	25	M33178.m	TRUE	reported	g22WMS	2	TRUE	0.468985	20.31325
3914	rs209338	A	T	A	T	0.078	0.072157	0.9651	0.954187	FALSE	TRUE	FALSE	ieu-b-508	5	1.61E+08	0.08116	486484	0.373966	Sepsis (28	Sepsis (28	Sepsis (28	TRUE	igd	0.0173	6.39E-06	2575	M32691.m	TRUE	reported	SiuD6F	2	TRUE	0.007833	20.31232
4258	rs4674860	T	C	T	C	-0.0284	-0.07636	0.4516	0.456382	FALSE	FALSE	FALSE	ieu-b-508	2	2.25E+08	0.033536	486484	0.022792	Sepsis (28	Sepsis (28	Sepsis (28	TRUE	igd	0.0063	5.74E-06	4274	M32800.m	TRUE	reported	Q7MVsa	2	TRUE	0.004732	20.31198
1958	rs6557244	T	C	T	C	0.0302	0.049243	0.9101	0.899887	FALSE	FALSE	FALSE	ieu-b-508	6	1.53E+08	0.055502	486484	0.374964	Sepsis (28	Sepsis (28	Sepsis (28	TRUE	igd	0.0067	7.32E-06	7353	M17805.m	TRUE	reported	ZDRoA2	2	TRUE	0.002756	20.31169
1988	rs1338783	A	T	A	T	-0.0622	0.079736	0.9417	0.935693	FALSE	TRUE	FALSE	ieu-b-508	2	1.07E+08	0.068661	486484	0.245517	Sepsis (28	Sepsis (28	Sepsis (28	TRUE	igd	0.0138	6.24E-06	7071	M18254.m	TRUE	reported	clvu6V	2	TRUE	0.002865	20.30952
8119	rs1202158	A	G	A	G	0.0311	-0.05344	0.3846	0.365727	FALSE	FALSE	FALSE	ieu-b-508	1	1.89E+08	0.034679	486484	0.12329	Sepsis (28	Sepsis (28	Sepsis (28	TRUE	igd	0.0069	6.00E-06	7054	M36098.m	TRUE	reported	hs9SZl	2	TRUE	0.002872	20.30951
8372	rs2081670	T	C	T	C	0.0284	-0.00564	0.1437	0.161291	FALSE	FALSE	FALSE	ieu-b-508	9	92887231	0.045432	486484	0.901262	Sepsis (28	Sepsis (28	Sepsis (28	TRUE	igd	0.0063	6.77E-06	3248	M36756.m	TRUE	reported	QsW4oR	2	TRUE	0.006218	20.30898
7794	rs2209436	T	C	T	C	0.138	0.115432	0.0297	0.029801	FALSE	FALSE	FALSE	ieu-b-508	9	17704139	0.098864	486484	0.242972	Sepsis (28	Sepsis (28	Sepsis (28	TRUE	igd	0.0306	6.62E-06	1383	M35451.m	TRUE	reported	GMRGAN	2	TRUE	0.014493	20.30892
3957	rs2184568	A	G	A	G	-0.0248	-0.00781	0.6762	0.665301	FALSE	FALSE	FALSE	ieu-b-508	10	26648294	0.035318	486484	0.824953	Sepsis (28	Sepsis (28	Sepsis (28	TRUE	igd	0.0055	6.01E-06	1766	M32709.m	TRUE	reported	YR8SB4	2	TRUE	0.011382	20.30887
4932	rs3780205	A	G	A	G	-0.1254	0.029206	0.0519	0.072942	FALSE	FALSE	FALSE	ieu-b-508	9	1.27E+08	0.06448	486484	0.650589	Sepsis (28	Sepsis (28	Sepsis (28	TRUE	igd	0.0278	6.60E-06	1017	M33178.m	TRUE	reported	g22WMS	2	TRUE	0.019615	20.30722
6993	rs1298120	A	C	A	C	0.0496	0.004864	0.0814	0.088358	FALSE	FALSE	FALSE	ieu-b-508	19	15467442	0.058614	486484	0.933861	Sepsis (28	Sepsis (28	Sepsis (28	TRUE	igd	0.011	6.16E-06	1567	M34416.m	TRUE	reported	q9OtaZ	2	TRUE	0.012809	20.30595
5948	rs1078328	A	G	A	G	0.032	-0.00171	0.2235	0.22824	FALSE	FALSE	FALSE	ieu-b-508	12	49146947	0.039631	486484	0.965522	Sepsis (28	Sepsis (28	Sepsis (28	TRUE	igd	0.0071	5.86E-06	5328	M33652.m	TRUE	reported	vYkdTv	2	TRUE	0.003798	20.3058
496	rs1690297	T	G	T	G	-0.1325	0.120565	0.0232	0.033358	FALSE	FALSE	FALSE	ieu-b-508	5	36369458	0.093022	486484	0.194942	Sepsis (28	Sepsis (28	Sepsis (28	TRUE	igd	0.0294	6.36E-06	7274	M00569.m	TRUE	reported	1TjBaz	2	TRUE	0.002785	20.30569
4715	rs1162079	A	T	A	T	-0.0857	0.034767	0.705	0.682526	FALSE	TRUE	FALSE	ieu-b-508	14	26656318	0.035998	486484	0.334141	Sepsis (28	Sepsis (28	Sepsis (28	TRUE	igd	0.019	6.64E-06	1017	M33178.m	TRUE	reported	g22WMS	2	TRUE	0.019612	20.30484
5460	rs2008827	T	G	T	G	0.4093	0.152112	0.0302	0.028014	FALSE	FALSE	FALSE	ieu-b-508	5	34030600	0.101825	486484	0.135214	Sepsis (28	Sepsis (28	Sepsis (28	TRUE	igd	0.0903	5.78E-06	171	M33391.m	TRUE	reported	EgMd1b	2	TRUE	0.10726	20.30479
8187	rs1106806	A	C	A	C	-0.0284	-0.00182	0.3883	0.393073	FALSE	FALSE	FALSE	ieu-b-508	13	1.1E+08	0.034321	486484	0.957686	Sepsis (28	Sepsis (28	Sepsis (28	TRUE	igd	0.0063	6.78E-06	2400	M36230.m	TRUE	reported	V5KJeR	2	TRUE	0.008396	20.30456
8106	rs4374185	T	C	T	C	-0.3303	-0.0492	0.5959	0.632637	FALSE	FALSE	FALSE	ieu-b-508	16	7248357	0.034618	486484	0.155278	Sepsis (28	Sepsis (28	Sepsis (28	TRUE	igd	0.0727	5.53E-06	122	M36097.m	TRUE	reported	RHr18S	2	TRUE	0.144711	20.30345
8308	rs1681886	A	G	A	G	0.0311	0.038729	0.7189	0.713004	FALSE	FALSE	FALSE	ieu-b-508	8	1.08E+08	0.037067	486484	0.2961	Sepsis (28	Sepsis (28	Sepsis (28	TRUE	igd	0.0069	6.37E-06	3346	M36585.m	TRUE	reported	pXZg13	2	TRUE	0.006035	20.30313
5335	rs1157863	T	G	T	G	-0.0523	0.008508	0.0162	0.177028	FALSE	FALSE	FALSE	ieu-b-508	1	1.57E+08	0.043701	486484	0.845645	Sepsis (28	Sepsis (28	Sepsis (28	TRUE	igd	0.0116	6.24E-06	1582	M33380.m	TRUE	reported	OHRfl5	2	TRUE	0.012686	20.30196
5581	rs4811103	T	C	T	C	-0.4766	0.009202	0.3103	0.292493	FALSE	FALSE	FALSE	ieu-b-508	20	49595815	0.045861	486484	0.840979	Sepsis (28	Sepsis (28	Sepsis (28	TRUE	igd	0.104	4.58E-06	60	M33423.m	TRUE	reported	MoEnO7	2	TRUE	0.259269	20.30103
5175	rs1755624	T	C	T	C	0.1361	0.087025	0.0315	0.049078	FALSE	FALSE	FALSE	ieu-b-508	15	46119716	0.077142	486484	0.259273	Sepsis (28	Sepsis (28	Sepsis (28	TRUE	igd	0.0302	6.43E-06	4625	M33195.m	TRUE	reported	Sl4STh	2	TRUE	0.004372	20.30086
1504	rs7908166	A	G	A	G	-0.0356	0.096464	0.9724	0.959337	FALSE	FALSE	FALSE	ieu-b-508	10	24328932	0.084088	486484	0.251303	Sepsis (28	Sepsis (28	Sepsis (28	TRUE	igd	0.0079	7.68E-06	5656	M12017.m	TRUE	reported	bbGtsK	2	TRUE	0.003578	20.29982
8160	rs1225608	A	G	A	G	0.0559	-0.03684	0.9155	0.927122	FALSE	FALSE	FALSE	ieu-b-508	10	20041146	0.064162	486484	0.565895	Sepsis (28	Sepsis (28	Sepsis (28	TRUE	igd	0.0124	6.69E-06	1780	M36131.m	TRUE	reported	DbYm5t	2	TRUE	0.011288	20.29981
5165	rs2377394	A	T	A	T	-0.1095	-0.00052	0.0675	0.116441	FALSE	TRUE	FALSE	ieu-b-508	17	77272610	0.052585	486484	0.992093	Sepsis (28	Sepsis (28	Sepsis (28	TRUE	igd	0.0243	6.84E-06	5064	M33194.m	TRUE	reported	xRUveq	2	TRUE	0.003994	20.29757
7437	rs1175889	T	C	T	C	0.0766	0.157324	0.0242	0.033804	FALSE	FALSE	FALSE	ieu-b-508	6	39839067	0.093658	486484	0.093002	Sepsis (28	Sepsis (28	Sepsis (28	TRUE	igd	0.017	6.36E-06	6234	M35189.m	TRUE	reported	54XZFj	2	TRUE	0.003246	20.29646
5722	rs730596	A	G	A	G	0.0401	-0.0722	0.9335	0.92704	FALSE	FALSE	FALSE	ieu-b-508	10	98172014	0.064608	486484	0.263792	Sepsis (28	Sepsis (28	Sepsis (28	TRUE	igd	0.0089	6.43E-06	5107	M33488.m	TRUE	reported	mZmDY6	2	TRUE	0.003959	20.29264
6247	rs768846	T	C	T	C	0.1284	-0.17336	0.9819	0.982424	FALSE	FALSE	FALSE	ieu-b-508	18	31410237	0.128058	486484	0.175805	Sepsis (28	Sepsis (28	Sepsis (28	TRUE	igd	0.0285	6.67E-06	6756	M33935.m	TRUE	reported	ELBTDc	2	TRUE	0.002995	20.29139
5148	rs9298802	T	C	T	C	-0.3291	0.226117	0.9866	0.995315	FALSE	FALSE	FALSE	ieu-b-508	9	19896420	0.25339	486484	0.372196	Sepsis (28	Sepsis (28	Sepsis (28	TRUE	igd	0.073	6.53E-06	1229	M33190.m	TRUE	reported	UkhAw5	2	TRUE	0.016268	20.29097
7181	rs8180157	A	G	A	G	-0.041	0.01954	0.3926	0.378971	FALSE	FALSE	FALSE	ieu-b-508	4	1.78E+08	0.034748	486484	0.573879	Sepsis (28	Sepsis (28	Sepsis (28	TRUE	igd	0.0091	6.97E-06	4515	M34535.m	TRUE	reported	z9BiJF	2	TRUE	0.004476	20.29049
3095	rs7728541	A	G	A	G	0.0455	-0.23359	0.0167	0.01854	FALSE	FALSE	FALSE	ieu-b-508	5	89632906	0.123714	486484	0.059009	Sepsis (28	Sepsis (28	Sepsis (28	TRUE	igd	0.0101	7.09E-06	7346	M32197.m	TRUE	reported	VZjwa9	2	TRUE	0.002755	20.28905
2862	rs527140	A	G	A	G	-0.0329	0.009053	0.7481	0.77921	FALSE	FALSE	FALSE	ieu-b-508	11	57500134	0.040295	486484	0.822234	Sepsis (28	Sepsis (28	Sepsis (28	TRUE	igd	0.0073	6.77E-06	1758	M27672.m	TRUE	reported	5cTg55	2	TRUE	0.011422	20.28858
3013	rs7638353	A	C	A	C	-0.1359	0.0636	0.5531	0.43051	FALSE	FALSE	FALSE	ieu-b-508	3	1.52E+08	0.033614	486484	0.058483	Sepsis (28	Sepsis (28	Sepsis (28	TRUE	igd	0.0301	6.48E-06	423	M31536.m	TRUE	reported	3nAuUl	2	TRUE	0.045975	20.28839
7197	rs7340748	A	G	A	G	0.0437	-0.00579	0.0539	0.096742	FALSE	FALSE	FALSE	ieu-b-508	3	1.51E+08	0.057253	486484	0.919467	Sepsis (28	Sepsis (28	Sepsis (28	TRUE	igd	0.0097	5.92E-06	4800	M34539.m	TRUE	reported	puA8ni	2	TRUE	0.004211	20.28796
8237	rs1783235	A	G	A	G	0.0347	-0.02168	0.7188	0.717846	FALSE	FALSE	FALSE	ieu-b-508	11	1.16E+08	0.037294	486484	0.560945	Sepsis (28	Sepsis (28	Sepsis (28	TRUE	igd	0.0077	6.59E-06	1908	M36468.m	TRUE	reported	vycAFr	2	TRUE	0.010532	20.2872
4425	rs1540603	A	C	A	C	-0.0676	0.10517	0.0122	0.013332	FALSE	FALSE	FALSE	ieu-b-508	5	17220275	0.147301	486484	0.475238	Sepsis (28	Sepsis (28	Sepsis (28	TRUE	igd	0.015	7.00E-06	1760	M32910.m	TRUE	reported	2OCBKh	2	TRUE	0.011408	20.28696
7122	rs907609	T	C	T	C	0.1592	-0.02732	0.1354	0.151327	FALSE	FALSE	FALSE	ieu-b-508	11	1857270	0.046528	486484	0.55707	Sepsis (28	Sepsis (28	Sepsis (28	TRUE	igd	0.0352	6.18E-06	241	M34516.m	TRUE	reported	7Ah2wy	2	TRUE	0.078235	20.28531
6034	rs2623476	A	C	A	C	0.0446	-0.04795	0.2405	0.262939	FALSE	FALSE	FALSE	ieu-b-508	15	39487576	0.037939	486484	0.206288	Sepsis (28	Sepsis (28	Sepsis (28	TRUE	igd	0.0099	6.56E-06	3962	M33782.m	TRUE	reported	875Pal	2	TRUE	0.005096	20.28523
4267	rs307944	A	G	A	G	-0.041	0.034867	0.205	0.206799	FALSE	FALSE	FALSE	ieu-b-508	19	54394301	0.044543	486484	0.433759	Sepsis (28	Sepsis (28	Sepsis (28	TRUE	igd	0.0091	5.95E-06	2711	M32802.m	TRUE	reported	m8vmvZ	2	TRUE	0.007432	20.28451
4142	rs1695866	A	G	A	G	-0.0509	0.104272	0.0523	0.057565	FALSE	FALSE	FALSE	ieu-b-508	16	11880407	0.071696	486484	0.145844	Sepsis (28	Sepsis (28	Sepsis (28	TRUE	igd	0.0113	6.26E-06	7250	M32757.m	TRUE	reported	TJV9Xb	2	TRUE	0.002791	20.28425
7002	rs1208353	T	C	T	C	0.0527	-0.00557	0.2656	0.324062	FALSE	FALSE	FALSE	ieu-b-508	1	1.78E+08	0.035728	486484	0.87601	Sepsis (28	Sepsis (28	Sepsis (28	TRUE	igd	0.0117	6.82E-06	4452	M34420.m	TRUE	reported	20uofq	2	TRUE	0.004536	20.27937
8070	rs6899809	T	C	T	C	-0.0554	-0.06004	0.0436	0.050506	FALSE	FALSE	FALSE	ieu-b-508	6	8875817	0.076335	486484	0.431559	Sepsis (28	Sepsis (28	Sepsis (28	TRUE	igd	0.0123	7.20E-06	4529	M35978.m	TRUE	reported	CN0wRR	2	TRUE	0.004459	20.27764
4595	rs1094153	A	G	A	G	0.0401	-0.07547	0.6584	0.636336	FALSE	FALSE	FALSE	ieu-b-508	5	41200358	0.034641	486484	0.029362	Sepsis (28	Sepsis (28	Sepsis (28	TRUE	igd	0.0089	6.29E-06	1751	M33165.m	TRUE	reported	RZt3uS	2	TRUE	0.011461	20.27741
7817	rs1248228	T	G	T	G	-0.1167	-0.04271	0.9753	0.976122	FALSE	FALSE	FALSE	ieu-b-508	21	24461998	0.109718	486484	0.697047	Sepsis (28	Sepsis (28	Sepsis (28	TRUE	igd	0.0259	6.59E-06	1640	M35472.m	TRUE	reported	oZU10n	2	TRUE	0.012228	20.2774
4403	rs1185269	T	C	T	C	-0.0446	0.059937	0.934	0.924858	FALSE	FALSE	FALSE	ieu-b-508	15	27303219	0.063527	486484	0.345433	Sepsis (28	Sepsis (28	Sepsis (28	TRUE	igd	0.0099	6.41E-06	2148	M32869.m	TRUE	reported	Rvim1n	2	TRUE	0.00936	20.27658
7800	rs7025875	A	T	A	T	-0.1843	-0.09151	0.9753	0.962896	FALSE	TRUE	FALSE	ieu-b-508	9	1.38E+08	0.091737	486484	0.318514	Sepsis (28	Sepsis (28	Sepsis (28	TRUE	igd	0.0409	6.54E-06	1383	M35451.m	TRUE	reported	GMRGAN	2	TRUE	0.014469	20.27569
6013	rs29766	A	G	A	G	0.1091	0.020581	0.0862	0.089192	FALSE	FALSE	FALSE	ieu-b-508	5	31632899	0.058287	486484	0.724024	Sepsis (28	Sepsis (28	Sepsis (28	TRUE	igd	0.0242	6.39E-06	831	M33751.m	TRUE	reported	Cxdkr4	2	TRUE	0.023874	20.27553
1322	rs2349945	T	C	T	C	-0.0347	-0.01562	0.3863	0.362576	FALSE	FALSE	FALSE	ieu-b-508	8	62168429	0.034773	486484	0.653197	Sepsis (28	Sepsis (28	Sepsis (28	TRUE	igd	0.0077	7.25E-06	1175	M02137.m	TRUE	reported	iRGqBE	2	TRUE	0.01699	20.27392
1131	rs9419004	C	G	C	G	-0.254	0.004944	0.186	0.273569	FALSE	TRUE	FALSE	ieu-b-508	10	1.35E+08	0.037329	486484	0.894637	Sepsis (28	Sepsis (28	Sepsis (28	TRUE	igd	0.0563	6.53E-06	496	M01640.m	TRUE	reported	o3qxzl	2	TRUE	0.039419	20.27197
2406	rs6005580	A	G	A	G	0.0446	-0.06954	0.7521	0.771127	FALSE	FALSE	FALSE	ieu-b-508	22	28104051	0.039797	486484	0.080594	Sepsis (28	Sepsis (28	Sepsis (28	TRUE	igd	0.0099	6.38E-06	1690	M21184.m	TRUE	reported	63ppuJ	2	TRUE	0.011867	20.27146
2050	rs7697449	T	C	T	C	0.0383	0.002871	0.5636	0.556689	FALSE	FALSE	FALSE	ieu-b-508	4	19898883	0.033822	486484	0.932346	Sepsis (28	Sepsis (28	Sepsis (28	TRUE	igd	0.0085	6.18E-06	1204	M18357.m	TRUE	reported	o7AvpG	2	TRUE	0.016583	20.26925
3965	rs7281501	T	G	T	G	0.0482	0.033639	0.9362	0.93514	FALSE	FALSE	FALSE	ieu-b-508	21	17658152	0.067501	486484	0.618238	Sepsis (28	Sepsis (28	Sepsis (28	TRUE	igd	0.0107	7.18E-06	1766	M32709.m	TRUE	reported	YR8SB4	2	TRUE	0.01136	20.2691
5137	rs2753980	A	G	A	G	-0.0793	-0.01456	0.6032	0.607363	FALSE	FALSE	FALSE	ieu-b-508	20	3603141	0.034208	486484	0.670442	Sepsis (28	Sepsis (28	Sepsis (28	TRUE	igd	0.0176	6.74E-06	1229	M33190.m	TRUE	reported	UkhAw5	2	TRUE	0.01625	20.26813
7147	rs9277380	C	G	C	G	0.0631	0.007762	0.7148	0.700217	FALSE	TRUE	FALSE	ieu-b-508	6	33050369	0.036341	486484	0.830863	Sepsis (28	Sepsis (28	Sepsis (28	TRUE	igd	0.014	6.78E-06	829	M34530.m	TRUE	reported	8plvGA	2	TRUE	0.023919	20.26533
5200	rs1710456	A	G	A	G	0.1077	-0.07285	0.292	0.287232	FALSE	FALSE	FALSE	ieu-b-508	2	87965359	0.051108	486484	0.154016	Sepsis (28	Sepsis (28	Sepsis (28	TRUE	igd	0.0239	6.56E-06	930	M33203.m	TRUE	reported	Sto7pR	2	TRUE	0.021368	20.26285
4936	rs3811321	A	C	A	C	0.1266	0.023752	0.9373	0.937807	FALSE	FALSE	FALSE	ieu-b-508	14	22481824	0.068873	486484	0.730197	Sepsis (28	Sepsis (28	Sepsis (28	TRUE	igd	0.0281	6.78E-06	1017	M33178.m	TRUE	reported	g22WMS	2	TRUE	0.019568	20.25815
5320	rs8011328	A	G	A	G	0.1081	0.10555	0.0129	0.021808	FALSE	FALSE	FALSE	ieu-b-508	14	91317813	0.114543	486484	0.356796	Sepsis (28	Sepsis (28	Sepsis (28	TRUE	igd	0.024	6.59E-06	1378	M33363.m	TRUE	reported	7K0MM0	2	TRUE	0.014509	20.25807
823	rs3748771	T	G	T	G	-0.0671	-0.32478	0.9877	0.990927	FALSE	FALSE	FALSE	ieu-b-508	1	2.31E+08	0.173889	486484	0.061796	Sepsis (28	Sepsis (28	Sepsis (28	TRUE	igd	0.0149	6.35E-06	1765	M01358.m	TRUE	reported	kSPQgd	2	TRUE	0.01136	20.25723
8304	rs9922902	C	G	C	G	0.0635	-0.03291	0.0778	0.242168	FALSE	TRUE	FALSE	ieu-b-508	16	88187607	0.039612	486484	0.406111	Sepsis (28	Sepsis (28	Sepsis (28	TRUE	igd	0.0141	6.45E-06	1636	M36559.m	TRUE	reported	g6l9hg	2	TRUE	0.012245	20.25713
4366	rs1117767	T	C	T	C	0.0743	0.018508	0.1109	0.125207	FALSE	FALSE	FALSE	ieu-b-508	12	69832067	0.050567	486484	0.714353	Sepsis (28	Sepsis (28	Sepsis (28	TRUE	igd	0.0165	7.01E-06	1988	M32863.m	TRUE	reported	cLOFYE	2	TRUE	0.010097	20.25688
799	rs1706683	C	G	C	G	-0.0707	0.04923	0.0202	0.01836	FALSE	TRUE	FALSE	ieu-b-508	18	58034591	0.125046	486484	0.693808	Sepsis (28	Sepsis (28	Sepsis (28	TRUE	igd	0.0157	6.24E-06	1755	M01356.m	TRUE	reported	ah1g5W	2	TRUE	0.011423	20.25556
2660	rs9477741	C	G	C	G	-0.0851	0.103559	0.0113	0.013029	FALSE	TRUE	FALSE	ieu-b-508	6	18425703	0.147823	486484	0.483577	Sepsis (28	Sepsis (28	Sepsis (28	TRUE	igd	0.0189	6.89E-06	1547	M22649.m	TRUE	reported	NAzwRF	2	TRUE	0.012936	20.24761
2140	rs1716919	A	T	A	T	-0.1825	-0.02253	0.9347	0.924906	FALSE	TRUE	FALSE	ieu-b-508	7	97501547	0.063808	486484	0.724056	Sepsis (28	Sepsis (28	Sepsis (28	TRUE	igd	0.0405	6.61E-06	698	M18497.m	TRUE	reported	90JFPc	2	TRUE	0.028269	20.24741
3549	rs1299971	A	T	A	T	-0.0153	0.00478	0.6299	0.630298	FALSE	TRUE	FALSE	ieu-b-508	2	51156549	0.034602	486484	0.890118	Sepsis (28	Sepsis (28	Sepsis (28	TRUE	igd	0.0034	8.38E-06	7373	M32504.m	TRUE	reported	MN0yyR	2	TRUE	0.002739	20.24451
3	rs2199619	T	C	T	C	0.0054	-0.0584	0.2787	0.269636	FALSE	FALSE	FALSE	ieu-b-508	2	28854958	0.037591	486484	0.120294	Sepsis (28	Sepsis (28	Sepsis (28	TRUE	igd	0.0012	5.23E-06	7372	M00053.m	TRUE	reported	4Smluz	2	TRUE	0.002739	20.24451
1079	rs1113001	T	C	T	C	-0.0081	-0.05318	0.153	0.154942	FALSE	FALSE	FALSE	ieu-b-508	3	43833177	0.046004	486484	0.247729	Sepsis (28	Sepsis (28	Sepsis (28	TRUE	igd	0.0018	5.09E-06	7371	M01604.m	TRUE	reported	hE3l2k	2	TRUE	0.00274	20.24451
1098	rs9557545	A	T	A	T	0.018	0.024315	0.032	0.029878	FALSE	TRUE	FALSE	ieu-b-508	13	1.02E+08	0.098556	486484	0.805131	Sepsis (28	Sepsis (28	Sepsis (28	TRUE	igd	0.004	7.83E-06	7371	M01604.m	TRUE	reported	hE3l2k	2	TRUE	0.00274	20.24451
3695	rs4447076	A	G	A	G	-0.0162	-0.01885	0.6152	0.599272	FALSE	FALSE	FALSE	ieu-b-508	10	88696381	0.033945	486484	0.578618	Sepsis (28	Sepsis (28	Sepsis (28	TRUE	igd	0.0036	8.58E-06	7369	M32587.m	TRUE	reported	uknv7G	2	TRUE	0.00274	20.2445
1641	rs3788706	A	G	A	G	-0.0117	0.007295	0.1302	0.13505	FALSE	FALSE	FALSE	ieu-b-508	22	46869170	0.04864	486484	0.880782	Sepsis (28	Sepsis (28	Sepsis (28	TRUE	igd	0.0026	6.10E-06	7368	M15140.m	TRUE	reported	jEcqMJ	2	TRUE	0.002741	20.2445
583	rs1010069	A	G	A	G	0.0081	0.015083	0.4623	0.456451	FALSE	FALSE	FALSE	ieu-b-508	1	16352937	0.033518	486484	0.652702	Sepsis (28	Sepsis (28	Sepsis (28	TRUE	igd	0.0018	8.58E-06	7367	M01110.m	TRUE	reported	0A31Sl	2	TRUE	0.002741	20.2445
592	rs180022	T	C	T	C	0.0117	-0.05018	0.8328	0.838948	FALSE	FALSE	FALSE	ieu-b-508	6	16295861	0.045231	486484	0.26721	Sepsis (28	Sepsis (28	Sepsis (28	TRUE	igd	0.0026	9.43E-06	7367	M01110.m	TRUE	reported	0A31Sl	2	TRUE	0.002741	20.2445
8599	rs1271385	A	G	A	G	0.0081	0.036858	0.6774	0.671411	FALSE	FALSE	FALSE	ieu-b-508	2	75950957	0.035648	486484	0.301158	Sepsis (28	Sepsis (28	Sepsis (28	TRUE	igd	0.0018	5.24E-06	7366	M37506.m	TRUE	reported	m2kaRt	2	TRUE	0.002742	20.2445
8611	rs2269282	T	C	T	C	0.0081	0.008923	0.6994	0.706092	FALSE	FALSE	FALSE	ieu-b-508	5	83334641	0.036536	486484	0.807052	Sepsis (28	Sepsis (28	Sepsis (28	TRUE	igd	0.0018	4.53E-06	7366	M37506.m	TRUE	reported	m2kaRt	2	TRUE	0.002742	20.2445
8634	rs9947662	A	G	A	G	0.0081	0.011494	0.2582	0.264065	FALSE	FALSE	FALSE	ieu-b-508	18	44860264	0.037799	486484	0.761058	Sepsis (28	Sepsis (28	Sepsis (28	TRUE	igd	0.0018	7.46E-06	7366	M37506.m	TRUE	reported	m2kaRt	2	TRUE	0.002742	20.2445
414	rs1479402	T	C	T	C	0.0306	-0.017	0.0137	0.01229	FALSE	FALSE	FALSE	ieu-b-508	3	61973121	0.151341	486484	0.910546	Sepsis (28	Sepsis (28	Sepsis (28	TRUE	igd	0.0068	6.85E-06	7365	M00063.m	TRUE	reported	Hdsv1L	2	TRUE	0.002742	20.2445
418	rs1772892	A	C	A	C	0.0081	0.014769	0.1768	0.178997	FALSE	FALSE	FALSE	ieu-b-508	13	26132841	0.043593	486484	0.734756	Sepsis (28	Sepsis (28	Sepsis (28	TRUE	igd	0.0018	5.30E-06	7365	M00063.m	TRUE	reported	Hdsv1L	2	TRUE	0.002742	20.2445
443	rs886282	T	C	T	C	0.0081	0.040405	0.1989	0.217944	FALSE	FALSE	FALSE	ieu-b-508	17	55188150	0.040809	486484	0.322133	Sepsis (28	Sepsis (28	Sepsis (28	TRUE	igd	0.0018	3.71E-06	7365	M00063.m	TRUE	reported	Hdsv1L	2	TRUE	0.002742	20.2445
3258	rs1420426	A	G	A	G	0.0081	0.015694	0.6202	0.618236	FALSE	FALSE	FALSE	ieu-b-508	7	37227330	0.034348	486484	0.64773	Sepsis (28	Sepsis (28	Sepsis (28	TRUE	igd	0.0018	4.16E-06	7365	M32348.m	TRUE	reported	DJeLDx	2	TRUE	0.002742	20.2445
1002	rs1280330	A	T	A	T	-0.0081	-0.00454	0.6752	0.672856	FALSE	TRUE	FALSE	ieu-b-508	11	96180609	0.035659	486484	0.898773	Sepsis (28	Sepsis (28	Sepsis (28	TRUE	igd	0.0018	5.97E-06	7364	M01564.m	TRUE	reported	IPUdyD	2	TRUE	0.002742	20.2445
1010	rs1703468	T	G	T	G	0.0081	-0.05468	0.2881	0.288189	FALSE	FALSE	FALSE	ieu-b-508	4	1.06E+08	0.036966	486484	0.139113	Sepsis (28	Sepsis (28	Sepsis (28	TRUE	igd	0.0018	7.90E-06	7364	M01564.m	TRUE	reported	IPUdyD	2	TRUE	0.002742	20.2445
1016	rs3193970	T	C	T	C	0.0081	-0.06054	0.5801	0.573165	FALSE	FALSE	FALSE	ieu-b-508	10	97072008	0.033857	486484	0.073768	Sepsis (28	Sepsis (28	Sepsis (28	TRUE	igd	0.0018	6.07E-06	7364	M01564.m	TRUE	reported	IPUdyD	2	TRUE	0.002742	20.2445
1027	rs7710027	T	C	T	C	0.0081	-0.04908	0.2886	0.29254	FALSE	FALSE	FALSE	ieu-b-508	5	55901945	0.036716	486484	0.181298	Sepsis (28	Sepsis (28	Sepsis (28	TRUE	igd	0.0018	9.16E-06	7364	M01564.m	TRUE	reported	IPUdyD	2	TRUE	0.002742	20.2445
1029	rs7919076	T	C	T	C	-0.0081	0.027466	0.3416	0.338936	FALSE	FALSE	FALSE	ieu-b-508	10	68328936	0.035565	486484	0.439944	Sepsis (28	Sepsis (28	Sepsis (28	TRUE	igd	0.0018	7.79E-06	7364	M01564.m	TRUE	reported	IPUdyD	2	TRUE	0.002742	20.2445
1036	rs9327706	A	C	A	C	0.0081	0.015553	0.5937	0.597954	FALSE	FALSE	FALSE	ieu-b-508	5	1.35E+08	0.034053	486484	0.647857	Sepsis (28	Sepsis (28	Sepsis (28	TRUE	igd	0.0018	5.61E-06	7364	M01564.m	TRUE	reported	IPUdyD	2	TRUE	0.002742	20.2445
1037	rs9372398	T	C	T	C	0.0081	-0.03835	0.5453	0.448303	FALSE	FALSE	FALSE	ieu-b-508	6	1.15E+08	0.033625	486484	0.254075	Sepsis (28	Sepsis (28	Sepsis (28	TRUE	igd	0.0018	6.74E-06	7364	M01564.m	TRUE	reported	IPUdyD	2	TRUE	0.002742	20.2445
3435	rs3806851	T	C	T	C	-0.0081	-0.00396	0.4918	0.48303	FALSE	FALSE	FALSE	ieu-b-508	5	31191910	0.033537	486484	0.905939	Sepsis (28	Sepsis (28	Sepsis (28	TRUE	igd	0.0018	9.85E-06	7364	M32452.m	TRUE	reported	8GtFpl	2	TRUE	0.002742	20.2445
3442	rs7545139	A	G	A	G	0.0081	0.001537	0.5822	0.580253	FALSE	FALSE	FALSE	ieu-b-508	1	1.11E+08	0.033894	486484	0.963829	Sepsis (28	Sepsis (28	Sepsis (28	TRUE	igd	0.0018	9.14E-06	7364	M32452.m	TRUE	reported	8GtFpl	2	TRUE	0.002742	20.2445
5260	rs1572948	A	G	A	G	-0.0126	0.017448	0.4296	0.421673	FALSE	FALSE	FALSE	ieu-b-508	13	1E+08	0.034136	486484	0.609255	Sepsis (28	Sepsis (28	Sepsis (28	TRUE	igd	0.0028	4.98E-06	7364	M33230.m	TRUE	reported	nzcTOn	2	TRUE	0.002742	20.2445

5266	rs7301800	A	G	A	G	0.0162	0.032802	0.2124	0.215043	FALSE	FALSE	FALSE	ieu-b-508	12	1.06E+08	0.041039	486484	0.424126	Sepsis (28	Sepsis (28	Sepsis (28	TRUE	igd	0.0036	8.73E-06	7364	M33230.m	TRUE	reported	nzcTOn	2	TRUE	0.002742	20.2445
6352	rs303610	A	T	A	T	-0.0117	-0.04595	0.2958	0.304449	FALSE	TRUE	FALSE	ieu-b-508	9	1.06E+08	0.036714	486484	0.210732	Sepsis (28	Sepsis (28	Sepsis (28	TRUE	igd	0.0026	8.88E-06	7364	M33960.m	TRUE	reported	o8RcxU	2	TRUE	0.002742	20.2445
6359	rs969948	T	C	T	C	-0.0117	0.059958	0.6023	0.590722	FALSE	FALSE	FALSE	ieu-b-508	4	27123087	0.034028	486484	0.078068	Sepsis (28	Sepsis (28	Sepsis (28	TRUE	igd	0.0026	5.74E-06	7364	M33960.m	TRUE	reported	o8RcxU	2	TRUE	0.002742	20.2445
7301	rs3127434	A	G	A	G	0.0198	-0.06156	0.489	0.489578	FALSE	FALSE	FALSE	ieu-b-508	6	1.67E+08	0.033305	486484	0.064552	Sepsis (28	Sepsis (28	Sepsis (28	TRUE	igd	0.0044	7.05E-06	7364	M35126.m	TRUE	reported	1haOJA	2	TRUE	0.002742	20.2445
719	rs1333926	A	G	A	G	-0.0081	0.035806	0.8587	0.857131	FALSE	FALSE	FALSE	ieu-b-508	16	49001156	0.04799	486484	0.455601	Sepsis (28	Sepsis (28	Sepsis (28	TRUE	igd	0.0018	6.52E-06	7363	M01301.m	TRUE	reported	w8TL71	2	TRUE	0.002743	20.2445
3072	rs6904596	A	G	A	G	-0.0531	0.017089	0.1218	0.125175	FALSE	FALSE	FALSE	ieu-b-508	6	27491299	0.050291	486484	0.734005	Sepsis (28	Sepsis (28	Sepsis (28	TRUE	igd	0.0118	7.36E-06	7363	M31787.m	TRUE	reported	XMp8YI	2	TRUE	0.002743	20.2445
3460	rs1293768	T	G	T	G	-0.0081	0.007715	0.3221	0.325517	FALSE	FALSE	FALSE	ieu-b-508	12	1.13E+08	0.035585	486484	0.828352	Sepsis (28	Sepsis (28	Sepsis (28	TRUE	igd	0.0018	9.31E-06	7363	M32489.m	TRUE	reported	hnljaD	2	TRUE	0.002743	20.2445
3468	rs2103340	T	C	T	C	-0.0081	0.039641	0.6181	0.621698	FALSE	FALSE	FALSE	ieu-b-508	16	8840025	0.034401	486484	0.249179	Sepsis (28	Sepsis (28	Sepsis (28	TRUE	igd	0.0018	7.41E-06	7363	M32489.m	TRUE	reported	hnljaD	2	TRUE	0.002743	20.2445
3472	rs2846444	A	G	A	G	-0.0081	0.043877	0.5854	0.586717	FALSE	FALSE	FALSE	ieu-b-508	9	1.14E+08	0.033931	486484	0.195965	Sepsis (28	Sepsis (28	Sepsis (28	TRUE	igd	0.0018	6.34E-06	7363	M32489.m	TRUE	reported	hnljaD	2	TRUE	0.002743	20.2445
3476	rs511319	C	G	C	G	0.0081	0.004227	0.3878	0.389314	FALSE	TRUE	FALSE	ieu-b-508	6	12251188	0.034395	486484	0.902185	Sepsis (28	Sepsis (28	Sepsis (28	TRUE	igd	0.0018	7.27E-06	7363	M32489.m	TRUE	reported	hnljaD	2	TRUE	0.002743	20.2445
3481	rs6886152	A	C	A	C	0.0081	-0.0439	0.5983	0.602336	FALSE	FALSE	FALSE	ieu-b-508	5	1.17E+08	0.034194	486484	0.19916	Sepsis (28	Sepsis (28	Sepsis (28	TRUE	igd	0.0018	6.12E-06	7363	M32489.m	TRUE	reported	hnljaD	2	TRUE	0.002743	20.2445
874	rs1083149	A	G	A	G	0.0126	-0.01836	0.2209	0.23537	FALSE	FALSE	FALSE	ieu-b-508	11	95947410	0.03949	486484	0.641907	Sepsis (28	Sepsis (28	Sepsis (28	TRUE	igd	0.0028	4.97E-06	7362	M01365.m	TRUE	reported	N1cZTs	2	TRUE	0.002743	20.2445
888	rs7016645	T	C	T	C	-0.0117	0.02748	0.253	0.257847	FALSE	FALSE	FALSE	ieu-b-508	8	80542027	0.038162	486484	0.471473	Sepsis (28	Sepsis (28	Sepsis (28	TRUE	igd	0.0026	8.44E-06	7362	M01365.m	TRUE	reported	N1cZTs	2	TRUE	0.002743	20.2445
3751	rs1005075	A	G	A	G	-0.0081	-0.0153	0.4668	0.473665	FALSE	FALSE	FALSE	ieu-b-508	15	93921835	0.033664	486484	0.649426	Sepsis (28	Sepsis (28	Sepsis (28	TRUE	igd	0.0018	5.84E-06	7362	M32634.m	TRUE	reported	7T2RUz	2	TRUE	0.002743	20.2445
3755	rs1116185	T	C	T	C	0.0081	0.019185	0.5897	0.597502	FALSE	FALSE	FALSE	ieu-b-508	1	75298782	0.034012	486484	0.57271	Sepsis (28	Sepsis (28	Sepsis (28	TRUE	igd	0.0018	5.41E-06	7362	M32634.m	TRUE	reported	7T2RUz	2	TRUE	0.002743	20.2445
3759	rs1412853	A	G	A	G	-0.0081	-0.01724	0.2693	0.273898	FALSE	FALSE	FALSE	ieu-b-508	13	84066105	0.037486	486484	0.645562	Sepsis (28	Sepsis (28	Sepsis (28	TRUE	igd	0.0018	7.23E-06	7362	M32634.m	TRUE	reported	7T2RUz	2	TRUE	0.002743	20.2445
3760	rs1744401	A	G	A	G	0.0117	0.048242	0.8636	0.861713	FALSE	FALSE	FALSE	ieu-b-508	4	41323426	0.048888	486484	0.323753	Sepsis (28	Sepsis (28	Sepsis (28	TRUE	igd	0.0026	7.81E-06	7362	M32634.m	TRUE	reported	7T2RUz	2	TRUE	0.002743	20.2445
3761	rs174578	A	T	A	T	-0.0081	0.085149	0.3433	0.352673	FALSE	TRUE	FALSE	ieu-b-508	11	61605499	0.034922	486484	0.014757	Sepsis (28	Sepsis (28	Sepsis (28	TRUE	igd	0.0018	6.73E-06	7362	M32634.m	TRUE	reported	7T2RUz	2	TRUE	0.002743	20.2445
4518	rs9686531	T	C	T	C	0.0081	-0.0155	0.119	0.119666	FALSE	FALSE	FALSE	ieu-b-508	5	10916640	0.051972	486484	0.765486	Sepsis (28	Sepsis (28	Sepsis (28	TRUE	igd	0.0018	7.74E-06	7362	M33132.m	TRUE	reported	dTXJa8	2	TRUE	0.002743	20.2445
6053	rs1244947	T	C	T	C	-0.0162	-0.00655	0.2067	0.209492	FALSE	FALSE	FALSE	ieu-b-508	17	11653008	0.041002	486484	0.873112	Sepsis (28	Sepsis (28	Sepsis (28	TRUE	igd	0.0036	8.10E-06	7362	M33821.m	TRUE	reported	eVYICL	2	TRUE	0.002743	20.2445
6425	rs1090664	A	G	A	G	0.0081	-0.02159	0.8721	0.872366	FALSE	FALSE	FALSE	ieu-b-508	10	14318491	0.050128	486484	0.666719	Sepsis (28	Sepsis (28	Sepsis (28	TRUE	igd	0.0018	7.66E-06	7362	M34062.m	TRUE	reported	RZyHKP	2	TRUE	0.002743	20.2445
6430	rs1288425	A	G	A	G	0.0081	-0.01013	0.1395	0.160046	FALSE	FALSE	FALSE	ieu-b-508	14	26724539	0.045584	486484	0.82408	Sepsis (28	Sepsis (28	Sepsis (28	TRUE	igd	0.0018	8.02E-06	7362	M34062.m	TRUE	reported	RZyHKP	2	TRUE	0.002743	20.2445
6446	rs5999780	T	C	T	C	0.0081	-0.02555	0.1528	0.166175	FALSE	FALSE	FALSE	ieu-b-508	22	35627919	0.045727	486484	0.576326	Sepsis (28	Sepsis (28	Sepsis (28	TRUE	igd	0.0018	7.21E-06	7362	M34062.m	TRUE	reported	RZyHKP	2	TRUE	0.002743	20.2445
454	rs1498350	A	C	A	C	0.0045	-0.01532	0.4954	0.501727	FALSE	FALSE	FALSE	ieu-b-508	1	67294143	0.03346	486484	0.647122	Sepsis (28	Sepsis (28	Sepsis (28	TRUE	igd	0.001	1.88E-06	7361	M00513.m	TRUE	reported	ohacnP	2	TRUE	0.002743	20.2445
461	rs4460629	T	C	T	C	-0.0045	-0.02073	0.5224	0.514288	FALSE	FALSE	FALSE	ieu-b-508	1	1.55E+08	0.033365	486484	0.534315	Sepsis (28	Sepsis (28	Sepsis (28	TRUE	igd	0.001	2.21E-06	7361	M00513.m	TRUE	reported	ohacnP	2	TRUE	0.002743	20.2445
682	rs1512342	T	C	T	C	0.0072	0.01031	0.3384	0.341357	FALSE	FALSE	FALSE	ieu-b-508	4	1.49E+08	0.035294	486484	0.770194	Sepsis (28	Sepsis (28	Sepsis (28	TRUE	igd	0.0016	7.01E-06	7358	M01299.m	TRUE	reported	gfM0a3	2	TRUE	0.002745	20.2445
3531	rs1863824	A	G	A	G	0.0117	-0.03063	0.613	0.61626	FALSE	FALSE	FALSE	ieu-b-508	10	88112939	0.03433	486484	0.372268	Sepsis (28	Sepsis (28	Sepsis (28	TRUE	igd	0.0026	9.64E-06	7358	M32497.m	TRUE	reported	244bZN	2	TRUE	0.002745	20.2445
1456	rs7896866	A	C	A	C	0.0108	-0.0809	0.2982	0.278498	FALSE	FALSE	FALSE	ieu-b-508	10	1.01E+08	0.03734	486484	0.030257	Sepsis (28	Sepsis (28	Sepsis (28	TRUE	igd	0.0024	8.34E-06	7357	M03141.m	TRUE	reported	y4mG6m	2	TRUE	0.002745	20.2445
1826	rs7102716	A	T	A	T	0.027	-0.00406	0.7654	0.759444	FALSE	TRUE	FALSE	ieu-b-508	11	79756660	0.039205	486484	0.917454	Sepsis (28	Sepsis (28	Sepsis (28	TRUE	igd	0.006	7.85E-06	7357	M15753.m	TRUE	reported	VDJYDI	2	TRUE	0.002745	20.2445
3198	rs8453	T	G	T	G	0.0144	-0.02011	0.158	0.156485	FALSE	FALSE	FALSE	ieu-b-508	1	1.15E+08	0.04569	486484	0.659892	Sepsis (28	Sepsis (28	Sepsis (28	TRUE	igd	0.0032	8.06E-06	7356	M32338.m	TRUE	reported	q5cuaP	2	TRUE	0.002745	20.24449
33	rs1179636	A	C	A	C	-0.0045	-0.0329	0.5356	0.535756	FALSE	FALSE	FALSE	ieu-b-508	7	75230392	0.033624	486484	0.327839	Sepsis (28	Sepsis (28	Sepsis (28	TRUE	igd	0.001	1.83E-06	7355	M00054.m	TRUE	reported	QTDAnh	2	TRUE	0.002746	20.24449
812	rs1237044	T	C	T	C	0.0081	0.004613	0.5836	0.578341	FALSE	FALSE	FALSE	ieu-b-508	17	7042246	0.034348	486484	0.893153	Sepsis (28	Sepsis (28	Sepsis (28	TRUE	igd	0.0018	5.10E-06	7355	M01358.m	TRUE	reported	kSPQgd	2	TRUE	0.002746	20.24449
829	rs6453594	A	G	A	G	-0.0108	0.001849	0.2196	0.242637	FALSE	FALSE	FALSE	ieu-b-508	6	73335690	0.038929	486484	0.962108	Sepsis (28	Sepsis (28	Sepsis (28	TRUE	igd	0.0024	7.01E-06	7355	M01358.m	TRUE	reported	kSPQgd	2	TRUE	0.002746	20.24449
836	rs9568232	T	C	T	C	0.0126	0.072289	0.1152	0.064291	FALSE	FALSE	FALSE	ieu-b-508	13	50089844	0.06841	486484	0.290646	Sepsis (28	Sepsis (28	Sepsis (28	TRUE	igd	0.0028	5.06E-06	7355	M01358.m	TRUE	reported	kSPQgd	2	TRUE	0.002746	20.24449
1146	rs1773910	A	G	A	G	-0.0198	-0.00663	0.942	0.935981	FALSE	FALSE	FALSE	ieu-b-508	6	94059632	0.068091	486484	0.922445	Sepsis (28	Sepsis (28	Sepsis (28	TRUE	igd	0.0044	8.09E-06	7355	M01644.m	TRUE	reported	IB0DTL	2	TRUE	0.002746	20.24449
1154	rs269194	A	C	A	C	-0.0081	0.020483	0.4386	0.438229	FALSE	FALSE	FALSE	ieu-b-508	8	75620766	0.033674	486484	0.543001	Sepsis (28	Sepsis (28	Sepsis (28	TRUE	igd	0.0018	9.01E-06	7355	M01644.m	TRUE	reported	IB0DTL	2	TRUE	0.002746	20.24449
3496	rs1373469	T	C	T	C	0.0081	0.06655	0.5235	0.520672	FALSE	FALSE	FALSE	ieu-b-508	4	1.62E+08	0.0334	486484	0.046315	Sepsis (28	Sepsis (28	Sepsis (28	TRUE	igd	0.0018	4.85E-06	7355	M32492.m	TRUE	reported	rZZFcE	2	TRUE	0.002746	20.24449
3514	rs7712191	T	G	T	G	0.0081	-0.02425	0.6607	0.660843	FALSE	FALSE	FALSE	ieu-b-508	5	1.25E+08	0.035311	486484	0.49231	Sepsis (28	Sepsis (28	Sepsis (28	TRUE	igd	0.0018	7.10E-06	7355	M32492.m	TRUE	reported	rZZFcE	2	TRUE	0.002746	20.24449
552	rs1482928	T	G	T	G	0.0081	0.019396	0.7912	0.790194	FALSE	FALSE	FALSE	ieu-b-508	15	81860965	0.041139	486484	0.637307	Sepsis (28	Sepsis (28	Sepsis (28	TRUE	igd	0.0018	4.42E-06	7354	M00606.m	TRUE	reported	r5smf7	2	TRUE	0.002746	20.24449
561	rs787496	A	G	A	G	0.0081	0.002116	0.1702	0.193064	FALSE	FALSE	FALSE	ieu-b-508	1	68083054	0.042454	486484	0.960256	Sepsis (28	Sepsis (28	Sepsis (28	TRUE	igd	0.0018	5.98E-06	7354	M00606.m	TRUE	reported	r5smf7	2	TRUE	0.002746	20.24449
917	rs7561255	A	G	A	G	-0.0072	0.012867	0.7086	0.71287	FALSE	FALSE	FALSE	ieu-b-508	2	1.81E+08	0.036873	486484	0.727133	Sepsis (28	Sepsis (28	Sepsis (28	TRUE	igd	0.0016	4.72E-06	7354	M01494.m	TRUE	reported	7JWNKI	2	TRUE	0.002746	20.24449
2072	rs6768610	T	C	T	C	0.0144	-0.0817	0.914	0.921035	FALSE	FALSE	FALSE	ieu-b-508	3	1.84E+08	0.062034	486484	0.187823	Sepsis (28	Sepsis (28	Sepsis (28	TRUE	igd	0.0032	7.08E-06	7354	M18369.m	TRUE	reported	ykBjIT	2	TRUE	0.002746	20.24449
2253	rs2807592	T	G	T	G	-0.0081	0.038957	0.6979	0.705466	FALSE	FALSE	FALSE	ieu-b-508	9	1.27E+08	0.037301	486484	0.296295	Sepsis (28	Sepsis (28	Sepsis (28	TRUE	igd	0.0018	4.45E-06	7354	M19934.m	TRUE	reported	HtcWcQ	2	TRUE	0.002746	20.24449
6305	rs1091371	A	G	A	G	0.0081	-0.00949	0.212	0.21444	FALSE	FALSE	FALSE	ieu-b-508	1	1.79E+08	0.040645	486484	0.815359	Sepsis (28	Sepsis (28	Sepsis (28	TRUE	igd	0.0018	8.90E-06	7354	M33955.m	TRUE	reported	mhHFnT	2	TRUE	0.002746	20.24449
6308	rs1165452	T	G	T	G	0.0081	-0.09155	0.1988	0.195036	FALSE	FALSE	FALSE	ieu-b-508	17	76703516	0.042194	486484	0.030023	Sepsis (28	Sepsis (28	Sepsis (28	TRUE	igd	0.0018	8.24E-06	7354	M33955.m	TRUE	reported	mhHFnT	2	TRUE	0.002746	20.24449
6313	rs1781486	A	G	A	G	-0.0081	-0.02129	0.7346	0.739811	FALSE	FALSE	FALSE	ieu-b-508	2	1.44E+08	0.038118	486484	0.576452	Sepsis (28	Sepsis (28	Sepsis (28	TRUE	igd	0.0018	8.40E-06	7354	M33955.m	TRUE	reported	mhHFnT	2	TRUE	0.002746	20.24449
6325	rs6714201	A	G	A	G	0.0081	0.020267	0.483	0.496068	FALSE	FALSE	FALSE	ieu-b-508	2	2.24E+08	0.033615	486484	0.546575	Sepsis (28	Sepsis (28	Sepsis (28	TRUE	igd	0.0018	4.41E-06	7354	M33955.m	TRUE	reported	mhHFnT	2	TRUE	0.002746	20.24449
6334	rs9880529	T	C	T	C	0.0117	0.029467	0.8762	0.863252	FALSE	FALSE	FALSE	ieu-b-508	3	1.76E+08	0.048753	486484	0.545566	Sepsis (28	Sepsis (28	Sepsis (28	TRUE	igd	0.0026	7.47E-06	7354	M33955.m	TRUE	reported	mhHFnT	2	TRUE	0.002746	20.24449
1960	rs9560579	A	G	A	G	-0.0153	-0.06119	0.5193	0.513367	FALSE	FALSE	FALSE	ieu-b-508	13	90861688	0.033437	486484	0.067226	Sepsis (28	Sepsis (28	Sepsis (28	TRUE	igd	0.0034	6.41E-06	7353	M17805.m	TRUE	reported	ZDRoA2	2	TRUE	0.002746	20.24449
652	rs503360	A	G	A	G	0.0081	0.023827	0.8799	0.87707	FALSE	FALSE	FALSE	ieu-b-508	11	1.06E+08	0.050908	486484	0.639753	Sepsis (28	Sepsis (28	Sepsis (28	TRUE	igd	0.0018	4.79E-06	7352	M01125.m	TRUE	reported	yw0clB	2	TRUE	0.002747	20.24449
1611	rs2259038	A	G	A	G	-0.0216	-0.08692	0.203	0.042143	FALSE	FALSE	FALSE	ieu-b-508	2	2.04E+08	0.082795	486484	0.293775	Sepsis (28	Sepsis (28	Sepsis (28	TRUE	igd	0.0048	7.51E-06	7352	M15122.m	TRUE	reported	VlnI8T	2	TRUE	0.002747	20.24449
1620	rs9878149	A	G	A	G	0.0117	0.019397	0.2674	0.269308	FALSE	FALSE	FALSE	ieu-b-508	3	14402491	0.037877	486484	0.608582	Sepsis (28	Sepsis (28	Sepsis (28	TRUE	igd	0.0026	5.48E-06	7352	M15122.m	TRUE	reported	VlnI8T	2	TRUE	0.002747	20.24449
5854	rs2838532	T	G	T	G	0.018	-0.02614	0.2993	0.297971	FALSE	FALSE	FALSE	ieu-b-508	21	45639866	0.036497	486484	0.473799	Sepsis (28	Sepsis (28	Sepsis (28	TRUE	igd	0.004	7.83E-06	7352	M33587.m	TRUE	reported	ssG6bF	2	TRUE	0.002747	20.24449
267	rs1822834	C	G	C	G	0.0108	-0.03212	0.0629	0.070214	FALSE	TRUE	FALSE	ieu-b-508	15	58635927	0.065775	486484	0.625363	Sepsis (28	Sepsis (28	Sepsis (28	TRUE	igd	0.0024	6.59E-06	7351	M00060.m	TRUE	reported	kqwl4V	2	TRUE	0.002747	20.24449
279	rs2285137	T	C	T	C	-0.0108	0.087947	0.9436	0.950358	FALSE	FALSE	FALSE	ieu-b-508	22	42980678	0.077139	486484	0.254242	Sepsis (28	Sepsis (28	Sepsis (28	TRUE	igd	0.0024	5.35E-06	7351	M00060.m	TRUE	reported	kqwl4V	2	TRUE	0.002747	20.24449
3577	rs3784934	A	C	A	C	0.0126	-0.01059	0.0856	0.085484	FALSE	FALSE	FALSE	ieu-b-508	16	75569739	0.05941	486484	0.858539	Sepsis (28	Sepsis (28	Sepsis (28	TRUE	igd	0.0028	5.64E-06	7350	M32518.m	TRUE	reported	V0ZCiU	2	TRUE	0.002748	20.24449
6070	rs2269543	T	C	T	C	-0.0162	0.003926	0.2719	0.25734	FALSE	FALSE	FALSE	ieu-b-508	22	45244930	0.038214	486484	0.918171	Sepsis (28	Sepsis (28	Sepsis (28	TRUE	igd	0.0036	8.66E-06	7350	M33822.m	TRUE	reported	Wic4uS	2	TRUE	0.002748	20.24449
7424	rs4530945	T	C	T	C	-0.0117	0.015271	0.732	0.735766	FALSE	FALSE	FALSE	ieu-b-508	7	42556319	0.037708	486484	0.685487	Sepsis (28	Sepsis (28	Sepsis (28	TRUE	igd	0.0026	9.98E-06	7350	M35186.m	TRUE	reported	UZfu6Z	2	TRUE	0.002748	20.24449
3143	rs841845	T	C	T	C	0.0108	0.04202	0.7848	0.788471	FALSE	FALSE	FALSE	ieu-b-508	1	43404713	0.040852	486484	0.303673	Sepsis (28	Sepsis (28	Sepsis (28	TRUE	igd	0.0024	9.21E-06	7349	M32315.m	TRUE	reported	4igzkL	2	TRUE	0.002748	20.24449
1214	rs6753621	A	G	A	G	-0.0117	0.048811	0.2183	0.224524	FALSE	FALSE	FALSE	ieu-b-508	2	2.19E+08	0.040125	486484	0.223796	Sepsis (28	Sepsis (28	Sepsis (28	TRUE	igd	0.0026	7.02E-06	7348	M01670.m	TRUE	reported	kjghbc	2	TRUE	0.002748	20.24449
7913	rs1235145	T	C	T	C	-0.0081	-0.0021	0.4209	0.43263	FALSE	FALSE	FALSE	ieu-b-508	20	8325689	0.034003	486484	0.950837	Sepsis (28	Sepsis (28	Sepsis (28	TRUE	igd	0.0018	9.08E-06	7348	M35675.m	TRUE	reported	D7fdyZ	2	TRUE	0.002748	20.24449
7937	rs4984992	C	G	C	G	0.0081	0.026692	0.6116	0.597358	FALSE	TRUE	FALSE	ieu-b-508	16	8624028	0.034151	486484	0.434462	Sepsis (28	Sepsis (28	Sepsis (28	TRUE	igd	0.0018	7.83E-06	7348	M35675.m	TRUE	reported	D7fdyZ	2	TRUE	0.002748	20.24449
7941	rs6851835	A	G	A	G	0.0081	-0.00432	0.5236	0.521827	FALSE	FALSE	FALSE	ieu-b-508	4	34967580	0.0334	486484	0.89717	Sepsis (28	Sepsis (28	Sepsis (28	TRUE	igd	0.0018	8.89E-06	7348	M35675.m	TRUE	reported	D7fdyZ	2	TRUE	0.002748	20.24449
1170	rs1173111	A	G	A	G	0.0081	0.008957	0.5958	0.590248	FALSE	FALSE	FALSE	ieu-b-508	4	14872737	0.033852	486484	0.791311	Sepsis (28	Sepsis (28	Sepsis (28	TRUE	igd	0.0018	4.32E-06	7346	M01645.m	TRUE	reported	d5nAn3	2	TRUE	0.002749	20.24449
3083	rs2817479	T	G	T	G	0.0126	0.018293	0.5278	0.536646	FALSE	FALSE	FALSE	ieu-b-508	1	2.46E+08	0.036388	486484	0.615166	Sepsis (28	Sepsis (28	Sepsis (28	TRUE	igd	0.0028	6.92E-06	7346	M32197.m	TRUE	reported	VZjwa9	2	TRUE	0.002749	20.24449
6254	rs6907864	T	C	T	C	0.0261	-0.09353	0.1108	0.103934	FALSE	FALSE	FALSE	ieu-b-508	6	24293324	0.054844	486484	0.088117	Sepsis (28	Sepsis (28	Sepsis (28	TRUE	igd	0.0058	5.79E-06	7345	M33936.m	TRUE	reported	jwA4B2	2	TRUE	0.002749	20.24449
7700	rs1252875	A	G	A	G	0.0108	0.044437	0.1278	0.127527	FALSE	FALSE	FALSE	ieu-b-508	6	1.31E+08	0.049843	486484	0.372641	Sepsis (28	Sepsis (28	Sepsis (28	TRUE	igd	0.0024	6.20E-06	7345	M35397.m	TRUE	reported	Y56mZr	2	TRUE	0.002749	20.24449
3215	rs485878	A	G	A	G	-0.0081	-0.04972	0.4238	0.418215	FALSE	FALSE	FALSE	ieu-b-508	11	1.26E+08	0.033954	486484	0.143124	Sepsis (28	Sepsis (28	Sepsis (28	TRUE	igd	0.0018	4.67E-06	7344	M32339.m	TRUE	reported	SVp504	2	TRUE	0.00275	20.24449
3232	rs9876174	T	C	T	C	0.0117	-0.06693	0.8297	0.840456	FALSE	FALSE	FALSE	ieu-b-508	3	31436131	0.045586	486484	0.142046	Sepsis (28	Sepsis (28	Sepsis (28	TRUE	igd	0.0026	7.00E-06	7344	M32339.m	TRUE	reported	SVp504	2	TRUE	0.00275	20.24449
4433	rs2009217	T	C	T	C	-0.0081	0.038648	0.7257	0.72465	FALSE	FALSE	FALSE	ieu-b-508	18	69116214	0.037346	486484	0.300736	Sepsis (28	Sepsis (28	Sepsis (28	TRUE	igd	0.0018	4.91E-06	7344	M32910.m	TRUE	reported	2OCBKh	2	TRUE	0.00275	20.24449
4438	rs2382376	T	C	T	C	0.0081	-0.04027	0.3053	0.317579	FALSE	FALSE	FALSE	ieu-b-508	9	12929692	0.036051	486484	0.263992	Sepsis (28	Sepsis (28	Sepsis (28	TRUE	igd	0.0018	6.50E-06	7344	M32910.m	TRUE	reported	2OCBKh	2	TRUE	0.00275	20.24449
4446	rs4084164	A	G	A	G	0.0081	0.038119	0.3942	0.394507	FALSE	FALSE	FALSE	ieu-b-508	11	62201458	0.034633	486484	0.271045	Sepsis (28	Sepsis (28	Sepsis (28	TRUE	igd	0.0018	4.04E-06	7344	M32910.m	TRUE	reported	2OCBKh	2	TRUE	0.00275	20.24449
2700	rs1760904	A	G	A	G	-0.0081	-0.00399	0.5414	0.540028	FALSE	FALSE	FALSE	ieu-b-508	14	20852029	0.03351	486484	0.905106	Sepsis (28	Sepsis (28	Sepsis (28	TRUE	igd	0.0018	7.90E-06	7340	M25459.m	TRUE	reported	ACJY35	2	TRUE	0.002751	20.24448
2714	rs7467889	T	C	T	C	0.0081	-0.00499	0.4331	0.431267	FALSE	FALSE	FALSE	ieu-b-508	9	1.05E+08	0.033707	486484	0.88236	Sepsis (28	Sepsis (28	Sepsis (28	TRUE	igd	0.0018	8.84E-06	7340	M25459.m	TRUE	reported	ACJY35	2	TRUE	0.002751	20.24448
3175	rs883352	T	C	T	C	0.0189	0.019028	0.7693	0.731256	FALSE	FALSE	FALSE	ieu-b-508	1	76733733	0.037772	486484	0.614424	Sepsis (28	Sepsis (28	Sepsis (28	TRUE	igd	0.0042	7.55E-06	7340	M32328.m	TRUE	reported	YGsb6D	2	TRUE	0.002751	20.24448
3049	rs349022	T	C	T	C	0.0297	0.046478	0.6757	0.679418	FALSE	FALSE	FALSE	ieu-b-508	2	2.23E+08	0.035735	486484	0.193388	Sepsis (28	Sepsis (28	Sepsis (28	TRUE	igd	0.0066	7.81E-06	7338	M31591.m	TRUE	reported	KltFxx	2	TRUE	0.002752	20.24448
3899	rs3737294	A	G	A	G	0.0081	-0.14587	0.857	0.859845	FALSE	FALSE	FALSE	ieu-b-508	10	1.12E+08	0.048121	486484	0.002434	Sepsis (28	Sepsis (28	Sepsis (28	TRUE	igd	0.0018	4.83E-06	7338	M32675.m	TRUE	reported	9FmPh9	2	TRUE	0.002752	20.24448
3732	rs1723646	A	T	A	T	0.0189	-0.01369	0.1577	0.162698	FALSE	TRUE	FALSE	ieu-b-508	8	1.21E+08	0.045437	486484	0.763145	Sepsis (28	Sepsis (28	Sepsis (28	TRUE	igd	0.0042	8.26E-06	7337	M32632.m	TRUE	reported	pIHzPT	2	TRUE	0.002752	20.24448
3735	rs1775098	A	G	A	G	-0.0162	0.073951	0.2501	0.260936	FALSE	FALSE	FALSE	ieu-b-508	8	1991386	0.038072	486484	0.052091	Sepsis (28	Sepsis (28	Sepsis (28	TRUE	igd	0.0036	6.38E-06	7337	M32632.m	TRUE	reported	pIHzPT	2	TRUE	0.002752	20.24448
3745	rs4780050	T	C	T	C	0.0162	0.017829	0.8238	0.81589	FALSE	FALSE	FALSE	ieu-b-508	15	33151160	0.042518	486484	0.674982	Sepsis (28	Sepsis (28	Sepsis (28	TRUE	igd	0.0036	5.89E-06	7337	M32632.m	TRUE	reported	pIHzPT	2	TRUE	0.002752	20.24448
2941	rs2257061	T	C	T	C	-0.0162	0.010269	0.6471	0.644061	FALSE	FALSE	FALSE	ieu-b-508	7	24740177	0.034951	486484	0.768902	Sepsis (28	Sepsis (28	Sepsis (28	TRUE	igd	0.0036	6.33E-06	7336	M27738.m	TRUE	reported	UtPBd9	2	TRUE	0.002753	20.24448
2948	rs8031609	T	G	T	G	0.0198	-0.03462	0.8038	0.797171	FALSE	FALSE	FALSE	ieu-b-508	15	1.01E+08	0.041493	486484	0.404121	Sepsis (28	Sepsis (28	Sepsis (28	TRUE	igd	0.0044	7.45E-06	7336	M27738.m	TRUE	reported	UtPBd9	2	TRUE	0.002753	20.24448
2950	rs9572687	A	G	A	G	-0.0162	-0.04932	0.7063	0.699456	FALSE	FALSE	FALSE	ieu-b-508	13	71784621	0.03636	486484	0.174991	Sepsis (28	Sepsis (28	Sepsis (28	TRUE	igd	0.0036	6.55E-06	7336	M27738.m	TRUE	reported	UtPBd9	2	TRUE	0.002753	20.24448
5610	rs1161712	A	C	A	C	-0.0081	0.050894	0.8238	0.804257	FALSE	FALSE	FALSE	ieu-b-508	13	83443386	0.042027	486484	0.225898	Sepsis (28	Sepsis (28	Sepsis (28	TRUE	igd	0.0018	3.78E-06	7336	M33442.m	TRUE	reported	B1Peb3	2	TRUE	0.002753	20.24448
5629	rs9500927	A	G	A	G	0.0081	0.063126	0.1534	0.167638	FALSE	FALSE	FALSE	ieu-b-508	6	32962882	0.044635	486484	0.157286	Sepsis (28	Sepsis (28	Sepsis (28	TRUE	igd	0.0018	6.56E-06	7336	M33442.m	TRUE	reported	B1Peb3	2	TRUE	0.002753	20.24448
794	rs1015633	A	G	A	G	-0.0153	-0.07033	0.8473	0.837495	FALSE	FALSE	FALSE	ieu-b-508	8	95643315	0.04572	486484	0.124004	Sepsis (28	Sepsis (28	Sepsis (28	TRUE	igd	0.0034	7.17E-06	7335	M01356.m	TRUE	reported	ah1g5W	2	TRUE	0.002753	20.24448
805	rs9291034	T	C	T	C	-0.0117	-0.01479	0.4068	0.42227	FALSE	FALSE	FALSE	ieu-b-508	3	1.93E+08	0.03372	486484	0.66101	Sepsis (28	Sepsis (28	Sepsis (28	TRUE	igd	0.0026	5.42E-06	7335	M01356.m	TRUE	reported	ah1g5W	2	TRUE	0.002753	20.24448
6165	rs9654430	A	G	A	G	-0.0189	0.051741	0.5156	0.495079	FALSE	FALSE	FALSE	ieu-b-508	5	75647750	0.033455	486484	0.121958	Sepsis (28	Sepsis (28	Sepsis (28	TRUE	igd	0.0042	5.92E-06	7334	M33884.m	TRUE	reported	aAWgRQ	2	TRUE	0.002754	20.24448
1048	rs1713197	A	G	A	G	-0.0216	0.027112	0.0607	0.034705	FALSE	FALSE	FALSE	ieu-b-508	5	1.39E+08	0.0908	486484	0.765251	Sepsis (28	Sepsis (28	Sepsis (28	TRUE	igd	0.0048	8.18E-06	7333	M01572.m	TRUE	reported	EOUAWy	2	TRUE	0.002754	20.24448
2614	rs2850874	A	G	A	G	0.0171	-0.00491	0.0817	0.083674	FALSE	FALSE	FALSE	ieu-b-508	18	75031899	0.06026	486484	0.935005	Sepsis (28	Sepsis (28	Sepsis (28	TRUE	igd	0.0038	7.56E-06	7329	M22481.m	TRUE	reported	bXpf0k	2	TRUE	0.002755	20.24447
2303	rs1097375	A	G	A	G	-0.0117	-0.00204	0.8139	0.796251	FALSE	FALSE	FALSE	ieu-b-508	9	38360890	0.04155	486484	0.960853	Sepsis (28	Sepsis (28	Sepsis (28	TRUE	igd	0.0026	8.25E-06	7327	M20699.m	TRUE	reported	Z3EKFa	2	TRUE	0.002756	20.24447
2307	rs1726729	T	C	T	C	-0.0117	-0.01391	0.7884	0.752624	FALSE	FALSE	FALSE	ieu-b-508	4	1.84E+08	0.038681	486484	0.719153	Sepsis (28	Sepsis (28	Sepsis (28	TRUE	igd	0.0026	6.68E-06	7327	M20699.m	TRUE	reported	Z3EKFa	2	TRUE	0.002756	20.24447
1301	rs6040877	A	G	A	G	-0.0081	0.070789	0.2715	0.291188	FALSE	FALSE	FALSE	ieu-b-508	20	11756767	0.03676	486484	0.054145	Sepsis (28	Sepsis (28	Sepsis (28	TRUE	igd	0.0018	4.01E-06	7325	M02132.m	TRUE	reported	uJFm2t	2	TRUE	0.002757	20.24447
1306	rs7012395	T	C	T	C	-0.0081	0.065817	0.3041	0.305262	FALSE	FALSE	FALSE	ieu-b-508	8	8538009	0.036331	486484	0.070054	Sepsis (28	Sepsis (28	Sepsis (28	TRUE	igd	0.0018	4.96E-06	7325	M02132.m	TRUE	reported	uJFm2t	2	TRUE	0.002757	20.24447
3671	rs312994	A	G	A	G	-0.0198	7.69E-05	0.5193	0.511267	FALSE	FALSE	FALSE	ieu-b-508	18	1899696	0.033478	486484	0.998168	Sepsis (28	Sepsis (28	Sepsis (28	TRUE	igd	0.0044	8.77E-06	7322	M32578.m	TRUE	reported	LPkMFr	2	TRUE	0.002758	20.24447
6372	rs1113348	T	G	T	G	-0.0189	-0.01893	0.2844	0.28919	FALSE	FALSE	FALSE	ieu-b-508	4	58000298	0.036857	486484	0.607514	Sepsis (28	Sepsis (28	Sepsis (28	TRUE	igd	0.0042	6.58E-06	7322	M33968.m	TRUE	reported	sgVOvT	2	TRUE	0.002758	20.24447
6373	rs1170940	A	G	A	G	0.0198	-0.03271	0.3576	0.3963	FALSE	FALSE	FALSE	ieu-b-508	3	22106135	0.034286	486484	0.340031	Sepsis (28	Sepsis (28	Sepsis (28	TRUE	igd	0.0044	5.63E-06	7322	M33968.m	TRUE	reported	sgVOvT	2	TRUE	0.002758	20.24447
7131	rs2076196	A	G	A	G	-0.0162	0.05569	0.1888	0.199771	FALSE	FALSE	FALSE	ieu-b-508	22	41895409	0.04174	486484	0.182137	Sepsis (28	Sepsis (28	Sepsis (28	TRUE	igd	0.0036	6.46E-06	7322	M34527.m	TRUE	reported	EWFGP8	2	TRUE	0.002758	20.24447
7137	rs659599	A	G	A	G	0.0117	0.017194	0.5251	0.533951	FALSE	FALSE	FALSE	ieu-b-508	2	12840575	0.033468	486484	0.607445	Sepsis (28	Sepsis (28	Sepsis (28	TRUE	igd	0.0026	9.44E-06	7322	M34527.m	TRUE	reported	EWFGP8	2	TRUE	0.002758	20.24447
6301	rs9556	T	C	T	C	-0.0189	0.009042	0.3103	0.320593	FALSE	FALSE	FALSE	ieu-b-508	9	1.03E+08	0.035885	486484	0.801057	Sepsis (28	Sepsis (28	Sepsis (28	TRUE	igd	0.0042	6.08E-06	7320	M33941.m	TRUE	reported	culqiU	2	TRUE	0.002759	20.24447
4063	rs3754909	A	G	A	G	-0.0081	0.01079	0.6615	0.651012	FALSE	FALSE	FALSE	ieu-b-508	2	20252352	0.03497	486484	0.757661	Sepsis (28	Sepsis (28	Sepsis (28	TRUE	igd	0.0018	3.73E-06	7318	M32740.m	TRUE	reported	YLshfP	2	TRUE	0.00276	20.24447
7857	rs1008945	A	C	A	C	-0.0126	-0.00916	0.5399	0.580007	FALSE	FALSE	FALSE	ieu-b-508	8	93555474	0.034387	486484	0.789987	Sepsis (28	Sepsis (28	Sepsis (28	TRUE	igd	0.0028	6.53E-06	7317	M35631.m	TRUE	reported	002wka	2	TRUE	0.00276	20.24446
7863	rs2161134	A	G	A	G	0.0117	-0.00938	0.6204	0.611039	FALSE	FALSE	FALSE	ieu-b-508	5	1.72E+08	0.0342	486484	0.783971	Sepsis (28	Sepsis (28	Sepsis (28	TRUE	igd	0.0026	6.93E-06	7317	M35631.m	TRUE	reported	002wka	2	TRUE	0.00276	20.24446
7876	rs7403602	T	C	T	C	-0.0117	-0.00294	0.5361	0.534877	FALSE	FALSE	FALSE	ieu-b-508	15	30297008	0.033809	486484	0.930746	Sepsis (28	Sepsis (28	Sepsis (28	TRUE	igd	0.0026	7.15E-06	7317	M35631.m	TRUE	reported	002wka	2	TRUE	0.00276	20.24446
1726	rs9321388	A	G	A	G	-0.0072	-0.00637	0.1772	0.196916	FALSE	FALSE	FALSE	ieu-b-508	6	1.33E+08	0.042001	486484	0.879484	Sepsis (28	Sepsis (28	Sepsis (28	TRUE	igd	0.0016	7.58E-06	7310	M15506.m	TRUE	reported	mVRmZO	2	TRUE	0.002763	20.24446
1836	rs1278316	T	C	T	C	-0.0081	-0.06751	0.2909	0.336607	FALSE	FALSE	FALSE	ieu-b-508	10	13089027	0.035526	486484	0.057376	Sepsis (28	Sepsis (28	Sepsis (28	TRUE	igd	0.0018	6.56E-06	7308	M15778.m	TRUE	reported	941o3l	2	TRUE	0.002763	20.24446
3930	rs1687678	A	G	A	G	0.0144	0.062608	0.1222	0.128787	FALSE	FALSE	FALSE	ieu-b-508	6	83219541	0.049935	486484	0.209916	Sepsis (28	Sepsis (28	Sepsis (28	TRUE	igd	0.0032	8.18E-06	7308	M32698.m	TRUE	reported	srqncz	2	TRUE	0.002763	20.24446
5662	rs1018947	A	C	A	C	-0.0081	0.017868	0.4262	0.432452	FALSE	FALSE	FALSE	ieu-b-508	2	2.19E+08	0.033731	486484	0.596311	Sepsis (28	Sepsis (28	Sepsis (28	TRUE	igd	0.0018	5.91E-06	7307	M33477.m	TRUE	reported	Aw8PBK	2	TRUE	0.002764	20.24446
5675	rs1338580	A	C	A	C	-0.0081	-0.07922	0.7023	0.686203	FALSE	FALSE	FALSE	ieu-b-508	2	1.43E+08	0.035983	486484	0.027699	Sepsis (28	Sepsis (28	Sepsis (28	TRUE	igd	0.0018	9.39E-06	7307	M33477.m	TRUE	reported	Aw8PBK	2	TRUE	0.002764	20.24446
5685	rs4141184	T	C	T	C	-0.0081	0.008946	0.2907	0.287272	FALSE	FALSE	FALSE	ieu-b-508	12	1.27E+08	0.036888	486484	0.808384	Sepsis (28	Sepsis (28	Sepsis (28	TRUE	igd	0.0018	8.46E-06	7307	M33477.m	TRUE	reported	Aw8PBK	2	TRUE	0.002764	20.24446
5693	rs6426492	T	C	T	C	-0.0081	0.00491	0.3336	0.337371	FALSE	FALSE	FALSE	ieu-b-508	1	2.28E+08	0.035435	486484	0.8898	Sepsis (28	Sepsis (28	Sepsis (28	TRUE	igd	0.0018	8.75E-06	7307	M33477.m	TRUE	reported	Aw8PBK	2	TRUE	0.002764	20.24446
5523	rs2276228	A	C	A	C	0.0081	-0.0335	0.2783	0.280017	FALSE	FALSE	FALSE	ieu-b-508	21	37377890	0.037101	486484	0.366526	Sepsis (28	Sepsis (28	Sepsis (28	TRUE	igd	0.0018	5.35E-06	7306	M33422.m	TRUE	reported	PLgjrT	2	TRUE	0.002764	20.24446
5530	rs40525	A	C	A	C	0.0081	-0.02915	0.7741	0.770737	FALSE	FALSE	FALSE	ieu-b-508	5	1.48E+08	0.039774	486484	0.463644	Sepsis (28	Sepsis (28	Sepsis (28	TRUE	igd	0.0018	5.94E-06	7306	M33422.m	TRUE	reported	PLgjrT	2	TRUE	0.002764	20.24446
5531	rs4396797	T	C	T	C	0.0081	-0.00866	0.7483	0.750797	FALSE	FALSE	FALSE	ieu-b-508	21	20897134	0.038534	486484	0.822206	Sepsis (28	Sepsis (28	Sepsis (28	TRUE	igd	0.0018	5.39E-06	7306	M33422.m	TRUE	reported	PLgjrT	2	TRUE	0.002764	20.24446
5534	rs550336	C	G	C	G	-0.0081	0.069693	0.2034	0.16674	FALSE	TRUE	FALSE	ieu-b-508	1	55553554	0.044722	486484	0.119145	Sepsis (28	Sepsis (28	Sepsis (28	TRUE	igd	0.0018	8.68E-06	7306	M33422.m	TRUE	reported	PLgjrT	2	TRUE	0.002764	20.24446
2755	rs278013	A	C	A	C	0.0081	-0.04014	0.8065	0.801466	FALSE	FALSE	FALSE	ieu-b-508	5	1.67E+08	0.041785	486484	0.336692	Sepsis (28	Sepsis (28	Sepsis (28	TRUE	igd	0.0018	5.51E-06	7303	M27256.m	TRUE	reported	mJwzsi	2	TRUE	0.002765	20.24445
2766	rs7956757	T	C	T	C	0.0081	0.040234	0.8055	0.801011	FALSE	FALSE	FALSE	ieu-b-508	12	28723004	0.041908	486484	0.337033	Sepsis (28	Sepsis (28	Sepsis (28	TRUE	igd	0.0018	5.03E-06	7303	M27256.m	TRUE	reported	mJwzsi	2	TRUE	0.002765	20.24445
2289	rs4556997	A	C	A	C	-0.0189	0.018831	0.1388	0.135865	FALSE	FALSE	FALSE	ieu-b-508	2	1.01E+08	0.048821	486484	0.699713	Sepsis (28	Sepsis (28	Sepsis (28	TRUE	igd	0.0042	6.40E-06	7301	M20675.m	TRUE	reported	CXe9rE	2	TRUE	0.002766	20.24445
2436	rs1319093	A	G	A	G	-0.0117	0.036161	0.3445	0.362455	FALSE	FALSE	FALSE	ieu-b-508	6	28411244	0.034704	486484	0.297418	Sepsis (28	Sepsis (28	Sepsis (28	TRUE	igd	0.0026	6.58E-06	7284	M21630.m	TRUE	reported	7eaO4H	2	TRUE	0.002772	20.24444
1075	rs7250750	T	G	T	G	0.0081	0.013428	0.8583	0.861084	FALSE	FALSE	FALSE	ieu-b-508	19	17371484	0.048358	486484	0.781257	Sepsis (28	Sepsis (28	Sepsis (28	TRUE	igd	0.0018	7.03E-06	7271	M01585.m	TRUE	reported	M7QiSM	2	TRUE	0.002777	20.24443
7487	rs1258801	T	G	T	G	0.0117	-0.01213	0.3744	0.377784	FALSE	FALSE	FALSE	ieu-b-508	14	62724837	0.034393	486484	0.724424	Sepsis (28	Sepsis (28	Sepsis (28	TRUE	igd	0.0026	8.57E-06	7261	M35253.m	TRUE	reported	VboRmj	2	TRUE	0.002781	20.24442
1596	rs1715056	T	C	T	C	0.0117	-0.00493	0.7256	0.731727	FALSE	FALSE	FALSE	ieu-b-508	7	27727849	0.037702	486484	0.895979	Sepsis (28	Sepsis (28	Sepsis (28	TRUE	igd	0.0026	4.61E-06	7258	M12626.m	TRUE	reported	17lClc	2	TRUE	0.002782	20.24442
1597	rs4978089	A	G	A	G	0.0117	0.008586	0.2125	0.225491	FALSE	FALSE	FALSE	ieu-b-508	9	26651364	0.040442	486484	0.83187	Sepsis (28	Sepsis (28	Sepsis (28	TRUE	igd	0.0026	8.78E-06	7258	M12626.m	TRUE	reported	17lClc	2	TRUE	0.002782	20.24442
2968	rs1097693	A	G	A	G	-0.0117	-0.03336	0.7662	0.755514	FALSE	FALSE	FALSE	ieu-b-508	9	8303735	0.038906	486484	0.391177	Sepsis (28	Sepsis (28	Sepsis (28	TRUE	igd	0.0026	8.61E-06	7257	M31453.m	TRUE	reported	vdTd9M	2	TRUE	0.002783	20.24442
2981	rs6920798	A	G	A	G	-0.0117	-0.04556	0.787	0.793106	FALSE	FALSE	FALSE	ieu-b-508	6	1.12E+08	0.041087	486484	0.26754	Sepsis (28	Sepsis (28	Sepsis (28	TRUE	igd	0.0026	9.51E-06	7257	M31453.m	TRUE	reported	vdTd9M	2	TRUE	0.002783	20.24442
7755	rs1024145	A	G	A	G	0.0117	-0.01877	0.5409	0.562195	FALSE	FALSE	FALSE	ieu-b-508	7	1.3E+08	0.033963	486484	0.580478	Sepsis (28	Sepsis (28	Sepsis (28	TRUE	igd	0.0026	7.64E-06	7256	M35439.m	TRUE	reported	DnuAGZ	2	TRUE	0.002783	20.24442
4136	rs1122836	A	G	A	G	-0.0225	0.033948	0.4543	0.439578	FALSE	FALSE	FALSE	ieu-b-508	11	68593258	0.033781	486484	0.314916	Sepsis (28	Sepsis (28	Sepsis (28	TRUE	igd	0.005	7.05E-06	7250	M32757.m	TRUE	reported	TJV9Xb	2	TRUE	0.002785	20.24441
3800	rs1016295	T	C	T	C	0.0081	0.014462	0.6424	0.628631	FALSE	FALSE	FALSE	ieu-b-508	15	1E+08	0.034575	486484	0.675731	Sepsis (28	Sepsis (28	Sepsis (28	TRUE	igd	0.0018	9.14E-06	7229	M32644.m	TRUE	reported	JAa2WD	2	TRUE	0.002793	20.2444
3804	rs1102682	A	T	A	T	-0.0081	-0.0105	0.3792	0.379095	FALSE	TRUE	FALSE	ieu-b-508	11	22905962	0.034502	486484	0.760985	Sepsis (28	Sepsis (28	Sepsis (28	TRUE	igd	0.0018	9.20E-06	7229	M32644.m	TRUE	reported	JAa2WD	2	TRUE	0.002793	20.2444
1359	rs3094600	A	G	A	G	-0.0099	0.027525	0.2637	0.271302	FALSE	FALSE	FALSE	ieu-b-508	6	31347144	0.037468	486484	0.462777	Sepsis (28	Sepsis (28	Sepsis (28	TRUE	igd	0.0022	6.21E-06	7226	M02730.m	TRUE	reported	XYa035	2	TRUE	0.002795	20.2444
1368	rs9436667	T	C	T	C	0.0135	-0.04199	0.1138	0.119654	FALSE	FALSE	FALSE	ieu-b-508	1	63405885	0.051663	486484	0.416361	Sepsis (28	Sepsis (28	Sepsis (28	TRUE	igd	0.003	7.30E-06	7226	M02730.m	TRUE	reported	XYa035	2	TRUE	0.002795	20.2444
6274	rs4328432	A	G	A	G	0.0207	0.083055	0.1812	0.202168	FALSE	FALSE	FALSE	ieu-b-508	16	57725231	0.041913	486484	0.047523	Sepsis (28	Sepsis (28	Sepsis (28	TRUE	igd	0.0046	5.50E-06	7222	M33937.m	TRUE	reported	QanGCI	2	TRUE	0.002796	20.24439
7550	rs1051986	A	G	A	G	0.0081	-0.0086	0.2313	0.225174	FALSE	FALSE	FALSE	ieu-b-508	15	34017054	0.039919	486484	0.8294	Sepsis (28	Sepsis (28	Sepsis (28	TRUE	igd	0.0018	7.04E-06	7217	M35270.m	TRUE	reported	Ct8V1n	2	TRUE	0.002798	20.24439
7568	rs2395911	T	G	T	G	0.0081	0.051928	0.7477	0.752855	FALSE	FALSE	FALSE	ieu-b-508	7	1.07E+08	0.038646	486484	0.179058	Sepsis (28	Sepsis (28	Sepsis (28	TRUE	igd	0.0018	6.76E-06	7217	M35270.m	TRUE	reported	Ct8V1n	2	TRUE	0.002798	20.24439
2353	rs209755	C	G	C	G	0.0099	0.023484	0.302	0.290763	FALSE	TRUE	FALSE	ieu-b-508	15	95740004	0.037372	486484	0.529759	Sepsis (28	Sepsis (28	Sepsis (28	TRUE	igd	0.0022	8.51E-06	7203	M21047.m	TRUE	reported	ISF1Cr	2	TRUE	0.002803	20.24438
2645	rs628238	A	C	A	C	-0.0144	0.030683	0.2548	0.261899	FALSE	FALSE	FALSE	ieu-b-508	18	3519661	0.0379	486484	0.418184	Sepsis (28	Sepsis (28	Sepsis (28	TRUE	igd	0.0032	6.03E-06	7184	M22548.m	TRUE	reported	mZtwX2	2	TRUE	0.002811	20.24436
8209	rs4654649	T	C	T	C	0.0144	0.017379	0.5518	0.5493	FALSE	FALSE	FALSE	ieu-b-508	1	2.29E+08	0.033602	486484	0.605023	Sepsis (28	Sepsis (28	Sepsis (28	TRUE	igd	0.0032	8.49E-06	7184	M36300.m	TRUE	reported	ZjeC9m	2	TRUE	0.002811	20.24436
4539	rs1768520	T	C	T	C	0.0333	0.165145	0.9177	0.895183	FALSE	FALSE	FALSE	ieu-b-508	19	28914976	0.055872	486484	0.003119	Sepsis (28	Sepsis (28	Sepsis (28	TRUE	igd	0.0074	6.83E-06	7166	M33138.m	TRUE	reported	IUpLAI	2	TRUE	0.002818	20.24435
947	rs9365106	A	G	A	G	-0.0117	0.027282	0.6824	0.682847	FALSE	FALSE	FALSE	ieu-b-508	6	1.6E+08	0.035845	486484	0.446591	Sepsis (28	Sepsis (28	Sepsis (28	TRUE	igd	0.0026	8.06E-06	7160	M01508.m	TRUE	reported	go526k	2	TRUE	0.00282	20.24434
5354	rs4624748	A	G	A	G	-0.0081	0.004264	0.2344	0.228241	FALSE	FALSE	FALSE	ieu-b-508	5	54021166	0.039866	486484	0.914813	Sepsis (28	Sepsis (28	Sepsis (28	TRUE	igd	0.0018	3.81E-06	7118	M33380.m	TRUE	reported	OHRfl5	2	TRUE	0.002837	20.24431
1112	rs6920504	T	C	T	C	-0.0135	0.025326	0.7729	0.751289	FALSE	FALSE	FALSE	ieu-b-508	6	1.16E+08	0.038841	486484	0.51438	Sepsis (28	Sepsis (28	Sepsis (28	TRUE	igd	0.003	7.53E-06	7104	M01638.m	TRUE	reported	8dp0Ac	2	TRUE	0.002842	20.2443
7513	rs486052	T	G	T	G	0.0117	-0.00814	0.6521	0.650258	FALSE	FALSE	FALSE	ieu-b-508	11	84135406	0.035391	486484	0.818061	Sepsis (28	Sepsis (28	Sepsis (28	TRUE	igd	0.0026	9.46E-06	7100	M35254.m	TRUE	reported	HfVgPn	2	TRUE	0.002844	20.2443
8262	rs2615697	T	C	T	C	-0.0108	-0.04583	0.8166	0.817584	FALSE	FALSE	FALSE	ieu-b-508	16	81425858	0.043203	486484	0.288742	Sepsis (28	Sepsis (28	Sepsis (28	TRUE	igd	0.0024	7.72E-06	7092	M36552.m	TRUE	reported	5CxMJz	2	TRUE	0.002847	20.24429
8268	rs7037029	A	G	A	G	0.0108	-0.03008	0.7501	0.730259	FALSE	FALSE	FALSE	ieu-b-508	9	1.32E+08	0.037813	486484	0.426369	Sepsis (28	Sepsis (28	Sepsis (28	TRUE	igd	0.0024	7.21E-06	7092	M36552.m	TRUE	reported	5CxMJz	2	TRUE	0.002847	20.24429
1506	rs1043581	A	G	A	G	0.0081	-0.01022	0.8067	0.780251	FALSE	FALSE	FALSE	ieu-b-508	9	5533450	0.040383	486484	0.800228	Sepsis (28	Sepsis (28	Sepsis (28	TRUE	igd	0.0018	4.01E-06	7081	M12067.m	TRUE	reported	O5x7RD	2	TRUE	0.002852	20.24428
5826	rs4781872	C	G	C	G	-0.0081	-0.03944	0.58	0.585807	FALSE	TRUE	FALSE	ieu-b-508	16	18147263	0.033887	486484	0.244482	Sepsis (28	Sepsis (28	Sepsis (28	TRUE	igd	0.0018	8.35E-06	7066	M33515.m	TRUE	reported	X9mul1	2	TRUE	0.002858	20.24427
5835	rs7684565	T	C	T	C	0.0081	0.011746	0.4972	0.51185	FALSE	FALSE	FALSE	ieu-b-508	4	1.18E+08	0.033518	486484	0.726019	Sepsis (28	Sepsis (28	Sepsis (28	TRUE	igd	0.0018	8.67E-06	7066	M33515.m	TRUE	reported	X9mul1	2	TRUE	0.002858	20.24427
854	rs1124520	T	C	T	C	0.018	-0.00048	0.1119	0.108095	FALSE	FALSE	FALSE	ieu-b-508	10	1.26E+08	0.054052	486484	0.992924	Sepsis (28	Sepsis (28	Sepsis (28	TRUE	igd	0.004	8.94E-06	7063	M01361.m	TRUE	reported	NIAcQ3	2	TRUE	0.002859	20.24427
857	rs1705622	T	G	T	G	0.0153	0.035763	0.1416	0.14834	FALSE	FALSE	FALSE	ieu-b-508	18	72767269	0.047075	486484	0.447437	Sepsis (28	Sepsis (28	Sepsis (28	TRUE	igd	0.0034	7.07E-06	7063	M01361.m	TRUE	reported	NIAcQ3	2	TRUE	0.002859	20.24427
868	rs7710912	A	G	A	G	-0.0189	0.015973	0.0728	0.072389	FALSE	FALSE	FALSE	ieu-b-508	5	83978954	0.064457	486484	0.804286	Sepsis (28	Sepsis (28	Sepsis (28	TRUE	igd	0.0042	8.30E-06	7063	M01361.m	TRUE	reported	NIAcQ3	2	TRUE	0.002859	20.24427
4288	rs434943	A	G	A	G	0.0135	-0.04593	0.3163	0.336217	FALSE	FALSE	FALSE	ieu-b-508	14	69314059	0.035276	486484	0.1929	Sepsis (28	Sepsis (28	Sepsis (28	TRUE	igd	0.003	8.57E-06	7063	M32814.m	TRUE	reported	z0OeTx	2	TRUE	0.002859	20.24427
1384	rs1759345	C	G	C	G	0.0144	0.058771	0.8978	0.877168	FALSE	TRUE	FALSE	ieu-b-508	3	1.79E+08	0.05141	486484	0.252968	Sepsis (28	Sepsis (28	Sepsis (28	TRUE	igd	0.0032	7.09E-06	7037	M02734.m	TRUE	reported	0y0dDD	2	TRUE	0.002869	20.24424
1390	rs2396461	T	C	T	C	0.0117	0.097294	0.121	0.159091	FALSE	FALSE	FALSE	ieu-b-508	2	2.28E+08	0.045701	486484	0.033258	Sepsis (28	Sepsis (28	Sepsis (28	TRUE	igd	0.0026	8.38E-06	7037	M02734.m	TRUE	reported	0y0dDD	2	TRUE	0.002869	20.24424
4335	rs7707678	A	G	A	G	0.0198	-0.02312	0.4165	0.417712	FALSE	FALSE	FALSE	ieu-b-508	5	1.5E+08	0.033913	486484	0.495333	Sepsis (28	Sepsis (28	Sepsis (28	TRUE	igd	0.0044	8.10E-06	6967	M32847.m	TRUE	reported	eYme29	2	TRUE	0.002898	20.24419
8045	rs917046	T	G	T	G	-0.0369	-0.03792	0.9531	0.951252	FALSE	FALSE	FALSE	ieu-b-508	7	96628907	0.078251	486484	0.627988	Sepsis (28	Sepsis (28	Sepsis (28	TRUE	igd	0.0082	6.71E-06	6960	M35854.m	TRUE	reported	EujB0p	2	TRUE	0.002901	20.24418
1420	rs1728001	A	T	A	T	-0.0234	0.002757	0.2083	0.255033	FALSE	TRUE	FALSE	ieu-b-508	11	97056516	0.041763	486484	0.947366	Sepsis (28	Sepsis (28	Sepsis (28	TRUE	igd	0.0052	6.91E-06	6941	M03127.m	TRUE	reported	JscEZw	2	TRUE	0.002909	20.24417
1435	rs7924591	T	C	T	C	0.0108	-0.0748	0.5985	0.601813	FALSE	FALSE	FALSE	ieu-b-508	11	1.04E+08	0.034124	486484	0.028388	Sepsis (28	Sepsis (28	Sepsis (28	TRUE	igd	0.0024	9.52E-06	6941	M03127.m	TRUE	reported	JscEZw	2	TRUE	0.002909	20.24417
8405	rs1163871	A	G	A	G	0.0081	0.012518	0.7783	0.784005	FALSE	FALSE	FALSE	ieu-b-508	15	58079462	0.040711	486484	0.758468	Sepsis (28	Sepsis (28	Sepsis (28	TRUE	igd	0.0018	4.62E-06	6940	M36808.m	TRUE	reported	u7lqaj	2	TRUE	0.002909	20.24416
8406	rs1167905	T	C	T	C	0.0081	0.011015	0.7521	0.757275	FALSE	FALSE	FALSE	ieu-b-508	2	2.12E+08	0.039034	486484	0.777797	Sepsis (28	Sepsis (28	Sepsis (28	TRUE	igd	0.0018	4.14E-06	6940	M36808.m	TRUE	reported	u7lqaj	2	TRUE	0.002909	20.24416
8410	rs1302247	A	G	A	G	0.0081	0.013446	0.8262	0.823616	FALSE	FALSE	FALSE	ieu-b-508	2	1.25E+08	0.043703	486484	0.758333	Sepsis (28	Sepsis (28	Sepsis (28	TRUE	igd	0.0018	6.50E-06	6940	M36808.m	TRUE	reported	u7lqaj	2	TRUE	0.002909	20.24416
8429	rs7129047	A	C	A	C	-0.0081	-0.06118	0.1751	0.179193	FALSE	FALSE	FALSE	ieu-b-508	11	10143942	0.043574	486484	0.160284	Sepsis (28	Sepsis (28	Sepsis (28	TRUE	igd	0.0018	7.21E-06	6940	M36808.m	TRUE	reported	u7lqaj	2	TRUE	0.002909	20.24416
2034	rs2511986	C	G	C	G	0.0153	0.004273	0.7684	0.765939	FALSE	TRUE	FALSE	ieu-b-508	11	57231559	0.039745	486484	0.914387	Sepsis (28	Sepsis (28	Sepsis (28	TRUE	igd	0.0034	7.63E-06	6939	M18349.m	TRUE	reported	M5qPs8	2	TRUE	0.00291	20.24416
2214	rs947678	A	C	A	C	-0.0117	-0.08003	0.5736	0.616272	FALSE	FALSE	FALSE	ieu-b-508	1	7492616	0.034365	486484	0.019863	Sepsis (28	Sepsis (28	Sepsis (28	TRUE	igd	0.0026	5.03E-06	6929	M19364.m	TRUE	reported	XkjMEx	2	TRUE	0.002914	20.24416
2902	rs934668	T	C	T	C	0.0252	0.017962	0.7739	0.705609	FALSE	FALSE	FALSE	ieu-b-508	17	75750768	0.036517	486484	0.622801	Sepsis (28	Sepsis (28	Sepsis (28	TRUE	igd	0.0056	6.78E-06	6830	M27722.m	TRUE	reported	rzVgUy	2	TRUE	0.002956	20.24407
2951	rs1049569	A	G	A	G	-0.0288	0.102797	0.9526	0.930456	FALSE	FALSE	FALSE	ieu-b-508	2	19693806	0.066315	486484	0.121107	Sepsis (28	Sepsis (28	Sepsis (28	TRUE	igd	0.0064	6.35E-06	6795	M30805.m	TRUE	reported	YHV70c	2	TRUE	0.002971	20.24404
1890	rs7260033	T	C	T	C	-0.0117	-0.00388	0.4757	0.477556	FALSE	FALSE	FALSE	ieu-b-508	19	35136617	0.033401	486484	0.907511	Sepsis (28	Sepsis (28	Sepsis (28	TRUE	igd	0.0026	8.79E-06	6782	M15990.m	TRUE	reported	15fe4N	2	TRUE	0.002977	20.24403
6243	rs1741285	A	G	A	G	-0.0351	-0.01721	0.4861	0.493088	FALSE	FALSE	FALSE	ieu-b-508	20	4116436	0.033661	486484	0.609117	Sepsis (28	Sepsis (28	Sepsis (28	TRUE	igd	0.0078	5.95E-06	6756	M33935.m	TRUE	reported	ELBTDc	2	TRUE	0.002988	20.24401
6800	rs1233196	A	C	A	C	0.0081	-0.00356	0.4969	0.494669	FALSE	FALSE	FALSE	ieu-b-508	4	7491167	0.033421	486484	0.915158	Sepsis (28	Sepsis (28	Sepsis (28	TRUE	igd	0.0018	4.00E-06	6755	M34359.m	TRUE	reported	0UotaJ	2	TRUE	0.002989	20.244
6806	rs136230	A	G	A	G	-0.0081	-0.01364	0.5971	0.594612	FALSE	FALSE	FALSE	ieu-b-508	22	31214382	0.033905	486484	0.687573	Sepsis (28	Sepsis (28	Sepsis (28	TRUE	igd	0.0018	5.83E-06	6755	M34359.m	TRUE	reported	0UotaJ	2	TRUE	0.002989	20.244
6815	rs2541164	A	G	A	G	-0.0081	0.00588	0.2961	0.302609	FALSE	FALSE	FALSE	ieu-b-508	9	1.32E+08	0.036321	486484	0.871381	Sepsis (28	Sepsis (28	Sepsis (28	TRUE	igd	0.0018	6.77E-06	6755	M34359.m	TRUE	reported	0UotaJ	2	TRUE	0.002989	20.244
2872	rs472847	T	C	T	C	0.0243	-0.02659	0.3494	0.398518	FALSE	FALSE	FALSE	ieu-b-508	18	55783897	0.034199	486484	0.436899	Sepsis (28	Sepsis (28	Sepsis (28	TRUE	igd	0.0054	7.14E-06	6715	M27710.m	TRUE	reported	XeaFav	2	TRUE	0.003007	20.24397
4182	rs9316990	A	G	A	G	0.0189	0.057669	0.433	0.441692	FALSE	FALSE	FALSE	ieu-b-508	13	59113604	0.033593	486484	0.086036	Sepsis (28	Sepsis (28	Sepsis (28	TRUE	igd	0.0042	7.24E-06	6712	M32759.m	TRUE	reported	9eIHr7	2	TRUE	0.003008	20.24397
2224	rs535902	A	G	A	G	0.0252	0.017999	0.9346	0.931757	FALSE	FALSE	FALSE	ieu-b-508	6	74195459	0.066448	486484	0.786486	Sepsis (28	Sepsis (28	Sepsis (28	TRUE	igd	0.0056	7.89E-06	6678	M19368.m	TRUE	reported	QaKPad	2	TRUE	0.003023	20.24394
7819	rs1411737	T	C	T	C	0.0243	-0.01538	0.7834	0.78052	FALSE	FALSE	FALSE	ieu-b-508	13	1.07E+08	0.041102	486484	0.708334	Sepsis (28	Sepsis (28	Sepsis (28	TRUE	igd	0.0054	5.82E-06	6595	M35472.m	TRUE	reported	oZU10n	2	TRUE	0.003061	20.24386
1937	rs6024831	A	G	A	G	-0.0189	-0.00768	0.9232	0.926118	FALSE	FALSE	FALSE	ieu-b-508	20	54938464	0.064042	486484	0.904516	Sepsis (28	Sepsis (28	Sepsis (28	TRUE	igd	0.0042	7.09E-06	6549	M16822.m	TRUE	reported	UNI018	2	TRUE	0.003083	20.24382
7459	rs1686598	A	G	A	G	-0.0162	0.005836	0.2149	0.219576	FALSE	FALSE	FALSE	ieu-b-508	2	7304656	0.040275	486484	0.884781	Sepsis (28	Sepsis (28	Sepsis (28	TRUE	igd	0.0036	6.32E-06	6543	M35193.m	TRUE	reported	BMMWXU	2	TRUE	0.003085	20.24381
7531	rs1115213	A	G	A	G	-0.0162	0.006989	0.8149	0.813518	FALSE	FALSE	FALSE	ieu-b-508	18	56849429	0.042854	486484	0.87045	Sepsis (28	Sepsis (28	Sepsis (28	TRUE	igd	0.0036	6.23E-06	6538	M35257.m	TRUE	reported	iphouU	2	TRUE	0.003088	20.24381
955	rs1165061	C	G	C	G	0.0081	0.042902	0.6907	0.709931	FALSE	TRUE	FALSE	ieu-b-508	17	46123698	0.036748	486484	0.243028	Sepsis (28	Sepsis (28	Sepsis (28	TRUE	igd	0.0018	5.99E-06	6523	M01558.m	TRUE	reported	AZK0p4	2	TRUE	0.003095	20.24379
960	rs1556880	T	C	T	C	0.0081	0.032622	0.2976	0.304854	FALSE	FALSE	FALSE	ieu-b-508	6	1.51E+08	0.036438	486484	0.37065	Sepsis (28	Sepsis (28	Sepsis (28	TRUE	igd	0.0018	6.04E-06	6523	M01558.m	TRUE	reported	AZK0p4	2	TRUE	0.003095	20.24379
966	rs2252585	T	C	T	C	-0.0081	-0.09508	0.7349	0.730914	FALSE	FALSE	FALSE	ieu-b-508	21	36241929	0.04215	486484	0.024091	Sepsis (28	Sepsis (28	Sepsis (28	TRUE	igd	0.0018	5.97E-06	6523	M01558.m	TRUE	reported	AZK0p4	2	TRUE	0.003095	20.24379
970	rs3813810	A	C	A	C	0.0117	0.059471	0.1334	0.134458	FALSE	FALSE	FALSE	ieu-b-508	1	4088339	0.04923	486484	0.227035	Sepsis (28	Sepsis (28	Sepsis (28	TRUE	igd	0.0026	5.73E-06	6523	M01558.m	TRUE	reported	AZK0p4	2	TRUE	0.003095	20.24379
974	rs4923082	T	C	T	C	0.0081	0.001173	0.2518	0.256695	FALSE	FALSE	FALSE	ieu-b-508	11	23288296	0.038204	486484	0.975496	Sepsis (28	Sepsis (28	Sepsis (28	TRUE	igd	0.0018	6.95E-06	6523	M01558.m	TRUE	reported	AZK0p4	2	TRUE	0.003095	20.24379
981	rs9354329	T	C	T	C	-0.0081	-0.01376	0.6322	0.611809	FALSE	FALSE	FALSE	ieu-b-508	6	66600184	0.034267	486484	0.688021	Sepsis (28	Sepsis (28	Sepsis (28	TRUE	igd	0.0018	5.10E-06	6523	M01558.m	TRUE	reported	AZK0p4	2	TRUE	0.003095	20.24379
6110	rs8104825	A	G	A	G	0.0153	0.032944	0.4384	0.438693	FALSE	FALSE	FALSE	ieu-b-508	19	50315423	0.033719	486484	0.328561	Sepsis (28	Sepsis (28	Sepsis (28	TRUE	igd	0.0034	7.29E-06	6506	M33871.m	TRUE	reported	mQD6DP	2	TRUE	0.003103	20.24377
4582	rs239430	A	G	A	G	-0.0153	0.063996	0.7871	0.771989	FALSE	FALSE	FALSE	ieu-b-508	8	37234473	0.039811	486484	0.107944	Sepsis (28	Sepsis (28	Sepsis (28	TRUE	igd	0.0034	8.78E-06	6404	M33163.m	TRUE	reported	YzGXkR	2	TRUE	0.003152	20.24368
4586	rs3886930	T	C	T	C	0.0117	0.051133	0.6	0.60798	FALSE	FALSE	FALSE	ieu-b-508	4	1.73E+08	0.034197	486484	0.13485	Sepsis (28	Sepsis (28	Sepsis (28	TRUE	igd	0.0026	9.18E-06	6404	M33163.m	TRUE	reported	YzGXkR	2	TRUE	0.003152	20.24368
5973	rs1713003	T	C	T	C	-0.018	-0.10564	0.9127	0.91286	FALSE	FALSE	FALSE	ieu-b-508	1	87986477	0.059359	486484	0.075116	Sepsis (28	Sepsis (28	Sepsis (28	TRUE	igd	0.004	7.79E-06	6279	M33666.m	TRUE	reported	dvWEtk	2	TRUE	0.003215	20.24355
2465	rs4433392	T	C	T	C	0.0117	-0.00041	0.3697	0.357152	FALSE	FALSE	FALSE	ieu-b-508	1	1.16E+08	0.034801	486484	0.990567	Sepsis (28	Sepsis (28	Sepsis (28	TRUE	igd	0.0026	5.10E-06	6180	M22030.m	TRUE	reported	ZNV0qR	2	TRUE	0.003266	20.24345
7069	rs133565	A	G	A	G	-0.0189	0.027424	0.3318	0.339732	FALSE	FALSE	FALSE	ieu-b-508	22	48639993	0.035163	486484	0.435456	Sepsis (28	Sepsis (28	Sepsis (28	TRUE	igd	0.0042	8.51E-06	6144	M34469.m	TRUE	reported	WMOqbm	2	TRUE	0.003285	20.24341
3295	rs4694161	A	G	A	G	0.0513	-0.06449	0.0297	0.026234	FALSE	FALSE	FALSE	ieu-b-508	4	74194326	0.104789	486484	0.5383	Sepsis (28	Sepsis (28	Sepsis (28	TRUE	igd	0.0114	7.19E-06	6119	M32379.m	TRUE	reported	MLimM6	2	TRUE	0.003298	20.24338
7265	rs2970366	A	G	A	G	-0.0126	0.011567	0.1664	0.17799	FALSE	FALSE	FALSE	ieu-b-508	15	60116817	0.043643	486484	0.79099	Sepsis (28	Sepsis (28	Sepsis (28	TRUE	igd	0.0028	4.95E-06	5885	M34912.m	TRUE	reported	W8lcT6	2	TRUE	0.003429	20.24312
7266	rs32487	A	G	A	G	-0.0126	0.01366	0.8291	0.826766	FALSE	FALSE	FALSE	ieu-b-508	5	55602029	0.044161	486484	0.757077	Sepsis (28	Sepsis (28	Sepsis (28	TRUE	igd	0.0028	6.25E-06	5885	M34912.m	TRUE	reported	W8lcT6	2	TRUE	0.003429	20.24312
7282	rs9807799	T	C	T	C	-0.0126	0.08398	0.1397	0.143387	FALSE	FALSE	FALSE	ieu-b-508	18	76118216	0.0474	486484	0.07644	Sepsis (28	Sepsis (28	Sepsis (28	TRUE	igd	0.0028	6.56E-06	5885	M34912.m	TRUE	reported	W8lcT6	2	TRUE	0.003429	20.24312
1797	rs1049327	A	T	A	T	0.0243	0.017637	0.8124	0.817382	FALSE	TRUE	FALSE	ieu-b-508	1	60592321	0.043308	486484	0.683834	Sepsis (28	Sepsis (28	Sepsis (28	TRUE	igd	0.0054	5.83E-06	5829	M15749.m	TRUE	reported	TR861N	2	TRUE	0.003462	20.24305
7646	rs2174753	A	T	A	T	0.0081	-0.03576	0.3905	0.397305	FALSE	TRUE	FALSE	ieu-b-508	13	69363379	0.03414	486484	0.294891	Sepsis (28	Sepsis (28	Sepsis (28	TRUE	igd	0.0018	3.58E-06	5768	M35326.m	TRUE	reported	jRytyJ	2	TRUE	0.003498	20.24298
7652	rs4765845	A	G	A	G	0.0081	0.005033	0.4785	0.482771	FALSE	FALSE	FALSE	ieu-b-508	12	1929958	0.03337	486484	0.880124	Sepsis (28	Sepsis (28	Sepsis (28	TRUE	igd	0.0018	5.57E-06	5768	M35326.m	TRUE	reported	jRytyJ	2	TRUE	0.003498	20.24298
2486	rs815198	T	C	T	C	-0.0126	0.007704	0.4745	0.464593	FALSE	FALSE	FALSE	ieu-b-508	15	46886109	0.033757	486484	0.819484	Sepsis (28	Sepsis (28	Sepsis (28	TRUE	igd	0.0028	6.02E-06	5676	M22032.m	TRUE	reported	xpe96K	2	TRUE	0.003555	20.24286
7891	rs1696708	C	G	C	G	-0.0207	0.044596	0.3734	0.374359	FALSE	TRUE	FALSE	ieu-b-508	17	39687632	0.034486	486484	0.195955	Sepsis (28	Sepsis (28	Sepsis (28	TRUE	igd	0.0046	6.34E-06	5629	M35669.m	TRUE	reported	gSecuE	2	TRUE	0.003585	20.24281
1091	rs510032	T	C	T	C	0.009	-0.07579	0.169	0.193497	FALSE	FALSE	FALSE	ieu-b-508	1	2.27E+08	0.042488	486484	0.074437	Sepsis (28	Sepsis (28	Sepsis (28	TRUE	igd	0.002	6.80E-06	5604	M01604.m	TRUE	reported	hE3I2k	2	TRUE	0.0036	20.24277
1021	rs588973	A	G	A	G	0.009	-0.01739	0.646	0.647252	FALSE	FALSE	FALSE	ieu-b-508	19	50736956	0.03489	486484	0.618173	Sepsis (28	Sepsis (28	Sepsis (28	TRUE	igd	0.002	6.80E-06	5603	M01564.m	TRUE	reported	IPUdyD	2	TRUE	0.003601	20.24277
597	rs532098	A	G	A	G	0.009	-0.02622	0.461	0.456883	FALSE	FALSE	FALSE	ieu-b-508	6	32578052	0.033456	486484	0.433235	Sepsis (28	Sepsis (28	Sepsis (28	TRUE	igd	0.002	6.80E-06	5601	M01110.m	TRUE	reported	0A31Sl	2	TRUE	0.003602	20.24277
4504	rs1771851	T	C	T	C	0.009	0.039008	0.77	0.765534	FALSE	FALSE	FALSE	ieu-b-508	19	6862498	0.039354	486484	0.321577	Sepsis (28	Sepsis (28	Sepsis (28	TRUE	igd	0.002	6.80E-06	5600	M33132.m	TRUE	reported	dTXJa8	2	TRUE	0.003603	20.24277
4517	rs932748	T	G	T	G	0.009	0.05038	0.878	0.881422	FALSE	FALSE	FALSE	ieu-b-508	1	63805122	0.052118	486484	0.333725	Sepsis (28	Sepsis (28	Sepsis (28	TRUE	igd	0.002	6.80E-06	5600	M33132.m	TRUE	reported	dTXJa8	2	TRUE	0.003603	20.24277
459	rs2521310	T	C	T	C	0.009	-0.05134	0.874	0.866911	FALSE	FALSE	FALSE	ieu-b-508	7	89352860	0.04925	486484	0.297216	Sepsis (28	Sepsis (28	Sepsis (28	TRUE	igd	0.002	6.80E-06	5598	M00513.m	TRUE	reported	ohacnP	2	TRUE	0.003604	20.24277
462	rs6502247	A	G	A	G	-0.009	-0.07682	0.119	0.119882	FALSE	FALSE	FALSE	ieu-b-508	17	13203562	0.053709	486484	0.152637	Sepsis (28	Sepsis (28	Sepsis (28	TRUE	igd	0.002	6.80E-06	5598	M00513.m	TRUE	reported	ohacnP	2	TRUE	0.003604	20.24277
3757	rs1165162	A	G	A	G	0.009	-0.02861	0.321	0.308426	FALSE	FALSE	FALSE	ieu-b-508	17	59580081	0.039849	486484	0.472717	Sepsis (28	Sepsis (28	Sepsis (28	TRUE	igd	0.002	6.80E-06	5598	M32634.m	TRUE	reported	7T2RUz	2	TRUE	0.003604	20.24277
3774	rs439401	T	C	T	C	-0.009	-0.0397	0.351	0.362706	FALSE	FALSE	FALSE	ieu-b-508	19	45414451	0.03472	486484	0.252836	Sepsis (28	Sepsis (28	Sepsis (28	TRUE	igd	0.002	6.80E-06	5598	M32634.m	TRUE	reported	7T2RUz	2	TRUE	0.003604	20.24277
699	rs8035304	T	G	T	G	0.009	-0.00237	0.81	0.812215	FALSE	FALSE	FALSE	ieu-b-508	15	93723925	0.043687	486484	0.956822	Sepsis (28	Sepsis (28	Sepsis (28	TRUE	igd	0.002	6.80E-06	5597	M01299.m	TRUE	reported	gfM0a3	2	TRUE	0.003605	20.24276
3471	rs2467261	A	G	A	G	-0.009	-0.0613	0.735	0.731587	FALSE	FALSE	FALSE	ieu-b-508	17	787784	0.037756	486484	0.104463	Sepsis (28	Sepsis (28	Sepsis (28	TRUE	igd	0.002	6.80E-06	5596	M32489.m	TRUE	reported	hnljaD	2	TRUE	0.003606	20.24276
6435	rs1938299	A	G	A	G	0.009	-0.02685	0.889	0.892421	FALSE	FALSE	FALSE	ieu-b-508	1	45744810	0.053807	486484	0.617773	Sepsis (28	Sepsis (28	Sepsis (28	TRUE	igd	0.002	6.80E-06	5594	M34062.m	TRUE	reported	RZyHKP	2	TRUE	0.003607	20.24276
132	rs6001129	T	C	T	C	-0.009	0.090273	0.134	0.131565	FALSE	FALSE	FALSE	ieu-b-508	22	22506340	0.049287	486484	0.067012	Sepsis (28	Sepsis (28	Sepsis (28	TRUE	igd	0.002	6.80E-06	5592	M00054.m	TRUE	reported	QTDAnh	2	TRUE	0.003608	20.24276
915	rs4646693	T	C	T	C	-0.009	0.007981	0.435	0.436411	FALSE	FALSE	FALSE	ieu-b-508	15	1.01E+08	0.034195	486484	0.815453	Sepsis (28	Sepsis (28	Sepsis (28	TRUE	igd	0.002	6.80E-06	5592	M01494.m	TRUE	reported	7JWNKI	2	TRUE	0.003608	20.24276
2060	rs200449	A	G	A	G	0.009	-0.01308	0.569	0.562024	FALSE	FALSE	FALSE	ieu-b-508	1	6726401	0.033624	486484	0.697283	Sepsis (28	Sepsis (28	Sepsis (28	TRUE	igd	0.002	6.80E-06	5592	M18369.m	TRUE	reported	ykBjIT	2	TRUE	0.003608	20.24276
2073	rs6797094	T	G	T	G	0.009	0.017187	0.611	0.596093	FALSE	FALSE	FALSE	ieu-b-508	3	1.81E+08	0.034038	486484	0.613592	Sepsis (28	Sepsis (28	Sepsis (28	TRUE	igd	0.002	6.80E-06	5592	M18369.m	TRUE	reported	ykBjIT	2	TRUE	0.003608	20.24276
809	rs1075227	T	C	T	C	0.009	-0.02991	0.552	0.55074	FALSE	FALSE	FALSE	ieu-b-508	4	18512526	0.033618	486484	0.373661	Sepsis (28	Sepsis (28	Sepsis (28	TRUE	igd	0.002	6.80E-06	5590	M01358.m	TRUE	reported	kSPQgd	2	TRUE	0.003609	20.24275
6314	rs1875897	A	G	A	G	0.009	-0.00952	0.257	0.264624	FALSE	FALSE	FALSE	ieu-b-508	8	4188891	0.038124	486484	0.802728	Sepsis (28	Sepsis (28	Sepsis (28	TRUE	igd	0.002	6.80E-06	5590	M33955.m	TRUE	reported	mhHFnT	2	TRUE	0.003609	20.24275
1151	rs2073805	T	C	T	C	-0.009	0.053434	0.407	0.410223	FALSE	FALSE	FALSE	ieu-b-508	11	3361087	0.037523	486484	0.154439	Sepsis (28	Sepsis (28	Sepsis (28	TRUE	igd	0.002	6.80E-06	5589	M01644.m	TRUE	reported	IB0DTL	2	TRUE	0.00361	20.24275
3513	rs7669416	A	G	A	G	-0.009	-0.06046	0.549	0.543518	FALSE	FALSE	FALSE	ieu-b-508	4	1.4E+08	0.03349	486484	0.071028	Sepsis (28	Sepsis (28	Sepsis (28	TRUE	igd	0.002	6.80E-06	5588	M32492.m	TRUE	reported	rZZFcE	2	TRUE	0.003611	20.24275
7712	rs3786099	T	C	T	C	-0.009	-0.06924	0.207	0.208212	FALSE	FALSE	FALSE	ieu-b-508	17	9864752	0.041025	486484	0.091466	Sepsis (28	Sepsis (28	Sepsis (28	TRUE	igd	0.002	6.80E-06	5587	M35397.m	TRUE	reported	Y56mZr	2	TRUE	0.003611	20.24275
7713	rs4269090	T	C	T	C	0.009	0.074064	0.226	0.226696	FALSE	FALSE	FALSE	ieu-b-508	3	1.03E+08	0.039796	486484	0.062734	Sepsis (28	Sepsis (28	Sepsis (28	TRUE	igd	0.002	6.80E-06	5587	M35397.m	TRUE	reported	Y56mZr	2	TRUE	0.003611	20.24275
787	rs577266	A	C	A	C	-0.009	-0.03006	0.637	0.637893	FALSE	FALSE	FALSE	ieu-b-508	9	29640242	0.034718	486484	0.386519	Sepsis (28	Sepsis (28	Sepsis (28	TRUE	igd	0.002	6.80E-06	5586	M01336.m	TRUE	reported	FAIrya	2	TRUE	0.003612	20.24275
3388	rs1004569	T	C	T	C	-0.027	-0.00084	0.691	0.691406	FALSE	FALSE	FALSE	ieu-b-508	5	30703934	0.036222	486484	0.981541	Sepsis (28	Sepsis (28	Sepsis (28	TRUE	igd	0.006	6.80E-06	5586	M32425.m	TRUE	reported	u5RLGY	2	TRUE	0.003612	20.24275
747	rs6766715	A	G	A	G	-0.009	0.005918	0.128	0.131017	FALSE	FALSE	FALSE	ieu-b-508	3	1.73E+08	0.049654	486484	0.905134	Sepsis (28	Sepsis (28	Sepsis (28	TRUE	igd	0.002	6.80E-06	5585	M01302.m	TRUE	reported	sNp2IC	2	TRUE	0.003613	20.24275
1049	rs2643891	A	G	A	G	-0.018	0.008543	0.165	0.169686	FALSE	FALSE	FALSE	ieu-b-508	1	2292352	0.044528	486484	0.847856	Sepsis (28	Sepsis (28	Sepsis (28	TRUE	igd	0.004	6.80E-06	5585	M01572.m	TRUE	reported	EOUAWy	2	TRUE	0.003613	20.24275
4445	rs3887893	T	C	T	C	0.009	-0.01526	0.617	0.606537	FALSE	FALSE	FALSE	ieu-b-508	16	16205501	0.034234	486484	0.655677	Sepsis (28	Sepsis (28	Sepsis (28	TRUE	igd	0.002	6.80E-06	5584	M32910.m	TRUE	reported	2OCBKh	2	TRUE	0.003613	20.24275
7938	rs6133587	T	G	T	G	0.009	-0.08295	0.719	0.893281	FALSE	FALSE	FALSE	ieu-b-508	20	902060	0.059241	486484	0.16146	Sepsis (28	Sepsis (28	Sepsis (28	TRUE	igd	0.002	6.80E-06	5584	M35675.m	TRUE	reported	D7fdyZ	2	TRUE	0.003613	20.24275

3227	rs785896	A	G	A	G	0.009	-0.01407	0.563	0.563712	FALSE	FALSE	FALSE	ieu-b-508	9	79179861	0.033639	486484	0.675864	Sepsis (28	Sepsis (28	Sepsis (28	TRUE	igd	0.002	6.80E-06	5583	M32339.m	TRUE	reported	SVp504	2	TRUE	0.003614	20.24275
6501	rs2076434	A	G	A	G	0.0342	-0.02567	0.8443	0.828861	FALSE	FALSE	FALSE	ieu-b-508	16	1572393	0.044529	486484	0.564241	Sepsis (28	Sepsis (28	Sepsis (28	TRUE	igd	0.0076	6.08E-06	5581	M34112.m	TRUE	reported	97JqFb	2	TRUE	0.003615	20.24274
2309	rs1755916	T	C	T	C	0.045	0.058183	0.027	0.159471	FALSE	FALSE	FALSE	ieu-b-508	10	83875533	0.049484	486484	0.239677	Sepsis (28	Sepsis (28	Sepsis (28	TRUE	igd	0.01	6.80E-06	5579	M20699.m	TRUE	reported	Z3EKFa	2	TRUE	0.003617	20.24274
5665	rs1083268	A	G	A	G	-0.009	0.065347	0.547	0.556277	FALSE	FALSE	FALSE	ieu-b-508	11	16843375	0.033609	486484	0.051856	Sepsis (28	Sepsis (28	Sepsis (28	TRUE	igd	0.002	6.80E-06	5573	M33477.m	TRUE	reported	Aw8PBK	2	TRUE	0.00362	20.24273
5666	rs1093661	T	G	T	G	0.009	0.007371	0.675	0.672254	FALSE	FALSE	FALSE	ieu-b-508	1	20342503	0.035658	486484	0.836231	Sepsis (28	Sepsis (28	Sepsis (28	TRUE	igd	0.002	6.80E-06	5573	M33477.m	TRUE	reported	Aw8PBK	2	TRUE	0.00362	20.24273
8380	rs1250935	C	G	C	G	0.018	-0.08452	0.161	0.198462	FALSE	TRUE	FALSE	ieu-b-508	4	1.9E+08	0.041797	486484	0.043168	Sepsis (28	Sepsis (28	Sepsis (28	TRUE	igd	0.004	6.80E-06	5568	M36776.m	TRUE	reported	VnYglx	2	TRUE	0.003624	20.24273
1773	rs2825115	T	C	T	C	0.009	-0.01125	0.568	0.565561	FALSE	FALSE	FALSE	ieu-b-508	21	20156686	0.033644	486484	0.738032	Sepsis (28	Sepsis (28	Sepsis (28	TRUE	igd	0.002	6.80E-06	5566	M15676.m	TRUE	reported	s1NfKN	2	TRUE	0.003625	20.24272
1776	rs6596867	A	C	A	C	0.009	0.011761	0.543	0.546072	FALSE	FALSE	FALSE	ieu-b-508	6	2012739	0.033593	486484	0.726266	Sepsis (28	Sepsis (28	Sepsis (28	TRUE	igd	0.002	6.80E-06	5566	M15676.m	TRUE	reported	s1NfKN	2	TRUE	0.003625	20.24272
5404	rs2980872	A	G	A	G	-0.009	-0.00575	0.461	0.473827	FALSE	FALSE	FALSE	ieu-b-508	8	1.26E+08	0.033637	486484	0.864253	Sepsis (28	Sepsis (28	Sepsis (28	TRUE	igd	0.002	6.80E-06	5564	M33389.m	TRUE	reported	Hf6XVM	2	TRUE	0.003626	20.24272
4073	rs6503078	T	G	T	G	-0.009	0.126951	0.283	0.280374	FALSE	FALSE	FALSE	ieu-b-508	17	7996640	0.038113	486484	0.000866	Sepsis (28	Sepsis (28	Sepsis (28	TRUE	igd	0.002	6.80E-06	5559	M32740.m	TRUE	reported	YLshfP	2	TRUE	0.00363	20.24271
4083	rs9038	T	C	T	C	0.009	0.005045	0.616	0.609475	FALSE	FALSE	FALSE	ieu-b-508	17	75495397	0.034159	486484	0.882594	Sepsis (28	Sepsis (28	Sepsis (28	TRUE	igd	0.002	6.80E-06	5559	M32740.m	TRUE	reported	YLshfP	2	TRUE	0.00363	20.24271
4084	rs930551	A	G	A	G	-0.018	0.016194	0.069	0.068236	FALSE	FALSE	FALSE	ieu-b-508	17	77905149	0.066168	486484	0.80666	Sepsis (28	Sepsis (28	Sepsis (28	TRUE	igd	0.004	6.80E-06	5559	M32740.m	TRUE	reported	YLshfP	2	TRUE	0.00363	20.24271
4086	rs9322898	T	C	T	C	-0.009	-0.01548	0.318	0.318016	FALSE	FALSE	FALSE	ieu-b-508	14	32829827	0.035836	486484	0.665866	Sepsis (28	Sepsis (28	Sepsis (28	TRUE	igd	0.002	6.80E-06	5559	M32740.m	TRUE	reported	YLshfP	2	TRUE	0.00363	20.24271
5348	rs208357	T	G	T	G	-0.009	-0.05907	0.25	0.247358	FALSE	FALSE	FALSE	ieu-b-508	7	2774789	0.038729	486484	0.127237	Sepsis (28	Sepsis (28	Sepsis (28	TRUE	igd	0.002	6.80E-06	5536	M33380.m	TRUE	reported	OHRfl5	2	TRUE	0.003645	20.24268
2545	rs1457192	A	G	A	G	-0.009	-0.00485	0.272	0.27238	FALSE	FALSE	FALSE	ieu-b-508	8	4682675	0.037467	486484	0.896966	Sepsis (28	Sepsis (28	Sepsis (28	TRUE	igd	0.002	6.80E-06	5474	M22177.m	TRUE	reported	2s6Sly	2	TRUE	0.003686	20.2426
3806	rs1173598	A	G	A	G	0.009	-0.01058	0.305	0.306144	FALSE	FALSE	FALSE	ieu-b-508	4	1.62E+08	0.036257	486484	0.770479	Sepsis (28	Sepsis (28	Sepsis (28	TRUE	igd	0.002	6.80E-06	5463	M32644.m	TRUE	reported	JAa2WD	2	TRUE	0.003693	20.24259
3811	rs12484	T	C	T	C	-0.009	-0.0081	0.427	0.420798	FALSE	FALSE	FALSE	ieu-b-508	7	2768626	0.033821	486484	0.810664	Sepsis (28	Sepsis (28	Sepsis (28	TRUE	igd	0.002	6.80E-06	5463	M32644.m	TRUE	reported	JAa2WD	2	TRUE	0.003693	20.24259
2195	rs7802565	C	G	C	G	-0.036	0.001533	0.024	0.026216	FALSE	TRUE	FALSE	ieu-b-508	7	23979820	0.10441	486484	0.988289	Sepsis (28	Sepsis (28	Sepsis (28	TRUE	igd	0.008	6.80E-06	5436	M19362.m	TRUE	reported	SkNEKw	2	TRUE	0.003711	20.24255
1234	rs1082430	A	G	A	G	0.009	0.016189	0.383	0.390503	FALSE	FALSE	FALSE	ieu-b-508	10	54011665	0.03428	486484	0.636752	Sepsis (28	Sepsis (28	Sepsis (28	TRUE	igd	0.002	6.80E-06	5422	M01769.m	TRUE	reported	rismUZ	2	TRUE	0.003721	20.24253
1381	rs151743	T	C	T	C	-0.009	0.035348	0.426	0.422633	FALSE	FALSE	FALSE	ieu-b-508	3	60407864	0.037024	486484	0.33971	Sepsis (28	Sepsis (28	Sepsis (28	TRUE	igd	0.002	6.80E-06	5370	M02734.m	TRUE	reported	0y0dDD	2	TRUE	0.003757	20.24246
575	rs167277	A	C	A	C	-0.0207	0.013462	0.781	0.779773	FALSE	FALSE	FALSE	ieu-b-508	12	78483079	0.040323	486484	0.738491	Sepsis (28	Sepsis (28	Sepsis (28	TRUE	igd	0.0046	7.92E-06	5344	M01107.m	TRUE	reported	EbPV1D	2	TRUE	0.003775	20.24242
8123	rs4141381	T	G	T	G	-0.144	0.084661	0.985	0.983478	FALSE	FALSE	FALSE	ieu-b-508	16	13927979	0.135472	486484	0.532016	Sepsis (28	Sepsis (28	Sepsis (28	TRUE	igd	0.032	6.80E-06	5329	M36098.m	TRUE	reported	hs9SZI	2	TRUE	0.003786	20.2424
1900	rs1041553	A	G	A	G	0.036	0.044494	0.167	0.178397	FALSE	FALSE	FALSE	ieu-b-508	19	6818701	0.043436	486484	0.305669	Sepsis (28	Sepsis (28	Sepsis (28	TRUE	igd	0.008	6.80E-06	5315	M16634.m	TRUE	reported	P9BdXh	2	TRUE	0.003796	20.24238
3860	rs9626993	T	C	T	C	0.0126	-0.01219	0.3485	0.339396	FALSE	FALSE	FALSE	ieu-b-508	22	47688227	0.034729	486484	0.725631	Sepsis (28	Sepsis (28	Sepsis (28	TRUE	igd	0.0028	6.07E-06	5211	M32651.m	TRUE	reported	DeuPwv	2	TRUE	0.003871	20.24223
3978	rs1177282	A	G	A	G	0.009	-0.00127	0.323	0.329967	FALSE	FALSE	FALSE	ieu-b-508	7	55655081	0.035549	486484	0.971494	Sepsis (28	Sepsis (28	Sepsis (28	TRUE	igd	0.002	6.80E-06	5199	M32729.m	TRUE	reported	XGRSN1	2	TRUE	0.00388	20.24221
6912	rs3825895	T	C	T	C	0.0207	0.005562	0.4752	0.472209	FALSE	FALSE	FALSE	ieu-b-508	15	1.02E+08	0.033421	486484	0.867825	Sepsis (28	Sepsis (28	Sepsis (28	TRUE	igd	0.0046	8.32E-06	5195	M34389.m	TRUE	reported	suiRIN	2	TRUE	0.003883	20.2422
6814	rs2281071	A	G	A	G	0.009	0.00642	0.346	0.342673	FALSE	FALSE	FALSE	ieu-b-508	6	25622212	0.035273	486484	0.855583	Sepsis (28	Sepsis (28	Sepsis (28	TRUE	igd	0.002	6.80E-06	5179	M34359.m	TRUE	reported	0UotaJ	2	TRUE	0.003895	20.24218
7681	rs2498680	A	G	A	G	0.009	0.026547	0.81	0.808881	FALSE	FALSE	FALSE	ieu-b-508	6	1.39E+08	0.042397	486484	0.531217	Sepsis (28	Sepsis (28	Sepsis (28	TRUE	igd	0.002	6.80E-06	5160	M35327.m	TRUE	reported	SquFiY	2	TRUE	0.003909	20.24215
6705	rs7795256	T	C	T	C	-0.0135	-0.08142	0.2434	0.240623	FALSE	FALSE	FALSE	ieu-b-508	7	53787288	0.039102	486484	0.037333	Sepsis (28	Sepsis (28	Sepsis (28	TRUE	igd	0.003	5.33E-06	5081	M34336.m	TRUE	reported	jrSSxL	2	TRUE	0.00397	20.24203
3415	rs6517442	T	C	T	C	0.0252	-0.0385	0.6652	0.677811	FALSE	FALSE	FALSE	ieu-b-508	21	39289946	0.03565	486484	0.28021	Sepsis (28	Sepsis (28	Sepsis (28	TRUE	igd	0.0056	5.94E-06	5080	M32445.m	TRUE	reported	qcc6f3	2	TRUE	0.00397	20.24203
1877	rs1076302	A	G	A	G	0.018	-0.00522	0.201	0.201447	FALSE	FALSE	FALSE	ieu-b-508	8	1461822	0.041637	486484	0.900246	Sepsis (28	Sepsis (28	Sepsis (28	TRUE	igd	0.004	6.80E-06	5026	M15990.m	TRUE	reported	15fe4N	2	TRUE	0.004013	20.24194
8452	rs1008284	T	C	T	C	0.009	-0.00274	0.393	0.401746	FALSE	FALSE	FALSE	ieu-b-508	12	24300902	0.034268	486484	0.936278	Sepsis (28	Sepsis (28	Sepsis (28	TRUE	igd	0.002	6.80E-06	5018	M37058.m	TRUE	reported	32p8qV	2	TRUE	0.004019	20.24193
8455	rs1076230	A	G	A	G	0.009	-0.00403	0.532	0.53101	FALSE	FALSE	FALSE	ieu-b-508	19	1244900	0.033427	486484	0.904062	Sepsis (28	Sepsis (28	Sepsis (28	TRUE	igd	0.002	6.80E-06	5018	M37058.m	TRUE	reported	32p8qV	2	TRUE	0.004019	20.24193
8468	rs1727198	T	C	T	C	-0.009	0.068693	0.556	0.556969	FALSE	FALSE	FALSE	ieu-b-508	2	19479000	0.03357	486484	0.04073	Sepsis (28	Sepsis (28	Sepsis (28	TRUE	igd	0.002	6.80E-06	5018	M37058.m	TRUE	reported	32p8qV	2	TRUE	0.004019	20.24193
620	rs9851393	A	G	A	G	0.0297	-0.01919	0.5199	0.521263	FALSE	FALSE	FALSE	ieu-b-508	3	54334723	0.033456	486484	0.566284	Sepsis (28	Sepsis (28	Sepsis (28	TRUE	igd	0.0066	7.38E-06	4934	M01114.m	TRUE	reported	fqHdM2	2	TRUE	0.004087	20.24179
948	rs1000413	A	G	A	G	0.009	0.059604	0.581	0.58388	FALSE	FALSE	FALSE	ieu-b-508	4	84671147	0.03384	486484	0.078179	Sepsis (28	Sepsis (28	Sepsis (28	TRUE	igd	0.002	6.80E-06	4915	M01558.m	TRUE	reported	AZK0p4	2	TRUE	0.004103	20.24176
5656	rs4807122	T	C	T	C	0.018	-0.0257	0.386	0.383945	FALSE	FALSE	FALSE	ieu-b-508	19	1586274	0.034714	486484	0.459163	Sepsis (28	Sepsis (28	Sepsis (28	TRUE	igd	0.004	6.80E-06	4599	M33453.m	TRUE	reported	OzEeLQ	2	TRUE	0.004384	20.24119
2470	rs7377975	A	G	A	G	0.018	0.033315	0.189	0.199781	FALSE	FALSE	FALSE	ieu-b-508	4	6200070	0.041719	486484	0.424539	Sepsis (28	Sepsis (28	Sepsis (28	TRUE	igd	0.004	6.80E-06	4558	M22030.m	TRUE	reported	ZNV0qR	2	TRUE	0.004423	20.24111
5280	rs4767064	A	G	A	G	0.0216	-0.02914	0.3478	0.369283	FALSE	FALSE	FALSE	ieu-b-508	12	1.14E+08	0.034579	486484	0.399325	Sepsis (28	Sepsis (28	Sepsis (28	TRUE	igd	0.0048	8.17E-06	4409	M33250.m	TRUE	reported	WIZI6U	2	TRUE	0.004572	20.24081
4265	rs1051101	T	C	T	C	0.0261	0.026326	0.2764	0.284335	FALSE	FALSE	FALSE	ieu-b-508	3	71346583	0.037132	486484	0.478328	Sepsis (28	Sepsis (28	Sepsis (28	TRUE	igd	0.0058	6.31E-06	4311	M32802.m	TRUE	reported	m8vmvZ	2	TRUE	0.004675	20.24061
7332	rs1006081	A	G	A	G	0.009	-0.0009	0.323	0.327922	FALSE	FALSE	FALSE	ieu-b-508	5	63156739	0.035983	486484	0.980117	Sepsis (28	Sepsis (28	Sepsis (28	TRUE	igd	0.002	6.80E-06	4081	M35137.m	TRUE	reported	Eq2siS	2	TRUE	0.004938	20.24008
5633	rs1243281	A	C	A	C	0.045	0.094173	0.9717	0.969438	FALSE	FALSE	FALSE	ieu-b-508	14	82261349	0.097359	486484	0.333407	Sepsis (28	Sepsis (28	Sepsis (28	TRUE	igd	0.01	7.17E-06	4004	M33443.m	TRUE	reported	G1JrRV	2	TRUE	0.005032	20.23989
7649	rs2873461	T	G	T	G	-0.009	0.005784	0.358	0.420154	FALSE	FALSE	FALSE	ieu-b-508	13	1.13E+08	0.033809	486484	0.864174	Sepsis (28	Sepsis (28	Sepsis (28	TRUE	igd	0.002	6.80E-06	4004	M35326.m	TRUE	reported	jRytyJ	2	TRUE	0.005032	20.23989
2527	rs2031504	T	G	T	G	0.0225	-0.01986	0.4374	0.43152	FALSE	FALSE	FALSE	ieu-b-508	1	1.07E+08	0.033675	486484	0.555368	Sepsis (28	Sepsis (28	Sepsis (28	TRUE	igd	0.005	8.14E-06	3838	M22175.m	TRUE	reported	IIlqyJ	2	TRUE	0.005248	20.23945
2370	rs2393374	T	G	T	G	0.0351	-0.02606	0.3095	0.308408	FALSE	FALSE	FALSE	ieu-b-508	12	1.18E+08	0.036266	486484	0.472323	Sepsis (28	Sepsis (28	Sepsis (28	TRUE	igd	0.0078	6.39E-06	3422	M21049.m	TRUE	reported	UDtLE4	2	TRUE	0.005883	20.23816
5196	rs1181503	T	C	T	C	0.1134	0.060964	0.0578	0.05341	FALSE	FALSE	FALSE	ieu-b-508	10	69299192	0.074718	486484	0.414549	Sepsis (28	Sepsis (28	Sepsis (28	TRUE	igd	0.0252	6.97E-06	3221	M33203.m	TRUE	reported	Sto7pR	2	TRUE	0.006248	20.23743
5905	rs1029659	A	C	A	C	0.0198	0.035451	0.1991	0.189893	FALSE	FALSE	FALSE	ieu-b-508	17	13888021	0.042689	486484	0.406287	Sepsis (28	Sepsis (28	Sepsis (28	TRUE	igd	0.0044	7.94E-06	2757	M33627.m	TRUE	reported	4RHaUX	2	TRUE	0.007291	20.23531
5909	rs1460191	A	C	A	C	0.0207	-0.04802	0.1654	0.182987	FALSE	FALSE	FALSE	ieu-b-508	18	50181192	0.043246	486484	0.26686	Sepsis (28	Sepsis (28	Sepsis (28	TRUE	igd	0.0046	5.38E-06	2757	M33627.m	TRUE	reported	4RHaUX	2	TRUE	0.007291	20.23531
5209	rs1113306	T	G	T	G	-0.0135	-0.01362	0.5241	0.540117	FALSE	FALSE	FALSE	ieu-b-508	4	1.76E+08	0.033576	486484	0.684917	Sepsis (28	Sepsis (28	Sepsis (28	TRUE	igd	0.003	6.74E-06	2683	M33204.m	TRUE	reported	ROvPWK	2	TRUE	0.007491	20.2349
4364	rs7129619	C	G	C	G	0.0297	-0.02022	0.6449	0.667583	FALSE	TRUE	FALSE	ieu-b-508	11	1.27E+08	0.035537	486484	0.569403	Sepsis (28	Sepsis (28	Sepsis (28	TRUE	igd	0.0066	7.25E-06	2656	M32857.m	TRUE	reported	MVAWzT	2	TRUE	0.007567	20.23475
8525	rs1718085	A	T	A	T	0.0837	-0.06297	0.864	0.858796	FALSE	TRUE	FALSE	ieu-b-508	12	5554548	0.048156	486484	0.190968	Sepsis (28	Sepsis (28	Sepsis (28	TRUE	igd	0.0186	6.76E-06	2560	M37112.m	TRUE	reported	P3GiAg	2	TRUE	0.007848	20.23418
5878	rs1083670	C	G	C	G	-0.1272	0.025311	0.326	0.299746	FALSE	TRUE	FALSE	ieu-b-508	11	4835606	0.03647	486484	0.487677	Sepsis (28	Sepsis (28	Sepsis (28	TRUE	igd	0.028	5.53E-06	102	M33610.m	TRUE	reported	tmg43v	2	TRUE	0.168281	20.23289
4411	rs3737137	T	C	T	C	-0.0306	-0.00063	0.233	0.229496	FALSE	FALSE	FALSE	ieu-b-508	20	60897721	0.039631	486484	0.987365	Sepsis (28	Sepsis (28	Sepsis (28	TRUE	igd	0.0068	7.99E-06	2148	M32869.m	TRUE	reported	Rvim1n	2	TRUE	0.009339	20.23115
6645	rs1706698	T	C	T	C	0.1974	-0.06551	0.0473	0.065167	FALSE	FALSE	FALSE	ieu-b-508	3	62699739	0.067841	486484	0.3342	Sepsis (28	Sepsis (28	Sepsis (28	TRUE	igd	0.0433	5.16E-06	75	M34322.m	TRUE	reported	16GdG9	2	TRUE	0.216984	20.22926
6621	rs4953342	A	G	A	G	-0.0432	0.046912	0.7182	0.704533	FALSE	FALSE	FALSE	ieu-b-508	2	46552047	0.037005	486484	0.20489	Sepsis (28	Sepsis (28	Sepsis (28	TRUE	igd	0.0096	7.20E-06	1766	M34314.m	TRUE	reported	wPKEWG	2	TRUE	0.011337	20.22707
7664	rs7706802	A	G	A	G	-0.0189	-0.00375	0.4621	0.536163	FALSE	FALSE	FALSE	ieu-b-508	5	18373608	0.033513	486484	0.910925	Sepsis (28	Sepsis (28	Sepsis (28	TRUE	igd	0.0042	7.06E-06	1764	M35326.m	TRUE	reported	jRytyJ	2	TRUE	0.011349	20.22704
7928	rs2344567	T	G	T	G	0.0225	-0.04541	0.2843	0.290763	FALSE	FALSE	FALSE	ieu-b-508	2	2.34E+08	0.036763	486484	0.216719	Sepsis (28	Sepsis (28	Sepsis (28	TRUE	igd	0.005	6.35E-06	1764	M35675.m	TRUE	reported	D7fdyZ	2	TRUE	0.011349	20.22704
447	rs1163838	T	C	T	C	-0.0171	0.107336	0.1875	0.185952	FALSE	FALSE	FALSE	ieu-b-508	15	29496888	0.043717	486484	0.014079	Sepsis (28	Sepsis (28	Sepsis (28	TRUE	igd	0.0038	5.06E-06	1763	M00513.m	TRUE	reported	ohacnP	2	TRUE	0.011356	20.22703
521	rs1841535	T	G	T	G	0.0252	0.036062	0.1482	0.254958	FALSE	FALSE	FALSE	ieu-b-508	3	1.32E+08	0.038304	486484	0.346462	Sepsis (28	Sepsis (28	Sepsis (28	TRUE	igd	0.0056	7.00E-06	1754	M00584.m	TRUE	reported	oBt02X	2	TRUE	0.011413	20.22691
7766	rs1262524	T	G	T	G	-0.0297	-0.01833	0.8274	0.872289	FALSE	FALSE	FALSE	ieu-b-508	20	55393483	0.050209	486484	0.714987	Sepsis (28	Sepsis (28	Sepsis (28	TRUE	igd	0.0066	7.16E-06	1718	M35439.m	TRUE	reported	DnuAGZ	2	TRUE	0.01165	20.22643
4107	rs335810	A	C	A	C	0.3722	-0.10427	0.9883	0.989047	FALSE	FALSE	FALSE	ieu-b-508	15	60368206	0.164398	486484	0.525936	Sepsis (28	Sepsis (28	Sepsis (28	TRUE	igd	0.0827	6.80E-06	1368	M32754.m	TRUE	reported	n0okFy	2	TRUE	0.014591	20.22583
8176	rs1889157	A	G	A	G	-0.0342	0.011945	0.4713	0.485584	FALSE	FALSE	FALSE	ieu-b-508	9	1.37E+08	0.034472	486484	0.72895	Sepsis (28	Sepsis (28	Sepsis (28	TRUE	igd	0.0076	6.44E-06	1657	M36134.m	TRUE	reported	ekMsfG	2	TRUE	0.012073	20.22556
4465	rs1019202	T	G	T	G	-0.0513	-0.07284	0.0878	0.092083	FALSE	FALSE	FALSE	ieu-b-508	2	2.1E+08	0.058765	486484	0.215131	Sepsis (28	Sepsis (28	Sepsis (28	TRUE	igd	0.0114	7.46E-06	1618	M33009.m	TRUE	reported	8RTEdM	2	TRUE	0.012361	20.22497
5341	rs1307915	A	C	A	C	0.0144	0.038016	0.5539	0.558594	FALSE	FALSE	FALSE	ieu-b-508	3	1.09E+08	0.033645	486484	0.258509	Sepsis (28	Sepsis (28	Sepsis (28	TRUE	igd	0.0032	5.80E-06	1582	M33380.m	TRUE	reported	OHRfl5	2	TRUE	0.012638	20.2244
8102	rs1689756	T	C	T	C	0.2875	-0.01294	0.8063	0.824987	FALSE	FALSE	FALSE	ieu-b-508	6	1.66E+08	0.044479	486484	0.771061	Sepsis (28	Sepsis (28	Sepsis (28	TRUE	igd	0.0638	6.70E-06	488	M36097.m	TRUE	reported	RHr18S	2	TRUE	0.039949	20.22324
7799	rs6698967	A	G	A	G	-0.3177	0.252176	0.9723	0.972306	FALSE	FALSE	FALSE	ieu-b-508	1	1.1E+08	0.102419	486484	0.013809	Sepsis (28	Sepsis (28	Sepsis (28	TRUE	igd	0.0704	6.49E-06	284	M35451.m	TRUE	reported	GMRGAN	2	TRUE	0.06691	20.2218
6084	rs1402168	T	G	T	G	0.1077	0.1075	0.8834	0.863216	FALSE	FALSE	FALSE	ieu-b-508	2	50536068	0.049063	486484	0.028447	Sepsis (28	Sepsis (28	Sepsis (28	TRUE	igd	0.0239	6.49E-06	471	M33835.m	TRUE	reported	crhBhm	2	TRUE	0.041332	20.2203
7432	rs1327695	A	C	A	C	0.0657	0.018787	0.3037	0.300715	FALSE	FALSE	FALSE	ieu-b-508	8	1337849	0.036567	486484	0.607423	Sepsis (28	Sepsis (28	Sepsis (28	TRUE	igd	0.0146	6.50E-06	1254	M35187.m	TRUE	reported	Zx7JfE	2	TRUE	0.015892	20.2177
6113	rs1199184	A	G	A	G	0.2732	-0.02871	0.0226	0.022423	FALSE	FALSE	FALSE	ieu-b-508	8	1.08E+08	0.11325	486484	0.799852	Sepsis (28	Sepsis (28	Sepsis (28	TRUE	igd	0.0605	6.30E-06	228	M33877.m	TRUE	reported	yDVi1f	2	TRUE	0.082094	20.2127
5009	rs6578873	T	G	T	G	-0.3042	0.014897	0.0535	0.542754	FALSE	FALSE	FALSE	ieu-b-508	11	7580388	0.03347	486484	0.65626	Sepsis (28	Sepsis (28	Sepsis (28	TRUE	igd	0.0649	2.72E-06	25	M33178.m	TRUE	reported	g22WMS	2	TRUE	0.467745	20.21235
5890	rs4291236	C	G	C	G	0.2588	-0.03453	0.0549	0.072201	FALSE	TRUE	FALSE	ieu-b-508	8	1.06E+08	0.06475	486484	0.5938	Sepsis (28	Sepsis (28	Sepsis (28	TRUE	igd	0.057	5.54E-06	102	M33610.m	TRUE	reported	tmg43v	2	TRUE	0.168126	20.21057
4859	rs1748101	T	C	T	C	-0.2574	0.040498	0.0237	0.063493	FALSE	FALSE	FALSE	ieu-b-508	3	15295234	0.068565	486484	0.554753	Sepsis (28	Sepsis (28	Sepsis (28	TRUE	igd	0.0572	6.78E-06	1017	M33178.m	TRUE	reported	g22WMS	2	TRUE	0.019523	20.21018
5172	rs954483	A	G	A	G	0.0616	-0.01083	0.8406	0.843276	FALSE	FALSE	FALSE	ieu-b-508	7	1.09E+08	0.046107	486484	0.814381	Sepsis (28	Sepsis (28	Sepsis (28	TRUE	igd	0.0137	6.81E-06	5064	M33194.m	TRUE	reported	xRUveq	2	TRUE	0.003976	20.20918
5239	rs1195499	A	T	A	T	0.121	0.34841	0.0131	0.01432	FALSE	TRUE	FALSE	ieu-b-508	5	1.73E+08	0.147269	486484	0.017991	Sepsis (28	Sepsis (28	Sepsis (28	TRUE	igd	0.0269	6.85E-06	1612	M33228.m	TRUE	reported	JwOXbQ	2	TRUE	0.012396	20.20817
6850	rs1177250	T	C	T	C	0.1134	-0.06916	0.1441	0.175124	FALSE	FALSE	FALSE	ieu-b-508	7	1.46E+08	0.044094	486484	0.116764	Sepsis (28	Sepsis (28	Sepsis (28	TRUE	igd	0.0252	6.92E-06	954	M34365.m	TRUE	reported	8eexD0	2	TRUE	0.020785	20.20755
5452	rs1117313	A	G	A	G	0.068	0.004158	0.6636	0.620087	FALSE	FALSE	FALSE	ieu-b-508	11	39018158	0.034291	486484	0.903479	Sepsis (28	Sepsis (28	Sepsis (28	TRUE	igd	0.0151	6.81E-06	528	M33391.m	TRUE	reported	EgMd1b	2	TRUE	0.036988	20.20299
4954	rs4379054	A	C	A	C	-0.2713	-0.09364	0.027	0.017872	FALSE	FALSE	FALSE	ieu-b-508	4	27129076	0.125775	486484	0.45655	Sepsis (28	Sepsis (28	Sepsis (28	TRUE	igd	0.0603	6.89E-06	1017	M33178.m	TRUE	reported	g22WMS	2	TRUE	0.019516	20.20273
487	rs6468437	T	C	T	C	0.1246	0.037922	0.8334	0.797878	FALSE	FALSE	FALSE	ieu-b-508	8	37590446	0.041532	486484	0.361206	Sepsis (28	Sepsis (28	Sepsis (28	TRUE	igd	0.0277	6.86E-06	1233	M00553.m	TRUE	reported	IFjXiL	2	TRUE	0.016145	20.20094
4563	rs4364244	A	G	A	G	-0.0463	0.057983	0.7874	0.797232	FALSE	FALSE	FALSE	ieu-b-508	4	22393873	0.041456	486484	0.161911	Sepsis (28	Sepsis (28	Sepsis (28	TRUE	igd	0.0103	7.29E-06	4817	M33144.m	TRUE	reported	cgXZoM	2	TRUE	0.004177	20.19794
7600	rs1698237	A	G	A	G	-0.2028	0.093684	0.0111	0.011105	FALSE	FALSE	FALSE	ieu-b-508	21	16730194	0.160431	486484	0.559253	Sepsis (28	Sepsis (28	Sepsis (28	TRUE	igd	0.0451	7.02E-06	1765	M35320.m	TRUE	reported	ijCk2X	2	TRUE	0.011326	20.19717
7107	rs1117179	T	C	T	C	0.3447	0.013306	0.957	0.950618	FALSE	FALSE	FALSE	ieu-b-508	12	56682816	0.077348	486484	0.863415	Sepsis (28	Sepsis (28	Sepsis (28	TRUE	igd	0.0761	5.86E-06	128	M34516.m	TRUE	reported	7Ah2wy	2	TRUE	0.138146	20.19639
6710	rs1021399	A	C	A	C	-0.0914	0.036685	0.2991	0.299947	FALSE	FALSE	FALSE	ieu-b-508	5	1.18E+08	0.036637	486484	0.316678	Sepsis (28	Sepsis (28	Sepsis (28	TRUE	igd	0.0203	6.80E-06	535	M34338.m	TRUE	reported	h4FB9a	2	TRUE	0.036509	20.19639
5161	rs1048551	A	G	A	G	-0.0427	0.022696	0.4042	0.401299	FALSE	FALSE	FALSE	ieu-b-508	20	15103600	0.034116	486484	0.505897	Sepsis (28	Sepsis (28	Sepsis (28	TRUE	igd	0.0095	7.29E-06	5064	M33194.m	TRUE	reported	xRUveq	2	TRUE	0.003974	20.19468
6732	rs1778496	T	G	T	G	-0.3165	0.059989	0.975	0.969724	FALSE	FALSE	FALSE	ieu-b-508	2	1.63E+08	0.09758	486484	0.538711	Sepsis (28	Sepsis (28	Sepsis (28	TRUE	igd	0.0703	6.76E-06	535	M34338.m	TRUE	reported	h4FB9a	2	TRUE	0.036503	20.19344
2995	rs1254053	A	C	A	C	0.1022	-0.05988	0.1129	0.11711	FALSE	FALSE	FALSE	ieu-b-508	7	48926609	0.051996	486484	0.249502	Sepsis (28	Sepsis (28	Sepsis (28	TRUE	igd	0.0227	6.74E-06	522	M31536.m	TRUE	reported	3nAuUl	2	TRUE	0.03738	20.19217
3301	rs9284892	T	C	T	C	-0.0382	0.03069	0.0588	0.058388	FALSE	FALSE	FALSE	ieu-b-508	3	5687674	0.071504	486484	0.667779	Sepsis (28	Sepsis (28	Sepsis (28	TRUE	igd	0.0085	7.03E-06	6119	M32379.m	TRUE	reported	MLimM6	2	TRUE	0.00329	20.19049
3312	rs1688548	A	G	A	G	-0.0355	0.07782	0.0207	0.028985	FALSE	FALSE	FALSE	ieu-b-508	4	31641006	0.100771	486484	0.439968	Sepsis (28	Sepsis (28	Sepsis (28	TRUE	igd	0.0079	7.48E-06	7307	M32393.m	TRUE	reported	Pub0X0	2	TRUE	0.002756	20.18755
995	rs1051473	A	G	A	G	0.0346	-0.12014	0.985	0.983972	FALSE	FALSE	FALSE	ieu-b-508	3	85798950	0.132737	486484	0.365432	Sepsis (28	Sepsis (28	Sepsis (28	TRUE	igd	0.0077	6.51E-06	7364	M01564.m	TRUE	reported	IPUdyD	2	TRUE	0.002734	20.18612
6039	rs756316	T	C	T	C	-0.0373	0.009606	0.6015	0.602377	FALSE	FALSE	FALSE	ieu-b-508	17	6068117	0.034206	486484	0.778836	Sepsis (28	Sepsis (28	Sepsis (28	TRUE	igd	0.0083	6.95E-06	3962	M33782.m	TRUE	reported	875Pal	2	TRUE	0.005072	20.18562
3404	rs1117727	A	T	A	T	0.0355	0.050742	0.8925	0.860009	FALSE	TRUE	FALSE	ieu-b-508	12	68925160	0.047953	486484	0.289981	Sepsis (28	Sepsis (28	Sepsis (28	TRUE	igd	0.0079	7.29E-06	5080	M32445.m	TRUE	reported	qcc6f3	2	TRUE	0.003959	20.18513
6371	rs1074283	T	C	T	C	-0.0337	0.074199	0.0697	0.0735	FALSE	FALSE	FALSE	ieu-b-508	11	48139198	0.063955	486484	0.245973	Sepsis (28	Sepsis (28	Sepsis (28	TRUE	igd	0.0075	7.82E-06	7322	M33968.m	TRUE	reported	sgVOvT	2	TRUE	0.00275	20.18453
8509	rs1317896	T	C	T	C	-0.0355	0.123431	0.8388	0.84671	FALSE	FALSE	FALSE	ieu-b-508	5	64263967	0.046334	486484	0.007723	Sepsis (28	Sepsis (28	Sepsis (28	TRUE	igd	0.0079	6.47E-06	4520	M37104.m	TRUE	reported	bR3g7d	2	TRUE	0.004448	20.18414
2859	rs9549759	T	C	T	C	0.0337	0.009161	0.4298	0.428865	FALSE	FALSE	FALSE	ieu-b-508	13	1.14E+08	0.03363	486484	0.785303	Sepsis (28	Sepsis (28	Sepsis (28	TRUE	igd	0.0075	7.25E-06	5673	M27531.m	TRUE	reported	v01zDr	2	TRUE	0.003546	20.18293
2209	rs6579773	T	C	T	C	-0.0472	0.052599	0.933	0.955962	FALSE	FALSE	FALSE	ieu-b-508	5	1.49E+08	0.081931	486484	0.520879	Sepsis (28	Sepsis (28	Sepsis (28	TRUE	igd	0.0105	6.47E-06	1654	M19364.m	TRUE	reported	XkjMEx	2	TRUE	0.01207	20.18273
856	rs1683001	T	G	T	G	-0.0328	-0.03151	0.9719	0.970156	FALSE	FALSE	FALSE	ieu-b-508	2	1.35E+08	0.098588	486484	0.749244	Sepsis (28	Sepsis (28	Sepsis (28	TRUE	igd	0.0073	7.41E-06	7063	M01361.m	TRUE	reported	NIAcQ3	2	TRUE	0.00285	20.18269
2870	rs1770991	A	G	A	G	0.0472	-0.08057	0.7757	0.788889	FALSE	FALSE	FALSE	ieu-b-508	5	17469690	0.04087	486484	0.048686	Sepsis (28	Sepsis (28	Sepsis (28	TRUE	igd	0.0105	6.41E-06	1510	M27710.m	TRUE	reported	XeaFav	2	TRUE	0.013206	20.1804
6896	rs7771614	T	C	T	C	-0.162	-0.00234	0.5	0.425589	FALSE	FALSE	FALSE	ieu-b-508	6	45975798	0.034009	486484	0.945054	Sepsis (28	Sepsis (28	Sepsis (28	TRUE	igd	0.034	1.85E-06	18	M34365.m	TRUE	reported	8eexD0	2	TRUE	0.557766	20.17993
8126	rs791232	T	C	T	C	0.031	-0.0633	0.491	0.471429	FALSE	FALSE	FALSE	ieu-b-508	12	92788929	0.033395	486484	0.058034	Sepsis (28	Sepsis (28	Sepsis (28	TRUE	igd	0.0069	7.63E-06	7054	M36098.m	TRUE	reported	hs9SZI	2	TRUE	0.002853	20.17911
5991	rs1329128	T	C	T	C	0.1277	-0.01928	0.0306	0.043665	FALSE	FALSE	FALSE	ieu-b-508	9	83634829	0.083931	486484	0.818297	Sepsis (28	Sepsis (28	Sepsis (28	TRUE	igd	0.0284	6.77E-06	1027	M33683.m	TRUE	reported	wSozpU	2	TRUE	0.019307	20.17895
8188	rs1302519	C	G	C	G	-0.0364	0.016167	0.8014	0.797313	FALSE	TRUE	FALSE	ieu-b-508	2	46383017	0.041726	486484	0.698422	Sepsis (28	Sepsis (28	Sepsis (28	TRUE	igd	0.0081	6.72E-06	2400	M36230.m	TRUE	reported	V5KJeR	2	TRUE	0.008344	20.17765
8539	rs3214031	A	C	A	C	-0.0301	-0.04832	0.7088	0.716581	FALSE	FALSE	FALSE	ieu-b-508	11	94965717	0.036972	486484	0.191249	Sepsis (28	Sepsis (28	Sepsis (28	TRUE	igd	0.0067	6.05E-06	6934	M37190.m	TRUE	reported	tiNuK9	2	TRUE	0.002902	20.17707
8398	rs4976017	T	C	T	C	0.031	0.031765	0.0472	0.049562	FALSE	FALSE	FALSE	ieu-b-508	5	66933195	0.077224	486484	0.680821	Sepsis (28	Sepsis (28	Sepsis (28	TRUE	igd	0.0069	7.79E-06	4317	M36802.m	TRUE	reported	njeQfy	2	TRUE	0.004654	20.17548
7739	rs4626843	A	G	A	G	0.0292	-0.01189	0.0341	0.040918	FALSE	FALSE	FALSE	ieu-b-508	1	35043869	0.08419	486484	0.887712	Sepsis (28	Sepsis (28	Sepsis (28	TRUE	igd	0.0065	7.39E-06	7007	M35431.m	TRUE	reported	tLfE4l	2	TRUE	0.002872	20.17507
5952	rs1692816	A	G	A	G	-0.0885	0.06558	0.974	0.970692	FALSE	FALSE	FALSE	ieu-b-508	11	35993611	0.100404	486484	0.513651	Sepsis (28	Sepsis (28	Sepsis (28	TRUE	igd	0.0197	7.22E-06	5328	M33652.m	TRUE	reported	vYkdTv	2	TRUE	0.003774	20.17395
6773	rs1791914	A	G	A	G	-0.0566	0.043184	0.2847	0.387628	FALSE	FALSE	FALSE	ieu-b-508	11	1.16E+08	0.03472	486484	0.213583	Sepsis (28	Sepsis (28	Sepsis (28	TRUE	igd	0.0126	6.63E-06	5270	M34344.m	TRUE	reported	enFm6p	2	TRUE	0.003814	20.17098
8095	rs1265023	T	G	T	G	0.5126	-0.02847	0.9469	0.947366	FALSE	FALSE	FALSE	ieu-b-508	4	88916501	0.074995	486484	0.7042	Sepsis (28	Sepsis (28	Sepsis (28	TRUE	igd	0.1139	6.82E-06	488	M36097.m	TRUE	reported	RHr18S	2	TRUE	0.03985	20.17094
3419	rs1100417	T	C	T	C	0.0274	-0.03738	0.9683	0.963752	FALSE	FALSE	FALSE	ieu-b-508	10	56012772	0.089246	486484	0.675349	Sepsis (28	Sepsis (28	Sepsis (28	TRUE	igd	0.0061	8.15E-06	7364	M32452.m	TRUE	reported	8GtFpl	2	TRUE	0.002732	20.17082
4637	rs4805580	T	C	T	C	0.964	-0.28054	0.9867	0.984138	FALSE	FALSE	FALSE	ieu-b-508	19	31052575	0.133536	486484	0.035653	Sepsis (28	Sepsis (28	Sepsis (28	TRUE	igd	0.2118	5.35E-06	76	M33173.m	TRUE	reported	04fkUQ	2	TRUE	0.214193	20.17067
6130	rs4785153	A	G	A	G	0.3758	0.220672	0.0201	0.023652	FALSE	FALSE	FALSE	ieu-b-508	16	48968166	0.110085	486484	0.045011	Sepsis (28	Sepsis (28	Sepsis (28	TRUE	igd	0.0832	6.35E-06	176	M33877.m	TRUE	reported	yDVi1f	2	TRUE	0.103878	20.16989
4358	rs1176113	A	T	A	T	-0.0301	-0.04278	0.6277	0.616279	FALSE	TRUE	FALSE	ieu-b-508	7	1.04E+08	0.034339	486484	0.212857	Sepsis (28	Sepsis (28	Sepsis (28	TRUE	igd	0.0067	6.27E-06	2656	M32857.m	TRUE	reported	MVAWzT	2	TRUE	0.007542	20.16769
3150	rs6926138	T	C	T	C	-0.0337	-0.00911	0.6583	0.62266	FALSE	FALSE	FALSE	ieu-b-508	6	39138337	0.034607	486484	0.792341	Sepsis (28	Sepsis (28	Sepsis (28	TRUE	igd	0.0075	6.26E-06	1765	M32319.m	TRUE	reported	9YFmUx	2	TRUE	0.01131	20.16717
2819	rs7804260	A	G	A	G	0.0292	-0.00618	0.4115	0.414243	FALSE	FALSE	FALSE	ieu-b-508	7	12608263	0.033837	486484	0.85518	Sepsis (28	Sepsis (28	Sepsis (28	TRUE	igd	0.0065	7.01E-06	2759	M27447.m	TRUE	reported	s1NdyU	2	TRUE	0.007261	20.1662
5887	rs2803893	A	G	A	G	-0.0621	-0.05061	0.7876	0.786588	FALSE	FALSE	FALSE	ieu-b-508	14	38659141	0.040797	486484	0.214774	Sepsis (28	Sepsis (28	Sepsis (28	TRUE	igd	0.0138	7.09E-06	470	M33610.m	TRUE	reported	tmg43v	2	TRUE	0.041305	20.16383
6044	rs6893667	T	C	T	C	0.0319	0.031163	0.551	0.538011	FALSE	FALSE	FALSE	ieu-b-508	5	1.37E+08	0.033479	486484	0.35195	Sepsis (28	Sepsis (28	Sepsis (28	TRUE	igd	0.0071	7.19E-06	1761	M33801.m	TRUE	reported	zhDCxK	2	TRUE	0.011333	20.16374
8332	rs877682	T	C	T	C	0.0795	0.023982	0.9622	0.963786	FALSE	FALSE	FALSE	ieu-b-508	7	78395400	0.090232	486484	0.790407	Sepsis (28	Sepsis (28	Sepsis (28	TRUE	igd	0.0177	7.00E-06	3977	M36589.m	TRUE	reported	27GADo	2	TRUE	0.005047	20.16366
3199	rs952045	T	C	T	C	0.0247	-0.08401	0.9494	0.943884	FALSE	FALSE	FALSE	ieu-b-508	9	73161881	0.07269	486484	0.247816	Sepsis (28	Sepsis (28	Sepsis (28	TRUE	igd	0.0055	7.34E-06	7356	M32338.m	TRUE	reported	q5cuaP	2	TRUE	0.002734	20.16278
7689	rs1098765	A	G	A	G	0.0346	-0.09443	0.1032	0.102227	FALSE	FALSE	FALSE	ieu-b-508	9	1.3E+08	0.054882	486484	0.085324	Sepsis (28	Sepsis (28	Sepsis (28	TRUE	igd	0.0077	7.52E-06	1375	M35331.m	TRUE	reported	GBnxhN	2	TRUE	0.014472	20.16223
4484	rs1291642	A	G	A	G	-0.0274	-0.0461	0.2058	0.207993	FALSE	FALSE	FALSE	ieu-b-508	15	97501800	0.041141	486484	0.262485	Sepsis (28	Sepsis (28	Sepsis (28	TRUE	igd	0.0061	8.06E-06	2773	M33131.m	TRUE	reported	uGKURO	2	TRUE	0.007223	20.16174
6644	rs159314	A	G	A	G	0.1866	-0.04782	0.054	0.054387	FALSE	FALSE	FALSE	ieu-b-508	17	32686084	0.073815	486484	0.517085	Sepsis (28	Sepsis (28	Sepsis (28	TRUE	igd	0.041	5.44E-06	75	M34322.m	TRUE	reported	16GdG9	2	TRUE	0.216412	20.16124
6983	rs7668227	A	G	A	G	0.0238	0.032623	0.9017	0.885003	FALSE	FALSE	FALSE	ieu-b-508	4	1.87E+08	0.052515	486484	0.53446	Sepsis (28	Sepsis (28	Sepsis (28	TRUE	igd	0.0053	7.86E-06	7344	M34407.m	TRUE	reported	X7I83Y	2	TRUE	0.002738	20.15969
8157	rs9479657	T	C	T	C	-0.053	0.052647	0.0711	0.072538	FALSE	FALSE	FALSE	ieu-b-508	6	1.54E+08	0.064869	486484	0.41703	Sepsis (28	Sepsis (28	Sepsis (28	TRUE	igd	0.0118	7.75E-06	2709	M36115.m	TRUE	reported	ZV1jWp	2	TRUE	0.007392	20.15891
5967	rs1182517	T	C	T	C	-0.0584	0.092938	0.0223	0.025436	FALSE	FALSE	FALSE	ieu-b-508	11	88522278	0.105961	486484	0.380435	Sepsis (28	Sepsis (28	Sepsis (28	TRUE	igd	0.013	6.84E-06	1764	M33666.m	TRUE	reported	dvWEtk	2	TRUE	0.011311	20.15795
6634	rs1165602	A	G	A	G	0.1074	0.015079	0.2014	0.229897	FALSE	FALSE	FALSE	ieu-b-508	17	76644567	0.039749	486484	0.704427	Sepsis (28	Sepsis (28	Sepsis (28	TRUE	igd	0.0236	5.45E-06	75	M34322.m	TRUE	reported	16GdG9	2	TRUE	0.216385	20.15794
5179	rs2490700	C	G	C	G	0.1208	0.004084	0.0343	0.227877	FALSE	TRUE	FALSE	ieu-b-508	10	7007631	0.039763	486484	0.918195	Sepsis (28	Sepsis (28	Sepsis (28	TRUE	igd	0.0269	7.35E-06	4625	M33195.m	TRUE	reported	SI4STh	2	TRUE	0.004341	20.15772
6931	rs1049251	T	C	T	C	-0.0238	-0.00413	0.1563	0.158041	FALSE	FALSE	FALSE	ieu-b-508	13	90072538	0.045787	486484	0.928173	Sepsis (28	Sepsis (28	Sepsis (28	TRUE	igd	0.0053	7.65E-06	5208	M34395.m	TRUE	reported	QVOPA3	2	TRUE	0.003857	20.15744
3040	rs7742715	A	G	A	G	0.0292	0.056274	0.356	0.364669	FALSE	FALSE	FALSE	ieu-b-508	6	1.65E+08	0.034648	486484	0.104344	Sepsis (28	Sepsis (28	Sepsis (28	TRUE	igd	0.0065	6.59E-06	1713	M31555.m	TRUE	reported	iQ5E3v	2	TRUE	0.011644	20.15727
2166	rs913342	C	G	C	G	0.0229	0.009318	0.1392	0.193644	FALSE	TRUE	FALSE	ieu-b-508	9	1.39E+08	0.042232	486484	0.825377	Sepsis (28	Sepsis (28	Sepsis (28	TRUE	igd	0.0051	8.19E-06	7369	M19323.m	TRUE	reported	yzKFKX	2	TRUE	0.002729	20.15639
6226	rs9864709	T	G	T	G	-0.0238	-0.03163	0.2158	0.222319	FALSE	FALSE	FALSE	ieu-b-508	3	1.95E+08	0.040254	486484	0.432045	Sepsis (28	Sepsis (28	Sepsis (28	TRUE	igd	0.0053	5.74E-06	4533	M33901.m	TRUE	reported	mQkhrb	2	TRUE	0.004429	20.15629
6111	rs1083231	A	G	A	G	-0.1452	-0.10678	0.9183	0.917299	FALSE	FALSE	FALSE	ieu-b-508	11	14922363	0.060761	486484	0.078855	Sepsis (28	Sepsis (28	Sepsis (28	TRUE	igd	0.0322	6.67E-06	228	M33877.m	TRUE	reported	yDVi1f	2	TRUE	0.081881	20.15557
3709	rs7783170	T	C	T	C	-0.0229	-0.02271	0.7251	0.736369	FALSE	FALSE	FALSE	ieu-b-508	7	70984103	0.037924	486484	0.549222	Sepsis (28	Sepsis (28	Sepsis (28	TRUE	igd	0.0051	7.96E-06	5955	M32593.m	TRUE	reported	6zVl1i	2	TRUE	0.003374	20.15509
6863	rs157009	T	C	T	C	-0.2438	-0.02258	0.3529	0.394738	FALSE	FALSE	FALSE	ieu-b-508	13	1.1E+08	0.034188	486484	0.509018	Sepsis (28	Sepsis (28	Sepsis (28	TRUE	igd	0.0512	1.92E-06	18	M34365.m	TRUE	reported	8eexD0	2	TRUE	0.557456	20.15464
7120	rs7549636	A	G	A	G	0.1145	-0.09059	0.271	0.269568	FALSE	FALSE	FALSE	ieu-b-508	1	4220301	0.037705	486484	0.016274	Sepsis (28	Sepsis (28	Sepsis (28	TRUE	igd	0.0254	6.36E-06	241	M34516.m	TRUE	reported	7Ah2wy	2	TRUE	0.077762	20.15229
3291	rs1705804	A	G	A	G	-0.044	0.083944	0.0554	0.060857	FALSE	FALSE	FALSE	ieu-b-508	8	27733012	0.069846	486484	0.229422	Sepsis (28	Sepsis (28	Sepsis (28	TRUE	igd	0.0098	7.33E-06	6119	M32379.m	TRUE	reported	MLimM6	2	TRUE	0.003284	20.15168
8544	rs7831994	T	C	T	C	0.1316	0.021486	0.0356	0.028908	FALSE	FALSE	FALSE	ieu-b-508	8	8137345	0.099633	486484	0.82926	Sepsis (28	Sepsis (28	Sepsis (28	TRUE	igd	0.0293	6.91E-06	1745	M37190.m	TRUE	reported	tiNuK9	2	TRUE	0.011428	20.15016
7166	rs1385234	A	T	A	T	0.022	0.080334	0.3159	0.310356	FALSE	TRUE	FALSE	ieu-b-508	8	96681968	0.036109	486484	0.026095	Sepsis (28	Sepsis (28	Sepsis (28	TRUE	igd	0.0049	7.00E-06	4925	M34534.m	TRUE	reported	iA78RM	2	TRUE	0.004076	20.15008
6655	rs4589435	T	C	T	C	-0.064	0.072109	0.7069	0.689659	FALSE	FALSE	FALSE	ieu-b-508	13	66297052	0.036247	486484	0.04666	Sepsis (28	Sepsis (28	Sepsis (28	TRUE	igd	0.0142	6.20E-06	248	M34322.m	TRUE	reported	16GdG9	2	TRUE	0.075708	20.14961
4194	rs798727	T	C	T	C	-0.0211	-0.07276	0.8227	0.809245	FALSE	FALSE	FALSE	ieu-b-508	4	1685137	0.042621	486484	0.087813	Sepsis (28	Sepsis (28	Sepsis (28	TRUE	igd	0.0047	8.00E-06	7313	M32761.m	TRUE	reported	QefVGc	2	TRUE	0.002748	20.14886
4597	rs1917331	A	T	A	T	0.0211	0.021538	0.8179	0.823386	FALSE	TRUE	FALSE	ieu-b-508	4	58193388	0.043783	486484	0.622782	Sepsis (28	Sepsis (28	Sepsis (28	TRUE	igd	0.0047	5.58E-06	7263	M33165.m	TRUE	reported	RZt3uS	2	TRUE	0.002767	20.14882
2897	rs1221633	T	G	T	G	-0.0211	0.026067	0.1744	0.200417	FALSE	FALSE	FALSE	ieu-b-508	6	1.1E+08	0.041705	486484	0.531947	Sepsis (28	Sepsis (28	Sepsis (28	TRUE	igd	0.0047	5.99E-06	6830	M27722.m	TRUE	reported	rzVgUy	2	TRUE	0.002942	20.14847
5716	rs3024981	A	G	A	G	-0.0862	0.187687	0.0147	0.026379	FALSE	FALSE	FALSE	ieu-b-508	12	57492099	0.104337	486484	0.072041	Sepsis (28	Sepsis (28	Sepsis (28	TRUE	igd	0.0192	7.14E-06	5107	M33488.m	TRUE	reported	mZmDY6	2	TRUE	0.003931	20.14846
3677	rs7866	T	C	T	C	-0.1033	-0.01836	0.0443	0.043286	FALSE	FALSE	FALSE	ieu-b-508	1	27272844	0.08246	486484	0.823848	Sepsis (28	Sepsis (28	Sepsis (28	TRUE	igd	0.023	6.95E-06	1726	M32578.m	TRUE	reported	LPkMFr	2	TRUE	0.011552	20.14844
1887	rs309293	T	C	T	C	0.0256	-0.00282	0.4315	0.43233	FALSE	FALSE	FALSE	ieu-b-508	2	7189354	0.033735	486484	0.933337	Sepsis (28	Sepsis (28	Sepsis (28	TRUE	igd	0.0057	6.90E-06	1756	M15990.m	TRUE	reported	15fe4N	2	TRUE	0.011357	20.14816
8315	rs7220100	A	G	A	G	-0.044	-0.03105	0.8574	0.854555	FALSE	FALSE	FALSE	ieu-b-508	17	6348823	0.047832	486484	0.516192	Sepsis (28	Sepsis (28	Sepsis (28	TRUE	igd	0.0098	7.50E-06	3346	M36585.m	TRUE	reported	pXZg13	2	TRUE	0.005989	20.14622
1055	rs884870	C	G	C	G	-0.0202	0.062597	0.0689	0.070805	FALSE	TRUE	FALSE	ieu-b-508	15	87789669	0.065143	486484	0.336594	Sepsis (28	Sepsis (28	Sepsis (28	TRUE	igd	0.0045	7.40E-06	7333	M01572.m	TRUE	reported	EOUAWy	2	TRUE	0.00274	20.14463
8349	rs1214340	T	G	T	G	-0.0328	-0.03756	0.2971	0.301388	FALSE	FALSE	FALSE	ieu-b-508	1	69278274	0.036441	486484	0.30271	Sepsis (28	Sepsis (28	Sepsis (28	TRUE	igd	0.0073	6.05E-06	922	M36738.m	TRUE	reported	mVQQcD	2	TRUE	0.021427	20.14461
4778	rs1516626	C	G	C	G	-0.3444	0.052753	0.0239	0.016951	FALSE	TRUE	FALSE	ieu-b-508	11	83540621	0.135436	486484	0.696904	Sepsis (28	Sepsis (28	Sepsis (28	TRUE	igd	0.0736	2.87E-06	25	M33178.m	TRUE	reported	g22WMS	2	TRUE	0.466909	20.14459
7781	rs807532	T	C	T	C	0.0202	-0.12054	0.0687	0.067779	FALSE	FALSE	FALSE	ieu-b-508	6	24556675	0.066865	486484	0.071427	Sepsis (28	Sepsis (28	Sepsis (28	TRUE	igd	0.0045	7.46E-06	7256	M35439.m	TRUE	reported	DnuAGZ	2	TRUE	0.002769	20.14457
2170	rs1187293	A	T	A	T	0.0202	0.055119	0.1999	0.199488	FALSE	TRUE	FALSE	ieu-b-508	18	41406224	0.04185	486484	0.18781	Sepsis (28	Sepsis (28	Sepsis (28	TRUE	igd	0.0045	6.61E-06	7251	M19324.m	TRUE	reported	1pj7ZT	2	TRUE	0.002771	20.14457
1788	rs1117428	T	C	T	C	-0.0615	-0.05819	0.0957	0.097258	FALSE	FALSE	FALSE	ieu-b-508	12	62408708	0.056307	486484	0.301389	Sepsis (28	Sepsis (28	Sepsis (28	TRUE	igd	0.0137	6.89E-06	5502	M15677.m	TRUE	reported	1CuWi9	2	TRUE	0.003649	20.14425
2369	rs2118838	C	G	C	G	0.0422	-0.00571	0.8319	0.840502	FALSE	TRUE	FALSE	ieu-b-508	2	1.96E+08	0.045575	486484	0.900368	Sepsis (28	Sepsis (28	Sepsis (28	TRUE	igd	0.0094	7.09E-06	3422	M21049.m	TRUE	reported	UDtLE4	2	TRUE	0.005855	20.14259
2268	rs744093	A	G	A	G	-0.0238	-0.13173	0.892	0.875145	FALSE	FALSE	FALSE	ieu-b-508	9	32700942	0.050987	486484	0.009775	Sepsis (28	Sepsis (28	Sepsis (28	TRUE	igd	0.0053	8.51E-06	1759	M19934.m	TRUE	reported	HtcWcQ	2	TRUE	0.011334	20.14226
5929	rs1079479	T	C	T	C	-0.0202	-0.01623	0.3454	0.339282	FALSE	FALSE	FALSE	ieu-b-508	10	1902639	0.035312	486484	0.6457	Sepsis (28	Sepsis (28	Sepsis (28	TRUE	igd	0.0045	6.82E-06	5080	M33637.m	TRUE	reported	tfLznu	2	TRUE	0.003951	20.14219
5873	rs6978556	T	C	T	C	0.1712	0.06016	0.0175	0.018089	FALSE	FALSE	FALSE	ieu-b-508	7	50497534	0.125701	486484	0.632227	Sepsis (28	Sepsis (28	Sepsis (28	TRUE	igd	0.0381	7.08E-06	824	M33609.m	TRUE	reported	h7B9gN	2	TRUE	0.023918	20.14198
6576	rs317279	A	G	A	G	0.0202	-0.01182	0.6525	0.626137	FALSE	FALSE	FALSE	ieu-b-508	2	76285465	0.034422	486484	0.731272	Sepsis (28	Sepsis (28	Sepsis (28	TRUE	igd	0.0045	7.62E-06	4908	M34245.m	TRUE	reported	pRwBKr	2	TRUE	0.004089	20.14191
5223	rs1265373	C	G	C	G	0.0202	-0.01531	0.3139	0.324795	FALSE	TRUE	FALSE	ieu-b-508	5	1.49E+08	0.035705	486484	0.668152	Sepsis (28	Sepsis (28	Sepsis (28	TRUE	igd	0.0045	8.14E-06	4665	M33221.m	TRUE	reported	8ZrV1r	2	TRUE	0.004301	20.14148
2334	rs2861960	T	C	T	C	-0.0193	0.013105	0.8651	0.871601	FALSE	FALSE	FALSE	ieu-b-508	2	80185514	0.050366	486484	0.794708	Sepsis (28	Sepsis (28	Sepsis (28	TRUE	igd	0.0043	7.98E-06	7366	M21044.m	TRUE	reported	Ym5rae	2	TRUE	0.002727	20.14001
877	rs1296779	A	C	A	C	0.0193	-0.05971	0.0794	0.07669	FALSE	FALSE	FALSE	ieu-b-508	18	70116989	0.06251	486484	0.339458	Sepsis (28	Sepsis (28	Sepsis (28	TRUE	igd	0.0043	6.13E-06	7362	M01365.m	TRUE	reported	N1cZTs	2	TRUE	0.002729	20.14001
606	rs1713101	A	G	A	G	-0.0781	0.017768	0.0404	0.047738	FALSE	FALSE	FALSE	ieu-b-508	1	70123406	0.078725	486484	0.821437	Sepsis (28	Sepsis (28	Sepsis (28	TRUE	igd	0.0174	6.88E-06	4934	M01114.m	TRUE	reported	fqHdM2	2	TRUE	0.004067	20.13852
3527	rs1280622	A	G	A	G	-0.1127	0.025975	0.024	0.258087	FALSE	FALSE	FALSE	ieu-b-508	11	1.34E+08	0.038233	486484	0.496893	Sepsis (28	Sepsis (28	Sepsis (28	TRUE	igd	0.0251	7.05E-06	1767	M32497.m	TRUE	reported	244bZN	2	TRUE	0.011281	20.13764
3062	rs1018596	T	C	T	C	0.0754	-0.07266	0.0527	0.050925	FALSE	FALSE	FALSE	ieu-b-508	2	2.36E+08	0.077052	486484	0.345653	Sepsis (28	Sepsis (28	Sepsis (28	TRUE	igd	0.0168	6.83E-06	7363	M31787.m	TRUE	reported	XMp8YI	2	TRUE	0.002728	20.13753
5465	rs904720	A	G	A	G	-0.1106	-0.0318	0.2414	0.340148	FALSE	FALSE	FALSE	ieu-b-508	5	1.74E+08	0.035507	486484	0.370428	Sepsis (28	Sepsis (28	Sepsis (28	TRUE	igd	0.0246	6.61E-06	528	M33391.m	TRUE	reported	EgMd1b	2	TRUE	0.036871	20.13687
7690	rs1730139	T	C	T	C	-0.0202	0.027288	0.147	0.149463	FALSE	FALSE	FALSE	ieu-b-508	3	59909673	0.047279	486484	0.563817	Sepsis (28	Sepsis (28	Sepsis (28	TRUE	igd	0.0045	7.17E-06	3007	M35331.m	TRUE	reported	GBnxhN	2	TRUE	0.006656	20.13672
5380	rs7145855	A	G	A	G	0.3181	0.0397	0.9691	0.960962	FALSE	FALSE	FALSE	ieu-b-508	14	57831323	0.087922	486484	0.651606	Sepsis (28	Sepsis (28	Sepsis (28	TRUE	igd	0.0708	7.07E-06	803	M33384.m	TRUE	reported	lOffrZ	2	TRUE	0.024522	20.13621
3821	rs4766159	A	C	A	C	-0.022	-0.06005	0.7548	0.778731	FALSE	FALSE	FALSE	ieu-b-508	12	3761997	0.040311	486484	0.136331	Sepsis (28	Sepsis (28	Sepsis (28	TRUE	igd	0.0049	8.37E-06	1766	M32644.m	TRUE	reported	JAa2WD	2	TRUE	0.011286	20.13544
8565	rs1337985	T	C	T	C	-0.0184	0.012246	0.627	0.627506	FALSE	FALSE	FALSE	ieu-b-508	13	73134912	0.034709	486484	0.724224	Sepsis (28	Sepsis (28	Sepsis (28	TRUE	igd	0.0041	7.64E-06	7333	M37203.m	TRUE	reported	3glIlv	2	TRUE	0.002739	20.1349
3030	rs1175877	T	C	T	C	0.0184	-0.03187	0.7686	0.770494	FALSE	FALSE	FALSE	ieu-b-508	6	1.54E+08	0.039745	486484	0.422655	Sepsis (28	Sepsis (28	Sepsis (28	TRUE	igd	0.0041	7.67E-06	7263	M31555.m	TRUE	reported	iQ5E3v	2	TRUE	0.002765	20.13485
7406	rs7115681	T	C	T	C	-0.0184	-0.03098	0.4669	0.409764	FALSE	FALSE	FALSE	ieu-b-508	11	42459273	0.034283	486484	0.366233	Sepsis (28	Sepsis (28	Sepsis (28	TRUE	igd	0.0041	8.18E-06	7263	M35160.m	TRUE	reported	eRox3e	2	TRUE	0.002765	20.13485
5177	rs2159190	C	G	C	G	-0.0552	-0.03039	0.8227	0.817525	FALSE	TRUE	FALSE	ieu-b-508	7	1.39E+08	0.043504	486484	0.484845	Sepsis (28	Sepsis (28	Sepsis (28	TRUE	igd	0.0123	7.40E-06	4625	M33195.m	TRUE	reported	SI4STh	2	TRUE	0.004336	20.13168
8434	rs1236525	T	C	T	C	-0.0359	0.063223	0.1707	0.205266	FALSE	FALSE	FALSE	ieu-b-508	11	1116034	0.041292	486484	0.125734	Sepsis (28	Sepsis (28	Sepsis (28	TRUE	igd	0.008	7.78E-06	6511	M36850.m	TRUE	reported	j6lLja	2	TRUE	0.003083	20.13147
2007	rs7663798	A	G	A	G	0.1966	-0.09915	0.0486	0.043527	FALSE	FALSE	FALSE	ieu-b-508	4	1.42E+08	0.082254	486484	0.228038	Sepsis (28	Sepsis (28	Sepsis (28	TRUE	igd	0.0438	7.23E-06	2433	M18281.m	TRUE	reported	3mLp7z	2	TRUE	0.008213	20.13083
6625	rs9345410	C	G	C	G	0.0835	-0.00821	0.9419	0.932731	FALSE	TRUE	FALSE	ieu-b-508	6	65423034	0.066595	486484	0.901831	Sepsis (28	Sepsis (28	Sepsis (28	TRUE	igd	0.0186	7.56E-06	1766	M34314.m	TRUE	reported	wPKEWG	2	TRUE	0.011283	20.13052
5476	rs679020	T	C	T	C	-0.0552	0.072533	0.9156	0.919289	FALSE	FALSE	FALSE	ieu-b-508	6	56284456	0.061363	486484	0.237191	Sepsis (28	Sepsis (28	Sepsis (28	TRUE	igd	0.0123	7.03E-06	3890	M33408.m	TRUE	reported	tWZWla	2	TRUE	0.005151	20.13004
7608	rs9362985	T	C	T	C	-0.0175	-0.04033	0.3161	0.317624	FALSE	FALSE	FALSE	ieu-b-508	6	93274584	0.035996	486484	0.262575	Sepsis (28	Sepsis (28	Sepsis (28	TRUE	igd	0.0039	6.48E-06	7359	M35320.m	TRUE	reported	ijCk2X	2	TRUE	0.002729	20.12931
8049	rs1254788	A	G	A	G	-0.0175	-0.06962	0.7663	0.766355	FALSE	FALSE	FALSE	ieu-b-508	8	9995515	0.039551	486484	0.078374	Sepsis (28	Sepsis (28	Sepsis (28	TRUE	igd	0.0039	6.32E-06	6901	M35977.m	TRUE	reported	NHnUo7	2	TRUE	0.002909	20.12894
8590	rs6082720	T	C	T	C	0.2245	0.174569	0.0112	0.014302	FALSE	FALSE	FALSE	ieu-b-508	20	2302087	0.141175	486484	0.216257	Sepsis (28	Sepsis (28	Sepsis (28	TRUE	igd	0.05	7.03E-06	1228	M37459.m	TRUE	reported	55F8jy	2	TRUE	0.016152	20.12727
8343	rs4846407	T	C	T	C	-0.0175	-0.0027	0.3452	0.353209	FALSE	FALSE	FALSE	ieu-b-508	1	2.17E+08	0.034947	486484	0.938312	Sepsis (28	Sepsis (28	Sepsis (28	TRUE	igd	0.0039	5.60E-06	5292	M36673.m	TRUE	reported	CD5lvR	2	TRUE	0.00379	20.12717
795	rs1111889	T	C	T	C	0.0202	-0.0227	0.4675	0.418275	FALSE	FALSE	FALSE	ieu-b-508	1	2.22E+08	0.033814	486484	0.502104	Sepsis (28	Sepsis (28	Sepsis (28	TRUE	igd	0.0045	7.16E-06	1755	M01356.m	TRUE	reported	ah1g5W	2	TRUE	0.011351	20.12716
8448	rs1758367	T	C	T	C	0.0633	-0.07458	0.9631	0.963006	FALSE	FALSE	FALSE	ieu-b-508	1	1.93E+08	0.088379	486484	0.398735	Sepsis (28	Sepsis (28	Sepsis (28	TRUE	igd	0.0141	7.57E-06	1467	M37004.m	TRUE	reported	EKCQye	2	TRUE	0.013552	20.12689
4321	rs4721104	A	T	A	T	-0.0341	0.073144	0.2583	0.259458	FALSE	TRUE	FALSE	ieu-b-508	7	12529753	0.038027	486484	0.054421	Sepsis (28	Sepsis (28	Sepsis (28	TRUE	igd	0.0076	6.93E-06	6250	M32846.m	TRUE	reported	lwF2Yy	2	TRUE	0.003211	20.12531
2275	rs1131718	A	G	A	G	-0.0166	0.01729	0.8565	0.852054	FALSE	FALSE	FALSE	ieu-b-508	10	1.35E+08	0.047615	486484	0.716511	Sepsis (28	Sepsis (28	Sepsis (28	TRUE	igd	0.0037	5.71E-06	7301	M20675.m	TRUE	reported	CXe9rE	2	TRUE	0.002749	20.12305
4602	rs7819602	C	G	C	G	-0.0166	0.075597	0.3909	0.386047	FALSE	TRUE	FALSE	ieu-b-508	8	10726842	0.034408	486484	0.028014	Sepsis (28	Sepsis (28	Sepsis (28	TRUE	igd	0.0037	7.57E-06	7263	M33165.m	TRUE	reported	RZt3uS	2	TRUE	0.002764	20.12302
1965	rs1709156	T	C	T	C	0.0332	-0.00189	0.1249	0.12368	FALSE	FALSE	FALSE	ieu-b-508	14	95363869	0.050805	486484	0.970302	Sepsis (28	Sepsis (28	Sepsis (28	TRUE	igd	0.0074	7.53E-06	7226	M17807.m	TRUE	reported	zd6sZl	2	TRUE	0.002778	20.12299
4287	rs2576722	A	T	A	T	0.0166	-0.00029	0.8553	0.834613	FALSE	TRUE	FALSE	ieu-b-508	2	55461823	0.044849	486484	0.994899	Sepsis (28	Sepsis (28	Sepsis (28	TRUE	igd	0.0037	9.92E-06	7063	M32814.m	TRUE	reported	z0OeTx	2	TRUE	0.002842	20.12286
6509	rs6596884	T	C	T	C	0.0332	0.007487	0.4477	0.413343	FALSE	FALSE	FALSE	ieu-b-508	6	2470233	0.033937	486484	0.825387	Sepsis (28	Sepsis (28	Sepsis (28	TRUE	igd	0.0074	6.91E-06	5581	M34112.m	TRUE	reported	97JqFb	2	TRUE	0.003594	20.12135
7789	rs1252270	A	G	A	G	0.2998	0.181041	0.0337	0.025686	FALSE	FALSE	FALSE	ieu-b-508	5	11567187	0.10895	486484	0.096574	Sepsis (28	Sepsis (28	Sepsis (28	TRUE	igd	0.0666	6.84E-06	284	M35451.m	TRUE	reported	GMRGAN	2	TRUE	0.066599	20.12081
3865	rs1257343	A	C	A	C	0.0377	-0.12242	0.087	0.092699	FALSE	FALSE	FALSE	ieu-b-508	10	1.06E+08	0.057743	486484	0.033992	Sepsis (28	Sepsis (28	Sepsis (28	TRUE	igd	0.0084	7.96E-06	1766	M32654.m	TRUE	reported	6lSQOP	2	TRUE	0.011277	20.12019
5268	rs9315938	T	C	T	C	0.0323	0.059851	0.0442	0.043119	FALSE	FALSE	FALSE	ieu-b-508	13	43293347	0.08221	486484	0.466596	Sepsis (28	Sepsis (28	Sepsis (28	TRUE	igd	0.0072	6.92E-06	7364	M33230.m	TRUE	reported	nzcTOn	2	TRUE	0.002725	20.11973
6291	rs1220578	A	G	A	G	-0.0323	-0.04629	0.0778	0.085778	FALSE	FALSE	FALSE	ieu-b-508	13	24730147	0.060369	486484	0.443193	Sepsis (28	Sepsis (28	Sepsis (28	TRUE	igd	0.0072	6.89E-06	7320	M33941.m	TRUE	reported	culqiU	2	TRUE	0.002742	20.11969
4404	rs1213747	T	C	T	C	0.0359	-0.02451	0.1187	0.118747	FALSE	FALSE	FALSE	ieu-b-508	1	44287148	0.052311	486484	0.639367	Sepsis (28	Sepsis (28	Sepsis (28	TRUE	igd	0.008	6.92E-06	2148	M32869.m	TRUE	reported	Rvim1n	2	TRUE	0.009288	20.11891
7433	rs4084390	C	G	C	G	0.0323	-0.01044	0.238	0.23261	FALSE	TRUE	FALSE	ieu-b-508	11	84230206	0.039716	486484	0.792614	Sepsis (28	Sepsis (28	Sepsis (28	TRUE	igd	0.0072	7.24E-06	5953	M35187.m	TRUE	reported	Zx7JfE	2	TRUE	0.003369	20.11843
8198	rs6533515	A	G	A	G	-0.035	0.04236	0.2112	0.202023	FALSE	FALSE	FALSE	ieu-b-508	4	1.11E+08	0.04154	486484	0.30785	Sepsis (28	Sepsis (28	Sepsis (28	TRUE	igd	0.0078	7.04E-06	2400	M36230.m	TRUE	reported	V5KJeR	2	TRUE	0.00832	20.118
3795	rs551488	T	C	T	C	0.0157	-0.01494	0.7412	0.740931	FALSE	FALSE	FALSE	ieu-b-508	3	1.83E+08	0.038078	486484	0.694823	Sepsis (28	Sepsis (28	Sepsis (28	TRUE	igd	0.0035	7.41E-06	7369	M32635.m	TRUE	reported	jJl9P8	2	TRUE	0.002723	20.11617
4089	rs1329561	A	G	A	G	-0.0314	0.010557	0.1636	0.164166	FALSE	FALSE	FALSE	ieu-b-508	9	82799227	0.045226	486484	0.815433	Sepsis (28	Sepsis (28	Sepsis (28	TRUE	igd	0.007	6.86E-06	7356	M32753.m	TRUE	reported	XlC6zq	2	TRUE	0.002728	20.11616
1594	rs1042685	A	G	A	G	-0.0157	-0.09018	0.8949	0.890721	FALSE	FALSE	FALSE	ieu-b-508	19	33096406	0.053403	486484	0.091266	Sepsis (28	Sepsis (28	Sepsis (28	TRUE	igd	0.0035	8.06E-06	7258	M12626.m	TRUE	reported	17lClc	2	TRUE	0.002765	20.11609
2036	rs2932525	A	G	A	G	-0.0157	-0.01221	0.8432	0.849777	FALSE	FALSE	FALSE	ieu-b-508	5	1.43E+08	0.047027	486484	0.795178	Sepsis (28	Sepsis (28	Sepsis (28	TRUE	igd	0.0035	7.55E-06	6939	M18349.m	TRUE	reported	M5qPs8	2	TRUE	0.002891	20.11583
560	rs7593385	A	C	A	C	0.0359	-0.1416	0.9685	0.990492	FALSE	FALSE	FALSE	ieu-b-508	2	1.35E+08	0.172781	486484	0.41247	Sepsis (28	Sepsis (28	Sepsis (28	TRUE	igd	0.008	6.87E-06	1766	M00606.m	TRUE	reported	r5smf7	2	TRUE	0.011274	20.11485
4649	rs787878	T	C	T	C	0.2768	-0.03403	0.6233	0.61428	FALSE	FALSE	FALSE	ieu-b-508	9	1.35E+08	0.034259	486484	0.320564	Sepsis (28	Sepsis (28	Sepsis (28	TRUE	igd	0.0609	5.51E-06	76	M33173.m	TRUE	reported	04fkUQ	2	TRUE	0.213726	20.1148
6096	rs4947639	T	G	T	G	0.0561	0.088035	0.1513	0.06749	FALSE	FALSE	FALSE	ieu-b-508	7	51842792	0.067206	486484	0.190222	Sepsis (28	Sepsis (28	Sepsis (28	TRUE	igd	0.0125	6.69E-06	1428	M33864.m	TRUE	reported	yr5h52	2	TRUE	0.013909	20.11393
7860	rs1710934	A	G	A	G	-0.0884	0.104718	0.9737	0.983317	FALSE	FALSE	FALSE	ieu-b-508	14	39979953	0.130762	486484	0.423232	Sepsis (28	Sepsis (28	Sepsis (28	TRUE	igd	0.0197	7.28E-06	1750	M35631.m	TRUE	reported	002wka	2	TRUE	0.011375	20.11294
7612	rs1111974	C	G	C	G	-0.0949	0.063772	0.9047	0.889025	FALSE	TRUE	FALSE	ieu-b-508	1	2.12E+08	0.053656	486484	0.234619	Sepsis (28	Sepsis (28	Sepsis (28	TRUE	igd	0.0211	6.69E-06	349	M35322.m	TRUE	reported	om3h9x	2	TRUE	0.054786	20.11275
2046	rs1772858	A	G	A	G	-0.0341	-0.00979	0.6211	0.640723	FALSE	FALSE	FALSE	ieu-b-508	1	2.01E+08	0.034777	486484	0.778431	Sepsis (28	Sepsis (28	Sepsis (28	TRUE	igd	0.0076	7.98E-06	2079	M18357.m	TRUE	reported	o7AvpG	2	TRUE	0.009591	20.11239
8136	rs1309827	A	G	A	G	-0.0305	0.03662	0.203	0.202825	FALSE	FALSE	FALSE	ieu-b-508	3	37232458	0.041506	486484	0.377625	Sepsis (28	Sepsis (28	Sepsis (28	TRUE	igd	0.0068	7.48E-06	7311	M36103.m	TRUE	reported	sjeaPC	2	TRUE	0.002744	20.11236
5500	rs1333677	A	C	A	C	0.0624	-0.04621	0.1736	0.151232	FALSE	FALSE	FALSE	ieu-b-508	16	48318728	0.046537	486484	0.32067	Sepsis (28	Sepsis (28	Sepsis (28	TRUE	igd	0.0139	7.39E-06	977	M33420.m	TRUE	reported	eTToDb	2	TRUE	0.020211	20.11174
674	rs9403794	A	C	A	C	0.0175	0.021066	0.5963	0.57521	FALSE	FALSE	FALSE	ieu-b-508	6	1.47E+08	0.034066	486484	0.536334	Sepsis (28	Sepsis (28	Sepsis (28	TRUE	igd	0.0039	8.09E-06	1679	M01284.m	TRUE	reported	E4hc9c	2	TRUE	0.01185	20.1108
6694	rs1696397	A	G	A	G	-0.0305	-0.01594	0.0772	0.164261	FALSE	FALSE	FALSE	ieu-b-508	19	30740078	0.045156	486484	0.724034	Sepsis (28	Sepsis (28	Sepsis (28	TRUE	igd	0.0068	6.13E-06	5081	M34336.m	TRUE	reported	jrSSxL	2	TRUE	0.003944	20.10994
613	rs4244808	T	G	T	G	0.0305	-0.02992	0.5307	0.540058	FALSE	FALSE	FALSE	ieu-b-508	11	2163110	0.033702	486484	0.37473	Sepsis (28	Sepsis (28	Sepsis (28	TRUE	igd	0.0068	8.30E-06	4934	M01114.m	TRUE	reported	fqHdM2	2	TRUE	0.004061	20.10971
7205	rs2172784	T	C	T	C	0.0323	-0.00097	0.8464	0.850434	FALSE	FALSE	FALSE	ieu-b-508	6	1.66E+08	0.046908	486484	0.983421	Sepsis (28	Sepsis (28	Sepsis (28	TRUE	igd	0.0072	7.50E-06	2545	M34674.m	TRUE	reported	plxOKz	2	TRUE	0.007846	20.10938
7124	rs1079737	A	G	A	G	-0.0148	0.063732	0.6912	0.692803	FALSE	FALSE	FALSE	ieu-b-508	1	2904056	0.03628	486484	0.078971	Sepsis (28	Sepsis (28	Sepsis (28	TRUE	igd	0.0033	8.16E-06	7322	M34527.m	TRUE	reported	EWFGP8	2	TRUE	0.00274	20.10837
1571	rs1027932	T	C	T	C	-0.0148	0.150907	0.9522	0.945662	FALSE	FALSE	FALSE	ieu-b-508	7	16154920	0.073447	486484	0.039914	Sepsis (28	Sepsis (28	Sepsis (28	TRUE	igd	0.0033	6.52E-06	7314	M12593.m	TRUE	reported	IBXgSU	2	TRUE	0.002743	20.10837
6766	rs749427	A	G	A	G	0.1032	0.009675	0.0625	0.075057	FALSE	FALSE	FALSE	ieu-b-508	9	864122	0.063737	486484	0.879351	Sepsis (28	Sepsis (28	Sepsis (28	TRUE	igd	0.023	7.04E-06	1649	M34339.m	TRUE	reported	RwGZ7c	2	TRUE	0.012062	20.10836
5554	rs1244356	A	C	A	C	-0.618	-0.02829	0.1	0.225232	FALSE	FALSE	FALSE	ieu-b-508	14	34697832	0.039854	486484	0.477746	Sepsis (28	Sepsis (28	Sepsis (28	TRUE	igd	0.1355	5.13E-06	60	M33423.m	TRUE	reported	MoEnO7	2	TRUE	0.257441	20.10829
4039	rs9440550	A	G	A	G	-0.0148	-0.04511	0.1936	0.206961	FALSE	FALSE	FALSE	ieu-b-508	1	1.09E+08	0.041227	486484	0.273923	Sepsis (28	Sepsis (28	Sepsis (28	TRUE	igd	0.0033	6.70E-06	6042	M32739.m	TRUE	reported	dYSC7n	2	TRUE	0.003318	20.10721
7718	rs6892541	T	C	T	C	0.0148	0.001804	0.1866	0.183955	FALSE	FALSE	FALSE	ieu-b-508	5	1.78E+08	0.046261	486484	0.968894	Sepsis (28	Sepsis (28	Sepsis (28	TRUE	igd	0.0037	6.54E-06	1758	M35397.m	TRUE	reported	Y56mZr	2	TRUE	0.01132	20.10566
4223	rs7870790	A	G	A	G	0.0287	-0.02881	0.309	0.302124	FALSE	FALSE	FALSE	ieu-b-508	9	96779553	0.036343	486484	0.427998	Sepsis (28	Sepsis (28	Sepsis (28	TRUE	igd	0.0064	6.97E-06	7330	M32786.m	TRUE	reported	rwF0co	2	TRUE	0.002736	20.10413
5635	rs1882545	A	G	A	G	-0.0148	-0.05314	0.6567	0.666769	FALSE	FALSE	FALSE	ieu-b-508	12	31943385	0.043384	486484	0.220138	Sepsis (28	Sepsis (28	Sepsis (28	TRUE	igd	0.0033	9.36E-06	4004	M33443.m	TRUE	reported	G1JrRV	2	TRUE	0.004998	20.10382
6115	rs1308282	T	C	T	C	-0.0152	-0.04677	0.8387	0.84672	FALSE	FALSE	FALSE	ieu-b-508	3	1537685	0.046686	486484	0.316475	Sepsis (28	Sepsis (28	Sepsis (28	TRUE	igd	0.0322	6.73E-06	176	M33877.m	TRUE	reported	yDVi1f	2	TRUE	0.103568	20.10287
2402	rs2073030	A	T	A	T	0.0287	-0.07949	0.7776	0.785464	FALSE	TRUE	FALSE	ieu-b-508	6	22569634	0.040696	486484	0.050775	Sepsis (28	Sepsis (28	Sepsis (28	TRUE	igd	0.0064	6.99E-06	5436	M21184.m	TRUE	reported	63ppuJ	2	TRUE	0.003686	20.10222
2893	rs3111102	A	G	A	G	-0.048	0.064968	0.0953	0.10949	FALSE	FALSE	FALSE	ieu-b-508	5	51132945	0.053511	486484	0.224708	Sepsis (28	Sepsis (28	Sepsis (28	TRUE	igd	0.0107	7.00E-06	1768	M27718.m	TRUE	reported	PHL57z	2	TRUE	0.011254	20.10126
5072	rs7944027	A	G	A	G	-0.0473	-0.02457	0.0252	0.022107	FALSE	FALSE	FALSE	ieu-b-508	11	1.33E+08	0.115492	486484	0.831512	Sepsis (28	Sepsis (28	Sepsis (28	TRUE	igd	0.0743	3.00E-06	25	M33178.m	TRUE	reported	g22WMS	2	TRUE	0.466371	20.1011
1671	rs7240261	T	C	T	C	0.0565	0.007507	0.562	0.555422	FALSE	FALSE	FALSE	ieu-b-508	18	5428938	0.033403	486484	0.822156	Sepsis (28	Sepsis (28	Sepsis (28	TRUE	igd	0.0126	7.52E-06	5606	M15335.m	TRUE	reported	a7RjFn	2	TRUE	0.003574	20.10022
4763	rs1332905	T	C	T	C	-0.3543	-0.12682	0.9711	0.979414	FALSE	FALSE	FALSE	ieu-b-508	14	1.03E+08	0.117723	486484	0.281356	Sepsis (28	Sepsis (28	Sepsis (28	TRUE	igd	0.0758	2.91E-06	25	M33178.m	TRUE	reported	g22WMS	2	TRUE	0.466355	20.0998
4225	rs994645	A	G	A	G	-0.0417	-0.08123	0.1146	0.119429	FALSE	FALSE	FALSE	ieu-b-508	9	19922228	0.051615	486484	0.115526	Sepsis (28	Sepsis (28	Sepsis (28	TRUE	igd	0.0093	8.18E-06	7330	M32786.m	TRUE	reported	rwF0co	2	TRUE	0.002735	20.09961
1600	rs729761	T	C	T	C	-0.0139	0.056119	0.2479	0.288123	FALSE	FALSE	FALSE	ieu-b-508	6	43804571	0.037181	486484	0.131208	Sepsis (28	Sepsis (28	Sepsis (28	TRUE	igd	0.0031	8.52E-06	7258	M12626.m	TRUE	reported	17lClc	2	TRUE	0.002762	20.09956
8440	rs4579381	T	C	T	C	-0.0278	-0.03301	0.6475	0.669335	FALSE	FALSE	FALSE	ieu-b-508	6	58357694	0.035549	486484	0.353067	Sepsis (28	Sepsis (28	Sepsis (28	TRUE	igd	0.0062	7.00E-06	6511	M36850.m	TRUE	reported	j6lLja	2	TRUE	0.003078	20.09892
2497	rs6596879	T	C	T	C	0.0157	-0.03462	0.6201	0.605144	FALSE	FALSE	FALSE	ieu-b-508	6	1.53E+08	0.034216	486484	0.316675	Sepsis (28	Sepsis (28	Sepsis (28	TRUE	igd	[illegible]	[illegible]	[illegible]	[illegible]	TRUE	reported	bjUU6r	2	TRUE	[illegible]	[illegible]
5222	rs1166977	C	T	C	T	0.0278	0.004535	0.138	0.165094	FALSE	FALSE	FALSE	ieu-b-508	19	20423698	0.044938	486484	0.919609	Sepsis (28	Sepsis (28	Sepsis (28	TRUE	igd	0.0062	8.01E-06	4665	M33221.m	TRUE	reported	8ZrV1r	2	TRUE	0.004291	20.09648

3666	rs1011177	A	C	A	C	-0.0462	-0.02293	0.7263	0.722766	FALSE	FALSE	FALSE	ieu-b-508	8	85128127	0.037406	486484	0.539854	Sepsis (28	Sepsis (28	Sepsis (28	TRUE	igd	0.0103	8.00E-06	1726	M32578.m	TRUE	reported	LPkMFr	2	TRUE	0.011522	20.09583
5923	rs2933259	T	C	T	C	-0.0278	0.012096	0.5499	0.535852	FALSE	FALSE	FALSE	ieu-b-508	3	1.49E+08	0.03358	486484	0.718692	Sepsis (28	Sepsis (28	Sepsis (28	TRUE	igd	0.0062	6.53E-06	4323	M33633.m	TRUE	reported	e36XGE	2	TRUE	0.004629	20.0958
7648	rs268290	A	C	A	C	-0.061	0.116565	0.0174	0.014962	FALSE	FALSE	FALSE	ieu-b-508	10	44598285	0.139345	486484	0.402861	Sepsis (28	Sepsis (28	Sepsis (28	TRUE	igd	0.0136	7.58E-06	1764	M35326.m	TRUE	reported	jRytyJ	2	TRUE	0.011276	20.09505
1978	rs878983	T	C	T	C	-0.0269	-0.0065	0.2282	0.240721	FALSE	FALSE	FALSE	ieu-b-508	2	20277748	0.038989	486484	0.8676	Sepsis (28	Sepsis (28	Sepsis (28	TRUE	igd	0.006	8.15E-06	7226	M17807.m	TRUE	reported	zd6sZI	2	TRUE	0.002774	20.09471
7219	rs1244373	T	G	T	G	0.0269	0.044402	0.1232	0.126822	FALSE	FALSE	FALSE	ieu-b-508	16	13402339	0.050198	486484	0.376407	Sepsis (28	Sepsis (28	Sepsis (28	TRUE	igd	0.006	8.29E-06	6381	M34761.m	TRUE	reported	AMtp1w	2	TRUE	0.00314	20.09398
4647	rs7082110	A	G	A	G	0.1386	0.039246	0.8649	0.84412	FALSE	FALSE	FALSE	ieu-b-508	10	34161048	0.047117	486484	0.404873	Sepsis (28	Sepsis (28	Sepsis (28	TRUE	igd	0.0309	7.10E-06	1574	M33173.m	TRUE	reported	04fkUQ	2	TRUE	0.012621	20.09358
2911	rs1735937	T	C	T	C	-0.0323	0.05967	0.261	0.267137	FALSE	FALSE	FALSE	ieu-b-508	2	5947841	0.038217	486484	0.118446	Sepsis (28	Sepsis (28	Sepsis (28	TRUE	igd	0.0072	7.85E-06	1227	M27728.m	TRUE	reported	WOp1XE	2	TRUE	0.016137	20.09239
5763	rs42789	A	G	A	G	-0.0278	0.001144	0.426	0.425135	FALSE	FALSE	FALSE	ieu-b-508	2	40845671	0.033895	486484	0.973065	Sepsis (28	Sepsis (28	Sepsis (28	TRUE	igd	0.0062	7.33E-06	2759	M33508.m	TRUE	reported	UMXHcP	2	TRUE	0.007234	20.09052
1366	rs832404	T	C	T	C	-0.013	-0.04824	0.8684	0.875442	FALSE	FALSE	FALSE	ieu-b-508	5	56267431	0.050473	486484	0.339239	Sepsis (28	Sepsis (28	Sepsis (28	TRUE	igd	0.0029	7.53E-06	7226	M02730.m	TRUE	reported	XYa035	2	TRUE	0.002773	20.08956
7823	rs1763928	T	C	T	C	0.026	-0.00122	0.2643	0.311773	FALSE	FALSE	FALSE	ieu-b-508	19	36520765	0.036008	486484	0.97299	Sepsis (28	Sepsis (28	Sepsis (28	TRUE	igd	0.0058	8.99E-06	6595	M35472.m	TRUE	reported	oZU10n	2	TRUE	0.003038	20.08903
8272	rs1158161	A	G	A	G	-0.013	0.003325	0.3437	0.348514	FALSE	FALSE	FALSE	ieu-b-508	1	1.79E+08	0.03503	486484	0.924385	Sepsis (28	Sepsis (28	Sepsis (28	TRUE	igd	0.0029	6.59E-06	6464	M36553.m	TRUE	reported	2Rk7nd	2	TRUE	0.003099	20.08891
6191	rs1544149	T	C	T	C	0.013	-0.05873	0.6652	0.662876	FALSE	FALSE	FALSE	ieu-b-508	1	2.46E+08	0.035587	486484	0.098867	Sepsis (28	Sepsis (28	Sepsis (28	TRUE	igd	0.0029	7.22E-06	6004	M33892.m	TRUE	reported	w7HgU7	2	TRUE	0.003336	20.08843
6192	rs167675	C	G	C	G	0.013	-0.00366	0.371	0.369917	FALSE	TRUE	FALSE	ieu-b-508	17	31019996	0.034576	486484	0.915676	Sepsis (28	Sepsis (28	Sepsis (28	TRUE	igd	0.0029	7.54E-06	6004	M33892.m	TRUE	reported	w7HgU7	2	TRUE	0.003336	20.08843
6510	rs7836759	A	C	A	C	-0.026	-0.01349	0.3393	0.341974	FALSE	FALSE	FALSE	ieu-b-508	8	28977112	0.035337	486484	0.702558	Sepsis (28	Sepsis (28	Sepsis (28	TRUE	igd	0.0058	6.61E-06	5581	M34112.m	TRUE	reported	97JqFb	2	TRUE	0.003588	20.08792
2086	rs1767870	A	G	A	G	0.0511	0.008208	0.1871	0.213491	FALSE	FALSE	FALSE	ieu-b-508	10	1.26E+08	0.043737	486484	0.85114	Sepsis (28	Sepsis (28	Sepsis (28	TRUE	igd	0.0114	7.88E-06	6970	M18394.m	TRUE	reported	Lt9awu	2	TRUE	0.002874	20.08665
4914	rs2804609	T	C	T	C	-0.2427	0.167915	0.0214	0.019012	FALSE	FALSE	FALSE	ieu-b-508	10	1.14E+08	0.123193	486484	0.172877	Sepsis (28	Sepsis (28	Sepsis (28	TRUE	igd	0.0541	7.13E-06	1017	M33178.m	TRUE	reported	g22WMS	2	TRUE	0.019405	20.08585
3097	rs9973135	T	C	T	C	-0.0251	-0.05172	0.0499	0.034305	FALSE	FALSE	FALSE	ieu-b-508	18	75858172	0.092123	486484	0.574477	Sepsis (28	Sepsis (28	Sepsis (28	TRUE	igd	0.0056	8.32E-06	7346	M32197.m	TRUE	reported	VZjwa9	2	TRUE	0.002727	20.08414
8356	rs1157846	C	G	C	G	-0.0251	-0.0198	0.8688	0.86279	FALSE	TRUE	FALSE	ieu-b-508	1	2.21E+08	0.048433	486484	0.682721	Sepsis (28	Sepsis (28	Sepsis (28	TRUE	igd	0.0056	8.40E-06	6876	M36754.m	TRUE	reported	f8bkFg	2	TRUE	0.002913	20.08376
8259	rs1698556	T	C	T	C	-0.0139	0.013298	0.5111	0.524817	FALSE	FALSE	FALSE	ieu-b-508	20	60350365	0.033571	486484	0.692024	Sepsis (28	Sepsis (28	Sepsis (28	TRUE	igd	0.0031	8.32E-06	1765	M36552.m	TRUE	reported	5CxMJz	2	TRUE	0.011263	20.08232
3958	rs2256321	T	C	T	C	-0.0121	0.012417	0.6891	0.68813	FALSE	FALSE	FALSE	ieu-b-508	10	56877760	0.036221	486484	0.731732	Sepsis (28	Sepsis (28	Sepsis (28	TRUE	igd	0.0027	5.53E-06	7363	M32709.m	TRUE	reported	YR8SB4	2	TRUE	0.00272	20.07822
1605	rs1083997	T	G	T	G	0.0121	0.020004	0.1872	0.186172	FALSE	FALSE	FALSE	ieu-b-508	11	8076174	0.043258	486484	0.643772	Sepsis (28	Sepsis (28	Sepsis (28	TRUE	igd	0.0027	7.91E-06	7352	M15122.m	TRUE	reported	VlnI8T	2	TRUE	0.002724	20.07821
7421	rs381227	T	C	T	C	-0.0121	-0.11179	0.287	0.298372	FALSE	FALSE	FALSE	ieu-b-508	16	73779636	0.036616	486484	0.002265	Sepsis (28	Sepsis (28	Sepsis (28	TRUE	igd	0.0027	6.30E-06	7350	M35186.m	TRUE	reported	UZfu6Z	2	TRUE	0.002725	20.07821
6552	rs1288686	A	G	A	G	-0.0121	-0.03733	0.3911	0.399548	FALSE	FALSE	FALSE	ieu-b-508	14	68948118	0.034086	486484	0.273413	Sepsis (28	Sepsis (28	Sepsis (28	TRUE	igd	0.0027	7.09E-06	7347	M34244.m	TRUE	reported	JyF0Sp	2	TRUE	0.002726	20.07821
6557	rs1883995	A	G	A	G	0.0121	-0.0298	0.6271	0.619113	FALSE	FALSE	FALSE	ieu-b-508	22	25215436	0.034359	486484	0.385716	Sepsis (28	Sepsis (28	Sepsis (28	TRUE	igd	0.0027	6.57E-06	7347	M34244.m	TRUE	reported	JyF0Sp	2	TRUE	0.002726	20.07821
6970	rs1105155	A	G	A	G	-0.0121	0.001802	0.5217	0.523959	FALSE	FALSE	FALSE	ieu-b-508	12	31890602	0.033411	486484	0.956988	Sepsis (28	Sepsis (28	Sepsis (28	TRUE	igd	0.0027	8.47E-06	7344	M34407.m	TRUE	reported	X7I83Y	2	TRUE	0.002727	20.07821
8390	rs9954696	A	T	A	T	0.0121	0.023242	0.7848	0.781317	FALSE	TRUE	FALSE	ieu-b-508	18	11441123	0.040845	486484	0.569342	Sepsis (28	Sepsis (28	Sepsis (28	TRUE	igd	0.0027	5.36E-06	7335	M36776.m	TRUE	reported	VnYglx	2	TRUE	0.002731	20.0782
2628	rs8056954	T	G	T	G	-0.0121	-0.04548	0.1734	0.166985	FALSE	FALSE	FALSE	ieu-b-508	16	56930229	0.044799	486484	0.310012	Sepsis (28	Sepsis (28	Sepsis (28	TRUE	igd	0.0027	8.27E-06	7329	M22481.m	TRUE	reported	bXpf0k	2	TRUE	0.002733	20.0782
5532	rs4524522	C	G	C	G	-0.0121	-0.08242	0.0858	0.088962	FALSE	TRUE	FALSE	ieu-b-508	5	27751456	0.058517	486484	0.158983	Sepsis (28	Sepsis (28	Sepsis (28	TRUE	igd	0.0027	6.92E-06	7306	M33422.m	TRUE	reported	PLgjrT	2	TRUE	0.002741	20.07818
5783	rs1683029	T	G	T	G	-0.0121	0.041657	0.3419	0.345266	FALSE	FALSE	FALSE	ieu-b-508	3	1.19E+08	0.035022	486484	0.234267	Sepsis (28	Sepsis (28	Sepsis (28	TRUE	igd	0.0027	5.55E-06	7269	M33510.m	TRUE	reported	WxGKnn	2	TRUE	0.002755	20.07815
7493	rs2479415	T	C	T	C	0.0121	-0.03413	0.5265	0.521503	FALSE	FALSE	FALSE	ieu-b-508	1	55498745	0.033515	486484	0.308542	Sepsis (28	Sepsis (28	Sepsis (28	TRUE	igd	0.0027	8.98E-06	7261	M35253.m	TRUE	reported	VboRmj	2	TRUE	0.002758	20.07814
7494	rs329497	T	C	T	C	0.0121	-0.02688	0.6894	0.69265	FALSE	FALSE	FALSE	ieu-b-508	2	64327592	0.036198	486484	0.457787	Sepsis (28	Sepsis (28	Sepsis (28	TRUE	igd	0.0027	5.67E-06	7261	M35253.m	TRUE	reported	VboRmj	2	TRUE	0.002758	20.07814
3832	rs9570627	T	G	T	G	-0.0121	-0.00425	0.8432	0.811274	FALSE	FALSE	FALSE	ieu-b-508	13	62435236	0.042932	486484	0.921228	Sepsis (28	Sepsis (28	Sepsis (28	TRUE	igd	0.0027	7.47E-06	7229	M32644.m	TRUE	reported	JAa2WD	2	TRUE	0.002771	20.07812
2343	rs1172360	T	C	T	C	0.0121	-0.00142	0.1817	0.239071	FALSE	FALSE	FALSE	ieu-b-508	4	1.85E+08	0.040035	486484	0.971643	Sepsis (28	Sepsis (28	Sepsis (28	TRUE	igd	0.0027	8.03E-06	7203	M21047.m	TRUE	reported	ISF1Cr	2	TRUE	0.00278	20.0781
871	rs961959	A	C	A	C	-0.0121	0.055619	0.1925	0.198841	FALSE	FALSE	FALSE	ieu-b-508	9	1.22E+08	0.041763	486484	0.182929	Sepsis (28	Sepsis (28	Sepsis (28	TRUE	igd	0.0027	5.26E-06	7063	M01361.m	TRUE	reported	NIAcQ3	2	TRUE	0.002835	20.07799
5237	rs1023380	T	C	T	C	0.0121	-0.02748	0.5881	0.587824	FALSE	FALSE	FALSE	ieu-b-508	7	29536564	0.03392	486484	0.417869	Sepsis (28	Sepsis (28	Sepsis (28	TRUE	igd	0.0027	7.69E-06	7063	M33228.m	TRUE	reported	JwOXbQ	2	TRUE	0.002835	20.07799
5245	rs227769	T	C	T	C	-0.0121	-0.01021	0.467	0.470141	FALSE	FALSE	FALSE	ieu-b-508	2	71858565	0.033381	486484	0.759717	Sepsis (28	Sepsis (28	Sepsis (28	TRUE	igd	0.0027	9.32E-06	7063	M33228.m	TRUE	reported	JwOXbQ	2	TRUE	0.002835	20.07799
2385	rs954050	T	C	T	C	0.0121	-0.04274	0.5912	0.582956	FALSE	FALSE	FALSE	ieu-b-508	7	97880226	0.03387	486484	0.207003	Sepsis (28	Sepsis (28	Sepsis (28	TRUE	igd	0.0027	7.68E-06	6999	M21127.m	TRUE	reported	WCjhS3	2	TRUE	0.002861	20.07794
6607	rs2377324	T	C	T	C	-0.0121	-0.03149	0.7038	0.704467	FALSE	FALSE	FALSE	ieu-b-508	19	45768946	0.036585	486484	0.389337	Sepsis (28	Sepsis (28	Sepsis (28	TRUE	igd	0.0027	7.39E-06	6994	M34306.m	TRUE	reported	WPXPUN	2	TRUE	0.002863	20.07793
6610	rs7080146	T	C	T	C	0.0121	0.011049	0.705	0.689482	FALSE	FALSE	FALSE	ieu-b-508	10	71367790	0.036105	486484	0.759588	Sepsis (28	Sepsis (28	Sepsis (28	TRUE	igd	0.0027	8.00E-06	6994	M34306.m	TRUE	reported	WPXPUN	2	TRUE	0.002863	20.07793
753	rs1016648	T	C	T	C	-0.0121	-0.04043	0.3157	0.324	FALSE	FALSE	FALSE	ieu-b-508	2	1.9E+08	0.035672	486484	0.257086	Sepsis (28	Sepsis (28	Sepsis (28	TRUE	igd	0.0027	5.29E-06	6946	M01303.m	TRUE	reported	3b8x0F	2	TRUE	0.002883	20.07789
763	rs690191	T	C	T	C	-0.0121	-0.00486	0.7821	0.779642	FALSE	FALSE	FALSE	ieu-b-508	18	74078079	0.040228	486484	0.903744	Sepsis (28	Sepsis (28	Sepsis (28	TRUE	igd	0.0027	9.23E-06	6946	M01303.m	TRUE	reported	3b8x0F	2	TRUE	0.002883	20.07789
2028	rs1162344	T	C	T	C	0.0121	-0.0064	0.6919	0.686686	FALSE	FALSE	FALSE	ieu-b-508	14	57407989	0.036162	486484	0.859597	Sepsis (28	Sepsis (28	Sepsis (28	TRUE	igd	0.0027	7.84E-06	6939	M18349.m	TRUE	reported	M5qPs8	2	TRUE	0.002886	20.07789
1545	rs2130791	A	G	A	G	0.0121	-0.00811	0.7132	0.712725	FALSE	FALSE	FALSE	ieu-b-508	10	71791351	0.036878	486484	0.825874	Sepsis (28	Sepsis (28	Sepsis (28	TRUE	igd	0.0027	7.96E-06	6864	M12129.m	TRUE	reported	iNzdgb	2	TRUE	0.002917	20.07782
6176	rs7655244	T	G	T	G	0.0735	-0.09959	0.0616	0.059779	FALSE	FALSE	FALSE	ieu-b-508	4	10568213	0.070316	486484	0.156668	Sepsis (28	Sepsis (28	Sepsis (28	TRUE	igd	0.0164	7.15E-06	4999	M33885.m	TRUE	reported	uCOy6o	2	TRUE	0.004002	20.07766
2425	rs4266604	T	C	T	C	-0.0121	-0.00973	0.7099	0.700127	FALSE	FALSE	FALSE	ieu-b-508	7	52978724	0.03641	486484	0.789243	Sepsis (28	Sepsis (28	Sepsis (28	TRUE	igd	0.0027	9.19E-06	6556	M21188.m	TRUE	reported	kvdnjc	2	TRUE	0.003054	20.07755
7468	rs6107820	A	G	A	G	-0.0121	-0.03072	0.5819	0.586682	FALSE	FALSE	FALSE	ieu-b-508	20	6486648	0.033891	486484	0.364637	Sepsis (28	Sepsis (28	Sepsis (28	TRUE	igd	0.0027	9.95E-06	6543	M35193.m	TRUE	reported	BMMWXU	2	TRUE	0.00306	20.07754
7533	rs1299717	T	C	T	C	0.0121	0.008197	0.5646	0.565204	FALSE	FALSE	FALSE	ieu-b-508	2	80373814	0.03365	486484	0.807544	Sepsis (28	Sepsis (28	Sepsis (28	TRUE	igd	0.0027	6.79E-06	6538	M35257.m	TRUE	reported	iphouU	2	TRUE	0.003062	20.07753
2650	rs1081081	A	G	A	G	-0.0121	0.000364	0.1837	0.187416	FALSE	FALSE	FALSE	ieu-b-508	9	17603496	0.043136	486484	0.993273	Sepsis (28	Sepsis (28	Sepsis (28	TRUE	igd	0.0027	5.20E-06	6517	M22649.m	TRUE	reported	NAzwRF	2	TRUE	0.003072	20.07751
2653	rs1316352	A	G	A	G	0.0121	-0.02874	0.8339	0.826937	FALSE	FALSE	FALSE	ieu-b-508	5	18662686	0.044362	486484	0.517101	Sepsis (28	Sepsis (28	Sepsis (28	TRUE	igd	0.0027	8.11E-06	6517	M22649.m	TRUE	reported	NAzwRF	2	TRUE	0.003072	20.07751
6288	rs817338	T	C	T	C	0.0121	0.062391	0.8271	0.825569	FALSE	FALSE	FALSE	ieu-b-508	20	62604201	0.044147	486484	0.157582	Sepsis (28	Sepsis (28	Sepsis (28	TRUE	igd	0.0027	7.06E-06	6502	M33939.m	TRUE	reported	PeKlgz	2	TRUE	0.003079	20.0775
3997	rs261332	A	G	A	G	0.0121	-0.03189	0.1695	0.209873	FALSE	FALSE	FALSE	ieu-b-508	15	58727325	0.041014	486484	0.436799	Sepsis (28	Sepsis (28	Sepsis (28	TRUE	igd	0.0027	8.88E-06	6497	M32729.m	TRUE	reported	XGRSN1	2	TRUE	0.003082	20.07749
4592	rs9472173	T	C	T	C	0.0121	0.017285	0.4953	0.502064	FALSE	FALSE	FALSE	ieu-b-508	6	43933256	0.03508	486484	0.622214	Sepsis (28	Sepsis (28	Sepsis (28	TRUE	igd	0.0027	8.68E-06	6404	M33163.m	TRUE	reported	YzGXkR	2	TRUE	0.003126	20.0774
2500	rs1235542	A	G	A	G	-0.0121	-0.01202	0.277	0.274047	FALSE	FALSE	FALSE	ieu-b-508	10	10144150	0.037639	486484	0.749402	Sepsis (28	Sepsis (28	Sepsis (28	TRUE	igd	0.0027	9.20E-06	5702	M22130.m	TRUE	reported	YDbJlz	2	TRUE	0.00351	20.07663
2930	rs7929077	A	G	A	G	0.0121	-0.0092	0.4899	0.490914	FALSE	FALSE	FALSE	ieu-b-508	11	1.23E+08	0.03346	486484	0.783251	Sepsis (28	Sepsis (28	Sepsis (28	TRUE	igd	0.0027	6.63E-06	5562	M27728.m	TRUE	reported	WOp1XE	2	TRUE	0.003598	20.07645
3849	rs4316494	A	G	A	G	0.0121	0.012175	0.4925	0.510585	FALSE	FALSE	FALSE	ieu-b-508	11	12664541	0.033587	486484	0.716985	Sepsis (28	Sepsis (28	Sepsis (28	TRUE	igd	0.0027	8.33E-06	5211	M32651.m	TRUE	reported	DeuPwv	2	TRUE	0.003839	20.07597
5171	rs8059937	T	G	T	G	-0.0605	-0.0149	0.8405	0.83077	FALSE	FALSE	FALSE	ieu-b-508	16	7588994	0.04458	486484	0.738241	Sepsis (28	Sepsis (28	Sepsis (28	TRUE	igd	0.0135	7.88E-06	5064	M33194.m	TRUE	reported	xRUveq	2	TRUE	0.00395	20.07574
4196	rs1223898	C	G	C	G	0.1605	-0.11472	0.0233	0.013013	FALSE	TRUE	FALSE	ieu-b-508	1	10605895	0.149527	486484	0.442963	Sepsis (28	Sepsis (28	Sepsis (28	TRUE	igd	0.0358	7.51E-06	1672	M32762.m	TRUE	reported	Q5IKLH	2	TRUE	0.011878	20.0754
1921	rs4665701	T	C	T	C	-0.0121	0.05279	0.4689	0.465969	FALSE	FALSE	FALSE	ieu-b-508	2	24702158	0.033538	486484	0.115476	Sepsis (28	Sepsis (28	Sepsis (28	TRUE	igd	0.0027	4.90E-06	4689	M16816.m	TRUE	reported	J1abbO	2	TRUE	0.004265	20.07511
4668	rs1027439	T	G	T	G	0.348	0.044882	0.9757	0.978042	FALSE	FALSE	FALSE	ieu-b-508	7	98411352	0.114846	486484	0.695942	Sepsis (28	Sepsis (28	Sepsis (28	TRUE	igd	0.0745	2.99E-06	25	M33178.m	TRUE	reported	g22WMS	2	TRUE	0.466035	20.07399
1961	rs1322254	T	C	T	C	-0.0941	-3.23E-05	0.0194	0.019502	FALSE	FALSE	FALSE	ieu-b-508	7	1E+08	0.120449	486484	0.999786	Sepsis (28	Sepsis (28	Sepsis (28	TRUE	igd	0.021	7.57E-06	7226	M17807.m	TRUE	reported	zd6sZI	2	TRUE	0.002771	20.07338
5867	rs1766502	A	G	A	G	-0.0628	0.009592	0.7991	0.789471	FALSE	FALSE	FALSE	ieu-b-508	2	1.07E+08	0.040829	486484	0.814268	Sepsis (28	Sepsis (28	Sepsis (28	TRUE	igd	0.014	6.87E-06	824	M33609.m	TRUE	reported	h7B9gN	2	TRUE	0.023837	20.07279
3908	rs1082918	A	G	A	G	0.0372	-0.0171	0.8267	0.816596	FALSE	FALSE	FALSE	ieu-b-508	10	18962541	0.043444	486484	0.69383	Sepsis (28	Sepsis (28	Sepsis (28	TRUE	igd	0.0083	7.99E-06	2575	M32691.m	TRUE	reported	SiuD6F	2	TRUE	0.007741	20.07207
4090	rs1690821	T	C	T	C	0.0466	0.102297	0.924	0.915118	FALSE	FALSE	FALSE	ieu-b-508	8	1.39E+08	0.059862	486484	0.087476	Sepsis (28	Sepsis (28	Sepsis (28	TRUE	igd	0.0104	7.21E-06	7356	M32753.m	TRUE	reported	XlC6zq	2	TRUE	0.002722	20.07183
1964	rs1693451	A	C	A	C	-0.0233	-0.01369	0.5653	0.572023	FALSE	FALSE	FALSE	ieu-b-508	9	16358278	0.033908	486484	0.686452	Sepsis (28	Sepsis (28	Sepsis (28	TRUE	igd	0.0052	7.36E-06	7226	M17807.m	TRUE	reported	zd6sZI	2	TRUE	0.002771	20.07174
5483	rs1164492	A	T	A	T	-0.0233	-0.03811	0.6891	0.68728	FALSE	TRUE	FALSE	ieu-b-508	16	11645013	0.036348	486484	0.294467	Sepsis (28	Sepsis (28	Sepsis (28	TRUE	igd	0.0052	6.85E-06	6797	M33415.m	TRUE	reported	xHSphe	2	TRUE	0.002945	20.07139
5916	rs1193482	A	G	A	G	0.0354	-0.02598	0.8122	0.81121	FALSE	FALSE	FALSE	ieu-b-508	4	1.72E+08	0.042824	486484	0.544019	Sepsis (28	Sepsis (28	Sepsis (28	TRUE	igd	0.0079	7.80E-06	4323	M33633.m	TRUE	reported	e36XGE	2	TRUE	0.004623	20.07018
[truncated: 628,162 more chars]
