# Supplementary material for: Mendelian randomization analysis reveals causal associations of serum metabolites with sepsis and 28-day mortality
Source: Sci Rep. 2024 May 21;14:11551. doi: 10.1038/s41598-024-58160-1 (PMC11109149; doi:10.1038/s41598-024-58160-1)
Supplement: Supplementary file 3 — Supplementary Table 3. [file 41598_2024_58160_MOESM3_ESM.pdf]

Supplementary table 3. The reverse MR analysis of serum metabolites and sepsis using various methods.

Outcome	Exposure	Method	Nsnp	OR (95% CI)	P-value	Global_test_pval	Heterogeneity_pval	Intercept_pval
**Carbohydrate**								
glucose	sepsis	Inverse variance weightec	5	1.02 (0.99, 1.04)	0.191	0.852	0.850	0.961
glucose	sepsis	Weighted median	5	1.02 (0.99, 1.05)	0.265			
glucose	sepsis	MR Egger	5	1.01 (0.83, 1.23)	0.920			
glucose	sepsis	MR PRESSO	5	1.02 (1.01, 1.02)	0.089			
**Lipid**								
heptanoate (7:0)	sepsis	Inverse variance weightec	5	1.00 (0.97, 1.03)	0.967	0.582	0.541	0.723
heptanoate (7:0)	sepsis	Weighted median	5	1.00 (0.97, 1.04)	0.843			
heptanoate (7:0)	sepsis	MR Egger	5	0.95 (0.76, 1.20)	0.721			
heptanoate (7:0)	sepsis	MR PRESSO	5	1.00 (0.99, 1.01)	0.964			
androsterone sulfate	sepsis	Inverse variance weightec	5	0.98 (0.90, 1.07)	0.679	0.408	0.360	0.698
androsterone sulfate	sepsis	Weighted median	5	1.01 (0.89, 1.16)	0.837			
androsterone sulfate	sepsis	MR Egger	5	1.18 (0.50, 2.79)	0.729			
androsterone sulfate	sepsis	MR PRESSO	5	0.98 (0.96, 1.00)	0.712			
propionylcarnitine	sepsis	Inverse variance weightec	5	1.01 (0.98, 1.04)	0.620	0.652	0.632	0.240
propionylcarnitine	sepsis	Weighted median	5	1.02 (0.98, 1.06)	0.408			
propionylcarnitine	sepsis	MR Egger	5	1.21 (0.94, 1.56)	0.227			
propionylcarnitine	sepsis	MR PRESSO	5	1.01 (1.00, 1.02)	0.570			
epiandrosterone sulfate	sepsis	Inverse variance weightec	5	0.80 (0.58, 1.10)	0.171	0.413	0.334	0.622
epiandrosterone sulfate	sepsis	Weighted median	5	0.74 (0.48, 1.13)	0.162			
epiandrosterone sulfate	sepsis	MR Egger	5	1.85 (0.89, 3.00)	0.718			
epiandrosterone sulfate	sepsis	MR PRESSO	5	0.80 (0.73, 0.87)	0.270			
1-oleoylglycerophosphoetha	sepsis	Inverse variance weightec	5	1.02 (0.97, 1.07)	0.411	0.078	0.051	0.780
1-oleoylglycerophosphoetha	sepsis	Weighted median	5	1.02 (0.95, 1.10)	0.560			
1-oleoylglycerophosphoetha	sepsis	MR Egger	5	1.14 (0.55, 2.36)	0.742			
1-oleoylglycerophosphoetha	sepsis	MR PRESSO	5	1.02 (1.02, 1.02)	0.621			
**Peptide**								
X-14205--alpha-glutamylty	sepsis	Inverse variance weightec	5	1.03 (0.95, 1.12)	0.454	0.991	0.992	0.918
X-14205--alpha-glutamylty	sepsis	Weighted median	5	1.03 (0.93, 1.14)	0.571			
X-14205--alpha-glutamylty	sepsis	MR Egger	5	0.99 (0.50, 1.96)	0.985			
X-14205--alpha-glutamylty	sepsis	MR PRESSO	5	1.03 (1.02, 1.05)	0.045			
**Xenobiotics**								
salicylate	sepsis	Inverse variance weightec	5	0.84 (0.66, 1.07)	0.157	0.523	0.465	0.402
salicylate	sepsis	Weighted median	5	0.85 (0.61, 1.17)	0.319			
salicylate	sepsis	MR Egger	5	2.15 (0.32, 4.45)	0.487			
salicylate	sepsis	MR PRESSO	5	0.84 (0.78, 0.90)	0.209			
saccharin	sepsis	Inverse variance weightec	5	1.14 (0.97, 1.33)	0.114	0.808	0.804	0.846
saccharin	sepsis	Weighted median	5	1.17 (0.95, 1.45)	0.143			
saccharin	sepsis	MR Egger	5	1.31 (0.36, 4.71)	0.711			
saccharin	sepsis	MR PRESSO	5	1.14 (1.07, 1.20)	0.068			
**Unknown**								
X-11787	sepsis	Inverse variance weightec	5	1.01 (0.99, 1.03)	0.467	0.938	0.929	0.585
X-11787	sepsis	Weighted median	5	1.01 (0.98, 1.04)	0.460			
X-11787	sepsis	MR Egger	5	1.06 (0.90, 1.25)	0.538			
X-11787	sepsis	MR PRESSO	5	1.01 (1.00, 1.01)	0.193			
X-12063	sepsis	Inverse variance weightec	5	1.03 (0.96, 1.11)	0.415	0.31	0.265	0.446
X-12063	sepsis	Weighted median	5	1.06 (0.96, 1.17)	0.240			
X-12063	sepsis	MR Egger	5	1.40 (0.70, 2.83)	0.411			
X-12063	sepsis	MR PRESSO	5	1.03 (1.02, 1.05)	0.515			
X-13435	sepsis	Inverse variance weightec	5	0.99 (0.95, 1.03)	0.624	0.649	0.628	0.570
X-13435	sepsis	Weighted median	5	0.98 (0.93, 1.04)	0.548			
X-13435	sepsis	MR Egger	5	1.10 (0.78, 1.56)	0.609			
X-13435	sepsis	MR PRESSO	5	0.99 (0.98, 1.00)	0.575			
