# Supplementary material for: Mendelian randomization analysis reveals causal associations of serum metabolites with sepsis and 28-day mortality
Source: Sci Rep. 2024 May 21;14:11551. doi: 10.1038/s41598-024-58160-1 (PMC11109149; doi:10.1038/s41598-024-58160-1)
Supplement: Supplementary file 4 — Supplementary Table 4. [file 41598_2024_58160_MOESM4_ESM.pdf]

Supplementary table 4. The reverse MR analysis of serum metabolites and sepsis mortality within 28 days using various methods.

Outcome	Exposure	Method	Nsnp	OR (95% CI)	P-value	Global_test_pval	Heterogeneity_pval	Intercept_pval
**Amino acid**								
methionine	Sepsis (28 day death)	Inverse variance weighted	5	1.00 (1.00, 1.01)	0.177	0.775	0.745	0.355
methionine	Sepsis (28 day death)	Weighted median	5	1.00 (0.99, 1.01)	0.428			
methionine	Sepsis (28 day death)	MR Egger	5	1.03 (0.98, 1.08)	0.311			
methionine	Sepsis (28 day death)	MR PRESSO	5	1.00 (1.00, 1.01)	0.248			
4-acetamidobutanoate	Sepsis (28 day death)	Inverse variance weighted	5	0.99 (0.98, 1.01)	0.406	0.31	0.198	0.644
4-acetamidobutanoate	Sepsis (28 day death)	Weighted median	5	0.99 (0.98, 1.01)	0.402			
4-acetamidobutanoate	Sepsis (28 day death)	MR Egger	5	0.97 (0.87, 1.07)	0.583			
4-acetamidobutanoate	Sepsis (28 day death)	MR PRESSO	5	0.99 (0.98, 1.01)	0.452			
cysteine-glutathione disulfide	Sepsis (28 day death)	Inverse variance weighted	5	0.98 (0.94, 1.03)	0.491	0.227	0.174	0.638
cysteine-glutathione disulfide	Sepsis (28 day death)	Weighted median	5	1.01 (0.96, 1.06)	0.710			
cysteine-glutathione disulfide	Sepsis (28 day death)	MR Egger	5	1.08 (0.76, 1.54)	0.697			
cysteine-glutathione disulfide	Sepsis (28 day death)	MR PRESSO	5	0.98 (0.94, 1.03)	0.529			
3-(3-hydroxyphenyl)propionate	Sepsis (28 day death)	Inverse variance weighted	5	0.95 (0.90, 1.00)	0.056	0.59	0.552	0.713
3-(3-hydroxyphenyl)propionate	Sepsis (28 day death)	Weighted median	5	0.97 (0.89, 1.05)	0.450			
3-(3-hydroxyphenyl)propionate	Sepsis (28 day death)	MR Egger	5	0.86 (0.55, 1.36)	0.570			
3-(3-hydroxyphenyl)propionate	Sepsis (28 day death)	MR PRESSO	5	0.95 (0.88, 1.02)	0.129			
**Lipid**								
glycocholate	Sepsis (28 day death)	Inverse variance weighted	5	1.00 (0.96, 1.04)	0.950	0.335	0.269	0.927
glycocholate	Sepsis (28 day death)	Weighted median	5	0.99 (0.95, 1.04)	0.695			
glycocholate	Sepsis (28 day death)	MR Egger	5	0.98 (0.71, 1.35)	0.922			
glycocholate	Sepsis (28 day death)	MR PRESSO	5	1.00 (0.96, 1.04)	0.953			
taurochenodeoxycholate	Sepsis (28 day death)	Inverse variance weighted	5	0.99 (0.95, 1.03)	0.698	0.373	0.284	0.896
taurochenodeoxycholate	Sepsis (28 day death)	Weighted median	5	0.98 (0.94, 1.03)	0.542			
taurochenodeoxycholate	Sepsis (28 day death)	MR Egger	5	0.97 (0.69, 1.36)	0.862			
taurochenodeoxycholate	Sepsis (28 day death)	MR PRESSO	5	0.99 (0.95, 1.04)	0.718			
3-carboxy-4-methyl-5-propyl-2-furanpropanoate (CMPF)	Sepsis (28 day death)	Inverse variance weighted	5	0.99 (0.94, 1.04)	0.764	0.359	0.293	0.704
3-carboxy-4-methyl-5-propyl-2-furanpropanoate (CMPF)	Sepsis (28 day death)	Weighted median	5	1.01 (0.95, 1.07)	0.765			
3-carboxy-4-methyl-5-propyl-2-furanpropanoate (CMPF)	Sepsis (28 day death)	MR Egger	5	1.08 (0.72, 1.62)	0.732			
3-carboxy-4-methyl-5-propyl-2-furanpropanoate (CMPF)	Sepsis (28 day death)	MR PRESSO	5	0.99 (0.94, 1.05)	0.779			
1-stearoylglycerophosphocholine	Sepsis (28 day death)	Inverse variance weighted	5	1.01 (0.98, 1.03)	0.544	0.371	0.319	0.985
1-stearoylglycerophosphocholine	Sepsis (28 day death)	Weighted median	5	1.02 (0.99, 1.04)	0.285			
1-stearoylglycerophosphocholine	Sepsis (28 day death)	MR Egger	5	1.00 (0.84, 1.20)	0.961			
1-stearoylglycerophosphocholine	Sepsis (28 day death)	MR PRESSO	5	1.01 (0.98, 1.03)	0.577			
X-13183--stearamide	Sepsis (28 day death)	Inverse variance weighted	5	1.00 (0.94, 1.06)	0.929	0.061	0.026	0.890
X-13183--stearamide	Sepsis (28 day death)	Weighted median	5	0.97 (0.92, 1.03)	0.353			
X-13183--stearamide	Sepsis (28 day death)	MR Egger	5	1.04 (0.63, 1.69)	0.898			
X-13183--stearamide	Sepsis (28 day death)	MR PRESSO	5	1.00 (0.94, 1.06)	0.933			
**Peptide**								
aspartylphenylalanine	Sepsis (28 day death)	Inverse variance weighted	5	1.02 (0.99, 1.04)	0.190	0.71	0.681	0.702
aspartylphenylalanine	Sepsis (28 day death)	Weighted median	5	1.01 (0.97, 1.06)	0.528			
aspartylphenylalanine	Sepsis (28 day death)	MR Egger	5	0.97 (0.78, 1.21)	0.799			
aspartylphenylalanine	Sepsis (28 day death)	MR PRESSO	5	1.02 (0.99, 1.04)	0.260			
gamma-glutamylphenylalanine	Sepsis (28 day death)	Inverse variance weighted	5	1.00 (0.99, 1.01)	0.395	0.49	0.433	0.547
gamma-glutamylphenylalanine	Sepsis (28 day death)	Weighted median	5	1.00 (0.99, 1.01)	0.924			
gamma-glutamylphenylalanine	Sepsis (28 day death)	MR Egger	5	0.97 (0.90, 1.05)	0.492			
gamma-glutamylphenylalanine	Sepsis (28 day death)	MR PRESSO	5	1.00 (0.99, 1.01)	0.443			
gamma-glutamylglutamate	Sepsis (28 day death)	Inverse variance weighted	5	1.01 (0.98, 1.04)	0.529	0.765	0.742	0.743
gamma-glutamylglutamate	Sepsis (28 day death)	Weighted median	5	1.00 (0.96, 1.05)	0.911			
gamma-glutamylglutamate	Sepsis (28 day death)	MR Egger	5	0.96 (0.73, 1.26)	0.788			
gamma-glutamylglutamate	Sepsis (28 day death)	MR PRESSO	5	1.01 (0.98, 1.04)	0.563			
**Xenobiotics**								
cotinine	Sepsis (28 day death)	Inverse variance weighted	5	0.96 (0.86, 1.09)	0.541	0.258	0.224	0.636
cotinine	Sepsis (28 day death)	Weighted median	5	0.94 (0.82, 1.08)	0.378			
cotinine	Sepsis (28 day death)	MR Egger	5	0.75 (0.30, 1.91)	0.593			
cotinine	Sepsis (28 day death)	MR PRESSO	5	0.96 (0.85, 1.10)	0.574			
**Unknown**								
X-11529	Sepsis (28 day death)	Inverse variance weighted	5	0.96 (0.90, 1.03)	0.246	0.071	0.032	0.456
X-11529	Sepsis (28 day death)	Weighted median	5	0.94 (0.89, 1.01)	0.084			
X-11529	Sepsis (28 day death)	MR Egger	5	0.79 (0.49, 1.26)	0.389			
X-11529	Sepsis (28 day death)	MR PRESSO	5	0.96 (0.89, 1.04)	0.311			
X-11538	Sepsis (28 day death)	Inverse variance weighted	5	1.00 (0.98, 1.03)	0.658	0.599	0.557	0.628
X-11538	Sepsis (28 day death)	Weighted median	5	1.00 (0.97, 1.03)	0.967			
X-11538	Sepsis (28 day death)	MR Egger	5	1.05 (0.89, 1.25)	0.599			
X-11538	Sepsis (28 day death)	MR PRESSO	5	1.00 (0.98, 1.03)	0.681			
X-11550	Sepsis (28 day death)	Inverse variance weighted	5	1.00 (0.99, 1.01)	0.524	0.1	0.056	0.430
X-11550	Sepsis (28 day death)	Weighted median	5	1.00 (0.99, 1.01)	0.472			
X-11550	Sepsis (28 day death)	MR Egger	5	1.03 (0.95, 1.12)	0.475			
X-11550	Sepsis (28 day death)	MR PRESSO	5	1.00 (0.98, 1.01)	0.559			
X-12465	Sepsis (28 day death)	Inverse variance weighted	5	1.01 (0.97, 1.04)	0.777	0.165	0.121	0.887
X-12465	Sepsis (28 day death)	Weighted median	5	1.02 (0.98, 1.06)	0.381			
X-12465	Sepsis (28 day death)	MR Egger	5	1.03 (0.77, 1.38)	0.863			
X-12465	Sepsis (28 day death)	MR PRESSO	5	1.01 (0.97, 1.05)	0.791			
X-13549	Sepsis (28 day death)	Inverse variance weighted	5	1.00 (0.99, 1.01)	0.608	0.505	0.448	0.714
X-13549	Sepsis (28 day death)	Weighted median	5	0.99 (0.98, 1.01)	0.262			
X-13549	Sepsis (28 day death)	MR Egger	5	0.98 (0.91, 1.06)	0.675			
X-13549	Sepsis (28 day death)	MR PRESSO	5	1.00 (0.99, 1.01)	0.635			
